# Transcriptomic analyses reveal comprehensive responses of insect hemocytes to mycopathogen Beauveria bassiana, and fungal virulence-related cell wall protein assists pathogen to evade host cellular defense

**DOI:** 10.1080/21505594.2020.1827886

**Published:** 2020-10-05

**Authors:** Jin-Li Ding, Jia Hou, Ming-Guang Feng, Sheng-Hua Ying

**Affiliations:** Institute of Microbiology, College of Life Sciences, Zhejiang University, Hangzhou, China

**Keywords:** insect immunity, hemocyte response, encapsulation, cell-wall protein, entomopathogenic fungus, *Beauveria bassiana*, virulence

## Abstract

Entomopathogenic fungi naturally infect insect hosts in environment. Fungal invasion and host immune defense are still in the progress of co-evolution. In this study, entomopathogenic fungus *Beauveria bassiana* and lepidopteran insect *Galleria mellonella* were used to investigate host cellular immunity and fungal strategy to evade host defense. First of all, genome-wide expression revealed the transcriptomic responses of hemocytes to insect mycopathogen, which dynamically varied during infection process. Enrichment analysis indicated that differentially expressed genes were primarily involved in metabolism, cellular process and immune system. Notably, cellular response involved a series of hydrolytic enzyme and antimicrobial peptide genes which were sorted together in clustering analysis. In *B. bassiana*, a cell-wall protein gene (*BbCwp*) contributes to fungal development in host hemocoel and virulence. RT-qPCR analyses indicated that infection by Δ*BbCwp* mutant strain caused the up-regulated expression of a series of immunity-related genes, including β-1, 3-glucan recognition protein, hydrolytic enzyme and antimicrobial peptide genes. Disruption of *BbCwp* resulted in a significant change in conidial lectin-binding feature and the enhanced encapsulation by the host hemocytes. After being treated with hydrolytic enzymes, Δ*BbCwp* mutant displayed a significantly enhanced sensitivity to osmotic and oxidative stresses. In conclusion, fungal invasion initiates comprehensive physiological responses in the host hemocytes. For mycopathogen, cell-wall protein plays an important role in fungal evasion of immunity defense and colonization in host. Our studies provide an initial framework for exploring more mechanistic details about the fungus–host interaction.

## Introduction

*Beauveria bassiana* is a well-studied entomopathogenic fungus (EPF) and is of great potential as biological control agents of insect pests [1]. The parasitic processes of EPF involve following basic phases: spore adhesion to host, germination on cuticle surface, penetration through exoskeleton, colonization in hemocoel, and sporulation on the mycosed cadavers [2]. Successful colonization and propagation are essential for fungal virulence [3], but may be limited by the host immune defenses [4].

The larvae of the greater wax moth *Galleria mellonella* are excellent host models for studying the pathogenesis of mycopathogens [5], and have been widely used to study the mechanisms involved in the interaction of *B. bassiana* and its hosts [6,7]. In response to microbial invasion, *G. mellonella* adopts two types of innate immunity, i.e., humoral and cellular immunity [8]. The humoral type mainly depends on the synthesis of defense molecules, including the reactive radicals of oxygen and nitrogen [9], and antimicrobial peptides (AMPs). In *G. mellonella*, most AMPs (e.g., moricin-like peptide and gallerimycin) show both bactericidal and fungicidal activities, and are induced by bacterial cells or compositions [10,11]. However, *B. bassiana* induces host to synthesize the antifungal peptides (AFPs) (e.g., gallerimycin) and lysozyme, but not antibacterial peptides (e.g., cecropin) [12]. The cellular immunity involves the host hemocytes which inhibit and eliminate the invading microbes via phagocytosis, encapsulation, and nodulation [13]. For example, in *Spodoptera littoralis*, plasmatocytes, and granulocytes contribute to the host cellular immunity to *B. bassiana* [14]. In addition to humoral receptors (e.g., peptidoglycan recognition protein), the surface receptors on hemocytes mediate the recognition of the invading microbes by direct binding to the adapters on the microbial surfaces [15]. Upon infection of the hemocoel, the cellular immune is activated more quickly than the humoral response, and acts as an immediate response line [17]. Relative to humoral immunity [12,16], the hemocyte responses to the invading EPF are still largely unknown.

To overcome the host immune defense, EPF develops a plethora of strategies [17]. In *B. bassiana*, the bibenzoquinone oosporein acts as an immunosuppressor and facilitates fungal development in the host hemocoel [18]. For evasion of host recognition, fungal pathogens remodel their cell surface in response to the host [19]. In the host hemocoel, *B. bassiana* develops into yeast-like hyphal bodies (*in vivo* blastospores). These *in vivo* cells modify their surfaces with carbohydrate epitopes and lots of proteins to counteract insect immune defenses [20,21]. In *B. bassiana*, LysM effectors disrupt insect immune responses, and protect fungal cells from hydrolysis [6]. Similarly, in *Metarhizium anisopliae*, a collagen-like protein MCL1 masks the immunogenic β-1, 3-glucan in cell wall and protects pathogen from insect immunological recognition and phagocytosis [22]. Although, more and more investigations uncover the strategies used by EPF to overcome host cellular immunity, and this kind of arms race is still in the progress of co-evolution [17]. The hemocytes of *G. mellonella* have a high ability to form melanotic capsules, which indicates that the cellular immunity plays a more important role in counteracting the fungal pathogen [23]. Thus, the combination of *B. bassiana*/*G. mellonella* becomes an ideal system to explore the cellular immunity responses to EPF in the fungus–host interaction.

More understandings of the molecular responses of hemocytes would be critical to wider adoption of EPF as biocontrol agents. In this study, we characterized the transcriptomic responses of hemocytes to insect mycopathogen with RNA-seq analyses. Many differentially expressed genes (DEGs) were uncovered to be involved in the host cellular immunity. Of particular interest, a virulence-related cell-wall protein (BbCwp) [7] was found to assist *B. bassiana* to colonize in insect hemocoel by evasion of the host immune defense.

## Materials and methods

### Fungal strains and culturing conditions

The wild-type and Δ*BbCwp* mutant strains of *B. bassiana* ARSEF 2860 were maintained as previously described [7]. Sabouraud dextrose agar (SDAY: 4% glucose, 1% peptone, and 1.5% agar plus 1% yeast extract) was used to generate conidia as initial inocula for fungal transformation, phenotypic assays, and immunization of insects. Czapek-Dox plates (CZA: 3% glucose, 0.3% NaNO_3_, 0.1% K_2_HPO_4_, 0.05% KCl, 0.05% MgSO_4_ and 0.001% FeSO_4_ plus 1.5% agar) was used to screen the candidate transformants. Ammonium glufosinate or chlorsulfuron was included in CZA media as a selection reagent when required. All plates were incubated at 25°C with a photoperiod of 12/12 (day/night).

### *Illumina sequencing the responses of* G. mellonella *hemocytes challenged by* B. bassiana

Conidia of the wild-type strain were grown on SDAY plates for 7 d and prepared into conidial suspension (10^5^ conidia/ml). The larvae were feed with artificial diet and reared as previously described [24]. Last-instar larvae of *G. mellonella* (~300 mg in weight) were used in this study. To observe infection dynamics, the time-points of sampling were set at 1, 2, and 3 days post-infection (DPI). Each treatment included 35 larvae from same batch, and each sampling point included two independent replicates. Aliquots of 5-µl suspension were directly injected into healthy larvae which were incubated at 25°C, and the uninfected larvae were used as control. The surviving larvae were sampled at the indicated time-points and bled. The hemolymph was immediately mixed with anticoagulant solution (0.14 M NaCl, 0.1 M glucose, 26 mM citric acid, 30 mM trisodium citrate, 10 mM EDTA, pH 4.6) [3]. The host hemocytes were collected via centrifuging, and kept on ice.

Total RNA was extracted from the hemocyte sample. cDNA libraries were constructed as described previously [7]. Briefly, mRNA molecules were purified by using magnetic oligo(dT) beads and used as templates to synthesize first-strand cDNA using random hexamer primers, which was followed by second-strand cDNA synthesis. The purified dsDNA was end-repaired and created the cDNA library. The resultant libraries were paired-end sequenced on an Illumina HiSeq^TM^ 2500 platform at MicroAnaly Gene Technologies Co. (Hefei, Anhui, China). Sequence data were deposited in the NCBI Gene Expression Omnibus database and are accessible under GEO Series Accession No. GSE146263. Sample at each time-point was replicated twice in independent experiments (biological replicates).

After filtering, the resulting clean reads were mapped to the *G. mellonella* reference genome on NCBI (BioProjects: PRJNA498111 submitted by University of Illinois at Urbana-Champaign, USA) using the HISAT program [25]. All mapped reads were assembled and retrieved against the gene database of *G. mellonella*. The new transcripts were annotated via BLAST searches against NCBI non-redundant protein database. All identified genes were quantified in terms of the expected number of fragments per kilobase of transcript sequence per million base pairs sequenced (FPKM) with the software program Cufﬂinks [26]. The DEGs between the paired comparisons (sampling time vs. control) were analyzed with the Cuffdiff method [27]. DEGs were considered between two libraries when the q-value (false discovery rate) was less than 0.05 and an absolute value of log_2_Ratio was greater than 1.

For functional distribution analyses, all DEGs were first annotated with Gene Ontology (GO) and Kyoto Encyclopedia of Genes and Genomes (KEGG) terms. The down- and upregulated genes were separately subjected to enrichment analysis, using hypergeometric test. A functional term was enriched when a corrected *P*-value was less than 0.05. To view expression pattern during infection process, all DEGs were subjected to clustering analyses with *k*-mean method.

### Insect bioassay

The effects of *BbCwp* loss on conidial virulence assayed with the intra-hemocoel injection method. Conidia were obtained from 7-d old culture on SDAY plates. In the previous study, conidial concentration was 10^5^ conidia/ml [7]. In this study, conidial concentration was adjusted to 10^6^ and 10^7^ conidia/ml. Aliquots of 5-μl suspension were injected into *G. mellonella*. The inoculated larvae were reared at 25ºC as mentioned above. The mortality was recorded every day, and the median lethal time (LT_50_) was calculated by Probit analysis. Three parallel replicates were performed in each treatment, and each replicate included about 35 larvae.

To examine the development of *in vivo* hyphal bodies, conidial suspension (5 μl, 10^5^ conidia/ml) were injected into the host body. The infected insects were cultured at 25ºC. The host hemolymph was sampled at 2 and 3 DPI, respectively. The hyphal bodies in hemolymph were quantified and indicated as the cell number per milliliter of hemolymph.

To observe fungal evasion from host hemocytes, aliquots of 5-μl conidial suspension were injected into the larval hemocoel, and the infected larvae were grown at 25ºC. Conidial concentrations included 10^5^, 10^6^, and 10^7^ conidia/ml. The hemolymph was bled every 6 h post inoculation. The interaction between fungal cells and host hemocytes were recorded under a microscope. The higher the conidial concentration, the shorter the time for killing hosts. Experimental duration for 10^5^ conidia/ml was 72 h, and the durations for 10^6^ and 10^7^ conidia/ml were 54 h.

### Conidial lectin-binding traits

The Alexa fluor 488-labeled lectins were used to examine the lectin-binding profiles of conidia with the previous method [20]. Lectins included concanavalin A (ConA), *Galanthus nivalis* lectin (GNL), peanut agglutinin (PNA), and wheat germ agglutinin (WGA), and were purchased from Vector Laboratories, Inc. (Burlingame, California, USA). In brief, conidia were wetted in 0.02% Tween80 and collected by centrifuging at 12,000 g. Conidial pellets were resuspended and fixed in 3% (v/v) formaldehyde for 30 min, and rinsed three times with PBS buffer (137 mM NaCl, 2.7 mM KCl, 8.1 mM K_2_HPO_4_ and 1.5 mM KH_2_PO_4_, pH7.4). The fixed conidia were buffed in binding buffer for each lectin and labeled with lectin for 1 h in darkness. Unbound and residual lectins were removed by washing five times with the binding buffer. Fluorescent signals of 2 × 10^4^ conidia were quantified on a CytoFLEX LX flow cytometer (Beckman Coulter Life Sciences, Indianapolis, USA) using an argon laser at the excitation/emission wave lengths of 488/530 nm. Each assay included three independent replicates.

### Immune recognition at early stage of infection

All the primers used were included in Supporting Information Table S1. The promoter TEF fragment was amplified with the primers TEF-F/TEF-R, and then TEF fragment was purified and cloned into the *Eco*RI sites of p0380-sur [28] (conferring resistance to chlorsulfuron) with the ClonExpress II One Step Cloning Kit (Vazyme Biotech, Nanjing, China), generating vector p0380-tef-sur. The coding sequence of *mCherry* was amplified by mCherry-F/mCherry-R primers and ligated to the *Xma*I site of p0380-tef-sur. The resultant plasmid (p0380-TmC-sur) was individually introduced into the wild-type and Δ*BbCwp* mutant strains.

The transformants labeled with red fluorescent signals were cultured on SDAY plates till conidiation. Aliquots of 5-μl conidial suspension (10^8^ conidia/ml) were injected into the host hemocoel. After an incubation of 3 h at 25°C, hemolymph was bled, and hemocytes were observed under a fluorescence microscope.

### Real-time PCR analysis

Real-time PCR analysis was used to evaluate the effects of *BbCwp* loss on the responses of host hemocytes. Conidia (500 cells) of the wild-type and Δ*BbCwp* mutant strains were injected into the *G. mellonella* larvae which were cultivated at 25°C. Hemolymph was sampled at 1, 2, and 3 DPI, and hemocytes were separated by centrifuging.

Total RNAs were extracted using TRIzol® Reagent (Sigma-Aldrich, Missouri, USA) according to the manufacturer’s instructions. cDNA was prepared from total RNA in a 50-µl reaction mixture using PrimeScript^TM^ RT Reagent Kit (TaKaRa, Dalian, China) following the manufacturer’s instructions. After dilution (1/10), cDNA sample was used as template for real-time PCR analyses. The 18S rRNA gene was used as an internal reference to normalize the amount of RNA template. All the primers were included in Table S2. The relative expression levels of target gene were calculated by the 2^−ΔΔCT^ method [29]. These experiments were repeated four times for same sample.

### Conidial germination after the stresses of hydrolytic enzymes

Lysozyme and chitinase were used to evaluate the effects of *BbCwp* loss on conidial resistance to hydrolytic enzymes. Conidial concentration in suspension was adjusted to 10^4^ conidia/ml. The final concentration of lysozyme was adjusted to 50, 100, and 150 mg/ml; the chitinase concentration was 2, 4, and 6 U/ml. The buffer without enzyme was used as control. After a 3-h enzymolysis at 25°C, conidial suspension was diluted by tenfold. Then, the resultant suspension (100 μl) was smeared onto CZA plates supplemented with different stress reagents, including 0.5 M NaCl, 1 M sorbitol, and 0.02 mM menadione. The CZA plates without chemicals were used as control. After an incubation of 5-d at 25ºC, the colony number was counted and used to calculate survival percentage.

### Statistical analyses

Tukey’s honest significance test (Tukey’s HSD) was used to determine the significance in the indicated phenotype among different treatments. P-values less than 0.05 were considered as significant.

## Results

### Dynamic transcriptomes of hemocytes after fungal invasion

To identify genes involved in cellular immunity of *G. mellonella* to *B. bassiana*, eight cDNA libraries (four time-points × two replicates) were constructed from larval hemocytes at 1, 2, and 3 d after fungal infection as well as the control hemocytes. Correlation analyses indicated that the correlation coefficient between two replicates were not less than 0.99 (Figure S1). The features for each library were summarized in Table S3. All libraries had a Q20 value of > 97.7%, and all Q30 values were greater than 93.3%. Among all libraries, the percentage of mapped reads to total reads ranged from 76.16 to 82.70%. In addition, 938 novel transcripts were identified and annotated (Table S4).

**Figure 1.**
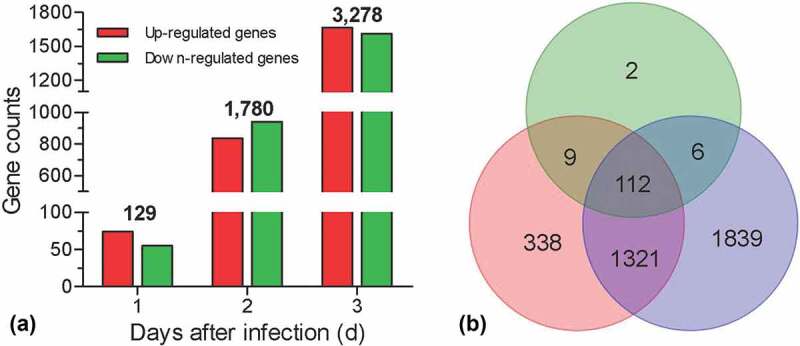
Summary of RNA-seq analyses. Global expression analyses were performed on the *G. mellonella* hemocytes challenged by *B. bassiana*. The hemocytes were sampled at 1, 2, and 3 days post infection, generation three libraries. The hemocytes without fungal challenge were used as control to screen the differential expression genes (DEGs). (a) Numbers of the DEGs. The number on the column indicates the total DEGs at different days post infection. (b) Venn diagram showing the number of overlapping genes among three libraries.

To identify the induced genes after fungal invasion, the pairwise comparison was performed between the sampling time-point and control. Relative to control, genes with FDR ≤ 0.05 and |log_2_Ratio| ≥1 were considered as DEGs. Our results displayed that there were 129, 1780, and 3283 genes that were significantly changed in hemocytes at 1, 2, and 3 DPI, respectively (Figure 1(a), Table S5-7). A Venn diagram showed that there 112 DEGs were overlapped among three time-points, whereas 2, 338, and 1839 DEGs specifically appeared at 1, 2, and 3 DPI, respectively (Figure 1(b)).

### Functional annotation and enrichment analyses of DEGs

**Figure 2.**
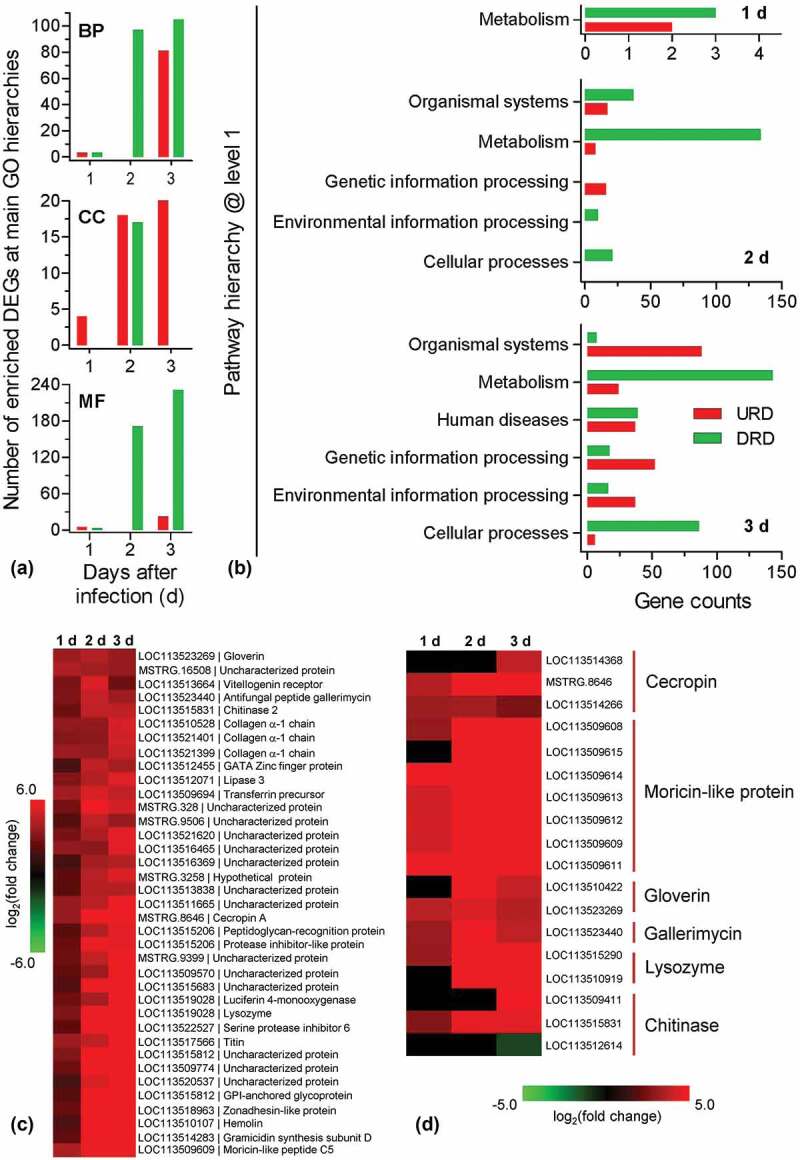
Enrichment and clustering analyses for the differential expression genes (DEGs). All DEGs at the indicated time point were divided into the up- and down-regulated DEGs (URD and DRD) which were separately subjected to GO and KEGG analyses. (a) GO analysis indicated that DEGs were enriched in biological process (BP), cellular component (CC) and molecular function (MF). (b) KEGG analyses indicated that the enriched pathways at main level. The pathway of “metabolism” was enriched at three time point during infection process. (c) Representative cluster from clustering analyses. All DEGs were analyzed with *k*-mean clustering. This cluster includes a series of immune-relate genes whose expression is enhanced during the infection process. (d) Heat map showing the expression patterns for the representative hydrolytic enzyme and antimicrobial peptide genes.

Following GO annotation, DEGs were enriched to different functional terms belonging to biological process (BP), cellular component (CC), and molecular function (MF) categories (Table S8). As illustrated in Figure 2(a), the number of enriched DEGs changed dynamically during infection process. The changing trends were similar between BP and MF categories. Only down-regulated DEGs were enriched at 2 DPI, whereas the up-regulated DEGs were enriched at 1 and 3 DPI. However, in CC categories, the up-regulated DEGs were consistently enriched at 1, 2, and 3 DPI, whereas down-regulated DEGs were only enriched at 2 DPI. In CC categories, the up-regulated DEGs had different expression profiles. For example, the expression of some genes (e.g., serine protease inhibitor and transferrin) was enhanced at 1 DPI; some genes (e.g., β-1, 3-glucan recognition protein) were induced at 2 DPI; some genes (e.g., cecropin B) were only induced at 3 DPI.

The KEGG classification system sorted DEGs into different pathways (Table S9). As shown in Figure 2(b), the enriched main pathways (level 1 in KEGG hierarchic system) and DEG numbers varied during the infection process. At 1 DPI, DEGs were only enriched to the category of “metabolism”. At 2 and 3 DPI, the category of “metabolism” constituted the largest group. Relative to 2 DPI, there was an additional pathway (human disease) enriched. At 3 DPI, the enriched DEGs were significantly over-presented in four main pathways, i.e., organismal systems, genetic information processing, environmental information processing, and cellular processes. At lower-level hierarchies, there was more detailed information. At 1 DPI, the up- and down-regulated DEGs were associated with taurine/hypotaurine metabolism and arginine biosynthesis, respectively (Table S9). At 2 DPI, the up-regulated DEGs were associated with protein processing in endoplasmic reticulum and sphingolipid metabolism. The repressed DEGs were mainly involved in metabolism, including many genes associated with amino acid metabolism, fatty acid degradation, glycosphingolipid biosynthesis and so on (Table S9). At 3 DPI, the induced DEGs were chiefly enriched in the category of organismal systems, including many genes involved in aging, digestive system, immune system, and so on. The immune system-associated DEGs included a large number of genes involved in natural killer cell-mediated cytotoxicity, Toll-like receptor signaling pathway (e.g., serine/threonine-protein kinase Pelle) and so on. These two pathways had some overlapped DEGs (e.g., Ras-related protein Rac1 and phosphatidylinositol 4, 5-bisphosphate 3-kinase). The down-regulated DEGs were mainly related to the category of metabolism, including amino acid metabolism, carbohydrate metabolism, fatty acid metabolism and so on (Table S9).

All DEGs were pooled together, and their expression modes were grouped with *k*-means clustering method. The results indicated that all DEGs were classified into 15 clusters (Table S10). As a representative illustrated in Figure 2(c), 37 up-regulated DEGs formed a cluster in which there some hydrolytic enzymes (e.g., chitinase and lysozyme) and antimicrobial peptides (AMPs) (e.g., gallerimycin and moricin-like protein). All DEGs related to lysozyme and chitinase as well as AMPs were summarized in Figure 2(d). All selected AMP and lysozyme genes displayed the progressively enhanced expression. Among three chitinases, only a chitinase-3 like gene exhibited a slight decrease in expression at 3 DPI.

### BbCwp *contributes to fungal colonization in host hemocoel*

**Figure 3.**
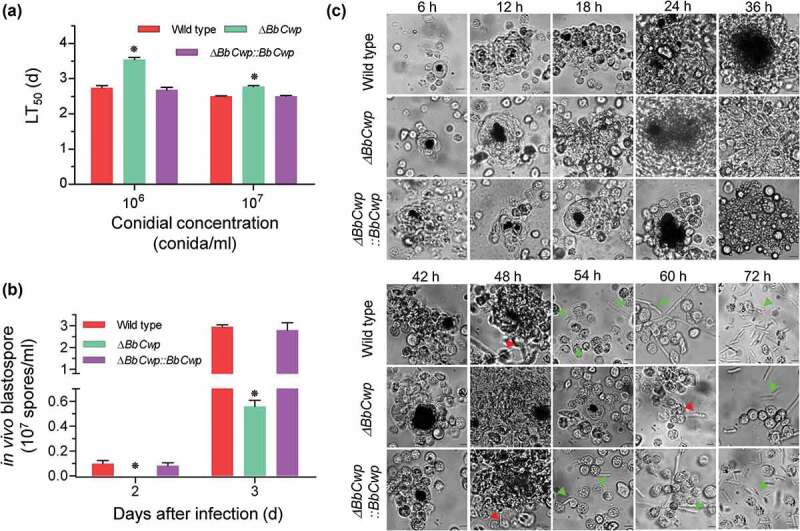
*BbCwp* roles in the fungus-host interaction. (a) Insect bioassay. Fungal virulence was evaluated via intra-hemocoel injection method. Conidial suspension (5 µl) was directly injected into the host hemocoel. The infected insects were reared at 25°C, and the mortality was recorded every day. The median lethal time (LT_50_) was calculated with Probit analysis. (b) *BbCwp* contributes to fungal development in the host hemocoel. Gene loss resulted in a significant reduction in the hyphal-body production. (c) Fungal colonization in the insect hemocoel. Conidial concentration (5 µl, 10^5^ spores/ml) was injected into the host hemocoel, and the hosts were reared at 25ºC. The host hemolymph was sampled every 6 h. Insect hemocyte encapsulation was observed after fungal invasion. After 48 h, the wild-strain formed detectable *in vivo* blastospores. However, the blastospores for Δ*BbCwp* mutant were seen at 60 h post infection. Red arrows indicate the fungal cells evading from hemocyte encapsulation, and green arrows indicate the free hyphal bodies (Tukey’s HSD: *P* < 0.05). Scale: 5 µm.

As shown in Figure 3(a), *BbCwp* contributes to conidial virulence. When applying conidial concentration of 10^6^ cells/ml, the LT_50_ values for the wild-type and complemented strains were 2.74 ± 0.06 (mean ± standard deviation) and 2.67 ± 0.08 d, respectively; whereas the Δ*BbCwp* mutant strain showed a moderate delay (29%) in LT_50_ (3.54 ± 0.06 d). When injecting more conidia (10^7^ cells/ml), LT_50_ value for Δ*BbCwp* mutant was 2.77 ± 0.03 d, with a slight decrease (11%), when compared to the wild-type strain (2.50 ± 0.02 d). The complemented strain did not show any significance with the wild type.

Previous study had indicated that Δ*BbCwp* mutant strain displayed a delay of 37% in LT_50_ value when using conidial concentration of 10^5^ cells/ml [7]. As shown in Figure 3(a), Δ*BbCwp* mutant strain displayed the delayed LT_50_ values at conidial concentrations of 10^6^ and 10^7^ spores/ml, respectively, when compared to the wild-type and complementation strains. However, the delay in LT_50_ values decreased with the increase of conidial concentration. Thus, we performed a further experiment to view fungal colonization in host body by using conidial concentration of 10^5^ cells/ml. As illustrated in Figure 3(b), no any detectable hyphal body was observed in the host hemocoel infected by Δ*BbCwp* mutant strain at 2 DPI. At 3 DPI, the mutant strain produced 0.56 ± 0.05 (mean ± standard deviation) × 10^7^ cells/ml, with approximate reduction of 80%, when compared with that of the wild type (0.56 ± 0.05 × 10^7^ cells/ml). The typical cellular immune responses (i.e., hemocyte nodulation and melanization) were also observed during fungal colonization process (Figure 3(c)). After fungal invasion, host hemocytes flocked together and form melanic dots. There were no significant difference in hemocyte response between the Δ*BbCwp* mutant and wild-type strains up to 42 h post-infection (HPI). At 48 HPI, the wild-type and complemented strains began to produce hyphal bodies from a cluster of hemocytes, and the gene disruption mutant escaped from the host hemocytes at 60 HPI. At 72 HPI, all strains produced a number of free-floating hyphal bodies. Increasing conidial concentration (10^6^ and 10^7^ conidia/ml) could significantly decrease the time for hyphal body formation, but the difference still persisted between the wild-type and Δ*BbCwp* mutant strains (Figure S2).

**Figure 4.**
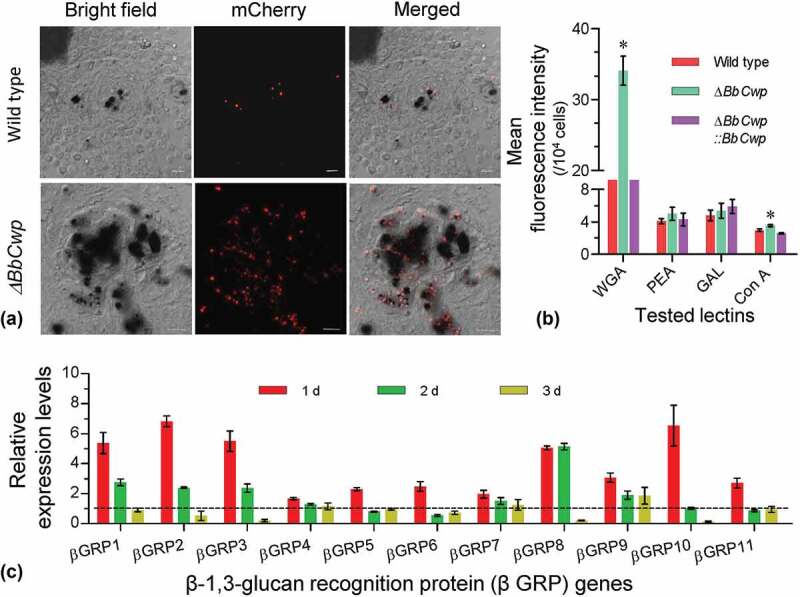
BbCwp protects fungus from host recognition. (a) Disruption of *BbCwp* resulted in an enhanced hemocyte encapsulation. Fungal strain was labeled by expressing the *mCherry* gene. Conidial suspension (5 µl, 10^5^ conidia/ml) was injected into host. After an incubation of 3 h at 25°C, hemocyte encapsulation was examined under a fluorescence microscope. (b) Conidial lectin-binding pattern. Lectins included concanavalin A (ConA), *Galanthus nivalis* lectin (GNL), peanut agglutinin (PNA), and wheat germ agglutinin (WGA). Δ*BbCwp* mutant strain displayed a significant increase in fluorescence intensity of WGA. (c) Relative expression levels of β-1, 3-glucan recognition protein genes (*βGRP*). In *G. mellonella*, there are 11 *βGRP* genes. Comparative analyses between the wild type/Δ*BbCwp* were performed at different time points during infection process. Gene disruption led to a significant up-regulation of all tested genes at 1 d post infection. Tukey’s HSD was used to determine the statistical significance using a threshold of *P* < 0.05. Error bars: standard deviation.

To view hemocyte encapsulation at the early stage of fungal invasion, fungal conidia were labeled by expressing a *mCherry* gene under a constitutive promoter. Upon entering into host hemocoel, conidia were wrapped by hemocyte layer after layer, and most existed in the melanic regions (Figure 4(a)). Flow cytometry analyses indicated that disruption of *BbCwp* changed conidial lectin-binding features (Figure 4(b)). Δ*BbCwp* mutant strain displayed a significantly enhanced ability to bind WGA and Con A, and the affinities to other two lectins were not significantly changed. In addition, Δ*BbCwp* mutant induced different transcriptional responses of host hemocytes (Figure 4(c)). RT-qPCR analyses indicated that the expression of 11 *βGRP* genes at 1 DPI could be more highly induced in hemocytes infected by Δ*BbCwp* mutant than by the wild-type strain. The induction modes among *βGRP* genes were different at 2 and 3 DPI.

### Conidial survival under the chemical and biochemical stresses

**Figure 5.**
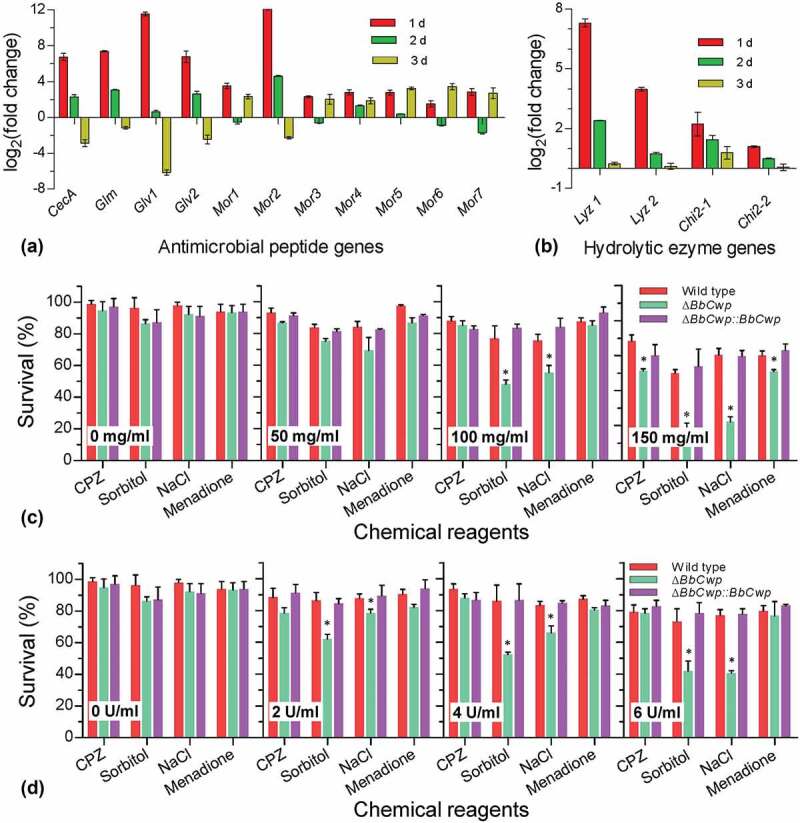
*BbCwp* roles in fungal adaptation to enzymolysis stresses. Relative expression levels of antimicrobial peptide (a) and hydrolytic enzyme (b) genes were analyzed at different time points during infection. Disruption of *BbCwp* caused the enhanced expression of tested genes at 1 d post infection. Lysozyme (c) and chitinase (d) were used to test conidial resistance to enzymolysis. The lysozyme concentration was adjusted to 0, 50, 100, and 150 mg/ml. The chitinase concentration was set to 0, 2, 4, and 6 U/ml. After being treated with hydrolytic enzymes, conidia were inoculated on Czapek–Dox plates (CPZ) supplemented with chemicals, using CPZ medium as control. After an incubation of 5-d at 25°C, colony number was determined and used to calculate survival percentage. Enzymolysis caused Δ*BbCwp* mutant strain to display the enhanced sensitivity to chemical stresses. Asterisks on columns indicate a significant difference between the Δ*BbCwp* mutant and wild-type or complemented strain (Tukey’s HSD: *P* < 0.05). Error bars represent standard deviation.

RT-qPCR analyses indicated that Δ*BbCwp* mutant strain led to altered transcriptional responses of hymocytes challenged by fungal cells (Figure 5(a), (B)). The expression of 11 AMP genes at 1 DPI could be more highly enhanced in hemocytes infected by the gene disruption mutant than by the wild-type strain. With infection processing, the expression of AMP genes appeared different trends. Similarly, five hydrolytic enzyme genes at 1 DPI were significantly induced in hemocytes infected by the mutant strain. Relative to AMP genes, this induction mode of hydrolytic enzyme genes only disappeared at 3 DPI, but was not reversed. Thus, hydrolytic enzymes were used as biochemical stressors to investigate conidial resistance to environmental stresses.

After being hydrolyzed with lysozyme (Figure 5(c)), conidia were stressed under different chemical stresses. Survival rates under different chemical stresses were not significantly changed when the lysozyme concentrations were less than 50 mg/ml. With increasing concentration of lysozyme, significant variation in survival rate were found on the CPZ plates included NaCl, sorbitol, and menadione. Similarly, chitinase treatment resulted in decreased survival rates on plates containing NaCl and sorbitol (Figure 5(d)). With increasing concentration of chitinase, the reduction in survival rate increased on the indicated medium.

## Discussion

*G. mellonella* and its natural mycopathogen *B. bassiana* is an ideal model for exploring insect innate immunity and fungal strategies to withstand [8]. In this study, we examined the dynamic transcriptomic responses of *G. mellonella* hemocytes against the filamentous entomopathogenic fungus. It was found that cellular immune response producing some molecules involved in humoral response. In addition, a cell wall protein (BbCwp) was found to assist fungal colonization in host hemocoel.

After invading into the host, *B. bassiana* triggers the cellular immune and its cells are wrapped by hemocytes. Fungal invasion dynamically remodels a wide range of physiological processes in hemocytes. In terms of physiological functions, the enhanced processes are associated with metabolism, cellular structure and function, organ system, immune system and so on. More detailed, the processes associated with immune system mainly include immune recognition, signaling transduction as well as biosynthesis of effector molecules. Although there are some similarities, these findings are different from those in transcriptomic responses of *Helicoverpa armigera* hemocytes to *B. bassiana* [30] and *G. mellonella* whole body to bacterial invasion [31]. The immune-related responses of *G. mellonella* hemocytes to *B. bassiana* are discussed below.

Pathogen recognition is essential for initiation of host immune defense and is accomplished by a series of pattern-recognition receptors (PRRs), including peptidoglycan recognition protein (PGRP), β-1, 3-glucan recognition proteins (βGRP), hemolin, and so on [32]. β-1, 3-glucan acts as a main component of cell walls in filamentous fungi [33]. In *Bombyx mori*, β-1, 3-glucan activates prophenoloxidase cascade, and is recognized by βGRPs [34]. Similarly, β-1, 3-glucan inoculation results in cellular and humoral responses in *G. mellonella* [35]. In *G. mellonella* hemocytes, no *βGRP* gene was repressed, and all up-regulated *βGRP* genes appeared at 2 days after treatment of *B. bassiana*. This indicates that, with the progression of infection, all *βGRP* are required for the cellular responses to fungal challenge. However, in the whole tissue of *Plutella xylostella*, not all *βGRP* genes are induced by infection of insect mycopathogen *Isaria fumosorose*a [36]. When infecting with *Candida albicans*, some *βGRP* genes are repressed in *G. mellonella* [37]. For human pathogen *Aspergillus fumigates*, human ficolins interact with glucan in cell wall and play critical roles in pathogen recognition [33]. Questions about whether there are other *G. mellonella* PRRs recognizing *B. bassiana* glucan are still open. *B. bassiana* infection also enhances the expression of PRR genes which are involved in recognition of bacteria. For example, C-type lectins (CTLs) associate with various sugar moieties (e.g., mannose and galactose), and play an important role in bacterial agglutination [38]. Two *CTL* genes were induced in *G. mellonella* hemocytes. In addition, four *PGRP* genes in hemocytes were induced by *B. bassiana* invasion. The similar results were observed in transcriptomic responses of fat body (*H. armigera*) and whole body (*Ostrinia furnacallis*) infected by *B. bassiana* [30,39]. Invasion of *C. albicans* induces the expression of *G. mellonella PGRP* genes [37]. However, most *PGRP* genes are repressed in *P. xylostella* infected by *I. fumosorosea* [36]. These findings suggest that the expression patterns of *PRR* genes varied with different fungus–host interactions.

The Toll pathway is primarily activated by fungi [40]. In the present study, we found that a spätzle gene was induced in hemocytes at the early stage of infection. At the late stage, Toll-like receptor signaling pathway is activated. In *Drosophila* species, spätzle protein functions as the ligand for the Toll-1 receptor and activates the expression of AMP genes [41]. This result suggests that *G. mellonella* hemocytes sequentially perform transcriptional regulation of Toll pathway.

In humoral immunity, there are two important processes (i.e., melanization and AMP formation) [8]. Melanization involves prophenoloxidase cascade. The prophenoloxidase complex consists of serine protease and its inhibitor [42]. In *G. mellonella* hemocytes, prophenoloxidase genes were down-regulated. Similar results were seen in the treatment of *C. albicans* [37]. Most serine protease (14/19) and its inhibitor genes (2/3) were up-regulated, respectively. As a result, *B. bassiana* infection causes significant melanization accompanied by nodulation. This complicated transcriptional response might be the outcome from the complex interaction between fungus and host. To the contrary, all identified AMP genes, including moricin, defensin, gloverin, gallerimycin, and cecropin, are enhanced at different stages of infection process. Moricins have a broad of activities against Gram negative and positive bacteria, as well as yeast and filamentous fungi [11]. Cecropins are another group of AMPs with antibacterial and antifungal activity [43]. So far, four types of cecropins have been characterized in *G. mellonella* [31]. When responding to *B. bassiana*, hemocytes mainly depend on cecropin A and B. As for gallerimycin, it is a peptide with sequence similarities to the antifungal peptides drosomycin. Its recombinant peptides exhibit activity against the entomopathogenic fungus *Metarhizium anisopliae*, but not against yeasts and bacteria [10]. Gloverins have first been isolated *Hyalophora gloveri*, and exert high activity against bacteria (e.g., *Escherichia coli*) [44]. Defensin represents another group of antibacterial peptides [45]. Although most AFPs in hemolymph are considered to be synthesized in the fat body [8], this study provides new clues for the hemocyte origin of AFPs. Fungal challenge activates the expression of bactericidal peptides. Accordingly, antifungal peptides (e.g., gallerimycin) are also induced by bacterial components (e.g., lipopolysaccharide) [10]. This suggests that there might be common pathways involved in insect immune response to different pathogens. Furthermore, the lysozymes in hemolymph function as important components of insect humoral immune response [8]. In *G. mellonella*, lysozyme belongs to the c-type family and exerts antifungal activity [46]. After fungal challenge, *G. mellonella* hemocytes increase the expression of lysozyme genes to counteract the invading fungus. In addition, fungal invasion induces the up-regulated expression of chitinase genes in hemocytes. Chitinases belong to glycosylhydrolases that hydrolyze the β-1, 4-glycosidic linkage in chitin [47]. The finding in this study is similar to that in mammals. Once recognizing the intruding fungus, mammalian neutrophils and macrophages secrete chitinases to eliminate pathogens [48]. In insect, the main function of chitinases is to digest the chitin-containing diets in gut and reuse the chitin-containing extracellular matrices during molting [49]. In this study, the expression of a chitinase-3-like protein 2 gene (*CHI3L2*) negatively associates with infection. There is very few information about its roles in insect. In human, CHI3L2 lacks chitinase catalytic activity and is involved in osteoarthritis [50]. This study implies that the member of the chitinase family may contribute to insect immune to invasive fungus in diverse manners.

In *B. bassiana*, β-1, 3-glucan, chitin, and protein constitute the main cell-wall components responsible for the cell-wall structure and function [7,51]. BbCwp is a cell-wall protein. Its loss has no effects on the *in vitro* blastospore development, but weakens fungal virulence [7]. Meanwhile, the loss of *BbCwp* results in the delay of fungal cells escaping from hemocyte nodulation. The reduction in virulence and the delay in evasion decrease with the increasing quantities of invading fungal cells. This study unveiled that *BbCwp* maintains the lectin-binding feature of cell surface and prevents fungal cells from nodulation by the host hemocytes. The *BbCwp* mutation exposes a large number of carbohydrate epitopes to lectin WGA which is the best investigated chitin-binding protein [52]. The *BbCwp*-mediated homeostasis of cell wall contributes to the transcriptional responses of the host hemocytes. Compared with larvae infected the wild-type strain, Δ*BbCwp* mutant induces a higher expression levels of a number of immune-related genes, including βGRP, hydrolytic enzyme, and AMP genes. This result is similar to those in the *B. bassiana* disruption mutant of *Bly5* gene whose products have affinity to chitin in cell walls [6]. Chitin could motivate insects to express AMP genes [53]. Thus, in normal state, BbCwp protein predominately masks chitin in cell wall and functions as a shield to prevent cells from host recognition and immune activation. Additionally, BbCwp protein acts as a coat to protect fungi from being damaged by hydrolytic enzymes. Without protection of BbCwp protein, the chitinase-attacked fungal cells are hard to proliferate under osmotic conditions. In addition, BbCwp protein contributes to protecting the lysozyme-treated cells from osmotic and oxidative stresses. Lysozymes are well known for its antibacterial activity, and their antifungal activities are related to disturbing the integrity of cell wall and membrane [46]. Insect hymolymph is hyperosmotic to fungal cells [54]. In cellular immune response, *G. mellonella* hemocytes produce reactive oxygen species (e.g., O_2_^−^) [55]. These findings suggest that BbCwp assists fungus to propagate in the host hemocoel under the combined physical and biochemical stresses. In *B. bassiana*, LysM effectors mediate evasion of host immune responses via preventing cells from chitinase hydrolysis [6]. *M. anisopliae* uses a collagen-like protein (MCL1) help cells to block the host recognition by shielding the β-1, 3-glucan in cell wall [22]. These findings suggest that it is a general strategy for entomopathogenic fungus to utilize a protein to shield cells from the host immune recognition.

In summary, we have unveiled a dynamic transcriptome of *G. mellonella* hemocytes challenged with an entomopathogenic fungus *B. bassiana*. These data massively expand the understanding of insect cellular immunity against natural mycopathogen. Upon fungal invasion, hemocytes dynamically regulate the comprehensive genes involved in metabolism and cellular functionality. Interestingly, hemocyte responses involve activation of the humoral immune-related genes, e.g., hydrolytic enzyme and AMP genes. On the fungal side, a cell-wall protein masks chitin in cell wall and assist fungal cells to escape from the host cellular immunity. Our studies provide a valuable framework for revealing more details about cellular immune responses in insect, and the reciprocal adaptation mechanisms behind the fungus–host interaction.

## Supplementary Material

Supplemental Material

## GEO reviewer link

Go to https://www.ncbi.nlm.nih.gov/geo/query/acc.cgi?acc=GSE146263

Enter token **mlstuwuclfedtwh** into the box
