# Supplementary material for: Transcriptomic analyses reveal comprehensive responses of insect hemocytes to mycopathogen Beauveria bassiana, and fungal virulence-related cell wall protein assists pathogen to evade host cellular defense
Source: Virulence. 2020 Oct 5;11(1):1352–65. doi: 10.1080/21505594.2020.1827886 (PMC7549920; doi:10.1080/21505594.2020.1827886)

Figure S1 Correlation degree between two libraries. Correlation analysis was performed between any two samples with Pearson method, and the coefficients between two replicates are indicated with white texts. CK: control group; INTxd: the experimental group infected with fungus at the indicated time-point; R: the independent replicate.

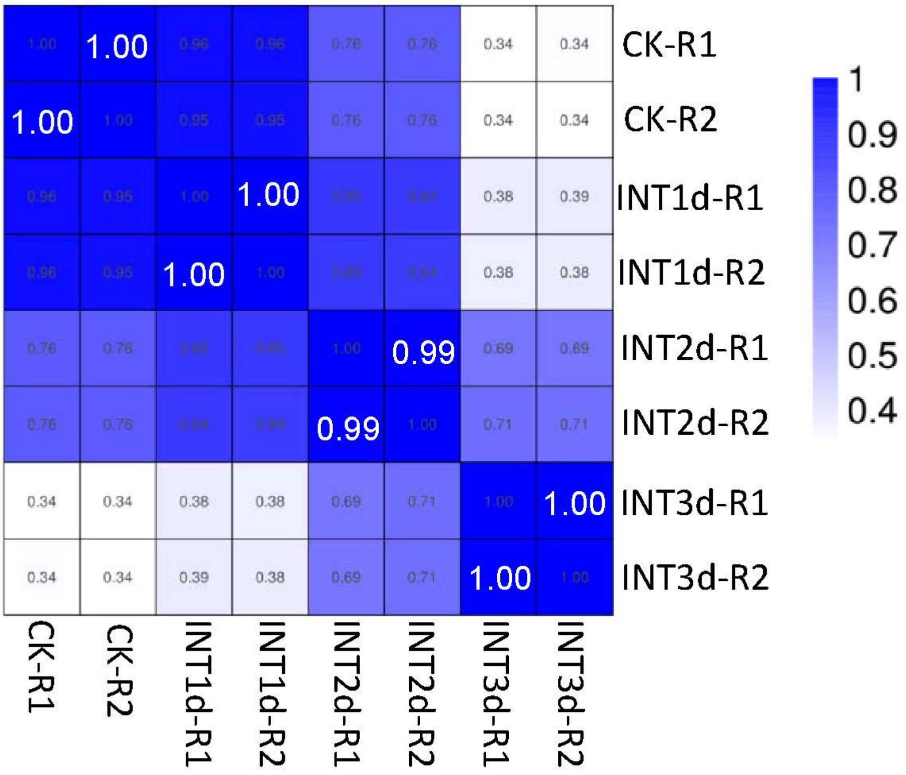

Figure S2 Fungal evasion from host defense and colonization in the insect hemocoel. Conidial concentration was adjusted to 10^6 (A) and 10^7 (B) cells/ml. Suspension (5 µl) was injected into the host hemocoel, and the hosts were reared at 25ºC. The host hemolymph was sampled every 6 h. Hemocyte responses and fungal colonization were examined. Red arrows indicate the hyphal bodies evading from hemocytes, and green arrows indicate the free hyphal bodies. Scale: 5 µm.

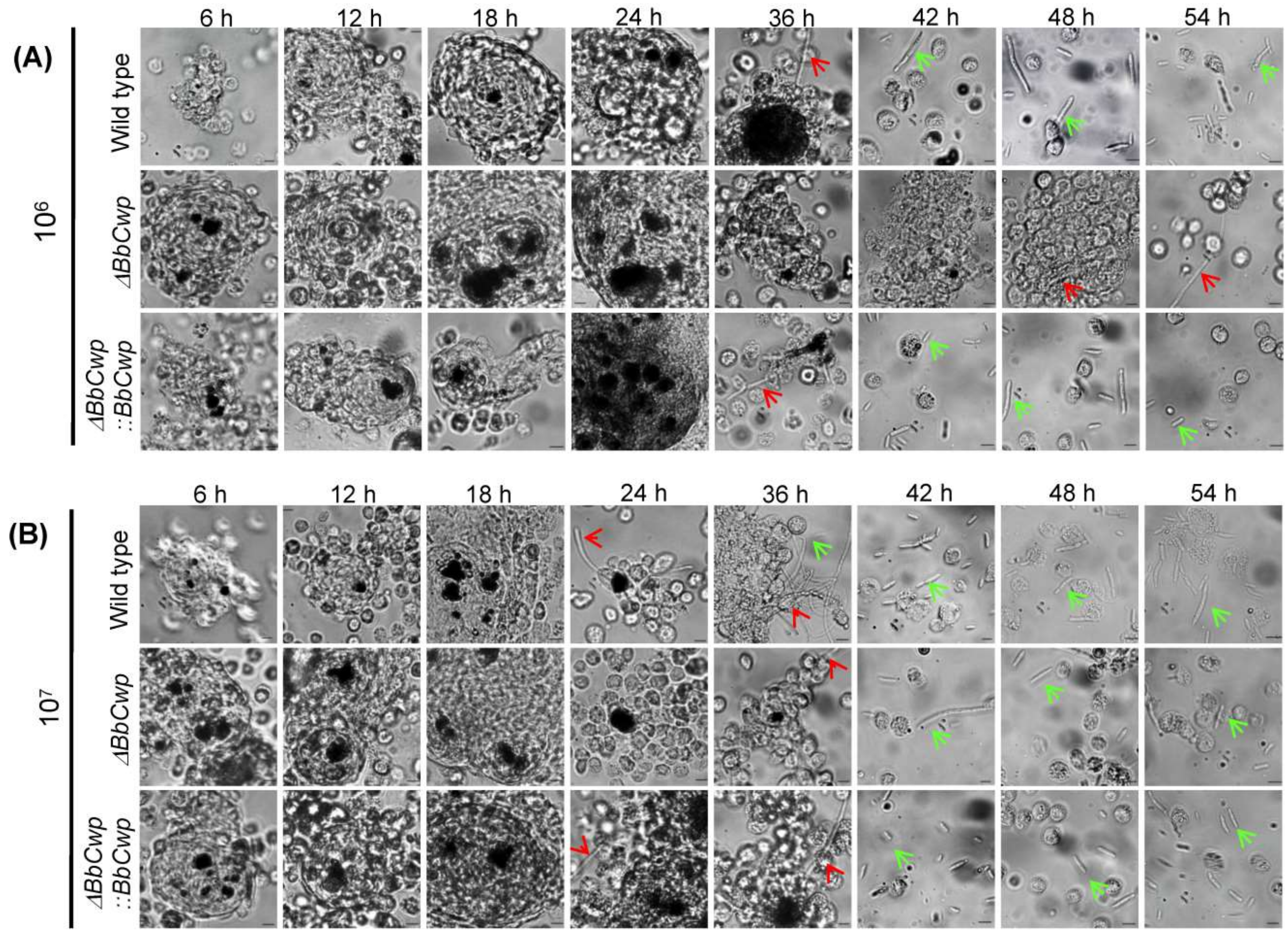

Supplement: Supplemental Material [file KVIR_A_1827886_SM8204.zip › Figure S1-2.pdf]
