# Supplementary material for: Transcriptomic analyses reveal comprehensive responses of insect hemocytes to mycopathogen Beauveria bassiana, and fungal virulence-related cell wall protein assists pathogen to evade host cellular defense
Source: Virulence. 2020 Oct 5;11(1):1352–65. doi: 10.1080/21505594.2020.1827886 (PMC7549920; doi:10.1080/21505594.2020.1827886)
Supplement: Supplemental Material [file KVIR_A_1827886_SM8204.zip › Table S1.pdf]

Table S1 Primer for plasmid construction

Primers	Sequences (5'-3')*	Purpose
TEF-F	GGCCACCATGTTGGGCCCGGCGCGCCTACTGCCGCAAGCAATTCTTTACGAGCAG	Cloning *TEF* promoter
TEF-R	CTGCAGGTCGACGGATCCCCGGGTTTGAAGGTGTTTGTGATTGAATGC	
mCherry-F	CAATCACAAACACCTTCAAACCCGGGATGGTGAGCAAGGGCGAGGAGGA	Cloning *mCherry* gene
mCherry-R	CTGCAGGTCGACGGATCCCCGGGCTACTTGTACAGCTCGTCCATGCC	

*: The underlined sequences are required for integrating the DNA fragment into plasmid via homologous recombination.
