# Supplementary material for: Transcriptomic analyses reveal comprehensive responses of insect hemocytes to mycopathogen Beauveria bassiana, and fungal virulence-related cell wall protein assists pathogen to evade host cellular defense
Source: Virulence. 2020 Oct 5;11(1):1352–65. doi: 10.1080/21505594.2020.1827886 (PMC7549920; doi:10.1080/21505594.2020.1827886)
Supplement: Supplemental Material [file KVIR_A_1827886_SM8204.zip › Table S10.pdf]

Table S10 Clustering analyses for the differentially expressed genes of insect hemocytes against *Beauveria bassiana*

Gene ID	Annotation	Minimal value	Mean value	Maximal value	Standard deviation	Coefficient of variation	NNR value	Cluster NO.
AT056_gr02	Uncharacterized protein	0.00	1.36	2.42	1.24	90.95	0.55	1
LOC113509069	Papilin-like	-3.06	-1.02	0.00	1.77	173.21	0.51	3
LOC113509072	Adhesive plaque matrix protein isoform X1	-3.51	-1.69	0.00	1.76	104.16	0.47	9
LOC113509073	Uncharacterized protein C6orf106 homolog	0.00	0.45	1.34	0.78	173.21	0.16	8
LOC113509076	Uncharacterized protein LOC106131149	-3.13	-2.09	0.00	1.81	86.60	0.47	9
LOC113509077	Uncharacterized protein	0.00	1.40	2.45	1.26	89.97	0.97	12
LOC113509080	Phospholipid-transporting ATPase VD	0.00	0.60	1.80	1.04	173.21	0.26	8
LOC113509083	High mobility group protein DSP1-like	-1.40	-0.47	0.00	0.81	173.21	0.22	3
LOC113509086	Glutamate receptor ionotropic, delta-1-like	-2.31	-0.77	0.00	1.33	173.21	0.16	4
LOC113509096	Hypothetical protein RR46_05082	-2.65	-1.63	0.00	1.43	87.45	0.78	3
LOC113509102	Uncharacterized protein LOC110371017	-1.76	-0.59	0.00	1.02	173.21	0.08	3
LOC113509106	Delta-sarcoglycan-like isoform X1	-2.54	-0.85	0.00	1.46	173.21	0.27	3
LOC113509107	Iduronate 2-sulfatase	-1.76	-0.59	0.00	1.02	173.21	0.08	3
LOC113509108	Cubilin	0.00	0.73	2.18	1.26	173.21	0.87	7
LOC113509109	Uncharacterized protein LOC106131182	0.00	1.02	1.79	0.92	90.53	0.16	1
LOC113509111	Uncharacterized protein LOC106139573	-1.62	-0.54	0.00	0.94	173.21	0.14	3
LOC113509113	Uncharacterized protein LOC106131089	-2.59	-1.42	0.00	1.31	92.50	0.28	9
LOC113509116	Uncharacterized protein LOC110372303 isoform X2	0.00	0.72	2.17	1.26	173.21	0.89	7
LOC113509117	Uncharacterized protein LOC110375215 isoform X1	2.64	7.85	12.17	4.83	61.45	0.34	14
LOC113509119	Phospholipid-transporting ATPase IA	0.00	0.95	1.44	0.82	86.61	0.36	1
LOC113509125	Troponin I isoform X2	-1.35	-0.45	0.00	0.78	173.21	0.23	3
LOC113509130	Low-density lipoprotein receptor-related protein 4	0.00	0.84	2.51	1.45	173.21	0.22	7
LOC113509135	Lipase 1-like	-4.69	-2.93	0.00	2.55	87.19	0.74	6
LOC113509137	Ras-related and estrogen-regulated growth inhibitor	-1.79	-1.12	0.00	0.98	87.11	0.64	9

LOC113509159	Transmembrane protein 60	0.00	0.63	1.90	1.10	173.21	0.41	8
LOC113509169	CD151 antigen-like	-1.51	-0.50	0.00	0.87	173.21	0.18	3
LOC113509183	Uncharacterized protein	-1.66	-0.55	0.00	0.96	173.21	0.12	3
LOC113509196	Uncharacterized protein LOC106136669 isoform X1	0.00	1.83	5.48	3.17	173.21	0.12	5
LOC113509198	Uncharacterized protein LOC105389192	0.00	0.87	1.39	0.76	87.18	0.29	1
LOC113509200	Zinc finger protein 700-like	-1.66	-1.05	0.00	0.92	86.94	0.75	9
LOC113509201	Caspase-3	-1.65	-0.55	0.00	0.95	173.21	0.48	4
LOC113509208	UDP-glucuronosyltransferase 2B10-like	0.00	2.84	8.52	4.92	173.21	0.38	15
LOC113509209	Sodium/calcium exchanger 1	0.00	3.43	5.17	2.97	86.61	0.47	10
LOC113509215	Lethal(2) giant larvae protein isoform X1	0.00	0.41	1.23	0.71	173.21	0.23	8
LOC113509224	Uncharacterized protein	-5.50	-3.66	0.00	3.17	86.60	0.64	6
LOC113509226	Ribosomal protein S6 kinase delta-1-like isoform X2	0.00	0.98	2.95	1.70	173.21	0.17	7
LOC113509237	DNA-binding protein D-ETS-6 isoform X1	0.00	5.36	10.35	5.19	96.80	0.77	14
LOC113509240	Lysine-specific demethylase 5A-like	0.00	0.70	2.11	1.22	173.21	0.90	8
LOC113509243	Methionine--tRNA ligase, cytoplasmic	0.00	0.92	2.77	1.60	173.21	0.05	7
LOC113509247	Neurotransmitter gated ion channel	-2.84	-1.44	0.00	1.42	98.53	0.40	9
LOC113509250	Bumetanide-sensitive sodium-(potassium)-chloride cotransporter isoform X1	0.00	0.49	1.46	0.84	173.21	0.08	8
LOC113509251	Uncharacterized protein	-3.15	-2.00	0.00	1.74	86.92	0.39	9
LOC113509252	Uncharacterized protein LOC106137745	0.00	0.67	2.01	1.16	173.21	0.63	8
LOC113509256	Venus kinase receptor	-3.15	-1.88	0.00	1.66	88.41	0.25	9
LOC113509259	Uncharacterized protein LOC101742154 isoform X1	-2.10	-0.70	0.00	1.21	173.21	0.07	3
LOC113509262	low density lipoprotein receptor adapter protein 1	-3.88	-2.26	0.00	2.02	89.24	0.65	9
LOC113509263	Uncharacterized protein LOC106139704	0.00	2.13	6.40	3.70	173.21	0.24	15
LOC113509269	Fatty acyl-CoA reductase 1	0.00	2.08	3.60	1.86	89.64	0.11	12
LOC113509271	Adapter molecule Crk	0.00	0.55	1.66	0.96	173.21	0.43	1
LOC113509276	Immune-related Hdd13	-1.93	-0.64	0.00	1.12	173.21	0.01	3
LOC113509291	Hypothetical protein KGM_207016	-1.21	-0.40	0.00	0.70	173.21	0.28	3
LOC113509292	Alkylglycerol monooxygenase-like	-5.62	-2.93	0.00	2.82	96.12	0.09	6

LOC113509294	Methionine--tRNA ligase, cytoplasmic isoform X1	0.00	0.83	2.50	1.44	173.21	0.23	7
LOC113509295	Uncharacterized protein LOC106142916	0.00	2.52	7.57	4.37	173.21	0.20	15
LOC113509312	Serine-enriched protein isoform X1	-2.65	-0.88	0.00	1.53	173.21	0.33	3
LOC113509314	Asparagine--tRNA ligase, cytoplasmic	0.00	0.51	1.54	0.89	173.21	0.02	8
LOC113509323	Uncharacterized protein LOC106130856	0.00	1.18	3.54	2.04	173.21	0.89	11
LOC113509325	Esterase FE4-like	0.00	2.13	3.47	1.86	87.62	0.31	12
LOC113509337	Uncharacterized protein LOC106135284	-1.49	-0.50	0.00	0.86	173.21	0.58	4
LOC113509342	Uncharacterized protein LOC106135284	-1.72	-0.57	0.00	0.99	173.21	0.44	4
LOC113509343	Structural maintenance of chromosomes protein 4	-1.66	-0.55	0.00	0.96	173.21	0.12	3
LOC113509350	Uncharacterized protein LOC110382040	0.00	0.50	1.51	0.87	173.21	0.05	8
LOC113509351	Ommochrome-binding protein-like	-2.05	-0.68	0.00	1.19	173.21	0.05	3
LOC113509357	Uncharacterized protein LOC110383549 isoform X1	0.00	3.50	5.38	3.03	86.68	0.40	10
LOC113509362	Hyaluronidase isoform X2	0.00	1.09	3.27	1.89	173.21	0.55	7
LOC113509364	Cytochrome P450 6B2-like	-6.37	-3.28	0.00	3.19	97.21	0.28	6
LOC113509365	Cytochrome P450 6B2-like	-5.93	-3.54	0.00	3.13	88.34	0.47	6
LOC113509366	Esterase FE4-like	0.00	2.01	3.48	1.80	89.64	0.45	12
LOC113509367	Ommochrome-binding protein-like	-1.71	-0.57	0.00	0.99	173.21	0.10	3
LOC113509379	Uncharacterized protein LOC106131730 isoform X1	0.00	1.40	2.11	1.21	86.60	0.79	1
LOC113509382	Venom carboxylesterase-6-like isoform X1	0.00	1.94	3.11	1.69	87.20	0.34	12
LOC113509386	Uncharacterized protein LOC110382337	0.00	1.12	3.37	1.95	173.21	0.73	7
LOC113509387	Uncharacterized protein LOC106140905 isoform X1	0.00	0.86	2.59	1.49	173.21	0.44	1
LOC113509389	Homocysteine-responsive endoplasmic reticulum-resident ubiquitin-like domain member 2 protein	0.00	0.42	1.26	0.73	173.21	0.21	8
LOC113509393	Serine protease easter-like	0.00	0.89	1.40	0.77	86.99	0.29	1
LOC113509396	Juvenile hormone epoxide hydrolase-like	-1.62	-0.54	0.00	0.93	173.21	0.14	3
LOC113509398	ATP-sensitive inward rectifier potassium channel 1-like	0.00	0.74	2.21	1.28	173.21	0.78	7
LOC113509403	Uncharacterized protein LOC101742334	0.00	0.51	1.52	0.88	173.21	0.04	8

	isoform X2							
LOC113509404	Uncharacterized protein LOC110371130	0.00	0.72	2.16	1.25	173.21	0.94	7
LOC113509407	Uncharacterized protein DDB_G0291812	-1.26	-0.42	0.00	0.73	173.21	0.27	3
LOC113509411	Chitinase 2	0.00	1.61	4.84	2.80	173.21	0.27	11
LOC113509412	GATA Zinc finger domain containing protein 1	-1.18	-0.39	0.00	0.68	173.21	0.29	3
LOC113509413	Hydroxylysine kinase	-2.83	-0.94	0.00	1.63	173.21	0.41	3
LOC113509423	InaD-like protein isoform X3	-3.52	-1.91	0.00	1.78	93.01	0.41	9
LOC113509424	Uncharacterized protein OBRU01_26608	0.00	0.98	1.62	0.86	87.87	0.22	1
LOC113509425	Uncharacterized protein LOC106110582	0.00	3.82	7.90	3.96	103.53	0.55	10
LOC113509430	Leucine-rich repeat-containing G-protein coupled receptor 5	0.00	2.31	3.66	2.01	86.99	0.28	12
LOC113509438	Tricarboxylate transport protein, mitochondrial	0.00	0.45	1.36	0.78	173.21	0.50	1
LOC113509439	Neutral ceramidase-like isoform X1	-7.84	-4.07	-1.73	3.29	80.94	0.90	6
LOC113509440	Protein peste-like isoform X1	-1.80	-0.15	1.34	1.58	1026.93	0.88	4
LOC113509441	Separin isoform X1	-2.59	-0.86	0.00	1.49	173.21	0.30	3
LOC113509446	Uncharacterized protein LOC106143208	0.00	1.83	5.50	3.17	173.21	0.86	11
LOC113509454	Uncharacterized protein LOC106709162	-3.68	-2.44	0.00	2.11	86.61	0.95	9
LOC113509455	Hemicentin-1-like	-3.38	-0.70	1.28	2.41	344.37	0.41	4
LOC113509459	Multiple epidermal growth factor-like domains protein 10	-5.02	-2.46	0.00	2.51	102.14	0.32	6
LOC113509468	Protein singed	-2.14	-0.71	0.00	1.23	173.21	0.09	3
LOC113509480	Uncharacterized protein LOC110378812	-5.36	-1.79	0.00	3.09	173.21	0.88	6
LOC113509482	Uncharacterized protein	-1.54	-0.51	0.00	0.89	173.21	0.17	3
LOC113509486	Nodal modulator 1	0.00	0.42	1.27	0.74	173.21	0.20	8
LOC113509487	Uncharacterized protein LOC106140707	-3.53	-1.18	0.00	2.04	173.21	0.68	3
LOC113509489	Organic cation transporter protein-like isoform X1	0.00	2.74	4.66	2.44	88.91	0.64	12
LOC113509492	Uncharacterized protein LOC106712877	-2.79	-0.34	1.78	2.30	680.62	0.62	4
LOC113509498	Kynurenine formamidase isoform X1	-2.15	-0.72	0.00	1.24	173.21	0.22	4
LOC113509500	von Willebrand factor A domain-containing protein 8 isoform X1	0.00	1.18	1.91	1.03	87.43	0.35	1

LOC113509501	Uncharacterized protein LOC106124574 isoform X1	-1.26	-0.42	0.00	0.73	173.21	0.27	3
LOC113509513	Vanin-like protein 3	0.00	1.14	3.41	1.97	173.21	0.81	7
LOC113509515	Uncharacterized protein LOC106138752	0.00	1.41	2.41	1.26	89.02	0.67	1
LOC113509538	Low-density lipoprotein receptor 2-like	0.00	2.86	5.50	2.76	96.41	0.86	12
LOC113509548	NAD kinase 2, mitochondrial	-1.48	-0.49	0.00	0.86	173.21	0.19	3
LOC113509550	Uncharacterized protein LOC106129482	0.00	2.24	3.68	1.97	87.71	0.12	12
LOC113509551	GTP-binding protein 2-like	0.00	1.40	4.21	2.43	173.21	0.05	11
LOC113509556	Transmembrane protein 135-like	0.00	0.45	1.35	0.78	173.21	0.16	8
LOC113509557	Circadian clock-controlled protein-like	0.00	2.30	6.90	3.99	173.21	0.03	15
LOC113509559	Uncharacterized protein LOC106142577 isoform X1	-2.49	-1.39	0.00	1.27	91.45	0.61	9
LOC113509568	Fat body acyl-CoA delta-9 desaturase	0.00	0.60	1.79	1.03	173.21	0.24	8
LOC113509569	Carboxylesterase	-4.03	-3.30	-1.86	1.24	37.72	0.53	2
LOC113509570	Uncharacterized protein DDB_G0277255 isoform X1	2.35	4.09	6.26	1.99	48.60	0.58	13
LOC113509573	Neuronal acetylcholine receptor subunit beta-2-like	-4.15	-1.38	0.00	2.40	173.21	0.84	3
LOC113509574	Hypothetical protein RR48_07415	0.00	0.85	2.54	1.46	173.21	0.19	7
LOC113509584	JH-inducible protein	0.00	1.70	3.16	1.59	93.87	0.48	12
LOC113509585	Uncharacterized protein LOC101747015	0.00	1.31	3.94	2.27	173.21	0.28	11
LOC113509588	DNL-type Zinc finger protein-like	0.00	0.60	1.81	1.04	173.21	0.27	8
LOC113509590	Protein toll-like	-2.17	-0.72	0.00	1.25	173.21	0.21	4
LOC113509596	LYR motif-containing protein 2	0.00	0.68	2.03	1.17	173.21	0.67	8
LOC113509598	Odorant-binding protein 11	0.00	2.08	6.24	3.60	173.21	0.34	15
LOC113509602	Leucine zipper tumor suppressor 2 homolog isoform X1	-1.85	-0.62	0.00	1.07	173.21	0.04	3
LOC113509605	Protein inturned	-1.31	-0.44	0.00	0.76	173.21	0.25	3
LOC113509606	Monocarboxylate transporter 10-like	-4.08	-2.21	0.00	2.06	93.22	0.96	6
LOC113509608	Moricin-like peptide A	2.88	6.39	8.85	3.12	48.78	0.57	14
LOC113509609	Moricin-like peptide C5	4.08	6.31	8.01	2.02	31.96	0.85	13
LOC113509611	Moricin-like peptide D	4.95	9.07	12.23	3.74	41.19	0.52	14
LOC113509612	Moricin-like peptide C3	4.20	8.69	11.74	3.97	45.70	0.46	14

LOC113509613	Moricin-like peptide C2	4.10	9.29	12.52	4.54	48.89	0.49	14
LOC113509614	Moricin-like peptide C1	4.86	7.75	9.78	2.57	33.17	0.62	14
LOC113509615	Moricin-like peptide B	0.00	6.23	9.54	5.40	86.66	0.35	14
LOC113509617	60S ribosomal protein L26	-1.17	-0.39	0.00	0.68	173.21	0.30	3
LOC113509622	Uncharacterized protein LOC106137572	0.00	2.20	6.59	3.80	173.21	0.14	15
LOC113509623	Uncharacterized protein OBRU01_17125	-1.45	-0.48	0.00	0.84	173.21	0.20	3
LOC113509626	Nuclear receptor-binding factor 2-like	0.00	0.42	1.27	0.73	173.21	0.20	8
LOC113509631	Carboxypeptidase N subunit 2-like	-2.86	-1.50	0.00	1.44	95.64	0.29	9
LOC113509632	Juvenile hormone binding protein	-2.71	-1.74	0.00	1.51	86.78	0.22	9
LOC113509636	Protein unc-13 homolog D isoform X4	0.00	0.63	1.88	1.09	173.21	0.38	8
LOC113509639	Odorant binding protein	-4.23	-2.16	0.00	2.12	97.83	0.89	6
LOC113509642	Hemicentin-1-like	-2.56	-0.85	0.00	1.48	173.21	0.07	4
LOC113509644	Uncharacterized protein LOC106137340 isoform X1	-1.85	-0.62	0.00	1.07	173.21	0.04	3
LOC113509658	Organic cation transporter protein-like	0.00	1.01	3.04	1.75	173.21	0.26	7
LOC113509667	Toll-like receptor 7	0.00	0.93	2.78	1.61	173.21	0.06	7
LOC113509668	Juvenile hormone binding protein	0.00	1.03	3.10	1.79	173.21	0.33	7
LOC113509675	Spondin-2	-1.98	-1.17	0.00	1.04	88.53	0.61	9
LOC113509677	Asparagine synthetase	0.00	0.46	1.39	0.80	173.21	0.49	1
LOC113509680	E3 SUMO-protein ligase EGR2 isoform X1	-3.58	-1.76	0.00	1.79	101.52	0.45	9
LOC113509684	Uncharacterized protein LOC106136946	-3.20	-1.89	0.00	1.68	88.66	0.41	9
LOC113509686	Glutamate receptor ionotropic, delta-2	-2.55	-0.85	0.00	1.47	173.21	0.28	3
LOC113509690	Potassium channel subfamily K member 1-like	-3.54	-1.93	0.00	1.79	92.65	0.43	9
LOC113509694	Transferrin precursor	3.82	4.47	5.10	0.64	14.37	0.33	13
LOC113509698	Organic cation transporter protein-like	0.00	2.55	4.18	2.24	87.68	0.37	12
LOC113509700	Methylthioribulose-1-phosphate dehydratase	-2.05	-0.68	0.00	1.18	173.21	0.04	3
LOC113509701	Replication factor C subunit 3	-1.79	-0.60	0.00	1.03	173.21	0.07	3
LOC113509707	Uncharacterized protein LOC106139395	0.00	0.79	2.37	1.37	173.21	0.43	7
LOC113509711	aminoacyl tRNA synthase complex-interacting multifunctional protein 1	0.00	0.86	1.39	0.75	87.37	0.27	1
LOC113509713	Uncharacterized protein LOC106136999	0.00	0.73	2.18	1.26	173.21	0.88	7
LOC113509717	Prefoldin subunit 3	0.00	0.47	1.40	0.81	173.21	0.49	1

LOC113509722	Gustatory and odorant receptor 22	0.00	3.28	5.51	2.90	88.45	0.44	10
LOC113509730	Lysosomal aspartic protease	-1.32	-0.44	0.00	0.76	173.21	0.25	3
LOC113509732	Hexokinase type 2 isoform X1	0.00	2.07	3.54	1.85	89.01	0.40	12
LOC113509735	D-beta-hydroxybutyrate dehydrogenase, mitochondrial	0.00	1.06	3.19	1.84	173.21	0.75	1
LOC113509739	Nucleolar protein 16	0.00	0.43	1.30	0.75	173.21	0.52	1
LOC113509748	Methionine synthase reductase-like	0.00	1.21	2.04	1.07	88.50	0.64	1
LOC113509755	DNA fragmentation factor subunit alpha	-1.32	-0.44	0.00	0.76	173.21	0.24	3
LOC113509757	Uncharacterized protein LOC106135696	-1.93	-0.17	1.44	1.69	1018.86	0.83	4
LOC113509765	Delta(14)-sterol reductase	-1.38	-0.46	0.00	0.80	173.21	0.23	3
LOC113509767	Organic cation transporter protein-like isoform X1	0.00	0.80	2.40	1.38	173.21	0.41	1
LOC113509770	Ribonuclease H2 subunit A-like	-2.27	-0.76	0.00	1.31	173.21	0.15	3
LOC113509771	Transposase	0.00	0.77	2.30	1.33	173.21	0.57	7
LOC113509774	Uncharacterized protein LOC106103079 isoform X2	2.59	5.35	6.78	2.39	44.76	0.69	13
LOC113509785	Transposase	0.00	0.96	2.87	1.66	173.21	0.12	7
LOC113509792	Rootletin	-2.87	-0.96	0.00	1.66	173.21	0.43	3
LOC113509793	Uncharacterized protein OBRU01_15671	0.00	0.50	1.51	0.87	173.21	0.04	8
LOC113509794	Ribosome biogenesis protein NSA2 homolog	-1.45	-0.48	0.00	0.84	173.21	0.20	3
LOC113509803	Dehydrodolichyl diphosphate syntase complex subunit Nus1	0.00	1.12	1.94	1.01	89.50	0.67	1
LOC113509813	Hypothetical protein RR46_13217	0.00	1.72	2.77	1.50	87.34	0.41	12
LOC113509814	Axoneme-associated protein mst101(2) isoform X1	0.00	0.80	2.39	1.38	173.21	0.39	7
LOC113509818	Regucalcin	-4.08	-2.04	0.00	2.04	99.82	0.86	9
LOC113509826	Uncharacterized protein LOC106104008	0.00	1.94	3.10	1.69	87.21	0.14	12
LOC113509827	Uncharacterized protein LOC106143557	0.00	0.92	2.77	1.60	173.21	0.05	7
LOC113509835	Uncharacterized protein LOC106135260	0.00	2.18	6.55	3.78	173.21	0.16	15
LOC113509839	Protein toll-like	-1.39	-0.46	0.00	0.80	173.21	0.22	3
LOC113509840	Kv channel-interacting protein 4-like	0.00	0.83	2.50	1.44	173.21	0.24	7
LOC113509841	Uncharacterized protein LOC106135211	0.00	2.04	6.12	3.53	173.21	0.43	15

LOC113509842	Uncharacterized protein LOC106135203	0.00	0.82	2.45	1.41	173.21	0.42	1
LOC113509844	Uncharacterized protein LOC106135184	0.00	2.85	4.39	2.47	86.70	0.73	12
LOC113509846	Testis-expressed sequence 10 protein homolog	0.00	0.41	1.24	0.72	173.21	0.22	8
LOC113509852	Inhibitor of apoptosis protein	0.00	0.41	1.24	0.72	173.21	0.22	8
LOC113509865	Circadian clock-controlled protein-like	-6.29	-3.39	0.00	3.17	93.50	0.33	6
LOC113509868	Uncharacterized protein LOC110377500	4.08	6.96	9.47	2.71	38.97	0.67	14
LOC113509870	Alkaline phosphatase-like isoform X2	-4.86	-2.62	0.00	2.45	93.53	0.23	6
LOC113509871	Acetyltransferase ACT13	0.00	3.17	5.38	2.82	88.77	0.58	10
LOC113509873	Uncharacterized protein LOC106135995	-3.30	-1.54	0.00	1.66	107.77	0.55	9
LOC113509885	Uncharacterized protein LOC106136933	-2.83	-1.72	0.00	1.51	87.78	0.09	9
LOC113509888	Toll-like receptor 3	-4.59	-1.53	0.00	2.65	173.21	0.92	3
LOC113509901	Slit homolog 3 protein-like	-2.87	-1.77	0.00	1.55	87.47	0.30	9
LOC113509932	Uncharacterized aarF domain-containing protein kinase 1	0.00	0.78	2.33	1.35	173.21	0.51	7
LOC113509934	Uncharacterized protein LOC106135020	-2.94	-1.63	0.00	1.50	91.63	0.15	9
LOC113509938	Uncharacterized protein LOC110377493 isoform X2	-1.42	-0.47	0.00	0.82	173.21	0.21	3
LOC113509956	Inverted formin-2 isoform X2	0.00	1.90	3.51	1.77	93.52	0.30	12
LOC113509965	Uncharacterized protein LOC110375196	-3.36	-1.12	0.00	1.94	173.21	0.62	3
LOC113509968	KAT8 regulatory NSL complex subunit 1-like	0.00	0.58	1.73	1.00	173.21	0.16	8
LOC113509972	Solute carrier family 25 member 35-like isoform X1	0.00	0.55	1.65	0.95	173.21	0.08	8
LOC113509976	Facilitated trehalose transporter Tret1-2-like	-2.05	-0.68	0.00	1.19	173.21	0.05	3
LOC113509985	Aldose reductase isoform X1	-1.29	-0.43	0.00	0.74	173.21	0.26	3
LOC113509986	Neutral ceramidase	0.00	0.64	1.91	1.10	173.21	0.40	1
LOC113509992	RAB6-interacting golgin	-1.26	-0.42	0.00	0.72	173.21	0.27	3
LOC113509994	Uncharacterized protein LOC101738673	0.00	4.56	7.83	4.07	89.24	0.44	10
LOC113509995	Tyrosine-protein phosphatase 69D	0.00	0.93	1.45	0.81	86.77	0.31	1
LOC113510000	Ecdysone oxidase	-4.04	-1.35	0.00	2.33	173.21	0.82	3
LOC113510002	Serine-rich adhesin for platelets	0.00	0.57	1.70	0.98	173.21	0.14	8
LOC113510005	Unconventional myosin-Va-like	-2.33	-1.44	0.00	1.26	87.39	0.19	9

LOC113510012	Cysteine dioxygenase type 1	0.00	2.34	4.81	2.41	103.02	0.48	5
LOC113510014	Sortilin-related receptor isoform X1	0.00	0.55	1.64	0.94	173.21	0.07	8
LOC113510025	Transferrin-like	0.00	2.53	3.98	2.20	86.92	0.34	12
LOC113510029	Programmed cell death protein 4	-2.31	-1.23	0.00	1.16	94.56	0.61	9
LOC113510031	Uncharacterized protein	-1.72	-1.04	0.00	0.91	88.09	0.77	9
LOC113510038	Uncharacterized protein	-3.36	-2.01	0.00	1.78	88.29	0.41	9
LOC113510042	UDP-glucuronosyltransferase 2A3-like	-2.54	-1.36	0.00	1.28	94.29	0.40	9
LOC113510044	Protein mahjong	0.00	0.48	1.43	0.83	173.21	0.10	8
LOC113510046	Phospholipid scramblase 2	-2.94	-0.55	1.30	2.17	396.74	0.47	3
LOC113510048	Uncharacterized protein LOC110381761	-1.98	-0.66	0.00	1.14	173.21	0.01	3
LOC113510051	Hypothetical protein RR48_06950	-1.82	-0.61	0.00	1.05	173.21	0.06	3
LOC113510053	Hypothetical protein KGM_202555	0.00	1.14	3.41	1.97	173.21	0.80	7
LOC113510059	Vacuolar ATP synthase subunit S1	0.00	1.44	4.32	2.50	173.21	0.04	11
LOC113510060	Nuclear factor interleukin-3-regulated protein-like	0.00	2.30	6.90	3.98	173.21	0.03	15
LOC113510062	Uncharacterized protein LOC110374899	0.00	2.12	3.38	1.85	87.07	0.06	12
LOC113510066	Uncharacterized protein LOC110382578	-2.05	-0.68	0.00	1.18	173.21	0.05	3
LOC113510072	Zinc finger protein 28 homolog	-1.42	-0.47	0.00	0.82	173.21	0.21	3
LOC113510074	Zinc finger protein 62 homolog isoform X9	-1.24	-0.41	0.00	0.72	173.21	0.27	3
LOC113510092	Zinc finger protein 100-like	-1.39	-0.91	0.00	0.79	86.66	0.94	3
LOC113510094	Gastrula Zinc finger protein XlCGF57.1-like	-1.50	-0.94	0.00	0.82	87.22	0.95	3
LOC113510095	Prostamide/prostaglandin F synthase-like	0.00	1.06	3.19	1.84	173.21	0.43	7
LOC113510107	Hemolin	1.95	4.61	6.11	2.31	50.09	0.62	13
LOC113510109	Suppressor of tumorigenicity 14 protein homolog	0.00	1.69	2.54	1.46	86.60	0.53	12
LOC113510111	Hypothetical protein KGM_213196	-1.38	-0.46	0.00	0.80	173.21	0.22	3
LOC113510114	Phosphatidylethanolamine-binding protein isoform 1	0.00	0.39	1.17	0.68	173.21	0.25	8
LOC113510121	Uncharacterized protein LOC106130105 isoform X1	0.00	2.20	3.67	1.94	88.22	0.35	12
LOC113510126	F-box/WD repeat-containing protein 11	0.00	0.63	1.89	1.09	173.21	0.39	8
LOC113510131	Tetraspanin-9-like	0.00	0.45	1.35	0.78	173.21	0.50	1
LOC113510135	Serine hydrolase	-2.51	-0.84	0.00	1.45	173.21	0.09	4

LOC113510137	Extradiol ring-cleavage dioxygenase-like	0.00	1.03	3.10	1.79	173.21	0.33	7
LOC113510138	Hypothetical protein OBRU01_12387	0.00	1.97	4.26	2.15	108.96	0.91	12
LOC113510143	Zinc finger protein 840	-2.04	-0.68	0.00	1.18	173.21	0.27	4
LOC113510145	Zinc finger protein 26-like	-1.19	-0.40	0.00	0.69	173.21	0.29	3
LOC113510153	E3 ubiquitin-protein ligase sinah isoform X1	0.00	3.38	5.92	3.05	90.16	0.32	10
LOC113510161	Serine hydrolase-like protein	-2.67	-1.36	0.00	1.33	97.73	0.46	9
LOC113510166	Serine hydrolase-like protein	-3.43	-1.94	0.00	1.76	90.54	0.38	9
LOC113510168	Uncharacterized protein LOC105383361	-1.51	-0.50	0.00	0.87	173.21	0.18	3
LOC113510171	Uncharacterized protein	-1.62	-1.00	0.00	0.87	87.51	0.83	9
LOC113510172	Gamma-butyrobetaine dioxygenase	-3.09	-1.03	0.00	1.78	173.21	0.52	3
LOC113510177	Transferrin-like	0.00	0.78	2.34	1.35	173.21	0.49	7
LOC113510192	Kinesin-like protein KIF3A isoform X1	0.00	0.46	1.37	0.79	173.21	0.14	8
LOC113510193	Zinc finger protein basonuclin-2-like	0.00	2.11	6.32	3.65	173.21	0.29	15
LOC113510198	TBC1 domain family member 4 isoform X1	-2.30	-0.77	0.00	1.33	173.21	0.16	3
LOC113510204	Uncharacterized protein	-2.13	-0.71	0.00	1.23	173.21	0.08	3
LOC113510207	Fidgetin-like protein 1	-1.39	-0.46	0.00	0.80	173.21	0.22	3
LOC113510213	GTP-binding protein 2-like	0.00	1.39	4.16	2.40	173.21	0.09	11
LOC113510218	Ras-related protein Rab-18-B isoform X1	0.00	0.42	1.27	0.73	173.21	0.21	8
LOC113510219	Meiosis-specific nuclear structural protein 1-like	-4.97	-1.66	0.00	2.87	173.21	1.00	3
LOC113510220	Gelsolin-like	-3.38	-1.89	0.00	1.73	91.21	0.34	9
LOC113510222	Neuroendocrine convertase 1	0.00	0.87	2.62	1.51	173.21	0.10	7
LOC113510223	Tafazzin homolog	0.00	1.09	1.75	0.95	87.27	0.31	1
LOC113510224	Vacuolar protein sorting-associated protein 13D	0.00	0.88	2.65	1.53	173.21	0.06	7
LOC113510234	Organic cation transporter protein-like	-5.90	-3.12	0.00	2.97	95.10	0.92	6
LOC113510237	Protease inhibitor 4	0.00	1.36	4.08	2.36	173.21	0.15	11
LOC113510247	Acidic amino acid decarboxylase GADL1	-3.73	-2.30	0.00	2.01	87.43	0.87	9
LOC113510248	Venom protease	-5.60	-2.67	0.00	2.81	105.28	0.21	6
LOC113510250	Neurexin-1	-1.94	-1.19	0.00	1.04	87.65	0.57	9
LOC113510258	Hemicentin-2-like	-2.76	-1.66	0.00	1.46	88.12	0.04	9
LOC113510273	Uncharacterized protein LOC106143525	0.00	3.51	10.53	6.08	173.21	0.58	15

LOC113510280	Acetylcholinesterase-like	0.00	0.51	1.53	0.88	173.21	0.45	1
LOC113510286	Kinesin-like protein CG14535 isoform X1	-3.02	-1.90	0.00	1.65	87.07	0.32	9
LOC113510299	Uncharacterized protein LOC106136821	-1.95	-0.65	0.00	1.12	173.21	0.01	3
LOC113510301	Uncharacterized protein LOC106131008	-2.71	-0.90	0.00	1.57	173.21	0.36	3
LOC113510306	Neural cell adhesion molecule 2-like	-1.72	-0.57	0.00	0.99	173.21	0.10	3
LOC113510310	Uncharacterized protein LOC110370405	0.00	0.65	1.94	1.12	173.21	0.49	8
LOC113510312	Carboxylesterase	-3.18	-1.78	0.00	1.62	91.33	0.51	9
LOC113510318	Coronin-7 isoform X1	-1.19	-0.40	0.00	0.69	173.21	0.29	3
LOC113510329	Uncharacterized protein LOC106130461	0.00	3.55	10.65	6.15	173.21	0.59	15
LOC113510337	Glutathione S-transferase	-1.70	-0.57	0.00	0.98	173.21	0.11	3
LOC113510344	Aldehyde dehydrogenase, mitochondrial	-1.41	-0.47	0.00	0.81	173.21	0.21	3
LOC113510351	CYP6AB47	-4.42	-1.47	0.00	2.55	173.21	0.48	4
LOC113510353	Uncharacterized protein ZK1073.1 isoform X2	0.00	0.79	2.36	1.36	173.21	0.45	7
LOC113510355	Uncharacterized protein LOC101745796	-1.94	-0.65	0.00	1.12	173.21	0.32	4
LOC113510362	Uncharacterized protein	0.00	2.53	7.58	4.38	173.21	0.20	15
LOC113510367	Uncharacterized family 31 glucosidase KIAA1161 isoform X1	-2.50	-0.83	0.00	1.44	173.21	0.26	3
LOC113510373	Carboxylesterase	0.00	1.47	2.33	1.28	86.99	0.93	12
LOC113510374	Esterase FE4 isoform X2	0.00	1.84	2.86	1.60	86.77	0.27	12
LOC113510386	Uncharacterized protein LOC106136737 isoform X2	0.00	0.56	1.69	0.98	173.21	0.12	8
LOC113510387	Transcription factor Y subunit alpha isoform X2	0.00	0.44	1.33	0.77	173.21	0.17	8
LOC113510405	ATP-dependent RNA helicase DHX34	-1.47	-0.49	0.00	0.85	173.21	0.19	3
LOC113510409	Chloride channel protein 2 isoform X1	-5.81	-3.87	0.00	3.35	86.60	0.68	6
LOC113510411	Uncharacterized protein LOC110380790	0.00	1.87	5.61	3.24	173.21	0.12	5
LOC113510422	Gloverin	0.00	2.96	4.97	2.62	88.48	0.85	12
LOC113510424	Uncharacterized protein LOC110375326	-3.32	-2.21	0.00	1.91	86.60	0.62	9
LOC113510435	Inactive hydroxysteroid dehydrogenase-like protein 1 isoform X1	-3.04	-1.90	0.00	1.66	87.12	0.26	9
LOC113510442	Synaptotagmin-16 isoform X1	0.00	2.08	6.24	3.60	173.21	0.34	15
LOC113510451	Lipase 3-like	0.00	2.96	4.83	2.59	87.61	1.00	10

LOC113510453	2-oxoisovalerate dehydrogenase subunit beta, mitochondrial	-1.48	-0.49	0.00	0.85	173.21	0.59	4
LOC113510461	Cysteine desulfurase, mitochondrial	0.00	0.39	1.17	0.67	173.21	0.26	8
LOC113510478	Apolipoprotein D	-1.54	-0.51	0.00	0.89	173.21	0.17	3
LOC113510482	Uncharacterized protein LOC106136572	-2.17	-1.35	0.00	1.18	87.35	0.31	9
LOC113510485	Protocadherin-like wing polarity protein stan	0.00	0.81	2.43	1.40	173.21	0.33	7
LOC113510498	Aldo-keto reductase AKR2E4-like	-7.20	-3.98	0.00	3.66	91.95	0.88	6
LOC113510501	Rho GTPase-activating protein 21-A-like isoform X1	0.00	0.49	1.46	0.84	173.21	0.08	8
LOC113510502	Membrane-bound alkaline phosphatase	0.00	0.76	2.27	1.31	173.21	0.64	7
LOC113510506	Bifunctional glutamate/proline--tRNA ligase	0.00	0.63	1.90	1.10	173.21	0.42	8
LOC113510512	Uncharacterized protein LOC106124870	-3.74	-3.00	-2.16	0.79	26.50	0.41	2
LOC113510517	Acyl-CoA synthetase short-chain family member 3, mitochondrial	-2.35	-0.78	0.00	1.36	173.21	0.15	4
LOC113510528	Collagen alpha-1(IX) chain-like isoform X1	3.40	3.96	5.03	0.93	23.62	0.45	13
LOC113510529	Protein phosphatase 1 regulatory subunit 3B-B	0.00	0.47	1.41	0.81	173.21	0.12	8
LOC113510542	Deoxynucleoside kinase-like	-1.37	-0.46	0.00	0.79	173.21	0.23	3
LOC113510546	TBC1 domain family member 4 isoform X1	-2.65	-0.88	0.00	1.53	173.21	0.33	3
LOC113510551	Cathepsin B-like cysteine proteinase	-2.31	-0.77	0.00	1.33	173.21	0.16	3
LOC113510555	Hemocytin	-5.24	-2.55	0.00	2.62	102.89	0.24	6
LOC113510563	Hemocytin	-5.04	-2.46	0.00	2.52	102.67	0.32	6
LOC113510565	CRAL-TRIO domain-containing protein	0.00	2.71	4.27	2.36	86.91	0.56	12
LOC113510566	Alpha-tocopherol transfer protein-like	0.00	2.26	3.94	2.03	89.86	0.19	12
LOC113510568	Heat shock protein 68	0.00	2.58	5.30	2.65	103.02	0.75	12
LOC113510571	Glutathione S-transferase epsilon 6 isoform X1	-2.02	-1.22	0.00	1.08	87.90	0.51	9
LOC113510572	Uncharacterized protein	-2.02	-0.67	0.00	1.16	173.21	0.03	3
LOC113510575	Uncharacterized protein LOC110380322 isoform X2	0.00	1.01	1.59	0.88	86.91	0.30	1
LOC113510592	Cytochrome c oxidase assembly protein COX16 homolog, mitochondrial	0.00	0.93	1.40	0.81	86.60	0.37	1
LOC113510598	Collagenase-like	-4.13	-2.15	0.00	2.07	96.49	0.73	9

LOC113510604	Pancreatic triacylglycerol lipase-like	-5.58	-3.14	0.00	2.85	91.01	0.25	6
LOC113510611	Glycosylated lysosomal membrane protein B-like	0.00	0.43	1.30	0.75	173.21	0.19	8
LOC113510614	Elongation factor-like GTPase 1	0.00	0.95	2.85	1.65	173.21	0.11	7
LOC113510615	Uncharacterized protein LOC106129672	0.00	1.87	5.62	3.24	173.21	0.96	15
LOC113510629	Krueppel-like factor 9	-3.97	-1.93	0.00	1.99	103.05	0.71	9
LOC113510631	Uncharacterized protein LOC106129727	-2.14	-0.71	0.00	1.23	173.21	0.09	3
LOC113510634	Cuticlin-2 isoform X1	-1.26	-0.42	0.00	0.72	173.21	0.27	3
LOC113510638	Bumetanide-sensitive sodium-(potassium)-chloride cotransporter	0.00	4.87	7.89	4.26	87.44	0.73	10
LOC113510643	Alpha-tocopherol transfer protein-like	0.00	6.08	9.64	5.29	87.03	0.27	14
LOC113510648	Ubiquilin-1	0.00	0.48	1.45	0.84	173.21	0.09	8
LOC113510656	Pancreatic triacylglycerol lipase-like	-6.48	-3.52	0.00	3.28	93.00	0.39	6
LOC113510661	Isoleucine--tRNA ligase, cytoplasmic	0.00	0.46	1.38	0.79	173.21	0.14	8
LOC113510662	Omega-amidase NIT2 isoform X1	-2.29	-1.25	0.00	1.16	92.94	0.54	9
LOC113510666	Fructose-1,6-bisphosphatase	-1.93	-0.64	0.00	1.11	173.21	0.01	3
LOC113510667	Solute carrier family 22 member 21 like protein	-2.50	-1.59	0.00	1.38	86.91	0.09	9
LOC113510668	Vacuolar protein sorting-associated protein 33B	-1.20	-0.40	0.00	0.69	173.21	0.28	3
LOC113510671	Uncharacterized protein LOC101747082	-3.54	-1.75	0.00	1.77	101.26	0.87	9
LOC113510674	Elongator complex protein 1 isoform X2	0.00	0.54	1.63	0.94	173.21	0.06	8
LOC113510675	Ribosomal biogenesis protein RLP24	-1.36	-0.89	0.00	0.77	86.68	0.94	3
LOC113510687	Tumor necrosis factor, Alpha-induced protein 8-like protein 2 A	0.00	0.51	1.54	0.89	173.21	0.03	8
LOC113510688	E3 ubiquitin-protein ligase RNF126-B isoform X1	0.00	0.95	2.85	1.65	173.21	0.11	7
LOC113510696	Monocarboxylate transporter 1-like	0.00	0.92	2.75	1.59	173.21	0.04	7
LOC113510698	Extensin-like	0.00	1.20	3.61	2.08	173.21	0.74	11
LOC113510700	G protein-activated inward rectifier potassium channel 3-like isoform X2	0.00	3.24	6.18	3.10	95.73	0.50	10
LOC113510707	Uncharacterized protein LOC101747082	-3.49	-2.75	-2.11	0.70	25.34	0.68	2
LOC113510710	Uncharacterized protein LOC110384236	0.00	1.31	2.04	1.14	86.79	0.65	1

LOC113510715	Hemicentin-2-like	-1.62	-0.54	0.00	0.94	173.21	0.14	3
LOC113510718	RNA polymerase II elongation factor ELL	0.00	0.62	1.87	1.08	173.21	0.36	8
LOC113510730	Uncharacterized protein C19orf52	0.00	0.47	1.40	0.81	173.21	0.49	1
LOC113510735	Venom protease-like	-1.42	-0.47	0.00	0.82	173.21	0.21	3
LOC113510737	Venom protease-like	-2.82	-1.73	0.00	1.51	87.55	0.30	9
LOC113510738	Uncharacterized protein	-5.52	-1.84	0.00	3.19	173.21	0.65	4
LOC113510744	Peritrophin 1	-2.81	-1.36	0.00	1.40	102.93	0.88	4
LOC113510752	Tropomyosin-2 isoform X2	0.00	0.54	1.63	0.94	173.21	0.06	8
LOC113510753	Hypothetical protein KGM_203118	0.00	1.03	3.10	1.79	173.21	0.32	7
LOC113510759	Protein fem-1 homolog CG6966 isoform X1	0.00	0.66	1.98	1.14	173.21	0.56	8
LOC113510765	Lon protease homolog, mitochondrial-like	0.00	0.49	1.47	0.85	173.21	0.08	8
LOC113510766	Uncharacterized protein LOC110376441	0.00	0.77	2.31	1.33	173.21	0.55	7
LOC113510773	Uncharacterized protein	-2.58	-0.86	0.00	1.49	173.21	0.29	3
LOC113510774	Uncharacterized protein	-2.26	-0.75	0.00	1.30	173.21	0.14	3
LOC113510776	Solute carrier family 22 member 21 like protein	0.00	0.49	1.47	0.85	173.21	0.47	1
LOC113510781	Uncharacterized protein OBRU01_02162	-3.99	-1.33	0.00	2.30	173.21	0.81	3
LOC113510783	Aldehyde dehydrogenase, mitochondrial	-1.43	-0.48	0.00	0.83	173.21	0.21	3
LOC113510785	Centrosomal protein of 120 kDa-like	-1.88	-0.63	0.00	1.09	173.21	0.03	3
LOC113510787	Protein unc-13 homolog A	-3.88	-2.02	0.00	1.95	96.25	0.70	9
LOC113510788	Phorbol ester/diacylglycerol-binding protein unc-13-like	-1.28	-0.43	0.00	0.74	173.21	0.26	3
LOC113510790	Endophilin-A isoform X5	-1.44	-0.93	0.00	0.80	86.76	0.96	3
LOC113510803	A disintegrin and metalloproteinase with thrombospondin motifs 7-like	-5.27	-3.51	0.00	3.04	86.60	0.61	6
LOC113510810	Sodium-independent sulfate anion transporter-like	-1.40	-0.47	0.00	0.81	173.21	0.22	3
LOC113510812	Uncharacterized protein LOC110373838 isoform X4	0.00	0.87	1.43	0.76	87.85	0.24	1
LOC113510817	Alpha-tocopherol transfer protein-like isoform X2	0.00	0.98	1.64	0.87	88.18	0.59	1
LOC113510820	Lachesin-like	0.00	1.46	4.39	2.54	173.21	0.07	11
LOC113510833	Uncharacterized protein	0.00	2.74	8.22	4.75	173.21	0.33	15

LOC113510846	Sex-determining protein fem-1	0.00	0.81	2.42	1.40	173.21	0.35	7
LOC113510847	Protein fem-1 homolog CG6966 isoform X3	0.00	0.77	2.31	1.33	173.21	0.55	7
LOC113510862	Zinc transporter ZIP3	-3.37	-1.57	0.00	1.70	108.42	0.56	9
LOC113510868	Uncharacterized protein OBRU01_00709	0.00	4.72	7.31	4.10	86.74	0.60	10
LOC113510872	Acyl-CoA Delta(11) desaturase-like	0.00	0.53	1.60	0.92	173.21	0.04	8
LOC113510874	Furin-like protease 2 isoform X1	0.00	0.55	1.66	0.96	173.21	0.10	8
LOC113510876	Endoprotease FURIN	0.00	0.48	1.44	0.83	173.21	0.10	8
LOC113510880	Uncharacterized protein LOC106129699 isoform X2	0.00	0.60	1.81	1.05	173.21	0.28	8
LOC113510881	Sodium-dependent nutrient amino acid transporter 1-like isoform X1	-3.61	-2.07	0.00	1.86	89.83	0.52	9
LOC113510894	Uncharacterized protein LOC106133593 isoform X1	-1.40	-0.47	0.00	0.81	173.21	0.64	4
LOC113510897	Tetratricopeptide repeat protein 7B-like	-6.04	-2.01	0.00	3.49	173.21	0.98	2
LOC113510899	Sodium-independent sulfate anion transporter	0.00	3.80	6.68	3.43	90.40	0.10	10
LOC113510904	Acid sphingomyelinase-like phosphodiesterase 3a	-5.33	-2.31	0.00	2.74	118.28	0.51	6
LOC113510911	Beta-1,3-glucan recognition protein	0.00	2.08	6.24	3.60	173.21	0.19	5
LOC113510916	Uncharacterized protein C630.12	-1.52	-0.51	0.00	0.88	173.21	0.17	3
LOC113510919	Lysozyme	0.00	3.74	5.69	3.24	86.63	0.42	10
LOC113510920	Microtubule-associated protein futsch-like	-2.59	-1.33	0.00	1.30	97.68	0.51	9
LOC113510922	Protease inhibitor-like protein	2.52	4.63	5.96	1.84	39.86	0.36	13
LOC113510923	Protease inhibitor1	0.00	0.69	2.07	1.20	173.21	0.40	1
LOC113510926	Protein mahjong	0.00	0.50	1.50	0.86	173.21	0.06	8
LOC113510927	Uncoordinated protein 58-like	0.00	0.62	1.86	1.08	173.21	0.35	8
LOC113510928	4-coumarate--CoA ligase 1-like	-1.58	-0.53	0.00	0.91	173.21	0.52	4
LOC113510932	Spermatogenesis-associated protein 20 isoform X1	-1.30	-0.43	0.00	0.75	173.21	0.25	3
LOC113510939	Venom carboxylesterase-6-like	-4.52	-2.21	0.00	2.26	102.48	0.69	6
LOC113510941	Alpha-tocopherol transfer protein-like	0.00	3.19	4.89	2.77	86.66	0.68	10
LOC113510944	Uncharacterized protein DDB_G0282133	-2.43	-0.81	0.00	1.40	173.21	0.22	3
LOC113510949	THAP domain-containing protein 4-like	-1.58	-0.53	0.00	0.91	173.21	0.15	3

LOC113510950	Uncharacterized protein LOC106131018	0.00	1.24	3.73	2.15	173.21	0.54	11
LOC113510960	Uncharacterized protein LOC106129749	-1.64	-0.55	0.00	0.95	173.21	0.13	3
LOC113510961	Lipopolysaccharide-induced tumor necrosis factor-alpha factor homolog	-2.00	-0.67	0.00	1.16	173.21	0.29	4
LOC113510988	Uncharacterized protein LOC106711668 isoform X1	-5.33	-3.10	0.00	2.77	89.28	0.31	6
LOC113510992	Uncharacterized protein LOC106136019	-5.69	-1.90	0.00	3.28	173.21	0.81	6
LOC113510993	Mitochondrial uncoupling protein Bmcp	0.00	0.54	1.62	0.94	173.21	0.06	8
LOC113510995	Nucleolar GTP-binding protein 1	-1.41	-0.47	0.00	0.81	173.21	0.63	4
LOC113511004	Syntaxin-16	0.00	0.41	1.24	0.72	173.21	0.22	8
LOC113511010	C1A cysteine protease precursor	-4.64	-1.98	0.00	2.39	120.77	0.87	6
LOC113511011	Calbindin-32	-3.56	-1.68	0.00	1.79	106.82	0.52	9
LOC113511017	5-oxoprolinase	-1.32	-0.44	0.00	0.76	173.21	0.24	3
LOC113511018	Tubulointerstitial nephritis antigen precursor	0.00	1.05	1.63	0.91	86.76	0.45	1
LOC113511019	Transient receptor potential channel pyrexia-like	-2.49	-0.83	0.00	1.44	173.21	0.09	4
LOC113511020	Uncharacterized protein LOC106135940	-1.55	-0.52	0.00	0.90	173.21	0.54	4
LOC113511027	Nucleolar protein 4-like	-1.63	-1.03	0.00	0.89	87.08	0.86	9
LOC113511028	Uncharacterized protein LOC106135978	-4.17	-2.51	0.00	2.21	88.04	0.67	6
LOC113511039	Solute carrier family 2, facilitated glucose transporter member 8-like	0.00	5.49	9.82	5.01	91.32	0.60	14
LOC113511042	Protein NDUFAF4 homolog	0.00	0.46	1.38	0.80	173.21	0.14	8
LOC113511044	Long-chain fatty acid transport protein 1-like	-1.54	-0.51	0.00	0.89	173.21	0.55	4
LOC113511046	Transcription factor SOX-15	0.00	3.46	5.20	3.00	86.60	0.46	10
LOC113511047	A disintegrin and metalloproteinase with thrombospondin motifs 1 isoform X1	0.00	2.70	6.14	3.14	116.13	0.76	15
LOC113511049	Glutathione synthetase-like isoform X3	0.00	2.52	4.05	2.20	87.28	0.33	12
LOC113511052	Derlin-1	0.00	0.52	1.57	0.91	173.21	0.02	8
LOC113511054	Protein LTV1 homolog isoform X1	-1.28	-0.43	0.00	0.74	173.21	0.26	3
LOC113511062	Flap endonuclease 1	-2.39	-0.80	0.00	1.38	173.21	0.20	3
LOC113511069	Chondroadherin-like	-2.57	-1.60	0.00	1.40	87.23	0.26	9
LOC113511074	Uncharacterized protein LOC106135838	-1.57	-0.52	0.00	0.91	173.21	0.16	3

	isoform X3							
LOC113511075	Uncharacterized protein	-3.56	-1.71	0.00	1.78	104.40	0.47	9
LOC113511083	Facilitated trehalose transporter Tret1-like	-1.71	-0.57	0.00	0.98	173.21	0.10	3
LOC113511087	Rhophilin-2-B isoform X2	-2.50	-1.63	0.00	1.41	86.68	0.19	9
LOC113511091	Protein CIP2A homolog	-1.47	-0.49	0.00	0.85	173.21	0.19	3
LOC113511100	GTP-binding protein 1	0.00	0.96	2.88	1.66	173.21	0.12	7
LOC113511103	CCAAT/enhancer-binding protein gamma	0.00	0.84	2.53	1.46	173.21	0.20	7
LOC113511104	Collagen alpha-1(XXI) chain	-3.24	-1.69	0.00	1.62	95.97	0.70	9
LOC113511106	Glutamate--cysteine ligase regulatory subunit	0.00	0.81	2.44	1.41	173.21	0.32	7
LOC113511112	Caspase-4	-2.15	-0.72	0.00	1.24	173.21	0.22	4
LOC113511122	Ubiquitin-conjugating enzyme E2 T-like	-1.57	-0.52	0.00	0.90	173.21	0.16	3
LOC113511124	Dual specificity protein phosphatase 23-like	0.00	0.78	2.34	1.35	173.21	0.48	7
LOC113511128	Uridine 5'-monophosphate synthase	0.00	0.41	1.23	0.71	173.21	0.23	8
LOC113511129	Protein toll	-4.54	-1.51	0.00	2.62	173.21	0.91	3
LOC113511136	Uncharacterized protein LOC110379930	0.00	1.33	3.99	2.30	173.21	0.23	11
LOC113511140	S-adenosylmethionine mitochondrial carrier protein homolog	0.00	0.54	1.61	0.93	173.21	0.05	8
LOC113511144	Protein toll	-2.17	-0.72	0.00	1.26	173.21	0.10	3
LOC113511154	Uncharacterized protein LOC106102871	-1.22	-0.41	0.00	0.71	173.21	0.28	3
LOC113511167	RNA-binding protein fusilli isoform X2	-5.94	-1.98	0.00	3.43	173.21	0.69	4
LOC113511175	Salivary cysteine-rich peptide precursor	0.00	1.43	2.81	1.41	98.33	0.98	7
LOC113511179	Transcription factor AP-2-epsilon-like	0.00	2.37	7.10	4.10	173.21	0.07	15
LOC113511182	ATP-binding cassette sub-family A member 3-like	-3.17	-1.81	0.00	1.63	90.37	0.21	9
LOC113511187	Hypothetical protein RR46_05501	0.00	0.49	1.48	0.85	173.21	0.07	8
LOC113511192	Uncharacterized protein LOC106718755	-2.76	-0.92	0.00	1.59	173.21	0.38	3
LOC113511193	Phosphoenolpyruvate carboxykinase	0.00	1.45	4.35	2.51	173.21	0.05	11
LOC113511203	Hypothetical protein RR46_07971	-1.85	-0.62	0.00	1.07	173.21	0.04	3
LOC113511207	Uncharacterized protein LOC106135765	-2.31	-1.35	0.00	1.20	89.17	0.52	9
LOC113511222	Uncharacterized protein LOC106135801	0.00	0.82	2.45	1.42	173.21	0.30	7
LOC113511224	Sodium/hydrogen exchanger 10-like	-3.86	-1.29	0.00	2.23	173.21	0.78	3
LOC113511225	Protein tyrosine phosphatase domain-	0.00	1.44	4.32	2.49	173.21	0.04	11

	containing protein 1-like							
LOC113511228	Uncharacterized protein	-2.33	-0.78	0.00	1.35	173.21	0.18	3
LOC113511229	Uncharacterized protein LOC106135922	-2.94	-0.98	0.00	1.70	173.21	0.08	4
LOC113511232	BAG domain-containing protein Samui-like	0.00	0.49	1.47	0.85	173.21	0.08	8
LOC113511254	Uncharacterized protein LOC106138012	-2.78	-0.93	0.00	1.61	173.21	0.39	3
LOC113511256	Uncharacterized protein LOC106138012	-2.78	-0.93	0.00	1.61	173.21	0.39	3
LOC113511274	Metabolite transport protein CsbC	-6.49	-2.16	0.00	3.75	173.21	0.73	6
LOC113511279	Facilitated trehalose transporter Tret1-like	0.00	4.47	7.89	4.05	90.64	0.41	10
LOC113511283	Trypsin proteinase T2b precursor	-5.01	-1.67	0.00	2.89	173.21	0.99	6
LOC113511286	TBC1 domain family member 16 isoform X4	0.00	0.41	1.23	0.71	173.21	0.23	8
LOC113511287	5-oxoprolinase	-1.28	-0.43	0.00	0.74	173.21	0.26	3
LOC113511288	Beta-1,3-galactosyltransferase 5-like	-6.51	-3.07	0.00	3.27	106.64	0.30	6
LOC113511289	Uncharacterized protein LOC106140597	-5.22	-1.74	0.00	3.01	173.21	0.92	6
LOC113511292	Ceramide glucosyltransferase	0.00	1.30	2.50	1.25	96.19	0.90	7
LOC113511295	Prolow-density lipoprotein receptor-related protein 1	0.00	1.41	2.28	1.23	87.43	0.72	1
LOC113511306	Uncharacterized protein LOC106139408	-1.38	-0.46	0.00	0.80	173.21	0.65	4
LOC113511307	Oxysterol-binding protein-related protein 3-like	0.00	0.57	1.70	0.98	173.21	0.14	8
LOC113511308	Lipase member H-B-like	-5.39	-2.82	0.00	2.70	95.80	0.04	6
LOC113511309	Hypothetical protein RR46_06210	-4.03	-2.33	0.00	2.09	89.63	0.88	6
LOC113511315	Three prime repair exonuclease 2	-1.89	-1.23	0.00	1.07	86.65	0.48	9
LOC113511317	Ras-responsive element-binding protein 1	0.00	0.43	1.30	0.75	173.21	0.19	8
LOC113511320	Pancreatic lipase-related protein 2-like isoform X1	0.00	1.38	4.14	2.39	173.21	0.10	11
LOC113511322	Sodium- and chloride-dependent transporter XTRP3 isoform X1	0.00	1.17	3.51	2.03	173.21	0.96	11
LOC113511323	Acylcarnitine hydrolase-like	0.00	0.68	2.05	1.19	173.21	0.40	1
LOC113511335	Kynurenine formamidase isoform X1	-6.51	-2.17	0.00	3.76	173.21	0.74	4
LOC113511336	Hypothetical protein KGM_201149	-2.07	-1.22	0.00	1.08	88.75	0.52	9
LOC113511343	Aldo-keto reductase	-5.83	-4.52	-3.23	1.30	28.76	0.57	2
LOC113511345	Uncharacterized protein LOC106127048	-5.24	-1.75	0.00	3.02	173.21	0.91	6

	isoform X1							
LOC113511358	Protein canopy homolog 1	0.00	1.44	2.20	1.24	86.66	0.91	1
LOC113511360	Uncharacterized protein LOC106139170	-2.36	-0.79	0.00	1.36	173.21	0.19	3
LOC113511363	Uncharacterized protein LOC106129217	-1.53	-0.51	0.00	0.89	173.21	0.55	4
LOC113511367	Uncharacterized protein	0.00	2.87	8.60	4.96	173.21	0.39	15
LOC113511378	Zinc finger protein 648-like isoform X5	-1.83	-0.61	0.00	1.06	173.21	0.05	3
LOC113511379	Zinc finger protein 628-like isoform X8	-3.45	-1.15	0.00	1.99	173.21	0.66	3
LOC113511386	Spastin isoform X1	0.00	0.51	1.54	0.89	173.21	0.03	8
LOC113511400	Bifunctional glutamate/proline--tRNA ligase	0.00	0.52	1.56	0.90	173.21	0.01	8
LOC113511401	Sortilin-related receptor-like	0.00	0.46	1.39	0.80	173.21	0.13	8
LOC113511409	Hypothetical protein KGM_212946A	-4.61	-2.20	0.00	2.31	105.00	0.66	6
LOC113511419	G-protein coupled receptor Mth2-like isoform X2	0.00	1.15	2.08	1.06	91.78	0.26	1
LOC113511421	Protein yellow-like	0.00	0.99	1.58	0.86	87.12	0.28	1
LOC113511427	Tyrosine-protein phosphatase 69D	0.00	1.09	1.66	0.94	86.66	0.37	1
LOC113511437	Bacilysin biosynthesis oxidoreductase BacC-like	-2.32	-1.26	0.00	1.17	92.73	0.51	9
LOC113511441	Insulin-related peptide binding protein precursor	0.00	1.06	3.17	1.83	173.21	0.42	7
LOC113511449	Sodium-independent sulfate anion transporter-like	0.00	4.63	7.55	4.05	87.57	0.49	10
LOC113511452	Uncharacterized protein OBRU01_13522	0.00	1.62	2.88	1.47	91.09	0.52	12
LOC113511455	Uncharacterized protein LOC106139975	0.00	0.84	1.30	0.73	86.70	0.36	1
LOC113511471	Hexosaminidase D-like	-1.48	-0.49	0.00	0.86	173.21	0.59	4
LOC113511473	Trehalase-2	0.00	0.41	1.24	0.72	173.21	0.22	8
LOC113511485	Serine protease Bi-VSP-like isoform X1	0.00	1.81	3.20	1.64	90.59	0.63	12
LOC113511497	Sn1-specific diacylglycerol Lipase beta-like	-1.64	-0.55	0.00	0.95	173.21	0.13	3
LOC113511506	L-ascorbate oxidase isoform X2	0.00	5.99	9.91	5.27	87.98	0.28	14
LOC113511510	Uncharacterized protein LOC106130779	0.00	2.45	4.98	2.49	101.67	0.68	12
LOC113511515	Uncharacterized protein LOC106132000	0.00	0.89	2.66	1.54	173.21	0.06	7
LOC113511516	Hypothetical protein KGM_209449	-1.97	-1.25	0.00	1.09	86.93	0.46	9
LOC113511521	Troponin T	-2.21	-0.74	0.00	1.28	173.21	0.12	3
LOC113511522	F-actin-methionine sulfoxide oxidase	0.00	1.38	2.32	1.22	88.31	0.64	1

	MICAL3 isoform X1							
LOC113511534	Multidrug resistance-associated protein lethal(2)03659	0.00	1.27	3.81	2.20	173.21	0.42	11
LOC113511545	Uncharacterized protein LOC106140795	0.00	0.39	1.18	0.68	173.21	0.25	8
LOC113511548	Uncharacterized protein LOC106132691	0.00	1.39	4.17	2.41	173.21	0.08	11
LOC113511557	Glutathione S-transferase 1-like	-5.71	-3.94	-1.68	2.06	52.27	0.94	6
LOC113511560	27 kDa hemolymph protein-like	0.00	3.06	5.52	2.81	91.87	0.71	10
LOC113511563	Extracellular serine/threonine protein CG31145 isoform X1	0.00	2.42	4.73	2.37	97.89	0.54	12
LOC113511577	Cation-transporting ATPase 13A3 isoform X1	0.00	0.68	2.04	1.18	173.21	0.71	8
LOC113511593	SH3 domain-binding glutamic acid-rich protein homolog	0.00	0.92	1.52	0.81	87.90	0.21	1
LOC113511595	2-oxoglutarate dehydrogenase, mitochondrial isoform X4	0.00	0.45	1.34	0.77	173.21	0.16	8
LOC113511598	Uncharacterized protein LOC106132252	0.00	1.00	2.99	1.72	173.21	0.21	7
LOC113511602	Sulfatase-modifying factor 1	-1.33	-0.44	0.00	0.77	173.21	0.24	3
LOC113511606	27 kDa hemolymph protein	0.00	0.50	1.50	0.87	173.21	0.46	1
LOC113511617	Antichymotrypsin-2-like isoform X6	0.00	2.19	3.35	1.90	86.64	0.14	12
LOC113511619	ATP-binding cassette sub-family G member 8	-1.39	-0.46	0.00	0.80	173.21	0.22	3
LOC113511622	Proliferating cell nuclear antigen	-2.18	-0.73	0.00	1.26	173.21	0.10	3
LOC113511629	Dual specificity protein phosphatase 14	-1.65	-0.55	0.00	0.95	173.21	0.48	4
LOC113511631	60S ribosomal protein L22-like	-1.22	-0.41	0.00	0.70	173.21	0.28	3
LOC113511632	Uncharacterized protein	-2.24	-0.75	0.00	1.29	173.21	0.13	3
LOC113511638	Uncharacterized protein LOC110378979	-1.63	-1.04	0.00	0.90	86.89	0.83	9
LOC113511640	luciferin 4-monooxygenase	-3.47	-2.30	0.00	1.99	86.61	0.74	9
LOC113511644	Protein FAM107B	-1.76	-0.59	0.00	1.01	173.21	0.42	4
LOC113511646	Uncharacterized protein LOC106133717	-1.97	-0.66	0.00	1.14	173.21	0.01	3
LOC113511647	Bardet-Biedl syndrome 1 protein	-2.68	-1.57	0.00	1.40	88.88	0.39	9
LOC113511649	Mucin-5AC-like isoform X1	-3.19	-1.91	0.00	1.68	88.34	0.84	3
LOC113511655	KRAB-A domain-containing protein 2-like	-1.80	-0.60	0.00	1.04	173.21	0.06	3
LOC113511664	Uncharacterized protein LOC110375286	-2.08	-0.69	0.00	1.20	173.21	0.06	3

LOC113511665	Cardioacceleratory peptide receptor-like	3.57	4.83	6.49	1.50	31.02	0.38	13
LOC113511672	Protein anon-73B1	-1.27	-0.42	0.00	0.73	173.21	0.26	3
LOC113511679	Sodium channel protein Nach-like	-1.35	-0.45	0.00	0.78	173.21	0.24	3
LOC113511682	Acetyltransferase	-2.02	-1.27	0.00	1.11	87.01	0.45	9
LOC113511683	Uncharacterized protein C2orf47 homolog, mitochondrial-like	-2.82	-0.94	0.00	1.63	173.21	0.04	4
LOC113511685	Pancreatic triacylglycerol lipase-like isoform X1	0.00	4.67	8.25	4.23	90.62	0.55	10
LOC113511695	Protein yellow-like	-4.04	-2.59	0.00	2.25	86.79	0.75	6
LOC113511698	Innexin inx3	0.00	1.05	1.80	0.93	89.44	0.67	1
LOC113511703	Uncharacterized protein LOC106143543	0.00	0.42	1.25	0.72	173.21	0.21	8
LOC113511704	Protein transport protein Sec61 subunit alpha isoform 2	0.00	0.44	1.32	0.76	173.21	0.51	1
LOC113511719	60S ribosomal protein L37	-1.42	-0.47	0.00	0.82	173.21	0.21	3
LOC113511731	Lambda-crystallin	0.00	3.08	5.85	2.94	95.37	0.67	10
LOC113511741	Uncharacterized protein	0.00	1.00	3.01	1.74	173.21	0.24	7
LOC113511743	Substance-K receptor-like isoform X2	-1.51	-0.50	0.00	0.87	173.21	0.18	3
LOC113511745	Blood vessel epicardial substance	-3.24	-1.08	0.00	1.87	173.21	0.58	3
LOC113511749	Uncharacterized protein LOC106133686	0.00	0.53	1.59	0.92	173.21	0.44	1
LOC113511756	Hypothetical protein RR46_01282	-2.36	-0.79	0.00	1.36	173.21	0.19	3
LOC113511757	MAP kinase-interacting serine/threonine kinase	-1.82	-0.61	0.00	1.05	173.21	0.38	4
LOC113511759	D-2-hydroxyglutarate dehydrogenase, mitochondrial-like	-1.99	-0.66	0.00	1.15	173.21	0.02	3
LOC113511769	PR domain Zinc finger protein 1	-2.77	-0.40	1.56	2.19	545.39	0.55	4
LOC113511770	Uncharacterized protein PFB0145c	-1.91	-0.64	0.00	1.10	173.21	0.34	4
LOC113511771	Uncharacterized protein LOC110384167 isoform X2	0.00	1.70	5.09	2.94	173.21	0.45	11
LOC113511774	Seminal fluid protein CSSFP041	0.00	4.21	8.28	4.14	98.49	0.48	10
LOC113511777	Meteorin-like protein	-2.93	-1.41	0.00	1.47	104.19	0.56	9
LOC113511796	Uncharacterized protein	0.00	0.46	1.38	0.80	173.21	0.49	1
LOC113511798	Ras-related protein Rab-3 isoform X1	-2.43	-0.81	0.00	1.40	173.21	0.22	3
LOC113511802	Zonadhesin-like	0.00	0.48	1.45	0.84	173.21	0.47	1

LOC113511809	Netrin receptor UNC5C-like	-5.38	-3.58	0.00	3.10	86.60	0.63	6
LOC113511812	Zinc finger and BTB domain-containing protein 42	-1.59	-0.53	0.00	0.92	173.21	0.52	4
LOC113511818	Caskin-2 isoform X1	0.00	0.58	1.73	1.00	173.21	0.16	8
LOC113511819	Uncharacterized protein LOC106130173	0.00	0.79	2.37	1.37	173.21	0.42	7
LOC113511820	Protein lin-28 homolog	-2.50	-0.83	0.00	1.44	173.21	0.26	3
LOC113511822	cAMP-specific 3',5'-cyclic phosphodiesterase, isoform I	-4.74	-2.52	0.00	2.38	94.74	0.83	9
LOC113511824	D-2-hydroxyglutarate dehydrogenase, mitochondrial-like	-4.57	-2.23	0.00	2.29	102.75	0.65	6
LOC113511829	PDZ and LIM domain protein 7 isoform X1	0.00	0.86	2.57	1.48	173.21	0.15	7
LOC113511832	Adenylate kinase 7	-2.13	-0.71	0.00	1.23	173.21	0.08	3
LOC113511844	Histone-lysine N-methyltransferase PRDM9-like	0.00	1.14	3.41	1.97	173.21	0.81	7
LOC113511849	Carboxypeptidase N subunit 2-like	-3.08	-1.03	0.00	1.78	173.21	0.12	4
LOC113511850	Uncharacterized protein LOC106133343 isoform X4	-3.32	-2.20	0.00	1.91	86.61	0.61	9
LOC113511854	UPF0691 protein C9orf116	0.00	0.96	2.88	1.66	173.21	0.12	7
LOC113511860	Uncharacterized protein LOC110377235 isoform X5	-2.92	-1.93	0.00	1.67	86.61	0.31	9
LOC113511874	Rhomboid-related protein 2 isoform X1	0.00	4.96	8.54	4.44	89.40	0.84	10
LOC113511881	Uncharacterized protein LOC106132083	0.00	2.02	6.07	3.50	173.21	0.47	15
LOC113511891	N-ethylmaleimide sensitive fusion protein	0.00	0.44	1.33	0.77	173.21	0.17	8
LOC113511898	Hypothetical protein KGM_210031	-2.53	-1.31	0.00	1.27	96.45	0.50	9
LOC113511902	Protocadherin-like wing polarity protein stan	0.00	1.09	3.27	1.89	173.21	0.56	7
LOC113511904	Serine/threonine-protein kinase DDB_G0267686	0.00	0.55	1.64	0.95	173.21	0.07	8
LOC113511908	DNA N6-methyl adenine demethylase	0.00	0.43	1.28	0.74	173.21	0.20	8
LOC113511912	Vitamin K-dependent gamma-carboxylase	0.00	1.87	2.81	1.62	86.60	0.28	12
LOC113511915	Adhesive plaque matrix protein-like isoform X1	-6.00	-2.00	0.00	3.46	173.21	0.70	4
LOC113511921	Bifunctional 3'-phosphoadenosine 5'-phosphosulfate synthase	-1.50	-0.50	0.00	0.86	173.21	0.18	3
LOC113511922	Isovaleryl-CoA dehydrogenase,	-1.92	-1.18	0.00	1.03	87.50	0.58	9

	mitochondrial							
LOC113511928	Polypyrimidine tract-binding protein 1 isoform X7	-1.47	-0.49	0.00	0.85	173.21	0.59	4
LOC113511934	Atrial natriuretic peptide receptor 2	0.00	0.92	1.44	0.80	86.79	0.45	1
LOC113511941	Uncharacterized protein	-3.30	-2.19	0.00	1.89	86.61	0.59	9
LOC113511946	Uncharacterized protein LOC106142506	0.00	1.22	3.66	2.11	173.21	0.66	11
LOC113511948	Transcriptional regulator ATRX-like isoform X1	0.00	1.87	3.17	1.66	88.71	0.19	12
LOC113511950	Pancreatic lipase-related protein 2-like	-2.18	-1.17	0.00	1.10	93.86	0.72	9
LOC113511953	Elongation of very long chain fatty acids protein AAEL008004 isoform X1	0.00	0.44	1.32	0.76	173.21	0.18	8
LOC113511954	Phosphatidylinositol N-acetylglucosaminyltransferase subunit Q	0.00	0.39	1.18	0.68	173.21	0.25	8
LOC113511956	Zonadhesin-like	0.00	4.15	8.21	4.10	98.95	0.47	10
LOC113511962	Uncharacterized protein LOC110375307 isoform X1	0.00	1.42	4.25	2.45	173.21	0.03	11
LOC113511964	Transcription cofactor vestigial-like protein 4	-1.64	-0.55	0.00	0.95	173.21	0.13	3
LOC113511966	Cystinosin homolog isoform X2	0.00	2.13	3.75	1.93	90.49	0.15	12
LOC113511975	Ribosome biogenesis protein TSR3 homolog	0.00	0.42	1.25	0.72	173.21	0.21	8
LOC113511991	Oxidoreductase GLYR1 homolog isoform X1	-1.38	-0.46	0.00	0.80	173.21	0.22	3
LOC113511994	Uncharacterized protein LOC106138389 isoform X1	0.00	2.57	3.92	2.23	86.63	0.40	12
LOC113512002	RNA-binding protein 41-like	-1.17	-0.39	0.00	0.68	173.21	0.29	3
LOC113512007	Fibroin light chain	-6.15	-4.76	-3.60	1.29	27.16	0.50	2
LOC113512008	Senecionine N-oxygenase-like	0.00	2.62	4.74	2.41	91.96	0.54	12
LOC113512011	Ribosomal protein S16	-1.25	-0.42	0.00	0.72	173.21	0.27	3
LOC113512015	Facilitated trehalose transporter Tret1-2 homolog isoform X1	0.00	0.77	2.31	1.34	173.21	0.41	1
LOC113512027	Uncharacterized protein LOC110379767	-1.52	-0.51	0.00	0.88	173.21	0.18	3
LOC113512029	Uncharacterized protein LOC106135547	-1.77	-1.03	0.00	0.92	89.41	0.95	9
LOC113512041	Hemicentin-1-like isoform X1	0.00	0.99	2.98	1.72	173.21	0.62	1
LOC113512044	Hemicentin-1-like	-1.25	0.24	1.97	1.62	672.43	0.94	3

LOC113512048	Uncharacterized protein LOC106716864	-2.13	-0.71	0.00	1.23	173.21	0.08	3
LOC113512055	Alpha-N-acetylgalactosaminidase isoform X1	-4.27	-1.95	0.00	2.16	110.70	0.93	9
LOC113512064	Uncharacterized protein LOC106135577	-5.61	-1.87	0.00	3.24	173.21	0.66	4
LOC113512070	Replication factor C subunit 2	-1.48	-0.49	0.00	0.85	173.21	0.19	3
LOC113512071	Lipase 3-like	3.06	4.17	5.26	1.10	26.37	0.25	13
LOC113512072	Uncharacterized protein LOC106140671	-3.14	-1.05	0.00	1.81	173.21	0.54	3
LOC113512073	Venom acid phosphatase Acph-1-like	0.00	3.31	5.41	2.90	87.65	0.44	10
LOC113512074	AN1-type Zinc finger protein 2A-like	0.00	0.88	2.64	1.53	173.21	0.07	7
LOC113512075	Uncharacterized protein	-3.28	-1.81	0.00	1.66	92.10	0.26	9
LOC113512082	Uncharacterized protein LOC106135578	-6.70	-3.57	0.00	3.37	94.50	0.40	6
LOC113512086	Hypothetical protein RR46_14329	-5.50	-1.83	0.00	3.18	173.21	0.65	4
LOC113512090	GAS2-like protein 3 isoform X2	0.00	1.14	3.43	1.98	173.21	0.84	7
LOC113512093	Hypothetical protein KGM_205821	0.00	0.41	1.22	0.70	173.21	0.23	8
LOC113512095	fatty acid-binding protein, muscle-like	1.48	2.02	2.87	0.74	36.79	0.83	1
LOC113512105	Uncharacterized protein LOC105391666	-1.32	-0.44	0.00	0.76	173.21	0.25	3
LOC113512108	Uncharacterized protein LOC106139412	0.00	3.11	5.81	2.93	94.18	0.63	10
LOC113512112	Carbonic anhydrase-related protein 10 isoform X1	-3.10	-1.03	0.00	1.79	173.21	0.53	3
LOC113512115	Uncharacterized protein LOC105561123	-1.87	-0.62	0.00	1.08	173.21	0.35	4
LOC113512118	Cilia- and flagella-associated protein 97-like	-6.37	-2.12	0.00	3.68	173.21	0.74	6
LOC113512121	Uncharacterized protein LOC110372977	-5.48	-1.83	0.00	3.17	173.21	0.85	6
LOC113512123	Cytochrome P450 49a1	0.00	0.73	2.20	1.27	173.21	0.81	7
LOC113512130	Protein abnormal spindle	-1.88	-0.63	0.00	1.08	173.21	0.03	3
LOC113512136	Myosin light chain kinase, smooth muscle-like	0.00	0.50	1.49	0.86	173.21	0.06	8
LOC113512140	Uncharacterized protein LOC106143264	0.00	0.65	1.95	1.12	173.21	0.50	8
LOC113512156	Trichohyalin-like	-1.93	-0.64	0.00	1.11	173.21	0.01	3
LOC113512196	Neutral Lipase	0.00	3.68	5.59	3.19	86.62	0.42	10
LOC113512197	Dynein heavy chain 1, axonemal-like	-2.95	-1.62	0.00	1.50	92.53	0.18	9
LOC113512212	Rho GTPase-activating protein 26	0.00	0.40	1.20	0.70	173.21	0.24	8
LOC113512228	Uncharacterized protein LOC106129208	-2.12	-0.71	0.00	1.22	173.21	0.08	3
LOC113512229	Uncharacterized protein LOC106139759	0.00	4.03	6.43	3.51	87.15	0.23	10

LOC113512232	5-hydroxytryptamine receptor 1A-like	-2.17	-0.72	0.00	1.25	173.21	0.10	3
LOC113512234	Multidrug resistance-associated protein lethal(2)03659	0.00	1.38	4.14	2.39	173.21	0.10	11
LOC113512243	D-arabinitol dehydrogenase 1-like	-6.26	-3.44	0.00	3.18	92.36	0.36	6
LOC113512252	Uncharacterized protein LOC106140275 isoform X1	0.00	1.90	2.86	1.65	86.60	0.26	12
LOC113512262	Uncharacterized protein LOC106138972	-3.43	-1.14	0.00	1.98	173.21	0.23	4
LOC113512275	Juvenile hormone epoxide hydrolase-like	-1.88	-1.12	0.00	0.99	88.57	0.72	9
LOC113512276	Kinesin-like protein KIF19 isoform X1	0.00	0.49	1.46	0.84	173.21	0.09	8
LOC113512280	Fatty acid synthase 2	0.00	0.60	1.81	1.04	173.21	0.27	8
LOC113512282	Na,K-ATPase beta-subunit	-5.61	-3.74	0.00	3.24	86.60	0.65	6
LOC113512287	Mitochondrial carrier protein ymc	-3.55	-2.94	-2.23	0.66	22.59	0.44	2
LOC113512289	Isoaspartyl peptidase/L-asparaginase CG7860	-2.23	-0.74	0.00	1.29	173.21	0.13	3
LOC113512293	Post-GPI attachment to proteins factor 2-like	0.00	1.05	1.74	0.92	87.93	0.25	1
LOC113512294	Inactive pancreatic Lipase-related protein 1-like isoform X4	0.00	3.66	6.64	3.37	92.16	0.21	10
LOC113512295	Pancreatic lipase-related protein 2-like	-3.46	-1.15	0.00	2.00	173.21	0.25	4
LOC113512296	Androgen-dependent TFPI-regulating protein-like	0.00	1.50	4.51	2.60	173.21	0.13	11
LOC113512298	Serpin B8-like	0.00	0.80	2.39	1.38	173.21	0.40	7
LOC113512299	Protein decapentaplegic-like	-2.40	-1.31	0.00	1.21	92.49	0.42	9
LOC113512301	Diapause hormone receptor	0.00	4.37	7.24	3.85	88.00	0.35	10
LOC113512308	Acyl-CoA desaturase HassNPVE	0.00	2.18	6.54	3.78	173.21	0.16	15
LOC113512315	Carboxylesterase CXE26	0.00	0.63	1.90	1.10	173.21	0.42	8
LOC113512321	Xanthine dehydrogenase	-4.17	-2.21	0.00	2.10	94.96	0.89	6
LOC113512326	Immunoglobulin superfamily containing Leucine-rich repeat protein-like	-2.11	-0.70	0.00	1.22	173.21	0.07	3
LOC113512330	T-cell activation inhibitor, mitochondrial	-1.21	-0.40	0.00	0.70	173.21	0.28	3
LOC113512331	Hypothetical protein KGM_208540	-4.79	-2.41	0.00	2.39	99.34	0.39	6
LOC113512333	Uncharacterized protein LOC106132298 isoform X1	-2.57	-1.36	0.00	1.29	94.96	0.40	9
LOC113512341	Alpha,Alpha-trehalose-phosphate synthase	-1.79	-0.60	0.00	1.03	173.21	0.40	4

LOC113512343	Endoplasmic reticulum lectin 1 isoform X2	-1.22	-0.41	0.00	0.70	173.21	0.28	3
LOC113512346	Sodium/hydrogen exchanger 9B2-like isoform X1	0.00	1.00	3.01	1.74	173.21	0.23	7
LOC113512351	Hemicentin-2	-2.99	-1.44	0.00	1.50	104.22	0.53	9
LOC113512354	ATP-binding cassette sub-family G member 4-like isoform X2	-4.07	-2.09	0.00	2.04	97.42	0.90	9
LOC113512371	Uncharacterized protein LOC106142703	-1.87	-0.62	0.00	1.08	173.21	0.03	3
LOC113512373	Down syndrome cell adhesion molecule-like protein Dscam2	0.00	0.45	1.34	0.77	173.21	0.16	8
LOC113512374	Cytochrome P450 9e2-like	0.00	1.53	2.60	1.36	88.96	0.71	12
LOC113512377	Organic cation transporter protein-like	-1.63	-0.54	0.00	0.94	173.21	0.13	3
LOC113512381	Uridine phosphorylase 1 isoform X1	0.00	0.69	2.07	1.20	173.21	0.40	1
LOC113512382	Synaptic vesicle glycoprotein 2C-like	-2.96	-1.86	0.00	1.62	87.11	0.21	9
LOC113512385	Endothelin-converting enzyme homolog	0.00	1.48	2.34	1.28	87.01	0.72	1
LOC113512388	J domain-containing protein	0.00	1.71	2.63	1.48	86.68	0.46	12
LOC113512391	Uncharacterized protein LOC106142706	-2.24	-1.41	0.00	1.23	87.12	0.24	9
LOC113512394	Progestin and adipoQ receptor family member 3	-1.37	-0.46	0.00	0.79	173.21	0.23	3
LOC113512396	Inactive pancreatic Lipase-related protein 1-like	-2.66	-1.52	0.00	1.37	90.03	0.12	9
LOC113512398	Solute carrier family 52, riboflavin transporter, member 3-B isoform X2	0.00	0.73	2.18	1.26	173.21	0.89	7
LOC113512401	Facilitated trehalose transporter Tret1-like isoform X1	-2.73	-1.50	0.00	1.39	92.24	0.20	9
LOC113512403	ATP-binding cassette sub-family G member 4-like	-3.41	-1.87	0.00	1.73	92.60	0.34	9
LOC113512416	Phosphoinositide 3-kinase adapter protein 1 isoform X1	0.00	1.08	1.80	0.95	88.22	0.59	1
LOC113512420	Gremlin-1-like isoform X1	0.00	3.42	5.69	3.02	88.14	0.31	10
LOC113512425	Multidrug resistance-associated protein 1 isoform X1	0.00	1.16	3.49	2.01	173.21	0.98	7
LOC113512426	CMP-sialic acid transporter 1	0.00	1.10	1.99	1.01	91.89	0.21	1
LOC113512435	Synaptotagmin 1 isoform X1	-3.41	-1.75	0.00	1.71	97.67	0.35	9
LOC113512446	Myophilin	0.00	4.65	7.60	4.07	87.65	0.50	10

LOC113512447	Uncharacterized protein	0.00	5.81	9.06	5.05	86.80	0.38	14
LOC113512450	ATP-binding cassette sub-family G member 4-like isoform X1	-1.64	-0.55	0.00	0.95	173.21	0.13	3
LOC113512452	Uncharacterized protein LOC106130842	0.00	2.59	4.84	2.44	94.10	0.56	12
LOC113512453	Uncharacterized protein LOC106130912	-1.26	-0.42	0.00	0.72	173.21	0.27	3
LOC113512454	Uncharacterized protein LOC105340758 isoform X1	-1.54	-0.51	0.00	0.89	173.21	0.17	3
LOC113512455	GATA Zinc finger domain-containing protein 14-like	1.84	3.40	4.46	1.38	40.54	0.78	13
LOC113512461	Limbic system-associated membrane protein-like	0.00	3.19	9.56	5.52	173.21	0.50	15
LOC113512465	Transmembrane protein 177	0.00	0.50	1.49	0.86	173.21	0.06	8
LOC113512473	Lipase 1-like isoform X2	-6.29	-4.19	0.00	3.63	86.60	0.73	6
LOC113512475	Aldehyde dehydrogenase X, mitochondrial-like	-2.06	-0.69	0.00	1.19	173.21	0.05	3
LOC113512476	Mitochondrial aldehyde dehydrogenase	-3.20	-1.57	0.00	1.60	102.16	0.43	9
LOC113512481	WD repeat-containing protein on Y chromosome	-1.71	-1.13	0.00	0.98	86.61	0.64	9
LOC113512485	Serine palmitoyltransferase 2	0.00	0.53	1.59	0.92	173.21	0.44	1
LOC113512486	Reticulon-3-like isoform X2	0.00	0.56	1.69	0.98	173.21	0.13	8
LOC113512490	ATP-binding cassette sub-family G member 1	0.00	0.97	2.91	1.68	173.21	0.14	7
LOC113512507	Glutamate receptor ionotropic, delta-1-like	-2.60	-0.87	0.00	1.50	173.21	0.30	3
LOC113512508	Acyl-CoA Delta(11) desaturase	-3.70	-2.20	0.00	1.95	88.55	0.74	9
LOC113512510	Homeobox protein goosecoid-like	0.00	2.02	6.06	3.50	173.21	0.48	15
LOC113512515	Delta9-desaturase	0.00	1.61	4.82	2.78	173.21	0.26	11
LOC113512517	Protein turtle	-5.16	-2.51	0.00	2.58	102.64	0.27	6
LOC113512525	ATP-binding cassette sub-family G member 4-like isoform X2	-1.68	-0.56	0.00	0.97	173.21	0.11	3
LOC113512533	Protein yellow-like isoform X1	3.13	6.18	8.93	2.91	47.06	0.81	14
LOC113512534	Pancreatic lipase-related protein 3-like	-6.10	-4.06	0.00	3.51	86.60	0.71	6
LOC113512540	Signal-induced proliferation-associated 1-like protein 1	0.00	0.50	1.51	0.87	173.21	0.05	8
LOC113512543	Xanthine dehydrogenase-like	-4.44	-2.54	0.00	2.29	90.04	0.48	6

LOC113512546	Protein yellow	-2.28	-0.76	0.00	1.32	173.21	0.17	4
LOC113512554	Uncharacterized protein LOC110370740 isoform X2	0.00	2.94	4.58	2.55	86.79	0.91	12
LOC113512556	Cadherin-86C	-4.47	-2.56	0.00	2.30	90.16	0.89	9
LOC113512558	Muscle-specific protein 20-like	0.00	3.13	5.78	2.92	93.37	0.61	10
LOC113512560	Serine protease easter-like	0.00	2.29	3.62	1.99	86.95	0.27	12
LOC113512577	Dorsal 1b	0.00	0.55	1.65	0.95	173.21	0.08	8
LOC113512587	Sodium-dependent nutrient amino acid transporter 1-like isoform X1	-2.15	-0.72	0.00	1.24	173.21	0.09	3
LOC113512600	UNC93-like protein MFSD11 isoform X2	-2.43	-0.81	0.00	1.40	173.21	0.22	3
LOC113512603	Uncharacterized protein OBRU01_14198	-1.23	-0.41	0.00	0.71	173.21	0.28	3
LOC113512613	Uncharacterized protein LOC101739854 isoform X1	-1.37	-0.46	0.00	0.79	173.21	0.23	3
LOC113512614	Chitinase-3-like protein 2	-1.36	-0.45	0.00	0.79	173.21	0.23	3
LOC113512615	Headcase protein	-1.60	-0.53	0.00	0.92	173.21	0.51	4
LOC113512622	Sodium-dependent nutrient amino acid transporter 1-like isoform X1	-5.82	-1.94	0.00	3.36	173.21	0.79	6
LOC113512633	CUGBP Elav-like family member 1 isoform X5	0.00	0.42	1.27	0.73	173.21	0.53	1
LOC113512634	Uncharacterized protein	0.00	5.16	8.61	4.55	88.26	0.89	14
LOC113512639	Serine protease easter-like	0.00	1.19	1.97	1.05	88.00	0.34	1
LOC113512650	Calphotin-like isoform X2	0.00	0.49	1.48	0.86	173.21	0.07	8
LOC113512653	Chromatin assembly factor 1 subunit B	-1.21	-0.40	0.00	0.70	173.21	0.28	3
LOC113512658	Uncharacterized protein LOC106114649	-1.41	-0.47	0.00	0.81	173.21	0.63	4
LOC113512665	Matrix metalloproteinase-25-like	-3.01	-1.55	0.00	1.51	97.23	0.32	9
LOC113512666	Adenosine kinase	-1.48	-0.49	0.00	0.85	173.21	0.19	3
LOC113512678	Clavesin-2	-1.45	-0.48	0.00	0.84	173.21	0.20	3
LOC113512680	Uncharacterized protein	0.00	1.85	3.11	1.64	88.48	0.49	12
LOC113512681	Salivary secreted peptide	0.00	1.88	5.63	3.25	173.21	0.94	15
LOC113512683	Uncharacterized protein LOC106129086	-4.74	-2.31	0.00	2.37	102.69	0.51	6
LOC113512684	Chloride channel protein 2 isoform X1	-2.31	-1.27	0.00	1.17	92.53	0.50	9
LOC113512706	Peptidoglycan recognition protein-like	0.00	4.27	7.12	3.77	88.23	0.29	10
LOC113512725	Tartan/capricious-like protein	-3.22	-1.07	0.00	1.86	173.21	0.57	3

LOC113512729	Uncharacterized protein LOC106713194	-1.31	-0.44	0.00	0.76	173.21	0.25	3
LOC113512735	Semaphorin-5A	-1.68	-1.09	0.00	0.94	86.72	0.70	9
LOC113512738	Triokinase/FMN cyclase-like	-2.61	-1.38	0.00	1.31	95.21	0.39	9
LOC113512739	Uncharacterized protein LOC106135661	0.00	5.29	8.41	4.60	87.08	0.75	14
LOC113512743	Testis specific tektin	0.00	1.87	5.62	3.25	173.21	0.96	15
LOC113512748	Cytochrome P450 9e2-like	-3.98	-2.02	0.00	1.99	98.32	0.77	9
LOC113512750	Serine protease 4 precursor	0.00	2.61	4.40	2.31	88.66	0.45	12
LOC113512758	Zinc finger protein 628-like	0.00	0.58	1.73	1.00	173.21	0.17	8
LOC113512770	Chymotrypsinogen B-like	0.00	0.64	1.93	1.11	173.21	0.40	1
LOC113512772	Uncharacterized protein LOC110371712	-2.64	-0.88	0.00	1.52	173.21	0.32	3
LOC113512773	Myrosinase 1-like	-3.44	-1.77	0.00	1.72	97.10	0.36	9
LOC113512779	Facilitated trehalose transporter Tret1-like isoform X1	-2.89	-1.42	0.00	1.45	102.13	0.50	9
LOC113512789	Uncharacterized protein	-2.11	-0.70	0.00	1.22	173.21	0.24	4
LOC113512790	Acyl-CoA synthetase family member 2, mitochondrial	-2.71	-1.76	0.00	1.53	86.67	0.16	9
LOC113512793	Cytochrome P450 301a1, mitochondrial	-2.25	-0.75	0.00	1.30	173.21	0.14	3
LOC113512799	Niemann-Pick C1 protein-like	-3.42	-1.76	0.00	1.71	97.22	0.74	9
LOC113512819	Myb-like protein I	0.00	0.41	1.24	0.71	173.21	0.22	8
LOC113512823	Uncharacterized protein LOC106136292	-5.22	-1.74	0.00	3.01	173.21	0.92	6
LOC113512840	Speckle targeted PIP5K1A-regulated poly(A) polymerase-like	-1.33	-0.44	0.00	0.77	173.21	0.24	3
LOC113512844	Uncharacterized protein LOC106708416	0.00	2.77	5.64	2.82	102.00	0.92	12
LOC113512851	Uncharacterized protein LOC107172115	-1.47	-0.49	0.00	0.85	173.21	0.59	4
LOC113512854	Uncharacterized protein LOC106136339	-2.11	-0.70	0.00	1.22	173.21	0.08	3
LOC113512858	4-coumarate--CoA ligase 3	-6.81	-4.53	0.00	3.93	86.60	0.77	6
LOC113512863	Uncharacterized protein LOC101744658	-1.97	-0.66	0.00	1.14	173.21	0.01	3
LOC113512866	Calcineurin B	0.00	1.38	2.08	1.19	86.61	0.73	1
LOC113512868	Uncharacterized protein LOC106135683 isoform X1	0.00	0.85	2.54	1.46	173.21	0.19	7
LOC113512870	Tubulin-specific chaperone cofactor E-like protein isoform X1	-2.11	-0.70	0.00	1.22	173.21	0.08	3
LOC113512873	MAGUK p55 subfamily member 5 isoform	0.00	0.61	1.83	1.05	173.21	0.30	8

	X2							
LOC113512878	Semaphorin-2A-like	-6.55	-3.14	0.00	3.28	104.47	0.29	6
LOC113512883	Uncharacterized protein LOC110384473 isoform X1	0.00	1.61	3.37	1.69	104.83	0.87	12
LOC113512885	G-protein coupled receptor moody-like	0.00	0.43	1.28	0.74	173.21	0.20	8
LOC113512893	Uncharacterized protein LOC106138361	0.00	4.15	6.71	3.63	87.37	0.26	10
LOC113512894	Prostaglandin reductase 1-like	0.00	1.05	3.14	1.81	173.21	0.38	7
LOC113512897	Fumarate hydratase, mitochondrial	-1.88	-0.63	0.00	1.09	173.21	0.03	3
LOC113512903	Uncharacterized protein LOC110369842	0.00	2.75	8.24	4.76	173.21	0.34	15
LOC113512906	Serine/arginine repetitive matrix protein 2	-5.38	-3.96	-2.82	1.30	32.92	0.46	2
LOC113512908	Promoting protein precursor	-4.31	-1.44	0.00	2.49	173.21	0.87	3
LOC113512911	Gastrula Zinc finger protein XlCGF57.1-like	-1.35	-0.45	0.00	0.78	173.21	0.23	3
LOC113512912	Methylmalonate-semialdehyde dehydrogenase	-1.33	-0.44	0.00	0.77	173.21	0.24	3
LOC113512913	39S ribosomal protein L49, mitochondrial	-1.35	-0.45	0.00	0.78	173.21	0.23	3
LOC113512920	Organic solute transporter Alpha-like protein	0.00	0.87	2.60	1.50	173.21	0.12	7
LOC113512924	39S ribosomal protein L33, mitochondrial	0.00	1.21	1.96	1.06	87.53	0.37	1
LOC113512927	Mitochondrial tRNA-specific 2-thiouridylase 1 isoform X1	0.00	0.48	1.45	0.83	173.21	0.09	8
LOC113512933	Uncharacterized protein LOC106136478 isoform X1	0.00	6.41	10.56	5.63	87.84	0.23	14
LOC113512935	Leucine-rich repeat-containing protein 58-like	-2.31	-1.35	0.00	1.21	89.00	0.31	9
LOC113512940	Uncharacterized protein LOC106136299	-4.65	-2.51	0.00	2.35	93.36	0.37	6
LOC113512946	Uncharacterized protein LOC106136501	-2.07	-0.69	0.00	1.20	173.21	0.06	3
LOC113512947	Uncharacterized protein LOC106142568	-1.94	-0.20	1.33	1.64	816.87	0.46	3
LOC113512948	Uncharacterized protein	0.00	1.57	2.87	1.46	92.56	0.93	1
LOC113512949	Tetra-peptide repeat homeobox protein 1-like precursor	0.00	1.95	5.86	3.39	173.21	0.66	15
LOC113512953	MD-2-related lipid-recognition protein-like	-4.20	-2.27	0.00	2.12	93.26	0.80	6
LOC113512962	Protein stoned-B-like	-2.33	-0.78	0.00	1.34	173.21	0.17	3
LOC113512968	Chymotrypsin-2-like	-6.51	-3.55	0.00	3.29	92.93	0.40	6
LOC113512971	Iron/zinc purple acid phosphatase-like	-2.86	-0.95	0.00	1.65	173.21	0.42	3

	protein							
LOC113512972	Speckle targeted PIP5K1A-regulated poly(A) polymerase isoform X2	-1.73	-0.58	0.00	1.00	173.21	0.09	3
LOC113512976	UDP-glucuronosyltransferase 1-6-like	0.00	0.60	1.80	1.04	173.21	0.41	1
LOC113512980	Uncharacterized protein LOC106121753 isoform X1	-1.95	-0.65	0.00	1.13	173.21	0.01	3
LOC113512981	Echinoderm microtubule-associated protein-like 2 isoform X1	-2.74	-0.91	0.00	1.58	173.21	0.37	3
LOC113512983	4-coumarate--CoA ligase 3	-3.25	-2.12	0.00	1.83	86.68	0.50	9
LOC113512991	Beta-amyloid-like protein isoform X1	0.00	2.80	4.62	2.46	87.94	0.70	12
LOC113512993	Fasciculation and elongation protein zeta-2	0.00	1.02	1.81	0.93	90.75	0.74	1
LOC113512995	Uncharacterized protein LOC106136292	0.00	0.44	1.31	0.76	173.21	0.18	8
LOC113512996	DNA repair protein REV1	-1.64	-0.55	0.00	0.94	173.21	0.13	3
LOC113512998	Hypoxia up-regulated protein 1	0.00	1.09	1.80	0.96	87.94	0.58	1
LOC113513003	Hypoxia up-regulated protein 1	0.00	1.16	1.96	1.03	88.73	0.64	1
LOC113513004	Sortilin-related receptor-like isoform X1	0.00	0.60	1.80	1.04	173.21	0.25	8
LOC113513006	Hormone-sensitive Lipase isoform X1	-1.48	-0.49	0.00	0.85	173.21	0.19	3
LOC113513007	G-protein coupled receptor moody	-3.12	-1.84	0.00	1.64	88.71	0.22	9
LOC113513009	Fasciclin-2 isoform X2	0.00	0.55	1.65	0.96	173.21	0.09	8
LOC113513010	Uncharacterized protein LOC101736409	0.00	1.18	3.53	2.04	173.21	0.91	11
LOC113513022	Uncharacterized protein LOC101746298 isoform X1	0.00	1.38	2.74	1.37	99.48	0.86	7
LOC113513026	Hypothetical protein KGM_210399B	0.00	2.48	7.43	4.29	173.21	0.17	15
LOC113513030	1-phosphatidylinositol 4,5-bisphosphate phosphodiesterase isoform X1	-2.38	-0.79	0.00	1.38	173.21	0.20	3
LOC113513036	Ecdysteroid-regulated 16 kDa protein-like	-1.93	-0.64	0.00	1.12	173.21	0.01	3
LOC113513037	Xanthine dehydrogenase/oxidase-like	0.00	1.18	3.54	2.04	173.21	0.89	11
LOC113513039	Xanthine dehydrogenase/oxidase-like	0.00	0.84	2.52	1.46	173.21	0.21	7
LOC113513043	Uncharacterized protein	0.00	3.70	6.65	3.39	91.65	0.18	10
LOC113513044	Zinc finger protein 436-like	0.00	0.57	1.70	0.98	173.21	0.13	8
LOC113513051	Parapinopsin-like	-3.73	-1.24	0.00	2.15	173.21	0.74	3
LOC113513069	Dual specificity tyrosine-phosphorylation-regulated kinase 1A isoform X1	0.00	1.00	1.57	0.87	86.90	0.46	1

LOC113513085	Cytochrome P450 monooxygenase CYP367B12	0.00	1.91	5.74	3.32	173.21	0.79	15
LOC113513087	PhosphoLipase C beta 1	-2.14	-0.71	0.00	1.23	173.21	0.09	3
LOC113513088	Wnt inhibitory factor 1	-5.50	-2.77	0.00	2.75	99.40	0.08	6
LOC113513094	Gamma-tubulin complex component 6	-1.38	-0.46	0.00	0.79	173.21	0.23	3
LOC113513096	Bumetanide-sensitive sodium-(potassium)-chloride cotransporter isoform X1	0.00	0.45	1.34	0.77	173.21	0.16	8
LOC113513100	Uncharacterized protein LOC110378060 isoform X2	-1.31	-0.44	0.00	0.75	173.21	0.25	3
LOC113513108	Mitochondrial sodium/hydrogen exchanger NHA2	-2.99	-1.57	0.00	1.50	95.79	0.72	9
LOC113513109	Beta-1,3-glucan recognition protein precursor	0.00	1.36	2.10	1.18	86.72	0.68	1
LOC113513111	Uncharacterized protein LOC106122485	-6.23	-2.08	0.00	3.60	173.21	0.75	6
LOC113513122	4-coumarate--CoA ligase 1-like	0.00	2.76	5.24	2.63	95.46	0.81	12
LOC113513127	Hypothetical protein RR48_08923	-1.57	-0.52	0.00	0.91	173.21	0.16	3
LOC113513129	Membrane-bound alkaline phosphatase-like	0.00	2.15	3.79	1.95	90.50	0.16	12
LOC113513140	Protein shuttle craft-like	0.00	0.81	2.43	1.41	173.21	0.33	7
LOC113513141	Cytochrome P450 9e2-like	-1.87	-0.62	0.00	1.08	173.21	0.03	3
LOC113513145	Zinc finger protein rotund isoform X3	-2.88	-1.83	0.00	1.59	86.96	0.19	9
LOC113513160	Mitotic spindle assembly checkpoint protein MAD2A	-2.38	-0.79	0.00	1.38	173.21	0.20	3
LOC113513163	AFG3-like protein 2	0.00	0.43	1.30	0.75	173.21	0.19	8
LOC113513171	Adenosine kinase	-1.50	-0.50	0.00	0.87	173.21	0.18	3
LOC113513173	Transcription initiation factor TFIID subunit 4 isoform X1	0.00	1.50	4.49	2.59	173.21	0.12	11
LOC113513174	Vacuolar protein sorting-associated protein 13C	-1.64	-1.04	0.00	0.91	86.91	0.77	9
LOC113513180	Protein TANC2 isoform X1	-3.56	-1.19	0.00	2.05	173.21	0.27	4
LOC113513183	Kinesin-like protein KIF20B	0.00	0.45	1.35	0.78	173.21	0.50	1
LOC113513184	Kinesin-like protein KIF20B	-1.56	-0.52	0.00	0.90	173.21	0.16	3
LOC113513185	Uncharacterized protein LOC106136069 isoform X1	-2.39	-1.33	0.00	1.22	91.30	0.37	9
LOC113513186	Uncharacterized protein LOC106137033	-1.99	-1.27	0.00	1.10	86.89	0.43	9

	isoform X1							
LOC113513204	Protein spaetzle	0.00	2.62	4.15	2.28	87.01	0.47	12
LOC113513214	Uncharacterized protein LOC110370951 isoform X2	-1.65	-0.55	0.00	0.95	173.21	0.48	4
LOC113513215	Cytosolic 10-formyltetrahydrofolate dehydrogenase	-1.96	-0.65	0.00	1.13	173.21	0.01	3
LOC113513223	Uncharacterized protein LOC110378268	0.00	0.73	2.18	1.26	173.21	0.86	7
LOC113513233	Baculoviral IAP repeat-containing protein 6	0.00	0.44	1.31	0.76	173.21	0.18	8
LOC113513236	Peptidyl-Alpha-hydroxyglycine Alpha-amidating lyase 1-like	0.00	0.71	2.12	1.22	173.21	0.40	1
LOC113513243	Glutathione S-transferase sigma 5	-1.88	-0.63	0.00	1.08	173.21	0.03	3
LOC113513247	Testis-expressed protein 2	0.00	0.52	1.55	0.89	173.21	0.02	8
LOC113513259	Uncharacterized protein LOC110381499	-2.38	-2.02	-1.53	0.44	21.67	0.84	9
LOC113513263	2-oxoisovalerate dehydrogenase subunit alpha, mitochondrial isoform X2	-2.44	-0.81	0.00	1.41	173.21	0.23	3
LOC113513266	Lon protease homolog, mitochondrial isoform X1	0.00	0.41	1.22	0.70	173.21	0.23	8
LOC113513268	Centromere protein S-like	-1.59	-0.53	0.00	0.92	173.21	0.15	3
LOC113513270	TBC1 domain family member 4 isoform X1	-3.39	-1.13	0.00	1.96	173.21	0.63	3
LOC113513273	Olfactory receptor 36	-2.24	-0.75	0.00	1.29	173.21	0.13	3
LOC113513275	Retinol dehydrogenase 13-like	0.00	1.83	5.50	3.17	173.21	0.86	11
LOC113513276	Uncharacterized protein ZK1073.1 isoform X1	0.00	0.67	2.00	1.16	173.21	0.62	8
LOC113513277	Rho GTPase-activating protein 7 isoform X1	-1.61	-0.54	0.00	0.93	173.21	0.50	4
LOC113513281	Uncharacterized protein LOC106137863	-6.61	-4.40	0.00	3.81	86.60	0.76	6
LOC113513282	Transcription termination factor 2 isoform X2	-1.46	-0.49	0.00	0.84	173.21	0.20	3
LOC113513288	E3 ubiquitin-protein ligase sina-like isoform X1	-1.41	-0.47	0.00	0.81	173.21	0.22	3
LOC113513289	TBC1 domain family member 4 isoform X1	-1.82	-0.61	0.00	1.05	173.21	0.05	3
LOC113513295	Uncharacterized protein LOC110378785	-2.59	-0.86	0.00	1.50	173.21	0.30	3
LOC113513297	Tricarboxylate transport protein, mitochondrial	0.00	0.47	1.40	0.81	173.21	0.49	1
LOC113513298	Serine protease inhibitor-like	-5.30	-2.48	0.00	2.66	107.20	0.32	6

LOC113513300	Carboxypeptidase B-like	-4.72	-4.03	-3.38	0.67	16.68	0.28	2
LOC113513306	NAD kinase 2, mitochondrial	-1.79	-0.60	0.00	1.03	173.21	0.07	3
LOC113513308	Uncharacterized protein LOC106143374	0.00	1.02	1.76	0.92	89.41	0.19	1
LOC113513310	Beta-1,3-glucan recognition protein precursor	0.00	1.66	2.70	1.45	87.56	0.70	12
LOC113513313	Aldose reductase isoform X1	-2.60	-0.87	0.00	1.50	173.21	0.06	4
LOC113513314	Tumor protein p63-regulated gene 1-like protein isoform X2	-1.77	-0.59	0.00	1.02	173.21	0.07	3
LOC113513316	Hemocytin	-5.26	-2.59	0.00	2.63	101.55	0.20	6
LOC113513318	Uncharacterized protein LOC106137281	0.00	0.58	1.73	1.00	173.21	0.42	1
LOC113513321	Inorganic phosphate cotransporter isoform X2	0.00	0.46	1.38	0.80	173.21	0.14	8
LOC113513324	GTP-binding protein 1	0.00	1.09	3.28	1.89	173.21	0.57	7
LOC113513325	Cytochrome P450 protein	-4.04	-2.10	0.00	2.03	96.64	0.88	9
LOC113513329	Vacuolar protein sorting-associated protein 13D	0.00	0.81	2.42	1.40	173.21	0.35	7
LOC113513334	Organic cation transporter protein-like isoform X1	0.00	3.44	5.87	3.06	89.03	0.27	10
LOC113513335	R3H domain-containing protein 2 isoform X1	0.00	0.45	1.36	0.79	173.21	0.15	8
LOC113513336	Fatty-acid amide hydrolase 2-B-like	-2.89	-1.72	0.00	1.52	88.50	0.36	9
LOC113513347	Uncharacterized protein LOC110378737	-2.10	-1.39	0.00	1.20	86.61	0.29	9
LOC113513352	Protein regulator of cytokinesis 1-like	-1.60	-0.53	0.00	0.92	173.21	0.15	3
LOC113513356	Uncharacterized protein LOC106130098	-1.43	-0.48	0.00	0.83	173.21	0.21	3
LOC113513360	Uncharacterized protein LOC106135284	-1.51	-0.50	0.00	0.87	173.21	0.56	4
LOC113513361	Trypsin-like	-2.38	-0.79	0.00	1.37	173.21	0.20	3
LOC113513362	Apyrase-like	-2.07	-0.69	0.00	1.19	173.21	0.05	3
LOC113513369	Uncharacterized protein LOC106129477	0.00	0.48	1.45	0.84	173.21	0.09	8
LOC113513370	Uncharacterized protein LOC106134371	-2.48	-0.83	0.00	1.43	173.21	0.24	3
LOC113513377	Hypothetical protein KGM_213212	0.00	0.50	1.51	0.87	173.21	0.05	8
LOC113513385	Laccase-4-like	0.00	9.18	14.58	7.99	87.05	0.56	14
LOC113513387	Uncharacterized protein LOC106107480 isoform X1	-2.23	-1.42	0.00	1.24	86.84	0.30	9

LOC113513388	Heat shock protein 90 cognate	0.00	1.40	2.39	1.25	89.23	0.98	1
LOC113513404	Aminoacylase-1-like	-4.03	-3.31	-2.69	0.68	20.56	0.16	2
LOC113513413	Uncharacterized protein OBRU01_00994	-1.22	-0.41	0.00	0.70	173.21	0.28	3
LOC113513419	Protein regulator of cytokinesis 1 isoform X1	-1.26	-0.42	0.00	0.73	173.21	0.26	3
LOC113513421	Larval cuticle protein F1-like	-5.23	-1.74	0.00	3.02	173.21	0.92	6
LOC113513424	Inverted formin-2 isoform X1	0.00	1.71	3.34	1.67	97.84	0.56	12
LOC113513429	Intraflagellar transport protein 140 homolog	-1.23	-0.41	0.00	0.71	173.21	0.28	3
LOC113513432	Uncharacterized protein LOC106137196	-3.31	-2.07	0.00	1.80	87.19	0.46	9
LOC113513436	Venom carboxylesterase-6-like isoform X1	0.00	1.18	1.90	1.03	87.31	0.36	1
LOC113513445	TBC1 domain family member 20	0.00	0.62	1.87	1.08	173.21	0.35	8
LOC113513450	Protein charybde	0.00	1.42	4.27	2.46	173.21	0.03	11
LOC113513452	Uncharacterized protein LOC106105275	0.00	0.73	2.18	1.26	173.21	0.86	7
LOC113513453	Bm8 interacting protein 2d-2	-1.43	-0.48	0.00	0.83	173.21	0.62	4
LOC113513458	Uncharacterized protein LOC106141359	0.00	1.29	3.86	2.23	173.21	0.37	11
LOC113513459	Venom carboxylesterase-6-like	-4.48	-2.11	0.00	2.25	106.56	0.80	6
LOC113513462	Zinc finger protein rotund isoform X3	-2.35	-1.50	0.00	1.30	86.92	0.15	9
LOC113513463	Filamin-A isoform X1	-4.73	-3.13	0.00	2.71	86.61	0.58	6
LOC113513466	Solute carrier family 22 member 6-like	-8.21	-5.46	0.00	4.73	86.60	0.85	6
LOC113513473	Alpha-N-acetylgalactosaminidase-like	-1.49	-0.97	0.00	0.84	86.67	0.90	9
LOC113513475	Galactokinase-like	-3.13	-1.50	0.00	1.57	104.89	0.51	9
LOC113513490	Glucose dehydrogenase	-2.71	-0.90	0.00	1.57	173.21	0.36	3
LOC113513491	Glycine receptor subunit Alpha-4-like isoform X3	0.00	0.58	1.74	1.01	173.21	0.18	8
LOC113513501	Atrial natriuretic peptide receptor 1 isoform X1	0.00	0.48	1.44	0.83	173.21	0.10	8
LOC113513503	Sodium/calcium exchanger 1	0.00	2.15	3.54	1.89	87.91	0.33	12
LOC113513507	Geranylgeranyl diphosphate synthase	-2.15	-0.72	0.00	1.24	173.21	0.09	3
LOC113513508	G2/mitotic-specific cyclin-B3 isoform X1	-1.97	-0.66	0.00	1.14	173.21	0.01	3
LOC113513510	Diacylglycerol kinase epsilon isoform X2	-1.80	-0.60	0.00	1.04	173.21	0.06	3
LOC113513511	Uncharacterized protein C6orf105	-5.25	-3.20	0.00	2.81	87.75	0.71	6
LOC113513526	Epidermal retinol dehydrogenase 2-like	-2.70	-0.90	0.00	1.56	173.21	0.03	4
LOC113513527	Uncharacterized protein LOC103314804	0.00	1.31	3.93	2.27	173.21	0.29	11

LOC113513535	Structural maintenance of chromosomes protein 4	-1.52	-0.51	0.00	0.88	173.21	0.18	3
LOC113513537	Uncharacterized protein LOC106099929	-3.08	-1.75	0.00	1.58	90.44	0.16	9
LOC113513542	Uncharacterized protein LOC106142389	0.00	0.50	1.50	0.86	173.21	0.06	8
LOC113513545	Echinoderm microtubule-associated protein-like CG42247	-2.76	-1.63	0.00	1.45	88.66	0.61	4
LOC113513548	Venom protease-like	-2.45	-0.82	0.00	1.41	173.21	0.23	3
LOC113513560	Chlorophyllide A binding protein isoform X1	-4.80	-4.45	-3.86	0.51	11.58	0.38	2
LOC113513562	Uncharacterized protein LOC106142695	-1.59	-0.53	0.00	0.92	173.21	0.15	3
LOC113513566	Uncharacterized protein LOC106139737	0.00	1.11	3.34	1.93	173.21	0.67	7
LOC113513582	Glutathione S-transferase 1-like	-3.33	-1.87	0.00	1.70	90.93	0.30	9
LOC113513583	Uncharacterized protein LOC110376907 isoform X1	0.00	1.47	2.36	1.28	87.30	0.73	1
LOC113513585	Hypothetical protein CAPTEDRAFT_227268	-1.52	-0.51	0.00	0.88	173.21	0.18	3
LOC113513586	Bardet-Biedl syndrome 1 protein	-2.99	-1.74	0.00	1.55	89.26	0.13	9
LOC113513595	N-acetylglucosaminidase	-3.79	-1.72	0.00	1.92	111.31	0.59	9
LOC113513597	Myrosinase 1-like	-3.74	-2.48	0.00	2.15	86.61	0.99	6
LOC113513602	Uncharacterized protein LOC110376805 isoform X1	0.00	1.28	3.84	2.22	173.21	0.39	11
LOC113513611	Uncharacterized protein OBRU01_06696	-1.63	-0.54	0.00	0.94	173.21	0.13	3
LOC113513612	Serine-enriched protein	-2.30	-0.77	0.00	1.33	173.21	0.16	3
LOC113513616	DNA replication complex GINS protein PSF1-like	0.00	1.22	2.14	1.10	90.36	0.75	1
LOC113513625	Uncharacterized protein	0.00	0.43	1.29	0.75	173.21	0.52	1
LOC113513628	Solute carrier family 26 member 6 isoform X1	0.00	0.89	2.68	1.54	173.21	0.05	7
LOC113513641	25 kDa silk glycoprotein Fibrohexamerin	-2.86	-1.71	0.00	1.51	88.30	0.35	9
LOC113513649	Carotenoid isomerooxygenase	0.00	1.88	5.63	3.25	173.21	0.95	15
LOC113513650	Sugar transporter protein 2	0.00	2.37	7.11	4.10	173.21	0.08	15
LOC113513662	Serine protease 1-like	0.00	1.37	4.10	2.37	173.21	0.57	5
LOC113513664	Vitellogenin receptor	2.64	3.58	5.13	1.36	37.92	0.78	13
LOC113513670	Sugar transporter SWEET1	0.00	0.40	1.20	0.69	173.21	0.24	8

LOC113513673	Uncharacterized protein LOC106141535	0.00	1.31	3.93	2.27	173.21	0.29	11
LOC113513674	Fibulin-2-like isoform X1	-2.41	-0.80	0.00	1.39	173.21	0.12	4
LOC113513679	Fibroblast growth factor receptor 3-like isoform X1	0.00	1.10	3.30	1.91	173.21	0.60	7
LOC113513693	Organic cation transporter protein-like isoform X1	0.00	0.88	2.65	1.53	173.21	0.07	7
LOC113513699	Microsomal epoxide hydrolase	-3.74	-2.31	0.00	2.02	87.40	0.88	9
LOC113513701	Uncharacterized protein LOC106136208	-3.88	-1.29	0.00	2.24	173.21	0.36	4
LOC113513706	Protein crumbs	0.00	0.48	1.45	0.84	173.21	0.09	8
LOC113513707	KRAB-A domain-containing protein 2-like	-2.33	-1.44	0.00	1.26	87.49	0.19	9
LOC113513720	Serine protease 7-like	0.00	2.06	3.79	1.92	92.97	0.24	12
LOC113513724	Metalloprotease	0.00	0.47	1.41	0.82	173.21	0.12	8
LOC113513725	Nucleolar GTP-binding protein 1	-1.37	-0.85	0.00	0.74	87.51	0.92	3
LOC113513726	Protein RRNAD1-like	0.00	0.74	2.22	1.28	173.21	0.77	7
LOC113513731	7,8-dihydro-8-oxoguanine triphosphatase-like	-1.94	-0.65	0.00	1.12	173.21	0.01	3
LOC113513732	Uncharacterized protein LOC106124838	-2.39	-0.80	0.00	1.38	173.21	0.20	3
LOC113513733	UDP-glucuronosyltransferase 2B1-like isoform X2	-5.82	-3.39	0.00	3.03	89.27	0.39	6
LOC113513738	Uncharacterized protein LOC110381944	-1.77	-0.59	0.00	1.02	173.21	0.08	3
LOC113513743	Kidney mitochondrial carrier protein 1	0.00	0.54	1.62	0.94	173.21	0.05	8
LOC113513750	Acyl-coenzyme A dehydrogenase	-3.98	-2.02	0.00	1.99	98.61	0.77	9
LOC113513755	Follicle-specific yolk polypeptide-4	0.00	0.60	1.81	1.04	173.21	0.41	1
LOC113513757	Group XV phosphoLipase A2-like isoform X1	-1.40	-0.47	0.00	0.81	173.21	0.22	3
LOC113513763	Interference hedgehog-like	-4.05	-1.83	0.00	2.05	112.26	0.75	9
LOC113513781	Uncharacterized protein LOC110372689 isoform X4	0.00	0.43	1.30	0.75	173.21	0.19	8
LOC113513782	Alpha-tocopherol transfer protein-like	0.00	2.34	7.02	4.05	173.21	0.05	15
LOC113513784	Membrane-bound alkaline phosphatase-like	0.00	3.86	5.98	3.35	86.73	0.28	10
LOC113513789	Superoxide dismutase	0.00	1.91	5.74	3.31	173.21	0.80	15
LOC113513791	Diazepam binding inhibitor protein	0.00	1.45	4.34	2.51	173.21	0.05	11
LOC113513793	Peroxisomal N(1)-acetyl-	0.00	0.97	2.91	1.68	173.21	0.15	7

	spermine/spermidine oxidase-like isoform X4							
LOC113513798	Protein croquemort-like isoform X1	-1.67	-0.56	0.00	0.96	173.21	0.12	3
LOC113513800	Uncharacterized family 31 glucosidase KIAA1161-like	-10.05	-6.20	-2.23	3.91	63.08	0.92	6
LOC113513801	Suppressor of cytokine signaling 2-like isoform X2	0.00	1.85	3.39	1.72	92.98	0.31	12
LOC113513802	Neuronal PAS domain-containing protein 4A-like	0.00	2.18	6.54	3.78	173.21	0.17	15
LOC113513806	Phosphatidylinositol 4,5-bisphosphate 3-kinase catalytic subunit delta isoform	0.00	0.62	1.85	1.07	173.21	0.34	8
LOC113513807	Lipase member H-like isoform X2	-2.58	-1.65	0.00	1.43	86.84	0.10	9
LOC113513808	Unconventional myosin-Va-like	-3.50	-2.32	0.00	2.01	86.61	0.77	9
LOC113513817	Beta-1,3-glucan recognition protein	0.00	1.15	2.07	1.06	91.45	0.26	1
LOC113513823	GPI inositol-deacylase	0.00	0.51	1.54	0.89	173.21	0.03	8
LOC113513832	Serine protease 7-like	0.00	3.79	6.77	3.46	91.21	0.15	10
LOC113513835	Cyclic nucleotide-gated cation channel beta-3-like	-3.60	-1.20	0.00	2.08	173.21	0.71	3
LOC113513838	Uncharacterized protein LOC106140978	2.18	3.57	4.28	1.21	33.78	0.57	13
LOC113513841	Organic cation transporter protein-like	-4.25	-1.85	0.00	2.18	117.71	0.87	9
LOC113513848	DNA-directed RNA polymerase III subunit RPC5	0.00	0.48	1.45	0.83	173.21	0.09	8
LOC113513857	Uncharacterized protein LOC106141788	0.00	3.87	6.71	3.47	89.68	0.08	10
LOC113513860	Phytanoyl-CoA dioxygenase, peroxisomal-like	-1.83	-0.61	0.00	1.06	173.21	0.05	3
LOC113513861	Phosphomannomutase 2	0.00	0.67	2.02	1.16	173.21	0.65	8
LOC113513883	2-oxoisovalerate dehydrogenase subunit alpha, mitochondrial	-2.15	-0.72	0.00	1.24	173.21	0.09	3
LOC113513884	Hypothetical protein KGM_204941	0.00	1.91	5.74	3.32	173.21	0.79	15
LOC113513888	Uncharacterized protein LOC110381073	0.00	2.15	3.75	1.93	90.00	0.14	12
LOC113513894	Fatty acid synthase	-1.80	-0.60	0.00	1.04	173.21	0.40	4
LOC113513897	Cytochrome P450 9e2-like	0.00	1.37	4.11	2.37	173.21	0.12	11
LOC113513899	Protein lethal(2)essential for life-like	0.00	1.41	4.23	2.44	173.21	0.04	11
LOC113513902	G-protein coupled receptor Mth2-like	0.00	2.57	4.35	2.28	88.70	0.41	12

LOC113513908	Apyrase	-2.30	-0.77	0.00	1.33	173.21	0.17	4
LOC113513910	Uncharacterized protein LOC101744654	-4.52	-1.51	0.00	2.61	173.21	0.91	3
LOC113513912	Short-chain specific acyl-CoA dehydrogenase, mitochondrial-like isoform X1	-1.36	-0.45	0.00	0.79	173.21	0.23	3
LOC113513917	Indole-3-acetaldehyde oxidase-like	-4.28	-2.84	0.00	2.46	86.60	0.66	6
LOC113513922	Pro-resilin-like	0.00	1.88	5.63	3.25	173.21	0.94	15
LOC113513928	Myotubularin-related protein 10-A-like	0.00	0.58	1.75	1.01	173.21	0.19	8
LOC113513931	Uncharacterized protein	0.00	2.89	4.63	2.52	87.19	0.84	12
LOC113513934	Cuticle protein 16.8	0.00	3.21	5.93	3.00	93.37	0.51	10
LOC113513939	Selenium-binding protein 1-A	-5.43	-3.00	0.00	2.76	91.99	0.19	6
LOC113513951	Fatty-acyl CoA reductase 1	-2.62	-0.87	0.00	1.51	173.21	0.31	3
LOC113513952	Sulfotransferase isoform X1	0.00	0.89	2.66	1.54	173.21	0.06	7
LOC113513957	Solute carrier family 25 member 35-like isoform X2	0.00	0.68	2.05	1.18	173.21	0.72	8
LOC113513965	Pancreatic triacylglycerol lipase-like	-5.28	-3.82	-2.29	1.49	39.07	0.61	2
LOC113513977	Protein Spindly	-1.46	-0.49	0.00	0.84	173.21	0.20	3
LOC113513980	Cytochrome P450 monooxygenase CYP9G18	-4.93	-2.69	0.00	2.50	92.66	0.21	6
LOC113513982	Galectin-9	0.00	0.49	1.46	0.84	173.21	0.08	8
LOC113513989	Histone-lysine N-methyltransferase E(z) isoform X1	-2.43	-0.81	0.00	1.40	173.21	0.12	4
LOC113513996	Cytochrome P450 4C1-like	0.00	2.24	6.73	3.89	173.21	0.08	15
LOC113513999	Proton-coupled folate transporter-like	0.00	4.27	8.33	4.17	97.70	0.48	10
LOC113514001	Cytochrome P450	-4.27	-2.64	0.00	2.31	87.38	0.60	6
LOC113514004	Hypothetical protein RR48_10059	-1.59	-0.53	0.00	0.92	173.21	0.15	3
LOC113514005	Uncharacterized protein LOC101742758 isoform X1	-2.30	-0.77	0.00	1.33	173.21	0.16	3
LOC113514009	Serine protease gd-like	0.00	8.00	13.34	7.06	88.20	0.45	14
LOC113514022	Sn1-specific diacylglycerol Lipase beta-like	-1.28	-0.43	0.00	0.74	173.21	0.26	3
LOC113514026	Hemicentin-2	-3.16	-1.58	0.00	1.58	99.90	0.38	9
LOC113514027	Mediator of RNA polymerase II transcription subunit 15-like	0.00	3.39	5.60	2.98	87.90	0.34	10

LOC113514028	Uncharacterized protein LOC106710656	0.00	0.69	2.08	1.20	173.21	0.40	1
LOC113514033	Uncharacterized protein LOC110381450 isoform X1	-1.49	-0.50	0.00	0.86	173.21	0.19	3
LOC113514040	IQ and AAA domain-containing protein 1	-2.58	-0.86	0.00	1.49	173.21	0.06	4
LOC113514041	Nuclear protein localization protein 4 homolog	0.00	0.50	1.49	0.86	173.21	0.06	8
LOC113514043	Nose resistant to fluoxetine protein 6-like	-5.62	-2.93	0.00	2.82	96.19	0.99	6
LOC113514053	Uncharacterized protein LOC106142918	0.00	4.01	6.68	3.54	88.15	0.63	10
LOC113514058	Voltage-dependent L-type calcium channel subunit beta-2 isoform X1	0.00	1.83	5.49	3.17	173.21	0.85	11
LOC113514061	Tyrosine-protein kinase-like otk	-3.21	-1.07	0.00	1.85	173.21	0.57	3
LOC113514062	Unconventional myosin-Va	-2.12	-1.36	0.00	1.18	86.77	0.30	9
LOC113514079	Uncharacterized protein LOC110374962	-1.45	-0.48	0.00	0.84	173.21	0.20	3
LOC113514080	Cysteine and histidine-rich protein 1-like	-4.95	-2.59	0.00	2.48	95.67	0.21	6
LOC113514085	Uncharacterized protein LOC106130373	0.00	0.62	1.87	1.08	173.21	0.37	8
LOC113514086	Talin-B	-1.88	-0.63	0.00	1.09	173.21	0.03	3
LOC113514087	Cryptochrome-1 isoform X2	-1.80	-0.60	0.00	1.04	173.21	0.06	3
LOC113514091	Ribosomal protein S6 kinase-like 1	0.00	1.08	3.25	1.88	173.21	0.53	7
LOC113514092	Acyl-CoA-binding protein	-6.13	-3.00	0.00	3.07	102.18	0.21	6
LOC113514099	DNA polymerase epsilon subunit 2	-2.50	-1.28	0.00	1.25	97.51	0.58	9
LOC113514100	Ferric-chelate reductase 1 homolog	0.00	2.57	3.99	2.23	86.76	0.41	12
LOC113514124	Collagenase-like	-5.03	-3.53	-2.44	1.34	38.08	0.49	2
LOC113514128	Laminin A	-1.33	-0.44	0.00	0.77	173.21	0.24	3
LOC113514133	Hypothetical protein KGM_206883	-1.51	-0.50	0.00	0.87	173.21	0.18	3
LOC113514137	Vacuolar protein sorting-associated protein 33A	-1.30	-0.43	0.00	0.75	173.21	0.25	3
LOC113514144	Serine/threonine-protein kinase WNK3	0.00	0.53	1.59	0.92	173.21	0.03	8
LOC113514145	Mitochondrial fission 1 protein	0.00	0.96	2.88	1.66	173.21	0.13	7
LOC113514146	Protein abrupt	0.00	0.84	2.51	1.45	173.21	0.22	7
LOC113514148	Uncharacterized protein LOC106139738	0.00	2.17	3.81	1.96	90.24	0.16	12
LOC113514149	Cytochrome P450 6B6-like	-4.32	-2.39	0.00	2.20	91.79	0.62	6
LOC113514150	Cytochrome P450 6B6-like	-2.30	-1.23	0.00	1.16	94.40	0.61	9
LOC113514152	Uncharacterized protein	0.00	6.51	10.60	5.70	87.55	0.23	14

LOC113514153	Uncharacterized protein LOC101736974	0.00	2.80	8.41	4.86	173.21	0.36	15
LOC113514156	Nose resistant to fluoxetine protein 6-like	-5.23	-1.74	0.00	3.02	173.21	0.92	6
LOC113514170	Zinc finger SWIM domain-containing protein 4-like	0.00	1.18	3.55	2.05	173.21	0.86	11
LOC113514174	L-xylulose reductase-like	-2.81	-1.85	0.00	1.61	86.62	0.25	9
LOC113514175	Uncharacterized protein LOC106134414	-6.49	-3.51	0.00	3.28	93.41	0.87	6
LOC113514176	Laminin A chain	-1.75	-0.58	0.00	1.01	173.21	0.08	3
LOC113514178	Organic cation transporter protein-like	-4.12	-2.32	0.00	2.11	90.87	0.70	9
LOC113514181	Hypothetical protein KGM_205793	-2.63	-1.52	0.00	1.36	89.69	0.12	9
LOC113514183	Uncharacterized protein LOC101737344	0.00	1.16	2.14	1.08	92.97	0.85	1
LOC113514185	Uncharacterized protein	-2.19	-0.73	0.00	1.26	173.21	0.21	4
LOC113514188	cGMP-specific 3',5'-cyclic phosphodiesterase-like isoform X1	0.00	3.61	5.59	3.13	86.74	0.33	10
LOC113514198	Ras-related protein Rac1	0.00	1.71	2.70	1.49	86.98	0.56	12
LOC113514205	Scavenger receptor class B member 1-like	-3.03	-1.81	0.00	1.60	88.25	0.18	9
LOC113514207	Aminopeptidase N-like isoform X2	0.00	3.62	6.74	3.40	93.81	0.27	10
LOC113514209	P protein-like isoform X2	-2.10	-0.70	0.00	1.21	173.21	0.07	3
LOC113514215	ABC transporter G family member 23 isoform X2	0.00	1.12	3.36	1.94	173.21	0.71	7
LOC113514216	Uncharacterized protein LOC106106086	0.00	0.55	1.64	0.95	173.21	0.43	1
LOC113514217	Uncharacterized protein LOC106130712	0.00	1.31	2.47	1.24	94.89	0.45	1
LOC113514222	Uncharacterized protein LOC106140037	0.00	0.41	1.24	0.72	173.21	0.22	8
LOC113514226	DNA replication licensing factor Mcm6	-1.63	-0.54	0.00	0.94	173.21	0.13	3
LOC113514238	THO complex subunit 3	-1.40	-0.47	0.00	0.81	173.21	0.22	3
LOC113514240	Muscle M-line assembly protein unc-89-like	0.00	0.49	1.46	0.84	173.21	0.09	8
LOC113514250	Proline synthase co-transcribed bacterial homolog protein	-1.93	-0.64	0.00	1.11	173.21	0.01	3
LOC113514252	Targeting protein for Xklp2-like	-2.00	-0.67	0.00	1.15	173.21	0.02	3
LOC113514253	Fatty acyl-CoA reductase 1-like	0.00	1.15	3.45	1.99	173.21	0.88	7
LOC113514254	Fatty acyl-CoA reductase 1	0.00	1.72	3.39	1.70	98.77	0.58	12
LOC113514256	Fatty acyl-CoA reductase 1-like	0.00	2.76	4.79	2.48	89.75	0.69	12
LOC113514257	Peroxisomal biogenesis factor 19	0.00	0.74	2.22	1.28	173.21	0.76	7
LOC113514259	ABC transporter G family member 23	0.00	2.85	4.62	2.49	87.41	0.78	12

LOC113514261	U6 snRNA-associated Sm-like protein LSm5	-1.63	-0.54	0.00	0.94	173.21	0.13	3
LOC113514263	ABC transporter G family member 20-like	-2.72	-0.91	0.00	1.57	173.21	0.36	3
LOC113514266	Cecropin A	2.44	2.92	3.22	0.42	14.34	0.92	12
LOC113514267	Bicaudal	-1.41	-0.47	0.00	0.81	173.21	0.21	3
LOC113514268	Homeobox protein MSX-2	-1.31	-0.44	0.00	0.76	173.21	0.25	3
LOC113514269	Large neutral amino acids transporter small subunit 1	-2.82	-1.43	0.00	1.41	98.89	0.92	9
LOC113514274	Rho-related BTB domain-containing protein 1	-2.19	-0.73	0.00	1.26	173.21	0.11	3
LOC113514275	Golgin subfamily A member 7	0.00	1.02	1.70	0.90	88.23	0.59	1
LOC113514281	HMG box-containing protein 4	-1.81	-0.60	0.00	1.05	173.21	0.06	3
LOC113514283	Linear gramicidin synthase subunit D	2.37	5.29	6.81	2.53	47.83	0.75	13
LOC113514287	Uncharacterized protein LOC106130694 isoform X1	-1.60	-0.53	0.00	0.92	173.21	0.14	3
LOC113514294	Uncharacterized protein LOC106130786	0.00	1.09	3.27	1.89	173.21	0.55	7
LOC113514303	L-threonine ammonia-lyase-like	-2.93	-1.68	0.00	1.51	89.89	0.11	9
LOC113514309	Sideroflexin-1	0.00	1.49	4.46	2.57	173.21	0.11	11
LOC113514316	Serine/threonine-protein kinase pelle	0.00	1.29	1.97	1.12	86.66	0.59	1
LOC113514317	Anoctamin-9	-2.12	-1.34	0.00	1.17	87.02	0.38	9
LOC113514336	Facilitated trehalose transporter Tret1-like	0.00	0.49	1.46	0.84	173.21	0.08	8
LOC113514337	Facilitated trehalose transporter Tret1	0.00	4.38	6.89	3.81	86.90	0.41	10
LOC113514343	Mushroom body large-type Kenyon cell-specific protein 1 isoform X1	0.00	0.58	1.73	1.00	173.21	0.42	1
LOC113514345	Nitrilase homolog 1 isoform X1	-1.23	-0.41	0.00	0.71	173.21	0.27	3
LOC113514348	Importin subunit Alpha-1-like	-1.51	-0.50	0.00	0.87	173.21	0.18	3
LOC113514368	Cecropin 2	0.00	1.24	3.72	2.15	173.21	0.55	11
LOC113514369	Equilibrative nucleoside transporter 1	0.00	0.39	1.18	0.68	173.21	0.25	8
LOC113514371	Uncharacterized protein LOC106133025	0.00	0.48	1.44	0.83	173.21	0.48	1
LOC113514379	Uncharacterized protein LOC106133038	-1.41	-0.47	0.00	0.81	173.21	0.21	3
LOC113514381	Uncharacterized protein LOC106138920	-1.87	-1.08	0.00	0.97	89.72	0.84	9
LOC113514387	28S ribosomal protein S25, mitochondrial	0.00	1.08	1.81	0.96	88.43	0.25	1
LOC113514391	C-terminal-binding protein	0.00	0.44	1.32	0.76	173.21	0.18	8

LOC113514403	ETS DNA-binding protein pokkuri	-4.69	-2.86	0.00	2.51	87.73	0.43	6
LOC113514407	Multidrug resistance protein homolog 49	0.00	2.76	4.42	2.41	87.21	0.62	12
LOC113514409	Uncharacterized protein LOC106136549	-1.57	-0.52	0.00	0.91	173.21	0.16	3
LOC113514411	Leucine-rich repeat flightless-interacting protein 2 isoform X1	-1.29	-0.43	0.00	0.74	173.21	0.26	3
LOC113514419	Estrogen sulfotransferase	0.00	0.65	1.95	1.12	173.21	0.50	8
LOC113514427	Uncharacterized protein LOC106130714	0.00	1.53	2.95	1.48	96.29	0.80	1
LOC113514429	Corazonin	0.00	2.14	3.69	1.91	89.39	0.84	5
LOC113514430	Serine/threonine-protein kinase	-2.26	-0.75	0.00	1.31	173.21	0.14	3
LOC113514440	Uncharacterized protein LOC106138911 isoform X1	-1.47	-0.49	0.00	0.85	173.21	0.19	3
LOC113514441	BTB/POZ domain-containing protein	-2.16	-0.72	0.00	1.25	173.21	0.10	3
LOC113514448	Scavenger receptor class B member 1-like	-5.00	-2.57	0.00	2.50	97.38	0.21	6
LOC113514450	Uncharacterized protein LOC110378527 isoform X1	0.00	1.95	5.86	3.38	173.21	0.14	5
LOC113514457	BCL2/adenovirus E1B 19 kDa protein-interacting protein 3 isoform X1	-2.08	-1.34	0.00	1.16	86.80	0.33	9
LOC113514458	Fatty-acyl CoA reductase 2	-2.11	-0.70	0.00	1.22	173.21	0.24	4
LOC113514463	Uncharacterized protein LOC106142077	0.00	1.91	5.74	3.32	173.21	0.79	15
LOC113514464	Uncharacterized protein	-2.83	-0.94	0.00	1.63	173.21	0.41	3
LOC113514486	Facilitated trehalose transporter Tret1	0.00	4.67	8.16	4.21	90.10	0.54	10
LOC113514491	Actin-binding LIM protein 2	0.00	0.73	2.18	1.26	173.21	0.88	7
LOC113514493	Zinc transporter ZIP13 homolog	0.00	0.83	1.27	0.72	86.65	0.39	1
LOC113514496	Transmembrane channel-like protein 2 isoform X1	0.00	1.04	3.13	1.81	173.21	0.37	7
LOC113514504	Uncharacterized protein	0.00	2.90	4.73	2.54	87.59	0.79	12
LOC113514507	Uncharacterized protein LOC110375939	0.00	2.71	8.13	4.70	173.21	0.32	15
LOC113514511	Carbonyl reductase	-2.88	-1.51	0.00	1.45	95.95	0.30	9
LOC113514518	cGMP-specific 3',5'-cyclic phosphodiesterase-like isoform X1	0.00	3.41	5.34	2.96	86.86	0.40	10
LOC113514532	Carboxypeptidase B-like	-4.57	-2.57	0.00	2.34	90.92	0.89	9
LOC113514536	Protein toll-like	0.00	1.07	3.20	1.85	173.21	0.75	1
LOC113514549	Uncharacterized protein LOC110381915	0.00	0.91	1.43	0.79	86.82	0.31	1

	isoform X1							
LOC113514551	FAD-linked sulfhydryl oxidase ALR	0.00	1.11	3.33	1.92	173.21	0.65	7
LOC113514552	Facilitated trehalose transporter Tret1-like	-2.88	-0.96	0.00	1.66	173.21	0.06	4
LOC113514563	Transmembrane protein 80-like	-1.93	-0.64	0.00	1.11	173.21	0.01	3
LOC113514576	Complexin	-1.77	-0.59	0.00	1.02	173.21	0.07	3
LOC113514594	Trypsin CFT-1-like	-6.22	-2.87	0.00	3.14	109.56	0.96	9
LOC113514597	Uncharacterized protein	-1.95	-0.65	0.00	1.13	173.21	0.01	3
LOC113514600	Hypothetical protein RR48_04984	0.00	2.04	6.11	3.53	173.21	0.44	15
LOC113514606	Insecticyanin-A	-2.75	-0.45	1.41	2.12	474.95	0.50	4
LOC113514608	Solute carrier family 2, facilitated glucose transporter member 1-like	0.00	0.48	1.45	0.84	173.21	0.47	1
LOC113514609	Aldo-keto reductase AKR2E4-like	-5.07	-3.18	0.00	2.77	87.10	0.66	6
LOC113514610	Uncharacterized protein LOC106134395	-2.05	-0.68	0.00	1.18	173.21	0.05	3
LOC113514618	Ankyrin repeat domain-containing protein 50	0.00	0.76	2.27	1.31	173.21	0.64	7
LOC113514620	G-protein coupled receptor Mth2-like	0.00	2.45	3.70	2.12	86.61	0.29	12
LOC113514621	N-acylneuraminate cytidylyltransferase	-3.10	-1.92	0.00	1.68	87.33	0.34	9
LOC113514632	Fidgetin-like protein 1	-1.79	-0.60	0.00	1.03	173.21	0.07	3
LOC113514635	LIM/homeobox protein Lhx3 isoform X2	-3.16	-1.05	0.00	1.82	173.21	0.55	3
LOC113514646	Proton-coupled amino acid transporter 2-like	0.00	3.89	6.03	3.37	86.76	0.46	10
LOC113514650	Prostaglandin F synthase 1	0.00	1.95	5.86	3.38	173.21	0.67	15
LOC113514651	Alpha-tocopherol transfer protein-like	-3.24	-1.66	0.00	1.62	97.58	0.77	9
LOC113514654	Uncharacterized protein LOC106131814	0.00	0.59	1.78	1.03	173.21	0.41	1
LOC113514656	ATP-dependent RNA helicase DHX57	0.00	0.93	2.78	1.60	173.21	0.06	7
LOC113514657	Cytochrome P450 9G3	-6.48	-2.16	0.00	3.74	173.21	0.73	6
LOC113514659	Protein mahjong	0.00	0.43	1.29	0.75	173.21	0.19	8
LOC113514672	Uncharacterized protein LOC110380322 isoform X2	0.00	1.62	2.83	1.46	90.16	0.53	12
LOC113514673	Uncharacterized protein LOC106140704	0.00	1.48	2.41	1.30	87.50	0.87	12
LOC113514675	N-acetyltransferase 6	-2.03	-0.68	0.00	1.17	173.21	0.04	3
LOC113514677	Uncharacterized protein LOC110380322 isoform X2	0.00	1.52	2.48	1.33	87.59	0.76	12
LOC113514680	Insulin-like growth factor 1 receptor	-2.85	-1.64	0.00	1.47	89.85	0.09	9

LOC113514685	Mitochondrial fission process protein 1	0.00	0.49	1.46	0.84	173.21	0.08	8
LOC113514690	Phosphatidylinositol 4-kinase alpha isoform X1	0.00	0.47	1.41	0.81	173.21	0.12	8
LOC113514709	Hypothetical protein RR46_03464	0.00	4.50	8.47	4.26	94.70	0.49	10
LOC113514718	Uncharacterized protein LOC106130386	0.00	1.87	3.25	1.68	89.67	0.20	12
LOC113514719	Protein phosphatase 1 regulatory subunit 14B isoform X1	-2.39	-0.80	0.00	1.38	173.21	0.20	3
LOC113514722	Hemocytin	-5.10	-2.47	0.00	2.55	103.39	0.31	6
LOC113514739	Kinesin-like protein KIF18A	-1.39	-0.46	0.00	0.80	173.21	0.22	3
LOC113514752	ATP-binding cassette sub-family G member 1-like	-4.44	-2.23	0.00	2.22	99.65	0.70	6
LOC113514762	Peroxisomal N(1)-acetyl-spermine/spermidine oxidase-like isoform X3	0.00	1.59	4.78	2.76	173.21	0.25	11
LOC113514763	Uncharacterized protein LOC106137312	-2.25	-0.75	0.00	1.30	173.21	0.14	3
LOC113514764	Lysine-specific demethylase lid	0.00	0.50	1.50	0.87	173.21	0.05	8
LOC113514771	Lipase 1-like	-7.13	-3.34	0.00	3.59	107.36	0.99	6
LOC113514777	Zinc finger DNA binding protein	0.00	0.72	2.15	1.24	173.21	0.98	7
LOC113514784	Neutral ceramidase	0.00	0.67	2.02	1.17	173.21	0.40	1
LOC113514799	Required for meiotic nuclear division protein 1-like	0.00	0.74	2.22	1.28	173.21	0.77	7
LOC113514814	Serine proteinase stubble	0.00	1.13	3.40	1.96	173.21	0.77	7
LOC113514815	Interference hedgehog-like	-4.15	-1.91	0.00	2.10	109.60	0.84	9
LOC113514818	Uncharacterized protein	0.00	0.67	2.00	1.15	173.21	0.60	8
LOC113514820	UDP-glycosyltransferase UGT46A3	-2.45	-1.62	0.00	1.40	86.62	0.14	9
LOC113514822	Lipopolysaccharide-induced tumor necrosis factor-alpha factor	0.00	0.94	2.83	1.63	173.21	0.09	7
LOC113514823	Inactive peptidyl-prolyl cis-trans isomerase shutdown-like	-1.44	-0.48	0.00	0.83	173.21	0.20	3
LOC113514830	Uncharacterized protein LOC106140625 isoform X1	-2.68	-1.52	0.00	1.38	90.29	0.13	9
LOC113514834	Cytochrome c oxidase assembly factor 6 homolog	0.00	0.64	1.91	1.10	173.21	0.40	1
LOC113514838	Protein N-lysine methyltransferase	0.00	0.90	2.69	1.55	173.21	0.48	1

	METTL20-like							
LOC113514842	Solute carrier family 23 member 2 isoform X1	-3.89	-1.30	0.00	2.25	173.21	0.36	4
LOC113514846	Calcitonin gene-related peptide type 1 receptor	0.00	2.76	4.81	2.48	89.85	0.66	12
LOC113514848	Bifunctional glutamate/proline--tRNA ligase-like	0.00	0.59	1.78	1.03	173.21	0.23	8
LOC113514851	Niemann-Pick type C2 protein Npc2-t01	-5.98	-3.98	0.00	3.45	86.60	0.69	6
LOC113514853	Uncharacterized protein LOC110381924	-2.10	-0.70	0.00	1.21	173.21	0.07	3
LOC113514856	Juvenile hormone esterase-like isoform X1	0.00	1.19	2.31	1.16	96.97	0.93	7
LOC113514862	Mitochondrial glutamate carrier 1-like	0.00	1.86	3.01	1.63	87.37	0.22	12
LOC113514865	LIM and SH3 domain protein Lasp isoform X2	0.00	0.56	1.68	0.97	173.21	0.42	1
LOC113514869	UDP-glucuronosyltransferase 2A3-like	-4.40	-2.48	0.00	2.25	90.74	0.52	6
LOC113514877	Cation transport regulator-like protein 2	0.00	0.50	1.49	0.86	173.21	0.46	1
LOC113514878	Polypeptide N-acetylgalactosaminyltransferase 2-like	-2.54	-1.49	0.00	1.33	88.98	0.13	9
LOC113514881	Uncharacterized protein LOC106142650	0.00	0.46	1.38	0.80	173.21	0.14	8
LOC113514885	Glycine N-methyltransferase	0.00	2.19	4.13	2.08	94.82	0.35	12
LOC113514887	Hypothetical protein RR46_05101	0.00	0.39	1.17	0.67	173.21	0.26	8
LOC113514889	Phospholipid-transporting ATPase VD	0.00	0.49	1.48	0.85	173.21	0.07	8
LOC113514891	Protein real-time	-1.39	-0.46	0.00	0.81	173.21	0.22	3
LOC113514897	Organic cation transporter	0.00	0.94	2.83	1.63	173.21	0.09	7
LOC113514898	Venom carboxylesterase-6-like isoform X1	0.00	1.15	1.81	1.00	86.97	0.36	1
LOC113514899	Hemicentin-1-like	0.00	0.97	2.90	1.67	173.21	0.14	7
LOC113514900	Collagenase-like	-2.09	-0.70	0.00	1.21	173.21	0.25	4
LOC113514901	Protein singed	-2.27	-0.76	0.00	1.31	173.21	0.15	3
LOC113514905	Protein D7-like	-1.29	-0.43	0.00	0.74	173.21	0.26	3
LOC113514906	Plexin domain-containing protein 2	0.00	0.53	1.60	0.93	173.21	0.04	8
LOC113514911	Prestin isoform X2	0.00	1.49	2.99	1.50	100.61	0.97	12
LOC113514916	Glutathione-S-transferase-like protein	0.00	0.58	1.73	1.00	173.21	0.17	8
LOC113514921	Aminoacylase-1-like	-1.51	-0.50	0.00	0.87	173.21	0.18	3
LOC113514927	Uncharacterized protein LOC106138571	0.00	1.73	5.18	2.99	173.21	0.52	11

LOC113514935	DNA-directed RNA polymerase, mitochondrial isoform X1	0.00	0.44	1.33	0.77	173.21	0.17	8
LOC113514936	Protein KIAA0556-like	-3.63	-1.21	0.00	2.10	173.21	0.71	3
LOC113514938	Uncharacterized protein LOC106139763	0.00	1.82	5.46	3.15	173.21	0.12	5
LOC113514946	Peptidoglycan recognition protein D	0.00	2.46	3.71	2.13	86.61	0.29	12
LOC113514947	Glucose dehydrogenase	0.00	4.73	9.29	4.65	98.31	0.72	10
LOC113514948	Glucose dehydrogenase	0.00	2.75	4.52	2.41	87.84	0.62	12
LOC113514950	Glucose dehydrogenase	0.00	1.56	4.68	2.70	173.21	0.21	11
LOC113514951	Glucose dehydrogenase	0.00	1.51	2.64	1.36	89.90	0.96	8
LOC113514953	Glucose dehydrogenase	-3.25	-1.08	0.00	1.88	173.21	0.58	3
LOC113514955	Uncharacterized protein LOC106134169	0.00	1.66	2.63	1.44	87.01	0.97	7
LOC113514967	Fatty acid synthase-like	0.00	0.58	1.74	1.00	173.21	0.18	8
LOC113514975	X-ray repair cross-complementing protein 5-like	0.00	0.64	1.92	1.11	173.21	0.44	8
LOC113514990	Protein I'm not dead yet	-1.29	-0.43	0.00	0.74	173.21	0.26	3
LOC113514993	Uncharacterized protein LOC105387960 isoform X1	0.00	2.36	3.82	2.06	87.46	0.19	12
LOC113515000	Uncharacterized protein LOC106134174	0.00	1.04	1.80	0.93	89.57	0.67	1
LOC113515012	Calponin homology domain-containing protein DDB_G0272472-like isoform X2	0.00	1.29	3.87	2.24	173.21	0.35	11
LOC113515023	Serine--tRNA ligase, mitochondrial	0.00	0.46	1.37	0.79	173.21	0.15	8
LOC113515031	Proline dehydrogenase 1, mitochondrial	0.00	3.46	5.20	3.00	86.60	0.46	10
LOC113515034	Uncharacterized protein LOC106129906 isoform X1	-2.62	-1.54	0.00	1.37	89.00	0.09	9
LOC113515043	Uncharacterized protein	-1.38	-0.46	0.00	0.80	173.21	0.22	3
LOC113515057	Glucose dehydrogenase	0.00	1.17	3.52	2.03	173.21	0.95	11
LOC113515060	Venom serine carboxypeptidase	-1.32	-0.44	0.00	0.76	173.21	0.25	3
LOC113515073	Myb-binding protein 1A-like protein	0.00	0.53	1.59	0.92	173.21	0.03	8
LOC113515081	Ceramide synthase 6-like isoform X2	0.00	0.49	1.48	0.86	173.21	0.46	1
LOC113515083	Fanconi anemia group J protein homolog isoform X1	-2.02	-0.67	0.00	1.17	173.21	0.03	3
LOC113515088	Uncharacterized protein	0.00	0.77	2.31	1.34	173.21	0.54	7
LOC113515096	Collagenase-like	-4.21	-3.28	-2.19	1.02	31.08	0.36	2

LOC113515108	Uncharacterized protein LOC106129912	0.00	0.45	1.35	0.78	173.21	0.16	8
LOC113515115	IAP-binding motif 1	-2.36	-0.79	0.00	1.36	173.21	0.14	4
LOC113515116	Prophenoloxidase subunit 2	-5.14	-2.18	0.00	2.65	121.75	0.63	6
LOC113515117	Pupal cuticle protein-like	0.00	2.71	8.14	4.70	173.21	0.32	15
LOC113515123	Uncharacterized protein LOC106116870 isoform X1	-2.67	-0.89	0.00	1.54	173.21	0.03	4
LOC113515128	Uncharacterized protein LOC110371709	0.00	0.56	1.68	0.97	173.21	0.12	8
LOC113515129	Cytochrome P450 6B5-like	-1.62	-0.54	0.00	0.94	173.21	0.14	3
LOC113515131	Uncharacterized protein LOC110371738	-1.69	-0.56	0.00	0.98	173.21	0.11	3
LOC113515136	Coiled-coil domain-containing protein 42 homolog	-2.13	-1.39	0.00	1.20	86.66	0.30	9
LOC113515146	Protein orai-like isoform X1	0.00	3.18	5.81	2.94	92.55	0.54	10
LOC113515147	Methyl-CpG-binding domain protein 4-like	-1.38	-0.92	0.00	0.79	86.61	0.98	3
LOC113515150	Mevalonate kinase	0.00	0.52	1.57	0.91	173.21	0.02	8
LOC113515158	GPI transamidase component PIG-S	-1.35	-0.45	0.00	0.78	173.21	0.24	3
LOC113515162	GPI transamidase component PIG-S	-1.54	-0.51	0.00	0.89	173.21	0.17	3
LOC113515164	Rhomboid-related protein 1-like isoform X1	0.00	1.03	3.09	1.78	173.21	0.32	7
LOC113515165	Rhomboid-related protein 2-like	0.00	0.45	1.36	0.78	173.21	0.15	8
LOC113515168	Fatty acid synthase-like	-4.02	-3.48	-2.49	0.86	24.59	0.44	2
LOC113515169	Fatty acid synthase-like	-2.54	-1.54	0.00	1.35	87.98	0.08	9
LOC113515170	Fatty acid synthase-like	-2.32	-1.36	0.00	1.21	89.16	0.51	9
LOC113515172	Fatty acid synthase-like	-5.40	-1.80	0.00	3.12	173.21	0.63	4
LOC113515181	Unconventional myosin-XVIIIa isoform X1	-1.70	-1.05	0.00	0.92	87.41	0.83	9
LOC113515187	PhosphoLipase A2	-5.69	-3.79	0.00	3.28	86.60	0.66	6
LOC113515195	Trypsin, alkaline C-like	-2.31	-0.77	0.00	1.34	173.21	0.17	3
LOC113515197	Rabphilin-3A	-2.58	-1.56	0.00	1.37	88.00	0.05	9
LOC113515202	Vacuolar protein sorting-associated protein 33A	-1.44	-0.48	0.00	0.83	173.21	0.20	3
LOC113515203	Neurogenic locus notch homolog protein 1 isoform X1	0.00	1.23	1.98	1.07	87.35	0.41	1
LOC113515204	Small G protein signaling modulator 2-like	-2.35	-1.27	0.00	1.19	93.47	0.52	9
LOC113515206	Peptidoglycan-recognition protein-S	2.20	3.93	5.39	1.61	41.02	0.38	13
LOC113515209	Citron Rho-interacting kinase-like	-2.67	-0.89	0.00	1.54	173.21	0.34	3

LOC113515211	Sodium channel protein Nach-like isoform X1	-2.40	-0.80	0.00	1.39	173.21	0.21	3
LOC113515213	Tetratricopeptide repeat protein 28	0.00	1.83	2.75	1.58	86.60	0.33	12
LOC113515217	Disulfide oxidoreductase	-1.67	-0.56	0.00	0.97	173.21	0.11	3
LOC113515218	Apoptosis-inducing factor 3-like isoform X1	-1.83	-1.03	0.00	0.94	90.78	0.97	9
LOC113515229	Vanin-like protein 1 isoform X1	0.00	1.38	4.15	2.39	173.21	0.10	11
LOC113515232	Bicaudal D-related protein homolog	0.00	1.45	4.36	2.52	173.21	0.06	11
LOC113515236	Uncharacterized protein LOC106133236	-2.75	-0.92	0.00	1.59	173.21	0.37	3
LOC113515237	4-methylmuconolactone transporter	0.00	2.07	3.80	1.92	92.99	0.25	12
LOC113515253	Mucin-17-like	-2.00	-0.67	0.00	1.16	173.21	0.29	4
LOC113515259	Uncharacterized protein LOC106132232	0.00	0.56	1.67	0.96	173.21	0.10	8
LOC113515265	Uncharacterized protein LOC105842577	0.00	1.02	3.07	1.77	173.21	0.67	1
LOC113515268	Zinc finger protein 91-like	-1.48	-0.49	0.00	0.85	173.21	0.19	3
LOC113515269	Diazepam binding inhibitor-like protein	0.00	1.59	4.77	2.75	173.21	0.24	11
LOC113515270	Unconventional myosin-Va	-1.57	-0.52	0.00	0.91	173.21	0.15	3
LOC113515283	Uncharacterized protein LOC106136012 isoform X1	-1.25	-0.42	0.00	0.72	173.21	0.27	3
LOC113515288	Nose resistant to fluoxetine protein 6-like	0.00	0.50	1.50	0.87	173.21	0.06	8
LOC113515290	Lysozyme	2.99	5.34	6.77	2.04	38.32	0.56	13
LOC113515292	Required for meiotic nuclear division protein 1 homolog	0.00	0.74	2.21	1.28	173.21	0.77	7
LOC113515296	RNA-binding protein orb2	-3.42	-1.14	0.00	1.97	173.21	0.64	3
LOC113515315	Diamine acetyltransferase 2-like	-6.10	-2.85	0.00	3.07	107.81	0.26	6
LOC113515318	Digestive cysteine proteinase 1	-2.75	-1.39	0.00	1.38	99.02	0.46	9
LOC113515320	DNA-directed RNA polymerase III subunit RPC7-like	-1.38	-0.46	0.00	0.80	173.21	0.22	3
LOC113515321	Uncharacterized protein LOC106134903	-2.88	-0.96	0.00	1.66	173.21	0.43	3
LOC113515324	Cytochrome b5	-6.06	-2.02	0.00	3.50	173.21	0.77	6
LOC113515329	Cadherin-23	0.00	1.07	3.20	1.85	173.21	0.45	7
LOC113515341	A disintegrin and metalloproteinase with thrombospondin motifs 3-like	-3.01	-1.00	0.00	1.74	173.21	0.49	3
LOC113515344	Uncharacterized protein LOC106142844	-5.36	-2.96	0.00	2.72	92.03	0.17	6
LOC113515347	Trypsin-1-like	0.00	1.46	4.39	2.54	173.21	0.07	11

LOC113515350	Aminopeptidase N-like isoform X1	0.00	1.63	2.81	1.46	89.46	0.86	12
LOC113515351	Aminopeptidase N	0.00	0.67	2.00	1.16	173.21	0.40	1
LOC113515352	Uncharacterized protein LOC106142867	0.00	0.47	1.42	0.82	173.21	0.11	8
LOC113515353	Aquaporin-like	0.00	1.08	1.76	0.94	87.70	0.28	1
LOC113515355	5-aminolevulinate synthase, nonspecific, mitochondrial	0.00	0.42	1.26	0.73	173.21	0.21	8
LOC113515359	Uncharacterized protein LOC106103767 isoform X2	0.00	2.17	4.21	2.11	97.15	0.43	12
LOC113515360	Uncharacterized protein	3.80	7.80	10.58	3.55	45.53	0.41	14
LOC113515364	ATP-dependent (S)-NAD(P)H-hydrate dehydratase	-1.45	-0.48	0.00	0.84	173.21	0.20	3
LOC113515367	Diacylglycerol kinase 1	-2.02	-0.67	0.00	1.17	173.21	0.28	4
LOC113515371	Cytochrome P450 monooxygenase CYP304F17	-2.82	-1.40	0.00	1.41	101.01	0.49	9
LOC113515375	Protein timeless homolog isoform X2	-1.46	-0.49	0.00	0.84	173.21	0.20	3
LOC113515377	Ommochrome-binding protein-like	0.00	0.44	1.32	0.76	173.21	0.17	8
LOC113515380	Extensin isoform X3	-1.61	-0.54	0.00	0.93	173.21	0.51	4
LOC113515392	Trypsin beta-like	0.00	2.46	4.68	2.35	95.55	0.48	12
LOC113515393	Trypsin beta-like	-3.47	-1.16	0.00	2.00	173.21	0.25	4
LOC113515395	Cytochrome b5-related protein-like	-2.08	-1.21	0.00	1.08	89.41	0.56	9
LOC113515396	Protein patched homolog 1-like	-2.86	-1.62	0.00	1.47	90.55	0.11	9
LOC113515399	Inositol polyphosphate 1-phosphatase	-1.60	-0.53	0.00	0.92	173.21	0.14	3
LOC113515402	Tyrosine kinase receptor Cad96Ca isoform X1	0.00	1.03	3.08	1.78	173.21	0.31	7
LOC113515404	Alanine--glyoxylate aminotransferase 2-like	0.00	0.70	2.10	1.21	173.21	0.88	8
LOC113515407	Serine proteinase stubble	0.00	0.82	2.47	1.43	173.21	0.42	1
LOC113515408	Molybdenum cofactor sulfurase	0.00	1.12	3.35	1.93	173.21	0.69	7
LOC113515413	Sodium-dependent multivitamin transporter isoform X2	0.00	0.57	1.72	0.99	173.21	0.15	8
LOC113515417	Aldo-keto reductase AKR2E4-like isoform X5	-1.69	-0.56	0.00	0.97	173.21	0.11	3
LOC113515418	Retinal dehydrogenase 1-like	0.00	0.86	2.57	1.48	173.21	0.15	7
LOC113515419	Myrosinase 1-like	0.00	5.54	11.16	5.58	100.72	0.73	14

LOC113515420	Phosphoserine aminotransferase	-2.64	-0.88	0.00	1.53	173.21	0.32	3
LOC113515424	Chaoptin	0.00	0.64	1.91	1.11	173.21	0.44	8
LOC113515438	Ras-related and estrogen-regulated growth inhibitor-like protein	-1.68	0.04	1.80	1.74	4217.85	0.87	8
LOC113515439	Retinal dehydrogenase 1-like	-5.85	-2.85	0.00	2.93	102.50	0.16	6
LOC113515444	Zinc finger protein Elbow	0.00	0.49	1.46	0.84	173.21	0.08	8
LOC113515447	Uncharacterized protein LOC106134655 isoform X2	0.00	1.93	5.79	3.34	173.21	0.74	15
LOC113515459	Sodium-dependent multivitamin transporter	0.00	0.50	1.50	0.87	173.21	0.05	8
LOC113515460	Carbonic anhydrase 7	0.00	8.57	13.49	7.45	86.92	0.51	14
LOC113515461	Cytochrome P450 monooxygenase CYP304F17	-2.51	-1.54	0.00	1.35	87.57	0.08	9
LOC113515467	Odorant receptor 85b-like	-3.30	-1.10	0.00	1.90	173.21	0.19	4
LOC113515469	Substance-P receptor-like	-1.32	-0.44	0.00	0.76	173.21	0.25	3
LOC113515470	Hypothetical protein RR46_01680	0.00	1.11	3.33	1.92	173.21	0.65	7
LOC113515473	Protein enabled homolog isoform X1	0.00	3.28	5.13	2.85	86.82	0.54	10
LOC113515476	Major facilitator superfamily domain-containing protein 9 isoform X1	0.00	0.41	1.22	0.71	173.21	0.23	8
LOC113515482	Phosphatidylinositol-glycan biosynthesis class F protein	0.00	0.88	1.36	0.77	86.71	0.34	1
LOC113515483	Uncharacterized protein	0.00	1.01	1.61	0.88	87.17	0.50	1
LOC113515484	V-type proton ATPase 116 kDa subunit a isoform 1-like	-3.25	-1.99	0.00	1.74	87.59	0.37	9
LOC113515485	Myophilin-like isoform X1	-3.63	-1.21	0.00	2.09	173.21	0.29	4
LOC113515487	Uncharacterized protein LOC106142908 isoform X3	-2.32	-0.77	0.00	1.34	173.21	0.17	3
LOC113515493	Uncharacterized protein LOC106143241 isoform X4	0.00	2.35	3.55	2.03	86.61	0.22	12
LOC113515494	tRNA (guanine-N(7)-)-methyltransferase non-catalytic subunit wuho	0.00	1.25	2.01	1.09	87.40	0.58	1
LOC113515498	Serine protease gd	0.00	2.10	3.29	1.82	86.87	0.08	12
LOC113515500	39S ribosomal protein L32, mitochondrial	0.00	0.92	1.42	0.80	86.72	0.32	1
LOC113515502	Whey acidic protein-like isoform X2	0.00	1.16	3.48	2.01	173.21	0.97	7
LOC113515506	Uncharacterized protein LOC110378016	-2.82	-1.49	0.00	1.42	95.35	0.29	9

	isoform X1							
LOC113515508	2-acylglycerol O-acyltransferase 2-A-like	0.00	0.56	1.67	0.96	173.21	0.10	8
LOC113515509	Katanin p60 ATPase-containing subunit A1-like	-2.50	-1.52	0.00	1.33	87.90	0.34	9
LOC113515513	Protein LMBR1L	0.00	0.42	1.25	0.72	173.21	0.21	8
LOC113515515	Androgen-dependent TFPI-regulating protein-like	-3.31	-2.02	0.00	1.77	87.77	0.43	9
LOC113515525	Uncharacterized protein LOC106134739	-3.93	-2.02	0.00	1.97	97.55	0.73	9
LOC113515542	Uncharacterized protein	0.00	2.02	4.09	2.04	101.23	0.57	12
LOC113515545	Uncharacterized protein LOC106134731	0.00	1.50	2.38	1.30	87.03	0.86	12
LOC113515552	V-type proton ATPase 116 kDa subunit a isoform 1	0.00	1.37	2.75	1.37	100.50	0.82	7
LOC113515554	Zinc finger protein Noc	-1.69	-0.56	0.00	0.98	173.21	0.11	3
LOC113515557	G2/mitotic-specific cyclin-B	-1.67	-0.56	0.00	0.97	173.21	0.12	3
LOC113515564	Uncharacterized protein LOC106103590	-3.33	-2.20	0.00	1.91	86.61	0.61	9
LOC113515565	Arylsulfatase B	0.00	2.60	7.81	4.51	173.21	0.26	15
LOC113515570	Coiled-coil domain-containing protein 58	0.00	0.49	1.46	0.84	173.21	0.47	1
LOC113515573	Ras-related and estrogen-regulated growth inhibitor-like protein	0.00	0.60	1.80	1.04	173.21	0.26	8
LOC113515576	Cytochrome oxidase subunit 2(mitochondrion)	0.00	1.13	2.01	1.03	90.88	0.25	1
LOC113515578	Meiosis-specific nuclear structural protein 1-like isoform X2	-2.67	-1.37	0.00	1.33	97.62	0.45	9
LOC113515583	Membrane alanyl Aminopeptidase	-3.40	-1.65	0.00	1.70	103.21	0.97	9
LOC113515584	Membrane alanyl Aminopeptidase	-4.27	-1.42	0.00	2.47	173.21	0.45	4
LOC113515596	Uncharacterized protein LOC106143246	0.00	1.59	2.55	1.38	87.26	0.64	12
LOC113515606	Carbonic anhydrase 7-like	0.00	4.89	7.61	4.24	86.78	0.76	10
LOC113515608	Lachesin-like	-2.77	-0.92	0.00	1.60	173.21	0.03	4
LOC113515610	Serine protease nudel	0.00	1.92	5.75	3.32	173.21	0.79	15
LOC113515614	Fasciclin-1	-1.60	-0.99	0.00	0.86	87.53	0.97	9
LOC113515622	Uncharacterized protein LOC106134691	-1.49	-0.50	0.00	0.86	173.21	0.58	4
LOC113515627	Protein amalgam-like	0.00	1.03	3.09	1.78	173.21	0.31	7
LOC113515628	Uncharacterized protein LOC106134633	0.00	2.27	3.72	1.99	87.74	0.13	12

LOC113515631	CD2-associated protein isoform X1	0.00	0.57	1.71	0.98	173.21	0.14	8
LOC113515641	Uncharacterized protein LOC106142856 isoform X1	-2.42	-1.53	0.00	1.33	86.98	0.25	9
LOC113515642	Tetratricopeptide repeat protein 21B-like	-1.98	-1.27	0.00	1.10	86.80	0.42	9
LOC113515644	Xaa-Pro Aminopeptidase 1	-6.15	-2.05	0.00	3.55	173.21	0.76	6
LOC113515652	Tolloid-like protein 1	-5.01	-2.61	0.00	2.51	96.11	0.18	6
LOC113515654	Geranylgeranyl pyrophosphate synthase	-1.51	-0.50	0.00	0.87	173.21	0.18	3
LOC113515659	Uncharacterized protein LOC106130344	-1.28	-0.43	0.00	0.74	173.21	0.26	3
LOC113515677	Uncharacterized protein LOC110383954 isoform X1	-2.17	-0.72	0.00	1.26	173.21	0.10	3
LOC113515680	Disintegrin and metalloproteinase domain-containing protein 10	0.00	0.43	1.28	0.74	173.21	0.20	8
LOC113515681	Serum response factor homolog A	-5.34	-2.34	0.00	2.73	116.99	0.49	6
LOC113515683	Uncharacterized protein LOC106143250 isoform X1	2.05	4.45	5.75	2.09	46.81	0.46	13
LOC113515686	Glutamate-gated chloride channel isoform X5	-1.65	-0.55	0.00	0.95	173.21	0.48	4
LOC113515687	Calcium-activated potassium channel slowpoke isoform X8	0.00	0.53	1.59	0.92	173.21	0.03	8
LOC113515693	Membrane alanyl Aminopeptidase-like	-5.01	-2.22	0.00	2.55	114.97	0.94	4
LOC113515696	Membrane alanyl Aminopeptidase-like isoform X1	-5.49	-1.83	0.00	3.17	173.21	0.64	4
LOC113515697	Aminopeptidase N-like	-4.17	-1.39	0.00	2.41	173.21	0.43	4
LOC113515699	Synaptic vesicle glycoprotein 2B-like isoform X2	0.00	1.01	3.04	1.76	173.21	0.26	7
LOC113515704	Glycerol-3-phosphate dehydrogenase	0.00	0.51	1.53	0.88	173.21	0.03	8
LOC113515705	Uncharacterized protein LOC106720737 isoform X2	0.00	0.96	2.88	1.66	173.21	0.13	7
LOC113515715	Transcription factor CP2 isoform X5	-1.64	-0.55	0.00	0.95	173.21	0.13	3
LOC113515722	Tubulin beta chain-like	-2.02	-1.34	0.00	1.16	86.62	0.35	9
LOC113515726	Nuclear hormone receptor HR3	-1.72	-0.57	0.00	0.99	173.21	0.44	4
LOC113515727	Hemocytin	-5.05	-2.40	0.00	2.53	105.53	0.39	6
LOC113515729	Uncharacterized protein	0.00	2.14	3.28	1.85	86.67	0.12	12
LOC113515743	Uncharacterized protein LOC106136462	-1.50	-0.50	0.00	0.86	173.21	0.18	3

LOC113515749	Glucose dehydrogenase	-5.41	-1.80	0.00	3.12	173.21	0.64	4
LOC113515756	Pachytene checkpoint protein 2 homolog	-2.08	-0.69	0.00	1.20	173.21	0.06	3
LOC113515761	Nose resistant to fluoxetine protein 6-like	0.00	0.77	2.31	1.33	173.21	0.55	7
LOC113515770	Uncharacterized protein LOC106114015	0.00	0.54	1.63	0.94	173.21	0.06	8
LOC113515772	Minus strand ecdysteroid 22-kinase	0.00	0.97	2.90	1.67	173.21	0.14	7
LOC113515775	Delta(24)-sterol reductase-like	-2.66	-1.48	0.00	1.35	91.74	0.21	9
LOC113515776	3-ketoacyl-CoA thiolase, mitochondrial-like	-3.80	-1.96	0.00	1.90	96.93	0.61	9
LOC113515777	3-ketoacyl-CoA thiolase, mitochondrial-like isoform X1	-3.14	-1.93	0.00	1.69	87.57	0.29	9
LOC113515795	Uncharacterized protein	0.00	0.85	2.56	1.48	173.21	0.17	7
LOC113515798	Arginine kinase isoform X1	0.00	0.61	1.84	1.06	173.21	0.41	1
LOC113515807	Irregular chiasm C-roughest protein-like isoform X2	0.00	2.39	4.67	2.34	97.85	0.53	12
LOC113515812	Uncharacterized protein LOC105398799	3.13	5.66	6.99	2.19	38.75	0.66	13
LOC113515813	Uncharacterized protein LOC106141310	0.00	0.68	2.05	1.18	173.21	0.73	8
LOC113515814	MAP kinase-activated protein kinase 2-like isoform X1	0.00	0.86	2.58	1.49	173.21	0.14	7
LOC113515816	Uncharacterized protein LOC106130383	-4.94	-2.71	0.00	2.51	92.41	0.20	6
LOC113515821	Serine/threonine-protein phosphatase 6 regulatory Ankyrin repeat subunit B	-1.93	-1.23	0.00	1.07	86.83	0.48	9
LOC113515831	Chitinase 2	2.64	3.87	4.51	1.06	27.44	0.35	13
LOC113515836	Uncharacterized protein LOC106137681	0.00	0.54	1.61	0.93	173.21	0.04	8
LOC113515837	Bifunctional glutamate/proline--tRNA ligase	0.00	0.60	1.81	1.04	173.21	0.27	8
LOC113515841	RNA polymerase II degradation factor 1-like	0.00	1.37	4.10	2.37	173.21	0.13	11
LOC113515847	Uncharacterized oxidoreductase TM_0325-like	-3.67	-1.22	0.00	2.12	173.21	0.30	4
LOC113515848	Uncharacterized protein LOC105841275	-5.38	-3.42	0.00	2.97	86.91	0.66	6
LOC113515849	Uncharacterized protein LOC110378293	-3.46	-2.27	0.00	1.97	86.63	0.72	9
LOC113515857	Collagen and calcium-binding EGF domain-containing protein 1-like isoform X2	-5.94	-2.88	0.00	2.97	103.33	0.19	6
LOC113515859	Acyl-CoA synthetase family member 2, mitochondrial isoform X1	-3.43	-1.96	0.00	1.77	90.22	0.39	9
LOC113515861	Protein lethal(2)essential for life-like	0.00	1.42	4.26	2.46	173.21	0.03	11

LOC113515865	Adhesion G-protein coupled receptor G2-like isoform X3	-3.51	-1.17	0.00	2.03	173.21	0.26	4
LOC113515876	Juvenile hormone epoxide hydrolase-like	-3.65	-2.24	0.00	1.96	87.60	0.75	9
LOC113515883	Twinfilin	0.00	0.47	1.42	0.82	173.21	0.48	1
LOC113515884	Fatty acyl-CoA reductase 1-like	0.00	2.24	6.73	3.89	173.21	0.08	15
LOC113515885	Uncharacterized protein LOC110381014	-4.67	-2.53	0.00	2.36	93.15	0.84	9
LOC113515889	2-oxoglutarate dehydrogenase E1 component DHKTD1 homolog, mitochondrial	-3.40	-1.13	0.00	1.96	173.21	0.64	3
LOC113515894	ATP-dependent RNA helicase DHX34	-1.62	-0.54	0.00	0.94	173.21	0.14	3
LOC113515899	Cytochrome P450 6B6-like	-6.83	-2.28	0.00	3.95	173.21	0.76	4
LOC113515902	O-acyltransferase like protein-like	-5.88	-1.96	0.00	3.40	173.21	0.79	6
LOC113515907	Chitooligosaccharidolytic beta-N-acetylglucosaminidase isoform X1	-5.40	-2.76	0.00	2.70	97.79	0.05	6
LOC113515912	GTP-binding protein REM 1-like	0.00	0.97	2.90	1.67	173.21	0.14	7
LOC113515914	Uncharacterized protein LOC106142761	-4.37	-2.22	0.00	2.19	98.32	0.74	6
LOC113515918	Protein mahjong isoform X1	0.00	0.48	1.43	0.82	173.21	0.11	8
LOC113515920	Cytosolic non-specific dipeptidase	-1.20	-0.40	0.00	0.69	173.21	0.29	3
LOC113515921	Ankyrin repeat domain-containing protein 50 isoform X1	0.00	1.41	2.18	1.22	86.71	0.86	1
LOC113515922	Uncharacterized protein	0.00	1.39	2.10	1.20	86.62	0.75	1
LOC113515939	Ski oncogene	-1.23	-0.41	0.00	0.71	173.21	0.27	3
LOC113515943	Group XIIA secretory phosphoLipase A2	0.00	1.17	2.08	1.06	90.92	0.74	1
LOC113515946	ADP-ribosylation factor 2	0.00	0.48	1.44	0.83	173.21	0.10	8
LOC113515952	Glyoxylate reductase/hydroxypyruvate reductase-like isoform X1	-1.72	-0.57	0.00	0.99	173.21	0.10	3
LOC113515953	2-oxoglutarate/malate carrier protein	0.00	0.52	1.55	0.89	173.21	0.02	8
LOC113515955	Uncharacterized protein LOC110373240	-3.50	-2.00	0.00	1.80	90.02	0.45	9
LOC113515958	1,4-Alpha-glucan-branching enzyme isoform X2	-2.77	-1.61	0.00	1.44	89.24	0.06	9
LOC113515960	Dyslexia-associated protein KIAA0319	-2.99	-1.61	0.00	1.51	93.55	0.21	9
LOC113515970	Ribosomal protein L11	-1.15	-0.38	0.00	0.67	173.21	0.30	3
LOC113515973	SET and MYND domain-containing protein 4-like	-1.26	-0.42	0.00	0.73	173.21	0.27	3

LOC113515980	Troponin C	-2.02	-0.67	0.00	1.17	173.21	0.03	3
LOC113515982	Glyoxylate reductase/hydroxypyruvate reductase	-1.36	-0.45	0.00	0.78	173.21	0.23	3
LOC113515986	6-phosphofructo-2-kinase/fructose-2,6-bisphosphatase-like	-2.11	-1.21	0.00	1.09	90.14	0.57	9
LOC113515990	Pancreatic secretory trypsin inhibitor-like	0.00	1.13	3.40	1.97	173.21	0.79	7
LOC113515992	Phosphatidylinositol 3,4,5-trisphosphate 3-phosphatase and dual-specificity protein phosphatase PTEN isoform X1	0.00	0.42	1.25	0.72	173.21	0.22	8
LOC113515993	Cytochrome c	0.00	1.29	1.95	1.11	86.63	0.55	1
LOC113515997	Protein FAM136A	0.00	0.53	1.59	0.92	173.21	0.03	8
LOC113515999	Estradiol 17-beta-dehydrogenase 11 isoform X1	0.00	4.84	8.81	4.47	92.36	0.74	10
LOC113516001	Activating signal cointegrator 1 complex subunit 2-like	0.00	0.61	1.82	1.05	173.21	0.28	8
LOC113516003	Serine protease inhibitor dipetalogastin-like	1.93	5.59	7.58	3.17	56.77	0.92	14
LOC113516004	PR domain Zinc finger protein 16-like isoform X1	-1.50	-0.99	0.00	0.86	86.60	0.88	9
LOC113516007	Monocarboxylate transporter 4-like	-2.93	-0.98	0.00	1.69	173.21	0.07	4
LOC113516012	Iron-sulfur cluster assembly 2 homolog, mitochondrial	0.00	0.42	1.26	0.73	173.21	0.21	8
LOC113516017	Zonadhesin-like	0.00	1.18	3.53	2.04	173.21	0.91	11
LOC113516019	Calcyclin-binding protein	0.00	0.49	1.46	0.84	173.21	0.08	8
LOC113516020	Dihydrolipoyllysine-residue acetyltransferase component of pyruvate dehydrogenase complex, mitochondrial isoform X1	0.00	0.95	1.56	0.83	87.84	0.22	1
LOC113516021	Fumarylacetoacetase	-2.96	-0.99	0.00	1.71	173.21	0.47	3
LOC113516023	Ribonuclease ZC3H12C isoform X1	0.00	1.17	1.83	1.01	86.89	0.50	1
LOC113516025	Histidine triad nucleotide-binding protein 2, mitochondrial-like	0.00	0.70	2.11	1.22	173.21	0.90	8
LOC113516030	Cell wall integrity and stress response component 3-like	-2.48	-0.83	0.00	1.43	173.21	0.10	4
LOC113516033	Glyceraldehyde-3-phosphate dehydrogenase	0.00	0.88	2.65	1.53	173.21	0.06	7

LOC113516035	Uncharacterized protein LOC106138830	0.00	0.76	2.28	1.32	173.21	0.61	7
LOC113516036	Uncharacterized protein LOC106138832	-1.52	-0.97	0.00	0.84	86.88	0.89	9
LOC113516037	Uncharacterized protein LOC106143445	0.00	4.46	7.83	4.03	90.35	0.40	10
LOC113516044	Histone-binding protein Caf1	-1.18	-0.39	0.00	0.68	173.21	0.29	3
LOC113516045	S-adenosylmethionine decarboxylase proenzyme	-1.30	-0.43	0.00	0.75	173.21	0.25	3
LOC113516048	Uncharacterized protein LOC106131020	0.00	3.89	6.03	3.37	86.76	0.46	10
LOC113516050	Pancreatic secretory trypsin inhibitor-like	0.00	8.45	13.55	7.37	87.21	0.50	14
LOC113516055	Serine/threonine-protein kinase RIO3	0.00	0.53	1.58	0.91	173.21	0.02	8
LOC113516058	Carboxypeptidase B-like	0.00	0.79	2.38	1.38	173.21	0.41	7
LOC113516059	Origin recognition complex subunit 4	-1.53	-0.51	0.00	0.89	173.21	0.17	3
LOC113516060	Zinc finger and BTB domain-containing protein 24-like isoform X3	0.00	0.69	2.07	1.19	173.21	0.77	8
LOC113516063	Frizzled-4	-3.08	-1.03	0.00	1.78	173.21	0.12	4
LOC113516066	Sedoheptulokinase-like	-2.66	-1.61	0.00	1.42	87.86	0.32	9
LOC113516072	Sialin-like isoform X1	0.00	5.32	8.47	4.63	87.10	0.71	14
LOC113516075	Glutaredoxin-C4-like	0.00	0.42	1.26	0.73	173.21	0.21	8
LOC113516076	Sarcoplasmic calcium-binding protein 1	0.00	1.70	5.09	2.94	173.21	0.45	11
LOC113516077	Uncharacterized protein LOC106134335	-5.00	-2.30	0.00	2.52	109.52	0.50	6
LOC113516080	Runt-related Transcription factor 3	0.00	0.42	1.27	0.73	173.21	0.21	8
LOC113516083	Uncharacterized protein LOC110383028	0.00	3.29	6.48	3.24	98.44	0.49	10
LOC113516094	Sialin-like	0.00	1.52	2.38	1.32	86.85	0.82	12
LOC113516097	U3 small nucleolar RNA-associated protein 14 homolog A	0.00	0.39	1.18	0.68	173.21	0.25	8
LOC113516100	Ribosomal protein L37A	-1.16	-0.39	0.00	0.67	173.21	0.30	3
LOC113516106	Monocyte to macrophage differentiation factor 2	-2.09	-1.27	0.00	1.11	87.90	0.44	9
LOC113516107	Protein atonal homolog 8	-5.69	-3.19	0.00	2.91	91.20	0.83	6
LOC113516109	Androgen-dependent TFPI-regulating protein-like	0.00	0.47	1.40	0.81	173.21	0.13	8
LOC113516110	Homogentisate 1,2-dioxygenase	-3.14	-1.78	0.00	1.61	90.53	0.48	9
LOC113516115	Inositol-3-phosphate synthase	0.00	0.57	1.71	0.99	173.21	0.15	8
LOC113516116	Uncharacterized protein LOC106138839	-3.27	-1.09	0.00	1.89	173.21	0.19	4

	isoform X1							
LOC113516117	Frizzled-10-like	-9.41	-5.01	0.00	4.73	94.42	0.74	6
LOC113516119	EF-hand domain-containing protein D2 homolog	0.00	0.92	1.45	0.80	87.00	0.28	1
LOC113516120	Neuropeptide precursor protein isoform X1	0.00	3.11	4.69	2.69	86.61	0.82	10
LOC113516123	Uncharacterized protein LOC106142239	-1.24	-0.41	0.00	0.72	173.21	0.27	3
LOC113516124	Uncharacterized protein LOC106142239	-1.35	-0.45	0.00	0.78	173.21	0.24	3
LOC113516126	Uncharacterized transporter slc-17.2-like	-1.97	-0.66	0.00	1.14	173.21	0.01	3
LOC113516128	Uncharacterized protein LOC110378115	0.00	2.07	3.58	1.86	89.55	0.43	12
LOC113516132	Protein msta, isoform A	0.00	6.70	10.34	5.81	86.71	0.35	14
LOC113516133	Cytidine deaminase-like	-3.29	-1.10	0.00	1.90	173.21	0.60	3
LOC113516139	Enhancer of split malpha protein	0.00	1.15	3.46	2.00	173.21	0.92	7
LOC113516140	Uncharacterized protein	0.00	0.86	2.57	1.49	173.21	0.14	7
LOC113516141	Protein drumstick	0.00	4.97	8.39	4.41	88.61	0.85	10
LOC113516144	Uncharacterized protein LOC106710224	0.00	0.74	2.21	1.28	173.21	0.40	1
LOC113516149	Uncharacterized protein LOC106142225	0.00	1.06	3.19	1.84	173.21	0.44	7
LOC113516152	brefeldin A-inhibited guanine nucleotide-exchange protein 3	-3.20	-1.60	0.00	1.60	100.12	0.38	9
LOC113516157	Insulin receptor substrate 1	0.00	1.36	2.22	1.19	87.63	0.81	1
LOC113516160	Uncharacterized protein LOC106138812	-8.59	-4.47	0.00	4.30	96.33	0.65	6
LOC113516161	Uncharacterized protein LOC110373188	0.00	1.32	3.97	2.29	173.21	0.65	5
LOC113516162	Uncharacterized protein LOC106138810	0.00	1.07	1.78	0.94	88.02	0.26	1
LOC113516163	Hypothetical protein RR46_04746	0.00	0.78	2.33	1.35	173.21	0.50	7
LOC113516166	Niemann-Pick C1 protein-like	0.00	0.45	1.34	0.77	173.21	0.16	8
LOC113516168	SWI/SNF-related matrix-associated actin-dependent regulator of chromatin subfamily A-like protein 1	-1.62	-1.04	0.00	0.90	86.78	0.77	9
LOC113516169	Glyoxylate reductase/hydroxypyruvate reductase-like	-3.66	-1.22	0.00	2.11	173.21	0.72	3
LOC113516171	Uncharacterized protein LOC106720945	-1.77	-1.16	0.00	1.00	86.65	0.60	9
LOC113516173	Ecdysis triggering hormone precursor	-2.48	-0.83	0.00	1.43	173.21	0.25	3
LOC113516174	Pancreatic lipase-related protein 2	0.00	2.02	6.06	3.50	173.21	0.47	15
LOC113516180	Uncharacterized protein LOC106134226	-1.19	-0.40	0.00	0.69	173.21	0.29	3

LOC113516187	Protein KIAA0556-like	-2.74	-0.91	0.00	1.58	173.21	0.37	3
LOC113516195	Cytochrome c oxidase subunit 7C, mitochondrial	-1.48	-0.49	0.00	0.85	173.21	0.19	3
LOC113516200	PR domain Zinc finger protein 16-like isoform X1	-1.65	-1.03	0.00	0.89	87.26	0.87	9
LOC113516205	Uncharacterized protein LOC101736819	-4.16	-2.19	0.00	2.09	95.14	0.92	6
LOC113516210	Troponin C-like isoform X2	-5.39	-1.80	0.00	3.11	173.21	0.63	4
LOC113516213	Uncharacterized protein LOC106142200	0.00	1.63	4.88	2.82	173.21	0.30	11
LOC113516219	cell wall protein IFF6-like	-4.44	-1.48	0.00	2.56	173.21	0.48	4
LOC113516223	Protein MIS12 homolog	-1.68	-0.56	0.00	0.97	173.21	0.11	3
LOC113516240	Chromosome transmission fidelity protein 18 homolog	-1.66	-0.55	0.00	0.96	173.21	0.12	3
LOC113516241	Canopy-1 like protein	0.00	0.96	1.50	0.83	86.84	0.30	1
LOC113516250	Inositol polyphosphate multikinase	-1.19	-0.40	0.00	0.69	173.21	0.29	3
LOC113516253	Sialin	0.00	0.70	2.09	1.21	173.21	0.84	8
LOC113516259	Fatty-acid amide hydrolase 2	0.00	1.92	5.75	3.32	173.21	0.79	15
LOC113516263	Carboxylesterase 1E	0.00	1.90	3.62	1.82	95.77	0.37	12
LOC113516264	MOXD1 homolog 1-like	0.00	0.54	1.62	0.93	173.21	0.05	8
LOC113516266	Hexamerin	0.00	0.49	1.46	0.84	173.21	0.08	8
LOC113516270	Uncharacterized protein LOC106709791	0.00	0.92	1.39	0.79	86.62	0.40	1
LOC113516277	Fatty-acid amide hydrolase 2-B-like	-1.27	-0.42	0.00	0.74	173.21	0.26	3
LOC113516282	COX assembly mitochondrial protein homolog	0.00	0.49	1.46	0.84	173.21	0.08	8
LOC113516290	Uncharacterized protein LOC106143382	-4.68	-2.58	0.00	2.38	92.08	0.34	6
LOC113516293	WEB family protein At1g65010, chloroplastic	0.00	1.83	5.49	3.17	173.21	0.12	5
LOC113516300	Tail-anchored protein insertion receptor WRB-like	-1.29	-0.43	0.00	0.74	173.21	0.26	3
LOC113516303	Leucine-rich repeat neuronal protein 3-like	-3.32	-1.11	0.00	1.92	173.21	0.20	4
LOC113516307	Sperm flagellar protein	0.00	1.27	3.81	2.20	173.21	0.76	5
LOC113516326	Cytochrome P450 monooxygenase CYP367B12	0.00	2.16	6.47	3.74	173.21	0.20	15
LOC113516327	Homeobox protein cut isoform X2	-3.80	-2.11	0.00	1.94	91.58	0.71	9

LOC113516328	Uncharacterized oxidoreductase SSP0419-like	0.00	2.02	6.07	3.50	173.21	0.47	15
LOC113516334	Uncharacterized oxidoreductase SERP2049-like	-2.46	-0.26	1.67	2.08	790.38	0.67	4
LOC113516339	Uncharacterized oxidoreductase SSP0419-like	-1.50	-0.50	0.00	0.87	173.21	0.57	4
LOC113516350	Proton-coupled folate transporter-like	-5.38	-3.58	0.00	3.10	86.60	0.63	6
LOC113516356	Sparc	-3.77	-1.71	0.00	1.91	111.64	0.60	9
LOC113516367	Atlastin-like	-1.24	-0.41	0.00	0.72	173.21	0.27	3
LOC113516369	Uncharacterized protein LOC106135296	1.74	3.29	4.23	1.35	41.17	0.91	13
LOC113516374	Prolylcarboxypeptidase	-3.25	-1.54	0.00	1.63	106.07	0.52	9
LOC113516384	RNA-binding protein EIF1AD	0.00	0.86	1.39	0.75	87.34	0.28	1
LOC113516387	Apolipophorin-III	0.00	1.11	3.33	1.92	173.21	0.66	7
LOC113516390	Superoxide dismutase	-1.43	-0.48	0.00	0.83	173.21	0.21	3
LOC113516392	Aromatic-L-amino-acid decarboxylase-like	0.00	2.08	4.08	2.04	98.15	0.45	12
LOC113516393	Defensin-like protein precursor	0.00	1.72	3.55	1.78	103.55	0.72	12
LOC113516401	Heat shock protein 68-like	-2.01	-0.67	0.00	1.16	173.21	0.29	4
LOC113516406	Synaptic vesicle glycoprotein 2B-like	0.00	2.92	8.76	5.06	173.21	0.41	15
LOC113516407	Talin-1-like isoform X1	-1.30	-0.43	0.00	0.75	173.21	0.25	3
LOC113516421	PRKCA-binding protein	-3.28	-1.61	0.00	1.64	101.90	0.42	9
LOC113516429	ATP-dependent RNA helicase SUV3 homolog, mitochondrial	-2.68	-0.89	0.00	1.55	173.21	0.34	3
LOC113516430	Uncharacterized protein LOC106142761	-4.68	-2.38	0.00	2.34	98.23	0.45	6
LOC113516431	Hemicentin-2	-2.90	-1.62	0.00	1.48	91.40	0.14	9
LOC113516440	Mucin-5AC-like	0.00	1.64	4.93	2.85	173.21	0.33	11
LOC113516444	Pyruvate dehydrogenase phosphatase regulatory subunit, mitochondrial	-1.61	-0.54	0.00	0.93	173.21	0.14	3
LOC113516445	Alanine aminotransferase 1	0.00	0.61	1.84	1.06	173.21	0.41	1
LOC113516446	N(4)-(Beta-N-acetylglucosaminyl)-L-asparaginase-like	-1.49	-0.50	0.00	0.86	173.21	0.19	3
LOC113516448	Neurogenic locus Notch protein-like	-5.16	-2.45	0.00	2.59	105.70	0.34	6
LOC113516449	Transmembrane protein 205	0.00	1.76	3.09	1.59	90.33	0.68	12
LOC113516451	Neuroglian	0.00	0.69	2.08	1.20	173.21	0.81	8

LOC113516452	Neuroglian isoform X1	0.00	1.23	2.32	1.17	94.99	1.00	7
LOC113516461	Scm-like with four MBT domains protein 2	-1.45	-0.48	0.00	0.84	173.21	0.20	3
LOC113516462	Adenosine monophosphate-protein transferase FICD homolog	0.00	0.50	1.51	0.87	173.21	0.05	8
LOC113516464	Translocon-associated protein subunit beta	0.00	0.42	1.26	0.73	173.21	0.54	1
LOC113516465	Uncharacterized protein LOC106132990	3.31	4.03	5.34	1.14	28.34	0.45	13
LOC113516466	Alpha-crystallin	-2.21	-0.74	0.00	1.28	173.21	0.12	3
LOC113516477	Multiple C2 and transmembrane domain-containing protein 1 isoform X4	-6.52	-2.94	0.00	3.31	112.53	0.34	6
LOC113516478	Tubulin beta chain-like isoform X1	0.00	0.48	1.44	0.83	173.21	0.48	1
LOC113516486	Apoptosis-stimulating of p53 protein 1	-3.34	-1.11	0.00	1.93	173.21	0.21	4
LOC113516487	Uncharacterized protein LOC110374577 isoform X1	0.00	0.70	2.10	1.21	173.21	0.85	8
LOC113516488	Membrane steroid binding protein	-1.38	-0.46	0.00	0.80	173.21	0.65	4
LOC113516493	Microtubule-associated protein futsch	0.00	0.83	2.50	1.44	173.21	0.24	7
LOC113516507	Fatty acid synthase	-1.92	-0.64	0.00	1.11	173.21	0.33	4
LOC113516513	Uncharacterized protein LOC106133772	0.00	0.74	2.21	1.27	173.21	0.80	7
LOC113516519	Serine protease snake-like	-1.30	-0.43	0.00	0.75	173.21	0.25	3
LOC113516521	Talin-1-like	-1.64	-0.55	0.00	0.94	173.21	0.13	3
LOC113516538	Uncharacterized protein	-2.23	-0.74	0.00	1.29	173.21	0.13	3
LOC113516540	Uncharacterized protein LOC106129968	-1.58	-0.53	0.00	0.91	173.21	0.15	3
LOC113516550	Nucleolar GTP-binding protein 1	-1.72	-1.57	-1.29	0.25	15.74	0.94	9
LOC113516554	Trypsin-like protein	-3.09	-1.03	0.00	1.78	173.21	0.13	4
LOC113516556	Cuticular protein RR-2 motif 59 precursor	0.00	1.60	4.79	2.76	173.21	0.26	5
LOC113516562	Ankyrin repeat domain-containing protein 65-like	0.00	1.20	3.59	2.07	173.21	0.94	5
LOC113516579	Protein O-mannosyl-transferase 2 isoform X2	-1.53	-0.51	0.00	0.89	173.21	0.17	3
LOC113516587	Short/branched chain specific acyl-CoA dehydrogenase, mitochondrial	-4.82	-2.28	0.00	2.42	106.16	0.53	6
LOC113516588	Tetraspanin	0.00	4.20	6.91	3.69	87.83	0.27	10
LOC113516605	Rho guanine nucleotide exchange factor 17 isoform X1	0.00	0.40	1.19	0.69	173.21	0.25	8

LOC113516614	Tetratricopeptide repeat protein 14 homolog isoform X4	0.00	0.43	1.30	0.75	173.21	0.19	8
LOC113516615	SUN domain-containing ossification factor	0.00	0.51	1.53	0.88	173.21	0.03	8
LOC113516617	Lectin3	-3.60	-2.35	0.00	2.03	86.67	0.79	9
LOC113516620	Junctophilin-1 isoform X1	-2.51	-2.05	-1.62	0.45	21.83	0.88	9
LOC113516621	Junctophilin-1	-3.62	-2.21	0.00	1.94	87.71	0.76	4
LOC113516624	Myrosinase 1	-5.51	-3.10	0.00	2.82	90.98	0.24	6
LOC113516626	Alpha-tocopherol transfer protein-like	-2.10	-1.13	0.00	1.06	93.58	0.80	9
LOC113516632	Glucose-6-phosphate 1-dehydrogenase	0.00	0.86	1.33	0.75	86.68	0.36	1
LOC113516635	Uncharacterized protein LOC110375481 isoform X2	-3.10	-1.03	0.00	1.79	173.21	0.52	3
LOC113516642	Homeotic protein spalt-major-like isoform X1	0.00	0.98	2.94	1.70	173.21	0.17	7
LOC113516645	NADH dehydrogenase	0.00	0.45	1.35	0.78	173.21	0.16	8
LOC113516653	DnaJ homolog shv	0.00	1.13	1.90	1.00	88.58	0.62	1
LOC113516654	Translationally-controlled tumor protein homolog	-1.25	-0.42	0.00	0.72	173.21	0.27	3
LOC113516656	Abhydrolase domain-containing protein 2	0.00	0.45	1.36	0.79	173.21	0.15	8
LOC113516663	Inner centromere protein isoform X1	-3.28	-1.09	0.00	1.90	173.21	0.60	3
LOC113516666	Monocarboxylate transporter 12	-2.46	-1.35	0.00	1.25	92.35	0.36	9
LOC113516667	Protein FAN-like	-1.72	-1.08	0.00	0.94	87.12	0.76	9
LOC113516676	Uncharacterized protein LOC106130524	-2.43	-1.36	0.00	1.24	91.08	0.33	9
LOC113516677	Long-chain fatty acid transport protein 4-like isoform X1	0.00	1.03	3.08	1.78	173.21	0.31	7
LOC113516679	Tyrosine-protein kinase transmembrane receptor Ror-like isoform X1	0.00	0.66	1.98	1.14	173.21	0.57	8
LOC113516683	JmjC domain-containing protein 4	0.00	0.47	1.41	0.81	173.21	0.12	8
LOC113516693	RNA pseudouridylate synthase domain-containing protein 2-like isoform X3	0.00	0.57	1.70	0.98	173.21	0.14	8
LOC113516703	Krueppel homolog 1-like	-5.48	-2.42	0.00	2.79	115.30	0.43	6
LOC113516705	Lipase 3-like	0.00	1.82	5.47	3.16	173.21	0.12	5
LOC113516706	Uncharacterized protein LOC106142978	0.00	2.81	4.69	2.48	88.23	0.69	12
LOC113516707	Uncharacterized protein PFB0765w-like	0.00	1.68	2.96	1.52	90.51	0.80	12

LOC113516710	Alpha-tocopherol transfer protein-like isoform X1	-1.68	-0.56	0.00	0.97	173.21	0.11	3
LOC113516716	DNA primase large subunit	-2.03	-0.68	0.00	1.17	173.21	0.04	3
LOC113516719	Slit homolog 1 protein	0.00	0.72	2.17	1.25	173.21	0.89	7
LOC113516722	Protein eyes shut	0.00	4.52	6.81	3.91	86.61	0.52	10
LOC113516725	Hypothetical protein RR48_09864	2.32	5.98	8.17	3.19	53.40	0.68	14
LOC113516752	Apterous A isoform X3	-1.63	-0.54	0.00	0.94	173.21	0.13	3
LOC113516758	Protein kinase DC2	0.00	1.03	3.10	1.79	173.21	0.33	7
LOC113516769	Uncharacterized protein LOC106136053	-3.43	-0.57	1.72	2.63	460.39	0.53	4
LOC113516771	WD repeat-containing protein 35	-1.26	-0.42	0.00	0.73	173.21	0.26	3
LOC113516776	CD109 antigen-like	0.00	1.56	4.69	2.71	173.21	0.21	11
LOC113516784	Buffy	0.00	2.84	4.51	2.47	87.05	0.74	12
LOC113516792	Calcium load-activated calcium channel	0.00	1.23	1.96	1.07	87.09	0.42	1
LOC113516799	Glucose transporter type 1 isoform X9	0.00	0.48	1.44	0.83	173.21	0.10	8
LOC113516800	Uncharacterized protein LOC110375454	-1.99	-0.66	0.00	1.15	173.21	0.02	3
LOC113516802	Uncharacterized protein LOC106143158 isoform X1	0.00	0.67	2.02	1.17	173.21	0.40	1
LOC113516813	GDP-fucose protein O-fucosyltransferase 1-like	-1.21	-0.40	0.00	0.70	173.21	0.28	3
LOC113516815	Nucleolar protein 10	-4.36	-2.41	0.00	2.22	91.94	0.58	6
LOC113516826	Uncharacterized protein LOC106143636	-1.16	-0.39	0.00	0.67	173.21	0.30	3
LOC113516829	Uncharacterized protein LOC106143591	0.00	2.19	3.42	1.90	86.85	0.11	12
LOC113516832	Trypsin delta/gamma-like isoform X2	-3.60	-1.96	0.00	1.82	93.06	0.61	9
LOC113516833	Protein msta, isoform B-like isoform X1	-1.55	-0.52	0.00	0.90	173.21	0.16	3
LOC113516834	Krueppel-like factor 10	0.00	0.48	1.43	0.82	173.21	0.11	8
LOC113516838	Protein tramtrack, beta isoform-like isoform X2	0.00	0.63	1.88	1.08	173.21	0.37	8
LOC113516846	Titin	-2.08	-0.69	0.00	1.20	173.21	0.06	3
LOC113516859	Acid trehalase-like protein 1	-1.78	-0.59	0.00	1.03	173.21	0.07	3
LOC113516864	Cell division cycle protein 23	-1.36	-0.45	0.00	0.79	173.21	0.23	3
LOC113516872	Large neutral amino acids transporter small subunit 2	0.00	0.48	1.44	0.83	173.21	0.48	1
LOC113516874	Nuclear hormone receptor HR38	0.00	1.92	5.75	3.32	173.21	0.79	15

LOC113516877	Protein tilB homolog	0.00	5.34	8.40	4.64	86.91	0.70	14
LOC113516882	Annulin-like isoform X1	3.47	6.66	8.81	2.82	42.32	0.63	14
LOC113516895	Maltase A1-like	0.00	1.87	5.62	3.25	173.21	0.95	15
LOC113516922	Pyruvate dehydrogenase phosphatase regulatory subunit, mitochondrial isoform X1	-1.25	-0.42	0.00	0.72	173.21	0.27	3
LOC113516924	Tribbles homolog 2-like	0.00	2.07	4.74	2.42	117.20	0.78	11
LOC113516934	Uncharacterized protein LOC106143149	0.00	2.62	4.37	2.31	88.21	0.45	12
LOC113516935	Uncharacterized protein LOC106133603	0.00	0.45	1.35	0.78	173.21	0.16	8
LOC113516936	Uncharacterized protein LOC106099788 isoform X1	0.00	1.33	2.27	1.18	88.94	0.82	1
LOC113516937	Alpha-tocopherol transfer protein-like isoform X2	-5.59	-1.86	0.00	3.23	173.21	0.83	6
LOC113516939	Uncharacterized protein LOC106143151 isoform X1	-3.19	-1.79	0.00	1.63	91.14	0.51	9
LOC113516941	Uncharacterized protein LOC106134039	-3.43	-2.24	0.00	1.94	86.68	0.68	9
LOC113516944	Cytochrome b5-like	0.00	3.62	7.15	3.58	98.81	0.41	10
LOC113516955	Uncharacterized protein LOC106099430	-2.66	-1.76	0.00	1.52	86.61	0.19	9
LOC113516959	Myosin-VIIa isoform X1	-1.44	-0.48	0.00	0.83	173.21	0.61	4
LOC113516972	Zinc transporter ZIP6	0.00	1.42	4.25	2.45	173.21	0.03	11
LOC113516974	NADH dehydrogenase (ubiquinone) complex I, assembly factor 6 isoform X1	0.00	0.44	1.32	0.76	173.21	0.17	8
LOC113516980	Zinc finger protein 131-like isoform X2	-1.34	-0.45	0.00	0.78	173.21	0.24	3
LOC113516985	Integrin beta-PS-like	-1.34	-0.45	0.00	0.77	173.21	0.24	3
LOC113516996	MKRN2 opposite strand protein isoform X1	0.00	0.52	1.56	0.90	173.21	0.01	8
LOC113517000	Protein slowmo	0.00	0.62	1.85	1.07	173.21	0.32	8
LOC113517005	Androgen-dependent TFPI-regulating protein-like isoform X1	-4.38	-2.91	0.00	2.52	86.61	0.63	6
LOC113517007	TBC1 domain family member 24 isoform X4	0.00	0.39	1.18	0.68	173.21	0.25	8
LOC113517010	Alpha-tocopherol transfer protein-like	-2.19	-0.73	0.00	1.26	173.21	0.11	3
LOC113517014	Uncharacterized protein LOC106143120	-1.81	-0.60	0.00	1.04	173.21	0.06	3
LOC113517016	Uncharacterized protein LOC106143663 isoform X1	-1.30	-0.84	0.00	0.73	86.71	0.88	3

LOC113517017	Peritrophin-48-like	-3.88	-1.94	0.00	1.94	99.77	0.81	9
LOC113517025	Uncharacterized protein LOC110380372	0.00	1.38	4.14	2.39	173.21	0.10	11
LOC113517026	Sodium- and chloride-dependent glycine transporter 1-like	-5.38	-1.79	0.00	3.11	173.21	0.87	6
LOC113517028	Centrosomin	-1.80	-0.60	0.00	1.04	173.21	0.07	3
LOC113517032	28S ribosomal protein S18b, mitochondrial	0.00	1.10	2.08	1.04	95.13	0.20	1
LOC113517033	Venom dipeptidyl peptidase 4	-1.40	-0.47	0.00	0.81	173.21	0.64	4
LOC113517036	Protein unzipped	0.00	0.54	1.62	0.93	173.21	0.05	8
LOC113517040	Collagen alpha-1(IV) chain	-5.31	-2.43	0.00	2.69	110.69	0.39	6
LOC113517042	Collagen alpha-5(IV) chain-like	-4.69	-2.17	0.00	2.36	108.90	0.68	6
LOC113517045	Protein anon-37Cs	0.00	1.00	3.00	1.73	173.21	0.22	7
LOC113517047	Neural-cadherin isoform X4	-2.03	-0.68	0.00	1.17	173.21	0.04	3
LOC113517054	Ral guanine nucleotide dissociation stimulator isoform X1	0.00	0.61	1.82	1.05	173.21	0.28	8
LOC113517059	Uncharacterized protein LOC110371035	-1.42	-0.47	0.00	0.82	173.21	0.21	3
LOC113517068	Integrin beta pat-3 precursor	0.00	2.45	3.85	2.13	86.90	0.27	12
LOC113517072	Uncharacterized protein LOC106143669	-3.91	-1.87	0.00	1.96	104.55	0.66	9
LOC113517076	Visual pigment-like receptor peropsin	0.00	1.72	2.66	1.50	86.71	0.43	12
LOC113517079	Tubulin polyglutamylase complex subunit 2 isoform X2	-1.61	-1.00	0.00	0.87	87.40	0.83	9
LOC113517089	Long-chain fatty acid transport protein 4-like	-2.17	-0.72	0.00	1.25	173.21	0.10	3
LOC113517091	Uncharacterized protein LOC106143133 isoform X1	0.00	1.46	4.39	2.54	173.21	0.07	11
LOC113517092	Homocysteine S-methyltransferase 1-like	-1.48	-0.49	0.00	0.85	173.21	0.59	4
LOC113517095	Aromatic-L-amino-acid decarboxylase isoform X1	0.00	2.27	3.61	1.98	87.08	0.14	12
LOC113517096	Hypothetical protein KGM_214113	-6.53	-2.18	0.00	3.77	173.21	0.73	6
LOC113517102	Venom acid phosphatase Acph-1-like isoform X2	0.00	1.26	3.77	2.18	173.21	0.48	11
LOC113517103	Venom acid phosphatase Acph-1-like	0.00	2.15	6.46	3.73	173.21	0.21	15
LOC113517115	Uncharacterized protein LOC106143014	0.00	3.01	5.08	2.67	88.61	0.85	10
LOC113517116	B9 domain-containing protein 2	-3.14	-1.98	0.00	1.72	87.05	0.38	9

LOC113517119	Uncharacterized protein LOC106133560	-2.29	-1.30	0.00	1.18	90.40	0.41	9
LOC113517138	Calcium-dependent secretion activator	-1.97	-1.27	0.00	1.10	86.76	0.42	9
LOC113517141	Tyrosine-protein kinase PR2	-1.34	-0.45	0.00	0.77	173.21	0.24	3
LOC113517143	Tryptase-like	-2.75	-1.46	0.00	1.38	94.70	0.30	9
LOC113517147	Aminoacylase-1-like	-3.89	-2.87	-2.21	0.90	31.23	0.52	2
LOC113517150	Uncharacterized protein LOC106110612	0.00	0.53	1.58	0.91	173.21	0.02	8
LOC113517157	Glutamine-dependent NAD(+) synthetase	0.00	0.60	1.80	1.04	173.21	0.26	8
LOC113517161	Coagulation factor-like protein 2	0.00	1.92	2.96	1.66	86.70	0.28	12
LOC113517164	Calcium-dependent secretion activator isoform X3	-1.77	-1.16	0.00	1.01	86.63	0.59	9
LOC113517165	GTP-binding protein 2-like isoform X1	0.00	1.41	4.22	2.44	173.21	0.05	11
LOC113517176	Acetyl-CoA carboxylase	0.00	0.73	2.18	1.26	173.21	0.88	7
LOC113517178	Uncharacterized protein	0.00	0.86	2.59	1.49	173.21	0.13	7
LOC113517194	Lethal(2) giant larvae protein	0.00	0.45	1.34	0.77	173.21	0.16	8
LOC113517197	Exportin-5	0.00	0.43	1.28	0.74	173.21	0.20	8
LOC113517198	Uncharacterized protein LOC106116396 isoform X1	0.00	2.18	3.48	1.90	87.13	0.09	12
LOC113517200	Uncharacterized protein LOC106131819	-2.16	-0.72	0.00	1.25	173.21	0.10	3
LOC113517202	Uncharacterized protein LOC110369978	-1.59	-0.97	0.00	0.85	87.71	0.98	3
LOC113517211	Nitric oxide synthase	0.00	0.94	1.51	0.82	87.28	0.51	1
LOC113517215	Cerebellar degeneration-related protein 2 isoform X3	-1.95	-1.07	0.00	0.99	92.44	0.94	9
LOC113517216	Alkaline nuclease	0.00	1.07	3.20	1.85	173.21	0.46	7
LOC113517217	Kynurenine 3-monooxygenase	-2.90	-0.97	0.00	1.68	173.21	0.44	3
LOC113517218	Aldehyde dehydrogenase family 7 member A1 homolog	-2.48	-1.56	0.00	1.36	87.09	0.09	9
LOC113517221	Potassium voltage-gated channel subfamily H member 8 isoform X1	0.00	1.88	5.63	3.25	173.21	0.95	15
LOC113517222	Uncharacterized protein LOC106133799	0.00	0.72	2.17	1.25	173.21	0.92	7
LOC113517225	Uncharacterized protein LOC106128213	-2.08	-0.69	0.00	1.20	173.21	0.06	3
LOC113517227	Uncharacterized protein LOC106138607 isoform X1	0.00	0.48	1.45	0.84	173.21	0.47	1
LOC113517233	Uncharacterized protein LOC106131872	-3.52	-2.14	0.00	1.88	87.82	0.60	9

LOC113517244	cGMP-dependent protein kinase, isozyme 1-like	-2.92	-0.97	0.00	1.69	173.21	0.07	4
LOC113517246	TNF receptor-associated factor 4 isoform X1	-1.44	-0.92	0.00	0.80	86.78	0.98	9
LOC113517249	Uncharacterized protein	-3.17	-1.71	0.00	1.60	93.46	0.60	9
LOC113517257	Uncharacterized protein LOC106134103 isoform X1	0.00	0.69	2.08	1.20	173.21	0.81	8
LOC113517261	Beta-1,3-glucosyltransferase	-2.26	-0.75	0.00	1.30	173.21	0.14	3
LOC113517270	Isocitrate dehydrogenase	-1.88	-0.63	0.00	1.09	173.21	0.03	3
LOC113517271	Serine/threonine-protein kinase Aurora-2	-1.76	-0.12	1.39	1.58	1284.66	0.51	3
LOC113517275	Cytochrome P450 18a1	0.00	2.45	7.35	4.24	173.21	0.14	15
LOC113517282	Argininosuccinate lyase isoform X1	-1.84	-1.13	0.00	0.99	87.52	0.67	9
LOC113517287	Failed axon connections	-1.39	-0.46	0.00	0.80	173.21	0.22	3
LOC113517288	Mpv17-like protein	0.00	0.46	1.38	0.80	173.21	0.14	8
LOC113517293	REPAT31	-2.44	-0.81	0.00	1.41	173.21	0.23	3
LOC113517298	Ecdysone receptor isoform X1	-1.34	-0.01	1.30	1.32	8870.42	0.72	8
LOC113517304	Proline-rich protein	0.00	4.31	6.75	3.74	86.84	0.38	10
LOC113517309	NADPH oxidase 4-like	0.00	2.45	4.81	2.41	98.09	0.56	12
LOC113517313	Protein outspread	0.00	0.47	1.41	0.81	173.21	0.12	8
LOC113517314	Cytochrome b reductase 1-like isoform X2	0.00	1.73	3.11	1.59	91.64	0.39	12
LOC113517316	Mediator of RNA polymerase II transcription subunit 13	0.00	0.44	1.32	0.76	173.21	0.18	8
LOC113517317	Mediator of RNA polymerase II transcription subunit 13	0.00	0.55	1.65	0.95	173.21	0.08	8
LOC113517323	Integrin Alpha-9-like	-1.19	-0.40	0.00	0.69	173.21	0.29	3
LOC113517324	Uncharacterized protein LOC106137858	-1.24	-0.41	0.00	0.72	173.21	0.27	3
LOC113517325	Ankyrin repeat domain-containing protein 29	-1.93	-1.22	0.00	1.06	87.04	0.51	9
LOC113517327	Nuclear receptor-binding protein homolog	0.00	1.12	1.95	1.01	89.70	0.68	1
LOC113517345	Protein claret segregational-like	-2.25	-0.75	0.00	1.30	173.21	0.14	3
LOC113517347	LIM and SH3 domain protein 1 isoform X2	0.00	0.93	1.53	0.81	87.93	0.21	1
LOC113517354	Sulfotransferase 1 family member D1-like	-2.80	-1.35	0.00	1.40	103.64	0.60	9
LOC113517359	Adenosine receptor A3	0.00	2.78	8.33	4.81	173.21	0.35	15
LOC113517365	VMP32 protein	0.00	3.57	6.12	3.19	89.17	0.17	10

LOC113517371	ATP-binding cassette sub-family C member Sur-like	0.00	3.23	6.42	3.21	99.45	0.55	10
LOC113517377	Kinase D-interacting substrate of 220 kDa isoform X2	0.00	0.39	1.18	0.68	173.21	0.25	8
LOC113517379	Rotatin-like	-1.24	-0.41	0.00	0.72	173.21	0.27	3
LOC113517382	Uncharacterized protein LOC106109383	-2.96	-0.99	0.00	1.71	173.21	0.47	3
LOC113517383	Phosphatidylinositol N-acetylglucosaminyltransferase subunit P	0.00	0.48	1.43	0.83	173.21	0.48	1
LOC113517391	Peroxidasin isoform X1	-2.65	-0.88	0.00	1.53	173.21	0.33	3
LOC113517398	Nuclear pore complex protein Nup155	-1.18	-0.39	0.00	0.68	173.21	0.29	3
LOC113517402	Serine hydrolase	0.00	1.45	2.74	1.38	95.06	0.88	12
LOC113517403	Serine hydrolase	0.00	1.43	2.80	1.40	97.78	1.00	12
LOC113517406	Guanylate cyclase 32E	0.00	4.11	6.19	3.56	86.60	0.41	10
LOC113517407	Sterol O-acyltransferase 2	-1.99	-1.18	0.00	1.04	88.71	0.66	9
LOC113517409	Transmembrane protein 132B	-1.76	-0.59	0.00	1.01	173.21	0.08	3
LOC113517412	Cytochrome P450 CYP306A1	0.00	1.79	5.36	3.09	173.21	0.70	11
LOC113517415	Uncharacterized protein LOC106134838 isoform X2	-2.10	-0.70	0.00	1.21	173.21	0.07	3
LOC113517421	Uncharacterized protein LOC101735991 isoform X2	-7.18	-3.08	0.00	3.70	119.93	0.45	6
LOC113517426	Phosphatidylinositol 4,5-bisphosphate 3-kinase catalytic subunit delta isoform	0.00	0.73	2.20	1.27	173.21	0.81	7
LOC113517432	Echinoderm microtubule-associated protein-like CG42247	-2.81	-1.59	0.00	1.44	90.75	0.57	4
LOC113517434	Coenzyme Q-binding protein COQ10 homolog A, mitochondrial	0.00	0.39	1.18	0.68	173.21	0.25	8
LOC113517439	CD63 antigen-like isoform X1	0.00	0.42	1.26	0.73	173.21	0.21	8
LOC113517441	Uncharacterized protein LOC106131879 isoform X1	0.00	1.09	3.26	1.88	173.21	0.54	7
LOC113517453	Sodium-coupled monocarboxylate transporter 1	-1.64	-0.55	0.00	0.95	173.21	0.13	3
LOC113517455	RNA-binding protein 18	0.00	0.49	1.48	0.85	173.21	0.07	8
LOC113517457	Fringe glycosyltransferase	-3.08	-1.59	0.00	1.54	96.85	0.30	9
LOC113517458	Nuclear transport factor 2-like protein	0.00	0.39	1.16	0.67	173.21	0.26	8

LOC113517467	RNA polymerase I-specific transcription initiation factor RRN3 isoform X1	0.00	0.58	1.75	1.01	173.21	0.19	8
LOC113517469	15-hydroxyprostaglandin dehydrogenase	-5.39	-1.80	0.00	3.11	173.21	0.63	4
LOC113517470	Alcohol dehydrogenase AD1	0.00	1.06	1.95	0.99	92.89	0.18	1
LOC113517474	Neuronal PAS domain-containing protein 2-like	-3.42	-1.14	0.00	1.97	173.21	0.65	3
LOC113517475	FGGY carbohydrate kinase domain-containing protein	-1.48	-0.49	0.00	0.86	173.21	0.19	3
LOC113517477	Uncharacterized protein LOC106131834 isoform X1	-1.48	-0.49	0.00	0.86	173.21	0.19	3
LOC113517478	IML1	-3.61	-2.86	-1.74	0.99	34.53	0.69	2
LOC113517479	Polycomb	-1.77	-0.59	0.00	1.02	173.21	0.08	3
LOC113517492	Inositol-trisphosphate 3-kinase A isoform X2	0.00	0.85	1.36	0.74	87.23	0.30	1
LOC113517495	Multiple inositol polyphosphate phosphatase 1-like	-1.45	-0.48	0.00	0.84	173.21	0.20	3
LOC113517505	Leucine-rich repeat-containing protein 70	-4.39	-2.06	0.00	2.21	106.99	0.90	6
LOC113517513	ATP-binding cassette sub-family F member 3	0.00	0.53	1.58	0.91	173.21	0.02	8
LOC113517514	CD63 antigen-like	-2.59	-0.86	0.00	1.49	173.21	0.06	4
LOC113517518	Cathepsin K-like	-4.10	-1.37	0.00	2.37	173.21	0.83	3
LOC113517522	Receptor-type tyrosine-protein phosphatase N2 isoform X1	-2.14	-1.19	0.00	1.09	91.35	0.62	9
LOC113517527	F-box only protein 7-like	0.00	0.64	1.91	1.10	173.21	0.43	8
LOC113517528	Ras-related protein Rap-2b	-4.82	-2.36	0.00	2.41	102.36	0.44	6
LOC113517529	Tyrosine 3-monooxygenase	3.82	8.58	11.23	4.13	48.15	0.44	14
LOC113517530	Patched domain-containing protein 3	0.00	4.15	7.62	3.86	92.91	0.33	10
LOC113517531	Uncharacterized protein LOC106138558	-1.44	-0.48	0.00	0.83	173.21	0.20	3
LOC113517537	Uncharacterized protein LOC106131769	-1.58	-0.53	0.00	0.91	173.21	0.15	3
LOC113517538	Uncharacterized protein LOC106138673 isoform X4	0.00	0.66	1.98	1.14	173.21	0.40	1
LOC113517540	Uncharacterized protein	0.00	2.76	8.29	4.79	173.21	0.44	5
LOC113517542	DNA-directed RNA polymerase III subunit RPC9-like isoform X1	0.00	0.44	1.33	0.77	173.21	0.17	8

LOC113517545	Prisilkin-39-like	0.00	0.60	1.79	1.03	173.21	0.24	8
LOC113517557	Proton-coupled folate transporter isoform X1	-2.76	-1.41	0.00	1.38	97.76	0.41	9
LOC113517559	23 kDa integral membrane protein-like	0.00	0.89	2.67	1.54	173.21	0.05	7
LOC113517566	Titin	3.67	5.64	8.76	2.74	48.50	0.79	13
LOC113517567	DNA replication complex GINS protein PSF2	-2.49	-0.83	0.00	1.44	173.21	0.25	3
LOC113517569	Sarco/endoplasmic reticulum calcium ATPase	0.00	0.44	1.32	0.76	173.21	0.18	8
LOC113517570	Uncharacterized protein LOC110369958	-3.61	-2.03	0.00	1.85	90.90	0.53	9
LOC113517575	Heparan sulfate glucosamine 3-O-sulfotransferase 1	-6.16	-2.05	0.00	3.55	173.21	0.76	6
LOC113517580	DNA polymerase delta catalytic subunit	-1.16	-0.39	0.00	0.67	173.21	0.30	3
LOC113517584	Uncharacterized protein LOC106713365	-4.00	-2.29	0.00	2.06	89.98	0.94	6
LOC113517588	Uncharacterized protein LOC106109243	-1.34	-0.45	0.00	0.77	173.21	0.24	3
LOC113517593	Sodium- and chloride-dependent GABA transporter ine isoform X1	-1.20	-0.40	0.00	0.69	173.21	0.29	3
LOC113517595	Tissue Alpha-L-fucosidase-like precursor	-2.31	-0.77	0.00	1.33	173.21	0.16	3
LOC113517596	Uncharacterized protein LOC106139924	-1.35	-0.45	0.00	0.78	173.21	0.24	3
LOC113517597	Ras-related protein Rab-37 isoform X1	-2.13	-1.33	0.00	1.16	87.19	0.41	9
LOC113517599	Integrin Alpha-PS2	0.00	1.54	2.34	1.34	86.62	0.80	12
LOC113517602	Uncharacterized protein LOC106137997	-3.75	-2.92	-2.34	0.74	25.29	0.45	2
LOC113517603	4-aminobutyrate aminotransferase, mitochondrial	-1.16	-0.39	0.00	0.67	173.21	0.30	3
LOC113517606	Uncharacterized protein	-4.85	-3.80	-2.03	1.54	40.52	0.62	2
LOC113517608	Uncharacterized protein LOC106133773	-3.30	-1.10	0.00	1.90	173.21	0.60	3
LOC113517610	Laccase-5	0.00	0.61	1.83	1.06	173.21	0.30	8
LOC113517615	Uncharacterized protein LOC106134414	-7.41	-4.15	0.00	3.78	91.24	0.60	6
LOC113517616	UDP-glucuronosyltransferase 2B1-like	0.00	1.05	3.15	1.82	173.21	0.38	7
LOC113517621	Uncharacterized protein LOC106141905	-5.60	-1.87	0.00	3.23	173.21	0.83	6
LOC113517628	Hypothetical protein KGM_202129	-6.22	-2.07	0.00	3.59	173.21	0.75	6
LOC113517636	Protein outspread-like	0.00	0.52	1.57	0.91	173.21	0.02	8
LOC113517643	BMP-binding endothelial regulator protein-	-1.51	-0.06	1.32	1.42	2335.55	0.85	8

	like							
LOC113517644	15-hydroxyprostaglandin dehydrogenase	-3.03	-1.51	0.00	1.51	99.94	0.39	9
LOC113517645	Alcohol dehydrogenase AD1	-3.57	-2.06	0.00	1.85	89.72	0.53	9
LOC113517647	Sodium-dependent dopamine transporter	-3.79	-1.26	0.00	2.19	173.21	0.76	3
LOC113517648	Leucine-rich repeat-containing G-protein coupled receptor 4	-1.96	-1.13	0.00	1.02	89.52	0.71	9
LOC113517650	Uncharacterized protein LOC110370027	0.00	0.70	2.11	1.22	173.21	0.90	8
LOC113517673	Lysosome-associated membrane glycoprotein 1	-2.07	-0.69	0.00	1.19	173.21	0.05	3
LOC113517675	Uncharacterized threonine-rich GPI-anchored glycoprotein PJ4664.02	2.13	4.88	6.62	2.41	49.41	0.70	13
LOC113517684	Uncharacterized protein LOC106131541	0.00	1.20	3.61	2.08	173.21	0.76	11
LOC113517686	Acetylcholine receptor subunit Alpha-type unc-38-like	-5.03	-2.35	0.00	2.53	107.93	0.45	6
LOC113517691	Solute carrier family 23 member 2 isoform X2	-2.70	-0.90	0.00	1.56	173.21	0.03	4
LOC113517694	SUN domain-containing ossification factor isoform X1	0.00	0.57	1.70	0.98	173.21	0.13	8
LOC113517696	Glucosylceramidase-like isoform X1	-3.25	-1.08	0.00	1.87	173.21	0.58	3
LOC113517700	Uncharacterized protein LOC106129404	0.00	1.66	2.99	1.52	91.71	0.47	12
LOC113517705	Uncharacterized protein LOC105389318	0.00	1.44	4.32	2.49	173.21	0.04	11
LOC113517706	5-methylcytosine rRNA methyltransferase NSUN4	-1.39	-0.46	0.00	0.80	173.21	0.65	4
LOC113517713	CD63 antigen-like	0.00	0.92	2.76	1.60	173.21	0.05	7
LOC113517722	P protein-like	-1.50	-0.50	0.00	0.87	173.21	0.18	3
LOC113517728	Uncharacterized protein LOC106139019	0.00	1.50	2.62	1.35	90.25	0.82	1
LOC113517732	Membrane alanyl Aminopeptidase-like isoform X1	-3.99	-1.33	0.00	2.30	173.21	0.39	4
LOC113517733	Cytochrome b5-like	0.00	1.10	1.97	1.01	91.62	0.77	1
LOC113517750	UDP-glucuronosyltransferase 2A3-like	-6.24	-3.03	0.00	3.12	103.21	0.23	6
LOC113517754	Ankyrin repeat and Zinc finger domain-containing protein 1-like	0.00	0.80	2.39	1.38	173.21	0.40	7
LOC113517757	Uncharacterized protein LOC106143497	-1.93	-0.64	0.00	1.12	173.21	0.33	4
LOC113517766	Hypothetical protein KGM_201298	-5.22	-1.74	0.00	3.01	173.21	0.92	6

LOC113517767	Protein arginine N-methyltransferase 9	0.00	0.90	2.71	1.56	173.21	0.03	7
LOC113517774	Uncharacterized protein LOC106127544	0.00	1.79	4.04	2.06	114.53	0.82	11
LOC113517776	Lysosomal thioesterase PPT2-A	0.00	0.44	1.32	0.76	173.21	0.51	1
LOC113517777	Zinc finger protein Gfi-1 isoform X1	0.00	1.78	2.75	1.54	86.72	0.36	12
LOC113517799	Phosphatidylinositol 4-kinase alpha	0.00	0.42	1.27	0.74	173.21	0.20	8
LOC113517800	NF-kappa-B inhibitor cactus	0.00	2.40	3.78	2.09	86.92	0.23	12
LOC113517801	Uncharacterized family 31 glucosidase KIAA1161-like isoform X1	-3.86	-1.29	0.00	2.23	173.21	0.78	3
LOC113517802	Fibrillin-1-like	0.00	0.43	1.28	0.74	173.21	0.20	8
LOC113517807	Laminin subunit gamma-1	-1.80	-0.60	0.00	1.04	173.21	0.06	3
LOC113517812	Leukocyte surface antigen CD53-like isoform X1	-3.36	-1.12	0.00	1.94	173.21	0.21	4
LOC113517817	Pancreatic triacylglycerol lipase-like	-6.07	-4.04	0.00	3.50	86.60	0.70	6
LOC113517820	Cell division cycle protein 20 homolog	-1.61	-0.54	0.00	0.93	173.21	0.14	3
LOC113517827	Alkaline phosphatase	-1.86	-0.62	0.00	1.07	173.21	0.36	4
LOC113517845	Uncharacterized protein LOC110376544	-5.53	-3.43	0.00	3.00	87.31	0.70	6
LOC113517846	Uncharacterized protein LOC106134054	-6.55	-3.53	0.00	3.30	93.68	0.39	6
LOC113517853	Dipeptidase 1-like	-5.08	-2.81	0.00	2.58	91.95	0.18	6
LOC113517854	Anion exchange protein 2 isoform X3	-1.24	-0.41	0.00	0.72	173.21	0.27	3
LOC113517875	Fatty-acid amide hydrolase 2-A-like isoform X1	-3.44	-2.23	0.00	1.93	86.71	0.67	9
LOC113517884	Serine hydrolase-like protein	0.00	0.63	1.90	1.10	173.21	0.41	8
LOC113517896	Polypeptide N-acetylgalactosaminyltransferase 2	-2.25	-1.46	0.00	1.26	86.73	0.26	9
LOC113517898	Uncharacterized protein OBRU01_11868	0.00	0.61	1.82	1.05	173.21	0.28	8
LOC113517917	Uncharacterized protein LOC106708587	-3.95	-1.32	0.00	2.28	173.21	0.38	4
LOC113517923	Protein phosphatase 2C T23F11.1 isoform X2	0.00	0.98	2.94	1.70	173.21	0.17	7
LOC113517925	Zinc finger protein 624-like isoform X1	-1.89	-1.16	0.00	1.02	87.49	0.61	9
LOC113517926	Phosphatidylinositol 4,5-bisphosphate 3-kinase catalytic subunit delta isoform	0.00	0.97	2.91	1.68	173.21	0.14	7
LOC113517945	Prophenoloxidase	-4.98	-2.08	0.00	2.59	124.65	0.72	6
LOC113517948	L-threonine ammonia-lyase-like	0.00	1.95	5.86	3.38	173.21	0.67	15

LOC113517959	Calcium-dependent secretion activator	-1.99	-1.20	0.00	1.06	88.04	0.55	9
LOC113517972	Serpin I2-like	-5.35	-2.49	0.00	2.69	108.08	0.33	6
LOC113517978	Globin 1	0.00	1.57	2.44	1.37	86.74	0.71	12
LOC113517980	Enoyl-CoA hydratase, mitochondrial	-1.28	-0.43	0.00	0.74	173.21	0.26	3
LOC113517982	Cancer-related nucleoside-triphosphatase homolog	-1.26	-0.42	0.00	0.73	173.21	0.27	3
LOC113517984	Uncharacterized protein LOC108734152	0.00	0.56	1.67	0.96	173.21	0.10	8
LOC113517989	Zinc finger protein 43-like	-2.37	-0.79	0.00	1.37	173.21	0.19	3
LOC113517990	Acetylcholine receptor subunit Alpha-type unc-38-like	-4.66	-2.16	0.00	2.35	108.83	0.69	6
LOC113517994	Facilitated trehalose transporter Tret1-2 homolog	-5.36	-3.57	0.00	3.09	86.60	0.62	6
LOC113518005	Glucose-1-phosphatase-like	-1.86	-0.62	0.00	1.07	173.21	0.04	3
LOC113518006	2-oxoglutarate dehydrogenase E1 component DHKTD1 homolog, mitochondrial	-2.44	-0.81	0.00	1.41	173.21	0.23	3
LOC113518015	Zinc finger protein OZF-like	-1.51	-0.94	0.00	0.82	87.26	0.95	3
LOC113518020	Uncharacterized protein LOC101739749 isoform X1	0.00	2.05	6.14	3.54	173.21	0.17	5
LOC113518031	A disintegrin and metalloproteinase with thrombospondin motifs 14-like	0.00	1.39	4.18	2.41	173.21	0.07	11
LOC113518034	Uncharacterized protein LOC106137789	0.00	1.85	2.87	1.60	86.75	0.26	12
LOC113518036	Phytanoyl-CoA dioxygenase domain-containing protein 1	-2.06	-0.69	0.00	1.19	173.21	0.26	4
LOC113518043	Lysine-specific demethylase 6A isoform X1	0.00	1.62	3.07	1.54	95.09	0.61	12
LOC113518044	Monocarboxylate transporter 5	-2.26	-0.75	0.00	1.31	173.21	0.18	4
LOC113518046	Lachesin-like	-2.61	-1.46	0.00	1.33	91.18	0.21	9
LOC113518050	Major facilitator superfamily domain-containing protein 12-like isoform X2	0.00	1.75	2.78	1.52	87.08	0.38	12
LOC113518067	Cyclin E	0.00	0.55	1.64	0.94	173.21	0.43	1
LOC113518072	Muscle M-line assembly protein unc-89	-1.56	-0.52	0.00	0.90	173.21	0.16	3
LOC113518075	Hypothetical protein KGM_202228B	0.00	3.86	6.71	3.47	89.77	0.08	10
LOC113518076	Uncharacterized MFS-type transporter C09D4.1 isoform X2	0.00	4.63	7.82	4.11	88.63	0.49	10

LOC113518084	Structural maintenance of chromosomes protein 6-like	-1.22	-0.41	0.00	0.71	173.21	0.28	3
LOC113518086	C-Maf-inducing protein-like	0.00	0.63	1.90	1.10	173.21	0.42	8
LOC113518095	Trans-1,2-dihydrobenzene-1,2-diol dehydrogenase	-2.80	-1.36	0.00	1.40	103.32	0.59	9
LOC113518096	Facilitated trehalose transporter Tret1-like	-1.36	-0.45	0.00	0.78	173.21	0.67	4
LOC113518097	Carboxypeptidase N subunit 2-like	0.00	1.54	2.68	1.38	89.75	0.67	12
LOC113518098	Immunoglobulin-binding protein 1	0.00	0.41	1.24	0.71	173.21	0.22	8
LOC113518101	Uncharacterized protein LOC106133521	0.00	1.74	5.21	3.01	173.21	0.55	11
LOC113518105	Ornithine aminotransferase, mitochondrial	-3.14	-1.56	0.00	1.57	100.47	0.39	9
LOC113518106	Peroxisomal acyl-coenzyme A oxidase 1 isoform X1	-1.63	-0.99	0.00	0.87	87.76	0.97	9
LOC113518121	Elongation factor 1-delta isoform X1	-1.54	-0.51	0.00	0.89	173.21	0.17	3
LOC113518128	Pancreatic triacylglycerol lipase-like	-3.29	-2.14	0.00	1.86	86.68	0.53	9
LOC113518138	Translation initiation factor eIF-2B subunit delta isoform X1	0.00	0.69	2.07	1.20	173.21	0.78	8
LOC113518142	Mid1-interacting protein 1A	0.00	1.57	2.43	1.36	86.75	0.73	12
LOC113518143	SHC-transforming protein 1 isoform X1	0.00	1.53	2.64	1.37	89.37	0.69	12
LOC113518164	Uncharacterized protein LOC106138152	-1.25	-0.42	0.00	0.72	173.21	0.27	3
LOC113518166	Uncharacterized protein LOC106138152	-2.09	-1.36	0.00	1.18	86.69	0.33	9
LOC113518173	Alpha carbonic anhydrase 8-like	-1.39	-0.46	0.00	0.80	173.21	0.22	3
LOC113518176	Dm0-like lamin	0.00	0.95	1.58	0.84	88.25	0.20	1
LOC113518180	Myosin-IIIb-like isoform X2	-1.45	-0.48	0.00	0.84	173.21	0.20	3
LOC113518189	Leucine-rich repeat neuronal protein 2-like	0.00	1.35	2.15	1.17	87.11	0.61	1
LOC113518191	PDZ domain-containing protein 2-like	-2.05	-0.68	0.00	1.18	173.21	0.04	3
LOC113518192	N-acetylgalactosamine kinase	-3.12	-1.58	0.00	1.56	99.01	0.36	9
LOC113518193	Dual specificity protein phosphatase 3-like isoform X1	0.00	0.61	1.83	1.06	173.21	0.30	8
LOC113518195	Peroxisomal acyl-coenzyme A oxidase 1	-3.22	-1.90	0.00	1.69	88.64	0.41	9
LOC113518198	Band 4.1-like protein 4	-2.84	-1.84	0.00	1.60	86.69	0.21	9
LOC113518201	Transmembrane protein 138-like	-1.65	-0.55	0.00	0.95	173.21	0.13	3
LOC113518202	Hydroxymethylglutaryl-CoA lyase, mitochondrial	-2.31	-1.21	0.00	1.16	95.83	0.67	9

LOC113518207	Sialic acid synthase	-3.42	-1.70	0.00	1.71	100.73	0.40	9
LOC113518210	Uncharacterized protein LOC110383836	0.00	0.69	2.07	1.20	173.21	0.80	8
LOC113518214	Uncharacterized protein LOC110373875 isoform X7	-6.41	-3.56	-1.78	2.49	70.02	0.98	6
LOC113518215	Nuclear pore complex protein Nup214-like isoform X1	0.00	5.42	9.26	4.83	89.04	0.61	14
LOC113518216	Mucin-5AC isoform X2	0.00	1.24	2.04	1.09	87.88	0.61	1
LOC113518224	Uncharacterized protein LOC106140166 isoform X1	-2.94	-1.56	0.00	1.48	94.65	0.67	9
LOC113518231	Uncharacterized protein LOC101738767	-5.77	-2.65	0.00	2.92	110.00	0.29	6
LOC113518232	Uncharacterized protein LOC106135508	0.00	1.24	1.90	1.08	86.65	0.67	1
LOC113518233	Serine/threonine-protein kinase 3 isoform X5	0.00	0.61	1.82	1.05	173.21	0.41	1
LOC113518240	Mitochondrial enolase superfamily member 1-like	0.00	0.92	2.77	1.60	173.21	0.05	7
LOC113518245	Tubulin gamma-1 chain	-1.44	-0.48	0.00	0.83	173.21	0.20	3
LOC113518246	6-pyruvoyl tetrahydrobiopterin synthase	-1.26	-0.42	0.00	0.73	173.21	0.26	3
LOC113518251	Larval cuticle protein LCP-30-like	0.00	3.12	9.37	5.41	173.21	0.48	15
LOC113518256	Long-chain-fatty-acid--CoA ligase ACSBG2 isoform X2	-1.38	-0.46	0.00	0.80	173.21	0.23	3
LOC113518258	Uncharacterized protein	-1.85	-0.62	0.00	1.07	173.21	0.04	3
LOC113518262	Tyrosine-protein kinase Drl	0.00	0.50	1.49	0.86	173.21	0.06	8
LOC113518263	Glycine-rich protein DOT1-like isoform X1	0.00	5.13	9.11	4.66	90.85	0.92	14
LOC113518264	Uncharacterized protein LOC110378921	-3.28	-2.17	0.00	1.88	86.61	0.57	9
LOC113518265	Uncharacterized protein LOC110383784	0.00	3.11	5.66	2.87	92.30	0.63	10
LOC113518274	Arf-GAP with SH3 domain, ANK repeat and PH domain-containing protein 1 isoform X1	0.00	0.40	1.19	0.69	173.21	0.24	8
LOC113518280	Carbonic anhydrase 2	0.00	1.67	2.93	1.51	90.30	0.45	12
LOC113518285	Uncharacterized protein LOC101747221 isoform X1	-3.07	-1.02	0.00	1.77	173.21	0.12	4
LOC113518288	Ras GTPase-activating-like protein IQGAP1	-1.61	-0.54	0.00	0.93	173.21	0.14	3
LOC113518301	Serine/threonine-protein kinase greatwall isoform X1	-1.28	-0.43	0.00	0.74	173.21	0.26	3

LOC113518303	Sorting nexin-17	0.00	0.43	1.30	0.75	173.21	0.19	8
LOC113518306	Alpha-catulin isoform X1	-2.50	-2.00	-1.70	0.44	21.85	0.73	9
LOC113518308	Cystathionine gamma-lyase	0.00	2.65	4.16	2.30	86.89	0.47	12
LOC113518309	Uncharacterized protein LOC105393001	-1.74	-0.58	0.00	1.00	173.21	0.09	3
LOC113518311	DNA helicase MCM8	-1.79	-0.60	0.00	1.03	173.21	0.07	3
LOC113518314	UPF0489 protein C5orf22 homolog	-1.35	-0.45	0.00	0.78	173.21	0.23	3
LOC113518330	Ras-related protein Rab-26 isoform X1	0.00	0.58	1.75	1.01	173.21	0.19	8
LOC113518331	Cationic amino acid transporter	0.00	0.56	1.69	0.98	173.21	0.12	8
LOC113518335	Nuclear-pore anchor isoform X1	-2.01	-0.67	0.00	1.16	173.21	0.29	4
LOC113518338	23 kDa glycoprotein Seroin	0.00	0.45	1.36	0.78	173.21	0.15	8
LOC113518339	G2/mitotic-specific cyclin-B	-2.09	-0.70	0.00	1.21	173.21	0.06	3
LOC113518342	Hydroxyacid-oxoacid transhydrogenase, mitochondrial isoform X1	-1.56	-0.52	0.00	0.90	173.21	0.16	3
LOC113518345	Protein disulfide-isomerase A6	0.00	0.46	1.39	0.80	173.21	0.13	8
LOC113518350	Uncharacterized protein LOC106138210	0.00	1.36	2.71	1.35	99.32	0.84	7
LOC113518351	Phosphatidate phosphatase LPIN2	0.00	0.67	2.02	1.17	173.21	0.66	8
LOC113518354	Nucleoside diphosphate kinase 7	-1.53	-0.51	0.00	0.89	173.21	0.17	3
LOC113518355	Protein rolling stone-like	0.00	3.65	5.72	3.17	86.85	0.50	10
LOC113518362	Protein rolling stone-like	0.00	1.95	3.38	1.75	89.75	0.49	12
LOC113518364	Myrosinase 1-like isoform X2	1.74	5.43	7.59	3.21	59.06	0.94	10
LOC113518369	Uncharacterized protein C45G9.7	0.00	0.95	1.50	0.83	86.90	0.29	1
LOC113518374	Lipase 1	-3.07	-2.03	0.00	1.76	86.61	0.41	9
LOC113518380	FAST kinase domain-containing protein 3, mitochondrial-like	0.00	0.74	2.22	1.28	173.21	0.76	7
LOC113518388	Facilitated trehalose transporter Tret1-like	0.00	0.83	2.50	1.45	173.21	0.23	7
LOC113518392	Nuclear pore complex protein Nup214-like	0.00	2.38	7.15	4.13	173.21	0.09	15
LOC113518394	Protein-cysteine N-palmitoyltransferase Rasp	-2.27	-0.76	0.00	1.31	173.21	0.15	3
LOC113518397	39S ribosomal protein L42, mitochondrial	0.00	0.46	1.39	0.80	173.21	0.49	1
LOC113518398	ARL14 effector protein	-1.38	-0.46	0.00	0.80	173.21	0.22	3
LOC113518403	Lachesin isoform X1	-2.73	-1.80	0.00	1.56	86.61	0.21	9
LOC113518405	Protein rolling stone-like isoform X1	0.00	6.36	9.59	5.51	86.61	0.30	14
LOC113518408	Protein tyrosine phosphatase type IVA 1	0.00	0.40	1.21	0.70	173.21	0.24	8

LOC113518414	Solute carrier organic anion transporter family member 3A1 isoform X1	0.00	0.63	1.90	1.10	173.21	0.41	8
LOC113518416	Uncharacterized protein LOC110375181	0.00	0.67	2.00	1.16	173.21	0.61	8
LOC113518420	Uncharacterized protein LOC110381236 isoform X2	-1.75	-0.58	0.00	1.01	173.21	0.08	3
LOC113518426	Uncharacterized protein LOC106137813	0.00	2.14	6.41	3.70	173.21	0.24	15
LOC113518431	Glutaminase	0.00	1.22	1.99	1.07	87.69	0.59	1
LOC113518433	Uncharacterized protein LOC106137789	0.00	2.74	5.38	2.69	98.30	0.83	12
LOC113518438	Peroxisomal acyl-coenzyme A oxidase 1	0.00	1.87	5.62	3.25	173.21	0.95	15
LOC113518439	Peroxisomal acyl-coenzyme A oxidase 1	-3.33	-1.94	0.00	1.73	89.17	0.35	9
LOC113518443	Sodium-coupled monocarboxylate transporter 1-like	0.00	1.14	3.43	1.98	173.21	0.85	7
LOC113518445	Sequestosome-1-like isoform X2	0.00	0.82	2.47	1.43	173.21	0.27	7
LOC113518448	Fructose-bisphosphate aldolase-like	-5.37	-2.69	0.00	2.69	99.80	0.11	6
LOC113518457	Protein AF-10-like isoform X1	-1.75	-0.58	0.00	1.01	173.21	0.08	3
LOC113518458	Solute carrier family 12 member 4 isoform X3	-2.00	-0.67	0.00	1.15	173.21	0.29	4
LOC113518462	Trypsin-like protein	-4.33	-1.44	0.00	2.50	173.21	0.46	4
LOC113518463	Trypsin-like protein	-2.83	-1.67	0.00	1.48	88.75	0.37	9
LOC113518465	Trypsin-like protein	-4.21	-3.30	-1.88	1.24	37.68	0.52	2
LOC113518468	Uncharacterized protein LOC110374098	0.00	0.43	1.29	0.74	173.21	0.53	1
LOC113518469	Uncharacterized protein LOC110374078	0.00	1.83	5.49	3.17	173.21	0.86	11
LOC113518476	Uncharacterized protein	0.00	2.24	6.73	3.89	173.21	0.26	5
LOC113518480	Solute carrier organic anion transporter family member 3A1-like isoform X2	-2.21	-1.30	0.00	1.15	88.91	0.55	9
LOC113518482	Uncharacterized protein LOC105842463 isoform X8	-1.88	-1.11	0.00	0.99	88.51	0.73	9
LOC113518483	Serine/arginine repetitive matrix protein 1-like	0.00	1.95	5.85	3.38	173.21	0.67	15
LOC113518490	Aquaporin PIP2-7 isoform X2	-5.51	-1.84	0.00	3.18	173.21	0.84	6
LOC113518491	Beta-arrestin-1 isoform X1	0.00	0.43	1.28	0.74	173.21	0.53	1
LOC113518496	Protein eiger	0.00	0.44	1.31	0.76	173.21	0.52	1
LOC113518497	Ankyrin repeat and MYND domain-containing protein 1	-2.69	-0.90	0.00	1.55	173.21	0.35	3

LOC113518500	Voltage-dependent calcium channel subunit Alpha-2/delta-4 isoform X1	0.00	2.97	5.21	2.68	90.28	0.89	10
LOC113518505	Uncharacterized protein LOC106129782	0.00	2.13	6.40	3.69	173.21	0.24	15
LOC113518509	Uncharacterized protein LOC106135505	0.00	2.13	4.06	2.04	95.54	0.36	12
LOC113518510	Transcriptional coactivator YAP1-like isoform X1	0.00	0.48	1.45	0.84	173.21	0.47	1
LOC113518514	DNA ligase 4	-1.16	-0.39	0.00	0.67	173.21	0.30	3
LOC113518516	Peroxisomal acyl-coenzyme A oxidase 1	-1.34	-0.45	0.00	0.78	173.21	0.24	3
LOC113518529	Voltage-dependent calcium channel subunit Alpha-2/delta-3	-2.43	-0.81	0.00	1.41	173.21	0.11	4
LOC113518530	Protein grainyhead isoform X1	-1.72	-0.57	0.00	0.99	173.21	0.44	4
LOC113518531	E3 ubiquitin-protein ligase MYCBP2	-1.75	-0.58	0.00	1.01	173.21	0.08	3
LOC113518534	Mitochondrial import receptor subunit TOM40 homolog	0.00	0.78	2.33	1.35	173.21	0.51	7
LOC113518540	Peptidoglycan-recognition protein LF-like isoform X1	0.00	0.55	1.65	0.95	173.21	0.08	8
LOC113518557	Dynein heavy chain 8, axonemal	-2.55	-1.60	0.00	1.39	87.11	0.25	9
LOC113518566	Serine proteinase stubble-like	0.00	3.22	6.68	3.35	103.97	0.68	10
LOC113518572	Acetylcholinesterase	-1.33	-0.44	0.00	0.77	173.21	0.24	3
LOC113518573	Uncharacterized protein LOC110383802	-2.09	-0.70	0.00	1.21	173.21	0.25	4
LOC113518576	Uncharacterized protein LOC106133529 isoform X1	-1.61	-0.54	0.00	0.93	173.21	0.14	3
LOC113518579	GILT-like protein 2	-5.53	-2.53	0.00	2.80	110.54	0.33	6
LOC113518612	Paired box protein Pax-1	-2.59	-0.86	0.00	1.49	173.21	0.30	3
LOC113518616	Uncharacterized protein LOC106138515	0.00	0.55	1.64	0.94	173.21	0.07	8
LOC113518618	Hemicentin-1-like	-5.91	-1.97	0.00	3.41	173.21	0.69	4
LOC113518620	Glucosylceramidase-like	-2.38	-0.79	0.00	1.37	173.21	0.20	3
LOC113518632	Hsp70-binding protein 1	0.00	0.48	1.43	0.83	173.21	0.10	8
LOC113518638	Uncharacterized protein	0.00	0.55	1.65	0.96	173.21	0.43	1
LOC113518647	Alkylglycerol monooxygenase-like	-5.07	-2.66	0.00	2.54	95.49	0.14	6
LOC113518648	Lipase member H-like	0.00	4.67	7.64	4.10	87.64	0.53	10
LOC113518649	Cytochrome P450 6B46	-2.62	-2.21	-1.93	0.36	16.28	0.97	2
LOC113518655	Lipase member H-like isoform X2	0.00	5.97	10.32	5.35	89.55	0.32	14

LOC113518657	Uncharacterized protein LOC106130394	-3.37	-1.12	0.00	1.94	173.21	0.63	3
LOC113518661	Toll-like receptor 6	0.00	0.76	2.28	1.32	173.21	0.61	7
LOC113518663	Crustacean cardioactive peptide precursor	-2.61	-0.87	0.00	1.50	173.21	0.30	3
LOC113518668	Phosphatidate phosphatase PPAPDC1B	-1.59	-0.53	0.00	0.92	173.21	0.15	3
LOC113518669	Transmembrane channel-like protein 7 isoform X1	-1.51	-0.50	0.00	0.87	173.21	0.18	3
LOC113518671	Titin	0.00	1.78	3.71	1.86	104.74	0.75	12
LOC113518672	Tkr	0.00	1.37	4.10	2.37	173.21	0.13	11
LOC113518681	Hypothetical protein OBRU01_15499	-5.57	-2.76	0.00	2.78	100.93	0.12	6
LOC113518684	Thyrotroph embryonic factor isoform X5	0.00	0.66	1.97	1.14	173.21	0.55	8
LOC113518685	Serine protease snake	-1.56	-0.52	0.00	0.90	173.21	0.16	3
LOC113518689	Acheron	-7.45	-5.85	-4.98	1.39	23.69	0.67	2
LOC113518690	Gamma-glutamyltranspeptidase 1-like isoform X1	0.00	2.98	4.95	2.63	88.12	0.93	10
LOC113518691	Baculoviral IAP repeat-containing protein 6	0.00	0.39	1.18	0.68	173.21	0.25	8
LOC113518692	Uncharacterized protein LOC106136531	-1.32	-0.44	0.00	0.76	173.21	0.25	3
LOC113518702	Uncharacterized protein LOC110378268	0.00	0.83	2.49	1.44	173.21	0.25	7
LOC113518704	Uncharacterized protein LOC106720002 isoform X1	-3.24	-1.08	0.00	1.87	173.21	0.18	4
LOC113518711	UDP-N-acetylglucosamine pyrophosphorylase	-1.64	-0.55	0.00	0.95	173.21	0.13	3
LOC113518712	Ubiquitin-like protein FUBI	0.00	1.95	5.86	3.39	173.21	0.66	15
LOC113518715	Uncharacterized protein LOC110375196	-2.61	-1.69	0.00	1.47	86.71	0.20	9
LOC113518736	Nucleolar pre-ribosomal-associated protein 1	0.00	0.39	1.17	0.68	173.21	0.25	8
LOC113518747	Saccharopine dehydrogenase-like oxidoreductase	0.00	0.78	2.33	1.35	173.21	0.50	7
LOC113518748	Serine/threonine-protein kinase dyrk2 isoform X1	0.00	0.49	1.46	0.84	173.21	0.08	8
LOC113518749	Hypothetical protein KGM_203921	-6.11	-4.07	0.00	3.52	86.60	0.71	6
LOC113518753	Ras-related C3 botulinum toxin substrate 1	0.00	0.48	1.45	0.84	173.21	0.09	8
LOC113518756	Uncharacterized protein LOC101737963	0.00	0.67	2.01	1.16	173.21	0.64	8
LOC113518757	Amino acid transporter	0.00	5.57	9.51	4.96	89.07	0.50	14

LOC113518758	Uncharacterized protein LOC106122557	0.00	0.99	1.67	0.88	88.54	0.20	1
LOC113518761	Kynurenine formamidase-like isoform X1	-1.32	-0.44	0.00	0.76	173.21	0.70	4
LOC113518766	Scolexin precursor	-1.73	-0.58	0.00	1.00	173.21	0.44	4
LOC113518767	Hypothetical protein KGM_208267	-2.36	-1.35	0.00	1.21	90.17	0.34	9
LOC113518775	Cubilin	-2.59	-1.72	0.00	1.49	86.60	0.16	9
LOC113518778	Uncharacterized protein LOC106136399 precursor	0.00	0.71	2.14	1.23	173.21	0.99	8
LOC113518791	MOB kinase activator-like 1	-1.30	-0.43	0.00	0.75	173.21	0.25	3
LOC113518792	ATP-dependent RNA helicase DDX18-like	0.00	0.81	2.44	1.41	173.21	0.32	7
LOC113518797	Uncharacterized protein LOC106133123	-2.17	-1.20	0.00	1.11	91.79	0.78	9
LOC113518799	DNA replication licensing factor Mcm3 isoform X2	-1.41	-0.47	0.00	0.81	173.21	0.22	3
LOC113518800	Chaoptin	0.00	1.82	3.46	1.74	95.60	0.40	12
LOC113518801	Glutamate dehydrogenase	0.00	0.56	1.68	0.97	173.21	0.11	8
LOC113518804	Peptide transporter family 1 isoform X1	-2.47	-0.82	0.00	1.43	173.21	0.24	3
LOC113518806	Protocadherin-15	0.00	1.00	2.99	1.73	173.21	0.22	7
LOC113518809	Pyruvate dehydrogenase E1 component subunit beta, mitochondrial	0.00	0.46	1.39	0.80	173.21	0.49	1
LOC113518815	NADP-dependent malic enzyme-like isoform X2	-1.60	-0.53	0.00	0.92	173.21	0.14	3
LOC113518819	NADP-dependent malic enzyme-like	0.00	1.10	1.92	0.99	90.17	0.71	1
LOC113518820	Titin	-1.79	-0.08	1.54	1.66	1981.40	0.99	4
LOC113518829	Glucosidase 2 subunit beta	0.00	0.52	1.55	0.89	173.21	0.02	8
LOC113518833	TAF5-like RNA polymerase II p300/CBP-associated factor-associated factor 65 kDa subunit 5L	-2.01	-0.67	0.00	1.16	173.21	0.03	3
LOC113518834	Mitochondrial pyruvate carrier 1	0.00	0.50	1.51	0.87	173.21	0.46	1
LOC113518841	Protein CREG1	0.00	1.25	2.14	1.11	89.29	0.70	1
LOC113518851	Iodotyrosine dehalogenase 1	-1.63	-0.54	0.00	0.94	173.21	0.13	3
LOC113518862	Carboxylesterase 5A	-4.77	-2.33	0.00	2.39	102.67	0.49	6
LOC113518876	Uncharacterized protein LOC106136779	0.00	0.61	1.82	1.05	173.21	0.29	8
LOC113518881	RING finger and transmembrane domain-containing protein 2	0.00	0.98	2.93	1.69	173.21	0.16	7

LOC113518889	Uncharacterized protein LOC106137666	-2.47	-0.82	0.00	1.43	173.21	0.10	4
LOC113518890	Uncharacterized protein LOC106134782	0.00	2.21	6.62	3.82	173.21	0.13	15
LOC113518892	Venom peptide BmKAPI-like	0.00	2.36	3.91	2.08	88.02	0.19	12
LOC113518896	Apolipoprotein D	-1.84	-0.61	0.00	1.06	173.21	0.04	3
LOC113518905	Angiotensin-converting enzyme-like	0.00	0.85	2.55	1.47	173.21	0.18	7
LOC113518906	Uncharacterized protein	0.00	2.49	4.34	2.24	90.01	0.35	12
LOC113518908	AarF domain-containing protein kinase 4	0.00	0.76	2.28	1.32	173.21	0.61	7
LOC113518909	Protein sidekick-1-like	0.00	2.06	6.17	3.56	173.21	0.39	15
LOC113518915	Uncharacterized protein	-3.31	-1.10	0.00	1.91	173.21	0.20	4
LOC113518916	Kynurenine formamidase	-2.57	-0.86	0.00	1.48	173.21	0.29	3
LOC113518918	Sparc	-3.56	-1.19	0.00	2.06	173.21	0.69	3
LOC113518925	2-hydroxy-6-oxononadienedioate/2-hydroxy-6-oxononatrienedioate hydrolase isoform X1	-1.78	-1.06	0.00	0.94	88.35	0.83	9
LOC113518936	Uncharacterized protein	0.00	1.38	4.15	2.39	173.21	0.10	11
LOC113518938	Crossover junction endodeoxyribonuclease RuvC	0.00	1.17	3.50	2.02	173.21	0.99	11
LOC113518948	Hypothetical protein RR46_03373	0.00	1.43	2.41	1.27	88.48	0.95	12
LOC113518959	Glutathione S-transferase delta 4	0.00	3.42	7.98	4.11	120.34	0.79	15
LOC113518962	Lipid storage droplets surface-binding protein 1 isoform X1	-2.81	-1.59	0.00	1.44	90.56	0.48	9
LOC113518963	Zonadhesin-like isoform X1	2.50	5.38	7.06	2.51	46.63	0.75	13
LOC113518992	E3 ubiquitin-protein ligase RNF19B-like isoform X1	0.00	0.58	1.75	1.01	173.21	0.19	8
LOC113518995	Protein zwilch isoform X1	-2.02	-0.67	0.00	1.16	173.21	0.03	3
LOC113519004	Suppressor of lurcher protein 1-like	-4.55	-2.20	0.00	2.28	103.74	0.69	6
LOC113519008	Protein toll-like	-2.59	-0.86	0.00	1.49	173.21	0.30	3
LOC113519015	Pre-mRNA-splicing factor 18	0.00	0.97	1.54	0.85	87.02	0.28	1
LOC113519016	Uncharacterized protein C6orf203 homolog	0.00	1.16	1.82	1.01	86.90	0.50	1
LOC113519018	Uncharacterized protein LOC110375652	0.00	2.20	3.78	1.96	89.40	0.14	12
LOC113519020	Proline-rich receptor-like protein kinase PERK11-like	-5.60	-1.87	0.00	3.23	173.21	0.66	4
LOC113519022	Proton-coupled folate transporter-like	-1.81	-0.60	0.00	1.04	173.21	0.39	4

LOC113519024	Microtubule-associated protein tau	-2.15	-1.29	0.00	1.14	88.21	0.40	9
LOC113519026	Protein unc-80 homolog	-1.43	-0.48	0.00	0.82	173.21	0.21	3
LOC113519028	4-coumarate--CoA ligase 1-like	2.62	4.44	6.81	2.14	48.24	0.59	13
LOC113519029	Lachesin isoform X2	0.00	0.46	1.37	0.79	173.21	0.50	1
LOC113519031	Peroxidase-like isoform X2	0.00	3.01	5.29	2.72	90.46	0.81	10
LOC113519032	Uncharacterized protein LOC106137634	0.00	2.68	5.31	2.66	99.03	0.77	12
LOC113519035	Uncharacterized protein LOC106137625	-5.36	-1.79	0.00	3.10	173.21	0.88	6
LOC113519037	Uncharacterized protein LOC110379989 isoform X1	-1.49	-0.50	0.00	0.86	173.21	0.58	4
LOC113519049	Uncharacterized protein	-1.28	-0.43	0.00	0.74	173.21	0.26	3
LOC113519050	Glutathione S-transferase unclassified 1	-5.63	-1.88	0.00	3.25	173.21	0.82	6
LOC113519053	Vacuolar protein sorting-associated protein 16 homolog	-1.69	-0.56	0.00	0.97	173.21	0.11	3
LOC113519055	Polyserase-2-like	-5.77	-3.74	-2.26	1.82	48.60	0.84	2
LOC113519068	Uncharacterized protein LOC110385045	0.00	0.58	1.75	1.01	173.21	0.20	8
LOC113519071	Arabinose-proton symporter-like	-2.85	-0.95	0.00	1.65	173.21	0.42	3
LOC113519073	Cyclin-dependent kinase 2	-1.79	-0.60	0.00	1.03	173.21	0.07	3
LOC113519081	Uncharacterized protein	0.00	1.50	4.51	2.60	173.21	0.13	11
LOC113519083	Mitogen-activated protein kinase kinase kinase 4	0.00	0.45	1.34	0.78	173.21	0.16	8
LOC113519084	Hypothetical protein KGM_213880	0.00	0.97	2.92	1.69	173.21	0.15	7
LOC113519093	Uncharacterized protein LOC106136406	-4.80	-2.25	0.00	2.41	107.26	0.57	6
LOC113519094	Anionic antimicrobial peptide 2	-7.61	-3.69	0.00	3.81	103.38	0.48	6
LOC113519096	Uncharacterized protein LOC106104211	0.00	0.72	2.17	1.25	173.21	0.90	7
LOC113519098	D-aspartate oxidase	-4.63	-2.33	0.00	2.32	99.54	0.52	6
LOC113519112	MATH and LRR domain-containing protein PFE0570w-like	-5.78	-3.06	0.00	2.91	95.01	0.17	6
LOC113519117	Cytochrome P450 9G3	-4.83	-2.54	0.00	2.42	95.29	0.27	6
LOC113519122	Uncharacterized protein LOC106130390	-4.45	-2.48	0.00	2.27	91.58	0.49	6
LOC113519133	Uncharacterized protein	-3.90	-2.53	0.00	2.20	86.69	0.85	6
LOC113519141	Sodium-independent sulfate anion transporter isoform X1	0.00	1.99	5.97	3.44	173.21	0.56	15
LOC113519144	Microtubule-associated protein futsch	0.00	0.80	2.39	1.38	173.21	0.40	7

LOC113519160	Retinoblastoma-binding protein 5 homolog	-1.31	-0.44	0.00	0.76	173.21	0.25	3
LOC113519163	Kinesin-like protein KIF23	-2.18	-0.73	0.00	1.26	173.21	0.11	3
LOC113519166	Procollagen-lysine,2-oxoglutarate 5-dioxygenase 3 isoform X2	-1.16	-0.39	0.00	0.67	173.21	0.30	3
LOC113519168	Fanconi anemia group I protein homolog	-2.46	-0.82	0.00	1.42	173.21	0.24	3
LOC113519171	Facilitated trehalose transporter Tret1	-4.54	-1.51	0.00	2.62	173.21	0.91	3
LOC113519172	Apyrase-like	-2.76	-0.92	0.00	1.59	173.21	0.38	3
LOC113519174	Deoxycytidylate deaminase	-7.67	-2.56	0.00	4.43	173.21	0.69	6
LOC113519185	Long-chain-fatty-acid--CoA ligase 4 isoform X2	0.00	0.58	1.74	1.00	173.21	0.18	8
LOC113519196	Cytochrome P450 6B46	0.00	1.07	3.20	1.85	173.21	0.45	7
LOC113519197	Cytochrome P450 6B46	0.00	0.88	2.64	1.53	173.21	0.07	7
LOC113519215	Uncharacterized protein LOC101735755 isoform X1	-1.34	-0.45	0.00	0.78	173.21	0.68	4
LOC113519218	60S ribosomal protein L38	-1.25	-0.42	0.00	0.72	173.21	0.27	3
LOC113519221	Lipase member H-like isoform X1	0.00	4.17	7.05	3.70	88.66	0.24	10
LOC113519224	Cytochrome P450 6B7-like	-3.03	-1.75	0.00	1.57	89.65	0.15	9
LOC113519225	Sulfotransferase 1C4-like	-3.08	-1.45	0.00	1.55	106.79	0.58	9
LOC113519226	Uncharacterized protein LOC106720246	-5.08	-2.72	0.00	2.56	94.07	0.13	6
LOC113519227	Uncharacterized protein LOC106130388	-2.40	-1.30	0.00	1.21	93.20	0.46	9
LOC113519229	Protein SYS1 homolog	0.00	0.39	1.17	0.67	173.21	0.26	8
LOC113519240	Autophagy-related protein 13 homolog	-2.13	-1.40	0.00	1.21	86.62	0.27	9
LOC113519241	Cytochrome P450 protein	0.00	1.35	4.06	2.34	173.21	0.17	11
LOC113519242	Cytochrome P450 9e2-like	-2.08	-1.18	0.00	1.07	90.58	0.63	9
LOC113519243	Trypsin 5G1-like isoform X1	-2.69	-0.90	0.00	1.55	173.21	0.35	3
LOC113519245	Protein FAM57A	0.00	0.56	1.67	0.96	173.21	0.10	8
LOC113519252	Exonuclease GOR-like isoform X1	0.00	0.74	2.23	1.29	173.21	0.73	7
LOC113519265	Multiple C2 and transmembrane domain-containing protein 1 isoform X5	-3.03	-1.60	0.00	1.52	95.12	0.25	9
LOC113519277	ATP-binding cassette sub-family B member 3	0.00	1.46	4.37	2.53	173.21	0.07	11
LOC113519287	Uncharacterized protein LOC106130324	-4.62	-1.54	0.00	2.67	173.21	0.52	4
LOC113519291	Transcription termination factor 2 isoform	-1.95	-0.65	0.00	1.13	173.21	0.01	3

	X2							
LOC113519293	Synapse-associated protein of 47 kDa isoform X3	-1.29	-0.43	0.00	0.75	173.21	0.25	3
LOC113519295	Zonadhesin-like	0.00	1.26	3.78	2.18	173.21	0.46	11
LOC113519298	Fatty acid synthase-like	-1.95	-0.65	0.00	1.13	173.21	0.32	4
LOC113519302	Semaphorin-5A	-2.61	-1.56	0.00	1.38	88.36	0.06	9
LOC113519309	Uncharacterized protein LOC105841534	0.00	2.42	7.27	4.20	173.21	0.12	15
LOC113519311	Myotubularin-related protein 10-B	0.00	0.54	1.62	0.94	173.21	0.06	8
LOC113519315	L-lactate dehydrogenase	0.00	0.96	2.88	1.66	173.21	0.13	7
LOC113519316	Uncharacterized protein LOC106129825	0.00	0.41	1.23	0.71	173.21	0.22	8
LOC113519323	Retrotransposable element Tf2 155 kDa protein type 2	-3.08	-1.03	0.00	1.78	173.21	0.51	3
LOC113519330	G-protein coupled receptor 179	-3.63	-1.21	0.00	2.09	173.21	0.71	3
LOC113519337	Globin isoform X1	0.00	5.61	9.23	4.93	87.84	0.46	14
LOC113519349	Matrix metalloproteinase-25-like	-2.71	-1.42	0.00	1.36	95.62	0.35	9
LOC113519350	Hypothetical protein KGM_206883	-1.73	-0.58	0.00	1.00	173.21	0.09	3
LOC113519360	23 kDa integral membrane protein-like	0.00	0.55	1.65	0.95	173.21	0.09	8
LOC113519366	G2/mitotic-specific cyclin-B3	-2.22	-0.74	0.00	1.28	173.21	0.13	3
LOC113519367	Protein SMG7-like isoform X2	0.00	0.59	1.77	1.02	173.21	0.22	8
LOC113519369	Protein takeout	-6.26	-3.85	-2.03	2.18	56.55	0.90	6
LOC113519372	Calcium/calmodulin-dependent protein kinase kinase 2 isoform X3	-2.19	-0.73	0.00	1.27	173.21	0.11	3
LOC113519377	Facilitated trehalose transporter Tret1-like	-6.10	-3.83	0.00	3.34	87.07	0.73	6
LOC113519382	Uncharacterized protein LOC106137147	0.00	1.76	2.91	1.55	87.98	0.57	12
LOC113519383	Protein THEM6-like	0.00	0.80	2.39	1.38	173.21	0.40	7
LOC113519387	Latent-transforming growth factor beta-binding protein 4-like isoform X1	0.00	3.01	5.94	2.97	98.55	0.75	10
LOC113519389	Forkhead box protein P3	-2.65	-1.53	0.00	1.37	89.75	0.11	9
LOC113519401	Protein pangolin, isoforms A/H/I/S isoform X3	-1.68	-0.56	0.00	0.97	173.21	0.11	3
LOC113519405	Uncharacterized protein LOC110373911 isoform X6	0.00	1.26	3.78	2.18	173.21	0.47	11
LOC113519414	Potassium channel subfamily K member 9-	0.00	0.44	1.31	0.76	173.21	0.18	8

	like							
LOC113519416	Hypothetical protein KGM_208609A	-2.74	-0.91	0.00	1.58	173.21	0.37	3
LOC113519417	Facilitated trehalose transporter Tret1-2 homolog	0.00	1.55	4.64	2.68	173.21	0.19	11
LOC113519418	Nuclease HARBI1	-1.28	-0.43	0.00	0.74	173.21	0.26	3
LOC113519419	Heat shock protein 68-like isoform X1	0.00	1.50	4.51	2.60	173.21	0.13	11
LOC113519422	Heat shock protein 24.3	0.00	3.55	8.34	4.30	121.13	0.82	15
LOC113519423	Heat shock protein 24.3	0.00	1.66	4.98	2.87	173.21	0.36	11
LOC113519432	Protein lethal(2)essential for life-like	0.00	3.03	7.31	3.82	126.08	0.53	15
LOC113519435	Tubulin beta chain isoform X1	0.00	1.99	5.96	3.44	173.21	0.15	5
LOC113519436	Protein lethal(2)essential for life-like	0.00	1.77	5.30	3.06	173.21	0.64	11
LOC113519437	IQ motif and SEC7 domain-containing protein 2 isoform X1	0.00	0.53	1.60	0.93	173.21	0.44	1
LOC113519438	Small heat shock protein 19.7	0.00	2.94	7.28	3.84	130.50	0.45	15
LOC113519444	Solute carrier family 41 member 2-like isoform X1	0.00	1.60	2.90	1.47	92.12	0.56	12
LOC113519445	Latrophilin Cirl	0.00	0.42	1.26	0.73	173.21	0.21	8
LOC113519448	Nuclear factor interleukin-3-regulated protein	0.00	1.20	3.61	2.09	173.21	0.74	11
LOC113519449	Protein lethal(2)essential for life-like	0.00	3.50	10.51	6.07	173.21	0.58	15
LOC113519454	Chloride channel protein 2 isoform X1	-2.51	-0.84	0.00	1.45	173.21	0.26	3
LOC113519456	Transcriptional activator protein Pur-alpha isoform X4	0.00	0.44	1.33	0.77	173.21	0.51	1
LOC113519466	Uncharacterized protein	0.00	1.40	4.21	2.43	173.21	0.05	11
LOC113519469	Uncharacterized protein LOC106135241 isoform X1	0.00	0.48	1.43	0.83	173.21	0.10	8
LOC113519474	Uncharacterized protein OBRU01_08063	-6.36	-2.12	0.00	3.67	173.21	0.74	6
LOC113519475	Ankyrin-3	0.00	0.61	1.83	1.06	173.21	0.31	8
LOC113519476	Hypothetical protein 2 - silkworm transposon mag	0.00	0.50	1.51	0.87	173.21	0.05	8
LOC113519477	Facilitated trehalose transporter Tret1-like	0.00	1.40	4.19	2.42	173.21	0.07	11
LOC113519494	Prohormone-3 isoform X1	-3.26	-1.09	0.00	1.88	173.21	0.59	3
LOC113519506	Uncharacterized protein LOC101746861	-2.78	-0.93	0.00	1.61	173.21	0.39	3

LOC113519511	Uncharacterized protein LOC106129042	0.00	0.51	1.54	0.89	173.21	0.45	1
LOC113519512	Uncharacterized transmembrane protein DDB_G0289901-like	-6.00	-4.36	-3.50	1.42	32.71	0.48	2
LOC113519518	Phosphatidylinositol 4,5-bisphosphate 3-kinase catalytic subunit delta isoform isoform X1	0.00	0.63	1.90	1.10	173.21	0.42	8
LOC113519521	Protein PFC0760c-like isoform X2	0.00	0.41	1.24	0.72	173.21	0.22	8
LOC113519522	Zinc transporter 2-like isoform X1	-5.82	-2.85	0.00	2.91	102.39	0.16	6
LOC113519524	Synaptic vesicle glycoprotein 2B-like	-5.92	-3.94	0.00	3.41	86.60	0.69	6
LOC113519526	Zinc finger protein 525-like	-1.67	-1.01	0.00	0.89	88.04	0.95	9
LOC113519530	Uncharacterized protein LOC108019554	-5.22	-1.74	0.00	3.01	173.21	0.92	6
LOC113519539	Uncharacterized protein LOC106138567	0.00	0.91	2.72	1.57	173.21	0.49	1
LOC113519546	Lon protease homolog, mitochondrial isoform X1	0.00	0.86	2.59	1.50	173.21	0.12	7
LOC113519548	Brachyurin-like	-5.37	-1.79	0.00	3.10	173.21	0.63	4
LOC113519549	Uncharacterized protein LOC106134838 isoform X1	-2.59	-0.86	0.00	1.50	173.21	0.30	3
LOC113519555	Krueppel-like factor 7 isoform X1	0.00	1.03	1.60	0.89	86.81	0.32	1
LOC113519558	Uncharacterized protein LOC106142421	-4.23	-2.85	-1.94	1.21	42.64	0.69	2
LOC113519571	Serpin B5-like	-4.02	-2.38	0.00	2.11	88.63	0.76	9
LOC113519574	Immulectin-2	0.00	0.71	2.12	1.22	173.21	0.40	1
LOC113519576	Autophagy-related protein 2 homolog B	-1.36	-0.45	0.00	0.79	173.21	0.23	3
LOC113519582	M-phase inducer phosphatase-like	-3.23	-1.08	0.00	1.87	173.21	0.58	3
LOC113519587	Serine protease HTRA2, mitochondrial-like	0.00	1.29	2.31	1.18	91.39	0.43	1
LOC113519591	Uncharacterized protein LOC106141788	-2.42	-1.30	0.00	1.22	94.24	0.49	9
LOC113519593	Myrosinase 1-like	-3.63	-1.81	0.00	1.81	100.08	0.47	9
LOC113519595	RING finger and SPRY domain-containing protein 1-like	0.00	0.55	1.64	0.95	173.21	0.07	8
LOC113519600	C-1-tetrahydrofolate synthase, cytoplasmic isoform X2	0.00	1.05	3.16	1.82	173.21	0.40	7
LOC113519601	Protein phosphatase 1 regulatory subunit 12A	0.00	3.99	6.15	3.46	86.69	0.31	10
LOC113519603	Uncharacterized protein LOC106136193	0.00	1.37	2.81	1.41	102.69	0.54	1
LOC113519604	Solute carrier family 35 member C2	-1.24	-0.41	0.00	0.72	173.21	0.27	3

LOC113519607	Transient receptor potential channel pyrexia isoform X1	0.00	1.99	3.45	1.79	89.64	0.12	12
LOC113519615	Uncharacterized protein LOC110379113	0.00	0.45	1.34	0.77	173.21	0.51	1
LOC113519620	Transcription factor kayak isoform X7	0.00	1.04	1.75	0.92	88.43	0.60	1
LOC113519622	Chromosome-associated kinesin KIF4 isoform X3	-1.18	-0.39	0.00	0.68	173.21	0.29	3
LOC113519628	Uncharacterized protein LOC106136198	0.00	1.96	3.41	1.76	89.84	0.14	12
LOC113519637	Lipopolysaccharide-induced tumor necrosis factor-alpha factor homolog	0.00	0.44	1.31	0.76	173.21	0.18	8
LOC113519639	Zinc transporter ZIP1-like	-3.03	-1.01	0.00	1.75	173.21	0.50	3
LOC113519642	Serine protease easter-like isoform X1	0.00	0.94	1.55	0.83	87.70	0.55	1
LOC113519654	Senecionine N-oxygenase-like	0.00	1.29	2.08	1.13	87.31	0.50	1
LOC113519655	Carboxypeptidase A	-6.90	-4.59	0.00	3.98	86.60	0.78	6
LOC113519661	Protein NDRG3-like	-2.91	-0.97	0.00	1.68	173.21	0.44	3
LOC113519667	RNA helicase Mov10l1 isoform X2	-1.37	-0.46	0.00	0.79	173.21	0.23	3
LOC113519668	Beta-mannosidase-like isoform X1	-1.32	-0.44	0.00	0.76	173.21	0.25	3
LOC113519674	N-acetylneuraminate lyase-like	-5.65	-1.88	0.00	3.26	173.21	0.66	4
LOC113519683	CXXC-type Zinc finger protein 1	-1.47	-0.98	0.00	0.85	86.61	0.92	9
LOC113519685	Uncharacterized protein LOC106129641	-1.90	-0.63	0.00	1.10	173.21	0.02	3
LOC113519687	Protein UBASH3A homolog isoform X1	-1.21	-0.40	0.00	0.70	173.21	0.28	3
LOC113519691	Hemicentin-1	-1.26	-0.42	0.00	0.73	173.21	0.27	3
LOC113519703	Uncharacterized protein LOC106136222	-3.90	-2.58	0.00	2.23	86.61	0.86	6
LOC113519717	Uncharacterized protein LOC110373446	3.29	8.26	11.62	4.39	53.16	0.37	14
LOC113519720	Zinc finger protein 541	0.00	3.12	5.85	2.95	94.37	0.61	10
LOC113519728	N-acylneuraminate-9-phosphatase	0.00	0.53	1.59	0.92	173.21	0.03	8
LOC113519736	Ecdysteroid-regulated 16 kDa protein-like	-2.67	-1.36	0.00	1.33	97.98	0.47	9
LOC113519738	Zinc transporter ZIP1-like	0.00	2.18	3.29	1.88	86.61	0.15	12
LOC113519740	Transcription initiation factor IIA subunit 2	0.00	0.48	1.44	0.83	173.21	0.48	1
LOC113519745	Uncharacterized protein LOC110373384	0.00	0.46	1.39	0.80	173.21	0.13	8
LOC113519746	Protein distal antenna-like	0.00	0.51	1.53	0.88	173.21	0.45	1
LOC113519751	Uncharacterized protein LOC106136278	-3.72	-1.24	0.00	2.15	173.21	0.74	3
LOC113519755	Uncharacterized protein LOC106136237	-3.23	-1.08	0.00	1.86	173.21	0.57	3
LOC113519756	G-protein coupled receptor Mth2-like	0.00	1.93	3.01	1.68	86.82	0.17	12

LOC113519758	PH domain-containing protein DDB_G0287875-like	-1.42	-0.47	0.00	0.82	173.21	0.21	3
LOC113519762	Elongation of very long chain fatty acids protein AAEL008004-like	-2.91	-0.97	0.00	1.68	173.21	0.44	3
LOC113519768	Calcium-binding mitochondrial carrier protein SCaMC-2	0.00	2.07	3.77	1.91	92.22	0.21	12
LOC113519771	Uncharacterized protein LOC105382408	-1.49	-0.50	0.00	0.86	173.21	0.58	4
LOC113519773	CncC	0.00	0.47	1.40	0.81	173.21	0.12	8
LOC113519787	Dynein beta chain, ciliary-like	0.00	1.56	2.59	1.38	87.97	0.66	12
LOC113519788	DNA replication licensing factor Mcm5	-1.80	-0.60	0.00	1.04	173.21	0.06	3
LOC113519789	1,5-anhydro-D-fructose reductase-like	-3.68	-2.44	0.00	2.11	86.61	0.95	9
LOC113519792	Uncharacterized protein LOC105387048	0.00	1.21	3.64	2.10	173.21	0.69	11
LOC113519793	ETS-like protein pointed isoform X3	-2.43	-0.81	0.00	1.40	173.21	0.22	3
LOC113519795	UDP-glucuronosyltransferase 1-1-like	0.00	0.68	2.05	1.18	173.21	0.40	1
LOC113519796	N-Alpha-acetyltransferase 40	-1.95	-0.65	0.00	1.12	173.21	0.01	3
LOC113519797	Zinc finger protein 879-like	0.00	4.03	6.62	3.54	87.77	0.18	10
LOC113519799	Serine protease easter-like	0.00	0.48	1.45	0.84	173.21	0.09	8
LOC113519802	Epidermal cell surface receptor	0.00	0.97	2.91	1.68	173.21	0.14	7
LOC113519808	Protein pellino	0.00	0.51	1.52	0.88	173.21	0.04	8
LOC113519810	Myosin light chain kinase	-1.94	-1.23	0.00	1.07	86.92	0.48	9
LOC113519812	Uncharacterized protein LOC106136143	0.00	0.84	2.51	1.45	173.21	0.22	7
LOC113519815	Homeobox protein MSH-D-like	-3.03	-1.01	0.00	1.75	173.21	0.50	3
LOC113519816	Unc-112-related protein-like	0.00	1.20	1.85	1.04	86.71	0.49	1
LOC113519817	Uncharacterized protein LOC110377460	-1.28	-0.43	0.00	0.74	173.21	0.26	3
LOC113519823	MATH and LRR domain-containing protein PFE0570w-like	0.00	1.20	2.14	1.09	91.06	0.77	1
LOC113519827	39S ribosomal protein L9, mitochondrial isoform X1	0.00	0.46	1.38	0.80	173.21	0.49	1
LOC113519837	Uncharacterized protein LOC105389589	0.00	2.39	4.44	2.24	93.69	0.39	12
LOC113519838	Myosin-I heavy chain-like	0.00	1.00	1.64	0.88	87.80	0.23	1
LOC113519841	SPARC-related modular calcium-binding protein 1	0.00	0.55	1.65	0.95	173.21	0.43	1
LOC113519844	Hypothetical protein KGM_202689	0.00	0.95	2.84	1.64	173.21	0.10	7

LOC113519846	N-acetylneuraminate lyase-like	-1.21	-0.40	0.00	0.70	173.21	0.28	3
LOC113519854	Protein serrate	-1.25	-0.42	0.00	0.72	173.21	0.27	3
LOC113519860	Muscle M-line assembly protein unc-89-like	-2.21	-0.74	0.00	1.28	173.21	0.12	3
LOC113519864	Bifunctional glutamate/proline--tRNA ligase	0.00	0.64	1.93	1.12	173.21	0.47	8
LOC113519869	Serine protease gd-like	0.00	5.32	9.52	4.86	91.26	0.73	14
LOC113519875	Tryptophan--tRNA ligase, cytoplasmic	0.00	0.45	1.34	0.77	173.21	0.16	8
LOC113519878	Papilin like protein	-3.03	-1.01	0.00	1.75	173.21	0.50	3
LOC113519881	Steroid hormone receptor ERR1 isoform X3	-1.42	-0.47	0.00	0.82	173.21	0.63	4
LOC113519887	Acetyl-coenzyme A synthetase	0.00	0.56	1.68	0.97	173.21	0.11	8
LOC113519888	Tryptophanyl-tRNA synthetase	0.00	0.60	1.81	1.05	173.21	0.27	8
LOC113519903	Hemicentin-1-like	-1.51	-0.50	0.00	0.87	173.21	0.18	3
LOC113519914	Vacuolar protein sorting-associated protein 13D	0.00	0.91	2.74	1.58	173.21	0.03	7
LOC113519915	Beta-hexosaminidase	-4.85	-2.17	0.00	2.46	113.59	0.65	6
LOC113519918	Sodium-dependent serotonin transporter	-2.13	-1.26	0.00	1.12	88.72	0.45	9
LOC113519921	E3 ubiquitin-protein ligase UBR3	0.00	0.46	1.38	0.80	173.21	0.14	8
LOC113519923	Cationic amino acid transporter 2 isoform X1	0.00	0.83	2.50	1.44	173.21	0.24	7
LOC113519931	Protein yellow	0.00	4.52	7.84	4.05	89.76	0.42	10
LOC113519932	Uncharacterized protein LOC105388803 isoform X3	-3.17	-1.06	0.00	1.83	173.21	0.55	3
LOC113519934	UDP-xylose and UDP-N-acetylglucosamine transporter isoform X1	-1.21	-0.40	0.00	0.70	173.21	0.28	3
LOC113519946	Cationic amino acid transporter 2	-6.75	-3.70	0.00	3.42	92.42	0.46	6
LOC113519951	Glutathione S-transferase sigma 5	-2.31	-0.77	0.00	1.34	173.21	0.17	3
LOC113519959	Uncharacterized protein LOC105842437	0.00	0.61	1.84	1.06	173.21	0.41	1
LOC113519961	Facilitated trehalose transporter Tret1 isoform X3	0.00	2.46	4.03	2.16	87.65	0.37	12
LOC113519981	C-MYC	0.00	0.42	1.25	0.72	173.21	0.21	8
LOC113519987	Uncharacterized protein	0.00	0.89	2.68	1.55	173.21	0.04	7
LOC113519988	Phosducin-like protein	0.00	1.22	1.89	1.06	86.78	0.51	1
LOC113519993	Facilitated trehalose transporter Tret1-like	0.00	0.64	1.91	1.10	173.21	0.43	8
LOC113519999	DAXX	0.00	0.89	1.42	0.78	87.03	0.29	1

LOC113520000	Gamma-glutamyltranspeptidase 1-like isoform X1	0.00	2.65	4.54	2.36	89.18	0.51	12
LOC113520001	Small heat shock protein	0.00	1.25	3.76	2.17	173.21	0.50	11
LOC113520013	Calponin homology domain-containing protein DDB_G0272472-like	-2.24	-0.75	0.00	1.29	173.21	0.13	3
LOC113520016	Dihydrofolate reductase	-1.96	-0.65	0.00	1.13	173.21	0.01	3
LOC113520017	Glycosyltransferase 25 family member isoform X1	-1.57	-0.97	0.00	0.85	87.56	0.99	3
LOC113520021	Zinc finger DNA binding protein	-2.65	-1.67	0.00	1.45	87.02	0.09	9
LOC113520028	Mesencephalic astrocyte-derived neurotrophic factor homolog	0.00	1.48	2.41	1.29	87.64	0.87	12
LOC113520040	Carboxypeptidase B-like	-7.96	-5.30	0.00	4.59	86.60	0.84	6
LOC113520054	GILT-like protein F37H8.5	-6.67	-3.65	0.00	3.38	92.63	0.44	6
LOC113520065	Diamine acetyltransferase 2-like	0.00	1.88	3.77	1.89	100.20	0.54	12
LOC113520067	Transcription factor GATA-5-like isoform X1	0.00	0.49	1.48	0.85	173.21	0.47	1
LOC113520069	Uncharacterized protein	-2.05	-0.68	0.00	1.18	173.21	0.05	3
LOC113520070	Uncharacterized protein DDB_G0282133	-2.54	-0.85	0.00	1.46	173.21	0.27	3
LOC113520073	Uncharacterized protein LOC106136669 isoform X1	0.00	4.31	6.51	3.73	86.61	0.44	10
LOC113520075	Beta-1,3-glucan recognition protein	0.00	0.52	1.55	0.90	173.21	0.02	8
LOC113520078	Adenylate cyclase type 2	0.00	3.97	6.03	3.44	86.63	0.41	10
LOC113520080	Androgen-dependent TFPI-regulating protein-like	-7.17	-3.52	0.00	3.58	101.89	0.42	6
LOC113520087	Ubiquitin carboxyl-terminal hydrolase 7-like isoform X1	-5.94	-1.98	0.00	3.43	173.21	0.78	6
LOC113520094	Adenosine deaminase CECR1	-2.99	-1.69	0.00	1.53	90.61	0.14	9
LOC113520096	Ester hydrolase C11orf54 homolog	0.00	0.55	1.66	0.96	173.21	0.43	1
LOC113520100	Bifunctional glutamate/proline--tRNA ligase-like	0.00	0.47	1.41	0.81	173.21	0.12	8
LOC113520101	Uncharacterized family 31 glucosidase KIAA1161 isoform X1	-2.43	-0.81	0.00	1.40	173.21	0.22	3
LOC113520105	D-3-phosphoglycerate dehydrogenase	-2.81	-1.77	0.00	1.54	87.03	0.14	9
LOC113520110	Niemann-Pick C1 protein-like	0.00	0.40	1.20	0.69	173.21	0.24	8

LOC113520116	UDP-N-acetylglucosamine--peptide N-acetylglucosaminyltransferase 110 kDa subunit-like	0.00	2.16	6.47	3.74	173.21	0.20	15
LOC113520120	Facilitated trehalose transporter Tret1-2 homolog	-4.37	-2.38	0.00	2.21	92.91	0.59	6
LOC113520122	von Willebrand factor D and EGF domain-containing protein	-2.80	-0.93	0.00	1.62	173.21	0.39	3
LOC113520126	Baculoviral IAP repeat-containing protein 5	-2.20	-0.73	0.00	1.27	173.21	0.12	3
LOC113520128	Aminoacylase-1-like	-3.97	-2.80	-2.02	1.03	36.90	0.64	2
LOC113520135	Zinc finger protein 816-like	-1.51	-0.91	0.00	0.80	88.04	0.96	9
LOC113520136	UDP-glucuronosyltransferase 2B10-like	0.00	2.11	6.32	3.65	173.21	0.29	15
LOC113520146	Hypothetical protein KGM_206883	-1.83	-0.61	0.00	1.05	173.21	0.05	3
LOC113520151	Uncharacterized protein DDB_G0283357-like	0.00	0.79	2.37	1.37	173.21	0.43	7
LOC113520153	Plasma glutamate carboxypeptidase	0.00	0.70	2.10	1.21	173.21	0.88	8
LOC113520159	Lysosome-associated membrane glycoprotein 2-like	-1.89	-0.63	0.00	1.09	173.21	0.02	3
LOC113520163	Cation transport regulator-like protein 2	0.00	0.57	1.70	0.98	173.21	0.42	1
LOC113520164	Programmed cell death 4a	-2.26	-0.75	0.00	1.30	173.21	0.14	3
LOC113520176	Facilitated trehalose transporter Tret1-2 homolog isoform X1	-3.95	-2.15	0.00	2.00	92.82	0.86	9
LOC113520178	Chymotrypsin BII-like	-7.13	-3.96	0.00	3.63	91.77	0.55	6
LOC113520181	NF-kappa-B inhibitor cactus	0.00	2.51	3.93	2.18	86.85	0.32	12
LOC113520195	Retinoblastoma-binding protein 5 homolog isoform X1	-1.51	-0.50	0.00	0.87	173.21	0.18	3
LOC113520202	Potassium channel subfamily K member 18-like	0.00	0.86	2.57	1.48	173.21	0.15	7
LOC113520205	Nucleoredoxin-like	-5.43	-3.17	0.00	2.83	89.23	0.32	6
LOC113520217	Membrane-associated progesterone receptor component 1-like	-1.96	-0.65	0.00	1.13	173.21	0.31	4
LOC113520220	DE-cadherin	0.00	1.52	3.04	1.52	99.74	0.88	12
LOC113520221	DE-cadherin	0.00	1.13	3.38	1.95	173.21	0.74	7
LOC113520233	Tight junction protein ZO-1-like isoform X2	0.00	0.82	2.47	1.42	173.21	0.28	7
LOC113520238	UDP-glycosyltransferase UGT40L1	0.00	1.08	1.77	0.95	87.84	0.57	1

LOC113520240	Eukaryotic translation initiation factor 2-alpha kinase-like	0.00	0.42	1.27	0.73	173.21	0.21	8
LOC113520242	Superoxide dismutase	0.00	2.15	3.47	1.88	87.35	0.29	12
LOC113520257	Hypothetical protein KGM_212484	0.00	0.84	2.52	1.45	173.21	0.21	7
LOC113520262	Uncharacterized protein LOC106131566 isoform X3	0.00	0.47	1.40	0.81	173.21	0.12	8
LOC113520264	Neuralized-like protein 4	-1.49	-0.50	0.00	0.86	173.21	0.19	3
LOC113520265	Neuronal PAS domain-containing protein 4B-like	0.00	2.16	6.47	3.74	173.21	0.20	15
LOC113520269	Uncharacterized protein LOC106131445	0.00	1.69	2.65	1.46	86.89	0.48	12
LOC113520279	Msx2-interacting protein-like isoform X1	0.00	0.42	1.27	0.74	173.21	0.20	8
LOC113520283	DE-cadherin	0.00	0.85	2.54	1.47	173.21	0.18	7
LOC113520284	Titin-like isoform X1	-4.54	-2.75	0.00	2.42	87.86	0.47	6
LOC113520285	Uncharacterized protein LOC106131566 isoform X1	0.00	0.69	2.08	1.20	173.21	0.82	8
LOC113520286	UPF0518 protein GF15501 isoform X1	0.00	0.40	1.21	0.70	173.21	0.24	8
LOC113520288	Endochitinase	0.00	1.08	3.24	1.87	173.21	0.78	1
LOC113520291	Glycoprotein 3-Alpha-L-fucosyltransferase A	-2.90	-1.55	0.00	1.46	94.34	0.24	9
LOC113520294	Protein trachealess isoform X1	-3.15	-1.78	0.00	1.61	90.67	0.19	9
LOC113520314	Ecdysone 20-monooxygenase	-2.69	-0.90	0.00	1.55	173.21	0.03	4
LOC113520317	Transmembrane protein 198 isoform X1	0.00	0.58	1.73	1.00	173.21	0.16	8
LOC113520319	Uncharacterized protein CG1785	-1.74	-1.02	0.00	0.91	88.77	0.93	9
LOC113520322	Hypothetical protein KGM_209029	-1.49	-0.50	0.00	0.86	173.21	0.18	3
LOC113520328	Uncharacterized protein LOC106131668	-1.85	-0.62	0.00	1.07	173.21	0.04	3
LOC113520329	Prostaglandin E2 receptor EP2 subtype	0.00	4.34	7.76	3.96	91.34	0.37	10
LOC113520348	Uncharacterized protein LOC106131594 isoform X4	0.00	2.25	4.90	2.47	110.08	0.94	12
LOC113520349	Uncharacterized protein LOC106131507	-1.89	-0.63	0.00	1.09	173.21	0.03	3
LOC113520351	Histone acetyltransferase type B catalytic subunit	-1.58	-0.53	0.00	0.91	173.21	0.15	3
LOC113520356	Deoxyuridine 5'-triphosphate nucleotidohydrolase	-1.64	-0.55	0.00	0.94	173.21	0.13	3
LOC113520358	Proteasome assembly chaperone 2	0.00	0.45	1.36	0.78	173.21	0.15	8

LOC113520363	Aurora kinase B-like	-1.75	-0.58	0.00	1.01	173.21	0.08	3
LOC113520364	Hydroxypyruvate isomerase	-4.00	-1.98	0.00	2.00	101.00	0.76	9
LOC113520366	Uncharacterized protein LOC110377214	0.00	1.69	5.06	2.92	173.21	0.42	11
LOC113520377	Uncharacterized protein LOC106131697	0.00	0.88	2.64	1.52	173.21	0.08	7
LOC113520381	E3 ubiquitin-protein ligase TRAIP-like	-1.21	-0.40	0.00	0.70	173.21	0.28	3
LOC113520387	Facilitated trehalose transporter Tret1-like	0.00	1.06	3.17	1.83	173.21	0.74	1
LOC113520402	Spondin-2-like	0.00	2.58	5.07	2.54	98.31	0.63	12
LOC113520406	Uncharacterized protein LOC106131448	0.00	2.51	7.54	4.35	173.21	0.19	15
LOC113520410	Glutathione-specific gamma-glutamylcyclotransferase 2	0.00	0.40	1.19	0.69	173.21	0.25	8
LOC113520421	DPH3	0.00	0.44	1.32	0.76	173.21	0.17	8
LOC113520422	RDH13	-2.70	-1.46	0.00	1.36	93.21	0.25	9
LOC113520423	Mitochondrial glutamate carrier 1	0.00	1.22	2.30	1.16	94.64	0.98	1
LOC113520429	Uncharacterized abhydrolase domain-containing protein DDB_G0269086-like	-8.92	-4.04	0.00	4.52	111.75	0.62	6
LOC113520434	Uncharacterized protein LOC106104179 isoform X1	0.00	0.59	1.76	1.01	173.21	0.41	1
LOC113520436	Uncharacterized protein LOC110379888	0.00	0.54	1.62	0.94	173.21	0.06	8
LOC113520440	Aminomethyltransferase, mitochondrial	-2.38	-0.79	0.00	1.37	173.21	0.13	4
LOC113520441	Proton-coupled amino acid transporter 1-like isoform X1	0.00	4.21	6.98	3.71	88.05	0.27	10
LOC113520443	Antichymotrypsin-2-like	0.00	1.97	3.27	1.73	88.15	0.39	12
LOC113520445	Activating signal cointegrator 1 complex subunit 3	0.00	0.63	1.90	1.10	173.21	0.41	8
LOC113520446	Dynein light chain Tctex-type 1-like	-5.22	-1.74	0.00	3.01	173.21	0.92	6
LOC113520458	Asparagine--tRNA ligase, cytoplasmic	0.00	0.42	1.25	0.72	173.21	0.21	8
LOC113520459	Alpha-tubulin	-2.73	-0.91	0.00	1.58	173.21	0.36	3
LOC113520464	Potassium voltage-gated channel protein Shaker	-3.07	-1.61	0.00	1.54	95.58	0.27	9
LOC113520465	Alpha-tubulin	-2.14	-0.71	0.00	1.24	173.21	0.09	3
LOC113520466	3-hydroxyisobutyrate dehydrogenase, mitochondrial	-5.00	-2.51	0.00	2.50	99.79	0.27	6
LOC113520467	Protein jim lovell	0.00	1.31	3.93	2.27	173.21	0.29	11

LOC113520468	Lysosomal alpha-mannosidase-like isoform X1	-4.01	-1.94	0.00	2.01	103.20	0.75	9
LOC113520474	Uncharacterized protein LOC106130527 isoform X1	-1.76	-0.59	0.00	1.02	173.21	0.08	3
LOC113520485	Ras-related protein Ral-a isoform X2	0.00	0.47	1.40	0.81	173.21	0.13	8
LOC113520491	DNA (cytosine-5)-methyltransferase PliMCI-like	-1.23	-0.41	0.00	0.71	173.21	0.27	3
LOC113520493	Uncharacterized protein LOC110375908 isoform X4	0.00	2.02	3.72	1.88	93.00	0.25	12
LOC113520498	B-cell receptor-associated protein 31	0.00	1.48	3.11	1.56	105.27	0.90	7
LOC113520500	Acyl-CoA Delta(11) desaturase-like	-4.03	-2.37	0.00	2.11	88.83	0.84	6
LOC113520503	Dorsal-ventral patterning protein Sog	0.00	2.90	4.90	2.57	88.64	0.92	12
LOC113520509	Protein lin-37 homolog isoform X2	-1.23	-0.41	0.00	0.71	173.21	0.28	3
LOC113520515	Uncharacterized protein LOC106132893 isoform X2	-2.11	-1.14	0.00	1.06	93.44	0.79	9
LOC113520516	Sarcoplasmic calcium-binding protein	-3.54	-1.99	0.00	1.81	90.92	0.46	9
LOC113520521	Protein bric-a-brac 1-like isoform X1	0.00	1.49	4.46	2.58	173.21	0.11	11
LOC113520523	Replication protein A 70 kDa DNA-binding subunit	-1.72	-0.57	0.00	0.99	173.21	0.10	3
LOC113520534	Cytochrome P450 49a1	-2.87	-1.74	0.00	1.53	87.86	0.11	9
LOC113520537	Uncharacterized protein LOC106132075	1.74	4.33	6.13	2.29	53.03	0.70	13
LOC113520552	Uncharacterized protein LOC106108538	-3.96	-1.76	0.00	2.02	114.45	0.69	9
LOC113520555	Cytochrome P450 9e2-like	-1.94	-0.65	0.00	1.12	173.21	0.01	3
LOC113520563	USP6 N-terminal-like protein	-1.51	-0.50	0.00	0.87	173.21	0.18	3
LOC113520586	Coiled-coil-helix-coiled-coil-helix domain-containing protein 2, mitochondrial-like	0.00	0.69	2.07	1.20	173.21	0.79	8
LOC113520587	Uncharacterized protein LOC110374731	0.00	3.15	6.31	3.15	100.29	0.63	10
LOC113520589	Translocon-associated protein subunit gamma-like isoform X1	0.00	0.43	1.29	0.74	173.21	0.52	1
LOC113520595	Target of rapamycin complex subunit lst8	-1.46	-0.49	0.00	0.84	173.21	0.20	3
LOC113520600	BMP and activin membrane-bound inhibitor homolog	-1.69	-0.56	0.00	0.98	173.21	0.11	3
LOC113520601	Rac GTPase-activating protein 1-like isoform X2	-1.67	-0.56	0.00	0.97	173.21	0.12	3

LOC113520605	Uncharacterized protein	-5.40	-2.72	0.00	2.70	99.21	0.09	6
LOC113520612	ATPase family AAA domain-containing protein 3	0.00	0.58	1.75	1.01	173.21	0.19	8
LOC113520619	Uncharacterized protein LOC106129264	0.00	0.42	1.26	0.73	173.21	0.54	1
LOC113520620	Solute carrier family 35 member B1 homolog	0.00	1.21	1.87	1.05	86.76	0.50	1
LOC113520629	Uncharacterized protein OBRU01_00156	-2.20	-0.73	0.00	1.27	173.21	0.11	3
LOC113520632	Eukaryotic translation initiation factor 6	0.00	0.73	2.18	1.26	173.21	0.88	7
LOC113520633	UNC93-like protein	0.00	4.11	7.14	3.69	89.78	0.22	10
LOC113520637	Serine hydrolase	0.00	0.52	1.57	0.91	173.21	0.02	8
LOC113520645	Acetyl-coenzyme A transporter 1	0.00	0.87	1.41	0.76	87.40	0.27	1
LOC113520651	Uncharacterized protein LOC110375899 isoform X2	-2.36	-1.43	0.00	1.26	87.81	0.20	9
LOC113520654	Protein hairy	0.00	0.64	1.93	1.11	173.21	0.46	8
LOC113520659	Facilitated trehalose transporter Tret1-like	0.00	1.70	2.76	1.49	87.45	0.43	12
LOC113520663	Hypothetical protein KGM_207817	0.00	0.61	1.82	1.05	173.21	0.28	8
LOC113520664	Prostaglandin reductase 1-like	-2.58	-1.29	0.00	1.29	100.13	0.62	9
LOC113520665	Macrophage migration inhibitory factor homolog	-5.27	-3.24	0.00	2.84	87.50	0.43	6
LOC113520666	Multidrug resistance protein 1A-like	0.00	3.03	5.52	2.80	92.43	0.75	10
LOC113520669	Uncharacterized protein LOC110374769	0.00	0.45	1.36	0.78	173.21	0.50	1
LOC113520670	Antennal esterase CXE13	-4.05	-2.13	0.00	2.03	95.49	0.91	9
LOC113520676	Transcription elongation factor SPT4 isoform X1	0.00	0.41	1.23	0.71	173.21	0.23	8
LOC113520679	Uncharacterized protein LOC106139261 isoform X1	0.00	0.54	1.63	0.94	173.21	0.43	1
LOC113520692	Tyrosine-protein phosphatase corkscrew-like	0.00	2.69	4.50	2.37	88.39	0.54	12
LOC113520694	Protein yippee-like CG15309 isoform X1	-2.34	-1.31	0.00	1.19	91.52	0.42	9
LOC113520698	Uncharacterized protein LOC106140812	0.00	1.49	2.63	1.35	90.58	0.76	12
LOC113520702	Uncharacterized protein LOC106130581	0.00	0.58	1.75	1.01	173.21	0.19	8
LOC113520705	Nitrogen permease regulator 2-like protein	-3.71	-2.20	0.00	1.95	88.58	0.74	9
LOC113520706	Cyclin-dependent kinase 12 isoform X4	0.00	0.47	1.40	0.81	173.21	0.49	1
LOC113520711	Valine--tRNA ligase isoform X1	0.00	1.06	1.89	0.97	91.14	0.75	1

LOC113520736	Uncharacterized protein LOC110380886 isoform X1	0.00	1.04	3.13	1.80	173.21	0.71	1
LOC113520740	Broad-complex core protein isoform 6	-2.74	-0.91	0.00	1.58	173.21	0.37	3
LOC113520741	MDS1 and EVI1 complex locus protein EVI1-like	-3.62	-1.21	0.00	2.09	173.21	0.71	3
LOC113520749	Facilitated trehalose transporter Tret1-like	0.00	1.95	3.16	1.71	87.43	0.12	12
LOC113520751	Uncharacterized protein LOC106132055	-3.67	-2.37	0.00	2.06	86.73	0.91	9
LOC113520754	Uncharacterized protein LOC110374696	-1.83	-0.61	0.00	1.05	173.21	0.05	3
LOC113520761	G-protein coupled receptor Mth2-like isoform X1	0.00	1.55	4.66	2.69	173.21	0.20	11
LOC113520762	F-box/LRR-repeat protein 4 isoform X1	-1.77	-1.02	0.00	0.91	89.60	0.97	9
LOC113520774	Clustered mitochondria protein homolog	0.00	1.10	3.29	1.90	173.21	0.59	7
LOC113520775	Ubiquitin carboxyl-terminal hydrolase calypso	-1.33	-0.44	0.00	0.77	173.21	0.24	3
LOC113520780	Dual oxidase	0.00	1.54	4.61	2.66	173.21	0.18	11
LOC113520787	Septin-2 isoform X1	0.00	1.88	5.65	3.26	173.21	0.91	15
LOC113520792	G-protein coupled receptor Mth2	0.00	2.98	5.00	2.63	88.46	0.93	10
LOC113520794	Multidrug resistance protein 1A-like	-1.97	-0.66	0.00	1.14	173.21	0.31	4
LOC113520795	Alcohol dehydrogenase	-1.33	-0.44	0.00	0.77	173.21	0.24	3
LOC113520797	Matrix metalloproteinase-14-like	-1.46	-0.49	0.00	0.84	173.21	0.20	3
LOC113520804	Uncharacterized protein LOC105380498 isoform X2	-8.13	-4.83	-3.11	2.86	59.16	0.73	2
LOC113520813	Tyramine beta-hydroxylase	-2.93	-1.67	0.00	1.51	90.41	0.12	9
LOC113520818	Phosphoserine phosphatase	0.00	0.73	2.20	1.27	173.21	0.80	7
LOC113520819	MutS protein homolog 4-like isoform X2	0.00	1.31	2.44	1.23	94.02	0.45	1
LOC113520821	Transcription factor 2 isoform X4	0.00	1.17	2.22	1.11	94.94	0.96	1
LOC113520822	Tyrosine-protein phosphatase non-receptor type 4	0.00	0.53	1.58	0.91	173.21	0.02	8
LOC113520823	Fibroblast growth factor receptor 2-like isoform X2	0.00	0.81	2.42	1.40	173.21	0.35	7
LOC113520835	Integrin Alpha-PS1-like	0.00	0.71	2.13	1.23	173.21	0.96	8
LOC113520836	Rho guanine nucleotide exchange factor 10-like protein	0.00	1.36	2.46	1.25	91.78	0.98	1
LOC113520837	Uncharacterized protein LOC106130822	0.00	1.11	1.70	0.96	86.68	0.45	1

LOC113520840	High mobility group protein D	-1.91	-0.64	0.00	1.10	173.21	0.02	3
LOC113520841	NFX1-type Zinc finger-containing protein 1-like	-1.97	-0.66	0.00	1.13	173.21	0.01	3
LOC113520844	Inorganic phosphate cotransporter isoform X2	0.00	1.21	2.00	1.06	88.06	0.61	1
LOC113520845	Multidrug resistance protein 1A-like	0.00	2.76	5.55	2.77	100.33	0.90	12
LOC113520849	WAS/WASL-interacting protein family member 2-like isoform X1	0.00	1.00	1.74	0.90	89.89	0.16	1
LOC113520850	Serine/arginine repetitive matrix protein 1 isoform X2	0.00	2.33	5.47	2.82	121.01	0.93	11
LOC113520864	Sortilin-related receptor-like	0.00	0.54	1.63	0.94	173.21	0.06	8
LOC113520869	GPI mannosyltransferase 4	-2.46	-0.82	0.00	1.42	173.21	0.24	3
LOC113520875	Sodium-coupled neutral amino acid transporter 9 homolog isoform X1	0.00	2.54	7.61	4.40	173.21	0.21	15
LOC113520899	Lipase member I-like	-4.60	-3.05	0.00	2.65	86.61	0.59	6
LOC113520900	Uncharacterized protein LOC106133892	-4.70	-3.36	-1.72	1.51	44.94	0.77	2
LOC113520906	Aminopeptidase W07G4.4	-3.67	-1.97	0.00	1.85	94.10	0.53	9
LOC113520924	La-related protein 1-like isoform X2	0.00	0.41	1.24	0.71	173.21	0.22	8
LOC113520936	Adhesive plaque matrix protein-like	0.00	0.92	2.76	1.59	173.21	0.04	7
LOC113520938	Uncharacterized protein LOC105842306	-1.76	-0.59	0.00	1.01	173.21	0.08	3
LOC113520941	Lysosomal alpha-glucosidase-like	-2.68	-0.89	0.00	1.55	173.21	0.03	4
LOC113520942	Ankyrin repeat and Zinc finger domain-containing protein 1-like	0.00	0.74	2.22	1.28	173.21	0.75	7
LOC113520961	Tubulin Alpha-8 chain-like isoform X1	0.00	2.02	6.05	3.49	173.21	0.16	5
LOC113520966	Hypothetical protein KGM_200275A	0.00	1.09	3.26	1.88	173.21	0.54	7
LOC113520971	Myrosinase 1	-6.45	-2.15	0.00	3.72	173.21	0.73	6
LOC113520972	Myrosinase 1	-7.93	-3.89	0.00	3.97	101.89	0.54	6
LOC113520987	Acyl-CoA Delta(11) desaturase isoform X1	0.00	0.45	1.34	0.77	173.21	0.17	8
LOC113520988	Aminopeptidase N-like isoform X2	0.00	3.71	6.55	3.36	90.58	0.12	10
LOC113520993	P protein-like	-1.63	-0.54	0.00	0.94	173.21	0.13	3
LOC113520996	Ankyrin repeat and Zinc finger domain-containing protein 1-like	0.00	0.78	2.35	1.36	173.21	0.47	7
LOC113521001	Uncharacterized protein LOC106129135	-3.58	-2.37	0.00	2.05	86.61	0.85	9

LOC113521004	Chaoptin-like	-2.15	-0.72	0.00	1.24	173.21	0.09	3
LOC113521007	Alpha-1,3-mannosyl-glycoprotein 4-beta-N-acetylglucosaminyltransferase A-like isoform X1	0.00	0.71	2.12	1.22	173.21	0.92	8
LOC113521009	Papilin-like	-2.73	-0.91	0.00	1.58	173.21	0.36	3
LOC113521024	Uncharacterized protein LOC106133696	-1.70	-0.57	0.00	0.98	173.21	0.10	3
LOC113521027	Laccase-4-like	2.91	7.87	11.32	4.40	55.96	0.31	14
LOC113521028	Uncharacterized protein LOC106133088	-2.16	-1.34	0.00	1.17	87.35	0.41	9
LOC113521031	Ig-like and fibronectin type-III domain-containing protein T04A11.3 isoform X2	-3.34	-1.59	0.00	1.68	105.40	0.49	9
LOC113521034	Inorganic phosphate cotransporter	0.00	3.75	5.83	3.25	86.77	0.28	10
LOC113521037	Protein nessun dorma isoform X3	-2.07	-0.69	0.00	1.19	173.21	0.05	3
LOC113521041	Lipoma HMGIC fusion partner-like 3 protein	0.00	0.70	2.10	1.21	173.21	0.85	8
LOC113521049	Voltage-dependent calcium channel type D subunit Alpha-1-like isoform X3	-6.66	-3.03	0.00	3.37	111.38	0.97	9
LOC113521050	BTB/POZ domain-containing protein KCTD1	-5.24	-1.75	0.00	3.03	173.21	0.91	6
LOC113521052	Uncharacterized protein LOC106139651	0.00	0.44	1.31	0.76	173.21	0.18	8
LOC113521060	Talin-B	-1.41	-0.47	0.00	0.81	173.21	0.21	3
LOC113521062	Protein boule-like	-3.17	-1.06	0.00	1.83	173.21	0.55	3
LOC113521066	Uncharacterized protein LOC110372636 isoform X6	0.00	0.46	1.39	0.80	173.21	0.13	8
LOC113521082	Phosphatidylinositol 4,5-bisphosphate 3-kinase catalytic subunit delta isoform	0.00	0.60	1.79	1.03	173.21	0.24	8
LOC113521090	Uncharacterized protein LOC106709604	-3.13	-1.04	0.00	1.81	173.21	0.54	3
LOC113521092	Cytochrome P450 6AB13	0.00	1.99	5.97	3.45	173.21	0.55	15
LOC113521102	Gastrula Zinc finger protein XlCGF17.1-like	-1.31	-0.44	0.00	0.75	173.21	0.25	3
LOC113521103	Uncharacterized protein LOC106130198 isoform X1	-1.64	-0.97	0.00	0.86	88.63	0.96	3
LOC113521105	Guanine nucleotide-binding protein G(f) subunit alpha	-4.91	-2.97	0.00	2.61	87.94	0.78	6
LOC113521110	E3 ubiquitin-protein ligase UBR3	0.00	0.52	1.57	0.91	173.21	0.02	8
LOC113521113	Protein cab-1 isoform X2	-3.22	-1.55	0.00	1.61	104.41	0.48	9

LOC113521118	Hypothetical protein KGM_206502	0.00	0.45	1.34	0.77	173.21	0.17	8
LOC113521121	Facilitated trehalose transporter Tret1-like isoform X1	0.00	1.04	3.13	1.81	173.21	0.36	7
LOC113521130	Uncharacterized protein	0.00	2.02	6.07	3.50	173.21	0.47	15
LOC113521135	Inorganic phosphate cotransporter	-2.58	-0.86	0.00	1.49	173.21	0.29	3
LOC113521139	ATP-binding cassette sub-family G member 1-like	-2.33	-0.78	0.00	1.34	173.21	0.17	3
LOC113521140	Metal transporter CNNM4-like	-5.71	-1.90	0.00	3.29	173.21	0.67	4
LOC113521144	Uncharacterized protein LOC106142945	-1.22	-0.41	0.00	0.70	173.21	0.28	3
LOC113521146	Uncharacterized protein LOC110380962 isoform X2	-1.30	-0.43	0.00	0.75	173.21	0.25	3
LOC113521147	Glucose dehydrogenase	-1.24	-0.41	0.00	0.71	173.21	0.27	3
LOC113521152	Ataxin-2 homolog isoform X1	-3.06	-1.60	0.00	1.54	95.98	0.28	9
LOC113521153	Methionine--tRNA ligase, cytoplasmic isoform X1	0.00	0.95	2.86	1.65	173.21	0.11	7
LOC113521154	Zinc finger protein 713-like	0.00	2.52	7.57	4.37	173.21	0.20	15
LOC113521169	Serine protease K12H4.7 isoform X1	-1.35	-0.45	0.00	0.78	173.21	0.24	3
LOC113521173	Chitooligosaccharidolytic beta-N-acetylglucosaminidase-like	-3.25	-1.68	0.00	1.63	96.69	0.30	9
LOC113521180	Protein N-terminal asparagine amidohydrolase isoform X1	0.00	0.45	1.35	0.78	173.21	0.16	8
LOC113521183	Potassium voltage-gated channel protein Shab isoform X1	0.00	0.63	1.90	1.10	173.21	0.40	1
LOC113521184	Neuropeptide receptor A10	-2.61	-1.64	0.00	1.43	87.03	0.08	9
LOC113521185	Purine nucleoside phosphorylase-like isoform X1	0.00	0.49	1.46	0.84	173.21	0.47	1
LOC113521192	Uncharacterized protein LOC106135037	0.00	0.90	2.71	1.57	173.21	0.03	7
LOC113521205	Chondroadherin-like	-2.67	-0.89	0.00	1.54	173.21	0.34	3
LOC113521220	RNA exonuclease NEF-sp	0.00	0.55	1.65	0.95	173.21	0.08	8
LOC113521223	FK506-binding protein 5-like	-1.48	-0.49	0.00	0.86	173.21	0.19	3
LOC113521224	E3 ubiquitin-protein ligase ZNRF1	-1.44	-0.48	0.00	0.83	173.21	0.61	4
LOC113521225	Neurofilament heavy polypeptide-like	0.00	0.40	1.21	0.70	173.21	0.24	8
LOC113521229	Prefoldin subunit 4	0.00	1.07	1.69	0.93	86.92	0.33	1
LOC113521232	Epidermal growth factor receptor isoform	-1.58	-0.53	0.00	0.91	173.21	0.15	3

	X1							
LOC113521234	Translocon-associated protein subunit alpha precursor	0.00	0.43	1.29	0.75	173.21	0.52	1
LOC113521237	C-type lectin 37Db isoform X2	0.00	1.31	3.94	2.27	173.21	0.28	11
LOC113521241	Laminin subunit alpha isoform X3	0.00	0.58	1.75	1.01	173.21	0.19	8
LOC113521248	Uncharacterized protein LOC106135118	0.00	1.31	3.93	2.27	173.21	0.29	11
LOC113521263	Protein extra-macrochaetae	-3.15	-1.80	0.00	1.62	90.22	0.20	9
LOC113521268	Catalase	-3.37	-1.63	0.00	1.69	103.68	0.46	9
LOC113521273	FIT family protein CG10671	0.00	0.52	1.56	0.90	173.21	0.02	8
LOC113521275	Carboxylesterase	-2.61	-0.87	0.00	1.51	173.21	0.31	3
LOC113521276	RNA-binding protein 40-like	-1.21	-0.40	0.00	0.70	173.21	0.28	3
LOC113521278	Arf-GAP with dual PH domain-containing protein 1-like isoform X2	-1.31	-0.44	0.00	0.76	173.21	0.25	3
LOC113521279	DNA replication factor Cdt1	-1.32	-0.44	0.00	0.76	173.21	0.24	3
LOC113521283	Guanylate kinase-associated protein mars isoform X1	-1.77	-0.59	0.00	1.02	173.21	0.07	3
LOC113521309	Black	3.82	7.61	10.16	3.35	43.98	0.43	14
LOC113521313	Glycogen-binding subunit 76A	0.00	0.62	1.87	1.08	173.21	0.36	8
LOC113521317	Glycerol-3-phosphate phosphatase	0.00	1.09	1.75	0.95	87.20	0.52	1
LOC113521320	Uncharacterized protein LOC106135015	0.00	1.52	2.90	1.45	95.76	0.78	12
LOC113521322	TPPP family protein CG45057-like	0.00	0.52	1.56	0.90	173.21	0.44	1
LOC113521324	Alanine--glyoxylate aminotransferase 2, mitochondrial	-4.47	-2.67	0.00	2.36	88.30	0.48	6
LOC113521331	Tether containing UBX domain for GLUT4	0.00	0.39	1.16	0.67	173.21	0.26	8
LOC113521335	Mitochondrial deoxynucleotide carrier	0.00	0.69	2.06	1.19	173.21	0.76	8
LOC113521358	BAI1-associated protein 3	0.00	2.53	4.23	2.23	88.37	0.36	12
LOC113521362	Multifunctional methyltransferase subunit TRM112-like protein	0.00	0.92	1.51	0.81	87.81	0.22	1
LOC113521365	3-phosphoinositide-dependent protein kinase 1 isoform X1	0.00	1.31	2.18	1.16	88.13	0.74	1
LOC113521368	Zinc finger homeobox protein 3	0.00	0.68	2.03	1.17	173.21	0.67	8
LOC113521372	Uncharacterized protein	-9.38	-4.99	0.00	4.72	94.51	0.74	6
LOC113521375	Cuticle protein 16.5, isoform B-like	0.00	1.92	5.75	3.32	173.21	0.78	15

LOC113521376	Lipase	-3.26	-1.09	0.00	1.88	173.21	0.59	3
LOC113521377	Alanine aminotransferase 1	0.00	1.83	3.09	1.62	88.54	0.52	12
LOC113521397	Collagen alpha-1(XVIII) chain-like	0.00	4.19	6.93	3.68	87.97	0.26	10
LOC113521399	Collagen alpha-1(IX) chain-like isoform X1	3.55	3.96	4.69	0.64	16.20	0.47	13
LOC113521401	Collagen alpha-1(IX) chain-like isoform X1	3.13	3.81	4.95	0.99	26.09	0.50	13
LOC113521406	Lipase member H-like isoform X2	0.00	2.05	6.16	3.56	173.21	0.40	15
LOC113521414	Condensin complex subunit 2	-1.61	-0.54	0.00	0.93	173.21	0.14	3
LOC113521417	Fas-binding factor 1	-2.43	-1.33	0.00	1.23	92.70	0.41	9
LOC113521425	Protein phosphatase 1 regulatory inhibitor subunit 16B	0.00	0.48	1.43	0.83	173.21	0.48	1
LOC113521427	Mpv17-like protein 2	0.00	0.51	1.52	0.88	173.21	0.04	8
LOC113521429	Anamorsin homolog	0.00	0.60	1.80	1.04	173.21	0.26	8
LOC113521431	Breast cancer anti-estrogen resistance protein 1	0.00	1.48	2.50	1.31	88.53	0.84	1
LOC113521449	Ribonuclease UK114-like	0.00	1.55	4.66	2.69	173.21	0.20	11
LOC113521451	Uncharacterized protein LOC101736230	0.00	0.91	1.40	0.79	86.68	0.34	1
LOC113521464	Uncharacterized protein LOC110370179	-2.44	-0.81	0.00	1.41	173.21	0.11	4
LOC113521483	1-acylglycerophosphocholine O-acyltransferase 1	0.00	1.83	5.49	3.17	173.21	0.85	11
LOC113521488	Acidic Leucine-rich nuclear phosphoprotein 32-related protein	-5.38	-3.58	0.00	3.10	86.60	0.62	6
LOC113521491	Uncharacterized protein	0.00	2.62	3.96	2.27	86.61	0.45	12
LOC113521492	GTP-binding protein Rhes	-2.43	-1.53	0.00	1.33	87.09	0.27	9
LOC113521494	Mpv17-like protein	-1.84	-1.21	0.00	1.05	86.63	0.52	9
LOC113521502	Uncharacterized protein LOC106135000 isoform X1	0.00	5.01	8.63	4.48	89.47	0.90	10
LOC113521506	Uncharacterized protein LOC106134940	0.00	0.63	1.90	1.09	173.21	0.40	8
LOC113521510	WW domain-binding protein 11-like	0.00	1.48	2.44	1.30	87.84	0.85	12
LOC113521536	Low-density lipoprotein receptor-related protein 2	0.00	0.45	1.34	0.78	173.21	0.16	8
LOC113521538	Kelch-like protein 10	-5.94	-3.14	0.00	2.98	95.05	0.91	6
LOC113521541	15-hydroxyprostaglandin dehydrogenase	0.00	3.03	5.29	2.73	90.02	0.78	10
LOC113521544	Palmitoyl-protein thioesterase 1	-1.94	-0.65	0.00	1.12	173.21	0.01	3

LOC113521556	Apolipoprotein of lipid transfer particle-i/II	-1.96	-0.65	0.00	1.13	173.21	0.31	4
LOC113521562	Protein KBP homolog	0.00	1.41	4.24	2.45	173.21	0.50	5
LOC113521563	Chemosensory protein 6	0.00	2.72	8.15	4.71	173.21	0.32	15
LOC113521564	Down syndrome cell adhesion molecule-like protein Dscam2	-4.68	-2.45	0.00	2.35	96.01	0.39	6
LOC113521566	RING finger protein nhl-1-like	-1.66	-0.55	0.00	0.96	173.21	0.48	4
LOC113521567	Peptidyl-Alpha-hydroxyglycine Alpha-amidating lyase 2-like	0.00	2.64	4.40	2.33	88.25	0.52	12
LOC113521568	Uncharacterized protein LOC106132613	3.69	7.30	9.11	3.13	42.84	0.50	14
LOC113521572	Chemosensory protein 10	0.00	3.50	6.55	3.30	94.15	0.31	10
LOC113521573	Chemosensory protein 2	0.00	0.88	2.64	1.52	173.21	0.08	7
LOC113521574	Ejaculatory bulb-specific protein 3-like	2.29	5.81	8.16	3.10	53.45	0.81	14
LOC113521578	Transcription factor jun-D	0.00	0.42	1.26	0.73	173.21	0.21	8
LOC113521579	1-acylglycerol-3-phosphate O-acyltransferase ABHD5-like isoform X2	0.00	0.53	1.59	0.92	173.21	0.03	8
LOC113521583	Uncharacterized protein LOC106132743 isoform X1	-3.91	-2.16	0.00	1.99	91.81	0.84	9
LOC113521590	Non-specific lipid-transfer protein-like	-3.17	-1.82	0.00	1.64	89.96	0.22	9
LOC113521596	Bardet-Biedl syndrome 2 protein homolog	-1.49	-0.50	0.00	0.86	173.21	0.18	3
LOC113521599	Serine--tRNA ligase, cytoplasmic	0.00	0.68	2.04	1.18	173.21	0.72	8
LOC113521604	Transcription factor Sox-12-like	0.00	0.68	2.03	1.17	173.21	0.69	8
LOC113521605	Transient receptor potential cation channel trpm isoform X8	-1.76	-0.59	0.00	1.02	173.21	0.08	3
LOC113521606	Enkurin isoform X2	-5.37	-1.79	0.00	3.10	173.21	0.88	6
LOC113521607	Acetylcholinesterase-like	0.00	0.67	2.02	1.17	173.21	0.65	8
LOC113521608	Uncharacterized protein LOC106132646 isoform X2	-1.31	-0.44	0.00	0.76	173.21	0.25	3
LOC113521614	Dihydropteridine reductase	0.00	1.80	2.88	1.57	87.21	0.46	12
LOC113521620	Uncharacterized protein LOC106132635	2.85	4.11	5.40	1.28	31.10	0.28	13
LOC113521621	Protein amalgam-like	-3.50	-1.17	0.00	2.02	173.21	0.67	3
LOC113521625	Diuretic hormone receptor isoform X1	0.00	1.06	3.19	1.84	173.21	0.44	7
LOC113521631	Methylcrotonoyl-CoA carboxylase beta chain, mitochondrial	-3.16	-1.79	0.00	1.62	90.50	0.20	9

LOC113521632	Uncharacterized protein LOC105390299	-4.04	-2.67	0.00	2.31	86.62	0.78	6
LOC113521634	ADAMTS-like protein 4	0.00	2.54	7.63	4.40	173.21	0.22	15
LOC113521635	Ejaculatory bulb-specific protein 3-like	1.77	3.04	4.14	1.19	39.35	0.78	12
LOC113521636	Ejaculatory bulb-specific protein 3-like	0.00	1.96	3.01	1.70	86.67	0.17	12
LOC113521640	Hexosaminidase	-2.36	-0.79	0.00	1.36	173.21	0.19	3
LOC113521642	Glutathione S-transferase 1-like	-3.94	-2.61	-1.77	1.17	44.60	0.88	2
LOC113521649	CHH-like protein isoform X2	0.00	5.91	9.63	5.17	87.58	0.31	14
LOC113521650	Uncharacterized protein LOC106139988	0.00	0.92	2.76	1.59	173.21	0.05	7
LOC113521655	tRNA selenocysteine 1-associated protein 1-like	-1.24	-0.41	0.00	0.72	173.21	0.27	3
LOC113521660	Alpha-N-acetylgalactosaminidase-like	-2.79	-1.58	0.00	1.43	90.39	0.10	9
LOC113521663	DNA replication licensing factor MCM4	-1.82	-0.61	0.00	1.05	173.21	0.06	3
LOC113521665	Centromere protein F-like	-1.74	-0.58	0.00	1.01	173.21	0.09	3
LOC113521672	UDP-glucose 4-epimerase-like	0.00	3.21	5.42	2.84	88.62	0.53	10
LOC113521691	Nicalin-1	0.00	0.39	1.17	0.68	173.21	0.26	8
LOC113521698	Semaphorin-1A isoform X5	-1.76	-0.59	0.00	1.01	173.21	0.42	4
LOC113521705	Actin cytoskeleton-regulatory complex protein PAN1-like	-2.18	-0.73	0.00	1.26	173.21	0.21	4
LOC113521709	Ras-related protein Rab-23	-3.84	-2.01	0.00	1.93	95.87	0.66	9
LOC113521711	Uncharacterized protein LOC106132549	-4.06	-2.22	0.00	2.06	92.76	0.97	6
LOC113521712	Chemosensory protein	0.00	3.41	6.05	3.10	90.88	0.30	10
LOC113521717	Uncharacterized protein DDB_G0286901	-1.44	-0.48	0.00	0.83	173.21	0.21	3
LOC113521718	5-formyltetrahydrofolate cyclo-ligase-like isoform X1	-2.52	-1.57	0.00	1.37	87.20	0.27	9
LOC113521719	Hemocyte protein-glutamine gamma-glutamyltransferase-like	0.00	2.79	4.26	2.42	86.65	0.64	12
LOC113521722	Protein dead ringer	0.00	2.89	8.67	5.01	173.21	0.40	15
LOC113521723	Uncharacterized protein	-4.06	-1.35	0.00	2.34	173.21	0.40	4
LOC113521734	Protein PRRC1-like	-1.61	-0.54	0.00	0.93	173.21	0.14	3
LOC113521736	Growth arrest-specific protein 1-like	-1.24	-0.41	0.00	0.71	173.21	0.27	3
LOC113521737	Uncharacterized protein LOC110379736	-3.28	-1.89	0.00	1.70	89.63	0.29	9
LOC113521739	Wiskott-Aldrich syndrome protein family member 2-like	0.00	3.69	6.10	3.24	87.96	0.13	10

LOC113521742	Cyclin-dependent kinases regulatory subunit	0.00	0.45	1.34	0.77	173.21	0.51	1
LOC113521746	Uncharacterized protein LOC106132632	0.00	1.47	2.31	1.27	86.96	0.95	12
LOC113521749	Selenide, water dikinase	-1.20	-0.40	0.00	0.69	173.21	0.29	3
LOC113521752	Uncharacterized protein LOC106132637	0.00	1.48	2.24	1.28	86.61	0.98	12
LOC113521756	Leukotriene A-4 hydrolase isoform X1	-3.40	-1.83	0.00	1.71	93.85	0.33	9
LOC113521757	Uncharacterized protein LOC106140333	-1.48	-0.49	0.00	0.86	173.21	0.19	3
LOC113521759	Activating signal cointegrator 1	0.00	0.56	1.67	0.97	173.21	0.11	8
LOC113521763	Trimeric intracellular cation channel type B	-2.07	-1.14	0.00	1.05	92.05	0.74	9
LOC113521764	Uncharacterized protein LOC106132753	-1.18	-0.39	0.00	0.68	173.21	0.29	3
LOC113521767	Alpha-tocopherol transfer protein-like	0.00	1.18	3.53	2.04	173.21	0.92	11
LOC113521776	Translin-associated factor X-interacting protein 1-like isoform X2	-2.11	-1.23	0.00	1.10	89.31	0.52	9
LOC113521779	Esterase FE4-like	-3.17	-1.84	0.00	1.64	89.43	0.44	9
LOC113521780	Esterase FE4-like	-5.23	-2.81	0.00	2.64	93.91	0.10	6
LOC113521781	Uncharacterized protein LOC106141718 isoform X1	-2.79	-1.39	0.00	1.40	100.22	0.48	9
LOC113521784	DNA repair protein RAD52 homolog isoform X2	-1.86	-0.62	0.00	1.07	173.21	0.04	3
LOC113521791	Uncharacterized protein	-1.53	-0.51	0.00	0.88	173.21	0.17	3
LOC113521797	Hypothetical protein CDL32_26650	0.00	0.67	2.02	1.17	173.21	0.66	8
LOC113521809	Vascular endothelial growth factor receptor 1-like	0.00	2.63	7.89	4.56	173.21	0.27	15
LOC113521811	Uncharacterized protein LOC106137262	-2.45	-0.82	0.00	1.41	173.21	0.23	3
LOC113521813	Uncharacterized protein LOC103309331	0.00	2.67	4.35	2.34	87.61	0.50	12
LOC113521819	Lipid phosphate phosphohydrolase 2-like	0.00	2.18	3.31	1.89	86.62	0.18	12
LOC113521822	Sex peptide receptor-like	0.00	1.62	2.80	1.45	89.57	0.53	12
LOC113521825	Uncharacterized protein	0.00	1.02	3.07	1.77	173.21	0.30	7
LOC113521828	Uncharacterized protein	0.00	1.78	5.33	3.08	173.21	0.67	11
LOC113521829	Uncharacterized protein LOC106716499	0.00	4.56	8.20	4.18	91.62	0.48	10
LOC113521833	Odorant-binding protein A10	0.00	4.68	8.90	4.47	95.57	0.64	10
LOC113521838	Uncharacterized protein LOC106132721 isoform X1	0.00	4.09	6.15	3.54	86.60	0.39	10
LOC113521852	Follistatin-A	0.00	0.62	1.85	1.07	173.21	0.33	8

LOC113521856	Uncharacterized protein LOC101742184	0.00	0.58	1.73	1.00	173.21	0.17	8
LOC113521863	Flagellar attachment zone protein 1	0.00	0.44	1.33	0.77	173.21	0.17	8
LOC113521864	ATP-dependent (S)-NAD(P)H-hydrate dehydratase-like	-1.27	-0.42	0.00	0.73	173.21	0.26	3
LOC113521869	Leucine-rich repeat-containing protein 49	0.00	2.43	5.16	2.59	106.68	0.84	12
LOC113521871	Zinc finger SWIM domain-containing protein 5-like	0.00	1.03	3.08	1.78	173.21	0.30	7
LOC113521874	Uncharacterized protein LOC110382845	-2.97	-0.99	0.00	1.71	173.21	0.47	3
LOC113521876	Ionotropic receptor	0.00	0.93	2.78	1.61	173.21	0.06	7
LOC113521896	Ionotropic receptor IR64a	-1.85	-1.18	0.00	1.02	86.89	0.56	9
LOC113521899	Uncharacterized protein LOC106139546	-3.76	-1.81	0.00	1.88	104.18	0.56	9
LOC113521907	Uncharacterized protein LOC106132537	-2.24	-1.34	0.00	1.18	88.30	0.32	9
LOC113521910	Cyclin-dependent kinases regulatory subunit	0.00	0.58	1.75	1.01	173.21	0.41	1
LOC113521918	Guanine nucleotide-binding protein G(o) subunit alpha isoform X1	0.00	0.42	1.25	0.72	173.21	0.21	8
LOC113521921	Uncharacterized protein	-6.04	-3.23	0.00	3.04	94.19	0.25	6
LOC113521922	Zinc finger protein 100-like	-3.29	-1.10	0.00	1.90	173.21	0.19	4
LOC113521924	Lipid phosphate phosphohydrolase 1-like	0.00	1.42	2.48	1.28	90.17	0.94	12
LOC113521929	Carbohydrate sulfotransferase 11 isoform X1	-2.16	-1.31	0.00	1.15	87.84	0.37	9
LOC113521930	Sodium/potassium/calcium exchanger Nckx30C	-2.81	-1.86	0.00	1.61	86.61	0.25	9
LOC113521931	BAG family molecular chaperone regulator 2	0.00	1.36	2.40	1.23	90.50	0.94	1
LOC113521944	Glutathione S-transferase	-2.04	-0.68	0.00	1.18	173.21	0.04	3
LOC113521949	Transcription factor HNF-4 homolog isoform X1	0.00	0.65	1.94	1.12	173.21	0.49	8
LOC113521951	Calphotin-like	-3.37	-1.12	0.00	1.95	173.21	0.63	3
LOC113521952	Aldehyde Dehydrogenase	-3.04	-1.51	0.00	1.52	100.65	0.41	9
LOC113521954	RecQ-mediated genome instability protein 2-like	-1.45	-0.48	0.00	0.84	173.21	0.20	3
LOC113521957	Uncharacterized protein LOC106711175 isoform X1	-2.01	-0.67	0.00	1.16	173.21	0.03	3
LOC113521961	Vegetative cell wall protein gp1-like isoform	-6.57	-2.19	0.00	3.79	173.21	0.72	6

	X1							
LOC113521962	Uncharacterized protein	0.00	0.67	2.01	1.16	173.21	0.64	8
LOC113521964	Uncharacterized protein LOC105842592	0.00	0.60	1.81	1.04	173.21	0.27	8
LOC113521966	Catenin Alpha-like	0.00	0.47	1.42	0.82	173.21	0.11	8
LOC113521969	Uncharacterized protein	0.00	1.30	3.90	2.25	173.21	0.32	11
LOC113521970	Zinc finger SWIM domain-containing protein 5-like	0.00	0.91	2.73	1.58	173.21	0.03	7
LOC113521973	Titin	0.00	1.75	3.67	1.84	105.27	0.78	12
LOC113521975	Dynein heavy chain 12, axonemal	0.00	1.03	3.10	1.79	173.21	0.32	7
LOC113521978	Uncharacterized protein	0.00	0.78	2.35	1.36	173.21	0.47	7
LOC113521980	Synaptic vesicle membrane protein VAT-1 homolog-like isoform X1	0.00	0.43	1.30	0.75	173.21	0.19	8
LOC113521981	Short-chain dehydrogenease/reductase	-5.15	-2.67	0.00	2.58	96.72	0.12	6
LOC113521982	Tctex1 domain-containing protein 1-A-like	-2.77	-1.83	0.00	1.58	86.62	0.23	9
LOC113521984	Uncharacterized protein LOC105842600	-4.51	-2.53	0.00	2.31	91.09	0.44	6
LOC113521986	Uncharacterized oxidoreductase YjmC	-3.32	-1.77	0.00	1.67	94.62	0.29	9
LOC113521987	Malate/L-sulfolactate dehydrogenase	-3.51	-1.17	0.00	2.03	173.21	0.26	4
LOC113521991	Gonadotropin-releasing hormone II receptor isoform X3	0.00	0.86	2.58	1.49	173.21	0.14	7
LOC113522003	Protein lethal(2)essential for life-like isoform X1	0.00	1.40	4.19	2.42	173.21	0.07	11
LOC113522005	Esterase FE4	-3.48	-1.86	0.00	1.75	94.05	0.63	9
LOC113522009	Phosphatidate phosphatase like protein	-2.36	-1.53	0.00	1.33	86.69	0.15	9
LOC113522036	Circadian clock-controlled protein-like	0.00	3.13	5.04	2.73	87.33	0.70	10
LOC113522048	CD9 antigen	-6.05	-2.02	0.00	3.50	173.21	0.70	4
LOC113522052	Fatty acid-binding protein	-7.03	-3.58	0.00	3.52	98.09	0.93	6
LOC113522055	Retrotransposable element	-1.29	-0.43	0.00	0.74	173.21	0.26	3
LOC113522063	RNA pseudouridylate synthase domain-containing protein 1-like	-1.79	-0.60	0.00	1.03	173.21	0.07	3
LOC113522083	Sodium-independent sulfate anion transporter-like	0.00	5.02	8.47	4.45	88.58	0.92	10
LOC113522092	Cytochrome CYP6AB14	0.00	2.55	7.65	4.42	173.21	0.22	15
LOC113522093	Cytochrome P450 6B6	0.00	2.70	8.09	4.67	173.21	0.31	15

LOC113522095	Uncharacterized protein LOC106136531	-1.45	-0.48	0.00	0.84	173.21	0.20	3
LOC113522098	Uncharacterized protein LOC106101275	-2.68	-2.54	-2.40	0.14	5.60	0.77	2
LOC113522101	Nuclear protein 1	-1.38	-0.46	0.00	0.80	173.21	0.65	4
LOC113522114	GrpE protein homolog, mitochondrial	0.00	0.58	1.74	1.01	173.21	0.18	8
LOC113522116	Ero1-like protein isoform X2	0.00	0.78	2.33	1.35	173.21	0.50	7
LOC113522118	Uncharacterized protein LOC106107037	-6.27	-2.09	0.00	3.62	173.21	0.74	6
LOC113522123	Heat shock protein Hsp-12.2	0.00	4.44	9.49	4.78	107.67	0.71	10
LOC113522127	Uncharacterized protein LOC105389520	-1.64	-0.55	0.00	0.94	173.21	0.13	3
LOC113522132	Carboxypeptidase B-like isoform X1	0.00	1.25	3.75	2.16	173.21	0.51	11
LOC113522133	Facilitated trehalose transporter Tret1-like isoform X1	-2.89	-0.96	0.00	1.67	173.21	0.44	3
LOC113522134	Zwei Ig domain protein zig-8-like	0.00	1.92	3.48	1.77	92.09	0.24	12
LOC113522135	Uncharacterized protein LOC106134417	-1.53	-0.51	0.00	0.88	173.21	0.17	3
LOC113522137	Uncharacterized protein LOC106133593 isoform X1	-2.17	-0.72	0.00	1.25	173.21	0.21	4
LOC113522138	Cyclin dependent kinase 4	-1.25	-0.42	0.00	0.72	173.21	0.27	3
LOC113522139	5-oxoprolinase	-1.82	-1.11	0.00	0.97	87.75	0.72	9
LOC113522141	Spermidine synthase	-2.88	-1.58	0.00	1.46	92.14	0.16	9
LOC113522153	Uncharacterized protein LOC106133695	-3.48	-1.16	0.00	2.01	173.21	0.66	3
LOC113522159	Uncharacterized protein F09G8.5	0.00	0.69	2.07	1.20	173.21	0.79	8
LOC113522160	Uncharacterized protein LOC106130779	0.00	1.44	4.32	2.50	173.21	0.04	11
LOC113522165	Lysine-specific demethylase lid	0.00	0.47	1.41	0.81	173.21	0.12	8
LOC113522179	Irregular chiasm C-roughest protein-like	0.00	2.02	6.07	3.50	173.21	0.47	15
LOC113522183	Cobatoxin-like protein	0.00	1.78	2.86	1.55	87.28	0.49	12
LOC113522196	Uncharacterized protein	0.00	2.26	6.79	3.92	173.21	0.06	15
LOC113522197	D-arabinitol dehydrogenase 1-like isoform X1	-6.49	-3.49	0.00	3.27	93.90	0.37	6
LOC113522198	Ras-related protein Rab-18-B isoform X1	-1.81	-0.60	0.00	1.05	173.21	0.06	3
LOC113522199	Uncharacterized protein LOC110380143	0.00	0.90	2.70	1.56	173.21	0.03	7
LOC113522200	Uncharacterized protein LOC106101275	-2.83	-1.74	0.00	1.52	87.49	0.11	9
LOC113522203	Leucine-rich repeat-containing G-protein coupled receptor 6-like isoform X1	-2.63	-0.88	0.00	1.52	173.21	0.32	3
LOC113522219	Biliverdin binding protein-1	-2.91	-1.75	0.00	1.54	88.13	0.34	9

LOC113522221	ATP-dependent RNA helicase DHX57	0.00	0.97	2.92	1.68	173.21	0.15	7
LOC113522222	CD63 antigen-like	0.00	0.68	2.05	1.18	173.21	0.73	8
LOC113522226	Uncharacterized protein LOC106136638	-3.80	-1.27	0.00	2.19	173.21	0.76	3
LOC113522228	FERM domain-containing protein 5	0.00	0.50	1.50	0.86	173.21	0.06	8
LOC113522232	Uncharacterized protein LOC106104845	-5.61	-1.87	0.00	3.24	173.21	0.83	6
LOC113522236	Cuticular protein RR-2 motif 134 precursor	0.00	5.41	9.75	4.96	91.75	0.67	14
LOC113522241	Fatty acid synthase-like	-2.26	-1.47	0.00	1.27	86.70	0.19	9
LOC113522252	Multidrug resistance-associated protein lethal(2)03659	0.00	1.18	3.55	2.05	173.21	0.88	11
LOC113522283	Uncharacterized protein LOC101743779	-2.31	-0.77	0.00	1.33	173.21	0.17	3
LOC113522287	Uncharacterized protein LOC106110164	0.00	1.36	2.71	1.36	100.00	0.82	7
LOC113522290	Outer kinetochore Ndc80	-2.36	-0.79	0.00	1.36	173.21	0.19	3
LOC113522291	Fibroblast growth factor 7	0.00	0.82	2.47	1.42	173.21	0.28	7
LOC113522293	Solute carrier family 25 member 35-like isoform X1	0.00	0.52	1.57	0.91	173.21	0.02	8
LOC113522294	UDP-glucosyltransferase protein 3	0.00	2.29	6.86	3.96	173.21	0.04	15
LOC113522295	Phospholipid scramblase 1-like	0.00	1.19	3.58	2.07	173.21	0.80	11
LOC113522299	Hypothetical protein KGM_205387	-4.42	-2.06	0.00	2.22	108.18	0.89	6
LOC113522301	Uncharacterized protein LOC106142083	-4.39	-2.62	0.00	2.31	88.36	0.99	9
LOC113522304	Fatty acid synthase	0.00	0.61	1.83	1.05	173.21	0.29	8
LOC113522311	Protein phosphatase 2C T23F11.1 isoform X1	0.00	0.59	1.77	1.02	173.21	0.21	8
LOC113522333	Growth arrest and DNA damage-inducible protein GADD45 alpha	0.00	1.96	3.48	1.78	90.78	0.17	12
LOC113522340	Uncharacterized protein K02A2.6-like	-2.12	-0.71	0.00	1.23	173.21	0.08	3
LOC113522346	G-protein coupled receptor moody-like isoform X1	0.00	1.46	2.40	1.28	87.78	0.91	12
LOC113522348	Mitochondrial import inner membrane translocase subunit Tim9-like	0.00	0.53	1.60	0.92	173.21	0.44	1
LOC113522349	Nidogen-1	-5.93	-2.65	0.00	3.01	113.88	0.34	6
LOC113522357	Kinesin-like protein KIF18A	-1.94	-0.65	0.00	1.12	173.21	0.01	3
LOC113522361	Zinc finger protein 57	-1.78	-1.07	0.00	0.94	88.14	0.81	9
LOC113522374	Tetratricopeptide repeat protein 30A	-3.59	-2.20	0.00	1.93	87.59	0.60	9

LOC113522379	Uncharacterized protein K02A2.6-like	-1.19	-0.40	0.00	0.68	173.21	0.29	3
LOC113522388	Uncharacterized protein LOC106141564	0.00	2.33	7.00	4.04	173.21	0.04	15
LOC113522390	Ceramide phosphoethanolamine synthase	-1.31	-0.44	0.00	0.76	173.21	0.25	3
LOC113522400	Uncharacterized protein	-5.50	-1.83	0.00	3.18	173.21	0.65	4
LOC113522413	UDP-glycosyltransferase UGT44A2	0.00	0.96	2.87	1.66	173.21	0.12	7
LOC113522415	AF4/FMR2 family member 1-like isoform X5	0.00	0.69	2.07	1.19	173.21	0.77	8
LOC113522419	Fatty acyl-CoA reductase 1-like	0.00	1.99	5.97	3.44	173.21	0.56	15
LOC113522425	Partner of xrn-2 protein 1-like	-1.31	-0.44	0.00	0.76	173.21	0.25	3
LOC113522438	Innexin inx1	0.00	1.38	4.14	2.39	173.21	0.10	11
LOC113522439	MFS-type transporter SLC18B1-like	-2.47	-0.82	0.00	1.43	173.21	0.10	4
LOC113522440	Uncharacterized protein OBRU01_10191	-3.70	-1.76	0.00	1.86	105.83	0.53	9
LOC113522442	Uncharacterized protein LOC106136992	-4.93	-2.13	0.00	2.53	118.93	0.68	6
LOC113522443	Serine/threonine-protein kinase GA29083	0.00	0.53	1.59	0.92	173.21	0.03	8
LOC113522448	Uncharacterized protein LOC106130957	0.00	1.03	3.09	1.79	173.21	0.32	7
LOC113522449	Cysteine-rich with EGF-like domain protein 2 isoform X1	0.00	1.52	2.63	1.36	89.67	0.71	12
LOC113522454	Glia-derived nexin-like isoform X1	-3.03	-1.01	0.00	1.75	173.21	0.11	4
LOC113522455	Uncharacterized protein LOC106136943	-2.52	-0.84	0.00	1.46	173.21	0.08	4
LOC113522456	Ras-like GTP-binding protein RhoL	0.00	0.94	1.58	0.83	88.26	0.60	1
LOC113522457	Serine/threonine-protein kinase polo isoform X2	-3.14	-1.05	0.00	1.81	173.21	0.54	3
LOC113522459	Uncharacterized protein LOC106129346	-3.13	-1.79	0.00	1.61	90.08	0.19	9
LOC113522462	Fizzy-related protein homolog	0.00	0.57	1.71	0.99	173.21	0.15	8
LOC113522463	Mucin-5AC	0.00	1.20	3.59	2.07	173.21	0.79	11
LOC113522466	Uncharacterized protein LOC106138412	0.00	2.37	3.99	2.10	88.54	0.21	12
LOC113522477	Uncharacterized protein LOC105388560	0.00	0.53	1.60	0.92	173.21	0.04	8
LOC113522480	Glutamate--cysteine ligase catalytic subunit	0.00	0.52	1.56	0.90	173.21	0.01	8
LOC113522488	Inter-Alpha-trypsin inhibitor heavy chain H4-like	0.00	2.24	4.20	2.11	94.58	0.35	12
LOC113522496	Kynurenine aminotransferase	-1.70	-0.57	0.00	0.98	173.21	0.10	3
LOC113522497	Uncharacterized protein LOC106138090	-2.88	-1.46	0.00	1.44	98.49	0.39	9
LOC113522500	Uncharacterized protein LOC106129400	0.00	1.88	3.70	1.85	98.31	0.47	12

LOC113522502	Carbohydrate sulfotransferase 4-like	-1.33	-0.44	0.00	0.77	173.21	0.24	3
LOC113522508	HIG1 domain family member 1A, mitochondrial-like isoform X1	0.00	1.41	2.12	1.22	86.60	0.82	1
LOC113522510	Division abnormally delayed protein	-1.35	-0.45	0.00	0.78	173.21	0.24	3
LOC113522512	Gamma-tubulin complex component 3 isoform X1	-1.27	-0.42	0.00	0.74	173.21	0.26	3
LOC113522517	Tubulin beta-4B chain-like	0.00	1.70	5.09	2.94	173.21	0.17	5
LOC113522525	Beta-carotene-binding protein	0.00	0.56	1.68	0.97	173.21	0.11	8
LOC113522527	Serine protease inhibitor 6	2.30	4.81	6.10	2.18	45.27	0.53	13
LOC113522530	Regulating synaptic membrane exocytosis protein 2	-1.71	-0.57	0.00	0.99	173.21	0.44	4
LOC113522531	Uncharacterized protein LOC106132906	-1.29	-0.43	0.00	0.74	173.21	0.72	4
LOC113522536	Argininosuccinate synthase	0.00	0.94	2.82	1.63	173.21	0.09	7
LOC113522542	Long-chain-fatty-acid--CoA ligase 4	0.00	0.52	1.56	0.90	173.21	0.01	8
LOC113522547	O-glucosyltransferase rumi homolog	-1.55	-0.52	0.00	0.89	173.21	0.16	3
LOC113522555	GTPase-activating Rap/Ran-GAP domain-like protein 3	-1.22	-0.41	0.00	0.70	173.21	0.28	3
LOC113522562	N-acetylglucosaminidase	-4.53	-2.18	0.00	2.27	103.81	0.71	6
LOC113522563	Sulfiredoxin-1 isoform X1	0.00	0.60	1.81	1.05	173.21	0.27	8
LOC113522572	Uncharacterized protein LOC110371012	-6.78	-4.17	-2.58	2.27	54.46	0.96	2
LOC113522574	MFS-type transporter SLC18B1-like	0.00	2.56	4.19	2.24	87.63	0.38	12
LOC113522576	Protein maternal effect lethal 26-like	0.00	0.60	1.80	1.04	173.21	0.26	8
LOC113522584	DNA repair protein XRCC2	0.00	0.62	1.87	1.08	173.21	0.40	1
LOC113522591	Multiple C2 and transmembrane domain-containing protein 1-like	0.00	0.45	1.36	0.79	173.21	0.15	8
LOC113522593	Neuronal membrane glycoprotein M6-a	0.00	0.45	1.34	0.77	173.21	0.17	8
LOC113522598	Uncharacterized protein LOC106139316	-5.45	-3.95	-2.55	1.45	36.81	0.57	2
LOC113522607	DNA replication licensing factor Mcm7	-2.26	-0.75	0.00	1.31	173.21	0.14	3
LOC113522612	Aquaporin AQPAe.a	-1.72	-0.57	0.00	0.99	173.21	0.44	4
LOC113522620	Peritrophin-1-like	-1.67	-0.56	0.00	0.96	173.21	0.47	4
LOC113522631	Beta-1,4-glucuronyltransferase 1	-1.87	-0.62	0.00	1.08	173.21	0.03	3
LOC113522635	Uncharacterized oxidoreductase SERP2049-like	-1.96	-1.19	0.00	1.05	87.70	0.58	9

LOC113522643	SET and MYND domain-containing protein 4	0.00	0.48	1.45	0.83	173.21	0.47	1
LOC113522644	Uncharacterized oxidoreductase SERP2049-like	-3.04	-1.01	0.00	1.76	173.21	0.50	3
LOC113522647	Protein takeout	0.00	3.45	6.59	3.30	95.82	0.37	10
LOC113522657	Fanconi anemia group D2 protein	-1.67	-0.56	0.00	0.96	173.21	0.12	3
LOC113522665	Uncharacterized protein LOC106139316	-7.01	-4.67	-3.30	2.04	43.60	0.65	2
LOC113522666	Protein archease-like	0.00	0.41	1.22	0.70	173.21	0.23	8
LOC113522667	Uncharacterized protein LOC106139316	-5.30	-4.23	-2.14	1.82	42.90	0.76	2
LOC113522672	Uncharacterized protein LOC106129314	0.00	1.88	5.65	3.26	173.21	0.91	15
LOC113522675	Uncharacterized protein LOC106118229	-1.66	-0.55	0.00	0.96	173.21	0.12	3
LOC113522676	Uncharacterized protein LOC106129334 isoform X2	-2.07	-0.69	0.00	1.20	173.21	0.26	4
LOC113522677	von Willebrand factor A domain-containing protein 9	-1.24	-0.41	0.00	0.72	173.21	0.27	3
LOC113522679	Glutathione S-transferase zeta 2	-1.26	-0.42	0.00	0.73	173.21	0.27	3
LOC113522681	DnaJ homolog subfamily C member 3	0.00	0.49	1.47	0.85	173.21	0.08	8
LOC113522683	Uncharacterized protein LOC110371118	-7.40	-4.43	-2.30	2.66	60.02	0.90	6
LOC113522693	DNA polymerase alpha catalytic subunit	-1.69	-0.56	0.00	0.97	173.21	0.11	3
LOC113522710	Replication factor C subunit 4	-1.59	-0.53	0.00	0.92	173.21	0.15	3
LOC113522714	E3 ubiquitin-protein ligase RNF180-like	0.00	0.41	1.22	0.70	173.21	0.23	8
LOC113522721	ATP-binding cassette sub-family F member 1	0.00	0.40	1.20	0.69	173.21	0.24	8
LOC113522728	Tubulin beta chain	0.00	5.07	8.77	4.54	89.62	0.89	5
LOC113522730	Uncharacterized protein LOC106138999	-2.74	-0.91	0.00	1.58	173.21	0.37	3
LOC113522731	Aquaporin AQPAn.G isoform X1	-1.54	-0.51	0.00	0.89	173.21	0.17	3
LOC113522738	Retinol dehydrogenase 11-like isoform X1	0.00	1.27	3.80	2.20	173.21	0.44	11
LOC113522739	Aldose 1-epimerase-like	-3.50	-1.77	0.00	1.75	99.14	0.40	9
LOC113522753	Uncharacterized protein LOC106138721	0.00	1.13	3.40	1.96	173.21	0.77	7
LOC113522757	Uncharacterized protein LOC106129350	-4.01	-1.90	0.00	2.01	106.22	0.74	9
LOC113522759	Surfeit locus protein 6 homolog	0.00	0.87	1.33	0.75	86.69	0.35	1
LOC113522760	Uncharacterized protein LOC106139325	-5.69	-3.00	0.00	2.86	95.39	0.13	6
LOC113522767	PAX3- and PAX7-binding protein 1	0.00	0.60	1.79	1.04	173.21	0.25	8

LOC113522768	Glucose dehydrogenase	0.00	0.52	1.57	0.91	173.21	0.44	1
LOC113522771	Rab-like protein 6	-1.47	-0.49	0.00	0.85	173.21	0.19	3
LOC113522775	Zinc finger protein 235-like	-1.49	-1.00	0.00	0.86	86.60	0.88	9
LOC113522777	Neurogenic locus Notch protein isoform X3	-2.78	-1.45	0.00	1.40	96.31	0.34	9
LOC113522778	Multifunctional protein ADE2	-2.23	-0.74	0.00	1.29	173.21	0.13	3
LOC113522784	Brinker	0.00	0.78	2.35	1.35	173.21	0.48	7
LOC113522785	Dual specificity protein kinase TTK	-1.67	-0.56	0.00	0.96	173.21	0.12	3
LOC113522786	Uncharacterized protein LOC106138639	0.00	1.36	2.45	1.25	91.69	0.54	1
LOC113522789	FGFR1 oncogene partner-like	0.00	1.01	1.54	0.87	86.66	0.35	1
LOC113522799	Uncharacterized protein LOC110374636	-1.53	-0.51	0.00	0.89	173.21	0.17	3
LOC113522801	Uncharacterized protein LOC109421171	-2.12	-1.18	0.00	1.08	91.65	0.65	9
LOC113522803	Zinc finger protein GLIS2-like	-2.42	-1.53	0.00	1.33	87.02	0.26	9
LOC113522808	Transient receptor potential cation channel subfamily V member 5	-4.18	-2.16	0.00	2.10	96.87	0.93	6
LOC113522824	Lipopolysaccharide-induced tumor necrosis factor-alpha factor-like	0.00	0.97	2.91	1.68	173.21	0.15	7
LOC113522828	Uncharacterized protein LOC110371440	-7.20	-4.39	-2.26	2.54	57.74	0.91	6
LOC113522838	Small integral membrane protein 8	0.00	0.99	1.63	0.87	88.06	0.21	1
LOC113522840	Glycine--tRNA ligase	0.00	0.53	1.58	0.91	173.21	0.02	8
LOC113522844	Uncharacterized protein	-2.57	-1.33	0.00	1.29	97.06	0.49	9
LOC113522845	Hypothetical protein KGM_202170	-1.34	-0.45	0.00	0.77	173.21	0.24	3
LOC113522851	Gustatory receptor for sugar taste 64f-like	-2.52	-0.84	0.00	1.45	173.21	0.26	3
LOC113522854	Glycine N-methyltransferase	0.00	2.32	4.11	2.11	90.87	0.26	12
LOC113522856	Vesicle-trafficking protein SEC22b-B	0.00	0.45	1.35	0.78	173.21	0.16	8
LOC113522860	Protein crumbs	0.00	0.84	2.52	1.45	173.21	0.21	7
LOC113522864	Outer kinetochore Dsn1	-1.41	-0.47	0.00	0.82	173.21	0.21	3
LOC113522868	Innexin shaking-B	-5.54	-1.85	0.00	3.20	173.21	0.65	4
LOC113522881	Uncharacterized protein	-2.43	0.08	2.67	2.55	3186.92	0.93	7
LOC113522883	Innexin shaking-B	-8.05	-3.77	0.00	4.05	107.51	0.99	6
LOC113522888	Uncharacterized protein LOC110383083	-2.90	-1.55	0.00	1.46	94.47	0.24	9
LOC113522889	Protein SMG7-like isoform X2	0.00	0.39	1.18	0.68	173.21	0.25	8
LOC113522895	Mediator of DNA damage checkpoint protein 1	-3.93	-2.57	0.00	2.22	86.67	0.91	6

LOC113522896	Neurogenic locus Notch protein	-2.79	-1.39	0.00	1.39	100.60	0.50	9
LOC113522899	Uncharacterized protein LOC106129443	-3.70	-1.23	0.00	2.14	173.21	0.31	4
LOC113522904	Uncharacterized protein LOC106127552	-4.95	-3.19	0.00	2.77	86.77	0.50	6
LOC113522905	Aquaporin AQPAn.G isoform X1	-1.95	-0.65	0.00	1.12	173.21	0.01	3
LOC113522918	Uncharacterized protein LOC106111100	-2.65	-1.67	0.00	1.46	87.00	0.09	9
LOC113522927	Uncharacterized protein LOC106129317	-2.59	-0.86	0.00	1.50	173.21	0.30	3
LOC113522928	Uncharacterized protein	-3.98	-2.49	0.00	2.17	87.15	0.81	6
LOC113522930	Zinc finger protein 395	0.00	1.30	2.32	1.19	91.34	0.85	1
LOC113522932	Uncharacterized protein LOC105383334	-1.36	-0.45	0.00	0.78	173.21	0.23	3
LOC113522936	CTL-like protein 2 isoform X3	0.00	0.47	1.42	0.82	173.21	0.48	1
LOC113522941	Homeobox protein B-H1-like	-2.28	-1.33	0.00	1.19	89.15	0.35	9
LOC113522943	Kallikrein-7-like	0.00	1.91	5.74	3.32	173.21	0.12	5
LOC113522950	Multidrug resistance-associated protein 4-like	-2.73	-1.54	0.00	1.40	90.88	0.14	9
LOC113522951	Sarcosine dehydrogenase, mitochondrial	-1.96	-0.65	0.00	1.13	173.21	0.01	3
LOC113522957	Choline-phosphate cytidylyltransferase A-like isoform X2	0.00	1.38	2.39	1.24	89.84	0.95	1
LOC113522958	Centaurin-gamma-1A-like	-3.44	-1.15	0.00	1.99	173.21	0.24	4
LOC113522967	Uncharacterized protein LOC110384273	0.00	1.18	3.55	2.05	173.21	0.87	11
LOC113522976	Elongation factor 1-alpha 2	-4.50	-1.50	0.00	2.60	173.21	0.90	3
LOC113522981	Matrix metalloproteinase-14 isoform X1	0.00	4.03	6.39	3.51	87.03	0.25	10
LOC113522982	Uncharacterized protein LOC106136966	-5.04	-2.91	0.00	2.61	89.68	0.28	6
LOC113522992	1-acyl-sn-glycerol-3-phosphate acyltransferase Alpha-like	-1.82	-0.61	0.00	1.05	173.21	0.06	3
LOC113522996	Golgin subfamily A member 4-like	0.00	0.86	1.30	0.74	86.62	0.43	1
LOC113522998	Zinc finger protein Dzip1	-2.28	-0.76	0.00	1.32	173.21	0.15	3
LOC113523000	Pseudouridine-metabolizing bifunctional protein C1861.05	0.00	1.56	2.37	1.35	86.63	0.77	12
LOC113523002	EF-hand domain-containing protein 1-like	0.00	0.63	1.88	1.09	173.21	0.38	8
LOC113523004	Stromal cell-derived factor 2	0.00	0.40	1.20	0.69	173.21	0.24	8
LOC113523008	Multidrug resistance-associated protein lethal(2)03659 isoform X1	-2.16	-1.15	0.00	1.09	94.48	0.95	9
LOC113523010	Acetylcholinesterase 1	-6.27	-2.09	0.00	3.62	173.21	0.72	4

LOC113523011	Surface protein bspA-like	2.29	2.70	3.33	0.56	20.75	0.68	12
LOC113523022	Uncharacterized protein LOC101740673	0.00	1.40	4.21	2.43	173.21	0.05	11
LOC113523023	GTPase-activating protein	0.00	1.09	1.66	0.94	86.64	0.38	1
LOC113523025	Carboxylesterase	-1.51	-0.50	0.00	0.87	173.21	0.18	3
LOC113523028	Uncharacterized protein LOC110384608	-2.84	-1.87	0.00	1.62	86.62	0.26	9
LOC113523029	Pancreatic triacylglycerol lipase-like	-3.82	-2.33	0.00	2.04	87.71	0.95	9
LOC113523033	Uncharacterized protein	0.00	1.96	5.88	3.40	173.21	0.64	15
LOC113523037	Titin-like isoform X1	0.00	3.77	5.88	3.28	86.79	0.27	10
LOC113523041	Uncharacterized protein LOC110371101	-2.68	-1.47	0.00	1.36	92.20	0.22	9
LOC113523044	Hypothetical protein KGM_204434	0.00	1.87	5.62	3.25	173.21	0.12	5
LOC113523050	4-coumarate--CoA ligase 1-like	-2.41	-1.45	0.00	1.28	88.08	0.39	9
LOC113523060	Adenylate cyclase type 8	0.00	1.96	2.98	1.69	86.64	0.24	12
LOC113523069	Uncharacterized protein LOC110378344	-3.97	-1.32	0.00	2.29	173.21	0.80	3
LOC113523072	Glypican-6 isoform X2	0.00	0.54	1.61	0.93	173.21	0.04	8
LOC113523076	GC-rich sequence DNA-binding factor 1	0.00	0.61	1.83	1.06	173.21	0.30	8
LOC113523079	Cytochrome P450 family 337 subfamily a polypeptide 1	0.00	0.79	2.37	1.37	173.21	0.44	7
LOC113523085	Hypothetical protein KGM_208650	0.00	0.46	1.39	0.80	173.21	0.13	8
LOC113523089	Neuroligin-4, Y-linked-like isoform X1	-3.39	-1.95	0.00	1.75	89.79	0.37	9
LOC113523090	Gibberellin 20 oxidase 2	0.00	0.96	1.48	0.83	86.74	0.43	1
LOC113523092	Oxidation resistance protein 1 isoform X2	-1.50	-0.50	0.00	0.87	173.21	0.57	4
LOC113523094	Formin-binding protein 1-like	0.00	1.06	1.81	0.94	89.20	0.65	1
LOC113523096	Phosphoglycerate kinase	0.00	0.51	1.53	0.88	173.21	0.45	1
LOC113523100	Uncharacterized protein LOC106136948 isoform X1	-1.78	-0.59	0.00	1.03	173.21	0.07	3
LOC113523104	Heat shock 70 kDa protein cognate 3 isoform X1	0.00	1.44	2.37	1.26	87.92	0.97	12
LOC113523106	Cytochrome P450 6B5-like isoform X1	0.00	1.24	3.72	2.15	173.21	0.56	11
LOC113523114	Interferon-related developmental regulator 2	0.00	1.40	4.21	2.43	173.21	0.06	11
LOC113523131	Uncharacterized protein LOC106113363	-1.86	-0.62	0.00	1.07	173.21	0.36	4
LOC113523133	Leucine rich repeat protein	0.00	0.83	2.48	1.43	173.21	0.26	7
LOC113523136	2-oxoisovalerate dehydrogenase subunit beta, mitochondrial isoform X1	-1.32	-0.44	0.00	0.76	173.21	0.24	3

LOC113523144	Oxidoreductase	-2.71	-1.80	0.00	1.56	86.60	0.20	9
LOC113523146	Lysosomal alpha-mannosidase-like isoform X1	-2.89	-1.62	0.00	1.48	91.21	0.13	9
LOC113523151	Cyclin-dependent kinase 1	-1.29	-0.43	0.00	0.74	173.21	0.26	3
LOC113523160	Neuropeptide receptor A6-B	0.00	2.05	6.16	3.55	173.21	0.40	15
LOC113523167	Uncharacterized protein LOC106140814	0.00	1.54	3.17	1.59	103.29	0.95	12
LOC113523175	Tyrosine-protein kinase-like otk	-1.53	-0.51	0.00	0.89	173.21	0.17	3
LOC113523183	EF-hand calcium-binding domain-containing protein 2 isoform X1	-2.54	-1.40	0.00	1.29	92.05	0.29	9
LOC113523187	Porphobilinogen deaminase	0.00	0.40	1.20	0.69	173.21	0.24	8
LOC113523190	Uncharacterized protein	0.00	4.34	7.92	4.01	92.48	0.39	10
LOC113523204	Irregular chiasm C-roughest protein-like	0.00	2.18	6.54	3.78	173.21	0.17	15
LOC113523209	23 kDa integral membrane protein-like	0.00	3.05	6.32	3.17	103.73	0.75	10
LOC113523210	23 kDa integral membrane protein-like	0.00	2.94	6.30	3.17	107.71	0.96	10
LOC113523213	Inactive tyrosine-protein kinase 7	-1.93	-0.64	0.00	1.12	173.21	0.01	3
LOC113523214	Uncharacterized protein LOC106125798	-1.36	-0.45	0.00	0.79	173.21	0.23	3
LOC113523215	Ryanodien receptor	-1.78	-0.59	0.00	1.03	173.21	0.07	3
LOC113523217	Glycerol kinase-like isoform X1	0.00	1.33	2.12	1.16	87.12	0.58	1
LOC113523219	Uncharacterized protein LOC106130432	-2.85	-1.58	0.00	1.45	91.77	0.15	9
LOC113523237	Serine/threonine-protein kinase MARK2 isoform X6	0.00	1.70	2.82	1.50	88.04	0.41	12
LOC113523239	GTP cyclohydrolase 1 isoform X1	0.00	3.68	5.60	3.19	86.63	0.42	10
LOC113523252	Three-prime repair exonuclease 1-like	-1.24	-0.41	0.00	0.72	173.21	0.27	3
LOC113523265	Protein bric-a-brac 2-like isoform X2	-1.85	-0.62	0.00	1.07	173.21	0.04	3
LOC113523269	Gloverin-like	3.58	3.85	4.27	0.37	9.54	0.57	13
LOC113523271	Neural/ectodermal development factor IMP-L2-like	0.00	2.35	7.05	4.07	173.21	0.06	15
LOC113523274	Uncharacterized protein	-3.68	-2.02	0.00	1.87	92.47	0.56	9
LOC113523275	Mitochondrial sodium/hydrogen exchanger 9B2 isoform X1	-1.53	-1.00	0.00	0.87	86.65	0.88	9
LOC113523288	Uncharacterized protein	-2.06	-0.69	0.00	1.19	173.21	0.26	4
LOC113523292	Calnexin isoform X1	0.00	0.56	1.67	0.96	173.21	0.10	8
LOC113523293	Pollen-specific Leucine-rich repeat extensin-	0.00	3.05	4.75	2.65	86.79	0.87	10

	like protein 1							
LOC113523296	Uncharacterized protein LOC106131367	0.00	1.18	3.53	2.04	173.21	0.92	11
LOC113523298	Cytochrome P450 CYP12A2-like	-1.81	-0.60	0.00	1.04	173.21	0.39	4
LOC113523301	Vascular endothelial growth factor receptor 1 isoform X1	-1.98	-1.10	0.00	1.01	91.67	0.84	9
LOC113523307	Uncharacterized protein LOC106131368 isoform X1	-3.69	-2.14	0.00	1.91	89.48	0.55	9
LOC113523315	Arylsulfatase I-like	-1.31	-0.44	0.00	0.76	173.21	0.25	3
LOC113523317	Multidrug resistance protein 1A-like	-3.39	-1.13	0.00	1.96	173.21	0.22	4
LOC113523321	Insect cytokine precursor uENF2	-1.30	-0.43	0.00	0.75	173.21	0.25	3
LOC113523330	Uncharacterized protein LOC106131362	0.00	0.47	1.42	0.82	173.21	0.11	8
LOC113523334	Uncharacterized protein LOC106721094	0.00	0.90	2.71	1.56	173.21	0.03	7
LOC113523340	Hdd1-like protein	0.00	1.66	4.98	2.87	173.21	0.37	11
LOC113523353	LIM domain only protein 3 isoform X1	-2.05	-0.68	0.00	1.18	173.21	0.27	4
LOC113523354	Uncharacterized protein LOC110370996 isoform X1	-2.44	0.04	2.56	2.50	6152.02	0.98	7
LOC113523358	Proton-coupled amino acid transporter 4-like	0.00	4.47	6.89	3.88	86.70	0.55	10
LOC113523359	Proton-coupled amino acid transporter 4	0.00	6.97	10.88	6.05	86.81	0.31	14
LOC113523362	Uncharacterized protein LOC106131717	-5.38	-3.58	0.00	3.10	86.60	0.62	6
LOC113523363	Protein sprouty isoform X1	-4.27	-2.17	0.00	2.14	98.49	0.86	6
LOC113523364	G1/S-specific cyclin-D3-like	-2.15	-0.72	0.00	1.24	173.21	0.09	3
LOC113523374	Cytochrome P450 CYP12A2-like	0.00	0.93	2.80	1.61	173.21	0.07	7
LOC113523375	Uncharacterized protein LOC106131388 isoform X1	0.00	2.27	3.47	1.96	86.66	0.22	12
LOC113523377	Bestrophin 1b	0.00	1.07	1.68	0.93	86.88	0.47	1
LOC113523378	Uncharacterized protein LOC106131292	0.00	0.98	1.63	0.87	88.19	0.59	1
LOC113523379	Uncharacterized protein LOC106131151	0.00	0.52	1.57	0.91	173.21	0.01	8
LOC113523382	ATP-binding cassette sub-family G member 1-like	-3.98	-2.04	0.00	1.99	97.69	0.78	9
LOC113523394	Nose resistant to fluoxetine protein 6-like	0.00	1.76	5.29	3.06	173.21	0.63	11
LOC113523401	Laminin subunit beta-1	-2.96	-0.99	0.00	1.71	173.21	0.47	3
LOC113523403	Phosphatidylcholine:ceramide cholinephosphotransferase 2-like isoform	0.00	0.48	1.45	0.83	173.21	0.09	8

	X1							
LOC113523409	Hemolymph lipopolysaccharide-binding protein	-4.10	-1.37	0.00	2.37	173.21	0.83	3
LOC113523410	Zinc transporter 1	-2.96	-0.99	0.00	1.71	173.21	0.46	3
LOC113523415	Protein yellow-like	0.00	4.10	6.90	3.63	88.47	0.20	10
LOC113523417	Cytochrome P450 4g15	-2.22	-0.74	0.00	1.28	173.21	0.19	4
LOC113523423	Potassium channel subfamily K member 18-like isoform X1	0.00	2.37	3.57	2.06	86.60	0.24	12
LOC113523425	Defensin	0.00	1.73	2.75	1.50	87.08	0.40	12
LOC113523439	30.3 kDa protein	0.00	0.63	1.90	1.09	173.21	0.40	1
LOC113523440	Antifungal peptide gallerimycin	3.02	3.80	4.65	0.82	21.51	0.52	13
LOC113523442	Defensin	0.00	2.65	4.05	2.30	86.65	0.47	12
LOC113523448	Proton-coupled amino acid transporter 4	0.00	5.35	8.39	4.65	86.88	0.69	14
LOC113523449	Uncharacterized protein	-2.95	-1.54	0.00	1.48	96.07	0.74	9
LOC113523460	Uncharacterized protein LOC110370969	0.00	1.54	2.98	1.49	96.96	0.77	12
LOC113523465	Transient receptor potential cation channel protein painless	-1.22	-0.41	0.00	0.71	173.21	0.28	3
LOC113523468	Nose resistant to fluoxetine protein 6-like	0.00	5.75	9.87	5.13	89.29	0.40	14
LOC113523473	Synaptotagmin-5	-3.84	-2.32	0.00	2.04	88.04	0.95	9
LOC113523474	Venom dipeptidyl peptidase 4	0.00	1.96	3.09	1.70	86.94	0.29	12
LOC113523476	Axoneme-associated protein mst101(2)	-1.49	-0.50	0.00	0.86	173.21	0.18	3
LOC113523482	Heparan-Alpha-glucosaminide N-acetyltransferase	0.00	2.49	4.31	2.23	89.58	0.35	12
LOC113523483	Golgin subfamily B member 1-like isoform X3	-1.52	-0.51	0.00	0.88	173.21	0.18	3
LOC113523494	Tetraspanin-17	0.00	1.30	3.90	2.25	173.21	0.32	11
LOC113523495	Cysteine--tRNA ligase, cytoplasmic isoform X1	0.00	0.81	2.42	1.40	173.21	0.35	7
LOC113523502	Uncharacterized protein LOC106131056	-1.43	-0.48	0.00	0.83	173.21	0.21	3
LOC113523505	Mucin-5AC-like isoform X1	0.00	0.62	1.86	1.07	173.21	0.34	8
LOC113523512	Uncharacterized protein	-1.98	-1.13	0.00	1.02	90.22	0.73	9
LOC113523522	Uncharacterized protein	-2.25	-1.39	0.00	1.21	87.44	0.26	9
LOC113523523	MAP kinase-activating death domain protein	-2.58	-1.39	0.00	1.30	93.54	0.34	9

LOC113523533	Uncharacterized protein LOC106131316	-4.10	-3.41	-2.96	0.61	17.83	0.11	2
LOC113523539	Glutamine-dependent NAD(+) synthetase	0.00	1.01	1.76	0.91	90.03	0.70	1
LOC113523540	Cytosolic carboxypeptidase 2	0.00	1.33	4.00	2.31	173.21	0.22	11
LOC113523541	Teneurin-3 isoform X1	-2.98	-1.46	0.00	1.49	102.34	0.48	9
LOC113523542	Uncharacterized protein PFB0145c-like	-1.63	-0.54	0.00	0.94	173.21	0.13	3
LOC113523547	Programmed cell death protein 6 isoform X1	0.00	0.87	2.62	1.51	173.21	0.10	7
LOC113523555	Proton-coupled amino acid transporter 4-like	0.00	0.79	2.37	1.37	173.21	0.43	7
LOC113523560	Uncharacterized protein LOC106131089	0.00	0.40	1.21	0.70	173.21	0.23	8
LOC113523568	Epoxide hydrolase 1-like	-2.42	-1.56	0.00	1.35	86.77	0.12	9
LOC113523571	Uncharacterized protein LOC110370405	-2.53	-0.84	0.00	1.46	173.21	0.08	4
LOC113523572	Uncharacterized protein LOC106123314	0.00	7.35	11.62	6.39	86.99	0.36	14
LOC113523582	Zinc/cadmium resistance protein	0.00	0.68	2.03	1.17	173.21	0.69	8
LOC113523585	Leucine--tRNA ligase, cytoplasmic	0.00	0.40	1.19	0.69	173.21	0.24	8
LOC113523600	Vesicle-associated membrane protein 7	-1.41	-0.47	0.00	0.81	173.21	0.21	3
LOC113523601	Phospholipid-transporting ATPase IA isoform X2	0.00	0.39	1.18	0.68	173.21	0.25	8
LOC113523605	Eukaryotic translation initiation factor 4 gamma 2	0.00	0.82	1.29	0.72	86.87	0.35	1
LOC113523607	Uncharacterized protein	0.00	2.27	4.33	2.17	95.94	0.41	12
LOC113523609	Spondin-1	-1.53	-0.51	0.00	0.88	173.21	0.17	3
LOC113523611	6Tox	1.64	2.76	3.93	1.15	41.55	0.65	12
LOC113523629	LIM domain only protein 3	-4.31	-1.44	0.00	2.49	173.21	0.46	4
MSTRG.10017	Uncharacterized protein LOC106138419	-1.73	-0.58	0.00	1.00	173.21	0.09	3
MSTRG.10058	Kunitz/Bovine pancreatic trypsin inhibitor domain protein	0.00	1.12	1.76	0.97	86.89	0.35	1
MSTRG.10067	G-protein coupled receptor Mth2-like	0.00	2.69	4.47	2.37	88.05	0.55	12
MSTRG.10085	Uncharacterized protein	-1.94	-0.65	0.00	1.12	173.21	0.32	4
MSTRG.1009	Uncharacterized protein	-1.86	-0.62	0.00	1.07	173.21	0.04	3
MSTRG.10131	Zinc finger BED domain-containing protein 1-like	-2.45	-0.82	0.00	1.41	173.21	0.23	3
MSTRG.10169	Uncharacterized protein LOC106134230	-1.69	-1.12	0.00	0.97	86.61	0.65	9
MSTRG.10207	Uncharacterized protein LOC106134231	0.00	1.61	2.53	1.40	86.87	0.73	12
MSTRG.10254	Uncharacterized protein	0.00	3.28	5.36	2.87	87.66	0.47	10

MSTRG.10267	Uncharacterized protein	-2.38	-0.79	0.00	1.38	173.21	0.20	3
MSTRG.1027	Acyl-CoA-binding domain-containing protein 5 isoform X6	-3.18	-1.06	0.00	1.83	173.21	0.56	3
MSTRG.10275	Azurocidin-like	-3.23	-2.14	0.00	1.85	86.61	0.53	9
MSTRG.10296	Uncharacterized protein	0.00	2.93	4.63	2.55	86.98	0.90	12
MSTRG.1036	Uncharacterized protein	0.00	1.99	3.50	1.80	90.28	0.49	12
MSTRG.10395	Eukaryotic translation initiation factor 4E-binding protein 2	-5.51	-2.79	0.00	2.76	98.91	0.07	6
MSTRG.10427	Uncharacterized protein	0.00	0.75	2.24	1.29	173.21	0.72	7
MSTRG.10470	Uncharacterized protein	-1.49	-0.50	0.00	0.86	173.21	0.19	3
MSTRG.10478	Uncharacterized protein	-1.51	-1.00	0.00	0.86	86.61	0.87	9
MSTRG.10481	Neural/ectodermal development factor IMP-L2-like	0.00	0.78	2.35	1.36	173.21	0.47	7
MSTRG.10541	Uncharacterized protein	-2.63	-0.88	0.00	1.52	173.21	0.32	3
MSTRG.10559	Uncharacterized protein LOC106135284	-1.79	-0.60	0.00	1.03	173.21	0.40	4
MSTRG.10582	Uncharacterized protein	0.00	6.96	10.90	6.04	86.86	0.30	14
MSTRG.10599	Xanthine dehydrogenase-like	-5.38	-3.58	0.00	3.10	86.60	0.63	6
MSTRG.106	Asparagine--tRNA ligase, cytoplasmic	0.00	0.96	1.55	0.84	87.26	0.51	1
MSTRG.10612	Transmembrane protein 205	0.00	1.33	2.53	1.27	95.36	0.48	1
MSTRG.10620	Protein shifted	-5.16	-2.56	0.00	2.58	100.73	0.21	6
MSTRG.10659	Uncharacterized protein	-2.83	-1.78	0.00	1.55	87.07	0.15	9
MSTRG.10707	Homeodomain-interacting protein kinase 2 isoform X1	-1.59	-0.53	0.00	0.92	173.21	0.15	3
MSTRG.10774	Uncharacterized protein	-3.45	-1.72	0.00	1.73	100.37	0.39	9
MSTRG.10824	Uncharacterized protein	0.00	6.52	10.09	5.65	86.74	0.26	14
MSTRG.1086	Hemicentin-2	-6.89	-3.33	0.00	3.45	103.46	0.36	6
MSTRG.10901	Uncharacterized protein	-1.99	-1.18	0.00	1.05	88.48	0.59	9
MSTRG.11014	Uncharacterized protein	-1.26	-0.42	0.00	0.73	173.21	0.27	3
MSTRG.11080	Uncharacterized protein	0.00	3.42	5.45	2.98	87.12	0.37	10
MSTRG.11123	Histone-lysine N-methyltransferase SETMAR-like	-2.85	-1.67	0.00	1.49	89.20	0.08	9
MSTRG.1113	Uncharacterized protein	0.00	0.61	1.83	1.06	173.21	0.41	1
MSTRG.11162	Uncharacterized protein	0.00	0.45	1.34	0.77	173.21	0.17	8

MSTRG.1123	Uncharacterized protein	0.00	1.02	1.79	0.92	90.29	0.72	1
MSTRG.11246	Uncharacterized protein	0.00	0.53	1.59	0.92	173.21	0.03	8
MSTRG.11269	G-protein coupled receptor 158 isoform X3	-3.08	-1.03	0.00	1.78	173.21	0.52	3
MSTRG.11278	Uncharacterized protein	0.00	1.50	2.42	1.31	87.28	0.83	12
MSTRG.11335	Uncharacterized protein	0.00	3.76	5.75	3.26	86.65	0.43	10
MSTRG.11336	Gamma-1-syntrophin	0.00	1.06	1.63	0.92	86.70	0.44	1
MSTRG.11341	Uncharacterized protein	0.00	1.36	4.09	2.36	173.21	0.14	11
MSTRG.11342	Uncharacterized protein	-1.38	-0.46	0.00	0.80	173.21	0.22	3
MSTRG.11349	Uncharacterized protein	0.00	0.84	1.33	0.73	87.07	0.31	1
MSTRG.11374	Sphingosine-1-phosphate phosphatase 1-like	0.00	0.66	1.98	1.15	173.21	0.40	1
MSTRG.11376	Uncharacterized protein	0.00	0.41	1.22	0.70	173.21	0.23	8
MSTRG.11410	Uncharacterized protein	0.00	1.42	2.15	1.23	86.61	0.83	1
MSTRG.11424	Uncharacterized protein	0.00	1.87	2.83	1.62	86.61	0.30	12
MSTRG.11460	Uncharacterized protein LOC106137858	-1.34	-0.45	0.00	0.78	173.21	0.24	3
MSTRG.11462	Endonuclease and reverse transcriptase-like protein	-2.17	-0.72	0.00	1.25	173.21	0.10	3
MSTRG.1149	Uncharacterized protein	-1.91	-0.64	0.00	1.10	173.21	0.33	4
MSTRG.11542	Hypothetical protein RR48_14652	0.00	2.02	3.13	1.76	86.73	0.23	12
MSTRG.11591	Uncharacterized protein	0.00	2.33	7.00	4.04	173.21	0.04	15
MSTRG.11622	Uncharacterized protein	0.00	3.32	6.34	3.18	95.71	0.44	10
MSTRG.11625	Uncharacterized protein	0.00	1.23	3.70	2.14	173.21	0.58	11
MSTRG.11687	Uncharacterized protein	-1.68	-0.56	0.00	0.97	173.21	0.11	3
MSTRG.11697	Uncharacterized protein OBRU01_03818	-2.76	-1.60	0.00	1.43	89.53	0.07	9
MSTRG.11809	Uncharacterized protein	-3.69	-1.23	0.00	2.13	173.21	0.73	3
MSTRG.11819	Uncharacterized protein	0.00	0.46	1.37	0.79	173.21	0.15	8
MSTRG.11854	Uncharacterized protein	0.00	0.45	1.36	0.79	173.21	0.15	8
MSTRG.11855	Uncharacterized protein	1.71	2.75	3.55	0.95	34.38	0.56	12
MSTRG.11858	Uncharacterized protein	0.00	0.82	2.45	1.42	173.21	0.30	7
MSTRG.11941	Uncharacterized protein	0.00	0.87	2.60	1.50	173.21	0.11	7
MSTRG.11981	Uncharacterized protein LOC110371971	0.00	0.78	2.35	1.36	173.21	0.92	8
MSTRG.11997	Uncharacterized protein LOC106139637	-3.67	-2.28	0.00	1.99	87.27	0.81	9
MSTRG.12030	Ionotropic receptor 4	0.00	0.75	2.25	1.30	173.21	0.69	7

MSTRG.12036	Uncharacterized protein	0.00	2.15	3.33	1.87	86.72	0.11	12
MSTRG.12050	Uncharacterized protein	-2.39	-0.80	0.00	1.38	173.21	0.20	3
MSTRG.12064	Hemocytin	-5.00	-2.41	0.00	2.50	103.89	0.38	6
MSTRG.12093	Mitochondrial aldehyde dehydrogenase	-1.64	-0.55	0.00	0.95	173.21	0.13	3
MSTRG.12111	Uncharacterized protein	0.00	0.45	1.34	0.77	173.21	0.16	8
MSTRG.1217	Pleiotrophin-like protein precursor	0.00	0.48	1.45	0.83	173.21	0.47	1
MSTRG.12191	Uncharacterized protein	0.00	0.48	1.45	0.84	173.21	0.09	8
MSTRG.12222	Adapter molecule Crk isoform X1	0.00	1.04	1.57	0.90	86.62	0.37	1
MSTRG.12258	Uncharacterized protein	0.00	2.66	5.12	2.57	96.40	0.80	12
MSTRG.12264	Uncharacterized protein LOC110379302	-4.40	-2.24	0.00	2.20	97.97	0.70	6
MSTRG.12319	Trypsin-like protein	-5.37	-1.79	0.00	3.10	173.21	0.88	6
MSTRG.1232	Uncharacterized protein LOC105380700	0.00	0.39	1.16	0.67	173.21	0.26	8
MSTRG.12417	Uncharacterized protein LOC110378902	0.00	0.70	2.11	1.22	173.21	0.89	8
MSTRG.12523	Uncharacterized protein	0.00	0.94	2.82	1.63	173.21	0.09	7
MSTRG.12598	Uncharacterized protein LOC106125623	0.00	4.46	7.19	3.90	87.30	0.41	10
MSTRG.12610	Uncharacterized protein	0.00	1.18	3.54	2.04	173.21	0.90	11
MSTRG.12634	Microspherule protein 1-like	-6.30	-2.97	0.00	3.17	106.75	0.27	6
MSTRG.12673	Peroxisomal acyl-coenzyme A oxidase 1	-5.98	-3.19	0.00	3.01	94.34	0.23	6
MSTRG.12744	Lysosomal Pro-X carboxypeptidase	-3.48	-1.67	0.00	1.75	104.52	0.47	9
MSTRG.12745	Uncharacterized protein	-6.07	-3.25	0.00	3.06	94.08	0.26	6
MSTRG.12781	Neuroblastoma-amplified sequence-like	-4.35	-2.10	0.00	2.18	103.61	0.89	6
MSTRG.12790	Uncharacterized protein	-2.87	-0.96	0.00	1.66	173.21	0.42	3
MSTRG.12804	Uncharacterized protein LOC106138099	0.00	2.96	4.79	2.59	87.41	0.86	12
MSTRG.12809	Uncharacterized protein	-3.90	-1.30	0.00	2.25	173.21	0.79	3
MSTRG.12810	Uncharacterized protein	-3.90	-1.30	0.00	2.25	173.21	0.79	3
MSTRG.12813	Uncharacterized protein LOC106107297 isoform X1	-2.50	-0.83	0.00	1.44	173.21	0.26	3
MSTRG.12820	G-protein coupled receptor moody-like isoform X1	0.00	0.85	2.55	1.47	173.21	0.17	7
MSTRG.12821	Trypsin-like	-2.78	-0.93	0.00	1.60	173.21	0.38	3
MSTRG.12888	Vacuolar protein sorting-associated protein 16 homolog	-1.54	-0.51	0.00	0.89	173.21	0.17	3
MSTRG.12915	Uncharacterized protein LOC110378268	0.00	3.45	10.35	5.97	173.21	0.57	15

MSTRG.12943	Nucleolar pre-ribosomal-associated protein 1	-1.77	-0.59	0.00	1.02	173.21	0.41	4
MSTRG.12967	Uncharacterized protein OBRU01_26429	-1.66	-1.04	0.00	0.91	87.21	0.77	9
MSTRG.12981	Mitotic-spindle organizing protein 1	-2.10	-0.70	0.00	1.21	173.21	0.07	3
MSTRG.12993	Uncharacterized protein	0.00	2.20	6.61	3.82	173.21	0.13	15
MSTRG.1301	Paired box protein and transposase domain containing protein	-3.48	-1.85	0.00	1.75	94.44	0.37	9
MSTRG.13027	RING finger and transmembrane domain-containing protein 2	0.00	0.93	2.79	1.61	173.21	0.06	7
MSTRG.13062	Uncharacterized protein	-4.57	-3.55	-2.39	1.10	30.93	0.33	2
MSTRG.13104	Unknown unsecreted protein	0.00	2.98	5.67	2.85	95.52	0.80	10
MSTRG.13181	DD34D transposase	-3.63	-1.98	0.00	1.84	92.94	0.50	9
MSTRG.13220	Protein CREG1	0.00	1.25	2.13	1.11	88.93	0.68	1
MSTRG.13222	Golgin-45	0.00	1.53	2.31	1.33	86.60	0.85	12
MSTRG.13262	Uncharacterized protein	-2.18	-0.73	0.00	1.26	173.21	0.11	3
MSTRG.13265	Uncharacterized protein	-1.34	-0.45	0.00	0.77	173.21	0.24	3
MSTRG.13268	Uncharacterized protein	-2.17	-0.72	0.00	1.25	173.21	0.10	3
MSTRG.13323	Synaptotagmin-5-like isoform X1	-3.15	-2.02	0.00	1.75	86.82	0.39	9
MSTRG.13326	Uncharacterized protein	0.00	0.50	1.50	0.86	173.21	0.46	1
MSTRG.1343	Uncharacterized protein LOC110374261	0.00	1.52	2.34	1.32	86.67	0.84	12
MSTRG.13498	Uncharacterized protein	0.00	2.16	6.48	3.74	173.21	0.22	5
MSTRG.13515	Vacuolar protein sorting-associated protein 13D	0.00	1.49	4.46	2.58	173.21	0.11	11
MSTRG.13671	G-protein coupled receptor 158	-5.03	-2.88	0.00	2.59	90.09	0.90	6
MSTRG.13678	RNA exonuclease 4-like	-3.67	-1.70	0.00	1.85	109.20	0.56	9
MSTRG.13719	Uncharacterized protein LOC110379302	-5.26	-2.40	0.00	2.66	110.67	0.41	6
MSTRG.13731	Protein takeout	-5.97	-3.96	0.00	3.43	86.61	0.74	2
MSTRG.13762	Plasma membrane calcium-transporting ATPase 1-like isoform X2	-4.52	-2.24	0.00	2.26	100.75	0.65	6
MSTRG.13777	Uncharacterized protein	-1.36	-0.45	0.00	0.79	173.21	0.23	3
MSTRG.13804	Uncharacterized protein	0.00	1.05	3.16	1.83	173.21	0.40	7
MSTRG.13866	Uncharacterized protein	-1.98	-0.66	0.00	1.15	173.21	0.02	3
MSTRG.1387	Tigger transposable element-derived protein	0.00	0.56	1.68	0.97	173.21	0.11	8

	6-like							
MSTRG.13883	Uncharacterized protein	-4.05	-2.43	0.00	2.14	88.19	0.79	6
MSTRG.13907	Hypothetical protein KGM_209630	-5.58	-2.67	0.00	2.80	104.57	0.20	6
MSTRG.13915	Uncharacterized protein	0.00	1.12	1.72	0.97	86.69	0.45	1
MSTRG.13930	Carboxypeptidase	-7.49	-4.20	0.00	3.83	91.17	0.61	6
MSTRG.13946	Bifunctional ATP-dependent dihydroxyacetone kinase/FAD-AMP lyase (cyclizing)-like	-4.17	-2.51	0.00	2.21	88.11	0.68	6
MSTRG.13956	Uncharacterized protein	0.00	1.42	2.24	1.23	86.99	0.92	1
MSTRG.14072	Endonuclease-reverse transcriptase	-5.40	-3.59	0.00	3.11	86.60	0.63	6
MSTRG.14076	Uncharacterized protein	-2.88	-0.96	0.00	1.67	173.21	0.43	3
MSTRG.14089	Ankycorbin	0.00	4.70	7.46	4.09	87.02	0.57	10
MSTRG.14140	Autophagy-related protein 2 homolog A	-1.43	-0.48	0.00	0.83	173.21	0.62	4
MSTRG.14256	Uncharacterized protein	-3.89	-1.30	0.00	2.25	173.21	0.79	3
MSTRG.14290	Uncharacterized protein	-1.68	-0.56	0.00	0.97	173.21	0.11	3
MSTRG.14312	Uncharacterized protein LOC110378431	-1.49	-0.50	0.00	0.86	173.21	0.19	3
MSTRG.14318	Uncharacterized protein	0.00	0.58	1.75	1.01	173.21	0.19	8
MSTRG.14322	Hypothetical protein RR46_08551	0.00	0.96	1.58	0.84	87.87	0.22	1
MSTRG.14364	Uncharacterized protein LOC106107598 isoform X1	-4.34	-1.45	0.00	2.50	173.21	0.88	3
MSTRG.14365	Uncharacterized protein LOC106107598 isoform X1	-4.55	-1.52	0.00	2.63	173.21	0.91	3
MSTRG.14376	Uncharacterized protein	0.00	1.09	1.65	0.94	86.61	0.39	1
MSTRG.14401	Uncharacterized protein	0.00	0.99	2.97	1.71	173.21	0.19	7
MSTRG.14446	Uncharacterized protein	0.00	1.25	2.09	1.10	88.33	0.64	1
MSTRG.14456	Uncharacterized protein	0.00	2.16	4.80	2.44	112.75	0.95	11
MSTRG.1453	Uncharacterized protein	0.00	0.60	1.81	1.05	173.21	0.27	8
MSTRG.14553	Uncharacterized protein LOC106118616	-2.36	-0.79	0.00	1.36	173.21	0.14	4
MSTRG.14558	Uncharacterized protein	0.00	3.11	4.66	2.69	86.60	0.83	10
MSTRG.14604	DNA-mediated transposase	-1.44	-0.48	0.00	0.83	173.21	0.20	3
MSTRG.14611	Uncharacterized protein	0.00	3.16	4.94	2.74	86.84	0.69	10
MSTRG.14617	Uncharacterized protein LOC106684797	-3.12	-1.91	0.00	1.67	87.65	0.36	9
MSTRG.14620	Esterase B1-like isoform X1	0.00	1.77	2.81	1.54	87.09	0.35	12

MSTRG.14641	Uncharacterized protein LOC106133263	0.00	0.69	2.08	1.20	173.21	0.80	8
MSTRG.14725	Uncharacterized protein	0.00	0.57	1.71	0.99	173.21	0.15	8
MSTRG.14756	Uncharacterized protein	0.00	1.58	2.86	1.45	91.96	0.59	12
MSTRG.14833	Uncharacterized protein	0.00	1.24	2.11	1.10	88.70	0.66	1
MSTRG.14999	Uncharacterized protein	-1.51	-0.50	0.00	0.87	173.21	0.18	3
MSTRG.15013	Uncharacterized protein	-2.01	-0.67	0.00	1.16	173.21	0.03	3
MSTRG.15052	Uncharacterized protein	0.00	1.35	2.47	1.25	92.71	0.96	1
MSTRG.15084	Uncharacterized protein	0.00	0.56	1.69	0.98	173.21	0.42	1
MSTRG.15112	Protein unc-13 homolog A-like	-4.14	-2.12	0.00	2.07	97.73	0.98	9
MSTRG.15148	Uncharacterized protein	0.00	4.34	7.32	3.84	88.57	0.33	10
MSTRG.15154	Myosin-11-like	-4.21	-1.40	0.00	2.43	173.21	0.86	3
MSTRG.15163	Uncharacterized protein	0.00	2.09	6.26	3.62	173.21	0.33	15
MSTRG.15168	Retinoid-inducible serine carboxypeptidase-like	-5.71	-1.90	0.00	3.29	173.21	0.81	6
MSTRG.15170	Beta-1-syntrophin	0.00	0.45	1.35	0.78	173.21	0.16	8
MSTRG.15180	Uncharacterized protein	0.00	1.34	2.74	1.37	102.55	0.50	1
MSTRG.15182	Uncharacterized protein	0.00	1.01	3.03	1.75	173.21	0.25	7
MSTRG.15207	Lysosomal alpha-mannosidase	-3.49	-1.77	0.00	1.75	98.55	0.39	9
MSTRG.15229	AF4/FMR2 family member 3	0.00	0.98	2.93	1.69	173.21	0.16	7
MSTRG.15234	Uncharacterized protein LOC106103208	0.00	0.87	2.61	1.51	173.21	0.11	7
MSTRG.15246	Uncharacterized protein	0.00	4.03	6.16	3.49	86.65	0.34	10
MSTRG.15252	Uncharacterized protein	0.00	0.44	1.33	0.77	173.21	0.17	8
MSTRG.15255	Mediator of RNA polymerase 2 transcription subunit 26	0.00	0.97	2.90	1.67	173.21	0.14	7
MSTRG.15283	Uncharacterized protein	0.00	1.08	1.88	0.97	89.70	0.68	1
MSTRG.15392	Uncharacterized protein LOC106130466	0.00	1.16	1.82	1.01	86.82	0.38	1
MSTRG.15449	Ubiquinone biosynthesis monooxygenase COQ6 like protein	-4.77	-3.17	0.00	2.74	86.61	0.57	6
MSTRG.15546	Aminopeptidase M1	-2.82	-1.48	0.00	1.42	95.81	0.31	9
MSTRG.15649	NFX1-type Zinc finger-containing protein 1-like	0.00	0.60	1.79	1.04	173.21	0.25	8
MSTRG.15715	Hypothetical protein KGM_203431	0.00	3.63	10.88	6.28	173.21	0.60	15
MSTRG.1579	BCL2/adenovirus E1B 19 kDa protein-	-1.93	-1.14	0.00	1.01	88.70	0.68	9

	interacting protein 3 isoform X1							
MSTRG.15794	Septin-4	0.00	2.23	6.68	3.85	173.21	0.10	15
MSTRG.15833	Bifunctional coenzyme A synthase isoform X3	-1.61	-1.05	0.00	0.91	86.69	0.77	9
MSTRG.15840	Cuticle protein CPH43	-1.18	-0.39	0.00	0.68	173.21	0.29	3
MSTRG.15853	Uncharacterized protein	0.00	1.56	2.85	1.45	92.57	0.63	12
MSTRG.1586	Transmembrane protease serine	-7.45	-4.51	-2.20	2.68	59.52	0.88	6
MSTRG.15873	Uncharacterized protein	0.00	1.97	2.99	1.71	86.62	0.18	12
MSTRG.15909	Uncharacterized protein	0.00	0.78	2.35	1.36	173.21	0.46	7
MSTRG.15912	Neuropeptide-like 3	0.00	4.24	7.24	3.78	89.07	0.28	10
MSTRG.160	Prostamide/prostaglandin F synthase-like	0.00	0.76	2.29	1.32	173.21	0.59	7
MSTRG.16063	Uncharacterized protein	-1.91	-1.16	0.00	1.02	87.77	0.61	9
MSTRG.16106	Protein PRRC1-like	-4.10	-2.25	0.00	2.08	92.37	0.66	9
MSTRG.1611	Aldehyde dehydrogenase family 1 member L1	-1.97	-0.66	0.00	1.14	173.21	0.01	3
MSTRG.16142	Uncharacterized protein	0.00	2.63	7.88	4.55	173.21	0.27	15
MSTRG.16169	Uncharacterized protein	0.00	1.58	4.73	2.73	173.21	0.23	11
MSTRG.16294	Uncharacterized protein	0.00	1.87	5.62	3.25	173.21	0.96	15
MSTRG.16298	Uncharacterized protein	2.30	2.83	3.43	0.57	20.01	0.76	12
MSTRG.16346	Uncharacterized protein	0.00	0.41	1.24	0.71	173.21	0.22	8
MSTRG.16392	Zinc finger SWIM domain-containing protein 4-like	0.00	0.97	2.90	1.67	173.21	0.14	7
MSTRG.16406	Uncharacterized protein	0.00	2.80	5.35	2.68	95.88	0.88	12
MSTRG.16416	Max-binding protein MNT-like isoform X1	0.00	0.52	1.57	0.90	173.21	0.01	8
MSTRG.16445	Methionine--tRNA ligase, cytoplasmic	0.00	0.59	1.78	1.03	173.21	0.23	8
MSTRG.16473	Beta-1,3-glucan recognition protein precursor	0.00	1.46	2.21	1.26	86.61	0.95	1
MSTRG.16486	Dihydroxyacetone kinase 2	-2.99	-1.64	0.00	1.52	92.45	0.18	9
MSTRG.1650	Uncharacterized protein	0.00	0.58	1.74	1.01	173.21	0.18	8
MSTRG.16508	Uncharacterized protein	3.52	3.82	4.13	0.31	8.00	0.63	13
MSTRG.16509	Chromosome transmission fidelity protein 18 homolog	-1.78	-0.59	0.00	1.03	173.21	0.07	3
MSTRG.1678	Venom dipeptidyl peptidase 4-like	0.00	1.81	2.76	1.57	86.64	0.37	12

MSTRG.1708	Uncharacterized protein	0.00	0.59	1.77	1.02	173.21	0.22	8
MSTRG.1742	PiggyBac transposable element-derived protein 4-like	-2.16	-0.72	0.00	1.25	173.21	0.10	3
MSTRG.1763	DNA-directed RNA polymerase III subunit RPC4-like	-1.51	-0.50	0.00	0.87	173.21	0.18	3
MSTRG.1768	Uncharacterized protein	0.00	0.82	2.46	1.42	173.21	0.28	7
MSTRG.1832	Uncharacterized protein	-1.78	-0.59	0.00	1.03	173.21	0.07	3
MSTRG.1868	Retrovirus-related pol polyprotein from transposon tnt 1-94	-3.25	-1.94	0.00	1.71	88.38	0.32	9
MSTRG.1873	Ras-like protein 2	0.00	1.08	1.66	0.93	86.70	0.44	1
MSTRG.1877	Uncharacterized protein LOC106139762	0.00	6.26	10.22	5.49	87.60	0.23	14
MSTRG.1883	CYP6AB46	0.00	2.42	7.27	4.20	173.21	0.12	15
MSTRG.1928	Protein crumbs	0.00	0.46	1.38	0.80	173.21	0.14	8
MSTRG.1964	Uncharacterized protein	0.00	1.43	4.29	2.48	173.21	0.03	11
MSTRG.202	Cuticular protein RR-1 motif 54 precursor	-2.10	-0.70	0.00	1.21	173.21	0.07	3
MSTRG.2103	Uncharacterized protein	0.00	1.44	4.32	2.50	173.21	0.04	11
MSTRG.2140	Uncharacterized protein	0.00	2.11	4.14	2.07	98.19	0.46	12
MSTRG.2260	Uncharacterized protein OBRU01_06617	-3.45	-2.03	0.00	1.80	88.91	0.45	9
MSTRG.2273	Uncharacterized protein LOC106137009 isoform X1	-5.97	-2.77	0.00	3.01	108.77	0.27	6
MSTRG.237	Uncharacterized protein LOC105385187	-2.40	-0.80	0.00	1.38	173.21	0.13	4
MSTRG.2454	Uncharacterized protein	-5.17	-2.54	0.00	2.58	101.56	0.23	6
MSTRG.2471	Uncharacterized protein	0.00	2.27	3.67	1.98	87.41	0.30	12
MSTRG.248	Uncharacterized protein	0.00	0.40	1.21	0.70	173.21	0.24	8
MSTRG.25	ATP-binding cassette sub-family G member 1	0.00	0.95	2.86	1.65	173.21	0.11	7
MSTRG.2506	Glypican-6 isoform X1	0.00	1.83	3.22	1.65	90.48	0.26	12
MSTRG.2528	PAX3- and PAX7-binding protein 1	0.00	0.50	1.49	0.86	173.21	0.06	8
MSTRG.2571	Uncharacterized protein	0.00	5.54	8.76	4.82	86.97	0.52	14
MSTRG.259	Uncharacterized protein	0.00	0.68	2.04	1.18	173.21	0.69	8
MSTRG.2627	Calpain-A-like isoform X3	-1.98	-0.66	0.00	1.14	173.21	0.01	3
MSTRG.2644	Uncharacterized protein	0.00	0.53	1.58	0.91	173.21	0.44	1
MSTRG.2751	Zinc finger protein jing-like	0.00	1.56	3.05	1.52	97.81	0.77	12

MSTRG.2773	Uncharacterized protein	-3.85	-1.93	0.00	1.93	99.65	0.63	9
MSTRG.2846	Uncharacterized protein	-3.04	-1.01	0.00	1.75	173.21	0.50	3
MSTRG.2858	otopetrin-2-like	-1.48	-0.49	0.00	0.86	173.21	0.19	3
MSTRG.286	Centaurin-gamma-1A	0.00	1.09	1.70	0.95	86.82	0.35	1
MSTRG.2884	Uncharacterized protein	-1.68	-0.56	0.00	0.97	173.21	0.11	3
MSTRG.2911	THO complex subunit 2	-1.76	-1.11	0.00	0.96	87.05	0.70	9
MSTRG.30	Uncharacterized protein	-5.95	-3.13	0.00	2.99	95.32	0.75	2
MSTRG.3030	Uncharacterized protein	0.00	1.68	2.84	1.49	88.73	0.44	12
MSTRG.3045	Uncharacterized protein	0.00	1.11	1.82	0.97	87.84	0.57	1
MSTRG.3046	Phospholipid-transporting ATPase VD	0.00	1.13	1.98	1.02	90.06	0.70	1
MSTRG.3078	Uncharacterized protein LOC106132165	-1.97	-0.66	0.00	1.14	173.21	0.01	3
MSTRG.3168	4-coumarate--CoA ligase 1-like	-1.67	-0.56	0.00	0.96	173.21	0.47	4
MSTRG.3184	Uncharacterized protein	-1.88	-0.63	0.00	1.09	173.21	0.03	3
MSTRG.3190	Uncharacterized protein	-5.17	-2.51	0.00	2.59	103.17	0.28	6
MSTRG.3191	CRAL-TRIO domain-containing protein	-1.76	-1.08	0.00	0.95	87.43	0.69	9
MSTRG.3195	Uncharacterized protein	0.00	1.96	5.87	3.39	173.21	0.65	15
MSTRG.320	Multidrug resistance-associated protein lethal(2)03659	0.00	1.23	3.70	2.13	173.21	0.59	11
MSTRG.3258	Uncharacterized protein LOC106140707	2.30	3.93	5.18	1.48	37.57	0.32	13
MSTRG.3269	Uncharacterized protein	0.00	1.31	2.21	1.16	88.58	0.76	1
MSTRG.328	Uncharacterized protein	2.76	4.53	5.86	1.60	35.23	0.32	13
MSTRG.3286	Organic cation transporter protein	0.00	0.50	1.50	0.87	173.21	0.05	8
MSTRG.3287	Tryptase	0.00	0.54	1.61	0.93	173.21	0.44	1
MSTRG.329	Uncharacterized protein	-3.26	-1.09	0.00	1.88	173.21	0.59	3
MSTRG.3337	Uncharacterized protein	0.00	0.97	2.90	1.67	173.21	0.14	7
MSTRG.3401	Fatty-acid amide hydrolase 2-B-like	-5.23	-1.74	0.00	3.02	173.21	0.92	6
MSTRG.3420	Laminin subunit gamma-1	-2.08	-0.69	0.00	1.20	173.21	0.06	3
MSTRG.3430	Uncharacterized protein LOC105286551	0.00	2.08	6.25	3.61	173.21	0.34	15
MSTRG.3449	Uncharacterized protein	0.00	0.67	2.00	1.16	173.21	0.61	8
MSTRG.3477	3-hydroxyisobutyrate dehydrogenase, mitochondrial	-4.31	-2.47	0.00	2.22	90.01	0.59	6
MSTRG.3514	Talin-1-like	-1.43	-0.48	0.00	0.83	173.21	0.21	3

MSTRG.3523	5-methylcytosine rRNA methyltransferase NSUN4	-2.26	-1.30	0.00	1.17	89.93	0.41	9
MSTRG.353	Gelsolin-like	-2.18	-0.73	0.00	1.26	173.21	0.10	3
MSTRG.3598	Uncharacterized protein	-1.61	-0.54	0.00	0.93	173.21	0.14	3
MSTRG.374	Serine protease HP21 precursor	-1.39	-0.46	0.00	0.81	173.21	0.22	3
MSTRG.3742	Uncharacterized protein	0.00	3.08	5.10	2.71	87.96	0.74	10
MSTRG.3769	Uncharacterized protein	4.59	9.25	13.02	4.28	46.29	0.51	14
MSTRG.3852	KAT8 regulatory NSL complex subunit 1-like	0.00	0.71	2.12	1.22	173.21	0.92	8
MSTRG.3892	Uncharacterized protein	-3.45	-1.59	0.00	1.74	109.60	0.57	9
MSTRG.3893	Uncharacterized protein	0.00	0.81	2.43	1.41	173.21	0.33	7
MSTRG.3949	Uncharacterized protein	0.00	6.21	10.17	5.45	87.68	0.23	14
MSTRG.3959	Cytochrome P450 9e2-like	-3.37	-2.16	0.00	1.88	86.80	0.58	9
MSTRG.398	Uncharacterized protein	0.00	0.52	1.56	0.90	173.21	0.02	8
MSTRG.400	AF4/FMR2 family member 3-like isoform X4	0.00	0.58	1.74	1.00	173.21	0.18	8
MSTRG.4044	Uncharacterized protein	-1.46	-0.49	0.00	0.84	173.21	0.20	3
MSTRG.4121	Uncharacterized protein LOC106103906	-2.26	-0.75	0.00	1.31	173.21	0.14	3
MSTRG.4164	Uncharacterized protein	-1.56	-0.52	0.00	0.90	173.21	0.16	3
MSTRG.4195	Uncharacterized protein LOC106138234	0.00	0.56	1.67	0.96	173.21	0.43	1
MSTRG.4246	Rotatin-like	-3.70	-1.23	0.00	2.14	173.21	0.74	3
MSTRG.4261	Facilitated trehalose transporter Tret1-like	-3.29	-2.18	0.00	1.89	86.61	0.58	9
MSTRG.4266	Uncharacterized protein	-1.25	-0.42	0.00	0.72	173.21	0.27	3
MSTRG.4280	Uncharacterized protein	0.00	1.40	2.46	1.26	90.58	0.61	1
MSTRG.4292	Uncharacterized protein LOC106141537	-2.29	-0.76	0.00	1.32	173.21	0.15	3
MSTRG.4305	Uncharacterized protein	-1.77	-0.59	0.00	1.02	173.21	0.41	4
MSTRG.433	G-protein coupled receptor 112	-7.27	-3.35	0.00	3.67	109.32	0.42	6
MSTRG.439	Uncharacterized protein	0.00	0.87	2.61	1.51	173.21	0.10	7
MSTRG.4413	Gelsolin-like	-3.10	-1.59	0.00	1.55	97.52	0.32	9
MSTRG.4433	Synaptotagmin-16 isoform X1	0.00	2.43	7.28	4.20	173.21	0.13	15
MSTRG.4449	Uncharacterized protein	-2.71	-0.90	0.00	1.57	173.21	0.35	3
MSTRG.4469	Ankyrin repeat domain-containing protein 50 isoform X2	0.00	1.61	2.49	1.39	86.75	0.64	12

MSTRG.4513	Phosphoglycolate phosphatase 1B, chloroplastic-like	0.00	1.58	2.38	1.37	86.61	0.74	12
MSTRG.4535	Uncharacterized protein	0.00	0.40	1.21	0.70	173.21	0.23	8
MSTRG.456	Uncharacterized protein	-2.56	-0.85	0.00	1.48	173.21	0.28	3
MSTRG.4568	Zinc finger CCHC domain-containing protein 24-like	-4.34	-1.88	0.00	2.23	118.39	0.93	9
MSTRG.4582	Galactokinase-like	-3.15	-1.85	0.00	1.64	88.91	0.22	9
MSTRG.4589	Uncharacterized transmembrane protein DDB_G0289901-like	0.00	0.90	2.70	1.56	173.21	0.03	7
MSTRG.4617	Uncharacterized protein	0.00	0.62	1.86	1.07	173.21	0.34	8
MSTRG.4627	Hemicentin-1	0.00	1.55	2.57	1.36	88.09	0.98	12
MSTRG.4654	OTU domain-containing protein 7B	0.00	0.58	1.73	1.00	173.21	0.17	8
MSTRG.4673	Uncharacterized protein LOC106129089 isoform X1	-7.09	-3.07	0.00	3.64	118.28	0.43	6
MSTRG.474	NADP-dependent oxidoreductase	-2.65	-1.44	0.00	1.34	92.91	0.26	9
MSTRG.4830	Uncharacterized protein	0.00	0.64	1.93	1.11	173.21	0.47	8
MSTRG.4848	Uncharacterized protein LOC106143306	-1.82	-0.61	0.00	1.05	173.21	0.05	3
MSTRG.4862	Myotubularin-related protein 10-A-like	0.00	2.46	4.29	2.21	90.03	0.32	12
MSTRG.4875	Uncharacterized protein	0.00	0.55	1.66	0.96	173.21	0.09	8
MSTRG.4880	Uncharacterized protein LOC106136113	-1.78	-0.59	0.00	1.03	173.21	0.07	3
MSTRG.4912	Uncharacterized protein	-4.39	-1.46	0.00	2.53	173.21	0.89	3
MSTRG.495	Uncharacterized protein	-2.38	-0.79	0.00	1.37	173.21	0.20	3
MSTRG.4988	Uncharacterized protein	0.00	1.60	4.79	2.76	173.21	0.25	11
MSTRG.5000	Rabphilin-3A	-4.38	-2.44	0.00	2.23	91.49	0.55	6
MSTRG.5071	Uncharacterized protein LOC110379667	-1.76	-0.59	0.00	1.02	173.21	0.08	3
MSTRG.5158	Uncharacterized protein LOC106135886	-4.03	-2.29	0.00	2.07	90.28	0.91	6
MSTRG.5166	Uncharacterized protein LOC106135993	-1.28	-0.43	0.00	0.74	173.21	0.26	3
MSTRG.5170	Uncharacterized protein LOC106135953 isoform X1	-1.40	-0.47	0.00	0.81	173.21	0.22	3
MSTRG.5201	Homologous-pairing protein 2 homolog	0.00	2.91	8.72	5.03	173.21	0.41	15
MSTRG.5275	Uncharacterized protein	0.00	2.92	6.04	3.03	103.69	0.86	10
MSTRG.5335	Uncharacterized protein	0.00	1.62	2.72	1.43	88.33	0.54	12
MSTRG.534	Lethal(2) giant larvae protein isoform X1	0.00	0.78	2.35	1.36	173.21	0.46	7

MSTRG.5341	Uncharacterized protein	-5.89	-2.91	0.00	2.95	101.24	0.16	6
MSTRG.5431	Uncharacterized protein	-1.73	-0.58	0.00	1.00	173.21	0.09	3
MSTRG.5434	Reverse transcriptase	-4.60	-1.53	0.00	2.66	173.21	0.92	3
MSTRG.545	Uncharacterized protein	0.00	1.01	3.03	1.75	173.21	0.25	7
MSTRG.5458	Hypothetical protein KGM_200275B	0.00	1.41	2.52	1.29	91.08	0.92	12
MSTRG.5472	Uncharacterized protein	-4.13	-2.32	0.00	2.11	91.06	0.80	6
MSTRG.5515	Multidrug resistance-associated protein lethal(2)03659	0.00	1.18	3.53	2.04	173.21	0.92	11
MSTRG.5530	Neural cell adhesion molecule 1-like	0.00	1.26	3.78	2.18	173.21	0.47	11
MSTRG.5540	Zinc finger CCHC domain-containing protein 24-like	-4.05	-1.35	0.00	2.34	173.21	0.82	3
MSTRG.5544	E3 ubiquitin-protein ligase HECTD1	-1.54	-0.51	0.00	0.89	173.21	0.17	3
MSTRG.5549	Uncharacterized PE-PGRS family protein PE_PGRS54-like isoform X1	-5.70	-1.90	0.00	3.29	173.21	0.67	4
MSTRG.5596	Reverse transcriptase	0.00	1.14	2.13	1.07	93.72	0.24	1
MSTRG.5604	Uncharacterized protein LOC106114752	-1.56	-0.52	0.00	0.90	173.21	0.16	3
MSTRG.5657	Poly (ADP-ribose) polymerase	-2.53	-1.61	0.00	1.40	86.88	0.22	9
MSTRG.5750	Endonuclease-reverse transcriptase	0.00	0.45	1.35	0.78	173.21	0.50	1
MSTRG.5836	Uncharacterized protein	0.00	1.83	5.49	3.17	173.21	0.85	11
MSTRG.5837	Uncharacterized protein LOC106132472	0.00	2.54	4.84	2.43	95.79	0.53	12
MSTRG.5902	Hypothetical protein RR46_00006	-1.54	-0.51	0.00	0.89	173.21	0.55	4
MSTRG.5918	Protease inhibitor 5	-2.63	-1.51	0.00	1.36	89.98	0.47	9
MSTRG.6005	Uncharacterized protein	-1.48	-0.49	0.00	0.86	173.21	0.19	3
MSTRG.6023	Uncharacterized protein	-1.43	-0.48	0.00	0.82	173.21	0.21	3
MSTRG.6081	Uncharacterized protein LOC105222572	0.00	0.58	1.74	1.01	173.21	0.41	1
MSTRG.6118	Uncharacterized protein	-2.48	-1.45	0.00	1.29	89.33	0.19	9
MSTRG.6172	Uncharacterized protein	-2.74	-1.57	0.00	1.41	90.09	0.10	9
MSTRG.6298	Hypothetical protein ALC56_14032	0.00	1.01	3.04	1.76	173.21	0.26	7
MSTRG.6349	Protein phosphatase 1 regulatory subunit 14B isoform X3	-1.27	-0.42	0.00	0.74	173.21	0.26	3
MSTRG.6381	Uncharacterized protein	0.00	3.82	6.54	3.41	89.14	0.02	10
MSTRG.6397	Protein turtle-like	-2.79	-1.49	0.00	1.40	94.02	0.25	9
MSTRG.6429	Uncharacterized protein	0.00	2.36	7.07	4.08	173.21	0.06	15

MSTRG.6451	Uncharacterized protein	-2.35	-1.34	0.00	1.21	90.40	0.35	9
MSTRG.6462	Menin	-2.05	-0.68	0.00	1.18	173.21	0.05	3
MSTRG.6498	Uncharacterized protein	0.00	0.46	1.39	0.80	173.21	0.13	8
MSTRG.6504	Uncharacterized protein	-4.03	-1.96	0.00	2.02	102.97	0.78	9
MSTRG.6511	Hypothetical protein RR46_14024	-1.81	-0.60	0.00	1.05	173.21	0.06	3
MSTRG.6537	Hypothetical protein RR46_13991	-2.77	-0.92	0.00	1.60	173.21	0.38	3
MSTRG.6595	Uncharacterized protein	-1.93	-0.64	0.00	1.11	173.21	0.01	3
MSTRG.6685	Uncharacterized protein	0.00	1.13	1.81	0.99	87.17	0.52	1
MSTRG.6737	Uncharacterized protein LOC106129042	0.00	0.64	1.92	1.11	173.21	0.40	1
MSTRG.6746	Laccase-4-like	0.00	4.32	7.41	3.85	89.25	0.32	10
MSTRG.675	Uncharacterized protein	0.00	3.16	5.02	2.75	87.03	0.67	10
MSTRG.6786	Uncharacterized protein	0.00	2.57	7.71	4.45	173.21	0.23	15
MSTRG.6790	Uncharacterized protein	0.00	2.23	6.68	3.86	173.21	0.10	15
MSTRG.6805	Uncharacterized protein	0.00	1.06	3.19	1.84	173.21	0.44	7
MSTRG.6831	Uncharacterized protein	0.00	0.41	1.22	0.71	173.21	0.23	8
MSTRG.6841	Uncharacterized protein	-2.19	-1.45	0.00	1.25	86.62	0.24	9
MSTRG.6905	G-protein coupled receptor moody	-2.81	-1.52	0.00	1.42	93.23	0.62	9
MSTRG.6923	Uncharacterized protein	0.00	1.86	5.57	3.21	173.21	0.96	11
MSTRG.6924	Uncharacterized protein	0.00	2.12	6.37	3.68	173.21	0.26	15
MSTRG.6925	Endonuclease-reverse transcriptase	0.00	0.49	1.48	0.85	173.21	0.07	8
MSTRG.6941	Hypothetical protein KGM_206686B	0.00	1.10	3.31	1.91	173.21	0.62	7
MSTRG.6946	Ankyrin repeat and SOCS box protein 3-like	0.00	0.71	2.12	1.22	173.21	0.93	8
MSTRG.7017	Uncharacterized protein LOC110372193	0.00	0.85	2.56	1.48	173.21	0.16	7
MSTRG.7116	Hypothetical protein RR46_09733	-1.58	-0.53	0.00	0.91	173.21	0.15	3
MSTRG.7150	Uncharacterized protein	0.00	0.61	1.83	1.06	173.21	0.30	8
MSTRG.7153	Uncharacterized protein	0.00	2.83	4.74	2.50	88.38	0.76	12
MSTRG.7165	Uncharacterized protein	0.00	0.57	1.71	0.99	173.21	0.15	8
MSTRG.7238	Short/branched chain specific acyl-CoA dehydrogenase, mitochondrial	-3.16	-2.09	0.00	1.81	86.62	0.47	9
MSTRG.7295	Trehalase-like isoform X1	0.00	1.36	2.15	1.18	87.00	0.76	1
MSTRG.7296	Diamine acetyltransferase 2-like	-3.03	-1.70	0.00	1.55	91.16	0.16	9
MSTRG.7313	Protein AF-10 isoform X9	-2.22	-0.74	0.00	1.28	173.21	0.12	3

MSTRG.7314	Uncharacterized protein	0.00	1.12	3.37	1.94	173.21	0.71	7
MSTRG.7335	Venom dipeptidyl peptidase 4-like isoform X1	0.00	1.82	2.86	1.58	86.86	0.40	12
MSTRG.734	Uncharacterized protein	0.00	2.34	7.01	4.05	173.21	0.05	15
MSTRG.7381	Facilitated trehalose transporter Tret1-like	-7.24	-4.82	0.00	4.17	86.60	0.80	6
MSTRG.7386	Uncharacterized protein	-1.56	-0.52	0.00	0.90	173.21	0.16	3
MSTRG.7389	Uncharacterized protein	0.00	5.83	9.62	5.12	87.89	0.34	14
MSTRG.7478	Glutathione S-transferase 2-like	-2.57	-0.86	0.00	1.48	173.21	0.29	3
MSTRG.7498	Unconventional myosin-Va	-2.59	-0.86	0.00	1.50	173.21	0.30	3
MSTRG.7518	Protein espinas isoform X3	-1.81	-0.60	0.00	1.05	173.21	0.06	3
MSTRG.7529	Uncharacterized protein	0.00	0.52	1.55	0.90	173.21	0.02	8
MSTRG.7531	Uncharacterized protein	0.00	1.98	5.95	3.43	173.21	0.58	15
MSTRG.7564	Protein amalgam-like	0.00	1.83	5.49	3.17	173.21	0.86	11
MSTRG.7565	Uncharacterized protein LOC106133343 isoform X1	-3.26	-2.09	0.00	1.82	86.80	0.48	9
MSTRG.7567	Uncharacterized protein	-2.00	-0.67	0.00	1.15	173.21	0.02	3
MSTRG.7576	Phosphatidylinositol 4-kinase alpha isoform X1	0.00	0.41	1.24	0.71	173.21	0.22	8
MSTRG.7593	Uncharacterized protein	0.00	0.46	1.39	0.81	173.21	0.13	8
MSTRG.7596	Uncharacterized protein LOC106135670	-6.49	-3.07	0.00	3.26	106.25	0.29	6
MSTRG.7609	E3 ubiquitin-protein ligase UBR4	0.00	0.46	1.39	0.80	173.21	0.13	8
MSTRG.7639	Vacuolar protein sorting-associated protein 13D-like	0.00	0.78	2.33	1.34	173.21	0.51	7
MSTRG.7643	Uncharacterized protein	0.00	0.59	1.76	1.02	173.21	0.41	1
MSTRG.7675	Uncharacterized protein	0.00	0.66	1.98	1.14	173.21	0.57	8
MSTRG.7690	Aldose 1-epimerase	-2.82	-1.67	0.00	1.48	88.48	0.06	9
MSTRG.7703	6Tox	0.00	1.90	3.48	1.76	92.86	0.63	12
MSTRG.7710	Uncharacterized protein	-1.50	-0.50	0.00	0.87	173.21	0.18	3
MSTRG.774	Uncharacterized protein	-5.82	-3.87	0.00	3.35	86.60	0.68	6
MSTRG.7746	Uncharacterized protein	-4.61	-2.46	0.00	2.32	94.40	0.41	6
MSTRG.7767	Uncharacterized protein	-3.40	-1.64	0.00	1.70	103.45	0.45	9
MSTRG.7791	Uncharacterized protein LOC106130198 isoform X1	-2.11	-0.70	0.00	1.22	173.21	0.07	3

MSTRG.7832	Unconventional myosin-Va isoform X1	-1.68	-0.56	0.00	0.97	173.21	0.11	3
MSTRG.7848	Uncharacterized protein	-1.30	-0.43	0.00	0.75	173.21	0.25	3
MSTRG.7865	Uncharacterized protein	0.00	4.24	6.86	3.71	87.40	0.30	10
MSTRG.7934	cAMP-responsive element-binding protein-like 2 isoform X1	-1.84	-0.61	0.00	1.06	173.21	0.05	3
MSTRG.7954	Uncharacterized protein	-3.49	-1.16	0.00	2.02	173.21	0.67	3
MSTRG.7956	Zinc/cadmium resistance protein isoform X1	0.00	0.85	2.54	1.47	173.21	0.18	7
MSTRG.7962	Uncharacterized protein	-2.06	-0.69	0.00	1.19	173.21	0.05	3
MSTRG.7974	Zinc transporter 2-like isoform X1	-5.01	-2.39	0.00	2.51	105.06	0.39	6
MSTRG.7976	Uncharacterized protein	-5.40	-1.80	0.00	3.12	173.21	0.63	4
MSTRG.8019	Talin-B-like	-1.49	-0.50	0.00	0.86	173.21	0.19	3
MSTRG.8065	Histone-lysine N-methyltransferase SETMAR-like	-1.33	-0.44	0.00	0.77	173.21	0.24	3
MSTRG.8101	Uncharacterized protein LOC107037501	0.00	2.13	6.39	3.69	173.21	0.25	15
MSTRG.8130	Hemicentin-2-like	0.00	1.18	3.54	2.05	173.21	0.88	11
MSTRG.8223	Cytosolic endo-beta-N-acetylglucosaminidase	-2.01	-0.67	0.00	1.16	173.21	0.03	3
MSTRG.8239	Uncharacterized protein	-2.95	-0.98	0.00	1.70	173.21	0.46	3
MSTRG.8243	Uncharacterized protein	-1.52	-0.51	0.00	0.88	173.21	0.17	3
MSTRG.8275	Uncharacterized protein	0.00	3.75	6.11	3.28	87.57	0.14	10
MSTRG.8330	Uncharacterized protein	0.00	0.65	1.96	1.13	173.21	0.52	8
MSTRG.8387	Uncharacterized protein	-2.98	-1.52	0.00	1.49	97.82	0.34	9
MSTRG.8392	Lysine-specific demethylase lid	0.00	0.42	1.25	0.72	173.21	0.22	8
MSTRG.8449	Uncharacterized protein LOC106140458	-1.82	-0.61	0.00	1.05	173.21	0.06	3
MSTRG.8544	Aminopeptidase N-like	0.00	3.21	5.69	2.91	90.70	0.50	10
MSTRG.8560	Reverse transcriptase	-1.32	-0.44	0.00	0.76	173.21	0.24	3
MSTRG.8578	Uncharacterized protein	0.00	0.56	1.68	0.97	173.21	0.12	8
MSTRG.8598	Huntingtin-like	-1.42	-0.47	0.00	0.82	173.21	0.21	3
MSTRG.8601	Uncharacterized protein	0.00	0.91	1.37	0.79	86.60	0.38	1
MSTRG.8602	Seminal fluid protein HACP040	-2.99	-1.89	0.00	1.64	86.97	0.25	9
MSTRG.8646	Cecropin A	3.54	5.35	6.35	1.57	29.30	0.45	13
MSTRG.8652	Uncharacterized protein	0.00	1.74	2.88	1.53	88.02	0.37	12
MSTRG.8654	Uncharacterized protein	0.00	3.78	5.93	3.28	86.88	0.25	10

MSTRG.8659	Fumarylacetoacetate hydrolase isoform B	0.00	2.41	4.03	2.13	88.39	0.38	12
MSTRG.8662	Uncharacterized protein	0.00	0.40	1.19	0.68	173.21	0.25	8
MSTRG.8781	Glucuronyltransferase I	0.00	0.45	1.35	0.78	173.21	0.16	8
MSTRG.884	Uncharacterized protein	0.00	3.33	6.31	3.17	95.16	0.42	10
MSTRG.8877	Uncharacterized protein	0.00	3.72	7.19	3.60	96.86	0.36	10
MSTRG.8891	Uncharacterized protein	0.00	0.48	1.43	0.83	173.21	0.10	8
MSTRG.8904	Uncharacterized protein LOC106138074	0.00	0.53	1.59	0.92	173.21	0.03	8
MSTRG.8967	Venom serine carboxypeptidase-like	-2.75	-0.92	0.00	1.59	173.21	0.37	3
MSTRG.8976	Uncharacterized protein LOC106142918	0.00	6.29	10.04	5.48	87.14	0.23	14
MSTRG.8981	G-protein coupled receptor moody isoform X1	0.00	0.66	1.99	1.15	173.21	0.59	8
MSTRG.9030	Protein toll-like	-1.70	-0.57	0.00	0.98	173.21	0.11	3
MSTRG.9041	Uncharacterized protein	0.00	4.22	6.93	3.70	87.75	0.28	10
MSTRG.9111	Uncharacterized protein	0.00	3.32	5.19	2.89	86.83	0.63	10
MSTRG.912	Hemocyte protease-1	-5.36	-2.63	0.00	2.68	102.06	0.18	6
MSTRG.9128	Uncharacterized protein LOC110373625	-3.50	-1.75	0.00	1.75	100.14	0.40	9
MSTRG.9150	Retinol dehydrogenase 14-like	-2.10	-0.70	0.00	1.21	173.21	0.07	3
MSTRG.9268	Uncharacterized protein	-2.01	-0.67	0.00	1.16	173.21	0.03	3
MSTRG.9286	Hypothetical protein RR48_00560	-6.70	-3.20	0.00	3.36	104.79	0.32	6
MSTRG.9305	Uncharacterized protein	0.00	1.21	1.89	1.05	86.84	0.51	1
MSTRG.936	Nucleoporin-like protein 2	-1.41	-0.47	0.00	0.82	173.21	0.21	3
MSTRG.9374	Phosphatidylethanolamine binding protein isoform X1	-1.33	-0.44	0.00	0.77	173.21	0.24	3
MSTRG.9390	Uncharacterized protein LOC106125039	-1.26	-0.42	0.00	0.73	173.21	0.27	3
MSTRG.9391	Uncharacterized protein LOC106133236	-3.42	-1.65	0.00	1.72	104.28	0.47	9
MSTRG.9399	Uncharacterized protein	2.57	4.46	6.21	1.82	40.85	0.32	13
MSTRG.9439	Uncharacterized protein	-1.47	-0.49	0.00	0.85	173.21	0.19	3
MSTRG.9470	Interference hedgehog-like	-3.53	-1.63	0.00	1.78	108.81	0.55	9
MSTRG.9472	Microtubule-associated protein futsch-like	-2.31	-1.49	0.00	1.29	86.74	0.24	9
MSTRG.9506	Uncharacterized protein	2.07	3.39	4.52	1.24	36.49	0.80	13
MSTRG.9516	Uncharacterized protein	-3.52	-2.12	0.00	1.86	88.10	0.57	9
MSTRG.9521	Glutathione S-transferase 2-like	-1.96	-0.65	0.00	1.13	173.21	0.01	3

MSTRG.955	Uncharacterized protein	0.00	0.52	1.56	0.90	173.21	0.44	1
MSTRG.9558	Uncharacterized protein	0.00	2.03	3.47	1.81	89.18	0.43	12
MSTRG.9560	Uncharacterized protein	0.00	1.95	5.86	3.38	173.21	0.66	15
MSTRG.9618	Zinc finger SWIM domain-containing protein 8-like	-1.84	0.04	1.96	1.90	4909.57	0.93	8
MSTRG.970	Ankyrin repeat domain-containing protein 50	0.00	1.97	2.99	1.71	86.62	0.22	12
MSTRG.9714	Uncharacterized protein	-2.34	-1.55	0.00	1.34	86.62	0.18	9
MSTRG.9757	Uncharacterized protein	-2.52	-0.84	0.00	1.45	173.21	0.26	3
MSTRG.9758	Uncharacterized protein LOC106142856 isoform X1	-2.51	-0.84	0.00	1.45	173.21	0.09	4
MSTRG.981	Phosphatidylinositol 3-kinase 3 isoform X1	-2.78	-0.93	0.00	1.60	173.21	0.39	3
MSTRG.9841	Uncharacterized protein	-1.42	-0.47	0.00	0.82	173.21	0.21	3
MSTRG.9855	Uncharacterized protein LOC106125039	0.00	2.80	4.55	2.45	87.50	0.70	12
MSTRG.9856	LIM and SH3 domain protein Lasp isoform X1	0.00	1.00	1.61	0.88	87.26	0.27	1
MSTRG.9867	Uncharacterized protein LOC106138072	-3.93	-1.99	0.00	1.97	98.71	0.72	9
MSTRG.9891	O-acyltransferase like protein-like	-3.67	-1.92	0.00	1.84	95.92	0.51	9
MSTRG.9899	Hypothetical protein	0.00	0.44	1.32	0.76	173.21	0.17	8
MSTRG.9902	Uncharacterized protein	2.21	5.82	7.73	3.13	53.80	0.81	14
MSTRG.9984	UDP-glucosyltransferase precursor	0.00	1.24	3.72	2.15	173.21	0.55	11
MSTRG.9989	Insulin receptor substrate 1	0.00	0.51	1.54	0.89	173.21	0.02	8
