# Supplementary material for: Transcriptomic analyses reveal comprehensive responses of insect hemocytes to mycopathogen Beauveria bassiana, and fungal virulence-related cell wall protein assists pathogen to evade host cellular defense
Source: Virulence. 2020 Oct 5;11(1):1352–65. doi: 10.1080/21505594.2020.1827886 (PMC7549920; doi:10.1080/21505594.2020.1827886)
Supplement: Supplemental Material [file KVIR_A_1827886_SM8204.zip › Table S2.pdf]

Table S2 Paired primers used in qPCR analyses for gene expression levels in *Galleria mellonella* hemocytes

Gene name	Gene tag	Sequences (5′-3′) of paired primers (forward/reverse)
**β-1,3-glucan-recognition protein**		
βGRP1	LOC113513310	CTAGAGACATCACCAAGT / TATCACATAAGTCCAGAAGT
βGRP2	LOC113513109	ATTACAGATGGCATATCC/CTTGGACATTCTCTTTCT
βGRP3	LOC113521212	TTAGAGACAGGAAGATTC/GAACATATTGGAGTATTATT
βGRP4	LOC113513817	GCTTTGATATTTACCGAAA/CAACTTAGGACTAATAATGAG
βGRP5	LOC113511574	TTCATTAGGTTCAGTATTG/GGGTTTAAGATATTAGGATTA
βGRP6	LOC113511574	TTCATTAGGTTCAGTATTG/GGGTTTAAGATATTAGGATTA
βGRP7	LOC113522106	TATGGAGGACCTGTTCTA/TGTAGTTATGGTAGTCTCTG
βGRP8	LOC113520075	CAAGACTATGGAAACAAG/GCTGTATTCGTGATAATC
βGRP9	LOC113510911	GAGTGGGTGGTGTAAATG/CAGTTGTCCTTGTCCTTC
βGRP10	LOC113521212	TTAGAGACAGGAAGATTC/GAACATATTGGAGTATTATTG
βGRP11	LOC113511574	TTCATTAGGTTCAGTATTG/GGGTTTAAGATATTAGGATTA
**Cecropin**		
CecA	LOC113514266	ATATTCCTGTTCGTGTTC/CAGCCTTAATGATACCAT
**Gallerimycin**		
Glm1	LOC113523440	TACAGAATCACACGACAT/TTGTAACCTCTACTCCTG
**Gloverin**		
Glv1	LOC113510422	TATTGGACCAGTCTAATC/ATATTCAATGACAACAGAG
Glv2	LOC113523269	TAATAAGAATGGCGATGT/ATGAAGTTGTGCTGATA
**Moricin-like protein**		
Mor1	LOC113509608	CTCTTTATAGGGTCAAATGA/GCTGCACTGATTACTTTA
Mor2	LOC113509615	ATGATGGTGATGGCTATG/TATTGATTCCACGCAGAG
Mor3	LOC113509614	TGCTCGCCCTGTTTGTTG/CACCAATTACACCAAGACCTTT
Mor4	LOC113509613	TCTATTCCTCATGTTCAT/AGACCCTTTTTAATAATTTTG
Mor5	LOC113509612	CTCTTTCTCATGATTATGG/ATAATTTTGCCACCTTTC
Mor6	LOC113509609	CGGTTTATTCCTTATGATTATG/TATAGCTTTACCACCCTTT
Mor7	LOC113509611	AGTTCTTCAATCTCGTAT/AATAATTTTTCCGCCTTT
**Lysozyme**		
lyz1	LOC113515290	CAGGGACTATGGTTTATTC/TTATATCGTCGGTCAAGA
lyz2	LOC113510919	TAGAGATTATGGCTTATTCC/TGACGCTTGTATATCTTC
**Chitinase 2**		
Chi2-1	LOC113509411	TACCTCACCTATTATTCC/TATGATGCTGTAGTAGATA
Chi2-2	LOC113515831	AGACTACCTGAACCTATG/CCATCTCTTCTACAATACG
**Internal control gene**		
18SRNA	LOC113514995	AACCTGTTAAGAGACTGTAT/GCCTGTGTAGTGAGTAAT
