# Supplementary material for: Transcriptomic analyses reveal comprehensive responses of insect hemocytes to mycopathogen Beauveria bassiana, and fungal virulence-related cell wall protein assists pathogen to evade host cellular defense
Source: Virulence. 2020 Oct 5;11(1):1352–65. doi: 10.1080/21505594.2020.1827886 (PMC7549920; doi:10.1080/21505594.2020.1827886)
Supplement: Supplemental Material [file KVIR_A_1827886_SM8204.zip › Table S3.pdf]

**Table S3 Summary for the clean data in RNA-seq of the cellular responses of *Galleria mellonella* to *Beauveria bassiana***

| Sample Name* | Clean Reads number | Base (M) | Q20 rate | Q30 rate | GC rate | N rate | Total Reads | Uniq-Mapped | Multi-Mapped | UnMapped |
|---|---|---|---|---|---|---|---|---|---|---|
| CK-R1 | 52,203,378 | 7,812 | 97.95 | 93.92 | 42.93 | 0 | 26,101,689 | 21,483,122 (82.31%) | 285,168 (1.09%) | 4,333,399 (16.60%) |
| CK-R2 | 46,744,456 | 6,992 | 97.79 | 93.48 | 42.33 | 0 | 23,372,228 | 19,328,573 (82.70%) | 231,339 (0.99%) | 3,812,316 (16.31%) |
| INT1d-R1 | 46,443,014 | 6,951 | 97.88 | 93.68 | 41.6 | 0 | 23,221,507 | 19,083,877 (82.18%) | 225,748 (0.97%) | 3,911,882 (16.85%) |
| INT1d-R2 | 43,218,246 | 6,471 | 97.75 | 93.38 | 41.33 | 0 | 21,609,123 | 17,641,731 (81.64%) | 200,310 (0.93%) | 3,767,082 (17.43%) |
| INT2d-R1 | 50,652,292 | 7,581 | 97.86 | 93.65 | 41.17 | 0 | 25,326,146 | 20,874,695 (82.42%) | 242,793 (0.96%) | 4,208,658 (16.62%) |
| INT2d-R2 | 47,745,144 | 7,148 | 97.79 | 93.44 | 41.15 | 0 | 23,872,572 | 19,575,227 (82.00%) | 243,900 (1.02%) | 4,053,445 (16.98%) |
| INT3d-R1 | 46,901,854 | 7,019 | 97.9 | 93.75 | 41.81 | 0 | 23,450,927 | 17,859,228 (76.16%) | 215,941 (0.92%) | 5,375,758 (22.92%) |
| INT3d-R2 | 50,607,450 | 7,574 | 97.95 | 93.85 | 42.47 | 0 | 25,303,725 | 18,465,227 (72.97%) | 190,012 (0.75%) | 6,648,486 (26.27%) |

*: "CK" indicates the control group with infection. "INT" indicates that the experimental groups infected with B. bassiana. "R" means the independent replicates.
