# Supplementary material for: Transcriptomic analyses reveal comprehensive responses of insect hemocytes to mycopathogen Beauveria bassiana, and fungal virulence-related cell wall protein assists pathogen to evade host cellular defense
Source: Virulence. 2020 Oct 5;11(1):1352–65. doi: 10.1080/21505594.2020.1827886 (PMC7549920; doi:10.1080/21505594.2020.1827886)
Supplement: Supplemental Material [file KVIR_A_1827886_SM8204.zip › Table S4.pdf]

Table S4 Sequence and annotation of the predicated novel genes

Gene ID	Annotation	Sequence (5'-3')
MSTRG.10017	Uncharacterized protein LOC106138419	TAATTAAAATAGAATTTATTTTAATTTTTCAGATTATCTGTATATTAATTCATCGAAATATTTTAAATGATTTATTAAATAC<br>AAATTTTGATATTTAGGTGATAGTTAAGTATAACTACAACGTAAATAACTATTTATATGGAAATATATATTATTATTTATTG<br>GATTCAATGTTTATAAAAATGATACATAGGTATTTAATGCAGATATTATCCATCCACTGCTGGACATATGCCTTCCCCATA<br>GACTGCCACGGAACACTGAAACGGTCCTAAGCTACCTGCATCTACTAATTTCCTGCAATGCATTTGAGATCATCGCTCCAT<br>CTAGTGGATGGTCATAATACACTGCATTAAAATTATATATATTCTTTTATTATACACACATATAATAATAACAATATTCGAT<br>CTGTATAATTAAAAAAAAATTAAATAAAATTCAACATAAATAATTTTATTTAAATAAAATTTATTTCTTATGGACAAATCA<br>GTTTTAACTAAAAATTATCTGTTTCATTTACACTTAACACGATGGATTACTTATATAGAATTTGTCATACAAGTACCTAAGT<br>TCTTCCATCCACACTATGTCATATGTAGCCGAGGTTACATTACATTCATACGCTGATATCATTCGGACACCCCTAGAACTGT<br>CCACAAAAATGTCTCCTACAACACTCATGAAGCAGCCTTCTTCAAAAACCACACAGTTCATTTTGTTTTTAATTCTATCCAT<br>CATCTCTGACATTATGAGTATATTAGGGTCCCC
MSTRG.10019	DNA ligase 1-like	GCACCGGCTTCTCCGACGACGACCTGCACAAGCTCAGTACCGCGCTCAAGGAACACGTCATCGACGCGCCCAGGAATTAC<br>TACAGGTTCGACGCCGCGCACCGGCCGGACGTGTGGCTGGCGGCGGCCTGCGTGTGGGAGGTCCGCTGTGCTGATCTGTC<br>GCTGTCGCCCGCGCACCGCGCCGCCCGCGGCCGCCTGCACCCCGACAAGGGACTCTCGCTGCGCTTCCCCAG
MSTRG.10044	Tyrosine-protein phosphatase 10D	AGGAGCCGCGCGACGACACGCCGCCGCCGCTGCCCTCGTGGCGCGACGTGCACCGCCTGCCGCTCTGGCCCCCCTACCAG<br>GTGACGGAGCCGTACTACCCGTTCGCGTCGTCGCCGGTGGAGGAGTTCACGATCGGCGCGGAGCGCTGCGAGGCGGCGGG<br>CCGCACGTACTGCAACGGGCCGCTCAAGCCCGGCACGCGCTACTACGTCAAGCTGCGGGCCTTCACCGCGCCCGACAAGT<br>ACGCCGACACCGCC
MSTRG.10049	Zinc finger and SCAN domain-containing protein 30-like isoform X1	TAGGTCCTTCGGTTCCGAGTGGCACAGTGGTGCCCAGTCGGACTCAGACTCAGACCCTGAGCGGCTGAAAGACACCCTGG<br>GCCCACCGCCCCAACAAGACGAGATCAGCGAGTCGGAGAACGAGTCCCCCGACAAGAGTTTCCAATGTCATATATGCCAT<br>AAGTGGTATTCCACCAGGGTTACGTTGAAAATTCATCGACGAGTCCACCAGAATCGCGGCGGTGGCAACTCGCGGTCCCG<br>CACTCGCTCGTCAGACAGATACGAGTGCGATTGTTGTAATGAAACGTTTAATCGGCGCGAGAAACTTTGGGATCATAAG
MSTRG.10058	Kunitz/Bovine pancreatic trypsin inhibitor domain protein	GACCAATTTTTAACCGACTTTAAAAATGGAGGAGGTTGTATATTTACTTTTTTTTTCAGTGTTTATTTTTTTTTTGTTTCAGA<br>TCGATGCAAAGCTCCGATAGACGATCGTGTTTGTTTTGGATTTCGCGGATACTATGCCTACGATCTTCCCAATAACAAATG<br>TATAGAATTACGCTTCGGCGGTTGTTACGAAGGACCGAACGAGTTCAAATCTAAGGCAGAATGTGAAAATACCTGTGTCA<br>AAAAATAAGACAAAAAAATAACATCTTTTTTTTTGTCTATAGATTTAATTTATTTTTTGAAGTAAGAACTAAAATTTGTGTA<br>ACATAATATTCCAGACAAATTA
MSTRG.10067	G-protein coupled receptor Mth2-like	AGGTACCGTAGCATCCGCGGTCTGCCTCACATACCGACGGACTCCAGAAAGAGTGGCAACATGACCAGCACTGCTATCAC<br>AGAAGAAAGCAGACTGTCCTCCGATGACCCTGGCGTGCGGTTAAAAGATTTACACTGAATGAAAGATTACGTATCACGTC<br>ATATTCATGCAAAAGAATCCTCATCAATAAATGTACGGCACCGATGTTGTCAAAATTTGCTAGTCAGTTTCACAACAAATG<br>CTTTTATTATTATCTTTAGCCTGTAGTTATTTTAAGATACTTTTCATAAAATTAAGATCATTGTTAAAATTTACGCACATTTA<br>CTTTGTATTTCATGAAATTATTATCGGTATTGACTATTTAGCAAATATTTTTTACTATAATTATAATATTTGTGTACTTTTAT<br>AAATAGTAGAAACAGGTACAAAACAAAACATTCTTTTTTTAATATCATGAAGAAAAAATATGTATATATATTTCTCAGTAC<br>GGGACACAGTTATATTATGCCTAGAGTGGGTTGTAAAAAAATACATTACGATCGCGTTCACATAACAAAATTTTTTATTTA

		TATTATTAGAAAAACGCTTGTATTTTCTACATAAAAGAATATAAATTTCATAGATATAGTATATAAAGTTCTACCAAATAC<br>CAACAATCACAACAAAATTAAGATAACTTTTTAGCTTGTCAAGATTGTGTTTAATATCAAAGCGTTCGTAACAAATAAATA<br>AAGGTGGCAAAAACTCGTGTACCATGTATGTTTTCGACATGAACGCGGTTTGTCACTTAATAATTTGTAGAATTGACACAA<br>ACCACTCTGAGTTATTGCTTTTTTCTATTGCTGCAGTTATTAAATGTAAATTAATACCTAGGTGCTCGTAAACGTATGATAT<br>TGTCTCTCTATGTGTTTAATTATAGGCTAGAATATATTTTTTTTAGTGTACAACTATATAAAAATTTTATGTATTAGTTATAT<br>AAACGATTTTCTATTATATAATAAATATAAGTGATGAATGATGTTCGTAATAAAATTAATTAAGTACGTAATCTATTAAAT<br>ACCTCAGTGAACCTAGAAAATAGAAAATGTAAACATATATATTAATTTTAATTAAAATTGTTTATTTACATCTCTCCTTGTC<br>TCCCACTACTTCTAAAACTACAGCAGAGGATATCTGTCATGACAGACAGACAGAATTCCACTGCATACTCCGCGAATTCCC<br>TGCAATCACACGACCTGCAGCTTTGCACCTACCATATGCATACGCAATATATAGGTCTAAGTGATGACCGCGTAAGTCGAA<br>TTAGTGTAAATCAGGGTTTAATTCCTTGTCTTTTAGCTCTTAGCTATGCATATTTCGCAAAAGATGTTCAAATACGTACATT<br>AGCTTAACAATAGTGTGGACAAAATGATTAATCTACAGTTGTTTGGCGTTCAAACATCGAATCGACGAGATATGAAAATC<br>GCGTAATTCCTAGTTCGCGTAAATTGAGGTAGCGTGAGTCAAGGTCAACATCTTGATCCGGCAGCTGGCGGGCATGCAAG<br>CAATAAAAAATAAAAATTTCAGGACATAAAAATCATTTTCACAAGTAAAAACAAATTTACCTAACCTGTCTTTACAGCTGA<br>CCATTTCGTCATAAATAACCATTGACATTATTTTCGTTAAATCCATAAAAATTCAGAAGTAGTAACTTTGTAGATATATTTT<br>ACAAATGTCTACTATTACAACAGACTTTAATTATTTGGAGTTAGTACAACCTCTTTTATCTTCCATTTTGGTTATCCAATTTT<br>TTATTTTAAGCGATGTACCTAATGAATATCAATAAATCCTAAAAGCATATACACTGACTTCTTTAGAAACTATTTAAGGCTT<br>GATTACAATACCTTTAAACTCAATCTAGATAAGATTTACCTTGTATTTACAGTAATCTTATGACTATTGTAAAATTTTTACA<br>ATTTCAATTTAGCATACTTTTCTTAAATTTGCATCTTTTCATATATCTATCGTGGGTTAAAAGCACGTCATTAATAGTATAC<br>AAAAAATACCTATTTAATTTTACTTAAAATCCAAGATCAATGTTTTTAAATTGTACTAAGACATTATAATTTGGTGTTACCA<br>AGATATATGAACTTTGTAAGTACACTATAATACATAGATAAGGTATATTAAGATAGACATATATGGACCTAACTGTACCTG<br>AATTTTGTATTTTTGTTCGTATTTTAAGAAGACAATCAGTTACAATAAAGTTAGTCCAATTAAA
MSTRG.1<br>0076	AT-rich<br>interactive<br>domain-<br>containing protein<br>2-like	CTAAAAGCCAACCCACTTATGGGGAAGGCTTATGTCCAGCAGTAGACAGTTAATGGCTGAAATGATGATGAATGAAAACT<br>ATTGTTGCTTTCAGAGAATGAAAGTGAATGATATGATAAGTATATTATGGGAATGTTTTTGATCATTGGATCATTACCTTCA<br>CAGATAGCTTAATACAAGGTTTTTTTTTATAGGACCCTCGTCTCCAATACTGAAAGCACAATTGACGAATAAACCAGCAGA<br>AGCTAAACCAGTAGTAACACAATCGCAGCCCCAACCACAGCAGCCGGTGGTCTCCCACCCGCACCTCTCCCAAGCCCTCG<br>AGTCGAGTCCGTCCAACACGACCCTCATAAAGCACCTACTCGCCCACAAAGTAAGCCCCGCACAACAACAGCATCAAGTA<br>AGTGCATACTAACAGTACTAACTTGTCTTGTGTGCGTGCGCGCTCGTAGCCATACACGGGTATTGTAGGGTTGTCAATTTG<br>TTCTGTGGTTTTTGGTAAAACAGAATTGGTTTTTAATGGGGTGAATGGTACGATTAATGGGGTGAAAACTTTTGCGCTATC<br>GGTATACCAGGTGAGGAT
MSTRG.1<br>0092	Ubiquitin<br>carboxyl-terminal<br>hydrolase 8<br>isoform X1	AGTGGGATGTAAAAGTGCATTACGCCCGGCCTCCAGTGGCCCTCAAAGGCGGTTACGAAGACTGGTTACTCAAGTATCCG<br>GCCTTCACCACGAACCCTCAAGCTGTACCGCCCAGGCAGGAGGAGTTCCTTGACGACATGCTCAGTGAGATTGACTATCCC<br>AACTGGTCTGACCTGGCCCCAGTGACTCCACCGTCGAGGCCCAAACCTCACCAACCCAAGCCTGGCGAGCCGATCATTGA<br>CCGCGCTAGTAAGG
MSTRG.1<br>0131	Zinc finger BED<br>domain-<br>containing protein<br>1-like	GTGTGGACAGACCTTAATAGGGGCGGCGCGGTACGGGCGGCGTGGCGCCGGCGTGGCGATACCAGCGCGCTCGGCGTTCG<br>ATCGCCAGTGTTATTCTACTCGCGCCAATGTCGGTGTGTATCGCGGTTGAAGCTTGCGCGAGAAGTTTTTTTCTGTTTACA<br>CTCTTCCTAAAGTTTTTCCCACAATGGTGGATATTGATAAGCATACGTTAATGAAGACTAAATTAAGGTATCTCTCCATAA<br>AAACGTTGCATTCCTCATCAAGAGTCAGTTTTTGTTGGAAGAAATTTGGATTGTTAATGATTACAGTGGACGGTAAAAAAA<br>GATTTGTACCATCAGATCAAGTGTACTGTATGGTCTGCTTAGAGAACGCCAAGAAGGTATATAAGGATCAGTTATTTGAAA<br>GTGTCAAAGTGAAATCATACAGCAAAAAAATAAGCACAGGAAACCTCATTAGGCACCTGAAGGAAGAACACCAATTATT

		CGAAGAAGAGCCTCGCAAACGTAAATCCCGTGAACCACATAAACCTCGTGAACCACGGAAACCTCGCAAATCTCGTGGAA<br>AACGTGAACCTCCTGAACTTAACGAACGTCATGAACCTGACGAATCTTATCAAGCTCATGAATCTTGTGAACCTCAGGAAC<br>CCTGTGATATAGAAGAATTTCCAAATTCCATTATAAGTTGCCCTAGCAGCTCAAGTAAAATCGTTTTTGAAATTAAACCGT<br>CTAAATGGAGCTTGGGAAGAGATCTGACATTGTGGTTTTGCCGTTCACTTCTATCGTTTGATTCAGTAAACGACGAAGCTA<br>CAGTTGAACTGCTAAAAAACTACAATGTTACTGTAACTGCAGAAGATCTTCCATCACAAACTGGCACGCGAAATTCTCTAG<br>ACAATATTTATACCTGTATGGTGGAATGCATAAAACAATCCATAGCAACGTCGACTTCAAGACATATCGCCGTCACTGCCG<br>ATTTGTGGAGTGATTGTTACAAACGCCACAGCTATATAACTCTAACTTTACATTTTATTAGTGATTCATTTGTTCTTGAAAA<br>TGCAACCCTTGCAACGCAACCCATTATAGGCTCCAAAACAATAGAGAACATATCTAATTGCTTAATGAATATCCTGCAAAG<br>GTTTGCACTTCAAGAAAAACACCTTCTCCTCGTATCTAATATAGGACTTGAGGAAAAGAAAGTTGCTAAATTATTGTCAGA<br>GAAAATCACATGTAAAACTCACTATTGCTTAGGGCAAGCCTTCCATAATCTTGTTGTTGACGATGGAATTAAAAATACGCC<br>TAATTTATTGAAATTAACAATAAAATGCAACAACATAATAAAAACATTAAGACTGAAATTACCGGAAATAGAAGATACAG<br>CTGACGAAGAACAAAGAATTATTTTAAGCTGTATTGAGAATGCTTGTCAAATTATTGAACATGACGAGAACCTATACTTCG<br>ACAATAACAACGAAATGGAAATAGATCACAATGGTGTAACTGAAGCAACAGTAGATGGAGTAGAATCTAAAAAAAAAAT<br>CTTGCCAACGCGTTGGCAAAGTTTACTAGCCTTGTTTGAATGTTTATATAACAAATATAATCGAATACCTATCAACCAATT<br>GCTAATAAAGTTAGAAAAATCCGATTTAATATTGAATGAGACTGAATGGCTATTAATAAAGCAACTTACAGATTTCTTGTC<br>TAAATTCCGCGATTGCGTTTCTATCATGAGTGTACAAAATACTTGCACAATCAATATGACTCTAGTATTTAAGATGGAAGT<br>AGCTGAAATTTTGGAATCACTCGAAGAGAAAGAAAGAATTGAGATATTTCATATGAAGAAAAACATGAAAACGAATTTAG<br>ACCATCGATTCCCAACAAATGAATTGTCTGTGGCCGCTACATTATTAGACTGTAGATTTCAGGCCATCAAAGAAGTAGACA<br>AATATTTGCAAATGCATAATTTGAATCGCGTATCGTTTTTGGCAAGTTATACAAGACAAATATTGAACACAGAGGATTTGC<br>ATGTAAATAAAATTAATATACCATCTCCTTCAACGATGGAGAACTCTTCCCAAAAAAAATCTTCGTCTGTATTAATGCAAC<br>TTGCCTTGAAATATGGAGGTGCTAGAGATTCCACTGAAGTGGAGAACAATTCTATTGAGACAGAGTGTTGTAAATACTTTG<br>CTGGGTGTGATCCGCTAGATTTATCTAGTGGCGATATACTAAACTATTGGAAAGAAAGAAAAGCAGCGTACCCCTGTTTGT<br>CCACATTGGCCAAAAGAGTACTGTGCACACCAGCGATTAGTACAAATAGTGAACGCATGTTTACTGTAGCCGGTCTAACCT<br>TAGCAGCAAAACGGTCGCATCTTTCTGGAAGTAATATCAACAAAATTATATTTGTCCATGACAATTATAATAAATTCGAAA<br>ATTGCATCGATAATCCTCTTATCGCAGTTTCGATGGATGAACCGACCTTCTAGATTTATGATCTCAAAAGATATTATATTTT<br>TATAAGGTATTTTATAATTAAAGAACTTATATATTTTTAAAACTCATATCATATAGCAC
MSTRG.10169	Uncharacterized protein LOC106134230	AGTAGAGCGTAGTAGTGGGGCGAAAGCAAATCGACTCCATTCATTCATTGACGCGCCGGCCGGCCGTCCAGTCGGTTGTC<br>ATTAATTTAATGAAAATCCGAAAAAGCTGTATATAATGAGTTTCGCGCGCATCTACCGACTATTCGACGATTAAAAAACGA<br>ACTCTATGCTAATTTATTTTGACTGTGTTGACATTCGTTTTGACAATTACAGCAGTTGTTTGTTCTGGTGATGCGATGTTGAT<br>TGTGGTAGTGATTTTGTAAAATGTGGTGAGGAGATGCTTGTGGGAGAACAGTCACCGGCGCCGCGAGTGGTCGCCGGGCT<br>GAGGAAACTACGACCAGAATTGACACCAGGACCCGTGCCCGCGCCTGCTGATCCCAACCTACGGATATCAGGTTTACTCC<br>GTCAGTAT
MSTRG.10192	Ubiquitin conjugation factor E4 B isoform X1	NNNNNACCCGCTGTACGCGTTCCAGCCCGGCTCGTGGGCCAGCGTGCGCGACGACACGCGCCTTTACTTCACCGCGCAGG<br>AGGCAGCCGACTGGCTCGAATCACTCAATAATGACCCTAATCACAAATGGCCAGAAGCAAAGTTCCAGACGATCTGTTGG<br>TTCTTAACACTTCACATGCATCACGTTGCTCTCATTCCTGCACTACACACTCATCAGCGTCGTATTCGGGCATTCCGTGACC<br>TGCAGAAGGTGATAGAAGAGCTAGTGGCTGCAGAGCCCCAGTGGCGCAACAGTCTATCTGCCTTCCGCAATAGGGAATTG<br>CTCCGTCGGTGGCGGCGACAAATTAAAAGACTACACAGGTCGAAGCAGTGCGCGGAGACGGCGCTGCTGGACGGCGAGC<br>TGATGCGGCGCAGCGTGCAGTTCTACGCGTCCGCGTGCGCGCTGCTCAGCGGCCAGCTCGACGCCGCGCGCGCCGCCTCCT<br>CCGCCTCCTCCGCCTCCTCCGCGCCCGCCGCCGCCGCCTTCCGCGCCCTGCCCGAGTGGTACGTCGAGGACATCGCCGAGT

		TCATGCTGTTCGCCGTGCA
MSTRG.10207	Uncharacterized protein LOC106134231	GAATAGTTGTTTAATGTTTATTTTCTTCATAACTTCAATTCTTCTTTAAAACACTATGGATCGCCTGTGGATACTTGTGGAAT<br>GGGTGGATGGAAATAACGTATTCCCTGATTATGGTGTGGTGAACGTGGATCCATTGACTTATAATGATTACGACTTGACTC<br>CTGGCAGAGTTATTTTAATTCGTGCCAGACACGAAACTACCGCTCGAACAGGAAAAATATTGACAATTTCTGAAAGTAAA<br>TACAACATCAAAGCTCACAAGAGATTATTACAAAAACAAGACAGTCAAGTAAAAAGCGTCCTGTCTATGTGCATGCAAAC<br>TATCAAAGGCATTAAATCAGACTCCATGTACTTACCTGGGATAGAAATGAGTTCGTCCCTCAGTTCCTTACCTGGTGCACC<br>TCAGATCGTTGCTGTTGATAATGACTCCACTAGCAGTGATAGCGACAGTGAGCCTCCCCGACGCTTATATTCGAAAAGATC<br>ATTAGGGTCTCCATCTGTTCATAAGTTAATCGATCAATCTGGAAGTAGCCGCCGTAATTCAATTGCTCATAGCAGTACTAC<br>AACAATATCGATGCATAGCAGTACACCTCTTTCAATGCTAAAACCAGAAGTATCGAACAAAAAAAAAGTAACAAAGGCCG<br>ACATAGCGACGCAGACAGAAAACCAAGACTCAGAGATAGATCTATTAGAGAATAATCTTAGAAAACTATATAGTTTATTT<br>TTATCAGTCATAGGACGACTTGAGATTCAAAAAGCTGCTGAAACTGTAATAATGGATCGAACGATCATGAGAGATTTGAA<br>AAAACATATTTCAAGCGAAGCCATCGCGAGGAACGTGAATAATATGAGTCTTGATGAAATTCTTGACGAGTTGAAAACTG<br>CAATGTCGGAGAGGGATCTGATAGCTCGCCGGCTGCAGGACGAGTTAGAAGCGACACCAGCTAAGCAGATTTGTCATGCG<br>CAGTCTAACGGGATTCCCGTAACACCACCTTTACAAGAACACATATATTGCGCGCAAACGATGAATAATGATATTGATAC<br>AAGAAATTCATCGAGTGATGAAATGGTATCAATAGGAAGCGGATATGCTACAATACCCGCTAGATTTTTAAATGAAATTG<br>ATTGGAACTCATATACTACAGCAACAAGGCAATTACTGAAGTCTGTCTTTTCACGCAAAGTTTTAGCAACGCATTCTCTTA<br>CTGGGAAACGATCACCGGCCTTTGCAGACAAGCCAGCAAAAAAGCGTTTAGACCCTAAATTAGTGGATGATATTGTTAAA<br>ACAGTATCATTGAGGTGTGGAGTCCCCGAACGGCTAGTAAGAAACAGCATTACCGTGAAATGTACAGACGAAGCCAAATT<br>ATACCGGAACCGACAATTCTACAGGCATGCGCTTCGCGAAAGCAGGCAAAATAACGAAAATATCTCACCGGAAACAAGT<br>ATTTCGTCAGTTGAATCTTTAGCTATCCACTAAAATATATACCAAAATGTATAGGTACATACTTTTTGTATTTTCGACAACA<br>TCTCAGAGTCGGTCTTAGCCGATGTGGTGCTCATGAGTCATCACTTTTTTGTTAGTATCCTAAAGAAGATCACCAGGGGTG<br>TCGAGTGAGTTAAACTACTGGAGAAGATATTGCATTGAAACGTGGCTCCTTGCGCCTCTTGGATCTCGGTGCCGATGGACA<br>CCGCACTACCTAAGATCTGTACTGATCTTATTGTAAAACAATTTTTAATAGTCAACATTGTACTTGTAGTTAGACTTTAATG<br>CTCAGTTTATTAATGAATACCAGCGGAGATTGTTTTATTCGTGTATAATGTTTTATTACATTTCAGTTATAACTTTATGGGCT<br>TATTTACTTTAGTAGTTCTGCTACCCATTGATAAATTGCGTTTCACTTATTCTATGTTTCAAATGGCATATGCGTTAAAATA<br>AAATATTATACATACTCTTATCCTGTTATATAAACTGCCNN
MSTRG.1027	Acyl-CoA-binding domain-containing protein 5 isoform X6	CACACCTACAAATAATATTAATTGCACACTATCAAAAGTACCTGTATGTATAAAAATACGGTATATTTTTTGCGTATATATT<br>AATAAATCCTATACCAATATAAAGTGACGAGATAAGTCGTGTAGAATGTGGCACGCATACCAGCTTTATACCTAGCCTTTA<br>ACTGTCCACCGCTGAACATAGCCTCCCCCATAAGGGGGCATTTGCCAGTATTTGCCACGCTTAGCAGGCGGGTTGGCAACC<br>GCAGTTTGGCTTTTGGTAATATTTTAAGAGGGACGCTGCTGCCCAAAGAGATATGGCCTGGAATAGAGCTGGCTGGAAGA<br>GAACATATGAATTAGTTGCACCGACAAGGGGAAACTCTTAAATACTTACATCAAGCATCCTGCGGTCGCCCTCGACTGCCT<br>TCCTCAGCGCCGCCACGTCAGCCTCCAGCCGTGCCAGGGTGCCGGGCAGCTGTTCCAACACTTTCAAATCTGGATACCAAA<br>CATGAAACCATTTTATTACACGACTGCCCAATAGTTTAAAATTGTGTTACAAAATAAACTGACCTCAATTGTGAAA
MSTRG.10275	Azurocidin-like	ATTGAATGTCAATGAATACGATCATGTAAGCTGTTAACTTGTTATGATATAACAAACTTACAAATAAACATCAATTTTAAA<br>AAATGTTAATTATAAGTTGTGTGATGCCGGACCAAACATAGAAACAAAAAAAAAATCTAAAAATTATTATAGTAGTATTG<br>TAAGTAGGAGGAATTATTAAAGATGGTGTTGGTGGATTAGTTGTGAAATCAAACATCATAAAGTCGTCATGTTCGATTACA<br>TCACGTGAATCATTGACACCGCATGTAAGAAATTTATTATATTTGCTCAAATCAAGAAATCCCACTCCACAAGGTCGATCT<br>ACGAGACGAGAAGTCATCATGCCTTGAACAAATCCAGAACATACAATAACTGTCCCTAG

MSTRG.10305	Anaphase-promoting complex subunit CDC26	ATTATGACACGACACGCCACAGTTCTACCAACAGATCAGGTATTACGTCATCGCTTAAAGAACGAAAACCAACTTACTTTC<br>CAAATAATCCTCAAGATATAGCATTTTGTTCACAGAATGATACGAAGACCTTTAACAGAAATAACATTAAAATTGGACGA<br>TGTACAAGACTATGAGAATTCTCGAAAAGATCAAGAAAGTAACTCATCTATAGTAGCAGAAATGCAAGATGATGCGTCTA<br>AATCTCTTGGTACCGGGCCGAAAACGACAGAAGAAATTCATAATAGAATTGGTTATGCTCCCAAAAAGAAGCCAAGTGGG<br>CCAAGTACTGTTTAAAATAGGTTATGCAACTTGGGAAACCAACAGCGCCGAGTAAACAATGCGCCTGCATGAAGTCCATT<br>CGAAATCAACACATTCCTTACTTTTTCTAATCATTAATAATCCATTTCACTTTTTCTAGCAATGTTGAAAAAAAAATTCAAA<br>GCTTCGTAATGTGATTTAATTACTGGAATAAAATAAATACGAGTAAAAATAAATGGCAC
MSTRG.10313	Uncharacterized protein LOC106108737	TTGAGAAATAGTAGAATAGCGTCATATGCAGATGATACCACTCTCACATTTTTTGCAGACTCATGGGAAGAACTATTTGAA<br>CTCGCACAGAGTGGGTTTAATAAAGTATCTCATTGGCTTATGAGTAATTCTCTTACTTTAAATGAAGATAAAACTAAATAT<br>ATTATTTTTACAATGTATAAATGCGCTAAAATGTACAATAATATCTACTCTATTTGCGCACACAAATGTAACTTTCCTCCAA<br>TATCAGATTGCAATTGCCCCAAAATTAACACAACACATCACATAAAATATCTAGGTGTTATAATAGATGCAAATTTAACAT<br>TTGAAAAGCACATTGATTCTGTTTCACATAGGGTGCGTAAACTTGTCTATATCTTTAAACAACTTCGAAATGTCGCAGACG<br>AGAAAACTATAAAATTAGTATACGATGCATTAGCAAGATCTGTCTTAACCTACTGTATTAGTACATGGGCTGGCTGTGCAA<br>AAACCAAATTGATAACACTAGAAAGAGCTCAAAGATTAATATTGAAGGTAATGCTGCATAAACCTCGTCTTTTTCCTACTA<br>TAAAACTTTATGAAGACGCAGAGAGGCTTACCGTACGACAGCTGTATATATTACAAATAGTCTTGCGCAAACATTGCTCTC<br>TATTATATGAAAAAATAATTATCGTCAGCAACACAAGAAGACACTTCCGAGTCTGTGAATCAGTTAATGTTAAGACCTCAT<br>TTGCGCAGAGATTTTACCAATTCCGCAGTGGAATCGTATATAACAGGATAAACAAATTATTAAACATCTTTAAATTTAGTA<br>AAAATTAATGTAAACATATTGTAACAAATTGGCTATTAAAACAAACTTATAACGATACGGAAAAAGTAATAGAAGTAATA<br>AAATTATAA
MSTRG.10318	Rap1 GTPase-activating protein 1 isoform X2	CTCTGCCGCGATGCCAACAGCCGACCAATCAAAAACTCAGTCGCCCGCCGTCTTTCGTTGGAAACGGTATTGTTCGATGTC<br>GCGATGTGATCGTTCGTATGTTCGGACTTTTTTTTTATCGATAACGCGTTTAAGAAAAAGATGTGCATTTTGTGGAAATGTG<br>CCTGTTTTCTATAGCGACTTTTGTCTGTTAAGGGATTTTATTGATAATAAAATCAATATTATTGCATCAACCGCTGGATTAT<br>TTACAAAGATATGGTTATCGTCAGCGTTTTTAGCAGGGGAAAAGGCGGTTTTGGACTGGACACAGTAGTACGACAGTGAA<br>CTGTAATTCTTAGTTAAAATTGTTATTTCGAAGTACTAATTGTGCGGTGATAAGAAGTCGAATGAAGTCGAAAATAGTGAA<br>GATGAAATATTTGCCTGCGGGCGGTCATAAGGAATCGTATACGTTACTTACCGCCAGGAGGAGATATATGAAGAAGCTAA<br>TGTAT
MSTRG.10347	Uncharacterized protein LOC106105123	CTACAATGTGTGAATACACGTAAGGATAACACATTGTTAGGTCTACGTTTTTTAATTCTGTGGTTTCTCCATACGATAAGC<br>TCGATTATATGAGGGCACGACCTAAAAATGACTATAAATATTGCACTGCTCTTTACCACAATATCATTTTAGTATCATTCAT<br>TGAATTGTAAACGTGTTTAAGTACTAAGATTCAATATGTCTTCAGTGGTAATCTTACTTTTGGTTTGTGCTACAACATGCCT<br>TTGCCAGCGTCCTTTCTATGCTGGATCCCGGCCCATCGGTTATCCAGATGTTCCATCGCAAACAGGGATATCCAACCGATTT<br>GGAGACACATCATTACCAGCACCGCTTAAAGGAGATGGAAACTCCGCAAATCGTCTGGCTAACTTGCCACAGGATCGGCA<br>GCCTATATGGTTCCTAAATAAGGAGCAATATGCTAACTTCTTGCAAAATCAACAAACGTATCCACAAAGGCNNNNN
MSTRG.10355	Hypothetical protein KGM_215481	ACTAACCACAACCGTAGCTGGTCGCGTACGCACCTAAATAATTATCTGGAAAAGCCGTGACGTTAACTGAACAAAATGTG<br>AACAGTAAATATTTGGGCAGTTTACAAGATAGCATGGCGAAGTTAACACAATTGTGGCAGTTATATAACGGAGCCTTTGG<br>CGGAATATAAAGAAAAAAGAAAATGTGGTCATATTCAAGCGTGGACATCCCAGGACCCCGGATGAGCCGCACAGATCGA<br>ATCAAAATATATTTATCCGAATGTATAGTTTGTTTTTGTATCGTCTTTTTTATAATATGCGTGGGACTGTTGTTATATTTATT<br>TTTTCACTTATATAGCACCGTATCCAATGCTGGAAAATTGACGGGTACTTTAGCAGAGAGCTGAGCCAAAACATACATAAC<br>ATTTTATCTATGACCAAAGTGTAATTTCATAGATATTAGTATAATGTTTTTTTTTTTTNNNNNNNNNNTTTTTTTTTGTTTAT<br>CTTAAATAGTAACTATTTTTAATATAGAATTGCTTACATCGACTCATCTATTTTTGAGTGTCTTAGTAAATGTTAAGTATTA

		GATAAGCATGAGCTTTATAAAGCCTTATTTCTTAAAATTATACCCCTAATAAAATTGTAGAATTAACGGCTATTTTGAGGT<br>AACTAGAAAAATAAAACATAAGAAATATCTTAAATATATAGTATAAATACGAAACTTTTTAACTAGGGGTGGATGGATAT<br>TTGTTAGTTTGTCACGTAAAAAGTACTGAATGGAATTAAATGAAATTTAGTACACACGTAGATAGGTAGGTAGGTATTTAT<br>GCAGGTGAAGCCACGGAATACGACTAGTTTTAAATATAATTCTAACCAGGCTTAGTGGGCTTGATTCAGTGACTTAATAAA<br>ATATAAAGACTGCTATTCAGTTGAATATTATTATTGAAGGAGACCATATGCGAAGGTTTTGCACCCTTATGTGGGTAAATT<br>CCTGTCCAAGACACATTATCATTATATTATCATAATAATGACGGAACGATAAAATATAAAGATCAGTTAACAGTCCGAAA<br>AATTGTGATATAGGTTGTATTATTTGTTTCAACTAGTTGTTTAGTAAATTATTTTTATGAAAAATAACCAAAACTGTGATT<br>TTGTTTATATTTGATGTTGCCAAGAAATAATTGTATTCAATAAAGAT
MSTRG.10395	Eukaryotic translation initiation factor 4E-binding protein 2	ATAGAGTTATTTGTTAACCTCTTTAGTCATTAAAACGTCAAACATCTCATAAGTGTATTTTAGGTATTAAACCGTTTTATCT<br>CTTTTTTTTGTGCTCAATTTCTAATTTGAAAGAAAATGACAAAACAGTATAATAGTAAAAATAGGCTTGTAGAAAGATTTT<br>AGTTTTTATAAATAAAGGGGCAAAGGTCTAATCTGCCGAAAAAGTATATGCACTGGCTACACCCGACTCAGTCCTTCTTGT<br>CTTCCCGTCCGACCAACAGTGAGAGTGCTTGTGAAAATTGAACTCTTAGTATTAAAATATAAAGTTGAAAATGTCTGCATC<br>ACCGATTGCAAGGCAAGCTACTCACAGCCAGGCCATCCCGTCAAGGAGGGTGCTCATTACGGACCCTGCGCAGATGCCTG<br>ATGTATACTCAAGTACTCCGGGTGGCACTCTTTACTCAACTACACCGGGAGG
MSTRG.10411	Vesicle transport through interaction with t-SNAREs homolog 1A isoform X1	AATCAAATCAATAAATTACGGATCTATATCATATTCTTAAAATAATTTCTTGAATAAAATTTTGATATGTTATTTTATAGAA<br>TTTACAGTTTCCTTTGTGCGTAATATTAATGTAGTTTATTGATTTAAGATAGGAACTAAAAAATGGCTACCTTAATTCAGTC<br>ATACGAACAACAATATTCAGTGCTTACAGCTGATATTACAGCTAAAATTGGCCGTTTGAAATCGGGAAACGAGGATAACC<br>GTGATCAGTTGACAAGAGAGATTCAAGCGAATTTTGAAGAAGCCAATGACTTGGTAAGATTGCATTTTTTTGTATAAAAAA<br>CCACAGCTTGGCTAACTCTGTCCCACTGGTATTTCTGTCTTAATGTGAAGATAACATTTTAGTCCTCCAGTTTGGAGATATG<br>GATGTCACAGTATGTTGATAGTTGTCAACTTGTTTCCATTCCAAGTTGCATAAAGAAATTATCTATTTATTGCAGTGTGGA<br>AGATTTCATATAATTTATCAAAATAATATCTACTTTTAAGTATTGTTTATTCATCTAGTACTCTATGGTATAGCTAACAAAA<br>CATTAAAAAAAGTGGAAGCCAGTGATGTAAAAAAATAACAGTC
MSTRG.1044	Ras-related protein Rab-21	CTGTACCCCACTTCAATTGAACTTTGATTTGTTGTCTATCAAACCATATCAAACTGAAAATGTAATTTGTTTCTTGCATTGT<br>TCGATATTCTCGCTCTACGCGCAAAGTAGCACGCAAACCTACTGATAATCGCATCAAATTAGATAGACTCTGAAAAACAAT<br>GTGACTCGTTTCGACACAGTGATTTTAAAATTATTAGTATACTCGTTGCTTTGAACAGTGATGTTCTTCCACTGCCGTAAAA<br>GAAAACAAAGTTGAAATTGTTGTTTTTATTTTTTTTGTTTTATTTTTATTTTATAATGTAAAGATTCTATTTACAAATGTAGT<br>GCATTGTATGTTTAAACTATCAAAATGAAAGTAATTGAAGCAAAAATTGTTGTTTTAGGATCCCAAGGTGTGGGCAAGAC<br>GAGCCTCGTGGTGCGTTACATCGGCAAGATGTTCTCCAAGCACATTTCGCCCACCATCGGCGCGTCCTTCTTCACGTGTAA<br>CATCAACGTCGACGACGCGAGGGTCAAAATGCAGGTGGGTGGCTTTTTTTAATGGGTGATAATGGAATGGTACCCCCGCTT<br>GCACTATCAGTATACGCTGGTAAGGCACCAGTGATAGATGATTGGTGACGATGAAAACAGGTCAGTTAGCCCATCGATAA<br>TCCAGGGGGAACCACGTGGAGGTCGGCTTAGCAATGGTGTTTTTAAATCCCATTATTAAAATATCCTTACCCCTGGTGGGT<br>ATGGATTTGTTTTGAAGATTTTTTAGTAGGTGAT
MSTRG.10457	TELO2-interacting protein 1 homolog	GGCAGCAAATACGAGATGCTCCATCTGGCGGGACTGAAGGCAATTAATGATATAGCGCACGCGTGCGGGCATGCGACCGT<br>GGCCGAGCTCATACGCTGCAACGCGGACTATTTCACCAATCAAGTAACGTTGAGGCTCAAAAAGGCGTGGAATAGTCAGT<br>CGGCATTGCAGATACTGTCTGTTGTAATGAAGTACAGCGACGTCTCTATGTCGGACTGTCTGTACAGCATTGTAGAAGATG<br>TACTAGTGCAAAGTTGCGATAAATATTACGAGAACAATCTATATG
MSTRG.10481	Neural/ectodermal development factor IMP-L2-	AGGTGCTACGCTCCGGTGAGCTTATAATCTCGCCGCTGCGGTGGAGCGACATGGGGGAGTTCACGTGCCACGCGACCAAC<br>ATGTTCGGCAGCCAGTACGCCAACACATTCGTCTATCCCGCTAAAGCGGGGTAACCCATGTCTATAGTAATCACACATACA<br>AGCGTGAAGCATACTTAAACTGGATGTTCCTGTTATTAATTAAGTTAAAGATAAAAAAACGTTAAGAGCCTTACACACGC

	like	ACGCGGGAAACGCTGCGTTTCTGTTACCAAACGCACGACCATAAGAACTTAAAAGTGTTATATTTTATAATATTTGAATAC<br>TTGTTCTAAAAATATTTAATATGATAAGGATGTTTACGCAAAGCAGTATGAAGGTGGCACTTAGACGTGAAAACAAAGAG<br>TGACTTTAAAAAAAAAACATAAAGGCGGGAATTGTTTTTTTTTTTATTTTCCCGCCTACAGACTAAAGCAGAAACAAAGT<br>ATAAATATGTTATGACACAGTGGCGCCCACATCGGGATAAATGAAAAGTAAACTGCAGGCAGAAAAAAATAGTTATCTCC<br>ACAGAGTATTGTACTGTTTTATTTGACAGATACGTTTTAAATGTGTTTTTAAATAGCATATCTTTTCAGACACGCGGTAATT<br>TATTATTGGAATATAATTTTTCATATTTTAATAGTGTTAAC
MSTRG.10489	RNA-binding protein 25-like	AGTACAAGTGGCGGCGGCGGCGGCGGAGGAGGAGGAGGAGGAGGCGCGACGTCGGGCGACAACTCGCCCATCACT<br>ATCAGCTTCAAGAACAAACGCAGCCCCTCGCTCTCCACCGACACCCCAAGCGAGGGCGCGGGGCGCGTGCGGCGCGTGCA<br>CGCGGCGTTCGCGGCCGACGACGACGACACGCCGCCGCACCACCCGCCGCCCGCCGCCACCGCCAGCACCGGCAAAGAC<br>AAAAAAGACAAGGACAAAAGTAAAAAGGGCGAGAGCGGCGAGAAGGAGAGCCGCAGCGCGGAGGAGAAGCGGAAACA<br>CATAAAGAGTCTCATCGACAAGATCCCCACGCAGAAGGAGCAGCTGTTCCAGTACAAGCTGGACACCTCGCAGATCGACA<br>ACACGCTGATGGAGAAGAAGATCCGGCCGTGGATCAACAAGAAGATAATCGAGTACATCGGCGAGCCGGAGCCGACACT<br>GGTGGACTTCATCTGCAGCAAGGTGGTGGCCGGCTCGGCGCCGCACCGCATCCTCGACGACGTGCAGATGGTGCTCGACG<br>AGGAGGCCGAGGTGTTCGTCGTCAAGATGTGGCGCCTGCTCATCTACGAGCTCGAGGCCAAGCGTGCCGGCCTCGCCAAG<br>TGACCGTCGCCACCGCCGCCGCCGCCGCCACCGCCGCCGCCGCCAGCACCGCCGCCGCTGCCGCCCTCGCACCATCACCAC<br>CACCACCACCATCATCACCACCACCACCATCACTGCCGCCACCACCGCCGCGACCAGGGCGTGTAACGTATAGTAAATAC<br>ATTACTAGACTGTGAGAATTTAATGACCGTACTACTTTGTGGCAGTTGTTTTGACTTTATCTAGAAATTAAATCAAATACAG<br>TGAACGCTCTATATAAGCAACCTCTCTATAAGCAACTATCTTTATATTTTATTTTCTCAAACATGCTTGGAAATGTGAGCAT<br>TGTTTTGACAATCTTTTTCGAGATAAATCAAAATTGCCGAATTCGTTATTCGTCATTGAACTTAAAAAGTAAAATTGTGTTT<br>TTGATTTCCGCGCATTGTTTGAGAAAAGTACTATACAAGCTTTGGCCATAACTAGGCATTGATAGACTCACGTATGTGTGC<br>CTCGGCTACGCATCGGCCCACATTTGCACGTTCGTCCATCAATACCTCAGTTGCTGGCCACTGCAGTAATGTACTATACAA<br>GCCTTGGCCACAACTAGGCATTGATGGACTCATGTATGTATGCCTCGGCTATGCCTCGGTCCACGTTTGCACGTTCGTCCAT<br>CAATGCCTCAGTTGTTGGCCGCTGCAGTAATTAACTATTATTCTACTCAGCTTATAGTATTTTTATATTCCTTTTTTCCCTAT<br>AAGCAACTTTTTTCAATAAGAATTTTTTTTATTGAAATTAGTACAAATGAGTTGCTTATATAGAGCTTTCACTGTATACAAA<br>TGATTAAAACTGACAGATAGAATTACGACTTGATTGAATCCCTTCTTTTTATTTTTTTTTAAATGGCTTCTACCATAGAGAC<br>AGTGGTGATTTTCTGCCAATGTTAGGATTAGGGGAGACTCCCAGTTAACTGATTATAATAGTAATTATAAATAGGTCACGC<br>TGCACCCGAATATGTCACCATAATAGTGCTAGAATTATAATTCTTAGTTGGTCCTGTACATTTTAATTTCTATATTTCATATT<br>CATAATTTGATGTAAGTGCGTTTGGTAAGGAGCGACCCGTGGCGCGGCGGAGGTGGCCGACGTGCTGCAGTATCAAGGAG<br>CTTATCATTTTTAATTAAATTTGTAGTTTGTTTTATAATTAATTAGGCCCCTGTCGCGGGCACAACTATTAAACAATAGTCA<br>AAATTTATTAAAATAATCGATTTTATTTTCAATTCATACTATTAAAATGAAACATGTATTTATTATTTATGTATTTATATGTG<br>AATATATACAATACATAGAGGTTGTACTTAGCCAATTTTTATTATGTTTTCTTCAAATCGAATGTGCATCGCACGATTAAAG<br>TGTGACGTGTCCGCGGTGCGTAGAGTCCTCTGTTTTACCGCCAGGGAACTACACGAACTATACACGCAGGATTCTACCTCG<br>TCTGCGCAGGTCCTAAGCTGTTAGTTTCCATATCGTAGTATATACTGTACAGTTACAGAAATTTTACATTATTTCATAATCT<br>GATGACGCGAGTTTCTGTCGTTTTTAGAATTTCGTACCAAGAAGGTAAGAGGAAACCCTTATATGTCTATACCCATCTATTT<br>GTCTGTCCGTCACGACGCTATATATCAGCAACTATTAATGTTATCAATTTGATATTTGGATTACTTGTACATATTGCTGGCC<br>CGTACATATTACAATGGAAGTAATTGAAAAATGTTTATTTTCTTTATTGGGGGGTTCAAGTGAGCTTTATTTTGTCCATTTG<br>AAAATTGATTTTTATTTTAAATAAATAAAAATAATTTAAATCGACTCTAGTAACCATCTTTCATTGTCCAGTGTATGATTTG<br>CCTTAGAGTTTGCATTAAATTATTCTTGACACTGCCTATTTTTGTATACAAATGTATATTTTCGTGGTAAATCTAAAATAGT<br>AAACGAGCTTTCTTGTTTATATAATAAAATATATTGTAAATCACCTAGGAACTAAGTTTGATCAACTAAACTGGGATTGTG

MSTRG.105	Fatty acid synthase-like	ATGCAATACGATGGATTTGGACAAAACGCTCAGTACAGAGACGACATGTAAATTTGGTCGCGGATCCACTTGTGTAATATAAGATAGTTGTCGTCTCTTAGTACTACATTTATTACGAAAACTGAGCATTACCGTGGAACCGCTATTTCTGTGGTAGTGGCGGACCGGACTGTTGTTCTTCGAATATTACATTTTTTTTTAAACAATTCTTATATAATATCTTTACTCTATTTCTTACTTCGAAGAACGAAAATGGTGCCTACACCTCAGGAACCCTCGCTGATTCAATCGGAGAAAGTGGCTCCGTTATCAGATGGCGATCGAATCGTTATTTCTGGCATGTCCGGAGCTTATCCCTCCGCGCATAACATCAAGGAGTTCTCCGATATACTCTACAATAAG
MSTRG.10507	Lysine-specific demethylase lid isoform X1	AGAAAATTTACATATAACATTTCAGCTGATAACTATCAACAGCTGAAAATAAGCATTTCCTATAAGGGAAGTATTGTTAGTAGTTGCCAGACTTAACAAATAAGCTGGCAACCGAATCTAGGCTTTTAGAAATGTTTTAAGAGGACCTGCCCCTCTACTATTACATTCCTTTAGTCGTTTTTTACGACACTCACAGGAAAGAAAGGGGTGGCCTATTCTGTACCGGGACCACACATACAATCGAAATAAATATACATTTTCAAATAATATATGAATATATTGCTTTCCAGGTGGAAGAGATGCAGTCCAAAGACTTATACCCCTATATGCATAGTGTGGAAGTGTTAGTGAAGCGAAGTCAATCTATTCCATTGAATTTATACGAGAAAGATCAACTCGCGGCTGCGTTGATGTCTGCAAAGGAATGGAAAAGAGGAGCCGCTGAAATGTTCCTAAAAAAGAGCTGGCCGTACTCGCTGCTAGAAGCTCTATCGCCGCGGACGGAGAGCACGTGGACGCCGCGGCGGCGTGGGCGCGGTACGACCAGCGCGGCCGGCGGCGAGCCCGCGCCGTCGTCGCTGGAGGCCGACCAGTTCCTGCGCAACTTCAGCGAGGACTCCACGCCCACCGAGATCGTCGCCGCCTTCAAGCAGGCCGAGCACCGCGAGCTCGCCGCCATCAAGGAGCTCAGAGCACGGAACATGCGAAAGGAAGTCCGCGCGTCGCCTACGAGTGCGGCGAGCGGCGCGGCCGCAGCGGTCACGTTTTGCCTGTGCCAGAAGCGGCAGTACGGCGTCATGACGCAGTGTGAGCTCTGCAAGGACTGGTTTCACGCGTCGTGCATCGCGGCGGCCAAGGAGGAGCGTGAGGAGTCGCCCGAGTGTGAGCGGGA
MSTRG.10513	Uncharacterized protein LOC105383894	CTTATGGCCAGTTCGGACCGGACTAAATGTAGATTAAAAAAAATGGTTTAATTTGTCAGTTTAATATATTTATTCGGTGTATGGTAAAATAAGAGAAATAACAAATGTTACTCGTTGTTTTTATCTGAGGTAAATCTTATGTAACGAAACACTGAAATAATTAATTGAATTCATAACTGTTATGTCAAAAATAAAAGAAACCATTCTTCCCTATTGATGGAAAAAACAGAACTAAAAATAAACAATTACAAAAACAAAAGTAATTTAGCGATAACGATAAATGAGATATTTATCATTCCAGTGTACAACACAGATATGAGTTTGGGAAATTTTATTAAAAAAAATCTTGTTGCGCTGGTAATGGTGCCCATGATTATCGGTACTCACTATGGATGGTATAAATTACAAGAAGTTGATAGCCTGGTATCAGCTGAAGAACGAAATAAGCTACCAATTACAAAGTTTATAAAAAGCTTC
MSTRG.10516	Nuclear pore complex protein Nup98-Nup96	GACATGTATATAACATTTATATTTACTGTCCACCCCCAGCGGCGTACGGCGTGAAGAGTCCGACTAGCGAGCTGGCGCGGCTGGAGCACCGCCAGAGTCATAATGTTCAACTGATGAAGGCATCCCTCTACGCAGATATAGAAATGGACGACGACGTGTCGGTGTCGACCGGGGACCAGCAAGTACCGCTGGCGGTACCCACAGAGGACCTGCCGGTTACTACGGGGCCGGCACCGGAGCCCTACCTCGACATCACGCCGCCTGCTTACACCGAAGAGGTGCCTATGAAGCCTCTCATAGTCCGTCCCTACACCATAGTGCTGAAGTATCACAGGAAGGTGCCGCCTTTTAAGGAGACCATAGCCGGT
MSTRG.10527	Serine/threonine-protein kinase RIO1	TTCATATGTATACAACATACACGTGTTTTATTTATCATAGTTACTCACGTATTGAAACAGAACAGTTCGTGTTGTTTTAATTGTGGAATGTCTGAAATTGTGTTTTTCAAAACTCATTGCAAAAGTTATTAATATTTAAATAATAATGTCTGACGTATCAAGCGGCCAGTTCAGTGATTATGAAGAAAACATAATTAACAAAGTTAAATCGGTACGGTTTGCAGAGTTGCCTACTTATAACAAGGGCAGTAGCAATGATGACGTATATTCGGATGACTACTTCTATGATTCTGATGATCCAACACAAAGTACAAAGAAGAAAGAGGGTATAAACTGTC
MSTRG.10539	Ubiquitin-protein ligase E3C	AGGTGTGCACGCATCTGTTGCGCGCGCTGTACGCCCGCGACGCGCGACTGCAGTTCTGCGGCGCGGAAGTGTGGGGCGCAGGCGGCGCGGGCGGCGTGAGCGGCGTGAGCGGCGCGGTCGGCGCGGGCCCGGAAGGCGTGGCGGGGCTGCTGGCGGCGGCAGGCCCGCTTACACGCTACTCGCTACGCCAGCCGCGCCGCCCACCACCGCCAGCCTCCGCCGCGCTTGCACTCTCACAAGAGGAGGGGCCTCCTTTAACAATCAAAGAATTAAGAACAGTGACAATTTTACGGGAAATACCATTCGTGGTGCCATTTTCTACACGAGTTTTAATCTTTCAAGGACTATTAATTAGAGAAAAGCACGACCATTGGTATGAATTGAACAATTTCAATGAAGGGC

MSTRG.10547	Uncharacterized protein LOC105397376	CCTCTATAAATATCAGCGTGCGTCGCACACATTTATACGAAGATGCATTTGATAAACTTAGCCCTAGTAACGAGCCAGATT<br>TGAAGCTGAGGCTTCGTGTGCAGTTAATAAACCAAGCCGGCGCTGAAGAGGCGGGTGTTGACGGCGGTGGTCTCTTCAGG<br>GAATTCCTTTCAGAGTTGCTCAAATCAGCTTTTGATCCAAACAGAGGTCTGTTTCGGTTAACAAAGGATAACATGTTGTAT<br>CCAAACCCTGGCGTGCATTTACTTTACGATGACTTCACGATGCATTATTATTTTGTTGGAAGGATGCTTGGAAAGGCGCTG<br>TACGAGAACCTGCTGGTGGAGCTGCCGCTGGCGGAGTTCTTCCTGAGCAAGCTGTGCTCGCGGCGCGAGCCGGACGTGCA<br>CGCGCTGGCGTCGCTGGACCCCGCGCTGTACCGCGGCCTGCTGCAGCTCAAGGCGCACCGCCGCCGCGACGTGCCCGACC<br>AGGCCATCATCAACTGTAAAGAGGAGCTCAGACAACACGCTAATGTTCTCTGCCCAACGTTGCTCGAACTTGTAGCGAAC<br>ACGCCGCGCGAGGAAGGAGGCAGGAAACGTCTCAATGGATTGCATGTGGATATTCTCGATACAGTGACGAAATGGTGTGA<br>AGACCCCGAGTGTGGCGGCGTTTTAATACCTAATTCCTCGCATGGCCACCTCAATACAGCCATAGAATACCTCGTGTGTAC<br>AACCATAGACATCGGGGAAAGGTTCCTCCTGCATCTCGTGGAGAAACTGATTGAGCTCTATGGTAATGACGTCACGGTGC<br>CGTGGAGGTGCTTCGAGGGGTTTCTTGAGGG
MSTRG.10557	zinc finger protein 79-like	GCGCGCACGGCTGCGCGCGCTGCGGCGCCGCGCTCGCGTCGCGCTCCTCGCTCGCCAAGCACGAGCGCACCGTGCACCGC<br>GGCCAGCGCCCGCCGCCCGCGCACGTCTGCCACACCTGCGGCCGCGCCTTCCGGGGCAAGAGTGTGCTAGTGAACCACGT<br>GCGGACTCACACGGGCGAGAAGCCGTTCGAGTGCGAGGTGTGCGGACGCAAGTTCACGCAGAAGACGGCCATGAGGACC<br>CACATCAACCTGGTGCACCTCAAGATACGGAGGCAGGCCAAGGTAAAGCCGGAAACGCCGCTGGAAGTCCGCGAACCCA<br>AAATCGATTTATTTACTAAGGAAGACCAACCCCTCGAGTTTGAAAGCTGGAATCGACCACAGATGGCGCCCTGCGACGTC<br>TATTTCCAGGTCACCGCTGGACCTTAGGAGTCAATGTCGACTGGGTTTAAGACAAGGCAGATTTACATTGAGTTATTTATA<br>TATTTTCAAAATGGCCGCCGTTACATCATGGCGGATTGTGCCTACATATTTTGTCAACCCTCTCAATATGGGTATCAAATAA<br>AAGAGTTTGAAGAATGGATAATGATAAAATAAATAACATGATTTTAAAACAGCCGCAGTTGTAAGATGGCGGATTAGTTT<br>ATAAAAACTTGTCCCGTATGGAAGTCACTTCATTAATACGATGTGTTGTCGTTTAAGTTGTGTGCCAACAGTGGCGCTACTT<br>ACGCAAAGTATTACGTACTCTCCGCATTTATATTAGCTTGTTTCAATGTAAATCTGCCTTTATAAACCACACATTAACTAAT<br>TTAAAAAATATAGGTTCCTCTTTACACCTCCAATTGTACCTTTACAATATGATCTCATTTGTACTTTTAATGGTACCAAACC<br>AGCGCCATCTGTTAGGTAGCAACTCAAACTTAGTTGCTAGCATGCAACCGATAGATGACGCTTATATTTAGTTTGCTAAAT<br>TAGTGTAGTAAATATTTTTGTACGCTTGTTTCGTGAATACGTCGAATTTTGTGGAACTATCTTAATATTTTCAGTGTATCCGT<br>CCCTTTCTTTTGAGTTAATTTCTTAATTTGAGAGAAAGTGACAAAATACATCACGAATTTTAAATATTTTAGTATAATTATT<br>TTTAATATTAATTGTGAAGTGCCCCGATAATAAGTATCCGTTCACATAGAGCGGCTGATCGCATTTAAAATTGTTTTTAGGT<br>AATTGTATCGTGGCGCAGCGGTGGTTACGTTGCATTGCGTTAATGAGGTTTCGAGTTTGATCCCCGGCCGCGCCAATTTAG<br>GAAACGCACTTTTCAGATTTGTCTCGGGTCCGAGTGTTGTGAGCGATGCCCCCAGATGTTCGACGCCCGGTTCCTCATATC<br>ACAGGGATTCCTGGAGGAGCAGACGATGGCGTGATCATGTGGCATTTATTAATTTTGAAAGAAGATTTTTTACAATTCTA<br>CCTTTTTGTTAAAACTAAATTAAGAGTGAATAACATTCGCCGTATGCTCCCGGCTTGGAATAGGCCACCCCTTCCTTCCCCG<br>TAAGAGGCGATTAAGAAGGCGTGAAATGGGCAGCAGCGTGCCTCTTAAAACATCTCTAAAAACTACGGTTGCCAATCCGC<br>CTGTCAAGCGTGTCAACAACTGGCTAAACTCCCCTTATGGGGAAAGCTTATGTTTAGCGGTGGACAGTTAATGGCTAAAAA<br>TATATGCTGTTACTCCTCGTTCGAAGCCAGGACACGTCGTTATTGTAAAAATAAATCTTATTTCAATGATTTAAAACTCATA<br>TTTCGGACACAATCTATATACGCTTTACTCAAAGCCAGCCTGTGTGAACGGGCGCTCATTCATTTTAAATATGTGTGACTG<br>AATAGGTTTTATTTGCATGCAATTCTCTCTGTATACTAGATACATTAACTTTTTGACCGCTCGCCTTCGCCTCGATCGCCGC<br>GCCCCCTAGCGCCCGGCTCAAATCGGCAACTTTATATTGTACTTTACCTGCGACTTCCAATCGTTATCCGTGAACCAATCTT<br>TATAAAACTTGGTACATACGCACTTTATGGGATGGAGAATGCTAAAGGCTACCTTTTACCCGGTCACGCGAAGTCGCGTTG<br>AAAAACTAGATTATAGTACAGTCTAAATCTCGATTATCCGGATATTCGATTATCCGGACTTATTATTCACAGCTCAATTAG<br>AGCTGTTTTTGAAATGGTATTATGTATTAATTGGGTAAACAGGTTTTATGTAATCCATTCTTTATATTTTGTTTTTTTTTTTAT

		ATCGTATTGTCTAAATGACGTATGTAAAACCTTTTACACAATAAAAAAATATTAAAATTTAATTATATATTGTTTGATTAAAGTTTCAAATCGATTTTCCGGATTTTCGATTATACGGAATAATCCTGGTTTTATTCGATTCGGATAATCGAGGTTGTGCTATAAAATTATTTTTTTATATTTCCATCTATAATGAGTCTTTTTATAACTAGTAAACTGTTCTATATTCTACCTTTATTTAATTACTGTATCCAATGTAGTGGGTCGTTGACTTAGATTACATAGATAGAGTGTCTCCATACAAATGCTTCGAGAAAAACGGATAAACTTTTTCATACAATTCTACGTGATACATAGTTACGAGTGATTCCTTTATAACTTCTATATTTACTTTGTCTTATAATTTCCGCATCTTTTTAATGTGTAAACACTCATTTTATTTATTAAACAATACGTCAAGATACATTGTGTGCGTAAGTATATAGAACACTGATAAAAACACGAGTCAATTTGACAATTAGCTGGCGAAAAGTTGACGTTTGAGGCTCGATGCTCTATCTATATAATATAAGACAGTTAAATATATGGAAGAAGCAAATATATAAAGGAGCTCCCTGTCGGACGACTTCGTGTGGGGCGATCAGAAAGTTAACATGGAAAATCGATG
MSTRG.10559	Uncharacterized protein LOC106135284	ATTCCAGATATCATACAAATTGTTTTACGAGTACGAGTGCGGTGAAGACGACACATACCAGTCTTATTTGGCCTCTGTGGGTATAGTGACGCCTCTAGCAGTGCTGCCGGCATTGGACGCCAAAGTGTCTGCTCGATTGGAGGACGCGCTGTGGAAAATGGCGGCCGACATCGCCATGCCCTCGTTTGTCGTCACCGCCATTGCAAGATTAGAGTTGGACGACCCATTCGTCGAAACATCAGGCAGCTTGAATCTGACCTCTCCGTACCTAGACAATTACTTCATCAAGATGGAGTTCCGCAAGGACTTCGCAGAGAGCGAGAACACAGTCGGCGGCGGCATACACATACACCAGGGTGACCAGGATAATTATGTGAGTGTGATGAAGTATTTATGGCACCAGAGTATTATAAAATAAGAAAAACTGTTTTTACCAACCCGATAAGGTTGAAAAAAGTAATGGTAACTCCGCCTGCGCTATCAGTATAAGCTGACGAGGACCAGTGAGAAACAATAAGTAAAGATAAAAGCAGGCCCAGTTGGCCCAGCGTAACAGTTCCAACAATGATTAACCACGATGGTTTCCAAGGGGAACGTATGGAGATTGGTTTGGTATTGTCTAGCCTTGAATCTAGAAATGAGCTATCAAATACAAAAAGGACTTTTAAAATCTAACCGCTAGTTTCAGAGATTATTGCGTTCAAACAAACATTTCCCCCGTATTATATTACTATATACTACTTATTTTATATATTACTATAGACGTAAATTGTACCGTAACTATGTGGAACGGCCTTTCCCGTAACATGTTTCCTTTATTCTATAACCTAAGATTCTTCAAACGTAACGTGAAAAAGCACTTAATAAGCCGTCATAGTACGACTAACCATTGAGAGTGGAATATTCTATTTTCGGTTGATCGTATTCATGGACCTTGTAT
MSTRG.10561	Hemicentin-1	GTTCCCCCAGATATCGTGAGCGATGACACATCTTCAGATGTATCGGTCCAAGAGTTGGAGAATGCAACACTGACTTGCAAAGCTACAGGACATCCTCCACCAAAGATTACTTGGAGGAGAGAAGATCACGAACCAATACTGCTAAAGAAACCCCTTTCAAGAGACTTCGACAAAGTACCGATATATTAAACATCATTTATCATGGGGAGTGCTCTGAAGAATTGTTCGGATTGATACCTGCTACAGATTTTCACCATCGTACAACCCGCCATAAACTAAAATTTCATCCCAACCATCTGGACGAGTGGCGGTCCTCCACCGTGCGTTTTTCAAGGCACTTTCTCCCACGCACAACCACTCTTTGGAATCAACTTCCAGCAGCAGTATTTCCGAACCGATACGACAAC
MSTRG.10568	Prominin-like protein	GCTGGCGGGCGACGGGCCCGAGGTGGTGTGCCAGCTGGTGCCGCCCGCGCTCATCGACGACCTCAAGACGCGCCTCGACGCCAAGGACGAGGACAACGAGTCCGGCATACACGAGTCCGAGTAAACGGCCCCACGCTCGTCGCCGCTCAGTTTTGTCTATGATTATATCTTTTTACATTTTATTCTCAAAAGAAGGTCACTTGTTTATCAACCTATTTATCTATTAGTTATTGGAGTGTTTTGTCTCTGTCTTTCGAGCTTAATTATCAGTTTGAAAGAGAGGTTCGAAACGTATTTTGTTATACTCATACAGTTTTATTGAACGAACGAATGAATGAAATAAATACGCTCAGGCGTTTCCTTTTTTTTTTTTTATAATTAAAGAGTATTTAACACGGTGTGAATGTTATTTAAAACCATCGACTTTAGTATTTTTAACAGTTTTGACGTATTAAATACATGTGTAGTATTAATATTTTGATGGAATTCATAATTGTATTAAATATACGAATTAACGGAGGGAACACAATTGTTTACTCAACGAATCGTATTGAATGTTTAATAAAAATATAATATTACGCACGTAAGGGACAGTCTTTTGCGTACGTGTTGTTTGCCAAAATGAACACTAGACAGACTATAATAAGAAGGACTAAACTACAGTCTTTATCTCTATATCACACTCCGGAAACTTAACACAAAAACGGCGTTAACGAGATGAAATTTTTTTAAAACGATGCGTCGTTTTTCTAATGATTATATTTTTCACAGGGAGGGGTTGAGGTAATCTTGAGGGACAGGGTTTAACCTGGATTCGTGACGGTATCTAGTGAAAAAAAAATATGCGTTAAAATTACACCAAAGACTGTCTCTGATTGACTCTAGTCTACGTCTTTACACCTTTAATATTTTACCATAGACAAGAAACTAGGTATTACGTGATTTTACCGTTCTAGTATTTAACTTTTTATATAATT

CGAATTTCGATGTTAGATTTAAGTGTATTAAGGCTTTCGCAGACGAAGGACATTTTGTGCGAGAACATTGTTTCCCTTTATT
TGCCTTGTCTTCTCGGACTTACAGATGCGCGGCATAAATTATACTTTGGTCTTAAAAGCCTTAAGATCAATGCTGCAGCGT
ACACTTGTTATTTAAGTATAAGGTTATTTTCTCATAGGGATATTTTCTCTGCGTACAAAATATCCGTCGCATGCGCAGCCGT
TTATAAGTAACTGGTTCCAGTAAGCAGCCATTTCTGTGTGGGCGTGCAACGCGAAATGTCTTCCATGGCCAAGAGCGACCA
TCCCTCGAGTCTGCTGTCCATTCTCGTAACGTATTCATATCAACTACCGCGTTTTATATACTATTTATATAAAATTATACTGT
TTTACTAATGTTTTAGCATAAATTTGTCCCCGAAGGGCAAGTCTACGTTTTATATCATCACATCTACCGACTATATCAAGTA
TATCACTAAACAATATATTATAAATAGAATATACAGTTAAATATAGTTCCGCTTTCGCTTGTATTGCCGCGACGGACGTAG
GGTATGACCGGGAAATAAGTTAGCAGAAATAATTGCTCGAAAATGAAATTAGCTATTGAAACATTTACAAGTAGATTATG
TTACAGGAGAATCTCGATTATCGGAATCAAATAAAACCCGGTTTAATGATATTCCGTATAATCGAAAATCCGGTTTAATGA
TATTCCGTATAATCGAAAATCCGGTTAATCGATGTCAAAGTCAAATCAAACAATAAATAAATTTTAATATTTGTTTAAAAT
TTTGTTTAGAAATAATTAGTTATTAATCTTTGACGTAATAAATCGCATTTTTTCATTGCGGCTAAGACCCTAATGGGCTTCA
GTTGTAACAACTGTATAGGATCAGATTCGTCCTGTTTTTTTTTCCGGATAATCGAATTTCGTATAATCCATTTTCGGATAAT
CGAGATTCTACTGTATTTGTTGGTATTACATTTGGAAACATTAGTCCGTTTAGGTTTAAAAGCTATTGGCTTCAAAACTTGG
CCAAAATATTACTAAGTTTATGTTGAAGATTATTATCACATTTTGTGACAATAAAATTGTAAGAAAATAGTTGTACCTAAT
GTCTCACTTGTTTATAATAAGGACTGCCATGCTATTTACTAACCTAAGCGAGACTTGGCCAATTCTTACCTCGGTCCGACGA
CAAAGTTACGTCCAAAGACAATCTACAACCGCTTGTGCCTCGCCAAACGTTTGCGTTCGGAACAAAGATTGGACGATAAT
GTGACACTTTTTAATACTTATACGCGAGCTTTATAGATCAAAGTGTGTGGCCAATGTTTGTCCCGAACTCGCAACGTTCAC
GTTTGTAACCCGTATACTATGATGCAACGCTTGTACAATGTTCGATCTCATAATATAAGTACATATATAGTCATACTGTAGT
GAACTTTTATACGGCGTACCGATTATCATTTGGAGGAGTTTTTCAAGTAGTTTTGATACTGTACAATACAGTAGCGTGGTTT
GTATATGCATTTACTGTTCTCGTTGAAAGTCCATGATTGCACTTTTGCAATAACAAGTAGGTAGGATTATAAATTTCGTTAG
TTATAAACATCTCGTGCTTCTGTCGTGATGTTGCCATTTGAGAATTTCGAAATTTTGATGTTAACCACTAAATACATTTTGT
AAAAATTATATTTTAGTAAATTTGATGATATATAGGCGTCGGATATTGATATTTTTTTAATTAAATATGAACTTTTAAATAA
TATTATTACAAATTTGTGTTAATTAATTTACTTATATATATTACTACAACACTGCAATATTTGCAGTGAATTTAGAAAATAG
TGCGCCTCTCATGAAGAGAAAAATGTCTGAATTTATAATAATAAATGTAAAAAGTAGTTTTTTTACATTTTTCTATATAGAT
CATTTTTTTTATTAATTTATTTAACTAAGTCAAGAAACGGGCAATTATGTTATTTGTATAATTAAAACAGTGATATAGTATT
TATCATATCAATAATTTAGTTTCATGATATTTGATACGCCACTAGATCGTATAGTTAAGAACTATATTCATATGAAGGAAA
TAACATTAGATAGTTTGCTCATAATATTGGGCCATAGAAAGCAAGGCTTTCTACACTACACACTAATTGGTGATGGAAATA
GACAGCATATCAGTATTGCAACATGTTATACAGGGTGCAAGGGATGGGTTGGCAAAAATGGAAACACTGAGTTCAGGCCA
TTATTCTGAACTGAAAACCAGTAGAATTTCTTTCCGGAAAATTTTAAATTTATTTTCTGCTTTTTCATTTAAAATTATGTAAT
TTATGATATGAAATATAATAATAATAATATTTTATAGTGAAATACTATGAAAGACTAAAAAATAGAAACGTAATACACGT
TTTAAAATCCTACCGCGACACGCAGAATCAGCTGCATAAGGTCAACGCCACAAGCGTACGAAAGTTACCTACGAAGAAGC
GCGCTACTGAGCGCTCGCTGTAGGTATCACGCGTCGTGTGGATCGTAAAATTTCAAATACATTGCGCGGCGTTCATACTTA
AGAATCGTTAATTATACGGCTGTGGTGCATTTCCAGTGGAAAAATAATCTGCCATCTGATTCAGACCGTTCACCTGGATTA
GCTGTTTTCGTTTTGGCCAACCTGTCCCTTACACCCTGTATAATGTTTTAGGGGCTTCGCTAGATAGGTGAACATATATAAA
AAATAAATAAGTACACGTACTTGTGAATGCTTTTTACATTTACCTATATACTCCTAAATTTGTATATCTACTACAATGTAGT
TGCACTGAAGTGAATTGATGTACCCGTGCTGCCAGTATCTCTCACACTTATCGTGTTAGCTAGGTAGGCATAATACAATTA
TACATAATTAAAAATTAGTACAATAAGTTTTTAATACTAATAACCTATACTTACAATAACTTAGTTAATACTAATATTAACT
TATCATATTTGATATGTATATCAATATGTCACATGTATGCATTTTATGTTTTTTTTTTTTTATTAAAAATCCATTGACTATTTT
TAAAGTTGTTTTGGAAATATACAGAATTAAAAATGTATTAAATAAAGCTTATTCTAAGCATATTCCAATTTTCAATGTAGT

		ATCATTAATTATATGCATACATGACGTGGGTTTTTCAATTTCACATTTAAATGGTATGTGACGAGATGTAACAAGAAGTTA<br>ACGAATATAATGTTGTGCCAATAAACTACTTAGTAAATATATTTCATTCTATTTTTATTATAATTGTAGAGTTTTTTAAATTT<br>TGCTTCTATTATATACAATGACTGAACAAAAAATTATATATCTCTTTTAGGTCATCACTTGTAATAATTGTACATTTATAAA<br>AAAAAGCTCATTACATGTTACAATTACTGTAATTTTTAATAATTACTACATTCACCTACATAGATAATTTATTTTTATCAAA<br>GTGTTTGACATCTCTCATGTTCTAATATTGTTCGTTTTCCTTAATATTCGACATATTGCATTATCAATTAAGATAATATCCAA<br>AGCATTAATTCTTGTGTCCGTAAACAGTATTTAAGAGTTGCCACGTTATGTCAACTGTACCTATGTTATATACACATCTCGA<br>TGTTTTAAATGTATACCTACACTGTAAGGTAGCTGTTTATACCTATTTGTAAGTAACGCTTGTATTATAGATGTTTGTCGGA<br>TTTATATTCATTACCTTGTATCAAGTTGTTTTATTTTCTAATATTAAACAAAATTGCGTTGTATATCCGAAGAAATTCTATAA<br>ATCCCATGCTATTTTTTTATACTGCTAAAATTGGTTAAAATGTTGACGATTTACTTGATGGATTAGTGGTGACCGTCATTTT<br>TTTTACATTGTCTCTCCTTAAAGTCGTTCAATGAAACACTAAGATTAATACAGTAGTATTTTGTAACGTGTCGAGATATAAT<br>AGTAGTGAGTTTACTCTGACTCGTGTCGGCGAACTGGCTGATTGTACGCCGAAAGTTGGTCCAATGTTGGCCGAAAGTTGA<br>CGCTCTGTAGACCGAAAGTTGACTTATTGGTCAACCAGGACGGAGTTGCAACCTGCCGACCGCTCATATTGTTGACGGTAA<br>CGAATTACATTATATACTTACCTCTGCGTGTGCTTGCGACACTGGGTGCATTTATTTTTGTTTGTAGATCTTGATTTCTTTTC<br>TTTC
MSTRG.1<br>0599	Xanthine<br>dehydrogenase-<br>like	AGGCTGTGGGAGAACCACCTCTGTTTTTGGCTGCGTCAGTATTTTTTGCTATAAAGGAAGCAATTAAATCTGCGCGAATCG<br>AAGCTGGTGTGAAACCAGATTTTGTGCTCCACGCACCAGCCACTTGTGAACGGATAAGAATGGCATGTGAAGATGATTTC<br>ACCGAAAAGGTGAAACCGACTATAAATAAAGAAGGTCAGCAGTGGAACGTCGTTGCGTAGGAATTAGTTGCAGATTGTTT<br>ATTCAAAATATTATTAAGC
MSTRG.1<br>06	Asparagine RNA<br>ligase	TGAGGTGAGTCAGTTCAGATCTGTCATCTCGCCAAATCTTCGCACCGTCTGTCATCATATTCCAAGTGTACATGTGCATATT<br>GTTCATATTGCGGTAAATGCAGATAAAAAGAGAAAAATCTTATAAATTTCTTTAAAATTGTCATAAACTGAAAGCACTTTG<br>CCTTTACGATGGCAGATATTGAAAAATTGACAATACAGGATGAGATTTACACGTCCGAGAAGAACGGAAACGACAGTACT<br>GGCAATGGAACATCAGAGAAACCGTTCAAGACTATCCTTCAGGCTATGCGTCACGCAGGCAAGGAGCCATTCCCTAAAAT<br>CTTTGTAGACGCCAAAGAAGAAGGCAAAGTATATGAACCCGCGGCAAAATCGCAGCTGAAAAAGATACAGAAAATATGG<br>GTGAGGGAAACCCACAAAGCGGCTGATAAAGCCAAAGCAGAGGAGGAGAGCAATGAAAAAAGACTGCAGAACCTTGAG<br>GAAGCTAAGAAAATAGTGATCCAAGTAGATCCAAATCTACCTAAAGCAAAGACTGTGAAAATAGCAGAGG
MSTRG.1<br>060	Pyruvate<br>dehydrogenase<br>(acetyl-<br>transferring)<br>kinase	GTAGCTAGCGTTTTCTTACTTATCTAAGTATTTTTCTCTTTGGTTAATTCAGTAGGTTTTCTCGTGTCAGTGAAACAGGTTCT<br>TTTAACAACGGATTCTGATTTACTTTTATTCATATTTTCTTGAGTATGTTTTTGATTGTTATTTAGTGGTCGTCACTTAACAT<br>TAAAGTTGACATATATTGCTGTTTTAATTTTTCAATCGACGAAATTTGTGTTTAATATTATACAAAATGAGGTTGGCAGGAG<br>CCATTTTCACAAATGTTACGAAAATGCTCGATTTTTATTCTCAATTTAATCCATCGCCGCTGTCTATTAAGCAGTTCATCGA<br>TTTCGTGCTGCGCTCTTGTCGGTGGAGCTCTTGCCACTCACTACGATGTGCAGCGAGTCTCTTCACCTCTTGATACGACACG<br>AC
MSTRG.1<br>0612	Transmembrane<br>protein 205	GGGGATAAGATTTGCTCGACTTATATTTCTGATCGCGGCGCCGAACCGCACAAACGACATCGGGCCTCGGTCGCGGACGT<br>GTCTAAAATTAAAAAAAAAAAACTATGTGCAACATATGTACACGCGATGTAATGAGCGCGGAGGCACCGACGCCTCCCCCG<br>CTACAAAATAACTGCACACTGGAACGTTTCAAGAACAAACACAACATAGTGACGGACCCCACTAATCCGGAGAAGCAGA<br>AGGAAGTGGAGTACCAGCCAGATTTGCTCGCTGTGTCCACGCAGTACACCAAAATTGCATACGATGTGTTCAAGCAATGT<br>ACAACGCGATTGCAGGAGTCCAAGGCATTTGTATTACCACCGGTTCAACCCGCTCATTAACAAGAATTTAAACGAC
MSTRG.1<br>0620	Protein shifted	CTAGCATATTGTAAAGCAAAAGCACAAATGTAGACAGTCTGTGTTGAACCACAGTTGTGTGCAGACACGTATCGGTGTTTC<br>AATTTTAAAAGATATAAACACGTTCGCGAAGTGTGCAGTGAGTGAAGTTTAAAAGTGTGAACGTATATGCTTTGTCTTCAA<br>TTAAAAATTAAAATAAAGCGTCCACAGATTAATTTATTATTTAAGGGGCATCGACTGTGACAATGTGGCGGTCGGCATCGA

		CAGGCGCGCGCTTGGTGGCGGGGCTCGCGCTTGCTATGTCGCTGACGTTAGGCGCCGGCCGCCGCGGGCACAGGGATCCA<br>GAGGCACGCCAGCACTCCGACATATCGCTGTGGATAGACGAGCGACAAGTCCGCATGTTCAGTGG
MSTRG.10648	EH domain-binding protein 1 isoform X1	GTAAAGAAAAGGACCTTTTTAGAGAGAAAGAGACGGACGTTGAAGTTGGGTGAATGTTTGTTTTGGTTTTTAAGCCAATTA<br>TTTATGGTAGTATTATAATTATTTGCAGGAACTGTTGGCGGCTGTCAACGAACGAGACAGGCTGGTGCAAGAGATGGATG<br>AACAAGAGAAAGCGATAGCCGACGACGACGCAATAGAGCGCAACCTCTCACAAGTGGAGATACAAAGAAAGAACAACTG<br>CATACTGCAGTGAACGACGCCAGTAGACGTTCGTCGGAGCGCGCTAGTAATGCTACGGATTATACTATTGGACGCGATTTG<br>GACAATGCATTTACATTAAACTGCGCAAAGTAGCGGTCGTAAGCCGCGGACGTGACTTTGGCTCAAGGTGCAAATGTGAC<br>CTTGGATAGAGAAGCGGACGTGACCTTGAAACAAGTTGCGGACGTGACCTTGAAAAAAAATCGGACGCGACCTTGGATCA<br>AGATGCGGACGTGACCTTGAACCAAGTTGCGGACGTGACCTTGAACCATCTTTGAGCGATCAGACGTCCAACAACGATCT<br>AGCTTAGGCATGGAAATTATTGCAATTTTTAATTTATTATTTTTTATTATCACAATCACATGTGAGCAAAGGATAGGAGTG<br>ATCTGCGTCCGCAGTGAATATTAGGTGCCGTTTAAAGTAAAGTTTATGTTAAGCGCACTAAAGTCTCTATTCGTCCGCTCTT<br>TACGGACCAGTGGACGGTTTGCTGCGATTCCATTCCACTCTTAGCTGTAAACAGCCGATGACTTACGAGTGAATCCGAACG<br>CAACACTTAAAGAGCCGACATATTGCGGCCGAACGTCAAAAGAACATTCTTCTATTCGGTTGATAGTTTTCATGGACCATA<br>TAGTCCCTGTGTTCGCGTAGATATAGCTGCTATTAAAAAAGTCTAGTCTAATGAAGAGACATAGAAGGAGTAATGTAAAA<br>TTTTATGTATCTTATGCTATTTCGCATGTTACTTGTGAAATATTCCAAGCCTAAGCTCGCTAGATGTTTTACACCCCTAAGG<br>AGAGAAAGAGACGGCAGTCTGTCCATCTCGTGTCCATCTGTGACATAGAGACGTCCGATAGTATGTTTGTAGTTAAATACT<br>TTATTGGAAATTCACACTAACGTTTTTGGGGCGTTCAGTTTGTTTGGACTTTTAAAATCGGCGGTTGGGACGCGCTAATACG<br>CAATATGGGTAGTTATATGATTTCTATATGTACGTTCTGTGACTTTGTTCAATATTTTACATATCTAAATGATATAAATATA<br>TTACACTATTGTGTTTCTCGATTTAATTGTTAACATATTATTGTATACAATAGGATGTAAAAAAATCCTTTTTTTAAAAAGT<br>CCTAGAGGAAAAGGTATTTGTTTAAGTAGTGATATCTACTTTTTTTGGGGTTTTATAATATATTATTTTTTTAATTTTGTGTT<br>AAACTTGATATTTTATGGATGTGAAATATTCAATGAATCGCAGCGTTTGAAACGCCCAGAAAACGTCCAGTGTGGACACG<br>ACATTACAAGCGTAATATTATTTTTAAAATATGTTTTCGGGTACTAATTAAGGCCTGTCATTAAAAAGACGGGTGAAGAGT<br>TGTGGACAGTCCATTAGTATATAAAAAAAAAAAA
MSTRG.10655	Kelch-like ECH-associated protein 1 isoform X1	AGCTGTTTGCTGACAGATATCGAACGTAATAGAGGCGTGCTGCGCGTTCTTGGAACGCCAACTGGACCCATCCAATGCCAT<br>CGGTATCGCCAACTTTGCGGAACAACACGGCTGCTGCGAGCTCAGACAGAAAGCCAATACATTCATCGAGAGACATTTTA<br>CGCAGGTTTGTCAAGAAGAGGAATTCTTACAACTGACGGCACCCGAGCTTATCTCTCTAATACGCAAGGATGAACTGAAC<br>GTTGGCGAGGAACGGGAAGTTTACAACGCAGTACTAAGTTGGGT
MSTRG.10676	Transcription elongation regulator 1 isoform X1	TGCAGCGCAAGGAAGGCGGCGCGGAGGCCGCGGCGGCGGCGGCGGCGGCGGGCGGCGACCGCAAGCGCCGCGCCG<br>GCAGCGACTCGGACGACTCGGACGCCGCGCCCAGCAAGAAGACCGCCCTCGACGAAGAACGCACGTCGGCGGCCGCGGC<br>GACGGCGGCCAGCAAGAAGACGAGCAACGGCACGAGCGGCGCGGGTGCGGCGGCGCTGGAGGCGGGCCGCGACGCCGC<br>CGTGGAGGCGGAGGTCCGCGCCGCCCGCGAGAGGGCGGTGGTGCCGCACGAGCTGCGCGTGCGCAGCTTCCTGCAGATGC<br>TGCACGAGAAGGACGTGTCCGCCTTCAGCACCTGGGAGAAGGAGCTGCACAAGATCGTCTTCGACGCGCGCTACCTCATG<br>CTCAACTCCAAGGAGAGGCGCCAGATATTTGACCAGTACGTGCGCGAGAGGGCCGAGGAGGAACGCAAAGAGAAGAAGA<br>ACAGGCTGCAACAGAAGAAGGTGGCCTACAGACAGCTCATGGAGGAGGCCAAGGTGCATTCCAA
MSTRG.10678	Hypothetical protein	AAAAGTGCTAATAACCAATAGTTTTCAGTTAAAACCACTTTTTACCGACCTGTTCAGTTAAAAGTACTTTTGACAGATTTTT<br>GCTAGTCAATAGTACTTTTGACTGGTTAAAAGTTTTGACTGCAACATGTATAATTGTTATCAATTATTCTCTGCTGAACATG<br>GTTGTTTAAAACGTCTTCATTCTATTCAGCAGGGCATATATTGAGTATTATCGATGGGTAATAGGTATACACCAATATCTAT<br>GTATTTGTTATTATCTATATATCGTGTTTCAACCGTGAAGCAGTTGTGTTTGCATGGCTGTGTTTCGGTTTGAAGGGTGGGC<br>ATATGGCAGTGAATTTACACGGTACAGAGGAATAACACCCGAGTCCTCAGGAATGGCAACGCATGGGAGGTATCATGGGC

GAAACAGTATACTCTGTTATGTTCATGGTACTGTTAATGTGTATGGTATAGGTGACGGTTACAATTTTCCATCAGGTGGGC
CGTCAGCTTGTTTGCCATTCTAAGTTGTATAAAAAAATTCATCTCTTCACAAGTGTACAATTCTGGATTCAGGCCAATTCAA
TTTTTAGTGAATACATTTATAATAACTTCATGAAGTTACTGTCAGTATAATGGGATTGTTTTTCATTACATTATTGGTATTGT
GAAAACATATTTTGTTTATTGACAGGAGAGAGGTTTTTCGAGCCACATAATGGCTTTAATCTAATCTCACAATCCCATACG
ACACATACGTTCGATATCATAACTGATAGCCATTGGTGAAATGTACACTTTGATGTCACTATATCTATTTTTCTATGGCGGT
AGTGTAGTTTAGAATTGTATAAAGACGTGGTATTTTTTTCTATTCTTACTAATTGTGAAGAAACACTCGAATTATATTATAT
ATTAATGAATCGTCCTAATTTTTTAATCACTTTAGTTGTCAAATTGTTAGTAAACGCTTCGTGTTTAATAATTATTTTTGTAT
TTATTTTTAATGGTAACGCAGCGATGTAAAAAATGAAATTTAACATTGTAACTGCTCGGAGAGGTTAATGGATAAATTAAA
ATTAATTAGACGTCGCTGTCTTCAAAAATCTACTAATGTATAGAACATATGAAAACAATTCCAGTGGATTTTTTTTAATGA
GTATTAATGGTATGGTAACCCCACCCGCGCTATCGGTATACACCGGCGGGGCACCAGTGAGGGATGATAGGTGAGGATGA
AGGCAGGTCAGTTAGCCCATCGTGACGAATTCAACATGAATTGTTCACGGTGGTTTCCAGGGGGAACCACGTGGAGGTCG
ACCTAATAAAGGGCATATTGCTAGCAGTGCGGCTGTGAAACTCCATTGCTAACAATCCCTTTCCCCCCCACAATTCCAGTG
GTGTAAGAATGTACGTAAATAAGGTAATACAGGTGTGCTAATACCATAGTGTGCCTACAGGGACACTGCGTATTGTGGAT
TGGGAACATTGAAATAAACTTTAAGTAACTTAAAAATCTTAAGATTGCTGTGATAAAACGGGAGGTAGGGGATATATATG
CGTACAGATGTTTTACATTGTGTTGATATCTAGACAGGGTAACTTGACGACGTTAAACTCGATGGCTTCAGTCGGTTCCAG
CTAATTGTACTTTTTTTTGTAAGCACGCATTATATTTTAATATAATAAATACCGGTTTAACTTGCGTGTAATTAGAACGAAA
GAGCTCGACTAGTTTCGGAATCGATTCGGGATCCTTAGTCATGAGTGACTGTGACCCGAGCACGAGTCTGATTGCGTGCTT
TAAGCCCACTGATTTCGAGTTGTAGGCACTTGATGAATAATCATTCAGTTACTAATAATTATAAGGTCGTTATTCTCATGAT
TAAGGATCACGAATTGATATCGAAACTAGTAGAGCTGTCTCGATCTAACTACACGCTGGTCAAACCGGTAGTTTATTACAT
TAAAATTGTACTTTTACTGGTACCGTACGAACCGGAGCGGTAACGTCGTCGTACAATTGAAATGTATTTAAAAAATACAAT
CCCGGGATACTGAATGGCAAATGCAACTGGCAATATATGCAGCAGCAGTAATTATGTATGAATTTATTATATAAAAAATG
TTTGAATGACCACAGATGCAAAAAATAAAATTGTAACTGTCTTCGAATGTAATTGTAATGTTTCGACGTCTATATAATGAC
CTGCGGGTCTACCATGAAATGAAATTCCGAAATTTCGGACTCAATTTCGTGTTTTTGTTCGTACCAAAATCGACACTATAA
AAGGAGGTTAAGATTTCATTTGTCATAAATCCTACCATATAAGTCCAAAGGAACGATACTTTAGTATTTTATATATTTTTAA
ACGGTACGTATCATGTAATTTTAACATCAAATTTGTGTTTCGTTCAGACCCGAAATTTCGGAAAACATTTCATGGTAGGCC
CTCTGTTCTTAAATTGTAGCATGCCCGACAGGGTATAACTGTTGATACTGATGTCAAATCCAAAGCAATGGTTACTGCTGT
ATGTACGTACACAGTTAGCAGTAAAATTCTATTCTATTCTATTCTATTCTATCAACCTACAGAAGGTTTTTTTTTAATGGA
TGATAATGGAATAGCAACCCCGCCTGCGCTATCGGTATACATCAGCGGGGCACCATTGAGGGATGATTTGTGAAGGTGAA
AAGGCAGGTCAGTTAGCCCATTGTGACGAATTTAATAATAAATTAGTCACGATGGTTTCCAGGGGGAACCGCATGGAGGT
TGACCTGATAAGGTATGTTGATAGCAAATAACCTCCCCATTACTAACAGTCTTTTTACCCCTCAAAAGATGGTTAGGAAGA
TATATAATATAGAAGTTTTTGGTAGAAGTTTAAATTACAATTGACTAAGGATGACATAATTTTATTTTGTTGCGTCTGTGTT
CATTAAAAAAATATTTTTAATTGTTTGTTTTAATTTATATATATATACTAGCTGCTCGGTAAACGTTGTTTTACCATATACAT
TATTTCTAGAACTATACATGAAAATATATCGTACTACAATTATGAGCGTAAAAGCGTGAAAATTTGAATTTGTATGTATTT
TTCAATGCTAAATTATAATAAAATAAATATAAAAAATTTTGTCAAAAAAAAAAAATTTAGAGGTGGGTCACCCCTATCATTG
AGGGGTATGAAAAATAGATGTTGGCCGATTCTCAGATCTACCTAATATGCACACAAAATTTCATAAAAATCGGTAAAGCC
GTTTCAGAGGAGTTTGGTAACAAACACCGTGACACGAGCATTTTATATATTAGATAGCTGTATATGTGATGTTACATTGCC
TTTGTCATTCGGGATCCCAGGATTGTAGTCTTCAAATGTAACTTATAAAGCATTCGAAACTATTAAATCTGAAGTAACCGC
AGCCATTGTGTAAACGGGCCACCTTCCATGTGTATTTTGTTTTGTGATAACTCGTCGTCTACTGCTGAACTGTGATTATTTTT
AATTAATTGTAC

MSTRG.10690	4-coumarateHypothetical proteinCoA ligase 1-like	AATAAAAGCATGATCATATTTATTTTTTTAATGTTTTGAATTTACAGCCCTCAGCGATATTAACTGGCTCTTCGACTATAAC<br>ATATATTCTGAGGCATGCAAAGAAATGCGATTTAACTTGCTTCGATGTAATTATGCTCACTGGTGGAAAGATTCATAAGGA<br>TCTAATAATGGATTTAAAGTCTCGAATGCGGAAAAATGCACTGTGTGTTGAAGTTTACGGGCAAACGGAAAGTCTAGGGC<br>CTGTCCTCCAGGCGAATATTAACGGACCATTAGGCAGTTGCGGAAAAGCCTCAGCGATCTCTCTAGTTAAG
MSTRG.10701	Hypothetical protein KGM_209264	ATAACAATGACATGTTTTGTTTGATGGATAGGGATGACGACGTACCTAAAACTTAAGTATATAAAAATTTGTTCAGAAAAA<br>AATAAACTGACTTAGTCTTCTATTTGCGTTTTTATTATATTCAAGTTAAAAGTAATGTTTACGGATGTGTTTATCAAATAGT<br>GGATAATATTTTTAAGCTTTACATAGTTTGTTTCTAGACTAAAAAAACCAAATACGCGTTTGAACATTTAAATGTATTTCGA<br>TATGGCCCGTGTTATTTTGTTGTTCTGCTTTATCTTGGTATTTGAGAGGGGCGTATTTGGAGTTCAGTTGTCTATTACTAATG<br>ATGGGCCAGCAGTGCGTGGTTCTTACATAACATTTGTAGCGTCTGTGACTGACTTTGTGACAGGGGAAAGCCTTAAATTTT<br>CCTTTTGGGATGACGCTCAACCACAGCATACTGCACAA
MSTRG.10707	Homeodomain-interacting protein kinase 2 isoform X1	GCAACAGCCCCAACAGCAGCAGCCGAGCGCAGCACCATCTAGTAACAATGCGCAGTCGAGCGGCCGCACGGGTCCGGCC<br>CCGCAGGCGACGCCGCAGAGTCAGAAGAAGCGATTGCTTGCGCTCGCGCAGCAGGAGGCGCAGGCCAAGCGCGAGCCGC<br>AGCCCGAGCCGCAGCTGCACTACGCACCGAGCGGTTCGACAGTACGCCAAACGAACGAGTACCAATGCAATCAATATGTT<br>CCGGCGCAACATAATAAAAGGGATAGTGTAAGTAGACTCTGTGACTCTAAAATATGGAAGATTTCACACTTCGCCAGAAA<br>GAAATATCTTCGATATTGAGAACCTATTATTTAGAACACTGAGAAAACAAAAATAATATTAACATAAGTCCACCCTTGCTC<br>ACAACATTTATTATTATAATTATATTATTTATTACATATAATTTACAAGTGCAATGAAGAATATAGAATACAACTTAATAAT<br>ATTTTTTCATTTTATTAAGTTGTATTATATATTTTGTTAGATTCAAATGAAACAATGGTAACTAAACATTGATGACTGAATC<br>AATAAATATTTTACTTAGATTATATA
MSTRG.10713	Carboxypeptidase	CAGATGTACTATATGTTTGGTTTCCTGTTCCTCGTGTTTGTGATTCTTGTGATCACCTGCTCCGAGACAACGATTCTGCTGTG<br>CTACTTCCACCTGTGCGCCGAGGACTATCACTGGTGGTGGCGGGCATTCCTCAGTTCCGGTTCAACAGCCGGCTACCTTTTC<br>ATCTACTGCTGTCACTACTTCGTCACTAAACTAAACATCGAGGACGCGGCCTCCACTTTCCTCTATTTCGGATACACTTTCA<br>TAATGGTATTCCTATTCTTCTTACTAACCGGCACCATTGGATTCATGGCGTGCTTCTGGTTCGTAAGGAAGATTTACAGCGT<br>TGTGAAAGTCGATTAAATTCAGTGTGGTACTAAATGTAAGATACAAAAGTATTGGTACAGCGGTGATGTGTGGCGGTGAT<br>GAAAGTTTTGCTTCATCATTTATTTTAAATTTATTGCTCACGTTGATCGCATTGATATCATCGTTAGTCATGGTAATGATCA<br>ATCGAAACTTTCATCTATGATGTTAGGTCCATATTTGCGTCAACACCGCATTTCCTAAATAATGGAATGTCATACATTATGT<br>ATTAGACATGTATTTATACCATTTTATACATGTTGAGATACATGTATAAAACTTATTAATGTTTGTGTGGATATCATGACAA<br>TCGTTTCCAAAAAATACATATCGCAAATACGATATGTACGATATTTTGGCGGAATCTGGGAGTCAAGTGATAGTATGTCTA<br>ATGTAATGGTTAATTTAACATGTGTAAATGTATTTGCATTTGGAGTGATAAATTTACCATTACATGTCATATAAACTTCGTG<br>AGTTGGCATTTTACTCTGTTTACCAGATCTTTATGTTTGTGCACACTCTACAAAATTGAAGACGACCATTCATCCGCGTTAA<br>ATAATTTCTTTTGTAATGTCAAGAATATAGTGAATAGTGGGGTCGATTCTCGTAAAAACAAACCACTCATTACGAGTAGCG<br>TAGTCCTATCCAAATTTTATATTTGTATACTTGTCAATCAACAAACAAAATGTTCCTGGAAATGGAATCAGGTAGGTGTCC<br>TTGTGTTGTGTTAAGTTTATAAACTATTAATAAATTATGCAAAATATTGTAAATTAGATATTATACATAATTAAATATTATA<br>AAATATTTAATACGAAAACGATTTGATGACATTACGTTTGAAAGACTTTAGAATCAGCATCATTCAAGAATTAGAATAAG<br>AATGTATAAACGATTGTAACGCTACGATGCCATTGGGGTTTATTACGGGAATTGAGCTCTATATTTAACGTCTAATCATAT<br>TTAATGTCATCAACAATCACATTAGGTGTTAGAAAGGTAACACTTGTATATTATGCGTTAATCAATATCGTAAATATGGCC<br>CGAGCATTATATACAAGTTTGCGATGTACGTTTATATCCGTAAAGGATTCGAATTTATGGTAGCTACAAGGAGCTAGTTGC<br>AATAGGGATGCGAGAGCTATCATAATCACTAGAGACTGCTGTACGCGATGTGATTTTATGTAAAAAAAAAATGAAAGTTT<br>ATATCGAAATTAAATCGATAAGTCACGTAAAATTTTTTAGTTGTCAATTTGTTTTCATATTGTTAGTTGTTAGATGTAAATA<br>AGTTTATGTAAAAGAGAAATCTTAATCGAATTATTTATTGTTTGTCCTCGATTCGTCTGCACAATCGATATCTGTAGTAATG

		ATAAGACGATAGATCCATAGACAGAGTATTAACATCAAAAGGCCGTACTTAAAACGTCAATTCGATAAAAATGAAATTTA<br>AATTACAATAATTTTTTTAATATTATTTCGGAATACATAAGGATCCTAAAGAGACTGGCCACTTTGTAATTGGAATAGTGT<br>AACAAATTTTAGCATAACATAACAAGATGTTTATAAGTTGTTGATGTGTTCTGATATCAGACTCCATTAGTTCATCTTCAGA<br>AGTGAAATATGTAATCAGAATATTTTAATTACAAATTAACAGACGCCATTGATTTAAGAAACTCAATGCCATCATTATATC<br>AGCTATTAACTGTCCACTACTGGACATGGCCTCTCCCTATAGATTGCCATAAAAAACGGTCCCCATCCACCTGCATTCATT<br>GACGGCCTGCAATGTATTTGAGGTCGTCGCTCCATCTAGTAGGGGGTCGTCCTACACTATGTATGCTTGTACGGGATCGCC<br>ACTCAACCTTCCCCATCGGTTGAAGGTTCTTCGAGCTATGTGACTAATTGATTGCCACTTCAGCTTGTTTATCCTTCGGCTT<br>AATGGTAGACAATTATGAGGCATATAGATTATATGCTTGAAGTACAAGCATGTCAAATGTTATTCAATGGTAACAATAAC<br>AACATGTCTCTGACTACCCGATTGGGATTATAGTCCTGAAATTATATTTATATTTAAGTATTCTTCAGACATCACAAGATCT<br>ACCGAGCCATAAAACTTAAGTCTTTAAACTTAACCACACATTGACTGAACTGTAAAATCAATAAATTTATAGAGTACCTAC<br>AACAAAAGTATATATATATATATATATATATATATATATATATATTCAAATTAGCTTTGGGGGTTGCCATGATAAAACTATAA<br>AGTACTTTACTTGCCATTTTTTTTAAATACCATGAATTTTATCCCACTCTTTCGTGAAAAGAACATCTATTATGGTACATTAT<br>TCACTAAGGGAAAGTAAGAGACTATCACTTTAGTTATGAATCGTGCTTTATCCCAAACGATAGTGATTTCATACTACACGT<br>GGAAGGATTTTATAAAAAAAAGTTATTTATATTAAACCATTAATCATATAGAATATTGCAGAACTGTTAATAGTCGTAGGTA<br>GCTAGTTTTTATTTTTTTCATTTTAAATAAATATTATATTGATTTGTCTTTTTTATATCTACTTGTTGCAAAAGATTCCAACTA<br>TTGTCTTTTATCTGAGGATTTAGTCATTGTTTCTAAGATTATAAAGCTCATATCACGTTTTTAAATAGAAAAATCTTGTAGT<br>ATTATCATAATGTAATTATTTCCTTTATATTTATGTCTATGGCAGCCCCTTAATTCTAAACATTTTGTAAGTATCTATATCCC<br>AGTAAAAAAATATTGGCTGTCTATAGGATCCCAATCATATTTTATATCGATAACACGTATTAGGGACTAGGTCTATCCAAT<br>TTATTATTTAAGTATATTAATTTAAGTGTGTGCATATTATGAGATTTAATTTTAAAATAATATTATGTTAAAAATTATGCTT<br>AACTGGAACAGTTGATTGTAAACAATAAATTGACTATTTTATAATAGTATTTTATTTTATTAACAGATTTGCAATAAGTAAT<br>GACACTGTAATAAATTTATAAATAAATAATAATGGCGCATTCA
MSTRG.10717	Pre-mRNA-processing-splicing factor 8 isoform X1	TTATATTAAAATTATTCACAAAAAAAAGTCTATTCTGTGGAAGGGGCTTCTATTTATTTTTAGAGACGCTATGTCATATTTAT<br>TGAGATTAAAGAAATACATCTTTGTTCGTCTTAGAGAAATGGGAATATACATATTGAATTATTGTAAGAAATATTAAATAA<br>ATGATTGAATATGGATAAAAGCCAATCATTTTCATCATCATATCAATCAAACAGTTATCTATGCTACAAATTTCATGCAGT<br>TTACAGTTTCTCGGCACTAGACTTATATTTCTAGAACAAAATGTAATCAAACTATGCGAAAAGGTCTTCTCTGTCTGCGGC<br>GAGTGTGGGTGCCGTGGCGTCCTCCATGGCCGCGAAGTTCATAAAGTGGGCAGGTCGGTGCACTTCGTGGTAAAATTCGC<br>GAGGGTTCCCCAACTGAACGCCATACTTCATGTTCGGATCGTGACGAACGCCTGTTAAATTACAACATTATTAGAAACAGT<br>AATAGATTTAATTCATCTTCAATTTAATGAAATTATTTCTGTACATTTATTTAATTAGTGAATTTGAAAAAGATTAATTTAC<br>AAAACTGTAGATAAACTTAGAAGAACAATATTATAGATGAGTTTGCAATAAATGTTATCTAAAAGCAACTATTCTCATAAT<br>ACATTTTAAAAAATTATTTAATAGAACTTTCATTGGACATATTGGATAAACTTGGAATATAGATGGTTAAAAATATGTGCC<br>AAAAAATAAAAAAAAAATATAAAATTAAATACATTTATAATATTTTAAGAATATTTTTACTCACCCATGAAATTGTAATTCC<br>AACTGCCTTGCGACGGAACCATGAAGTATCCAAGGAATCTATCTGACAACAGCATTTGCACCCTTTCATAATGACTTGGTA<br>GGTATCCTTTGGGATTGTTACCCCTATCTGTGTTCCGGGCACCCCATTCATACCCACTGGGAGTGAGTTTGTATGCAGTGAG<br>CGAACACGAGCCAGGGGTGAAG
MSTRG.10719	Uncharacterized protein LOC106139651	GGAGGATCATGAGGAGGTCGATTTGATCCCATTGCTAGCAATATGGGGTATATTCGTGTTTCAACCGTGAAGCAGTTGTGT<br>TTGCATGGCTGTGTTTCGGTTTGAAGGGTGGGATGTGGCGTGAATTTACAGGGTACAGAGGAATAACAACTGAGTCCTCTG<br>GAATGGCAACGCATAGGGGGTATCATGGGTGAAACAGTATACTCTGTTATGTCCACGGTACTGCTCATGTCTATAGGCGAC<br>GGTTACCACTTTCCATCAGGTGGGCCGTCAGCTTGTTTGCCATTCTAAGTTGTATAATAGGGTTGTTAGTCTTCATAGCTAA<br>TAAAACTTATTTCCCTACCAAGTGCAGTTTGTCACAATTAAGGATATAGGGACAATCTAAATTACTCCCAGAATATTATCG

		CCCCACATTCACATATCTCCTTATGGCTGTTGCTGTATGTGCGCAGGTCGGGCACCAGCACGCTGCAGCCAGCGCCGGCCC<br>CGGCCCCGGGCGGCTGCCAGTACTGCACGCACTGCAACTCGCACATACGGGAGTACAAGCACGTCGCCACCACCGATGAG<br>CATACGAACGAGCGCGTGCTGTACCCGACGCCCGCCAACGAGACCAAACTGCTAACGCCGCACGAGCCGCACCCTGGACA<br>CGCACCGCATG
MSTRG.10724	SET and MYND domain-containing protein 4-like	AGGTGGCGCACGAGTTGAGGAAACTGTCAGAAGTGATGCTGGAAAGGATCGTGCGATCGCCACAGAATCCAGACTACAG<br>AGACTGGTGTTTAGAAGCCCACAAAATAATGAAGAAGGCCGTACAACTAATGGAGCTGAACTACGGCTCCTGGGAGCCCC<br>TGGTGAAGAGACTGAAGGAACAAGAGAGCCTGACGGCCGCCCTGTTGGCTGACATCAGAACGCCAGACGCTGCACAGGG<br>AGTACACCACGACCTGCATTATAATCTCAAAATATAGTTGTACAATAAACAATCACTTGTATGAAACTCTTCTGGTAAAAA<br>AAAATACTGGCATATAAACTCAGATAAAGACACTCTATCTATGTACATTTATATTGCAGAGCCGCGTCTGAAATTTGAATA<br>TGGAATTAAAGATTCGCGCCAATACATTCACAATTAAAAAGTGAGACGGTGTGTTCCATTTTCGAATAAAATATTTGGAAC<br>ACACCACCTTATGAAGTCACTGCCAATTTTTACTCCATGCATTATAATAATTATATTAATCAATTCAATAATTCAATTAAAT<br>GTATAAAATACAAAACATATTGCAATAATGTTCGTAATCGAGAAATCGGCCATCTTTATGTTTGATATTTGTCAAACGTAT<br>ACGTGAAATAATGATATAAACACTTTTCTAAATTGTCACTGTAAATATTAAGGGAACGCCTTTGGGATAGTTAAGAAAGCC<br>GTCTAGGCACACGTAGGACTGTCGTCAAAGCAATAAGTAGGTAATGAAGTTTCATTCTATTATTTCACGTGAACGCGCATC<br>GCTACGAGTATTTCTTAATAAAGTGATCACACCACGGTCAATTTTGATATTATTACATACTTGCAAAATATTTTTTATATCA<br>GCTTGTATAGAATATGCTTCGTATGCTAATGAAATGCAACGTCTGTGACGTCACATCGTGCATTGCGTTGTGCAAGCTACT<br>TGCATTAAGAGGGCTTATCACCATAAGACTGGCAAATTCTTTATTTATTTGTCATTATTTGCACAATTTTCATATATATATA<br>TATATATATATATATATATATTTTTTTTTTTTTTAATATAAACTATTACAATATTTATTCCAGACACGAATGTGAACGATTCC<br>AATGTGTGCTTTTACACCGCGACACTGTATCGTATGATTATATCGTCACGATATTATAGAACAATAAAAAAAATTACATTG<br>TTGTAGTACAATTCAATCGTTCATAAACTGCAATAAATAAAAATATATGAATACTCATTCAAATTTCCATGCGAAATTCTC<br>GCGGAAAATTAATAGTGGCTCCATACAGTATACAATGTCCTCCCTATTGTATCGTGACGATAATATCGTATAAAATTGTAC<br>CGCGGTACAATACTGTACGATAAATTATAAAAGTGTGTCGTGACATCAATAATAATCATTAATAAAATATACAATGAAAC<br>TATTATTCAGCATTTTGTGTATTTGACATTTGTGTTTTTTTTTATTTATTTATTTATTTATTTATTTATATAATGATTAAACTG<br>GT
MSTRG.10737	Ornithine decarboxylase antizyme	AGCGGCGATGGTGCGTCGCTGAGCGCGGCGGGCAGCAAACGGTCCGCCTTGTCGGCTTCCGACGCCGAGTGCTTCTCGCT<br>GTGCCTGGGCGCCGGGCCTCTGTGGTGGTCCTGATGTCCCTGCTCATGGCTCAGCACCGCCCGGAGGGGTGAATAGCGGG<br>GCTTCGAGCCCCGCAACACCATCCACACCCACCCACGACGACAACAACCGTGAGTAACATCTACATTCTCATAACAGAAA<br>TCCTTGTTCTAGATTTATAC
MSTRG.10743	P daughterless isoform X2	GTTGTTGTCGCAGAACGCAACCTGAACCCAAAGGCGGCATGCCTGAAACGACGCGAGGAGGAGAAGGCGGAGGAGGCGC<br>CCAAGCTCCTCGCCGCGCCCCTGCAGCACTACCAGCCTATGCCGGGCATGGGCCAGCTGGGCGGGCCGGGCGCGGGTGCG<br>GCGCCGCAATAGCGACGCCCCGCCCGCCCGCACGCGCCGCCCTAGCGCCGCCCGCCGCCCGCCGCCCGCCGCCCGCCGCC<br>CGCCGCCCTGCTCACTAACTAGCTGTGTGTGTATTACTCTTCACTATTGTAAACTACGCCAATGTTTCACCTTTCAAATGCG<br>TGCTATCTCTTTTTGCTCTAGCAGTTAAAAAAGGATGGCATGAGTTTTGATTCTCTCGTCTGTGAAGGTTGTTGAATATGTC<br>ATACGTTAGAGTATAGTAATTGAAATAAATAGAGAAATCATTTGTTTAAATGAATCTGAGTAAATTTTTGTCGTCGAATCA<br>TTTGTGAAGTATTTAATAGTTTTAATCTTGAATTTTGTAATTCAAAATTGAAAACTTTATAAATTTTTGTTACGCATAAACT<br>CTTAGTATGTTATTGTGAATTTCGATGCACTACTAGTACTGACGCGTTAACCTTTTTACCAATTATTGATTCATAGTCACAC<br>ACCTGGTCCAAACCAGTTTAGTAGTGCACGATGTCACATAAACTATCGAATTATAATTTATGTATTATTTTATTGTACACAA<br>AATTTCATTTGTAACATTTAATATAAATAATTTTAAAACTTAAAAGAAAAAAGGAAAACAAATAACATATAAAGTATTTA<br>ATATACCATTTGGTAACATTGATTTCATACGTACACCTCGCAGAGCGTCATACACACATGGCTAGTTAAACACGATTTGTA

ACTTAAAAGGCATTGATTACTTAATTTGTAACGTACTAGTCTGTGACATGTTTTGACATATTTGTCATTTTATATAAATCGG
CGACTGAATAAGGACGAATGCAGAATAAAACAAATTAACGACTATGACAAAGTCATAAAATATTACAGAATGTAATATG
ACATTCTAGTAACAAGTTATTGTTACTACGACAAACTCATAAAATTATAGAATGTAATATGACATTAAAGTAACAAGTTGT
TTTTAATAATGAAATTTATGTATTTTGTATAAGTTTTAGAATTTAAATTGGTATATTATATTATTATAATTATGTAGTTTTTT
TTTTTTTTGCTAATTGCAACGAGTCGCGTGTCAACGCGTGTCACGGACGGTACAGTACAGTACAGTAGATTTGGTCGATAC
GACGCGAATGTCCCAAAGAGCAAGCAGTGCGTAGTGCCACGTTCGGACTATATAGTACGTAAGTGTGTATATTATTAGCG
CTAGACATAGTTTTCTATACGCCACGAACATCGCGTGCAACATGAGATTCGAACAGCGTTGTCTGCCCAAAGACAAAGAC
GGAGAGCCAGATTTTGTAAAATATTAGACTTACATCTATGTATAGGTATATTATAGTTTAATCTATCGAAGAACATATTTTT
AATTACGATTTTAACTGTTCTTTTTGCTGCCATAAGCGTAGTTAAGGTCGGTCCGGGCTCCGTACTCGCATAACAATGTTCG
TATTCACCGTCGCCTCTTTGCCATTCTATCTCTGTTTTTGTCGGGCGAAAACAGATGATTTTCTACATCATCCCCAGGGATC
GTTAAGCGCTTGACAATAACGCGTCGGGTACGGAGTCCGTGATCGGAGAATTTATGATTATGCATATTATGTGATTTCTTT
TGAATGTGGACATGGAAGCGGTGCGAATTATGGTTTGATATTTATTTGGAAATGTGTGGGGTTTGGATGTAACGTAGAGTT
CTATTGCCAGGCGATGGTGGGAACTCCACTATGAAACTTGAAACGTCATATTCTCAATTGTGAATAAGACGTTATGGAATT
ATACTTTTTGTTTTACGTTTAAATTATAAAATTTGTTTAATACAATTAATTATGTTCAACGTAATATAGTTAAGCGGAAAGT
AACATTTGGTGTAACCGGTGGCGGTGAACGAGTGGTATTAAACAGTCCCAGTTCGAGTATCAGTGAATACTCCAGTATTAT
TTATTTATTTATTTACATTATAATGATTCTTCTATTGAGAATTATATATGGTGCAGTATAAAAATCAAATTTCCACACATTTT
CAAACGATACACGAATTTTATTATCTGTTTTAATTAAAAATAAAATGGCTGCTAACTTAAGGATGTACTTAAGGGATATTA
TTATGTTTTGTCTCTCTTTTTTGTACCAAAACTCCAGTGGATATAGTTGATATATTGCGGCTTATCTCTCATCGCGTAGACAC
GTTTTACTGCGGGTCCGGGCGACTTGCCTTAGTATAATTTTTTATATACTTACGTTTTAGATTTATTTGATAGCTGTTTGACA
TGAAATCATGAGAAATATGACTTTAGAATGGTGTACTTATAATGTGAGACAATGACTTGATATCAGAAACGTATTACATTT
AAATGACGCAATACTTCTATACATTATAAGTGGAATGCGGCCTTCTTTATAGGCTTTTATTTATTATTTCTATTTATGACAC
GATACGAGATTACATTAAGTAGTCTGTAACCATATATAATTATTTGTAATGAATTACCCGTGCATGTGTGTGTCTAGCTTGT
GCACCTCTATATATACATATAAATCGGTTGCTGATTCTAAATCATTGTCTGACATTTAATTTAGATGTCAAACTGTAAACAA
TAATGATTTATTGATTCATAAAGTCTTAAATAATTTTCACGAGCCATACGTTTAAGTATTGTACGAATGTAACTAGTATTCG
ATTATGTAATGTAACAATACGATTATGTAATGTCAATTTCAAATATATATTATAAAGTTTGATTATGTGAAGTAAATAAAC
AAATCTTAATGACTACGTATGAAGTTATATTATTAAAATTTTATTATGAATTGTGATTTATTATTATTATATATTTTGAACTT
TGATTATGTAATATGACATTTGATTATGACATTTAATAATCAGCTATTTTAAATGAAACTAATATTACATTATGGAATGAA
GAGTTTGATTATGGATTATGATTTTTGAATGTAATTATAGTATTGTGGTGTTTAATTTTTTTTTTAAATTTGATTATGTAATG
GTAAAACTCTGTCGATAGTGATGTGACCCGTTATTGTTTACAGTGTGTCGTCCCAACACTCGATTGCATAATCTTAATTTTG
TATATAAGATACGATAAAGCTCAATGTAATTGGTTATGTTTATGACAAAAACTTGACAAATTCGTTTAATAAATTATTATA
AACAATACATTTGTGTGGTAATCAAAACGAATGTGCTTCGTGTTGACTGTATATTGTATTTGTTGTTGCTACAGCTGTGTTC
ACGCTACTTGCTATCGCCCCCGTACCAAGAGGTTCCAAAAAGTAACAGGACAATAAACTAACAAGCAAAGTGACAATTCG
TTTGTTATAATATTTTTACATAATGTAATACATTGAAATTACTGGGTACAGAGGTATAATGTTTTTGCATGAACGTCACACT
GTTAGAAAGAAATAAGAAAAATAAGCTCTTTTGTAATAATAGCGTTTTAAAATTAGTACATATACATACAAACATACTTTG
ACTTGTCCTTCTCTAAACCCTGTTTCTAAAAATTACAAAATTTAGAGATAGGTTTAGAAAATGACTAAAGTGTCGAGCCGA
GTAACCTGAACACAACTATAGGTGAACAGCAAAATACTAAAAAGAAAAAACAAAACAAAACTATGACAATATTTTCGTTT
CATGACATTAACTAATACGGAACCATTTCTCCCAATTGTAACAATTACTTTGTGTACTGGATCAGTAATCCAAGATTATGA
GTAAAGTACATAAGCGCTAGTTGTTTCACACCATTTAGTAATTAATTTTCACTATAAATAGTTTATTGCACTCTCGTATATG
TAGGATCAACACTGGATCAACATTGGATCAACTCTTAAAGCTGTTGACACACTCGGAATATGAATGGAAAAATTTATAACT

		CTTTGTACAGTGTTACCAACTCCCTTTCCACTATTCGTGGCTCTCATACACATATGGTAATACATGTACGATGGTGTAATAA<br>TCCCCTAAAATGTATTATATAATTGTAAGGTTGATTTTTCAGTTGTACTAGACATGATATTTGTCGCTCGATAGATAACAAA<br>GTTGATTATAGTGAAGTTGATTGTGGATGCCGTATCACAATTGTTGGATCAACTTATAATCGACGGTATTTGAAAGGATTT<br>ATTTATCAAGCGCGTCACAGTAGTCGGGAAGGGTATACCGAACCGAACGGCCGTGATGTTATTCGTGAAACTGAACGACT<br>TTCAATGAGTGGAAATTGTTTTTGAATTTTCCATGCAAATTGCACGGTGACCCAATTGGTTTTGTATGAAAGGTTCAAAGA<br>TCTTTCAATTTGGACCATTTCGCTTGGGGAAAGTCGTGCAGTTTTCTGAATACACTACGTTTTGGCTTGACATACTCTTTTTA<br>ACTCTTTTGTATTTAGTGTAAAAATCTTATAAAAAAAAAAAAACCTAATTCCCTAGACGTAGTATTACTTATTTAAAACAG<br>TTGAACATTTAAAAAATGTACAAAAAAAAAATTGTAAATAATACGCAATATAATATTATATAATACATTAAATTGTAAGTTG<br>ATCCAAGGTGGCTGCGGTACAGATGTCGCCTGTGTCGGGCGCAGGCGCAGGCGCAATACAAACCCGAACACCACACACGA<br>AGTTCTTATTTATAGAGTTCAAATATTTTTTTAGTAATCTTAGATGTAAGCACTTTGGAACCCTTTAGTTAGATTAGTTGAC<br>TTCTTCCAATCGATTAAATTTAAGTTTTTTTTTTTTATTATTATTATTATATAATTTTGTTGTACGTACGCGCGGTACCGAGG<br>TATCCATATTCGATATTCAGTAATTTATTGCAAAACGCCTATGAATGGTCGATATAGCAACTACCTTGTTTGTAATCCTGGA<br>GATTATGTACAAAATGAGGCTTTTTTGTTATTTTTTTTTTTCCATTTTTTAATGGTGGCAACACTGACGTGTAACATAGAGC<br>ATTTGATATTTTCTTATCAGTTCAAATGCATAGATTAAAATCATATAATTACAAGAAAGGTTTAATTATAAAAATGAATAA<br>TATTAATTAAATGTGCCATTCATTTTGCTAA
MSTRG.10745	Ubiquitin-like-conjugating enzyme ATG3	ATTCAGGGTTTTATTTATTTATTGTTTTATTTAATTATTTTATTTCTAGGAATCAAAGTTCCATGAAACAGGAGTGCTGACTC<br>CTGAAGAATTTGTGGCAGCCGGAGATCACTTGGTGCACCATTGCCCTACATGGCAGTGGGCCAAGGGCGAGGAAGCGAAG<br>ATCCGGCCCTATCTTCCAAATGATAAACAGTTTTTGATCACCAGAAATGTGCCATGTTACCGCCGGTGTAAACAGATTGAA<br>TATTGTGAAGACAATGAGAAAGTAATAGAGTCAGAACATGATGAGGATGGTGGCTGGGTGGACACACACCACTACGACA<br>CAGCTGGGTCGCCTGCATTAGAAGAGAAGGCTTGTATTTTTTTGTTTCTTTATAGTAATACAATTGCTCCCATATTCATATA<br>AAAGGCC
MSTRG.10746	Uncharacterized protein LOC106125039	CACTAGATTTCCGCAAAAAGTGGCCGAACGATACTTCGTACTTCGCATGTTTTCAATGGCACATGGATTGAAAAAAAACAT<br>CCGTAATTGATTAACTACATTTATGGTATTTGTTATACATTATTGTTATATATTTAAATTGCCTTTATTACTATAATTAAACA<br>TTTAGATTTAATTTAATTTAATTAATTATCCATATTTATTATACCGCTAATTCTGATCTTGTCTTAGTCTATGGTAGTTTCAT<br>GTTTTTGTTTGATAATCTGTGGCGTATCGCCGTGCCGCCATTTTGTGTCACATTATAAATAGTGGAAATAGTGGACATAGTG<br>GACGGAAGAGTGGTCGGAGATCGATGGCCAATATGGAAGGTTGTGGTACTTCTGGCAGTAACAGTGGTGGTCAGTCTTCG<br>AGTGTTGGCAGTCGCACACATACTAATGGTGGCGGAATTAAACGAAACGATTAGAATCAAATAACCAGTGGTATAGACTT<br>CGCCAAGACAACACCAAGTTCAGTAGAAAATTTATTACTTGTTAACGCTTCTTAATAATTTCTTCCACGCACAACCACTCTT<br>TGGAATCAACTTCCAGCAGCAGTATTTCCGAACCGATACGACAACATAACCTTCAAGAAAAGAGCATATTCCTTTTTAAAA<br>GGCCGGCAGCACACCTGCAATGCCGTAGGTGTTGTGGGTGACCATGGGCGGCGGTAGTCACTTACCATCAGGTGAGCCGC<br>ATGCTCGTTTGCCCCCTGTGTTGTAAAATA
MSTRG.10757	Uncharacterized protein LOC106125039	ATTGGATCGGTATCCATTTTACCCATGCCTACATCAAAGGATGTTTGTTGACGGTTCCAGTACCGCCGGCATAGAAATAAA<br>TATATATATTTTATAAATATATGACATTAATAATTGAATATTGTAAATAAAAATGTGTATATAAATTGTAAATATATTTTTT<br>ATTAGTATATAATTCAAAAATGTTTCCTTAATTAACGAAATGGTATATTTTGTTGTAACAATGTAATGGATGGGTGTAACC<br>GCTTGACTTCATCATTAAATGTTCCATAAAATGCACACCACTTGTTCACAAAACCGTCAAATGTAATATAATAAAATTGTA<br>GTCACACGATGTTTTCACATGGGAGATGTTGTAAGGAGAATTGATATGAGAGGTCTTAATAAGGCCAGTATATGTCGAAA<br>CTAGAGCTGCCATCACCTGAATTGCCGGCACCGAAAAATCCCGGACATTCGGGCGAAATATGGGCCTTTCCCCGGACACTT<br>CGGAGAACAATTTTTTTTGTAACAATAATAAAAAAAAAAGTTATATAAAAACCAAGCTGAACTGTGGATGACTAACGTAAC<br>TAAGATTTATTTCAATTGATCGCCTTCCTATAAAAAAGTGGACAGGCCCCAGAACGCCTTTCAAAATCCGGGAAATTCCGG

		ACGGACGGCAGCCCTAGTTAAAACAATATTATTTTTGACATTTTGCCTGTCTTTATTTTCGTTCCGGTATATACACAGAAAG<br>TTATATTCCAAATTGGACGTCGTTATCGCAATTTTATTTGATGCCGGAACTATAATAGACAAAGCAAAGTTATGAAAAATA<br>TTAATATTAATACAGGGTGTAAAGGACAGATCAGCTGTGTGAGGTCAAAGCCACAAGCGTACGAAAACTACCTACGAAGA<br>AGCGCCACTGACCGCGCGCTGTAGGTATCACGCGTCGTGTCGTGTGGAGCGTAACATTTCAAATACATTGCGCGGCGTCGA<br>CACTAAGAAGCGTTAACTATACGGCTGCGGTGCATTACCGGCGGGAGAACAATCTGCCATCTGATTCAGTACATTCATCTG<br>AATCAGCTGTTTTCGTTCTGGCCAACCTGTTCCTTACACCCTGTATATTTAGAATCTAATATGATTTAGTTCTCTTAGTTAAA<br>GAAATAAAGTTTAAAATTACTTGGAAGGACTCTAAGGTGAATGAGCTAATCTTAACAGAGTTATATTAGCTTAGCTTAGCT<br>CACTATGTGGTTTACACTCGACATGTCAAATTGTTTGCTAATTATGGCAAGTGTAAAAAAAGGTTTCCACTTTAGGTTTTTT<br>GGTGGAACTGCCAATTAGGAATTTATCACGGGGAGTGCTCTGAGGAATTGTTCGGATTGATACCTGCTGCTGATTTTCACC<br>ACCGTACAACCCGCCATAAACTAAAATTTCATCCCAACCATCTGGACGAGTGACGGTCCTCCACCGTGCGTTTTTCAAGGC<br>ACTTTCTTCCACGCACAACCACTCTTTGGAATCAACTTCCAGCAGCGGTATTTCCGAACCGATACGATATCGTGACCTTCA<br>AGAAAAGAGCATATTCCCTTATGAAAGGCCGGCAGCGCACCTGCAATGCCGCTGGTGTTGCGGGTGATCATGGGCGGCGG<br>TAGTCACTTACCGTCAGGTGAGCCGCATGCTCGTTTGCCCCGTGTTATAAAAAAAAGGTATTTTTCTTTTTTTATATAAATA<br>CAGGGTGTATACAGTGAAGTTGTAAATTGTATGTCGATACGAAATGTGATGTTTGCTAATTTGTACTTACACCAGATGTCG<br>CTACGTGTTCGTGCGTAGGTGGCGCCCTCTGGAATCATGTGTGTGTAAGGTCGCCACTGATGCGTTGTGACGTTAACTCCT<br>AGTAGTTGATCGTTTGTTGTTGACAATATTGAAAGGTTTGTCTAAAGGGATACGTTACACTATTAAATATATAGGGTCATG<br>TATTCATGAGAGTCATACTTTATTTCAGAAAACTTACCCCCTTATTCATAAAAACTGAAACCGTTTACAAATCTATTAACCT<br>TTAAATGCCTGAAGTTTCAAATTTGATACCGCGGTTTGCATTGTTTCATTGTTAAATAAATTTAAACATTTTGATCACCGTA<br>ATGGCTAAAGTATCAAAAATGATACCAAATGTTTTTTTTTCCAAAATAAATATATATATAAAAATATCGTTTTGATTTTTTT<br>TACTACTTTTTTTTATACAACTTAGAATGGCAAACAAGCTGACGGCCCACCTGATGGAAAGTGGTAACCGTCGCCTATAGA<br>CATGAGCAGTACCGTGGACATAACAGAGTGTACTGTTTCGCCCATGTTACCCCCCATGCGTTGCCATTCCTAAGGACTCAG<br>ATGTTATTCCTCTGTACCGTGTAAATTCACGTCAGATCCCACCCTTCAAACTGACACGCAGCCATGCAAACAACTGTTTCA<br>CAGTAGAAACACGAATGTGACAGAGGTGGCCAAGCAATCGACTTTTGTACAAAGTCTCCCTCTGGTGACTACTACTTTTCC<br>CCATTAGTGAACACAGTAAAGGGTACACTTATACATATTTTTAATTCGAATTAAAAAATC
MSTRG.10777	Uncharacterized protein LOC106101235 isoform X1	GTCGCATTCTATTGTAGTGTTGATTCACGCAGAGTGAAGCTATATAGATATTGGTTAGGAAGCTACATAGCGGCCTTCACA<br>TCGTTATTTCTCCTGTCTCTTTTCCTCCTACTAAACTTGCTCTGCTTCAAGTGTTGCTATTTCGCTCGTTCGCCGTTAATTGTA<br>ACGCGACAAATAAAACGTAGCCATCTTGTTAGTTATAAACGCCGCGTGATAGCCAACTCGTTTGTGTTATAGAAAACTGTA<br>TATTTAACTTGTTTTTGGCGGTGTTTTTGATTATCATGTCTGAGATACCGAAAATTTGGGACTACTTCGAGAGAAGTACAGA<br>TAAAAATAAAAAGAGAGCGTTGTGTAAAATTTGTGGAAACTATTATTCATATTCATCAACGACGGGTAACCTAAGAGTTC<br>ATTTAGCGAGTAAACACAGCACCGACATTAAGGTACCGCCGAAGAAGGAGCGTCCGGCGCATTATGCCCCATACGGGAAA<br>TACTCCAAAATCATATGGGGTTACTTCTCGCAAGTCGAAGGGGAAGATAAAGAAGCCGCCTGTAACTTCTGTGATAAACA<br>TGTCTCGTACCGTACAACCACGGCCAATTTGAAAACGCATTTACAAAAGAAACATCCAGATGAGTACACCAGGTTCCTGG<br>AACTGCAGCCGCCGGATTTAGACGCTGTCAGCAAAGAAGTGTACTTGACATTGAGCGATGAAGATCTGGTAACACAGTAA<br>TCTGGGGCTACTTTGTAAGGAATAATGAAAACAAACTAGGGATGTGCAAATTTTGTAAAAAATGGCTATCGTACAAGACT<br>ACAACGACTAACTTGAAACAACATTTGAAATTAAAACACACAGATGTGTTCTATGAGATGGATAGAAAATTACGCGCGTA<br>CCGTGATATGGGCAAGGAAGAGGAATCCCACGAGGAAG
MSTRG.10785	Poly(U)-binding-splicing factor half pint	CCGCCGCCGCCGCCGCCGCTGTCGTCCGCTGAGCTCGCACTGACGGCCCGCTGTGTGCTCGCTGTTGCAGACGCCGCCGCC<br>GCGCCGCCGCCGCCGAGCCGCAGCCCGCGCCGCGACTGCGCCTCCTAGACTATACTAGACTAGACTCGATCGCTCGTAGCT<br>CGTTCTCGAAACCATCCTCAGTGATCAGTGTTTCTGCTTTCTGTTTCAGGCTAGATTTGTAAGTTAGGTTATTGTGCTATAC

		GCTTAGGCGTGGTGCCGAGTCGACTCCGGGTCCCGGGCAGCCTCCTCGCCGCCGGTCCGGTCATAATGTGGCTTCATCTCG<br>GCGCCCATCTACGACTTGCAGCAGATTGGAGATGTGTTCACAGGCCCGGGCGCCAAGTGCTCGACGCTGCCGGCCATCCT<br>GGGCGGCTCGCTGCCGCGCCTGGCCGGCGACCAGGCCGACGCCGTCGCACGCGCCAAGAAGTACGCCATGGAGCAGAGC<br>ATCAAGATGGTG
MSTRG.10795	Protein lingerer-like	AGGTGTTGGAACTGATGGAGATGACATGTCGCACAGAGGAGGAGGTGTGCTCGGCGCTACATGACTCTGACAACGACATG<br>GTAGCCGCGTGTAATCTACTCCTCGAGGACACACAGCGATTGCAGGGAGAGTGGCAGACTAATGAGAAAAAAAAGAAAA<br>AGCCAGCGCCCGCGGCCGGCAACGGCGAGCTCGAGCCTCGCGAGCCGCGCAGTCGCTCCGGCCCGAGGCCGCGTATGTAC<br>CATATTTC
MSTRG.108	ADP-ribosylation factor GTPase-activating protein 1	AGGTGAACAACCGCAGTGGCTGGAGCTCGCTCGGCGGCACCCCGGATGTGCGTCGCGCGAGCAATTCCGGCCACTATCAG<br>TCGCAGAGCTATGGTGCTATGAAGAGTTCCACTTCCGAACCTTACTCGCAATGGAATGAACAGACCCAGCCGTGGAAGGA<br>GTCCGTGACGACCCGCAACAACTTGGACGCGCGCGACCTCTCCCAGGTGGGCGTGTCCAGCCCACCGCCGGACATCAAGA<br>GCATCACCATTAAGAAGAAGAGCGACGACGACTCGTGGGACTGGCTCAACAATTAAATATCCCCTCGTTGCAGTTTGGAG<br>ACACACAGAGACATCCTTTCATGCTACAGTTCAATTCTATAAGAGAAGCCAAGTTGTGGACATACAACCTTTCGTCCCTCC<br>AAATTGTACATTACGTGAGTTTATACGAGATTTATTCTTATTGTACAGTACAGCGAACCCCACACTGACGTGTACAACGTA<br>CAGATACTATCGCACGTATATAAATCTGATTTCATTATTATATCTTAAGTTTCACGTATATTTTTTAGTTATGGCAATTATTT<br>TTTTTTTTTATTTACGTATAGTGATATTGTACTATTAAATGTGAGTTCAAATTTCAATTGAACAACTTTCAATATAATTAAAT<br>TGTCTTCTTGTACAATTTAGTAGTATTAAGTGAGTGTGGCGCCCTCTGCTATTGAAGGATTTAAGACAACGCATGGGGGGT<br>ATCATGGACGAAACAGTATGTGTGTGTACTGTTAGCTCCATGGTACTGCTCATATCTATAAGCGAAGGTTTCCACTTTCCAT<br>CAGGTGGGCCGTCAGCTTGTTTGCCATTCTAGGTCGTATAAAAAAAAGCCAGCCGGTATGAAGATCGGGCTGATTAGTGT<br>ATATAGCTAGCAATGTGACTACTAGACCCAATTTCTAACAATCCCATTGCCCCCCACCAACATCAAATCATTACCCCGCCT<br>GGTTACTAGTGAAGAATGATAGATGAAGATGAAGCATCTCCGTTAGCCCATCGTGATGAATTCCACAATAATTAACCACG<br>ATAGTTTCCAGGGGGTACCACATGGCGGTCGATGATGAACTAATTTCATCATCTGTTATTGTTCACTAGTGCTAGGCAGTA<br>TTAAGCAAAAGCCCAAGCAGGGTAACTACTTCATTTTCACCCATTAAAAATAAGACCTGTTAGGATATATTAGACTGTGAG<br>CCTAATGATCATACTTTACGCAGTGTCAATAACCTCCGCCTCAAATTTCCCGCTCATTCTTCGTACTTTTATTCTAAATCCTT<br>TACTGTTCAGGCAGTTCGCCTATGGAACTCTCTGCCTACCTCTATTTGGTGCGCTTAAACTCTTACTAGCTTCAAAACATCT<br>ATTAAATTCGGCATATTAAATATGTATGGATGTGTGTGTGTGTGTTTGTATTTGTATGATAGATAGTGCGCCGTCTATCCTT<br>CGTCTTTTTCTTAATGTCCTCTACCCAAAGGTTGTCTGGAAGAGATTGCTTAGCGATAATGCCGTGTTTTGTATCACTTCTC<br>ATTAATATTGACTGTATTTTTTATTTATTTTACTATGTGCAATTAAGTGGAAAATAAATACAATATATTGCTAGCAATGTGG<br>CTGTGAGACCTTATCACTGCCATGTATGTACAACAGCTTTATTTACCCTGCTCAAACTCAGCCAGTGACATTTTTATAGCAT<br>CGAAACGTGGACCTAAAAAATTCAAAGGTTCTGTACTGGAAATGGGGTCCTCGGGCCGTATCCACGTATTATAAAACATC<br>GCTATCACCTCCCATGTAAAGCGCCACACTTTCTTAATTTATCATCATCTGTGACTATAGTACATATAATCCTATATGTTTA<br>ATAGTGCAATATACTCCTTAAACTGAGGGTACAATGTCGTTCCTAGAACGCGTTATTATAGCAAACATTTAATTATTAGTTT<br>TAATCCACTGTAAGTTTATTAAATATATTTTCGTATGCATAAATGTTAATTATAAATGTAGGTGTAAACCTTTCAACATCGC<br>ACTCATATATACTTATGTACGTCACTATTAATATAATATAGCTGTGTGAAATATTTCCCAATTATGTCCAATCAGATGACAG<br>TGTTATAAAAAATTCTACTTTGAGCACCATTCACACAACTCCGAGTCTTTCCAAGTTCCTGCTCGTTCGAGCTATGTATCCC<br>ATCAAATATGTGCCATCGATTACTCATGTCACTAAAATGCTTATGGTCCTAAAGAAACAATACTTCATCATTTATACATTG<br>ATATGTAAATCTGTCGTTATTATTCTCTTTTAGTATGAGTTTGGGTAATATAATTATAGCAATATTTATATCCCTTTCAGACC<br>TGAACGATACGGGCGTGAGTTGTAACTTTAACAAATTCTAAAACCGCGCGCAATTC
MSTRG.1	Translin	AACACTTGTTATACTATGGTTATTATTGATGGACAGGTTATTAAGTTTGAAGAACGATCACCTTCGCAAACGCTTCGACGC

0810		GCTCAAATACGATGTGAAGAAGATCGAGGAGGTGGTATACGACCTCAGCATCCGAGGGTTGCTGCCCAAGGCCGAGCCGG<br>GCACCTCGTAGTCTGGATCTCATCACCACATCACCGGTTCCTGCATTGAGACTTGAAAATTGCGTACAGAGTGTGTCGTGT<br>CAACAGATTTTGATTTTGTAGAAATACCGACTTCAAAAAGTAGGAGGTTCTCGTTTCGGTTGTATTTTTTTTTTATGTTTGTT<br>ACCTCATAACTCGGTTATTTACAATCTTATCAGGAATTTTTTTTATCTGACAGGATATACATACAGATAATCTAATATATA<br>AAGTTCTCGTGTCGCGGTGTTTGTTACCAAACTCCTCCGAAACGGTTTTACCGATTTTTTGAAATATGCACCCGATATGCAC<br>ACAAATATCGGGTAGGTCTGAGAATCGGCTAACATCTATTTTTCATACCCCTCAATGATGGGTGACCCACCTCTAAATATT<br>TTATTTTTATTATATTATGACTTAGCATTGAAAAGTACATACGAATTCAAATTTTCACGCTTTTACGATCAACCTCTGTTTTT<br>TTAATAGCGATTTTTATATGCCGCAATAGGATTGCAAGATAGCAATCGAATGTAATAATTGTAGTAGGATAAATTTTCATA<br>TATAGTTGTCGAAATAATGTATATGGTAAAACAACGTTTGCCGAGTCAGCTAGTATACTAATTGTAAAATTAAAATAATTT<br>GTTTTATCCCAGTTAAAGTTTTTTTTTTTTTATTATTATGGCTCATATACCATATGGAATGGTATTTAATTTCAAATTTAGTA<br>AACTATCATGATGAATTCAACAATAATATAATCACGATTATTTTCAGGGAGAACCGCGTGGAGGTCGACCTGTGGGGTGT<br>ATATTGCTAGCAATGGAACTGATAATCCGCATNNNNNNCCCCCCCCCCACCGCAATGGGTTGTCATAAATGGCCAGAACT<br>CGATATATAGTTATCGAATATTACTCATTTCTAATTACAAAAACTATCTGTTGTCATGCACACGTGTACTTAAAAAAACTAT<br>TTTGTCAAAAC
MSTRG.10822	Coronin-6 isoform X1	AATGTAGCGGCTGTAGGCATTACCAAATATGGAGTTCGCTCGTTTTCCCAACCGTCCTCCCTTCGATTCTTAGTAGCTCTCT<br>ATTTTATTTTCTATAAATTATTGAGTTATTTTCAAATGATTCAATTTTATTCTCTTGTGTAATTAATATAATTTCCTCTTATAA<br>ATAAAAATGTCATTTAGAGTGGTTCGTAGTTCAAAATTCCGCCACGTTTACGGGCAAGCCCTGAAAAGGGAGCAGTGTTAT<br>GATAATATAAGAGTATCGAAAAGTTCTTGGGACTCTACGTTTTGTGCCGTGAACCCCAAATTCCTGGCGATTATCGTGGAG<br>TCCGCCGGCGGAGGCGCCTTCATAGTGCTGCCACACAACAAGGT
MSTRG.10826	RNA pseudouridylate synthase domain-containing protein 2 isoform X4	ATCCGAGAACACGCGACAACAGTTCATTATCAACGATTACATAACAATGATCACGTACAAGTTGATAGTAAATTCATTTTA<br>TTTCGACGCGTTCTTTTGTGCTAGGGTTGTGCCAGCACAGAGTACAGGGTACTAGTGAAAAGTGTTTAATAAGATCAATGT<br>TTGCACGATAGTGTCAGTGGTGTTGTGTGGATGTGGTTTGGAAGCTGATCGAGCTGGCGTGGTACGGGCCCGGCGGGGGG<br>GCGATGCAGGCGGGCGGGGGCTGGTTTGTCCCGTGGACGATGGGGGTGCAAAAGTTTGCGGTGGCACTCCCGATGGGCGG<br>CGTTGTGCCACTAGTGCCCCCGTTGCTGGCGGCGCTGCCACCGGCCCCGCCGCCTCTGCGCCTGATAGCCCCGCCACCATT<br>GCCGCATCAAGCTCAGCCTGGGAGAAAGG
MSTRG.10847	Methyltransferase-like protein 13	TCGGCCTCTCGTGTCCGCCGAAGCAGTTCCTCGAAGAGCACGTGCTGGACAATGTTAAGAGCATACTGACAAAAGATGGT<br>AATTTTATACTGAATCTAGTGTGCAGGGACAAGTGTCTCCAAGAGTCCATACTTGAAACCCTAAAACAACACTTCACACAT<br>CTGGTGACGGTAAAATTGTATGAAGAGGTGAATGAAATAGTTTTCGCCACGAATTCCAAGAGAGCATACTCTATGGACCA<br>TTTCGAGGAAGCCATCAAGAATCTCAACACGACAGCTAGAGAGAAGAGGCTAGTGGATGTCAGATGTGTTGATTTGAAAG<br>ACTTTATGCAGTCAGTCACTGTGATGTCATAGAAAATAAATTGTCTCCTAGTTCATAATGCACCTCATTGGCGTATTTAAAA<br>AAAATCCAGAGTGAGTTGACCTTGTAGACGAATGTTAAGCGAATCCATTAATGGTATTGTTTTATTATTATTTTTAAAATTT<br>TTGAAAAATTCACATCTCCCCTCTTAAGGAGAGGAGATGGCAAACTAACTCCCGGCAGAAGCACAGTCGGCCCGGGTTCA<br>GGGCCCCAAGGAGAGAAACCGGCGTACAATGCGGGCAGAATCTAAGAGCACCGCTTTCTGCATCTTTCCCGGGAGTGAGC<br>CGCCTCGGAATCCCAACCGTCTGAGATGTTGTTCCAGACTGGTTGGGATAAAACCGTTCACCGACATAACGATTGGAATTA<br>TTTCGGCGGATGTCACCCCCCACATTTCGGTCACCTCGTGAGCCAGGTCGAGATATTTTCTCTTTTTCTCAGTTATTATTATT<br>GAAAAACTAGCTTTTTCAAACAGAATGACCTGTGGCACTGTGACTTTTCCATGATCTTTTGTGCTCGCCCTTTTTTAAAAAA<br>TCGCTGCTAATTCCTATCAATGATAGGGATTTTAAGATGTTTTTTGAAGCAACGTTTTTTATCTCCTCAGGATATAGTGTGT<br>TTTCCAAAGGAGATATATTGTTTATGTATCAGTGGGCTACAGCCACACATTCAGTGCTGCGGCGTTTCTTTAAAATGGAAT<br>CATTATCAATAGCAAGTTCATGAGTTGGAATGAGACATCGGATGTTGCTATCTTTTTCTAGATCAGGGATTCACTAGAAAC

		TCATCTAGAGGGCTAGGTTTTGTAAAAAGGCGCATTATAAATACAAAATTAAAAACTTAGCTCGCAACTTGGGGTGGGAA<br>TGGACCTTCTTCCCCCCTTAAAAAAGCATATAGTGTACATAGATATATTTGATATCTGTATAATATTATGTAAAAAATATTT<br>TTATTCAATATAAATTGGTTCAATCAGCAGCCACACTCTCGAATCTATAATTGGTATCAACCCATTCTAATGCCAATGACA<br>ATTTTGTACTACACTCATGAAACTACAATTAAAAATTAAGTTAGAATTGGGATCGGTTTGCAATAGTTTGCATTGGTAAGT<br>TGCACTAATATGAATCTATCAGGACAGTATGGAACCACTATGTCGTTAGTGTTGCAATTCGAGTGTGAAACGTTACAATTC<br>TAATATCAATTTATTGTAATTTATTTCAAGAGTAACGCCACAGTACTTATTGTAAATTGCGTTCGAATATATAGTTTGTTTT<br>CTATAGAACTTCTACATACATACATACCTGTCACGCCTGTCTCCCATTTCTATAGAGCTTCTTTTAGAGAGTAATCAAGCGT<br>TGTTGCAATTATTACAGAAACACCTTTGAAATTAAATTAGTAACAAATTGAAATGATCAATTAATCAAATTTTGGACCGAC<br>TTCTTGAAGAAGCAGGTTCTTAATTCGGGTATAAAATGGTTTGTTCTTATTGTGCTATTTGGATATATATATATTTTTTTTT<br>TTATTAAAATCAGTTTAGTAGTCCTTTCTGAATTAATATGACTAGTTTTGGCGCAACTGACGTTGTTTTTTTTTTTTGTAGAA<br>CATAATTATATTTGTACCAAAATTAGATATAAGCACTTTTCTATGAGTGGTGTTTAATTGTTCGTTTGTTTAAATATTTATTA<br>AATTCTATTGAAATAGACTATAAATTCTTTAAAAAAAAAGGGAACAAAGATATTTTGTTTGAGAATATTGTTTCAATTTTG<br>GAGTCTTATACAGAATCGACCTACACCTATTGATTTTGACAGCTTATTCTAATAATGCGATAATTCCATACAATTGGATGC<br>GATATACAGTGATATCGTTAAAGAAAATGTTATGTGAAATCTCTGATCTCAAGAGAAAGTACTTTCTCTAGAGGTCATAGA<br>TATGCCTCAGTATTTTCTGATATTGTTTAAGTTTTAAAATTGTATATTTTTGTACATACTTTTATGTTACTGTGATAAAATGA<br>AATGAAACAAGAG
MSTRG.1<br>086	Hemicentin-2	TTCCGCCATTAACTGTACACATGGTGGGTTCAGACCAGCCTATATCAGCGAGCAGAAGATATGACCTTCTATGCCAGAGTT<br>CAGGGTCCAGACCCCCAGCTTCCATCACGTGGTGGAAGAACGGTCATAGGATAAATAATGCTAAGGAAACGCTATCAACA<br>GATGGGAACACGACTACTTCAACAGTGTCACTGCAGCTTACTAAAGCCGACACTGGAGCAAAACTTGCGTGTAGAGCCTC<br>AAACCCTCAGATGCCGTCAGTTGCACCATTGGAGGATGACTGGACATTGGATGTGCAAT
MSTRG.1<br>0913	Fibronectin type-<br>III domain-<br>containing protein<br>3a	CTCGTACGTATGTACCAGTCCGCTCCGCGGCGTCGACTTATGCGCCGGTGTGTCGCATACACCCTCGCCCGACCGTTAAGT<br>GTCCGACCTCTGTAACCAACAGTTTATGACATTCGACAACCCCGCCCTGTGAATTTTTCTTAGTTGCATTTCGATCAGTCCG<br>AGGAGTGTGTCAGCCACAGACGATTTGCGTCGGTGTCGGTGGCTCGCCCGCGTTTATTTATGTTGTCGTTTTGGTGTGGTGA<br>ATATTTGTGTTGTACGGACGATAACCGTATTATCTGTGCGATCGAGTCGAGTGGATATTTTTAACGATCGACGTCTATTTGC<br>GAAGGCTGAGTGTGAGAAGTGTTCGAAGCGGATGCGGCGCAAGCACCTGCGTGCGATGCGCTGCTAGAGGCCGCGATGGT<br>TGGCGTGGGCGTCGCGGAGGGGGGCGGGGGTAGCGGCGGCCCCGCTGACAGCTACTACGGCGAGTACTACCCCGCGGAG<br>TCATACTACGTGCCGCAGGAGATTTGCCCACATCCGCAGCAACACCCGCAACATCCACATATGTGTACAGTTCACACTGAA<br>TACGCGGGAATGCCAGTGGTGACGTCAGCGGCGATGATGCCGCAGTTGATGCCGCAAGTAATGGATGAGAGCTTGCGGCA<br>CTA
MSTRG.1<br>10	C-factor isoform<br>X1	CGAGTCGCGAACGTTCGTCTTGCTAATAGGACAATATTATCAGATCGTATCAAAGGATTTATAATATTATTAAAGCGTAAA<br>ACGTTACTCAATATTGTCATTTTGACGTAAATGACAGAGAAGGGAATTATCACAACTGTAGGTACGTAAGCACAAGTTGTG<br>TAGGTATACGAGAGTTATCTTTCGATCAAACGTCAAGAACGCAGGTCGAGAAAAAAATGCAGTCCGTTCTAATTACCGGC<br>GCAAATAGAGGACTGGGCTTGGGGATGGTAAAATATCTCCTAAAACAGAACAAGGCTGAGAAAATAATCGCAACTTGTA<br>GAAGCCCGTCTGAGGAGTTACAAAAGCTATTACAAGATAACAAAAATCTTCACGTTATACAACTTGGTAAGTAAATATTTT<br>TATGTTAATTGAAACGTAGAACTTTGTATAGAATTCCTTATATGTACATATATCTTATCATAACAAAACATCACGGTCTGTC<br>TGTTTATTACAACACGTC
MSTRG.1<br>101	myb-related<br>transcription<br>factor	GTCCGAGCGGACTGGAAAACAAAAACAAAAAAAAGGCATTACAAATCCAGCAACATGCCAGAGGTACTGGTGGAGGGCC<br>AAATAGCAGACTAGCCCTCACTGTATTAGAGGAAAGGGTGTTAGCTCATATGGGTGTATCTGCTGTAGTGGACCAGTCTGA<br>AGTACAAAAGCAAGGATTTGAAATACCTCCACGGCAGGAAACTCCACCAGTAACTTCACCAGAACCTACAGCATTCCCAG

		AAATAGAATGCGCCGAAGGAGGACACAAACTCCATTCGACAGGGTCGCGACCCAATTCGCGGCTGTTGAACAACGTAGCC<br>TGGAATTAGAAGAGGCGCGTCTATGACATTATCAAGAACTCTTTTTCTCCATATTGTTGTCAATTCTATATTTCACATATTA<br>TTCTTAATTGTTACGCCTGCAGCGCAATTTCTTTAAACCTGCC
MSTRG.11093	Hypothetical protein	GGAGCCCAAGATTGTGGCCAATGACGTCGATCTGTACCATTCCGCTCTCTTACATAGACGGAGAGTGAGATACTGATCAA<br>ATTTTATTGCTAGATGACGTTTTTATACGAAATAAAAAAAAATTTATGACAAGCCTAAAAGAAAGAAGATACGTCTTACTG<br>CAAGTTTGTCATAAAAAAAATCAAATTTGAAAAAGGGAGACTCGAAGATGGAACTACGAAGTTACTGCATTTGATTTTGCTT<br>TTGTTTCTGATTTGTTATTTAAGTGGGTATATAAACGATATTGATAGTATTCAATATTTATTATTTAACCTGTAATCATGTCG<br>CTTACAACTGAAGAATGCACCAGAATCCTCACAATGTTGGAAACCGGCGGGAGTCAGCGCGATAGAGCCAGGATTGTCGG<br>TGTGTCAACAGTACAGTTTTCCGATGTTACAAGGAAACTGGCTCTAACCTACGTAGACCAGGAACAGGCAGAACTAGATG<br>TACCACAGCACGAGAAGATGGATACTTGGTGAACACCATGCATCATAATCGCTTTTTCACAGGAACTGAACTTACAAATC<br>GGTTTTGACGCCTCGGAGTAAGTAACCCAAGCGAATGGACAGTCAGATGACGACTTGCTGAAGTTAACTTGAAACCATTT<br>AGACCTGTAAATGGGCCTAAACTGGAATGTGGTGATTATATTTCAAGATTAAGATATGGCCAGAACCATTTAAATTGGACT<br>ATTGAAGATTAGTCGAAAGTTATGTTCTCCGACGAATCAAAACTTATGCTCTACAAGTCTACAAGCACGATGGTAGAAAG<br>AGTGTATAGAAGACGAGGGGAAAGATTAAAGCATGCATATAAGAAAAAGTAGCCTACAGTGTTAAGTCTGTACTTGTGTG<br>GACCGGAATTTCATCTGAGAGTCGCATAGAACTGGTGTTCATAGAAAACGGATCTTTAATTAGCGTAAGATATGTGGAAG<br>AAATCTTTAATGAACACGTTGGACTATTTATGGTCAATTTGGATGAGGATGCTATTTTTATGTATGACAATTCCCGACTTCA<br>TACAGGTTTCATTGTGAATGACTACCTACAAGAGATGAATATTACTCGTATGGAATGGTCAGCACGCAGCGCGGATATCA<br>ATCCGATCAAGCATGCATGGGACGAGCAAGAGAGATGCGTAAAACAACGAAACTCTCCCCTAATCACGTTTCGTGAGCTC<br>AAAATTGCTCTCACAGAGGAATTGGAAAATATATGCCAAAATCGTATAAGAAATTTGGTGTACATGCTGTACACAAAGTT<br>TCTTATACGATTACAGTTTCTTACAGTTTAACAAAGAGACAAAGTATTAAGGTTCAAGAAAATACATATTGTTGTACAAAA<br>ATGCAATGTTTCCGATTTTTGTTTTAAGGCGAGATCAGCAGTTTTTTTCTTTATGTAATTTTTAATAAATAAAAT
MSTRG.11123	Histone-lysine N-methyltransferase SETMAR-like	AGGGATTGTTAGTAATGGAGACTGAGAATCCCATTGCTAGCAATATACCCAACCAGGTCGACCTCATCGCGGTTCTCCCTG<br>GAAACTATCATGGTTAATTCCTGATGAATTCATCATGATGGGCTAATTGGCCTGTTTTCATTCTCGTCTATCACCCTTCACT<br>AGTGCTCCGCCAGCGTATCCCGTTAGTGCAGGCGGGATTACCATACCATTTTCACCCATCAAAAAAAAAATTGAAATGAA<br>ATGACAAAAATAGCAACAGATGGTGTATCTTTCGTCCTCCTTTTACTAATCATATTTGCATAAGAAGAAAGGAGGCAATAT<br>TAGTTCAGTCAGAAAGTATTGCGAGAAAAAGTTTTTATGTATTTGAGACTGGCCAATTATAATTTATTTAAAAATATCATT<br>GCTTCTAAGTAAAAATAGGTGATGAATTCTGGATTTATCAATATGATCCAGAAACAAAGCGCCAGTCGACGGTTTGGGTGT<br>TTAAAAACGAATCAATGCCTACCAAAGTAAAGCGACCTAGAAGCGGGGGGAAGATTTTAATCGCGTGCTTTTTCAAATTG<br>ACTGGCTACTTGATGTCAATTCCTATACGAAATCAAAGAAGCATTAACGACGAATGGTATACTATCAAATGCTTGGCAGA<br>AATTTTCTCTGTTGTGAGCAACAAGCGACTAAACATAGGGTTCCGCGGGCTACTGCTCCACCACGATAATGCGCCCGCCCA<br>CACCGTAATCAAAACGAGGGACTTCCTAGACAGCACACCGGTCAAACTCATGGGTCACCCTTCTCCAACCCTGATCTAGCA<br>CCGTGTGACTTTTTATTCCCAAAAATAAAAAATCAGCTCAAAGGGATACAATTTTCGTCGCCGGATAATGCGCTCTCAGCG<br>TTCGAGCAGGCTGTAAACGAGGAGTCTGCGGCGGACTGGAAAAAGTGTTTCGAAAACTGGTTCAGACGCATGAAACTTTA<br>TATAGAGGCTGGTGGAAAGTACTTTGAAAAGAT
MSTRG.11253	General transcription factor 3C polypeptide 5	CTAACAGGAGGTTAGTCCAAAGTACAGCGAGCACTCAACTTATCGAGACATCATGCCCTCCTCTTTATGTTGTGATTCATC<br>CGTACTCTGAAAACAATTGTAACAGTTTTATTGAGTATTATCGAACATTTTTGTATTACTATGAAGACTTCAAAAACAAAA<br>AGAAATGGAAAAAGTTGAGGCCAATATGCAAATGGTACTCAAACATGTGGTCACGGCATGTCTCCTCGCCTTTGGGTGGC<br>AGCCAGCCGCGTTTAGGGTGGCAAAGGTAACCAGGTGGTGGCTCTTTCGCTAATAACTCTTCCACTTCGGGCAATTTTATA<br>TCGCACAACTGTAACAAGACGTGATGAATTCACAATAATACAACCATTTAATATCTAAAATTAATCATTTTACAAATAAAG

		CCAGTTTTAACAGTTTTTCTTTTTATGATATACTAGCTGTAACTTGTCGTAAGTTACAGTTAAGTTATGGGCC
MSTRG.11269	Probable G-protein coupled receptor 158 isoform X3	AGGTATCTCGTCGAGGACCAGCGGAGGGTTTCGGGGTGGAAGGTGAAGAAGAGCCTACATCTGAAGAGGTCAGGGCCGA<br>GTTGAAGAGGTTGTACGTTCAACTAGAGATTCTTCGCAATAAAACGCTTAGACGCGACAATCCGCACATCAGCAAACGAA<br>GAGGCGGTCGGAAACCGCCGCATCGACGGTTTTCCTTGCAGGCTCTGCAAGCGCGACGTGGTGCAAAAGGCAAGAGCGGT<br>GGGAGCGCTAGCGTGGTGGAGGCGAGCGAGGCGGGCGACGTGTCCCGCACGCCCGAGGACTCGGTGTGCTCGGCCGAGG<br>GGCCCTCGCACTACACCGACGGGGACACGCAGGCGTGATCGAGGACCGCGCCTCGCCATCACTATCACGACGTTCACCTG<br>TAGATCACGTACTGTAATATTTTACATCACACCGTAGGAGTAAAGAAACCGGGTAGAATTGACTTGCATATACTTTTCTTT<br>GTGAAGGTATCAATGTCATCTCTTCTCTTCAGACTTTGCAGATAATTTCATAAATTCTTTCAAATTTTAACAAATAATTTCA<br>TAACACGACGTAACTCACACGACACAAATGCGATTAAACGAGACGCACAATTTTGTTGCGGTGTCTATTGCTTGCAAGCCC<br>TGACTGATTTAGTTCTAAATAGGATGCGAGTCATCACCACAACAATGTAGTTATAATTATTGAATCCAAGAGAGTGTCAGT<br>TCTACCTGAATATTTATTATTATATTTAGTTCTTATGATTACACTTTAGCAGTCTAGAGCAGCGATTCTTAAAACAAGTTCC<br>ATGGAAACTCGTCTCCTTATAGATCACAGAGCCATGCAAAGGGAGGCGTTTTATACAACAACCGCTTTTGTAACAAAATTA<br>ATTGTCCCAAATATTAGAACTTAAAATTGGATTTATTTCAAAGAAGGTTTAGTCCCTCATAAGTTTTTAAAAGCTTGACCCG<br>AATGAACATATAGTTGATAGTGGGTATACTTAATAAATAGTACGGACCAACAGTACGCTTTCGTATTGATAAATTTATACG<br>AGTATACTCATACAGTATGTATCGTATCTTATATTTCAGTAAGCAATATGAAATTTATTTCAGTATCTTTAAATTAATATAC<br>AATATATGTTTTTTTTTTCAAAATACAAAAAGAAAATTATAATTGAATCGGTTGCCTTAAAAGTTTAGGAACCGCTGATCT<br>AGAGAATTTTGGAGTTTACGAAAGCCATTCTCTTGACAAACAATAATTTGCACTGTTTCTTTTTTGTCAAAATTTTATTTTA<br>AACATAGAAAAGGCTTTTTAAATGGAGTTCTAATATTATTATTTATCAATAAGTTTAATCAATTGCTATTAAGATTCAATAT<br>TTTTTAAGGCCAAGAGGCATTCAGAGAATTCAAAATATTTTAGAATGCTGTTTACAGATTAACGTACGACCCGATCACATT<br>GTATAAAACATATTGTTGATAAAGTCTAAGTAAACTGTTTCTTACTTAATTTTTATGATATATATACATTTATACATATAGA<br>AGCAATTACGTTATTTGTTTTTTTATTACGTTTTCTAAATAGATATTTTATGTTGTATACATACAAAAATTGATGAGATTTCA<br>GAATGTAATAATGTCACTAACAGATAGAGTTTGACCGAAAAATGATATCAATAAAGAAGAAACCTTAATACCGATATGGG<br>AAAAAAAGAAAGTCGATTGGTAGAAAAGATCTGTTTAGTGGAGACCAATATCCATAAAGGGTTGGAATGTTACAGGAAGT<br>AAGCTATGATAGAGAGTGACAAGATGTTAACTACAGCATTTTTCGTTTCTCTTAAAGTGGAGTTAGCCAAATAGCCCACTT<br>GTTGTAACTCCTATTTCTTTCTCCGATTTTAAAGTTATTTTTTTTTAATTGAACAAGTAGTATTCTCATCAATAGTATTCATA<br>CTACTTTTTTTTTATTTATATTGAGTTTTATTTTTTAGAAAATATCATAGTCTCATTTCTCTTGTAATCGTTAGTACCGACCAC<br>TGAAAATAATGCCTTTATTACATTGTATAAAATTTTAGACATTATTTGTTGCGATAATTCAGGCCATGAGTGGTAGTTACTC<br>ACCCGAAGGCATAGTAAAAATCACTAGGTACCTATATCGCAAATTCGAGTTGTTAATAAACATATACTTGTAAGTAGTAAT<br>TTAAGAGCGTTTATTAATAAGTTTACCTAAGTGTAGCAACGTCAAAGCTACCTACCTGTCAATTTTCAAAAATAATTGATC<br>AATTTTCAATATTTCTGTTTAACTATTTCGTTATATTAGCCGCTAATTGTACATTGTTGAACATAAGCGTCCCACATAAGTG<br>CTGATTTTGCTAGTGATTGCCATGTTCGGCAGGCGGGTTAGCAACAGCAATTAGGATTTGGTGATGTTTTAAGAGGGACGC<br>TGCTGTCCATCTCTCGATTTAAGTATCTTCCCTTAATCGCCTGTTACAACACCCACAAAAAAGGAAGTAAAGGGATTGGTT<br>ATTTAATCATAGAATAGATTGTACACAAATTTCTATTTTGAACCTTTCATCTAATATAGGTATATATATATTAGATGCAAAA<br>AATCGATTTTTAAATCGATTTTTCGCCACCAAAATTCAAATATTCAGTGACAACTTAATAAAAATCTTATTGCATGATGAA<br>CATTCCAGTTCGTTATCAGAGCACTTTATAATTAACCAGTCAGAAATATTGAAACATATTCAACTGTTTTAAAAATCTGAC<br>AGCTGATTGCGACGTTGCCACACATGAGTAAGCTTCGATACGAACGCTCTTAAGAATTGTGTTAAAGTATTACTAATTTCA<br>TTTGTCGCTAGTGAAATTATTTATTACGTAGTGAATTGTTTGTATAGGAGGAGAGTTAGTACAATAATTTAATTATTACGTA<br>TTCATAGGGAATAGCGGTGATATTTTATACCTGTAGGTATTAGCAAGTGGGTTCATTAATGTTGATAAATGTATCCAGGAA<br>TGGTGTTGTTGCTGTTTTATAAGCTTTTTTACCCTGAATATGTATGTCATTTATGTATTTCTGTAAATTGCTCGAACATTGGC

		TTGTAAAGTTGTTTCATTTAAATACGTGTATAAAAATGAATTTTAATTAATAAATCATTAACTTATTTCACATGAAGTATAG<br>CTAAATTAGTATAAAAAATAAACATCCAGAAAAATAAAATACCTGAAATTTTGTGTAAGTATTAACTAGTCGATTTTTTT<br>TTATTTTTAATAATAAAATTGTTACCTATAGACTATTTGTAATTTAATGGTATCTATCATTAGTCTGTTAATGTACAAGTCC<br>ATAGAGTATTTATTGTATTGTTGGATGTATGATTGTTTTCTATTCTTAATAGTAAAACCAAATTCTTTAATATATTTCGAATT<br>TCTACTCTTTATTATTGAACTATACATACTCATGCTAATAAACTACTCAGGCCTGCTTTCCAAAAATTCAGAGCAAAATGG<br>GTCCACTCAATACTGATTCTGTAATACAATATTTTTTTTATTACAACCAATAAATTACATTTAACATGTTGAGGAGAATTTT<br>TGTGTCACTTTTCTAAAGGATTTTTTGTAATCATGTTTTGTATATTATTATGTATGTTTTTTTTTTATTTTCTTACCTACGAAT<br>AAGGTTTAAGATACCTTATTCTGCAACTTTTTCACAGTTATATAATGTGTATAAAAGTTATAAAATAGCTATAAATAATTGC<br>AATGAGTTAATAGAACGCGACTGATAAAAAATAATAATATTATAATATACATATTTTTAAATAACTGATGTTCTTTAACAT<br>GTAGACAAGTCATAATCGATAAAATAGCTAGCACAAC
MSTRG.11322	Dynein heavy chain	AGGTGCGCGAATCGTGGCAAAGCTACGAGTTGGACCTTATCAACTACCAGAACAAATGCAAGATTATCCGTGGCTGGGAT<br>GATCTTTTCAACAAAGTCAAGGAGCACATCAACAGCGTGGCTGCTATGAAACTCTCGCCATATTATAAGGTGTTTGAAGAG<br>GAGGCACTGACATGGGAAGAAAAATTGAATCGCATCAATGCATTGTTTGATGTATGGATTGACGTTCAACGACGATGGGT<br>CTATCTCGAAGGTATCTTCAGTGGATCGGCTGACATCAAAACCCTCTTACCTGTTGAGACAAGCCGCTTCCAAAGCATCAG<br>CTCTGAATTCCTGGGGCTAATGAAGAAAGTTAGTAAATCTCCGATGGTAATGGACGTACTGAATATCCCGGGTGTACAAC<br>GGTCGCTGGAGCGACTCGCCGACTTACTTGGCAAGATACAGAAGGCACTCGGTGAATACCTTGAACGGGAAAGGAGCAGT<br>TTTCCCAGGTTCTACTTCGTCGGAGATGAGGATCTGCTGGAAATTATTGGTAACAGCAAAAATATTGCTCGCCTTCAGAAA<br>CACTTTAAGAAAATGTTTGCAGGCGTCGCTGCTATTATACTGAACGAAGATAACACAATCATTAACGGCATTGCTTCTCGT<br>GAAGGCGAAGAGGTATACTTCTTGTCCCCTGTTTCGACCATAGAGAATCCCAAAATCAACTCATGGTTGTCTATGGTGGAA<br>CGTGAAATGCGCATGACACTCGCGTGCCGACTTAAAGACGCAGTCGCCGACGTTA
MSTRG.11336	Gamma-1-syntrophin	GACGGATGCACGATGTCATGACCGTCAGTCAGTGACCACTTCAACTTGGAAGAGGTACTTACATCGGTACCTATGTAATTA<br>TAATTATTGGTTTACTAAAAATATAATACATTTTGTTTTTATTAATCGTAATTATTCAAAATATAGAAATAATAAAATGAAG<br>AACAACGATTTTTTAACGGGATCAAGGAAGTGAGTGTATGTTTAATGAGTGTTTACGAATTGTTTTGGTGCCATTCACCTA<br>TATTGATTTATCATGCCAGTGACCTGTTTTTCGTGTTTGTAATGTGTAATACATAAATATTTAATGAAACCATTAGACGGCT<br>GCATACAGATGTTTTGGGATGAACCTGATATGAAAACTGTGATACGGAATTGTTAATTACGATGGGAGAGCTAGCTTTTAA<br>ACCAATAAAATATCTGATAGAGCTTAGCTGAAAACTTGTAATAATATTTATAATTCGTTATGGTGTGCATTTTTCACAATG<br>ATGACGCCAATTGAGGAGAAGAAAGATGAAAAGATCCGCACAGGCATGGTGCAGGTGTCGGACGGCAAGTCGCGGCCGC<br>GGCCGGCGCGGCTCGTGCTCACTATGGACGCGGTGACCGTGCAGCGCGAGGTGCCCGTGCCCGCGCACCCGCCGCGCGAC<br>AAGAACGTGCCGCACGCCAGGG
MSTRG.11374	Sphingosine-1-phosphate phosphatase 1-like	CTAGTAGTTCTGGTTCCTCGTATGAAACTGATACATCGTCGGAGAGTAACAAGGACGAAGTGGAGTGCACGATAAACAAC<br>AAATTTTGGTACTATGTCTTCGTAGTGGGCACCGCCTTTGGTGACGAGATATTCTATGCTACTTTTATACCATTTTGGTTTTG<br>GAACATCGATAGTGCCGTTGGTAGAAGGGTTGTTTTGGTCTGGACGGTCGTTATGTATATAGGACAAGGCTTCAAGGATAT<br>AATACGATGGCCTAGACCTGGCTATCCTGTTAAGAAGTTACAGACAAAATGGGCAATAGAATATGGTATGC
MSTRG.1141	Tyrosine-protein phosphatase non-receptor type 61F-like isoform X1	AAGCGCCGCGCCCGCGACGCCGAGAAGTGGCACGCAATCAAGAGGTCGCTCTATAAACCACTCACAATCACTTTTGGCTT<br>CTTAATTGCCGGCATTTTCATTTATTCATATGTCAAGCACTAGCCACATTTAACACGTATCTCCCATCAATAATTTGTGTAA<br>TTATCACCAACACGAACTATCCGATTCAGTGATTTGCTTTCGTCCGTATTGATACATTTGCGATGTTATTTGTTTATTCTTTT<br>TATTTTTTTCTTTATTTAGTTTACTATCGAACTAATTTTTTTTTTTTTTATTTTTTATTTTTTTTTTTTTTGTTATCGTAAATGTTG<br>TTAATACACGAAAGTCTATTATTGCATTTGTTTAAACAGTAACCACCGTTGCTTCTTACATATTAATTTCCGGCAGCTCTTT<br>ATTGATTTAATTACTAGTTATTTAGCGTTTTTATACTTAACAATTTAATGATATATCATTAATAACTGAAGCGTATGTGATA

		CGGTATCATTAATGTAACTTTACACCATGTGGGTAAATGTTTAAGTATATAAGGGTAGCAGAACTCTGCGCTATACAACGA<br>TAAACGTAAATAGGTGTATGAAACGATGCAAAATGGATAGTGTGCATGAAATTGAATAACAATTCTCAGAGGAGTCAATT<br>TAAAATATGGCCACAATTCTTCGAACCTAGTGTATGTGAATGTGAAAAAGACTCCATTTTCCGCGCTTAGCCTCTCATTTG<br>GGCCGTTGTGCGTATGAGTCGTGGCTAGTGCAGCCAACCTAGCTTCGCCCAGAAGCGCAGAAGCTTCCTGGGTTCTTCACA<br>GACCTCACGGAGCGACCTCACCGTTTCGAGGATTTCTAAACGTTGATGGGCCACAGCGAACCTGGTGTAACCGAAATGGG<br>TTTTACACACCGAACCGAAATTGGTTCCGAAGTATTCTTCCTTCTTTATTCTTATTTCTTTCCTTCCTTTGGAAGTATATTTTT<br>TAAATGACTCTTTCTGCTAAATAATGTTTTCCACTCCGATAATATGTTAAAAAAGCAATTTATATTTTCTTTTATTCTATTGA<br>TTAAACGTGAAATGGTAACACAGCTTGTGCCGTTTGTTTACGCTAGCAGAGTGAAGGGGGACAGGTGACAGTGAGGTGAG<br>ATCAGTTGGCTCGTTGTAGATGTCGCCCTGACAGGATATTTTGTCATCATTAGTGCCGCTTGTTCTCGCTACTCACGATACT<br>AGCCAACAACCTATATTTTATTAAAGAGAATAAAAAGAATTAGCAAAGAATTATATATTGTATGTAACAATCAACACCCA<br>TATATGAACATCTAGTTACCATAGCGACCTAAACACAGATATATAACTACACTGATTTAAGATCACTAGACGTGCTACATA<br>CTGGAAGTGCTTCCATAACATATTTTGAATAGCTTTTTGGCCTTTACACGAAGATCAAAGTGATCTATTTTTTTATTTTCTTC<br>CTTGATAAATGTTACTCGATTTCATTAACATTATCACTGGGTTTTTAGTTGATATACCTATTACGGTTAATCGTGTGTATTCA<br>GCAGATTATGCACTTTAATGAGAGAATAATATTGTATTTTTGATTAAAGTAACTTTATAATTAAGATATGATTGCATGAGC<br>GTGAACATTGTATGTTGTATGCTCATACACCATGAAGTGTCTTTTGTTACACCAATTAGTATGTTAGAAATAAAACTATTTC<br>GTTTCTCCATGTGACACATTGACAGGGAACTTTGTCCATCGAATGTCTAACGACTTCTCTTTTTGAATTATTTAAAAAATCA<br>TAAAATGCTAAAGGAATTAAAAATATGTAAAAACAAAAAAAAAATATTGTATTTTGTTATTCTATTTTTTTTTATGACTGA<br>TTATTAAAAGATCTTAAATCGAATTTAGAAGAAGTTTATTTTATATATTCACAACTGAATAGACAGCAAAATATGTTTATT<br>CCTCCTCAATCGATTAGGGATGTAACTGGTACTTGGTTACACAGAGTCGTTTAATTTCAATGACCACTAGTATGAACTACA<br>AAACCCAGCTTCTTTGAATGAGTGAATAAAGAAGAATTGGTACTTCTACAGGAATTTATTTCCACTATGCATTTTAAATAA<br>CTCGGATGCATTCAGTTGTACATTTATTTCCATGCTAGTGTTGCCACCAGCG
MSTRG.11423	Death-associated protein kinase related isoform X1	ATTTGAATGGTACGTTGCGTTTTTCAAGTTCCCTCAACGGCAGAAACGTGTACGGCGAAGTGCGGTCGACTCGTCGATGCG<br>CAGCTGTCCCGCCGCGCTTGTCAATTTTGTTTTGTCGAAAACGTGTGTGTAAACAATAATTTATTACTTTTCATTACGGTTA<br>ATGATGAAGTGATACAAATGTTTGTTTTATAATCGTGTCGATTTACAGTGGAGAGTTTGAATGAAACCGTAACCGCTTCTC<br>GCAATGACAAGGGAGAGCACTCTGTTACCTTTTGAGAAACCGGAGGCATTAAAATGTTTCATGTGGTCTAATTGATGCCTT<br>TTTTACATGTATAAGTGTATACGTTGATTGTAAGAACTATTGTGAATCGTTTAGTGACATTTAAAAGTTAATCATTTCCGTT<br>TCGACTCGGTCGTCAGTCGTCTGCACGTTAAAAATGAGTGTTTTGAAAGGTTTGAGCAAAGCTGACTTCACGACAGCGAGC<br>GATGGCCTGCTGGAGTTGCCGGAGGAGAAGCTCCGCAGCATCATGCGCTCGGAGCCGATCACTGACGTCTACCACGTCGA<br>ACAAACGCCCTTTGCAAGGTATGAAATATGGCGGTGTAAAATGGACTTCTAATGGTAAGCATGGTGTTGTGAAACGAGAT<br>TCCGCG
MSTRG.11460	Uncharacterized protein LOC106137858	CATTCCTGAGGACTCAGGTGTTATTCCTCTGTACCCAGTAAATTTACGCCATATCCCACCCTTCAAACCGAAACACAGCCA<br>TGCAAACACAACTGCTTCATGGTAGAAATACGAATAGGACAGAGGAACCCAAGCTATCGACTTTTGTACAAAGTCTCCCT<br>CTGGTAAATAACTTCCACTTGATACCTCCACGCGTTCCTAAGAAAAAGAGGCTTGACAGGACGGACGAACGGACGGACAA<br>CGAAGTGATCCTATAAGGGTTTCGTTTTTTTCCTTTGAAGGTATGGAACCCTAAAAACTAAAAATAACTAAAAATTAATTA<br>CTTACTAAAAATGGTCAAAAATACTCTTGGACTGTTGTCAGTAGGTCTGTTAGCTCTGTAACCAGCATAGAAGTACCATGT<br>ATCTCCCATTAGATTCGCATTCATTACTGCTTTGGTCATGTTCCATACAATCAATTGCTCAGCTTCCGAGTTAATCACTACC<br>AAATGTGCTCCTTCAGCTTTGCACTCCGCATAGGCCTGGTCCCAAGTGACCCTAACCATAGGGATCTTATAACAGCTTCCG<br>ATATCTCCTTCGTACTGATAATCTGAAAATGCATAAATGGAATTATATTAATGTCTAAATAACAAATATACTATACCATTA<br>AGCTGATTTGCCATGTGGTACTGTCATAGAGTATGCTAAACATGAGTATCGTAAGAAGCGACGAGCGAGAAGAAGAAGGA

MSTRG.11462	Endonuclease and reverse transcriptase-like protein	GGGCGAGGAATAAGTTACCTTTAACTGTCCACTGCTGACCATAGGTTTCCCCCATAAAGGGAGTTTTGCCAATAGTTGCCA<br>TGCGTGGCAGACGGGTTGACAACCGCAGATAGACGTTGGTGATATATTAAGAAGGACACTGTTGTCCATCTCTCGACTAGT<br>TTCCTTACTCACCTTTTACGATACGGGAAAGAAAGATCAAGGAAGGTGGCTCACACGGCATTACTATTTTTTTTTCAAAT<br>GAATGAAAATAGGTGATAACCTCGCCTACGTTAACGGTACACGCTAGCGGGGCACCAGTGAGGAATTATAGGTGAGGATG<br>AAAACAGGTCAGTTAGCCCATCATGACGAATACAACGATAAATCACTCAGTCGATAAATCATCCAGGGAGAATCGAGTGA<br>AAGTCAACCTGATAGGGATGGAGTCTAACCTCCCTATTACTAACAATCCCTTCCCCCATTATTAATTTATTGTAACTATGAA<br>TAATATATTTATTTACCTTTGCCGTAAACTTTACACTCAGCATCGTAGTAGGCATCTTTCGCATCCACTTTACAGACAAATG<br>GCATGTTTCGAATGCAGCCGCCGGTCTCGATTTTTCCTTGACGTGTAAGGACGTCGCAATTGTACATTTGATTTACACCACT<br>GTCTTCATGATATGATGGCGCTTTAAAATTTATAAAATAATGTAAATAATATTACCATATAACAGTCCTCTACTAGGCATA<br>GATTTAGATTTATCTAAGAAGCATAATATATTTACTCTAATTGCATAATATATAATTTAAATTAATACAGTTAAAATAAAC<br>CAGACAGACTTTGTACAAGTCGTTTGCTTGGGTACCTCTGTTCCATTCGTGTTTCTACCATGAAGAAGTTGTGTTTGCATGA<br>CTGTGTTTCGGTTTGTAAGGAGGATTGTGGCAGTGAATTTACTGAGTGCAAAGGCATGACACCTTGGTCCTTAGGAATGGC<br>AAAGCATAGGGAGAGTATCATGAACGAAACAGTATACTCTGTTATGTCCATGGTACTGCTCATGTGTATAGGCGACGGTT<br>ACCACTTTCCATCAGGTGGGCCGTCAGCTTGTTTGCCATTCTAAGCTGTATAATAAAAATATTTACAATTAAATTTTTATAC<br>AGATGTCACTTTAAACTTCACAAATATTAACCTCAGAAAAAATAAACATTAGGCATAAATAAATTTGTTTCAAGCAGTTAT<br>TCAGTTGTTTTATGTTATTAATGAGGAGATTAGTTAAAAATATAATAACAAGAACTTTTAGATAATTTAATTTATTCTCGTT<br>ATTCTGTAATTTCGTAATAAAATTTTATGGCGTAAGACATCTATTTCAATCAGTGAGACCAAATACCTGATTGCCGCATTTT<br>ACCCACCTGGCATCCTCGTTACGCATTATAGGTATACTAATGATTGCTATTAACACGTTCACAAACACTC<br>CTATAATAATTTATTAACAAGATGTATTATAATAGTTTGCATTAAATAATAGTATTAAAATTAAACTACTGTCTCATTCTGA<br>ATGACTCATTCAGTAAATAAGCACGTATGTAACATATATGTATGTATGTATGTATGCTTATATCTAAAGTTAAATTTTGTAA<br>TGCTCGGTCGGAGTAATTTAGGAAATAACCTTTTCGGGCGGACACAAAAGCGACGTTTTTCCAAACTTCGTCAAAATTCTA<br>TGGACAGCGGCGCTGGCGGTGATTACTATGCACGGCGTTGCAGATTGCGTTGCCTCACTATGCGTGTACAACGGATTGTGC<br>TCAGAGGAATTGTTCAGTATGATGCCCACGGTCACTTGCTATCATCGTTCGGCTTGCAATCGACAGGGAGTTCACCCTCAT<br>AGCCTACAGTCTAAATGGTCGTGTACAGCGCGATTTCAGAGATGTTTTCTCCCACAAACACTCAGGTTATGGAATGAGCTC<br>CCTGCCGAGGTTTTCCCAAGAGACTACAGCATAGGGTTCTTCAAATGGGGGGTAAAGAGGTTTCTTCAGGGTCGCCAACA<br>CGCGCGTAATACCTCTGGTATTGCAGGTGTTCATAGGCTACGGTAACTACTTACCATCAGGTTTAACATAATTATAGTTTTC<br>TAGTTCCAGATTAGCAGTTGCCAATTGACTTATACGCATACTTACCAACGAATCACATTTGCACATCAAGGATATCATGGA<br>TTCCGATTCATCACTGCGTAGTCTGTATACTGTGTCCGAAGACACACTCATCTTGTAGTATACGGCCTTACCGGCATGTGAT<br>GCCCATGATACCCCTTATGTGTTGCCATTCCAGAGGACTATGGTGTTTTCGTTCCCTATAATTTCACTGCTATATCTCACCCT<br>TCAAACCGAAACACAATCATGCAAGTACAAGTACTTCACTATAGTAATGGATGGAAAAACTAACCCATGCCAAGATTTTA<br>TTTTACAATTACAGAAAAATTAAATCCCATTTTCTTTGACTGTAGTTACTATTTTCCTTTCTCACTTTATTTCTTTATTAATTT<br>TACTACATTCGTCATATTTTGTTGTGGTTCTATGTATATATTTATATGTGTATGTATATGTAGTATTATTATTTGTATAAGTA<br>ATTTAATAAATAATATGTATTTGAAGTTTTTGTGCATTATAGGTTTTTTATTTTATGTAAAAAAAAAAAATATTAATTCACG<br>TTTACGCCACCTATCCACTTCTATATAACGTTCTCCTTTAACCAAAGGTTGTCTGTAAGACATAGTACCCTTGCGACAAGAC<br>CGCTTTCTGTACCTTTATGTGTATAATTTAATCTTTACTTTGTGCTACTGTGTTTCACTTGAATTGTTTATATGTCATTGTTTA<br>TTTTTATGTTTTTGGTACAATAAAGAGTTAAATAAAAAAATCCTGACAGGTTTCATAAATACATAATTAAAAAAAATATTT<br>ACAAAATATATTATTAACTTGAAATTTCATTCATTTTGAAATTAAAAATCTAGAAGAATATGTTTGGTTGTTTGCCACCTCA<br>ATAGTGTAAAAAAAAAAGAATAATTAAAATAGTATTTCCAGCAAGTTGAATGTCTTAGATACCTACACAATTGCTAGAAT<br>AATAAAATTCTATTTTTCCAACGACATCTCTAAACTCAACAGGTGGTAGTGGGTAGAGTACAGCGGATAACAAATTCCGCA

		TACTTTTTATTTGAACGGTTGATTATATAATGTTAAATATCAATTTGTCTTTGAGCACGAATGTCATCGCTATTAAATTAAA<br>AATTTTAAGATTGTAGATGGAGGGAGTTTTATTTTAATGGGTGATAATGGATTATCAATCCGACCCCTCGGTGTATACCGA<br>TAACACCGCCTACATTATCGATATACGCTGACGGAGCACCAGTGAGGGATGATAGATAAGAATTAACGCAGCACAGCTAA<br>CTTATCGAGACG
MSTRG.11542	Hypothetical protein RR48_14652	GTGACGTAGACCCTTATAGATAACAAGGAGATATTTATAAGCGCGGAATATAAGTTCACATATTATTTGAGTACCAGACTT<br>GAATAAAAACGTAGTGCGAAACTCAAAAAATAATTGATATAACCATGAAGTCGATACTACTTATTTTTGTTGTGATTAGCA<br>TCGTGGTTGTAGGGAGTGCGAGACACATTCAATTTGACGTTGTGTCCAGTGGCGATGATGCCGAGTTAAATATCGACACCA<br>CAGAGAAAATTGCGGATATTCATGATACGTCTATTGAGGAAGTTCGTAACATCAAAGCCTCACGATTCGTTCCAATAAATA<br>ATGTTATATTTTTCAAGAAGCACCCACCCTGCGAGAAGGATGGATTCCAAAGAGATTTGTTCGGTATATGCAGGGAAGTTT<br>GGTAATAAATAAACAGATGTTATGTTTTATTATCGTATAAATAAAATCTACTAAATTCTAAGTTCTCG
MSTRG.11543	Hypothetical protein	ATACGCGAATACAAAGTATATACGTCGAGTTTTGCGATAAGTAAAGTTCGCGGACATAGTACTTCTCTAGCTATATAACAG<br>CGGAGCCCGCGAGATCTATATTAGTTTGCAAACCAATTTAGTGGCCGTTGGTTGTGAGTGTGTGTTTATAAAGTAAACAAT<br>ACATTTCCGTTCAAATACCATGGCAGACAACGATCGTATTGTGTATCCTGATGAAGTAGAAACATCGAAGATAGAGGCAG<br>CGAATGCTGAAGACAAAACTACAGGTGGTGACACAAACACAATAGAGGAAGCACCTCAAGATATTGCAATGAGAAATAT<br>GATCACTGTGCCTCCAAACTGTCCAGCTGGCTACGAGTTGTCCGCTGATGGTGTGTGCCGCGAAGTCTTCAAGTAGCCGGT<br>GTGACACCACTAATATTGGAATTAAATTTAGTAATTTATTTTTACGACATTAAAGTCTGATAAACTATGGAATCTGAGATA<br>CATAAAAAATAAGGCGTTTTATAAGCAGTTTTTTAATTGAAGTGTTTAAGTGTATTTTGTTCAG
MSTRG.11610	Uncharacterized protein LOC105118166	CAAAGCTTACACCAAGTCTGTCGTAACAAGTCTTTTTAATGGCAATCGGTCATTTTTACAACCATTTCTGCCAGCGCCAGC<br>GTCATTTACAAATAAGTTTAGAAAAACGAAGCCTCAGAATTGACACTATTGGCTACCAACACCCATGCATTTATAACTACA<br>AAATTTAAGTAGTAGGATTCATTATATTTCACAATATATCATCATCATCTCATCAGCCACTATATGTCCATTGCCGAACAC<br>TGCATTCACTGACTCCCTGCAATACATGAAATCATCTTCATCTAGTAGGGGGTCGTCCTACACTGCGTTTGCCCGTACAGG<br>GTCCTCACTCAAGAACCTTATATATACTCCCAAAATATATAATTATTTATTAATATTGTATATATACTGTAAACCCTAATAA<br>AACGCAGGCTATTATTATCGGGAGCCCTAACTATATTTCAAGAATTTGCTGGGAAAATCTACCTAACATAATGTTCGACGG<br>GATATCTGTGGACTATAAAGACTTTGTAAAGGATCTCAGAATATACCTTAACAGAACGCTATCCTGGAGCACCCAAATAA<br>CAGAGATTAGTAGGAGGGTTTTTGCGATTGTGGGCTCACTCAGAAGGTTACGTAATTTTTTACCTATGGCCACTAGAGTTG<br>CGCTTGCTCACTCCCTTGTCCTTCCTGTCCTTGACTATGCTGATACCTGCTACATTGATCTCACCGAAGACCAACTTAATAA<br>ACTTGAGCGTCTCCAAAACCTGTGTATTAGATTTATATTTGGACTTCGTAAATACGACCACATCTCTAATTTTCGCACTCAG<br>CTCAAGTGGCTCCCAATACGTCGACGCCGAGATTGGCATATATTATCTCTTCTTTATTCCTTTCTATTCAATCCTCTTACACC<br>TCACTATCTTAAAGAGCGATTTATATTTCTCGGTGAAGTGCAGAGTCACGCTTCGCGTACCTCAAATAGTCTCCGTTTAAA<br>AACCCCAGCACATACCTCCGTGTACTACACCAAATCCTTTTCCGTTCAGGCAGTGCGCCTATGGAACTCGCTGCCACCGTC<br>TATACGGAGTGCTCAATCCTTGGCTTCGTTTAAAGAACAAGTCAAATCACATTTTCTTTCACTGTAGTTACTCGTTTCCACT<br>CCTTTG
MSTRG.11639	Uncharacterized protein LOC100569569 isoform X1	AAATCCCCTTTAACTTACTAGTACGGCTAGCTTTTTATTGAAATTCAAAGCTTGTTTTAGCTTCATATCATATAGGGTTTATT<br>TTACGAAGTAATAAAAATATGGGGGATTCCTTGGTGCTCCTAGCGTTAATAATTATCACTTTCTAGGGAATTGCCGCAAGC<br>GTGACAATCGAAATATTTGACAAACGTTGACTCATTAATGTTTTTCACTTCTTTTAATTAAGACATAATATTTTAGTGCTTA<br>CGATTTCTTTCATATTCTCAACTTATTATTTACGTGAGACGTAAAGCCAACTAATTGGAACACAAGACCATAGTAAAAATA<br>CATAAAGGTTAAGTCTGTTCTTTTTTACCTCAACATAAACTGCTTGTTATATCTACTTTCTGGCATTTTTTGTACCCTACATG<br>AGTTCGTGAAAGAAACAGCCATGCCAAAACCTGCGCATCCTGTATGGACCAATTTTAATGTTATTGTCAAGACTGGTAACA<br>AGGACAAATGGGCTCAATGTAAGAAGTGCAAGAAAGAGCTTCAAGGTATCCCTGAGTGGCTTTGTTAACATATGAGTATT

		TGTCCAAGACCAGCAGCTTCTAGCTCAATTTCAAGTACAAGTTCATCAACAACAGTTTCTAGTACTCATGCTGCACAACAA<br>ACAACTTTAAGTAACGAATCAGTAGCAACAACTAAGCGCTCATTGTCCACTATCGACTTTTTAAAAAAATAAGTAACTGTT<br>ACATCAGAAACTCATGCGAAAGAAATTGATTTAGCGATTGCAAACCTTTTCTATGCCACGAATACGCCCTTTTTGCACGCC<br>GACGATATTAAATTTAAAGAAATGATCACAGCGCTTCGTCCCGGCTACAAATCTTCTACGTCTCATCAAATAGGAAGACGG<br>TCATCAAGAAGGAAGGACTTAGAAGATAGATATGATGACATTTTTCAAAAACGGTATTTATGTTTTTGGACGGTTGGTCTA<br>ATGTCCACAATGAGCCTATAGTTTGCTGTAGCCTCACGACACCTGAAGGGCAATCAATATTGGTTGGTACCATTGATACGT<br>CAGGCCACCCTGATACTGTGGAATATTTGAAAGGAAGTTGCCACGAATGCTATCACTAACAGTAATAAAAACTTTAACGTT<br>AATGTTAAGAGTTTCTTTACTGACAATGCCGCTAACGTTACGAAAATGAGACACGAACTATGTAAAGATAACGAAGACAT<br>AATTCAATATGGTTGCTGTTGCTGGAAATATAATTAAGGTAGTAAAATATTTTCGGAATAAACATATACCTGTAAGCAGGT<br>ATGGTGCTGCTGGTGGTAAAAAGTTGGTGATGCTCATTACCGTCAGATAGAATACCACGAGAGATTGTATTCGGTCTTACC<br>TTGACAATCGTGGAATACTGGTGCAAATATGCCAAAATAATAAGAATGAAATTGATAACGACATAAGTAAAATCGTACAT<br>GACACACAATTAACAAACAAAGCAATTCAACTGCTGTTACGTTTGGACCCAAATGCTAGAGCCTTAGACAGGACCCAAAG<br>GGATGGAACAACAATATCCGTGGCAGTAGAAATGTGGCACCGGCTTGAAAAGGAATTTGAAAATGAAATACCAGAAGTT<br>AAAAATTTATTCCAGCGCAGACATGATATGGCGTTTGGGCTCTGTTCATTACTTGGCCAATATATTGGACCACCGGTTTCTG<br>GGTAAACAAGCCAACCAACGATCAAAGAGACAAAGCTTTTGGTGAAATGCAGCCCGATCATATGCCTACTGTAATGGCAT<br>TAATGACACAATCATGTTAATGTTTCCCAAATATTTGTTTGGGTCACAATTTGTTAAACAGACCTCATCACCATTGGAATGA<br>TGGAAGCTCCTAGATATGAACTCAAACGATCCGCGTTGGCTCAACTGGGATGGCGAAGAAGCGTTTCAGGCACTCTGTAA<br>ACAATTACTGTCAGCAGTAGCGTCAACGGCAGATCTTGAAAGAATTGTCTCTACCTTCGGCCTGGTTCAATTAATGTTTCG<br>TAATCGATTAGAAACCCCAAAAAGCGCATAAATTGGTTCATATCTTCAAGACTTTTAACCAGTCTGCAAGGGGCGTCCATA<br>GTTTAGCTGCAACTCCGTCATACAATATGGACTGGATTTAGAATCCCAGTGTGAATGTCAATAGGGACATCATAGAAGAA<br>ACAGAAACAGAGGTCGATTGGGACTGGGAGCCAGATGATGATGTCCCACTCATAGATCCCGACGTTGAATGATTACTTTT<br>ATAATTACAAATTATTATTAAAATTTGTTGATTTATCTCGCAAAGAAATTAAGATTATTATTATAATAATAGGTACTTGTTT<br>TTTTAATTGATCATTAAAAAAATGTTTCTCAAATAAACACTAAGTAATTCTTTTGTTAGTTGTTAAAAGTTTTTTTAAATA<br>ATAAATGTAGTAATAATTTG
MSTRG.11654	Sodium- and chloride-dependent GABA transporter ine isoform X1	GCTTGAGTCTAGTTGACATCTGACAGATCATCAGCAACAGCTCCATTCCTCTATTTATAGTATCTATCATCTATTCCTAAAA<br>TTCCAGACATATGATAATATGGAAGACTAATCACCACTGTCTCTGACTTTAGCCTTTTCCACAACGGAACAAACGTACTAA<br>AAAAAATCATCTATTCCTTGTTTATATACTGTTTGATTTGTATGAATCTTTACTTGAGGGAGGTTTTATTAGGTAATCTAATT<br>TTCCAATTTACCACAAACCATTTTAAGTTCATAATAAATTTGTTTTTGTATTTTGTGTACTATTCTTTTGCGCTTCTAATTAA<br>GTCTTGTAGAAAGTCATGATACATTTAATGAATATGACTGTAACTGTAAATAAAAAAGAAATAGATAAATTAGCTATGGA<br>AGTAAACGTACATCCAACACATATGGTTAAATTTATAGAGGAATTTCAAAAACGAGCAAATACAACACTGACCAATTTAA<br>TTGAAGATCGTGGGGAATCGAATGAAGATATAGAAGACGAAGATAATGATGGTGATGAAGACGAGGAGGAAAATATACA<br>TGAGATCTCATTACGATCACGACTGCATACACTGGTATACAGAGCAAGGAGAATGAAGCGATAGTCCATCCTAGAACCTG<br>TGCCCACGCGGGATACACATGCTGCCGGTAGCTAGGTGGTGTGTAGTCCACTAGACTGGCCACCCACAGTGCTAGTAGTA<br>GGGCCGGTGTTGCCACTAGCCAGCAAAATCGGAAGTAGAATGACGGACGTCGACCTGTAATATTATATATTTAAATACAT<br>AAATACGAGT
MSTRG.11678	Hypothetical protein	GATAAAATTGTCAATTGGTTCATCGTAAGGAATTTACTACGAATTAACAACGATGGTTACCAGAAAGATTGCATGGAAGT<br>CGGCCTCACAGGGTATTTGCTAATCTTCTCATTGAGAAAATAAGAGATTGTTGAATACCAACACATCGCCCATGAAACCAA<br>TCCTGACAACGATCGCAACATATATAAAACCTGAAAAAGAAAAAGATATTAAGAGTTAATAATTGTTTTCAAGTATCAGA<br>TTTTATAGGAGCTATAGAATTTACAAACTAAATGTTCTATTATGCTTATGGAATTAAATGAAGGATATAGTAAGTAATATT

MSTRG.11697	Uncharacterized protein OBRU01_03818	TCTAAAGAAATCCCACCATGTCAAAGTTTGTAAACATGTTTGTGTCTGTTACTCAAACACGTTTCAACAGATGAACGGATT<br>TTAATGAAACTGGGTGGGCAGGTTATAAAACTAATATAAATAAATTCTTCTTTTTGATGTGATGA<br>AAAAAGATATTGCGTAATAGGTAACAAACAAACAAAAATAAAGGCTCTTATTTTTTAACATCAGTTAAATGAGGGCAGAA<br>AATTTTAGACTTTAAATGAAAATTTTTACAATTTTAAAATAGTATGATATAGTTCCATAAAGAAGTTATAACACAGTGACT<br>AGTATATAGTACATATTAATATATAAGCTGGGGAAAGATTTGCATCGTGTTTTGGTGGGAGCGCCGTCAGCGCCCATGCGG<br>GGCCGTCCAACGCTAGGACTGTGTGAGCCTCGCTAAATGCCACGCCGCCAGGTATCGCGCGAGACAAACACCATAGTGTT<br>TCGGCCTCACCCCCACCACCAGCAGAACACACCATAACCAATGAACCTGATAAAAAAATGTTCTCCATTGTAATTCACGA<br>GGAAGTCAAAAGTATTGTTAGCTATTACCATTATAGGCAACATAGCAATCAACAAAATCACTATTTTCAAATTATATTAGA<br>ATTCAACATGGCAGGAGGCAAGTTTACCACTTCAATATAATACTTTATTTGTTAATGCAATAGAAGAATAAAAATGTAAAA<br>TTTCTCCAGAGAACAAAAATATGTAGGTGACTATTTATTTTTTTCCATAAACAACAATACTTATATTGATGTTTTTATATAA<br>AAGGTAAAAATCTGTTTAATCAGTGACGCAAGAGGTATAATACTTTACTGAAGAGTAACAATGTGTGAGGTCATGAAATT<br>TTTTACATGAATAAATTGGTAAACAATAATTTAAGGAGAAGAATAAATGCAGAATAGTTGTGTATGTATAGTAAGTCTAA<br>AGAGAAGACTGGTGTATAAAAGTGGTTAATTACTACTTTTCGTCAATAGCGCAACCAGTATTGTGCTATTCTAAATACTAT<br>AAAATAATTTACAGTTCCATTCGCTTTTAACAATAGGTAAAGATCGAATGTATAAAACGCACGTCATAAATTAACTATTCT<br>AAATTAAGAATTATCAAATTTATTTTTACCATTTTCATAAACAGCACTGTGAACTTGTTTCGGTTTAAGTACTGAAGCGTTG<br>GGAGTGAATCCAGGCGGTAACCTCACGTGAAGAAGTGTCAGGTATTGCGGCCGTTGAGATGCGCCTTGACTCGCGCTGTA<br>ATTTCACAAAATGTCATATTATATAAGGAACTAACTACTAAATTACAAGTTAATAGACTTACTAGTTTAGGTCATGCATAC<br>AGAAATAAAAAATAATTTTAATTAAAAAAATTGTCAAATGATTTTGAAATTAAATTCAATTTTCTTTTTAACAAAAAGAAA<br>TTTATAATTAATATTATGCACTATAATTAATATCAGTGTCACGATGGATTCTGTGATATCCATAGTACTGTTAATGTTCATA<br>GGCAATTATTACCAGGAATACTATCATTGTATATCACTTCAATTGGTGGTTTGTATTCACACAATTAAACTTAAAAATGTA<br>AAGTAATAATAAAATTATTTAAAAGAGCAACACGCAAGTTTCTTGCTCGTTCTTCTTGTGATGAGACAGCTTTCCGAACGA<br>GCGGTAGATAAAAAAAAATGTAATGACTTTTCAAAAGTGCTTCAATGTAAGTTTATTTGAATAAATGATTTCTATTCTATT<br>CTATTCTTCAAAACAAAAAAAAATTTACTAGCCTTTGTGTACAAATACTTACTCCCCACTTCCAGTACTAAAATACAATCTT<br>GCACCAGTTTGTGTAACTGCCACCAGATTTAAGTGTTCAGATTCTGATTCATCCACAGCAGATATTGCTACAACTGGTTTG<br>AAATTATTGGCTTCTAATGTCCTAAAAAGTGATGATAGTTATAGGTTTAGTTAGTATTTATCATCATTATATGTATATAAGC<br>TAATACTAACAACAAAAAATGTGTATATTTTTGAAATATTAATTTGCATTACATGGCATTTTGTGTAATGTGGTAATAAAA<br>TATGAACTTTTCTCTCTTTTCACTCCCAGGATATTCTAAGAGGCTAAAAGTTCCCCTATTCTACAAGTTCAATACATAAGAG<br>TAAGAAAAGGAAATTGTGTAAATCTTTTTGCTACTGACATGACATAATCAGGCTAATATCCTCCTCTTATGACGAGGCACT<br>AGTGCCAAGCAGTGAGATGTTAAGAAGGTATTATTATGATGAGAACCCGGAACAAAATTTTAATACATTCTAAAAATAAT<br>AAAACCACTAACTGCAAAGATATTGATAGTATTATTGAAACTTGTTTGAAATTAAAATTATGACTGGTTTCACCTTTATGG<br>CTATATCAAAGATCTAAGATTATCTTCATTATTTACATCTCACGATTATACATTATCACGGCACATAAATATTACAGATCTT<br>TGAGAATGTCATAAAATGATAATCAGTTTGATATTATATAAAATATAAAAATTGTAAAATTATATTAATTTCTTTTACAAC<br>AATTATAAATAAAAATAAGTTTATTGTATTTTTTTTTATACCACTGAGGTGGCAAAAAAGCATACGGCCCACCTGATGGTA<br>AGTGGTTACCATAGCCTATGAACACCTGCAATACCAGAGGTATTACGCGTGTGTTGCAGAACCTGAAGAAACCTCTTTACC<br>TCCCATTTGAAGAACCC
MSTRG.11708	Histone-lysine N-methyltransferase SETMAR-like	AGTGATACCAAATAAGATTCAGGTTTTCGGGCTAAATCCAATAAAAAGGAACAATAATATGAAAACTTTGAAAATTTATC<br>TAAATTAGTACATCAGTAATCAAATTAATTATGAGTGAATAGAAAAGGTGCGAGGACCAGTTTCAATCGTTTTCTACCGTT<br>TTCCGCACTGATGTATCAGTCCCTAGCCCCTCCCCTTCAAGGTTGGGATAACTGTCAAAGATGATAGTTTTTACACGGCA<br>AACCGAATTTTGTAATAAATAAAATAACCATTTATTCTATTCCAACACAATATACATTCTGTGAATCTTTGTTGTTATTTAA

		ATTACAATAGTCGCTTTTTTTGCATAGGGCCTCTTTCTTTTCACCTAATTTCTATTTTGGCCTTTCCTCATCCACATTAATTAC<br>TTTTCCACCACTAGTGCGTTTGCCCACCAATTTTGCAACCAATGTATTAATTATGCTTGTAGAATACATCTGTACAAGATAA<br>GGAAAACTTGAAATAAAATCCTATATCAAGCCACCTGATCAACATGACAATAGTAGGCTCTAATATCATCATTCCCGAGTA<br>GTAGTCGTAGATGATATCCTTCTCTTAAGAGAGACCTGTGTCCAGGGGTGGGACATTAATTTGTAAAAATAGTAAAACCAA<br>ACGTTCACGGAGCTTTTAGTACTGCTGTGCTGTCAGATAAGAATCTAATGCCAATACCATTTATTCCTAGCTTATGTACAGC<br>TGCAGCAGCCTCGGTTGTTGCTACACATTCGCATTGGAATATTGAGCTGTGTATACCAAACGGTATGTGCATGTTTATATTA<br>AGGTCTAAGGAAAAGATTCCGGTTCCTGAACCATTCTCAGTTTTGGAGCCATTGGTGTATATGCGTAATTTGTGTAGTCCA<br>GATTGATCCTTTTCCTCTTCTTTTATTTGTACTTGAAAAAAAAAATATCTGATGCCCTCACCAAAATAAAAAACAGTTTTCA<br>GCTACAGGCAAAAACATATCTGAGAAATAATTTCAAATATACTAATGGTTTTATATTCACATAATAATTGTACACCTAGAA<br>AAATGGAAAAAGTATGTGGACAGAAACAAATGAGGACAAAAACAAAGGAAATTACAAGAGAGAAAGAGAGTTTATAAT<br>TTTTGGTTTGCAGCACATGTTTTACAAGAAGGAAGATGAAATAAATTTGAATATGTTTTAGAATACATTTTAGATATGTAG<br>GAGGGCTACACTGAAAATTTAATAAATAAAAAAAATATTCAAATTTTGAAAATGGAACTCTTTATGAATAATACTAAGTA<br>TTTATATACTTTATTCGTTTGTCATCTGTTTTACGGTTTGGCGGGAAATTAAAAATAGTCTGTGTAACATCCTGAAACAAAC<br>GGAAGAAAATTTTCATACGATGATTTTTTATAGCTTTTGGATGTCATCTAAGTCAACAAAAAGTTATGATAGACTACGATT<br>GACCTTTCACGATGAAATGCCCGTCTCGTGCCACTGTTTACAACTGGTTTAATGAGTTTAAACGTGATCGCACTAATCTTAC<br>CGATGATGTGCGTGAAGAACGGCCTTCTAAGGCGATGACTGAAGATAACATCAGTGTTGTGCGGCGTATGATAGAGACTG<br>ACAAAAGAGTGACCTATCAGCAGATTCCGGCAAGCTTAGGGGTTGGTAAGAGTCAAATGCACAAAATCCTCCATGAACAT<br>TTGGCGGTCAGGAAGCTTTGTGCCCGGTGGATACCTCATAATTTGACCGAGGCTCAAAAACTCCGTTGTGGTGAATGGTGC<br>CGTGAAATGCATGAAAGATTTAATGGAGGTGACTCGAATTCTGTGTTTGACATAGTTACGAGAAACGAAAGCTGGATTTA<br>CTGTTATGATCCCGAAACTAAAAGACAGTCAGCTCAACGGGTGTTTCCTACTGAGGAGTTGCCAAGTAAAGTGAAGAGAG<br>GTCGAGGCGTGGGAAAAAAGATGGTGGCCTCGTTCTTTGGAAAGACAGGTCATTACGCGACAATTGTTTTAGAGGATTGA<br>AAAAACAGTTACTGCAGAGTGGTATAGTAACAATTGTTTGTCACATGTTTTGGAAATAGTGCGTGAGAAACTACCTCGCAG<br>TAGAATCCTCCTTCACCACGACAATGCTTTGTCACACACCACCAGGCAAAAGATCGATTATTTGGCGATCTCAGACTTAGA<br>ATTACTGGGTCATCGACCTTATAGCCCTGACCTCGCACCTTGTGATTTTTATATATTCCGAAAAATAAAAGAAAAACTTTG<br>AGAACAACAATTTATGGACATCGAGGAAGCTGTGGCTATGTTTCAGAAGGCAATCAAAGAGATCCCTAAGGACAAGTGGG<br>CAAAATGCTTTTCTCAGTGGTTCTATCGGATGCAGCGATGTATTGACATGAATAGACACTATTTTGAAAAGATAAAAGAAA<br>GGTAAAAACTAATTTAATCAGATTTTGAATTTTTGAAGACTTTCAGTTTGACCCTCATACCATGGTCATACATTTCCAAAAT<br>ATCACCTCCTTTAATGATAATTTAAAATGTGTCATATATTTATGTCTTTGTTTATTTTTTTAGTCCAATAGAAGCTTAAAAAT<br>ATAGTAATTGTCAGGAT
MSTRG.11743	Transmembrane protein 132B isoform X3	CAGAATGAAATTTACGGTGTGGATTTAAATTCATTATTTAAATACACTGCGAACGTTAACTTACCTTTGATCTGCGATAATC<br>GATTATCGTCCACGTCAATTTCTAATGGATTCTCCGGCATCCACACAGTGAAGCGAGCTAAGCCAGTGTAAGTGCCGTATT<br>TGACAATCACCGAGGCATTCGACGAGCCTCGTATCTCGGATCCATCCACATAAACTGAACTGCATGATGATGATACCTAGA<br>GTCAAGGGGCAGATAAGCAGTATTCATCCGTCAGTCAGCGATATGAAGAGGTTTAAGGTCGATAGAGCGTCCAGCCATGC<br>TCTCTTAATAGGTCTACTAATGATTGATAAATCAATTACTATTTGCAGGGTGTAACAGA
MSTRG.11781	Ras-related protein Rab-37	GGGTTACACCAGGGACAAGTCCCTACACGGTTGAACATTGCACACAGATATGAAAAATATGCGGGATATTCAACCCTAAA<br>GCTACGCTGCGTTTCTCAATGTGTCCTACATTTACCGATCTGCGAGATAACCTCAAAATTGTGAGTTTTTGACCTGTACAGA<br>TCATGTTATTGAGTCTTAAATGTTTCTCTGACGTTTTAAGCTGTATAATATGTTAATTTAGAGGTAGCTTATAGTGGATATT<br>GTGTTATTATCGTTAAGTGACGAGTGTACGAGCGTGTGTCGAGTTTCGGGCGGCATTCGTTTTGGATTACGGATTTTCGTCA<br>CTCATATCTCGTACACAGTGATGTGGTACGTACAATTGCAGGCAATAGTTGTTGATTTATGATAGTTTGTGGTTATAATTAA

		TGTGTGGATGTGAGGGAGCCGTCGAGAAATGAGCATGACAACGGACGACAGTATGAGCGATGACGTGTTCGAGGATGATT<br>CGTTGCGTGCGCCAGCGCAGCGAAGCACGCCCTCTCCAACTGGTTACAGAGATTACCAAGCTCCCGCGGAGACCCCAGCG<br>CGTACGCATGTAGACGACGACGTGCCTATACACAAG
MSTRG.1<br>1861	Uncharacterized<br>protein<br>LOC106140923	AGGCGTCAGCAGCTCACTATCAAAGTCAACTGTCAGTGTTAAGTGCGGAGAACGATCGTCTTCGCGGGCAACTGTCGGCT<br>CTGTCGGCTGGGCTGGGCGGCGGGGAACACGAGCGACGGCTCGACGATGTCGCGCAACAAGTCGTGCGGGCCCTGCTCTC<br>GCAGAAGAGCGTACGCGAAGAGTTGGGATGCGCCCGCGCAAGGGTACGCGAGCTCGAAGCGCAAAATCGAGCGCTGAGT<br>GCCCTCCTGGTGCGGCAGCTACGCCCGCAGCCGCGGCCCTCGCCCGCCACCCCCCTCACACCACATACACCTCACACGCCA<br>AAAGACTNNN
MSTRG.1<br>1862	Hypothetical<br>protein<br>RR46_02912	TTGCTAGTACTTGTCATGCTTGGCAGGGTTGGAAAACCCAGTTAGGCTTTTTGAGATGTTTTAAGAGGGACGCTGCTGCCC<br>TCGCCCTCCCTTAGTCGCTCTTACGACACTCACGGAAAAGGACTGTCGGATGTATCGCTCATTGCTGGCCAGACTCCGAAC<br>AGCTGGACAGACATGGGGACACTTGAGTCGGCTCAGGAGGAAGGTCCAACGGTGGTTAAAGGGAATGGTGCCGCGCAAG<br>AAGAAGCCACACCTAAAAGCCACTTCGGAGTTCTTCATATAGGTAGCTTGAACATGAAGTCCAAGTCATGTTACGACAAA<br>TGTTTGGAGTTAACGTTTGTGATGTTGGGCGTCGTTACTATTTTCAAATGCTTTGGAGTGATCGTAGTGACAATATCGACCG<br>CCATCGCGACCAAGTTGTACAGCAGCCAGCAATCAGGGCGGCTGGTCGCCATGATTACGGTGGCGGTGACGGCCGCCATT<br>GCTTTGTCGGTGGTCATTTACGCCATCGTCGGGGTCTTTAGGAAGTACAAGAAGCCACTGCATGCGACCACTATAGTTCTC<br>ACGATACTCGCGATTCTGCAAGCGATCATCAGCGGTGTGTCAGTGAAGGTGACGACAGAGGATGAGCAGGTCTTGGACGG<br>GTCTCTCTCCGAGTCGTTTCAACTTGCTATGGAGGACAATCCGAAACATATAAAACTGTGGGACACCACTAACCACGACCT<br>TAAATGCTGCGGTTTATACGGCGCACAGGATTATAGAACTGTGCAGGCGAGTTTCTTCCCTCCGGAGGTGCCAATCTCCTG<br>CTGCCCCACATATGACCCCACCAGATCCGAGCTGGTACAAGAGAGAGAACGGGAGATCTGCAAAGCTCGAAGAACGTACT<br>ACACAACCGGCTGTAGACAACCTGTATTGGACATGTTTAGGGAAACGTCTACAGTCGTTTTGATCGTCGTCGTAATACTTA<br>TTGTACTTGAGATTATTCTAGCAATATTAGGAGGAATGTTATACAAAAAACAAAAGGAAAGGAGCCGAAGAAATGCTGAC<br>AATATCGAAGAACCGGCACCGAACACTTGATTTCGAGAAAGATCTTATGTACCTACCGTAAAGAAATTGTTGATGTCTGA<br>ATTTTTTAAAAATGTTATAGCTTACCTATAAACTTACAAAACAATATAAAAGAAAAGGGTCAAGTGTCCGCATAATTTTTT<br>TTTGTTGTATAAAATGAAATAGGTTGTTTTCAAATTAAATGAAAATCTTATAAATATGGACTTTAGGTTGGTGCAACTAATT<br>AAGATTGTTTTTGGTAATATTAATAAATTATTGTAAATTATCTATATGGTAACTTGTTACCCATTTAATTGAACAAAGTAAA<br>ATTAAATCCTTACTTGTTATATATAATAATGATTAAACCAAAATCACAACGAATTAAATAACATCTATCTATTTTTAATTAA<br>AATAATTTGATAAATAAATTGAAATTAGACATTTATGAATTAAGTAGATACAGTTGGCGCCACTAGCGCAAAAACTAGTT<br>AGTTTTTGGCATGGGATTTGAACAAAAAAGTGTCACTTATATTTTAGTATGTATATTGAGTAATAAAAAGATACTATAGTA<br>TTATATTTAAATGTAAATCTAATTAAGCTAACAAATAATGTAATTAAATTAATATTTGACAATTGAGATCGTAGCAAGTAC<br>AGCGCCAACTAGTGATAATCAACTGAATTTCCGTTGTAGATTTTTATCGCAAGATCTCAGCGTGTGTTATACACGTTAAGA<br>TTTTATCTTGACTGATGTTATTAAATATGCCTAATTGCTTAATTAAAGAATGGATTTTATTTTTAATTCGATTAATAAAAAT<br>GATAAGACTGGCATTAATTTTTTAATCATTACTTTAGAGCCATCTATTAAACTTTTATGATACTATAATAAAATTTAGTTAC<br>TGCTATTCGCCACTTATAGTTATACACATAACTTAATAGATGGCGCTGTTTATATTTTATTTTAAAACCCCTATACAT
MSTRG.1<br>1869	6-pyruvoyl<br>tetrahydrobiopteri<br>n synthase	GACAGTGACAGCGTCAAACACAAAAATTTTGACTTTGACTGACATACCGTTTAATTTAATTTGACTTTCTTTCACCTTACAA<br>AAGTTTACAAAACTTATTATTATATAGTAGATATTCTTTTACCTTTTTATTTGGTGATCACAACTTATAAGTACTAGAACGA<br>TTATAAGTGAATAAAATAGAAATGGCATTACCAATTGTTTCCATAACCAGACGTGAAACCTTTAGTTCTTGCCATAGATTG<br>CATAGTCCATTTTTAAGTGATGAAGAAAACAAAAAATTGTATGGCAAATGCAACAATCCTAATGGACATGGCCATAATTA<br>TGTTGGTGAGTTATTGAATATTTTT
MSTRG.1	Alkaline	CATTGGAGAACCACGAGGCCGAGCAGTATTCGTCATGCGCTCAGTTTTATTGGTTTGGTGCTTCCTCTTTACTGTGCGGTGT

1888	phosphatase	TCCCTGAGAGCTGATCAACAATATTGGAAAGATTTAGCAAAAAAGGAGTTAAAAGAAGCACTAGAAGTCAAATGGAACC<br>TAGAAAGAGCAAAAAATGTTATTATATTCATTGGAGATGGAATGGGACCAAATACAGTGACCGCGACGAGAATATATAAA<br>GGCGGTGAAAGTCATCG
MSTRG.1<br>1893	Structural maintenance of chromosomes protein 5-like	GGTAGTGTAAAAATTAAAAGATGTCTTTAAATAGTTATTCCATTTTCAGCTACCTTGGTTTCTACACGTACCTTATAGAAAC<br>AATAACAGCGCCGGACGCGATCATACGCTACCTGTGCAAGCAGTACTCAATACACAGGATACCCATTGGCAACGATCACA<br>CGTACAAGAACAGCGGCAAGGTGCCGAATGACATCACATACTTTTATACTGCGAACCACCGCTTCACTGTCCGCGTGTCCG<br>CGTACAGCGGCGCCAAGTCCAGCTCCACCATCGAGATCCGTCCCGCGAAGCTGCTCGCCAATTCACTCGACGTCGACCAG<br>CTCACCAACTACAACACACAG
MSTRG.1<br>1896	Uncharacterized protein LOC106710199	TGTAATACCACAAAAGAAGTAATAACAGGCATTACGCGCGTGTTGCCGACCTTGAAGGGACCTCTTTATCCCTTTTTGAA<br>GAAACCCATGCTGTAGTTTTTTTGGAAAAATCTCGACAGGGAGTTTATTCGATAACCTGAGTGTTCGTGGGAGAAAACATC<br>TCTGAAACCACGCTGTACGCGATCATCTAGGTTGTAAGATATGACGGTAACCTTCCTATCGATGGCGAGCTGTGTAATGCT<br>AGAAGGTGGCTATGGGTATCATATTGAACCATTCCTCCTGAGCATATTCATTTATGTACAGCAAGGACTGATTATTATTAA<br>TAATTTTTTTTTAATAAATATTATCAACTCTGTAAATTATTAAAAGTCAATATTTTCTCTATGCTATGATTTATTTTTTTATC<br>AACAGTTCAGGTGTGATAAGACAGAATTGAAAGAGTTATTGGAGTTGGCGGGACATGTCGTCTCCTGCACCGTTGTTCACG<br>CTCATTCCAGATATGCCAATGTTATGTATAGTCACCCCTTGGAAGCCGTACAGGCTATATCAATGTTAAATGACCGGAAAC<br>TCTATGGTCTCAATTTGAAAATTACTATGATAAAATATCCAAATAACACAGTTCTCCTACCAAGAGGTCTTACTGATATAG<br>GCAATGGTTTTGGACTTAATGGCAAACCTTTAAGGGACGTTGCGAGAGAATATGAAAAGTTTGTAAATAAACAACCTTGTT<br>TGATAAATAAATCTTTATTCAAGTCCCTTGATTCGAATTGTGGACAAAATGACAATGACAATCGACATGTTAAAAGAGAA<br>AAACGTTGCGATAAAAAGGAAATAAAAGAGGAAGGTAATAATTGTGAAGTATCAACAAAAGCGATGGAATTTGATAATA<br>TAATAGCCTTCATAATTAAAAATATGGATGATATAGTTGACAGTATCAATATGGACTGTGGTAGTGATATTGCAGAGAAA<br>AAAAGCATCAACAAGGAAAGTAAACGTGAAGATAGTAATTCTATTAAAGAATCTGATAAAGAAGCAACCACCCAAGGCA<br>AGGATCAAAGTATTACAGACGATCAAAAAGACAAAGTAAATGTAGCCAAAGCAATACCTCATAAATCAGATAATAATCTA<br>TCAAACTTGTTACCATCAATGTCTACAAAACCTGTTGATAGTCGTTATGAAGAGACACAGAATACGCAGAACACTTTTACG<br>GCGTCACTACAGCCACCGACACAAGGAATTAGTCCCATAGGAGAGAAGGTAGAAGATGTTAAGTCACATGGTTCTATTAG<br>TACCATGGCTGAAACCAGTGGGTCTATGGGTAGATCTAATTGGCCTATGAGTGGGCCTGCTGGACCAATAGGTCTGTTTGG<br>TCAAATAAATGGTCCAGCATGGCAGATGAGTTCCAATAGTTCCATTGGTGGTTCCAGTTGGTCCACAGGTGGTGCAAGTGG<br>TTTAATGGGTGGTCCTACGGGTAGT
MSTRG.1<br>1901	RPII140-upstream gene protein	TAAAGCTTACTGGCATCACAATGGATGATATTCGAAAGTCGTTGCATAAAATTAAAGAAGCCAGAGAGGATCAATATAAC<br>CAAGCATTAGAGAAATCAGCTAAAATAAAGAATGACGATCTGACAAGGTATCATCAAGCTCTAATAGAAGAAAAAGGAC<br>CACTGAAAATAGAAGAATTAAATTAGAAAATAGCATGTATATAAATAAGTTAAAATTTGTAAATAAGAATATATTAATAT<br>ATGTAAAAATACTTGATTTTCTTAATAAACTGCCCCAAAAAATCTCAATTAAATAAAATCATTAGATAAATACATAAATAA<br>ACAGAAATGTATTGCTTTACATCAGCTTACAAAAATGTACTAGGTAAATAGACAAAATATTGTCTCAGCTTTATCTGAAGT<br>TAGCATATCACGACGCTGGTTTTCATCGTATACATTTGTTATAAGTAGGATAATATAAGAGGCGAGTATTAATAGGAGTAA<br>ATCTGAATTGCTGACAACTGTCACATTTGTAATATATGCACCCAAATCACAGATGAAAAAAATATTGTAATTAATTGGTTG<br>CAAAAATTTACTTATATGGAGATTGAAAATCTTATTGTGGTGATAAAATAACTGGATCATGTATATATAAACACACACGCA<br>CTCACACACGTACGCATTTGTGCACCTACTCACACGCACTCGTTCGTCTTTCACTTATTCTCTCACCCATGCTAGTACATAC<br>ACGTAGATTATAATTTTTTTCTAATGTTATTTATTAACTTGTATACTCTTATCTTCATAAATCAAATAAAATAGATGTCTTTG<br>AGTTTGGAGCCTGGTGATCTGTAAGACGAGTTCCCCTAGTATCGACACCAGTCCCTCAAAAAGATTGTTTTAAATTGTAAC<br>CTATATAGCTGTAAATTTTATTATGTTGTTATTTTGTGAGAG

MSTRG.11902	AT-rich interactive domain-containing protein 5B-like	GATACCGTCATTATTAAGGTGATATATGTTGATTGCAGAAGAGCAAACCGGTGGGTCGCAGCTCGCTGCGGGCCGTACGCGTGAAGCCAGCGCGGCCCAACCACGCGCCCGCAAACACACCGCCCATTCCCCCCCTCCGGCCGGAGTCGGCGTCGTCATCGCCTCAGCCGGTGGTAACCACCTCCACAGTCGGGCCGCCCATCACCAACTTCGGGATCCATCATCCGCCAGCGCCTATTAGCGATGATGAAATTGTGGAGGTCCCATACAAGCCGAAGACACCAGAGATAATAGATTTAGACGAGTACCCGGAGAGTCCGCAAGCCGTCAAGAAGAAGAAACTGGATATCCTCAAAGAGAGGGGTCTGGAGGTAACCGCCATACCGACGCCCGCCTGGCCCGGCCCCGTGCCCCTGACCACCATCAACCCCATCCTG
MSTRG.11921	Protein lingerer-like	GCAGGTCCCTGCGGCGTTACCGCCCGGCTACGCGTACTTCTACCAGCCACCACCTACCACATACCAGTATGGTGTATATCCCACGTACGGCGGCGGGTCGAACGTGGGCGGCGTGGGCGGCGTGGGGGGCGTGGGCGGCGTGGGCGGCGTGGGCGGCGTGTCGGGCGCGGGCGGCAAGGTGTCGTCGTACGCGGCGCAGCAGCAGGCGCCGTACGAGGCGGCCGACTCGTACAAGGCGGGCGCGGGCGCCGCGTACGCCGCGTCCAAGCCGGCCGCCGCGCCCGCCGACCTGGCCGGCGTCTACGGCAAGGGACACGTCGCGCTCTCCAAGGTCAACAGTTACGAGAAGGCGACGTTCCACAGCGGGACGCCGCCGCCGTTCGGCGCCGGCTCGCACCTGTACATCCCCGCGCCGCCGCACCACCACCACCACCACGCGCCGCAGCACCAGGT
MSTRG.11946	UDP-glycosyltransferase UGT40Q1	CAAATAATAATCAGACGACACGGTCGGACAACGCGTTATACACAAACTTTTGACCACGTTAGCTAACAGTGACTGTGATCAATTTAATAAGATCAAGAAAAAATAAATAAATTGTTGATATAAAAAAAAATGTTTAGGACCCTTATTTTGTTTTCGTTACTGAGTTACGTGTGTGCATACAAAATACTTGTAGTATTCCCTTACCCGGGCAAAAGTCATACAATTCTTGGGAATGGATATGTCAGGCACTTGCTCAAAGCTGGACATGAGGTCACATATATAACTGCTATGCCCATAAAAAATCCACACAAGAATCTGCGACAGATTGATGTATCAAGTAACATAGAGCTGTTTACGTTCT
MSTRG.11949	Protein ERGIC-53	TGATAAATTCCATGCGTATTTTGCAGATGCCGGCGCTGCCGATCGGGCGGCACGACTGGGACGCGCTCGTCGCCAACAATCAGATCATGATTAACACCATCGCCGAACTCAAAGGGTTCATTATCGAGGTGGCGCGTAAGTCAGACTCGCTGCTAACCGCCGGCACTGGCGGCGGCGGCGGCACCGCCATCAACCCACAGTTCCTCAACGAGATGCGAGACTCCATCAACCAGGTCAAGCAGACCGTCGCGGGAGTCGCACAAAGG
MSTRG.11964	Leucine-zipper-like transcriptional regulator 1	CTCAACAATTTTGCGACGTGACACTACTGGTTGGTGACGAACAAACTCCCATACTGGCCCACCAGGCGATGTTGGCCGCACGCTCGCAGTATCTCCGGGCTAAGATCAAGGAGGCTCGTGAAGAGTTGTCCAAACGTATCGCATCTGGTGAGGAGAAAGCATCCGAAGTATACTCGTATAAGGCCAAACCTCAACTAACAGTTAAACTACCAGAGGCCACGCCCGAAGCTTTCCAGATGGTGCTCAATTATATATATACTGACAGGATAGATCCTACAGAAAAAGATGAGGACCCAGCTTCACCTGCGACGATACTGCTGGTGATGGAGGTGCTGCGGCTGGCGCTGCGGCTGAGCATCCCGCGGCTGCGCGGGCTGTGCG
MSTRG.11972	CD2-associated protein isoform X1	TATATCCCATTCTCAACAAGTACTCAGAAAAAACCGCATCCGTCAAAGAACTAACTACGTCTAGGATAAGTTTGCACAAGGACAACATAACTAATAAAGAAAGTAGTACGCAGAGTACAATTACACACACAAACAAGAATGAGTCAGAAAAAGTGATTCACAGTGAGACTACCACAGATGGACCGCTAGATGGACAAATTAAAAAACCCCCCGTGCCGACGAAGAAAAGCCCCACGATGTCATCCGGTGTCGGCGGTTTGCTCAGCGGGCTTAAAAGCAAAATGTTGTCGTCGACTGATAA
MSTRG.11973	Mannosyl-oligosaccharide glucosidase	AGGAAAAGGACGTGAACTTTGTACCATTTCAAATGCTGGTCGAGACCCCGTTCGTGCTTGACGTTGTGTACGCGACGGAAGATCTGCCAGCGCCCCCGTTGAAGGGGGACGAATATACTAGGGTGTTAGAACAGAAGAAGAGCTCGTTCGACGCCGAGTTCGAGGACAAGTTCCAATTGGTCGCGAAAGGATACTCGGAAGAGGACATAGCCATCGCTAGAGCGGCCATGTCCAATATGATTGGAGGCATCGGTTACTTTTACGGCGCTGGTCGCGTCCGATCGCAGTATACCAGGGAACCAGTACCGTACTGGAGGGCACCGCTGTATACTGCTGTACCTAGCAGATCGTTCTTCCCTCGCGGGTTCCTCTGGGACGAGGGTTTCCACGGGTTGCTGATCGGGAGCTGGTCTCCTGAGATACAAATGGACATTGCGGCACATTGGCTCGACCTCATCAACGTGGAAGGATGGATACCTCGCGAACAGATACTAGG
MSTRG.11976	Cathepsin O1-like protease	ACGGCTGTCGAGGTGGAAAACCGTCAACTGCTCTTGAGTATTTGAAAAAGAGTCGCCTGCCAATCGTCACTGAGGAACAGTATCCTCTGTTGCTGGTAGACCAAACCTGCAAAATCCGTGGCCGACCGAACGGCGTACTGGTCACAGATTACACGGATTTG

		TGTAACGCACAAGAAGAGGAGATGCTTCGTCTACTCGCAGAGCATGGCACGCTCATAGCGGTTGTGAACGCCAAACTTTG<br>GCAGCACTATGTTGGCGGAGTGATACATAAGGGCTGTCTGT
MSTRG.11981	Uncharacterized protein LOC110371971	ATGGCCTACACAACCGTGGTATCCATTATTCAGAAACCTCCTTGTCTCTGAATTAATAACTTTTTACCCTGAGGATAATGTT<br>TTATTATTTAATTCCAGTTATCAACGTTTAAGCTCAACGCTTACCCTGGTTGCAGGAGTGTTATCAGGAAAGCATTATCAAG<br>GAGAAGTTTGCCACCAGCCTCTATAGATATAATGTTAGCATCATTATCTGATAGCTCAATTAGACAATATGATACTTGTTT<br>AAAAAAGTGGTGGAATTTTTGTAAAANNNNNNNNNTTAATTTCTATGAAGCATCGATACCTATCATAATTTCTTTTCTTAC<br>TGAGATATTTAATGACGGTTATCAATATGGAACATTAAATTCAATAAGGTCGGCGTTATCCTTGCTTTTAGGCCCTATTTTA<br>TCAAAGGATGATAGGATAGAAAGATTTTTCAAGGGAGTGTATCGATTGAGACCACCTCTTCCGAAGTATAACGCTACATG<br>GGATACCAATGTTGTTTTAGATCATTTAAGCAGATGGTGGCCCAATGAAGAATTGTCATTAGATAAATTAACAAAGAAAA<br>CCATTACTTTAATAGCATTAACGACAGCTCATAGAATACAAACTCTTTCCAAAATTAATATAAAGAATATAGAATTTTTCA<br>ATGACCATATAGTGATTAAAATTCCTGACTTCCTTAAGACGTCACGAAGAGGTTCTAAGCAGCCGATTATTATTCTTCCTTT<br>CTTCTTAGAAAAGTCTGCTATCTGTCCAGCAAAAACTTTAGATTGTTATCTAAATAGAACAATTCCATTAAGGGATTCTGA<br>TAATCTTTTCATTGGTGTCTGTCGACCTCACAAAGCGGTAGGCAAACAAACTCTTAGCCGATGGGTGAAACAGACATTAGA<br>CGAATGTGGACTGGACGTATCTGTATTCTCTGCGCACNNNNNN
MSTRG.11991	Rab3 GTPase-activating protein regulatory subunit	CAATAGCATATGTCACAACGTAGTTAGCCTACGTTTTTGTTTACCTTTTTAATTTTTTATATCTATCTCAATACTAATTATGG<br>TGGGTGTATTCTAAAAAAAAAGAAGAATAACAACAATGAATATTTCAAATCTTTGCTGCTAAATCTTTTCTCCATAAAATA<br>ATGTCGTGTGAAGTGGAACAGATCGCTGACTGCGGTGATATTTCAAGCATAGCTCGAGCTCTCTTTGTACCAGAAAAGGA<br>AGATACAAATTGGCTATCTCAATGTTCAGTATCATTATCTTCCTGTGGGAACCTACTTGCCATTGGTAACAAAAACCGTCT<br>ATGCCTGCTCACTTCACAGTGGATAAGTTCAACAGACAGCAACACATTTTTAATTGTATGGAGTGGAACACTACCTGCAGA<br>CATAACTGTAGTTTTAGCATTACCCATCTGTCCATCACAACAATCTTCACAGAATGGCCCAGACTGGTTTTGTATCATAGTC<br>GGTTTTAAAAATGGCAGTGTCGGTTTCTACACTAACACTGGCCATTTGTTGCTACTAGAAAAACTTGATGACAAACCTGTT<br>ATGAGAATCTCATGCCACACTGGAACATATGGAACATTACCAGATGATGTACATATTCTGTTCCAAAATTGCGAGTGTATT<br>ATTACTGGAGCCAGCTTATTTCAGACATTAAAAAATGCTAAAGCTCAATTAGCTAGAGGTAA
MSTRG.11997	Uncharacterized protein LOC106139637	TAATCTAAATCCGCTATTTTGGATAGAGAGAACTATATCTTTGTTAAAAGAAAGACGTGATGCTACAAAGTCCTTTAATTT<br>ATTCATTACAATTAGGTGTTGCCAAGCCTGCTTCACGAACCGTAATAGCTGGATGGATCAGAACGCTGTTAAAGGAAGCC<br>GATATAGCAGCTACTCCAGGGAGCGTTCGATCAGCTGTAGCCTCTAAAAGTTGGCTAGAGAATCATTCTGTTGAAGATATT<br>CTAGCTCGTGGGAATTGGCAATCTGTTAATACGTTCCAACAGTTTTATAGAAGAGAAGTTATTAGGAGCGATAATTCTGAT<br>ACCATTACTCGATTGTTTAATCCAATTACTTGATTTATCATGGGTTTCGTTTGGTACAGACTAATAATTAATAAAATCATTA<br>TAATTTAGTTATACTTAATTTTGTTAGCACAAGTACATGAAGAACATAATAAATATTACATATATTTATTATGTTACCTAAT<br>TATATATAAAATAAAGACGAGTTTATTTTTGCTGTAATCTACTCATTATCACATTGGCGTTTCAATTACTTATTATTCAAAG<br>TATCTAATTATATAGAGCAAGCAGGTATGGCATTGCATATACACCCCTAGTTTCTATTTCGATAACTTAATGCTTCACAAGT<br>CCTGAGCTTTAAGCCCACCTTGTACCTTTGTGTATTAAAATTTAATTAAACAAAGAAGTAGTGGCGTCACCAGGCAATAAC<br>AAACAGGTCTCAATGTATAAAGCTTCCAGTGA
MSTRG.12000	Heterogeneous nuclear ribonucleoprotein 27C isoform X2	GGTTTTGGCTTCTTGTCTTTCGAAGACGAGATCTCCGTAGAGCGTGTGACCCAGGAGCATTTCATTAACCTCAATGGAAAA<br>CAGGTGGAGATCAAACGCGCCGAGCCTCGCGATGGATCCGGCAAACTGGGCGCTGGAGGCGGCGGCATGGGCGGTGGTG<br>GTATGAGCGCGCCCGGCGACGCGCCGCAGGCCGGCCAGTGGG
MSTRG.12026	Transmembrane protein 189	GTTAAATCTTTACAAATCTTTGTAATCTCAATTGCCAATAGTGTTTACGTGTTTTGGGAATAACTATGGGAAGGTCAAGTGT<br>TCCGAATTGCGGACAAAGGACAAAAAGGAGCTTTTCAAGCAATTGGAAGAGCTCAAAACAGAACTGACTAATCTCCGTGT

		TGCTAAAGTTACTGGGGGTGTGGCTTCAAAATTATCAAAAATATTCTAAAAGTTTGTATCCTGCTTCCAAAATTCATCTCTA<br>CTCCCCATGGACAAATTTATTAGAAGTCCAAGTAAGTTAAGGAAGCTATGGTGACTTGTAGCCATTAATT
MSTRG.12030	Ionotropic receptor 4	GCGAGCGATAGAGCGGACATGTCATACATGGCTCATCCTCACTGTATCGTGCTATGTTGACGCGACAGCGTGTCATTACGC<br>AACTATGATTATTATTATAATATCAAGTATAAAATTCAAATTTTACTATATTTTTGTCAACGACTATTAACAGCACAATGCG<br>ATGGCTACTGGTATAGACCTCATAATCTCGACAGTATGCAACGCCACATTTTGCGAACCGATCTATGACAATCCAATTTTA<br>GAAATCACAAAGAAAGAAACCGATTCGCGAAATTTAATTAATTTCATCAACGGAAAGCATCTGAAAGTCGCAACCTACGA<br>TAATGTTCCTCTGAGCTGGACGGAAAATGGACTAAATGGTTCTCTCGTCGGTCGAGGGGTAGCGTTCGTAATCCTCGACAT<br>ATTGCGCCAAAAGTTTAATTTCACCTATGAGGTAGTAATACCGGAGAGAAACTATGTGTTGGGAGGAGATAAACCTGAAG<br>ACTCCTTAATTGGGCTTGCTAACAGTAGTGTAAGTATAGTGGGTAAATTGGTATGATGTTAAGGTCTACATGTTATAAATT<br>AAAACTATAATATGTAGACTTTAACTAAAAAAGTAACTTTAACTAAACTAGCT
MSTRG.12046	E3 ubiquitin-protein ligase UBR4	AGATAATGGAACGTATACTATCAGTAGCTGCGAGCGAGAGTCTCGAATCGTTCCTACAATTTTCGCTGACCTTTGGTGGAC<br>CGGAATACGTGCAAGCTCTTCTTAACTGCACAGAATGTCCAGGTATTCGCAACAACTCAGTTGCGCTGGGCCACCTGACCC<br>GCGTGCTTGCCGCCCTCGTATACGGCAACGATCTCAAGATGGCGATGCTTGTGGACCACTTTAAACCGGTATTAGACTTCG<br>ATCGTCTCGACTCAGAGCAATGGACCGAAGAGGAATTCCGCATGGAGCTGTTCTGTGTACTGTGCGCTAACATAGAGAGG<br>AATTCCATTGGTGGAACATTGAAGGATTACTTGATATCGTTGGGAGTTGTGCGCGACGCTTTAGAATATATAGTGAAACAC<br>GCGCCGTGCGTGAAGCCGA
MSTRG.12064	Hemocytin	GTGACAGTACATGCGTGGGTGGTCGATGGGAGTGCAAGCCGGCAACTGAAGACGACATCCAGAACTACCCGCCCGCCGA<br>AGACCTGAAGAGCAACTGCAGTGCTACCGACCACATGGAATTCACCACATGCGTCATCGCTGAACCTCTCACATGCAAGA<br>ACATGCACTTGCCCCCGTCGAAGACCAGCGAGGAGTGCAGGCCAGGATGCCAGTGCAAGAAGGGATACGTGCTGGACAC<br>TAGCTCGAAGAAGTGTGTGCTGAGCACGCAGTGTCCGTGCCACCACGGCGGCCGTAGCTACCCCGACGGGCACGTCATGC<br>AGGAGGAGTGCAACACTTGTGAATGCAAGAACGGCAACTGGTCGTGCACGACGCGGCGGTGCGCGGGCGTGTGCGGCGC<br>GTGGGGCGACTCGCACTTGACCACGTTCGACGGCGCCGACTACGACTTCGAGGGCGTCTGCACGTACCTGCTTGCCAAGG<br>GCGCTATGGACTCTAACGATGGCTTCGACGTCGAGATACAGAACGTCCCATGTGGCACTACAGGAGCGACATGTTCCAAG<br>TCGATCACCCTTAAAGTGGGAAGTGGGGATAACCAAGAGGTGGTCTCGCTTACCAGGAACGCGCCTCTACCTGATGTCTCT<br>AAATTGAAAAGGATCACGCTGCGCGAGGGCGGCGCGTACGTGTTCGTAGAGGCGGCGTCGCTGGGCGTGCGCGTGCAGTG<br>GGACCGAGAGTTGCGCGTCTACGTCACGCTCGACTCGCTGTGGCAAGCCCGGGTGAAGGGTCTATGTGGCAATTATAACT<br>CGGACCTTCGTGACGACTTCCAAACCCCGTCGGGTGGTGGCATCGCTGAATCCTCGGCGCTTATATTCGCCGACTCCTGGA<br>AATTGAAGCCCACGTGTCCAAAAGCGGAACCCGTCACCGACTACTGCAAGCAGCGGCCGGAGCGTACCGAGTGGGCGGT<br>GAGCACGTGCGGCGCACTGAAGCGGTACCCGTTCTCGCTGTGCCACAGCGAGGTGCCGGTGGAGCGCTGGCTGCAGCGCT<br>GCACGCGCGACGCGTGCGCCTGCGACGCGGGCGCTGACTGCACGTGCGCGTGCGCTGCGCTCGCTGCCTACGCGCACGCC<br>TGCACACTACGCGGCGTCACACTGCGCTGGCGCACCCCACAACTCTGTCCAATGCAATGCGACGGCGAATGTTCCAACTAC<br>GATGCGTGCATGTCACCGTGCCCACTGGAGACGTGTGACAATACCATCGACTACGCCGACATCAAGGGCAACTGCGAACA<br>GGACCCGTGTGTCGAAGGTTGCAAGCCAAAGAAGACTTGTCCAGAAGGTTCTGTATACAGTAACAGTTCATTTATCGAAT<br>GCGTACCTCGCGCCAAATGCAAACCATTATGTATGACCCTCCCTGACGGAAAAGAGATACTGGAAGGAGAAATCATTGAA<br>GAGGATGCTTGCCATACTTGTCGATGCTCCAAGAAGAATAGGGTCTGCACTGGCCAACCGTGTTCTACAATAGCGCCTAGA<br>CTGATTGAGAGTACTACATCAAAACCGCACGATGAGCCACTCAAATGTGTAACCGGGTGGACAGAATGGATCAGTAGACA<br>TACGCCGGAAGCTGGAACGAATGGAGAATCAGTTGAAAAAGAACCACTACCGGAAATTAATGAATTGCAAATCGGCACG<br>TCGATGTGCAGTAAGGATATGATGACAAAGATCGAGTGTCGCACGAAGGTGAACCATCAGAGCCCGAAAGAGACCGGGC<br>TCGACGTGGAGTGCAGCCTCGAGCGCGGCCTCCTCTGCAGGGAGACCGGCGGCACGTGCCCAGACTTCGAGATACGAGTC

		TACTGCGAATGTGAAGAACAATTCCAATGCTTGAATGCCGACCGTCCAAGTTACGCACACCCAAAGAACTGTAGCCAGTT<br>CTACGAATGCACGCCAGACCTGCTGAACCCGGACAAGCCACACACCGTGCTCAAGTCGTGCGTGGAGGGCACCATGTACA<br>ACAACAACACCATGGTCTGTGACTGGCCCGCCAACGTCATACCACTGCGACCCGAATGCGGCGCACTTGAAAGAGGTGCT<br>GACACAACGCCGTTTATTCCCGAAACAACCGTGACATACCCTACAGTTGGCCAGTCAGTGTTGGAGACGACAGCGATATG<br>CCCACCGGGATTCGTGTACTCTGACTGCGCGTACCCGTGCGACTCGCTCTGCGACCACTTCAAGAGCACCCTGCAGAACAG<br>GAGCAGGTGCATCCCTGGAGAGAAATGCGTGGAGGGCTGCATACCTTCTCAATCCTTTTGCGGTGGTGCTACCAGATGGC<br>GCGACGAGAACACTTGCGTACCAATAAAGGACTGCACATGCTCTGACAATT
MSTRG.12068	Dipeptidase 1-like	AGTGAGAATGGTGAATCCGTGCGAAAGGCGTGCAGTAGCATGCATACTCCTCGCCATAATAGCGGTTGTGGCGGCTGCAT<br>CGTACGACCGTGAGAGGCTGGAGATCGCGAAGCAGATCTTGGAAGAAGTCCCCCTCACTGATGGTCACAACGACCTTCCG<br>TGGAACATCAGGAAGTTTCTGCGGAACCAGATCAACGATTTCGAGCTGGACACCGATCTCACCGTCGTGGAGCCGTGGTC<br>GATCTCCAAGTACTCGCACACAGATCTGCCGAGGCTCAGAGAAGGCATGGTCGGGGC
MSTRG.12074	Craniofacial development protein 2-like	NNTGTATTTAATAGATGCCATAATAAATTTAGACGGTTTTTGTTTATTATATTCGTACATTTTTGGAAGTGTATTAAAATGA<br>TAATGCCCATTTGTAATAACTATAGCAATATATGCCCTAATATTACCAATATATACTAATACCAATCGTAATGAAATGCCT<br>TATGTGATATCCATCGTGTCTGACGCGATGATTAGCGAATTGGTTGCTATTTTCATTCAAAGTTTTATATTTTATTTATTTTC<br>AGTTTATTTTCGATATTTTTAAAGGAAAGCTTTATAAAATCTTGTAAATACAGGCAACAAATTGGTTGTTTTTAGTATATTA<br>CGCTATTGTCTTCAACAATATTGTTGTAGAATGGCAACGCATGGGGGGTATCATGGGCGAAACAGTATACTCTGTTATGTC<br>CACGGTACTGCTCATGTCTACAGGCGACGGTTACCACTTTCCATCAGGTGGGCCCTCAGCTTGATTGCCATTCTAAGATGT<br>ATAAAAAAAAGACATTTTAGTTTTTTTTTTGTCCGCGGGAATAGTGTTGTCTGCCATGTTGGTCTCACATAAATTTTAATAA<br>TATTTTTTCTAGAAAATGTATTATAATTTTTTTTTGTCATAAAATTCAACAAGCATTTCTAAAGTAGAATGAGATAGCACAA<br>AGTCATTTGTACAGTGAACGCTCTATATATGAAGCAACTCACGTGAACCTATTTCAATAAGAAAATCTTATTGGGAAAAGT<br>TGCTTATAAGGTAAAAAAGGAGTATAAAAATACTTACCATTTTGTAAAGTATTGTTACTTATTTCTTTTATTAATTAAGCTG<br>AGTAGAATAAATAAAATATATAAAGAATGTTGCTTATACAGAGCGTTCAATGTATTATAGCACAAAATACTTTAAAATAT<br>GTTTCAAGATAATAAAAACGATTTAATGTAAAACGTTAAAGTAGTTGCAGCGCTATGATAGTTACATTAGTATATTAGATT<br>AAAGAGCGTTGATTGAAATAATAAAGAGTAGAAGAGGCAAAATGCTGGGCCACCTTATACGACATGACAATTTTTTTAAG<br>AGCATAATAGAAGGAAAGATAGAGGGCAAAAGAGGAAGAGGAAGACCGAGGATTAACTACATGGGACAGATAAAGGAA<br>ACACTAGGCGTCATGTCGTATAGGGAGATAAAGGATATGGCCTGGGATAGAGCTGGCTGGAGGAAGATGCATGAATTAGT<br>TGCACCGACAAGAGGAAACTCTTAAATGAAAATGAATGAATTAGATTAAAGATCTACAAAGGAACGCCTTGGAAACATTG<br>TTTTACGTCATGTGTGAACACAAAATATTTTGTGAATATCTGAAGATAAAATGTAGGATCTTATCTTTCTGAAATCCGACA<br>AATATGTCCACAATCCGTATTATATATTAGCTAATTTATGAAGATAACATAATGAGCCTATATAGACATTACAATGTGTAA<br>ATAATTATATATTTAAAATACACAATCGGAATTTAACGCGATCTTAAAGCCTGTAAATATATACCTGCTTTTTATTGCCGA<br>CGTTTCGACGTGGATTACACCACGTCCTGTGACAACGACGTATTTTTTTTTATACCACTGAGGTGGCAAACAAGCATACGG<br>CCCACCTGATGGTAAGCGGTTACCGTAGCTATTGTATGTAAGTACTATTTACCTGTAAAGGTTTTTACGTCGTTTCAAGTCT<br>CGATTGTGTATTTAAAATATATGTGTGAACAACGGGAAAGTTTAAAATTAACTACATATTTGGAAAATTTCATTTATTGTT<br>ATCTACTAATGTTCGCATACGACGCGCCCGATGTAAGCCTCAACACCAATATGTTCTATTATTATTATTCGTTATTAGTATT<br>ATTATGTACTTAGTCAAAATGTATCCTAATTTTTCTTTACAAATACTAAACACATAACTAGTTATAATGAAAAATAATAACT<br>TTATACTAACAGAGTTTGTTTTGAACCTACTTCAAAGAATTATAATGTATTTTTACATTTAATTAAATTGACGTTCCTATAA<br>CGTATGTTTAGAGGTGAGAAGTAATATTACAATTCAACTATATATTGATTTAATTTAATTTATTCACAAAATACGAATTAC<br>ATCGTCTATTTGTTAATATACTATGTATATGTGTGTATATGAGACTGAAAAGTGGCGCTATAGTCGACCTGTCACGTTTTTC<br>CGGCTTCAACAATTTGTGCAGGCACAGGACGTAGTACCAGCGGCCCTTATACCTATTTATTTCAAAGAAAAAATAGTAGG

		ATTTTAAATTATTACTATTGTTACTATACAATATGCATTAATTTTATTAAAATGCTGATATTCTGTAATGTAGAAGCGAGAT<br>CTTTGCAAAGACTTACGAGCGTTCATCTGTCAATTTTTAAAAATATTTAAACGTTTTTCAATGTTTCTGATTGGTCATTTAA<br>AAAGTGCTCTGTCAACGAATTGGAACGTAATAACTAAAAACTTTGCACTTAATACATATTGGGCTTCAAAATGTTTTTAAT<br>TTGCTCGTGTCTGTCATTAAGAGGCAGCCATTTAAGAATGGAAGGAAAGATTTTTTTAATTATTTGTTTTTGAAAATTGGT<br>AGATTGCAATTGCAATGTTGGCAACACTTTGGTAAACTAATTAAAGTTATAAACGCTCTTAAAGTTTCTAAATACATTTTT<br>CCTAAATCTTTTATATACGTCTTAAACTTGTGTATCTTTGGTCGTGGATTTTGAAGGTTCCCAAAAAGTTGCATTTTTTATAG<br>TATTAGTTAGTTTTTTTTTCCAATGTTTTATTGTGGTCACAAGGCAGGCCCTCTAAAGCAATAAAGTATTGTTATATATATT<br>GTGATACTGGTTTCGTTATAAATTTATATGATATTGCAAATATGTAAAATAATGTACATATATCATGTATCTTTAGAATTAC<br>TTGCCAAAATTGTTAAAATAATCCATGCTATAAGGTTTATATGTGCAAAAGTACATTTTGTCTTAAAGTCAGAGACAAAAA<br>ATAAATGTTATATTTGTGAAATGGGGGACTTTGAGAGTTTCAGACTACGGTGCCAATAGGTCGTTTAAAAGACTTCGAAAC<br>AGGACTTTCTCGATTATTTTATGGATTTGTGTTATGCAATATTTTTGCTTATTCTCAATCGATTGTTGTAATTCTTTTTGCAA<br>TCGACAGAGTTTATTGCCTTGTTGGCGTTATGTTTTTGTTAGGATCAAAGTTGCCGTTTTGTGGGGCAAAATCTATCGCACA<br>ACAGTGTGAATTGTCTTGGATTTTTTTTTTAAATTGGAGTCTATATTTAATACTTTAATATCCATATAGTTTACTCATTTTTC<br>ATATTTTTTTTTATTTTATATTATTTTACAATATTGCATGTGTATAGTTTTATATTTATAGAGCGTAGGTGGATTATTATTTC<br>ATTGTTTTTTTAATTCAACATAAATGTGTCAGTTTTCAAAAGTCATCTCCATAGACTTTTTTTTTTATTTATGATGACTTTGG<br>CGAAAGCCATCAGCCAAAAAGTAACAATTACAAGGTAACATCTCCATAGACTAAACATGCATAATTTTGTAAATATTTTTT<br>TTTATAACGATCTTAAATCTATATATTTGTTCGTTTACGTAAAACAAAGGCGATTCCACTACATTTTTTTTTCTAAAAAGC<br>GAAGATCCGGAGAGCTAATCCCTTCGGTAAAATATAATAAATATCTAACTTCTACTCCTTTGGAATAAGGCAAACACTTTA<br>ATAAAGTTATAACTCATGAAGAGTGGAATTCGTACTAATAAATCAATAAACAAATAACAGATCTGTAATTCTATCTGAATC<br>TTTCAATTATTATTTAATTATATATATATATTTTTTATTTTGTAGTACGTTGTATGTACACATTGTTATTTT
MSTRG.12093	Mitochondrial aldehyde dehydrogenase	GTTATTACAACCCTGCGTCAACTCTAGCGAAATGAAACCGGTTTATCAACATTCTCGACTGATAGTCATGGCAATGAGTTT<br>GGTGTACATAAAATTTTATAAATAGAATCGTAATAAAGACATTTAGAATTCAAACTAAATTACAAAGTATTAAAAATTCTA<br>TCACTATTTCAAATTAAATTTATTAATATATTTATATATAATCATATATAAAAATAATATAAGTTAGTAATATTTAAAAAAA<br>ATCTTATTTCGATTTTCGAATGGAGCAAAATATAAGTTCGATTTATGATAATAGGAACGCAAATGCCAACAACTGTACAAA<br>TAAGGAAATTTATATCAATTGTATAAATGAAAATGAGCGTGAAATCTTTAATCCGTATACTTACTGATATTATAAATGTGA<br>AAAAGACTATATCCACTGTAATATATTAATGGTTCAGTATATAAAGTTTCCTATGTGCTATGTTAGGATATAACCTGCCCTT<br>ATACCAAATTTAATTCAAATCCGTTTAGTAGTTTATGCGTGACAAACATCCATCCATCTGTGCTTACAAACTTTCGCTATTA<br>CAATATTAATAGGATAGTAATGTTTGTCTACAGGTATATGTGGTCAAATCATCCCATGGAACTTCCCGTTGCTGATGGCTG<br>CGTGGAAGCTGGGGCCGGCGCTGGCGACGGGCAACACCGTTGTGATGAAGCCGGCTGAGCAGACACCCCTCACCGCTCTG<br>TACCTCGCGCAACTTGTCAAGGAAGCTGGTTTCTCACCAGGCGTGGTTAACATGCTGCCAGGATACGGAGACGCCGGCGC<br>GGCCATCGTCGAACATCCCGATGTGGATAAAATTGCCTTCACTGGTTCAACGGAGGTGGGCAAGCTGATCCAGCGCGGCG<br>TGGCGGACAGCTTGAAGCGCGTGACGC
MSTRG.12102	Neuroblastoma-amplified sequence-like	CCGCGCGCCCGCGCCCGCCGCGCTGCACTGGTACTACGTGCAGAACTGCCTCGACGGCGGCGCCACCGCCCACCACGAGA<br>CTGAAGTGGTCCAGAAATGTGCGGAGGAGCTGGTGTACAAGGACACGCCGCTGAGCGTGGCGTGCCTGCTGCGGCCGGCC<br>GGCGAGTGGGCGGCGTGCGCCGAGCGGGCCGGCCGCGAGGGCGCGGGCCCCGCGCAGCTGGTCAGCCGCAGCGCCACCG<br>CCGCCTCCGCCGTGCTGTACGCCACGCTGCTCAAATGCAACGCGCCCGACTTACGCGATAACGTCTATCTCGCTAAACCCT<br>CCACGATGGCGCGCACGACGTTGAAACAAAAGAACGCGTCCGAGGAGCAGTTGGAGGTGATCCGCCAGTGTATAGAGAA<br>GCTGAGCGGCGCCGGGGACGTGGAGCAGGTGCGGCGGCTCGGGTACAGCGTCAACGGGCTGCTGTTCAACGCGGACGAG<br>GACTACCGCACCGAGGTCATATACCGCATCGCCAGGTGCGGCGACAAGGAGCACCTGTCGGCGGCGCTGTCGCTGGCGGC

		GCGGCACGGCGCGGACACGCTGCTCGTGTGGCTGCAGCACGCGGCCGCCGCGCCCGGCCTGCGCCTGCACGCCGCCGCGCTCGCCGCGC
MSTRG.12107	Microcephalin	TTAACCAGTGACAGTGCGCCAGTGAATAATGACCATAATACTAGTGTTAGAATAAATAATCGAGTGATAGATAGGCTTGGCAAAAATGCACGTACCTTCAACGCTTTAGCTGGGGCCGCTCGCGGCGCTAGAGTACTGTACGCACAGTGGGCGTTAGATTCCTTGGAAGCCAAGCAGTGGTTACATCACTACGGTTACGAAGTGCCACATTTGAAGAAAATTTCACTAAAAGCACGCGTCGAACGTACAGCTCTCGGGAAGTGTCACTCTGAATACGCATACGACATCTTTAGTGGCATGCGCGTACTATTAAAAAGTACAGCTGAACAAAGGGACGCCGCGAAACAACTGCTAGAACTGTGCGGCGCGGTTGTACAAGATGGCGGACACGCACAAAATGGCGGCAGTTTTGACATCACTATCGGCGCGGGAAATGGTGAAGTGAGCTCGAAGTGGGTGTTCGATAGTGTTGCTTCAGCGAGAATGAGGACTATAAGGCGTTATGTGGTGAGTGGGGTGGGTGGTAGGGATGTGGGGACACAAGAAACGATTAGGTAATGTGCGGTGCTCAGTATTGTTGGGTAATATTATTGTTATAATGTCTATATACCGACTCAACAATGAATAATTATTGTTGTATATCTATAATCCAATATTTAATGTCGAAATTAAATGAATTTATTTTGCTAGAATGTAGGTAATTTTAGATGATAGAGTTTTATATTTTCTGTGTACATTCTGCCTCTATGTCGGCGTGCAAATTTTTAGCAGTATATATAGG
MSTRG.12117	5'-nucleotidase isoform X1	ATGGAATCGTATTTTTGAATTATTGTTTTAATTTGTTATTGTTTATTACGTTGCACTGTCAATTATATTTTCTGCTATTCCTGTCTACGCTCATTGAAGACTAGGCAGCATGGTTCCCATCTTAGCCTGTTCCACAACGAAACAAAAAAAAAAGTCTACGCTCGACTGCCATCCAACGACCGATCTGACGCCATCAGTGACTTCACCAGGTACGTCGACGCTGGTCGACTCTATTAAGGAGCTCCATCTGGACCGGAGTGCTGCTTGCGCCGCGAACGACGCCATAACTCGGACTGTCTTCACTAAAATCATTGTTCAACTGATGTGATTCTCAACTTTTGACTTAGAATTCACAAAGTAACTACACAGTTGTATAGAAACACCTTGTCACCATAAATTCGTTCACAGTTCATCAGTATTGTGTAAACTTATAAATATACACGATTTTGTTTTTTTTTTTACTTTGATTATAATATCTTCATCAGATATTTTGTAAAACTACAAACAGAAATGTGTAAAATACAAGACTTCATCATTATAGTGTAAAATTGCAAACACTATTTTCTAAAACTACAAACAGAAATGTGTAAAATAAAATACTTCATCAGTATTGTGCAATATGATACACAAGATTTTGTAAAACCATAATCAGTAGTGTAAAATAATATACTTCATCAGTATTATGTAAAATTACACAAAAAAATTTTGCAAAATTATAAAAAAAAATATGTAAAACAACATACTTTATCAGTATTGTGTAAAATTATACACAGTATGTTACAATGTATAAACAGTGACATTGAGAAAATAAATCATGTATCGGAATTATTGCAACTTTTGGAATCATCCAGGTGCAATATTTCCCGCTTGGCTCAATAAATTTAAATGTCATACAAAAATCTAGATGGTAATTGTATGTAGCATAAAAGTTGAAGTTATTCCTACTAACATTTTACAGAGGGATAGTTAAAAATGCACAATTTTATTTAAAAATATAATAAAAAAAGTTTTTTGAAATCATTGATGAATATTCCCTTTATATAACAAACTGTTAAAAGAGAATAATAATAATATCATCATGATCTTCCTACCCTTATCCCAGTTATTATTTGGGGTCGGCGTAATATGATTTTATCCATATTACTAAAGGATTTAGCTTATAGTTTTACGACCGGTCGCCTGCCTGACGTCAACCCTCTTTGGGAGAATAATAATAATAATTGATACATATATAGCTCATATAGGTCAGAGGCTAACATTACAATATATATTTTATTCATGTAAGATTCTGATTATAAAACAAATACATTGCTACAATAATCATGCTTTATTGTATGTGGTATTTTTGATAGAGTTCATACATTTCATACTATTCTGTGATATTCATAATGCTGTTTATATTCAGCCTGTCATAACCACTTTACATCAACTCCGATCATGTCACTATCAACCTATCTATTTGCTGTTTAAAGTTACATAAAAAAACAACTTTAATGAAATAAATTTGAAAATAGAATATTCAAATATTAAATGATTATTATTTTTAAATACCTATTATAAAAAAAAAAAAAAACAGAGTC
MSTRG.12118	5'-nucleotidase isoform X1	CGACAGTCGATCGAATTTGACAAATAACTGACAGTTATTATATATTCCGTTTGTGGTGGGTGTTTTAGTTCGATGCACATAACAAAATCCATCCTTAAAAATCGAAATATAATTTTATATAAGATTTCATTATTATTATAACACAAAAGAAGACAATTAAGAGGCTTGTCAATATTTAAAAAATCAATTGTTCCATTTAGGAACCTGCCACTCTGTTAGTGAAGACAGTCCGAGTTATGGCGTCGTTCGCGGCGCAAGCAGCACTCCGGTCCAGATGGAGCTCCTTAATAGAGTCGACCAGCGTCGACGTACCTGGTGAAGTCACTGATGGCGTCAGATCGGTCGTTGGATGGCTGAAACAGGCATGATATCTGTTATTGGCACGAAAAATGGGGCACATCGCACGTTTATATTCGAAACACCGAAATTGATGACATCACAAAAACTTTTGGATGTTTAAAAAATATATTTACTTTTAATGAATTTATTTTAATGCAATTTTAAGGGGTTGTCTATAATTTTTACTATATAAATTTGTGATGTTATCAATAAATTATGTCATATTTT

MSTRG.12127	ATP synthase-like protein	AATTATTCATTATTATCTTAAATAATTTAATTCTCAGTTTTTGAGTTTACATCTGTATTCATACCCTTGGTATTCCTTGGTTTTTAAATAAAATCACTTAATAGTACATCACTGGCGCAAAGTACATTTTTTGAGACATTCATAAATTTACAAATTTATAAATTTTGTCTTTAAAAAAAATGTTATCACGAGCAGCTTTGCGTACAGTTGCTGCCCGCCAGTCCGCGTCGACGGCGCTGGTGGCGCGAGGCTCCGCCACCGACAGTGGTGTGCGTGACGAGAAGAATTTCCCTCGTCCCGTGAGAGGGGAACCCGGAAAAGTCAGGCTTGGATTTATCCCTGAAGAATGGT
MSTRG.12150	Actin cytoskeleton-regulatory complex protein PAN1-like isoform X3	CGTTCCACGCGGCGGAGGAGCCGGTGACGGGGGGCGGGTACCTGGCAACGGTCCGCTGCGTTAACCCGCCGCCGCTCGCCGAGCGCCGCGTGTCCGCCAGCGACGCCAACTGTCCCGCTGAACTCGAACTGCTCAACAAGAGAAGTAAGACGACGTTCCCGATGTACACGGCGCGCGCGACGCTCGAGATCGGCAGCGGCAGCGTGTGCTCGGGCCGCGCGGTCACCACCACCACGGCGTCCAACCAGGTTGATTCTGCTATACCGTTTAGTTGCATCACAACACCAGAGGTTGTGACCAGTTGCAACACGACATCTTCCACTTCGCAGTCTGCGGTTGCTGTCGATCCAAAGCCGCTGCAGTTGGGCTCCTGTTACCGCGCGGTGGCCAGCCTCACGGCCGCGCTCCCCACCCCGGCACCCGCCTCCGCTCCTGCCCCCGCCTCCGCTTCCGCCTCCGCCTCCGCCACCGCCACCGCCACAGCCGCCGCCCGCAGAGAACAGCCTCGCAGTGTAATCGGCTACAACGGTCTCTCGGGACCGCGGCAGCACGGGTTCATTGTCGCGTCCGCACCAGAAGAACACAAGAAATCTCTAGACGAAAACGGCAACGCAGTACAAGGAATCAGTTCTCCCAGTCTACACAAGAAACCTAGGAGAAAAAAGTCTTCCAAGAATGAGACAGTTATCAACTTGCATCAAATAGACGGCTACCAAGGCAACAAGGACT
MSTRG.12155	PAB-dependent poly(A)-specific ribonuclease subunit PAN2 isoform X2	ATTCGCCATAGGACGACACCAACTGAACAGATGTCTCAAGTGTAACAAAGAGGAAGAACGCGAGTCGGTGGTGCTAGCGTGCGCGCTCCAGTACCCAACCGTGGCTGGACGTGACGGCGCTGGCAGTAGTGGTGAGGTCCGCGGTGGGTTCGTGGAGCTGGTGCGAGCGTCGCTAGCCGCCCGCCGAAGCACGCCTGCCTGGTGTGAGCAGTGTGGTCGCTTCACCCCCACCACCCTACGCGGGCGAATCGTCAGACTACCGCCGATTTTAGCTATAAATTGCGGAGGAGTAACGGCACATGAAAAGGCATATTGGTCAAAAGGAACTCAAAAAGATACGCCGGAAGTGGTTAAGCGAGGCGGTAGCGGTAAACCATGTCGCTATGGCTTACACTGTGCGCGACCAGGCTGTAGATTCAAGCATCCTGATAGACCGCCATCAAGCCAGGCGACTTCATCCAAGAGCCCTCAGGAATCGCACTGTGTATTGCCTCAACATTTGCTAATTAGATTGCAGTCTGATGGTGACGTTATCATCAATGATAAGGT
MSTRG.1217	Pleiotrophin-like protein precursor	GGGGAGCCGGGGCGATATGCGTACAGGACCGAGTATAGTGGCTGGTGCAGGCGGATCAATAGGCGAGCGTCCTGCCTCTCCCGAACTGCCGATCCCCGACCCCCGTCCCCAGCCCGCCACTCTGAGGCCACTCGTTAGACCGACGCGGACGCCTCGGTTCATCCACCACGATTAAACTGCGAACTCACGAAATGGAGAACAAACATTGGTGGTGGTTGGGTGCGGGGCTGATTCTGCTGTCTGTCGCCGTCGTGTCCGCTGACGGCGAGGTTTGGGAAGAAGGCGACCACGAAGTCCTTGTCCGGAGTGAGAGGGGAGCTAAAAATAGAG
MSTRG.12191	Hypothetical protein	ATTTAACTTTGACAGTTGGGTTTAATCACAATTTTTTCGAGTGCTACTTATCGAGTTATTATTTCTTTAATTATTAATTTAGTAAATGTATGTGTACCTTTTTTTTTATTTATTTACTTAACTATCTACATAGCATGAAGTAAACTCTCTGATCTATGATGGAGAGACATATTTTATTCCTTACTTCAGTGGAAGAACCTGTACAGTCTGAAAACAGTATACCGGCACAAGATCCACTGAATGGTATTGGAATACAGGGGAATAACTGTTATAGATTAAACAACAGTATCATATTTGCTGAACCATTAAATTACAGCAAGCAAATAAAAGAAGAGAATACAGATGGCCAAATGACGACATTCATAGATAATGTGATAGTACAGAAAACACCTGGTAAAAGAAATAAATATCACATGTATATTGAAGATTTATCAAATCCCACATGTCCACAGGTAAATTAATCTATTTGTATTCCACGGCTTCACTTGCATGAATCAGTA
MSTRG.12204	ATP-binding cassette sub-family A member 2-like	ACCCTATAGAGGATGAAGACTACCACCATCGAATAATTCTGCAACACCCGAAGAGCCAAGTCATGACTATGGCGTGGGCTCTCACCGTTCTCTTCATTATCCAGGCGTCGTGCATCAACTTCGTGTCGCTGCCGAGCGCGGAACGGATATCTGGGGCCCGGCACATCCACGTGATGTCGGGCTGCCCGCCGCCGCTGTACTGGCTGACGTCGCTGGCCGCGCACATGTCCGTCACGCTGTTCGGGCTCGTGCTGCCCGCGCTCGCCGCCGCCGCCGCCCTCGACACCGACCACACGCTTCACC

MSTRG.12208	Ancient ubiquitous protein 1 precursor	TCGATGCTGACGATCAGCCCATCACGCAGTTACGTTTTGTACCACAAAGTGAGGGCGATCTTCAAGCGATGTACTCTGCGA<br>TGAGTCAAGGGCAGGCCCTCCACCCAGACCCCACAGACGAGGTGGACGAGGACGACCCCTACATGGACGGCGAGGAGTT<br>CGACGAATGTATGTATTGATATTGGCATAAATAAAAGAACCAC
MSTRG.12218	Methylosome subunit pICln	CAGACAATATAGGTTTGGTGTACAATCAAATTATATTTCTTTATCGTACACCTGTAGATTCATAGACACTTTTCTTAATTTT<br>TTTTCAGGTGATAAAGATGAATATGAATGGCCAGTGGGAGGGGGAACTGAACGGGAAATTCGGCCACTTCCCCTTCACAT<br>ACGTCGAGTTCATAGACGACACTGTGACCAGTTAAACCGGGGGTAACTGTCCATAATACCTGGACAAGTACGTTATAATC<br>CATTTACAGTTATCTATACTCAGACCAACTGCAGAATTATGGGTGAACAATTATTTGTATAAATATTGTCTATAGACAAGG<br>GCCAAATGGAATTATTTTCACGTTGTAGATAATTGTTAAGTCACCACAACGGCAAAAGACAAACGCCTCTGACTATCCCAT<br>TATGGATTATAGTCTTGAATAATTTATATATATTTTTTTAAATAATTCATATTCTATTTTTTTAATAGAAGAATATTTGAG<br>GGCATACAGAAGATCGCAGACTGAGTAACTATTTTAATTTTTGTTCTTTGAAACTCTCTTCGAATTTGTCAATCACTACACG<br>CAGTGCGCTTCGAGAATGCATTTCTAAATTCACTATTTTTCAGCCGTACCAAAGTAAAACGATCTATTCGTCATTACAGTTA<br>TCAGGCCACCGATATAAAATGATACTTTTGACGTGCCATGTCTTTCTTGTGCATGCGGTAGCGTAAACGTTTAGAAATTAA<br>ATCCTTTGTCTCTTGTCTAGACTGCATTTATCAATAGTTCTTGTATGTTTTTAAACAACTACAAAAAAGAGAACTGAATTC<br>GCCTTTTTTTATATGTGTATATTATACATACATTACCTTCGATGTTTTTAAACTAATATTTATTTTAATTAAAAGGTTTTAGT<br>TTACTTTGTAAGTTAGAAAACTTTAGGGACAATTTATAAAAAGGTAAATGCAATTTCCTTCTCAGAACGAGAATTTTATAC<br>TAAAATTCCTGTGTAAATATTTATTTTCTTGAACCTTAAACGACGTGAATCATTCCGTTATATAAAATATCCCTATTATGTT<br>AAAAAAAAAATAGTGAGTTTTAACTTACAAAGTAAACACTCGAAACCTTTCAAACATCTGACGCTAAAACAAACCGTTAT<br>GTGAAAAAGATAGATAAATTATTACTATTATTATCAACTAAGTATACTCACTGAACCCAACAAATGAGAATATGAAACTC<br>GTGAGTGGCATTTGTCATCTGAACAATTTTGTCACTAATGTCAATTCCATCGGGACATAGACACTCTTTATTATTTCCTCCA<br>TAAGATTACAATAGTAAACTTATTATTGATACAAACAATTACAAGAAAATATTACACTGAGAAAACAAAGGGCGACCTTA<br>TCGCTAAGAGCGATCGTTTCCAGACAACCTTTGGTCATATATGCTTTATTGCACAAGATATTATAAAGAAAAAAGATTATA<br>CGTGTAAAATTAACAGTACGGAATTGTATTTATTACTTAGAAATTAGTGTAAACAAAACACGATCCCAAAATTGAAACTTA<br>GCGGAATTTGTCACGTTGACAAATATCACTCATGTGTGTGAGCAGTAAAACAATTTTTTTTAGGTGTCTATTTATTTCGTGT<br>TGTTTCTAACAAGCGTGTTATTAACATCAAACTTTTAATAACTTATCCTTTATGCAACCATTTGTATGGAATAATATTTAAA<br>GAAAAAAAAAATGCAAAATGGTATAGTAAAAATTTTTGCACTTAGATCTATATAATTAATTCATACAAATACTAGCATTTC<br>TAAATTATATTTTGTATTTCTAATATTGCTGTCGATATATCATAAACACGCACAAAATATGTATATTACACATTTAAATTTA<br>ATAATTTAAATAACATTACTGCGTCACGAAGGATACTGCTTGTTCACACCATAAGATACACATGAATTTATGTAAATTTAT<br>TGCACTTAACACATATGTGGAATAACTATAACATAGGTACCTATTATTGATAGAACACCACAGATATATAATGATTATACG<br>AAAAGATTGAAATTTGTACGAAAATAAATAACAAAAGTTGCACAAAAGGTTAAATTTTTAGTTAATTTTTATAATTTTTTA<br>CTGTGTCATAAATAGTGATTTTATAATATCGAGTACGGTTTACATCAGAAGAGGCGAGTCGACGCGTGTACGGGCGCATGT<br>TATTTAAAAATGCCTTCCGCGTGAACGACCAAGTTCCAACTTATACAAGTTTCATGCAAATATAAACTTTAATTCATTATA<br>GAAACAATTTAATTTTCTTTGTCATAATCTAAAATGTGCTCTCACGCTCGTAGACGTGCTGTTCCGTGTAAATCTTTGAAAA<br>ACACGTTGGTTCTAATATTTCTGCTGCACTCACGGTTGCATGTCTAATAAGCATTTTTTATTAAATATAAATATAATTAATA<br>CTATGTACATCGAAGCTTCTTGACTGCATGAATGCTATTTAACGATTTTATACTGCAATATGGATGTAACGAGATATATATT<br>ATGTCAAGATATATACAAGACAATGTGCTGGCGATTTACGTCTGACTGGAGACAAATTGAAATAAATATATTTTGCAAATA<br>TA
MSTRG.12222	Adapter molecule Crk isoform X1	CTATGTTAAAATTTCGCTTCGGCAAATGCTACGGTGACAATACAAGACAAATTCTACGGGAAATACGCTCAAAAGGATTA<br>TTCAACAAGCCTTTAGAGTATCGGCCGGGAGACAACTACGAGCTGGAGAATATGCCGCGTCACCGCAACCCTCAGGAGGA<br>CGTGTACGACGGGCTGCACAACCGACAGACGGCATACATGACCGGCTACACGGGATACGTGCCCGGTATGAACTTCAG

MSTRG.12234	Protein FAM166B-like	ATCGACTGATGAGCAAAGCAGCACGGATTCCCAGTTCATGGAATTTAAATCTTCAATAAACTCTGTCATATTTACAGTTTA<br>TTTGTATTTATATATACAGTTTATTTGCCAGAAACATAGTTACCTCCATAATTCGTGACTTGGGTATTATATTTGACTCAAA<br>ATTGCCATTTAGACAACATTATGATTAATTAGATGCAAAAAAAGCAAATAGAACTTTGCGGTTTATCTTTAGAACAATTAG<br>ACCTTTTAATAACTCGCTTTTTAATAATTTTTAACTATTTTGTCACTCCATACATCACTAGTTAGAAGTTCATTTGAGTACTG<br>TTCTGTTATTTGGTCTCACTTCTATCAGAACCACGAAACAGTATACTATGTTATGTGCATGGTACTGCTCATGTCTATAGGC<br>GACGGTTATCGCTTTCCACCAGGTGGGCTTTTTTTTTTTTGGTGGGTGAAAATGATATGGTAACCCCGCCTGCGCTAACGGG<br>ATACGCTGGCGGAGCACCAGTGAAGGGTGATAGATGAGAATGAAAACAGGCCAGTTAGCCCATCATGATGAATTCATCAG<br>GAATTAACCATCATAGTTTCCAGGGGGAACCGCGAGGAGGTCGACCTGGTTGGGTTTATTGCTAGCAATGGGATTCTCAGT<br>CTCCATTACTAACAATCCCTTTCCCCCCCCCCACCAAGTGGGCTGTCAGCTTGTTTGCCATTCTAAGTTGCATAAAAAAATAA<br>ACATATATACAAAAGACTCCAATCTTTTCTCTTTATAATAAATAACTTAGTATAGCGTAAAATGTTTATACACTAGCTTTTA<br>CCTGTGATTTCGTCGGTGTGACCGCGATTATGTTTACTGGCCAGGATTAAAAGTACCTATCTAAATTCCATAGTAATTGGCT<br>TAATTCATTGAGAACTACGGAACCCTCGGCTTATGAGTTCAACTCGAATTTATTTAATCTCTTTCAGCTATGCCGTTGACTG<br>TTACGAACGTCGCTGTAGAGAAGACGCACTTAGACAGATATCTACTGGAGTTTGAAAAAAATGCAATAAATAGACACAAA<br>AGGGCAACATGTCGGTATGTAGAAATTATACCCCAAATGCATCTTCATTATCTTCTTTTCAAAATTGGGTAAAAATGTCAT<br>TGCATCCCCGCCTATGCTATTGGTATACATTACCAGTGAGCAGTGATAGGTGAGGATGAAATCAGAACAGTTGGTGATGAT<br>TACATCAAAAATCAACAACAATGAATACCAAAGATGACCGCATAGAGGTCAAACTCACAAAGTATATTGCTAATCATGAG<br>ACCGTTAATCCTTGTCACCAACAATCTCTCCTCTTCGTCTCACACTTCGTCATTTCTATCACTACAAAGCAACACTCTCCCA<br>CGGGGGGAAAAGGATTGTCAGCAATGGAGTTTGACAGCCCCATTGCTAGCAATATACCTTATTAGGTCGACCTCCACGTG<br>GTTCCCCCTGGAAACCATCGTGAACAATTCTTGTTGAATTTATCACGATGGGGTAACTGACCTGCTTCATCCTCACCTATCA<br>TTCTTCACTGGTGCCCCGTCGGTGTATACCGATAGTACAGGTGGGGCTACCATTCCATTATCATCCATTTTAAAAAAAAAT<br>ACTCTCCCACTTCTGGACATGGGGATATTGCCCTAAAACGACATCGTCCCAGATATCTTGGCCGAGTGTTCTTTTTTTAAGA<br>TTTTGTTTTCTTAACAGAGGGAGATAGTATAAATGTCTGCTTTTGGACCTCTGTTCCACTCTTGTTTCCGCTGTTAAACAATT<br>ATTCTTGCATGGCTGTGTTTCGGTTTAGAGTGTAGGAAATGACAGAGAAACTATTGAGAGGTTCTGGGAGTCGGAAAAGC<br>CCCATCCACATGTAAATACTAATCTATTGAAGGGCCAGTGGCAAGATATTTACTGCATTATCACAGCCCGAGAATTATGGT<br>GATTTTGTCTTTCCTTCATAGTGGCTCTGTATAGGGTGCTACTCGTGGTATGGCTCTACGTGGGTTTCTCTATTTAGAAAAG<br>AAGCCAGAGGAAGACTTTGTACAAAAGTCGATTGCTTAAGTACCTCTGTCCCATTAGTGTTTCAACCGTGAAGCAGTTGTG<br>TTTGCATGGCTGTGTTTCGGTTTGAAGGGTGGGATATGGCGTGAATTTACTGGGTACAGAGGAGTAACATCTGAGTCCTCA<br>AGAATGGCAACGCATGGGGGGTATCATGGGCGAAACAGTATACTCTGTTATGTCCACGGTACTGCTCATGTCTATAGGCG<br>ACGGTTACCACTTTCCATCAGGTGGGCCGTCAGCTTGATTGCCATTCTAAGTTGTATAAAAAAAAAAGAAGCTACAAACAAT<br>TTAGAAGCTAAACAACTTAGTCCAGTGTCATTCATGGTGCTGTTCATGTCTATAAACGATAGTTACTTAGGCATCCATTTTA<br>AGATCATGTTTTCCATTATCTTAAAAATAGTTATTTAAATATTACTTTATTACATAATAACAGTAATAATTTATTTTATAAA<br>TAATATAATTAAACTTACCCAGAAATGATCAGTGGCAATGTCGCGACGGCACGTGCATCGGCTTCGACGGCAAGTGTGAC<br>GGCGTCGTCGACTGTCCGGAC
MSTRG.12247	Pattern recognition serine proteinase precursor	TGTTATTGATCAATGCTTGACACTTTTGACTTCTGACATTAAATTATGTCAATGTTCTTTTCTAATTTAGAGTATTAGATTAA<br>TATAAACGATATTTTATTTGCTAAACCAAACTCAGTTACGAGTTACAAGAAAACCTTTGAATATTTCTCTGAGTAAAAGTT<br>AATACATTAAACGAAACAATGCCTCCAAAAACAATGTTTGAAGAAGAGGTCTTTGTATCTGAAGAAGAAGAAGAATGTTT<br>CGCTACTTTAATTTATAAATTAAGATATCAGCAACAATTGGCGAAAAGGGATGCCTTGATAAAATCAACACAAGAGTTAG<br>ATGAAGATATAGCTAAGATACAAGCAAAAAAGAGGGTTTTGGTTAGCCAATTACTAAGCGGCCTTGTAATGTATATAAAC<br>ACCTACAAAAAAACTGTCTAAATTTGCATTGGTGTAATAACGAAAATTATTTGTAAGCATAACCATACTATACTTTTAAGT

		TTTTGTCTTTTGATGTTGAGTTAAGCATTCGCTTTACGACAGTTGTTTAAATTTGCCTATTGCTACAAGTAATAGACATTTTT<br>ACCAAAAATGACAGATTTTTTTAAATTCTATTGAGGCTTAGGTTAATAGGATTCTTTACAGTTTGGGAAAGTATTTTATAAT<br>TTGTAATTGTAAATTTTCTTGTTTATGATACTCATGTTATGCTAAAAAGCTAACATTAATTAATGAATACTCAGTCTTTCTC<br>AAAATAATTGCCCATTTATATTTAGAAATATAAGACAGTATTTAGTTTTATCCATAAACTTAGTATATAATTGAACCTATTA<br>CAGTATTTTAATAGTGTGGAGTCATATATAATTGGATAAGCATCAGTGTTCTGAAGAAAAGGTGACAAAAAGCAGGATAT<br>CTCTTTATATGAACTTATTGAGCCATCTACCAGAACAAGTCTTTGTAAAGCAAAATCTGCATTTTCTTCAGTGCATAAATCA<br>CAGTTTGATGAAGTCCTCAGTTTGATTTTATATAAATGAAATGAAAAAAGTCCATTTGAATCCTATTAATTGTTGTGATAA<br>ATTTCCTGGAAGGATCTTTTGACGTGAAGTACCATGATTTACTGGAAATAGGTTTTTTAATATCGCCATACCATTTTCTTT<br>ATTTTCACTCCACTTACAATTCCATTTACTGTACATTTTTCTTTTAAACTATCGAAATCAACCATCGGTACTAAAAGAAATT<br>TATGATAATTTGTCAACAACTTCATTTCCAGTAATGCCGGAGTGAGATGGGACCCACATAAATTCTATATTTTTTTCTGATG<br>TATAGAACAGTGCTGTACCATGCTCATACTATCGTTAGATGATTTATCGATTCTGTATAGGATTGTAAATCGAAACTCAAA<br>ACTTGCAATTTTATTTAACAATTAATAATTTTAGTAGTTAATAATTATTTATTTAACTTCAGTACACAAGTAATAAATTGGT<br>ATATTATGTAATAATTGTGTTTTAAAAGAATTATATTGCAGAGCAAAGAGCTCAGCCGTGTAATAATATTAAAAAATTTAT<br>TTTATTTTAAAATTGAATTATTGATGCTGATATTATTTTATTTTTTGAGTAAGTGGTAATTGAAAAATATTTTTAATATTTTG<br>TTTTAATTTATATAATAAATTTATATATAATTATTATAGCTGCATATGTGACGACCTGTTGCTTTTATCAATTTGTTGAATCA<br>ATACAATCGATAAAATGTCATTAACACTTATTGGCAATCTCTTATGAATCTCTTAACTTCAAGATTTGAAGATGTTCACGTA<br>TTTGTTATTATTTTAATTTGAATACTTAAGTCTTCTGAATATTAATTAGTTGAAATGTGATAATTTTCTTTGTTAAATCGACA<br>AAGATACAACATATTTGTTGTGGTTCTAAATATTGTAGTTAAATAAGTGAAATATTTTAAGCATATTTACACGTGAAACAT<br>GAAATATATTTATATTACATTCAGGTAAAGCATACAAAGAGAGTATTAACAAAATTATTTTGTTAATACTCTCGCCAGCTT<br>CAAAACAGCTTCAAGCTTATGTTGTGCAAATTATTGTCCTATTATCTCTCTCGCTTTAACCTACAGGTTATTGTATTTCCTTT<br>TTTTTTACATTGCCAATTTGTATATTTTATAATGTAAGGTTTTATCTACCTCGGTAAATGTATAATAACTGAAATAATGCAT<br>TTTTTTAGTACGATTGTTCCGTTATGGAACAGGCCAAGGTTGGAGACCATGCTGCTTAATCTTCAGTATGATAATTTGTCTG<br>GAATTGAAATAATGCATAATAAACGTCGTAAAAAACAAAA
MSTRG.1226	Hypothetical protein	TGTTATTGATCAATGCTTGACACTTTTGACTTCTGACATTAAATTATGTCAATGTTCTTTTCTAATTTAGAGTATTAGATTAA<br>TATAAACGATATTTTATTTGCTAAACCAAACTCAGTTACGAGTTACAAGAAAACCTTTGAATATTTCTCTGAGTAAAAGTT<br>AATACATTAAACGAAACAATGCCTCCAAAAACAATGTTTGAAGAAGAGGTCTTTGTATCTGAAGAAGAAGAAGAATGTTT<br>CGCTACTTTAATTTATAAATTAAGATATCAGCAACAATTGGCGAAAAGGGATGCCTTGATAAAATCAACACAAGAGTTAG<br>ATGAAGATATAGCTAAGATACAAGCAAAAAAGAGGGTTTTGGTTAGCCAATTACTAAGCGGCCTTGTAATGTATATAAAC<br>ACCTACAAAAAAACTGTCTAAATTTGCATTGGTGTAATAACGAAAATTATTTGTAAGCATAACCATACTATACTTTTAAGT<br>TTTTGTCTTTTGATGTTGAGTTAAGCATTCGCTTTACGACAGTTGTTTAAATTTGCCTATTGCTACAAGTAATAGACATTTTT<br>ACCAAAAATGACAGATTTTTTTAAATTCTATTGAGGCTTAGGTTAATAGGATTCTTTACAGTTTGGGAAAGTATTTTATAAT<br>TTGTAATTGTAAATTTTCTTGTTTATGATACTCATGTTATGCTAAAAAGCTAACATTAATTAATGAATACTCAGTCTTTCTC<br>AAAATAATTGCCCATTTATATTTAGAAATATAAGACAGTATTTAGTTTTATCCATAAACTTAGTATATAATTGAACCTATTA<br>CAGTATTTTAATAGTGTGGAGTCATATATAATTGGATAAGCATCAGTGTTCTGAAGAAAAGGTGACAAAAAGCAGGATAT<br>CTCTTTATATGAACTTATTGAGCCATCTACCAGAACAAGTCTTTGTAAAGCAAAATCTGCATTTTCTTCAGTGCATAAATCA<br>CAGTTTGATGAAGTCCTCAGTTTGATTTTATATAAATGAAATGAAAAAAGTCCATTTGAATCCTATTAATTGTTGTGATAA<br>ATTTCCTGGAAGGATCTTTTGACGTGAAGTACCATGATTTACTGGAAATAGGTTTTTTAATATCGCCATACCATTTTCTTT<br>ATTTTCACTCCACTTACAATTCCATTTACTGTACATTTTTCTTTTAAACTATCGAAATCAACCATCGGTACTAAAAGAAATT<br>TATGATAATTTGTCAACAACTTCATTTCCAGTAATGCCGGAGTGAGATGGGACCCACATAAATTCTATATTTTTTTCTGATG

		TATAGAACAGTGCTGTACCATGCTCATACTATCGTTAGATGATTTATCGATTCTGTATAGGATTGTAAATCGAAACTCAAA<br>ACTTGCAATTTTATTTAACAATTAATAATTTTAGTAGTTAATAATTATTTATTTAACTTCAGTACACAAGTAATAAATTGGT<br>ATATTATGTAATAATTGTGTTTTAAAAGAATTATATTGCAGAGCAAAGAGCTCAGCCGTGTAATAATATTAAAAAATTTAT<br>TTTATTTTAAAATTGAATTATTGATGCTGATATTATTTTATTTTTTGAGTAAGTGGTAATTGAAAAATATTTTTAATATTTTG<br>TTTTAATTTATATAATAAATTTATATATAATTATTATAGCTGCATATGTGACGACCTGTTGCTTTTATCAATTTGTTGAATCA<br>ATACAATCGATAAAATGTCATTAACACTTATTGGCAATCTCTTATGAATCTCTTAACTTCAAGATTTGAAGATGTTCACGTA<br>TTTGTTATTATTTTAATTTGAATACTTAAGTCTTCTGAATATTAATTAGTTGAAATGTGATAATTTTCTTTGTTAAATCGACA<br>AAGATACAACATATTTGTTGTGGTTCTAAATATTGTAGTTAAATAAGTGAAATATTTTAAGCATATTTACACGTGAAACAT<br>GAAATATATTTATATTACATTCAGGTAAAGCATACAAAGAGAGTATTAACAAAATTATTTTGTTAATACTCTCGCCAGCTT<br>CAAAACAGCTTCAAGCTTATGTTGTGCAAATTATTGTCCTATTATCTCTCTCGCTTTAACCTACAGGTTATTGTATTTCCTTT<br>TTTTTTACATTGCCAATTTGTATATTTTATAATGTAAGGTTTTATCTACCTCGGTAAATGTATAATAACTGAAATAATGCAT<br>TTTTTTAGTACGATTGTTCCGTTATGGAACAGGCCAAGGTTGGAGACCATGCTGCTTAATCTTCAGTATGATAATTTGTCTG<br>GAATTGAAATAATGCATAATAAACGTCGTAAAAAACAAAA
MSTRG.12264	Uncharacterized protein LOC110379302	ATTTTGTAAAAAGACTCACTCTGAAATATTACCATAGTATTCAGTTTAAATTAATGTTCTATTCATATATAGATATAAAATT<br>ATACTACATTTACCTTTACCACCTTATTATACCATAATTAATTTGTAGGTACAAATCGATTTACATATTACGATGTAAATCA<br>ATTTGTGGGTATAATTTAATTATATAAAAATAAATGTGACATGGCGTCCCGCTGCAAAGAACACATCAGTCAACATGGCTA<br>CAGCTATGATACATTTTACGAGATCCAAACTGATACGAGAGCTACCAAAGATCCTGACAAATCCTTTGAAATGCATCTAGC<br>TATTCAGGCATCTAGCAACGGGCACATATTGCTGTCTCCAGTCAGTCGACCCGGATCGGCCGACCCTGTTTATGAAATAG
MSTRG.1229	Hypothetical protein AB894_15350	TTTTACTTTGTATGTATATCAATTATTACTAGTACCGTATTCGTGTATTCCGACACTTCCTTAAGGTTGATTGGGGGAGAAT<br>GCAAATAAATATAAATAAATAAATACTTTTAGTATGAAGTCCGCCTTGTACACATACATGTGCTAAAGAATAAATGAATA<br>AAGAACAATCTCATTAAATTAGATACCCATCCTTAGATGGCTTCTTTTTATTTAACTCGAGCACCGCTAATTCCTAACATCT<br>AATTTGTAAAAAGCAAAATCGTACTGCTTTTTCCAATTTATGTCGAACCTGGCCTTTTTTTACATTACAAAGATTGCTTTTT<br>ACAATATTAGTTGATGTGGCCGTGTTTCACACAATTTGATAACTTATATATAAATCGATTATTAAAGATTTTGACATGAATG<br>TCTTTGCAAAAAAATCCGCAATGTTAAAAAGTGTGACCAGAAGATCTTTTAGTGTTCTAGAGTTCACCTTTGGGGAGAAGCA<br>ATTGAGAGACACGATGAGGCTCTCGCTGTATCTCTGTACGTTTCTAAGGCATTTGACAGGGTTTATTTTTTTTAGTAAATGA<br>TAATGGAATGGCAACTCCGTCTACGTTAACGATATACATTGCCAGGACATCAGTGAGGGATAATAGGTGGCGATGAAGCA<br>GGTTAGTTGGCCTATCATGACAATAAATCAATCCCAATATTTTCCAGGAAGAACTGCATGGAGGTCGACCTAATAGGGTAT<br>ATTGCTAGCAATGGGATTGTTAATTCCCATTACTACTTAGGGGTAGGTAGTAAAGGGAATTAACAATTCTCTCACTGCATT<br>TGACAAGGTTTGGCATAACCATTTTTATTGAAACGCTTTCTTCTTTCTCAATTTCCGAGAACCTGATTACGTAACTTTCGCA<br>AAGGACGATCAATAAGAGTTGTCATCGGCGATTGCTCGTCAGACCTTGTAGATACCAGTGCCAAGGTACCCCAAGATCGG<br>TTCTATTTAAAACATTATTTTGCCCTATATATAGCCTGCTTAAGCTTGGAATTAAAACTATTAAGTCCAAGGATAACGTAAG<br>CCTTAGATGGTATGCTCCCATGATAAATAAATCAGACCACCCATTTCCTCAAACGAAAAAAGTATAAGCGGCCGCGTAGTT<br>ACGCAGTTTTCACTGCCAGACCAAACTTTGAAATATTCTTGAAATCTGTAGATCCACGTCAAAACGGCGATGACGTAATAA<br>AGCAGGTCATAAATAGTGTCAACATTAATAAGTATAGGTTTAAATCGAGTCACCAAAATAAAAAAGTTACCATGAGCTGC<br>AATTCAAAACGACCACATAGGAAGAGTGCAAAAAGCAAGCAGCTAGAAATGCCGCTATAAAGTATTGCTAAGCACAACG<br>ACACAGATAGAGCGCATGACGCCCACCCAACACAGGACCGTCTAAAAATTATTGGAAAAACAGAGTCGTTTGAAAACTTC<br>TCACAAATAATGGTCAACTAGTTAGATCTGTTGTTGCAGTTTTAAATCCTGAAATCGAAATAATTGAAAACCCGGAGTAGA<br>CAATTCAAAATGTAGTCGCCATAAAAATAAGATATTGCCGTTTTGCGTTTATTGACGTCTATTTGGACAAGACTTTTAATAT<br>TGAAGATTCCATTATCTAGGTGCTAACAATTATTGTTCTATAGATACATCAAACTTTATTATTATTGGAAACATTAACGCCA

TTAGTCGTTGGATACGATACAGATGATGTTAAAGACTTTCGTCTTATATATATTATCTTCCGACTAAACATGAAAGTATTAC
ATGAAGATACACAGTCAACATTATATGTTTATTGACAAGGGTAACACTAACCCAGCATCATTGATATTATAACTTGCTCCG
TCCACCTTCTCAGATTACAGACTGGCATGGGAATCCAACACTTTGCTCTCTCTTTGATCATATACTGATGACTTTTGCTATT
GGTTTACACAGAAATTATTAAACTGATACGCTAACTCCACCTCCACACGTACTTACAGTATAGCAAATGCAAATTCGACCA
TGTTCCAGGAAACCGTGAAAGTCCAACAAACAGCAAAAAATATAAATCCAAATGCAATCTCAAATATTCAAAAACACCAA
GACTTAGAAACCTCAAACTCAAAACAAAACCAGAAAGATAATAGAAAAATTAAAGGCTGAACTCTAATAGCTTATTGTAT
ACATCCTCATCATCCCCATCATCTGCAAAATAAAACAGAATAATATTAAAGAACAATTGCTGACCTCAAATAATATTAATA
TTGCGAAAGTGAATTTATTTGTTTCGCTTTCACGTAATTGCAGAATCTTGGTATAGAGATAGATAGGACCCTGGAGATGTT
ATAGGCTATTTTTTCTCCTGGTTAGTGAATATATCTCAGTCATGCGGACAAAGTCTTGTGTAAAAGCTAGTAACCATAATTG
GAAAAGTTCTGTATAGGAGAAGAAAAATAAATAATGTGAGAAAGAAGCTATAGGATCTCGAAGAGAAGTGCGAAAAACA
TACGTGTAAACCTGCTTACAGATGAAAATGGAATCATCTAATGAGTCTGTCACTCCTAGCTGATACATTCTTCCTAATAGA
CGATTCATCTAATGACACTCAAATAATGCAATAAGATAAAGGAATAAATTCATTAATAAAAAGTAAACCAATCACAAATA
AACAAATAAGTAGAGAGACTTTGTTTAAAAGTTATTTGCTTGCATACGTCTGTCCCATTCGTATTTCGACCGTGAAACAGT
TGTGTTCGCATGGCTGTGTTTCGGTTTGAAGGATGGGATATGACAGTGAATTTACACCTTAGTCCTCAATAATGGCACCGC
ATGGAGGTTACATGTCTGCTTGTTTCCCATAGTTGTTGAATAAAAAAACTTCATTCACAAAATACGAATTATATTATAAAA
CACTGATAGTTATAAAACCTAAGAAAGTAGCCCGAACGGACGGGTTTACAGTGGACATCTACCAAAAAACACATGAAATC
CATCGAGACATCTTTGTTGGCCGTATTCAATTAATATCTTTTTGTAAGCTATTTTCCTTTAATTTGGAAGAAAGCATTCATT
AAAATTATCCCAAAGGCTGGTAATAACTGTTATACTTATTTAAAGGTCCCCTTCCTCTTATGGGAAAAGTTCACTGCTTTAG
TATTTTATAAACAAAGGCTCCTTGCAGCCAAATCCATATAGCTTTTCCAAGCTCCAAGCTATTCCAAAGCGAGGCACCGAA
GATGCACTGTCAAATGTCTTAAAAAATTAGTGATAACGTTAAAGTTAAGGCATAGCCGTTATGCTCACCTTGGATATCCAG
CGAGCGTTTGATAATGCTTGGTGCGTCAACAAGCATTACCAAATGCCCTCTGTCACTTCACGTCTGGACAACTTTTAACAC
TTTACCAAGCAAAAGTCCGGTCTTGCATGGAGTATTTTCCTCAGTTGTGGTATGGATCTGCTCAATATCAGTTTAATACTCT
GGGCTCTATTGATCGACGTGCAAAAAGATTCATAGAAGATGAGGCACTTATTGTTAAGAAGTTCCATAGTTTCGGTATTCG
CCGTATGATGGTTTGTTTATTGGTATTGTACCTAGTATCCTATCAATGAGGCGCAGGGATTTTTTCATCCTTATACGGTTGA
CATTCTACCTATTGAACGATAAAACTAAGTGCTTGGTACAGTGTTGATTTCGTCGTTTCTTATACGAACAATCAAGGAGTG
GAATTCTCTTCCGGCGTGATCTAAAAACAAACACGCCGAATGAAGATGGGGTTTTATAATGCTAGAGTGTATAGGCTTCAT
TTGAGTGTGCGTGTAACATTTTAGATCTCATCGTCACTTAAGATCAGGTGAGATTATAATAAAACTAGCTGAAATAAAAAA
ATCAGTATAAACTATAATATACGCGTTTAATAAATAAAAAAAAAAAAATACTACACATTCATACAAAGGCAAGTGCAACGG
TTTTCTCAAGGATCCAATATAATCGATCCAGTAAGCACATAATGTAATTAATTTTCGTGGTTTTCCAATACAATACTTTTTT
TAATTCAAGGTTTGAAAATCTGTTAATGAAATTATCATAATTATAAAAAAAGAATATTAAAAAAAAAAATAACAATAATGA
TATTTCATTTAGTTGTGAATTTGGTTTTTATGGTGAAAATAAATAAAAAAAGAAAATTACTTATAAAACTAAAATCCCCCC
CGCGTCTGTCTGTATATTCGCGATAATCTTAAAAACTACTCAACGAATTTTTATGCTATATTCATTTATCAATGGAGGAATT
CCTTAGGTAGATTTAATTATGTATTTGGTTAGGATTTGTAGGAATTGATTGAAATATAACGATGATTGTTAAAAATGTCGG
AAAACATTAAACCGCCTGAGAGCGTTCATCGATCAAGCTGTTTAATCCGTTTAAGATATAACAATATGATGTATAGTAGAA
CTGTTTCACTTTATTGGGGTCAAAAAAAAGTCAGCGAGGGCATGTATCTATATTATACAGAAAGCTTTCTTTAGCAATTTTT
GTATTTAAAAAATGAATACAAATTATGTGGAGCTTTCGAAACCATTTAAATGAAAACTTTGTCCTTATCAAATTAAATAAT
TGTAATTACCTTTTACACTACACCATTTAATATATATCATATCCGGGTGGCAGAGTTGCGGCGCGAGCCTTTAAATGTGTTA
TCCGCTGATGACCTATACAGCTTCACATAATGCGTACTTTTTTTTAATGTAATGGTAACTCTGCATGCTCTAACGGTATACG
CTGTCGGGGCACCAGAAATGGATGATAGGTGAAATGAAAAGGCAGGCCAGTTAGGCCATCGTAATGAATACACCAGGAA

MSTRG.1 230	Uncharacterized protein LOC106710629	TTCAACACAATGGTTTACAGGGGGTACCACGTGGAGGTCGACTTGAAATGGTATATTTTTAGCAATGCGACTGGTAGTTCG<br>CATTACTAACAATTCCTTTACAGAATAGGTACGTGTGTGGAAGGCATTCCAAGACCGCAAGAGGGGAGCGACAAAGTGTC<br>AACTGTTACCGATGGGTTATGGCGTATGCCCAAGGTCATCAGAGCGGTTCAGATCCTAGATTAGGTTGCCGTACTTTGAAC<br>CCCGTCGAGGAATTGAAGGTCGGTTATATTTAAGGGGGGAGGTGTGAACGAGATGTGCTAAGATACGTATCTGACGCTGC<br>AGGAACGGCGTGGTGGGGGCGTCGTCTCCGCCGGCGGGGAAGGTGTCCTTTTTGCGAGTGTGCTTGTGTGCGTGTCGAAAT<br>TTTGCGAAAGAAGGAGAGGAGGGTTGTCAAATTTTGGCAACGAAGTAGTGTCGACACTATTTTATGAATTGCTTGATCGTG<br>AGGAATTTGAAGTCGCGGTAGAAATCCGCATTGCGTACCTACCATGTAGCGAGGTGACGCAAGAACTTGGTCTAATTTAC<br>CTGTAGTCGATTTAGATAAGCGCGCTTTATAGCGGTTATTAATGAATGGGTAAAAACAAATATATATAATTTATAAGATAA<br>TCATCTTTATATTTTAACTAATTTACACAAAACCGTATCGAGGAATCACTGTGTACACAAATAAAAATCGTATGAAAATGA<br>ATTTAGTAGTTTTTGAGATCATCGCCAACATACAGGCAGACAGACGCGGCGGATGATATTGGTGTTAGTGTACATACCAAC<br>TAACACAGTTGGTGTTTGTAGGCGTTCATATCTATATATTATTTATTACATATGTATTATATAAAAATCAAAAATATATACA<br>ACAATAAAAATATGGTATTCTAAATAGTTCATAGCATTCTTGCGTCCCGAATTATTTTACTGTGGATACTGTATTATTTTAA<br>TAACATCAAATACTTTTTTATGCCGTTACAGATTTGTTTTAATTATATTATAAAGCGAATTTGTTATTAAATGTTTATTTTAC<br>TTCCACTCATTTTTTGCATGTTCATGTTTAAAGAAAAGTTCTTGCTACACAACGGGTGGTTATCTTTGCGCTAAACACTATC<br>TAAATTTATTTATTTATTATTACCTGCAGTTTACAGATGATCAAACTCTAACACACTACAGGTAATTACAATATGTAAGGCA<br>CGAAACTTACGTACGATGAAACGGGAAACTTTTAAAAAAAAATACTAAATAGTAATATTAGTAAAGATTTGCATGGATTTC<br>AGCGCACAATCTAACTTAGGCTTCTCGTATATTAACTGTGAATTTTGTAGTGTTAGACTAAGTGATATCTGTTATTTTATGT<br>AAATATGCATCCTGAAAAGTTGGTTTTTGAGATACAGTAACACAATTTACGATTTCAATAAAATTCTTATTACATTTTTCTT<br>TTCACAGCTCTTTTGCTGCGTATATGTAGATAGGTCAGGCGCTATCTTTGCATTATATCTGTAATTTAAGGTTTTATACTAG<br>TACATTAAAAAAACCGACGAACTATTATTCACGTACCCATTATTATTATTTTTAATTAATAGTCCAGTGTTTAATTCGTGGC<br>TAAGTAGGAAAGGATTACTCAGAGTAAAACGGTGAGAATACACGGAGGTAATGTTAAATGGGTAATTATTTAATTCAAGT<br>TCCTAAAAATACAGTACAATAAAAAATACGATTAACAAGTCTAATAATAATTTGATTAGTTGATTAAAATACTTTTGTTTA<br>ATTCCACTACACATTAGTATGCGTACTGCTGGTTTTTGTTTTTGTTTTCTTAATATCAATTATCCAGTAATATGGACTATCGT<br>AAAGTAACACCTTTATCAAAATCTATAATGTGCTATTAACTGTAAGGATAACTTGACGTAATAGAATAAGGAAAAAAATC<br>GAAACCATATGGAACAGACGTTGAATAAAAGGTATAAATAAGGTAACGTCCATTTGACGTCTATATTATATGATTTCGATT<br>TGATCTCAATTCGATTGCGTCAATATGTCTCCGCGTTCTGTAGTTTTAGTAAAAGATAATAATAAATTACCTATTTAATATA<br>TCTACAGTAAGACAGAATCTTCAGTCAGACAGAACAGTATATCTAATGATATGTGGCCTTAAAAAACTTTGCTTAATTTGA<br>GATATCATATGAAATCTCATAACATTCTATAAAACGATTGTTTTTTTTTTAGTTTTCTTATATTTTTATATGAGATTTGGTGT<br>GCATTTGTTTCAAAAGAACTTAATGAAAACGTATCCAAGTCTGTTGAAATTATACAATCAATTAC<br>GTTTTGAATTTATAAAGTACCTATTTTTACATATAAGTAATCATATTTTTAAATAAAATATAACTTTTTTAAATAAATTATA<br>ACTGGCCAGTCTCACATACATAAAAAGTTGTGTGAGTGACCTGGTATTTATGTAAGTAGGTGCACATTGTATTAATATCAC<br>AAGTTGACGTTAAGTTCAGTACAAGTTTTTTTTTTATCTTAAAATGAATTTAACAATGTTTTATTAAGACGCTAGTTGACAA<br>AATTTGGAAATGAGTTTAAAAATATAGCTTATATATTTACCCTAAAAATATATTATTTAAAAAAAAAAGCCATTTTACCAA<br>GGAATTTTGCGTTTTTAGTAATGAATTCAAAATTGACATCGAAAAAGGCCAAGATTGTCATGGCGTTTTGAATGACGTCAC<br>GCCTCGTAAAACATTTGTATTTGTTTATGACAGTCAATTGTTTTTATTCAGAAATAATGATTCTCTCATATTATAAATACTG<br>TGCAATGCCCCAATGTAAAACACAACGATAAAAACACCGAATAAATTATTCATATACGTTCCTAACAATAAGCAAATACG<br>TAAAAAGTGGTTAGAAATAGCAAGTAGGCAAGATGCTTCTAATATATCAAATGACTGAACACTATTTCTCTGAAGAACAT<br>TTTGATTATCGTATTATGGGAAAAATCTCACAAGTGCGCATGAAGCCAGATTGTATGTCAAAGAAATTCGAATGCCAAGA<br>AAATAGAAAGAAACGTACATGCAGCGATATCAAATGACCATATATGTTGAAAAAACAAAGAATGTCAACAATCACAGAG

		TGAACCAGAACAAAATATAAGTAGTACATCAAGTTCATCTATCAAGGATACTTCAACAAGTTCAGATTTTCAAAAAGTTAC<br>AGGAAGAGGATGTACCTGAAGCTTTGTTTGCACTGACAGCAGATAAATCTGTTCAAGTATTAGTAACACACAATTTCAGA<br>AGTAAAGCTGTCCAAACTAAAATAAAATCAATAGAAAAGGCAGTATCATTTTTGAAACCATCACAAATATCTGTCTGCAC<br>ATCCCCATTCAAACTAATGAAAATTATAAATTCAAGACCTG
MSTRG.12319	Trypsin-like protein	TGGGTGGCGCGGTTACTGATATCTCCCGGTACCCCGAAATAACTTCCCTTCTATATTCATGGAACGGTGTTATATTTAGCCA<br>AGCGTGTGGCGGGACCATCCTTAACCAAAGATCCATCTTGTCCGCCGCTCATTGTTTTGTTGGTGATTTAGTCTCTAGATGG<br>CGTATTCGTGTTGGATCGTCTTACGCTAACAGCGGTGGTATTATACATGGCGTCAGTAACATCGTTCTGCACCAAAATTAT<br>AACATAGTTACTCAGGACAATGACATTGCCATTCTTCATACTGTTTACAATATTGTGTATAACAATTTTGTCAAGGCTGCCA<br>GTATTCTAGGCCCTAATTTCAATCTTGGTTACAACGAAGAAGTTTGGGCTGCTGGATGGGGCGCTGTAGCGTATAATGGAC<br>CTCCGTCGGAACAGCTTCGCCATGTTCTAGTGTGGACTGTAAATCATAAAGAATGCGCTGATCGCTACAGAGAAGTTCAAT<br>TAGACGTAACTCCCAAC
MSTRG.1232	Uncharacterized protein LOC105380700	AGTAATATTCATTCTCTTTTTTGGTTTTTTTTTTTTGTAAACTCAACTTTTCTTTGAAAAGCAAAGCTGAGTTTCTTGCCCATT<br>CTTCTCAGCGGTAATTGCATTGTGAATGGGCGGTAGAGTCAAATTATTAAATATTAATTATTTTTATTTATCTATTCTAAAA<br>ATTACAACTATGACATTATGACAATTTTAGATATACTTAGAGCATAAAATATAGATATATTCTATTACATGTTATATAACTG<br>TTGGTTCATATATACCTAAAAAAGAAAAAAAAAAGTAGTAACAGTAAATCTATTTTTCGATGTTACATATCTAAGTATAGGT<br>AATTGTTTTAATGTTTCAACGATTTTATTAAAAGCCGTGATAAGATTATTTATCGAATAAAACAAATGAAAAAAACACATT<br>TTTGCAATCTTATAAGCAAATCGAGATATATATTTCGAAAAAAAAATAATTTAGAAAGCACTACATGAGCCATAGTGCTA<br>ACATTCTACAGAGCTGTTTGATAATAACCTTACTACAGCATAGGATAACTGTAGGTTAATTATATAAGAAAAGATATATAG<br>GTTAAAAGAGTTCCCTAGTTTTAGAAGTTGCGTGTTTTTACGCAAGCGCTGATGTTGAGGTTCTGGGCTCGAACCTTGGCCT<br>AAGCCGCCGTACACTGTCGAGGGATGATAGGTCAGATTTAGTCAGAGACGATTGGTATTTTGATTACCAGGAAATGCGTG<br>TGATGATCTTCGAGGCGATCTTTGAGATGGTTTTGGAGGTGTTCTCTGAGATGGTTTTTGAGGTGAACTTCGAAATAATTTA<br>GAGGTGATCTCTGCGATGGTTTTTAGGTGATCTCTGAGATGGTTTATGAGGTGAGCTTCGAAATAATTTAGAGGTGATATC<br>TGCGATGGTTTTTAGGTGATCTCTGAGATGGTTTATGAGGTGATCTTCGAGATGGTTTTTGATGTGATCTCTGATATGGTTT<br>TTGAGGTGATCGTTGATGTGATCTTCGAAATAAATAAAGCAAGTGAAAAAAGCAGATGAAGGAGAGCATGACATCATTAA<br>GGACGCTAGTATTTGTGGGCACGCGCTTTTGCGCATGCCGTTTTCGAGGTAATAGTAGTAGCGATAGTTTTCATGACTATG<br>ATAGATTATTGATGAGGCTCTCCTTCGGGATTAAGGTCCATGCAATGTGTTACATTCCACAATTGATTGCTGTATGGATGTA<br>TTGATGAGATCGTATTATGAACATCTAGATGGAACTAAAAAAGATAAGTGTGGGAAGATTATACTTCAATTAACTTCCTTC<br>TGTGTTGCGTGTTTTAAAAATAGGAATACTATAATTATTGAATTTGCTTGCATGTTCGAGTCCAACTAAATTACCTACGTAT<br>TAATTATGCCAGTCAATCTTTGGCTGTACGTGTGTTCATTATTTAATTTAGTTGGATTCGAACAGTAAATAGAATATAAGTA<br>ATTATTTATACTTTTCGAATTTAGCCAAATTATGTACGTTCTTATAAATCCAGCCAAACTGACTGTGCGTGTGTTCATTTAG<br>TTAATTTCGTTGGATTCGAAGAGTAAAAATATTATAATACTTGAATATAAATATATAGATTATAAATTAAGGCAGCCGATC<br>TGCGGCTGTGTGTGTTCAATATGTTTTTATTATTTGTTTTATTTGCTAAACAACTTCGACGTTTCTTTTTAACAAAAACAACA<br>AATAATAAAAAAATATATGACAAGAAATATTGTAGTAACATAATGATCTCAGTAAAAGTCATACCAAGATTATTTAACGA<br>TAATAAAGCACAAACCAAAACAAAAAATCCATTTTCGATTTTCCGTTGCAATTCAAACAGTAACATTATAAATGAAATATA<br>TTCAATAGCAGAATTAACCAGTATATAGAGCATTATAAATAAGTGATAGCAATGTAACATTTATGGAAATAGTGAAAAAA<br>TTGAAAGCCAGATTTAAATAAAAAAAAAATCATGGATCACATTAAAAAGATAGTATTTAAAATAATAAAAGAATAATTTA<br>TTAACCTTTAGTAAACAAATGTCAATAGGAAGCCGGAGTGTGATCGAAATTATTGGCATTCTTTAAGGAATACTACAAGA<br>GAAACTATTTTTTTTTTCTAAATAGACATTAGCTGGATATGAACAAAAAAATTTGTTGATTTACAACTCATGTTTTTCTAGA<br>AAAATGTTTTGTTTCACGGATAGAACGCTCTTATAAGCATGTTCATATTATATTAATATCATCTCGTATTAAATATTACTAC

CAACATTTTATTTTAAAATATACTTAATTGATAAGTAGTAGTTAAGTACTTAATAAGCGCTACAACATTTTGTATTATATAA
CGCTGTGGATTGTTTTTGTTACCAGAGAATTAGATATAAATTACAACAATAACTTCGTCAACAAAATCTGAATTTACGACC
TCTTATACCAATGTAATAAACTATTAAACTACGACAATGCTCTCAAAAATAAAATAAAGGGAAATAAGGTTTACAGCCAA
AAACCTCGCCCCTCACTTCTGCAAGACCTTATAATGAAGCTAATTATAAATGAAAGATAACCTAAAATATATAACAATGCA
TTGCACGAGATTGCTTATTATTAGTTAGGCTTAACTTACAGTTGTAAAATGAAAATATATACTAATTATGGTAAATTTGGTT
TGACCAAATAAACCAAATATAAATTTATGGTTTTCTATATAATAGAAACGATAAATGTATTCATAACAACGGATGCGATCC
GAAATTATTCTATATAATACTATAAGTGTCAGCCTTTATCAAGAAATCTAGCTGTCAAACAGTATTGCCAAAGATGTGAGT
AAAATCGAATTTGTGTTTTCTCGATATCAGGGATTTGATTTCTTTCAATCGATTCTATATGGAGTTTCTTGTACCGAAATAA
TCCTATTCAAATTTAGTGTTTTAGATTTTAACCTAATGAAATTGTTACATACAATTCGGACAATATTAGCATTTGTACATAT
TAATATTTTGTACTTTATATCAGCATAATTTAAAATTATATAATTTTTTGATATAAAAAATATGACCTACTTCAATCGTAAT
ATAATTAATATGGAAACTAGGCATCCACATCAAAATAAATTCTATCGATTACAAAAAGATTCACAAAAATCCATTCAAAA
TTAGTATTGATATCGAGTAAAATACTTGCAAATAATCAGAGAAACAAGAAAAAGACATTTTCGTTACTCACATTTTTATTA
GATATTTTTAACGAATTTGAAATTACTATATTTTTTACATTAATATTTTTATTAAATAAGAAAAACTATTAGCTATTGTGGC
TATTATTTTTATTAAAATTAAATATTATTGAAAAAATAAATTATTTTATTCAATGTTATAAAATAATAAAAGTAAATATTTT
TAACACAAATTTTCAATATTTTTAACAAATAAGAAAAACAATATCTTTAATCAATATATTTTTTAACGACATCAAATAAAA
CTAAATTACATAAAACATATAAACGCAATAAGGCGCGTGTCAAGGACAAACATTCACTAGAATAAAAATTTTACTTAAAT
AGATTTCAGGCGTAATTTTGATTAAATCCAGGCTCTTTTCGGAATTGTAATAAATCAAAACGTACCAAAACCAAAACTCAG
CGTATCACTAGGTATAGAAATGAGATTAAGTAAATTGTGCCAAGAAGTTTGCAGAATAATTTATGCACAAGTTTCTCCGCT
GTTATTCGCATGGCTTTCTCATTCGGGACATAGCACTGCTAATATTTCAATAATTTTTTCATTGCACGTCACACCAGCTCTT
GACGAAGGGTTACAAGTGGACGCTGCTTGCGTCAATTTTCGTAAGACCTTTGATGCCATTGACCATGATTTTCGTCTGGAG
AAACTAGCAGCAACTCGAAAATTATAGTAATTCTTTGCCAACTATTTATACAAAAGATAGCAGCACTTGGAATATAAGGG
GAAAGTCTATGAGCCTTACTTCAAATTCTCTGAAGTAAGCCAAGGAATTACCTTCGACGCGCTGGAATTCATATTCATGAT
TAATGAAATATAGTAAAAAAGTCAGTCGGACTGCATTTGTCTTTTTCATTGTCATTTGGATTGCTGAAAAGTTTAAACCTAT
ATTGCTTGTAGTACTTTGTTGATTTGGGATCTACCCTGGATGGGCAGTGTTATAAATATAAAATATTTTCATAAATAAAAG
ATGTTTTAAGATTAATATTATTAATTATATATAATATTTTAATATTATATTATTCAATATTTTAATTAATAAGAGAAATAAT
ATTTTTTACATTAATATTCAATATTGTTAACGAATTAGAGGAACAATATTTTCATTCAATATTTTTAAGAGAATTAGAGATA
GTATATTTTTAACCCTAATATATATATACAACAAAAAAATAAACAATATTTTAAACTAAATATTAGTTAAACCATAAAAAT
TAACTTATGACGATTATTAAGTATTAACATTTAACGAATAAATAAATTCAACCATTACACCCAACTTTACACAGTAACATA
TGGTTGCCTATAAAAACGGCCGCAAGTAAATATAGCAGCATCTAGTTTTATTCTACATGATGTTGTTGTAGTAATAGGATC
TAACTCCCTATAACCTGTATTACAAAGTGTGATAAAACCTCTACTAAACGTAAGTTTATTCTTTTTCCTATACGACATCATA
AAAAATAATAATTCCCAACTGGCAGCCCTAGGTTGCGCCTAACCGATAGCAAGTAACCCGGATACATTGTATTTATTATAT
TTTAATTCATCTTTCATTTAAACAAGTTTTATAAAAAATCTTATACCTGTAAAACTCTGCGCCGGAATCTTAGGTTATTTCA
TTTGTATAAATAAATGCATATGATGAATGTTTTATCTTTAATAAAAATAATACGTTTATCTACATTTTATAATAATTTGTAA
TGGCAATCTAACTCTCTGGATTACAAACTAACTTTCTGCAAATTTTCACCAAAATTAGTTACTATTTTTATATTTACTTTTTC
CTTTGACAAGTATTGAAAGGAAATTATAAAGATAATGGATATGACATTCACATTTTGATTTTAGATATGCATTCATCCTTGT
TTCACTGTATAATTAGGTATAAAATAATGAAATAAGTAAATGTTATTCAGCATTTAATCATAGAATAATATTAATATAATT
TCGGAATTCGTGATTATTTTATTATTGAATCAAAAATTATATTAATTACTTATAAGACTAATCGGTTACAGATCCTAACATG
TTAGTTACTAAATCTTTTGATTTTTCATTAAACTTCAGTTAAGTATTACGTTACATTATTTTTTTTTACGTATGAATTTATATT
CATAAAAATAAAATTGTTGATTCTTTTTAAATAGGACAAATACTTTGTTATATTATTTTAAATATTCTATGAATATAACTAA

MSTRG.12344	RNA-directed DNA polymerase from mobile element jockey	TAATACGAATTTCAATGTACACAGAGCGACAAAAGTTTACAAATCGAAAACCAATCAGAGGCCTCACCACATAGTACGTA<br>ATATATTATTCAAGGTAAATCTGTATAAATTGAATATTAAATGTTGTACATTGGTTAACACAACAGATTAGAATAGCCCGA<br>TCGATTTGGCCAATAGTCCGATAATCCAATTAAGGATTTTAGAGAGAGAAAATAAACAGAATATCCACTAAACGCTTTATT<br>ATTAGATATTAGTCAAATAGTAGCTATAGTAGTAGCAGTAGTAGTTGTTGTAATTAATCTAGTGGAATCAGTAGGTATAGG<br>AGTAGATATAATTACACAACTCAACCTATCCCCTAACTAATCAGAGCTATACTGAAGAACAAACAGACATACTCAAATTA<br>AATTTTTTGGGAATACATATTGAACATAAAAATACTCAGCTCGATACAGCAAACATACTTGAATATTTTTTTTTTTAGAAAT<br>ACATATTGTACATAGAAATACTCAAATCAATACGGTTAAACACATGAATATCATCTTTATCGTTTTGGTTTTCATCAATCAA<br>TTCTATGGTGTAGGCCTCCTTCAATCTTCTCCTTTTTAATCTATTTTAGGCAATCAAAGTCGAATAGTACCCCGTTAGTCAC<br>CAGGTCATCTTTCTATCACATCTATAGCCTCACTATGGGGTTTTTACAATCCCATGGTCTTCACTTCAGCACATATTTGCAA<br>TATGAATCAACTTCGAGCTAAATATTTTATAAACATAAAAGTTTATATTGTACAGCGATCGAATCTGCTACTATTGGAAGA<br>CATGTCATTGATTAAAAAGTGATACCACGAATCGAAATAGTTCAATATCCGCAATAATTACTCTACTGCTCCACTAGACAA<br>AATGCAAACGAATCTATCGATAGAATCGCTTCGGTCAGCTCTCACATGAGAAGAACAAGACGCACGTCGCCACCCCATTC<br>AGTCTAATGGGCTCACTAGTGATAACAATTCTGTCAATAGATTCATTTATACTTTGTCTAAAGCGGTTGGTTAGTTGGTTCT<br>TCTTTTTCTGTACTTTGTCGCTCTGGTTAACTCTCTATATGTTATAGATGTAAGAAAAAAAAATAAAAAAAAGTAGAAT<br>AAATATATTTAAGGTATGCCTGGAAGGAATATTGGGAAAAAACAAGTACAATAAAAGGAAGATTTTATCGCCTTATACAA<br>CCCGATATTCCTTTCAAACCATGGTTTTGTAAATTAAAGCTTGGAAAGACCGCAACAACTATTATAACTCGTATGCGTCTA<br>GGTCACGTATGCACACCTTCTCATTTATACAAGCTTGGTATTGTACAAAGTTCGTCGTGTAATTGCGGTGCTGAAGTGGCG<br>GACTTGAATCATATATTCTTCTCATGTTCTAAATTTGACCACACCTCCCTTTTTAATCATCTCCGATCCATCCGTGTTCCATT<br>CCCAACTTCTATTCTTACTCTACTTTCTTCCAATGATAACAATATTAAGATTTAGTTATATTACATGAATACATTTATAGTTG<br>CATCAATTGCTTTTCCTATATATTTATAAGTATATTCTCATATGTACAAGTATATCCTTCATATCCTATAAATATCCTAGACC<br>TAATCCTTATCCTTTCTAAATTCGTTTGTGTTAGGTATTTTTATTACTATTATCGTTTCCCCGTTTTGTTCTTGGCAAAATACC<br>CTAATATCGGGGTGAACGCCACAAAAAAAAGAAAAA
MSTRG.12368	Superkiller viralicidic activity 2-like 2	GGCCTGTGCCATTCTTTTAGCTTAGTAATTACTTGCCCTGTGAAATGAGATTTACTTTGTTGTTTCATTCACTTTTATATTTT<br>ATGGTTATTTATTTCCATGATAAAAATATTTTAGAAAATGGCTGATATTAATAGTTTATTTGATTGTTTTGAAGAGCCGGTA<br>CAGAATGAAGCTTCTCTTAATTTTCCGAATATTCAAAACTGCAAGGATAAATTAGATTCCTCATCTTCAGCGAATAAACGG<br>TCACACGAAGAGGTGGAATATGTAGATACATCACAGGCAAAAATACCCAAGCATGAAGAGGATACATTAGAACTGATTA<br>GTGACATTAACTTGGAACTACTCAGTTCTCAAATAGTTATACACACTTTAGAGACAAATGAAGGCTGTACACATGAAGTGG<br>CCATACCTCCTAACTATGAATATGCACCACTTACACCCCTTACATCTGAACCCGCCAAGCAGTATAGTTTCGTATTGGATCC<br>ATTTCAGAAGGAAGCCATATTATGCATAGACAATTTGCAATCTGTATTAGTTTCTGCTCATACCTCAGCTGGTAAAACAGT<br>GGTTGCAGAGTATGCAATTGCACTTTCACTGAAGAACAAGCAAAGAGTGATATACACAACTCCAATTAAGGCACTTTCCN<br>NNN
MSTRG.12385	Hypothetical protein KGM_203723	CTGTTTGGCGTGTTTGGTTTGTTTGTTATTGTACTGTCAGTCGGCGTGCGCTAGAGTCATTTCATTTATTTATTTACAAATAA<br>ATATAGAAGCTAATTATTATAATATAACTAAAAAATATGAATTCGAATAATAAAAGTACAACGAAAAATCAAAATAATTC<br>ACAGTATTCAAGTTATTATCCATGGAATTTGTTTACACAACGATCTCGTACAGAAAGTGAAAACAGTACCAGCTCTACGGC<br>TTCACAGAGTTATCAATCGGGTACACAATTCGAATCCCTGCCAAGGCAAGCGAACAAAAGCAATGAGGAGTATTTATGGA<br>TGATGTGGCGTTCCTAATCGAACGCGCACTGCCAGCGAGCCTGCTCCACTCGGCGGCTGCACACGATACAAAACAATTATT<br>AGTTGTTACGTGCTATAATTATGAATTGTTTGGAAATATGAACCACTGTCCCAAATAAAAAGTGAAACCATGTTAGAGCAA<br>GCGATTTTACCTACTTGGTTTCTGATGGCGTGGTTGGGCGCTTGTATACTTTTTAGTCCTTTCGCAATCTTCTTCGTGGTCTG<br>TTTACCTCAGCTGCCACTTTTAGTCGTTAGAAGGGCCTGTCACATACCGTTTGAATATATTTGACGTT

MSTRG.12417	Uncharacterized protein LOC110378902	AGCTAGTATTATGTACACACGAAAAAAGGATATTTATAGAACAACACGTACTTCACATTCATTCTCATTTATATATTTTTGATTATTCAATGACAGAATGTATAAAAGAAAATTTCAAAGATAGAACCACTCAGTTCATAATGTCAAATCTCCGATGTCAAAGTGTACAGACAAAAGCGTTTTCAAAATGTATTATACAATTCATAGAAATGCGTAATAAGGATTGCTTTTTATATGTTTTTAGATGCTGACTTTATTAGTTATTGTGGCGGTATTAGTGTTGGGCTATCGACCAAAACACATGACGACTGTCGTAGACAATGTTGGCCCCAGTCAAGTACTGGCTAACGGAGTGTGGGGCTTTAGTCATAACTGGGATGTAATCTTGGAACAATATGGAGTACCATTCATGCACGGTTGCATACGTTTCCTATACGCGATTTATGTGAGGCTCACTTTTACAAACACGGCAATATTCCTTATCAAATATGTGCCAACTGGATTGTATATGTACAAATTAATTAAAAGAATTTATGTAACTTCGAGAGAAAGAATCAAACAGAAACAAATAGAATTGGAGGATATACAGC
MSTRG.12418	Uncharacterized protein LOC110378902	AAATAAAATGTAAGCACACACAGCTAATTAGCTGCAGAAGGAATGCATCAATTAAGTGATGCAGGCAATTGCAAGCTATCCAATCTCGTCAATGGTGCGGTAGGGGAAATTGGTCTTTTGATGCATTATTCTGGTTAACGCGTTACTACACTACACCTTTGTAGGTAAAAGGATCAAAGGAGTAATTTTATTCCATTTTGAGTGTTTGTTTGTATTGGTGGAATAATTTATAACCCATATTCTGAATTAACGTGTAGGATAGTTTCTTTCATAATTTATGCAGACAAATTCGCGAATTACAACTTAATAGATAACAATATACGAAAAACAGATCCAAATAGCGAAGACAAAGTTGTGCGTGCGTGATGCGGAAATGACGGAGCGCGTGGAGGAACTGCGGCGCGGGGTTGAGCGCCTGCGGCGAGTGCGTGCGCGGGTGCGTGGTGTGATCGCGTCCGACGACGCGTTACGGCAGACACTACACGTCGTGCACGCGCACGTCGATACTAGCCCCGACGAAGATCGAGACTTTATAACAGAACTGCTGAAAGATTTGAAATGCGACCAAGTAGCGATTTCAGAGAGACAACCCGAGAACTTGGAGTGTGGGGAGCATGTACAGACCGAAGCCAACACGTCTGAGAACAGCGTCTACTCGTCGGCGAGCGATGTGGAAGTACAACAGGATTACCGAGTCAAGATCGTCAGGGTCACCAATGTATATAAAGTCGCATATCTAAAGCACTATATTAAACAGAAGAGACTACGTCGCGCGACTAAACAAGCGCTGCTGAAGAAGAAGATGGAAAGCATAAAGAAACTTCTGGAAGATTGGCAAAAGACGCTCAACATGGTAATCAATACCAAACTGACGCTACTGAATATGGATTCTCACGTTGACGCCGCCTCACAAGAGGCCATGGGAGATTTTTCAAAGCCCAATTTAAGAGAATCTTCAGATTCCGACAGCGATTTTAAGAATGGAACAGAAATTTGCAATGATGAATATAGTCTGAGTTGGACTCAGCATCCCTATCGTAACTATGCTTATGAGGATATCCCAGAGACCGCCGTATCTCGGGAATTTTACGATACTCAAGGGTTCGTCGCCGATTCAACATTCCCATTTTGTAATTATGCGAAATCAAGCAAACTTTGTACACTAATGGAAGAAACAACGAGTCAAGTGGCTATTGAGGAGATAAAACACGATGACGACGAAGATAGTGTTTGCCAG
MSTRG.12451	UPF0585 protein CG18661	CGATTGTACACTACCGTTGTTATGTTTTTTGTATTACAGGATTCCAAATCGCAAAGAATTTAAAACCGTCTTCTCGATTGAACGGTTTTATGTTTCTTGCTTTCAATCCTTTCATATGGGGGAAAACGGATTGTAAGTAATGGAGTCTAATTTCCCCATTACAAACAATAAACATATCACGTCAACCTCTACGAGGTTCCTTCTGGAAACTATCATGATTAAATCTAGCGGAATTCGTCATGATGGGCTAACTGACCCGCTTTCATCGTCAACTATCATCCCTCACTGATGCAACGCCAGCGTAAACCGATAGCACAGGGTTCCGTATCTCCGAAGGAAAAATGGAACCGTTATCATCACTTCATTATCCGTCCGTTCATCCATCCGTCTGTCAAGACACTTTTTTTTAAGAACGCGTGGAAGTATAATGTTGAAAATAATATATACTCAGGTCTACAAACTCTTAAAGGAGTGATCAAACTTCTAAATCGACGCAATCAAAAGATACGGCTGTTCATGTTCCATTTATATCCCATATTTTGACATTCACAAAAGGAATCAAAATCTATAGGGTACTTCCCGTTGAACTAAAATCATGAAATTTGGTATTTCACAAAAAAAATTAAATTTGATTTTGTACGGAATACTCGTTGCGCGTATCCGACTCGCGCTTGACCGGCTTTTTATACCACATGACTCCATCATTTATAAACCGGTTTAAAAAATTCTTACAGTTACAAATTATTCTAAAAGGGTATATTTTCAAATTGGTCCTACAGAAATTTTATCAAATTTTTTTATAGAAAATATAAAATATTTTTAAATATAAAAATTAACGAACGAACATTGTACTGCTTCCGCAGAGCGATTTAAAAATTATAATTTATAGTTGGTTACATTTTTTCCTCAAACATGCTTGGAAATGTGAGCATTATTTTAACAATCTTCTTCGAGATAAATCAAAATTGCCGTACTGGCCCAGATATATGCGGGCAGCGACCGGCAAAAGTTCTATAAAAATCCATATCTGTCGTGTTTTGCGGCCAGATAAATTACTATGTAAACTCATATCTGTCCTGTTATTTAAAAATGGAATGACTTTTACTTCGAAACATGGCTACCAAGGAGGTAACTAATAAAAGGTTTTATTTAAATTTCGAACTGGCTTGCAAATAGAAAGCTGTTTATAGAAAAAAAAAAAACAAAACATTATGGCGC

		GTGAAATATTATTATTGTTCATTATTCGTCATTGAACTTAAAAAGTAAAATCGTGTTTTTATTTCGGCGCATTGTTTGAGA<br>AAAGTACTATACAAGCCTTGGCCACAACTAGGCATTGATAGACTCAATCAATAATTCATCAATGCCTCAGTTGCTGGCCGC<br>TGCAGTAATGTACTATTACCACTTAATCATTCCGTTCGTAATTGGCTTAGGTGGTAAGTTATGTTGTGATCTCATTTTCATA<br>AATGTGGGTTTTTTAATTGTATAAATATAACATTTCAATATTTAAACTTACTACATATGGATGGGAAAACATAAAAATGCA<br>CATGTCTAATTTATCAATATATTTAAATACTTTAAAGAAATAAATTACAGAGATGGATTACATTCATAATACAAACATAGT<br>ATTGAAGACATTCTTGTTTCAATTTATTTAATTACTTACTTAATTTTTTTTCCAAATTAATGTTTTATTATTAGCAGGCATTT<br>CTACAGTATCTATAAGAGACAGGCTATTCTCTTCCCCTAATTTTATTAAATCATTAATATCACGAATACCCCAAGATGGTTC<br>TCGAGCTTTAAGAGCTGCATTGAAATCAATATTGCTTTGAGGAGTTATTACTCCATCTTTACTATAAGGCCCATAAGTGATC<br>ATAAGAGCATCAGTTTTAAGATACTGACCTGCATTTTCAAATAGTCCAACAGTACATTCATAAGGGCTGATATGCATCATA<br>TTGGCACTGTACATATAATCAATACTATTATCATCAATACCATAACAATTCAATTTATTTCTTATATCAAGCAGTAATGGGG<br>GTAACATATTTTTGGTTGGGCATGTTTGAGCATAATAAGTAATACTCCCAAATAAATTTTCATCAATTTCCGATGGCTGAA<br>ATTTTACTCCAGGGAAATAGGGTGCAAAATGAAATAAGTGTTGACCAGAACCAGATGCTATCTCCAGAAATATAGGACTA<br>ACATCTTCTATTTTATCAATATCACAAATTATGAATCTTTTTAAAACTTGCAGTATTGGTTCACTATTTCTTGTTGCTGCTGG<br>ATATATTAACTTTTCTCCTTTGATACTGAAAAAGATATTTGATTTGTACAAAATATTTAATTATGTAAACACTTAAGGCTAA<br>TGTAATCTAACATCATCATCATTTCAGCCATTATCTCACCATTGTTAGGCATAAACC
MSTRG.12491	ATP-dependent 6-phosphofructokinase isoform X2	GCTTGAACTCTGTACGGAAATTTTCCCCGGTGACAAATTATTTTATTAACATGCTGTTCAAATTACGGGCAATGTAACTTTT<br>TTTTCACATAAATTAACTTAAAGATTGTCATTATCTATACTCTGTCATTATTATTTATTATAAACAGTGAAGTCCGATTGGA<br>TGACTGATAATGTATTGTAGTAAAAGCCAACACACTATCGAAGATATTACGGAATTTTAATAATTATCCACGACCACCTTC<br>TTCACGAGTTCAGCAGTGATCGGCTTCCCTTCGCGATCGATAGCCCCTTCCGCCACGATAATGATATTCAACCGTTGACCA<br>GATTTCCTCTCCTAAATAACATTATTAAAAACAATTAGTAGGCGATAATAATTGCAACATTGCAACATGCACATCAACAAA<br>ATTAAAATTATTTGTTTTTCAAAAATAATCAAGACATATATAAATAACATATAAACAGATTAAGAGATACTGATAGATTTT<br>CATAAAAATCGAATAAAAAAAAGCATTTACATTTGAATTTTAACATGCAAGGATTATGTTTTGTCTAACAATGATTTGCCT<br>TTTTGATGCCTTTTTTGATGGAACAAAAATAAGATATTATGTAATTAACTAAAGATTATATAATAGATAACTTGGAAATGT<br>TATCGAACATTAGAATCAATGCCATACTACTGCTGTGCAGTCAATTTATATGAAGAGGAGCATGCTAGGAAGGCCGAAGG<br>CCTTAGTAGGGCCATCGGCCTTCCTAACATGCATGACATGCATGCATTCCTAACATGGGGAATAGCATCGCTAACATAGAA<br>AGAAAAGTGGAAAGATATTCGGAAGTCAGAAACTTTATAATCATCTCCGCGCTCATATACAAAATCGAGAATAGATTAAG<br>TATTAGAATAAAAATGAAATAAACGCGGACGAAATGAGAGTAAGAAGGAAAATCTGTGGAGAAAGAGTTATATAGTGTG<br>AAAGCATGTTTAATTTGAAAGTGCAAATAGCATAAATGAAAATAAATATATATACATATCTATAGATGGGGAAGGTATAA<br>AAATCGGATTGAAAACATGAATGCGAAAATAGAAGGATGTAATAGTATCGAAGTAGACCTAGACTGACCTATTTACTGAT<br>CATGGTAGATTAGTCTAGGTCTACTTTGATACATGATGGAATGTTTGGGAAAAATTCAAATATTACATGCATAAGTTATAA<br>AAGTAGATAAAGAAAGAGATATAAGAACAGATACTTTGGGCACTCGATTCTCCTTAAATACACTGTAAAGGACCAGGTAT<br>GAATATAGAAAGATATACATAAGACAGAAGAAACACATAAAATAATGAAGTTCAGAAAATTCTTAAAACAGTACAATGT<br>ATATGGTTAAAATAATGAAAATAACTCAAACAACACACAACCACTATGGGTTTTAATATAAACTATCACCAACAATCCAA<br>AATCGTAACATAATTATGTATATTAAACAATTTAAAATATATTAAAAATTAAATAGTTTAATTAAGTGATCTAAAAGCCTA<br>TTAAACTTTATCTTTACACTGCAATAGATAAATTGTGTGTTATATTACAGTGCCACTCAATTAACCCTTCATACCTGGTGTA<br>ATCGTTTACATAGCTTTTCAACCCAATTATAGGGAACAGGGTCCTCGGGAATGAAAACTGCATCTGCTTCGCTCGCCAGAG<br>CAGCCACTAATGCCAGATAACTGCAACAAGCAGTACCCGCATAATGGAACATGCAAATGTCGCTCCCATATTTAGTTTAA<br>AGCGTTTATGAAAGATGTAGCCATATGACAAACAATTTCACGCAAAATTTTAAAATTTCGTCTAAGTATAAAACCAACGG<br>AATTTGCCAATTCAAGATGGTGAAATGGTAAGTTGATATTTAGTGATCCTGCTCCCATATGAAAAGGTAAATTCCTTGCCT

		CAATTAACTTTTCACACAACTTTTGGTCCCAATTGAGGGGCGCTGGATCTTCACAAATAAAACTATAAGTAGCATCCGTAC<br>ACAAGGCGGTGTAAACTGCAAGAAAACTAAAAAGATACTTGAATTATAGAAGATATGAGTGGAAGGCGGCGTGCATGGA<br>CAAATTATTTACATACAAAAAGGGAAAAGTAACATCCACAAATAACTGAGCTTGCCTGTTCTAACTTATTACAGAGTTTAT<br>TTTTCCAGTCAACTGAAGGTGGTGCTTCAGGAATAAAAACAAAATCCGCTTCAGATGCCAGGGCTGTGACCACAGCAAGA<br>TATCTAAATAAATAAGTGTGCATTAGCTTTAAGTAAATTTATTGCTGAATTGACTTAAAATACACCTATTCCCTCTACTGAT<br>AGTAATATGATTCAATATTGCCAATATGATAGAACCTCGATAACTTAAATTTAAAGGAAAATACTGAAAATTTCAAGTTAA<br>TGAGACTTCAACTTAACAAGTAATTAAGTAGTTCCAC
MSTRG.1<br>253	DD34D<br>transposase	ATAGATATAATTGCTAAATACGAGGTTTGTCCGGAAAGTACGTATAAAAGTTTTTTAAAAATTTATTTTACAATTATTCAG<br>GTAAATCAATTTTATCCCCTTCAAAGTACTCCCCCTGTGACATAATGCACTTGTGCCAACGCCGTTTCCACTGTGAGAAGG<br>CTCCTTGAAAGTCCTCTGGTTGTAGGTTTCTCAGCTGCCCCGTCGTAGCTTTTTTTATCTCATTTATGTCCCCCAAATGCCGC<br>CCCCGAAGTACCAATTTGGTTTTTGGGAACAAGAAGAAGTCACACGGGGCCAAATCCGGCGAATAGGGGGGGTGTTCGGT<br>CACCGTAATGAAATTTTTACTCAAAAACTCACGGACAACAAGAGAGGTGTGGGCAGGGGCATTGTCGTGGTGAAGAATCC<br>AACGCCCCTCTCGCGCCAATTCTGGACGAACTCGTGTCACACGAGCTTTGAGGCGTCTGAGCACATCGACGTAGAATTTTC<br>CATCAACTGTCTGGCCTGTGTTCGTCGGAATGTGAAGTGGAAGGACGTCCTGAACGTGGGTCATCTTGAACGCTTTCTCGG<br>CCCTCTCTGAATCTTTTTAGCCACTTGTGGACGGTTGGTTCCTTCACAGCATCATCCCCATAAACTGTTCTGAGATCGGCAA<br>AAATTTCACGGCCATTCTTGCCGAGTTTCGAGAGAAACTTGATTACGACGCGCTGCTCTTCAACGGAAGCGGGCTCCATGC<br>CGACGGGGTTTCGTTAAGAGGTAACTTCACAGATGGCGTGACAAGCCAGTTCGCACCGCACGGCGGTCAGACCAGAACTG<br>AAGCATTGTTAGGGACATAAGGAAGGGGTCCTGCGCTTACCTGGCCTCCCCACTCCTCTTTCTCGCATTCCAGAACACTTTT<br>ATACGAACTTTCCGGACAGACCTCGTACTTTGTATT
MSTRG.1<br>258	N(G),N(G)-<br>dimethylarginine<br>dimethylaminohy<br>drolase 1	TGGTCTTTCTACTATGAACAGTCTCCTCTGAAGTCTCAGTGTCTTAGTATTGTCAGTTCATGAGGTGTATTAACGGTATATA<br>ATGTCATTTTATTTTCCCTTAGATTGCATCTAAATTATTCTAAATTTATCACCTATCAGTGCATGTGAAATACTTTTACAAAA<br>TAGACCGGGCTTTGGTTATATGAGTGACAGAGGTGTTTATTGAGATAGGAAGAATAATTATTGAAGGACAATGGATTTTA<br>AAATGCATGAATATACTCATGCAATAGTTGGAAAAGTGACACCAGTGTTGAGACTCACAGACAAAGCTAATTTGAATGAT<br>GCCCGGCGACAACATGATTGCTATTCGAGACTCTTGAGAGAATTAAATGTTGAAGTGATAGAAGTGGACATTGGGGGCAC<br>TTTTCCAGAGAATGTCTTTCTAGAAGATGTTGCTATAATATGCCATGGTATAGCACTGCTGCCAAAGCCATCTACTACAGC<br>TGATGAGCTAAAAGTAA
MSTRG.1<br>2598	Uncharacterized<br>protein<br>LOC106125623	TATGTTATTCATTATTAAAAGAAATGTCTGTACAAATGAGACTAATAGAAGCTAAAACTTTTAAATTATGGGAAAATTCCT<br>TAGATTTACACATTATATATTCATATCAAACGACTTACTTTAAATACGAGTAAATACAGTAGTTACTTTAGATAGTTATTAG<br>GAATCGCTATTTTTTCAAATGTTGCACTACATTACATAATATTTATATTAAATGTACAAATAACTGTGATGTGAAAGTGAC<br>AGTGATGTGTGAAATCTGTCTAATGTAAATGAAAAGGATGACAATTCAATAAATGAAAATGGAATGACAGCCCCGCCTGC<br>GCTAACAGTATACGCTGGCAGGGGAACAGTGATGGGTGATCGATGAGAATGAAAACAGGCCAGTTAGCCCAGAACCGCG<br>TGGAGGTCGACCTGGTTGGGTTCACCGGATGAAACAGAACCAGTGCTGACGGTACTTCGAACCCGCGGAAGGACTTACGG<br>AGCGGACTTTCGCCGTCTCCTGCCCACCGGATACGACTTAAATATTATCTAAGAGCACCACGAGGTGGTTTGAACCTCAGC<br>TCGGGCTGCCTTATCTCAACCCTCACCTGAAAGGACAGGAAGGTCGTTTCCATGACTTTAGCTTATATAAAATACACTAGA<br>ATTGGGCTAACGTGTCTACGTGAAACTTAACTGGAAATTTTTCTAATGCATGACAAAGAGCTTTGCTAAGAACTTAATAAA<br>CAATAGAATATAAGTAAATAAGTATACCAACCTGTAAGTAATTATTTGTTCACTATATTATAAACTAAATATTAATTTATT<br>ATAATAATGCATATCAATGGTTTTATCTAAATAATAGCATATACGCAGATATCAAATAAATGCATCTTATTATTTTTCCCAG<br>GTACCTAATCATAACTGATTGTTCTCGTACTTAATTAAATACCTATCTACATTATTACAGCTTAATTAAATAAGATAAAGTG<br>CAAAATAAATAAATACTTATATACGAACTGTGCATACAATATTTCTTAGCTAAAGCAGATAATTGAGTCAGTTGTAATAAT

		TACCCTCATATCTGTTTAATCTATCTGTAACAATTGTTGTAAGTACCATAATTAGATATTATTTTATATATTTATTTTACTTA<br>ATAACTTTTACTTAATAACTTTTACTTCTAATAATACTATTTTTAAAGCCATGTGCCAACCGTCGCTTATAAGTCTATATCTT<br>ATGAATATTTTGCTAAACTAAGATATTATCTTATTATCCCTACCAGAAATTCCACAATCTTCCATATGAACCATTTATCTCC<br>GACTGCCATTTTGAATCACACACGTTTTGCGACACATTTTTCGTCTCTAAACGAGGTATTTTCAGGCAAATTATTATACGAG<br>TGTTGGCAGTCACGCACATACTAATGACAGCGGAATACAAAATCGGCGGCTCTGTCACATGCTGGCTCGACGGTAAAGAA<br>GTCTACTTCACCAGTTTTCGTCTTGAAGATTTCTGCTCCGTCTTTTAAGCAGAACTAGCAGCCATACTCAAAGCCATTGAGA<br>CAATGTGCAAAAAGCCCAAATTTCAAAACGCAAATATCTTAAGTGATTCAAGATCAGCTTTAGAAAATATCGCTGATCCAT<br>GTCCACTCCATCCGCATGGAACTCCGTCAAAAAATCGAGAATCTCTTGGAACGCGGTGGAAAAGTCAAATTCTACCGAGT<br>GAAAGCTCATATAGGTATAAATGGTAACGAACGCGTCGACAACCTTGCCAAAACTGCGGCCTTGAAAAAGAAAACAAAA<br>CCTATATATGATCGGTTTCCTCAAACGTACACCAAAAAACTAATGAGAGAAAAATCACTAGATATTTAGCAAAATTTATAT<br>ACAGAATCGAATACAGCAGCTGTAACAAAAATGTTCTTACCTAATATCCGCAAAGCATATAAAATAGTCAAGATTATAGA<br>TATAAATAACACAATAACTCACCTCACCGGACATGGAGGAAACCGATCATATCTTCATAGGTTCAAATTGGTGAATAGCC<br>CCAACTGTCAGTGTGACAATGAAACAAACCATACAGCATTTACTGTTTGACTGCCAAAGATTTGCTAAAAACCAATATGA<br>GTGTGAAATCAGAATGGACATAGCACTATCAGAAAACAATATAGAATACATATTTGAAAACAAGCAAACTAGGGATATAT<br>TATTTAACTTTGCATTTTTTGCTGAGGATCGTGGCAAGGGAGAACAACGCAAGAAGAGTGGACTAAATACCGGAGACGAT<br>AAAGTTTATACGTTTACTTAGTTATTTATGCACCTAGTTTTACAAATTTATATATACATAAACTTATATATATAAAAAGAGG<br>AGAAAAAATCCTCAGTTCTATACACAGATATATAATATACAGACTAAAAGATTATGCGACAACCTTTTAGTCTGTATTTAT<br>ATATCTGTGTATAGAACTGAGGATTTTTCTAAACATAGCGAC
MSTRG.12634	Microspherule protein 1-like	ATATAGTTGTTGACACAATTCAGTAGATAAAAATTATCAGCGCCCATTTAGGTACAAATAACTCACACTATATACGCATGT<br>GTGGACAGTTTGCGGTTCACAACGAAATTTTATGTTAAATATATTAAAAAGGGATTGTGTTAAATGATAATACTTTTATAT<br>TTACACACAAGTTATAAATTATGCTAACTGTATGTTACGTGAGGCCACAGAGGTTAAAGACAATAGAGTAACTTGTGTTTC<br>GTATCATTCGTTTCGAGTCATTGAATATGTCTAGTTGGTGTTGTGTCAGGATAACATTTACCTACACATTACCTTGTAAAGG<br>CTGTACTGTCTGATCGGGCAATAACTGATATTGTTTGAGTAGTTGCCAATGGGCCATGAGTGACTTTGCTGTACGAGAAGG<br>ATAGAACACATGTGGGTTTTGTTCAAGCAACTCCTGGAACTTTTCAGGCTCTGGATGTGAAGTCTGTAAAATAAATAAATA<br>AAATCCTTTTACTTTAGGCATACCCCATAGTTTACATTACATTTAATTCATTAGTACAATTTAAAATTAAAATATTGATATT<br>CATGATTTTTATTTAATTATTGTAGTATATCTAACATACTTATTTTACAATTAATAGTTCAGCTTATTTATACCTAGTAATAA<br>CTGAAAATTATTT
MSTRG.12673	Probable peroxisomal acyl-coenzyme A oxidase 1	GTTTCCCCTGCATGAACGGCTTGAGGTGCTTGTGGAAGGAGCTGTTGACGGGCTCCGCGTTGAGGGGACTCTTGAGGGCCT<br>CCTCCATCAGGCGCTCGTACACGCGCCCGTCGTACGCGCCCAGTGTCGAATTCAGAATCTGAATATAAAAACAACTGCGA<br>CTGATGTCGGACGGCCGAGTAGCGGACAGCAATAATTGCAGCTGCAGATAACTGCATCGCGGCGTCATTTACAATATTCA<br>CCCTCACTATAACC
MSTRG.12683	PiggyBac transposable element-derived protein 3-like	TTAACCCTTTCAGAACTGGCGTGCACAATTGTAGACATAAAATATAATTTAAAAAATAAATATTAAACTCTCAAGCTTTCC<br>GATTGAAAAGGGGTTGTATTCATTTGTTCACTAATAAACTACTCAATTTAATACTACTTACGCCATTTCTTAGCATATTCTA<br>GCGTTTTTCGACAAGTTGCGACTAGTGTCCGTGCGTGATTTGAACTGTACAGATGCAAAAACACCAATATGTCCTCATTGG<br>AGGCGTTACAAGCATTAGATGAAGGGATCTTTCTGACTTATTATTATCATCTGATGACGAAGATAATTATTATGGAGATTC<br>AATTCGTCCAGCAAATTGAATCTAGCAGCTAATGCTAGAATTAGCGCAGATGTTATTCACCAAAACTGAAGGGAGTTTTTT<br>CCACCTGCATTTGATGACAATGAAGGACCTAGCGAATTGTATGACGGCGTCCCTGCTTTACTTTGACAATTATTTTTCATCA<br>TACCATTTACTACAAATCCTAAAACAAAAAGGTATTATGGCCGCATGCACAGCTCGTGTTAACAGATTTGTTAAACCACCG<br>TTTTTAAGTGACAAGGTAACAAATAAGAAGCCTAGGGATTTTTTTCAAGAAGTATATAGCAGAGACGGAGATGTAACTGC

		TGTAAAGTGGTTGGACAATAAGATTGCCCATTTGGCTTCCAATTTTGTTGGTATAGGTAACAAGGACTTAGTAAAGAGATG<br>GTGTAAAAAAAAGAAGTGATATATTGAGGTTGCGCGACCTGAGGTCTTAAAGCGCTATAATCATGCAATGGGTGGCGTTA<br>ATTTATTAGATCAACTTATGAGCTATTACAGAATATTTATAGAATCAAAAAAATTTACTCTACGTATGATATTTTATGCATC<br>GGATGTGGAAGTTCCTCAGGCATATCGAGAATACTAAATAGATTACGACTTATAACGGCACACCTAAAAAGCAAGGACTT<br>ATTTTACTTTCGTCGTCGCTTGACTGACAGTTTGGTGCTACAAGATAAATGTTCTACCACCAAAAGAGGTCAAGCAAGTAG<br>CAATAGTAGCTCAAGGATGCAAATTATACCAAGAAGGCCAGGAGAGATTCGCCCTGCAGCTGAAATAACTCGTGACGGTG<br>TTGGGCATTTCCCTACACATGACGATGGAACTGGGACTAGGTGTAAAATGGTGAGATGCAAGGGAAAATTCCGTGTAAAA<br>TGCAAAGTTCATTTATACCTAACCAAAGACAAGAATTGCTTCTTGATATTTCACACATAGATAAAGTTTTTTTTTTCCTAA<br>TTATTGTTCTTACTGTGTTTTTGTATTTGTACCAATGACTGATTATGCCAAAAAACTAATAAATATTTATTTTATCTCATAGA<br>AAAAATTTAACT
MSTRG.1<br>2722	Hypothetical protein KGM_201411	ACAGCGTCGCGCGCGCAGTGTTCGCGGCGCCAGCGTCACGATGCGCGTACTACTGTTGCTCGCTCTATCCACGGTGTGTCT<br>GGCCGCGCCACAACTATCCAGGTCCAATCTGCAACATGGATATAATGACTCTGATAGTCAACCGTTCATCGAGGAAGTGG<br>TCGTGGAATCCACGCTCCATGTGAAAAATCCCATCGCCGATCGACAAGCACGCACCAACACTAGAACTAACCTGCAAGAT<br>GGCGCCAGGGTCGCAGAAATGAAACCTAATCCTTAATTATATTCTAAGGACTGTAGCTTCGATAATTTATGCACCCCTCGA<br>TGCACTTTTAATAACATTTTACGTAAAATTAAATCCATATATCTAATAACACAAGGACATTTTATAATGAAAAACTGTCAA<br>AAATCTGAACACCTTTCTGAAAAAAAAGAATACTATAAGAAGATAAGCTGACCCGGTAAAAGTTGTTCTACCATATATTA<br>TATATATATATTTTTTTTTCTAGATAGCTATCATAGATCACCTGGCTAGGTGATTAGGGTGTCCCTATATATATCATATCGC<br>TATATGAAATGTACTTTACGTTCTACTTTCATATCTAAAAAATTTCATACAAATCGGTCGAATCGTTTCGCAAAAGTTCGAC<br>CTCAAATACTGTGACACGGAGTTTTTATATATAAAATTTGAGCTTCCCATATGACGCGTTTAACTAAATATAAAACAGTAA<br>CACTTTATTCCATGGCTGGACCATGACATAAATAAAATGTTTTATTAATAAGAATAATGGACTATTATTACGGCTTCAATTT<br>ATTCCCAATTTTAACCTGCATTTATATTTTATTAATAAAATTATTTTATGTTTTAATTATATTATGTTTTAATGTATTTTTAAT<br>AACTACCGGTTTAAGTTACATGTATTTTAGTTGAGAACAATATTACGAAGAAGAAAAATTTGATTTTTTGTTAGTAATGAA<br>TAAACTAAAAAAAAAACCTTACCAGAAAAATTCACTTATCTATAGAAAGCTACATAATCAACGAATAACTAGGATCCTGT<br>ATTTTTTTTTTAAAGTAAGAAGTAGACATGGGTCCCTTGTATCCTATTATACATTATGTAAGTAATAATTATATCCCTTAAT<br>AAAGTGTATTTACTTTTTTTTATACAACTTAGAATGGCAAACAAGCTGACGGCCCACCTGATGGAAAGTGGTAACCGTCG<br>CCTATACATATGAGCAGTACCATGGACATAACAGAGTATACTGTTTCGCCCATGATACCCCCCATGCGTTGCCATTCCTGA<br>GGACTCAGGTGTTATTCCTCCGTACCCAGTAAATTCACGCCATACTTTAAATACACTATCGGAACTGAACATCATGAACAT<br>TTTTTCTATGTAAATTTTTTATCATATTAAGTTCTGATAGTGTATTTAAAATATATATAAACAACGCAAATGTTTAAAATTA<br>ACTAAAGTGTATTTAATCTAAAATACTTTAAGATCGAACCTGTGATTAATATATATTAATAAGCTATTTTTTTTTTTTATTTG<br>TTACTTCAGCAATATAATAATAAATTAATAAAACAATAAATGA
MSTRG.1<br>2744	Lysosomal Pro-X carboxypeptidase	CTAAGACGATCCTCAAACTATTACATAAATTGTCTTACAATTAAAGTCAATAATTATCAGCGTCGACCTTCCCTTCAATTGA<br>CTGATAAAAGGACTATAATGAATCATTATCACCGATCCAATGATGACTGAATCATATCGGGCAGAGAGCCAATAAAAGTT<br>GCGACCACGCCAGCGGCTGTGTATATTGTGCTATCAATTGGAACCCGATACAGTGCGCGTGCGCCGGTGCAAAATGTGGA<br>GGGCTCTCTTTTTACTGGTGGCGCTGCTGGCTACAGCCAAATCTGAATACAAATACGAGACGTTATGGTTCAAAGTCCCGC<br>TCGATCACTTCGGATACCAGAGGAACGAGACCTTCCAAATTAAGTATTTGGTGAACGAAGATTATTGGGACAGAGGAAAT<br>GGTCCGATTTTCTTCTACACAGGGAACGAGGTAAGAACAATATATAATAGTCATTATTGCACTTAAAATAATGTATTTATA<br>AATGTTGCGAGCAAGGGTGGACTTATCTCTGACAGAGATCTCTTCCGGTCAACCCAATACAGTGAGAAAGCATTTCGTTAG<br>CGGGCAGCTGAAGTGCTACAACTATTAATCTCCCTCAGTTTCCATAACTAACACTATTATCCCACCACCCATTATCCCCCCC<br>CCCCTAAAACCTTATGACGAAAGAAATGTTAAGCTCTATTTACAGGACCAATTTCGATATGAACGAAAACAC

MSTRG.12772	Ribonucleoside-diphosphate reductase subunit M2	GGATTCCCTACTAGTTGGTAATTATCGTGTTTCAAACGTAACCATTAACCGTGCTCATGAGTAAGGACATTTCTCGAGTTCG<br>AAACTATCGGCATCCCCATATTTAAAACATATAAGCTCGTTGCTGAAGGTCAAGCCTGGCATGAGACCTCTTTTCTTCAGC<br>CAGAAGATGGATGCAAAACTACCAGAGAAGAAAATGCCTTCAACTGCAGCAAAGGCAACAATACGCTCGCCAAACGTAG<br>CGGATTTGCTTGCGATCCACTGCAGTGCCCAGTCAGCCTTCTTCTTCACGCATGGCAGAGTCTCAATAGCGTTGAACAGGA<br>AATCCCTAAATTTAAATTAGTCATACATTTTTTGCCTTTAAAGATTCTATTATACATAGTAATAATCAAATACTATAATAAT<br>ATTTAATATTTATCAAAAACAAGGCATGCTGTGGTTGCCTATAAATGTAAGAACAGTAAGATCTAATCTACCAGTGTACTG<br>TATTGAAATGTTTAGCTGTATCTTATATCGGTAATCCTATAAATAAATAAATACAGCAATGATTTATTTATTGTTAAGTATT<br>AATGTTCTAATTTCAACCAACTTTAAAAAAGAGGCTCTAAATTCAGTTGTATTTTTTTGTGTGCTGTTATTTCATTACTTAGT<br>AGTAAAATGATTTGGAAGTTGTTTTTTATCTGAAATGATATACTTACAAATTGATCCCATAAATTTTTTATCAAAATCAGTT<br>CAGTAGTTTAGTTTTTAAATCAAAATAACTGGTTTCACCATAATTAAAGTCAGTTTTTTTTAGTAGAAC
MSTRG.12781	Neuroblastoma-amplified sequence-like	GCGAGGACAGCGGGTGGTAGTGCAGGCGCACCAGCAACGGCAGCCGCCACACCGACACCGCGCCCGACCAGTGCAGGCA<br>CACCAGCTTCGACCCGTCCGGAGACATCACCATTTGCACTATGAACTCCTGAAGCGCGACCTTGTTGGCGGCGCGCGGCAG<br>GCCGGCGACGTAAAGCGTGTGGTGCGGCGCCGCGTACAGCAGCGCGCCCAGCCCGGCCGCGAACCGGAACGTGTGGTGC<br>AGCTTGCAGCCCTCCGTCTGCGACACCACGTACCCGCGCAGCTTGCCGTCGTGCGTCACCACCGCCACCTCCCAGTTCCTG<br>CACCCATCACAACACCATATGACAATACTTTCATAATTCGAACCATCACAATTTGATCGTATATTTATTTTCATTTTAATAT<br>AAAAAAGGCATCAGAAATACTTTTACTATTTCTAAAATTTACATTTTGTACATTTACATTTTTTTTTCTCACAATTTACCTTC<br>ATATATTTTGATAACTTAATGTATGTAATAATATAATTATTTTAAGTTCTTAAATATCTATTATTGTGCCACCACATTACTG<br>ACCTTAAATATATTATACTGACCATTTATTATCTTTGACTCGAAGTGGCATAAATATTATGTCAGCAACAGCATGTGTATTG<br>TCGGTGCACTCTAATCCACCAGGTCTTGAACAATCCTAAACAGGATTAAAACAAATATGGTTTAATAAAAATAAAATTTCA<br>TAACATTAAAATAAAAATTCAACAATAAACTAATACCCTAGTAACAAATGTAAATATTTTAGAGCATCTAAGAGAAGCAA<br>AAGAATTTATATCAAAATATAACAGATAAAATAAATTAATAAAGTATTCATTTACACTACATATGAGACAACATAACACA<br>AAAAAAAAAAAAAATAACCAGAAATATTTATAATTATGAACATCTTTAAGTAGGTACTTTAAAAAAAAAATACTTACAATTG<br>GTATGTTAAAGAGATTGGAGGCTGTAAGGTCAAAGAAGCTCACAACCCCATTGCCATAGGCGAGAACCAGGAATGAACA<br>GTCAGGACTCCATACCAATTTACGCCACTGTGGATATGCATCACGACTCACTACAAGAAAAAAAAAAAAACAAAAATAATA<br>AGAACTCTTTATTATACTGGTTTAAATATATTTAATAATATAACATAAGAGTTAACAGACAATTTCCAGATACATATTTCTT<br>AAATATAATTTATTTAAGAAATGTGTATAATGATTGCAGTTAAATAATTAAATCATTTTTATTAAGTTGTTCATTAAGGACA<br>GGACTTAATAAAAAGCAGACATCTATGATATTTCAGACAGCTTATATGCTTGACTAAGGCTGA
MSTRG.12804	Uncharacterized protein LOC106138099	CACGAGGCCGGCACACGGGACAACTTGAAGACTCACTCTTTGTTCGCACATCGAACTGAGCACCCATTTTAATCATAATTG<br>GACACTTTAGGGAATTTATATTGCAATTTTTATTATTAAATTTAGTAAAATTTCGCTTTCTTTTGAACGCTCCAGTTGTCGTC<br>CTCGTAACTGGCGCATGCAGTAGGACAGTTGGAGTCAGTGCTCGCCGCAGTCGGTATCCAGCTCCGACAGTCCCTGCTCCT<br>ATTCGTAGCCACTGGTTGAGCTGTCAAGCTTTCGAGTGTCCTGACTTTGGGAGACGAAGCCAGAGATCCAAGCGTAGATAC<br>AACCCGAGAACAAACGAGATGCTGCTCATAATTAAACCTCAACACCGAGCTGGAACCAGATGACAGTATGAACTACTCAA<br>ACGCCGTACTCTCCGAGGATATAGCACGGAGTATGACGGAGACGAAGCAGGCGGAGGATCCCGAACGCGTCACAGACAC<br>GCTGTTCATAGACAACTGCGATCACAAACAACTCAAAGGTACTCAGACTAGAGCCTGTTTCGATAGACCTTAAAGAAAAA<br>GTAACCGAGAATTGTTGAAATTATCTCGGGGACACAGTGTAAATGGAATTAGTAAATAAATAGTGAATAATCCAAACATT<br>TGAAACAGTGCATGCATGAAATAGAAACAGCCAAGACAAATCTGTGTGCAAAACCGAATTAGTTAAGAAAGAAAACGAG<br>TGGGTCGTCCAGAAAGAGAAGATTGATATAAACGAATTCATGCTGCCCAATTTCAGCGAAGAAGAGATTAAAAGACTTAA<br>CATAGAAGGTGACAATCACTGTGAGAGATGACGAACCATGTACCATTTCGCTAGAAAGAACGATATAAAAATGCTATTCC<br>CGTGTGATCGGTGCGAGAAAATATGCAGGACACTGAGCTGTCGCTGAAGTTGCACAACGGGAAACACGAGAACCCCAAG

		CCGTTCAAGCGTAAAGTGTGGAAACGCAGGATTATTGGAGGGCGGTGTGAAGTCTGCAAAAGCTCATTTGAGCGAGACAC<br>CGGTCGGAACCGATAAACGTTCTGCATAATTGATACATCGAAATAGTTTTAATGCAATATTCACGTGACTAGCAATTGTT<br>TTTATACATTTCAATCGTATTACTGAATATATAGTATAATTATTTATTTTATTTTAAACATGCAGTGGCAAGATCGTGTCTA<br>ACGTTAGACTAGGTGTCCATGTGACTACTGCTGGTCTTCAACGAAATATCCATGGACGAGATCGTTTAACCACCAATATTT<br>CTCACCAGAGGTAGACTTTGTACAAGTCGTCTGCATGGATACCTCTGTCCCATCCGTGTTCTCGCCGTGAAGCAGCTGCGC<br>TCGCATGGCTGTCTTTCGGCTCGAAGGATGGGGTATGGCAGCGAGATCACAGGACACGGAGGCATAGCCATAGTCCTCGA<br>GGGGGGAAGGGATTGCTAGTAATGCGGACTGTCGGCACCATTGCTAGCAATACACCCGAACAGGTCGACCTCCACGCGGT<br>TCCCCCTGGAAACCATCGTGATCAATCATTGTTCAATTGGTCACGATGGGCCAACTGGCCTGTGTCCATGGTGTCCCGCCG<br>GCGTACACCGCTAGCGGAGGCGAGGCTGCCATGGACACCACCACGCCATTAATTCGTGGTGGATTAAAATAAAATTAATC<br>TGATAGGAGTGGTTTAAGTATATCATTTTAATATAGTTTTCTTTTTTCTTTCATTTTATTTCGTTTTTAGCGTAAGGCATTAG<br>TAAGTTTCCCACTTGCCCGCTGGGGCTGGTGGGCCGGTGGGCTGGTTTCCTATGCAGAGGATCCAGGTAGATGATATGTCG<br>AGTAAGATTATCCATTTTAATTATTTTACTTCTTTTTTTCTCTAGCGTAAGGTATCGAAAACATATATATATATAACTAATGTA<br>AGTAGTTTAAGCATTCCATTTAGTATAGTTTTTTTTTTTTCTTTTATTTGTCTTTAACGTAAGGTATTAAAAATATAAGATGT<br>CTTATTTTAAGATTGTGGGTACGATAATTGTTTTTATATTGTGTAGCCTCCG
MSTRG.12811	Endonuclease-reverse transcriptase	CGTCGTGCGTGCTGGAACGGGTGACGGCGCAGCGGCCCGCCGCCGCCGCGCCCGCCGCCGCCGCCGCCGCCGCCGAC<br>ACCGCGCCGCTGCTCATACACCACCTGGCGCCGCCGCGCGCCTGCTGACCGCCGCGCCGCGCCCGCCGCCACCACCACCA<br>CCACCACCACCACCTGCCCCTCATACTGCTGTAAGCCAACACCTACATATATATCACAAATATAGATGACAATAGATGAGA<br>ATTTTATGAGACTAAACACGCCATGTTATTTATACTTTTTGTCTTATAAGAGCTATTTATTGATAGCTATTAATTACTTTCAG<br>TATTTTCGAATATGCCACCTGTAGTATAGTTTATATATAATAGATTTTGCAAAACCTAGACATTTTTTTAATATAAAAATTA<br>ACGAGTCCAATTAAGCTCTTTTGAGCATACTTACAACCAATTGTCGAGCAAAACAAGTAGCGACATCTATTGACATCTATA<br>TTTGTAATTATCACTAAACTTCACAAAATAAACACGACGGTCATTTCATGAAATGCACACAAAATGTGGGCTTCCTCGACA<br>CAGTTATGTCACACCTGGGCGGGACATGGATCCTTTGACTTGTTTATATAGATTTCAAGATTTAGACAAGATGAATATATA<br>TATTTCGTAACATAATTTAAGCCTTTGTATTGTATCAGTCGTGAACTGTCCACAGCTGAACATAGGCCTCCCCCAGGCTTTG<br>ATTTTGTCTAAACAAATTGATTAGTGCAATGAAATATGGTAATTGTGTAATCCTGAGACTTAAACCATTTTTTTTTTCATCA<br>TTCATTCAACGTTTATCACATCTTTATATTTAAATAGGTAACCTTCGCCTACGAAAAACCGCTTTTTATTTTTCTAATTCTCA<br>GAAAATTGAAAGTGTGTTTATATTTTCCTAAATATACACAACATTCACTTATTACATTGCACTGTCTATCTTGAGTTCACCA<br>GTTGTATGTGTTTTAAAAAACACAACTAAAAACGATCCCAGGCTAATAATCCAGTAGACTAAAACTGAGTAAATTGTTCTT<br>AATTTTTTTACATGTTAAAAATATTGTTATCAATTGAACAGTATGGCTTTTTTTAACATGATTAAGTTATAAACTATAGAGA<br>GTTTTAGATACATTTATGAAATGTTAGTTACTAATACGTCGTCCATCTTATTATTTTCTTTTTCACGGTTGAACTACTATCAA<br>GTTCCCTATTTCTTTAAACTTGTCTCTTAACCTATTATTTAAAGTTTTGCGATATTATTATTTTTGAATCCATCATAAACAAA<br>ATTTTTAATAATTTTGACTTAAATATAATAGAAATTTAATTTATCTGTCTTTTAACTTAAGAATAAAGATTTCGCTATTAGT<br>GATATTAAATGAAAATTTTAATCATAGAGATATATCATAGTGTTTTTTTTTTTATATAATTTGTTTTTTCTTATATAAATTGC<br>AAATTTGTATTCCTCGATATATGGGGTTGATCATAGAGGACTTATTTCAGATCATAGACATCTTATTTTACCATCCATTGTT<br>ACCTATGTATCACAATTTAAATAAATAAATAAATAGAGATAGGTTTAAAGAAATGCCACTTCATAATCGACATCAATATAT<br>AACAGATATCATTGTTGCGAATATAGATAGTGACATTTCTAAATTCTATATCTTAAATGGTCGATGTTCAATATGACTGAT<br>AAATCCTATAATAATTCAGGTCACTAATCGAAATAAGAAACATGGATATATATTGATGGTAATGCGTATCACTACCACTCT<br>GGTCAAGTATCGATTTATGTATATCGATTATCGATAAAACATCGAATCGAAACAATCGTTTGAACATCACTAATTTATCTG<br>TTGAACTGTCATCGAGGAATAATAAGATGGGCATAGTACAGTTTGAGAATTTTCACGCAAATAAATATATAAAATATTAGT<br>GTTGAGCCACAATCGTGTTAAATAGCTGATCAACGTGAACTTTCATTATAATCGTCACTGTAAAATACTATCCGTCCTTTTC

CAGTCTCAGAAAAACCCAATAGTTGACTTTTGCTCTTCGGTGATATCATTTGACGTGTAGTAGAAGTCTTCTAAAGGCATTT
GTAGAAAAGAAAATCTATGAAATTGTTATTTGTAATTAACTATTATTTTTATACGTAATTGAATGTGAAATTTCGATTGCTT
CCGATTTTATTCAAATCGATCTTTTTATTCGATTGATTAATAGATTTTCGCTTTTTGTAACGTTTGTATTTATAATTTATAAG
TTAAAAAAAAATGCAATTTCATTTATATACAATTAAGCATATATAAGTTTATTATAGATCTCAAGCAATTCAAAAGAATCTG
AAAATTAAAACAATTTATTGCATTAAATGAAACGAATTTGAGACGTGGTGATAATATTTGTTTTATAATGATGAAATTATC
AAGGCGATAATGTCTGCTATGAATTGACTGCTATATTTTAACATAGCAACAAAGATCAATAAATCTCTGATAGATATTTGG
TATTAGTTCAATTAGAAAACTGATCCATTTGTGAGATGTGAAGAGAATTGAGGGAAATTTAATAAAAAAATAAGTTGTAAA
TATCGACTTAACAAAACTGAATAATAGGTATATCACTATTTTACCCAAGTGTTGTACGACATAAATTCAAAAGAATTTTTA
TATGCAATGAGTTTTTTTTTATAATCAAATTAGTAAGAAAATCATATTAACAATTAAATCAGATTAATTTTGACTTTTTTAT
AATAATTATATTAATTTATATTTTTATATACTTACCATAATTGCTATGTAATTTGCGATACTTTATTGTAATATAATACATAT
AATGTTAACATAATTACAAAAGAGTTATATAGATCATGGAATTGATAAATGAAACATGTATTAGATTATAATGTATAATTT
TTGATTTTATTTCAGTATAATAATAATAATGCATATATGTAATAGTCTTGACAAAGTTTGTTTATAAATTTATATGTAATTC
AACCTACTTTTTCGGTATTTAAGTAAACCAGCGTTGTTTATAATGTTTACTTTATGTAACGTTTAACAAAACTGTGACAAAC
GTATAAAGCTACTAACTACCCCACCTATGATCACAGTCGTGATCATGTTTTTTATGTTATCTTTTAGTATAAATACCTACCT
AATTATTGATATAATAATATTTAACAATTTTTCAAATTTAAATTGATGAATGAAAAATCTTCCTACCCTTATTCCAAGTATA
TAAATTTTGGGGTCGGCGTAACATATTTTTATAGATAATACCCAAGGTTTGGCATTTAGTTTTACGACAGGCCGCCTGTCTG
ACGTCAACCCTCCTTGGGAATTTAATTAATGAATGAGAGATGTATGATGCACCTGACAATATGTCGTATACTTTTAGTGTG
TATAAAGATTTTTTTATGTTACGCTGTCGGCGTATGCATGTAGTAACAATGTACTTCCTTGGCCAGCATAAGCCCTAAGGCT
CCAAATATCTACAAAGAGGTCCACCTCTATTCGCACACAATCTTTGGTTTTTTTTAACCAACTTCAAAGGAGAAGTTCAAT
GCCTATGTTGTACACAGCTTTAAATAATTATTTCTTAATTGAATAGGGTTACTGAATTTAAGTTGGTTTCCTTATTAACATT
ATTTAGTGTTTGCTTTTCAGTCTTGGAAAGATATGCCTGTTCAGAGGCAATACTGTAATATTTTTACTGCGTTTCAGCGAAA
TTTTTCTCTATTTTTCAATACCGCAAAAGCGAGATTGTAAATGCGAATTTTGCGGTATCTAAAGGGGAATCCCCCTATCGA
ATAGCACTCGCACTTTACTACCAGTTATTTTGTCTTGCAGTTTTAATATACTCCCGAGTGCTATCTGTCACAAATATCGCAA
CAGAACTATTGGGTTTTTTTTTTTAATTTGTGGCACAAGTCAAGGTTAAGCTGACTGACGTAATTATTAAGCTGAGCATGTA
TAATTATTTATTAATTCTGCCAGATTTACAATTAAGTCGTATTATTTTCACCTAGGGGTTCTTGCTAATATCCATTCATGTTG
TAACTATAACTTACCATACAAATATATCACGATACATATCTTGCCCCACCATTTAACAGAGAACCCCACCCCAATGTTAGT
AAGACTGGCGTTAATCATTATTTTATTATTTATAATGTTGGCAATATGGGGCGTCATTTAATTTATCGATTAGATTACAAGA
CTTATTGAGTTATAATAGTCTTTATGAACTTTTATAAGAATCAATTTTGTGTGAAGTCTTAAAATAGATTTAAGTGTTTTCA
TTTCCATAAATAAGTTCCTAATATTTCTAAATTCTCTTATTTCATTCTACATATGACGCCTCCTATTGCAAACGACGTGTTTT
AATTTAATTGGCTTTATATTTACTTAGTTTGTGATAAGTTGCAACTTGAAAACTTGAAAGTGCTAAATTATGACGAAAAGA
AAGCAATGAAGTAAGCATTAATTTTAAACTGATGTAAATAAAGTGATTAAAAAGTTGTTTAGCGCTGTATTTGTCTTTAGA
ATAAATTTGAGATTCGTGCCATAGGAAACGTTGGTGATAAAAATTTAAAATTTTTATAGTCTACTTGATTTACATCAAGTG
ACATGAATACACCATTAAAGTATGAAATTAGTTTCCATTAATCACTTGTATTGTGAACATCACTATTTAAACTGGACTATTT
AAACTAGGAATAATCCTAATCAGTTTATGTAATTTTGTGTCTCAACTATTTTCAATTACTTGCCAGTACGTATAATATTTTC
TATGTGTTACCATAAATGAATAAGTTGATTTTTGCAGAAGAGTTATTTAAGCGGTTCAATTACAAGACTATGGACGCCGTG
TTCTATGGCCAAAAAAACACATATAGATTTGTGCTTTTTTTCAAAAGAGTACGTTATTACAGAGAGGGTATGAGAATAAAA
AAGTAAGCCCGCCTGCGCTGTGAATATACACTAACAGAGGGGGTATAGACAGCACTAGAGAGGGTCTCACCCTTTCGCGA
TGATAAAAATAAAAAGTTACTTTGGACTTACTTAGTAAGAGCTACATAACGTATGTGTGTAAAATTTCAACTTTTACAACC
AACACAAACACATTCACAATCTGACACCGCAATAAAAGGGATACTATCTATCTATCTATTTAAGAGCTACGCTCTTGTCGG

		TGGAGCTCTTGCCACTCACTACGATTTTTAGCGAGTCTCTTCACCTCTTGATACGACACGATACCAACTCCTGCCTTGATCTGCCTGATGTAACTTTGTCTCGGTCTCCCTCTTCCCCTCCTTCTTTCTATCTTACCTTCTATTATAATGTTCTTAACTTCGTCATGTCGTATCAAGTGTCCCAACATTTTTCCCCTTCTGTCCTCAATAGTTCTAATAATTTGTCTTTTTTCCTTAACCGGACTTAAAACAGGACCTCCTCATTGCTAATTCGATCGAATTAAAAAAAGGGATACACCAACATTAAATTAAAGGGCAATAGAAGACAGGAAAAATTAAGGAGTGAAAGATGAAATCATTCATTATTAAGAATACGCAGAGGGGGCGTGGGCAGATTAAAAAATATAGTTAAAAAAGCAAATAAGTAAGATTAACATGGCGATAACTAGAAAAATACGTATGGGCAAAGAGGGGGGATGGCAAAATAAGGTTAAGAAACTATGGTCCAGTGAAATGAAAATAGTTGCAGCGCGAACAAAATTTGAGACTAGATGGGAATATTGTTTATGGCAACCCTCTTATGTACTACAAAAAAACATGTACAAATGCTTTATATTGTCACTATCGATCAATATCATAATCAATGGCGTAGCTAAGTTGAACAACCAATTAGCCGGTCAACTAACTTTCTAGAGTTATCCGTGGCCCCTTCTCCCCCTAGCTACGTCAATGATTACGAATTAAAAAAAAAATTCTGAACAATAATTGTTTTAGGTAATTTTTCTAAGGATTTTTTTGTTTCAAAAACTAACAGTTGCATCAAATCTATTGATTGTACATAATTACTTGTTAGTATCAAATCGTACAAGTAAAATTCGAAACATTTGAATGTATATATCAATGAAAATAATTGTGATCAATTATCATGATAGCGTGTATGAAAGATATATCAATGGGAGATCATATTTTATTTGATTAACCAATGCCATGTGCATATCACCATACATAGACTAAGTAAAGTTGCCAAATGTATATACAGTTATAATGTAAGATAGCTTTAGTATTCTCGTCTGGATGTCGGCAGTTTAGGTTGTGGACACGTGGTGTTTAGAATATATATAAAACAGACATATAGATTGTATTATTATTAGAACAAAACAAATAAGTTCGTTATGTTGGTAACACTTTATTGTACAGTAAAATCAACGTTATTTTAGATTTATTATTTATTTATTTTTTCAATTTTATATGTTACCAATCATTTCCTTTATATCTACCTAAATACAAGGTTTACCTAATTCATATTGGAGATGCAATAATATATAAATAATTAAATATCTTTACCATAATAAGGTAGCGAGTAATCTAATGTAATAACTATCAATTGTGCTCGTGTCAGCCGCTTATGATTAAGGGTCACGTATTGATCCTGAAACTAATAGAGCTTTCTCAATCCAATTACACGTAAGTGGAACCGGTAGTTATTACATTAATATGTGTGCTTACGACACTTTTAACAATAAGATAACGAATGTTGATCGGCCTAAATGAATTTCAAATATATAATTAGCCATGAGTCCACAACTAGAATAATAGAATTAATTGTAAATGAATATTTAGCGGTTTTAGCGAGGTTAGATTCAGTAGAGTAGAGTTAACTATTAGACTATGCGTTCATTGTCCTTCTCGTGTAGTTTAACGAACGTCACGCCAGCTATACATTTTATTTAATTAACGAAACTAAATGTCTGTTGAAATTTGATGTACGTATTAAAATATATTTTCTAGACAAAAA
MSTRG.12813	Uncharacterized protein LOC106107297 isoform X1	GGTGAGGATGAGCCGAGCGAGCAGGAGCAACGACTGCGGAAGCTGCGCGGACAACGCCGAATCTCCCGCTCACTGCTGTCGACGGACGAGACCGATACTGACGAAAATGACATTGTGCATACTTCGCACCCGACTCCCAGACGCCACTCGCACCGCGTACGCAAGCGGCCTCACAAGCACCGCCGTATCAAGAACAAGTTCATAGTGAAGTTGCTGCGTACCCTCATGAACGGCACGGAGGAGCATCTAGAGCTGGACATACACTGCAAGCACAAGAAGCTGTACCCCAATAACAGCTTCGTGAGGGACCTGGTCAGCATACTTAACGAGAGGAACATCAAGGTGATCAACAACGGGCGCGTGTTCGAGCGGAACAAGGTGATGGAACTGTCTCGCGGCCACAGCCGGCTGCAGCACACTCAGGGCGAGACCGAATGGCCTCCAAAGAAGCGCAAGC
MSTRG.12820	G-protein coupled receptor moody-like isoform X1	TGTATTACTTAAAATTACAGGTAAGTTCGATTACGACCCCGAGTTGGGCACGTGCTCCATTATGCCTGATGAACGAGGGCGTTCATCAAAGACTGCTCTCTTCGTCATCGCCTTTATTGTACCCGCTCTGCTCATTTTCATATGCTACGCAAGGATATTCTGGGTGGTGCACAGCTCAGAGCGAAGAATGCGTGAACATCAGAGATCACAGCACACAAGTCCGGGCACACTAAATAATAGCACTGCAGACAAGCGATCCACTATAAAGGATAACAGGGAAACAAAGGCGAGACGAAACGAGTGGCGGATCACGAAAATGGTTCTAGCCATCTTCTTGTCCTTCTTAGTATGTTACCTGCCCATCACCATAGCAAAGGTTGCCGACA
MSTRG.12821	Trypsin-like	TTATTATACTATTTTTGTCCTAGGAATTCTCTCTGGAGGAGTAGACGCGTGTCGTGGTGACTCTGGAGGGCCGTTAGCCTGCATGACTGCCGACAGGTGGCAGCTGCACGGGGTCGTGTCGTGGGGATCAGGATGCGCGCGGCGGTCTCGCCCGGGGGTCTACACGCGGGTCGCATCATACGTCGACTGGATAAAGTACACTGCCGCCTCCTTGGGGCATAAGATCGCATGATTATAGATAGCCATAGATTAAAAATAAAAATATTGTAACAGAGGAAAAATACTGTAATGTACGTGTTAGAGCGAGATACATATACAGGGTCAAAACACGCTGGTCTCTAGTGGTTTGACGGGAGTTTTGAACGAAACAAATTTGATAATTGATGAAAGAATAAAAAATAG

		TTATTAAGGGTGGGTAATATATAACAATATATATAGATTTGTTATAGTTACAATATCTATATATATACCACAGGGAGACTG<br>TACAAAAGTCGATTGCTTGGGTACCTCTGTCCCATTCGTGTTTCAACCGTGAAGCAGTTGTGTTTGCATGGCTGTGTTTCGG<br>TTTGAAGGGTGGGATATGGCGTGAATTAACTGGGTACAGAGGAATAACACCGGAGTCCTCAGGAATGGCAACGCATGGTA<br>CTGCTCATGTCTATAGGCGACGGTTACCACTTTCCATCAGGTGGGCCGTCAGCTTGTTTGCCATTCTAAGTTGTATAAAAAA<br>AAAATTGGTCCAATAAACAATGTGATAAAAATCTGTTATTCAAAACATAATATTAGATAACAAACATTTAACATATTGGA<br>AAATAGATATCCGACCGATATGGGCGATATCGAGACGGGCGATGACCTCTT
MSTRG.12837	Nicotinamide riboside kinase 2	CACTTTACAATTATTATCATCCATAAAAAAAAACACTGTGCAGGTACTACTTTGTGCTGGAGTACGGCGAGTGCGTGTCGC<br>GGCGTTGCTTGCGGCTATACGACCCGCCCGATGTCGCGGGCTACTTCGAGCGCTGCGTGTGGCCCGAACATCTCAAGTACC<br>GCGCGCAGATCGAGCAAGACAAACGGGTGCAAATCTTGGACGGCACGCGACCGGACATCTACGACGTCGTCATAGCAGA<br>CCTCAACACTCTGGGCCTCACCGAGATACGATAAGACTTCAAATGTTAAGGGAGTAAGCAGTGTAGTGTTTGCAAATCATT<br>ATGAGATTGAAACAGGATCGAGATGTGTGAAAATAATTTTAAAATCCTAAATGTACATTTGAATATTTTAAAATACTAGCT<br>TTTACCTGCCACCTCGACCGTTTGTCCGAAATATATTCATTTACCGGAATTAAAA
MSTRG.12862	FERM, RhoGEF and pleckstrin domain-containing protein 2 isoform X5	GTGCGAGTGGTAGCGTGTGCGCGGGCAGTGCTCTGGCGGGCGTGTGCTGGCACCGGGCCACCTCGCTGGACAGGCAGCAC<br>CTGCACCGCGCGCTCAGGACCCAGCTATCAGGCCACCTGTTACGCAAGTTCAAGAAGTCGCCGGGCTGGCAGAAGCTGTG<br>GGTGGTGTTTGCCGCATTCACGCTATACTTTTACAAGAGCTGGCAGGATGAAACGCCGCTCGCTTCGCTGCCGCTGCTCGG<br>GTACAGTGTGGGCCCACCATCGGAGAGCGACGGTATCGACAAAGACTTTGTGTTCAAGCTACAGTTCAAGAACCATGTAT<br>ACTTCTTCAGAGCGGACAGCTTGTTCACGTACAATAGGTGGACAGAAGTGCTGCAAACATCAATGGTTCAACATAGCAAT<br>TAATTTAAGTAAATTATTCCAAACTATAGCTACAAAAATTGGTGCCATAATGATGTTGTGTTAAAAAATCATTTAAAACTG<br>AAGCAATATTAATCTTGCCCATAATTATGTATGTAATGTCTTAGTCCAGATAAAAATAAAGCTGCTAAGAATAAGTTAAAG<br>TGATTTTGAAGTGATCAATAGTTTAAAATATAGAAATGATAGCTACTTAATTCATGTTTCTGAATCAGACTGCTTATTTAAT<br>GTAAGCTGTTTATTTGGTTTGCTTTCCAATTTACTGTCAAACATTCTGATTGCATATCTGTTTGACAGTGTAAACATTTTTGC<br>TCACAAATGCCTTTATTGTGATATGAATACTTGATATTATTATATATTACTAGTGGATAAAGGCAGGCTTAAGAGGACAAA<br>CTGACATTGTTTACCTTTCATGTTTCAAACAGAAATATTTATTTATATATTTCCGATTTACAGGGTGTAATAGAATATGTTA<br>ATACTATATTTATTAAACAAATATAATGTTTCCGTCTATTATTGTACGCTTACTGCCCGCTAATAATTAATGCTCCTTTCCAC<br>TCAGGGTTTGCTGGAAGAGAACTCTTACAGAGTTAAGCTCGCCTGTTGTACATCTTTATTTTATATATATAATATGTTGTAA<br>TTTTTTCTGTGTACAATAAAGAATAAATAAATAAATAAATTAAAATTTGTGTAGATATATATTATACATAATAATGTATAC<br>TATTTAATAACATAAAATTGAACAATTTTTTCCGAATTGAAAAATTATTTATTAATATAATTATTATTTATTTATATATTTTT<br>TGGAAAACGTACAGCTGAATTACAGTCAATACATAATCAAATTTATATCTATGTTAAGCCATGAATCGTTTTCACATTTAT<br>AATACACAAGAGTACCTAATAAATGTTTGATGACTTAAATTATATAGATAGAGTGTTTCCATAAAAATGCTTCGAAAAAA<br>ACAGGTCAATATTTTATCCATGTGTTTGTGTGTGTAAATCAGAATACTAGAATGTATAGTTATTACTAAATATGTATAGAA<br>AATTTCTATTGTAAGGTTAAATCTATACTTATAATAAATCTGTAGAGAGGTCAATTCTGTACATGAAATATATTTCCAAAAT<br>AACTATCAGGGGGTGATTAGTGATCGATACTGATGCCAAAAATGCAATCAGTAAAGTTTTTGTCTGTCTGTCTGTATGTTC<br>GTTATAGAAACAAAAACTTCTCGACGGATTTTAACGAAACTTGGTACAATTATTCTTCATACTCCTGGGCAGGTTATAGTA<br>TACTTTTCTTCACGCTACGATCAATAGGAGCAGAGCAGTGAAGGGAAATGTTGGGAAAACGAGAGAAGTTACTCCATTTT<br>TAAGCTTCAGTCGCGTGTGCAGCCTTGAAGGACACTTTTTAAAACGTAAAAATGTCCACCCGTGCGAATCCGGGACGGGC<br>CGCTAGTTAAAAAGTAAAAATAATATACGAGGTCTGTCCGGAAAGTTCGTATAAAAGTGTTCTGGAATGCGAGAAAGAGG<br>AGTGGGG
MSTRG.12888	Vacuolar protein sorting-associated	ATTCACACTGCAGGTACTGGTGGCTGCGTATCCTCACACTAGCAAAACTAAACGACTGGGACGAATTGGAACGATTCTCG<br>AAGTCAAAGAAATCCCCAGTCGGTTACGAGCCGTTTGTGGACGCCTGTCTCAAGCATGGAAAGAACGATGAGGCGCTGAA

	protein 16 homolog	GTATTTGCCAAAATGCCGAGATGATATTAAAGTGAAATACTACGTAAAAGCAGAGTTCTACGAAGATGCAGCACAAGTGG<br>CATTCGAACAAAAGGATAGAAGCGCACTAATATTCGTTCAGAGCAAGTGTCCATTACGAGAAACAGTAAAACACGACAA<br>AATATCTAGTCTGATAGAACAATTAGGAATTAGGAAGTAAGCGTAGTTATTACACATTATTATATATTATAATTATACATC<br>AAATCACATATATTCCTATAACTCTGTATATATCTTGTAATAATAGTTTAGCCACGTAAAATGTATATATATTTTCTAACG<br>GAGCTCTGCCGGCTACCGATCTATGTTTGCTATTGAATTGCAACTAGATTCCAAAATATAAAATTAAATAATATATAATAA<br>CATTTAAGATTGACATTTATATATATATTAAAAAAAAAATTGTGAAATGGCCTGCGTTCAAAAATATATAACAGTATTTCG<br>ACGCATTATGACGCTTCAAATCCGAGGCTCGAATCCAACGAGACAAAAAGCAACTCTACATTTGGCTGTGCACGGAGAAT<br>A
MSTRG.12891	E3 ubiquitin-protein ligase UNKL	GCCGGCGCCGGCCGCCGCGAGCTGCGCGGCTTGCCCATCAACACGCTGAAGGACCTGCTGGTGCAGGCGCGCGCCGACTT<br>GGAGGAAATCGAGAAGGTTCTGTACCTGGAGACGGCTACCAAGTGTATGGTGTGCGAGGAGCAGCCGCGCAGCGTCACG<br>CTCGGCCCGTGCAACCACTACGTGCTCTGCGAGGGCTGCTCGGCTACCACCAAGGAGTGCCCCTACTGCCAGACGCCCGTG<br>CAGCAGCAGCATCACTAGTAGGGAAATGTTTGGGCGTTGCCAGTTGTAGGTTGCATGTCGTGCTTCGTGGTGTGTAAGTCC<br>CGTTCGGTAGGGCGGATTAAGGTTCGCAGTGGTGTGCTGCGAGCGGAGGAGCGGTCGCAGCGAGTAACACGTTCCGCTCG<br>CTCAGCCGCTCGCCGGGTGGTCGCTCTCTACTTACGTAGTCTGACGAAAGAGTAATCCCGCTGGTGTTTCGGGCTATTTATT<br>GATCAAAATAAATACTTTCCTTGATATTTTTATATATGGGTAAAATGGGGTCTAAAGGGGTATATAACTCGATAATAACTA<br>TAGGATATTTAATTTTAGTGAAAAGAGCAAGACTTATTCTGGTAAAATTATTGGATGAAATTAATAAAGCATGAATTAGGA<br>ATTTTCGAATTAACTGCCACTGTCATTTACATGAATAATAAATATAAGATAGATATTTCCATACCTGTTTGCCGCTATCCTA<br>TATAGGTAGAGAAGAATATTAACCAATCTCGTACACATCTTAACAAAGCCAACTCTTTATATCTACTCTGATGGGTAGCTT<br>TAACACTAACTACAAAAATATTAATAATATTAACCAATATTCAATTATTTAGAAGCTATCTCTGCTTTTATAAATGAATTAG<br>TATTCGATTTAGTGGACTTGTTGTAATCATAACACCAAAGTCGTATTGTATATCGTTACAACAAGAGGCGTAAATTTTGTAT<br>ACACATATTACTAAAATATGTATTGAATATTTTCATAAATTATAAATGTATTTAGTGCATGAATAAGAAACATGCTGTAGA<br>AGTTAACAAAGAGGATAACTATGCTTTTAAACTAATTTCGTTTATTTAGGTCATGTGTCAGTCGGCTCGTTGCTTTCACGGA<br>CGTGTGTTTAATTACAAAATAAATAGTATAGCTATTTTTGACATTTATGAAGTCTTGAGCATAACCTTTTGTGGTCGTGCTT<br>TCAGCAATTATCATAAAATTTATAGCATTCGTATAGAATTGATTGTATTGAGAGTAAAATGGTTTTGATTGTATTATATGGA<br>TGTTGATGGAAGCAAGGCCACGGTATATCATTAGTGATATAATTGCAGTAGATTTAGTTTTGTATGGATATTAAAACAGAT<br>TATGTATGTTCCACATTTATTATATATAACAATATAATCGGAAGTTGTTACTTGTTATGTTTCTACATTTTTAATTGAACAA<br>TTTGTCTGTAGGCGAAAGTGTGAGGTTCTTCAGTTGTTTTAATGCACAAGTTTTGTTTTTCTCGTTCACGTCAAAGTTAGTC<br>ACCTAAGGACCAATATTAATATATCGAATATTGTAAGTAAAGAAGTTACAGGCATCAACTCTACTGCTCTTCGCCCCATTT<br>TGGGAATTCTAAACCGATTTCGATATCTGTATTTTTGGAATGATTTTCTGTAATGAACTACAGTGTGTAATGAATATATAGT<br>TGGGTGATACGAATGGACGACAATCTGTTTATATTATGTGAATTAGATCTAAACTGTATTGATGCAAATGAGGGTACTGAT<br>CGAGTTGTTCGTTTAGATCCTACAGCATTTTATGTTTACATTCCCAACTAATAATTAGCTATCTCTTTTTTTTTTAACTCATG<br>GTGTGGGTAAAGGTAGTGATTGCATTTAGTGAAATTATACCAACCGACTAGACCTCCCGTGTTGGAGGACATTTTTTTTTT<br>AAATAGCGAACAATGTTTTTTTTTTGTACAATAATATAATGTTTGTGTGGGCTAGGCATTCAAAATGGAACAAGTGTAATC<br>TCACCATTAGGTATTTGTATTTAACAAATTTGCACAAGCCTATTATAAGCAATCAATTGGGTGTATAGAGAAGTAGCGATC<br>TTCCCTTATATACGCTTATTGACAATCGCTAATGCAAAATAAGGCAAGGTAGGCGTGCGAATTTTTTTTTTTATTTTTTTA<br>TCTCTTTCCGAGTACCGTCTTTTGGGTCAGCATCACAGAGAATTGTCAGATGGGAATTCTCGGTCAAATACATCACGTATT<br>GCACCGTGCGGTGCATCTATGTATTTCTAAATACTATACGAACGCATTTTGTATAACTTTATTTCAATTTTACTATTTTAAA<br>AATATTATAACAATATATATATATATATATTTTGAAAATCTTGAGAAGTTTTGTATCGGTATATTATAATAGTAATAACAA<br>ATTAAACTTAGTATTAATGACTTAGATATAAGTAGTGTTTTCAAATTGTAATGGACATTATTTTAAGTGTGTAGCCGCATCG

		ATGTATAGTAAATCAGAAGCAATATGACCACAGCCGCCTTTTACACAATATACAGATCGGTGTTTGAGCGGGAGTCCGCG<br>GGGAGCGGTGCGAGCGACGAACGATCTATACTAATATTATAAAGAGGATACATTGTTTCGTTTGATTGTAATGTATAATCT<br>CGAGAACTATTCAACCGATTTAAAAAAAAAATCTTGCACCTATAAAATGTTATATTATCAACGACGTACATTTTCGGAAAT<br>TCTATCTACTAGTATGAAGACGGGTCGGATCGCTAGTTGCGTATTTTGTTTTTGTATGGCTAAATAAGAACCTATTATCATA<br>TAGAAATTTTGCTTGGGAAGATTTATTTATTTATAAATTAAAATTTTATCACAGGTGATTTTCTAATATTAACACGACATGG<br>TATGAAATTACAATAACATGTTCAGAGGTGAACAAGAGAAGTTGTACCGTGTGCCGCGTGAGTTCTAACGTCAAATGGCG<br>ATCCGAAACAAACGGAAACAAAACGGAACTTTACGTCAATAAGGTCGATTCTAAGGGGTCATGTCTCGAAACATCTCTTA<br>ATCTAATACTTTAGTAGTTACACGGAATCCCTGTTTTATTGAGCATCATAAACTAAATTTATAATAATTTTAATTTTTTTAA<br>AAATTTAGTTGATGTTGGTACTTCGAATAAAGATTTCGCTGTGATATGAAATTGAATTTTTAATTTTGAAGATAGAATTAG<br>AGAACGGACGCCTCAGAATCGACTCTGTTGTTTTAAAGCTTATAATAAAATGAAACGCAGTGTACAAGTATTAGAATTCAT<br>GTCGTGGACTCTCGCTCCTTAATATTATGTCGACATAGCACATTTTGTATTGTTAGAACGCAATAATAGATGACTTGTGGA<br>ATAAAGTCAATAGTGGAAAGAAGTGCTGATTTTTTTATTAATAAATATACAATAAAATTCGGCTAGAAATAAAAGAATTG<br>CACTCGAGTGAATTAAAAGAAATGTCTGTAGTCTCTTGTGCAATAATATGTTATTAAATACGATCATGTCTCCTCTTACAG<br>AAGCATATTAAAATGGCTCCCGATTCGTCAACGTCGTGATTTTCATCTTTTATCTTTCCTGTATTCTGTACTGTTTAATTCCT<br>CCTTCCCATCTTATCTTTGTGAACGTTTCACTTTCCTTGACCCCACTGGCATGTCCAGTAGGTCGAGTCTCCTCATGCTATAG<br>TGGATCATTTACGGTTCATGCGGCTAGACTATGGAACTCTCTTCCACTGCATATTCGTGAGAGTCAATCGCTGAATATATTC<br>AAGAGCACATTAAAAGAGCATCTT
MSTRG.12898	Uncharacterized protein LOC106134371	CCGCCACGTCCGTAGCTCCAGGCTGCCTATGACAAGTGCTAGTGTCGCTCGCGTGCTGTGCAGACACGCATCTAAGTCATT<br>ACCCGAAGAGGAAATTGAGGACATAGCATCAAGGTTACGATTGAAATTAGCAGCAACCCAAAGCCGGATGTGGCACGTG<br>ATAGAGCTGATGGAGAAAGACCAGGAGGAGTCAGTGTCCAAACAGATTCACATGTTACCCAGCAGAGTGACACAAGCAC<br>TGAGCAAGTACAGGAATAAGAATATGAGGCCGGAGGT
MSTRG.12902	Uncharacterized protein LOC105207208	CTTTTATACGAACTTTCCGGACAGACCTCGTATAGTGGTAGTGGGATTTAACTAAGTTTTAAGTTAACTAAGTTAAGGATA<br>GGTCCTTATAATCACTTAGTCCTACGTATTTACAGTTTTATATGTGTTTTATATAGATATGTGTGTTAGTATGGTATATTTGT<br>ATGTATTTATGCGTATGTATGTATGTATGTGTATGTATATATTTGGATATGTGTGTTTATTTATGTATGTGTATATGATTATG<br>CTGTCATAGATATTATTTCTTACAGATCTATTTCTTTTAACGCGCCACCTACATATTTATGCTTTAATTCCTTCTACCCAAAG<br>CTTGTCTGGAAGAGATTGCTTAGCAATAAGACCGCCTTTTGTACTACACTATACGTATTGAAAATTGTTCAACAAATCCTG<br>TTATGTTGGTGCAATAAAGTGTTAAATAAATAAATAAAACCTTTCAAAGGATTTTAACACTGCTGACCGGGAATTCCGTCA<br>CGGTTGAAATCTTTTTAATTAAACTAAAATTGTGATAAAAACCTACAATAAATACATTGAAAAGTATTTTTTTTGTTCATGA<br>ACGCTTTGTAATCCAAATGGTGGGGGATTTGCTAATAGTTGCCACACAGTGCAGGCGGATTGGCAACTGCAGTCAGGCTTT<br>AGAGATGTTTTAAGAGGGTTACTATTGACGCGTGGGCATGAGCCCACGCTCTACCTTAGTCGGCTCTTATAACACTCACGG<br>GATAGGAAGGGGTGACCTATGCCAGGACCACACGGTCAACTTATTAGTTTTCCTGCTTTAAAAATCGACATAGCGAGTTTT<br>TTTATGGCTGATAATGGAATGGTAACCTCCTGCGCTAACGGTGTACACTGGCGGAGCACCAGTGAGGGGTGATAGATGAG<br>AATGAAAAGGCAGATCAGTTAGCCCATTGTGATGAATACAATTGGAATTCAGGACGATGGTTTCCAGGGGGGACCACGTG<br>GACCGGACTGAGTACATTGCTAGCAATGCGACTGGTAGTTTGCATTACTAAAATAATCCCCCCAAGGGGTAACGAGCAGA<br>CAGCAAGTCTGTAATAAATTTTATTTTGGACTATTCCAGGGTGATAAATGCTGCGTCAAACGTGATTTGCTTGGTCGGCGG<br>CCGGAGCAAACTGGCGACAAGGCGAGGAAAGACGACGAGAAGAGCTAGGCTGCATATAAAATATATTCTGTTATATATA<br>ATAGAAAAATCCCCAAAGGCTCTGGTCAGAGCTTCTTGAATTGCTATAGAAAAAGTGATGCGGTTTTTGTCTTTACATTAC<br>ACACAATAAAACTGGCTGGCCAGTATTTGTATATTCTACTAATCTTGTGAATGTTAAACTTTGTAAAGATGTTTGAATGTAT<br>GTTAGTCGATCACATGTGAACGGCTGAATGGATTTGAATGAAATTTGGTAAATGGGTAAGTTATATATATATATATGTATA

		ACTTTAAAATTAGGATATAACTGTTTGTACATCATTGATACATAGAATATATATATATATATATATATATATATATATATATATATATATATATATATAGTAATATATATGTAAAATATGCAAGTATATATTATTATATCAGGTGGCTCAGGACCGTTCCTTATGGCAGTCTATGGGGGAGGCCTATGTCCAGCAGTGGACGGATAATGGCTGAAATGAGTGTTGTATTAATTATATATATTATAATATAAATTTATATACCTTTATATATATTTATTCTATGTACAAATGATGTACACGGTTGTGTACTGTCCCAAACTGTCATAACATCATTTGGGACACTGATTGTTGGATCATCGTTGTTTGTAAAATTGTATGGATATTGAGACCGATTCTGACGCGCCCTTTTCTTAATTTTCTTTTGATAGTTTATACATTACTGTATTATGGATAATCATAGATTAGATTTATAACAATTTTAATTTTAAAATACATTAGAAATTTACCCCTATTATTTTAGTTTATTCATAAAAACGTTAAGTCTCATACACTTATGTTAACAGTCGGAACTGTTTTGTCTCTTTCTTCTGTGTAAAATTCCCAATTTGAAAGTAAGTGACAATATTCTTGTCAAGAATTCTTGAAATGATTAAGAAGCTGGGGAAGAATTGAATTTTAATATTGTATTGTCGCCATTTTTAATTTCTGCTCCGTGAAATTGTGTATACATTTGTGGTTAATATTTATTTATTTACACTTTATTGCACCAAGTAAAAAGAGAAATAACAAATACAGTAAATATTAATGAGAAGTGATACAAAAGGCGGCCTTAACGCTAAGCAATCTCTTCCAGACAACCTCAATCCGGGTCTTCTTGCCCGCCTTTAATAGTGTAGGCGGGTGAGGTAGGGGTCTCGGGGTAAACCCCTTTACCCCGGCTGCATCACCGGAGGCAACTACCTCCGGGTCACTGGGTTTTGAGCCGGCATACACTGGTGGTCTGTGTATGCCGGCCAGACAACCTTTGGGTAGAGGACATTGAGAAAAAGACAAAGGGTAGGTGGCGCACATATATATATATATATATATATCGTATATACGAGACCATGTTTTGATTTTTCTTTGGTCTCGGATTTCTTCTGATCAGAAAATGATGGAAGCGATTAATAATTTGTTAAGTGTGTCCGCTGCACCGAAGTCTCTATCTTTTACTTGTGTTTGGGCTCAGCTAAACAGTTGCACGTCGACCCATTGGGATCTTGTACATCCCTCTGTCTTACATACGTACACATATCTGTCACGCTTGTCTCCCAATAAGGTAGACAGAAACAATGGAACGTCAAATGCTTCGATTCAAACAAGCATTTTATAGATCCTATGCATTACCTTTGCGATATCTCGCGGTTTAAGATATCTCTAGTCCCCTGGCTCCGGATAAGCCGTTCCCAGTAGACTCATACGCATACTTGCCAACGAATCGTAGTTGCACATCAAGTGTTCCATGGATTCCGATTTCTCCCCGCACAGTCTGCACAGTGTGTCCGAAGATAGACCCATCTTGTGTAGTATATGCCTTACTGGCATATCACTGGCATGTTCAGTTAAGGCACCCATTATAAGTTTTGCCTGTTGTCTGCCACCGCTGTAGATGAGTTTATTCCATCCCTCAGTCTTGTTGTTTAAGAACTTTTTAGAATTTTTTTATACAACTTACGAGTGGCAAACAAGCTGACGGCCCACCTGATTTAACAGAGCATACTGTTTCGCCCATGATACCCCCCATGCGTTGCCATTCCTGAGGACTCAGATGTTATTTCTTTGTACCCA
MSTRG.12915	Uncharacterized protein LOC110378268	CTGTAGTAAATGATGTCATGAACTTGAACAAGATGAATCAGAATATTTGAACTCTTGGATCATCACTCTGCAACTGATATTATATAGGTAAATATATTTTATAAACATAGTGTTTCAAAAGAAGTTAATGAAAAGGCAACCAAGTTTTACTGAAGTTATCATAGAAACAAATGAAAATTGTTACTTATTTCATTTAAGGAGTGTGTGAAACTTTTTTGTAATGTTTATTTTTACAGTTTTTTTAGTGATAGAGGAAGTCTTCAGCGACTTGTTTTATGTTATTGACGAGCTGCTCAACCTCGGGCGGTGCCCCTACCAGCTGCACATCAAAATGGAGCGGCTCCCGTTCAGGTGGCGGCGCTTCTAGTAGAAGCGAGTTCGGCCGGGACGCGCGCTCTCCTTCTCCAGACATTATCACCAGAAATCATCAACTTTCATCACTGGCTCCACCTTACAATTTCACTTTCACAATAACATGAACAATTTATTCGAAGCACTTCACCCATTAGCTCACTGAACACATAATGATTCTGTGTGCTGCAGTGGGCGGAGAAATCACTTGACACCGAGCTCTTATTGTTTTATTATTTACATTCATTTGATCAATTTAGTCCGACAAGCGATCGGTTGTCGGGTCGTTTCAATTAATTTTATTCTGATTGGTTCATGTTGTACAGGAACGTATATTATGAACTTTAAAGATTGTTTTATACTTTATAGACATAAAAATCGTTTTCTTAATTTACGAACAAAATAATGGAGAAAAAGAAAAATTATAGATATCAATATAGACAGAAACCAACCAATTTCGCCAATTTGACAATTGAATGATATGATTGCCTTGACATCGAGCCAAAAACAGACAAATTTGATACACAGATTAAAAAAATATGTAACTGTATAAGGTCGTACACGATTAAGTAATATGAATTGTAGACTGGAAATAAAATAAAAAAAACTTTGTACAAAA
MSTRG.12943	Nucleolar pre-ribosomal-associated protein 1	GCATACTCGTCCTAAAAAAATAATTCTCCCGCGTAACTCCTCTTGTTCATTTTCTATAAATAAATTAAATTTTTCAAGTAACATGCAGAACCTGTGAAGTACGTGATACGCTGCTGCTCTCATTGACGGACAATGAGAACTCAACGCCATTACTGGTAAAGACAAGAGGCCAGTCCGCAATATCTTGAAACATGAAGCTACAGACCCTGGGGCGAGGAGGTGGCTTAGGAGTGGAAACAAGAAAACTGGATCATATATACTGTCATCATCGTCTACATGGGCTACCGATGTTACGTCATCTTTAGTGTCAACCATCACCTTGT

MSTRG.1295	Peptidoglycan-recognition protein-LF	CGTGCAAACCTTTTAAAATTAAATCTTTAATTGCTCCTTCATTATCCTTAACCGTTTTAGTTTTAAAAACGCCATCAGCATA<br>CATTTCTAACAGAGCATTTTTCAAAGATATACCAAGCGATGATTCATAATTATAATTAATATTTGCAGGTAACTTATATTCG<br>TATTCCAGTTTTTGTGTCACTGGAAAATTCTTCACAGTTGTCTCTATTATTTCTTTATCAAATAAATTCAAAACCCTGTTAGG<br>TGTAGGGTGCCCCCATAATGTAGAAGATCTATTTTTGCGGACCGCATAATAATTTGCTGCTGAATCGCCAAAAACGTAGGG<br>TTTATATTCATTAACAGGTTGACCATTACTTTCATAAAAATGCAATATTGCCAGTATAACTCTATCACAGGGACTTCTGGTG<br>GCATGATATGCACTTAAATACACAGGTATTTGAGAAGCTTTCATTACGCTTTTGTTTAATTGTATCAGCGTATATAGGAATT<br>CAGCTAATCTCGATTTTATCTCGGAACTGTAGTGACTAAGCATCACATTCAAAAACTCCGAGTGTGATGTAACCATGTCAA<br>ACAACAACACAATATCTTGATGATCTGGTGGATACAACAACTTTACTAGAGTACTTAGTAAAAACAAGAGGTGAGGTCCG<br>CCTGTGCTTTCTTGGTCTACCGTTTTAATTTTCAAAGAATCTTTTAAAACGGCTTTACAAAAGTTTTGCCAAGTTGAACTCT<br>CCACAATCTTCTGGAATTCTGTCTTTCCTCCTAAATTAAAAACTGTAATGGATTTAGATATCTGAATAACATTGTAAATACT<br>AGATACAATTTTGATTAAAATATTGCTTTCTTTATTTTCTTTTAATGCCAC<br>GTGGAACATTTGTATCAGCCGGACGCGAAGTACGCACTCGCTCTGTAATAATTGAAACATATTTTATATTTTTACTTCCAAC<br>CATATATTTATATATGAAATAAAAATCTACATAGGCTGAGCGAGCCAGTCCGTCCTTGAAACTATCCTCAAGTGGTTTGGC<br>GCTATCAACAAAGTGTCCGCTGAAAATCAAGTGTTCATTATAAATAAACATCATCGTGACATGCATAGATAATATTTTACA<br>CTTAACTTTCTTAAATATGGATTATATATCAAACAATGTAACTAAAAATTTAAAATGATACCTCTGCAATAAAATTCACAG<br>TCATTTCAGTGTTCATATAGGGGATTGTTAGTAGGGTGAATTAGTTTTTACAATTACGTAATTAATCACTAGTTATCTTATA<br>TCTCCATTTATGTCATATATTAAATTACCTGGCAAAAGATTAAATTTAATTTAATAAACTACAATATAACTTTTAATACATA<br>TTTAATTCACAAATTGGTTTACTGTTAATATATTATATTATATATTTTTTTATATATTATATAAAAAAATCTCCAAATATCTG<br>TTTATTATAGTTTTTAATTTGGAAATTTTATTTTACAATATCTCCTCAAGATAAATTTCATTTTGTTAGGAACAAATATATGA<br>AGAATTCTGAGAAATTAGAAATTCTCACACATTCTAATTCAGAGGAGTTTTTGAATGTACTAGCTTAGCAATAACTGTTGT<br>AAAATTATAGTAACAATGCTGCCAATACTATTACAATTCATGAAATTAGTACTCAACCAAACATGAGAATAATGGCACAG<br>CATACTAATTGATATTTATTAAATTTTCCTCTGGCAAATGTAATAAACATTTTTATTTTAAATATTACATTCTATGTTATAAG<br>TTGTAAGTAATGCTGTTTACCAACTTTTTCTAGAGCATGTTAATACTTATATAATAATAAATCAAAAGGTTAGATGGTATAC<br>CTTAAATAAGATGAATGGAAGTGACCGATTACCAATGAATGTGATATTTGACACCCAAGTTAAAAAGATGGCATAAATAC<br>ATGAGTTGAGAATCATACTTAATTCTTTATACCACATCTACAGGGATATTTTAACACAACCCTTTTCCTTAATTAATTGCAT<br>ATATTAAAGTCTAATAATAAATCCTCCATACCTTTAATTGTACTTATTGTGCAAAAAAGAAATACAAACTACCAACTCACA<br>GCTTAAGTATTTTCTCCCATCTTACCTACACTTATACCACTCACTCCATGGTTTGAGTAGGTGCTTATTTAACTCCATTTTAA<br>GACTTATAAAATTAATTTTATAAATAATAAACAACTCGGGTTCTCTATTAAAAAAAAAAATTAATTAGTAATAATGACTTT<br>TTTGCTGATACTATGTTAATTTTATAACTACCAATCTGAGAAAATGTATACCTTCTAATTATGAAATTGCCTACTACTGTTT<br>TAAACTTAATTATTATTTTTTAATTAATAATTATTATTAATTAAAATTAATCTTCCTAGTTTTGGGAAAAAAATCACTTTCCGA<br>GCAGATGTAGTATAAGAAATATTACATATGCAGCCAATTGAAACTATAGGATCATACCTTTCCTTTTAGCATTTTTTGTCAT<br>GCGATTTTCGAATTCTTCAATTGTACTAATCGTGGAATGGAATTTTCTTTTCTTATCAAAGTCCAAGGTTTGAGGTGTTAGT<br>AATGAATATAACATCAATATTATTGTTAAAGGTACCATGATTGTAAACATGATCAATTGAAAATTGTCATGACATACTCCC<br>ACCACCCTTGTATAGTCTGAAATAATAAAATCATAATGATTTACCATGTGATCTATGTTAGGAACGTTATGCATATTTTTGC<br>CCATAAGGAAAAGTAAGTTTGTAAGTCATTTTACTAAGAAGCAACACATTCTCTTCGTATAATATGTTAGTTATGTAATTG<br>TTACTAACAATTTGTATAATTAAGGTATTCTATATATTTCTTTGTGAATCACTCCATTTCTGAAATATATCTATCTTTTTCTA<br>AGTCCCGGAAATGTTTGAGCCTATTTTTATATTATCTCGGAAACGGAAACTAAAAAATTTCACATGACCAAAGTTGATCTT<br>CTCAGAGAAGCCTATCCAATGAGCGCATGACAATAGGATTATTGTAACATCAAAAAACTCATCAAAGTCCTTGGCCCATA<br>ATGGGCACTATGGGTCAAGGACTTTGATAAGGATAATAAGAAGGATAGGATATACTCTAACCCATTATACACTTTATCTAG

		ATGATGTATACAATACCCAGAAATAATACAGAATACCTGCTGGTTCATAGGATATATGTAAAATTTATT
MSTRG.12952	Coronin-6 isoform X3	ACCAGATAGCGATATGGAACGTGGGCACTGGCGAGGTGCTGATCACGCTGGACTGCCATCCGGACCTCATCTACTCGGCC<br>TGCTGGAACTGGACCGGCTCCAAGCTCCTCACCACCTGCAGGGACAAGAAAATTAGAATAATTGATCCACGCAAGGGTGA<br>AGTAGAGTCGGAGGCTATTGCCCACGAGGGCAGCAAGGCATCCAGGGCAATTTTCCTCAAACATGGCCTCGTTTTCACCA<br>CTGGGTAAGAACATGACACATAGCCAAAAAAATTAATTCCATGCAGTAAATAAAC
MSTRG.12967	Uncharacterized protein OBRU01_26429	TGAATTGTCAAAGTGAATAAACCAAAATATCATTTTGAAAGTTATAAATGTCATAATATAATGAGAATTTTGTTTGTAAAC<br>ATACCCCTTGAAGATTACGATAATCGATTAGTCATTTACTTTTTATAAGTTTGTAAAAGAAATGTAATTATAAGATACCCA<br>CAAGAAGAACATTGCCACTAAAACTATCACAACAATTTACACAACAGCTCACTTCTTCATGACGCGTTTAGAACGCGAGG<br>AACTCGTACCAGCCGAATCTAAAGTCTTCGAGGCGGCCAAGGAGTTTGCTGACACGGATATAACGTTTTCAAACTAACAC<br>GATGCCTCTTACCATCTGAACCAATGATAACCACGATGCCATTTCGTGTCCAGCATTTTGTGATACCGAAGTGTTTCCGTGC<br>CTTGCTTTTGTAAGAAACGCGGACAATGTAAAATCCGCTATTTTTAAGTTCGGTTTTTGCAGACCAAACTTTGTCTCACATC<br>AGTACAACAAAATTTAACTAAAATTAGCCAGAGTTTCCTTTAAGACATACGGCTAATTCGATGGGTTCTGCTTATATCTTCT<br>GGCGAAATTGGCATCTTAAGTTTCTCAGTGAGAAAGTTGGAGACTAGTATGAATAAGCCACATTCACTATCCTCACGAACA<br>CCATGTAGCAATAGGGTTTTGCGACGACTGTGCATCTCCAAGCGATCCTGTTGTTGCCGTTAAAGATTGCTCATTGACTTTG<br>CCAAGACAAGAGACTTAAATTGTGTAAAATCTGTTGATAATGAACTTATAGTGACCGGAGTAGCACTGGCCTTATCTAATT<br>TGTCTTGAAATGCTGCCATCCTGGTGTTAAATGCACCATTATCTGTTCCAAGGACTGTTGCACAGACTCCATTGTTTAAAAT<br>AAAAATATTAAATTGTCAAATTTTTTTTTTGTTTAGATTTTTAAATTATTAAGTATTTTTACCTTCCTTAGATTTATGATACC<br>TATTATACCATGAACCGACAATTCGGTTTTATCTTGTTGCTTTACATGTCTGCATATGCGGAACAGGGGATCATTAAGTTAC<br>ATTGTTATGTCAATGAGGATACATGGAATAATTTTAGTATTCCCTTATTAGCTCCTGCTTTAAATGAATTCTATATATTTAC<br>GCATACAATGTATCTCGATGTATTATTTAATAAATAAAATGAGTTTTTACACATTAAAAAGATACAGAAATTATAAAAAGA<br>AATAAATATAGAAGTTATAAAGGTATTACTCAAATTAAGTGTAAAATGTATACAAAATTCCATAGTGATTGGTAGAGCCG<br>CTAAGGCGCTATAAGGTGATAAACAAACACATTTATAATCGCCAAGATTATTTACCTGTTAATTAAATATTCCTTGAAAA<br>ATAATCTCTCACGTTACACTCTACTCTATGAACTAGACATAATTAAGTCACTTTTTTATCTTAAATAAAATTGAAATTAAAA<br>AAAAATTGGTCTCCTCCATTCATCCACATCCTGCCATTGCACTGTAACAGGAGTGGCAAACTAACACGGTAACATGATAAA<br>AACTGCATTACAGATACGTTATCAGTGTAAAGTGATATTTGATAACCCAAATTTATGTGCAATCTAGTTTTTTTTTATGGAA<br>TGGCAATCCCACCTGCGCTAACGATATACGCTGGCGGGGCACCAGTGAGGGAGATGGGTATAGAGAGGTGATATATATAC<br>GTATCAGATACAGTTTTGGCCAAAATTTTTTCCCCTAGTGTCTCGGTTAATTCAGCAGCCAATCCAGAAGATGTTTTCTTAA<br>GATCTTTCAAAGAACTTCGCGTTATCTGTCTGTCTTCTCTTGCAGATGTAATTTGTTTCTTTTCACTTCGTTTCGTCTTCGTGT<br>CTTGTGGTCATCTTGTACCTATTTAAGGTATACCTTACAGTACTTTTTTTTTATTTTTATTTTATACACTGTAATTTTATGTAC<br>TGTGAGGACATTTCCTAATCTTGGCATTTTCACTTTTATTTTTGCCTTCTTCAGACAACAACTGGATTTTTAATTGTTCCTCG<br>TTACTTATTTCACGGCGCTTAGGCACTTTTCTAGTTTTTTTTTATGAACCGTCTCACGTAAAACAACGACCAATTTACAATA<br>ATTTGTTGACAGTTCTTCACACTATTATGAATCGTAGTAAACTAAAAATTTCCCTAAAATTCGACTGTCGACTTACCTGGAA<br>ATGAGTCACATCTATTGTGTTTATAGTTTTATGACATAGTGAGATGTTTTGATTGGCTCATTTTAGTCGTTAGACGCGTATT<br>ATGCGACAACAGACCCAGTACAAGTCAATTTTTAGTGGTTTTTTTCTAAAAATTTCTATAGATTTTTAGGGTGGCAAATACT<br>TTGGGGTAAGGCTGTAGAGGCATCTATGATTTTAAATTATTCAGTGTTTTAATGCATTTATGACTTTTTCTAAGTGACTTAA<br>GCTTGATTGTTGCGGATTTAACTTTAGACGTTTTTTAATATATTTTTTTTAAAAGGTAAATCCTAGTTTAATTTACCAATAAA<br>ATACTGACAAAAATATTGAGACAAATAAAATATATGAATGACTTTGATTGGTAGTTACTCGTTTGGACATATTTCGTTTAA<br>TTTCGCTACTATTAAATTAACAAAACAATTTTTTTCGGGTGTCCACGATACAGGTTCGCCTAGTGTGGGGCCCAGTGTATCT<br>TGTTTTTGTCAAATAATAAATTTCCATTTCTAAAAGAATTCTAACACCCACAACACACTTACTCATGGTGTGGGGACTAGG

		TTACTAGTTTATTTGTTGTTTTAGTTTATATGTAATAAGTTATAATAGTTGCCATGCTTGGCAGGCAGATTGTCAACCACAG<br>TTAAACTTTGATGTTTCATGAGGGACGCTGCTGCCCTATTGTTAGTAATGGAGACTGAAAATCCCATTGCTAGTAATATAC<br>CCAACCAGGTCGACCTCCTCGCGGTTCC
MSTRG.12977	Endonuclease and reverse transcriptase-like protein	NNNNNNACAGTGGTGGCCCTCATACACTGCGGCCTCACCATGCTGCGGGAACAGGACAAGATGCCCGGCACCGGCGGCGT<br>GATGACGACAGCCCTAGCGTTCAGCAACACGAGCCTCATCCAACGTCTCAACGAGAACAATCTTAAATTCCAAATAGTCG<br>AGAATAAATAGAGGGAAAAATATGATTTCGGTGCCTTACGACGATGATCACGTTGGGAAAGTGTTATCCTTTCAGACCCA<br>AGATTTTTTTCTTTCAGATTAAATATATTTTCGTATTTTGGAGCCAAGCCATCTTGCCGGCACGCACGAGACTACTGCACCA<br>CCTAACCGCTAAACTGATTTCTTTTTGGCTGGTTTAGAGTCTCACTATCATGTTATTTCAGCTTCGTGCGTAAAAATGAATT<br>GTGCGCGGTTTTAGGATTTTTTAAAGTTACAACTCGCGCCCGTATCGTTCAGGTCTGAAAGGGATATATGTTGGAGATATT<br>AAAAAGAGAAATTTTTCACATTTCAAATTTGATAACTATTTATATATTATTATGGTTATAATTCAACTAACCTCTAGAAATT<br>AGTTATATATCTATATTTTTATACTGTAATGTTTATATGTAACTGGAAAACGGGTATTTAATGAACTTAACTATATTTGTCG<br>TGTCTTACTTCATTCAGAACTTTTGTCCAGTTATTTATAAATATATATATAAAAAACTAACATAGATTAAAAAAAAAAAGA<br>AATAATAAAAATTATAAATTGTATCGCTGTAATGTAAAACGGCTTCTTATTTTAAAATCTCCCGCCATTGTGTTGCGGAGA<br>ATTGCATTTAGTTTTACAAATTGGGTATAACAATATCCAAGGATACAAGTTCTAATGTATTGCATGTCTCACAATCAAACT<br>GCCATAATATTTCACAATTAATATTTTAAAATACATTAACAAATATACTGAGCTTTGACTATGTAAGCAATATAAAATAAT<br>AAAAGGCAGTTATATTCTCTTGAGAGTTTCTTAAATATAATATATAACCAATTAGTCCCACTGTTGGAAATGAATATCTCG<br>CCATTAGGGGACGACGTTAGCACCCCGACTGGACGGATATATGTAGCACGAAATAGCATTTATTACGCATTTTTTTTTTC<br>CTTTTGAAACATGTGTGATTAAAATCTTGCATTTAAAATATTATATTAGGAATTCTACATCTTTATTTTATAATTTTACAAAT<br>TTTTATTTTATTTCACAATTTAATACGAATACCATACATACATATCTGTCACGCCTGTCTCCCATTAGGGTTGGCAGAAACA<br>ATGGAACGCCAAATGCTTCGATTCAAACAGACCTCGTTTATTTTCGTTCTTTTTCAACAGAAATTAATTGTAAGCATTTATT<br>TTTATAACTTTATTATTAATAGTTGCATGTAATTGATAAGATATAATTTTGTTATTTTTATATCACCAAAGCCTAACAGTAA<br>CACGATCTGCTATTTATTTTAAAACGAATATTGTGAATAAAACAATGATTGTGTTAATAACATGGTTAGAAGACTTTAATG<br>TTCCGTATTGAAATAAACACGGAACTATTATAGTTTCGCATTTCTGTCTATCCGTCTCTTTTTAGAAGTGATATTTTTAATTA<br>GCATTTTTTTTTCATGGAATTTAATGTATAAGTATTCTGATTAAGTTAGAATCAATAAAGTGTCCGACAATTTGAATTGAG<br>ATATATCTACAAAGCTCGCCATCGACAGGGAGTTCACTATCGTACCTTACAGCCCAAATGGTCGCGTACAACGCGGTTTCA<br>GAGATGTTTTCTCCCACGAACACTCAGGTTGTGGAATGAACTCCCTGCCGAGGTTTTCTCAAGAGACTACAGCATGGAATT<br>CTTCAAAAGGGGGATGAAGAGGTTTCTTCAGGGTCGGCAACGCGCGCGTAATACCTCTGGTATTGCAGACGTTCATAGGC<br>TACGGTAACCGCTTACCATCAGGTGGGCCGTATGTTGGTTTGCCACCTCAGTGGTATAAAAAAACTCTTTTATTTGATAAT<br>GTTCTCTCTTGGTAATTTTTAATTTATTTAAGTTACTAATTAATACAATAATCAAAATATTGTTTATGATTCTTGTACAGTTG<br>TTTTCGAGTTATATATCCAAGAAGTTTGAAAACCAGCATAATATGAATTATAGAAATGGATAAACCATGAAATTTATGAAC<br>GGTTTTAGTTTTCAGACCTGTCTACCCCGTAACGGAATGATGGATAGATATATAGATAATTCGAAAATAACTAACTCCCAT<br>GAGAAAATAACAATCAATGTTGATAGAATTGTTTTAGAGAAGGAAATACTAGATGTCGCTATCTCATTCTCTTACGTTGAC<br>TCGCCAACGATATCGATTCGATGGATAGATTCGTTTACCACTCGCCTCGGGGCCGGCTATCCAACTTCTAAGGATGTACTT<br>ACATTATATTCTTGTTCATTATAAATTAAGTGTAAGCATTTATTTTTATAACTTTATTATTAATAGTTGCATGTAACTGATAA<br>GATATAATTTTGTTATTTTT
MSTRG.12980	Hypothetical protein	GTATTTTTAAAAATAACTTATAAAAAAAATATTTCTTGCGTGTGATACTTAATAGAGCCTCTGTATTCCAGAGTGGCAGAC<br>GACGCGAGGACAGGCGGCGGCAAACTGATGATGAGAGCTCCGTGCTAGACAGCCAAGACGTCTCCAGTGCCGATAGAGG<br>TCTGTACACTTTATTATCTACAGACACCAATGGCGGACACTAGAACCACAGTGTAAGGAACAACAGTCGTCCAACACTTCT<br>AGCATGTCTCCAACGAAATGCGACAACAGGAGACGCGTCGCTATGTTTAAACTTTAGATTATATAGATAGAGCTTCGAAC

CTCAAAAACGTGTCAACTTTTTGTCAGCTGACTCGTTTTTATCAGTATATACTTACGGATACAATGTATCTTGACGTATTAT
TTAATAAATGAAATGAGTATTTACACATTAAAAAGATGCAGAAATTACAAGACAAAATAAATATAGAAGTTATAAAGGAA
ATCACCCATAACTATTTATCACGCAAAATTGTATGGAAAAGTTGACCCGTTTTTTTTTTTACGAAGCATTTGTATAGAGACA
TTGTATATAATGTAAGGTTTAAACGTAGCCGTTGGACGATTTATATCAGACTTCTATAGACTGTGATAATACTTACTCTGTT
TGTACACCGACGCGGTAGCGAAGCGAAACTTGGGTAAATCTGGTTTTACTGCACTATCGTATAATATAACGCGTTATATTC
AAAATTAAAATTCAAAATTCAAAAATCATTTATTCATGTAGGACATTTTAGCCGCTTATGAACGTCAAATTTTTACATTATA
ATTTTATTTTTTTCCCTACCGTCCAGTTCGGTATATAGATCCAATTGAGAAGAACTGGCAAGAAACTCAATTGAATTACTTT
TTTTTTTTAAAGCCAGTTTATTTACAATGTATATTTATATAGGTGTCTATGTCTAACATTAAATGAAAATTCAGTGCATGAA
CAACTATTGCCATGCACACTCGTCATTCAAGTAATCGTTTAGTGAGTAATAGGCTTTGCTCAGTAAATATGATTTAACAGA
TGTTTTGAATTTATTTAAGGGTAATTCCAACATTGAAAGTATACATAATTTATATTTATTTATATTTTTTGAGGAACTTAAA
AATTTACTCACAATTGTAGTTCCCAATGGTGTAGCTGAATTAGTTGCATCGACAAGAGGAAACTCTTAAATGAAAAGAGA
GAGAATGGTGTAGTCAAAAGCGTTTAGCGATAGTTTTTAGTTTTATAGTTGACGTATGTCTCAACTTAGACACGTTATGCA
TAACGTATAAAGTATCGCCAAAGCGAGCACTGTCGTACTATCCTATTTTCGATGCGACTCACAGCACTTTTGTCATTATTGA
CTTGTGATATCATGACTTTTAAAAGTAAAAAAAAAAAAAAACTATGTTGCCAGTTTTGAATTTTTTTTACATTTTGTTTTTGA
TGCGGCTGTTCATTATTATAGAAATTGAATCAATCGATTTATCATTATTATAAAAATAATAGGCAAATTACGCGTTAAAAG
AGGTAATTGCGGAGTTTATTTTTTTTTTAGTAAGATATTTACATAAAAAAGTTTATTAAAATAAATCTATACTATTGGGTAC
ATGACTAATGTCATTATATTTTTTCTTTAACATGGACGTCTTTTCGAACAATAAATTTATCTTATTTATACTTGTTTCGCTTG
AATGCTACATTAGTGCGGTGTGCAAACGGTGTCAGCGATGTTGATTGAATCAAAATCATTATTTACCAAGTTCAAAATGAT
GGACTGTAAAGACATTGTTCATTATTTTTTTTTCTGACAAATATTGCATGGTACAAATATTTTGCGGAACGTTGAAATCTGA
AAATATTACTGAATGTGCTAGTTAAAATGTAGTGAGTAGTGCTCTGTTAAGTTTATTATTATATGTATCTATTGCATTCTAC
TATTTATATATGTTTATATATATATCTATATATATGTTCATGTTTGTGCACCTTGGTTTTATTACATTTTAATTAGTACATGTT
GAAGGCGTAATGCAGTAGACAGACGAATGGGACTTTCCTTTTTATTGTTATTATTTTTATAACTCGGGACGAATGTTCGTTT
GCTATCTCGACGGAGGCGGTACTTTTCTCTGGTGACGATTTCTTGCTGCTCTCTTATTAGTGCAATAATACGGTGAAGGTAC
ATACTGACATAATTTAAGTGGATTGTTACTTACAATGAATGACGTGTTCTAGAAGTTAGTAGGTTTATAATCAGAGATATT
TTAAATTATTTGCATATTATTGTACATAACGGATATTATTGTTAGAAAAAAAAACTTTTAAATCTGGTGCTCATTACATAATA
AGTAACTGGGTATTTTGAATTAAGGCTTATAAATATTATGACGTGAACTGTGACATCACGTGTTTATGAGAACACGTGGCA
AATTTTTTATTTTGTTATAATGAATGTTGCGTTTTTACTGTGAAATATAATAAAATGATTAGAATATTTTTAAACAATTTGT
ATATTGTAAATAATAGAATTAATTTTTTTTTTCTTAATTTACGTGGTACTGACATTTACCGTTTATTGTTATTTAGCTTTTTTG
ATTTTTTATTTTTTAATAATAAAAAAATATGAGAATTTGACAAATAATGTTCTTATCTATGTTATGTTAGAATATTTATTTTA
AAATTAGAGGAATACGGGCGAGCGAGACCGAGAGGAGTCGCGTGCTTGTCCCGAATACACTTTTGTCTCTTGAATGATTA
CCAATAATAGCGTATTGGTGTCGCGGAAACGCAACTATATCGGGTAGCAAACGGGCGTTTCTCTCGGTGCGGTAAAGTTA
GAGTAGTGGCCGGGCCGCGGTCGGAGCGGTCCGGTGCGGTCCGGTCCGGTCCGGTGGAGTAGAGGCGGGTAGGCATGAG
ATGTCGTGTGTCCAGAGTCCGCCACGTCCGACGAGGGCCGCGCCGCGCCCGGGGCCGCCGCCGCCCGCCGCCGCCGCCGC
AAGAAGCAAGGTACGCACCTCCACGCACCGCACGCACGCACACGCACCACACGCACGCACACGCACACGCACACGCACG
GCACAGCCTTAGCCTCCCTAGACGAGTCGATATCGATCAAGTCAATGAAATAGACGAGATGGCTTCGTCCGGTGTCTCTTT
ACTTTTTTACGCGACCACTAATCAAAACAATTACGTCGATAGATTTGTTTTTATTTTTTTTTTATAATTTTACTTTTGTCTAG
GGGTGCAAAACTGTCGTTGTCTAGTATACTCTCTAATATTTAGAAATTTAGAGAGTGACGTCCTAGAATCGACCCATCAAC
TCGGTATTCCTTTCTGACGATACTTTCATCTCAAATTGCATAGAGTGATCATATTTGATTCTTATCGACCTCATATATAAGTT
TTAGAAGTAGATAGATTAAGAAATTTTATACACAACATAAATCACAATGCAGAATAAACATGTGAACTTAGCAATTATTG

		CTTTTACATGCTCACATATGTATATTTCACACAGATATAGACGCTGCAAGTGTCTGCATTACTTGATGTTACGATAATCATT<br>ATCGCTTACTTGTATATGTTTTTCGACGTAAATTTTATGTAGAAACTGCAATCGAAAAGTAAAAGTTTGCGCGTAGTATAC<br>ATTTGCGGCGTCTATTCAAATCCTTTGATATGTCACAACCACATAACAAATACTTGACACACTATCTACATTGCAGATTTGA<br>TTGCCAAGCAATAAATACAAAATGTATTTATCTATATGTCAGATCGAAGTAAGTTCTCATTTGCTTTATGTCATACGTAAAT<br>GTGTGTAGCATGTCTATTGTGCATATGTATGTGTTACTATTGTAGAATATTTACTGCCATTTTAGAAATACATTTTATTTTAG<br>TTTTTTTTCTATAGACCTGTATTGAAAAATCTATAAATTGATTTAAAAAAAAAAAATCTAAAGACATCACATAAATATAACG<br>ATTTCATAAGTAGTTTTTCTGTGAGTTATTTTTTAACTCTTGCCATTTAGTTTAGCAAGCGGATTCAGCCGAGAAGAACTGG<br>CGAAAAATGTTAATATTACTTTTATACTTGGATGGAAAAATAAAACAGATATTCAGTTTTTGTTTTTATTTATTAAAACTTT<br>ATATTCATAATGTTGCATGTTGCTAGACTTGAACATGTCAATCTTGCGTACCTCTGCCTGTAGTCAGTATGTAGAGCATGCA<br>TGCCAGTGTAACCAACCACACGATGTAAGATTTTCAGATTTACCGCTAGGTTTTGTTAACGTGTCGAGTCGACGGCGATCC<br>GACTGTAAATAATATCATGGCACAGCGGCTTGTTAACACTATCATGTTGTGCTTTAGAGACGACCGTCTCAACGCTATCTC<br>CATAACATCCAACGAGAAAACCCTTACCACCATAGACATATAAATATTGCGTTGGTTGTATTTATATATTTATGGACGGTT<br>ATGACGTTTGCGTTCATTATGATTTGTTGAACATCGATTATAGTCGATTTGGAAATACACTTGTGAATAAATTTGGAGGTTA<br>TAAATGATAAGAAACGATGTGGACATTATCATTATACATTTTTCCTTTTTTAAAATATGCATATGTAAACGTTTTTAGCTTG<br>ATAAATAAAAAAGTAAATATATAAAAGTCAAAATTGGGGATGATCTGTTTTTTTTTTTAATTATTAGCGTAAGACCAGTGT<br>GTTCCTCTAAATTTGTCTACGAGTGTAGTTTCAAATTTAAATTTCGAATGCGACTTCAGTGTTCAGTGTTAGTGTCGATTGT<br>GGCATAATGTACTGTTATCGTTTCGCAGGAGGTGGGTGGAAGAACGGCGTGAGCGGCGGCGGCNNNNNNNNNGCCGCCA<br>GCGGACTTGACCCGACAGCCCTGACTTGACGCCGAGCGAGGCAGACATTGTATCCTAACGTTACGTACGGCGGCGCGGCG<br>GCCGCGACCACCCGGCCCCGCCCGCCCCTAGACTTACTCGTATTATGCTTTTATAAGAGATTCGTGATTTGTATTCCTGATT<br>ATTCGATAAATATGAAAAATTAAAGATGTGTACGTCGCTCCCCGGACGATACGCGCCGATCGCGGCCTTTCGGTACCTTTA<br>TGGGAGATCGGACATAGCGCAGTACATACGTGTGTCGTAGCCTGTACTCTGTGGTTATGTGATTTCCTATAAAATAAACAT<br>TTCTGTACATATTTTCAATAGTATTTTTATTTGTACAAACACTCGACCCTTGTTCTTACGCTAGCGATACTCGGTAAATTTAT<br>ACATTATATAAAAATATTACGACTAGTGATACGTTTACAATCGATCGCGCTCTACACTAGTAATACTATATTGGAAGATAC<br>CTCGGGGCCTCGGGTGGGGAAGAAGGCGACACACATATTTTTCGTGACACTAAAAATTTCTTAAATTCTACAAGTGAGATT<br>CCGTTGACATAGTCTAAATTTACTATTTACATGTTACTCTACAATATCAGTCATTATGAATAACTTATACGATACAGTGATA<br>TGCATTATTGCCGGGAATATCACTAGAAAACGGGAGGGTGTTACTATTTACAGATATTTTCTCTTTAAGAATGTATACATT<br>GATCTGGTTGTACAAGTTGATATGTGTTAACAAGAAACGAACCGTGTTACAATAATATATCTATATAGGCCATCAGAAATT<br>TCGCTCGAATTAGATTAGTATCTAATTATCACAATTCCATATTCCATTATATTAATTTCAT
MSTRG.12981	Mitotic-spindle organizing protein 1	CGTCAAAGTCACATTGACATTTCATAGTGTTTTAATGTTTCTTTAAATTTTTCAAGTTGTTCTGTATTTACTTGTTTACAATT<br>TATAATCATATACAGGTACCTATTATTGAACTCTCTCAGCACTAAATAATTATCTAAAACACAAATAAAAAAAAACACGTC<br>AAAAGGATTTAAAATGACGTCGGAGAAGAATGCCCAGGTGGGGCAAGCTCGGGAAACATTCCAAATGTTATACCAGATAT<br>CGCAACTTCTGTCTACTGGTTTAGATACTGAAACATTGTCTATCTGCATTCGGCTGTGTGAGCTAGGAGTGGACCCTGAAG<br>TGTTAGCTCATGTTATAAAGGAAATTAGAAAAATGGGAGAAAACACTGTACAAAACAAACCTGCCATTTTACCACAATGA<br>AAACCAACAATAGCTATATAATTGATATTATACTTCAGATAGCTTCTGAAAGTATTATTTATAAGGATGTGTAAAATAATA<br>CCTAAACATTATTGTTTACCTGAAATTATGTTTAACATATTCTAATAAAAATCTAAGTACTTTATTCTCA
MSTRG.12992	WD repeat-containing protein 44 isoform X1	TATGTCAAAGTTGTCAATTCAGTTGTTGAACGGGTCGAGTCGATTTCAACTTTCTTTTGCAAAAATTAACTGCTCATCATAT<br>ATGATAATGTAAATAAGTTGCCCAATATAGTGGTCAGTGATCGTCGCTACACGTTTATTGTGTCACAATGGGCGACAGTAG<br>CGATTCAGACGAGTTTTATGATGCCGAGTTCACACCCATTGACGGTTCAAATATAAAAGATCTGCCAGAGGGGTCTGAGA<br>AAAACCAAAGGCAAAAGAATTCGGCAGTGAAGCCGGATTCACCATCTGTAGCATTGAGTGTGTCAGGCACTGAAGTTCCT

MSTRG.1301	Paired box protein and transposase domain containing protein	CCAGTTGAGGATGACAGGACCACAGTTAAAAGTGTAGTACAGGGCCGCAAAAGATTCCAAGAGTTACGGAGGTGCATGC<br>AAACGGAAGATGAAGATGATGATATCCCAGATACTGTGGATACAATGGACCAACAAACATTTGCCTTGGAACACCCATTC<br>AAGATTATAGCGCACGACACAATGAGCCTGCAAAGTATGACGTCACTAGGCAGGATCGGCAGAATACTGAGCGGCGCTGC<br>CGAATCGCATCCTAACATTCGAGACACAGCCCCACCGGTGTCGACACCATCCAGGGAGCCGTCGACGATATCCGACGACC<br>CTCTGCCTGCAGATAACAGGAATCAGAATGACAAACTGCACGGCTCACAGTCACTGTCAATAGCGGACAGCGACAATCTG<br>AGCGTGTGTACGACGGATACTGACGAATTTCGGTACGTGCGGCGCGCCACCATCGACAAGCCGCAGCTCGACT<br>GTGTGTATCGCGATACCCTTTTCCATATACATAGTGTAAATACGTTCCTTTTTACCGCCCAAACACGCAACGATCCACACCC<br>AGCTCGACCCCAGGACTTGTGAAGGATGGTGAGAACCAAACTGATGAGTTCCCCGTCCGGAGTCGGCTACAATATTGTAT<br>TACATGGAGACATATCTATGTTTGTATTTGCAGTACAGTAAGAAAATTGTTTGTTTATCGAGAGCAAGTTAAACAGCGACT<br>GATTTCACGAGCGCTATGACGCTCGTCGGCACTCAAAGGGTTAAATAATGAAGAGAAGATAGTCGCAACCCGGAGGCTGC<br>CGGGATCTACAGAGCAGCAGTGACCTAGGAGTGGGGAGCTCAATCCTGTTTGACGCTACGTTGCCAGTATGTCGAAATGA<br>CAGAGCTAAAATGCCCCGGGCTTGACATAATATCTAAAAAGATAACGCGAAATTAGCCTAGAAAAGAAGGATTGAGCGC<br>CGGATCTCCCAGGTTAAAAATTCTAAATGGCAGACTGCTCTGCTTCGGGAAGGACAATATTCGCCCAAGAATTATGCACCC<br>CGTAAAAGAGGCCTTTGCTGGAACAGTTACACGTCTGTTCCAGCCGAACTTTGGACAAAAAATTACGGAGTACATCGATG<br>ACCTAAAGGAGAATATCCCCGAATGGGAGAAGTGACTTTGATGGTACTTTAAGAAAGTCGAGATGTATCAAGAGTGTTTT<br>TATGAGTGATCAACTGAAGTTCTATAGGAACTTAGAGTGCTCAAAATTGCAGCTCACAGGACAAATACCAGAAACAGCCA<br>ACTTCGTCACATTTTGGCTACAGCTGTGGTCTAAACCTGTGGAGTGCGAGAAAAGCGCATGGATGCATGGTGTCGAGGAA<br>ACATGCGTTCGAATAATACCGATGCAACCTACATTACCGGTCAAAAGTTTGAGACCACTATAAAAAATATAAAAAAACAA<br>ATAAAAATTAATATATTTAAAACTTTATGAAAAACAGGTTTTACTTTAACGTTACACACAAGATTTAAAATTCCAAAATTA<br>CATGAAGTCTTGACGAATACAATGTTCTAAAGAAAACACACACAAGAGAGTTTAGAGATTTTTCATCAAAATATCTTCCTT<br>TGCATTTTATGACCCGACCAATCTTTACTACATGCGAGCAATTAATTTGTTTATAGTCTCTTGCGAGATATTCCTCCAGCTT<br>TATTCTAATGCATTCCACATAGTTTGCTGTGAAGTAGGACATTGTTTTCGCACGTTTTGGTCAAGCTCTTTCCACAAAAGCT<br>CTATAAAGTTAAGATCGGGGATTTGGGGTGGCCATGTCATGTTTTTTAACGCCTGTTCAGACTGTTTTTTTTTTCTAAATAG<br>CCCCTACATAATTTTGAACTGTGCTTTGGATCATTATCTTGTTAGAAAATAAATCCTTAGCCGATTAACTTTAGTCCGGAGG<br>GAATAGCATTTTTCTCCAAAATTACTTCATAATGCTCCTTTTTCATAATTTATTTAATTTGGACCAGATTTCCAGTCTCGTAG<br>CCTGAGAAACAACTCCAGACTGTAACAGATCCGCCACCATGCTTGATAGACGGGACTACACACTGTGGCATCATCTTTTCT<br>TCGGTGGAACATCGAATGAAAATTTTTCTTTTTAAACCGAATATTTCAAATTTAGATTTGTCACTTCATAGCACTTTATAAA<br>AATCTTCTTCGATCCAGTCTCAGTCCAGAGCCCATCAGCAATGTGACCAAACAACTTGGCATCTCGTAATCGTCCTTACAC<br>TGCAGACACTGACACAGGTTTTTCCCATACCATACCATAATAAATTTTAGAAACATCTTGATTGTAAGAACACGAAATCAA<br>ACGAATTGCGGATATTTGTAGATAACCCAAAAAAATACAGAGTTTAAATAAACATTAGAAATATTTTAAGAATTTAGAACA<br>ATTGTTTTAAAAGTTGATGTAACATAAAAAAAAAAAGGATTAGTTGCGGTAATGCGGTAATAAATTTTTTCGATCGATTATC<br>GGACTATGGCTCACATTGGAAAATATGGAAACAATGTTTGTTTATTGCCCTTTTTTTGGCTTATATACAAAACAATACAAT<br>ATGTAGGTGTGCAGATTACTTTTTATCGCCAATATTTGGGAAATATTTAGTAACAATAAAAGTTTTTTATTAGTAGTCTCAA<br>ACTTTTGACCTGCGTGCATTTCCATTAAGAAGGAAGACATGGTTGCCGCAATTCGCCGGGCTTCGGAATAGAAAAGTCCGA<br>AGATCGACATATTGCAGCGCTACTGGTTGAAGGCGTTCTCGTGCAATACCAGAGTGGCCTCTAAAATAAGGATACTGTGTA<br>GGAAACCATCTTCATATCGCTCCATCAATTGTCACAATATATAAAAAATTTACATCCGTTCTGAACGCTAAGATCTCTCGT<br>AATGTATACGAAAATACTTATGCTTACAATTGAGGTAGGGGCCGTAACCGCGAATCGAAGAAGCTGCTCCTTATTAATACC<br>GTCACTGACCACCCTCCATATAGAACGGATCACTAAGTCTTCAGTCATGAGGCTGTATATTCTGCGGAAGGCTAGTGGCAT<br>GCACAACCGCGAGATTTACAGCCTTAGGAGATATTTTCTAAAGAAAAAAGACGAGAGTATGCATAGAGAGGTCATAGAAT

		GTGACAAAAGATTCATTCCTCATTCTTTAACTAAAGAAGACTGGCAAAAACCCGAAGTACAGGTTACCTCTCAACGGGAG<br>GCCGTATGGAAAGACAAGAGCTGCATAGAAGACTGAAAATTCTTCACGAGTCATACTTCAACACGAAGACCACCGTGCAC<br>TAGCTACAATTCGGTGATCTCTTTGGAGAGACTGAAGGGTTTGTCTGTGCTATACAGGATCATGTGGTTAAGACGAACAAC<br>TACCGAAAACACATTCTAAAGAATGACACTATGGACATCTGTCGGAGTTGTCGCGATCCTGGAGAGTCATTCAGACATTTA<br>CTTTTGGGTTGTTCAGCTCTTGCCAACAATAAGTACTCGCAGAGACATAACCAGGTAGTCAAAATACTTCATCAATAGTTA<br>GCTCTCAAGTAGGGCCTCATGAATGTGAGGTTGCCTTACTATAGTACGAGCATCAGCCTGACCTCTACAGCGACAGCATCA<br>AGTTCAATTGCGAACGATCCATCATCACCGACAGGACGGTTTCGGACAGTTTGTGTAGAAAAAACTACTTAGAGAAACAG<br>TGATTAATGTTATCAATAAAGTCATTTTATTAAGAGAATTCATTAATGACTTCATTAAATACCTTAAGACAAGCAAGGAAA<br>TACTCCATGCCACGATTCTCCAGGAACCTTTGGCAATTCTGTGGGGTCTCGTCAGTCACATTCCACATAGTAGACCATGCT<br>ACCTCCAGTACGTCATCACATATGCGTCGCTCCAGCCGGT
MSTRG.13027	RING finger and transmembrane domain-containing protein 2	GTCATTTATGTAAGTACCTATGTCAAAATTGCCAAATCAAAATAAAAACTTCACCTTCGTGCTGTGCTTCGTGCTCACAAA<br>TAAATAATTATTTTGGAAATGTTATTCCTACATTAAAATCAGACTATTTAAAAAAATATATCATATTAAGTATCCAGTGAT<br>GGCTCAACCGAACCAAGTGAATTTAGATATTGAAGAAGATCCACCTGCAGATGGAACACCACCACGTAACAATCCTTTGC<br>ATAGATTGGACTCTAATTCTAGGCCTGTGCCTTCAATTAGAAATAACCTGGGCCTTGGAGATCGCCTAAATAGTGTTTTTA<br>GAGAAATCAGGCCTTTTGTCGAAAATGCTCGAATGGGAAATAATCCAAGGTTATCATTACCAACATGGCTGCCGAGACAT<br>ACACCGGGGACTCATCAAATTGGTGAAAGTAGTACACAGAGACCACAAAGCTCAATTGCACATGTGAATTTGGGACCAGG<br>TAGCCAGACTTATGGGGTCACAGATAGAGGTACACCACCATCGCAGAGAACACAAATACTGGGCCACGAGTTGAATACTA<br>GTATATCACATGACTCCAATCTCTCAGAGCAAGGGCAGGATCATCCAGAGATCAATGTGTCAGTGAACCCAGCTAATAAT<br>AATAACATACATGATAATGGCGATAATGAAAGTCAAAGTGATGATGGTACACAACAGGTTTTGGATGTTAGAACAACTTT<br>AAATGTACTCATCCGTTATGCACCATTCTACATAATACTTTTCATAAAGTTCATGTATGATAGCAGAGAAGGACTGTTTAC<br>ATTCATTGTATTGCTTTGTACATTTGCACATTGCAACACCCTTGTGAGGAGGGAACATGGGAAACAAATGAATAGGAGCAT<br>AATAGCTCTGTTCAGTGAGCTTGTGTTCACAGGCAGTTGTATAGTGGTTGTGCATTTCCTGTTCGGCCATGGGAAACTGTTG<br>CCTAATGTGGTAATGTTCCCGGCGTACACTGACTCCATAGATGTATGGGAGCTGCTCTGGCTGGTCGTGTTGACTGATTTG<br>ATAGTCAAGATCATTACTGTAGACGTAAAAATACTGGTCACAATGATGCCTGCTTTCTTGTTGCCGTATCAGAAAAGGGGC<br>AAAGTATACCTTTTCACGGAGGCAATTTCCCAGCTATACCGTTCCCTGCTCACCATACAACCTTGGATATTCTTCCTGATGC<br>AATCGTATGAGGGCTCCGAGAAGATGATGGGCATGTTCTTAACATCGCTCTATGTTATATCAAAGGTTATCGATGTGATCA<br>CCAGGTTGCTACTCTTCAAATATGCTACATGGACTCTTTTGCAAAGTGTGGTAAGATAGTTTCCTACAGTGTTAATTTTTTT<br>TTAATATATTTACTATTTGCC
MSTRG.13104	Unsecreted protein	CGTGTTTCAGATAGGGTCGTACTTGCCAAATATGAGTCACAGGCGATATAAAATCACATCGTCTTAGATTCCCGCATCATT<br>CCGTATCAGACCGCAGCACGCTACTGAGTGTAGATAAATAAATAAATTAATCAAATGTTTGTGCAAGGTGTTGACGTTCTA<br>AGTTCTACATTGGAAAATATTAGTTTGGACATTCAAAATAAAATGCAGCCAAAAACAAATAAGACACCTGTATGCCCGAA<br>GGGTCAACTACCTCTTCTGCATCTAACACCAGTTGGGGGTGAAAAGAATACCGATGTTAATAGCCAACAAGAAACAGAAA<br>ACCCTTGCCTTCTGAAGCCACACTACGACATGCCGCCCACGCCACCACCACAAACCAACCCACAATCCTTTGAATTCTTTC<br>CACAAAGAATAGGGTAGCTACTCTTTGCTCATTTTTAGAAACGACATAGCTTTTAAATGGCAACCCGCCATTTCGCCAAAA<br>CATGGATCTTTACAACAAAGTCGTCAAATACGCTTCGTATCGCTATTACAGTGAAATACGCAAATGCCGGAGACGTATCGG<br>TGGATCTTTTTGCCAAATGTGAGCCAAACTACGTCAGTGGACCGACATCTCGTGTTTATTTTGCTCAAACTTTTGTTGTGAT<br>TGACGTGAAAATGTATTGTTCTATAATAGAAAATTTATTATAATGTAACGCAGTAATGTTCCTAAAATTGATAGTAATTTC<br>AATATTATATTTTATAAGTTATTGCCAATATAGTCTTAGTGATATTAAATCCAGCAATAAAAATCTAACTAAGTAGATGTT<br>AAAGCCTACGAATGTTATGATTTGATATATTAAAATCTCCATTAAGTAGGTACATACTAAGTACTATAATTATAAGTATTA

		TATTTGGGTTACTAATATTTAATAACCTATTTAGTACATATTAAGTTATTCAATTATGAAATGAGTTATTAAACATTCATAATAAAAATATACCAAATAAATATATTAAAACA
MSTRG.13161	DD34D transposase	ATTTTTTTATACGAGGTTTGTCCGGAAAGTACGTATAAAAGTTTTTTAAAAATTTTATTTTACAATTATTCAGGTAAATCAATTTTATCCCCTTCAAAGTACTCCCCCTGTGACATAATGCACTTGTGCCAACGCCGTTTCCACTGTGAGAAGGCTCCTTGAAAGTCCTCTGGTTGTAGGTTTCTCAGCTGCCCCGTCGTAGCTTTTTTTATCTCATTTATGTCCCCCAAATGCCGCCCCCGAAGTACCAATTTGGTTTTTGGGAACAAGAAGAAGTCACACGGGGCCAAATCCGGCGAATAGGGGGGGTGTTCGGTCACCGTAATGGAATTTTTACTCAAAAACTCACGGACAACAAGAGAGGTGTGGGCAGGGGCATTGTCGTGGTGAAGAATCCAACGCCCCTCTCGCGCCAATTCTGGACGAACTCGTGCCACACGAGCTTTGAGGCGTCTGAGCACATCGACGTAGAATTTTCCATCAACTGTCTGGCCTGGAGGGACGAATTCTTGATGGACAATCCCCCGAACATCGAAAAAAACAATCAACATTGTCTCGGCCCTCTCTGAATCTTTTTAGCCACTTGTGGACGGTTGGTTCCTTCACAGCATCATCCCCATAAACTGTTCTGAGATCGGCAAAAATTTCACGGCCATTCTTGCCGAGTTTCGAGAGAAACTTGATTACGACGCGCTGCTCTTCAACGGAAGCGGGCTCCATGCCGACGGGGTTTCGTTAAGAGGTAACTTCACAGATGGCGTGACAAGCCAGTTCGCACCGCACGGCGGTCAGACCAGAACTGAAGCATTGTTAGGGACATAAGGAAGGGGTCCTGCGCTTACCTGGCCTCCCCACTCCTCTTTCTCGCATTCCAGAACACTTTTATACGAACTTTCCGGACAGACCTCGTCAATTAGTGCA
MSTRG.13162	Uncharacterized protein LOC105286551	GGCAAGAATGGCCGTGAAATTTTTGCCGATCTCAGAACAGTTTATGGGGATGATGCTGTGAAGGAACCAACCGTCCACAAGTGGCTAAAAAGATTCAGAGAGGGCCGAGACAACATCAAGAATTCGTCCCTCCAGGCCAGACAGTTGATGGAAAATTCTACGTCGATGTGCTCAGACGCCTCAAAGCTCGTGTGGCACGAGTTCGTCCAGAATTGGCGCGAGAGGGGCGTTGGATTCTTCACCACGACAATGCCCCTGCCCACACCTCTCTTGTTGTCCGTGAGTTTTTGAGTAAAAATTCCATTACGGTGACCGAACACCCCCCCTATTCGCCGGATTTGGCCCCGTGTGACTTCTTCTTGTTCCCAAAAACCAAATTGGTACTTCGGGGGCGGCATTTGGGGGACATAAATGAGATAAAAAAAGCTACGACGGGGCAGCTGAGAAACCTACAACCAGAGGACTTTCAAGGAGCCTTCTCACAGTGGAAACGGCGTTGGCACAAGTGCATTATGTCACAGGGGGAGTACTTTGAAGGGGATAAAATTGATTTACCTGAATAATTGTAAAATAAAATTTTTAAAAAACTTTTATACGTACTTTCCGGACAAACCTCGTATAA
MSTRG.13174	Hypothetical protein RR46_03807	AAATATTTGCTTTCAGGCGTAATGATACAAATGTCAAATGGCAAATCGCAACTTCTGTTCAAGAGGTATACATTCTTCAAACACTACCGCAGAAAGAACTTTAGTGTACGGTGGAGTTGTACGAGATTTCCCAAATGCAAAGCCTATATTTTCGCCGACGAAGACTTGTTCGTGATCAGTTATGTTAATGAACATAACCACAGTTGTGATCCTCTCCGTCAAATTTCCGACGCTGAAATAATTTATACGATAAATGACAAAGAAGTTCTTAAATATCGAGGACATTACTACACTCGGGTTCCGGGATCGTCGACAAATCGATGGCGTTGCATAAACACTTCAATTTGTTACGTAACTCTCTGTTTTAACAACAATTTTGAGCTGAAAAAGGAGCCTAAAGAACATAATCATCCATCAAAGGAGTTCTTAAAACTGTACGACGGGAAATATGATTCAGTTAGGTGCTACAAAAGGCGTCAATAATATAAAAAATATAACTTCAAAAATGCTAACATGGTGGAACAACTTGGATTTGGTTTTAGCATCCGAAATACAAATTCACAAATAAACAATAATACCAAGTGTCCGGTTGAACCTGTTATAGACTTTTTTGATTCTGTGTAATTTAACAGCTAAATTTTGAAGATTAAATCCTACACATTTTTATCCTAAATCATATATCACATAAATCATGACAACTATTTGAAATAAATTTATAGTACATACGAGGACTTATGTTGAACTTTAAAATATTATAATCATAGTAGTGTACTATGTAATAAACCTGAACCTGTGTCTGAAATCTGAAGAAATGTTGTTATGCCTAAACTTTAAAGACCTTTTGATATTTATTTGTCATACCATGAGATTATACTTGCTGTATGTTAGCTGTATTTTCATATTGAAAGAATGATACTCTAGTTGCAGTTTCAAGGTAAAATTAAGACGGTATAACAGAATTGAAAGAATGTTCTAGAACTCGGAACACCATTAAATTATTATTTCTTTAAATTTGGTTTAAATTTTTTTAAC
MSTRG.13181	DD34D transposase	AAATAAACCTACTTTCTTAGAGACTTTACAGATTTATAACACAAGTTCTTACTTGCTCTGATTATTACAAAAGCTTTAGCAAATAGGTGGTATCTGCCATAAACTGTAGTGTAAAATTTTGTGCCAAATAGTCACTCAAAGTAGGTACAAAATTTCGTTCGCTAAACTAGGTTTAGTAAGTGATACCCTGACCTGCTTTCTTTTTTAATCCTCATCTATCATCATTCGTTTGTACCCAGCCAACATAAAATAGTAAGGGTTAGCATTTTATTTCATCCTATTATATGTCTTAAGTTTTATACTACTATTGCAAGGAAGATGATTATT

GCACCAAAACAAACCTTATCCCAAAAAATGTGTAGTAACCGAGCTTCGGATCCTTGATTCCTTCTATTCGAGAGCTTCGTC
CATATTTCTGCACGAAAACCACAGCTGCATCAGGCTCTGGCGGAGAAGCAGGATTACCACCCACCACAGCATCAGCTGGC
CACCATAGGCTCGTCATTACACCACACCATATCCACAACCATACAGATCTCATTTTACCAGCGAAATAAAATAAACTTTAT
TTGTTACCTACTGCAATCTATTTTTAACGGATGTCCTGTGATATCACTTCAATATTTTAGTGGAAAAAAAAAAATACGACTGG
GCGGATTATTGAATTCTAAAGCGCTTACTGTTTATTTCATTATCTTTATTATTTTTGATCATGAAAAAAGCTAATTACTTAGT
AAAGATTACCTAAATTTAATTTAATATCTCGTTTCAAGTTTCTTATATTGGATAAAATTGGAATGGTAACTCCACCTGGGTT
TTGGTTGTGATGATAGTACCATTTACTCAAGCATATGCGCCATTTTATTGTCAACCACAAAAAATCCTTCAAGATGGAACT
TTACTTTTTTTTTTCAAAGTGTGAAAATGTAGTAGCGACTCCGTTTGTACTGTCGGTATACACCGGCGGAGCAACATTAAA
GGATCATATGGAATCATGAAGACGAGGATGAAATAGGTGAGTTGACCCGTCGTGACGAATTAAATACATAGTTATAAACA
TCGACATTTTGTTGGGGGGAACCGTGTGGCGGTTGACCTAATATTGCTAGCAAGGAGGCAGTTTTTTTAATATTTAAATAT
ATATATATATATATAATACACAGTATAATTTTTGCCTTACTCAAAAATCTCACGTGGTTTATCCAGGTATCAGCGAACCGCT
AGTTAGATCCCATTAACAATCTCTTCCCCTTGAAGTTTGTTACGACGTCGCCCACGCTATATCCAATATCGATATCGATATC
GGGTACGCCTTACCTTTATATTTAATAACATTATATTGAGGGGGAAAGGAATTGTTAGTATTAAGGTCATTGCTAGCCCAC
ATCCACTTGGTTTCCCTTAGAAACCATAGAGATTAATTCTTGAATTCGTCACGATGGACTAACTGACCTTATCTTAACCTAT
CATCCCTAGCTGGTGCCCCCCCAGCGTATACCAATATAGCGCAGGTAGGATAATCATGCCATTTTCACTCATTAAAAAAAA
ATTGCTCCTGCGCAAATATATGTGTTGGAATTAAACATTTATAAATTAAACATATTTTCAACATTTGTGCCAAATAAAGTGT
TTTTGCGGGGAGTTTTACTTCATTATTTTATTATGAAAAAATAATGTTGCTGAAGTTCATCGTATTTTAGTGGAAGTATATT
TTTTTTTATTTATTTATTTAGGTTTACCAACAATTGTTTCCACAAAATAATGTAAATATATATACAATTACGGAGCTTAGAA
CTAAGTGTAACAATTTTTATAGGCAAACACAGCATATATTGATACATGTATAATAATAAGTACGGAGTATATAGTGATCAT
GCTCTAGCTAAGCAAACATGTTGGAAGTAGTTCGCATGTTTTAAAACTGATGATTTTGAACTGAAAGACAAGGAGCGACC
TGACCTGTCAAAAAAATTTAAAGATAAAGAGCTGGAGGCATCGCTCAACGAAGACTCATATCATACACTTCAAGAATTGT
CTACATCATGCATCATTGGAAATCGACTTATCGACGGTAGGAATACGTCTTAAAATATTAGTTATAATCCATAAGGAGGGA
CATTGAGTCCCATATGAATTGAAACCAAGGGGCATGAGAGGCATCTTTTGACATGTGAACTGCTGCTTCAACGGGAGGAG
AGAAATGGTTTTTTTTGCTGGTGATAAACATCCATTACAACAATCCGAGCGGCAGAAAGTCATGTATTAAGCCCGGCCAAC
CATCAAGATCAGCGGCAAAGCAAAATATCCATGGGTTTAAGCTCCTGCTATTTATTTGGTGGGATCAGAGAGGTGTGACTT
ATTATAAATTGTTGAACCGAACGAAACTATGACAGGGGATCGCTACCGACTGCAGTTGATGCGATTGAGTCATGCAATGA
AAGAATAATGGCTGAAATACTCCGAAGAACATGACAAAGTTTTTTTGCTGCACGACAATGCTCGTCCACGTTTCGCAAAAC
CGGTACAAATGTACCTAAAAACACTTAAATGGGAAGTCCTACCCCACCCGCCGTATTTTCCAGATATCGCTTCTTCAGATT
ACCATTTGTTTCGTTCTCTGAGCAGAAGTTTACTTCTTACGAAGAGTGTCAAAAATGGGTCAATTCATGAATTGCATCAAA
AAACGAGGTGTTCTTCCGACACGGTATTCGTAGCCTGCCCGGAAGATGGTCAAAAGTAGTAAAAAGCGATGGGGAATATT
TCCACTAGTGTATTTTTTTTTTACTTTCCATTGATAAAAATATTTTAATACATTAAAAAAACGCTACGCATTAATGCCAACAT
CTAATAGATAATTTATTAACCGGTTGTTAATAATTTTTTCTAAAGTTTTCGAAATGGATGTTAGGATTGAAATTGGTCTGAA
ATTTTCGGGTTTGTTACCCTCTTCTTCTTTGTGCATTTGCGTTATAAGAGCCTGCTCCAAAGCATTAGGGAAAATCCCCTGT
TCACATTATAGATTCAATAGATGGCATAACAGAGTTACAATCATTTCATGTGCTACTTTTAAAATACTAGTTTGTAATTCAT
AAATTCCAGTTACAATGTCCCTTTTTAACTCATCAGAACTGAATGTGTCTCAGCAGGGTCTGTGTCGTATAGCACAAATGA
AAAATTAGGAGGGTTTTCAGGCTCATAAATATTAGGATCCTAAATAGTGAAGGAGGCCACCTTAATTTTATCTACCTGTAC
CTTAACAATATTGGAAAAATGATGTGTGTTGTGTTGATGCCATCTCCAGTAAATTCAAGTCTTGTAATTTTTTAGGATTTCT
TTGAGCAATACAGTTTACAGTATCCCATAATTTTTGGGTTATATTGTACATTACTAAGTTTTTATCTCTTAAATTTTCGTTTC
TGATTTTTAATCAAATTATTACAATGGTTACGGTATCGTTTGTATATTATTTTTAATATTTCATTATGTGGATCTTCATTTTTT

		TTTTGCATATTATTGTAATTTCTAATACAAGGTACCAATCCTACTGTCATCTAGGGTTTGAGGATTCTATCCAGGTTGTCTG<br>AGTTTGCTTTCTGCTTTAGCAATGACACAAATACTAGTATAGTATTCTATTATCTGTGGCAATGAGTTCGCCTTTTGTATAT<br>GCACATTCTATATAAATACTACTTTTATACATCTCTACTGTGCACAATAAAAAGTATATTATTATTATTCTTTATTTTACAA<br>GAATTGTAATTAAATTAGTGACTAATAAGACACTGTTGAATTCTATTAATTATTTATAAACGTGTTCGGATTATTTAATTTA<br>ATCAAATCCAATTCTATAACTAATATATTAGATAGCGGTTGCCCAATATATGTTATCAAAGTAAAGAAACCGATTGAAATC<br>GATATACATAACAGATATGTATGAACAATATCGAAATTATGTAACAAAAAAGTGATGTGTTGAATGGGTGACTCGAGCCA<br>TCACTTTTTTTTTATCTATATTTAACTTTCAATATTGACAAAATATTTAGAAAAGATCTTACTATTTTGTGATAATTTTAAAG<br>GTATTTAAGATAATTTTGTTAAACGGAGTTGGCAACATTTTAGAACGCTTTTTTTTCGTCTTAGAAAATATTGAAAATTTAT<br>TTATTTATTCTTTCATTACATAATATATGTAATTTATTTACAGTCAATTTTGTACTCTACGTAATATTACGTTACCTGCGTGT<br>ACAGAATACGTCCATAAAATATAATTCTAAAAACATAACTTGCACGATAATGTTGCTAGTGCATTAAAGTAATTTATTATA<br>TTGTGGAAATAAAATGAACAGTAATTTCTGAAAATAGGTAGATATATATTTTGTAACGTGAT
MSTRG.1<br>322	DNA-mediated transposase	ACTGAAACGACGTTAGACGATGCACGGCTTCCGTCCTCGCACCGCTCTGCCGCCGTAAGAGTGGGGGGTGTTAGGTTCCAT<br>TTCCGTTACGGAATTTTTACATTTGGTCTCCGCGCTCAAGGCCCGCGATAAAAGCTATACAATAGCTTAAAAACAATTGAT<br>TTGCGCGTCATTACTAAGGTGAAATTCAAATGTTTTAATTTACCTGTCATAGTTTTTGGATTTTTTTTTTGTCAAATTAATGT<br>TATGAAGCAAGTTAGTGACTGATTCAGATTTTATGCGACTGTTTTCATCATACAGCGACGCCATTTAACAAGAGAGGAAAT<br>GTTACGGACAGTAGGCTTGCTCCAAACGGGGGCTATTCAACGGACGGTTGCTGAAGCGATAGGAACAAAACAAAATGTGA<br>TATCAAGATTACGGTTACGTTGTCGTGCTACTGGTGAAGTGGCTAAAAGACAACCTGGCGGGAGGCGCATTAAAACTCAC<br>CGGAAAGACCGATTTTTGTAAGTTGCCGCGAGAATGCAACTAAATGTTACCGCTATGTAGTTAGTTCAAAGGCTCTAGTAA<br>GAGCACCAGTTGCTTGTGAGTGACCACCACCACCAGTGAGAAGAAGATTGCACGAAGTTAACCTGCATGCTCGCAAACTA<br>CTTCGTATTCCAGCGCTGCGTCGAGGAAATCATGGCCAAAGGTTACAATGGGCCCGTGAACATTTGCTCTGAGAGGATATC<br>CAGTGGTCTGTGGTACTGTTTACCGATGAGTCCCGATTTGGTTTCCATCCTAATTCACGAAGAGTACGTATATAGCGAATTT<br>CTTTATGGTTTGGGCGGGAATTCATGTAGTGGTAGAACAGACCTCGTTTGAATCAGAAATACCATGATCGCTGAAAAATAC<br>TGTAACCAGGTGCTAATACCAATTGTCCATCCCTATCGACTACAAATGAATTCAGACTCATACAAGATAACGCTCGCCCGC<br>ACACAGAGCTAGTCGTGACCAACTGGTTACAGAAGTACGATGTATCGGTCTTAAGCTGGCCAGCGCAATCACCAGACCTA<br>AACCCCATAAAGCACGCGTGGAACATGCTGTAAAGAAGGGCTTTGAAGAATTTTCCTGCAAACATTGCAACGGAAGAGCA<br>ACATTTTTTGCATCTAAGCAGGACTTGGG
MSTRG.1<br>3220	Protein CREG1	GTGAAGTTTGCCATGAGTGGTTATTGATAGAAAATTACTAATAACATTCGAGATTAGCGAATATACTTGCTGTTTTCCGAG<br>CAATCGAGATGCCTGCGATGTTATCATTAGCTAATAAAGACAACATAGACGTTTATTGTTTCCCTCCGGACTTTCCTATCAT<br>CTGCTTTATAAACACATTTCTTATGTAGGCAAGTACGGTCATTCTTGTTTGGCACTAGTCTTGTGGCGGACGTGACGCGCTA<br>GGAAACAAATATTTTGAATTTGTTCATATCTGTTTATAAATAAATATTGTTCAGTGTATCTATTTGGCAAACATACGAGGTA<br>TATTCAGAATGAAGGCAACATTGTCGATATTTGTTTTGTGTCTGCTGAGTCTACATATAGAAGGTAAATGGAGTCCAGATT<br>TCGACGAAAGTCGAAATGAATACAGAAGCTATCGTGATGCAGCGAATGAGGTTTCGACCCGTCGACCAAATTATAACATA<br>GTCGATCCACCAGACCATACCAAACTGATTCCAATGGCGAGATACGTGCTTCATAATACTGGTTAGTAGCTAATAATTAAA<br>TGTACACGCAATAACCGTTCGAC
MSTRG.1<br>3222	Golgin-45	AATAATAATTTANNNNNNNNNNNNNNNNCATTTTGTAGCTAAAGATTATTAATAATATAAAGGTGATTAATAATATATACA<br>TGAAGATTTATTAAATTGTATAACTTATAATCAATCGTATAACTCGATCATAAAATATGAATCACAATATTTTAAAAATCG<br>ATATTCGGTTGATTATATTTGTCATAAGAGAATTATTAGACTCCTTATACTTTTTTTCTTAAATATCTCCAATGTACAGATAT<br>TATTTACAATTTTATTATATTAGCAAAATTTTATGATTCTAATTGATAATAATGAAATTTATACCAATGCAATATTTATAGA<br>AGTTGAACCTTTCCATTGCAATGTATGCATGATGCAACCGGCCGTACTGTGTTCCCACTGTTGCTGTATGCTAAATGGACC

AGTGCATTTACTGGTACCTCATTCACCTTTCTAACTTCTAAAGGCATAGCCATAACCTAAAAGCAATAAGTTTTAATTTAAA
CACATATACAATTAATTGAATGATGTATAAGACAGTAACTTCCAAATTAGGAAAGTTAATTGGGTGCCTGCTCATATATGT
TGCAGATTATTATAGTTATCACTTATAGGTGTACCATAGAGAAATATGCACTTCTCAGCCATTGTCAATCATAAAGGTGGT
ATGTATTTTGTAATTATCAAAAAAATTTACTTATTTACAAAACCAATACATTTATTTTTTATTTTTGAATCCTTTATTTTCTT
AAACAGCAGTGGACAAACTGTTGCTTTTCATTAGGATTCAACAATTAGAATGGGAAAGTAGATTGAATCACATATAAAAT
ATTGATAGTATGAATGTATAAATATGTATGTTGTTCAATGATATAATGACACTAAGAATAACCTTTCTATCATAGCTTTTAT
TTCAATTGATTGAATCATTTGGGATAATGTTTAATGCCCCTTTTGAGTTAAGGCAATAATAAGAATATATTCTATAGCCATA
AATAATGATTTATAATGCCCTTAAGAATTCACAATTTTCCATGGAACATAGTCAAATAGGTAAACTTGTCAATTTTAATAT
ATTTTGTTTATATAATCTTCTCATGATAAAATATCCTCAAGATATGATAAGGAGTGACTAAAGGAATAAAGTAACATACAT
AGTGTTCGCTTCCAATGTTGAAATAATATATAGGAGTGTATAATGGTAGTATTATGATTATAAACGTTATAAATAGCACTT
TATAAACTTACATTTTCAGCATGCTTTTCTCCATCAGTAGCCGAGGGTAACTTATCTAAATGACTGATATCTTGTTTCTCAA
CTGTACTACTAATATTTTCTGACAGTTTCAAGTTAACTGAGGCCAAATCTATTATATTAGTAGAATGTGGAGTTGTAGCATT
GAAATTCAAGTTACCAATGGGACGGTTGTAAGCTCTGCTATTGCTCATGTACTCAGAAATGGTGACATTTTCAAGCCACTT
TTCATGTAGACTGTACAAATTTTTGTATGTACACAACATCATGTCTCTTGATCGGCACCTTTCATCCAACAGTTGCCTTATT
GCTTGTTGTAGATTGTCAGTCTTCTCAGTGAGCAATTTTTTGCATTGTGCTAATTCTTCCACCATAAGACTGGAAGAAATTA
ATTAATAATTAAATCTTTATTACCTGTTTTAAGAGTTATTTTAAAATTGCAAATATAACAGGTAACTAAATTAATAATGGGT
TATTATGGACAAATCTATATATATTCTTTATTGCACTTAAACAATATATGCGATGATTAGTTTAACAACTTGTTAGCAAGGG
AGGACTTATTTTTGAAAGAGATCTCTTCCAGCCAACACAGTGTAGTTAAAGAGAAAATAATTTATCTATATAGGCCTATTT
AAGTAAATATATATATTTATAAACCATAATGTAAAACAAATGTAATAGCTTTAATAATAAAAATAAATAGTTTAAATGTTC
AAAGAATAAAATGGCGTGGCTTTGGTAATTCATTATTCATTAAAAAATTACACAACTAAATATTAAGTTATTATATTCATA
TATACTTTATATATAAAATATTATACCTACATATAAAATATATACATATATATTACTAGACATACTATATTATTAGACATGC
TGAATATGCTAAAATAGGTACCTGCTGGCTAGAAATTTACTTCTCCATACTTCACACTGTCCTGCCAACCATTCTGTTTGTT
CCTAACAAAGAAAACATTGGATTATTTTACAAAATAAACATAGAACTTCTAGAAATTTCCATTAATGCAGGCATCTAATGA
CATATCATTATTGTATAGTATAGAATGAATATTACCTTTTACAAATAATATAAGTTACATGTCAATATGTCAGTCTATTGTT
ATGGCTAAACTGTTAGTCCTTTTAATTTTTAAGTTAGATATTCTATGGGGTAGAGAAGTAGGCACGATTTAGAAGACTGTC
TAGTTGAAATATGGTGATAAGGTATTTAATAAGTAAATAAGTGAAATAAACTGACACAGAATTATATAGTATATTTTATAT
CATAGATTATGTCACATAATATCCCTTTAATACCTGATGGGTAGAAAGATGTTGTGCAGAGTTTAGCAAAGCATTTGCTAA
GTGTTTTTTATCTTCATTAAGTGATTGTACTTGGGTCTCTAAATCTTCTCCCATTGATGCTACTAACATAGTTTTCAATTCCT
TATTGACCTGAAAAAGCATAACATTTATATTGTGTTTTATTTATTTATTTGGAGTTTGCTTTAATATATAAAAGTTGTTTTAA
AATAATTTCACTACAATCTACAAACTTTTTAAGCAATACACTTCAGATTTAAAGGCATGCATATTTCTTATAATCATAAAG
ACAATTCTTTTTAATCTTATTGAGAATTACTATCATCAGAAATAAAGGACACAATTGAACTAAACAAGGAAATAATTTCTA
TATATTTATACAAAATTAATTACTGATAATAAAAAATATGATATAAAGTTATAGATTGTTAGTTGATCATGTAATCTAATG
CATTATTCCTTTTACTCTCACAGGTTGCTACCAATCTCAAAGGTTCTATTCTTGCTGATTCAGGGGAAGTAATTAACTGACT
AGTTTTTTGAATAGATGAAAACAGAATACTATCCTGACTAGACTAATTGAGGATGAATTCATCAGAATTTTGCCATGTTGA
TTTTCAAAAGGACTATATTGAAGTTGTCTTAGCAGAGTACTTTATAAATAATAAGGCTTATAATTTTCATTACTAAGAATTC
CCTCAGACCTAATCTAATATTTTTACTTTAACAGAAAAGCTATGACATTTATGATAATATTAATTAATGTTAAATTGATACA
ATGTGACCCTTATCTATTATATTGTAACTAGATCACACCTGTGCTTGCTGCTTCAACTGCTCTTTCAAAGACTCATTTTCCA
ATAATAATTCATGTATCTTTTGTTGTCTTTCATCTTTCTCTAGGATTTGTTTCTCTGTATCTGATGATATACTTTTGTCACTAC
TAGACAACAGTTTTGGTCGTGGTTTAAATTCATTTAAATCTGTGTTCATTTGTGACATCTGAGAAATTAATGTATTGAGATC

		CATATTGTTCCTAGATTTCTTTGATCCTTTTGATGCAGACTGAGCTTTGATTGGTTTAATAGCTGCTGTGTAAGGTTCATATG<br>GTACAAACTTAGGACTTTTAGAATGTAAATGGGTCTTCTTCTTTTCAGTTTTAATTATATTAGTAGGATACACCTCGATAAG<br>GCGAATAAGTGGTTCCTTACATTTACTTGTCGACCCTTTAGTTGGAAATATCGCATTTGTAACATTTTTCTGAACTGTTTCT<br>ATTGTACCTTCATCAGATTCCAT
MSTRG.13296	Uncharacterized protein LOC106125039	TTTCATCCCAATCATCTGGACGAGTGGCGGTCTTCCACCGTGCGTTTTTCAAGGCACTTTCTTCCACGCACAACCACTCTTT<br>GGAATCAACTTCCAGCAGCAGTATTTCCGAACCGATACGACAACATAAACTTCAAGAAAAGAGTATATTCCTTTTTAAAA<br>GGCCGGCAGCACACCTGCAATGCCGCTGGTGTTGTGGGTGATCATGGGCGGCGGTAGTCACTTACCATCAGGTGAGCCGC<br>ATGCTCGTTTGCCCCCTGTGTTGTAAAAAAAAA
MSTRG.13310	ATP-dependent RNA helicase DDX46	AGGTGAACTAATAGAACAGAACCAAGATGGATTGGAATATTCATCAGAAGAAGAAACTGAAGATATCAAAGATGCTGCT<br>GCAAACCTTGCGTCCAAACAAAGAAAGGAGCTGGCCAAAGTGGACCATGCTAGTTTGAATTATATGCCATTCAGAAAATC<br>TTTTTATACTGAGGTTAGCGAATTATCAAGAATGACGGCAGAAGAAGTAGAAGCATATAGAACTGAATTAGAAGGCATCA<br>GGGTTAAGGGCAAAGGTTGTCCTAAGCCTATCAGAACGTGGGCCCATTGTGGTATCAGTAAGAAAGAGCTAGATATATTA<br>AAGAAGCTGAGCTTCGAGAAGCCTACACCTATACAAGCTCAAGCTATACCCGCTATTATGTCTGGAAG
MSTRG.13315	T-complex protein 1 subunit delta	CGCCGCCGCGCTCGCGCGCGCCGCCATCGCCGCGCCCGGCGCCGACCACTACACGCTGCGCGCCTACTCCGACGCGCTCG<br>AGGTCGTGCCCTCCACGCTCGCCGAGAACGCTGGGCTGAACCCCATCGAGACGGTGACGGAGCTGCGCGCGGCGCACGCG<br>GCGGGCGGGGGCGCGGGCGCGGGCGCGGGCGTGAACGTGCGGCGCGGCCGCGTCACCGACATGCGCCACGAGCACGTGC<br>TGCAGCCGCTGCACGTCACCGCCTCCGCGCTCGCGCTCGCCACCGAGACCGTGCGCGCCATACTCAAGATCGATGACATCG<br>TTAATACTGTGAACTAATAAGAAAAAACAACCATAGATTTACGTTCTTTTAGTTTATTGTTTTACTTAACATTTTATCTAAT<br>AGTATTATGATACAACATAAATGTCAGCCTATATGTGATAACAAAGTAATTGATAACTGATGCCTATTTATAAAATTTATT<br>TTGCAATATAATTAAAATGTAATCTTACATTGAATTTAGTGCTTGGCTGACTGATAAAATTTTATTTTCTTTGAATATGTTA<br>TTAATACTAATTATTAGCTACTATTTAATGTAATTCTTTAAAATAAAAGAATTAAAC
MSTRG.13323	Synaptotagmin-5-like isoform X1	AGATGTGTATGTGAAGGTGTATTTGCTGGTGAATGGCAAAAGAGTGAAGAAGAAGAAGACGAATAGGAAAGAGATAAAC<br>AATCCCGTATGGAACGAGGCGCTTTCTTTCAGCTTGCCCTCCGGGAATTTACAAGAGGCTTCTATCGAGGTATGCGTATTG<br>ACTGGTGGCAGCGGACTGGTGGTGGGCTGGTGCTGCGTGGGCGCGGCCGAACCCGCCGCTGAAGGCCGCCACTGGGCGCA<br>GATGGCGCACGAGACACGCAAGGCCGTCGCCATGTGGCACACGCTCAGATAGACTGCCGTCATCACACACCGTTAACACC<br>CACATGGCTAGCCGTATGCTGACGCACGCAGGAGAGGTTGCATATAAGAACGGTTTTTTTTCGGTTGATCGTCATAGGCAT<br>ATTGAGGAAAAGAGCGGGGGATGGGATCCGTTCCACCTGGAAAGCATCGTACTGAATTCCAGAGGTATTCATCACATTGG<br>GTTAACCGACCTGCCTTTACATTCTCACCTATCATCCCTCACTGGAGCCCCGCCAGCGTATACCGTTAGCACAGACGCGAT<br>TACCTTTCCATTATCAGCCATTAAAAACATAATAAAAAAAAATTGTTTACCAGAGAGACTTTGTACAAAAGTCGTTTACTT<br>GGTACTTCTGTTCCACTTGTAATTTTTTCTGCCGTTAATCAGCTGTACTTGCATAGCTGTGTTTCGGTTTAAAGGGCTATGTC<br>AGTGAAATTACAGGGTACGGAGGCATAACACTATAGTTCTCAAGAATAATAATGCATATGGGTTTTTTTTCAATGGATGA<br>AAATGGAATGGTCAGTTAGCCCATCGTGACGAATTCATCTTAAATTAACCACGATGGTTTCCAGGGGGAACCGTGTGGAG<br>GTCCTTAAAAGGTAGACTAAGGTATTTATGGTATATTGTTAGTAATGGGCCTGTTAGATATCATTACTGACAATCCCTTCCC<br>CTCCTCGCATAAAGGATACCATGGGTATCATAGTTTATTGTGATAACCGTGGTGCTGCTCTTGTCTATAGTCGATGGTTACC<br>ACTATTTATCAATGGACCGTCAGCTTGTTTGCCATTTTAAATCTCATAAAAAAAGTTCAGTTTTCAAAATATGAAATTTTCT<br>ATGCAACCTCATCAACCGTTTAGACCCCAGACCGTTCATCGTGGATGAAGCATAACATTGATAGATCTGTGTATACTGCAA<br>CGCAGATAAATGAAACACCAAACGTATGTCAGTTCTGAGACTTTATTTAGGTAATAAATATGCAACTAGTCAAACACTGAT<br>TTGTTATTTTCAATTTTCTGACACCTAGTCATTTACCTTCCTTACGTAACACCATACATTCAGCACATATGTATATCTCTTGT<br>GTCAACTGCCATACTCCCCACTGTACTATATTGCAGTTCACTGCTGCGTCTCAGCATACGCCTAGCCTTAATCTACTAACAC

		CTCGGTAGATTAGTATAAATTGTTATACATACATATGGATTCTAATCTGACAACTTCGTGCACAATACTCTTATAGCGTGAC<br>AACGTATGGATAACGGTATTGTAATGTAAGAAGGTATATTATATTGTCTATGGGGCGCATTATTTATGGATGGACGTCTTG<br>CAAACTCTGAGAAAATTTAGCACAAGATGTTGACTAACTATCCAAGGTGAAATCGAAACGACTAAAATGCTCGTAAGCCA<br>ATCCTGTTGTTCTTACATTAAAATATAATGTGTACTTGTGCAACTTTTAACAGTAAATGTTTATTTTTGGTATAACTTGGCA<br>ACATTTAAAAACTAAACAGACGAGGTGTTAAGTCTGATTACTAGATATAAGGCAAACTAAATTACATCTAAGCCTTATGCC<br>TAAAGGCAATTATGCAATAGCTTATCTATAATCTATTATAACTATTATCCAGTCTTAACTGTGGGCTCGCTGTACATAATAT<br>ACTAATATCTTCTTTCTTCCAAACTATCATTTCATACAGCCTTTTTGGCTATTTATAAGTATTGTACAATAAATATACTAACT<br>CTATTAATCACTCATATATGTACATGTACTTATCTTTAATAGATTAATGGCTCCTATGCTATGTAAATTATATTCAATAGTA<br>GTTTTAAATTCAAAAACCTAAAAGACCCCGAGTATTTTTATCATATTTATAATATTACAAAGAATATATAATATTTTATATT<br>TATTATATTGAAAGTCAAATCAAACAAATATGGCAATACATTTGAGATTTTTATGTGTGAAATCCTAGAAATAATCTATTG<br>AGCTTGTGGAACACCAAACAAATCTTATGAATTTGTAGAGTTTTATACGAGTATATTAATTTTGTAATATTAATTTTAATGA<br>TCCTTGTCCAGTTAGTATTATTGGAACATGGATGGAAAAAGGATGCCGCGCCCCATTAGTAGGTAGTTAGGTGAAGTGCTG<br>ACCAAACTAAACATAAGTCCTAAACCTAAATGCCTCAGTCGGAATCCGTCGAGTAAAGCCGAATCTGTTTTCATACCCAAG<br>GCTGGCCTTAATGTACATGGCGTCCCTGGGTAAGTTTTAGATGTCCTCCCCACACTCCCGATGACGAAATCTTAAAAAGAA<br>AATTATTATTCATTTGAATGTCGTCCACATTGGGTCCAATATCTATAGGTACGTTGGGTTTCTAAAAAAAGGTTACCACTAA<br>TTTTTCGCCCATTTCAAGTCCACCGAGTTTGATATATATTATTAAGTTCCTGTAAAAAAAGTTTATCGTCCATTTTGATGTCC<br>CTACACTTGGCTCGATATCGCCAACGTCGAGTCCGATGTCCATAGATACACTGGGTGGACCTTTAACAACCTCAGTGCCAC<br>TGGGCGCCTCCCGACTAGTCTTTGAAACCTTTACGTACTCGTATTGTTAATACCCTCTGCCCCTTAACCTATGTCCAATTTTT<br>GCCTGTTTATTTAACGGCCTAGTATATTAATTGTTACATAAATGGAACACACTAGCAACCGGGGTGTTATGTTGAAAGTAT<br>TCATGGATGACTAGCAGCTTCCATCAGATGGGCTGTTGGTTCCTTTGTCTTCCTAAC
MSTRG.1<br>3385	Hemicentin-1	AAGACGCGCGCGTACCGAACGTACTGTCCGCGCCATAATTGTGTTCGAAGTTTAAATCGCGATGTGATTTGTTTTTATATTT<br>AGAAATTTAAAAAATATATGTAAACTTTTTAAAATAAATAGCCAGTGATTTAAAAGTGACTGTGACTTTATTTAGTTAAGA<br>AAAGAATAATGCGATTTTTTGTTTCTGTAATAAAGTAAAAAAGACACTGGGATAATGTTCGATACTTATTAATTAGGTATG<br>AATTGGTGAAATAAATAAGAAATCATGAAAATGAAGATGATTTGGAATTACATCACGGTTTCGCTGATGTTGCTTTTTATT<br>AAAAATATCGATGCGATACAGCGATTTGTGGAGATGCCAACGTATACAGAAGTCAACCCGGGTGAAGATGCTTTGCTCAA<br>GTGTAGAATATCGGACAAAAAAGGCGTTTGCTCTTGG
MSTRG.1<br>3391	Hypothetical<br>protein<br>KGM_200100	TTTGGTTGCTTGCAGTTTCCTGCGGCGTCCCAGAAATGGCTTATGATCGGCGGTTGCACGCCCCGCGGCGGCGTCTCCGCC<br>GGGCAGCCCGGTTGCCGAGATGGCGCCGCCGGCCGCGGAGGTGGAGGCGGAGGAGCCGCCGCCGCTCGAGCTGGCGACC<br>AGGCCGAGCCCGGAGCGGCCGTGCACGCTGCAAGTGGAGGCGGCCGCCGCGCCCGCGCCGCTGGCGACACCTACGGCGG<br>CCGGCGCAAGCGCACCCATGTCGCCCGCCGGATCCACCATCTCGCGCCGCGACTCGCAGCTCACCGACCGCGGCTTTTTCG<br>ATGTCAAGTTCTACCACAACAAGCTGTGGTAGATACCACCGCTGCCGCCGCCGCCGCCGCCGCCGCCGCCGCCGCCACGC<br>GACGCGCAAACGCTCAAACTCCGTGTGGTATTTATAGAAGTTTATACAACGCCTGCGCGGTATTATTATTGCTAGGGAAAA<br>TAAGACAGTAACGCATTAAAAATTTTACGCTTCACATGGCCGTACCGCCGCGCCTGGGCACCCGAGCACGACAGTGGCGC<br>CACCGCACGTCCACGTCCTGCAGCGGATAGGACACAGTTTATTTAATTAATTAATTTATTTATTTATTTGTAACAATTATTA<br>ATTTATTTGCTCGAAATGTGATAATGATAATGATCTTAAATATCACTGATCTACACATTTCGCGTTATAGGCATGCAAATTT<br>AATATTATAATTTATAGGA
MSTRG.1<br>3414	Glutamate O-<br>methyltransferase<br>-like isoform X1	TAAAAAAAAAAAAAAAATTTGAAGATCGAATTTAATTTTCCATCAACACTGCACAAAACATTATTGAATAAATTAATATTTC<br>TGGTTCAAAAATAACACTTCTTTATCTGTCATGGCCTTAAAAATTTGAAAAAATTCAACTAAAGAAGTAAAAAACAAAAA<br>ATGAGTTCAAACAGTGTTGTTTCATCTAAAAGCTCGAAAGAGAGTTCGAGACCGGGTGCGGGAAATACAAAACAAGAAG

		ATAGCACAAGTCCAGATTTTATTTATGGCGGGCCCTTTATTAAAGTAGAGAAACCGGAACCGTTTTTTGATACAGCTACAC<br>CTGTAAACCTGCTACTTCAAGGGACATTTAAAAAG
MSTRG.13416	Uncharacterized protein LOC110377987	ATACATTTTGGCGCCAATAATTTAATAGCTTTAGACGCAATGATATTTGTGGCACATAATATAATATTAAACTGCAATGAT<br>ATAAAGTTAGTAAATTAGCTGTTACAGACAGGCATAGATAAAATTAAAATCTTTTTCTACCATTGCTCTTAATGCGCGTAT<br>TCTTCAGTGTAATGGTTGTAGACTGCAAAGGGCGGCCATTGTTTCTGTTGCGAAGCACTGGACAGTATGTTCTGCAATATA<br>AAAAAAGAGGATTATCCAGGGAGGGGAATTTGATGAGCTTGGTATAACGTTATACATTGGTGAAATTGCACGAGAATGGC<br>TTGGATTCGGCAGTATGGGAGATTGGAGGGGTGAACGACTTATTTGGGAGAAATCTGGTGATACATTACAAGAACTGGAA<br>GTCATCAAAGGAGCTTCAGCCTTAACAGGTGTTTTATTCTCATATCCTACGTTTTTATTGGGAAACAGTTCATAATACACAT<br>TCTGTGTGGAATTTTTTTGTTCAGAAAAGTTGCGATCTTTTGGGGGTGTATGGGAGAATGGTAGAGGCTCATCATTTTGCCA<br>GATTGTCTTAGCCAACTCCACGTCCTGATCCCATGTTCTTGGAGGTGATGTTTGGACTACATTAGGATGCACTGGCTGTGCT<br>GTGCTCTGGAACTTTCTACCGACTATAGGACGGTATCTTCGCATAGGTGTATCAGGGAATGTGCTAGGATGTGGCAGTCTG<br>GTTAAGTCAGGAGATTGAATAGTGGAGATGTGAGGAAAAGAATCTGGAGACATTGAGTCTGCAGAGCTGGAAGATCTGTT<br>GACAACTCCTGAGCTCATCGGTGATGATAGAAAGCTGGTGTTGCTTTGTGCACTTGGTGATGGTAGTAAGTCTGAAATAAT<br>AAAAGTATTGTGTATTTTTGATGAAATATATGTATATAGGTAGTTTACTCTAGGTAATTTTATACTTTTCATTGGTATAATG<br>AATTATATAAATGTAAATTGAATCCATGGAGTAATTATAAATCACAATAAGATATACACAGCTTGTATTATTTTCTGTCTA<br>CATATTACCTATGTAATTAATTTGAGAGAATCATTACTACAAAAGTATAGAATTGATATTGATTTATTTCATTCAAATTAAT<br>TTTTAAAAAGTTATTAAAACAGCATTTATTAACTAAACAAAAATTTGGGTTGGTCGCCTGGTAGCGACTGCATTCAACATA<br>ATAATTATTGTGGTGAATTATTTATACTGTATGTTTAATTAATTAATAAAATATATTATTTATGCATTATTTATGTAAGTATG<br>TATCTATATAATACAATTTTATCTTTGGCTTTTAAATTGGTTACACAGGTTACACTCTGGGTTTGTTGCACGCGTCTGTGTA<br>GCCTAACTTCAGGCTGCAGCTGAAAATCAGTGCTTTACTTTTAAGTAAAACCATCACTGAGCTGTAGCCTATTTGAGTCAC<br>TGCGACACACATGGCGAAACCATATTTTATCTTTTGTTGTATATTTTAAAAAGTGTAAATAATTTGATTGTATAAATTCATT<br>TGTAAATTTTTGTCATAAATGGTTCAAATAAAAAGTTTATCTATCTATTTTTTTTTAATCGATTTTGAAAAGGAGATTCTTAA<br>TACAGCTTTTTTTTTATGTTTGTTACCTCATAACTTCATCA
MSTRG.1343	Uncharacterized protein LOC110374261	CCTCGCCACCTTCAACGACCTATGCGAGAATGCAGGCGACAACTTGCAGTAGGATCGCCGGTACCAATGATATGTGGCGG<br>CCGGATTGTGTTTGCTCAACAGACGATAGTACAAAGCCAGTATCGAGGCCAAGGTCTAAAATCAAACCGGCTTTCCAACA<br>CGGAGAAACTGCCATTTATTATGAACCAGCCGAACACGTTCCTAGTGAAGGTGTATCATTCGTCTATCACCCATTTATTATT<br>TTGAACCCGACATTTAGACGACATTCTGTTTCTAACACCGTGAATCCTGCAATAAAAATAACCTCTCATATTGAAGATAAC<br>ATAAAAGAAGAAACGAAACCAAGTACAAAATATAAGCGAAAACATAATTTTACACAAAAATTACAGACCGAACCAGAAG<br>AACGACCTCTATATCTAGATATTGCTATCCAAGACACAGTAAGAAGTAGATCTTCTGTCAAGGAATTTAGTAGTATAGATG<br>ATTTACAAGTTATTGAACAGAACTTACTAGATTGCATAGATCTTCATGTGTTAATTTACTCAAGAAAGAATAATAATATTA<br>CAAAATTAAAAGAAATTTGGGGAGATAATAAATTTAAAAAGTACGACAATTTATCTAATTCTGATTTAATGATCAAGATTC<br>AAAGTAAGTGCGGCCAACCAGCAAGTGTCATAAAACAAAGTTTAAATGAAATAAAAAAAGCATTACCTAGAAAAAATGC<br>TACTGAACACAAGCCGGGTCAAAATGATAAATTTGATGAAATAATAACTGAGCTGCCTAAAGCAACAGATATAAAAGGGT<br>CTTATTTTACAAGATCAGATGAATTAAGTTCTTCTACAGTCAAGACAACTAGCGCAGAGAAAACACAGTTCGAAGAAAAA<br>ACTACGACTAGTAAGTATTTTTCCAAGTCACTAAATAATTTATCAAAAAATAATATAAAATTAATATCAACTAGATCAACA<br>GCACTGAATGCTTCAAATGAACAGAATGAAATAAACCAGCAAACATTAACTTCAACAATAGAACCAACTATTCAATATTA<br>TACAGAAGATAGTGTAACAACTTCGTACAATAACAGACACATTACCCAAAGTTTTAGTAAGAAAGATAGTTTACTTTCAGT<br>TAGTCCTACAAGCAGTGAAGCTATTCCTGAAAATGAATATTTACCAAAAAATATTTTAAAGGATAGTAATATTTCCAATAC<br>AAGTACAGAGCATTTTTTTTCGGATTTACCTAATAATCATTCAGATAAAGATATAAACACTATTGGGTTTTCCTACACTGAT

		TCTCATGAAAATATTTCACCTGCGGAAACTTCAACAGAGAATGCAGTTATAGATCATAGCAATATTACTAAAAACTACCAA<br>GATTTGCAGAGTGAAGGGAGAGTTCATAAAGAACACATATTATTAAATAGTACAACTACTGTAGGTGATAATCGAATATT<br>GTATCCGGAAGGTGAAATGAAAATACCAAAAGTAATAGTTAAAGAATATCCGAATGATACTGTTATTAGTGAAATATCTG<br>GTAATGAACTATTACCTAATAATGATTTTACCTATTCAACAATTCAACCAATCTTAAATATCTTAAAAGAAAACAATACAT<br>ATTCTGATACTAATTTTACGTTTGGTGTTCACAAACCCATAAATAAGAATACTGAAATAGGATCGACAAAATCGTTTGGAA<br>TACAAGGCAGTCCACGGCCAGAAATAATTGGCAAACTCTATTTCCATTTTAATAACGAATCTATTCCGGTACACTTCGTTC<br>AAGAATCCGATGGAACTATTAATGTGGGTTTAGATGGAATATCATTGTGTGATGAACTAAATAATAGTGGGCGAAATAAA<br>TCTGTTCTGTTGACAGTTTTGTGTAATTGCGCACGATCGCCAAACTGTGCGAGTACTACTATAAACTAGTTAAGTGTATCAT<br>ATGCTGCATTAAAAATGTTTAGTTCTGGATTCTA
MSTRG.13435	Germinal-center associated nuclear protein	ATCTATTTAACAGGAACGCTTTACTAGTGATGAGCCACGCGTACAATAGCAGGCAGCTGGCGGTGCCCACTGCTGTACTCA<br>GGCAGTGGCTGCGGTTTCCCAGTGATGCCGATGTCATCGCCGTTTGCAAACACTACGGATTGCAGGCTGAGGGCGCCACTG<br>TGCGGTTCTCTAAAGCAGACTTCAAGACTGAAATTGCTACTCATGAACCAAAAAAATTATTATTGCACGAGGAAACATTA<br>AAAATGGACTTGGAAAATATCTTTACCTATAAAACACAAAAATAACCAGTATCGTCCGATAGTGCTTATAATACTAAACTG<br>CTGTGATACTAGTGTTTAAAAATAATAAAACTACTTTAAATATTATAAAGGAA
MSTRG.13465	Transcriptional regulator ATRX homolog isoform X1	CAGGTACAAGCAGAACTCTGAACGCGCATACCAACTGCAGCAGTGGTACGAGTGCGGCGGCGTATGCGTTCTAGGATATG<br>AGATGTTCCGCAACCTGTCTGCTGACAACGTAAAAAAGTTCAAGAAAAAGATGCTGCGGATCTTCCAGGAGAGCCTCGTT<br>GATCCCGGTCCTGACTTGGTAGTTTGCGACGAGGGTCACTTGTTAAAGAACGAGAAAACGAGCCTGTCGCAATCTATGAA<br>CAGAGTGAAGACGCGCCGTCGCATTGTCCTCACTGGAACGCC
MSTRG.13469	Uncharacterized protein LOC106101723 isoform X2	CTCAGGAATGGCAACGCATGGGGGGTATCATGGGCGAAACAGTATACTCTGTTATGTCCATGGTCACTTATATACTGCTCA<br>AGTCTATAGGCGACGGTTACCACTTTCCATCAGGTGTGCCGTCAGCTTGTTTGCCATTCTCAGTTGTATAAAAAAAAATGA<br>TTTGATAAACAGATTTGAATGAAATTTGGTACAAAGGTAGGTTATGTCCTAACTTAACAGATAGGATGATTTATACCCAGA<br>GGTATTGATTTACGCGAGCAAGGCCGTGTGAAACAACTAGTAATATTATAAATATAGAGAGACTACAGTATGGGTTTTATT<br>AAGGTTTCTTCAGGATTGGCAACGCGCACGTGACACATCTAGTGTTCCAGGTGTTCATAGGCTTCAGTGACCGTTTACCAT<br>CAGATGGGCTGTATGCTTATTTGCCAAAATCGTGGATAAAAAAATGTGTTTGTAATATTGCAGGCTAGAGCGACGGAAGG<br>GGAGCCGGCCGTAGCTAAATTGAAATCTCAAATAAAAAAATTAAAGGAGGAGGTGGGCGCGTCGTCGCGGCAGGAGGCA<br>GCGCTGCGGGCGCGGCAGGCTTCCGCCGCCCTTGCGCTTCGCCGCGCGACGGCCCTCGCCGACGTCTCGCTACGCGAACTA<br>TTGGCCGGTCTGGAGCAACTCAAGACGTTGAGTTCGACGCTCGACCCGACATGATACACGGCTACCGAGGACATCACATC<br>ATAATATTATGCTTTAATTAATACCGAGTTCCCCTATATTTATGTTTGAATTATCCATAAACATGTGATCCATGGATGATGA<br>TATGTGATGAATTTTTAATCTGGAAGTG
MSTRG.13478	Phosphate carrier protein	CAGGGTCTCTGCAAGTTCGGCCTGTACGAAGTGTTCAAAGTGGGCTACACCGGTCTACTGGACGAGGAGACCGCCTACAC<br>GTACCGCACGCTCGTGTACCTCGCTGCCTCCGCCTCCGCTGAGTTCTTCGCCGATATTGCTCTGTCGCCGCTCGAAGCAGCT<br>AAAGTGCGTATCCAGACCATGCCCGGCTTCGCGAACACGCTCCGCGAGGCGTGGCCCAAGATGGTCCAGAACGAGGGCTA<br>CGGCACATTCTACAAGGGCCTGGTGCCACTGTGGGGCCGACAGATCCCTTACACCATGATGAAATTCGCCTGCTTCGAGAG<br>GACCCTCGAGTTGTTATATAAG
MSTRG.13489	Protein IWS1 homolog isoform X1	TGGCTACTGACTCGCACATATTGTACATACTACCACGTGATACTACTTGTATACTACTTACCGCTACTACTATTCACTTGCT<br>AATTTTTCATCGCGTCGAGTCCCGATAATATGAAGTATCATCGCATCGTTCATATCCCCGCGTATAATCGATGATTTTGATT<br>ATTTTGTTTGTCCGTACCCGTCGATAGATATAACACAACGGTAAGTTTCATAATAAATAGATTATTTTGTAACATGGACGA<br>CCGCTCGCCCTTCCAGGTGACCAAAAAGAAGATGACCCGCTACGAAAAGCAGATGAAGCGGTTCATAGACCAGAAACGC<br>ATGCGCAGCGGGTCGCGGCGCGCCGTCGAGATCTCCATCGAGGGCCGCAAGATGGCGCTGTAGGCGGCCGCTGGCGCGTA

		CTGCGAGGCGGCGAACTAGTAGCTTGGCGTCGGTGCCGGCTAATTAAGATTATCTTGAAAAATAGCGCGTATATGATTAC<br>AAAATCCTGTGGGAGGGTCCCGTCGCATGTGTATATAAAATATTACATGCGTTGCAAGTAGGCCAACACTCGCCCCTTGGA<br>ACGATCCGATACCGACTAGTCGTGTAGTCAACCCGTTTATCCCGCTTGCTGTACGGATGAGATTGTATGAGGGCGTGGTGT<br>CCAGCATTACATTACATTACATATACTATTACTTTACAATTGTATACTGTATTTGATAATGATAAATGTCATATTGTTCAC<br>AACACACGACCATAAAAGATGACTCGATTAGCTTTATAAATATATATTTATATTTACAATATTATATTACATTGAAAAATG<br>CAACCTTTCATTTTAACTCATTTTAAAATATTTTCCATTAATCTGTCCTCTTCTTCTTATCATGTTACAAAAGTTACTTATCA<br>ATAACACGCTGGGCAATACTATTGTTATCATTCCTCTAGACTTTCTTTTAAATATATATGTTTCATGTAAAATCATAATTTC<br>CTTCGATCATTCTGTCCACCTGAACTATATTCTATGTGTTGTGGTATGCTGCGGGCGACGGCCGTGTGTGCACGCGGTGGC<br>AATTTAATATTTTGTGTGAATAGTTCTCTTAACTGCATAAGTTACGTTTTAGTAATTATTTCGTTGTGGCTTCAGTGACCCGC<br>AGTATGTTGCGCCGCGTAGAGTATAAAACGCAACTCAAACATAGCTGTCGTATGCACGAATACTACATTAATGAAAAGAG<br>AAGTTATTGAAAGTTTAAATTATTTTAACCTCAGTTACAAATTATAAAAGTTTAATTTATTACATTTAGTCGTGTTTCATGA<br>TCGAAGCTCGACAATTATTCAAGCGCACGCCCGACAATGGTCCAGCTCACAGTCTATATGCATTGTCTACGATCATAGAAT<br>GTTTCATGGCGTGTACATTGTACATCCTCACTGACAGCCGAAGACGGACTGTGTCCAGATATTGTGATGTGTCTACATGTT<br>GTTTTATATAATGTTTTATACTTATTTATATATTAATTTGATTGTTATTGTCTAAGTGTGGAGCCGTGTCCCGTAGGCTAGTT<br>ATTGTAATTAGATCAACGCACAACTTGGACACTTGAAGTAACACGTGCGGAGCGTGTTTCGTTTACTAATTACGTGACGGC<br>GGCGACGCTCGCTCGCGCTCGACACGTGCACGCGTCCTGCCTGCGTGTGTGTGTATGTGATGTAAACTATTGCACTTGAAA<br>TTAAATCTACAATCCTATATCAGCTATAGCAAGTTTTATAATAAGTTTAGTTGATGATGCTCTAAAATATATCAAAATGGC<br>GCCTCAGAACCAACTCGTGTGTTTGTCGCTGGTCAAGAATTTCGGTGACGGTCAAGCCTGATTGAGCGGCCGCTATATTTG<br>CTCGCTGCCTGAATCGAGCCACTGAGTGCTTGACACGCGCTCGATTCTAATTTCTACTTGACGCGAAGACTTGATCATCATT<br>TCACTTTGATAATAATACATTATTATTTAATTAAATAAGTAGAATAAATAATTGAGAGAACTATAACCATAATTTATATAA<br>GTTTATAAATACATATATGGAGCCACTTAACATGTGTATGAAGCGGCTTGATTAAGCCTATAAGTCGCTAAATGTACGTGC<br>TGGATTGAAGCTGCTGCTGTTGCTGCTTGACCGTCTCCGCAGCTTGTCGTGCGCGGGAGTGGCGGCCGTCGTCCATCGCGT<br>GTAATAATTGTATTTAAGTTGAAATAAACGTGTACACACAAC
MSTRG.13496	Aprataxin and PNK-like factor isoform X1	GTTGTTGGCAACATAGTGTTTGTGTTTTGAAATTGTATCTGATATCATCTATTTTTTGTATATTTATTTTTAATTAATTAACT<br>GAACTAATTTCAATTCTTCAACTTTTTTTGCGTACATTATAAATTCATGAACTTATTATGTTAGATTATTTGTGATAAAATG<br>GTTTTTAAATTAGTACGGACCGATTCTGAAGACCCATACAAGATACAACTTGATGTAGGGACGCATTTGATTGGAAGGGGT<br>AAATTTTTAAATTGTGATGACAAAAGAATATCAAGGAACCATGGGGAGTTACAAGTTGATGAAGACACAGTCATTATTAA<br>AGCAGTAAGATATGAAATAGTGTTCAATTCTAATGTCTCTTTGTAATATATTAGTTGAAGCCTCTTACCAGTTCTTCCTGGG<br>CGGAACTGTACACTGAACTAGAT
MSTRG.13506	Ubiquitin carboxyl-terminal hydrolase 32	TACGTGGTGAAGTTATTGAGGTCTTTGCTTCAAAATATTCGATTTGGACCGAGACGGGGTATTGAATAAGAAGGAGCTGAT<br>CGACATGGTGGGCATCCTGTGCACGGTCGCCAACGAGTCCCTGAAGAACCAGGGCTCGAGGGCGTCGACCCCGTCCGACG<br>GGGAAGACTCGGACACAGAGAAGGGGTTCGATCCTGAGGTGATACTGCTCAACCTGCGGGATAAGCTGGTGAGCGTCCCC<br>AAGGATGGCAGGAAACCGGTGTTCCAACTCGGGCCTAGTGCTGAGGGGGAGGAGATCATTGTTTTTAAAGAGG
MSTRG.13515	Vacuolar protein sorting-associated protein 13D	AAAAAAAGTACCGATCGTGTGTGAGATCGTGTTGTCCGTGGACAGTCATAGTAACAACGATATGATGGTTGGTCGCAGCA<br>TGGACACGATGTCGGGCAGCATCCGCGGCTCGGAGCCGACGTCGCCGCGGTCGCCCACGTCGCCCACGTCGCCCACGTCG<br>CCCACGTCGCCCACGTCGCTGGCCTCGCCGCCGCCCGCGCCGCTGCACCCGCACGCGCTCAACCACGCGCTACACTCGCTC<br>ACGTACGCCGGCGCCGCTCACAAATTTGAAGAGGGGGGCCGCACTAGTCCCGGCCTTCGTTGGCCCGGCAGTTCGAGCCA<br>TGCCGGCGGGGCCGGGCTGGATGGGGCTCGAGCAACAGAGACGGACCAGGGTGCAGCGCGGGCGCCGCCTGGCCGCG<br>CCCGGCCTCGTCGACTCGGAGGCGCTCATCGCTGTTGAACTCTGCTTCGTGAAGGGAGAAGAGGAGGACCTCAGGATTGC

		CAACATACTCTTCAACAACCTTGATATAATTG
MSTRG.13520	ATP-binding cassette sub-family B member 7	CCATCCTCAAGGACTCGCCCATCATAGTGTTCGACGAGGCCACCTCCAGCCTGGACTCGCTCACCGAACACGCAATCCTTC<br>AAGCTTTGAAAGCTGCTACCGTAGGTCGTACATCTATTTGCATAGCGCATCGACTGTCGACCGTCGCGGATGCGGACGAGA<br>TTTTAGTCCTTGAAAACGGTTCGATATCGGAGAAAGGCACCCATAAAGACCTCATCAGTAATCCAGCATCACTCTACTATA<br>GACTATGGGAGAAGCAAAACAAAAGTGCTATACCAAAATGAAACGTACCAGTATAATAACAGGGATCATGTTTCGCCAGC<br>GAGATGCGGCAAACGCTGAATGAAATAGTTTTGTATCGATTCTTTTATTCGCATCGAAACTCGAAAGTGTTAATCGAATAG<br>AACCGTTGTTTTGTATGGTATAAAAACTAGTGTGAATATTTAAACATAAGTGTACCATGTAAAAAGTATGCAAAATCGAGG<br>AAAACTTGAAGTATCAATGCGTTAGGTGGTTTAATTTGTATGATTTTTTGTTTTTTTTTTTTTTTTTTTTTTTTTTTTTTTTTG<br>TAGCATTTATCGCAGTTTTTTTTTTTCTTTTTAAATTATTCCAAATTTGAATTTCAAATATTCATTCTATGCATACAGTATATTT<br>ATACTAGTCGCATTGTACTCAAAGAATTGCAGATTCTTTTATATTTATTTTAATTTTTAATATCGCCCCGTACTTAAAAAAA<br>GTCGTAGTACTGGCTAATGACCTATTTAACATTTGATGTCCATTCCATAAAGGTTTATCGCATTTAGAGTATTAGAAAAAA<br>GGGCAGTGGGTAAATTTTGTAAATACATTTTTTCCTTTATAGTATACAATCTGGTATCCCGATTTGATAACAAATTATTTGT<br>CTCGTTGTCATCTCATTGGCTTGTTATTTTAAATTTTTTTTTTGGCTTTTCCTTTTTTTCCAAAAATAATTCTATTATGTGCAA<br>TAAATATATGTAATATGTGTAATAAATCAGATAACATATTTGGATTGTGTAATTCATGAAATGCATTTTATGTTTACATTGT<br>ATTTTTTATCGACAGTTTGTAAAGTTGACTTCGTCATAAAATGCTGTGAAAATTACTTATAAATGTTAATATTATATACCAC<br>CACAAGTGTGATATTATAATAAGTTATTATTTTAAAATATTAATATCTATATATTTGAAAGAATCAAATTCTTTGGTATCGT<br>GCATATTAATGGATCTATCATCACACCATTTATGGGATAAGCAGATAGAATAATTTTCTTTTCTTTTTTTATACAACTTAGA<br>ATGGCAAATAAGCTGACGGCCCACCTGGTGGAAAGTGGTAACCGTCGCCTATAGACATGAGCAGTATCATGGACATAACA<br>GTTTATACTGTTTCGCCTATAACACCCATGCTTTTTCATTCCTGAGGACTCAGGTGTTATTCCTCTGTACCCTGTAAATTCAC<br>TGCCATATCACACCCTTAAAATCGAAACACAGCCATGTAAACACAACTGCTTTACAGTTGAAACACGAATGGGACAGAGG<br>TGCCCAAGCAAACAACTTTTATATAAAGTCTCCCTCTGGAAAAGTGTCGTGGACAAACAACTTTTGCGTGTTTTCAAAGGT<br>GGGATATGACAGTAAAATTACAAGGGACGAAGTTGTAATTCCTTAAGCTGGATCCACACTATAGTCCCGCTCTCGGTCTGG<br>TATTATTATACAAGAAGCAATGTTTGTATTCATACTAAAATTCAGTCCTGTTCTGAACAATTTCGAACTGTTTCAACTGACC<br>ATACCAAGAGTGGCCCAAAACTGATATCATAGTGGGAATCCGGCCTTAGTTATCTGGAATAGTAACAGATGAATTATAGA<br>ATTTAACAGCATAGTATGTTTTACCCAGGCATAGGTAATATGGATACCGATCCGATATCTATATATTGGGATATTGATGGT<br>TGATAAAAAGTCATTACCTATTTCGATATCACCCATATCGG
MSTRG.13523	Cyclin-G1	AGGTACTAGGGCCTCCGCGCCCGGTCGCTCAAACCGTCGGGTCGACGAGCCGTGGGGGGATCGGCGCGGGCATACACAAC<br>GACACTTAGCCGCTGCTTGCTCCTGCGCACGCTTAGGTGCCGATTCACCCGCCGCTCGCATAGCCCGATCATGTTGAGAGC<br>GGGCGCGAGGCACCCGGATGCTGACGAGCTCGAGGCGTCCACATCGCGGCAGCACGACCAGGACGTGGACGTGCAAATG<br>TACGCGACCCTCAACGAGTACCTGCAGCTGGAGCAGAAGTTCCAGCCGAGACTGTGCCTGCCTGCCGGCTCTGAGGT
MSTRG.13527	Hypothetical protein KGM_208274	AGACAATTTTGTTTCAGCGGAAATGATAACAACGGTGCAGGGCAGACAGTTTTTAAAATATAAAAATTATTTTTATGGTAA<br>GACTCCTTACCTGAAGAGAAGGTGGCGGTGCGTGAACACAAGACGTTGTTACGCGTCTATGTCGGTTTCGGAAGACCTAA<br>CTGTTATCAAAGAGCCATCGCCTCACAATCATACTACCAAACAGTTTATTATATTGCCTGATGGTAGATACCAATTCATTA<br>CGCAAGTTACAAATTAATTTTATTTGTTATACAACAATATCCTAGCTTTAGTTTTGTAGTACCAACACAGAATGTGAAAGC<br>AATAACAATGCTGGAGGGCAGGCGGTTGCTGTTGTATAAAGGATATCTCTACAGTAAGACAGCAAATTTGAAAACACGCT<br>GGCGGTGCATAAATACGAATAATTGTTACGCGGGTATTTGTGTTAAAGAAGAGGACGAAACAATTCTTATCAAGGAGTCA<br>CCGACGCCCCACAACCATTCGCCCCGAAAGATTCGAAGAATGTCAAATGGCAGGTACACGTTCGCGAAAAGAAGCTCCAG<br>TCCTGTCTCGATTTATACAAGATATATTACACTAATGCATAGATGAATTGTGTTAGACTAATTTTTTTTTTTTAGTAATATGT<br>CTACGAGTACATAAACCGTAAGCTTAGATTATACAGATAGAGTGTGACAAATGAAAAACAAACGGACCCATGTTTAAAGT

		GGACACAGATGTCCTCTGATATGGATTTTGAAGGCAACCATATATTTTATACCAGAGGGAGACTTTGTACAAAAGCCGTTT<br>GCTTGGGTACCTCTGTTCTATTCGTGTTTCAACCGTGAATCAGTTGTGTTTGCATGGCTGTGTTTCAGTTTGAAGGGTGGAA<br>TATGGAATTTACAGGGTCCAGAGGAATAATACACCTGAGTTTTTAGGAATGGCAACGCATGTGGCGTATCGTGGGCGAAA<br>CAGTATACTCTGTTATGTCCATGATGAGGTGGGCCGTCAGCTTGTTTGCCATTCTAAGTTGTATAAAAAAAAAACATATAT<br>ATTACGTATTCACATTTTCTAAACCACA
MSTRG.13528	Endonuclease and reverse transcriptase-like protein	CAAGATTAAGGAAGTAAAGATGTTTTAAATTTGACTATGTCTTGCATTAGATTATAATTATATAGTTGTACATGGATTGGG<br>CCCAAGCTATTTACCATCTTCAATTGTTTCTTTTAAATTTTTTTTCTTTTTTAGTATACGAAATAATATTGTCTGGCGGCAAG<br>AAGTATTTAATGCTCAACGGCTATACATTCACGAAGGACCACAGGGTTATCCGTCACTTAGGGTACAGATGTACAAATACC<br>ACAATATGCTATGCAAAGATCCGCGTAGCAAACGATTTGACGCTGCTTAGTGACATCCCTATACATAACCATCGACCTCCC<br>AAATACAGTATCCGTAACGGCACTTATATCAAATTGTGATGGATGCAATTCTCCTAAGTTGGGTTTTTTTTTTAACTCAACG<br>CATTTGGACAAGTTAGTTATTTTTTTGCTTGTAGAAATAAATAAATGGTTTTCCAAATGTAGTTGTTCTTCGTGTTTTCCAA<br>ACTTTTTGTAATGTTAAACAAATTCTCGTTTGAAAGAATCGTAATTTTAGATTTGCTTTTTATTTATTCATTACATGTTTTTA<br>TCAATTCTGTGTCTAATTTAATAATTCTTTTTTACCATTCGTATTGTTTACTCCTTTCAGTTAGTCCCATATTAATTATGCCAT<br>TATTAAGTCGACGTTTTTTTTTTGTGAACATTTTTTTTATGTCACTTGGAGTACCAAATAAGTTGACAATCCACCTGATGGA<br>AAGTGAGAACTATTGATTAAGAGATGAACAATACTGAATACGCCCACGACCAAGGGCGTTTACTTTAGTACCCCATAATT<br>ATTACAAAAGCTAAGTAAATCATTAAATTCAGATTACATATGTACAACACATTTATAATCTGGATTTCTCTCTTTATGTTGT<br>ATATATAAAATGATGTGATCTTAAAACAACATTATGTTTTGCGCAGGCGATCCCCAAGAAATAACATTGATTAATGGGAA<br>AAAGTTTATTTGTACAACGGTTACACATACAGTTTCGGGTTCACGAACCGAATCGGCCAGCGGTGGCGGTGTAGCACAG<br>GCTGCAAGGCCCACATCGTCGTTACCAGCAACAAGGAACTGGTTACGGCCACCGGGGTCCACACACACGTCGCACCCAAT<br>TACCACTTGTTGTCAAATGGCAGATATATGAAAGTTAATAAGAATTGAAGTTCACGCCATGAGGCATGCGTGCGTGAACA<br>GAGTGATAACACGCCTTACCATCGACAGGGAGTTCACCCTCATACCCTATAGCCTAGATGGTCGCGAACAACGCGATTTCA<br>GAGATATTTTCTTCCACGAACACTCAGGTTATGGAATGAGGATTCCGAGGGTTTTCCAAGAGATTATAGCATGGGGTTCTT<br>CAAAAGAAGGGTAGAGAGGTTTCTTTAGGATCGACAACGCGCATGTAATACTCGTACCCCTGGTATTGCAGCCGTTCATA<br>GGCTACAGTAACCGCTTACCATCAGGTAAATCAGCCCCACCATCGACATCTTACAAAGATATGTCGTATAGATTCGTGACA<br>TCTGAAAGAGGAAAACAAATTTTGCTTTGCGGAGGTTATAGATACTACTTAAAAAGACACACGCAAACCGGACGCACGTG<br>GCGGTGCGTTAGAACTGCAAATTGTTCAGCGAAATTGATAATGGACTATCATTCTTACATAACAAAACAAGACCCGCATA<br>CTTGTGAGCCTGATTTCTTAAAAAATGAATTCAGCGATTTTGTCGAAGTATGCAAAAAGAAAATGTTTTCCGAACCCCATA<br>AATCGGTTCCGAAGATATACAAGGACACGGTCGCCCAATGGGCACAGCTGGGACTGGCGGTGCCTACATTCGCACAAATT<br>AAAAGCACCCTGTATAACAACCGTTGGAAACGAATTTGTGATCTTAGTAACACTTTGTTCCCATCTTAACTTTAATACTCAT<br>TGGTTTTGATTTTTATATTATTTTCTGCGAATTTTTTATTCGCGTACTTTATGTTTCGACGTATACTGTTATCATGCATTTTTT<br>TACATCATACATGTTTCATATAAAATCTACAAAAAAATTTCGTGCTACAATCCACAACTAAACTTAAAAGTGATATGATACA<br>AGCCATAGATAAAGCGCGCGCATCGCACACAACACATTATGTCGGGCATAGACAAAAACTTCAAGTTATAATTTTAAATT<br>AGGAATTAAAAAATCTATTAAAGTAAATCATTTTATAAATGCTGGACAAAGATTCAGCATTACACGTAAGCTGTACACGTT<br>TTAGTATAAATATACTAAGTATATTTTAGTATATTTATAATAAATGTACTTTATGCTAAAACGTGGATCGGCTGAAATAAT<br>ATTTCTTAGTATTTTTAGAATACTAATTAAATACACTGTATTACGAGTACTCTATTCTTGTTAGCTTCTTAATGTAAGTACTT<br>TAAGGTAATACATAATTGCTTCATGTTTTAAACAAATAAATA
MSTRG.13535	Hypothetical protein KGM_208251	AGATATTGCAAAATTATGCATCGATAAAGTCATCTCACATAATGCCCAGTATATAAAAGGCACAACATGCAAACCGCAAC<br>GTAAAAAGAACAAAGTACACTTCGGTTTATCCAGTCGTGGTCATTTGGTAGTGCATGTGGACGGGTTTAAATTCATCAAAC<br>ACGAGTCGTACAACAAGAAGATCAGGTGGACGTGCTGCAAACGTATACCATTCGGCTGCAAAGCTAGCATCACCACTGAA

		AATACAGTAATTGTAAGATCTTTCTTAGACCATAATCATTCATAGTATCAATTTTATATGACATTCAACATTTTTATAGCGA<br>TTAATTATGTAGAGTCACGAGCTATAATATGTAAACATTTGATAATTTGTTAAATAATATTTTTAATATTTTTTTTTGTAAA<br>AGTATAAAAAGTTGTACGTTAAAATATAAACCATCATTGCATTGCCTTTTTCCATTTATTTGGGATCGGCAAAATATATCTT<br>CATTTACCACAAATATCTATCAACTGGTATTTCCGGGCTTACTCTTTTATGCATATTACCTTTCACACTGTCTATCCACCTAG<br>CGATAAAATATAAATGTTTTTAAATAATAATAATACGTGACTACTATGATAATACTATGGTACTATCGTTATTTTAATTAGT<br>TCAATGAACATAACATGAAACAAGACAAGTAAGCTTCAAATATGATCACAATTGTTACTGTATTGTTAATAAATATTATAC<br>AGCCGTGAAAATAATTCAAAAATTTAAATAGCTTTCAAATAGTCGTATTTTAGAGAAGTTTTCTAAAATATCCTGGTATTT<br>GTGATACACTTACGTGTAATATTAAGTAAAATTGTTCATTTTAACCATTTTCTATTATTGTTTAAAGTCGTTAAAATATTTA<br>ATATCTATGCTAATGTCACTATGACAAAGTACTAAAATTTACGTAAGTATATAAACATACAGTCTTTTGCTCATGTAAACA<br>TTCTCTAAAAAAACATTTTGTTGGCTTTAAAACCTTAAAAAAAATGTCGCAGACAGTTCTAGCATTTAGATGTTTAAATAT<br>CTATTCATAAATATTTAATGTTAGTCAGTCATAACTATCTGATTTCATGATAAACGGAACAATTATGATTATATTAATAATA<br>TTTTTTAGTTCAGATATATTCCGATGTAATTTCCAACCTGAATTTTCTAATCATTCCTCGGATTTAGTCAATTAAGTAGTTAT<br>ACTAAGACAGTGCCTTTTAAAATAATAATGTGCCTAGTAAACAGAAATGATGCATAGCTTTAAAGTATTTAAAAGAATGA<br>TTACATAACTGTAAGTTTTTTATAATTATAATAATTTAATGTTTTTTTCATGGTTGAATATACAATGATAAATATATTTATTT<br>TTCTTT
MSTRG.13544	Modifier of mdg4	GTTCTCTTATTACGTTGTGTTTATGAAGTTAGTTATTATTATAATTAAAACTTTGTTCATTTTGACACTTTACAAAAACGGA<br>ACGCGCCATAAGTTTAGTTGTAAACACTTTGGTTATGTCAAATAAATTTGTTTACTATTTTCTTATTTTGTAATAATTTAGGT<br>GGGTAACTTAATTAATATGGTAATGGTTAAAGTATATAATGGACATAAAAGTGCTTTACTTGGTAAAACTTAATAGATAAC<br>ACCCATACAGCTTTGTTGTGACAACTCTATAACCTACAAATGTTGTGTGTAGTGTTCTTACTTGTTTACTAGTGGAATATGT<br>GTTATTTTCTCAACAAATATGTTTAGTGTAGTGATTTTATCCTATAATGTGAGATAGTGAAGTGTAATTTGGGTTCCTGGTT<br>GCAGATCACAGATTTGCCATACGAATTCATACCAACTCCGAAGGGGAATCATCTCATAATGCTAAATAGTTACACTTACTC<br>CAAAGATAACAAAAGTCGCAATTACTACTGTTCGAAGAAATCAATTGGTTGTAAGGCGCGGATCAAGTTGTTGGACAACG<br>GAAAACTTATAGTTGGAGACAGTTACCACTGTCACGAGCCACCGAAATATGTAGTGACCTCTTCAGGAAAATATGTAAAA<br>GTGAAGTAAAAATGCATATTTGTTATTTTGTAATCCCTTGTTTTTGGAAAATGTATTTTATTTTTCTGAAATAAAAAAGAAA<br>ATGTATCCATAAACGTTGCCACGTTCTAATAAAATGCTTTTTTATAAAGTTGTTTTTCATTTTAATGTTCTATGGTTTATCAT<br>TAAATATTAAATTAAACATTATATAGTAAAGTACAAATAACTCATTATATTTAATGAAATTATGTAATGAAGTGATGCCGT<br>AAGAATACAATATGTGAAATATTGTCTTTAATCTCAAGGATGAAGGTGACTAAGGGGATAAAGTGTAATCCACCAGTGGA<br>AGAGAATGGAACTTATTAATCCGTTAGCATAAGTACAGTAAGCTAATATTATCCTCTTAGCTGTGGCCAGT
MSTRG.13545	Hypothetical protein RR46_03798	CAATTATACATATTTAAATGTATAATAAACTGGGCATAAAATTACAAAACGTTTATTGTAAAACAATAACTTCCAAGCTTT<br>TCTAAGGTAAGTGTTAGTCGTTATCAAAAGTACTATACATGAACGTTTACAGATTTCCAAAACCCAGTAAACACTATTGCA<br>TAGATTGGATGAATAATGTAGGAGGTCAAAGCAAGTTGAATACATTCGCCACGCAATGTTTATAGGATTTTGAAATTTTTA<br>GTGTTCATTACATTTTAATGCTGTTTCACTCATGCAGAGTGTAAATTCATTCCAACCGTACAAAAAATGAACTTACTGATGT<br>TAAATGGGTACACATAGTCGTAAATGGATTTTTTTTTGTGACACGGGGGCAAACGAGCATGCGGCTCACCTGATGGTAAGT<br>GACTACCGCCGCCCATGGTCACTCACAACACCAACGGCATTGCAGGTGTGCTGCCGGCCTTTTATATATCAAACAAAAAA<br>AAAGTAGTAACTGTTGGGTTCGTGTCAAGTTGAATGACAAGCATAGTATTATAAGCGCAAATTAAAGTCATATCCACCGTT<br>GAATAATATACTGTTATCACGGGGACAGTGCTAGGAATATTATTAATTTGAAAATGAACTTAAACTTGACAATTTGTAGTG<br>TCAATCGAGACACTCCACTTGGTGGAGAGTGAATATGGTCTCACTGTCCGTCAAGTGTTCGTGTCTCATGGACACCATAGA<br>ATATACAGTGTCGTTCAAGGTACCCTTAACGTGTTTATTGAGATATAACACCGGTGGGTTATCAATTCATTCCTAATCCAA<br>AGGGACAGCCCTTGATAATGTTGAATGGGTATACCTACAGCAAACACAGCAAGAGTTTAAATTATTACTGCTCAAGAAAA

MSTRG.13546	Uncharacterized protein LOC106130143	AATATAGGATGCAAAGCTCGACTCAATCTAGCGTCAGATGGAAGGATAAAGAAGTGGTTCTGTTTCCATGTTCACGATCCT<br>CCTAAGTTTGTGCTCACGTCGTCTGGACAATTTTTAAAAATATAATTTTTAGGTTTACGTGAAAAGTACAGATAGGGTTGC<br>CAAAAAGTTAGATATTTGCTACCTCTTGCATCGAACAAACACATTGTCATTCTCATATTGTTATGTACCTAAAAATCAAATT<br>GTTAGGAACTTAACATTCGTGGAATACTCTTTCTAATTACTACTTTATTTGTCATGTAAGAGTAGATTTACTGTGTAGAGAT<br>TAATAAAATGTATGTAGACATAAGGATTTATCTCTGAGAAAGCAAGTCTAATCAACTACACGTTAAGTGCTTGGTGTAATC<br>TACTTATTGGTAATTCCCATATGTGTGTTACATTGTGTGTTTGCTTTAACTAGAAGTTTTAAAAGTTAATCTCTTGGCGAATT<br>GTGTGTACTCGCACTTTTAAGACAGATTTACACTGTAATGCCGGTCAGGCTTCGATCTCGATCTAGTAAAATCTTACATAA<br>CTTTGTGAGCTTCCATACTATTAGATAGTTCAGTTCCATTCCAGTTCAATTTCGATACTTACTCGAGATTGGACCGAGAACG<br>AACCGTGATCGGCATTATAGTGTCATACTAGTCGTACAGTTTGGATACGATTGCACTTTGGGCTTAGCGGCAAGCGGCATC<br>ATTATGATGTCAATGAGCTCTCTCATCGGACATTGCTATCTCATTTTATCTATGTTTAGAGAGTCTATTTTTGAAGCGACTT<br>CCAAAAGAGGTTCGTGTAAATTGTATGCGTCGCCAGTACCTTTTTTTAATTGGCTGTATACACTGCCGCTAGTCTAGATTTT<br>ATGTCTATGTTGCGCAAGTATGTCATTAATACTAAACTGATTTTCACAATTCTTTTTTTAAATCAATAAGGTTTACTCCTAT<br>GTTAGTCCCATGTTAATTTTGTAACGATTAAAGCTGTTTTTATTTGTGGGTAAAAGGTTTGTTTGTTGCGAAGTAAAATTTA<br>ACTGTGGACATGTAATTTGTTGTTCTAAATTCAAATTAACGAACGTGTCTACGACGGTACATCTCGTACTTCATTGGTGTCT<br>GTATGTATTCAGGATTGAAGTCGCTTATTTGCGCCCCTTTTTTTTAATGGGCGAATACGGAATGGTATACCAAAGAGTGAT<br>GATAGGTGAGGACGAAATTAGCCCAGTTGGTTCATCGTGATGAATTTAACAAGAATTAAGAAATAACAAGTGGGAACCAC<br>GTGAAGGGTGACATGATATGGTATATTGCTAGCAATGGCGTTTTGAGACCCCCATCACTAACAAATACTCTCTCCTTAACT<br>CACGACGCCTTGGATACATAAATAAACGGTAGTAGGTACATTCCTTCCTGTAAATAGTATCTGGGGTTCGACTCTGAGTTA<br>GACCAATTTATATTTCGGTCTTTGATATGGTCAGACGAAATGTGTAATTATGGGACATTTATCATTTTTTTCGCGCGTCTTG<br>CCGCCAAGCTCTTGCTTATAGTAGCGTAGTAGTATCCAGGCTGTTAATTACCTGAATTTGTTTAAGATGTTCTTGGTCAAAT<br>ATCATGTATTTACATTATAATATACAAATCGTCTTAGGTGCAGATGTAATACTGTCTGCCCATAGAGTAG<br>CAAATTTACTTACTCCAAAGACAGTAAGAGTCGGAACTATTACTGTTCGAAGAAGTTAATGGGATGCAGGGCGCGGATCA<br>AATTATTGGAAGACGGAAAAATCGTAGTGTATGACGATAAACATTCCCATGACCCGCCGAATTATGTAGTAACCTCTTCAG<br>GAAAATATGTAAAAGTAAAGTAACTGGTTATCAGTTTCTACCGACTCGCAAAGGTCACCTGTTACTGTATATGGGTTATAC<br>ATATTCAAAAGACAACAAAAGCTCAAATTATTACTGTTCCAAACGATGGCGTGGATGCAAGGCCTGCGTGAAGATCATTG<br>AGGGTGTGATTGTAAAGGCATACTGTAATCACTGCCACGAGCCACCTCGTCGGTTTTTTAATTAGCTAAAGAATTAGCTGT<br>ATATGTCAGTGATCTTTAAAGTGTGCTTTGCTGGGCTCTGTAAGGTTTTTGCGAAGGATCTGCTGTAATGAGACCCAGAGA<br>GCAAAGCATATCATATTTTGTCTGGTGGAAAAAAATTGTTAATAATGAGGTTTAATAGCCCCATTGCTAGTTATGTATTTTA<br>TTTTATTTTAATGAGTAATAATGGAATGGTAAACCCCACCTGCGATATCGGTAAACGCTGGCAGGTCACCAGTGAAAGATT<br>ATAAGTGACGATGAAAGCAAGTCAGTTAGCCCATCGTGACAAATTCCACTAGAATTAACTACGATAGTTTTCAGGGAGAA<br>CCGCGTGGAGGTCGACTTAATAGGGTATATTGCTAGCAATGGGTCAGTCATATCCCCTTACTAACAATCCCTTTCCTCACA<br>ATATTCTCTATTACGTCAACATCCACGCAGTTCCCTGTGAAAATCATCGTGGTCATTTCTTGTTGAATTCGTCTCGATGGGT<br>CAACTGACCTGCTTTCATCCTCAATTGTCATCTCTCACGGGTGGTCAGTGCAATGTTGAGAAGTGGAAGCATATCTCATGG<br>AATCGCGGAATATCCGCGTGAATCATAATATAAAATATGCTCTGTTTCAAATTAGGATATAAGCTATTCATGTACCAAATT<br>TCATTTAAATCCGTCAAGTAGTTTTTGCGTGAAGAGTAGGAAAAACATCCAATCATTTATCTACACTTTTTCTGTAAAAATA<br>GTAGTAGGTTTTTGATAAAGCTAGATTTTTCTTATTTTACATACATTCATACATACATATCTGTCACGCCTGTTTCCCATTAG<br>GGTAGGCAGAAACAATGGAACGCCAAATGCTTCGATTCAAACAAACCTCTTTCGCTTCTTTCACATTCATCAATCTTTTCAT<br>ACACGCTCGCCGGTTACGGGTACTTTCAATTTGGCCCTTCTTCAGCACGTCGCCTATTTGGTCAACATACGTCCGTCTAGGG<br>CGACCCCTACTGACATCTCCATTCATTCTTGCTCGATAAATCTCTTTCGTAATTCTTCTTTCATTCATTCTCTCCAAATAACC

		AAACCAACGCAACATTCCTTTTCGATTTTGTCACTACAACGTCTTTCAGTCCACACTTCTCTCTAATCACATTGTTTCTTA<br>CACTATCTTTTAATGTAATACCATA
MSTRG.13575	Uncharacterized protein LOC106130136	TTTCATCCAAAAAAAAAACTAAATAGATATGTAAATATATGCTTTTATTTTATATTGCCGCATGGTCCCATGGCATAGAA<br>TGGGCCTTTTCCCGTGGGTGTTGCAAGAGGTGATTAAAGGAACTAAATGATTGACAGCAGCGTTCATCTTAAAATGTCAC<br>AAAAGCCTATCTGCAGTTGCCAACTCCTCTGCCAAGCTTAGCATCTACTGGCCAAACCCCCAATAGAGAGATTATGTTCGT<br>CAGTAAACATATAACGGTTAATATGATAATACCTTTTATTGTTTCTTCCAAAATAATATTTTATAAAAAAAAGTACAAGAT<br>AAAAATCAAATAGACAGAAAACCTCCCACACAATTGGCATATATATTTAACGTTAGAATCGTTTCGGTATTGCTCCTGTGA<br>GAATGAAAAAGACGTTGGACGTTGTTATCTCATTCTATCTCATGAATTCGCGATAACGATTCTGTCGATAGATTTCTTTTGC<br>CACTTGTCTACAAAGTTTTTGCTTCACATATTAATAATATACCTATAGATAATACGACATCGCGACTGTCTCAGTCTCAGCT<br>CTTACATCTGTAGTTTACGCCAATTTGTTTTTACGTTCGCAGCTAAATACAAATTAATCAAACTAAAGGACAAAACAGTGA<br>TATTATATAATGAGCACACTTTTTGGAAATCGACGGCTCGCTACCGCTGGTACTGCTCTAAACGGTCTGTACTCAAATGTG<br>GTGCCAATTTGAAGGTGAACGATGACGGAGAAATTGTCGACGCGTACAATGAACATAATCATCCGAAACCAACGCTTGTC<br>AAGTTGTCTGACGATATTTACTACAGAGTTTAAAAGATTTTTATTGTTACTTAGAACGTCAAGCACTGTCCGTCCATTTGGA<br>AAATGGTAATCGTTGCCTATAGATACGATTTTAATACGTACTATATGCATTGTATTAATATCATGTGCTGAATGTAAATTCA<br>ATGTTTATATTTTGTAGTGCTCAAAGGATTTATCAGCAGTTCTGTGAATTATGAGCTATAAATTTGCAATTTGTATATTTTT<br>GTAACAATAAGGGTGTTCCAAATGTTAATGTTATGTTTCCTTAAATATTTGTACTATATATTCGATTTTATATGAATGAATT<br>AATGAAAACTCTTTGTAAAAGCACGTACATTGGTAAATCTTAAGTTTTACCTCTTTTTTTTCTTACTTACTTAGATCTTAATT<br>TTTGTATCTTTGGATTTACTAATGCGAGTTGGTAAATGTGAGGTTTTTCTAAAAATAAGGTAATTTTTGTTTCTTTCACCAA<br>ACCAAACCTACCTTTCTGATCTTTTTTTTTCTTTACAGTGCTAAACATAATTTTAGACATGTTCAGTGTGTGATATTCGTAAC<br>TCACATTGGTGACTCCTAACATTTACTGGTAAAACTTAAGACTCACCAATGTACGGGCTTTTACACTAACATATATGTTATA<br>AAGATTGACAATATGTACACAAACCAAAAATAATTTTTAATTACATAAAATTAATTACATAAAAGCAAGACAAAGAGGCA<br>GCATTTGGTGGCCTTATCGCTGGATAGCGATCTCTTCCAGATGAAAATGTAATAGCACACCTGCCTATGCTAACAGTAAAC<br>AGTGATAAGGCTCCAGTGAAAAATGAAACGAAAAAGATGCATTCAGATAAATTCAGATTCATCAAACTAAATTACAAAGA<br>TGCCATCTTGTATAAAGATCAAGTTTTCACGAAAGCTTCGTCGCGTTATATCTGGTACTGCTCGGGCCGATCTCAGTATGG<br>ATGTAACGCCAGGCTGGTGATGAATGACGAAGAAAAAATAGTTGAGGTCTATGCAAAACATAAGCACCCGAAACCTATAC<br>TTAAGAAGTTGCCTGACGGAGCTTATTACAGGGTACGAACAAGATAATATGACAGATAAGAGAAGCAGTCTACGATCTTG<br>TAAATGGTCATCCCTTAAAAAACCTCTTTAGTTTGTAAAAATTTATTATAAACCTTGAATTACTGTAA
MSTRG.13576	Modifier of mdg4	AGATAAATTCCAAATCATCAAAATAAATAACAGAAATGCAATTTTGTACAAGAGACAAGTTTTCGCGAAGGGTTCCTCGC<br>GCTATATCTGGTACTGCTCCGGGCGGAATAGATACAGATGTAACGCCAGACTGGTGATGAACGATTGTGATAAAATCGTT<br>GAGGTGGACGCAACACATAATCACGTTAAACCTACACTTAAGAAGATGCCTAACGGGGTTTATTACATGGTACGTGCTGC<br>AAGGACTAGATAATGTGGACAATTTATTAAACAAAGCAAATTACTATCTCGCAAATCATTCAATCCAAAGTAATTTGTTCT<br>GTTGTTAAAAATAGTGTATAAAAATATTTCTACATAGTGTTTTTCTAAGAGCCCAGATGCACTGAAATTTATAAAAGTAAA<br>CGAGAAAACTGTGATTTTGTATAAAGAACACACTTACTCGAAGGCGGCATCTCGTTACGGTTGGTATTGTTCCAAACGTAC<br>CGTACTCAAGTGTTCAGCCAAACTGAAGGTGGACGATGGTGGGATGATTCTTGACGAATACGCAGAACACAATCACCCAA<br>AACCAACACTTATGAAGATGCCAAGTGGTGATTATATTAGACTTTAAAACAATTTAGAAGCCCGTTAGTACGTAGCCTGTG<br>TTACTCTTCATATGCTATTATAAACAATAAAGAGTCTTATTAATAAATTTAAATATAAGAATGGCGCAATATGTAGATAAA<br>GGAATCACAGAAAAGCTGATTTGCCAAACCTATATCCACTATAAAGCAGATATAATTACCAATTATTATTATAGCATAATT<br>AATAACCCATTTCGACAAAAATTAAAATAAGAAACATATGTGGGTTTATGTATGAAAAATAATAAAAAAAAATGAAATAT<br>AGATAGTTAAATAGATAAACTTGGTAGAATAACAAAAATAAAATTACAAGAAAAGGTCAAATATAAGAAAATAATGGGC

		GGCCTTATCGCTAAAAAAGATTTCTACCAGGCAATCTTTGGATGTAGAAAAGTAAACTTCTTATAGTTTAGTGAAAGGCTT<br>GATAAAATTCTATCCGTTTTTGAATACATTCTATATTTTTCTTCAAACATTCTATACTTTTCTTTCAGCTTCTCAAATGTTGA<br>ATGTAAGAAATCCCTCTGTAGGATAAGTCCGCC
MSTRG.1<br>3580	Uncharacterized<br>protein<br>LOC106130136	AGATTATGCAGATTCGTATCAATTTATAAAGCTGGGCGAAAGGAATTTAATTTTATGTAATGAATACACATTTTCAATGCA<br>AGGGGGCTCGAAATATAATTGGTATTGTTCTAAGAAGACTCGTCTACGGTGTCCAGCAAAACTGAAACTTGATAAAGATG<br>GGAAAATTATTGAACTTTACTCAGCACATAATCATCCGCAGCCTAATCTTATTCAATTGTCAAATGGAGCCTATTATAAAG<br>CATAAACATATAGTTCGTTTCATTAGAATTAAATAAAAAGCTAAACAAAATCAAGGTTTGTTAAAATTACCAGAGAAACG<br>ATCTCTAATTGAAATTTGTAACAAATTACAATTAATCAATTGTTGATTGGACATTTGTTATCATTATATCAGCTGTTATCTC<br>TGCCCGCTGCTAAACGTAGGCCTCCCCCATAAATTGGGGGGTTTGCCAATAGTAGCCACACTTGGCAGGCGGTTGGTAACC<br>GCAGTTAGATCCTGGTTATGTTTTGAGCGAGACGTTTCTGCCTATCTCTCGACACTTAAGTTCCCTTAGTCGCCTCACACAC<br>ATACTTACAATATAAGAACTAAAAACAAATTTAATGTTTATATATATAAAGACTCTGTACTAAAGCATATTATTAAATAAA<br>TGACTATTTGACTATTTAAATGACAAAATCCATGGATTTGAATATAGTATTAAAGTGTATTGAATTGATTGAAATTGTAATT<br>ATTGTAATTGTTTAAATCAATATTATTTTAATGTGGTAATTATTATAATTATAAATGACGTATCATAAGAGTCTGTCACAGA<br>TGTATATGATTAAAGATGTTTTAATTGATTGATTGATCATACCGACATCCAGGGGAAAGGAAGAAGCGGCCTATTCTATGC<br>CGGGACCACACGGCGAATTCATTCATTCTTATTTATGGCATTTCATTCATTTATTTATTCATTCGTTTCATTTTCTTTTATTTT<br>TATTAATGGCAACAAACAGTCATTTACAAAATATCAATACAATATACATTACAGTTTTACAACAATATACCTTATAGTGCC<br>AATAATAGTATTTTAAATATAGAGTTATTATTCATGAAGTCCCATGGAAACACGCAAGAAATTTTATAACAAATCCAAAGC<br>AACATATCCGTTACAATAAGTGGTGACTTTATTCCATTATCATCCACTAAGCTAGTGTTGCCGATTTACCTGACAGTATTCC<br>AG
MSTRG.1<br>3589	Hypothetical<br>protein<br>KGM_208282	GTATTGTTAAAACTGATGATTAGTAGCCCCATTGTTAGCAATATACCCTATCAGGTCGACCTAGTTGGGTTTATTGCGATTC<br>GGTTGATATTGTCCAAAAGTTGACGAGGAGAGAATGCTATATATCATTAAGTCTTTTGTACCAAATTATATATGCCATTTTT<br>GGTAATGTGGCCAATAAAGTATAAATATTGTTACAGATATAAAATTCAAATTCATCGACTCATTCCGCGGCGGCCTTCTTC<br>TTATGATCAACAATTATACTTTCTCCAAAACGAACAGGAACTCCAGGATCTGGCTGTGCTCCAACAAGACATCTTATAGGT<br>GCAAAGCTAAAGTTAAATTAGATCAAAGCGAAATCATAGATTGTGATTTACAACATGATCACCCGCCACCCAATTATCAT<br>GTGACTGAAACGGGACAGCATATACCTATTTGAGATTATCTTCGGCTCACTGACTACATTACATCGCGGACTGACTAATTC<br>GCCATAATTTTAGTGCATCATGACATATATAAGTTGGTATTTTTTTTTTTGGATAAATATTTTTCTCTTTAATTCACTTATTG<br>TACGTCAAAAAGTCGATAAATGTTCGAAATAATCAGAAAAAAAAAAATAATTTCATAAATAGTTTATTGACACAAAAAAAG<br>GTTTCCGTTATTTTTAAATTTATTCATGTTATGCGTTATACGTTTAAAAATAGAACATAGTAAGGCTTAAAGTGAAATTAAT<br>TTGGTTGCTCCTACTTGAATATCATTCCTATTATATATATTACATAAATCATTAATTACATACATACATATCTGTCACGCCT<br>GTCTCCCATTAGGGTAGGCAGAAACAATGGAACGCCAAATGCTTCGATTTAAACAAACCTCTTTCGCTTCCTCCACATTCA<br>TCAATCTTCTCATACACGCTCGCCGGTTACGGGTACTTTTAATTTGGCCCTTCTTCAGCACGTCGCCTATTTGGTCGACATA<br>CGTCCGTCTAGGGCGGCCCCTACCGACATCTCCATTCATTCTTGCTCGATAAATCTCTTTCGTAATTCTTCTTTCATCCATCC<br>TCTCCAAATGACCAAACCAGCGCAACATTCCTTTTTCGATTTTTGTCACTACATCGTCTTTCAGTCCACACTTCTCTCTAATC<br>ACACTGTCATTAATTAATTTAAAAAAAAATTGTATACATGAATAAATTGATGTTTCAATTACCATTATATTTTTCTACAAGT<br>ATCAGAGCATGCTGATAGTTCACTGGGAAGGAAACATAAGTATCTAAATAATGTTAAAAATTAGATATATATAATATATG<br>ATTTTTTTAAAAACGATAATTAAAATCATTGTGAATTATTTAAATTTAAAGACATTAGAAAAGCTTTTTTGTTATTTGTTTT<br>AATTCATTTTTTTTTAATCTAATAAATAGTTATTAATAATAGAAAGATCTTTTATTCCATAAGACATTGTTTAAAATAAAAG<br>CATTCTTAACTAAAAA
MSTRG.1	Hypothetical	GTCAGATTTAATATCTTCAAAATGATAAACAAAACAGCAATAATTGAAATTATTGTTAAAAATACTGCTTATTTTTTATTTT

3591	protein KGM_208284	TCGCTTAAATATTGATATGTTTTCGTGTAAAATAACACATATTAAAAGTGAAATTGTGATAGTTCTTTACATAACCTAAAA<br>ATACTGTTACGTTGTAGAAAGTTTGCGAAACAAGTGTTATTAGCTATTAACAAAATGGAAATACATTTTTAAACGTATTGT<br>GTAAAAAAGTATATGTGTCCAGATGGTATTTTATATATACGTAAAGTATTTGATGGCATAGTACCGCGCTATAGCTGGTCA<br>CGGCACGGGAACGCTATCATTCTACTAGGAGAATATAGATTCAAGAAACGTTCCAAACACATAAACCAAAAGCAAGAGA<br>CACACTGGGTGTGCAACAAATGCGATATAGGTTGCAAAGCTAAACTGACCACGTTAAACGACGCTATAGTAAAAATGTAC<br>AATGTCCACGACCATAAAGGATCAAGAAAATTAAAAACCTCTTGATGGTATTGTATTTTGTTTTCTAACTATTGTATATAG<br>AATGAAATCGAAATCGTTAGCTGTACGTTAAAATAAGTATTTACTAAAGCATGTTTGTTTGTTTGAAAGTGCTAATCTCTG<br>AAACTACCAGTCGGTTATAGACTATTCCATATTGCGATAACACTACGGGCGATGCCACAGGATATTTATACGCGCCATATT<br>TATCCACCTGTTTCTTTGGGCAAAATATTTATTTTCGCTGATCCAATACTCCCTTAAAAGAAGGAATGAATTTTTAGAAATG<br>TGGCTGCCATTGCTAGTAGTATACCTTATCAGATCGAGCATCGACCTCCACGCGGTTCTGTCTGGAAACTGTCATAATTAG<br>TTCTTGTTGAAATCGTCACAATAGACCAATTGACCTATTTTGATCCTCACCAATCATCTCTTACTGGTGCCCCGCCAATACA<br>AGTATACCGATAGCGGAGTATAGCGAACAGGCGCAATTGCTAACCCAATTTCATCCATCATAAAAAAAAAACACTCCCAT<br>GAAAGAACTCTTATGATTTCGATTTCAATCTATATATAAATTAGGTAAATTTATGTATGTTTGTTGGTAAGCTAAATTAATT<br>TAGCCTACTTTTTTTAAAACTTTTTTTTAAATTGGACACGAAATACTAAATGATGCTGGAATCGAGACTTTTCTGTTTACTTT<br>GTTTCTGTTATTTTGTAATTAAGGATAATTTGAAATAATGTTTCTTAATTGGCGAGTGGTTTTATTTTTTTTTTTACTGATAT<br>AGTAATAATTTATCATAAATTTATTTGTTCTCTTGTATTGTAAAACTTTGTAAAAAACACATACTAACAAGAACAATTACG<br>ATTGTGTGGGTTATAAATCATTTAGGAAAATATCGCGTATTATGTGTCACTTTAAATTCACACATAATACTTGTCAACTAAG<br>AAATTTTACTTCAAATTATCTTTAATTACAAAATTATCAACAAAAACCACAAGTCTCGATTACCTTAAGGTTTTTAGGAAA<br>AAAATTATGAAATTTGTCATTTAGTAAGTCGCATAATTTTAGCTACAATCAGAAACTACAAAATATCTTTTTACTTGTTAAT<br>AACACTTGCTATATGGAAGCAAGTTAAATATTTTTTTTTTTGTATACAAGGATCAAGAAAAATTACAAACAATGAAGTGCA<br>CATTGAGCTTATAACAATAAATTCAGCAT
MSTRG.13594	Uncharacterized protein LOC101735705 isoform X3	TGTGGTTGTCCGTCTTCACGCCGTGAAATCTATGGTTGCAATTGTGTCATTCAAAGATTGTTTACTATTTTGGGAAATAATA<br>AAATTTTGTTGTATATTTTGTTTAATGTAATAATTTAAAATGAATACTACGGTATTAGCTTATTAGTATTACAATAATGGAA<br>TATTAGTTATTGATATATTTTCAGTGTGTTACCATTTCAGTTTCGAACGTGTTTACGTCAAGTGTTTTATGTGATAATAAATA<br>AAGGATGTGTTATTAATGAGTGATTATAAATAAATACAGTGTGATTCAATGGTGGAATGTTGTGATGTGTGCAGTATTATC<br>TTTTGGAGATCTCTACATTGGGGAAAGTTACTGTACATGTAAACGAATGCAGGTTGTCAACGAGAGTACCAGTTGTAGGGT<br>CAATAAACAGCAACGTCAAGGGCGAACTCCATTTCATTCAGACGAAGAGTGGCCATCTACAATTGATATATGAACCGTAC<br>ATATTCGTGACAGACAGTGTGCGCAGTGGCAGAACGTTTTGGAGATGCATGGAGTACGGGAAACGGTGTCGGGCGAGAAT<br>TACGTCGAAGAATAACGTGTTGCGAATAACTAACCCGGTGCATAATCATATTGATAACCACCTGCAAAAGATCGCCCGGA<br>AATACGAACAGGGCGAGGTAGTCACTTGCCCCACTGTTTAGTACACTTTGATCTCTAAATGAGTGTTTTTTATTTTTATTTT<br>AGCTGTATATTTTGTGTGTGATCGTTTTAAACATACAATCATGTCTTTTTCCATAGCTAATAATAACATGCGTTCATATTTCC<br>TAGAATTTCTTTTTATTTTCATTACATTCATTTTTTACACAAGGAACTAAACGTAAACCCGTTTAGTCGAGAAAAGCTAGTT<br>TTTTTGTGAATTCTGTTATCTGAACATTTGTCATTTTTTATTTCCTTTGTAAAGTTATTGTAATGTATTTATACTTTTTACATG<br>TTGACTATAACACACAACAGAATATTAAAAAACATTCTTTTTCTACTAGGACTTGAATATTTAATGTGTCTGTTTATTTTTG<br>TTTGTTTTTATTTATGATGAGGAATTCAATTAACTATTTTTGAAGTGAAGGACTAGGACAATTATTTTACTAATAAGCAATG<br>TAATTGACGCTGTCGTGTGTCAGACTTTTTCATTTTAAATCTTGAAATTTTGTTAGTTGTATTATATCGTTTCATAATTCGAT<br>GTAGATATTTAGTTGAACCTACTAAAGATTCTTCTAATTTAATTGTCAAATTGTAAGGTAATCACAAAAAATTAAGTACTGT<br>AGTTACATGAAAGATTGTGATCAAATAGCTATAAGAACATAATTGTAATGCATAAATAAAATAATGAATTATAATGAATA<br>TTTTATTTTTTTAACTCTCTGACGTGTTATTTGCATGGATTGTAATATTTCCATTGAGTATGATGGAACAGGTGTTTCGAGA

GACGTGCGCTACACGGTTACGAGGCGCGGAGCACCTGCCCTTCTAGTGGAGGGATACGGGTTCGTTGCTCGGAAGCGGCG
CGGTGCTCGTGTTTACTGGTCCTGCCGCAGACGCACCCAGGGCTGCCCGGCTCGGGCGCTCACGTACGAGGGACGACTACT
AGCGAGGGTGGCGGTACACAATCATTCACCGCATACGCCATATATAAACGAACACACGCGAATAGAGAGTCTGATTGAGA
CTATTTCATCCAAATGACTTATTATATAAATAAATGTATAATAAAATTCGACTTTTTTATTTGTAAGCATGTGACTGTGGT
TTGGAAATCATGATTAGTACTTAACCAGCATGACTTGTCTGTCTGGGATGTGGTTATGTATAGTGACGGGGGTGGTGTTTT
ACAGTGGAGTTCGTGATATCGAATAGAGGCCGGCGACACATGAGACTCGGTGGGTTCTCCTTTTATGCTGAGAAGGTGTTC
CCCGAGAAGAATAAGGTGCGGTGGCGGTGCACGCGGCGCACGTGTCGGGCGTACGCGCACACTTTGCACGACCGTATCTT
CGCTCTGAGTAATGTGCACTCACACGAGCCCCCGGCGGTGTTACCGCCTGCAATTGCTACCGCTACCGCTGCCGCCGCCGC
CACCGCCACCGCCACCGCTACTGCTACCGCAGCCGCTCAGCGCGCCTTCGCGCAAGCCGCGCTCGCCGCTCAAGCGAAGG
CTATGCTCGATGACTAATCCACGCATGGCTCGAATAATTATCTAATACTTACGACCGATACCAATACCTTATCCGATTCTTA
AAATAAGAGTGATAATGTTATTCTCATAATAGTTATTCATATACTTAGTATATTAACACATTACATTACAAAAGTACTATTG
ATTGTAAAAAGAAAACAATGAAAAGACGAAGGCAAGAATCTGTAAAGGAGTACAATTGCTTTAGATTTATTCTATTAAAT
CAATTACCAGCATAGATAGACCAATCAAAAGTACTTTTTACTGGCTAAGATTTTGGCTTTAACGTCTACATATTTGGAACG
ATAAGATGCTATTTTAGCTTTAAATTGGATTTATGATTTATTGGCGTTGGTCGTGTGTTGCTAACTAAAAAGACTAACAGG
GTGCGAAAGAATATTCATGAACAGTTATGAGCGCATAGAGAGATGTGTGCTCTGAATCTGAAACTGGTTCATTTTTGTGAA
GTCTATTCCTAGTGTAGTCTATTTTGTTATACTTGATAAAATTAGTAAATAATCTTGGCTTGTCTTTTCTACGAATTTAGCCA
TATACGTGTCTGACACAGTTTATATATAATTAAGGGGAAAGCTATTGTTGTACAAATGATTCTTTTGTCTGCTTTATTATGC
TCCAATATTTAAAAAAATACAACAGAAAATAGTCAGAATTTTTTTTTATGTTTTATTATTTCGATAAAGATAGGCCAAGAT
AATTTACTAAATATGTCATGTATAAAAAAAGATACGAGTGCTGTACTTACTGACAAGAGAGGTTGTGTTGAAGCTGATCTA
GTCAATGTTTTTAAATAGAATTTATTTCTATGGTAACTTGCACAAATATGTAGTTGTAAATGAACTACATGTCTTCATGTTA
AAAATGTCATCTAATGTACCTAATACCTATTGTGCTTGTCAATACTAATGATCTCAGTAGGTTTAACCATTGAGTTTAACCT
AATCTCTACATACACGTGGTTTTAATAATAATTGATAATAATTCTGTTAGAGCGTAATTGTAATGTCACTAACAATTTTAGC
AATAAATTATTAGATAGTTATAATCCAGTATCTGCTTCTATTGGTACTGTTCTTCGTAGTAGAATGTGTGTTTAAGAGAGAG
CTTATGTCAATGGTAATAATAACCTGATATTAACTATGTCTAGTCATATAGTAATATAATACAAATAATATCTAATTTATAT
ATATTTTTCGCGTAATGTCAAGTGAAGTTGAACAAATAAATAACATTATTTTTGTGGCGTTGAAATCTAACAATTGTATGT
ATATATGTATTTACTGTTCTAAAACCAATGTGATTAGTTCCAATAATAATTAGTTTTAACAAAATAACGCTTTACATTGCAT
GCCGTCGTTGGTCAAATTGTCGTTGAATTTAAATAATGTGGAATTGTAACGTAACTTTTGTTTCAATATAATGTTTGTTTCT
GTAGAGAAGTTCGTGCGATTGTGCGTGGGTCCGGGCGGGCGGCCGCGTCTTTGGGTGCGCGGGCGCAGCTTCTACGCAGC
GCATACGATGCGGTCAGGCGTCGTTCGCTGGCGGTGTACGATGGGTGGCTGCGGGTGTAAAGCTTATACACAGAAGGGCA
CGCTACACAAGCTCGTCGGTGAACACAATCATACCACGCGTTGCGGTCGGCGGAACGCTGCCGCGAAGCAGGTCAAGTTA
GAAAATTCCACCAGCCTTGCGCCATCGCCGCCGACGCCGTTGCTTTTACCGCGGATGCCCCGTTTCGACTTCCATAACTTCG
ACCCAAATGCTTGGGTGGTACGTTATTAGTGAGAGTACAAATAAGGTTATATATATAAGTCATCGAGCCAGTGAATTTGAG
AAATTATAAAAGATATTAGTTTTATAAATTCAAAATGATGCCCATCTTTCTTTTGATCTGATCGATTCGATTCATCAGGCTG
TTAATATCTATTTAAAAAATATATTTTGGAATGAACTATTTTGATAAATTTTATCAATAATGGTAGCTGGAAAAGCTAGCT
ATAGCGTTCAACTTCGGAGGTGATTCAGATTGTTCCCAGTTAAACTCTTTTTTTTTTGTGAAGGAGTGGCTGAGGTAATCAA
AATTAGCTTCCCTTTGTCTTCAAATTGCTGTCCAGATTGGTAGATTTAGTTGTAATCGTGACATAACGTTCCTTCACTAACT
GGTCATTTCCAATGTTAAATGTCTATAAGTTATTACATTTTAACCCTTAAAAGCCTGATTTTTTAAATTAAATTAAAAAATA
TATAAGTGTATCCTTTACTGTGTTTACTAATGGGGAAAAATAGTAAAAAAAATCAAAACGATATTTTTATATATTTATTTTGA
AAAAAAAAAAAACATTTGGTATCATTTTTGATACTTTAGGTATTACGGTAATCAAAATATTTAAATTTATTTAACAATGAA

		ATAATGCAAATCCCGGTATCAAATTTGAAACTTCAGGTATTTAAGGGTTAATAAATCTCGGTATGTGTTACTATTGTTCAA<br>AATATTCGTAATATTATTATTAAGTTCAAAAAATTTAACCTGCCTATAATCTATGATTTATGAATTCGTGCCATGTAGTTAG<br>TTTTTATGCTAGTCTCAAATGTTGTAATATTTTATGTATGTGATTTTTTGTGATAATACACTCATAATCATGTAGATCAGTCA<br>ATAAGATCTCATTTTATTTAAGTGCCAAAATATCGTATTAATGTGAGTCGTGTAGTAGGCATAAAATCACAGTAGGTTTTA<br>GTAAATAAGATATGATGAACTCCTTATAGTTAAGAGATATAGTCAAAATATTTTTATGCATGTGACCAGTTTTATTTTGATT<br>TAGTATAGTACTGTGATTTTCCTATTATACTGAATACATTTTGTTATTATTTTATTCGACTTTGTTTTATAGCAGAGATAACA<br>AAATTGAATTTAGAAGATAATTGTCGAAACTATGTTTAGTATCCTTGGATATGATCTCTAACTATATTACGCAACGAAATA<br>TTAATTTTTACTCTTATTTATTGTAAAATATTAGAGTTTTATTCAAGTCTTAGATTAACTGAGTGCTCACTGTTGAATACTTG<br>TTGCCAAATATTTAGTTCAATGTATGTGCCAAATAGGTATTTAATTGCTCATTCGACCTATCTCATAGAGAAAAGTATTTGA<br>TGATCACTATATTTATTACATTACCAACCTGTACGTTATCTTCCTATTAGATATTTTGCATCTTGAAACGATGAGTCGTGTA<br>TGTGTAATTGTGGTCAATAAAAAAACAAATGAC
MSTRG.13602	Hypothetical protein RR46_03780	GGAGGGACGACCTGAAATGCAGAGTAAGTGGATGCAGAAGGCTCAGGACCGTTCTGTATGACAGTCCACAGTACAGCCTT<br>ATGTCCAGCCGTGGACGAATAATGACTGAAAGGATGACGATGATTATCCTATCTAATAGAGTAACTAGAGTAATTGGTTTT<br>TCCAAAATAATACCAGTTTCAATTAATTAAACTGCTATGTTTTGTTTCAAAGAAACTGAAATTAAGCTGATACCGACACGA<br>AGCGGNNAGAAGTTCCTCATCATGTTGGACGGGTACACATACTCACAGATTAACTATTCGATCCTGTGGCTATGTTCGTCG<br>AAGGGCCAGGGTTGTAACGCGCGTTTTCGACGCGGCAAAGATGGAAACATAATACGTGTGAATACCGTGCATAATCACCC<br>CCCACCTAAATATGTTTTTCAAAATGGCAATTATTACAAAATATAATATAAAGAAAAGAAATATTCTGAGCCACGGCAAG<br>ATGTATTTTTTATATACCCAATAATATCTTTAACTAGAATTTTAGGGAGAAACTTGCGTGTGTATGTATCTTTTCCTTTTCTT<br>TACGATTGTATGAGTGCACAGGAAGTAAAAGTAATTTGGTACCATTCATGCGTTGCCACCCCTGAGGATTAAGGACGTCAT<br>TGCTCCTTCAAATTGCTTACACAAAAAAAATGCGTATTCTGCTTCGGTA
MSTRG.13607	Uncharacterized protein OBRU01_12726	AGTTCCGGTATACACAAAATCGCGATATGGAAAACCGGTTATACAATTTGGAAAATACCGTTTTAACAAGTGGGTCGGCT<br>CGAAGGGTAACAGAGCTCGCTGGTATTGCGTGAAAGCCTGCAAAGGCTGCCACTCAAAGATCGTCACTTTGAATGATGAA<br>ATAATTTTATTCGACTCTGAACACAATCATTGAATGTAATTTCAGATTACAGTCAGAGGTAGTGTTCACGACAAGTTCGCG<br>CGGCAATCCTGTTATCGTTATGGATGGATACCGGTTTAATAAGTGGAGTGGTAGCAGAGGACCAAGAGTACGGTGGATCT<br>GCGTCAAAGACCATTACGGATGTCGGGCTAAACTTACCACGGTCGATCACCAAATTGTCAAGTGTTTAAATGTGCATAATC<br>ATTCTTGAAATATGTATATACATTAATTCTAGTCTTATATGGAATTAATTTTTATTTGTGATTACATTATAAGCAATTTACTA<br>ATTGTGAATTAATATTAAAATATGAAAATCATCTATATAACTAAAAGTAAATTGCCATATGTATGTATTTATGTGCGTTTTC<br>AAAACGACCGCATTAGTTTTGACAATTTTCTTATTGTATTTGTAATTGTCAAGACAAAGTTTATACAAAAGAAAAAGTTTT<br>GAAAATTGCACGGAAAAATGAAAAAATCTGAGACAAAAAAGAGAAAATTTGGTTTCGTTTTTATTGCTGACGTATTGGCA<br>GCCTTTATATATTTCTTTTTATGTTTTTTTTTCTCTATTTTGTTTTCGTTTTCTCTTTTTTGCACTTATTTTTATATTTTATTCTA<br>TATTTTCATTCGCAAGTCTTAGAAACAGTATTTTTTATTAGTACACCCGTGCAAAGCCGGGGCGGGCAGCTAGTATTACCTT<br>TCCGCTTCAAAATTTTTCATTCACACCGTCATTGTTAGAATATGGTTGTCATATTTAAATAATACATTTTGATAACACGTTTT<br>AAAAAAATAGAAAATGTCAAATGAAGTGTGTTCAATTATGTCGTATATTGACTGCTGTATAAGTAAGCTAATTACGATCTT<br>TTATAAACTAGATGGCGTGACCGTTTAACCAGTTGTGTATTGAAGAGTATTTTTCAGGGGAAGCCTGGCCCTTTGAAACCT<br>TTATTACAATATATATCCACCAGAATAGGTTTTGGGGTGGACATTAGCCAATTCTGGGGCGGGCACTCCCCGGTTCAAGCC<br>TAAATACGCCTATGGGTTTAATACACTGTAAAACCGTTTGATTGTAGTTAGTTCGATCTCCGTGTAGTACAAGTTGTGTTCA<br>TAACTATGCCTGTGTTGGTCTATTTTGATGTATTTACCAAAAAATATATTTAAATATACCTATCTGTTGTTTTGTACCTATTG<br>TACAAACTCGACTTAGTTTAGGACTAGATGGCGCTGTGCAGTATGCCCAATATTTGTTTATTCTACATGCATTTCAGAGCCA<br>ATATTCACTGTCTCTCGGTACGGGAATCCCGTCATTATATTAGGATGTTATCGATTCAACAAGAAGGCTGGCAAAGGACCA

		AAAGGACGATGGCTGTGTGTCAAGGAACCCACTCATAAATGTCGAGCCAGACTCATTACCATCGACAATACAATTGTCTA<br>CGAACATAATGAACATACTCATTAGTAACGATAGAAGTACCACGCGCAATAGTACAAACGTTTGTATAAAAATTTGCAGA<br>TTTTGTTATAAAGAAAACTGAAGGGAAAAGTGGCAATGACAATGTGCTTTTATAATATCTATGGTACTGTTCACGGCTATT<br>GATGACGGTTATATTAACATATATATATATATATATATATATATATATATATATATATATATATA
MSTRG.13617	Hypothetical protein KGM_208245	AGTTCCGGTATACACAAAATCGCGATATGGAAAACCGGTTATACAATTTGGAAAATACCGTTTTAACAAGTGGGTCGGCT<br>CGAAGGGTAACAGAGCTCGCTGGTATTGCGTGAAAGCCTGCAAAGGCTGCCACTCAAAGATCGTCACTTTGAATGATGAA<br>ATAATTTTATTCGACTCTGAACACAATCATTGAATGTAATTTCAGATTACAGTCAGAGGTAGTGTTCACGACAAGTTCGCG<br>CGGCAATCCTGTTATCGTTATGGATGGATACCGGTTTAATAAGTGGAGTGGTAGCAGAGGACCAAGAGTACGGTGGATCT<br>GCGTCAAAGACCATTACGGATGTCGGGCTAAACTTACCACGGTCGATCACCAAATTGTCAAGTGTTTAAATGTGCATAATC<br>ATTCTTGAAATATGTATATACATTAATTCTAGTCTTATATGGAATTAATTTTTATTTGTGATTACATTATAAGCAATTTACTA<br>ATTGTGAATTAATATTAAAATATGAAAATCATCTATATAACTAAAAGTAAATTGCCATATGTATGTATTTATGTGCGTTTTC<br>AAAACGACCGCATTAGTTTTGACAATTTTCTTATTGTATTTGTAATTGTCAAGACAAAGTTTATACAAAAGAAAAAGTTTT<br>GAAAATTGCACGGAAAAATGAAAAAATCTGAGACAAAAAAGAGAAAATTTGGTTTCGTTTTTATTGCTGACGTATTGGCA<br>GCCTTTATATATTTCTTTTTATGTTTTTTTTTCTCTATTTTGTTTTCGTTTTCTCTTTTTTGCACTTATTTTTATATTTTATTCTA<br>TATTTTCATTCGCAAGTCTTAGAAACAGTATTTTTTATTAGTACACCCGTGCAAAGCCGGGGCGGGCAGCTAGTATTACCTT<br>TCCGCTTCAAAATTTTTCATTCACACCGTCATTGTTAGAATATGGTTGTCATATTTAAATAATACATTTTGATAACACGTTTT<br>AAAAAAATAGAAAATGTCAAATGAAGTGTGTTCAATTATGTCGTATATTGACTGCTGTATAAGTAAGCTAATTACGATCTT<br>TTATAAACTAGATGGCGTGACCGTTTAACCAGTTGTGTATTGAAGAGTATTTTTCAGGGGAAGCCTGGCCCTTTGAAACCT<br>TTATTACAATATATATCCACCAGAATAGGTTTTGGGGTGGACATTAGCCAATTCTGGGGCGGGCACTCCCCGGTTCAAGCC<br>TAAATACGCCTATGGGTTTAATACACTGTAAAACCGTTTGATTGTAGTTAGTTCGATCTCCGTGTAGTACAAGTTGTGTTCA<br>TAACTATGCCTGTGTTGGTCTATTTTGATGTATTTACCAAAAAATATATTTAAATATACCTATCTGTTGTTTTGTACCTATTG<br>TACAAACTCGACTTAGTTTAGGACTAGATGGCGCTGTGCAGTATGCCCAATATTTGTTTATTCTACATGCATTTCAGAGCCA<br>ATATTCACTGTCTCTCGGTACGGGAATCCCGTCATTATATTAGGATGTTATCGATTCAACAAGAAGGCTGGCAAAGGACCA<br>AAAGGACGATGGCTGTGTGTCAAGGAACCCACTCATAAATGTCGAGCCAGACTCATTACCATCGACAATACAATTGTCTA<br>CGAACATAATGAACATACTCATTAGTAACGATAGAAGTACCACGCGCAATAGTACAAACGTTTGTATAAAAATTTGCAGA<br>TTTTGTTATAAAGAAAACTGAAGGGAAAAGTGGCAATGACAATGTGCTTTTATAATATCTATGGTACTGTTCACGGCTATT<br>GATGACGGTTATATTAACATATATATATATATATATATATATATATATATATATATATATATATA
MSTRG.13626	Uncharacterized protein OBRU01_13767	AGAACCTAAATACACGACGTCGCGGCAAGTCAAGCCGGTATACGAGATTGCATTATACACGGTATCCCGACGCGGACGGC<br>CGGTAATACAAATTGGTATTTACCGGTATAACCGGCACAGCAGATGCAAGGGCTCCAAGGTGCAGTGGCTGTGTTGCAAA<br>TGGGCGACGTCGGCGTGCAGGGCCAGTGTTACCACCATCGATGGTTACATTGTGAAGTCCCTTAACAAGCATAACCATTGA<br>TGTGAAAAATAGTGTAAATATTTTGGGGTGAAATTAAATCTTCAGTATAATTCCGAAATTTATTTTCCGAATCAATACGTC<br>ATCGCAACTTCCAAGAAAAATATATTCTTTATTTAAAGGCGGGGTGGTACATTTGCAATTCCTCCGGTGTCGCGAGTGCTT<br>ACAAGCGACAGTAGTCACTGGTCATCAGACCCTGTATACAAAACGTCCAGATTTGGGAAGCCAGTGATAGAAGTGGGAGA<br>ATATCGCTACTATCATTATTTGAAACGACAAAGCGGCATGAGCATTTGGAGATGTGCAAAATGGACCAGTAAATATTGTC<br>GCGCCACGCTATCCACTGCGAACAATATAGTTTTGAAAACTACGAATTTCCATAATCATTAAGTAGTATTTTTTTTTTATAA<br>ATAAAAAACCATTCCCAATAATCTATCTGTGCATATGTTTATCTTCTCTAGTCTGTAATTGCAGTTGTGAGTAACACAGTTC<br>GGATGCTTCAGACCACTACGTATAAAGTTTCACTGATGGTGTCACATTATTATTTTTAAAACTTATATTTCATCTATTAATG<br>ATGTTTGCATACATACACAATGGGATTTTTTTTAACTTACTGTGTTTATCGGGTTCATGGCCGTAGTTAGGATGGGGACAGG<br>GGATTGAGGGGCAATGTCTTACCTTAAAATGAAATACCCCAAGAAAAGTAGACAGATTGTTTTTCGAACTTATTTAATTAT

		GGCTTCTGTCTTACCAAAAAGTTCTTCCAAAAAGTCAAATTTCGAAGTTTAGAATCACAGTAAATTAATTCCTGTACCGAG<br>TAATGCCCTTTTTTACATAATATTTTATGAAGTTGATATCGGAATGTCTTGTTCTTCTCTATGCTTTTCAAACTATCAGGTGT<br>TTTATTATACATTTTAATACTGCGGGCTGTTTCGAAACATTTCTATTTGGGTTATTGGCAAATCTATTTTTGTTTTGTGTCGG<br>TCATTTATAACAAATTGGAATAAATCTATATGTATGTATGTTACAGTAAGTAAATAGTGAAAATTCTAACCCTCGTGAATA<br>ATTTTAGAGAAAAAGGACTGTTGTATTATACAATGTAATATAAATGATCACAATACTCGTTGCGTTCTGTATATACCAGTT<br>CGATTCTCGTCGAATGTAATCATATTATCTAAAATAGAATTTAAAAAAAGTACACTTTTAGTTCCTTAGTCATAAAAACAT<br>TAAACGTCTCATAAACCTGTATTTTAGCTATTGAAATCTTTTATCTTTTTCTTTTGCACTAAATTCTCAATTTGAAAGAATGT<br>GACAGAAATACTTCATTAGCCAAAATAGGTTTGTAGGAGGTATTAGTTTTTATGAAAAAGGGGATTATTGTATATATATCT<br>TAGTATTTATAATATTATTCATAATTGTACATTGTAATCTATAACGTCCAGGGTTGTAAAGGCACTATTCTGGTTTCATTGC<br>GCTCGTGGACCGCCGTTTGTTTTATGTAGAAAAACTTTAAAGGGAGATAACTATAGTCATTCC
MSTRG.1<br>3636	Hypothetical<br>protein<br>KGM_208244	CTAAATAGTAGTTTGTAGACGCCATCTATTTAATTATGAAATTAATGTTAATAATAAATCATATATACGTAATTTTTATATG<br>TGCTCATGGACTAATAGTGTAGGAATATTCTCATCGAGCTATTTATAGTGTCACAGGTGTAACTGTGTACTTTGTTATATCC<br>ATGACATTTAATGTCATAGATGACGTTCTCATTTACCATTCTAAGGTGCATTAAAAAATCTACATATATTTATCAAGGGCA<br>CCGTCTAATATTTATTTTATTTTACCAGGATTTCCAAAGAATGTCGGTGATGGACAACATCTGTGGTATGGAGGCTACCGA<br>TACTCAAAGAACCGAAGCAACGGTCCCAGGATATGGTGGAGCTGCTACAAAAGAAATTCCATGAGTTGTAAAGCCACGCT<br>GCTCACATTTAATGAACCACGCTTCGGTTTGTCAAATCGTGGAAATCCGGTTATACTAATTGGGGAGTACCGATTTATTAA<br>GGAAAGGGATATTGGCCCAAAGACTAGATGGAATTGTGTGAAGAAGCGGTCTCAGCGGTGCACGTCGTCTCTCATGACTT<br>GCGAAGGTGTTATTCTTAAGACCGTGGGCAAACATAATCATTAACTGCTGGTATGAGTGAGGTAGAAAAACATACGGCTA<br>GGCTGATGGTAAACAATTGCTGTAATGGATATAGATATAATACATATAACGAGTGTTGACATCAATTCTTTTTTTTATACC<br>ATTGAGGTGGCAAACAAGCATACGGCCCACCTGATGGTAAGTGGTTAAATTAATTCAATTAATTAATTCTATTTTTAAACA<br>CACATATTCGACCACAGATTATGAAGATTTATCGACAAACTCGATGTCAATACATTTATAAGACATCTGAACATTGTTGCT<br>TGCAATTTGTATTCTTTTATGACCAGACAAAAATTTTTGCCCAGGATTATTTTACTGTACTTAAAAGCGTGAAAAATATAAA<br>TCTACCCAGTTGAAGAATTTTTACCCCTAAATTTAATTTCCCTTTATGTTTACCTCGTCTTTACACACAATGTTCTTCGGCGT<br>GTATTTTATTTAGTTCGATTATTAAAAAAAAACCTTTTAAAAGTGGGCACAATATGAATTTTCCCCTTTGAGCAAAATGTAC<br>GGCCATTTTGCACAAATGTGTGCGCAGACTTACGACTATATTTGAAGAGAAGTGATCATTGGTCGTGGGTTTTTCAATCG<br>ATATTATAATTCGATAGTTGGTATTTGATATTTACATTTAGTCAGATTGTTTATTATATGAATAGGTGAAATAAACTTTTAG<br>GTCAAGAGAGAGAGAGATTTACTTTATAATATATTTATAAGACATATAGTGTTGAAGAAGGGAACAAAGATAGTTATTTTAA<br>ATTATATTTATATTTCAATAATTTTTATTTTCACAATTTACTTTCATTTTTTAACGATGTCTATATGTATATTTATAAAGAATT<br>TTATGAACTTACTTTTGTTTGTCGTTTCCGACAATTTTTTCTTAACAGGAC
MSTRG.1<br>3638	Hypothetical<br>protein<br>RR46_03785	AGAACATACAAAGCCATTCTTCTCGACGACTCGATACGGCAATCCGTTAATGATAATCGACCATTATCGATACAACAAAC<br>GCAGCGATAGCCGGGGCCCTCTCACTAATTGGGTTTGCACAAAAAAGGATCGCCAGTGCCGCGCTACTGCCGTCACTATG<br>GGAGAAACGTTAATAAGACTTAAGAACTTCCATAATCACTGACAGAATTAAAACCAGTATTCACTAAGTCTATATGCGGT<br>CAGCCAGTTATTGAACTAGGCGGTCACAGATATAATAAGCGCACTGACCGTCATGGCCTACGTATTACCTGGCGCTGTGTT<br>AAGAGACGGAGTTTGAGTTGTCCTGCTCAAATTATCACTTACTGCAACGAAGTAATTGAAATAAAAAACTGCCACAATCA<br>TTGAAATCCGTTTCCAAAGAATCAAAATCAGTATTCATCAAGTCTTTATGCGGTTATCCAGTTATTGAACTAGGCGGTTAC<br>AGATATGGTAAGCGCCCTGACCGTCACGATCAACGTAATACGTGGCGCTGTATCAAGATACGGAGCTTAAGAACTGTCAT<br>AATCATTGAAAATTGCTGCCCAATATTGGTTATATATAAATTGAATTAGAGGGATTAAGTATACAATACTTATTTAATTGA<br>TTTTGTTTAAGTTTACATGTACATAAAATAATTAACTTAATAATAAATTTTAAGATTGTTTTTTGTTTTCACAGTGTTTACTT<br>TTATAAATCCTATTTGCTTTATTTTTCTATATTTTTTTTATGACTAAAATATTCGAATATATTTTTAGAATTGCGTCCGATATT

MSTRG.13653	Modifier of mdg4	TACTAAGTCTATACTCGGTCAGCCGGTTATTGAACTAGGCGGTCATAGATACAACAAGCGCAGTGACCGTTTTGGTAGACG<br>AGTCACGTGGAGGTGTGTGAAACGGCGACGACTGTCATGCACAGCGCAGTTGGTCACTGTTGACAGTGAGGTGGTAGAAA<br>TTAAGAACTGTCATAATCATTGAAATGTAGTCGGAGTTTTGAATATTACATTGTACATATGTTGTTGAATTGGTTGTATTTA<br>TAGTGTATTTTGGCTAAATTATTATGATGTATAAATATTGGTAGTTTGGTAGTAATAGAAAGGACACTATTAATTCCTGCC<br>TTTTTTTTCAGTTGTTTTTGCAGTAAATAAAATGGGAAAACGAGTAGCCATCTACGAAGGGTATACGTATTACTTTTGTGGC<br>AACATACGTTCGAAATCGGTCATAGGAATGTGGCGGTGTAGGAAAGGGTATCCCTGTAAGGCACGCTTTAAAGCTACCGA<br>ATCTGGTGTAGTTGTGGGTATCACTACATTACAACACACGCACGAACCACCACAGATTGCCATTCACAATGGCGTATTAGT<br>AAGGCTTATTTAAATTTGTATTGTATTTGTAAAATAAAATAGTAATTAGTTCATATACATGTTACTGTATACTGTACTTGTG<br>GCTTTACTAATATAACTTGTAATTAATAGTCGACTGTTAATAATCCATATAAAAATGTTGCATGCCAAACTGGCTGAATGT<br>TAAGGTTACACAAAATGTATAATATTACCTGAACTTACCACATTCATCGCAATAAATTTCATTTCAATTTAATTTCATTGTA<br>AAAGTCCGCACATTGGTAAATCTTAAGATTTACCAATGTGAGTTGTTATTTGTGAGGATTTAAACCAAACCTAACATAACC<br>TGTCTTTCTGACCACGAATACAAATATCACACACTGAACGTGTCTAAAATTATGTCTAACACTGTAAAAGCACGGTAATTT<br>TTCCTTTCTATGCTTACATAAGAGACGTGGCAAATAAAAAAAAATCAACATAAAATTCAAATGCATATATTACTGTACAGA<br>AAGATAGGTTAGGTTGGGTTTTGTTTTGGTGAAAGATACAAAAATTATCTTACTTTTATAAAATCCTCACGTTTACCAATTC<br>ACATTGGTAAATTCTAAGATTTGCTGGTAAAACTTAAGATTTGCCAATGTGCTAGCTTTTACAGTAAGATATACACGCAAA<br>ATATTAAATAAATGAGAGGTAAAAATAGTAATAGAAACCAAAAGAGAGATATCTGTATAAGGAACGTTTTATAGCTACTG<br>ACGCTGGTAAAGTTGTGAGACTCGCTACATTACGACTACGTAATATACGGATTACTCTTCACAATGACGTACTGGTGAAGC<br>TTACTAAAATTCAATTCGTACAAAATCAATCCGGCAAGCAGTTGGCGATTGTCGGTGGTTACACGTTCTACTGCAACGCAA<br>GAGGCACCAAGACGAATATCTGGAGGTGCACCAGATGGGGCTCGTGTAAATGCAGGTTTATTATGACCCAGATCGGAGAG<br>CTTGTCACTGGCCACTTTGAGCATAACCATGCACCTCCTAGTTTTATGATTAGAGATGGGCAGTATTACAGAATTTAAAAA<br>ATACTGTATTTTAAATTTCGAACATGTTCGACTACAGTTTCATCTAAATGATGGTGTTACAAGATGGAGC
MSTRG.13654	Mod protein	TAATTTTTTTTTATAACACAGGAGGAAAACAAGCATGCGGCTCACCTGATTTTCAGTTCGTGCTCAACCAATCTGGGAAGC<br>AATTGGCTATTACTGGTGGTTACACATTCTATTGCAACGTGAAGAGGAGCACGACGAACACCTGGCGATGTACCAGATGG<br>GGTCTGTGCAAATCCAGGTTTATTATAACCAAGCAAGGTGTTCTGATTACTGGCAACTTCGAACACAACCATTTGCCACCC<br>AGATTTATTATAATAAGACGGGAATTATTATAAAATTTAAAACAAGTGCAGTACGTGCCAAACAGAGGAGGAAAAATTAT<br>ATGTATATTGAACGGGTACACATTCTACCGGGATGGTAGAAGAAAAGCTACGGATGTATGGCAGTGTACGAAGTCTGGAA<br>ATTGTAAAGCGAGGTTCATCACGACGAAAGACGAGTACATTTTACGAGCTAATATCGACCACACTCATCCACCGCCTAACT<br>ACAAGATACATAAGGGAGTGTTATATAAAATATAAAATACATTTATTATGATTAGCTATTTCACAATTAGTTATTAATTGC<br>GAAAAAAATCTATATTAAAATGGTGTTCTGACTGGCGGTGTCCAATTAGAGAAAAGCTACAGATGTATAGCAGTGTACGA<br>AGTCCGGAAATTGTAAAGCGAGGTTCATCACGACGAAAGACAAGTACATTTTACGAGCTAATGTCGATCACACTCATCCA<br>CCGCCTAACTACAAGATACATAAGTTGCATTCATACCGAATAATAGAGGAAAATTAATGGCAGTAATGAATGGATACACA<br>TTCTACCGGGATGGAAGAGCAAAAACTAGGGATGTGTGGCAGTGTACGAAGACCGGACGATGCAATGCAAGGTTCACCA<br>CAACGAAAGATAATATTTTGATAAGAACTCAACTTGACCACACGCATCCACCTCCTGTATATAAGATACATAAGGGGGTG<br>TTATATAAAATTTAAAACTCATTTATTTTACTTAGTTTATAC
MSTRG.13655	Uncharacterized protein FLJ37770	AGTGCACTGGTTACGAGGTCTGTCCGGAAAGTTCGTATAAAAGTGTTCTGGAATGCGAGAAAGAGGAGTGGGGAGGCCAG<br>GTAAGCGCAGGACCCCTTCCTTATGTCCCTAACAATGCTTCAGTTCTGGTCTGACCGCCGTGCGGTGCGAACTGGCTTGTC<br>ACGCCATCTGTGAAGTTACCTCTTAACGAAACCCCGTCGGCATGACTTTTTGAACCGAGTCATCACTGGAGATGAGTCTTG<br>GTTCTACGAATTTGATGTCGAATTAAAATCCCAAAGCAAAGAGTGGAAGACATCCGGGGAACCACGAACAAAAAAAATCA<br>AGAAAATCCCGCTCAAATGTCAAGACAATGTTGATTGTTTTTTTCGATGTTCGGGGGATTGTCCATCAAGAATTCGTCCCTC

MSTRG.13671	G-protein coupled receptor 158	CAGGCCAGACAGTTGATGGAAAATTCTACGTCGATGTGCTCAGACGCCTCAAAGCTCGTGTGGCACGAGTTCGTCCAGAA<br>TTGGCGCGAGAGGGGCGTTGGATTCTTCACCACGACAATGCCCCTGCCCACACCTCTCTTGTTGTCCCATCTGCTAA<br>CACCGCCGCCCGCCGCCAGCGCACGGCCGCTCAGCCAGCCGCCGCCCGCGTGCCAGTGCTCCACGCGGCACTTGCAC<br>CGGTACTGACCACAAAACTTAGAGCTGGATAAGCTTCGCAGTCAGTTGTACCAGCATCACAAAGAGGCGGCCCGCCGAGC<br>CATTCATGCATCGTGTCCTCGCAAGGCAAGGCGGTCCAGCGACGAAATATCGCCGCCGCCGCTCTCGCTGGTCCGGCGCCG<br>CGAGCCGCGGCCGCGCCGCCCCACCATCCCCAGTCGTAGTCGTCGCAGGAGAACCACGCGTTCGACCACACCACGGCACT<br>TGCGTTCCAGGCCGAAGCCAACGTCCTCCAGAACTGACGTAACGTGATCGAGTTGTTTTGTTGAAAAACGATGAGTCGGG<br>GCGGTTCCGCTATGAAAAGCACGCCGAGGTGAAGTTGGGGCGCGCCGAGTGCGACCGCGGCGGGGGGCGGCGACAGCGG<br>CGCGCGTGCCGTGGCGCGAGCCAGCGCGGCGGCTCGCGTCTCGTCTGCCGCGCCCTGCTCTACTAGTTCCGCCAGCGCGCG<br>CGCCGTGTGGACTGCCGCTGCTTCATGTTCGCGCAAATTCTAAAAGTAAAATACAAGTTTTAGTTGTAAGTAATTAAACGT<br>ATTTTTAGATAGACAAACATTAATAACTAATTGATTTAATTGTATTTTCACCCGACTTCAAAAAAAGATGTTCTCGATTCTA<br>TTGTTTTTTAAGTGTTACAAGGTTACACCTCGTAACACCGTCATTTATAAATAAATTTGAACAAATCTTTTTGCTTGAAAAA<br>AATTTTTTTAATCTTTCAGTTAGGTCCCATAGAAATTTTATCAAAATCGGTTGAGTAGTTTTGTTTTTATATCAAAATAACT<br>AGTTTAACCGCGATTGAAATCGGTTTTTTTGTGAAACACGATCTTAAATTATATTTAATATGCAAATGTATTCTATGGTAAA<br>TTATACCCAAATTTAAGTTAGTGAATTTGTAGCATTTGCCTGGTAGATAATTTACTGCGTAAAAAATATAATGTAAATAAC<br>AAACGTTAAGTCAAAATCACGTTCTAAAATAATTTAAAATATCAAAACTGTATTTGAATGTCTAATCTAATCCAATACGTA<br>TATTCATACTAAATCTATCCCCTATTTCATGGAATTCCCAAAATCTATTCCTATGGGCGTCAACATCACAGAATGAGTCTAC<br>CTATATTAAACATTTTAAGGCCCTAGAGAATTTGACCGTGCATTAGAGTCAGGTCAATTTCAATAGAATTCTTAAAATCAA<br>CGCTAATCCATTTTTATAGTCCCTGCGACAAATAAGTACTAAAACATAAAATTTAATTAAAATTCAATGCCATTACATGAC<br>ATTGATTGATTGAACTTCACGAACGTTAAATGGTATATACTCGCCACTTTACAAGTATCGTTGCCACCTACTTAAAATATGT<br>CAAAACAACAAAAGCTGATATTTTAACAGCTTTTTAATATATTAATTGATACTAAAATATATTATACCTACATACAATATG<br>CCAAGCTTGCGTTTTACAACCAATTTCCCATATACCTACTACAAAGAGTTTTTAAGCGAAATTAATTTCTACAATCACTTTC<br>AACAACAATTCTCACTGAAGAAATTTCACGTCCGCTTAACGGGGTTAAGCGTCGGGCGCCAC
MSTRG.13674	Venom protease-like isoform X2	ATTTCTTTTTACAGAGGAAACGTGTTCTACTATAGATGATCAGATCGGTAGTTGTGTAAGCCTGTTCCAATGTGAGAAGTA<br>TTTGAAGGTTGTTCAGGAGGCCGGCTCCAGCCCGGCGGCTGTGCAGCTGTTGAGGAAAGTGCACTGCGGATTTGACGGGA<br>ACAACCCTAAGGTCTGCTGTCCACGACCAGGCATCCCTACCGGGCCACCACCGTCGGTACCCCCACCACCGCCACCAACA<br>CAACC
MSTRG.13678	RNA exonuclease 4-like	CAAGTTAGAACGAGAAGGGATCGATTTTAACATTGTACAAAGAGATGTGAAGAGATTAATAGCTGGTTGTGTGGTAGTTG<br>GCCACTCTCTAGACTACGATTTTAAAGTTCTGAACTTGGATCACCCCGATGAAGACGTGATAGACACATCCAGAATAGATC<br>CGTTTAAGTCTCTAACAAATAAAGGGCACACGCCAGGTCTCAAATTTTTAGCTCAGAGGTTTTTAAATGTCAATATACAAC<br>AGGGTCAACATAGCTCCATCACCGATGCCAAAGTTGCCATGAAACTGAGTTTAATGGCTATCCCCAAGCGGCAATAGTTAT<br>CCCCGAAAATCGACAATGTCCCCAAAAGTTCGATAGCTGTCCCCGAAGTTCGATAGCTGTCCCCGAAGTTCGATAGCTGTC<br>CCCAAACGTCGGCTGTATCCAAGCGGTGACAGACTGAGGGCGGCGTCGCGTTTAGTGTCCGTGGGGCCCTAAGCTACATC<br>TGTGATGGGGCACCTAGTTTACGTTTAAAATAAATTATTATATTTCAAAATATTTACTTTAGAATTACAAATTGATTTTTTG<br>TTATTAAATTTTTAATAATTTGTGTGTAACATTGATAGAAAATTGAGTTCCCACTTTTCCATTTATTTAATTTTTTAATCAAC<br>TCTTTCCATTTGTGGCAAGAGTCCATTTATGAGGGGGCCGGGGCGTTCTTTGTGGTTGAAGAACTTAGGCTATGTGAAGAT<br>GGAAGATCTATGAAGATGGAATCAGACATGAGCCTACCATGAAAAATTTTCCAAAATTTCGGGGACGAACGAGACAGAA<br>ATTTTAAGTTAAAATTACGTAATGTTTAACAGTATGTTTAAAATTATTAAAAATGATAAAATATCGTTCCTCTGGACTATTA<br>GGATAGGATTTATGATGAACGAAATGTTAAGCTCCATTTACTGGCTCGATTTTGGTACGAACAAGAATACGAAATTGAA

		CGGAAAATTCGGAATTTCATTTCATGGTAGACCACCAGGTCAGTTGGCCCCTCGTGATACTGATGATTGGGGAACCGCGTGGAATTGTAAGGGTGTATTGCTAATAATGGGTC
MSTRG.13683	Integrator complex subunit 7	CAGCAAGCGTCAACCGAAGCGGCAAGTGAAAGGTGTTCAAATCACCGTCACCGCCACTCCGCATCCACGAACCAATGAAAAGACCGTGGAGCTAACGAACATCCCTCCAGTGTTGACCGCCGTACAGACCGTGACGCCGGTCCGCGATTTCTTCTCAGCACAGCAACTAGTGTCCGTGAACGCGCCCGGTCTCTACACGGTGGCAGTGGAGGCGGCATTCGTCGACGAAAACGGCGAATTATGGAACACTGGACCTAGGACATCTATTGTTATCAAGGTAGGC
MSTRG.13687	TBC domain-containing protein kinase-like protein	ACGGTCATCGCGCGCTCAAGCGGTTGCTCAAAGCCTGGCTACTCACAAATCCACAGTATGTCTACTGGCAAGGCCTGGATTCACTCACCGCACCATTTTTGTATTTGAATTTTTGTAATGAAGCTCGCGCTTTCGCATGCTTGTCAGCGTTTGTTCCAAAATTTCTGCATAAGTTCTTCTTGAAAGATAACAGTTTTGTGATAAAGGAGTACCTAGCAAAGTTTTGGCAGATGACCGCCTTCCACGAGCCCGAATTGGCTACCCATCTGCACGAGATAAACTTTGTGCCAGAACTGTTTGCAATACCATGGTTCCTTACTATGTTTTCAC
MSTRG.13702	Thioredoxin reductase 2	ATTTCCAGATGCAACATCACGATGGAGCAGCTAAAGAACACAGTGGGCATCCACCCGACGGTGGCGGAGGAGTTCACGCGGCTCAACATCACCAAGCGCTCCGGGAAGGACCCCAACCCCGCCTCTTGCTGCAGCTAAACACCACGCGCGGACACACGTGCGTTTCACGATGCCTTCACACTACTAAATAAAATTGGTCTTAACTCCCTTCACGATATGGTTTAATTTTAAATGTCAACAATGACTTTATCATTTGATCAGTGGATTTACTTTCCTGACTCTACTACGGACGAGAGGATATTATTATCCAGGCGGCCCGGTATAGTGTTTTTTTTTATTTCGAATTTTACTTCTCATTCTTTGCATTTTTCTCGTATTTGTTGACGTTTAAAATAAAATACTAATATCAATGTAGTTAAATTGTATACGTAATCTGAAGGAGTGATTGTATCGTGAAACGCTGTCCTACGCTCTGTTGGTATACGACACTCGTTTCATGCAATGTAGACGGATGTAAGGATACAGTAATGTATAGATTACCCTATTACGTAACTAACATGTCGTGAATTTACTGGGTACAGAGGAATAACACCTGAGTCCTCAGGAATGGCAACGCATGGGGGGTATCATGGGCGAAACAGTATACTCTGTTATGTGCATGGTACTGCTCATGTCTATAGGCGACGGTTACCACTTTCCATCAAGTGGGTCGTCAGAAAAAATAACAAGGAGTAAAATTATTAAAGAAAATGTTATAATATACTTATTCTTATTAAAAAATTTTCATTACCTTTTTTGTTGACAGTACTTATATATTAATTGAGCTAGCAAAAATTTCTAAGTCACAATGTCCATTATGAAATACCGCTAATAAACATTTGTATAAAGTGGTGAAGTATTCAGTACTAGTAGATTTATTTGATTCAATTTGTGCTTCCTAAATAAGGTACAAACGTTATCGCTAGCAAGTCCATTAGATACAATGAGATAGTGACGTGCGCTGTCTTTTTCTTTCTTATGTGAGCGCTGATCTAAGTGATTCTGTCAAGAGATTCGTTTTTACCTTCTCTAGGTGGCTTGGATAGCCTATGGACTATAGTTGATATTTGAAATTGACGTTGTAGTACTTATTACCAATTTTGTTGGGTCAAAACATCTGTGATTACGTTATACATATAGTAAATACTAGATACTTGTGGATTTTTCCATATAAATTAGAAAAAAAAATTATCAGTAATATTTCAAAATTGCCTCCAATTAATATAAAACAAAAATACTCAAAATTGCATGAAACTTGTGAACGTTGTCTGTAGTTATTGTTAATGGGAGTCCCGGAGAAGATGCTAGAAACAATGGTATTACTCAAGATTTTAAATGTGTTGTCTATTATAAAAAAAATATATGTCCAAGTCAGCAGGGACGTTAGAAAAGAGATATCTGTTATAACTGTATAATTACTATGCCAGAGGCAAATAATATAGTTGTTAAATTGCTAATAACTACTGCATTTCAATTAGTATTTCTTCCTAACATTATTGATCTAACAACCTCGCCCTGTTTAGTTAACTTTTTCATTGTGTAATGTTACATTCAAGATTTGAATTTAAAAGTAAAATATATATAGATTTGTTGTGAACAAATTGTTAATAAGCCGCGTACAGATATTCTGTGACATGTAATGTGTATGTGTATGACATGCAATGCAATTAGTACGTAAATTTAACGGACGGCGCGCACTATTAAAGAACTGAGATTACAAAGGAATGTTCCAGATGTCATCATGGCAAATTGTATACTAATTTTGATGTTAATTTTTTTATTAAAATAATTATATATATACAGTGAATGCTCTACATAAGCAACTCATTTTTACTACTTTCAATAAAAAAATTTTTTGGGAAAAATTGCTTTTGGGGAAAAAGGAGTATAAAATTATCTGAAAAATATTTTTACTTATTTCTTTTACTAAGCTGAGTAGAATAAATAAAATATGAATAATATTGCTTATAGAGAGTTTGCTTATATAGAGCGTTCACTGTGTGTGTATAAATATATATATATATATATATATATAGAAACTATTAATTGACAAAATCTTAGGCGGTATGTTGATGTCCAATAATACAAACAACAATACAAATAAATTTTTTTATTAATTGAATTAATGTTTCGATTTGTTACCAATTTCATTTAGAGACTGTTCTGTGTCTACTTTTCCATTAATTTCAATTGGAATAGACTGTAGTGTGCGCGTGCGCATGACAATAAATTATACGGACG

		GTTCAAAATATATGTACAAGGCTATATATTATATATTGTTAAATAAAATTAAATAACTAAGCGAGCAGTTTTAGTACAGTATTATTTTTATTATTTATTTCTTCAAGCTGTTTGTTACAATCATTCTCTTCAAACACAATTAGACAACATTTGGGGCACACTGAACGAACAATGGTTACGTTTATTTCATCATTTTACTTTGTATGTGATTTTAAACTTTAATAAAGTGATATATTTTTTATGATTGT
MSTRG.13705	Kinase D-interacting substrate of 220 kDa isoform X8	GCCTCATGAACGTGCTCTACATCACAGGTCGTCTTCTCAAAGCGTTCCAAATAGAATTCAATTGGTACCAATTAGCATCTTGGGTGAACCTGACCGAGCAGTGGCCTTTCCGTACGTCATGGATTATCTACCATCATGAAACCTACGAAGAGCATATCGACGATTCAACTTCACTCAAACATATATATGAAAAAGTAAAGCCTATGATAAGTGCGCTGCGGGAAGCAAGCACGTTGATGGAGTTAGACCGAGATGAGCGCAAACTAGAAGTATTCCTCAGCTTCCACCGCACTACGCTCACAGCTGCTGACCTCAAAATATTCCTGCCATTCACCATCAATTTGGATCCGTACATCAAGAAGGTTATCAAAGG
MSTRG.13707	Zinc finger protein 91-like	GAACAAGAAATTCCTATAAAGATAGAGCAAGAGTTAACCGAATCTAGTCAAGTTGCTGATCTGGACTTAAGTAGAGAGAATCCGATGCCAGTACCAGCCAAACCCAGGAGGCAGAAGGTTGTCAAAGAGGGATTTACATCTAGGATGGTCCAAGAAACAGACGAGTATGTAGTCATCAAGTTAACGAAAGAACAGGTGTTACAAGAGATGCAGGAGAGATCCAAAACGGTGGAGTACCAGAGGACGTTGTTCAAGTGCGAGAAATGTGTCAAGGGGTTCAATTTCGAGGATGTACTGAGGAGTCACATGGTGAAGCACTCACAGG
MSTRG.13711	Lysophospholipid acyltransferase 7-like	AATTACTGTTTTAAATAGATTTATGTTTTTAAACAGGTTTATATATATATAATTTATGATAAACCTTAAAAATACATTAACGTCTCACTTCTGGGTACTGGTCTCTCTCCATAAGAAGAGAATATAACTAACTTTTACTTTAATGAAAAGACAAAAAAAAAATACACACAGTTAAATTGTTTGTTGTTTTTAATTTTTTTTAAGAAAAATGTTTAGTTTCCATGGTAGAATACTTTTAAAAGAATGGTAAAGATTGGTAACGTGTTAATCCTATATAACATAGTCCCTGGTACCACTAAGAGCAAATATTCTTATAATTAGACATAATTTTAAACAATGTATTGGCCTTCTAAGATCAGCTGCAATCATAATTTAATTCTGATTATAAACAATATTTGTAGGACTGATGAAGAAGCTAAAATTGCTCAATATGATTTTAATACAGTTGAATCTATGGATGTGTGGGGATGCGAGACGGTAGTTACATTGCGTGATTCGATGAAGGTATGGAATAAAGCAGTGCAGTACTGGGTGGCAATGGTTGTGTACAAAAGGTTCCCTATAAAGCCTTTAAAGATTCATGCAGCGTTGTTCGTTTCCGTAATATGGCATGGAATTCACGCTGGATATTTCTTCTGCATTTACTTCTGTCCGTTTTATTTGATGGCAGAAGATATATATTACAAATTATATTACAAACCCG
MSTRG.13719	Uncharacterized protein LOC110379302	CTATAGTAATCCAACCATGGGAACCGCACGAACAAATCAAAATGGAACTCCTTAATCCAAAAGATCTTAAAATGCAAATTTTTTCAGATATTGATACGATTGTGAGGAATGAATGGCAAGTGCGTTGGGACCAACACGTTCTCCCCTCGTTAAAATGGAATAAATTGTATCCGAGCACAAATAATGAGACGAACCAGAGTGATAAATTTGTGATAACCCTCAAGCTGAAACGAACGGCTACCTCTCATATCATCGTGTTCTACACTCCGTTATTAGTACTAATAACGTTCGTGCTACTGTCATTCTGGAGCGAGCCGTTGAAAATGT
MSTRG.13731	Takeout/JHBP like protein	CACGAACCACGACCGCGTACTGACCTCCCGCCGAGGCGCTGTGAACTCTTTAGCCAATATTAACTTTTTAATTTCAAACCTGTGCGCAAGATGTTTTTGAATTGTATATTTTTAATTTTGACATACACGTGTTGTGTTGCGACCGCGGCGAATGATTTGCCGGATAACTTCCAGCGATGCCGACAAAAGGACACGAAATTGAACGACTGTCTCAAGTCGGCTGTTCCCGATGCCCTTAGGAAGATGAAG
MSTRG.13742	Cleavage and polyadenylation specificity factor subunit 5	GAAACACTATCCATAATGGCGATTGTTTGGCTTTAACGACGCTACTATAAGTGTAGTGAGTGGAGACAACTCCTATTTTAATTTAGAGCTATATTAAAATTGACTTATAAGATTTTAAAGTTAAATAATTGTTTGTAAATTGTACACAATGACTGCAGTTCAAGGCGGTCCCGGCAATAAACAGTGGCCAAGGCCCGGCATACAGCATCAAAATAGTCAAAGTAATCCGAGCTTGACTTTGAATAGATCAATTAATTTATATCCACTAACAAATTATACATTTGGTACAAAAGAGCCGCTGTTTGAAAAAGACGCGTCGGTGCCTGCAAGATTTCAACGGATGCGAGAGGAATTCGCGAAAATTGGCATGAGAAGGTCAGTGGAAGGTGTTCTCCTGGTGCATGAGCACGGTCTACCACATGTACTGCTGTTGCAGCTTGGAA
MSTRG.1	Plasma membrane	GTGTGGCTGTGCGACGACAGCCGCTCCACGTCGATGTCGTGCGGCGCGAGCGTGTCGCGCGCCGCGTCTGCGTAGTCGAT

3762	calcium-transporting ATPase 1-like isoform X2	ACTCGACGAGTGTGAGAAACGCTTCGACAGCGCCGTCTGCTTGCGCAGCGCCTCCGCGAGCGCCGCGTCCGCCAGCGAGCCGCGCGAGTCCAGCCCCTGCCGGAACGCGTTCACCACGCGGATCTGCCACCACCACCACACCGCGCGGCACCGAGTAGTGTGAAGATGATGCCGGCTTGTGAGTTCACACCTACCGCTGTCACAAGCATTTTGCCAGAGCCTGGAATGTAATGTTAAATAAGAAAAATACAAGGAAACAAGAAGAATTCCGGATATACATCGCACTCGCACATCAATCAATCAAATCAAGGCGGGCTGAAGG
MSTRG.1 3769	ATP-binding cassette sub-family D member 2 isoform X2	GGCAATCCTGCGACCGATCCATCAGTGGAAGAATTTCTATTTTGACTTTATTTTCAGTGTTTGTTTATATTTTATAAAAACAATCACAAGTGAAAATATATATTCTAAACAATTTGAATAGTCCATAGAAACAACGGTGAAAATTTTTCATAATCCTTTCAATAGGTTTTTGTTGAAATTAGCGTGTTACGGATAATAAGCAAAGATTTCCGGGTAACATTTGACAGTGCAGTCAGTACATCACAAAATTAAAATGGACGAACGACCAGAGACGACCATCGCAACTTAATAAAGAGGAAATTTAGTAGAAATTTTATTAAAAAAAGAAACAAATAATATATCCAAACTAATCAGTTCCCGATATCTGTGATTTAAAAATAAGTAATAACGATCTCTAATTAAAATATAACATAATTTAACACATTTTAATATAAGGATACAACAATATCGATTATAATAATAGAAGAAATATCACAAGACAAACAGAAAAATGCCAGCGATTTTCTCAAAAATTCGAGAGCAGGCGTCAAGGGTGCAACCCACAGTGGCTCTGGGCGTCGGTGTTGGGGTGGCCCTAGCAGCCTGCGCTCTATACGGGACCAGAGACAAGAAGCAGACAACACCATGCAACAATAATAATAATTATAAGGT
MSTRG.1 3817	Rho GDP-dissociation inhibitor 1	ACTCTAAATCACTTAAATTTAACAGTTGAGTTTTTCTGAATTTTTTTTCATTTTCTCATTCGAAAACAAACGTAATGACAAATCCAAGGTAATGACTGATCCCAACATGGCGTAGCCAAATAATAAGTTAAAGTAGTTAAGTGAAAATTAACCTTAAAGTTCCAAGTAATATAGTATATTTTGTAACTGACAAATTACTTTTATTACATGCTCCACTTTATGTTCTATTATTTAGTAAATATTAGTCAATGTTTTGTCAACAATTTGTATATTTTATGTTTTGTAGGAGAATTCGCATGTACAGTCAGAAAAAATAATGTCTGGTAACTATTCATTGCTGTAATGTATCTCCTTTTTTATCACATTTTATTGACATGAAAATATATATATATATATATATATATATATAAAAACATTAATACAACATAATACTGGTGACAAATATAAAGCTGAAATAAAAAATGACAAATTAGTCTAGATTTAGTAATGGAATCTAGATTTAGTAACTGGCATTGAGAGAGAACTTGAATAACTAAATTTTAGTATCAGGACTATCACAATTTTTTTTGGTTTCTTTTGTTTTTTTGTCGTAAAAAATTATTAAGAGAATTGTGCTGGCGTTATTTATAAAAATATCTTCCAGTATAAATATATCACGACAAAAAAAAAAAACAAAAAATAACAAAATATAAAGTTATAGAAGTTTTTTTTATACCACATAGGTACATATATACAATACGCACATATATAGAGGTTTCGTTGATTATAACTCCACGTACGCCTTCATTAATCGGTGATTGGACACCGAAAGCTGTATTGTAATAGCTGGATCAAAATTATAATGTTGTTATAAAAAAAATGACATTGGAATAGATTTCGAGACACCATTTATCTTTACCTCTCCCTAATATGTTTTAATTTGATAATTCCACTTCGGAACAACAGTTTTACACCTACCGTCGTAATTTAAATTTGGAATAAATAAATCTTTTAAATAAATTTTGTACACATTTCTTTTATTCATTATTGTATACAAAAAGGATTTTATTAGAATAATAATTAAACATAATATCGCACAAGGGTGAGCTTATCTTTAAAAGAGATTGCTTCCAGCAAACCCAATATAAGATAAACTGACAGGGAGACTTTGTACAAAAGTCGATTGCTTGGGTACCTCTGTCCCATTCGTGTTTCAACCGTAAAGAAGTTGTGTTTGCATGGCTGTGTTTCGGTTTGAAAAGTAGAATATGGCGTGAATTTACTGGGTACGGAGGAATAACACCTAAAACCTTAGGAATGGCAACGCATGGGGGGTATCATGGGCGAAACAGTATACTCTGTTATGTCCATAATACTGCTCATGTCTATAGGCGACGATTACCACTTTCCATCAGGTGGCCCGTCAGCTTTTTTGCCATTCTAAGTTGTATAAAAAAAGAGTTCATTAGTTACAGGCATACATGTGTACCAATCGACTTTAATGGTAGAGTTAAAAAATAAATAAATAAAAGATTTTTACAATTGAAAACATTTTGGTCCATAGAATAAAGATTCCCCTGTAATATCTAATTGAAATTTTAATTTTAGACATGCGAGTAGGTTAAGAGAAATGGCGTCCTAGAAACTCCATTATATGTCAAAAATAATTTAACACATACAAAAACAAATTTGCTGTTCATTTGACGAAGCGTTTATTGAGGGACACTATTTAGATAACGTGAATGATGGATATTTGAGAATTGTGAAGGTATTAGATATTTGACTTGGTTTAATTTTTCCACGTGTGGACAAACGTCAGCGCTTGGAACAGCTAAGAACCAATTTAATAATATTTCTTTGTAACCAAATGCTTTTATAAGTGTTAGACTGTTTAACTATAGGTCACGACATATTATGTATTTATCCGGTTTTACATATGCTGATGTACTTTTACATATCCCCATCGATGGATAAAAATGAATAAGGTATAAACAAACCTAAACCAAACCCTAGGCATTAGCCTAAAATACTAAATAATCAAATTCTAGTTAC

ATATTAAAATTCCTTAAATACTAATACTAATTATAAGTAATCCCCCAACGCACCACACCGAGGCAGCGCACCCAAGATACT
GGCAACATTACCTCTTTGGATTGCCAAACTTATCTTTTGGGCAAAGTAGCTACCAGCTCTTGGGTCACCAGTAACCTCGAT
GAGGCGCGCTGCGATCGTCCGATTCAATTTTTATCGTTACATATTAAAAGTCGACGATATGTCCAAGTAAAAGTTTTTTTTT
AAAGTATATAAAAAAATTACCCTATTAAGTAATTAAAAAAAGACAAAATATTTCTACTTATAATGTTGATAAACATTTGCA
CTTACTCTGCATGCTTTAATAATTTTATTATAATTAAAAAAAAAAAAACTTTTAAATTAAAATGCAAGTTAATGAGACAAGA
CGCCATTACGAGGAAGTTGATACTTATCGAAATTAACCATATGTTATATATACAATGTTGCCAACATTAGGCACAAATATT
AAATACCAACCTACAGTCACCAATCAAGTTATGGGCCCAGCACCTCTGTTGAGTCTGATAGTACGGTTGTACTACAGATAT
ATCTGTATATATATATATATCTGTATATATATGTGTATATATACAGACTGTATACAGTTGTAGTTTCATCATAAGCACATGG
ATAAAGTTGTTAAATTTTCTTGCTGTTGCTACCATCCTTTGTGTTTATTGTCCATTGATGGGGAAGGGAAATATAATAAAAG
TCATTCTTTTACCGACTTTGTATGCATAGATTTCATAGTTTATAAAACTTGACACTTTTTTTTTTAAAGCAAAATGAGTCGTG
CCAAATAAAACTATTTTCTTATCAGAATAATTGGTAAGAAAAATTATTGTTTTTTTTTTTCTGTGCAAACATTTTTAATATTA
TTCAAACAGTAACTGTGATTTGAAAAAATATTTGTAGTATCTTTTTATATACTTGGCATATTTATTAAACTATTTCGATATTT
TTTTTTAATAAATAGAAAAAAGGCATTATTGGCATTGGTTTAATAAATTAGTAAATTGACTTAGTAAAAAAAAAAAGTAA
AAATATATTTATTATCAAATTATGTACAAGCATAAAATTTATCATTGATTCCTACATATAGAAATAACAATCAGCTTTCTAG
TTATACATATTTACCTACATAATTATGCCAAGTATAAAGGTACCTACTGAATGCATTATGAAATCTATGCATATTTGAAAC
ATAAATGTTTTAAACAATACACAAGCATATATATGACAAATAGTTTAAACATTAGTATTTATGCTATCAAATATATAAAAC
TTGATCTGCTATCAGGACCAGTAGAGTTTTACAATATCTTATATGGTTTAGGTACATTGTACATACCTACTTTTGTAGGTCC
CGACCATTGACAGGACAATTCGGGACACATTTGTCAAAGTTTTTTAAACTCTGACTTAGTAACCATCTAGTGGGATTTATG
ACTCAGTATATGTGTTTGATCCTATATATTTTATAGTGTAATATACTTCACATAATATGTAGTTAACGAATTTAACTGCGCA
TAGAATAAGATAAATTTCAATCGAAAATAGGTGACTTGTTTGTGATATGTAAGTTGTTGTGATTGAAAGTTGATAGGTACA
TTTATAACACGGGCTAGCTAGAACACCTTTATCTAGAGAAGGACAGGTATGTTCAAATTTTGTGCGCATTTAAATTACAAT
ACAAAAAAAAAAGAATTAATTGACCTCACATTGACTTTGGAAAATTATTTTCTAATATTAATAATTAAATAGGACAATTTGA
AACCACAAATGAAACTTCCAGCATGTTTTCCAATTGCTAATCACATGTGTCAAAATTTGGGGTCAAATCTTTTTTTTTCATT
TAACCGTTTATTAACTATGTTATTGTTTTAATAATAAATACGTTGTTTGAGTGGTTCCAATGAATATTGAAGTCACTGGCTG
AATATTATTAAGTTAACAATACGTAATTTAGCAAAAAAAAATATTACAAAACATTTAAACATCAAATTTAGCTAATCATTGT
GAGCCAAAGAATTGTGGACTCGGTGTGACCTCAAAGATTGGTTAAGCGACATCTATCGGATGTATGTTTTGTTATATTTAA
CGGCTGCGGGTGGCTTTGTTGGCATTAAGGCAATATTGTGCATAGTTATTTATTTTCTATAACATAATAGTTTTAGATTAAG
ACGATTATCAACTTATGAATGTTTGTTGCATGCCTGTTTGACATTTTGTATGTTGACAAATATTTAATTATTTTACTATATAT
ACCTATTTTAATTTGTTAAGTTGCCGCCTTCGATGAATTTATTTATACTTGACTTATTTAGTAACAAACTTGGATCACTAAA
TATGCCAAATATAAATAATTTATATTTAAACACTGATTTTAACATATCGATGCGGCACATTCAAGTTGTATTAGAAAAAAA
AACGTAAAAGGTTTATTCAAATTTGTAACATGTTGTGTTAACGTAATTATTATCTATGGTTTGTCTGTCTGTTTATTCTAAGT
TAGTTATTAAATGTTTTATTGTTTTTATACTAAGCACTTTTAGGCCATAGTGATTCTATAGAAGTACGTCAGATGTGCATTG
TGAGTTTTGTATGCTTTCAATTTTAAATGATTATACCAAGAGAAATGTTAATGATTTTGCTTGATATTCTAATGACACATTT
AAAATTATTTAAAAATAACACTGATCACAGCAATACTGTACCTTTAGGTATGTATTTTAATAAAATTGTTAAAATATGCAA
CTATTTTAAATTTATGTAAAAAGAACATTTTTAATTGTCACAAAATATTATTAATATTTCGTTTTTAAGGACCATCGTCTAA
ATTTACTATTTCATGTATAAACACTGTGTGAAGTATCACACAATCATTTTATGCTTACTTTTTTAGAAACGCCTGTCATAAA
ATGTCACGGTAATAAATAAATTTATGTAATAAAAAAAAAAATAAGTAATCCTATCAATTCACCCTAGATTTTATCGTCAAAG
TACAAATCTTTTGACAGACTCGTTTTTTACAATTTCCCATATGTCCAGCTAGGTATTGCGTGTAGGTACAGGCGTCACGATT
TTATAGCATTTTACATTTTTTTTCTAATTGTAACCTCTATTATGCATTGTCACGAATAAACAATGTAATCTCG

MSTRG.1387	Tigger transposable element-derived protein 6-like	TTTTAATAGTATTAATGCAAATTGATAAGTATCTAATAAACTATCTTGACTGCAAAATTTTTATTTACCTGCCATTTATCAT<br>AACAGAAAAAAAAATTGAAATATAGTGGCATCCATTGTAATTAAGTTTTTTCCTATCTTTGAATTATGCTTAAATACATAA<br>TCTCGTTACTTATTCCTAGACGAACCTTGTGAAAAGTAAAAGCATCCTATTAGCTGTGTACCTTTTAAAGTGTTCTTTGCCT<br>GAAGTGCATAAGTCTGTAATTAAATTATAATATTTTAAATAGTTGATCGCATATAAATATGTGTTATACATAGGTGTAAAT<br>GATTTAGGATAATATACTGTACAGAGTGTCACAGAAAGGGCATTCAAATTCTTCGTTGATATACTCGTATTAAGAAACTGA<br>ATAAATTAATCCACGTCTGTCAACGAACAGGACGAAAGTCTGAAAAGGAAAACTGGTTCTTTACACAAGATGTGCTCTTTT<br>TTTATTGTTTTAATGTCTATGAATTGTTACAATTGATTTACTGAATTTTTTTTTGTGTCATAGTATGAATAGTACCTACTGAT<br>TAGTATAGATGGAATTAATTGCCAATGACCAGTATGCATTACACAGTTATATTTTTGTGCATTGGAAAAAGGTATTTAGTT<br>TCGCTTATATTATTGCTAAATTTCTCAATGTTTTGCCCTTTCCGTTATGCCTTGTAAGTATATATACCTAATTGTATCGACTT<br>CGTTAAACTTAATAAGTGTTGCACAATGTTTTTTTACTGAATGCTATTTTTGTTGTATATGTAATGTTTATCGGTTACTTTAG<br>AATTGGCTGTGAACATTCTTTGCCATTATTTGTGTAAACAGATCGTAAAATAGATTTTATTATGACATAAAAATATTCTTAA<br>GTTCAGTTGTATCTATTTAAAAATGTTTTAGTACAAATTGTATGTAATATTTTTTTTTCAATCGAGAATTGTTGATTAAATTA<br>TAAAGAAAACTAAATATATAAAATGTATTTAGTTGTTTGGCCTCTGTGAGAGCAATTTTGTTGAGAAGATAATTCCTTGAA<br>AATATTTAGCATTCCGAATAGACTACCTACCTTTTATTGTAGAAATAATGTCAATAGTTTAGATAATATGCTTGTTATTTTG<br>TCAAAATATAATGTTATTTGATATTATATTATTCTTGTTGTATGTGACTGTGGTAAAAATTGCCTAAAACTTTTGTTTTTGTT<br>ACATAAATTCGTAAATATATGTAGTAGCTAGGATGTAACATATTTGTTTACTTTCTTAGGCATTTTTTAAAAAAAAATTGAT<br>AATTACGTTCAAGGATATATTTCTGAGAAATATGTCACTGAGAGTAGTATCAAATAATTATATTCTCAATTCTAACTCGATT<br>ATTGTCTACCCATAAAACATTTGTTATTATTACTCCTTAACACCTCTTTTACATTATTCCAGTAGCATTAAGTACAATACTTG<br>ATGATATTATTGTAGTTACATAACAGTCAAGTATTATAACAGTCTTCAAAAGGGGGTAAAGAGGTTTCTTCAGGGTCGGCA<br>ACGTGCGCGTAATACCTCTGGTATTGCAGGTGTTCATAGGCTACGGTAACCGCTTACCTTCAGGCGGGCCGTATGCTTGTT<br>TGCCACCTCAGTGGTATAAAAAAAGTAGTGATGTACTGGATCGTTAATAGTGTAGATCCGCGGTATGTATGCGGATCTCTA<br>ATTTTCACTTTATCTCAGTTGGTATCAATGTTCTTTTCAATGGATGCCAACGGTAATCCCCTCTGCGTTATTGGCATATGCTG<br>GCAGGGCATTAGTGGCGGATGGTTGATAAGAATAAAAGCAGGTCTTACCCATTACGAGTTCATCAAGAATTTATCATGAC<br>AGTTTCTAAGGGGAACTGAGTGTATATGTATGGATATCAACCTATTGCTGTCAATGGGCCCGCCAGTCCCTACTACTAACA<br>ATTCCTTATCCTTCATGAAACAAAACACTGTGTTATACAAACTATATTTCGAGTTGTACTGTGCGCGTTTTTTTTTAATTGA<br>GATAACGTGGAACAGTACAGATTATGTTTATATGGTTAGAGTAGTATTCCTTTAAATAGTAAAACAATATCTAAATGAAGT<br>TTAGATTTAATATTAAAAAAAAAATTTTTATTAAAAAGATACAGATATACGGTACATCACTTGACTGCTACTGGGAAAATG<br>TAAATCAGACATTAAATTTTCTTATTGTAATGCCTTAATATTTGTATCCTTAGATAGTAGATGACCTAAAATCTGACTACAT<br>TTTATAATATGAGAACACTAATTCAGTTTATAAATTACAATTTCGTCATGGGTAAATGACGTTAATTTTAGTGATAATTATG<br>GACGGCAAAGGTAAGGACAATTATTTTATATATTCATGATGGCATATATCGTCATAGTCGTAGAATACCGACGGATATTTA<br>TATGTAATGTTTGTCAGATCCGTCAGTATTCTACGACTATGACGATATGCGCAGTACAGTATAAAGTTAGATCCAAATCGA<br>TTAAAAAATAAGGACCCTGTGAACAAAGTTGCAATCCCTTTGGAGTATCTCTCCAGTGCGCTCTATACAAATCACAGTACT<br>TGCCAATTTGTTAATAATTAATCAATTTTGATAATAATTTGTAGACAAAATTTAAATATAAATTAAAAATTTTGAGGGAAA<br>TAAGGTGGGGTAGTTGGTGCGGGGGTAACGGCGAATTCGGACGCCACTAGTGGTGCCTTTCGCGGCATCATTAGACATAG<br>TGTCTCTAATCTGTGGATGCGGCGCGAAATGTGGGAAGTTTATATGAATTTTACGAATACTTATTATATGGTAGCTATTTAA<br>CGCTTTTTGTCGGACGTTTTTTCATACACAGAGATGGTTTACTCCTTACCTTTACCCTCCATAGTAATATTGTAAGTAGGTA<br>CCCAAAATACTTGTTTATATGAAATATATGAAAAATAATACAATATTGTAAGAAAATATTTTTAAATCTCTGTTCAAACAT<br>TTTTGGTGCTTATTGTGTAATTATTATAATTTTCTAATAAATATATAGTTTTTTTTATTTGTTTTCAAGTTCCATTATTTAGAT<br>GCACAGTAATAATAATATTAAAGTTGTTGTTCTATTTAAATATGCGATTGATGTAGGAAATGTACTTAACTGCACTTATTTG

		TGATGCACAATATACATATATTAGGTATATACAGTGAAACCTGGTTAAGTGGGCCGTGGGTATGTGAGAAACCTCGGCTAT TTATACTTACAATGAGGTCCCGACACATTTGCATTGAAAGATCTTTGTCACAAGAAATTTTTAAACCTTGTCAATTGAGGTT TGAAAGCTTAGCTTTTACGATGTTTACTGGATTGGAACTTTGTCGTCCTGGTTTGGGCAAGTTATAATACGTTTTTTTACAA ACATTTCAATTTCTTCTATACTCACTTTGTTTTTCAATACTATCTAAGTGTTTGTATCGAAACGAAAGTATAATTCGTTAACA GCCGCTGATAAAATTGTTATTGGATTATGATAGTGGAAAGTCGCGTGACGTACTATACACTATAATTAATTTACAAAAGTC AACTCTTTATATAATTATTTAAAATAAGAAAAAAACTTGTTCTCAGTAGATTGACGGAAAAGGAAAACTATAACGAATAC GACCAGCAGAGTATCCGAAACTTGAAAATGTCTCTCAACATGGATAAAAGAGGTACGGAATAAAAATATTCCTATAATTA AGACAACTGTCATTTTAAACCTACCTATTTGGGGAACTGGATGAATGAGAAATCTTTATAAATGATAAAAAATACTAGGTC CCTTGAGATCCAGTTATACAGGTTTCATTGTAATTAATATCATATACTTGTTGTCGACGTATACTTTCTTCTATTTATACCTA TTAAGAGAGTATATATATTCTATGGATAAGTATAAAATACATCATTATATACATATAGCCAGCAATAAATGTCTACAAGAA AACTTGATATACTAAATTTTAAGCTTTGCCTCTATTTAAGACTTTATTTATACCTAAATATACTTAAAATATATCCATATGT ACTTATTAAACATTTATAGTACTTAGTACATGAGTTGTATATTCTTATATATATATCTGTTGGCACAATATACGTAGTTATA CTTATGTGGGCTTCATTTTGTGCTTTTGCAGTAGGTATATATGCTGGTGCGAAAATATTAATTATTTTTGGATAGTAAATAT ATAGTCAAGCTTGTTGGAATGGTTACAGTAAACAATAGCGCACTTACTGCCTGCTACAAATAATATTTTTATACTGCCTTGT TTTTTATGTTATATCTAATTTAAGAAAATAGAAAGAAAGAATGACATTTAATTTTTTGATGAAACACTCAATTTAATCCTCT CGTCTTTTCAGTTCCCTTTAATTCAATAATTAATGACAAATATGATTGTAATGCATTGTTTAATATATTACCTAAAGATTAT CCAAATAAAGTTATTTAAATGAAAAGGGTTAAATGAAATTGTACTCGAAACTATATGCCTACCTACTTTCATGTACCTACT AATATAAATAACATCGCAATAATTGTTAGCCGATTATGTTACGTCTATTCATTTGTTAAACAAACTATTTAATCTAATATAT GTCTTCGTATGTTATTGTGTAGGTAAATAGTTAGGTGCAACTTTTTTTGAGAAAGTCTCGTGAAAAGTTAGTCATATTTTTT GATGTAGTATGTTATTAGAATGACTAACTTGTATCTACACTAAGAAAATATATTTATAGTAGGTGTGTTACAACAGGGACC TAGAATCATTCTGATTGGACGAACATACAGTTGATAAAAATACGTTGAGGTAAAATAGGTACTAGACAACGTAATTATTT AATAATGTTACATTAGAAGCTGGTAAATTAGAAGTCACAAAGATAATTTATGAAAAATATCTCGGTGTTTTATCACAAATC AAGCGACATTGACATCAGTAAGGTACATGTATGACTTAGTCGAGATACTCTTAGATCCTGATTTTTTTCTTTAAATAAACTG TTAAGTTCTGAATTACACAAACATCTCTAAATAGACCTTTATAAGATGCTCAATTTCTTGTATATACCTAATAAAACATTTA TAGTATCGAATTTTATAATTATTATTATACTATTAAAATTCAAAAATATGTGTATACAAATGGGAATAGGTTTATTAAGGT GTTTACATAATTTTGATAAACTTTTTTTTATTATAGGTAATAATGCCCTGTTATTTGAAGCGTATGATACTTTTATATATTCG GTTTTATGTTCATAAGTACGTGTTTATTTTTAATAATGAATATATAATGTACCTAATGTTGCCGATATTAGCGTTGCTTATTT AAAAATTTAAGAGTATTGCGAAGGACATTATTTACCGACGAATATTTTATACATGGTGTAGCAAAAAGAATACCTAACAA TCTGAAAACTAGCAAGTGACGTTCAAGAAGGTGAACAACTTTATACAAGAATGGATCGGTAACAAAAACATTTGAATCTT CAGACTAGTTCACGTGAATTGTCTGAAGAAACATATTTTTATTTCAACCTTCGAGCTTGTTTACGGTAAGTTGTTCATTTTA TTGAATGCTAATGTTGGATTTCAAAGATATATCTAGATAGTGTCGAGTGTCAAAAACTGTTGCAAACATTTCTTGCAAACA TGTCTTGTCAGCGCGTACGAAAACCTCAAAGTAAAATATTGTTAACATAGCAAATATAAATAAATTATATGAGGACTTATG TGCTAAAAAAATACTACTTACTAGATTGAGCAATATTAAAAATACGCCATTTTCTATCAAACCAAGTCAATCAGGCAGTTT TTGGCGAAAATAAATTCTATCTAGATGTTTTAGACCTAAAATTTCAGACTGTTGTATCCTTTCTGCTGTACCCGATAAAATT GTACCCACTGACTTGTTTTCATCGATGTCTATAAACATTACCAGAGGAATAAAAAAATATTGGCACTAAATTTGTGTAGTG ATTTAGCCGGTAAGGGATGTTATTGTGATTGTATGTTGAAACGAACATATCATTGAAAGTGATTAAATAATAATGCTATTG AAA
MSTRG.1 3878	1- phosphatidylinosit	GACTCGTTAGAATTGGAAAAGAAAGAGATCCTGAACAAACTTGCGTGTTCGCGGAAAGAAGACGTGAAAGCGCTTGCCA AGAAGACCAACAGGGATAAGGATGAAATACTTAGGATAAAGAGGGAAATACATTCTCAATCCGTGGAGCGCGGCGTGGC

	ol 4,5-bisphosphate phosphodiesterase classes I and II	GGAGTGCTCGCGGCTGGAGCAGGCGTACGCGCGGCGCCGCGAGGCGCTGGCGCGCCGCCACGAGCGCCTGCGCGCGCTG<br>CTGCAGAAGCACCGGGACCAGG
MSTRG.13881	DNA-binding protein Ewg	AAAATCACACCGCGCAGACTCGTACAAAATGGCGCTTTGTATCTATGAAGTATGTACTATTCGTCCACCCCGTTTACCGAT<br>CGATACTAATGGAGGATTCGGATTTTTCGGGGTCCCTTGTGCACTCTGTGATTTTTGTGAATGCAGTGTCTGGTTAAGTGAA<br>AAAGTCTTGAAAAGTGTGATTCTGGAGCAGTTACATCACAGTGAGTCCAGAAGACTTCAATTGAAGCCGCAAGATGTGTT<br>TCAAATCACGCAGGTTTTAATTGGAGTATACTGAGCGTGTTAACTATAGGCGTGCGAGTTACGCTGCTAACCCTCAGTTTG<br>TACTCCAATGGTCCTGGAGGTGAGGTGCCGCGAACAGGACCACGACACCGACTTTGACATCATGAATGCCGCCGTTAGCA<br>CAGAGTCTATGGATATGGCAGAGGAGGTAATGCTTATTGGCACTCTTCTTTTATGAATACCTTGTGCCCAC
MSTRG.13907	Hypothetical protein KGM_209630	CCTCCAGCGGGGACAAGTGCCCACAGTATGTATGCGTACCTCCGCCCGAGCGGCCGGTGTTCTGCAACGTGACAGGCCGC<br>ACCTTCACCACCTTCGACGGTGTTGAGTACAAGTATGACGTCTGCTTCCACATCCTTGCTAGAGACAACAGGTTCGATGCC<br>TGGACTGTGCTCATCCGTAAGAAGTGTCGTTTAGAGGGCTGCCAAAACTATCTGATCGTACATCAGGACGATCAGCTGATC<br>ATGGTGAAACCGAACCTGATGATAGAATACGACAACTACGAGTACACCGTCGAACAGACATCCAAGATCTGTTTCCAGAA<br>GAACAGCTTCGATGTGCACCGACTCGGTAACGGACTGTCAATCAAGTCCAGAAAATATAACATCACTGTACTTTACTCGAG<br>CGATGGAGATATTAAGATTGGGGTGAG
MSTRG.13913	PiggyBac transposable element-derived protein 3-like	AAATAATTCAATTCCCAATAAGTACATCCTTATTACTGTATATTCAATACATGCCATAATCAAGAACAAGCCTATGAAAAC<br>TTTTAACTCGCCATTTGTTATTAGCAGACTCTTTCCTGTTTTATAAACGGCTGTTTGATTCGTTTTATCTACGATCATATCTA<br>TTAGCTCATCATCAAAGTATTGAGTAAAATAGTCTAATTTTGAAAAATTTGTTCTGTCTTGTATCGAGTACGATGGCTCAGA<br>AAGCATAGGAAGGTTTTTTGTGAAATTCTTTACAGCCCATGTAAACTGATTATTTGAACTGCTGCCACCGCGAGCACTAGT<br>AAATCGCGGAGTGCGCTCACATCTTCTGCTGTAATGTGCATGACTACGGGCTCGCATGTGCGATCGATCTCGTCCTCGACT<br>ACTTTTCAGAATTGGTGGTGGGCTCTTAGACTCTGATTCTGAGCTGGTACTGGTTTCACTTGCATCATTCCTATCTGGAACG<br>AACTCATTGCCAGTATCCTCTCCTGTAAAAGAATCTCCCTCGTCGTCTTCGAAATCTGATTGATATAGTATCTCTAATATTT<br>GACTATCTGTGAAAGGGAAAACTGTGTCAGCACAATGGCGGAGAATAGATAGAAGGAAAATAATTAAAGGTATAAATGC<br>CCCCCGTACTAGTATTAACATGAAATAAAATCACTAAAAAACCATATAAACGCTTATAGTGCTAGTATTAGTACAAAAAA<br>ATAATCACTAAAAAAGCATATTGATATTGACAAAATTACGATAAAATAATAGTGTTTATATAAACTGCATCCCTTTGTCCC<br>GATAAAAAAACAAAATGTCGTAAATATAACACAAAGATTTAAAAGATATAAATTAATTAAACAAACCTTTTATCGTTGTA<br>CTTACTATCGCTTCGACTTAGAATGACAGAAGAACGAAAAAAAAATCCCTGTCCATTGAAAGCAGTGCACTGCCACAATA<br>CCAAATATATTTACAAACTAAACGAC
MSTRG.13918	Uncharacterized protein LOC110380377 isoform X1	GAAATTCTAGATGGACCCGTTTCTCTGGTAAATTGGAATTATGTTTATTTGGATTAAGTGGCCATTGAGATGAATCTAGTTT<br>TAAAAATGAAGGCCCTGACCACCACAAGGAAGAATTGTTTAATAAATCTGCTCTTAGACCTCGGGATACTAAGTCAGCTG<br>GATTTGATTTTGAGGGTACATAACGCCATTCGTGAGAACCTTCCTGTATCTCCACAACTCGGTGCTTAACAAATGGTTTAA<br>GCTGACTTGGTGAAGAAGCAATCCAGCCTAAGACGATGGTGGAATCTGTCCAAAATACACAACGACTGAATTCAAGAGTA<br>ACCGATGTAATCACCTTCTTGTACAGTCGTGCTCCTAAAAGCGCGCCACAAAGTTCAAGTCGTGGGATTGTAGTAATTTTA<br>ATAGGAGCTACCTTATTTTTGGAAACCAAAAGACGGACATCAATTGAACCTTCGCTAGATATACTTCTTATATATACACAT<br>GTACCATATGCTTTCTCCGATGCGTCTGTAAATATATGTATTTCTGTGTTGACTGTACTGTTATTTTTAATACACCTAGGAAT<br>CTTAAGATTATTCAGTAATGGTAAGAACTGTACAAATTCTGACCATATTTTTTCTATGAGAGGGGGAACCTTGTCATCCCA<br>AGAGCATTTATTTATCCACAATTGTTGCATTAATATTTTTGCTTCCACTATACATGGCGATACAAGGCCTAATG
MSTRG.1	Catenin alpha	TCACCGATCGTGGTGTGGCGGATGAAGGCGCCGGAGAAGAAGCCGCTCATCCGGCCGGAGAAGCCCGAGGAGGTCCGCG

3923	isoform X1	CGAAGGTCCGCCGCGGCAGCCAGAAGAAGCAGCCGAGCCCCGTGCACGCGCTCGCCGAGTTCCAGAGCCCGGCTGAGAG<br>TGTTTAAGGCAATAAGGCGAGCTTCGGAAACGTCGTATTTATTTATATTTAAAGTAAATTCGGAAGCTCACAACAAAATTG<br>TATTGTGATTGTAATTTCTTAAATTTATAGCTCATTTATTAGTATTAATATATATATATATATATAAGGCCCATTTCATATTG<br>AGCGAAGTGCGGATAATAATCAATAAAACAAAATAAAAAAAAAAAAAATATTGGCAGAAAGTTACATAGTTACAACAGAA<br>AATACAGTAATGTTTTTAAATATAAGAAGTGTCTCTTATCAGGCAAAAGGAACTGGCTCAGCTGATGCTGCGGGTGTCCCA<br>GTGCAGTGTGTACAGCTGCACAGCGCTGGTATACAGCTAGTAGTGTATTGAATGACTTAAAAAAAATATATATATAAAAA<br>CTGTTTATCATGAAAAGTTACAACACAACGTAACACATCTAAATGATATCTTTAAATTTTATTTATTTATTTACACATTACA<br>CTGAACAAACTTTTCATTTAAATAATTTATTAGATACAATGTCTTTTTAAGAAAATAATGTAAATTATACATGCTACATATG<br>ACAAAAACAACAGAGAAATTAAGATTACACAATTATATAATAACGTTATTACTTTATTTTGAAATTAGAATAAAAGAAGA<br>AAAATATATTAAAAAAACAAAATGAATGTAGCAGTATTTGTGCAAAGGACCTTGTTCACTACACTATTAAACTGCACCGA<br>GTATTTTTGAGCTTTATTAATCATTTTAATACAAAAAAACAAATCACTATATTGTAATGGAACATTAGAATATATATCGCG<br>ATGCAATATTCACCTAACGCCATCTATGTGGTCAATAGTGAACTAATTTTGCTGTTTACTGAACAGATTTAACTCAGCATGT<br>GTCGCAGTTTCATATCTGAAGCTACGCCATTGATTTTCAGTAGGCCCTAAATATGTAAATTTTTATTTGACATATTAATCAC<br>TGGCTGCGCTTCGTTCGATCTGGAACGGGCCTAATCGGCCAAAATTGTATTATTTTGCATGCATTTTTATACTGTTATGAAT<br>ATTAATAATTATAGTGAGTTATTAATGAAAGTGGGTTGCAATTTTGAAAATGCTCTATAAAGAAATAAACTGTTTCCTGCG<br>GTTTCGCACGCGGATCTTTTTAGCTCTTATTTTTATATGGGAATGTATGTTTCATGATTAGTAATCTTTCATTCTTTTTTAAT<br>ACAATCGTTTTTAGAAATTTCGTCTATCTATCTGTCTGTTTGTTCCAACATCACACAAAAATTACTGCACCAAGTCGGATGC<br>GGTTATTATAGTTGTATTTCTAAAGGTGTAACTTAAAAACTGATTTGATCTTAACACATCATCTATAAGTGATCTATTTCAT<br>TGTATTTCCAACTTCTGAAATCCACGCGGTGCGAACTAGCTGGTAAAAAGGTTGTAAACCATAAAAAGTGTACGGTCAAC<br>ATTGCAACTCACTTTAGTGCAAGTCGGTTAAGGAGTAACGAAAATTTCTACGTAATAAGACAGCCAAATTTTTATAGTTAA<br>CCCACGTATGGTACAATAATACTGTAGGTATTAAACAGTTAAAATTAACTAAAGCAAGGAATTATGGAAAAATACTGGTA<br>TCACGTTTGGTTAAGTTAAGAAAACGAATGAGGCAACTTCAAACATGTGATTTCAAGATGGCAGCTAGATTTCTAGCCATT<br>TTTCTCTTTTTTATAAATAAATTGTTTAAATCTAGCACGCGTGAGTGACATTTGTCATATTGACAAATATCGCTTTAAACTT<br>CACGAATTGTAATCTCAGTATTGGGAACGTTCTAGAAGAAGCGCAGAGCGTTGTAGCAAATCGCAGAAGGTGCCTGAAGC<br>TGCTAAGATCAGCGCAGACTAATGGAATCGTGGTGAACATCAATTGATTTGAAAATTACACGATCGAAGTTTATTATTATT<br>ATGCTTACTAATGTTATAATTTAGCTGCATGGTCCCGTCATAGAATACGCCGCCCCTTCCTTTCCCGTGGGTGTCGTAAGAG<br>GCAACTAAGGCAGCGCCAGGGATGGACAGCAGCGTCCCTCTTAACCCTTCTTTGCATTACATTGGTGAATTAAATCGAGGT<br>GATATAATTCGTGTTCAAAATTAAAGGAAAAATGGTTGAAAAAATAAATTTGACAGCTATGGTTTTTTTTTCTGCAATGG<br>AAATTAAAAAGGTGTCCAGACAAGATCAACTTAAAATGAACTTGTTCTTTTATATAATTTTGGACATTCAATGTTATGATG<br>GAAATTTCCACCATTATGCAAAGAAGGGTTAAAACATCTCCAGAGTCTAACTGTGGTTGCCAATCCGCCTGACAAGCGTA<br>GCAACTAGTGACTCCCTAAAGGGGAGGCTTGTGTCCAGTAGTGGACAATTAAAGGCTGATACGATGATGATGATATTATA<br>AATGTGAAAATGAGTTTGTTTGTTTGTTTGTTACCTCTTGGCATCTTTACAGTTAAGCGACCGCTATGAAATTTTGGTATAC<br>ACGTACTATATGGGGATGGAGAAAAGGATATAGGCTACCTTTTATCGCGGTCATGCGGACAAAGTCGCGAATAAAAGCTA<br>GTATTTTATTAAATGAAACTACATTTTTTCGAAATGGTCCTAATATTTTTTCTTTATGGGTGAAAATGGTATGGTAACTTTG<br>CCTGCGCTAACGGGATACGCTGGCGGAGCACCAGTGAAGGGTGATAGATGAGAATGAAAACAGGCCAGTTAGCCCATCA<br>TGATGAATTCATTAGGAATTAACCATGATAGTTTTCAGGGGGAACCGCGAGGAGGTCAAACTGGTTGGGTATATTGCTAG<br>CAATGGAACTGGTAGTCTGCATTACTAACAATCCCTTCTCCCCCACAAATTTAAAGTTAAATTTGTCATAATGACAAATGT<br>CACTCATGCAAGCGATAGGGTTTATTTGAAACTAATCCATCGGCATATTGTACGTAAGGTCTAATTAGCGGAACTTGTATA<br>ACACATTAGAACATTTATATAACGCAGTCGAGTAATATGATAAGAGTGTTTACGTCGTAGTGTGTTAATATCGAAATTGTT

		TTTGAAAAACAGTTGTACAATTTTAAATTATAATTTTTAGTCTATGATTAACGTTTCAGTTATCTACCTATTTTTGAATAATT<br>AGTCAGAAACAATTGTGTAGTTTTTCTTGTTTAAGAATTGTTCCAATATTGTGCACCGCTGTATCCGCTGACATTGACGCTC<br>CTTTCCACTTTGGGTGTTGATGGCGGAGAACTCTTGCAGAGTTAAGCTCGCCTTTGTACGTTTGCTCACCTCTGTCATATTT<br>TTTTATGTATTTATCAATGTGTACAATAAAATATAAAAGAAAATAAATTGATAATTGTTAAATTGTTTTTTCGAAAATGGA<br>CAGGTAACTGGGAAGTTGCCACACTACGGTGTAAATACACACAGTGTTCGATCCCAACGATTTATCAAAACAGCTCACAA<br>TTTGTAATAAGTAGAAACGTAGATTAGTCTTGGATAACTAGTTTGTAAGAGATATAGCAATACTGTGGGCGTTTGATAAAT<br>ACATACCATATATGTATAACATTTGTAAATCATAAAAATAAACAAAGTTATTCCAATATATTTTTACTGAACCGCTTGGTT<br>GTCTTGCGGAAGTTCATCATCATCATCACCACTTCAGCCCTTATCTCACCACTGTCACAGATAATATAATACTGTACTAGTA<br>CAGATAGAATGTCTTAACCGTTTTGGTGCCATTCATTTTTACTATCTACTTAAAAATGCCAAAGAAATCTTTGTCGTGTTGA<br>TGTCATTTACCATTGTCTCTAACCGAAACCGTATCTCCTCCGAAAATGATCCAATTTTCATAATTTTTCTACAGCGAACTAA<br>ACAGCGACTGATTTCACGAGCGCAACGGCATTCGTAGTCACTCAAACGGTTAAACAAAAAAGTGGAAAGCCCCAACGCGG<br>CTTATTCAGCGATTTTATTCTTAGTTGTTCATATGCCCGCTCGATGTCGCTACTTTCCTAGTCGTTATTAAATTATTTACCGA<br>CGACTACTAGTTGAGTTAGCAACCAACAGATGGCGTAAATATCTGGCGTTTGCTAACGTTGTGGTATATTGTAGCCACTGC<br>TGAACGTAGACTGTCATAATAAAGAAGGGGGCCGTCCCTTATGACAGTTTACCACTTTACGGAGATTTTGCTAGCAATTGC<br>CACGTTTGACTAGTGATGTCTTCAGAGGGACGCTGCTACCTATACCTCGGTCATTAATTTCCCTTAATCGCTATGTCGGGAC<br>CTACACGTCAAGTATTTTTTTTTATACAACTTAGAATGGCAAACAAGCTGACGGCCCACCTGATGGAAAATGGTAACCGTC<br>GCCTATTGACATGAGCAGTACCATGGACATAACAGAGTATACTGTTTCGCCCATGATACCCCCCATGCGTTGCTATTCCTG<br>AGGACTCAGGTGCTATTCCTCTGTACCCAATAAATTCACGCCATATCCCACCCTTCAAACCGAAACACAGCCATGCAAACA<br>CAACTGCTTCACGGTAGAAACACGAATGGGACAGAGGTATCCAAGCAATCGACTTTTGTACAAAGTCTCCCTCTGGTAGT<br>AGCAAGGATAGTTAAAAATTGTATCGTTCTTGCCATGTTTCTTTGGGAAAAAATATTCAATTTCACACTTACATTTGTCCAA<br>CAGTGGGACATTTTGTTTATTTTTATGATAAATTTTAATGCTAGTTATTTATTTAGGATTAGGATAGGTTTATACACGAACT<br>TATTAAATACAAAACATATATAACGATTTATTATTTGCCTTCATAAAAATGTAAAACCTTAAGCTATATGGATAGAGCATC<br>GAGTGTAAAAACCGGTCAACTTTTTGTCTAAGAACAAAGAATTACACAGAAGGAATGATAAAATAAAAACTGTAACGTAC<br>ACGGTTGCCTCCTCTCTTATTAAGTTTTTATTTTACCGTTCCTTCTGTCTAATTACTTGTTCTTAGCTTTTGACAACTGATTTG<br>ACGTTTGATTTGACATTTTGACACATATCTTTTTTAAAATCAGAACGCGCCATTTATATAGTGTTGCAAGTTAAAAGTTTGC<br>CAGGCCCAGTCGGTTTTCTAAATTTGAAACGGCCACTGTTTTTTAGATTCACCAGGAGGAAAAAATATAAAATACATATGT<br>ATAGTTTTTTATTTTATACTTTATTGCACTTAAAAGAAAGAAAGATACGGCGATTAGTTTACCAACTTATTAGCAAGAATA<br>GGTTTATCTTTAAATGATTTAAAAACGATAACATATGATAAGTCTACATTTCAAAGCATCAGTATTATCAGTTCGAATCGTT<br>ATGCATTGTTTGTCTTGTCCTTATTTATTATATTATATATTTATTTTTCAACTTACAATGTAATTTCTCCCATAAATTTAATAA<br>TTATTTTTTATGGTAACCCTTTTGGTGTACACGTGGAAAAAAAGTTCACTTAATATATATATATATATATATATATATATTG<br>TTTCAATTAAACCTTCAAAAAAAGAGTATATTCCTTTTTAAAAGGCCGGCAGCACACCTGGCATTGCTGGTGTTGTGCGTG<br>ATCATGGGCGGCGGTAGTCACTTACCATCAGGTGAGCCGCATGCTCGTTTGCCCCCTGTGTTGTAAAAAAAAACTTTATAA<br>TATATATATAAATTTATATTCATTTACTATATTAATTTATTTATTATACGATTAGATACCTTGTCTCTTTCAACTTTCCTGAT<br>CCCGTAATCCTGTCCCTAAATTTTCCGCCACTATATTCCCGTTTTGTGTTGAGCCAATTTCCCTGATTTTTAATAAAACTTGA<br>ATGGCTAATGGCTACCCTAAAAAGCCAAAGCAAAGAAGAAAAAAGTTGACCAGTTTTTAATGAAGCAATTGTATGGAAAC<br>ACTATGTTTATAATCTAAGTGTAAAAGCTACAATTAGGGGAAAGAGGGGTTGAAGTAGTCGTTTAATACTAGGGTACAAG<br>TAATATCCCATGCC
MSTRG.1<br>3930	Carboxypeptidase	AAACAGTATTGACAGTAATAAAATGGCGCTCTGTAGAATTGTATTTGTGCTTGTTTTATTTGGTTTGGTATCGGCCAAACTT<br>AAACAATACGATGGTTTCACACTATATGAAGTTGTAGTAGAAAACACAGAACAGGAAATGGTGCTTAATAAACTGGAGAG

		TGAGTTAAATTTGGATGTGTGGTCTCGTGCAACGCCAGAACAATCGGGGCGGATTCTTGTATCAAAAAGTGAAAAGACTG<br>AATTTGAAAATGAACTTTCCACAGCTGGAATAAAACACACAATTGTAGTGGAAAACATGGAAGAACTTATCGAGCTAGAA<br>CGAAAACAATTGTCAGAAGCTGACGAAATGAGTGCTGGC
MSTRG.1<br>3946	Bifunctional<br>ATP-dependent<br>dihydroxyacetone<br>kinase/FAD-AMP<br>lyase (cyclizing)-<br>like	ATCTGCCAGGGTATACTTCTAGCAATGGGACTGGTAGTTGGCATTACTAACAATTCCTACCCCCTGATACAGATGTTAGGT<br>ATCTAATTTTTTTTCTTTCCATCAGGTTGAAGCAGTAATTATAGGCGAAGATGTTGCATCTAGTCACAATAAGACTGGAGG<br>AAGAGGCATCTGTGCTGAAGTTTATTTATATAAGATTTGTGGTGCAATGTCAGCGAGGGGCTACGACTTGTCGAGTATCCA<br>CAAGACGTCGATAGACGTGAGCAAGTGTTTTGCCACGCTCGGCGTTTGTTTGAGCGCCTGCTCCTTGCCTGGTAAGTATTC<br>AACAGTAAAGCTTTGGG
MSTRG.1<br>3958	Transcription<br>elongation factor<br>B polypeptide 3	CACTTCTCATTAATATTTACTGTATTTGTTATTTCTCCTTTTACTTGGTGCAATAAAGTGTAAATAAATAAAATAAATAAAT<br>ATATATGTATATTCTTTGTATAGGAAAAAAACAGTAAACCTAAGTAGGTATATGAGGTCTTGCCCTAGCAGTCGTTCTATA<br>TATTAGTATCTATTTGGTAGATATGTAGTAACATGACGTGAATGGCAGCTGACAATTTGGCTTTTTCATGTAAATAACTTTA<br>TTTGCAATCAAACACAATCTAGTTTGCAATGTTCTGTTGAACCCTTGAGTTGAAGCTCAAGACTAGACTTGTAAATTTTGTA<br>AATTCCTCTACTACTATTGTTGCAGATTTTAAAATGTATTGATAAGTTATTCAAGCTTGATGTCACTGTCCAACATCTGCAA<br>GACACAGGGGNNNNNNNNNNAGTGAATGCACTGTGGGAACATCCAGGTGATGTAGGCGAGGCCTCCCAAGCTCTGGTCT<br>ATAAATGGAAGTGCATGGTCGCAGCTGAGGNNNNNNNNNNTGGAGAATATCAACGTGAAAAACATAATTACAATTATAA<br>AAACGATGGCCAAAAGTATAATAAAAAAGTGAATGGCAACTATAGTGGAACCAAAAGAAAATATCGAAGCAGTGAGGAT<br>GAAGAACATGATATAAAGAAGAAGAGAAGCTCTGCCTCTAATGGAGATTATTATAACGGA
MSTRG.1<br>3973	Unconventional<br>myosin-XVIIIa<br>isoform X1	CGGCAATGCGTCACTGCGTTTGGTACCTGGCAAGCACGTATCCAGCTCAGGGCTCCAAGCTCACGCCCGAACGATTCGAC<br>GCGGCGTTCGATGTGCTCCATACCTTTGGATCCTCTCGCACGGGCTCGAATACCCATGCGTCCCGCTTCGTCTCGCTGACGT<br>CACTCGACTTTGACGGCGGCGGCGCGCTCGTCTCCGCCTCCGTACAGACGCTGCTGCCGGATCTTAGGCCGGGACAAGCTC<br>CGCTAAGAG
MSTRG.1<br>3998	Enolase-<br>phosphatase e-1	CCGTCAGTTTATTCGCCGGTCGTGCTCTTTCTCGTGCGTGGCGTTCAATAAATGCTTTTTTTCCGTGGTGCGGGGAAGTAAC<br>ATACCGTGAAAGGATTTCTAGTCAAATAAAATCGAATACTGTATAAAAGTACTCCTTTTATAGGCAGTACTAACGTAACGA<br>AATGGCGAATGAAAGTAATGAAATCGGCAGCATCATCAAGAAATGTAAAATTCTTTTGTTGGACATCGAAGGAACAACAA<br>CATCGATCAGCTTTGTTAAGGACAAGCTTTTCCCTTATGCTGAAGAGAATGTGAAGCAGTTCCTGGAAAGCAAGTGGGAA<br>GAAGAGAAAG
MSTRG.1<br>4026	ORF2 protein	TATATATATTATTATTGAATATATGTACAGTTTTGGTAGATATTTAGACAATATTGTTTGATCGGAAATATTAGACTTGGT<br>TTAGTGTATTCTAATGTACCTATACATTTGTAGACACAATTGTTCAGGATTGTAATTGGCTATAGCAAAATTTATTCATTCG<br>CCCCCCCCCCCCCTCTCCTATTAAGGAGGAAGTAAAATGTTAAGATACTACACTGCATTCGCCCTGATGAAAACTTAAGGCT<br>CCTAATCTACTATATTTCTTTATTTTTACTATTTACCTCTTGATGGTTACCACTTGAATCAGAGGGGCCGTTAGTTTATTTGC<br>TACTCTAAGTAAAAGAAACTGTATGTATTTTGTATACTTCAACTTCGGCTAGATTTCCTACATGAAAATTATGTCGAAATA<br>AGTTGAAGATTGACAGAAACAGCATCATAGATTATTATCTATTTTATGATCTGTATTTATATTATGTTACCTATCAAAATTA<br>ATTCCCTTAGTACAATTATATATTTATAATTGTACTAAGGGAATTTAATTATCTATCTATGTTTGCCATTTCTGATTGTCTCC<br>TTCATGCGGTAGTCTTTTTCAGAGTGAGTACAGTGCTTATTATATACAGTATTAGCGGCCCGGTACGTGCTTCGCTACGTAT<br>AAAGTGAAGTAATAAAAAAAATTAATTTTAAATGACCGCATGAATGACATAACAACACGCTTGTACTGTCAACACTGAAA<br>AAAGTTTCAATTTGACAGCACAATTTCCATCTTAAAAAAGTTTGAATTTGCACAAAAATCCCCATTAACAAAATGATCCGC<br>TATTTGTGAATTCAATTGTTACAAAAAATGTGATAAAGAAGGACCGCTCGACCGATTTTGTGCAAACTTCACATAAACCAT<br>CTACTAAATAGTTCGAACAAAGCTTAAAGATTTGGTTTCGATAGGTGAGCCCGTTATTGAGTTATAAAATTACAACGAAAA

GTGGCATTAATTTTTATATATGTATTGTAATTTTTATATATGATTATAGGTATTGGGATCGTACTGAAGCGCCACTTTTCTA
AATCTTTCTTTAAAATTGAAAATTTCAATTTGATATCACAGCGAAATTTTTACTCTAGGGACCAATATGAACAAAATTTTTA
ATAGAATTAAGTGAAAAATATTCTAAATTTTGTTTGTGATGGTCCATAAAACAGTAATTACGCGAAACTATCAAATTAAAA
TATTAGGATAAGAGATAGCTTTAGAAAAATAGACTTTTCAGTATCAGTCCCAATAATTCAGTTTTTAACATTACAGTAGAA
AGAAAAAACATGCCACACTTGGTTCTCTCATTATTTATTAGCTAACAGGGTGAAATCGGCGTCGTTAGTTGTATGTAAACT
AATGAACATAGGCCTCCCCCATAAAATAGACTTTTGCTAGTAATTTCCATACTTGAATGAAAATTGTTGGGTACAAAACTG
CAGTTACGCTTTGGTGATGTTTTAAAAGTCACGCTGCTGCTTATCCCTGGACCATTAAGTTCTCTTAGTCGCCTCTTAGGAC
ACCCACGAGAGAGAGATGGCTCATTCCGTACTGAAACCACACGACTAGATATAGAATATATCTAACCACATTATGTTAATT
AAAAATTCACAAATGTCAAAAAATTTAAAGCCATTCTTACTGAAATGCTGGACGCGTCTTTTTAACTCGCTTCAAAAAGAC
GAAGGTTCACAATTTGATTGGATTATTTTTTAAATCTTTGTTGCCTCATAACATCAGTTGTAAACCGGTTTGGAAAATTCTT
TTTTTTTTTGCCTGAAAGGATGTGTAAATTTTTTTATTTATTTATTACATCCACTGGGTGAGCCTTACAGTCTTCCAATACGC
TCACTCAGGAAAGCATGAGTACAATTACATATAAAAAGGAATATTACACAAAAAAATAACAAAAATAATATAAAAAGAAT
TTACTCAAACAAAAAAACGTCTGAACACAAATACTCTGTGGTCACCCGACGAAACTCCAGCAAGTTGGAGGCAAATACATC
CAATTGCGGATACTGGCACAAAATATCATTGAGTAGTTACAATGCAAGAGATAGGGGAGCTTGTTTAGCCAACTCTGTTCT
TCGTAATGGAACAAATTTTATCGGAATCGGTTCAATAGTTCAGTTTTTAAATCAAAATAACTAAATATTGATTCACAATTG
ATGTCAGTTTTTTCTTTTTTTTTTATGGAATACGATCTTAAATTATAAAATGGTAGGCTCAAAATATTTTGTTGTTTTCGACC
CATAACTCTTTAGTGAAAATGTTCAGGTTTACTGATAGAAAACTGTAGTAAAAAGATGCTACCGATTTCACCCTGTAGATT
GAGGTACATGCGTACTCATATATGTCTATAAAACAATGTATATAAAGCGCATTTTATAGATGTCTGCAAATAATTTGAACA
CGGATTTAGTATCACGTGATGTGCGCCATAACAGCGTCTACAGCTCAGGCTTCGTTATCTTTTGTGTCGTTCAACTGGTTTG
TCGCGAAGTAAACGCTAAAACAAGTCTAAAATAATTCCGATCACACATCTCATAGTTGAAAACTGTGGGCGTTATCCTGCC
TTCACTAGTTCTTATAGCATAGTTTAAGAAATTATTGGCGTATAGCGTGTTGTGTTGTGAAGGAATACTAATCGGTGTTACA
CCTCGGCGGATTCGTTTCCGATATTAAGCTGCATTTCACTTGTGATATGTTCTGTTAGTGCATTCGAAATATAAATTGTTTA
AGTCTCTTTTCGAATAACGACCGGGTAAATGTCTAGAGCGATCAATTTTAGATGTATTGACGATCTCATTTTATTGTAGGG
AAATAATGGATGTAAGTAGCTCAGCTACCTTAGGAAAATGACAGACGAAGCGCTCGCATGAGAGAGAACAAAATGCCAA
GCGTCGTTGCATCGTTCTACATCTTGCTAGCGTTAGCGATAACGTATCTTATCAATAGTTTCGTCTACTATTTGTCTAGGGA
GGCTGAATCAATCTAAATCCACTTTACCATCAAAATATATGTCGCATAAGCATGTATTGCATAAAATTTCTAGAAGGCCAA
TCTAAAAGATCTTTTCCTATTCTTTTAGATTAAAAAAATGCAAATGGTTTTGTAAATGATGAACTTAACAAAAAAAAAAAC
CGAATAGAAAACCTTATTCTTTTTTGAAGTTGAAAAGACGTTCTGACTTACTGACTGATCGATATACAGTCTAAATTTAAA
AATAGTATATTTATGAATTTTTTAAATCTTCATGCGAACGGAAGATAAAAAACGCTCAAGTGATTTGATGATCCGCGGTAA
ACACATTGATCCCACATATTCAACAGTGCGGTATAGCCCTGAGATAACCTTTATAGTTGTCACTCTGTCGATATTTTCGTCC
GGTCTCATGTACCATTCTTACTGCCAGAGTGGAGGCGGCGTGACATTGAACATCAAACAGTAGACGTAACATACATCATGT
CTGCTTTTTGACCCACCTCACTAAATTTTGTCGTACGCTGCCAGGCATCGTAATTATTTCGTGATAAACACTATTACTGGCG
TATTCATAAAAAAAAACAACAACGTCCAATTAACCTATTTTATCAGACTGCTTTTTCTCTTAAGAGCGTTCATTACGAAGTAT
GGCAACGTCGCAATTGTTTGTCAATCTTTAAAAATCATTAAACATTTTTCAATGATTATAATTGATTATTTAAAAAATGCTC
TGTTAGAAAACTAAAACGACACTAATAATTTTCTTTTTCTTAAAAAGTTTCTTCGTGTCGAGGCAACTAATTCATGTATTCT
CTTCCAGGTCATTTCATTGGCTATATGACATGACAGTATTTTATTTATTTGTCCCACGTCTCGGACATGTCATGTCTCGGAC
TTCACCTTTCTCTTTTACTCTCTATTTTTCTTTCTAGAATGCTTTTTAAAAATCATCATGTCATGTAAGGTGACCTCATATTA
ATAAATTACGTTGCATATATAACTACTACTAAAAACTGTGCACCTAATTGATATTGTACTTAGAAATTTTTCAAATACATTT
TTGCCATCAAAATTTAATTGTTCAGTAAAAATTTTATAACAATATTATTCGATTATAGCACGATTCACGTTTTATTTCACTA

ACAGATAACTTTTTAAATAATCAGTCGTAAACATTGTCATTTTAAAATTGTTTTTAAAATCAACAATTAGTTGAATTAATC
AACAACGTTGCCACACGTTGGCAGACTAAAGTTATGATACGCTCTTAATTTGAAGATAAATTGATAGAAAATTACAAAAC
ATTTTATGATTTAATAATTTAATAAAGAATAATATTAATGAATAATAATTAATTGAAATACTTTGCAGGTTTTGTATTTATT
AATAATACAATTTTTTTTAATTTCTTTTTAATAATTTTACTTATCTAATTACAATACAACAATAAATGAAAGTTAATATAAA
ATTCCTTAAATACTAATACTAATTATAAGTAATCCCCCAACGCACCACACCGAGGCAACATACCCAAGATACTGGCAGCAT
TACCTCTTTGGATTGCCAAACTTATCTTTTGGGTAAAGTAGCTACCAGCTCTTGGGTCACCAGTAACCTCGATGAGGCGCG
CTGCGATCGTCCGATAATACAATTAAACTTTGAATAATGAAAATAAGGATATGATATAACACAGTCATGTTCCCTTACTTC
TCTAAAAAAAATATTTCGGTTTGCAAATTAAGCTTAAAAAATTAAGGGTTTTTATTTTAATTTAATTTATTTACGTCCGTTAA
TTTTGAATAATTCTGAAGCTTACAAAACGTTATCCATTTAGTTATTTAAGAATCCTAGTGGTTGTAACTGGAGATTGTTGGC
TCTTCTAATACTCTTCGGTAGTGTGTATATAAATTCTTGAAGGCTTCTATGAGTCCATGTAATAAATATTTAAGCGTTCTTT
AACAGCTTTATTAATAATAAAAATTGTAATTATAGTTTTTTTTTTTTTTTTAATTAAACATTAAATACGATCGAATCATAAT
ATGGCAACAAATCTTTTAGTTTTAATTGCGTATTTTTATTGACTGCAAAAGTGCTAATTTTGTTGGAATGCCGTATAGACTG
ATTTTTCTCTCCGTAGCATATCTCGAATGCATGTAACATTACATATCCTGAGATTTTATTTTTGTAACATAAGCGACCTGTT
CAAGCAATATTACACAGATCAAACCATATTTAATAATATTTTAATTGCTTTAATTAGGATGGCTAGAATTATTGTTATTACA
ATATCTTTATTTAACTCAACTGTCATTGTAAGGCGGAGGAGTGATATGTATTTTAATTTTTTATCTACATTTTAATAAAAAA
ATAATACTAACATAAGTGGAACAGAGACAAGCAAACCATTATATAAGTTGTACTGTTTTTCATTTTTTTCATTGGCTTATTG
AGGAGCTTAAAACATATTTCAATAAAAAAAAATAGATTATGTATAATAAATACTGTTATGTTGTAATAAAATTTGAGAAGA
CTAAGTCTGCAGAATAATCGCTGTAGTCTAAAAGATCAGCAAGCCGATTAACAGTGCATATAGACTGAACAGATAACTGG
TATAAGATAGGCTCTGATCGGGCCACTTCATAGAAGCAAATGCTTTTTCATACAACCCGTTCTAAATGGAGTGACCTCAAA
GACTTTTCAATTACTTGACATATCTAGCCTAAAATTGCTTTATGACAGTCTTGTTCATTGGATGATGAACAGTTGATATGAT
AATAAAAAAATATAAATTAAAATAATGCTTCTGAGTTGCTCAATAGCCAATGAAATTGTCCATACTTCATAGATAACTACA
CTCATTTATTTCACCGAAGTATCATATAACAAATAGACATAGAACCAAATAAAAATATTTTTGTAAAAGCTTTATGTTCCT
AGACAAATCTTATGTGATTATTTTATGGCTGATTTGTTTGCGTAATAATTGTTTCTAGTATTATATATATTTAATTGGTACTC
TTAGGTGTTCGTAATCATCTATTTATTTCATTTTTTGTATTATAAATTCGAAAGTACATAAAAGCATTTTTTTATTGCAAACT
ATTTATGAAAATCATCAAAACTAACAATATATTACCGGTCACCAAAAGTAACTATAAATATTCATATAAATTGAAAAATTT
ATTATTATATACTTTAAGAGATGGCTCAGTTATAGTAATGGAATGTTTTTATAATTTGCCGTTAGCGCACCACGGCAAGGC
ATGATTTTATCGTCACGATTTAATAGGAATGCATAATTATTATGCATTTTTTTCTATGGACGATAATATTATATCATGCACA
TAAAGCAACTTCATAGAATTGCATAATGCATACATTATGCTTTCTTATATCATTATGATAAATTCGTGCTTTGCTGTCTTGC
AATGGCAAATTATAATAGTGTCCATAGACATTATGCTACATTATTTTTAATAAGAAAATCGTGAATATATTTTTTTTAATAG
CTACAGATTAACGGGTAATATTAAATAGTATACCACAGAGTCCAAACCATAAGTTTTAATTTCATCCCATACTTATAGCTT
TTTAATTAAAGAGACCTGAAAACTATTAACTATTAATTTATAAGCACTTAATTTTTTTATTTTTTTTTTTATTAATGATTTAAC
GACCAAGAAATTTAATAATCAGAACGAGGTTGTAACTTTTAAATACAGTATTATTAGTGGTACACTTCATAGTGTACCACT
AATTTCCATTCGGCAAAGAGGTATCCACGATTTTCTATATGGAATTAAAAAGAACAATATCGTCCCTTCGCGCCATTTCTT
GATGTATTGTTTTATAGTTAATGCTGCGACAACGGGCTGTGCAAGCGTTATTTAGATTGATTAAAAGTCATTCAAAGCAAA
TTTACGAATGTTTTTTTATCGATCTAAAAGTCGCTTGTGTGTGTCCTTTTATATTTATATTTTTTTTAAAGGGTAACACAATG
TAACTCCCACGCGATTATTATAGTGGCAGTGTATATTTCAATTGTTTATTATATAAGGTCGCTACTAACATCTTCATTTACT
TAGTGATGTCGCCATGATGTAATTAAAAAAGGCGGACATATCAGGTTATTCAAAGGTATAAAACTGTGTACTTTGAAATAC
AAAATATTGGAAAAGCCTATAAGTAAATGGCGCTGTTAACAATTTATTGGCGTCATAATTATTTGTTTGTCTTTCAAAACA
GTTTTGTTGGCAAAAGTTGTGTGGTTGATAGGATTTTTGAATAAGTTTTAAAAAATTGAATACAGGACAATTAATTATTAA

TAATATTAATTATCCCGTATCTATGATGTTAGTAAAAAATATATTGAAAAATAACCTTACTCTATATAAATTAAATACGTG
GTCAATGTTAGTGAGGTCTGCGTATACTGACGGAGTAACGACGAATAGCTACAAATTGGTTGCAAGCAGGGTATGGAACG
TCGAAACAAATTTGGACACCGGAACTAGTATTGATATTGCATTCCGAATTCATAAATTTTCTAATAAAATAAAAAAAAGAC
AAAGAACTAAAAATGTTTATAATTAATAGGCTATTTGACAATATAAAAAAATAATAAATTATTGCCATCCGCATAGTCTAG
GGGTTTTAAAAGCGGTATGCATAAATCTACAGAAAATTATGTCATTTATTATGCTTTATGCATAATAAATGACATAATCCA
AAAAATATCGTATTATAATCTGGTATCTCGTGATCCAGTTGTCTAAAATGTATTATGACGAAACATGGAAATAAACAACAT
GTCTGTCATAGATGTTTCTTTATGTTCTTGGTAGCGGTGCGCAAATCCGAATGATTTCGTTATTCGAATAATTAGTAGTTAT
TTTTATTTATTATTCGATTAAATCATTGAAAGCATCAGTGAAATATTCTGATAATTTTATTGTACTTACACATGGCGCGTAC
AAAACTCACTAAGTTGTCGCCTCCATGCCACTCCTAACACCATCGAGAGCCGTTGTTTGTTTTATTATACCTATTTTAGCTG
TATACTAAAGTGGTTAAAAACGACTTTCCTAACGAATTGACCTATTCGCCGCTGCTCCCTCATTCTGTACTGACCCTAAACT
ATACAAACAAGATATAACTCGTAATTTATCTGATTTTATATATCTAGTAATATAACTGAGATATTACTGGATATCAAAACT
AAAATGTCGACAGTGGCGCTTTAATATCATACGTGCATGCGTAGTAAATGTAAAGATACGCTTTATTCCCCCATATGCAGT
ACCTGCACTTTCGTTTTGTAACAATATACAAATTACAATAGTTTATAGGGAATTTGTAATTAATGTATATGTATTTTATTGT
GCTAGTTATTTATTATTTGTACTTTTGTAGATCAATTCGAATTGACTGCGCTTAGGGAGATTATAATAACGTAATGTAAGTA
TCGCACCCTAGTTTAAGGTTATTTGTAGAGATCGTTAGCGACGGAGTACAGATCACGTGTGATGGTGAACATCATCAGTAC
AGCGTCATTGTATATTATCATTTGTATTGTTATAGTAGAGTAGATGGTGGAGATATGACAGAGAGCGTCTCGGTTCAATTT
TTCTTTGACGTCACATTGTCTGAATCCTACAAAACTGGTTGATAATAGTTACATTCTGTGTTCGTTGTTAGCTTTTAGGGTTC
CGTAAGAAAATATTATTGTACTAACGATAATCGTATTTGTTAGTCTAAGTATGAAGAATGAAGTGGCAAATTGATATCCGC
ATTAATAAAATTATGCAAATAATACCACTGTTCAAAGTCAGAAAAGGAAAATAGCTTAATTTCAAAAGTATTTTGAGCAG
TTAAGGCTATGTTTTGTTTTTACCTTAAATTTAACTTAAAATAATTTTTGTTGTATCAAAGTAGTAATTTTAGATAAATTTTT
TTCAGGTCCAAAACAATAAGGCAAAAATATACTCTTAATAGGAATACGCAGTATTTTAACGGAAATAATAAAATAAGGGA
AAATGTAACTAAAGCAAAGTCAAAAATTAATTGTAAGAGAAGTTTTTTTAATTTAATTTATGTTGGTTCGGAACCCTAAAA
ATACGTTTTTTATATAAATCCGAGCATATATGTATTTAAATTTTTTAAATACATATATGCTCGGATATTGATTTCCAGGATT
TTGACATTTATTTCATACATACTTTTACTTACAGGTACCTTTCAGATTTTGGTAGGATTTCAGGATTTTTGGGTACTATTTAT
TTATTATTATTTTGTTGACATTCGACTGACTTATTACATTTTACTTATTTCTATTTCCTTATTTCTAGTTGATTACATTAACAT
TTATATTGCCTGAGTATACTCAGTATACTTATTTAACATAATTTCTATTTACTATTTTTAATATTTTTATACTGTATTCTAGA
AATTTCTTACGCTTCTTTTGGCTAATATATAGGAATTATGGCTAATAGCCATTATTTCTATATATTCTTCTTCGTCTGGTTTC
ATTTCAATTTCAATATCCAATTTCCTTCGCATTTCCCCGAAACGAGGACATTTTGATATTATATGTTTAATCGTCTCTGGTTG
TGTATGATCACAAGGGCACGACGGGTCCTCCCGACACCGAAAACGGTGTAAATACGCAGCGAACCCACCGTGTCCCGTGA
GTAATTGTGTTATGTATCTGTTTGCTTGAGTTTTACCGACAATCCTGTACGCCCTCATTACATCTGGTAAAAACATTTTGGT
TCCAGAAGCCGTCCCTCCCGACCGATACCTCTCGTTCCATTCCCCAATCGTTCGATCTCTAATCAAACGCTTGACAAATGA
GACGGGACACCGATCGTAGTCGGACCTTCTCCTCAACTTTAAAGCCGCCTTTTTTGCCAATAGATCCGCTCTCTCATTTTCT
TCCCACTCAGCATGTACTTTAATCCAATACAATTTTATTATTTTATTTTTTTCTTCCGTTACAATTTTATGCTTTTTTCGGAAT
TCTACTGCTAATGGGTGAGAAGTTCCAGGATTTATAACTATCTGTATTGCGGCCATAGAGTCGAAGAGAATACCGAACGTT
GCCGCGCGATTTTCTAAAGCTACCCCTATCGCTCGATTTAGAGCCAAGAGCTCCGCCTGATAAATAGTGCAGTGTGGTTCG
AGAGCAAGTTTCAAGGACCTTGATTTCCACCTCCCTGTTCTACACTGATAAGGCTGCGCCAACCCGGCCCTCAATCTTGCT
CCCGTCAGTGAAGATTCTCACTTCATTTTGTTCCCTCACCGCACTATATTTGTCTTCGTCCATCAGATATTGGAACTCCAAG
CTCCTTTGTTTCGCGAGATGAGGTCTCTCAACTGCTGGGGTCATCCGCTCGATCTCCCTGGCCCTCACTAGGTCGCCGGCAC
ACCCTCTCCTCGTCTCATATAACGAAGCAACTTCGTGTATGCGAAGATCAAGAGGGAGGAATCTTGACAACACAAGCGTG

		GAGTCCAGGGAGACCGTTCGATACGCCTGGCATATTTTTGTGCAAATCCCCTTTGGACAACGTTCAGCTGCTTCCGAAAGCTTAGCTTAAACACTAAAGGTGCCCAAGCACTTGCCGCGTACAGAATTATCGGTTTGACCGCCGCTGTGTAGATTGTTCTTATACACTCTGGGTGCACATAAAAAAATTAGGTTACCACAAATTTTATATTTTATATAACAGATATAAACAATAAATTGGATAATAAAAATACCGAGACGCTACGTTTATTCTCCTTTGTATTAAGGTTTGAGATCTTGTAAGCAACAGCACGTAGGTGTTGTATGTGTTTTTACAGATTTTGTGAGTAATTGGGCATAAAATACAGTTTAACACTATGACTAAACCTCAATTTGATGGAATATGACAGTATACAATGTGGTAAAATATCTTATAGGTACATATATAGTAATGCAAGGGTAAATTGCCTGATTTCTTACCATCAAACGTTAATGTCATATCGTAATAGGACCATATTCTTATTATCGCAACTCTGGCATATGTACCATTCCGGATAAGGGTAAATGGTTTGGACTGTGTAAAATGTAGGCCCATAGAGATGGTTATAAATATTAGTGTCGGTGTTTTAGCAGTTCACGAAATGTGGTAAATGTAACAAAAATGTGTAATTGCCGAGTAAAATCGTGTTGTTTATTTAGCGAAGTCCGCGATTATTGGAATGATGCGTATACATACATTTGTATCAATGAATCATTTGTGCATATTGCGGAACGCGAAACAGACAACACAAATTGCTCGTTTTAATTGAAATAAGTTAGAAAACTGCTACCTCTTGAATGCCTGAAATTGTTCACAGTTTAATGTCCAATGCGTGTAACATGATGATGTTAAAAATGAAATAAACATGAAGAAATTTTGACTCTTATGCTCGACTTTTACTTTCACGTGTCTCTGTGTGTGTGTTAGCATATTTTGAATAATTCATTCCGTGTGTTCATACGTTATACGATCTTGGCGAGTCATTCAGTAGCTCGGTAAAAGTTTGGAAGTGGTCCCACTACATTATCAGTTCATGTATTACAATATAGATGTAAAATTATCTACGTATAATCCTATTAGTAATATTAATTGAAACTGTGACAGGATGATCATTAAAGAGAGATAAAGAATAAGGA
MSTRG.14048	Hypothetical protein WN55_03031	AAATAACACAACAATACGAGGTTTGTCCGGAAAGTACGTATAAAAGTTTTTTAAAAATTTTATTTTACAATTATTCAGGTAAATCAATTTTATCCCCTTCAAAGTACTCCCCCTGTGACATAATGCACTTGTGCCAACGCCGTTTCCACTGTGAGAAGGCTCCTTGAAAGTCCTCTGGTTGTAGGTTTCTCAGCTGCCCCGTCGTAGCTTTTTTATCTCATTTATGTCCCCCAAATGCCGCCCCGAAGTACCAATTTGGTTTTTGGGAACAAGAAGAAGTCAC
MSTRG.14051	Uncharacterized protein LOC110382591	AATATACAGTGGGTTGCCAAATTAATAGGGACAGTCACATTTTTTATAATTTTTGTTCCTCAAGTGCCCCTGATTGCATTTTTAAGTGGTATAAGTGTGTTTGAAATATGTTTTTATGTTACCAGTATCTCTGTAACTCAAATTTAAAGTGAATTTCAAGTGCTGAGAGATTTATTTATTTTGTAAATATATTTTAATAACGAGAGAGACACACAGTCGTCCACACGTGTAAAAGTGAAACACGTAAATAATTCTTTTGAACAGTTCATTTGTGGTTTTGTGAAATAATTATTACCTGCAACTTTGGCTATGGGTCGAGGGGTAGATTTGTCTCCGAGAAAAAAATCTGAGATTAAAACCTTTCTCCTAACCACAAGTCATTCTCAGAGAAAGATAGCAGAACTGGCCGGCGTATCTAAGTCTGCTGTTAATAAAATAAAGGTCAATTTAGACCAGAATCGGCCACTTTCGCCCAAAAGAAAAGGCAAATGTGGCAGAAAA
MSTRG.14072	Endonuclease-reverse transcriptase	GTGTAAATGCGATTTTATATTTATTATTCTTTTACTATCCAATTTTTAAACACATTTATGGCCTAAAATAATAAATTACGATTTATAGTATTAATAATAATAACCATTTGGCGCTTATTTCATATATTAAGTGTTGTAAAGGTCGCCACTCCATCTAGTGCGGGGTCGTCCTACATTGCGTTTACCTGTACGTGGGCTCCATTCAAGAACCTTTCATCCCCACCGATTGTCGTTTCTTCGAGCTATGTGACCAGCCCATTGCCACTTCAGCTTGCTTATCCTTCG
MSTRG.14075	Uncharacterized protein LOC106113188	AGTTTCGACGGCTACTACGAGAGTACAAGAAGAGCTCAATGCGACGTGCAGTTCACGCTCGGCCAAGGGTTGATGGAGTGCATGAACGATGCAGTCTCTAAGTTATTTAGCAGCGAGCATGCCCGCCGGGTGTTGACTGTACCCAGCAAGATGTCACTCCTGGTCTACATGAGTCAGTGAAATCTCAAGATGGTGATACCTACTGAACCTACGGAACCTGCCTGCCGATAGATAATATGATGCGATGCTACTCCAGTACAAGAACACATTTGACATGGATAAATTCACTTTGCGCGCCAATTCTTTATCAAGAGACTGGTATGTTTCTTTTAAATGTCAGTTAATAGATTCATATTATGTGTTGTAATATAAATAGTAAGAAATTTATATTATACGTACTATATACATACTACATGTACAAATGATTATGAAATATTAACAGGAGCATGATGTTACATGTTTTGTTGTCCACTCAGCACTACTTCACAATAAATATGCATTTACTATTTACAAAAAACTTATACCAAATCATTTAATAAGGGATGTGTGAAAAAAATTCTCTAAAAACGTTCAGCAATGTGTTAACTGATAAGTTGTTTTTCTTAACTTATTAAATAATAATAAATATAAATAAAAGAAATATTACAAGATTTGATATAAGAATAGACGATAGAATTTTTAAAAATCATATAAGAAAACGGCGTGAATTATGGTCCCAGTCTTCAAC

		GTCTACATCAGAATACAGATATAGGCAACTTATTAAGCAAATCATCAAGTGTATATGGCGTGACATACGCCAGTTTAATAT<br>TGTATACAAAGTGTTCGCCAGAGATTTGACTATTGGGCAAAGCCGACTTACAAAGTTGAAGAACGCTACCGATGATATAA<br>ATTTCGCTACATCTGAGATTTTGAGTGAGATTGTATGGACAGCTGTACACAACGTCGTAAACACCGCATACCAATGTAAAT<br>AACGACCTAAGAGCTAAAGTCACCCGACACTACCGATAATATCTCAGACGTCAGTTTGCAGGAGATTTAAATAGCTCTCTA<br>ACAGTTGCTTAAAAACGGCAAGGCAGCGTTTAAGGGTGTCACTTTATCTTTTGGAGGCGTCTTCTTACACTGTGTTTGCCCG<br>TACGGGCTTGTCACCGAAGAGCCTTTCTTCCCCACCAGTTGTTGCTTCTTCAAGCTATGTGACCAACCCATTTTCACTTCAC<br>CTTATTTATCCTTCTGGCTATGTTAGAGACTGGTTATTTTACGGATATCCTAAGTTTGGATTCTGTTACGTAAAAAAACTCT<br>GAAGTACCCCTCTCCATAGCTTGGCGAGTAATTTGAAATGTCGGTCTCACCGGTGTCGGTCTTCACGTTTGAAAGTTGGCTT<br>CACCCAATAGCCAAATCTCTGGCGAACACTTTAGAGCCTTAATTTCACTTTATTGCGAGTTCAATGCATGAATAGTATAAA<br>ATGTCCTCTAACACTGTTCCCACCTCATCGTCGAAATCTGTTACGGCCGGCGCATATACCTGTGTAACTTTCAACACTCTTT<br>GGAATCAACTTCCAGCAGCAGTATTTCTGAATCCATACGACATCATAACCTTTAGAAAAAAGAATATATTCCTTTTTAAAA<br>AGCCGGCAGCACACCTGCTAAGCCTCTGGTGGTGGTGATCATGTGCGGCGGTGGTCACTTACCATCTGATGAGCCATATGG<br>CTGTTTGCCTTCTGTGTTATAAAAAAAGATAATCCTATAATATTCCAGTGTAACTTATTTACCTCTTCTTCCAGCTTAACAA<br>TCTCCTCATCCGCCCTCAAAGTTTCTGTATTATATGTTGCCAGGTCTAGTCATTTTAGCTGGTAGCCAAATTACAAATGAAG<br>ATTCTTAGCATCTCCTGCCCCACCATTACCATGACAGCTGACGTGTTTACTCTTCATCAGCCCTTAAAGTTTGTGCATTATA<br>TGTTGCCAGGTCTAGTCATTTTGGTTGGTTGCCAAATAACTGCCGAAGATTCTTAGCATATCCTACCCCACCGTTACCATGA<br>CAGCTGGCGTGTTTTCTTTTTATCAGCCCTCAAAGTTTGTACATTATATATTGCCAGGTCTAGTCATTTTGGCTGGTAGCCA<br>AATTACTGCCGAAGATTCTTAACATCTCCTGCCCCACCGTTACCATGACGGTTGACGTGTTTACTTCTCATCAGCCCTCATA<br>GTGTGTACATTATATGTTGCCATGTCTTGTCATTTTGGTTGATAGCCAAATTACTGCCGAAGATTCTTAGCATCTCCTGCCC<br>CACTGTTACCATGACGGCTGAAGTGATTACTTCTCATCAGCTCTCAAAGTTTGTGCATTATATGTTGCCAGGTCTAGTCATT<br>TTGGCTGGTAGCCAAATTAATGCCGAAGGTTCTTAGCATCTCCTGCCCCACCGTTACTATGACGGCTGAAGTCTTTACTTCT<br>CACCAGCCCTCAAAGTTTGTGAATTATATGTTGCCGCTTAAAATTTTATAAAGGTCACATATGAAATACTATTTTCACCTAT<br>AGGCAGAAACGCCACAGTACTAGCTTCTTCCGTTAGATCGCATTCAAAGTCGGGTGGCTCGAATTATTGATGGTCAGCACC<br>TTACTGTCCGACTCGACACACTGAAACTGTGTAGAGATATTGGAATTTTGCGTAATTGGCACTTTCTTCCACGTACAAACG<br>CTCTTTGCAATTAACTTCCAGCAGCAGTATTTTCGAACCCATATGACATCATGACCTTCAAGAAAAGAGCGTATTCCTTTTT<br>AAAAGGTCGGCAGCACACCTGCTAAAGCTCTGGTGGTGCGGGTGATCTTAGGCGGTGGTGGTCACTTATCATCAGGTAAG<br>CCACATGCTCGTTTATCGTCTGTGTTATAGAAAAAAAAAGATAGTTCTATAATATCCCAGTGTAACTTCTTTACCTCTTCTAG<br>CTTCACAGTCTTCTTATGGGCCCTCAAAGTTTGGCCGTTTTACCTGGTGGCCAACTTTTGCACTATATCTAGACCTGTTAAT<br>ATAGCTCGAGTTCTTTGAATATTGTTTACATAACACTCGAACAGTACGTTATTGTTAGTACTAGATTGTATCAAGAAGTCTA<br>TACCTTGAAAATAATACAAGACAAGTTAGGCATAAATCTATTTGCTCTCTTCTTCGGAAGTGAATTACTTCTTAGTACCAG<br>CAATAACCTTTCCTGTCATCAATGATACTAGTAATCAGTAAGGCAGTCTACCATACACTTCCAGGTGGAGGTCGATATCCT<br>TTTTTTATATAGGAACATGCATCATGCAGCTTCATACGTCTCTTTCGGGGCGTGTGGTACTGACTGATGATGACTTGATCCT<br>TCTTTATTATAAATCGATACACTTTTTATTGGAATCTCCCTATTGCATTATCTATTTTTTCATTTGCAAATGTTCACTTTAAA<br>AATACTACATCATTATTACTACTGGGTGGTACCAAAAATCTATAGCCTCAAAATGTAAGACCCCATTTGCGGATCAATTAA<br>TATACAAACAGATTTACTATACGGGGTTATAAGATGAGAGCCAATTTTCGTTTGAAAATGTGGAAAGTGGCTTAGTGTGAT<br>TTTGAAATATTCTTACTATGTATTACAAATGGACTCCTTTCAGGCAATCCTTCTACTTCCACATTATATTATTTCCAATCGAG<br>ATTTTGTGAGATG
MSTRG.1<br>4089	Ankycorbin	GGAAATAGTGGCTGACACCAATATACATTTACCGTAAATAGCTACGTCAACGGATCTTCCAAAGTACAAGTTTGTAACCAC<br>TACCGCGAATATCTAATATCACTTTTACGACTAATTCATATTAATAAAATACAAAGTTTCTATAGCAGCGTCGTTAATTTTA

		TAAGTGTGTGTCACATCATTCAACACAACGACAAGTTTGCGTGTTGCAAACTAAAATAGTGTTATTCACGTGAATTACATA<br>GGAGAATTTGTGTTTATGAAATCTCTGTAAAGTAGTTGCCTATAATAGGCCACAATTAAAGCTCTAGCACAATAAAAATGT<br>AAATAAAAACCAAAGACTAACATGTAATCGTGTTACATTTTTGAGCTAATTATATAAAATACGATGACTGTTTATGGTCAC<br>CATTACCGAATACAAGTTCTGCAATGAATTAGAAAAAAATCCCTGATCGACAGTTGAGTGATAGTCAATTCTTAAGAATCT<br>GTCTCGGTAGCCTAATTTGTAAGTTAATAAATATCAACTCAAAACCAAATTTCCTAATTAATTTTAATAAAATTACCTATCT<br>TCTATTAAACCAAACCATCTGATCACGAAACTGTGAAGATTTTAATAAAGCAAACATGAAACCGGGTAAACAATCAAAAG<br>ACACAAGAGCGAGTCAGTTTGAAAGATTCGTTTCCTAAATTGTGTTGATCAAGTCGAATAAACTGCGATCTCATTGATTAG<br>ATAAAACAGACAGTCTTTTTATCTACGTTATGTAGATCATCGTACATACACTTACCAGTGGCCCAATGTACTACAGTGTGA<br>TGTTCATCGTCCCTGGCATTGACATCAGCTCCGGCCGCCAATAGCGTCTGGACCAGAGGCACGTTTCCGTGAACCACAGCA<br>AGATGCAGCGGTGTAAGGCCGTCAGCGTCTCGGGCGTCGGCCAGTGCCGGCGCGGCCATAAGCACGCGGTCCGCGCACGC<br>AGCACGGGCAGCTTGATTGGCGCCCGCGCCGCTTGAAGCGCAGTAGTGTAACGCGCAACGTAGGCCTCTATCACGGATGC<br>GCACTGAAGCTGGCTGCAAGTTGTGTAATACACGATTATAAATTAATAACTTAATGTTATGATTATACGGTATTTTGGTGT<br>AATGCACCAAAAGGTGCAATAAGAATCTGCAACACTATCATAGCCCCACAATTACAATAATACGATTTAAAGTTTTAATAT<br>GAAAAGCTATGTTATGAAACATTGTTTTTTCTATGCATATATGATAGTTATTTAGCAGGTTAAATAATATCAGAATCGATA<br>AGTACTCGTAATTAATAGTAATAAGATCTAATATAGAATTTAGAAATGTTTCAAAAACCTCTCATTTACATACCTCTTATTT<br>CCTGTTTACATTATTCATCAGTATTATTCTTTTCTTTTTTGTTTATAATGTATAAGTAAAAAATACATAATTAAGAAACACTC<br>AAACATTTACAGGGCTTACAATTTAACGAAGACCGTAACTTATATAGTAAAACATACAGATTTCAGAATTATAAAAGATTT<br>CCTTCAATGTCTTGATGGAATAAAAAAATATTAAATATAACTAGATCATTGAAACATTAACTGCAATTGGGCCAACAATCA<br>CTAACTTTCTTTTTATTTCATAGAAGTCTGACAGCAACTTTTAGAATTCACGAACAATGTTACCTACTTACTTGAGGACTTA<br>GAATTAATAACGATTGATGTCGATTTATGACATGTATGAATAATGATCATGCCGTAAACAAGATTTGATACTTTGACCCTT<br>ACTAAAATGTACCTTAATAATTAATTGACATGGCAAGGATTGATGCAGTCAACACGTGCGACATGACTTCGAGCGATATTA<br>ACTTCACACGAACTAGCGTGAATAGCTCCATAATAGCTTATAGTGTAGCAGTCGATTTCTACACCTATTATATTGATTGTGC<br>CCCGCACCCCCGTCGACAGTGCCCCGTGCCCAGGTGCATTTCCTTGAAAGCTTTCAATTTGATAGTGTTTATATCCAATATA<br>TTTTACTGACATCTTCGATGGAGAAGCTGATGATATGAGATGCCATTGCAAGCGGCACCAGCCCTCATATGTGTACTTGGA<br>AGGTAAGATGGCGCCAGCGTATCAACGCAGGTGAAGGGCCACACAAGCAGGTCGCTTCATCCTCCAAGATAGCATGGACA<br>TCCCCTCAGGTTTCTTGCCGTCACCACGCGTCAAACCAGGAGGTTCTAGGATAGCAGGCACACCTGCAGTGACAAGGGCC<br>CGACGGAGAATATCATTGATAGCTGAGCGCAGCAAGCGCTCAGCACTTTTAAACCAAAAGATGTCATGGTGTCATAAAGC<br>CTTAACGTTGTCCCCATACCTACAACGGTGGAAAAGATTCGTTATGACTCACAATCTGAGAGAGATTAAGAGTCTAAACGT<br>AGCGTCATCCACTAAAGTTCCGATATTAGGTGAGAAAATTGTGTGTAACCATAAGCCATATTTCCATTTGGACAACGCGAG<br>TAGGCGGGTGCTCTCAGCGATATTCTGGGATTTCCAGGCCTCCATTCTGGATAGGCAAAGAAACTTGAGTCCATATACGGT<br>CGTCTTAATACCACACATTAAAGTTTGAATTTAAAAGCCGAATTATTATTATTTATTACTCCTAGGCTAAAAATGGCTAGTC<br>CCAGAGGGTCGTCCTTTTGACAATCAACATCCGAGAGTAAAGTATCATTCAAATACATAAGTTTAATAAATTTGCTATAAC<br>ATTGAAAAAGGAAGTTTGAAACGTGGGTTTTTTTTTGTTTTATTTGAGTTAACAGGGCGTCCC
MSTRG.14140	Autophagy-related protein 2 homolog A	CAGACCACTATACCTACCAATACGGACGGTTCTCACCCTCGGAAACTTCGCCGTATCCAGCAATCTGATCGCCGAGACGAA<br>CACGTCGTGCTTGCGGTTCCTCGCCCAGGAGTGCACCTTGTTTCTAATGCATGTCCACGGGACGAAGCCCCATGTGGCGGT<br>GCCGCAAGACGATGACAAACTGCCCGACGTGACGAAGGATTACGTTTGTGTCGTCGACGTCGGATTGTTTGAATTGTCCGT<br>CAGGAGGGAAGATAAAAATAATGCTCCCAGTGAGCAGCCGCAAGTGGACCTGAGCGCATCGAACAACATGGTGAGCCTG<br>TATACGTGCTGGGACTCCGCTTCGGCGCTGTGCCGCCTGCTGACGTACGCGGCCGCCGACGGTGACTCGCAGCCGCCCTTC<br>GATCCCTCCTCGCGGCACACCAGCATCTGCTCAGACCAACCGTTGGAACAGTTAGTTGGTTTAGACGATCGTCCTATAGAA

		GAAATAAGAGAACTATCACCCAATGAAATTCAGCAAGTCAATGATTTAATGGCCGAAGCAATGAAGGAAAGCCCAAATA<br>CGACACTCGAGGAAGAAGACCTCAATAGTTCTACAGAAAAAGAAGGTGTCGAGATATTCTTTTTCCCCGACGAATCGAAT<br>ATGCGGCAGAGGCAAACGGATCTTATAGATGTAGATACCGAATCTAAGTCATTAGAGTACGAGGACTTCCATAGTGGAAC<br>ACAAGAAAACCAAGACTTGAAGCCAACAAACGTGCAAGTGGCGCAGGAACTTGGCGATCCTACCTCCACACCTANNNNN<br>NNN
MSTRG.1<br>4141	Autophagy-<br>related protein 2<br>homolog A	CTTTCCCCCCGTATTGCCAACATTACGTAAACTTAGATGGACTCGGGCGGCGATGGCAGCAACACAGACGACGAGTACTG<br>CATAGTGGAAGCGCAGGCGACCGAGGATGACGACCTCGCGGAGCCCGAGGTGCGCTGGTTCGGAAGCGGGCCCCCGCCC<br>ATGATCGACAACCACTTCTCAGTGCCCGCCGCGAGAACTGATGTGCTCCAAGCGCCCAAGAGCTTCCCTACACCGGTTTAT<br>AGGTACAGTTTATGCGAAATGAGCATAACATGGCATATGTATGGCGGAAACGACTTTAGATCGGCCAACGAACCCTCATC<br>TAGCAAGAAGACCGTTACTATAGATGTGGATCCTCGCAAACAATCGCCTTTAGCAGTCAAACGCAACAAAGAGTACGAGC<br>CATACGAGAGTCCACGGTCGGTGAACATTAGTACAGCTGGTGTGAGCTGGACCGCGGGCTCCGAGCGTGTTCGCACGAAT<br>GTTCGCGCGGCACGACCGAACAGAGCCGAGCTCAGGACGCGGGGTGGGCCCGGCAGGGATCATTGTACTTGTGTTAAATT<br>GTGTCTCACAAAGGTGAAATTCCAATACGACATATACCCAACGGGAGGCGTCCACGCATCTCGGCAAACATTGGCCATCA<br>GCAAAATAGAAATCCTGGACCGGCTCGAGTGTAGCAACATTAACAAATTACTCAGTCAATACAAACTGAAAGACGAACCT<br>GAAAGGAAGAATGCTCATATGTTAATAGTAAAAGCGGTGCACCTACGGCAGGAGCCGGCTCTGTCTGCTCAAGAGTGCTG<br>CCTGAAGGTGTCACTGTTACCGCTCAGATTTAATCTTGACCAAGACACACTCGCTTTTTTGGTTGGATACTTCTCCAAACTA<br>GGCACCGACGAAGNNNNN
MSTRG.1<br>4285	Integrator<br>complex subunit 1	CTCGCTGGACACGAGTACAGCATTCAACTGTAATGCGTACAGACTTGAACTGATCTGCTTTCATCGTCACCTACTGGTCCC<br>TTACTGGTGCCCCCCGGCGTATCCCGATAGCGCAAGCGGGGTAACCATTCCAGTAGCACCCATTAAAAAGACACAGAGA<br>CTACGCATACGCCTTTTTAAAAGACTACGCCCCTTTTTTAAAATGATCTTAAATACGCCAGTAGCAATATTAATTGAGTTGC<br>CTGTACTGATACTTCTAGTCATAGTCTATTTATTTATTTTATTTATTATTATTTGACAAAATTACAAATACAAAATACAATG<br>GGCCCCACACTAGGCGAGCCTGTATCGTGGGCACCCGATAAAGATTAACAAATCACAAATGTGTTGTAATAAAAAATACA<br>TAGTGGGCGGGTATTAATATAAAAAGGAATATAAAATTGAAATAGTGAAAAAAAAGTAATAAAATCAACAATATATACA<br>CAAGCAATTATAAATATTATGTCCTTAAGAGAAACAAAGACAGAATAGACAAAATAGGTCACTTTGGAAGCGATGATCTA<br>AATCTATCAAGTTTATAATTAGTTTGACATTGTACTGTACAAATTCACTCTAATTTCATATCACACTGCGTCCGACAATGCA<br>GGGTCGTCATGTGTCGACGGGATTTAGGGCTACGCCTAAAAGTAACGAAAATTCAGTAATATAATGACTTTTATTATATTA<br>ATAAAATGCATTTACATTATACTTTTTTATTACATCATGAATAATGCTCATAGTTGTACATTCATACTGCCATAATTATAAT<br>CTTACCAACCTAAACAAAACCGGCAGGTCTGGACGTGATACTGGAATGCCTGGACTCATCACGGCCGGAGATCACACAGT<br>CGGTGCTGGACAAGCTGCCGGACCTAATTATCGGGATGCAGGAGCACGCCACCCTGATCCTGATGCGTGTGTTCGAGCTG<br>GGCATGAAGTCGCGCCACCCCGTCGAGCAGTGCATCGCCAAGTGCGTCACCACCATCAACCTGCACCGCGGCTGCTGAGC<br>ACCACCGTGGCTAGATGCCACTAGTCTTAACCCTTCTTTGCATAATGGTGGAAATTTCCATCATAACATTGAGTGCCCAAA<br>AATTATATAAAAGAATAAGTTCACTTTAAGTTGATCGTGTCTGGATACCTTTTTAATGTCCATTGCAGAAAAAAAAACATA<br>GCTGTCAAATTTATTTTTTTAACCATTTTGTTTGAATTTTGAACATGAATTATATCATCTTGACTTATTTCACCAATAAAAA<br>AATTATGCTGAGAAGGGTTAACGATTCATTAATGTACCACTGTTGAATATAAGTAAAAAGTACATGAAAAATACAAAACC<br>AACTATGCTAGCCGGATATAAGGTTTTATTGCAAGATTTCCTTCTCTTGCACTGATGTGGCGTGGCTTGCACACACTGGCGT<br>GAATTTACTGGGTACAGAGGAGTAACACCTGAGTCCTCAGGAATGGCAACGCATGGGGGGTATCATGGGTGAAAC
MSTRG.1<br>429	Uncharacterized<br>protein<br>LOC105118166	TACAAAAGGCGGCCTTATCGCTAAGCAATCTCTTCCAGACAACCTTTGGGTAGAGGACATTGAGAAAAGACAAAGAGTA<br>GGTGGCGCACTATTATATAAATATTATCATATACAAACATATATAGATACATACATACATATATACACATACATATATAAT<br>ATATACACAATTAAATATATACATAACATACATACATACATACATACACATACATATATAATATATACACAATTAAATATA

MSTRG.143	RNA-directed DNA polymerase from mobile element jockey-like	TACATAACATACACATACTTAACAGCATACATACAACAGAAAAAAATGATGAAGGAAAACGAAGAGAGAACACAGGAAA<br>ATAGTACTAAAGTGAGAGGTAGTAAGATTTAACAGAGGTTTTGAAGCTAGTAAGAGTTTGAGCGCACCGAATAGAGGTAG<br>GTAGTGAGTTCCATAGACGAACTGCCTGAACAGTAAAGGATCTAGAATAAAAGGAAGAAGAATGAGTGGGAAATTTAAG<br>TCGGAGGTTATTGACACTGCGTAAAGTATGATCATTAGCCTCACTGAGAAACAAGAATCGGTCTTTTAGATAATCAGGAGC<br>AGCAGGATTAAAAAGGATTGTGTAAAGAAGAGACAGGATATGCAGATTACGACGAAAACGGATAGAGAGCCACTTGAGT<br>TGAGTACGAAATTGAGAAATGTGATCATATTTGCGAAGACCAAATATAAAGCGGATACATAAATTTTGAAGACGCTCAAG<br>CTTGTTTAATTGCTCTTCGGTGAGGTCTAAATAACATGAGACGGCATCAT<br>ATTTTAGAATATCTCCACACAGATGCTTCGAAAAAAGCGGGTCAACTTTTTCATACGATTGTGTACACGACAGTGTTACCA<br>AATTAGAAATTATTATTTTAGATTAAAATAAATAAGTAAAGGTCTTAACTTAAAATATTATTAGGAATAAATAATACTTTA<br>TAACATTTAACTTTATTTATTTATTTATAATAATGCGTAGACAAACTGATTCAGCTCATGGCGGTAAGATTCTAATACGTGT<br>ATAACATTCAATGCTGTCTGTTTGTATTTCATTATATTTCACTATAAAATATTATTTCTTTATATTATGACATGACATCATAG<br>ATTAAATAAATAGATAGATAATTTATTTGTATTATTTACAACGCACAAATAACATTAGATATTGGTCCATACTTAAAAAAG<br>AAATAAAACAAAACATATACAAAAAAAATTACACGATTATAAGGAAAAATAAATAGAGAAAGAAGTATGAAATTTTCGG<br>AAAAGAATTTTTCATTGCATTGGTTTTCAGTTCAGAACCATTATTCTAAATTCAGACCATGTTTGAATTCAGAATAGTTCAG<br>TTAGAATCAGTTTCAGTCTTTGCCAAGCTGACTGAATGAATGATATGAATTCGTCTACATATGCACACTGTATTACATATAC<br>AGTGTGTATATGTAGTCGAATTCTCTTTTTGAAATTACTTGAGAATGGTGGCTGTTTTGTTAATAATTCAAGTAATTGTCTC<br>GTAATAGTGTACTTTAATTTGAATAACATTAAATAGTTTTGTTTGTTTGTATTAAGAACTCTGTATTAATATATAATTAATA<br>TGTATTAATATGTAATTTAAGACTTTACTGTACAATTAAGTGTCGCAAACTTACATAAAATAGTTTGCATATTACTCAATAA<br>TATCTCTGTGTACATACATACGTATATATTATATTATGTAAGAAAATAGCACAAAATACAAATAATAATAAAAATAAATTT<br>ATTGTTATTAAAAAAAATCCACCTCATTACGAAAATTTTAATTTAATTGTAGGGATTTTTTCGGATACTTGCAACACTAAAA<br>AATGGCGCGAATTTAAAAAAATAAAGTTATAATTGTATTCATGATTAAAATGGAAGATACCAGTTATAAGTGATGTCTTTA<br>TTAGTTCTTTTTTTTTTTTCGTTTTATAATTATTACTCCATTTTATTGCGTAATAACTAGTTTTAATTAAATATAGACACATCA<br>AAATACGTTGTAAGTATAACAATAGTAATAAAAACGTTTTGATAATTAGCTGACAAAAAGTTGACCCGTTTTTAAAGCTCG<br>AGGCCCTATCTATATAATCTAAGTTTAATACTTAATATAACAGTAAAACTACGATTACAATCATAGAATTTACAATAATAG<br>TAACATTCTATAAAATAATACGCGAATTTTACTGACAAATCTACTAGTGATTTGCATTTGATATTCTGTAAATAAAAATGG<br>AACACAATGCTGTTTCTTAAAAGTAACATCGTATTGAATTGGTATAAATGTACTAACCATCCTTAGAAACTCACTTTCGTTT<br>TCAAAACTCCTTTCACTTGACAATCGAAACTTGTAGAATATAGAAAATACATAACGTGAATATTTAAGTTAAATGATACTG<br>AAAACGAAATTTACTGTGACTTCAATACAAATTTCTACAGAATAATATTGTAGATTAATTGATTTATATAATTTTGGATTAC<br>AAACGGTAGTTTCCATCTTGATTTAGTCATCGAAAATTCATCTAATAGCAGTGTGACCATTTGTGACAGATTTCTGCCATTT<br>TTATTTTTAAAAAAGATGTGTTAAAATTTTTACTCGGTTAGTTTCATGGGCTTCTAGAATGACGTACACACTGCTTTGAATT<br>GTTTTTTATTACAAAATTCTAATAACAGCGGTAGCCATGTTAAAAAATGTCCCTCTGTGAACCTAATCATTGAGATAGCGT<br>TTTGGCGCGCGAGAGACGCGGCGCGGTCTCTGATTGGCCTACGCGACGCATGCGCCTAGAGTTACTTTTTCGGGCACGAAT<br>CTGCCACAGACAAAATTTAGGAGCGAAATTTGGCAAAATGGCAGCAATCTGTCACAAATAGTCACACTGTCTAATAGTAA<br>ATCTTTAATGTAATAATAAATCAAGTACGATTTTGTTTTGCGTTAAAATTTTATGTAATAATAAATGTTTAATGTAATAGTA<br>TATAGGAAATTACATTCATTAATAAAATAAATACTTTTTTTTTTATTTTGGACTACGAATAAATGTAATTTTGATCTCAATT<br>GTACTCTATTGATGTTTTATGTAACAGTAAATAGGATATAACGAATTCATTCTAAAATAAAATCAACTACTAACATATTAA<br>TTGATTGGTTTTATTTTGGAATTGCAAATGATTGTATTTCTGATATCGATTCAATCATCGATGTGTAACTAAGTGTTAACAG<br>GTAATAACAATAATTAATTGTGCTAAGAATAGATTAAAAAACACGTTTTCGATGCGATGTCGCATTGTGAACCTGGCTGTA<br>ATACACGAAACCATCACATGTTTTTTTTTTTGTTTACTTTCAATGCTCGTAATATTTTTTATGTTACAATATTTTGTAAAAGG

ACAGGTCCGTTTGACTATAGAACTGACATATTTATACTCACTTACGTACTCAAAGACATAACCTATATCTATCACCAGCGG
ATACAAATACAGGATCCAAAAAAAGTAGCAATATAGTACAGTGCATGAGATTTCAACAGTACGGTCATAGTAAAAACAAT
TACATGAGACCCTTTCGATGCGTTAAATGTGCCGGACCTCTCAAAACTACATATTGGAATATCGGGAAAAATGTTCCAGCT
CTACGTGCGCTTTGTTCAGGGCAACATCCAGTTAATTACAAAGGATGTCAAGTTTACAAAGAAATAAGAGCAAGAAAGAT
GAAAAAAAACACTCAAGAGATCGCAAAATCCTAATAAACTAATTGACATAACAAATAATAAATCAGAATACCCACCATTA
CGTAAAATAAACAAGAAACATGATCCAAAAGAAGAAGTTTATATGAAAGAGACCCAGAATAAAATAATACCTGCATACA
GCGAGATAGTAAATAAAATAGAAACTAATTTTAAAGTGAACACACGAAAAGATCAAAAAACAAGAACAGATGACTCTAT
CTAAGTCACAGAGAAACAACCGTACCGACTGTGAAGGTAACGCAAGTAAAATAAATTCACATGAAACTACCACCTTACAT
AGAATCGAAGAGCTATTCTTGAAAGAGTCAGAAAAAGTCGACCGTATCTTAGAAATCATGACTTCACTGATGAATCTTGTC
ACAAAGCTAATCAGTAAGAATGTCTAAAATGGATTTAAAAATAGCAACATGGAACATAAACGGCTTACTTTCCAATAGTG
ACCATTCTAATAAAAACACATAAATTTGATGTCATCCTCATATCAGAGACACACCTTAATAATAGTAAAACATATAAACTA
AAAGGCTATGATATCTATAATACTAACCATCCTGATGGGCAATCGCATGGTGGTACAGCCGTTATCATTCGGAATAACCTA
AAACACTACCTGCATAATAAAATAGAAGAAAAATACCTGCAAGCTACAACAGTTACTGTTATGAATTGTAAAGGAACATA
AACATTAGTGCCATATATTGTCCACCAAATGAAAAAATCAGCGATAAAATATTTGCTACCTATTTCAAGACATTGGGTCCT
AGATTTGTATGTGGAGGTGATTGGAACTCGAAGCATACATTCATATAGACCGAGTACAACAAGAGGAAGACACCTAAAAA
CTTGTGTTGATAGATTGAAATTACATTCAATGTCAGCTGATGAACCAACTTACTGGCCAACTGATATCAATAAGACACCTG
ATTTGCTTGATTTTTATATAGTGAAAGGATTGTCCAATTTATATACCGTTATGGAATCATGCCTTGATGGCTCTTCTGATCA
TACTCCAGTAATAGCTACAATCAGTATTGTCATATTAAGGAGAAAAAGACCCTTAACCCTGTACAATGAAAGAACAGATT
GGGACGCATTTGCTGATTATCTGGAAAATAGAATTGATCTGAAGATTAATCTAAAAACTAAAGAAGATCTAGATGATGCG
ACATTTTATATAACCAACCTAATACAAGAAGCAGCATGGCGTTCATCCCTAGACCTGGAGGAAAAAGATCAGGCACCGTA
TATAACAGTAAAGATACGGGAAAAAATTTGTGAAAAAAGAAGACTACGTAAAAAGTGGCACCAAAATAGGAATATGTTT
GATAAAAAAGCCTATAATAGATCAGCGAAAGAACTTACGTTGATGATTGATTTGCATGTGAACAATAACAAGGATAACAA
GCTTAAAAGATTATCTTCCACAAAAAAGGATAATTATTCATTGTGGAAAGTGACGAAAAATTTTAAAAGACCAATTCAAC
ACATACCACCACTTAAAACAAAAGCTAATACGTGGGCCCAAACAGATGGAGAAAAGGTTCAATTATTTGCTGAACACTTA
AGGGAAGTCTTCACTGCAAACCAGTCAGATATGATAGGATTTAAGGAAGAAATAGATAGGCTCTTGAATAGTGATCAACA
ACTTTCTCCTATGTTGAAACGTGTCACACCTAGGGAAACAGCGGGATATATGCGCTATCTAAAAAATAAAAAAGCACCAG
GCTAACTTGATAACAGGAGAAGTGCTCAAAAAACTGCCCAGAAAGGCTGTGGTATTCTTGACTATGATATTTAACGCTATC
TTTAGATTGTATTACTATCCTGCGCTCTGGAAAGTGTCACAGATGTGCATGGTTCGGAAACCCGAAAAAAGTCCCAATGAA
GTTACATCATATAGACCAATAAGTCTCTTGCCTACAATGTCAAGTCTTTGAAAAAATATTACTTAATAGATTGAGACCAGT
CCTAAATGAGAACAAAATCATACCTAATCATCGGTTTGTATTTAAAGAAAAACACTCAAAAATTGAACAGATGTATAGAG
TTATACAAAAAGTGAGAGAATCGTTTGAAAAAATAGATTATTGCTCTGCTGTAATCCTCGATATAAAAGAAGCTTTTGACA
AAGTATGGCACAAGGGGTTGTTATATAAACTTAAAACACAACTACCTAATTCATTTTATATGATTTTGAAGTCTTATCTTGC
AGATAGAATATTCCAAGTTAAATCTGGTGAAGCTTATTCAAAATTCTACAATTTATGTGCGTCGGGCCCGCAAGGGTCCGT
TCTTGGACCTGTACTATATTCTATATATACGGCGGACTTACCTGTGACAAACGATATAACAATAGCACTTACGCTGATGAC
AACATGCTTGGCAAGTGACACAGACGCCCATGTGGCATCACAGAAAGTTCAAGCACAATTAGATCAAGTTGGTTATAAAT
GGCGAATAAAATGCAGTACAGAGAAATCCCACCACATTATATTCACATTAAGAAAGGGAGATGCAACACCGGTGCAAAT
GAAGGGACAAAAACTACCAAAAGTAACAAGTGTTAAATATCTGGGAATGCATCTCTGCTTGCTCAGTAGCCAGACATTGA
AATTTTTATTTATTTTAAACGCCTACTTGAGATTGAGACTCAGAATATTGCAACCGGCTTCTCTTTCCGCACCGTTAAAAAT
TTTTAAAGTTTGTTAGTAGGGGATTTTTACACCCTAACTGAAAATGTACCGAACGGTCCTGTCTGTAGAGGTGAAATTGTC

		TATCGTGCAGGTCGTATATATATATATAATTTTATTGTCAACTTCATTTTTTTAGTCACTAGAACCAAATACTGTTTTATTACAATTTATTATATACTAATATTAAATACTTGTGATTCATGTGAACGAAAATTATGCGGTATGTCATTCCCAATGTCACATTCAAAGACTGATTTATGTACGAATGCACAGCGCCGTACAAACATTGTCAAACATTCAATTGTATGAGACTTTATTTTGTATTTTTCCCCTAAATCATATCTTTGGTGTTAATAAATAAGCCACTGGTTGTACATATATTTCTTTTATTTCCTCTTATTAAATGTATAACATAATAATTACATCATACTAGTTCTGATTGGAATTCAAGTATATACAAATACAAAACAATAAACTCTTTAAACGTAACAATTTTAACACGTCGACAACCCTGTAGTGAGTTGTTATATTTTGCATGTTGTAGTATGGTAGAAGAGGCAAAATGCTGGGCCATCTTATACGACATGACGATTTTTTAAAGAGCAAAGTGGACACTGGTGCCTTGCCAGTGTATACCATTAGCGGAGGCGGGCCATACCATTGCATTATCATCCATTAAAAAAAAANNN
MSTRG.1430	Uncharacterized protein LOC105118166	CTCATGTTATTTAGACCTCACCGAAGAGCAATTAAACAAGCTTGAGCGTCTTCAAAATTTATGTATCCGCTTTATATTTGGTCTTCGCAAATATGATCACATTTCTCAATTTCGTACTCAACTCAAGTGGCTCTCTATCCGTTTTCGTCGTAATCTGCATATCCTGTCTCTTCTTTACACAATCCTTTTTAATCCTGCTGCTCCTGATTATCTAAAAGACCGATTCTTGTTTCTCAGTGAGGCTAATGATCATACTTTACGCAGTGTCAATAACCTCCGACTTAAATTTCCCACTCATTCTTCTTCCTTTTATTCTAGATCCTTTACTGTTCAGGCAGTTCGTCTATGGAACTCACTACCTACCTCTATTCGGTGCGCTCAAACTCTTACTAGCTTCAAAACCTCTGTTAAATCTTACTACCTCTCACTTTAGTACTATTTTCCTGTGTTCTCTCTTCGTTTTCCTTC
MSTRG.14312	Uncharacterized protein LOC110378431	NNNCTTGTTCAAAAATTTCACTAAGGAATTTAAGTATCTTGGGGATGGATAACTCGTACACATTTTTCCCATTGCAATAAACCCACCATTTTTTAAGTGCTACTATGTACGAACTACTGCTTAACGGTGTTATCTGATCAAGAAGTTATAACCTTGATAGCCTCCTCCGGTATTAATCTCTTCCTAAACACTTCCCTGATAAGATGCAGGCTCCCAGGGAAAGGCTGCTGTGCAAGGGATACGGATCTCTGAAAGGTGATATCAGTAAATATTTACGAGGGTGAAATATTATGATTTGAGATACAGCCATATTATTAATAAGAAGATACCAAAGTTAAGATTTCCACATAGGAAAAACCAAAATACCGGTTGCTTTATAATATTAAATTTTTTTAATAGTTTTCAGGATTAAAGAAAAGGGTGGAAATGCATAAAACAATGTATAATTTGACCAGTCGACTGTTAATGCATTAATATTTACTGCATATGGGTCTCTGTGCCAGGAAATATATTTAGAACATTTAGTATTGGCTCTGGTTTCAAAAAGATCAATAGATGGATTCCCTAATAATCTAGTATTTTTTTAAAGGCTTTATTGCATAGCTCCCATTCAGAATCTATATTCTGTACCCTCGAATTAAAATAAGCTTCTATATTTTCCTTAGATTTGATATATGATGCAAACAACCAAATATTTTATTTGACAGTACAAAGACCAAATGGCATACAGTTGAACTCATACAATATACTCTGAAATTCAAATCTAAGAAACTTTTTATCTTTACCAATTGAGATTAAAAAATATGCATCTTTCAAATCTATAGTGCACATATAATTATTTTTATGAATTAAACTACATAAAGTTCTCATATCTTCTATCTTAAAATGCTCAGGTAAGATGAACTTATTCAATATTTTTAAATTTAAAATGAATCTAATTTTGCCATTTGGTTTTGATACTGTAAATATTTTTGATATAAACTGTCCCGGTTCATGTTTACATTTTGAAATGACATGTAATTTTAACAATCTTTCAATTTCCTCTGTTATACCTAAAATTTCTTTAGGCGAGAATTTAGAATGTTTTGGTATTATATGTTGAATAGGGGGTTCTAAGAACGGAATATCATATCCACTAATCCATGACAGTATAAGTCTATCTTGAGAACCCAGGAAGCTTCTACAATGGCCCAAATGAGAGACTAAGTGCGGAAAATAGAGTCTTTTTCACATTCACATTGAAGTCTATCTTGAGTTATTTTTTCCCAACAGGGAACAAAATACTTAAGGCGGCCAGCAAAAACTTTTACCTTCATGATTGGCGCACAGGCCTCCGTGCAGGGTGGTATGCTTTGGCTGCTGGACGCCGCTGCTGAAGTCTCACTGAGGTCCTCTGCTGATACCTCCCCTCCCCCCCTCCTTGTTTGTATTGACCTTGGCGAGGAGGGCCTTGATAGTTTCCCAAAAGCTGTATCCCATGACTTCGATTTTTGTTATTGGGCTTTTGAGTATTAATATTAGAAGGTTTCAAAATTGAAGCCGCAGATTTCTTAATGGCATTAGCCGATTTAACATTTTCATTGAATTTTTCTCCAAACAGGAAACTATACCGAATAGAACTATCAACGGACTTTACTATATTTTTGTCTAAATTGGCAGCAATAAGTTTTCTGCGTGTGACTGTATCTTCCCAATGCAAGTCACTTAACAATTTTGTCACATCGGTGAGAATGGTAATAGTGTTAATTTTATTAAAATTACCCTTTACTAAGGTGTCCATTATATAACTGATAGTAGCAAGGGCAGTACCCAACTGCTGTTGCCGTTCCTCTACTTTCTTGTCTCTTGCTTTTACAAGTTCTGTCAGGAGACCCTGGAGCTCCACATTTAACTTGGGAGCATCTAACAATTTACCATTTGCAGGTATTAAATATTTTTTAATAATCTCTTCTTTTATCTCTTTCTTCAAGCCACAGGTCAAAATATTTTCAAACCTTTTCATAATTTCTGGATGTATGTCTTCCCCATATACTTTATCAG

TGTTAAAGGTGTTACCGAGAGCTGACAATATAGACTCATCCAATGTTGGAATAATGTTTTCATCTGTAATAAAAATATTTT
CCTTAATTGGAAAACATGTTCTATAATTATGAAATGCTACCAGCATTACATAACACAGGCATTAAAATAAGGCCACCGAA
CTTATTTTATATAAAAAATAAAGAGAACTTCTCTTTATATAAATATAAGTACGTCACTCGACTGGAGTACGTATTGTATCA
AGTACCACTAGTTACAAAGGGACCAAACATTACTGCGTACCTATGGCTGAAAATAAAGAATTCAGATAAAACATACCTTG
ATTTTGTTCTGCTATAAGTGAATCCACTACAGTTAGATCTGCACAATCATCGGTTTCAACCTCCACCGACTCCACCGGTGGT
TCTGGTGACGTCGTAGAAAAACGTGATTTACGACGTTTTTTATTCAGATTTCTCTTTAATTTCGTAATTTTACGTTCAATTCT
ATCCTCATCATCACGATCTCTACGCCTTGTAGGCATAATAATAACTTTTAATTAGTAAAAAACTTAAATTTTCAATATTTAT
TTGAAAAAGATTGACACTATTTTCACACAACGTGAAAATAATGAATGAGTGACATCTGTGAAGTTGCCAGTGCCTTGTTTT
TTACTTTTTAATGTAAGCATTCGCTATTATCTAAATAACACGAAGACACAATGCGGAGATTGTTGAAACTACAAGTGAATA
TATGAAGAGTGTCTCGGACTAAGCATCTCGTATAATTCTGTTGTATACAGCGCTTTACTACTAACTTTGATTATATAGATAG
AGCATCGAGCTTCCAAATTTGGTCAACTTTTTGTCAGCTAATTGTCAAATTAGATTACTCTTTTTTTAATCAGTGTTCTATAT
ACTTACGTACATCGTACATATGTAGATATATCCAATGTATCTTGACGTACTATTTAATAAATAAAATGAATGTTTACACATT
AAAAGATGCAGAAATTATAAGATAAAATAAATATAGAAGTTGTAAAGTAATCACTCTTAACTATTTATCACGCAAAATTG
TATGAAAAAGTTGACTCGTTTTTCTCGAGACATTTCTATGGAGGCATTCTATCTTAAATGTCTTTAACACTAAACTTAAATG
TTTTTTAATGAAATTATTCATTTAAATTTAAGAAAGATCAATAATAATTAATGAATCAATTGACAAAAAATTATTAAATTA
ACAAAATATTGTTTACCTATTTGTGAACTCTTAATTAAATTAAATTATTTCATTAAGAAGTACAAGTGTTTACTAAAAAGCT
AATAACGCGAACTATAAATGTTTCCAATCGACATTATTGGCTACCCAGTCATGTATAGTTTTGAAAACGGTGCAAATGTAT
GACTGACATGTTATATTCTTAATAGCTGATGTGTACCGTCAGCTAGAACCACAAGCCAGGTTGCTTTTTAAAATTGTATAA
TTACTTAATTTTTTTATAAGTCACTGTCGTGCTTAAGAAAAAAAAAAATCATAAAAATTTCATTATTTTTATCGCAACACACA
GTTGTCTTTAATTGAATGAAAATGGTAAATCCCGACTGGACTATAGGTATAAACTCGTATAAACGGGAGGGAGGACAGAT
GAGAAAGAAAGCAAGACAGCCCATCGTGATCAATTCACCAGGAATTAAGGAACCGATGGTTTCCAAGGGAGTCGCGTGA
AGGTCCACCTGATAAGGTTATATTGCTAGCAACGGGGTCATTAATCACCAATTGCCCTCTTTCCCTCTCAATTCAATTATAT
TGTATCTCAAAGATATAATGAGGCTATTAGCCTATTACTAAAAAGTAAACTTGTATAGGTCTATGTTTCGAGGTTTTTCTTT
TATTTTTCTAACGCCAACGTATTCACGTTTTTGTACACCTAGCCTACTCTTATGACATCCCGGGGATGAAATGGATACCGTA
TCGATAGTTATGTTTTGTACCAGCTTCAAGAAGGAAGTGGCTCTCATTTTTTACATTTTGTTACACTTTGTATATGACACGC
ATTAAAATTTTACATTACAATAAATAATTTTTCTTAAGCACGACGATAATAAATAGGTATTCATTCCACTGCAACCACCTG
AGAAAAAATATCAAATATATCATTTTATTATTTATATAGTTGTAATTATGTTTATACAAGAGCCATATGTTGGAACTTAGGT
CATAATTTATTGTTTGTAAAAACCAAATATGCAATCTTTGTGGGTATATACACGTTTGATATAAGATTAAAGGTCTATCCAT
ATCACAACCGACGGTGAACGGGCGTACGAGGCAAGTCTATTATAAAATAATTCTTAATATATTGAGTGTTTCATCGTTCAT
CTTGATCAGACCGCAGCCAGACGGAGGAAATGACGATACTGTCAAAGTACTGTATTTTTTATCAATATTAAACTTTGCTTA
GTTGGCACTAGATGGCGTTAAATGCACTTTTTCGCCCGGCTGTTGTAATATGGGTAATCTCTATGATTTCATTGTCATAATC
AAGTGAGTATAATAATATATTGCGTTACATACGTGTTTATTAATTGAGTTTATTTATTAGTTGTAAATTAACAGAATAGAA
ATTGACAATTTTTAATCTAATTAAACGTCACTTTCCTAATTTGATTTTGCATTTTTATTCTGAAGTAAATTAAAGGTATATAT
AATATATTATTACACAGTTTCTATAGTTGTGTTTTGATACTTTTTGTAATTTTAAATTAATAATAATTTTTATTTTTCTACAA
CTTTATGGGTACAATTGATCACTTAAGATGCCACAATGTATAGATATATATTTTTATTCTTCTTCTCTAACTATTACTCTTAA
CTCTCTTTTTCTCTCTCTGATCTCTGTTAGCAAGCCCTTGTAGTTTGATTTTAACATATCCTTAAGATATAATTTACATAAAA
ACGAATACAAAATCCGGGATTTTCGCGAAAGCTTTTTCTCGTATTAGCTTTATTTTGTTGTATTTTTCTTTAAAAAAGAAAT
AATTGTGACAAATGAAATCACTATGTCAAAGATAGATCTAAGTTATTTTAAGGGGAAACTGAATGGTTCGAGACTGACTC
GTGTTACTGCAAAAGAGCTTATAAAAATTTAATTACATGTACAAGCGACACTTTATTATGGTGGTTTGTCCGCTCATGAAG

CTCTGTGAGTAATATCTATATTATTAAATAAATAGTTGTATAAACGTCAAAAACACCATAATTCTATGCCGCCAGTGTGAG
ATACGATCTCTCCCATGAGAAAAGAATTGCAATAACGTTTTCTTAACTAACCGAGTCAGTTCCCGACTGTAAAGATCCATA
GTTTTAACATTATTTAAAATATGCCTTTCTGCCTAAGATATGTAATGAAATATTTAATTATGGGCACTGTGGACTTTGCATC
GTATTGGAAGCTTCGCTTAACAGCTGTTCTACGACTTCACATGCCATCGTGACAAACATTGCATTTATGTGTATAGAATTTT
GTAGGATATTTTAAGAAAGCAATGGCTGTCTGTACATTCCATTGTCTATCAATTTTGGGTCATTAATAACAAGTCGAAGAT
TTTTCAGTTTTAACTTTTAAACCCTAGAATGATTTCTAATTTTTTTTTAATTTTCATGAAACCCAACATCTATGATATTAAGC
TCGCGATTCACCCTGGCTTCACATGGGTAGATAGGTAGAAAAATAGATAAAATAACCCATATTACTCGCTGATGTTGTAGC
TTTTTATAGATGACACAATTTTTAATATAAGTATTTTTTAAGTTTATCTGTTACAAAGAAGAAATCACATTTTTACTCTTTAT
AACCTAATATTCGTTTGGATAAAATTCATAGTGAACTCTTGTCTTCGTATCCATTCGTTCCATGTTATAAAGAAAGAAATGG
CATTCTTAGAACCCGCACTAGGCACTCTTAAACTTCGGTTCTAAAATGCAATATACAGACAAATATTGATTTAAATGTGAG
CTTCCTATCGCAAACACATAACTATTCATACTATTCATAAGCATGCAAACATAATGACAAAATATTCGTAATATCAATACC
CTACTGAGTGCTAATTTGTCCACGAAATCTTTAGGTTCCCTATCTCTAGATCTTATATTTTTAAATAAAGAACATACTAAAT
GAATTAAAATTCTGTAGTCACGTTCTAAATTTTACGTTACTTTTTTTAAACTATACGCAGCAGCTTCGCAAAATTAAAGTCA
ATTTGTGACGTCAATTGGTACTGGCTGTTGAACGATGCATCTAATAAATATTTACTCCTAATTATGTTTATAGTATACCAAA
TGTATTTATGTTTGCGCGTGAATGAAATGTAAAGAAATTACCAGGAATTTTACTTAGTAAATGTTTGACTCTTGTAAAAAC
AAAGAGCTTCGAATTCAGTTGCTGGTGATGATCGAATAATTAATTATATTAAATAATTCTTGAACAAAGACGACATTTTAG
ATTAGAGCACCTGCTTCACCCCTCACATATACGAGTAGATCTTGCATAAAACAAAATTATATTTAAAACGTACAATTAAAG
AGTTAAAAAAAATCGTTTTGTGCTAACTTACTGAACATTGTTTGTCACAGCAAATATCAACTGACGTAAATTTAAAAAGGT
CGATACCATATTACACTAAACCTTTCGCTAGAAATACGTACATAAAATATCTAAACATATCTATACATTTCGCACCCAAAA
CAGTAGGCTTAAAAACTCTACTTTACCCATGTCATAATAAGATTTGTTCTGAATAATCCGTCGAGTTTAATTAGCATCTCGC
ACGGAAGGCGTATTTATAAACGGACTAGTATATTTACATTGGCGGGATGTAAAAAGTAAATCTTTCTATGATTCGTGTGGA
ACGGAGTTTTTAACTTCCCAGGATACATACGACCCCTGAACTAGTGGCAAGCGAATATCGGTAGACGCGTTCCTTTAAAGA
ATATTGCGTGTTTATTTTCCATAACTACATTGTTTTGTGTAAAACTAAAAATGTAAGACTTCAAGCTATTTCTATGCCAAAT
TTCATCCAAATAAGTGTAGCAGTTTAACTTTAATAACAACGTATCAATCAAACTTTGTACAACAAACTTTTTTATACAACTT
AGCACGGGTACTAATTTCCATTAGGCGAGATTGACGGTTCACCTATTGGAAATTAATAACCGTCAGGCAATCGTGATCAGA
GGGCCTACCATGAAATGTTTTCCGAAATTTCGGAGCCGAACGAAACACAAATTTGATGTTAAAATTACATAATACTTACCG
TTTAAAAACATTAAAAATATTATTCTTTTGGACTTTTATGATAGGATTTATGACGAACGAAATGTTAAGCTCCATTTACTGG
GCCGTATTAGGTATAAACGAAAACACGAAAGTGAGTCCGAAACTTCGGAATTTCATTTCATGGTAGGGCCCCAGTACCGT
AGTCATCACTGGATTAACTATAACACGCTCACGCTCCCCTCCCGTACCCAGTAATTTCACTACGCTCATATTAATAATCATT
ATACGGACATTAATTACTTGCTGGTTCAATTCGTGCTATCCTTGCTTACATAAGATACTTGTGCAACTGAAAAAAAGGGAC
AGCATGATTTAATAGTTCAGAAATAAATAGACATAAAAGGCTAACCTTAATAAACAATATTGTAGGCATCGCATATGTTGT
TTCAAGTTGAATCAGAAACCTGTTAACTATTTTAATATAGAAAATAAATACTACTGGACACTAGGTTTTGTATTAAAACTG
CCATTTATTACCAGACATCATTGATAATTATAAAAAAAAAAAAACTAAGATTTTAAAGTACATTTCATGCATACTTACATAT
TTCATACTTCATATTGAATGTGGCTTAGCTACGCTATTTGTGATGGTATATGGATCTATAAGAAATTAATCTAGGTTTTGGT
ACAATACTAAAATAAAAAGTATAAATAACTAACTTTTATACTACTACTCCTCCCTTATAAAAAATGAAAACCTACGAATCT
TTTATAATTATTACAGTATTTTGTCACTTTCTTTCGAACTGAGAATTTAGTACAAAAAAAAAAAAAGAAACAGTTCAATAGT
TAAAATAGGTTTATAAGAGATTTGACGTTTTTATGAATAAGAGTGTAAATCTGAAGTGTCTTATTGCATTAAATCTGTTTTA
CACGCATATATTATTATTAATATCACTCTTGTATCAATAAAATTTGTTTGACAATAATTTTGCTATTTCTTTTTGTGTAGAAG
GTATAAGTTTTAAGAGTAATTCTAAAATCCATTAATTACCCTATTCATGAAAACATTTACTGTATACCTGTTCAAAAAAAA

		ATTATAGTAAGACTTTTTTAAATATGTAAATTCTTTAAGGTGGTCACTAAACAAAATTAGACTACAAATACTAGATGGCGC<br>TGGAATCGAGAATTGTGGTTTTCGTTGATTATTTTGTAATCAAAGATAATTTAAAGTAAAATTTTTAAATTGGCAAGTGGA<br>TTTTTATTATTATGTGAATAAACTTACTCGCAAATTTAGAAATTTTGTTTTATATTGTTTGTAATTGCAATATAATCAACAA<br>AAACCACACGTCTCGATTCCAGCGCCATCTAGTATTTCTTGTTTAATTTTATTTAGTGACCAACCACCTTAATATAAATAAT<br>TTTAATAAGATACGAGATAATACCAATGTGTAATTAAGGTCTAAATACCAATGATGTCTGGTAATACACACACCAAGTGA<br>CTGTTCTACGGCACGCAAAAATAATGATTGTGATGGACGATAAATATTTGATAGGGCTTAACAATAATGGTGAACTTCTTA<br>AATTAATATCATGCATCAACACCAATTTTAAACGTACTTATAACTTGTATATTGATACGCGATACGCTTCTGGCGAGTAAA<br>CTTGTATTTTTATAAAATTCGTAAACAGTCACTTGCTACCGTTACATCTTACCAACGTTTCAATGTACTGCCCTCAAATTAG<br>CGAATTATTTGCAAAGCTGTTCAACAAATTGATAAATTTTGTATAAATATTTATAACGATAATATAACTGTTGAAAACAAA<br>TATTGTAACAGAGGACATATTGTAAGGTGCTTATGATTATCGTTATAATACAAAGTTGTGCATA
MSTRG.14322	Hypothetical protein RR46_08551	GCCAGAGTAAGTGTCACTAACATCACTACTGTCACTTAATTTTGCTTCTGTTTATCAATGGCATGTTACAAATGAAATTGTT<br>TAAAAAATTATTATAGTTATTGACATATGAACAATTATTTGTTTTTGCTTCAATAATGGGTAAGAAGAATAAGTCTGGTTC<br>AAAAGCCAATGTATTTAAAGTAGCAGGAGCTAAAAGTTTGAAAAAAACAAAGGCCAAAGCAGTTAATTTGGGATTAAAA<br>AATATCAAACAGAAGATTTTGGATATTGATAAACAATTCTTAGAAATAAACAAGAATCCCAAATCGAAAGAAAAAGCTAA<br>GCCCGAAGTAAAGCCAGTGCAGAAAAAGCCTGAAAAAAATAT
MSTRG.14353	Trifunctional purine biosynthetic protein adenosine-3 isoform X1	GACGAAAATGTCATGCACGCTGACAAATGCCGCGCGTGCGTCCGCTCTCATTAATCGTGTTTTTTTTTAGAAACGAAAAT<br>ATAATCGCTGGCGAGGTTAGCCTGCGCTATCGGTATACGCTGACGGGGCATCAGTGAGAGATGAAAGCAGGTCAAGTGGC<br>CCATCGTAACGAATTCAACAAAATTAACCATGATGATTGCCAAGGGGAACCGCGTGGAGGTCGACTTAATAGAGTATATT<br>TTCTTTTAATGGATAATAATGGAATGGCAACCCTGCCCGCGCTAACAATATACGCCGACGGTGCACCAGTGAGGGATCAC<br>AAGCAATGAAGAGGCAGCAACTCTTCAACGACAACAGTCTGGCCAGCATCGCCGAATTTAGCTTCGGACAAGATCTCGTC<br>GACAGCCTGGCACGCCTCCTCTTTGGTAGCAGCGACCACTACACCTTTACCAGCAGCCAAGCCTGATGCTTTCACCACGAG<br>AGCTGGGTATGGAGCGCTAGAAACAAAAACGTAC
MSTRG.14364	Uncharacterized protein LOC106107598 isoform X1	ATTTCATTTCGATATCGATTTACAGGCAAAACTTACGATGAAATAATTCGTTTACTGCAAGGCTATATAGAGAATGAAATA<br>GACATGAACCAGGAGAGCACATGCCAGCAAAACTGCGTCAATTACAAAGTAGCCAAGAACTATGGTTGCTTTAAAGACCA<br>ATTTTGCTCACAGCAACCCAAGTGCAAAGGCACCATACTCAACTGTACGTTCGTCGAGGCTGATATGACAATTGCTGCCGC<br>GAATTCCAAAAGCCATCGTCGTTACGAATGGATTCAGTATAAAACCGGACGGACGCTTGGGAAGCAAAACAAAGAGAGG<br>TGTGAAAATAACGATAAATGCCGGAAAAATTTCGTAACCCAACATCAGGTGAACTCGTGGTGGCGCTGGTTGTTCTGGCA<br>CTGTTCATACTGCATGTGCTTGTGCGACGACCCAGAAAACTCTGACCGCTACTTCAGTCTCAAGGAAGTCACCTCCGATAT<br>TCGAGAAAACAAGGTTGTAACCGGAATTCGTCTGGTGAAACTTGATAAGGTGTTCCATTTTCAAATCAGCGAGAGCACCCT<br>TCATCAAGAGGGATTCGTCAGTCCGGGATCTTGGCTCCCGATAGAAAGAATCAATATTGACCAACAACAAAATGGCGTAG<br>ATTACCACACATTGAGTCACATTGCAAGAGCTATCGATCTAGACGATGTGGTGGCGCCGACCGGATGGGTTTTGACCGGA<br>GTTAGGTTTCGCATGCTCGGCTCGCATCTGAACCTGTACATCCGGATCACAAAATTCAACTTCCAAACTGGCCGTCTGTCTT<br>ACAACAACAGTATGTGGTTAGGCAACGATAACACAGACGCAAGCGAAAATCCTAGGAGCCGTTTGAATCTGAACCGGCCC<br>AGTCTACCGACACGGAGCGCTGTTGCGTTGCCAGTGGACTCCAAGCACGACCAGTTCATGGAGTTCACACACAGCGACTTT<br>GACAATGATGCGGCGCAGAGCACTGTGCCGTTCATCGACATACAACCACTGCAGCCATATACTCGCGGAGTGCTGCTGAG<br>CGGCGCGGGCGTGTCGCACCGCGGCGCGGTGGGCTCGGGCGGGTTCGTGGCGCTCAAGCTATTCACCTATGACTACGCGC<br>CGTACGTGCGCGCCCGTCAGCCGCGCGAACCCAGCTAGCGCCCCGCGACCCACCACGCTCAGGGCCACACTAGTGTTCGC<br>GGGACCTCCAGTATTACTCATAAAGAACGGGGGGGGGCTCTTATATCACAGCTACATTACCGCATCGTTTTCAATGTTTAA<br>CGTGTCACGAGTTCCAATCAATAGATATGGAATTGCGCGCAAAAATGCGTTTTAAATATAATCACTTATATTAACTTTACT

		ATTATTTTATAGTACTTTATCTTATTCTCAATTTCGTGTATAATATAGTATATGTCATATATGTGTAAGTATATTTGTTATTT<br>ATATATATATTTATTCGTTTATATATGTAATTTTAGTATGTATGTATATCAATAATTAGTAGTACCGCATTCGAATATTCTG<br>ACACTTCCCTATGGTTGAGGAAATGCTTTTAGCATTAAGTCCGCCATGTACACATATTTGTACTAATGAATAAATAAATAA<br>AATCACACAATTACTTCTTTATGTAATACTAGTTTTTACCCACAACTTCATCCTGATGATGGAAATATGTCAATTGACCGGA<br>ATCCTTATGAGAACAGTAAAAACAGTAGCGTTATGTCCTTCTCAATTTCAAATAATACATGTGTATCAAACTTCATGACAA<br>TTAGTTGAGCCGTTAAAAAAGGTCATAAACAAACTCACTTGCGCGTTTGTAATTACTAAGGAATTTAGCTTATTTATTTTAA<br>TAAAATTCTATAGCTATTGTTAGCGGTATCTCCTATTTAAGACGCCGCACCACCCGCCGCAACTATTAAAATGGTCGAAAA<br>CCAACGCATCGGCCATTTTGGTTGTCAGGCACGCACTCAAACAAATATCCGAATATAATAATAAAATTATATTATTATCAC<br>ATTATTGTTAAATACAGAAATCTTGTATAAATTGATCTATTTAATTATACGAAACATTTGGTCTTAGTTTTTTTTTTTAATTA<br>AAAATACGATATATTGTTAAGACTATATATATACACGCCAACTTTTAAACTAAGCCCAATGCAAAAATTGATATTTTTTTTT<br>TATATCTATGATTAACGAATCCCGCT
MSTRG.1<br>4365	Uncharacterized<br>protein<br>LOC106107598<br>isoform X1	NNNTACAAGTGTCTCCACACCAGCTCATCTACGACTTGTACAACACCATAGCCCTGGCGGAGATCAAGGGCTACGTCATG<br>ATGCAGTTCTCTTGGATGCTACTGAAGATATATCGACCAGGCAACATAACAGAAGAGGTAAATCAGACGAGAGTGCAATA<br>TGAAGGGGACGCTATACAGATAGCAGAGAAGACTGGAAATGCTTTGGCTAATGCGACAAGGGAACTGTACCGCTGCGAC<br>CCACTACAACATATAGAAGGGCAAACTTACGATCAAATAACCCGTCTAATGCAAGGTTATATAGAAAACGAAGTGAACAT<br>GAAACATGAAACATGAAACATGAAAACACATGTAGGCAAAACTGCGAGTATTACAATGTGGCCAAGTACTACAATTGTTT<br>TAAGGACCAATTTTGCTCGAGACAACCTAAGTGTAAAGGCCATATACTTGGTTGTTACTTCGTCAAGTCCGATATGACTGT<br>GTGTACTTCGAGCTATAAAAGCCATCGTCGTTATGAATGGATTCGATATAGTAACGGACCCAAGTTTGGAGAAGCAAACA<br>ATTGTACTCTTCAAAAAGGAAATACGTACCAAGTGAACTCGTGGTGGCGCGGGTGGTTCTTGCACTGTTCATACTGCATGT<br>GCTTATGCGACGACCCAGAAAACTCCGACCGCTACTTCAGTCTCAAGGAAGCCACCTCCAATATTCGAGAAAACAAGGTG<br>GTAACTGGAATTCGTCTGGTGAAACAGAATAATGTATTCCATATTCAAATCAGCGAAGGTACACTTCTTAAAAATGGTATC<br>GTCAGTCCCGGCTCTTGGCTCCCCAACAAAATAATCAAAATAAATGATCAAAACATGAAAAACGGCATAGATTACCACAC<br>TCTGAATCATGGGACAAGAGCTATCGACTTAGATGATCTGGTGGCGCCAGCTGGATGGGTTTTAACTGGAGTGAGATTTCG<br>AATACTGGGCGCACATTTGAACCTAAATATCCGGGCAACAAAATTAAATTTTGAAACAGGCCACCTGTCAGAAGACAGCA<br>TGTGGATCGACAACGACAACACAGATGGAAGCAAAACTCCTAGGAGTCGTTTAACACTGAATCGGCCGAACCTACCGACA<br>CGGAGCCTTGCTGCACTGCCCGTGGATTCTAAACACGATCAATTTTTAGAGTTCACACACAGCGACTTTGACAAAGACGCT<br>GCGCAGAGCACCGTGCCGTTCATTGACGTGCAACCACTGGAACCGTATGGACGAGGAGTGCCTTTAAGCGGCGCGGGAGT<br>GTCGCACCGCGGCGCGGTGGGCTCGGGCGGGTTTGTGGCGCTCAAGCTATTCACCTATGACTACGCGCCGTACGTGCGCGT<br>CCGTCAGCCACGCAATCCCTACTCACCACAACCATAATGCTCGCATCTCTTTATCAGATTTATCAATGTAACATAGCGTACT<br>ATTTTTCTTAAGTATATTA
MSTRG.1<br>4379	Uncharacterized<br>protein<br>LOC110378894<br>isoform X1	TGCTCCGCCAGCGTATCCCGTTAGCGCAGGCGGGGTTACCATACCATTTTCACCCATCAAAAAAAAATATATTAACTAGTG<br>CAATAAATTATATATATATATATACTTACTGGAAATGATATTAGGCGTCTTAGAAAACTCATCTCCATCAGGGTAGGTAAT<br>TTTTACCTTATTTATAACATTTTGATTCATTACCAGACACATACGTATTGATAAAATGTTATTTTACACCAATATCACGATA<br>AAGAAAAGTATCTGTATGTACTAGATTATATATTCCACACAGTTTATGTAAAACGTATGCAAGCAGGTTGTATCTGAATTT<br>GGTAGGATTATACGGCTGTATAATATTTTCTATACACTAGCCTGCTATGAATTGATAACAATAGCAACTGATGGTATATCT<br>TTCGTCCTCTTTTTACTAATTATATTTGCATAAGAAGAATGTAAGCAACCATATGATTTACAGTTTTTATTTTATCATTCCTT<br>CTAAGTAATTCCTTGTTCTTAGGCCTGAATACAACGAGCTATACACGATATGGTCTATTAATTAAATTCCGAAATCTTTGTA<br>AAAAGTTGCCAACCTGTATCTATATAATTTAAGAATAGGATGGTTTTTTGGATGTGACGTGTCGTGTATATCTATGATACTA<br>ACATACTACCAACTTCCATACAAAATTTACGGACAAAGATGAACTGCTCAGCATCTTGACTACGATCATGCAAACTGCGAT

		AGGCGAAAATTGCACGATAATATCTTAATTTTAAATATCATTTGTATTATATAATAATTAAAGCTTAATTAGCAAGCAAAT<br>AATAATATACGTTGATGCTGCCTCTGCAGAATGATTTAGGAATTAAATGGGGAGCATGATGGATTTCCCAGTTTTATGGTG<br>AAAAATAAATACTTCTCTTTTAATAATAATTTGCCGCCGTATATTTTATGCACACAATATCACACACAATGGCACAATATC<br>AGTAATAGTTATAGTATATACATATTAATTCTGTGATCCAGAAAGTTATTATGTTACTTTTTAAAATTACCAATCGATCACA<br>AAGTAAACGTGCAATCTTTACACATTGTTACTTTATAGGCAACAAACCTGGCGACAAGCCACCTACTACCCTACACTTACT<br>TACACGGTACGGTGGGAGGAGAAGGAATTGTTAGTAATGAGGATTAGTAGCCCCATTGCTAGCAATATACCCTGTCAGGC<br>TAACCTCCTCGTGGTTCCCTCTGGAAACTCGTAATTAATTTATTGATAGATTTGTCACGGTGGGCCAACTGACCTGCTTTAT<br>CCTCACCTATCATCCCTTACTGGTGCCCCGCCAGTGTATACCGATAGCGGAGGCGGAGATACCATGCCATTATCATCCATA<br>AGAAAAAAAAACACGGTACGGTTATACCGAGGAGGTGATTCCTTTTAAATACTGTGAAAACACTTCGCTAGTAATTGCTA<br>AATAAAAAAAAATGGTATTACGATTACGAAGGACGATTGCTATATAATTCTGACAAATAGCGCCATACATGATCAAATAG<br>CAATAGATAGAAATAATTCATGATATTACTTTAATTTCTAAATCGCTCTAAAGTGGCAGTCTTGAGATTTATATTAATACAT<br>AATATTTGAAAAAATAATATTGACAACAAATATCGTGCAATTTTCCACTTCGCCAACAGTTTTTACAGAATGTTTTATCATC<br>AAAGTATCTAGAATCATTTTTGACATTTGAAGGTAGTTTAATATCTACCTTTCAAATGTCGATTTTTATTAAACGAAACGGT<br>TATAGATCGACAACCATGCACTACTTTCAAACCTTACTACATCACCACCCCAGTTAGACGTTGTGATCACATCGTTATCAG<br>TTCGAGAATTATTTCAAAATTATTTCGTTGGAGAGATATCATCAGAAGAAACTAAGGTAGGTGGTAACATATGGATATATG<br>GAACAGGTGGATAATATTTAACAAAATAATTTCTACCCGCTATAGTACAAAGTCACTCCTATTTCGGCGTGATGTAAATGA<br>TATATCTTTTTGGTGGCACGCTGGATGATGTTTAGTGGGACATAATGGTAAGATCAGGCCGACCTTCGTGTGGTCCCTTCTG<br>GCAATCATCGTGATTTATTTCTGATATTTTTTTATGGATGATAGTCCAATGGTGACCACCAGTGAGGGATGTTAGATGAGG<br>ATTAAAAGGCAGGTTAATTAGCCCATCGTGATGAATACAGTTGGAATTCAGCATGTTTTTCGGGGGGACCACATGAAGGT<br>CGACCTGATACGGTATATTGCTAGCAATGGGACTACTAATCCCCTAATAAAAAATAATAAAGTAAGTGTAGGGTAGTAGG<br>TGGCTTGTCGCCAGGTTGATTGCCTATAAAGTCCATTCTTGATAATTCCATCATGCCGAGCATACTGAAATGTATTCGTCTT<br>CGCCTGTCATCTTTCACTGGTGCTACCTGCATATACCGATAGCGCAGGTTAAGTGGGATATCATAGTAAAGGGTTTTTTGT<br>ACATTCCAAATCGTACTAATCGTCGTAATTATTTTATGATAATATTTTTGAGTAATTATCAATAACGTTTAAAAACAAACAT<br>AGTTATGCTATCTGATTTTTAAGTACAGTTATGGCTCAAAATACGGATTACCGAATTGTAATAAAATATTAATTGTTAAGC<br>GTCTATTTGATACAAAAAAACGCAATAAATATATACATTTATACAGGAAAATGATAATAGATACAGTCATTTTATCACATT<br>CCTAGTCCTACATATTTAAATATTTTTTTATTCAAAATAATAATAATAATTAAATATAAAAGAAATCGATTATATATGGAA<br>CCCTCGATATGTAAGTTGAACTCTCACGAGCTTATTTTTTTTTTATTCAGGTTCGAAAATGTCTTCAGAAACGCTATCACCA<br>AATTCAATACACATGATGGAATATTTACAATCAATGTTTGATGAGGCAGTTAAGACAGCGTATGTATTATGCATCAATGAT<br>GAGCTTCCCTCGAATGAAAATAGTAATAATGAAAAGGCAAATGCACAGCCACGATTAATCCCGTACACCCCTCCACCAGG<br>TAATTCCATTAACAGTACTCTCGAAACTGAAGGCAACACTGGACCATATGAGTTAAACTCGATTGAAAAAACAAAAAATG<br>GTTATACCAAAAAATATTCAATCAAGATTAATACAGAAAAATCAAAAAAAATCATGTGTAAGGACTTCATTCGAGGAATT<br>TGCAGAAGGGGAAATGATTGTATTTATTCCCATGAACTAGATTTAAAACAACTGAATGGAGTCTACAAGTTTTGTCGCGAT<br>TATGTAAAAGGAAATTGTTACAGAAAGTTTTGTATTTTCGTTCATGCTACCATATTTGAGAGAGAAAATTACTTTAGAACT<br>GGTTTTTTGCCACCTCATACTCTGAGACACTTGAAAGAAAGAAATGTAAGGCGCCCGTAATGTATTCGAGTAGGTCCATCG<br>CGTGAAAATATAGAGTCTGTATATGCAAAGATACCTTCTGATTTATACGCATGGTATATAAAATTCTAAATATACATTAAT<br>ATAGCTTCATCGACCACCGTACTAAAACATAAAACCTATTGTATAGGTATTAATTATCTTTTTATTATAGTTAAAATTAACT<br>AATATATATATTTTTTAAATAC
MSTRG.1<br>4380	Uncharacterized<br>protein	TTATAATTAGTTTAAATCAATAGAGAATTGGTCTAAGAATGTAGTGTGTCTCTTCCTTTTACTACTTGCAAAGTATACCAAG<br>TTATTACATAGAGATGGATTTTGCATTATTCATATTTGTGGCAGGATATCTTCAATATACAATTGGCGAATACGTTCGAGTG

	LOC106100376	GAAAATCTATTAAGCATGCAACAGGGCATAGGCTGGCCACACTCTTTGACTGTCGTTGAGGGACACCAAGCTCTCATAAAGCTAAGCACTACATTGGAGAACCAAGAGTCGTGTGAAGTTACAACTCCGGCTGGTGAAAGATTTAACGTACTACATCCGCCTAACAGCAGGTA
MSTRG.14389	Facilitated glucose transporter member 1-like	GGGTATGAACGTGAGGCTGGCGTTTGCTGTAGTCTCGTCAGCTTGCTGGTCAGCCTTCCAGCATGGGTACAACACTGGCGTGCTGAACGCTCCACAAGCGGTTATGTCCGAATGGTTGCACAAATACGCGCTATCTGGTACAAATCTAACCATTGACGCCAACTCAGACCCGAAAGTGACTTCGGTGTGGTCTGTGGCGGTCTCAATATATTGCGTGGGCGGAATGATCGGTGGGGTCATCACTGGAATCATAGCTGACAG
MSTRG.14446	DD34D transposase	TTTAATTTGAGCTGTCAAAGTGAACACTATTTAGAGTTATTTTACAATTTTTTTTATTATGATAAAAATGTCGAACTTTGTGCCAACAAAACAACATTTGCGGGAAGCTTTGCTTTTGTTGTTCAATTTAAAGCGAAATGCAACGCAAGCTCATGAATTTCTTTCTAAAGTTTATCCTGATTATGCTCCAGAGATTAGAACATGTCAAAAGTGGTTTTCACGCTTTAGAAGTGGAGATTTTGATACAGAAGTTAAGGAACATCCAAAAAAAATTTTGAAGATGAATAATTGGAAGTATTACGTAATGAAGATCCGTGTCAAACGCAAGAAGAAATTGCAAAATCATTGGGAGTAGATCGATCAACCATTTCCAAGCGTTTGAAGGCAGCAGGATTTATTCAAAAGCATGGAAATTGGGTCCCACATGAATTAAAGTCAAGAGACGTCGAGCGCCGCTATTTCACTTGCGAACAGCTCCATCAAAGACAAAAAAGGAAGGGATTTTTGCATCACTTTGTCACTGGCGATGAAAAGTAAATCCATTACGATAACCCAAAGCAAAAACAGCATATGTGAAGCGCGGCCAACCAGCACCATCGACATCAAAGCAAGATATTCACAGATTTAAGGTAACACTGTATTTGATGGGATCAAGAGGGTGTTGTGTATGAACTGCTGGAACCGAATGTAATCACCCTACCGACTCCAATTAATGCGTTTGAGCCGAGCATTGAAGGATAAACGGCCAGAATATAAGCAGACATGATAAAGTAATTCTTCAACATGACAATGCTTGGCCACATATTGCAAACATCGTAAAACATACTTGGATACACTTCGATGGGATGTGTTACCCCACCCGCCGTATAGCCCAGACATTGCTCTTTCTGACTACCATCTTTTTATATCGATGCAAGATCATCTGGCTGGTACATGCTTAACTTCTTTTGAGGAAGTAAATAATTTGTTCATCACATCAAAAAAATGAGGCATTCCTTCGGCGCAGAATCCGTATGTTGTCAGAAAGATGGGAAAAAGCTATAGCATCTGATGGC
MSTRG.14460	G-protein coupled receptor Mth2	GGATGGAAGATTGTTCATCGAGCTGTCTAACTCAATACCGCCGTATACTCTGCGAGAACCCGACAAGTACTGCATCGATACGTTTGTCTCCGAAGATGTAAATGGGGTGAAAAAGTCCCAAGTCGATGCGCTGGTCTGTTTCGCCGACGAAGCTGGAGGGCATCATTATGCTTTGAGCAGCTCATGTATGTTAATTTCCTGCGTGTTTATCCTGGCGACGGTGGGGGTCTACGCATGGTTACCGGAACTGCGCAACTTGCACGGCAGGGTGCTGATGGCATACCTCCTGTGCTTGTTTGTGGCGTTTGCATTTATGGCCACTATGCAGATTCTGCTGCCGTTGAAGATAATCACCAGACCTTATTGCATTGCAATGAGTAAGTACTTT
MSTRG.14485	Exportin-5	GCGTGCACGCTGGCGCTGCCGGCGCTGCGCTGCGCGCTGGCGGGCGGCCGCGTGGGGCCGGCCGAGGCCAACGGCGCGCTCGCCGCCGCGCTGCACGCGCTGCGCACGCACGGCCAGCACGACGCCAACCAGGCCGCGCTGCTCGCGCTCGCCGTGCAGGCATACGAGACGCTGCGGCCACGGTTCCCGGGCGTGGCAGCGGTGCTGCGCGAGATCCCCGACGTGGACGAGCATGACCTGCACAGGCTCGACGAGAAGCTCGCCGCCAACACCGCTAAACCCTCCAAGATCGATAAAAGCAAGCGGGACCTCTTCAAAAAAATCACCTCTAAGTTAATTGGTCGCAACGTAGGACAACTATTCAAGAAAGAAGTGTCGATTTTGGACTTGCCAACGATGCCGGCGGTGGAGCGACCGCGGCCACTCGAAGCAGACGGTGTAGCATTTGAACGGTTGTTTGCTGGCGCGCCCACGTAGCAACTAGCAGCTGCAACCATGTCTCATGATATCCCATATAATGTATAATAATTATATCAATGTAATATTAAATTATGTTAATTATTCAA
MSTRG.14488	Spectrin beta chain, non-erythrocytic 2	AGGACTCGTTCCGCAGCGTGCTGCAGCACGGCGACGCGCTCGTGCGCCAGGGGCATCCCGCCGCGCAGGAGATCCAGGAGAAGTGCTCGGCGCTGCTGGAGGAGCGCGCGCGGCTGCAGGCGGCGTGGGGCGCGCGGCTGGTGTCGCTGGACCAGCTCATCGACCTGCACTGCTTCCTGCGCGACGGCAAGCAGCTGCACGACCTGTGCGCCGCGCAGGAGGCCGCCCTCGG
MSTRG.14492	Hypothetical protein	GGGCACATTGATTCACGGGGCACATTGGTACAGTATCTATATCTCAAAACCGATAAACGCTACACAGTCCGGCACATCACTAGAATGCTCAAGGGCATTCACAAGAATGACAGTTGTGACACCGTTCGCCGCCAGTTTGCCATTGGACCTCAACGGTCACTA

	KGM_201209	ACGTGTTGAAAGTGGCGTGCGCCGCTGCCTACAAAAAAAAAAAAACGTGAAATGTAATTGTGTCTCAGAAGGATTCTTTTA<br>GTTGTTTTTGTTATTATTTGTTACTACAGAAAATGGTGCAGTTGTACGTGCACAAACCCAAAACTGATAGAAAAAATATCA<br>ATGAAGAAAACATTGAAGACGCAATACGTGAAGTATTGTCAAAGACTTTATCTATACGCAAAGCAGCCGACAAATATAGC<br>ATCAAAACTGAGACCCTACAACATTGCATAGAAAAAGCCAGAAAATCATTTGAAGCTACCAGAATATTATCTACAAATGA<br>AGCATGCTCTTCATTACAAGCAGAATCTTCTTTATCACAAGCAGGTCCATCTTCACCACAAATAGCACCATCTATGGAGAT<br>GGCACAAATAGCGTCATCTTCAGCACACACTACTACTGCAGCTTTATCATCGCACACTGCTACAGCTACTTCATCAAACAT<br>CTATGGTTCCAAGTATACCGTTGCACAAGTTTTTTCAATCGAACAAGAAAAGGCATTGACTCAATATTTATTGAATTGCAG<br>TAAAATGCACTACGGCCTTACATTGAGACAGCTTTTGACTTTGGCATACGAGTTCGCAGAATCCTCAGGGTGTAATTATCC<br>AAAACCCTGGAAAAAAAACAAATGCGCCGGAAAAGATTGGGCAGCTGGCTTCAGGAAGAGAAACCCAGAATTAAGTCTA<br>CGAAAACCAGAAAACACAAGCGCAGCAAGATGCTTTGCTTTTAATAAAGCTGCTGTAGCTCAATTTCATGATAATTACGA<br>ACGCATAATGAGACAGTACAATTTTACACCCGATCGAATAATAAACTTAGACGAAACAGGCATAAGCACAGTTCTTTCTA<br>CTCCTAAAGTTATTGCTGGGAGAATACAAAGACAGGTGAGACAAATAGTTTCTGCAGAACGCGGAGAGTTAGTTACTTTC<br>TGTGGTATTATTACTGCTACTGGTTCTTCTCTTCCTCCAGTTTATGTCTTTCCAAGAGTTCACTACAAAGATCACTTCCTAAA<br>TGGAGCTCCTGATGGATGTCTAGGGTTGGCAAACAGAAGCGGCTGGATGACATCAGAATTGTTTATAAGAGTTTTGAAAC<br>ACATTCAACGCCTTACTTCGAGCAATAAAGATAACCCAATTCTAATCATTTGTGACAACCACGGATCGCACATTTCAAGAG<br>AAGCTGTTAACTACTGTCGTGACAACGGTATTGTTTATTTGAGTCTGCCCCCACACACGTCACACAAATTGCAACTTCAAA<br>GGCAAATTGAAGATAGCTTTCAACGACTGGCACATTCAAAATGTTGGAAAGACTTTGACCATATACAACATTGCTGAACT<br>GTCAAAATTAGCATATTTGGAATCATTTACACCAAAGAATATCATAGGTGGTTTTTCCAAACCTGGAATTTGGCCAATTAA<br>TAAACTGATATTTGGCGACGACGACTTTGCACCGATAGACATTTTCAGCACAGGTTATCACGATTTGACAGATGAGAACAC<br>TCACAAAACACAAGATTTACATTTTGTAGACTCGGAAACAAGTCAAAATAATGAAGTTGTTGATAGAGAACAAAATAAAA<br>CTCCTACTTTGGATACCGACCCAATTTCAGTGTCTGATGCTGACGATTCTCTACATGTTTCTTCATCACAGGCTATGCTTAC<br>TCCTGACGTAGTTAGACCTTATCCAAAAAAGGCGATTACTGACAGTGTCTTAAAAAGAAAAGGAAGAGAAAAGGGACAT<br>ATCAAGAATATACACTGACACACCAGAAAAAAATAGATTAGAAAGCTTACGTAATGAAAAGGACAGGAAAAGAGAATTA<br>CAAAAAGCAAAACAGCATGCAAAAGAATTGAAAACAGCGAAAAATCTGCTGGGGTTAACTGAGCCAAAAAAGAAAAAG<br>AGAGTAACTTCTTTGCCTGCAGAAATATATATATATATATGAAGTCGTGATGACGAGTGATAGTGAAGAGATTGAAATGC<br>CATCGGAATCAGAAGAAGAGGTAATTAATGAAGAACCAGTTAATCCTGAACACATAAATATTGGAGATTTCCTACTAATT<br>AAGTTTGAGAAGAAGAAAACTGTGATTCACTACGTTGCCAAAGTCGTATTAAAGTATAATGTGACAGAATATGAAGTATC<br>ATATCTCAGAAAAAAAACAGGGTCTTATAAGTTCATATTTCCAATTGTGGAAGACAAAGCAAGTGTGGATGTTAGAGATG<br>TAGTTTTGCAATTACCAAAACCAACTTTCTCCAAAGGCACATCTCGAACATCGTCGCTTTATTCATTTTCGGTAGGTTTAAC<br>TCGATATAATATCCAGTAGATTGTTTTGTAATTTATATGAGAGTTTTTATGATTATTTTTGTTAAATTTTTGATGTTTGTTTA<br>ATTTTTTAAGAAAACTATTGTTTATTTTGATAGGAGGTTCTAATATTATATTTTCATTAACAATATTCAATGTTAAAGTCTG<br>ATTACTTAGTAAGTATATAACTGATTATATAATTAAGAAAATAACAGAGAATTAGTCGTGTTCTATGA
MSTRG.14553	Uncharacterized protein LOC106118616	CAGGGCTATGAAACTAGTTAGTGAATTCGAGGACGAGTACATTCGAGGCTCACTGGTGACAGATGGCACCGAATACGGCA<br>TTTCCGGGAACGTCACCAATCGAGAACCTCTCGAAATGGTCGTAACCCTAGTGCCCAAAGGCCAAGGCGAGTTGATAGAC<br>GTCCGTATGAAGTTCGAGTCGAGCCCCACCGTGTACGGTATGTCGGCGCAGCTCACGGGCGCGGTGCAAGCCACCATACA<br>AGCTAAAGCAGAAATGGAGACCAAGTTCACCGACATCAACCTTAAAGTTAGTTTTTTCTTTATCATCATCATCGTAACAGC<br>CTTTAACTGTCCACTGCTGAACATAAGTCTCCTCCCTTGGAGGATTTTGCCTGCAGTTGCCACGCTTCGCAACCGCAGTTAG<br>ACTTTAGTAATGGTTTAACAGAGACACTGCTACCCATCCCTCGACTATTAAATTCCCTTAGTCGCTTTTTACGACACCCACG<br>GGAAAGAAAGAGATGAATCATTCTATGCCGAGACCATACGGCTAAAAATGTGGACGTTTCGAGTTTTAAATCAAACAGAA

MSTRG.14570	Uncharacterized protein LOC101735678	AGCTCGACATGCCAGTCAGCAGGCGCTTCGCCAACATGGGCCGGGTAGTCGGCATTACGCTCGGCTGTTTCCTGGGCATGA<br>CGCCGCTGCTGTTCAAAGACGACGAGAAGGTGCCCGACCCCAAAGAGGAAGAAGTACAACCAAAAAAGTAAACACGGTA<br>AAGGTGGCGCTGGCGGCCATCGTCGCGACACTGCACTAGTTTGATTGACACATACACACACACACATCACATCTTTGACTG<br>CCATGTAATGTCCTAATAACAATTTTTTATTTGTTTTAAATATATTACTTTACCAAAAAGTAATAATTCTAATGCTGGGTAC<br>ATACCTGCACTTGTCGTCATGAAGCAATTTTTATTCTGTAATATCGTACTATTGCCTTTACCCCCTACAGCAAGTGGCTGAC<br>GAGTCTATCGGCAGATATATCTACCGCACACCAAAAAAGAATTACACATCGCACATTGATATCCTGTTCTATCTGGATCTA<br>AATCTAAGATTAAGTAGATTCGTTTGCCAAATCTTTAAGAGGGCTGGGCACATTAGCGACTTGTTAACAGTTAACGAATTA<br>AATTAACTAACTAATAAAAATTATTAAATTAACTAACTTTTAACAATCTAAGAACAATAGTTGCCTTAAACAATGCTAGTA<br>AATTAATACGTAGTCCTAATTTCCAATACAATAATTGATAATGGCTGATTTCAACCATACGTACCTATTATTATACAAAACT<br>TCGACACGACAAATGCTGGTGTGGCCCCAGCATAAATATCCTACGACTCTTGATATTTCGATTCGATGTCACGTGTGTGAC<br>GTTATTATAATTTTGTTTTAATTTAATAATTTTAATTTATAAATGTAATGTCCACGCGAGAATGCGTTTATGTATATACATAT<br>AGGTCTATATTAATATTTGCACGACTTCTAAATAGGTTTTTTTTTTTATACAACTTAGAATGGCAAACAAGCTGACGGCCCA<br>CCTGATGGAAAGTAGTAACCGTCGCCTATACACATGAGCAGTACCATGGACATAACAGAGAATACTGTTTCGCCCATGAT<br>ACGACCCCATGCGTTGCCATTCTAAATAGGTAGCTAATTGCATTTGTGAATTGAATGTATAGTGAATCTTATCAAGTGTTTA<br>AAGGTTTAATTGTTGTGAATAGCAATAGTACTTACAAATGTATTCATATTTTCGAAAATAGCACAAATTATAGGTACAGTT<br>GTAATAATATATATATTGTAAATACCTTATTAATTAACAGTGGCATTATTATTATTTTGCTGCAATAATGTACAAAAGTGTT<br>TACATTATTATGTGTGACTGTACATATGTAAAGTCATATGTTTTTTATAATAGATCAAACAAACTCATTAGTGTTCAACAGA<br>CGGTGATTCTTATTTATTTATTTATTAAACTTTAATACACCTAGATAGTGTAAATGGCGGACTTAACATCATATGGCGTGCT<br>GTCTACCAGTCAACCGTAGACAGGAATAATAGAAAATATAATTGACGATGCGACGTGATGAACAATACTAAATTAAAAAA<br>AAATATTTAAAAATAACACATATAATACTCATATATTCATATATATATATATATACATTTCCATTCGGATACAAGGAAAAC<br>ACCAGCATTAGACAAAGCAAAATGAATACCATATGTTTTAAATATCGACAAGAACATTTAAAATACCTATCACTTTTACTC<br>TTGCTAAAACTGATCTACATGGTGTGCTACGCTCCTATATACACGCGTGGGTTGTTAAACTATATACTGGTGTATTCCCACA<br>AAGCTGCTGCACTATCCACAGAGAAATTATTGCCGCCTCACTTTATTGTGCATTATTGAAGTGTGTGTATTAAATTATCCAA<br>TCTGGTGGTCTACCATGAAATAAAATTCCGAATTTTCGGGCTCAATTTCGTGTTTTTGTTCGTATCGAAATCGGACCAGTAA<br>ATGGAGCTTAACATTTCGTTTATCATAAA
MSTRG.14593	E3 ubiquitin-protein ligase MGRN1	AAACTTTGAAATTACCAGGCGGCGGAAATATTAAATCGTTGCAACCTGGAGCGCAGCTCGTCCACAAGCCTGGGCAGTAG<br>CGGCAGTAGGAAAGCCAAGAGTGGCTCCAAGGATGACATCAAAAGCGCCCGCGACAGGTCCGTGGCGCACGCGGTCACA<br>CCTGAGTTCCGCATGTCGGTGTTACTGGCGCGGGAGGAGGAGGCCAAGCGCGCTGAGGAGAAAACCGCTGCCAATAGTCC<br>CCTGCTCTACAACCACAAGAATACAAATGGCACTGATAAAATCTCCAAATCATCTTTAAGCTTGGAGTATCCGACGGACGT<br>GGGGTCGGCACAGGACGGCTCGTGTTCGGAGGACGAGGGCTCGCTGCGTGCGGACCACTCGCCGGCCGCCGCCGGCCGCC<br>CGGACGCCGGACACGACGTCGACCCTGACGGCGAGCCGGACATCGACCCGGACGCCGAGCCGGACAGCGACGCCGA
MSTRG.14597	Uncharacterized protein LOC101745796	TATAATATAAAAAAAAAACATAAAAATATTTCAAATTTCAGAAATGAGGATTCTCAAAACAAATCTGATCATTCAGACGA<br>AGAGCGAGACAGACAGAATAAAACAGAACGAGAAGCTTCCAAACGTAAGAACGAGAAAGGAGATACTCAAGACCGGCC<br>TGAGTCGTCCAAACGCCAAAAACGAGATCCCAACACTAGTTCGTCGTCTAAATATACAAACTCGTCCGACTCTGATTCAGA<br>CAATGATGACAATGATCAAAGTAATTGTGTTCTTATAAAGAAATTCAAGCTAAATGGAGTCAATTTCAGCAAAGCCACCA<br>CGATTAAACACAATACAGATTCGGAAAACGGCGAACAGCCATCGACATCAAACTGCCAGAATGCATCTACCGATTCCAAG<br>CCAAAGCTCGAAAGTTACGTACGTTTCCGTCAAAACGAAGCGCCTGTATTTATTAATTGTCAACTATTTAATAATGAAAAT<br>AGAAATGGAAATGCAAATTCTAACGTTAACAATGATATTAGCAATAATGGAAATAGTAATGCAAACAGCAATAGCAACA<br>GTCATGGAAACAACTCCAATAATGAAATTAACAACGCTAGTAATGAAAATATAAACGGTGATAATGAAAATGCAAATAAT

MSTRG.14598	Uncharacterized protein LOC106715637 isoform X2	GAATATAGAGATTTTGAAAATAGAATCAACGATGGCCCCCCGTATAGGATAGTGCAATACAATCCCCAAGATCCATTACA<br>CCGTATACCAAGAGCCCATATAGTCTATGTCAATGTTAGAGAGCAACGCCACAATGTATGCCGTTTGACGATACCAAACG<br>AAGCGCCAAACGCCCACGAGCCTAGATATTGTGTGACCAGGTTACCTCCGACCAGGGAATCTGATCCGTCAACGTTAGTC<br>ACAGACATTGCGGTGCAGCATTTCGGGAGAGCTGAAGGGGAGGATGTAAGTTACTTCAGGATCGGACCGCCCGGTCCTGA<br>ACTAATGGGGAGGCCCAATGTGTCCAACCTTAGGGAACTAACAATAAACGGATACAGGAACATAACGGATCGGAGTTTGG<br>TACATCTAGCGACGGCGGCGCCGCATTTGAGACGGATAGATTTCCGTGGTACAAGGGTAACGCAGAGAGGCGTGGACGAT<br>TTTAAGAGCCTGCGTCCAGATGTAGAAATCGTGTTTAGTGAATTTGTAGAGAAAGAATAGGGAGCTCACGCAGTATTTCGT<br>GAAGGCAATATACTATGTAGCAACGTAAACAGCAAATAGTGATTTGGAAGTCTACAGTAAGCACGGCTAATATCGGTACT<br>GCTCTGATATCTATATACTGGGTTTCTGTGAACATCGATGTCGGAAAGGTCAGCACCCATTACGATATCGCCCAAATCAGT<br>CGGATATCTCTATTTATTTCCATCGGCTTTTTAATTTTAAATAATTGAATAACGGATATTTAATATATCCGTTATTCGGCTAA<br>TACATAGATATAGTACCTACAATATACACATATTGACTTTGATGGCGGGAAATAGTTTTTTATTATTTTGCCATCTATCAA<br>TATGAACTATGTTTCAGTTTAGTGCTGTATCGTTTACGTTGTAACACAATAGCGTCTTTATCGATTTATATGCCAGTGAGCC<br>TCCTTACGAATTAGTCGCAATACGGATTTAGCATAATGTGCGTATCAAGCGTATATAGCATGTATCAAGCTTAGATAAAAT<br>ACATTTAGATAGAGTTGTCGAGTGTCAAGTGCACACAGAAAATGGTTTACGTGTTTTTTTTTTTTTTTTTAAATAACATTGC<br>GCGCCATTTCTGTGTTGCCAGCAATGTTTCGCTTATTTATTGTTTTTAATGTTATTCTAATATATGTATATGCAATACAGGGT<br>GATATCAAAATCGTAAGCATCCATTTTAAGGAGTGTTCTGTATAGGGGGAGGGGGGCTGTCTTTTTACCCACGGTTGCAAA<br>TAAGCTTTGCAAAGACGTGTCTAAGAGTAAATATGCCCCATTTTCGTAATGCCCAGTTCCCGACTCCCTACTCCTGGAAAA<br>TAAACTTAAATACATGGTGTTCTACCGGCGAAACTCAACATTTTCACCAAAGGAACATGGGCGAGAATCAATTTTTTAATT<br>GCCTCCCATATTGACGGCGCATCCAACAGTCAAGTTAGTATTATTCAATTTTTTTTATAGTTGAGTGATATTGAATTTTTTT<br>ATCTACATTTCTGTATTAATATATACTAGCTGTATCCCACGGTCTCGCCCGTGCTTGTATCGTGATATTGTAATTGCCCATA<br>CCTTACTCAAGTAATGAGTTATTAAATAAACCCAACCGATTGTTCCACAGATTGAGTGTTTAAATATATATATATATATAT<br>ATTATTCACATAAGAATAAAAATATAAAAGCCGGTCTGTTTTACGCAAAGCATTTGTATTGCGACACTCCATCTAGATGCC<br>ACTAACCTAGTACATAATTTATGCGAATTTATTTTAAATATGAAATATCAAACATCGGACTGGAGAGATCTCAGATTAATG<br>CTAATGTTAAGGTAGAACTAAACGCATAATGGCGGACATAGGCCGCCATTTTGTTTCAATAAATAATATTGTACTATTATT<br>ATAATTGCAATTATTTTGAATTTAGAATGTAATAATTTTATTATAAATTAATTGGTTTAAAGATATATGATTATAATAAAAT<br>TTTGATATAGTAATTTTTTATTGTTTTTATTTTTCATCAATCCAG<br>GTATAATGAGAGAATGGCGGCAGGTCGCAAGAGTCTGTTCATATTCCTCATGGGCCTCGCGTGGAAGTGTCGCTTCAGTAT<br>GCCGCTGCTGCTGAGCGGCACCGTGGCCATACTGGGCCGCGCCTGGATGTTCCAGATACACGTGCGCACCGTCCGCAGGC<br>TGCACGAGAGCGAGCTCGCTTATTACGATGATGAAGAGGAGCCAGATGACGACGAGGGTTCGTGGGAAACAGAGTCCAC<br>AGACTCTTGGGAGACCATCGTGGAGGAGGTCGCTTACGGGTAACGATTGTCGTCCTCCATTAAAATGTAAACATCAGACC<br>AAGTACTATGGTTAAATAACTACAATACCTATTTATATTGACATATTATCAACTATGATTGTTGAAGAAACTGGCTGGAAA<br>TAATTGATAATTTTGATCTCTGTATTAGTGCTCTATGGGAATTTGAACTTAACGAAAATAATTTATGGTGTTCGAAATTTGA<br>TTTGCTTTAGGATTAATGCACACTGCGATTTTATTCTCTTAATAATCTTAAATATAAAATATAAGGAAAAAAAAATCGTAA<br>ATCACAGTGTGTGTTACGCTTACATATTTATTTTTAATTTTACATATCTTACGTAAAATTTTCACAAGTAATAACAATTATT<br>TCTTTACGGCATGGAGTAAATGCATGATGCATTAGAAATGCTGTACAAGAGAATGATTCTTTTTATTGATTATATTAAAA<br>TTATAATCATTTTGGTTTTAAATATTGTTTTAAATTGTTAAAAGTCCTAATAGCAGTCAATGTCACAATATGCATTAGTACC<br>TTCATACAAATGTAAATATGATTAAATGTATATTTATACTGTAAATTATAACATTAGCAGATACAATTTATTCATCCAAGA<br>CATAAACACATCAGTGATTACCAGTGACATTACGACATCTACAAGCCACAGGTCAAAACCATTTAATATCTATATAAATAT<br>GTTGTAAAGTGGTCCGTAGACGTTCAAGCAGCTGGAAT

MSTRG.14604	DNA-mediated transposase	TATAAACATAGTTGATAATGAGTGATAATGGTATGCTAACCCCGTCTGCGCTATTGTTATACACTGGCGGGGTATCAGTGA<br>GGGATGACAGATGAAGATGAAAGTTAACCTACTTTAGCCCATCCTAACGAATTAAACTAGAATTAGTCACGATAGTTTCC<br>AGGGGGAACCACGTGGAGGTCGACCTGACAGGGTATATTGCTAGTAATAGGGCTATTTGACCCCATTACTAACAATCCCTT<br>TCCCCCCAGACAATTGTTATTAGATCCTGGTTACGTTATTTTTAGCGGTCATGGTGTTTCTAATCTAAACGAGGTCTGTTCT<br>GCCACCGACATGAATTCCCGCCCAAACCATAATTACCCCGCCATGGTAAGGATGGACTTTCTGAGGGTGTTGCAAACGGCT<br>CTTACGATCAGGAACTCGCCATACACGTACTCTTCGTGAATCAGGATGGAAATCAAATTGGGACTTTTCGGTAAACAGTAC<br>TCCAGACCACTGGATATCCTCCCAGAGCAAATGTTTATGTGTCCACTGTAATCTTTGCCTACGATTTCCTCGGCGCAGCGCT<br>GAAGTACAAAGTAGTCTGTGAGCATGCGGGTTAGCTTTATGCAGTCTTCTCCTCACAGGTTGGTCACTTACAAACAACTGG<br>TGCTCTGACTGGAGCCTTTGAACTAACTGCATGGCCGTAACATTTGGTTGCATTCTCGCGGCAACTTGCAAAAATCGGTCT<br>TGCCGGTTATTGATAATGCGCCTCCCGCTAGGGTCTTTCAGCCACTTTACCAGTAGCACGATAACGTGACCATAATCTTGA<br>TATAACATTTTGTTTTGTTCCAATCGCCTCAGCAACCGTCCGTTAAGTAGCCCCCGATTCGAGTATGCCTACTGCCCGTAAC<br>ATCTCTCTTGTTAATTTGACAAAAAAGATTTCTAAAAATTATGATATGGTATTTAAAACATTCGAATTTCACCTTAGTAATG<br>CCGCGCAAATTAATTGTCTTTATTTTTTCAAATTCAAATTTAGAATCGTTAAAAAAAACCTACAAAATATTTTCAAAAAAA<br>ACAAATGGTCAGTATATTTAGAATTTAAGAACTTTATATGAAATATCCCTAGACGGTGTTGAGGTGTGTATTTGTTATATA<br>CTTATAACACAACATTTTTTTTTGTCATACTAAATACTACTAAATCTATGGTA
MSTRG.14613	Fibronectin type-III domain-containing protein 3a-like isoform X2	GGTCCAACAAATGGTGCAGGAGGTCCTCAACATTACTATGGTGCAGGGTACTCACCTCACTACCATCACGTGCCTCAACAC<br>CATATGCAGCATTCTCCACCTCCATCGGTCTTCCAAAAAGATGAACGGACACAACGGCAATATTCCAAGCTTAAACAGAA<br>ATTGGAACGTAAACAAACTAATAGAAATAATGGTGTAGTGGAATTGAATTCTGGTGCGAGCACACCGTCGCTGTCACCGC<br>GCAAAGAGTTAAATGGCCGCGGTGGAGGCAGCGGCGGCGCCTCCTCGGGCGCGTGGTCCGAGGGCGAGGGCTCCTCGGC<br>CGGCGCCTCCGTGCAGGACGACGACGAGAACGACACGCAGGCTGTGTTAGATATGCTGTCCACGATACGCACACCACAGG<br>TGAGCGAAATAACTTCTACGAGTGCCCTAGTGCAATGGAATTCACCCTATCCAGATGGAGTGTCAGTACCTAATTTGGAAT<br>TAACATATGACCTGCTGCTTGCGGAACGTGATGTCAAACGATACAAAGCAATTTATAGTGGACCATCGC
MSTRG.14614	Zinc finger protein 567-like	AAGAATGAATCTGAATTCTTTTAAATTTTAAAATTTCAGGCATGTGTGAAAATAAATATGCAGACAAGAGTGAACTTATTA<br>AACATTTCGAGATTCATGTTGATAATAATTATTGCCAAATATGCTCTGAAAAGTTTAATTGTTGGCCACAACTCTTTAGTCA<br>TCGACTTGTACATTTACCCGAAAAAGGGGGCAAATGCCATATTTGTGGCAAAAGATATGCAACACCCTTATATTTGGAGTA<br>TCACTATAGAACATTACACTATAATGGAGAGACTATGTTACAGTGCAGTCTATGCAGTCGTAGTTATGGTACACCAAGGAA<br>ATTAAGGAGCCATATGCGGAGTAGTCACGCTGAAGTAAAATATATATGTGATTATTGTTCTAAAAGGTTTATTGAGAAAA<br>GCACACTGAGAACTCATATTAGATCCCATA
MSTRG.14617	Uncharacterized protein LOC106684797	GTGTATTTTATGCGATCGGTATAGAAACAGAAACCATGATAACATATAAATGAAAAAATAATAACTTACCGTAGATATGT<br>GTACTTCGTCACTTAAAAAACTCAATAACTCAAACGACCAAGTCTTCGGCTTGTAAACATTAGTAGACGAATCGTTGAGTG<br>CTCTTGATAAAATAACTTTTTTCAGTTCTCTTCTATAGTTGGATCGCAAAGTGTTTATTTTCATTCTCACGTCATTCACAGTT<br>GCCGCGGGTTTTATTAACTTGTACACTTGTAACAATGTCTGCAGAGCTTCGTGCCGTTGATATTTGTTAGAGTACTGTGGAT<br>GTTTCGAGTCCCATAGTATTGTTACAAGTTGGCCTGACTGGGAATCGAACCCGGGACATCAATTGGGTGGGCAACGTGACC<br>ACCACTACGCCACGTAGGTCATTAACCTTTAAATACCTGAAGTTTCAAATTTTGATGCCGGGGTTATAAATCGCATTGGGC<br>GCCTACAATCGATTTTAATATTTGGCTGTTTACATGTTACCAAATGTTTTTTTCAAAATAAATATATAAAAATAT
MSTRG.14619	Hypothetical protein ALC57_15604	ACAGGGTGAAGAGGGTTTAAAAGCATCTTCAAAAGAATACTTGTGGAAGCGATTTTTTTTCAATAGCTTCTCACTAATTGC<br>GTATCTGTATTGAATTTTTTCTTAAATTTTTATTTTAAATGAACATGGACGAACCTAGTACAAGCAGTAGCAATGATACTGA<br>AGAAAAATTCTGTGAAATTTGCCTTTTAAATACGTCAGAAAGACTTGTGCTGGTGACTTCAAAGGGTAAAGTAGGTTTAAA<br>AGCAGCTTCAAACGAAAGACGTGATAAATTGTTTTCAAATATGGATAATTCAAAAACTGTGTATGTTCATCATACGTGCAG

AAGTAATTANNNNNNNNNNNGGAAATATTGCAGCAAGTGCAAAAAGAGCTGCTGTAAAAAACAGTGTAAGTCCAACAAAG
AAAAAATTGAGAAGATCAGGTACAAGTTCAAGTGTTGGTTCAGCAAGTGATGTAAGTGCTACTCTTTTTGACTGGGATAA
AAATTGTTTTATCTGCGATAATGAGATAAATACCTTACAAGAAAAAAAAAAAAGCGTCTGCAATGAAAAAGAAAACTTCGA
AAGTTCAGTCTTTAGATTTTATACAAAATTTAGCAAAAATGCTTATTATATTCAAAGACGACGAACGTCGAGAGATTTTGA
AGCGCATTCACAGCGCGGAAAATCATGTCATTCTAACAGAGGGCAAATACCACGAGGATTGCGTACTAAAATTAAAAAAT
GAATATAAACTTTTTACGAGAAGTTCGCAAACCCCTTACTTTGACAAAATCAGCGAAGCAATGGAAGAAATCTACGATTTC
ATGCTCTCTAGTGAAGAGTGTCAATTTACTATGACTCAACTAATCGAAGCTGTACAGATATGTGATGTAATTCCACATGAA
GATACTATAAAAAATCATTTAAAAAAAAAGGTTTGGAAATCAAATAGTTATTTCTAGCAGAATAGGTGGTGTAACGTACGT
ATGCTTTTCAACTACATTGTACGATATATTGACCGACTCATGGCGAAAGCAAAAATCAAAAACTATCGAAGAGGAAGAAA
ATGCACTCATTGACTCTGCTGCCGAGCTCATACGTAGAAAAATTAGAACCGTCATTTGCGATGTCAATCAGTATTCACCAA
GTGATAAAATATTAATTAATGTTAACGAGAGCATTCCGCCTCAATTGCTCAGATTTTTAGAACATATTATTTATAAAGATA
AGCATCATAATGAACAAAATTTTAATTGGTATTCAAAAAAAATTACATCTATTGCTCATGCAATAATGTCCTCCGCTCGAC
CTAAAAGTTTTATTTCTCCACTTCAACTTGCTACAGGTGCTACGCTCTACAGAAAATTTGGTTCGAAAAAGATGATTGAATT
ATGTTATAATCTTGGTTTTTCGTGCAGCTATGCTGAAGTTCAATTGTACGAAATTTCAGCTGCATGCCAAGACGAGAGACT
TCTAAAAGAACCATTCCTTCAAATCGTTTCAGATAATAGTGATTTTAATGTTTGCACAATAGATGGACGAGGAACTTTTCA
CAATTTAGGATCGATTGAAATAATTACTCCTGCAGAATGTTTACAAGCAAGAAAACCCATTAAACGTTTGCGTAGTTCAGA
AGTTCCTCTTGAATCAGAATTGGTGGAGAAAAACCGAATAGATATTCAATTATATACTAAAAAAACAGGAAGTGGTTTGG
GTATCATTAAAGTTCAAAAAATTAGTCCTGAACCGAAATTTACAATAACCATAATCGATGTGCTGAATGTCTTATGGAGTT
ATTTCAAGTATGCAAGAGATCTCGATTTTTTGGGTTGGAATGGATTCATGTCTATGCTATGTGCAGAAAACACTAATTATA
AAGTGTCTAAAATAAATTTCTTGCCATTTATTAATGGACCCCCAAGTGATTACAATACTTTATTCACAGCTTTAAACAATGC
TGCTGCAATTGTTTCAAAAGAAGGCATGAAAACCTGCATCGTAACATTTGACCAGCCTTTATATATTAAGGCTTGTGATAT
TGCCGAGACTTTGGTTTTCGATGACGTGTTAATAGTTGTGCGACTCGGAGGTTTTCATCTCTTAATGTCTTTTATGGGATGC
ATTGGCACTATTATGGAAGGAAGTGGAATTAAGGAAATATTTTCGCTCATCTTTGCAGAAGGATCAGTTGATCAAATTCTA
AATGGGCATTCGTATGCTCGTGCAGTGCGAGCTCATTTTATTCTGCCGCAGGCATTATCGCTTCTAATATTTGATGAATTAA
AAAGAGAAAACAATGTTGAGTTTCAAGAATTACTTGATAATGAAGAGTATTTTACTTACGAAATGAATTTTGAGAAATTG
AAGTCTAATGAGCACTTAAAAAAACTGAATGAAATATTTCAATCGAAACTGGATGAAATTGAAAACAGAGGAAAAACAT
GCAAACTGTGGATATTATACTATAAAATGGTATCTTTACTTAAAAAATTTCTTGCAGCTGAAAGAATGGGTGACTGGGAAG
CGCATCTGAATTGTATTGAGCTTATGATACCTTTTTTTCACGCCGCTGGGCATTTCAACTATGCAAAATCTGCTCGACTTTA
TCTTCAAAAAATGAGACTTTTGAAATTAATTATGGATCCACGTGAGTTTAAAAAATTTACCAAGGAAGGATTTTTTACTTC
CCGGAGGTCCAATGAGTTTTATGCTGGTATTTTCTCCGACCAAACCATTGAACAAACTCTGATGAGAGCAATGAGCGTAGA
AGGTGGACCGTTCAAACGAGGCACTACGGAAAGCACTGTATTCAAATGGATCAAAGGTATCATATACACGAACGATGTAA
TTGAAGCATTAGAGAAATTTTGTGATATCGCATTTAATAAAAGCTACCAACATGTTGACGCGAGAGATGCGAGAATCAAA
AAAGATAAGAAAGACGTGTTGGTTTTAAAAACATTTTTGTTGGAGCACAATCCATTTGAAGACATTGATCACCTAAAAAA
CATTGTAACAGGGTTGATCGGCACTGATGAGATAAATTGCTACAACGCTCTGGCGATTGGCATCGAAGCCATGAAGAGCA
TCGATGATATAACTTTTAATGACATAAAATTGTCCAAAAAAGATAAAGTTATCTCATTAATTGGTGTCAATAGCAAAGTCA
AAATTGACGATAAAATTGTGCCTATAGATCCACTACTATTATTTCAACGTATTTGTATCATGAAAAAGAGTAACGAAGANN
NNNNNNNTACCTGAAATATGAACTGGCACCATATCCACTTTCATTATTTGACGATATTGGGATGCGAAAGACAAACAAA
TCTATCTTATACTCATTATTCGAAACCCAGGACATTGTGATCAACAAGGAAGCATCAGTGTACTTTATTGATGGAGGAATG
TTATTATATAGAGTAAAATGGCCTTCGAAATGTAATTATGCAGACGTTTTTGACAGTTATATTTCATATTTAAAAAATCATT

		TTGGTAATAATATTACGGTAGTTTTGATTGTTATGATAGAGAAAGTAATAAAGCATCAGAGAGAAATCGTCGAGCACTG<br>AAAGTTGCTTCAAAGGAATACCAATTTACCAAAGATATGCCAGCCAATATTAGCCAAGATAAATTCTTATCGAATTATAAA<br>AACAAAAGACGTTTTATTAAATTTCTTATGGAAGAATTGGAAAAAAAATCTATTAAATGCTGCCAAGGTGAAGGTGAAGC<br>TGACGAACTAATTGTTGATACAGCAGTATCATTTAGTACTGACTTGACAAAAATAATTGTTGCAGAAGATGTTGACATTTT<br>AGTGATTTTAACTGCCCGGGCTACAGAAGACGAAGAAATTTTATTTTTAAAATTAGGCAAACAAAGAGTGCAAACAGTAA<br>TATATTTATCGAAAAGTTTGGAAATAAAATACCCAAATAGTTCGAAATTTATTCTTTTTGCACATTCATTTACTGGGTGTGA<br>CTCAACTTCGGCGGCATATAATAAAGGCAAAAAAATATTTATGGATCTTTTGGAACGAAGGCAAGATTTGAGAAGTAAGG<br>CAGAGATATTTTTGAATAATACATCTGAACTCGAAGATATTTTAGAGGCAGGTAGATATTGCA
MSTRG.14620	Esterase B1-like isoform X1	TAATTGTAAAATAGAAAAATACAAAAATAATATCATTTGAATTGAACAAAGAGAGTCTGCACTAAAAAAAAACAATAATA<br>ATAACAACAACAATCGTAAAGCATAAAACATTTGGTTTCGTTTTTGATGGCAACATTAAAACTTACAAATCTAATTGGGCT<br>TAAAAGGCCTAACATCCAGAGCTGTAAAATATATCATTAAAAATATTTATACCTACATATACACACACAAACAAACACAC<br>ACACATATTTACATTAAGAATTATATGTGGGTATCACCTATCCATGTTAAGGAACATTAATAAAAATGCCAGTCTAATTTG<br>TTATAACTAAGTTATGAAACTAAGTTAGTTAATGAACAACGACACTTGTTATAGAAATTAAGAATTATTTTTCTTTCTACAA<br>AACATTTAAATTCTTCAAAATTACTTTTTTGGGTCATGTTATTTTTTTTTTTATCTAGACGCATAGACGAGTCAGAGGTTTTTT<br>GTTGATTTGTATTAACATATAAATGTTTTAAAAATCATTATTCTTAAGAAATCTATCTTTATAACTTTTTTTTACTGAGGTAG<br>TTTTTACGAACCCCCTATAAAGGCTTCCTGGTCCCCTTAGGGGTCGCAATCCATATGTTGAAAAACACTGTCATAAAATAT<br>TCATAGAAAAAAAAGAACTACAATTACGGGGTACGGAGGTATAGCGTTTTAGTCCTTAGAAATTGCAACGCGTGAGGTAC<br>CAATAATGTATCAATAGTGTATAGATAGAATTTTTTATCTGATCGGCTTGTTTACCATACTTTATTGAATAAAACAATATAC<br>TTTGTTATGTCCATGGTACTGCTCATGTCTATAGGCGACGATTACCACTTTCTATCAGGTGGGCTGTCAGCTTGTTTGCCAT<br>TCTAAGTCCCATAAAAAAAGTAGTCAAGTCCTGAAAATGCTCAATTAAACAACATGATAATTATGAAGAGATAAGTAATT<br>TACAAATATTTTTTTATTTTTTTTTTCAGTAATCCAACACCAAATAATAGTCTGAATGTGAAGTGGGAGCCATTCACCCGTA<br>CTGGGAAACAGTACATGAAACTGGAAGAGCCGCTGTGTGTGGGACGCGCTGCCAACCGCGACAGAATACAGTTTTGGGAC<br>AATCTGTATTGCGAAGCAGGGCTGCCTTGTATTGGAAATTAACCCTATGTTTTGCCTTGTATGTAATAGTCACTATCAATTA<br>GCTAGAATAACAACCGCAAAACAGTTCTATGATAACAAAATAAAGAAAAAAATCCGTAAGAAATTTATGAAAAACTTAC<br>ATTTGGAGCATGGCATTGTATGGGTGTGAAACATGGAATGACGCAAAAAGAAATAAAGATGCTTGGGGCATTTGAGATGT<br>GAAAGGATAAGCTGGACAGACAAAGTAACGAGTGAGCAAGTCTTGAAAAGGGTGAATTAGAAACGAATATTAGTAAAAA<br>CAACAGAGGCAAAATGCTTGGGCCATGACGACATGACGATTTTTAACCCTTCTTTGCATGATTTTTTTTATTGATGAAATA<br>AATCAAGGTGATATAATTCGTGTTCAAAATTAAAGGAAAAACTGTTAAAAAAATAAATTTGACAGCTATATTCTTTTTCCG<br>CAATAGACATTAAAAAGGTATCCAGACACGATTAACTT
MSTRG.14625	Uncharacterized protein LOC101735738 isoform X3	TACAAGTGTCTCCACACCAGCTCATCTACGACTTGTACAACACCATAGCCCTGGCGGAGATCAAGGGCTACGCCATGATGC<br>AGTTCTCTTGGATGCTACTGAAGATATATCGACCAGGCAACACAACAACAGAGGCGAATTCAACAAGATCGCATTATGTA<br>CAAAGCGTGTTACTGACCGCACAGGCCACTAGGGATGCATTGGCTGATGCCAACAGAGAGCTATACCGTTGCGACCCTTT<br>ACAACACGAACGGGG
MSTRG.14632	Uncharacterized protein LOC106130565 isoform X1	CACTGTTCAACGCGTGGCTCGGTCTCGGCGTGGGTCAAGTGGAGGGTCAGTGGCACGCGGCCCTCGTGTCGACGGCCCGC<br>GGCGTGCCCGACCCCGTCACCGATATACCGCCCGCCTTGGCCACCATGAAAATTGTTGTACCCACCGTTGGTGGATACACA<br>TCGAGCGGCAGCGGGCGCGGGACTGTCCGACTCGATGTCGAACGCCCAGTACAGATCGAAGTCTCAGAAGACAGGCTGCG<br>TCGTATTAAAGGCATCCATGCACTGATTGAGGGCAGAGTAATGAGTCCTGTACAGGAGTTTCAGACGCCCGTGGTCAAAC<br>TACCATTCTTGTACAAGTTCCGACGTTTTATGG
MSTRG.1	E3 ubiquitin-	GAAAAAAAGAAATAAAAGAATATATATACAAAAAAAAAGAAAATATATAAAAGAAATAACATTTTTCATATCGTGAATG

4634	protein ligase CBL-B-B isoform X1	TGCCGCAGTAACACTAATATATGTAATGGCGTGAATTTACTGGGTACAGAGGAATAACACCTGAATCCTCAGGAATGGCA<br>ACGCATGGGGGGTATCATGGGCGAAACAGTATACTCTGTTATGTCCATGGTACTGCTCATGTCTATAGGCAACGGTTACCA<br>CTTTCCATCAGGTGGGTCACAACTTAGTTACTAATTCACATGGACAACGGCACAACTAGAAATTTATTCTCATCTACCACC<br>ATTTGTTAAGGTCACATTTTGTATTAATCAATTGAAATGTTTCAGGATTCGGAAGGTCAAGGGTGTCCGTTCTGTCGCGCG<br>GAAATAAAGGGCACTGAGCAAGTCGTCGTGGACGCGTTCGTTCCTCCGCGACCGCCCAACACAACTAGTGATGTCAAAAG<br>CGCAAACACAAAGACTGCCGTGAAGCCGGTATCGTCGAACTCATCAGGGTCGTCCGGATCGTCCGGCTGCAACGGTTCTA<br>GTGTAGATGTAACTATATCGAACAACTCCAACGGTTGGTCAAGAAATGCCACGTTCAATGGACTCACCGATGACATAGAT<br>GACGCTGAGGTAAATTGATTTATATTTACATACGACTTTCTTCTACATAAAACATGTATGAAAAAGTTTGCCTCACAATGA<br>CGCCTAGACTACTAAACGAATTTTTATATGGGTGATTTTCAACAAGTGCATTTTCTGTCTAGTGTCGGTGAAATTTCTTTCG<br>TTTGGAGTCTTCAACAAAGATGGTGTCCCCTAGAAAGTATTTTATAAAATGCTTCCTTCTTTAAATTCCTATCGAGAAAAG<br>GTGAAAGCACTGTTGTAAGATCCACCAAAGTTGGACAGGAGGGGGTCGCATTAGCTGAAAAAAATACCAATGTAATATAG<br>CTATTATAATATATGGTCACCCAAAATAACCAAAAATACGTC
MSTRG.1 4641	Uncharacterized protein LOC106133263	ACAGAACCACAAAATGCCTGCGTCCAGGGACAGCCGGTGTCAGCGCTCAAGTAATGCTAGCAGTGGCATCGCAATCCAGG<br>CGAGGGTGTACCACCCTGCCACAAATCTAATGCCAATCACCCCACCGCGTTCACCTAATGGCGTATCTTCCAGCTTCCCCC<br>GGCCAAATACCACCAATTCCTGGTCACGCCAGAACAGCTACAATGGAAAGTGCTCATGACTACATCAAGCCGAAGATCCA<br>ACACGTTTAATCAGTAGCTGAATTCCCTACTAGTTTAATGATCCCTGATAACTCAATGATAAATCAAGATCTTGCATTTATA<br>TCCTAATAATTATGTACTAAATAAAAGTAAAATATGTAGAAATATTAACATATGAATTCTCCACTATAGTGGTAGTCTTCC<br>TCCCCCTCCCTCCCTCTTAATAAAAAAATAATAAATATTAATTGACACGAGCGTGATTAAACAGTTACATTGGAAAATCCA<br>CGACTTTATTCTAAACAGGATACACTTGTATGCTTCGGAAGCAATGCTGATGTTACAGCGAAACGTGAGTAAACATACATC<br>AATCTATATAAATAGTATAAAATAGATACTGTAGTAAACGTGGAAGATAATTATCATTATCTGAGGCAAGATACTTTTGAA<br>TGCTTCAAAAACAATGATAAATCAAGCTCATGTAATTATACTCCAAAAGCATATACGTATGTATAACTCAGCACCATAGAA<br>GACTCATACTTCAAGAGGCTCGTAACTATCTCAGGCAGGATGCTCTTGTATGCTTCGAAAGCAACAGCACTCAGTAATCAG<br>TTGTTGTACACCAATGTATTGGAAAACGATGGTTTACAATGGAAGATATTCGTGACATTATCTGGCAGGATACTCTTGTAT<br>GCTTCGGAAGCAATGCTGAACCTATCACTCTCAGTTGCTGTCTATTTGTCAAAGAAGATTATAGACTAGTCTGATAACTGT<br>TTTGATGTTAATTTTGGAAAGACAGTATCAATTAATTTATTCAATAGCTGCTCAAGAAAGTTCCGCTTAATTTTGAAGCTCA<br>AGATTTGCAGTGGGACAATAAATATGTTATTTATAGGACAAAATAGAGTTTGATATTTTTTATTACTTTATAAGTTAATTAG<br>AAGCTTTATATAGTGTAGTTAGTAAAAATTATGGACTTCGATACTGAAACTTCTTTTTTAATAAAATGGTATCAAATATTAT<br>AAACTCAATACAGATGGCGCTATTCCTAATTTTATTTCTGGGAGTATTAAATTCTTAGTAGAATAGAGAAACTGACTAAAT<br>TATCAATGTGTAACTGAAATAGCTTGGTTTAGAAATATAAAATTTTCCTCTGTAGTTCTTTGTATAACATAAATGAACTTTA<br>AAAAGGCTCAGGGCAATTTTTGTTAATATTATTTTAATATAATGTTTTAGCGCCATCTATCTAAGATTTTTTTAAGTTGAAA<br>AATAGATTAATATTGGGAAATTAATAGTAAATTTTAAGTTGGAAGTTTAATGAATTGTTTATCTTATTGGATAATTTACTCA<br>AATCGATTGTCCGTGTTACCACTACATTTAATTTCATAATAATTATTTTAGAATTTATAAATATTATCATAATTAGATAATA<br>AACGTGCACTTGTATTGACTTGTGTAGTAACAATTTTTAGTATAAAAGTATGTATTAGTAACTATTATTATGACTAGTATAA<br>TTTTTTTATTCATACTCGTATTTTTATTGTAATAAGGAAAATAAAAATAAAATGTTAACGAGACGAATCTGAATACGTTGTA<br>TAAAGCTTAATTTGTTTTATATACCAATAAAATAAATATGTTTACGACTATAATCCCTTATGAGGTAGTCGGAATCATTTGT<br>CGACTGTTACCATTGTGGTGAACGTAATACTTGACGTGTCCACATTTTTTCGAGTAGATTTAATAAATTAATAAATATTATC<br>TATATTACAATAATTAAGATTGTGATAATTTGTGTGTAATTTGTGTGTAAAATAAAGTATAAATAAATAAATGAAATTGTG<br>TTCCACAAAAAAAAACGACTTTAATTATGGCAAAACCAGTTATTTTGACCTAAAAAGCAAACTACTTAATCGATTTTAATA<br>AAATTTCTATGGAACCAATCTGAAATTTTAGATTTTAAAGAAAGATTTTTCAAATTGGTTTATAACTAACGGAGTTATAAG

		GTAACGAAAATGAAAAAAATACCATTGGATTTATTTTTAAAGACGATTTAAAATCATATAAAAAATTCTTGTTTGCATAC<br>TAGAATAATTCACAGGATATCACTGTCTTATCATATATCGAGTTTCATACAAGAGAAAGTTATCGAATATAAATTGCCAAA<br>ATGCACTTAACTGATTGTTTTCATATTAAGGCTATATATAAATTTTATATTTAAGTAGGTATCTGCATTTATTATAATATTTT<br>ATTTGTTTAAATTTATAAAAAATATTGATGCATTTATTTGTAGTAAAAAATATGTAAATTTTTTTTTTTATGTATTGTATCAT<br>CAATATTATATATTATAATGTTGAATATTTTAAAAGGTAC
MSTRG.14662	Fat-like cadherin-related tumor suppressor homolog	CCGGCCGCGCGCTCCTCCACGCTGCCGCTGCACGTGCTCGTCGTCGACCCGGACGACGCGCCTCCCAGGTTCACTTCGAGC<br>GAAGTAGTCTGTGAGGTGTACGAAAACGAGTCCGAGGGCAGCGTGCTGACTACGATCGAGGCGCGCAGCGCTACGACTCT<br>GTGGTACTCGCTCGAGGGTGGCGAAGGACTGTTTCGCCTCAATCCCGCCGCGGGTCTACTAACTACTGCCGCGCCCCTGGA<br>CTACGAGACGAGGGATTTTTACAATTTGACCGTTGTCGCTTATAATATGG
MSTRG.14670	SID1 transmembrane family member 1-like	ATAGACGTACCTTTATCCTTAGGAGTTGGAATTAAAAAATATATTTATAGTAAAACTCCAATAAGCCAAGCAATGACAAAT<br>AATTTACTATCAGTATCTGATAGATAGGGATTATTGGCCGACACAGATACCCGTGATAACACCGTATCGCTCCCCCATTTC<br>CATACACAACCATCCATTTTTGGTGCACAAGTGTAAATATCGTAGAACGTTAGTCATTTAACATTAAACAATATCCGAGTG<br>AAGAACACGTGCGTGGTTAAGTAGATATAAGGAAAAATATATTTAAAATGTTCTATGAAGTTATATGAAAATGTTTATAAT<br>TAGTGTGTGGCTAATTTCGATAGCAGCGGGTGGTGTGTGGTCGCTGGAGCAAAATGTTACAGTAATCAGAGAGAGGTTGG<br>AATTTGATCAAGAGTATACGTACACCGTACAATACAATACTGAATATATTTTGGAATTTGTACCATCAGTCGAAGATGGAA<br>AGTGGCCGAGTCGAGTGTGGGTCAGTGCGGACGGCGTCGATACTTCGCGCCCTCTACTGATCACCTACAGACAAACAGCT<br>GGTAAGTATATCTTACTAATATTCAAATTTGGAAGTTTGTGAAAATGTTTGGAC
MSTRG.14676	Cytosolic non-specific dipeptidase	ATTACAGCCATGTCTGATTTGATTTATTTAATGGGTCAACTAGTAGACAAGGACGGCAAGATACTAATTACGGACATCTAC<br>AAATCGGTAGCACCGTTAACGGAAAGTGAAAAATCTCTTTACACAAATATAGATTTTGACCCCGAGGCATATAGAAAATC<br>AATTGAGGCCCCGAAGTTGGCACATAATGGTGTAAAGGAACAATTGCTGATGCATAGGTGGAGGTATCCTAGCCTATCCT<br>TGCATG
MSTRG.14687	E3 ubiquitin-protein ligase UBR4	AGCCGGTTCGTCATTTTTACTGACATTTACAGCTGACTTCATAATCAGAATCACATACTCTCGAAAATGGCCGCTTCGGGG<br>GGTAATATTGAATGGTCAACGACTGTAAAACCAATTTTAACGGCTCTTTACGGCCCATTTTTCGACAAAAATGATGTCGTT<br>GAGAATATACAAGCTATTATTAAAAGTGAAAGTGAGTTCCAGAACCATGAAGACGTCTATGATCTATTTTACACATGTTTC<br>GCCTGCCTATCTGCACACTTTATCAGCAGCAATGCAAATAATTTACCAAGTGATCAGTTGAGCACAGCTCGTGAAGCTTTC<br>CGGATTATACTTAGCCAAATATTGAAGAAACTGTCTGAAGCTGGATTGAGCTGTGATGATAATTCTACTACATCTGCTCCA<br>AATGTATCAGTCAAGCAGCTCCTGCTTCCAATTGTGGGACTGTGTGGCATTGATGGAGGGGGCTATCCACAGTCTGACTTA<br>GTAATATTGACTTCTTTGTTGAAAAATGCAAAGTTACCAGCGCATGTTAATGTGCCAGAAACACAAACAACTAAATCATCA<br>GTGATACCAAGCCCGATCACATCACTGGCATCACAAACTACCAGTACAGAAAAACAGGCACCGTCGAGCGCACAGCGTCG<br>TTCCGATGCTCTTTTAGAACAATTGACGATACCCCTAAGGAATCCAGTCGCTGCTGTGACGTCATCGCAGGCGCAACCCCC<br>GGCGGTACAGTCGCCGGGAAGCAAGGATAGCATTGAAAGCTCCAAGGATGGCACTACACAACAGGCTGCCATTGATATA<br>AAGAAATGGATGGCAACGACGTGCGCGTCCCTGCTGATGACCCTCCGCGCCGGCTCCGTCGTGCTGAAGGTGTGTTCCTCG<br>CTGCCCTGTCTGCAGCGCTACCTGAACCGCTGGCAAGCTGCCAAGGACTCAGCTATGGCGCCGCAGTTCAACTCGGATGCG<br>TCACTTCTCCACTTCCTAGTCAACGAGGTGCATGTGTGCCGGCTCGCGTTGTCGCTGCCGTGTCTAGAGCCTTTCACGTCGG<br>AACGTATCGCCACGCTCAGTGATGTCAGCACGGCTTGGCTGCTATGCGCCACTGCACAGGCGTCCCTCCATCCAAAAGATG<br>AAGAGAGTGAACAACAAACAGCGACACTGGTAGAGAGCGCTCTTA
MSTRG.14688	Transmembrane protein 94	TATGTGTGTGTGCAGATGACGCGCGACGCCGACGTGTCGCTGTTCACACTAATCACAATGTCACGCCACTTCATGATGTGC<br>TTGTGGAACAGCACGCAGTTCTGGCTTTGCAGCTCCTGTTTTATTGCTTTATTGCAGGTGGGGTCGCTGTGCGCGTTCCTGC<br>CGCTGTCGCTGAGCGCGGGGGGCGCGGCGTGGGCCGGCGTGGTGTGCGTGCCGCTGCTGGCGGCGACGCTGGTCTTCGCG

		CCGCTCGACGCAGCAATCATGCAGCGCGCCGCCACTCGCCCGAACCTCCATATGACCGCGCAGATGGCGGTGTTTGTGTTC<br>TGGTGTTACGCATGCAAGTTCCTACCGT
MSTRG.14748	ATP-binding cassette sub-family E member 1	AGTTACTTAGGTATGTACCTACATAATGCCCAAAACATAAGTAATGAAACTTTGGGTAGAATTGTCCATCGCGTCATCTTG<br>AATAGGTATTCCAGTTTCAGTATGGCCTAAAATATTCTGTAAAAATTTGCATACGTATACTTGCCTATAAACATGACGTCTT<br>AACTAGTATTTATGTAGGTGTATGTATGATGCAAACTATCATGCAAAAGAAGTGAATGTTGTGAACCGGTTTTGTGTCTTA<br>CACGAACGAGACAACGAGTGCATCGTTCGTTTTATTAAATATCTTCCTACCTAGGTTATCTCGATTGCGCGGGAATTTCGG<br>GAATTCATATTGTTTTTGTTTTACCCAAGAACCATCGGATGTATTTACCCTACAGGGTACTTAGCATCGAAGCGAGTTGATG<br>CATTCCAAACTTATCAGGTTCGATTTCTATTTTTTATAGCAATAACACCGTCAAACTGTCACCATGCAGAGACCTAGTTTGT<br>ATTAGGACAAGGCTAATGAACGCTTGTTAATTAATTACCAAAAACACTTAGCTGAGCAGATGAAGCAAATCAGTTCAAAC<br>GGAAATAACTATATATACAAGTAGTAAGCAATTAATAAATGTGCTTTATGGCTCATTTTAAAAACTGTTTACGTAGGTAGG<br>TAAGTCATAATTTTCAATGCTATTTTCCTGAAGAAATATAAGCAGCATAATGCAAGAGTTAAGCATAAAAATCGATAAAGT<br>TGTTGCATCTTTTATCGAATATATCATGGGGAGTGTTCTGAGAAATTGTTCGGATTGATACCTGCTGCTGATTTTCACCACC<br>GTACAACCTGCCACAAACTAAAGTTCCATCCCAAACAATTGGACAAGTGGCGCTCCACTACCGTGCGTTTTTCAAGGCACT<br>TTCTTCCACGCATAATTACGCTTTGGAATCAACTTACAGCAGCAGTATTTCCGAATGATACGACATCATAACCTTCAGAGC<br>ATATTCCTTTTTAATATGCCGGCAATACACCTGCATTGTCTATGGTGATGGTGCCCATGAACGGCGTTAGTCACATACCAA<br>CAGGCCTGAAATTGTTTGGATCTCTACGGAAGGTGATTCCGAGCAGCTCAAGGAATCTGTTCATGCCGTTCAGCAGCGACT<br>GCGGCGCGTGTGCTGTCGCGTGACTTGATGGCGTTCCCTCGAAGACTATGACGCGGTCCGCCAAATAAGTGGCCATTATAA<br>AATCGTGCTCGACTACGAAGCCCGTACGTTTAGCGTGCAATATGAACCTGAGAACAATTTACTTTATAATTTAGCATTTAT<br>TGTCTGTATAAAAATGTTAATTTCTTTAGAGAAATAAATTTAATATGAACAATATAAAGAAATAGCACAACCAGAAAAAT<br>ATTATTTTAAATAGTGTTAAAACACAATAGTCACCATATTGTTAGTTTTGTAAGTAATACAATTTATTAGAAATTTTAAAAT<br>AACTTTAAAAAATGTGCC
MSTRG.14754	Glutamate receptor subunit 1	CTATAGAAGAGAAACCGTTTGTATACGCCCGAAGAGTAGAATCTGAAACTGATTGTATTGCCGAAGAAGAAATTCTCTGC<br>CCCCATTACAACGCAAGTGACGACTTTGGTCAACTTTACTGCTGTAAGGGATTTTGTATGGATCTGTTAAACAATTTAGCG<br>AAAGTTATCAACTTTACATACTCATTAGCTCTATCTCCGGATGGACAATTTGGAAATTACGTAATACGCAATTTCTCAACA<br>CCTGGTGCCAAGAAAGAATGGACAGGATTAATTGGAGAACTGGTTTATGAGCGAGCAGATATGATTGTAGCCCCTTTAAC<br>AATAAATCCAGAGCGAGCAGAATTTATAGAGTTTAGCAAGCC
MSTRG.14891	Hemicentin-1	GTGGCTTATTCTGTGCCGGGACCTTATGGGGAAGGTCTATGTTCAGCAGTGGACCGTTAATGGCTCATATAATAATATAGC<br>TACATTGTCAGCTTGTAACATAGCCTATATTTTCGAAATTTCTGTCTAACCCGTGCGGAGCCGGGGCGGCTTGCTAGTTTAT<br>AATATATAACTTATAGTAACCTAAAATTATATTTGACAGATGGCAACTATACATAGTGGCGTGTGTAATCCTGGTAGGCAA<br>CGTTGTGCCCGCGCTCTACTTCGGCTACGTACACCAGGCCGCCACAGTGAAAGTGGTGCCGCTCCTGCGGGACGCTGTTCC<br>TCACAACAGATCCTCCATCGCCTTCCTGATGCCTTGCCATTCGACGCCGCTCTACAGCCACCTCCACATAAACGTGACGAC<br>CCGCTACCTGAACTGCGACCCGCCAATCGACAAAGTGGGCGAGACGCACGAATCGGAGGCGTTCTTCAACAACCCGCTGC<br>GGTGGTGGCGCGCCGAGTACTCGACGCGGGCCACGCCCACGCTGCTGGTGATGTTCGACCGGCTGCGCGGCCGGCTCGAC<br>GCGCCGCTGGCTGCCTACACGCTGCTGCATCAGCTCCCGCACACGCAGGTC
MSTRG.14906	Cytoplasmic dynein 1 intermediate chain	GCGTGGCCTTCAACCGCGTGTCGTGGACGCCCAGCGGCTCGCACGTGTGCGCGGGCGACGACGCCGGCAAGATATGGGTC<br>TACGAGCTCGCCGAGCACGTGTGGCAGCCGCGGCACGACGAGTGGAGCAAGCTGGTGTACACGCTGCAGGAGCTGCGCC<br>ACAACCAGGCCGACGACGACGACCGCCTCGGCCTCGCGCTGGCGCCCAGCGGCCCGCCCTCGCTCACCAGCCTCACCTCG<br>CTCGCCAGCAACCCGCTCAGGTAACTGGCCTGGGACGTGAGATGCGGCTGGCGACTTTAACATCACACACGACCTTACAT<br>ACTCGTCTTTTTACCCACTACACAGGGGAATAACCTCACTTGGACTAGACGTAAATTAGTAGCTTAAACTAGTCTTTGATA

		AGCACCAGCGCTCTGCAGCCTCAAAGAGGTCCTGTGTGCTGCGCCACCTTTTCTTTTCATTCATTCATCTTTTCTACAGTACTTAGATACCAATATCATTATTAAATTTAATAAAAAAAAATTACTATGATATTCAACGACAGTTTTAGGTCTAATTTCTCAAAGATTATAGTAATATAAAATTTTTTGAAAGAATGCAAGTATTACATAGGTAAAAAATAGGCTGGATTCTAAGACCATTTCTTTAAACTTATCTTTTAAAATTTGATAGCTTCGCGACATTGCTGTTTATTGACCATCATAAACTGCATTTCTAATAATTTTGACGGATATCTATTAGAAACTTAGTCCATATTGGTTTGTAGAATAAAAATATCGATGCAATACTAAATTGTATGTTTATTATAGAGATAGGTTTAAAGAAATAGCGTTTAGGAATCAACTCCTTTATCTCTCTTACAAAGAAACACTAATAAATTGGTTGCTAAATAATATGTGAAAATTTATGGATATGACATTAAATGTCAAATTAGCTTGATTTTTTAGGGAAGGAAATTTGTTGATAATGTATAATAACAGTTGATGTACAATAATTAAAAAAATAAACTGAGACTGTTGATGCACCGTTTACACAGGACAATGCACAAAATGATGAAATTGAACTTGAAATTTAGAGAGCTCGAGTGTTTACAGCATATTTAAACTGATTCTGGATTATAGAGATGTTTAAGAGATTTTGATTAGGGAGAGATTGAGTAAAAATATCTTACAACAAAATATTTATGGTTAGCTGGAACAAATATGTTAATATGTATCTATGTGTTTAAAGCTTAGCTTCGTACAAACTTGATCAATACTATTTTATGAAGTTATTTAATTAAATATTGAATTTATTGATAGATAATTGCATTTATATCAGCGTATCTATCTTTATCGGACAAAATGTGTTAAGTCCATAATTGTGAATATTGCAGTGTAATACAATGCAACTTATAATCTCTCTTATTCGTATTTCTAGAAATCATTAATATCTGATCAAAAAAATAAACTATGGAATAGAAGTTGAGCAATGACGGGATAGGTGTTGAAGAAGTGACTACACATTTTAATCGAGTCTTATATATGTATGAAAAGAAAAAAATGGACTGGCAGCGAATACTAGATTTGTCCGAAGTGGCGAAATTGCTAGATAGGATGTTGGTTAAAGGTGCAGATAAAATAAGCTTACGAATGTTATTGGCAATTATATTACTGATAGAAAGAGAACCGCTATTAACATGAAAAGAGGTGCAGATATTTTATTTTTCTGTGCAGATGTTAAGTTAGAATAAGTTTCTGTTATAAAGATGTATGTAATTATTGTAACACTGTTTAGTCATTAATTCGTTTTACATTTATATTTATACGAAATGTACTGGATATATTTATTAAAAGACAGGTTACTTAGTATTATAATAATAGTATTGCAGATAGTGTTCTGTGCTTTAACATCAATGAAATAAATGCTTAATAAAT
MSTRG.14907	Peptidyl-prolyl cis-trans isomerase CWC27 homolog isoform X2	GTAGTTGTCTCTATGATCTGTGTTTCTTGCTTTGTTTGTATTATAATTAAAAATCAAATTTGTTTTATTAAAATAAATATAATGGTATCCTTTTTATTTCCTTAATAACAATAAATTCACCTCTACACGATAATTACATATGAAACAATATGAGTAATATATATACATTCAGGAACCGCCCGCATTAGGCAAAGTATTACTAAAAACAACTGCTGGAGATATAGATATCGAGCTATGGACGAAAGAGGCCCCTAAAGCATGTCGCAACTTCATACAATTGTGCATGGAAGGATATTATAATGGCACAATATTCCATCGTGTGGTACCAGGATTCATTGTGCAAGGTGGGGATCCAAATGGGGATGGAACGGGTGGAGAGTCTGTATATGGAGCCCCATTTAAGGACGAGTTCCACTCTCGGCTCCGGTTCAACC
MSTRG.15018	Glycoprotein-N-acetylgalactosamine 3-beta-galactosyltransferase 1 isoform X2	GCATTATTTAGAATGTGTAGTAATTTATATGAGATACTATTTTAAATCTTATAATAGTAAGCAAGAGACATCAAATAAAATATATAGTTGCAATTTTTTTATTTTTCCATTATGATAATTTAGTCCACGTTATGTGTGATTTCAATGACATCAAAGTATTAAAACTTACAATCTTATTGACATTTTTGACATCACTTCCCTTCTACCCCTTCGTCCCTCTCTTAAGCCGTCATTCTTCGTATTTTTGATGGTTCTTAATTTGTTGTTATCGTTTTTTGGCGTAATTTTTAACAAACAATGTACCACATTTTTATTTGTATTGCTAATTTATTGAGTTAAGGTTGATATCTCTTAAACCCATAATGTTTTTTTGTCTGTTTAATAGGAAGATCTAATAAAGTAGCAGAGATGACGGCCGATTCTCGGGTGATGGGACGCCGTTTTGTGCTAACATTGGTGATTGGAGTATCTGCTGGATTTAGTGTTGCGTACATATTTTTAACCTCGGCTGGATACAATCGAGATGTAGTTTGGTCGTCCTACAGG
MSTRG.15101	WD repeat-containing protein 44	CGCATCGTTCGAAATTCAAATGTATATACAGTGTAAATGTTCTGTGTGTTGTATAGAAGGGCGCCGAGGGTGGCATGGTGTCGTCGTCGCAGACGGGCCACGTGCTGGTCAGCGCAGACTTCAACGGCTGCATCAAGGTGTTCGTACACAAAGCCAAGCCCAAGCACAGCTCGCTGCCCGCGTCCGCGCTCGCGTAGCATCAGCACGCGCACGATTCTGCCAGAGACTGTAAGTAAAATGTATTCCGCGGCTCTGGTATCAGTTGTGCCCAATAGAAGGAAACAAATTTTTCGAACTGTCAAATTGGAAAATGTAGTAAGGATCTTTTGTAAATGTTAAATAAATTACGTTAATAATTATTTATGTAAATGGCAGCTTTGTTTCTGTCCACTATAATA
MSTRG.15104	Ribonuclease 3	AAAATGCGATAATTTTTGCAACTACCAGTCGGTTTTGAAAATTCTCTTCTGCATTTTATAGCTCATTTTTGAGAATGATTATATGTTATACAACATCACGATAGAACCACGAGCGAAGTTGTGGGATACAGCTAGTCATAAAACAATTTTGTTCAGTTTTATG

		TCTTTATATTTCAGGTGTAGTATTCAAACACATTCGTTTACTGGCCCGGGCGTTCACCGACCGCTCAGTTGGATTCACCCAT<br>TTGACGCTGGGCTCCAACCAGCGTCTAGAGTTCCTAGGCGATACAGTGCTGCAGCTTGTTGTCTCTGATCGGTTGTATAGA<br>TACTTCCCTGATCACCACGAGGGACATCTTTCGCTGCTCCGCTCGTCGTTGGTGAACAAGCGCACGCAAGCGATGGTGTGC<br>GATGACCTTAACATGTCCGCGTACGCTATATACAACAACCCCAAAGCAAAGCCGACCACTAAGAAACATAAAGCCGATTT<br>GCTCGAGGCATTTTTGGGCGCTTTATTCATTGATAAAAACTTGGAGTATTGCCAAGCATTTTGTAACGCATGCCTGTTCCCG<br>CGGCTGCAGGAGTTCATCATGAACCAGGCGTGGAATGACCCCAAGTCCAAACTGCAGCAGTGCTGCCTCACGCTACGCTC<br>TATGGAGGGCGGCGAGCCAGATATACCCTTATACAAAGTCATCAAGTGCTTGGGCCCGACGAACACGCGCGTGTACGAGG<br>TGGGCGTGTACTTCCGCGGCACGCGGCTGGCCTCCGCCTGCGGTCACTCCA
MSTRG.15105	Uncharacterized protein LOC101742417	CCGCCGTCCCCGATCCACCCTACGAGCCGCACCTGTTGCCGCAGCTCGTCGACACGCAGAATGTACGCGGCCGCGTTGTAA<br>AGTTGGCAGCTGGATGGCATCACTCATGTTTGGTGGATAACTATGGCACCATATATTCTTGGGGCCTAAACTTTGATGGTC<br>AATTGGGTAGTGGAGATAGAAAACAAGTGCAGATTCCTACCGAAATAAAAATTCGAACAGAACAAGAAGCAACTGAAAA<br>AACTAGCAACTCCAAGTTGCTAGAAGTAGAAAATTCCGACGTGTACACCAAAGTGTTGGTTGCTTGCGGTGGAGACTTCA<br>CGGTATTTGTGGATGAGGATGGGAAAATCTATGCGAGTGGCAATACTCACTTACAG
MSTRG.15112	Protein unc-13 homolog A-like	TGCAGTGGCTCAACGAAAACGACGATGTATCACTCGAATACCTCAACGGGGCCTTCAATAGAGATAAACGAGATGGGTTC<br>CAAAAATCCAGCGAGCATGCCCTCTTCAGTAACTCCGTAGTGGATGTGTTCACGCAGCTGACACAGTGTTTCGACGTGGTC<br>TCCAAACTGGAATGTCCAGATCCTGAGATCTGGAAACGGTATATGAAGAGATTCGCGAAGACCATCGTGAAAGTCCTCGT<br>CGCTTATGCTGATATTGTGAAAAAAGAATTCCCTGAGCATTTGAAGGAAGAGAGAGTGGCATGTATTCTTATGAATAACAT<br>CCAACAACTGCGAGTACAATTGGAGAAGATGTTCGAAGCGATGGGAGGTCCGAA
MSTRG.15147	Uncharacterized protein LOC101746464 isoform X1	GATTTACCAATGCGCACACTAACGGTGGAAGAATGTGAAGCGCTCTGCGACAATCTGCCACAGCGTCGCGTCAATATAGA<br>AGTGACAGGGCTCCAAGTGCGGGTGCATCCGTGGGATCACTCGCCCCACGAGCGGTCCACTGCGCCCCCGATCGTGCTCG<br>ACCTTGAACTGCCCAATACCACCGTTCTCATAAACGGCCCCTTATATCCACACAGGGTGTGCTCGGCGGCCTGTCAGATGC<br>CGGACGACAGCGGGCCTTTATGGCAAGGGGCGCGGTTACATATCTCGGCCGCAGTATCGTCGCTGGCTGTACGCATAAAC<br>TCTCCGACCGAGTGCCAACGGCGGCCGTGCGCCCACCTCGACGTACGCCTGCTGCTGCACATACTGCTCAACAGAGAGTTC<br>TTCGAGCGACGGGAGAGCGTCAATTTCAGTTACTCGTTGAAAATACGTGAGGCGAAGGTGTGCGGTTCAGCAGCACGCCT<br>GCAGGCCGCGTGCCACGTGCCGCTGTCGTTGGTACAGCAAGCGGCCTCCACTGCACTGCGGCACACCTCGCTTGCCAAGG<br>ATGCATTGAATGACTCCGAGTGCGTGGCCATCGAGCTGACTGCGGAGGAG
MSTRG.15154	Myosin-11-like	AGGTGCAGCCGTGCCGTTTACGGTTGATAATCTAATTAGTAATTGGAATGATGGCTTCCCCAACTATCCGTTAACTGCAGC<br>GGACCTCAAGAAGCCTCATGCACTGATGGGTGCACTTTTTCTCGTGTTCGATAGACTAGGCATTGATCGAGATGCTGTAAT<br>GATGCCACCGCCTGAAGAGAGTCGCAATGAACACACAATCTACTACTGGGATCTGTTGCCAATCATCAACATGACTCGTGT<br>CATCAATCATTTTGTAGCAGTGCTTCCACAAATGAATGTCAACATCACATTAACACACT
MSTRG.15157	Kinesin-like protein KIF13A	CAGGAGCCCAGCGAAAAGGCCCTCGATCCATTCTACGAGTCGCAGGAGAATCACAACTTGATCGGCGTGGCTAACGTGTT<br>CTTGGAGGCACTGTTTCATGACGTCACCCTGGACTACCACACGCCCATCATCAGTCAACAGGGAGAAGTCGCTGGTCGACT<br>GCAGGTGGAGCTAAGTCGTGTGGGAGGACAGTTTCCACAAGACAGAATTTGCGAGGCGGCCTCGGACAGTTCGGGAGATA<br>TGAGAGATGACGATGATC
MSTRG.15168	Retinoid-inducible serine carboxypeptidase-like	GCAAAAGATATGCAATTATGTCATGGCGTGTACGGATATTGAAATTCAATCAAAACTAAAATGTTTATTTAAAACAACTCT<br>ATGTACAAAAAGTGTTGATAACTATTAATAATATATTTACAGGACAAATATTATGGGTAGATCGGTTGAAGTGGGCTGGC<br>GCGGAAGAGTACCGGAAAGCACCTCGCCTTCCTATTTGGGAAGATAACACACTGGAAGGGTACTATAAATCATATGGAAA<br>CTTTAGGTTCTTTTGGATTAATCATGCAGGACATAGTGTACCAAGGGACAATCCTGCCGGGGCAAATGCTGTTCTACGCGA<br>TATGACTTCATTTGGATAATACCAAATTATCTAATAAGCATAGTCATATTTTTTTATTACATATTCGTAAAGTCATTTTTAT

MSTRG.15170	Beta-1-syntrophin	TATAGTTATTAACCTAACACTATTTACCTAACACATTTATCAATTACTGTCATCGTGGCAAAATATAGCACTCAATATCAAT<br>TTAAAGTTTTTCGAACAAAAATGTTCGAAAAAATAATCTTAGTAAAATATCAATTTATTTAGACAAAATTATAATCCAAT<br>ATATAATATAGATATATACATCAGTACTTATTAGTTTTGTTTTTATTTAACTTAATGTACATTATAAAGGTACATATGGTGG<br>ATATAATATCTAAAGGCATTCTCTATCAGTCAACCATGGGGCAAACAGTTAGTGGCGTTACTTTAAAATAAACACAGCGAT<br>AATTTTAAATAATAAATAATTGAAAATAACTTTATACAAATATTTACAACATTATATTTATATTCAAATAAATACATAAAG<br>CAGATCATAAAAAATATTATCAATAGTTTAAGCAGTAAAAGAAATGTAAATAGAGAATAACTAAAAAGTCAGTACGATGT<br>TCTGGAAAGTAAGCAAGAAATTCCTAGTACAATGACAT<br>GGCCGCGGCGGCGCGCGCGCTCTACCGCCGCCCGCTGCACGCGCTGCGCGCCTCCGCCGACGACGACCACTCCGCGCT<br>CTGGCTGCACTTCGCCGACGATGACACTGTTGAGTTGGACATGGAAGGCAGTCCGAAGCCGGCGGTGTTCATCCTGCATA<br>ATCTATTGTCGGCGCGCGTACACTCGCTGCCGGGCGGCGAAGCAGCTGACGTCAATCCCCTATAACCCGAGCCACGCCACT<br>AGCAGCCAACAAATGGCGGTAATCCACTTTTGAATCTCTAATTATTATCTGATTATATTACAGAGATAAATCTAAATAGAT<br>GTCGCAGGCGGGCCATATTTTTTTGCCACTTCAAGGGGTGTTTTCTACGAGAATAGAACAGGCGCTTATAGTACGTAAACC<br>ACGCAGACGATGGTGTTTTCAAACTATTTTCACATTAATTTTAAAAATGGGAACATCTGTTGTCACATCGGTAGCGATATA<br>TTTAACTACGATAACAATAACTAATATAATAGCTTAGTTAAATACGTGGGAATCCTCGTAAAACTGATTGGAAACCTATAA<br>TTCTTTAGTAATTATAGAGACGTTCTTGATGTTCAATTACGTGTAATGTAGCTAGAGTCCGATTAAGAGCTTTCTATTCAAC<br>AAGTACAAGTGAACAAATAGGTACATTTGCGGCATCTATTTAATTATTTCTGTTGATTACTCTGTACACGTAAGAACTTGT<br>GAACATGTTTAGAGTAAACAGGTAGTACCTACTCATACAAGTCTTGAAAAGATCGACTATAAAGACAAGATTCTGGAATA<br>TGTAATAATTATGTTGTCATGTTTACTCCTAAGATTTAAAGCATGCTTTAAGTTATATATAGAGTAGCAAATGATAGAATTA<br>TGTTCGTTTAAATATAGACAGTCAACCGAAAGTCAAACAGTAGTCGAGGAACTGTTTGACTCTTAAATTTACAGAACAGA<br>AAAACAGGAGAAGTGTAAAGTAGACAAATTGCGTGTTATATAATTAATTATAATTAGTTCTAGAATAATCAAATATTTGCA<br>TCGTTGTAGTTGACTTTAATTTCGTAATTGTATCACCTCGCTGTTATTAATGAAAGTAAAAGACTATTTTGAAAAACGCAAA<br>ATCTACAGTCGTGTAACAATTCTGTTAAAATTTATTTTCAACAACATTTGACACCAATTTCTTTCTTAAACAAAGTTTATTA<br>TATTGAATACTATTATTGTGATATATAAGTACACACACATGTATATATTAAAATGTTACACTATAAAGTAACATTTTATTGT<br>AATATTCGTTTAAACTTCTCTATACGAGTCCATATTCATATCAACATATCTGCGGTGGCTCAGGAAATATTTAGGAATATAT<br>TTGCCATATCATATTATGGTATTGATCTGCCTATTTTCTATATATTCTTTATAGAACTACCTATTAGTATCTCAATAGTATTT<br>TTTAACATTATTTGTGAATTTGGTATAGACATGGAATGGAGGACATTGGTATGTATTTACATACTTACACAATAAATATGC<br>TTGATATGTTAATGCTATATAAATACAAAATAAAACACCAACATGTAGGACATTATTACAAAATTGTATTAATTTATCCTC<br>ATTATAAATTGTGATTACCTTGTAATGATAAAATCAATTAATTATAATTAATGGGATTGAAAGTAATTGGAAAAGTATCTC<br>CGATATCAATTATTATCACTATCGTAAGTATGTCTACAGATAAGTTAGTGCTGTTTTTGTGAAGAATTAAACAATATTATTT<br>GAATAATTTTCATAGAGTAATTATAGATGTAACGGTATAAAGCGTCGTTCACTTAACGCCGTGCGGTCTTATACCACCGTA<br>TCATCAGAGACGTACCTTCTATTAGCCGCGTTCATACATTTCGGGCCATCTATATGTACATAGCGAGATTTCTTGTTTAATA<br>CAGAATAATCGATAATTGGAACTAGTTATTCAAAAAACATCCTCTTTACCTGTAATGTCCTAAGAGCAGTTTTTTTTTTTC<br>ATAACTAGCTAAATTATTGTAAATTATCTTTTTGTTTTTTATTTTAATATTTTCGTTACGTTTAGTAAAATAACTTTAATAGT<br>TATGCCAAAATATCGTCTAGGAATCTAATAGCAATGATGATTGATACATATGTTAAAACATGCATCGGAACACTGAGGGC<br>CGACATGCACTTGACATAGAACTATGTAAATTAGTATATACAAAATGTACGCAAGTATTTTTGCATCCATCCCTAACGAGT<br>ACTAACAGCGCCCGACGCTGGAGTCGTGTGAATGACGCCTTAGTATCAGAAGGTTGATTATATTTGTTTGAAAATTTCGAA<br>GTGTGAATCTCAATTCAAAATTTTTTTAAGTTTAATTATGATAGTTGAGAGCCATTTGTATAATATTTTTTTACCTATACTTT<br>TTATACCTACTTTTAGTATTACGTTTAGTGATTTCCTTAAAAACACTAATAATCTCATACCCGTGACTTGGTTGAATGCATA<br>AGTATGTTCGAATTAGAATTGGCGAAATACTAGTAAATTTTTGTTCAGGATTATTTTACGTTATAATTTGCTTTTAATTATT

ATTAAAGATTTGCAAGTGTCTTATGCAAATTAGAATTATACTCGTTTCTGCCTTATATTGATGACTAGAATAGATGTAAATG
ATTATGCGTTTACGACTTTTTGTACTATTATAATACTTTAGATTTTTGAACGGCTTACTGATTTTTAAAGCAAAAAAGAGAT
TATCTATATATGTGACGGCGACTAAAAAATAATGGCGTCACTAATCGGTCCGCGCGGGTACGAAAGTTCTACCGTATATTT
ACAGTACAATAAGCTGATATTAACTTCCAAAGAATTAATCTCATATAATTATTTTAAATTTGTATATTATGCGAGTGTGCA
AGTGAAATAGGGGTGGCGGCTTACTCGTCTAAAACTATGGCGCAGTTATATTGTAACAGTTTACTCTAGTGGCTTAATGTA
ATACGGATATATTCAACTACTACTATAAAGCAACTAACTATACTTTTATACATAAATCGAGTTTGTACCGCGTTGGTAATTT
TTTGATTATGCTGTGATTGCATAGGTTTGAAATTGGTATTTTACAAATTCTAATATCGGAAACTCCTGTTAAATAATTTCAA
TAAAAAAAGAATGAGACTTCATTGGAGTAGTCTTTTAGTTTAAGGTGTAATCAACTTGAATTGGATTACTTATACATTTTTG
TTTTCAGATTTCTTTTTAGGAAAGATGTGTGTACATGCTAATGTTGTTTCGCTCATTTTACATGCAATAAAAAGAAAAAAAT
ATATACGGCGCCATCTTTGAGACTTAAGCCGGCATCCCTGTTGCAGATATTTTTCTAATATGTATGTAGACACATTGAAAC
ATTCCCAGAATATATTTAATTAGAATTATGCTTTAATTTTCTGTAGTGAGTTATTTATAGCACAGAATGAAGGGTCGGTAGC
TCGGGTTGTATATTGAAGTATGTTACCTATCATTACTGTTTTGATTTTTCAGGGTCTTTGCTGATTTTATATTAAACTGTAAA
GTTCTTATTAAAAAGTCATTACTCGTTTTACGGATTCTTGTAGAAAACTGTCAATTAGATGAAGAGCAAAAAATGAATCCT
CTTGAGAATTATCAAATTGGTGACTTTAGATATCTGGTTCTAATGAGTATCGTACTTTGTTAATAGCTACATTGTGTTTTTTT
TTTTGCCGATTTTAAATCAGAGTCATTTAACTGTTTTTCTTTTTTTTATTTATTGTATATATTACGCCATATCATTACAAATTG
TGATCGGATTTTGACGGCTTTCTATTTAATTGAAAGTACTTAGGTTTTCAAAATTTAGTCTGTGTTAGTTTTTTTTTTAAGAA
AATTCTTAAATTACCTCATGAATATTTAAAGACTCAAATATTATGGTAATGGTAATTCTATGGTCAACTCGAGGCGCTGTA
CATCTCGATGTCTTATAAGCTAGCTTAAGGTTTCATGTACATTACGTAGTTAATAATAACGGTCGTACGACTAAACGGGCA
ATATGTGAAGTGATAACGAATTACTTTTTAGATATATTTATTTTATTATCAGAAATTTACATATTTCGTTTAAAGCTATTAT
AGGAGTTTAGCGTAATATTGTTTACTCTGTACTCAGTATTTGTAATTCATATCTTGCTGACATATTTTGTACTTTGTAAACTA
TATGATTGTATATTTAATAACATAATACACCTGTTGTCTAACAAATGGCACTTTATCGACTAATGACTACTTGATATCTAAA
TAATGATATCCTCTTTAGGAAGGAGTACTTCATCCGTTTGTGACGTGTATGCGGCGGGAGAGGCATAATATTCCTCTTTCGT
TTGTTGACTCGTATTGCTTTGTAATAAATATTTCATTTCACTAGTGGAACACACTTTTGTGAAAGTATTTTACTATGTTTGTA
ATTTTAAATATTTGATTTCACTGTATATTAATAGAAATAAGTCAGTTATTGTTATTTTATGTTTAATGTGATTGTAGAGAAA
AAAAAATATATCTATTTAACTAAACTGTCAAGTAGACTCTGATTTCTGTCTTTTTTTAAATAATTATTAAGTTTAAAAAAAA
ATATTGTTTATATTTGCAATTATACAAAGTCCGTTGTTAGACAATATCCGTATCTATATACACAACAATGTTTAACTGTAAG
TCTTTCCATTATTATAACTTATCAAGGAAAATGTAATCATCGCTAGGTTTAAGTAGATATCTTCGAATAATGAAGCTCTTTA
AACTTACTCTAGTTACCGTATCTTGTAATATTTCGAAACTAAAATTATTAAAGAACGTATGTGTTACCATAACGAATATTTA
ACACAAAGTGTGGCCATATCACTAACTTGAAACTAATGCTAAGACATAATAACTTGTAATCTCCTAGAATTTCTACTGGTG
ACATTTCTTCGTATGTTTTTTTATCTTTAACGTAATTACTGTTATATATAACTAAATAAATTAAGAAAATTTTTAAATGTATG
TTTTAAAAGGTAAAAATTGGCTTTCATTGTATTGGCTAACGCTTACAGTGTTTTTTGCGATTGCATGAAATTGGAGAATTAG
TTCCGGAATGTGACCGTAGAGGTTAGTTCCGGGGTTAATTCCAAAATCAGGCAAATACTTTATCTGTATTTTTTTTTAATAA
TATCTAATGATTTTATTTAAAAATTGGGAATACTAAATGCTAGTTACGTTCAAACACTGACATATTTTAAGCAGCGTAAAT
AAGTGCTTTTATTTACTTATTTTTATAAAAACTAATACGGGTCATATTTCTGAGAAAGTCTTTATTATGTTTTATTATATACT
TAATTGTAACTATTTAGAGACTAACCTGATTGATATTAATTATTATTACTTTTTAAAAATATACTTTTTTTTATTTGTAGTAC
TTCTTTAATTATTATTTATTTGATCATAAACAATAAATTTGCATAGGCATAAATCATTAATTTATCTCAATTTATATAATGTA
TAATTGGTATTTGTATAATACTTTTGTAGTATCTGAAAATGCTTTACCCGGCATAATATTCGTTGGTGTAATATTTATTATTT
CCTCTTCAGGCAATTGTTTTTCTGATTATACCTGAAGAAGCAATGGAAATATTACAACAAAAACGATGTTATATTTATTGTT
TGTGCTCAATTTATTTATTTAATCCAAACTATTTAAAAAGCTTTGTTAATATTGGTATATAAAGTTCTGTCATTAAGAAGCA

		TTAAAAACAACGAAG
MSTRG.15196	Actin-like protein 6A	GATACGCGCTGGTGAGCGCCGCGGTCCGCTCCCCCGTGGGAGGCGACCACCTGGTCGCGCAGGCCAAGAATTTGCTCAACTCTATGCATATACAGATGCTGCCTTTATACAGTATACAGAGCAAGGAAGTGATCAGGGAGCGTGAACGTGCCAGGTACACGCTGAAGACCCTACCGGCTGGCCTGACGCTGTCTTGGCAACAGTATATGCTCAAACGACAATACGAAGATTTCTGCCACTGCGTTGCCCAGGTAACGGGAGAATAGAAAATATGCTTGGATTGTTTCATATGTAAAGTACATTCATTATAGCATTAAGTAATAGAAGAGACCAAATTCTGGGCCACCTTCTACGAAATGTTATTATAAAGAGCATACTAGAAAAGAAAATTAGAAGGC
MSTRG.15206	Uncharacterized protein LOC101735752	AGGTGTGGGGCTGCGGTGACCAGGCGTCGCGGGAGACGCAGCTCGAAATTAAGAAGTGGCAAGTGCGAGAGGCGGAACGACAACGACAGGTAAAGTTATCAGCAGCCGACTGGATCGAGCACCCGGACCGCTACTTGCTGGAGCTGGCTGGGCGGCCGCAGTACAACAACTCGGCCAGCTAGTCCCACGAGCCCCACTAGTGGGCACAGCTGGCTGACGACACTTGTTGTGTACAACTCGTGCACCGTGCACTAGTGATGAGAACGATGAAGTGTACAAGTGGCTCTCCGAGTTATGGTCAAAATAAGAATAGGAATGTCAATGTCATTGTCAATGTCAATGTCAACTGTCATTTGATAAGTGCGGTTTGCAAAGAAAATTTGATGCCATGTAACTTCTCTTTATTATTTTACTAAGTATTTATAATTCAAAATTATAACTATTCTAAAATAGTTTTTGTGTACTTAATCTATCGACAGTTACTTAGAAGGGTTTGCAAAAACAAAATAAACTTTACAAGTTTATTTTGTTTTAAATAAGTTCACTGATTCTAGAATTTATTTGAAAGTATTCTGAAATGCATAATCAAATATTTCGTAATAGAATAGATGGAATGCATAATTCACATAGTGTGCTGCTCGTTGTATTTACGACATTG
MSTRG.15207	Lysosomal alpha-mannosidase	GGCAAGTCATCTGATATCTTCACCGGTATATTGTACAATACATACTCCCCGCCGCCCGGATTTTGTTTCGACGTGTTGTGCAGTGACGAGCCTATAGTAGACGAGGTCAACTCCCCTTTGTACAACGTCGACAGGAGGGTGAATGAGTTTCTCAGTTACGTCGAGACCCAGTCTCGAAGGTACAGGACAAACAACGTGATATTGACCATGGGCGGTGACTTCACATACCAGGATGCCACCATGTGGTACACAAATCTGGATAAATTGATAGAGTATACCAATCTGAAAGCCGCCAAAGAGAATTTGAACATAACTTTGTTCTATTCAACTCCGAACTGCTACCTTAAAGCTGTGAAGGATGCCAAGCCATGCAAACACAACTGCTTCACGGTAGAAACACGAATGGGACAGAGGTGTACAAGCAAACGACTTTTGTACAAAGTCTCCCTCTGTTCAACAGTGGACAGTTTTGAGTTATTTGATGATGATGATGATGATCACCAGCATTACCGATAGACTATTATATAAACCATAATAATATAAACCAGGGGTTTCCAAACTTATTTTGTCTACTGCCC
MSTRG.15218	mRNA-capping enzyme	CTACACACAAAAAGGCTGTATTTGTACCTGGAGTAAGAGGCGTGGAGCCCTTCAATACTCAGCCACGCCTCAGCGAAGTACAGAGGAAGACACAAAAGTTTTGCGAATGGGATAAAACAGGGTTCCCAGGTGCGCAGCCTGTTTCTATGGATGTAACAAATTTGAGGAAATTACATGAAAAACCATACAGAGTTTCATGGAAAGCGGACGGAGTGAGGTACATGATGTTAATAGACGGTGAGGGAGAGGTGTACATGTTTGACAGAGACAACTGTGCCTTCAAAGTGTTTGGTTTAAGATTCCTGCACAGACATGATCTGAGAAGACATCTAAAGGATACCTTGCTTGATGGTGAATTAGTTATAGACAAAGTGAAAGGTCAAAACATACCAAGGTATCTAGTTTACGATATAATAAAATACGAGGGAGAAGACGTCGGCAAAATGGCGTTCTACCCCACGCGGTTGGAGTACATTGAGAAAGAAATTGTTAATCCCAGGTATGCCAC
MSTRG.15226	Protein p270	GGTGCAGTCGAGGCATGTTGGCTCACCGTATCTCAAATTGGCTTCAATTAACCGGTCCAGCATACACCGTAGACACCGCCTGTTCAAGTTCACTGTACGCTCTTGAACACGCCTTCAAAGCCATCCGTACTGGTCAATGTGACGCTGCCATTGTAGGCGGGACCAATTTATGCCTTCATCTCATTATATCGCTGCAGTTTGCCAGGTTGGGTGTATTATGCCTCGATGGTAGGTGCAAGAGTTTCGACGACAAAGCTAATGGTTACGCTCGTTCAGAAACTATCGCCGCATGTTTACTTCAGAAGGCGAAAGACTCTAGAAGAATATATGTCCAAGTGCTCCATGCGAAAACAAAC
MSTRG.15229	AF4/FMR2 family member 3	GCAGACGTCGCCACGGGTGCAGCCCGCGTTCACAGCGCCGCAGAAACGCGGCAGACCGCCCAAGGCGCGCGCGTTGGAGGAGCGGCCCGCGAAGAAGAAGCGCGGCCGCCCGCCCAAGCAGCAGCCCGCCGCCGCGCCCGCGCCCGCGCCCGCGCCCGCCGCGCCCGAGAGCGACGTCGAGCTGGACCTGCCCCAACCGCACGCGCAGGACAACAAAACACACATCTTCCGAAAGGTTTTTACGCCCAAAAAGGGTGACGAATGCGGTGGGAAAGGGGGCAAAGGTGGTAAAGGGAAAGGCGGGAAGGGCAAGGGA

		CAGGTGACAATCATAGAGGTTCCCATGACCCGAGAGTCTGCGGATGATGACGACCGCAGGAACCGTGAGCGTTCCACGGA<br>ACGGCGGAACGAGGAGGCGATGGCGCGTGTCTCGCCTCCTCAGGAACCGGAAACCCTCACCAGAAGACGAGAAGCGGAC<br>CGAGTCGCCAATGAGAGGATACAGGAGCGTGTATCTATCGAGCGGGCTGACCACCGGCGCTCTGCT
MSTRG.15234	Uncharacterized protein LOC106103208	CAAAGTCAAATAAAAATAAACATTATTGTTTCTGTCTTGCTAACTATTGGGACTTCCAATACATTAAAAATACTTAATTTAT<br>TCAAGGATATTTCTTTTATTTTCCTCTTTTAACATTAATGATTGTAAAATATGCCTGTCAAGAATATAATAACTTTGTGATAT<br>GAAATATAAAAGATAATTCGTAATAATGTCGGTGAGAGACAAATGGGTCCGCCGGTTTTCGGTGGATGAAACCTCAAGAC<br>ACAAGAATGGTTTTACCATTTATAAAATTACCTCCGTTCTGTTCCCCATTGAATCGCCAGAGGCAGTCACTGTAGTATCGGT<br>ATGGAAGCGATACAGTGATGTTCAGCAACTTCACAAGAACATGAAATTGCTGCATGCTGGTCTACACTTGCGGGGGACTTT<br>CCCTATTCTTCCGAGGTTCAGTTACTTTAAACGGTTTCAGACAGAAGTAATAGAGGAGAGGGCCAGAACAATCAAAACCT<br>TGCTAGAGTTCATAGCTG
MSTRG.15248	Protein MON2 homolog isoform X1	CTTGTTATCTACGTGTGTGTTAATTTTACAGTTAAATAATTTAAACGCGGGTGAAGCCGCAAGATACAGTCAGTAGTCAAT<br>AAATCTCCTACTTCTTCTTATTTAAATGATCGTAGGAAGTTAAATGGGACTATCGATATCGATTCTGTACAACGCTAGAAT<br>GTTAGTCTAGAATGAATCACCAGCAACCTACATATTCCCGCAATGGTGATATAATGCAGCTTTGACCTTATTGGTCGAACC<br>GCCCATGCGGTATTTGAATAGACCAATCACAAGCTCGAATTCAAAGCGTCGCTCCCCGTGACACCGACGCTATATAAGTCG<br>TTTCAACGGATTTTCCGGCGTTATTATTCGTTCGGCAAGCCTCTTTACCGGTCGCGCTGAACGACGACTGACATTTATTTAA<br>TTAACTTATTCCTACGTTAAGTTCAACAAAACTCAATTCAAAATGTGTGAGTGCAAGTGTGAGAGTGAGTCAAAAAGCAG<br>ATATAAAAACACATCAAAAGTCGGTTAAATGGCGCTAAGAGGTGTGGTACATAAACTCTTATAGTAGTACAATATGAGTG<br>TGAGTGTGAGAGTACAAATGTGTGAGTGACTCACTGAGACGGATGTAGAGGCACATCCAGAGCCGGTCGAGTGGCGCCAA<br>GTCGGCGTGGTGCGGCAGCTCGGGACACAC
MSTRG.15255	Mediator of RNA polymerase 2 transcription subunit 26	AGCGATCAGTTCGACGGCTCGGTGGCACTCCTCTCACTTCGCCAGCCCTCTCCAGGGTGGTGAGCCCGGCTGTACCCAGTC<br>CTCGCCCACCATCACGACCTGCATGGGGTGGCTACGAGTCGGACTCACAAGATGTCATCCTGGTGGACGATGACCCGCCC<br>CCGCCCGCGCCGCCGCCTGCACCGTTCAAGTCACACATACCACCTGTAAAAAGGACACTATCGCCGGCGAATCCACACGA<br>TGAAAAGAAAGCGAAACGAGACAAAAAACCCAAGAAAAGAAGGTGAGTCAGAACTATATTTTTACTAATAAAATGGTAA<br>CTGGACTATGTTTATACTTGGGGGATATTTTTGAAAAATTAATGTGTAATATAAATATAATAACAGAGTGTAATATATTTA<br>CATATGGTTTATGACCAATAGAT
MSTRG.15264	Two pore calcium channel protein 1-like isoform X3	ATAAATATGTAAGTCAAGACTAATTAAATAAAACAATGTTGTGCGTTATTATGAATTTGAAAAACTTTGTTGTGTCCAGGT<br>GGATGTGACGTTTATTGGTTCAAGACCACGAACGAAGGAGGTGCTGCAAAGGAGAATGTATCAGTCTGATATTCAGAAGT<br>GGCTTGCTGAGGAAGACGAAAATGAAGGTATGCCCCCATCGACTACGCTAACCTCAAGCGAGGACCCACTATCTCCTCCT<br>CTCATCCACCAGCCCGAAACGGAGTCCAACAGTCAACTTCAAACCGGCTACCAGTTGTAGAACATAATTCTGAGCGCGCT<br>GTTCGACCGGCTCGCCTTCATCTGGGACGCGGTTAAGACTTTATGATGGAGTATATTCCGATGGTTCTTCGGATCATTCCAG<br>GCTTGAATAGTGCAATTATCTACATAGGATTGAATGTGTGCTGTGAGGTATACTCTGAATATAATTATATAGCTATCTAGC<br>AGCTTGATGTCGATAAAACAGCAAGTACATTCGCGGTAAACAACTTGAACAAAACCATAGACAATGATGCTTGCGCAGTA<br>CATAGTTGTGCAAGCAAAATTGTTACTGCGCAAATCGTATTGTGTTAAATGCTCAAGTTATTTGTCGTAGTCTATAGAATC<br>GTTATGTTCATAGACAATTTTTAGCAGCTTTACGAATACATAATAAATATATTGTAATATTTTGTTTATAGATATATTATTA<br>TAAATGCAGTACTGTTTTAATATTTTATATAATAACAATTAATAAATAGACACATACCTAGTAATAGATAAATTGTTTAAT<br>GCCTTTATTTTGATTGACAAAATTGGCATATTTGAAAAAAGTATACTTGTAAACATTATGGTATAAATAAATTCTAATTTTG<br>AAATTGAAAATCTGCTACAAGTGTAGAAATTAGAGTACGTAAATTGTAATAAATTGCCAGATAAATTTTTTTGCAAATATA<br>TAAAAATAAAGGTTTGTATGGTGCATGGATGTATAAAATAATATATTTAATAAAGTATTTCGAATGGTAACAACATCTGTG<br>ATAACGGAATCATGATTGACCAATGTTTTAATAGAG

MSTRG.15272	Replication factor C subunit 1 isoform X1	GCAACATCAACAACAACAACAGCAACAACGACGACAACGATAACATAATAATAAAAACATCAACTACGACAACAACAAT<br>TTATATATAGTGGTTTTATAATATGCATATTTACAGGTAATACCGGTAAACAAGCGGTCACTAAGAAACACGTGTTGGTGA<br>TGGACGAAGTGGACGGCATGGCCGGCAACGAAGACCGAGGCGGGTTACAAGAGCTGATTTCTTTGATAAAAGCGACATCT<br>GTGCCCATAATATGCATGTGCAACGACCGTAACAGTCAGAAGATGCGCAACTTCGTCAACTACTGCTACGACCTGAGATTT<br>AACAGGCCCAGGGTCGACCAAATCAAGTCAGCAATGATGACGATATGCTTCAAAGAGAACATCAAGATTCCACAAGAGG<br>CGTTGACTCAGCTAATCGTGGCTTCCAACCAGGATGTCAGGCAGACCCTAAACCTGCTGTCGATGTGGGCTGTAGACTCGG<br>ACCTCACTGACATGGAGCGATTGAAGAAAGATGCCAAGACTATAAAAAAAGATATTAAATTAGGGCCATGGGAAGCTAT<br>AAGGAAAGTGTTCACAGCGAAAGAGCACAAGACCATGTCGATACACGACAAGAGCGACTTGTTCTTCTACGACTACAGCC<br>TCGCGCCGCTCTTCGTGCAAGAGAACTACCTGCAGGTGG
MSTRG.15279	Vacuolar protein sorting-associated protein 13D	CGGAGATCACGTTCGACTTCCACCGCCTGGGGGTGCTGCTGCTGCGCGCCGCGGTGCACGACGGCAACCTCGTGGCGAGG<br>AAGATCGCCACCGCCACCGTCGCCGAGGCCAAGATACAGGCCACCCTCGACGGGTGCAGCGTGGAGGCGGAGGGCTCGC<br>TGGGCGGCGTGCAGGTGGTGTCGCTGTGCGACGGCGCCGGCCTGCACACGCGCGTGCTGTCCGCCGGCCGCGCCCACCAC<br>CCGCAGCAGCCGCAGCACCCGCAGCACCCGCAGCACGACGCCGCCGCCGACGACGAGAAGGCGCTGCTCTTCCACGTCAC<br>CAGGAGGCTCGACACGCCGGAGGGCGCCGAAACACCAGGTGTCCATTTAACTATTGACGTTTTCCCTACACATACTGTATG<br>AGATTCTTCCAAAACGACGTATTAAAGTTTCAAC
MSTRG.15295	Mitochondrial ribosomal protein L54	TTCCAGTTAAAAGATGACAGCGAGTACCCTGAATGGTTGTGGACGCTACGCCTAAAGCCTGTACCTATAGAAGAGTTGGA<br>CCCCAATACAAAGGAGTACTGGCTGAGGGTCCGAGCTGCTGGCATGAAGAGAAACAACAAGTTGAGATCGTTAAGAAAA<br>TTCTAGAAGTGATACAGTTGTATGATGAACTAGTTGTATTTGTAGAAATCTTAGTAAGAAAGTTAGTAAATTTGTAAGTAG<br>GGGTTGATTCTGAAGCGCCATAATCTAAAACTAAAATTTATGAAAATATCCTCATATGTATTTCTATATAGGAATAAGACA<br>TTTTAATCGTCAGTAAGAACTATTTTTGTCTGTCTAATTGGATATTAATCCAAAAAAGTCACGATAGTCCAACTGACCTGAT<br>TCTACTACACGCGGTTCCCCTTGGAAACTATCGTGACTAATTTATCATTGAATTCGTCATGATGGGCTAACTGACCTACCTT<br>TTCATCTCACTGGTACTCCGCCAGTGTATACCGATAGCGCAGATAGGGTTACCATGCCATTTCCACCCATTTAAAAAGAAC<br>TGACCTGGTTTCATCTATACCTATCAATTATTAGTGCCTCATAAATAACCATGCAGAGCACAATTTCATTGTTATCCATATC<br>CATATGTTATAAGATATATTAAGATAATAATTTAAAAGACATATTTAGAGGATGACCTTAGAATCAATTCCATTGTACAAT<br>AATTATGAAATAGGATAAACTAATAGGGTCTTACACTCGAATGTTACATCGCAGAAGTAACTACAGTGTCGCCACCTATCA<br>GTTGTGGCCGTCGTTAAAAAGATATAATTTTAGCTCAGTATACATACATACATACATATCTGTCACGCCTGTCTCCCATTAG<br>GGTAGGCAGAAACAATGGAACGCCAAATGCTTCGATTCAAACAAACCTCTTTAGCTCAGTATA
MSTRG.15297	Guanylate cyclase 32E	AGTGGCACTGGTGTTGGAGTATGCTGATATTATTAGCACTGGATAGTTCACAAGGCGAGAAGTATACCCTGGGATACTTAA<br>CTGGCTCAAAGCGCAGACCTGGCGACTACAGCTATTCAAGACCTGGACGAGTTATTTCCGGGGCGATATCAATGGCAGTA<br>GAAGAAGTTAATGAAAAACTGCTAGGACCAATGGGGCATTCTTTAGATTTTGTCGTAGCTGAAACTTACGGTCAAGAAGA<br>AGAATCTATAAAACAAGT
MSTRG.15303	Integrin alpha-PS3-like precursor	AGACAATTAGCTGACAAAAAGTTGGCCCGTTTTCGAAACTCTATGCTCTATTTATATATTTTAAGGAGAATAATTATATATT<br>TGTTTAGGATACAGCGTTGAAACAGGCGTATTTGAAGAGAATGATACAGTACTTTATGTGGGGGCGGCGCCGAGGTCCAA<br>AAATGGACTGGGTCAGGTTCTAATATTTGACCCTCCATCACAAGAAACACAACCATATAATATAAGACAGAAGATCATTG<br>GACCCCAAATTGGTGCATACTTCGGGGCTTCTCTCTGCTGCGTGGATATTAATAATGATGGCAGACTGGACCTCTTAATAG<br>GAGCTCCTACATTTGTCAAGAAAGATGGCGGATTGAACTTTGACCAGGGCGCAGTTTTCGTCTATTTAAACCATGCCAAGA<br>ATGGTACGTTTTCCTTAGAAGAGTCTGGTTCCGTGTTAGGATCAGAACACAGCGGGTCACGTTTTGGAATGTCCATAGCCA<br>GCCTTGGTGACGTCGATGGAGATGGTTATGATGATATTGCAGTTGGTGCACCATGGGAAAATGAGGGTTCTGGAGTTGTTT<br>ACATATATCAAGGTAGCGTTGAGGGATTGAAAACTCGATATGCACAAAGGATCCTTATGGAAAAAGCTCTTGGTTTTGGC

		ATGTCTATTTCTATAGGATATGACGTTGATAACAATTTTTGTAATG
MSTRG.15319	Protein singed wings 2 isoform X1	AGATCACATCGCTGGAGGCGGTGGGCGACGACCCCACGTACGAGCACCTGCGGCAGCTGATCGTGGACTATAACGACATC<br>GTGAACATCGTCGAGCTGGAGGGCACCAAGTTCATTGACAACTTCATGCTGTTCTCCATTGTGCATAATAAACTTAAAACT<br>ATCCATACTTACGTGCTGTGGAACCGGTTCGACACCAAAGGCCCTACATTCCTAATAGCGGGGAATCGCATTCACTGCGAT<br>TGTAACACTGAAAAAATGCTCAAGGTATGTATTTTGCTTACAGAATATTATAAATATGTCTGTGTACTGTAATGATAAATA<br>AATAACCCAAAATCACAATCTTGTACAAAAAACGAAATCGTACTGTTTCATATCTGTGCCGTTTCTGTTCTCTGTATTGTGT<br>AAAGAAATATTTTAGACATAAAACGCTGGCTTTTTAGAAATTGGT
MSTRG.1532	Protein furry	AGGGAGTACCCGTGCGACCACTACACGGCGATCATCAACGTGACGCTAATGTGCACGGGCTGCCCGCGGGCGCCCGTCCA<br>CGAGAGCGCGCTGCAGCTGCTGCAGCTGCTCGACAAGCGGTTCTTCGGCACCGTCGGCCCGCTGCCCGCCGACGACGACG<br>CCGGTTCGGAGAAAGGTCGGGGTACACTGGACGCGCTGCTGTGCACCACCTACTGCCGGTCGCAGTTGTACCTCTCCCGGC<br>AACTGTCGCAATTGCACCCGGAACTCACAATGCCCATGTTCTCAGAGATCACAGCGCGGTTTCAAACGGCTCGCACCGAA<br>GTCCGGCAGCTGCTGCTCCAGTACCTGCTGCCGTGGCTCGTCAACATCGAGCTGGTGGACCCCAACGTGCCGCCAGCTAAT<br>CCGCTCTCGTACATACAGGTAAGGAACTAAGGTATTACCTTCACATTAAATCAGCATTGTGACGATGCAACGAAAAGGAT<br>TTCATAAAATTACAATTACTCATTATAAGTAATTAATAATATTGATTAACTATTCGTAATCTGGTAAACCACGTCTGTCGTC<br>AGAAAGGGGCGCCACTTTCACACAAGTAGTTCACAGAAGTTCACTAAGTACTAGTCCTCAAAAATATGTATTCTCATTTCT<br>TAGGACTGATTTAGGCTCTGTATCCACTCAAAGTGATATAAAAAATACAATACAAAAACCGCGT
MSTRG.15377	Endonuclease-reverse transcriptase	GTGTCACCGTTTAGTTGACTTTTAGTACACAAATAAAGAGCACGTCAGTTTTTAAGCTATTCATCTAATTTTTGACAACAT<br>TCAAGGACAAATTAATCCTTGAAAACGAGATAAGATTACGGATAAATGTCAGTGGAATCTGGATGTTACTACACCGTGCT<br>ATCAAGAGAGTCAATGAAGATGGAATATCTCTGCATAAGGCCGCCCAAGAGTTTAATATTCCATATAGTACTTTGCAAAA<br>AAGGTATCGCAGAGCAGTACAAACCAATCCTCGTCTCGGAAGGAAACCAATATTTTCTCCAGCTCAAGAACAAATTTTGG<br>CTGATCATCTCATTCACATGTCGAATCTTTTTTACGGCTTAGACTCCATGAAATTTAGAAAATTAGCATACGAGTGTGCTGA<br>ACAGTTGAAGATATCTCATAATTTTAATAAGGAACTTAAATGTGCGGGACCAGACTGGCTTAAAAGATTTTTGAAAAGAA<br>ATACTAATATCAGTGTTCGCAAACCTGAGTCAACTAGTATTATTGATTATAGATTTTTGTATGAAGCGATAATTGTTTTTAA<br>TTACATATAATTAACTGTGATTTTGCATGTTCCTAAAGATAATATAATTATATTTTGAATGAGAATAAGTTTAGTTTATAAA<br>CACATGAGGAAAATGTATAATTATACAATATTTTGTTACATATTGTTATTGTGGTTACCATTACCCCGGTGGATCTTATTGC<br>CCCTATTTCTGGTTCTAATTACCCCGTACTCTTGGCAATGGGAGCCAATGTTACTTTTTTTTTAAGTAAGAATAATTTTAATT<br>TTAAGTATGTTTATTTAATTTCTATATTAACAGGATAAGCCTGATTAGTTACATTATAATTAAAAGGAAGAATTAAAATAA<br>AAAGTTATTAATACAAAAAGTTATTTAAGTTTTAGCTTAAGTGGCGCCAATTCCCCCGTGCTCCCC
MSTRG.15392	Uncharacterized protein LOC106130466	GGTCAACAAGAATGATCGTTTAACGTTCTAAACTAATAATACAATACAAAAAAAAATCAAATATGACGTCCATATTGACT<br>TTGAGCAAGCAACAAGAAAAATTAATTGAGTTAGTTAAACAGAGACCTTTACTGTACGATTTATGTGATGTTGATAATAAA<br>AATCGTGTTAAAAAAGCGAAAGCCTGGCTTGATATAGCAAAGGAAATGGGCTCGAATGACGTTTCTCTCTGGCGCTACAA<br>ATGGAAGGGCCTGAAAGATAATTACACCAAATATAAGAAATACAACGAAACTTCCGCTGGACAAGCATATAAGAAGTAC<br>AAAAATTGGCCTTGGGCTGACAACATGAAGTTTTTAGATAATTATAATACTGCGAAAAAGACAATAGAAATCAGGCGTAT<br>TAATCAAGATGACCTCGCAGAAATGTATAATCGGGATTCACATCATAATGCGGTTATTGAAAATTCTCCCGTTGAAGAATT<br>CCAAGATTCTGGCGAAGGTTTCGAGAGCATTGACAGTTCAAAGATTAGCGGAACGCTACAAAATAATCCACAGTTACCTA<br>ACGAGAGATGTGGCTTAATTGAGAAATTAGATGGAACGGATCTTTTCTTTTTAAGTTATTCCCAAACTTTCAAAACACTGC<br>CAAGTAGAATGCAAACTATGTTGAAGTTGGAAATAGCGACGTTATTCGCTAGATACGAGACGATTATTAGTGACGGACGC<br>CCTCCTGAGTCTATAAACTCACCGCAAACAATGAGAGAGGTACACGAGAATTATGACGAGGAATCTCTAAATGACCCTTT<br>GACTGACGTCTCTGTCAAACAAGAAAGTCCCTGGGCTTAAATATTTGACGCGTTC

MSTRG.1<br>5449	Ubiquinone<br>biosynthesis<br>monooxygenase<br>COQ6 like<br>protein	GGTCAACAAGAATGATCGTTTAACGTTCTAAACTAATAATACAATACAAAAAAAAATCAAATATGACGTCCATATTGACT<br>TTGAGCAAGCAACAAGAAAAATTAATTGAGTTAGTTAAACAGAGACCTTTACTGTACGATTTATGTGATGTTGATAATAAA<br>AATCGTGTTAAAAAAGCGAAAGCCTGGCTTGATATAGCAAAGGAAATGGGCTCGAATGACGTTTCTCTCTGGCGCTACAA<br>ATGGAAGGGCCTGAAAGATAATTACACCAAATATAAGAAATACAACGAAACTTCCGCTGGACAAGCATATAAGAAGTAC<br>AAAAATTGGCCTTGGGCTGACAACATGAAGTTTTTAGATAATTATAATACTGCGAAAAAGACAATAGAAATCAGGCGTAT<br>TAATCAAGATGACCTCGCAGAAATGTATAATCGGGATTCACATCATAATGCGGTTATTGAAAATTCTCCCGTTGAAGAATT<br>CCAAGATTCTGGCGAAGGTTTCGAGAGCATTGACAGTTCAAAGATTAGCGGAACGCTACAAAATAATCCACAGTTACCTA<br>ACGAGAGATGTGGCTTAATTGAGAAATTAGATGGAACGGATCTTTTCTTTTTAAGTTATTCCCAAACTTTCAAAACACTGC<br>CAAGTAGAATGCAAACTATGTTGAAGTTGGAAATAGCGACGTTATTCGCTAGATACGAGACGATTATTAGTGACGGACGC<br>CCTCCTGAGTCTATAAACTCACCGCAAACAATGAGAGAGGTACACGAGAATTATGACGAGGAATCTCTAAATGACCCTTT<br>GACTGACGTCTCTGTCAAACAAGAAAGTCCCTGGGCTTAAATATTTGACGCGTTC
MSTRG.1<br>547	Serine/threonine-<br>protein kinase<br>tricorner-like	CATTTCTCTTATAAATTTATATGAATAGAAATTAAAACGCGATTGACAAAACTTCGCAATAAATTCTTTCTCATCTATCGTG<br>AACATAATGATTGTAAGATTTCGCGACAAATTATATATTTAACCAATACGTGAATGGCAATTTTTCATTACTCGACAATAA<br>AATGTCGTACACAGATTAAAGCCTCCATTTCGAGAAATTATCGAATTAATAAATAACCTTATATCATCCCAAAGTTCTAAA<br>ACCGACCTCGCTTACATTTTTATAATGTATCCACGATACACACAACTGACTTTTTTGCTATTAACCATTTTTAATGTCTTCTG<br>TCAATGGCATCGTAACTTGGTTTAATTCCATCTTGCCACCAATAGATAATCTAATTTTAAAAAAAAATTTATTAATATTGCT<br>CGGCTGTTAAAATGTTTTTATCAAATTTTCATCGTGATTAATGGCTGGCAGTAGTGATGTGACGGTACCTTAATTCAGCCGC<br>CGCCTTCTTATAAAATGTAGAGACAATCGAACAATACTTTCAAATCCAATATGTCAGTGGCGATGACAACCTCTAAGTGTT<br>AATCAAATATTAATAATTACCATCTGCCCTCTATTACTATCCATGTGGTCTAAGCATCAATCATATTATCTAAATATACATT<br>ATCAGGTATGGAAGAAATTGTTCGGCGCCATTTTCCCATACTTATGCAAGAACTTCGACGTCGGAAATACGTCTCGATGTA<br>GTGACCGCAGGTACTTGTAGTACACCTGTGCTCACCAGATCGAGACGTATCTCGAAACGGCACGGTTTTGTTCCACTCTAT<br>CAGTGTACTCACGTTGATACGTGATGATTATCACGTATAGTCTCTGGCCGAGCATGAAGTGTATATGTATATGAAGTAGCG<br>AGATGATGATGACGGTGGATCGTTGGTGTGTTGCAGCGGCTGCGCCGATCTCGCAGGAGGGCGAGGCGGCGTATAAGGAC<br>TGGGTGTTCATCAACTACACGTTCAAGCGGTTCGAGGGCCTCACGCAGCGCGGCACACCCGCCAAGAAGTGAGCCGCGAG<br>CCGTCAGCCGCCAGCCACCAGCCACCAGCCGCCAGCCACCGACCGCCGCCACAGCAGCGATAGCAGCGACATCCGCCCAC<br>TGGGCCACTGGGCCGCTCGCTCGCCCCGTGCACTACTCGACCATGTAGCTTTATTATATATTTACTGACTATTCAGTGAAA<br>TGTGAATAGTTGTATAAAATAGCCTAGGGATCGACGTGTATCACTGGAACGGTGTAGGGAGGCGCGAGCCGCCCGCGCCC<br>CCTGGAGGTCCAAGGGAATGTTCTTTAGCTAATTATTATTTGACTATCGCTAGTGCTCGTTATCGTTTGTGGTATGTTGAGC<br>GAGCGAGCCCGCTCGGAGTGGCGCCATACAGTATGAACGTCGCTTAGTACGCACCGCTACCACCCGGTAGCCAGGAACAC<br>AGTACAAACGTAATATTTGAATCGATGACCATTAAGCTATTCTCTAATTACGTATTATTTTAATTATAATTATGTCCATAAT<br>CTCCTTGTAGGTCAGCCGAGGATAGTCGTATGCACAGTTGAAAGTTAACTGTCAAAATAACCTGTCGAATCGAATGTAATT<br>AAATGCGTGTGTGTGCGCGCGTGCGCGCGTGTATGTTTGTGTGTGTGTGGGTGCATGTTTGCGTGGGCGCGTGTGTGCGCG<br>CGGGCGCGTGTATGCACGTATATGAGTGTGCGTGCACGCGTGCGACTCGGGTCGACATCGCCACTCATGTTCAACTGCAAC<br>CCTTCGTATTCAGAACCAAAATGTGCACTGCGATGTGTTCACTTTGTTATTATATTAGTATTCATATTTCGCGTTGATTTAG<br>CTGTTCACGTATATTATTATTCGCTCGCGTAAGATTTAAAGTTTAACAATCAAATTGTGTTTTTGTAATGTATAAGAGACCG<br>TAGTGGTTGCGTGAACCCAGTTTTTAATTGAATACATTACGTAAAGTTGGAGGGCGACCCGTTGCGGGCGTTGCGCCCGCT<br>CGATAACATTGCAAGAACTTAATTAACAGTACACTAATTAATTAACAACATTTCTTTTATACTATGTTATGTATGTTTAAGT<br>TTTTACTGCTAAAATCTCGATTCATCGATACTTACGGACGGAACATTACCAAATTAGTTACTGTGCGCGACAGCGTAATTA<br>TAATGGAGCGCCATCTCTCGTATAAAACCTATACCTTGCCTTGAGAATATATGGCGTATTTGCAGAATGCCTGTGAATTAA

		TAATATTGTTTATGCAGTGTGTACTGTGACGCGTAGCCGTCCTGTCCGTAGCGCGCTCCGCCGCCGTGAACGCGAGACG<br>TTAACGAGACGAGACGCTCGCACCAAACTTCGCACACCGCACACCGCACACCGCACACCGCACACCGCACACCGCACACC<br>GAACACGCGCTCTGAACACCGAACACCGAACATCGCACACCGCAGCAAACCGCAGCACAGCACACGCCGAACATCAGAC<br>TCCGCAGCGCAGCACAGCACAGCGCACTACCGGTTCTTCTCTTATCTCCGTACTAATGTCGACATGGTTTTAATTTACGGTG<br>ACGCAATTAAAAAAGCAATCCTAAGTTTGTGCGCCGCGTAGTACGATGCGCTTATTTTGTATAACTACGTGTATATATAAT<br>ATTACGCAAGACGCTAACTATCGAACGTTCGGTTCGAGTGCAATAGAATGCCGTCCACTCCAAATTATTTTATTGTCCAGG<br>AAATATTCAGTACTCTTACAAATGTATGTTATTGTTATATTTTGTGCAATAAATGCAGTCGTCGCATGTGTTGGTATCTGGA<br>AGAAACAGTTCTCTGTGGAGGTGCTGTGATTGCACGGCGCACTACTTGAAGCGCTACTGAGCATTTCTAAAAGCTATCGTC<br>AAGTCTCGCGCTCGTCACGACGCCTAGCTCTGCTCGTATGCAGAGAGATGGTTACCGCAGTCCGCCTGTATGTACCAGCGG<br>TATTAAATATATAAGTTATGAAAAAAAACGGTACATCATCACTGTATTAGCTTGCCATATCGGTATATGATGACATTACCT<br>AGCTACTCTACTACTAGGACGCGTCGCGTGATCGGCGCATATTAACCCTCTGACCGCGTTTTTTTTCGCTTCATTTCCGCAC<br>ATCGCTTTGACAACAAATAGCAGTACGAGTATTTAGATATCTAGCGAGATTCAGAGAAACAATATCGGTCTAGCAAAACA<br>AACATCGCGGGGCAACAAGGTCAATAAACATAAAAAATGCGAGCACGTCGAAATCGCGGGATGCCCGCTCAGAAGGTGG<br>ACAGATCGGTGTGAAATATTATATTCACTTCCACGTTGAAATACATATAAAGATAATAAATCTCATTCTTCGATGGACTTC<br>TCGCAGTTTCGAGCGCTGCGACGCAGTGCAAGGATGTGTATAGATTTATTATTTACAATCAGTGTAACATCACAATTAAAT<br>TTAGGTCATTATACTACGCAAATAAACGCCGGGCAGCGGCGGCCGCGAGCCGTGTGCCCGCGGGACGGTGGCGTACGCGA<br>ACAAAGTCGCGGGTGCAGCGGACACTGGCTCCGCGAACGCTGTGCCTGTTCGGTGCCTGTCGGTTCACGGTACGATACGA<br>CACGGCGGCCGCAGACGGCAGCGCGGCTACCACTCGAGCGACTTCCATTCGCACACTTACTGTAGTGTCGAATGCTTCCGC<br>TATTCCTCATTAGATTTACCAATTAAATGGGTTTACTGATAGAAGCGTTTAGTATTGTTATGTTTATTACGTCGTAGATTAT<br>TTTCGCCACGTAACATATTTTAAAATCTCTCTATTAAGTTGTATAGTAATCGGTGTTTTCTTAAGTTTCGAGCGGCGACCTT<br>GTATGTCGAGCGTGGTGGGCGGCCAGCGACCATCGGCCGCCTTGAGATGAGACATACTGTTTGAATATAGCCATCGCTGG<br>GCTCGCGGCGTGGCTGCCAGGTCGTACGTGAGTAGCGCTATGTGAAAAACTACCTAACTAATAATGCTTGTTCCTTTTTTT<br>GTTACGTCTTGCAATAAATAATAAGGAGTATCCACACACTGATTTGCGGTGGGCTGTACGAATTCTTATCATCTATTGGAA<br>GGCGCGCGACGACGAGACGCGCTCCGTGATGACGATGATGGCGAGCGCCAGTTGATGGTCGAGCCCGCGATTGCTTTGTT<br>CCGCGCGTGTGTCGTCTTGCCATTAGGACAATGGTCGTCCGCTGCCACCCGGTAACGCTGCGCCCCCTAGTGTGCCCGCCA<br>TCAGCCGACCATCTCTGTGTTCATATCGATAACCGAATACCAGTGACGAGGGTAACACTCCTCCTAAAACCATTTCTCTCT<br>ATATTTCGGGCGCTTACGTTTACATCACAATTTAAACTTATTCGTTAAATATTATAAACTGGTTGAAATAATGCAAATGTTC<br>TAGTAATACTAGCTAGGTACTAATTTCTGTTTAATAAAGTACATGTAAAGTACCTAAGTACACATTTAGCGTAGTCGTTAC<br>TACATTGTAAATGTATGACATGCTAGCGCGGCGTGTGGTCGGGCGACGGTCGACGGTCGACGGACGCCGGTCGCGGACTA<br>CTTGGAGTGTAGTTGCGTTTTGCAGAGCATAATTCTAGAACGGCTACTTGCATCACGTAGGAGATACGCAGAGACACTATT<br>TGATAGTTCGTTAGATATTTGTATATAAAGATTAATATTGTAATATCACGTAAGTAGAGCTACATGTCTATTTTCTACTATT<br>ACAAACTGAGAGTTACTCTATTAGTATTATTATCCCATAGGATGTATACCAACGCAGACGGAGTTCATCAACGAATGGCCG<br>ACCGGAGTGTCGAGTCGGACTCGCGTTGCTACCAGTGCGAATTACTTTGCTATAGAAAACAATGAATATGAGAATACGTTT<br>ATTGATCAGTTAGTGGGTACATTGGTACCGAGCGCGGACACGGCGCGTGCGGTACGGCGGCCGGGCCCGGAATAGATG
MSTRG.15484	Uncharacterized protein LOC106135198	GAGCTGTATGTAGCATATAATCGTTTAATTAGCGTTAAAATCCTCGGCCCCAAGTCTACTATAAATGAAAACGTTCGCTTA<br>TTTACGTATGCACGGGACAAAGTCTCTACTATATATGGATACTGACATATTGCTTCATTATTTTATTACAACCATTGTTGGG<br>CCTGTAGAAGATACGAAGACGGAGAGAGCATTAGTTTTTACACCGCTTACAAGGCAATCAAAATATAGAAGACACATCAG<br>GTCAATGTCCGATGTACAGAGCAGCACACCATACAAATTACCTACCAAGTCATGGCATGGAAGCAGTCAAAATATGTACG<br>ACTCTGACACTAGAGCGAGGAGGATAAGGTATTACAACAAGAGGCTGTTACAAACAGATTCGTCAGATTCGAGTGACACC

GAGAAGTTGTACAGGAAAATCGTGAGTTTGAGGGCGAAGGTCATGAAACCTTACGATTCTATGAGGTTTCTCTAATTATAT
AAGGATCTGATAGTAGTTATATTTGAGGCTGTACATTATAGTTTTATTCTAGATGGGATCACATTTGAATTGCACAATTTTC
TAGATGAAACACTGTACGATTTGACTGGCACAAACCGATGCAAAGTACCTAAGTATTAATTTTTTTCATAAATTATGATCC
CAACGTGATACAACTATAAATGGACTTTATAGTGTTTTCCTTTTGAGACTGTGATCGAATTAGAATAGGTGTAACTATTAG
TCTAAGAAACCTAAGAGTAATTCTAATGATATATCTATATAATATATTTAGTGTTGTTTAAAAAAGAGTTTAAAGAATATT
TTTATCGTCTATAAAATATTGAAATGTTAACAAATGCCATACGTTCGTTTTGCTCTAAATCTGTTATGTTTTGATTTGCCAA
GATCACTGAAAGTTTCGAAATCTCACACAACGCATATTATTTAGTTATACTGTAATATTAAGCATTGTGTTATTTACGATAA
ATATTAATGTTTTAAGTTTTAAATGAACAATAAAGACCATTAGTGTATATTACACACCATAATATTATACTTATTTATAATC
ATAAATAGTTTAAGTTTTTACAAATAAGATAAATTTAAATTTACATGTAGGTAAGTGGCTACCTAATATCTATTCAAAAGC
AGTTTTTATCCAAAATATTTTTTTAATGTCAATTTTAATAAATTATTGTATTATATTACAAAGATATACGATAAAAATGAGA
TCACTCGATTATTACTAAACTATACAACTATGTTAAATATGTGTAGACTTTCTGTCTATAGCGGTATAGTTAATTTGTAATT
ATTTTTGTGAACATAATTTAGTAATTTTTTAACATTACCGATAATAATAATTATTTGAAAAAAAGCTGGGTATGTTCAAAGT
TTCTTTTTCCATAATTGAAGATATTTTCATTCCTTTATCTCTTATTAAGATAATTTTAAGAGGTAAGAAAAAAAAATTGTGTA
AATGTTAGAGGTGTAACATTCAATAATTTTAATATTCCGGCGTACCCGGTAAAACTACGAATCACTCATAGAAAAAATGGT
ATACTGATTAAACAAAAAAAAAAAAACAATGATAATAGGTATAGTATTTGACATAAATAAAGACCAAGGTTTGGATTCTCT
ATCCAGTAGAGGCGATTTAGTAAATGAGCTTTTCTAAATTAAGTAGACTCTGAATGTTCTAGGTCTGATGACCTGACTTAG
TTTCTCATATTTGATTTAGATACTCGTATAATAGTTTTGAAGTAAAAATTCAATAAAATTTTAATTGACTACACAAGTGAGT
AGTCATTTGCACTTTTTTTAATGTCTAATTAGTTATTTATAAAAATGAAAGAGAACGATAATATTAAAATTTAAAATTACTT
AGTACTACTTAGTACTACGACCCAAATAAGACATACTTAGCTAAGACTGACGCCTATAAAAAGTGAGACAGGCAAATAAG
GGATAGCTATTTAGTTTATTTTTCAGCTGTAGGACGTTTTACAAGTTGTATATTTTAGGAATAATCGAAAGAGATCGTCCTA
TATACACATCTACCTACCAGAAACAAGTTAGTAACTCTTGTAAAATTTGTACCTAATCATATTGTTACCAATTCCATATTAA
ACATGTATAGGTACTTAGGTGTACTTACAGTACAACCAACTTATCTGAGAAATACAAAAATAAATAATCTGTCCGAATCTT
ACGCACACGAAGTTAATAATATTAGTGCATTAATAGATAGATAATATAATAGTGCCTTTTCTACTAATGCCTAGTATTATTT
GAATACTTATTTGAAAATATATTTTTATTTTAAAATTTCGTGTGTACTAGATACAAGTTGTTTTTGTCTATTATAGATTATCC
ATTTGGAAAATGTATATAAACTTCGAGCATTATTTTTAGTTCTTGATATAGTTTTAACATCTTACAATCTTATAAGGGCTAT
CAAACTTATAAGATTACATTTTAACGATTTTTATATTTATAATTATCTAGATACGATAATGTACACATTTGTATTTACAAGT
ATGTCTGTTTGTATTAGTATTAATGTTTTCTACATATATATAATAATATGTATATGTAGGTATGTATATTAATGTATTTATGA
AAACTAATTATGTATATCAGTTGTCTAGAAGCCCTAGTACAAGCTCTGTTTTGTTTGATTTTTTTTTTAGTACGTTTGTTCCG
TTGTGGAACAGGCTAAGGTCTGAGACCATGCTGCCTAGTCTTCAATATGATAATGTGTCTGGAATTCTGTTTTGTTTGAGAC
TAAATGACATAACAACCCTGATGATGATTTTAATTTTTTTATCTATTTTTTTTTTTTATTATATGTACTTACAGTTACATTAAC
TGTTTGGGATTTGAAATAATAGATCTTTCTGTAATATTCTTATGCCTTTTGATTTGCTTGCTATATAAATTTATTGTAACATT
GACAGTAAATTATTTCTATAGCAGACAATATATTATATTTATTTACTAAAAATGATTGTTTTATTTATATCAATCAACTTCA
AAAAGAGAGAGAGAGGTCCGGAGCAGCAAAGCTAAAATGAATCACTCAGTTTAATAAGTTGATGTAATTTTACAAGCTTT
TTATTCAGTTCACACTTGTGTGTATAATTGTTTATATTTATTTAGTCAAAATCTTGCAAAATTAATTTAAATTAGACTTAAAT
AAACATACCCCCAGGACCAAAAAGAGCTAGAGTAACTTTTCGCTATGTTTATCACATCATTATCGTATCGGCCTTTATCTG
TTCACTGCTGAACATAAACCTCCTCTTTAGGAGATTTTGGCGGTAATTGCCACGCTTGGCAAGTAGATTGACAACCGCAGT
TAGGATTTGAAGACGTTTTAAGAAAAATGCTGTTGCCCGTCTCTTGCCCTCCCTTAGTCGACATTCTCATCGACAGGTCTTA
CGACAACTACGGAAAAGAAAGAGGTGGCCTATGCTAAGACCACACATCAAGTTTTTCATATGATCAAATATGTATATTTG
GTATTTGATAATTATTGCCTAAAAAGATAAAAATGTTTACTATATTAAAATATCATTTTTACCAAATAGGAAACACAGTAG

		ATATATAAAAAGATTAAAGATTTTCTAGATTTTTCTATTCACCCCACAAATTCTATTAACCCTATTCTACGG
MSTRG.15514	DNA replication factor Cdt1	AAAGTTTAGTATTTTTAAACTTATTTTAAGACTTAATGAGATGTGATTCAAGAAAAATAGAATCTACACACATGATTTTAT<br>CGAAATTGCAACAAATTCTAAAAAAAAATTAAAATTTCATTTGTTTGTATTAAACTATAAAAAGGTCACTAGCAGACACAT<br>TTGCCTTAAAACTGCTGTCTAATTTTGAAAGAATAATATTGATTGCCAATACATTTTTTCTCTCAGTAACAAAAATATTTCT<br>TAATGTCCTTGCAAGATCTGGCAGACGGCTATAAACTAAATACTTTTGTTCATTTTCTGAAGATCTTGTCATGGCTTCAAGA<br>GCCTTGGCAGCTTGTTTTGCTTTGACTTTTTCCAATAATGAAGCTGATCAAATTCCGGGTGCCAGCGAACTAGTTTATTATC<br>AGGTATGATCATCGGAGGATCCAATGATGACAAGTATTGAGCATGATGTTTTTTCACAAGTCGAAGCAATGTCTCATAAAA<br>GAATCTACGTCTTTCAAGAAGGACACTCGGATTCATAACTTTAATATCATTAGGAAGATTGGGCG
MSTRG.15546	Aminopeptidase M1	ATGAAACTGTAACCCCGCTCGCGTTATCGATATATACTGACGGAACAACAGTGACAATAATTTCATTTCACGCTAAAATAA<br>GTAAACTTCAAGATCAGTCATTTATTAAAAGATTTTAAAAATTTTTCACGAGCTTATCTGTTTTGATCTATAGAAAGGAGA<br>GAATTGTTAAATACTGTACCTCAGCAGTATGTGTGTGTCCGGCTTGTACAAGCGCGAAGCAGTCGTCAAGTAAGCCGAGA<br>CGATCGAGTGGCGGCAATGAGCCATCACGGACCGCCGTCACCAGTTGAGCCAACATCGACGCTGGGTACCGTGTACGATA<br>GTATCCCACCTAATCCAAACAAAAAGAACGCATCAGGATACACAGGAATATAGGAAGTTAATCTAAATTTTAAAGTGGTA<br>TACCTAACTACTAACATATAGATGTCTCATTATATTTCATCCTATTTAATATTATAATTGCGAAAGTTCTTAAGGATGGATG<br>GATGTTTGTTCCGCTTACACACAAGAAATATTTGATGGCTTTTGCATAACTTATACATCAGAACAGCACATTGGCTGAAAT<br>AAAAAAATACTAGATAAATTGGCCGAAAAATCTATATATGTAAAATTCTCATGAGTCAGTGTTAGTTGCCATACTGCTCCG<br>AAACGATTGAACCGATTTTGATGAAATCTTATATGTATACTCGGTAGGTCTTAGAATAGGTTATCTATTTTTTTTATTTCCTT<br>AAGTGATAGGGGTGGCTCACCCCCAAAAAAATCCCTAGAAATAATTTATATAGCAAAACAACGTTTCAGTGCCGGGACAG<br>CTAGTATAAAACTATAATTTTCAAATAAGTTAACAAAAATCCTGCCATCTAAGAACAATTTCGGCGTATGCTACATTAACA<br>ATTACAATTACACAAAAATAATGTATGGTGGAATTGTAGTTTTCTTACTCCTATTACTAATAAAATTGTAACTAGACTATAT<br>ATATATATATATATATATATATATGAGGATAGGAGAGATATAGACTACATTTATCAATCAAATCCATGTAATCAATCTTTA<br>GTAAAACATGTGGGAGTGAACTGCGTGGTGTCAAGACGAAGTCGTGGGCATTATATAGTTCTTGAGGTTATTTTTATTTCT<br>TTATTTATTATCTTTGAGAGAAGGTTACAGCTATAATAACTTAATAATTAATTATACACGATAACAATTAGCTAATTAAAA<br>AGTACCACACTATCATACATACTATCTTTATCGAAAGAGCCTAACAATAGAGCTGTGTCATACACACAATATATTTTATGA<br>TTTCTGTGATATTGTTCATGTTTATTATATCTGGTGATTTGTTACATTTGTTATTTATATTTCTTAAGAGTGATTAGTAATTTC<br>CCTTAGGTGAGGCGTGTCGTCAGCTAGTTTGTCATTTCTAATAATATAAATAGGTTGATTGACAGACATTAATACTTACAG<br>TGCCAGGATTAAGCTTAACCCAAGAGTTTTCAGCAACATTTTCTAGGACTACTTCTTGAGTTCGCTTTTCCAACACAGTAGA<br>AAGTGCCACCTGAAAGTGAAATATCAAATGACTTATTTAGTAGATATTATATAGGAATAGTAAATTAAAATAATTACGGC<br>ATAAATATTACAGTAACCACAAAATATATGAATGTTACTAAAATGACTTTTTTACATAATCATCACTGACGCTCAACCACT<br>GGCATGTGCAAATAACGTCATATTCACAATCTTCAATGGCATTATTTTCATGCAGCAACTTTTTTCGTAACTGTTTATACAA<br>AATGTAAACATTATAGTTGAACATGGAATTATGATAATATATCTAATCTAATATTTAATCTCTACATACATATATATATCAA<br>ACAGATATATGAGACATACAGATGTATGAGTTAAAAATGGGTCAATGTTAAGTTGCATTTAATGTTATGTTTGATAAGGTA<br>TCTAACCTTAATAATAAATTTGTGAAGAAAACATTACATCAATACCTTAGAAGGTTGTTCCTGTGTAGAGATAGTGATTGG<br>TACCATCCAGAGAGTGTCATCAGCTTGGCTTCCATCTGCACAGAACTTTTGCTGGGTCAGTCTGAGTACTCGATTTGAACCT<br>CTTTGTTCTGAGCTCACCTAGAAGACAAAAACCATAATTAGCATGATCATTCGTGATTGAAATAATGTAGGAAAATATTAG<br>TTGATATCAATCTGCATTCACATAAATGTAAATATATAAAAATCCCATGTCATAACTCATTCATACAAATCTTCGAAATCA<br>TTGAACCGATTTTAATAATAATTTGTACAAACACATTTTTTGCTCCAAAACAAGATATAGGAGACATTTTATATCCCAACA<br>AAAAAAATATGTGTGCGGCAGTAGGGGAGGCAAAATAATGTTTGTCAACCCTGCTAGTTCTAGTATATTAACAAAAGGTG<br>T

MSTRG.15616	Protein deltex	TTGCAAAAACCAAAAATCAAGCCTGCAATTGTCAATTCCACTGTCAAAGTCAAAACATTACAAAAAAGCCACTAGTATTA<br>GAATTAATTAATTATAATTAATTGTGAATGAAGAAATGAAACACACTTTCACAACAAAATTGTTTGAAATTGTCGTCTTTT<br>TATTAGTGCTTGATAATATTTTGTAGTTGTACTAAATTAACAACCATATCTTGGGCGAGTGACAGTGCAGTGGAGTTGTTTG<br>CATACAAGTTAAAACTTGTGTTGATTGTGTAGTATTGTTCAACAATTTATATTTCCGCCTTTGTTATCACAGAAATGCTCAA<br>TATAATTGAGCAATGATTGTTTGTAATTTAAGAAATCATATAAACTCCACTCAACCGCTATTGGATAACTGGTGGCGGCAC<br>AGAGGATGTGGCAGAGCGCGGGTGGAGGCGGCGGGCACTGCGTGGCGGTGTGGGAGTGGCGCGGCGCGGAGGGCTGGCT<br>GCCGCTGGCGCCGGGCGTGAGCCGCACGCTGGAGCGCGCGCACGCCAAGCGGCTGACGCGCGTGGCGCTGGCCGACGCC<br>GAGCCCGCGCTGCGCGGCCACCACGTGAACCTGCGCGCGCTCAGCATGAGCCGCGAGCTGGCGGCCGGCCGCGCCGCCAC<br>CTGCGCCG
MSTRG.15649	NFX1-type zinc finger-containing protein 1-like	ATTATGTAGGAGTAACCATTATCAATTTTTTGCTTTATCATTCCTCATTTCAATTTTTGTTTTTGATTAGATAGTCTATCTGA<br>ATAGTGATTATCTTTACTTGTGGATTGTCTGTTTGAGTTGTACCTAAGACAATACGACAGGCAGACAAGATTCTAAATTAG<br>AAGTAGTAACTAACGCCTATCGGACTACTCACACTTATTCAGCTCAGCTTCAGACATTCAACTTATTCAATTGTGAAATAG<br>TTATCGGTATATATAGGCGGGGCACCTGTGAGGGAAGATAGGTGAAGATGAAGCAGCAGTAATATCTAATGTAAGAAGA<br>ATGCAAGCGCTCGTGTCAGCTCGCTTGCGGCCACCTCTGCCGCGGGTTGTGCGGAGAGCGTTGCCCGGACATCTGCGCCCA<br>CTGCCGCCCGGACACCTTCCCCAGGGACTTCCTCGGCGACGAGTTCGACGAAGATGCCAAATTCGTACAGCTTGAGGACT<br>GCCCTCATGTCCTTGAAGTAGACGACATGGATAACCTTATGATGGGAAACAGTAAGAGCATCGCCATACGTACTTGCCCCT<br>TCTGTCGCAAACCTATAATTAATACACAGAGATATAAAGATATTGTCAGTCATTTGTTTGTTACTGATATAAATCCAATCA<br>AGGAACGAGCGTTTGGAAATATGGTTATTAATGACAAACGGAAGGAACTCACAGATGCAGTAACCATTATGAGTGTCAAA<br>TATGATCCAGCACTTAGAGGTAACAAAAGAGCTTTTAAAGCAGTAGTACGGCTTAAAAAAACAATGTCTCTATATTCCAG<br>ATATATCTGATAATGAAAAGTAAAATAATATCATACGTCACCCAAAACATACGATTTCTCGTTTGCTTTCAGCATGTACAG<br>CCATTAACAGCCAAGCTTTTGAAAATTCGTACAATAAATCGATCGCCTTTGTAAGTCCTGAAGTAATAAGACCACATTCAA<br>AGGCAGGGCCAAGAAAAAGCCCGAGAAAGGGAAGAAAAAAAGGTAATACTAGAATTTTAACCGACACACCTGAGAAACT<br>GGCAATCGAAAACAAATGGGAAGCTAGAAAACGCAAAAAAGAAAAGAAGATTTGCAAAGTTAAGGCCAAAGTAGTGAA<br>ACGTAACTTAAATTTACAGGAGCAAGAAGTAACAACATCAGAAGACGAAGAATTGCCGCTATCTCCAGACTCAGACTTAA<br>GTATGTTAGATCAAACAGATGACTTTCCTAGTGAATCCGATCATGAAGATAACACTAATGCCTCAATCATCAGTGGAGATT<br>GGATAATAGTTAATATAGTCTCGCAAAAGAATCTAGTACATAGATATGTTGGACAAGTTCTGAGTGAAAGCCAAAAAGGA<br>TATGACATTAAATTTGCTAAAAAGATTAATGATCGATTTTTCAAGTGGCCTTCTGTAGATGACATTTGTGGTCATGGCTGG<br>AAAGAAGCCATATAGTATATTTAAAAAAAGAAAAAGAATGATTTGAATTTTTGAATACCATTCTTGTATATCTTTGTGTAA<br>TGTTTTATAACTATAATTTTGATATGTAAATAAATATAAAAAACACTAGTGGAACTATAGTGATATCATTTTGGAGTACAT<br>CGTCTAATTAGTGCTATAGCTAAATATCAAGTGGTAAAGAAACTACCAACTCTAAAAGTGAAATCATCGTCAAGACGAGT<br>GATTAGCTTTAAATTCCCGAAATCATTAAGGAAGTTCAAAATTGAATAAATGAAAAAGAGATCTGAAGACATTATTTTGTT<br>TTGATGTTATACATAATTTGTTCCATATTTTTAATTTTGTCCTTTAAAGAAAGTTACATTATTGATTAAAGTTTAGTAAAATT<br>GAGGGCAGAACGGCTTATGTTATTTATTATGTTAAACTGTTACAAGTCACAAATAAATCAATGTTATAATAACA
MSTRG.1569	Echinoderm microtubule-associated protein-like 2	CAGATCGCACGACAGCCGCCTTGCGGCCACTGGCGATGACTTTGGTAAAGTCAAATTGTTCGCTTACCCAGTCACGCAACC<br>AAAGTCGCTGTGCCACCAGTACGGCGGCCACTCGTCGCACGTGACGTGCGTGAGGTTCCTGGCGGACGATGCGCGGCTGG<br>TGTCCGCCGGCGGGCTCGACACCGCCGTCATGCAGTGGCTGCTCGACTAGATCGCCGTTACCACCTCACCACCCTCCCCAC<br>AACCACTTCACCACCCTTCCCACAACCACTTCACCACCCTTCCCACAACCACCTAACTACCCTACCCCATCCCAATTATTAC<br>CTCTATCACAACAACCATCCTCTTCACATCCTGGACACTTCATATACTAAAGTCGTGATTCATAGTCGTGACTACTATGTAA<br>AGAACTAATGTTTACCAAGTGCAGTGCGTTCTTTTATTGAACGGTCGCATTTGAGTCACGGAGAGAAAGGTGATGTCAACT

		GAGGGAAAGGCCTTAAAGTGAAGATAATATTTCTTTATACTGTGCCGCTAATCTGTACCGCAACATTAGCAGAGGCATAG<br>AGCATAAACGTCACAGGGAAATCAACCCATAAGTTTAATACAAACTAAAATAATTCCCAATTATTACGAATTACATGCA<br>AACGGCATATTGATATTAATTCACATACAAATATAGTGTAACAGTGAAAATAAATTTGTATTATTATTATTTGTGTCCAAG<br>TACAATAAGAGAAGTCCAAGTACAAGAAGCGAACTTTATGTAAAGTACAGTAAAGAAGCGAACTTTGTGTACCGTACATA<br>CATTAATCATTGATTATAGATCAATATACAAAAAAAAAAATGTCCTTCCAGACAAAAAATTGGAATTACGTATTCTATACAA<br>AAAAAAATCAGCAATTGCCATTATAAAATATTGATTTAGCCGGTTATACGTATATAAATATTTTATTAATTCATTAGAATA<br>AAAACAAAGTTTTAAAAACCCCATATTTTTTTTGTTTTAAATATCGAAATATTATTAAGTTGTTAAACAATTTGACAACACT<br>AATGCAAAAAGTACATATTTTTAATGTATTACCTTTTAAAATACTTTCTATTTATTTATATATTAAAATATGCATAATTTTTA<br>TTTTTATTTGTTAACTAACAACATTTCACTTCTCGGACCAATCCGGCAACTGTTGCGAAAAAGGAATCTTGCTACGTAACTA<br>GTTGCCATAGATTTCTTTTTCTGTTCGACTAAATTTTATCATGTTTTTTTTTTTATTTTAGTTAAAAACACATACTGGGTACT<br>AGATAATATTACGAATACAGCTTTAGCGACTGATATTAGAGGTGAAAAAATGTCATGGAACAATTTTATACGACGCTTTAA<br>CCTAAATATAAAATAATATACATTTTACAATAATTACACAATAATTAAAAGCCTAATAAACGCCTACATTTACTGAATAAA<br>GTATTTTCTAATTTCCATACGTCTTTTCGATATTAAAATCGTGTTATTGTATATGTAAATAGATACTGGAATCGGAACATTG<br>AATAAATAACAATAATAAAAAAAAAAATAACAAAGTTTGTCAACGCGGTATTAGGTAAATGTACAGACAAAAGTGTTTTTA<br>TGTTTAAACTATTTTAAGTGCAAAAGTACAATTGTAATTTTATTTTATCATTTTATTTTAATAGACTGTTTAGTTATTTTTTTT<br>TTTATCGCTGGGTAAACTCTTACTAGGCTAAAAATCATAATTTTATATTTATATTCAAAATAACGGAACTATGTTGAAAAA<br>AGTGCGCGAAAATTAATCTGTATAATATACAGATTATTTCGCAGTCAGTGTGTTTACCCTTAAAATATGATTGCGAGCTTA<br>AAATGCAGGTATTTGTGTTCTAAACACAACCATGCATTGGTTAATTTGGGTGTATGTTTGTAATTCATTAATAGTATGTTTT<br>TATCAATGATACTAACACTAACTTGATGATGTACGGTTGTGAAGAGATTTCTTTTTTTATAATAGATAGTGTTAAAATGTTT<br>TTTTTCTTTATATATATATATATATATATATATATATATATATATATATATATAAATCCTGGCTAATTGAAATCAATTTAATCTT<br>CTATAAAACGTTGGAATATGATTTTTATATTTTAATAATGGTGTTTTTTTTTTATATTATTTTTATTAATTTCTTAAACAATTC<br>TCTAAACAAAATATAAGATTTTAATAAACTTTCTAAAATACAATTCGTGAATTTGTCAATTATTCTCAAATTATTCTCACAT<br>TAAGTAAGAGTATAGCAACACTGAGAACAATTTCGATTATTTTACACTTGCAACACTGCGAATATTCATGATATGTGATTG<br>AGCTTACTAAGTGTAGTATTTTAAGCGTAATAATTATTTTGATGGCACCTTTTAATCTGTATTCTAATGATTTAAGTTAAAA<br>TGATCAACACTACTTATTAAGTTACTTATAAGTTTTATGTTATTTAATGTGGTTCATAAAATAGGTACGTAATTAATTTTAA<br>TAATTAATAAAGTTTATTGGTATATGTATCAATTGTAAAATTCAAGCAAATTACATGTTTATTCTTAAACCGTAGTAATTTG<br>CTTATTATTTAGTCTTTTTTTTTATACAACTTAGAATGGCAAACAAGCTGACGGTCCACCTGATGGAAAGTGGTAACCGTCG<br>CCTATAGACATGAGCAGTACCATGGACATAACAGAGTATACTGTTTCGTCCATGATACCCCCCATGCGTTGCTATTCCTGA<br>GGACTCTGGTGTTATTCCTCTGTACCCAGTCTACATTAATCACTAAATCTATGTAGGTACGAGGAGAAATTATATAAAAGA<br>ACAAGTTCACTATAAGTTGATCGTGTCTGGATGCCTTTTTAATGTCCATTGCAGAAAAAAAAACCATAGCTGTCAAATTTA<br>TTTTTTTAACCATTTTTCCTTTAAGTTTGAACATGAATTATATCTCCTTCATTTATTTCACCAACACAAAAAAATTATGCAAA<br>GGGTTAATCACCCATTGCGACATCGCTCATACCAGTTGGATTTCTAGTATATCTGTTATTGGCCCAATATATGTATAGATAT<br>TGTACACTCTCACCACTAGCTCCCTCTATACTACGTTAGACGTACCAAATTTACTAATTTTATGAACCACATTACCTTTTTA<br>AGATTTCGTGACACAATCCTTTGTACATCGCGGTTTCGTTAGTAACCTAGTCACCGGTAGGTATTTCATTCGTGTTTATTTA<br>ACAATTACTCTCATTCCATAGTTATAATTATTTAGCACTAAAATAAGCTCTGTAAATAAATTAATTATACGATGGTTATCTC<br>CGTCTTTTTAAATGTGCCTCTAATAATAAAAAAAAAAATATTATTAAAAAA
MSTRG.1<br>5715	Hypothetical<br>protein<br>KGM_203431	AGGGTTTTGAACCAATCTGACGTCGGTTTCTTTGGGATCATTTTTAGTGTCTAATATTGTTAATTAGCGGAAATGAAATTAA<br>TCTACGTGTGTGTGTTACTGGCTGTGCTCATGTGTTTAATCATCGCTGATGCTTACCCTTTCCGAAGTGGAAATATAGATTA<br>TTTGGAGGCTTTGCGGAGAATGCAACAAATACCCCAATGGCACTGTATGCGATATCGACGGCTTCAGTTGCACGGCCATTG

		CAACCGATGGTTAATGTTGAGAAGAACTTGATATCCGTAACCAGTAGGACAAATAGTACTCGATACTTATTCTATGAATGG<br>ATATAACATTCTTTTAAATGTATAAAAGTTTATTGTTTTGCGATATTATCGAGTAAAGCTTTGTCTTATAGGTGTACCCAGT<br>GTGTATCCCATATATTTCTATGTAGTAAAGCTAGGTGATGAAAGAATTAAATTTTTAATAGACTTATTTTTCATAGATAGAT<br>CACCTTAGCATAGTACAGACGTAAATATTTAAGTAAATATGTAATTATAAAAGTACAACATAAATAATATTAGGCATACTT<br>AGTACGTACTTACTTATTGTTTAGTAGGTACCTTAATTATAATGATATTTATCTATATATTATAAATTGTTATTGAACAGCT<br>AATTATTTTTTATTGTTATTCTATTTATTTTAATTTCTTATTCTTTTTTTTTTATAAATTAATGTGTAATCATTCCAGCAGAGA<br>TAGAAATATTAACTATGTACTGCAATAATATACATTGTGGTGAAAGAACTATTAATCATCTGTGAACATATTGAAACTCAA<br>ACTGGCAGGGAGTAAATACAATTAAATTTGGTATTAACATCGGATTATCAACACATTGGGATAAAACTTACAGACAAAAT<br>TTTTTTTTCATTATGTGTACTTTCATTATGATTTACTAAGGATCTAAATACAAAGACACCGTTGTTGTTGCATCAGTTGTAAC<br>TGTTAGTTTCCTGTAACTGGAAACTAGGACAATTCCAGAGCGTAGCGGACTAATCAGAAGAGGGACACGCGGTATAACAT<br>GAGACCACAGTCGTTACAACTCTGACTCACAACAGCTTTATATCTTTTATGAAACTATGAGATTGTTCGAGAAACTTGTTG<br>TATCGACTTGTTTGATTCTAATAGAGGTATTATTAAGCAACTACTTTTATATGAGTTTTAAATTTAATTAATTGATCAATAT<br>TTAACATACATACATGATAGACACTATATAACATTATACTTAAAAGAAAATAGGTAAATTGTGCATCTATATTAATATCAC<br>GAGATAATATAAAATTTGTTAAGTTCTAATGTCACGATAGTGATATTTGTGAGCTAAAATATCAAGCGAAAGGTGTGCTTC<br>TGTGTGTCACGTCATTTGTATGATGCAACAAAAATAATTCCATGAATATATTGTATAGAACCTTCGAATATTTATTTAAATT<br>TAATGGTGTCTTTTGTTTATCTTTTCATATATGCTATCATAAACGATAAGTGTAGAATTCTGTACTCCGCTGTTGACATATG<br>GTCCTTGAGTTTGTTTCTGTTCTATTCCCAAATTAATCCTTTTGTATTTTTTTCTTTCTTGTATAGACAAAACAACATTTCTTT<br>TTTGATTACGTTTGTCCATTAAAATGTTACTTATAAATCTAACGGTAAAATAGAAAATTAACTCGATAAAGCTTATTAGAC<br>TGGCTCCAACAAATAATCCAGTGGTACCGCCAACAGACACTGTAAAAAGTATTATTATCTAATTTAGTAATAGTTTATATA<br>AAGTTAATATAATTTATTAAATATTTGTAACTCACCGACTAGGTCTAAGCGACTCCTTACGACATTACGCTTGAACCTCTCT<br>GCGGGTATATGTGCTAGAAC
MSTRG.1579	BCL2/adenovirus E1B 19 kDa protein-interacting protein 3 isoform X1	CAATTGTCCCCACAGAGCCACTGCCTGTCCGTGCGGCGCTCGTGCCTGTTCAGCCGGGGCGCGGTGGCCGCCGTGCTGGTC<br>ACCAACCTCGTCTCCCTGCTGCTGGGAGCTGGCATCGGGGTGTGGTTGAGCCGGAAGGGCATGCTGCCGCCCAGACTCAT<br>AATCCTGAACTGAGCAGCTACCAAGACTGAGTCGGAAGTGAGCGCTCGTAGTTGGAATGGCCTGAGCATCTTCTAGAACG<br>ATTGGAACTGTATTTTGGACTAAAGAAAACTCTTAAACTGGCTATTATTTTACGTCAAGATCTCGGAGTTTATTGTTAACTA<br>TATAAAATTAAAACGTACAGTCAGAGTGATAATCATCATACGGCCCGCCTGATGGTAAGCTGATAAACTACCCATGCAGC<br>CTATGAAAGCTTGCAACACCGTTGCAGCTCTAGCAGTCAAGATCACGAGTCGCAGTACGCAGTAATGTTATCGCCATATCT<br>CGCCCTTCGCGCCGGATCACGACGCACGGGCAGGTATAGGTAGGACAGAGGTACTCGTGTACAGCTAATGGTTACCACTC<br>GCCATCAGGTGGGCCGTGAGCTTGTTTCTCAGAGATGTACAAGAAAATCTGATCGGTCTCGGCCGTTCCACCTACGTATAT<br>TCACATTGTCATATTGTTTAGGAATGCTAATAGAAATATTATTGTGTAATGATTATTTATATGTATAACATATTTATTGGGC<br>ATTTGGCACGTTGGAGGTAGCACATTCGATTCCTAGTTAGGAAACAAATATGTAATCGTACACATATATGTGAAAAAGTA<br>GCAATCTGTTTAGTATGCCAAGATTTTTTTTGTACAAAAGTTACTCTCGTTTCTAAATTTTTATACATTTTTACTTTGTACTA<br>CTATTTATGGGTATACCCTAGTTTATATTTATAGACACCATCGTACCGGCCTTTAAACATACATTGCTTAACATAGGCCTTT<br>TCCACAAGCAGAGATTTGTCGATAGTTTTCACACTTGACAGGCATATCGACAACTGTAGTTAGAGCTTGAGTGATGTTTTA<br>GGAGGGAATCTACTGCTTTGCCTATCCCTCCACCGATAAGTTCCCTAAGCCTCTCACGGTACTTATAGGAAAAGAAGGGGG<br>TTGTATTTATATAATCTAAGGGTTGAACCTTAAAATTTAAGATAAATGTAGACAACGGGCAAAAATACTTCTATCATCTAT<br>GGATAGTGTTCTATTATTGTCTTAGTATTAAATACTGATATATTTTAAGAATTGTAAGTCATAATTAATAAATACATGATTA<br>GCTGAAGTTATAAGATTGGCCATTGTGTAAATTACAAGGGATCCTAATTTAAATTAATCTGGATCCCAAATTGGATCCACC<br>GGATCAGAAACATTTTATATGGACGAGTTGATTAAACTGTAAAGAGATGCACAGATATGAAGCAAGAAAATGCAAGATAC

		ACATACAAGCCAGTATACTTTTGTTATTTTTTTGTTTTTTTTTTGTTTTAATAAAGCAAAATATCATACATTTGAAATGATAA<br>TTTAAAGATAAAAGAATTGATTAGAAAACTGTGTGGAAATATGCAGGTCTGTAAGTGTATTTAATTTTCTTTTTTTTTTTAT<br>TTTTGTATGAAAATAATCATACATTTATTTTGAATGTGTTTTTTTACATAGGGATCCAAATTTTGGACCTTATATAATTTTTT<br>AATAATACTATTTTCCATTAAACAATAATATTTTTGTATCAGTGTGGCTGTAACCATAGATAGGGAAAACATATAAAAAAA<br>TATATAATAATAATAATAAATAAATAATATTACATATTTAGGATCCATGCCACTTTTGTATTTTCCTATTTCTCAATTTCTCT<br>ATGATATTACAGTTTTTCCTGTCAATTGTTTATTTGAAGTTGGTCTAACGAAATTGACACTGTAGGATTTTATTTTAAATGA<br>TTGACAGGAGAGATTTACCTTATCAATATTTTTATATACTAGTCCGTAAGCTTATGGAAGAATTTATGTTTTAAACTTAACT<br>GTGAATGAATAAGGAAAAAAAATTCTAGCTGCATTACTAAAAAAAAATTACAGTGGATCCTTATGTTCTCTTCCTAGTTCCTC<br>TGTGGATCTACAGTAACTTTTTTTTAAGTGCGCAATGTTGGCACTTTCTATTGTTTTAATTCAAAATTAGCTTCGATTCTAGA<br>ATGCTTAGGTTTTTTTTTTCC
MSTRG.15794	Septin-4	ACAGGTATACCAGTTTCCTGAGTGTGACAGTGACGAAGATGAGGAATTCAAACAACAGGACCGCGAGTTGAAAGCTGCGG<br>CGCCATTTGCCGTAGTAGCGGCCGACACTGTACTGGAAGTGGGCGGGAAAAGGGTACGCGGACGACAATACCCCTGGGG<br>AATCGTCGACGTGGAAAATCCTCGACACTCAGACTTCACTAAGTTGCGAACAATGTTGATATCAACACACATGCAAGATTT<br>AAAGGATGTCACCCAAGACGTTCATTACGAGAATTTCCGCGCGCAATGTATCTCGCAAATATCACAACACGCTATGAGGG<br>AAAGAGGAAAACTTAAAAGGGATTCAATGGGCAACAACAACGAAGTAGTTTTTACTGACACTGA
MSTRG.158	Cyclic nucleotide-gated channel rod photoreceptor subunit alpha	AACCATTAGGTAAAAACAGAAAGCGTATGAAGGGCAGATGTGGTGAAAAATATGTGACAATATGTTTTTTTTTTGTTAAA<br>ACATTTTTGCCAATTCAGGAGTTTTTAAAGAACAATCGCGTATCAAAGAACCTGGTGGCGGTGGTGAACAAATACTCCCTG<br>CACGTATGGCGAGAGTCGCGCGGCGAACAAGTGCCGGACCTCCTGAGGAGTGCGCCTCGCTGCCTCAAGCTGGCCGTCAT<br>GTCTGCCGCCTACCTCGAGCATCTGACCAGTAACCCTGTCTTCAAGAACTGCGAACCTGCTTTTCTGAGACAACTTGTGGG<br>ACATTTACAGTTATACATTTATAATGAAGTATGAAGGTCTAAACCGCAATACACCTTATGCACACACATATCAGACGCTCA<br>CCAAATCTCGAGTGCTCACGCTACACTTGGCTGATTGGGAATATTTACTCGAACATTTTCCTCAAAGTAAAAACTTGATTTT<br>TAAATACATGCAGTTTGATAATGATCACCCGGATAAACAAGGAGATCCAGAAGTAATAAATGCAGACCCTTCTAAATTCA<br>TAGCTGAAGATTTGAAAAAGGAACCAAGTTCACTTCGCGTTAGGCCTCCT
MSTRG.15810	Cysteine-rich with EGF-like domain protein 2	AACTAATATGATTACTTACCTAACCTATATATATATACATATATGCAAAAAATTAATATTTTATTCAAACTAATTTTACGTA<br>CTTGTTTTTTTTCCCTGCCGCTAATTTTCAATCTGCTTTATTTTGTTTACGTATTTGAAATAAATTTTATTAAATAAGAAAA<br>TATCATCTGAAGACAGGACAGTTAACGACTGTCAACGTTGTAAAATATTAACAGACTCTTTTAATTACTGGATTGACAAAA<br>CTTCTCGAGGAAAACACGAAGGTGGTGATGCTGCTTGGGAAGAAGCTAAATTGAAGTCTTATGCTCATAGTGAAATACGT<br>TTAGTTGAGATACAGGAAGGCCTATGTTCAGAATTAAAGGCACATGAAAGTGATTGTTATATCTTAGCAGAGAATGCAGA<br>ACAGTTTCTAGAGAATTGGTGGTTTAGAGAAGATCCTAATTCTGTAGACTTACATACATGGCTGTGTATAGAAACTCTTAA<br>ATATTGTTGTCATAAAAATCATTTTGGAGATAACTGTTTACCTTGTCCTCTTGATACACAGAACCAAGTCTGTGGAGGACA<br>TGGTAGATGTGATGGTGATGGTACTCGTTTAGGAAATGGTACATGTATTTGTAACAAAGGCTATATAGGAGTATTATGTAA<br>AGATTGTGCTAAACATTTTTTTCAAGATAATGACTTATGTAAGCCATGTCACAAGGCTTGCAATGGCTGCTCTGGTGACAG<br>TGCTGCAGCTTGTAATACATGTAAAGTTGGCTGGAAGCTAGAGTCTGGTGTGTGCACAGATGTAAATGAATGTTTGGATAC<br>AACCTTGTGTAAATCTACCCAATTTTGTATTAACAAAGAAGGCTCCTATGACTGTAAATCATGTGATGCATCATGTAGAAC<br>ATGTGCAGGTGCTGGACATTCAAACTGTACATCATGTGAATCTAATCATGTGTTATGGAGTGGTAGATGTTTAGATGATAA<br>ACATAAGAGTAACCTTTTAAGAAGTACTATAAAAAAATTAGCCCTCTATTTAGGATTATTTGTAATAGCTTTTTTTATTCTC<br>AGGAACTCAAAAACATTGGCATCACTTGTAATTTTAATCATTACGATATACATACATTATTCTGAGAAAAATTCAGAAATG<br>AACATTTTACATGTACTTTTAAATCTTTATGTGAATTAATTGTACAATTTTTTTTTTATATGAACTCCAATAAAATTCTGTTT<br>ATGTATAATTGTTGGATATTTTTAATATGCCATACTTATTAAACAGGCCTTTTTCCTTAGTTTTGTGCAAAAGATTTGTTTAC

		TCCGAACAGTTAACTTTTAATTATTTTCAATGACATGTAAATAAATGGACAGTTTATTAAGAATATTTTGCAATTTTTTATG<br>GATAAAAGTAGCTTGTACTTTAGAAAACAATCTGCCTATGAGGTTAATTCTGAGGCAGTATACTAAACCTATATCTCTTAA<br>ATTAATATTTAAATTAAATAATTTTGCAAAGTTACTGTTTTATAGGTTCGTGTGCAACGTGAATCTCCCTTATCATCTTAAA<br>ATCCCCTCAATCAAACAGTTCATGAAGGAAGTGTCTCAACACTACTTCGTTGCTGTTGAGTCCCATCCAAATCAGCTTATC<br>GTAAAATCGGTCAGCTACACGCCCAAGAAGAATGTCACACCCCAACGTCGCTCAATGACTATGGACTTGTTAATAATGCTT<br>TTTTTTATACAACTTAAAATGACAAACAAGCTGACGGCCCATTTGATGGAAAATGGTAACTGTTACTTATGTCTATAAGCA<br>GTACTATGGACATAACAGAGTGTACTGTTTCATCTGTGATACCCTTCATGCGTTGCCATTCCTGAGGACTCGGGTGTTGTTC<br>CTCTGTACCATATACCTCACACACTGTAAATTATTCAATACATCCATTGATAATCTCACTTCATCTATGTATTATATTCTGAT<br>CTTTATTCCTTGGAGAAAATTATTAAATCTTATGAACACTACATAGATTAAGGACTGTACCTGTGGTAATACTATGAATCA<br>TTACAGATTGTTATACCTACCACTTTATGATATGCTTTATTTGTACATCTATTTTTTCCACACTACTATTACTCAAGTAATAA<br>AAATTGAAAAACAAGATAAAGGTGTATTAAAAATACATTTTATTGCAAGTATGTAAACTACATTTTAGTGTCATTTATATA<br>CAAAAATTAAATACAATGTTTGATATAATAAACCAAATTGTGTAAATACTTTATAATACAGGACACTTATTACTAACAATT<br>AAAGATAAAGTTAAATAATTATATAAATTCATAAAGAAACAAATGCCAAGTTAAAAGGTAACATATTAAAGACCTTTTGA<br>TTTATTAAAAAATTCACATATGTTATTAACACATATTTAAGTATAAACAATATTTATCCTTTCTTAACACTCATTAATTGTG<br>ACAAAGGGCCCATAGTTAATCATTATATTCTAAAAGACAAAAGTAAGTTAATAATTGGCAATAGTTAATTTGGCAGCAGC<br>AAGCATAGGTATACATTTTCAGTCTGAATAAATAATTAATGCTTAATTATTATCAATTATTGATGGTGC
MSTRG.15833	Bifunctional coenzyme A synthase isoform X3	CTAGGCGGAAGCGGAAGTTATATTCAATCATAGAATGCTGTAGGTACCTATTATAATAGTTTGTTTATACCAATTAATTTA<br>TAACGCAATAATTCAAAATTAATATAAACCAAAATTTCCATTACTTGTGTGTTAATGATTTCTGCGACAGTATTTAAAACTC<br>TGTCTTAATTTGAAAGAATGGGAAACCTGAGTTGAAGCTAGAACTTAATACTAAATATAGGCGTTATTGTAATTAATATTT<br>ACACACAGCATCAGTGAAGTTCTAACAGTGCATTCATCACAAAAATATATGAGACAATAACTGAAAACAACAGTACAGCA<br>AACGATACACCATATGAGATGTTACACATTCTAATGAGGTCCAAAGGTTGAATGTTATCAAATTTTTATGCAATAAAAGTG<br>AATGATAAAAATACTATTTGTTTATGGAACTGTACTAAACTGAAGTGGAATGAAAAGAATGAATCGTCCTTCGACTGAAC<br>AGACAATAACAGAAATAATTTACATATTTATACTATGTTCTTTAATATGAATGAAGAACCGGGATACATTTTTTACACCAC<br>AACTTTAACATTACCTAAATATATGTAATACAATCGGA
MSTRG.15840	Cuticle protein CPH43	ATTAATCAATTTTTGGCAATATTTTTTAATTAAATATAATTTGGTAAATTTGAATAGAGACATTTTATCTATATAGGGGAGA<br>AAGGGATTGTTAGTAATGTTAAGGAATTGTAAGTGGGCTAACAGCTCCATTGCTAGTAATATACCTTGTCAGGTCAACCTC<br>CATATTTTATTTGTATTTTATATAAAAATGATCATGTTTCATTACACCAGCGCGAAGTAGAGACAAATCGATAGTATTTTAT<br>ACTTTTTTTATAAGCACAATCGGTACTCGTGAGACTACTCGATCTGAAAAAGGTGGGTGATAAAATAATGAGTTTGAAGAT<br>ACCTACTAAAGGTACGTATCAAACAAAATTCATACAACATGACTCGGTTAATGTGCAAGAAGCCATTTTGTTCTGTACCAT<br>AGCCATGGCCATAGACAACATGAGTGTATAAATATCGAAACGTCTACAATAAACGGGCTATTCAAGTGCTTCACGTATTCC<br>ACTATTGTATATAGTGTTTTATAAAGTTTTGTCCAATATGGGGCTATTGTCCGCAATCATAGTTAACGTCGTTGGTGGCACT<br>GTTATCTATGCCACCGGAGGCCTGGGAATTGGCTTAGTGGCTCCTATGTTGGGTTTTGGAAGTGCCGGAATAGCGGCAGGC<br>AGCACCGCCGCCGCTGCCCAGGCGTACTACGGCAATTTGGTCGCCGGCAGCATAATTTCGAAGCTAACTGCAGCCGCCAT<br>GGTTGCCCCCACTCCATGATAAGCGAGAATAGTCTGCTGTCATTCCTGAGTATTATTGTAAAATAATTTCAGAATTATTTTT<br>ATTAAAACTCGGATAAATAATAAATAGTGTTTTATTATTATTGACAAATAAAACTACTAGTTTTAATTGTC
MSTRG.1586	Transmembrane protease serine	CGTGTACGTGCGCGAACATGTAACAGTTGTTATGTAGTGTGTATGTTGTATTAGTGTTGTGTAGTTCTGCGCAAATCTGTTT<br>TGTTAAGATTTTTATTAAAAAGTAACTTATCGATATTTTTTTATATTTGAAGAGTTCTTCTGTTTCGTGGTGGACCTTGGTGG<br>TATCCTGTCGACATTCCTTGAGTTCCTTCAGCAGACCGCCCTCGTTGAGCTGGAAGGCACGAAACGTCTGCGCCACGCTAT<br>GCATATGCATACGTCCAGCCCTGGAGCCCGGGTAGCCTGGGACCGTTACTGGCGTGCTTCTATTCACCCACCTGGGCAGTC

		CATCTTGCCATCGCACATATGTGATTGCGAGATGCACCGCTGGTTTCCGCAGTGCACGTCGCCTGTCCGTTTGCAGACGCG<br>ACAGTTGAGTTCGTCAGATCGGTCTGCGCAGTCCACGTGCCCGTCGCAGCGCCAGTCGTGTGGGATGCAGCGTTTTATGTC<br>GCATTGGAATCCAGTGGGGCATACTGGTGAAGATAGTACTTAGTTAAAGTATGCTGGTGATTGAATGTGGTAATGAAGTA<br>AAATTACAACTTGTCAACGCCTTAATTTATATCTCATAGCCAGGATATTGTAAGAGGCGATTAATATATAAGTTTATGAGA<br>AATAGAAGGATAACTCGAAATAAAACCACGCGCCATGTTAACCCTTCATTGGAAGTTTTTTTTTTTTTTGGTGAAATAAAT<br>CAAGGCGATATAATTCATGTTCAAAATTAAG
MSTRG.15912	Neuropeptide-like 3	TACACTGCTTTACACCATACAAGATTGTTTTGTTCGCTCTCGTAGCAGCATGCTTCGCTGCCCCTAAGCCTGGTGCGCTGTT<br>GACATACCCCTCATCAGCCGTCATTTCACCAGTAGTTTCACCAGTTGCGAGTGTCTCCAGCTACTCGACCGGCATCGTCCAT<br>GGCTCCCCATTGCTGTCCCTTGCTCATCCACCACTTGTCCACCCTGTTCATCCAATCCACTCCAACATTGCTCATCCCGTTTT<br>TATATAAATAATTTAACCCGCTAATATGTTACATCGGTGTATAGTCGTATTTTTTAAGAAGATTCAATGTTATGAATACAAG<br>TAATTATTACATTTTCTTTGAAAACAATTATACACCATTAAATTGTATATAAAAAAGGTCGTTTATATATTACTAGTTATAT<br>TGGTGAAATGGTTTATGGTGCCAATATTTTTATTCATGGAACATTTTACCATAATATTCTTTTTGATGAACCAGACAGTATA<br>TTATCACTCCGCAAAATTTTAATAAAACCTGTCCTATAGTTTTTGCACGGTCTTCTTATTTTTTTTTCAATTATGTTTAAAG<br>TTTTACTAAATTTATTCAAATGGATTAATTTTTAATTACTTGCTATTATATGTATATTGTAAGTTTATGCTTCCATTAATAGC<br>GTAAATATCAATGGTTTCAAGTAATTGAGTACGGTAATACCCTCGAAGATTGAGTGAACAATATTGTTTGAAGTGTAATGC<br>TAATCAAAATATTAATGAATTTGGTAGATGATAAGCAAATATAAACACATACGTACTTGTTACAAATACAACTGCAACAT<br>ATTCAAAAGAAAAAAAATGAAGGACGTTTTGTTATTAAAAGAGTGTTGTGATAACTTATTGTATATATAACAGGGTATAA<br>ATAAATACATTGCAGCAAAAC
MSTRG.15948	Uncharacterized protein LOC105386840	ATAATAAGTTTGATTACTACTAAAATACACTACTACTAGAAGCTATGATTCTATGGTATAAGAACATTGATTTAATTAGGC<br>TTGGCATAATAACGGGCAAATTTTGGTTATTGGACGAAGTAGACTACCAACGGAAGTTTTTAATTCAACCACCCTAACAGT<br>GCCGTCAGAACCTGGATAAACCTTTGTTACGCGGGCTGTCTTCCATCGAAGCGGTGGCAAAGCGGATTCCTTTAGCAAGAC<br>CAAATCACCTACTTGGACATTAGGTAGCCTAGCAAGCCATTTGGGTCGTTGTTGCAATGTGTGAAGGTATTCGGTTTGCCA<br>ACGCTTCCAAAAATGCTGATTTATTTGACGAACCAATTGCCATCGTTTTAAAAGATTAGATTCTGTCATTTCCTCCTCAGGT<br>AATGCCTGAAGGGGTTTACCGATGAGGAAATGGCCCGGTGTTAGAACATCCAAATCGTTTGAATCATTAGTTATAGGGAC<br>TAAAGGGCGCGAATTTAATGCAGCTTCAACCTTGGCAAAAATGGTAGCTAATTCTTCGAAAACCAGTGAGCGATCGCCGA<br>TAACTCGAGCTAAAATAATCTTGGCAGAGCGCACGGCGCTTTCCCACAAACCGCCCATATGTGGACTGGCGGGAGGATTC<br>AAATGCCAAACTACTTGTTGACGTGCTAAATGTTTGTGAATTTCTTCATGTGAATTTTTAATGTGCTGAGTTACGTTCTTTA<br>AATAATTTCGAGTACCGATAAAATTAGTACCGCAGTCACTAAAAACGTCGCTGGGAAGACCTCTACGAGAAACAAATCGA<br>TCAAAGCAGGCTAAAAATGCTTCAGTGGACAACTCTGAAACAATTTCTAGATGGACGCACTTAGTCGTAAAGCATACAAA<br>AATACAAAGATATGATTTAACAGATTTGGCGTTTCGAATTCGAGAAGTCTTTAAGGAAAAAGGACCTGCGAAATCGACTC<br>CTACTTTTTCAAAAGGTCGACACGACGTCACTCGATGAGATGGCAAATCTCCCATAAATGGTTCAATGGACTTAGGTCGAG<br>ATCTATAACATCTGTGGCAACGAAATATTTGTTGGCGGATTATTCGACGCGCTGACAAAATCCAATAATTTTGTTGCAATA<br>AATTCATGAGGGAATTTAAACCTGAATGCAAAAAAATATGATGATAATGATCGATTATTAACCGTGTTACATGGTGAGTTT<br>TTGGAAGTAGAAGCGGATGCTTTGCGTTGTAGGGTAAATTTGAGTTGATAAGTCTTCCACCAACTCTAATGAGGCCGTCCT<br>GATCAAGAAATAGGTTGAGCCTTAAAAGACAACCCTTGAGAGGTTTGTCCTCACGTAACCTTCTAGAAGTTTCCAAAAACT<br>CCGAAGACTGAACTAGCCGGACAATAGTGTGCGTGCTTGCACACAACTCGCATACGCTAAGAGGTCCAGTTCTTCGTTGA<br>GAATAGTTACGCAGGTTGTAAATAAAGCGCTTACACCAAGCTACCACAGACTGTAACTTGGGAAACGAGCTGAATTTTGT<br>AAGAGCAGGAAATTCATCAACCGTCTGAGTCACATAGACTTCAAACGGTGAAGACTTCATGCCAGGTAATTCCGCTTCACT<br>TAGGTCAGGAATGTTATCTGGCCATGTTGCGTCGTCCAGCTCAAGCCATTTGGACACCACCATAAAGGATGATCAACTAA

		TTCAGCAGGACTGAGACCTCGACTGGCTGGATCAGCTGGATTCATCATGGACGGTACATGCTTCCATGTAACATCGGGAA<br>ACAGATTTTGAATCTGGGATACACGATTACCCTCATACGTTTGAAGTTTATGAGGAGAAGTTTTAAGCCATGAAAGAACTA<br>TACTTGAATCGCACCAAGCAACAACACTACTAATTTTGACTTTGACATTTAAGATGTCAAATACGTGTTTTGTAAGACGTA<br>TAAGTAAAAGTGCACCGTTTAATTCCAATTTTGGAATGGTACTGAGCAGAGTCAAAACTATGAGAACCACTGACAACGTC<br>GTCTACGAATGTGTCCTCTAGCAACACTCTACAAGCCTCAGGATGCTGGCTGGAATTTATGTGAGCCAGCTCATGAATGAC<br>ACGAATAGCTTGAAAAGCAGAGCTGGTGACGCCATATGTTACTGTGATCAATTCATATTGCTTCAGCGGCTCATCAGGTGA<br>TGCACGCCAGAAGATAGTTTGATATTTACAATCTTCATTATGCATACGTACCTGGCGGTACATTTGTTTTATGTCACATGTG<br>AAGACAACTTTATGGATTCTAAAACGCAAAATTATATCTTCAATACTTTTTTGAAGTTTGGGGCCAGACATAAGTATCTGA<br>TTGAGGGAAACACCAGTGGAGCTAGAGGCCGAAGCATCGTATACAGCACGAAGTTTTGTGGTGCTACTATGTTCTCGTAG<br>TATGCCATGATGCGGAATGTAATATCGCTCCCTTGAAACAGGAGTTGATACTTCACGCATATGGCCAGCTTCAATGTAATC<br>TTTCATAAAGGCTACATATAATTCATGCAATTTGTCATCTTTTAACATACGGCGCTCTAAATTATGAAATCGCTTTAAAGCG<br>GTAGTGTGTGTTTCTCCTAAAGGCTTGTGATCTACTTTGAATGGTAACCGTACAATGAAACGACCTGTGTCATCTCTCGTGT<br>GGGTGTCA
MSTRG.1595	BTB/POZ domain-containing protein 9 isoform X1	AGGTGTTCCACCTGGTGCACCTGGAGTGCCCGGCGCAGGTGGAGGAGGCTCGCGACGACCCCGCCGTCAAGAAGCTGCGC<br>CCCGCCGACAACAAGCTCAACCCGCCGCCCGCCGCTGGCACCGCGGCCGCCGCTGCCGCCTCCGCTGCCGGCTGTACGAC<br>ATCATCTCAACAAACGACTGCGAACAACGAGAGTCGATCAGTGTCGCCGCCGAGCTCCGAGGCGAGTGCTGCAACCACCG<br>GTGGTGGCTCTGGTAACGCGACTAGCGGTGGCGGCGGCGAGCCGTCGACGTCCCGCAGCCGCTCGCTGCCGCCCGCCGAG<br>ACGGCCACGGAGGCCGACGAGCGGCTCGAGGAATGATGCACGCACGCGCGGCCTCACACACGCGGCCTGTGTCCCCCCCA<br>CATGCATGTCTCTAATGTGAACGCGCCGAAGGGGAAGGTAACCAAGTATGTTATCACATACGCCTCGCGTCGAAGTAATT<br>GTGACTAATAATAGAATTGCACATCGCCGTGACCCCCTAATGACTTCAGACTGACTGATTACATCCTGATGATGCAAATGT<br>GTCATCAGAAATTACCAGAGAAATATAAAATAGCGGGGCAGAAGTTTAATTAGACATTGATATAGAGAAATGTACCAAGA<br>TGCACAATCGGAGATTAAAAAGTAGCACCGAAAATGACCATTTTTTATCCAAAGCACTTTTTTGTAACAAGCCAATGATGT<br>TTGAAGTAACACGCTAAAATTTGTACATTATAAAATTTTGTTAAAATTTTTAAATTATTCGTAATAATCGATAATTTATATG<br>TATAGGAAAGTAATTTCACGTTTGTGATAATTATTTGATTTCATTGACGTTTTATCATACAATAACTATTAATTTGAGACAG<br>TATGAAGCTGAGCTTGTATATAATTGTGAATCTTGGGTCTTTTGTATATCAATGATGTACGTCGTTGTTTTATCTTAGGAAT<br>TAATGAAGTATATTTCTGTTATTAAATATTTAGTGTAAAGCCCACTGAGTATCTAAAATATGGTGTAAATATCATAAGCAG<br>CCATGACTCTCTTTTATTTATTTAGGGGATATTTCTCTGTCTTCTAAAAGAATTATAAATTTACAGAACAAACTGGCACCAT<br>ATTAAAATGGATTTTTTTTTATAGATTTTCTCCAGTGTTACTTTATGAATGAAAACAGTGCCATAATGATTGCCTGAACATT<br>CTGCCTTCAATATACAGTAATTATTTATTTATGTATCGTCTTGTTCCTTAAAAATATTCTCAGGTTGTTTCATCGTAAAATCT<br>ATTACTTATGTTGTAGTTTAGGATTTATTATCGCATGTAATATTGTCATAAAAAAAACTATGTTGTCATAAGCAAAGCTTAT<br>CGTTTTCACAAAAAAATGGTTGTGTGATAATAAAATTATTGTAATGTAAGAAATAAATTTCTATAGGGCCCTTGAAGTAAGA<br>TATTCTTGAAGTTATTTACTCTTTTGTCACTGCGTAAAAGTAAATGCTAATAACAGGGCTGAGTAGTGTATCTATTCTGTAT<br>TTTATAAAAAAAAAGCTCTTTTATTTAAAGACATTGAATTCATCTTATTGACATTGAAATAAGAATCAATTTCAAATGTCA<br>ACAACATTTGTTTTGTGAATATTTGGAACAATGTTAGGGTCGATTCTGAGGTACCATTCCATTCAATTCATAAGGTCCTACA<br>TCCATCAATAATATTTTTTATAGTTTAGCAAAACTATTGTATATGGTATAAGACCATCAAAATTGACCTACTAAAAAAAAG<br>TTATCATAGAAGTATTGATTTGCTGTAAAATCAATTTGAAATTATATGATTTTTAGAGATCGAGAATGGTGCTTCAGAATC<br>AACTCTGATGTTTTTACTCATTGATCATATAAATGCCTACTCATCAGGACAATATCACAAAATATGGACTGAAAATTTCAT<br>ATGCGAGATGCAAGAATTATTTTTAATGATAATTTTATAATCTTTGTATTTTAACGAAATCTCAAATATTATTTTATTAATT<br>AGCACTAAGATTCTACAATCATACAAGTTTGAATGGTTGAGCTACTGTGATAATGGTTTGAAATTAATTGATTTGCAAATG

		TTTAGACGTTTAGGATATAGAGGACATGAGGAGGATACATTGAAACTGTCAATCATTTGTAACATTTCATAATATTAAAGCTTTTAATAAATAGTATTAATCTGG
MSTRG.15957	Dedicator of cytokinesis protein 9 isoform X1	ATCCAACCATTTACTTATATTTAAAAGGCCTATAGACAATAAATTGCCGGAGGAAGTATACGACATTGATGCCGAAATCGAAAGCGATCATGAAGCTCAAGCCGCTAAAGTGGACATCGGTACCAAGGAGGGGTACCTGCTCAAAGGACCCGAAATCGGCTCAGATAGAATTTTCAGTAATCTCGCTTCCAAATCGTTTAAAAAACGATACTGCTCAATGGTTAGGGAAGCAGACGGCACATATATTCTGGAAGTGTATAAAGACGAGAAGAAAACGGACACCAAGCTCACCATAGTCATGGATTTTTGTTCAGATGTTGTTAAAAATCCGAAACGTGGGCGTTTTTGTTTTGAATTGCGAATGGCCGGTGGGAAAGGATACACATTTTCAACTGATAATGATGAAGAGATGAACGAATGGATCAACGCTTTTAACGCAGCACTAAAGAAAAAACTAGATAGTCAGGAAGGTCATCAGAATGATGAAGTTTTAGATAAAGACGCGGATGTGTCCTTGACAGCTGAACCGCCACCTCCAACGTATGGCACGCTCAAAGGATTAGAGCATAGCATGAACCCGCTCCTGATGCGGTACTCGCGAGAGACCGACCTATCAATAGCCGCGGCTCGCAACGAGTGCCGCGTCCATATATTCAGTATGCCGTATAAACGGACACCCTCTCCTGAAGCTCAGTTAGAACCATTCAAAGAACACTTTGGCCAAAGAATATTGCTCAAATGCGAAAGCTTAAAGTTTAGGTTACAAGCGCCGATAGATGGCGACAAAGAACTATTGTGCCAAGTGGAGCCTTATTTCACGCACCTTGCGCTGTACGACGCGAGGAGCGGCAAGAAACTTACAGAAAACTTTCATTTCGACCTCAACCACGCCGCGGTCAAAGATCTAGTCAAGGAGAACGAGTGTACAGACTCGTTAATAGAGAACTTTAGCTACGACGTTAAATTAGATGTTACACAGATTCCCGAAGAGTGGTACAGAT
MSTRG.15985	Uncharacterized protein LOC105842644	ATTCAATAAAAAAAATATGATAACAAACATGGCGCGATAGAAAGAAGCGTCTTCGTATGGATTTGTTTATTAGGTCAACGTTTGTATAAGGGATAATTGTCTTTACAGATTATGTCGTCACATACAAGGGGGCGGAATCTCCATGTTAACGTGCGGAAAAGGAGGTGCCGGGGCGAAGGCGCTCGGCGGTAGACGCGTGTGCGGGTGATATGCAAGCGGCGCCGGCGCGCTACGTGTCAGTGAGCGAGCTGTATTCTACCGACGAAGTGGAGCTGTTGCTTGACGAGATTCGCGAACTGGAGCCCACGAGCTGCCGCGGCGAGGACGTGCAGGACTTCGAGATCGCGGGCAGCGGGTGCGAGGGGTGCGGCGGCGGCGGCGGCGGCGGCG
MSTRG.160	Prostamide/prostaglandin F synthase-like	TCAGTCCACAGAGCTGAGAAGTTTCTGGCAAGATCAGAATGTGGTCATCATATTCTTCCGTCGTTGGGGCTGCTTGCTTTGCAGGCTGTGGGCTAGAGAGTTGGCTGAAATAGCTCCTGTACTGCAGAATAATAATATCAAGTTGGTAGGCGTGGGCGTCGAAGAAGTTGGCAGTAAGGAATTTGTCGATAAGAAATTCTTTGATGGAGGTATTACAGTTTTTTATTATCTACTATTATAAATGCTCCCCGTGTGGTCCCGGCATAGAATAGGCC
MSTRG.16030	ATP-binding cassette sub-family A member 3	CCGACGCCGCTGCCGCCGCCACCACCGCCACCATCAGCGACGCAGATGAAACGGATGGACGCGAAGTGAACAATTTGAAAGAAAAATTAGGAGCATTATTCAGCGATAATGTACCGACAGACGAGCATAAGACGATGCTCCGGTACCGTATAACCGAAACGAGGAACTACAGCGAACTGTTCGATCAACTAGAACAATTGAAAAGTAGCTGCCCGATCATCGAGGATTACTCCATCACGGAAACGACGCTGGAGGAAGTATTTCTATCTTTTGCTAAAAACAATAAAAGTAATAAAAGCGATAATCCTGTATAAGTGCGTAAACTATGGCAGTGGTACTGCCACTACAAAAATATACGGCTTTCCTTCAGCACTCATTGTAAACGAGTGCGGCTACGTGTCGCCGCCTGCGAGCCGATGGCTATTTAGATATAAACGCCATCTATCGTCTAATAGATTCACTATTGTCCTTGCTGCTTTACTATATTTACAGCTAACATGTGCACGACATTTTTCTATATTCACCTTTATAACTGTACTGCAAAAACACATCACAAAGAAAGAAAGTAAAATAAATGCGTTCGAAATACCGCGAAGAGATCGCAGGGTGAGGCCAACAAGCAGACATAACGCAGTAGCGCGTGAACGAATTTGTTCGAAGGAAAGGGTCTGGTGTAGGGATGACAATTAGCAGGTCAAGTTGGTTCGGACGTGTCAGGAGTATATTGATGATGATAGATGGTGACAAAACCAGGGTTGCCAGATGCAATTTATGATTTTTACCCAACTGTCCAATGTTTTTTCTCCAAACCCATATAATTTTCATCTCTTTTACTAAATCACATACTTGTTTCATTTATCACTTTGTTTTATCCTATCCACACTGCGAATGACGAAATTAAAAAACGCTCTGTCATATCTATATCAGATTCGTTAAATTTCAGCTGAACATAGACACGTAATTTTCCTTTTTGAATGGGTGATGATAATGAAATGGTAACCCCGCTTGCGCTATCGGTATACGCTGGCTGGGCACCAGTGAGGGATGATGAGGTGACAATGAAAGCATGTCAATTAGCCCATCGTGACGAATTCCATTAGAATTAACCACGACGGTTTCTGGGGGGGAGCTACGTGGAGGTTGATCTGATAGGGTGTATTGCTAGCAATAAGGCAGTTACATCCCATTGCTAACAATCCTTTTTTTTCCCG

		GCCACTCGCATTAGGGGTAAGGAGTTGCCATTAGTCGTCACGCTTTACTTTACAGGCAGTTTGGCAACCGTAGCTAAACTT<br>TGTTGATGTTTTATTACGGGCACTGCTGCCCATCCATCGACCATTTACCTTAGTCGCTTCTTACGACATGGTAAAGGAGGGG<br>AGAATTTTATTCATATATTTTATTGTGTTTACATACCACTATAATGTTTACAAGTACCAAAACTGTGAAATATAATTGTCCA<br>AATTTCACTAAACCAATTCACTGTACTCCAAAGATTTCTCCAACAATTTTATTGGTCCAAATTTTTGCGAACACAATTTTTT<br>ACCCAAAATTGCAAAATGAAAATACCCAATCTGGCAACACTGGACGAAACATATTTTTTATGCGAAAGTGGATGAATGTA<br>GACCAACTAGTTAAGTACAGACTGATGGAAAACCACTGAGCCACCAGAATCGGTGTGTCTGGAGAACTACCCATACCCGC<br>GGACCCAATAGAGCTACTGCTCCAAAAAGAACTGAGGGCCAACTATGAAATATTATGTAAGAAGCATTTAATGTATTCAT<br>ACTACAGTCTAGTCCTGCTCTGATCAATTTTATTTTTTTTTTATTATGTATTAATACCGACTGAGGATAGGGGGGTGGGGTG<br>GGGGGCACTATAGTGTGGATCCGGTCTTATTTAGAGTATTTTAATTATTATAATCTTTCTGTTGAGAAAGCAATCCACACAT<br>TTCTGTGTTCACACATAATATGACAACTTTATTAGTCTATGTTTGATGCATATAGAGTGATGCTCTCTGGTAAATGTAAAAA<br>AAGAAATATGTCGCCTCTAATACATTTTGTAACTTAGAAATATTAATAAATGTATAAATATGAATAGTATTTTTAAATATA<br>ACAATAAATACCTCAAAATAATAATAATTTAGGTATAATAAATATAAAAATAATGAATTAACCATGATAGTTTCCACGGG<br>GAACCGCGAGGAGGTCGACCTGGTTGGGTATATTGCTAGCAATGGGATTCTCAGTCTCCATTACTAACAATCCCTTTCCCC<br>CC
MSTRG.16064	PiggyBac transposable element-derived protein 3-like	TTTTAACTTTTTTTCTTTAATGTAAATACTAAACATTTTGAATCGAAGTTTAGCCGAAGTCCAAATAGGCTTTTTCTAAAGG<br>CGATAAATTGGTAATGGCATACACAGAGTGTACAGATGCGTCATCTATTTAAATCCATATCTGTAGCTACACTATGATTGA<br>CTACGTCATTAACGTAAATATTTGCTATGGAATTTGTGAGGGCGTTTGCACATATAAACTATTTGACCGGTTTTTTTAATGG<br>AGTGAAAATGGAACAGGCGGAGAACGCCTGCGCTATCGGTATACGCTGGCGGGGCATATTAACCCTTTCAGAACTGGCGT<br>GCACAATTGTAGACATAAGATATAATTTAAAAAATAAATATTAAACTCTTAAGCTTTCCGATTGAAAAGGGGTTGTATTCG<br>TTTGTTCACTAATAAACTACTCAATTTAATACTACTTACGCCATTTTTTAGGATATTCTAGCGTTTTTCGACAATTTGCGACT<br>AGTGTCCGTGCGTGGTTTGAACTGTACAGATGCAAAAACACCAATATGTCCTCATTGGAGGCGTTACAAGTATTAGATGAA<br>GGAGATCTTTCTGACTTATTATTATCATCTGATGACGAAGATAATTATTATGGAGATTCAATTTGTCCAGCAAATTGAATCT<br>AGCAGCTAATGCTAGAATTAGCGCAGATGTCATTTACCAAAACTAAAGGGAGTTTTTTCCACCTCAATTTGATGACAATGA<br>AGGACCTAGCGAATTGTATGACGGCGTCCCTGCTTTACTTTGACAATAATTTTTCATCATACCATTTACTACAAATCCTAAA<br>ACAAAAAGGTATTATGGCCGCATGCACAGCTCGTGTCAACAGATTTGTTAAACCACCGTTTTTAAGTGACAAGGTAACAA<br>ATAAGAAGCCTAGGGGTTTTTTTCAAGAAGTATGTAGCAGAGACGGAGATGTAACTGATGTAAAGTGGTTGGACAATAAG<br>ATTACCCATTTGGCTTCCAATTTTGTTGGTATAGGTGACAAGGACTTAGTAAAGAGATGGTGTAAAAAAAAGAAGCGATA<br>TATTGAGGTTGCGCGACCTGAGGTCTTAAAGCGCTATAATCATGCAATGGGTGGCATTAATTTATTAGATCAACTTATGAG<br>CTATTACAGAACATTTATAGAATCATAAAAATTTACTCTGCGTATGATATTTTATGCATCGGCTGTGGGAATTCTTCAGGCA<br>TATCGAGAATACCAAATAGATAACGACTTATTACGGCACACCTAAAAAGCAAGGACTTACTTTACTTTCGTCGTCTGTAAC<br>TGTCAGCCAAGCGACAGTTTGGTGCTACAAGATAAATGTTCTACCACCAAAAGAGGTCGACCAAGTAGCAGTAGTAGCTC<br>AACGATGCAAATTATACCAAGAAGGCCAGGAGAGATTCGTCCTGCAGCTGAAATAACTCGTGACGGTGTTGGGCATTTCC<br>CTAAACATGACGATGGAACTGGGACTAGGTGTAAAATGGTGAGATGCAAGGGAAAATCCCGTGTAAAATGCAAAGTTCAT<br>TTATACCTAACCAAAGACAAGAATTGCTTCTTGGCTTTCCACACATAGATAAAGATTTTTTTTTCCTAATTATTGTTCTTACT<br>GTGTTTTTATATTTGTACCAATGACTGATTATGCCAAAAAACTAATAAATGTTTATTTTATCACAAAGAAAATATTTAACCA<br>CGTTAATTCATTTATAATTTTGTAATAAAAATGTTTTTAAATTAATATTATTTCATTTCAGAACAACTTCGCATACCTTATTC<br>TTATTACTATGTAGGACGGGAGTCCACATTTGTGCACATTAAGAAAAAATAGATTTCCTCGAAAAATTTAAAGCTAATTTT<br>TTTTTATTTATTTTTTTTATAGTCTCCTTATTATACTTTAGATACATGGAATTTACCAAGTCCATCGGATGAAAAAAAATAAT<br>TCAGTTCTGAAAGGGTTAAG

MSTRG.16106	Protein PRRC1-like	GTTACAAATAAGCAAATATGTATCCTTCAGTCCACTCGCAACCATGACTATCGCAACGTAGTTTAAACGTCGAGTGAGCTA<br>TTGACTACTATTACCTGTAAACTAGCAGCCATAACAGAGCCTATAGTAACGCTGTAGCCGGTCTGCGCGTGCGCATAGTCC<br>GCGGGCGTGGCAGCCGCCAGCGCCGCGAGCGCCGCCGCCGGCACGGGCGTGCCCTGCGACTGCAACTGCAGCTCGATACC<br>CAGTGAAGGCTGCGACAGCACCAGCAACGATAGCTCATACCATCTACAAATATAAGCCATTGTCATGTTATAATATGGCT<br>AGTAAACCCTCTTATAAATAAAACTTAAGATCTGTCACAAACACATTTTACCTATTTTCAACAAGAAAACTTTCTTTAAAA<br>TTAATAATTTAGCGCAAAATAAAAAGACGAAGC
MSTRG.1611	Aldehyde dehydrogenase family 1 member L1	GCAGCGAGCCAACTGTGGAAATCAAATTCTTCGGCTCCAGTCTCTGGGAAGGAGAATACGAAGCAGAGGGGGATAAACT<br>GTTGATTCCAAATTTAAACAAGCCGGCGGTCATACACGAAGCGGGCTTGTTGATCACTGCCAATGATGGTGTAAAGCTAA<br>ACGTTCAAAGACTAAAAGTGAATGGTAAAATGATAAACGCGCAAAACTTCTTTAAGGCGAACGATAACAAAGTAACATTA<br>GAATTGACAGTGGATGAGAAACAATTCGTCGAGAGCGCCCGAGATATCTGGAAGGCGATCTTACGGATAGACATTGACGA<br>GGACACGGATTTCTTCGAATCTGGCGCCGGCTCCATGGACGTAGTCAGATTAGTTGAAGAAATCAAAGATTTAGCAAACA<br>TAGAACTCCAAAATGAGGATATATACATGAACACAACATTTGGTGATTTTTACACAGTGGCCATCACAAAAGCTAGAGGC<br>GGTTCAAATAAAAAAGAAATAGTATACGAAGGTGTAGAATTAGAGGTTAACAAAATGAAAGTTAAATTTCCAACGCAGTT<br>GTTTATAAATGGGGAGTTTGTTAATGCTGATAGTGGGAAAACACTGACCATCGTGAACCCTAGTGACGAAAGTGAAATCT<br>GTAAAGTGCAGTGTGCGTCCGCGGCTGATGTTGATAGAGCGGTGCTGGCCGCTAAGAAAGCGTTTGAAGAAGGAGAGTGG<br>TCTAAGATCAGTGCCCGGGAAAGAGGCCAGTTATTATTCAAATTAGCAGACCTAATGGAACAACACAAAGAAGAACTAGC<br>GACCATAGAATCAATAGACTCTGGCGCAGTTTACACATTAGCCCTCAAAACCCACATAGGAATGTCTATAGAAACATGGA<br>GGTACTTTGCGGGCTGGTGCGACAAGATCCAAGGCAGTACAATACCTGTGAACCATGCTAGACCCAACAGAAACTTGACA<br>TTGACAAAAAGAGAGCCAATCGGTGTTTGTGCGCTGATAACTCCTTGGAATTACCCCCTGATGATGCTGTCATGGAAAATG<br>GCGGCATGCCTGGCGGCCGGCAACACGGTCGTCATGAAACCAGCTGCTGTA
MSTRG.1613	Nucleolar protein 6	GGGCACAGTGGAAAATGGCCGGGCGATATCGAAGCGTTCCGTTGTCTGAAAGCCGCGTTCCACCTGCAAATAGCCGAGCG<br>TCTGAACAAACAGTTCTCACTGCCCACTCAGGCATATCCGACGCACTTCGACGTACTCAGAGACGGTCTAGTATTCCGACT<br>AGAGATCGCTCACCCCAAGGAGATAACGTTACTAAGAAGACAGACAGAGAATGGTGTGGTGAAGTTCAAAGAGAGCGAG<br>GAAAGCATTCAATTGCATTATGATACTGTGGTACTGCCGAGGTTGAGAGGTGCTTTGCATGGGCTTCACCAGAAGCACGCG<br>TCTGTGGGTCCAGCGACGTGCTTGCTGAAGCGCTGGCTGTCAGCGCAGCTGCTAGGTTCGTCGCTGCCGCCGGTGACTGCC<br>GACTTGTTAGTGGCAGCGGCTACGCTGCGCTGCTCACCACTGCCTTCCACCGTCACGCCGCTCGCGCTACTTGCTCGCGTCC<br>TGGCGCTGCTCGTCGACACAGACTGGGCCCAGGAGATCGTCGTGCTCGACTTCAATGACGACTTCACACGTAATATATAAA<br>ATATAAATAAGTGCCACATAAGTACTCCAAGCGTCTGGTCCTTAGGTTATCCCATATTGTGAGGTGAGGTACCAGGCAGGA<br>ATATTTCAGGGTATAGCCCAGACTAACCGGGTCAACGTGGGGGGAAAGGGATTGTTAGTAATGAGGTTTAACTGATCCAT<br>TGTTAGCAATACACCCTTTCAGTTCGACCTTCACGCGGTCCCCCCTGGAAACCATCGTGGTTAATTCTAGTGGAATTCATCA<br>TGATGGGCTAACTGATTTTTCATTGTCACCGTCATAACGCAGCCGGGGTTACTATTCCATTATCACCCATTAAAAAAAATC<br>GGGCCACTGTTTCTCAGTAAGTTAATAGAATATGAACTGGGCTGTTCCAGTAAGCAAATTAATTATTTTTTGTCGAAGAAC<br>TTCCATTCCTTCTCGTCCATATTTGTTTTATGTTTTTCAGTAAATTAATATTAATAATTGTATTTATAGAAAATATCGGAAAT<br>ATTTATATTTTTATATTTTCACTGATATTTTAGAAGTTTAACTAAATCACAAATAGAGTAACAGCCCACCTAATGGAAAGTG<br>ACTAGCGCCTCTTGACATGTACGATTCCGTACTGTGACCTCATGTTTTTAAATAAACGCTGTCATTCCTGAAGACTAGGATT<br>TTTTTTATACAACTTAGAATGGCAAGCAACCTGACGGCCCACCTGATGGAAAGTGGTAACCGTCGCCTATAGACATGAAC<br>AGTACCATGGACATTACAGAGTATACTGTTATGTCCATGGTATTTATTTAAACTTTAATGCACTGTGAAACTATCTACATTG<br>GACGGACTTAACACCGGACGGTGTTCTCTACCAGTCAACCATTGGGC
MSTRG.1	Serine/threonine-	TATAGTTTATCTATATACTCTTTGGAAATTATTCTCTAATCTATGAAAAAAAATTTCGAAAAAATCAAATCTTGTCATTGTA

6199	protein kinase PRP4 homolog isoform X1	AAATGAAATAATATTAAAATATGTTTTCTAAAATTCTAAAGTATAATTTTATTATGTGTTTATTGATCACTTTGAATAGCTG<br>TACAATATTCAAGGAAAATTTTATTTAAATTTGTCTAATTGATTGAAAACATGGCAGACCAAAAATTAAAAAGCAAATCAT<br>CCAGAAGTATCAGAGAAGAATCTAAACCAGCAAAAAGACATTATAAAGAAGACACCAAATACCGTTATGAGTCTAAAAC<br>TCCACCATTACCTACATCTAGTGTTACTATGGAAGAACTGATAAAACAAAGGGAGTCTCTTAAAAAAGAATTGAAAAAAA<br>TAAATTCTAAAAATTATTTGGACACCAAAGTAAAATTACAGAAACATAATAAATATGATAAATCAAAAGATAATACAGTT<br>TTAAAGTACAAACGAAAACATAGCAGTGACCAAAAAAAAAGATGACAAAAATATTGTAAACACTTCACCTGACCATATAG<br>ATTCAGAAGATGAAGAAAGTATTATAGAACAAAGAAGGAAACGGCGTAAACAACTTTTAGAAAAATTTATTACATCATCA<br>CATGAACACAAATCTGAAAGTATAAAAGAAGAAAATATTATCGAAAGTGCCAGTAGCAGTAATGAAACTGATAATCAAA<br>TACAAAAACCTCAAATAACAACTGTTACTAAAGAAACAAGAGATATGTTCTCTGAAAAAGATGATTTTATCTTCAATAATG<br>ACTCTGAATTTGTGACACAAGATAATGAAAATAATACACAATTAATTGACAACTGGGATGATCCAGAAGGATATTATAAC<br>ACGCGAATTGGAGACATTATTGATAGTAGATATACAATAAAATGTATATTAGGTCAAGGCGTATTTGCAAGCGTTGTTCGT<br>GCTCATGATATTAAAAATGGCAACAAAGATGTTGCTATAAAAATAATAAGAAATAACGATCTCATGTATAAAACTGGTCT<br>TAAAGAATTATCTTTTTTGAAAGAAATCAATGATGGAGATATAGAAAATAAATACCACTGTGTGAAGTTTATTAGGCAATT<br>TATGCACAAAGGACATCTTTGTTTAGTGTTGGAATCTTTACACATGGACATGCGAGGTGTTATAAAAAAATATGGCAAACA<br>TGGTCTCAATATGAAAGCGTTGATGAGCTACAGTAGACAACTAATGTTAGCTTTGCGACTTTTAAAAAAACTTGGTATTAT<br>ACATGCGGATGTAAAACCAGATAACATTCTTGTAAACGAGAAAAAAAATATTTTAAAATTATGTGATTTTGGCTCAGCGTC<br>AAAAGCTGAAGATAATGACGCCACGCCATATTTGGTGTCCAGATTTTATAGAGCGCCGGAAATAATATTAGGAATACCGT<br>ATAAACACGGTGTAGATATTTGGTCAGCCGCATGTACAATTTTCGAAATGGCAACTGGAAAAATACTGTTTACTGGAAGCT<br>CTAATAATAAAATGTTAAAATGTTTTATGGACTTCAAAGGTAAATTTCCAGCTAAATTACTAAGAAAAGGTAAATTTAAAG<br>ATCAACATTTCAACTATAATAATAATTTCCTTCTTCATAAAAAAGATGAATTGTCTGGTAGAGAAAAAGTAGTTGAAATAA<br>GTAATGTTTGTGCCACTAAAGATTTACACAGTGTACTTAAAAAATCAGCTAATAACTTAACTACTAAAGATGAGAAGAAA<br>TTAGGCCAACTGAAAGAGTTCTTAGATAGAATTTTAGTATATGATGCTTACCAGAGAATGTCTATTTTGGAGTGCCTAAAA<br>CATCCTTTTATTCAAGAAGAGTTAGAAAAATGATATTAAACTGAAAAGCAATAAATAAATTTTGTTGAGGA
MSTRG.16392	Zinc finger SWIM domain-containing protein 4-like	CAGGACAGCCATCCTGCGATCGCAGACATCCACTGGGCGGTTGCGCTGGCACACTCGCTGGGTCGCGCCGAGCTGCAGGC<br>GATGTTGCCGCTCCTCGTCAAGAACGTACAGTGTGCCCCTGTGCTGTCGGACGTGCTGCGGCGGTGCTGCGTGGCGGCGGC<br>GGGGTGCTCGCGCGCGCGGCCGGCGCCGCCGCGGCCGCCGACGCCGCTGCGGCCGCTGCTGGAGGCGGCGCTGCGGGCCT<br>ACGCCTCCACCACGCACGCGCGTCTCGCGCACATCTCGCCCAGGCACTACGCGGACTTCGTCGACTTTCTCGGCAAAGCAC<br>GTGACACGTTCGCACTCGCCCATGACGGTCCGCACCAGTTCGCGGCGCTGTTGCAGGAGATCAAACTCAAGTACAAGGGC<br>AAGAAGAAGCTCATGTTCCTCGTCAAGGAGCGCTTCGGTTGAATGCCCACTCGTCCACCGACATATTATGGCATTTCATTT<br>TTATTCCAATGCGACATGTCCAATAAAAAAGTTAAGTTTGACTTTTGAATTTGACAGGTGTCACATTTACAGGTGACATGA<br>CTATGTTTTATCGTTATTTCAAATTCGGAATTCGCTTTAGAATTATTATTTTTTTAATATAATTAGTGCAATTGTTTCGTTTA<br>AATTTTTACTTGTTTTTTTGTTGACACTGTGCAATAGAAAACCGTGTATATATTTTTTTTGTAATCACAATGTGACAATACAT<br>TATGTAAAAAAGAAATATGTCCCAAAATATTAGACTACAGAATACCATTGCGGTAGACAATTACCTCATGTATGGTACTGT<br>TAGTGTACCCGTCGTGTACCAAACCAGTCTATTCTAAAGTATTATTTTATTTAATTACTATAGCTAACCTCGTGACGTCATT<br>TACTTTGTAAGTAATATTGTAATGATCTCAATGATTCCATTTGTGTAAATAGCTATAGGGTTATGTAAATATATGGCAATGT<br>AAGATTAGTACTGAGAATTATAGTTGTTTTAAGTAGTCCCTTTGCGTTTTGTGTCCGCGACGATGTTTTATATAATCTGTAA<br>ATTGCTGATCGATTCAGTATTCGTTTAAGCTTTACGAACACAAAACGCAGAGCGTTGGTAGGTGAATAGTGCGCCATTCTT<br>ATAGCGTCCAATAGCAATGGATAATCATTATGACACAACACCAAAGAAATATATAGTAAGTCCAAGTGTGAGATAAGCAA<br>TGCCATCATTGTTTCAGTATTTTCGAGTATTCGATACATATAGTTATTTGTTTTTAACATAATTCCTTATCTACCTTTTAGTA

CTATTTTCTTATTTAATTTTCTTCATTTAAAAATGCTTGGGTGAAACGAAGTTAAAACTATATCATTTATAATGTTTCCATGT
ATAATAAAGGCATATTATATAAATACAACAAACAAATATAATAACCTGGAATCCAGTGATGTATTGATGAACATTCTCAC
AATTTTGAAAACATGATAAAATGTTTGTTCCTCAATCATGTTTAAAAATAGACTTGAATAAAATTTAAAATACAGACAAAC
CAGTATATCAAAGTTATACACAGTGTAGCAAATTGATGTAATTTATGTGAGCAAAGCTCCAGGATGCAATTAGTAAAGAA
ATAAATTAAAAAAAAACTCTTACATCACAATAAAAGGTTATAAAATTATTCTCCTATTTTCACTTTACATTCAGTTTCGCCT
ATAGTACCACTGGGTTAAGATTTAGCATAACTCCTTCCTTTAAACTTGTCGTTTCGAAATACAAATCTTGTCATATTTTTTTT
TATCACATTTTTATATTTTTTTTTTCTGAAACTTCTTTAATCCATTTTTATTGTGACTTCACCTGCATTTATGTTTTTTTTCTCT
AAACATTATTTTCAGCCAGTTTTTAACTACATACTTTCTCTACTTACCGCCGTATTTTCTATAGGTTTAAAATTTATCAAGAA
GTGCATTTATAAAGATAGAAAAATAATATTTAAATATAGAAGGCGAAGTTTGGATAGTGTTCAGAGGTTAAGGCCAAATA
AAGGCGTAGTGTGTAAGCTTAAGGAAGGACTTTTGTACTTTTTGTGGTGTGAAAGAGGGTTGTGCAAATCGAATATGCATA
TATTCATATAGCCGTTTATTATATTATCATATTATGTATTTTATAGGACCATATATATAAATGAGTCTATATATTTTTAATCT
AAAAACATTACATTAGTAGTAAAAAAGTTGATAAAATTTAATTGAAAATCACTGCATAGTATAAAACAAAGTCGCTTCCC
CTCTCTCTGTCCCTATGTATGCTTAGATCTTTAAAACTACACAACGGATTTTGATGCAGTTTTTTTTTTTTTATGAGAGTGAT
TCAAGGGGGGCGTTTGTATATATAATACATGCATAATATAGAAGAGAAACACTGATAATTTTAGAGATTTCTAATGCGATA
ATGTGCACTCGTGCGAAGCCGGGACGTGTCGCTTGTATAATATAAACGAAGGCCGTGTTGTCCCGGCATAGAATACTCTAC
CCCCTTCCTTTCCCGTGGGTGTGGTAAGAAGCGACTAAACGATGACGAGAGATGGGCAGCAGCGTCCCTCATTAAAAATA
TCTTTAAAGTCTAACTGCGCTTGCCAATCCGCCAGCTAAGCCTGGCAACTACTGGCAAAACTTTATGTTCAGCAGTGAACC
GGCTGGCATGATAATGATAAACGATTCCTTTTTTCTACTATGTAATTACGATCTTATATATATGGTATCTATAATCAAATAT
ATATATGATAATATAATAAAACCCTATTGATATACAATATCAATGAAATCTACATGTACTAACAATTAAACCTAATAACGA
ACTCAATGGTGCAGTGCTTAGAATTTAATAAGGTCTCGAGTTCGATTCTCTTTAGTTTAACCCTTGAAGCCTGATTTTTTAA
ATTAAAAAATATATAAGTGTACCCTTTACTGTGTTTACTAATGGAGAAAAATAGTAAAAAAATCAAAACATATTTTTATGT
ATTTATTTTGAAAAAAACATTTGGTATTATTTTTGATACTTTAGGCATTACGGTAATCAAAATATTTAAATTTATTCAACAA
TGAAACAATGCAAACCCCGGTATCAAATTTGAAACTTCAGGTATTTAAGGGTTAAATAAATTTTTCTAAGTTGGCTCGGAT
GTTGGGACTCTCAGATATACCCACCAAATGTTGATGAAGGTTTTTTTTTTAATAAAGGAGTACCCTTGTTTTCACATATCCT
TAAACTACCTAAATACGGAGTATACTCTGTTATGAAAATAAAAGAATATTAAGAGTATTTGAGAACAACTCACCCAGACT
CGAGACAAGAAAAAGTCATTAGTGACTAAACTAATATTCGAACTGTGACCGCATCTATGCGGTGTTACAAACCGCTGTGC
CGTTGTGATCGTCAAAACTGCCATGCAAACTAATAAAATGTCAAGTAACGAAAATATTTGCCAACCCTGCAATGTGTCAA
ATCAGCAAGAGTGTAATAAAAATAAGAATGAGGTGTTATTAAAAAAAAATAACTTGACATTAGTTTATTATGTTAATAGT
GTGTAAATATAAAGGGGATTGTTTAGTTAGAGCGCTGAAATACATCTTTATAGAGACTTCCAGTATCTTCCCATTCAGAGG
TTCATTTAGATTTATATTTAAAAATATAAAGATACATAATTCCAATGTACCATTGTAACATAGAAACGGTTTTAAAAATAG
TTTTTTTTTTGTCATCCATTCACAATATAAAAACTCATTTAGTACGTTTTACACCGAACGGTCGTAGTACGTTGAACCGCAT
TTTTATGAATTTTACATTATTGTAAAAAAAAATATTTGAAAGTCCCTTTACTATAATGTAAAATTAAATAGTATGAAATGTA
TACAACTAAGATAGATAGATAAACACTTCATTTGAGCCATTTATGACACACGTAAATTAACAATTTCATACCATCAATTTA
ATTACACTTTTTTTTTAATATAAAACAAAAAATAAAATACGAAAATATGTTTTCACCATGAGTGTCGCAGTGTACCAAATA
GGCTACAGCTCAGTGATAGTTTTGCTTAAAAGCACAGCACTAATTTTCAGCTGCAGCCTGAAGTTAGGCTACACGGACGCG
TGCAACGAACCCAGAGTATAACCAAGTTAACAGCCAAAGATAAAATTATATAGATACATACTTATATAAATGATGAATCC
AGTTGACTGTATAGCTTCAAATCAGATTTGGAACTATAAAGATACGAATTTATTTTTCCATTTTGGAAAAGCGCTTCAGTG
CACTCCGAGACGCGCCGTTAGGTGTAAATCGTACTTTAGTGAGAACACATAATTTCAAAAATCCGTCGTATAAGTGGTCTA
TCGTCTGTCTTTATGTCATTTATTTGTAACATTGCAATTTAGTCTTTATACCATTGCCAAAAATAATAAATACAATAAATTTT

		AATATTAATTGCATTTAGCGATTTTTATAAAATTAAACTGTTAAATTATTTGTCAATAAAAGAAAATTAATGCGGCCATCA<br>CAGGATATCTTTACCTATTCTATACTGACTTCGATGATTATTTTTGTAATCTATTTTTTAATTAATGATTTTTTTTTAACAAA<br>ATAATTATATGGGATATAGACCACTTTTTGACGTATCTCATTTGTAATTGTTATTAAAAATTAAATTACCGCATTGTCTGTT<br>ATATATATATGTCCGGCTTTGATTTACTGCATAATATAATATATCGGCTTCGCCTTGAACAATAGATCTTGTATTACAAATA<br>TACACAATCGGCACTATACAGTGAGGCTAAGTGCAGACTGAATCATTTTACTTAATCATAAAAAAAAAAAAATTAAATCGA<br>AAGAATTTTATATATTACATTTTCTATATGTAAATTAATTTAGTCTGAACATAGTCTAGGTAAATAAATGTGTAACAATTAT<br>TTTATAAAATATTAATTTGTCTAATAATGCAAATAAATTTGATATCGAGCAATAATTTTGTAGCCTTAAGCCGGTCTGCTGT<br>AACTGTATTTTGGGAGTACTTTATAATTATATAATGCTAATTTATTATTTTTGATATATTTACTGGATTTCTGATTGTTTATT<br>ATAAAAATGGAGTTCGTTGACCTCGAGTTTTTTTTTAAAAAGAATAGTAATCGAACTTCGTATTTGGAAAAACGTTGAATG<br>ATATTACACGCCTATATAAACGGTAAGCGCTTATATAAGGCAAGCCGTATGCCTTCACTAAACAACAAAAAAAAAAATAAC<br>CAACTTCATATTTATAATAATTGATCACTAGCCATTATTGTTTTGCAATATTTTAATGTAAATGACTTGCCAAGTTGTAACT<br>CCAAGCAAATAAATATTTGACCTCAAGTGCCTTTAGGCAAGTCCGCATTGGCGACAGACCGGCTTTGTTTTTTTTTTATTTG<br>TTAGCTGTCCAATATTCCGATCTGTGTCAAATAATCGTTTTTAATCATTGTGTTGTACGTGTAACGTTGACACCCTTCAGTG<br>CTGGTTTTAAGATGGTGATAATTTCAATATAAATAATTGAAAAATA
MSTRG.1<br>6404	RNA-directed<br>DNA polymerase<br>from mobile<br>element jockey-<br>like	GTCAGCATAGTGATAAGTAATGCTCTCTTAGACGACGCTTAAACATGCCTATACTTGCCGATTCCCTTATTTCGGATGGCA<br>GACTATTCCATAGTCGAATGGATATAACTGTAAAGGAATTCCCAAAAGCCTGAGTATGATGTGGTGGAGTTCTTAGAAGT<br>AATCGTGTCTGAGGGCGAAGTGGTTTATCTCTTGAATATAAATATTCATAACGCTCGCGGAGATAACAAGGAATGGAGGG<br>ATTATGCAAGATATTGTAAAGAAGCGTCAGTATATGGACATTCAATCGAAAACGTGAAGGTTTTTTATTTCTATTTTATTAT<br>ATTTTAATTCTTTTCGATATTTAGAGACATGATCAAACTTACGCAAACCATAAACGAAACGAATGCATAAATTTTGTAGAC<br>GGTCAATTTAGAAAATAACTCTGAAGTGGCGTCAAGAAAACATACATCTGCATAATCCATAAGGGGAAATAATAATGTTT<br>GGGCTAAATTGACCTTTGTTTTAATAGGTAGAAAATTTTGTAGACGTCTGAGTGAGTGAACAGTCGCATGTACTTTTTTACT<br>TATCTGGTTTACGTGAGACGTCCAGGAAAGATTTCTGTCAAAAAGTACGCCCAGATTTTTAACGACATTTGTATATGATAT<br>TATTGTCCCCTCATACACCACAGATGGAACAGTCATACGATCTAATAAATCTGTCATGTATCTGCTTCCGATAATAATAAC<br>TTGTGATTTTGTTGGATTCACTAATAGACCATATGCATTTGCCCATCTTGAAATGTTTACCAGATCATGATTAATCAAGTCT<br>ATAGCAGTACCGATATCACAAATGTCAAAAAAACTGTACAGCTGCAAGTCATCTGCGTATAGATGAAAGTAGAATGAAAA<br>TCGTCCTG
MSTRG.1<br>6413	E3 ubiquitin-<br>protein ligase<br>UBR4	GAGGCAGAAGGCACCAGTGGTGCTTGGAAACCAAGTTCCTTGGAACGACTAGCGTGTGCAGCGCTAGAAGCGCTTGAACT<br>CAGCGCTGGTGCTATCCCAGCCGTGACTGCGGCGGGCGCGACCAGTTCTACAGCCGTCAGCTCCACGGGGGCTGCAGGGC<br>CGGCGGCAGGAGAGATCGCGGATTGCGGCGCGGACGCAGCGCGGCGCCTGCTGTGTGCTCCCGCTGGCGCTCATCTGCAT<br>GCTCGTGCGCAGTGTTTGCTGCGCGCGCTGCACCATACACACTATCACCAGTACAAGGACCAAGCGATCCTCAAATACGTA<br>TGTACATCGTTGCAAGAGTTGCGCGACACAGCGGACGCACGTAATATCGACCCTGAGGCGTACTTCCGGCTTGTACTGCTG<br>GCTCGCGGTGTGGCGGTGGCACGTCCACAGCATCTCGTCAAATATAGCGCTCCAACACCACAGAGACCTGCTAATGG
MSTRG.1<br>6416	Max-binding<br>protein MNT-like<br>isoform X1	CATAATACCAGACAGTTCAGTGTTTATATAGTTGTCTCGTTCGCACGGGTACTTTGGTCTCGCTTACGTGGTTACTTACAAC<br>TCCGACTGAGTTATGTTTTCCTGGTAGTTTACCGCCACGTATTGCAGCCGCCGACGAGCGGTTGACACGGGTTCGACAAGT<br>ACACCGACTGGTGCGAGGAGGAGCCCAAATCGCCGGTTTCGGTGCGGGTAGTGACGGGATTGTCGGTAACACCGCGCAGC<br>AACGGCGTGGTTAGACCAACGTCGCCGGTGCCGGTGGCACCCCCTTGCACGCCCGCGCCCTTGCTTGCCGCTGCCGCCCCT<br>GATCCGGTCTCCTCGTTGGCTGCGACCGCCCCCGCTCCTCTTGACTTCGTACAAGAACATTCCAGGCCACGAACGACGAGT<br>AATAGTCATGGG
MSTRG.1	Uncharacterized	AGCTGCACCGGTGCGCGCCGGACGCTGGCTGCTGCTACGACGAGGCCGAGGTGTGCGCGCCCGTCGCCGGCCAGTATATC

6421	protein LOC106132305	GAAGTACCATTCTATTTAAACAGAGCGAATGGCAACATGAGCATGGCACGAATGCTGTTTTTCAACCACACGCAGTGCGCTTGCGTGTCCCGCGAGACACTGCAGAGCACCGTGCATGCTAGAGTGGAACACCAGAAGCGAGAGAACCACGAGACGAGGAAGGCAGTGACAGAACACCAGAGATACGACCGGCAGCCGACTGAGGAGCCGGCTTTAGAGAAAGATGAGCCCACCGCACCACCCCAATTGAGAAGGTCAG
MSTRG.16445	Methionine tRNA ligase	TGCACAGGAAACTGGTATACCATCAGACGTGTGGCGGTTCTACCTGGCCAGCATCAGGCCGGAGACATCGGATTCCAGTTTCAGTTGGACCGAATTGGGAACCAGAAATAACTCGGAATTGCTGAACAACCTGGGCAACTTCTGCCACCGTTCGCTCAGTTTCTGCGCGAACGCGTTCAAAGGCGTTGTTCCCGAGATCAAGCCCACACAGACGGACTACGAGCTGATGGCGTTGGTGAATAGAGAGGTCGCTGGTGAGTTGACACAC
MSTRG.16455	Kinesin-like protein KIF20B	AGGTTAACAGCACAATAGCAGAGATGACCTGCCTAGACAGTACACAAAATTCGACAATACCTTCGAGTGCTGAAGTGATGAAACTTCGCGCGGACAATGAGAGATTGCATTTCGAACTGGTTCAAGCGCAAGCCCGCAACAAGGAGCTGCTGGCGAACATGGAGGAGCGGCAGCAGCAGGCGGCCGCCACCATGCGCGAGCTGGTGGACGACGCCAAGGACATGACGCGCCAGTACTACGAGGCGCAGCTGGCTGCACAGCGCTCAGAG
MSTRG.16464	Cytoplasmic aconitate hydratase-like	CAGACCGTTTGCCGTTCAGCATCAGGGTGCTCCTGGAGTCATGCGTCCGCAACTGCGACAACTTCCAAGTGCTGGAGAAGGACGTGCAAAACGTTCTAGACTGGGAAAGGAACCAGGCCCAGGATGCTGTGGAGATCGCCTTCAAACCCGCCAGGGTTATACTGCAGGATCTGACAGGTGTTCCGGCCGTGGTAGACTTCGCAGCCATGCGCGACGCAGTCAGAACCCTGGGCGGCGACCCTCAGAAGATCAACCCCATATGCCCAGCTGACCTGGTCATAGACCATTCAGTGCAGGTGGACTTTGCTCGCAC
MSTRG.16468	Autophagy-related protein 2 homolog A	AGCAACATACCACTCCGGCCAACTGGTGTAAACGTCCAATGTAAACCAATGAAACGGGCCGATTTCCAACTGATTGAGGCTCAATTACTGGGCAACTTGGAGAAGCAGAGCGCGAGCAAACCTTACGTTGGAATGAGTGGATGGTCTGGACCAAGTTACGAAGAGAGTGACACAGAGAGCGAGAGATTCCACCCGATGATACCACGCGGCGAGATGACGGAGAGCTTTAATTCATCAGTCAGTTCTATGAACACCAGCATGACATCCAGCGTCAACATGCCAGGGTTTCAACCACGCATCAAGCGAAATAAAAAAG
MSTRG.16473	Beta-1,3-glucan recognition protein precursor	AATGAACAGTGCTTAGTATTAATCAAGAACGAATTATCCAAAATAATCCGCGTAATAATTACTTACAGGATTATCCATTCAACGTATATTTGTACGATAATGTACATGTGAGAGACGGCAAATTGATTATTACGCCAACAACACTCGAGTCTAAGTACGGCGAAGATTATGTGAGACAACAGCTGGATTTGACGCAGAGATGTACAGGTACAATAGGGACAGCTGATTGTACGCGTGTAGCGTCCGGACCCATAATCTTACCCCCAGTCATAACTTCCAAGATCAACACCAAGAATAGGTTCAGCTTTAAATATGGAAGAGTGGAGGTCAGGGCGAGAATGCCGACTGGTGACTGGTTGATACCAG
MSTRG.16486	Dihydroxyacetone kinase 2	TAATATCTCATTTGAAATTGGTGACTGGTGACAGAGTGACCGTGATGGTGAATAATTTGGGAGGGACCTCTATCTTGGAAATGAACATAATTGGTGCTGATATCAAAGAATATCTAGACAGCAAAAAAATAAAGCTAGAACGTCTATATTCCGGCCACATGAAGTCCTCCCTTGAAATGCATGGATTCAATATTTCCGTGCTGCATCTGAACAAGGAACAAGGTGACTTCTGGTTGGGGTTGTTGGATGATCCCACCACCGCTCCAGGATGGCCTGCGGGCGTCCTCTCTGTGGGTGACCACGACAACATTAGAGATGACGAGACTGTACTGCAAAGTTTT
MSTRG.16502	DNA ligase 1	GATACCATTCCATTATCATCCATTAAAAAAAGACCTAAATAAGCCAAAACAACGCCTATTTTAAGTAAATACATGACAAGTAATCACCGAGCTTGGGTCCGTTTAATTAATTTTATCATCGTGTTACCCATTATCTGTCCAGTGCTGAACACAGGCCTCCCCCATAGACTGCCACAAGGAACGGCTCTGAAAATTTCACTGCTATACCTCTTTCTAATATACAACTGTATACCAGGCGAGTGGCAGTTCGCGACGTCCAAAGACTGCAGCACAATGGAGGAGGTGCAGCAGTTCCTGGACGAGGCCATCCGCGGCTCGTGCGAGGGGCTCATGGTGAAGATGCTCACCGGCGACAACTCGCGGTACGACATCGCGCGGCGCTCCCACAACTGGCTCAAGCTGAAGAAGGACTACCTGGAAGGCTGCGGCGACTCCCTGGACGTGGTGGTGATAGGCGGCTACCACGGGAAGGGGAAACGCGCCGGCGTGTTCGGCGGCTTCCTGCTGGCCTGCTACGACCCCGACACCGAGGAGTACCAGTCGCTCTGCAAGATCG
MSTRG.16509	Chromosome transmission	GACGCAGTTCCCGACGTGATATCATCAAGCAGCGTGTTCTACCAATACAGGGAAGGGTTCAACAACGCGGTGCGTCGCAGCGTTCGAACCAGAGACATGCTCTAGCCGTAGACACATAGTTTGTTTCAATAAAACTTAATGAAAATGTAAATAAACTAATA

	fidelity protein 18 homolog	ATTAATCCGACCATAAGGTAATCTATTGATGTCAAATAGTAGTACATATTTAAAATCACTCGCCGTGTAGTCCCGGCATAA<br>AAAGAGCTACCCCTTCCCTTCCTATGGATGTCTTAGTAGTCGACTAAGGGGCCTTAATGGTCGAGTGGTGGCAGCAGCGT<br>CCTTCTTAAAACGTCACCAGCTTAACTGCGGTTGCCAATCCGTATGTCAAGCCTAATAACTACTGGCAAACCTCCCTAATA<br>GGGAGAGACGTATGTTTACTAGTGGACAGTTAAAGAATGATGATGATTAAAATCGATTAATTGTTCGATATGTTGGTAATT<br>TCAATTTTGAGATTATTTTTGTAATAGTTTCAACAAAACTTAGTTGACTTTCATTCAGTTTCATTGAAGCAGGCTTATAATTT<br>GCATATTAACGTTTTGTAAACTGAAACATTTTCCTTTCAAACTATGTACATATAATGTTTGTTGTTCTGTAGTACATACAAT<br>TTTGTTTTGTTTTTAGCTGTAACATACAACACTGTTGTTTTTTCTATTCATTAAAATTTAAGATTCAAATAGTCTTATTTAAA<br>TAATTTAAGATTTAAATAAATAAAAAAAAAAACCGACTTCAGTTGTGGCAAAGCAAGTTTTTTTTTTTATTTAAAAACAAA<br>CGATTGAACCGATTTTGATGAAGTTCCTTTGATAATTTTTATATATATTTGTATTTTATTTATATTTCTTATATTAAATATCT<br>AGTGTATCCTACACTTACAGATTAGTCCAAAGAAATATTATATATATATTATATATGAAAGAATTTTCCAAATCCGTTCGT<br>AACTAAAGCAATTATGAGGTATATAAAATATGGATATGACAGTGAATTTACTGGGTACAGAGGAATAACACCTGAGTCCT<br>CAGGAATGGCAACGCATGGGGGGTATCATGGACGAAACAGTATACTCTGTTATGTCCATGGTACTGCTCATGTCTATAGGT<br>GACGGTTACTACTTTCCATCAAGTGGGCTTTCAACTTGTTTGCCATTCTAGGTTACAAAAAATAAAAATAAAATAAACCTT<br>TTTTTGAAGTCGATTGAAAATCTGTCTGTAAAATTAAAGTCAGTTTAAAACAACAGATGGCAAACAATACGAAAGTCAGC<br>CGCGAACTCAAGTTGTATATATATTTGCAGGGAAACGTTGAAGTCTTACTTTATTGGGCAGTAAATTGTAAACTTGCGTTAGT<br>TAGCACACGGAGACGGCGGCGCACCTGAGCTTGCTAACTTTGTTCGTAAATCGTCCACGATAGAGATGTATAGATATAAT<br>ATTAAAACATACTGCAGACTCGCCCCGGCTTCGCATAGGTTTTCCAAACTTCCATAATCCACGCATACAAATATGCAAAG<br>CTAGTACAAAATAAATACAGTGAATTATATTCAAACGTTTGTTTCCTTATCGTTTACGTATTTAAACATGTTTAAACAAG<br>TATACAAATACGTGTTACGTTTCTTGTACATTCTAGTAGAATTTAATTCAGTGTACCTTTACACTCGAATAGCTAAAAATCT<br>GTTATATACCTGATATATATTTTAAGGAGATTAAAATAAATG
MSTRG.1664	Serine hydroxymethyltransferase	AAATAATCATAACCGAGATAGTCATTCACTCTTTACTAAGCCTGTAATAGGAACAGCGAAACGTCACCGGCTGATTGTTAT<br>CCGTCAGTTAATGTCACTCATCGAATTATGCTATTGCCATAGAGTTCATATTAGGTTATCGATAAATGATAAATACAACTA<br>TTGTTTTCACGAATAAAAGCATAATTTAATGAACAACAGAGAGAATAGTGTCTAAAATATATAAGTTTTAACAAATAAAA<br>TGCGTGTTAAATTAGTAAATACATATATTTTTACCGGAACTCGCAGATTTAGTGAATTTTCATCACAGTTAATAAAGAAAC<br>CGGCAAGAAACCCACTGTCAAAAATAAACCTTCAGGAGAAAATTACTTTAAGAAATTTTAGAACTTCAACAATGAGTAAT<br>CAGCTCCTAAATAGCAATGTATGGGACAATGATCCTGAACTTTTTGAGCTCATTAAAAAGGAAAAACAACGGCAACAGTC<br>TGGTCTTGAGATGATAGCTTCGGAGAACTTCACTTCAGTTGCTGTCCTCCAATGTCTGAGTTCCTGCCTCCACAACAAATAT<br>TCTGAAGGAATGCCTAATCAGAGGTATTATGGTGGCAATGAATATATTGATGAAATAGAAATCTTAGCACAGAAACGCTG<br>TCTGGAAGCTTACAGGCTCAATCCAGAAGAATGGGGAGTCAATGTCCAACCTTATTCAGGTTCCCCAGCAAACTTTGCAGT<br>CTACACTGGTATTGTGGAGCCACATGGTAGAATTATGGGTCTAGATTTACCAGATGGTGGCCATCTCACTCATGGCTTCTT<br>CACTCCTACCA
MSTRG.1678	Venom dipeptidyl peptidase 4-like	AATAAATATTCAACGTATTTTGTTGCATATTTCAGAACGGTGCATTGGTGCGAACTTGGGCCGACAATAGCCAGTTGACAT<br>CACAATTGGCAGACATAAGCAACTTCCCTGTTACTTTCCGTGTATCTGTACCATCTGAATCTGGACTGCCCAATGCCGATGT<br>GCTCATCCAAGCTCCACCTGATTATGCACAGCGAACAAATGTACCGCTGCTGGTTTATGTCTACGGTGGTCCGGACACGGC<br>TCTGGTGACCAAGGAGTGGTTGCTGGACTGGGGCACGTCGCTGGTCAACCGGTGGAACATCGCGGTGGCGCGGATCGACG<br>GCCGCGGCTCGGGACTGCGCGGCGTGGACAGCGCGTTCGCGGTCAACCGGCGGCTCGGTACCGTCGAGATTGAAGACCAG<br>ATCAACGTCACCAGATACCTGCAACAGAGTCTGCCCTGGCTCGACAGCAACCGCACGTGCATATGGGGCTGGTCGTATGG<br>CGGGTACGCGTCGTCGCTGGCGCTCGCTCGCGGCGGCGACGTGTTCCGCTGCGGCATCGCCGTCGCTCCCGTCGTCGACTG<br>GCGATTCTACGATACCATTTATACGGAGCGATATATGGACACTCCTGCTAACAATCCACAAGGCTATTTTGAGTCGTCACT

		TCTTACAGAGGAAGCTTTGGAGTCGTTCCGCGGTAAACGTTACTTCCTGGTGCACGGGACGGCGGACGATAACGTTCACTA<br>TCAACACGCCATGCTGCTCTCGCGCCATCTGCAGAGGCGAGACATATACTTCGAGCAAATGAGTTACACCGATGAAGGGC<br>ACGGTTTAATCGGAGTACGGCCACATTTGTACCACGCATTCGAGAGGTTCCTGCGTGAAAATTTGTTGTAATATATTGTAT<br>TTTTAAGGATGGGTAATATTTATCAATATCTATATATTGCGGTTACAATATTGATACATATATTGACCCAATAACAGATATT<br>TTAGATATCGATTATGTAATAATATCGAAAAAAAGAACCCGCTCGTAATGAATATAGGTTCCTGATTAGTATGACCGGTAT<br>CGTAATGGGTGGTCACCAAAGTCCAGAAGATCAATATATAGATATCAGATATTACCCATCTCTATGTATTATAGATAAATT<br>ATTTATTGACCGACAATTTGAAAAAAAATTGCCCACGTTTCATTAGAAAATTGAGTATTAAAATGTTTTAAAATTTATATT<br>GGAATGTTCCTAAAATAATTTAATTCACGTGACACTGAACATTGATATAAAAAAAAAA
MSTRG.1693	Diacylglycerol O-acyltransferase 1 isoform X1	GGTGCCAAATCATCTCATTTGGTTGTGCTTCTTCTACCTGTGTTTCCATTCATTCCTGAACTTGTTGGGTGAGCTGCTGCATT<br>TTGCTGATCGTAACTTCTACGGTGACTGGTGGAATGCAAACAACATAGCCGTTTTTTGGAGCAGTTGGAACTTACCGGTGC<br>ATAGGTGGGCTGTACGCCACGTGTACATACCTATCACGGAAAGAGGCCATAGCAAAGTTGTTGCTAGTGTTGTTGTATTCT<br>GCATATCGGCTTTATTCCATGAATATTTGGTGAGCGTACCGCTGCAAATGTTCAGAATTTGGGCGTTTTTGGGCATGATGGT<br>GCAGCCACCGTTAGCTGTTATATCTCGCTTCGCGGAAAATAGGTGGGGCCCCCGCTGGGGCAACATCATCGTGTGGAGCTC<br>GCTCATTCTTGGTCAGCCACTCGCAATCATGATGTACTATCACGATTATGCTTTGGCTCATTTTACACCAACACAAGAACA<br>ATAAGCGTCATTTATAATATCTAATTGAAATTATTCCATGACAAATATACAGAAGTTACGACCTGATTTATAATATTTCGTA<br>GTTACTGTAGGTTGCAACGAATTCGTATTCAGCCAATGGCATTAACGAATCATTTTTCCCTTCGTCTTTATTTTTATACTGA<br>AATAAGTTGATTGCAGTACATTCTTCGCTAATAATAATTTCGAATTTATTTTCAAATTATATTATGGCTACATTGTTTACCA<br>AATGTAGAAAGTAATTATAGTATAGATTAATTACGTGGTTTACGTTTTTTACACTACATCTACAAGGGAAATATAAGGCGG<br>GAGCGGGGAAAAATAGGCTTTGAAAGTTATATAAATTGTAAAAAAAAAAAACAATTATTATAATCTCCTCAGGCAAATTGT<br>ATTGGTCATGTAACTATTAGACTTTTTGTGTATTTATTTCCATAATGCTCTGATTTTGTCAATACAATAGCTAAAGTATATTT<br>TTAAAAACTATGCCATAAATAATCATATGTCTATTTGCTGTTTATCTACATTTTTCTGTCTAGTTTTTTACAAGGTAGATGA<br>AGTGAACTGTTCTGTCTTTATACACATTTATATATTTTGTATAACATTTACCATTTTTTGAGAGGTATAATGAAGAATTTTCC<br>TTTTTTATTCATTCATTCCAATATAAAAAAAAAAATGTGAATTTGCTTTAATTTCTTCTTCGCAAAGATCACGATATATTTTCA<br>ACTCTATTATCCAAATGATACATTTTCCGAGTAGATATAGTAGTTAATAATTTATAATTAGAACAAAAAAAAAACTGACTAA<br>ATCTTTACGCAGTTTAAGACAAATACTTGTCTCTTTCGATTGAAATGGAATCATTAAGTCGATTCTCAGTTTGCGAAGATAT<br>TGTATAATATACAAACAGAAAAAGAAATTTGATTTGAAAACCTCTCCATTTTGAAGTGAGTTATATAATTAGGCACAATTA<br>ATTTATTGTTCGTCGAATGATTTCACTGCAATATGCAGTAAACGAAATGTCGTCAATCAGTCGGCAGTACATAGTTATGTA<br>ATTGTAATTCACAAATGTTTTATTTATACTAATTATTAATACTATTTTACTGTTTTTTTTTTTTATTTTTTTTACTTTATACCAT<br>TGTTGTGAAATTGTTGCCTGAATTTAATTAATGAGTGCTATGAATAAACATTCTTATAGATTATTTAGTATTGTAGTTAACC<br>TAGTATTTGAATCGTTTTTAGATATAACAGGGAATTTATTATGGAATTATGTTTGCTATCCAATAACTACGTTTTGTAATAT<br>TTCGTTATAAATATTTTCAGAATAATGCCTTGAGAATACAAAGAATAATTATGTTAAGCGACAAAAAACGTGCCAGAAAA<br>GTAGTTTAAGAATTATTTTGTTAATTGTTATTATGTGGTGTATATCTATTTACGTAGTTAGTGGTGTATAGTAACAAATTTA<br>TAATGCAAGGCGTTGGATCCATTAATACACAGCTTTTTCGCATGATAGTAACATAGTAGTATGATATACAATTAAAATAGA<br>TAATTTATTCAACTACGCTTTAATTAATAACTAATGAATGCCTTTTAGGTTTTAAATATCACGGCAGAATATATTTATTTTT<br>ATAAATGCATTTATATTTATTATAGTTAATTTTTAAATTTATTACATATCATTCCTATGAATTTGGATTATGAATTTACAAGA<br>AGCCAACGCAAGACTGAATGTGCCTATTAGAAAATATAAATAGTGATAAGTGAAACTTGTCGGCAAAACGGAAATTTATA<br>TTGTACATAAAATACTTTTTAATAAGTACGCATGCATATACATTGTTAAATATTTATTATATAAATATTTTTTATATTAAAT<br>GGATAATGTAAGGAAAAATGCACACGGTATATTGGACTGGATCGACAAGTTGCTCTTCGGTACAGTTGTAGATAACAATC<br>AGAAAAACATCTAAATAAAAGATAATAATTTGGTATAAAATAGATACACAACTTGTATTAGTTGCTATCACTTTCTTATAC

		TAATCTTTCTATTTTTAACTCTATCACTAGTTATATCTAATTTTCCTGTCGCCGATAAAACTAATAATTATATTGTTTTGTTTAGTGTTTTACCGTTATCTACAAGAAATAATTGCCAAATATCGCGT
MSTRG.1697	Sin3 histone deacetylase corepressor complex component SDS3	GGTGAATCGCCAGTTCGCGAGAGTAGCGATACATCGGAGACGCGCGTGGAGGACGGCAAGTTGTTGTACGAGCGGCGCTGGTTCCACCGCGGGCAGAGCGTCTACGTCGAGGGCCGCGAGGTGGCGCGCTTCCCCGGACAGATACACGCTATCACTGACGAGGCGATCTGGGTGAAGAAACAAAACCTCGAGCGGTTTCGAATATACATATCTCAGTTAGCGCGCGGTAAAGTGACGTTAAAGAGACGTGCCTCATAAGGCACCGACTGTACAGTGAGAGTGAGGTTTGTGAGCGGTCTGCGGCGACCGGACCAGTGACCAATGGCGGAGTGCGTGTACCGGCCTTTAGTACAGATGCTAGAGTGTGCATGACCTTCGATACAGTGCTGTCAAATCAAGAATAGGACTGTCAATGTCAACTGTCACTTGAATGGTATGCATTAGTTTACCATATTATTTAGCATGCATACTTTTTCGTATAATGTACATTATTTTATTGAGCTATAGCTTATAATCCTTATTTTTAATATTTTATTTATTTATTTTCTCTTTTTTTTACACTAAACTCAATTTGAAAGATAGTGACTGAGCATTCTAATTTATTATGGATTATGGCAGTATGGAGGCATTACCAGTAGTTGTTAGGCTTGGCAATCTGGCAACCTGGCAAAGGCTTTGGTAATATTTTAAAAGGGAGACTGCTGCCCTTCGACTTTTTTTTATTAAGTTTCCTTAGTCACCTCTTACGGCATTCATGTGAAAGGAAGAGGTGGCGCATTCTTTGCTGTGACCACAGGCAAACGGATTCACCTTACCATGCCATAATAATTATATTTTTTAATATTTTATTTATTTATTATTTATGGATTACATTTTATGCCAACGATATTGGTATATTTTTAAAAAGTGTCGCTAATTGCGTTAATGTAGAAAGTGCATTCTCTTCATTATTTAATTAATTTTATACTGACAGTGTAAGCGCGCACAATATGGCGGAATGTAATTCCTATTCTTATTTTGATAGTGTTGTATTGATCCGATGAATTTATCATGATCTATAAAATTGAAACCTATGAAGTTTCACAGCACTGATTTTTGTTTGTGTCCTTGTAGATTACTGTAAGGTATGGTTTATTTTCACAAAGTTCATATGATGACAATAATTACTTGATAATTATTTATAAAATTTTTATGTTTACTATAAATACCATTGTTTTAGAGAATTTGACAAGATGAACTATTACAGTGACATGCGAATGGATTTTTTTATAGTTATTTTTTTTACATTTTAATTTTATCGTGAACATTGTGTTGTGAATGAATGTTTACAATTAAAACACAAAGTTATTAAGTAACAGAGTGAAAATTTCTTTATCATACTGGAGAATGTATTTGGAATGTTCCAGAAAGAATTTCAACTGGTTTTCAGTTCCGAATAATGGTCAGAATTCACTGTTTTAGTTTTTGCCAACCTGTCCTTTACACCACGTTTACACGTTTATATATATATGTAATAACATATAATTATATAAAATAAAATCATATAATTTTGACCTGTAAAAAAAGATATAATATATCGATTAAGAAAAAAAGTGGCCAAATTTGAAAATATTACACTAAAACCATGAAGAGTTTTCTCATATATCATTGATAAGTATTAAAATGCTATTTTCACTCTGTCCATGGAATAACTTGCCCATTAATAATTTAATTATAAAAAATGTATAGATGAACGATATGAAATTTATTTATTTATTAAATTTTCATGTACATAACAGAAGTGCATATGGCATTACAATATGAATTCATTTTACTCCATTTATGAGATAACCTTTTCCCAAAACAATTGTTGAAATCCCAATCCCAAAAATATTGCTTGTAGTTCTATGGTGGTTCTAGTCAGATTATAAATGCATTATCTTTAACATATCGGTCTCTGTCTATACCACAAGAGATCCGGCATGAAGGTATTATTGATTAGATAAACCCAACGTTAATTTACGAGGGGAGGTTGGTTGGTTCGCAGTCTCAGGCAGCATAATGAGTGAAAATATATTGTTTTATATATTTTCCCACATCCCTTTAAGTAAGTAATCAATTAACATAATTCCCTCGCTGTCTTTGAACTTGTCTAGCTTTGAACCCGACTTTTCCACTTTAAACTTCTTTAGTGTCCTTTCTCCCTTTTTATCCACTGCGTCGACTCTTGATTAGACTTTGGATCATACTGACGAATCCAGATCTCTTTAACGGTGACAATACGGTTACAAATTTCGTCCAAATCACTCTGATATAATTCTCAAAAAATTACAACTTTTCAATCGTCGTTGCTTGTCGAGCGATGTGAGCATTCATGGTACCCAATGCTCACAATTTTTTTTCCATGTAGAATGTCTCCAATTCGTTCCTTACTAATCTGTAGTTCTTCTGTTATCTGGCACAGTTTAATTTTTCGATTGGCCAGTACTATTTGTTCGACTTTTTTAATCTATACTTCGGTCGCTCTAGTTAATGGGCATCCAAGGCAAGGGTCGTCTTCACACGTCTTTTTTCCTCGTTGAAATAACCGAGTCCATTTTTTTACTACCGTATATGAAGGTGTCTAATCCTGAAG
MSTRG.170	Iron regulatory protein 1	ATTTTATAATTAACTATTTATTAACCATTTTAAAATCTGAAATCACGTCCTGTTACAGACCGGACGCGTTGAACAAGAATCAAGAATTAGAGTTTGAAAGGAACAAAGAAAGATTCCAATTTTTAAAGTGGGGCGCCCAAGCGTTTGACAACATGCTTATAGTGCCACCCGGTTCTGGAATTGTACATCAGGTTAGTCCTCTGTTATAGCGAATATACGGCCC
MSTRG.1	Hypothetical	AGAATTTGACATTGACAGTTGTATATATGAAAAAAGCTGAAAATGGACGACCGCTAGATTCCCGTTTGGCACGTGAAGTT

733	protein RR46_01430	ATTTTTTTATTTATCTATTTAAATTAAACAAACTACCGTCAAACTGGGTGCCAAAACGGTTGAAAGATTCCTAAGCCTCGC<br>GGACGACAATTTCGTCGACGATGTCAACGACGACCTGAAATGTTTTGTGGCGGAACGAAATGTTGACAGTGTGCTGGTGTT<br>TCCACCAGTGAGCAACTACCGGAGGTTCCTCATCCACCGGTGCGTCGAGGAGCTCGCCCGTCCTGATCTCGCCACCTTCTC<br>CATTGGCTTCAGCGACGAGCGACGGACCGTTGTCTGCTACCGTGACAGGCTACTGAGTGATGAGGCTTGCGCCCGACCAG<br>CCATGGGACTGACACACATGGACCGCGGCGATGAGGCCATATCACGTTCCG
MSTRG.1738	Histone-lysine N-methyltransferase SETMAR-like Protein	AGAGAGGAAACGGCAAGAAGCCCTTCACAAGGAAGCGGCATGGGAGGGCTCGCTCGCCAACCAGTCGCTCGACGCGTGA<br>GATTCAATCACCAGTCTGTAGTATTATAAAGAACCGCACCGATCCTAATATTACGTTAGTGTAAAATGCTCTCAAAATTAT<br>ATTGTCTACGTAAAAGTTACATTAGTTGTACACATAGATATTTTTGTAACAGAAACCTTAGTAGTATCACGCCCAAGGGGT<br>TTCGGTCCTTATGCCAATGTGTCATAAAGTAGGTTTTTCAATTAGTTCATAATTCTCTTCGCTTTTAGTATAATTTTCTCTTG<br>TGATTTAACTTACGAGCTTCCCATACGACTGATTTGACTTGGATGAAATAGTTTTTATGCCTTTCTTTGAAATACATCACAC<br>ATAGTTTATAATTTTAAATAAATCTCCCAATGTTTTATTATTATAAATAAAATGATATATTGAATAATAAGAAAGTGACAT<br>ACACAACAGTAGTTTGTGAAAGGTTTTAATCGTTTAATTCTTTAGCTAACTTCGCATATGACAAGATCTCTTTCATTTCATC<br>TTTAAGATGAAATCGCTCATAAAATATATTGTTATAGTATTTTAATACCAAATATTAGTATTTTTTATTATTTTATAGTTTAT<br>TAATTTTATAGGCAATGTATTTATAAATATGAGAGGATTTACTTTTTTTATTATATTAATGCAAACAATCATACGGCCTGCC<br>AGATGGTAATATGCTGCGGTAACCTGCGAGCATCACACACGCGTTGAAGAGCATTGTGATATGATTGAATAATTACAAAT<br>ATTAAAATACTGAATTACAATACTCGGTAATTTGCCTAACAAATCTGATCCTGCTATCCGGAATACAATATCGCATGCTCC<br>TTACCGACAGAAGTAGGTAAAACAGATATATCCATATATACGAGGGGAGGTCAAAAGTTCGCGGAATGAAGGGGAAGGC<br>GGTTGAAATTAAACATGAAACTATTTTTTCTTTTCAATATATTCTCCCTTAACCTTAATACATTTTTCGTACCTCTCAAATAA<br>TTTGTTTATACCATCGAAAAAACAGGTTTAAATTTGCTGTCAAAATGCAGACTTGATTTTTCTTCATCGTCGCCAATTTTTTT<br>TCCACGCAGCTCCTTTTTCATTTTTGGGAATAAATAATAGTCACTGGGGGCTAAGTCTGGACTATAAGGTAGGTGGTCTAA<br>TTTTTCAAACCTGCATCAGTGGATGGTAGCCGTCGCAACATGGCACATGTGGACGAGAGCGTTGTCGTGCAAAAGGAGCA<br>CACCTTTTGACAGCTTTCCTCTTCTTTTTTCCTTGATAGTTTCTTTCAATCTGTCCAATAAAGAAGCGTAGTTCTGTCCCGTT<br>GTAGTTACACCACGATCTTTACAATCGATCAGCAAAATACCTTCTGTATCCCAGAAGATAGTCGCTATGATCTTTTCGGCC<br>GATTGTGACACTTTAAAACTTCTTCAGTGTTGTGCCTTTATTATACCACTGCATTGACTCTTGCTTCGACTCAGGTTCATAGT<br>GGTGAATTCAGGTTTCATCTACAGTAACAATCCGGGCCATAACTTCTTCCCTACTCTCTTCACAGAACTCCAAAATTTGGCG<br>AGAACACTCGACACACTCACTTTTTTGAAGTGACGTGAGCATTCTTGGAACCCATCTTGCACTGACTTTAGTCATGCCAAG<br>ATGATGATGTAAGAATATTTAAAATATTTGTTTCGGATACTCTAACCATTGCTGCAAGCTGCTTCTTCTTCAGCCGGGTATC<br>TTCTAATACAAGTTTTTCTACTTTTGCGACGATTTCCGGTGTGGTGGCCTTAATTGGCCGTTCGGAGCGGGGGTCATTTTTA<br>ATAGATTCTTCTTTATTTGAAAAGATCATGCCACTTGTAAACCGTGGTTTTACCAGGGACCGCGTAAACTGCTAACAGCTC<br>TTGAAAAATCGTCTGAGCGGATTTTTCTTTCTTGGTAAGGAACTTAATAAAGGCGCGGTGTTCATTTTTCTCCATACTCGCT<br>GACTTCTTCCCGATTCACTTGTTTGCAGGTAGATAACTCAAAAGTCAATGACACGATTTGGTTCAAACTTGGTATATATACT<br>CTTAACGAGGTGCTTAACAAGTGACTACCTGGACGAGTAAACCAACTTCCGCCAACCCCTCCATTCCGCGCGAACTTTTTG<br>ACCTCCCCTCGTATTACTCTATGTTTCCTATGATGAGTACATTATATGAACCTCGGATCGTTGATTACTCTGTGACAAGCAA<br>TAAATAAATATAATCCTTAATAAGTATAAAAAGAAATCTTATAAACGCCAAATGTCTCTATAACTAATCAGTTGTTGATTA<br>GTCTCCAATCATAGCATGTTTGTAAAGATATTTGGCTGTTTGTTACTTTATCACACTAAAATTACTAAATTAATTTTAATGA<br>AATTTTATACACACATGAACTATATCCTGAATTATGACATAATAACTATATTACAGTATGATTGCTTAATATCCTAGTTATT<br>GTAGACAAAACTGCAGAATCACTTAATTTTCTATGTACAATTGAAACTTCATTTATTTTAATACATTTATGTTTTGTTGTGA<br>GTTATTTGTGTGCCTTTGTCTCTAGTCTCGGTGTACAGAGCGACTTGAAACCTCTTGGCGTTAAAATTATTAGTCGAAATTA<br>GATATAGAGATACCTACATAGATTAGAAAGTGTATAGATTTATTGTCTTTGAAACATTAGATACGTTTCTAAATAGAAATG

		TGATTGTCTATGGTATTGTAAAATTAGTTTTGATTATTGTCTAAGTTTATTTTATATGTATATAGATAAAAGGTATAAAATTTGTTTTTAAAATTACCACTGTTACTTTGCACGAAGTTTTTTTTATTATTTTTGTCAATATTAATATGCCATAGACAAGTTTTTAATTTTTGCACATTTGTGGAACATGTCTACTGTTTCGAAGCATCGGAAAGTAATACATATGGGTTACAGTATGGAAACTATGCTAGACATCGGTGGATCGATGTTAGACTATTGCTAGTTGTGTTCAAAATTTGACGTATCCTGATGTAAGGTATATATTTAATTTATATTTAAATATAGAATCTGATGACAGTTAACAGCTGTCAGCTTGAGATGAATACGGATTAGCAATAGATATTTTTTTTAATGTACATCACAATTGTAGGAATGAGTTAATATCGTAACTGAAAGTTGATTTTTAATATAAGTTATCGAAACTTAAAACTGAAGTACTTTTTTTAATGGTTGACAATGAAATATGACTACGCTA
MSTRG.1742	PiggyBac transposable element-derived protein 4-like	TCAGTACCTGCTCTCTCAGTATCCACCGAGCTGTCAGCGTGAGAAGGAGTTCAGATAAGAATGGACTGGTTCATTACAGATGAAGAAATCAGGGCGGAGGTAGAGAGGTTGAACGCCGAAGATTTATGGAGTGACGATGAGGACGACAATCGACGACCAGTGGAGAGGGATGAGAGTACTTTGTTACAAGAAATTTGCAGTGATGATAAGGATAATTTATTAGAAAATGATGACAACCAAGACGACGAGTTTGAAGTTTTGGTAGAAGATGAAGTTCCTCAACCTGATTTACATTCTGATACTGGCGACAGAAATTTTATTCTCGGTAAAGACGAAGAAACTATTTGGACTGATACTCCTCTACTGCCCAACAGGAGCAGCAGAGTACCAGCAAGAAACTTGATTACACATTTACCAGGAGCCAAAGGAACAGCTCGCACCGCTGAAAATGAGTTGGATTTTTTTATGCTTTTCATCACTAATGAAATGGTTGAGAAAATTGTGCTCCACACGAACGTGAAAATTGCTGAAATAGCAGAAAACTATACTACCCCACAATCATTCACAGTACCAACAAATGAAAGAGAATTGAAAGCTCTGTTCGGTTTGTTGTTCTTTAGTGGAGTATTGAGAAAATCAAACCTTAGACTTGATGATCTATACTCCCGTAAATATGGACCACCGATCTTTCGAGCTGTGATGTCAAAACAGCGATTAGAATTTCTACTATCTTCACTATGATTTGACTCGATTACTACTCGTGTAGAGAGAAGTAAAACTGATAAGTTTGCAGCCTTTCGAGAAATATGGGACGAATTTTGTGATCACTGCCAGAAGAATTACACTCCGTCCGAGTACCTTACGATTGATGAGACATTGTTGAGCTTTAGAGGTCGGTGTGGATTCAGGATGTACATACCTAACAAACCCGACAAGTATGGGTTGAAAGATATATCTCTTTGTGATGCAAGGACTTTTTACTTTATGGGAGGTATCCCTTACGTAGGAAAAGGTACAATCACCCAAGAACCTGGCATGCAGATACCTACTCAGTACGTGCTGAGTCTAACAGAAAATGTAAAGAACACTAACCGTAACATCACTGTTGACAACTGGTTTGTTCATACCAACTGGCGGAGAAGCTAAAAGAGCATAAACTTACCTTAGTTGGTACACTAAGGAAAAATAAGAAAGAAATTCCACCATCTTTCATTTCAACGAAAAGAATTCCGCTGAACACTTCAAGGTTCTTGTACCAGCCAGATAAAATTATAGTTGCTTTCAATCAAAAACCCAGGAAAAACGTCATCCTATTATCCACAATGCACGATGATGGTGAAATAAATGTCGCATCTAAAAAACCAGAAATAGTAAGTTCTATAATTTGTTAAAAGGGGGTGTTGATGTTTTTGACCATCTCTGTCATTCTTACTCTACTAAACGAACGTCAAGAAGGTGGCCAATGAGGTATTTTCATGGTGTCCTTGATGCTGCTGGAATAAACAGCTATGTGCTGTTTAAACTAAAAGGAAATAAATGTACTTGAGCAGATTTCTTGAAAACATTGGCTCTTTCCCTAGCCAAACTTCATATGCAGGAGAAAATGTATAACTCAAGACTGCCTAAAGCACTTCGAAGAGATATTAGACTTGTATTGGACCTTGAAGAAGAAGAAGAACCCTCCTCAATGCTACCTGAACAGCCACAAAAACAAAGAAGGTGCTTCTATTGTCCTCGCAATCAAGATAAGAAAACCAAGTTTCATGTGCTAAGTGCCAAAGGGCCATATGTAAGAAACATGAAAAAAACATTCATGTTTGCCTACAGTGCCAAGTGCGATTTGACGACTAGCATCTTGATATGCGTTCGTTCATTTGTTTTTTATACATAAAATGTTCATACCTCAACAGTGTTTCGTTTTTCTGAGATCAAACTTTTTTATGTGGGTTTTGTTCCTCTTTTTGTTCAACTTTGTTCAACTTTTGTTCCTTTAATCTAGTTGAGAATCATTAATGATTTAAAGTATTGTATTTTTTCGTTACCTATATTCTAGTTGATTTGTATTCACTTATTTTTTGTCTATTCAATTTGACTGAAAAAAAGGCTGATTTTTCTTTCTTTTTTTTTCGAAGTTTGTTGGTTGAAATTAAAATTAATATATATGTTGAGACATTTATATAGTTTATATAAATATAAATAAATACTGTTTATCCAATTTGTAGTTTGTTCATGGCACCTGGAGAATCAAAACTCGTAGAAAATGACGGGGGTTCAAAAACACCCCACCGTAAGGATCGTGCATTAGCACTTCACTGTAGGGATAGAGGGT
MSTRG.1763	DNA-directed RNA polymerase III subunit RPC4-	ATTCTGAGCTCACGTAATTAATGGATGCCCCCTTAATGTAAATAGGTAAGCATTTTTTTTAACTAATTTAATTTTAAGTACAGAATAAAGAATCTTTTATACAGGTACTGAAAACTTTTCATTCTTGTTTGATAAAAATATTAATTAATTTTTTGTTCTTTATTGTACACAAAAAGACATTTTATTAGAGTAATAATTAAATAATATAATGCACAAGGGAATTTATCTCTAAAAGAGATTTCTTCC

	like	AGCATACCCAATATAAGATAAACTTACAGTTTATTCATAGTTACAGGCATACATGTGTACCAATTGACTCAAATGTTATAGTTAAATATAAATAAATAAAAAACTGATATCACTTATCTGTAGTTTGGAAAAATACTACAAAGTCAGTTTCTCATATTATAGGCTGGTTGCAAAACCAGACGAAGTCAACTGTGAAGGTAAAACAGGAGGTGGCGATCAAGAGCGAGCCTGGAGACGATGTAGACTGCGCCATGGCTATCGAAGACAAAAAACCATTTGTAGATATAAAGCAGGAGGTATTCGAAAATACAGACGTGGTGAATTTACTGAAGAGTGATCAGCCAACGCTTATATTGCTACAGTTGCCAGATACCTTGCCAGGGCGGGGAGGTAGCATGGAAGATGATGCGCCGCGACGGAAGCAGACAGATCAGCCGTCTACTTCCACGGGGGAGAGCAAAGAGGAAAAACCGGT
MSTRG.1773	Centaurin-gamma 1A	CCAGATCCAGACTGGGCGAGTTTGAACCTGGGAGTGCTGATCTGCATCGAGTGCTCGGGTATACATAGGAATCTTGGATCCCACATATCACGCGTACGCTCTCTCGACTTAGACGAGTGGCCGTTGAGCCATGTTAGTGTAATGGTGTCAATGGGCAACACTCTCGCTAATTCAATATGGGAGGCGGACTTAAGGGGCCATATTAAGCCAATTGCGACGTCGAGCAGGGAAGAGAAAGAACG
MSTRG.1778	AP-3 complex subunit sigma-2 isoform X1	AAAAAGTTTTCTATTCGGAATTACATGGTCTAGTATCTTGACGTGTCTCGCTTTGCTTTATATCTACGTGCGATCTATAAGAATCAAATTTTATTCAATTTTTATTTTTTAGTCTATTGCAAACTGAAAAAGAAATAAATGATTTAATTTAATACTATTATTATTTGTTTAACTTAAGTGCGTTAAAATGATTAAGGCTATTTTAGTGTTTAATAATCATGGAAAACCACGATTGTCGAAATTCTATCAATATTTTAATGAGGATATGCAGCAGCAGATTATTAAGGAGACCTTCCAGCTGGTATCCAAGAGAGACGACAATGTCTGCAACTTTCTAGAAGGTGGCAGGTACTGGCTCATACAATTTATAGGAC
MSTRG.1803	TELO2-interacting protein 1 homolog	TGTTGGCAGCACTGCAAGATGTTGCCACATGCACAAATAATCCAGGGCATGCTGTTGTTGTGGCTTCAATAGATGCCATTCACCGCATCCTATGCCTCACAATGCAAGATAAGTTCTTAACAAAGAAAAACGAAGTAACCGTTGAGGATATAACCAAAATGGTGAAGGAAAAAACCAAATTAGCAAGCGGTGATAACAAAGAAGTAAGCAGTAAGGGTAATTAGTAATTTTAGAGCATTTGAATTTTTTTACATAGATTTATTATTTACCTTTAATGGAC
MSTRG.1812	Rap1 GTPase-GDP dissociation stimulator 1-B	AAAGAAATTATACCCTTTCAGACCAATCTACGAGTGTGGAACGCGTGGAAGCTGATATAATAATAATAATAAAATTAACATAGAAACATAGCATATTTTAAATACTGTATCATCAACATTCTCATACCTTATTTTTTAAACTTACGTACAAACACAGTAAAGTCAATTTCTCCTTATACATGTAAAAATAGACATATCTTAATTACATTTACTTGATTTGACATCAATTTATCTTCCAGGTGGATGGTTGTGTGGCAAGTTTGGTGAACATGTTAGTGGCACCTCACTCTGTCATGCAAAATGAGGCCATACTAGCGCTTACTCTCCTAGCCATAGAATCACTTAAGAAAACCACCCCCCAAACAGAAGAGCCATCAGATTTTGATTGCGAAAGGAGTTTTATTACTCAGCTAATAAAGTCGGAAATTGGTAAACACGTATCTGTATTAATAGAGACAAACTGCGCGAAAATGCCAGTGGAAGTTGCAGAGAATTTGCTCGCATTCCTCGATATAACGTCTAAGAAGAACGAAATAGGGCTCGACTACAAGGAGGCGAAAGTCCACGAGTCCTTACAGAAGTTCATGGATTCTAGAAAGGATTTAAACGAGGACCTGCAATCTTGCATCGCGGCACTTGTGGTCACCATTTCGGATAATGGCAAGTCGGATTAATTTCCGATAATAAGAAGAGAAGGGAAGAAGCATTTGATGAGACAATGAAGTAATTTGACCTCATCTGAAAAATAAATAACAGCATGAAACTCGCAATAAAAATTGGCCCCAATTTGATAAAGGCCTGTGTATTTTGTTTTAATTTTTTTTTTTTCTAATATTTGTCGAGTGTAACATTGGCGTCGATACTGAAGCACCCTTTCTTTAAACCTATTTTTAATATATTTCAATTTGAGTTTACAGCGAAATCTTTACTCCAAGCACCAAAATAAACTAAATTTCTATTAGATTTAAGTCGAAATTATTATAAATTTATTTCATGGTGGTCCATATAACAGTAATTTCACGAAACTAACAAATTAAAATATAAAATTTAAAGAACTGGCCTCTCAGAATCGACTGCAGCGTGATGATTGATTATTTTTTGTTAGCTATATATTTTTTTCTATTATTTGATTGTGACATTCGGTTAATTTTGAAAATATCATTTTCAAAAAAGCGATATCTACACATTCTTCCAGACATATTGTCAAATGTGTAGACGTACTGTACATTTATTCAGTTTTTGTTTAGGGGAAATTTAAAAAAAAAAAACATTTATTTAGTATGGTAAGAACAATTCTGCTTGTCATTTATTGAAGGTATCTCTAAAATTCTTTATTATTATAGATTTTTTACATTCAAATAATGGAAGTTTAGAGTTGGCGCACTTATTTCTTTATTATTAGTGTACATTAACCAAAAGTGATAAAAAAAAACATGAATATAAAATAAAACTACAGTAATCGACTACATGTCGTCATATTTACAATAATATTCAACTTCATGTAGTTTTTATTTATATTATTCTGAAGAAGTTTGCTTTAATACATTGTAACGAGGTCACAATATATTATATGATTATAAAACATATACCTACCTAAAGGTGTATTGATCGAAACTAAATACTAAATTGT

		AACTATTTAAATATATTTCATAAAGCATCGCTTTCCTAAACTTCTAGCTAGTCATAATTTTAATAATATAATTACTATTAGT<br>GGTTTTGCTAGAATTTTCCTGCATTTAAACGTGATGTAAATTGTTAAGCGATAATTCGGAGGACAGTAATATCTGTTGTATA<br>AACTTTCATCTTCAATTTATTTAGATATGACTAATCATATTTTGAGATGTTACTGTTTTAATGATAAGTGATAGGAATGTTA<br>TTTTTGAGATACTCTCTGTTTTCGCTGTACTTGTTGTACATTCCAGCAAACATCAGACAAGCTATTTATAATTATATTACAT<br>ATTCCTTTCCGTAAAACTTGGATGCTTGACTAAACACGAAACTTTGACTTGTTGTGAGAAGAAGATGTGTGTGTATTGTAT<br>GACTGTAACATTTATGATCAAGTCAAAGTTATACAGTTAAAAGTTATACACATGCCATTTAAGAGTTATTCTTACATGTGT<br>ATACTACACAAAATATAAAAACATACAAACACTATACAACACACATACACTACACAACCCACACACAATTTCACAACACA<br>CACGACTACACTACACACACACATACACAGTGTATGTGTGTGTAGTGTATTCGTGTGGTATGTAGTGTATGATACTACT<br>ACTACATAACACACACTTATACAACACACATACACTACACAACATACACATACACTACACAATACATACACACTACATAA<br>CATACACTAAACAACAACCACAGACACTACACAACTCTCATATGTGCAAAGGGAGAGAGAGGAACTTCGGTAGGACTAA<br>ATGATAAGAGTCAACACTTTCACAGAAATGTTTATTTTTAATTAATTACTTCTTACATACTCATTATCGAATTCTTAGGCAA<br>ATCAAGCATCCATGGTTTTCACTCATTGATGGTTGTGTTACCACATAACAAAAGTGAAACAGCAATACTGACGGAATACTC<br>AAAAGTACAATAAAGTCACCGTTTTAAATATTTTGTAAATACGCTTTAGCCACAGTTGAGGCGATTAGATATGAAGCTATA<br>AGCAGCCACTTTATTATCAAAGAATCTTAAAGAATTAGTTTAGAAAATATGTTTCTTGTTCTAATATCTAATTTAAAAAAA<br>AAAAAATTCACAGCAACAAATTTGTAGATAAATGCTTCAGCGTCGTATTCGATCGCCTCGTCTGAGATCGGCCTTAAGGAT<br>GCCATTTGTATCAGTGCCGCTTCAACACAGCAAGCGATTTGTCATTCAAATTTCAACTTTAATTTGTAGTGCTTACAATACA<br>TTATCATACAATGTTCTACCATGAAGCGTTTGAAATTTTGTTATACTGAAATGTATTATATTGCATACATCTTAAATGTCAA<br>ATTTAATAAGCTTTCTGATATTACACCACAATGTGGACTATACCGTAACAAAGTCTCTCTAGTTGATCGTGTTTTATTGATC<br>ATATTGCCTCTGATATCGCTCAAGATGGTGTGGTCATAGCGTGCTGTAATAAAAAAAGCTACTGCAGTGTTTCCCAACCTT<br>TTTGTGCCATGCCCCACTTGACCATTTCTCAAATGTTTATGCCCCCATACATATTTTTTCCCGACAAAGTTTACTTGACGACT<br>CTATTCGCCCAGTTGATATTTTAGTAACATCAAACTAAATTATACCTAATAA
MSTRG.1819	Uncharacterized protein LOC106125039	GGGTATAACATTATATTTCAAAATGAAACTCAATGATAGTTTTCACGATCATGGATCTCGAAATATGACATGAACACATCT<br>GAAGTGAACACATTTATGAAGTACCTAAAACGACAAATTATAGAGCATATATATATATAGCGGGAATTTTATTTTATGAAC<br>TATTTTTGTCGTTCCCGACAATTCTTTCGACTTTTTAAACTCCCACTACACACACAGACACACACACATATATATATATATA<br>TATTAATATGTATTCCTAATTATAGTATAATTGAGTTCATGAATATTGTAACAAATTTATGCAATGGTATATCCTTTCCATC<br>CAATCCTGCCCTCCTAGACAAATTGTAAAATAATCTATTGATATATATGTTATCGCTACCGCGTGCATGTGATAAAGTGAG<br>ATAGCAACATTCGGCATTTGATTCTGTGTGCGCTAGCGAAAATGGTAGATTAATTTGCTATTATCGAGAGGCTGTGTACTG<br>AATGGAACAAATGTTGATATTCTTAAATCTATTATACTATTTCAAGATATAAAGTCTCGATTTATTCCATCTTGGATTTATT<br>ATTGTAAGAATTCTTTTTTATATTTAATTATTTAGAACAAATGTAAGTGTAAAATAAATCATTAAAAATGCTAATATTTGTA<br>ACGTCTAATTCACAGAGCTTTAGTATTTTTTTTTTATTTTTATTTAATAGAAATATCTCTTACTATTTTTTTTTTATACAACTT<br>AGAATGGCAAACAAGCTGACGGCCCACCTGATGGAAAGTGGTAACCGTCGCCTATAGACATGAGCAGTACCATGGACATA<br>ACAGAGTATACTGTTTCGCCCATGATACCCCCCATGCGTTGCTACTCCTGAGGACTCAGGTGTTATTCCTCTGTACCCACTA<br>AATTCACTGCCATATCCCATCCTTCAAACCGAAACACAGTCATGCAAACACAACTGCTTCACGGTTGAAACACGAATGGG<br>ATAGAGGTACACAAGCGATCGACTTTTGTACAAAGTCTCCCTCTGGTGAATAACTAGTAAATAACTTACATGTACCTATTC<br>CTATAAGTTTATTTTTTGCAAGATAGCGTTTTGGTTCATACTCGTTGGAGACAATATAGTAAGTTCATAAAATTCCCGTTAT<br>ACATATATATAGAGACATTGTTAAAAGATGAACATACATTGTAAAAATAAAAAATAATAAAATATTTAAGACTTGTTTGT<br>ATGATTTGGAATTCAAACATTTTGAGTATTGAATGACGAATGTGCATGGTGTTAGTCGATCATGCGCGGGCTTTTAACGAC<br>CATGAAATTGTAGACATAGTACATTTAAATAAATACACATAATGTTAACACAGATCTAAAAGAGTACCTGTTTAGTTTCTT<br>CTCAACAGATAACAACCTTCCGAACCTTGCAGTCATTAACAATGACGATTTAAAAGGTTGCTTGTAAACAAATCTTTGAAT

		AAAAATGATTTTAAAGTATTCTGCGGTCCATTGTATGTGAACTAGGATGCCAGGAATGTTCTTTTGTTCCATTGAGTACAA<br>AAAAAAATGTTTAATGATTTTTGTGAGAGAATAGATTAGCAAATGTTTCCGTTTTAGCTTTATAGTATTTGTAATAAAAAA<br>TCTATGGCAATGACGATGATTATGGTTTGTTTCAATGATTTTAATGTGTCGGGTATTAATTAATATAAAAGTTAATTTGTAG<br>CTTTTTTTTTATTATATTTTACATTTTATATATAATAAGACATATAATAAGGCTTCTGAGGAATTGTTCGGATTGATACCTGC<br>TGCTGATTTTCAATACCGTACAACCCTCCACGAACTACAGTTTCATTTCAATCATCTGGACGAGTGGCGGTCTTCCACCGTG<br>CGTTTTTCAAGGCACTTTCTCCCACGCACAACCACTCTTTGGAATCAACTTCCAGCAGCAGTATTTCCGAACCGATACGAC<br>AACATAACCTTCAAGAAAAGAGTATATTCCTTTATAAAAGGCCGGCAGCACACCTGCAATGACGCTGGTGTTGTGGGTGA<br>CCATGGGCGGCGGTAGTCACTTACCATCAGGTGAGCCGCACGCTCGTTTGCCCCCTGTGTTGTAAAAAAAAAGCTGAAAC<br>ATATAAAAATCGACAAAACAATTATCTTAATCAACTTGCCACATAGTTGATTCTAATTTCCTACCAAAAATATTCAAACTG<br>CCACAACATCCAAGAACTAAAGGACAGTGTCTAAAAATATATAATATAAAAAAATTCCATACGAGTAGATGTTCTCGCTG<br>GGGAGTACACTCTTTTGTTCGTGCACGCATCGTGATGAATACACCTGGAATTCAGCACAATGGTTTTTAAGGGGGACCACG<br>TGTAGGTCGACCTGACAGAGTATATTGCTTGCAATGGGACTGGTAGTTTGCATTACTAACAATTCCTTTCCCCCTTGGCATA<br>CGCAGTTTGGCTTTGGTGATGCTTTAAGATGCTGCTGTTCATCTCTTGATCATTACATTCCCTTTGTTGCCTCTTAAGACATC<br>CATGGAAAAGGAAGGGGCGACTTCATTATATTGTCTACGAATTATTCAATTAATTAACAAATTAATTATTAATTAATTAAC<br>TAACTAAACTAACAAATTATAAATAAATTTAAATTGTTTTAGCTATTAAGGACTTTTAATTGAAATGAAACGAATTCAATA<br>TTATGAAATATTAACTTTAATGCTTTGGCGTCAATAAATTATAAATAGTAAAAAAAAAGCTATGTAAATTGATATTTAGAA<br>TACATTCATGTTTACCTAAGTAAATAAAAGAAAAAAAAAAATAGTTTAAAAATGTTATTTTGTGATATTTGCATTACACATA<br>AACGATTGCATAATACTTTGGCGTAATTTGATCAAATATGAGCTGACGTTTTATAATTGTTTTTTTTTTTATATTATACATT<br>AAAAACACTCTATGTACAAAATTATTTAATTTAAAATCTCACACACATTCCTAGTAAGGTTCATACATAATATTATTAGGC<br>GTATATTTTTATTTTCACATAGTTTAGGTATTAGAAATTTGTACAGGTGAAATCAGAATTATTAGCAACCTTTTAAGAGAAT<br>ATTTTAACAGTAAAACTCAATATTTTTCAAAAGAAATATGGGTCGATAAACAAGTTTAGAGCTTTCCATACTAAACTGACA<br>GTAGGACGTGGAGTGTTTGTATGGGAAGCTTAAAATAATGTCTTTCATTTTCATAGATAGAACAGTCCCTTAAAAGAGTAA<br>CGATGTATACACAGTCATATTAAAAATTGTAAAGACTTGTACTTTGTGGCATTATTTTATTTGAATTTAAAAATCTTTGGAA<br>TTGTCAGTCGCAGTAAGGTACCTTATTTTAAAGTACTAAAATCTTTAAGTACTTATCTATGATTACATACAATTACTACAGT<br>TTTA
MSTRG.1835	Protein SMG8	AGTGACAGTAGCATACCGATTTAATGTCAGTTACAATAACAAAATAAATTTTTAAATAAATAATTTCCAATAACTTTGTAA<br>ATAAATGAAAACAAACAATATTTTCTCAGGTTCACTTATGTTCATTGAATAGTTACGGATATTATATACAATATATAGTATT<br>CCAGTTGAATATGAAGAGATTTACGATAAATGATATACCGGAATTTTCAAAAAAAGAGCGGATTGTGGTTGTTGGTGTGA<br>TAGGAAAGTCTCCATATCGGTACCCTAATAAAACAACCCCACTTTTGCCCTCAGTGAAATATGACGAGAATGGTATTGAGT<br>GCCATTGGGATGAGCATAGTGGTGTGTTATACCTGCATGCAGTCACATACCTGGACACACAACGCCTGGTTGCCCTAACAG<br>AGACCCTTGATGAAAATTCAAAGACCACAGAGAAAGATGCTGATGCTGCACACTGGTTGGTCGCTTCAGGAGAGCTAGCT<br>GCTGATTCCTGTAAAGATATAGCTTTGCTGTTCCACCTTTGTCACATTGTGGTCCTCTCTTCACCGACTTCAGTGTTTG
MSTRG.1847	Kinesin-like protein unc-104	ATAATGCAGAATTCGCTGCCGGCGTGAAGCAGTCGGCGAAGATTTCCTTCGACGACGACGTGGCGCCGACTCGCTCGCGA<br>CTCAAGGCACTCACCAATGTCGAGAAGAACAACGCGATCAACCTGGAGGATCGTATCGCCGATCAACAGGACTCCAACAT<br>CAAAATCGAAGAGCTGGCGGTGGGCGAGTGCGACGCAGACAGTGGGCGCGGTGACAGCTCATTGGCATCTGAGCTGAAG<br>GAAGAGGAACTGCCTGAGCACCTGGCGCCGGGCCGCGAGTTCACCTTCCGCGTCACCGTGCTGCAGGCGCACAGCATCTC<br>CACCGACTACGCGGACGTCTTCTGCCAGTTCAATTTCCTGCATCGCAACGAAGATGCTTTCTCGACAGAGCCTGTCAAAAA<br>CACCGGAAAGAATTCACCTCTCGGATTCTATCACGTACAAAATATCACGGTACCTGTAACGAAATCATTTGTCGAGTACAT<br>TAAAACGCAACCGATCGTTTTCGAAGTATTTGGACATTACCAGCAGCATCCACTACACAAGGACGCGAAGCAGGACGGGC

		CGGTGGGCGGGCGCACGCCGCCGCGGCGCATGCTGCCGCCGTCGATCCCGATCTCGGCGCCGGTGCGCAGCCCCAAGTGG<br>GGCGCCGCCAGCGTGGCGCCGCCCTGCTGCTCCTCGCACCTGCACTCCAAGCACGACCTGCTCGTCTGGTTCGAGATCTGC<br>GAGCTCGCGCCCAGCGGCGACTACGTGCCGGCGGTGGTGGAACACAGTGATGAGCTGCCATGCCGGGGGCTGTTCCTGTT<br>ACACCAAGGCATCCAGCGCCGCATCCGCATCACCATCTTGCACGAGCCCTCCTCTGACCTTCAGTGGAGCGATGTGCGGGA<br>ACTAGTCGTTGGCCGTATCCGTAACACGCCGGAGGCCAACGAGGACACATCCGACGGCGACGAGGACGGCGCACTCTCGC<br>TCGGCCTATTCCCAGGCGAACGGCCCACGCTCGACGACAGAGCCGTCTTCAGGTTTGAAGCCGCTTGGGACAGTAGCTTAC<br>ACGGTTCACCGCTACTCAATAGGGTGAGCGCAAACGGAGAGGTCGTCTATATCACGCTCAGTGCGTACTTGGAGGTACGT<br>TGAAATATTTGTTTTAAGGTTTATAT
MSTRG.185	Peroxisomal N(1)-acetyl-spermine/spermidine oxidase	CAGTATTTCCACGCAGTAGCGCCCACGCCACAACCTATGGGGCAGTACGTGACACAGAGGCTACACGAGTACTTGAGAGA<br>AAAGCACCCGTCAGTCCTCTCGGACCAGGACTTCATTGACGAGTTCCTGCGTTTTGTAAACCTGTTTATATCCGGATACGA<br>AGCGACCACCAGCTGGAACGACCTCACCTCCAACTGCAGGTTCAGGAATGTGGAAGGTCATCCGTACGTGAGCTGGAACA<br>GGCATGGATATAGGACATTCTTTGAGATTTTGTTG
MSTRG.1851	Serine/arginine-rich splicing factor 1B	CAGCTTGTTTAGCGTACGCCACTAGGTCAATTTATATAAGTGTACCAAATGTAACACTTCCTTCAAGTACAAAGTTTGTAA<br>GCATGCCTACTGTCAATTAGACATTATAACGAGACGCCATATTTTATTGAGTGATACATGTAACCCGACGAATATTTGCGT<br>CGAAAGTTGTAACGCGAATGGAATTGAAAATCTTGTTTAGCTATAAGTAAATCTTATTAAATTCATATCTCTTATATATAGT<br>AACTAATTACTATGTCCGGTGGAAGCGGTAACAACAGAAATGAGTGCAGGATATATGTCGGTAACTTGCCTCCGGATATC<br>AGGACAAAGGACATACAAGACTTATTCTACAAATTCGGTAAAGTCACATTCGTCGATTTGAAGAATAGAAAGGGACCACC<br>ATTTGCTTTCGTAGAATTTGAAGATCCCAGGTGAGTACATTGCAAAATGGTATAAAAATATGTAGAGTTGTAATGCAAAGA<br>CGCACATAAATATTTAAACATCGTTTTTATGTCCACT
MSTRG.1860	Hypothetical protein KGM_204126	CAGACTGTTCGTGATATCCCAATTCCCGTCATTATACCACTTGGATGACAGAAAAGTGACCAGCGACCAACGCCAGGAAG<br>CGCAGCGGCTGTACAAGCGGCCACTGCTGGAGCGCTTCATCAGCCCGCGCTCAGGCGGGCTCGGGCAGCTGGTGCACTCG<br>TCTGTCTCGTGGAGCAACGTCCAGAACAAATTGCACTCGTTGTGGAACAAAGAGCCGGAGCGTAACAGGAATCTACCGAT<br>ATAATTCTATTAGGCTATAAAGAAATGGAAATTGTGCTGGGTTAAATGGATGAAAATTTGTTATGGTATGTACTTTTAACG<br>TCTTCCGTCTCGCTCACACTCCACTCGGAATACCTACGTACCTAAGTACTTCAAAGAAGGTAGTTTGTAAACTAATTAAGA<br>TTGCACGGAGAAAAATGGAAGCTAATTAATCTGAAATATTAATGTATTCTGAAATAATGTTACAAATACTATATAAATTTT<br>CCTGGTACTTAACAATTATAATAAACTAGGCCCTCTGTGCATACACATTTGTATCCAAACTGATATATTTAACATACCTTAT<br>ATAAGTCATTAACCTTTCACTGCTGAACATAGGCCTCCCCCGTAAGGGGATTTGCCAGTAGTTGCCACGCTTGTCAGACGG<br>GTTGGCAACTGTTAGATTTAGGTGATATTTAAAGAGAGACTGCTGTCCATCCCTCGACTGTTAAGTTCCCTTAGTCGCCTCT<br>AATAGCACTTTCGGGATAGGTAGAGGTGGCCCATTCTATGCTGGGACCATACAGCAATATTTATAATAAATATAACCTAAA<br>AATTACTTACCTGGTAAAGTAATCAGCTTCCTAGACAAGGTACAAAAAGTATTGTTACCGCTAACGAGTCTATTAGGTAGA<br>ATTTTTTTTTATACAGCTTAGAATGGCAAACAAGGTGACGGCCCACTTGGTGGAAAGTGGTAACCGTCGCCTATAGACATG<br>AGCAGTGCCATGGACATAACAGAATATACTGTTTCGCCCATGATACCCCCCATGCGTTGCCATTCCTGAGGACTCAGGTGT<br>TATTCCTCTGTACCCAGTAAATTCACGCCATATCCCACCCTTCAAACCGAAACACTGCCATGCAAATACAACTGCTTCACG<br>GTTGAAACACGAATGGGAATATAGAATGAGATAGCGACATGCGCTGTCTGTCCTGTTCTTTCACATGAGAGCACCAATTG<br>AAGAGATTCTATTGATAGATTCGATTGTACATTGTTTACTAGGAATGAGGTTCGTATATAAATACTATTATTGCTACCTACC<br>TATGGTTCCGACAAACTTAGGTATAGGAATAAACTTGTTTAATGTGTTCAAAAAGCATTTAATATATTTATATAATTTATAT<br>AGGTTGTGATTGTTATCGAATGTTCAAATTTTTTAAAACGTTGAAATATTATGTAAGTAAAACAAACATGTTTAAATACTTT<br>CCGCTTGCTAAATTTGTGTACTATATTTTTATCCATTTGTATATAACTGGAAAGACTATACGACGGCTCAAATTAGGGATTA<br>AGTACAGAAAAAACTGTCCAATTATTATTACAAGTGAACAGTAGAAAATTAATTAAAAGTTTTCCTATTGTATTTAATTTC

		CAGTTATTTAATTTTTCTAATACTACTTTTAATGTACGCCCTCTGATGGCAACAGTTTTTCGGCCATCTTAGGTATCTGATGT<br>GACATACCGCTTTTTTTTGTCAACATTCTCTAGTTAAATATAAGAAATATACGTAAGCTTTAAAAATTGTAAATATTTTATT<br>CGATTCAAAATATTATTGTTGAGTAATAGCTGTCGTAAGGAATACTCTTAGATTATATAGATAGAGCATCGAGCCTCCAAA<br>ACGGGTCAACTTTTTGTCATCTAATTGTCAAATTAGCAAATTAGTTCAAATGACTCGTTTTTTTATCAGTGTTATATTTAAA<br>AATACGTCAAGATGACGTATTATTTAAAAAATTAGTGTTTACGCATTAAAAGACGATGAAATTATAAGACAAAATAAATA<br>TAGAAGTTATAAAAGGATTACTCATAACTATTTATCACGCAAAATTGTATGAAAAAGTTGATCCGTTTCTCTCGAAACATT<br>ACTATGGAGACGCTCTATCTATATAACCTAAGGTCATATTGTATTTTAATTACTTATATTCACGTTTTAAATTCGGGGATAC<br>CCGACTAGTTTCGAACTCAATAGGAGCCCTTATTCATGAGCGCAAGTTCACGGCTGTGGTGCGAGCAGCACTGCGCAACC<br>AGCTCGCTGTTTGCCGTGATAAAGCCAACATAAATAGATACAGAAATATTACTAATGTTATTTTACGACGGGTAGATCTCT<br>AATGATATAATTACGCTATTTTGTC
MSTRG.1868	Retrovirus-related pol polyprotein from transposon tnt 1-94	TGTGAAACTGCACATGAAATGTGGCAAAAGCTCTATGATATATATACGAGCAAAGGTCCGGCAAGAAAGGCTAGTCTATT<br>AAAACAACTAATTCTGAGTAGATTACAAAATGGCAATGATATACGTGAACATTTAAATAAGTTTATGGATATAGTTGAAA<br>AGTTAAATCAAATGGACATAAGTATTCATGACGATCTACTTTCAGTTATGATGCTATACACCTTGCCGCCAGACTATGAAA<br>ATTTTAAAGTAGCGATAGAGTCGAGAGACACGTTACTTAAACCTGAGGATCTTAAGGTTAAAATTATTGAAGAATCAGAA<br>GCGAGAAAACATTTATCCTCTGCAGATGCAAGCGCAAGTAATGAAGAAGTTTATTATGCTTTTTGCAAGATATGTAAGAA<br>AAAAGGACATAATACTAAAAACTGTAGAAAGAAGTCATCATACTACAAAAATAAGACGAATATAAAATCAAGTCATGAT<br>AGCAAAAATGAAAATAAAAATAATATTGCGATTTGTCTATATGTATCTAAGGAAGAAAACAAAAAAGAATGGATCATAG<br>ATAGCGGATGCACTTCACATATGTCTTATGAAAGGAATATATTTCACAATTTAGAAGACACTTGCAAAACGTTAAAATTAG<br>CGTCTAACGATGGTGCTGCTGAAATAAAAGGACAAGATCAAAGCAAAATTCATCACTGGCACAGGAAACTTGGTCCTATG<br>AATGAAAATGACATGAAGTTTGCTTTAAGAAATAAAAGCCTAAAAGGTTTAGAATTTGGTTTAAATGAGAAACTTGGAGA<br>TTGTGAAATTTGTATTCAAGCCAAAATAAAAAGACTACCATCTAAAGCT
MSTRG.1870	TATA-binding protein-associated factor 172	AGAAGAGAAAACGGAGCCAGAGGACAACGGGCGGCTGCGATGCGAGACCTTCGACTTAGAGAAGGTGCTGCAGAATGGT<br>GCGCACCTGATGGGCTCCGAAGGTCACGAGTACGACATGGAAGAGGAGGCTATGTCCGCTGCAGATATGAAAGATCGATT<br>ACTGAAGCAACGACAGCAGTTAAACGCTCGTCTGGGGCTCGACATGGCACAGCAGCTCGGCGTCGACCTCACCTCCGTGT<br>ACTCCAACGAAGACTTGTGTCCTGTCAAGCCGCAACCTAATAAGACTGAACCGCCTAGGAGGCCTATACAAGAACTAGTC<br>CCGATCGTGTCATCCAAAACATTAAGCTCCCGTGAGATGAACCTCGCGAAGCGAAAAGCGCGCGCGGCGTTCAGCAAACA<br>GAAGTCTAGAGACTGCGAGGAAGGCCCGTCAGCGCCGCCCACGCCGCCGATTGAGCCGGACAAAAAGAAGATTAAGTTG<br>GAACCGCCTGATGAGTT
MSTRG.1873	Ras-like protein 2	AGGTATCACTAGAGGCGGCCCAAGCGCTCTCCCGCCAACTGAAGGTCCCGTACATTGAGTGCAGTGCCAAAGCGCGGATG<br>AACGTGGACCAGGCGTTCCACGAGCTGGTGCGGCTCGTCCGCCGGTTCCAGGAGGCGGAGAGGATCAACAACAAGTCCAA<br>CTACACGCCGCACAAGAAGCGGACATGCGTTATGTTGTAACGGAAGACTGTTGTAAGCTTTAATTACGTATATGTATTATA<br>TTAACCAACTCCAATTCGATTGTACAATGCGGACTCCGAGTCGGAATCGTTAGAGTTGTCTTAAGTTGTTCTATTGATTGAA<br>ACTGCTGTTCGATGCACAATAAACAAGTAGATTTTTCAATACTCTAATTATCAAAATAACAAAAATTGTACAAGTGCCAAT<br>GATAAGTTTATAACCATAATAAAAATAAATGGATGGTGTTGCCACCTACAAGAAAAAAACGCCTTATAATACAGTGTGAA<br>TTGCCAGTAAAGAAAAAAAAGGTACGTGGGTAGAAAATGATAAAAAAAAAATGAAATCTTGATATTTTCGTATTTTTTTA<br>ATTATGCTTTAGTGGTAATTATTTATATCGCTTTACTAACCTTTTAAATTCGGAGATGTCCCGACTTGTTTCGAACGCGACT<br>GGACTAACATTCGCGACACAACCGTGATCGGCGCTCATGAATAAGGCCTCTTATTGAGTTAGGAACTAGTTGGGAATTACC<br>GAATTGAAAACTTGAGTGAAACCGATATAAATAATTCCAATATGATTCACAGTAGTTATTATTTAACATACTGGCGTTGTT<br>GGAATATGATTTTTTATAATTAATGTTTTTAAAACAATATGCAATTAGTAAATTGCCAGTAAAATGGCTAATTATAAATATT

TTAAGCTTTCCATACTAATTGTGCATTCAGCCCCAATTTTTTTTTTTTTATTTCTTTGGAATATAATTACAGTTTGACTGGTAG
AACATTAAGAGAATGCTAATGATTTCGATCCAGCCTGTATTTTGTAAACTGACAGATGTTGGTTGATGTTCACAAATTTGA
GTTATACAGGGTGTGCAAGTGCAAAATAATTAGTAATACAGCATTATAGGGGTTGATTACTACGTACTGAAATATAAAAT
CTTAACATATTTTACATATGAACGCACTTTTATGTCTGGTCATAACTGTGGGTCTACCATGAAATGAAATTCCGAAATTTCG
GACTGAATTTCGTGTTTTTGTTTATACCAAAATAGTACCAATAAATGGAGTTTAACATTTCGTTCGTCATACAATACTATCA
TAAAAGTCAAAAGGAACAATATTTTAACATTTTTAATATTTCCAAACGGCATGTATTATGTAATTTTAACATTAAATTTCTG
TTTCGTTTGGCTCCGAAATTTCGGGAAACATTTCACGGTACGCCCTCTGGTTGGTCAAAAACGTATCTGCTGCCTCAAATAT
ATCTCAAATTTGAAAACAACAACGGGACATTTGGAAAAGTTTTTATATACATATAAATATGATTTTGATAGCTCTATAACT
AAAAATAGGACGCAGAAGCGGTATTAAACAGGTGTTAGAGTCAGCGCATGCTGTAACCAGTACTGTTAGAACGATTGTCT
AATGCAAATAATATGCGTACTCAGGGAATTGTCGATATATGCGAAAAGTTTACATGTTAGATTTAAGAGCATCGAGCTATC
ATACATGTATATCCACCCTTTGTGTTCTTTTAATTCAGTTTTGACAATTTGATTTCTAATTTATTCTCTCAAAAAGCAATATT
AATTCTTAGAAACAAATTAGTTTTGCTATATTTGTTTTTCTATTCGATAATGCACCGCTTACGTATTTCTCGGTTCGCCCAAT
GGTTAATTGGTAGAGAACACCGTCCGGTGTTAAGTCCACCAAATGTACATAGAATTTTACTGTGCGTTAAAGTTTAAATGA
ATAAATAAATATAAATATTTTTGCAATTTTAGAAGCAACAAGTGATCTAAAATGTTTGGTGTGTCCCAATGAATTATGTGA
CAAGACTGAGACTTTTTTTTTTATTTATTTATTTAGCATGCAAAAATACATTAAAAAAACTGGATTTTTAGTTAAATCGTAT
ATAAATACGAAAGTAAGTGTTAACGCTTTGTTTCTGAATGGCTAAACCAATTTCATAATTATTTATCATGAAGTATAGAAC
AAAGAAACGGAACTCTTTTAGAGTTAAGCTCGCCTTTGTACGTTTACTTACCTCTGTCATGTTTTTTTATGTATTATTAGTGT
TTACAATTAAATATAAATAAGTAAATGAATCCGGCATAGTATAGTCCACCTTTTCCTTTCCCATGAGTGTCGTAAGATGCG
ACTAAGGGAGGGCGAGGGATGGGCAGCAGCATACCTCTTAAAACATCTCCAAAGCCTAAGCAAAGTGTGGCAACTGCTG
GTAAAATTCCCAAAGGGGAGGCTTTTGTTCAACAGTGGACAGTTAAAGGCTGGTATGATGATGAAGTAAATAAAGCCTGG
CCTTCAATATGTCCCGGATACACAATGTTAAGGCCATATCAGATACATAATAAATGGTTATAATTAAGATTTAGGCAGTTA
AAGCTTAAATTTCATAAAATCTATAACTGGTTTTTTTTTACACAACTTAGAATGTCAAACAAGCTGACGGCCCACCTGATG
GAAAGTGGTAACCGTCGCCTATAGACATGAGCAGTACCATGGACATAACAGAGTATACTGTTTCGCCTGAGAACTCAGGT
GTTATTCCTCTGTACCCAGTAAATTCACGCCATATCCCACCCTTCAAACCGAAACACAGCCATGCAAACACAACTGCTTCA
CGGTTGAAACACGAACGGGACAGAGGTACCCAAGCAATCGACTTTTGTATAAAGTCTCCCTCTGGTAAGGTGCTGTATTCT
CAAGCGGTTGTTAAAATTAAAATAAAAGAATATAAAGGGTATAATAGAAATAAATAATATTAAAGTGTTAAATGAATAAT
ATTAAAGTTCGTTGGTTACATGTGACCCGAGTTGCTCGATCTATATGTAATAGATTGAGCTCATTGAGGTGTAGAGGCTTC
AAACTTGTATTGGGCTCCGAAAGAACTTGTCAGTTTTAACTATGAGTCTTTTTTATTATTTGGATTATCATAAATAGATAGA
TAGACAGATACTCTTTATTGTTTCCTCCAAAAGATTACAATAGTAAATCATAATGTCTATATAATTATAGTCCATGATCAAA
GACTAAGTACTGCTGTACTAATAAAGAAAATAGATAATGAAAAAAAAAATACAGGCAACGGAAACCTTTTTTGTATTTAGC
AGTTTTTTTTTTATTATAGTGTCTAAAATAGAAAGTTTGTTTTATAGTTCTTCATCTTAGAATAATAATGATTAGATTTGAAA
AAAGTAGATTTTTGGCTCCAATATAAATTTGAAGCCATATTTAACACCATATTTGGTAATCAGATGTCATAGTGCACAACT
GTTTTTTTTTTTACAAAACTATGACATCTCATCCCCGAATAGGTGTTTAATATTTTTATTCTGAATAAATATTTATTTTTTGT
ATAATTTCCTTTATAGGTTGTATAACACTGGAGTGAATTTGTACTATACATAATTATATTTATACTGAACAATGCAGTTACA
TTTTGTTTATATCAATAGTTACATTATATTTTTGTATTCACACGAGTGATTTTTGTTTATATTCCTTATACGATAAGGTATTT
ATTTTGGTTAATGTTTTAGGTTATTTTTAAGTAATGTTTAGCCAGTATCTTTTCTGTTTTTTTTTTCTTATTTTCGGTTGAAAT
ATCATAATTGTTTTTCACGATAAAATTTAAAATCTTATATACAAAAACTCCAAATGTTTGTTTTTATCTGCCCTAGATTGAC
AAACAAAACGTCTGTAATCATTCTTAATACGTCGCACTAGCTTAACCGATAACAATAATATATCAAATATTTTTTTTAATCT
TATCAATATTTGTCATAGATTATGTTTTGGCAATGTATTATAGAATTTTCGATTTGTCTATGGTGCCATACAGAAATCATAA

		TAATGTCACCAAATAGTTCTTAATATACCTATCTGTGGCTTTAAAGTTCGAGTAACCGTTTTCTAATTTTCTTTCATTTATTA<br>TAATTGTTCTTTGCGGGTGAGGGAAGGAGACGGAGAATAACTTTTTTAATTGTTGATCATTTGGAAATAATACTAGATGTG<br>GCAATAAACAGTATCTTATAAGACAAATGTGGAATACACTTTGCAGTGATTGTACTATTTTCAATGTATTCCTTAAAATGT<br>GTTACATATGCTTAATTCCGCCATGTTTACTGTTTAATTGTAAAATTATTTATATAAATCATTAATTAAATATATAAATAAT<br>AAGAAAATTTAACAATACAAATATGGAACTATGCTAAAACAAAGTTATTAATGTCAATATAATAATATTACAATAAGTAT<br>GCATTTGAAAAGGTAATACTAAATTTTAAATTAAATTTCTTAACTTTTTTTTGTACAGTTTCCTTTCGTCACAGACGAAGAA<br>CATGGTAACAGAGTACTAAAGTTAGCACATATTTGATGGTGCTGCAAAGCATAGCTACTATATTGCACAATTCTTGTAAGC<br>TATGACAGGATCTAATCCTCTATTAATTTTTGTTCACTTTTTTTTAATTCTACAAGCGAAAATGCACCAATGTAATGTGGTT<br>AACTCGAATAAAATATTTCCGAAGTAATTATGATGTGTTTGAAATTGAATATCTAAATTTAATGTTAAAATTATAAATGGT<br>AGAGTACATTTTCGCTTGTAACTGTAGTGTTATAATTTGAAGTACCCATTAATACAGTTGTTAAAGAGTTTTCAGTCGGCAA<br>TTGTAATAAAATTTAATATTTATCGCCTAATTGTGTAAAATATAAGCTGTAGAATGTATAAGAACTGTAAGAACGACGTGC<br>AGTTACGTCATGAAACCAAATACTAATTCGTATGTTTTCTTTAATATTCTTCCGACAGTGATTTACTTTAATTTAGCGCGTA<br>AAATAGTTTTGCGAATGGTCTCCCAGTGGGTAATTTAATAGAATAATAATAATTATGCTATAATTGTAATTTACTGACATG<br>TTTACTAAAGATATGATATTGTTAGAACAAGGATAAAAATAATTAACTCATAATTGTCCGAAGACAAAACTGGAATATCA<br>TATTGAATAAAATGCGGGCAAAATAAATAATTCGAAGCCTTCAAAATAATGATCATTTAAAACTAGTTTAAATAAATAAA<br>ATATTTGTTTTCTAATGTAAACTATTTTTCGAGTAATAGTTGACAGCTTGAGGTCATTGACATTCCGTTCTGATTTATTCCTG<br>TTTTGTCTCTTTTGTTTCGAACTAGTTCTGATACAAATAAGAAATTTTGTTTCCCATTTGTTAGATTACGATAATAAAGTGA<br>CGAATCTGGAATTTTCGACGTATATATATTTATATAATCATATCAAGACTGCTATGTGAATTTGGAGTTTCACATAGAACTC<br>TTGCAAACAATAGTAAATTAATTTTAGTAAAAGAGATGTATATTTTTATATTGTAGTAATTGGGCTTAACGCAGTTTTTGCT<br>AAGCATACACATTAGTTGGAGGCAGACACGTTTTGATATGCATTTTACAAATTATTTAAATGAATTTAGCTTATGACGTTG<br>TCGAGGACAAATGGTTTTATTAGCTTTCAATGGATAACATGTGTAGATAAATATACTTACCTAATTAATTTCCTTAATTAAA<br>CACTCGTTCTTTGTGATTTCACTTTTCAATCTATTGTTTTTTTTATTCATCTGGCATATGTAGTAAATAATCTTTGTTTTTTTT<br>TTTAGAAATTATTAGAAACGAGCCATTTGTGCTACACAAAAAAAAAAACTGTCCTGTTAAGAAACGAACTATAACAAATTT<br>GTATATATGTATATATATTTAATTTTATTCAGGCATTTAAAGATATGATAAATAAAGCAACATATCTTGTTCAGGGTTCATG<br>TAGAACTCTGAACTGTTGGTAATAAAAAAATATATATAAAAATACATAATACATAAAATCTTTTTATTCTTTATCAAAGAA<br>AATAAATAAAAAAAAAAATCAAAGATTATTTATTAATATACCGCCTACTTATTAATATCCTTGTAATTTGCGAATTGGACAT<br>TATTAAAGTTGAATTTTTAGCCTATTGTATTGTTTTGGTGCTGATCTATAAAATTTCAATTTGCAAATGCAAGTAATTCTGTT<br>AATAGTTTAAATGTATGATTAATACATCCAAAATTAAAAGTTGTTCGACACCCCCCCCTCTAACCTTTGTCCACGGTGGAC<br>ACAATTTGTGTCCTGGTGCAATAAGTCTACGAAAAGCTTTAACTACGGGCCCCTGCTGCCTGTTAATTAACTAAACCACAA<br>ACTGTTGTCCGGCTTATTTTATTGTATTATGTAATAATATACATTATGTAACTA
MSTRG.1877	Uncharacterized protein LOC106139762	CAAACAAAAAAAAAATTCAAGTTGCTTTACAACATTAAATCATTCATTTAAAAATAATTTATATTTGGTACAGACTAATATT<br>GTTAAAGTTTTAGTTTGTTTGCCCTGGTTGCCTTGATTAAAGAATTTATTTATTTATTATTTATAAATAAGTACAACCCAATT<br>GAGAAGCACTTTTTGAAGTCGGTTAATAATGACGATGTGCCCTAGAATTTCACTATAGTAATATTACAAGCTAAAAATAAC<br>AAGGACAAATAAAAACAAACGTACGGACGGCGCGTTTGTCGGCTTTTATGTTATATTCGGTCATAAACATGACAATGTTCG<br>CAAAAAATAATAAAATTCCATGGAATCGTCGTCCAAATCTCATGTTGCATGATTCACGTACATATATTCAAAAGTACGTGA<br>TAACGTTCCTGTGCATGTCAGTGAGCGCTGCACCGCCCGCCCCGCCGCGCCTCTACCCGCAACCAGCGCCTGCGCAGCTAC<br>AGCAGATCAATCTTTATGACTACTATTTGGATAATCCAGAAGTAGGGAACTATTACAACCAAATACAACAGAAACAAGGT<br>A
MSTRG.1	CYP6AB46	GGGGAGCGACTGGGTTTAATGCAGTCTTTGGCAGGACTAGCTGCGATTCTATCACGATTCACGGTCTCACCAGCTCCGGAA

883		ACCAAGCAGTATCCGAAGGTGAACCCTACGTCAGGCATCGTACAAAGTATTCAAGGCGGTTTACCACTTTATTTCAAAGA<br>AAGAAAAGGGACATGATTTCGATTATACTATGCAATGTTAATAATAGTGGCCGATCGAACTTTCGGTTTCGGTTTCGGCTA<br>ATTTCGGCCAGAAATTATACTTTCGGCCTTAGTTTCGGTTTCGGCCGCGCCGAAACTTTCTGTGTGTTTGTGTTGTGTGTGT<br>GTCTAATTGAAAGTTGTTTTTTTTTTTAAGTTTACTTTCAAGCGTAAGTCATAATTTTAACATGTGTTTACATATAAATATAT<br>ATGACATATTTGTTACATACTTTAATTAGTGTTTATTATTACCCTTCAAATATCTGTGTGTTTGTGTAAATAATTGTATAAAA<br>ATATACTAGATTTCAGTATAGAAGAAAAATTTTAAATATGTTCCAAAAAGTTTCGGTTTTTGCCGAAATTGAGACCAAGGC<br>CGAAAGTTGGGTTTCGGTTTCGGCTGTAAAACCTGTTTCGGTTGGACACTAGTTAATAATACGAGTATTTTAGAATAAAAG<br>AATGCAAATATGTAAACAAGTAATGGGTAATTGTGATCGTCATTCATCAGGCGTTAATTGTCCACTGTTATGAATATAGGC<br>TTCCCCTTATCGACTGGTTTTGCCAGTAGTTGCCATGCTTGGCAGGCGGATTGACAACCGCAGTTAGATTTTGGTGATGTTT<br>CAAGAATGTAATATTATGTATAATTTAATCCGTTTCGACAGAAATTTATTTAAAATGTACCAAGTTTTGTTTAAATTATCAA<br>AGAAACACTAGGCATGAGTAGTATAGATACCGATCCAATATCTACATATATATTAGGTTCTCTAGATTTTTATGGCAGAAA<br>AAGGTTGACCCATTTTGATATCGTCCATATCGGCCGGATATCTATTTTCGAATATGTTAAAAGTCTTTTATCCAATATTATT<br>TATTGAATAACAGACTTTTAACGTATTCGTTATTGGACCAAAACATAGATACTATAACTAAAGTATATAAAAATTGCTATA<br>CATATATTACCAACTTCTAAGAAAATACTCTCTAAATGAAATATACGAATAAATTAAAATGTTTAATCAATTAATTTTATT<br>GTTAAAAGTTTTGTAAGCACACATTATATTTTAATTTAATAATAAAAAAGCTTGACTAGTGCTCCGTTACAGTCACTCGCG<br>ACTAAGGATCACGAATTGATTCCGAAACTAGTCGAGCTTTTTTCGATCTAATTACACGTAAGTTAAACCGGTTGTTATTAA<br>TTTAAAATAAATTTATTTTAACTTTTTAATTTTTTTTTGATGAGTGAAAATGATATATGTAGTAACCCCGCCCGCGCTAACG<br>GGATACGCTGGCGGAGCACCAGTGAAGGGTGATAGATGAGAATGAAAACAGGCCAGTTAGCCCATCATGATGAATTCATC<br>AGGAATTAACAATGATAGTTTCCAGGGGGAACTGCGAGGAGGTCAACCTGGTTGGGTATATTGCTAGCAATGGGATTCTG<br>TCTCCATCACTAACAATCCCTTTCCCCCTAACTTTTTAATATTAGATATTAGTAGACTGTCTTATGGTCTGGTTAATTATTTA<br>TTTGTTTATACTTTCATTAAATTTAGTGTTCCGGGGAGAGTTGATGTCAGACAGACAACCAAAAGTCAAAATCTTGAGTAT<br>TATAAATACAAATATATTCATCATCGTCATATTAGCTTTTAAATGTCCACTGCTGAACATAAGCCTCGCCCTAGGGGAGTTT<br>CGCTAGTAGTTGCCATGCTTGGCAACTGGATTGGCGAACGCAGTTCAGCTTTAAAAATGTTTTGAGAGGGACGCTGCTGTC<br>TATCCCTCGCCCTCCCTTAGTCGACTCTTACGACATCCAGGGAAAGGAAGGGGTGGCCTATTCTATACCGGGACCAGATGG<br>AACATTTAAAAACATATTAGGTTAGTTTTTGTTAAGTTCTAGTAATACACATCATACGTTTTTCTAATTTCAACATTAAATC<br>CAGCACACTACTGTCCAAAATAGGATAAGTTTGTGTCTTAAATATCAGTGCCTTCTTTAAGTAGTTTTTTTAATGGATGATA<br>CTGGAATAGCAACCCCGCTTGCGCTAACGGTATATACTGGGAGGAAAGGGATTGTTAGTAATGAGGTCAACCTGATAAGG<br>TATATTGTTAGCAATGGGGCCTCTTGTCCCATTACTAACAATCCCTTTCGCCCCAATTTAAGTGGTTAGTGCAGTTAGAGTC<br>TTATCATTTTAATATTTAACCAGTAAACATGGTAATATGATTTTATTTTCCGATTATGAACTGTATCATAGTCTTACTAGAA<br>TTCAGTTATTTATCTTTGAAATACAAGTATTAATCTATGAATTATGACTTTAATTAACAATGTATATATAAATAATGAATTA<br>TTACAGAGATTATTGTATGTATGTAGATAAAGATTAGCAAAAAGTGCTGATTCATTTGTAATTCATATCTCTTGTAAAACA<br>CTTTTATATTATATTTTATATATGTATTATTAAAAAAAGTGACGCTATTCTTATGTTGTTTAATTAAAAAAATATATTTAAAT<br>AGACATTTGAATGTATAACGCCAGAACGTATATCTTAAAGTTTTAATTTTTGTTTTTGTTTTTAACAAGCAAAGCTTTACA<br>CAGAAAAAAAAATTTACATTCGAACATGGAACCTTTTTTTTAAGTCGATTAAAAAAGGCGGCTTCAATTGTCAACAATTAA<br>TATGGTACCAATAGAGGAGTAATGGGGGG
MSTRG.1885	Chromatin complexes subunit BAP18-like isoform X1	CACCAGACTTTTCAATTAATCAGTTTGTTCTTGCTTGCTTAATAAATAATACTTTCAATAAAGATTTATATATACAAAATTA<br>AATAGGCAAATTGTGTGATAAATATTAATTGATAAATAATTATTTAGTACTGGTTACGGAACTGAAAGTGCAACAGATTTC<br>GTAGATATTCCTGCAGCAAGCATGAACAATTCGGCAGCAAAGGTCGGAGAGATCTTCCGAGAGGCAGGAATTGCTTTCAA<br>TAAACTCTCAGAAATGACTATGCTGTTACACCCAATGGGGGATTCACAACCAGGGTATGTCTTAGTTGAGCTATTTTATTT

MSTRG.192	Centaurin-gamma-1A	ATTACATTGTCTACAGGCAATGTGTATATTATGTATATAAAATTGGTAATCTATACAGCCATCTGTTTTGCAACAATATGAA<br>GCAATTGAATTGAACTTAATGGTATTCAAACTAAATGTTGTGTACTAATGTAGGTATATATAAAAATAGACTGCAGATGTT<br>AACTGTATACAATAGTGGGACACCTGTGAAGGATGAAATGATGTGGTTCAAGTTAGGCATACTTTTTTTAATGGGTGATA<br>ATGGTATGG<br>TAACTGTTGAATTTTTTTGGCACATTTAAAAAAAGACGTATAAAAGAGCTTATTTACAACTCTATTTAAATGAAAAATGAC<br>TCGAATTTGAATAGTAATATAATATTTTAATTTATTAAGGTGAAATCAAACTGGGGCTAATTTTTATTTCTCTTTTACAGC<br>ATAACGGCAACCCGAACTTTCCAGACGCGGACGGTCACACGTGCGTGTTCTACGCGCGAGCGGCCGCCAACCATCTCTCA<br>GGCATCCCCACACAGTACCCCAAGAACCTCCAACTCACCTGCCCCCTGCACCCCAACCCCCCGGCGACCTCCACGAACAGT<br>TCCAGCACCAGTTCCACGACCTCCACGTCGAGTAACAAAAGCTCTATAAAGAATCCGGAACCGGAATGTAACTGTATCAT<br>ATTCGAGTTACTAAACGCTCAGAGTGCTTCGAATGCGCTAGTGGAGTTGCTTATAGGACTGCAAAATAACCAGTACATGA<br>ACGGGTACGACGGCTTGAACGGCAGTGGTATAACAAGTTCGTTCAGCCACGTGACGTTGGGCCGGCCGTCGCTGGGTCCT<br>AATCTATCGCTCACGAACGGTAAGTGCGGCAGTATTGTTTAAACTAATTGTTTAAACAAACTGTTTGGAATTTGTGATCGA<br>TTCTCTAAGAGATTGCAGCTATATAGTTGGGTGCATCGAATTTTGAAACTTAACTTGAAGGTAATAGAGTCTCTAGTGCTA<br>AGCGGTCAGTATCGTCATTTCTTTTTAAATCTATCACGAACTTTGTTCTTCAAGTCATTATAAACCTTAATTTACAGATTTA<br>GAATTTATATTTTAATGTTTTTCTACTTATCGTAGCATTACCTTTTTTTTTTTAATGTAGATCCTAAATACGTGGAATATTTG<br>ATTGGAATGTTTTGGCCTGACGTTTTTAATCCTACGATTTCCAATACTTTATCACTTAATATTACGTACGTAATGTATTTGTA<br>ATGATAACTGACTGAAATGTTAGGAGAGAATTAATGTTATGTAGTTACCAAATTACTTAAATACACAACGTTTTTGTGCCC<br>ACCATAGGTCATGGGGACTTTACATTATCGAATAGTCATAGATTGTGAGCAGCTCTGCTCAATTTGTTGATTACTTCTGCGA<br>AGCTGCTAAGAGATTGTAAATAAAAATATGCGACTGACTGTCCAGTGCGCACTACATCTGGAATGTAATTCAAGTAATTCT<br>AAAAAAAAATCAACGGTCAATTTCATTAGTGAAATTTAGAAAGAAATATTTCGCAAAACCCGCGTTATAGTTCAAATATTT<br>GGAGAAAGTTATTTATTAATTTAATTTAGTTTTAAAAGTTACGGTTTAGGCGAATGTTAGACGAGCAAGCGGGTTATTGTA<br>AGTATAGTTAACCGCCCTGATTTTATTCTGGATTGGACAAAGTGCTGTATTACAGTATAAAGTGGTTTATGTAAATAGTTAT<br>GCAACTACAAGTACAGACAGCAAAAAAATTCTTTCACCAATCTCTGTGTTTATTCATAACTGTGAGTTTTTATTATGGAAA<br>AAAAACATTTTTACATGTATATTTTTGTATATATATCAAATTTGCGAATTTAAATTCACAAAGCCCGAAAAATATGTCGTCG<br>ATATTACGATAGCTCATGGAAAATGTATACGTTAATTAGACAATTTGATGGGCAAAAAACTAGTCTAACACGGAAGAACA<br>ATCCGGAATTTACCGGAAAACATGTCGGTATGTATTTGTATAATGTATAATAATGATAAGGAACAAAATGAATGAAATAT<br>GAATTTGCTAAAACTCCCGATTGAGTTCCAACATTTCCGAATAACAGTAAAATAGATTTAGGTATTTATAAATTATAAAAT<br>AGTTGCAGGATCTCAGCTCAACGCTACTTAGGGTAAATTAGGGTAATTTATATTGATATTGTTGCTTACCCTTCATCCCTTG<br>GGTGTATTTGTGTACTTTGTTACTTGTGTATTTGAGTACAATATTTCTTCTACGTAGATAGGAACTTATTTCTGCGTTATAAG<br>ACGTAAACAATACGTAAATACAATGTAGAAGTTGATGTAATTTTTGCTGCAAGAATTTTCTTTCCAAGTCTGCGTTTTATGT<br>TAATTTTGTAATAGAGCATTGGAATTTAGTCAGGTGTTCGATTTTGCACTTTAATGTAATGTCAAAGAGTTTATAATTACTT<br>GCTAGCATGTTTTTATACTGGTTCTTATTAAAAGAAATAGCAGTCTTTTCTAGGAGACTTGAAAAGTAGAAAAGAATGCTT<br>TGACATATTTTGCTACTAGTAGAAAAAAAATGGTAGTGAAACTATTACAATGTATTGTATTACTTGTAGTAATTGTTTCATTT<br>TAAACAGTTAATATAATTATATAAATTGAAAAGAGTTGTAGCAGAGCCATCTAGTGGAAATTACGGCAAGTTAGTTACAA<br>ATAAATTAAAATACATTCCATTAAGATAAATGTTTGCCCAAGGGTCAATATAAAATAGTTGATGTGATATACCTAAGGTTT<br>TCCCGTAATAAGCCAAATGTACTGAACCCTCCTGTGCCTATTTAGGTAGTTTCGCTTGATTACCTAATAATAGATTGAGAG<br>CCTTCATAGATGAGAGATCTAAAAGAAACCATTCATTTATCGACTTGATTATAATGTCAAATTAGAAAGTACTTCTTATTTC<br>CTCTCAAATATCAACCTTATGATGTTAAATATTAAGGTTCGATGTTAAATGGAAGAAGTTGAATTCAAAGTACTTGAATTA<br>GTTCTTGATCTATTATTGAGTATTTCTGATTTATAATAAAACTTCTAATGCATCTATTAGTGTAATTTTGCAAATGTAGGAT

		AAATTACATTGTAATTGGTTTAATTCAATCAATAAGTTTTTATTGTAAACGTAACTTTTCAGTCGGCTACATAGCATTCTGT<br>ATTTTATTCTCTTCAATATAATAGTTAGATTTGTTTGTTACATCTTTGTGTAAATCGATCGATTGAATATAATATTTTTTCAT<br>TGTTACAATAAAAAAAAAAAATCTACTTTTATTCTGTTAAAAGTTATTCGTGAAGTTAGCTCGTGTACTTTAATTATTACAAT<br>GTCGATTAAATCGTATTTCTTTTTACTTAAATTTTGTAAAAATCTAAAGGATTGTTTAAGTTTTTTTATGTAAATGTTAGCAA<br>TAACCGATTACCGACGTCAGTATTTGATGGTATTGTATAGAATGCACGTTTCGAATAGCGCACTTTTTCCTTTTAATGTAAA<br>ATGTATTTTTAATACTTATGTTGATATTTTATTGCTAATTTTAAATTCGTTACCGACACCTTGGTTCACAGTTTTATTGACAA<br>TTATTTTTTTAATATGTTTTTTTTTTGTTTGTGTATATTTTTCTTTTTTGTACAGGCCAAAGAGCTTACAATGTAAATATTGTA<br>TATTGCGGCTACGAGTTC
MSTRG.1921	Protein FAM102A isoform X2	AGAAATGTGAAGATTTGCCTGAGCCCAATGCGGTGGGTAATCAATATAATGACTGGCCGGTGCCTGTTGCCGCCTGTGAAT<br>CGATAGCCAGCTTTACTAATGACTCCGGCAGCTATGCGACTGTGCCACGACACGCCCGTTCGAAGGCGATGCAACCCTGAT<br>GCCAAAATAATCACTCCATATTTACTTGATTAGTATATTTTTAAATATTACCAAATTCATTTACAAGTGCGTTGTTATTAA<br>CACACATGTGAAGTGAAGTGTTTGTGTTGGTGTGGTGTGTTTGTATCTGGACTGATCCGATCGGAGCCGGATTTGTGTAGT<br>GCGGGTCGGTGGACCGCGGCCCGATGTTGGCGGCGTCATGGCGTTCATGACGAAGAAGAAGCGATACAAGTTCGGTGTGC<br>AGTGCTGCCTCGAGGAGCTGACCGAGGTGCCGTTCGTCTCGGCGGTGCTGTTCGCCAAGGTCAGGCTGCTTGACGGCGGC<br>AACTTCCAGGACCACTCCAGCAGAGAGGAGGTGCGGAACCACGCGGTGCGCTGGAACGCGGAGTTCTCGTTCGTATGTAA<br>GATGTGCGCCAACGCGAACACGGGCGTGCTCGAGCCCGCGCTCATGAGGGTCTCCGTGCGCAAGGAGCTCAAGGGTGGAC<br>GGTCGTACCAGAAGCTGGGCTTCTGCGACGTGAACCTGGCGGAGCTGGCGGGCGCGGGCGAGGCGACGCGGCGCTGCCTG<br>CTCGAGGGGTACGACCCGCGCCGCCGCCAGGACAACTCGGTGTTGCGACTGCGCATCAAGATGAACATGATCTCAGGCGA<br>CCCGCTCTTTAAAGTTCCGGAGCGTAAGCAGGACGTACCAGAGACCAAGGGCGGCGGCGACAGCGGGTCAGAGAGCGCG<br>GCCGGCGCCGCCGACGACGACTGCGGCTCCTCCACAGCTAGTTCCGGCTTTGGCTCGCTCACCAAGAAGAAGAACTACGA<br>GGGTTGCCACTCGCAGCAGCTGTCGTCACTGCCTTCGTACGAGATGCCGTCGCCGGATGCGGACGAGCCCCCGCCGCCGCC<br>GCCGCCGCCGCCCGCCGCACTCGTGGCCGAGTACAC
MSTRG.1928	Protein crumbs	CGGCGGCGGTGGGCGCGGGCTGTGCGCTGGCGGCGCTGGTGGTGGTGGGCGGCGCGCTGGCGGCGCTGGGCGCGCAGGC<br>GCGGCGCAAGCGCGCCACGCGCGGCACGTACTCGCCCTCGGGCCAGGAGTACTGCAACCCGCGCGCTGAGATGATCACGC<br>ACGCGCTCAAGCCGCCGCCGGAGGAGCGCCTCATATAATTTCACAAGACTCGGCATCATCAAGGGCACGGATACATTGAA<br>TCAGATAATGAGACTCGTCGCATATCAACCAAATGTGTATGTGTTGTGTGGCTGTGTGTGTGTGTGTGTGCGCGTGGATGG<br>ACGCGTGTGTGCATACGCTTGTTATTGTTCAAATGTTTGTTGCGCGTGTGCGTATAACGGTGTACCTGCGTTTTATATAGCG<br>ACGTGTGTTTGTATGTGTGTGTGTGTGTGTGTGTGTGTGTGTGTGTGTGTGTGCGTAATTTAATAATTAGTTTTAAGGTTCA<br>CAATAGAGACGTTTTTGTTATAAGTATTGTGAAACTGTACGCATGTATCACAGCGGGCGCTCACTCTACTGACACGATGAC<br>GTGAAGCGAGAAACAAATATATATATAGTAACAATTTTATATTAGATAAAATATAGTATGTACTTAGAGATTGATGTAATG<br>TGACTAACAGACATGACACGTGCATGATTATTTATTATTATTATTATACGGAGACAGAGGAACGTTGTGCCTGAAATTGCA<br>ATTTTTAAACTTTGTACTTACAATTTATTAGATTAAGAGGGAAATTTTAAAACCTTAAATATTTGTGTGTGAAAGGATTTTG<br>AGCGTGGTACTAGCAGAGACCAGAGGGCCTACAATGAAATATTTTCCGAAATTTCGGGCACGAACGAAACAGAAATTTGA<br>TGTTAAAATTACATAATACATGCCGTTTAAAATGATTAAAAATATTAAAATATCGTTCCTCTGGACTATTACTGGATAGGA<br>TTTATGATGAACGAAATGTTAAGCTCCATTTACTGGCTCGATTTCGGTACGAACAAAAACACGAAATTGAGCGCGAAAATT<br>CGGATTTTCGTTTCATGGTAGACCACCAGGGTACAGTATTACAAGCAGCGTGACAGCCGACCGGCTGAACGAAACGGTAC<br>TTCGTCATTCATACAGATTGTAACATTCAGAGGTACGAGCATACTTTTTAATTGTAGATTGTATTAAATATATGAATTTACA<br>TATATTGCAACTGAAGGGCGTACACTTCGAGGCGGGTTCGACTTGTGACGTGACAACAACGCTGCAACTTTAAAAAAATG<br>TCAAAAGCATGCTTTTGTAAACGAAAAAAGTTATCGTAGAGTTCAATCTATGGTATCTAATGATAAATGTAATGTACATTT

		TACTAAAAAGTGTCCAGCCACACACACACGCGTGTATTTTTTATATACAAATGTTATATGTTGTGGCGGTTGTTAGCTGTG<br>ATAGTGGTCGGCCCGCCACCGCCTCGTATTCTTAGCGTATGTAAGTGTAAGTTGAAATAAAAAGCGTGCAACATTCTAAAT<br>TACATTGTAGTGAAGGCGGCTATCGCCCGTCAGTCCCGCAGCGGCCCCGCACTCGCGCCCCCCCACACCCTACCCCCCTCC<br>CCGCCGCCACCACTCGCGTATTGTAAATACATATATTAAAAATTATATTTAAATATATTACATAGGCGACGTTCTAACGAT<br>ACATAGACAATTGTTCATATTGCGTTTACTCTAAAAGATTATAGTAGAAAATTCATTACAAATACAAACAATTACAAGAAA<br>TGTTCATAGTTCATACACTGACTCGTCGCTAAAAGATTTATATAATCGGGTCTTTTTTTTTTTATACAACATAGAATGGCAA<br>ACAAGCTGACGGCCCACCTGATGGAAAGTGGTAACCGTCGCCTGTAGACAGACATGAGCAGTACCGTGGACATAACAGA<br>GTATACTGTTTCGCTCATGACACCCCCCATGCGTTGCCGTTCCATTCTATTGCACACTGGTCGCACCTATAGACACTCCGTT<br>TGCGTTTTATGTAATCCTTTTCTGTATATTCGCAAAGATAGTACTGTGTCTGTAAGTCGGTTTATTTGAGACCACAATCTTG<br>CTTTTTTTTTCTTTGAGACCGTTAAATTTTAACATAAGAATGTGAAACTGGCACTAAACGCGGAGCTAGTTGTGGTATTTTC<br>ATGTTAAAAAATAAGTAATTTATATCTACATCTACACTAATATTATAAACAGGAGAGATTTGTATTTTTGTATGTATGTTTG<br>TAATGAATAAACTCAAAAACTACCGATTTCGAAAATTCATGTGTCCAACTATTATCGTTATGTCACAATAACCACTTTTTA<br>AAATGTTGTAAAAATGTCCACCCGTGCGAAGCCGGGACGGAACGCTAGTATTATATAAATGAAACGATTATAGTAATTTG<br>TACTGTGCAGTAGATCATTTGGTCCGTTTACTTTCCCAGTTTGATATAATTACAATAGTGAAATGTTAAATATAATAGTAGA<br>GTGGCGGTAGAGTAGCGGCGGGGTGCGGGGCGCGGGGTGAGGGGCGCGGGGCGCGGGCGCGGCGGAGCGGCGAGCCGT<br>CCGTGCCTTAACCAAAAGTCTGTATATTTGAGAGCTGTATACACTTAGCAATAAGTTAACATTTACTATGTAAACGTATTA<br>ACAACTGTAGTATTAAGTACAGTGAGCAGATCCACTCGCCTGGAGACTAGCGCCGCGCGGCCACCAGGGGGCGCCTACTT<br>ACTGCGCAACCTGTAGTATTATTGTCCAGTACTTACACGGAACTTGTACTTACGCTTTATACTTACGTTGCTCAACACACAT<br>TCCAAGGCCCGCGAGGCTTGTATTGTATTGTTTTTATTGAATGAAATCTGTAAAATATAAATTGTAGACGTTTGAAGAAAG<br>CGCGACCGTACGGTGTAAATATCACGCACCATCACAACTGGACACGTGACACGCGTGCATGGTGTATTATATGGTGTATA<br>AATATACTTCTTTATTCCTAAACTATCATAAACTCCTGGTAAACTTTTAACTAAAAGGCATTGAAAATGTGTCAAAATCAA<br>AGTAGTAATTTTTTACGATAGAGACAATAAAATTTCTAAAGACTGGAAATATCCATGGGCACAGATTCATTTTCTGCGTGT<br>TCTGTAATTAAAAACTTATTGGCCTACCCTCTTTTGAAAACGTATCATTGTACGCATGTTTCTTAATTTATTTTTCCATACAT<br>TTTTCAATTTCATTCTCACGCCATGTTTCTTCTTCGTGTGGTCGCTCCTCGACTCGTTCGCGCTTTCTTCACACGCTAGTGAT<br>AAAACGTGTAAAATATTGTGATACGTATTTTTTTTACAATCAAGTTTTATTATTCGATTTTTTTTTTGTATTCGTTGCTGCAA<br>ATAATATATTTCGGCAGCTCAATTTATATCAAATTGTTATTTTACAATAATAAAATAAATGTATTGTGTACATTCCCATATT<br>TTAGTAGATAGAATATTATATTAAGACATTACATAATGAACTATGATCAAATTTATATAATAATTGTACATAGCCTTATGT<br>CGTATTCAACTGCATTCGGAAACTCACTGCAAGCAATGTTACCGTCCACAGTAATTCTTGTTCTACATGTGATTAGTTCGTG<br>TGTTATTGCACGCCAGCCTATTGGCCTATTGACTCAATAAAGGGACTTTCTATTGCTAA
MSTRG.1971	Endonuclease and reverse transcriptase-like protein	GTCATTAGCGATCAAAGATGGTGTCAGAAGAAAAAACACAAAAAAAAAATCTTAGTGGAAATCAAAATTACAGTAAATAT<br>GTAGGTTTGTAATTTCAGAAAAAGATATTAAAAATGATCCAGATCATATTAAGGCAATAATCCAATTAGATGTACCAAAG<br>AATATAAAACAGTTACAGAAGTGCTTAGTTTCAGACGACTTTTTTGATGCCAGTATACAGTCTAACGATAATCGCTGTAGT<br>CAGTTATTTTCCTGAAAACTACTGAAAACACGTTTGAAGATGTAGCAGCTGTCAAAAATGATATCATCTTTGAGGTTATTG<br>ATAATCAAATAATTTCTGCAGTGGATAAAGATATGATTATGTTAGAGAGCGTAAATGATTCTATTATACTTGCACCAATTT<br>CTCACTCAACTCCTTTTACCAGTCTTCAGTTTCTAACGGCAATAATAGTAGAGAAGTACCACAGCCTCAGGTATCGACAAA<br>AGCAACTAAAAACAGAAAAACAGACGGACGTAAACAAAAGTTTCCACAAAGTAGTATTGATTTTGATAAGATTGAAGTAT<br>ACGATCCCGACCCAAAGCCAAATACAGCAATACCTAAGAATCGGAAAAGAAAAACGCCAGCAAAAAAGCTTCCAACAAC<br>AGCTGCAGCAAAAATGAGTTTACACAAAGATGCAGTTGAGGTAGTCGATTCTCCACAGAAAACTATTCAAGGTAGATGTA<br>GTACCAACACTTCAAATGATGGATCACAAAAAGACAATAGTTTAAATATTCGGGTCCATGATCAGGACTGTATCCCTGCA

		AGGATATCAAAGAGTACTAAGAGAGCTGAAATAACTGCCTTTAAAGATATCCACTTACCAGTTCTATGGCCAGAGGGCAA<br>TAGCTTCATCAACAGCAAGAATGCAAGATCTCCGTGAATTATTGAAGTCTGTACCGTCTGACTGTCATGAATTTTACACAT<br>TTCTAAACAATGTCAAGTCTTCGGATTTTATTGATGATGTTGAGGGGTTTGGAAGTGCTATCGATTTCAATATACAAGAGG<br>AATGAAGACTGTACTTGAATAAATCCCTCTTCCTTTACAATATTTTTTGTTGTCAATTCAAAAAGTAAATTCCTTGTCAACT<br>CTTTGATTTAAAAACTCTTATTGATAATTATTTTTCACGCCATTTTTGACAATTATTATTGGTTATTTGGGCGAAACAGTATA<br>CTCTGTTATGTCCATGGTACTGCTCATGTCTATAGGCGACGGTTACCACCTGATGGTAAGCGGTTACCGTAGCCTATGAAC<br>GCCTGCAATACCAGAGACATTACGCGCGCGTTGCCGACCCTGAAGAAACCTCTTTACCCCCCTTTTGAAAAACCCCATGCT<br>GTAGTCTCTTGGGAAAACCTCGGCAGGGAGCTCATTCCATAACCTGAGTGTTCGTGGGAGAAAGCATCTCTGAAACCGCG<br>TTGTACGCGACCATTTAGGCTAAAAGGTATGAGGGTGAACTCCCTGTCGATGGCGAGGCCGTACGATGATAGAAAGTGGC<br>CGTAGGCATCATATTGAATAATTCCTCTGAGCGCAATCCGTCGTATAAGCGGTAGAACACGCAGAGTGAGGC
MSTRG.1973	Spectrin alpha chain isoform X4	GTAAGCATGAAGGTCTGGAGCGCGACCTGGCGGCGCTGGGCGACAAGATCCGCCAGCTGGACGAGACCGCGAATAGGCT<br>GATGTCGACCCACGGCGACTCGGCGGACGCCACGTACAGCAAGCAGCGCGAGATCAACGAGGCTTGGCAGCAGCTGCAG<br>GCGCGGGCCAACGCCCGCAAGGAGAAGCTGCTCGACTCGTACGACCTGCAGAGGTTCCTGTCGGACTATCGTGATCTGAT<br>GGCGTGGATCAACTCGATGATGGCGCTGGTGAGCTCCGACGAGCTCGCCAACGACGTCACCGGCGCCGAGGCGCTGCTCG<br>AGAGGCACCAGGAGCACCGCACGGAGATGGACGCGCGCGCGGGCACGTTCCAAGCGCTGGAGCTGTTCGGGCAGCAGCT<br>GCTGCAGGGCGGACACTACGCCAGCGTCGACATACAGGAGAAGCTCAACAACATGGGCGACGCGCGCCAGGAACTGGAA<br>AAGGCGTGGGTGGCGCGGCGGCTCAAGTTGGACCAGAACTTGGAACTGCAGCTGTTCTACCGCGACTGCGAGCAGGCGGA<br>GGGCTGGATGGGCGCGCGCGAAGCGTTCCTGGCGCCCGTGGACCACGCCGACAGCGCCGCCGAGCCGCACGCTTCCGACA<br>ACGTCGAGCAGCTCATCAAGAAGCACGAGGACTTCGACAAGGCCATCAACGCGCACGAGGAGAAGATCGCCCAGCTGCA<br>GACGCTGGCTGACCAGCTGATCGCGGCGGAGCACTACGCGGCCTCGCCCATTGACAGCAAGCGGAGCCAGGTGCTCGACC<br>GCTGGCGGCACCTCAAGGAGGCGCTCATTGAGAAGAGATCCAGGTTGGGAGATGAACAAACACTGCAGCAGTTCTCGCGC<br>GATGCCGACGAGATGGAGAACTGGATCGCGGAGAAACTGCAGTTGGCCACCGAAGAAAGCTACAAAGACCCGGCGAACA<br>TCCAGTCGAAGCACCAGAAGCACCAGGCGTTCGAGGCGGAGCTGGCGGCCAACGCGGAGCGCATCCAGTCCGTGCTGGCT<br>ATGGGCGGCAACCTCGTGCAGCGCGGACAGTGCAGCGGCTCCGAGGACGCTGTGCAGGCTCGTTTAGCTTCCATTGCGGA<br>CCAATGGGAATTCCTAACGCAAAAGACGACCGAAAAATCACTAAAATTAAAGGAAGCCAACAAGCAGCGCACATACATC<br>GCCGCCGTCAAGGATCTCGACTTCTGGCTTGGAGAGGTGGAGAGTTTATTGACATCGGAGGACTCTGGAAAGGATCTCGC<br>GTCGGTACAGAATCTTATGAAGAAACATCAGCTGGTCGAGGCCGACATTCAGGCGCACGAGGATAGAATCAAAGACATG<br>AACGCGCAGGCGGACGCGCTGGTGTCGAGCGGGCAGTTCGACAGCGCCGGCATCGGCGCGCGCCGCGCCGCCATCAACG<br>AGCGCTTCGAGCGGGTGGCCGCGCTCGCCGCGCACCGCCGCGCGCGCCTGCACGAGGCCAACACGCTGCACCAGTTCTTC<br>CGCGACATCGCCGACGAGGAGTCCTGGATCAAGGAGAAGAAACTACTGGTCGCTTCGGATGACTACGGGCGTGATCTGAC<br>CGGCGTGCAGAACCTGCTGAAGAAGCATAAGCGACTAGAGGCGGAGCTGGCGAGTCACGAACCAGCGATCCAGGCCGTG<br>CAGGAGGCGGGCGAAAAGCTTATGGACGTGTCCAACCTGGGCGTGCCCGAGATCGAGCAGAGGCTGCGCGCGCTGGCGC<br>AGGCCTGGGCCGAGCTGCAGGCGCTGGCCGCCGAGCGCGGCGCCAAGCTGCAGCAGTCGCTCGCCTACCAGCAGTTCCTC<br>GCCAAGGTCGACGAGGAGGAGGCCTGGATAAGCGAGAAGCAGCAGCTGGTGGTAGTGGGCGAGTGCGGCGACAGCATGG<br>CGGCGGTGCAGGGGCTCCTGAAGAAGCACGAGGCGCTGGAGGCGGAGCTGGCGGCGCGCGGGGAGCGCGTGCGCGAGCT<br>GGCGGCCGAGGCCGAGCGCCTCGTCGCCGGCGGCAACCTGCACGCCGACGCACTGCACCACCGCAT
MSTRG.202	Cuticular protein RR-1 motif 54 precursor	CCCGCCGCAGCCGCGACCACAGCCGCGGCCGCAGTACAGGCCTGCACCACCACCGCAACCGCAGTACAGGCCCGCGCCGC<br>AGTCTGCTCCCACACCACCTAAGCCTACATTCTTCGCCGGCGCAGCGCCAGCACCAGTACCGGTCCAAGACAGCTTCTTCA<br>GCCCTTCACCCCAGCCGGCCAGGCAGTACAAACCGCAACAGGACTTCAGACCAGCACCGCAATTCGCTCCCAAACCCCAC

		ATCGAGTACCAAGACTACGAGGAACCAGCCCAGCCCAGATTCCCTTCTGCCAACCAGTTCGCTCAACAAAGAAGCTCGCAGCCGTTCTCAATGCTGGATCAGCTCCTGAAAGAATATTCCCTGCCTCAAGGAGGAGCAGCTCCCCTACACGATATCACCTTTGGATCCTACTAATTTAGG
MSTRG.208	Squamous cell carcinoma antigen recognized by T-cells 3	TCGGACAACCCAAGAGGGACGCGGGGGGCGGCATGCGGCGAACACAATTGAGCAGTTTCATACCGAGCGTTCTACAGAGACCATCAACCAGCAAAGCAGCGACCAACGGCGACCACACCAACGGGGAGAAGCGGCCGCTCAACAACAGCGACTTTAGAAACATGCTGTTGAACAAATGAATAGTAGTAAGGCAACTCACTAACTCGTAAGGAATGTTAAAATATTTTTATTTAGAGCATTGTAG
MSTRG.2081	Ras GTPase-activating protein nGAP isoform X4	GTGATAGGTATACACCTAGATAAAGAACAGAGCGACAGCAATATTGGCAATTCACCAATTTCAATCAGTTTTTGAATGCGAGTGTATGTTGTGGTTGTGTTATCGGTTAGAGTATTTTTGTGAATATCACGTGTGTAAATGTTTTTGTTATGAATTAGATACATTAACTATAGATAACTGAAAAACATCAATTTTATGGACTTTTATATTATAGAAGTTGGTGAAAGTAAGGCGCGGCGGTCGAGGACCCAGCAGCTGTGACGGCTGTTTCAATCTGATCGACTGTTACTAGTGTACCTATAGGATAGTGGAATATGTAAAGTGGCAATGTGTATAGTCGTACCTACCAATACACATTTGGTAGTGTTTGTTATTGTATTGTGAAAGTGTGCGAAGATGAAGGTCGATGATTTGCTGGTGGTGATCCCGTACCCGTGTCGTGCTGAGGGTTGGCTTGACCGATGCGATGTAGCGACAAGTGCCGTGGGCGGAGCAGCGGTAGGGGTGCCACCATCCTGGGAGCCGTTTTACTGTGTTCTGCAGCAAGATCGGCGGACCCTTACCTCGTATA
MSTRG.209	Dual serine/threonine and tyrosine protein kinase-like	AGAAGGCATCGCATGTGGCCAGGCGTGGATCGCCTCCGGCCGCGATGGCGGGTGGGTGCGCGTGGTCGCGGCGCGTGGCCGCACGCGTGCGCACCATGCGCAGTTTAGTGCGCGACACGGAACGCGCACTGCGCGATGTTGTTGACATACTCAGCGTTGATCTGGACATATCTGTAACAGTCCATGAAATCGGACACTTGTCAGCCCGCTCCACAGCCTTGGTCATATTCGGCACCAGTCCATCGGCGCGAGCACGTCTACTCCACTGTCTGCTGGGCAGGAAGCTGCTACCGGAACCGACTCCAAGAGGCTGTCGATGGCTGCGTATCCAGTACGGTAGTACGACGCAAGTGCACCTCACGCTCGGCAACTCGGAGTTCGAGTTTGTCGAAGAGTTGGTATGCAACAAGGGACCATGGGAGACACTGCCCATAGAGGATCTACTCCGCCAGGACCAAACAGACCTGGCCACGATGCTAGAAGTGGAATTGAGCAATACATTCCTGAAAGATGGCCTCCGAATCGTCGTGCCCCCCGATATCTGCGACCCTGGATCTTTAAATCTCGAAACCCTTAAGAAAATGCACTCCGAATTATACTCCAAACGGGATCTGATACTCAAAAACTTTAATCCGGTCTACTTATATGCCATAGACAAATTAGGGAAGAGTGTGTTCAGTGAAAACTTTGTCTTTGGTTCTATGGTGACGTCACACAGTGAGGAGGATTTTTGGAATATGTTTAATTTGTACAGTGGGAACAATGGTGGTAGTGATAAGAGTGTTGTGCCTGATAAGGAGATGGTTGTGGAGGGGGGCGGGGATGATGCCGTGTTCTCGCCTGAG
MSTRG.2105	Dynamin	AGTGTTGTTGTCGATCTTCCATTGACCGAATATCGATGCGCAGATGCTCGAAAAGACTACGGACGCGAGCATGTCGTGGCTTTATGTTGACGTCATGATTGATTTTCCTTACAGTGACTGGGAGTTACTTGTATGTTATTTGATATATTATTGCTTGGCCGCGAATGTTCAACGACTAAAGATCGGCCTAGTGTGGCATTAGCTATAATCAGATTTTTTTTATGTTTGGCACAATTAGTAATCATTCAGCATAAATAAGGTTCTCGATGGTTCCTAAGCTAGTCGAGTATCAACAAAAATACACACACAAAAAGATGTTAAAATGCTCATCGAATTACACGCAATAAAAAATTTGATGTTGATACTGAAATAATGCGCACTATTTTTAAAATTTCAAAATAATAAAATCAGTTATAAAAATAAAATAAAGAAGAGGTTGTTTTATAGTTCTGCGAAAGAAAAAAAAAAAGTTGCTGACATGGTTAATTTAATGAGATACAACAATAATATCCAACATATGTGTAATCAATTTATACTATATTTTATTCTATGTGCCAAATATAAAAAAAAGATTATATAACCTGTTGGCATATAAATTACAGTAGACACTTAGCAGAAACTAAAATTACGCAGTAATAGATTGCACAGAGTATTTGTTATTTTATGCTGTGTTATTTAGATAAAGAGACATGCCAAGATTTTTTATTATATTTGCCAAATAAAAGAAAAAAAATCTGCCTTTTAAAATTTTTCAAACGATTTGAAAAACAAGATTTTCTAAAATGAATAAATAACAACGCGAAATCGAACACCTGTAATTTATGTCTGTGATGGTTTTTGCGGGTCGTCACTGACGACTGATCGTGTCTTTTGACACAAAAAGTTGTTCATAGGTCTGGCGATATCGGGCAAAATACACAATGTTTCGTGATGCCCTGTGTTGTAGTACAAATGCTGCTTATTGAATTCACCGTTTAGTTCGTAAATCCATAGACTGCGGGGTTGACGTCACTGATGTGCAACCTAGTCTATACCTGTGTATAATGTGAATTCCAAA

AGTAGGCCTAAGGGTCTGGTTTGTGCTTTATGTGTTGTACTTCGCAAGAGATAGCGTACGGAGTTAGTAGAAAACAGGCGT
ATTTGTTTTTGTGAAATGGATTCGTCGATATGTGTGTATGTTTGTTTGTATTCTATTAAGCTTTATCACCGCAGTATATGAAT
CATTAGATATGTTGAGATAATGCTACAATTAATTGGGACAATGACAGTTAAAAAAATATTAATCGATTTATTGTTTTAAAT
ATCAACATAATTAAAAATATTTCACCATTTTGAATAATTATTTTCGTTAGTTTTTATATTGTTAGCATTAAATTCGTGTTCCT
TTCTATCTCTTTGTTGACAGATCAATGTTGCCATATTGCTAATACTTTGACGTTGGCAGGTGCACTTATAATCGAATTTACT
GTTGAAACTCTATTTAATTCTAATTTTGCGTTGTAATATAGCGAACACTACACGTATATGATAAAATGTTTTCATTGGCATA
TTTTATTTTTATAATTAAAAGTTATTCCTTTAATATGTTTTATGTAATACCTTCCATGTTTAGCAGATCAAAATATTTATTTC
TGACGTAATGAATACTATAATGTTGTAGTACAATTTATCTGTTGCGATTCGTTCGTCTCGTCTAGATGATATATTTAGGATA
TTTAGGAAAGCTAATATTTTGTCTAGAAAATAAGAGTATGCCATCTTTTAGGTACAATATGCCTGGTGTGCCAGTTTTGCTT
TTGGTTTTATATATTAATACATTTACTAATATATATAATAGAGTAAATAATAGAGGCATTTGCGTGGGTAATGTAACAGCC
ATAATTATGCATTTAATAAGGTATGCTTTTATATTTATCATATTTCACCTATGTATGTATCATGTTTTGTATGGGAAGTTAAT
ATATGTTACAAATATTACCTAGAAAAAGAAGTCAATATCAACTTGACGGTAGAAAAATTGAAAAGGATTTGTATTTGTTTG
TGCTAATAATGTTCTATGAGTAAATATTCTAACATCATTTATGTATATATGGTATTTAAAAATGTACTGTAAAAGATTAGG
ATAATAAGATTAAATGCGTTTTGAACTGATGTGAGAATAGTGATATGATTTGTAAACTCTTCGATGAATAAGTTTTTTTTT
TATTAAGATAAGAATTATATCTATGCATGCTTCATATTCAAAAAGATTTTTCGTATAAACAAAATTTCGTGGCAGCCACGA
ATTATTCTATTTATTCCTCTATAGATTTAGTTCAATAAATATCGTAGCTGATATTGTTGTAGAGCACTTGTAGAAATACCAA
TTTTTTCATGATTTTGCAGTACATATTTATATATCATATACAAAGGTAACCATTAGCACAAGCAGCGCATACTGAGCTGTAT
GGTTGTTTTCGCAGGCGGCGCTAAACGTGCTACACGGCGACCTCACTAACTAACTAACTTAAGTTACGTACACAAATAAGG
TACTAACTCTTATGAACTTGTTAACTTAGTACATTGGTTCTACGTTTCAGCATGCGGGACCTAACGTTGTGTAGTTGCATAT
ATCAATTGATAATATCGAAATGCCTGAATGAGCATACTGATACATTTTTGACATTTGCATAATTTTGAATATCCAATAAAT
AAAGTTATATTAAACATAAAATATTGTAGTAAGTTAACAAATGTATCATGAAGTTTATAAATAATTATACATATGTTATAT
ACAATATTATTAGTCATGTAATTGATACTTATGATACGTCTGAAAGAATATTGCTTCCATAAAAATATTAAATACATAGTG
AAATTTATGAAATAATCTACTTGTAGTATACAAATAGTAAATTCTGCTTATTATTCTCCTGCAGGCAGTCATTCAAATGAA
GTCATCTGTAACCACGTACAGTATAGTACAGTAATATTACTTAGAATATATTTTTTTCAATATTATTTTGTTATAAATATAA
AGGTTGTGGACTCATCGAAAACTATAAGGGCTGCCTGCAGCGAGACGATGTTTCAATATTATAGTCCAAATAGGTGTCTAT
TCGATATATACTATCTTTTACTTGTACGATGAGCTAACGAAACGAGGGACAATGTACGACATTACAGTCGTGTTCTGGGAG
TGCGGTGGTGTAGTGTCAGAAAACATACTGTCGTGGTAAAATCGATCGTCTATATATTACTCAATAAACGAATAGCTCTTA
AATTACGTATAAACAAGTAAACATGAAATTAGTACCGAATTATTTGCTTCTAGATCTTTAAAAACGAGGTTTGACCTCTTA
TTGTACCAAAGATAATAAATACCGAGTTCCTAGTACACGACAGTTATATTTTTTCTATAAATTTATTTTAAAATATTTTAAA
GAGTTATTATTAAATAAACACTGCATGTGTTAGCTTTAAAAATAGTGTGTGTGTGGTCTTCATAACGCCTATGTGTCCAATG
TTTTTCTAGAATATAAAATACTTTTTATTTATATGTTAATTTGAACTATATTTGAAGGTTATTATTGCACTAATATAAATGTT
AGAGTGTTTTCTTTTTACATGTCATTATTAACAATCTCCATGTGTTAACTACCTATACTTCATCTTATTTTCTTAGTAGTGTA
TTTTTGTAAATGTTAAGTGTGAATGAAATATATTTTATATACCTATTTAAGTACATTTTTCACAGTTGCCCTGTAAGAATTA
TATTTTGCGCATATTGTACATTCGTTTTGTATTAAAATATTTATTGCGGTTACATTACCCCGAAGTGTCTTACAGAGAAGCT
GTGAGGTTTTCACTCACTTGTATGCACTTGTTGTTGTTATTACGTATATATAAAGCAGCGTCTTTCTTTGACCATTTCATATA
CATGTCATCAATCAATAGAAAATTCAATATCCCAAGGCTCTACAAATATTTCTGTGTTGCAAAATTAATTATTTGCAAGTA
AATTACAATTAATTTTCTGTAAATTATTTAGATTAAAAACGAAAAAGTTTAGTAATCCGTATTTTATATTAAAAAAATAGTT
TTCGAGAAAAAAAAAGTTTAGAATGGCCTACCCTTATTGTACATATTACACACATACATATATAATATGGTTTACATGTTTC
GTATATTTCCTCATATATATGAGTTAAAATAATAAAAATACTAGGCGGCCGGGCGGCGGGAGTGCGGTGGGCGCTGCCAT

GGGCGCTATGAACCAGATGCCGCCGCACATGCGCCAGCAAGTGAACCAGGCAGTCGGGCAGGCAGTCACCAACGCGGCC
ATCAACGAACTCAGCTCCGCCTTCGCAAGATTCAATCGTCCGGTCCCGAACGTGCCTCCCAAGTTGCCCGAGCGACCGCAG
AATGGACGGCCATTTTGAACACGCCTTCCTGTTGCCGCCTTCTTATTAGATGCATTGTATATAAATCTCTCCTTTTAATTTGT
ATATGGCTAAATTTATTTAATGCTTAGTATGTACGAAAATGTAATAGGTATTTTTTATTTGCGCCCTATTCAGAAATTTTAC
GCCTAAGGTGTAAATTAATATTTATTCGAAAATGAAGTCCTATATTTTATGGTAGCTAAATAGTTATCAATTTGTAATATGG
GTAACTTTTCAATTACAACGCTATTTCTTACCATTTCTATAGACACGTGTACTGATGTATTATGTATACTGGCGAGTTATTG
CATTGACCACTTATTTTAGGTATAGTATTAGATCCCACTGCTTGATTATGCTCATCTATTTTTTTTTTTTTTTATCAAAGTGC
ATTCTTTATTTATAAATTAATAGAGGGTCCGCGTATCTCCTCTCAATGTTCTAAACCTTATTTTTACCCAATTACCTATTAAA
AGTCCAGGTAATTTGATTAATTTACTTTTGAAATTTAATGACGTTAATGTTTAAATGTTACACATAAAAATATACTTTACAT
GTGTAGTTTTTTTTTGAGACTATATTTTGATAGACAACGGGGGAAAACGTATTATTTTATATATTATTTGTAGGAAAAAAA
AACAGATGAGTGTTGGTTATCATGCAGGGGGAGCTTAGGCTATTATAACTGTCTTGAAAATGAAAAATAAACTTATCCAA
ATGCTAATTATGTGTAAAATCTTTGTCAATGCTTATTGTCTTAATTACTAAACATGTTTACTAAACATATGAAAAATATAAT
TTCATATTACAATGGTCACAAGCTATAGACAAAATAAGGGCATTCTATTGTCACCTAATAAAGTAGGCAAACTGTACAATA
TTTTGTGTATACGTATTTTTGATACCTATTTCGTACAGTGTGTACATAAATAAATACATACTTGACCATCTTAATTGAATAT
TGTTAGATACAGAATTATTCATATTTTTGTATTCATTCAACTACACTGTGATATATCGTATTTGGTAAGTGATAACGAATTA
TGTAAACATACGATGTAATTGAATGTAATTATCATATTTGCACTAAAAATACACAAGTCGCAGGGTAGTGATGTGTATAAA
TAGAGAAGACGTCGTTATAGGACACAGATTGCATTATTTGTTGCCTTATTTACACATGTCTAATTGGATTTAACTCCAGATT
GGATAAAAAGGGCACTTTACGCGATTCGTATAACCTGTCAAGTGGGAGCGTAATATGAACGACCTTGCACAGGTTTGCTA
ATGTAACAAATGAATCGCATTATGATTTGTATAAAGTGGGATTTTAAACACAGTAGTTTTAAGATCAGTGACTTTGAAATA
AACGAGGACATGGCGTTCAAACGCCACGTCGCTATAATTTCACAATTTTGAATGCCGCAAGATAGGTTCAGTTTAATGTAG
GGATGCGGCCTTAGTTTTTTTTTTCAATAAAAATATTCTATCTTTATGTCTAAATCTAGAAAAAATATAATTGGATTTAACA
ATGGCCATTGGTCCACTAAGGATTTTTATGTAACATTTCTGTGTTAACATAAAATTTACATCTTTTTATCGCATTTTCCATTT
GTAGCCCATAATATAATGCATAGGAATGTATTTACCACCGCTATCATATTAATATGTTATAATTTGACTTTATATTCATAAG
TAATTAATTCAGCATCATTCGTGTTATATGGTATTTACAATTGTTTTGCGTGATCAGTATCAGAAACTATACCTGTGCATGC
AATCTCTTTTTAGCAAAAAAAAAAAAATGCCACTAAAAAAATATTAGAAACGTTTTATACGTTCCATGTCATAATAAAGATA
ATATTTGATAATGAATGAAAAGCGAAAACATACCGTGCAATAAGGTAACTTCAAAAGACTTAAAATATGATGCTGAAAGG
TTGATGACGCAGGTCTTTGAATGTTTCTTTCGTCCTTCGTACTTCCTAATAGTTTTTTTAATAATAAATAGGCTTACAGCTG
AAGTCTCCGAGGTGGTGATAGGTGCTCAATAATATTTAAAACCCTGTCAGCACACCGACTTCGCGATCGCGAACGGCAGT
AGCGAAGCGAAATGCTGGTAACTAGCTCTATTTGCAGGACAAATAATGATTTGCTTTGTCCCTTCATTATCACCGTTCGCA
GTGCCTGTGTGCTAACGGCGAAAATTGCTCGAATTTCGTTGAGCATATCTAATTACAACAGATTCATAAGTTATAAGTTTA
TTTTTGAAATTATTTACGCTACATTTTTAAACATTGCGTTTAACAGGTGACGTATTTAAGTGACTTACAAGGAGGTTCTCAT
TTCGTTTCTAATTTGTCTTGTGTTCTTCCGAACCGAATATGAACTGAATTTGATATTTTTTTTGCTACTGATAGGCTTATTAA
CTCTAGCTATTATTTTGCAAAGCAAAATCTTATTGATAAATGTACAAATATTATAAAGCACTAAATGTAGTATAAATGTTA
TATAACTATAACAATAACCTCGGCAATATTGTATTGTTAACGTGACAATAGCTAATTAAAATGGAATTAACAATATTGTTG
TATTGGAATGTATAATTATTATATATACAAAATAGGAATAATAATCCGAAATTCAACATTAGTAACTGGATATGCACAAAA
CTAAATAACTTTCCGTAGAGATCGATTTCTTGCGAACTGATCCTAGATGAAAGTTGTTTAGTTTTATGAGTTGAACTTCGAA
TTGTTACGTTTCCGTTTATATATGTACTTGTATTATTGGTAAAAGTGAATATTGTAAATCACTGTAGTTGTTATTGTACTCGT
ATCGTGCATCACAAGGAGTATTTGAATGGTGTTTGTAGTTACTATGTTCGACTGATATTGTGATAGGGTAATAGAATGCTT
GTGATAGTGAGGTGCTTATATTGTGACATGCACCTTCTAAGAGATTGTAACTTTTAATAATCGTGTTATTGTGGCATTTAGT

		ATTATGGTCTGGCGTGTGGATTTCGTTCAGATAATTTGTTGATATTTTTACCACCATATACTTATTCGTAGGCCTACTGAAT<br>TCAGCATGTCTACAAAATATAATTTTGAAGCAAGTACTAATAAAAAATATGTGTAAAAAAAAGAGATTTGCTCTAAAGAT<br>TCTTTCCGTCCAGATTTAATTCTCCTTCCTCGATTTTATTATATATATGATATTACTGATCTGAGCAATGAAAATGTTAAATG<br>GATTATTGTAATCGAACATGGTTTAATCCTTATTTCATGTATTTTGCATGTATATATTTTTTTTGTCAAGTGTCGGTTTGCAC<br>TATCATTTTAAAATGCCCACTATATTGTAACAGGCAAGCAATACAACTAATGGAAAATGTTAGACATGCAGAATTCAGGG<br>AATGAACTCAGAATGATGCCATTGGCGTGCTTCATGTGTTGTTATTCCTGAAATTTAGTGTATGTTAGTAGTAGGATTATTT<br>CTTTTACAACGAAACAATACAGCACCTATGTCCGTTTGTTTAGGGCCCCCTCGTGGGTCTTTGGTGAGGTATACAGGGAGG<br>GTGTGGGTATAAATGATTCATGAAAAGATTGCAATAGGACCACGAGGGATAGTAAAATCTATTTTATCTACTGGTAGGAT<br>AAATTATTAGGACCACGAGAAAAGACCTAGCTCGCGGATACGGCTGTATAGTGGCGCACGCGCTTTCCTGCCCGTCTATGT<br>ATATCTCTACGCACCTGCGGAGTGACGTATTTTATGTTCTTTTTTAATATGATATTTTTGTTTTTCTTTTTTTTTTGGCATATT<br>AACATAAAAATATTCTAGTTATTTTTTTGCTTATGTACTGTTATCTCGTGTATATGTATATAAAATTATATTACTAAAAATG<br>TTTAGAGTAAAAGAACAAACTATAAAATTCTGCGTCATTCCGCAGGAGTTTGCTGATAATTATGTTGAATAGACTTCGCGC<br>GAATGGAAAATGCTTTAACAGACATTGTTGCTTAAGTGATGATAGAGTTTTGTTGTGTTATAACTAAATATTTTTAATACAT<br>TCCCGTATCTCCAATTCACACGCCAGCTATATGTGTATATACATGTAAAAACTGTAATATGGACCTGTTTATACTTTGTGTA<br>CTGTTGTATGCAACAATAAAT
MSTRG.2107	Reverse transcriptase	TTCGTAGTCATAACATCCGTTTGGTTTTTCAGTTTCATATTTCACCGATGTGGCTAAATGGGATAAGGATCGAGCTGATCAT<br>TGTTTTTTAGATTAATAATTTCGGTTGTTAACGGTGATTTATATCACTCGAGAGTTAATCGGAAATAGAGCCCTAAGTGTTT<br>GAATAGTACACCTACATCATTATTGAATCCTGCCTTTCAAGTTGAAATAAATATAAGTGGGCTGTATACTCGTAATACATG<br>AAACATAAAAACATAAAATTGTAATTTGAAAGCTGCTAAATGGAGAGAAGTAGTCTTATGGTCTAAGTTTAAAAAATGTC<br>CGAATACAAAAGAAGAAAAACAAAAACAGAAGCATTAGTTTATTTGGGCCCGCAGTCGTTAACTCTCCACTGCTGAAGAT<br>AAGTCTTCCCTATAAACAAAGTTTTGCTGGTAGTTGGCGTGCTGTTTTTGCCACGCTTGGCAGGCGAATTGACAACCGCGA<br>TTTGGCTTTTAATGGCCCACAGGAAAGAAAAAAGTAGCCTATTCTATACCGGACCACATCTTAGATAAGTATTGTTATAAG<br>CTTAAATTTTACTTGAAGAATCAATTTTCATGGAGGGTCAACAGCCAGACGGGGTTGCATACTCTTTGTTCCAATCCTGTCG<br>ACTAGCGAACTAGCGACGCGACGACGTTGCACGACGTGGCAGGACATTACCCTTCTCATCTGTTATTTACCCGTGAACTTA<br>CAGTTTTCATAAATAAATATAAAATATACGATGTATATAGGTAAATATTTTAATTTTATATATTCATCCTGAAGTGGCATAT<br>AACTTGGCTTCTTTGTATATTCTGCCATCTGTATAATGGATTGTTCTATTGGATTATTTGACATGATTCTTAATGCCGCACCA<br>CTTACAATTTTAATATATTAATAAATTTGTTACTAGAATTGGTTTTATTCGTATTTTGTAAGATATTGAGAAATCGTTGTTTG<br>GGTCACTAGAAGCGGCACATTTAAGCATATATTGTCGTAAGTGCTGTTAGTTATAAATAAAAATCATGTATATTGTGAGAT<br>TACTCTTGAAATTAGTAAAAATAACAACGATAAAATTAGAGACGCCTCCCCACATGGAGACAGGCCCCGGCGAGATTCGA<br>ACTCGCGATCTCCTGTTTACTAGACAGGCGCTTTAACCAACTAAGCCACGGCGCCCTCATTACATATGTTTCAAATTTAGA<br>AATGTATTATCATTATCCTTCTCATGTTTATACATTACGTTATATTACATCTATGATTTATATATAAATAGGTAAATGCCCTT<br>TGATGATTGTCCTTTTCTTATACCTTATGTACTCAGAATTTTGATTCTCAACAGGTAGGTCTTAAGATGAACATAGACAAGA<br>CTAAAATAATATCTAACATCTGTGTCACACCTATACCAGTTACGATTAAGAACATTATGCTCGAAGTTGTCGACGAGTATG<br>TATGTATACCTGGGACAAATTGTCCGAATAGATAAGTTCAAATTCGCCCGAGAGGTGAATCGACGAATCCAACGTGGCTG<br>GGTAGCTTACGGAAAATTACGCCACATCTTTTCATCTAAAATACTTCAAAGTTTGAAGACAAAGGTTTCAACCAGTGCGTG<br>ATTCCAGTGATGATTTATGGAACCGAAACATGGTCCTCCACGATTGTCCGTATGTAAAAGCTTCAAATTACTTAGCGGGCT<br>ATGGAGAAGGCCATGCTCATAGTTTCTTTGCGTGATAGAATCCGAAATGAGGATATCCACAAAAGAACCAAAGTAACTGA<br>TATAGCCCGAGGTATAAGTAAGCTGAAGTGGCAATGGGCTGGTCACAAAGCTCGAAGGATCGACGACCGAATGGAAAA<br>AAGGTTTTTGAGTAGCGAGCATTTACGAGCAAACGCAGTGTAGGAGGACTTCCTACTAGATATAGAGTGACGACCTTAAATA

MSTRG.2134	Uncharacterized protein LOC106129326	CACTGCAGGGAGTCAGTGGATGCAGGTGGCTCAGGACCGTTGCTTATGGCTGTCTATGGGGGAGGCCTATGTCCAGCAGTGGATAGATAATAGCTGAGATGATAATGTACATATTCATATTATTATTATAAAATCAAACATGTTGTCACACTGAATTTTTTTAAATAATAGTAGGTATCGAGTATGAATAGAAGTTGGTAAAATAATCGACGAATCTGTTTCTTGTTTTTCAGAGTTGTCGTTCAAAATAAGCTTACAGAACCAAGAATTAGTCTTACTTAACACACTTGTCTGATTCCAGGAATATGAAGCTCGGATGTATAGTTGTCCAATGGGATATTATTATAATTACAAATCCGTATGGCCAGAAAATCCATGTGGTCTCGCCTCAGAAGTAAACTGTGTAGGCCGCTCATATAATAATACCGAATGGAGAGGCAATTGCGACAATATCTTTCATGACATTTTCCCATTCGGAGATATGGAATGTGGAAAATACGTAGTCTGCGACCAAGGCACAGCCATCACGATGGCATGCCCAGAGGGCCTTGTATTCAATGCTAATAGAAAAGCTTGTGACTGGCCTGTGAATGTACCAGCATGCAATCCCGATGTATTCCGTGGATTCACCTGTCCTGAGCCCATGCGAGATGACAGAGGTCGAATGTCAGACATTATTTCAAAATTCAGATACGAACATGATTGCAGTAAATTCATAGCATGTCAACGGGGTTACCCTCGCCTTCTAAGCTGCGACGCTGGATTTTACTTCAGTGATGTAACTAGAAGCTGCGTCCATGCTGCTCTTGTACACAATTGTATACCCACACCACC
MSTRG.214	Epsin-2	AGGTCTAAACGTAAGGGAGAAAGCGAAACAGTTGGTCAATTTGTTGAAGGACGACGAACGATTGAAGAACGAGCGAGCGCGAGCGCTTAAAGCGAAACAGCGGTTCGCGCAAAGCGCTAGCGCGTTCGGCAGTGACGGCGCGCTGGACACGCCGTCCAGTCCAGGCTACCCCACGATGAGCAGCAGCGAGTCGGCGGAGTGGGGCGGCGGCGGCGGCGGCGGCGGCGGCGGCGGCGGCGGGCGCGAGGCGGAGCTGGCGCGGCCGCTGACGGCGGGCGAGGAGGAGCTGCAGCTGCAGCTGGCGCTGGCCATGTCGCGCGAGGAGGCCGAGCGCGACGAGCGCCGCCGCCGCTCCGACGACGTGCGCCTGCAGCTCGCCATCAGCCAGTCGCAGAACGACTTCCAG
MSTRG.216	ATP-dependent RNA helicase DDX46	GCGCGGCGCCTCGCCTCCCGCATCAACCTCGCCAAGGGGCTCGGCGCCGAGCACAAGGGCGCCACGCAGCAGGCCGCCGAGGCGATCCTCAAGGGGGCGCCGTCCGCGCACACGCTTATTACGGCTAAAACAGTAGCGGAACAGCTGGCAGCTAAATTGAATACGAGATTGAATTATCAGCCTCGAGATGAGAGTGCATCGGAGCCAGCCGAGGAGGTGTTCCGCAAATATGAGACTGAGCTGGAGATCAACGACTTCCCGCAACAGGCCCGCTGGAGGGTCACTAGCAAGGAGGCGCTAGCATTGATCAGTGAATACAGCGAAGCTGGCATCACAGTGCGGGGAACGTACGTCCCGCCTGGCAAGACACCACCAGAAGGCGAACGCAAACTGTACCTCGCCATCGAAAGCTCCCAAGAGCTCGCTGTAGCTAAAGCGAAATCGGAAATTACGCGCCTCATCAAAGAAGAACTGCTCAAGTTACAGACCTCTGCTCATCACATGATAAACAAAGCCAGATATAAAGTTATATAAGTGATGAAATAAATGGAATGTATCTAAAATAAAATATGATT
MSTRG.2191	Integrator complex subunit 8	TATACAGTTTATTTTAAAGTTAGTCCTATTTTCACGAAAACAATTTAATGTTGACTCTCGTCAACATAACTTTTTAGCGTCAAGAGTGTATTGTCAGTGTCAGCTGTCATAAGTCAGATGTGCATATATCATTTTAGTGGTTGTGGTTATAGTTGTATTTTTTGTTGCTTTTGTCTTCTCTGATTTATAGTTTTATTATTATTGTTATTTTTGTTAATTTTAGCCTTACAATTAACAAGAAATCTAGAACTAATAGTTTTGTACCATCAATGTTTACCTCAATGGTAAAAATAATACCTTATAATAGTGTTTGTTGATTTAAATACTTATAAAAATCATGGATGTGGATCTACTACGGCCGGGCACTGTACCTATATCCCCTGATACTATATTATGGTTCGAATTTCTACTTAACGAAGACCTCCTCAAGAAACATCTTAGTAAACCCAATCCAGAACCATCACCATGCGATTTAATAGAAGAATTTCTATCCGTGGACACAGACAATGGAAATTCAAAGCCAACAAGTGAGAGAGTGGTGGATGTAGATGCACCACCAAGTCCACCCTCTGAGACTACACTGACTCCTTTGCAATTCAGTCTTAAAGAGCTGGCTCTTAAAATTCTAGCACTTAAAGTGGCAGCAACTCTTCACTGGAATTTAGATGTGTTGGAAGCTAAACTACCTCCTCAAATCCAGCAACAGTTAATGCAAGATCTAGTTTACATGGCATCTGATTCAGCATTTATTATACCACCACAGGAGTTTTCACAGGATTTATTGCATAAACCTCAGGTTCAGTTTGCCTTGACTTTGTACCACAGATGGATACTAAGATTTCCTGTGAAAGCTGCATTATATGCTAAATCCACAAAAATGTCCTATATGCATGTGCCCGGTATACAAGCAGATAGTGGGTATACACCATTAAATCAAAATTTTGAAAAAATCTTAAGAATGTCAGAGCTATCTCGAATGAACAGTATCAAATACTTAGAAGAAATATTATCATACTATGAACTAACTCCAGAAAGCCGCAGAGAGCCAAAAAAAATTAAAATTCCTGTCATGGATGCATTTGTCCACCTTAATGAGGATTCCACTGATATGAATCATGACTGGAATGCTGGGAAAATT

		GTCATCAATCAATATGAATTGGCAATGCAAATTCACTTTGACCTCTGTTATAATTACTTTTTCTATGGTCAACATGACATGG<br>CTGCAAAACACATACTTGGTTGCAGAGAAAACTCAAATTTATTGGAAAGAGAGATATCTTTATATGGATATGGTTCCCACA<br>AGGTTATGCCGTGGGGAGATTTCTATTATGCAAGTGTTGCCAAGGAAGATA
MSTRG.2209	Spastin	GGTAATTGTAGTAAGTATAAAGTGGTATACAAAGCGAATCTAATTGAATTATACATTATATGCATATAAGATTTCTTATAA<br>CGATGAAACGTTTTTAACGTGACAATTTTTCAATATAATTGCGAGTATTTTATTTAAATTCGTGGTCGAATTGAACAAAATG<br>GTCAAAAATGAAAATTGGCAAAGATTTTACAATTGATAGTTGCAATATTATAATGACTTTATTTGCAGATTATTTTGTCATT<br>TTTGAAATATTTTAATTAATAAGGTTAATCATAGATACAAGGTATTATCGACAAGAGAAGAGCCAGAGAATTACAAGTGT<br>GCAATTGCACTTTGCCGTGTGTACACTTGAAGATTTATTTTTATGACGTTAAAGAATAAGCAGGTAACGAACATTTCCATA<br>ATTATATTTTCAAATATTATTAATTACGATAGTGTTCCGGAAATAAGTATTTTTACGCAGGTATTTTAGCTAGAAAATTGAA<br>TAATACTATTAATACTCATATATCATGTCATAAAGTAGAAATGCAATGCTGTTATAAAATTGTGTGTTCTGTAATTGGATAC<br>ATTTAAAAAAAATATCGTCTAATAATCTATTAAGATTATATTGATATAAATGCATGTCTCTTGTATTCACGAAGAAATATCT<br>TCCGGATTATGAAGCATAAAATAACTATTTTCATAGGCGCTAACTGGATATTAGAAATTAATTGTTTAAGACAATTTAAGC<br>TCTGATGTACACATCTCTTACACCTTTGTTTTAATGTTTTGAAATAATATTCCAATGTGCAGCATACTACCAGAAATCATAT<br>TTCCGTATTAAATAAATACTTATTTGTTAGCGTCACTCGTAATAAGCGTTGTAGCTGAAAAGTTGTTCATAAGAAATATAT<br>AATTGTAGTGACGCTGAATACGCAATCTATGAGATACTATATGAAATATGCAATCTACGTATCGTATATAAAAATTGAAAT<br>CATTATATATGAAAGATCAATTCATCAGTGTGATTATATTAGATCAATTATCAGTAAGAATTATAAAACACTACAAAATGT<br>ATATGAATAGTGACATAGCAAACTTAATTAATAATTTAGCAAGAGCCTGATCGTATTACTGATTCCGGCACAGAATATAAT<br>ATAATATTCTCGCTACAGTGGGAACCTACCCGTACTACTAAGAGGAGGATACCCGCGGTCACTTATGTTATTAATTTTTCTC<br>ATCAGTACAACAATTTATGTTGTAATCGTTGTAATAATTACAAAACAAAAGTATACATTTTTTATAATAAATTTTATTCATT<br>AAGCCATCATTACCTACTCTTCCGTATGAGATTTGTTCAGGTAGCTAGCGTAAATGGTAATGTAAAGCATTTGGTGAATAC<br>TAATCACAACTAAAGTTGAGCCTGAATCTTTACCTATATTTTTGTTAACATATTGAATATGAATCATAGCAACTGATTGAA<br>GGCAAATAAATATATATATATTTTGTAATATGTACTTTCGACCATTCGTTTCACTGTGGGTATCCGTCATTATGATAAAGGC<br>TATAATCTCTAATGAATATGTAGATGATAGTATTTAGGTACATAAAGAATATAATAAGAAATGTTATGTATAATTTATTAG<br>CACAAGGCCATATTTAAATAAACGTTTAAATCTAAAAATTTGATTTTATGTAGGCATTTATTAAGAATGAACTGTAGAACT<br>ACAGGAAAGGTAGAGAATTGGAATGCACGCCAGTAAAGCTTTTCTTCTAATTACTCACGAAAAGCAATAGTTAATATATT<br>AGGTAAAAGTTTATTATGACAGGACAATATAACAATCGATTAGATAAGCATTTTAAAATTGTGGCTTGAGATGACACTGA<br>CTCGCTCATTATCTCGATTTCTTTAAAAATATTTTACAAATAACAACGAGCATATTATGTATGGTTGCTTCATATTTTTCTTT<br>ATATTTCAGACGATGGATGAAAAAATGCAAAACTAAGAAGAAATTGGCAAAACTTTTTATACATTGGATATTCTTCAAAC<br>AACTGATTTACAAGAATCTGTCCAGCGACGTAGAAGCGTATTAAAGATTTCCGGTTATAATAATCAAACATATTAATGCAA<br>TAAATCAGCACAAAAACAGGCGGCACTTTTTATATTTTTAAATAATATGGGTATTACATGTTAAAAAAGGTGTAACAATAA<br>TAAGCCTATTATATAATGATTACATTTTGATACAGAATAGTGTACTATCGAATTGTATAGATCGCTAAGGGTGGGTTTTGA<br>ATAGTCTACTGGCGGCAGATGTATAAATCTGTCACTGATAGAGACACAATTTGTGTGATAGAATAATGATGGGTAAAAGA<br>AAAATCATACTTCGAAAGCGAAAGCATAACAGCGTTACATAGCGTTGCCAGATTACGTATTTCTTTCGCAATTTTGGGTAA<br>ACATTTTTATGCTCGAGAAATTTTGGAGAAATTAAATTATTGGGAAGACTTGGAGAACAGTGAAATGGGTTTGGAGAAAT<br>TTGGCAAAATGTTGTGGACGGTCTGATCGAAACTTTGAGATAATCTAGGTAAATCGTAGTTTACCTAAATGATCGCCTTAA<br>TTTATGAACTATAAACCGCAACGCTAAAGCTTATTACTTATAAGCAGCAAATTTTATTTCCGAGTGAAAGTTTGCCAAAGG<br>ATATGTTATTTACGTAACCCATCCCCTAAAACAACTTATATAAATACTTAAATAAATTATTATAGATAATATTAGATATTGT<br>GAACATATTGTAACATAGATATTGTGGTTGCATAGCAATATACAAATTGCACGCGATTTTATTTTATGACACAGTTATTATT<br>AAGTTCTTTAAACTGACATGACAATCTCTACAAGTAAAAAAAAAAAAAAATGGAGGTATATTCACACATTCGTTTAAGTTAT

		TTCGAAGATGCTTATCACGTAGTTTGTCTTCATTACCTTCATTTATAGTACATAATGAATTGTGAACCTTGAATTCATTCTC<br>GAATGAGAAATGAAGTTTTAAACGACTAGGTAACTTTTTTAACCACCCAACATACCTTTAATATCATAATCATTACCATGA<br>TCATAAATCAGCTGGTCCTTATGTAATACGAATGTTGATAATCTGTTTTCATGGACTCATTTATTGACAGTTTTTTTTGATG<br>GGTGAAAATGGCATGGCAACCCCGCCCGCGCTAACGGGATACGCCGGCGGAGCACCAGTGAAGGGTGATAGATGAGAAT<br>GAAAAACAGGTCAGTTAGCCCATCATGATGAATTCATCAGGAATTAACCATGATAATTTCCAGGGAGAACCGCGAGGAGG<br>TCGACCTGGTTGGGTCATAGAGATTCCTGTACAATGGTATTATGCTCTAAGGAAATTACTTAAACTATTGGAATATAGCTA<br>AAGTCATCACGCTGCCTAAGCCCGGGAAGAACATATTTGCAACCGGAATCATACTGGCCAGTAACGTTGCAGCATGTGTC<br>AAACGTATGTTGTTCCTCAAACATCTACGTCCGCACGTGAAACTATAAACAGAAGAAATTCGGCTTTAGAGCAGAGCATTT<br>AACATTACTACCAGGGGTGTTAGTTGAAGTTTGCCAGTTTTACTAGTAGATGTCGCTAGCAATATGTATAAATTTCTATGAT<br>TTGGCATGTATGTCATTTTTTTCTATTGACAATAACCTACAAAAATACGCAAGTCACATTCCGCCAAAATAATAGCGTTTGT<br>ACTAATGTTTACCTGGTGTAGTAATAATGTTTACCTCAGTGAATATGTCGAGTTTCGTTCCAAGTAAATCGCATTTGCAGCA<br>TTCGTTGTTTTTCTTATTTCATCAAAAGAAAAAAGCAGCTAAAGCTCACCGGTTGCTGGTAGAAACTTATGGTGAACATGC<br>TCCAGTGATTAGAACATGTGAGACATGGTTTCGTCAATTTAAAAGTGGAGAGTTCGATTTGACAGACAATGAACATCCTGG<br>TGGTAATTTACCAAGGTATAATCACATATGCCCATAATAAGAGAGAACAAGTTGAAGCATTTCTGATCATGGGAAAGGCG<br>TTCTACCGCGTCTGGCACAACGGTCTGCTGTACAAGTTAACGCTTATCCAAATGGTGGCTTCCTTCCTCGCAGGCAACGTA<br>GTCTACGTTTTGTCGTCTATATCAACAAAGCATGTAGATTGTGGTACATCATCACGAGCGAATACTCTAGAAAGGAAACAG<br>GGCCTAACAGAACTTCACGCTGGGTACCATGACCGGCGTGCACAAATACGTGAGGAATGCCACTCTTAAGAGGACTTTGA<br>GATTCTCACATTGGGCGCACACAGAAGACTTAGTCATAAGGGAGATCGCGCGTGTAGATACGTCCGACTAGGACCACCTA<br>AAGGACCTAGCGTTGCTGAATGCGTGGCCACCGGAGTCAAGACCGTACGCGCACAAATTTACTGTTCACCCGAGTACAGA<br>CGATGCCGCTCCTTAGCGAGAGTGATAGTGCTATTAGGCTCATTCATACGCTGTGCCTCCCCCGTACGGGATCTCCCTCGG<br>CAGGCGAAGACTGAGTGAGCTGGATAGTACAAGAATTAGAGGACCATCGGACTATGTTCACTTTGACGAAAACGATTTTT<br>ATGTACGGTGGTTGACACATACCGTCTGTCTACCGAAGAAGGGTAAACTGTTGCCCTTATTCGTTAGTCCAAACTGGTTTT<br>CCGTACCCCGCAAAGGACTGAGAGGACACTGTTAATATATTACCAGATATACTTAAGCGATATACGTAAGCATGTGACAG<br>GTCACGGGAAAATCGGACAAAGGCAGCAACGCCTCATCAAAGGACAGTCGTGACGCCGAGAGTAGTTATATAAATTACC<br>ATTGTATTACATGTCTATTATTTTCTCTAGGCAAAAAAAAAAAAAATCTAGCGTCCTATTAATGAATACTAACACTAGTTTTA<br>GGAAGGGGTTACGTTAATTAACGTATAGTATCACTTCTTTTACAAAATAGCGCTAATCGCCAAATCCCGATGACTCTTCTTT<br>TTCTTCATTTTTATCTGTCACTGTCAAAAAGATGTTCAGGAACATTGGCGTAAACTTTAAAATGCTCTTGTAAATAATATTC<br>AGACATTCAACAAATTTTTATGTATACATAATAAAAAGAAAGTTTTAAACATCATCCATGACAAAGGTAGTTATTTTTGAA<br>TAAACATAGTTCACTAAAA
MSTRG.2<br>260	Uncharacterized<br>protein<br>OBRU01_06617	GTTAGGACACTACAAACGCCATTTTTGCTGTTATGTTTTATGAGTCTTCATAAAAGACAGAAGATAATAGATAACTTTATG<br>AATTACTATACTACGTACCTATTTATTCCGTAAAATATGCAAATGTTTTGGGATAACTAACTCTACGAACTAAGTTATAAA<br>ATATAATTACACCTAAGATATTCATACTTTTTGGAGTACTGCGACAATACTATGGCGTTTCTACTATTGCTGTCAAATGGTT<br>TTCTTATTTGCTAATCAGACGCATAGAATGTTTAAATTGTCAGTTATTTTATTTCACTGTATCAATTATAATGCTGTTTTCAT<br>TTTGTAGTTGTGACGAACCCGATCAAAATACCCATTTCAATGACATGTTTCCAAGTTCAAATCAATTATGGGGAATGTTAG<br>GTACTATAGCAGCAACTGTTGCGTGTTGGATATTTTACACAAAGATAATAATAAAAAAAAATACAACATCAAAACAAACCA<br>CGAATCATATCCACTAGCTGTAGAGGACGTATCTAAACCGACAGTAACAATTGAAATAAAACGCTACATAATAATATTTC<br>ATTTTATTAATGTATAAAATACAAAGTAATATGGCTCATTATAATTCATAATAAAAATAAAATTTAAAATTTCCCCAGCAC<br>CTTCTACAAC
MSTRG.2	Uncharacterized	GCGAAGGATGTTTGCCATGAATTTAGTAATGATAATCTCAGTCGGTCCGGTGCAAGTCCACGAGGATAATGTTGCGAATTA

273	protein LOC106137009 isoform X1	TTTGTTTTCTTCTATTGTTATTAATAACAATTGCATTATCTGGGCCGAAACTGCACTATCGGGAGGAGATGCCAAAGGATAT<br>CGATGAAGAGATTGTGATAAGAAAGGATGTAGAGGATTCTCCGGCATGGTTGGAAGGTGGCGGATGTCCTGCTGGTTACA<br>AGAGAGACTTTAATGGCGTTTGCCGAGAAGTTTGACGAAAATACAAGATTTTACGAAGGGATAAGAGGTGCAATGAATCT<br>CCAGGTATCCAATTGGTCGAGGAGGGGGTTTCCGACGGCGATGACGGCGAGGGAGGCAATTTTGGAGATGGCGCCCTGGA<br>AACGGTTGACGTATCCGATGATCACTTCTTCAGTTATTGCGTTGACGATCGCGTCGAGGTCGTCACCACCACCGATCACTC<br>CGGCGAGGTACGGAGATATCCAAGTCGAATGTCAGACCACGCAGAGACAAGTCGCTCCTGTGCACAACGAAGTCACCGA<br>AGCCTGAAGCAGCGATTCTCTGGAGCTCCAGATCCAAACTGGAAGTAGAAGATAATTTTATTTATACTAATAATACAATTA<br>ACATTTTCATTGCAATAGAAAAAGAAAGAAATTTTGTATAAAATTCGTCACGAATGTTATCTCTGTATCATTCACAACCTT<br>CTCCTCAAAGTTGTGATTGCATTTTAGGTTACGAAAGCATAAGACCTTAGTCTTTAAAAATAGAATAACAAACAGAATAGC<br>AAAGGCGACATGGTATATTCTGTGTTATCCATATCCACTTCGATGCAACGTTAATTTATATCTCATTTCAAATTGCATAGAG<br>AATAGGTATCTTTCATACTGTTTTTTGTCTAAGTAAAATAATTATCATAATAAAATGAACCCATATTGAAG
MSTRG.2 286	NAD(P) transhydrogenase	AAATCCTCATAATTTTCCACAAAATGCATCAAAGTAAATGAAATAATCATATCTCTACATAATTTATTAAATATAACTTAA<br>AAGCAAAGATTTTTTATAAAAAAAAACTTGTTGGTGGAGAATGTGTTAATCAAATATAAGAATATTATAACAATATTAAA<br>ATATTACCTTTTTGAAGAACCTGTATATCATGGAGAATATAGTAGCACTTTCATCACTAGTGGCTGGTGGCAGTGCCACCT<br>TACTCGGTGCCACCATCGTATTACTTGCCTACAACAGTCATATTGGTTTTATTTTAACAAGGTGTCTCCGGGACGTACCCGC<br>CGCCCATGAGAAGCAGGCCGCCGACTGCCGTGATGCCCGACACGGCGTTGGTAACTGACATCAACGGTGAGTGCAGCGCG<br>GGCACCACACCCCAAACCGTATGGTATCCTACCACGCCTAACAAAAATACACTGTATTTAGATATTATCGAAATTAATAC<br>CCAC
MSTRG.2 320	TAR DNA-binding protein 43	CGATACAAATCATCTACCTTGGCACATCACTATTAAAGATTTTATTCAATCTAATAGATATTAATGGTGGGCTAAAAGGAA<br>ACATCCAAAACCGTAAAGCTATCTAAAAAAAAATTCAATCATTTTGTTCGCTACGTTGTGTCATATTCCTCTTAAAAATATT<br>GAAATATTTGTCGATAAATTTGGTGATAGTTTTTATTAATATAGTGTATTCATCATGTGTAGTAAATCTGATAACTATAATG<br>AAGATTTATTTGATTACATTAGAGTAAGTGAAGATGAGGATGATTCTAATGCCGTGGAAATTCCTTGTGAAATGGATGGCA<br>CTTTACTTTTGTCAACGCTTGTAGCGCAATTCCCCGGAGCATGTGGTTTAAAATATCGTCACCCTGATAGTAAATCTGTTCG<br>AGGTATTCGTTTAAGTGATGGAAAGCTTCACCCCCCTAGTGAAGTTGGATGGGGAAAACATCTATATATTTGCGTTTTTCC<br>TAAAGAAAATAAACGTAAAATGGAGGACGTATCTCCTGAAAATTCAGCAGCAAAAACCAAGCGACTGGAAAAGAAACTT<br>ACGTGCTCTGATTTAATATGCCTAGGTCTACCTTGGAAATCAACTGAAGAGTCAATAAAACAATACTTTGAACAGTTTGGT<br>GAAGTTGTAATGGTTCAACTAAAAAAAGATAAAAATGGCACTTTTAAAGGCTTTGGATTCATTCGCTTTGCTACTTATGCT<br>TCACAAATGAGAGCTCTTGCACAAAGACACAATATTGATGGTCGTTGGGTGGATGTACGTATCCCAAATTCCAAGGAAGG<br>TGTGGTACCACAAATGCCCTGCAAAGTTTTTGTGGGAAGATGTACTGAAGATATGACTGCAGATGACCTCCGTGATTATTT<br>TTCAAAGTTTGGGGAAGTGACTGATGTGTTTGTTCCTCGACCATTTCGGGCGTTTGGTTTTGTTACATTCCTAGATCCTGAA<br>GTTGCACAGAGTCTATGTGGAGAGGATCATGTTATTAAAGGAGCATCTGTATCAGTATCTAGTGCTGCACCAAAGATTAAA<br>AGTAAATCTAACCAAAACTGGAAAGATGATTCATATGGATCTAATAATTGGGACGGAAATAGATCAAGTTCTTCTGGGGG<br>ACCAAATAGCACCAGTGGGAGCAGTAATATTGACTCACTTAATATGCAAAATCTTGGAATCAATCCAAATGGAGGTCCAC<br>CAAATAATTTTAATTTGCCTATAAGTCTTGTTGCTGCAGCACTAAACCAAGCTGGGTGGGGTGGTTTTCTTGGTGGTCCAG<br>GTAATTCTGGTCCTAATAATTGGCAAGGTGGTCCACCCCAAGGCAATAATTCTGGAAAAAACTGGAATAGCAACCAAAAC<br>TGGGGCCAAAATGGCCCACCTCCATCATGGAATCAAGGAGGTGGTAGTGGCCAAGGATGGAATGGAGGAGGAGGCAAAA<br>GTGGCAATTGGAATCAAGGAAATTGGTCAAGTGGTCAAGGCAGTTGGAATGGTAACGGTGGTGGTGGTGGTAGCTCTGGC<br>TCTGGCAACGGATGGAATAATAAGCCACAGACATGAGGTGCACAGAAGGCTTACACACTGTAATGAATACAGTTCATGAA<br>AAGAAGTCATAGGAGTGATACAAAGATAACAGGATCTGGGTGCCTTCTTTTTTTTTACAATGGTCTAAATTGTAAAGAAGA

		ATAGAGAGAGAGAGAAAGAGAGACACATTATAGAAAAAGGAAAAAAAGTGTGATTATGTTATAATTACTATTAATCTTGT<br>GAGGGTGCCACCCTTTGGTATCAAGAACTTCTAGGTCGCCTTCATTGTACACAACCTTTAATCTAGCTTACTGGATAACAG<br>CTATACATACCCTTACAAGCATGAACCAAGCAAGTGACAAACATTTTTAGAAAATCCTAACATGAGTTTGAAACTCGGGA<br>GGTTGGGATTAGGATTCCATCTTTAGACTTCTGGACCATGTTCAAGCACTGAGATGTCACTACTCTCAGTATGATTATGAA<br>GCATATTAAATTGTTTTTTTGTAGTCTGGGCAAAGTATATAAAAATATAATCTTTAGTGTTTTTTTAAATCACTTCAATAT<br>ATTTATTTAAAAACATCTAATTGCATTATTAATAGGTATAGACTAAATAGGCTAAAAACTGCAAAAGGTGGTTTTATTGTT<br>AAACAAACCATCTCCAAAAAACCTTTGGGAACAATATTAATAAAATATTCACAAAAGCAACTATTAGCTTTTACGCGTAA<br>CATTATACAAGAGGGAAACTTTGTACAAGTTGTTTGCATGAGTACCTCCATCTTAGCTGTTTCTGCAGTGAAATAGCTGTG<br>CTTGCAAGGCAGTTTCTGTTCAATATGGTAGCGAAATTATAGG
MSTRG.235	Uncharacterized protein LOC106138469	TAAAAATAAATATACCTTAAATTCTATAAATTTGTCCATATTATAGAGGGTAATATTAACCAGTATTTCTGTAATGTTTTCT<br>AGAATTAAGTGTCAGAGGCACGTGGTTTTTGCATTTTTGTATTATATTGTAATGTGAAGCAAAATGGAAATACAAAATAAA<br>TTTCATTCTTTGAGAGAATTAGAAGAATATTCCTATCGTTTTTTACAACATATAGCTAAATCTATGGCCTTGCCTTCTAATTT<br>TAAGAAAGTCTACTTGATCCAGTTAATACATGCCAAACAGTTTGGCACCATGGATGAGGTGAAAAGTATTGTGAAGAAGG<br>TGAAATTGGAACGGCTGCAACTGTCACAGGTCAGGAAGAAAAGCAGTCGCAGGACATCATATACTGTGCAGGGATCGGA<br>AATATCTTCGACCAGCAATAGTCACTCGCCACCTATAACGATCACCCCAAAGCGTCCAGGTCAAATTATGCCGTGCAGCTC<br>GGATTCACATCAGACTGTTATGAACTACCCTCGACTGAAAGCCCCAGAACCCAGAGTCGTGCAAAGAACCTACCCCTCCC<br>CGAAGAAATCAAGACGAAGCGACAG
MSTRG.2356	Nuclear receptor coactivator 7 isoform X1	AGCATGTGTATATGTGTGTCGCATATTGTTGTTAGTTGTCAACGGGATGTTTATGTAGACCGTGTAAGGCCCCCCTTAAGTG<br>GAGCGAGAATGGCGCGAGTTCAACGAAGCGAGTACAGCGCTTATAGACGAGTATGAACCAAAGATTAAGTACGTAATTGT<br>ACGTACGTAAAGTACGTAGCCATTGGCATATTAAGCAGTAATATTTATTGTATAATGATTGATAATCTTCCGGAAAATTAT<br>GTATATAATCTATGACATAATTTAAAAACAAAAAATAATACTTTATTAACTGTAACTATCGTTTAGACTCGTCTGTAAGCG<br>CGCTAAATGAAATAGAGCCTCGTATGCATGGAAGAAGTATCACTGTAAGATTATAGTAATGTACTAACTGTGGAGGGCGT<br>ATCACCGATTCCATGCATACCTTGGAGAAGACGCTGGTGGAATTGTGGCTGTCTGCGGAGCTGGGCAGTGACTCCGCCTGC<br>GAGCTCTGCGGACCCGGCGCCCCTCCCTCGCCAGTCAGACCCTCTACCCACGACAATGGATACGACAAACGTTTGAGCATC<br>TGTCAGAAAAAAAAAATCCTTACTAATATGATAAATGTAAGTTTGTTTGTTACTGTTTGGTGTCTTACGATGACTGTTTAAC<br>AACATATTCCGGACATACGGACGATGTCGCGCTAAAAGTCAGTAAATATATAAATATTGAACCTGCAACTAGTACCAGTA<br>TTAATAAAAGGCTATGGGTCCATGGTTTGGGTCCAAATCCCGCCCGTTTCGAAAATTTTCAATTGCACAATAATTATTGTTT<br>AGAAATATGTATTATTATGTAAATTGGTTAAAAAAAATGCATTCTTGAAATATGAAAATCATATTTTAATAATACATATTT<br>CTAACCAATTTGCAAGTAGTTATTTTTATTTTTAAATAAAAAAAGGACTTGTTTAAATAAACTTAGTT
MSTRG.237	Uncharacterized protein LOC105385187	ACCAGATTCAAAGTAAACCGAGATGTGGATCAAAAATGGAATATAAAAGCCAATTGTGTTGTAAAAAAGCCACAGCAGG<br>GAATGCTGAAGTCTAGTAATTTCGACTTTAAAAGTAGTGAAAATAGATGTCCTTATTCCGTACCTAGTACTAGCATGAACT<br>CCACGCCGGCGGTGCCTGCGAGACGTCAAAGAGTTTTGAGCGGCATTTACCCCATTACGTCCAAAGAAGTGGCCCTAAAA<br>AAGAGCTCATTCGTACAAAGCATTGGCTTCAGACACAGAGACGGGAAGATTTCCAAAATAAACGCCCTAGTACAAAAACC<br>TAAAATACCAATAAAACATTCTGTAGAGAGTAGTAATACTCAAAACACCTGCCGCGATCTGGAAGTTACTGTTCAAGATA<br>TAATCGACTACAACGTCGAAACACAAACCAATATGGACCCTGTATATTCAGCCACAGAAACCAATATGCCGTCCAATAAT<br>TATCTTTATCCGACTTTGACAAATATTAATGAGGATTTTGAGAACCATGCGTACTATAGCACATATGTGGCACAAACTCAT<br>CACGATGGTCAATATTATAATGATCCGTGTAGCAGTAACTATTATCCAAGGGAGGCATCTACCAAATTGCCACATATAAAT<br>GAGGTATTCAGCAAATTTAACGATATACAAAGTGATTTACGTGAACCAATTTATGTACAAGTGAGCGAAGAGGCAGACAG<br>AATAAGAAATTTCCCGTTGTATGTAGCGAGCACAACGAACAACACCAGTTACCTGGAGTCGTTGTACAACTTCAGGGGTC

MSTRG.2<br>423	Small G protein<br>signaling<br>modulator 3<br>homolog	AGTTTGTGTTGCACCAGCCACTGCTGAAGGTGGCTACCACCTCGAATACAATAAGCACATACAGTACACCTGTCATGGCGT<br>C<br>GTCAGTGATAACAAGTGACGACAAAAACAAAAACCTAAACAATAATGTAACAAATTGTGTAACACTTTTGTCCATTATAA<br>AAAAAAAACGCCATTCCAGTCATCGAGTCTTCTTTTTTGTGGAATTATAATGACATGGTAATTCCTCCTGCGCTACCAATAT<br>ACGCTGAGAGGACACCAGTGAGAGTGAGTGATGATAGATCCCAAGAAAATAAGTGATGTTTTACAAGCATATCTCTAACA<br>CCTTCTTTTAGTGGCTGATTTTGTGTGTCCTTCTTTAATGGCAGCTCCCATTCTGGATTCAGATTAAAACCAAATTGAGATA<br>GTATTCTTAATTCACATTTAATCTGAACCCAACCAGGGGAGTTTATAAAAGACCATGGATGGTACCATTTTTGGACAACAT<br>CTACACAAGAACATAAAACTTCTAACCACAAATGTAGAGCTTCTTCATTTAAACCCATACATATTAATGTGCGTAGCTTTA<br>CATCCATTTGAGCATGAGCATTGTCATGGGACAAATTGATAGCTTGGACACATCTATATAACAATTCTTCAGGTGTTAAAA<br>CTTTTCCATCTTCATCTAAACGATAAGTCTTGCATAACACCAAGCGACTGTAAACAGAATTAAAGTCTTTTTCTACTTCGCG<br>TGATGAAGCCTCTTCAATAAATAGCCATGGGTGGCAGGGACCATCTAAAAAACTAGGCTTTTTAATTCCATGTAATAGAAC<br>TCTTTTGAAAGCAGGAGCAAGAACACCTCTAACCAAATGAGCTGCCTTTTCTGTAACAGAATCATCTCCAGCTCTGCTATA<br>TTTAACTCCTTTCTCATCTAACAATTTGACAAAGCGGGCAGGAAACCAACCACGGAGGCCATTCAGTTCACCAATCCAACA<br>GTGCTCATCTCTAGAACTGATAACTTCAATGACATCATTCTTTCTAAAGCCCAATTCATCTCCATCCCTCCTTTCAAAATCC<br>AGTAATGCTTTTGCTCGTCTCCTTTTACTCCTGGATACATTAACATATCTTTCTCTATCTGCAGCATGACTCTGCATTGTGTA<br>GTCAGCAGTTAGTTGAATTTCAGAAGCCAACTGTGGCTCTAGTGCCAAGAAGTGACGAGCAACTTGTAATATTGCTTCTCT<br>AAGATCAACTAATATTTCAGTTTGCTTAACATTTTTTGAAATAGCATCTTCATTGTTACTATCCTCTCCAAAGAGAATTGTT<br>TGAATAACTGACTTATTCCTTTTAATTTGACGTCTCTGTAATTGTTGTTTAGGGAGGTTGGGGACTGCGTTTGGATTTCCAA<br>CTAGAGCACCTTGATCTGCCATAAGGTTAAAATGGGAAACAAAGAACATTATAGGAAATGTGCCAAATGTGGCAGTAGTC<br>CAGTACAAGATCGAAATCTGAGTTTTCATAGATTTCCTCAACCTGGTAAACACCGGTCGTTGAAGGCGCGTGTGTGGGCTA<br>AATACTGTTATCCAGATGAAGATTGGTCAACTGAAAAGTCATTGAATGATTTATACTTGCAACATAAGATGTTATGCAGTA<br>AGCATTTTGATGACTCATCATATACCAACAGTTCAAGAACACGATTAACCAATTTTGCCATTCCAGCTGATGCACAAAACC<br>TATTGTTAGAGTTACGCCAGTTAGAAGAACGTGAAAAATCCTGTGAAATATTGGATGATGCCCCTAACCATGATATTAAGG<br>AAGATCAAGCATCAAAGGTGTTTAAAGAAATATTGAGAAAGCTAGACATCAATAAAGCTGCTGAATTAACTCCTAAAAAG<br>CGTAAACTATACCAAAGGTGCACAAGGAACTTGCATGAAATAATGAGGCTAACAAAAATTCTTAGAAAACAACCTCATGC<br>CCTTTTACAAGCATTAGTATCAAGTGAAAATGTTAAACAGTTGATGGACAAAAATATATCCGGTTCAATTGCTTTGCTATT<br>GCAAAGTCATCTTGTAAAAGCTCCAAGAAAATTTACTGGACGAAAATGGTCTATTGAAGACAAAGTTTTGGCTTTGAGTAT<br>ATATAAACGATCAACTACATGCTACAGATTACTAAATCGTCTTATTTGCTTGCCCAGTGAATACACACTAAAGGCGCTTTT<br>AAATGATGTCCCAATAAAATGTGGCATAAACAATAAAATATTTGATACAATCAAAAGAACCGCAGATAATCAAATAAGCT<br>ATGAAAATCTTTGTATTTTGGCATTTGATGAAATGCCAATACGAAAGAAATTATCATATAATAATGAACAAGATATAATTA<br>ATGGTTACCAAGATCACGGTACCCAAGGTAGATCTACAGAGCTAGCATCACGTGCATTATTATTTATGGCCATTGGTATTA<br>GAAAAAAATGGAAACAACCACTTGCATATTATTTTTCTGGTGATTCTATTACGGCGGACGAATTATCTGTTCTTATTAAGG<br>AGGTCCTTGAAGCTAGCTTCCAGGCTGGACTCATTATTGCAGCTACTGTTTGTAAGAATATTGCTGTGAATGTGAAAGCTT<br>TAAATTTACTCGGTTCATCAGTAGTAAATCCCTTTTTTTATTTTAAGAATCATGAAATCGTTACAATTTTGGACCCCCCTGA<br>TTTACTTAAAAGTTTCCGAGATAACTTTATCAAATACGACATTGATTGCACTCAACAGCAAAATGATGGGACTCAAATAAC<br>AGGTATAGCAAAGTGGAGCCATGTCAAAGGATTTTATGAAAAGGACCATGCAAACACCAGTTTTGTTTTTGCACCCGTCTT<br>AACTAAAGATCATATTGAACCAAACTCCCAACAAAAAACTAAACTCACCCTAGCTTGTCAAGTGTTGAGCCATTCAGTTGC<br>GGTAGGAGTTCTCGTGAAGGTTGCAAATAAGGAGCTGCCACAAGAAGCGACGGTGACTGCTCTGATCATCGAAAACATGG<br>ATCAATTATTTGACGCATTAAATGGCGATTCACCAGACAGTAATCGTGGCAAAAAGTTATTAACAAATATGACCAAGAGG

		AGTTCTCATTTTGAACATTTTGAAAAAATGAAGAGAATGTTTCGAAAGTTGAAATTTATCGGTGCCAGCACTAGACCGATG<br>TGTCAAGATGGTTTTGTAAGAACGATTAATGCAGTGGAGCATCTTTTTGCAAATTTAAAAAAGAAATATAAAGTAAATAGT<br>TTGTCCACGAGAAGACTCAACCAGGAGCCTCTTGATAATTGTTTTGGTTGCATACGCAGCTACTGTGGGCGTAATCAAAAC<br>CCAAATTCAACACAATTTGTTGCAGCTCTTAAAACTGCATTTATTGAAAATTTACAACACGGCAGCCGCAATAATAGCTGC<br>AGAGATGACAATAACATTGTACTGAGTGATTTAAAATCTTCATATATTAAAGATATTAATGAACAGTCTTCTATTATTAAT<br>GTTGATAAAGACCAACATGATTCTTCTGTTCATGTGGACGCAAGTGAATTTGGAACACCTCAATGTTATGGTGAATTACAA<br>ACATGTGCATATGTTTGTGGGTATGTTATTAAAAAAATAAGATCAAATTGTAAAAAATGTAAAACTGCAATGCTTGTAGAT<br>CAAAACATTGAGGTAAGCCATTTGTTCAATTTTGTTGAATACAACTGTATTAACACCAGTTTGATGTATATAAATAATAAC<br>TTCATACAAACAGTCGCGTCTGCTACAACTATTGTTAATAAATATCTAGATGAAGAAGGTTATGTTGATGAGTTAAAAAAT<br>AGACTAATATATATACTAAAAACACAGTTGACATTTGATTGGTTACATACTTGTTCTAAGCATTTTGATGCAAATCGTATG<br>CACATTATTGTGTCTGTTATATTTTTATGTGTTAAAAGCTACTGTACCATAAAAAATAGAATATTTGAAAAGGAAATATTA<br>AAAGAATCTTTATCTTGAAATATTGCAAAACCTTTAAAGGAAAGCAAAGTGACGTGAAATATTAAAGAATTATATATAAC<br>TAAATGATATTATGAACATAATATTGCTATTAGTTAGTTATATGAATACCAAAGTTATATTTATTTATTCTCTAAGAATTAA<br>CATGATACATGTATACCATTAAAGTATTAAGACTAAGTTAGTAGGTAAATGTTAGATGTTAAAATTATAAATGATTCAAAT<br>CATAACATTAATTTATAAACCAAGTAAAGAACTGATAGCAGTAAATTATAATAAGCATAAGAATTAGATATAAATAATAA<br>TGCCAAAATTTAATATAATAGAAGTGATACCATGTAGACTTGTATAACAAATTAAAATGGACATCCAAAAATACTCTATTG<br>ATGTTCTATTTGAAAAGGGAAGGGTGCAATGGGGTCGAGTATGAGGTGCCATTTCTCTAAAATTACCTCTCGATGTTTTGA<br>TTTAATAGTTTTACAAAATTACTGATTTAAACATCAATAATTTTGATTAAAAATCTATTAAAAATTAACTGCCGGCCTCGGC<br>TTTACATGATAACATAATGTGATTATAGCACAACTGCGATCAATTATGTCAATATTTTTAAAGATCCATAATCATCTATCTC<br>ACTCAGTTACTCACTTTTTTACGCAGAGATTTAATGACCCAACTCTTTCATTTTACATATGATTTTATAAACATTTTCTTTCA<br>TAGAAACATTTCAAACATTTCCCTAATAGTTATAAATACAACAAAAAAGAATTGGCAGAATAGGTGCAGCTGTTCTGAAG<br>TGACGCTCTTACCTATATTCAAATATTGATTTTTATTAATACAAATTTAGCTCATATTGGTCCTTAGAATAAAGATTTCGGT<br>GTAGTATTCAAATTTTAATTTTGAAGATATGTTTACAGAGTGATTGCCTCAAAATCGACCCCAATGCCATATTACATTTTAT<br>CATAAAAATACTCATTATACCTTAATACTTATAATAAGGGGGAAAGGTATTGTAAGTAATGGGGAGGCTGGTACACATTG<br>CTATCGATATACCCTATCAGATTGACCTCTACGCGGAAAGTGGAAACTGTCGTGACTAATTTATCGTTGAATTCGTCACGA<br>TGGGTTAACTGACCTGCCTTTTCATCCTCACCAGTCATCCCTCACTGGTGCCCTGCCAGTGTATATAGATAGCGCGGGCAG<br>GGTTACCATTTAATTATCATCCATAAAAAAATACTCATAAAAAGTCATACATAGGAGTTACTGTTTTAACTTACAAAGTAC<br>TGTATACTTTCCTATTTTTTTTTATAAATCTCGTTTTCTACATAACTGTATGTATATCTATCTGTATTTATTTCTGTATTAAAA<br>TTTTTCTACAAGAAGTAAGCGAAAAATAAAAACTAGATTATGATAAT
MSTRG.2495	Anoctamin-9 isoform X1	CGTGATTGACGTTTGGTATCATATTTGACATCTGTTGACTGAAAATTTCGCAAAACCAAAAATATTTCCTTTATCCATCCAT<br>ACTCCTCCACTTTTATCTTCACTTTCTATTATATATATTCCCAAGATTTATTTAATATAATTGGCGGGTATTTACAATAAACA<br>CATTTTATAGCCACCGTACTTGTAATTACAATAGTTGGAAAATCTGATTTTTCCTTTCTTGCCGATCAGTGACTAATACAGT<br>AATCGCTCCGAACAGGCTACGTATTCGGATATGACTATGTGTTAAGTGCTGTGGGGTCTAAGATATTGTACCTCGGCGTGT<br>AATGTCAGACGGTGACCCATCACTTGAAGAGGACGTTCCGAAGCCTCTGTTGGAGAGAGCAGAGGAAAATGTGTTTACGC<br>CGAAGATAGCTACTCCGATATATACTGGTGGAGTGAAGCTTCATCATAAAAGGTCTGACGCGGCGGAGACCGAGTCGTTC<br>CTGAATGACTCGCAGCGCAACGATCTCGCTTTCAGACAGTTATTCCAGGATGGAGATGCGGAATTTCAACAGTGATCCAGC<br>CTGGCTTTGCACGGGTAGATAGAATTTTCAAGAATAATACTCATCGAACACTTCTCATTGGCAATGAAGCTGTCTGTAGGT<br>TAAAGAAAATTTAAAATTGTTTGAGTTTATTTATTTCAAACAAATCTTTTATCTTTATAATATTATTACAGAAAAAGGCACA<br>CAGAGATTATTTATTGATTATGATTGATTGTTGAATATCATAATGAACAAATACAAATGTGATCACTTGAATGAATATAAT

		GTGATTGAATGAAGTTCACACAGAATGCAACAATGTGGTATGACCTTAACTGTGATACTTGTTTAGCATCAAATAAGTAA<br>TTTATACATACTGTTAGGGACTATATTTCTTATATGACTCACAGGAATGTTAACAAAATTTATTTATTTATTTAAATAACTA<br>ATTAAGTTAAAAAGAAAATAATTATGGTACAAAAGACAGACTATTAAGTTTGTGCTAGTAGCATTCTCTATCAGTTAACCT<br>TTAGTGTGAACAGTCAATCATGAATGCCATACTTTATTATTGGGATAGATAAAGAAAATAACAGTTGATAATGTATACCTA<br>TATACAATACATAATAAATTTATACTTTAAAATGCCAC
MSTRG.25	ATP-binding cassette sub-family G member 1	ACTCTATTCCCAGTATGGAAGCCCTGAGGAAACAACACCCTCTGTGCGAGCTCAGAAACCTGGATCCTGGTTCATCGGATG<br>AGGAGACCTCTCAATCCAATCAGAGATCGGTACTCATCAGACGAGGATTCCTGAAAGCGAAACGTGATGCGACAATGACT<br>CACCTGCGCGTTATCGTAAACGTATTAGTAGGTCTCATGCTAGGAGTATTATTCATCAACGCCGGCAACGAAGGCTCCAGG<br>GTTTTAGACAATTACAACTTACTTTTCGCCATCTTGATGCACCATATGATGTCACCGATGATGTTAACTATCTTAACTTGTG<br>AGTATCTCGACTATAAACTGTTTCCCATGTCCCGTAATAATTTTATTACGCTCTTCGAATTAAAGATTAAAATATTTGCGGT<br>GTGGTCCCAGTATAAAATAAACCACCTGTTCCTTTCCCGTGGGTGTCGTAAGAGGCGACTAAGGGGGTTTATAGTGAAGA<br>GATGCGCAGCAGCGTCCCTCTTAAAACATCGCCTGCCAAACGTGGCAACTACTGGCAAAGCCCCCC
MSTRG.2506	Glypican-6 isoform X1	GCAAAAGCAGTCGGGTCGCGACCATCATGGATCGTAGTTAGGTTGCAGTGAATCGTGCATATGGCGTATATATTACCGCTT<br>ATTCAATTTCAATTAAAAATCTTTAAACGTTATATAAATTTACGAAACCTTTGAATAGTTAAGTGTTTAACTTTAACACAAG<br>TACTCGTTATAGGAACATGGCTGTGAAAAAGTGTTATTGTGGTAAACTGTCCCATACCAAAAAATCGATTTTTGGATTAAG<br>TATGAGTAGCCAAATATTGTATTTACTCTTTGGTCTTGCTTTTATTGCATCTGTATCTGCAGCTGAGACTAATTGTACCACT<br>GTGAGAGATATTTTTAATAGACGAGGATTATTTACAACTTTGGAGCTACAAAAGGAGCCAAATTCAGGTAAATTCGCAGT<br>GAATCACGAACTTTTTGTGAAACTTTTATAAATGGCCAGTAAGTAGGATCAAGGAC
MSTRG.2520	Probable glutamateHypothetical proteintRNA ligase	ATGATTGCCTCTATGATGTAGAGCTAAAGTGACAGAATAATACTAATAAATTATAATATTAACAATTGAAATTATTATTAA<br>TTGAACAAATACATAATATGCTTTGGAATTATTAAATAAATAAAAAGTGTTGCCGCTTTATCTCCAACCTGTTACATTCTTC<br>TAAAAGGTCTGGGTTGAGACGGCTTGGATGTGATGATATCTTGTCGATCTGGAACTGTATCCCAAAGGGAATATATTACAA<br>CCAACAGAATTTTCAGGTTGCGGCATTTGTTGTCATACTTTGGTACTCTCTGACTCTTCACCGCATCTCTGCGGAGTACATT<br>CAGACGGCGCTCTGTACAGAAACATTTGTATGCAGAACCATTCTCCAGCAACTTTGTTATGTGATCTCTGAAATATTTTTTA<br>TTTATTAACAAACAGAAATGTTCATAATTATAGAGAAAGTTCATTGCTTAAAACCTTTATGCAGATTTTCTTGTATATAAAT<br>CTATACACAGAAAAACAATGAAATAC
MSTRG.2521	Kelch repeat protein	ATAAAATAATCTGAAATAATATATATTTAATTTACGACGTTAATGCTGCAGCGGTAGCAACAGTGTCTCACCCGATGATCG<br>TGGATTCAATCCCTAGACAATGCAAACATTTGTATTCAAATTTCTGTTTGTATTGGCCAGCGTGTCTACATGATTTATTTTA<br>AAAAAAAAAAAGTATGCCTATCAGTTATCTAGCTCCCATAAGCTTATTCTGTATGATAATGATGTCCAGTGATTATTATTT<br>ATTAACTTTGCCTTCAGCAACAAAGTTACGTTATACACTTTTATCAAATAGCAAAAAGATACTAAATATGTCAAGTGAGAA<br>CGATTCTATACCTATATATTATATAATGCAGTTAGATTATGCAGACAACTGGTTTTAGAAATTTTACCCGTGTTTAATTATG<br>AAATATTTAATTTATCAATTTCATTTTAATAGTACTCTGTTTTAATGGTACTGAAACGTATACAAAAGAAGACGCTTCCAAA<br>AAATATATGAAACGAGCGAGTTTCGGGGACCAAATTTTATTATTTCTGTTCGACGTGCCATCTACTTAGCTTTTATTTAATC<br>TATGAACATGCTGTCGTGTGTAGGGTTACCATCCGTCCAAATTTCTCCGGGCATGTCCGGACATTCGGCCTTCCGTTCGGTA<br>TTTCAGCATGTCCGGATTTTTTACTTAATATTCCTGTCCCTTAAAAAGTGCGCTACGCGTATTTATTACTTCATTTTATAAAT<br>ATCTAAACCTTTTTAAATGATCAAGTTTTTTGTAGTACTCTTTAATTTTTTCTAAATGGTAACCCTAAACATATGTCACTTCG<br>AATTCACGCTTGCAATAAACGTTAAAAATGTAGCGTTTTAGTAATGTGACTTATTAGAAACGTGTGAATGAGTTTCAATGC<br>ACGCCGCCGTACAGGTAGAGCGTAGAGCGCTGCACACACATCATGGCCGACATGCGCGGTGATGGACCGCCGCGTCTCCT<br>GGCCGCCGCGCATGCGCCGCCGACAACGGGATCCGAAGGGGTCGCGGCCCCCCCTAACTTCATTGTGAATACCTCGTCGG<br>TCACCACTGAGGAAGCAGCCATGACGCAGGCGGAGCGGGCGGAAGGCCCGCGGCCGGAGGGCTGCAGGCGGCTCCAGGT

		CCTGGTGTCGAGGCAGAAGCAGTGCAGGTCGTCGTGGTAGCGCGTCGCGGCCGTCGTCCGTGTACCCGCCGAACACCA<br>CGAGCCGCCGCCCCACCAGCGCCATGCGGTGCCCCGACCGCGCCGACGGCCCGTGCGGTGCTGACACCTGTCGGGAATTT<br>AATTTTTTTATTTTTACAAGCTTTTATCAAACACTATTGACTTCATATGAGTGGATAGAAAGATAAATTCGTGTAATTTTT<br>AGCTTTAACATGCATACTTTTACTAGGTATTTTATTAAATCTTTATTTGGTCTCTTAGCTCACTGATACCATTTAATATTAAG<br>ATTACTATGTATTTATACATAGTGTACTGATTGTACAATGTGTTTTAAGGTACCTTGGATTACATTGTTGTAATTTTTGTAG<br>GGACCTCTAATCTATCTATACTTATAATAAATCTGTAGAGAGGTCAATTCTGTACATGAAATATATTTCCAAAATAACTAT<br>CAGCGGGTGATTAGTGATCGATACTGTTGCCAAAAATGCAATCAGTAAAATTTTTGTCTGTCCGTCTGTATGTTCGTTATAG<br>AAACAAAAACTACTCGACGGATTTTAACGAAACTCGGTACAATTATTCTTCATACTCCTGGGCAGGTTATAGTATACTTTT<br>CGTCACGTTACGATCAATAGGAGCAGAGCAGTGAAGGGAAATGTTGGGAAAACGGGAGAAGTTACTCCATTTTTAAGCTT<br>CCGTTGCGTGTGCAGCCTTAATGGTCACTTTTTAAAATATAAAAATGTCCACCCATGCGACGCCGGGACGGGCCGCTAATT<br>TGTTTTAAAATAATATATCTAGTATATATTCCGTTATTATCTATTAAAAAAAAATCTCCTCCAGATCAATTCACAGAGCTAA<br>CCACAGAATGTGTAACAAATTTTACCTTTTCCCATTTTTTGTCGGCAAGTGAAAAACACCACAAATCCTTGTAATGATAGA<br>ATTGCGTCTCTGATGGACTTGTGAACTCGCCGCCAAACACCCACAGCTCACCCCTGCAAATATAACAGTAGATACATTAGC<br>ATAGATAAATTTAAATACCTTGGTCACCTGGTGACCACCAGCCTTAGAGACGATGTAGATATGGAGAGGGAGCGTAGGGC<br>TTTGTCAGTCCGAGCCAATATGTTGTCCTGCCAATTTGCGCGCTGTACAAATGCAGTAAAAATAACACTATTTAAAGCGTA<br>TTGTGCATCGTTGTATTCGGCGAGTCAGTGGTTCCGGTATACTCCTCGGCTTGCAATGCCTTGCATATCCAATTTATTAATA<br>ATTTCAGGTTGCTGTTGCGGCTCCCGCGCTTCTGTAGCGCCTCAGCGATGTTTGCCGACGCTCGCACAGATAGTTTTGACGC<br>AGTCTGGCGTAAAAAAACAGCTTCGTTTTTACGTCGAATACGCGGGAGTAACAACAGGATCCTGAACATGTTTGTTAATAA<br>ATTAGATAGTCCTCTCGTGAAAATGCTCGCCGAGAGGACTAGATCATTATTAGTGTATTAAGTATTAACATAGTTTTTAAG<br>TACACTTACTAACAAAAATGGATCCAAGTAATCTAAAAAGAAATAAATAAAAAAAAATAGATAGCAAGAAAAAAAAATTT<br>CACTCATCAGACCAGTGAATATTATCCGCCATTTATAATGAACTATCTACTTTGCCCGCTACTTCGTACATGTGGTTTTTGG<br>AAGCAGAAAAACCAATTAAATGTGCCATCTCTAGATGACATATTAAGATATACAAGACATACAAGATCTTGAGTCAATAC<br>CTTCAGAAGCACACAACAGGAGCAAGCAACAGTGAAAACAGCATCAATTTTGATGCAGTAGTGTTTATATAACGCCAGAG<br>CAAACATACAGACAGACAAACAAAATTTCTAAAAATCGGCTTATCATTCACCACATAGGTGAAAATGGAATGATAAGCCT<br>CCTTGCGGAAACTATTGTGGTTAATCCTTGTTGAATTCATCACAATGGTCCCAAATGAACTTGTTTCCACCCTCACTACAGC<br>CCCGTTAACATATATGAATAGTCCAGAGAGCCTTACCAAGCCATCCATTATAAAAAATTAATAATGAAATGAAATTTTAAT<br>TTATATTATTATATAAATAAATTATGCCTTATAAAAATACCTTTTATGACCTCATAAAAGATTATAATAGTAAGCACATTTA<br>TTTATTACAATATTAAATTGAATGGGTACCATATAGGCATACTTATTGGCAGGTGTGGCAACCATCTGATGGGCGCTGCGT<br>GGCGGCGGGGCCCCAGGCGCCTTCACTTGCTTCCATGTGTTGTTTACAGGGTTATAAAACAACAAGTCATTGTAAACTTCT<br>GTCTGAAATTAATATTTTATTTTTTTAATGGATCGACATTTCTATGTGAAATTGTTGTTACTTTGTATAGGCAATGGTCCTCG<br>TTTCAAGTTTATGTGGAGCAT
MSTRG.2528	PAX3- and PAX7-binding protein 1	CGGCGCGCTGCGGGACCGCGCGCTCGCCCGCCTGGCCGCGCTGGCGCACCTCGTGCTGCAGCACCTGCACACCGACAACC<br>CCATGCACCTGAAAGCTCTTGAACAGGCCAGAAATCTTATAGCAGAAGCCCGCGCGTTAGAATGACACAACGTAGACTGG<br>ACTTCCAATGAAAATACTGTATATTAATTAGACTTAATCCCTCCTAAATATACAACGTCACTGTTCTAACCATTGTCATTTA<br>CAATCACGAATTTATTATTAATCTATTTCAAAAAGTTTTACGCCATGCGATATCTTGGCCAATTGTGAACGTAATTTAATGA<br>AGATTTTTTAGGCTGATCCATACTATGTATTTAAAAATATAAGTTTTTGTTTAAAATGATAAAAATAATCGACGTTATAAA<br>ACTTATTTGATACCATTCTGTAAAAAAGAAGTCCAGTGAGAATATATAATAATGGATGGATGGCTTTTTGATAATAAAACA<br>AGTGAAATAGTGTCATATTTTTTATTGTTTTGATTTTTATAACTAAGTGTCGCTTGACACATTTTATCCCGAAGCCAAAAAC<br>TTAAGTGATATAAGAGAACTATCAGATATATACAATCAGAAAAGTTTCGACTCGGATGTAGTCACAATGATTAGTAATAA

		AACTAAGAGGTCCAATACAATCGAGTCCGTTCCTGAGCTAAGACCTGTGCATATTGTAGTTAAGATTACCCATTGTCACCAATTCTACAATGATACAATCAAATTTCTAATTCCTCTGGTCATATTCAAAACCAACTTGAGCTTAATCTATGATCGTGTAATAAGTGCTTTATAGGCGAGCGAGAGAGCAATATTTTCTTACTATCGTCACAGCTTAATAAGCTGTATCGTCTATCTTTGCCTGCCTACAGGACAGATTTAAAAACAACACCAAGACCTTAGATCAAAAACCGGCTCGGTTAACTGATAACACTATGAATTCGTTCCAATATTATTAAATTAACATTTATTATGCTTTTAGTATAAATATGGCATCAAAAGGAGAACGCATTCTATACGAGTGAGTTTATCTAAGCAATAGAAAAAAACTTCCTCTATTTTTATCAGGGTTAATACGCACTGGGACTTTTTTTCACATAGTTATGTATGGATAAGTGTCAAGTATAGATTATTTTCACGTGTGTAGAAAATACAATACGTAAAACTTCATACAAGTTCCAAATCTTACGCAGAGAATATGCGAAAATTCGCTCGATACAATTTTTCAGTGCGTATTTACTCTTAAGTAACACCGATCCAATCCGGTTTCGAGGACCAACAAAATATGTCAATTCTCTATTCACAATAACTCTTTATCAATGCGTTCCCGAAAATGAAAAACATGTAGGTAAATCTATTAATCTGTTTTATATATACATGTTTCTAAGTCTATAAATAATATACATAATGATATAATCAAAGTTAACTCATATTTTTGTGGTTTACATACTTAATATTTATGTTAGTTGTATTAAAAATACTAAGAGGTACATACTCTTACGCAATGTCTTGCTTCATTTACATGTGTTGTGTTGTAAAATTTTTGATGATGGACCGATTTTGACGTAGGTTTCATTATATTTAGACTGAAAAAGTTACTTGATTTTACACTAACATTCCTCT
MSTRG.2530	Probable glutamateHypothetical proteintRNA ligase	GTGATCGCCCTGGCAGGGTATATTGCTAGCAATGGGGCTGTTAGAACCCATTATTAATAATACTTTTCTCCCCTAAATTTCAGGAATTTATCCACTGGCATTGGTGAATTATATAACATTGTCCGGTGGAGGTTTCGACCATGTACCCGGCGCGGGGGTTAGGTTGAAGGACATGGACGAGTTGGCGGAGGAGTTCCAGATCGACAAGATATCATCACATCCAAGCCGTCTCAACCCAGACCTTTTAGAAGAATGTAACAGGTTGGAGATAAAGCGGCAACTGCAAGATGAGAAACTCTCGAAAGATTTATCGAGTAAGCTAGAAAAATTAATCGAAACCAAATATCCAGAACAGAAGAAGTATGTCACAGAAGAACACATAAAATCCGTTCTGAACTGGGCCACAGCGAGGGTGACAAGGATAGAAGATTTAGCCAGTTCAGAGTTCGGTTTCCTGTGGATTCTACCGTCTAACACCGAGATAGATTTTGATAGGAACCTGTTAATCAAATTGTTAGACACACTGGAAAGAATAGAAAGGTTTGATCAAGAAAACCTTAAGGATCGCTTGAGGGAATTCTCGTCAACTAATAATGTGAAGTTTCCAACACTTATGAAAACTCTCAGGTCAGTGCTTAGTGGGCTCAAGGAAGGGCCGGGCGTCGCTGAAATGATGGCCTTGTTGGGCAAAGCTCAGTCGTTGGAAAGAATAAAGGCTGTTATAC
MSTRG.2561	AlanineHypothetical proteintRNA ligase	TGGGCTGCGCCGGAGGAACTATCCTCGCCACGTGCCGGGTGCCACAGGAGTTGGTGAGCCCGTCTTTCAGCGCGGAGCGGTGGCTGGGCTGCGTGGCGGCGGTGTTCGGCGCGCGACTGCTGACGCATGCGGACGCGGCCGATCGCACGCTGTATGCAGAGATGGCCGGCGCTAAGGTGAGCCTGATAAACTGCGAGCAACTAGTGCAGGACGCGATGCGTGTTGCGATCAAGTATGCGCAGGCGCACGTCAAAGACAATGGCGACCGTAGCGCCACCGGCGGCCACACCACCAAGAACAGGCAGCAGAATTAAAGTTAATTATCACAGAGCGCGGGATTTAATTGGCGTTTTATAGAATTTGGATATTTCGTTTTCTTTATAGTTTGTTTTAATAAAAATTAATATGTAGGCAAACGAATAATTGGCCCGGCCATAAGTTAATTTGATGATGTCCAAGTAGTGGCAGCTATAAAAAAATCGATTGAATGTTTAGATTTGTCAATAACTACCATTTCCCAAAGAGGGTTGACGTCAGGCTGGCGGCCGGTCGTAAAACTAAAAACCAAATCTTTTTGTAATATGGATAACATCATATTACGCCGACCACAAATGATTGGGATAAGGGTAGGAAGATGATGAATAACTACCATTTCTAACATAACTCACATAACTAACCATTCGTGTTTCTACCGTGAATCAGTTGTGTTTGCATGGCTGTGTTTCGGTTTGAATGGTGGGATATGGTGTGAATTTACAGGATACAGAGGAATAACACCTGAGTCCTCAGAAATGGCAACGCATAGGGGGTATCATGGGCGAAACAGTATATTCTGTTATGTCCATGGTACTGCTCATGTCTATAGGCAACGGCTACTACTTTTCATCAGGTGGGCCGTCAGTTTGTTTGCCATTCTAAATTGTATAAAAAAAAATTACTAGCGATTCAGGCTGGCAATACTAATAATAGTATAAAATATAATAATGTAATAAAATAATAGTAGATGAAATTTTTTAAAATAAAATATATTTCACTTCCTAATAATGTATATTTCTATAGGTGAAAGAATTTTTAAAATCAATTAAGTAGTTTTTGAGATTATCAATTACAAACATATTTTTCCACTTTATAATGTTGTAAAGTATAGATTTTATTTAAAAAAGGAAAGGATTACTCTGTGATAATTGAAAAAAAAAAATTGTTACGTTTTCATTAAGTTTTATTAAAACAGACTATATGAGTAGATATTTCGTACATATTCGTTGTTACTAATAAATATGTCCCCTAGCGAGG

		TCATTTCTCGATAAGTTCAGTATAATTAATTTACAGAGTACAGAGGTATAACACCTGAGTCCTCAGGAATGGCCACGCATGGGGGGTATCATGGGCGAAACAGTATACTCTGTTGTGTCCATGGTACTGCTCATGTCTATAGGCGACTGTTACCACTTTCCATCAGATGGGCCATTCTAAGTATCATAAAAAATAATCTTAATTAATGTCTGTTTTAAAAAATAGATAGCCTTTATTCAGCAGTGGATTAATCGGTTAATTTTAAACTTTCGCGTTGTTTACACATATGTATTTTAAAAACATTATCCGGACTTAATGCAATGATTTTTTGGCAGCTAAATAGTACCAACTTCGACTGTAGTTACTGCTCTTTTATTGCCGACGTTCCGACATGGATTGCACCACATCGAAATAAAAAACAAAAACACTCCGTTTAGTCCCGATAGTGAATTTAAAATATGTGTAAGAAACGGGAAAGTTTGGGCAAATGTCTAAAATTTTTATGATCAATACAGATTCAATAATATTTGGAATTGTGAAATATAGTCATTAGAGTAGAGAAAAAAAAATCGATTTAATCGAGTAGTTAGTAATTAAAAAAGAGGATTGAAAATTTCTCAACAAAAAATGGAGGTAAATTTTAAAACGTCTTTTACGAGTTTGCTCAGAAGTTGATGTCATGTGAGATTAATTAGTTTTTGTGTTTTGTAAATATGCGATAGATATTAATAACCGGAATATTTAAGTTTTATAAACACTAAAACCAGATAAAGAATCTCTGCCTTAAAAAAAATTCGTAAAAGTAATATGGGTAAAATAAAAATATATTTATTATCTATGGTAATTTTTTTTTCGCCTTTTCGAACATAGATACTTAATGTTACTACATAATCTAACTTTAATTTAGAAATTGAGTTTTCATTAGTAATTATAATCCAGTTAACAATTTTTTTTTTATTAATTGAGATTCCATTACAATTATACGACTATGACGTTATAGCACATATCGTCATAGTCGTAGAATACTGACGGATCTTTATATGTAATCTTTGTCAGATCCGTCAGTATTCTACGGCTGTGACGATATTATGGATCAAAATAACTCGGAGATTAGTTAAAAAAATAAATATACATTTTTGGCAATTATAGTATTTTATCTTAGATATTCTTTGCTGACAGCTTTTAATATTTATTACTTTAATTTTATTTTTATATAATGACTAACGTA
MSTRG.2565	Uncharacterized protein LOC108759490	CGGATGTTTTGTTTTAGGTCATTAATTGACCGCGGGTTAGAGTCGTACACTTTAGCCTTAAGGTAGCCCCACAAAAAAAAATCCATTGGAGTCAGATCCGGGCTACGTGGTGGCCAGGAAATGTCTCCTCTCTTAGAGATAACTTTGTTTGGAAAAAGTTGACGGATAACGGGCATAGCAGTATTGGAGGTGTGGGAAGTTGCTCCATCCTGTTGAAACCATGTTCGGGCATTAAAGCATAAGTAAATCTTTCTTCAAAATCCGTTGTAACGATGTCCTGGATACGTTTAATGCACTGGAACGCTTACGAGTTGACTGTGTAGGATTTTCACGAATAGATTGTGCCACTGTGTCGATGTTTTGTTCCGTACGTGATGTCCTTGGGCGACCCAACCGCGGTTTATCTAACGTCGAACCGGTCTCCTCGAACTTAGCAACCCAATTCTTAATCGTTTGGAGAGCTGGAGTGTCATCAAAGTTGTGTAAACCTTTGTGTTCTCGAAATTTACGCCTCGCAACGGTTGCCGAATTGTTGTTTTGATAAAACTCGCGCACACAAAACGCACGGTCCGCTCCGGTGAAACGCTCCATCGCGACTAAATTCCCAGAAACCGAAGTTACTGCCCCCGCTTCCCCTGCACCGCTCACGCGCGCCGTTTTCGTTTAATTCAAATTTTACTTTTCTAAGGACTTTTGGGCCACCCTGTACAT
MSTRG.2573	Serine palmitoyltransferase 2	AGGCTCGAGAGCATGGGCGTGGTGGTATTCGGTCACCCGGACTCGCCGGTGGTGCCGATGCTGGTATACACGTTCAGCAAGATGGCGGCGACTGTGGAGCTGCTTACAGAGTGCGGCATCGCCACCGTCGGCGTCGGCTTCCCCGCCACACCTCTTAACAAGGCTAGAATACGGTTCTGCCTGTCTGCGGCGCATACTCGTGAGCAGCTGGACGCGTGCTTACGCGGCATTCAGAGCGCCGCCGACACCCTCGGCCTGCGGTACTCGCGCCGCCCGCGCCCGCCGCACTAGCCGCCTCGCCCGCCGCCGCTCTACCTCCTACTTCTCGTATTATTATTATTATTATTGTATTATTAATAAATTATTTTCACAAGCT
MSTRG.2578	Regucalcin-like isoform X1	ACTCCACGCCGGACGCAACTGGGTCAAAACAAAAGCAGCTAAACTCTTTTTTTTATCAATTAACAATAGCTAATAAAAGAATTTAAGTAACGAAGACGTAATGAAAGTCAACATATTAATAGCAGTGTTTTTAACTGTATTGATTAATATAAATGCCTACAGATCGCCTGCACCGCTTATAAAAAATATATTCCGTAGCGATGGTCACTTAGAGGGTCCTCATTGGTCGACGGAGGAGAATGCTCTCTATTTTGTAGACATAACAGGACAGAAGGTGCACAGACTGGACGTACAGACTGGCAATATAACTACCAAATATATAAGT
MSTRG.2593	Neutral alpha-glucosidase AB isoform X1	CGAAAACGTATCTATTTGCGTTGCGTATGTATGAGAATTATTATTTTAATTCGTTTATTATACACGGCAGAACACATCGGAAGGCACTCTGTACATTGACGACGGGGAGACCTACGATTACAAGGAGAAGAAGTATATTTACTCAAAAGTGAAGTACACACCAGAGGGCATTACTTACTCATTCATCGACGACAGTGCCTCGTATCCCACCCGATCGTGGGTGGAGCGTATCGTCATAGCGGGCATAAAGACTCCGCCAAAAACAGCCAAACTCAGCCAGAACGAGAAACAAACAGCCCTGCTAATGACGCTGCACAAAGG

		CAATGATGTGCTCGTAATAAGGAAGCCTGGTGCCAGCATGGCTAAACCTTGGAAGATAACGTTCAGTTATTAAAATTGAT<br>ACATGTGCATACTTACTGGTGTTTTAATGAAACTTAGATATGATATTAGGTAATTTTAAATTAATACAAATGTTTTAGTATG<br>TTGTTACCAGCTGTAAACACAAATTTGTTTGTGCTAGATTACTTATATACAAAATATAATTTTGTTTAACTGTGATCGTGCA<br>ATAGAAATACCTTTTCAAACTTTCACATACATAAAAGGTCCTTTAAATATAAGAGTTTGTATTATTAATTAGTATTTTTAAC<br>ATTATATGAAATAAATACACTGCCAGGTCCTAAACTAATATAGATTTAAAATCCTATACATCATGTTTGTTAATAAAAATA<br>AAAAAAATATTTAACTGGATTTTGTGAATTAACTACGTGAAATTAATTTATATGAAATTTTATTCAAATAATTGAGTAAAA<br>GCCAAATTTTCACATTTGTGTTCATTAAAACGCCAATAAGAGATTTCCCGACTTCAAAGAAGTCTTATGTCGTTGAATGTAT<br>TTCGATATGGGGAAGCCGGTGGTGTGATTTGGGGTGTAAACAGCCAAGATTGTCAATACAACTTACAGTAGATTGTATACT<br>CTAACTGCATGTCAAGCTCGGTGAGTTGCCACACTCCTGTATTTTTTTTTTACGGCTTGGTAGCCTGCGATATTTGTTTACA<br>ATGATAACAATCGAATTCAGCACGTTGTTGACCAAGGGGACCGGTGTGGAGGTTTGCTAACGAAGCATATACCCTGTTAA<br>CAGTAAATTACAGTAATAGATAATTCACCCACCATCCTTATTATGCTATATGAAATTACTAGTTTTACTGGCCACTTTATTT<br>CCTCCCCTTTAAAGGTTGGTATAAAACGTAGTAGATATCCTTTTGTGCAATTCTCATACCAAATTTAATCAAAATGGACTTA<br>GCCATGAAAACCAAGCAGTTACTTTTATAAAATAGACAAATTATAACTTTATTATTTATTTATTTAACTTGTATTTTTTAAA<br>CTTATTTAATTACAATATTGATCAAGTTTGAGATGCGAATTTTGCATAACCAACTCTCTTATAAGCTAGGCAAACAATATTC<br>GGACGTAATTTACTATGACGTCCCACTTCTATAATGTAGTATAATATAATGTGGCAACTCATATTAGTCCCCTATGCCATTG<br>TGAAAGTTTAGCCAACTTTTTTTATACTTTGATATAGCAATACGTTATTTACAATCCAGTCAAACGTAGATTCAATGTCATA<br>TAGAATAATATAAATATAGTACACAATATGGTAAGATAATAAAAAAAATAGTATGACACATAATATTATGTATACCCTGT<br>TTTTATATATACTTGGCATATTTATAGTATTTAAACAATTTACACACCTATTAATTTAATCATTAAAAATAAACACTAATCC<br>AAAAACATTGTAATAAAAAAGTAAAACAAAGTGCAATTATTATAAATAATATTATTTAAAATTAATTAAAAAATATTTAA<br>GCCGTTTGTTAATTTTATACGCTGTAATTTCCGTAAAAAAATGTCAAATTGCCTTTTATATTAAATATGCCAAGTGAAACAG<br>TTGTTGATGCATAAATCTCGACGATGTCTCATTTTTGTCACATTCGTGACAATTTTGAATATGTTCTTGTCATACTTAGTATT<br>AAGTTTCTTTCAGATTTAAGTGACGTTTTATCTAATTGAATAATTGAACAATGTCATCATTTTATACAGTTACTTTAATGTTT<br>TTTTTTTTATTAATCCAATTAAGGTCACATTATTGTCGAGATATATTCGTAATTCTCGTTAGCTGGCAGTGAAATGGTTAAC<br>TGGTTGTTTTGATTCACGCATTTTGTTTAGAAATAAAGTCAAGATCAAAATCAATTGTATACAAAAATAGTAAACATTTTTT<br>GTTTCTAAACAATTTGCGGTGGATACTAATTGAGCTTTGAGGGTATTAGTATTTTCTTGACGGGTACAGAAGTTTATCATG<br>CAGAGTGATAATAAAACAATTTTAATATTAA
MSTRG.261	Actin-like protein 6B	GGTATACGCTGGCGGAGCACCAGTGAGAGGTTATAGGTGAGAATGAAAAGGCAGGTCAGTTAGTCAATCGTGATGAATAC<br>ACCTGGAATTCAGCACATTGGTATCCAAGGGTGACCGCGTGAAGGTTGATCTAACAGGCTATATATTGCTAGCAATGGATC<br>TGGTAGTCCGCATCACTAACAATCCCTTTCTCCCTTATCTAGAAGCAGGATGTTGGCGGTAGAGATAATGATGATGTATCT<br>TGTTAACAGCAAGTGAACGGGGGCAACGAGGGCGGCTGCGTGTCGGCCGCTGAGTGGGACGCGTTCAAGCAAGAGATGC<br>TGCGTGAAATGCGACAGCAACTCAATCAGGCCAAGAAAGAGATTATAGACGCAATGAAGGCAGAGTTTGCGCGCAGATA<br>AAATTAAAGCGCGCTACCCGAGTGAAGCTAGTCCACCGCGCCGGATACCGGTGAATAACTATTTTACGTCTTAGTTATTTA<br>ATGAATAGGGGATCACTGTTACCCCACTGTAACGACCGTGAACTCAGTAGGAGCAAACACGGGGTCAACGTGACCACAAC<br>TTGAATATAATTAATATATTGATCGAGACGAATGTAAGAATAACACCATTTATAGCTAATGTTATTTGGGGTAAGTTACCC<br>CACTCAATAAGGGGTTATTATATTATCACTTTTATTATATATATTTATAGGATCATATATTGCATGTTCATATGTAATACAT<br>TAATGTAATTCGAATATATGACTTGTTTGTCACACATTTTGGAAAAGTGCTTATCTATTATGAACTATGCTCCTATAAATAT<br>GATAGTGTGATAACCTCTCAAGGCATTGGAGGTATGCATATGAGGTCTATTCTACTTGTTTTTTAGTATAATTTATTAATAA<br>TTTGTAATTTAAAATGCTTTTGGTCTAATGTAAACCCCCATCGCTATTCCAGGAGTGAAAAAAAAAAAATTATAATAGTTAC<br>CACTGTTCCATGAATTTGATAAAGTGGGACAATGTTGAATGAACAGACAGCCTCGCCTATTATATTATTAATTATAATAAA

TGTAATTATATTATGCATTGACATCTTACTGTCAATGGTTAAAAAACGCAGGATTTGGGGTAACTTTGATTATGTTACCCCA
AATTTAGCGGTTCTACTGATCCTGTCTTAATTCTGAATCTAATACTATCTAATTATTTAAATATTTTGATAATTATAATTCTT
TATTAAAAATTAAAAAAAGATACAATGGTTCTAAAATGTCGCTTTGGCAAACAATAATAATTTATATATCCATTTTGTATA
ATATTTTTATTTATTTAATTATATCACGCATTTCGTGATAATAGGATAGATTGAGAACTGAAATTACGAAGTATGGTGACG
CAGATGAGTAATAAATACTTCTAAATATATTGAAGATAAAGTATGATGACGTAAATTAACTAATAAATAATATTATATCTA
GTAACTATACAGGCTGATCGAAATCGTTAGCTTCCTTCAGTTAAAGATGATGTTCTGTCGAAGTATTAGGTCGAAAAACAA
GAAAAAAATGTTTAAAGCGTACTTTGATATAAATAATCAGTTTCACCACAAAAAAAACACCCTATATAAGGATGCTAATG
ATTTCGATCTCAGCCTGTATATTTATATATAATTATAATAGCAATTTGTAATTAATTTTTGACTATGCAAAGGTCCTAATCA
AGACTGTACAAAATGAAGATTGAACAAAATAGCCTAATTTCTTTTTTTTTTATAAAAATATACGTAAATACACGTTCGTGT
GTGGGTGATGACGTGTTTTTTAAATTTACACAGTTTTTTAGTATAATTTATACTAAAATATTGTTTTTTTTTGCACTAGTACT
CTATCAGAGTTTAAATTTAATTGTTCTAAAAAGAGTTTGACAGCTGGTTAATTCGTCGTGGTGAGTTCAGTACAAATCAGT
CACGATGGTTATCGACCTGACAGAGTATAATGCTAATGGGGACTAGAACCCCCATCATTAAAAAATCCAGCTTAAATACC
TTCCACCCAAATAATAATACGATACATGAAAAGTATATAGATACGACAGGAATGTGTCGAAAATGCAATCCGAAAATGAC
CCTAATTATTGCCTCTAATACTTGCCTAGACTTTTAGAAAAATTAATTAACCATCTCTAGTACTAGTAGTGACATCTATATT
TATGCTGTCATTGGTTTTTTAAAGATGAAAAAGAAAGTGGTAACCCCACCTACGCTGTCGGTATACACTAGCGGGGTACCA
GTGATAGATAGATAGGTGAGGACGAAGTCAGAGCAGTTGACCTATCGTGATGAATTCAACGGGAAAATAAATATGATTGT
TGGCAAGAAGGAGCTCGTTGAGTCCACCTGATGGGGGTATATTGCTAAGCTTACTACTATTGGGGTTGCCATACTCCATTA
CAGATAATCGGTAAATACGGGAGTTATTTACCCACCAGATATTTCGTTTATAATTTAATCGTTTAACGTATCTGGTAAAAG
TAATTGCTTCCAACAAGTTCGAAGAGTTAAAAATATAAAATAAACACTAAATAAGATAATTGTTCACCAGTATTAGCTCGG
TACAATGTGATGATGATGGAGATGGTGTAATAAACTAAGTTGCCAAGCGCTGTGGCCTCCGACAACGATGTGGCAACACA
GTAGTGTGTTAAGTGTTGCCATCTTATCTCGGTCTACGTATAAGTCGTGTTTACACTGATTTAATAAATATATATAAATAAT
GTTCTATTAACGATTAACGATTCAGTGGTTGTTCATAAAAGTACAAGATACGATGACTACGGTAAAATATCGTGGTTCTTC
GTGTTAAAGTTGAACGGTAAAATATCGTGGTTCTACAAAAGAGTGGGTATTTTGAAACTTAGTTATGTTAATAATGTGGAT
GCAAATGTAGATTCAAAGCTTATGATTGATTTTTTTTTAATTCAACTTCTGTATTTTTTCTTCGTAATTTGGTGTCCATATAT
TGTGGGTACCATAAGTAACTACTATCAAAATGTTGCCATATTCAACCATGTATCAATAGTTTTTTTTTTTGCAAAATGTAC
TGTAATTTCTTGACAAAACAAAAACGATATGAAAATACGAGTAGTTGAGAGACTTATAACACATTTTTTTTTAGTTACGT
GGATTATATTTTTATCATTTTTTTTTTAATAATCGCTTTCAATGTGGTGCTTTTTGATTTCTAAAATTAGAGAATCTATTCCA
TATATTTTATTTAATTTATGGTCGTCATATCAGTGTAAACACGACCTAACAGAAGTTGCTAGGAATAGTGTTGCAACATAC
AGTTGCAATACGTTTCCACGCATTATCGATTACGACAGACCGAAGTCGATTTAAAAAAAACTAGATTTAGCGCACGAATAT
CGAAGCATAAAAAAAGTTGTGTTTTTTTTAATTTTATACTTAATAAAGTATTTATTCGGATACGTATTATGTACGTAGTGGA
TAAGAGTTCGGATATTTTACAAAAGGAATCAATATTTACTATTGGGGTTAGCGGATATTTTTTTTTTCTTTATTATTTATATA
ATAATAAAGAAAAAAAGAAACTTAAAAATTATATGTATAAAACGCAATTTTGCAAATAATATTCGATTTTGTTGAAAATTC
TACAGAGACTATCGACCGACATATAATTAAAAGTAACAGTAAATGATGAAGTATCATTAATAAATCGATCTATAATAATA
ATGCCTATAAATTTGTTCGATTAATATCGATCAATTCGTTAAATATAATCGAATTAATTGGAGTAATTGGGACTCTTTTGTA
TTACATTTTGAAACGACTGTATCAATCGAGCAACTTTATAGACAATTTAATGGGTAACAATTTTTTTTTTATTATAACACCA
ATAGTAAATAATGTAACGTTAAAAATTTATATATATATGTGTAATATTCATATAGCGTCAAGTTATATAACTAGTATGTAA
CTCGATATTTATTGTACTGTTTCGCGTAATTTGTACGCGACAGTATAGTGTTAAGACGTACCTATTACAGGATATATTTAAA
TTGAGAACTGTTAAATGTCACTGTCACTGGCTGGTGACATATGGATTTTATTCCATATTCAAATTCTTATTTAATCTATGGC
ATAGTTCTGAAAAAGCGCGCGAATTTTAAAATAGCCACATTTTAAGGTTACTAATTGAATGATTTAATAAGTTATCGATTT

MSTRG.2616	Protein enabled	TGAAATATTGCGAAAAATAGAAACAGAATTTTGAAAAAAATGCAAAACATAGATTAAAACATTTAAAAGTTCTTTGCCAA<br>TTTAATTATGCTCTGTAAATACTGATAATATACTATGTAATTGAATATATGTAATATTCTATGCAATGTATATAAAATCTTT<br>TGCCGGGTAAAATTGACATATTTATGGGCACCCTATCTTCCCATACATTAATGTCTTCCATAATAAATAAGTTTAAAAAAA<br>TATTTTCAATACATTTTGGTAATGTTTTTAATCTGGCAACAATGATCCACGGTAACTGCTGTAATCGTTTTGGCGGGAAATT<br>ATTACTTGTTTTTTTTTTATACAACTTAGAATGGCAAACAAGCTGACGGCCCACCTGATGGAAAGTGGTAACCGTCGCCTA<br>TAGACATGAGCAGTAGCATGGACATAACAGAGTATACAGTTTCGCCCATGATACCCCCATGCGTTGCCATTCCTGAGGACT<br>CAGATGTTATTCCTCTGTACCCTGTAAATTGAATGAAGTGAATCATAGTTCAGAGTTTAAGTCAATCTTATTTTTTTTTAAA<br>CTTGATATTTTATTATTAATTTATATTATGTTTACAATTTATTGTGTATCATAATTTATGACCGTCTGTAACTATTGAAAAAA<br>AAAATATCTTAACCTTATTTTTTATTGAAATTTCTATTTTAATATCCACAAAAATCTTACCTAAGTTTAATTTAGTCTCAAAT<br>TAATTAAAAAGAAAAAATAAAACAAAAATTTCATATAAACATTCCAAACGCATTGTCCAGAATCATAAAAAGCCGATTGA<br>TTGAAGTAAAAATTTATATATCCAACCAAAATACTACATGAAACGATATTTTTAACATATTAGGGTGTCCAATACACATAA<br>AAGCAGAACAAACAGGCCAAACTAGTTAATAAGTGCTTCACAATTCGCTGATAAATAGCAGGCTTTCGAAAAAGCGTTTA<br>CCCGACAAAAGGTATATTGATATATTATATGAAATGTATCATGTAACTATAAAGGGAGTACATATATGTAGCAGTACAGG<br>CATTTGGTTTACAAATTACACGATTTCCGCTTTAAATTGTATTAGCACCTGGATTTTTAACATAGACATAGATGTACGTAGA<br>TGTCGCAAACGCATGTGCTACAAATAAGACCCCGATTTTAACTAAATATCAATTCAGAGTCATTTTTATTTTAATTTTTTTT<br>TTTCATTATTATTTTCTATTTCGTAAAAAGGGTGATTAAAATATTAAAGTATAAGTTTACGTGGTCGGACAAATTTACAGAC<br>AGAAAAAAAAATCACTAACGTATTATGGCGAAAGCAATAAGGACGTAGACAATAATGGTGAGAGATTGATTACTAATAA<br>GACGCTTCTAAGACCAACTATTCACAAAATAAATTGAAAAACAAAATAAATTGATTAATTGATTCAAGTAAATTTAAATTT<br>AAGCATTGTAATCGTCTTACTAAGTTCTCAATACAGGTCTCAATCAGGACAGGCCCAGCCTAGTGTAGGAAGCAATGATA<br>AATTCTGTGCTAATACAAACATACTTTGTTTAATAAATATATCTTTATCTACCTTATCTTTGTCAATACCAAATGTTACAAA<br>CAAAACAGTTATATACAACTAACTAATTTGGCGAAACGCTTGCCGGTATATAAATCGAGGCACGTGCGTCATCTCTGTCCA<br>TCTATGTCTATGGTTTTTAGTTATAGGTACACGACTGATCGAGCATTCCATTCGAACGTGGTGTTGCCATGAACTTTACAGC<br>AATATTGATTTTGTATTAATGGTTCCAAGTATTGATAATGAAATTGCAACCTTACTCCTATTTAAATTGTATGTTTTTACA<br>ACGTTATCTTATAAATACAGAAATAGATAATAATTGTTTTCATGATTACATTTTTAGAAAGAATGGCAATGCATGGGAGGT<br>ATCATGGGCGAATCAGTATACTCTGTTATGTCCATGGTACTGCTCATGTCTATAGGCGACGGTTACC<br>GGTATACGCTGGCGGAGCACCAGTGAGAGGTTATAGGTGAGAATGAAAAGGCAGGTCAGTTAGTCAATCGTGATGAATAC<br>ACCTGGAATTCAGCACATTGGTATCCAAGGGTGACCGCGTGAAGGTTGATCTAACAGGCTATATATTGCTAGCAATGGATC<br>TGGTAGTCCGCATCACTAACAATCCCTTTCTCCCTTATCTAGAAGCAGGATGTTGGCGGTAGAGATAATGATGATGTATCT<br>TGTTAACAGCAAGTGAACGGGGGCAACGAGGGCGGCTGCGTGTCGGCCGCTGAGTGGGACGCGTTCAAGCAAGAGATGC<br>TGCGTGAAATGCGACAGCAACTCAATCAGGCCAAGAAAGAGATTATAGACGCAATGAAGGCAGAGTTTGCGCGCAGATA<br>AAATTAAAGCGCGCTACCCGAGTGAAGCTAGTCCACCGCGCCGGATACCGGTGAATAACTATTTTACGTCTTAGTTATTTA<br>ATGAATAGGGGATCACTGTTACCCCACTGTAACGACCGTGAACTCAGTAGGAGCAAACACGGGGTCAACGTGACCACAAC<br>TTGAATATAATTAATATATTGATCGAGACGAATGTAAGAATAACACCATTTATAGCTAATGTTATTTGGGGTAAGTTACCC<br>CACTCAATAAGGGGTTATTATATTATCACTTTTATTATATATATTTATAGGATCATATATTGCATGTTCATATGTAATACAT<br>TAATGTAATTCGAATATATGACTTGTTTGTCACACATTTTGGAAAAGTGCTTATCTATTATGAACTATGCTCCTATAAATAT<br>GATAGTGTGATAACCTCTCAAGGCATTGGAGGTATGCATATGAGGTCTATTCTACTTGTTTTTTAGTATAATTTATTAATAA<br>TTTGTAATTTAAAATGCTTTTGGTCTAATGTAAACCCCCATCGCTATTCCAGGAGTGAAAAAAAAAAAATTATAATAGTTAC<br>CACTGTTCCATGAATTTGATAAAGTGGGACAATGTTGAATGAACAGACAGCCTCGCCTATTATATTATTAATTATAATAAA<br>TGTAATTATATTATGCATTGACATCTTACTGTCAATGGTTAAAAAACGCAGGATTTGGGGTAACTTTGATTATGTTACCCCA

AATTTAGCGGTTCTACTGATCCTGTCTTAATTCTGAATCTAATACTATCTAATTATTTAAATATTTTGATAATTATAATTCTT
TATTAAAAATTAAAAAAAGATACAATGGTTCTAAAATGTCGCTTTGGCAAACAATAATAATTTATATATCCATTTTGTATA
ATATTTTTATTTATTTAATTATATCACGCATTTCGTGATAATAGGATAGATTGAGAACTGAAATTACGAAGTATGGTGACG
CAGATGAGTAATAAATACTTCTAAATATATTGAAGATAAAGTATGATGACGTAAATTAACTAATAAATAATATTATATCTA
GTAACTATACAGGCTGATCGAAATCGTTAGCTTCCTTCAGTTAAAGATGATGTTCTGTCGAAGTATTAGGTCGAAAAACAA
GAAAAAAATGTTTAAAGCGTACTTTGATATAAATAATCAGTTTCACCACAAAAAAAACACCCTATATAAGGATGCTAATG
ATTTCGATCTCAGCCTGTATATTTATATATAATTATAATAGCAATTTGTAATTAATTTTTGACTATGCAAAGGTCCTAATCA
AGACTGTACAAAATGAAGATTGAACAAAATAGCCTAATTTCTTTTTTTTTATAAAAATATACGTAAATACACGTTCGTGT
GTGGGTGATGACGTGTTTTTTAAATTTACACAGTTTTTTAGTATAATTTATACTAAAATATTGTTTTTTTTTGCACTAGTACT
CTATCAGAGTTTAAATTTAATTGTTCTAAAAAGAGTTTGACAGCTGGTTAATTCGTCGTGGTGAGTTCAGTACAAATCAGT
CACGATGGTTATCGACCTGACAGAGTATAATGCTAATGGGGACTAGAACCCCCATCATTAAAAAATCCAGCTTAAATACC
TTCCACCCAAATAATAATACGATACATGAAAAGTATATAGATACGACAGGAATGTGTCGAAAATGCAATCCGAAAATGAC
CCTAATTATTGCCTCTAATACTTGCCTAGACTTTTAGAAAAATTAATTAACCATCTCTAGTACTAGTAGTGACATCTATATT
TATGCTGTCATTGGTTTTTTAAAGATGAAAAAGAAAGTGGTAACCCCACCTACGCTGTCGGTATACACTAGCGGGGTACCA
GTGATAGATAGATAGGTGAGGACGAAGTCAGAGCAGTTGACCTATCGTGATGAATTCAACGGGAAAATAAATATGATTGT
TGGCAAGAAGGAGCTCGTTGAGTCCACCTGATGGGGGTATATTGCTAAGCTTACTACTATTGGGGTTGCCATACTCCATTA
CAGATAATCGGTAAATACGGGAGTTATTTACCCACCAGATATTTCGTTTATAATTTAATCGTTTAACGTATCTGGTAAAAG
TAATTGCTTCCAACAAGTTCGAAGAGTTAAAAATATAAAATAAACACTAAATAAGATAATTGTTCACCAGTATTAGCTCGG
TACAATGTGATGATGATGGAGATGGTGTAATAAACTAAGTTGCCAAGCGCTGTGGCCTCCGACAACGATGTGGCAACACA
GTAGTGTGTTAAGTGTTGCCATCTTATCTCGGTCTACGTATAAGTCGTGTTTACACTGATTTAATAAATATATATAAATAAT
GTTCTATTAACGATTAACGATTCAGTGGTTGTTCATAAAAGTACAAGATACGATGACTACGGTAAAATATCGTGGTTCTTC
GTGTTAAAGTTGAACGGTAAAATATCGTGGTTCTACAAAAGAGTGGGTATTTTGAAACTTAGTTATGTTAATAATGTGGAT
GCAAATGTAGATTCAAAGCTTATGATTGATTTTTTTTTAATTCAACTTCTGTATTTTTTCTTCGTAATTTGGTGTCCATATAT
TGTGGGTACCATAAGTAACTACTATCAAAATGTTGCCATATTCAACCATGTATCAATAGTTTTTTTTTTTGCAAAATGTAC
TGTAATTTCTTGACAAAACAAAAACGATATGAAAATACGAGTAGTTGAGAGACTTATAACACATTTTTTTTTAGTTACGT
GGATTATATTTTTATCATTTTTTTTTTAATAATCGCTTTCAATGTGGTGCTTTTTGATTTCTAAAATTAGAGAATCTATTCCA
TATATTTTATTTAATTTATGGTCGTCATATCAGTGTAAACACGACCTAACAGAAGTTGCTAGGAATAGTGTTGCAACATAC
AGTTGCAATACGTTTCCACGCATTATCGATTACGACAGACCGAAGTCGATTTAAAAAAAACTAGATTTAGCGCACGAATAT
CGAAGCATAAAAAAAGTTGTGTTTTTTTTAATTTTATACTTAATAAAGTATTTATTCGGATACGTATTATGTACGTAGTGGA
TAAGAGTTCGGATATTTTACAAAAGGAATCAATATTTACTATTGGGGTTAGCGGATATTTTTTTTTTCTTTATTATTTATATA
ATAATAAAGAAAAAAAGAAACTTAAAAATTATATGTATAAAACGCAATTTTGCAAATAATATTCGATTTTGTTGAAAATTC
TACAGAGACTATCGACCGACATATAATTAAAAGTAACAGTAAATGATGAAGTATCATTAATAAATCGATCTATAATAATA
ATGCCTATAAATTTGTTCGATTAATATCGATCAATTCGTTAAATATAATCGAATTAATTGGAGTAATTGGGACTCTTTTGTA
TTACATTTTGAAACGACTGTATCAATCGAGCAACTTTATAGACAATTTAATGGGTAACAATTTTTTTTTTATTATAACACCA
ATAGTAAATAATGTAACGTTAAAAATTTATATATATATGTGTAATATTCATATAGCGTCAAGTTATATAACTAGTATGTAA
CTCGATATTTATTGTACTGTTTCGCGTAATTTGTACGCGACAGTATAGTGTTAAGACGTACCTATTACAGGATATATTTAAA
TTGAGAACTGTTAAATGTCACTGTCACTGGCTGGTGACATATGGATTTTATTCCATATTCAAATTCTTATTTAATCTATGGC
ATAGTTCTGAAAAAGCGCGCGAATTTTAAAATAGCCACATTTTAAGGTTACTAATTGAATGATTTAATAAGTTATCGATTT
TGAAATATTGCGAAAAATAGAAACAGAATTTTGAAAAAAATGCAAAACATAGATTAAAACATTTAAAAGTTCTTTGCCAA

		TTTAATTATGCTCTGTAAATACTGATAATATACTATGTAATTGAATATATGTAATATTCTATGCAATGTATATAAAATCTTT<br>TGCCGGGTAAAATTGACATATTTATGGGCACCCTATCTTCCCATACATTAATGTCTTCCATAATAAATAAGTTTAAAAAA<br>TATTTTCAATACATTTTGGTAATGTTTTTAATCTGGCAACAATGATCCACGGTAACTGCTGTAATCGTTTTGGCGGGAAATT<br>ATTACTTGTTTTTTTTTATACAACTTAGAATGGCAAACAAGCTGACGGCCCACCTGATGGAAAGTGGTAACCGTCGCCTA<br>TAGACATGAGCAGTAGCATGGACATAACAGAGTATACAGTTTCGCCCATGATACCCCCATGCGTTGCCATTCCTGAGGACT<br>CAGATGTTATTCCTCTGTACCCTGTAAATTGAATGAAGTGAATCATAGTTCAGAGTTTAAGTCAATCTTATTTTTTTTTAAA<br>CTTGATATTTTATTATTAATTTATATTATGTTTACAATTTATTGTGTATCATAATTTATGACCGTCTGTAACTATTGAAAAAA<br>AAAATATCTTAACCTTATTTTTTATTGAAATTTCTATTTTAATATCCACAAAAATCTTACCTAAGTTTAATTTAGTCTCAAAT<br>TAATTAAAAAGAAAAAATAAAACAAAAATTTCATATAAACATTCCAAACGCATTGTCCAGAATCATAAAAAGCCGATTGA<br>TTGAAGTAAAAATTTATATATCCAACCAAAATACTACATGAAACGATATTTTTAACATATTAGGGTGTCCAATACACATAA<br>AAGCAGAACAAACAGGCCAAACTAGTTAATAAGTGCTTCACAATTCGCTGATAAATAGCAGGCTTTCGAAAAAGCGTTTA<br>CCCGACAAAAGGTATATTGATATATTATATGAAATGTATCATGTAACTATAAAGGGAGTACATATATGTAGCAGTACAGG<br>CATTTGGTTTACAAATTACACGATTTCCGCTTTAAATTGTATTAGCACCTGGATTTTTAACATAGACATAGATGTACGTAGA<br>TGTCGCAAACGCATGTGCTACAAATAAGACCCCGATTTTAACTAAATATCAATTCAGAGTCATTTTTATTTTAATTTTTTTT<br>TTTCATTATTATTTTCTATTTCGTAAAAAGGGTGATTAAAATATTAAAGTATAAGTTTACGTGGTCGGACAAATTTACAGAC<br>AGAAAAAAAAATCACTAACGTATTATGGCGAAAGCAATAAGGACGTAGACAATAATGGTGAGAGATTGATTACTAATAA<br>GACGCTTCTAAGACCAACTATTCACAAAATAAATTGAAAAACAAAATAAATTGATTAATTGATTCAAGTAAATTTAAATTT<br>AAGCATTGTAATCGTCTTACTAAGTTCTCAATACAGGTCTCAATCAGGACAGGCCCAGCCTAGTGTAGGAAGCAATGATA<br>AATTCTGTGCTAATACAAACATACTTTGTTTAATAAATATATCTTTATCTACCTTATCTTTGTCAATACCAAATGTTACAAA<br>CAAAACAGTTATATACAACTAACTAATTTGGCGAAACGCTTGCCGGTATATAAATCGAGGCACGTGCGTCATCTCTGTCCA<br>TCTATGTCTATGGTTTTTAGTTATAGGTACACGACTGATCGAGCATTCCATTCGAACGTGGTGTTGCCATGAACTTTACAGC<br>AATATTGATTTTGTATTAATGGTTCCAAGTATTGATAATGAAATTGCAACCTTACTCCTATTTAAATTTGTATGTTTTTACA<br>ACGTTATCTTATAAATACAGAAATAGATAATAATTGTTTTCATGATTACATTTTTAGAAAGAATGGCAATGCATGGGAGGT<br>ATCATGGGCGAATCAGTATACTCTGTTATGTCCATGGTACTGCTCATGTCTATAGGCGACGGTTACC
MSTRG.2627	Calpain-A-like isoform X3	GAAACTTATCAGTCCAGTTTTCAGTCAATGGTGTTAGTTTATAACGACCGGTCACTTTAACTGCGAAATATTTCTTTATTTA<br>AAAAAAAAATAAACAGCGAAAGTGTAATGTGACTGAAAAATAAAAAATAAATGTAGATAATTAAAAAGAAAATAAAACA<br>AATATTGTCGATAGTTTAAACAATTGTACTACAATAGAAATAAATAGAAAAATCAATCGACTCAAGGACATACATCATGG<br>TTAAGCAAATTAAGAAAAGTATCTCAATCGTTTAAAACAATCCACTTAAGCTATTTACTTAAGTGCAGTTTGAAATAGAAA<br>CGGTAACAGTCATAATGTCGAGCGAAGTGCAAGAAGACACATTTTTGAAATTGTATAAAGAAGACGTCGGGAATTTAAAG<br>ACATTCTATTTAAGTGCTTCTCTGACGGCTGCAGCGGATTCATGGAAGAACAAATCTCTCATTGCATCTCCTAATCTAGGA<br>AGAATGTTGAAACAAACAAGCAAAGTCTCTCCTAAAGATGTGTCTATGGAACCAAATTCTATAACACGCATGTTGGATGA<br>TTTCAAAGAAGTTAAATTACAACAATTAAGTTACCACTCGAGTCCTTTTTGGGCCACGAAAGTAGACACTAAAAATTCGAT<br>CCCTGAAGAGACTTTAACACTGAAACAAGTTACTGTAGTTTCAAGAAGTAATTATGTAACGAACAAAGCTTTAGTGTTTGA<br>TAGAGAAACGATTGCGTCCCCAAAAGTGACTTTGAAAGATCATGCGAAATATTCACCAGAAAATGTTAAGTTCGCTGTGT<br>CTGAACATAAGGTA
MSTRG.263	Uncharacterized protein LOC106129949	ATAGGTCTTTCGAATAATGAAATATTTTAATCTTATTGTGTTATTTCGTTGTAAACATGTTGCGAACAATAACATTGGTCGG<br>TGTGTTTTTGGTTGTGGTGAACGGGCAACTCTCCACTCCAGTTAGTACTTGCAATTCGAATCCGGGTCAACCACCAATTAA<br>CACATACATCGAGAATTGTGTCAGTCTCCCGTGCTTGCTGCCACAACTCCAGAATGTTGTTCTGAACATAGCATTTAGAGC<br>TC

MSTRG.2633	Calpain-B-like isoform X3	ATCTTTATCAACTACAAAAAGAATCAAAAATTCAGTCGGAATTGTTGAAGTTATAATAATATCGCATATTAGATACATAGA<br>TTACCTTATTACTACGTTTATGCATAAAATCTTAGCATAAAATAAATAGCCGGTTGATTGAGAACCGGTTGTTTTTTTGTAC<br>ATATTTCGTGGATTGAAAGAAAATCCTACGAATGAACGAATTCATCGAATTAAAAAAAAAGATTTGCACAAAATAATTGT<br>GGAAGAATTATTTATTACGGAATAGGAAAACCTTATAAAAAACACAGGATTAGGAATGAGCAACTTCAATATGGTATTCG<br>AGTTCTGCAGACACACTCAGATCACACAGAGTATAAAATCAATGTGGTGGTTACGTCACTGCTTGCAACAGTCTGTGTAGT<br>TACAATAAAGCAACTAATTTGAGGAGACCTCTATGATTGTGAAACAACGGAATGTAGAACTGTTTCTAAATTTATATACCT<br>AATTCTTCTCCTGTCAGTTTCCATTCAGTGTTCGGGTATGTTTGTCAACCCTAAAAATGTCTTAGCGGTGTGTAAAAAAGAC<br>AGACTGTAGTGTAGTAGTGTGTGTGAGACCTCGCAATGGCGGGGCAAAATGGTGGAAATTTTGGCTCTATTCTCCAAGGA<br>GCCGGAAGACAGTTGTTAAACCAGGGCGGGCAAGCGCTACTAAACTATGGTGCTCAAGCTCTGGGTAATATCATTAATGA<br>GGTGTTCCAGAAGAAGGAGGTGGAACAGAAGAGGTTCCTGCCGAGCATTAAGAATTATAAAGTG
MSTRG.2677	Uncharacterized protein LOC106130397	AGGAGAGTCAAGAAGGGAGGGTTCGACAGAGGAGCAGACCGCTCCAGATGCTGATGTCGACACTAGTGATGCAAATACG<br>AAGGAAGCACCTGACAAGGACAATAACTGGCGTTCAGCGGCACCTGGCGGCCGCGGCGTGAACCGCGCGGAGAGCTGCC<br>GCGAGCGGGATGCGCCGCGCCGCGGGAAGCACCGGAACGCCTCGGATCCGAATCGTCTTACAGCGCACAATAACCA
MSTRG.2698	endonuclease and reverse transcriptase-like protein	GTTATATTGTCTTCCTTTAAGAATAATCATAGGAATACATTATTATTATATCATTATAGAAGTTTAGTGATGCACAGTGCCA<br>CTTTTGATAACGTTTGTAATAAAAAAAAAAAACTATCGCCTGCTCGATATAAAGGTTTTTTTTATTATCATTTTAAATATGAC<br>ATTCTAAAATTTATCTTTACTGACTACTACTAAATGTCCTATTTTGACCTTAAAGTATGTCTCATTTTAAAGATGAATTGCA<br>AATTCTGGAATATTGTCGAAAAAAAAAAATAAAAGAAGGAAAAAGATGATTTCATAGTTTTCATAATTGAGGTCATTATTA<br>AATAATTTGTATTGAAACATTGAATAGGATTTTGTTATAACTGTATATAAAATCTATCAGTTACGTATTCAATGTAAAATG<br>ATAATAATAAAACTAATAGAAAATTTGACAGCTCGCGAAATGACGAGATGCGAGAAATACATCTTTAACCCTCCGCCAAC<br>CGTATAGGACGATATCGTCCGCGCCAAGTTTCGACCTGATGCATATCTTTGTACGAAACCCTTTTTTTCGCTGGCTTCGGAC<br>CCCTCTGTAAAAAAACTTATACACGCGCGTACCCCACAGGCACAAGCACGTCAGTATCCGTTGCAACATCGTTGAGTATGA<br>ATGTGTGCAAGTTTGCAGGCGGACGATATCGTCCTGTACGGTTGATGACGTTACTTTTACGAATATTTAGGCATACATACA<br>TACATACATATCTGTCACGCCTGTCTCCCATTAGGGTAGGCAGAAACAATGGAACGCCAAATGCTTCGATTCAAACAAACC<br>TCTTTCGTTTAGGCATATGAAATATAAATATTTTTCATTGCATTATCAGTGTTCTGAATGAAAATTTAGTGTAATAATTGTT<br>AGATGTAATTTTTTCAGAATTTTCATTTGTTATTATGGCTGAAGCCTTACACGAACATCAGTATTATCTACTAGTGAATCGG<br>AATCTGAAATTGCATGTAAAAGTGAACATCACTCCTCCGATAACCAAGAAATTGAGGGGGGAAAGGGATTGTTAGTAATG<br>AGATTTAACAGCCCCATTGCTAGTAATACACCAGGTCGACCTCCACGCGGTTCTCCCTGGAAACCATAGTGGGTCAATCTT<br>GTTCAATTGGTCACTATGGGCCAACTGACCTGCCTTCATCCTCACCTGTCACCCTTCACTGGTACTCCGCCAGCGTATATCG<br>ATAGTGCAGACGGAGCCACCGTTCCATCTTCACTCATTAAAAAAAAAACAAGAAATTGAAGAGGAAGGTGAATCTAATCC<br>TGATCAACCTCCACCAGCGAAACTTATTTAAATACTCTTTTTTTTTCTGTTATTCTCATATTATATTGTAATTTAAAAACCAA<br>TTTTGTATTAATTTCTTAATAATAATTATATTTTATATATAATTATTATTAAGAAAATACTAATCCAATAAAATACTTATTTT<br>CTTGATAAACGTTCCTTTTAAAAATATACCATTTTCGGGTGTTAAAATAAATATATTTTTTTGAGTGGTGTAAGAGCTTATG<br>TGAAGAATAAATCTTCAAAACATTACTTCTTAATACTAATTTATATCCAAGGTATCGATTTTTGAATGAAATCTTAGAAATT<br>ACGGTTTTTGTAGAGATTACAAAATTTTATTTCACCAGGGGGTAAAGTATTCAAGTCAAAAACTATGGAAACAGAAAAAA<br>GAAATCTAGAGCATATTCCCCGATTTTTTTGGAATTTTTCCCTACCTTGAAGTAGGTTATATTATCGTCTAGGACGATATCG<br>TCCGTGCCGGTTGACGGTGTGCAAAAAAAAACATATGGTTGGCGGAGGGTTAAATATATTTGATACTCAGTTATGTAATAG<br>TTTTAGAAAACTTTGAGGCTGTTGTTATGTATAAAAAATACAATTTGATAGCAAAATATGTTTTACTGTCTGTGCGTTGCTA<br>TGTATTCTAAAAAAAAAAAATCTTCAAAATAGTGCCTTACAATCGAATGGGTGCTTGTTAAAAAAAAAATGCGTTAATGCAA<br>CTATTTATAATAAAATTAGTCGCCATTTACTTTAAATATAATATATCAAAAGATTAATTGTACTTATATTTAGATTTTAGAC

AAATTAGAATTTATTTATTATAAAACATTGGTTGTATTATACATTGTAGTTAGTTTTGTGTTGCATATATGAAAAGCGGGTC
TAAATGTCTTACTAAATTATGTTTCATGCCACAATGTAAATATCGCCATCAATTACCATAGTACACATAAGTAATGTAATC
AGTATTAATACAATATCCTAATTTATATGAAGGTCTAATTGTAATGACACATCTTGGTGAACTTATTAAGGCCCATGATCT
AAACGTTTTTTCAAAAATCCTCACATTTAAATGTATGTAATAATAATCAAATTATAAAAAGGTTATAAATATGGGGTGTAT
TCAAAATTTTTAGCCTTTTTGTTGTTTTTTTTCCTAAGAATAAATCTGAACATTTTTTTACAAAAAAGGTCAGGAAAAACAA
ATGTCAATGTATGATTCTCCGAGAAAGTATCGTATGTTTAGTTTCACCGGAGAACACTATCATTCTCCATGACTTTAATTTC
ACCCAGTTTATTTAGTAACTATTCGAAAAAAAATATCACTTGAATAACATACATACCATGGGGTCGTTAATAAGTTTGCCAA
GACAACAAATTAAATTCAGTTACGGGATCTATTTGCGATGTAAGTTAGCTTTAAGTTAGTAAATCGCGACAGACTGTTAAA
TATTCTGCGAGTAGATATCGGTTTGTTATTCGTTAGCGGTAAGCTGTTGATTTTCCATTATAACCTTATTGTCATAAAAATA
AATAACTGAATAGAAGTGACACTGCTGGAAATAATATTCATACTTGGTTCATATCCGGCTCGAAGGACCATAAGGCCTGTT
AATCTATAACCTAAGGTACAATTCTCAAAGTGTGTACTCCTAATTATTTTATTAATAAAAAACTGCAGGAAAGTTGCACTA
AGCTGCACGTACGAAGTGACAACACGATACTTTATCTTATAAATTTAAAACAACCCTAATACAAAACTGTATTAGATCGTA
ACATTAAAAACTACACGCATGCGCCTTTAAATCTTTACTGCACAAAAGAGAATGGAAAATAGATAATAGAAAAAATATAA
AAAGTTCTAAACAGGAACAAGTCTATTCAGAATACAGTATCTAAAAGGAAGAAGTGTAGGTACATTTTGCCGCCAATGCT
GCACTGGTAAAGTTCATCAGTGTCACTTTTATCTGGATATTTATTCTTGTAGTTATGTTGAATCTCAATCTGGAATTGTTAG
TCATCATTGGACTCAATTTTTACATATAAGTTGGTATATTTGAGGCAGATTCTGATGTCATTCTCTAAAACTATCTCTATTC
CAATGAACATTGATTTTTTATGATATGATTGTCTCCTTTAGTCTTTCTCTCTATTCTCGTCTCTTCTTCCGTTCTATCTATAGA
GATACTGTTTTTTTTTTTAATATTAGTAAACTATTACAGCAATCTTCATGTATTTTTACGCATTAAAAGTCTGTAACTGTGTT
TGCGTCATAATAAAATAAATTCGAACAGCCAATGTGCTGCCAAGGACATGCATCAGAGAAAGACATTAATTGTCGATCTA
TTAAGCTATTAATTGAACATTATCGTTTCATGTGTATCTAATTTCTTTTTTACAAAATATAATTGAATGGGATTTTGAGAAT
AATTTTAGAGAATGAATTAATTATACAGGTAGATAAACTGAATTTCACTGCAATAGTGCAAATTAAAAAAAATATTTCAAC
ACTTAACATCTCCGTCATAAATTATACAGGGTTAGTTGTAATACCTTACTTATCAAACCAAACTCCGATGATAATTCCTAA
AACTTGAACAAACACCTCTCAAGAAACGTTACGCTAGCACAAATCTCAAAATTATTTATTTAACTTTTCACAAAGTCGCTA
TTTTACGTTTCCGAAATAGACAAAAAGTTCCAAAAACAATTTTGCTTTTATTTAGTTTCATAAATAGGTAGATACTAACCCT
GGTTTTCGAATTGGTATTCTCATTACAATAACAGAACTATCTTAACCATACATTTAATATTTAATAACTATTGTACTGTCTT
TTTTTTCATATCTGAAAGTGTTAATAGGCAAAAAGGAATTCTCAATGGATTCGGTTATAATGCACTTTAGCTGTTGCTAAAT
GAGATCCTGTTTGCTATTTATTTTCATGTTTACCTCTAGGTATAATTCTCATGATTTAAGTTTCCACTAAAGTAACCTTTACT
AACAGAGATAGATCTTGCCAATAAAATGTTAACTATTTCTCATATATTATACTTATACATAAATACCTCTATAATGTACAG
CTAAAAGTTGTAATAAAATTAACGTTCTTGCAAAATAACAATAAGTTAAACTATGGTATTATCGATTCTATTGTGAAAATA
TGCATAATTAATATTTTTTATTTCAATTTATTGGAGTTGATTCTGTAAGAGTGGGTAATATATAGCAATATCTACATTTTAG
TTACAATATCTATATAATGGTCCAATAACGAATATGTTAAAGGACTGTTGTCCAAAAAATATTGTATAACAGTCTTTAAAT
ATATTGGAAAATAGATATCCGACCGATATAGGCAATATTCAAAGGAATAATGACTTTTTCGGCCATTAAGTCCAGAGAAC
CCGTCCCGAAATGTCCGAAGGAATTAAAATTTCAATTTGATATTACAGCGAAATCCGGGCCATTGTGATCTAATTTTATAA
TAATTTCGCGAAATAATAAAATTTAAATATTAGGTTTATAGAATTGTCATCTCAGAATCGTATATTTTGTATTTCCCCAACT
AATTACACAGTCTTATTAATGGATTTGTAATTAAATATTCATCTTCACATAAATTCCCACTAGTCATGACTAGTTTTTGGAA
TGTTTCACTTATGGGCGGGAGCACCTAGATACCAGCTTGAACCCTTCGAGTCAGGGCAACGACGAGCTATGAGAATTGTC
GATGATTTCAGACTTACAAGCGGTATCGAATCCTTAAGTCTTAGGAGATATTTTGTCCACTCTGCGTGTTCTACCGCTTGTA
CAACAGATTGTGCTCAGAGGAATTCTTCAATATGATGTCCACGGCCACTTTCTACCATTGTACGGCTTGCCATCGACAGGA
AGTTTACCCTCACACCCTAGATAGTCGCTTACAACGCGCTTTCAGAGATGTTTTCTCCCACGAACATTCAGATTATGGAATT

		AACTCCCTGCTTAGGTTTTTCTAAGAGATTACAGCATGTCGTTATTCAAAAGGGGAGTAAAGAGGTTTCTTCAGGGTCGGCAACGTGCATGTATTGCAGACGTTCATAGACTATGGTAACCGTTTACCATCAAGTGGGCTGTATAATTTTTGTCATATATCATTTATAGTATCAATGGTATAAAAAATAGCCGAAATATTTTTATACGCAACATTTTTATATTTTACTAAAGATATTTATTTTATGAATTATGCTAAACGGCGTGTAAGCACTTGCATCTCTGTCTAGCAAAATGTCTGGGTGATTAAATTAACCACTTTATACTATTATAATATGATTATTATTGGATTACTATTGTTTATTTAACAGAGTTTAAATGTCTTATATAGAAATGTATGTAATAGGTCTCTAAACGTACTTAGTTTTAAGTACCTACGTGTAGAATCGATAATATTGTAGCC
MSTRG.270	Nodal modulator 2	AGTAAACTAAAAAAATATTTTCATAATGCCTTAAAAAAACAATCCTTATATTATTAAATTAATTGTGCATATTTACAACACTATGGTTTTATATCCAGGTAAATGGTGCACTTTCCTGCCGCCCGGCGTGTATACAGCCAAAGTGGATGTCAGCGAACAAGAACAGAGAGATGGTTTGCAATTTTACCCACTCACTCAAAAGGTGTCGGTAGTCAACGCGCCAGTAGATGGCATCGTGTTCTCACAATTGAAGGGTCGGCTCACCGGAAAGATAAATTGCATAGTGCCCGCTGACTGTGCATCGTTGACAGTCACACTGCGAGCGCTGTCCGTCGACGGCGGCTACGTGGGCCAGCCGCTCACTACTGTTGCTAAAA
MSTRG.2708	Trithorax group protein osa	CCTGGGGCGGGGCGTTCCCACGCGCCCCGCAGCCACCCTCGCCGCAGGCCCCCGGCCCCTGGCGCCCGCCGCCCGCCACGCAGCCGCCGCCCTCTTGGCCGCCGTATCAGCCGCAGACTGCAGGCGGGCCGGCATGGGGCGGAGCTCCGCGGCCGCCGACCCAGGACGCATACCCCTCCGCTGCGCCATCTACTGGTTCTGGTCCAGGGCTTGGTCAGATAAAACGAGAATTGACATTTCCCCCCGAGTGTGTGGAAGCAGCAATGCCGACTGGAGAAAAGCGGCGGAGATTAACGAAAGCCGACGTTGCGCCGGTCGACGCGTGGCGTATTATGATGGCGCTCAAATCGGGTCTGCTCGCCGAAACTTGCTGGGCATTAGACATATTAAACATTTTACTCTTTGACGACAACTGTATAGGTTATTTCGGGCTACAGCACATGCCCGGTCTTCTTGACTTACTTTTGGAACATTTCCACAAGAGTC
MSTRG.2714	DNA repair protein XRCC1	CGACTCCTCGGACGAGGATGACTACCGGCCCGGCCAGCTGTTCGCCGCCGTGCACGCCGCCTCCGCAGACACAGGTGCACAAATAAGAAAAGCTACGTCACAAATACTGAAGAATATATTAGACTCCTCGAAACTAGTAAAGTCGCCCATAATTAAAAATAGCTCCAAAAAACAAAATATCGCAAATGATGCACCGAGCACTAGTCGAGACAGGGACGGCCTCGTGTACACGGACGAGGACAACGAGCCTCACAGTAAAATAGATCAAGTTGTAGAGAGACACCGGCATGAGAAGAAAGACAGGGACGATGCACCCAAAACAAATGTGAGGGATGGTGACAGCAACAAGAAACATAATAAAGATGTTGCCAAATTAAGTGTTAAACATGAAAAGAAAGAGAGAGACGATGCATCCAAAACGAATGTCAAAGATAATAACAATGACAAAAAACATAAAGATGTTACAGATTCAAGTGATAATTCATCTAAAAGAAATAAACATAATGCGCATCATACAAAACATTCTGACAATGGTAATAAACGCGACTCGATGAAAAGAAAACGTGACGATGACGAATCGAGTAGCAGATCGCCACATCATAAGACGGCCAAAGATAAATCATTTGACGCTGACAACGGGGGAAGCAGTAATCCCGACGAAATTTTATCATTGCTTAATGACGATAACGCGGCGCAGCTGGCGCGGGTGCTGGCGGGCGCGTGCTTGGTGCTGAGCGGCTACGTGCACCCGCAGCGCGCGCGGCTGCAGCGCCTCGCGCGGGCGCTGGGCGCGCGCCTGCTGCCCGCCTGGGGGCCGGCCTGCACGCATCTCATGTAC
MSTRG.2717	Prominin-like protein	CAGGCGTGGTGCACGTGTCCGACGGCCACTTGGAGCTGCCGTCGGTGCGCAAGGAGTGGCGGCCGCTGCTGGCGCACTACGCGGGGCCCACCGCCGTCATCGTCGTCGCCGTACTCTTCGCCGTGTCGCTGCCGCTCGCCGGTCTGTTTTGGTGCTGCTGCTACTGGTGTCGGTCGGGCCGACGGCGGCGGCCCTTCGACCGCAAGTATGACGCGTGTCTCAAGGGTCTGCTCGCCATTCTCCTCATAGCTCTACTGACGCTCTTCCTGTTCGGCGTGGTGTGCGCGTTCGCGACGGAGGCGCAGCTGGAGGCGGGCGCGGCGCGCGCGGCGGGCGCGCTGCGCGCCGGCCTGCGCGACGCGCACGCGTTCGTCAACGCCACGCACGCGCACGCGCACTGGCTGCTCGTCGTCAACTACGGCGAGCTCGAGCGCAAGATGGACGCGCTGCTCACCGGTGCGGGCATGGCGGTGTCGGTGCAGCTGGGCGAGTTCTCGCACGCGGTGTCGGTAACGACGCTCAACAAGATGGTGCAGCAGCTGGACGGCGTGCAGGCCGACCTGCGGGCCGTGCAGCACCTCACCGCCACGCTGC
MSTRG.2729	Conserved oligomeric Golgi	AGACGAGATCCCTCAACTGGCTGCGTGGAACAACAACGACGCTCTCTCCACGGACCTGCCAGATTTCTCCTTATCGCCTCAGGAGTACATTACGAAGATCGGACAATATCTGATGACGCTGCCCCATCATTTGGAGATGCACCTGTCGGAGAAACAGGCTC

	complex subunit 7 isoform X1	CGTGGCAGTTCCTTTCTGAGGTATGCATCCACACATGCGACAAGTACGCTGAGAAGGTGCTCAATATACGAAACATGGAC<br>GCGCTTGGAACGAAACGGTGCCTCACTGATATCGGTAAGAACCCATTATTATCAATGCTGCAGATTTATGATGTCCTGGTA<br>CCACGACGGACTCTTGTATACAATTTTTTTTATACAA
MSTRG.2751	Zinc finger protein jing-like	GCCCGCCGGCCAGCACACGCTGACCCTGCACGCGGCCCTGCGCGCTACCCGGTACAACGCCGCCGCCGACCGCACCGAGG<br>CGCTTGTCACGTACTTCCCGCCACATGTAATCGAAGACGAGTGGGTGCCAGAGAAGGAGGTGCAACGCATCAAGAGGGTC<br>GAGATAGCGTCGCTGCCGGTCCACACGAAGGTGGTCGTGTACGAGCAATTCTGCATGTCGTACAAGGCCGCCCCGGTACA<br>AGCGGCGCCCGTGGCCCCTCCAGTCGCTTCAACCAGCACTACAGCACGGCAGTTGCCAGCGCGGCACTGCACGTCGTCAC<br>GCGTGTTGTCCAACTTGCTCGCCAAGCAAAAGCGTGCAGGTGAGAGACTGGAGTGCGTTGCTGAGGACCCCGCAGCGTTG<br>TACGCGGCAGCCGCCGACCGCGAGCCGGCCGTGAGCGGCGCCAAGAAGAGGAAACTCGGACGTCGCTACGGCGGGAACT<br>GCTTCTGGCCGTGCGGTCGATAACTTCGATGCTCTTTTGGCTCGCGCCGCATCGCCACGCGACGGCTCGTCTCTAAGTGGT<br>AATTTCAAGGTGGATTTAGATCTTACGAACGAATACTCGTTTTTCTCCGTTTTGGAGGATTTTATTTTGATTTTTATTTCGCA<br>ATCAATTTTATCTACATTATGTTCTCGCGCTGGTTCTGTTGTTCTATTAGTTTGTTGATCTAAATCCACCTTCATAATTACGT<br>TTGCTCTTCTATAGCAAAATAAGTTTGCATTGTTTGTGGCGTAATGTGGCGGGTGACGCGGCTCGAGCGGACAGATGTTGA<br>TTCTCCACCGGGCGGGTGGCCGACGATATCCGATTTAGGAAAATTCTCGCCAAAGATTCGTTGACGGCCCGCGACGCGGTT<br>CATAGACTGCTAGTTGATTGTAGCAGACGTAAATTTATTCACAAGACTGATGCTTACTTTTTTATAATGATATTGATCGGTT<br>TGTCGGCGTTTTGAGCTTTATCGCGTCCGCGCACGGGCGGATCTCGCTCGCCGAATGCGACGGGTTCGGTTACGAGAAAAT<br>ATGTAAATAATTAGTGCGATGTGTAAAAAATATTAGAGTGCCCGGTTCGTTTCGCTAATCGAGTTTTGATGTACAATAGAT<br>TCTATTCGAGTGTTCGATTTAGTTGGACGTGTCCGAGTCGCCGAACGGGCGAGTAGTCTGAATACTTTAAGTTAGTTTAAG<br>GAGTAGTGTCGCAGTAGCGACACAGTCGACCAGCGATACGCATCACTGCCAACCTGTACATACTAGTTTGTATTGAATCGT<br>GATTCGGTTTAGCTTAGAATAATTTGCGATCGTCGCTTTATCGATGTGGTTTCGTTTAGTTGTAATTACCCCGCGTCTCGAC<br>TTGCTTTCGCTTGAGGTGCCAGTACGGATGGTCTCGGATGCAGAGACATTGATACGGATAGACGACGCAAGTGACTAAAC<br>GTGTAACACTTATAATGCAGTCACTAGTTACTTCATTATCATTGTTCTGTTTAATTCCGCTGCCATTATTATGTGTATCAAA<br>GTTTGATACGGAGACTGATCACCACTAATGATAGAAGTCTAAAACTTCCCTACCAATAACCGATCTCCAACCGTTATTAAA<br>TCTATACTTATCTATATCGTGTCAGTTGTTCACACATACTAAATGTAGTGGAAATTAACAAAATAATGAAGTAACCAGTTA<br>TATGGACCTTCCTACAACAACATATAATTCAATAGAATATACTCTTGCAATATTACATCACACACGCTATTTATTGTTAGCA<br>AATAGCGACAAAGTTCTTTAAAAAAATAATCGTGTACTTGTCAAACATTTAAAATCAATAAAATATATGCTTGCTATTACA<br>TTAAAAACGTTGTTTATTGTTAGCAAATTGCATAAAATAATATTGCATTTGTCCAACAATTAAGATTGTTTAATGTAAATTC<br>TACTCAGTATAGGAGCAGGGATTAAAATTGGTACTAGTAATACGAAAATCGGCCACATTACCTGAGATATCCACTTCACAT<br>GACTACATAAGTAGCCAATTTAGCCATAGTCGAAAAAATTTACCATAGTCCAAGTAGGCTCTCTCGAGGGTACTAAGTACC<br>AAGAACAGTTGCGCCATCTATCTCCATTCATGTCTGTGGTTGGGTGCGTGACCACGAATGTAGTATTCGGTGTACCAACTC<br>TTCCGTCATCACTCACCAAGTGCCTCGACACAGCACAGGATATACCAATAACGATGTAGTTAGTGTTCACTTAATATCAAG<br>TTGTCCCGCGTTAGTCATATCAAACGAAACACACACTTAGCGTTAAGGTTTATAGAGTTCAATTTTGATATTCGACGTTTTC<br>GAGTTCCAGTTTTTGTGTTCCAGTTTTATTGAAGCAATCTCGACGACGCTTTACAGAATCAACAGCTGTCTACGTATTTATG<br>TCCAATGTAGTTTTCACTGCCATTCATGTTGTGTTTAGTTTTATTGTACTTTAGCATCAACTGTAAACATTTTTTTAAATTAC<br>ATGATATGTTTAGTAAATAATCTTGACACGTCTTTTTAATGAAATTAGTCGTTTTTTTTTTATAATGCCCTGTAAAAAATTAT<br>ATAATACCAATAATGAA
MSTRG.2787	Hypothetical protein RR48_06353	GTTAGAGTTATCAATAGTCAAACGACAACGTGAGAAAATTTATCTATTTTTATTTTTTCTCTTTAACATTTATACATTATTTA<br>CGTTATTTAAACAATTTTTATGATTTCATGCTGTTGTAATAAAAAGTAATATACAATGACTGCTGAAGTAGCAACTCGTTCT<br>AAAGATTGTACGCCGTGCAGACTATTCGGAGCCGCTGGTTTGGTGGGTATTGGCGGATATTTAGCAAATGCTGCGTGGAA

		AAACAAAACATTTGCCGGGAAATACACTGTCTCTATTCTTTCCTTTGGTTTCATCTGGCTTGGTATACAGAGATATAAACA<br>GGCATTTCCTTTTAACAAAAAGGAAACTCAGAGTTAAATGATTAAGAACAACTCTTATTAGTAAAAAGTATATACTGTTAA<br>GTATATTACATATATTCTGAAATATGTGATAATATATCATTAAGTCTTCAATGTAAAATAAAACATAATTGTATATCAACA<br>TGTGACCATTGTCGCACCTTTTTGTTATATAATACTTATGCAAGGAGTGGAATGAAAGTTAAATATTTAAAAGCTATTAAA<br>CCACAGATGAACACAAAAAGTATTGTATGTTACTGGCAGAGCACATAAAAAAAATACAGGTTGAACACCAGCTTAAAAGT<br>TTATAATGTTCATGATATATTCATAATTGTGTTGGAATTCAGTATTGTATTGGAATAATGAAATTGTAAAACATTTATAATT<br>AAATTTTCTCAGTTATCGCTCCAAAATGTGATATCTTTGATAACTATGGTCGAAATTTCTCGAAAAAATAATAATAACTCA<br>AGACAAGATTTGATTGATGGTGAAATTTGAATTCATTCAGTGATTATTGAGTTCTTGATTCTTGATTTTATTACTAACATAA<br>TAAAATTATAACTAGACAATCTGTAACATGAGAGGTATTTAAGCGAAATTAATATGAGAGCTCTTAATAAGACCAATTAA<br>CGCC
MSTRG.2840	Cytoplasmic dynein 2 heavy chain 1-like	ACAAGCTCAGGAGAGCACGGTAGTGGGATTTGAGGGTATGGAGAGGGCGGAGGACGCGACACTGGAGGTGCCGGTGTAC<br>AGTAGCGAGGCGCGTGAGGAACTGGTGCTGTCAGTCCGCGCCCCACTCGCGCGTTCTTTCCCTCGCGACGAGGCGCTACTC<br>AACGCCGTGGCACTTTTCATCGCCCCTGTCGAATCAATCTAGGAC
MSTRG.2858	Otopetrin-2-like	GATTAGAATAGGATTATTTACTTATCTGGATTAATTTGACATTAGTCATACACAGTAAATAATATAATGTAAACAATAATT<br>CAGTAGGCAATTTTAGAGTTTTGTTATTTTCCATGCACTCCTGTATAAGAATCATTTGTAACAGCAACTTATTTTTATTTTCA<br>TTCCAATTCAATAACAATTATATTATAATAAACTTAGATTGAAACAACTATGAAAAATTATAGAGTAAAGCAACTGATGAT<br>ATAATATTACAGCGGAGCGAAGTTGTTTTGTTTTAGTGAACGTTTATCGACTAATAGTGGGTTTTAATGAAGCGCTCGTTG<br>GACTCACCTTTCATCCTCAGGCACGGAGAGTAGCGGCTCAGACCTCTCCCGCCAATCACGTCGCTCCTTCACCGTATTTTG<br>ATTAGATTTATCTTTATTTTTCATTTTTAAGAATACTCTTAACTCTATTTAACAATTCGTATAAATCACCCGATTAAAATTCA<br>AATGAACAATATGATTGCATCATCAGCTCATTATAAAATCCTTGCTAATAAATGAAAACTATAATGTAGGTATATAGATTG<br>TAAAACCATTTGTTAAATCTTCAGTATAAGCACACAGTGTCGATAGTCGTGGTGCAAACCATGGACTGTGCGCTAAGGCCC<br>CTCTGAACGGAAGGCGGATGTTCGTGAACGGTGGAGGCGACAGAGGCTCGCGAGTGTGGTGACAGCTCGGACAGGAACC<br>GTCGCTGCGTTGCGGCACATAATGCAAGCGCCTGCGATGGCCGCCGGAATGCTGCGATGATCGCTGACGGTCGTCTATGTG<br>TTGATGTAGACATATTAGCTAGCCAGCTAGATATTTACAGATTTAATTCTCTTAAATTTGAAGAGGCATTGTACAGTTTATG<br>TCTATAACGTAATATATACTGAACCCACCGATCTGACGTGACCGGGCTGACGTGCGTGCGTCATCAACATCGATGTCATAG<br>GTACACACAAACTATAGATGTTCTCATCCATACGTAAGTTTATACCGAAATTTGTACCTGGGTGTACATTTACTATAGTAAT<br>ACTTCTCGCTTTGTATGAGTACAATCCCAATATCTATTGATATATGTAATTAATATTATTTATTTGTATGTAGCGCAGAACA<br>TGGGTTATGAAATTTATTAAATGCC
MSTRG.286	Centaurin-gamma-1A	CTCACTCCGGCCGAGTTCGTACGTATCGGCTGAGACGACGCGGCATCGGCGCCGCGGCCACCACACGACGTTTTAATATCA<br>TAAACAGTGAACCAATCCTTACACAGCAACAGGACTATGATACGTATTACGAATACTAATTAGGACCTGCACAGAACAAT<br>AGTTGTGACACAAAATCTAAACAAAACAATAGAATCGAGATTCCGGTCAGTGCCGACGGAAGTGAGTATTAGAAGCTGGA<br>GAAAATTTGGAAACAAAAATTTGCAAGTGATTATTCAATAGTGCATCGTATTGTTCGAGATCCACGACCTTGTAATGTTAA<br>ATCGCCCATAAAATTACGATTGGTCATAGTTTATTACATAGCGCAGGTGAATCGGAGTGGCAATGATCCAAATGACGATA<br>ATCAAGACGATATAAAACTTTTGAGTGATGTAAACGTTGGAAATGAAACAACTTTTCCGTTTGGATGTGATGGCACTTCTG<br>TTCAAGAAACTCAAACAAATCAACTGAGTTGGAAGTACATGGGAAAGTAATTGTCTTTTGTTAAACTTGGAATTGGTAATG<br>ACAATGTTGGCAGTGAAGAATTTCTTTATGCCAGAGAAGAGGGTGGATTCCCCGAGGTTCTCTCGAATGCCGGAGGCTTTA<br>ATGAGATCGATTCGGAGGAGGTCCCTGAGGGTGAAGAGGACGAAATCGCTTGTACTCGTCAACGAGAAACGGAAGTCCG<br>ATTCATTCGTAAACAGTCAAGAATGGACGGCGGCGAGAGGAGTGACAGAGCTCAGGGTCGGTGTGCTGGGATCTCCAGAC<br>AGTGGGAAATCTGCTTTAGTACACCGGTATTTGACTGGAGCTTATATGCAGGAGGAGAGTCCAGAAGGT

MSTRG.2911	THO complex subunit 2	CAAATATCTAAATAGAATTGCTGTTAAATTTCTGAACATATCTGTCAGTCACGTATTGCATTATTGTTTTAAATTATTATTT<br>TGGTAAAGTCTGTTATTTACAATAATAACGTAATATAAATCCTTGACTGAAAGATAGAATGCCAAATGCTAAGCACCCAAT<br>TAAGTCAGGTACCTAAATAGATATACAATTTTATTATACACCGTTTGTGGTCAACACATGTTTGTCATGTGAACATTAATTA<br>GATACGTAGAAATGGGGCTGTAATCACGACGAGTAAAGGAGCATTGGGCCCTAACAAACCTGTAGTTCTCGTAGCCGACG<br>TGGTCGTTGGCCTCGGTGAACTGGTTGGAGACGCGGTACTTGGTGACGAAACCGGGGTAGTGTGCGCACTCGGCGTGGAA<br>GGCAGCGCGGTCGGCGTGCCAGCGCATGGCCGTGCCCAGCACGCGTCGCAGGAACTGGCCGTAGCGCGCCGCCTCGCCCT<br>CCGAGCACGACATCACCGAGTACGTGATGTCGCAGAACAGCTGCACCGCGCACGCCACACCACGCGGCCACATGCAACCA<br>CACCGCCGATGACATGAGCATCATTGTCGTCGACACCAAGCAGCGTAGAGAAGTTGGGCGTCTTGAGTGAGTGCACCGTG<br>TGCACGAACTCGGCGCAGTAGAGCGCGTCGGCGGCTGTGAACACGCAGCGTGGGAACAGGCACAGCTGCATGAGCCGCG<br>TCACGGTCTCGTTCTTGGCGGACTTGGCGGCGCGCGCCGGGAACCAGCCGCTGCACTCGCGCTGCAGCCGCGCCGACACG<br>CGGGACACGTGCTCCTGCTGGCGCCGGCGCTCTTCCTGCAAACACACGCCAAGCGGCACCTGCGAAGGCGGCCAGCGCCT<br>GCAGCCAGGGCGGGTGGGCTGCGCCCGCTGTCCCGCGCCGCGCCAGCGCCTCCAGCAGGCAGCAGCCCAGCACATCCAGT<br>GACAACGATGTCAGATACTTCAGCGACTCCACCACCGGACCGATCAGGTTATCGTATGTTTGAATCTGAATCCATAGAATA<br>ATCACCACATATAATCCTTGGAGAACAAAAGACCTATAATTTTTATAGTTGTTCTCGAGACATATTCCAATAAATTGAGTC<br>ATTTCATACTCTTATATTGTAATACTATTC
MSTRG.2944	Tyrosine-protein kinase Btk29A isoform X1	GGAGTACTTATGTGGGAAGTATTTACATGCGGTCAGGTGCCATATGGTCGAATGAAAAATTCTGAAGTCGTGGATATGGT<br>ACAGAAAGGTCAAGTTCTTGAGAAACCCAAAGGCTGTTTGAACGAAATATACAGTGTGATGCGGGCTTGCTGGAGACATA<br>TGCCTGACGAGCGGCCGTCATTCCGCGTGCTCATGGACAAGCTGACGACGATTGAACGGACCGTGTTGGCCGATTGACTG<br>CGGCTACTGTTGCAGTTGCTGCATCCCGCTGCGTCGCTACCTGCTCCTCAATCGCGTTCTCACCCCACCGCAACCCTGCCTC<br>GCGCCCGTCGCTTGCATGCCGCATCGCCAGTTTGAGCAGCACCGTCGGTGTATCCACTCATCTGTACACTTGCTGGGGCCG<br>TATTTACTGTAGGGTTGTAAAAATCGCCCAACCATGAGTATCTTCACAGAACTTATGTGGCCAATTGTAGGTACACTCACG<br>TCGCGATGTAATGAGGGGTGTGACAAATCATGTTGGCAGTTTCACATAGTGTGTTAAAAACTAGATAATGTCGGTTCAGCC<br>TATGCATCTGTATGTGTGAGTGAAGCTACAATGGACCTATATAACTGGGTTTCTGTCGGGATATTTATTCATTTGATTCAAC<br>TGCAGTTAATAAAATGCCGTCCGTATCGTAACTTCACTGGTTGAAAGGTCGTCGCACAATGACAATTATTCAATTAATGAT<br>TAAAGGGTATCGTATATTGTATATAAATACAATATACAATAATATCTAAATCAATTTACTGGTCTGAAGACGCATTTTCGG<br>TGTCAGTAAAATGTTTTGCAAACTTTTAATTTAAAACTCACTTTGTTTCCGATCTTTTCTTATTATCTCAAATTATAAATATG<br>ATTTTATATTTTTTGCAATATATGTGTCAGATACGTGAAAATAATGTATGTATATAAGATTATAAAATTGTAATTAATTTGT<br>AGGAAGACAATTGAGTATTCAGTTATTAATTATGACTTCATGTGTGACAACCTTAAACACGAATGCGTACGTTTGTTGGTG<br>GTTGGAGAGAAGTGTAAGAAGAAGTATATATAAATGTCGGGGGTAGTGCGCATTTGCTTTATAGCAACGCTCCTATATTGC<br>AACTATTAATGGGTTGTTTAAACCCATTGACACTCATTGAAGACGTAAATAAAACGTTAATCATTGAGCATTATTTTATGA<br>TACTTCATAATTGGCGCTAGTTATATATACATATATGCAGTCTTGGACGAAAGTATTTGCTACCTTAGAAAATTACAACTG<br>GCTTAAAAAAAAATCCAATTCCTGAGGCAGACATGCAGCCCCAGACCATAATACTGCCTCTGCCGTGTTTTACAGTCGGCA<br>CCACACACTCCGGTATTCTTCTTCAACCTTCTTCGCCAACTCTGCGTCTCACGTAAGTGGCACCTGCAATTCCAAATACCTG<br>AAATTTAATAAAGTGATAAAATTTTTGAAAATACGTCTGTTAATGAACCATTAACTTCATTACATCTTAAATGTATTTAATT<br>AATATTAAATAGTTTAAATGACATACATTAAAATTAGATCTCAGACGAACTGATATTTGCCCAATCTTCCTCGGTGTAATG<br>TTGATATTCTAAATACCACTGCAAACGTAAGTTCTTGTTATTCTCAGACAGCCATGACTTCTTCCTTGCCTTGCAACCTCCT<br>GCAGCCTGCATCCTGGAGCCTTTAGTAGTTCCAGCAGATTTTTTTACCCAGCGTCTCGCTTAGTTCACCAGTTAATTCAGA<br>TATTTTCTTACGATTTTGCAAAGAAGTTCGCATTAACTGTCTATCTTCTCTTGCAGATGTAATTCTTTTCCTTCCACTTCGCT<br>TCAAGATTTCGTGTCTTGTGGGTATCTTATACCGATTTAAGGTATACCTTACAGTACTATGAGGACATTTCATGGTTTTTGC

		AATTGGCAAAAATCAATGGCAAACTTTTTGCCTTCTTCAGACAATAACTGGACTTTTTAATCGTTCATCGTTACTTAATTCA<br>GGGCGCTAAGGCATTTTTCTAGTTTTTTATGAACTGCCTCACGTAAAACAATAACTAATTTACAATAATTTGTCGACCATTC<br>TTCGTACTATGATGAATCGTGGTAAATTAAAAATTTGCCTAAAATTCGATTTACCTGAAAATGAATCACATTTATTGTGTTT<br>AGTTTTATGACGTGGTGCGCTGTTTTTATTTATAATATTATTTTAATCTTTAGATGTATACTATTCGACATTACACCCAGTAT<br>CGATCAATAGATAAGAAATTTCCAAGAATTTTAAGGGTGGTAAATACTTTAGGCTAAGGCTGTATTATTATAGAAGAGTCA<br>CATTATAGGTAATATATGGATATCCCAGGGGAAGCCCATATCACATAGCTGTTTTCTGTATAACTAGCTCCAATTTTAATGT<br>AATAACTACCGGTTTAACTTACGTGTAATTAGATCTAAAAAGCTCGACTAGTTCCGGAATTAATCCGTGATCCCTAGTCAT<br>GAGCGACTGTGACACGAGCACGAGCGTGACTGCGTGCCTCGGCCTGCTGAGCGCGAACTGTTGGCGTTGTAGCTCCAATTT<br>GCCTTCAGTTGTGCAAACTGTAACTATTTGTGACGGTCATGTGTTTACATTCGTCGTAATGATACACAGAGAAAAATGGAT<br>TATATTTTGATTGTAAAATTACTATTAATAATGTGATGAAGATGGGTTTATTCCCAATATTAACGTTTTTTTATTGAACATT<br>GACAAAATTAATGAACACTGACGTTTTGGTAAACATTGCATTTTAATTTTTTTATTACAATTTATTCAACGTTTTAATGAAA<br>AAGAGCTATTTTAGAACAGGGTTTTGTATGAATAAGATTTACTAATAACTATTATCTACATCGAATTTGACAATAAGCTTA<br>TTTTAACCAGAAATAAAACTATTCCATTTTATGTCTGGTTAAATAAGTGTTTACTAATGTAATACTTACATATGGAATTGAA<br>TGAATTTTTTACAATCCGCAAAATGGTTTAGTAATTAGTTTCGTTTATAGATTCAAAAAACTTGTAGGTCTTCAATGTGCCA<br>ATTGAAAGAAGTTCGAATTGAAGTAGTTTGTCGCAAACATGCTACGTCCAGTGCCAGACTTTTTGTTGACAATGTGATGTA<br>CTTATTATGGGTCCTATAGATGGAGCGGTAATCGTAATGATTAATAATTCGATTTTAAAATCTGGAGTTATTATATATGTGG<br>CGATTTTGTTTTTCTCATTTTTTTTTGTCGAATACCCCATCACAAATATCAAAGAATAACTTTATAAATATACGACCTAAAG<br>CTCGTTAATTATCAGGCGCAAATTTGTTTGCCACTTTTTGGGCAGACCTGAAAATATTTAGCCATTTACATTTTATTTTTATT<br>AAATCGTGCGAGACCGATGTAAATAGATGGTTACGATTATCGTTATAATTTATCGCCGTTTGTATGTATATGTGAGGCGTA<br>AGTTATTGGCAAGAAACAAAATGAATGTGATAACATGTTGAGCCTAATTGGAATTGGTCAACTTTATATAAATAAGTCTTA<br>AATAACAGTGTAGTCTAGCAAAAGAAAAAAATCTGAGCGGATCTCGAGTTGCATCTATAACTCTAAACGATTGTCTAGTC<br>CGCACCGCGCATAGTTCGTGTATGAGTTTTAATACAGTTAGGAAGTAATGGTAGTCAATCAGATTTATACGGTGATATTAA<br>TAACTTCTCATCCTCATATCATTACCTATCAAAGTTATAAATGTATATACAGTACTGTAGGTATTTAATAGCTTAGATATTA<br>TTAAAATTTGTAAATTTAATGTTTTATTATAAAAAGGTATTAGAGAGTTAATATTCAGTTATTGCGATTTCTCATTGATTCA<br>TGTAAATTAGACCCCTCATTTCTTTTTTAAGCTTGTCCCGTATATTTTTTAAACAATGCAGTATTCATACATTCCTTTTAACT<br>AGTCACGTTTAGTGATGTGTAATCAAATGTTTGTTTACACATTTGTGGTAATTTTCAATTATTTTCAAATATTATTCATTTGT<br>GTTAGTGGAGTGTATTAATGTTTTAAGTAATATTTCTTTTATAACACTGAGGTGGCAAACAAGCGTACGGTGCGAATGGAG<br>CTTATTTCACTTAGGTTGAGTAAATTTCGTCCCAATAAGGCAATGAAAGAAATGGTATTATATATTATGAAAGAATTTGTA<br>TATTGAATGTACGATGGTGTTGGGTCGCAATTTACTTCGCTGTGAAAGGGGCCTTAGTTGATACGGCAATGACACTTCTTA<br>AAATGTGCCAATTATTCCACACAACCTATATAATAGACATTTAGTACACTGTTTAAGGGATTTACGACTGAATATTATTAA<br>ATTTTGAGTGAAGGATTTT
MSTRG.2<br>99	Transmembrane<br>protein 161B	GTCGTGAATCGTCATGACATTTCTAGTCTAGAATTCTAAATAGATTTTACTAGTCTATACTAGTATTACCTATTTTTTATCAT<br>TTTTATTTATTAAAGATTTTTCTTATGTATATTAAAGCATGTAGTAGAATGCATTCCTTTATAAACCTTAGTTACTCTATAAT<br>CTAAATTTAAATTTATTCAAATTTACCATTTTAACACAAAGAAATAACAGGTCGAGCCGGCGCTGCGCCATGTCGAGGTAG<br>GCCTGCAGCTGCCGCGGCATCAGCGCCAGCCGCAGCACCACTGTCCCCACAACCAGCACCAGTCGCAGCGTGTCGAACGC<br>GTCTGATGACATGCTGCGCAAAAAAAGCACTACCAGTTAAAATTACAGACAAGCAAATTGGCAATATCATTCACTGACTT<br>ACACAGAAGACAATCCTTGGTTCTCCAAGAATTTCGATGCATTTTCATAAAAACTGTCATATGCAGGATCCACCCCGACTT<br>CCAGATTAGTGTCATCAACAATCAACACCATCATAGCAATCAGTAGAAAAATGCAAAATGCTACAATGCATGTCGATCTC<br>TCAC

MSTRG.3	Superoxide dismutase	AGATTAAAAAAACTGGACATATGCCAGTACGACATATACTACACAAGATGGGTCTATCCTTGGACACACTGTGCAGACTG<br>TACAGTAAGGAATAGGAATCCATTAAATTCTTGATGTGCAACTGCGATTCGTTGGCAAGTGTACGTATGAATTTAGTGAGA<br>ACGGCTTATCCAGAACCAGGGAGCTACAGCTATCTTAAATACGTTAAGTTGCTGTGGCTAATAGCGGTTTTGAATGGCAAG<br>ACCTTGCAAGGAGTCCCAGGATACGGAAGGAACCTCATTATAAAAACTTTACCAGCC
MSTRG.3032	Rab proteins geranylgeranyltransferase component A 1	ATATAATTGTAACAAACGTGCGTGAAACAAGTAAACAAGATTTTAGTTACGTCACAGATAACATTATATATAGGGAAATT<br>GTTTACCGTCGATGTTATCAACGTTTAGGCTTATTACACCAGTACACGAAGCGACGAAATAATTAATTGAGTTTGGTGCTA<br>AAACAAGTGACCTTCTATTGTTACGTGACATCGAGGTAGCCGGTCGTCGGCTTCTAATAACACTAACAACAAATTAACCAA<br>GTGTTACGTTATATTAGACATCTAAGAGTTATTCTACTGTAGGGTCAAAAGTGTTGTGTCTATATATAATCGATATGTGTTA<br>TTAATTATCTTATATCTGTAATAATATGTTTAAAAATTAAGATTGAGTTTTAAAAAAAAATCGATTTTAATGGTGTCATAAC<br>CAGTTATTTTAATTTAAGAATTAAACTACTGAACCGATTTTGATAAAAATTCTACCAATCTTTTATATATATGCGGAAGAG<br>ATTTTCAAGCAAATCTGTCCCGGTGAGGAGTTCCTGCCACGTGCCCCCGATCCAGAGGAGATCGTGTTTGAAGATGACGTC<br>ACCCACGGCCCGGAGTTTCGGCGCGAGGATGACGATGCGGACGGTGACGCGCCCAAGGAGTAGTCGCGTGCCGCCCTCTA<br>GCGAAACAATTCCAACAGTGCGTTTGAACGTGCCACCCTAACCGTATCCTACAAATGACGGAGCTCTCATTATGTAACGTA<br>GCTTTTGTTTACATTGCACAATATCACAGTGTAAAGAATCTAAAGCCCTTTGTACACGGGTAGAGCAAACAGTGTAGTCGC<br>ATAGTAGAGTACTAACTGTACCAACATAGACTTTTGGTAGAGTATTAGAATTTGGAAGAGTAAAGTAGCTAGAATTAGGG<br>GCAAGTCGCCACATGACTTGTCTTCCACATCCCAGTATCCTGTCTACTTTACCGTTTTAATTTGAAGATACACTGTTTATTCT<br>AACAAAATATAATAGTTTAGAAAATTGTTCTAATTAAATTACAGTACTGGGTATGGTTAAAGCTGTAAGTTAGTACACTTT<br>ACTGTTATTTCTACCTACATGCCCTTCCTTTTACATTGGTTCCATGCAACTGGCGATATGGGTTGATCTTGGAGTGGGGGGC<br>TGAGTAAAGCTGTTGTAGATTACTGGACTGTGATATAGCAGTTTAGTACTCTATTCAGATTTCTATTATATTTGTAAGATAC<br>GATTATTTACACTGTGGCAACACTTGCAGATGCTATTAATATCGAGCGCGGACTTTACATTTCTAGGTGTGGTGGCGTTAT<br>AATTACAGACACGGTCAAAACGTACTGCATAGTTACTAAGGTGTAATCTCAGTAAGATTTATACGCTTTGGTCGCTTGACT<br>TCGTGCATTCCGACTGGTGCGTGCGGTTGGTGCACGTTATACGACGACTCCGGTTGTGCAAGACTCGTAGGGGTCATCGAA<br>ATTATCGTCTGCTGAGTTTACTATATTATGTTAGTATAATAAACATAATCTCAATTTTTTTTATAATTGATAATATCATGATT<br>AATATAGTCTAAGTTTAGTAAACTTAGCAGGTCGAATTGATGTTGTGGTCGCCACATGTGACGGAGGCTTCAATAACTTAG<br>CTTGGTTGTCTTGATGAAAGTGAGCAAGATAAACTCATAGCCAATGTTAGTAAAAGTATGTATTCAGTTGCATTCAAAGAG<br>ATATGTTGATTGATCGAAATTATGTCTATAAAAGAAATTATGTCTAAAAATATCACGAGGCAAGCCTACGATTATCACGGT<br>TTAGGCAAGCTAAAATGTTTATTAATATATTTTTGTGATTTATTTATTTATTTATCAAAATGAGGTATAGACTATAATATG<br>GACAATTTGTGTCTGGCATGCTCTCTATCGAGCCTATGGTGGCACAGTGTTTGAAGTGAAACCAAAGTATTATCACAGGTA<br>TTACAATCTATTTTTCCTTACTTGTACTATTAGGTAAACAATTTTGATCTTTTTGCGAGAATATCCATATAGAAGTGAGATC<br>TAAAAATAAATATGTACTCAAAATTATAATAACATAAAAATAATATTTAGCATTCCCGAATGCAGTATTTGTTTTAAAATA<br>GTTTAAAGAATGAACTACTATAATATAAAGGATAATAAATATTTAACAAGAATATTATATTTTAGTAATTTGTTAATTTTA<br>AAGCGTTCTGATACGCCGAGATTGATTACCTAATAAGTCCAGATAAATAATACATTCAAGTGTGTACGAGGCGCGTGCAA<br>TGTTGGTAGGTAGGTACTTATAGTTAGCTAGGATGCAGTTTTGAAATGTGTAGTACATTTTGGACGCACCAGTCTAAGGCA<br>TAGATTGTAGCACTATTATTTTTACTATTGTTTAACTTAGCACTTTTAAATGATATTTATTGTCAGATGATGATTATCATGCC<br>TGCGCAGCTTGTGCATTTATAATTCCAATAAATTGTGATTATTAGAATTA
MSTRG.3046	Phospholipid-transporting ATPase VD	ATCAGTAGAGCGGTACGTGCGAGTGAAGTGGCGGGATGTCCGCGTCGGAGACATTATTCACCTGTCGAACAATGAAGCCG<br>TGCCGGCCGATATGGTGTTGTTACATTCCTCAAACCCCATAGGTATCTGTTACCTAGACACGTGTAATCTCGACGGGGAAA<br>CTAATTTGAAACAAAGAATAGTGGCGCCCGGTTTTAAAGACAAGCACGCAGAGTTCAATCCATTAAAGTTTAGAAGTACC<br>GTAGAAGTGGAAAGACCATCGACTAAAATTTATCGATTTACCGGCACGATTTCACATCCGGACGGTGCACGAGTACCGCT

		CAACTCGGAACATCTTCTTTTAAGGGAATGCACGATCAAAAATACAGATTACGTAGAAGGCATTGTTGTTTACGCGGGAC<br>ACGAAACTAAAGCTATGCTCAACAACGGTGGTCCGAGATACAAATGCTCCAGTTTAGAGAAAAAGATGAACACGGACATC<br>ATTTGGTGTGTCTTGGTGTTGCTTTTTTTATGTTGCGCCGGCGCAGTTGGCTGCAAAGTTTGGTTAGATCAATACTACACAC<br>CTGACATGCCGATGTATAAATTTGTCCCTTTTGTTCCATACGATGCAAAACCGGCGTACGAGGGTTTATTGATATTTTGGAC<br>TTACATAATAGCTCTTCAAGTCATGATACCTGTGTCTTTATATGTCACTATAGAAATGACGAAACTCCTCCAAGTGTATCAT<br>ATACATCAAGATGTAGAAATGTACGATCCAGCAACGAACACTCGAACCGAATGTCGAGCGTTGAACATAACCGAAGAATT<br>GGGGCAGATTAACTATTTATTTAGCGACAAAACAGGGACTTTAACAGAGAACAAGATGGTGTTCAGGCGATGCACGGTAG<br>GAGGCGTGGACTATGACCATCCGCCTGGTCCGCCTGCTGAACCTTCGTCAACTCTGCCGCCAATAGTCACACCCATAACTA<br>AAGTGTCGCCAAATAGAAGAATGCTTCAACACTTGCTAGACAGTAACGATACCCAACATACACAGAAGGTCCGCGAGTTC<br>CTGTTGATTCTGGCAGTATGCAACACCGTGGCGGTGAGTCAACCGCACGTCGACACCATGCAGATGAGTGGCTCTCATTCC<br>GGCGACCAGTATCCCGATAAGCCGAGTCGTTCCAACGGCACACTCCGATCCAATGATAAGTACGCGCGTCTGACCGAATC<br>TCGTTCTACGACGCCCTCGCCGCCGCCGTCCACGACTTCGCCACTCAGGATTCGCTTGCCGAAATTGCCATTTGTATGCCGA<br>GACGATAACAATAGCGAACCGAGCACATCTACCGAGCAACAACTAGCACGATACGAAGNNNNN
MSTRG.3078	Uncharacterized protein LOC106132165	CTGTATTGCTGTCGGACACCAGCGAGACTTTACTGCGGTGTTCGTGTAACAATTACGACGGGCTCGATGTACACGACACAC<br>CGCTACTCACCTGTGTTTACTGCAACACGCAACAGCACGCGGTGTGTTTCGGTATACTGGGCGGGGAGGTGGCGCGATTCC<br>GTCGGCACTGCTGCTGCGCCTGCGCAGACGACGACCGCGCACGCACGCCCACAGATCCTAAACTCGTCCATCTCTCCAGG<br>GTGAAGAGGGAGAGTATGTGCATGTTCCGTCGAGCGCTGGCGTGGTGCAGCGAGTTGGTGGCGGATGCAGGCGGCCTGGT<br>CTCTGTGAGCGCGTCTACATTGACGTCTCAGTTGGCCCTGAGACCGCTATACGGTCCCAAACTCCTGAAGCTATTCGCCTC<br>CTACGGAATCCTTGCACATCAGCCCGAATTAGACATTTCTACACCGAGGCAAATAAACCAGGATCAACTGAAATTAACAA<br>TGGAGAAATTCTTCGACAGCCAAGGTAACATCGTAGATCGACTTCTAGCCGAAACTTTCGGTTCTCAGGAAAGTCAACCG<br>GATCCAGTTGGGGAGGTTCTTGGCCCTTTAGAGAAGGTGAGTCTACAGAATGCCTCTAAACTCGGCAGGGTTATAGACAA<br>TCCTATTGATTCACAGCCAAAAGCCGTGGAAAATTCAACCTTGCTGCAATACAAAGAAGCTCTCATATCGAATTACGAAA<br>GAGACAAAGAACTACCTCTGAGCGGCTCTCATAACCCAATAAAAGATGTCGAAAGTCTAGGGAAAAAGACTTATAAGAG<br>AAAATTGAATGAAAACGAAGAAAAACCTAAGCTCAAATCGGGTGTTAGAACAAAGAAAGCCAAGAATAGAGGATGAATG<br>TTAACTGAGAGCTTGTTTTCCCTAAATAATTCACTTTGTTATTTGTTGTTTGTATTGTTCTCCTTATAAGGACATTTGATAAC<br>TACTGTTTATTATCCATTTCAAAATATAAATGTATTTTACTGACCTATTATACTAACTATAGTAGGTAAATAATAATAAAAT<br>AAAATATATGTGAATATTACAGAAGGAATAAAAGTGTGTTTTTATTAGTTTTGAAAAAAATATCAAAAACAAAAACAAAA<br>AATAAGTCTAAATTTTATTCACAATTATAACTTAAAATTACATATTGAAATATCAATTTTAATAACTTTTAGGTGTCTATTC
MSTRG.3117	ATP-binding cassette sub-family A member 3	GTATAAGAGACACATAAAGGTAATTTTATATCATATCCATCTACAGAACCCAGGGCGTGTAAGTTCAACTCGCACCTGGCC<br>GGTTTTTATTTTTAATCCACGTAACTTGAGCTTGGTTTCTGCTAGTTTCATGTTTGACCCAAGTGCGTGTATACAGGTAAGT<br>GCTTCGGGTTGCTGGGTGTGAACGGTGCGGGCAAGTCGACGACCTTCAAGATGCTGTCGGCCGAAGAGTGCGTATCCCAC<br>GGCCAAGTACGAATCGGTCACACCCCGCTCTCACGGTGGTCTACTCAGATTTGAAACCTCGGAGGCTGGGGATTCAACTTT<br>GGGATTCCTATTGCTACTACTATATTCTGTGAGGTCCAACTCCCGCATTCCCCTGGAAACCGTCGTGTTGAATTTCAGGTGT<br>ATTCACCACAATGGGCTAATGAACTAGCCCGCCTGCAGCAGGTGGGGTTACTCTAACATCCATTAAAAAAACTAGGGATA<br>TCTAAACCAGAATCGAACACTATCTGTCTGAATATTGCATCTATTTTTTTTT
MSTRG.3136	Uncharacterized protein LOC106124541	AGACCAAAAATCTTAAAACGTCCCATCGAGCCGCGAGCTGACGAGGACAATTTGTCGGTTCTGCTCGACTCGCTGGGTGC<br>CGGTCTGGTGATTAAAGTCTTCGGCTCTTTGCTGCTCGAGCGGAAGGTCATCGTCATCAGTGATCAGCTCAGCGTGCTGTC<br>GTCATGCATGGAGGCGCTGCAGTGGTCGCTGTACCCGCTGGTGTGGCAGCAGCCGCTTATCTCGTGCATCCCGTCCGCCAT<br>ACAGCGGGACGTGCTCGAAGCCCCCTTACCGATCCTTGCTGGGATGCTCACCTGCAAACCGACAGACACCACCATACATG

		AGGGTATGCTGATAGACCTGAGGCACCCGTCCAAAGTGCTTCACTGTCAGGGCGACGAGTCCACCATACTGCCCACCGCCAGTTACAAGACATTGAAGACGGCTCTGCAG
MSTRG.3140	Autophagy-related protein 2 homolog A	AGGATATCCTAACAACTCTGCCTATGGGGTCAGATTGTATATCGCCTGCCTCCGCATACGAGTTACATGAGGTGTCCACAGCGTTCTTCGAACATCTGCAGCCGTTCTCAATATCAGACGGAGGAACGAAAGAGTTTTGTGTGGCCAATGAGGCTCTCGATAACGCTACGAAAAGAAATCATTTGAGACTAATAACATCCGAGGTGACACTAGATGGCAGTGACAAGACAACTTCACATGGCAACCAGACTCTCTGTGAAGCATCAATACGGCATCTTCTACTAAGGGAATGCCTCTACACATCGAACGACAACAAACCACAAAGTTATGACTTGATACGGTTTGACCAAAAAGAGGATGATGAAGATACTGCCAAAAAATCGTCAGCGTCTTCCAATGCAAATGTAAAAATCAATTTCAAACAAACTTCCAAATATATGAGAGTCTCTGGAGAAAAGAAACTAGTTTATCCAACGACTGAAGTGGTTGTAAAATGTATGCCATGCCACTTAGACGTAGAGCTAACAGTGATTGAACGTATGTCGGCGACATTCTTCGGTGGGTCGACGGCGACCAAAGCTCCCGCTGCCACGACCTCACACAACCAGTTCAACTTCTCACTGCACTGTCCTAATCTCAACGCCATACTAAGGTTCCCTATAGCAGATCTTCGGCCGTCAGTGGACCATGAGACGCGACGTGTCCGAACCGACTACCTCTTGTTCAAATTCCAGAATGTGACAATAGGATCACAACAGTTACCCAGCGTCCGTCCTCTCCCTACCACCATCACCCTTAAAACCACCATGCTGGATTTGTATTATTATGAAAATGACAGTTTACCAGCTACTCACATAGCTCGTACAACAATGAGCGATTCCTCTGAAGGGAATATTTTAAGCGGCAACACTACAACCCTTTTACCGACAATATCTTTAACATTCCAACCGAGCAAAACAAACAAAGGCCCATTTGACGATGTCGCAAGCTTCGAACCGGCAAACATTAACCCAATGACAACGTCCATGTACATAATGCACAATCTACACAGTACACAACCCAGTCCGTTCAGTGGCAAGAAAATGGCGCATCAAAGCTTTACCAAGCACGAGAGCGATAATACACAAGACCGTGAAGAAGAATTAATTGTTCCTGGCAATGATGAAGAAATGGCCGAATTCACCGCTAGCGCCATAGAAACTTCATCAATACACTTGGAATTCAATTTGCCTGTATTGAGCCTGCAACTTGAATCAAAACAACTCTACGAGATCATTTACAACCGGATCAATTCAGACTTGCTGTTGTGGGAGCCGCAGCCATTCGATCCGTACGAAATCAACCCGCTCATGTGCGCCTCTATATATCCTGCGTTTGGCGCCTGCAAGACCACTGGCTGTG
MSTRG.3168	4-coumarateHypothetical proteinCoA ligase 1-like	AAAATCATCATCATCACATCAGCCGTTATCTGTTCACTGCTGTACATAGATCTCCCCCTATTGGGGGATTTGCCAGTAGTTGCCATGCTAGGCAGGCATTGGCACACACAGTTAGGCTTTGTTGATATTTGAAGAGGAACGCTGCTGCTTGCCCCTCGACCGTTAAGTTTCCTTAGTAACTTCTTATGACACTTGCGGGAAAGAAAGGGGTGGCCTATTCTATACTGGGACCACACGGCGAATAACCTGCCACTTTTGACCCAGAGAAGACTTTCGCGTGGCTGGTGAGTACGAGCGGCACCACCGGCACATTTAAAGCAGCAGCAATTACTCACAAGTCAATTTTGAATTTCATTCGCATACACTTTAACAGAAATGTGGTAAACATCAGGAGGAACAAGAAAATATTAACGTTATCACCAATCCAGTGGATAACTTGTATTTCTGATATTATTTACACACCAGCAACAGAATCTATTAAGCTGCAGACTTCGTCGCCATCTATGGAACACATTATTGATATTATCAACGAATACAAGGTGCT
MSTRG.3174	Uncharacterized protein LOC106125039	ATTCTATTCTATATATTTTGATAGTCTAAAAAGCTTCTTAAATGTTTAAGATGCAATACCTCCTAGTCTTTTTCGAGGGCACCGTAGCAATGAAATTGATGATGATGATGATGATGAATTTATTTGCATGATTCCTAATAATTTAAGGCTTGGAAATGAATTAGTAGGGCTTAGTACGTAGTACGTTAGCTTGATATGTGATACCGGATTAGATTCTTGAAGGATTTGAAACCTTTTTATGAAATTAAGCATTTTTTTTAAAGCTTTTATTTATTATTATCTGTGGTAATCAAATTTTAGGAATGAATTGATCACTTTTAATATAATTTTATCTCAATTTTAATAGGAGTTAAATTGAAATTAATTTTATATGTTTTTAGATTAATATATTAACAATTAAATAACCGATACTATCCGACTCGATCCATTGAAACTGCGTAGAGACGTTGTATCTCTGTACATCTTCTACCGAATTTATAATGGGGAGTGCTCTGAGGAATTGTTCGGATTGATACCTGCTGCTGATTTTCACCATCGTACAACCCGCCATAAACTAAAATCCTATCCCAACCATCTGAACGAGTAGCGGTCTTCCATCATACGTTTTTCAAAGCACTTTCTTCCACGCGCAACCACTCTTTGGAATCAACTTCCAGCAGCAGTATTTCCGAACCGATACGACATCGTAACCTTCAAGAAAAGAGCATATTCCTTTTTAAAAGGCCGGCAGCACACCTGCAATGCCGTTAGTGTTGCGGGTGATCATGGGGCGGCGGTAGTCACTTACCATCAGGTGCGCCGTATGCTCGTTTGCCCCCTGTGTTGTAAAAAAAAAAAGACGCTCGCAGATTTCCGAATGTTTAAAAACATATTGCCACAGAGAAATAGTCGTTATGTTAGTAGTAATTTAAATGTATTATCGTTTACCGAGTTTAGTAGATTCCTTTTTTTGTTGAGCGTTCAACACTTTAGCTTTGG

		CAACATTGCAATTATCTGTCAATTGAACATTTTCTCAATGTTTCTTATTGATTATTGAAAAAGTGCTCTACTAACGAACTAA<br>GGCGTTATGAAAGATACCATCGCATACAAATTCCTACTAAAAACTCAAAATTTACATGTATTTCGCTTCGAAATTTTACAA<br>ATACTTTTTTAATATATAAATCCAAAATTTTTAATGACATCTTAATAAGAATATTATTGCATAGTTAACGTTTTAGTTTGTCT<br>ATTAGAACACTTTTTAAATAATCAGTAACATTGACAATTGTTTTTTTTTTTTTAATTGACAGATAGTTGCGTCGTTGCCACA<br>CTTCGTTTAGCTGAAAATAATTATGTTTTAATCAATGGGATATGGCAATGAAATTACAAGGCACAGAGATATAACACTTTT<br>GTTCGGGGGAAAAAGGGATTATCAGTAATGCGGACTATCAGTCCCATTGTTAGCAATATACTATTGTCAAGTTGAACTCCA<br>CGTGGTCCCCCAGGAAACCATCGTGCTGAATTCTGGGTGTATTCATCACGATCGACTAACTGACCTGCCTTTTCATTCTCAC<br>CTATCATCCCTCACTGGTACCCGTCGAGACGCCGGACGGGGTTGCCATTCCATTAAAAAAAAAACAACATTTTTGTCCTTA<br>GTAATGGCAGCACATAAAAGGTACTATAGGCGACATAATATACTCTATCCATGGTACTGTTCATGTCTATAGGCTACAGTT<br>ACCACCTTCCATAAGATGGGCCGTCAGCTTGTTTGCCTTTGTAAATTGCATAAAAAAAAAAAGCTAAAGTTATGAACGCTGT<br>TAACATGGCGGTACATTATCATGATCCTAGCTATTCTCTATCCACGGTTATACTTTTTGAGTAGGGCAATGTGCCTATCGTA<br>GGCCCAGTAACTTTTTACGTTTCTGCTCAGTATTAGATTTAGTTAATGTAGCTTAAACCGGTTGGCCTTTCGACTTAATTTA<br>TATATATATATATATCATAATAATAAATTCCTCGCAATATTAAAATACAAAATTCGATATTAGTTTAGAATAATTTTACATT<br>TATTTATTCACTGACATTCATATGGTGTCGGTTTAATCGATTTAGTTAGTTTTTCTCTCATTAGTTTTTTTTTTGTTTTTTTTT<br>TAATATCTCGACTGAACGAAACGCATATTTTAAATATAAGATTGTTTTTACGATACACTTTTTTTTTTGCTTTGACAGCAAA<br>TTCTCATGCACTGATGTTTATTATTTTAAGATTTATAATTAGTTTAATGTGAATTTTACTACTTAATAATATCAATTTAAATA<br>TTCAACTATAACTATAGCAGGTGATGGTAAACATCGTTAACAAACTGAAATTGTCCAAGTGATGTAGGTGCGCCTTCGCCA<br>TGTTAGATTGACATTTGCATGGGCGTATTTAAGATGGTCGCCGGGTGGGTTGTGCCCACCCCAGAATTATCCAATGCCCAC<br>TCCACGATGTGAGTGTAGCTTTTTATTTTGCCC
MSTRG.3<br>175	Uncharacterized<br>protein<br>LOC105388564	CTCATAATAAGATGTATTTTAATTTATTCCTTTTTTTTTTGGTGTCTGTAAATTTTTTAATAACAATAATAGACCCGATAAAA<br>GTTGATGCGTCATTACGCACACGTGAGTTCCAATAATCTATCTGTGAATAAAAATTCCATCAATTACGAATTAACAATTGA<br>GTGAGTTTGTAATTTGACAAGAGAAACTAAGGAAAATTTGCCACTAACAACACATTTACCAAAGATACGTTATTGGAAAT<br>AATTTTAAATGTAACAAATAGCAATACTAAAAACAACGATGGTATTCGAGAAATGTAATATTATTATACTGCCTGTATCCC<br>GCGGCTTTACTCGCTTAACTTAGTAATTCAATGGTCTAAGCCGGTGTTTCCCAACCTTATTTGTGCCACGCCCCACTTGAAC<br>ATTTCTAAAATTTTCATGCCCCCAAATTTAAATTTAGCACCGATATCTCTCATATCACTCGTAGCATTGCATTTTAATGGCC<br>CGGAGCAAGAGGGTTAAAATCGCTTCGAGTCCATTTTTTACTTGCCCTTTTCATGGGCGTTAGCCCCACGTTGGGAAACAC<br>CGGTCTAAGCGTTATTGACAAACATTTTCCGCCCTTATTGTTTCCAATTTTTTATTTTTGACACGTTAGTGCATTAATCATTG<br>TTGGATGACAATGATGATCATCGATTCAGTGTTGTCCACGACGCTGTTTATATCTACAGTCAATAGTTGCCATTCCATCAGG<br>CGGGCCGTGTGCTTGTTTGCCATTCTATGTTGTGTAAGTGTAAAGAAAACTATCATCGCTGTATTTTATAATTGTTATAAAT<br>AAAAATTAGGAAATATATAACTGTTTCCAAATTGTGCTGCCATTTTTAAATGAATGAAAACTTAATATAAGTACAGTGAGA<br>ATTTATAAAATAAACTCGTGTTATATTTAAATGTAATTTTTATTACAAGAATGTTATTGCGGAGCGGTGGACTGTGATGAG<br>GTGATGAAATGGTGTAGACACGGAATTTAATAGCGAAATAAATGTATTAAACACCAAGCAAGGAACAGAACAATATGAT<br>ATATGTCAAAAAATATAAAATTTAAATTAGAAATAATAAGAACATTCTAAAAGTAAATGTATATAAGCAAGTATAATATT<br>GTCTAAAGAGTTTGCATCGGCCGGTCGAGGCTCACTCTCTAATAAAATTAGTTTGCGAACTGTTCTGCGCAACGTACCGCC<br>TATGGTGAAGACGTCCGCTACGCGGACCACTCCGTCCTGGCCTGGGTAAACTTTTACTGTTCTCCCTAGTGGCCAGAGGCC<br>ACGAGGGAGAGTGGAGTCCACGATAAGCACCACATCATCCTCACATACTTTTAACGCCTCTTTGT
MSTRG.3<br>18	Serine protease<br>snake	GAGGGATGTTCAGCAGCGTCCCTCTTTAAACATCTCCAAAGCCTACGTGCCAACCCGTCTGCCGACCGTGGCAACTATTGG<br>CATAACCGCCCAATAGAGGGAGGCCTGTGTGACTAAATGTAATTGGGAATGGAATTCATTGCAGGTACAAGGCAAAAGAG<br>CATTCCAATTGGCACAAAGGCCACCATTATGAATGCAAAGTATGCGGCGCCAAATTCTCAAAATCAACAAGCCATTTGAC

MSTRG.3183	Hypothetical protein KGM_203000B	CCACGTGAGGCTGCAGCATCCGTCGGAGCACGTCTGCGATATATGCGGAGAGTCATTCATTGGGGAGAATGGGCTAAGGA<br>TGCACCACAAGAAATCCCATAGAGATGTCAAGTGTTTAACGGACGAGGCAGCTGTCTGCGCGCTGTGCGGGGTACAATTC<br>GTTTCAGTGGAAGCTTTGAATCGGCACACGGACGTCACTGAGAACGGAATATGCGATCCTAATTTGATG<br>GGCATTATTGGTTGTTTAGTTAAAAAGCCAATACTTGATAAGGCTATAGCCGTCCCAGGCCAGTAATGTAGTGCGCACGGT<br>GCGTGTACGCAGGTGGGGCGCGGCGCTGGCGATGGTGCGCGAGCGCAACGCCGAGCGGATCCTGGAGCGCGGCGAGCTG<br>CCCGACGATGACGTCACCAGCTTGCTGCACGTCTACTGCAAGGGGCTCGCGCAGGCCAACCCCGACATGGTGGCGATATC<br>CCGTCCCGTGAACGAGCTGTCGCAGAACTTGTCCTTCATGATGGAGAGCCTGTACAAGGTGTCGGTGGCGCTGCAGCGGC<br>AGGAGTACGGCTGATCACCCCGCTCGCCCCCCGTGTCCCCCGGGCCACCCCGCCGCCGCCCCGCCCCCGCCACGCCCTCGG<br>CACGCTCGCACCAAAGCATTCCCCACCCGCACGCGACTGACGTGATGTTGTTAAGCGACTGATGTCAGTCGTGTCCCCACT<br>AATTATTTGTATTGTTATCAAACACCGGCTAACGAATTGAACACGATTCTCATGTCACGCTGTCAGTTTTATATAAAACCCC<br>CTTATACAAATAAGGAGGTAAAATAAAAGATTTTTTATTTTCATTAATTGAAATATTTTTTTAACGATTACTCTGTTTTGTC<br>ACTCTTTCCATCTGAGAATTCAGTGTAAAAGAAAGAGACAAAACCATTCAATAGCTAAAGTTTATTTGATGTTCGTCGTTT<br>TAGGTTTATAAAGGGAAAAATATGTACCGGCAGATGTAGAATAGAATTTACGTCTTGCATCACATAACCAAATA
MSTRG.3185	Uncharacterized protein LOC106107368 isoform X1	TGAAGTTCAGTTTGACAGTTCTAATGTAACTAGTGGTCATCGTATTTTTTTACCTCCTTGCATTTTGATACCAAAATATTAG<br>TTATTCGCAAAGTGGTATTACGTGAACAAGATGAAAATAAGAGCCCACGCACTTCGAGAAAGGTGGAATGCGCACCGCTA<br>GCATCGACTGACATGTCAACCCGCAGCGGCGCTAGCGCGCTCCGGGCCCGGCGCGGTCACAAGGAGCGCTCCAAGAACTT<br>CACCGAGCTGGAGAAGCGGACGGTGCTGGAGCTGATCGCCAGGCACCGCGACGTGCTCCGCCAGGGTCGCTCCAACAACG<br>CCACCAACCGGAGCAAGCAGGTACTACTGCGTGTGAAATTTCTAGTCGTCTCTGTGTGAGCTCAACAGCGTCACTGTCCGG<br>AGTAAGCGAGTACTTTTCCGGCCATCTCCAG
MSTRG.3191	CRAL-TRIO domain-containing protein	AGCAGTCACTTTCCGACATCCTCGCACTACGTACATTTGCGAGACAGTATCGGTTTTATTAACGAAAACAATATTTTAATTT<br>GTGTAAAACGAGTGAAACAATCGTTCTTCTGACACCTTGATATTTAATCTACCAGGCTGGCATAATGTTCGAGAGCTTCCT<br>CGAAATAGCATTCGAAGCCGAAGTGTTGAATACTGAAGAGAATGAGGAGTTCTTAGACCTGGCTGCGGAGTTATGCAATG<br>AGAATATAGCCACAAGATCAGAATCGCTCCAGGAGCTGCGGGATATGATAAAAG
MSTRG.320	Multidrug resistance-associated protein	GGGTCAATCTAGAAGAAGCCAATAGCGCTGTGGTCAATGCATTCAAAAATCAAAGTCTAATAGACGGTAGCTATAAGTCC<br>AGCGCCACTGGTTCTCTTAACCTCACCAGTCAACTGAACACAACTGTGAATCAAACGTCCGTAAGCCAAACCACCGGGTC<br>GAATGTCGCCTCTGATCAGAACAGCTTAATGAACAGTGTCTTAAAGGAGAACATAACTTCGTTTCAAAACGTGACGTTCCA<br>AGATCTGCCCAGCAACTACTTCCACCTGACCCGGGAGCAGTGCGTGTACGTGTACGCCTCCCTGATCCTGGTCTGCATCTT<br>CCTGACTTGGAACAAGCTGGTCGTCTTCTACAACACCTGTATCAGGGCGTCCGTCTCGCTCCACGACTCCATGTTCAGGGG<br>TGTAACGAATGCGCCGATGTGGTTCTTCCACCACAATCCGTCCGGTCGGATACTGAACCGGTTCTCGAAGGACATGGGCCA<br>AGTCGACACCCTGCTGCCCGTCGCCCTGGTCGACTGTCTCGGTTTCTTGTTCGAGGTGCTCTCAATCCTGGTGGTGGTGTGC<br>CTGGTAAACTGGTGGCTGCTGGTGCCAACGGCTGTGGTGGCGGTGTTGCTGTACCTGCTCCGAGGATTGTTCCTGTGCACC<br>AGCAGGGAGTTAAAGCGGGTTGAAGCTATTGCGCGCAGTCAGTCTCTCAACCACGCGGCCGCGACCGTCAACGGCCTGAC<br>GACGATTCGCTCGACGCGGGAACAGCAGCGCACCCTGGCGCGCGAATTCGACAAACTCCAAGACCTGCACTCCTCGTCGT<br>GGACCCTGGTGCTCACCACCAACCGGGCGTTCGGCTGGTGGATGGACATGGTCTGCTGTCTCTACCTTGCGTTCGTCACAT<br>TCAGCTTCTTCTTGTTCTCCGGTGACGACACGCTAGGCGGTAACGTGGGCCTGGCTGTTACGCAAGTGATAGGTCTCGTGG<br>GAATGTGCCAGTTTGGTATGCGACAAACCGCTGAAGTGGAGAACCAAATGACCTCAGTGGAAAGAATCTTGGAGTACCAG<br>AATTTGCCGCCAGAGACCCCAGTAGAGATGAACAAGAAGGCTTTAAGAGCTAATCACCCGGAGCTGGACTTCGACAAATG<br>GCCCAGTAAAGGTGAGATCGTCTTCGAGGACGTGAGTCTTCAATACCAGAGCCCCCCAAAGAAAGAGGAGCCGGTGAAG<br>GCGGGCGAAAAGCCTGAAGAGCCAG

MSTRG.321	Centaurin-gamma-1A	AAGGATTCGCTGCCTCGTGGTCACCACACGCTTTCAGCGTCATCCCATCACCTTCACCGCGGAGCACAATCCTTCGATGCC<br>TCATCAAACAGCAGTGGCATCTCATCCACCCACGTCGTCGAGATCCACAGCACAAACGGATCAGATACCTCATCCCGATG<br>GGGAAACTCGCTCGGGAGCCACGGGTCCTCATCGGGCCTGGTTTCCATTGCGACGGAGAATAATAACGTCAAGTTCACAG<br>CTCCACACTGTCTGGACAATCTCCAGCCCATCAAGGATCCCAAAGATCTGCCCACACCATCCTCTACGCCGACCACATCGA<br>GGAAATCCAGGAGAAGATCGAATCTCTTCACGCCCTCGAAAAAGGGTGATGACAGATTGAAGAACGGCGAGCTCGGTTCT<br>GGCCGCGCTATTCCACTAAAACAGGGATATTTGTATAAGAAGAGTAGTAAAGCTTTAAACAAGGAGTGGAAGAAGAAGT<br>ACGTCACTCTTTGCGATGACGGCAGGCTGACGTACCATCCAAGTTTGCATGATTACATGGAAGATGTGAATGGTAAGGAG<br>ATATCATTGCAGTATGTGACAGTGAAGGTACCAGGACAGAAGCCCAGAGGGTCTAAATCTATCATCACAACCGTCCCAGG<br>CTACAATGGTTACAGTAGTCTGGACATCCATGACA
MSTRG.3224	Signal transducing adapter molecule 1	CTCTCTAATCTGTGTTAGGTACTTGCTCTGTGGTAAATGATCATGTATATTGTATTTCTTTATTGTCACGTCAAATAAACAA<br>TTATTTTGTATATAAATTTCTTTGTTTCTATTGATAACGCAAACTATCATAAAAAATATATGAAATACACTTTTTATTTCAA<br>TGAGTGTCTATTCGTTAATTTGTTATTGTTAATGAAGCACAAATAAATTACTATTATGGGTTTATTTAGTACCTCTTCGCCG<br>TTTGACCAAGATGTCGAGCGCGCCACGAACGAGAACAGCACGAGCGAGGAGTGGGGGCTGATCCTGGAGATCTGCGACC<br>GCGCGGGAGCGTCGGCGGCGGCGGCNNGGCGCTGTACCCGCGTGTCGAGCCCGCGGAGGCCGCCCCGCCGCGCAAAGTA<br>CGAGCGCTGTACGACTTCGAGGCGGCCGAGGATAACGAGCTGACGTTCCTGGCCGGGGAGATAGTTCACGTGACTGACTC<br>CAGCGATCCCAACTGGTGGAAAGGCTACAACGATCGTGGCGAGGGATTGTTTCCAGCTAATTTTGTGACATCTGATTTGAC<br>GGAGCCACGCCCTG
MSTRG.3229	Uncharacterized protein LOC106718292	CCCAGAGAGCCTATATTAACTACATGAATCAGCATATAAAATATACTATGTGCTAAGATAGGATATAAACTACTCGTGTAC<br>AAAATTTCATTCAAAACTGTTCAGTATTTTGATTACATATGTGAATGTGAAAAAGACTCTATTTTCCGCGCTAAGCCTCTCA<br>TTTGGGCCGTTGTGCGTATGAGTCGTGGCTAGTGCAGCCAACCTAGCTCCGCTCAGAAGCGCAGAAGCTTCCTGGGTTCTT<br>TACAGGCCTCACGGAGCGACCTCACCGTTCCGAGGATTTCTAAACGTTGATGGGCCACAGCCTCACATTCCAGGATTACGC<br>GAGCAGCCGTTTCTTCGGCGTCGAGACAGCCTCGACATAGAGGGCTGCCTGTGGCTCCTATTGTGTATAAATGTTTGTTTA<br>ATGGACAATGGCCCATTATTGTCCCTATCATTATTTTGAGGTTTTTTCTATTTTTTTTTTTATTCTTTGATTACATAACAAAC<br>ATACAAACTTTCAAATTTATAACATTAGGAGGAAGTAGGATTTGCTATTTTGTTCACATTTTTTTTTTTATTTCTATATAAAT<br>ATTTATTACTTAAGCAAAGTTAAACAGTTAAGCGATTTGACTGAAATTGGGTACACAAGTAGGTTTTGTGAAGTTACCATT<br>AGTATATATTTTTTTATAATAATAATAATATACTTTATTACTCAGACTACAGTTTGGTCCATTTTGTTAGTAACAATTGTCTT<br>AAAAACTATGTTAGTAAAAAAAGAAAGCAAATTAATTAAAGCAGTTCACTCTGTTGTGAGCCAGCCGACTAGAACAGCAT<br>CTGAATGAAATGCCCAATTATAGGCGAGTTATATTTCTCGCCTATTACTTTTAGAATGCTGTTGGTGCTTGTCCTAACCCTG<br>TGTAGCAGGGGGGAAAAGGATTGTTAGTAATGGAGATTGAGAATCCCATTGCTAGCAATATACCCAATCAGGTCGACCTC<br>CTCGCGGTTCCCCCCGGATACTATCATGGTTAACTCCTGATGAATTCATCATGATGTGCTAACTGGCCTGTTTTCATTCTCA<br>CCTATCACCCTTCACTGGTGCTCCGCCAGCGTATCCCGTTAGCGCAGGCGAGGTTGCCATACCATTTACATCCAATAAAAA<br>AAAACCCTGTGTAGCAATGATGCAGTTTTTTCTTTTTATATATACTTAAATGTATTAGGTATTAATTATAATGAATAGTTTA<br>ATAAATAAATGTATTTAAGATCCATGGTGGAGAGATGGACACCACAGTTTAAAATATATACGTAAAGGTACAAAAGGTTA<br>CTGGAAGCTCTGCCGGTAGTTGGTGCTCTGCGGATGGTTTGGCAACCGCAGTTAGGCTTTGATGCTTTAAGAGGGACGCTG<br>CTGTCTATCTCTCGACCATGAAGTCCCTTAGTCGCCTCTAACGACATCCTGACTTACGGGAAAGGAAGGGGTGGTCCATTC<br>TATGCCGGGACCACACGGCTATAAATGTAATTAGTATTAGTTTTTTAATAAATAAAGTTATGTTTACATAAAAAGTATTTT<br>TACCATAGGGGGACTTTGTACAAGAATGGTATGCTTGGGTACTTTTGTCCCACTCGTATTTCGTCTATGAACTAATTGCTTG<br>CTAGCTGTTTCAGTTTGAAAGGTGATATAGCAGTGAATGTATAGGGTACAGAGGTACAACACTAGTCTTGGGGAGAAATT<br>GTTAACCTTTTTACACAGA

MSTRG.3238	Peroxisome assembly protein 12	TTAATATTGTTGTTGTGTACGTTGCAGCCCGATGAACGCGCCGTAAGATGGAAGAACAAGTGTCCAATATGCCTTCAGAGC<br>TGGAAGATTCCCACCGTGCTACCAGTCTCAGGCTACATCTTCTGCTACGTGTGCATCTCGCGGCACGTGCGCGCGCGGGCC<br>GCGTGCCCCGTGACGCGCTGCCCGGCCGCCGAGTCCTCGCTCGTGCGCCTCTACGTCGACTGATCGCGCGCGGACTCGCTG<br>CTCACTCCATCCATGGATGACACAGTATGGGGGAAAAAGCTGTAGTGGAAATGGAACACGCACAATTCGTTAACTGGACA<br>TTCGCGACGTCTTACATAATTCATACTCCGCTTACCACTCGTACATGACACATCACTCATAATTCACTTGGCACTCGTACAT<br>GACACATCACTCATAATTCACTTGCCACTCATACATGACACATCACTCATAATTCACATACCACGCGTACATGACACATCA<br>CTCATTATTCACATACCACGCGTACATGACACATCACTCATTATTCACATACCACGCGTACATGACACAACACTTATAATT<br>CACTTGCCATTCGTACATGACACATCACTCATAATTCACTTACAGTCTATATAAAACACAACACAGAATCCATTACCTTAT<br>GATGGCTCAGATCGTATTCTACCAACAAATGTACGAAGTAGTCTTGTTATGTAAAATCTTTATTGTACATAATTTACATTTA<br>ATTCATTCTTAAGTCGAACAAAGCCGGTATTCAGCTTAAATTAGATCTGAAAAGGCAAGTTATTTCAAGTCTTATGTAGCT<br>GAACAATTTTTTTTTTTAATTCACATAAAGTTTCGTGGTATCTTTTATGATATTTTATCAAATTCTGAATTATGACTATTTAC<br>TTTTTGGTATATAGGTTAGCGCTTTATGTAAGGTACGTATAAAAATATTATTAATACGATGTATTGTGTGTTAATAAGGGA<br>GGATGCAATAATACATGTATGTATTCGAAGGTGCTTGCATTTATATTCGCTTATGAGAGGGTAGATGGCGCCTGCGTTTTG<br>TTTAATAATGTTTTTAAGAGAGATTAAAAAGTATCAGTTGTCGAAATGTACGCGATCTAAGAAATCGTAAGTTTTTGATTT<br>AAAAGTTGTATACTTAAATTGGTATATATAATGTAATTTGCAATATAATTTGAACAATAATGTGAAAAGAATAGAAAATA<br>AATCAGTTATTTTTTAATATACAACTACCCTCTTATTCATAAAAAAAAAGTCAAACTTTGTTTTAGCTATTGAATTGTTGTCC<br>TTTCTTTTGTGCTAAAATCTCAATTTGAAAGAATCAAAACAGTTTTCATTATTTTTATTTATTTATTATTACAAAATCCATAG<br>CTTTTAAAACATATAATATTTTAGTGTTATTTTAATACTTGCAGTCAATTACGGATTTATAATTTATAAAATATTCCTTATTG<br>AGAAGTAAGCCTTCACAAAGCTGATATTATATTTTTTTTTATGCAACTTAGAATGGCAATCAAGCTGACGGCCCACCTGAT<br>GGAAAGTGGTAATCGTCGCCTATAGACATGAGTATTACTATGGACATAAGAGTATACTATTTCGTCCATGACACCACCCAT<br>GCGTTGCCATTCCTGAGGACTCAGATGTTATACCTCTGTACCCTATAATTTTCAATGTAATATTAAAATTGTCATTCCTGGC<br>ATACCTGCGCCTACATATGATTGTGATAAAACTAGATTATAGTGTTATTTTATATATAAAGGTTGTATACCTACTTAGATTA<br>TATACATAGAAATATATAGATTTGTATGGAGACATCCATAGAAATGTTTCGAGAAAAGTGGGTCAACTTTTTCATACAATT<br>TTGCGTGACAAATAGTTGAGTGATTCCTTTATAACTATATATTTATTTTGTCTTATAATTTCTGCAATTTTTTAATGCGTAAA<br>CACTCATTTTGTTTATTACATTTAATACGTAAGTATATATAGAACACTGATAGAAAAACGAGTCAACTAATTTAACAATTA<br>GCTGACAGAAAGTTGCCCCGTTTTTGAGACTCGATGCTCTATCTATATAATCTAAGGTGGTATTATTAGGTGGTATATTTTG<br>AATATTAATATTGTTAATTAATTTTAACGAATTATTATAAGTACTCTATGACCATCGCACGAGGAACAGTAAAATTGAGAA<br>TAATCTATTTTTGAAGTCGGCTAAAAATAAATGTGTCAACGCCGACAAAAAACGCCATTTCACAAGACTTTAAAGGGTTTG<br>TCACTATTGTATATATTTATCCTTACACTGTGGTATCGAACAGGTATCTTTGTACTCTCAATAATTCTCTAACAGACTGTAC<br>TTATTATCAAAAAATTATCTATAGGTAAAAGTGGCATATTTTTTCCCATTTATCCTTACTGGACACACAATGGTAGTGACAA<br>GGGAAAAAAATTCCGCTCTTACAGTTGGTCGGGACAAGTGGTATTGGCCAAGTGCACTGGGCCCATAACAGTGTAATACT<br>TAATAGTTCCAGTGCCTTTGTTATTTTTACATTGTTACATATTTATACATTGT
MSTRG.324	Rab GTPase-binding effector protein 1 isoform X1	GGTGATTAAATCGACAATGAGTTTTACAACCGGTGGAAAAATTGTCCTGTATGGGACATAATGTAATCTGATGTACAAATA<br>GTGGTATTAGGCAATAGTAATGTATGTGACATTGCGTGACATGACCGCCCGCGGCGTGTGGCAGGAGGTGTCGCTGGCGC<br>CGGCCACTTTCGTGCGATCGCTCGCGCGCAAGCTTGGCGCCGACGCCGACGCCGACCAGCCCGCTAAGAAGGGCGCGGAG<br>GACAGCGAGCTGATGCGGTCCATAATCCAGCCGCTGAACGACGAGATTGTGGTGCTCAAGGGCAAGCTGCGTGACACCGA<br>CTGTCAGCTGCAGGAGGCTCTG
MSTRG.3241	Sin3 histone deacetylase	ATTTAGTGTCATAATATGATGCTTTTTTGTGCAGTTTGACGTAAAACAACTTCAATTTGTTCCAATTTCGTTGTTCTGTCTTA<br>TTAGTTTTTATATGAATTTCTTTTTACGGGTGACACAACTGGTACATTTTACTAGTTGTTTGGATAATTAACTATCGTCCGCG

	corepressor complex component SDS3	TGTCCAGAGTTTGTTTCTGAGACGGTGATCATAGTCAATCTGTATGCAACTGTTACGCGTCGCCTCGACTATGTCTTACCAAGGATCACCATATTCGGGCCCGGGAGATGAATATGACTTCGAGGATGACGGGTACGACGACTTGGACGAATATAGGGATCAGGAAGACGCCTTACCACCACCGCCGTTGGACGACAGTGATGAGGACACAGAAGAGGCTAGTGAAACAGATGTACCCAAGAATGATGAACCACTTGAAATCAAAGAACAG
MSTRG.3250	TELO2-interacting protein 1 homolog	GATAAATTAAGCGTGACCAAACGATCACCGGAATGGTTCGCGATGGCCGGCGAAAGATTGGTGCTGATTACAAAAAGCTTGCAGACCCTGCAAACGCACGAGCATTATAAAGTCAGGAAGGAGTTGGCCGTGTACTGCTCAAGAATCCTCATCGAATGTAATCGAACCATGCAACCCTCTGTGCCGATAGCGTTAGACATTCTGATAGCACTGGCGAAGGACGAGTACCCGCTCGTGTCCGAGTATTGTTCGAAAGCAGTCGACACGTACTTCAACGAGGGCTCCGAGGACGCTAAGATGAGGATAATGGATCAACTGTGTGATAACTTTTTTGCTACATTGAATAGTTTGCCCAGCATACTCAACAATATAGACGATGCCAGAAAACTGTCGGCGCTGAACTTGCTGCACGGGTACGTGGAGGCGCTGTGTTCAGGTGAAGCCAGCCAGCAGAGGCTAGGCCGCGCGCTCAGCGGCTGCAGCGGTCTGCAGCGGTTGTGTGCCGCGCTGCAGGCCGCCGCTACACTGCACACCGATCTAGCGCTGTTGAACCATCGTACAGCCACAGATGTGGGCTGGACGTCGCCGGGTGGTGTCCCGTGGCGCCGCTTGCGGCACGTGGACGCCGCGGGCGAGGCGCGGCTGCGGGCCGTGTGCGGCGCGGTAGGGGGCGCTGCGTGCGCGGCGCTGCTGCTGGACGCGCTGCTCGACACCCTGCAGCAGACTCGTCCGCCAGAGGTCGCCTGCCTGCTCAACTGGATGGCCGCCGCTCCAAAATCCCCTATCCACCTAG
MSTRG.3256	Pre-mRNA-splicing factor ATP-dependent RNA helicase PRP16	ATTTGCAAGTTCAAGACGAGGCTTGGACATAGACGATCCAGAATGGCAAGAAGCTGAGAAAAAGCTGGACAGGGCATGGTACAACATGGGCGAAGGTGAAGCAGACGAATCCGATCCATTTGCGGATACGAGCGCTGAATATATCGCTCGGAAAGAGGAACAAATCGAGAAACGGCGTAACCGCAAGGTGTCAGCACAGCGGCAGCAGATAGACAAGGACAACGAGCTGTGGGAGCGAAACA
MSTRG.3258	Uncharacterized protein LOC106140707	CTTGAAGACTTTATATAAAAGTTGCCAAGCAACTATGTTTAGTTTGAGTATTTGGGTTTAAAGGGTGGTGGAGGTATGACAGCATAACATCTTAGTCCTCAGAAATGTCAACTTCTCAGAAATATCTTTTCATGTCTATAGACGATGGTTATTTCTCAACAGGTGAGCCATCTGTCGATGTTCCTGGTAGAATTTCCAAAAAAGTTCCCATAATTTTTAAGAAAGCAGTTAATAATATTGGACTGCATGTTATTTCCAGTTCGACCTGGGCTGCCAACCCTGGAAGAGGACGATGATCGGATCAGTGCATAACCTCGGGCTGGTGGTCTCGTTTCTGTTGTCCGGCTTTATATCAGACAGGTACGGCCGAAAAGTTGTTATAATAATTACACCTCTTGTGGTTGGCATCGTAGGCCTGATAAAGTCCTTCTCGGTCAACTATTGGATGCTACTCGTCTTTGAATTCCTCGAAACTGCACTAGGATATGGAAATGCTTCTATGGTTTTGTCACTCGAAACCGTGAGTCATAAGCGCCGTGTGATCTTCTCGTGCATAGCTGATATTTTGTCCAGTTTTGGAAGCAGTTTCTTGGGTCTCATAGCGTGGAAAATTCCATACTGGAGGTATCTGATGCGAGCCATCTACGCACCGCTTCTGGTGGTGGTGTTCTACATTTTCTTGGTGGACGAAGGCGTTCGATGGTTGCTGGCACATAACAGAAACGACGAGGCCGTCAGAGTGCTGAATAAAGTCGCAAAAATTAACAATATCACTCTATCCAACAAGTCCAAAGAGACATTGATCAAGATTTCACACCAGAACAGTAAACCGAATCAGGTTGGTACATGCATCATTTTCTATACATATTGTCTACTGGGCATAGGCCTTCACAGATTGCTAATGAAAGGTTTGAGTCAATTTATAATAATTATAATAATATATCCTTTTCAGCTGTTAATTGTCCATTAAAAAAAAATAAGTCTCCCT
MSTRG.3261	Uncharacterized protein LOC106124541	AGAACATGGCGACGTGTCACCGCCGCCGCGCGTCCTCCGCGTCATCGACCACCGTCGACGAGGGCTTCGTCGACGCCCCACTACCCAACTTTTACCAACTATTCGAGGAACGCGTCCGCAACAGAAGCAAAAGTGACTCTCACAAGAATTCCGAAAGTAAAAACTATAGAAGTGCTGTGAATAAAAAAGTGAAGTTATTAAAAAGTAAATTAAGAGACTTGATTACATAAAAAGACGTGTTGAAGTGTAATTTATTGGCTATGTCTACATTATGGTAATATTTTTTTTAAACTGACTGTCAAAATGAAGTCTCGATTCAAATGTATTTTTTTGTGTATCACTCGGTATCTTTCAATCGATTTTTGAAAATATATTCAGTAATAGTCCAGTTTTTTTACAACACGGGAGTGGACTCTTTCGATTAAAAAAGAATCATTAGTATTGGTTCCCTATTGGCCAATATTTAATATATGTTAAACAAATACAAAAAATACAGTCGATTCAAACTTCTTTTTACGGTTGTTTGAAAATGTAATGCTTTAAGTATAAACTATGTTCAGTTTAAAT

AAAAAATGTGTATGTTTACGACTGTAATCCTTAATGGGGTAGTCAGATAATCGGAGCTTATTCGAATACATTAAGACAGGG
CGTTTATGGTTTTCTCTTGCCATATAATGTCCTCTTGGAAAAATTTTATGTCAATGATGTTTTCCACTATCAGAATGTTAGTC
AGGAATTTAAATGGACTTTTTAGTGATATATAAACTGATGACCTTTTTGAGTTGACAATATTGACATTTTGGAATTAGAAA
ATTCAATTTTTTCTAATTTCTTTTTATGGAATAAAGTAAACTGTACAATAATGGAGTTAGATTTTCCAGACAAATATTCTAT
CATGACTTATGAGCACCGCAAATACTTTTTTATACAGCTAACTACATTCATGACAATATAGCAAACGTTATAAAGTAACCT
GTGTCCAGTGTTAATCGTTAATATTTTTTAATTAATAAATACAAGATTTTACAGGAGACAAAAATATATGCTAAATCTAAG
TTCTAAATATGTTAAAGTAAAGAGTAATAGTAGGTCTTCATATCAATTCACAATAATTTTGATGTAAAAATTATTTCTCTAT
GTAACATAGAAAAGGAGATATGTATGGTTGGAAAAGATTTATACAACTATTTTGTATACGTTGGTATTTATTGTTGGTAAA
CCGTCGTTATGTCCTTCACCAAATATTACATACTAGTTTTACTCCGTGGCTTCGCTCTTGGTCGTATTGTGATGTTGCCTATA
ATCTTCCTCTACAAATAAGCTATCAAATGCAAATAGAATTTATAAAATCCGACTGGTAGTTCTAGAGATTAATGTTTTTTA
ACAAATAAATGGATATTCGTGTTTTTACCGTGAAGCAGTTGTGTTTGCATCCCTGTATTTCGGTTTAAAGGGTGCGATATGG
TAGTGAATTTACAGGGTACAGAGGAATAACACCTGAGTCCTCAGGAACGACAACGCATGGGGGGTATCATGGGCGAAAC
AGACGGTTACCACTTTCCATCAGGTGGGCCGTTAGCTTATTTGCCATTCTAAGTAGTATAAAAAAAAACACTTCCACTCTTT
ATTACATATGTCTAATTTTTTAAACATTATTTAATGTGCACCAACAAACAAACAAAGTTACTCACATTTATAACAAAAAGG
ACATTTGTCAAATGATTTGGTTTTCCATTCGTTGTAAGTTTAAAGAACCTTTTCAAGCATCGGAATGTTCTAATTGTTTATC
AAAGTAATTTTGTATATATATATATATATACACTACATATATCTATATGGCAATCTTTAAGAGAGATATGTATGTTTAGTAG
TGGGCAGATAATGGCTGAGTTAATGCTGATAATGGCCGAGTTAATGCTGATAATGGCTGAGTTAACGATGATGATACTAC
ATATCATTGTAAACTGCATTTATTTGTATCCATTTTCTTTCCTCTCTCTCTTTACTTAAGATTTGATATTTATTGTTTATTTGG
TCATTTATAAATGAATAGCACTTTTTTAAGGATGGGGATATTGTTACGAGGTTGTCGACCAGTTCATAATATTAAAAAAAA
AAAAAAACTATGTTTAGTTTAGATATCTCATGCTTTAATACAAAAAAATTTTTATTAATGTAAATTGTCAATGAAATACTTT
GTGGATATTATTTTTTAAGGCATAGATTTCTTGTATTATTGAATCTCTAAATTTTAATTTAATTGTAATAGGAACCGAATTA
GTTTTGTAACATTGTATCTGTATATTGCCTCCCTTCCATTCCATTGATTTCTTGTATGATAAATAAATGAAGTTGTATGTGTG
TAGCGTGACTTTGAAGTTATTCGCTATATTGAATCACTAAGTTCTTTACAGGGTAGGCAGAGAGAAGCGAAGTGATAGTTT
TGGCAATCGGTATTTTAAACTGAAAATATAAAATTTGGAATACAATTACGTAGCAGATGATGCGGCGATCTCCACATTGTC
TGTTTATTCATAAAAACATAAAAATTTTTATAAACCTATTTTAGTTATTGATCTGTTTTGTCTCTTTTTTTTGCACTAAATTC
GCCCTGCCGGCCTTCTGACTATGGCGAGTAATTTTTAATTATTGTAATTTTTTTTTATTATGTACTATGTTAAAGTAGTTTTG
AAGTAGTTAACAATATAATGTAAATGTAAAGAGACATTACTAAATCAACTCTAAGACAGTTGACACATGTCCCAACAGTA
GTAGAAATAAAAAATCGAATAATTGATTGAACGTTTCGATTATTTATTAATTTCATTTATTTATTTATTTTATTTAATTCTTT
ATTATACACACCAGTTATTTATAAGAATGTGACAGAAGTAATCAAATGTACAAAGACGATTAAGTAGTGTTTGTATAAAA
ATTTCAACAAAACTTATTTATGTTTTCAATAAAGACTATTGAAAGAGACTGTAGTACAATAAATGCAAGAACAAATATACA
AATACAGGATTTGTCGGTTAAATTCGACTACGACGTTACAAGCGTTTGTTGGAGATATCCGCTTCGAGCCCTTAAAAGATG
GATGACGATGTTAAAAGTTAATAGACGCTCTTGAATTCCATAATTTATTTGTATAAACTCGTACATATCATTTTATTGCCTA
TTGCTATCTAAACATTTGTTAAGTCCGGTAGCCATTTTGCTGTTTTAGTAAAGGGAGTTCTGTTTGTACATGTGATATAGTT
AGTGTTAATTATTTGTTGTATATTCCTTCGATTATTCTAATAATAAAATCGTTTACTTATAAAGTAGTTATTTGATTTGACGC
ATATAAAGTTTTATTAACTGCGTTGAAATTAATCTCTAATCAGAGGGGCTGAAAAACTAAAGTTCTTTGCGTAAGTGATAA
AAAAAGAATATAAAAATATAAGAAAAAATCAAAATACTTCATGACACTTTATAAATATCAACAGATTGTCTACGATTAAC
AAGGCACGTCCTTCCAAAATCTAAAGAATTAAAATTGTCTGAGTAAAAATATTTTCAAAGTTTTGACCAATGCTCTTATGT
CCGGATCGCTACTAATGACGTCTTCTCACAAACATAAACAGAGCACCACTTCTGTATTAAAGTAACAATTTGGAGATGCGT
GCCACGTTAAAGTAGTCAATCTATACCTAATTAATTTCTTGAAAGCTCGAGTAAAATGTTAACAAAACCAAAACAGGATA

		CCTAACGGATTACATTACGTTCATTATCTATGCTAAAAAATTAACAAAAAAAAAAAGGTATGTTCCCGCCAAAAATATTAC<br>CGATATTTATATATCATATTAAACAATGCCCCTTTTTTCTATCATATCACTGTATGTAAATTTGGTTTAAAAATGTAATTTCT<br>AAATATGTTAAACAGATTTTAATAATGAATAAAAGTGTTTTTTTTTATATGTTTGTTCCGTTGTCGAACAGGCTAAGGTCGG<br>AGACCATGCTGCCTAGTCTTGAATATCTAAAAATGTTTAGTCCGATAGTCATCACTTCACTTTAAAATCGATTTAAAAAAA<br>AAAACTTTTTTGATGCTTAAGGACAACTACGCATACAGCCAAATCTTGAAGTAATTAGTGCTTAGCATAACTGAAATACTG<br>TTATAGAACATTCGAAAATTGTTAATGTTTTTTTAATAATGTAATTATTTGTTAGTCCTATAATGAAATGTAATATATATAT<br>AAGTAATATAGTGTGTTTGTACTCTAAGTAGGTCC
MSTRG.3265	Uncharacterized protein LOC106107368 isoform X1	GAACGAAAGAGAAGCAAGACGAGCAAATTAAACACGACAGCGGAACGGCAACAGAGGAGGAGAGGTTGTATTTCAGTG<br>CCAAACGGCAACACGCGATATGGGAGCACGAGGCCAAAATGAAAATCTTAAACATGGAGCTCAGACAGAAGGAGGAAAT<br>ATTTTCATTGCAAAAACAACTCTTTCTCATCGAGTTAAAGTTGAAAATGGAGTTTTTGGAAAAGGCTGGCTTGAAATGAGG<br>GTTGAGGAAAATTTAACGGCGTCATATAATTATATATTACAGTCAGTGCCAATAAAGAGAAACCATTGTTGATGTAATTAT<br>AAATAAATATGATGATATATGTTAGTTATTTACATATATAAGTTTTACATTAGAATGCGAAAAATACGTAAGTTAGACATA<br>TACGGAAAAATAAATTCGACACAATATGATATTATTAGGGTATATAAAGATGAAATATATACAGTACAAACGGATAAATA<br>ACGAGATATAATAAATATTACCTCAGAACATACAAACATACAGTGTATTGTAAATATATAAGCAGTAGTTGTAACTTTTTA<br>GAAAACATTTTTATATGAGGGCGTCTTACACCTTCTTTTATGTTGTTTTTGTGTCAAAACGTTGCCAGTGACATTATATTAA<br>AAACGAGAGGAAAATTCAATTTTCGAACAAATTTCTTTGTCCTTCACATTACCCAGTCCGCGATGCTCAATTTTGAACCTG<br>GC
MSTRG.3269	Hypothetical protein	ATAAAGGTTTAATTCTTTTATAAACTACTTTTTTTTTATCTTTATTTATTTAGGAACTTTTGTCCTTTTAGGCACGTCAGTCCC<br>GTCGTACCTTAATCAATTTTAGGACATCATTGTATATATTTTAACAATCAAAAGTTCACAATTTTACAAAATTTTAACAATC<br>AAAAACACCACCAAATCAAATAATATGAATGAAAGGAATGTCGTGGAACAAAAAAAAAAAACTTAATAATCTATGGTCC<br>GATGCAGGCCTCAAGGCAGGTAGATCTACATTAAAACCTACATCCAACCATATTTAATAACCATAATTAATATAGCCGTAT<br>GAGGCGGGCGCACCGTACGTGGCTCAACAAAGGAGAGGCCTCGGCACGGGAGCCGCGGTCGGTCTAGGCGTGGGCGCGC<br>TGGCAGCCGGCGGGCTGGCTGGGTACGCACTCGGCGGTGGCTTCAGCAGTGACAGTCCGCCGAGAGAGGAGGTAAACGT<br>GGTGGAATTCTCCGAGAGCGCCGATTTCGGGGGCGACGACTGGATGGACTGAAACCGGGGACACAACCTTCACAATGTTT<br>CCATAATCGTCACCCTTGCGATCCTCTTCGTACTCATTGTCATTAAGTTCACTTTAATAGCATTTACACAGATTGGATTATG<br>GCTGTTTGGATGAACGACTCGGTTGTTCATATCCCTATCAATTTTTTTTTAACCACAGAGAATTATCCTTTAGAGCGCTCGT<br>AAGTTTTGCTTATGCTTTTTTTTTAATGGATGAAAATGGAATAGTGACCCCGCCTGCGCTATCGGGATACGTTGGCAGGGC<br>ACCAGTGAGGGAAGATAGGTGAGGATGAAGCAGGTCAGTTGGACCATCGTGATGAATTCATCAGGAATTAACCATGATAG<br>TTTCGAGGCGAAACCGCATCGAACTCGACTTGTTAGGGTATGTTACTACTAATGAGTCTGTTGGATCTCACTGCTAACAAT<br>CCCTGTCCCCCCTTTGCTTATGCACTTTTCCAAAGTTTGTTTTTGGTTTTACTTATGGTAGTGGTATCATACTGAAGACTAGA<br>CAGCATGGTCTCCGACCTTAGCCTGTTCCATAACGAAACAAACGTACTAAAAAAAAAAAGGAAGTGTGGCGATGTCGGAAT<br>TATTTGTCAATGTTAAAAAAAAAAAATTAATTATTTTCATTATTTCTGACTAATTATTCAAAAAGAGCTATGCTAAATAACTT<br>AAACGGTAATCATAGAATGCATTGCATATAAATCTACCACTAAACTCTTTACACCAATTTCATATTTTACTCCGACATTTTT<br>TAAATACATTATTTACCATCACAATCCAATTTTACAATTACAACTTAATAAGAATATGATTGCTTAAATAACGTTTCAATTC<br>GTTTGTAGAAAACTCTTAAATAATCTACCAGAAAAATATTCAATTGTTTTAAACCAATTTCAAAAACGAGTTTTTTTTATTC<br>AATGGACGGAATGGTAACCGCGTCTGTGCTATCGATAAACGCTGTCGGGCCACCAGTGAGGGATGAAAGGGCAGGTCAGT<br>TAGCTCATCGTGATGAATACACCTGGAATTCAACACGATGGTTTCCAGGGGGGACCACGTGGAGGTCGACCTGATAGGGT<br>ATATTGCTGGCAATGGGACTGATAGTCCGCATTACTAACAATCCCTTATTAAAGTGGAGAACAGAAAGCGATATTAAATA<br>TTAAAATACGTATAATAAGCAGAACAATATGTTACAATCAACAAGATGCTTAGCAACCAACATTGTCATCGATAGACACA

		GATAATTAGGTCGTATATCTACAACTCATACTGCTAACATCCCTTTCCCTCCAAAAGAAAGAGGTTGTCTATTTTTTTTTTT<br>TTTATATATATATATTTGTTACCTCATAACTCCGTCAGTGGTAAACGATTTGGAAAATTATTCTTTATCTAAATATTGGTCCCA<br>TAGAAATTTTATTAAAATCAATTTACAACTTTAGTTTTAAAACAAAATAACTTAATTTGCCACAATAGAAGTCGGCTTTGT<br>TTGTGTCAATCTAATTGTTATTGAAAAATTATATATAGTTACAAAGTAGCCAGACTTCGTTATGAACGCTATACTCTGTTAT<br>GTCCACGGTACTGCTCATGTCTATAGGCGACGGTTACCACTTTCCATCAGATGGGCCGTCAGCTTGTTCGCCATTCTAAGTT<br>GTATAAAAAAAAATCTATTCTCTACGACATATTTATTACTGAAATGCAGACCTGTGTTTTTATAATACAAATAAAATTAAAT<br>GGAGTTTCCAACGCGTACATAAAATTGTAAATTTTGACCGTGTAAGATCTCAGTCGTCTATAAGCGAGATAAAAGACAAC<br>GCCGAAATTAAAAATAAAAAAATATATCGGTTTTCCGATCTCCATTAATTTTTAAGCGTCTTGAAGATAAACGAGTAGTAA<br>TCTATTTTTTTTTTTGATGGTTGAAAATGGAATGGTAACCCCGCCTGCGCTAACGGGATACGCTGGCGGAGCACTAGTGAA<br>GGGTGACAGATGAGAATGAAAACAGGCCAGTTAGCACATCATGATGAATTCATCAATTAACCATGATAGTTTCCAGGGGG<br>AACCGCGAGGAGGTCGACCTGGTTGGGTATATTGCTAGCAATGGGATTCTTAGACTCCATTACTAACAATCCTTTTCCCCC<br>CAGTAGTAATCTATTAGGTATTAATTTTCCATTTGCAATATAAATTAAATAAATATATTACATATTTTTCAACGAATGGATG<br>CGTTGTGATACGGGATTGTTCTTTGTGCCAGCTATGGATTAATTTATTGCTCATAATTATGATTTTTTTTTGTTATGCGTGAC<br>CACGCACGGCGGTACGATCGTACCGCCAAACCGCGATAGTACTTGTTTATTTAAAATATTTCACTGTAACACATTTTAATA<br>TAATCAAAATCGTGTAATAAATTGTTGAAATTAATGATAATAATAAATTTTATCTAAATGATAAAGGTTAAATAAAATTAT<br>TCAAATATTTAAAATTGATATTGATCTATTTGGCGCGATTTTATTACGTAGCAGTATTTTTAATGTATCGATTTAGATACTA<br>GGCCTTTGGCCATATCCTATGAAAGTTGCCATTATGAAAATTTAAAGAACGCGACGGATATATTTGCATAAAAATAGCCGA<br>CCAAAATAATGGGTACGTAATTTGTAAGTGTCTACCTATGTATACTTTGCCCTGCTTTTATAAAATTAAATGTTCAATTAAA<br>AAAAAATCTAGTGTAGCTAGATTAAGGCTTCAGCGAAATTAAACAACAATGCTGAAAGTTGTCGGTAACTAATGTTCTTTA<br>AGAAAAAAATAATATTTTATCTAGATAAAGAAAAATTGTTAGTCTGTGATTTGTACCAGTATTAAAAAAAAGTAATGGTAT<br>TTTTGTTTTTACAAACATGCGATTTCATAAAATAATAACAACATTTTCACAAAATAATAACAGTACAAGAAGCGATACCGC<br>CGATTTCTGTTTGGCAATTTCTAAAAGTTTTTTTTAGGTAGGCCTGGTATTGCAAATTGTGCCTTAGTTATACTGGGGTTGA<br>AAATCGTATATTTCACAAATTATGGATAATAATAACACATCTGCAATAAGTCATAAGGCAGATAGCATCTATTAGATATTG<br>GACTAATATTTATACTTATTCTGAGGCAACATTTCTCTAATCTGAATCTATCTTTTAACTTAATATTTTAACTTGATTATTAC<br>TCGGTGTTGCTGTTTTGTGAACTATCATAAACTAAATAATAATTATGACCTAAATCTATCAGAAATTTAGTTAATATTGATT<br>TTTAAAATAAAAAGATTTTGCTGTGATATCAAATTGAAATTTTAATTTAAAATACATGTATAGAGAAACGATGCCTCCTAA<br>ACGGTCCAATTATTTCTGTTAGTGGGTGTTTTACGTTACCTATATATAAATATAATGGGATTATATATCGCTGTTAGCGATT<br>AAGTATTACTGGTTAGTTTTCTAATTTATTTTTATTTTTTTGTCATATTGTTGATTGTTGATTATTTTTGTAATATTAATATCA<br>TTTTCTAATCATTTGTTCATTAATCTTTATGATGAAGATATGAGGAAGCAAGTACAGATTATTAGGTGCTAGAAATGTAAA<br>GTGTGTATTTCAGATTTAAATATATAGAAATTGTTGTTAAATATATATAATATGTAAATTTTAAATAAATTATATCACTTGT<br>GTGGCTTAACACGTTTGATATTATTTCTTGTGTACGATTGTAAAATAAATATGATGCAAAATCTA
MSTRG.3274	Glutamyl aminopeptidase-like isoform X1	AATTAAATGCTGATGAAGATTGGTTGAAAATCAATAACAATCAAGTGGGCTACTACAGAGTGAACTACACTGAAGCCATG<br>TGGGAGAAATTAGCAAATTTATTGAGAAATAAGTCGCTGCAGTTAACAATATCGGATCGAGCGCATTTGTTGAACGACGT<br>ATTTGCTCTGGCCGAAGCACGCTTGGTGACGTACGGATTGGCGCTCAATCTGACCACTTACCTCACCGTAGAAGAAGACTA<br>TGTGCCATGGGAGACGGCGTCACATATATTCTCTTCATTGAGGAATAAATTACTTAATACACTTGCGTTTGACCCTTTACAG<br>AAATATATTCAGCATTTGGTGAAACCTTTGTACGAGAAACAAACTTGGGAGAGAACACAGATAAGTGTTATTGAGAGGTA<br>GGTATTATTATTAAGCTAGT
MSTRG.3286	Organic cation transporter protein	TAGTTTAGCAACCTTCCTATATCATGCGCTACACGTCTCGCTAAAGATCTTTCAATGTAGATTGGTGAAGAATGGGTCTAA<br>TCGATGTTGTTCCTTTATAATAATCTATGCAAAGAATATTTTTAAATATACATCGCGATGCGTCGACATAATTACAGTTGTA

		GTATGATACGGCATAGTGGGCACCGAGCTTTAAAATTAAAGGTCTCTCTATCACAAATCGATGCCGACAGTGATATTCGGC<br>GCGATGTGCCTCATATCCAGTGGACTGGTGCTCCTACTGCCGGAGACCAGGAACGTCAGACTCCCTGACACAATCGAAGA<br>AGCCGAGGAGTTGTCTCGAGTGAAAAGAAAATCGCCCGTTGAAAATGAGTTGTGAGAAATTTAGGCCCTTTTTCATTGTTC<br>CTTTATTTTAATTTTTTTTTTATTGCAATCTATACCCATTATTTAGTTTACATGCATGTTTAATAAGTCTTGAGTCTCGTTGTA<br>TGTAATTTCCTTTATTTGCATACCTACTTGATCATGAATTAAATCCGTTACCTCAAGGTTGTCTTGAAAAGATTAATTTCAG<br>CAATAAGGTCGCCTTATTGTACATATAGAACTGTTTCTAAAGTGTCTATCTATTGTGTTCTTTTCTTATATTTATTCATTAAT<br>TTTGCTTCAAATCAATTCTGTCAATTTAGCATTGCATTATGCAACGTACATAAGTACTTATATGAAAGTATAAATATACATA<br>TTTTTGTATTAAAACTGTGAGTTAAAATGTTATTTATTTAGGTGCTTACCTACATATTGTAAATGGTAAAATTGTTCTGGTTT<br>TTTACAATAATGAGAGAAAGAGAGAGCAGAGTTAAAGGCAGTTAGTGGTTACAGTACTGTTTTATAAATAGCCTCCTTCA<br>AGCAACAATAATATTGTTGATTGAATGAGGCAAGGAGGAGGAGGAATCAAATGTACTTTTTTACATATTTTATAATTTGAA<br>TAACACGATTATATATATTTATATATGTATAACTAAATTACGCTGTCCGCTTCAAAGCTCATTGTTTCAAAAGTAATACAAA<br>TGTAAACCAGGTATCTGTACAATATATGTGAATATAAATAAATATATTAATATTAATTTACAATGCAGTCACATTGAATTA<br>ATCATACGGAAATCGCGTTTAGAATTCAGATTAATAAAAATATAACCTTATATTGTTTCAATTAAGTCTTAATTTTAATTGA<br>TAAAGCTCGGATATAAAGTTGATAATGCAAAGTTCATTGGCCCCATTGTCTTTGATCTGAATAGCAATAGGTGTATATTAC<br>TAAAACATTGTTACGATCAATTCGACCTTAAATGTTGTTGCTTAAGTCGTATATTGCAATAAATTAATTTTCTCTAATGTTG<br>CTGACCTTGACTATGATAATGAACTTGACTTAATATACATATACTTTATATACATGTTTGTATAACAAAATTGTAAAGTAA<br>ATAAAAACATCAAGACTTCTTTTTAGAAATATGTTTTTTTATAGAAGTAGATAAAAAAAGGGACACATTATGAATATGATT<br>AATGTGACAAAGTATTTTTGGATTTTTGGAACCTACACGATTCACCATAAAAGTCGTACAAAAACATTCATAAAAGAGAA<br>CAATTTAAGTTACAGCATTATGTGCAAGAACTTCGTTCATTTTATTATCTCTTTTTGCCAGCAACTCAATAGGTCTTCATAA<br>GCCATTGTAACTTTTGTAACAAAGTTGTATTATGGATGTTACAATAGAATTGAAGAGGGGCCGTTCTACAGACATGTAGTA<br>AATTGTAACTTTATGTCGCATCAACAACGTACTTAACTCGAATGCCTGTCATTATTCTTATATTTATTTAATGAATATCATT<br>AATATCTCCAAACGTATTATGTATAATATACTTAGTTATATTACAAAAAAAATCAATTACCGGAATTACACTCTGATAAAA<br>CATCGACTAAAATGGCAGATCCCTTGAATAGACATAAGAAAGTCTTAGAGGTAATAAGTAAGAAAAAATTTTGTGTGATTT<br>TCTTGTAAACAGGGTATGAAGCGATGGTTTTGTTATATTAGAAGCGTGAGAATATAATATGTAAAAAAAAAATTATATTAA<br>TAAAATATGTTTGAACTTTCAAATCGCATAAAGACGTGATATTATTTATTTAAAAATAATAAATTTGCCTCCGTAGTCAAA<br>CCTATTACTTGATACTTATACATTTTTATGAGTAATCAAACAAAAAACGTCTGAGGGCATAGCCCTAAAACATACTTAAGG<br>CGATCAGCTTATACCGACAGCGCAGACGGGGTTATCATTCCATTATCATCGATTTAAAAAACCTACCTGCCACTAAGTCAT<br>GATGATTATCAATGAATATTAGTATCTAAATTAGATACGTTTTATACTATTTCCGATTTTGTTACATTGTGTTTACTCTTCAT<br>GTAAAAGTTGGTGTAAAATGTAAAAACGTTTATAATATAAATATATATATAATATTATGTAAACATCTGGAAGTTCAAAAA<br>CTTATAAACTCCGTCTTATTTATAAAAACTAAAATATTCTGCAAACCGATTAGAGTGTTTTGTTACTGTTTTTCACATTGGG<br>AATATAACGCGTAAGAAAAAGACAAAACACATCTACAGCTATATTATTCGGTTTATTAGACGTTTGATGTTTTTATAAATA<br>TGCAGATAAAGCTGGTGACTGACTTGCATCTTTGGTCTTAATGTAACATGTTACGATGCGAATATTCGATTCGCCATAGTA<br>TTCGATACTACCTGATAAATTTACAGTAGACTGAATAAAATATCAACCTTGGCAATAATTTCCATAATGAAAACTACAACA<br>GTATCACAGAGACTCAGTTGTTGAAACTATTTTTGAAAGATGTCGATTGTAAAATCACTAAATTGTAATATTATCGAATCT<br>TATATATATTTTAAATTTAAAAATCGACTAGTTTCGAGAACATAGGCGACCCTCAATCATAAGTGGTTAAAACGCGAACTC<br>AAACCTGACTAAAACTGAATCTGATTTCATGTATTAAATCTCCGCAAAAAACATATTTTTTTTACAATTGGCAAATTTGTT<br>GATAAATAATAACATCAATACAAA
MSTRG.3287	Tryptase	CGCGATGTACCAACCGCCAGTGTGACTGACTCGGTCTCACGGAACGCACAACCTTGCGCGTAACAGGAAAAAACCTTAAA<br>AAAATTATAATAATAATAAAAAAAAAAGTGAACTCGATGCGTTTCGCGCTTTTTTTAATCTGTGATGACATCATAGTGTAG

		GATTTTTTGTTTTAAATATTTAATATACCTAGATAATATAATAAAGTGATTAACTTCGTTTGCCATAGTGTTATATGTAGTGTTTTTTTTTTATTGTGTTTCGGATTTAATGCTTGTGTTAAATTGAAAAGTATTTAGGTCAATTTAAAATGTACAAATCACTAGCAATACTAGTACTGTTATCCGCTGTCTATGGTCAAGATACAGGAGGCGGTGACCTCGAGTCAGTCCTCCAGCAGATCTTCGGCAAGCCAGGAGACAATGCTGGCCAGGTCGGCACCGTGGCGCCTGTACCCAGGCCGTCCCCGACGCCATCTCTCGGCGACAAGAAGGGGGAGTCATCGACACTGGTGCCTGGGGTAGTTTCTGGAAATGACGATAGT
MSTRG.3291	Armadillo repeat-containing protein 6 homolog	CAGAAAAACGCTTGCTGGGCCATCCGCAACATGGTGGCCAGGTACCGCGACGAGAACTGCAAATTCCACGAGCTCGGTGTCGAAGAGCTTCTCAACGATGCGTACAAAAAGTTCAGCGGCGACTTTGGTTTCGATATAAAATCCGCTTTAAGAGATTTAGAATGCGATGTGAAACTAGAGGAACAGTGGAAAGGGAAGGGCGTTCAGATGGAAAAATAAACAAGACTAGCAGCCCACTCCGACTTCGTACGAGTTTTTAAATACAGCTAATGTTATTCGCTTACAATGCAACTTTCTATACATGAAAGGATTTTTAATATCGGCTTAATTATTATTTTCTTATTTTTTCATTCCAAACAATAATTAGATTTGTCCACTTTATAATATCGTTTTAGACAGATTTAATTATCCAGCAAGCATAACAGCGAAAAAACTAATGCTTCGCAAAAAAATATAACAATCTCAATCGTGACAAAACAGACCGATGAGTAACGATATATTTCGTAGACACATTAGACACGATACACAGAACATGTGTCTACAAGTAGACACGTGTATCTGTGTATTCAACTTAAGTAGTCACAATAATATGGGACCAACATGGGATGGCTTTTATTCGATTCTAAAAAGGAATTATTAAAATCTGCAGGGATTACTACTATAAGTTATATATATATATAAAGGAGTCTCGGCTTTGTGATACACGTATCAATTTTAATAATATTGACCAATTTTATTTTATAAAAATATTTATTGATTTCAATATAAAATCTTTATTTGGAG
MSTRG.3356	Rho guanine nucleotide exchange factor 18	CCAACACACGCGTACAGGGAGCTCGCCCGCCCCTCTCCCCTCACAGCAACAAATTAATAATAAGGCTACTAGAACAAATACATACCCGAAGTTACCGGAGAAATTTCGCGTTCGGTCGCCGGAGACAACCCCGGTGGCGACGGCGGCGGCCGCGCCCGAGGAAGAGGTCATATACTTCTGACGGCCACCGCCCGCCGCGACGCACGCGCGCTCCTGAGACTGCCGCCTCTCGTAGGCGCACGCGGCACACGCGGCACACGCGGCACACGCGGCACACGCGGACGCCGCCCATCGCACGCGTACGCGGACGTAAGCATGCGCACGCAATATGCACACATTCGCGCATAGACTCTACACGACGACGCGCCCGCGCACATATGTGTACTGGTATACGCAATATTATTTTGTTCTTTCAATATTTTAAACTCTACCCACACAGATCTATACCGTATATGTGAAATGTTCTAAATTTGAATTCTAAATCCGTATATTCACTAGATAAGAATCGATTGCAAACTGCAACATGTTGCGGAGCATGCAACGCAATATGTGTAATTTTTGTATCATAAAAGAAACCACAAAAAATGACATATATCTTAAACTTCAGTCGTTATCTATTGAATAAATGGCTTTCCATAGTCAATAGAGACCTCTTAACGAAGTAACAAACGAATCTGTGGAAACATATGTTATCGCTACGGAGTCTGTGAGATAGAGCGAGATGGCTACCTCTATTATTTCACCCTTTCTCATGTGTGCATTGATGAAATTATATTTAGCACAGATTTGTTTGCTACTTGTTTAGGAATTAAGGTCTAAGGTTACAGTCTATTAGTGATAATACATAGGTATGCAGGCCCAAATGTATGAAATTTCAATATTAAATTTATAAATGTAATTTTGTTATTCAATCAATTTTATATCATTTGGAGTTTAACTAACTAGTAGTATAACTAATAGATACATCCAAATTATATGTACTTTCAAAACTCATGTTAAATAATCATAACAGTTATGAATATTCACATTAAAAAAGGCATAGAAATATTAATTGTATATTAGACAATGATAATCATATTGCGATATAAATACTTTAATTTACACCTTTGTTTAAAGTGGTACAAACCAAATGTTATCAAGGCAGTATTGTATGACAAATTTCATAGGCAATACAAATATATTTTTTTTAAATGATTATTTAAAGAAACACATTTTAGATAAAAACTATCACTAAAAATTTCAATTATTGCACACGCTAATGTAATATCATTCAAAATACATCTGTTATCAAAATTTCAAAGACAATTTCATAATAATCTTAAAAAAATCGTAAAAGCACTTTCTAAATTATCACTGAAAATTATTTGAAATTGTTCAAATATTTTAATGTAATAACTATCGGTTTAACTTACGTGTAATTAGATCGAGAAAGCTCGACTAGTTTAGAGACCACTCGGGACCCTTAATCATGAGCGGCTGTGACGCGAGCGCGTGACTGACTGCGCGCCTTGGCCCGCTGAACAGTTCCGAAACTAGCCGAGCTTTCTCTATCCAATTACACGTAAGTTAAATCGGTATTTATTGCATTAAAATATGTATAATGTGTGCTTACGAAACTTTTAACAATAAAATTGTGCAGATATGTTTGAATATTGACAGAAAACAAAGACATTGCCAGTTTCAACACAAAAAAACAAACCCTCTTAAAACGTTAATTGTAACGCTCAGTACCTATTACATATTTTATCTTCGATTTTACTTTTATATAGCACTATTAAAAAAATACTATGCTATTTATTAGGACGTAACTCTACAAAAATCCTGTTGAAAACATTGTTTTGTCCCTTTCTATCAAGTTAGGATATTGGCTCAAAAGAAAGAGACTACGCATTGCAATAGCCAAAATAAGTCTAGACTTTTTACATTGGTGACGATATCTCAAAA

GTGTTTCTCGATTCTAGCTTATTTCTTTCTTTTAAGTTAAAAATTAACCTTGTATGAAAGAGACGAAACATTTGAAACTGAA
ATAGGTTTGTTAGACGTTTAATAATTTTAAAAATAAATCGGCGTCGTCATCGCATTCTCAAGATGGCCATAGATAAGTTAT
AATGATATTTTACTCATTATACACAATTTTCCCCATAAACCTATTGTTTTCACTTCCTTTCAAATTAAGAATTTAGCGGCGA
GAAAGAGACAAAATATTTTAGTAGCTAAAATAGGTCGATGTTGCATGTGTTTTGATAAAGGAAAGTTTATAGCAGTTATAT
AATGGTATATCTGATTTAATTCGAGTCACTGATTTTATATTTCGTAGCGTCACTAATTCTTTTTTTTTTTAATAATTATATTA
ATTGTCAGTATTTTAGCAAGACTCCATCTTGCAGAGGATAGTCATGTACTATCAATTTAGTATAATTAGAATAAATTTATG
ACAGTCACCGCTACAAAAACGAACTCACAGGTCTTTTTTACAATATTGACGACCGTGGTCGTGTCGTTTAACATGCTACTT
CACTACGACCATTTGTATTTTTTTAGTATGACCACAGTATAAGTAATAGAAAGAAGGTACAGTATGGACACTCAGTATCTG
ACCTCTAACTGTGACTACAGACGCATACATTGTCACATGTACACACAGTAATTGTCTATGTAATTGTGATAAGCATATATC
GCAATGTTTTTGTAGTGTGCTAGTGTCCAATGTGTGGTGTGAGTGTCTGTATGTGTGTTATATGTATTATAATTATGTATTT
ACATAATAAATGTACACATAATACAAGCTATTCTTATTTTGGGTCTAGATGGCGCTGTATTTAGGCGTTCAAAATTCGTCTT
GAAATTTCAGATAAACTTAAAGTGTATTACTTTACGCTTTATAAGCTATACTCCCGTTAGTCTCATATTAACGTATTTATGA
GAGACCCCGTCATTTGTTGGTGTATGTATGTTCACTTTTAAAGTCACTTTTGTGCTTGTTTTTTATTATATTACTAGTGATCC
GTCTTCGCAATCTATCTAGAATTTTCGAATATAATATATGGCCTATTTTTCTCGCTGATAACGATGCTTTCTACAGGTGAAA
GAATTTTTAAAATCAGTTGAGTAGTTTTTGAGTTTATCCATTACAAGCAAACAATCTGAACATTTCTCTTTATAATTTTAGT
GTAATAGCCCACCGTGAGGAATTCCACTAGAATTCACCACGATGGTTTCCAGGGGGAACCGCGTGGAGGGTAACCTCATA
GTGTATTATTAGGTCCCATTACTAACAATATCATTTTCCCCGGTGGCCATAGTTTCTAAACATAGAATCAATGACTCAAATT
AATTATCTGTATATAATACCTATTTAAATGAAACTGTGAACGCTTGTGTTAAGGTTTAAAATCCAATGTACATAAATAGCA
ACAGGAATTGTCAACATTTACAACTGCCAGTCTGAGTATTCGTGTGTGTTATTGCAATAAGTTAAATTGGGTTCAAATCAT
AAGTCTTATTAGGTATAATTTTATTTACATAAGTTGTGTATCATTGCTTGTAAGAAGTGGCACGTGTTAGATGGATTTTAAT
ACTCTTGATCAACTATTCGTATTATCTATGATTAATTATTTTTGATACATTCCTGGAACTTAATTCCTAACTTAAGATAAGT
ATAGAAGAAAGATACGTACGTATATAATTTAAGAGAATTCATACAGTAATGTGGCAAATATTTCAAATATAATAAAACAA
TTTTCAATATTTCCAAATGATTATTTTAAAAATCTATAGTAGCATAACAAAACGTTAATTTTGCAATAATATTTTGTCACGG
GCTTTTTGGTATTTGATCATCGTCATCATATTAATTAGCCTTTAATTGTCCACTGCTGAACATAAGCCTCCCCCTTGGGGGG
TTGACCAGTAGTTGCTACGCTTGGCAGGCGGATTGGCAACTGCAGTTAAGCTTTAGAGATGTTTTAAGAGGGACGCTGCTG
CCCATCCCTCGCCCTTCCTTAGTCGACTTTTACGACACCCACGGGAAAGGAAGTGGTGACCTATTCTATGCCGGGACCACG
GTTATATTGTAATTTGATAAAATTGCTAAAACTCCAAATATTATTTAGTATAGCAAAGTATTCAGTGATAGATTTATTTGCG
TAATATTTGGGAATATTGAAAACTAATTATTTTTTAATAAATTGACAAACTATAGACATTGCTATACTTTTGTATGAGCCCT
CTTAAATATATAATATTTTTTTAATTTTATATTTGATAATACATCCTTTGTCTACTTTAATTGAAGTCTCACCAGTGAAATAA
GAACCCTGTTTTTCTGGATATTTTAATATTGCGACAAATCGTACAATCGTGCCAACATAGAGAATTTACTTTCAAGACCTTC
TAAAGTTTGGAATCTAGAAATTCCCAAATTAGGGTGTTTGAATTTAGGAGTAAAAGTATGTGTAATAGGGTTTTAAGGGGA
ATTTTTTTGCAAATAATTTTCTTTTTGCAATTTTGTGTCACATACTTTGTGAGGTTTAAGTAGAAGTTTACAGCTTATTATGT
ATTATTTTGGTGATGTTGTATAATTGTATATTGGGTAATTTTTTTTTTATTACTGGGGGTTTTTACAATTTATTATTTATTCGA
GGGATCTAGAGGTATTTATTTGTACATGGTGTTTAGAGGTAAATATTTTTAAGAGTTGGCAACCCTAACTTCAGGGTTCTTA
TTTTGCTGGTCGGGTGTACTTAATATTTTTCAATCGTGTACTTAATTGGGACCATTAGAAATGCATAATTGATTTTAAAATT
AGAATACGGAAATTGTTTTACGTATAGTACAAATCTATATATTTCATAAAATTTACCCGCGAAGATTTTAAAAAGACATTC
CAGTCAATCGAAAATGATCTCTATAACTCTTATTATTATAGTTTTTATTTTATTGCGACCAGTATTTTTATGACACGAGGCA
GTTAAATTTATTCTTTTTAAAACTAAGTCGAAAATCTATGTCATATATGTGTAAAAAATATAACATTTTACATTTTTTTTTAT
CATTTAATAAACATATAAAAGTGGTTTGCTAATACATGGTTTGTATTCGATACCTAAATGTTGGAATTATATACTACCATTG

		TGTCACAGATGTAAATTGAAATAGATGTCGTAACTATATAAACTTTGGGTGTCAATTTTTTGACAACCTAATATGATTCAATAAATCCTATTTAGTGACGGCTTAATATCGATTTAGGGTTCCTACATGATATTTATGTCAAAAAAGTTCAAGAGCTTAATCTTCTAACACTGACTGCATTGTAAATAGTATTTATGAAAAGATTTACTTTCGAAAGCGCAGAGATACAGGATGAAATTTAAAATATAAGCATCCTTTTAAGGAAACATTGTATTATCCCAAAGATGGGTCAAAGAATGACTTAATTTTTTTTCGGTGCGTCCACTTGTTAGTATGGGTTTTAAAATTTAGTCTAGAACGCTTCTTAAAAAGGACGCAAATGTTTCAATTTAGTTTAGTAGTGACGTGCTTAGTTTTCTATTAACCAATTGATTGCGTCATCTATTTCTTATTGTGTCTGTGGTTTATATTAAATCCAACATATACTCATCGCTGTTTGTATATATTATATCTTGTATAAATATAAAATTCTACAATAATTCGGGTATGGAAAATATTTACTGGAAAATCGGGGGCGAGCTATTTGAAAATAATGTTATATGTAAATCAGTTAAAAGTAGTGTGTAGCTATGGAGCGATATAGGCATAATATTGTTACATTAGATATTCTGTAATCCTCATTGTTGAGTCTTATATGCACTACGGGCAAGGGTGAGCCGTGTGATACAAGAATGTTAATGTAATTGAAAGGACTTCGGATTATAATTTTTTTTTTATTATTTATGAATCTATCTCACTGCTGGGAAAAGTTCTGTACCCACATTCCATCTAATTTTATATGTCCGTACTAGTTGTTAAAAATAAATACATTTATACTAAGTATATTTTTCATACTGTACCGTATTTAACTGGGCCGTATCGAGCGTAGTGTATTAACTCCTGACTTTGTACGCAAATTGAAGTTACGTAGTATATCTGTCTCCCTAGACAAGGGGCAAATGAAACCATGTCGATAGATACGTTTTCGTTACCACGCACACGATATAGAACGGGACATCAGATGTTGTCTTATTCTATTTTATGCAGTAGCGATAACGTATCTATCGATAGATGTGTGCCATTTTTCTAAGGGGAAAGGGTTTTAGGTGATTAAATCTACAATAGTTCGTTTTGTATTAGGAGTCTTTAATAAGAGCATCTCGATGTGTCTTGAATAATTCAAGAAATGTTACAACTGTTTAGTAATTAAACTGTTGTTAAATAGATTAAACATTTTTGTATATTTATGATTATTTTATATATCTGTAAATGGATATATACATATTGCAAATATATAGATGACAATATATTTGAATAAAAAATTCACGGTAGAACTGTATCAGTACTAAAAAATACCAGTGCGACATTATGAAATCGCCATCAATTTAGATATATACGAGAAGTACTATTTAAATTTGTTTTAAATACATAAATTACATATTAAATAGACATAATAAATATATACAATTTTAATAATGCCTACCTGACGCCTCGTAAGGAAATATTAACCTCGGAATATGATAGCACGCATTTTTTTTTAATAAACACAATGATACGTCTCTGTTTTTAAATATTTAATACCATAGACATGGATGTAAATAGATGTCGCACTCTGTTTCAATGCCTACACTACATTGCGGGTAATGTGGGTGATGATTCGTATGTGCCACTTTATTCATAAAAAACTTCAACCTCTCATAAACCTATTTTAGCTGTTGAACTGTTTTGTCTCTTTATTTTACACTAAATTCCCAATTTGAAAGAATGTGACAAAACAGTGTAATAGTTAAAATAGATTTGTATAAGATTTCAGTTTTTATGAATAAGCGAGTAAAGCTCTACTATCAGATATGACGACAGAAAAAATAAAAAAATGCAAGTCGTTTGCAAAAGGTGGTATTTCTGTAAGGCTTTGATAGTAATAAATAGTTCTGTAACATCACAATTAGTTTAATATATACGTATTTGCGTCGTCTATTTATTGCTATGTCAATGGCTAATACTGTTATTGAAACACTGTTTTGTCTTTACACTGTTTAAACTAGAAACGCTATTGTATATCGATTGTGAATCGATAATCGATTAATGTTAATCGTATATCGATTATAGTGAACTGTTATTGGTGTAAATATGTTGAAATGTTTATTAAATATAATAGAAAATGTTATATTGGCTATTATCAAGTTATAATGTAATAAAGTTCCGAACAGATATTTGTATGTTTTAACGTGTATTTATATTATGTATGTATGTGTATTATGTAACTTATGTACAGGGTGAAGTCGTCATTCTGTCAAGGAAGTGTTCTGCTAAAAACATTAATTATTATTACCAAAGAAATATGGATCGAAAAACGATAAAAAAAATGTGCCTCCCATAATAATAACAGTTTTTTTATGTTTGTTCAGCTGCTAAGTTAGTTATTTATTTACTCTATTGCATTACTTAAAAAAAGGCAAAAGGCGGATTTATTGTTAAACCAACCTTTGGGTAGAGGAGAAAAGAAAAAAGATGGTAACTATTTGGCCCTAGAAGTGTATGTAGTAATAAATCGCTCATGTTATAAAACATACATCTAAATACTTATTATATATTACGTAAATAAATAGATATATAAAGAGATATACATATTGTAATATTAAACATATATATATATATATA
MSTRG.3357	Rho guanine nucleotide exchange factor 18	CATAGATAGGATTTGAAGAATTTAATGAAGTTTCAAAGTATTTTCAATCACTAAAAAAGTTATTTTATACTTTTTTACATTCGTTTTTCAGTGTTTAGCAAACTTTAGCATTTCAATCAAAGTTATTCCATTACTTTACAAGTGTATGCAAGTATATTATTGTGATGTATCGGACAAAATAACTGTTTATTGTTTTGTGTAGGTGGCTACATGGTGCCAATGTGAAAGTGTATTCGCATAAAATTTAGATAGTACCATTGTTTAAAACTTTCCGGTTTACTTGGGAAAATAGTGTTTAATTGTGACTTACACAAGTTATTGTGACGGCAATGTGTTTTAAAGGGTTTCCGAGTAGTTTTTAGAAATAATTTGTTTGACATTGGCAGTAATGATGAACGCCCCTCCTCAG

		GATAATGTGGGCCTCATATCATCAGACGAAGGCGGCGGCAGCTCGGACTTGCGCTCCGACAGCAGCGAGGACGAGGTGACAGAGCACGTGACCCGGTCCACGCCACCAGCCAACATGGCCGCCGCCAGGGCACAGGTGCCAACAATAAGCGTGACTCCCCACAGTCCGGGCGTGCTTGATGACAGCATACAACAGCTCAGACGGCTGCATGTAGCCGTGCAGCGGATGCGGGCAGCGCCGCTACCCTTATTGG
MSTRG.3368	Protein lingerer-like	ATTTATAAGAATAAAAAATTTACAAATGCTGCAACACTAGGAAATCACGTGACCAGCTTGCGTTTGAGAGCGCATTCACAGCTGCTTGTAAATAGAGGCTGTAAATATAAAAAAAATAGCTGATAATCATGAGTTTGGGTGCGCGCGCCACCAAGGGTGGTGCGAAAGCCGGCGGCGGGAAGGAGGGCAAGCACGCACAGGACAAGGGCAAGGGCACAGAGAAGCCACAGCCGAAGGAGAAGATCAAACCACAGG
MSTRG.340	RNA-binding protein	CCACTCTACAACCGCAGTATCGGCAAACGTTCATAAAACGTTTAGACGTAATAACCTTATAGTGTCACTTCTCTCCTTATTTTAGTATTTAGACCATATAAATTTTCAATTTTCTTCATTTTTAGTTGTTTAAGTGTTAAGTGTAACATATGATGTTTTAATAATGCAAAGTGCCGCATCAGTATACGCATTTTGAAGCAATTACGTAATGGGAATTATGCTAGAGAAGTTTAAAGTTTTGCCCTGAAATCCAACATGGCCGCTGACACGGGAATGGATACTTGCCCTAGTCCGGAAATCACGGACTCAAGGAAACGACCTCTTGACGGCGATTCAGAAAATGGAGATATCAAGAGATCCCACTTCAGCTCCGTGCAAGACTTGGTGACGGCCTTGCCACTGGCCAACGGCCATGGCAATATAACGTCTCATTTCGGTGAGTCGTCAGTCAACACATTGTTCGCTCCTCATTAGCTTTGTGGTTGTTGCGGCGCGCATGCCTCACGCGCGCACTCGCTCACGCTCATCACCACTGCTGATGCTCACCGTCCATCGCACCCGGCAGACCAGTCATTGGTGGGGGGCGGGGGGGGTGAGCCAGCAGCTTAGGTGTCAGCGTTAACTTCGTCCATCACACACCGTAAACATCGCGTCGATGTTGTGAGCCATGTTGTACCTAGCCTCACATTGACCCACTCATGGCTTAGTCTTGGCATCGTTGGTGTGACCATCACCTAAATGTACAAATATCGACCATACTTATTTGGTGTATCTATTCGCTACAAACTCTTCTTGAAATTTTTATTTCCCATCTGAACGC
MSTRG.3401	Fatty-acid amide hydrolase 2-B-like	AGGGGGCGCTGCTAGCGTGCGGCGCGTCCGCCCTGTCGGTGTCGTCCGACATCGCCGGCTCCATCCGGCTGCCGGCCGCCTTCTGCGGCGTCTTCGGACACAAGCCCACGCCAGGTTTAATATCAATAGAGGGTCACATACCGACACTGAGCGACGAGAACTATCCGCGGTTCTTGACCGTGGGTCCGATGACGCGCAAGGCTGAGGATCTGCCCCTCATGATGAACATCATGGCCGGAGAGAATAGGCACAAACTGCAGCTGGACAAAC
MSTRG.3416	Histone-lysine N-methyltransferase	CAGGTTCATGAACCACTGCTGTGAACCAAACTGCGAGACCCAGAAGTGGACAGTGCTCGGTGACGTCAGAGTTGGACTGTTCGCCATCAATGATATACCAGCGAACAGCGAAGTGACATTCAACTACAATCTGGAATGCGCGGGAATAGACAAGAAGCGGTGTTTGTGCGGCGCGAAGCGATGTAGCGGCTATATTGGAGCTAAACCGAAACAGAATGACACCCAACAGAAAAAGCCGAAGGTGGCGGCGAAACGTGCATACAACAAGCGGAAAAAAGAGGAGTCGCCATCCGTAAAAAACAA
MSTRG.3420	laminin subunit gamma-1	GTTGATTTCTACTATTTCAGGAGGCACACAAAATAAGAATAAGGCATCAAAGGCAAAAACTGAAGCTGTCAAACTGAGAGATGAGGCAGACAAATTATCCACTCGAGTAAAAGGCACGGAGACACAAATTAAGACCCTGGAAATGCAGGCGAATGAGAGTATGCAGTTGACAGAGGACGCTAAAAAGAAGGTGGGACTCGCGAAAACCGACACCCGCGAAGCTGAGAAACAAGTATTGAAGGGCCTCGAAGATCTGCAAACTATTAGAAACGAGCTGGCGAGTCTTCTCGACCTGGATGATAACGCTTTGGATAATCTCGAGAGAAGCCTGGATGAGGTCGAAGAAAATTTGCAGGCGGAAAATCTGGCAGGCAAGATCAAGTCTCTCGAACAGGCCAAGAATAATTATCGCGGCTGGATGAAACAATACCAAGCCGAGTGCGATCACCTCGAGAGCGAAGTGGAAAATATTAAATTTATACTCTCACAACTGCCAAATGGCTGTTTCAAAGGGATCACCCTGGAACCGACCGAGGGGCACAGTAGAAACCAGATAGGCCTTTAGATAAACTAGTTTTAAGTCCGTAGAAGATAAAGCATTCGATTCGAAGGTTATTCGAAGGCATTCACCGCGCTATAAGCCGAAAATGACACGTTATGGACTCAGAGGACGGTGTCATGTAATCTGATGGGTATTCCATAGAATCGTTGTTCTTAGGGATATTCGCAAATGATTTTTCGTATCGATCGTTCTTAAACCCTGAATACGGCTGGTAAAACAGCGTTTGCAGGCTTTGATAATTATCGTAGTATAAGAGATCGTTTGGAGAGCGTTGAATGTAGAACTTAGTGTTAGATGCGTTATATTTGGAAAGTCTTTTCATGTGAGATGTTACGTTTGTAACAGAATCAGTCGTCGGTACACAATTGTCGAATTCGTTTAATGCAGACGTCAAAAT

GTTTGTTAATTTTATGTGAATAGAAAACAAAATTGTCTGTTACGTGCGTTTGAAGGAAACAATGTAACGGATTTCCGGGCG
TACACGATTGATGGATTAATTCTATTTATATAAATCAGGCATTCGTCTCGCAGAAACCGTTAGCATTAGGTCTGTATAAAA
TTTTATATTTAACAAAAGGCAGGAATTAACAAGACTCTTTAGGTAAACTGTGACAATTTACTTGAATTTTTAAATTATTAA
ATTTTCTAGTTTGCCTAACAATAAATTAATAATTCTTTATTAAGGTTACCAGAACCCACTTTTTATATTTTACAATTTATTAC
AATTATTATTTACCGCAAATACGGGATATATGTTGGATTTCCAAAGAGATACCGAAGCCTTTTAAAATTGGGAATGACTGA
AAACTGACAATTTATTACAATTACAATTATTATATATTTATAATATTTACAATTATTTTTATAGTTAGTTTTAATTATTATTA
TAGTTGGGTTCTTGGCCACAATGGTTCATGCAGTAAGAGACCAGCCTAATTGTTAACATGGCCAGAATACAGTTCTGTATC
TATAAATGATCGAGTTAATTCCTGTCTCTTACAAAACATAAAACGTACAGCAGTTTAAAAGTTAATACTGTATAAAATTTG
TTTTGGCCGAGCTCGTAACATACAAGTAGGCAGTCGAGGTTCACATAGTTCCCAACTATCTAATTAAATGAAGACGCAGTC
GTTTTCGAAAATACTGATGTAAAATTAATATAACCGCAAATATGGGATATATGTTGGATTTGCAAAGTGATACCGAAGCCT
TTTAAAAGAATTTTAAAGAATTGGGAAAGACTGAAAACTATTGATGGAATACTATTTCATGACAACTATAGATATAAAAA
ATTCAATGCAATGGATTATATGATATCGTCCGTACCTCCACGATGTGTTATTAGTATCTTTATGAAATAATAATTTATTTAT
AATAAATATGATGAATAAATCTTTGACCTGCTTAATTAATATACAGCATTTGTCTCCTCATTAATTTGATTTAATGTAAATC
ACCAAAAAATTAGGAAATTAAATTAATTACGTTTTAATTAGAGTCTTCAAGGAACTGAAGGGACGATTTAAACTGATTTTG
TAAATAAAACACGAGGATATTGTAAAATTATTTAATTACAAAATTCAATATTGTATAAATATTAAAATAATAAGTAATCTC
GATCGAAAAAAAAAACTTTTTATATAAATTACAATTACATATTATTTTTTGTTCTTTACAATATCCTCGTGAGGTTTTTTAT
ATGTATACTTATAAAAGCAAAGTTTCAAACTCTTCAAAATTTATAAATATATTATAAATAGTTCTATAACCGGATTTGTTGA
AATGCTTTTGTATTTTGTTATATTGACAATTATTATTTAAGCTTTATTTAAGGTCTAATTCAGGTATTGTAACTACAGACTAT
TTGTATACGAATCAACCGTGACTTGGCAACATTTTTTTGTAATTTTACACTTTTGTACTCTTACAAAGCTTGATTTGAAAAA
TAAATACGTTATCTTCGACTATAATTAATTACTATTGAGATTATTTATCATTTTAGTATATATTTTGGCTAAACATCCATGTA
ACTGTACAGGATATATAGCTCGAATAGATGGGAAATCAATGTTCTAGACTTAGATATAAATTAATAGTAACAAATTGCATT
TATTTTTTTACTTATCTATAGAGTTTAGAAAAGAGATTCCTTACACAATTGTCTCTTTTTTATACTATTTGTTTTTTTTTTATC
TAGATACAATATTTAGTATTGTCATAGTAAATATTTTTTATTTTTCTGGTAAAGCTTTCATTAGATACATATGTTGTATAAA
AATACTCTTAATATAAAGAAAGAAAAAAAATAAGTTTCTTATAATTACTTAAATAAGTATAAAGAGAAATAAAAATTATTA
GCATCCTTTTAAGACAGTGTTATATCAGTGAATCTTATATATATATATATAATCAATTCAAAAAAGAAAATAATATTTTAG
CGAAACTAACAAACAAAAAATTTATTGTTTGTTAGTTTCGTTAAAATTCATAAATGGCTTGTCAAATTTATTTTTTTTTTCG
TTTTATTCGGTATAGTTGTGGTTTAGAAGAAAAAAAAAAAACGTAAAAACCCCTATAATTTAGCTACTTTGTATTACTTTAA
ACAGATGGCGTTGTAAGTGATACGAAATTCACAGTCATCTAGTCTTAAAGAAAAGTCATAAAGAAATATGGGTAAAAAAA
ATTTTAGCCTCCCGTACTAATTTGACAGTTGAACTCGGTGTTTTTTTAAGTTTTTTTTTTTTTATTTTTGTTTTATTATGTATT
TTTGCAAAGAAAACCTTTATGCCATATTCACGCAACGCCGGTCACGGTTCGTTCTCGATCCGACCTCAGTCTGGTAAAATA
TTTTTATATAAAAAAATAATTGTCCATTCGATGTTCATATTACATATACAGCCCAGTTCCGTTCCAGTCACGATCAATATCG
CAGTATTTCTATTTGGTAACACTCACTTGTACACTATTATGTATAATTAATATTCTAAAATGCCTTTTGTGTACAATAAGGA
AAAAAGAACTCTGACTGAGAACACTGACCGGCGTCCCAGTGTGAATCTAGCCTTAGTTTACTGGTAGATCGGTCTTTAAA
GGATTCTAATAATTGCATTTTTACCTTATATAAATAATATATAATACAAAATGGATGAATTGTATGAAGGAAGACTTGGTT
TTATGAAAGTGATGAGTGAATACAGAGACGACTGGTAGAGAAGACTGGAAGGGGAAATATGGTAAATCCTATTGCTTTGA
TTAGGGATGGGTAATATATGAGTAATATCTATATATTTTGATTACAATAATCGCGGTGTTTGTTACCAAATTTCTCCGAAAC
GGCTTTGCCGATTTTTATGAAATTTTGTGTGCGTAATGAGTAGGTCTGAAAATCAGCCAACATCGTTTTTTCATACCCTTCA
ATGATAAGGGTGACCCACCTTTAATTTTATTTTTTGACATATTTTTTTTTATGCTTTAGCATTGAAAAATACATACAACTTTA
AATTTTCGTGATTTTTATATTACGTAATATGGTTGCAAGATAACAATCGAAAATAATAATTGTAGTACGATTAATTTTCATG

		TATAGTTATATATATATATATATATATATTAATGATCATTTTTCCGTCATCAATATCTACATACTCTATATGTAGATATCGG<br>AGCGGTATTAGTATTGCCCATACCTAGTTTTTTTAATGGTAAAATTACAAAAAAATGTTACAAGTCAACGCCTAGTAATAT<br>AAATGTGCAATTCTCTGACAATGATGTTATGAATGATATATTTACTATTTTCTTTTTTGTCATCATTCGATAATTCAATCCCA<br>AACGTATTTAATACCCATAGGTATATTAAATGGGGGCCCAAAGGGTCGTGCCTCCCTCTATCTGAAAATTTATCTTAAATA<br>TGTCAACATCTATTATTATGTGCATGATATAAGAGGCGTGTATGATCTTTGTATGGAAGAAGTGATAATTATATAGGTACT<br>TACGAATATACTGTAATAATTTAAAAGTAAAATCTTTTTTTTTTAAATGCGTGAAAAATAAACCATTTTTTCTGTATGCCCC<br>TTAATTATATTTAACCTTTAAACAAGTTGCAAACGGAACTATCGGTAAATCTATTTCCGTTACCTTGCACATTGGCGAGAA<br>AAGGATAATAGAGCACGCTGTCTCGTTCTGTTTTATGTATGCGACAGCGAAAACTTGTCTATCGTTGGTTTCATTTGCCCCC<br>ATATCTAACGGGCTTACATTTAATACATAGACAAACTACACCTTAACTCAATGCTTTAAGGTTTAGTTTACTAAGATATTGT<br>ACTTATATCATAAGAACTTTATTATAAGTAGATATAAGTATTTATATTATACCTTATATGCATACTAATCATTAAAATCCAC<br>CGCAACTCGAGCCCTGAGTTAATTAACGATTTACTTGGCAATAGCTTTAGGTCCTTGTAATAATAGACCAGGGGGTCTACC<br>ATGAAAAGTCCGAATTTTCGGGCTCAATTTCGTGTTTTTGTTCATACCGAAATCGTCCCTGTAAATGGAGCTTAACATTTCG<br>TTTGTCATAAATCCTACCATAAAAGTCCAAAGGAACGATTCTTTAGCATTTTTTTATATTTTTAAACGGTATGTATTATGTA<br>ATTTAAACATCAAATTTGTGTTTCGTTCGGATCCGAAATTTCGGAAAACATTTCATGGTAGGCCCTCAGGTCACAGACATA<br>CACAGAGGCTACTTTTTCAAATAGTATTAGTATTAAAAACAATAAATCAATATAATTATTGCTCTCAGCTTAGATTAAATA<br>CAACTAGATGGAGCATCGAGTCTCAAAAAACAAATACGGTCATCCAACGGTTGGTTGACGGGTATTTAAAAAAAAAAACG<br>TTAGAGCTATTTTTGTAACTGTCCATAGAAGGAGTGTTAAGTTTTCAGGGTTAATTTATGTATGTGTTTCTTCATGACACCA<br>TAACTGCTAAACGCACATACAGATTTGGAATTTAAACGTCATATCGAGTGGTGTTGGATGTACATTTTGTGAAAATATTTC<br>TCTCTTTGGAAGAACGGTCGTGTTTTTAATTCGCGGTGCAAAATTCACAGTGTTTTAAATAGTGTCTTTATTATTTTTATATC<br>CTTATTCACGCACACACCGTCGAATCGAATGCCCACGTACGTATCGTCGACAATAAAAAAATTACTAACTCAGTAACAAA<br>ATATTTGGTCTAAATTCTAGCGAAGCGTTTTTTAGAGAAACACTCCATCTAGGTATTGTTAATCTTGAATTTATGCAATGAC<br>GAAATAAATGTCTCACCAATCCGTGTATTCGAAGCTCACAGTGTAACAGATGTTATAATAATTATTATGTACTATATATCA<br>GATAAACATTTTTTTTTTCTAGACTAAATTAAAAATGGGTGCCGCAGTTAGCACGTTT
MSTRG.3424	Uncharacterized protein LOC106130565 isoform X1	AGTTATGCGTCACACAACGGCGCCCTACAAAGGCGTCGGAGAGTCGAGCCGTCGATGAAGATGATGAGTTCCAGATAAGT<br>GCAGCAGCGATGGCCCGTGTGGCCCGGTATACTGGTGCGGAGGGCACGACCGCAGCGGAGGCCCGCGTACTAGCGATGCT<br>GCCCGCGCCCGCTCGCGCACACGCACTGTACGCAACGCTCACTAGCGCCATCCACCACAACGCCGACACGCACTTCCCGCT<br>CTTGTGACGTAGCCGTGACGTCACCGCGCACGTGGACCAGTAAGTGGTAGATAAACCCCGCTTGGTTCCCCGTGCCCCGTG<br>TCTCCATTACGAGTTTTGGTATACTGGCAGTAGGGACGTGGGCTACTGAGTAGTGACTCGTCTATACTTGCATTAAGGGCA<br>TGTACATGGAGTAATGAATGATTGATAGACTGGCCATGACGGTCAGTGATGACACATTCCTATACGTTAAATGTATGATAC<br>ACGATTGAATCCTCCTCGCCAAGGTCACCTGTGTCAAAATGTTCGCTTTATTTAGGCGTCCACGTCATATTTAGGATTTATG<br>CATAACTTTTTTGAATACTTACTGAGACTGGACAAACCATATGATACGAAAGTTTAAAAGTGACTACGACGTGATTTGAAG<br>CCAGAAAGGTTATCTCAGCAGAAAGGCCTAGTTCTTGACCGTGTATCACGCATTTTAGTCTATGGCGCTGTAAAATATTCC<br>TATAAAGCGTAACTAACGTATTGTATATATTATAAATTTATGTATGTACAGTGAAAGAAATTTATGAGTGCAAGAATGAAA<br>ATGTTATATGAATTAAAAATTATTTAGGAAGTTTTAATTGATGACGGTATGGTTTATTGAAAAAATGCAGTTGGTTGTTCTC<br>TAAACTGTAATATTGAAACACTACTCGGAGTTTAATGATACTGTGGCAAATCGTGTCTATGTACATGATCATAACTCATAC<br>ATTCATTACGTAAGCAAATGATAGTCACGACTAATCATACAAAATAATACTGTGGTTTGTACATACGTGCAGTCCAATATA<br>CCGTATCGCCCTTCGAGCCGGATAATATTACATTAATTGTGCGTTTTATTAGATATACAATTTTATTTGCCTACCGAATTGA<br>CTGAAGAAAACTTTTTCGTTTTCGAAGAATTTGTAATTTGCTTTGATGTCTTTTATTTTTATTTTTTTTTTTTTGTTATTCGAA<br>TTTTAATCCATAGTACTCTAAACATATCATTTGAAAATATTAACGTAGGTAGTACCTGCCTATCAAGAGTGTTAGTTTTATT

MSTRG.3429	Uncharacterized protein LOC105286551	CAACTGTGTAATTTTTATATCTAATATTTGTTGTACATAAATTTTATTCAATAACAGTTCTGTTTTAATGAAAATGTCAAAA<br>TTGTGTTTACAAACTTTTATCAGTAAGGCCGTTTTTTAACTTATAAAAAAAAAGAAAACATTGATAGGCA<br>AGGTTTCTCAGCTGCCCCGTCGTAGCTTTTTTTATCTCATTTATGTCCCCCAAATGCCGCCCCCGAAGTACCAATTTGGTTTT<br>TGGGAACAAGAAGAAGTCACACGGGGCCAAATCCGGCGAATAGGGGGGGTGTTCGGTCACCGTAATGGAATTTTTACTCA<br>AAAACTCACGGACAACAAGAGAGGTGTGGGCAGGGGCATTGTCGTGGTGAAGAATCCAACGCCCCTCTCGCGCCAATTCT<br>GGACGAACTCGTGCCACACGAGCTTTGAGGCGTCTGAGCACATCGACGTAGAATTTTCCATCAACTGTCTGGCCTGGAGG<br>GACGAATTCTTGATGGTGTCTCGGCCCTCTCTGAATCTTTTTAGCCACTTGTGGACGGTTGGTTCCTTCACAGCATCATCCC<br>CATAAACTGTTCTGAGATCGGCAAAAATTTCACGGCCATTCTTGCCGAGTTTCGAGAGAAACTTGATTACGACGCGCTGCT<br>CTTCAACGGAAGCGGGCTCCATGCCGACGGGGTTTCGTTAAGAGGTAACTTCACAGATGGCGTGACAAGCCAGTTCGCAC<br>CGCACGGCGGTCAGACCAGAACTGAAGCATTGTTAGGGACATAAGGAAGGGGTCCTGCGCTTACCTGGCCTCCCCACTCC<br>TCTTTCTCGCATTCCAGAACACTTTTATACGAACTTTCCGGACAGACCTCGTATGTG
MSTRG.3430	Uncharacterized protein LOC105286551	ACACATACGAGGTCTGTCCGGAAAGTTCGTATAAAAGTGTTCTGGAATGCGAGAAAGAGGAGTGGGGAGGCCAGGTAAG<br>CGCAGGACCCCTTCCTTATGTCCCTAACAATGCTTCAGTTCTGGTCTGACCGCCGTGCGGTGCGAACTGGCTTGTCACGCC<br>ATCTGTGAAGTTACCTCTTAACGAAACCCCGTCGGCATGGAGCCCGCTTCCGTTGAAGAGCAGCGCGTCGTAATCAAGTTT<br>CTCTCGAAACTCGGCAAGAATGGCCGTGAAATTTTTGCCGATCTCAGAACAGTTTATGGGGATGATGCTGTGAAGGAACC<br>AACCGTCCACAAGTGGCTAAAAAGATTCAGAGAGGGCCGAGACACCATCAAGAATTCGTCCCTCCAGGCCAGACAGTTGA<br>TGGAAAATTCTACGTCGATGTGCTCAGACGCCTCAAAGCTCGTGTGGCACGAGTTCGTCCAGAATTGGCGCGAGAGGGGC<br>GTTGGATTCTTCACCACGACAATGCCCCTGCCCACACCTCTCTTGTTGTCCGTGAGTTTTTGAGTAAAAATTCCATTACGGT<br>GACCGAACACCCCCCCTATTCGCCGGATTTGGCCCCGTGTGACTTCTTCTTGTTCCCAAAAACCAAATTGGTACTTCGGGG<br>GCGGCATTTGGGGGACATAAATGAGATAAAAAAAGCTACGACGGGGCAGCTGAGAAACCTACAACCAGAGGACTTTCAA<br>GGAGCCTTCTCACAGTGGAAACGGCGTTGGCACAAGTGCATTATGTCACAGGGGGGAGTATTTTGAAGGGGATAAAATTGA<br>TTTACCTGAATAATTGTAAAATAAAATTTTTAAAAAACTTTTATACGTACTTTCCGGAC
MSTRG.3477	3-hydroxyisobutyrate dehydrogenase	CTTCGAAGGAGGCGTTGGCAGCCGCAGCTAAGGATGGTATCACAGGCGCTAACTCCATCGCGGCGGCTGTAGATGGCGTG<br>GATGTGGTAGTCTCCATATTGCCCAGCAACAAAGTGGTGTTAGACGTTTATCTTGGAAATGATGGCGTAGTTAAACATGCT<br>CCCAAAGGCTCACTCCTAATTGACTCCAGCACGGTGGACCCGAACGTGCCCAAGCAGATATTCCCGGTGGCAATTGAGAG<br>TGGAGTAGGCTTCATCGATGCACCGGTATCGGGAGGTGAGGACGTCATATTGCACGTTTAAAAAAATTTTTGCTTTTAAAA<br>TAGAACCC
MSTRG.3482	Uncharacterized protein LOC105381275	GAGGACATATGGGCGGACGTGCGTGAGAGCGCGGTGCAGTACAGCCCGCCGCTGCTGGCGCTGCTGCCCGGCGCTGTACG<br>CGACCCGTCGCTCGTGCACCACATCGCGATGCTGCTGCACCGCCTCATCGACAAGGCGACCGTGAAGGCGCTGTGCGACG<br>TGTGCTTGCCGGAGACGTGTGTGAAGGCAGTGCGTGCGGCGGGCGGTTTGCCAGCGGGCGACGGCCGAAACCAATTGCTG<br>CGCCATCTGTACGAACTGCTCGATCACTTCACGCTCAAGGCACTTGCCGCGTCCCACGCCACACAG
MSTRG.3496	Src substrate cortactin isoform X1	CGATAACTGTCAATGTCACTGTGATGACGCATAGTAGGTGGTAGGTTCTTCGCCTTTTTAAATTATTTTGTTTTGTAACTAT<br>ATTTAAACCCTAATCCTACCTAATATAATTTGTCTAAGAACAATAAATAGAGATAATTTTGTTCAAAAATATTTTGGTACTC<br>CCTATAATTTTACTCAAACAAAAGTGAGTGAAAGTGTTGTGGGAAAACCGGCTCGCCTATAACTGCAGTATGTGGAAAGC<br>TGCAACAGATGTGGCAGCCCCAGCACCAGCAGAGGCTGACGATTGGGAGACAGATCCCGATTTTATCAATGATGTCACAG<br>AGCACGAGCAGCGATGGGGCCCCGGAGGGAGGAATGTGGAGGCTATTGA
MSTRG.3514	Talin-1-like	TAGAAATCGCGCTCAAGTTTCATAGCGGATGAGAGGACCACTGCGTATCTACATCGAGCGTCGTTCCTGGCCACCGCCTCC<br>AAAGCCAGCCAAGCGGTCGACCAGGCGAAGGACATAATTAACACGGAGTACCAAGGTCCCAAGATTGATCAGTACGGCT<br>CTGAGCTGTATGAGTTTGAGAAGGCTGCCGCTCAGAAGATGGGCAAATTGAATGCAGCCGTCGCATTTGTCTTAATGGCAC

		AAGCAGATCCTAAGAATGTGGACTATTCGTCTGCTGTGGTCTCTATGAACACCATCAACGAGCTTGTTCCGGAATTAGCTA<br>AAGATGCCCAGGCGTTAGCGAGCTGTATGGAAGGTGATCAGAAGAAAGCATTTTTGGATGATATAGCCAAACTGTTTGAT<br>GCCACGAGCCAAGTGTGCTCTCTAACAAGCATCGCTGACCACGAGAAAGTTCAAGAAATTTCGAACGTATACTCAGACAG<br>AGCTAGGAAACTAGTGTTCACATTTACAC
MSTRG.3516	Zinc finger and SCAN domain-containing protein 30-like isoform X1	CTCCAGAACTGTCCAATGATACCCCAACGCCGGCTAAGAGGAAGCGAGGTAGACCGCGTATCGACAAGACGAGTCCAGA<br>GTACCTAGCCAGCATGGCTGTCAGAAAACAGGCGAAAGAAATGGCTGCAGTTATGAACAACAGAACGTCTTCAAGTGATG<br>CAGGCGAGAAACGGAAGCGAGGTAGACCAAGGGTGGACAAGACCAGCCCTGAATATCTGGCCAGGAAGCGAGCTAGGG<br>AACTTG
MSTRG.3521	Acetylcholinesterase-like	GGTTTTCTCTCGACTGCTGACGAGAACTCACCGGGCAATTATGGCATCCTGGACCAGGCTATGGCACTCCGCTGGGTGTAC<br>GATAACATCGCCCCGTTCAACGGTGATCCATCTTCAATCACGCTCTTCGGTCCTGGAGCTGGTGCCGCGTCAGCTGGTCTC<br>CTCGCTGTCGCGCCGCAAACTAGGGATATTGTGACCAGGGTTATTGCTCAGAGCGGCTCGGCGATGGCTGACTGGGCGTTA<br>ATTCAGGACAAGTACAGAGTGCAAAACACATCTCTAGTCTTCGGTCGGCTGCTCGGCTGCCCCATAGACTCCTCGTGGAAA<br>CTAGTCAACTGTCTCCGCCAGGGCCGAAGCTTCTATGAGCTCGGCAACGCTGAATTTCAGCCACAAGTCGGCTTCATACCC<br>TGGGGTCCAGTGCTCGAGAACAACTTCACATTTCCTGGTGATGAGTGGTACGAGGGTTGGAGGGAGCGTGATTGGCATTTC<br>CTTAACTTGACACCCGAGGATCTCATACAACGAAGGCAGTTCAACCCAGCTCTACAGTATATGACAGGAGTGACCACTCA<br>AGAGGCCGCTTATTATATTTACAACAATGACTCGTTGGCACCAAGATACGAGATCAGCGAGGGCTGGTTCAACCAGAAGG<br>TGGCAGAGCTGGTACTGCGGTACAACTACACGCTGAACCCGCGCGGCGTGTACGAGGCCATCCGCTATATGTACACCTAC<br>CATCCCGAGCCGCACAATATCAGCGCCATCCGGGACCAGTATATACATCTCCTCTCCGATTTCCTATACCGCGCGCCGACT<br>GACAAAATGGTGAAATTACTTCTCGAGCAAAACGTGCNNNNN
MSTRG.3523	5-methylcytosine rRNA methyltransferase NSUN4	AACAATATGGATATACTCCTTTAGGAATGCCTTGACACTGGTGCGCGTGGGCGGTGCGGTCGTATACTCCACGTGTTCCTT<br>GAGCCCGATTCAGAACGACGGCGTCGTACATATGGCGTTAAAGAACGCCTTCCAAGACCACTCCATAGTGGCCGCTGTTA<br>AGGATCTGACAGCGTCTTTCGCCGGTTTGAGCAGCACATTGCAGCTTGGTTCGGGCGACATCGCCCCCCAGTACGGCCAGC<br>TTGTGGTCCCCACCATTGGTGCTAATTTCGGTCCCTCGTATATTGCTAGGCTTGTCCGTGTCAAATAAAATAAATGAAATAA<br>AAAGAATTTTAAAAAATATTTAAATAACCTTAATTTTATTAAAAAATTCGAATACATTTTGAATAATCTTTTATAATTCTT<br>GCTCAGTTCGAAATAGAATTTCAGCTGAGAAGAACGAGCGAGAAACTCGGCAATAAATAGAAATGTGTATATTATATTCC<br>TCAGTGACTGTATACATTAAATTATAACATTTTCT
MSTRG.353	Gelsolin-like	CAGAGAAGTTCTTCACAGCCCTCGGCTCTGGTGATCTGGACTCGGTTTCAGACGCGTCTGAAGGTGGTGATGATGAGGACT<br>TCGAAAGAGGCGATATTAAGGAAGTATCACTCAGTGAGATCTCAGACAGCAGCGGCAGCATCAAGGTGACCAACTTGCCA<br>AAACCTTACCAGCAGGATCAACTGAACACCAAGGTTAGTTTTAACCGCCACAAACTAAAGTTTCATCCCAACCATCTGGAC<br>GAGTGGCGGTCCTCCACCGTGCGTTTTTCAAGGCACTTTCTCCCACGCACAACCACTCTTTGAAATCAACTTCCAGCAGCA<br>GTATTTCCGAACCGATACGAC
MSTRG.3554	Methyltransferase-like protein 13	TCATGGGCGGCGGTAGTCACTTACCATCAGATCCCGTTCGTGTCGCTGGGCAGCGACGTGGGGCGACGCGTGAGGGTGCA<br>CGAGGGCCGCTCGGCCGTCTCCGGCGACTTCGTGGTGGAGGACGTGGACGTGGAGGGCGCCACGCACCGCCGGCTCGTGT<br>TCCTCGACAACCAGTTCCTGGTGCAGTCCGAGGCCAAGTTGAAGACGATAAAAAAGAAGAACAAACCAAGAACGGTGGT<br>AGACTTCGGCCACATATCGTTCTACCACTCGTTCATGTGTGTGGGCGTGCAGCTCTCGCGGGGCCCCTCCACCAGGGTGGC<br>GGTCCTCGGGCTGGGCGGCGGCAGCCTCTGCATGTTCCTCAGGAAGTGCTTCGATGGCCTCAAGGTCACCGCGGTGGATAT<br>AGATCCTACTATGCTGGACGTAGCCAGGAATCACTTCGAGTTGACCACGGATGAAGACCTGCAAGTCCAAATCAAGGATG<br>GCCTGGACTTTTTGAAGGACGAAGCCGTGGCTGG

MSTRG.3604	Hypothetical protein	GATGAATTCATCATGATGGCCTGTTTTCATTCTCATCTATCACCCTTCACTGGTGCTCCGCCAGCGTATCCCTTTAGCGGAG<br>GCGGGGTTACCATACCATTTTCACCCATCAAAAAATATATTGCTTAGTAATGGGGAGTTTAGATCCCATTACTAACTATCC<br>CATTCCCCCCTGAGAGGCTGATTTGTATGATTCATTTTGAATAATATGGTCTGTGCGAATGTCGGTTAAAATATGTTGTTTT<br>TGTGAAAAAAAATCTTACATTCATAGTCACGTGTCACTTGCCACAAAACTTCACTGTAGAAACAAAATTGACACCAATTAG<br>TAAAAAAGAAAAAAATTAATTATAACATGTAAATAATAGGTCGTAAATAGACGGACAGACCATTGGCATCATTCATTTTT<br>AATCGTCTAATGATAATGATTCGTCAATGTAAATTGAATTCAGGTGGTATTGTTGCACTGAAGTGTTAATTTGAACGTGAT<br>TTGAGTTTAAATAGACTACGCGTTAATTCTCACAGTTTCATTCGACTTTAAAAAAAAAAAGAGGTGATAAGTTCACCGTGT<br>GTTTCTTAACATTTGTAGCGTATGATTTATAGACTGATTTTAATGATTATTTTTTTTTATCACACACGTCGTCGCTACTTTGT<br>TATCGTATACTGTAATTAGGCGTGTAACTAATAAATTGGCCGCCTGTTTGAATAACAAATGAATTCGTCAATAGATACGGT<br>TTCGTTAGCGTACGCATAGGAAAGAATGAGACGCCTCTTGATCATGTTGTTGTCTCGTTCTGTCTTACGCAGTCGGTAGCG<br>AAAGCATATCTATCGATAGATTTGTCTGTATGAGTCATAAGAACCGGCTAAGGGGTGACGAGGAATGGGCAGCAACGTTT<br>TTTCTTAAAACATCTCCAAATCCTAATTGCCGTTGCTAATGCCTGCCAAGCATGGCAACTATTGGCAAACCCCCCAAGGGG<br>GAGGCTCTGTGGCCCTTATGTTCAACAGTGGACAGTTAAAGACTGGTATTTTGGGTATTTTGGGGACAGTTAAAGGCTGGT<br>AAATTCCCAAAGTGAGCCTTCCTTTTAACAACTTTGTTTATATTTAAGTTGGACAAGTTATTATTCTCCTTAGGAAATGCGA<br>AATTTATATTTTAAAATACATATTTTAACACTTCAGTGGCGAACACAATGCTGTGGTCACACCAAACGCGCGTTTGGAGGT<br>TTAAGAGAAGTGACAATGTCACAGAGTGACTAAATCGTGTCGCAATGTGACATAAGGTCTGTGATATGCTTTGCAATTTTT<br>TTTACTTATTTTATATATATATGTCTTATTTTCTTTTCCAATGTGTCTTAGTTGTCTAGGATTAATACTAATGTTAAGATTATC<br>GATTTTTTTTAAAGCAAAAGTGTTTGGAAATATAATTTTTTTTTTACAATAAATTGTTTATTGATTTGATAATAAAAAAAAA<br>AAAAAACTATTAAGGTCTCTGTGAGAGCCTTACTTGATTTTTGTTCGGTTCAATTTAGAATTTTTTTTAAACTGCGGTACGG<br>TTCTTTTTAAATTACGCGGTATTGTTATATACAGCAAGAGTGGCAATTAAGCATATGGCTCGCCTGATGGCAAGTGAGCAT<br>TGCAGCCTAATAAATTCCAGCAATATCAGAGGCTTTTACCAGAGGGAGACTTTGTACAAAAGTCGATTGCTTGCTCACGTC<br>TATAGGGAACGGTTACCACTTTCCACCAGGTGGGCCGTCAGCTTGTTTGCCATTCTAAGTTGTATAAAAAAAAAAAAGGCAT<br>TCAGGAAGTAATTGTATTACCTTTTTTGCTTGTAAACTGTATTCGGTTAGCTAACTGATACATCTATGTATATATAAATTGG<br>ATTATTTAACTAAACAATATTTCTTAAATTGATCCGATTGAGAATCAAACCTTGTACTTCATTGACGCAAAGCATCATACTC<br>GCCACACCACAATGGTCGTCTACAAACAACTTGTTTTTTTTTTTTTTTATTAATGTTTGTAGTATTTTTTTTTTTATTTTTAAT<br>TTAGTGTGACCACAACTTGAGCTCCATTTGAGAGCGCACGCTCAATTCAGTTTGAAATTGTTAAGGAAAGGAGATGTTGCA<br>GTTTGACGGTCTAATTGTACATTCTAAATATATATATATACATATACATATATATAAAATTCTCATGTCACGGTGTTTGTTA<br>CCAAACTCCTCCGAAACGGCTTTACCGATTTTTATGAAATTTTGTGTACATATCGGGTAGGTCTGAGAATCGGCCAACATC<br>TATTTTTCATTCCCGTCAATGATAAAATCCCGTCAAATTTTTCCATTTTTATTTTATTATGATTTAGCATTGAAAAATACATA<br>CAACTTTAAATTTTCGTCGTTTTACGATCAACCCCTTTATTTTAATTGCGATTTTTATATTTCGTAATAAGTGGTAAAACAAC<br>TTACAAATTTACATAGGTAGAATGGTATTTTGGAATGCCTTGTTCGTAAATAGCTTTTTTATTTGTATTGTTTATAAACGTA<br>AATCGGATATGGACAAAAAGAGACCATGAGTTGGAGATGTCACTATCTCATTCTATTTTATTATTAATATTCTTTTTTTTTT<br>ATACCACAAAGGGAATGCCTGTAGGGTTTTTTTTTGATGGATGAAAATGGTATGGTAACCCCGCCTGCGCTAACGGGATAC<br>GCTGGCGGAGCACCAGTGAAGGGTGATAGATGAGAATGAAAACAGGCC
MSTRG.362	Neuroblastoma-amplified sequence-like	AGGTTAGAATTGGAACTTGAAGGCCTCCAACAAATGGGCGCGTCTCTGAAAGAACACAACCACCTGACCAACCGCTGGCC<br>TTCTACCGACGACGAGTTCTCCGACGCAATAACTACTCCGGTTATAGAGAATAAGGATTTAATAGCACCGCCGCAAACAG<br>AGAAGAAACCACTCCTCAACTATCTGCTTGATACGTTCCAGAATAAATTTCCTTTTGG
MSTRG.3682	Alkaline nuclease	AAAAACGGTCGCGCTGGTAGATCACGTAGATCAAAGCAAATAGCAGTAATTTGCCCAAAATTGACGCCGTTATGCTTGGC<br>AATTTTTTTTCCTGCAAACGCCGATTTTTGCTCATCAGAATTCGGAAATGTTAAAACTTTTCTGTCATCACGAGCTTTATAC

		GGAGATGATGCTGTAAGCTATGTGCAGTTTAAGCGCGATGGCAAATTATATATTATAAAATGCAAAATATGTCCTTTCTGT<br>GCAATGTGAAGACTGCATAGCTTCACAGGGCGGTTGTAAGCATGCAATAGCTTTCTTAATATGGGTTCATCGCCGAAGCGA<br>GGAGCCCTCATACACATTTGTTAAATGCTACTGGAAAAAAAATCTTAATTATCAAAAATTAGTAACACCATAAAATATATG<br>ACGGGCAAAGATTTATCTAAGGGCAGCCTTCTCATACTGTCAAACTCATCTGTTTTTCTGAAGTTTTAGAAGGAAAGAAC<br>ACAAAAATTGAGGATTGTCAATTCCTTAAGTATCAACCCACACATAGTGCAAGTGAAATACAGGCTTTTTCAATGCATCAG<br>CTTATGTGGAAGTTTAAAGAGAAATTTCTAGATAATTTCTTGAAAAATGTAAAGATATCTGCATCTTTAATAACAAAAGTT<br>CAAGAGGAAACAAGGGAACAATCCAATAGCTCTCTTTGGCATGAGCTACGGTATGGACGAATAACCGCATCAAGAGCATT<br>TGAGGTTAGACGCTCTAAAAAGAATGATGGTACACTTGTGTCTATAATATTAGGGGCAAATACCTGATACTCCATCAATGG<br>GACATGGACGTGTCTTAGAAGGTCAAGTAAGGAAGACTGTGGAGGTAAAATTGAATAAAAAAATTAATAAGTGCGGACT<br>AATATTAAATATAGATTACCCAATGATTGCTGGTTCCCCAGATGGCATATTGGAAGATGGCTTAATTAAAATTAAATGCCC<br>TAATAGTGTAAAAACTTGTAAAAATTACATTGAGAATGATGAAACCACACAAAAGTATTATACTTAAATGCAAATACAAA<br>TGTATTTAACTGGAAAGAGAAATTGTTTATTTTGTGTGGCTGATCCTGATTTTAGCCAAAATAAAGTGGTTGAGGTATTAA<br>TGGTTCAATATGATCCAAAATATATTGAAAATATAATCAAAGATTTGTTAGATTTTTGGAAAAACAATATTTATCTTTTATT<br>ATATAACAGTGTAAAACTAAAATAAAAATACA
MSTRG.3<br>694	Cyclin-dependent<br>kinase 8	CAAATACAGTAATTATACTTATTGTTGTCTGGTACCTAGATATTTGGGCAATAGGCTGTATATTTGCTGAGTTACTTACATC<br>TGAACCTATATTCCATTGTCGGCAAGAAGATATTAAAACGAGCAACCCTTACCATCACGATCAGCTTGATAGGATATTCAA<br>AGTAATGAGTTTCTCACGAGAGAAACACTGGGAAGACATTAAGAAAATGCCAGAACATTCCACTATAATAAAACATTTCA<br>AAAGATCAAAGTAAGACAAGTTATTTGTTAGGGAC
MSTRG.3<br>74	Serine protease<br>HP21 precursor	CAGGGCGACAGCGGATGTCCACTCCAAATCAAGAGTAAGAAAATCAACTGTATGTATGTCGTAATAGGGGTGACGTCATT<br>CGGAAAGGCTTGCGGCCAGCCCGGTGAGCCTGGCATATACACCCGCGTCGTTAACTACGTACCCTGGATCGAGAGTGTGG<br>TTTGGCCATAAGAATAATTAATTTAAAATGAAAGAAAAAAATAAATGAAACATTGCCAAAAACAAAAAGGGTTACATTAA<br>TAGAAAACAAACCAGAGGGAGACTTTGCACAAAAGTCGTTTGCTTGACCACCTCTGTACCATTCGTGTTTCTACCGTGAAG<br>CAATGGCGACACCTGATGGAAAGCGGTAACCGTCGCCTATAGACATGAGCAGTACCATGGACATAACAGAGTATACTGTT<br>TCGCCCATGATACCCCCCATGCGTTGCCATTCCTGAGGACTCAGGTGTTATTCCTCTGTACTCAGTAAATTCACGCCATATC<br>CCACCCTTCAAACCGAAACACAGCC
MSTRG.3<br>798	Solute carrier<br>family 22 member<br>7-like	TAAACTGTTTTTATTTCTTACTTATTTATTTTAGGCTTACCACAGTTTGGACGATACCACAACATGCGTGGGAGTACTATCC<br>ACGTATAACATCAGAATGACAGTTTTAGTGTCATTATTATTTTGGTGTTTATATTATTTTCAATGGAGTTGTTTATATACAC<br>GAATATATAGCAAAGAATTTTCAAATATTACGTTACTCACGGTTCTTTCTTTTGTCAGCGTACTTGGATGTTTAATAATAAT<br>ACTTAAAAAAAACTGAAACTAAGACAATTGCCTCTGATAGTTATTTTATTGACAGGCGCTTTCACTACAGGAGATTTATTT<br>GTTGATCAAAAAATATTACGCAATTTCCTTTCTTCAATTGCATTAGCATTCCGTCTTGTTGGGCATGCATTGATCCTTAACA<br>TTACACCCCGGTATTTTGCCACTAAAATACGAGCAACTATCTTGCTATCACTAACTAACTATCTCGCTGCCGGTCAACTGG<br>GAGCTATGATTAGTTACTTATTAATCGTGTTTCAACCAATTAATGATGCCACTATCGTGGCTATAGAGGTAGCAGTCACAT<br>TGACTCTGGCGTCTTTGCGCTTCATATTTCTTGATGTAGATGGCAGAGAACTGCCGGATGTTCTGGAAGACATGGATTATTT<br>CTCAGAATTATCGAAGCCACTTCGCTGGGCATCACAGAGAAATAACTCGCCAACTAATGAGGAAGTGGAAATAAGAGTTT<br>ACTCATTCGGGAGCACTGGACACGGTTTATCACAAGGCTATTCAGAAGAAAGAATCCCGGCGCAACGAATTGGCTTCACA<br>AGAATATGGCCGGTTTTGATTTATAATTTTAATCGTATTTTCCGTCGAGGCACAACATGTTCCTAATTTGAAAAGGAGTGAT<br>GATAAAATATAATCTGTTTTAAAAAAACCTAACGTAAAGGTAGTCAATTTTTTAAGAATTATTTTTTTAAGTATCGAATCA<br>AGTAATAAATTACTTTTATTTGAATTACTAATGGTCAATAACACTGTTTTGTTCGTGTTAATTATTCGAGTGGTAATAATAT<br>ACAGATAATACTACAAATACAGTAATATAACAAAATACAGATAAGTCTTTTTTAAACCCTTTTGCCTATGAAGTCAAAATA

		GATTTAAAGACACCATAAT
MSTRG.380	Bipolar kinesin KRP-130-like	CCTCCGAGACTGACATATACGTTATTAGAAAGCAGCAACAAAACCAAGGAAAAGAGAACCCCAACAATTCCGTAACGTAC<br>AAGAAAACCACCAAAATACCAGCGCCGTCGTCGCTCAAGAAACCACTCGTGGAACGGAACGGTATCAACTGATATCGCAC<br>CATGGAACACGGCCTCGCGGGCGTAACGGCAGAGTTTACATCGTATACGAGGATCCGGGTTCGCTTCTCGGTCGGACCAA<br>ATTATGACTGGCTTCAGGATTATGAAGGATTGTATTACAAAGTTTATTTGGCCTTTCAAATGCCAAGTTTAACATGTTAATA<br>GTTATATATATAATATATTTGGTATAAAATAGTATATGTATAGTTCACAGCGAACAATTAGTTAGGAATCATAGATTCAAG<br>TCGATTGCCTGGCTACCTCTGTCCTATTTGTGTTTCAACCGTGAAGCATTTGTGTTTGCATGGCTGTGTTTCGGTTTGAAGG<br>GTGGGATATGGCGTAAATTTAAAGGGTACAGAGGAATAACACCTGAGTCATCAGGAATGGCAATGCATGGGGGGTATCAT<br>GGGCGAAACAGTGTACTCTGTTATGTCCATGGTACTGCTCATGTCTATAGGCGACGGTTACCACTTTCCATCAGGTGGGCC<br>GTCAGCTTGTTTGCCATTCTAAGTTGCATAAAAAAAAGACTATGAATGTTACGCACATTGCTCGCTAGATGCTTTTGTATGT<br>GAAATCAATACCATTATGATTTATAAGTGGTGGGAATCAATGAGATCGCTTATGTAGAAATATGATAATTTACCCAGGTTA<br>TTTTAACATGTGGTCCCGGCATAGAATAGGCCACCCCTACCTTTCCCGTGGGTGTCGTAAGAGACGACTAAGGGAGAACG<br>AGGAATGCAGTGTTCCTCTTAAAACATCTGTAAAAC
MSTRG.3852	KAT8 regulatory NSL complex subunit 1-like	ATGGCGACTGTTGGATGTACCTGAAGTGCCTCTGAATAATGGCATCATGAAGTCAAATAGAATGTCCATAGAAAGTGAGG<br>AGGAGGACGTGTCTGAAGCAGCGGTGCGCGAGCGTCACATCCGTGCGGAACACCGCGAGAAGACGCGGTACGCTCGGCG<br>GCCGCGCGGCCCGCGCCGCCAGCCCGCCGACGCGCCGCCTGCGCCAAGTGAACCGCCGCCGCCGCCGCCGCCGCCGCGGG<br>CCGCCACGCCGCCACCACCGCCGCCACCACCGCCCAACCACGAGACAGTGAGACCGTATACACCGCGACAATTCCCGCTC<br>CCGGAAGAGACGTACGCGGACATGTTGTCGGCGATGCCTGCTGGACACGCCTGGCCAGACGACCCTGACGACTCCGGTGC<br>CCCTGACGACACCAACGTCCCTGATGACTCAAATGCGCCCATCGGCCCCGAGTCTGACACGCGCTCAGGGTCGACTCTGTC<br>GCCCCTGTCTCCACTTTCGCCGACCGCCTTTGAAGGCGACGATCCCGATGACATCGAGTGGAACCCGGAAACAGAAAAAA<br>CGGAACGACGGAAAAGTGCTTTTAGGTGAATTTGGCTTATCGTTCACTATACGGACTTTATTATTATGCTCTATATAAACCT<br>TGCCATTTCTACAATTTAAAAAAAATAAATAATTAATGTTACCATTATCGCGGTGGCGTCGAAATAATTTTTGTATTTTATT<br>ATTTCTATTTTGAAACCGTTAAACATGTGTAATGGACTTTGCGTCATGCACACAAAAAGGAAGCCAATTAATTTGAATCTT<br>TATCCAAATTGGAGAGTAGTTGAAGTAGCTGAAACATTTTCAAACTATGGTCTATTATCTACAAGTTGCTGTGACATTGAA<br>ATGATTATATGTAACATCTTTTGTTGATTGATTTTAATGTTGTGTTTGAACCGCTATAACAACTTTGTTTTAAGCTTGGATAC<br>TAGAAATAATTAATGTTTACCAACAATAACGGTTTAATCAGTATATGCAAGGTGAAAAACGAGATTTTGTTCCAATTAAAT<br>ACTTATGAAATAGTTAATTTCATGAAGTTTGTAGTGGTCTTTTTATTCAATATCATGAATGATATAAAAAAAATTATCCACT<br>ATAATTGTGTTAAGCTTATTTTTCAATTTTGTTTTACAGTAAAACAGACATGTTTTCGATCTCCTGTAAATAATACACATGT<br>CTCGCTCGTACATGTTGCGTGCCACCATTACTGGATGGTGATTAGAAAATATGGCACCTCTGTTCGATATTTGTAAATATA<br>AATGTATATTCATAAGTTGCTAATTTAGTTATTTTTTTTTAGCGATAAGCACCGTGAACCTGACGACAATACAAATTGATCA<br>ATTTATTCGCACTTTAGTTTACTGTACATACATGTTATAAATGAGAGAAGACAAATTGAATTTGTTTTTATTTTTGCAATTG<br>TGTTTTTACAGTGTAAATTGAAAAAATACCTATACTATTTATCCGTTCATCTATAATTTCAACATTTCTGACATCCAATGTA<br>AAATTATCTTGTATGTGTATATAAAAAAAATTGAAAAAAGTTACCGTCTTTGTGGGTTCTTCTCATTAGATAATATTCCGAA<br>CTCAGCACTAGACTAGCAATAAAGTGACAATTTGAGCTTGTAAACAAGTCTACTGGAATAATATATTTCTATTCTAACCTG<br>GAATGTTCGTAAGAGATGTGTATATTCTGTAATGTCCGCGGTCCTTCGTCAATTCATGTCTATAGGCGACGATCTTCGTTTT<br>CCATCACGTGGCCCATCAGCTTGTTTGCCATTCTAAGTATCAAGATATATCTATTTATATAATCTATAAAGAATAGTAAGTG<br>CAATGTATGCCTTACTATTTAATGCGACGTAAGGCTTGAAGAAATTTAATAAGCTAAATTATTCAGATTTTAACAAGATTT<br>GTTGGATACAATTAGAATTTTGATATTTGTTGATGAGATTTTAGTTATTTTATAACTTCTTAAAACCCCATTACTGTGGTCTT<br>CATATATATTATTTAGAAATTGTAAATTTCATAACAACAAAAGTTCATTGTATACAATTTTTTTTTTTTATTATACTTTGGG

TGTTTTTTCAATTACTCTACACACTGCAATATTTTTATTTTGCTCCATTGGAGTTGCATAAAAATATTTCGCTTTCAAATTGT
CACATTATTTTTTTTTTCAGTTTAAGAATTTATCAAATAATTAGATTTTGTAAGGTTTAATGACAATGAATATTAATGAAAT
GCTATGGCCATGTTGGATTGTACTTTGTGCAAGTATTTTTTTTTTTTATTTCGAATCCCAAATTTTAAAGCTTTATCGTGTAT
TAGATTTAATTTAATGTGTAAGCATGAATTATTGTGTATAAATGTGTAAAATATTCAAATGAAGTTAATGTGAACATTCAG
TTTGTCGTCATATGAATGACACACAGTGTCTTACATTCCTATGTAATATATCAATTCATTTATAAGTGTAATGTTAATGTAA
TTTAAAAATATATATATTTTCTTTTGATAACAAACTCATTTCTGTTGCTTTTGTATTTTGTACATTTATTATTAAGTTGAGGT
GTGCATATTGTTTGTTCGAGTATGCATAGATTAGTGCTTTATGATAATTTTTACAATTACTAGATTTTATTATTATGCCCGG
GTCCCGCTAAGCGGTTTATTTTAAGATTATTGGGCACTGAATAGAAACTAATAGACTGGCTAAAATTGATAATACGAATCT
CTTTAAGAGGGAATACTTAGAGAAGTGTGAAATAAGCGAGCGTTTTGGAATTTTGAGTGATTCATTTATCATATTTATCGA
ATATAAAGTTATGGAAATGTATACACGAGGAAGTTGTAAACAAGTGCATTGTTTTGTTAGTGTGCAGTATCAGCGTTACCT
TTTGCTTAGAGCATTCAACGCTAGAGGTCGGTAAGGCTTTTTTAATTGGATCGCTTTTTATAGCTGTCTGGAGTTTGCTTGG
CGTAAATGGGATGCGATATACATTCTATGATGTTTTTAGAAATTGTTTAATTTCTTATAATACAAGGTAGCTTGAGGATCA
GTCAAATATTTAGCATTTATATATTTATAGAAAAGGTGTAGTAAAAAAATTAATTGATTTATTGATTCGCTACACAAATTTAT
TGATGTAAAATTTTCCACGATTATGAAATATTAATCCATAATAGCAATGTGGCAGTGCTTAACTGATCTCTCGCCTTGAAT
GCTCTCAGATTTTCTTTTTAGACTATCAGTGTTAGATTTATTTGCAATTTTTGTATGTACGGACAACATGACATTTTCTTGGT
ATAATTGAAATTATAGTATTTGCACATAGAATTTCCAAGCCAAACAATATTTTGTTGAATTATAAGTTTATTTTTAAACTCG
ACAATAGCGTTAAAAGCATTCATAACCAAGTATGACAACGTCGCAACTATCTGACAGTTTTCAAATACATTTGAACATTTG
TCAATGTTTTGTGATTGGTTATTTAAAAAGTGCTCTGCTAGTGTTTATCATGCAATAATATACTTATTAAGTTCTCGCTGAG
AATTTGGATTATGATCGTAAAAAATATATTTGAAAAATTCCTTAGTAAAACATGAAGTAGGTACATTTATTTAATTAATTT
GTTGAATTATTGGTAGATTTATATGCAATGTATTTTATGATAAACGTTTTAGTTCGCTAGCAGAGCACGTTTTAAATTATAT
GTTAGAAACATTGAAAAATGATAGTTTTTTTTTTAATTGACAGATAAATGCCATTGTTACCACATTTTTGTAAGCAAAAGTT
ATGAATGCCCTCATCACCGGTGATTATGTTACATATAATTATTTATATCTTGACAATTCTAGGCATTAAAGTTTTCTGTCTT
CCCTGATCGACTCTAATGTGAGGATACTGACAAGAGTTGTATAAAATATCCGTAATTCATGTCATCGTAGCAATCACCCAA
GTATTAGACCGGTTTAGTGAAATGAGAGAGAATTATTTCAAGCCACCCATTTGTTCGGACTATTCGTGCATTTTTAAATGCT
ACTAATTATCATCATTTGTGACAAAACAATTTATATGTACATATATAAGAGACAGGTTTACTTTGTGTGTCGTCGTTGTCCA
CCCAGATGTGTTTTGTACTTTACCCTCAAAGAGAACTGAATTGTAAAGTGATATGTAAAACTAAATTATTAAATAGGTTGA
AAATTAAAAACCCAAAAACAAAAACAATACATACAAAGAAACGAATGGTGGCGCGTTTTTTTTTTTTATTTGAGTATTTTA
TACCTACTACTCCACATACTTTGGACGTTGTACAGAAATTGTGAATTTATTGCTTTTTTTTTTATTGAAAAAGCGTATTATCC
AAAACACTGAACAAATTACACACGCTACAACAAGTTATGTAACGATAAATAATAGTATGTATTGGGGTTAATGTTACACTT
AATGTACAGACATTCAGCGTTTCTAGATAAAGTATTTGGTGGTTTGATACGAGCAGTAAAAGCAATAGTGAAAATTATACT
GAATGATGTGTATGGGTTTAGACTTTGTTAGAAACCTATCACGAAAATATCTGATTAGTCGCCTAATATTAAAACATTCGT
ATCATGTCAACAACAAAGAGTAGTAGTATATTAACTGTCTGTCAATTTTAAAGAACAGTGATCCGACAACAGAATAAATC
GGTGATCATAGAACATGTATATACCACGAAAAATAAAACACTTAATTCGTATTTGGCGTTGTCTGAAGTTTTTAAATCCTT
TTTGCCAACAAACTTATTGCCCTATTAATATTTTAATCCACTATCCGAAGAGCTTTTTAATAATCAATGTTTGTTTTCAAAA
ACAACGTCGTAATTAGCTGTTAATTTTCAAAAATTAACAGCTAATTACGACGTAACTACATTTTGTTATGAACATGGTATG
ATAACTTTTAAAGGAAATTATAGCAGCTGTCCTCAACACAATGTCTTATATTGACCTAGTCACACCTTTGAAATGTTTAATT
TAGTTTCAATGCCAAGTCTCCTTACAATTCTTTTTATAACAATAAATGGATGAGTGATAATGAAAAATGCCAGGAATTTCT
ATAAAATTGCTGTAGGCGTATTTTTTTTTAATTTTTATTTAAGATTGGAACGCCACGTATTCAGATAATGTAACGACTTCTGT
AATTGAAAATAAATATGATAATTTAAACCAAATAAAGTCATTTACTTTGTATAGTTTGTGTTGCGTTAAGATCGAAGTATT

		GTAATATTTTTCTTATAAGAAGCCAAAGTCTTCTTGTTAATGTAAGTAGTATAGGTTTTTAAGGTGACCTTTGAACCAAGCGCTTAGTACAAGTGCTTGAGAAAACAAAACTGTAACATAAAAAATAAAAGTTAAATTCTG
MSTRG.3900	U6 snRNA-associated Sm-like protein LSm1	TTTGAAGACTGACCTATGTTCTTTGAGACTGACGTTGACGACTGAAGTCGATCAAATCAATCTTTCTCTATATCTCTCTAACCTGTGAATCGAATTTATATGAGCAAAATAAAAAAAATTAAAATTATATACCGTTAGCTTATTTTTTTTTTTGCATTTTTTATGGATATTTTTATAAATATTTGATTAATTAATCACTCACTACTGTGAAGTAGTATAAAATTTATGGTGATAAAAAGAAGTCGAAAACGTGAATTTGGCATTTTCGTAATAGACAAACAGCCTACACTTTGGATTGGAGTCTCGCTATTCCATTGAGAGTTAAAAATAATTTGTATTTTCGTTAATAAATAAGTTAATATGTCATATAACGTGAACCCATTAGCTGGAACTGCTCATTTGCTTGATGAATTGGATAAGAAACTAATGGTGCTGCTCCGTGATGGGAGGACCCTGATTGGATATCTGCGTTGTGTCGATCAATTTGCCAATTTAGTATTACATAGGACAATTGAAAGGATACATGTTGGCAAAGAGTATGGGGACATACCAAGAGGCATCTTTATTGTGAGAGGGGAGAATGTTGTTCTTCTAGGTGAAATTGTAAGTATAATAAAAAAGCTGCTTT
MSTRG.3911	Chymotrypsinogen B-like	GCAACATTTACAAGCACGACGTACAAATGCACTTAGATATATCAGTGGACAGTTGATATCATAGTTGACCTCACGTATAAATATTGACATTGATATAAATATCAAATCTAAATTAACAAAGTATAAATTTATTAAATTGTGTCAAATTTCACAAGAAAATGATTCGAATGACAATAGTATTTTTTTTACTAGCAGCAGTGAATAGTGTACCACAAGATAGAAGAAATTGGACTCTTCTTGTTAATCCGTTAGTTTCTGCACCACCATGTAAAGGATATACAAATATGTACATGGAATTCGAGCCTGGTCTGCCACCGGATGAAGAGAACTCGTATAAAGTTTATGTAAACGAGGAACTGCCCCTGTACAGTCAAATCAAATTAAAATTTGATTCTGAAGCTACAATTATATTAAGCAACGAAACTGAAAATTTTGCTAGAGTGCATGCTTTCGAAATGCGTTTTAGTATTCAGTTTTTCAAAAAGGCCGACCGAGCAGTGTTCCGTGTCAAGGGACCGCCCAGGGACAGGGACAATCCCATATATGACAAGTGTCGACATCAATTATGTAGAATACTGTAAAGAGCCACTAACAGCTGTTTTTGATAATTACCTTGTCGGGCGTAAAGGTACAGCTCATATTGGGCTGTCAACTCCTGATGATGGTTATGGCAGACGGAAGGTTGTCCACACAGAGCTGATCGTCAACGGACAGCCTACCAAACATGGAGACTGGCCATGGCATGGAGCTATTTATCGACTGGACAGTTCGAGTCTTCGCTATATATGCGGAGGAACATTAGTTTCTAAAACTTATGTCTTGACTGCTGCCCATTGTACCACCATTAACGGCATTGCTGTATTACCTGAAATATTGAGTATTATATTTGGGAAGTACAATTTGATCGGCGGTGATACAGACACACAAGAAAGAAAGGTAATATATTTTTATGTATTTTAGAATA
MSTRG.3920	WD repeat-containing protein 7	GTGTGGCCAATATAACTATATTGATTAGCTGTCAGTGTCATTGCTGTCACTTTGGTGACAGCTGATTAATGCGAATTAATTTTATTCATTTTATTATTTATGTTTAATTTTTAATTACATTAAATTATTTTACATCTTTGTCTCCTTGTAGACCCCGATGGACTCACAATAATTGATAGTGATTGTGGTTTAATCTCTTGTCTTCGCGGACACCAGCAACAATGCCAGGCACAAACCTCATTGTGCCTGTTGTACTCTGGGGCAAGCATGCACCCACTCACTGCATCTCATCCGTTTACCTCTCCCGAGACCAGAAAACACTCGTCACTGGCTGCTATGATGGCCAGATCTGCATTTGGGGTGTCCAACCTGAGACCCTGAAAATGATACCGAAGTGCCTGCTGGTTGGCCACACAGCACCTGTCACTTGTTTGTCCAGAGCCTCTATAATACAAG
MSTRG.3953	Stromal interaction molecule homolog isoform X1	AGGTTCACAATTGGACAGTGGAGCAAACAGTCGAGTGGTTGTCCCAGTCAGTGGATTTACCACAGTACAAGACGTTATTTTTGCAACACAAAGTTACCGGTGCAACACTACCCAGACTGGCAGTAAACAACATGCAGTATTTGAGCAACGTACTAGGCATCAAAGATCCGATACACAAACAGAAGCTCGCTTTAAAAGCTATGGATGTGGTATTGTTCGGCCCGCCTAAAGG
MSTRG.3959	Cytochrome P450 9e2-like	AAGGAACTTTGAAAAATACTTCTATTGAAAAAGATGACGTCGGATTCGCTACAACAGAAGAAACCCTGAAACCAAGAAGTGTTACAAGGAAATGGACAGAAGATGAAATAATCAGTCAATTGTTTACATTCTTCGCGGCTGGCTTCGACAGCACGGCTGGAGCCATAACTTTTAGTGTCCACGAATTGGCCGTAAATCCAGAGGTCCAGGAAAAATTGTACCAGGAGATCAAGGGATTCGAAGAGTATGGCAAAACTTTGTCTTACGAGAACGTAGCACAATTGAAGTATATGGATTGTGTGATCAATGAAACAATGAGAAAGTGGCCACCGTCGATTTTTATGGACAGGGTATGTAGTAAACCTTACAAACTACCATCACCTAGAGAAGGTGGAAAGCCTTATCAATTGAACCCCGGCGATATAGTATACAATATGGTGAATGTCATACATATGGACGAAAAATTCCATCCTAAACCCGA

		AGTGTTTGACCCTGACCGCTTTTCTGATGAGAACAAGCACAAAATACAACCCTTCACCTTCATGCCCTTTGGTATGGGACC<br>GAGAAATTGCATCG
MSTRG.3978	Alpha-centractin	CACGGTTTAATCTGATGTCATTCATCATTCATTCATGTGTCACTTTTTAGAAAATTTACCTAAATGCTTTTACCACGAATTAT<br>ATATTAGTAAAAACTCATTGAAACGTTAAGATCTCGTATAGAATATAAACAATGGAGTTAAACGACGTGATAGTCAATCA<br>ACCAGTTGTCATAGACAATGGCTCTGGTGTCATCAAAGCTGGTTTTGCCGGCGATCAAATACCGAAATGCAGATTTCCCAA<br>TTAGTAAGTACATATTACTTTGTACGTAAAAAAAATAT
MSTRG.400	AF4/FMR2 family member 3-like isoform X4	GGTGACTGGCAACAGGGACCATGTTAGCCAGCAGATCCAGCACAAGCTCGGCGACTACCACCTCGCACAGTCCCTCATTG<br>ACGATCCCAGCAAGTCCATCGGGATCTACGCCGAGCCACCCAGTCCGGCGCCATGTACACGTCGCGAGAGCGAGTTCAAA<br>AAACCAGCGCACGCGCCGCAAAATGGTCGCCTTCACCATCGGCCGTTTTACAACAAGAGCGAGGCGACTGGCTCAACGGG<br>AACATCAGCGCATCGACCGCCGCCTATCCGGATACCAAACGGTTTTCAAAATCAGAACCAACTGGATAGCAGTCAACCGC<br>CTATAGAAAGCATTTTAAAGGAGATGAAATCTCTACCGACGCCTTTATCAGTGATAGCGGCGACGCCACGCAAAGAGTCG<br>GATAATAAGTTCATCTTCAACCCTTACACTAATAAGGTGCAAGAAAATCCACAACTCATGACCAACGATCTCAAAACACC<br>CCTTCCGAAACCTGGTTTAGACAACCGGATATCAAACTCGAGGAGTTCAGTCTCACCCGATGTGAACAAGGACCTTGGGTT<br>ATCAGAGAGCGATGACGAAGTCGCAGCCACCACCACCAGTATAGGGCCGTTATTGTCGCCCATTGGCTCCGCCAGCCCCA<br>GTTCGGGCAGCTCATCACCCTCGGACTCTGAGAGCGAGTCGTCGTCGGCGGAGT
MSTRG.4005	Hypothetical protein	TTATCTCTGACAGAGATCTCTTCCAGTCAACCCAGTACAGTGAGAAAGCATGTCGTTAGCGGGCAGCTGAAGTGCTACAAC<br>TATTAATACATAAACAAATATACAGTTAAATAAAATATTAAAATATATAATAAAATATTACACAAATACATATAATACCTA<br>CCTACTTATATAATAAATGTTAAAATAAATGAATATAGTACTTACATTAGAAATTTCGTTTAACGATTTTAAGATTTGACA<br>GTTCAATTAGGAAAATACAAAAAAAACAAAATATTACTCAAAGGAAAGAAAAATGTATAATGCAAATAAATTGTATTCAT<br>TTTGCAAAATTCGCTTTTCTTATAAAAAAAAAAGGTTAATAATCCGAATTGTGACGTTTGTAACACATTGAATGATGTTCA<br>CCATACGATATCCGAATGTACGTAATACAAAACACAAAGGCAAGTATTGATACAGTAATATAGTCTCAATAGACGGGAGA<br>TTGGTTTGTTTTCAAATATATACGCAGAGCCACACTCCGGAATGGCGAAATCTTTAATTACTATATTTTTGTATCGATAATT<br>CCCTTTATCAAAATAGACTACTAATATATTATGTATTATTTATTTATGTATTAATGTTTCAAGTATGTGAGTTGTATTTTTTA<br>AAATTAGGGCACGATGAGGCCGACATATCCTTATAGGATAAAAGCCTCTAAATAAAAAGATAAAAAAATATATATATTCA<br>TTTATTAAAAAAAAAAAAAAAATTTGCAACAGTATCGTTTACGCGGGCCATTTAAAAATAAATCTTTTTAATCGCTCAACT<br>TAAGTGCCGGTTGTAACTCGAGTATTTTATACGGGGACGGTTATTTTAGATAGTGTGTGAGTGCCCTAACATTCGCTCCGC<br>GGTACAACAGTGTACAGTTGCCTTTATGAACCTGATCGTCGGGGGGTTTTTTTTGTTGGATGAAAATGGAATGGTAACCC<br>CACCCGCGCTAACGAAATACGCTGACGGAGCACCAGTGAAGGGTGATAGATGAGAATGAAAACAGGCCAGTTAGCCCAT<br>CATGATGAATTCATCAGGAATTAACCATGATAGCTTCTAGCGGGAACCGCGTGGAGGTCGACCTGGTTAGGTATATTGCTA<br>GCAATGGCACTGGTAGTCCGCATTACTAACAGTGCCTCCCCTCTCTATTGATAAGATCTTCTCCTATGATGGTCCTGGGGG<br>AACCATCGTGCTGAATTCTAGATGTATTCATCACGATGGGCTAACTGACCTGCCTTTTCATCCTGTGATGGTCCCTTTAAGT<br>AATACCATTTTTTACATTATTTCACTAGATTAAAAATACCATAAATAAATATGGAAAGCGGGTCGTTTTCCATTCTCTCCGC<br>TTACCCCATTTGATAGACGTGAAGTGTATGTATGGATGTTGGACATACATACCGCGATCAGATTTGTAAAGGTTTATTTTGT<br>GTATGTAGTTAAAAATATGTTTATTAGATGGATCCAGACTATATATATATATACTATATGTATATGTATAAATTCAAAAAG<br>ATATCCTCATCCTATGTATTTTTAACCCCGCCTGCGCTAACGGGATACGCTGGCAAAGCACCAGTGAAGGGTGATAGATGA<br>GAATGAAAACAGGCCAGTTAGCCCATCATGATGAATTCATCAGGAATTAAGCATGATAGTTTCCAGGGGGAACCGCGAGG<br>AGGTCCACCTGGTTGGGTATATTGCTAGCAATGGGATTCTCAGTCTCCATTACTAACAATCCCTTTCCCCCGATCGTATTAT<br>CGTTGTTTTTTTTTTTTTATAAATAGTCTGCATCCGTCTATAATATTGTAAACAATGTAAAAGCAGGGTTCACGCTGTATG<br>CGCAGCTTGTATGCAGTTAAGAAATTGTCAAGGGCTGATATTATATATATATAATTAGAAATATAAACCATCTATTACA

MSTRG.4022	Hypothetical protein RR46_09673	ATTTGACAATGACATTTGTACTCTAATAAATTGTATTGTATTGTATTTGTATCTCAACATAGCCCGTTTCGTGCATTTATATT<br>GTTTTTATATTTCCTCTATCACAATACTACAATTTAAATTTAAAATATTACATTTTATTTCAATATTGTTACATTGAAATATT<br>TCCTGTATAGAAATCTTGTGCTTTTATTAGAATCTGATCATTACACGTTGTTAGAAAGTACATACTGTACTCATTGGTCAGG<br>TGAATTGAATTGTAACTCTTTCTAAAATGGGTAAATGTATAGTGAAAGAATGCAACTCCCGCACTCAAGTGGACAAAGCT<br>AGCGACGGAATTAGTTTCCATACGATCCCCAAAGATCCAGTACGTCGAGAGAAATGGGTGGAAGTGATTCGTGTCCTACG<br>CAAGGATCCCAAATGGAACGCGACAAACACAACCGTCATATGTTCAAAGCACTTTCGAAACGAAGACAGGGTTTTGTCCA<br>AGTTGGACAACGGTCGTATCTATTTACTAGACGATGCAGTTCCTCTGCTTGATG
MSTRG.4027	Hypothetical protein	GTGTAACGTAACGATTACTTGCTCTGTAGACTTGTATCCAGAGCCGTAAAACATATATTTAAAAAGTGATGTTAGTTCCA<br>TAAATATTACAGGAATAATATTAATTTAAATAATTCACTTTTGTAATAAAATATTATATACTTATAATAATGTCCGACGTTG<br>AAGAACCGCCGCCAAAAGTTTCAAAACTGGAGGCTGCGACGGAATTTGAAGAGAGTGTCGGAGAAATGGAGGAGGGTCA<br>GGAGGAGTGGCTCAGTGAGGGTAACCTCACTGATGAGGAGTACCATCCGGCGGTGAGTAGATTGGTTTGCTAAAAAATTA<br>AACTTATTGTTAGCCATGTGGTCCTGGCATAGAATAGGCCACCCTTTCCTATACCGTGGTGTCGTAAGAGTTGATTAAGAG<br>AAGGCTAGGGATGGGCAGCAGCGTCCCTCTTAAAACATCTGGACTGCGGTTACCAATCTGCATGCCAAGTGGCAACTACA<br>GGTAAAACCTTCCAAGGGTAAGGCAGTTCAGTAATTTTTCTTTATGGGTGAAAATGGTATGGTAACCCCG
MSTRG.4057	Uncharacterized protein LOC110380818 isoform X2	CAGTTCTTCACTGGACCTCTATTTAGCGTTAAAATTCACCATGGGAATGGTTTAAATAAAACACGCATTTATTTGGAAGTG<br>GTGGGAGTACTGACGTTTGCATACTTGGTGTACCAGTACTGGAATCTGCTCTTCATGGGGCAAGCCGAGAACGACCTTGAC<br>TGCCGCCACTCTACGCGGTCTCGTTCACGCTCCAGGTCATGTTCGAAGTCAAGGACGAGGTCCGGTAGCTACCGAGACTAC<br>AAATTAGAAGAGAAACATGGCCGCCGAGGACGGAAGCCGCATCGACGATGCCCTGACTGTAGCCTCTGCAATTTTGACGA<br>TGAAAATTAAGGTTACAGAATATGGCACAAAAGGCGGCAAAATTTAATAAAAGAACTGTCAAATGTCAAAAGAGTTTTTC<br>TATTCGATAGCAGGCAAAGGAACTAG
MSTRG.4069	EP300-interacting inhibitor of differentiation 3-like	CTTGATTGTTGGTAATGGGAATAATTGTTTTTGTAGTCACCCCCCTCTTGTTTCTGTAGTTTTGACAAGCACGTGATCCGTTT<br>ACACCTCCCCCTGTAAAAATGTGTCAAAAGACAATCAGCTAAATTGACGTTTGGCCCTCTACTTATATAGTCAAAGCTATT<br>ACCGTTGAGGCCAGGCGGTTTGGTACATTTTTGTATTGTACAGACGAGGAGTACGCGCTGCCGTTCGTGAGGCCGGTGTCC<br>CAGGAGCACGAGGACGCCGCCGGGGACGAGAACCAATTCATAGTCTCCTTGGATACGGCGCGGTGGCAGGAGCTGATAG<br>AAGCATTTAATATACAAAAACCTTTGATGGTACTTAAGCGGCGCTGATGGTGAACGGCCGCCCGCAGTCGGGAGCTAACG<br>TATATCTAGTATATTTATACTTATTTTTTTTTAATGGGTGAAAATGGGATGGTAACCCCGCCAGCGCTGTTGGTATACAGGT<br>ATGGTTGGCTGGCGCCGCACCAGTGAGGGAACATAGGTGAGGATGAAAGCTTGATGAAAGGATGCAAGTCAGTTAGCCC<br>ATCGTGACGAATTCCACAAGAAATAACCATGATGCTTTCCAAGGGGAACCGCGTGGAGGTCGACCTGACAGGGTATATTG<br>TTAGCAATGGGGCGGTTAGTCCCCATTGTTGCCAATATATTTACAAATTATTACAAATGCTCTGACTACAGTTAAGTACAG<br>TCTTATGTTTTTGAGGTTATAGTTTAAGTTATATTATGGAGCTCTTGTAATAGAAATAAAAGTTAATTTATTGTAATTTTTGT<br>AATAAAAATGATTCGTTTTATAAGGCTTTTTATTATGAATTTTTCAAAAGGTTTAAAATAACTGTTGTGAGTTATTTAACTA<br>TTATAAGATGTTAACTCCGGCCATGCTATCTGTATACGCCGGCGGGGCACCAGCGAGGGATGATAGGTGAGGATGAAAAT<br>GCAAG
MSTRG.4095	Hypothetical protein	GGGATTGTTAGTAATGGAGACTGAGAATCCCATTACTAGCAATATACCCAACCAGGTCGACCTCCATGCGGTTCCCCCTGG<br>AAACTATCATGGTTAATTCCTGATGAATTCATCATGATGGGCTAACTGGCCTGTTTTCATTCTCACCTATCACCCTTCACTG<br>GTGCTCCGCCAGCGTATCCCGTTAGCGCAGGCGGGGTTACCATACTACTTTCACCTATCAAAAAAAAAAAATAGTGAGTG<br>CAGAGTACCTTTGATGCTAGACATCATAATTAAAATAATCTTCATGTCAGTATGTGTGTTGAACTATTGAGGACAGAAGGG<br>GAAAAATGTTAGGACACTTGATACGACACGACGAACTTATGAACATTATAATAGAAGGCAAGATAGAAGGAAGAAGGGG<br>GAGAGGGAGACCGAGACATAGTTACACCAACTCCTTCTCTTCAGAGACTCGCTGAAAATCGTAGTGAGTGGCAAGAGCTC

MSTRG.4104	ATP-citrate synthase	CACCGACAAGAGCGCAGCTCTTAAATAGGATAGATAGATAGAGTCAGTATGTGTTGTTTAGGTCGCGTGTGCTTATCCAGC<br>AAGTAATGGTATACTTAGTAAGGTAATGTACCTAGTTATCAATAGAGCATGAGAACTCACAAGGTTTTCTAGAAACCGGC<br>ACCCCAATATACAATTAAAAAAAATAAATGTTTTGTTATTTTTTTAAATCAGTTTTATTTATTTATTCATTAGTATGTGTAC<br>ATGGCGGACTTAATGCTAAAAGCATTCTCTCCCAGTCAACCATAGGGAAGTGTCAGAATACTCGAATGCGGTACTAGTAA<br>TTATTGATATACATACATACTAAAATTCCATATATATATGTATATAACAAATATACTTACACATATATAAAATATATTATAC<br>AAGAAATTAAGAATAATATGTGGTACTTAGTGTTTAGATAAAGTAAAAAAGTGAGCATGAATGTTTTTTTAAAGACGGTT<br>TAGTCGGTAGTCTCTCAATGGTATCAGGAAGCGCGTTTCAGAGCTCTGATGAATGTAGGAAAAAAAGATTGTTCACAGCTT<br>TATTTCTGAATTTAAAAATTCATCTTGTTACGCCGTTTACAAGTGAACAATTTCACTTGTTTCCTACTTGTGAAATCCACG<br>CGTAGAAGCTAATAACAAGGAATTACATAGAAGAAATGATAAAATAAAAACTGTAAATCATATGGTCGCCTCCTTTCTTC<br>TTATGCAAATATAATTAGTAAAAAGAGGACGAAAGATACACCATCAGTTGCTATTTTTTTTTCTTAATTCATAGCAGGCTA<br>GTGTAGGAAAATGTTTGATATGTCAATTATTTGTCAAAGACAAATTTTACATTACTAGATTATATATTCGACACAGTTTATG<br>TAGAAAGTATGCGAGCAGGTTGTATCTGAATTTGGTAGGGTTATACGGCCGTGTAACATTTTCTATACACTAGCCTACTAT<br>GAAGTTTTTATTTTATCATTCCTTTTATGTAATTCCTTGTTCTTAGCGTAGAAGTAGCGGTCAAAAACTAATAAAGGCAGAT<br>AGGTAAAGACCTATTTTATTTTTAATTAAATTAAGTACTCAAATGTAATTCTTCAAATGATTTTTTTTTTAATTATTCAAAG<br>AGACAAAAGATATGTAATTTTAGTTATTAAAGTGTATAAAAAAATACATTAAAAATATACAATATTTTTATTAAATAA<br>NNNNAAGGTGTCGTCGCCGCCGCCATTTTTTGATCTTTTCCGCCATTGTCAGCTATTCACAGCCGAGGAAGGCAACAACTA<br>CATCAATATGGGATCCATTAATGCACTGTTCGTGCTTGGCCGCACCATAGGCCTTGTTGGCCACTACTTAGACCAGAAGAG<br>GATGAAGCAACCCCTATACCGCCACCCATGGGACGATATCACCTATATGTCGCCGCTGAATTGAGCGGCAGCACGCGAGA<br>TACGTGTCAGCATGCGTTAACATGCGTCAGCACCCGTTAGCATGCGTCCGGCGGTCAGAGGTTTGGTTAGTCGACCGCTGT<br>TCCCTTAGTTCATAATAGGTTACATGATTGTAGATAATTTTCTATATTTAATTAATTGTAATGTAAGAAATTGTGTGTAGGG<br>CTTTATTGTGACAATTTTAAATTTATTTTGTATGTGCCGCGTTATGAAAGAAGAAAAAAATGCATGCGTGTGAATAAATTTT<br>TGTCTATGTCATGCTGTAAAATTATATAAAATTGCATTAAAAATATAAGAAAAAGAAAACATATTCTTTATCTACCTATTG<br>AATATTATTCCAATTTCAAATATGCAATTTTAAAAATGGAATTAGTAATCTTAATTTCATTTTATTATATACCATAATTATG<br>AAATGTTCGATCAATAAAAAAGTAAAAATAAAAAATATATTATCTATGTGCCTCATTCTTTTTTTAACATTGCATACATTA<br>AAAAAAAACTAGAAATATGACTGTAAGAATTAAATTTACCTAACAATATTTCTATTTTTGCGCTTTATTTTTATTTTTTTAA<br>TATATCCTTGAGTAATAGACATAATCTTATTTTTTACTTATTGTTTTCATTTCTCATATGGGACATAGATAAATAACCTATTG<br>TTCTAAATTTGAATTTCTTTTACAACTTGATAAATATCCAAACCTCTGTACTTGTCAACTATATAATAATTCTAGCTAATAA<br>TTAAAACGTTTCAAGTCCCAACGGTGGGCACCGGCCCTGTAACTTCTTAATAAATACACATGTATTATTTATTTTTCTATTA<br>AATAGACAATGCTCAAATCAAAACATAAGCAAAAATAATGCACGTGTAATACGTTCCAATAGAACTTAATGTAAATGTAA<br>TCCAATTGTTATTAAATATTAACATAGAAATAAATTTGGTAACAAATGGATACATTCAATCCATTGTTTTTTTTTTATATTT<br>GGCCGTCAGCAGAGTGTCTATAGGCCACCCCTTCCTTTCTCGTGGGTGTCGTAAGAGGCGACTAAGGGAAGGCGAGGGAT<br>AGGCAGCAGCGTCCTTATTAAAACATTCCTAAAAACACTGCGGTTGCCAATCCGCCTGCCACGAGTAGCAACTATTGGCA<br>AAACCCCTTAGTGGGAGACTTTTGTTCAACAGTGGACTTAAAAATTTGATATGAAATTGAGTATCTTATGATCGGGTCAGT<br>TTTACATTTGTTTACGTTTTCATTCCCATCATCATGATATCAGCCGTTAATTGTCCACTGCTGGACATAGGTCTATTATTATT<br>CCTACTATTATGAGGTTTTGTCAGTAGTTGCCACGCTTGACAAGCGTATCGGGCTGTATTTAAGCTTTAGTGATGTTTAAAA<br>AGAGACATTGCTGCCTATCCTTCGACTATAAATTTCCCTTATTCCCCTCTTACGACATTCACGGGAAAGGAAGTGGTGGCC<br>TGTTCTATGCCGGGACCACATGGCCAATATTTTCATTAGTTCTATTGAAATGGACTATATTGATGTGATCACATCTGTATTG<br>TCAATTTTCAAGTATTTAATTAATCAGTTTTCAATATTCCTGAATGATTTATACAAAATGTGATCCGGAGGGTGTTATATAT<br>TATATTATAGGAATATAAGAGCAAATATTTTTTTATTACTTAAAAGCGAATAGCGAACAAACTACGGTGCTGCCTCTGCAG

		TGCGATTTAGGAACTAATCTATTACTATTATGAATTCTCCTTTTAGTCACTAAATTGTAAATTGTGCTGTTTGTCATAGAAA<br>TAAACATTAGGAAGCATTAAATTTGAAATTCGTTACAGGGAATACATTAATTTGAACAAATGGTAAATTATGTTTTGTTTT<br>GTCAAAATATTAACAAATTATTGTTTCGATCCTTCGAGTCAGATTATTCGGCTCGAAGGACCAAACCGAACTATAGTTTGT<br>TATCATACACCGGACTTATGAAATAACGAACTTTAAGTTCGTTATTTTACAGTTACATAAGAAAAATTATGTTAATTAATT<br>ATTAATTAAGTTAATAATAATAATAATTTATTGCTCCATTAAGTTTACAAAATAAGATTTTTAATCTTATATTATTCTGTTTA<br>GTTTATTAACTTCATCGAAACATACTATCGCGTTTCGTCGTCTTGTTACTGCAAGCATTATGATAAATAAAAAAAAAACCC<br>ACTACATTAATGCCATAGACAATATTAAAAAAATGTCTGTGGTCACCAATTTTTTTTTATTGTAATACATCCTATCCTATTA<br>TATTATGCATATAGTATAAGTACATTAAAGATCTGTTAAATTTAAAAGATATGTTTGTTCTGTGTGTGCGAGCGAGACGGC<br>GAATATGTGTACATTTCTCACAAGGGATAATGGGGTTGATTTAGTTTTTTGTTTTTTTGTAGGAAAAATGTTAGTGTAAAT<br>TGGATGTAACATAGAAAAACCCACAGAACGCATTATACTAGTAAATAAATAAATAAATACGATTAAGTGCACTTTAAATG<br>TTTTTATATGTTTAAATATAATACTGTAGAACTATAACAAATGTATTAAAATTGGATGAATAAAAATATGTAGCAATTAAT<br>CTAAACCAATTTTGAATGCCTACGAATTGAAATTTTTTTAACATAAAACTTTGCAAATTTAGATTACTTACATACAGAAG<br>GGGCAATAGCCCCTTATTCATAAAAACATATCTGCATTTATTTTTATCTAAAATTATAAAAAAAATGGGGGGGTCGTGTCC<br>TGGAATAGGTTGAAAAACACCGCTCTAAGTACATCCATGGTAATTATACTAGTATAGTTTTTCCCGCTCTCAATCCATTGCC<br>GGTCTGCCAGTACGTTTAATACTGCATCTATGCTTCCTACATCAAAAAGTATTTTGAAACACCTTGTATAATTTCAAAACAG<br>TCTATTGTGATAACTTAATTTCTTTCCTGTACTTATGACTGCGACATATATACATATGATCATATATATGATAGCATAATAT<br>GTTTTAATAAAATGGTAATCAAGTTTAGTTGAAATTAAACACAGACTGGAATTGGTAACAAAACAAAATATTAGATTGTTT<br>TTTTTTTTTTATTTATATTAGACATCCACCGAGTGTCTTGTGGTTGTTTATGCATTTTCTTATATATATATATTTTTTTATTAA<br>TTTCTATTGAAACAAACCATGGACATTAACATAGTCACTGGCCATTTTATTGTAATTAAGTTGTTAATGTATTTACTGAAAT<br>ATATTTTTTAACAAAA
MSTRG.4<br>106	ATP-citrate<br>synthase	TGGAAGATGATAGGTGAAAATGAATGCAGATCCAACCATCAGCCATATGACGCGAAGCCAGCGCCCCATAGATCTGATAT<br>CTAATTAGTGATAAACAAACAGACAGTATGTCTTCGAAAGCGATATACGAAGCGACCGGCAAGGACCTCATAAATAGACA<br>TATAGCGCCGGGAACGGCGTTGGTGCCGAGTCGTTTCGCTACTTTCGAACAGAGCACAGTGTGGGAGGACGAGCTAGGGA<br>AGAACCCATGGCTCGCCAAGGAG
MSTRG.4<br>121	Uncharacterized<br>protein<br>LOC106103906	GAAGGCATCGTTAATAATGGGAATTGGTAGTCCCATTGCTAGCAATATACTCTCATGTCGACCTTCTCGTGGATCCCACTG<br>GAAACCATAGTGATTAATTATGGCAGTCTATAGGGAGGCCTATGTCTGACAGTGGAGCTTTTTGAAGGTCACGGGCGATG<br>GCTTGGCCTTCTTCCACACTCTGCTCTGGTGTCAGCACCACCTCAAGCTCCATGAAGTGTCCAAGACCTTGAACGGAGTCT<br>ATGTGAACGCGTGTTTGACCGACCATGTATAGTTTCCTATAAATGAAATAAGTGCCAAATAAAAGTTGCTATAAACAGGA<br>AATAATTGAGTGCAGAAAGAGATATTTATTTGGTAGATTCCAATAACAGTCTCAACAGAAACAAAAACAATATTTTTCCTA<br>ATGGATGGGCAGCGCCCCTTTCAAAACATCACCAAAGTCTAACTGTGGTTGTCAATTCACCTGCCAAGCGTGGTAACTACT<br>GGCAAAATCCCCAACAGGGGAGGCCTATATTCGGCAGTGGATAGTAGACAGCTGGTATGATGATGATGATATTACAGAAA<br>CAACTGGATGCGGCAGATGGTGATGAAGAAGGTCAGGCTGACATGTTACTTTTTAGTAGTTGTTAAAAAAATTACAACTTT<br>TTTATTTTTTTTTACCAAACAATTGGACGATTTGCATACTAACCTTTCTTTAACGACTCGACCGCGAGAACCCAAACATTT<br>CCTTAATAATTCGTCTAATTGTTTAGCTTTCTGGCCATCAACTGCGGAGAATTCAACCAAATCGTAGTCAGACAGCTTCGG<br>ACCTTCTTCGTCGTCCCGAT
MSTRG.4<br>160	Hypothetical<br>protein<br>KGM_205375	AGTGCTTTATATTATTCTCAATTTCGTGTATAATATAGTATATGTCATATATGTGTAAGTATATTTGTTATTCATATATATTT<br>ATTCGTTTATATATGTAATTTTAGTATGTATGTATATCAATAATTACTAGTACCGCATTCGAGTATTCTGACACTTGCCTAC<br>GGGTGACTGGGAGAGAATGCTATTAGCATTAAGTCCGCCATTTACACATATTTGTACTAATGAATAAATAAATAAATAAA<br>ACACAGCCAATAACGGATTTTGTTTACACAAACCAAAATTATAATTCCAAAGCGAGGGTGTAACAACCATGGGGTGTCCA

		CAGATGCAGAGCAGTCTTGCCCAGGGCATGTTCTTGGCTGGTGGACGAGGGGCGTACGCTGGAGCCGCGCTGCAGGGTCC<br>CCTAGCTTATTTGGAAAAGACCGCCACTAATATAGACATGGTGGGACTTGGGGATGGAAGGAGGTGACGCGGTCGCGGGA<br>GGGGCAGGGGTCGGGGCCGCGGCAGAGGTCGAGGTCGTGGAGCATCCCCTGAATATGGCGGCAGCATGACGCCGCCATA<br>GCGCGCCCCTCTCCCGGCGCACACGGACGCACACACACACGCCCCCACGTCCACATGCACACACAGCACACACACACAGT<br>ACGGCGCAAGCGCAGACACTGGTTTTTTATAATTTTACTGGCTTTGTAAAAAAAATTTGGCTTTTTATTTTAACGACTGCCT<br>TAAAAAAAAGGAGTGTATTGTTTTCAGGTTCATGTATGTGAGTTTATTCCGCCATAAATTTTAAATGCCTGTAAAGATTTA<br>GATGTATGAGATATTGTTAAAATCCTTACATCATCCTGAGCAATACTGGTTATTTTTTTTTTACTTTTACTTATTTTTTTTTA<br>AAAAAGGCAGTCGTGTTTTTTTTCTGTGTAGCTTGTTTTTTTATGTTGGTGTTGGTAGTAGGTACTCTGATATGATAGCCGA<br>CGAGAGAGTCTAAGATACAGACATCACCATTTCATGGAAATCTTCAGGAATCATTTACCTACAAGCAGATTTTTTTAAATA<br>CAGCACTAA
MSTRG.4171	Hypothetical protein	GGATTGACAGCAGCGTCTTAAAACATCTCCAAAATCTAACTGTGGTTGCCAATTCGTCTGTCAAGCGTGGCAACTACTGGC<br>ACAACTCTCCAAACGAGACGTATGTCCAGCAGTGGACAGTTAAAGCTGATATGATGATAATAAGTATATGAAACAGTGTC<br>ATCAGAGAGAAGTGTGGACTGAAAGACGATGTAGTGACAAACATCGAAAAAGGAATGTTGCGTTGGTTTGGTCATTTGGA<br>GAGGATGGATGAAAGAAGAATTACGAAAGAGATTTATCGAGCAAGAATGAATGGAGATGTCGGTAGGGGCCGCCCTAGA<br>CGGACGTATGTCGACCAAATAGGCGACGTGCTGAAGAAGGGCCAAATTAAAAGTACCCGTAACCGGCGAGCGTGTATGA<br>AAAGATTGATGAATGTGGAGGAAGCGAAAGAGGTTTGTTTGAATCGAAGCATTTGGCGTTCCATTGTTTCTGCCTACCCTA<br>ATGGGAG
MSTRG.4182	Dermal papilla derived protein 13	ATAGGTTCGAATGCGTTTTATTTCTTTTACTCACCTGTGTATACTTTAAAAAAAAATTCAATTTTATGACATATCTTATGGA<br>ATATTTTTAATAACTTCATTAGGAACTAAAATTTCACGGTCATTTCGTTTTGTAAGTGAAATACATTAATTACGGCTTATTA<br>ATATAAGCCATAGATCGTGATAATGGTATCATTACATAAAATTAATTATTAATTTCTGTGTTGCGTATTTTGTCCGTGTTAT<br>GTGTAAATATTACATGATTGAGATCTAATTACATGGGCGATAACCAGCGGAGAGTGCGACCTGAAGATATGGCCACTACG<br>AGTACCGCAGTAACCACCGTCACTCCACAATCGCAAGAGCCTCAACCATGGTCGTTGAGGTGCGATTATTCTACGAAGGT<br>ACCGGCTCTATTGACCGGCATCAATGTTTTGGACCTGTCACGGGTGATAGCTGGACCTTTTTGCACTTTGACCCTGGGTGAC<br>CTGGGTGCTAACATTATCAAAATTGAGAGCCTGGAAGGTGATGAAGCTAGGAAATGGGGTCCTCCATTCATAAAAGACTC<br>TACGGAT
MSTRG.419	Alkaline nuclease	CTTCGCTGTAAAGTTTATTTTGTTTGGTAAACTTTGTGTTGTGATTCCAAGAACAAGAACTTTAAGTGGAGAGGACTAAGT<br>GGTTTTACGATCTAGCGATACACATAGGTACTGATAGATGCAGGTGGATCATTACTCAAATCCTTGTTCGGAACATTAGAT<br>TCGAAGGACGCTATTAAATTCTCAGAAGCTATCGATGATGTACAGTCAGACGAAAAACAATTAGCTCATTTAATAAAAGA<br>AAATATTCATGTTATAAAGTCTACAGTATCTACCTTTAATGACACATTGTACAAAGTCACTGAAAACGAAAATAGGTTAAA<br>TAAAAATTTTGATATTATTAACAAAATGTTCGAACAAATTACAAATTCTAATGATAAGCTAGAAATAAAAACCCACTTAAA<br>TTCTACGTTAAATTGCTTTAAAAGCATAATTATTACATTGTCGTTCGATATAGAAGATATTAATAACGCAATACTATTTAGC<br>AAAATGAATATTCTGCATCCGACTGTACTAAGTCCACATCATATGTATAATGAGCTCGAACGCAATACAAATAACTTGCCT<br>AAGCATTGCGAACTCCCTGTATCCTTATTACTACAAAATATACATGATATCATAGATGCATCTAAACTAGCTTGTTATTATC<br>ATAACAGTAGAATTATTGTAGTTATAAAAATAACGCTCGTCCTTCCTCAGCTTTATAATCTTTACCATGTAATACCGATTCC<br>AGTGCCGTATGATATCTCAAAACCTGACAACTATGCGCTAATCGCGCCAACTGCTCGTTATGTGACAATAACAGCTGATCA<br>TTTGTTTTATTCATTTGTTGACGATGTTTCCAAGTGTAAGCTAATAAGTGATAAATATTATGTGTGTGATTTAAACAACGTG<br>TTTTCTACTATTGCGCAGCCAATGTGCGAAATAGTGTTGATAACGGAAGTAGTGAGTAAATTGCCAAACTCTTATGAAGTG<br>AAACTTTTAAAGGGTAACATAGACACGTTTCATAGAATAAGTGACAATCGATGGATCTATGTCCAATCGGAACCCGGGAA<br>ATGCCACATTGCCTGTGACAATAATTCCAATAATTATAATGAATTACTGCTTGGTATAAGTATATTCAAGTTAGAAGAAAA

TTGTAAAGCATTTTTCAAAACTCTTCAGTTTATGTATAAGAATAGCATAAGGTTAAATGTAAGCGCTACTACTGAAATTTC
AAATTTCAACCTCATCGAAGATGACTGTTGCGAAGAGAATAAACTAAATAAAACCTTATCGAAATTGCCTTTCGTAAAATT
AAATAATGTTCATAATTTCGACTCGTTTTTGTATGCTAGTGTTCATCTAGACAGTTTCGAAAAGGAATTAGACAAGTTAGA
AAATCTTACACATTTCCAAAAATATAGTGTTCAATATATATCTGTAAGTTATGTTGTTTAAGTTATATTTCTATTACATCTAT
TATCATTCTATTATATAAGAGTAGAAAATTTATGTGTAAACGAGATTCGTTACGTTATTTTGTTCAAATATTTAATAACTGT
AGCAATAAACAGTCTGGTTCTAAACAACCTATACAAGTTGTAAGCACTATTGATAAAGCAAATAAAATAGCCGACTCTTC
AGATACCGAAGAACTCCAAAAAGAAGAAGAAGAGATCCAGCATCCAACTCCATCCCAGTATCAGAATTAAATAAAAACA
ACATTATATTTAGTTAAATAGTTAGATATTATAGAATCACTGGGTCACTGGTTCAGCTTAATTTTACTATTTGTCTATTCTTA
GTATTCACTTACTTCTAATAATTTTGAATGTAACTCTTGCATAAATAATAGTTTAGTTATTACCAACTGTTAATTACACTAG
TTCATTTAGTTTTCTATTTATGTTTTATTACGAATGTTTTAACTATGTTTAAACTTTAACTTTGCTACGTTGTTATATAGCCGT
GGCTATGTACAATATACGAATATAAAAGTGCTGAACTGCATTTTCGAAACAGTCAACGGTTAACAATAATGTATGCGTCAT
ACCATCCATTTCCAGCGATTTTTCGATATTTTTTAATTCAGACCGGATTTGAACCTCAGACGAACAAGACGCTTTACAAATT
TTTATAATAAAGACTCGTGTTAAATAAAATAATTTTCTTTTTAAAAAGTCTCATCTGACCTACCCCGTAACAGTACCTATTG
AATACAAAAAGTTAACATGTTGAGTATCAGTAAGTTTAGTGCATTCAACATTTGTGTTTTGAAAGAAGTGGAAGAAAAAA
TGAAGTTATAATACACATAATTTAATGTATTTTTATATTTACAGAAAATGTTGTATTTCTAAGTGGTAAATTTTTTTTAGTCC
TATTGGACGGTCTGCACATCCCTCTCGAGCCCAAATAGAAACTCTATTAGCCTTTCTGGAGGGAAATCCCAGCCTGGCTAA
GGAGTTTTCCAAAGTACCTAGTGCTCGAGATGCGACAAGAAAGAAATGGGAGAGATTGACGATGACGTTGAATAGTATAG
GAGGAAGTGTGAAATCCTGGAAACAGTGGACAAAGGGAGAACAATCTACACCTCAGAATGTCCAGTTTCCTTCAACACAG
GATACGCAGATAGTTGATATTCCAGAGGCCTCTACAACTAATATCTTGGAAGCAACAAATATTAATATTCCCATATCATTG
TATTCCGAGGCGAATCCAGTTTGTGTTGCTGGAGCGTCATATTTAAGTGATAGACCAAGCCTTGTTATTTCTGTATCAGTGA
CTCGAGTAACAGCCGATGTTGATATCCTGCCACCCTCGGCCCCACAACCTCAAGATCTCCAACACGCCCCCACAACTTCAA
GAGTTCAACTCGCCAGTTCGCCGCACGCAATCACCACGACGAACAACTCCACGCCGTAGGCGGCGATTGATACCTACACC
TGCGTCTCCACCTTCTCAGAGGCGGTCGCAGCTTACCAGCCTCACTGAGCGCTTCCTGAGAGTGGAGGAGCAGCGATTAGA
ATTAGATAGCAGAAGTGTAGCTGTATCAGAAAATATGGCTAGACTAATGAGGGAACTCTTGCAGCGAGACCAAGATAGGA
GTCATGAGGTTAGCCAGAGATTGCTGGCTGTAGGGGATGGTTTAAAATTGTTAACCGAAGCATTAAAAAATAAAATGTTG
AGAAAAACAAGGTCTTTTATTTTTATTGTTTTGTGACAAAATGTTTGTTATAGTAAATATATATATTTACTCCCTAAGAGAA
CGCCATAACTGATTTACTAGCATATCTCTGCTTGATTGACCCATTGCCAACTCGCTCTGAGAGTGGGTCGATGGCAACGAC
CCGCTGAGACGCGTGCAGTTGCACGAAAATTTGCTTCTTCCTCCTCATCATGACTATCTAGAGTCGGTGCAGGTAAACCAG
CTCTATTGTAAATGTTGTGCAGGACACAACATGCTATCACTATTTTTCCGGCTGTATCAGGATGGTAACCGTGGTGCACAA
GCAAACATCTAAAACGTCCCTTAAGTCTACCAATAGTTCGTTCGACTGTGTTACGTGTTGTTGCTTATAGTTTATTGTAATG
TTCTTCAGGTGTACCCTCAACAGCGTTAGGAATAGGAGTCATCAAATAAGACCGTTGAGCATATCCTGGATCTCCTACAAC
AAACATTAAAACCGGTTAGGTCAAATCTTTATCATTAGACAGAAGTTAGAATCAACTTTATTTATTTGATCTCACCAAGTA
AATATACAGTTTTACCAGCACCTACCAATTCTTCTAAATGGTTTTTTATATCACTATTATCAAAGATAAAACTGTCGTGTGT
GGCACCACCATAAGTAGCGTCTACATATTGAGATCCGCATCTGTGTACTGAAAGATAAAAATCTGCATATAATAGACATG
ATGACAACAAGTAAATTTCATTGAAATTATTTGTGTGTATATTATTACAAAATTCCTAATTCTAAAACTCTGATTCTTTTCA
TAATTAAAAGCAAGATGAATATAATAATCGATAAAATAAAATCGCTAAATACGGCAATCCGCCATGAAATGTTGAACGCC
AATCAGAGCGCATGACGTCACGTTAGAGTTCTTTCTCTTTGTTTACATTTGAATTACTTAAGATAATACACACTCGCTCGCT
TTTTTATTAAATACGCCGTTATTGTTTTGTTATTAACTTCAATTTCTTTTGTGTAAATTGATTACGCTAAATTGAGTCATGGA
ACAAGGATTTACAAAGGCTTCCAGCGCAAATTTACCAAGAATCGACTTGATAATGCTAGGAACGTTTCTGGCATCAAACA

		AAGACTTTTGTTCAGCAGAATTTAGAAATGTTAAAACTTCCATGTCTGCTAGGGCATCTTATGGTGATGATGCTTTAAGCT<br>ATGTTCAGGTCAAACGTGAAGGAAATTTGTGCACAATAAAAGCTAAAATCTGCCCAGAACATAAAGTGCATGCCAAGTTG<br>TATGGAGTGTCTTTGGTAGTCGACGAACAAGAAGAAACAGTTAAGTCAGTAGAATGTCATGATTGTGTGGCTTCTCAAGG<br>AGGCTGCAAACATGCCATGGCATTCTTAATGTGGGTTCATCGCAGAAGTGAAGATCCATCTTGCACATCCATAGAATGCTA<br>TTGGAGGAAATCCAAACTTTCTAGAGTTGGGAGTACCCTTAAATACATAACTGCTAAGGAATTATCTAAGGGTAGCCCTAG<br>CTTACCATATGATACCGAAGTATTCGAAAAGTTTGTAGAAGAAAGGAAGAGAAGAAGGTTAACCGACTGTGAATTAATGA<br>AATACCAAAAGGACTATGTTTTTGATAGACTTCTAAGTCTATCAATGGACAATTTAGTGTTGAAATATAAAGAAAACTGTT<br>GTGATAAATTTTTGGATAGTGTGATATTTTCTGATGATGACATCAACAATGTAGAAGAAGAAACAAGGGAGCAACATAAA<br>AGTAAGCTTTGGTATGAATTAAGGTATGGCAGAGTGACCGCTTCAAGAGCATATGAATTCAGTAGGTGTAAAACCAGTGA<br>CGGCACTCTGATGGCAGTAATAATGGGTGGAAAAATTCCAGACACAACAGCTATGAAACGTGGTAGAATTTTGGAGGATG<br>AAGTGAGGAAAACTGTAAGTGCCAACCTGGAAAAGAAAATTAGAAAATGTGGGTTATTCTTATCTAAAAATTATCCCATG<br>CTAGCGGGTTCACCTGATGGGATCTGTGAAAAAAGTATAATAGAAATTAAGTGTCCTATGAGTCAAAAAACAGTTAAAGT<br>ATATGTAAAGAATGGGAAGCCAACCCAAAAGTTTTATGTGCAAATGCAACTTCAAATGTTACTAACAGGACATAAAAAAG<br>GCTACTTCTGTGTAGCTGACTGCAACTACAGTGCCAATAAGAAAATAGATATGGAAATTATGTATGATGAAAAATATGTAT<br>CAGATTTTCTTAAGGTAATAGTCCCTTTGTGGAAGGATAATGTATATCCAGTG
MSTRG.4<br>195	Uncharacterized<br>protein<br>LOC106138234	AATGTACCGTAGAAAGGGGCTATTTGCAAGTCGTGGGGTTAAATGGAATTTCTTAAATAAAAGATTTGATTATCTACGTAT<br>ATTTTTTAGTTTAGCAATGAGTAGAATATTTGTTGTCACTTGGTAACACAACGACTATTGTCAATTTTTTTTTAATTTTTGAG<br>GTTATAGTTAAGCATTACACGAGGCAGCAAGTGTTTAAAAAAAAAAAGAATTTTCGCTCTTTTTCGTAAGTATTTAACTTT<br>CGTCTGACGCATGAAACATAGAAAGGGAAAATCAAGAAAAAATCATGGTGCGAACATATCAAAGGAAATATACCAACTC<br>ACGTTGCGGTAAAACACATCCACCATATCTAAGTTATTCTAAAGAAAGTTTAGAAAACGTAATAAAAGCCATTCAAAATG<br>GTATAATGAAATTAAAAGAGGTTTCCAAAAATTACAATATTCCAATGAGCACTTTGAGTAGGAAATCTAGAAACAAAAAT<br>TGCACGCAAGAAAAAGCGGGGCACCCAACTCTATTTTCAATAGAGGAAGAGAAGACTTTTGTAGAATATATACAATTACT<br>GAGCCATTGGGGATTTCCTTTAGATACAACTGACTTACGAGTTTTGGTACTCGACTGTGATGATAAAAACAGTAAATATTA<br>CATTGTATTTATCGAAAATATTAAATTTGATGGACAAAAATATAATTATAGTGTTAATTTTTACAAAACTATTAAAAA
MSTRG.4<br>2	T-complex<br>protein 1 subunit<br>gamma	GCATTGTCTTGCTTGACTGCCCACTTGAGTATAAGAAAGGAGAGAGTCAAACCAACATCGAGATACTTGGTGAACAGGAC<br>TTTACAAAACTACTGCAATTGGAGGAGGAGCATGTGCAGCGTCAATGCGAGGACATCATTGCCCTCAAACCTGATGTTGT<br>ATTTACAGAAAAAGGCATATCAGACTTGGCACAGCATTATCTTGTCAAAGCTGGCATTACTGCTATAAGAAGATTACGCA<br>AAACAGACAACAATCGGTTGGCGCGCGCTTGTGGCGCCACCATCGTCAACCGCACCGAGGAGCTGAAGGAGAGCGACGT<br>CGGAACTGGCGCCGGCAAGTTCGAGATCAAGAAGATCGGTGATGAGTACTTCACATTTGTTACCGAGTGCAAGAATCCCA<br>AGGTAGGATTGTTAT
MSTRG.4<br>201	Uncharacterized<br>protein<br>LOC101745670<br>isoform X2	GTTTCCAACGAGCCGCCTGCTGAAGAGGCGATGGCTAGACGCAATAAAGCCGACCAGTAAAATAGATACGAACTCCAAG<br>GTATGCAGCGCTCATTTCGGGAAGCATGACTACGAGAGGGTCCGCGGAAAGGTCAGGCTCAAAGGGAAGGTGGTGCCCTC<br>TATTTTCAACCACATCATTACACAAACGTCACCTGAACAGAATTTACCCGATTCCACACCTAAGACAGATGACCCGGATAA<br>AACTGTGACAGTTGCTGAAGGAACCGCTAAAGTGGCACAAGAGACTGAAGAAGTCATTCAGAATAATGGTAAACAGGTCT<br>GCAAGGAGACTACAGAAACGACCACAGAAGTTACACCTGATACTGTGGGAGTTGCTCAACATACTGCTAAAGTAACTCAA<br>GAGACTTCTGAGGTGTTACCTCAAAATACTTCTAAAGCAGTCTCTGAAGATACTTCTAAAGCGGTCACAGAAGATAATGTA<br>CAGAAAATAAGTAAAACTATACAAAATAAGAGTAAAGATGCCACAAAAGTTGCTGAGCCACCAATAGTGATGACAGATG<br>CGAACAAAGTCGTCGAAGGTGCAACAAAAGAAGTCAGTAAAGAAAATAGTTTAAAATTGACTCACTGTACTGCTACTAGC<br>AAAGCAGTCACTCAAGAAAATACTATAGTTATAAATAGTCCAAATAAATTAATGAGGGAACCAAGTAATAGAAGTACTTA

MSTRG.4208	Tripeptidyl-peptidase 2	CAATGTAGATAGAATCATAAACAATACGCCGTCAATAGATATCAACAAAGACGACATTGAGGACATATTGACGAATTATC<br>ACATAAAGCAAACGAAGCCGCTGCACAGGATGTTCGCGGAGTCGGAACGAGCGAGCGGCGGGCACGCGGCCGTCATAGA<br>GATCAGCGAGACGGAGGACAGGGCGCCCGTGTTCATCGAGGTGGCAGTGAAGGGCGACC<br>GGTGATGCACTACGGCGTATGGCACTGCTTCACCAACAAACATTGGGGGCGAGCGATCAAACTGCTCAACAAGATCCAAG<br>AGGAGCGGCCTTCAAAGGATGCGGAGGAACGCCTCGTCGAGGCGTACGAACAGTTGGGGTGGAATTACCTAGCGAAAAC<br>TACCCAGTTGTCGTTACCGATCAAGTACCCTTCGGCGTACCGACCCTTCTGAGGGTGTGTGCACGATCACTAATACATGCG<br>CGGTAAGCGCCTGCGCAAGCGGCGGCCATTTTGTCATAATTTCCCTTTCATGTAATTACGTAACACGTGTTTTTAGATTTT<br>TTTTATAGATGACATTGACAAGCCCTGTCTATCCCTTTCAGACCTGAACGATACGGGCGTGGGTTGTAATATTAAAAAATT<br>CTAAAACCGCGCGCAATTAATTTTTACGCACGAAGCTGAAATAACATGATAGTGAGATTTTAAACCAGCCAATAAGAAAT<br>CATTTTAGCGGTTAGGTGGTGCAGTAGTCTCGTGCGTGCCGGCAAGATGACTTGGCGCCAAAACACGAAAATATATTTAAT<br>CTGAAAGAAAAAAAATCTTGGGTCTGAAAGGGCTATGTTATCGGTTGACGCTGTTCAGGTACCAGTAGGTGATGATAGGT<br>GAGCTCAATTGGTCCATCCTGAAAGGGAGTTTTGTCCGCTTGACAACATGTAAGTCGTTCGCGCAATCCATTAACCGTTTC<br>GGATATCGACTCGTGATGTGGTAGTGACCGGATTTAGGCAATAGGCACGAATAACACTTCCTATCTCATTGTCAGACAACA<br>AAAAGGGTATTGAAACATGTTGTTTTGAACCTACAAATTGTTGGGTTCGAAATTTCTGGAACTTTTTTTTTTTATATAACTG<br>CCCCCTTATTCGATAACCATAAAGTTATTCAATAGATTGAATTTGTTTTGTCCTTTTTGTTTTTAGGTTAATTTTTATTTTGTG<br>ACAAAGTGACAAATCATGCTAATACCACAGTATAACGAATAACCAGTGGTTCAACGTCTTTGGCGTGTTACGAACGACTG<br>CATGGCAATAAGGGACGCGTCGCTTTGTCTAGACGTGTCCACTGGACGACTGGTAGGAAAAAAATTAAGTTTTTTAATCT<br>GTGGTTAAAAAAAATTTATTATAATTAGGAATTAGATGGATAATGTTTACGTACTGATACTTATTTTAAACATGTTAATAC<br>ATAATGGTAATGTGTAATCGATTTGTTTGTTTTGAACATGTATAGTTTGTCGTATGGCTTGTAGAGAACGCGGTACAGTGTT<br>CGTTGATCGAGATTATATAATAAAAATTTTTTGTATTTTATTAAAATTTCATATGAAATCGCCGACAATTCGGGAATAATTA<br>CTACACTAGTAACCGTAGTACTAGTATGGTGATTACTAATATAACAATTAGTAATTATTATATTAGTCATTACTAATTATTA<br>TATTACTGGTTACTAATTATTATATTACCGATTACTAATTATTATATTACTGATTACTAATTATTAATTACCATATTAGTAAT<br>CGCTAATTATTATATTAGTAATTACTGTATTAGTATAGTAATTTTTAATTATGATATAAATTATTACTGTATAATTTTTTTTT<br>CAAATTTTCTCTAAAGTCATCCATCCACAATTCGAGAGTAATTACTATATCGACAATTACTGTACTAGAATAGTAATTGTTA<br>CTTAAAATTAGTACTAATTAATAAGTAGTAATCACTATATTAGTACAGTAATTACCAGTATAGTATGCTGTTCTGGCACAG<br>TAATTACTATATTAGTATTAATTAGTCCTAGATACGATTCTCGGCTGATCTCATATGCCTTCGTTGATACGGTCGCGACCAG<br>AGCATGGTCAGGTATAGAGTGTACAGCTTTGCCCTTGGTTTCGCGTTAACAAGATAATAGATACGATTCGACGCTATTATT<br>ACGTCTAAGTATAGCAATATTGTTGATATTACGTAAAACATCATGTTATAGTTATAATTAATACATTTATGACGCATTTTCT<br>GGTTGTTCATATTATATGAAATGAAACCAATATTAATATATGAGTTATTTACCTTATCTTACACGACTTAAGCATTTTTTTTT<br>AATTATTTTTATGACGTGGATTTATACAGGTGAAATAATAATCTTTATCCTTCATCCATTCTTTTCCTTTGTTTAAAATTCGA<br>ATGATAAGTATTCGAAAAAGAAAATATAAACCTGTTTTATACTTTAACATTTTTTTTTTTATTTTTATGAAATAGTTGTTCCC<br>ATGGTCTTATCTGTGTGAATTAAAAAAAAAATTAATGGTAAAACGTAACCTAGATATTAAATAAAGATATAGTCCGTCTGT<br>GAATTTAGTTCAAAACAATTTAAGCAGTCGTTTAAGCATGTTTGTATGACAAACATCCAAATGTCTTTACAATCATTTTCGT<br>CATCATTACATCAGACGTTACCTGTCCACCGCTGGACATAGGCCTCTCCCATACTGCCGTAAACACTGACCCCAAGTCACC<br>TGCATCCACTGACTATCTACTATGGTATTTGAAATCGTTGCTCCGTCTAGTTTCACACTGCGTTTACCTGTATGAAGTCGCC<br>ACTTTAAAAACTTTCTTCAAACTAAGCCTATTACAGTTCATTTACTTTTAATAAACATTACCATTACATTTACCAGAGGGAG<br>ACTTTGTACAAAAGTCGATTGCTTGGGTACTTCTGTCCCATTCGTGTTTCAACCGTGAAGCAGTTGTGTTTGCATGGCTATG<br>TTTCGGGTTGAAGGGTGGGATATGGCGTAAATTTACTGGGTACAGAGGAATAACACCCGAGTCCTCAGGAATGGCAACGC<br>ATGGGGGGTATCGTGGGCGAAACAGTATACTCTGTTATGTCCGTGGTACTGCTCATGTCTATAGGCGACGGTTACCACTTT

		ACATCAGGTGGGCCGTCAGCTTGTTTGCCATTCTAAGTTGTATAAAAAAAATACATAATTATTGTATATATATACAATAAT<br>AATACAAATAGATTCAAAGGTTATATTTCATTATGTAAATAAACGAATGATTCAACTATGCCACTGTAGTTATATATTTGTT<br>AGTGTTAGTTAATGGTAAATATTGTGATTTGATTTGCTTACATGTAAATCATGCCTTGATCGATAAAGTCGCCTTTACGTGC<br>CAGTTTTGATATAAATGAAAAACTAATTATGATATAATATGATAAAACTGAAATATGTACGTGTCCAACCATTGATCAAGT<br>AGAATACGACGCAAGTGTTAAGTGCGTAAGTACTTTATAGCTTACTAAAGTGTGATAACGTCGGAATTGTACGTCGATTTT<br>CAAGAACATTTGATTAGTATTAGATATTTGCAATTGATTATATAAAAAGTGTTCTGATTGGTAATTAAAATGTATTTATTAA<br>TTAAATTGACATTAATTGATATTTTCTACTTTGGGTTTGTTGAAAGAGAAATCTTGTGTAGCGTTAACCAGAATTTATTTTA<br>ATAATCTATTAGTTTGCTATCAGAGCATTTTTTTTAATAGCAAATGCAATGAAATGAAATTGATAAAATAATAAAAACAA<br>ATGAGAGATATTGAAAAATTTTTCCACTTGTTCCTGTAAATTGACACATAATTCCGACGTTGTAACACTTAGGTACGAACG<br>CACTTAACTATATATCAACTTTTGACACATAATTCCGACGTTGTAACACTTCGGTTCAAACGCACTTAACTATGCGTGTATA<br>CCAAATTATAATATACGCATTGTAAGAGCATTCGCCGTGATTGATGCTTCCTTATAGAGAAATATGCAATGTTATTTATGT<br>GAAAACACATTTCGATGGAGTCGAACGGTGACGGCTTCTGGCATTTTTGTTTTAAGCGCAAATTGTACGGATACAAAGGCA<br>TATGCGTGACGAATGAACGTAGGAGGCACTCAATTGGATTGTATATATTTTTTTTTATTTTACCCTATGGAATATCATCGG<br>TTATTCCGAGACCGATTTTCATTTATTTACATGAGAGGCATTCGCTGCTGTATTGTTGTTTTAAAGATAAATATATTTCACG<br>CGAAGTACATATTGCATATGTAATAAATTTCATTTCAAATGCCACTTAATGCAAATATGCTGGAAGGACGCAAATGTATCG<br>ACCGCCCCCTTGTTTTCGAGTTTGTACTTTTCTAGTTAAAAATGGATATGAAATTTCAAATTTCTTTTGTTTCATGTATCAAT<br>ATTGCCATGATACATTTGTGAATATGGAATATGCGATTGTATATGTATAAGGGGGAATTTTATGAAGTCACTATGTTGTCG<br>TTTCCGACAATTGCTTCGTCTTTATCCGGATGTCTTCGTAAGGGGGCGTACGACGCTTTGCGTCGCAGCGTCACGTGTAAAT<br>TTCCCATTATACGTATATATAGACATGGTTAAAAATGATAACATAAGCTCTATCAATAATATTGTCGTGAATTATGTTTTTT<br>TATTAGTATTCTAGCTACAGGGTGCTGTTCTGTTGTGTGCTCAAAAGTAATAAGTACCTAAGTAATCTACGGACTAATTGTT<br>CCATACGTTCCACTTTATTTCGACAGTGGAATGGTAACCTTATTACTTTTACAGTAAGGTCGTAATTCCGTTTCCCAAAAA<br>TGAGATATAGGTAGTATTTTCTTTTGTAACAGTTTATTTTGGATTTTAGGTTTTGTTTTTCGCATTTTTTATATCTTGTTGCAG<br>TCAATGCTATTTAAGACGTTTTGAATGTACGTATGTTACGTTATATCCTTGAACCTACCCGTAATAATGGCGAAAAAATTTC<br>TTTCAGTGATGTCATACATTTCTATAAAAAAAATTATCAATTTTGTAATAATCAAAATACTTTTTATATTCTCTAGGTTGAT<br>GTGGATGATATTCCTTTTAGCAATAAATCCTATATCCATAAAAAAAATAATTATTATAAAAAAAAAATGCTGGCAAAATAT<br>TTTTATTAATTTTATATAAATTTATTGTTATATTACGCTTAATAATATATAATTATATAAATCAATATTTCGACAATTTATGA<br>AATAATTGGTCCGTAGGTAAAAGTTTTATTTTTAAATTTGCAGATTACTGAATATTGTAGTTCTGTGATGTCGCTTCTATAT<br>CATTATGTGATTATATTAAATAGTATGTAATTTTAAACAACTGCCGCATATGTATTAGTATATTATATTAAAAATGTAATTA<br>AATAAATAGGTATTTATTTAAATTAATCAAAAGGAATTTTTAAAATGCGATAACAAGCTATCACAGAATTATTTAATTCGG<br>TTTATATTAAGTATATATATATATTTTTTATTTTATAGTGTAATGCAATGTAAACCTCATTAGTGTTATTGACTAAATGAAATG<br>TCAAATGTGAATTATGTAATTGTCATTTTCGTAATATATCGTACTACGATTATGTATAATCGTACAATATCGTAATATCGTA<br>CCATTGTACCGTCACGGTATAATAGGAGAGCAGAATGTATGCAAATGTATAGAGGCGTGTCTAAATTTCCATGCACGTCCA<br>ACGTATCGTTGCGGGGATTAAAAACGTTTGTTAAAATTGCATATATTTTGCTCTCCTATTATATCGTTACGGTAAAATTGTA<br>CGATATCGTATCATAGTATGGTATATTATAAAAACGACCAGTGGGAATAAATGCAACGAGTTTACCAACTGTGGAATATTT<br>TAATAAAGAGACGGATGTTACATTTGTACATGATAATAAATGCTTTTTTTTCTCTAAAATTACTCGCCTTATGCGGACAGT<br>AGTCCTGATCATTGAGAGCAAGGGATATTTTAAAAGTTACTATATGGGAACATGGTATATACCATACGTAGTATTAGCTCT<br>AGGGTTTTTAAT
MSTRG.4212	Protein phosphatase 1A-	GCAAGCGTTACTGGATAAAATATACAGGGAGTTCTACCCGGAGCACGCGGACAGCTCGGAGACGTTCGACTGTCCAGAAC<br>TTACAGACGCTTTTGGAGCCAACTGAATGTCGTGCATTGCTGGGCCGAATCGAACACTATTGTGAGCCGGTAAGGATCAA

like	ATTGAAATGTCCATGAACTGTCACTGTGACATTAGATTCATTGTGTTGCCACCTAAGTATATTTTTTGTCAACGGCAATTTT<br>ACCAAATCAATCATATCATAGTGTGTTTAAATTGTTAATAAATCGTTTATTAAATATGTTTAACTTTAAATTTGGAGAGATA<br>TAAAGAGTTTCTCTTCATATTAAAAAAAAAAAAAATATAAACATGAACATCTTTAAAAATGGAAAATCAAAGTTCACACAC<br>GAAAACATATTTTCGGCCAGTAATAGTGTACGATTTGGTTTTATTTAGCAGAATATTTTTTTTGTAGTACAAATCGAATGGA<br>ATTTCAATTACATTACTTTAAATTGTTATGAAAGATGGCAACACTGTCTCTATTTTTTGTACATAAAAATAGTCTGGAAACC<br>ATAAGTAATGTAGTTGTAATTTTATTTTGATAACAATTGGATGCAACTACCACTTCGTCTATGTTGATTGTCATTAATTTTA<br>AATCATCGATGGCTTTGTACAAAAATCTGCTATAAACGTGACCATAAAATAGTCTATAATTGTAGTGTGTGTCTATGGTAG<br>ACCATAGAGATGCAGTAATTACAATACGGCCTTGTCCCCTGTGCTAAGAAGCGCATTGTCGCGATTTTAATCAGGGGAGCC<br>GTATGTACCGTACTGTTTTAGCTGCTGCTGTGGTCGAGTGAGGTTTCCTTTGAGTTATAGACAATTTTTTTTTTTCACCGTTA<br>GAGTAAAATGTGTCAGGTGTTCCACAGCGGGATTGTTTGTTTTTACCAGTTTGTAAATCGTAATTGTAAATGTTGATGTGTG<br>GCACAAATCTCCATTTCCTTTCTCCTTGTAGAAACTAACCAATCCTGTCATTAAAATAGTATTGATATAAAGTTACATTTGT<br>ATAGAGTTAAACGGGTTTATCATTTGGTATAACAGTCTCCATTTTGTTTTGTAGTTTAGATTATAATTTTTCGTGTAATGTCA<br>TAATATTAGATGTGTAATATAGAGATTGGAGTAACATGTAATATTAATAAAATAAAATCCGCTTCATAATTTATAGTTTTTT<br>CAAACCTATTTGTTTCGTACTGTGTAAATTCTATAGTGTAGGCTTCAAAATGGCCAGTTTGCAGTTTATGGAGTTATTATTT<br>TATCATTGATAAGGAAAAAATCCAAGATTCATATACGGATTCAAATGCTACATCTTAAGACTATTTTGAATTAAAATTTAA<br>ACAGTAAGACGTGCATCATGTCGTCGCTTACTTACTGCATAATTTGATGGAATACGCTCTGTTTTCAATCCCGTCTTTTTAT<br>TTTCTAATCGCACATCTTGGGTTTTTTGTATATCAATATATTTTACATAAAAAATATTGTGTTCTTTAAATGTTCTCTTTGTA<br>TTTTTTAAGCCTGAGATAGCTGCTTGGGGTTTCACAAGTTCTCGACTTAGAAATAAATATTTAAAACTGGCTATACTTGTGT<br>GAAATGAAATCAACTGTTATACTTTAAGGCCGCTTACATCTGTACCTGTAAGTTTTATTCACGTAACAAATTTGTGATAAAT<br>ATAAAATGTAAACACATGCGAGTGACTTATCTTTAATTGATCTGTTATGTAATCATGCGGCAGAACTTACGATAGTTACGT<br>AACATGCATAAAAACTATAGATATAAAGGCGGTCTAAGGTTAGTTTTTTTAATTCTTCAAAATTAAATAAATCTTTTACAG<br>TCGTAAGTGATTTTATGACATAATATATAACTGTCTTGACCATAAACACTTACCTCATCCATGCATACATATCTGTCACGCC<br>TGTGCCCCATTAGGGTTGACAGAAACAATGGAACGCCAAATGCTTCGATTCAAACAAACCTTCGCTTCTTGCACATTCATC<br>AATCTTTTCATACATGCTCGCCGGTTACGGGTACTTTTAATTTGGCTCTTCTTCAGCACGTCGCCTATTTGGTCGACACACG<br>TCCGTCTAGGGCGGCCCCTACCGACATCTCTTTATTCTTGCTATAAATAAATCTCTTTCGTAATTCTTCTTTCAATCCATCCT<br>CTCCAAATGGCCAAACCAACGCAATACTTCGATTTTTGTCACTACATCGCCTTTCAGTTTACACTTCTCTCTAATCACACAA<br>TTTCTAACTCTATCTTTTAATGTAATACCACACATACTTCTCAGAGCTCTCATCTCTATAGCATTCGCTTCTATACATGGGTG<br>TTGGTGTCAGTACTCCCTGAATTATGGCAAGGCGAGCTTTGCTCGATAATACATTTACCCCCTTATTCGTAAATACATCAAA<br>CCTGTCATAAACCTATTTTAGCTATTGAACTGTTTTGTTCCTTTATTCTATGCTTAATTCCAAATTTAAAAGTGACAAAACA<br>GTATAATAAAGTAGATTTGTAGAAGGATTTAGTTTGCATGAACAAGGGGTGAAAATTAAAAAAAAATATATTTGTCTATG<br>AGTTATAAAATTTTAAAACTCTTATCAAAAAAGTTCAATAGTTACGCCGGTAAATAATTATATACTATTAATTGTCTAATTG<br>AAATTTCACACAAGTAAATCCTGTTTTAATATTTTATTTCTAAGTCTCGGATATTGAATATTATCGCTGAATATAGTACATT<br>ACAGAGTCCTCTATGAATATTACTAGTATTTTTTTGTACATACAATATTTTTATGCTATCAAGCAATAGTGACTATGAAAAT<br>TGACAGTCATAAATATTAACACATTTTTTTTATTCATATTTTAGTTTAAACATTCGTCTTAATTGGGAAATTGGCTATTTAGT<br>GGGATTAACAATTATTTCTTCTATTTTTGTGTTCTATATAGATAAGCTTTGTTTTCAAAATTGAATGATGCGCTGGAGAAAT<br>CGATTATTGCAATTTATTTTTAAAACTTAATTTTTTTTTCATATTAGAAATATTAATCTATGTATGTCACTTCGGTACTCGTT<br>TTAACTATAGACATTAACAGTTGGAAATAAACTATTTGATATTAAAACATTAGGATGAAACTGCAATAATATGGAATTTAG<br>TACTTTCAAGGGCGTGGCCAGAACTTTTTCAAGGCGGGGGAAGAGGTTAGGCAATGGAAATTGATAATGTGCTAGTTTAG<br>TCTGAAAAAAATCTTTATTAATGCAAAACATATGTGTTTGAGCGAAAACACGTGATGATAATTATTGTATAAATGATTTTA

		AAAGTATATAACTATATCTATACCTGGCTAACGTCCTTGTGTAGTTTAATTTTAATTCTCCACGTTTCTTTTAAATCTTAACA<br>AATTTTCTATTTTTTTTTTTATTCTATATAACTTAAAGTGCCCATGTTGCAAGACCACAAATTCCTATTCGAAATAAATACC<br>AAAAATGATTATACATAAACGTTTTATTTATTTCTCTTCTAAAAAGAGAGATAATTAAAAGAGAATAAATGATGGTAAATA<br>CTTTTGTACCTGTTTGAATTCATGTTAGTGTAACTTTGAAAAATAATGTATACATAGATGCATATAGCCTCATATGGGGTTA<br>TTACAGTGTCATACATATACAATCATATATCTTATTATATATTAAAAGGAGTATCATATATTCAAAATATTCCTTCTAATGT<br>TTTACGATCCTACAAATGTGATACTGTAATAACCCCAAAACCCCGTTGATATATGGGGAAGTGGGTACATAAAAATCCTGA<br>CCTCTAATGGAGCGAATCAGTGGGTGCAAAAGGGATTCAGAGCAATGGTTCTGTAACTATAGTCTGTAACTATGCTAACA<br>CCTTTATTAAAGGTACCAGTACATGCTCATGTGCCACGCACGGGTGCTCGAGTTTGCTCAAATACATTGCAAAACCTTAGA<br>GGTTTACACTCGGAATATACATCGCTCTATTTATGAAGCCATTGTGAATAAATGTAGAGACATTGATTTATCTGAAATTAA<br>AAAGTTGTACCGCTTAGAATTAATTTATTGAGGTAACTCGGTTTTTATCCAGATGTCTTAGACAGTAATCAAAGATTTTCTT<br>TAGTCTACGCATGAGGAAAAAAAAAACTCTGAACATTGCAGCCTCGTTCTTGTTCATGGAGACAGTATCATATCTATGAATA<br>CTATAAATAATATCCTTTGCTACTAATTGGACAGTGTAATATACTCCTTATTTGTATCAATATATGTTCACAAACACGTAAT<br>AATAGCATTTATTCACTTTTATGCTTCTAATGAGTATGTACTGTTTACCTTTATATACATATAGTAGATTTATCGTTTAAAAT<br>ATATATAAATATACTAATATAACCTTATAAAACACTGATAATCCCATATTAAAGTGTCTACGTACATGTTGTTGTCAAATA<br>ATATGCTATGGTTTGGAATTCGAAGGCAATTTACTGTAAGACTAGCAATATTTTCATCTATCGATGAGGCAAATGTTGCTA<br>ATGAAAAACAAAATTAAAATTGGAACAGTTTTGTTTTTTATTATAATTCCTTTCTGGCCAGTTTAGTCGTACTAATGACTTG<br>ATGGAATAACAAAATAATAATCTAAGTGAATTAAATATTTTTGAATAAAAAAATTTATATTTCTTTACCTATTACCGTGAA<br>TAACGCTTTAGACTACATCAGAAAAATTGTTTAATTTTTTTTTCAAATTTGGTAGATAACAAACGTCTTACAGGATGCGCCT<br>TCATCATAATATTTTGGTAAAATGCAATCTTAACACCCTACAACACCATTGTGTTTAAAAATATTTCTTTTTTACCGATTGC<br>AACACATGTTATTTTTTTAATACGTTATCTTGTGATTCAGACATCGGTTGTAATAGTTGAGTTTGTAAAAAGGGATTCTCAT<br>GTCATTTATTAATAATTTAGACTTTTTTACACATTGAAATTCACATGGAAGTCACGTTATAGATTCCATGTGACCCGTGGAT<br>ATTATTATGGAGGTACTATTTAGGATATGTATAAGAAAAATCGAGATAATAGTTTTTGTCAGTCCTATCTCGGTTTTATTAC<br>TGTAAAGTTTAAATGTCATATTACTGTATTTAAATTACACGGTTTTAAGTATATAAAGGTTTCGGTGACTGTGCAGTAAATA<br>TTAACAATATGTATACTATATGAACAAGAAGCTTATAATCAGGAGATTGGGCTATGAATACGACATTGTTTTAAACTTGTA<br>TGACCATCTACTGTCTCGCTTATGATACCTTATTTGGAAAGAGTAGCTAATAGAAAAAGGTTTGTCTATTCCACTTATTCTC<br>TGAACAAAAATTTCGAGACAAATATATTGCATGTCAATTTTTTAGCGATCAATTTCGTGTTCATTGCAATGTCCGTTTATAA<br>GCCTCTTGGTAAACTAACATAAAGAGGAGATATGTACCTGTAACAGAATATTTCCAAATCTCTCTTCCCTTTTCAACCCATT<br>GTCGTGCATATACCATTGTATGAATATTTATTAATATTTTTAATATAATATGTACTCTCCTACAAGAATTCTACAATATATA<br>TAATAGTGCTATAATATAGGTTTATTTGTAATTTAGATAGTTGTGTTGGAATTGAACCAAATATTACCGGCATTGGGTTTTG<br>AATTGGAATTTTGTCCATCCTTGGCGATAAGTAGGTTGTTTTTTTTTTAATTATTATTATTTAGCTGTCAATCGGCGTTGTCT<br>TACGAAGTGTGTCTCGTTATTATCTAGTAGACGCTTAATGAACAATAAATACCTGTACCTTACCAATGGTTATTTTATTTTT<br>GTTTATGCCCTTCATTTTTTTTTTTGTAATGGACCTCGTAGACTGTCAAGTGGCTGAATCAAGTATGTATATTTTGTATGACA<br>AATATTTAACACTTACAAAGCATTATACAAAGTAATATAGGTGTTTTACTCGTGTAGTTCTAAGGATGTTCTACTCTGTCAA<br>ATTTAATACTTTTAATACATCATGTAAGTTTGGCAAATTTAGCAGATTTTTTTTTGGCATTGTAGCCTGCTGGCAAAGACA<br>AAGAGACAAAGTACTAAAACATGATCCGTATACATATATGTATGAGATAATTCATAACTTTAAAAAATTACAAGTTCTAA<br>ATTTAAAATAATACCAGATACAAGAGAGAACTAAAAGAAATATTAACAATTTTAAACTGTAGGTATTAGAGTAGAAATGA<br>CAATTCCCGTCTTCCTTCATCAATATTTTGGGCTGTAACTGTACTTTTTTAGTCGTTGAAATAATTGAGGTTATTGACAAAG<br>TCCTGAGCAGCCACCTGAGCCTCGAAGATGGACCAATTCAGAGCAT
MSTRG.4	Nuclear pore	CAATTTCTCGACGTGACTATTGGATATTGGCGCCATTTTATCTGTCAAGTTGTGAAGTGTCTAACGAAAGTTGCAGTCTAA

227	complex protein Nup88	ACAAAACAAGTTGAAATTTTAATAAAATTCGCAATGGATTCTCGTTTATATGAAACTAAATTGGCGAACCATAAAATATTT<br>CATGAAATAAAAGACGGTCTAAAAGAGATTCCCAATGGAATGTTGTGTAATTTATTAGAATTAAAGGACGATGTGTTATA<br>CGCTTGGAATGCTGCGAAAAATTGTTTATTATGTGTGAATCTGAAGCAATTGGAGGAACACGGAGACGAAACACAATACC<br>AGGACCGAGACATAGTTACATC
MSTRG.4238	Uncharacterized protein LOC106129134	GAATTTTTCATTCCATAAAAAAATATATGAATTTTTCGAAGTGGGTTTTGAAACTGGAATTATTTCATTACTTGGCTCCATT<br>TGAAGTAATTCGAAGTCTGTCACTTAATTTTTATATTATTTTATGTTTATCGACAACAAAAATATATATCATAGCATCAACG<br>TCTGGTGAAAATTCTCGCTTACGTCACCGCGTAATGTACCGGTAATGAGAAATTATGTGGTAATTTTTGAATATTGATAAG<br>CACGTGGCTGTGGGTTATGACAAATCTGCTCAAGCGCGCGAGTATACAAAGCGTATGCGGAAAAGAGTCGCAGAAGCTTT<br>GAATTCGTTGGGAGAAGGAGCTTTTTAAAGACTGGAAGGATGGCCAAATGTAAATATTGCAAAACAATAAATAACGAATC<br>ATAATCAACTTGTATTGTAATAAAAATATATTTACAGTATTGGTTGGATTATAAAGCAAAACTAAAAAGAAGAGTTGCTGC<br>AGTACGTCTGTGTCATAACTGTACTGGAG
MSTRG.424	PiggyBac transposable element-derived protein 3-like	CAATCACCTTTTTTTCAGTCGTCTGTATAGTTTGTATCGTCTTTTGTTTTTTCTCTAACTGTAAAATTCTAGTGTTTACATGT<br>GGAAACAAGAATTAAAACTTTTATATTTTCATTCATTTCGCCCTTGTTTCTTTGTTAATGTCATGATCGTCAGATAAATTCA<br>AAATATGTAAATTAATGTAATCGGATCTGCTTTGTTGTGTAATGTTCCCCCAAGCGAAATAAATTCAACAAAATGGTGGCT<br>AAAATTATTTTATTTTGTCTTTGAAATATAGTCGTGTTGTAATAGTAAGGTTAAATTGTTTGTGGTTTTTAAATTGTCTTTCG<br>ACATATTCAGTGATTGGTTCAGTCAAAACTAGTATTGTGTTTGAAATAATGTCAACATTTAATTTATATTGTTCATCTGCCT<br>TCTGGCAAGCCTTCCTACGACTGGACAATGTGAACATTTTAAAATATACTTAGGTTGAAAATTAAGTTAAAGCTAATCGAA<br>ACCAATGATGAAAGGCAAATAGCGATGCGAATATAATTGTCCTAGATCGTCTGAAAGTCGATGGAAAATTAAAGTAATAT<br>AAATATTGTCAACTATAAGAAAATATTTTACATTCCAGCTTTTGGTTTTATTTACTGGATAGCTCTGGAGCTGTTTCATAAC<br>TTCAAACAGGGTGACAGTGACTATCCCTTAGGGATAAAGAGAATTGTTTTTTTTTCTTTGTCTTGTCCTGTGTTGATATTTTT<br>TTTTATTTTTTTTTGCAATTATGTATGATATTTTTATAAATTTGATTTCACCTGTTGTTGTGTTGGGTAGGTATGATACAAGT<br>CTATTTCTAAATGTTGTAATCAAATGAATTTTGTGTGATTGTTGCACTGAGGTAGTTAAAGGTCACTAGTTCGAATGGATAT<br>GTGGACTGTAAATATGAATTGAATGAGTGTATCTAGATAATCCTCATCCAAATTAGTGAGTAATTGAAACTCCACCAACCT<br>TAAGGCTCACATTCACCACGGATGTATGAAGCGCTGCTGCTAAACACGCATGGCAATCCATTAGCACTACACACTTTTAAT<br>TCATTCCTTGTTTCTTTTGTGTTTGACATGATACAAATCAAGGGTTAATAATTACTCTATTATTATTATATTAGAACAAATG<br>AAGAATATTGCTTATAGTATTGTGTAATGTTATCCAATTATAAACAAATTAAGCAGCAAAAAGCTGAATGAACGGACGGT<br>GCAGAAAATTTTTTAGTAAGACTTGGTCGCCAACAAAGCGTCTCAAAGTTCGTTCACAGTGTGTACTGTGAACCGATGTCG<br>ACTGACTGAAACTGATGTCGTAGATGGCTTGAGATAAGCCGGCATGTTTATATAAACATATCTTGTTACTAGAGCCTTTCA<br>AAGGCTGCGCGGAAGGAACTTATGGGATGCTGTTAAACGGAAACCATGTACCACATTATAGGAGGGCTGTCGCAATTTCA<br>GCTTTGCATTATATGGAAACCGCATTCTGAGAGTTCTGCATTATAGGAGGGTTGGTTGTATTCAAATTAGAAAATATATAA<br>TCACATTGACCACAATTTAGTTTAAGAGGCAAATACCAGATTATTGGGATTGTCTTTATGGTTAGACCTTATAGTTCCACA<br>GGCATAAGTTACTTCTGTTTTCAATTTTTCTAATAAATAGATGTAAAAAAATTATCCAATAACTTTCTGTTTACCCTATTCTT<br>TTTCTGTTAAATCTAGGACAACTTTTTCACCCTGTGAATATCTGCAAATTATTCAGCTATTTCTTATTTTTTTCCTTGGTAAA<br>TTGTAAAGTTTTAATAAAACCGAATTGGTCAGAGAGGCAGCATAGCTTGTATCCTCTTTTTTCGATTTCATAGGGAGATAT<br>GGTTTCATTATCGACCTGTTTTTAATTTGGCCATAG
MSTRG.4246	Rotatin-like	AGACTGTCGCTTATGGTGGATGATTATCTCGCCTTCCCCGAATGCGCTATTAGCTGTGTATCCATACTAAATAGTGTCTGTT<br>TCGTTAGTCGCCACAATTTGATGAAGATACCAGATTTAGCGACACTATTGATTAGAGTGATCCTGGTGTTCCCGGCCCACG<br>AGTCTGTGGTGGCGATGTCGGCGCAGGTGTTGTCGCTGACAGCGTGGGCGGGTTTCGCGCTACAGGAGCTGTCAGAACGG<br>CGCCGTGTGCCCGCGCTTCCCGCCGCGGTCTACCAGCGAACTGTGCTACCATTCGTCGCCAGCACTTACTGGCATACCAGT

		CCCAATGCAGAGCATTCTTCTATAG
MSTRG.4261	Facilitated trehalose transporter Tret1-like	GATAGTGCGTAGACACTGATTCTGTGTGAATAGTTCTTGAAACTTTTGTTTGAAATATGTGAATATATTTTGTGTACCCTTCTTACCTGGTATGGGTCTTAAAATTTACATTCGGCTACACGTCTTGCAAAATAAATACGCTTGATCCATTATTTATTTAAATAAGTGTTGATATCACAAAATTATCAGTGACGACTCAGTGTCACTCTTACTCTGTACAATAATGGAACAAATTTCATAGTAATTATTATTAAACGAATGGAAGAGAATAATTGTATAGTCAAGTAATTAAAACTTGTGGAAATGGAGCTTCGAAGATGCATAACACCTTTTATTATACAATGTTTCGTATCGGCAGGAGTTACTTTAAATATGTCTGGAATGGGACTGGTTATGGGATTCTCTACGGTTCTGCTCCCCCAATTACAGAAACCAGATTCAATAATACAAATAGATGAGTCATCTGGATCTTGGATAGGGAGTCAGTGGATGCAGGTGGTTCAGGACCGTTATGGCAGTCCATGGGGAAGGCCTATATTCAGCAGCGAACAGATAGAGACTGAAATAATCCCTGAAGGACTTGAAAACTGTGCTTAATGACC
MSTRG.4265	Regulator of G-protein signaling 7	AGGTGGATAAAAAACGCGATAAATTGGAGCGTAAAGTGCTGGACAGCCAGGAGAGGGCGTTCTGGGATGTTCACCGTCCAATGCCCGGGTGCGTTAACACAACCGAGCTGGACCCTCGGAAGCTGTCCCGCATCAACGCACTGAAGGCCAAGAGGCTGCGGCAGTGTCAAGAGCTGGTCCTGCAATACAACCTCAAGAACAATCCAATAGTGACCATCACGAATCCACAAGAGAGCGAAGAGGAAATACAGAAGAAGAATTCGGACAACACTGTCAAGGAGAACGGTG
MSTRG.4279	Heterogeneous nuclear ribonucleoprotein L	AAAAAAAACTTATACCAGTTGTTCATTTCCGATACTTCAGGTCATCGTCCGGTCTAATGGAGTTCCAGAATATATCGCAGTCCGTGATGGCGATCATGGCGTGCAACCACGCCACTATTCAACACCCTGGTGCGAAATTCCCATTCGTAATGAAGCTGTGCTTCTCGTCGTCGCGGCAGATCGGGCAGGCCAAGGGCCCGCAGAAGAGCTACGACCAGAATAAGCAGGAGAGCGGCCAACGGCAGCCGCAGAACAACGGCATGGCCGAACACGAGTACTAGTAAGATATCGACCCTATACTATAGTTGATAACGTGCAAATTCGAACACGAACAAGTGGTTATAAAAAAGACATGTAAATTGTGTTTTAGGCACGACGGACCAATGTTATGTCGGTTACGGCAGTGCTGCCAACCGCGAAAGTGGAATTCTCTGAATGTTACCAGCTCGAAAAATATTGTTGAACATAGTAGATTTTATTTATAGTTGTCCAATTTTCTTTTTTTTTTTTATTAGTCGAATTTGTAAATGTTTTCCAGCACAATATAATATAATAGCGACAATTATAATATACTAGTATTAATTTTACTATATTAAGCGGGAGTTTCGGCCTAAATTCTAATTCTGTTACACGATAATTATGTTGTTTACCGTATAGATTGTATATTTTATAATTTGTCTATTGAAAACTGCAGCTTTTACACAATTTATCACAAATAGACTTATTCTCTAGTCAAAATTACATAGCATAGGGTATACGCCAACATTTTTTTGAATAAGTATTCCAAAAAATAAAAATACAACACCACAAAATAGACTTTATTATAAAATACAAAGAATATTTTTCACCTCTTTGACAGGTGACATCATATCACACGATTGTTTTGACCAGTGTCTAAGAGCAAGGAAGTACATAGAAGGAATGATAAAATAAAAACTGTAAATCATATAGTTCCCTCC
MSTRG.4283	WD repeat and FYVE domain-containing protein 3 isoform X1	GTGTTGCCAGCACAAACAGTGTCAATAATATACCAGCTCGGACCACACTATGTGGGCACGTTACAAGCTCCAGTGCCTCCTGGACCTAACCAAGAGCCTTTAGCTCCTCTGGTTGCCGAAGAAAAAGTTGTATTCGGCATCAATGCCCGCGCTATGTCACAATTAACTCTTGCCAAAATCCGCAAAGTTTACAGCAAAAATGATAACAAAGCTATAGCAAAGCAACTGGGCATGTCCTCGCACGAGAATGCAACGCCAATAAGGATATTGCACAACTCCGCAGGACATCTGATGGGTCCGGCGAGATGTTTGGGCGGGACCGTAGTGGGTTATTTAGGTGTGAGAGTGTTCTGTTCCAAGCCTGCTGCAATCATGATTGACACAGTTGGCGGTTGTTCAGTATTATTAGGTTTAATTGCTATGGCTCAAGATGTTGAGTGTTTATACGCGGGAGTGAAAGCTCTAGTGTGCGTTGTTCGCTCGAATAAGTCCGCTCAAGCTGAGATGGACCGCCGGAAAGGTTATCAAACTCTCGCAATGCTGCTTAAAAGGAAAAAGCAGTTGCTCAATTCGCATATATTGCATCTCATCTTTGGGCTGGTTGGCACTGTTGACAGTCAAAAGGAAACTTCGTCTATACCTAATTTGACTGCTTTTCAGGACTTAATATGTGAATTAGATGTATGGCTAGGTGCACCAGGCGGATTAATAAAATCTCTACTAGAACATTTACTCGAGCTCGCAACAGAGACTGCGCATCGAACGCACAATCTTCGCACAATGAGGGACTTACAGCTAGTTTCCAAACTTCTTTATATTATTAACGACGTGAAAGTCGTCAGCACAAAGAATGTTCTAATTCAATTATTAGCAGCATTACTAGGGGGACAACCGCGACCAAACGATTTATTATGCCTTGGCCAATTCATGGCTTATACTCTGCCTCTTCCATCACAAACCGAAAAAGGTATTAATCCAAAAGAGAGTGATGATGAAAAAGAGAGTGAAGGTGAACAGATAATCTTGCGTAATAAGTGTTTTAATGTTTTACACGGCTTATTGTTTACCGCCCGT

MSTRG.4291	Uncharacterized protein LOC106141537	ATTTGGTCAGAATAATAACAAATCAGCAGTGTATCAGTTCGATTCCTTTAGTTTTGTTTATAAATACGAAAAAAATTACAA<br>AAATATAATGGAACGTATAAAATACATTTAAAACTTATTCTATGTCGATAAATGTTTTCAATAAATAAACATATAGTCACC<br>TAAAATGTCTAAAGACGATGAAAACTTTTCCCATCTGTGCGTATTTCCCACTGAAGTCCTACTTTACATTTTTACGTATTTA<br>CCTGCGAAGGATTTGACCAGGTGTCGCCAGGTGTGTCTGAGGTGGAAGCAGATAGTAGACAGCATACTCAAAAAAGATTT<br>ACTATGGCGAGATTTTTGCAAAAACGATTTCAATTCCATTTACAAAATTGCTAGACACAAGGGGCGCTTTGGTCTTCGTTG<br>GTGCAATATTTACCGCTCCCTGTCTCTGTGGCCCAAGTTAACGCTAGCTAAGGAGACTCGTGATGAGTTCGCGCCCGCTTC<br>ATGCTTCATGGACGAGATTCGAGACTTTGTTGTACTGAGGGATGGGATCATAGGTGTCCAGAAGCGGAGTTGTATTACTTA<br>TTATGATATAGAAACTTTAGAGCGTGACGAAAGAGGCCCCATCAGGGGGGACTATGCTAGATATGCTGAGAACGAGCACA<br>CTATTGTCATACTCGGGTATCATTTACACCTTTTCATCATTCGTAAATTTATAAAGAATCCTTTTTTCGAAACGGACATAAC<br>CTTTGATAATGTTAAAACATTCATACTCATTAATAAGGAAGTATATTTTGTTAGTATGGAGGATGAGATATTCGTATGTAA<br>CTTAGACGCAGATAGATTAACCAAGACGTTTATTAAAAAGACCGAAGATGGAGTCATGACATTCGGATACACAGACTGTC<br>TCCATATATTGACATTGCAGCGTAATATTTACAGTCTTGTCAACCAAGATATAGTTTTAACATGTACACTTCATGAGGACTC<br>TAATATACTTCATGTGCTTAACCAGCACAACCTTATAGACAGTATGGATTGGCGCGTCTATATTCAATGGATGTATTTACTG<br>AACCACACATTACCACGGGATCCTATCCATAATATAATACTTGTCAAATCATATGGTGATGTCTTTTTTGTCGGATCCAACT<br>GGGGTGTATTACGTATATACTACGCTCCGTACAAATCTGGACAACTAGATTTATTCAATTCGGAACCAGTGAAACAGTACA<br>ATTTTGTGGAGCGTCGCGACTGTCCAGTCCTTGCCATGAGTCCGATCCTTCAGGTCGATGTGCTGGAAGCAGAGGACGGTC<br>ACACAGTCATCGTTGCAATGCCGAAGAAGATAGCTGTGTTGAACTTCACACATAACTTCAAAAGGACTGCTTCGGTAGCC<br>ATGCTGCCATATTCGGATATTCAAAAAGTTAAGGCATTAAAAATTGAAGAGTCTACATAAGCTACAGCCTCCATTGTACCT<br>CGGTCGTTTATATTCATTTGCTTATTATATACGTAATTATTCATTATTTATCAATGTGAACAGTCACGGATCATCCGCTGAG<br>TACGAGATGTCAAATAGATAGATAGATAGATAGAGAAACTTTTTATTTGAGCCATTTATGACACACGTAAATTTACAAATG<br>AGTTTGTACAATCAAATTATTTACACTTTTCAAAATATACAACAAAAGATAAAATATGAAAATATGGTTTCGCCACGTGTG<br>TCGCAGTGACCCGAACAGGCTACAGCTCAGTGATGGTTTTGCTTAAAAGCAAAGCACTGATTTTCAGCTGCAGCCTGAAGT<br>TAGGCTACACGGACGCGTACAACAAACCCAGTAAACATAATAACATACTGCAACACAGTAAAATATATAAACATACAGTA<br>TGGTGTTGCCGATCTCCCGCCATCGGAATATTGGTACCTGGACGACCGAATAGACGCTTTTATTCCGCGGCGTTACGTCTA<br>CGTAACGACCGATCTTCCGTACTATGTGACGGAGTATTAATCGCATGTAGCTTGACGTATTGGCTCGTCGCACGCGGATGA<br>TCGGCGACCGAAGGTCGGCCTAACGTAGCGTTCGTCATTATATAAATATAGGCAAGCTTATTACCAATTTAAAATTGGACT<br>GTGAATACAAAACTGAAGCCAATAAATTTTAATTCAATGTGACCTTTCGTATTGTTTTTTTACCTTGCTCGCACTTGTAGTA<br>ATAGATAGAGAGACTAAGTAATATTTAATTCTTCTCTAGATAGCATCAGTTTTGTGTTTACAGATTAGATAGAGTACTTAA<br>CGTTTTAAACTTTTTGAGCATAAAATGTATTAAATTAAAAAAAAAAAAAAAAAGAAACTATAATTTGCAAATACCCATATTC<br>AAGGTGAAATTAAAGACAGCATCTAATGCTTATGTTTCAATTTCGCCTATATAGGATTATATAGCTACACATATATTACCTT<br>AAAGGTAATAATAATTAACCAGATAAATATTTTTTTTTATGTTATTGTTTATTTTTATGTTTCTGGTGCAATAAAAAGTTGA<br>ATAAACAATTAATTCAGTGCAGGAAAATTGTGTATTATATATAATTTAAAGAAGAAGTTATTTTATGTTTTAGTAAAATAA<br>TTCTAATATGTTTATTAAATTTATTAAAAATATAATTGTTGTATTTGTATAATAAATAATTTTTAATTAAAAATGGTACATT<br>GGGTACCTAAATTTCCAATATGGATTCCTTTCATAAAAATCTAATTTTATTGTCTTTCCTTTTGATTTAACAGTTTCCTCTTG<br>TCGGTGCAACTAATTCATGCGTTCTCTTCCAGCCAGCTCTATCCCAGGTCATATCTTTGACCTCCCTATACGACATGACACA<br>TAGTGTTTCCTTTATCTGTCCCATGTAGTTATTCCTCGGTCTTCCCCTTCTTCTTTTGCCTTCTACTATGTTCTTTAAAAAATT<br>GTTATGATGTGTAAGTTGGCCCAGCATTTTGCCATTTCTATTCCCTATTATCTATTTTAATTTTGTGGTATAAAAATAAAAA<br>CAAAAAAAGAAATCAACTGTGCAGCCGAGCTGTTACAAAAGCTACAAAAAAAAAAAAAAGAAATTTAAATAGCTACTTAC<br>ATTAATGTTTTAATTTTAATCGATGTTTTGTTAAGTACAAATGTTTGATTTGACAAATTGAAGAGTTATATATATTGTTATT

		ATTTACCATATCATTTATTATTTAAATATTGTAT
MSTRG.4292	Uncharacterized protein LOC106141537	CTTTGTGAAAAACTATACTGCCTACCTAAGTCTAATAAGTTCTTTTTTTGTTTTTATTTTTATACCACAAAATTAAAATAGAT<br>AATAGGGAATAGAAATGGCAAAATGCTGGGCCAACTTACACATCATAACAATTTTTTAAAGAACATAGTAGAAGGCAAAA<br>GAAGAAGGGGAAGACCGAGGAATAACTACATGGGACAGATAAAGGAAACACTATGTGTCATGTCGTATAGGGAGGTCAA<br>AGATATGACCTGGGATAGAGCTGGCTGGAAGAGAACGCATGAATTAGTTGCACCGACAAGAGGAAACTGTTAAATCAAA<br>AGGAAAGCACATCGACCTGAAGGATCGGACTCATGGCAAGGACTGGACAGTCGCGACGCTCCACAAAATTGTACTGTTTC<br>ACTGGTTCCGAATTGAATAAATCTAGTTGTCCAGATTTGTACGGAGCGTAGTATATACGTAATACACCCCAGTTGGATCCG<br>ACAAAAAAGACATCACCATATGATTTGACAAGTATTATATTATGGATAGGATCCCGTGGTAATGTGTGGTTCAGTAAATAC<br>ATCCATTGAATATAGACGCGCCAATCCATACTGTCTATAAGGTTGTGCTGG
MSTRG.4296	Protein groucho-like	ACAGAGGCGGTCGGTGTCACCGCACGAGCGGGAACAAAAGTACCGTCCCCGCTCGCCCGTCGAGCCCGAAGCGCTGGAC<br>GTGAAGCGCAGAAAAGAGGAGAAGGTACACAGTCATGACAGTGATGCTGAAAAAAGTGACCAAGATCTTGTTGTAGATG<br>TAGCCAACGAGGAGGAAAGAGGATCTCCAAGTGTTCGCACAGATGGTGGCGAAAGAACGGAAAACGGACCCAGGCCACC<br>GTCCAGATCGGGTTCCTCGTCAAGTCGATCCACACCCAAAAGTTCGAAAGACGAGAAGCCCGGCACGCCATCTGGTGCGA<br>AGCTAGGCCGCGCGCCCACGCCGACGGGTTCCGCACGGTACGGGTTCCCCTCGTATCCGTACAGAACGCCCGCGCCCGCC<br>GCCGCCTCCGCGCCAGCTCCAGCGTCCGCGCCCGCTTCCGCTCCGCACGGGTACGACTCGCATATGCGTACTAACGGCATC<br>GCTCCGCCCAAGTC
MSTRG.4306	Etoposide-induced protein 2.4 homolog	AGCGGGTGCGTCTTCTCAATATTCTTCCCCGTGTTCATTTTGAGCGGGAATGAGGCCACGCCGGTTATTGGAAGTGAGTAT<br>CCTCTACGGCTGTTCTCGCCCGTGGTGGCGATGTCCAACGGTTTGTTCCGGGTCGCACGACGCGGGGCGGCCGCCGTTAAC<br>CGGCGGTGATGCAGCCACTCCGCCGGTGATGCGGCTGCGCCCCCGGTGATGCAGCCGCAGCGCCGGTGATGAAGAGGCAC<br>CGGCGGTGATGCAGGCGCCGATGGTGCACTCGTTGCTACCAGCGCTCAAGGCGTGCAGTATGTTGTCGAAATAATAACAA<br>ATGGTGCGGTATGAACTGTGTTGAGTATGGTTCGGTCGAGTGCCGCTCGGCTAGCGGTGATGGATATATCAAATCATATTT<br>ATTTAACAGTTCATTTTATATTTGCCTCTACAATACAAAACGCTTATTCCGCAGCTCGTCCGAACTCGTCAGAACTGGTCGC<br>GCTGGCTGCGTAGAGGTTAAGTTACAAATATTGATTATACTTTTATTATACAATTTTCAATATTTTTTTACTCATTGTGTAA<br>GTACATACGGATGTGTTTGTACGGTACATCTGTACCCAAATATTTTTAAACGTTGACTTTATAGAATTGTATTACTTAATGT<br>AACCGAAGTCATTAGCTCTATTTTTAAACGATAATAATGTACCCGTGATATAAAATACGTTAGGACTAAATTTAAGCAACA<br>GGCAATGCCCCGTCGCCGTCGCCGTCGGAGGTACAGTGTAGCAGCTATCGTGACTGAGGATCGCTTGGATATTTAAAAAT<br>ATGGCTAATAACTTCCATTTTGCAGTGTGACTGCTATGAAAAGATAGTAGCTATTTGTTAAAATAACTACTATTCTAAAAG<br>TATAAGTATTGCTTGTTGGTTTGGTCTTTTAAACGGCTGTGTGTGTGGTGCGGACGGTTCTCGGATCGATGACATATCAAAT<br>ATTAATTATTGTAAAAAAAAAAAAAAACTGGTTGCTAAATTAATTGCTATATGATTGACCCATTTATAGAGTATAATAGACA<br>CGTGAATATCAAGTTTAAGATATTTAATTGAACATTTCCAATCCGGATCGAAGGACCATCGGTTCGGTTCTGCAGAATTTA<br>TTAAACGTGGCGAATTAAAACTATAATACTATTACCATTAATGTCTATAGTGATCAGTGAATAGCAAACCGGTGTTAGTGT<br>TGGTCGGACGTCCGCACCGGTACGTGTAGTGGGCGGTACGCGCGTTCCTGTCGTCACACAGCGTTAGCTATCTCGCTCATA<br>CGCCTCTTTATATTATTGCCACTCATTATCGCAGTCATATATTTTTAATGTCTTAAGTCACATGCCTTCTGCTGTGCGTGCAC<br>AAAATTAGTCACGATTCGTGAGGTGCCGAGTCGATTTTTATGTTACTAGAGTGTTAGGTACCTACTTGTTAAAAATGATTC<br>GGATCCTGACTCGAGTTTGAGGGCTCGACAAGTACTAAGTATGAATACCGATATAAACAGTACAGTAGTTTTCAACTGATT<br>TCTAAAGCTTTTAATAAATGATTAGTCATTTAATAATTTTCCTTTGACAGTCAAGAAAAAAAAAAAGAAAACGAAACGAAG<br>CAAGTACTCTATTCGAGTTTCTAATTATTAGTGATGTATTCGAGTCAGAACTCGAGTCGGTTCGACTCGGCATGCCACTAG<br>CACGGTGCCTGTTTACGGATTGCAAGCTGCTTGCTTGTACCTAAAATTCATCGTATGTAAATATTTTTATTTGCCTCAGTTT<br>ATTTGTTTTTTTTTTATCTAAATGCCATGTTGTTCTAGTGGTACGGTTATGCGTGTGCCTCAGCGCCTAGTACATACGTGATT

		TATCACACGCCACTATTTTATGCGGGTATATATAGTATATATATGATTTGTAAATAAGTGATATTGATTGTATTTTTTTAA<br>ATGGATGATAATGGAATGGTAACCCCACCTGCGTTATCGGTATACACCGGCGGGGCACCAGTGAAGGATGATAGGTGAGG<br>ATGCAGTAGGTCAGTTAGCCCATCGTGACGAATTCAACAATAATTGTTCACGATGGTTTCCAGGCTTATTATTGCTAGCAA<br>TGGGGCTGTCAAACTCCATTGCTAACAATCCTTTTCCCTCCTATTGATTGTATTGTTATGACAGAGTTGTGCAAATCAGCGT<br>CAGTGCGCACTTTTATGGGGGTACTTGTTAATTTTATATATAATTTCTTAATCATTAGTTTTGGCTTTGTTGGGGTAAATTTT<br>AGGTTTCATACAATAGATCAGACGAAGTTAGGACAGTCGGCTATAGAAATGTGTTTAGTTTCGGATAAAACAGGGTTGAT<br>TCACGCATGGCGCCAAATTTCTTTATATAAGTTTTAATAGAATAAAGCGTTACGTGTGAACGTCTAAATGTAATTGAAAAT<br>GGTAACAAATTGTAGTTATAGATTGTTACGTTAACATGGCGCGTCCCTCCAACTTACTCCAATACACACGTGGTGCTCTGTT<br>TGTGTTCAGAGGGCCTACCACGAAATATTTTCCGAAATTTCGGGGACCAAGACCAACGAAATAGAAATTTGATGTTAAAA<br>TTACATAATACA
MSTRG.4310	4-coumarateHypothetical proteinCoA ligase 1-like	GCGACTCACCAGTCCGTGTTCGCAGCAGTGATACCGTTCTTCCACATATTCGGTTTCAACATACTGATGTTGAGCCAACTCT<br>ACCTCGGCTGCAAGCTTGTCACCCTGCCTCATTTCAAGCCGGATCTGTTCCTGCAGACGATCGCCAAATATAAGTCCGATA<br>TCTTATTCATGGTGCCACCTATCGCCGTGTTCCTGGCGCGGCACCCGGCGGTGACGGCGGCGCACCTGCAGTCCGTGCGCT<br>CCATCATATGCGGCGCGGCGCCGCTGTCCCCCGCCGATGCGGATGCTATTGTCG
MSTRG.4326	Uncharacterized protein LOC110380267 isoform X3	GATGAGTTGACAGTTCTTGCGCTTGTCCGTCTTATTTTGGAATCTGAGTTTTCTAATCCTTGCTCGTCCTTGCTGTTTGTTAT<br>TATTTACCAAATGAAATCACGGGAAATACTTTTTGGAACAATTCCAATGAGTATTTGTTTATATCAACCGGCGTATTAAAC<br>TTTTTCCGTGGCTTCTTTGTCTGAGGTTTATTTTTGGTTCTCACGCGGTGTCAGAGTGGAAATTTGGTGTATTTTAGTGATTA<br>TTTCTGAAAATGGGCTCATTGTCTGCCGATGCTGATGTTAACATTGATATGAACGACAGTGGTTACGTCCAAGATGAAGCA<br>ACCCCACAGAAGAGCTCCAGCTGTCAAACAAGTCTCCTCGACCTCTCTGATGATGTACTACTCTACATCCTCTGGTACTGTT<br>CACCCATGGACCTGAAGGCTCTTGGATTG
MSTRG.433	G-protein coupled receptor 112	ACTCATTCTTTTTATTTTTCATTGTTAGGGGTTCTTATGAATTACATGATAATCCCGGATTAAGCTGCAGTCCGCAGTGCGA<br>GGGGAGACGCTGTAATGCGACGCGCCGTACACCCTCTGAAGAAGCGTACGGAACCAAAAGTCCCTGGTGTCTTTATTGCC<br>GCACACAATAAACAAGAAAAGAAGGAACCCTTGTATGGTCACCGTGATGGTGAAGAGGTAAGCGGCGACGATATTGAAA<br>GAGAAGAGTCCAAATACCCACGGCAAGCCAAACAGGAACACTAGCACAAATCCTATACGAACGCATTTGACGGTCTCTTG<br>AAAATCGTCCACGTTTTGTTTTCCTAATTTTTTATTACATGTACTGGCAGATACGGCACCCATTATATAAAAAAATGATACG<br>AAATTCACTATCAATATAAAAGCAATAGGTGCGTAAACGGACAACCATAAAGCAAGGTTTGAGGGATAGCAGAAACTGC<br>CACTGGGTGTCATCTCCTCAAAACGACCAACGTAAGACTTCGGAGAAGCTATAAGAAGTATACTGATGATTAAAATAGGA<br>GCTCCCCAAGAGAAACACGAAGCTATCATAACTTTATGAGACATGTGCCTTGCGTATGCATATTTAATTGCAACCGTATCT<br>ATATAGGCAGTAATTGCTACCATCAACATCCAAAAAAATGAAGCTAATAAAGAATAATGCAAGACAATACCTGTGAGTAC<br>ACATGATACACTATATTCATCATATTTCATATACACGATAACTATAAAACAAACGGATAAAACAAATAATGCAATACACA<br>GTTGAAGCCAAATTCGATTTCGAAAATTCGATCGCCAAGATTTAAACGCAGCAGCTGTAAGTACTATCAAAAGAATGCCA<br>AAAATAGATAAAGAACAACCGATAATAGACAATATTTCCAATATTTTTTCATTGCGTTCAGAGAAGACGGGCCGAGGAAT<br>TAAAATTTCAGCGAAATGTGTGAGATGATCGCATTGACATGTGTCCAAAATTGTCGGATTATTTGAACGGATAAAAGAGC<br>ATCCCTCTTGAGACCAATATCCAGATCCATCCTCCAAGAATGCCCAATAACCGCAACTTCGCCTCATATCAC
MSTRG.4350	Coiled-coil domain-containing protein 186 isoform X4	GAAGCTGAATTAAAGGAGAGTCAAGCGGCACTTATAATGTGTCGGCATGAAAAAGAAGAATTGGACCGACGGCTGACCA<br>CTATCACACAGCAGTTCGAAGTGTGCAAGCGCGAGCGAGACAGCGCTAGCAGCGCGCTCGCTTCTTCCACCGCTGAGGTG<br>GAACAGCTGAAGCATCACAACTTGCGACTGGAGGAGGAAGCGGCGGAGCTGGCTGCACTCCGAGCCCAAGCCGCGCTCG<br>CGGACACGCTCAGCGCGCAGTTGCAAAGGGAGTGCGAGCGCGCGTTCAGCGGAGGAGGCACGCGCGCGCAGCGCGGC<br>GGCGGCGGAGACGTGCGGGCGGCGC

MSTRG.4356	NUAK family SNF1-like kinase 2 isoform X7	CCACCGGAAGAGGTCGTGAGTGCGGCCAATCGCGGCGTGAAGAAGGCCGACAGCGAGCCGTCGCACTCGTCCGCGCCGCT<br>CGACCCCGGCGTACTGCTGCAGGACGCTAGAAGAAGTCTACAAAACTCGATGGCAAAACTGGCGGAAGAGAAAGCAGGC<br>GAGGAGAACCGCAGACGCGCCGCGCGGACATTATATCATCAGCTATACGCAAGCCTCCGATGCCATACGGCCGCTCGTC<br>GTCGGCGGGCGTGGCTACGCTGCTGTCGCCTGCCACCACTCCCCCCACCCCACCCACACCCGCCGCTGCTGCCGCGCCACC<br>ACCCACCCGGGTGTTCCGCACTGAGGCTCAGCATCGTGTGGAAGATCATCGGCCGAGG
MSTRG.438	Pre-mRNA-splicing factor Slu7	ATCTTTTTTAAAGGCAAGGACATAAATCCCCATATACCACAGTACATATCATCGGCACCATGGTACTATGGTACTTCTGGA<br>CCCACACTGAAGCACCAGCGGCCACAAGAAGACAGGGAAGCTAACTTCACAAAACTGGACAAATACTATTACAAAGGAG<br>TGGATACTACAAAAGTAGCTACGAAATTCAGAAAAGGCGCATGCGAGAACTGTGGGGCAATGACACATAAGAAGAAAGA<br>TTGCTTAGAGAGACCGAGAAAGATTGGAGCGAAATTTTCAAATGCTGGTATAGCTCCGGATGAGTACCAACAGCCTAATC<br>TGAACCTCAGTTACGATGGCAAGCGGGACAGGTGGAACGGTTACGACCCCGCACAGCACAAGGCCATTATAGAGGAGTAT<br>CAGAAAGTTGAAGATGCTAAAAGAGAGTTACGGGCTCAGAAAC
MSTRG.4385	Mitochondrial genome maintenance exonuclease 1-like	TATTAACAGAATTTATGTGTGTTTAGAGTTCTGTAATAGCCTATACTTATTTGAGACAAGAGTAATTTTATGAAATTTTATT<br>AAACATTGATGAATGTGGAGGAAACGAAAGAGAGATAAACCGACACTGATTGAATGGAAAAAATCTGATAAACCACGCA<br>AATCTATATCTCTGACCTACGACAATCCAGTACAATTGGCAGCTTATTTCGGAGCTGTATGCAATGATCTTAACTACAAGC<br>ATTTGAATGTGCGTGACGCCTTACTCGTTATTGCATATACGGACGGCTCCAAAGCCGATGCCTACCACCTAACCACTGATA<br>AGTTGAGAGAACATTGGGCGCAGTGGTTGATAAGGCTAGAAGACTATATGAAGAAACATGGCAACGCTGAAGAGAAAAT<br>ATTGAAAGGTGGCAAACGGCTGTTCGAAGAGGACATAGAGAATCTCCAGTAATTGTTG
MSTRG.4407	Frizzled-2 protein	CGCCCTACGAAACGAGCATTCTCGATTCGTCGTTCATTCATTCAGTCAGTCTTCACACGTCGTATCGCTTGTACGTGTTCGC<br>GATAAAATAAATACGTAAACGCCGAGATTATTGTTTCAAACATTCGCTCAGCTGATCAGTGACAAAAAGTATCTTACGCCA<br>TCACTAAAAAGAAGCCACATAATTAGCTAGTGTTGTGAATTCGCGAGTGAGATACCAAAAGCAGATGACTATCGAAAATA<br>TCTCGAGTCGAATGTAAAAATATACGCGTCGCCGTGGAGCATTCACCGCGTGTGACTGATCGATATTTTATTTAAATGATA<br>ACATGGATTCAATTAGGTGTTGTTTTAGAAGACGGTGAGATATAGTGACTAGTTGCGTTTGTGTTGTGTGCGACGGGGTCG<br>GGACGTCGCGCGGTAGAAAGCGATGCCTGTAGTGTAGCCGACATGAGTACGAGCAGATGGCGCGGCGTGATCGCACTGGC<br>GACGTTGATGCTGGCGCAGGCGCACGCGTCGCTCGCGCTGCACCAGCCGCGCTGCGAGGAAATCGTCATCCCCATGTGCC<br>GGGGTATCGGTTACAACCTCACCTCTTTCCCTAACGCCCTCGACCATGACACGCAGGAGGAGGCCGGACTTGAGGTGCAC<br>CAATACTGGCCCCTCGTCGAAATAAAATGCTCCGCTGACCTCAAATTTTTCCTGTGCTCCGTGTACACACCCATATGCATCG<br>AGGACTACGCGAAGCCGCTGCCGGCGTGCCGCAGTGTGTGCGAGCGCGCCCGCGCCGGCTGCGCGCCGCTGATGCAGAAG<br>TACGGCT
MSTRG.4413	Gelsolin-like protein	TGTCTCTCAGTTCTCTAGTGCTCGTACTACAAGCTATGCTCAGTTTGGGCCTAGATGGTGCTGTGCGATTTGGCCAGTGGTA<br>TTTATTATCATTAATATCATTTCAGGAGAAGGCCGAAGCCATGAACAAAGCACAACAGTACCTGAAACATCATAATTTACC<br>TCCATGGGTGCACGTATCGAAGGTACCAGAAGGAATAGAACCGACAGCGTTCAAACAGTACTTCGAAGCGTGGAACTGAA<br>ATGTTATTATATGTACATTTTTACAAGGTTCATATATTTTTGTTACTTTTTCTATGTTCATATATTTTATATGTACTTACTACA<br>TAAAATGCACCATTAACACTAACGGAGCCATTAATCTTCAGTATTTCAAAATGCGGTATTAATTGTATACATATAATTATG<br>TATACATTTGTTTTTATTGAGTTCATATATCTTTGTTACTTTTCCTAAGTTCTAATAATTTACTTAGTCTATTACGAAGCGTG<br>ATTAAATGTATAAATAAAATATATATATATATATATATATATATATAAAGTATTGTTTAGTTTGTATAATTTTAATTGCATTAT<br>TAAAACTTCATTATTATTGTCATTAATATATCAAAAATATTCTAACAGTAATTAGAAACTTGGTATTAAATATTATCCATTA<br>AAATATATGTACCTTTTTATTAATTAAGTTCATATAATTTTGTTAATTCTATATAAGTAATAATACTGCATTTGTATTTCATA<br>ATTCAGATATTTCGAAGCTTTATATAAATTGGGGGATGTTCAAGAAATACGTAACGTAATTTAGGGGAAAGGTCTTGTAAA<br>ACATTACGATACGTTACACGAACGGGAGGGGGGTTCGAACAAAATGTTAAGTACATTTTGTTTATTCAATTAAAAACTAG

		AGAAATACAATCTCCTAAGCCGTCAACCCTTATCTTTTATATTTTTCGAGAAATCCCTCGAATATATTGTACGTCATTTTTA<br>TTTTGTTTTCGTTGTCAGTATTCGGTAATTTTGCGATTTTAGGTAAAAATGGTTACTTGGGTGTTACATAACGTTTAGGGAT<br>GAGGAGGGGAGTACAGAAAAATGCTACAGCACGTTACATGTAGGGAGGGGGACAAAAATTTACAAAAATTGTACTACGT<br>AAAACTTGGACGTCCGCTTGTAATGTATTCATTTATATTTTTATTACTAAGTTCATATATTTTTGTTAATTTTATAAGTTCAT<br>ATAAAGTATTGCATTTGTATTGCATCATTGTTACTAGATGATAAAAGAAACGTGATATTTTTATAATTAAATTTTAAATGCA<br>CAAATAATTTTTGTTTTAGTAAATAAATTATAAAAAAATAATAATTATTAA
MSTRG.4418	Recombining binding protein suppressor of hairless	AGGTGGACAAACAAATGGCACTTTTAGAAGCGGATGATCCTGTTTCGCAACTACATAAATGCGCATTCTACATGAAGGAC<br>ACAGAGCGAATGTACCTCTGCCTCTCCCAAGAGCGTATCATACAGTTCCAGGCGACACCATGCCCCAAAGAGCCAAATAA<br>GGAAATGATCAATGACGGCGCTTGTTGGACGATCATATCAACCGATAAGGCTGAATACCAGTTCTATGAAGGCATGGGAC<br>CTGTCAGGTCCCCAGTGACGCCTGTCCCTCTGGTACACTCATTGAACCTAAATGGCGGCGGTGACGTGGCTATGCTGGAAC<br>TAG
MSTRG.4430	G-protein-signaling modulator 2 isoform X1	AGCTCGGCGATGTGCTTGGCATGGCGACAGCTCAGATGAACATCAGCGACCTCCGGAAGGTGTTAGGTATGCCGGCCGAG<br>GCGGAGTCACCGCCCGCGCCCTCTATAGAAGAGGACAGGACGCCGCGGACGCCGTTCAACGCTCTGCGGGTACGACGTCA<br>GAGTATGGAGCAACTGTCGCTTATACAGATAACACCAGACAATAAGAAAAAAGAGTCCGAATTAGACAACGAGAAGAAA<br>CACGAAGTGGTCAGAACGGAATCCGAGGAGAACCCGGTGGATTCGTTCTTCGACCTGCTCAGCCGGTGTCAGTCGGAGCG<br>GATGGACGAGCAGCGCGCCACGCTCAATGCGAACAAAGAGAACCGGCCTAAACCCAAGGGGTCCAACAACAAGCTACAG<br>AGGTCGGCCAGCAGTGGGAACAAAAACCCAAGTCCAAACGACGCAATGCTGGACATGATCACGGACCTACAATCGGAGA<br>GGATGGACTCCCAACGCGCCGAGCTCCGTCCCGTGCCCAAGAAACTCAGCAAGGAGAGTAAAGGCACCTCCCTGCCAGGG<br>CTCCGCACGCAACAGACTACCTCCACGAAGCCGGAGGACGACTTCCTGGACATGATCGCTC
MSTRG.4433	Synaptotagmin-16 isoform X1	GCTCAAACAATCAAAGCAATGCGCACCGAGTGAAATAACAATTGTATATATAAAAACCTCTCAAATTGTCTCCACATCCTC<br>AAATTGCTTTTTTGTTACCTGTTCTCCTTGTCTCTCGCATAATTCCTGCCAGTGCCGCTCTTCTTCGCGGCCAGAGGGGCTGT<br>GTCCCAATGAGAACCAGCCCACCATTTCGTTGCGTTTAACGCTACGTCGCCACCAAACCGAAACCATGAGAGTAACTTCAC<br>TCAATTGGAATAAAGCAACCTGATGAAAACGAACAACAAT
MSTRG.4435	ATP-binding cassette sub-family A member 3	ATTATGTGCTAAGTTAAGATATAACCTCATGTACCAAATTTCATTAAAATTCGTTCAGTAATTATGTGAAAGAGTAACAAA<br>CATCCATCGTTACAAACTTTCACATTTATGATATTAATAGAATTACATATATAATTGCTGAACAACAATTATTTTTCTATTC<br>TACAGCCGTCGCAACAACTGAGTTACTGCCCACAATTCTTTGGACTGGACGAGTTCCTCACCGGTAGGGAGAACTTGGAGC<br>TGCTACTCATTCTCCGTGGATTCAACGACGACGATATAGAGCGAGAGGTCCAACACTGGATTGATATTGTCGGGCTGTCGA<br>AGTACGCGGACGTGGCGGTGTCGATGTACAGCGGCGGGTGCGTACGACGTCTAGGCGCAGCGGCAAC
MSTRG.445	Zinc finger protein 568-like	CTTTTGTTTACGTTGTATAGAGACCATGTACTTTCGTTAATCTATATTTATTGATAATATGTCACAAAATGTATGTTGTTTTT<br>GTACAATTGAATTATTGTTTGTTATATTTAAAAGTGTAATTTAATAATATAAGTAAACTTAATTAATTAGGATTATATGTGC<br>TGTATTTAAATATAACATTAATTAGAAAGGTAAAAGCTAAGGATAATACAAATCTTACCTGCCTGATATAGAAAGAAGTC<br>AGCGCACAGAAATGGAAGGCTTTTGTAGAGGATGCCTAGTACAGTATAATGATCCAGCTGAACTCCTTCAATATACAGAG<br>AAGAATAGGAGACTTTTCGTCTACTGTACTGGTTTACAGGT
MSTRG.4466	AP-1 complex subunit beta-1-like isoform X1	TTTTAAAATGTTGGCAACCCAGTACTACGCCTACAAATCTTGTCAAAAAGTGTCTCGTCTTCAAGTTTTCTGTTTATTTTAA<br>GAAATTTAAGAATTTAGAACATTAAGATTACATTCCAAAGAGGGCGTAAAAATTAAAATATTATTCGATATCTAAAAGGG<br>TTTATCAAAAAGTAGAAATACCAGCAAAATGACGGACTCTAAATATTTCACTACTACGAAGAAAGGAGAAATTTTCGAAC<br>TAAAATCAGAATTGAACAGTGACAAGAAAGAGAAGAAGAAAGAGGCAGTAAAAAAGGTCATTGCTTCAATGACAGTGGG<br>CAAAGATGTCTCTGCATTATTCCCAGATGTTGTAAACTGCATGCAAACAGATAATTTAGAGCTGAAGAAATTGGTATACTT<br>GTATCTTATGAATTATGCCAAATCGCAGCCTGACATGGCAATTATGGCAGTCAACACATTTGTAAAGGACTGCGAGGACTC

MSTRG.4<br>467	Unconventional myosin-XVIIIa isoform X1	GAACCCGCTAATAAGGGCGCTGGCGGTGCGGACGATGGGCTGCATCCGAGTGGACAAGATCACAGAATACCTCTGTGAGC<br>CAT<br>CCCGCGCGAAGACCGCAGCGGAAGCGGCGGCGGCTGCGGCCGCGGCTGAAGCGACCGCGGCGCGGGACGAGGCTCGCCT<br>GGCGGCGCGGCGAGCCACCGCTTTACAAGAAGCTATCGCCGGCGACCTCTCCAGCCCCGGGGACTCCAGGGATACTGATA<br>GCGATAATGACAGCTACAGTTCCGATGAGTCAATCGGAACCTTCTTGGCAAATCACAAACTGAGCCCCTCGGCGCCCTCAC<br>GAAACAGTATACAACTCGATTTACAAAAATCCCAGACGCCAGAAGGCAGACAGAGCCGCACAAGCAACAGTTCCAACTC<br>CGGCACTAAACAACTGAGTCCCACCAAGGAGTCGTTCGCATAACAGCCAGCCGCCGCTGCCGGGCTCCTCACGAGCGCAC<br>TGCCTGTCCTCTGCGCTGTGTTCTGCTCGCGGCAGCGTCACGACTCCGCGCACGTAGTCCTCGAGTGAGCTCCGAAAAAAA<br>ATAAACGTCAGACTGACGGTGTCCATGTGACATCTGTCAGTCGGTGGCGCGAAATTTAAATTTCGAATTTTATGACGTCAA<br>ACCATCGATATGATTACGATTGGAATGCTTGATAGGATAGAAATGGTAAATGAAGTAATTTATCACCGAACAGCAAAGAC<br>TTCTTTAGAACAATATAGAATAAAAAATGACTCCTAAAATTAGACTGCTCAATAATCTTATCTACATGCATTATTTATAAT<br>AGATGCTACGGATTTTAATATTTTCAAATATTTCATATTATAAGCTTTTACAATCACAAAATGTACGTGTTGTCGAGTGTTA<br>GTTAAGAACAGATATTCATTAGTAGCCGATTATGTACTCATACGAACATTATTATAATGTAATAAAGATAGAATGCATTCC<br>AAAGTATACACGAATAACATTCGGCTCGCGTGTTACCAGGCGCGCCATTTATATACAAATCATTTGGAGGGTATTTTCGCT<br>AGAAAGCGTGTCCTAATTTTCGTCACGTTCACTAGCACCATTTATTGTAGGCTTTGACATTAATTTATTTAGAGTGTAGAAA<br>CGGTAGAAAGTTTTTGACGAGTTAGTTTTGTTGTACTGCGTACAGCGTGCCAGTCAGTATTTCGACGGCCGAGGCGGACAT<br>GACTTACAAAATAATATTAGGTTCTTTGTTATTTAGTTAGCTAACTTTTGTACATAACTAGATGGCATATACAGTTTATTAT<br>TTAATTTGTAATATTTGTGTGATCAGACTGTTATACTATGATTTATTTGTTACAAGTGAATTGTTGCTTTATAGTAGATCATC<br>ATACATATCTGTTAGGTAATATTTTTTTATTAATTAATAATATACCTTCGACTGTTTGTTTAACTGTAACTTTACTGAACATC<br>ATTATTGGAATTGTAATCTTTATAAAATATATTCCTTGTAATTGTAACTCCATTACTTAAAAATCATCATTCAATCTATACT<br>ATATACATATATATGTGTCTGTAACCACAAGTATAAAATTTTACTATGTGTAATGTATGATAATGTGATGAAATGTTACTG<br>CATACCTACTGGTTGTAAAATGAAATTATTTAATAAAGTAACAGAAATGTA
MSTRG.4<br>469	Ankyrin repeat domain-containing protein 50 isoform X2	TAAGTTTCCTAATTACTTAATAATTTTGAGTTTTAATTAAAGAAGTTAATGTGTTAACAGTTGCTAACATTTTAACGGCGCT<br>AAGCTTTAACAAACTAACTGTACTGATATCACATTTAGTTCACACTAAATCCTATTTTTTCAGCACTATTTGAAATTACGTT<br>TATATGTTTAAGACTGGATTCTTACGTGGGGGTAGATTGTCTAAATCTATAAATTAAATTAGGATAGTAAACGTAATAAAT<br>AATAATTAGTGGGAAATTACAATGAATAGGCGTCGAATATAAGGTAAGATTACGTTCATTTGACGTCTATTCGATCTTTTT<br>GCTCCGAAATATTGAAATACCCAAATTTAACCTGTAGAATAAATCCCACGTAGTCAATTTCACTCGACCACTTCATGAGTA<br>TGACGGTATAACATATCAGTTAGTATTGTTTCATGCGTGTATCCGCTTGGGTTTTGATATTAATGACAAGACGGTCTTCTTA<br>AAAAGTAACAAATATTTTATTCTGTCTACAGTAAGAGCAAGAGGAACGGTATAGTCACAAATCCGGCCATGAGACTCGTA<br>GCAAATGTGCGAAATGGACTAGATAATGCTGCGGCCAATATTCGGCGCACCGGCGTGGCTCTGGCAGCCAGTGCCAGTTC<br>CGCAAATCCCGCAGTCAAAACGAACGCTTTTCAGTGGAGGAAAGAGACACCACTATAATACAACCTAAGGTAGAACGAA<br>ATTGATAAACGACCGCTCCGAAGAGCGTGGATACATTTCCAAATATTTGGATACATGTCCAGTGGGTGGCTCCTTAGAGTC<br>CAATATTGGATTGCGTCCGTGGTAGAGATAGGGAATAATTTGCAATAATAAAAAATTATAAATATTTGTTACTAATAAGGT<br>AATTATTATAAAAAAAATTATAAATCTATAAAAATAAATACTAATTATTAAAAATTGGCAATATTTATTGAACTATAGATA<br>ATAAATATACGAGTATTTACTTAGAAATTATTCGAATAATAATTATGTACTTATTCATTTAACATAATAATATTATTTACAA<br>CCTGTTTTTGATTAAAACATTTCAACGTTACACAATATTTTAAAGTAGTAGTCGGTTTAGTTTACAGATCCAACATTATCGT<br>GTCTATAAAAACGTACGTTTAAAATATTGGATCCAAAATTGGACGATTGGAGCCAACCTTTGGACAGTCGAGTAGACACG<br>ATGCAAAAACGAACGCTTAACGACTGTAATGAATATTTTTATTTTTTTATGACCATTCTTTTTATATTTGTATACGACATAA<br>AAATCTTGATATCATTTTTGTTTATCACTTTTGTACCTGATTATACATTATTTAATTTCAGTACTTAAGAATATGTACAAAAA

TATAGTTCAATTAAACTATTGTGTACATTTATCGGCTCAAGTGGTATTGAATTCAATACTCGGATGATGCGAATGTATGTTA
TTTATTAGTTCTCTGTTTGAGATCGTTATAAATTATAATTTTAACCATATCGATCGTATTATCTTTCTATAGTCCAAGAAAA
GGCAACAAAAGCATTTCAATCTATTTATTAAGATTTCCAAATGCAAGCATTTAAAAATATTTTTAAAGAATGTTATATGGG
ATTACAGTCTGTAGTTTATAAACACAATAGATGGCGCTTATCTCAAGTTTAAATTCATTATGTAATTCGAGAAGTCAGTTC
GGATAGCTAAGAGAACGGATTCAAGAACTTTTTATCTACAATAAAAATAACTTGCGTCTATAATCCCTAATGGAGCGGTCA
GTGGTATGTGTCGATTGTTACCATTGTCGGTTTATATAATAATATAATGTATTTCATAGGTACATAAGTGTTAAACAAAAC
AACGAATATTTTTTTTTTATTGTATACTCTCGAACATCACTGTAGAAGCAATGTATTAAACAAATATAGCATACAATTGTAT
TATTTAGAAATTAGTTTGTACGAACAATTATATTTAACTGGGTGATGTTTTAAAGAGTGTTTGTTTAAAATTGTAACGTCCT
ATCACAGTTAAAGGTGCTGTATAAGGGTGTTTAGTAATTTTATTTTGGTCAGTAATCATTTAACTAATTGATGATTAAACTA
AACTAATCGATCGATTAGTTTAATATTTAAACTAAAGTTAGACTATGATTTTTGTATTTACATATATAAGAAGAAGAGAAA
CACATTATATCGTTTAGAAGTTTAAAAATGGGCAAAGATTTCTGACCAAAGTAAAATGATATTATCAAATTGATTTTGAAT
AATAAATCAGTATTAAATATTATGCATTCAATATAGTATCTTCAAAGTGAATAGTACAATGATAAAGAAGCCTGATCTCAA
GTGAACAAAGCTGTAAAGTTTTTTTTTATTAGAGAAAAAAAATTTTTCGTAGCTAAATTTAGACGTATGATGCATTTAATAA
CATGTTTGTATATTGGTATATACTAGTCTCGGGACGTGCGTACGATTTCGTCAGCAAGTTAATTTTATATTAATCATCATTT
TTAAATCATTCATGTTGATGGCTATATTAGTTGATTTATTTTTTTCTATTTTCCACTTTCTTTTTTAATATTGTAAAGCGCAAT
GGTTAATTGTAATAAACAGAGGAACATGTATTTATTATTATTTCTTTAAAGGCTTCCTTTCCTATATGGTAAAGGTTGTTTA
ACTGGTTCATGTTTATCATAATCTACTTTTTAACGATTGATAAAAATATGATCAGTTTGAGCATAGAAAAATATAACTTGTC
AAAGTACAGAGTGGACCTTGCCTTTTTATTTTTGTCTGCGTTTTGGAATTATTTTGAAACTCTCTTGAAACAAAAAAAAAATA
AACATTTTATTTTAATGATTCTTAAAAGTTTTTTTTATCATTTAAAATTTTATCGCAGTATGAAACGAATTATTTCGTAAAAC
GTACCATTCGGATACTTTTTGTTAAATAAAAATAAAGTGTGTCTTAAGTAGGGGGAAAATTTGCTGCATGACCACCAGATT
TGTCGACATACCTTTTTTTTATTATTATTATTAAACGAAATCTACGCAGTCTGAATGTAATGTCTGCACAAATATATTGGGA
CAAGTATGTAACATAGTGTAGTTTATGAAGTTTTTAATATGGCAACACCAAACGACGACGACGAACGCTCAAAACCTTAG
AGTCTAGAGCCGAAAAATATTGCGAACGAATGTGTGTTTGTGAAAATTACAAGTGGCTTGCACTAGCTTATATGTTTTGCA
TATATTAGCAAGTGCTTCCAAAATTATGGGAGAATGTGAATGAATATATATATATATGTCAACACTAATATATAATTATAT
CCATCTTCAGGTTTAGTTCCACATGGGGTAGGCAGAGAGAATACGTAATACGTAATAATAATCGATATAATACTATTTATT
GCTTATTATAAATTTAAATTATAAGTCAGCAATCGATGTATCAGTTTAACGTTCAGAGGATTTTTTTATGAAAATTAAATC
AAATTGAGCTCTCTTGTTTGAGTAAAGAATTTGTTTTTTGTTATTCATACAGCTGAAGGAATACAATTTTTTTTTATTTTTAT
TTATGACTGGCAGGCTAATAACCTTATATTAATTAGTCATGTTTTCGAATGTATGGTGCAATGATTTCTCACTTTTGTTTAA
AGAAAAATACTCTTGAAGTTAGAGTCAAAGGAGGTTGTGTTATGTTTGAATTAGAATAAAAATGTATTTGCTTATGGTACG
TTGGACAATCGCCAAAAAATGTCCCGGTAATGACAGATATAGGTATGTAGCCAGTTTTCAATATCACAATTGTTTTTAAAT
AACAGCTCTTTGATTTAGTCAATTGATAGCGAAATATTATATTTTATATTCCAAAACGGCATTTATTAGTAAGACTTTCGAA
TAACTTCTCCTCCATGATATAAATGATATAGATTATGATTTAATTTCCATTTAATATAGCGTGTAATGTAAAGACTTAATAA
TGTAATTAGTAACGTACTAGCCACAGTAATTGAACAAATGCGTGTAAAAATATTTCTTTTCTGTAGCTTGCCGCGTAAATG
TGACTTTAAATATCACAGGACATATTACACAGCGCCATCTAGCTCCACTAAATGAAGCAGAGTTTGTACTATAGGTACTAA
ACAACTCATAGAAATCCTTTTTTTTTCTAAATAAACTTTTTTTTTAATTCCTTTGTTGGAGTATTTTCACACTTTAGTTTCGTG
GTTGGGTCATTGTGTGTTACGGTTAGAGTGTGGTTAATCCATCCAACCTAAACTTTTCCAGAAGATCACTGGGATTGCCAC
TAGAAGGCATCCCGGTATTCTAAAGATCTACTCCAATTTGTCGATCACGGTCCAGCGTAAATGTAATAACAATATTAAATT
TCTAAAAGAAATGTTACCAGTGTAATCAAATGTAAGTCTGACTGTATTAATAATTTATTTAAATACAATATTATTTGCCTTT
CGTCGAGTTCAGTAGGTAGTTTCAGCTGTGAGAAACTGGCAACACACTCAGATGTAACTGCGAAAAAAAAATTTGTTGTAA

		AGTATTATAAAAATAATCTATAATAATTCATTGTATATTTAAGACATACTTTTATTTACAGAGAGTTTATACCTATATAAAAGCGATGTGTGTGTGAAAAGTCGATTATCACATTTATGCGGCAGAGTCATTATGAAGTCATTACGAAATTATAACAAAAAAAAATAATAATTAGAATTGAAAACAAATTCTCAATGGGTAGTTGTGGTAGTTTTTTGGAATTATTAGATATATAAAAAATGAATGATTGAAATAACGCGAGGCTGATAAAGAAACGATGTGTCCGTTTTATTTTTTATTTTTAATCTAGTGTTTCTGAAACATCGATATTTATGTAGACTATCGATAAAAATACTAAAAGTCATAATAGATATTGTAAAAAAATTACGTTACGTCTTTATAATGTGTCGCTTTTCATTAGCTTTATTAATAATAGAAAATGTGTTAATATTATTTTATTAATAATGTAATTGTTAATTGGCAATCAAGAAGTATTTTTTTTGTAATTTTTTTCTAATTTATATGTGTTATTAATATCGATTTACCAATTAATCGATAATTATTTTGATGTTTATATAACATCACTATATCGCGTGTCATATATTGAGGCTATATATTTGAATATTAATTGGAAATGAAACGTATACAAAATATAAAGAAAAGCAATTAATGATAATGTACGAATGTTCTTATATTGTATCGTATAGTGAAGTTAAGCTGTGATCAAACGAACGCGGTAAAGGACTGAACGCGGGACAATCTGCCATTTATAATTCGTTTTAGTATATTATCATTTAATACTGTTTTTTTGATATAAAATGTGACAATAATACATGAATTATGAACTATTGGTACTAACTTAATATCTGTGTTGATGTTGCAAGGTATAGAGAAGGAAGGTATAATAAATAATGACGAATACAAAATATTTGTTGTCCCGCTGCTTGTTCGTATCCGCGTCGGTTTGGTCAATGCTTTGCCAGATATTATAGTGATATTTACTTTATATTGTAATCACGAATCAATTCTTGCTATATCGAGTCAATAGTTTAGTTATTTTATGTGGTTTATGGTATTGATAGAATTTTTTATTAGGTGACTATTGCACATTAAATATATATAATTGGTGCTTATTTTTTTTTGCAACCGTACAATTATACTTATGTTTTATTATAAAGTTATTATCTGAATTTGAATACTCTAAAATTACCTTCGATCTAAATGGGACGTCAATTGTTAAAAAGAATAAAAAAAAACTAGTCATTAACATCATGATTTTTTTTTGATGGGTGCAAATGGTATGCTAACCTCGCCTGCGCTAACAGGATACGCTGGCGGGGCACCAGTGAAGGGTGATCAACGAGAATGAAAACAGGCCAGTTAGCCCATCATGATAAATTCATCAGGAATTAACCATGATAGTTTCCAGGGGGAACTGCGAGGAGGTCGACCTGGTTGAGTATATTGCTAGGAATGGGATTTTTAATCTCCATTATTAACAATCTCTTTCTCCCCATCAGATCATGATTAGTTTTTTTTTTTCAATATATGTTGAATATGTTGAAAAAAATTTCAGAAAATGTTGGTTGACTCCTATTCTATTTAAACAAAATTATTCAAAACTCTCCTGGTCATGTTATTTTTACAATTTGTATTGATATTTTGTAATATAATATGTATCTATACGTTTTGTACAAATTAATGTATGTATATTAACCAAAATGGCGGCCTCTTTCAATGATTCTGACAACGAATGTATATCTATTGCTAGTCTGTTAACAGATATCTATCATAGTAAAGTAAAGGCGAGCATATTTGCTAATCTTGTTACTTTTACTTTATCCCCCCCCCCCTCTTTAGAATACAAACGTGAAGATCATCATTATGTTCTTACCCTTATCCCAATTATTATTTAGGGTCGGCGTAATATGTTTTTACCTATATTACAAAAAGATTTGGCTTATAGTTTTACGACCGGCCGCCTGCCTGACGTCAACCCTCTTTGGGAATACAAACGTGAAGATATTCTATTTATTTAGAAGTTAAAATTGCAGTACTCTTTTAACCCATTTAATTAGTAATAGCCATAACATTTAGTTCTGGTATTCCTATTTATGCACACAAGAATAATGGTTTGTCTATTTTTAGAGAAGCCTTGTAGTGAGATACTCTATCTAGGTCTTATTAGTCTGTGTATCCTTCAGGTCTAATCTTGTATTTTTTTTATACAGCGATATATGCTTTCTTTCTCTGATACTCATTAGTGATGAGTACGTGTAAAAGAGATGCACTAACGCTGAAACGAAAGGAAGGTGTGTATAAATTTATGTTTGTTTTATTTAATTACAAGATATTATGTATGGAAATTTTATATATTATAAATTCTATCAAAATCGTAAACCAAATTGTCTGTAGTTTTACATCGATGCTTTATTGTGTATATAATTAAATAGTATAACAATATTTATTTATTGAGTCACGCCCATGTAAAACTGTCTTTTTTTTTTGGACATTTAATTTTTATACTTTTTCGACAAAATGAATTAAATTAAAAGTAAAAAAGGGCTATTTACAATATGTACTTTTGTCTACATTTTTTTTTATTAAGTTAGATGGCGTAACAAATTTTGAATGTCCAGAAAAAAGCGGATTGGGTTTAGTTTTTGTTTTGTATTTTGCGTGTGAGTTTGGTGGCATTGTGATGTAGCTGTATAGATTACGGTGAATTTGTACGAATAGTGTAGTGAGTGCAGTACGGTATAACTGTGTTGTGAAGAAATATATTGAAATATATATAATTT
MSTRG.4481	Leucine-rich repeat-containing protein DDB_G0290503	AGTTCCCACAGTTACCTCTTATGACACCCACAGGAAAGGAAGGGATGGCCCATTCTATAGTCTATGTAATCTAAAAAAAAATGCATATATCTGCCATGTAATCTTAAAATAACGATTTCAATGTGTCTCTAAGTGGATTTATTAATATAAAATACTTATATACGTGTCATTTACAGATATATACAAAACCAGACTGGCTGGTCGGCCGTCTTGGAGTCGAGCAGTTTGCTATCGGAAGCGCTGGATCCAGCAAGAACACCATACGCCCTCACTACAGCCTTTACGCTGAGCTGCGTGTGCGCCCTCGTCGAGAAGGCGTCCACC

CTCGTCAATACTCCGGAGGCGACGTCCTACGAGCGGCAATGGTATAAATTCCTGAAAACCGTGGCGAGGTCCCTGAAAAA
CACAACACTTACTGTCGCCAAACCGGTAAATCCCCTGCCAGAGACAAAACTCGTGTACCACTTGAGGCAGCTTGAGTCTA
GAAGTACCATAGAGATCCTGCAGAGGGGGAAAACCGCGGCGGAGTTCGCGGATCTGACGAAAAATGATGTCTCCGAACA
GTTAGTTAAGCATCTGTGTTGATGACTAATTGTTAGATAAATACTGGCCACATCGACGGTGGTAACTTGACTAGAATATGA
AGATTGGCCAATTTGCATGTTGGCCATATTAATCTGATACTTGGTAAGTAGGTATTGACTAGATTGGTTATGGCCATGGTC
AAACGTTGGCCGCTTTTTCTACCGATTTGACATTAACTGGCCACTTTTTTTTTTATTTCAATATCTGACCATTTTTGTCATTT
GTCTCTCACGGTTTGGACTTGTCGTTGACTGGTTATTGGACAATTGGCCGACCGACTTGGATAGTTGTAGATACTGATTTTG
TATTATAATTTGTATGAAGTAAAAATTTCTGCCTTAGTTGTAATCGATTAAATAATTCGATTCGAATAATCGGTTCGGGTGT
ATTTGTAATATAGTTTCAATAAATTCAAATGTCCACGGCCGATTTACCTGTTTTACCGATTTCTGTGTATGTGAATTCAATC
TTGATTATCGAGTTTGTAGATAATGGTTTGGCCTATGTGTAGTTTGGTCAAACGTTTGAACAAATATTGGGGGTTAGGGGG
GGGGGGATTTGTCTACTTTTTTTATCGACTGAAATCAAAATGAAAACTATTAGCAATGATAAAAGTGTGTATAAAGCAATT
TCTTTTTTTTTAAATGTCTAATTCATGTCATCTTTATTGGATTTGTAAAAAATAAAAAAAATATAAAAAAGCCAATATTATT
ATCTAGGACGTCATTTGTTAAAAATCTCATTGCGCGAAATGGCACAAGCGAAAGTACGTTACGTATTTTTATTTCTTTTTAT
CAAGTATATAAAGAGGATGGTCGAAATAGGCGTTGTTTTCTAAATATATAATGTATAATTATATTGGTTATGTATAAAACG
TAGTTCTCAATGTATATTGTATGTATATTTAATTAATTAGGAACCTGAGCAATCGACTATTAATAAACATTAAAGTAACATT
AAAACATTTTTCTTTTCAACCCCTCCCTTGTTAAATTTTTGTATAGTAAAAAAAAAGACTAATATTTGTTAAAGAGTCAAAT
AATATTATAAATAGTTTTTCACCTTTCAATTTATACGTACTTATAATGGATTTTATAAAATAAAAAAAAATAAATAAAAAA
AAGGTAAAAAAAAAATTATAGATTTGTATTTTCAAGTAATTGGACATCTTTCCATTTTTATATAAAAAGTTAACACATTTA
ATTCGAGGTATATGACTTGAAAATATTTCTATGCTATATTTTAGGAATTAAATATAAAGAAACGCTATGAATTTATCTAGA
TAGCTGTAATTATTGTCCGCCATTTTGACATGACTATATGAAGCTGAAAAATGGCGGGAAAGCTTAATTTTTTTTTCTTTTT
TTTTTAAGTAAAACCACGTAGTCGATTGCTCAGACCATTCTTATACGTTTAGTATTGTCATTCTGATATTAGTTTAGTATAG
AATGACGTAAGTAATGTTGCTTCTGAATGAGTCTGATTCAATGTAACTATGTCATGAATGTTAAAGTAAGCATTCTGAAAG
TAGCGATAATCTATAGAGATAGACAATGGTGATTTGTCGCCCCGTGAAAGTAGCATAACCGATTTTTATTACATATTTGAG
CTTTTACGCAGGTCTTAAGGCGACAAACTGGTCGACCAGTCGACGTGAACCACAGTCTGTCTCTTTATATTCTTGCTGTGTT
CGGAACGCCAACTTTTTAGATGAGTTTCAATTTATAACGTTTCGTTTAATCATAATATTCAGATAATAAATACCGAAGTAG
TATATTTGCAAGGTAGCACGCGCAGAATGTGTTGAATAGCCGCGTTGTAGACTGACACGAGTTACTAAACTATTTTTGTAA
TAAAAACATATTTTGTTTGACCGAATTTAGCGACAAGAGCTTGTGCATAACCGCTAACATTGATTACCTAGCTTGATCGCT
CAACAGCTCAAAGTCCTTAACCTAGAAAAAAAAAAAACTGTCACATCATATTTATGTAGGTGTATTTTGTATTGGTTCCTGA
TATTCATACCAGTCGTAAGTTATCCCAAAGTGAAAGATATTCTTTTGGGACAACCGAAATCGTACGTAGTGTAAAAGCCAG
TGTTGCCATGTACAACCACAGTTTTAATCGAAAATATTCATATATGGCAACGCTGGCACTTTAAATCTATTATTTAATCATT
CATTTATTGTAAATAATAAGAACAGAAATAAAAAAAAAAGTTTTATTCATTTAGTTAGTTGTGTAATTTAATATAAAATGCT
TTTTAATTATTTAACTAAAGCGGTCGTTACTATATTAGTAAAAAATAAAAAAAAAAATCCATAGAATTTAAAAAAATATGGA
GGTATATGTTTCAATCCATAAAAATTCTTGGATGTACATTATTATATGTTAATTGTGGCAAGAGAAATCACTTATAGCTTGC
GCGTTCGACTACATTTTGAGGATTGGTTTTTTCGATGTTCAATTATGTTTTTTTTTTTTTAGATATTGACTGTGATCGGCTAT
GTTTTTAAATTAATAAAGTGCACGCGTGCTGCACTGCGATCTCGTTTGTGATATCTGTGCAAAAAAACCAGTCCTCACTTTA
AAAAAAAAATGTTAAATATTGTCTATAAGTTATATCGAATCTATGAATTTAATTGCAGTTTTTAGGCATTTTGCGTGTACGT
TATACTAAAGTTCTGCCATACATTATGATGTGGGTCGCTTACGTTCTCTACACTACGTTTTTCCTTCGCCCTTTTAGAATAA
AAGTAATTAATTACTTTACAAGTAATATTATTTATAATAGCATATAATGACTTTACAGCGGAGTTTTATATTTCTAATACAT
TATAGTTACGTCTTATGTACTATAGAGAGCTATTCGCAGACTTGGTCTTTTTTTAAGAGGTGTAAAACATTGGTTAGGTTTT

		TAAAGATTGTAATATGACTATAGGCCGGATAATTTCGTGTTTCACAAGGCAAAGAAAGCTGTATAATTGCTTCTTTGCCTG<br>TATTTGTTTTTATTAGTTTACTAGCTGTCGTTTAAATTAAATAATACGTTTGTGCGTATGCGATATCTGTATATGTACACAC<br>CTACGTTAATGAGATTGCCAGAATTTTGTGGCATCTATTTTTATTCTTCCATGATGGAAAATTTTATTACGTCATTGTTGTT<br>TCGTACCCACGTAACATATTCCACACTAACATGTTATGCGCAATTTGTATTCATAGAGGATTTTGTACATTATGCAAGTGAC<br>TTTTATGCAAGTTGCTTGTAACTTGTATATTAAAAACAATTGGCTTGCAAGTTGTATGTTTACGTAGGTTAATTGCTCGCGA<br>GTTTACAAGTTCTGCTTGCATTCAAATTGCTTGCATTTGGATACAAGTCGCAAGAAATTCTAGAATTTTCGATTTACATAGA<br>AATTTTGTAATTTGTGAAAATAATAAAATTTTTGTCACGTTACACAGAGATATTGTTTAATTAATTTAATATATAATAAAAT<br>GGGCAACAATGGCAATGGATTACAAACTTGCATTATGCATGCGAATTAAATTGTCGCCTGCGAATCCATCTTGCAAGTGAC<br>TTGTATCTGTGACGTAACACTTGCATAGTTACTACATTAGAAAAAAAACGTGTTTTCATAAATAAATATTGTTTGTAATGTA<br>AAGCTCTGCTAAATAAATACATAGACTATAAAATTAAAACGCTTATGACTGGCGACATCTGGATTTTCTGCCAAAATAGTA<br>AAATGTTTTTTTAAATAATTACTTTGATACATAATGATGGATACAGGCAAACAATCATTCAGTTTATTTGTGTGTATATTAA<br>AATTTTTGGCCTAGTTGTATAAAGACTTACGTAACGATCTTGTTCCGTTTACTCGATAAGTTTGTATGGAAATCTTAGTTTC<br>CGAAATTATTCGTTAGAGGCGCTGCTAGGTTCGATACAGACCTACTGATGTCTATATATATATGAATGAATGAATGAGTAT<br>ACTTTATTTGCATTATACAATATAGGGAATAAACAAGACAATTTACAAACATAACTATACAATTGTATAAGTACAGTAAGG<br>CGGTCTTATTGCTGAGAGCAATCTTTTCAAGACAACCTTATAATGTACTATATATAATGGTAGGTAATGGACTATATATAT<br>ATATATATATATATATATATATATATATATATATATATATATATATATATATATATATATATATATATATATATATATAACGAGAA<br>TTTAAAATTTTATTAACTTAATATCTTTGTCGAAAAAATTGTCGATAACGTCGTCGTAATGTCGTTGGGAAGTTTCGCGAGA<br>CAAAGCATTGTACTTACCCCGAAGAAGAATTGCTGATGGAGTGGAAACAATTGACGGAAATTTTATAAAATATCCGTTAT<br>ATATAGACATCGTTAAAAATTTGCTCAAGTTTTGAAAGTAAAGGTTATTGAGTAAACGTGAATGCAGGTAAATTTAAATAC<br>ATGTATGACTTACAATAGCCACTTAAAAGGCCGGCAGCACAACTGCAATGCCGCTGGTGTTGTGGGTGATCATGGGCGGC<br>GGTAGTCACTTACCATCAAGTTAGCCGCATGCTCGTTTGCCCTCTGTGTTGTAAAAAAAAACTCAGATGTTGCCAGTTTAC<br>CAGTAAAAACCTCGATAAATGTACAGTAACCCATCACCTAATACATTTTGTATTGCGATGGCATATTTGTTAATAAAACAT<br>TTTTTTTGTAAATTTAACACCTTATTTCAAGTTTCATAGACGAAGGAGTGACAGTTTACTTTTTTTTATACTATTAGTAGTTT<br>TTAGTTTTTTTAATCAATAAAAATAGAATTTGTTTATAGTAACACTTTCGTTTGATTTTGGCGCCTAAAATATAT
MSTRG.4<br>482	E2 ubiquitin-<br>conjugating<br>enzyme	AATGAAATGGAAGTAGACGCATCGGATGAGAAGCTAGAGTGCTTGGAAGAAGTACTGAAGGCGGACGCGAACTGCCCCA<br>GCATCAAGATTGAGCCCAGTGGAGACATGTCACTGCCAATGGCCAGTAGTCAAGAAGACACCGTTACAGAGGAGAAAAA<br>CGATTCAGGGATCAGCCCTGAGCCGACAAAGGAAGATAGCTCACTACTGAACGGTGACAGCAAGTACTCGGACGGCAAC<br>GACGATGACTCCGACAACTGGGAGAACACGAGTAGCGACGGCAGCGACACCGACAGTGGCGCGACGTGGTCGTCGCGGT<br>GCTCGTCGGCGGCGTCGCAGCGCGGCAAGCGTTCGCCGCAGCTGGCCGTGCGGCTGCTACGCGGNNNNNN
MSTRG.4<br>483	E2 ubiquitin-<br>conjugating<br>enzyme UBE2O-<br>like	GGAAATTATATTTTATTTATTTATTCATTTTACACTTTATTGCACCAAGTAAAAATAAAAATAACAAATACAGTAAATATTA<br>ATGAGAAGTGTAACAAAAGGCGGCCTTATACCTCATGATAATCCACGTAAATGTATCAACCTTTGAAATTAGATTTCCAAT<br>CCTCCGTTTTGCCATGAAAAACGAACAATCAGTATTAAATAAAAGTTATTTTCGACAGGATGGAACCATAGAGATGGGCA<br>TACCATCGACCCAACTGTATCCGATACACCATTTGGATGGTCAAGAGTGCTTTCCCGGCGATTTCGTAGTTAGCGGCGCTG<br>CCAATATTGAGGAGAATCAGCAGCTGAAACACAGAGAGTATGGCGTGGTGCAACGCGTAGACCACCACGGCCGCACCGC<br>CATCGTGCACTGGTACCGGACCTACACCAGCGCGGATGAGCCTGTTCCCCAGATGCTGAACGAAAGCGAAATGAGCGTTT<br>ACGACCTGAAGGACCACCCAGATTTCCAATATCGACCGGGGACAGTGGTTATACGAGTGGCCAACTTCACTGGCGAAGAC<br>GCCAACTGCACCGCCGGCCAAGTCATAGACAACTTCCCCACCGGCCGCGTCAAGGTCTGGTGGGTGGATGGGCACACCAG<br>CATGTGCTGGCCACAGGATCTTTACAAGG
MSTRG.4	Baculoviral IAP	GCAGATGTGTTAGCGGATAGTTTCGGTCTGGCGAAGAGTAGCAACTTTAGCAAGCCGAAAATTATACCACAAGGTCTCAC

484	repeat-containing protein 6	AAATATTATGAAAGATATGGTGGACCAGATGACTGCGGAATCTGAGGACTCGGAGTCTGAAGAGAAACTGGAGCAGTAC<br>ATTTTTGTACCATTCAAGAAGGAGAGCAAATCGAAGACTACTGTTGATAATAGTAACAG
MSTRG.4485	Uncharacterized protein KIAA1109	ACCGAATTATGCACTGTAATCTACGAATGCCCTCCAATGAAATTGAACTCGCTCTGACGTGTTTCCTATTAAAAAAGCGCG<br>ATTAAAAATTTTGCAAGTAAATAGTATCAACATACCTGCTTCTTTCTTTGTTGCACTTGTAAAAGATACAGCGATTAGTTAA<br>AAACTTGTCAGCAAAGGTGGACTTATCTCTAAAAGAGATCTCTTCCCGCTGGTTATATTTTTTTTTTTCATAATACATTACA<br>GCGGTGTAGAGACCAACCTAGTGCTGGCAGCTGGCGTGGGTGACTCCGGGGAGACGCTGCTATTGGCTGATGAAGACAAC<br>CGCCTCGCTGACATCATTGAGGGTAGTAGCTGGAGCTCCATGGAGAGCGAGCCTATCAATGGGAACGGCGCGCGACCTAG<br>CTCGAGCGGCGGCGGCGGCAGTGGCGGCGGCGGTAGTGGCGGCGGCGGACGGGCGGGGTCGTCGCAGGCGGGCGGCGTG<br>CAGCGGCAGCGGTCGCTGCCGGCGCGGCCGTCGCAGTGGGCGGACGACGCCGAGCTGGACCGCCTGGCCGACCTGGCGC<br>ACCTCGCCGACCTCGACCTCGTGCAGCTGCGCCGCCGTCCGCACCACGACCTGGGCGCTCCGTCGTCAGAGGGCGCCAAT<br>GCGGATGGCAAACAGGGCAAGAGCAAGACCGCGGAGCCGAACATTGATT
MSTRG.4509	Glutaminyl-peptide cyclotransferase-like	CTTGAGAGGAGAGCGGGGAGGGGTACTCACTGACAGAAGGACGAAGGTTGTCTGATGTGTTGTCGTTTTTCTAACTTGTTT<br>TTGAAATGATTCAAATAATCTGTGATTACTAATTATTTTTTGTACTAATCGTATGGATTGTTCAATCTAAATAATTAATATT<br>TATTAAATTAGCGCGTTTTGTCCTTATAAAGTTAACACAAAAATATTTAACTAATGAAATATATATAAGAAATCACGTTAA<br>TTATATTTAAAAGTGCGATTTTTGTAGATAATATATTAAAAATGATCAAGCACTTGGTAGTGGCCGTGTTGTGTTTTACGTT<br>TGTAAACACTGATGGGGCATTAAAATTCTACGAAGAGAAGAATTTCCATGAAGCAAAAGAATTGACTGATAATGATGTTA<br>TATCTATAGCTAGAATGTCAAATATGGATGATTTTAAATCTATATTAAATGAAATTTTAGTGCCACGAGTTGTCGGCACTC<br>CAAACCATGAAAAAGTTGGCAACTACATAGCAAACGAGATGAAAACGTTGGGTTGGGATGTCACAGAAAATGTTTTCCCT<br>GACCAGACACCTGTATTTGGAACATTGAACTTTAAAAATATCATAGCAAAGTTGAATCCTGATGCAGATAGATTTTTAGTG<br>CTCGCTTGCCATTATGATAGTAAATATACAA
MSTRG.4513	Phosphoglycolate phosphatase 1B	CCAATACAGATAGGAAAGCCAGGAAAAATATTTGGGGAGTTCGCTATGAAGCGAGCAGGCCTTTCAGATCCGAGCAGAAT<br>ACTCTTCATTGGTGATATGATCGAACAAGACGTGGGATTAGGCAAACAGACAGGGTTCAAAACTCTCTTAGTGTTGACTAG<br>CCTCACGAAGGAAGACATGTTGAATCATGAAACAATAAAACCTGATTACTACGCCAAATCCTTGGGGAGTATAGTGCCAT<br>TACTGAAATTTTAAAATAAAACATCATTGAATTACTTAAATGAAATTCAGTTGAAACATGTAACTACTTAACAGCATTTAT<br>TGACTACTATCTTTTACTCGTGACTTCGTTCACATGACCTGTCATTGATAAAGAAAAATTATATCCAATTTATGTTCTTCCC<br>AAATTGTATGTATATACCAAATTTCAATTGGTTATTAATCAAGTGTTAAGTTGCCAAATTGTAACAGACAGACTTTCACAT<br>ATATAATATCTGTGAGGATCGTCTTAACTGCATTTGTTTATCAGTAC
MSTRG.4525	Modifier of mdg4	AGAATATGTATACATCATGTCAAAGAAGAGGAAGCGGCTGCTTAAACTGGATGATTACACATATTACCAAAAGCGTAATA<br>CTAATGGGAAAATATCATGGGTATTTTGTTACTAAGATCGCAGCGAGGGAGACCACTGTTACAAATAAATGGTTACACGTT<br>TTATCAGAAGCGTGTTACAAATCAGAAAATAAGATGGGTCTGTTCAACACATCACAATCGGAGGTGCAGTGCGGCGGTAT<br>ACACGATCGATGATCAAATAGTTAGAATGTACACGATGCATAATCATTAAAATATTCATCGTAAATTTAATATTCTAAAGG<br>TGATTAATTGTAAAGGCTTTGTCTTTAGTACTATGTTTTTAAATATGAAACACATTGTTAATATTACTATATATACAGCAAT<br>ATCTTAAATTATACAGATATAGCATCGAACCTCAAAAACGGGTCAACTTTTTGTCAGCTAATTGTCAAATTAGCTTTTTTTT<br>AACAGCGTTCTATATGCTGACTCATAAAATGTATCTTGATGCATTATTTAATAAGTAAAATAAGTGTTTACGCATTTACCA<br>GAGGGAGACTTTGTACAAAAGTCGATTGCTAGGTACCCAACCTCTGTCCCTGTGTTTCGGTTTGAAGGGTGGAACATGGCG<br>TGAATTTACAGGGTACAGAGGAATAACATCTGAGTCCTCAGGAATGGCAACGCATGGGGGGTATCATGGGCGAAACAGTA<br>TACTCTGTTATGTCCACGGTACTGCTCATGTCTATAGGCGACGGTTACCACTTTCCATCAGGTGGACAGCTTGTTTGCCATT<br>CTAAGTTGTATAAAAAAAAATAAAAAAATGCAGAAATTATAAGACATAATAAATATAGAAGTTATAAAGGAATCACTCAT<br>AACTATTTATCACGCATAATTGTATGAAAAAGTTGACCCGTTTTTTGCAAAGTATTTCTATGGAGACACTCCATCTTTAATA

		AAGGCGGTATACACAATTTATGGCAAAATAATGAGAATGTATACAACTTGATCTCAGCTGTCTATTGCCCACTGGTGAGCA<br>TAAGCCTACCCTAAAGAGGAGGTTTACCAGTAGTTGCCACAATTAGCAGATGGCTTGGCAACAGATTAATATAAAGACA<br>TAGCATTATTTTACTAAACACAGTATGACGGTGTTGACACTTGATAGCGTGATAGTGATAACATCAACACAATTATTAAAG<br>TATTAATTTAAAACTCTAACTAGGATAAAAGAATGTGATGATTCTAAAGGTCTCATCTTAAATATTTTTTAAATATAAAAG<br>ACCCCATAAGTGCTAAGTGTAGTTTGGCGGTATATACAATCGATGGCGTAATATAGAGCAATATACAATACGCACAATCA<br>TTAAGCTTTGATCTAAAACTTAAACTTGGATCGAAGAGTGTGATAATTATCATGGACTCGTATCTAGTTATATTTTATTATA<br>ATAAATATTAGATTAACAATATATTTTATTGCAGTAAAGTCTATAAGTCTGACCAAATTAGAACAGCATACTATAACGTAT<br>TCTTAAGAATTGATTTAGATTATGTTTATGTACAAAAATCATGTATACTCATGTATGTATGTCCATTGACGCTAATCTCCGA<br>TATTCGTTTCGTAAATGTGTATAATTTGCTCCATCTGGAGAAATGATAGTTTACTTAGATATGACTATAGTTTTTAATACG<br>AAAAATTGTGACAATATTGTAAGTCTTATAGGTATAGGTGCCTTGCAAGTGACGTAAATAAACATCTCTTAAATTTTATTA<br>TAAAATTAATTTATTATTACTTATATCGAAGTTTAAGAAGAGAAATAATTCCATGGGACAGATAAAGGAAATACTAGGCG<br>TCCATGTCGTATAAAGAGATCAGAGATATGGTCTGGAATAGAGCTGGTTGGAAGAGAATACATGATATACTTGCCCTAAT<br>AAAAGGAGACTCTTAAAAAAATATATCAAAGTATTTTAATAATATAGTATAGTTGCCATCTCCTCTCCTTAAGAGGAGAGA<br>TGAGAATTTGTGTAAAAATTGAAAAATAATAATAATCTGCCCGCATTGTACGCCGGTTTCTCTCCCTGGGGCCCTG
MSTRG.4537	RNA-binding protein 25	CGGCGGCGCGACGTCGCACACTCGTTACATAGACACACTCACACATACACCTAGTTTATCCTTAGAGGGAGGCGCGCGAG<br>CGAGAGAGGGAGCGCGAAATGCGCGAGAGGGAGAGAGAACGGGAACGCGAGAGGGACAGGGAGAGAGAACGCGAACG<br>GGAACGCGACCGGGAGCGATCGCGCGAGCGCGAACGCGAGCGCGAGCGCGAACGTGACCGCGAACGCGAGCGGTCGCGC<br>GGCCGCAGCCGCAGCGAGGCGGACTCGCGCCGCGAGAAGGAGATGGAGGAGGAGGCCAGGGAGAAGAAACGTCTCGAG<br>AAGAAGGCGCGGGAGAAGGAGGCCGCCTACCAGGAGAGGTTGCGCACGTGGGAGGGCCGCGAGCGGCGCAAGGCCAAG<br>GAGTACGAGAAGGAGCGCGAGCGCGAGCGCTGCCGCGAGGAGGAGCGCGAGCGCGAGGCCAAGCGCCTCAAGGAGTTCC<br>TCGAGGACTACGACGACGAGCGCGACGACCACAAGTTCTACAAGGGCAAGGAGCTGCAGCGGCGGCTGGGCGCGCGCGT<br>GCGCGAGGCGGAGGCGGACGGGCGCGAGCGCGCGCGCGAGGCGGCCGAGCTGGCGGCGCTGCGGCGCGCCGTGTGGGCG<br>G
MSTRG.4540	Uncharacterized protein LOC106136884	GCTGCTGGCGCTGGCCGCCGTCGGGCCCGCCGGAGAGATGCTCATAAGCAAGGACACGGCCGGGGAGATACTGCGCATGT<br>TCAGCGAAGCCCTGCTGGCGGTGGAGCCGCCCGCCTGCACCGGCCGCGAGCGCGCGCAGGTGGCGCACACGGCGAGCCTG<br>GCGGCGCAGCTGCTCACCACGCTGCCGGACGCGACCTGCCCCCACTACGACGACCTAGTGTTCAGCCTTTTCCGGCTCGAT<br>TTACTTGTACAGTGTAAATGAATTGGACATACCGAGAATCGAACACGCGGCCTCGCTGTGCCCTTATCTACAGTGTGACGG<br>CAGCCTGGCGGAGCTCACCGCTCAGTTATTCGACTTCCCTACCTCCCCCACCGATACCGCCGCCGCAGCTCCGCTCGAAGT<br>GTTCGCGTTACGGTACGACTGCATCGTCGGCAAACTGAACTGTCCGTTTGATGACGACAACGAACATATCAAGAGAGTCG<br>TCGATGAATCCGCCGAAGCTGAAGTCGAAGAGTTCACGAGAGACAGCTTTATAGCAGATGCCACCAAGATTCTGTTTCGT<br>GCTATGTACCTGCGAACAGTTTTAATGCATAAAATTTCGTGCGATAATGACGATGACGATGCGCCGGAAGAGAACAGGCG<br>CAGCACGCTGTTGGACGATAAGTACTTGTGCACGGAGTTCACCAAAGTGCTGAGAGACTATGCGATCATAGATTCATTGC<br>ATGAAGGTTATGCCTTTTGGCTACATTATGAGTTAATTGAAGAAACCAAAGCAAAATTAGATTTTATACTGGAAGACATCG<br>TTCGAGGAACGTCGATACCTACACGAAACAGGATATTAGCTGTTCTAACACAACTAGCAGCTAAGGAGGGCTATTACTGG<br>TCATACGCGAGACGGTGGTACGATGTTAAGATGAAGGAGCTGGTCGAGCCATCGGAGGTGGAATC
MSTRG.4556	Hypothetical protein	TCAGACGTATATGTCTGACATGGCTCATGTAGAACTTAGCGTCAGACGTATATGTCTGACATGGCGATTAACGTGTTAATG<br>TACCCTTTTTGAAGAACTCCATACTGTAGTCTCTTTTGATTAGCGTCGGTACCCAGTGGATATTACTGTAACTTATACACCG<br>CGGTGGTATTAAGTTCGAATCTAGCCTACACCAAAAATATGCGTGAATTTTGATTTCCTACTTATTTCTGAAAACTATAATT<br>TTAATATAAAGCACTGTAAGTTATAACATACTGTATAACTTACAGTAGTTATGAAACTACAGTTGTGTTTTTACGTATTTTT

TTTTTATGCATCTTAAAATGATAAACAGTATTTTTTTTTCTTATCTGCATGAAACATGCATCGTACTGTCTCTTAAATTGGAA
CGATTAGAATGCATAATTTAAAAAAAAAATTCATTAAAATCAGTCGACCCGTTTCGGAGATTGGCGCGAACGAACATGCAG
TTCTAATATATAATGGTATAACAAATCCTATTATTGAGAATTCTAATAAAAAACTTGAGGGAAAATTAATAAAATGTTTGA
TATGAATGTCACTTTGATTTGTTGCGTGGAAATGTTTTATTTTATTTTATTTTTAAATTATTATTTATTTTTTTTATAAAATTT
ATGTGTAATTTTTTTTTATATTGTGTCGTTTGTACTGTTTAAGTTCTAATGTCTAAGGACTACTTAATATAACACGTAAGGGT
GTCCACACACGATTTATATACATGTCCTAATCGTCATCGTCATCATCATTTCAGCCATTACCCGTCCACTGCTGGACATAAG
CCTCCCCCATAGACTGCCATAAACAACGGTCCTGAGCCACCTGCGTCCACTGACTGCCTGCAATGCATTTCAGGTCGTCAC
TCCATCTAGTGGGGGGGTCGTCCTACACTGCCTTTGCCCGTACGCGGTCGCCACTCAAGAAACTTTATTCCCATTTTTATAA
AGACTAAAAATTACTATTTTAACTGTTACAGCGTTTTAACACTTTATTGGAAACTAGCGACCCGCCCCGGCTTCGCACGGA
TGCAATGTTGATACTAAATAGCGTCGCCAGCCCCCCATTACATTACTACATCACATTAGAAACCTGTAAAATTATCAGTGT
TTCTCTACAATATTATGCATGTATTATACATATAAACCTTCCTCTTGAATCACTCTATCTATTAAAAAATACCGCTTCAAAA
TCCGTTGCGTAGTTTTAACGATCTAAGCAATACATAGGGACAGACAGACGGGAATCGACTTTGTTTTATACTATATAGTGT
ACTAAGAAATAGATAAAATACTTTAATAGTTAAAAAATGTTTGTGACGACGTATTTATAAATCAGGAATCATGATCAATCA
GGCCGTTGATTTTGAAAGTCGCGTGTTGATACAATTATGTGGTGCATTTCTGACTTGAGAATGTGCACAGGTAATGACAGA
TAGATAGATAATTTATTTGCATTACTTACAGCACACAATTTACAATACAAACATACAACAATACACAGTAAAAGAGATAA
TAGGAATAACATAGAAGGAATGATAAAATAAAAACTGTATATCATATGGTCGCCTCCTTTCATTCTTATGCAAATATAATT
AGTGAAAAGAGTACGAAAGATACACCGTCAGTTGCTATTTTTCTCAATTCATAGCAGGCTAGTGTATAGAAAATGTGCATA
TACAATTTTTGTTCATTTACATTACTAAATTATATATTCGACACAGTTTATGTAGAAAGTATGTGCAAGCAGGTTGTATCTC
AATTTGGTAGGGTTATACGGCCAATAAAACCAAGTGGCATATCTTGATGGAATTTGACTTCTACGGACTAGTCGAAAGGC
AGATGCGTTTTTATATATCTATCCATCTCTAACGCATTGGCAATGTTAGAAATACAACGAGCAGCGCACTATGTGATTTAT
GCATTCCATCTATTCCGAAATCTTTGTTATAGACGCTCTTAAAAAAAAAAAAAGAAAAAACAAAAGATAGTGGACTGTCAA
ATCTAATCGTAGTGTTTCCATATTCTAAAGATATGGAATAGATATCAAATATCATAGATAAAGATGATGCATTGTATATTA
ATTAACAAATTCAATAAATCGCATTACATCGCCCTTAATTTGCTTTAAACGAGAGTGCATCGCGCCCTTTCAAGACAAGGA
CGATAATCCCTTTTTTATTGAAAACAAATTGTACAAGATAAGATATTATATTTATGTTATTTCAATAGGTATTAACATGACA
GAGAATTAATGTTATGTTACCAGTAGTCGCCACACTTGGCAAGCGTGTTGGCAACCGCAGTTGGGCTAGAGATGTCTTAAC
CCTTAAATACGAAGTTTCAAATTTGATACCGGGGTTATAAATCGCATTGGGCGCCTACAATCGATTTTATAGTAATATTTG
GCTGTTTACATGTTACAAAATGTTTTTTTTTTTGTTTTTCAAAATAAATATATAAAAATTTATTTATAAATATTTGATTTTTT
TTACTATTTTCCTCATTAGTAAACAGTAAAGGGTACACTTATATATTTTTTGATTTGATTTAAAAAATCAGGCTTTTAAGGG
TTAAGAGTGACGCTGCTGTCCATCCCTCCCCGTCCATTAGGCCTCTTACGACACCCACGGGAAAGGAAGGGGTGGCCCGTT
CTATGCCGGGACCACACGGTAATAAACGGTTGTAATATTAACAAGGATATCTGCAGTGTCTTAGGTATTATTTTCAAAACG
AATATGTTATTGATTCGACTGTATTTTTTTATGTTTTACCCTTCTTAATATTTTTTAAATGGTGATAAAGTTTTGATGATTCTT
TTTTAACTGCAATGTATTCACTGCCATGTTAGGCTCATGCTAATTCTGCCAATATTGTGAAAAAAAAAAAATTATTTATTTTC
ATTGTTAATATTTATACTTAATTACTAATTTATTAGATATATAATTTTAAAAATTTACAGAATTATACTAATTTATATATTAA
TTAATTGACGCGAATTTAATGGAATTGAATTATATTATTATGTTAATTTTATTACTTATGTCCGATAATTGTTACGCCTTCAT
TTGGAAGTCTCCAGGGTCAGTACGGTGCGTACATCGCAGCGTCTCGCCTCGCACTCGTCCTTACACAACTAATTCTTCAAC
CAATTATATATAAATGTATAAAAATGGATATAAGTATAAAAATGGATATAAGTTTAAAAATGGATATAAAATTTAAAAAT
GGAATTATACTTATATGTAACATACAATGTACTTTGTAAACGATTCTCAATTCAGAAAGCAGTTAGTGTAGGGTTGCTCTC
AGATAAAGGAGAAATGTTTTTATCTTGTAGTTAGTGATAGTTTGTAAGCGTAGCGATCAAATTGTTCTAGTTAGAATGAAA
TAGTAACCGAATGTTTAATCTAATAAAGTATTTAATTGAA

MSTRG.4557	Axis inhibition protein	CGCACACGGTGGTCGTGGTGACGTTCCTGGACGAGAACGTGCCGTACCGGTTCAAGGTGCCCGCCGCGCCGCTCACGCTC<br>AAGATCTTCAAGGAGTACTTGCCCAGGAAGGGCAATTATAGATATTTCTTCAAAACGAACTGCGCAGACCTAGATACGGT<br>GATCCAGGAGGAAGTGAGCAACGACAACGATACGCTGCCCATGTTCGAGGGGAAAGTCATGGCGCGCGTCAAGAGCATA<br>GAGTAGCCGCTCCACTACCGCGCCCTGTCGTCTACAGCTCGCAATAATGAGTCACAATTCACGTGTAATAGACTAAGGAAT<br>CGATTTATACAGACAAATACAGTGACGCTTGTAATACGGGTGGAAATATTCCCAACTATTGTATTCCGAATACAATTTTTA<br>AAATTTGGAATATTCATGCGATTCTGTGTTATTCATTTGTTTGAACTGCCAGATTTTTGATAAGATTTTTTAAAAAATGTT<br>GCCATTATCTTGTCTTTGAACAGAAATAAAAGTAGGCGTAGCGTAGACGGTATGCCGGTTGGTTTTGTTTTTTTTTTTTAT<br>GTAAAAAAAATAAAGCCTTATTAAATTTCTTTGACTTCGAATTAAATTACCACAGAAAAGGCTACTTATGCAAAATAGATT<br>TTATATTATATACCATAGGTATACATTCTTTTAAGTTGCAATAAATAGTACATTCTTAAATATTTAAATTTCACTTATTTTTC<br>GTTTATTACAATTCTTATTTATTTGTTACAATTAATTGAAGAACTAAATAATGTTTAATATAATTTTTATTATTGACAAATAT<br>AATTAATATACAGTTTTTTTTATTCTGCTTTATATTTTTATTGCTCCGACGATTTTTAAGAAAGTAAAAGTGATTTACTTTTA<br>AAATGAAAGAAAGCATATGTATTATATTAATACTTGTAAAATAACATGTAGAAGACAGGGCGAGGCTAGATTTATTATAG<br>ATATATTTATTTATAGCAATTACGATCGCCACATCGTAATAGCCGTAAACATGTTGTAAAATAAAAATTCGTTCCTCGCGA<br>TGCTAGCCGCGTGTGTGCACTAGTTGACGAAATGGACTTTTTTATTAGTTACATTACATACATTTCGAATTTTACAAAGGTT<br>TCGTAAGATTGTTCCAGCGTAATATCATTTAAAAGGATTTATATTCGAGATTAGTTAATGTACCGAAGCGTTTCTACTTCTA<br>CACACGTTAACTTTTATACTTAGTGGAATTGATCTTATATATTATAGACAATAGTTTTGTTTGATGCCCAAATTTGATCAAC<br>CAACTTCAAAAATTGTTCTTAATTAAAAAATACCTTAATTTTGGGTTTAATCGAGTGAGAAAGAGGGGGGGAGAGTGAAA<br>GTTACTACGCGGTTCTCAGTCTCTTACCATCCCATTCACTCTCTCTCTCCCTCCATTTTCGTCACTTCCCCACAAAGTTCATC<br>GATGCATACCATTTCAGACAGCTAACTTATATCATGCGAACTCCACTCTGTAGATTTTGTTGTCATTTAAATTTGAACCATT<br>ATTCCAACGCTTTTAAGTTGAATTACTTACGTATTTATATAAGTTGGCTGCTTGAATGTTTGGAATAGTTTATAAGTGTCGT<br>TTCACCTCTCCAATGCGGTGACGTTTGTAGAAATAGAAACGCTCCTGCGTTTGCCAATTGTCCAACCACAGAGCAGTGCAC<br>GACCGGCGTTGGTGTTCTGCATTATTTTTTTACTTGAACTAGATGAGTACGCTTACTACTGTACGATATTTTTTTTAATGCGC<br>ATTTTTTTTTTATAATATACCTAGTGTTACTTGAATGACTGCTTAGTTTGTATATTTTTTTAAATTAATATTTTAGAGTATAA<br>ATGAGGTGTTCTGTTTGGAAAATATAAAAATTATGTTAAAAAAAATTTGGCATTCGGAATTAATGCGACGAGTTTAATATG<br>AATTTGTACAGTTAAATACAGAATGTGTTGTTTGTTTTTGTTTCTATTTATCGTTATATCTGATTTTATTTTTTGATCCATCA<br>ATGATAATAAATCATTATTAATATTAGCTTTTGTAAGAATATATGCACATCCAAAAGAAACGACACATTCTATGTTTAGCT<br>ACCGGTTCATTCGAAATTTCAAAACAATGCATGATGGGATCTCAATCAACTATACAATCTATGAATTAAATACAATAAGAG<br>GAAGGTAATGTAAATTATTTCCGTTCTGCAGCTGTGAGTAGGGGTAATTAGCGAAATAGAAGTAAGAATAATTTGTTTATT<br>CTATCTCATAACCTGAGCAGTTACATGTGTACCAATGCTTTTGCATCGGAGGGAACGCATTTACGGAGAGAATTTAAATTT<br>ATATACGGTTTCAAAGGAATTTTCTTTCATGAACTTTCTGGCTGTATGGGATGAGCTCTCTGCCAAAAATTTTCCTTAGAGA<br>CTACAGTATGTGATTCATCATAAAGGATATACTATGGTTTCTTCAAGGTCAGCAACACACACGTGACACCCCTAGTTTTAC<br>AAGCGTCTATAGGCTACAGTGACCGTTTACCATCAGACGGGCTGTATGTTTGTTTGCCACCTTTGTTGGTACAAAAAAGAA<br>ATGCAGAAGATTGATAATATTATCTATTATTATTCACATATACATACGCATAATAATCACGAATCGTTATATACTTTTTGCG<br>TAAATCGTTACTTGCTACGTAACTGTAATTTTACAGGTAATAAAATGTAAACATCCACTAAGAGCAGTTGTTAGGTATTCC<br>TACAGTTTATAACAGTTTTTATATAGGCAAAATTATACGATATTGTATTATATAAAAATAATAATACATTAAATTCTCTCCG<br>TAAATTGGGTACGACTCGGAAACTCGTCCACCTGTTTGACTTCAAATACATACAATAATGTTCCGAATTAGATCGCTTCCA<br>TTTTTTACATTTTAAATCAATAATTTCGTTGGCTGTTTGAACGAATGTTCGGAGTGTTATTGTTTGTAAGAGTGACGTAATC<br>TATGACGTAACTGCATCACAACACGTTGCTACTGCGAAATCATTGCCAGACTGAGTTTCCCTAAGTCAACTTAGTTACGAT<br>AACTAGTCTATGTCCCCGCGCGCGTTGCTCCGCCGACCGCAGTCGTGTCGCTAGTGTCGTGGTCGTGTAGTAGCCGTGTCG

		TGGTCGTGTCGGCGTCGCACCGCGCGGGAACGTGCCCGTTAGTACTTACTATTTCGCATATTGTAAAATATAAGTTAGA<br>ATGTCATGTCCGGTCCAGTGTGCAGCCGGCGGCGTCACTGCGTACACTTGTACGAGGATTTGTTACACTTTTCATGTATTAT<br>ACACTCGAGGACACAAATTTATTACATATATACACATATCTGTCACGCCTGTCTCCCATTAGGGTAGGCAGAGACAATTGA<br>ACACCAAATTATTGTTGCCTCAAAGTATATTTTTTACAATATTTATGTTCGATTATTAAATAGTAATATTATTTATAGAATT<br>AAAAAAAATGTCTTCTAGTATTTTTATCTTTTAAAACACAACTTTTATTAATGCAGTTATTAACGAATAATTTTTTTCTTGA<br>ATTTTAAACTTCTTAAATTTTAAATAAATTCTATAATTCTTTTTATGATTAAAACAAACAATGGCTGTCATCAGAAAATAAT<br>CTATTAATTGTGCCTTTGGGTGTATAATACGAAGAGCTGACCACTGTGTTTGTTAGGTTTCTAGTATTGAATAATATTGGAA<br>TTGAATGTTTGTTGTCGCGCTGGTGGGCCAAGTGTCGCGGCCCCGCCGCCGACTGCACGCGAGTATTAGAGGGGCGCCGCC<br>AGCGAAACGGGTGCACTATAACGAATGCACGATCTTGAGAATCGGGTGCGAAACATGTGCCTTCAATGTTGATACATTTTT<br>CATAGTTGATGCTTCTATTTAGTCGTAAGTTAACACTTGTAAATATAGACGTAAAGCTACTTCGATTCGAAATCTATTTTTT<br>ATTAATTTAATTAAACAAAACATTTTTTTTTATCTATTTATTGCCTAAGATTGCGTTGTGGGTCCGCTCCAACCGTCTCAGA<br>CACGTACAGTATGAATTGTTCTGCTTCTACGAGCTTCTATTTGAAACGGTAGTACAGCCAGCCTCATGATAGGAGGCAGGG<br>TAGTTAACAATATGTTGCGTATTGAAAATATTATTTCACCGAATCATGTACTTTTTCAACTTAAAACTCATTGGTTACATAG<br>ATATTTTTTATGTATCTGCGTTTGGAAGGTTGTGAAGTTGTTGTTTTTAAACTGTATCGAACATTTTACTTGATGACAATTAT<br>TCTGATAATGTAGTGTCATCTATTATGAAGCTGACTGTATATGAAACCAATATATCATCTATCAACATAATGAATTCTTGTA<br>TGTTTCTGTTTTTTTTTTCAAGATCTATATTTGTGGTTGCGACCTAGTAATACTACAAAGATTTGCAAAAGTAATACCAATT<br>CTGGATGCGGGTAACGATTATAATAGAAAAGGCCCTCTTTAATGAATTGTAATTGATGATATTAGATTTACTAATGTATTC<br>CTTCGCCTACTTTTAAGTAGGTATATTTAAAAGAAATTTTATTTTGAGAGTGATTTCTAATCATTAGTAACACGTTCAATTT<br>GCAAAGATTGTTTGTTCATACAATCTCAGCTAATAAATGGTAACATTAGCAAATACATTGCATCGCTACAAATTTTATTTG<br>ATATTCATCATTTTGAGATACGATACGATGATAAAATGGACAGTGTAGACATGAATTTATCGTGTTGTCATAAACACATAC<br>GAAACTTGGTAGAAAATTGAATAAAAATATAACATGTCGGTGTCTGAGACGACTGAGAAACTGCTTCCTATAATGTGTGG<br>TTGCGGAAACAATATGTGTAAATGGAGGAAACTCTGTTTATTCTTACTTGACATATTTGTTAAACATTTTCGATATATCTTA<br>TTATGGTATATTATTTTACCTTATTGACCACATTACAAAAAAAATTATAGTACGAAAGGTGAATTTAATACTATATTACAG<br>CATTCTCTATCAAGCAATCTATGGGAAACAGAAGTGTTGAATTATTTATTTGAAAGTAGACGGAATAATCAACAACTAGTT<br>ATATAAATTACAATAAATGACAAAAAAAAAATTTTTTTGTCTATTTTCTGCAATAAAGAGACATCCAAGACCGTTTACTAA<br>TATTGTCAAGTAAGAAGAAACTAGTGTATTATACCAGTTGATATACATAACGCAATAAGGCGCTTTAAATATAACAGCGTC<br>TCAAAGTACCCTACAATAAGTCCCTAAGGGCACAAAGTATGTCGGAGTTAAAATTGCTTGTTAAATTGTAAAAAAAAGAA<br>TTATAATGTTTTCCATTTAGAGTGTATACGAATGAAAATTAAGCTAAACGATTTGTAATAAAATATTGACGTACTAAAGTA<br>TTATTTCAATGGAACAATGTAACGGTCATTAATAGTAAAAGAATTTCACTAATCGGACTGTATTATTTTTTTATCTAGCACA<br>ATGTAACGATTAAAAATTAAACAGAACTGTGCCATATA
MSTRG.4561	Uncharacterized protein LOC106130173	GGTGGACGCCGACCCGGACGTGCTGCCGCGCACGGACAACTTCGGCCTCAACTACATGGCGTTCGTGGAGCTTTGCGACG<br>AGACCACCGTGCCGGACTTCCTCAGGCTCGCCTTCAACTGCTGCATTTACGAGCCCAAGGCGCGGCCACTATTCCCGGAAA<br>TAGTGAGCAAGCTGGCCGAGGTCAAGGAGGGCCTCGACGATGCCGCTTGGGGGTGCCACACGCCAAACAACTCCTACGAA<br>AGCGAGGAGGCGGACTCGTCGGGCCCGCGCTCGTGCCCCGGACTGTACGCGTGGCGCCGCCCGCTGCGGTACAAGGCGGA<br>CGGCAGCCCGCGGC
MSTRG.4567	Neuroblastoma-amplified sequence-like	GCGCCCGCCACCTGCTCACCAGGCTGGCGCGCGCGCTCGCCCTCAGAGATGAAAAACCTACACAACAAGATTGGGCTGAA<br>CTGCTGAAAGACATCCTTGAGTTACAATCATCGCTGTTCACTTGCGTCTCCAAAGAAGAGTGTTACGAGATATACGCGTCA<br>TCGCTGTTAACATCAGGCGATGCGGCGAGCATCAAGTTGGCGGTGGAGGTGGTGTCGAACGGCAGCGGCGCGGGCGACCG<br>CGTACAGCTGGTGTCAGCCGCCGCCCGCGAGTACTTCAACTCCGCGTCCTCGCTGGCCGACCCCGCGCTCGACCTCGCTAG

		ATGCTGTTTAACATTGATAAAGGATGACAACGAGGAAATAAAAGAGGAGCTGGATCTCATTGCAGCTTTACAGATATTGA<br>ACGCGTTTGGCTTGACTATTCTTCCCATACAAGTTCGTCTCTGCGAGGACCGATTGACCTTGATCGAAGATTGTTTAAAACT<br>GGACCCTAATGCATTCCTGGCGAGTCATAAACTGCTCAAACTAGCCAAGCTGTTAAGAATCGCTGGCGATGATGAACAGA<br>CGAGGGAGGCGGCGGTGCTGCAGCTGGTGGGCGAGTACGCGATGCGCGCGGGCAGCGGCGGGCTGGGCGCGGCCGCGGA<br>CGCGGCGC
MSTRG.4568	Zinc finger CCHC domain-containing protein 24-like	AGTGTACAAATCACTATTGGCAGTTATTAAAAGTCCCTTACATTTAATATATGATCGGAGTATATAAATAAGCAAGCTAGG<br>AGCTACGTGCTTTTAAATTGAACTGAACCTGTGTACAACAAAGTGCAACAGAATTTCATTTGTGAAGAAGTGTAATAAATT<br>ACAATGTCCAAAAAAGAAGGCAAGGGCTTAACACCATACCAAGGCAATAGGCGATGCTTTGGCGAATACCGTTGTACAAA<br>GTGCAACAGAATGTGGATGAGCGCCAACTCCTGGGCCGACTACGGACAGCAATGCACAACTTGCAAAATTAACGTATATC<br>CTCACAAACAAGTTAGTATTTAACTGTTTGGTTTTTTTTTTATACAATATATATTTTTTATATAACTTAGAATAGCAAACA<br>AGCAGCCAGCTCACCTGATGTAAAGTGGTAACCGGCGTCTATAGACATGAATAGTGCTGTGGACATAACGAAAGTATTGT<br>TGGCAATCGAAACACCACGTGCCAATGGCAGAACAAGAAAAAACGGCACCTGGCAACCCTGTTGTCGTATCCCTACGTTG<br>GATCGATAATGGCGAGATGTTTAAATTACAGAAACAATGATTCATAGTGTTCCCATTACGTATTTGTTACCATTCGTCTGTT<br>TATAATAATTACGCCCCGTATAAAACTAATGCTCCTTTGTTTTTAAGTTTTGTTTGGGGGCATTGTTTGATTGAACCGTGAG<br>ATGAACACTTTGGCTATCCCGGGCCATATCCTTTATCTCCCTATACGACATGACGCCTAGTGTTTCCTTTATCTGCCCCATG<br>TAGTTAATCCTCGGTCTTCCTCTTCCTCTTTTGCCCTCTATCTTACCTTCTATTATGCTCTTAAAAAAATTGTTATGTCGTATA<br>AGGTGGCCCAGCATTTTGCCTCTTCTATTCTTTATTATTTAATCAATGTTCTCTTCTCGTTTACTTTTTATTTATTAATTGT<br>GGTATATAAAATTATAATTGAGTTAATGACTTAATCATCTAATTCAGTGTTTTAATTATGTAGAATTGTTATGTAGCTAATA<br>AATTAGTTCATTAAAGACTTGTATCTAATAGTATACTGTGTCACCAATGATACCCTTCATGCATTATCATTCTTCATGACTA<br>AGGTGTTTTTTTTAAAGGGATGAAAATTGAATGGTAACCCCGCCTGCGCTATCGGTATACGCTGGCGGGCACCAGTGAGA<br>GATGATAGATGAGAATGAAAGGGCAGATCAGTAAGCCCATCGTATATATAATTATTTTAAATATCTTTTATGTACATATA<br>TACAATTTTATTCTTTATTCTTTATTGTACACAAAACATATTGTTAATCACAATTCATTATAAACATAATGTACAAAGGCGA<br>AATTATCTCTAATAAGAGTTTTTTCCAACAAAACCTTTTAATGTAAATTGTGTTATTTAAAAAAATAAAAATAGGCAGTCA<br>TACGATCTGCATATATTGTATAAACACATAAGATATGTATACACAAAGAATAATACAAACTAAACATTATAAATTACATA<br>AAATACATAAACCCTGTAAACATACACATATACTTACACATACATACAGACACATATATACACATACATATACATAAACA<br>TATATAAAAAAAATCATTAATTTTATATAAAATTAATGTTTAATATAAAAGTGATTCATGAAAA
MSTRG.4582	Galactokinase-like	AGAATTAAAACGTCTCAACTGTAATAAATTAGTTATAATGAGAGCGCAGCATGTCATCGAAGAAATCTACCGTACCATCG<br>AAGTTGCTGAGAGTTTACGTCGGAACGACTTGGATCGGGTCGGCAAATTATTCTTCCAATCGCATTATTCTCTAAGCAAAT<br>TGATGGAGGTGTCTTGTCCAGAGTTGGATGAGCTCGTTGAAATAATGAAGGACGTACCAGGAGTGCTGGGGGCACGGATG<br>ACAGGAGGTGGCTTTGGAGGGTGTGTCGTAACCTTGGTAAAGAAAGATCAAGTCGATAATTTGAAGAGAGCTGTAATAGA<br>AAATTATAAAGGGAATCCTACATTTTTTATATGTCAGCCAAACGATG
MSTRG.4589	Uncharacterized transmembrane protein DDB_G0289901-like	ATCAATATTTGGCAACAGCGCCATGGCTATTGCAAGGATTGTCAACTGTATCGTCGATAAGAGAACAGATCTGACTCCAGC<br>TGTTTGCAATAACAATTACGGATCAAAATGGGGCTGGCCCAAATACACCGGTGGCTACAATAACGCCGAAGACGTGCTCA<br>AGTTCTACGACCTTCTGAAAGTCCGCCCTCAAATTGACGATCTCGTCGGAAAGTATACGAATGGAAGAGGTGTCGGCTCTC<br>TTACAGGCACCAACAAAATTATTGTGGAGTTAGTGAACGAATTGGCCCGACTACTCGAAAATAACGACAACAAATGGAGC<br>CGCGACGACTTGGTCACAGCATACGACAAGGTTTCCAGCATGATGGGTCTGCTTGGTAACAGTTATAATGACGGACTTGGC<br>GATGATCTCGTGGATTCCATGATCGATTATTGTAATGACGGTTTCAAGTCCCGAGGATACTCTGGTTTGGATTCGTTGGGAT<br>CGGATATAAGGAAGAGAATATCCGGATGCGGACGCTCCGACTTCAATGGCGCTCTGAGATTGGCATCTGACGCCAACAAT<br>AATATCAGTTCCATCGGCGACTCTCGCAATAATTTAGGCATTGACGACGTTAATGATGGAATTGACAGCGGTTTAGATAGA

		ACGAGCAGTGCCAGTAGTAATGCAGCAAGAGTCGGCGATAGTTCCGACTA
MSTRG.4614	Dolichol-phosphate mannosyltransferase subunit 3	CAACTTGACATTATTGACATATTCACAGTTCGTGTTGATTCATTAATATTTTTGTTTTAGAAATATTGAAACAAAAATCTTAAATTTTCGTTTTGGGTTTAGTTATAAATTATAAATAGTTGGTTGTGCAGTGAATAATGCAATATTTCTGTATTGTGTTAAATCTTTAATATGCACTGTCTTATGGGATAATTAGATAAAATATTACTAACTACCAGCATTCCATTAGTATTTATATGTTATTTATATCTTTTAAAAGAACAAAATTTGTAAACAAGAACCATGACTAAGCTAATAGAGTGGATATCAGTCATCTCAGCGGTGTTTGCTGTATGGTACTCTTTAGTAGGAGGATACGTGAAGCATCCCGCAATAGAAAATAATATGACATTAATATTATTTTCGCCTTTAATATTTATTGTATTGTTTGCCATATATGCACCCACTGTTATTCTTTATCGTGTTTTTACATTTAATAATTGTGAAGATGCAGCCAAAGAGCTTCAACAGGAAATACTTGAGGCTAAAAAAGACTTACATGAGAAAGGACTTAGGTGGTGATTGAATCATTCCAGTTGTTTAAATCTAAG
MSTRG.4627	Hemicentin-1	GCTACGAAACGCGGCGCGGAGCAGTCGAGTGCCACAAGCGTATTCGTTATAATCTATCGCGAAATATCGGGACTATAAATATTGAGATTACTTGCGATTTTAAATAAACACAAGTCGCTGTATATAGATTGTTCAGTGCTTGTAACATATTCAAATATCAAGAGAAACCTTTCATGAAGTTTTCTTTGTTTTGAACTGTGATGGTAATATAAAACAAAATGGGGGAATAAAATAAAAAAAAGAGAGAGAGAAAAAAAAAAAACAAATAAACTGGCAGTGAACGTAATGAATGTACGAAACGACTCTGTTCGAATGAAAATGTACTGTTTTGGGTGATATAAGGTGAATTGTTCATGAGGGCGGTATGAGGAACGAAAGGACATGCATCTGGTCGTTGCTTCAGGCTCTCGTGCTTTTACCCACTTTGGCAGTCAAAGACTCTCAAATGACAACGAAGAAGTTTCGGAAGAGCCCGAATTTGAACGCCCAATCGGAAATCACACTTTCTTCTTAGGCAGAGAAGCAGTGCTTGGTTGTGCTGTTTCCAATCTTGGAAAGCATAAAGT
MSTRG.4654	OTU domain-containing protein 7B	CTACAAAATACTCAGACCGAGCCAATTTAGGTTGTTTCCTAAATGGCTTGACCGAGAATTGTCCGCGATACCTCATTTACCCAAAGCAATGTAGGTACCTACTACACATAAGTTCTGACTATATGGACACGTGATACACAGGGATGAGAAGCATGTTTTAAAAAAGGTATTAAGAATGAATGTGGGTGGATATAAGGACAACGGAAGGCCTAAAAGACATAGATGGATGAGTGAAAGATGATATGCACACAAAATTTCATAAAAATCGGTAAAGCCGTTTCGGAGGAGTTTGGTAACAAACACCGTGACACGAGAATTTTATATATAAGATTTAATTCCATTCAGACAATACAAAAATAGGCTCACACACGTTCTTTAAAAATTAACATAAATGTAATTTATTTAACTTAAAATACATCATCTTTGTCACACATAGTGTTGACTATATGTGATCAGATAAATATCAAACTATATGGCTGATCTATTGAAATTTTTACTTATAAATAATTATGTACTCTTTATATAGGAAAATATTCAGTATTATCCTCAAATGCTCCTAACAATTTCAGAACGTTCATCGAAAAGGATCTCCTAGAGACGGCCACACTGGTGACCCTCGAGTCGGCCGGCATCCTGAACTGGTGGCGGCACGGCCGGGTGCCCGGCGCGCCGCGGCTCGGCACCGGTCGCTCGCAGTTCTACGCGGAGCCTGACCGTACCGGTGCCAAAGGGACCACCGGCGCCGGTGAAGTCAAGCGTGACCGGACTCTTTACCTGAGCAAATCCACCTTCTACGACGACCGTCCGCCGTCACCCAAACCGTGCCGCGCGCCACTCTGCATGTACTACGGTAGCCCTGCCAACGGGGACTATTGTTCGCGCTGCGCCCGCTTGCAGTGACGCCCCGCCTGATTTCCGCGCCTATAAGTCATTACTGCTGTCTGTAACAATGCCCCGTCTCCACGGGATTACTGAATCGCTTGCAGTAATGCCCTGCTTTCAAGAAGTTTAACTCCTAATGGGTTGAACTCCTTGAAAGTGGTGAATGGATTGTAAATAATCAGGATTTGAATCTCCATTGTTAGCAATATATTCACCATCATTAGGTGGACCTCAGCGCTGTCCCTCCTGAAAACTATCATAATAAATTCTTGATGAATTTGTGGTGATAAACTGACTGACGGGCCTTATCCTCACCTGTCATCCCTCGCTGGTACCCCACCAGCGTACATCAATAGCACAGACAGGATTACCATTCCATTTATCGTCTATATGCAAACCCTGATTGATGAAAGGTAATAGATGCGGATAAAGGCAAGTCAGTCAAGAACATTTCATCAAGAATTAACCACTGCAGTTTCCAGAGAGGATCGCATAGAGGTCAACGTGACATATATTCCTAAAAATGGCATTACTAGTCCTTATTACTAACAATCCTTCTCCCCTAAAAACCTATGGAATAACTGCAGCCACCTGCTGTAATGATCTTCCATGCTTTCACTCCTCCCATTTTACTCCATGGAAGTGATGTCATCTACGATATCATCTCCATCTTTTGCACTTCTAACCCGGAGTTAATATTTGATGGCGCGTGTTTGTTTGTTTTTAAGAGGAAGAATATAAAAATTGGCGACATAGAAAACAGGTATCGCGAGGACGATGTCTAGCTACCTTTACTTGGTATTATGTTGAGAAACATCGAGAAAATGTTAGATATTGTCCACGTCACGGGTAGCTAATTGATGTATCATTTTAGAATCAAACCCTATAGCACAAGT

		AAACTACAATGGAATTGTGGGTTTTCGTCTTCTATAAAATGTATTAATTACTTCCTCGCGTATAAAATTATTGTTTAGTTCA<br>GGGGTTCCCAATCTTTTTCATCCCGCGGCGCAGTTACAAATGTAGTATTTTGTCACTTCGTCCGACCTAGCTATTTGGTAAA<br>GATAATCGAAAGAAATAAATACAATACCAACAACAATAAAAGTTAGGTTAGATAGATAAGAACAATAAACATTAACAAT<br>ACGATGGGAGCGTTCAAAAAAGGTACCCGCGAATATTTGTGGTTTCAGATAATAATAATAGAAATAGAGGATCGACTCAC<br>GGCGCCCCTCTTTACTTTTTACGGCGCATCAGGATGCAGACGTTCATACTTTGGGAACCCTGGGTTTAGTTACTAAGATAA<br>ATTTTTATTAAACTATAAATATAAGCAAATGCTGTACGCATTCACCTACTGTTGGTTACGTTTTTATCTGATTTTTATTTTTA<br>TTTTGTTTTATAATTTTAACAATAACAATTATAGAAAGATTGTTAAGTTTATAACATGTTGCTCTATCTACAGTGAAACAAA<br>GAAGGCTATTTTTATGCTGCAAAACCTACTAATATAAGTTAAACTTAGAATCGCTTAGTGTATTAGTATTTAACATTACATA<br>AATCATGCAGAGTACTTACCCCCTAATCATTTTGTCATTTAATGACTTTGTTATTTAGTTCATAAAATTGTTCAATCCTGTTT<br>CCTTCACCGTCACCTTATCAAGTTCATTAGAGATGTATGATAAGTGGTAACACAAAGATAACACATTTATATTTTCTATCA<br>GGATATCAATAAAACATACTCTATGACTGTTGTTAATGTAACAAGTGCCAAAAGTGGCTTCTAAATGTATTTAAAGTATTT<br>GAAGGCAAGTGAGACTCCGTGAGAATGATATTATAACTAATGTGTTCAAAATTCAGTGTATCACGCTGAATGGGAAGTAA<br>TTTTGGACAATATGACGCGAGCTACGTAGAAAATTTCATATGATTTAGATGGAAAAAGGTTACATACATATTTGTACTAGA<br>TGAATAGATGTTGAGAAATTGGATCTACCGTAAAACACTTGTGAGTAGTAATAGATTGTTATTGGCAGAATCGAAACCTA<br>ATAAAGCTGACGACTGACCTGCATTGTTAAACATATTTACAAGGCATATTTAGTATACAATATTGTCAATTTCTTAATTATT<br>TCGTTAAAAAACAATACTGTTAACTAAATATGCTAAAAAAGTGTGATGTACGTTGTTACAATTTGAATGTTCATGTAGTAA<br>CAATATGATAAAATAGGTCAGTTAGCTAGCTTAATATATTTTTATAATCAAATCTCTTTTTATATAAAATCAATGTTTTACT<br>CATTTGATAACCACGACATAGTAAATTGTAATTTATGGTGTATCTATTTTAATCTGAGTTCTAAATCTTGGTACATATTCAG<br>TTCAGACATTAACTTGGAACCTCTATTTAAGTTAAAAATCTCAATTGTATATATTTTTCCTTTCAATCCATCTACCTCAAGG<br>CAGAAGAGTCCTAACCTGTTACAAGCATTAGACATTAAATAAAAGCATCAAAATACATACAATATAAATACAAAAACAAT<br>AATTGTCATCCACTTGGACAAAACCTTATCATTAATAAACTTACTAACATACTATCTCTATATAAAATTTTATCAAATGTTT<br>GAATGTAATATCAAAAGAATAAGAAATATTGGATAGAAGTGGCACTCCCGCGAATTCAAAATGTAATCAGTGAGGGTATC<br>TGCTGGGCTTAACGTCCCTATGTAATCAGGGCTGGCTACTCAGTAATACAATCCTAGGTTGCTAATCGGGACGTCACAATA<br>GCAGGTCACGCATCTTTATCATTTGTAGATATGCTGCAATATCACCCTGTAATTTTTTTTATAAAGAATGCTTAGCAAATTA<br>TTTTAGTATTGTAACTTACAATTAGAAAAGTACAGGTTTATACTGTGTTTAATTTTAAATCATATTAATATTTGTAATATTA<br>ATTACATACCCCTATTCAGAATTCGGCATCTGACAGGAAGAATTGATTTTAGCAGCAAAATCTCTCACAAAAATAGTCAAA<br>TCAGTGTCACTGCTTCCTAGATATTTTATTCTTATGGTAATGTGCAAGTGGATAGAACTAAGTAATTGTCTGAATTCAGGGT<br>AATTATTGGATGCACTAACTTTTTAAGATATTACTAGTGATATTAAAGTCTTCTTCACGACATCGTTAATCAGAATATAAGA<br>GCTTGGAGTGCACTCTCCGTCCTTAATTTGCTCGGTTAAGAAGATATTGCTGTAAAAAAAATATTTTTGATCTTCTAGTCCT<br>GTATTTTTAATATAGATTTTAGGATATTGGGTGATTTTAGGAATAAGTGATTTATGAAGTATTAAGTTTGTTATGAAATCAT<br>TTTCGTTGTATGTATCACAAATACCTAACGACGTTAAGTTTCCCCACCTAATTTATTGGTATCGAGTCTATTCTTTGTCTCTT<br>GATTTGCTGTCACTGAGTTTCTTGTTATTTTACTAACGAGCTTAACATTATTAAGAAACAATTTATTTGTAAAGCCTTAAAG<br>ATCTGTGAATAACTTGTATGTGAAAATGAGACAACATGACAAAATATAAATTTATAATACATAATAATATATTCATAAAA<br>GTTATTTTATAATATATACTTTGTGTAAGAAACCCAAAAATCTATATATATGTAAGTGTAAAGAGCTCCGTTAAAACAGAG<br>GATAAAAACATTATATTTTTACATTTGTGTAACGATATAGTCAGCATATCATGTTTTGTAGAAATATATATTATTATTAT
MSTRG.4667	Dolichol kinase	NNNNNTGGCGTGCCCGCTTTTATTAGTGCCGCAGCATGCGGGCGCTGAGCTCGAGCTGCTGAGCGGTGTGTTGGCCATCGG<br>CGTCGGCGACACTGCTGCCAGCTGGTTCGGCTCGCATTACGGCTTCAACAAATGGACAGGAAGTAATAGAACATTAGAAG<br>GCACAGCTTTTAATATTTTGTCACAAGTAGCTACAGTTTACGCTTTGGAACTCTTTGAACTGTTGCAAGGGAACAACGCTTT<br>AATCCGTACAGTTGTGGCGGCGACGGCAACTGGCCTAGTGGAAGCGAAAACTGATCAAGTTGATAACTTAATCCTACCTTT

		AGTTTCACTCATAGCCTACCAAGCGACTTGGTTCATATGTTGATCGAGAAAAGGTGCTCTTTACGACTCTAAGGATTCCAT<br>AAGCATTTATTAATCATTCCTTTTAGATAATATGTTTTGTTATTTATTTTACTACCGTAAATGGACTGATACGTTATAGCCTA<br>TTGAAGCTTATATTATATTATAAATTAAAAATATTAATTAAAATTTAATAATATTAAATAAAACTAGTATGAATGAATGGA<br>AAAATTTTAACATTATGAGCCACTACTTTGCGGTAGATGGCGTTATAGCTAAACAGTAAAATATTGTCTACTTATAATATT<br>ATCATGGAATTATTATTATTTTTATTTTAAATTTACGATATCTCGGCTTAAAACCGGTTTTACAGGTGTACACGGGCCTAAG<br>CCAAGTGCATAACTGTGTGCTATGTTTTTTGTATAAATGTAATTTGTCTGGTAATCTGTTATTATATTATTATAGCTTTATCA<br>TGGAATTATCAAAACTAAATTAATCGATTTTGTTATTATATGATTTGGAAATTGTAGTTATTATAGATTATATCAATAGATT<br>TTTACTTAAACTAGCAAACAAGTTGACCAACCACCTGGTGGAAAGTGGTAACCGTAGATTTAAATATAGATTTTAAATTGT<br>CATTACATGTTGACCAATAACCCAGTTTTAGAGGCCACTTATAACTGTTGATTATATGGCGTTATATTATGGATCTCATTAT<br>TTTATCTAAAGTCCTTATTTGTTTATAAAAGAAAACAACAAACCTCCATGTGCTAATGTCTATATTCAACAATCTATTTGTG<br>GTACTAATATATTTATAGTAGTATAAATATTTATTATTAAATTGGAATTGTGATAAAAAGTATAGACTAAACATAGAAATA<br>TTTTTTTCAAATATACCTTATTGTTAAAATGTGATATTGACATAAAAGTTGATGTCTATGATTTTTCGAAAAGAAAATGCCA<br>AAGAACTGGGAATGTACTGTTGTTTTTAGAGATTACTTATTGATTGTGAGCTGTATACTAGAAGATGCCATATCATATTTAT<br>TATTTTCATGACTGGAATTATTTGTATTATTACATACCTATACTTATTTACAATTGTTACAAATTGACAATATGTTTTTACTT<br>TTATAAAATAAAAATAAAACTGAAAATGTTAATTTTATTGAGATCTGTGTTGAAAACAAAATTCAATAATTCAGTGATTTC<br>CATAATACACTATATTTACACCAGAATAACATCACTGTCTGTACTTTGTTCAGTTTTAATAACACTGTCATCATCTAAAAGC<br>TCAGTCTTTATTTCATCATTAATTATACTTCCATTAGCTACATTTTCATTAGTTACTTCAAGTTTTATTACTTTAGATTTAACT<br>TCGATATTATCCACATTATTCTTTGAAACAACATCTGAACTGTCCCTTTTATTTTTAATCAAAATCTTTTTCTTCTTCACACC<br>TTTACATTTTGCTTTGAAAATTTTTTCAAGAGCTAACACATCTTTTCTCACTAAAGTCCAGTCTATGACACAAGATTCGGCT<br>TGTATAGTTAAATTATCTGCTTCGTTATATGTTGGTATGTCAATGTCTAATAGATTCATAACTCTCTCCAAAATATCATCAA<br>CATAATAATTTATAACTAAATCTGATTTATTATCCTG
MSTRG.4<br>673	Uncharacterized protein LOC106129089 isoform X1	CCGAACTTTATAAATGTAAAAATGAGTTTACTTGTTTTCAACGGTTTAACCAAAACACTATAAAATTTGATATACACGTAC<br>TATATCAACCATATGGTACGGAGAAGGACATAGGCTACTTATTATCCTGGACAAGTGATGGTTTGATGGAAAATGTATCGT<br>GAATGTATTTTGGATTATCATCATTATAAGCAGTCCCATGATGCCATGGGTCAAAAAGTTGACAGGGAGCGTCAGGAAGA<br>TCGAAGCCCGCCATGGTTACAAGAACCCTTTATCATTTCTGCTGCTATCATACTACTGGTATTCCTGGCAGCGACGCTGGTA<br>TATGCCACACCGCCGACATTCGGGTCCAAATCGGTGCAGCTGCCACTCTTATAGTCTATGTCCAACGTACTTCAAACTCAT<br>TTCGACGTCCACAAACGGGCGCCTACCTTCCTCTCTGGCCACACCCTCACAGCGTAGAATGAAACATCGCTCGTGTATCGT<br>CAACGCTCGCTCCGCATTACAAAACTTATTCAAGAAGTCATTTGTATCATCTGTTGGCACCAGTTTACGAAGATCCAGGCT<br>GCAGATACCTATTAATATACATAATTATTAATTTACTTAAAATTTATCATTATTTATTTATTTATTATTATTTCTCACCACAA<br>ACAATTAAG
MSTRG.4<br>703	Unconventional myosin-IXa-like isoform X1	NNNNNTCTTCCGCGAGCTGCCCGAGCCGCTGCTCACCTTCGACCTCTACGACGACTTCATCCTTGCCGCCGAGATCTCCGA<br>CCCGCAGGAAAGGGTATCAAGTATATTCACGATATTAAAGAAGTTGCCTAAACCTAACTTCGATCTAGTGGAACGATTAA<br>TATTTCACTTAGCCAGAGTAGCGCTGGGCGAAGATCATAATAGGATGGGACCGAACGCGCTGGCGATCGTGTTCGCGCCG<br>TGCATCCTGCGCACGCACAAGGTGCAGCCCGCACAAGACTCGCTGCACGACATCGCGCGCCAGACTGCCTGCATCGAGGC<br>CATCCTGCACGACAAGATGCGCACCGTCAGTATACCACGCAAACTGCATACCACATATACCAACACACGCTGCAGTTTTTG<br>TCGTGCACTATGATTGCACT
MSTRG.4<br>725	Dynein heavy chain	GGTGCTACGTCCGCGTGCCAATGTAGCTACAGAAACAGGCGCCGGTGGTGAGCCAGCTCAACCGCACTCGTTACCTATGG<br>ATGCCGCTGATGCTAACGCAATCGCTGAGGTGAACCTGGCATATGAAAATGTAAAAGAAGTAGATTGTTTAGACATCTCT<br>CGTGAAGGATGTGATGCTTGGGAAGCTGCTGTGCGTCGTTATGAGGATAGAATTGATCGTGTAGAGACTCGTATCACGGCT

		CACCTTCGCGACCAACTCGGAACCGCCAAGAACGCCAACGAGATGTTCCGTATCTTCTCTCGATTCAACGCGTTGTTCGTG<br>CGGCCGCACATCCGCGGTGCCATCCGCGAGTACCAGACGCAACTCATACAGCGAGTAAAAGATGATATTGAGGCTTTGCA<br>CGAGAAATTCAAGGTTCAATATCCGCAGTCGAAATGCAGCCGCCTATCTCTAGTGCGCGACCTCCCACCTGTGGCCGGATC<br>CATCATATGGGCAAAACAAATTGATCACCAACTTACGGCTTATTTGAAAAGAGTTGAAGATGTTCTAGGTAAAGGATGGG<br>AGAATCACATTGAAGGCCAAAAACTAAAAGCTGATGGTGACAGTTTTCGTCTGAAACTAGATACTCAAGAAGTATTCGAT<br>GACTGGGCTCGCAAGGTGCAGCAACGTAACTTGGGCGTATCTGGGCGGATCTTTGCCATAGAATCCGTGCGTGCTCGATCC<br>AGCAAAACAGGCACTATACTGAAACTAAAAGTAAATTTCCTTCCCGAAATTATAACCCTGTACAAAGAAGTGCGCAATTT<br>GAAAAATCTTGGATTCAGGGTACCACTAGCAATAGTGAATAAAGCACACCAGGCCAATCAACTGTATCCGTTTGCGATCT<br>CTCTTATTGAAAGTGTACGCACTTACGAGCGAACTTTAGAGAAGATCCGTGATAAGGCATCGATCATCCCGTTGGTTGCTG<br>GGCTTCGGCGCGACGTACTGAACCAAGTGTCGGAGGGCATGGCGCTTGTATGGGAGTCCTATAAGTTGGACCCCTATGTTC<br>AGAGACTCAGCGAAGTTGTGCTGCTTTTCCAAGAAAAGGTGGAGGACCTTCTGGCAGTAGAGGAACAGATCTCAGTAGAT<br>GCGCGGTCTCTGGAGACGTGCCCGTACTCAGCACAGAGTCTGGCCGACATCCTCAGCCGTCTACAGCGAGCTGTCGATGA<br>CCTTTCGCTGCGGCAGTACAGCAACCTGCATCTGTGGGTGCAACGCCTCGACGAAGAAGTCGAAAAGAGCCTCGCTGCTC<br>GCCTGCAAGCTGGAGTGGAAGCCTGGACCGCCGCATTGTTAGGCAAAAGTAACGAAATGGATCTCTCAATGGATACATAC<br>TCGCCCGCTGAACCCACTCACAAGCCAGGTGGTGAACCACAGATTGCTCGCGTAGTGCACGAGGTTCGTATAACAAACCA<br>ACAAATGTACCTGTTCCCCTCACTGGAGGAGGCCCGCTTCCAACTCATGCGACAAATGTTCGCGTGGCAGGCAGTTGTCAC<br>TAGCCAACATCGTCTCCAAAGCACCAGGTATCAAGTGGGCGTGGCACGTGCGCAAACAGCTACATATAGAAATCTGCTGA<br>CTAAACTCCCTGGAGGTTCATCGCCGCTCGAAAACGCATACGACGCCATCGAACAAAAAATTTCACAAGTTCGTGAATAT<br>GTAGACGAGTGGCTACGGTACCAAGCTCTATGGGATCTTCAACCAGAAAGCTTATATGGTCGCCTCGGAGAAGACATTAC<br>CTTATGGATAAAGTGTCTGAATGATATCAAGAAATCTCGCACAACATTCGACACGTCGGACACGCGACGTGAATATGGGC<br>CCGTGGTGATCGACTTCGCCCGTGTCCAGAGCAAGGTGGCGCTCAAGTACGATGCCTGGCACAAGGAGGTTCTTGGCAAG<br>TTCGGCGCACTGCTCGGCGGGGAGATGGTCACCTTCCACTCGCAGCTCGCCAAGTCACGCAACCAGCTCGAGCAGCAGAC<br>TATCGAAGCGGCATCGACCAGTGACGCTGTATCTCTAATTACTTACGTACAGCAACTAAAAAGAGAAGTATTGGCATGGG<br>AGAAGCAAGTTGACATATACAGGGAAGCCCAACGTATTTTGGAAAGGCAGAGATTCCAGTTCCCGGCACAATGGCTTCAT<br>GTAGACAACATTGAAGGTGAATGGAGCGCTTTCAATGAAATTATGCGCAGAAAAGATTCGTCCATTCAAACCCAGGTTGC<br>ATCCCTTCAACAAAAGATTGTAGCCGAAGACAAAGCGGTTGAAGCTCGTACTTTAGAGTTCCTCACTGAATGGGAACGCA<br>GCAAGCCTACTGATGGCTCAACCAGGCCAGAGGATGCTCTCGCTAGGCTCCAGGCAATGGAGACCAGGTATACCAGACTG<br>AAAGACGAGAGGGACAACGTTGCAAAGGCTAAGGAAGCTCTTGAACTACATGATACAGGTAGCTCAGTTAACAATGAGC<br>GCATGACTGTTGCTTTGGAAGAACTGCAAGACTTGCGTGGTGTGTGGTCCTCGCTTAGTAAAATATGGACCCAAATAGATG<br>ATATCCGCGAAAAGCCTTGGCTGTCCGTACAGCCCAGGAAGCTCAGGCAACAACTAGAAGCGATGTTAAACGAACTCAAA<br>GAGCTACCGGCTCGACTTCGAATGTATGACAGCTATGAATATGTGCGGAAATTGCTGCAGTCTTACACAAAGGT
MSTRG.474	NADP-dependent oxidoreductase	GCACAAGTCACGAAACTGAGTGGTCATGTCCCGCCAATAAACGACGGATGAGTGAACTCGCCCACAAATACGTTCTAATT<br>CTGCTAAATGAGAAAACTCCGAAAGTTCATTGTGAAAAATGCCTTCAAAGACTTCTTACCGGAGCGTTCAGATTTCGAAAT<br>AATAGAGGAAAAGCTCCCTAAACCGAGGCCCGGGGAATTTCTCGTAAAAGCCGAGTACATTAGCGTAGACCCGTATATGA<br>GGTCCTTTGCCAGCGAACAAGAGCCGCCATATGAACAATTTGGCTTTCAAGTCGGCAAAGTGATTGAGAGCAAAAATGAC<br>GAATACCAAGTTAATACATATGTCGTTTCCCACTCGGGCTGGAGAGATCACGTACTGCTCACCGACCATCCAGACGATATG<br>TTCAAAATAAAACCTTACAAACCCGAAATAGGGAATCTATCTTTATCATTAGCCATCGGAGCTTTAGGTATGCCTGGAATA<br>ACGGCCTATTTGGGTCTTCTAGAAATATGTCAACCAAAACAACATGAAGTGGTTTGCGTTACCAGTGCGGCGGGCGCGGTC<br>GGGTCTTTAGTGGGACAAATAGCAAAACTTAAAGGCTGTACAGTTATTGGTTTCACGGGTTCAAATCAGAAAGTGCAGTTT

		TTGAAACAAGAATTGGGCTTCCACTATGCTTTCAATTACAAAACGGATAATGTAAAGGAATGTTTAAGTACCAGTGCTCCT<br>AACGGTATTGATTGTTTTTTTGATAATGTCGGAGGAAATCTAGCTTCTATTATCATGGAAAACATGAACGAGCTCGGCCGT<br>GTTGCTATCTGTGGATCAATAAGTACATATGGACAGGAATCGTATCGACAGAGAAGACCAAAAACCCGTGTGTCAGTTCG<br>CATTGAGGCTTTCAGCTTTACACAATGGAATTTGTCGCAACAGTGCGCCGCGCTAACAGATCTGAGAGAATGGCTGCAGA<br>AAGGAATCATCAAAGCAAAGGAAACGGTCACAAATGGCTTTGAAGAGCTCCCAGATACACTTATTGCCATGCTGAATGGA<br>GACTGCGTTGGCAAGGCCGTCGTTAAAATTTAATATTCCTCTTCTTTGTTTTTAGCTTTAAAATAAATGATAACAATAATAA<br>AGGACTTCAAATAAATCTAAAGATCACATTATTTCGTTCGGCCTGTGGCTACGTTGTTTCAGACTTATTTACATAATAAATT<br>GTATTGTTGGTTTTCTAATGTTTTTCAAAGTATAGTATAATTACACAGGA
MSTRG.4848	Uncharacterized protein LOC106143306	ACAGAGACAGTAACAAATTCTCCTTTACGTATAAAAAATACGGGCATTGGTGTCCCAGTGGAAGATATGGAATTCACCCA<br>GAATCGTCTCAAAGGTCTAGTAGCCGCGTTTGTATATGACAACCGTCAGAAATTCCCCAACATAAAAGCGGAGGAGGCGA<br>TAATGCTACGTTTAACTTGGGACAACGGAGACGCTGAAAAGTGTAAATTTTACTTGTCCGCCGTCGCCGGCGCTGAGCATT<br>TCGACGAGCAGTTCTCGTATTGGCCCCTCATGTGTGCATTAAGGAAATTGCAGCTTAAGAAGATAGACGAAAAGTCCGTTA<br>TGAAAATTGCTAAGAAAAAAAAACAAAAATGGCGTCCGCTTGGCGCATATTCTGCGGCAGAATTATGACGAAGTTAGACG<br>TGTGTGGAAATATTTTCCGGACGCCGAGGATTTCGATACGTCTATCGGTCTTACTGTAATGTCGCCGGAATTGGTCAACAT<br>CTTTCTGAAAGTTTGAATTCCGATTGTCTACTTTGTGGTTATTGACGATATGATTGGCAAAATGTGATATACTTTTGCATAT<br>TAACAGTAATACATATGGCCTCTTTAATCTTTAGTATAACGGTCATATATTTTATTGTATTTTTTGTCTCTTTTTATTATTGTT<br>TAATTAATAAAAGAGTATTGCACTTGACCCATTAAAGCTTATTTTTATTAAA
MSTRG.4862	Myotubularin-related protein 10-A-like	AAACCGTTAATAAAAAAAAACACATAACTGTCATATTCTAGGAGTTGTGTTCTTAGGTCATGGATTTTATCAAATTTTATG<br>TTTATCCTATTGGAAATAAAAGTAGTTTGTGCTAATTTTGCAAATAATTATGTCATATTTAACTCTCTCTGCAAGTTAAATA<br>GATCCATATGTGATGGAAAGGACTAACAGACACATGGAAAGTTTTGTGATTACTGTGATGTTATCAATTTTGTATTCAATG<br>TATACGAATCATGCGGATTATATTTAATTCAATTATTAATCTAGTACCTTTACTTAATTTAGAATAAATATCATAGAGTTTG<br>TTAAAGTGTTTTTTTTGATAAATATTAAAGTGGGTCAGTGCAGCTCGAAAGTTTTATGATAACAATGAATGAGAAAAAGGT<br>GCAAAACTTCACAAGTTACATCAATTCACCTGTGAATAATGCAGAAAAGAATGGATCCATACAAGAAGTGAAACCAAAAT<br>TATTAAATGGTAAGCATTATCATTC
MSTRG.4868	Bloom syndrome protein homolog	AGTTGACCCCAGATGGTTGCATTCATTGACTTATCGAATTTTGTAGGTGTTGCCGATTCTTAAATAATGAATTATCATTTTT<br>TAGTATTAGATCTTTAGACAAAGTCTCTATAATCTGTGCTAAATTGTTATGAATTTAATTTTGTTCGTTTTTATTTGTAATTT<br>ATATTGTTTTTAATTTTTGTAGCTATAGCGATTAGTGCAAATGTGTACTATAATTAAGAAACAGTGAAACTACCTATATTAA<br>TTCTTTGTTATTTACTTGAAGTTATGGCATCCAAAGGCATGGATCCAAAACAAAAATACTCTTTATTAAATTTTATCAAAAA<br>AAGTAGTCGAGATTCAGAATCAGAATCAATTCAAAGCGCCAACAATCAACAAATAATAGAGAAAAAATCCAAGTTTGTTT<br>TTAAATCAAAATCAACAAGTACTGCAAACTGGACCCCTCCATTTAAAAAGAACTCAGAAAATGTGTCACAGTTGGCCAAG<br>GAACCTTCGTTATCACCAAGTATTTTGGATAACCGAGTTGAAAACCATGTTAACATTGACAGGAATAAAAGGACTAATAA<br>TGCACCTCTTAATGCATTTGATGAATATGACGATATGGATTGTTTTCCATATACCTTCAACGAAACATCTAATACACCAATA<br>TCAGAAAAATGCAATAAATCTATAAGAATAAGTGCAACACCCAGCCCTCAAAAAAAGAAAATAGAACAAACCAGTTCAG<br>CGAACACGAAAATTTTGAAAACACCTAAAAAAAGCAGTCCCAAAGAAAATGCCGAGATCAAAAGCCTGAAATTGGATAC<br>GTCTTTGAAAAATTTCTTGTTAAATATATCAGAGAATCCTTCTGTACAGAAAGCGAAATTAGATAAAAGTGCCTTAAGTTT<br>ACAAGATCTAGAGGAATGCAAAACATTATACATAGAGATACTAGAGAAAGTTTTCAATGCTTTTGATAGAATACCGACTA<br>GAACCAAAGAGAAATTACCAGGATATAATAGTAAGATATACTCACATTTAAAATATTGGAGATATAAATTGAAGATGGCT<br>ATACAACAGACACCCAGTAAAAACACAAGTAAAAAAGCATCCAAGACCCAAAACGACAGCAAATCTGTTTCTACTATTGA<br>ATCGAATCTCTCTGCATCACCATCATTGTTAAATAATTGTGAAGATGATGATTTAGATGATTTCGATCTTAACGGTACCCAA

		GTTTCTGTGAAGAAATCTGTTAAGACCATATCTGAAGTTCCAGAAGCAAGAGGAAGTATTAACATGAATTACAATGCTGG<br>ACCCAGTACAAAACTAACAGACAAGACAGTGAAGAAGTCACTGTTTTGTGATTCATTTTCGACTTGTACCTCTACAAAAGA<br>TTTAGATATTAAAACTGAATGTGATGCAGGCAACTTGGATACACCATCCAATGTTAAAGGAAAAGGTAAATTTGTGTTCAA<br>AAGGCCATCAAGAGCTGCCACAGAGGAGTCTAGTAATATGCCCTCTACAAGTCCCATAATTTCAAACACTGCTGAAAGAC<br>TAAAGAATGCGTTGGGAACTTTAAAACCTTTGGTGGACCAAGAACCACCAAAGGTGGTGCCAGTAGCAAACAGTTCAGTA<br>AACTTCCAACCTCCTCAGCTGAGCAAGAGCTCTTTACTTTCTATGACTGATACAGTGCTTGAACCTTCAAGTGGTGATGATC<br>CTTTGGTTGATGATGAGTACATGGATGAGTATTCAGTACCCATTGATATAGATGCTGATATTGAATTTACGCATCCGTCAA<br>GTGAGCCTATGAGTATAGACAGTGATCATAGTAAAGTTGCTGAAGTAGTCAACGATAAAGAGATACCGGTGGACGAGGAT<br>GGGTGGCCAGAATACAGGATGGAAGACTTTGAGGATGGTAATTTTGTATCGGAGAGTAAAGAACCAGAGGTGGTGAATTT<br>GATGGAACACTCAATGGTTGAAGACAATAAAAAACCCAAATATGAAGGAATGGGCGACTTCCACGAGGGCACTAAAAAT<br>GACGGTATCACAGGTGAATTTGACGGTATGAACTATCCCCACTCATCACTGATGATGGAAATGTTCAAAGAGAAGTTTGGT<br>CTAAAGACATTCCGTCCAAACCAGCTGCAGGTGATCAACGCGACGCTGCTTGGACACGACTGTTTCGTCTTGATGCCAACC<br>GGAGGAGGCAAATCTCTGTGTTACCAACTGCCGGCCATATTGACACCGGGTGTTACCATCGTGATATCGCCCCTCAAGTCG<br>CTCATACTGGACCAAGTCAACAAGCTGCTCTCATTAGATATCCCGGCGGCGCACTTGAGCGGCGACATATCGGCGGCGGA<br>AGCGGACGAAGTGTACCACAAGCTGTCGATGCGCGAGCCGCTGCTGAAGTTGCTCTACGTCACGCCGGAAAAGATTAGCA<br>GTTCGCCAAAATTTCAAGATGTCCTCACTGCTCTCTACTCGAGGGATAAGATTGCTAGGTTCGTGGTAGACGAGGCGCACT<br>GCGTGTCGCAGTGGGGGCACGACTTCCGGCCCGACT
MSTRG.4880	Uncharacterized protein LOC106136113	GTGATCAGTAGTTTCTGTTTACAGTCAGAAAAACTTAATATTAGATATTTTATGTAATTTTTTTTTTTTTACGAGAATATG<br>GCAAATTTCTTTATTTATTTAAGTTTCATTAATATTTTTTCTTTTGGCTGTTATTATTGTGTCCAAGTGAAGCACGAACGTGC<br>GTTAATCCAATATTACAACAAGAAGTACGTAAGCCACGCTCAAGCCTTCACCGGCCGGGCCAGACGCCGGGAGCCATATA<br>GAACTAATATTACAATCACTACGTTGCACACATGGCAAAACAATGTCACGATGAAATTGATATATAAGGACAAACAGTCG<br>GCTAGTGATTTTTATATATCGGTAAAATTATGTGATGCAGCGAAGTTCGCTTGGATAATTGACTATGTAAGCACTTATGCG<br>GACTTCAAAGTAGCCTGTCCCTTCCCTCCGGTGAGTATACTGGTGA
MSTRG.4891	E3 ubiquitin-protein ligase HECTD1	GCAACATTTTAAAATTTTGAGTAGATATAACTAACGATTTAAAAGCTTAATTATGAGTATATATTGTGTTGCAAAACCGTT<br>TTTATAATTGAGCACGTTTTACGCCGGGGCTGTAATATTGTGTTATCCTCATGAATAATATCCAGTTCTACCTACGTAAAAA<br>TTGCCGGCAAATGTTAGAAAAATAGTATAAAAACTTAGTTTTTTTTTCATTAGGATGACGATGATGGACATCTGATAGTT<br>TTGGGTATCGCAGAGGAACTTATGGTGAAAGCTGCAGATATATATTTGGAACAGTTCGCGCGTCTTGGCGTGTTTAGCAAA<br>GTGGAGGCATTAGCTGCAGCGCCTGCGACACTGCTCTCGTCTGACGGTGAAACAACTGTAGTTGCAGGAAGCGGCGAGGA<br>TGCATCGTGCCTGTCGGCGGGCGTGGCGTACTCGTGGGGCGAGTGGTCGCTGTGCCGCGGCCGAGACGCGCTCTACGTGT<br>GGAGCGACGCGGCCGCGCTCGAGCTCAGCACCGGTAGCAACGGCTGGTTCCGCTTCCTGCTCGACGGGAAGCTCGCCACC<br>ATGTACTCCAGCGGCAGTCCGGAGCACCAGACCGATAATACAGAAAACCGTGGCGAATTTATAGATAAACTCCAAAGGGC<br>AAGGGCTTCCGTAAAAAATTCTGTCCCACAACCGATACTTTCAAAACCAGGATCAACAAAACTTATTTTAGGTAACTGGGC<br>ATTATCATGCAAAAAAGAAAAGGAACTTCAAATACATAATACAGATGGACAGCAACAAACTACTATATTAAGAGAGGATT<br>TGCCAGGCTTTATATTTGAATCCAATCGAGGAACAAAGCATTCGTTCACAGCTGAAACATTTTTAGGTCCAGAGTTAGCCA
MSTRG.491	Rab GTPase-binding effector protein 1 isoform X1	GCGGTTGGGCGAATCGTAGACTTGTCACGAGCGATCCCTCCAATATGAATCATAGCCGGTCCCGCTTCTCGGCAAAAA<br>GACACGAGCCGGCAGTGCGACATGTGCGCGAACTACGAGAAGCAGCTGGTGTCAGAGCAGGCGGCCGCGGAAGCGGCTC<br>TCGAACGCGCCACAACGAACCACCGCGCGCTCAAACTGGCGACGGAGGAGCTGGAGGGCGTGCGGTCGCTGCACGACGA<br>GACGGTGCGCGCCGCGCTGCACGAGCGCGAGGACGCCGCCGCCGCGCAGCAGCGCGCCTTGCACACCGTCACCCACC<br>TCACCGTCGACCGCGAGATGCTGCAGCGGAAACTCGACACTTTAGAGAGAGACAACGCTATGTTGATCGGTCAATACACG

		AAGAAGGCGGCCGAGATGCAGAATGAGATTATAGACTTGCCAGACAATGTCGAGGAGCTGCAGGAGCACATCCTGCGCG<br>TGCGCGAGCAGCTGATCTTCTGCCAGATCGGGCGCGAGGAGGCGGCCGAGGCGGAGCGCGGGCTGCGCGCGCAGCTGCT<br>GCAGCAGGGCGCGCTGTTCCACCAGCAGGAGCAGCAGCTCGCCGCCCTGCGCGCAGACCTCA
MSTRG.4911	Stress response protein NST1-like	CAGGTGAGCGCGATCGAGGTGTCGCAGAAGTTGGAAGCGCTCCAGATACACAACGGGGAGCGGCACACCCACGACCAAA<br>ACTTAGACAATAAGGTAAACGAAAACAAAGAAAACATAGAGGTAGAAGCAAAACCATCGAAGCCCGAACAGAAATTAGT<br>AGATAATAAACCAGTAGAAGAAGTGAAGCCTGTCGAAAAAGAAACAACACCGCCCGTGGTGAAAGAAGAGAAGCCCACA<br>GAGAAGAGAGAAGAACAAAAAGTGGAAAACAAAGAGGAGCAAAAGGTTGAGAAGAAAGAGGAACAGAAAGTGAACGA<br>GAGAAGGGAAGAGAAGAAGGAACCCTCGCTAGAGAAGACTGACGAATCTGAAATGACAGCATCGATGACCGCGCGGCGT<br>ATCACAACCGCGGAAGAAGCGAAAGCAGCACTGGCTGAACGCAGGCGGCGAGCCCGCGAGGAGGCCGAGCGGCAGGCC<br>GAACTCGAGAGACAGAGATTGCAGCGCGAAGCGGAGGAGGAAGCTGAGCGACAGCGGCTGGAGGAGGAGCGACAGCGG<br>CGGGAGGAGGAGGAGGCACGGGAATTGGCGCGCCTGCAGCGGCAGCAGGAGGAGGAGAAGCTGAGGAAGGCTATTGAG<br>GCGGCGCAGCAGGCGGAGCGCGAGGAGGCGGAGCGGCAGTCGCGGTCGGAGGCGGAGCGGGCGGCGCGCGCGCGAG<br>GAGGAGGACAAGCGCCGCGCCGCCGAGCGCGCCGAGCGGCTGCGGCGCGAGGACGCCGAGCGGCAGGAGCGCGAGCGC<br>GTCGCGCGCAAGCAGCGCGTCGAGGCCATCATGGCGCGCACGCGCCTCAAGAAGGTGACGCACACGCACCCGCACCCGC<br>ACACACACACGCACACGCACACGCACAGCAACACGCACGTGGCGAGGACGCCGAGCGGCAGGAG
MSTRG.4914	Serine/threonine-protein kinase MARK2 isoform X6	AGGTGTTAGACGCCAATAACTGCGATTACGAACAACGCGAGAGGTTCCTCCTACTGTGCGTGCACGGGGACCCTAATGCG<br>GACTCCCTGGTCCAGTGGGAGATCGAAGTGTGCAAGCTGCCGCGCCTCTCGCTCAACGGAGTGCGCTTCAAGCGAATATC<br>CGGCACCAGCATCGGCTTCAAGAACATTGCGTCTAAGATCGCCAACGAGCTCAAGCTGTGACTGCCCGGGAGCGCCCCGC<br>GCCGGCGCCGCCGCCGCCGCCTGCCGCTCGCGCCCTCGCCCTCCACGCCCGACTCGTCCGCGC
MSTRG.4929	Protein FAM122A	CATATTGCGGCGATTTGACAGGTCAGCTTCGAGCCACTTTTTAGCTTTCTGAGTAGCGTATTTTAATTTTATTTCGTTATCCT<br>TTCATTTACGCGTAATGAGTAATTTCTATGTAGTCGTAATGGTAGATTTCTTGTTAAATTAGATTGTTTCTAAGTGCACTTA<br>CAGTGTTATATAAACTGTAGAGCTAATAGTGTCGGCAGTCCCGATGTGTATAACTACATAGTACGTCAGTGATCGAATGGT<br>GTGGCTGTAGATAATGGATGTGGATGCTCCTAACATATTGAAAAGGTGCAGCAGCGCACCTCTAATCAATGAGGCGGCCT<br>CTACGGCGGCTATTTCGCCTACGACCAACATAGCGCCCAGGAACACGTTCTTCAGCATGTTCAGTAACAACAACGTGTCGA<br>GAACGCGCCGCCACAGCGGCAGCTTCAGTCCCCTGGTAGCGCCCGTCACCGTCACTAACTCGTGGGGTCCCCGGCTGACG<br>CCGCGCGTGAACCAGCTACGCGCCG
MSTRG.4958	RNA polymerase II degradation factor 1-like	CACTGTCACACGCGCATTCATATTAGTGCGGTGGAAAGTGCGCGGGAGCGCGTGCGCAGTGTGACATGAAATTTACGACG<br>ATACTCTTGCTGGTGGCTGCCAGCTACGCAGCTGAACTAAGACCGTCTAGAAATAAGCAAAAAGAAGAAACACAGGAAG<br>ATAAAAGAAGAGGTGTCGAAGGAAATGCCGGTGTTGACAAAAGGGCGCCAGTTCTTTCTACTGTATCACCATCAGTCACT<br>CCTGGAATCGAATACGCTAATACTAAAGAAGCACAGCAGGACAGATCGCCTGCACAG
MSTRG.4977	Triglyceride lipase	TGTGTGTAATGTATCAAAACTATTTTTTTTTCTTAGCAATTTTCGAGTGTATACGCGGAGTGGAGTCCTTAGGCGCTGACTT<br>CGTACTGCCAACATGCAAACATTTCTTCAACATATTCCACCCGTACGACCCTATAGCTTACAGAATCGAACCCCTGATCAA<br>TCCGCAACTGAAAAGTATGAAGCCGTATCTCATCCCGCACCACAAAGGCAGGAAACGGATGCACTTAG
MSTRG.4978	Uncharacterized protein LOC110373864	GTAAAAAACAAAGCACTGGCAAGTCCACAAATGTCACTACTCATTATTTTAGAACGTCCTGAAAAAACTTTGCGTCTGTC<br>TACAACTCTCAACTTCAGAAAAGAAGGGTTGAACAATAAAAAATTGTACAGGCTGGATGGGCGCTTAGTGTTTCGGACTA<br>AGCATCTCGTACAATTTCAATAGAACTTCACTTCAGGTGAGTTCGTTTAATTGTACAATTATACGAGATGCTTAGTCCGAG<br>ACACTCTTCATATATTCACTTGTAGATATTTAGAGGTTGGGAGTTGTAGCAAGGCTTATAGGCTAGCAGTAACAAAAAAAC<br>TCACTATTAATTCACACAAATAACATATATTTTCTTCACAAATTAAATACTTATTACGAATAAAACATTACTTATTCAATAT<br>TCTCTTAAAACAATCGACATACAGAGAATGCCTAAACAGACTATATTACACACAGCCTAAGCAAACATGTAATCTGTGTTA

		GTTTGAAGAGATCTATTATAATAATTCGCAAATACCTTCGAACCTATAGACCAACCTGCAGTCTTAAAAATACTATCTAGA<br>GTTACGCCTTCTCTAAACGCTGAGGAAGTAGAGGCATGGTCGTGTGGAATGGGGGGTAATTTTTTATATCCACACCACTA<br>TCCGACATTACTTGTCTTATCCATCGACTTATTGTTTGATTATCGACATAATTATGAG
MSTRG.4<br>980	Dynamin-like 120 kDa protein, mitochondrial isoform X1	GTGTACGCGGCCGACACTCACGTACGACGAGGTGGTCGCCGTGCGCGACAACCTGCGCCGGAAGGGCATCGAGGTCGACA<br>ACGACTACATCAGGGAGCTCTGGAAGCCTGTCTACTTGAAACATTTCCTGCAGAAAGCGCAGACGAGGGCTCGCGATTGC<br>AAAAAGAGCTTCTACCTGTACAGTCAACAGAATGGAAATGGAG
MSTRG.4<br>990	ER membrane protein complex subunit 8/9 homolog	CTCCAAGGCCTAACTGTGGTTACCAATCCACCTGCTAAGTGTGGCAACTACTGGCAAAACTCCCCAAGGGGGAAATTTATG<br>TTCAGTAGTCGAAGGCTATGTTACGTTAAGGGCTGGATAAAGGAAAACAAATTACTGTATACACCTTATGCATTTGTAACA<br>GTGTCGCATTTACTAGAACGGGGCGTTCAAAAAGATTTAGTGGACTTTGATAACTATTTGGACGACCTGTCACAAGACTGG<br>ACCAACCTGGGCATAGAGAAACTGATTGCGAGCATAAACGCATCCAATACAATTGATTGCAAGGAAAAAGATTATCGATA<br>ATGTAAGATTTAACTTTGTAGTTCAATTTTAAGTAACTATGGGATATAACTGTATATTATAATAAGATGTTATTTTCAAAAA<br>GTTACTGTATGAATTTCATTCTACTATTTAAAAAACATACAAAACTGTAATAAAGAAAACTACCTAATCATCCATAGAATA<br>AATCAACTAATTCTATGTAAGTGTGCCACATTTTATTTGAAAGAATAACACATGAAGTTGGCATATAAGTTATACATAAAT<br>TTAAAAAATGTGTGTGTGATTTTTTTATTTGTATTTATGATCTACAATTTATTAAATTCCGTTACAAATCCATAGATAAAAA<br>TAAATATGTTTTAAGAAAAGTTTGAGTTTGTTCGAGTTACCTATATTCTTAGGGAGTAATTGTATGTTTCACGTGGATTGAA<br>TGAATGAAGTAATATTTCTCAATTTCGGTATTTTTTCCAAATTCACCCGGATGTCGTGTTTCTCGCTAGGTTATTTAAGTTG<br>AGAATTATCGGAAACAACACTTGGTAAGCTCATTAAATTCCCGTTAATTTATATGTATAAAACAATGTAAAAATAAAAAC<br>ACATGTTTCATAAATAGTATATAACATGTAAAAGTAATTTGTTCTAAAATTGTTATTTTATGCAATTGTTGGTTGTTGGTTA<br>TTTTCATTCCTTAGTTTGAAATTATAATAATTATAATCGTAACTTATGTACTATATTTAATATTTTCAGTTGTTATTTACAAA<br>ACAAATTATACTACTTATTACAGTAAAATATTGTGAATTTATGACAAGC
MSTRG.5<br>000	Rabphilin-3A	TAGATCTCCCATTCGTTCCAGGGAGCAGTATATGTGCAACCTTTGTGCGGAAACCAGAGAAATGTGGAAGAAGTCGGGAG<br>CGTGGTTTTTCAAGTCACTGCCAAAGTATATACTGCCAGAAAGGAGAGCTACTGGGCGGTACAATGATTTGGAACTCGCG<br>AGATCGCTACAGGACGGTGGAAACAGCAGCAATGATGGCAAGACAGATTCGGAGCCGGGCTCACCATTAAGCATCCATG<br>CGCCCTCCTTCTCCAGACAGGCCAGCAGTAATGACAATAGAAAATCCATAGGATCACCACATG
MSTRG.5<br>005	Tubulin polyglutamylase TTLL5	ACTTGCTTTTAATAGCTGACTTTTAAATGTTACTACTAATATATTCCATAATTTCCAGTCCACATGTATCATCAGAAACGCC<br>AAATCCGTCTACGTCAGGCCTTCAGACCACAGACGCGGCAGACAGAGACAGCATGAGAAAAAATGAAGAGGAAACAAGT<br>AATGAAAAAGAAAATGAGGGGTCCCTCTCAGACTGGATATCTGGGGGGCCAATCGGCTCGAAGGGAGCCGTTCTCGTGTT<br>CAGATCATGTGTCTTAGCGTCTAGAACTCCATCCATGGAGTCCACTACGAAAACTGTAGGGAATAACACTGGTACAACGA<br>TATTATTGAACAAAGCCAAGAATATGGCTAAAAGACTTGTAGCAGAGCCCCATTCCTTTAATGTAAGACCAAAAACAATA<br>ATGCACGACGTAATGGAAACGTTTGGTAATAGCAATAAGTATTCAGGGCAGGCGTATGTGGTGCGGCATGTTAGTGAAGC<br>GGAACTACCTCAGACGGTGTCTGTCGAAGAGCAGGGTCACGAGTCGGTCGAGCACAAGAGAGATATAGAAGATGTACTC<br>AACGATTTAAGCAAGATCACTTTAAAGAAGCACCTGAACATAGAGAATACGACGGCGAATATAAAACCTCCCGAAGAAC<br>CTCCTCCTCAACCCAAAGACTTGAAAGAAGATAAGAAAGTGAAGAAGAACAAGTGCAAGAGTAAAAGAAAGAATCCGCA<br>GACGCAGCGCGCTTCGTCGCCGCAGTTTGACAGCGATGACTCCGAACATATGCTGGTGAAGGAGGTGGACAGTATAAAGG<br>T
MSTRG.5<br>009	Nuclear pore complex protein Nup133	GCAGATACATGCGGGCAGCGGCAGGCAAAAGTTGTAGAAAATCCATATCTGTCGTGTTTTGCGTTCAGATAAATTATTATG<br>TAAACTCATATCTGTCCTGTAATTTAAAAATGGAATGACCTTTTACTTCGAAACATGGCTACCAAGGAGGTAACTAATAAA<br>AGGTTTTATTTAAATTTCGAACTGGATTGCAAATAGAAAGCTGTTTATTGAAAAAAAAATATAAAGGAACATTATGGCGC

		GTGAAAAATTATTATTGTTCGTTATTCGTCATTGAACTTAAAAAGTAAAATTGTGTTTTTGATTTCCGCGAATTGTTTGAGA<br>AGAGTACTATACAAGCCTTGGCCACAACTAGGCATTGATGGATGTGCCTTGGCTACGCCTCGGCCCACATTTGCACGTTCG<br>TCCATCAATGCCTCAGTTGCTGGCCGCTGCAGTAATGTACTATTAATTACCACATCACCAATTGGTTCACCTTGGTGACAG<br>ACGATAGCGTCGCTGGCGAAGTTATGCGCGGTGGCTTCGGAGGACGACTGGTCAAAAAGCAGCGCCTTCGCCGAGGCCGA<br>GTGGCAGCTGGCGCTCGCCGACCACGTGGCGGCGCTGCCCAGCGAGCTGCGGCTGCGGCACGGGCTCGGCGGCGACGACT<br>CCGACGATGGACCCATGCCGCTGCCGGAGGAGCTGGTGCAGATTTACATAGAATCGGACAGCAAGACGCTAACAGAATAC<br>GACTATAAGAAAGCTCTAGACCTCACCGAC
MSTRG.5016	Conserved oligomeric Golgi complex subunit 4	AGGTAGAAGTAGATGAAGCTTTAGCTGGTGTGCTGTCACGGGCTTGCACACTGGAGGGCCGACTACGTACTGCCAGTCAG<br>GCGTACACAAAGCTGGGAGAAGTGAAAACAGATGCACAAGTGGCTGCTGACATGGTAGATAGAACTGCAGAACTGGCGA<br>GAGATGTCAGTGCTAAAGTACGCCAACTAGACTTAGCTAGATCGCGTGTGGCCGAGTGTCAGCGGCGCGTGCACGACTTG<br>ATAGACTTGCAGCTGTGCAGTGCAGGCGTGGAGGCGGCCATAAAAGCTCACGATTACGAAACGGGCGCGGCGCACGTGG<br>CCCGCTACCTGTCCATGGAGCCCGGCTCGCTGCAGGCGGCGCGGGCGCGCGGCGCCCCCGACCCTCGCCACACCATGGAG<br>CGAGCGGCCGCCACGCTCGGGGAACATCTCGTACGCAAATTCGAGGAGGCAGCAAAGAAGGAAGACGAAGCGGCGGTGG<br>AGCGCCTGTTCAGGTTGTTCCCCCAGATCGGTCGCGCTGACGACGGAGTCGATTTGCTCGCGAAGTATCTCCTTACACAAG<br>TAAGTATTATTATATTAGAATCGATCATAATCTTCTTGCTTTTACTCCATATTATTTTGGGTCGT
MSTRG.5029	Uncharacterized protein LOC110372846	AGCTGTCATGGCCTTCGCCACAAAGAGGCGGTGGATATGAATTTGCTACGACTGAGCAGCTCGCAAGACATATCGATGAC<br>CTTCCTTTAACAGCGGCGATATCATTACCTCTTTATACGCTACTCAGGGAGAAACTACAAAGGCACCTTGCACTTGTACTG<br>AAACAGTCCACCAGCGTCTCGGAAGTGGTATGCTGCAACCCTAGTCTGCTAGAAGCTTACAG
MSTRG.5031	Serine/arginine repetitive matrix protein 1-like isoform X1	GTCAATATAATCTGTGGTCAATATATTAAATAAAAATGTCGGACAATGAAGATTTATTGGGCGATGATCTTGGTGATCATA<br>GCCTTGCAGATTATAATCTGGGAAACGAAGAAGAGGAACAACTTCTAGCTGATGACTATGAAACTGGGTCTTCACAAAAT<br>GTGCCAACTTCGATTGGTCCGGGTTATGGAAACGGCGTTGACATGGTTACTACGGAGTATACTGGAAATGGCATCGATTAT<br>GGCCAG
MSTRG.5071	Uncharacterized protein LOC110379667	AGACCTACCTCTTTTATTCCAGTAACCAAAGTAACTCGTTCCTTAGATCTGACTTTTATGAATCCTATTTCATTTTTAGTACT<br>GTAAAATAATAATGCTTCGGGACCTGCGAACAATAAGCAAAACTATTTTGATTTGATATTTGAAAAGATTGCTCTTGATTG<br>ACTAGTTCTAGATTGATTTGAACGAAGTATTATTATTGTGATCAACTGTATGTGTTTAATATAAATTTCTTTAGTAAAAAAA<br>AGTTGTCATAAACAAATTAAAAAAAATGCTTTTTCAATTATCACATTTATTGAAAGATGTAGCACTACAAAGACCATACAT<br>CTGTAGTAGCAAGTCATTGTTTCGCTTAGTCTCTTTGAATTCCATTGTCTGTGCCAACCATTGAAGTAGCTTCCAATGATGT<br>TCACAAAGTTTATTATTTTTTATAAACAACTCTGGTGGAAAATTCTGTAATGCTGTCTTTAGTGTCATTTTTAGTTTTGGGTT<br>CAATGTAAATACGTAGAGCAGCTGCAACTGCGGCATATTTAGAATACCAGTTTGATTTTTGAGAATAAATTGAAAGAGGA<br>ACGGGGGGGTTGTACAAAATACATTTAATTAGGGTCAAAATAATAAACTTTATTAATGAGAACAATCAAATATAATAAAA<br>ACCAGAATCTTTATGAAAATGCCTAACTAGAAAATTCCAAAAACTATATTGGCCTTAGGATGCAGATAATTTGGAATATAG<br>TATTCCAAATTACATTCACATTATTTCTTTGTCAATTGCTTACACTTTTTCTTAGAAACTAATAGATGTCTTGTCTTTGTAAC<br>AGTTTTATTAGTCTTTAGTCCTCTAGGTAATAATTTTTTTTTAAACAACCTAATATAACATCTAATATTAAGTAATCAGTCA<br>CAGATCATACATACATCACAGTGTTTAGTTTGAACTTATAATTTACTATTCTAAGTTACTCTTGCAGTAATCACAAATATAA<br>ACACCAGATTCATAACCACCACAAGCACCGTGAACTCACAATTCGCACATTAAGCACTATACCCATTCCTCACCCCTTTAA<br>TCCTCGCTATATTTTCCGTTACAGAAAAAGCACTCATCAGTAGGGACTGCAGTCTCATTTCTCGTTGCTACATTGTAGGAAG<br>AGTCTGTGTTATTTCCAGATATACCTTTAGGATTCGAATCTGATAAATCACTGTAGGTCATATTTCTTTTCCTTGTCTTTTTG<br>CTATTTTTTTTTTCTTGCCTTTTTCCTTAATATTTTCTCTTTGTGGTCTGTTTTCTTTATCACAGTTTTTAAATTGTGCATTTTG<br>AAGATATTTTTTATAAGAAAAACTTGAAATTATTGCCGATCTTGCTGGCTTACAGCCTCTTTTGCTAGTTTTCTTTTTTATTT

		TGTTGGGCACGGGAGAACTATGAAAGGAAGTAAACAAGGGAGTGAATTTCTGTTCTACTATTTGACGGCCCTGGCTCATG<br>GTCATTGAAATTCATCAATCGCAAAGAAGCAACTGTGGGAGAAGCGAGCGTCGTTGAAATAGTTTTTCAATTTTCATGCTT<br>TTCAAAATCTGCAGCAGCGGCCATACAATCAGCTTGAATAAATATATTTCGATTAAACGGATATATGCCACCGACTCTAAA<br>ACCATTAGCAGCAATTTCTCCCGTTTGACACTTGATGTAAGCTTTTCCGAACAGCTCAACAACATCATATGGTGTTAATGGT<br>CTATTAGTTTCTCTGAGAAACACATGAACTTCTTCACTATAATAGGATTTTAAAGGTACCATGAAAATGCGGTCTAGCGGT<br>TGAACTTTATGCGTACAATGAGATGGTAGTGATAGGATCACATAATGTTGCCATCTATCAACTCTATTTTTTTGGTGTCTGT<br>AATGGCCATCGAGTATCAAGAGCACTGGGGATGCCTTTGTAAGTTTCACGTATTCCATGAAGTGTTGAAACCATTGCGTAA<br>ATAAATGAATTTGGATCCATTCTGACGTATGTGCTTTTCCGATAGAACCAACTGGAGCTTCTCTCATTAAGGTATCGGACA<br>TATTTTTACGCGGGAAAATTATCATAGAAGAAACAAATATTCCTGCGGGGCTCATACAAATTACAATAGTTATTAAGGACC<br>CTCTTTCGGCTGACGCATTGCTCAGATTTGTTTCTTCACTTTTCGTGCTTATAATTTTTGGTATTTTAGTCTAGACTATGACG<br>AGACCACTCTCATTAACGTTAAATATGCGCGCAGCAGGGAAAGACTTATCAGCTTGAACTTTTTTAAGATTATCGTAAATT<br>TCGTCCATTCTTTCTTTAGTAAATCCATTAGCTAGTGCTTGAGAAGTTCCAGTTGGTTGTTTTATGGACAAAATTTCCTGAT<br>GATGCTTTAACCCTCCGCCAACCGTGTGGTTTTTTTTTTGCACACCGTCAACCATCATGAACGATATCGTCCCAGACGGTAA<br>TTTGATCTACTTAGGTAGGGAAAAATTCCACAAAAAATTCAGGGAATATGTTCTAGATTTCTTTTTTCTGTTTCCATAATTT<br>TTGACTTGAATACTTTACCCCCTGGAGAAATAAATATTTATAATCTCTACAAAAACCGTAATTTCTAAGATTTCCTTAAAAA<br>ATCGATATCTTGGATATAAATTAGTATTAAGAAGAAATGTTTTGAAGATTTATTCTTCATGTAAGCGCCTTTCAAAAAAATA<br>TTAATTGGATCTTACACCACTGGAAAAAGTATATTTATTTTAAACATTTCTTTTAAGTTATTAAACGCTTCATTGTGATTAT<br>CCTTCCAGTGAGGGATGATAGGTGAGGATGAAAAGTCAGGTCAGTTGGCCCATCTTGACGAATTCAACGATAAATTAGTC<br>ACAATGGTTTCCAGGGGGAACCGCGTGGAGGTCGACCTGATGGGGTATATTGATAGCAATAGGGACTAATCTATCCTTTA<br>CTAACAATCCCTCCCCCCGGGGCCTTCTTATTGGGGCCCAATTTAGGGATCCGACAATAGGGTCGCTGTTGCTTCTTGCACT<br>GCCATCGGCGTACGGTGTTTCGATTGCTAACAATACTTGTATTAAAATCTAGTATAAAATATATACTAACTTGATACGTCA<br>TCTGGGTGAGGTTTAATAGATTGGTTTTTCTACTTTCGAAAAGCCATATAGTTAAATATTAAAATCATATAGTGAACTAAC<br>CTGAAGCAAAACAGAGATATCCGAGCCTATCTTCAGTATAGCCTTCATCACAGCTACACTTAAATCTGCCATATAAATTGT<br>GACAAGTGTGCGAGCACAGGGGAATAGCTGCTGCACATTCGTCAACGTCTTCGCACGTATTTGTGGTGTAGTTGTACTTCA<br>AATGGTACGGCGACCCATTGCATATGCAAGAGGGACCATAACGCTCAGGGATGCAGCGTGTGTTATTGCCTTTACACGGG<br>TTGTCGGCGCAGCTCGTGTTCCCTGGAATAACAATAAGGATTTTTGTTCTTCGACTTCAAAATAATATGTGAGTAAAAAGA<br>AATAAAAAATAATACCATCCCTTCCTTTCCCGTGGCTGTTATAAGACTAAGGAAACTTAATGGTCGCATGATGAGCCCTCC<br>TTAAACATTTCCAAGGCCTGTGTTTGCCAACTTATCTGCAAAACGTGGCAGCGACTGGCAAAACGTCCCTTATGGGGGTGG<br>CTGGTATAATAATATGATGAGAGGAAACGGTTACTGCTTTCCATCCTTTCCATCGGGTGGGCAATTTAAGTTGCAGTAAAA<br>TACTAGGTAGGTAGATACTAAATATCCAATCGTTATTTCATAAATTACTAGGCGAACAGAAC
MSTRG.5075	Hypothetical protein	CCCCCAAAATTTTACCATATTTTTATCTGCTACCACGTGATCGGAAACTATCGGAAATGTATGATGACGTAATTTGGAAGT<br>AAACAACATGTTTGTCATTAATATTCCTTTGCATTTATAGTAGTGATGCGCGAATCGAAATGGTTTAGTTATTCGAATAATT<br>AGTAATTACTAAATTTATTAATAAACTGCTAAATCAAAAACTTAGGACAACTATGTGACAATATTGAATTCTAACCTCAAT<br>CATAAATAAAAATGTGTTTGTGTACTTCAACAGAAAATAGTTGAATCATTTACTATAGCATTAGGATTTCTATGAATAAGC<br>TGTAACAACTTCTTTTTATACGACTTAGAATTGCAAACAAGCTAACGGCTCACCTGATGGAAAGTGGTAACCGTCGCCTAT<br>AGACATGAGCTGTGCCATGGACATAACAGAGTATACCGTTCCGCCCATGATAGCTCCCATGCATTTCCATTCCTGAGGATT<br>CAGGTGTTATTCCTCTGTACCCTGTAAATTCACGCCATATCCCACCCTTCAAACCGAAACACGGCCATGCAAACATAACTG<br>CTTTACGGTTGAAACACGAATAGGACAGAGGTACCCAAGCAATCGACTTTTGTACAAAGTCTCCCTCTGGTATTTTGTGTA<br>TTTATGTATTTTGTATGGGCTTTTTTTTGGTACATTAACCAATAATTTTCCAGTGTATTTATAGTAGTATTTGCGCTCATTAG

ACCATTGATACGTTTTTTTCTGTTGGGAAACATTAAACATTAACAAAAATAAATATTGACCATACTCGTGTATCACACCGG
ATGATTATTTACTTCCATGTGACGTCACTGACGCCATATTGCTTGAAGGTTGCGTTTCGGCGGCCCATTTAAATTATAGTTT
TTATTTGCTACATTTTTGGAAAATGTGTAAACACCACTATATAGATGTTTTCTAGAAACCAATCAAATAGTCTATTATTAAT
ATTAATAGTATTATGGTAATATATATATATATATATATATATATATATATAAAATAGATAACTATATAAAGTGTGCTTAATAC
AAACACAAAAGCAATCATGGAACGCGTATGAAAACGCGTCAAGAGCTTAAGCTCTATAAAATTTAATTTAATATCGACTT
AATGATAAGATTTTCTATAGAAATGTTGGCGATCGCTGAGATGTCACTTTGACAGTGACATTTCTGTTTTTTTTTTTTTTTAT
TATACTTATGTAACCACCCCATCTAGAGTGAACTCAGAGACAATATGGTCCATGAATGTGATCAAACGAAAAGAGAATGT
TCAACTCTCACAGTTGGCTACACTATGGTGGCCAAGTGATTTTTTACGCTACGTTTGTAATCGAATTTCATTTCAGACGGTA
GCGAATTTGCTATGTTGCTCCAACTGGCCTAAACTTCGTCCTACACTGGTGCCCCGCCATTAAAATCATTTGCATAAGCGG
GTGCCCCATTACATCGTCATCCATTAAACAAATTATGTATAAATAATATCGTGCTAAAATAACACCTAATATATTGCAAAG
AATTATCTCGCGACCATGGCCGGAGTGGTTTGGTGGAAACTTCGAGATAACCTATGATTGTTTTAACTCCATGTAACGAGA
AAATTTATTCATAAAAAAACGTTAAACATCTTACAAACCTATTTTAACTATTGAAGTGTTTTGTCCTTTTTTTTGTTCTAATT
TATTTTAAGTGAAAGGCAGTGACAAAAAATCTTTAGTTAAAACAGGTTTGCAGCTTAGCTTGTTTTAGTTTTTATGAATAA
GGAGGTAAATGTTAAATTTTTCAAGCTGCTTTTAATTTTCGTCGACTATAACGCTAGTGTGTACGTACGGGAAAGCAGCAT
AGGCGTATTTAAGAGGGGGTCGGATGGGCTGTGCCTACCCCAGTATTGTTTGGTGCCCACCCAGGATGCGAACGGAGCTT
GAAGCGCAGACATGTCTGAGAGTATTAATGTGCCTTTATTAAGGGGCTGGATCCCACCTGGAGAATAATCTTAAATACGCC
AATGAAAATGGAAGTAAACTATAGACTTGTAATAAATCTTCTTTCTTTAAAGCACCTATCAATAGAATTATATGCTAATTT
AACCTACTTATTAAGAAAATAGCCTTATGCAAATCCAAAAAATAGTTGTTTTCTGCATTACAAGCTTCAATTATTCTTATTT
TTTTTTTTTATTGTACACAGATATGGATAAAAATTCATTTTCATGAACGCATCCATTGTGTGTAATCGTAAAAATAATATG
GTCGATTTATATATAAATTTTGACGAGAATGCTAACGCTTGCATCATATTTCATGATTCAGGGCCCGGGATAATCATTCTCG
TTAAAAGTATACACTTAAATCTAATCCGGTGACTTACAATTAAAATATATTTATTAAACGTTTTAAAGTTGACATTATTTAA
ATTGGCAGCAGTTGCATTCTAAAGGAGCTGCGGAAGATAAAAGATTAAAATGCAAAAGAATTGATAAGTTTTTTTTTCTTC
TATAAAATATATAATCTGTAGAAGTGTTTTCTCAGTACTTTGTATATAAATATATCTTATGTGGTATATAACAGAGCTCTAT
TCTTACCAACTATAAGAGATGCATGCTACAAATTGTTTTTGTTTTCGAATAATTAAAATTTACGTGATTCAAGCATGCACCT
GAATAAATTTGTTTATTTCGCAAAGGAAACGCAATGTGCGGATGAAATAATTAGCTCTTATTATTTTTTTCTTAACAAATAT
ATATTGTAAATATTTTTTTTTTAAACGTAGACTAGTGTAATCAGTGCCTATATGGGAACATTTGTTTTGTCTATGCCCGCAC
CGTTTAGTGTGAAGTGAGTTACAATTGCAATATAGGTGCTGATCTATTTGAAAAAATTATGTAGCAATTTATTCAAATACA
TACATATATTATTTTTAAGACATGTATATGCTAAGTACAATAGATTATCATGGAGAGGCAATGTGGCGTGGGCTATGAAAG
CTTTCGTGGATTAAATATTACCTTAAAACGCTGGTTTGTTAATATTTGGCTATTTATTCATTATATTTGTATTGGTCAGAAAT
GATCAACAAATCTAAATTGAATTTTATTTTAGTAGGCCTATTCAAAAGATCTTTTAAGTCGTTATAATGTTTGTTGTGAATT
GTACTGTATTAATTTAAATCGATATAATTAGCAACATTTTAAAGCTTCTCTCTTACCAGTGATACGTGTAACGAACGAATCC
ATCTATAGATACGTTTACGCTACCATTTAAGGTAGGTAGTGGCGGCATACTTTCCGAGTCCAGCGCTTTCTAAATTATGTTA
TGTCAGCAACTCTGTCGTCCTTATTCATAAAGACTAAAGCGTACAAGAATAACTCCCCTCGGGTTGCAGGTGTTGGGCGAC
GGTAGTTGCTTTCTATTAGATGGACTGTCAGTTTGTTTGCCTCTCTAACAATAAAAAAATTACTACAAACCTATTTTAGCTA
TTAAACAGTTTTGTCACTTTCTAATTGGGAATGTAGCATAAAAGAAAGAGACAAAACAGTTCAGTAGCTAAAATAGGTTT
GAGATATTTAATGTTTTTATGAACGTTCTCAGCGTTGTATCTATCGACAGATTCGTTTGTTATTTCACGTGCAAATTTCCAA
AAAATGTGTCTAAAAATGACAAAACGTTTTAGTCTCTCATACTGACACGACGCGTCCAGATGTCTAGTTTGTATAGAGGCT
ATATACAAGGTGTAAGGGACAGGTTGGCAAAAACTGAAACGGTGAATTCAGACCATTATTCTGAATTAAAAACCAGTGAA
AATTCTTTCTGGAAAATTTCTAAACTTTTTCCTGTATTTTTTTTCATTTTAAATCATGTAATCTATGATATTATGTCATAATA

MSTRG.5084	Uncharacterized protein LOC106130423	TAAAGAAATAATAATATGTTATAATGAAACTAGCTGACCCCGTAAACGTTGTTTTACCACTTATTGTGAAATATAAAAATC<br>GCGATTACAATACAGGGGTTGATCGTAAAACGATGAAAATTTAAAGTTTTATATATTTTTCAATGCTAAATTATAATAAAA<br>TAAAAATAAAAAATTTGTCAAAATAATAAAAAAAATATTTTGGGGTGGGTCACCCTTATCATTGAAGGGTATGAAAAATA<br>GATGTTGGCTGATTCTCAGACCTACCCGATATGCACACAAAATTTCATAAATATCGGTAAAGCCGTTTCGGAGGAGTTTGG<br>TAACAAACACCGTGACACGAGAATTTTATATATTAGATACTATGAAATACCAAAAAACTACAACATCCTTAAATTTTACCG<br>TGACGCGTGGAATTAGCTGTGTAAGGTCAACGTTACAAGTGCACGAAAACTACCTACGAAGAAGCGCCGTTGATCGCGCG<br>TTGTAGGTATCACGCGTCAAATTTTAAATACATTGCGCGGTGGCAAAACTAAAAATCGTTTATTAATATGACTGGTGCATT<br>TCCAGTGGGAAAGTAATCTGCAATCTGATTCAAACCATTCATCTGAATCAGTTGTTTTCCTTTTGACCAACTTGACTCTTCC<br>ACCCTGTATAATGTACAAAGATCTTTGCACCAAAATGATAAAATGACATTAAAACAGTTATTATATCAAAAAGTTACAGAT<br>ACCCGTGTTTTAAAGCTGTGATGATGTTGGTTTAGCTCAGGGGTCTCCCAACTTTCAAGTCTTAAAGCCACAATGGTTTTTC<br>CAGTATGGTCCAAGGGCCAAAATAATAAACATTGGCTGTCGTGGGCCGGATTGATGGCACTGGCGGGCCAGATTTGGTAT<br>GCAGTCTGTAGTTTGGAGACCCCTAGTTTAGATCATTAACAGTGCCTACTTTTATGAGTTTTTTTTTTTGTTTTACGCACTAA<br>ATACTGTAAGTGTACAACACGAATGTCTAGATTAGTTGGATATGCTGGTATATTGTAAAAATATGAAATTGTGGATACATA<br>TTACATTGAAAATAATTGCGTTTTCTAAATCATATCATATTGTCGAATTACACTTAATCTCAAAGTGTAACTAACATACTGT<br>TTTTCTATTATTATTATTATAAAATTTTTTCATCACGCTAACTTGTGTAAATTTAATTTTTATTTTATAAATTTATCGAGAAA<br>ATATTTATTAACTGAAATTATGATAATATTGCCTATACAAAATTACATACGAGAGTGTCTATTATTTGTTAATGTGGTGAAA<br>CATATTTCTGGAGCGTGTCGTGTGTTCGGGGAAAGTGTAAAATATTTATTGGATAGGTTTTTGTAAAGTAGGCACTTAGTA<br>TTTTTACAGTGAATTTTAATTCTCTCGAAGTGTTAATAGATTTTGCGATAAATAAGTTAGATATATTAAAAATATAGCTCAT<br>ACGGCGGCCGGAATTCGTTTGTCTATTATATATAACTTATTCGAAAATATACAATCCATGACTCGGGAGTTGCGAGCATTA<br>TTTTATGTTAGACACAAAGGTCTATTGTGTAAAATCTCCTTCAGATTATATTGTATTTTTATTGTAGTTGATAAATTCGGGA<br>CGTCCCTGAAATGGCGTATTTTTATGTAAAAATTGCCTATAAAATGACGTCGTAATGACGTGTGCGCACGTTTCGGTCGCG<br>TCCCGATAGGATAATTATGTAGATTAAAGACAACTTGCTAGTAAACATAGGCGACTTATAATAAATGGTTGCCTTATAA<br>GATATTTTCAATTTCCTTATTATTATTGTAGACCTTGTGAGATCTGACCACATATTTCAAAAAACGTTAATCCTTACACAAT<br>ATGGAAATAGTGAAAATAATAATATATAATTTAAATAGAATAAAAAATGTATTATTAATAGTGAATAATTAAAATAAGGT<br>TATGTTATAATTTAAACAGGGTTTGAAGCTGTTGTCCAAGTCAATTGCGACTACAAGATATAATATGTAAGATAAAATTAT<br>GATTAGTAATAATAGGTCTCGCTATATAGTAAATGCTTTCGACATTACATCACTCAGATTGAAAGTTCCGGTAGTGTGGCA<br>ACGTCGCAGTTATGTACCAATTTTTTTTTCTAATAATTAAACAATTTATATTATAACGTTTCTGAAATGATGTTGGGACTCC<br>CGCGCTTTTGTTGCGCATCAGAAATTTTCGCTCAAGTATGTATTGATGGTTTCCATGCCATCATTCGTCAGAATGGCGCGTT<br>CATAGTGAGAAGAATGCGTGGAAGTGGCAACACCATCTTTAAAATGTCAGTTGAAAATGTCCATGCACCTGTGATGATAG<br>GATGTGCATTTCGTCAGGATGATGACTAAAACACAAAATAAGTATTTTCTCGTTAGTGTAACGTTCTTTACTAATATAATTT<br>TAAGTTTGTATAAATAAATTTGTAATTGTTTCTAATTGATTATTTAAGAAGTGCTCTGATAGCGAACCAAAATGTAAATCAT<br>TGAATAAGTTTTGATACCTATCACTAAAAGTGTTGCACTCACTTCGTATTTTGCTTCGAATGTATTCAAATACATTTTTTGCT<br>ATCAAAATGCGTATTCTCAGATATAACTTTATAAGAAACTTGTTGAAATAATATTTTAGTTTGCTAACAATCAGTAAAAAA<br>TTTATGAAATATTATAATTGAAATTATTTTTCAAAATTCATTCGATGTAACCGAAAGATGTTAGATACATAGTTGTTTCAAC<br>CATAAGATAGTCTGCTGACGTCAATAGTTAGTACAAATAAAAAATCGATCAGTCGAATGAATGTTTCGACTTGTTATCGAT<br>TTAATTTAAAAACTGTTTCTGTGATAATTTCAATAAAATTTCACGAAATAAAATACAAAAATTTATTATATTTTCATTACGT<br>TCTATTGAAATAAATTATGTACTTATTTATTTATTTACATCTTTATTGCACCATATATAAAAATAATAAATACAGTTAATTA<br>TTAATTATAATACCAACGTTGCCATACTTCGGTAAGTTTCGATATGAACGCCCTAAACTTTTATTTTTCCAGTGTTTTAAAA<br>TAATGCAATTGGCTTTAATCGCGTAACATTCTATTTAGTAATTTACGCTTATCCTTAAACAAAGGTTTTTTATATAAATAAA

		TGTATAAAATATATAGTTATAAAATAGTTATTATTATTAATACTGAGTTTAGGTAATGAAACAGAAACGAGATGAAATATA<br>ATATATAATATCCACTGGATTATTACAAATGTATAGCTTAAATTAAGATTATAACGAAATTGTTCCAAAAATATATTCGTA<br>GTGTAGCTAATGATTGTTATTTTTTTTTTTTTTGTAATTAGTTTTAGTGTCTGTTTACATGTTGTTGTAATTTATTAACTTTTT<br>AGAAATATGTATGTAGAAAAAAATGTTTTGAATTAAATTTGTTGAATAAAAACGAAGAAAGATTTGTACCGTGATTTGGA<br>TTGGTTTGTTTGACCTTTGACTTTGTAAGTAGGGTTACCATTCGTTCAGTATTGTCCGGACTTTGAAACTTCTGTTTGATGGA<br>GTTATTAATATTTCCGGACAAGATTATTTAATAATAATTTTGGTATTCAATTGCTCATTAATTACGCACACACAGTAATGAC<br>ACTCTTAGGTGTCATTTGTAAAAAATGTCCAAACTTTTTTTTAGCAGTCCGGCTTTTGTCAGGACTTTATATTTTGAAATAG<br>TAACCTTATTTACAAGTGAATGATGACTTTCAAAAGTATTACGTATATGGTATTCTCCATAAAGTAAGAAATGAAAAAAAA<br>AAGAAATCTGAAACTAATATTTTGTTTCAAAAAAATGTTTTTTGGAAGGGATTTTTAGAAAATTATACCTATTGTCGTCAA<br>AAGCTACATTATTTGCGATAAGAAGGAACTACGATGATTGTATTCAGCATCACAAGGTTATGTTATGCTTAAAAAACATCA<br>TAATCTGTTCATTGAAGTGCAACGAACAGTTTGAGACAAAATTGTGTTTTCATAATTTGTAATGAAATCTACATGAAATAA<br>ATACAACAATTACGACAATTTATATTTTAAATTTGAAAATTTATGTTACAGTAGAATCTCGATTGTTCGAATCGAATAAAA<br>ATCAGGGGTATTCCGTATAATCGAAAATCCGGATAATCGATTTCAAAGTCAAATCAAACAACACATTATTATTTTAATTTT<br>TTTTTAAATACATCTTTTAGACAATTCGATATAAAAAAAAAAATACGAGCTATAAATATATTTGATTACATAATACCTATTC<br>ACCCATTTAATACATAATATCATTTCTAAATCTGTAATTAGAGCCGTGAAGAATAAGTCCGGATAATCGAATATTCGGATA<br>ATGAAGATTCTATTGTATTATTTAATTTAGCTTTTCTAAATAATTTCAATAGTAATTTATATTTATTAATTAATTAACTATCT<br>TGGTGTAACTTCTATGAACAGATCAGCATGTATTATTGATTTTCACTTAAAATTTATTTTTCTATATATATATTTTTTTTATT<br>ATTAGATGTAGTTAATGGATTTTGTGCTCAAATAGTGAATTTAATGTGATAGCTTTTTAGATAGTACTTGTAAATGATGTCA<br>TGTTAGTTAAATCTGACATTTCTGCGGTATATATCCTCTTTATATACCTATATATTATATACAAATCTTGTAAGTAAATAAC<br>AAAAAAAATTGTAATGGATTATTATAATATAGTCGAATTGAACATTCTCATTTTTGAAGTTGGCTAAGAAATGTAATTATG<br>TAAGAATTATACTCTGTACGTTATAGCACAGTGCGAATGACAGGTTATTAAGATCACAAAAAATACGGATTCACTAAAAC<br>ATTTGACTTTGAGAAGTACCAATTGTTATTATTATTTTTTTATGTTTTGGAGGCGAAGTTGTACTCAATACAATATTTTTAA<br>ACTGAACACTGCCATAACTGTCAATTGTCACACGCACCGTGCTACAATGCACATAATATAAGAGGTAATCTACTGCGGCTT<br>GTTAATAACACTAAAGAAATATCTATGTCGTAAGCCAGATCTTAGAATTGATCAATTGATGTGACCAACCATTCATGAAAC<br>TCCCAACGCGTCTCTAACGGTGGACCATTATTATATTAGTCGTTAACTCTTTAGGGGGGGGGGATGCCAGTAGTTGCCATG<br>CTTGGCAGGCTGATTGGCAACCGCAGTTAGGCTCTGACGATGTTTAAAGAGGGACGCTTCTATCGATCCCTCGAACATTAA<br>GTTCTCTTAGTCGCCTCTTACAACAAGTCGGAAAAGGAGTGGCCCATTCTATGCCGGTGCCATACGGCGTAATGGTGGAAT<br>ACTGTCTCATGTAACTATATTACGATATGACCACTAATCTTTGACCACATCACGAAGAAGCCATGCATTTAGCTGTTCATAT<br>TGTCAAACGTTGATTTATTCATTGACGTGGCCAAGTTGCATCATGACGTGGGCTGTAGGCATGGGTGATATGGATGCCGAT<br>TGTGTATCTATAGATTGAATTCCTCTGGATATTAATGGCGGAAAGTCAATACCCATTTTGATATCGCCCATATCAATCGGAT<br>GTCTATTTTCGAATATGTAAAAATTCTGTTATCTAATAATATTTTTTGGATAACATATTCGTTATTGGATCAGTATATAGAT<br>ATTGTATTTAAAATTACCAGAGGGAGACTTTGTACAAAAGTCGATTGCTTGGGTACCTCTGTCCCATTCGTGTTTCAACCGT<br>GAAGCAGTTGTGTTTGCATGGCTGTGTTTCAGTTTTGAAGGATGGGATATGGTGTGAATTTACTGGGTACAGAGAAATATC<br>ACCTGAGTCCTCAGGCGTTGCATGTATCTTTATGTAG
MSTRG.5108	Nuclease HARBI1	GACACTTCCCAAGGGCAATCCAACCAATCCAACCTAAAATCTTTTTAAAAAAGTTACGCGTCTTTGTATAATTTTTTTTTTA<br>TTGTTCGAAACGCATTTTGAGGGATCCTAGCTTTAGCGTCGATTTGCTATCATTGTATTTTGCGCGCTTCACTTATCTACAA<br>ATTTCCAGATTGGTTGAAAATTTTAATATCAATCATTGAATTATAAATCTATTAATTAAATATTTTAGTTCATCCTGCTTAC<br>CATCATATTCAAATAAATATTCTGTAAATACATCATTCTATCACCGGATTGAATAATACTGCAGAAGACGGCAGAGGTGTT<br>TTGGCTCTGTTTGGTGAAAAGGCTGATAAAGAAAATGAGAAAGAATAACTTCAGTCTTGTCTCAAAACATCTCAATAGAA

		TATATCAATATGAAAAAAGCTGACAGAAAAACATTTTTTATGCAATATATTTGGGAATATGAAAATAATATGTCTGAAAA<br>CTGAGAACAATGATCTTTATTTGGAAAATGCATTATGCATAAAGATAAAATTGCAGAGATTGTCAGTTTAGAAAATTTGTT<br>CTACATAAAGAAAAAAATCAAAACTGTGCTGGTTTAGAAAATTTATTATTTATTAACAAATTATTGATGAATACTGTTACT<br>TAGAAAAATTTTCATTTTACAAACTTGTAGATACAGTTGGTTAGGTAGATGGCAGAGGTAACAAATAAAAATGGCTGCTTA<br>CTACAATGTTTCAAGTCAATTAAATTAAAATTCTCTTATTCTTCATATAAGTATCAGCATTCCATTTCTGTTTATAACAAATT<br>GAAGATTTCTCTGTATCTGAAAGATACTATCTAGTTATGCTTATCATGTGAATGGCACTAATACAAAAAACAAGAGTCCTG<br>AAAAAAAGTTACATATGAAGTGAAGAATGAATTGAAATGAAGAATCAACAATATGGCTTTTTAAAATTCCCAAATCATTG<br>TCCTTAATACCAGTGTGATGGTAATTTAGAAAAAATGTAACAAATGATTCAAAGAGTGGAGTAACAATTGGAAAATTGTT<br>TATTCTTTTTACTCTGTCCGATTGCATACAAATAGTTCATTTAGAGATTAGTAATGCTGACATGGAAATTATAAGTGTGGAT<br>GCCAGCTATGGAGGAGCTACTCATGACTCCTTCATTTGGACAAATCATCCCCTAAGACAGCATTTAGAACAGTTAAGCAGA<br>AATGAGTCCATTTGGTTACTTGGTGATTCAGGATATGCTCTTAGAAAGTATATAATGACTCCCATTACAAATACTGTCCCA<br>GGGTCACAAGAAGCACATTACACCAGTGTACATGTGAGGACAAGAAATGTGATTGAGAGGACAATTGGACTCTTGAAAGC<br>TAGATTCCGTTGCCTGCTTGTACACACTGTACGTCATTATGCCCCAGAGGTGGCAGCATCTATAGTGAATGTTTGTGTGGTA<br>TTGCATAATATGACGGTAAAGTCTAAAATACCAGCACCAACACTCACTGATGAATATGCTACATCAGAGGGCACAATGCA<br>GCTCCATCATACTCACCATGATCTGTCTCAGTCTTCAAGTGCAGAATTAAATGAAGGTTTAGCTGCCAGGCGCGCCCTCGT<br>TTCTCGCCTCTGGAGTTCTCGAGCTTCTACAAATAAAAAAAA
MSTRG.512	Myosin heavy chain	CTGCTTAAAATTTGTAATTGAAATCAAAGTTAAGTCAACGGACAAGTTGTTTGTGTCCCAATGTGCACTCCTACAGTTTTA<br>AGCTGTTTTACTTTTTTCTATTAACTATATCATCAGTATGTTTACTTAGTTAATATTATTAAAGTAACTATTAAATTAGTCTT<br>ATTATAAATTCCAACAAGAACATTTTCTTACTAATTATATCCAAACTTACGTGGATTCGGGCGTCGACCGGTTCATCCCTCC<br>TACCCGTAATAACCGTTGCTGTTCTGTCAGCCGCTCGCTATCGTCATTTTGTACGTACTATCGCCGTGCGCAGTCACGGCAA<br>ACGCGTGGTAAAAGTATAACGCGGGGTGATTATTATAAAAAGCCAAGTGTTGTGAGGTGCAGTGCCGGTCTCCGCGACCG<br>TTTAATGTGGTATACAGTGCCCTTGTTAGGTTATAGTAGGTCGGGCCGCGCGGCGTGATTAGGCGGCACGATGGTGGACAT<br>GGATAGGAACGATCCGGAACTCCGGTACCTGTCCGTTGATAGGAACAGTTTCAATGACCCAGCGACGCAGGCGGAATGGA<br>CGCAGAAGCGGCTGGTGTGGGTGCCCCACGAAACCCAGGGCTTCGTGGCGGCTGGGATCAAGGGGGATAGGGGTGATGA<br>AGTGGAAGTGGAGATTGCTGAGTCGGGCAAGAGAATGTTGGTGCCTATAGATGATATCCAGAAAATGAATCCACCCAAGT<br>TTGACAAAGTAGAAGACATGGCTGAGCTGACCTGTCTCAACGAAGCCTCAGTGCTACACAACATCAAGGACAGATATTAC<br>TCTGGATTAATTTATGTAAGTTGCTGTCCATCATTCTTTCTTTATCAATTAGTAAAGTATTCCTTCACACGTAGGATTTTTAT<br>AATTATGCACAACCGGGTTTTAATTTATTAAAGATTAAAAAAAAAAAGTTTAATGGAAACTTAACTGTCTTAGGATTAGCT<br>TTATTTATTTCACGTAATGCAAAACTATATTCATCATTTCACTATACCGAATTCATTCTAAGATACGTATTGTTATTTTGAAC<br>TAGCGTTGTTTATTTGATAACGCATCTTATTTTATAACTTGAGCAATCACAATTCTGTTCCCAAACCACACGCATGAAATCT<br>CAGCTGAACGTAGTGTCGATGCTGCGGCATAGAACCGGTCTCTGTGCCGTTGCCCGTGGCATTTGGATTATGGGTCACAGG<br>TTCCACGCCGGTAGCCGTCCGATTTACAATTTAATATCCTCTATAGGATGGTCATGCGATTATCTTTATATACAGAATGACC<br>TCCGTAGAGCATTTGGAAGGCATCTTGCTTTGCGTACATGAGAC
MSTRG.5158	Uncharacterized protein LOC106135886	ATTTGATTAAAATTGCCGTTCGTACTGCGATATCATAGTTTGGGTGCCGAATGGTTAGCACTAGGCTCAGAACAACGATCT<br>ATTAATTTGCCTGAAGATGCTTTGGGGTCTATCGTGAAATGAAATTCTGAATTTTCACACTCAATTTCGTGTTTTTGGTAAT<br>ACCAAAATCTTTTCAGTAAATAAAGCATTTTCGTTCGTGATAAGTCATACCATAAAAGTCCAAATGGACAATATTTTAATA<br>TTTTAAAACGGGCGTCCCGTTTAGAGATGAAACACGTGTTGCAAAACGTGTGCGAACAAAATGGCGGTCGAAGATTGTAT<br>GGAGGGTTGCGGAACTTCTGATAGTAACAGTGGTGGTAACAAACTAATGGTAACGGAATTAAACAAACAAATTATGGTAA<br>ACCTTTGCACGATTTTTTTATTAGTGAAATAAATCAAGGTGATATAATTCATGTTCAACATTAAAGGAAAAATGGTTAAAA

AAATAAATTTGACAGCTATGTTTTTTTTTTTGCAATGGACATTAAAAAGGTATGCAGACACGATCAAGTTAGAGTGAACTT
GTTCTTTTATATAATTTTTGGGCATTGTTTGTTTTAGTTACAATTTTCCAAAACAGGTAGTGAACCATAATTATGTATGGAT
GATAATGGACTGCTAACCCTGCGCAGTAATAAGTGAGAATGAAGGTCGACCTGACAGGATATATTGCTAGCAATCCGTTC
TCCCCAATAAATTACGTTTTACAAACATGAAAATTTTAAACAACATTTAGAAAATTTATTTAATTTGTCATTAAAAATATCA
ATATATGGCGGCTGAGTAATCAGTCATATTGTTTTAGTCCTCACCTGCTGCCAACGTGAAGGAACAGTTTTCGGATACATC
TAAATTCAAACTTAAATCCAATTTATTCCGTCTCTGCTCGCGTCTTGCTTCGTAGAATTGTCGAAGAGTAGCTGTTGATTCT
TGAGGGAAGTCCAGAAAGTCTAAAAAAAGTTTAATAGTCTAATTCCAACCTTTTCATACAGAAACATCACGATAGGTAAA
AATAGTTAATTTTAAACCTACATATCTGCTTCTTTATTGCCGAAACGTCGGCAATAAAAAAGCAGGTATAGAGGTACTATT
TACCTGCAAACGTTTTTTCATTACGTTAAGGTCCGATTGTGTATTTGAGAAAAAAGTAAGCAATGTTGTATGAAGGTTCAT
TAATAATCTTTCTTTTCTAATTTTTGTAAAAGGACATAGCCTGTTCAGCCTAACTACCTTTGTCACCTATTAAAAACCAAAT
AGATTTTTTTAATCAAATAAAAAATATTACGAATTTTTCAATAAATTATTCATTATTACTTACTTTTGTACCTTTCATTTTCA
TCTATTTGCACATCCTTGCGGAACCCACCGAAAATATTAAAAAGACAGTCTTTAATATTGAATATCTTTTTTTCTGTGGTGG
CTAACGCCACTAACAGCCTTTTCAATTCTTCTCTCTTGCTTTCCTAAAAGGAAAGAAATTAATATTAAGCAATTAATTAATA
AAAACACGTGATTTTTATTAAAAAAAAAAAAAAAAAAAAACAGGGACCTACAATAGAGCCCTGAGGTACGCCCATTTTAATA
TGAGATGCATGAAAATATATCCATTGCTATTTGACACGTAATATCTTAGAGTAAAAGACAACAAATGGTAAATATTATAA
AAAATATACATTGTGTTTGTGACACAGAGATCTTGATTTCACGATTACACAGTTTATTCTCTACCAAAATTGTATGGTAGTA
GAATTAATAGGGCGAGACAGAGGTGCAAATTTCTTAGCACAAAATCTAGCTCTGCTGTGGAATCCTAGCCTATACTCTCCC
TCTAAATATGTATATGCACTTGATACAAATATCGCAAACTCGTGCTATTCATTGGTAAATTTATCACAATACTCACGATAG
CACGGACCACGGCGGGTAGGAGGCGGTCAGTGGGAGCGGAGGAGGCGGGTGTAGGAGAGGCTCCCGCGAACTCCTCCTG
GCGCTCGGCGAGGCGACGCAGCAGCCCCGCGAGGCAGGCCGCGCGAGCCGATACGTACGCACGTTCGCACCTCACTACCG
CTAACTCGTGCTCCTCTGCGCTTGACAGGAGGTGCGCGGGGGAGGACGTGCGGCGTGGCGGAGGCGAGTGCAGCCGCTAC
GGCAGCACGGCGCTCGCACAGCAGCCGCCACAGACTGTCGCCGCGACACTTCACCAGCGACGACACGTGCTCTTCTACAA
CTAAATTACATTTAGATACTTAGAATGTGTTGTCCCCCTGTGTGGATACAACTGATGAACACGCGACTGGATTCGCGGCAG
ACAGAAGCGAGCATGAAGATACTGGTGGATTACCTTGACCTTGCTGACACATCAGTGTCATACATTTCTCCAGTTTCTCCT
GGAAAATAAACAGACTTGTTTTATCTGTGTTAGCAGTAAAAAGACCTCGTCACTATATACACAACACATATATAATAATAA
TAATATTCTAAAAATGATAGGACTGAGTAGATTCTTCCAATATCCTCAAATGCGGCAAACTGTACTAATCCAGTAGTGATA
GTTTTCTGCTTTAACTTAGACTAAGTCCTCCTAACAAAATGAGTTACTGTAAATAGAACTGTTTGTTTGAATGGAAGCATTT
GGCGTTCCATTGTTTCTGGTTACCCTAATGGAAGATAGGCATAGCAGATATGTATATATGTTACAATAATAAATAAGTAAA
TACTACACACTCAAAATAATAAATAAATAATAATTATAAACATTACACTATGTTTAGTGTATCCCATTGCATCCTTTATGTG
TTGCCAGTCCAATAATATGTCCTATTAATATTATAAATGCGAAAGTTTGTTATTCTTTCAAGCAAAAAGTACAAAATGGAT
TTGAATGATATTTATTTAATGATTAATTATATTAATTGAATGGTATGTTATATCCTAACTTCGCAACTGGGATGCTTTATGT
ACAGATTCATGTGGACGTAGCTGCGAGATGTAGCTAGTATAAAATGACAAAGGCAACACACATGCATTAGTTATTATATA
TGCTGTAATTTTACAGGTGTATTACAGCAAAATATGAATTCCCAAAGATGGTTGATGTCAGGCAGGCGACCGGTCGTAAA
AGCTATAAGCCACATTTTTTTGTAATATGGATAAAAAACATATTATGCTGACTCCAAATAATAATTGGATTAAGGGTAGAA
AGATGATGATTAGAGCAAAACAAAATTCGAGTACTGCCATTAAGTAAAAATGGGAATAAAAGAGATAGAAGTATCCATG
TTTTTAATTATGTACGTACTGAAACATTGACTTCTTCATCAAACGAGCACAATGAGTCAGCTATTGTCTTGGTCTCTTGAAG
ATTGTTCTTCTTAGCTTTCAACTCCTCTGCAACTTGCTGGAGCAGCCACACGCGCCGTTGGAGATCCTGAACGCGACGAAC
AGAGAGGTTGCGTTGGGCCACTGAAACATAATATTTAATAGCATTAATTCGGGGAAAAAAGGGATTGTTAATTGTAGGAA
GTAGCAGCCTCATTGATAACAATATACCATATTTGGTTGACCTCCACGCATTTCCCTCTGAAAATCATCATGATTAATTCTT

		ATTGAATTCGTCATGAAGGGCTAGGAGACTATATTTTGTGCATTACAGCATAGTACATGCAACAGTTTTATACTAATATAA<br>AATTTTAGAAAAGAATATATCTATATGACAGATGACATCCGTCAAACCTTAGGGCCTTACTGGAGTAGAGTGGCAGAGGA<br>TAGAGTTCAGTGGAGAGAGCTGGAGGAGGCCTATGCCAGGAGGCACACTGAATTGTGAAATATAATAAATAGAAAGGGA<br>AAATATGTCTTACTAGGGTTATAGTGAATTCGGAGTAAAAGGGCTTATATAAGTAGTACTAAATAATTATAATAAAGTATA<br>ACTCATTATTATTATTTCAGCCATTAACTGTTTACTTTTGAACATACCATAAGGGCAACTTTGGCAGTAGTTGCCACACTTG<br>GCAGGCGGATTGGCAACCACAGTTATGCTATTAGATATTTTAAGAAGCTGCTGCCCATCCCTCTTCCTCCCTTAGTTACCTC<br>TTACGACACCGTTAAGGAAAAGGTGGCCTATCCTATGACAGGGCACACACAGTGTATAATTCATAATAAGCATTATTCTAT<br>TTATTTATTTGTGAATATACTTACTCTTTATTGTTATTTTGTCAGTAATTTGCTTAAGTTTTTCATGTGTATCAGCATTTTTAA<br>TTTCAGCTGCTTTTACTTTGTCCCTTAACTCAGTCTGCTGATGCCAAAGCATAAGCTCTTCAGGCATCACAGCATTTGTATC<br>TTGCTGTAACAACATACCATAAAATAAAATAGAAAAAATATTAGACTACATTGATGGCTTTAAATCATAGAGGTATACAC<br>ATATGTAAAATTTATCAAGAGTGAAAAAAAAATTTGTAAAGCACCAAATTTAAGACAAATTTGTATATAATTTAGTATTAAG<br>TTGAAATTTCTTTTAGAATGTAATCATTTATTAAACAATGTAATAAATTAATAGAAAAACATTTTTAATTTAACTAGTTATT<br>TATATTACGCTGTTGTTTAAAAAAGTCAAGTATAAAAATGTAGTCATAGAAATTACAGTACATACCAAGGCATAAAGCCTT<br>TTGCCTTAAGGTCCACAACACATGAGAGGTACTGTAGTTGTCACTGTATGTTGTGATGTCTATGATGTTAATCAAGTCTATT<br>GGTGATGTTTAATACTTTTTATTATATATTAATTAATCAATAAAGGTTAAGTTATTAAAAAAACATCTAAAATAATAATGTT<br>TCACCTCATTGAAGGGACTTTTCTTGCGCCACTTCTGCACATCATCACAGAACACTTGTAGCCTCTTTTGTTTGATGCCATT<br>CTTGGGTTGAACATGTTCCATAAGACTTCTCCACACCATATAATACTGACCTCTGCACATCC
MSTRG.5166	Uncharacterized protein LOC106135993	CTCTATTCGTTGAGTTTACTTGTCGCGACGCGGCATGGCCCCTCTTTAAGCTTATCCTTTTTATTTTGTAACACCGTTTAAAT<br>ATTTGTTTTCCAATATAAATCATATAACATTTTCAACACTTTAAGTGAATTATCGAACAATAATACGAAATGGAGTACTCA<br>GATAATATAATGAAGTTTGAATTTGAAGAAGAGGAACTTATTTATGAACATCCTTCGAATTTTATTGCAAGTAATTTTAAT<br>TTTCCTTTAGTATACACTACAAAACTAGACGAAATGGTTATATTAACTAAAGAAAATGTTAATAGCTTATCAATTGAGAAT<br>ATATCGAAGAGAATTATTAAAGAAAGTAAACCCAAATTTATTTCGTATCCCAAACAGGAAAGAGGCCTATATGCATATAC<br>AAATAAAAGGGCCTATAATAAGTTAGTAAAAAAAAATAGAAAGAATAAATACAATGTCTAAAACAAAACCAGTGAATTCC<br>AGGGATTCGGAAGAATATACTATTTATCAAATGTCTCCGGAAGCGGACGAAGTTCATTCGTATGTTATTAATAGAAAAAA<br>AAGGACTAGAAAGCCTCGTAATCAGAGCCAACCATATTATTTTGTTTTCGCAGACAAACAACAGAAAGATGTAGAAGTTA<br>TATCATCTGACGAAGATTCCGATGATCATTCTCATATCCGTTCTATTTTCTTAAATAGCAACGAAGAGTCTAACGATATTCT<br>TCCGGAAGACGATGTTATTATTGAAGATCCAAATGCCAACAGTGCCTTACCCACTCGTATCAAACCTATAAAGAATATCGT<br>ACGTTTCCAGAGAATACAAATAAGTCATAATTCCAATATAGATTTTAGTGGTTATGAAATATCTAATAGTTATCTCATTTTC<br>ACATACATATGTACGGATTACAGAGACATTTATTATATTGATGAACATTACACACTCAAAGAACATTATCAGCTCAATAAC<br>ATAGATCCAAATGAAGAATTTATAGATTCCTCGAAGACTGATTGTGTGTATGAAATTTACAATTGCTGTTGGTATAAACGC<br>GAACTAAGGGTACGACAGTTATTCCAGAACGTAAGAGATAAATCAAAATTGAGGTTTGCAAGTGTAAAACATCCTTGCAT<br>TCCGACTGATTGCGTTTGTTGTTGTAAAATAAAGACGGGAAACAATAGCAGTCATATCGCACAAGAATTTTCAATGGTGAT<br>AAAGGATAGAATAGAAACTGACACTTGTAATTCAGCTACTAGATCCAATACAAGTAAATGGAACCGTCACCTTTTAAACA<br>ATCTTGGTATTACGATTGGCTACACCATTAATTCGGCTTTACGATATAAACCAAAAATACCAAAACGAACAATACGAATTA<br>AAACAAAAAAAGATTATCGCCAATTTACCCTCTTACAGATTTTTCTGCGTTTCACTAATGATGTTGAATATTTTTACCTGAA<br>TGAAAGTGGGTTAATTCAGACACATGTATGTGTACCTAATAGTTTTGTATCCAAATTATATCTTAAATTAGAAACAGAAAT<br>AATAGACAAAGGCAAATACCGGTGTAGTTCAACAGTATGCTGTTGGTGTAAATATGAACAATCTCTATCCAAATTAGCAC<br>GTTCTGTATTACCAAGTGTCTTAAATTTTTTAAAAAAACCACATACCTGCGGTCACTATTGTACCTGTTGTTGCAAAGGCTG<br>TATTAATATCAGTAATAAAGTGGCAAAATTTATGCCTATAAGTTTGACACGTTCTGCAAAATACACAAATGGAGCAATTAA

		GAAAAAAATACAATCAATTAGGGGTCAACAAAAGAACGAAGTATACACTTCTATCCAAGAGGAGCAAACTGGAGGTCAA<br>GTCACACAATCAAATCCAATTCATATTGATCTAACTGAAATGAAATCGGAGACATCCTCTGTAGATACGTTCGATATTTAT<br>GAAACGTTTAAAGACCTGAAACTCAATATTGAACAAAAAGGAGAAGTAACTGCCGTAACAAGTATACGTCCTAATGAGTT<br>GTCGACTAAGAATCTAAAAATCCTCGCGCACATTATTAGTAATGCTCAAAAACAAATAAAACAACTCGGTATTATGGGGA<br>CCAATATGGTAATAGATAACATATTTAATAAAGACCCAATTTTACTTCCTCAAAATATACCATCATTACTTGAAAATAGAT<br>TGACTGATAAACCCGCAACATCTAAAACGTCTTCAGTTGATAACAATACATATTCGAAATCGACTACATTTGTACATAATA<br>AAAAAGATCTACATGTATTTAATAAGAAGGCCCAATATCAAAACTATGCTACCATAATGGGTCAGAAGACAGAAGAATAC<br>TACATATCCATTACGGCACTTGAAAATAATTGCACCAGACCAGAGCTCCGATCAAGTTTTGAAAATCTCACAACATACCTC<br>GACGTTGAACAAAAAGTCGGTACGCGGAACAAAAATATAAATGTTACGCACATTAATAAAATAATACCAACTATATTGAA<br>ACGAAAATCAAACCAGGGCATATCGGACTGTGTGAATGCAAAGCACCCAAAGAGAACAGAGAGACAGCAAATACAAACA<br>CAACGGGTTACAGTACCGAGCGGTGTTGATGTAAACCAAATATTAGAAAACGTTCAAAAATCAAGTACGATTAAAACCAC<br>AAGGTTTTTACAACCAATTACTCTACAGAAGCCTAGTACGGTTACACAAGCGCCGCAGCAAAGTTCTAATCCGGTAACAA<br>ATGTAACGTCGCTGCCCTCAAGGCTATTACAAATTTCAGGTACATCTTTAACATTAATACCAGTGACTTTTATGCCGACAG<br>ATCAAAATATTAATAATCAATTGCTTGTGAATCCAGGAACATCCGCTATTGTAGATAGTAATCAAACCTCATTAACACCAA<br>CAGTTCAACAACCTATAAATTTTTTAATCGCCCCAAAGGAGGTAGCTATACCTGTTATCAATAGTAATATACCAACAACTA<br>CACAGATTGCAACCAAGCCAATTGTTATAGATGTAACTAATAGCCCAGAACCTAGTACATCTAATTCCACTATCATTCCTA<br>ATCCTAAGGACACATCTGAGGACCCTGAATGTGTTTTAGGCATTTGAAAGTATTACTGAGAATATTATTTTACTAAAATAC<br>GTTGGATTGGATACCACACAATAGGTCTGGATTCATCAAATCACATGCAATTTTTTATTTTTTTCTTTTTATTTTACTTCTTA<br>TAGTAATTACATAGTAAAGAACATTAGAATAAATAATTTATTAATTTTTGACTGTTCATTGTATTTTCTTTTGACCATTTAT<br>ATATAAGCATTTGTTTTTAAATTGATTTTTAATTATAACTTTGAATTTTTTATATTTACCTTCTTTTTTATAAAAACGTCAAC<br>CTCTTACAAACCTAATTTAGCTATTGAAGTCTTTTGTCTTTCTCTTTTGCGCTAATTTCTCAATTTGAAAGACAGTGACAAA<br>ACACTCTAATAAAGGGGTTCATATTTTCCTTTGCCTATCTTAATAAGCATTTGTGAACAATTTGATTTTTAGTTTTAAAGTT<br>GGCATGATTTTTTTCTTACATTTAATATCTATGTATTCAGATACTGAGCTGAATGCTTTTTTTTTTTATTTGTTCAGTGTTCGA<br>CACTTCCCTTTTTTTACTGTTTTAATTAAAGCGTCTGTGAAAAAGTTATAATTTGATTTTGTAATTCATGCAATTTTTTATAC<br>TTACATACAATACATTGCGTTCTGTGACTTACCTCTCCC
MSTRG.5170	Uncharacterized protein LOC106135953 isoform X1	GGACCAGCAACAGTATGGCGGAGTTGGAGAAATTCAAAAGGCGGTTGATCCGCACGGTGGCCAGGCTCCGACAGCAGAA<br>GATACCAGAGAACGAGCTGTGGGGACAGTTGCAGAAGAATACCAAGTGTGATTGCCAGTGGATGTGGAACCGTATGAGA<br>TCGTTAACAATAAAGCGATTAAAGCGCTTACTCTTAGCGGTGGATCGGCAGGAAAACATCACGGCGGTAGCTCGCATGAA<br>GCTCACAGACTGGATGATGTTAGATTTCATACTTGTACACGAGAAAGTCGACCTTACCGGAGCGGATCTGAACCTTAAGTC<br>TGAGCCATTGTCTCTATTGGAGCTGTTCTCGCTGGTGCAACGGTTCGGCGTGGAGGGACGGTCTGGAGACGAGCTCGCTTC<br>CGCCTGGACCGCTGCCACAGTTTACTACAATAGCCAAGGGCGCCAGTGCTCGCCGATGTTGCTGCAGCGCCGGTGGTATCA<br>AATGAAGGAGCATACGCGGAACAGATTTTACAATTTCTGGTTTACTTATCGCGGAAACGCAAAATATCTGAGCGAAGCCC<br>GCGCGCAACACGATCCAACTAAATTACAAATGGCGATTGCGAAACGGTAAACATTAACAACGTCATTGTTATGATCATCA<br>TTGTGATCAGGTTGATTATCTAATATGATGATAGGTTTAGTCGTAATGCATCCAAATCAAAAGCGTAATCCACACGTGATC<br>ACATCAACTAATTTTCTAATTCATATTATTTTTCAGATACCCCCATATAATTACCATGCCGTTTCTTCCATGGGAGGAATTA<br>ATAGAAAAACGGCTTGCCATATTACCAGAAGAATTTGAAGAAAAAATGAGAACACTACATAAAGCTTCAGATCTTCCCAA<br>ACCCTGTAATGAAAATACACCAGGTTTCGTAATAGTCGAACCAGAAATGGAAGTGATAGATCTTGAGGTAAATTCCGAAT<br>CAGAAACTGAATATGATGAACAGGATATGGTCACAGAAGCGCAAAAAAATGTTGGGCACGACTGATATAGATGGCAAGTC<br>GCATGACTCTAACTCAACTGTTGCAGCAACTGAATTTAATTTTGCTAAGACTTCAACATCTCAATTAACGAGCGTTAAATT

AGAAGTACAAGAAATAACTACAGAGGAAGCAGATGATTCCAATTGCGAAGATTTGGCGACAACTCTTATACCTATTAATG
ATAATGCAATCGGTGACATTAACACTTGCATTGAAGATCCTCAAGGTGTTCATGAAATAATCAGTGAAGAACAACAAACC
ACAGTGGAATCTTGTACAGATAATTATCTAACTGGTGACGTAAAAATTAGTAATCAATTATTTAGTGAAAAAGAACAGTCT
ACAATGAAATGTTTCACAAATAATTGCTCAGTTAGTGATCTAGAAAGAGTTTCTGAAATAATCAATAAAGAAAAACAAGT
CACAGCTAAATATCTTACAAATCCGACTGGTGTCAATCTGTTAGAAATTAAAAGTGAACCACTTAGTAACGAAATTAGTAC
AAATCCACTAGAACCGATAATTATGCCTTTAATCACAAATGTTTTTGGAAATGTTTATGTTGAAAATGAAGTATTACCTAA
AATAGCTGATATTTTATTAGAAAATTGTAAAGATGCATCTGTTGTGACGGAAGATACTGTAAAAGTATCCGATATTAAAGC
AATAAAAGAACCAACATTAATTGAGGTAGAGAAAATAGTAAAGAATGAAATAGAGGAAATGGATATAGAAGAAAATATT
ATAAGTAATAGTGACGTTGAAGAGATCCTGAATACTCCTATTGTGAAAGATAGAATAAATATCAAAAAAGAAGTAGATAA
TTGTGAAGATACGCTGATAAATACAACATTAAAGGATAACGTTGCAGAATTGGCCGATAATGGTGTCCCGTTATTTGATGA
TAATGAAAATATAGATACTCATATCATACCACCATCTCTTAATAACGAAGAGAAAGAAACTGCAAAATTTGACCTAAAAC
TTCTAATGAAACCTGTGGTATATACAACAAAATTAGATGATATGCATTATTTTAGCTATAATGAATTTGTACATGTCAAAT
ATATAACAAATATAAATGACATTATTATAAAAAGTAAACCTATTAGTAAAAGGTCGATCGATAATTCCCTAAAACAAAAT
CCCATTGATTCCAAGTTTGACCTAGAAGATGAGTATATGAATGATGATTTATTCGATAATAATTCTTTAGGTAATGAAAGC
TCTTTATCAAGTGAAAGTGAAAATAATGAAGTACCAGAACAAATTAAAGTTAGCATGTCAAGTGCTTTATTGCAGAAACC
ACGCATTCGTACCTATAATTTCATAAACCTCTGCAAAAATCCTGATTTTAATACTAGATTAAAAAGGTTAACTCTAGGATTT
TTAAGCTCTACACGTAACAGGCAGTTAATAAAGACTTGTAAACCAATGACTATAGATGTAAACAAAGATTTCGAATCTAA
ACTTATAAATAATACTTTGTTTTTAAAATCATGTTCAGTGCCAAACAATGGTAAACCAGTAAGTGATATTTCAATGGAAAC
TAGTAATTTAGATGAGCAAACTACAACTATTGTCCCTTCTGCTATGTCTGTTCAGAGTCTTATAAATAACACGAAAATTAA
TATGCAAGATTTGTTACCAATATCACATCCAAATGTGAATAGCGGAGCAAATAAATTGCATAGACAAGAAATTTGTAAAA
ATCAACAAGATTTATGTAAAAGAAAGAAAATTATTAAATTGTCCGATATCGATACGGCAATAGTAACGCCGATTCATATT
AGAGAAGATAATGAAGAAAATGAAATAGTTCCAATTCAAATTGGCTCCCAAAGTGTCCAAATAAGTTCAACAGAAAGTCA
GCGAAATCATGTTCCTACTATTACACAATCTATTATCGATGGTTTCAATACAGCTGAAGATTCTGACCATCGAAATAGCAA
TATTGATGCAGGAACTATTTGCATTGACTTTCCACCAAAAGAACGAAGACCTACTAATTCAAATTTGATCAAAGAAAATAG
CAGTATAAAAGAAAACACATTAAATGCGACAAAGAAAGGAAAACCTATCGAAAAACCTATTTGGACACCAAGATTTGCA
GCTTCAATTAACTGGTACAAAAAACAAAATACAAAACAGCAAGAGTCGCAAACTCTTCTAACTGTGGATACACTTAATAA
AATGTTTAATGTAATTTGCCCTGATAGAGATACAACTACTAAAAAGTATAAAAAAAATAGTAAAAAAATTTCTAAAGCTTT
TAAAAATCAACAAACGGAATTGCAACAGAAGTTAGATGAATTTGAAAAAATTTCAAACAGTAAAAGATTAAATGCAATTA
ATAAAGATGAGAATAATCTTTCAAATGCTATCAAAAAAGATGGTACTATCGATAGTCACATGTCGGCTACAAAATTACCA
ACATTTGGAATACAGTTAAAACCGAAACAGTCTTTAATGGTTATCGACTGTAATAAAAATTGCTGTTGGGCTCGTCAGAAA
ATAAACGAAGTTCACTTAAAATCTATTCAAAAACATAACCATCCATTAAATCAATGTACCTGTTGTTGCAATAAGGATTTG
GACGACTTTTTAATTAAAAAAAGAAAATCTACTACTTCTGAATCTGCAGTTCTCTATGACGATGATGATTCAAGCCCGAAA
AAAATAAGTAAAAGCGTTGCTCAAAAGTTAACCCCTGATCTATGTACATCTTCATCTATTGAAGTGAAATATAAAGACTCC
TCGAAGTTACTTCCAGTGTCTGATATTATAGATGTCGACAATTATAATATAGAGAGCTCTGTAAAACATAATTCGATTCAA
CCATTACCTAATCATGTTCCACTTCATGTGTCAAAACTTCCGTTTACTCTTATAAGGAGGTCTAAAAATAAATTAGTACCTG
GACATGAATGTATTTTTAATAAAGGGCCAAATAAAATACCAACACTACCGCTTTATAATTTAAAAAAACAAATGGTTATTA
ATGGACAAGGACTACAAGACCCACAATTTCCGAGTTCAGTGACTTTTAATAATAAAGTTGTGTTTATCAACAGTAAACATG
AAAAAGGAAAAAGGAAACGGAAACAACGAGTGGTTCTTACTGATCCATTTGATGTTAGCGATAAGCTACCAGAGCAGGT
AGACGATAGCCATTATAATTCTTCTGATGATGAACCACTCGCAAATAAATCGAAACAAGTGAGAAGAGAGGATACGAAAA

		ACAATAGTGCAAATGTTGAAAGTGAAGCATTTGGCATTTATAATGAAAATCGTAGTATAAATACAACGCAAACTTACAAC<br>TTAGTGAACGAATTACCCAATGAAAGAATAGTGAACAGTCTGGAATCAGAATCGCACCCATCCGTTTCTTTAAAATCTGAA<br>GTCGATACGTTGCATGAAGACTGTATTCTAGGCGTTTGAAATATAATTTATATAGTATAAACAGGAGGATCCACAATTTAA<br>AGCATCGACAATATTACTAATGATTTTTTTGTTTCTATAAACTTTGGTAACATTGTTGAGGTGAACATCATGTTTATAGATA<br>TATACATCATAAATATACAAGTAGTAGGTATAACGTACTCTCTACATTGTCTTTCTTAACGATTGTATCAGATATATGTTTA<br>CTTTTGGCGTTTATTTATAATTGTATTATTGTATATTATTTTGTAATCTTGCTATAAATGATGAGACCCCAATACAATATCAA<br>TATAACTTTATTTTGTCCCCGGCTTCGCACGGGTGGACATTTTTACAACATACAAAAAAAGTGGTTATTGTAACATAACGA<br>TAATAGTTGGACATATAGTATCATTTACACCTTGAGTTACGTGACTAAAGTTTTAGTAGGGATCCATAATCTTTTCTAGCAA<br>TAAATATAGTCTATGTTATTCAGGGTGAATGTAGCTTTCTAATGGTGAAAGAATTTTTGAAATAGGCAAAGTAGTTTTTGA<br>GTTTATTCATTACAAACATACACACAAAAATACAAATCTTTCCTATTTATAATATTAGTGTAGATGAATACTGGTAAAGAG<br>GATGGTATAAATATTTATATTCTGTTTACACTCTTCATTATTCTGACTTACAAAATTTATTTATTGTTCTATGGATATAAATA<br>TCATCGTTGATTTTACATTTGTACCAAGATCTAGCTAGAACTAATTATTTTATGTACAAGTAAACACATTCGCGTGCGTGTA<br>CCTCAACTGCCTATTATTATGTACTTAATTATCTTATTAGTAATGGTTTCTCTTTATCGAGATATAATTTAAGTTATAGTCAA<br>GTTAATAACTGTATATAATTATGATAGTATAAGATCTTTGTGATTTAATATGTAAGTAGGTAGGTAGGTAGATATTAAATA<br>AACAAGGAGAATATGAAGATATTTCGGAATTGATTGTTTACATCTATGCCCAATAATTTGTTTAATAACACGATTTGTTTTA<br>TTCAAGAAAATGTTCTACGAGTATGTGCTCTAATATGGATACTTTAAAAATTTTGTAATTGAAAATTCCTAAACATTAGGT<br>AAACAAATTAATTGTACGAATAACGTAAGAGAGTTTGTTTTAAAAACTTGATACGGACAAGACTTATTTAGTTGATATTAA<br>TGTGACTTATGATTTGTGATTTATTTATGTTAAAAGTAGTATAAACTGTGTTGAATATGCATAATTTACCAAACAATTTTAA<br>TGTTTAACATTTAAATTGAGCGGTGATCATTTTGATTAACTATTACCTTAATTATTTCATATAATTTTTTTGTTTATCCATTG<br>TAAGCAACGCTCAACCTACGTGCCCGATATTATAGTTAAATAATATTACATATTTTTGACGACTTAAAATTTTATTCATTAT<br>GCGATCACTTACCTAAAATTCTGTCGGCTATTTTGAAAGATATGTTTAAATAAAAACAAAGTGCGCCATTTTTTTTAGTGTT<br>GCAAGTAATTATATTAAAAATTTCCCAGA
MSTRG.5<br>201	Homologous-<br>pairing protein 2<br>homolog	GAAACGTGCACGTGATATCTCTAGTGTTGCAGGCGTCCATAGTTTACAGTAATTTACTAACAGACAGTCTGTACGCTTGTT<br>TGCAACCTTTGTGGTATAAAAAAGATCATAGTTTTGTACGTCTCTGTTATGTTTTTCTGTATTCCTCCCTTCTCTATTGGGTC<br>AGATTACGACAATCAACTTAAAAATCTGATGCAGTCTATAACCGATAGTACAAATACATTAAAAACAGCTGAAATAAATC<br>TAAAAAATCTTACCTCTGCTCCTACTACTGAAGCAGCTATAAATGAAATAGAAGAAATTAAAAAGCGTTTAGAAAACCAT<br>GAAATCAAATTGAACAATTTAAGAAGTTCAACGGAGATTATAAATCCAGTTGAAAAGAATAAAATTCTGAATGATTTTGA<br>TAAATTTTTCAAGGAATATAGAAAGCGCAAAAGAATCTGTAATGACATGCTCGAAGCAATATTAGAAGGATATCCAAAAT<br>CCAAGAAGACATTGCTAGAAGAGCTTTGTATAGAAACGGATGAGATGGTCAATTTCAAAATTGTTACTAATTCTTACTAAC<br>CATTAGTGTTTTTTTTTTTTTATAATAAAAACAAAAATTAACGCTCAAGTTCTAGAACATTATATTTCTAATGTTATTCTATT<br>TTTCACTTTCAAGAGCAATTTGTAAGTAAAATTTTGCATTTTAAATAAATATTTATGAATA
MSTRG.5<br>247	Stress-associated<br>endoplasmic<br>reticulum protein<br>2	AAATTGGTTGCATAGATATCATACGAACTTTTTTTATTCTGTGTAACATAAACTTACGATTTTCGAGATTCGTAATGACGTG<br>TGACATTTCGTTATTGTTGACGTTTTCCTTCCATTAATAAAATCTTCACTTTTTACATACGTGTCAAGTGAGCTTGTACGCGT<br>TCGGCTTTAAAGCCCTAATTATAAGATATAACTGACAAATATATTTCAAATCAATTAAAATGGCGCCAAAACAGAGGATG<br>CGTATCGCCAACGAGATCGCCAGTAAAAACATCACCATGCGAGGAAATGTGCCCAAAACCACCAAGGTTAAAGACGA
MSTRG.5<br>253	E3 ubiquitin-<br>protein ligase<br>MYLIP	ATTTCGGTGGCAAACTTAACGTAGTATGGCCGTCGTCGGGAGGTATTCACAGTTTTACTTCCATAAAGAATACGCGATAAA<br>CATTGCTCAGTGCAATTTACGAAAGACATTTTTAAATAAATTCTTTACGAGAGTGAGATAAACAATAATCAATAACCAAAA<br>GTGTTAAAAGTATCGGCACGTTTTATAATTACGACCTCTTTAACTATTTTACGGTTTTCACCTAAATAATCAAAATGTGGTT<br>GATAAGTCAGCCCAATTCTGTGATCCTTGAAGTGAAGGCTGATCCTAACTCTATTGGACAGGAGTGTCTTGAGAAGGGGG

		CAAACGAGCATGCGGCTCACCTGATGGTAAGTGACTACCGCCGCCCATG
MSTRG.5 289	Prolow-density lipoprotein receptor-related protein 1	GGGGCGTGGGCGGGCGCGGCGCTGGCGCTGGCGGCGCTGCTCGCGCTCGTAGCCGCCGCGCTGTACCTCTTACACAGGCGGAAACACGGCGCGTTCGTGCACGCGCGGCTGGCGGACAACGTGGAGATCAACAACCCCATGTACCTGGCCGGCGACGACGAGCCCGAGCCGCGCCCCGCGCCCGCGCACCAGAACGGAGGCAACCACTTCGCCAACCCGGTGTACGAGAGTATGTACGTGCCGCAACAGAACCCCACGGAGGAGCACGCGAACTTGCTGGGCGCGAGCGCGGCGGATGGCTCGTCGCCGCCGCCCGAGCGGTCCGCGCTGCTGTGAACATGCCGCCGGCGTGCTGCCGCCACGCGGCGGCTGCACCACCACGCCACGTCGCCACCGCGCCCTGCTCACTGTACACGCGGCGGTTTCTCTTAGTTTTAATTATTGTACAATGATTGGCATCACGTACCATGATTCTGAAAAGAACAAAATGACCTATAGGAATGTAGTATTAAAAAAGGATAGTGTCTAAATATCCCATCTTGCTCAACTATGGAGAGAGCTTAAAGTTTTAAAAATTATTGGTCCAAACAATATTTTTTTCTATTTAGGGAGAGGTCACTTTGTTTTGGAGTTAAATTCATGATGGGCAGTTACTTTAAATGCTTTAAGATAATGCTCAATATTAGAACGGCCCTAACTGATCATGTTTTACTTGTAATGTAGGGGTTTACTCTCCATTGAATCTAAATACATGACATGAACATACATGACGTGAACATACATGACATGAACATACATGACGTGAACATACATGACGTGAACATACATGACATGTTTCTCCTCTATATACAGCGAATAACTACATCTTGCCTTTATGATAAAGTTTATTTATTATAAAAGAAATAATTTATCGACAAATCGACATTATAATGTAGTACATTATTTGTACGGCGCGTCGCACCGCAACCAATCCGTACTGCGTACCTATAATTATAATAGGTAATTATAGTTGTTGAGCGAGCGACCTTGACTCAAACAGCCACGTAATTTAATTTATAAATCCTACTTCAAATTATGACATTATATTATTACATTATTTCAAGGAATAATTTATTTTCGCTTTTATTATTTGAGTAAATATATTGAGACATTAATTTTCGTTAATTATAGAAAATACATAATATACCTGC
MSTRG.5 297	Hypothetical protein KGM_213353	TGAGGACTCAGATATTCGTCCTGCTTATTCTAGTCGTGGCGTGGATGGGATCTACATCACCCATAGACCCCAAAAGACAGCTCATCAACCGACCCAAAGTGGATATCTACCATAGACAAAGACGCTCCCCGCAAGATGTTAAAGGAGGGAGTCGAAATGCAGTAAAAGGGGGTCACACGCCACCTACGATACATCCGAGAAAAGGCGAGTACGTGTGTGGAGACAAGGTGTGCAAGCTCAAGCCGGGGGAAGTGCCCAAGAATTGCAACGGAGTGTGCCAGTACCGGGTGGCTTGATCAATAAAAGACTTTTGGAAAATATTACAAAAATTTTGAAT
MSTRG.5 323	Uncharacterized protein LOC106102800	TCTAAGTTGTATAAAAAAAAATCACAAAGAGAGGCAAAGTCTCTCAAACCAAATAATATTAATATAGATTAGAAGTTACATGAAACCGTTTGTTTCAGTATCCTCCCTACCGAGCCCGACGACGGCGGACGAGGATGAGGATGAGGATGACCCGACACCTACCCAGGGCCGCGCCGACGCCGACGATGACGGTCGTGTGTACAAGAACCCCAGGAACTCTCCGTCGGTCGACTGCCCCAGGGATGAAGTCCAGGCTACTATGCTGGGTCAGAAATGCCTGCGAAAGTGTTCCTCGGACGAGGACTGCAAAAGTAAAAAGAAGAAATGCCTGTGCGATGCGGCTTGTGGCATGTCGTGCATCAAACCAG
MSTRG.5 33	Maternal protein tudor-like	CTCGAGTGAAAAACAACTCGAATCCCATCACTACGATAATTGTTTGGTGTCTAATCAGACACTAGTAATCACTAGACATCAGCGAAATTTTTAAACTTTATTGTCTGTGTTAAAACTTTATTTTACACAAAAAAACAATTTTATCCTCATCGGATAAAGTTGACAAAATCAAACAAAAATGAGTTTCGTAGATCTTGCAACCTTAACTTTTCACCGACGAAATGAATTAGAAGCAAGGATCAATATCTTTGTAGAAAAAAGCGAACATGCAATTAGTGAAATAAAGAAATTGCAAGTGCAGGCTCAGGAGTTTGTACAAAAGTTAAATTGTGAACCAGCAGAGTTGAAGGAACGCTTCCTGTCATGCATGAAACAGCTTGATGAATATACATATCTCATAACAGACTTCAATTTGTGTGTTAAAAAGCTTGAAGAGAACAGCATTGTGGAGTACACACCAGTGCTAGATACGATGAGGAGCTCGCTACCGACTCCAACGCCCCAGGCTCTCAATCTGATGGACGTTCTCAGTGAGCCGATGCCTTCTACATCTACAGCTTGCCCAAACCTCTGCCCCTCAGAGAAACCGGCGCAAGAGAACCGACGTAAAGGGGAGCAACCCCTCATTGAGTTCTCTAGCACTTCATGCTCTTCTGAGAGTGTGCCCGTACACAAGANNNNN
MSTRG.5 34	Lethal (2) giant larvae protein isoform X1	CGTCACGTCACTACGTTAGTGTGGGTTGACTCGCGTCTAGATTTGTAATTTGTGAGTGCTTTTTTTTCATGTTGTGTGTATATCTAACGTCGTTTTTGTGAGTTGTCTATCAAATTATCTTTTTATTATTGGATTTCTTCACTAAAACTGCTCAAACTGTCGTGTTCAGGCTGGCAGAATGTTAAAATTTATACGAGGGAAGGGCCAGCAGCCCTCTGCCGAGAGGCAGAAACTTCAAAACGAGCTGTTTGCATTTCGTAAG

MSTRG.5345	Epidermal growth factor receptor substrate 15-like 1	GGTTGATCGTAAAACGATGAAAATTTGAAGTTGTGTGTATTTTTCAATGCTAAATCACAATAAAATAAAAATGGAATTTTT<br>TTAAAAAAAAAAATTTTTTTGGGGAGTGTCACCCTTATCACTGAGGGGTATGAAAAATAGATGTCGGCCGATTCTCAGACC<br>TATCCAATATGCACACAAAATTTCATAAAAATCCGTAAAGCCGTTTCGGAGGAGTTTGGTAACAAACACCGCGACACGAG<br>AATTTTATATGTTAGATATATATGTCATAAGAGACAGACTTACTAAAATTATACAACATCATTTAATATCAGGTACTGCTC<br>ATGCTCGTAGTTGCATTACGTTTTGCCTGGTCTGCTTGTGTGCCCGCCGTCACACATGAAACACCATACTTCCTGTTCCATT<br>AAGAATTGTTTACATTTATTTTACGTATATTTTTATTTATTATTTTTATTTATTACCTCGTTTTCTTCTTAACAGTAACAGTGT<br>CTGTATTATACAATAACTATTTAATATTCTCTAATATATTCTATGGGCTCGGTGTGGTCATTGGTGGTGTGGTACAAAGGTG<br>GATTTATGGATTACATTTCCTGTTCTTCCAATTTTTGTTCGGTGTTTTGTTTTGGGAGTAGCACAAGTGTTCCTAAAAATACT<br>TGTGCTACTTCCACAAAACCTTTGGAAGTACTACAACTGTTTGCTCATTTACCAAATCTTCAGCATCAGCAAAATTCCTTGA<br>GAGTATTTATAATACTCTTTAATTAATTTAGTGATTTATACTAATCACTAATCTTATTTTAGCCTAAATCCACTTTGAATTCT<br>GTTTTACCCGATTATAAATTTTATAATAAGAGAATTTGCTAAATTTAAATATCGTTGACCACGTCGCGGCCGGTGTATCATC<br>TAGCATCGACACGTTGACCACTAACATGCTAACCACTGCTCGTGTGCTTGTCCCCACATTGCAGCGCTAAGTGGACGCGGC<br>GGCGGAGCGGGCGACACGTGCGTACACTGTATCTCTCTCCCTCACACCCAGCTAACACACACACACCCCTCATTCACACAA<br>TTTGTGTTGCGTCCAGACCCGCGCCAACACTATGGACTTCACCGAGGACCCCTTCAAGGACTACAGGTACGAGGATCCGTT<br>CAATATAGAGGACCCGTTCGCTGACGCCGTCGACTCAAACAAGACTGACATGTTCACGAGGCCTATGTCAGTGGCCGCCG<br>CGGGCTTCGAGACCGACGACTTCTTTGGGACCACCCCCACCGCTAACGGCACCTTCACGCGCCACGACAAGCGCAGCGGC<br>CGCGCGTCCGCTCCCCCCGTGGCGTACGACCCGTTCGCCACCCGCTCAGACTTCAAGACGACCGCTGCCCCCACAGATACC<br>AACAGCAATACATGGGCGGCGTGGCCGAACGACGACTGGGCGACGGACGACGCCTGGTCCAAACCGACTAGCCCGCCCA<br>AGGACACGTGGTCCGCCAAAAACACTAACTCGACAAGTACCGACTGGAAGACCGATAACTGGGCGAACAAGTCTGAGAA<br>CTGGGCGGTGGACTGGGGCTCTGACCACAACAAGAACCTGAACGAAACGTGGCCCTCGAACACGCTGCCCAGCAAAAAA<br>GAGAAGTCGCCCAAACCGGTCAAGTATGCCAGGTCACTGGTGAACACGATAGGCGGGATAGGGCGGAGTAGACAGAATA<br>AAGAAAAGAAGTCGAAAGCGACATCATTGGAGGCCCGCAGCGCTGAGTTCTTGCCGTCAGAGGAGCAGCAGTGGGCGTG<br>GGCGGCGGCCGAGTCTAAAAAGCTGGAGGACGAGGCCGAGGCTAGGCGGAGGCAGGAGGACGTCGAGCTTCAGCTAGCT<br>CTGGCGATATCGAGGCAAGAGAAGTGACCTCGATGCGGCATCGAGCTGGATTTACTCGGTAAGAAAGGTTTGGACGTCAC<br>GATGCAGGAGTCGATGCGTTTTTGTATTCGTCCATCTTTGTTTCACGATTATGTACCTAAATGTACAGTTAATTTGATATAA<br>TAGTAAGTGGTCAAAGTAAGAATAGAAGATCGCTGTAGGAAGGCCAATGACCTTGACCTGTCGACAGGTACTTGAAAAGT<br>ACAGTTTGCTGTATAATTTGAAAGGAAAATAATTTTGTATATATAACATTTATTTAACTATTAGAATTACTTTTGACAGCTA<br>AGCTAACAATATGGTGGGCAGTTAGCCCTATTCTTACTCTGATCACACTATAATTGTTTTGTATTAAGAAAAAGAGATTCA<br>GCATTCCACGAACTAGACAGTTTTATTACCATCATCAAGAAGAGACTGGTAGGTCTTGTTTAGTCTGTGCAGCCTCAGATC<br>TTGGGTTCGTATAAATGTCTCGATTGATTGATCTTAATCGAGATATTTTATCGATACCATTTTGTAATACTCGATATGTCAC<br>TCGAATTTTGATATTATGCCATAAAGTAATTATTGTTTTTTTTTGTTAATTTTTATTTCTATGTAAATAAATGTATCTGAAAT<br>TAGTTAACAATTATCAAATTTATTTTTTATAATGGCAACACTGAACCGCTTATTTGTATTTAGATAAGGGCGGCACGAGTTA<br>ACTTCTGATGGTTGCAACAGAATCGGCATTGACATGTATAGATTAAAAACGTCTTGTTATTTTATTTTTTTTATGCTTTTAAT<br>AAGCTTCGAATTAATGTATCACTAGTCAAATTGTCGTAGGGGTCTCAGGTGAATCACAGCATATTACGTGGTAAAAATTAA<br>CGTCGCAGCTCGGTACAAAAATGCCGTCGTGTTAAATATTATATATATATATTATGTAATTAATAAATTATGTAATTCTTAT<br>TTTAAAAATTACAAGCATTTAATATTATTAATATGTTAAATATTGAAAATTTTGTTCCTAGTATCTTTTATATGGTTTTTTTT<br>TTTTATGGTGTAAATCAAGAGAATAATCCGTTTAAATGCCAATAAAAATCTTTGCGTATTTTTAAAAGATTATTGAATATAT<br>AAATAATCCTAATACTATGCAATTGCAATACATTAATTGTTTTGCATTCATTACGTTTTACATAGAAAATAATTATGCATAT<br>AATATTTCATTATAAATTTGAACTCGATGAAAAGTTCATTTGGATTGTTTTTTTTTTTCTCCGACTGGCCGGGAGTCATTGG

AATCCAAATTACGAGGATGTAATTCAACCTGCGAGGGCACATATATTTTTATATGCACAATATATTATCCTAACGCCCTCT
ATAATCAGTTGTCAGAGGGTAGAAACTGTAAAAATTCCGTTGAATACATTTCATTAATCTTTCGGGTAGACAGTTCGGTCA
CCGTTAATTTACATTTTATTCTTTGTGGTTACAATTTAAAGTACTGTTACACAGTCTATGTGTAAAATTATTTACACATATCG
TTTGATTTATACACAGTTTTGTACATCGAATGTGTAACATTATTTACACATTCGATTTGTTAGTATTTTTTGTTTAAAAATTG
TTACTAACAACATATCGAATATTGATTTTCTGTTCAATTCTCAAATCTTTCCTCTAGAAAAGGTGCAAACAATCTCTTTCTT
TAGACTAACGTCGGTTATCGCTCATATGAGATGGAAGGAGACGGCGCATGTCGCCATCTCATTCTATCTCATGAACTTGTA
TTAATAGATTCGTTTGTACTTTTGGCTTGTTTCCTCCATTACGTTTGTCCCTTAAAAATCTTATATTATGCCTAATTAGGCGT
ATGCATATTTTAATAATATGTATTAAAACGATGTATAATATAAATTTAAATCATTATATTTATAGTAATTGCTTTATTTCGA
TATTATTATTATTATTATTACATGTTATTTTGATTAATTAATTGTATATTTTAGATTTGTCGTTTATTCGTCGTTAACTATAGT
GTAACAATGTATAAATATTTGTTTTTTTTATAATATAAACGATTCCAATGTCTGTCCGAGTACAATTGTTGTGCATTTCATG
TGTTTGTTTTGTTTTTGTTATTATATACAGTGTGTGCTGAATGTGGAATGAACGGTTCGTTGTATATATATATATATATAAATG
GCTGTATTCATACAAATATACAGTTGATATTTAAGATATATTTAATACTCGACGTAGTAATCTATTTTCTCTTCAATAGTTA
ATAGTTTGCTTTTTTTTTGGTACGGAGTGAGTCTTGTAAAAATAATTATCAAAATCGGCTTCACACTTGGCGAAGATATCGC
TTAACGTACATATACAAAAATACAATCGAGAACCTCCTTTTTTGATGTTGGTTGAACATTATATTTAAACGAATAAAATAT
AAATAAAATTGGAAAATATTTTATTTATATTAGCAACAAGAGGCGTTTGTATGAATGTAGCCGTACACATGTAAAACTATT
AAATTATTTAGAGTCGCAACATACTCAATCACCCATACATGTCACACACTATTGTGTAAAAATGTATAAAAAAAACTTAAAT
ATGCAAGTATATTGTTGGGAGTCTGGACGGTAGCTAGCGCGGCGCATTTTATAGTCTGTAGTGCCTTCCTGTTTGTCATCGC
CTCTTTCTTGTATTAGCGTATGATAAGGCGATTGGAAAAAGACGCAGTTATATAGCTTCAAATCCACTGCTCATGATCTAT
CTTTTTGAATGTTCTGCAAACAAGTGACTGTTCTGCAAACCAATGTACCCAAATATAGAATAATAAGAAATTAATGTGAAC
TATTGCATATGGTTTAACGTAAATTGTACGCGAAACGTAGATTCCTTTCGGTAAATAAAATGTTCAATAAAATATGACAAA
AAAAAATTGAATAAAATTCATAATTTTCTACCACTTTGGTAGTATTTAGTTGCGTAGTGTGGCAACACCACTTGCTTACGTA
CGCACGGGCCCTACGCGCCGCCTCCGGTACAATAATTACATTAGCACACTCTTGTCGAGATATTTAAAAAAAGAACTAAGT
ATTTATTAAACATGCAAGTGCCTCTGTGGCATACAATTTTTTTTTTTATGAATATCTCCACAAGTAGTTAGTTTATAGGTAA
TAATTATGTAATTATAAACTAAAACATGTAACTATAATTTAAATACCAGTGTGTTTATGGGTATATCCTAAAAGTTGTTATC
TCTTTCCTAGGCGCTGTTGTTTGTGGCTCGACGTGCCGCGCGGACACCTTTACGACTGTTCGGTTTTTATTTCGACCGCTCG
TTCTTGATATTTGAGAATATATTTTGCTTAAATAGAATTATGTAGTAAGGTACTTATACGACTTAATATATAAACGTAGCTA
TAACGAATCTAAAATGTTTAATATTATTTAAAACGTTTATTGTGTTGTTGGAATTTATAGATATGTCTATTTTCGCTAAGTG
TTCTCAGTCAATTGATATTCACGTATTTATCGATAGTTTCTTATTAGATAGATATACTTATATCCGATAGAGTCCGCGCGGT
GCGTTCGACCAGTGTCTGTTATCAAGTATACAATATAATGTACTTTCAGTGATAGCGGAATAAAATAAACTTTAACTCTTG
ACGTTGACGTGTATATGTATATTTAAAGCTAATACCTAAATAACGGAATGAGCAAATTTGATATAATACAGGTAAGCTAA
GTCGGTAGTGACGGTGCGCGGGATTGTATTGGTTCACTGGTTAGTCGTAATTTGAGCGTTTCGAAGCTTTAAATATGCTAA
AATATAGCTGTTAGTATGAAAATTTCCATACGTTATCATAATCGCTCATAACAATCGGTTAACTAATAAGTAGGAGCCGTC
AAGAGCTGTTTCGTTCAGTATTACTCGAGACCGCGCGCCTTAGCTTGTAGTATAGTGGAATTATCATTAATCGCTTAAGTG
GAAGGGGTCTTAGGGGTACGTAGGGGTGACCAGGTATAGGCGTTTCGGTTATTGGACCATAGTCCACAAAACAATATGGT
TCTATGTTTGTAGCACGAATGTAAGGGAAGTATGTTATGGTAATAGGCAATCCCAAAAGAAATATCACTCAACGTAGCAA
TTTATCACATCTGTTGAAAGTATATCCATTGTTGTTGTAACCATAGATTTAAAGGCATACATGTTTTGCAGTGTCGCTCTTT
ATGGTCGTATGTTATGATTAGCCGATTTTATTCACCGCTTTTTTCATATGTGCAACGAAATATTTTAGTGATTACATTTCAA
GTGTCAAGATTGTTCACGCCGTGTTTGTGTCCGTTCACGGTCCGGGCCTATTGTACATGATTGTGCTATGCTATTATTAGTA
TGGAGTCGTAACTTATGAAATTTGTTAAAATGTAATAATAAGTAGAGAAGGATTAGCACTAGGAGGTTTAAGTTCCCCTAA

		TAATAATGAGCACGATCATTGTGTAATAGTCTAACCCCTTATAGCTTGTTTTAGCTGTTGAATCGTTTTGTCTCTTTCTTTTA<br>CGCTAAATTTCCAATTTGAAAGAAAGCGACAAAACAGCGCAATAGTTAAAATAGGTTTGTAGGTTTTTATGAATAAGGGG<br>GTAATGGTTTTCAAATATACTATTATGTTTTAATAGAATTTTAACAAAATTATAAAATTTTGGCACAATTACAAATCAAAA<br>ATATCATTAGTTCCTTTGTTATTTGTATATAATTTTTTTTTTTAATTATACTGGTTCAGTAAGTATATTATACGAAGCTGAAT<br>TCTTGTGAGTTTCTACTTATAAATTTGTCTCAAAGTATCTCAGTGATGCTGAGATAACAGCATATATGTAGTTAAAGTTTTG<br>AAATCGGCCCTAAATTTACTGTGTAGAGTAAAAGTAAAATAATTTGGCACAACTGTTATTTATAGTTTAGCAAATGAATTA<br>TTATCTGTTTATATATGGTGGCGATGTCATTGAGGCATGGGAGTGTGGTATTGTAGTCTGGATAAATATATTAGTAGCTAG<br>AGAAATTCGACCCATTTAATTTAATTTTTGGAAAATTGCAGAATGCTGTTCGGTTCCACTTATAATTAATCGCTTGAATAAA<br>TTAACAAAAAAACGAGCAAAGTATTATATTTGCTCTCAAAAATGTGAAGATTTAGAATGGCAACTTATCGACGTCTATAA<br>GATATTAGCCGGTACAACATAGACGCTAAACAATATCGAAGTACAATATATACATCATATCGCATGCGTTCCGTTTTGCGC<br>AGGTGCACGGGACGCAAGGATAGTTCCCGTATACAATAACTAGATATCATTATAATCAAAGTTCTTGTGATCTAGTGACGT<br>GTATAGATATCTCTATTTATACATAACGGTTTTTGGGGACTTTTTCCACATTAGAACATTCTTTTTTTTTCCACACTTTTTTTC<br>TGACTTTTTTTTTATAATGGCAACTATGTACCTAATCTGTGGTATTAATGTATATTAAAATTTTATAGATTTTTATTTTTATC<br>TACTTCTTCGAGTGTAGTGATTTGTGAAAAAGGCCCTTCGAACTCATAAATTATGCATATTGACGCGATTCTTAAATGTTAG<br>CTTAAAACGTTATTCACATATCGCAAGTTTGATTGTCGCGTTTTCGCTTCTGTAAAGTTTACGTTTTTGTAAGCTGACTTAA<br>CGACTGCATTTTTTACAGATAGTTACATTTATACTTATATATTTTTAAGCGTAAGTTACACAGATGACGGAGCGGGAACGT<br>TCTAGGTCCGAGGTGTCGCATAGGCGGGCCGAAGTGCAGTGTTAGCGAGGCGGAATGTACTTGGATCGGTTAGAATTCCT<br>TATCGGTTAACAAGCCGGAGCAACAACGAAACGGCAGAGTTAATTTAGACTGTATAATAATTTTACGCTTATTAATATAAG<br>TACACTAGCGACATTCGTGAGTGTACTAAATTTAAGTGATAGGTAATGTTGTGATTATCATCATAAGTTTTTCTTTCGTTCG<br>GGCTAATATTACAATACCTCGCAGCTGCACACTGGTCTTGTCACGACAGACAAACCCTGTGTTAGATTAGACCAGTAGTCA<br>GTCCGCCAAAAGCGCTGTTTAATATTTCATACTAATTCAGACAAGTCCCCCGGCCTTGAACGCTCCCGATCTGTATTCTGTG<br>TAGGTTACGTTTCAAATCGTTATATACCTATATAATTACGCGAATACATACTGTTTAATGCACTTCGTTCTAGTTAAAAAAA<br>GTTAGACAAATTCTACTAGGTACTTATATGGTAAAAATTAAATTAAATGTTTATATAAATAAAACAAGTTGTGTATGTTTG<br>TAATAATACCTATAGCGTGTACGACCAAATAATTTTATTATTGAATGTGGGTACTCCGAGATTAATGTTAATTTGTAACCG<br>GCTATGCCAATTACGACCTAATTTATATATATGTAAACAATTTCTTTCGATGGCTGTGTTTTGTTGCGACTTTTCTTTTTGTA<br>CAGTCCTAGTAGTTTTCTATTTTGTGCACTAGTCACACTGGCTATTTTTCGGGCGGCAAAATGGCTAGAAGCCAAAATGGT<br>CTTGGGCTCACTTTATCTTTGAAGCATGTCTTGCGTCCTGCTAGATCAGTAAAAATTATTTTAAGGGTTCTACTCACCTATC<br>GAATAAAAAATAATAATATTTTTAACGCATCCACAGACTATTAAAATTTGAACTAAGAAGAAACTTGATTAATTATATAAA<br>CTTAATAAATCAATACTTTAATAAACGTATGTAGGTATAGTTTTTAATGTAAAGCGTTATATTACAAAACAACTTGTTTTTT<br>TTTGTTTAAGTCTATTAGTAGGGATTCAAATACAAGTAAATTATTGAGTTCAGTGCCCATGAAACCTGGAGGGGTGAGTGG<br>GACTGCTAGGTATTAAGACAATTAAAAGCGAGATTTTGAGGGTTCAGTGTGTCCGAGATCGTCGAAAACTAGCCAGAAAT<br>GTGCATATGTGACTGTATTTATGTATATATTTTTTTTTGTTATGCTAAGAGGGAGCCAGTCGGGTACCATCGTTCGTGCATT<br>CGTCATTACCTGCTGACATGTATTCATTGTAATGAAACTATGATATTATATTAATATGAAAACAAATTCCTGAGATAATAA<br>AAGGGAAAATATTATAAA
MSTRG.541	Hypothetical protein	AGGCGAGCCAGTCGGTCTATGTGCGCAAGTTGCCAAGTTCATTGTTCACATTGTGCATACTTAATATGCAACGTAGGTGGC<br>GAAACAAACGAATACGTTCCGGCAAAACAGGAGCATCTGGCGGAAGGAACAACCGAGCGTTTCGTGTCAAGGTGAACAT<br>CGTTCGACGTGGTTTTGGTCGGTATAATGCAGATATTATGATTGTTTATTTGGATAAATCACGTGTTTCATACATTTGTCAT<br>TGATATATACGTGATATGAAGTAGTATTTTGCATATATTGATTTGACTTGATATTCACAACGATAGTCACTCTGTATTCATG<br>ACTAAATTAAAGTGTTGATATACCACCCCATTACATTATATTTATTTATATTTTATAATAACTTGAATACAATTTCCATTATT

		TATATTATATTATTATTCATATTGTTAATATTTTTACTCATTTAATAATTTAATTATATTTTAATGTTTGTCAAACACAAGGC<br>ATGCTGTGGTTGCCTATAAATGTAAGAACAGTGAGATCTAATCTGCCAGTTTATTGTATTAAAATTTAGCTTTATTGTAGCT<br>GTATCTTATGTTGGTAATCCTATAAATAAATAAATATATAACATGGTAGTTGAAACCGTATTATCAATTTGCATTTAGGTA<br>GTAAATTGTCAACTGTCTGACCATTAATAAATATGTTATAAATATCATCATCATTTCAGCCATTATCCGTCCATTGCTGGAC<br>ATAGGCCTCCCCCATAGACCGCCATAAGCTGCCGTGAGACACCTGCATCCATTGATTTCTGCAATGCATTTTAGGTCGTC<br>ACTCCATCTAGTGGGTTTTATTTAGTAGGTGATAATGTAATGGTAACCCCACCCGCGTTATCAATATACGCCGGCGGGGCA<br>CCAGTGAAGGATGATAGGGGAGGATGAAGCAGGTTAGTTAACTCATCGTGACAAATTCAACAAGAATTGTTCACGATGGT<br>TTCCAAGGGAAACCACGTGGAGGTCGACCTAATAGGGTATATTGCTAGCAATGGGGGTGTCAAACTCCATTGCTAACAAC<br>CCCTTTCCCCCCTCTAGTGGGGGGTTGTCCTACACTACTACTACTGCGTTTGCCCGTACGGGCATACATTATAATACATATG<br>GTTATAAATATATGTATATACGTCATTTTTTTTTACAACACAGGGGGCAAACGAGCATGCGGCTCACCTGATGGTAAGTGA<br>ATACCGCGGCCCATGATTACCCACAACACCAGCGGTATTGCAGGTGTGCTGCCGGCCTTGAACGAACATAATTATTTATAA<br>CAGTAAACATTTTTATTAATGCTTAAACACTTGGCAATTTAACCAAACTTTGAAC
MSTRG.5<br>42	Actin	NNNNNATGGATCGGTGGATCGATCCTCGCGTCCCTCTCGACATTCCAGCAGATGTGGATCTCGAAACAAGAATACGATGA<br>GTCTGGCCCATCAATTGTGCACAGGAAGTGCTTCTAAACTTCAATATAAGCAGTCGCCGCCGCATACATGCAGAAAATCCA<br>CGAACCGACACCCTGGGTGCAAGGCGACGGTCAAGGGCGCGGCAGCCGGCGCGGATCGATTCCCTTCCCCCTCCCCCATC<br>CACTCCCCAAACCGCCGTTCGATGCGTTCACTCGACTAGACATGCCCCATGTGCAAACCAACTCATGCAGTCTAATATAGG<br>TAAATACCTAAAATGCTTTACGCTTGTATGATTATAATACTATTATATGATTTAAGCCATGATTTATTGTATTGTATTGTAA<br>AATCTTTATTGTACATAATTCAAATTAAGCACCGATAAATGATTTAATATACAATGGCGGACTTATCCCATAAAGGGATCT<br>CTTACAGTCAACCTTTGAGAAGCTGAAAGAAAACTATAGAATGTATAAGGGTAATAACCGTAGAATTAAAGATAAACTAA<br>ATAATACAAAGTAAAAATAGAATATATAATACAATAAAAACAGCTCTATATACCTAC
MSTRG.5<br>426	Zinc finger<br>protein 668	ACAAATAGATTTTTGCCCGGTCCGCAGAGGCTACCTGCTGGCGCACATCCGCCGCTGCCACGCGGCCGCGCCGGCCGCCA<br>ACGGCGCCGCGCCGCCGCCCGCCGCGCCCAGTTCCGCACCCACGCCCACCTGCGACCGCTGCGGACGCACATTTGGTTCA<br>GTGGCGGCCATGAAGCGACACGCTCGCGTCCACCGGACCGACCGCTTCGTTTCACAGGAGGAAGGCAATGACTCCCAGGT<br>GGACGGCGAGGGTGACGACATGGAGGTGGAGGCGACCGCTGACCCCGGCACCGAGGGGGAGGTCGAGTACCTCGAAATG<br>GAGGCCCTCGATGATATGGAATACCGAGAGTAGACCACCAGCTCGCGTTAGGGAAATGTTTAAGTCATTAGTTTCATTGG<br>ACTTCAAAATTTGCTTCTTAATTATATGTGTATAGTTTTACGTTTACAATTTTGCTCTATTTGTGAACCGATTTTAATGAAAT<br>GGTTTATTTTTGTGTTACTTTCATACAAATCGTGTATGGATGTAAGTTTTTTTGAAGAACGTCTTAAAATGCAGGTTTTGGT<br>GTCGATTCTGAGGTGTTATCTCTATAATTTTTAATGATTATTTATATCGGCTGCACTCACGTAGTAAGGACAGGGTTGTCCG<br>ACTTAGTTTCGGACTCGAAGCAGCCTTTGTTCATGAGCGCGGCTCACAACTGGATTGCAGCATTGCGTAGATAGTACGCTG<br>TTTAAATTTTTTATTTGATAGTAAATATCAAAGAACTAAATTTAATGTAGTTTGTAGCTATCTCATCAAAAACCTTTTTTTTG<br>TCAAGAATTCTCGCAACTCAGTTTATAGTTTTGAGGGCAAAACAATAAAATAAGTTGTGACCATTTCGTGTTACTGTGGCG<br>CATTCACGAGAAATTATGTAACTTCTTTTATGTACTTTTGTTTTGTTTGTTTTTTTTTTTTTAATGTTTTATTTTTAAAATCAC<br>CTCAGCCATTATCTGTCCACTGCTGAACCTAGGTCTCCCCCATACTCTGTCATAAGGAACGGTCCTGAGACACCTGCATCC<br>ACTGACTGCCTGCAATATATTTAAGATTGTGTAGCCACATATAGTGGAGGGGTGGTCCTACACCGCGTTTGTAGTAAAATC<br>TATTAATTAGTGCATTATAGTTTCTGGAATAGAGATTTAACTGTAATATAAAAATTAAATGTTTGTTTTATAAATATGGAAT<br>GATACTTTAGAATCGACTCAAATATATTTTCATCCAATTCATTCGAGTCCTGTTGAAGTATACATACATACATACATTATGT<br>ATATAGGAGTATCGACTCGAGCGTAGTCCTAATAGGAGATTTAATTTAGCTATCCTCTATTGTAATATAGATGAAATAAAA<br>TATATTTTGCAATAA
MSTRG.5	ATPase family	TTTATAACAAGTTTACTATTGTTATCTTTAAAGAGTGTCTTGAATCTATAATTGTGATTAACGAGTGTGCCAGTGTTATAAA

432	AAA domain-containing protein 2-like isoform X1	AAATTGATAAATATTAATGAATCATTTATATTTCTTTCAGCCCAACAAAATCCACAGAGAAGGCAACTACAAAAACATCACCAAAGAAAAGAAATTCAGGACATGATACATCAAACGGAGATGGCGATAAAGTACTGATAAACAAGACTGAGTTAGATAACGTTGTATATAAGAATGCGAAGGCGTTGAGGTCAATAGGTCTGGAGGCGCTGTTGGACCTAGACGCTCAACTCTCAGCGGTCATACGAGCTTACTCCACGAAGCACGATCGCACGAAACTGCCGCACGAACTTCACGCCATTGTCAATAGGTACCTGCAGTTAGCGAAGAAATGAAGGCGAATGCGACGGAGATAATGTGTTCAACGCTTACTGTAAGGACATCGAATTTTAGATAGAAAGGTTTGGTCCATGTTTTTAGAGTAAATTTGTTGGTGTATTTATTTGGATGACGTCGGCTATGTTGAGTGAATTCGATTGTTCGAGGTGTGTCGTAGAGATGTCGCCTGTTAGCGCGCATTTGCTGTCCACTTTGAATTTTTATCATAGTAGTATTAGTGAGATTGTTTGTGTGTGGGTGTTGTCAAATTATTGAAGTTTAGGTCATGTTGTTGAGGGAATCGACTGCTAACCAGTTGCTGATCAATTAGCAATCACGATTTAGTACAAGGATAGGCACGGCGTTAGGCACACTAAGGTAGTATGTGTAAACAGAGCCTTGAGAAACTAACTCAGTTTCTCAGTCGACAGATGAAAATGGATTATTCTAACATGTTCGGGACTGGGTGTTTTGGATTGGATATTCGTCGTCTACTTTCTCATAATACAGGGAATCCTATTTTGAGAAGCAGGCAATTAGATGGTGTAACTGTATGGCACTTAAAAATGTTTGTATTTGCAGTTAAAATAATGTTTAGTCATGGATGTTGTGATGTGAGGAAACAGTTATGAATGTATGTGTTAATTGTTTACAATAAATTGTGTTCATTCAAAGTGGAGAGCCAGTATCAAAGACAAATACTAATTTGATTTTAGAAAACATATTTCCTTACATATTTTCTTTAAATTACTTGACAAGTGATTGAAGATATAAACGATTTTTTAGGATAGAAGCGTTCCTCGGAAGTATTGTTTCTTCTAAAAAAATACTGCCATAGATAAATTAAGATCGTAATTAAGACGATATCAGTTTGTTATATTTGATTGATCGATGAATGAAACAACTAAAATTGTTTGAGTATTATTATGTATAAAGAAAAAAAAAACTGCTTCGAAACACCTTTGTCAAACTACACAGGTATAATTAATTGAATGTAAACCATTCCTAATTAGCCAAATTGCGAGTGAGAAATATCAATAAATAAATATTGTGTCCGGTTATTTATAGGAAAGAAAGAAGTAAGAATAGGTGGTCGTACAAGATGGAGATAGAGCTATATAAACGACTATTTTTCCTCTCTTTCTTTGTACTATATCGCCGGTCACTAAACTAGGTTAGGTTAATCCGGTTGTTAAATTGCCCGCGGTCTGTGATATGTAGAGGAGGTTATATAGTGTGTACATAACATTTAATCAAATACTATTTATTTTTCACTCGTACGCTAAACAGTTTCATCTGTCGCGGTTCCACATTTTATGTGTAGAAAAATATATCCATAAAAGACTGAAAACAAGCCATTTGTGTACAATCAGCTTCGTAAAAATATTGCATAAGTTTCGGTTTTATCTCGATATTATCATTATCATTATATCAGTCGTTGACTGTCCATTTCTGAATATAGGTTGTTGTCACGCTTGGCTTGGCTAGGAGGGTTGACAACTCCAGTTTGGCGATATTTTAAGAGAGACGCTGCTACCAATTCCTCGACCATTCGATCATCGATCTATATGAAGCTGATTGTACAGATACGAAAGGCGTGACTGATTTATTTATATTTAAGTGCACGACGATATCAATTAGTTGAAATTTTTAATATTTTTTATAAGCTAATTTCTTTATAAAAATGTTGTTACGTATGTGCAAATTGTACTTCGATTGAGAGTAATTGTTGTTTGATTTGATCGGTTTATGGGCTCAGTGGACCGCGATGGGTGAATATATTTATATATTTTTATCATAACGACTTATATATGTATGTCTATGAATCAGTGCAAATATACAATTTGGCTTTTTAGGTGGGAAAAATTAAGCACTGATTTTATTAACTTATACCGATTACCAAATAAATTTAAATTAACCACAATGTTAATCGTAGTTTAAGATTATATGTGCTAACAATTTTTTTTTTTTTAATAGTTAAATTATAGTGAGTTTCAAGAGTTCAATTCAAGTTTTTCGAGTTAGTATCTGATCTAGGTTAATGTCTTGTGGACTCATATTTAATGACTTAGATTGGTAACCAATTTAAAAAAGGAGATTAGATATTGAACTATAATTTATTTATTGATTCATCGTCACATCAAATGAATTTATTTTAATAATAATTTTTTAACTAGTATCGGTTCTATATATGTATTTTTTGAAGTTTGAAGTTGCTTTGTTTATTTGAAATAATACATATATATTATTGCGCTGCCAGAAAAAAATTACAGAACATCTTTTTACTATTTTACAAAATCGAAATTATTTAGTGTTAAATAAATAAATTACAAATACTAAAATCGAAAATATAAGTACCTATACTTATTTATAATTTGTATTGTAGTTACACAATAATTATTTGATCTGATAACCATAGGTGTTCCCCTATAACAGTAGGATAAAGTTCATGTTTTACATGATGACATCCCATATTATCTCTCTTTGTCATTTGTAACCGAAGTTCTATGTAAGAAGAGAGAATATTTGGTAACTTGTGTCTACAAGGTATTAACCTAGCACGATGTCTGTTAAGTACTGTTGCCATATTGTGCGACGTTGTATTGTGAGAGCTAAGTATGTAGTTAAATTTATCGATAAGTCGGAGTGTAGTTTCGCAGAGTTTGGTCTGAATCGTGAAGCTTATAATGGAATAGAGGTGGTGCCGTGAATCCCAAAAGCCGATAAATTTGGACTCGTAATTTCTCGCTGTTTTCCATCTCGCTTACACTCTAATCGCTGTAATAAAGTAGCTGAAAGAAGGATATGTTAATTAACATTGAAATATTTTCCATCTCACTTACGTTCAGCGGTGA

AGTTGAGACAAGTAACTACGTCTTTTAAGGCCAAATTTATTAGGTTCTATTCTGTGTTCACTGTTCACGTTGCAATCTCTAT
CTGTTTTTATGGATATATTACACTATCAAATATACATAAAAAAATATATATAAAATTAGCTCTGATACAAGTATCTATGAG
AGGTGAGCATAATATTCAGAATTTGACATACTTTAGCTATTAAAAATAATTAAAACAAAAAGGAGAAATGAGAATTTATC
AATTTACTAAGATAGCGATACACCTTCGATACTGGTAAAAAGTTTTTCATTATCTTCTGTTGGTAGGGAACAACAAATGAG
AGTAGTTGTATATTGCCACCATTCTATATTTATAATTTTAAAATATACCTATGATCAACGTATTTGACAGTGTAATATTTCT
TTAAGCATTAGAACACATTACTCAAAATAAATATTTATACAGAAACTAATACTATAGTAAGTACTACTACACGTGGATGTA
GCGACCTCTAGTGGCTTGAATAAAGAAACATTAAAATGTTTCACGAATTGTGTTCTTAGATTCAGTTGAGGGCAAACTTTG
TGAACTGTACTGAAGAATGGTTGTTGTCTAATAAAAGATGTCGATTTTTAAATGTGTAAGATCTGTTGATTAGAGTTAGGT
CTGTCGTGGTTCTGCACTGTGAGACTGTCCGAACGTTTCAATGCAACATGTCCGTTAACCAATCATAAGAATAAATATACG
GGTAGAACTAACACTTCTGTAAATTCAATAATAATATTTATAGCTTGATTCGATAGAAATTAATGAAAAGGTTATTAATCT
TGTTTTTCTTGAAAAGATTGCTCACAGCAATAAGACCGCCTTACTGTACTTATACAATTGTACCGTTGTGTTTGTAAATTGT
CTTGTTTATTTCCTATATTGTATAATGCAAATAATGTATACTCATTCATTCTTGGCTAAATAATGAAACATTCTTTAAATGA
AAACAATTCGAGACATTGAATCATTTAATATATTTTTTATTTCTTTATCAGCAAACTGTTTTATAGTTGGGTTAATTATTAAT
TTGTTTACTTACTTTACCTTTAGTTTATTTACACGTTGGTAAAATATGAAAATATTTGTGACAACCCTCTGCAACTTATACG
GGGATTGAAGGAAAAAAATAGTATATTTTTGCAGTTTATATTTAACTTATGTGTGATTTCTACCTACTCTGTTTATTCTTAT
GGACAAACGATAAACAATAGTTCCCCAATCAGTCCGAGCCAGAACTCTGTTCAACAAATAGTGGACAGAGTCGAGTGCAA
CCCAACTCGTACTTTAACTTATAACTGTTTAAGGTCTATATTTAAGGAAATTATTTATTGTTGACAATATAATGTAATGTAT
ATAAGTACGCACTATTTTCAATTGACTTTGTACTTTAAATCTCACAGTTTAAAGTCTATAATGTACTGTATTGGAGTACACA
CTGCCGCTCGGACACATTGTACACATTTTCTTCAATTGTATGTCTAGTTTCTTAACACTATATTCATATCTCTATTTTTTTA
TTGTTTATCAAATTTCTTATAAATTGATTTCGTAATTATCGGCACGGTTTAGTTTACATAAATTTATAAAGCTTATTATAAAT
TATTACCTAGTTGATTTTTTTATATTTAAATCAAATAGTACTTCGTTATTCTTATGTTTTTTACTTCATGAAACTTACAGTTCT
ATAGTTCGTCAACTTTGTATGCGTCGCTCCTGCGGGGACGAAGTGTGCAGATAGTGCTGTTCTCTTGCTTGTTTTACCATCT
GTGTCCCGTACATGTTTTACTTCGCCGCCTGCACACCCCATGCACTCGTTAAGGAAGCTACAAACTAAGTTGACGTGTTAT
AGTAGTCTATTTTTTCATTATATTTCAGATTATTTTTGTTAGAGGCGCTGTACGTATTTCCGTATAAATATTTAACGCATTTG
TTACCACTTATCGCATTCGCCTCTGTAAACCCATGGAAAAAAAGCTGATACAATAATAATTGATAATGAGAGAGAAAATG
TACTTAATTGTATTCAATTACTGTCCGATTTATTTTTATTTGCGAGGTCCAATGGAGGGATGAATATGAGTAAAATTGATAC
ACAGGGTGAAACAGAATCAACCATATTGAATATTTCCACCAAGTAAACGTGACTCTAAAATTGACAATTTACTTTTTCCTT
TGTTCAAAACGTTCAGCTTTTGAGTTAGTTTAAAAAGCTGAACGAATTCTAAATTTTCTGTCGTTTTTGAATTTTTTATTTCA
CCAAATTTTTGTCCTAACACGTTCGATAACATAAGTTCAACTTACCTCTAAAGATTTAGCTTTCATCCTGTGTAAAGGTTGT
ACCGAAAAAATTTATAAACAAGTTTCAAGTTGACAATTATTAAAGCTGCTTAATGTAAATTGTTGAAATCTAGCAATTTTTC
CCCAAGAAACATGAGCTATCTTCATCATCTTTGCAATGCATCCAATGCCTAACCTTCAAGAAAAGAGCATATTCTTTTTTA
AAAGGCCGGCAGCACACCTGCAATGCCATAGGTGTTGTGGGTGACTATGGGCGGCAGTAGTCACTTACCATCAGGTGAGC
CGCATGCTCGTTCGCTGTAAAAAAAATGCCCACTGAAATAAGGCACCTTCATATTTCATAACAAAGTATGGCCACGAGCCT
CCTACATCCATCATGTTTCTGAAACACTGAAGGCATTTCATGGGCTAAATTATAAAAATAAAAATAAATGTACCACGTGAC
CGCGCGGTTTGCGTGAAAGTTTCGAAATCCCTCATATATTGACCCATGTTTCTTGGAAAAAGTTTGAAGGTACTCCAAAAT
ATTATAAACAGGATAAATGGGAATAAAGATATTGCGAATATGGTATACCCATGGTAACGGTTTTCAATTTTAACCCATCGT
AATATTGTTGAATATTTTATAATGATATTTATTTTGTACATGCTTTTTTTTATTTATAAATTTACTTTTTAAATTCATATACGT
ATTAATACTTACTTGTTGTAACCACTCATTGTTTACAGCTGATGTTTTTATTTCCATTTTTAATTTAGGTATATGGGGGACAT
ATTCCACTATCGAGTATATAGGAATGTATGAAGCATTCGCGAATTTTAAAATGGGCGAGTGAAGTCAATTATATATATAATTA

		TATATATACATACTCTTCATTGTATCTTCCATAAGATAATAATAGTAAATTTATTATAATTCCAAACAATTTATCATCTATG<br>AAGCTGTTTAAGTGGTTTCTAATTTCTATTGCTTTTTATTCTGTGTATGGAATCTATTAACGTAGGTAGGATCACATGTGTTC<br>CAGGTGTGATAGTAGTTGATGTTGGGATCATTTGGTGCGATCTGTTTTTATTTCTAATCTATATTTAAGATTCTTATTATATT<br>AATAATAAAGTATAAATTTAAACGCTATATTGGTACGATAGTGTAATAAGTCCCTTACGCTCTCCATACAGGACCTTTAGT<br>TTTTGTTGATTTAACACGATGGAAATAATAGTAATGTGTTATAAAGAGATTTATCAACAAAACAAACCCGTGTGATATTTA<br>TATAAGAAATGAAACACGTCAAGTGGATTATCAGTAGTTACCTACATCCAAAATGGAATCAAATTCTATGCCATAGAGTTC<br>GCAATGTTGACATGTTTAATGTGTATTTTTCAATTAATCTGCTTGCAATTGCTCAATACGGAGTGTATGTCGGACATTTTGT<br>AAACACTCACAATATCCACCGGTTAGTGTAATTTTGTAATAAAATTGTATTTAAATTAAACAAAATAAAAGGGTATGATTA<br>ATGTGATATCACATTTTGTGTTTTTTTT
MSTRG.5434	Reverse transcriptase	AGATAAATGGGCGTATATAATGCAGTGTACTGTGATCCCTTTAAGCACATCTACAGTTCTGTGTTATTGCCTTCTTTTACTC<br>ATCTAAATATTATCCTGCAATAATGTTTAAACCCTCGACGCAAAAAGAAGCATGTTATAAGTTTGACCTGTCTGTGTGTCT<br>GTTTGTGCATTCTAGTTCCTAAACAAACGAGTCGATTGTGATTTTTTTTTGTTTGAAAGCTGAGGTAATGGAAATTCCTTAG<br>ATTATGATCGTTAGCTGCATTTTATGAAGATCTGTTCAGGTGTTTCGGGAGTTACTTAATTTTTTTTCTATTTATAGCCTCAT<br>ATTCAAGAATCCGTTTGCGAAGAGCGTGCACCTCATCGCTGGATATCTTTGGCCTTTTCCGAGGCGGCTCATCGATACGTT<br>CAGCGCCCGCTGTTCCGCCGTTGAGGATCCCGCCCGGTGCCGCGCTGCCGCCACCGCTAGCGCCGACGCCGCCGCCGCCGC<br>CGCCGCCGCCTCCGCCTCCGCCGCCACCGGCGGAGATCCCGATTCGTTCCACACCCTGAATATAATTCATTGTAAGACTCT<br>AACATGATCTCAATATCATATTACCTCACTAAACTACAATATTATCAACTTACTAACTTATCTATGTTACAGTTGTTGTCAG<br>TATAAGTATTACTCACATTTATATACATATGTCGTATCATGTATGACTTTTCAAATTGTATATGACGTCACTCTTGCTAATA<br>AGTAAGAGAACGGACCCACCCCTACACCACGTTTTAAATTACAAACTTATGAACTTTTTTTTATATTTTTTTATATGGATAT<br>CCTTTCAACAACAACATACACAATAAAAAAAGGCAGATGTAAAACTATGTAGAGTATGTGTATTTTTTTATGAACGACTAA<br>CGACCCGCCCCGGCTTCGCACGGGTGCAAAGCCGCACCGCTATGTCCTTGCATTTTAAATCTGTAATGTCTTCGGAAATAT<br>TCATTTAAAATATATTAAATGGACAATGTATTTAAGGTACTTAACCCTAGAAAGATAATCATATCAATTAAAATATTAAAA<br>ATTAATGCGGTATTGCTTAAAATCACTTCGTAAATAAGCCATTTTTTCCCGTAAAATGTTATTGTTAAAAAATGGTTATTGT<br>GGATTATCCTTAAGAGATAGACATATACCATCAGGGACTTTTTTGTTAATCTTTTTAAGGTGTACATGTACTTACAGTACAT<br>TAATTCTTTATAAAATAATTTACTTTTTATTTTTGATTTATCTCGTCGGGTTCAGCCAGCGTTTGCAATGTAAGCGCAAAAA<br>ATGTGTTAATTTGCGACATCACATTAGAGACCTCTAAATTTATCAGTGTTTCTCTACAATATTATGCATGTATTATACATAT<br>AACCTTCCTCTCGAATCACTTTATCTATTACAAAAAACCGCTTCAAAATCAGTTGTGGTGTTTTAAAGATTTAAGCATACAT<br>AGGGACAGACAGCGACAGCGACTTTGTTTTATACTATGTAGTGATAATGGAATTATAGCCCCACCTGCGCTCTGGGTATAC<br>GTTGGCGGGGTACCGATGACGGATGTCAGTTGAAGATGAAGCAGGTCTATGAGCCCATTGTGATCAATCATTAACCATCC<br>ATCAAGAATTAACCATGATGGTTTCCAGAGGGTACCCCGCGTGAGATCAACCTAGGACTTTGGGTATATTGCTAGTAATGG<br>GACTGTTAGATATCATTACTAACAATCCCTTTCTCCCCCGGATATGTGTATTAGATATGTAGATACATAGCAAAGAAATGA<br>TAAAGTACACAAATATCTTTGTAGAGAGCATGTCTGGCTAAATTCGTGTTGCTAACCCTGAAAAAGGCCCGACGGTATTCC<br>TTCAATTATGCTTAGGACCAGCGCGCCAGTGTTAGTCCCGGCTCTTACGCCTTTTTTGGCTTTGTTACTAATCAGGTTTTGG<br>AAAACTGCTCTGGTATATCTAATCCCTAAAAAGGGATTCTCAGATTCAGCTAACTATAGGCCCATAGCTATTACTTGCTTA<br>TTCTTGAAGGTTATGGAATCTATCATTAACCGTAAACTGCTCCGAAACCTAGAAGAGCACCAGCTGATCTGTGATCGCCAG<br>TACGGCTTTCAACAGGGACGCTCGATCGGTGATCTTTTAGCTTATTTAACACACCGATGGGCAGTGGAAATCGAGAATAAG<br>GGATAGGCCTTAGTTGAAATTACCGAAAAACCGCTTGAAATTGTATAAAGTTCAAATCAGGTCACATACGGAATACTGTTC<br>TCACCTATAGGCAGGAGCGCCACAATACCAGCTCCTTCCGTTAGACCGCATTCAAAGTCGCGCAACACGAATTATTGATGA<br>TCAGTATTTACTCTCCAACTCGATCTACTGAAACTGCATAGAGATGTATTTCTGTACATCTTCTATCAAATTTAACATGGAG

MSTRG.5458	Hypothetical protein KGM_200275B	TGTGAGAGATTCTTTGGATTGATACCTCAGCAGCAAGCATTTTTACCAACGTACAACCCGCCTCAAACTAAAGTTTCATCA<br>CAACCATCTGGATATGTGGCAGTCTATCACCATATGTTTTTCAAAGAACTTTCTTCCACAACCACTCTTTGGAATCAACTTC<br>CCGCAACAATATTTCTAAACCGATATGACATCATAACCTTCAAGAAAAGAGCATATTGTTTTTTAAAAGGCCCGCAGCACA<br>CATACAATGCTTCTTCGTTGTAGGTGATCATGGGTGGCGGTAGTCACTTAACATTAGGTGAGCCGCATGTTTGTTCCCCCCA<br>TGTTATAAAAAAAATCTCAATACACTCTTTTCCAAAAAAAAAGGGAAGGGAGGATTAGCAACATATTTATTAGTATAAAC<br>ATCTTATCAGGTTGACCTTCACATGGTTCCCCCTGGGAACCATAGTGAAAGGAACCATATATATCAATATCGTACACGGCT<br>CAAGTGGCTCTCTATCCATTTCTTGAGGAGAGCAATTCTTATTTTTAACGCAGTACTGACAACCTCCAACTCATAATTCCTA<br>CTCATTCATCCTCTTTCTACTCCAAATCCTTTTCCGTGCAAGCAGTTCGTTTGTGAAACTCACTCCCTACCTGTGAGTTCAAT<br>CTTTGCACATTTTTAAAAACTTAATAAAATCCCGCTACCTATCCTTATAATCATTTACCAGCTCTATGGCAAGAGGTGTGAT<br>AGTAAAAGGGTGGCAATGATATCTGGTCATATAATTCTCTGGAATACTCTCCATTGTACAGCCAATAGGAAACGGATAAA<br>AAGGTAACATCCTGTCTCAAACAAAATTGTCTGTTAGAATGGGTTTATCGACAACTCATATCACTTGACATTATACAGTCA<br>AGTGGAAGGAGCCAATATTGGGGTGTTCCTGCCCAAAGATGTAAACAATATTCCATATGTCAGTGTTTTATTTGTCTTTCA<br>GAAATCTGCCTGCAGAAATTAGCTGCAAGATCTTACGACTATAACACAATTGTATTATATTTGTTCATTTTGAGCGTTGTCC<br>ATGTAATTAACATAAATATATTTTTTTTTTTTATGTATTTGTCCTACAAATCTTTATTCAGT<br>GGAATTGTTAGTATTGAGATCCAACAGACCCATTACTAGCAATATACCCTAATAGGTCAACCTCCACGCGGTTCCCCCTGG<br>AAACCATCGTGGTTAATTTTTGATGAATTCATCACGATGGGCTAACATCCCTGCCTTTTCATCCCCAATCATCTTTCACTGG<br>TGCCCCACCAGCGTATCCGAATAGCGCATGTGGGGTAACCATTCCATTTTCATCCATTAAAAAAAAAACTGACCTGCCTTT<br>TCATTCTCACTTATCATCCCTCACTGATTCCCCGCCAGTGTACACCATTAGCGCGGGCGGGGTTACAATTTCATTATCATCT<br>ATTAAAAAAATTTAAAATGTTCTCTCCTCGTCTATTCTTCCAATTTGTTTTAATGTCGACTGTTGTGAGTCAATGCTCTATC<br>AATATGTGCTAATCCGAAAAGTTTTTTTTTTATTACACGTCTGTATTCACGAGTATTAATTGCCTTTCTGTAATTAAAGTG<br>AATGAGTTCACACGCTTACAATTTATTAACTTCAGACTAGAAGAAGTACAAGTTTTAGTAAAAGATCACTCCTGAACAATA<br>ATTAAAGATTCCCATGAAAAAAATCCGACTTCAGTTACGAAGAAACTATTGAAACAATTTGAATAATATTTCTATGTGAAC<br>AATCTGTAAGCTGTAAGCTTTCCAATATAAAAATAATATTACAAATCGCTTTAGAACTAACAGATTTATGAGGTAAAGTTT<br>CTTCAGGGTCGGCAACGCACGCGTAATGCCTCTGGTATTGCAGGCGTTCATAGGCTACGGTAACCGCTTACCATCACGTGG<br>GCCGTATGCTTGTTTGCCACCTCAGTGGTATAAAAAAAAGAGGTAACAAACATACAAAAAAAAAACCGAATTGAGAACAT<br>CATTGGACAGTGATTTCGATGTACAAATATGTTTACAGGGAATCAGATAACCCAGTGCCATTGGATTCGGAGTTATGTGCC<br>GAGATATTGAAGAGTTTAGAGGACGCGCCCGACTCTGATGATAGACCAGAGTGCGTGGAGCGGTACCGCGGCGGCGGCA<br>GCAGCGAGGCCGGCACCAGCGACACCAGCGACGCGTCGCCCGACGACGAGCGCGACAGCCGCCAGCCCGGCGATCTCTC<br>CAGCAGGAGCCGAGCGGGGTCGGGGACTAGTACGACGGCGGGCGCGGCGGCGGCGGGGAGTGGTGCGGCGACGGCGGG<br>GAGTGGTACAGCGGCGGCGGCGGCGGCGGCGGGCGGCGCGGCGTGCCGCTGGACGCTGACGGGGCGCGGCGTGCGGCTA<br>CGCGCCGACCTGGCCAGCCCTGAGGACGTCACGCTCGACGCAGACCGCCACGTCGGCACCTCCGTCTGAGCCAGCGCGCA<br>CGGCCCTATACGCACGCTACATAACTTGACCATACTCCCTCTGGGCTTGGCTACAAATATTGAAAATTAATTATGCCCATG<br>TGAAATTTATAAATGGATCGAGCATAAGTTGTCGCTTGATTATGCATTTGCTTCTCTTATGATTAACTTCATGAGGGTCAAA<br>TAATCTTCGTTAAAGAAATAGATTTTGAATTAAGCTGTACTTATTTCGCTATGTTCACGCAATATATTAGTCAATTCCGACT<br>GATATTTAGCGATTTTAAATCAAAAAAGTGTACCATCATTCACCATCCAAGATTTTTTTTTATATTTGGATCAGTTTGGAA<br>TAAAATCTCACATTGTTTCTAATTTGAGCTATATTTTCAAAATTCATACAAAAAGTGTCTACCGTTCAAGCCTCTATATACT<br>TATAGAGTATGTTCAAGTTATTTATCGCGAAATGTAAATGTGTTTCAACAAATAAGAATACTCATTAAGCTGGTTAGCTCT<br>CTATGTAATAACAACCTCACATTTGGTGATATAATTGATCAAAATAGTTTATAACTCACACTGCCTACATTATGAGCCTAA<br>AATACGTTCGGCTAGCAATAATATACCCATTGTATGTAAAATCTGATCGTTACAGTATAATTTCTAATAATGTTAACGCAG

		TAAATGATGAAAGAATTTTAAGATTCCAATATATTATCAAAATATAATTTTAATCACTCCTCTTTATGGCTTGTCTTTATTT<br>ATTTTTATAACATACAAATTTAGTCTTTAATTACTCTTGCCTACAGTTTATATTTCGTTGTACTGTCTATTTGTGTCAAAAAA<br>GATTTTATTTTAATATATACTTAAGACATTGTATGTCCATAAGGTCGATATTAGATTGCATTTGCTTTAGTTCGAGAGAATT<br>CTAATAGACGTCACAAACTAGAGGCTCGGTATAATAGGAAAGCTATCTTAATTCTAAAAGTTAAGGTTTAATATCGCGTAG<br>AAACTTTCAATTGCTAATATTAAGAGTGATTAGAGTGGAATTGAAAAGACTCTGTTACTATACCTCGGAACAAGTGCACTT<br>TGTCTCAAACAACGATAGTGCTTGTATGTAAAGCTGTAAGGTTTATATTCCTTCGTCTTTAAGTGAGAGTTATAGATATTCG<br>ATTGAGCATTTAAAAAAAAAACTATTCTCGCATTTATTAAGATCTCTTCGCGTTTGTTTGAATTAATTTTATGTTTGTATTA<br>GAACAACGGCAGCTTTTGTAACAAATTATACAAATTATTATTATAAACTAATTAACTTATAAAACTTAATTTTTGTGCATTT<br>TTTCGTGCCATTTCTATTTCTCTAATAAAAAAAAAATACTAACTACAAACATTTCAACTCGCAGCGTTGATATATAATTTAA<br>AAAGTATTCGAATACACCGGGTAAATACTTTTTTATAAACATCATTTCGAACTAAAATATTCGGTACTAATGTTAATAAAA<br>TTGAATAACTTTCCCCAAAGAGTCGAGAAAATATTATAAAATCATTCGTACCAAATCGATTGTGTTACCGTGCCTTTTGTAT<br>TTCGAATTGCATAAAAAATTGACGATTCAAACAATTTCTTTTTTATATCCGTTCCGTTATACGTTGTATTCGATTTTTGAAA<br>AAGACAACACGACCGTCTAATCTCAAATTCTTTTTCCCTCATTCATAAAGTCAATATATCGGAACAAAAAATTTCAACTGA<br>AAAGGGATTATTCTTGTTCTGTCATTCGTAAAAATGTTGCCATCTCTAATAAATAAGCATTGCTATTAAAATATTAATTTGA<br>GAGTGACGAAACGTACTAATTGTTTATGTTTGTATGAATGATACAGTTTGACTACATGGTGTTTACGGGCCAATATTGAAA<br>GTAGTATATTTAACTATTTAAACACTGTTAAAATCATACAAATTACTTGTACATTATATCGTCATAGTCGTAGAATACTGAC<br>GGATCTGACAAAGATTACATATAAAGATCCGTCAGTATTCTACCACTATGACGATATATGACTTGATAGTTTAGAACCTAA<br>TATGGCTCTAGTTGCAGCTCTCTATCACATGATGCTTTATTTGCTATAAGCACATACAATGTTAGTTGATACGCTTCAGTAT<br>TAGGCGACATAGTGTGAATTTTATTTCGTATTGTGCACGTTTAGTGTGTAAATAATATATTGTGCCTATATTTAGGTAATCA<br>CGTAGCGTATAGACACTGGCGAATGGCTTAAGTATGCTTTTAATACCACGTAACTAGCGCAGTAACTTTTACGTACAAAAT<br>ATACATTTGTCTATGTGTATGCACGACGGGAATTTAACTAACTTACTGTGTTTTTTGTTTCCGACGACTGTTTCGATTCTATC<br>TAAAAGTCTATAGGGAGCGTACAACACTACGTCACGGCGTATCGCCTCGCGCCGAAACGACTGTCAAAAATGACAAATAT<br>AGTTGTCACATCAACCGTTAACTGTCCACTGCTGAACGTAGCCTCAGCCATAAGGGTTTTGCCGGTATTTGCCACGCTTGA<br>CAGCCGGGTTGGCAACCGCAGTTTGAATTTTGGTGATGTTTTAAGAAGGACGCAGCTGCCCATCATCATCATCATATCAGC<br>TTTTAACTGTCCACTGCTGAATATAAGCCTCCCCCTAAAGAGGGTTGGGAACCGCAGATTAGTTTTTGGTAATGTTTTAAG<br>AGGGACGCTGCTGCCGATCCCTCGGTCATAAACTCCCTTAAGCGCCTCTTACGACACCCACGGGAAAGGAAGGGGTGGCC<br>TATTCTATGCCGGAACCTAAACGGCTACGGCATACACAAACAAAAGTTCGTTAAATTCCCATTGTGCATATATAGACATCG<br>TATTAAAAAATTAATATAATTTTAAAAATAAAACATTTTTTTTGTGTGTTTGAGAAATTTGTACCAAGAATAAAGTGGTGA<br>TAAACAATACTACATTCAAACGTTACTAGCAACATGGTGATGAATGATAAACTAGTATGCGCGACGTATTTGCTTGCCGAT<br>TGCGTGTTGAGTGTAATTTCGCTGACTTACCGCGTTAATAGGTAGAATTTACGTATAAAATAAGTTATTTAAAATACAATT<br>CGTAACAAAAATAATAATGTACAATAATTTAGTATGTATTTAACACAAAAACCTTTTTATTCCAATAATTTGTTATTTTATT<br>CCTCCTTAAGAGATATCCCAAGGTTTTCAAGTGAGACAAAAAATTATATACCCTTAGTGTTGCAAGCGCTCATAGACTACA<br>GTAACCATTTACATCCAGATGGGCCATATGCTTGTTGGTCGTATTCGTGATATAAACATTTTTTTTTTAAATATATTCTAGA<br>CGCTTGTTTCGAACTGTATCGCCGATTCAGTCACGTACATATTATGCAAATAACTCGTTACGTGAACTGCCTACAACCAAG<br>AGATGTCCCAATATAATTGTGTTCAGCCGTATTATATTGTTTTATCGTGTGAATAATAGTTTAGTTTTACAATAAGCCTAGC<br>TGGCTTGACATGCATTTTGTCCCACCACCGCCACCTACTGGGCTCATTTTTTTACCGGAATAAAAAGTACCCTATTCCTCAA<br>GTTGGAGTTCTCAAGAAAAAATTATATGTACGTTGAAAAAAAT
MSTRG.5<br>462	Dual<br>serine/threonine	TTCCAGGGAGACCGCGTGAAGGCCAACCTGTAATCCCTCCCACCCTTCTCCACTAGCGATACACCCTAGAGCAATACACCT<br>TATCAGATCGACCTCTGTGCTGTTCCCCCTGGAAACCATAGTGGTTTCCATAAATCTTGTTCAATCGATCACTATGGACCAA

	and tyrosine protein kinase-like	CTGTCCTGCCTTCCTGCCTACCTCACCTGTCACTCTTCACTGGTACCCCGCCTGCGTATACCGAAAGCGCAGGTGGGGTTAC<br>CATTCCATCTTTGGTCATTAAAAAAGAAAAAAATCTTCTCTGTACCCCATAATTTTGTAATCATATTTTCATCCTTCAAAAC<br>GCTGTTGCATAATCGAAACCTAGGTAGGGCAAAGGGACAAACATACTCATGCAGATATTTTGCCCCGTCTTTCTCTAGTAA<br>CTTTCCAGAATGTGCCCTAACATTTGGTAAATGCCACATTCCAGAATTGCCTGGACCTCCATCAAATTAAAGAGATCAACT<br>CAAACAGCCAGGTTCTCTTCGTTCTCTTCTCTGACAGCATGGACCTAGAAGAGATCACAAGAGATAGAAATGAAGCGACC<br>GAGTCCACCGAGGATGCAGTATACAGTGTTGAGGATTTGCCAGAGTCGGATCAAGTACTGCGAGGAGTATTAAGACAAAC<br>ACAAGAATCTCCAGTAAGCAATATATCCATAGAGAATAGAAGACTGCTAGAGGAACAATTAGGGTTTATGAACGATTTAT<br>TGGATCAATGGGAGTTATTGTGT
MSTRG.5484	Peroxisomal N1-acetyl-spermine/spermidine oxidase	GGATCCATGGCACGAAGCCCAGCATGGTGTATGACGCGGCCGTCCAGAACAATGTCACAGTACTGTCCCAACAGCTGGAC<br>ATGATGCTGTTCAGGTCAGATGGGGAGCAGCCCAACAGTGCTTTGATAAATGAACTACTTGTTTTTACATTACAGGTAGTG<br>GATGAACCACCCTCCACAGCGCAACCTCTTGGCCAATATATAACTCAAAGG
MSTRG.5504	Epsin-2	GATGCCCCGTGGGCGGACGAGATGCAAGTGAACGTTTCCGGTTTGAGGAGAAATATCAAAAACCTCGCGCACAACTATT<br>CCGATGCTCAGGTCAAAGTGCGCGAGGCCACATCGAACGACCCATGGGGTCCGTCGAGCACGCTCATGGCGGAGATCGCC<br>GACCTAACATACAACGTGATGGCGTTCACGGAGATCATGCAGATGATTTGGAAGCGGCTGAACGACCACGGCAAGAACTG<br>GCGGCACGTGTACAAGGCGCTCGTGCTAATGGAGTACCTCATCAAGACCGGCAGCGAGAAGGTCGCGATGCAGTGCAAG<br>GAGAACATATATGCGATACACACGCTCAAGGACTTTC
MSTRG.5512	Methylosome protein 50-like	TATGATTCCAAACGAACAAATGTTAGTCAACGTTGTCAAAAAATCAATTGTCAATGATCATCGAACATGCATTAGAATTTT<br>GCTATCTCAAACAAATGAATTTTTTAAACTAAAATTATTATTACTTTATACATACGTTTATTACTGTAAGGAGTGTGCCAGA<br>ATTTTAATATGGAGAATAGCAATAAAATTGTTCCGCCGCACCTCAATGCTGAAGTTTACAGAACAGATACCACCGGCACC<br>AGCACAATCTCTTATTTAGATTATATTCAAATTCACGAGGACAGCAGCGTGCTAGTAGGACGCTCAGAATTAACTGGACGT<br>TACTGGAACGGTGGTGCTAGCGTTTATAAAACTATTGAAGAAGGTCAAAAATGTCTTAGTGAAACAAAAAATAGTATATA<br>TCTGACAAGTGGTACTGCAGACG
MSTRG.5515	Multidrug resistance-associated protein	CCAGATTATTAATACCAGTGTGCTTAGAAAGATTAATCAATTACTTCTCGCCGACCCACGCCGGCATCTCTGACAGCCACG<br>CGTATATGTACGCTGCTGGCGTAGTGGGACTAATGGCCCTCAATGCAGCCATGTTACACCCAATGTTGTTGTGGTTATTGG<br>AGATGGCGTTGAAGATTCGGGTCGCGTGTTCATCGCTCATATACAGGAAG
MSTRG.5530	Neural cell adhesion molecule 1-like	CAGCCCGACCTTCTTCACCAAGGAACTGCACAGCCAACCGTGTAATCCGGCACAGTGAAGCGTACCTAACAGTGAAGTGC<br>ATAGCAGGGTACGACGGAGGGCTCACTCAGCACTTTACACTGGACGCTCTCAGTGACACCGGCAGGGTTCTGGTCAACAC<br>CACCGCTGGACAATTGGATCTAGTGGTATGGCTGAACATAAGCTGGTCAGATTTGGAGACATTAACAGAAGAGGAGGTTC<br>TGGCTGTGACGGCGCGTAACAGCAAGGGTGCTTCCGAGCCTGTCCTGTTACGAGATCTGGTCTTCAGAGACGCAGCCAAG<br>AGAACTGAACGCACAAGTGAAGCGAGCAGCAATTTCCCCGCGGCCGCTGCACTAGCCGTGCTGGCAGCACTGCTCACGGC<br>CGTCGGCGCCATAATTGCACTGGTATTACGCAGACGCAGCGACAACGCAGCGAACAGCAAGCGACCATCGCAGAGCGTG<br>GTACAAGTCGACGCTCAAGGTCGGAGGTACCTGATAGCGTATCCAGCGCCAGCAGACAAGTTGGAGACCAAACCTGATAT<br>TTTGAATCCTAAGTCTGACAATGAACCCCCCCGTGTGGTGTTAGAATCGAGTGACAGCAAGAACTATACCATCAGGGAGG<br>CGGGAAAGGAAGCACACAAATCGGCATACAGCCCGCCGCCGACCGACGATGAAATG
MSTRG.5533	CCR4-NOT transcription complex subunit 3 isoform X4	TACAGATAAAAAGGATAAAAGAAGACGTTGAATACTACATAGACTCATGCCTAGACCCGGAGTTTGAGGAGAATGAGTA<br>CATATATGACGACATCCGAGGGCTAGACGACATTGATCTGAGCGGCGTGGGGCTGCCCTCCTCCGCCACCACCGACAGCA<br>ACAACAGTAACCACTCGCCGGGATCACCCACCAGTATACTATCAGGTACAAGTCCAATCGCGTCACCAACGTTAGACATC<br>CACAATCACTCAACGGACTCCACGGAAGTTGAGAAGAAGAAGAAAGAAGAAATCGTAGCGAAACCGGTAAAACCACTGC

		CGCTCCGAGTGGTGAACGCCAGCCTGGTCGGTAATAGCACGTCTAACGTTTGTTCCTTGCTCAATAACTCCGCAGCATCCA<br>CCAATAGTATAAACAATGCGGTATCGGGCGCGTCAACGCCGAGCAAACAGCCGGCGCCGAGTCCGCCCCACCAGCCGCAC<br>CCGC
MSTRG.5<br>536	Protein sprint	GAAACCATGTTGAACGAAAATGACTGTCCTCAAGAAGTGATGTCGGAGAAAATTGGACATAATTTCATACTAGCATACAA<br>GAGAATAGATGCCAAAATCGCATGGCCCCAACAAGCGCTGCTCTCCTATCCTTAAACAAAAAAAATCTCAATAATCATTA<br>AGTAGGCAAAATCTCTTTAAAAGTTTACAAAAACCTAATTTTTTACTATAACGAAACTTTACAAAAGGTTGTCGAATTGCA<br>TTCAGTATTGTAATGTAATTTACACGCTTTGGAAAATCACTACAAATTCTGTAACAGCAGAAATAGAAGTGGACACGGAAT<br>ACCATCACTGGAATAAAAACTAAACAAATGGAAAATTATTTAATTTTAATAGCAAAATAATGACATCGGAAATACAAGCG<br>ACAAATACTGTCTCGAAAATGTATTTTTGAAACATACATATAAAACAACACTAAAAAACATAGTTTTAATTACACTCTTTC<br>ATATAAGTACAATTGGTAAAATACAGGAAGTACCTGTGGAATAGTGCAAGTGTTCTAAATAATAAACATTGATTCCAAAT<br>TCGACCAATTAAGAAAAATAAACAGTATTACAAATTAAACCATTACTTCTAAGAAATGGAGTGATTTTCTTAAATTCTGTA<br>TATCAGCATGAATGTTATTGGATTATAACGTACATATATAGCTTATATTTTTATCACACAAACAATAATAAAGTGATATTTT<br>CTAAGATATTACCCAATTAATTAAATATGCTATTGTTCTCATAAGCTTACATTACCTATAAGTACATCGCTAAATAGGTAG<br>GATTTTTAAAATGTCACTAATGTCTGACATATTACTACGTAGCTTTTTTACTCAATAATTAGTAAGTAATTTTGTATAACCT<br>GACTGAACGCTTCAAGTGTCTGCTGACACTGATATTGTACATTTTGTATGACGTGTGGTTTTAATTCGATTTTGTGTGATTC<br>TTATACGCTAAATGGGAGATTTACAACTTACCGATTTTTATTGTTCCATATTATGTAGTCAACGGAGTAACACTTATCCAAC<br>TATCTTACAAAGGTAGTTTCTTCAAGTGTTTATTTAAATGTTCCTAAATTCGACGTGCACAGGACTCGCTAACCAAATTGTT<br>ATATATATTTTTTTCCTAATTTTAATATTTTCCCAAAATATTCTCTGCTGTTTTGTTTTAACTTTTAAAGTATTGGTATTTAGA<br>CTTTTTAATACTAACTTGTTTATGAACTGTGATGTAATTTGTATTAGTAATGTAAGGATTTGTATTGATTTTGTATATTCGTA<br>GATACGTTTCAAAGGCGTTAATATAATTTATTTATATTTAATCATAAGAGAAATATAAACCGTGAAAGATAAAGAGAGTA<br>ATGAATAAATTGCAGCCTACTAAATGAAAAAAGTGTATTTTTATGCAATAATTTTAATACAAAAGTAACATCTAAAGCTAG<br>CTTAACCTATAATTAACACAAATATTTGAGGCTAAGCAGTTTATAACTTGGAATGAAGAGGAAAACAAGAAGAAAAACAA<br>AATATGACCAATATTTTGTTTTCATTACACCGCTAGTTTTTAATTATTTTATTTACATAGAGAGATTAATAAAAAAATTAAA<br>CAATTTCAACTTATTGTTATTTTTATACAACGAAAACAGCTGATATTTATTCACTATTCTTAACAAATACTGTTTAGTTCTAC<br>CTTTACCACTAAACCAGCGTCAACGTGGGATATCTGTGTGGCATTTAAATTTAATAAAGCAGTATTACAACTTACATTAAT<br>TATTCATATTTATTTGAACAAAATTGATATGTCACAAATGAAGATGCCGTGGTATAAATTAAAATGCCACAAAAATAAAA<br>AAAAAATGTGATCAACAGTCATTTAAAATACTACAGAATTTTTTTCAAGATTATCATTCATGAGATTACCACATAATTACA<br>ATTTATTTTAAATAACACAGGTATTCCATGAGATTAAAATTAATTTAAAATACCACAGATAATTCAGTGGTAATGTAACTG<br>AATACTATTTTGAGTCTAGTTTGTGGTGTTACAAAGGTAGACTAAACACGAGCAGCGGTGAGCATACCGTTTGTATTTATT<br>ACAGTCTTGTATTTTAAAGTTAGTTATATAACACGTATATTGTGTTTTGATAACATTTTCAGTCATTTGTGAAAAGTAAAGG<br>AATTTTTACCAAATCTAAAA
MSTRG.5<br>540	Zinc finger<br>CCHC domain-<br>containing protein<br>24-like	ATTTTTAAATGTTTTGTATAATTCTAATTTCAGAGTCCACTGGAAAAACCTGATGGATTGGATAAAAGTGATCCAGAAAAA<br>AACCACATCCAATCGCTGTGTGAAAAATGTCGTACACTCGGTCGCAATTGTCGGACTTCATATTACTAATTTCGAGAATTT<br>GCAATGTGGGTAATGTATTTGGGTCACTGCGACACACTTGGTGAAAGCATGTCTTCTTCTCAAAACTTCATCTTTTGTTTTA<br>TATTTGAAAAAATGTAAATAATCAGATTGTATAAACTTATTTATTAATTTACGTGTGTCATAAATGGCTCAAATAAAGTGT<br>CTATCTATCTATGTGTTTACCAATCATAGAGATTATATAAATAAATATCGCTACTTCATGTAAATGTTTACCCGAGTAAACT<br>CTAAAAACTGAGGGTGGGGAATATATAGCAATAACTATATATTGTAATTACAATATCTATACATATATTGGTATAATAACT<br>ATCGGTTATCAAATATATAATATCGAAAAGAAAAGAAAAGAAAAGAAAAATCCGCTCGTAAGAAATATAGGTATCCGAC<br>TGGTATAGGCGATATCGCAATGGGTGTCATGAATGTCCAGAAGAAGTCAATATATAAATATTGGATATTATCCATATTTTG

		GTCTATAAAGGTACCAAATATCTAACAAGGCGAAATTTCCAATTGGATTTTTTTACACGACTGCCCAAAAAAAAAGTTAA<br>TTTTCTCAGAGTTCATATATGTACTTAGACTTTCTTTATTCCACAATAACTTTTAAATACTACGACAGATTTCAACTTATGG<br>AATATTGTTAAAATCCTTACATCATCAAGTAACATGGGCTCTTAGATTACTATATAGATAGAGCATCGAGTCTCAAAAACG<br>GGTCAACTTTTTGACAACTAATTATCAAATTTATACTTACGCACATAATGTATCTTAACGTATTTATATTTAATTACTTAAT<br>AAATAAAATAAGTTTTTACGCTTTAAAAAGGTGCAGAATTTATAAGAAAATAAATTTAAAAGCTGTAAAGGAATCACTCA<br>TAACGATTTATCATGCAAATTTGTATGAAAAAGTTGACCCGCTTTTCTCGAAGCATTTGTATGAAGACACTCTATCTATATA<br>ATTCAAGCATAGGCTTTCTTTTTTAAATAGTTCTTTTTCACAGGCAGTTATGTCTTTTAATTAGCGGAGCAAAAATTTACTT<br>GTGTACACGTAAACTGACGGCCCACCTAATAGAAAATAGTAACCGTCATCCATAGACAAAGCCTTGGTGATCAAATTTTC<br>ACACTTACTTTGCTGTCACTTATCACTTCCATCTGTTTTCTATTACTGTAATATCGTTTACAGAAACATTAACATTGACACTA<br>ACGCAAATCGGAATATTTCGTATTTAGTTTACAAATAATACATGTGCATGGAACTAGTTTATATCAGCAAGCTAAACACAC<br>ATATAAACACTAGTTATCTGTGGAATGAAAAAAAAAGGTTTTGTTTTTACTGATCTAGATGATAGTTTACGGAATTCCATA<br>CCACTGCTTAGTCCATTATATTTTATTTTGTTTAATTCCACTACTATTATATTGCGTGAGACTACCAACATTAGAAGATTGA<br>CTACCATTATTATTAGTAGAAATTCTACCATTTTCCATCACCGTCATCATATCAGCCGTTAAATGTCCACTGCTGAACATAG<br>GCCTTCCCCCATTAAGGAAGTTTTGCCTGTAATTTCCCTGCAATGAATTTACAGGGTACAGAGTTATAACACCTGAGTCCTC<br>AGGAATGACAACAAATGAGGAGGGCGAGTGTTATGGGTGAAACAGTATATTCTGTTATGTCCATGGTACTACTCATGTCT<br>ATAAGCGACGATTACCACTTTTCCATCAGGTGGGCCGTCAACTTGTTTGCCATTCTAAGTCGCATAAAATAAAATAAAGAA<br>AAGTTGCTATGCTTCGCAGGCGAGTTGGCAACAATCATTTAAGATTTTGGTGATGTTTTAAGAGGGATCCTTTAATCGCTTC<br>TTACTACAC
MSTRG.5544	E3 ubiquitin-protein ligase HECTD1	GTTATATTTTTCATCACACTACAATCAATAGGAGCAGAGCAGTGAAGGGAAATGTTGGGAAACGGGAGAAGTTACTCCAT<br>TTTTAAGCTTCCGTCACGTGTGCCGCCGGGACGGGCCGCTAGGAAAAAATATTACTAATCACAAACGTTGTCAAAAGCTA<br>ATAATAATTATTGTGATCTATGTACCTATGGAAAAACATTTAGAGCTAATATGTATTTTCTTTTATTTTACAGTCAACCATG<br>CTTTAAAAACGCGAAAAACAGCTTATTTACTAAAAACTATCCAGTTATTTTGAATTCTTCATTTCAGGAGCTAAGCATATA<br>AGTCATACATACCCACATACAGAAATGCGAAAATAAGACTCTCCACGGCCCAATGGCTAAGCTTAAACATCAACTACTAA<br>GCATGAAGTATGACTGGTATGATGGTAGTGGTCTAATAACATAACCGGTAGCATAACTAGTGACATAACCGGTAACCAGA<br>TACAAGGAACAAGACCAAGAACTGTGAATAGTAATCGGTAATTAATGAACATTGAAGAATGACCGACCACAACAAATGT<br>AATTGCGCGTACCGAGCAAACGTGGACAAGGAACAGGTCATCGGTAATAATTAGCTATGAACGAGTGTCATTTTTTCACCT<br>TCAAATGGCAAACGAAATGGAAAGTAGTAACCGTTGCTTATAGACATGAACAGACATTTGACATTTGTCACATGTACATG<br>TTAGCCATCACCAATCAAATGATTAGTGACAGCTAATATGCGACCGGTGTCACCGTGCGTCACAATCAGCTACACACCTGT<br>CGCGGGTATAGCCGAGCTTGGGCTCGGTGTACTGCAGCAGGTGATCGCGCGTCCAGGCAGGTCCACGTTCGCCGCAAAGC<br>AGTAGCCGCAGCTCGGCCGGCGAGAAGGCCCGCAACCGCCGCGGCGGGAATACGGCACCAAAGCCACGACGGAACGCCG<br>CCGCCTGCCGCCGGATGCCGTCGCGCACCATCCACCGTGCCGACGCCTCCGCGTACGCTCGCGCGTTATGTGGTCCCACCT<br>AATGGCCACGCAGAGGGGGAAAGGGTGTGAGAGGATTTCACGTACCGTCCAGTAGCACTGGGTGATGCATCGCAGAGTC<br>GATGGAAGCAGCGGGCGCAGGCGCGTCGTGCAGCCACATGCCGAGGTCGGCGCGCTGCAGCTCGCCGCCCACCAGCGCGT<br>AAAACTCCAACGTCGGGCCCAGCCCCGTGCCCTCCTCGCCCGCGAACTCCACCTCCAACACCGACTTGCGGCCCGCGTGCA<br>CGCGCATCACCTGCGTGTTCGTCCGTAAACACGACGTTAATGTCGATAAATAATTTGTATTTGCGACAAATACTTTATTGGT<br>AACGATTGTCGTGACATGTAAACAAAATTTATATAAACGCTTTACACGTTCGATGGAAACACAATCTTAGACATATACGTA<br>CATAGTGAATACTCAAATTTTCTATTTAGTGGTTCTTGTCAAAAGTGTGAAACTAGCGTAATTGCGTAATCTTAATATTAAA<br>AATTCTGTGTCAGAATATTCTTTCACATTAATATCTGAAACCGCTCAACGTGTACTCCTTACTGCCGATTCTATATTTTAAC<br>GCGACGTGACTCAACAATATCTTGCGTGTAAATTAGAAACATTTTTATCCGCTGAATTAATGGCAAATAGCAAGTACATCA

		AAGTAGTACGATCCTGTCTATATATTAAATAATTTACAAATAATAAAGGCACACCTCTTTATTATTTGTAAAATATTTATTT<br>AGAATATATATTATTATTTTAGTTTAATTATTTACAAACGTGCATTTTTAACAGATGCGAAAACAATATCAAATAGATCTCG<br>TTCATTTCCTTCATATTCTATTCAAGCTCATTTGTTCATGATTTGCTTTAAATATTTCAAATTAGGTTATAAGTACAGGGCCG<br>TCTTTACCCGGGGCTTCGACCACCTGTGCAGCCGCACCCAGGCGCGAATTCCTAGGAGGCGCCGAGTACTAGAAATCAGA<br>TTTTATTAACCTCTTAACCTCATTTAATCGTCGTTTCTTAACGTTTAAAAATCTTTAC
MSTRG.5<br>549	Uncharacterized<br>PE-PGRS family<br>protein<br>PE_PGRS54-like<br>isoform X1	GTAAAATTAAAATAACACGCTGTAATCCTACGCTTTCGTGTACATCAAGCCGATATCTTGTAATCTATATGTAATTATTATA<br>TATCGTCGATTAATCTGATAATATTGTTGAAAAACATATATATATATGTACTTGGGAGGATAAACAGATGAATTACAAGAA<br>AGTGACACGTGGAACTGCTGTGTAAAACAATAAAGTGACAATAAGTCTGAGCGCGTCGACCAGGCGCCATGTACGGAAA<br>ACTTTTATTTCTCTCCGCCCTGGCACTCGCCCAGGGTAACATCTTACCCAACCCGAATGCTGTGGAAGAATGTGACGAGTG<br>TCCCCCAGATATACACTGGCTGTTGCCCCACGAGTACGACTGTACGTTGTTTTACTACTGCGAGTATGGACTTAAGTGGAC<br>AGAGCCAAGACAGTGTGC
MSTRG.5<br>559	Mothers against<br>decapentaplegic<br>homolog 4<br>isoform X2	CGCAGAGCCTAGCGCCGCCTCCTGCGCCGCCGCCGCCGCTCGACGCGCCCGCCCTCCACCATCATTACTTTAATGGCAACC<br>CCGGCGGTTTGCTATCGAGTCAACCAGCGCCCGAGTACTGGTGCTCTGTGGCGTATTTCGAATTGGACACGCAAGTTGGAG<br>AAACATTCAAAGTGCCATCAAGTAGACCTAATGTTACTGTTGACGGCTACGTGGACCCATCAGGTGGCAACAGATTCTGTT<br>TGGGAGCACTTAGCAATGTCCATAGAACAGAACAGAGCGAACGCGCGAGACTGCACATCGGCAAAGGAGTGCAACTGGA<br>CCTGCGCGGCGAGGGCGACGTGTGGC
MSTRG.5<br>576	Vacuolar protein<br>sorting 26 vps26	AAACAATACATAGTAAGACGCAATATGCCATCCTACTAAATAGATAATTGAACTGAACATGTTTTCAATATATATTTTATC<br>AGAATTTGGAGTTATCGCCACTCGCCGTTACACTTCTTATCGTATGGGCGTGTACTCGAGAACAACTACCAAGAGGTACGT<br>TGCGAGCAGGTATTCGAGGCGATGCGCAAGTGTTGCATAAAATACAAGCCTTCGTCTTCGTCTCTAGTTTGTGAAGGTTAC<br>GATCTCGAGCCTAGACAGTTCGCGCCCGTAACTGATAGACCGCATAAGAAATAG
MSTRG.5<br>583	Mediator of RNA<br>polymerase II<br>transcription<br>subunit 26<br>isoform X1	AGTATATCTATAAAGTGAGTTCTCCTACTTCTTCTTCTAGGTGGCGCTAGTAGTAGCTGAGGCGAACCCCGCCAACATCTTC<br>AAAATCGACATCACCCCCGAGGAGGCGCAGCAGTATCTGAACAATCCGCCATTCACGGAGCCCCAGCTGTCTGGACGGAC<br>AGCTGTCTTGCCACTGGTTAAATACAACGATCCTAGGTTTCGGGCGGCCGATGCTGGCCCCACTCTAGGCCACTACTGGAA<br>GAATGGACGCGAGATTGAAAACACTGAGGA
MSTRG.5<br>587	General<br>transcription<br>factor IIH subunit<br>2	GAAAATTCTTCTTTTTATCTGATATCCTTTCAGATAAAAATATTTACATATTGATCCCATAGAAATTTCATCAAAATCGGTT<br>CAGTAGTTTAGTTTGTTAAATCAAAATTTTTTTTAAATACAATTATTATTAAATTTTATTGTAATTATTTATATTTATTATT<br>AGGTTATGTATTTTTTTCAGTTTATCAAGTATAGCTTTGGTCGGTGAGCCATCTTTACAAAACACACTTGAATATGCAGGGA<br>GGGTTCTGAGACCATTGCCAGCACATGCATCAAGAGAACTCCTAGTATTATTTGCATCACTTACAACTTGTGACCCTGGTG<br>ATATAAATACTACTATTCAAGCCCTGAAAACAGATGGAATCAGATGTTCTGTAATTGGACTAGCAGCTGAAGTAAGAATTT<br>GTAAAAAAATTGTGTCAAGACACTGGTGGTGAATATGGGGT
MSTRG.5<br>594	PAX-interacting<br>protein 1	GTTAAAGCAGATCGTAATTAAATAAAGAAGAAACTAAATACGATTATATGTAATGCAGGAGCGAACAGCGCTTTGGTGGT<br>TAACCCTAAAACGAAGACAGCTCTGGCTAATATGTTGAACATCCGGCTGCAGGGCGGCACGCCCGCGCAAGAACATGAAC<br>CTTCCGCCGCTGGAACGCTCAGGTTGATGACCGCTGCCCACGCAGTGAGGGCGCCGGCGAGACTTCTGCTCGGTCCCCAAC<br>ACCATCCGCATCAACAGGCAGGCGGCGTGGGCGCGGGAGGGCCCGTGGGCGTTGGGGTGGGGGTGGGCGTAGGTGTGGG<br>CGTGGGCGTGGTGGGCAACAAAGTATACGCTGGCGCCGTGAGACCACCGCCACCGCGTGCCCAGTTCTACGGACACAACC<br>CTAACTTAAAGCTCCCGCCGGACCTTTTCCTGCTCGGATGCGTCTTCCATATCGTGGAGTACCAGCAGTCGTGGGGCCAGG<br>AGCGCGTGTCGCGGTGGGCGGAGGCCATCCAGCGGCGCGGCGGCGAGGTGGAGGCCTCGTACTGCGCCCGCGTCACGCAC<br>GTGCTCTGCGAGACGCAGAAACACGGCGTCGTCATGCAGGCGCTGCGCGACGCGAAGCGGTGCGTGACGGCGTACTGGCT

		GTCGGACGCGATGG
MSTRG.5595	Stromal interaction molecule homolog	ACAGGAAAGCGCAACTACAGAAAAAAGAAATTTAGAGAAAAAACTAAGAGAAGCTGGCGACACGCCGCTTTTGAATTCG<br>GCATCGTCCGATTTAGAAGTTTCTCAGCTAAAAGCTGAGATTGAGATGTTACGAGCAGAGTTGCAGCGCGCCGAAGGCGA<br>ACTGTCAGACAGGTGCTGGACACCGCCGCCCGGTTTGCAACAGTGGTTGCAACTCACACACGAAGTTGAAAACCGCGCGT<br>ACCTCCGCAAGAAGCAGCAAGCTGATTTACAGCTGCAGCAAGCTAGGGAGGCG
MSTRG.5596	Reverse transcriptase	CTATATTGTTATCTTTTAGACTTTCGCTTTGCAAGGTTTATGATATACATGATATACTATAAGGGACGGTAATTGGATGACC<br>CATTTAAGTAGTAAACCAGTGTAATGGGGAACTGGTCTAATTAGAATTCTCAGGTCAGAATGAGGTCTGAGAATTCTCAG<br>ATGCCCAATGTTCCTTCCAAGAGACAGGAATTGATGCGATCATTCAACTATGATAATTTACCTGATACCTGAACCTTATGA<br>TCAAATTAATTCCCGTATGTTATAAACCATAAACTTAATTGTGTCACATTAACAATGTAGTTGTGTGATGTCACAGTGCTTC<br>TTATGTGTATTGGTTTATGATTCCATCATCCATTTCCATCATACAGGTTCACATTATTCCTTCGCCTATCTTTGGTGATGGGT<br>ACCAATTGGATCTTTGAAATATTGGGCGCAATTGTCGATCTACCTGACTGGTTCGAGAACTTGGTGGACGCATACAATGTT<br>CTCGTCGGTGTGTTCATATTCTTCGTGTTTGTATTCAAAACTAATATATTTCGTAAAATTTTCAAAAGATGTTGAAGAAATC<br>ACTCCGACAATACATATTTAAACAATATAGAAGTAACTAAGGAACTCTAAAATTAATTAATAGTTAAATTGCATTACAAAT<br>GTCGTATATATATTACATGAGATAAATCTATTTCATACTGTGGCAAATATGTTCTTATAATATAAACACTTGTGTAATTGAA<br>ATATCTTCAGTGCTGATACGGCCGTACAGATACTTCTTATCAACAGGACGTATGCAAATGTGTATCCAGTTCCTAAAGGAT<br>GGAGACGTGCCGACTCTATAAACTGTCGCTCTATTTCGGTCATCTCTATAACTTACAAGATTATGTAGCGTATATTGAAGA<br>AAAAATTCCTAATGTATCTTAAAGCCAATGATCTTTTCAGCGGTCGTTATATGGATCGTTAATATGGATTTCGCAAAAATC<br>GGTCGACTGGAGGTCTTCTTTTGTATCTACTTCACTCACATTTGAAGCAAGACATTCGAGAACCATAACTGGAGCAAGACT<br>AATTTCATTGAATTCAATGCCTCTAGAACAGAGGACCACAAACTACAAATTAAACACAGCGCACCCAACCGGATAATTGC<br>GATGGCCGTTTTAATATTCTGTATATTTTTTCTACTCATGCACTCAGTTAAAAGTGAAAAGTTGAAATGTGACGATAGAAA<br>CTTTACAGACAGTATGGGTGCAAATATAAGCGGAGATTATTGTAAAGAGAAACCGTGCATACTGACGAGAACACGAGGTT<br>TCTGTGTTAAAGTGATATCAAAAAAATCGGTAAAATGTGCAAATTTCACATCGCTCCTTGCTAACACGACCGACAGCGAAT<br>TGTTGCGATATCAATATGTCTACAGATACAATTATACAACAGATGTAATTGAAAAAAGCAACAAAACCTTTCCAGAAAGT<br>TTCCATTTATTGCGACACAAGAACTCGAACATAACCCGAAATTGTTTTACGGATTCCACGGTGATGGATTATCAAAATTTT<br>ATTCCATATATTCTAGAGGTAAGTGTTTTTACCTGTATTTTGC
MSTRG.5601	Endonuclease and reverse transcriptase-like protein	ATCAATATACAGAACTCAATGTATAAATAATCAAATATTATTCATATCTATTGAATTATTTTATTTATTTATTTATTTATTTA<br>TTCATTAGTACATATATGTGTACATGGCGGACTTGATGCTGAAAGCATTCTCTCCCAGTCAACCGTAGGCAAGTGACATTA<br>CTAGCTGACCCCGCAAACGTTTCGCCATATACATGAAAGGAGGCCGGCTTCGTCACCATCCCTACTCCGTAATGTAACTGA<br>TCGATTGGTAGTTGATCAAATATCGACCGGTGAGGTTTCATGATTAATTTAGATGAGTAATTATAAACTATACATGAAAAT<br>TATATTCGGTTGCCATCTTGCAACCCTATTGGGGAATTTAAAAAATGCGATTAAAAAACAGGCGTTGATCGTGAAAGGGTG<br>AAAATTTGAAGTTGTGTGTATTTTTCAATGCTAAATCATAATAAAATAAAAATAAATTATTAGGGATGGGTCACCCTTATC<br>ATTGAGGGGTATGAAAAATAGATGTTGGCCGATTCTCAGACCTACCCGATATGCGCACAAAATTTCATAAAAATCGGTAA<br>AGCCGTTTCGGAGGAGTTTGGTAACAAACACCGCGACACGAGAATTTTATATATTACATGGCTTTTTTAGACGTACATGT<br>CAGTGTTTTGTTGAACATTGTATAACGGACTCTATCACCTTGGCTATAAAGCAGTAAATTGTACGAAATTTCAAAATTACA<br>CGATATTGTGCCATTACATTATTTTTTTTTTTGCGTATTTTAATTAATGAAAATGTATTTTGGAATGGAAGTGTCACTATGTA<br>AGTGTTCTACCCGCTTGTACAACGGACTGTGCTCAGAGGAATTGTTCAATATGATGCCCACGGCCACTTTCCACCATCGTA<br>CGGCTCGCCATCGACAGGGAGTTCACCCTCATGTCCTACACCCTCAATGGTCGCGTACAACGCGGTTTCAGAGATGCTTCC<br>TCCCACGAATGC

MSTRG.5602	Endonuclease and reverse transcriptase-like protein	AAAAAGTTACATTAATATACCCTTTTTGATGAACCCCACGCTGTAGTCTGTAGGGTAAACCGCGGCAGGGAGTTCATCCCA<br>CAGCCTGAGCGTCCGCAGGAGGAAACTCCTCTGGAACCGCGCTGTACGCGACCATTTAGGGTCAAGCGTGTGAGGATGGA<br>TTCCATGTCAATGGTGATGAAAATGACGTTCAGAGCAGTGTTTTACTGAAAGTATGGTATGGTATATGGTAGAATCGATTT<br>TTTCAATTCAACTTTGCCGACAGAACATTCCGAAATGATTCTAACGCAGTGAACGCTCTATATAAGCAATTCATTTGAACC<br>GATATTAATAAAAAAAAAAAAAAAAAAAAAATTGGTAAAAGTTGCTTACATGGAAAAAAAAGGAGTATAAAAATAGTTTGCC<br>GTCTGGAAAATATTTTTACTTTTTTCTTTTACTGGCTTAGCTGAGTGGAATAAATAAAATATAAAGAATGTTGCTTATAGAG<br>GGCGTTCACTGTATTAATAATAATAAAAATTCTTTATTTAAAGATTACAAACATCCATTTTTGTTAGTAACAATTGACTTAA<br>ACTATGTTAGTAGTAGAAAAAAAAAAAATACAAAATTCATTTTATTGCCCCCATTAATAATTTTGACCCACAATGAGAGGAT<br>CGGGCTATCGTATTTATTGCATAATGTAAACAGTGTGAAATTGGTATTACCATACCAATTTTTTTTATAATCACATATTGAC<br>AGATTGAGTTTTTAAATAAAAATATTGACGCGTGTATGGGGGTCATAAAATTTACAACCTAACCTCTCCCCCACTCCCTTA<br>TTCATAAATGTCAAACCTCCAAAAGCCTATTTTAGCTACTGAAGTGTTCGTTCGTTTGTTTGTAACACTTTATTGTACAACA<br>AAGTCTAAAATTAACATATTTCTTAAAAAAGAATACAATGGCGGACGTATGCCATAAAAGGGATTTCTACCAGTCAACCTT<br>TAACCCTTAATTACCTCAAGTTTCAAATTTGATACCGGGCTTATAAATCGCATTGGGCGCCTACAATCGATTTTATTTTAAT<br>ATTCGGCTGTTTACATGTTACCAAATGTTTTTTTCAAAATAAATATATAAAAGTATCGATTTGATTTTTTTTACTATTTTTCC<br>GCATTAGTAAACACAGTAAAGGGTACACTTGTATATTTTTTAATTTGATTTAAACAAATCAGGCTTTTAAGGGTTAAATGG<br>CTGATAGAAATTGTGAATTGGCAGGAACAAAAAAACAGCTAATAAAAAAAAATAGATAATTTATTTGCATCACTTACAGC<br>ACACAAATAACATTAGCCATTGGTACACATTTAAAAAGTAATAAAACAAAACGTATACTTAAAAAAAAAAAAAAAAAGAA<br>AGTACATAAAAAAGGTAACACAATTTCTCGTGCGAGTACCACAGTAACACAAAATGGTCACGACTCAGTTTATTGCTTTGT<br>TTTTAAAAGAGGTTTTGTCTTTTAATTTCTCAAATAAAAAAAAATACAGTTAAAATAGGTATCTAGGTTGTTTTAGTATTTA<br>TGAATTAGGTTATACGTACACAGATTAAAATTCAGTCGACCCTTTTTCCCTCCAAAATTGCGTAACGCATACTAGTTCTTAC<br>AAATGTACGTTTAGGATCGAAACCTTTAAACTTCGCAGACAAAACACGTCATTTATCATCTACGCTAACCAATAATGGATG<br>CATTCTTCATTACCAAAGTATTGTAAATGCGAGTATATTCCTCGGATGTATGGGCCATGTCCTTTGTGTACGATCTTTGTGT<br>GTGTGAACAATTCGTACTTTTTAAAACAAATTTTATGTCGACAACTATTGGGGTCCATTCTGAAGCGCCATATCTTAAATAT<br>TATAATTTGACAGTTCCACGAAATAGTTTTAATGAGACATCATAAACTAGATTTGAAATAATTTTGACATAAATCTAAAGA<br>TTTCGCTGTGACGTCAAATTGAAATTTTTATTTTAATGACAAGTTTAGAGAAATGGCGCGTCAGAATTGACGGCATATATT<br>TTTATTTTTTTTTATTTTTATCGTTTATGTAATTCTTTATGGTACGTTACGCGAATAAGGTTTATGTAAAGAATACGATTGC<br>CATAGACTAAAAGTCCTTTTTTTTACAATTGTACATTATTATAATCATGTAAAATGTATATAATAAATCTTTAACGAATAAT<br>CGATTGGACTAGTGTAAGATTTGTATGTCCTTGTGACGTAGGAGTAAGATGGGATGATATGTAATAGACTTATTATAAATA<br>AAAATCTTTTAGTGATTTTTGCAAAGTCTAATTTCTTCCGTTTGCTCTCTATAAATTAAAAATATAAATTAATAATATAAAT<br>TAATAATATTTTTTTTATAGTTGTTTGTTCTTACAGAAAAAAATACTGGC
MSTRG.5604	Uncharacterized protein LOC106114752	AATAGAATAGAAGAATCACTTCTCCCCCCTGGAAGAGACTAGTTAGTTCTATTAGACTCGTATCGGCTTATAACTCGGAGT<br>TCCAGAGTCACGGATTAGCATTCATTAATGAGCCCTTACACGTATCCTTTTTCATGAGGAGTAGCCCTTCTACGTTCCTTCC<br>TCTACCAACAAAAAAAAAAACTGGGGATAACACTGGATTGTTAATAATGCGGATTGTTAGTCCCATTGCTAGCAATATAGG<br>TACATTGCTAGCATATTAGTCAGGTCGACTTTCACGTGGTCCCTCCTGGAAACCATCGGGTTGAATTCTATTATCAGGCATT<br>TAAAAAAAAAACATAATAACCCTATTTTTTTTTCTATCTTTACAGACAATTAGCAATGACCACGAATTTCTCTGCAAATCTT<br>ATGAATCCACCGAAATTAGTTCCTCTCAAGACTGAAGAAGAGAAAGGTATTATTCTTGATAAATTGATAACTCCAAATCTA<br>CTTAAAAATTTTGAACTATATGTAGTATTGAAACCTAAAACAGATACTATGTCACAAATTAAAAATAATGATAATGATTCG<br>GGAACGATCGATGATACGCCTCTCATATGTCTAAGCGATGATGAATGTACAAACTCTAGTTCATCAAAAACAAACTCTACT<br>AGCCAACATTCAACGACGCAAATCAATCGATCAACAAGCAGCAAAGATGCGTGCGACCTGATCGATTTATATGAAAACAT

ACTTGTGACGGCTTCAGATGTGCAGGACCATTCTAGAGCAGTCTACGCAAGTTTCTTTGACAAATATGAAGATTATGACAA
AGTTAATACAGTCTCAAACGATAAACGTTACGCTGCAAATACGCAAACAACTGAATTTAGTAAAAATAACACGAACATTC
CTTATAAAACTCAAAATACACTGATCGATTATTGTAATAGACCAATCGTAGAAGAAAACGTTCCAAGAGCACGTATGGTT
GATCAGATTTTCGACGATCTATTCGAAAAACAAAAAACGTTAATACCGAAATTAGATATGGACAGATTCGACGACATAGA
TGAAGAATTGATAGACAATGTTTTATACGAGAAATGTGACGAGAGCCTTCGCATAAAACAGCTTAAAGATAACGCTGAAA
AGTTGAGGGAGACTGTGAAAGGGATCGCAAAAGATACAAATACGGTAGTTCGGACATCACCTGCGTCGATTAAAATTTCT
CTGAATAGTTATAACATGTTAACACTATACAATTCCTTAGTGGATACAAAATTAAAAAGTAAAATTGATATACAAAATCCG
TTGAGGTATTACGAACCCGCCAAATTTTGCTATCCAATCCAAGGTGCCGGTTTAGACATTTTTGAATTTAGAACGCTCGCA
ATTAAAAAGAATTCAAAACAATACGCCAAAAAAAATATCCGCGTTAGTGACGAGGATAAAAGGAATTTTACTAAAGGTCC
AAGTTTGGATGAAAAGACGACTGTGCTACCACAGATAATGAAACGTTTGATTGCGAAAAAAGATGAAATGAACAAAGAG
AATAACTGGCATAAAAGACTCATACTAGACGACGGTACGTGCAGTAAAAAACCAGAAATGAGACTGGCTCATAACAAAA
ATGAAGTCATGTTGAATCCGGAGATTTTAACAGAGGACTGTAACAGAGATAGACTAAGAGCATTACAGACTCGACGGATG
TTTGAAAGTTTTGTTCATACCGGCAATATTATACCTTAAGAACGTACATAGACAAGGACAACAAGGAATTACATAGTAGG
AATGATAAAATAAAAACTGTAAATCATATGGTCGCCTCCTTTCTTCTTATGCAAATATAATTAGTAAAAAGAGGACGATAG
ATACACCACCAGTTGCTATTTTTCTCAATTCATATCAGGCTAGTGTATAGACAATGTTTGATATGTCAATTATTGGTCAATG
ACAAATTTTACATTACCAGATTATATATTCGACACAGTTTATGTAGAAAGTATGCGAGCGGGTTGTATCTAAATTTGGAAG
GGTATATGGCCGTATAACATTTTCTATACACTACCCTGCTATGAATTAACAATTTTATCACTCCTTCTATGTGATTCCTTGTT
CTTAGACATAGATATCTGACAATTTTAAAAAACAATTGGACATTTTACAATGGTTCCGACTGATCATTTAAAGTGCTCTGC
AAGCGAATTAAAACAGGTATCATGAAATAACATTCGTATAAGAGATTATGTCACAAAAAATTTGGATTTCGATAGCAAAA
ATGTATTGGAATAATAAAAAATTGTGCCCCACAAAAAAACCGATTGTAATTGTGACAAGACTAATTTCTTTTTGTCTATAA
ATTAAACTAGTGAACCGATATCGATAACATTTCTACTATCTGCAAATATATTCTATCAGATAAAGAAAGAATTTTCCAAAT
CGGTTCACGACTGATGGAGTTATAAGGTAACAAACAAAAAAAAAATACAATCGAAGTGAGAATCGTCTTTGTTTTTAAATC
GATTGACTATATATATATTTTTTTTTATATCTTTTTTATTTAGAGGCTTTTATCCTATAAGGGTATGTCAGCCTCATCGTACC
CTAATTTTATAAAATACGTCACATACTTAAAACATTAATAGATACATAAATAAATAATACATAATAGTAATAATACATAGT
AGTCTATTTTGATAAAGGGAGTTATTGATACAAAAATATAATAATTAAAGATTTCGCCATTCTAGAAGATTGATAATATAA
AGTAGGTGCAAATATTTTTAGTTCTAGTTATATATATAATCTTGTCTTAATCACTAGTGTTGCTACTTTATTAATTTTACGAC
TATTAAATGTTTTTTTTTTTTTTTGCGAAACAAAACTCCGGTCGACATTTAAGTTATAATAAATAGAGAAGAATGGAAAAGA
AAAACATACTACTCTGGCCACAAATAATAATTGGGACAAGGGCAAAAAGATTTTTTTTTATGCAACTTAGAATGGCAAAC
AAGCTGACGGCCCACTTGATGGAAAGTGGTAACCGTCGTGTATAGACATGAGCAGTACCATGCACATAACAGAGTATACT
GTTTCGCCCACGATACCCCCCATGCGTTGCCATTCCTGAGGACTGATGATAGATAGATAGATAATTTGTTTGCATTACTTAC
AGCACACGAATTACAACATATACAGATATATATAAAAAGCGAGATAACATAAATGATTAAAAAAAAAATACATAAAGCC
AAGTACAATTTTCCTTTTGCGCGTGTGCCATAACAATACAAAATGGTCACAACTCAGTTTATTGCTTCGCCCTCAAAAGAG
CAGAGCGCTGATATTCTGTTGCGGCCAAAGGGGATAGGGTAACAATGAAACAATACTACCAGATTATCCATACAGTAAAC
AGACAGATATGACAGTCTATCTAGCAGACAAAAGGAACAGAGAGGTAACTTGCTGGAATGCCTTAGCTTTATGTATTTTTT
TTTATCATTTATGTTACCTCGTTTTTTTTTGTATATCTATGTACTTTTTTTTATCATGTTATCTCGTTTTTTTTTATATATCTAT
GTTGTAATTTGTGTAAGTAAGTAATGCAAATAAATTATCTATCTATCTGATATTTTGTTGTGGCCAACAGAATGATGATGAT
GATAAAGGTATATTTGATCATCTAGTTGACAACTAAATCTTATATTGCCGCCTACCGCCATCTACCAGATGATGCATTTATT
CTTATTGAGATAAAAATTTATACATGTGTATTGTTCGCTACACTAAAAATATACATTTGCATTTTTTTATTAAATTCATTTA
AGTGTTTATATATATATATAATAAGATAAAATGTAAGTTATTGTTATATTATTATACAAGTATTTTTTAATAATAATAAAAA

		TGAACTAACCTGTCGCTCGCTACATCATACACGTGGATTTCAGAAATAAAAAAAATCCAATGTAACAAATCGCTTCTTGTT<br>GGCGTATCGAGATGAAATATACACCAGATTTTAAAATAGACCTGTGAAAACCGTACCTGATTTGGAGTAGTAGTTTTTGTA<br>TAATGCTTGAACAAACAAACATTTTTTATTTATTTATATATATATTAATGTTATATTATTATTCATTTACTTTTATTATTTAC<br>ATATGTAGGTATATAGAGCTGTTTTTATTGTATTATATATTAAATTTTTTACTGGGTATTATTTAGTTTATCTTTAATTGTAC<br>GATTATTACCCTTGTACATTCTACAGTTTTCTTTCAGCTTCTCAAAGGTTGACTGTAAGAGATCCCTTTATGGGATAAGTCC<br>GCCATTGTGTATTAAATCATTTATCAATGTTCAATGTGAAATATGTACAATAAAGATTTTACAATACAATACAATATTTTTT<br>TTTTAAATTCTCGTTTTGGATTCTACTGCTTTTTATAGGACCCCCCACATATAATTTTTTTTTTTTTTTTAAACATCTATCATG<br>TACAAATGTCGTGCATTTATAAATTTGATATAATATTCTTTATTGTACTAAAGCATACAGTAATAATAGCAATTAGAAACA<br>ATGTAAACAAGGAAGAACTTATCTCTAAAAGAGATCTCTTCCAGTCAACCCTACACAGTGGCGTAGGAAGTAGTAGGTGG<br>GCAGCAGTCGTGTCAAACAGAGAGCTTAATAATAATATTAAATATTTACATAATTATCTATATTTGAAATATACATGTCTA<br>TAGGCGACGGTTATCAGTTTCCATCAGGTGGGCCGTCAGCTTGTTTGCTATTCTAAGTTGTATATAAAAAAAATACTTTATA<br>AATTTATTAAAAAAAAAAAGTATGCTGCCTTTTTGGTACGTAACTGCGAGTAGCAGATTGTAAAATTACTTTTTTATATTAT<br>TTTTATTTTTAAAACTTACGTTCTATTTTTGAATTAATGTCTTTATACACACATACTATTTTACATTATAAATACACTATATA<br>GTAA
MSTRG.5 608	KRAB-A domain-containing protein 2-like	CTGGCAATTTTGTTAATTTATTGTCATCGTAATGTTTTCAAATTATATGTAATAATAGTCTAATCTTTTATTTTAATAAATTA<br>AATAAACAATACCGGCATAAGATTGTAGGGATTGACCTGAGACGGAAATGGAACATTTTGATTATGAGTTACTTAAAGTA<br>CACTTTTACAAACTCGTTTCACACCGTTTTAAAAACAATTCTATAGTTCGAGCCACATATCCGTGGTCAAGAATATCTTATG<br>ATGGTCTTATCACAGTGATTGAGAGGTGCAAAGCGAAAAGCAAGGAAGAAAGGAAAAAGACTCCATTTACAACATTCGA<br>ATTGCGTGTGTTAGCAGGCTACGATATATTATTCGAAAATGGTCGGAAGAAGCTCATCCTAAAAAGAAAATCGGCGACAG<br>ATCCGATTTTAAAGGTAATCCCTGACGATGAGAGCTTTGATTTAATTGCGTTGACACACATTGAAAACAATCATTGTAACC<br>AAGAGAAACTTCTGCAGTTACTAATTAAAAATTACAGTATTAATATTATGTGCGTGGAAATATTTTTCCAACTTCACACAA<br>CTTGTCACTGCAATAATCCAGTTAAGAAAAAATCAGTTAATTTCTATGATGAATGTTATATTTCTATTATTGATTTGAGACA<br>GTATTTTGATGACCCTTTCAATTATATAATAATGTTGGTGGATAAAGTGACTGGTTTTGCAATTTTGAGACCAATAGTTAAT<br>ATAACTGAGACATATTTAACCATTGAATTCATGAAGATCTTCACCACTTTTGGTGCACCAAGGTTGTTGAGAACGAGTAAT<br>CATCTGGCGCTCCATACAACGGCCATGAACAGAGTAAACAAATTGTGCGGTGACTTTGATGTTGTAGTTACAGCGTGTGAC<br>GATTCAAATCTTTCTAGACACAACAAGAAGGTGATATTGATGTTAAAGGAATGGATAGTGTCGTCCAATAGCAGATCTTG<br>GGCGGCTGCTATTCAAATGCTGCAGTGGCAGTTAAATAATGCACCTGATGATGTAGTGTTTCGAGGTCCTGAGAACAGGA<br>GAGTTGTTCCATACACTCTTAATGCTACGCCCACTTGGACGAAAGTCGAACACACTGGGAAATCTCATGCCTATTTAGATG<br>CATTAATAGAGAAGATTATCAGTACTAATACTGTATCTGAGGTGAATGAAATTATGTCTGATGTTGATAATTCAGATAGTG<br>ATGATACTATACTAAAATGTTTTGTATGTCATCATGAAATTTCAAATAAAAAAACCTGTAAAAAATGTATGAAACATATTC<br>ATGAGGTTTGTAGTCAAATATCAGTTGTTAACGGCAATTTAGAGTTTGTATGTACATTGTGTGCCTTTAAACGACCTGATA<br>ACAGTAATAAAAGTGATGTACCTTGA
MSTRG.5 633	Ester hydrolase C11orf54-like	GCCAGATGTGTTATGAGGTTGCTAGGCTTTATTACTAACAATCATCTCCCCCGTGCGCAGGTCTGGGCGGTGCGTTCGCAG<br>TGCACGAGGGCCGCGTGAAGCACCACGTGATGCCGGGCTTCTCGAGCACGCCGCTGTGCAGCGACCAGGACGTGGACACC<br>TGGCTGCACTACTTCGACATGCGCGCGCCCATCGTGCACCTCGGGACCCTCGTCACCGGCGACCTCGACTTGGACCTGCGC<br>GTGCAGCACTTCCACGGGTACAGCGCGCACGGCGACGGCGGCCACTACCACGGCGACACGACGCCCGACGTGGTGCACTA<br>CGAGGGCTACTTCGCGCTCGCCTCCGCCG
MSTRG.5 657	Poly (ADP-ribose)	TTTTTTTTAATGGGTGATAATGGAATGGTAACCTCGCTTGCGCTATCGGCATACGCTGGCGGGGCACCAGGGACGGATGAT<br>AGGTCATGATGAAAGCAGGATTTTGCTTGTTAGTGCAGCAGTAATTGGCCGATTTGGACACCATGTCGGCGAAATAAATA

	polymerase	CCCTTGCCGAACATGTAGCCGGTGACGGGCGCTTCGGGCGGCGCTATACGAAGACCTACAGAGAATAATAATAATTTATA<br>GCCGTTACG
MSTRG.5670	Afadin	CATAGAGACCCGCGTGCGCGACGTCTAGCCGAGCAGCAGGCGCGCGCCACCACCACGCCCATACCAGAACAACTCTCTTT<br>TAAGGAAAAGATGAAAATGTTCGCACTAGAGTCGGGCGAAAGCTCCACGCCCAAAGATAAGGTAAAAATATCGCGCGCA<br>CAGCGGGACATTGATGCAGTACATTAACGACCACTCAGCTGTTATTTATTAAAACAGATTATATTGCTATGTTACTCACTG<br>GACTTTAAAATATTCTTTAAATTCAGTATTATAATATGGTATAAACACAACTATGAATTGTTATTAATGATACAGAAAGTA<br>GACGATATATCGATACAACCCTTATGAAAACATCCGTCTTTTGAAAGTTGAGAACAGCGAAGCGGAATCGCAAGAGCCTT<br>TTTACTAATGACATATATTCACAAGCGTGGTCCACGGATATGCCTTCACTGAAATATAGCACCAATTTTACATGTCAATCT<br>AGATGATAAGCACTCGGTCAAACGGATAATAATATATTTTTATGGTAATGGATTTTAATGGACGTTTACGTGTCGCCTTAG<br>CAGCTAGTGCAATGAGTTTATTGTGACAACATATTGATGCTAACGATAAATAATTATTTGGTTATATTCCAAAATGAAATA<br>TATTTTAATTATATTGTGTACGAACGGACATATTGACGGCACGTTCGGAACTGTAATTTAATTTTTCTAAATTACGTGCCAT<br>TATAATACTATGTGTAAACAGTATCATTTTTATACTTATGATAGAAATTTATTGTTAGAGAGGACCAATATAATGATTCCCA<br>AGTCGGCGGGTGCCTTTTTGAGTACCTTAGTTAATATTATTACATATCCTACGTGATTCCTAAACTGTGCTGTCCAACTATA<br>TTTTTTATTCATGTCTACTTACATCTATAAGCTTCGTTACATTCCAAAGAGTATTTATTTCTTAATTTGAATTGCTATATTAA<br>GAATCATTCCTATAAATCTTCTGGAATCCATTCGTTATGTGTATAACACAGTGTTTATGACGTTTATACTCTTAGTAACATT<br>TTATATTTCATACTATATTTGACTTTCAACAAAAAGCAATAAATCATTGTGAAGCCTTTGTTGTATATAATAATTTAATGTT<br>AAAATATAAGCACATCTTTGTGCCAAAACGTATCCCCTTGTGGCAAAGTGAGCGTTCACGGATTACTCCCTACTTAACGGC<br>ATCCACAATTTTAAGGTCACAACACTGGATAGCTTGAACTCGACGTTGATTTCTGGAATGTATGAATGTCGACTTGTTGCA<br>ATTTCCTGAGGGTCGAGTTCGTGCTAACTGGTCGCTAAGCAATTTTATATAGTGAACATCTTAAGGCACCAATTATTTTATT<br>GTATATACTGTAGGTAGTGAAATCCCTATAATTATTACAAGGTTTTGTATACCTGTTTGTATATGTAAATGTTTATTTAAAG<br>ATTATATCCAGAGTTTAATGTGGAGAGGAGACGTGGCCTACTTGGATAAATTAGTTTAGTTCCTGGGGGATATTTTAACAT<br>TTTCCCAGTATTCACCCAACGATAGTCAATTAGCAAGAGATTGAGATTTATTTGTAGAATGGAATATTTTTATGGTAT
MSTRG.5750	Endonuclease-reverse transcriptase	TGTATGTGTCAGCTGTCGTAGTCGAAACAGGATTGATTATTTTATTTTTTTAGGGTTTTCGCAGTTTTTAATCACATATCAA<br>AATAAATTTATAGAACATACAAACGAATTTAAAAATTCTATCTGAATCCTTCCTACAAAATATTTATTTATAAAATCACAA<br>ATTGCGAAATTGTATAAAAACGTATGAAGTAGATACCATGATTATTATTAGACATTTTAGACGTTGACGTTGTGTAATATT<br>AATAAGAATTATTTTTAATGAAAAGACACTATGTTACAAAAAATAAGAACTGTACATTTAGTGAGTAGGAGAACATTCAA<br>AAATTTTGGTCATAAAAGAGAACCAATACCGATGGTCACTACTCTTTGGCATGGATTCCTAACATTTACTTTCATTGGATTT<br>GGAATTGAGTGGAAAAGAATATCAAAATTCTTTTTTGGTGAAGAGTATACTGGATCTGAGGAAGAAATATTTGATGTTGTC<br>AATAATTCCAGTATAAATTAAGATGTATGAGCTTTTGACATTTGTTTATATTTAGATATTATATTAATACAAATATTCTTAA<br>GCACTTAGAAAAAAAATTTAATTATTGAAATGTAACATCCAGTAGGTATTAATTTAAAAAATAATAAATGCTTATATGACA<br>GATTTGGAATATTTTAAAATTTTATCTTCATTAATTTTAAATTCTCACATTTTTGACACATATATTAAATACACTGTTGGGAC<br>TTAACATGTACTATTAATGTGCTACAAATAAAATTTCATCACATTAAGTCCCAATAGTGTATTTAAAATATTTATCCTCATT<br>ATTACTTACTACTTAGTTGAATATTGCAACTTCATCATCCTTTCAATGTTATCAATGCAGCCATTATCCATCCACTGCTTGA<br>CATTGGCCTCCCGCATGGACTGCCATAAAGAACAGTCCTGAGCCACATGCATCCACTGACTTTCTTGGATGCATTTGAGGT<br>TGTCATTCTATCTAGTGATGGGTCGTTGTACACTGAATTTGCCTGTAAAGCTAAAAACACTTTGATTTGTCGATTCGAGTTG<br>AAAATTCTCAGGGTTTTTGATAAGGGTATATGAAGGTCAAACCATTGAGTTCATCAGATTATACTTTTCAAGCCTAATATT<br>CAGGTTCAATCTACCTTTTATCATTTGGTGATCATTTTCCATTTTAAATCTATTGATCACTAAGACATTGAATAAATGGCGA<br>TTAATCGTCATGATGAAGTCTATCTCATTTTTTGTGTATCTGTCAGGGCTAGTCCAGATCCATTTCTGTGTAGCTACTTTAG<br>GAAATTGTAGTTCATCATAAAGAGTCCTTCCTCTATGAAGATAGCCAACTGCTATCCCCTAGAATTCCCTGCATGATACCG

		TAGTTCCCCATTATCGTCTTAGCGTCTTTCCTTGTGCCTAGTTTAACGTTGAAGTCCCCTATCTGTATCAGCTACGTAATTAT<br>TATTTAAGTTTATACCTAATGCCATATTAGACCGGATTCATGAAGAGTTCCAATAGGAATGATAAGAATTAATGGAAGTAA<br>ATACTTAGAAGCAGGCAAAGCCGCGGGAAACAGCTAGTAC
MSTRG.5837	Uncharacterized protein LOC106132472	CATGGGCGAAACAGTATACTCTGTTATGTCTATGGCACTGCTCATGTCTATAGGCGACGGTTACCACTTTCCATCAGGTGG<br>GCCGTCAGCTTGTTTGCCATTCTAAGTTGAAAAAAAAAATTATCCTGAATGTGTATCGTCACGTTACAACTACTGAAAGTT<br>CTACATTAAGATACGATGATATATTTTTTTATTTTAATTTTACTTAATACTAAAATCCTAATAATGTATCCTTTGTTTTTTCA<br>GTTATAGTAGGTAGAGTACAAAAAACTTTCAGAGGCGCCGACTTCTACGACGTATATAAAGTGAAAATTCGCAATATATTT<br>AAAGCAACATCAAAAGCAGTCGTTGTACTTAGATCTGGCCGCTTATTTACAGCATCTCAAGAGTCTATGTGTGGTGTCCAT<br>CTCCAGCCCAGAGAAACTTATGTCATCACAGGCAATGTACGACATTTACAAGCTCATATCAACCTCTGTGGGTATACGAGA<br>AAATGGCGCGACGTAACGCCTCGGCAACGGAAAGGATTCAGACTCCTATATAAACGAGGCTGCACATGCAAAGTGCACAT<br>GATGCGACGACGCACAAAATCGCCGAACGCTTGCGTGGCGAACTACGACGATTGTTACGAGCGACATGGTATTTGTCTAC<br>ATGACCGTGATGGACGCTGCCGTTGGACAAGAGCTCCAGCTCTCACGAGATGCACGCAGGCACATTATTTCAATACCACT<br>ATGATGATACCATGACTTGATTAAAGGAAACCGAATCGACTAACTAAAAATATAAAAATTTGCCTACATTTGTATATCTAG<br>ATACCACACAGAATGATGGGTTCGTTTACTATTTTTGTATCAAGCAATCGAACCAAAAGTAAGATATTTAAGATGACAGTC<br>CGTAACTACGTTATTTTTGGTCAGCATATACTTTGTTATCATTAAGGCGGAATATTTGGAATCTATAAACTTCTACAAAGTT<br>TTTTTTTTTTAATAATTGTAATACTGATGGTATACTGTTAATTAATAATATTTATTGTCGATATAAAATGTCTAGATAATTCT<br>AATAAGAAAGTTATCAATACATAACAACCTAAATGTAGTAGTTCTTTTTTACAGATTGGCAAAGTATCAAAAATTGTGACA<br>TAAATATACGAGTATAGGTGTTGAAAGGACTAAATATTATAATTCGGTTCGTAGTACTTTCACGGACTAAAAATAAATCAA<br>TATCTTTGTCTGAATAACAATAATAATACAACAAGCCATCATTGTTGAATTGTCAACTAACCAATTCATTACCTACCTATAA<br>TAACTATAGTAGTTACTAAACTGTAATTTACTTTGCAAGGCAGTAAAAAAGCCGCGGGACCTGATAAATGTCTTTCAAAGG<br>AAAATGGCGGCGTTAGAATACACTGATTTGACATTGTGTATGAATGGAGGCACTTTCATAGTGCTATGCACATTATGTGGT<br>CATGAAAAGTGTGTGAATGGAAACAAAATTTTAATGTAACGAATTTGTATTTAAATTATTCTTCATTGTATTTTTGATGATC<br>TTTATTGTTGTTTTGTTTTTTGTCCTTCGAGACAAAAATATTTAACGTTATTTTTACTTTATTTAGTTTCTAAGAAAAGAATT<br>GTAAATTAACAAAAAATAGTGATATTATCATATTAGGTTAAAATAAAT
MSTRG.5883	Uncharacterized protein OBRU01_00666	GTTTCTTTTCATTCCAAATAATACGTTCATTCCAAATTTCACAACGATTGTTTGAGCCGTTAGGGGAATAAAAGAAACAAG<br>TAGACTCAATTTTACATTTATAATATTAGTAAGGATTACTATATATATTTTAATATAGTGACTGTGCATTCATCTTTGTTCG<br>AGGCCCACCATTTCCTTAAACAGCATTCCCACGCGCAATCGGCACCTGCATGTGGTGTCCTATTTGGTAGCTCTGACGTCA<br>GAAGTCATTGCATTGTCTCATTGGTTTAGTTGCACAGCAGCTGTTTGTACTAAATTGATATACGTCTATTAATCTAACGATC<br>CATGCGCTGTGGACTTTCTAGATACGGGTTGGGCAACCGACGCGGAGCGATAAGGCCATCGCTTCCCGCGCCTGTCTCCCC<br>TTCCTGGACCATATCCACGGCACCCAGCGCTGACTCCGATACCAGCGTGGGGAGGCCCACGCCGCTTATGCCGTTCAATGC<br>GCCTACTGATAACACTAAAACAATATAAAATTACACTCGATTAGATACATTATCACTGAAAACGTACAACAGACAAGATA<br>AAGATACAGATTAAGGACTTGACTTTTGAGTTACGATATGTAATTCCAAATTAAACAATGAGAATTTTAGATAAGTAACCA<br>CGAATTTTTGTTTAAATAATTTACAGTAAATGAAACAAGCACCTATCTATGTTATACACAAAAACGGGGCTGAAATATGTT<br>TATTAAACAAATGTAAACATTATGAATGGCCGTACTATTTATAACTAGATCATCTGCTACAATCATTTGGTTTGTTTGTTAT<br>TATATACATAAAATGTATTACGCATTAAGTTTATTCATTATTGAAAAATGACTGTTAATTCAAAAGAATTTAAATGAATCA<br>TTGGTGATGATGTCATCATACGTGATGCGGTATTAAACGTACAGATTTGACGCTGAGCTGTACAAAAAATGTGACTGTCTT<br>AAATAATGAAAGAATTCGGCCAAAAGATTTTGAAATAAAACTTAAAATTATTGACAACATTTTAAAAACATCACATACAT<br>TTTGAAATAAAATCTGATATTGAAAATATGCACAATATTTTAAAACTATTTTATAATTTATGATTTAATTTCTCATCACATT<br>CACAATTTGCATAATGCTGCTTGCACTATACATATATAATGTTGTAATAAAATCAGTTTTTGGTTACTAAATTTACAATTAC

		ATTATTGCTAGTATTTTTTACTAATCGGTTATCAAATCGCAATTACCAAAACCCAAATATGTTGAAAGAATATGTATGCAA<br>TATATAATAGCACACAACAATATGGATTTTTGAAATTGAATATTTTAGGATGCAGCTTAAAAGGACATTTAGTAGATTGTA<br>CAGATGTAAAATTTTATTTAATACATATTGGAGTTATTTTAATAAACATACAAATTTTAAAACACCAAAATGTTTCTACTTT<br>CTGAAAACAGCAAACGTAACTGAATGAAACCTAATGTCGTATGGCGATAGGTGCAAAAATTAATGCATGCACTATAGCAA<br>TGCAGACTCCTAAATCTCTACAAAACTGGAAAACTGTTACATGCAACCTAGAAGTAACAAAGCAATAAATTAATATTTAT<br>AGTGTTGTACAATTATTAATATATACGCCTCTGTGTAAAATATATATCGATAAGTGTCATAAAAGATATTTTTTTACAAAA<br>AAATTTTCGTTTGAAATACCGCGGGTCTTGTTAATACTTCTTATTAATATAACCTGTAGTTATTCTGTAAGCATAAATGGAC<br>AGGTATTGTTTGACGATGACCTCATTTGTTTTGTAATTTTGCTGAGATTGCGAAATCACTAACTGCACCGGAGCCTCATTCT<br>CTAGCACCGCTTTGCTGTCTCATAATTTTTTTCTCTACACTTAGTGTTATCTGCAGTTAAGGTAGTTAATATTCACCTACATA<br>TTAGAATACTTACCTATATGCATACATTAAAATTTGTAAAGACACAGTTGCTCTTTGAACATTTTGGATTAATATTAATTTT<br>ACATTTTCTTTTAAATTAGAGCACAATTATTTACAAATAAACTAATTTTAAACTAGTACTTTATTTTTTAACGACCTAATAT<br>AATAAATAAGAAAGGTCCTTAAATGACGACCGCGCACGGACAAACGGACAAACGCAGTGTAGGACGATCCCCCCACCCC<br>TCCAAATAGATGGAGTGACGACCTCAAATGTATTGCAGGAAGTCAGTGGATGCAGGTTCGAAACCGTTCATTAACTTTAC<br>ATTTTTTTAAATTTTGTATGGTGGTCTATGGGGGAGTCCTATATATGGATATACAGTGGACAG
MSTRG.5902	Hypothetical protein RR46_00006	GTTCGAAGCGATAGTTTGCGTGAATTTATTCGGTATATTACACGTAATGACAATCCATCGCATGTCTGCATATGAATTTAAT<br>ATTCTGAATACAAAATAGCTATTAATCGTTTCCATAACACTGGAATGAGAGGAGATGGCAAACTAACCCCCGGCAGAAGC<br>ACAGTCGGCCAGGGTTCAGGGCCCCAGGGAGAGAAACCGGCGTACAATGCGAGCAGAATCTAAGAGCACCGCTTTCTGC<br>ATCTTTCCCGGGAGTGAGCCGCCTCGGAATCCCAACCGTCTGAGATGTTGTTCCAGACTGGTTGGGATAAGACCGTTCACC<br>GACATAACGATTGGATTATTTATATTATTGTTAATTATAAACATACATGAAAATATCATATGTAAAATTATATAGCATTGC<br>GTAAAAAATATGTCATTTATTATAAAGTTTTGTATTTAAAAATAACTGTCTTTTTATTTTATGTTAAACTGTCTCTTTTATTT<br>ATTGGTAGATATAAATCAATGGTTATTTATGAGACACAATTTTTTTACATGTAATCGAAATAATTATGCTGGAGGTAAACA<br>CCTCTTAGTATAGTCATTTTAACGAGTTCTAAATCTACATAATTAATGTAGGTTTCTTTACACAATATATAAAATATTGCTA<br>ATTAGACGTTTTAAAATTCATCTTATTTACATGTCACATGTCACTATGTCATATTGCAAATATAAACAGGAAAC
MSTRG.5908	Calcium homeostasis endoplasmic reticulum protein isoform X2	GAAAAAATCAATTTATATTTATCAAGATAAAAATCAATTGATATATATCGTGATGTATATCGGCGAACCCTGATCTTGACG<br>TATTATTAAATAAATAAAATTAATATACATTAAAAAGGTGCAGAAGTTATAAGACAAAATTAAAACTATTTATCACGCAA<br>AATTGTATGTATAAAGTTGACCCGCTTGTCTTGAAGCATTTGTATGGAGACACTATATAATCTAAGCTCAATCATGCTGATT<br>TTGCTACATACATGAATAATATTTTTTTATCCTTATTTATAATGCAGCCAGTTACTTTTTTTATAAGTTGTATTTATTAATCG<br>CATTCGTTATTTGAAGGTCGCAGGCGGTCTCGATCCCGATCTCGTTCGCGGTCGCGCAGCCGCTCCCGAGAGCGCGAGCGT<br>CACCGCGACTCACCACGATCTCGACGTGTCGAACGTTCCAGATCACCAACACCGCCTAGCTTCCTTGGTGGAGCTTACCCT<br>ACTACATCTAGTGGTTTAGATCCTAGTAACAAAGGACACCAACTCCTGCAAAAGATGGGTTGGAGTGCAGGTGGCCTTGG<br>CGCTTCTGGTCAAGGCATTGCAGAACCCATCAGCGGAGGCACAGTGCGTGATAGGCAAGATCAATATAAAGGTGTCGGAG<br>TGAATTTGAATGATCCTTATGAAAACTTTAGAAAGAACAAAGGTGCAGCATTTATAACAAGAATGAAAGAAAGAGCGTTA<br>GAACGTTCGTCGTGATCACGGTAGTGTCGTGGCTCGGTGTAAATGTGTACAATTGCGATTGTGTGGTTGAGATATGACTTA<br>TTTTATCTAAATCATCGTAAATAAAATATATGAATTTTA
MSTRG.5917	Hypothetical protein	CACAGAGAAAGTAATTCAAAGTAATTGCTACATAGTTTCTCTAATCTGTGAATACTTTACAAGTTACTTCATTTCTTGCTAA<br>TATTTGAGTTTTGTTTACTTGGTATTTGTTTATATTTATTGATAAACAATTTAAATTAATGTAAGAATTGATAGTGTTTGACT<br>AAAAATTTGTTGGTTTTTGGTTTTATTTGGCTTTTAATAATTGAAGCTAAAGCATTAGTGTTTTTTAATATTTTTGTTCATTG<br>TGGTGTTGGACGTTCATTAAAAAACTTGGTGCATTTTACTGCATATGCAGCAAAATTTGTGGGAAACACAAAAAAACTTTG<br>TTAGAAATGTCGGACAATTTTATTCCACTGTCTCAAAGTACTCCCGTCCATGGAAAGTGGTCAGCCCAAAATCACAATCAA

		AGATATCAAAACAATCGACATCACAATGGTCAAAGACGCGGTGGTTACAATAACAGATGGAGCAATGGTTCTCAAAGGA<br>ATAACAGTTATGAGAGTGGGAGTAACAGCAGTTATGGACAAGATAGCCGATCCAATATTGATGCTTACCTCCACCCTTCCA<br>TGTTACAAGATCCATGGAGCCAACTACGACAGAAAATGAAATGAGTGTGTGATATAAGCTATAAGTTATTACCAAATATG<br>GCAGTGATATGTACATATACTACATTTTAGCGCTGCTTAGAAATGTTTTGATAATGGTTGTGCTAATTTTAATAAATATTGT<br>CTAGGTTAAGCAAA
MSTRG.5918	Protease inhibitor 5	AAGCAGCATCTGGGTCATAAACTAGCCAGCGATAAATGAGCGAGGTGAAGGTTACCATATAAACGGCATCGCGTTGATGA<br>TAACACTGTAACAAGTTGTAGAGCGATCAAGGAAGATGAACGTCTGGATTCTTGTTTTGCTTATTGTTAATGTGACGCACT<br>GTATGGGAGAATGCCCATCCGGAGAATACAATCCCGGACCAAACTGTACCTTCGAAGAGATTTGCGGTTTACCAAGTTCA<br>CATACGTACCGAAGACACTATTGCGATTGCTGGTGCAATCCAGGCACCAGAAGAGACACTATAACTAATGCTTGTGTAGA<br>AAAATGTCCTATTTACTCTCCTTATCATTTATGGAATAAAGTGTAACATTTTAGCGGAAACAAATGATTATTATCACATTTC<br>TATATTACTATTGAATCTAGGTAACAAAATGAGGATATGTACGACATAAATTCTATAACTGTATCCACCGGATATCAACAA<br>GTGCAACTCCATGAAGGATTGTGTTACGGTAAATATTATATATGTATTATTTTTAATGAATTAATGAAAAAGCAACGCCAT<br>TTTCGCTAATGATATACGCTGGCAGTGTATCAGTAAGGGATGATAATGATATATATAGTTTCAAGGATCCTGTGGAGGTCG<br>ATCTGTTGGACTTTGTTGCTAGCGATGGGACCTATAACCCCTATTGAAAATACCTTAAACGAAACATCACGTTTGTTTCTAC<br>TTATTAATATCATGATAATTCATGCCACGAGTAATAATGATCATATGTTATCAAATTAAGTTAGACTAATAATTTAAACAG<br>TACTTACGTTAGTTACTAGTGTAATTAATTTAATAATTACCTACAAACTATGAATTATTATTTTTTTCTAAGCATTTTGGTTA<br>ATATGCTTGAAATAGAAAAATTATTACAGTATGTTTTAAAGTATTTTTACGGTTACCACTTTTCATTAGAGGCGTCATCAG<br>ACTGTTTGTGATATCAAGTTGTAAAAAGCATTGTTATGAACTTTTGCTTAAGATTTTAATCTTAGATACTTACCCCTTTATTC<br>ATAAAAACTAAAACAAGCTAAAAAAATTAACTATTAGAACAGTGTTTGTAATTGAGAAATTAGTGCAAAGGAAAAAAA<br>AAAACATTTCAATAGCTAAAATAGATTTCTTCATAACGTTTGACGTTTTTATATATGAAGAGGATTAGTTGCATGTGAACA<br>ATTTTTGCCAGTGTATTTTGTCTTTTTTATTGTATTAATTTTCATTATTTTTATTTGATCTGTCTTACTATTAGATATTATAAA<br>GGTTAATGTATAAATGAGGCAATAATCGTGACAGTGGTCGGCTAATAAAAATGTTACTGGTAATACCATAAAGTTTAAAA<br>TTATGAGTAATATTATTATTATGTAAAGTATTTTAGCTTGGAATAATCATTTACCAAGAATTCCGCGCCGTCCATAATTGAT<br>TTCTAAGTGAAATAAGTGAACAAATAATGTTATTACTCAAGTATATATCATGTATAGAAACAATAAGCATTCTTAATGTGT<br>CTCATAGCACATAGTGATGTGGAAATAACAAAAATACCTAATAGGATGCGCATAAAATATGTTCTCACGACGTTATTGTTT<br>TTCTTAGATGTACTTACGATTAAAAGATGACTAATTTGTTTTGTATTATTGTTTTATACCTACATGATTGATATCGTTATGTT<br>ACAGTAAAATCCTGATAAAATTACAATTTAATGGAGTTTTTGCATAAGTTTACTTAAATAAAATCTATTTCGATAGATTGA<br>TTAACTTTAAATTATAAGTTTTCCGTATTCACTATGGTTTTTTTTTTATTTATAATTCACTTATATACCATATCATATCATTGT<br>CTACTGCTGAACATAGGCCTTCTCCTATTGGAGGGGTTTTGTCAGTAGTTGTCACGCTTGGCAGGTGGATTGGTAACCGCA<br>GTAAGGCTTTGGTGATGTTTTAAGAGGGTCGCTGCTGCTGATCCCTCGGCCATTAGAACCTAAACCTTAGTCGCCTCTTAC<br>GACACCCACGGGGAAGCGGTGGCCCATTTTATTTCTGAACTGGGCGGAGCTGGGTTGGCTGTACTAGCCACGACTCATAC<br>GCACAACGGCCCAAATGAGAGGCTAAACCCGGAAAAGAGAGTCCAATTTACATTACATTACATCTATGCCTGAACTATAC<br>GACAAATTCAGGTAGGAAAGGATTATTAGTAATGCAGGCTACTAATCTCATTGCCAGCAGTATACTCTGTCAGG
MSTRG.592	Serine/threonine-protein kinase/endoribonuclease IRE1	CTGATTTATGACAGTTTCTCTAATCTTTGCAAGTTATGAACATAGTCTCTGTGATCTGTGGTGAATAATCGTGATTGTTGTT<br>TGTCAGTGTATCTTCCTATTTAGTGTATTTTTTTCATATTATTCATTTTATTATCTAAAAATTTTATTGTTATAATAAATGATA<br>ATGGAGTTTTCATTATTGAAGTGTTTTTGGTTTAATTAAAAATGACCGTGATGAAGTTTTTGTTTCTTACATTGTTTATTTTC<br>GAGTGGTGCGACTTTGTCTCACCAGAGGCTAAAGGTGGACAGGAAAGTACTGAGTTATCTCAGATATTGGATGACCGGCC<br>CTTACTATTTGCCACGTTAGGGGGTGGCATGGTCGCTGTGGAACCTTTAACCGGAAATATTATTTGGAAACTAAAAGATG
MSTRG.6	Uncharacterized	GTGAGTTGAAGAATTGGAAACTGAAATTACATATACATCAATGGAAAAATCTAGCTTATGTCCAGAAAAAAATTTAAAAAA

081	protein<br>LOC105222572	AATCAGGGCTGTTTACCGGCGTAGCGTATAATTTTGACCGTTATGTCGAAACGAGTAGTGGTAAGGATACTTTACACGACA<br>CGGTCTGAATTATGTATCAAAATATAGATTTAGACACAACTGCTGAACCTGAGATGTCCATAGCTCTGAGTGAAAACACTG<br>GAAACATTTCACTGAATGAAGAACGAACAACAACGGAAGAATCGGAGATGTCCGAAGCTCCGTTTGAAAACATTTCTCCA<br>AATAAAAGAAGAAAATCAATTGAAGAACATGACATGTCTGAAGTTTCAAGTGAAAACATTACACCGGGAAAAAAATGAA<br>GAAAGAAGAAGAAGAGCATTTGAGGAAATAAGTTTTGATGAAATTCTATATCCTAAAAAACCTAAAATGACGAATGAATT<br>GCAATTATCGGTGGATGATTTACAATATGTAGACTCCGGAAAAAACACTGAGATTGATACCATATGGATGGTTAGTCATGC<br>TTTGGGATTACCCAATGTACCTATGTGGGTTGGTTTTAATAGTCGTATTTATGATCATGACAGTCCACAACAAATCATATCT<br>TACTTGTACTTTGCTAGCAAATGGTTCAGTTAATGGATTTATAGACGGGAAACACTTTAATCGCTGCAAAAGATTGCATCC<br>TTTGGTAGCATTAGGACTGGAAATACTACATTTTAAATCATTTTTACAAAATAATAATATCATTTTGAGTAATGATGTGATA<br>GGGGAAGTTGAAAGGCTTCAAAATTGTGAAATATTTGCATTTGATATTGAAAATGAAAACCTGAAAGAGCTGATGAATAA<br>CTACAATATATATAAGCAACAAACTCTTAACGGGGAGCATGGTAAAACAGAACAATTTTATTTAATTTATATAAATCTTCT<br>ACATCATTATTTGGACTTATCTCGAAGTATTCATACTGGAAATTTTCAACTCTTTAAATCAGTATTACCAAAAATTACAAAT<br>ATATTTTTCATATGTAATCAACAAAACTATTCGAGGTGGACGGTCAAATACTCTGATAATTTACAAAAAGTTGCCGAAACT<br>CATCCTGATTTATATGAAGGATTCCAAAAAGGTTGCTTTGGAATTAAAAGAACTACCAAGCCATTTTCAAGACAACCTATT<br>GACCTGGTACTTGAGCAAACCATAAATGCAGATGCTGCGAGAAGGCTGATCGGTATTATACACTTCACAAAGTCAATTTC<br>GGCACGACAACGGTGGGCTCGAAATCATGACTTGACATCTACAATAATTAGCCAAGTTTATCTTGAATTAGGACTCCACAG<br>TCATCAAGATGTTTCAGCTGACTTAAAAACCCATAATATCCGGAAAAACAACAAGCAACTGCAAAGATTTATTGCTACTTT<br>TGATCAGTTTATTAATCCATTTAGTTTAGATGTGCCTAGAGACAAATTACTTAATATTTCATCAGGAAAGTCCGCATCACCA<br>CCAGTAGAAGAATTTTTATTAAATATAGAAGCTAATAGAGATAACCTTCGAAAAACATTTATTTCTGAGTGTCACGCTGAT<br>ATAGGAAGATTTGAAAAACCTATTAAGAAAATTTCTATTGATAATTTTTCTAAAGATTATTGAAAAAAAGAAAAAAATAA<br>ATGTTGGTGGAAAAATTCAAGAAGTAAAAATTCAAAAGGATTTGTTTGGACGTATGCTGGGAATCTCATTAGATTACAAG<br>ATTGACTTTGCAAAAATATTGTCTTACCCGATTACTTCAGTACCATTGTCAATGTGCCATTTGATATTTTGTAGTTATCTTTG<br>TTAGCATAAGTTGTCAGTTTTTAATCATTTAAATTGTTATTTTTTATAGTTCTTTATATGATGTATCTATAGCTTGTAAGTGA<br>AATAAAGCATTTTATTGTGTCGAATCCTTATTACCCTCAAATAGAAATAAGAAAAAAATTTAAAAATGCTAGTAATAAGGAT<br>GATTATAACAAATAAATAGACGCCAAATGGTGCCGCTCTGAAGGGTCACGCGTCCCTCTCACACTCGCCCCA
MSTRG.6<br>102	Uncharacterized<br>protein<br>LOC106137928	NNCTCCGCCACGCCATCCCTTCGCCAAGCAGAAGACGCTCGCGGAAGGTCAAATCCACTCGGACGCTCCGACCCCAACAA<br>GCAACACGCCGCCCGAAGAGGAGTCCACCGATTCCTCGCTAATGGACGACGAGAACGTGCCAAAAGCTAGGAAGCGACG<br>CATTCATCTCCCGTTCGGTAAAAAGGCTAAAACGCCTGCGTGACGTCGCTGCCGCCAGGAGCTGCGATCGCTCTGCCCGCA<br>ATTGCTTTCTAGCCGCGGCGTCTATCAAGTGAATGCACAATAAGTGTCGATTGCAACGGTCATGACGTAGCATTTTACATC<br>GATATTAATATTTAAATAGATTAACGCCCTTATTCCTAAAAAATAAAACGCCCTCAGATACAGTTTGCCTATTATATATAG<br>GGTTTTGTAATTTTCTTTCAAATCAACAAAAAAGGTTATTAGATGTTTCACGTTTTTATGAATAAGGAGCAAAATCGTTTTT<br>ATTTTGTTTTTGTACAATTGTACGCATTAATTGATATAGTTAATTATAACAAATTACTATTATTTATACAACAATAAACTTG<br>GCAATTCAGTAACAAATAGCTCGCTGGCTCTGCATTATTATTTTGTATGGCTATATTTTACATAAATAAGGAATGAAGACT<br>TCCACCGGAATCTGTAACTAGTCGGGCAACACCGAAGGCGAGTACCGTATATTAGTTAACAATATCAATTATAATTATTTA<br>AATTGAGAATAAAATTAATAATATATTGTTTAACATAGCTCAAGTTGCCAATCGCAAAATTTATGTAATATTATAGTAGTT<br>TTCCATCACTGGTATGGTAGCTTGTTTTAAAAAAAAGGCTCGTATTTTTCTGATTTCTTAAACATAATTTTTTAGTATTATCT<br>GCTGTTTTTAAAGATTCTGAACTCTAAGTATCAGATTGCCTCGGTGCAATCCTTTCTATATATGTATATATATTTTTTACGTA<br>TTATTTGCACGTTTAATAAAATGCTTAGTCATGACTGTTCGGTGATCAATTATGGCTGAGCCATACAATTGTCATGTCGAAT<br>TGCAATCAATAAATTCATATTTGTAAAACAATGTAATTATATAAGACTACATATATGACATCGTATGTCTTCCTCATAGTTA

AATTTATAAAAAAAGTTTTTTTTTCATAACCCGGTTTTTGATTGTGTGTAGTTCGTGCTGGCAACATTTTATTCATTGACTGA
ATAATTTATTTCGACTTTTTATAACGTTCAGATAAACAAGGCAATTTTAACCCTTCTTTGCATAATGGTGGAAATTTCCATC
ATAACATTGAATGCCCAAAAATTATATAAAAGAACAAGTTCACTGTAAGTTGATCGTGTTTGGATACCTTTTTAATGTCCA
TTGCAGAAAAAAAAAAACATAGCTATAAAATTAATTTTTTTAACCATTTTTCCTTTAATTTTGAACATGAATGATATCACCTT
GATTTATTACATCAATATAAAAAAATCATGCAAAGAAGGGTTAATATTATTATTTACAGGCGATTTGATTTGCCTACTGTTT
TTAATGTATCAACAACACAAGTTGATTTCTGTATGAAGATATCCTAGGAATTCCATACTTTATTTCTATTTAGAACTTCATA
TATTGAAATATTACACCTAATATGTGTATTATTGTTTTTATTTTCATCGAGTACTAGACTTAATACAAATTGGCAACATATT
GCCTGCTTCCATTGGCATCAATTATCAAATATTATATATAAAATATTTATCAAAAACATCAATGAATAGATAAATAATAAA
AATACAGGGGGATTAGTGTCACTACAAAAAAAGGTATGTTTATACATACTTTTTTAATTAAGTGTATATAGTGGACAGCTC
CATAAGGAATTGGGATAATTCGACTTTGATATGCATCTCATTTGTCCCAATTGAAAGTAAAATAAAACTGTGGAACTTTTG
GAGTATTCCAACCTTCTTATTCATAACTAAAACGTTCTACAAGCCGACATTAACTATTAAGATGTTTTGTCATCGTCTTTCA
TATTGAGAAATTAGTGCAAATGAAAAGGACAAAATTCTTTAATAGCTAAGCTTATAAAGGTTTGACGTTATAAATAAGGA
CTTATATATACTTTATAGTAATATATAGTCATGTAATAACGATGTCCTGTTTCAATTTGAGCAATTACGATTACATATGAAA
ATGTATTAGATGTATTATTTGCGATAATAGATTTATATACTATTTCCCCAATATATATATATATATATATTTGTAGTTACGTATC
TTTGTACATAAAAGTAATTGTCGCAAAGGGCTTCGAGCCAGACAATTGTGACTTCTGTCATGATTTATCGACAGAGGTAAT
AACGTATGGGTGGCCCCAATATATACAATGAAACGCTGTCAATTTGGAATGTTGTCTATTGAAATCCATTTCCTAAATTAA
CAATGTTATCCTTTACATCCCAATTTTAACTAAAGTATAACTATATTATATAATTTGGTATGAAATACCAGTATTTAATTTT
TATTTGATATGACTGCCCATGCTTTCATGTATGTCATATGTGGATCATTGTATATCATATCATTTCTATGATATATCTATTAT
ATGTATATCAATTTCATTACTTATGAGTATGAGATACAAGCCTACATTAAGAGGTCGTCTACACTAAGAATCGAGAAAGTC
CTGATTTCCTATCAAATTTTTGTTACTAGCCTGCGGGAATATCTGCAGTTTCGCTTCTGGTACTATTATATCTTATTGCCATG
AAATTTTTGGAAATACCCAAATAATAATAAATAATGAATATTGAATAATAAAGTAGGTACTTTTTATTTGGCGGGAGATTG
CGACGTTATTCTTGAGCAAAGGAAGTTTGTTATTATTGCCTCAAATGTATTATTCAGCTATGGCAACCCTAAGCATACGTC
AAATCAACTCGAAAGTATTAAGGTTGTACAACCCTAATATTTTAATTTGCCTCGATTTCAAGTTGACGTAGGAAAAACGTT
GACATGTGACTTACAGTTATTATTTTTGCGCTATTAAATTATGAACACTCTGGAAACATAGAGAAAATAACAATAATTGTA
ACTTAGATTATTTCTAGTGAGTGTTCATAATGACGTGTGTTTATGTAGCTCGTTTTTTATTCACTTGTTTGGTTTGATGTGTT
GTATAGGTATTATTATTATTAAAAAAAAAAAAAATAGCTATAAGTCACTTGTGGTAGAAATTAAACGTTGTCCTACACATGG
GAATCCTACTTTTACCCCGGTAAAAAAATCCTTTGTCTCGACTTTCTATGTTTTTAAGAGCGTTTACCGTTTACTTACCGAA
GTGTGGCGACGTCATAATTATATATAAATTTTTAAAAATAATTTATCATTTTTTAATATTTTTTATTATTTTCTTAAGTGTTT
TGCTAGCGAACTAAAACGTGAATTATTATCTTGCCTTTCCCCATTTATTTAGAGTGGGCGCAGTATGTCTTCTTCTTCCACA
AAGCCCTGTCAGTTGTCATTTCCGAGCTCACTGCTCTTTTATCCATATCATCTTTTACACAGTCCTTCCACCCTTTTTTTCCA
GTTGTCCCCTGCCTTTACATCCATCCACTTTCATAACTGTTTCTTTGAAACATGGCTTTCGTTTCTGCGCATAGCATGACCAT
ACCAAGATACTCTATTACTTCGCAACTTCTCCGTTGCGAATTAAAACAGTAATTATATCTAACATGAAAAATTTTATACCTC
ATTATATTTTGCGTTTAACAAAATACATATTTGCCATCAAAATTTAAATTTTCAATGACAGCTTAATAAGAATATTATTGCT
TGATTAACGTATTAATTCACTAGTAGAAGACATTTTAAATAACAATCAATCAGAAACATTGAAAAATGTTAAAAAAGAAT
TCAATTGTCTTTGAAAATTATTTATAATTGACGACGTTTCCATAGTTCGTTATGAACGCTCTTAAGGCGGGAGTTAATCCTC
ATATATTTAAAGTCCGGTAAAAAAAAACCTCTAAATTTGTACACGTATACGTGTTGTGCGAATAAGTGGCTGATATAGAGGA
AAGTTTGCTTTTACCGTGTAACCGCGGTCTATCGTTTATAATGGTCGCCGAATCGCGTCATTCCCTGACCTTAGTATAGCGA
TATTGTTCTTAGCTGTACGTAGATTAATTTAAGGCAATTTTGGTGCTAAATTAACATTTTCAGACTGAGACAGTGCACGGCT
CAAAGTTTTATAGAAATATTGATTTTTCATTAATTTACTGTTGTTATTATGAAAAGGTTTGTCTTTCATCGTCCACGGATTTA

		TCATAAATCCGCAGTTGAAATTGTAAGTTAAGCTATGTATTCACTAGGTTATGATTGAGGCAACACATTATATTTATTTAGT<br>ATTGTGGAATTGCCATAAGTCAAAAATCCCTAAAGAAAATAAAATCATCAACAAACATTATTTTATGATATATTTACCCTT<br>GTCTAAGGGAGGTTCATTTTTTCTAGATAGCAGAGATTTGCATCGTTGTTGCGCATCACCTAATGAATACATACAATGCTT<br>AGTATATAATTGAATATCAACTTGTAAATTATGGATAAACCTTAAAAATAATTGTTGTATAATTGTGATCATTCGATAAGC<br>TGCCATATTTAAAAGCTGCATTAGAGTGGTTGTGGCACTTAGATTTTTTGGTATTTGTTCAATAAGCTTAAGCACCTATTCA<br>GTGTTAAAGAGAAAAAAATCTAGAAACCGGTTACGTCAATGCAATCGAGCGTAAGAATGCATGCACTTAATCGAAATTAT<br>TAGTGTAATAGTGAAATGCCTACATTATTTATTGAGTACAAAAAGAAATATTGACATGTTGAAATGTCAGGTTATTTGTAA<br>TGAATTATAGAAAAAATGTAAATGATCTACTTTGTGAAAGTTTTTATAATCCATAAACAAAATATCTTGTCCAGATTTAAA<br>CTTGGTCAAACGTCAAAAGATGTCGCTTTTCAAACGAAAGCAATAATCTTTTCACACATATTTGAAAAGAGAAAACATTAA<br>TCCCGATAGTTCGTAATACAGTAATCTCCTGATTACTTGGTATTTTTAGAAACGAAGATAACTTTATTGTGATTATTTAGCG<br>ACTTCTTATTTACATATATATTATGTATATAAGTTATCAAATATAATTCTAAAGATATTTTGTCAAAACTTACTAATCTTTAT<br>CTTTAGCTTGTGATCGTCTTATTTGTATATACACCTACCTACTTAGTAATTTGTTATAAATGTAAGCGTAAAAATTATTAAT<br>TCGAAATATGAGGACGATCTATGTTGAACAGTGGTTGTGATGAATTAATTTATTATAAATAAACTTTGTCAAGTTGAATGG<br>ATTTGATGTTGGTACATATATATATATGGATATATGGATATGGTCTCAATGGTCTATATAAGAGTAATAATTATAACCTACT<br>CCATGCTGGTAAACCTTCTGATTACAACTAGTAAAAATTAAATACGTCACAAAGTAGATCTTTATTTAGATATTATACTAT<br>AGAATTTTTTTTCACAACTGTATATTTTATGAACAGATCTGACCGCGTAATGTTAAAAAGACAAATCGTGATACCTAAATA<br>TATTGTTACATCTTTGAAAATTAATAAAGTGATCTTACGAA
MSTRG.6228	Hypothetical protein	TTGGCTGTCAAAGCTGTCATTCATATCAATCAGTCAGTACCACATAATTTAAGTACGCATACTAGTTGTGTTTCATTTTATT<br>TAAAAAAAATCAAAAATAATTAGCAAAATATCTAAGCTTTTTTTATTATAATTATTAACCGATTGAAGGAATGCTATAAAC<br>TTTTTCGTATACTGGTGCCAGAGTGAATTATTAATTTTTATCTATCTAATCAATCGGCGGGTAGAACAGATACCAAAATTCC<br>TCCAGCTTCGGCGACACAAGAATCCTCTCTCCGGACTACTAATTGGTGGTCGGAGATGCTGGTGGCGTGCGAGGCAGGCG<br>AGGTGTTCGCGCTGCTGGAGGGAGGCGCGGGCGGCGCCCGCGAGGCGGCC
MSTRG.6229	Unnamed protein product	GGACGGACTGCGCTCGCGGTCGGCTGCCGGTGCCGCGTGGCCGCGCGATTTCGTATCACCGCCGCCTCCGCCCCGCCCTCG<br>CTTTGAATTCAGTAGAGCCGCCGCTGCGATATTTACGAATCTGTTCACGTTTGTGTCGCCCTCGGCCCCGGGCCGGCAGTG<br>CGCATACTTGTAAACGGCTCCCTGCAGCTGTAGTTATGCGGGTGCCGTACGATTACTGTTAGATGTTACGATGTCTATAGT<br>ACATATTATTATTGTTAAGTAGATAGTATTGTACGTTTCTATTGTCGCTCGTTTTACTCGCTCGGTCGACTGGCCGCCCGAA<br>CGTGTCTGTACATAAATATATCCCTTACCGAATGAACTGACAAACCTTACCGATCCATCCATATACCGTGGGGATAGTTGA<br>CTGTAAAAACTAGCGATCGTATTGAGACACAGGTCATGGAGATGCAGCCCCGCGTTATACCTGCCACCCACATACATGTC<br>ATGTTAACTTACCATCAGGTAACTGTAGAGTCAATGTCAATATGCCACGCCAGTATCGGAAAAATTTCAATAATAAAAAAT<br>AGCCGATGATATGTAGCTTTCTACGTGTCATATTTTTTTTATCGTATATGTTTTGTTTGAGTATATCTATCACAAACAAAAA<br>CCCAAACCTTTATGTTACGTTATATAGGTATAGCAGAAAGATAAACGATACAACAATTCGTCAATATAAATTTTATTTTAT<br>ACCTTATATTGAGCAAAAAATGTAGACACTTTCTGAGCAAGTATTGTATAAAAACTATTTATTCTGTAATAAATCCATTTC<br>GAAGCTAATTAAAAAAAACCGACTAATATCCTGATCACGCTTTACCTTTAAGGATAATTTCGCTAAGGTAATCAATGTCAT<br>TATCTCATTTCGTTTATAAATTATGTATTTGACATTATAGTTAAAAATTAAGAAGTGCTGTTCGTATGTCAGCCACCTCGAC<br>GCACTAATCTGTGAATTCACATGAAATTGATATATAAATGTGAAGTTCTAATAATGTAATTTAAAATATGAGGTTGTTGAA<br>ACATTGCGCCGTCACTGATTCTACTTGTGTTGTCGATATCATGATTAGTTACTTAGTTATGTTTAATATGTACGCTTTTATTT<br>AGTTGTTAAATAATTTGAAATGTGTTTATTTAACCTTCTGTCCTTCTGTGTTGATATCAATTTTTTTCTCAATCAACTATGT<br>AGCGGTAACAAATGTATTTGTAATTTATTACTGTGAGATCAACATATACACTAGCGCTCGAAAGTGTTTGGCCACCTAGCC<br>AAATACATTTTGTAAAAGTCTGATTGAGTGAAACTACCTCGTAATTATTTTAACATGTTTATAGCCATTTTTAAAAATAACA

		AGAATATAATTTTAAAATTTAGGACGCATCTATGTGTCCACCTTTATTTTTATTGACAGCTTCACAAATCTTCGGCTACCTA<br>GCTATTAATTTTTCCAATGTTGTCTTGATTATTTTCTGCCATTTCTCTAGCAGTTTTTTTCCACAAGTCTTCCTAAGATGTGG<br>AACAACGTTTTCACACCTGTCTATGCAGCTGAGCCCTCAATAGTTTAAGTCAGTGGACTGTGGAGGCCATATCATGAGCTT<br>CATCACATGTTGTTCTTTTTCTTCTAAAAAGTCCTTGCATAGTCTTGACGTGTGTTTCGGATCATTATCATGTTCAAATATAA<br>ATCCCTGTCCAATTAAATGGGTTCCACAAGGCACTGCACTGTCCTCCAATATCATTTTGTAACCTTCTTTTTTTTTAAGATTC<br>CTGGTATTCGCACAAGATGTCCTACAGCAGAGCCACCAAAACAGCCGCAAACCATCACAGACCGTCTGCCGAGTTTAACT<br>GAAGCCACAGTACACGCGTCATTCACATTCAAACATTTCATATTTTGATTCGTCCATATAGAGAACCTTCTCTCGATCTGCT<br>ATTGTCCAGTTCTTGTGTTCTCACGTCCAATGAAGTCTCTTTTTGTTAATGAATTTATGAACTTGTTTTAAGACCGCTACGCC<br>TCCCTTGCGTCCTGCGCCACAAAGTCTTTTAACTGACTGCTTTTTCACTACCCGCATTAAACTGTGCAGCTATTTCCGGTGC<br>AGTTAATCGTCTTATTTCGTTTGAAATTAATAACTATACTCAAATCTTCACTCGAAGTTGTGGCTCTCGGTCTGCCTGAACG<br>ACGTTTGTCTTGGTATCTCTTATAGTCTTGAAATTATACGATTACTTTTACCGGATTTATGAAGACCAATTATTATGCCCCT<br>GATCTGATCTCTTGGGAATATTCTTTAGTTTTACCCATTTTCTACAAAAAATAAACTCAGAAGTGAGAAATAAAAAAAAAA<br>TTGGTGGTAGACTTTAAGTACTGAGCTACGGAGAGCGTGTTTTGCGTTGTTATACAAATAATGGAACCTAATAAATGATCT<br>CTTTAAAATGTATTGTTTGTAACCAGAACAAAATGCAGGTAACCAAATACTTTGGAGCTCTAGTGTACATACACTGCACTG<br>TATGTACGCCACTGCACACTTCTGTGTTGTATACACATTTTAATTGTCAATAAAAATCAAATGTAAAAAAAAAAATTGTGAC<br>TATTTTTATTGATAGGACATTGTGTATCGGTCTAGATCAGTAACAGAATACGAATAAGTATGTAATTAGAAAACAGAATAC<br>GTTTATGAGAACAGAGACAATAACAATAGTTTCATGGTGTTGCGGGCTGATTACTTTTTAGATCCTTTCGATAAAAACATG<br>TTTAATTTTTTTTTTACTTTGTATTTGAGTTAAGGTGTACTTGACATTATATCAAATTAGTCGTTAATTACTGGCCGTCGCAG<br>GTACTGTCGGTGAGAGCGGTCAAAACGTGTTTTCGATAATTGACCGTCAATCGAGACGTTACTATACTGCGTACGTGTGTA<br>TGTAAATGTTATGGCGGCTCTTAGCGTATGACCTGCCGAGTTCGATGGTGTTGTCTTATAACATTTCTGTATGTATGTATGT<br>TACATTTCCGCACTGGTTGTACACCACCTCAAATGTCAACCTTCACACTTCTGTATTTGTATAAAATATATATAATTTGATA<br>TTGCAACGCTGTTATGTTACTGTAAGGCTTACTACTATTACTGACGAAGGAGGTGTTTTAGAATTGTTTCATACTTTAAACT<br>GGAATATTCGTTGTGCGCTTATTGGTAAAATATATGCTTCGCTAATGGAGCGTGTTGCAACGCAACAGTGAGACTTGTACC<br>AGTTGTTATATTAGAATAATTACATTATTTATATGTAGTACTTATGTTGCATTGGAAAAATAGGATCTAATAATTGTATTAG<br>TAACGGACCAGCTCCGGCCGAGTGACACGGGGTTCCTAACAAGAGTGTCACGTCGTGCCTCGCTGATAATTGTATAGATAT<br>ACATGTATTATATGTAATTGTATAAAATGATACCTACAATAAAAAAAAAGATATTTTGTATTAAATTATAAAGTATGTGGT<br>GCGCCTGCGTGTAGCCGCTGATTGTTATGCTACATACATATTGTAATTTTTTCTTTTTTATAATTAAGTTTGTACCTACTATG<br>TTTGTATGTAAAACAAAGTCTGGGCCTCTATTGAAACATCTCCTTTAATTGTGATTAAAATATTATATTAAA
MSTRG.6257	Interleukin enhancer-binding factor 2 homolog	CCTCGCCGCGCAGGACGCCGCCGCCGCCACCGCGCAGACGCTTCTTCGCGTGGTGGCGCGCGGCGGGCATCGATACGTGC<br>TGGGTCTGCAGGCCTTCGAAGGCGGAAAGGACATCTCCACAGAGATCACTGTATGGGACGGAGTCGTGGTCTCGCCTCTA<br>GCGCCCGCCTACCAAGAGACGCCCGAGCCTATGGACACAGATGACAAAGACGACGAGGGCGTACAAGACGGGGTGGCGG<br>CTTAGATAAGGATACTGATTTAAGTGAACTAGATAAGACAATTATATAATAAATTTATAATTTACTACCA
MSTRG.6286	Ubiquitin-conjugating enzyme E2 W	TGCTCATGTCTATAGGCGACGGTTACCACTTTCCATCAGGTGGACCGTCAGCTTGTTTGCCATTCTAGGTTGCGTAAAACA<br>AAAACAAAAAAGCACAATTTTGAATTGTATCCCTTGCAATTTTCAGAAGCGGCCCCCAGACAACTCATTCTATGTTAAAAC<br>CTGTAACAAAAACCCTAAGAAAACAAAATGGTGGTATCACGACGACTCCGTGTAACGTCACGTCGCGGCGCTGTATCGAG<br>CTTGGACGATAAAATCTCAATATGTGATAAGTGTTATAACGACGTGGTGTGTGTACATAGCGAGTGAGTGCCGTGGTGTGC<br>GGACGTTGGACTAGCGTTGCTTATTGCGTTGCCCTGTGCCCAGTTCGGACGCCATATTGCTGCGGAGCGACACCTGTCGGA<br>AACTTTGACTTCGTTTGACGGATTGAATTGTAAGTAAGCTGATATCCAACCCTTTGAGCTAGATATACACTGGCAGACTGT<br>AATGCATCTCATACTATCCAGTTAATACATTTAAGTCTTTAGAAATGGCATGGCATGAGGAGTATCATGGGCATCACAGTG

TATTTTGTGACATCCATGATATTCTTGTCTATAAGTGACGGAAACCACTTACCATCAGGTGGGCCGTCAGCTTGCCAATTTA
ATATCATAAAAAAGAATGTCTTGTCGTGTAGACAAAACAGTGAGTCCCTAATTGTCATAGCATAATATGGGACATGCCAA
AACCAGTGATACTCTGATATGAATCCAAACGACACAGTAGTGTTTCTAGCTTTTGGTTGTTTACCCAGGGTCACCACACGA
CACGACAAATCGCATTGGCGATAATGAACGCTGTACGTTTTTGTTAACCTTAAATTCCCTTAACGTCGTATTATTCTGTCAT
TACTAAACTGTTTATACTTGTATATGGGAATCATAAAATATATGTGAAAAATTTAACCTTCTGGTAACTCAACAGAATTCA
AACCCGTCTCTCAGCTGTACCAACTAGTATACTTAACCGGATCTCGACAAACATCACTACAGTAGAATTACTAGTCGGTAG
TTACATTTATAACACGATAGATGGCGCCTGTATAGATTTATTGACGTTTGAAAGACTTTGAAACATAATTCAAAATTTAAC
ACTTGATAGTATTTTTTTTTTTTTATTTAATTGAGACTACACTTGGCTCAGATGTTTCAGAGTTGTAGATTCGAATCCTTTTA
CATTGAACTCTAATACATTTTAAATGGTACACAATTGTGAGGCGATAAAAAAATCACTGTCATTCTGTGGTGAACTGTGGA
AACAGCCGAAGATAGGTGGCAAAGATGCTTATTTTAAATTTGTTACTAATTCCTCACGCTCACATACGAGAAAGACAGCA
CTTCCGTCCAATTTTGTTCTCTCACCTGTAAACTATAAAGCCCATTTTGCTTTGTTTAAGACTGAAATGTAAACAGATTGGA
AATGATATGTTTGCCACCTATCTACAAGCCTGGGTGTGGACCCATAAGCTCTTAAGCAGTATTAATTCGACCGTATGGTTT
GTGATAAACGGAATTACAACCTTGGTATAATGGTATAAGGTTGTAGTTCAAAGTCGGACATTAAAATTGGTGATTATACAG
TGTGTACTTGAAATATTACGAAATATGTAAACAATTCAAGGTGTATTGGTGTGTTTTTAGTATAAGCTATTTCTTTTTTCAA
TCGGGTAACTTAGCCCATTTTAATACATAAATAGGTAATATAAAACAAAGAATTATTATACATAAAATTTTATTAATTTCA
AAGGAAATCTTTATTGACAATATATAATTATAACATTATATCCGTGTATATAAAATACGTATTATTGGTTAGTTCCTATCAC
GTTGGGACATAAAATTATATAAGATAGGTATCTTAAACTTAATTTTAGTCATGGTAACATTATTAACTAGGATGCAATGTT
GCCAATATAAAAAAGAATATTACAATATTTAGGATCACAACAAATACAAAATATTACAATAATTGTATAAAAATAAATGT
ATTGGTCTTCTCTAATTGAAATAATAATACAATAATTAATATAAACTATCCACAACAACAAAAAAAAAATTAAAAATGTGT
GTTTAAAACCATACTAAAAATTTTTGCTTGGGGTGTAGGAGATATTTCGTCAATTCATTTACACATTGTATAACTTATAGGA
GTGTAACAAATATACAATCACTGTAATGTGATCTATAATATATTTAACTGACAGTGATGTTTAAAAAAAAATGCAAATTGT
ATATTGAACTGGAACAACTGTTTAAAGATTTGCATTATTTATACATGAACCTGGCAACACATACATTGTATATTGAATTCT
ATTCTATATTCAATTTTAAATATTTCTCAGCTGTTTTTTTTTTCAAACAGCTCTTTATTGCACTAAAATTGTAGATTAAATTA
CAACTAAATATTATGTTGCATTTTATTTTTTATGGTTGGCACTATTACAATAATCTTACGAATTATTTTTATTGAATGTAGTC
ATTTAAAAAAATAAAACAAAAACACTTATAGGACATAATTTTTTAATCAATTAATACAAAAATATAATAAGCTTTTTGTTT
ATTTTATGGACTGTAGATTTTTTAAAAAGATATCCTAGGATTGGTTTACTAATTGTGCAATATATATAAACGCTAAATATGA
CCCATATATGGTAATAAATCTATTACTTTATAGTGATTGGTGCAACAACTTGTTTCAAGGCTTGGGCTTGTCTCTGAAAGAG
ATCTCTTCCAGCCAACTTAATGCAGTGAGAGTCAGTAATTAGTTGGTAATCTAATAGTTGTAAATATACAAAATTAAACAA
GAAATGTTAGATGACGCTGGAATCGAGACTTGTGCTTTTTTGTTGATTATTTTGTAATTGAAGATAATTTAAAATAAAATC
GCAATGTTGTGTACAATGAAAGAATATAAAAATAATTATAACCTATTACTATTCAAGTAAAAAATCAATAAAACCACTCG
CCACTTTAGAAATAACGCCTTAAAATTATTTTTACTTACAAAATAATCGACAAGAACCACAATTCTCGTTCCTACCGCCAT
CTAGTATTTCATGTCCAATTTTGTTTAGTGACCAACCATCCCAAATACAAAATAAATAAATTATTTGCTGTCAAAGATTTGC
TGTCAAATTATAGACATCTCAGTGATAGTTGCATGATTAATGTTGTAGTGTTATACAAAAGCTATTTATGACAAATTCAAA
TTTTTTTTTTCTAATATGGCTTCCATCTTAAAGAATGAAGATGAGAAATATTTAAAATTTAAAAAGTAATGCAGACGTGCA
GTTGTGCAGTATTTTGAATGGTTATTTTTTTTAAAATTTGATGTTGTACTACAATCAGTCATTTCAAAATTAAAATAATTAA
CTTTTTTTTAGCTTATATGATTGTAACGATCTCTCTTTTCTTTTTTTTTTCTTTCAATGGACGATAATATAATGGTAACCCCGC
CTGAGCTGTCAGTATATGCTGATGGTGTACCAGTACTAATTATTGTTTAAACAAAATAAAAAAAAATACCAGTGAGGAATG
ATAGGTGAGAATGAAAGGCAGGTCAGTTAGCCCATCGTGATGAACACATCTGGAATTCAGCACGATGGTGTCCAGGGGGG
ACCAATTGGAGGCTGACCTTGTATGGTATATTGCTAGCAATGGGACTGGTAGTCCACATTACTAACAATCCCTCTCTCCTCT

		CTTTCTTTTTAGGTACTTTAATTTACTGTTTATTACTATTAACTAAACTAAACTTACAACTAAAAGTTGTAATCATACATAGT<br>TTTTGACTTAAAAATCCAATTTTATTGTGGCCTAAATATGTATTAACTTATGAGATATCATCCAATGAATTCACTTGAATCA<br>TTATAGTTTTAAAATAAAAATTATAGTGCCACTTGTACATTGAAATTTTATATTTTAATTCAAACATAATATATATAAAATT<br>AAACAGCTTTAAATCAAATAAAGATGAGAATTGACTTTTCCATTGTTTTCTGTCTCGCTCTTACTCTTAATTGAGGCAACTT<br>AGGTTAAAAAGAGATGCAAAATAACAATTGTTTTCAAAATCAATGTTTTCAATTAGTTGAGTAATAGTAAATTTGAAAACA<br>ATTTTAGTTTTCACATTGTCATCTTCATTTGATTTCAAAGCATCTTGGCAAGTAATGGAATTGCAACGCCATCTACCGAATT<br>TACAGTCAACAAAAAAGTATACAAACCATTATAACATTCAAGGCAGTAATAATCCGACGTCGAGCGAAGCCAGGCGATTA<br>GCAATAGCAACAACTGTGTATTGATATAAAAGACCTTATTTCGTTTTCAGTCATTGACCTTTTTAGTTATATTTAATCCATT<br>AGAATTATATTTTTAACTGGTCTCTATTTATGCGATATTTCATGTCTTCAAGGCTACATGTTCCCCGTCACGGAATGCTGTT<br>GCTTCATAAAAGACTCATATATTTTTTTTTTATTTTTTTTTATTCAATTAATTTATATTTAAATGTGAAGCGTTGAAGAAAAG<br>ATTTTCTGTTTTTAAAACCTGAATAAATGGCAAGGTATTTTATTTCTTTATACATATATGTTAACTCATGGGAAAGAATGAG<br>ACAGCATATCCGTCGTCTAACCTCCTTGTTGTTGATAAAATTAAACATCTAATAAACCTATTTTAACTACTGAAGTGTTATA<br>CCTTTTTCTTTCATACTAATTTATCGATATGAAAGAAAAGGACAAAATACCCTCTAATGGTTTATAGGAGGTTTTATTTATT<br>AAATAGGAGAGTTCAAAGTCTAACAAACAATAGATAATGTTGCCTAATTGTAAAAAGTAAATATGGAAATTGAATGTAAA<br>CAAACAAGATCATTGCCATTTATACAGGCACAATAATATTATAATTTTAACGAAGGCATAACGGATTCTTTATAATTATTT<br>AATAGAACTTATTTGCTATGTAATATTTATCTTTTTACTGAATCAATATAAAATATTTCATTGTAACTTTACATAATTGTAC<br>AACAAAAATTGTAAGTACATAAAACAATAAGCGACTACTGCCAAGTTGTTACATTCATAAAGAGGATTACTTTCGTCGCA<br>GAAAGGAACGTCTATGGTTCCTTTTAAAATGTCAAATGTCATTCGAATGTCTAGTCGATATTGTTAACAATTAGACCAAAA<br>ATGTTTTACTAAAACATACTCTGTCTATATAATATTGACTTTGGTAATATACAAAGAAACAAAGCGTTAGATTGATATTCG<br>AATCGGTGTACGTGTTTGTTTATTTGTAGTAACGTAAAACTCACTCTAATCAATGCAATGTAATCGCCATATAATCTAATTG<br>ATTTAGTACAACTGTATAATGTTTGAGATATAACTGCGGACTATTAAAAATAACAGCGATCACACCATAATTGAAACATAC<br>GCATGTATTTTTTTTTTTACAAATGTGAAAAAATATACAGCCATCACGCTTATTTGTCAACCTTGTGAAAAAAAAAGAGGC<br>AAAGGACTCAGCGTATACAGCTACAGTTTTACATAGAAATATAAATACTAAATACAGCCAGAAGAATATTATAAAAAATA<br>TTATGAATTGGATGCATACGTGTTGTGCTGTGTAATAAGCGACCTGACACATTGAGTGCTTCGCTTGTGAACTTTTTATTCG<br>TCCGGGGCTATATTATAAAAAAAAAATTATTTCATTATTGGTTAGATTCCTTCACGTGTAACAATAACGGATGCTTAGCAA<br>TTTTTTCTCTATTTTACTTATTCCTAACCAAAAGTTTTGTTTTTAAAAAAGTATGGGTGCTATCAATGTTTGTAGAGAAAGA<br>CATTTAATTAGTTTTCTATTTACTTCATAGCTCGCCTTTTTAAAGATACAACGTATGTTCGCAACACATTCCTGTATTGATTA<br>ATAATTCATAAATTTCGGTGAAACGCACCTGTATATGTTATTTTTTAAAATATGCATTGTAATTGTGTATTATGAGAAACGG<br>AATCCGCTTAATTTATTAAAATAAAAATATAGTTAAACATAA
MSTRG.6298	Hypothetical protein ALC56_14032	CTTTTCTACGAGATCTGAGGATGTGTCCATTATGTAGTCGACGAATGTGTGCTGATTGTGAAGACTGCTGACTCTCTGTTGT<br>TCTTTAAATATGTCTACTATTTTATACCTTCGCCTTGTCGTCGGTGTTATACGAAGTCTGATAAAAAAATAACCGTTATTTT<br>CAAATTTCGTAAGTTACGTATGTTCGATTTTCGCATTTTTTTTTCTGTTATGTTGGTACACATGTCTGTTATATATGTTGGCA<br>ATATCAGCCATTTTAGATTGTCAGTTTGTTTTTGACAGATAAAAAGGTTAGTCGTGTTTTGGTGTGCTCTTCGATTTTTTGCT<br>ATTCGAAAAAATGGATCAAAGAAACTGTGTTAAGTTTTGTGTAAAAAATGATATTAAATGCGCAAGGACATTTAAAATGT<br>TAACTGTGGCGTTTGGCGAGTTTGCTATGAGCAGAACACAAATTCAATCGTGGTATAACCGGTTTAAGGAAGGCCGAGAA<br>GATGTCAATGACGATGCTCGTCCTGGTCGCCCGAGTACGTCCACAACCGATGAAAACATTGAAGCAGTGAAGAAAATGAT<br>TTTGAATAATCGTCGAATCACTATCAGAGAGGTTGCTGATAATGTTGGCATATCGTTCGGCTCCTACCGTTTGCGATACCA<br>AAAAGCGCGTTTTAGAAGTGTTTCGAGGATTGGAAAAAACGCTGGCATAAGTGTATTATATCTGGGGGGAGATTACTTTG<br>AAGGGGACAAGATGAGAATGAAAACAGTATTGATGAATAAATAAATACTTATTGAAAAAATTTAAAATAACGTTTATTTT

MSTRG.6 300	Uncharacterized protein LOC106125039	TTGATCAGACCTCGTATTTAATAATATATAATGTTTATCAATGTTATGTTTGTGCGATGGCAACACTAGACGAGGCGGCCA<br>TATTGTGGAGATGATTAAAAGATCTGTCAAAAGCTTGACACGGTCTTAGTTCACTCTAATTAAATTAGTAATAAAGTAGTG<br>AACTTAAATACATAACTAAAAGTCGGGATATAGTAATTAATCGATTTTGACTGGTGAAAATGTGTCCTATATGTCCGTTGT<br>CCCAAAATAGCTACAGTAAGTTCTTCACGTTTAGTTAAAATCATGTTATTTTATTTATAACTAGATGCAAGCGAAGGCGTG<br>AGGGTAGTAGTAATTAACTTAATTTGTTTTTAACGCATGTTAAAATGTTTTGGTATCCAATTCTTGTGTATTTCGTATTTAA<br>ATAAACAATAATGTTTATGAATATAATAAAAGAGTATATTCTATTTATTTTATACAATAAGTTTCTCATGGGTGGCGTCAC<br>AGCTTAATGCTCGAGGGACGGATAGCAGTCTCTCTTAATAATAATAATTATTTGCCAGAAACATATTTAATTAATATG<br>GATACTCTTTATTGTTTCCTCCAAAAGATTACAATAGTAAACTTATTATTTAAATTAGTTTAGTAGTTTTTGCGTGATAAAA<br>AGGTACACACAAAAAAATTCTAAAAATTATTATTTCGGCATCTATTAGTCTTATTAAGACGTCTTAAACACGAGATTTTCAA<br>TTCTGTTGTGTTTTTTTTTTATTTATTACCTCATAACTCCGACAGTTATAATTTGATTTAAAAAATATACTTACAAATTGGTC<br>CCATAGAAACTTTAAGAAAATCACTTCAGTAGTCGAGTTATTAAATCAAACTGGTTTTGAAGTAGTTTTTTTTTTTTTATCT<br>GTGGAAAACAATCTCACATTAGGTATATTATTAAGATGGGTATAAAATTATGTAAGTTCACATATCATTTAAAATTTCATC<br>CCAACCATCTGGACGAGTGGCGGTCGGCCACCGTGCGTTTCTCAAGGCACTTTCTCCCACGCACAACCACTCTTTGGAATC<br>AACTTCCAGCAGCAGTATTTCCGAACCGATACGACAACATAACCTTCAAGAAAAGAGCATATTCCTTTTTAAAAGGCCGG<br>CAGCACACCTGCAATGCCGTTGGTGTTGTGGGTGATCATGGGCGGCGGTATTTATTTACCATCAGGGTTCGGAATGTTGTT<br>ATCTTCTGAGAAGAAAAAACAAACTGCAAATCAACAGG
MSTRG.6 305	Pre-mRNA-splicing factor Slu7	ATCGGTTTAGTACTTAAGGAGTTATGAGGTAACAAACATAAAAAAAATACAATACGAGAACCTCCTGTTTGTAAGTCGAT<br>AGGGAACTTAAAGTTATTTATATATTTCAACAGATCCTACAGCGATGGAAGATGATGACAATGCTGAGGAAGACGAAGAC<br>AAATACGTGGATGAAGTTGACATGCCTGGAACTAAGGTGGATTCCAAACAGCGTATCACGGTACGCAACTTGCGTATCAG<br>AGAGGACACCGCCAAGTACCTGCGGAACTTGGACCCTAACTCGGCGTACTACGACCCCAAGACACGCTCCATGAGGGACA<br>ACCCTAATCCTAATGCTACAGAGTCGGAGTACGCGGGCGAGAACTTCGTGAGGTTCACGGGCGACACTTGCGCGCACGCG<br>GCCGCCCAACTGTTCGCGTGGGACGCGCACGCGCGCGGCCTCGACGTGCACCTGCTGGCCGAGCCTACCAAGCTGCAGCT<br>GCTGC
MSTRG.6 314	etoposide-induced protein 2.4 homolog	ACCGACAGCGCAGGTGGGGTTACCATTGCAAGTTCATCCATTAGAAATAAAACACTCTCTGATTACAACATTAGCAACAT<br>AATGATTCTACATTGTTCTAATAGATGCATATTAGTGTAACTTACTAATGCAAACTGTAGTAAAAGATCCCAATTCTAAT<br>CTAATTCCTAATTGTTGTTTCATGAACTGTCATTGGCATTAGATTGGATTGGAAATGATATTAGATTCAAGATTAGATCTAC<br>AGGTGTCTACAGTATCAACTGCAGTCGTGGTAGTCTGTACATATGTAAACCGTTGCAGATTGTCCTCCGTGTTCTATTCTGG<br>AAACCATCATGTTAAATTCCAGTGAAATAACAAGGAACTAAATGTCAGTCGAAATTAAAAAAAGATGCAAATAAATTTGA<br>TAAATTATCGGTTTGATACAATGAAGTAATAGATAACAAAATATCGAGCAGGAACACAACATGGAGAGTATAACAAATTT<br>CTCCGTCTCACTAATAAAAGGCTTCATAGACAGTTTACGAGGAGTGACCGTTCTTTTATATTTAGATAAAGAAATAAATGA<br>GAAGGCGTTAAGTCGCTCGCCTCGAATAGAATCTGGAAATACACAATCTAAAGAGAAGCAGACAAAAGTGAAACCGTGA<br>GAGTTATATTTTTCTGTACTTTGCACTATAATTATGTAATGAAATAGTATCATTGATTTAATGACCAATGTTTATATTATAA<br>TATTCTAATTGACTCGGCGTTTGCTTCGCCTTATCGCATCCGAGCGATGATGAAGCCGAGGTGTGGAGTTCAGCATCTCAG<br>TTCGATCGTCAATGATTCCATTCACTGAAATACTTCTTCAATAATATGTATTCTCTTTCCTAAACTATTATTATTATTTGTGA<br>ACCACCCTTAAGGGCTAACTGACC
MSTRG.6 349	Protein phosphatase 1 regulatory subunit 14B isoform X3	GAAAGAGAGCAGCGGCGGTGGAGGCGGCGGCGGCGGCGGAGGGGGTGGGAGCAACTCGCAAGAGGTGGAGGTCGACAT<br>AGACGAGCTGCTCGACATGGACAGCGACGATCACCGCCGGCGGCACCTTACCACATTATTAGCGGATGCCAAAAAACCAC<br>AAAAGGATGTACATAAATTCATCAACGAACTTTTAGACAAAGCGAAAACTCTGTAGATTAATAAAAGGAATCTTCATGCG<br>GAACTTCTGCGAGCTGTGAAGCTGGACGCGACAGCCATGGAGACGCGGCTCCTCATTAGTTGTAGGCAATATACATAAGT

		GATTATTAGGTCGCGTGTGACCATTGGTCGTGTGACGAAACCGAATGACAATTGTTAGGTCTTTCTGTTCCTCACGGTACTC<br>TGTGCGCACGCAAGTTGTGATATACGTATGTATGTCTGTTTTGCGAACATGTCGAATGAGCTGCTTGCTTGTATTGGTGACC<br>AATAAATGACGTGATACTGCCGCGTTTAAGACTCGTTGAAATCGGCTTCGAAGTACACTATACGGATAACATATTGAGCTT<br>TCAATGAGTTTCATTATCTGCTTGTATTATTTGTATAATTAGTCAGATGTCAATATGTAGCTCACTTCGGGTTTTGTTTATCC<br>GAGGTTAGGTTAGATTGGCAACTAAGACTTGAAGATCGAATAGCCAGACCAAGATGGACCTTCGATCTTTGAACACATAC<br>ATCTAGATGTACCCGTAATCGTAGTCCGATCTCGAGGCCACGTCATATTTAATAAAATAGTGTTAGCTATCTAATTCGGGA<br>AGTTCTAGTAAACGGTGCGCGCCGCAATTATTTAGCGACGCGAATACGATAGGCAATTAAGATTTTTAACTTTGCGATGTA<br>TTGGTAATAGTTTCTAGTATTAATATTGATATACAACGTGTATATAAGGACAGTGTTGACACGAACGAAATTTATAATTGA<br>GAAAGGTGAACCATCACTTGTTCTTACTTCGCAAGGCATTATATAGACTTATGTGGTAATAAATATACGTATATAGTAAAG<br>TTTAAGTAGTAAATTAATGTTTAATTCCGAAATAAATATAGGAACGCGCAATAAACATATCATGCCGGCGTGATGGAGTG<br>CCGTATAGGTCTTGTGTCGTGCGGTCGAGGCAGTGGATGAACATAAAAATGGCATCCGATTTCTTCGAGCTGCTGCATTGG<br>TTTAATTTGTACCTACTCCACGTACAGTACATAACTACGTATTAGATCTGTAGCCCGACTCCCATAACCATTTTTTTCACGC<br>TCATTCTCGAACGTTGTCCATTGAATTATGTACTCAAAGACGCCGATAATTATAAAAAAGTACGATTGAAATTCTAATCGT<br>GTTTTTTCGTTCTTATTAGATCCGATTTAGTCCATGTTCTATTGAAATGCGTCCAGGTTAATATTATACACCATTTTTAGATA<br>GAACTTCAATTGGACGTCATCTCAACTTAATATTTTGTTTATACGGGAATCGGATTAGTGACGGCAGTGTGCGTACACAAA<br>CTCTGAACCAGGGAGTCTACCATAAAATGAAATTCCGAATTTTCGGACTCCATTTCGTGTTTTTGTTCATACCGAAATCGGC<br>CCAGTAAATGGAGCTTTTATCGTTCGATAAAAGTTAAAAGGAACAATATTTTAACATTTTTAAACAGCATGTATTATGTAA<br>TTTTAACATCTAATTTGTATTTCGTTCGGCCCCGAAATTTCGGAAACATTTCATGATAAAACCATAGATGCGTCTGTTGCAT<br>ATGAAATTAGTAAAAACAAAAGGCCTCGTTCCATGATTTCGTGCCGGTTGGCTGGCACGCAGTTTGTTAGTTCTACTGTAA<br>CTTACAGTCTGTAAGTTACATCTGTGTGTTAGACGTAAGGTTAACTTACAATAAATTGTTAAATGGCTTGTATAGTAGTTAA<br>TCAACCGTAATTGTAACGCTGTTTGAATTAGGTACATTTTTGGCGCGAAATAAGCGTCGCAGCAGTGATGTCGGCTGCGTT<br>ATACTATATCTTGTATCGGTTATACTTGTATATTTTTGATACGAGTACTTTTAATTTTTTTAATTGATTAGATAGTTTCGAAT<br>AAACTCGAACAAAGTAATTATTCAGTGTACCAAGGTGAATTAATCGCATTATCTTTCTACTAAAAAATAAGATGTCATTCT<br>TTGGTCAAAAATAAGGTTTTATACTGCCGGGGTATTGAACCTAATTTCAAAATAATGATTGTTACAATATAAGAATAAACA<br>TTATGATTATTTTTATATGTTTACACAAAAGTTGGATAAACGATAAATTTAATAGTTTTTATTTTAATAGACATTTGACAAT<br>TTGCCTAAAGTACTGGCCGGTCGCATTGCTAAGAATATAAAAAAAATCTAAGTTTAAATTTAAATTTTTGAATGTAGGTAT<br>ATCATAATGTAAATAGTCGTACAATCACTGCTGTGCAATTACCTATTCAATTGCTACTTATATAATATGTACCTAATTATTA<br>TTACAGCCTATGTCTTATTAAATTAGATTACTGTGTAAGAATGGCCATTAACTTTTAATAAACAATTCGAGATGAAACTCG<br>ACGAATATCTACCACTGTCGGCTGTTGAAGGCATTTTTATAATAAACAATTTTACTTAAAAA
MSTRG.6390	Endonuclease and reverse transcriptase-like protein	CAATACGCTATTTGACACTGCTACGAAATGTGGTCATTCAATAAGTTTCAGGTGATGTTCCAGCGTAAAATTCTACCCTATT<br>GAGCACCTCCAAAATTTTGGAAGCTATCTTAGCTTTTTCCTCCAGATGACCGCAGAATTGGACGTCGCTAGATATATCGAC<br>GCCAAGAATCCCCAGAGTCTACTTATTATCAAGGGAAGTGCCCTGAAAGTGAGAAGATACGACAAACGACTATATAATTA<br>GTAAAAAGAGGACGAAAGATATACCATCATCTTCCACGCACAACCACTCTTTGGAATCCACTTCCAACAGCAGTATTTCCG<br>AACCGATATGACAACGTAACCTTCAAGAAAAAAGCATATTCCTTTTTGAAAAACCGGCAGCATACCTGCAATGTCGTTAGT<br>GTTGTGGGTGATCATGGGCGGCGGTAGTCATTTACCATCAGGTGAGCTGCATGCTCGTTTGCCCCTGGTGTTGTAAAAAAA<br>AAATATGATTGACAGTTTTTATTTTATCATTCCTTCTATGTAATTTCTTGTTCTTAGGTGTACCCTCAAAAATTATCTGTCAC<br>GTGAGTGTAATGGGCTACTGTTGGCTTATTCTCTTCCAATATTGTTACCTTTTATAATGTAATTCTTATAAAATAAAATATTT<br>GTTACAGTGGATATTGCTCTTTAATTAAAAGTTATGAACAATAATATGTGCAATTGTGGCACAGATATATACAGATATTTG<br>TGTGTTTATGTTTTCATCATGCTGTGTACGCATTATTTTGAAGGGTTAGTTTGAACAAAACATTATTGTACTGTATAAATTA

MSTRG.6395	Uncharacterized protein LOC105846225	ATGTAACATTGTTAATTCTCTGTAATGTGTACCTATTGAAATAAAATAAAAAAAAATTGTGATTTAAATCACATTCTCCTA<br>AATTATCGTCTTTATAAACATTCTGTTCTTTTCAATTCTTTCCGTATTCTCGAATCTACTTCTGATCCAGCATTATTGATTTTT<br>AATAATTAAGGAGTATATAATTTGATTTCCTCTTTTATTTTTAGCAATAATATTAAGTTGTAAATTCTCTCAGTTTATGTACT<br>TTACATATTCATATATATTTTTAAGGTTAAATATATTGTAATTTCTATTTAGGTATCTGTCCTTTTCCTTATATTATCCACTA<br>GCGGCCCGTCCCGGCTTCGCACGGGTGGACATTTTTACTTTTAAAAAGTGGCCATTAAGGCTGCACACGCGACAGAAGTTT<br>AAAAATGAAGTAACTTCTCCCGTTTTCTCTACATTTTACTTCCCTGCTCTGCTCCTATTGATCGTAGTGTGATGAAAAGTAT<br>ACTATAACCTGCCCAGGAGTGTGAAGAATAAGCGTACCAAGTTTCGTGAAAATCCGTCGAGTAGTTTTTGTTTCTATAACG<br>AACAGACAGACAGACAAAAATTTTACTGATTGCATTTTTGGCATCAGTATCGATCACTAATCACCTCCTGATAGTTATTTT<br>GAAAATATATTTCATGTACAGAATTGACCTCTCTACAGATTTATTATAAGTATAGATAGAAGATATAAATTATTCAACTTT<br>AGTGATCTGTATCTTAAATTCCGTCATCCAAAACTCGCTGTATTAAAATGCCCTTCCC<br>TATTTCCACAGTCTTGTAGAAATTTTATTTTATGCTGACCTTTTCTATTTAATGCTGCAGTACGTTCCTCAGCTATTCTTTTCT<br>TTTCTGGAGTGTCCGTTGCTATCATGCTTTTACCCAATTTTCTACCTTTCCGTTTGTTGGAACGAGGACCAGCCTTTAGCAG<br>AGGACGGAACATTGGTGGAGTGATGATATTCTCTTTTGATTGATGAATTTCGTCTTTGCATTCTTCATCTGGTTGCTTTGTT<br>GGTGTCACGTTTTGGTAGAAACTGTGATGTTCTTCTGATAATATAGATGGACTTTCGCGATCAGGGGTAAGAGCGTTATAA<br>AAACTTGAACGACGTGTCTTTAGATGAGTCGAATGGGGTTGCTGATTATTTTTATCTAGATTTACAGAAGTTTCAGCATCA<br>GGGGATTTTTGTGACACTACGGGGCGATCTGTCACGCTACTTGGCATGAAATCTTCATCGGAGAATATGTCCTTATTATAT<br>GGAAAAATCCCACATGTTGCGAAAGCTGCCGATATATTTAGTGGAGTCATAGATCTGGGATAAGCATGACCAATAAATTT<br>TGCTATTTCGTAGATTGTAACTGGTCTTCCTGGGTTTTGCAGCATCCATGACTCAAGTGCGGAATAATAATATGTCTTTAAA<br>GGGCCCATCAAACCTACATCTAAGGGTTGTAATTTGTTAGTAGTGTGAGGATGAAAGGTAAGAACTACAACACCCGATTG<br>TTTCGCTAAGTCTAGAGCCTCAATGGACAAATGTGACTCATGATTATCCATTATAAGCACAGATGGATTTTCTGGTGTCGT<br>GCTTGAATACTTAATGAAATGTTTCATTACCTCTACGAATAAGTCACTAGTCATCCAACCTGAAGGACTCGCAAGACCTAA<br>ACTACTTGGAGGTGCACCGTGCATCATTAAGTTTTTAAAGTTTTTCCGAGGAAATATTAAAGCAGGGGGCAGGGCATGTCC<br>TGTGGCACTAACGATACAGCATGTAGTGACAAGAGTGCCTTTTTCTCCACTTGTCACTTTACCTACTTTAGCTCCTGTTGGA<br>GCCACAACTTTCTGTGGTTTCTGGACAGTTGTTGTGGCGGTTTCGTCAAGATTAAATATTCGAGTTCCATCCACGAATCTAG<br>GCTCCCGTTTTAGCAATTCCTTCAAATTATTAAAAAATATTGTCACAGCTTCTCGATTGAATGAGGTAGCTCGTGCAAGACT<br>TACAGCTTCTGGTTTCTTTACTGAAATATCAGGATGTCTTTTTTTAAAGAAACGAAACCATTCCAAACCTGACATTTTATCC<br>CTTTCCCATGAGCTTGGCATTTTAATGCCATTAACCTTTGCCATTTCGTACGCCACCTTCCTACATTCTTTGATGGTCAAACC<br>GTAGAACATTAGTGCACAGTCTTTATAGTATTTTAATAATATTTGTTCCTGTTCTTCCGTAAATACTTTATTAACATCATACC<br>TCGGTTCAAGTGATATATTATCACCATGTTCACGTTTTTTTTTAACGTAACGGCGCAATGTAGGGTAGGGAATTTTAGTGGT<br>TTTCGCCGCCGTCCTAATGGAGCTACCATTTTCTACCATTAATAATGCTGACATCATATCGTCTTTTTTGTGTTTTCCAATTT<br>TCCTTTCCTTATTTCTTGGCCTTGGCATCCTAAAAGAATCAAAAATATTATTGATTTTCAGCACTACACTGTTCAATACACA<br>CACTATTTAACTTATAATATTTAAAAAAAATAGGTTGTAGGCTAAATTGTCACGAAGCAATATGCTCATAAAACGTCTAAA<br>ACTACAACAATATCGACGCCATAAAGCTTTTGGTGCTAGAATTAATAACAAAATATATAAAAATAGAGACACTTACATGA<br>GAATTCTATGTTTTTTTGGCGAGAAACAAACAAACCACTGTCACACGTCACACTCAAGCCGTGGCCACCGGTTAACTACGG<br>GAGAGGGGAAGGGTGCCTACGAGAAAAGTTTTAACACAATCGGTCGAATTGGCAACCTCGCAGTTTTTTCAATATCGTGG<br>TGGTTCAATGTTCCTGGTTGGTTCACTGTTCAACAACAGTAGGGTATCATGACTATTATGTATACTAATATTTACTTGAATA<br>TTTTTAATTTTTCTGATCTAATATCATAATTATTTCTATTAATTATAAAAATTTGTAGATTAAAGAGTGATGTGGCTTAGTG<br>GTTTAATACATACGCACAATGGCCCAAGTGAGAGGCTAAGCGCGGAAAATTGAGTCTTTTTCACATAGTGGTTTAATACGA<br>GCAGCAGCTTTAAAAAATAACAAAGTCTGTACTAAATATTTGTGCAATTATTTCATTTTCTGATTTATTCTATGTTAGTTAA

MSTRG.6<br>397	Protein turtle-like	TTATATTACTTATCTATTATTTACCTTCTTAACAAACCATGAATCTAAACACCACAGTGGAGAAAACTGCATTGTAATTGAA<br>TGTGCTTGACAAAATGTAGGAAAGTCCTATAATGAGAATAATCATTAAGTATAGAACTTTACTAAACTGTAGACGTGAGTT<br>TTTTTGAAATTGTACATAATTTGCTAAGCCAATTTAGATGAATCTCGTATTGGTGAGGTGTTTTATGGACTGGCTCGCTGGC<br>CACAATGACGTAGCGGCGGATGTTCATGAACTATCATATTTGGAAGTTTTCAGCAAAACGAGAATATAACAAAAACTTTT<br>GTAGTTTTAGTATAAGTATACATATGCTTCTTTGTAAAATTCACGATTCATATCATATGTGACAAATACTGTACTTATTCCT<br>GGCGGTGTGGTAGGTCATTGTATGTAAAAGTAGGTCTTTTTTAAATCTAATACATCCCACCAATGAAATTGTATTATAGTG<br>CCTGTCTTTCGCGCGAGATGGCTATTAAACTCGATGGTAATGTGATTCCTCTGAAGTAGCTATTCAAGCTCTCAGCAGGAG<br>GATTACTGCTAATGTATGGTATTGCATACATTACAACCAAAAAAATTGTTCGAATTTTAGAACAGTAGACAATACATAACT<br>GCTCTGTATCTATACCGTATTGTAAAGTAGATCTGAGTCAAGCAACCGATATTCTTATGCCTGACAGTTTATTATTTGAAAA<br>AGATAGTTGTTATTTTTCCTTGTTATGGCTGTGTTTAGCATTTTTATGAGTTTATCTGTCCATTGAGTAGGCATCTTAAAGTT<br>AGTATACTATTTTGTACAGTCCCGTAGTCAGTGTAATTTTTAAGCAATTGTGCCTGCGACTCGGTCCTTCAGTAGGTACTTA<br>GTAAAATAATGCAATAATCTTGCCGTTATTTTATTAATTTTGCAGTCTATAGGTTACAGTGACCGTTTACTATCAGACAGGC<br>TATCCTTGTTTACCACCTTCGTGGTATATAAAAAAAAATAGTCTTATAATTCAGGGGTACATATACGAATCTGTCTACGGGA<br>CTCTTTCTTACATAATAATTATAGTATAAGTATCAGTAGTTGATAAAATCTTGTAAATTGGTTATAAATCAGCAATAACAGT<br>TCGTGTTTCTGAGACACCTTAAAAAACTTGTTTATAATTTATTTAACTTAGTTGTCGTTATCTATTGTATACAGTTAAACAG<br>ATCAACTATAACATAACATAGATTATTGTTAGTTGGATTTTAATATGATCTTATATTTTCTATATTTTTTTTTAATTTTTATTT<br>TTGGTATATGTTTTTTTTATATTGATTCTAATGTACCTGATTGTCAATCTATAGTTTCAGTTGTTAGTTACTCGCAGTCATTT<br>TTATGACTAAAATACCTACTTAACTGATTTGATGTAAAATGTTATTGATCAAATTAATAGTTATGTCATAGAGTTAAATTTT<br>ATTAAAATATAAATATACGGTTAGGTAAGTATGATGCAATTTAATAATTTTGTTAAAAAACTAGGTTGCTCCGTTGTGTGC<br>TATTTAAGAATATGCGTTGCATAGCATTAATCATGTATTTAATTGCAAATCACAACATGTATAAGATTGTATGGCCAGTGT<br>CAGTGCGCGAGGGAGTGCGCGTGCCCCGTGTCAGCCGTGTAAGCAAAAGTGCTCCCGATGCTATCTCCAAATAAACAATA<br>TAGTGCACCCAATAAAGAAAAAAATAATTATAATATTGGAAAATAAAAATCTTATCGTATTATGTAAAGTGGCAGTGTAT<br>GTGTATGTTCGTTATCTAACGGGGGTCTGCTGCTCTCTCGGACTCGCTTTGAAGGCCTTTGTCACTGTGGTGTACGTCGACC<br>GGTCGGTTGGACGACGGGCGCGGTAGGTACACGAGGCAATACGCAGAAGAGGCAAAGTACACAAAAGGCGTGCGAAGA<br>GCGCGCGTGGGGCGAGGGCTCTGGGGCGGAGCCCCCGGCCCGCGGCGCCCGCACTTTACCACTAAGCACGGGACAATAA<br>GATGTGTCGTCGAGACGGCACGTCAACATATACTTTACAGTCTGCGAGGCGCGTCGCGGGGACGAGCCTTCCTCTACTGAA<br>CAGAAACACACACGGTAGATGAGCAGCGTTGTATTGTAAGAGTGGAGTGTGGACCGTCGTACCGTTTCGCCTACCTTCTTC<br>TCCCACTGCAAGACGTACGGCACCGGGATGTCTTCGGGGAAGTCCACCTGACAGTTGAAGACGACGCTCTCGCCGAGGAT<br>CGCTGTGATGTGCACGGCATCTTGGTGGTCTTGGTGGCAGGGGCAGAATAAAAAAAGATTGTGGTACCTGGTAGATTGAC<br>TAGCAGCAGCAGAAGCAAGCCGGCGGCGATGTGCGGCGGTTGCACGGCGCGCCAGCCCATGCCTGCGCTCCCATGTCACG<br>GCCGCGCGCGGCGACTCATCGTTGATGCATCACCTATACAAAAATACACATTTTTTTGACACCGTAGAAACCTAGTATAGT<br>CATTTAAGAAGGATAATATTTTGCAGATCAAACAATTATAAATGCGAAACTTTGTGCGAATGAATGTCTGTAGTTTAGGAA<br>TTAGAATATTTACTAGATATTCTTACATACTTCAGTTTGTGATAGATGACAGATACGTGAGATTAGAAAACGATTATCGGT<br>TCAGTGGTCTAGGTAAAAACAATAAAACTAAAATTTGTTTACAAAAAGTAATATAAAAGAAAGTGTGTTTCATATCGTTTT<br>GTTATAATTATAATCCAAAATGAATATTTGTTATTTTTTCATGCAAAAACTACAGAATGGTTTTGAATAAAATTGTTATACC<br>GCTAGATATAAACTGATTCGAGTGGGCAAAGTCGCGCAAGATATAGCTATTATTTAATAATGTTAAGATTTGCGGAAGAA<br>AAGAAAATTGACGTTCGCCAAAACAGAAGCATGTTCTATAGAATACGACTTATTGTACTTACTTCTTAAAGTACTCGTATT<br>AATATATAAAGCTACGGACGCAAAGAAACAATCTGAGATTTCAAGGTCTTTTTAACTACCAATCGAAATTAAATTAAGATT<br>TTGGTAATTTTAGGTTAGTGGAGAATATTCCTTCTACTTACATATCCAAATATCTTAAAAGATTTCGTCGTTGTAAAGGATA

		CAGCAAAATTTTGACTTTATATAAGTTCGGTTTACGTCTAAAAAGTAGGTGTTGTGAATACATAGATCGACGTGACTTTAT<br>TTGCTTGTTTCTGTCTGTGATATCTTATCATTCAAACAAATTCAAAACAGATATTTTTTATTTGAAAAATGGTTTAATATA<br>AATGGTTTTTAACTATATTTGGTGAACATGTGACTTCACCTTTGCTGCACTTTGGAGACATTTTGACAATTTTCATAGTTTTT<br>CCATAACTATATTGAGATTTTTTCTATTCGAATCAAACTAATCGATAAAATAATCAAATCAGTTATAATTAACCCAAAAAT<br>AAAACAG
MSTRG.6<br>447	Endonuclease-<br>reverse<br>transcriptase	CAGAGTACTTTTTGCGAGCTGCTCCGGAATTGGACAAATACGTAATTGCTGTTGTGAGAATGTATATAAGTGTATAGGTAT<br>CTATCATCTATGTCCAACAGTGAATAGCTGTACGCGTAAAATTTGTGTATAAAGTGTTTTAATATTAAATATGAATAAACA<br>AAGTGCATTTATTAATGAAGAAAGTAATGAAAAAATAGTGAAAGAAAATGATTAACTGTATGTAGAAGCTTCAAGTTGCT<br>TAGCGAGGTATAGAGAGGGCTATGCTCAAAGTTTCTTTGCTTGATAGAATCCGAAGTGAGGACATCCGTAGGAGATATAA<br>GGATGAGGTCCAAAGTAAGCAATATAGCCCGAAGGATAAACAAGCTGAAGCGGTAATGGTCACATAAGTCGAAGAACGG<br>ATAACTAACGGGGAAGAAAGATTCTTGAGTGGCGACCCCGTACAGGTCAACGCAGTGTAATACGACCCGCTACTAGATGG<br>AGCAACGACCTCAAATGCATTCCTAGAAGTCAATGCAGGTGGCTGGGGCTGTTCCTTCTGATAGTCTATGAGGAAGGCTTA<br>TATACTCGTACAGCAGTAGACAGTTAACGGCCTAATATGATGATGATTTACATACTATTTACGAATACAATCATTTATACA<br>ATTATGATAGTCATTAAGCGTAATAATATTCGCAAATTAAATAAGTGAAAACCAAAC
MSTRG.6<br>462	Menin	GATAGTCAAAAGTTACACAAGGAATACAATTAACAAAGAAATATTTTAATCGCAAAAGTGACACATTCACATTAATCAAT<br>ACAAATTATTAATTTGGAGAATTAAAATTTTATCAATTGTTATAATTAATAGTTTATCACATTTTCTTATATTAAAAAAAAA<br>GACAAACATCAACGTTCTCGACGTGCTCGTTTAGCACGGGGAGTTGGCGTGCACGGACTGGGCTCATCATCGTCTCGCCTC<br>TTCATTGGAGCTTGTGGACGTTGTAGTTGACTCTGAGCCGTTAACTGAAGCTGTAACGCATGGGTATTTAACCTTTCGGCTA<br>ACAGCAATGGTTTTAGAGCTGCCATTTTATGGCTATAAAGGGTAACTCGAGGTCTACTATTTACTGCTGCAGTCAACTGCT<br>CACGCACACTCATTTCAATATTTTCAGAATTATTATTCCATTTGTCTTTTTCATTCTCTCTTTCTTCTTTTTCTTCCTTGATAG<br>AAGAGTCTTTAAATTTATCACTGTGTTCATCATTGTTAGGACTACATATAATGTGTACCTGTGCTCGGACTTCAGCATCAAA<br>TTTAGATATGGTGCTAACAAGAGGCTTTGCCCATCCAATATGGAGAATTGGGGTCTGACTGCCTTCCTCCCATTTGCAAAT<br>GCCATCATAAAATCTGTAATCATGAACATTTCAAATTATTTAATAATTATGTTTCACAAAAGAACACCATATCAATTGTGG<br>CAAAACCAGTTATATTGATTTAAAAACTAAATTACTGATTTTGACTTAGTTTTTATGAGATCGTCCCACCTCCTTTTAGATT<br>AAATAATAACTTGCAAAATCAGTTTACAACTGACAGAATTATGAGGAAACAAACATAAAAATAAAATAAACCCGAATTAA<br>AAACCTTTTTGATTTTTTATCAAATAGTTTTGTTTATATGATTTGGAATATTTTGTTATGGAACATAGAATGACAAACTGAC<br>TACCCACCTGATAAAAAGATATACACAATATATAGAAATGAACAAAACCATGGATATGAAAGAATACATTGTAATGCCCT<br>AGGTACCTTTTGTGTGTTGCCATTCTTGTGCACCTGTTATACCCCATACCTACTCTGTAATTTCACTAAAATAAATAAACTA<br>TTAATAATATTAAATACTACTGCAACTATTTAAATATTATTCTTTATTCTTTATTGTACACAAAACATACTGTTAATCACAA<br>TTCATTATCTTAATACTAGCTGTATCTTGCCGCTACATCCGCCTAAAAGAGTACATAAAGTACCTTATGTGTTAAATTTGTA<br>TATAACCTACCTATTTATCAAATTCAATTCAAATCCATCCAGTAGTTCTTACTTGAAAGAAAAACAAACATTCATCAATCCT<br>TACAAACTTTTGCATTTATAATGTATTAAAGATTGTAGTAATAATTTATAGGAAAATTATTAGTATTTATAGATCCCTTTAA<br>TGATGATTGAAGAAATAAATTATCCCTGGATCCATATGTAGATCCTGATTGTGGATTATGAACTGAGATTATATATTATAT<br>TTAAATAATTACATATTATTTATAATAAAAAAAAACTATTTTTTTATATTTTACCTAAGTAACGAAG
MSTRG.6<br>511	Hypothetical<br>protein<br>RR46_14024	TGCTCGTCTTGTTATTCAACATATAATAATATAAAAGGATTTCGAAATCATAAAAATGAAAATTGTTTACACGTGATAATA<br>AAATATTTAGATAAATAGTGATTTGTGAACAAAAATTTAAGAAATAGTGATGTGACATTCAAGTGATGTATTCGAAACAA<br>ACTATGCAGTTCCTACTTGTGCCTAGAAGACAATTGAATATTGAATAATGGAAATATATTAGGATGCCTTGAATATTAATG<br>AAGAAACCGAAGATGGGAGCGCATGGTTCAAAAGAGAAGCTATACAGAAGTAATTCTGAGAAATATCCATCCAAACTAC<br>CGGTTCCTAGTGACAAAAAATATAAGATAGCGGTAAATCGAGAAAGATTCGGGTCTTTCGGCGCAAGAGGGAACGGCAA

		GAAACGGGAGAGCGTCCCGGCGCCAACGAACCCCGCCCGTCCTCCACCTACTCCGCCACAACCTCCCAATGTTCAGG
MSTRG.6537	Hypothetical protein RR46_13991	ATTCGAACATGTGTTACGTTTTTATTGCTAATTTATTTATTAATTTTTAAAATACTTCATAGTCAAGTTTAATATTATCAGTA<br>TTTAGATATCATAGGAGGTGAGCGCGCTGGGGCGGCAGCGCCGGCGCGTGCTTGCCGGTTGCGGCGGCAGCCAGCTTGCG<br>GGCGACACCACTCGACACACGGGCGCAGATCGAAAGTTCGATGCGAGATCGAGCGCGCATATGTAAATATCGGCACATTT<br>ACAAGCGAAAGGATTCACCAGAGCGGCGCACTAACGCAGTGCTGCGCGGGAACCAGTCAAGTGAGATTCCTACGGCCTCC<br>GATAGAAAAATGTCGAACGAGCTTCGGGTCCATCACTCGGCGCGGGTGTGTGATACGCAACGCGGCCACTGCTCGACTGC<br>CGCCCTCCTCTCACCGCCACAGTGCTGGTATATTTATCGCTTAATTATTGAACTCGTCTGTGTAACAATTATGAAGCTCGAC<br>GAGTGGTAGGCGCTGACTGAGGGTATACGGCGCTACCGCGTCTGCGCTTGCGCATTGCGTCACACCGTCACACGCAGCAC<br>CGCACGCCGCCACTACATGCTGAGGTCGCACTGGTACATCATCAGCTGCCTCACCGCATGGATGGCAGCCTTCGAGTTTTA<br>TCACAATCAATCAATAGCCAATCAACTTCATTTTATACATTATACGAGTACTCACTTCTTTTTATACATTAACCTCCAGCT
MSTRG.657	Nudix hydrolase 8 isoform X2	ATTAGTGGTCATAATTACTGACTCATTACAGTTATCAAATATTAAATTTAAATAAAGAGGCCTCTAATTGTTTTTAAGCCAA<br>ACACGAATATCTGCTAGCAGCGAAGTTTCGATAAAAAAAAATAACAACACAGTCACTTAGTTCAGTGTCGCAAAGTAGTT<br>GCCAGTTATGTTTGGGTCTCTACAGTCTTCTGTTGCGTTGCTTTGAATTATATTTTGTTTTCACTTTACCTATCAAATACTAT<br>ACCATACGCTATGTCTTACGCGTTTCTCATTGTTGTTTCCGTGGCTATTACTGTGAAATTGTTATTTTTAACTTTTTCTATGT<br>ATTTCACATTGAAACAGAGACGACGTTCAGCAACTCTGTCTGATCAACAAACTACAAGACGAAATAGGTCTATGGCGATA<br>AATTGGAAGCACATACCACGGATGCTGTTACCGAAATCATTATATTGCAGCGATGTAAACAATAGTAGTAATTATACCAAT<br>AAATTATGATTAATATAAACCTACTTTTTGTATAATTTTTAGTGATAATCTTTTGCACTAAATAGGTAGGCTGTGCAAACCT<br>ACCTACATGTCATGTCAAAGTTTAGAGAATTGACATAAACAGCTTTACAGATAATATTTGAAAATTTTATGACACATTTTTT<br>GTAATAATCGATTTCACAATAACAATTCATGACATGTATATGATAAAGTGTAGGATCTATTTTAACAAATACATCAATATA<br>GCTCAGTTGCTACATATAGATTGTTATAAAGAATGATATCCGCGTGGCTAGGCCAGGGTTTAAAATAAGCACTAATTTTAA<br>ACACTGATCTAGACTTGCGTCATCTCAAACTGAAGGTAGTATACCTTCAGTTTCAGATGACTTATATCCATATACCATTAAT<br>GGAACTGTCATTATGACAGACCCATTAATAGTTTCTATAAATATTATTCAAAAAGTTGAACTCAACAATGTACTTATATAA<br>TTTTACAAGACCTAAAAACTACAATAAAAACAATCATTATTTTATATATTACTTGTATATTGTCAGCTTTGTATCTTTACAT<br>AATACTTAGCTTTATGATTGTACAAAAACTAATATAATAGGAAATGTTTGTATTTAACTATTATGTAATCTAATATTTACAC<br>TATTATATTATGTGCTATTTACATTCTTTCATTTTCATATTTGTCTACAATAACTATTTCTTGAAATTCACATACTTCCCAGT<br>CATCAATGGTTAATCATAATCTTCTACTAATAGAAAAGTCATATCTCTAACAGCTGTCGGCCATTTTACAGTCTTTTTTTGT<br>AAATCAAGTTTTAAATTTTTATTCTTATATTGGAGAGCTTTTTTCACAATCAATCTATTAAATTCATGTACATGTGGATGAT<br>TAGTATATTCTGATACATCCATCCATTTGCAATCCTTAACTTCTCTTGGAGAAATAACTATTTTGTCCGAGAGTGCCCTCAT<br>CATTAACAGCATATAAATATCAGAGTTGCCAAACATCATATTATGTGTGTGTCTAAATGTAACCAAAGATATAAAAGCTGC<br>TTCCACGCCAGTTTCCTCTTTCACTTCCCTTACAGCTGCCTTCGCAATGTCTTCTCCTACAACATAATAGTATTGCATTTCAA<br>TTTGTTTATTTATTTATTTTTCTTTATTGACCACATTACAAAAAAATGTCTGGTACAAAATGCGGACTTAATACTATATAGC<br>ATTCTCTCCCAGACAACCTTTGGCTAAAAAGAAATTTATTGTGCAGTTATTATATTTAAGTATCACAACACATACAATGGA<br>GAACTACTTTATTTATTTATTCATTAGTACAAATATGTGTACAGGGCGGACTTAATGCTAAAAACATTCTCTCCCAGTCAAC<br>CATAGGGAAGTGTCAGAATACTCGAATGTGGTACTACTAATTATTGATATACATACATACTAAAATTACATATATAAACGA<br>ATAAATATATATGAATAACAAATATACTTACACATATATGATATATACTATATTATACACGAATTTGGGAATAATATAAAG<br>TACTACTTCACATTATGATTAAATAAATATACATACATGAATACATTAAACAAATATTACATAAATATACATACATACTTA<br>TAGGATTCTATACAATAACACAAATAAACAATGATAGAGATCAAAAGTGAAATTGATGATGAAGATGATGAACTGCTTTC<br>ATAGATTTTCAAAAGATATTCGTATTTGTTCACATATCATGACTATAACCAACGCACATGTCACA

MSTRG.6610	Uncharacterized protein LOC106130853	ACAAAAAGACACCGTTCCTTTGGATATATAACATTCCGACAGCACCAGGAACCCGCTACCTTGCCATTTATTACTGTGACT<br>GATGCCGACAAAGAAAATATATCAAAAATATCATTGGAAAACGTGGACAGATCCCTGATAGTACCGGAAGAGCAGAGTA<br>GTGTTGAGAAAAATCTGATGACAATGCTAGAAGAAGCTGGCTTAGTTGACGTCACCACGGCTAGAGTTGTAGAACAGACA<br>CAGGAGATTGCGAAATCTCTAGAGAAGTCTACACGAAAACATGGAAGCGATACGTCGGAGACGCCGCTAGGCAGTCTAG<br>ACAGAACCAAGGTCTCTCTTGGAGATTCTGACCGAACTACAGACTCTAAGCGATTTATACACGACCAATGGGGAACAACC<br>GGAACAATGGTGAAAATTTTGAATTATAAGAAGATGGAGAAGAGGCCGTTGACAGTCCGCTATCTCATCTCGAAGGGCCC<br>GGTGCTAGCTGGCTACAAGTGTATCATTGCTGCTCGTTGTTTTACTTCAATACTCA
MSTRG.6641	Apoptosis-resistant E3 ubiquitin protein ligase 1	GCCGTCCTTGTTTATTTATTTTTCGCGAATTTAAATTTTCGATATAATAATGTTATTATGATTAAATAAATATGTACAAACA<br>AATTAACTATTACGGCACTACACTGTTTCTATGTTTAATTATTTATTTAGCGCGCTTAGTATTAACTGAAATATTTGAAGAC<br>GAGAGTTTGCAAGTGGACGAATGGTTAGAGCAGAACGATCTAGCAGATTACAAGAAACTATTCAGAGATTATGGTATCTC<br>CACTCTGTCGGGATGTGGTTCGCCGGACCACCTGGACCGGTTGCCGGAGCTCCCGGCGGCGGACGAGAAGCGCCTGCGCC<br>GCGCCGCC
MSTRG.6657	Uncharacterized protein LOC101743642 isoform X1	AGGCTACTCTATCTAAACTAGTAGTAGTCCGGCGATCGACTGAGGCGGTCGAGTGCGGCATTGCGGAAGTGATATGCGGA<br>TATGTCGTTGAGGGTTAAAATGTTATTGGAACGATTCCCATAAACGCGAGCGCGACTGTATCGCGGCCGGGTGAGTTTAGT<br>ATCGCGCTAGAAATCGTAAAGAGTCGTGCGAACGGAGGGCGCAGCGCTAACGTAGCGAGTGCCGCGGCGGAGGACCGGA<br>CCGAGCCGAGGCTCAATAATGCTCAAGTTCCTGACTCACAAGCTGCGGACGCATTCGTTGAATGAGGAGAACGTTTCCGA<br>GAAGGTGGAGGAGCGGGACTCCGGCACTGAGTCTGACGACGAGGCGGACCGCGAGCCCGGTGA
MSTRG.6694	Protein sel-1 homolog 1-like	CGAGAGTCCTTTGGGCCTGATCTTCCTGTCAGGCGACTCAATGTTCCTGTCCAACTGGGACCTTTACCTCATCACGGCGCTG<br>CTCGGGGCGCTCGGAGTCGTACTTTACCTGCGTCGACCGCGCCCGCAGCCGCAGCCGCAACCAGCGAATTGAACTATAAA<br>AATGTGATATATGTGTTACAAGTGACATGAAAACGTGAGGACTTGACAAAATAGGAGATACTGAGAGTTTCATTGATTGA<br>GTATTCAGTACTCATAACTAGAAACATGATAGGAGTCTTACGATGCCGTTATCGAAAAGGCCTATCGTATGCGTCTCACGT<br>GAGTTTAATGTTTTGTACAGAGAACTTATTAATTTTAATAGGATTTGTGTATATTATTAGGGTTTATAGACGATACAGCTCA<br>CTTTTAAGCTCATATTGTAAATAGATTTTAATCATCAGGGCTGGATTAAAATTTGGACGATTTTTAACCAATCTATGCTTTA<br>GAGGCCTTAGTAAGGTAAACAATTAAATGTTTCTTCAAATGTAAAAAAGTGTCGAATTTAATTTACCTTTTTTTTTGCGGAC<br>TCAAGGAATATTAGGCTGCGCATGGATTCATATATAAGGCTTTTGCCTTATATATGAATCCAATATTTAATCCAGGACTTAT<br>AATCATACCACTCTGTTCTTAAATAATTTATTTTGCACATAATTAATTTTTTTTTCATATTTAATTGTTGTTGAATAATAAAT<br>TATACAAAATGTAAAAGAATGAAGCTTTATTTTTTATACAACAAATGTGGCAATCGAGCATTTTTCGCCTGATGGCGATGG<br>ACTACTGCACTCCACAAATGCTGGCAACACCGGAGAAATCCCTCGGCATGCTGACGTTGAAGTCTCAGTAGCCAGTTTACT<br>GCCTTCATTCTATTGGGAGGTGTTTATACCGCTTTTAAAGTAAATTTTAGAATTGCGACTACTTTTTTAAATTTAGAACCAT<br>TCATGCTTGTATAGAAGTAGGTAATAAGTAATTGTACAGGTCGATATTGGTTTTTTTTTTTTATTAAGCTCACGGTTTAGAA<br>ACTATGTCGAAATGAAGAGGCTTTTGATTTTCCTTTAACCTTAATCTTCTTATCAATAAAAGCTAAACAAATCTATTTTTAA<br>CTATTATACTGTTTTGTCACTTTTATTTAAATTGGGATTTTAGCGTAACAGACAATATCTAAAAGTTTTCTTAGAGGCCCAA<br>TTCATGGATTTGCTAGCGATAACGACGAATTAATTAACCCTTTTTTGCATGTTTTTTTATTGGTGAAATAAATCAAGGTGAT<br>ATAATTCATGTTAAAAGGGGAATTGTAAAAAAAGAAATTAGCTATATATTTTTTTCTGCAATGTACATTAAAAAGGTATCG<br>ACACACGATCAACTTAAAGTGAACTTGTTCTTTTATATAATTTTTCGGCATTTAATGTTATGATGGAAATTTCCACCATTAT<br>GCAAAGAAGGGTTAATTGATTAATACGCATAAAATGTAGTTTGACGTATTATTTAATAAATAAAATGAGTCATTAAAAAG<br>ATGCAGAAATTATAAGATAAAATAAATATAGAAGTTATAAAGGAATTAATTACTCATAGCTATTTGCCACGCAAAATTGT<br>ATGAAAAAGTTGAGCCGTTTTTCTCGAAGCATTTCTATGGAGACACTCTATCTATATAATCTAAGATATCTAAAAACTTCG<br>CTAGCAAACCAAATAATTTTTTATCACTACTAACATACATACATATCCTTCACGCCTAATCTAAGGTCTTCTGTTTATTAGT

		ATACATTCTGTTTAAACAGAGAAAAATGGTGAGAATAAAAAAACTACTAAAGTTTTATAGATACGCCGTATCAATTGTTTA<br>GTTTACATTATCATCAAGGCATACTTTCATGACATAAATTCGTTACCAACTAAAAAGGGTCGTGTTAATATATTTTTTATTT<br>AAAAAATGTTACCTATTTTCTGTAAATATTCCATAATTTTTATATCATAATTGAATGTGTGATTTTGTAAATAAATAGCAAT<br>TTTCTATTTATG
MSTRG.6699	Glycogen synthase	CTTGGACATACGTATTTGACGTTTCATAACCTACAAAAGTACAAGAAAACTATTTTAATTTGAATGTTCTACCCGCGTAGA<br>CCGGTATTGTGTCGCGATGTCTTTGTAAATAATTACTGAAGTAGTTATAAGACCAATAAAAACGATTTAATACTAGTGTGG<br>AGAATATTTCTGTGATAAAATACCGTACTTTTCAAGTTAGTGGTCGGTATACTTTTGGCTGTCTGTTTTCTAATTGGTAACA<br>ATGTCGCGCGACAGGACATCACGGCGGTTTTACAGGGCGGAGTCGACCCACGACCTAACCTCCTATCTGGACCGGGGGCA<br>GGCAGCCATCGACGAGAACCGGTGGACCTTCGAGACCGCGTGGGAAGCTGCTAATAAAG
MSTRG.6701	Myosin heavy chain	ACAACAGCTCAGCAAGAGCTAAGATCGAAGCGGGAACAAGAGGTGGCGGTGCTGAAGCGCAGCTTAGAGGAGGAGGCG<br>GCCGCCCACGAGGCCACGCTCGCCGACCAGCGGCACAAGCACTCGCAGGAGCTGCAGCAGCTCAACGAGCAGCACGAGC<br>AGATCAAGAAGGCCAAAGCCGCGCTGGAGAAGGCGAAGGCGAGCCTGGAGGCGGAGAACGCGGACCTGGCGACGGAGC<br>TGAAGGCGGCGGGCGCGGCGCGCGCCGACGGCGAGCGGCGCCGCAAGCAGGCCGAGGCGCAGCTGGCCGACCTCGCCGC<br>CAAGCTGCAGGACGTCGACCGCACCAGG
MSTRG.6702	Uncharacterized protein LOC101746464 isoform X1	ACCGTACCTGCGTTTCGCTGTGCAATGGTGCACCCAGCCGGTGGCCAACTCGCTGGACTTCGTGGGCGTGTGGACGGAGCC<br>CACGGCGGTCGCGGTCGACCCGCTGCTCGTGGCGTGGCTCGCGTACAAACCGATCGTCAGACCCAACACAGAATCTCAAC<br>CAACTACGCTCCCTACGAAGCCTGTTTCAACGCAATACTTCTTGAGACGCCGTACCACACCACCTTCGTCAAGCGGTCGCG<br>GTGCAAGCAGGACGGGTTCTGGTGCTGAGCTGGTGCACGTGAGGCCGCGGAGCCTGGGCTCGAGCAGCGAGCCCAGCGA<br>AAAGAAGGAGTCCAAGCCGCCGCCGCAGGCAACGAAGCCGCCCGAGCCTCTGTGGAGTGGTGAATGGTTGCTGCGCGCG<br>CACAGCCGCCTGAAGCACATGCTGGTGAGCGTGGAGCTGGGGCTGGTGTTGGTGTACGTGACGCCGCGTACCGCGTCCGC<br>GCTGGACTGCGCCACCGTACGCGACGCGATGGAGAGGCACGCCGCCGACCACAAGCACGTCATTGCCTTCAGTCTCGGTC<br>GCCTCTCTATGCACTCGAGCTCGATGACAAAACATCTGTGGCAGGACATAAAACACGATGGCCCCACGTTCGTCATGAGA<br>AAACCGGATTATAATCCGGACGACGATGAGTCGTTCCCGTGGAAGATGCGGCTAGCGGACATGTCGTGTTACACGCTGAC<br>CGCCCGGCCGGTCCCCGAGCCGCTTGCCCGCGACCAGGCCGGCGGCCTCCGCTCGCAGATCAAGATGGCGGCGGTGACTC<br>CGCGGACGGTGCTCGAGCTGGTCACCACCACCGTCAC
MSTRG.6737	Uncharacterized protein LOC106129042	TACATCTGCACTCGTGCGCATATAGACGTCTTCGATCTGTCTACCAGGCTACTGGAACACATCCAACATGAAAGGGTTAAT<br>AGCGTTCTTGGCCGTCGCCAGTGTGGCCTCCGGCGTCCAGTACAATGGATTACGAGTTAAATTCGGCTGGTCGGACGCCCT<br>CGCTGATGTGGAGTACTTCTACAAAATCCCCCGAACTATTAACGACGCTGAAAGTGAAGGTTGGAAACGCACAGAGCGTC<br>CTCCTGGAGCTCTTAGCGTGCTTAGGATGTACTGTCCCCCGGGGAGAGGCGTGTGTG
MSTRG.6746	Laccase-4-like	TATTCCGCTTTAACGTTTTCTTTTTGTTACAGCAACCTCCTTGGTGACCTACGCTGGAGAGCGCTATGACTTTGTACTTGAT<br>GCCAACAATGAGATTGATAATTACTGGATTCGATTCCGTGGTCTTATGGACTGCGATGAACGATTCACAAAAGCAAAACA<br>AGTGGCGGTACTTCATTATGAAGGGGCGATGGAGATGGAACCGGAAGGAGACCCGACGTGGGAGGAGCTCCACAACGAC<br>GGATTAGTAAGTATATATATTTTTAATATAAAAATGTAC
MSTRG.6749	Fanconi anemia group I protein homolog,	GCGGAGTCCAATTCAGTGGAGCTCTGTCTCTGTCTATCCACTTGTATCTACAACATAACACAGGGGCTCACTGAGCTGGAG<br>ACTATTAGGAAACACCTGAAGGCGTGGCCGAAGACCCAATTGCTACGAGCACCATTTTTGATAGATGTAGCGCTCGCATT<br>GTCTGACAAAGGGCCGGAATTTAAAGGTGTTTGTTTAAATGTGATCAAATCTGCAATAGAACATCATATTATGGACGAGCT<br>GCGAAGAAAAGAGTCGGCGTGGTCTCGCTCTGTATTGCCGCCCGACGTCGACGTAACCAATATGCTCAAAGTACTCACCA<br>CTGAAAGCGTCAACCAAGTCAATTTGACAGCAATGGGTCTAATCAACCTGGCGTTGTCACTACTGTCAGTGTCTCGAGCGA<br>AACCTACTGCGCAAGCGTGCTGGTCGTACGGCAAATTGATTCTGGTGCGAGTGAGCAAGTTAATACTGGAAACTGCACCG

		TACATACTGACGCAATTGGGAGACAGGTTGGCCGGGGACTCTAATCAGCCGCAATATGCAGACTGCCTGCAAGTCCTGTG<br>CAAATTAGCACCATTGTCTGTAGAGCGCTGCAGCCAACTGCGCACTATCATCGAGAACTGCCAACCATCGGGCAACGATT<br>ATACTA
MSTRG.6770	Coatomer protein complex subunit delta	CAGGCTTACTGACGCTGCGTATAAGCAATGAACAGTTTGGGCGCATACACGTGCACGTCGACAATAAGGACACCAGGTCT<br>TTGCAGTTACAGACCCACCCGAACGTGGATAAGGAGTCGTTCAGGTCCCAGGGCGTGATCGGGCTGAAGCAGGCGCAGCG<br>GCCGTTCCCGCTGCACAGCGACGTCGGGGTGCTCAAGTGGCGGCTCACGGGCGCCAACGACGACAAGCTCGCGCCGCTGT<br>CCGGTAACTGGAGTTCACTTGACATGATGACGTTGATGATATTGTTTGCTAAAGGAAGGATCCTTTTAAGGGAGAATAGAA<br>ATAATCTTTATTTGAATAGCGTCTTCTACCGCTTGTACAACGGAT
MSTRG.6785	Multiple epidermal growth factor-like domains protein 6	GTGAATGTATACAGGAGCTAGCTGTGTGGGACGCGAACAGAGAGATGTGCAGACTTTTCGCATCTATTGGTGAAGAGTGC<br>GAAGTGAACAGTGACTGTTTGGCCGGGGAGATGGAGATCCAGTGTTTGGTTGGTGATGACGGTACAGGCTACTGCGCGTG<br>CCCGGATGGCTATATCGAAGTGGACGGCTTGTGCTTGACCACTGGGCTTGACCTCGGAGACCCGTGCCAAGCGGACATGG<br>AGTGTACTGAAACAGCGAACACTGCTTGTCTCAACGGAGTCTGTTCCTGTG
MSTRG.6860	Serine/threonine protein kinase Akt	TGTGGTTCGTATTTCATAATTAGTTTGCGTTGTGTACGAGCGCGGTTGACAGTGAGTTGTGTGGCGATCAGGTGCCGCCGC<br>CGTTCAAGCCACAGGTGGAGTCGGAGACGGACACGCGCTACTTCGACTCGGAGTTCACGGGCGAGTCGGTGGAGCTGACG<br>CCGCCCGAGCACGACGCCGGCCTGCAGCGCATACAGGAGGAACAATTCCCGCAGTTCTCCTACCAGGACATCAGCTCGTC<br>GGCGCACTCGGCGCTGTCTCACCTGTCGCAGTCCGCGCTCGCCGACCGCCACCAGTAGCCGCCGCCCGCGGCCCTCGGTAG<br>CCACTAGTGACAGTCGCCCCTGGCCGCTCGACGCTCGGCTCAGTCGTCCCTGGCCATCTGGCACTGGTGTCAACCCCCTTG<br>GCCGTCCGACGCCGTTGTCAAGCCCCTAACTGTCCGACATCGTTGTTAATCACCCCTGGCTGTCTGATATCAGTAATAATC<br>GCCCTCGACCGCCCGGCACTGGTGTCAATCATTCCTGGCCATCCGGCATCGATGTCAGTCGCCCCTAGTCCGACACCGATG<br>TCAATCGCCCGCTAGTCGTCCGACGCCAATGTCAGTCGCTCCTGGCCGTCCGACATCGATGACAGTCACCCCCTAGTCGTT<br>CTATACGAATGTCAGTTTCACCTGGCTGTCCAACACCAGTGTCAGTCATCCTTAGTTGCCCGACATCAATGTCAGTCGCCCC<br>GGGCCGTCCGACACTAGTCCGGCCACGGGCGACCAGGTCAGTCACCCCTGGTGAACTACCGCCAGTTTTGCCACAGTGCC<br>AGTCAACCTTGGACGACTGGATGTTACAAGCTTGTGGACAAAATAAGATTCTGTCAGTGATATCCCTAATAAAAAAATTGC<br>AAAATGTATAAGTTTAACATAAGCTTTAGAATATTCGAGTGTTCTCTGGACGTAAATCTCATGCATTTTGTAGTTTTTTTTT<br>TTTTTTTTTTTTTTTTTTTTTTTTTATTTTATTAGTGATACCACTAATAAAATATTACTTGGGGTACAGGCTTAAATCGCTCC<br>CGGCCGACTGCCACTCGAGAAGGTTATAAACAAATAGGTATCACAGTATAAATACATATTGCCAATAAATTTGATCTTGA<br>AAATCAGTCGTCCTCTTTCTCACCATCACCGTTGAGTGTAAGAGAGATAGAGAATAATTCCATGTTTAATTGTGAGGTTGA<br>GAATGATGAGTAAGCGGGACAGAAGACGCGAAACGTCATTCGAGGTCAAATGTATTGGATGTTCCACTATTATCCGATTC<br>CATTTGGTTATAAACTTCTGTTTAAATTGTATCTTTTTTTTTTTGTTATTTAATACTGGCATGTTCTCCCCCGCCCCCTGTCGC<br>CCCATCATACTTCTGGAACAGAAGTATCTCAAGGTGGATTTAGACTAAGCGAGCACATGCTCTATCTTTCCTTACTTTTTAT<br>TTAGTCTAAATTGTTCGCCGATGCGAACTATTTCGTCGCTGTTCCTTTAGTCTAAACATAATACTTTTTCTATTCTCATTGTT<br>AAGTCTAAATCCACTTTAACAGAACACGGATGTAATGGACTATGTGTTTACATGTATATATAATTGTACCTTAAACCCCTTT<br>TATCTTTGCCGGCTCGATTTTGTAAATAATACATTTATGGTATTATGAAGTCTTTAATAGTAAATAAATAGGATATATTATT<br>ATATATAAGATTATGCAGTTCGTATATTATTTTTACTGCGCATTTTAATGTTGACTTTCACGAAATCAGGAACAGTCACGTT<br>CGCGACCAGCCGACAAAGGTAAACGGAAAGGGTATAGAACACAGACATAACGTAAACAGATGTCGCAACTACATTATAG<br>CGTGCCAAGTTGGCACCTTGAGAAATATTGCAAAGTTCTGTTACTCTATGTAAGTTTTAAACCGGAATTCTTAGATGAAAT<br>TCATGTCGAAAAATTTGGGGGATACGAAATACTTTTCATATACAGATAGTGATTTAGTAAGGGTTTTGTTGATTGATAAAT<br>TGAAACGCCACAGTAAATAGAAATAATATGGCACGCTTATTTTTCACAAAGGTTGAAGTTGCGTCGTCTGTTTACATAATG<br>TCTGCGGCATAGAAAGTTAGTACGCGGGGCGAGAGGGGAGGTGCGGTGAGGGGGACACGGGTGCGACCGTACGAGCGGC

		CACAATGCTC
MSTRG.6905	G-protein coupled receptor moody	CTGTAATAGATTGAAAGTGCTTTATGCTACTGTATTTCACTTAAATTTTCTAATTGTCACTGTATATTTTTATGTTTTTGGTG<br>CAATAAAGAGTTAAATAAATAATATAATATATGCATCGTTACAATATCATGGGGCGATTTTATCTAATAGATATCAACGAG<br>AGCAACATGACGGTGATACGGACACTTCCTGAGCAGTTGGAGGTGATAACCTCGCGCATCGAAATGGCTTATCAACAAGC<br>ACACACACACACACTTGGCCGGAGTAAAGTGTCAGTCGCGGACAAAGCCGAACGCGCGGCCACTTAAATGGCCCCTACAT<br>GTGAATTTTGTACAAATAATGTGCTGTAAAAAGTTAAAATGGGTGACTTGCTACCTAGTATACCTAAAAGCACGTTTGCTA<br>GTGAACTAGATGTGCATAATTGGAGTGTTGTTGGCCTGGACGAGTACAAGACAGACGAAGCGCTTGCAGCGGTTGAGCTA<br>TTCAAAGATTACCCTGAAGCGTTGCTGAGGTTCGCCTCTGTGTGCTGTGTTTTGTTCATGCTGGTTGGCATTCCGGGCAATC<br>TGATCACCATCGTTGCTTTGGCCCGCTGTAAAAAGGTAAATACAATTGTTTATTACTAGCTAATACCCGCT
MSTRG.6925	Endonuclease-reverse transcriptase	ATAAGATAGGATTTCTTTTTTTTTAATGGGTGAAAATAGATTGGTAACCCCGCCCGCGCTATTGGTATACACCGACGAGGC<br>ACCAGTGGAAGATGATAGGTGAGAATGAATGCAGTCGACTGCATTCTAAATTATTATTACCGCGCAACACGAAACGGATC<br>CGGTTGGAAGCGGGGGGACCGGTATGGCGGCCATGTTGAGACGTGACGTCACGTGACGAGTCAGCGGAGCGTGCGGGAC<br>GGCAACGCTGCCTGCCTTCTGCTCACTCTGCTGTGTTGCCGTTCATTTCTGTTGTGTTAATAATAATGAAGGCTCATCGCTT<br>TTTGTATGCGATGCGAGGATCGGTGAAAAAACAGTTATGCGGCCAGGTAAACTACTATGACGAACCAGTGAACACTGTGC<br>GCGACGCACACTAATAAATTCCAAAATGAATAAATAATAAGACGTTGGAGAGACTATTATAACATTCATTTTTTTCATAGA<br>TTCAATAGATACTTCATAGTACTCATAGAATAGTAAGATGACGTCGCTTAGGATTAAGAAAAAGAGACAAATTATAAGAA<br>CTATTGAGGACAGAAGGGAAAAAATGTTGGGACACTTGATACGACACGACGAACTTATGAACATTATAATAGAAGGAAG<br>AAGAGGAAGAGGGAGACCAAGGCATAATTACATCAGGCAGATCAAGGAAGGAGTTGGTGTCGTGTCGAATCAAGAGGTG<br>AAGGGATTCGCTGAAAATCGTAGTGAGTGGGAAGAGCTCCACCGACAAGAGCGCAGCTCTTTAACAGGATAGATAAATA<br>GATAGAGGGGAAGATAATTATGAGGAAAGGTTAGCTGTAGAGCCAAATGGAATATTGTATGGAAGATATTAGGAGAAAA<br>TTGTTCCAAATAGCAGAGTCAAATTTTGCCGAGTTTTGATAAGTTATTGATTTGAGTTTTTCATTTAAAAAGACATTTAATT<br>TTGTTAAATTCAAGAATGTGATATGCACACAAACCAATAGAAGTATGGTTTTATGCCGAAACCGAAATATACATTGCGAAT<br>GTCATGTTTGTGCTGTGTCAGTTAACTGTGACACAAATAAAAAATATGAATAATTATTTCATTAGAATTATTTTGTTTAATT<br>TTGCTGGCATTAGTATGTGTGTGACTGTCAACTCGCAAAGATTGACCACCATTAATAACCGTTCAGCACTCAAATAGCGAA<br>TAAACACGGTTAACACTAGGCTCCGAGCAGCGGGTCTACCATGAAATGAAATTCCGAAATTTCGGACTCAAATTCGTGTTT<br>ATGTTTATACCAAAATGGGATCAATAAATGGAGTTTAACATTTTGTTCGTCATACATCCTATCCTAAAAGTCCAAAGGAAC<br>AATATTTTAATATTTTTAATATTTCCAAACGGCATGTATAATGTAATTTTAACATCAAATTTGTGTTTCGTTTGGCTCCGGA<br>ATTTCGGAAAACACTTCATGGTAGGCCCTCAGGGCTCTAATAATTTGCCTGAAGATGCTTCATTTAGAGCACTTATCGCAA<br>AACGTGCGTGGACA
MSTRG.6941	Hypothetical protein KGM_206686B	TTTTTTTTTACATCGAGCGAGAATCACCTTGACGAGTTATGAGTTTGAATCTGGCGCACACCGACGATTAGTCGTGAAATTT<br>TGGTTGCGCGGCGCGGCGCAACGGACAACGCGCAGTGTTTGCAAATAACGAATGAATGAGCGAGTGAGCTGTCTCGACCC<br>CAGCGTAGGGAAGACAGGTCTCCGCGGGGTAAAGGGAAGGGGGAATGACGTAGACAGATGCGCATTCGTGCCGGGCTGT<br>ATACAAACAATACGATGGACGTAAATAAACACAAGATAGAATTATTGTGAACTGCTTTATGTTACTTGTAGATCTCAGTGC<br>CTATTCTGTCTGTTATTAACAAAAGCTATTTACTACCTTAACTGTAGTTGTATCCATCTATTGTAATATTTATTTAAACCTGC<br>TTTCTATGTGGTCATAAAAATAATAATAAATACCATACCAGCGTTACTTAGATGTTGGCGAAATATTTAAAGAATCTGCCC<br>TACTTTCTATACATAAGATATACCTATGTGTATTACGGAAATAAATCGAGGGCGCTAATAGTTCTACCTGTCTAGGTTGAA<br>GCTTGTAACGCATAATTATTTTCGTAGTTAGGTAGTACCTATCTGGAAAATTACTATTAAAATAGCTTGAATTTTTTTTTAA<br>TTTACTCAAATTATAACTAAGTACTTAACAAAATATTGGGGCTATGCATTTTGGAATACTTTTTTAATAAGTGTTATATAAA<br>TTTATAATTAATACTCCC

MSTRG.6946	Ankyrin repeat and SOCS box protein 3-like	AAAGTGTTAAGTTACAATAACCTATGTGCCAAATTTTTTTCAAATCCAGTGATTTAAAACAGCATGATTTCTTCACATTCTG<br>ACATCAAGGGACTTTCCCCACCTGCAGCCAACTCATTTATATCAGTGATGAAAGGGAAAATATTTTGGACAGTTTCATAAG<br>AACCTTTACAGCAAGCTAAATGGAATGCAGTCAAAAGGCTCGGTCCATTTTTTATATCAGCATCTGCACCTGCATGTAACA<br>ATAATGTAACAGCTTCATGTTGAGCTGCCAATGCAGCATCATGTAAAGGAGTATCACCATCAAAATCTTGAACATTAATAA<br>TTGCACCATATTCTAATAGTAACTGAATTAACTCCACTCTACCCTGACCACTAGCTATGTGTAATGGTGTAACATCTTCAGT<br>GCTTCCATAGTTGACCATATCATCTGCACTATCAAGGAGAGCTTTAATAGTCAAAAGAGAGCATTGTTGCCGACAAGCCAG<br>ATATAATGCAGTATATCCTTCGTGTGTCTCTGCAAGTGGTCTACAGTCAGGATGTTGTAAAATTAATAACAAACAATCATA<br>ACTGTCCATATTTGCTGCTTCATGGAGACAGGTCCATCCTCTATTGTCTGTACAATTTGGATTAATTTTTTTAAGTAAGCAT<br>CTGACTGCTTCTACATCATTATTTCTTGCAGCT
MSTRG.6976	Serine/threonine-protein kinase SIK2	GGTTTGCTTGTTCCACGAGTACTCAACTCGATCGACGATCGACTGGCTGGAGTTCAGTACCCGGTTGACAGCCGGTGAGGC<br>GGCGGCGTCACGCGAATCCCTAGCTTATATTACGCGCACGCCGCCTCTGCGCGATTTTTCTAATATAATGTAAATATTTTTT<br>GTATACACGCTTTTTGTCATAAGTCTTAAATTACCGTGCGTTTTGGAATCGTACCTCATCCTAATCGAATTAGGTTCAAATT<br>GTGCTATTATGACGGAACATCTACGTTTGATATCTGTCTACACTGTATAGTATTGGACCCCTTATATTAAATATCGAGTAAT<br>AAAACAGTTATGAATGAATAAATGTGTATGCACGCAGCTTAGTGAACTTGTGAACTTTAAAATAAGTTAAACCTACCTATG<br>TAGGTATAAACCGATCCAACACCTATTTATAATAGTGTTTAGATGTTAATTCTTATTTGTCGGCGTTGGTGAAAACAACTG<br>ACAATCGCAAATGGATAAACAGAAGTGATTCATTTAGTACCCAACTATTTAATGTTTACTTAGTTTTTAAATCACCAGATTT<br>CTCTGGTCAACTATGTCAAGTCTGGTGATTAACAAGTCTACAATGGCGGATCGTGAACGTCCTGCAAAAAACCCCATTAGG<br>GTGGGATTTTACGACATTGAGAGGACCATTGGCAAAGGGAATTTTGCCGTCGTTAAGTTAGCACGACATCGAATTACGAA<br>GACNNAGGTAGCAATTAAGATAATAGATAAATCGCAATTAGATGCCAGTAATCTTCAGAAGGTTTACAGAGAAGTGGACA<br>TTATGAAGAGACTGGACCATCCTCATATTATTAAGCTATACCAGGT
MSTRG.6994	Mitogen-activated protein kinase kinase kinase 9-like	AGGACATCGAGGTCATTAAAGAGAGTGTATTGCAAGAAGCGAGACTCTTTTGGGTGTTGGAGCATGAGAATATAGTGTCA<br>TTGAAAGGTGTGTGTCTAGAGGAACCAAACTTGTGTCTGGTGATGGAATATGCTCGTGGTGGGCCTTTAAATCGAGTTTTG<br>TCAGGTAGAAAAATTCGACCTGACATCCTGGTGGACTGGGCGATACAAGTAGCTCAAGGAATGGAGTATCTTCACTGTGG<br>AGCACCAATGTCACTCATACACCGAGATTTGAAGAGTTCAAATGTGTTGCTCAGCGAAGCGATACTACCGGACGACACGT<br>TGGAAGACAAAACACTGAAGATCACCGACTTCGGATTGGCTCGTGAAGTGTACAAAACGACGCGGATGTCCGCCGCGGGA<br>ACCTACGCATGGATGCCGCCAGAGGT
MSTRG.6996	ELAV protein 2	TTTTCACTGTTTTCTTTCAGCTGTTTCACAACCATAGACAAAAAATTAATATATGCCACGACAACAAAATTTATTAAAATTA<br>TGGAATAAAAAATTGACAGAAACCAAAAATATTTAAACACGTGCAAATAAAATTATTAATTTATTTCTTACATTGTATTT<br>CGATAAATGTTGTATAAGTTGTTGTTCATTCATAATTAATTATTATTATTTTATTGATGTTCTGGTAACATTTTGAACGCAA<br>ATTGTTCGAGCCGATCCGACGACAAATTGAGTGATGATGAACTTGTGAACCGGCGTTGCTTAGCATTTTCGTGTGGTGACT<br>GTAGTTTTGTGTTCTTTCTTTGTTCTTTATTATACATGTTGCAAATGAATTAGTCAATTTCTTATAGTGAACGTTTCATTAGT<br>ATTTTGTTTTTGGTAGTGTAGTACGTAAAGAAAAAGAACAATGTTAAATAACGAAATGGAGAATGGCGGCGGCGACGCCA<br>ATTCCAATGTGTCACCGACCAAGCTCATGGTCAATTATATACCCGAATTGATGACGCGCGACATGATGTACGCGTTATTCT<br>CTGCGATGGGCAAGATTGAAAGCTGTAAATTGATCGCAAACAGAGGTTATGGTTTCGTGGAATATGAAAAACACGAGGAC<br>GCTGAGAAGGCGCGGGCGGCGTTCAACGGCCTCCTCATGCAAGGAAAGACATTAAAGGTGTCATTCGCACTGCTCAATCC<br>AGAAAATAAAGTCAGCCATAAACCCGACACAGAATCAAACCTCTACATTAGTAATCTACCTCCTGACATGACGTTGGAAC<br>GACTTAACAGCTTGTTTAGTTCGTTTGGGGTCATAACCAATAGTCGTGTATCACAAGGCATCGGTTTTGTATGTTTTGAAAG<br>CCGGCAACAGGCAGAAGAGGCGATAAAACATTTAAATGGACAGACGCCAATACCTGGAGCTGGTGCTATAGTCGTTAAGT<br>TTGCTAACAAGCCTAATGCAAATAAGAACGCACCGCGACCGGCTCCGCGGGTCGGTGTGGGCGCTACGCCTTTGGCTTAC

AACGGAACTGCGGCGGTGTTTAACGGGACTACTCCAGCCGCGTTTAATGGCACGAACCTCAGTGCGGCATTTGGAGCTCG
ACCTGCCGCCTTCGCACCTGGATTTCCTCAAGCTTCACCACCGCCACTTCTACCGTCACCCGGTAAGGCCCTTCCTTTCATC
AACAAGGGCCAGCAGCGCTTTAACCCAATGGCTGCAACTAACCATACACCACTGCCACTGCTAGGAGCACCTGCCAGCCC
TGTTCCATTGCTGGGTGCACCTCCAGCTCCACATCAAACTACAGTGTATGTATACAATATTGGTGAGGATACCGAAGAGCT
AGCTTTGTGGCAGCTTTTTGGCCCATATGGAGCAATTGACTCTATAAAAGTGATCAAAGATCCTGAAACTAAGAAAAACA
AAGGTTTCGCCTTTGTTAATATGAGGGAGTATGATGAAGCTGCAATGGCTATTCAGGCACTCAATGGATACACGCTCAATG
GTCAGGTTCTATCTGTTAGCTTTAAGACTCAGAAAAGAAATAATTAGGATGTGGACGGACGTTATAAACGCCGTTTTATTT
GTGATCATACCCGGGCCGAAATCTTAAAAGAAATATTTCATTCCAATCTCAGAATATTTTTGTAGTCGAGAAGGAAGATCT
TCTTTATGCATTTCATAGTCGCATAAGTATTTTTGTAAGATTTAATATAGTTTTATATTTGCAAAGAACATGACTATCTGTG
AGTTTAGTTGGCTTGACAGTATCTTATATGTGTATCCACTTTTTATCATATAATATGGTATTATATTATTGGACTATGTATGG
ACCTGTCGCAGTATGTATATCTGACCATAATGGGAGTTAGGGCACGCCCCGCCTCGCTTGTCGCGTGCCCTGGCTCCTGTC
TTAATCCTCCAGATATTTTATTTTGTGTAAACAAAACATTGTATGTTTTAATCTTTAATAAATTTCATGTTTAAATAGATTTT
AATGTATATTACTCTATTTTTTTTCTATATACTTATCCCCTACCTATTGTGTCCTTATTTACTTACGTTATTTATACATAATAA
TAATAATAATAATAATCCATAATATAATTATTTGTGGCAGTAGTTTAACATGTACATAATATATAATTGTGTGTATTGTCTG
GCGGCAGGTCAGCTGTGTAAAATTGCTTAAATATTGCATGTTGTGTAGTTGATGAGCAAAAATATTTTTTATTTGTTTATCT
TACCTCAGGGTTATAGCTCTCGTTATTGTTTTTCCCGTCCTCAGTTACTTATCCAATATTATTTTTGGCGCAATTGCAAGTCA
GGCCTTCTAGTTTCAGAATTATCATGTTTTGATTTATAAACGTATTTATTTCTGACACCGTCATAGACGGTTGTTACGATAG
CTTTAATTTTGTAATTAAATAAGTATTCCAGTCAGTCCACTAGTGGTGTAAAATACTGTGTATTAGGTACATAATGGAAAA
TTTTAAATTCCTGGTTAACTATACGTTGTCGTTAGGGGCGTTCTATAGGACGTAAGATACTAACTTATAGGATAGATATTCC
AGTGATCTGTAAAGATTGTTCTATTGAGCTAACCTATAAATGAGTGAATTTTGAGACTGCCACAAATGTAGATAAAGCACT
AGATGCCTATTCCTCCGTGCCTCTTACTTGGACTATTATTACCAAACAACAGTTCTAAGAGTGAAAGTAGAAAGTAAGTAT
AACAACGTGACACGGTGGTGAGTAATTTTTTTTAATTAATAGTAATTGGTAGCGTCGTCGCTTAATCCCTAGAATTTGTAGT
GGACGTAGGTAGTGTATTCTTTAAAAAAAAAATAACCTATTTGTTTATTTTACGAGCAATTGTGCAAAAATCATTAAAGGAA
GTTACGTGATGTGATTAACCGAAGTTGCGTTGGTGTAGCGATAACAAATAGCACGGGAGATAACATTGCTCATTGTGCCTA
CCTACTGACTGATAACTAAAAAATCTACTACTATACTGTAGGTCAATGAAACCAAAGCACTTGAAAAACATAAGTACCTC
AAACACTACTATTCCATATAAATATAAATCATAAAAAAGAATGGTCAACAATCTAGGAAAGTTTATATCGTGTAGGTCAA
AATCTTGTCGTGTCCCTTCAAGGTTAAAGCCACGGATGAGAACAAAGGACGTCGCGCCATCTAATTTAGCAAAATATCAA
ACTGACGTACATCAATATTCGCTTAAAATTAATATATCTACTACAAGCTCGTTAAAACGAGTGATGGGTTTATCAGTAGTT
TTAGTAAGTAGGAGTACAGGACAGCAGATTTTAGGGATGGAATATTTGGGGCCAAATTCGTTTTTAAGTTATAATTTCATT
AACAAAATTGTAATTAATATGAATGTCAACTAGGATATCGAATTATAGAGATCAGTAGGAGCAACAAAATTTGATTAGCT
TACTGGTGGCAAATGACTAAATCCATTACTAGTCAGGATGTATGTTATTTATAGTTTGCTACCGAATTCTATCAAAGGAAG
ACTTAGTACAAATTCGCATACATGAGGATTTATCTCTATCTCACATATTACTATCGCCATACGAAGTCTATGCTTTCTTACT
TATGGCCCAGTTTACTGCGATTCAAAATCTTCAAGAGTCTAATTACAACACTCTTAGTGTTTAGGCATTTATAGGACGCGA
TAGCCGCTTACCATCAGGCGAGTCGTATACTTGTTTGTCACTTTCATGGTATAAGAGTCAATTTGACTATTATTTATATTCA
CTGCTGCCACTCCTTCAGTTAGTTTAACAGTTTTTTGCTATTTGAAGTAGGTTGGTGCATATTAATATAGGTACGCTTTTAG
TATCATTAATTATCATCGTATCATATTTAGAAGTAGTGCGAATTTTTTGTTTTGTGAATAAAGCTTAATATTTGCCTAGTCT
ATGCTGATATTTAGTTATGTTGCTCACTTAAATATCTTAAAATTATTTCGAACTAGTTTGTCTGCAAATTCATACAGGTGGA
TTCCAAAAGTAGAAAAACCAATGTAATACTACTTCTAGATGGCATATCGAGATAAAACATATATCAGATTTTATGTTAAAC
CTTCAGAAACAAAACTCCGAGTCGGTCTGTGTGAACTGACACTATGAGAAGTTTATTATGCTTTTCAACCGATTTCAAAAA

		AAGGAGTAGATTCTCAATTACGTTGTAATTTTTTTATGTTTGTTACGTCATAAATCCGTCGTTTATAGTTTTTTTTTATATAA<br>ATCGGTTGAATTGAAAAATTCTTTTTTTTTTATCTAAAAGGTAAACTCTCAGATTGGTCTTATAAGAATTTCATCAAAATAC<br>TTTCAATAGTTGGGGTTTAAACCGAAATAACTGGTTTACTACTATTAAAGTCGGTGTGTTGTTTTTTTGATGTCGGTGTCTC<br>GTTTTAGTTTGGCGTCAAGGATTCATAGGCTACCTAGTGTCATATTAAAAATTAGTTCAGGTATACGTAATGCATTTATAAT<br>AAAGAAAACTTTTTATATTTTCTAATATTCTACTTGTTCGCTACCCAACACAGATAACTCGAAACTATTCAATTATAAAATG<br>TTATTTTTAACTAGTTTTTTCTTTGTTACTTCGTATTTTAGAAGTAGGAAAAAGTTTAAACTAGGTTAAAATGACGTATCAC<br>GTATAAAATATGCACCAGATCTTAAGTTAGATCTCCAGGAACGTAACAGTGAAAACTGCATCCAGTTTCAAGTAGTAGATT<br>TTGCGTGATATTGGGACAAGCATACAGACAGACAAACTTTCTAAAATATTGGTTTTATTACAACCTATTATATTTTCTTATA<br>TATCTCATGTACAGACGTCATCTAGTTACAAGTTTTTTATATTAATAAGATAATATTGTTTACTAATTTAACACAGGATAAA<br>CTCCATATAAATTCTAAATTAATTGGGGGAAAACTTGTGATAGGTGCCAGGTTAGGCAGAGATTATTTTGAAATATAATTT<br>TGAAACTTTAGTTCCTTTTAATTTTGACTTGGTAGTTCATATTAGCTTTCGACACATAACATAGTCATGTAAGAATCTAAAA<br>AAATATAGGTATAAACTGCATAGATTAAAATTAACTGAAGTTACAGCTCAATAGATACGAGAATTTGAATTTGGGAAACA<br>GCAAAAACAACTTTGTATCTTGAGAACACTTGAGCCCAGGATAATTAGCTGAACATCTTTTTTAATGGCATTAACTATAGT<br>CCGACATGGCTTTGATCGTTTAATATATTTTTCTTATTCAACACAAACTTTAACCGCGAAGACATCAATTGACCGGGGTAGT<br>GAATTATGTTGTACGGAACATATTTTGATGTTATTATTTTTATGACGATTAAATAAAACACTAAAGTGTCTAGAAGTAGCC<br>AACAAACTCACTTTCATATTTATAAAATACCTAGTAAGGAGACTTAGCAGGAATATTTCGTATTTACCTACTAGAGTTGTC<br>AAGTTTCCCATTTGATGACGCGAGTATAGAAATCAAGTGTATGTAGATCTGAATTACGTTGTGTTAAACACACAATGCCAT<br>TACGGACTGTTCAAATACTCGTCGCCATAAATTGCCTTAATATTAAATTGAATAATAGCAGAACATTTATCATATACATAC<br>TTTACACTAACTGTTTGCCGCGATTTCGACGTGATCTCTTGTTCTATGAGAATTTTTAAGTATGAAATTTCATATTTCTGTTA<br>CAA
MSTRG.7017	Uncharacterized protein LOC110372193	CAAAGGTATACACTTGCACGCTACTGTCGGAAAAAAAAATATATAAAAAAATTGTATTAAGTTACTGTTTTAATAAAACCT<br>TAAGGCTTTACTCATAAATTGCCGTCTCAATTTATTAAAAAAACCTAAACATACAATTATCTGTAATTTATATTTTATCATA<br>AATAGAAGAGGCAAAATGCAATGTGGTTCACCACCTTATACATGACGATTTTTAAAGAGCATACTAGAAGGAAAAAAGAG<br>GGCAAAAGAGGAAAGGGAAAACCGAGAAATAACTGCATGGAACAGATAAAGGAAACAATAGGTGACATGTGTATAAGG<br>AGGTCAAAGATATGGCCTAAAATAGAGCTGGCTAGAAGAGAATGCATGAATTAGTTGCTACAACAAGAGGAAACTCTCA<br>AATGAAAAGAAATAAAATATAGTAGTATATAAATGCCGTGCATCACCATTAAAATTAATAAATGCTGTATTTATCTTAAGT<br>GTATGTGAACACACGATAGACTACATGATGAAAGCATAAAATAAAGATCGTCATGAATTGTCAAAACATGTATTTAACAT<br>TCGAATTTAGTGTGACTTCATCACATACTTGTCACTTATAAAATCTCGCTTCACGTTCTAAGGTGGCGGTGGCGGCGGCAC<br>GGGAATGAGGAGACTACATGCACTGCGTCCACTGCGTCCCACTGGAAATATCAGATCGTTCGTGGAGGCGGACGGCGGGT<br>ACCCGGGCGCCAACCACCATGACCCTGGCTTGCTGTGGACCGGTCTCGGACGGTAGACTGCCCAACCCTCCCTTCTCACAG<br>CACAGAGCCAATTTGCCAAATTTAGAGTCCACATACATTAAAACAACGCCCAAATTACCTGCAGACTTAAAACAATTAGC<br>CTTTATATATAGCCCTACCGGAATCAATTTCAATAAAGGGATTAGTTCGTCAAGTGAGAAGAAACACTCGGTCAGACCTCC<br>CCAAGTGAAGATTCGTAGTGAGCAGCCGAATTATAATTTGATATACGACGGTATAACATTGTCAGATATTGTACTAGCTTT<br>ACATGAATATTGTTATTTGTGAATTTTTGAAATTAAAAGATATTTAATAGATCATTGGATATGGAAATCATATTAATCCCTT<br>CTCAAAAATAAAAAGCAGCATATATATGTACAATCGTGTTCTAACAGTCGTCAATGCTTAAATACATACTAAATATAAGTC<br>TTATTATAGATTATTGGCGTCAAATTTTAACTTATTTTGATCTCCGTTTCTAGTCAATTTATCATAGTAAAAAATTGTACCTA<br>TTTGTAATTTGACGTCTAAAACTTGGCTAAGATATACACTCGACTTGAAACTGAACCTCCTTTAATGTCTTTTGAAGTATTG<br>TTAAATTGTAATGTTAGATTAAGCTAAGTATACTAAACAAAAATCAGTATTTTCCAGTAATTAATTTCGTATTTGTTTCAAT<br>ATTAATAAATAAAAAAAAATTACCACCTCCCTATAAAATTGCATTTCGAAGTAGTTATCAAAAATTATAAATAAAAGTTAC

		TTTAACTTATTTGAACCCTTGTCCGTAATTAGAAGAGTTTTTCTCATTTTAATAGAAG
MSTRG.7<br>027	Uncharacterized protein LOC106136340	AATAAAAATCGCATCTGGCAACGCTTGTTAGCAATATGTAGGACTGGCTGGGCCATTCCTGTTTTTAAATGTACTATGTTC<br>ACACGTATTTAATGTAACTTGTGGCGAGATGTATTTTAAATAGAATATATGTTCTACATATTCATTTTAAGGAAAACGAAA<br>TTTTAATTGGTATAGAGTGTGCCTATGTATTGTATATAAAGCTGTGTTTTGTATTGAGAGATAAAAGGATATAGAATGACC<br>TGGAGAAATAAATTATCCTGTTCATTTACGCCGGTTACAAAATGCACTATAATAGGATTTTATTCAATTGTAATTTCATTGC<br>TGTTGATAATGGCGTCAATTGTATATTTATGTGAGCAAGAACAAAAGAAGATAGAATTTTTGCAAAAAACACTTCACGGT<br>AGTACGAACAAAATATGGATAGACTTCGTAATTGTGATGACTTTAACTTTGTCTTCGCTTTGGATGATCTTCTCGGGAACA<br>CTAATCATTGGCGTAGTAAAGAATTACGTCGGCTTTGTGTTATGCTACTTCGTCTATGGGATATTTATTGTAATAGCATTTA<br>ATCTTGGTGCTCTTTTACTATTGTTACACATGCATTGGATTATCGCCGTAACCGAGATTATAATGTCACTGTTTACGTCAG<br>GATGCTGGTAATAATTTATGACGTCTATGATTTAATGCAGCGGGGTAAAGTCATCAATACTAACGAACAAAGTTTGCTGGA<br>CGAAGAGCCGATATAGAGGACTGCTACCAAAAACTTACAATCTTTATCAAAAGATTTAATGAACACGTAACGAAATTTTG<br>TTAAAATTATGACAATAATATCTTTAAATTAAATTCGTGACTATATATGTACTATAGATCATAAGACAGATGTAATGCATT<br>TTCCTCTCATTTGGGCCGTTGTGTGTATGAGTAGTGGCTAGTACAGCCAGCCCAGCTCCGCCCAGAAACACAGAAGCTTGC<br>TGGGATCTTCATAGGCCTCACGAAGCGACCTCACCGTTCCGAGGATTTCTAAACATTGAGGGGCCACAGCAGGTCATAAG<br>ACAGTCTCATAGTCGAGTCAAAAAATGTATATTATTATCAGTTGTCAACCGTTCATTGTTGAACATAAGCTTTCCCCATAA<br>GGGGAGTTTTGCCTGTAGTTGCCACGCTTGGCAGGCGGATAGGCAACCGCAGTTAGGCTTTGGAGATGTTTTAAGAGGGA<br>TGTTGCTACCCATCCCTCGCCCTCCCTTAGTCGGCTCTTACGATATCCACGGGAAAGAAAGGGGTGGCCTGCTCTATGCCG<br>GGATCACACAATGGTTAATTAATTATTTGTTTGTTCTCATTAATATCTGTTGAAACAAGCTATGTATACACAAATAAGTACA<br>GGTATATATTAAATTAGTTACTTGACTCCTGAGATAAAACGGTTTCGATTTGCTATTTCACTACAGATCTTATGTAAATTGG<br>TTGTAAGTAAATGCAGCCAGTGCTGCACTGTTAGTACTAATGTTATCTAAATAGGGTTGCCAATCCTCCCGGATTACCTGG<br>GAGACTCCGGTAATAATGGTTAACCTCCCGACCCCTTGAGTGAAATAGTTTCTCTGGTTTTTTTCTAAAAATATTGATTCAG<br>ATAATGACAAATTGTACGATTAATTGGGACTTTTGCGTGATATGCAACCACATCTCGTTCTTTACGAAATTGAAGGATAGA<br>AATGCCTGTCCTAAATTGAAAAAAATGGTCACGTTTCTTTCCATTCCTGTCTCTAATACTTCATGCGAGCGTGTATTCAGCA<br>TGATGAAGCAAGTATGGAGCACAGAAAGAAATCGAACGAATATTTCTTTAGTAAAAACAGAGTAACGAACAAACTTAAG<br>TACTAACGAACTTCGATTTTTCATGTAAAAACTTTTAGATGCTACTCGAAAACAAAATAGATATAAATTTAAAAATGAATA<br>CCTCCTCCTGTCTTCGATAAAATTCTCCCAGGTTGGTTCTTTCGAGAGTTGGCAACCCTATATGTAAACTACGCATTTTCAG<br>TTGAAGCATTTAAAAAAATCCAAAGATTTTATTTTAAGCTAATGAAATCCTCTTTTCTTTTCGAAACTTTTTTGAAAACTTT<br>GTAGGTTATGATAAATACAAATACTGATAATAATAATACCAGATGGAGACTGTACAAAAGTCGTTTGCTTGGGCACCTCTG<br>TCCCATTCGTGTTTCTACCATGAAACAATTGTGTTTGCTTGGCTGTGTTTTGATTTGAAGGGTGGGATATGGCAATCAATTT<br>ACAGGGTACAGAGGAATAACATCTCAGTCCTCAGGAATGGCAACGCATGCAGGGTATTATAGGCAAAACAGAATACTCTG<br>TTATGTCCATGATACTACTCGTGTCTATAGGTGACAGTTACCACTATCCATCTGGTAGGCCGTTAGGTTGTTTACCATTCTA<br>AATTGCATAAAAATAAGTATCTATATACTATGTTAATCTGAAAGGTAAATACGTAATCATGAAAACTATTTACTTGAATTA<br>CTCCTAATGATAATGAACAGGACAAATAATTTTATTGTAAGTATAAATTAGTTATTAAATGTAAACATTAAATAATGCATA<br>ATTGAAAAGAAAAATTCTACATTGCAAGAAGTATTTCGAATCAAAACTCTGTCATCTGAATACAGATGATTGATGATGATA<br>TAAATTATTTTAAAACTTTTTTACATATCTGGTTAATCAATACTGTTGTAAGACACCTGTATATTATAAATATATACCTTAT<br>AAAAAATAAATTACTATAAAAGTAGATATACCATAATCTACAACACCTGTTGCATTTCAATCAGAATAAACAATATTCAAC<br>ACGAATTTTCATAATCATATCAGCTTTTAACGGTCCACTGCTGAACATAAGCCTTGCCATGCTTGACATACGGATTGGCAA<br>TCGCAGTTAAACTTTAGAGATGTTTTAAGAGGGACGCTGCTGCCCATCCTTCACCCTCCCTTAGTCGGCTCTTACGACACCT<br>TCGGGAAAGGAAGGAGTGGTCTACTCTACGCCGGGACCACACGGCATTTTATTATCTAATAATTATAGCGTACTTAACTAT

		TACTAATTTTAGTTCTATTGAGGCCATAAATAATGTAATAAATTTGTTTTGTAATCAATACTCCGATATAGTCGATAATCA<br>GAAATTAATTGTTAATTGTACTGTTGATTTTATACAATTACGTTAATCAGTTTTGTTGTTTCATTACTTATAGTTATAATCGA<br>TACTCTTAATAACATTGCATTGATTATTTATCGATAATTGTAATTGATTTAACTAGGTAAAACTTTATTTTTTATTTAATATA<br>TGCGCGCGTTAACTGTCCACTGCTGAACATTATTTTGCCGTAGTAATTTAACAGATAAACACGTTACATTATTAACATTGTC<br>TATAAATATAAGTGAGTGCATACTGGATGGAAAAAATTAACTTTCCTACAACTTGATCCAACGGGATTGTAACTCACATAT<br>CTCTACCCGGTCCGACTCGATAGTTTATATTTCCAGTTATACACCTGATTGGATACTATTTACCATATTCAGGTTAAATTTG<br>TCCATCTAGTATATTTTAAGACTCCCAAATTCAGAATTAGCGTTTAATAAAATTAATATAGACATATAATAAGTATCATAA<br>GACACCAAAGATATGTTTATTTGTTTAAATTTGACGGTAATTAGGAATCTTTTTGTAATAAT
MSTRG.7098	Ubiquitin carboxyl-terminal hydrolase isozyme L5	TGTTTAAATAATCAATTTCAATCTTGAATCACACACCCAAGCGTAAATGTCATTTTCAAGAAATAAGACAATGCCTTGTAA<br>ATAAAATCGTTTCTTTTGCTTGTGTGCTGTACGTAGTTTTTACTTGTAGGTTAAGTGACTTGCAAATGAAATGTTTTTTACAC<br>AATTAATATCTGGATGATACAACAAATTTAAATAACCTGCACTACCTTTCGCCTATCACATAATCAAGATGGAATCTGCCG<br>GAGATTGGTGTCTCATTGAGAGTGATCCAGGTGTTTCACACAATTGATAAAAAAATTTGGTGAGTGTATTAGGTATAAGT<br>ACTTTTGTATAATCATATATCATGTTTGGTATGTTTTTTTTTCTTAGTAAATAGGTTGCTTGCT
MSTRG.7099	Fatty acid synthase-like	GCAGGTGGAGCACGCGTACGTAAACACTTTGACTCGCGGCGACTTGTCATCGCTGCGGTGGATCGAGAGTCCGCTGAGAT<br>TTGCCAGCGAGATGCAGTGGCCGCCGCACACGGACCTGTGCCGCGTTTACTACGCACCGCTCAACTTCCGCGACATCATGT<br>TGGCCACCGGCAAGCTGCCGCCGGACGCGCTACCGGGCAACCTCGCCGGACAGGAGTGTATCCTGGGGCTCGAGTTCAGC<br>GGGCGGTCGTCGAGCGGCGCGCGTGATGGGCATGGTGGCGGCGCGGGCTCGCGACCAGCGTGGTGGCCGACAAGG<br>GGTTCCTGTGGGAGGTGCCTGCGGCGTGGTCGC
MSTRG.7102	Uncharacterized protein LOC106143034	AGCTGATCGCGTTATCCAGAACCTCGGACGTGATAGGCGCCGGTTGCGCGTTCCCAGTCAACCTGAACGAATTGGACCCTG<br>AGTGTCAGTCATCGTGCGGCCTTGACTCACCTCAAACATACGAGAACCAAGAACCGTTAGAAAAGACAAAACCAGATAAA<br>GAATTGAATGAAACTTTCGCCAGACCTCTAGAGCCCTTACCCAAGGATAAACTACCTAATATCCCGGTCCTTGGAGATGGG<br>GAGAAAAACCATTCGTCGTTTAAAAATGACAATGACTCTTTCAAGAGTCAATTGCATGACGAAATTATAGCCGAGTCCAA<br>TCTTTTAGACTCTAATAAACAGAACGGAACAGAAAGTGCTAGAATGAAACGGGATATAATTCAGGCCCTTGGTGTAGTGA<br>AGAGGACAGCTAGGCAAACGAATCAAGAGGTACTGAAGACTAGCTCAGAGGAAAATTTTAGCGATGATGAATGCGAGAA<br>AGTCAACGTGGGCATACTGTCCAGCCAAGCGATAACAAACACCTCTATCTTCGTGGAGACCGTTTGCCCCAGGGTATCATA<br>TACCTTCAACTACACCGTAAGGGTGTCTCACTGCATGAATTATA
MSTRG.7106	Uncharacterized protein LOC101746464 isoform X1	CAGAAGCCGTTCTTCCTGTCGGTGGAGGTGGGCCGCGAGACGTTCGTGAGCGGGCAGCTGCAGGTTTGTCCGCGCGAGCC<br>GGGCCGCTACGCAGCGCCGCCGCCCGGGCAGCTAGCGCTCGGCCACGCCGCGCACATCGCCGTCCAGTGCAGCCACAAGG<br>TGGCGCTACTCACGATGCACTACGAGATCAAAGAGGAAATAAACGTGCTCGGCGTCGGCTCCACGTACATGCTCCTGAAT<br>AGGCTCGAC
MSTRG.7110	Uncharacterized protein LOC110382705	AGCCCGACACACGTACCTCTCACATCCATTTAACTAGTAAGCTACGAGAAAGCCCATTTACAGTATCAACTATGAGAACAT<br>CGATCAGTTGTAATTATTTCTTTCATCGTCGCAGACGCGGGAAAACTAATGTTAAAAGAAAGCGGAGAGAAGACGCAGCC<br>GACGACGCCGGCTGCGTCGAGCAAGCTCGCAGGGCAACAGAAGGGCGTCGTGCTAACTGCAACGCCGGTCAAGACGCCG<br>CCCAAGCCGGGCGTCAAGATATTCACTATCAACAACAAGCTGCTCGCCATCCCCAAGGATAACAACAAGTTACCCATCAA<br>GAAGATAATAAACCCACAGGATATGCAGATCGTGAACGTGGATGGCACGCAGGTGAAGTTCGTGCAGCTGGCCGC
MSTRG.7116	Hypothetical protein RR46_09733	AATCAATTAAAAACAAAACGGAGTGTTTACATAGGTACCTTAGGGACATTTTTAAGTACCTATTTACGTACTCTTCTTAAC<br>AAAACGATCAATATCGACGTCTTCGTGTCAATGCAATTTTTGTGACTCGAATTCTCCATAAATCACTATAGTTAAATAGTA<br>CGAAATTTACTTTAACAAAAAATAAAAGCTTTCTTGTACGCTTCTAACTTTATTGACATAAACAACTACTAGTTGCACATA<br>ATCTGAATGTCTCGACTTGCGACTACTACACAGGCACCAGAGGTTTTAGTTAAACCAAGCAAAATGTTTTCTTACTGGTCA

MSTRG.712	Transcription initiation factor TFIID subunit 1-like	TTTAAGATGAAAGCAGGTGCTCTGCTGCTGGTGCTTGCTCTCTACATCTATTTCCTCTTATTTATCAAAACTATTTACTTAA<br>AGAGCAAGAGGCTTGATCATATTATGGCAATGACTATGGAGACAGAAAAGTTAATGCACAAGGTGCATACAATTGATGAA<br>AACCTGGCCATGAAGATAGAACGGCTCGATGAACTATATGAAGATTATTAGAGATGTATTTGTGTAATTGTAGACAAATT<br>AATAACGATGTACCTATTAATTTGCCTACAGTAACAAACAAACATTATTGCCAAGTTCTTCTATGTATACCATGTGGAGAA<br>ACTGTTGGACAGCAGATGGCACATTACAAATATGATTAGGTACCACTGCATCATTCAATGTACACTTGTACTTACTCATAC<br>TTAAATATGTATATACTGCACCTTTGTGGATCTGAATCTTCACACCATTTGGACTTTACATTCATCTCATGATCTCTCTTACA<br>CAAAGGTATACTATAATCTCACCAGTTGTTAATTCTCTAACATATCTGACTAAAGTTCTGTGCATTTCTCTGCATTTTTATA<br>GTTTTTGGCTCAATTTGAATTAAGACTTTTTTATTGTGTTTGTAGTGATTGTTGACATATTTGCCAAGACTGTCATAATATAA<br>TTTTGTTAAATTTGTTGTTATATTAACACACTGGCTGTTCCCGCGGCTGTGCCTGCGTGATTTGATAAAGTCTCCTGTGTATT<br>ATGTAAGGATATAATCTACCCGTATAGCAAATGTCATTCAAATCCATTGTCATCATTTAAGTGTAAAAGAGTTATTAATAT<br>TTATATAATATATATATATACCTGCCATCGGCCCTTCTACTGGCTGTAATCAAATCTGTTTACGATATGTATTTAGTTTTTAG<br>GATTTTCTCGTCCATTGACGTGCCACCCATTCCAATACGTCTATAAATGGTCTACTTATAATGAGGCAAGCAGGCGTGGTG<br>TTTTCAGATGGAACGCATCCCCACTCGTATCTTCAGGAAGCTTCACAGCTAACTTGATGGTACATTATGATATTGAAGTCG<br>TTGCTTGTATGTTATACATGACTTGTTCATTTCGGCGACGTTCGAGTCGCCGAAAGTCCCGATCAACGATGTCTCCGAGTGG<br>CCCGATCTCGGTTGGGATATTTTCATTATTGTTGATGTGTCTCTAATTTATTTTAACATTTATCCTACGGCAAAATTTTTTT<br>TTGTTCATATTTTATTATAGTAGAATTGTCTGAAGTGTCGTGCACGATAACTGAACTATAACATTTTGTATGTGACGATATT<br>GAATTAAAACACGAGTATTTCATACGTAACATTTATTGGGTATTAACTCGGTATAATGAAGTCCACAATTTGTTAACAATA<br>TTTTATTTATATGAATAAGTAAACATGCAATATATATATAGATTAATCTAAGATGCCATAATTATTAAACTATCAACGAGA<br>GGAAGGAGTTGGTGTCGTGTCGTATCAAGAGGTGAAGAGACTCGCTGAAAATCGTAGGAACAAGAAGGGCTGCACATCG<br>GGAAGATGGCGCATATGGTTGAGCACGTCCTCGAGCAATGAGGAGAGCGTAACAAGCGGATCGGTACGGCGTCGCTCAG<br>CCGGCCGTCGCACCAAATAGTCACAAGCCTCGTTCGCCCCGCCGCGTCGCTTGCCACGAGCCCCCATACCCCCCATACCAA<br>AACCACCACCCGAACGGAAATCTCCGCGGCGGGACCCACTACCACGACGCC
MSTRG.7140	N-acetyllactosaminide beta-1,3-N-acetylglucosaminyltransferase 4	ACAAGCTGGGCATAAAGCGGCAGCACGCCCCCGAGTTCTACAACAAGAAGGTGGCCGCCCACCCGTGCGCGGTTCAGAG<br>GGGAGTGGCCATACATATGGTGAGGTATCACGAGCAGTTCGACCTGTGGCGGAAGCTGTTAGACGGGAAGACCAAATGCG<br>CCAGCTAGTGAACAGCGATGCGATAGTTTTGCTGAACAAATAATGTTAACGGAACAATACTACTTTTAATACTGACTTACA<br>TAGTCATAGTGTAGCCAGACAACTTCGCTCGCAGCGCTACTGCGTTATTGACATACAGGTATTGAAGAAAGAGGGGCAAC<br>TCTATTTTTGGATTAGACGCACTTTTCTCTTCATTACCACAAAAAACGTGTTTAAATACAAGACTCAACCTCAAAAGATGGT<br>AACCGTGATCTCTAAACGCGTACAAGTCTTATTTGGTGAGGTATAATTTACGTTTCGTAAGTATTAATCATGCCTCCTTACT<br>TTCCAAGCACCCAGCGCTGTCACTACGCAGTACTGCGTTCGAAGTTGTCAGGCTATAGCTTTGTTAGTCGCACTATATACG<br>TATAAGTAATTATTAAAACATTAAAATTGTGTAGTGCAGTTTATTAATGCAGTCGATGGCGTAGGCTTTAAAATAATTTAT<br>GAAATCTACAAATCTAAGCCGTTATCCGGTTATGCAATTCCTGAATGGAAAATGTCATTTGTTATATGCCATGCCTTACAT<br>GTTTTGGAGTTTTACTCAATTCGCTTTTGGCATAGACAAGAGTTCTACTGGATTTTTTTGCCCCACACGTGGGCCATTAAAA<br>AGCTAATTACATATTTTATAATTATAAAGTAGAAGTTAGTGATTTTTATGGGTTTTTTATGCCATAAAATTGCAATAAACGT<br>TTATCTATTTATATTACAGTTTATATATATATATATATATATATATATATATATATATATATATATATATATATATATATA<br>TATATATATATATATGTGTGTATAATTTTTAAATTGTAAATAATATGATTATATAATATTTATCTAGACACGATCTGCGACA<br>GAACAAATATAGGTACTATTTACATAATAGTGATGTCATAAACATCTTTGCGAGATTATAGCTGTATATAATTGTTTACTTT<br>GGCTCGTCAATAAAAATAAAAGTAGTATTGTTACCAATGTCTGAATCTCGCTGGTTTAATTGTTTCTTTTGATAAAAAAAA<br>AATAAATAAATCTGATATTGTACATTGATATATGTTTTTATTTTTTATTTAAATCGAAATGTATTTTTTATAATCGATTATTG<br>CTATTGCGTATAAGCTGTGTGATGTGTGAAGGAACATAGTTACAAATGTTGCCATAAAGGTAAAATTGTATCAAAATATTA

		TTATACCTATATGAAATTGTTAGAATATTGTAAAATAGTTATAAATGTTTCTATTTAAAGTACAAATGCGTGCATGTAGTGC<br>ATGCGGCTTGTATATTTTGTATGCGAGGCGACAGTGCTTTATAGTTACGTCAGAGTATATGCCATAATGTTACCCCGCCATT<br>GTGGGTTCTAAAAGATTACGTGTGTAGATTTTCAGAGGGAGACTTTGTACAAAAGTCGATTGCTTG
MSTRG.7143	Serine/threonine-protein kinase RIO1	CAACTCTAAGTCATTTATGTCAACATACACGTGCTTTATTTAGGTTATCATAGGTACTTACCTAGTGTCAGAAAACAATTCG<br>TGTTGTTTTATTTGTAGAATGTCTGAAATTGTGTTTTTCAAACTTCATTGCAAAAGTTATTAATATTTAAATAATAATGTCTG<br>ACGGATCAAGCGGCCAGTTCAGTGACTATGAAGAAAACATAATTAAAAAAGTTAAATCGGTACGGTTTGCAGAGTTGCCT<br>ACTTATAAGAAGGACAGTAGCAATGATGACGTAGATTCGGATGACTACTTCTATGATTCTGATGATCCAACACAAAGTAC<br>AAAGAAGAAAGAGGGTATAAACTGTC
MSTRG.7158	Intraflagellar transport protein 140 homolog	GTATCATTTTACCACATTTTATACAATAAATTTATTTTGAACATAAAAATCATTTGTTTTTAGCATACAGTCTGAACTCAAG<br>TGATATTGTTAACAATATCTTCAGATTTCACAGTAATAAGATTATTTTACCGTGTGTTGATGTTGGTTACAAAAATTGGTAA<br>CAGCCATTTAAAAGAAGTCCTATTTGTGAATTTAAAAAATTGTCCAATTATATTATTATGTCTTACATCATTAATAAATGTA<br>TTCTTTACTGTATTCAGGTGTGACAAGTGGACAACAATTACTCCGCGCGCTATCCGGTCGATCTGGACTGATCACCGAAGT<br>GAAAGTACTTAAGTTACTCCTACAGTTCGCTGCTACTCATCCAGATGCCACAAAATTTGAAGAACAGTTAAGAGCATTACA<br>AAAACGACCTGAAGAAGAAAATAACTCCACTGATTCTCCTGAACAAAGTATAGAAGAAATAATATAAATCATTTATGTAC<br>CTGCAATATTTAAATGATTATAGTGATGTAATGCTAAGAGACGATGTTCGCGTACAGTGCCTTGCTGTGGGAGAAGTCTAT<br>GTGTAGCAGTAGACTGCTTAGGATAATTATAATGACGTAAAGAATAACAATGTAAATACTGGCGTCGGTTCTTCTCTAAAC<br>CTATCTCTAAAATTTAAATTTCAACTTGATATCACAGCAATATTTGTATACTAAGAACTAACTAATATGAACTAAATTTCTA<br>ATAGATTTAAGGCAGATTATTATAAATTAAGTCTATAATGGTCTATTAAACGGTAATTTCGCGAAAGCATTAAAGTACTAG<br>GTTAAGAGATAAGTTTAGAGAAATTATGCCTCAGAATTCGACCCCACTCTCACAAGTTGTACAA
MSTRG.7163	TBC domain-containing protein kinase-like protein	TGCTAGAATCAGGGTTCAACGAGTGCATATTACTGTTCTCGGACTTGCCTGAAATTGATATTGGGGAATGTGTCAAGGAGT<br>CTATAGCCATGTGCCGCAGTTCTCCGCGCAGTATTAGTTATAGAAGGTTTACCAACGAAGCGGAATTAAAAGACCCCATG<br>GACGTGGTGGAAATACCAATGGAAACATTGTTCACGGAGATTTGTCCAAGGATCAACCTCTCCGATTTCTTCTCATTGATA<br>TGCCAAAACAAGTGTTGTGTTATTGACATAAGGTCCAATTTGCTATACGAGAAGAGTTGCATCGAGGGCAGCATCAACGT<br>ACCGTTCAGCGGAGTGCAGCTGGGTCACGTGTCGCAGCGTGCACTCGGTCTGCCGGCGCAGCAAGCGCTGCAGAATGCTG<br>CTC
MSTRG.7165	Hypothetical protein	AAGAATTTGCCACAGGCCCCAAATGGTATAGTTACGCCACTGAGCGTAGGCGGAGTTACCATGACATTTTCACCAATAAA<br>AAAAAACTTACTCCTTCATCCACTCATACATATTGTATGTAATGCAGCAAAGTGGTGGAGAACGGCGAAGAGATGTGGAT<br>ATTCAACGGCGAGTACTGGAAGGCGCGAGAGGCGGGCTTCAAAGACAACACCGCGCCCAGGATATGGTGACCGCGTCTG<br>CTCATACACCGTGTACCGCCGAGCGAGGTGCTGTGCTAGAGGTTGTGTCTCGTCGCTTCCCGCTTCTAGAGCTTTACACAG<br>TAGCCTTTATTATAAATAACTACTACCACATTGTACTGTTCGTTGGACGGGACATATACCCGTATATACAATAGGGTGCGT<br>GTACCAATTCACCACAACCTTGTACACTGTTATATTAATATCATGTTTATTTGTGAATCAATTATGTTGTCGTTTGACATCTT<br>TTTTTATTATTATTATTATTTATTAATTTATTTACAATATTTTTATTAAAAAAAAAAAGTATTTAAATTATGAAATAGTGTCA<br>ACTTAGATTGTTATCCACACATCTCTGGGAATACATATTTTAATTTGCACTTGGCCTTGCCTCACCTTACGGTAAGTGGTTA<br>TAAAATGTAAGATTATCCGCGCTTACCCCGAAATTACTTAACTGTCAACATAAAAAGACCTAAGTTGTGTTTCCCCAGGAA<br>CACAGATAAAGACATTGACATTTGACATTTGACGTTTGACATTTTTATTAAAGTGTCAATACCAATGTAACCAAAAGGCAA<br>TTATAGTAATGGCAGCTTGAATTTACTGATTTTTTTTAACTATAAAATAATATATAATATTGATTACTTAAAAAAGCTGTTA<br>TGTTAAATTTTACCAACTACTTACCTTAGTAATAATCACCTTATTTACTGATTGGTATACATTTTTTTTTCATAAATACATTA<br>ATTAAAATAATAATAATTATATATATATATATATTATAATTTACAGCTATATTTAACAGCTATTGCTGCAGATATAGACTGT<br>CGCTTTTGTATAATTTAAAATTATTTTTAATTTTACAAGTACAATTTACAATACTACATTTGTGGTTGGTTAATTGGTACATT

TACCTTATTAATATGGATTCCGTATAGTAAGTGTGGAGATTTGAGAGTTTTACCAGTTTAATTAAAATTTATAATATAATTT
GTTACCATCGTAGTAATTATCGCATTATTCCTTTTATACGACATTGTACGAATGTATATTATGGCAATTATTTGTTGCTTTCA
AAGTAAACAAATTTCAACCTTATTGTCTTGTGCTATAATTGTTAAATTAATTTTACTTGGCAATTTTATGCATATATGCTTA
ATATACTTTTTAGGTAATGACATATGATAATAAATAAATTATATATATATATATATATATAGCTAGACGCGCCATCTTGGT
ATGGGAGACTCAACTTTTATTTTTAATCTATGTTTTTTTGGAAAAAAAAATCACATTCACAAGTCGAACGTCCTTTAAAATGA
TGCATTTCGATATCACCCTGTATATGAGGAGTCATACTGTATAAGTATGTACGGAATACTGAAATGTATGGACAAATCACT
GTATCTAGTATTTAAAAGGACAGTCAACACAAAGTTACCAAATTATACTTGTCACAACACAGGAGTTGGCAATGCAACGC
ATCTGGCTGGTCGTATGCTGGCTTGACACGTTCGTTGTATAAAAAATAAAATACATATAGGTTAATTATGATTTTACGTCAT
TGACACTTAAAATGCCTTATCTTAGCTCTCTCTCGCACTTATTACAAGCTGGATTAACATAGCGTGAATGATATTTGGAACT
GTGAGAGTACCAGCATGGATTACTACCTTACGGCGAACGAAATAAGGTCGTTATTGCATCCTACAAAGTATAAAATTAATT
AAAAAAAAATCCATCCGTATTTCTCATCCTTACTAATATTATAAATGTAAAAGTGAGTTTGTTTATTACTCGTCGCTTCAAC
GAGCCGCTCATCATAAAGTTTTCATTATACAAATAGTAGCCATTTTGCATAGTAAAGAAGGACATAAAATGCTGTTTCATT
CTATTCAAATAATATGCCCCAGTCATGCGGACAAAGTCACGATCATGTCTTAAATATTTTAAAACAAAATTCCAAAAAAAA
AACGAAAAAAAAAATATATTTGCCAATTTTTAATCATTTTTTACCGCCATATTCATTATATATGTGTATATATACTATAATAT
ATAGTCCATAACGACTGTATGTATATCTGTGTACCCCCTTATTTATAAATCTTAAACCTACTAACAATATTTTAAGTATTAT
ACAATTCTGTCACTTTCTATCAAATTAGGAATTCAACACCAAAGAAAGAGACAGAACTTCTTAATAGCTAAAGTGGAGAG
AGATTTGACGTTTTTATAAATAAGGGGTATTATTATTATTTAGCTTGTTGCACTCTCACAATTAAAACTCTATAAAGCTGTC
CAGACTACACGTTACGCCAAAAAGCAGGACGTACGTACATCCAAAAATGTTACCTTTATCTGTGACATTCATAAAAAAAA
TTATATTTCATACTAAAGTCCTATTTAGAGGATTACAAGCCGGGCAAATTGCCCAAAATAGTAAAATGAGTTCTTTGCGAT
TTAACATACATGGTCATACCCGAAACGGCAATATGACATCATAAACCGTCATTGGTTATTACTAACTCTCCTCACATACTC
GTTTGCCTTCATTCGCATTAAAAAAAATTAAATGTTGGTACTCTATACATTGTGATAGTAAATAAAAAAATATATATAGAA
TTATATTGGAATATTTCGGCAATTCCATAATTTCAGTTCAATTACATCGTTGCGTAGTGCATACAATTTTAAAATTGACCTA
GTGATAAGTGAATGCGTATGCGCAGCTACAAAATCTAACATACTGTCTAGCCAACCGTTGTGGACGTTAGTGCGTGGCCTA
ACTGTGGCTCGGTTGTCATAGACTATTGTAATTGATAAGTTGTTCATCATCATACCAACTTTTAACTGTCCGTTGCTAAGCA
TAGGCTCTTCCCTTCCCCCTCCCTGGGAGTTTTGCCAGTAGTTGCTACGCTTGGCAGGTGGATTGACAACCATAGTTGGGCT
TGGTGATATTTTAAGAGGGACGCTGCTGCCCATCCATCCATCCATCTAGACAGTTAAGTTTTTATTGCTTAGCAATATTTCA
TTTTTTTTTTAAATTAATTATAACATAATGGTAACCCCGCCTGCTTTGTCAGTAGACTTTGGCGGGGCACCAGTGAGAAATG
ACAGATGAAATGAAGTCTGATCTTTCGGCTCATCATGACGGCTTCGCCAAGAATTAACCACGGTGGTTTCCAAGGGACAA
CACACTGTCGTCCGTCTAATAGATTACATGTTTATGGCTAGCAATAGTCCCATTAATCCCATAGACAATCGCCCCCTCCCTA
CCTTGACCTTGTTTTTATACCACTGAGGTGGCCGTACCGTAGCCTATGAACGCCTGCTAATACTAGGGGCATTACGCGCGT
ATTACGTTTTATTAGAATCGTGTTACAGCTTTGTCCCCAATGATTGGTTAAGCAATATGCCTTTAGATCTTGTTGCTATGGA
TACGGCTTAAGCGTTATCTAATTTTTTTTTCGTTTTATTACAAAAAAAAAAGTATAAATTATAAGGTTTTAGAGTATTGTGG
CTGTGAAATGTTTCCATTAATGAAAGGATAATGTTAGATCTTTTTTTTTTTGTTGAAATTGCCTTATTAATATAATTTTCTTT
TCTTGCCAGTTATAGATTGATAGGTGTCATAGACAAATATTGAAAAAGGGAACGGGTAGTCTATGAAAATTTGGTTTTGAC
GTAATATTTCTTGACTTCACTGTTTGCGTTGTGACTTCTACATGGAACTTTATTATATCGATTTTACAATATTATTTAAAATA
CTTGTGTTGTATTTTAAATAATATATAGTCGTATGACTTCTTGATTTAACGTTTTGAAAAAAGAATCATATGACTCTGCTTC
TTGAAGGATTAGCCTACTAAATTACAGAATAAATGTGTTTGCTAAATCTATCATTCAAATTTTGGGCTTATCCAAATGTCTA
ATGAGACGAGGCGTATTGCAGAGCTTTTTCACAATTGAAACATCGTTTTGATGACGAAATAAAGTACAATTTTATACATCT
GTAACGTCATAAATGTGCCGCTTATATAGGTACATTTAAAGGGAAAGTGGCGGGTGGAACTTGCCCACCCCAGAATTCAA

AATTTTTTTTAAGTCTAATTTTGTGGAACCATCACCACAAGATGTGTATCTCTGAGCGCTTCTAATACAAAACCAGCTCCTA
AAAATAATTTTAAATATGCCGATGTGCTTATAAATTTAACAATTTATAGAAATGACATATTAAAAAAAAAAGTAAAGTTGT
ATTGCTTTTTAAAATGTTGATTTAATAAATACATATTCTCTTGTCAATTAAATATTGGAAAGTCAATCGCAATATAGTTGGC
ACATAATTAATTAATATTGCACAATATTAATTAAAATATATTACAATATGGTGGTGCAGGTCATTAAATATATAAAATAGT
AATGCAAGAGTTTATAAAAAAAAATGTAGAGAGAGGTGGATTGACAAATAAACGTGCTGTACTTGGTAAATTCGTATAAGT
AAGGGATATAACCTTGGTCGTTGTTTGGCTTGGTATAATGTATTTCACGTCACTACCTCTATCTACATTATGTACAATTAAT
TAAATTATCTCGTCTTATAATCACCTTTATTTACTTCCCAATGGTGCTGTGCATGTGTGGAAATTGTGTAAGTGTGCGTGTC
CGTTATTGTTTTATTTTAGTTTTCATTTTGTATTCTGTACAATTCGAAATTTTAATAAATGCGCGAACCCATTTGATGAGCAA
AAGACACAGGCCTAAAGTGTTTTTTTTTTATAAATCTACCAGTGAATGTTCTGTCAAGTTAATGCTTTCATTAATAACGATT
AAGTACAGACCAGCTGTTTTTCTTTTCCTTTTTTTTATTGTTATTACTATCAGTTATTCTGTAGCTTAGATGTAACAAAGCAG
TATATTTACAGTGATTATTAGTAATAATACATATTTTATGAGCACCCAATATTTGAATACTACTATAAGTAGTCGCCATTAA
AAACTATATAGTTTTAATTTGCAAATGACAACTTTACACTAAGTTTATAATACCAGTGCCCGAATATAAATGCATATTATG
CGACTCGTTTAACGTCTAATGAAATCAAACCTTAAGACATTAATATAAGGCTTAATTTAATCAGGAATTAGTTCGAGCACA
GACCTAGCAATGTTTTTTTTTTTTTTTTTATTGAAAATGTGTAAAAGACAAACGGTTAAAGGTTATAAATCTATAAAAAGTTT
ATTTTATAATATACTCTATGCCTTCTGCTTCTAGGTCGAGTTTGAAAGCTTCTAATGATAATTATCTATTATAAACTACATG
GATGTTGTTGCTAGATAAATTATAGATATATGTTTAGATATTTGTATATAGAGGATTAGTTTCGTTTTAAAAATGTTGTTGG
ACGTTTTTATGTTGCAAAATGTAGATATTCGCGCCAAGTATATTACGGGATAGGCAGAGAGGTGTTTAAATTTTATGTCAA
CAAAATTAAATATTTTACAATTAGGATAACTTTAATTAAAGTTATATATAATGTCGATGAAATTCAATATATTAACAATAA
ACAGAATTGTTACGTTTTACGTTCATATTATAGAAAATATCTACTTATTTAGTAATGAATAAGAGTCAACGTGCCCAAGTA
CAATACCTACTTACTTTGTTGTTATATCTTTTCCTTTTTTGAAAATTTGAAGCCAGTGACGACAATTATGTTATTAAAAATA
ACATGTTCCATTTTCAAAATAAAAACGTTCAATGTGATTTATTTTTAAATCAGCCATGTTTGCTCTTATAACATACGGGTTT
TAGGTTGTTTTTTTTTTTATTCAGTATTTTGTCGGGTCACATTCTTTTTTATGAATATTAAACACTTGTCACTTTTAAAAAAA
ATGACAATAAAATGTTTTGTGTATAAAAATAATAATATTGTTGTAAAATGTTATATTAATGAAATTATACTGATGTATTAA
TTATTAAATATTAACTTTTATGAGTTTAAAATGTAATTAAAAAATAATGTGAACGTGTATCAATCGGCTATTAAATCTAAC
AAAAAAAAACAAACAATTTGACAGGTTAGGTCCTAATGATTGACTACATATGACTACATATTTTGTTAGATTTTCTAGTTT
CGTACACACTTAAAATAATATTGTTTTCAAATTATTTTTAAATGTATAATGAAAGACAAGTTCATCAATTATACTGTATATA
ATACGTGGTTATTTTATTCATTTCAGCTATTAATTGTCTAATACTAGGCCTCCCCTAGAACGCCAAAATGGTCCAGAGCTAC
CTCCATCCACTGAGCTTCTCCAATCCATTTACATTTGTCTTATACAGAAAATTTAGTAAGAATTCGTCTTAACTTACGTTAG
TCTATACATGGTCGCCACCTGAGAACCTTCCTCCATATATATGACCTCAAATTGACCTCACTTAGTCCTTTAAGGTCTTTCT
TCTTCATTATGCTGTTACGTCATGCAGTCAAAAGCTTCCGATACCAACTAGACAGCTACCAGCTTACTATGAACTCATCAA
TTATATCAGGCTTTAACTGTCCACTGCTGAACATAAGCCTCTCCCTTGGGGGGGTTTTGCCAGTAGTTGCCACGCTTGGCAA
GTGGATTGGCAACCACATTTAGGCTTTAGAGATGTTTTAAGAGGGGCATTGCTGCACATCCCTCGCCCTACCTTAGTCGCC
TCCTACGACACTTACGGGAAAGGAAAGGATGGCCAACTCTGTGCCGGGACCACATGGCCATATAAATTTATACGAGACAG
ATTCTATATAGCTCAGAAAGAGACAATTAATTCTTTTAATCACGTTTTAAACACTGATTATAACCTTCTTTCACCGCAGCGT
ATTAGTACTAATTAATTTATCAGAGGGAGACTTTGTACAAAAGTCGATTGCTTGTGTACCTCTATCCCATTCGTGTTTCAAC
CGTGAAGCAGTTGTGTTTGCATGGCTGTGTTTCGGTTTGAAGGGTGGGATATGGCGTGAATTTACTGGGTACAGAGGAACA
ACACCCGAGTCCTCAGGAATGGTGGCAACGCACGGGGGGTATCATGGGCGAAACAGTATACTCTGTTATGTCCATGGTAC
TGCTCGTGTCTATAGGCGACGGTTACCACTTTCCATCAGGTGGGCCGTCAGCTTGTTTGCCATTCTAAGTTGTATAAAATAA
TAATAATATTACTATAGCATTTTTTTGTCTGTGGTATTAATATGAGAGGTTTAAAAAGATTTTTGACATATGACGATTGTTT

		TTTTTTTATTTTTTAATGGAAAAATATTGGATTTCAATGTCAGAATACTCCGTTAAGAGTAAGCAAACAATTTTAATGTCAC<br>CCAACTCATTTACAATCATACAAAAATCGCTATGTGTTTATTGTATGAACTTGTCTTCATTAATACGATGTAATTATTTTAA<br>TACAGTTCCGTTTAAAATGAGTCTATGGTTAACAATTTAACGTAATGGTTACATACGGTGGTTAGGATTAAAAAGTAATAT<br>AGTCTATTTATCATTATTCAATTAATTTCTAATAACATGTGCCTATGTATATCTGTTACTCTAATATTTTTATGTACTTAATT<br>ATTTTAATATATTTTCCCTTCACACCTAAAATAGGAAGATTTACAATGTAGACCTCCACGTTGCACGGTGACGTCTGCGACT<br>GTAAAGACGCCATTCTTTAGTCATTTTCAAGTCGCTAAATGACCGAAAAATGACGTCACCGTACTACTTGAACCGCCATAC<br>TGGTCTAATTGTAATAGTCACAACTATATTACATGTACAGGTGGAAATGGAAATATTTGCATTATTTTTAAGAAATCTTCTA<br>CCAGTCAAATTGAACACTGTTGAAACAGCGGTCAAAAAACGACAAAAATTAAATATTCTCCATAACTACCTAATATTTGTG<br>CACGCCCTGTATATAAAAGAACACTCTTGGAACACCCTTAATTTCAATTTCAAACCGTATATGGCAAAATTCATGCTTAAC<br>TAATGCAGATCAAATTTACTCATTAAAGAAAATTAATACTATAAAACAGTAATAAAAAAATATGTAATGCTGGCTGTTATAG<br>TTTTTTTTTTTTCTTTTTTTTTTTTAATATTTAAAATACAAATTGGCAGTTGAATAAAATATTTATTTCGAATACTATAATATT<br>GGCTTTAAAATTTTTAAAATAGTCAATGTTCCTGCCTTTACAACACAAGCATTAATTCTGTAATAATTCAGCTCCTAAAACT<br>GATGTAGTAGCGTAGATGGCTAATACGCCAGATCGTTATGGTTATGGCATTTTGAATCATATTTTTATTGTAATAGGACTC<br>ATATATTATACTACATATACAGTTTATCGTACAGCGCCATCTACTCCGAAACTATACAGAACTTGTTCTATCTACTCTTAAT<br>TAGGCCACGCCGGCATACATAGACCAACAGTGTATGCCGGCTGCAAACCCAGCGACCCGGAGGTCGTCACCTCCGGAGTA<br>GAAGCCGGGGTAATGGGGTACGCCCCGAGGCCCCTACCTCGCCCGCATACGCATCATCCAGCGGGCGAAGATAAGACCCT<br>GTTTTTGTTGTTTGTCTTATTTAGGTATATATATTCAAATACATTTTCTTGCGTTAAACATATATAATATGTAAAAGGACGC<br>AATTACAGTCTAGACGTATTAAAAACAGACGCTCCTAGGAATACAAATATTGTTTCTGTATGGGAATAAACCCGCAAATAT<br>CAGATATACTTGAGGCAGTCTGTTACATTACTTTTTTTTTTATTTTTTTTTCCTATACACTGACTTTTACATATATAAGTAAT<br>AGACACTTTTTATAGCTGGCCACTACTGCTATCACGCTTTTCTGTGAAGCCTTAAAAAATCTAAGCGCCATATTTTTATCTA<br>CAGCTTTGGACAACCATAGACAATAGAATTCATAAGTTTTAATTTTATTTACTAACGCTTAAAAAGATTAGTAATATTTAA<br>CAATGCATTATTGTAATTACTTATATAATCGAAATTATAATTCAATGTACTACTTATTTTTAAGTATTTGTTTTAAAATAAA<br>TTAAAAATTTTTAAACTGGCATACAATTGTCAGAAATATAATAGGTTATTTAATTAGTCACTCGTCACTCGAGTATTTAAA<br>ACATTAGGACCTGCACAGACGAGTTACAGTCTTTAGACAGTTTTTTTTTTTTTTCATAAAATAGAAGTCGCGAATTGATATA<br>TCAATGTTTAAAAAAGTTGTAAGATAACTTACCTACCAGAGAGAAATATTTAAAGTTGGATTATTTGATGAAATTTAATAT<br>TTTAATGGACAAAAAGTACCTACAAGAATTGTCGCTCGTCTACGCAAGTCGTTAGTTTAACCTGTTATTTATTTTTATATTT<br>TCATCACCCCTAAAGACGTAAAATACGCTCATTTATTGTTACTTATTTTTTTTTTTTAATTTTGTTACATAAAACACTTAAGT<br>GAATTATGCATAATAATAGTATTATTGGGATGTTATTTAGTTATCAATTTTTATCCATTTTCATTTTGGTTTTACAGTATGCG<br>AAGATGATATTAATGTTTTAAAATATTTTAAATTACCGGTCTGTAACATATTAAAATATTATGACCTGTA
MSTRG.7182	Nose resistant to fluoxetine protein 6-like	GGTCGACTGCAGTCCCGAGATCTCAATATCGAATACCAGTCTGAACCTCTGAATTACCCAAATTCACGTTTGGATTAGATA<br>ATTAAGTTTGTCTTCTACAAATAAAAACTCACTTCAATTGTGGCAAAACCAGTTATTTCTATTTGAAAATTAAACTAATATA<br>CAAATTACCTTCTTATGGTTTTTGACTATAGTTTTGTCATCTGGAATAATTTGTAGTCATTATATAGTTCTACGACCAGACT<br>GGGACAATTGGATAGTAGATGCCTTAATCAATTCCTATATGAGGCCACTTTGGGCTTTGTCTATTTGCTGGATGATCTTCGC<br>CTGTACGCATGGATATGGAGGTCCAATTAACTGGATCCTGTCACATCCTATGTGGAAGATATTGGCAAGATTATCATACAC<br>CATGTATATTTTCCATTTTCCACTTATGTTTGTTGTCAATGGATCCTTAGTAACTCCTCTTTACTTCAGCGATGAATTCGCA
MSTRG.7183	Lipopolysaccharide-induced tumor necrosis factor-alpha factor	TAATATATGAACGTAAAAGTTACTGATAATTACTGACAATACGATCGCGTGTAACATTTGGATGAACTCAGTTATTAGTCA<br>ACGCCGCAGGAGTCGGACGCGTCCGTGACTACAACAAGTGGTCGGCCATTTAATTCTGAGTTTTTGTGCTGTCCATAAAAC<br>CGGTTCAAAATGAACATTGCACCAGTTGGCCCTAAACCAACTCAATTGATCTGTCCGTCGTGCCATTCGACGATAGTGACT<br>AAAGTTGATCATAAATCAACAACAAAAACACACATAATTGCACTAGTTTTGTGCCTATTTTTGTAAGTACTTTTGCAAAAT

	homolog	ATGTAGTTCAGTAATGATTGTGTCCCACAAAAAAAAAAAAACTGGCTTCACTTGTGACAAAACTCGTTGATTTGATTTAAAA<br>TGAAACTACTGAGCCGATTTTGATAACATTTCTTTGGGATTAATCTATAAAGCACATATGTCCTTTCAGATAAGAAAAAGA<br>AGTTTCCAAGTCGGTTATTAACCGATGGAGTTATGAGGTAACAAACATAATAAAAACACAACTGAATTGAGAATTTACTTC<br>TTTTTGAAGTTGGTTGGAAACAGTGTAGTGATCGGTACTTGTCGAAATAAATCTCGTTGCTTCTTCATCCTACAGTTCTATA<br>TGTAAAAGTATATATCTATTGCCTGCTTGCTCCCCGTCAACAGTTACGCTGCCGCCTTGATCCACCCACTGCGCCATCAGCG<br>TAAATCTCATGTCTGACAAACGGCGCCGGTCGTCCTGTCCCAATTTGCTGTCGGGTACGCTTTTGTCGCAAATTAGAAAAG<br>ATTCAAGATTGATATACAATAAATATTATAATGAACCTCGTTTCACCGTATTCAAGCTCTCTTAAAATAGCTCTCTTTAATA<br>ATCTTGGAATGCGTGGGTACTTTTCTTAAAATTACTCTTTTTAAGTATTCGATCTTGGAATAA
MSTRG.7206	Intraflagellar transport protein 122-like	GTTAATAGAATTGGCCCGTAAGCTGGATAAAGGTTCAAGCGAGTCATTGCGCTGCGTGGCAGAGGCGCTGGTGACTGCAG<br>GCGAGCCGGTTACAGCCGCAGACGTGTACCAAAGGCTTGGAGACTATAGAAAAGTAGCGCAACTGGCAGTGTCGGCGGG<br>TGAGTGGTCGCGAGCTTTCACGCTGGCTCGTGAACACGCTGAATGTAGGCGGGATGTGTACTTGCCTTATGCACACCGTAT<br>GGCACAGGAGAATAAATTCGTCGATGCGCAAAAAGCGTACCATATGGCGGGTGAGACGGGTACGGCCATGCGTGTGTTCA<br>CAATACTGGTGGGGAACGCTGTCGCCGAGGAGAGGTTCAGCGATGCTGGATACTTGCATCATCTACTGGCAATGCAGTGC<br>TTAGACACAGCGGC
MSTRG.7210	Hypothetical protein KGM_202897	CGATTTAAAGTTGAATGTTACGATTGCATGTAAGCTGTATCCCGCTGCTTTGCTTACGTTGATTAGAACAAAGTTTTACTAT<br>ACGTTAATGTTCGACATGGTCTATTTGTGTGCGAAATTTTGCTTAAATCTGTTCGACCATTTAAGCATGATTAAGTAAACAA<br>ATATGTAACGAAAATGAAAAATTTACGAATTGCCTGACGGTAAGCAATACAATGTTTGAAGTCAAGTCGGATCTTGACAT<br>CTGGTTGGACTACCACCTACCACTTCGGTTGCATGCTCCCTGATTACTCTATGAACCCTGAAGCGCTGCGGATGCTGAGCTT<br>CCTCTGGTGGACACTGTTTATCAAGATATTCGAAATGATGGAGACCATGTTCTACATACTGAGGAAAAAGTACCGGCAAG<br>CGTCATTCTTACATATTTATCACCATATCAGCACTTTTACATTTGTCTGGATGGCTGTAAAATATGTTGGAGG
MSTRG.7216	Max dimerization-like protein	CTTTGAAATAAGCTACAATTTCTCTAAGATTTATAAAGAGTTTGGAAGAAAGAGAAAAACGACATTCGAGCCACAAAGAG<br>CAATTAGCGAGAGAGCAGCGATTCCTCCGGCGGCGGTTGGCTCAGCTCAGAGCGTCCCCACGGGACATCACCTCGCGAAG<br>CCTCTCAGAGAGTTCGATGACGCACGCGCACGCCACACGAGCGGACTCGCTCTCATCACTCGAGTCATCATCGGGCGTATC<br>CACCGCTGAGCGATCACCTTCATCCGTCTCCGAAAGCG
MSTRG.7238	Short/branched chain specific acyl-CoA dehydrogenase	CGATCTGTATTCCGGACTGCAAAGCGCCGGCGCACGCACGTCTACACGCGCGGTAATTGCAAATAGAAATCACAAAAACT<br>ATGTTCCCATTACGCCGTGTCGTTGGCAGCAAGATACTGGAGCAATGGCGCGCTCCGGTGGTAGCTGGCGCGAAACAAAT<br>GAAGAGCTACAGCTCAGAAGCATCAACGCCTCGACCTTTGTCCATGTTGACCGATGATGAATTGGCGATGAAGGATACAA<br>TTAGAAAGTTGGCCACCGAACAAATATTACCACTGGTGAGAAAAATGGAGGATGATCACCGCATCGATGATGGAATCAGA<br>CAGTTGCTGTTCGATAATGGT
MSTRG.7248	Uncharacterized protein LOC110385835	TTTGTAGGATCTTTACCGGGTTTAATTACTGGTAGGTTTATACGATTTAACAAAAGTTTATTAACTTCATCTATAGGTGCAT<br>GTTGTTCACCTGACGGAGTTGATTAAGAGTTCTTTGTGTTTATTTTAATAAAAGTCGGCAGGTGATAACTTCCTAACGGATC<br>ATCATGAACCGACCACTCGCATTTTAATGATATGGATGGGGATACCATAGTTAAATCAAGGCCATTGGGTCTCCAAATATG<br>CGAGTCAACAGTAGTAACTTGCTTATCATTTAATAGTACCAAATTACATTCATCTAAACAATCAAGAATGTCTTTACCTCTA<br>GTATTAGTGGAGGTACAACCCCACATAGTATGATGTGCATTTAAATCTCCAGATATGATAGTGGGTTTAGGAAGAGACTCC<br>AAAAAGAACTTAAATTTGGCTTTAGTAAAACTAGGTGTACTATTGGTAGGACAGTAAATACTGGCAACTGAATACCAAGA<br>ATTATTAACTTCAATACTAATAGCTATAGTTTGCAAAGAATTGTCGAAAAAAGAGTGAAGTTTTTATATCTAATACTATT<br>ATGTATAATAATCGCTACTCCATTGTGGTTATTACCACAATCATTTCGTTCTATACAATAATCTTTAATTTTTAATTTGTAAT<br>GTGGTTTTAGCCAAGTTTCCGAGATGAGAGCAATATGAATATGATGTTCATTTAAAAATTGTGTAAAAATATGTCGATTGC<br>TCAGTAGGCTCTGAGCGTTCCACTGTATGATAGTTAGTTGGTCTCGATTGAATTTTGTGAAATAGGTGTATTACCAATAGTA

		TCATATAACACATTCAAAACACTTTTAGTGTTAATAGATGTGTTCTTTGTGTTATGTAGTGAAATAAGTTTGATTATGCTTT<br>CTATTAAAAGTGATAAACTCTTGTTATCAGAATTCAGAATTGAAATAAGGTCTACATTTTTTTAGGTTTGTTACCCAGTTT<br>AGGAAATATTTCAGTGCTAAGTATGTCTCCAAAAGAAGCAGGTCTGAATTTAGATCTATTTTCAGCAATTTTCTTTTGTTTA<br>ACAGGGCATTGCGGTGATACACTTGTGTGATTTCCATTGCAGTGTACACAAATAGCGTTTTCCATTCCAGTTGTACAAGTTT<br>TATAATTATGCTCACATTTGCAGATACTGCAACGCTCAGAATTCTTACAGAATTTAGCTAAATGTCCATACCTCAAGCACC<br>AGTAGCATTGTTTAACTGGTGGTATATATGGAGTGACATTGTGGCGCCAACCTCCAATATCCAGATATTCAGG
MSTRG.7258	Sprouty-related, EVH1 domain-containing protein 2 isoform X3	CCTGTCGCGCAGTCACGGTGTGTTCATTGAATAACCGGGCTGTTTTATTGACGCAAGCGCACTGAGGCCCAACAAAGGGCC<br>GAAATAAGAACGACTTGGAAGTGAATCTGATGTATTTATGTCATAAAAATGGTGAAATCTGTGAAGTGGCGAGTAGAAAT<br>TAGGATATTCGACGTGTCGAATCATGACCGAGGCATCTGAAAATGCCTACTTGGTGCGCGTGCGTGCGCAGGTGATGTGCC<br>GCGACGAGGGCACGGGCGGCTGGGTGGCGCTGGGCGGTGGCGGGCTCGCCGACGTCATGGTCGGCAAGCGGGCCCGTCA<br>CGCGCACCGTCCCACAGGTTAGTACTGTAGATAGATAGATAGATAATTTATTTGCATCACTTACAGCACACAAATTACAAC<br>ATATATAGATATACATAAAAAGC
MSTRG.7270	Serine/threonine-protein kinase ULK3-like	GTCAAAAAACTGTCAAACCAAAAGTCAAAACAAAACATGACATTTCTTCTATGCCATGAGGAAATTCTTATCTTTTAATAA<br>AATTAATTAATTTCTTTCACAATAAATGGCTTTAAAATTACGATAATGTCTTTTCCGAAAATAGAGGGTTATGTGGTGACC<br>GAGAAACTCGGTTCGGGGAGCTATTCCACTGTATACAAAGCTTATACGAAGGTTGGTGCCCGCTCAGTAGTGGCGGTGAA<br>ATGTGTTGACAAGTGTAAGGTGAAGCAGTCGGGCACGGCGGTGGACAACCTGATCACGGAGATACGCCTGCTGAAGACCC<br>TCGCGCACCCGCACATCGTGCAGATGAAGGAGTTCACATGGGACGACAAAAACATCTACATTATAATGGAGTACTGCTGC<br>GGCGGCGACCTCTCCAAGTACATACACCGATATGGCAGGGTGCCGGAAATGCAGG
MSTRG.7272	CCR4-NOT transcription complex subunit 3-like	TAATTATGACCGAAAATGTAATACAATAAAATATATTTCCAGCAAATGGAGAGGTTCAAAGTGGTGGAAAGAGAGACAA<br>AAACGAAGGCATACTCTAAGGAAGGTCTCGGGGCGGCGCAGAAGCTCGACCCGGCGCAGAAGGAACGGGAGGAGATGGC<br>TTCGTGGCTTGTCTCATCTATCGATGCACTTAATTTACAGGTTGATCAATTTGAGTCTGAAGTAGAATCACTGTTAGTTGGT<br>AAGAAGAAACGACTGGACAAAGAGAAACAAGACCGTATGGAGGAGTTGAAGGTGAAGCTGGAGAAGCACAGGTTCCAT<br>ATAAAAAAGCTAGAAACCCTGCTGAGGATGCTTGACAATATGTCTGTTGAGGTAGAACAG
MSTRG.7285	Protein ERGIC-53	CTTTTAAACTAGTTAACATATTGTGAACGTACGTACCGGCTGCGCGCAGGCGTTCTGGTACACGACGCAGCGCGGCGAGTA<br>CACGGGCGAGGTGTTCGGCTCGTCGGACCGGTGGAACGGGCTCGCCATCATATTCGACTCCTTCGACAACGACAACAAGC<br>ACAACAACCCGTACATCATGGCGGTCGTCAACGACGGCACCAAGGTCTTCGACCATAAGAGTGACGGCTCCACGCAGCTG<br>CTGTCGGGCTGCCTGCGCGACTTCCGCAACAAACCGTTCCCGACGCGCGCCCGCGTCGAGTACTTCCACAACACGCTCACT<br>GTCTACTTCCACAATGGT
MSTRG.7295	Trehalase-like isoform X1	GACTCGTCATGTACAGAAAACGGAAAGAGAATATTGTTGCTACTGGAGGAGAAGATTTGAAACTTCTATCCCGGAAGCCC<br>TACACGGAACTACAGAGCCTCAATGGGGCATCGATCCAGCGGCAACGGTAAACCCTCCCCGGCTTGAACACTTTGAATAA<br>ACATTTGAAATAGAATGTCCAACTACTATTACAATTTGTATTAAAAAGTATTAATTTATTTTGGTAGTTGTTCCGCTTTTTT<br>GTTATTAGACCACAGAGTTGAGTTTTGTAGAGTGACCATTTGATATCGAACAAATTTTTATATTTGATATTGAACAAGACA<br>GCCCAATGGATTTTAACATCATAGAATATATTATACCGCCCACGTTACCATGTGGCTTCATATGTTATGTCACTGGCATTAG<br>AGATTGCACTATCAATTTTCAGATCTAAGCGTACACACGAATTGTTTTTGTCGTCACAATAAAAATAGTGGTGACCCAAAA<br>GAGTTACAGTTACCATCAATTAATGTAATGGTACGTTTATTTATAAAAACGTCAAACCACTCATAAACCTATTTTAGCTATT<br>GAACTGTTTTGTCTCTTTCTAGTTTTTCACTAAATTCCCAACGGGAAAGAAAGTAACAAAACAGTGTAATAGTAAAAATAG<br>ATTTGTAGGAGGTTTTAATTTTTATGAATAAGGGGGTAATGCATAAGTTTTAAAACAGTGCACGGTTGCTAAATGTATTTG<br>TTTTGTAACATCACTATTTATAATCTATCTCTCATTGTTGCAATTAGGGATAGCGAATTAGTTCATAAGGCGTCTCTATTAA<br>TTCATCTCTGTGTCATTATATACTAGTATTACAAATTTACTTCTTCCTTACAATCATTCAGTAGTTACCTGCTCCATTTATTT

		AGAAAATAAATGTTATTGTTAATACATAATAAAATAAAATTAAAAATCGGACATTTTAATGTATAATGAATTGTGAAAAA<br>ACGTTTTGACATTATAAATGTGTAAAGGCAGATTTACAAAAGGTCAGTACTGAATTCTAGCTCGTAAGAAGTCAGTGCTGA<br>CTCGCCACTGCCATAACCTAAATTGTGACAGTACAGTACTTTCGCGTCAGTGGTGACTTACGCACCTAAATTAGTATACCA<br>TTGTTGTCGTGACTACTAGCATCAATGAGCCAGTGTCAGTCAATTTACTGACTTTAAACTCTAGAGAAGTCAAATGCTGAC<br>ACGGCACTACCATTGTGTAAATTGTAACAGTACTGGCGCGTCAGTGCTGACTTACGCTGTGAATAACTCAATGTAAATTTG<br>TCTTATTTGTATAGTACCTACCAGTTTTATATGTGCTGTGAATGGCTAGCATTGTAAATATAGACATAAGGTTTTAATTGTA<br>TATTTTTAATTTATAAATGTAGTTTAAAAGAAATAATATTTAGTGATGTGACATGGTCAGACAGGCTCTTTAAATCCATATT<br>TTTTATTTTCTTTTATTTTGAACGATCTAATGAAATATATATATTTCATTTACATTGTAAGTTCCATGTCTTTAAATTTTTTTA<br>CATGACATAAATTATTAAAAATTATGGCAAATAAAAATACTATCAATTATATTTTATTGACAATTTTATTACAATACTCATA<br>ATAAATCGTATTATTCATAAAAAGTTGGAACTTGACCTTGACTTATGTCATTATGTTAGTTATTTTAAATTAAAAATATTCT<br>TAAAAAAAAAGATAGGAAACCAATAAATCTATTTTAGTATATAAGTTTTATTATAATGAAATTTCAATAATTAAATTGAGT<br>TAATGAAATGTCTATTTATGTCTAGTTCTAACACCTAGATAGAGAGTGTCTCTCTACAAATGATTTGACAAAATAGCCAAC<br>TTTCTACTTAGTCATTACTTAGTTTTTAATTTCTACTTAGCTAACTTAGCCAACTGTTCATATAGTTGTCTTGATTTTTTTTTA<br>ATGTTTCTTGACGCTCGACACTATCGTATTTATTTGTGTTGTAACTTTTTAAATTAAACGTCATTATTTATTTATTTAAACTA<br>CTTTATTTACCAAATAAAAATATGGTACAAAAGTCGGAATTAATGCTATTAGCGTTCTCTACCAGTCAACCTTTGAAATAC<br>AGTGAATCACGAACTCAGTACATCATAATATTGACTATTTTATGTTATTTTAAATAATATAATCAATATATATATATAAGTG<br>CCAGAGTCTGTAATTATTTTTGTCACATCACTATAGTTGGAAGTTATTAGCTCACTATGCTTATTAGTTCATTACATTTATTC<br>GTTCATGAACTGATCATTTATAAGTAGACCATTTTTATGGACCATATCAATTATTTTTATAAATTAAAATAGTGA
MSTRG.7296	Diamine acetyltransferase 2-like	GAGGGACGGGCAGCAGCGTCCCTGTTAAAATATCTCAAAAGATTTAATTGCGGTTGCCAACCCGCCTGACAAGCGTGGCA<br>ACTACTGGCAAAACCCCCCAATGGTAAACACTGTAAAAAACGGCCCCCAGTCCCCGGTGACTTCGCTATTCCTGATCACGG<br>TAGCGGCCACGGTAACGGTGGAGCACGGGGTGCTAAGAATCTCCGGCAAAGATTGCCCGTATGCAAAAACTTCAGATTAC<br>TCAGCAGGTTATGGAGAAGGTAAACACAGTGTAGGACGACCTATTGACTATATTTATACTTATATTTAGACTATATTTATA<br>CGATGCATTGCATGGAGTCAGTGGATGCAGGTGGTTTAGGACCTTTATGGCAGTCTATGGGGGAGGCTTATATGTAATAAT<br>GGACGGATAATGGCTGAAATGCTGATGATTGTAGGTGTACGATACTGTGCGCAGATGGCGCTGCAGGAGAACGTGCACCG<br>CATCGACTGGCACGTGCTGGAGAGCAACGCGGCCGCGCTGTCGTTCTACGCGCGGCTGGGCGCGCGCGACATGCGAACCA<br>GCGAGGGCCGCGCCGCGCTGCGCCTCGACCGCGCGCGCATACACCTGCTCGCGCACCACACGCCGCCCGCCCCCGCCTCC<br>TGATTCGTCAATACCACTCTGTTTTTGCATACGTTTATTAACAAGTAACTTTTGCCCTCGACTTCGTTCGCATGGAGTTCGCT<br>TATACTGCAAACCCCCTTTTTAAACTTCAAAGGTAAAAATTAAACAAATAAAGAAACACTATTTCTGGTTTCAAAAATACC<br>TGTCTAGCATTTTTACATGCAAAGTTCTCCATTTCACTCCCTCAGGGTGTCGGTTCAAATGTGATTTTTAATGAATGTAGGA<br>ATACGTACTTTCTGTCTTTACCCTCTTAGATGTTAAATTTTAAATCTTCCTTTCTTATTGTTCACCTACGCTATATGAAAAGC<br>CTATGTGCAAAGTTTCAATCAAGATTCCATTCCATTCAGCAGTTTCCACTGTGCATTCATATACGAGTCAGTCAGTGAGCAT<br>CCACTTTTACATGTGTATTGTATAGAAGATTTGGCTTTCCAACGAGTTACCAAAATGCGGATAC
MSTRG.73	Uncharacterized protein LOC110372752	GTGTAGACGTGGGCTAGATGATATTTATATGTATAGATTCAGAAAGAGTGAAAGAAAGAAAGATTTGGTACGAGATTTTG<br>TAATTAATAATATTTACAGTAAATAATTCCAGATATAATAAGTCGCGATACAAAATATAGTTCGGTACAAATGCTAAGACA<br>ATAACATTGTCAAGAACGTCACTATTTATTTTTCTGTAACTATGTAGGTTTGAAAGAGGTTGGTAGCCTACAAATTTATTTC<br>CAAACATATTCAGAATACTATTGCCACTCCCGCGTATTCTATGTAAGAGAAAGGCTATCTTTTTATTTAATCCCTACTTTGA<br>AATTATTGCACCCTTTTTAAAACTACAATACAATACTTTTTAGAACATACAAAGTAATAAAATAAATTTAATTAACTATTTT<br>TTTAAATACAGATGTCAAAAATACAGACAAGTTCGAGGAAGTCGCGTTGCAAATAAGTCGAGTCGTCGGCGACAATGGTC<br>TCAACTTGCTCATAAACAATGCCGGAGTCACGACAAAGTACACGAAGATCAACTTGGTTAAGCCTGAACAGCTTCTGGAG

		AATCTAGCTGTTAATACTATTGCACCAATCATATTGACTAAGACCCTGTTGCCACTACTCAAGAAAGCCT
MSTRG.7313	Protein AF-10 isoform X9	CCAAGAATAGCAACATGTGAGGAGTACTGTGTTACTGAATAATCTGTGATGCCCTTGATGCTGTTCATGTCTACAGATTATGGTTACCATTTTAAGGTTTCACTTTATAAAACGTCGTCTTTTTTTACCATTTCAATAACCTAAATTACATTAAAAAAAAATAACATTCCAGAAACTGCTACAAATCAGAATCTAAGCAACGTGCGTCGCCCACCACAACCCACTCCACCGCCAATATATCAAGAAACAATTAAAGATTCTCCGCCGAGCTCGCCCAGTTCTGAACGCCCATTAAAACCCAAAATGGAATCAAACAGGATAGGTGTGAGCTGCACTGCGCCGCATATGCTTGGCAACGAGTTGAATCCGGAGAGTGGCGCGGCGGCTCGCCTACAGGAACAGTTGAGCGCAGAGCTGGCGGCGCACGCGGCGGGCTCCGCTGGCGTCGACCCACTCGTGCCGCCGCCGCTCATCAACAAGGCCGCGCCAAACTCCAGTAGCAGCTCAGGACGTGGCAGCTCATCGGGTGCACAAAGTCTCGACCAGCTGCTTGAGAGGCAATGGGAGCAGGGCTCGCAATTTCTCATGGAGCAGGCCCAACACTTTGACATTGCGTCCTTACTGTCGTGTTTACACCAACTGCGCACGGAGAATGTTCGATTAGAGGAGCATGTGGGCAACTTGTTGCAGAGGCGCGACCACTTGCTCGCGGTCAATGCGCGCCTCGCTATACCGCTCACGACCGTGTCCGCTGGACCGCCGGAGCCAAGTCGGTGTACGCGCGAGAACAGTTCGCAGGTCCGCCCGCCGCCACCACCCGTACGTACCTCGGAGACCATCACGT
MSTRG.7335	Venom dipeptidyl peptidase 4-like isoform X1	TCACAATTGAAGTTAGTTTTTTTTGGTACTCAATCTTCAATTATGTGTAAAATTACAGGCCAATCTTGAAACCAGTAAGTTTCGTGACAAACGACACGATCGCGCTGATATGGACAAATAGAGAGCAGAACAACTTAGTAGTGGAACTATGCACATTCGGGTCCACTTGTACAAGGGTATTTTCTTATAATGAAACAGACGGATGGGTTGATAACATCCCACTGATTTTCAATGAAGCAGGAACGGCCTTCATTACTATTTTGCCACAAACCGTCGGAGGTGTCCGTTACAAGCAAATAATTCAAGTGTCCCAAGCAACAGGCGTGTGGTCTTCTCAAAATCGCATTAATACCCAGAACACGGTTCTTGATATACTAAAGTGGACCTCTGATGATGTCATTTGGTACAAGGCCACAGCTACAAACGACTCCTCTGAACAGCATGTATACAGTGTAAACAGCAGTGGTGCAGTATCCTGCTTTACTTGCAACATTGGTGATGAGAACAGTTGTCGTTACAACGATGCATCCATCAGCGATGGCAACCTGATCACTATCACCTGCGGTGGTCCAAATATACCACAAGTGTTCATATACAATCTT
MSTRG.7345	Enhancer of mRNA-decapping protein 3	TGTGTCTGCTCCGCAGGCTCCGGCCGGGCCTCGATGATTTTTAAGTCTTTTATATCTGCAGCACTACAAACAAGTATTCAAGCGTTTTAAATCGTATAAAATTTTCCACAAGTTCATCATACACTTTTATACTTACTTTAGCGTAACCTGAGCTTTAGGATATGGAAAACCATTTCGAAAGGCTTTAGTGAGAGTTATTGTTGTACCATCTGCTTCTAGAATAGTGCCTTGGTAGCAACCAAGAGGTTCACCACAGTTGACCGACACGGCATATCCAATCCAGTTCGACATTTTTTACCACACATGTGTCAGGATTGATATGTAGTGTATAATTCTTCTTCCTCTCTTTGATGATATGTCCTTATTTATTTTGAATCGATATTAATAATAACAACAAAACAATCATATCCCACACTTGGAAAACGGTAAGATGACAGGACGGGTACAGAGAATCTATAGGATTTGTCATACAGTATCTGTCAAGCAAACTGTCATGTCAATGATAGTTGTCATTATTAATTACTTTTCAGTTCAAAAATCCTTGTTTTAGAATCTAGTTCAATCAATTTTCAGTCAATTTCATTTAATTTTCATTCATTCACTTTCGGACTTTCAGTTGCATTTCATTTGTACTATACATTGCTTGTGTGGAAATTGATTGTTATCTAATAATTCGTTTGAATTATAAAATAAAAATACGTGATTAATTGTATAATATAAATTAAAATACTTATTTTCTATTATGACTAAATTTGTAATTAATTAGGGTGAACAAAATAATAATCTGTAAGAGAATAAGACTACTAATTCATGTTCCCTAGGAGGCTTCCCACTGATCTGTCTTCATACATTCACACTAA
MSTRG.7357	Sterile alpha and TIR motif-containing protein 1	CTAGTTTACTGAAAGTCCATCTCCAGCTGCGAGGTTTTACAGTATTTATAGACGTGGAAAGATTAGAAGCCGGCAAATTTGACAATAACTTGTTACAAAGTATACGGCAAGCAAAACATTTCTTATTGGTCCTTACGCCAAACGCACTTGATAGATGCAAGGACGATACAGAGCAAAAAGATTGGGTTCATCGGGAGATAGTGGCAGCGTTGCAATCACAGTGCAATATTGTACCAATTATCGACAATTTTGAATGGCCAAATGCGGAGGAACTTCCAGAAGACATGCGTGCGGTTTGTCATTTCAACGGAGTACGATGGATACACGACTACCAAGATGCATGTGTAGAGAAACTAGAAAGCTTTCTACGCGGAAAATCAAATCTCGCGAATCGTCTTGAGGGACCTTTACGAGGACGTGAAGTTCAAACACCAGGCACAGCCGCCATAGGCCGGGGACCGCCCAACTACCAGCGTATGACCTCTTGCGAGAGTCGGGGCAGCGACAAAACGGATTGACTAGAGCCGCCCATACACACACTGCCGCCTGTTAGACCACGACGAAGCCGTCAGTCATCACTAGAAAAAGCTTTACCAGCAACATCACAAAGTTGTGACCAACTTTCTAGTTATAGTAACGCATC

		AATAACCACATCACCAGAAAAATCGCTATACAGCCTACCTGAAAGACTATCGGAAAGTAGTGACAATATAGACACTAATG<br>AAATGGATAAAACTTCACGTAGGTGCCGCAGCTGCGAGGGCATATTAGATGCAGACGAACAGACGCACACGCAACTTTCT<br>TTGCCTACGCCTTTAGACGCCGCCACGCAGACTAAGCGAAAGAAGAATTTCATGGACAGATGTGTTAATAAAGTGAGACT<br>GTGCTGATGAACGGTATTAGACATGCGGAAATATGTTACTGCAGAGTGTGTATGGAACACGACTGCCTTATTGGACTTTTA<br>TCGAATATTTAATCGAATATTTATGTAAATTTTAATTTTAAAGTACAAAAATACGCACAATGTGTAAATATTTTGTTTATGC<br>CATTATAAATTAAGTTAGCTTGAACACAGCAATGTTTTACATATTGGTACATAATTAAAAAGATGTAATCTCAGTGTTGAG<br>TGCATGCGAGAAATCTGTGACGAAGTAATCTTGAACTGGGTAAATAATTGCCACCCTGCTGTGTTTTAAGAACATCGCACG<br>AGGTGGTAATGTCAGAAATGCCACTTTATTATGTAGTACGGCAAACTGCTTACAATTCTTGACTTAAATGTTTTATTTATCT<br>CTCTATCTCTTCACTCTCTATGTATAGAATTTACATAATGAACTTATGGATGTATTTTTTTGTTTTAATATCTTGCCAATGAA<br>GTATTTTTAAATACAGAAACTATACGAAATTATAATTTGAATATTGTGATAGCTTAGATCTTTTATGTTGTATAAACTATTT<br>GCAAAAAATGAAATTTAAATATAATGGAAATGTCGCTAGTACCTAATTTATTTTTTCAGCAATATTGAACACAATTTGTAC<br>CTTTTTAAATTGTATAATATTTTACCCTTAGGTTATTCTTTAATTATTTAATGTAGTCATTCACTTTTAAGAGTAACCACAAT<br>ATTTACTCAGGAAAATATATAAATACTTATATTTATGTACTAGCTAGCTCTAACTGC
MSTRG.7358	Uncharacterized protein LOC110371346	GGGAAATATCATTAGATGGCGCTTAAATTAATACTTAAATAAAATTGACAGATAGCCGAAATAGAGTTGTTTTTTTAACTG<br>ACTGGCTGCCATTTGCTCTTTTATTATATTTGTGTTAAATAATTATTTCAGTATTTGATTTGATGTAGAAAGTTTAAAATTGT<br>TTGCAACTTTAATGATAAAAATAAATGTTTTTTTTAAATTCAACAAAACTTAGGTCATTGAATCTTGTATTTAGATGTAGTG<br>TTAAAAGTAACCTATTCATTTACCAACTAAAATTGGTGTAATTTTAACCTAAAAAGTAATAGATACAGATTATAACAATGG<br>ATAAAGTAGCAGTTGATCCGAAATTGTTTATTACCTGTCGTTTATGTTTAGAGGATATGGGGCTTTACCAAATAGTTCCAG<br>CGGTTCAAAAACAGATAAAATTTTGCTTTGATATTGAGGTG
MSTRG.7365	Rho GTPase-activating protein 21-A-like isoform X1	TATTCCAGGAGAGCCTACAAGATCCTGTGAAGTCGCGGCAGTACGACTGGAGTCCATCATCAGAGCGCAGCCGGGCTGAC<br>ATCGACCGGAGTTTCAGCAAGGCGAGCTCTGACAGTCCACCGTTTGGACGTATCAATAAGAGATTAGGAAACTCAACGGA<br>GACGCTTCTCTTCCACAACAATGTTCGGAGACCAAATCAGCTCCACCTGCCGGCGTCCTCGTCCGGGGACAGCTTGGACGT<br>GGAGATCAGGGAGCGGGAGCCGAGCCACAGCAGGAGTAGGAAGTCGAAATCCATGATGGCCACGAAGAACAGGATGAG<br>GTCACCTGTACCGGCTCCCAGGCTGATGCTGCATCAAACGCCTGAA
MSTRG.7381	Facilitated trehalose transporter Tret1-like	GTGCCAGACCAACCAGCCAGCTCTTCGAGTGTGGACGTAGAAGAGGGCTCCTTAGACACTCCTAGCCCTCCAGAAATACC<br>GGAAAAGAATGGCCGACCAAAACAACTGCATCGGATCGAAACAGTGCCAGACATAAAGCGGGACACGTCTGGTATCACC<br>ACCCAGTACATTGCGACCGGTATAGTGAACTTGGGAGCGTTTGCGGCTGGCGCTTGCATCGCGTGGTCATCGGCAGCGCTG<br>CCCCTGTTGACGACAGAACCGTTGGAGG
MSTRG.7440	Kinase D-interacting substrate of 220 kDa isoform X1	CATAATTTCTCTATCTATTATAGATACTAGAACTACCTTTCGGTGACTGGGAATTGTTCAAGCTCCTGATTATAAATCTACG<br>AGAAATAGAGGTCAACATGCCTACTAATGCTCCTACAGTGACTGTAATATCCGATAAAACAACCGAATCGGAAGCAGACA<br>ACATCAAACCAACGAGACCTTCTTTGGATCATCAGCGTAGTCGGCCGTCCAATGTCGAAAAACAGGTG
MSTRG.7463	Uncharacterized protein LOC106137928	GGAATTTCTATTTAAAAATGTATAAAAACATTGCATACCAAAAATCTAAGTTCGATTTAAAAAAAAATTGCATAATACACG<br>AACAGATTTCATTAAATCACTTTTAAGTTCAAAATTGTTGAGATTTCTGTGAACATTGTTTACACATTTTATAAAGAAAAAA<br>ATAATTATATTTTCTTTGCACGTTTGAAAGCCTTTTACGATTGTGATTTTGTTTTATTTTGCATTACTTTCCCAAATGCCACC<br>AATCCTTAGTGTTGCAAGGATTTTACCTTGGATTTGATTCCAGTGTTAATATGGCAACAGTGATGACACGACTGAGGTAAG<br>ATAATTTAGTTAGATTTGCATCATTTATGAACTAACATTCACCATATATGCTTATACATAATCTTTTCTTTTATTCGGGATAA<br>AAATATGTGTAGATTATTTCATACATGCTTATGATAAGTTCATAAAACTTTCCTAGAGAGAATTAAAAAACGAATCCATTT<br>GTAATTATATTTACACTATCACATACATGAAAAAGAATGAGATGGCATGACTTGCGTCTCATTCTTGTAAGGCCATTACAG

		CTTAGAATCGCATAGTCTCGATTCGTCAAAATTTATTTGAAGTTACTCGTTACTTGAATGTAAGAAACAATTTCAGTCGCA<br>GACAACTTCGGATGGAGGGAAGATTCATCAGGCAGTGAGGATCCTGACCCACCACATTATCCATTGGACTTGAGACTCGC<br>TGAAGCAAGATTGCAGTTAGAAGAGCGGCTCGCGCAGGAGGCGCGAGAGAGACGGCTAGACGAGAAGCGGGACGACAA<br>GAAAGTGTCCACCGAGGAGTTGCGAATGAAGAGCACCATAAATGATGTGAGGAAAAGTCCAGTCGATGACAGGCGGAGG<br>GACCAGGTGCATTTTGTGCTCGGCGGGGACAAGTGAGTCA
MSTRG.7<br>467	Protein C19 orf12<br>homolog	AAAATAAACAAGAACTTCGTTCCTTTATCATTAAGTTTTATTGAAATAGATTGTAATTAACCAATTACAGCATTAATACCG<br>CTGAGAGAGATCTGGGAGACCGTGAAGGAGGAGTTAAAAGCTTTACTATACATAGTATACAACTACCTGCGTGAGTTGCA<br>CCCTGATGACTACATACAAGCATTCAAACTTGTAATGGCGTGCACAGAAAGTCGACAGCAGTGTGTTTACTTAGTAATTGA<br>CTTCATTGCTCATATATTAAGACGCAAGACTTGTATTAAGATGCCAGATATAAATGATTTGGGTTCCGGTGAAGATCAGGC<br>AACACAGTCAAACGCGAAACCAATCCATAAAGTTAAGCCACAGAGGGCGCCAGGGTCGACAGAAAAGGATAAATTGAAA<br>GGGAGTCATGCACCAGACGCGTCCAGTTTAAAAAACACTCGTGAAACGCAATCATTAAATGATAAAACGGAGCCATTTTT<br>GGCCCTTGTCGCTGTTAAATATAAACAGTACTCGCCTCGAACTAAAGCTATAGTAGAACATAGGATACATAACATTTTGTT<br>TGAAGCCGACATGGGTTTGTTCGACGAATCGCCAAGTACTGCAAATACAGTAAATAG
MSTRG.7<br>478	Glutathione S-<br>transferase 2-like	CGCGGCGAGTCGCGAGTCGAGTATTTACCGAGTGCGCTTAGCAACAAGCAACAACAAACATTCTGTTCATTATTCATCCCG<br>CCGGGCCGGCCGGCAAACCAGTCTTTCGTTCCAAACCACCAAGCGAAGTCATTCAGTCAGTTACTGTTCATAGCAACAGCG<br>GGAGTGTTGATATTGTTGTTTTAAATCAGTTTATATTCGCTTTTTTTCTCCTCATTTTATTAATAGAAAATGCCGAAGGCAGT<br>GTTCCACTATTTTCCCGTGAAGGCTTTGGGCGAACCCGTCCGCTTGCTGCTTGCGTATGGCGGTCAGGAGTTCGAGGATGA<br>CCGTATTCCGTCTGACAAGTGGCCAGAATACAAACCAA
MSTRG.7<br>497	Modifier of<br>mdg4-like<br>isoform X1	AATTGCCGCGTTACTCCACTTCGAATAGAGGTTCATTACAATTAATATTGAATGGCTACATTTATTATAATCATCACATCGC<br>TAATAAAGGACGAAAACGTCGCTGGAGATGTATAGACTATCGTACTATACAATGTCCCGCGTTTATAGATACTGATGACG<br>AAGTCATAGTGAACAGGGAATGCTTACATCGTCATCCGTCCCATAGAAATAAAATATTGAAGAAAGCAAACCAAAACTTA<br>ATATTTACATCAATAACTAAAGCTACAGAACAAGACTCTGTTTGAACTAAAATACAGTGACTACAACTAATTTGTATATTT<br>ACTGTATTTAACAATTAAAACATCGTAATAATAGTCA
MSTRG.7<br>498	Myosin-Va	ATTGTATCACTAACTGGGTATTTACAGGGTATAGAGGAATTAAACACCTGAGTCCTCAGGAATGGCAACGCATGGGGGGG<br>TATCATGGGCGAAACAGTATACTCTGTTATGTCCACGGTACTGCTCATGTCTATAGGCGACGGTTACCACTCTCCATCAGG<br>CGGGCTGTGAGCTTGTTTGTCATTCTAAGTTCCATAAAAAAAAACTAACTTCAAGGGTTTTATTTTAGTCGTCTAAAGTATG<br>TTTATTTGTTCCAAACACAGAAACCATGGTTCCAAGCACTGAAAAATGTCACGGTGAATCTAAAGTCGCTCGCGTCGTCCA<br>ACTACGGATCAGATGTGGACATTATTAACGAAGATGGAGAACTGGCGACCGCTTACGAAGCGCAGAAGGGAATTAACAG<br>ACAATTGCAAGAGGAGCTGACAGCTGAGAAGAAGTTTATTCAGAGCACATTACGAAAACCAAAGCTGAAATCGACCGG<br>CTCAGAGAAGAGAATGAGAAATATCAAAAGT
MSTRG.7<br>503	T-complex<br>protein 1 subunit<br>gamma	AAATGTTTCAGGCTTGCACGATACTGCTGCGTGGCGCTTCTAAGGATGTACTTAACGAGATTGAAAGGAATCTACAGGAC<br>GCTCTACATGTTGCTAAAAATTTGGTGGTGTCGCCGCGGCTGGTGGCGGGCGGCGGCGCGGCCGAGATGGCCGTGTCGCA<br>GGCGCTGGCCGCGCGCGCGCCGCACGCCTCGCCCTACCGCGCCGTCGCACACGCGCTCG
MSTRG.7<br>516	Zinc finger<br>protein 91-like	ATTCACACCGGTCTGAAGCCGTTCAAGTGTGAGATGTGTGGCAAGGGATTCAAGCACGCAAGCGAGAAGCGGGCTCACAT<br>CACGTATGTGCACCTCAAGAAGCCCTGGCCGAAGAGGACGCGCGGCAAGCGGCGACCGGACGGACGGACGGGACAGATG<br>CAGCCGCAGCCGTCCGCGCCAACGAGCGCGAGCGAGATGGACATAGCGCAGCCGCTGTGGCCCGCGTGCGACCAGAAAC<br>TGGGCGAGATGAACCCCATAATGGACGATAAACCTATGTATTATAATTTAAAGATATAGTGAAGTGTAGTTTTAGTGTCCA<br>CACGTGTGTAAAATATATTAGAATACACTGTAGTGCCATTGCTTGCGGTGGGTTCAGG
MSTRG.7	Protein espinas	CCTTTTTATAATATTTAAAAAATTGAACTATTCGCAGTTTCCATTTGATTACGACGCGAACGGATTATTCAATTTTGAAAT

518	isoform X3	TTTATAAACAATTAAACCTATTTGTGATAAATAAATGTGTAAAGTGATAAATGTGATATTATCAGTTACATTGTAGTGTGC<br>AAAAGTGTTATGATATTGAAACGCTGAAAGTAAACGCAAACGTTTCAATCAAATATTTAAATGCCTCCGGAACAGAGTCG<br>AGAAGGCATGCTGTGTCTGTCGAGACACGAAAATTGCCCCTGGTCGGATGCCCTCAAATCGGCGGATGTCAACTGGAATG<br>GTCTGCGTTACACCTGTCTGAAAATATTTCACCTGTACTGCAAGGTGTTCGATAAGGAAGATGTCTTCGATATGGTTATTA<br>G
MSTRG.7537	Fanconi anemia group I protein-like	AAGAATGGAGATTAATAATCCTATTTTTATCAAAATTAAAGAATTGGGTATTAAAAATAGTAAAAGGGAAGAATTGCGTG<br>AATATTGTAAACTAAAAATGGAAGAGATGTTACCGGTTTTATCTAGACGTATTATATGTAGCGATGCTGCCGATTTGTTGA<br>ATTGTTTATTCAATGGATTGTCAGAGAACTGCGCTATTTCAGCTAGGAATAAAGTTAAGGTAATAGATATAGTGCTACAAA<br>CAATGAGAAAAGAGTCTACATCTATCAGACACTGCAGTGAGGTTGTCAGTAGATTGTGCTTAGAATTATCGAGGATGCCA<br>GCTGATGACTTGGTAACATGGTGTGATGACAGTGTCCAGTCCATTGTCGAAGATGCTGATGTTAATATGATATGGAGAGAT<br>GTACTGCCCGAGTGTTTGTACGTGCTGGCGTCACATATTAATATAAAACATTGTGGTACAGTTATGACCACCACAGAGTAC<br>AAAGAACAGTGTGTACGCACATTATGCCAGTGTCGGTGGAAGGAGGGACAACTTGTACAGTTAGCTGCTATGTTTAAAGA<br>TATGCAGTTAAATCGCAATGATCATAAACAAGTTGTCAACAAATTATGCTCTTACATGACAGATATATCCCTGGAAAATCT<br>ACCACCATTAGTTTATCAACTGCTCAAGTTATGCAAGACAAATAATGTGGAAATAGTTATGGCGCACCTAAGTCATTATTT<br>TAATGGAAGACTGTATAGTAAATTCGAGCCTC
MSTRG.7551	AP-1 complex subunit gamma-1 isoform X1	CAGGTGGCCGCGTGGACGATTGGCGAGTATGGTGATTTATTGGTGTCGGAGGCTAGCAACGCTATATCGATGGTCGACGA<br>AGACGGCTTTGATGATTTCACTCGTCCATCCGAGGAGTATGTGATCGACATATACCAGAAGCTGCTGTGGTCCACCCAGCT<br>CTCCATCACCACCAAGGAGTATTTACTGTTATCATTGGCGAAGCTCTCGACTAGATTCACCACGCAGCCCAGCCAAGA
MSTRG.7563	Lens fiber major intrinsic protein	TTTTTCCGTTTATTGGAGCATAATTTTTACAATTTTATTACAGGGCGAATTGTCCGGCGCTAGCCTCAATCCGGCTAGAAGC<br>TTGGCTCCAGCGATCTTCCACAACTTCTGGCAGGATCATTGGGTGTACTGGCTGGGCCCGCTGGGTGGCGCAGCGCTGGGC<br>ACGCTGCTGCATCGCTACGTCATCGCGCCCCCCGTCGCCACCACTCGCGCCGACGGCAACGGCGTCCCCGAACGACTGCCG<br>CTGCAGGACAAAACCGCCGTCTAATCATGGTCCAACTGCCATGGCCCCGACCCGCTCTCTTTCCCTCTCCCATGTCCACCCC<br>TAGCCCAGGGCTAAGGCTGCTAGGACAGGGCATTTCCAATCAAAATCGCCAACGCCATCGATAAAACTCATCGAAATTGC<br>TTTAACGTTTAAAATCCCGATCGACGAACGAGATAGCAACAGTCTCGTCTGCGGCAACACAACATTAAGTAGTATGGAGT<br>AGCAGGTCAAAGAATACTTTGTAGTACCGACATTATTATTTTGGAACATTCGTAATCTCGCAAGAAAAATGTTTTTGAACC<br>AACGCTCTCGATTAATTCGTTTAACACTCATTTAGAGGACACACAGGGCAGGCAAATCGTCAACATGGTTCGAAAACTAGT<br>TGAACCAACCAAACATGAGATTTTCTTTCTTCACCGACTTCAATCGTGGCCAGACCCTTTATTTTGATTTAAAAACAATGTA<br>CTGACAGATTTTGATAAAATTTCTAAGGAACCGCTTTGAAAGCATATTCTTTGAGATAAAAGTTTTTTTTTTCGTTTTACA<br>AATGATAAAGTTATGAAGTACAACCGAACTCTGAAGATCACGCAGGGACGAGAGGACACGAAGCGAATTGTTTGTTCCAT<br>ACTCCCAATTGAAGAATTACGAATTGCTGGTCAATCGATGAGCACCAAATTCCTCATTTTTAATTGGCAAATGGGCATTTT<br>TTAAAGCCGCGGTATACCTACAGACAACTCGTACGTGGATCAGGATCTACGCGTAGTCATTAAGATCTCATACCAGACTTG<br>TTTAAAAGTCACCATCACTGTAAGACAAGATCTTTTTATATTTTTAATATTGTTTAGTTATTGTTAATTAAATAATACTTTAC<br>GATGAAACGACATTATCATTGTTTAGATAATTAGATTGTGCACGTATCCCGCCTGAAGGCAAATACAGTGACTACAAACA<br>GCGGCTTGACTGGCATGATTACATTGAATCGTCTCGATGGAATGGAATATCTTAATATGTATTGTATGTATATATGAACCC<br>TGGGTCATGATGTTTGTTCACGCTAATCTCTGTAACCGCTCGAGCGGTTTTCATACAACTTTGTAAATATGTATGGTTTGAT<br>TCAACTTGAGAGATACGGTTTGATCTTCACAGTATGAACTCGGATAGAACGACTGCATCCGCAACTCGTTAATTCGAATTA<br>TGAAATTCAGTATGTGGTTAAAAAGAATTTCGCAAATATAGGTACTTATGAAGATAGAAATTTAGTGGTTTCACATTCCGT<br>GTATGCAGTCACGGAAAAATATAATTTACAAATCGAATCAATATTTAGTTATTAATATATAATTAGTTGTGGTATCATAAC<br>GTACAAAATAATTACTACACGTAACGTTACACTGATATTAAAAAGTTAAATATTTTATAATATCAGTGTAACGTTACGTTG

		ATATTTGTAATAGAAAATGCCATTAAGAATGCCAAGTACACTTGAAAATATTTGTCAGTCGCCAAAGAACTTAATAAATGTGGTAAACTGTAATTACTTACATACTCGAAATTAATTGTTAATTTAATTTAATGTTTAACAATTTACAGGGTATCTATTTTATTCTCTGTTTATATTACTACTCAATTACTCAAGTGTGAATAAAGTGATGTAACAGAATATAAGCTAAGACTGAAATGCTTAAATGTAAGGACGTAACTAGAAGGTACGTTGGTACGTCGACACCGACTGCGCCTGTTGAACATTGAACCCCGAACCCAATAGCATCTGTGTCGGTCTTTTTTTTTGTTAAAGTAGCAAGCACTTTTATAGTTTCTCAGAGTTAGGACATAAGTATGATGTAAAACGTGCGTGTAAGGTGACCCCATTACACGAACAGACAATCTCAAATATGGCGTGAAAAATAATATAGATAGATCAGGATATCAGGACAGACCCTGTCAATAAAGTATCTGAAATTATGATGTACAATTGTTGTGTTATATACTTTAAGTTACACTAAGAGACAAACGAGTCTATCGACAGATACGACATACATGATAGTACAAGACACTAACGTCGTCTTGGTCATCCTCATGCGTGAAGTTGCGAACGCGCATCTATCGACAGATTAGTATGCGACAGGTTCTGCTTGCTGACATGCTGAACTGCGTGAGAAAGCGGCC
MSTRG.7564	Protein amalgam-like	GCGACCAACTCTCATGGTGTACACAACAAATCCGTGGAACTGGTCCAGAGGCCGGTCATCGAGGAAGTAGAGGCTGATGGCCTGAAGCTGGTGTGGAAGGTCCACTCGCACCAACCATTGGAAGACATCCGCATCCAAGCATTCCCTTTGAATGGAGACAATGCTGATCCTACCAACATCACTGTGCCCCTGCCGGTAACAAGAGGCAAAGTGTACGAAATGAGCCACGAGGTCAACGACCTCCCAGCTGGCCAGTACGAGGTGATCGTCAAAGTGAAGAACAATAAAGAATGGAGCAGAAACACGAGTCCAGTTGTCCTCAATATCGCTGAGCAGTCCATGCTTATTCAGCGTAGTTCAG
MSTRG.7565	Uncharacterized protein LOC106133343 isoform X1	CTTACTTGGATTCGAGCAAAATAATTACGTCGGTTCTATCGAACACAGTACGATTGATTTAGTACCAATAAAACTGGCCGGCGAATACTTTGCAGAAGTGTCCTTCGCCATACATGGAGAATTGAGTAGTTACTTCTCGTTGTTAGAAAGTGACTCCATTATTACCGTAATACTGAATGGCCCCATCCCTGAAGAATCGATACCCGCCAACCGGATCGTAGTACTTGAGATATACGCAACCGCGGAAAATGCCTTACCCGCAGTGGCAACGATAATACTAGAAGTCGTGTCTGATGACGAGTCTATTCCCACTCAATTGGTGTTTGCAGAAACTTACTATGTCGGATCTTACACCATAGACACCGGTCTCGAACTGCAAAACTCCATATCTCTGTCCGAAGGCTACGACGAAGACGTCGAATTCGATCTGAAAGGAGACGATTCACAATATTTCGCGATAAGTAGAGATGGGAATTCCATCACATTGAATGTGATCAACACGATACCGCCTGCCGTCTTAGAAAACAATAATCAGCTGCTGTTTGTCATCCGCGCTCAAAAACCTGGAAGCACAACTGCAAGAGCAAGTGTCATAATTTTACTGCCCGACGAGTTTAAAGATGGCAACTTACTTGGATTCGAGCAAACTAATTACGTCGGTTCTATCGAACACAGTACGATTGATTTAGTACCAATAAAACTGGCCGGCGAATACTTTGCAGAAGTGTCCTTCACCATACATGGAGAATTGAGTAGTTACTTCTCGTTGTTAGAAAGTGACTCCATTATTACCGTAATACTGAATGGCCC
MSTRG.7576	Phosphatidylinositol 4-kinase alpha isoform X1	CAGGTCTATGGCCCCAAGAATGGTACACGGGTGTGAAAGAGATAGCTGTCAAATCACCCTACCTCGTCTCCCAGACGTCGCTGCGTTCAGAAATGCGCGAATTACAATACACGTCAGCTGTGAGGAATGATTCCGTGTCCTTGAATGAACTTCAGGAGTTGAAAACACAAATATTGAATTTACTAGATCACCCGCCAGATGTCACTGTTATGGTGAATAAGTTGACGTTCGCGCCTTGTATGTACCTACTTAGCGTGTACTGGTTAGAGACATTGAGAGTATGCAACTCGCCAGAACCAAGTTTGCAACCGATTATAGAATATTTAAGTGATACAGCACTCCAAAAAGACAAGAGCGGCATGTGGCAATGTGTTGCCAGTGTTGGTGACAAAGTATTCCAAAAGTTTCTGGACGTGATGTCTGACAAAGTGAAGGACGAGTCAAGAGAACGAGAACTGGAGGGCCACGCACAGTTCCTGCTCGTCAACTTTAATCACATACACAAGCAGATCAGACGGGTGGCCGACAAGTGGCTCGCCGGACTGGTTGATAGGTTTCCTCACCTTCTATGGAACTGTCGGGTGCTCTGGTCTATGCTTGACATTCTTCAAGTGCTCAGCTTTAGTCTTGAACTAGACGCCAACCAGGAAACGCCGCTGCTTAAAGTCCCAGGCACCGATTTCACCTTGCAATTACAAGATACGTTGGAGGGTAGAGAGTCCATTGTAAAAGACTTCGCGGATCGTTGTCACGGTATAGTACAAGAGGCGATGAAGTGGGCGCCACAGTCTACACGATCGCATTTGCAAGAGTATTTAAACCAGGTCCCCAACTCGACGCTGTGGCACCACTGCGGGCTGGCGCTGGCGACTGGTGCGCTGCTGGGCGCGGCGGGGCTCAACCGCATGTCGGCGCCGCTGGCTCGCGCCGCGCTCGACAAGCGGCCGCCCTGCGTCACGCACGACTCCGCCGCCCTACTCGCCGTCGTCTCGCAGCGCAGCCGCTACGCCGGCG

MSTRG.7586	Serendipity locus protein H-1	ATTTCCAGACTTCAACTGATCTCTTAGTGCACGAGACATGTCATAATAAACATGTATGTTATGGGTGTAATTCCAAGTTTG<br>ACACTTTCAAGCTATTAGTGAAACACAGAAAGAACTGTGATTCCATCGCCACAGAGGCCAAGAGACCTTTAATGTTGAAA<br>GATGTCATGAGGTCTAAAAATAGAAAATCTGAAGACAATGAAGAAGAAGAAGAGGAGCATACAAACTCGTTGCAATGTA<br>AACTTTGCAACGAAACCTTCACGGATGATTACTACATGAACATACACCAAGAAATACACCATTCAGCCACAAGTCAGTGG<br>ACAGCGCCAGTTGACACACCCACAAGCATGGAGAGTGAAGTCCTGAATACTAGTATTACTGAACACAATTAGTGATCAGC
MSTRG.7596	Uncharacterized protein LOC106135670	CACCGCCGGCGAGCAGTCGCAACAACACCCACGCGCTGCGCGGACACCGACATGGAAGCACTTATGTCATTGCTGCTGTT<br>GATCATCAGCGCGTGGTCACAAGGCGAGCCGCTCACCGAGGCGGTACAGCGCCGCAGAGACTCGTTCGGTGTTTACGCGA<br>GCGAGTTCGCGCACGACTACGAGACGTTCGCTTTCGCTGCCATCCCGTTGCACTCGCCGCTGCCCATCCTGCATATCCTGA<br>CCCCCACGCCGCTGAGGCTGCCGCTATACAAGCACCCACGCCATCCAACACTCAAGCCAGCGGACTCGACTCACCAGCAC<br>ATATACGCCCCATCCATGAATCACCTGCTATACGCCACACCTTTCCCTGAGCGAGAGGAGATCCAGACTCCAGAGACGAT<br>ACTGTACGCGCGGCCCAACTCTAACGGAGGATATACGTACAGACGACGACCGGTCAATAAACGGAACGCGTCAAAGAAC<br>AACACTTTGAAGGAGCCAGTGATCATTAGAGTACACAAGTACAGGATTGTTAGAGACTGATGAACTACTTGTATAAGATC<br>GGTATACCGTCGGTCGTTGTACGATATTTATCACGGTGAAAAGGTTGTGATGAATTGGTAATACATTTCTCTGCAATTTGA<br>ATAGAAATTGCATTTGTTATTCTCGGTACTAGTAGGTGGCGTTTTAGTATTTTTTGTTTTACTTTAAAATTGTAGATACCTTA<br>TGAAGGTAAAAAAAATTATCAAACCAACCAGGC
MSTRG.7597	DNA repair protein XRCC1	GTCAAACGAACAAATCTGAATTGTCTGACAATTTGTGAAACTCGAAAGTTGTTTGTTTTGTTGAGAAATAACCTCAAAGTA<br>ACAGAAAAGAATCTCTTGTAAATAAACAAGACAAACCGGGGACTACGATAATACATTGCTGAACGAAACTAAATATTTTA<br>TTTTACAATTAAAATAAGTCATTTGTGATAGAAATATGCCACGAGTGAAAATTGATTATGTTGTGAGTTTCAGTAGCGAAG<br>ATCAAGACAATCAGGCTAACAACTTACTGGCCTGGGAAGTAGGCAAGAAGCGATGGCTGTGTAAGAAAGGGGAGTCTTC<br>ATGCTCAGTAGTGCTGCAGCTGGCCCGAGCTGTTCAGATATCAGCGATACACATTGGTGCATATCAAGCTGCATTAGTGGA<br>AGTTTTGGCCGGTAGGTCTGAGACGC
MSTRG.7604	Sprouty-related, EVH1 domain-containing protein 1	GGGTCTATAAAGAAGTTGCACGGGTACAGCGGGCCGCCGCCATTGCCGGGAAAGAAACCCACGGAACCGTTTCAAGCGA<br>GGCTCAAATGCAGACACTGCCACGAGTGGTACCTGGAGAGCGCCAACCGCGCCGGCGCCTGCGAGTACGCGCCCGACTGC<br>TGCCGCGCCTGCATCGACGCCGTCACCTGCATCAAGTGCGC
MSTRG.7608	Eukaryotic translation initiation factor 4E transporter-like	AGAAAATCTAGAATATTACTAACAATTGAACAATAATCCTCGATATATCAATCTGTCCCTCCTCTCTCTGGGGACAATTTA<br>TAGCCATCTCTGTTCCAGACTTCGGTAATAATAGAATTGTTGTATACGGTATACAGATGGGCGGTTCGCCGCTGCCCGCGG<br>GCGTGGGTCCCGTGGGCGGAGTGGGGGCACCGCACCTAGTGCTGCCGCCGCACCAGCCGCTGCGGCTGTCGCCACTACCG<br>CACCCCGGCGTGCCCGCTCGCATCCCATCGCCGCGCGAGTTGGTCGCGCACACGCAGTCCATAATGCAGGGCGCACTCAT<br>AAAGAAGAAGCTCGAAGAACAAAAGGAGAACTTCCGCCGGCGGCACGAGATGCAGCAGCAACCGAAACAGTCTACGCCC<br>ATCTCTTTCACACCCACCTCGG
MSTRG.7609	E3 ubiquitin-protein ligase UBR4	TATTGGACATCCCGCCGGATGCGGATGACGAGACAATGGTAGAACTGGCAATCGCATTGTCCTTGCAAGAACAACCGCGC<br>CGCGCCCCGCCCGCACCCTCTGCACCACCGCCACCAGCACCTGGCTTCAGTGACGTCACTGCTTCACCGCCAGGCTCGGAT<br>GATGAAGGCAGTACAGCAGCAACGGACGGAAGCACATTACGTACCTCACCAGCGGAGCCACCTGGCTCAGCGGGCTCCG<br>AGAGCGGCGGTTCCGGCGCCGACAGTATCGGTGGAGTATCTGGACGTAGTAGCGCATATGCGGGCCCGTCGACGTCGTCG<br>CAACCCGTTGCGTCGACGTCTCGCGCGATCGAAGCGGAGACGGAGACGAGGAAACTGCACGCGTTGAGGCTGTCGTTGCT<br>GTCGGCAGCATTGGATGCGTTACCATCTCTACGGTCATTGCCAGGAGTTCGAGCCATACCGTTTGTGCAAGTAGTCCTCAT<br>GCTGGCCGGAGATTTAGATTCGAGCGTGGAAGGCGACCGTGTGGTCCTCGAACGACTTTTAGAACTGCTCGTGAACGAAC<br>TAGACATTCAGGCCGAAGAGCCGGCTCCAAGCGAAGAGCGTACAGACCGCCGTGAACTGCAGCTGGCGATCATGCGCTTG

		GAGCTTCTGGTGTCGGAGCTTGACATGCAAGAGGAGACCTCGCCGCCCATACACGAGAGAA
MSTRG.7618	Pre-mRNA-splicing factor ATP-dependent RNA helicase PRP16	ATACTCAAGCCAGTGGTGTACAGAGCACTTCGTGCACGGTAAGGCGATGCGGAAGGTCCGCGAGGTGCGGCAACAGTTGC<br>GCGACATCATGCAACAGCAGCGACTGCCGCTCGTGTCGTGCGGCACGCAGTGGGACACTGTACGCAAGTGCATCTGCTCA<br>GCCTACTTCCAACAAGCGGCTCGTCTCAAAGGCATCGGTGAATATGTAAACTGCCGTACGGGCATGCCTTGCCACTTGCAC<br>CCCACGTCGGCGCTGTTCGGCCTCGGCTCCTCCCCCGACTACGTGGTGTACCACGAGCTGATGATGACCACCAAGGAGTAC<br>ATGCACTGCGTGACAGCAGTGGACGGACGCTGGCTCGCTGAGTTGGGGCCTATGTTCTTCTCTCTAAAGGAGACTGGCAA<br>ATCGAACCGCGACAAACGCAAAGAAGCCGCAGTGCACCTACAGAGAATGGAAGAAGAGATGAAGCAAGCGGAACAGAA<br>GATGGCAGAAGAACGTCGCAAGCGCGACGAGGAGGTGCCTCTGAAGCAGACCGTGGCCACGCCGGGCCTGAACACGCCC<br>AAACGGACACCGCATCGGCTGGGACTTTGAATGGAATGGAATGGAAATTGCCGACAACTGGTGACGTGGAAGCACTTATA<br>CTGAACCTCATTCAAATATGGAAACAAGAATTTAAGGCGTATTTAAGGGGGGTTAGTTAGGTCACCCCCCCCCCTTGCTGC<br>AATGAGAGCAGTTTTTAACTATGTGCAAAGACATGTTTGAATGTATATAATGTGCCTTCTAAAAGGGCTTAGCCCCGGAAA<br>AATAATTCCTTGTTTTTTTTAATATTGAAGATAAATACCATTTTTTATACAACTTAGAATGGCAAACAAACTGACGACCCAC<br>CTGATGGAAAGTGGTAACCGTCGCCTGTAGACATGAGCAGTACCATGGACATAACAGAGTATACTGTTTCACGCCATATC<br>CCACCCTTCAAACCGAAACACAGCCATGCAAACACAACGGCTTCACGGTTGAAACACGAATTGGACAGAGGTACCCAAGC<br>AATCGACTTTTGTACAAAGTCTCCCTCTGGTAAAAGCTCAAGCCAGTACAGTACCCATCGTCATGTATAGGTCATCATCTG<br>TCCACTGCTGGACATAGGCCTCACCTATAGACTGCAGGAACAATGCTGAGCCACCTGCATCCACTGACTCCCTGTAATGCA<br>TTTGAGGTCGTCACTCCATCTAGTGATCTAGTAGGGTACTAGGGTGTAGGGATCTACCTACACTATATAGTATACAGTATA<br>TATACATCATATAGTTAATAATATCATAATTTTATATACCCATAGGTATATAAACTTATTATGTACATGATTAAAAATAATA<br>ACTGTTTTTTGAGTTCCTTTTAACACATTACAATATTCCGAAACCCACACTAGAATATAAAAAAAAAAAATCTGATTTAAAA<br>AGCTTGTAAACAAACATATTTAAACAGAACATTATTTCGCTTGTATTCTTATTTTAAAGATTAAAATCTTGTTTTTAAAAAT<br>TTACAAATTCCGAAATATTAGTTACAATAGTTGGTAACACGTAAATGATACCTCCAGTGTTGCAAGTATACATTGACAAGA<br>CCAGAGGGCCTACCATAAAATGTTTTCCGAAATTTCGGGGCCGAACGAAACACAAATTTCATGTAAAAGTACACGATACC<br>TACCGTTTAAAATTATTAAAATATCGTTCCTTTGGACTTTTATGGTAAAATTTAAGATAAACAAAATACTCCATTTACTGAG<br>GCGATTTCAATATGAACAAAAACACGAAATTGAGTCCGAAGATTCGGAATTTCATTTCGTGGTAGACCCTTTGAGCACCAT<br>CAATAGAAAGAACAATATACCAGACATTGCTGTCTTATTTTATCTTTGCAGACAGTAGCGAAAACGTGTCCGGTTACATTC<br>GTTAGCCACTGACCCTTATGTCGCTTCAATTTGTTAATTAACCTATCTTATAAATTCCCAC
MSTRG.7636	Ribonuclease Z	AAGAAAACAGAAAAAGTATTGCAACAAGTACAGAGAAAGAGCCACTGCTCTGTTGCATATATTTGTACTTTAAAGAAACG<br>TCTAGGCACTCTGGACCTGGCGAAGTGCGTGGAGTTCGGCGTGAAGCCGGGTCCGTTGCTCGGTCAGCTGAAGAATGGGC<br>AGGACGTCGTGCTGCCGGACGGCACGCTCGTGCTCAGCAAAGATGTGAAGACGCCCGACGACCCCGGTCCTGTCTTCATT<br>GTGTTGGAGGTGCCAGAGCTGTCATACTTGAAGGAGTCGGAGTTCTCGGCGCATTTCGATAACGGCACCAACCCGCCGGA<br>GAATATCCCGCGTCTCGTCGTGCACTACACGCCGCCGCACGTGTTCAACCATCCCAC
MSTRG.7639	Vacuolar protein sorting-associated protein 13D-like	CTGGGTTATAACGTAACCGCGTTGTTGTCGCGGTACAGGTGCCAGCTGTGGCGACGCGGCCCCAACGACCAGCTGATCCA<br>CGAGGGCAGCTCGCCGCCGCAGCCCACCGACGCGCTGCTCACCGACGAGGAGGCGCTCTCGCCACACGCCATGGTGCTGG<br>ACATCAGCGAGCCGGCGCCGCGGCCGGGCGTGGCGGCGGCGCTGCAGCTGCGCCGCGCCGACCCGCGCCGCGCCTCCACG<br>CAGCGCTGGCGGCTCACGGCCGACGGCCGCATGGCCTGCGTGCACTCCAACCTCTGCCTGCAGCCCGCCGGCGCGCACGC<br>CGGCCTCATGGCGCTCACGCCCGGAAACCGCGTAGTGTTGGGCCTGGTGAGCTCAGGGCGCGGTGGGGATTCGTGTCCCC<br>CCGAGCAGGCGGTGACGTGGCAGACGCTGCGGCCGGGCTCGGGACGCCTTGACGTTGCGCTGCTGGCCGACGGACCTACC<br>CGCGTGCTGCGCATATACGACCACGACGCACCGGAATCTGGCTGGATCGAGAACGAAGAGGAGTCGGGGCGCGACAGTA<br>GGAATGCGAGTGGTAGTGGCAGTGGTAGTGAGCGGGCGGGCGGGCGCGAGGTGGCGGCGCGCGTGTGCGTGGCGGCGCT

		GGGCGTGTCGCTGGTGGGCCGCGCGCCCGCCGGCGAGCTGGTGCACGCCGTGTTCTCCGGCCTGCTGCTGCAGCTCGCGCT<br>CGCCGACCACCTCGCGCTCGAGCTCGCCGTCCGCCACATGCAGTGGGACAACCAGCTGCCGGGCACGCCGAGCCCGGTGC<br>TGCTGT
MSTRG.7<br>660	Zinc finger<br>protein 93-like	AAAATATTTTTAACAAATCTATATTTCTAATTTTAAAAGTAAATAATTATTAAGTATTGACAACGACTGTTTACAACGCAG<br>AAGCCGAGGTAAATATGAGTATCACAGACAGAAGCACCGCGGCGGGGCGCAGTGTCCAACATGCTCGTGCGTGCTCGCGA<br>GCAACGCAGCGCTCGTCGCGCATCGCCGGGCGAAGCATGCACGGTTACCACAGCGCGAGGATGCCAACTTTTATTGTCAA<br>CCGTGCGGGAAATTTTTCAAAACAAGCTGCAGTTATAGGAAACACCAGCGCACAGCTAACAAACATCTCGACGTTAGCAC<br>GTTGAAGTAAGTTTTATGTATATTTTACAGTACATTTGATTATATAGATATGTTAAATTGAAAATGTTTGAATCTTGAATTG<br>TGTCTAGTGTATTTTTCTATGCCATATGCACGCCTCGCCC
MSTRG.7<br>674	RNA-binding<br>protein 25-like<br>isoform X1	GTGATGGTGGGCGCGTACGGCGGCGGTGGGCGCGGTGCGCTGCTGCCGGCGCCGGCGGCCGCCAAGGTCATTTCGCGTCC<br>GCCCACCAGGAACAACGGTGCCAAGGATGCTGACGGCCCACCCGTCACCATTTTCATAGGCAACATCACGTCGCGCGCGC<br>CCGACCCGATGGTGCGCGCGCTGCTGGGCGCGTGCGGGCCCGTGCTGTCGTGGAAGCGCGTGTCCACCTTCGGCTTCTGCG<br>AGTTCGCCAGCCCCGAGGCGGCGCTGCGCTGCGTGCGCCTGCTGCACGAGCGTCCGCTCGCCGACAAGAAGCTGGTCGCG<br>CGCACCGACGGCAAGATGCAGGCGCTCGTCGACACCTACAAGACCGAGCAGCGCTCGCGGCTGGGGCTGGCGGAGGAGG<br>CGCCGGGCACGGAGGCGGCCGGCGACGGCTACCTGGACGCGCGCCAGCGCTCGCTCGACGAGGCGGCCGAGCGCCGGCT<br>GCGGCAGATCATGCAAGACTACCAGGACGACATCAACAACTACGAGATGCTGCAGAAAGAGGAGGCGATCTCGAAAACG<br>GCCCGCGTGCTCGAGGAAGCGGACATATCGGAGGAGAAGCGGGACGTGATCCATCGTGAGATCGGCAAGTTTCGGGAGA<br>GCATGAAGGAGGAGGCGGAAGTGGTAGTACGGCGCAAGCGCGAGCCCAAGGACGCCGACAAGGACAAGGACAAGGACA<br>AGGACGGCACCAGGGAGCGCGAGCGCGAGCGGGACCGCGAGCGCGACAGGGACCGGGACCGCGCCGACCGGGACCGCG<br>ATCGCACCGACCGCGATCGGGACCGGGACCGCGACCGCGACAAGGAGCGGCGCAGGCGGAGCACTTCCAGGACAAAGGA<br>CCGGCGCGATGAACGGGACAAGGAGCGCGAACGGGAACGCGAAGAGAGGGAGCGGGACAGGGAGCGCGAGCGCGAGCG<br>CGAGCGGGAGAAGGAGCGGGAG
MSTRG.7<br>678	ATP-binding<br>cassette sub-<br>family B member<br>7	TATCGTTCGACTCTAATTTCTTCACTTGTCAGTCAACCATAATGGCGGCTTTACTGCTGTCAACCAAGAATTTCAGTTATTT<br>AATAAAAAATCATGTAAAATTGAACAAATCATACTGTATTAATAATTCTACGATTCTAAATAAACCCCAGTTCAATTTAAA<br>ATGCAAACAGTGTGCCGCCGCAAGTCATTTCAGGTATTATTCTACACCGTCTCCAAACAACGCTGGAAATACAGACCCAGC<br>GAAAAATGTTTTGGCAGCAGTACTGGCAAACAAGCCAAAAACCGGAAAAATACCTGTTGGTAAGTTCAAATAAATGGTAA<br>CATTTATTATTTTAATTGGAAGTGAAATATTTTATGTTATAAAATATGAGGCAGGC
MSTRG.7<br>683	Aspartate<br>aminotransferase,<br>cytoplasmic	AGATTGGGCACGCCCGGCAGCTGGGACCACATAGTGAAGCAGATCGGCCTGTTCTCGTACACGGGGCTCACGCAGCGCCA<br>GGTTGAGTACCTAGTGCAGGAGTACCACATCTACCTGCTCAAGTCGGGCCGCATCAATGTATGCGGCCTGAACCCCAGCA<br>ACATACAGTACGTCGCGAGGGCCTTCCACGACGCGGTCACCAAGTTCCCGGGCGATGATGACGCGCGCGCCAAATTATGA<br>TACAGCAACACTGCTATGACGTGATGGTACGTGATGGTATTATGGTGGCGGTTGCTTGTTCTATACCAAAGTAGCTGAACA<br>ATTTTGACTCTTGTTGTAATTGAATAAGGTTTTATTTGTAAATATTTATTTATATTTTTGTTACAATGTTTAATAAACACCAC<br>ATATAATATCAACATGACTGTTAATAACTTGTGTGTCTTCATACAGCTACATACCTTAATTAATGGCAACAAAACATGATT<br>TTGATTGCACCAGATTGCCTTAGACATTTTTATGTTTTTGGTGCAATAAAGAGTTATTATTAAATATTTTTATAAAGTGGCA<br>CACAATTAATTATTTTAATTAAAAGGAAAATGGCTCAGTGTATGAACATTTTTACGACAGACTATTTTAGCTCCTCTAGGT<br>AAGTGGCCATTGAATCGGTATTGCTTACATGTGATAGAATGAGACGGCAATATCTGGCATATTGTCCCTTTCTAGTGAACA<br>AAAACGTATTGAATCTCAATAAGATTCAATAGCAATATGAAAGTTTAAACTTTAACTTGGTATGATATGTATATTATTTATT<br>TCATTAATATTTTCTTAAATCTATGAATTTAGATATAAATTGAAAAACATCATTTGCATCTTAAAAAGATTATAAATAAAA<br>C

MSTRG.7686	Dolichyl-diphosphooligosaccharide glycosyltransferase subunit STT3B isoform X1	CAGGGTTAAAAAACCCGATGAGTTCAATCGTCCACGTTTGCCACTGTCAAAGAGAACAGTTTCCACGAAAAATATGATCT<br>CGAAAAAGACGTCAAAGAGGAGAAAAGGCCTATTAAAGAACAAGCCGAATGTGGTGAAAGGAAAGAAGGTCGGACGGC<br>TGGAGTGACCGGCCCGCCAATACACCGACTAATGTTTATATGAGTGGTAATATTGCATTGGCCCCTCGAACCTTCCAAAGT<br>ACTGAATTGTGAGTAATAATAATGGTTTGAATAAATTCTAAAGTGTATATATATGTATGTGAACACTATCTGTTCGTCCACT<br>TTGAAATCTAAAGTTTTGTTTAAAAGTGGAACGATTTTCAAATAAAGAAATCAAAGCCGGAAATATGTTATTTTTATTAAC<br>AAATTTCATTGTAATTTAATGAATATTTTAAACAAGTTCCAATTTTTGGGCGTTTTGCAGAATTATTCAGTTAGAATGAATA<br>GTATAATGGATTATATATCATAATTAAGAATAGTATTATTTAATAGGATTCCTGATTATGATATATCAAATTATTTTTAACT<br>AAACTTTACATACCTGCATTGCAGTGAAGTATTTGTAAATAATTAGATGTCAGGCCCAGTTTATGATTCTCATATCACGTTC<br>TTATTTCAACTTGATCACTATCAACTGTAATGGAAACTGTATATTATTTGTCAGTATGATTAACGTGTTTTTAGTTTTAGTTT<br>TTAGTAACACAATAGTTACAGCCCTCTCATTGTTTATACGCGCTGAACAACCTATAGTTTTTATTACAACTTCAATAACTGA<br>ATGTATGAGAGAATGTATAGAGTTGATAATACTAAAACTTTACCATGTTTGCAAGTCAGTACTTGTAAGGTTTATATTATA<br>GTCTAATCTCATTGTGTCTTCAAAATCTGAAGAGGATTCACTGCATTTCTACCACTTATAGGAAATAATGAACGACTGACA<br>CCAATTCTGTATTTAGTCCATTAGATAAATGGTTTCTTATTACGTAGGTGATTTAGTTCAAATTGGTTTGCACTGCGTGCAT<br>GACATGAGCATCGAACAGCCGTGTGCTCCAGCAATAAGGTTGCTACCAATACTTTAACGGTAACGTATCGAGGCAACAGG<br>GAATTTAATAATCGATTCAAAACTCATATTTAATAATTAAGAGTGTATGTAAAGTAAATTGTTACTTACTGAAATGTTTTTT<br>AAAAAAAATATGTAAATAGTTAAACGAGTGTAGGTGGCAACCTTATATAACACGTGATACAATAGTCAAATTATTCTCGC<br>GCAATAAAATAAATGGAATAAGAAACTATTTTGTAAATTCTAATAAATTCTTTATTTAAGATATAATAAATATTCTCTTAT<br>AATTATGATAACATTATTATAAAGAATTGTTAATTTAATTAATTTTACAGACCTGTTTCTTAGTTGGACAAGTTTTCTCATA<br>TCTCAAATAAGCTAATTAAATCACGTGGCACATCTCATGTTATGGCATTCTAAAACAGTGTTCGATAAGTGTAAATTTACA<br>ATTGCTACGTCCATTTTTGTAATAATTGAATATGTTTAACATTGTTTGTTATATAAAACTTGGTTTGTTATTTTCAAATTACA<br>CATTTTGTTTATAATCGTTTAGTTTGCATTGCTCCATTTTCGATGAGCCAAAAGAAGCGAGCACAGATTGGTTTGGTCTATT<br>GTGTAAAATTATAGATTATAAATAAACATGTGATAAATTGACAGTAAATTGAGTTGGCAGTTCTTTTACATTTATCTACAA<br>AATTAGACTCAGGATTTGTATTGTGTTATTAATGAAAATGTAAGTCCTCAAATACATTGTCAGGTATACCAGAGTAACCTG<br>TGAAATTGCTCGAATTAGTCGAAAAGTAAATAAATGTTACGAAACCCGACTAAAATGAAAATGGAGCAATGTAATTAACG<br>AAAAGAAAGTAGTTATATTAAGTCCAGTAATAATTATTTGGCAGTTTTGAGACTTAAGTCTTTTTATCAAAATATCTTCGCA<br>ATAAATAGAATAAAAATGTTTGTTCCTATTACTTTTTCTGTCTTATTTTATACGGCCAATTAAAAAAAAAAAAACACCATTT<br>TTTTTATTAAATATACCAAGTATAAGCAAAGATATACCTCTCAGTACTTGTAACTTAGTGTACTTTATGACGCCCAAAGTGT<br>TATTCATGTCTATAGAGGCGGTTCCTACTTTCCATCAGGTGGGGTGATCAGCTTGTTTACTCCCAGTAGTATTAAAAAGATA<br>TTGCTATATATACTCTATTTCAAATATGATGATAAATATAGCTGAAAACTTGTAAAAGATAATTTTGATACATTAATAGCC<br>ATATCCGCAGGTTCCCTGCGAATTACGATAAAAAGTAATCTGTATTAACCAAGGATATGTGTGCTATCAGGCTTACGCTTA<br>CGTCACAGCCGCTCATGATTAAGGATTACGATATGATCTCGAAACTACTCGATCTAATTACACGTAAGTTAATCGGTAGTT<br>ATTACATTAAAATACCAAGGATATGGGTCGTTTGTTAATTACGTACAAATGTGTTCAACTATTTCAGTGTGATTAAGTAAA<br>GAAACACCTGAAATTTTTACAAATAACATTAGGATGGTAATTTCGACACTTTAAATACAAAATATAATTGAATAGTAACAC<br>TTTTAAAATAAATTGCTAGTTACTGGTTAACTCTGAAAGATAACTCGTCTATAAGAATGACGTTAATTCACAGTATTATATT<br>TGAAATGGAATACTAAATGCCGATGTTGGATATTAAAAACAATAAGTCTTTCAACTGCTAAATAATTATATTAATAATCGC<br>ACCTCGCTTCATTTCAGTACTTATCGCATTTTATTTCGGTACCACGAATGTAAAGATGTATAAAGTATTCGTCTGTGAAATG<br>CAATAATATTTTTATGACTATATTTGTTATAGAATGTATGTAATTGTAACTACCAAAGTTTTAATACATTTGATAGAATGTC<br>TTCTAGTTTGTATTCCGGTGTTGGGTGAGTCAGTAACCTGTTATCTACATGACGGACCGTTCTGTTCCTATGTACGAGACGA<br>ACAATGTAATTCTTTTTTTCTTCAA

MSTRG.7688	Required for meiotic nuclear division protein 1 homolog	AAAGAAGTGTTACTTGCAAGAGTCGTGCTTTGTAATGGTGCCAAATAAGGATAATTCTCTGGAGAGGTATACCTTCAGCCA GGCGATGGCGCAGTCGGCGCGGCTGGGCGCGTGGGAGGCGCGGCTGGAGGCGCTGGCGGCCGCCGTCAGCTACCACACG CGCCGCATGCGCCGCACCGGCATCGCCCAGGTCGACAAGAAGGAGGT
MSTRG.7690	Aldose 1-epimerase	GATCATACTGCGTCGCTACCATAGACATTGTATCAGTATAAAAAAAAGTACTCTGTACTTAATATAGGTCGTTACACTTTT AGTTACACTTACGTTATCAATCAAACGTGGCTATCTTCATTCTATAAATGCCACATGCGTTATCAACTTCACATCATTGTAT TATATTATTTGCGGTGACCGGACGACGTTCTAACAATTTAAGCCATGGTGAAGTTAAATGAAGAGGTGTTTGGAACATTTA AGAAGGATACCGTAAAGAAGTTTACGTGGACCACAGAAAGCGGATTCTCTTTGTCTGCTATATCCTACGGTGCAATAATAC AGTCCGTCAAGGTAGGTTTTCATTATCACCGAATTTCTCAGTTGCTTACCGCGGCTTTATAATTACTCCTCTCC
MSTRG.7703	6tox	TATCGTTGTTTAAATATCGCATGTGGCCTCGAATTTTCTAAAACATTACATGGTAGGTCCTTAGAACTCACTTACATACATT CCTTACAAATGACTTGTACAAAGTCTCGCTCTGATATAAGTAATGTACACAAACTTGAAACTTTAAAATCTTATCTCCTTAG ATAATAATCTAAGTGGGTAAAATTAGCACTTAGTAACGGCACAACGCCATTCCCAGTGCATCCGTGACGTGTTGGTGAATT CCCGATATCACGTACCTAATATACCAAGTCCTGTATATATATGACAGGAAAATCTTCCCTCTCCATACATTTATATTGCAGT CATCAAAACAATCATGTTAACGAAAATAATTTTAATTGTTTCCCTTTGTACTGTGAACGCGTTGTTAGTTAACGGTTCCGCT GTTGGTCATAATGACGCAGAAGAAACCAAAGTTGGGCTCGAGAAATGCATCTACTCGAACTGTGTCTCATTTTGCAATAGT ATAGGTTATATATTTGGTAATTGCATTGGAAATTCCTGCA
MSTRG.7706	Ribosomal protein S6 kinase alpha-5-like isoform X1	GTTTCTAGTGAGAAAACGTTGTGGTGTAGACGAAGGCAAATTATACGCGATGAAAGTATTAAAAAGGCATCCATAGTAC AGAAACTGAAAACTGCCGAACACACCAGGACGGAAAGACAGGTTCTGGAAGCAGTGAGGGCATGCCCCTTTCTGGTCACT CTTCATTACGCCTTCCAGACTGATGCAAAGCTACACCTAATCTTAG
MSTRG.7721	S-phase kinase-associated protein 2-like	CACTATTCTTCTACATTCTTTTTCTTTTGTCTATTCCTAATCGCGTAATCAATTCATTCACTTATATATTTTACTTAGGTTGAA CTGCAAATTTTGTATCAAATAATCAATTTTAAACTGTCGCAATGGGTGATAAAAGTACCGAGGATGATCGTCCTGTGCTAG GAACTTCAGATGAACTCGACAGTCCTAGAAAACGTATGCGCTTGGACAATTCGGCTCGTAAAGGTTGGAGTTTAACTGATC GAAAGGCAGATCACGAGGTTCTACAAGAGATGGGTGTCAGTCTGCTAGATCCTGACGACTGTGAGGCGTCTGACGTGGCC ACTTTTGACAACTTG
MSTRG.7746	Hypothetical protein	GCCATTCTGATACTTGTCCAACTACAAGTGTTTACTAAACGTCGCGCGTTTAGTTCAGTGTTAATTTGCAAAATTATGAGAC GGAAGATCAAAACGTTGTATATATTTGGTCGCCGAAGAAGTGTGATCACTTTTTTTATTATATTCATAGTTTCAATGTTGAG TGTGTGCCGGTGGCTGGCTCAGATTGGTAGCAGAGCGTTGTTGCTCCCATCACCACCAGACCAAAACCTGGAGCACCTTAG GAGGAACAGGAGTCTAAAGTATTACATCGATAAGACG
MSTRG.7750	Hypothetical protein KGM_204629	GTGCGTTTCAGTTTGAATAGTGGGATATAGCAGTGAAATTACAGGCTACAGACGCACAGTGTCACTCCTCAGGAATGGCT GCTACTTTATTTTCATGACGGCCACGGTGCTACTAATGTTCATAGGCAGCGAGCGCCCTTACTGTTACCTGGGCCGTCGGC CTTGTTTACTACGCTCCCAGTTGCACAAACGACCCCCGGTCTCGTTTGATCGAGGCGAAACCGGTAGGTAGTGAGTGAGTG ACGGTGTGGTGTGGTGTGGTGTGCAGCTGCTGCCGCCGCGCGCGCTACCAGCAGGCGCGGCTGGAGGCGGCGTTGTAC GCGGGCGTGCCGGCGCGGCCGCCGGCAGGCGGCGCCACCACCTTCGTGGCGCCCGAGGAGCCCGACGAGCCGGCGGCCG ACCAGCCGGGCGCCGCCGCCGCCGCCGCCGCCGCCGCCTCCGCGCTCGTCACGCTGCCGCCGCGCTCCAACGCGCTGGCG CTCGTGTACTGCGACGCCGGCGCCGCCAGCTTCGTGTCGTACCTCAGCAAGCTGGCCATGCGGCCGCACGACACCTTCTAC CTGCTCACCTGCACCTACACAGAGTGACCGCGCCCCGCGCCAGCCCCCCAGTGCTTGTACAAAATGTAAATTGCCTTCCCC GCGAGGACGGAGGACGGAGGACGGAGGACGGACTGCGAAGATTGTGGGTGGAGAGTGTATTGTTATATGCTATCGTGCC CGTACTTGTGTGTGTGTGTATGTGTGTGTACACGTTGGCAGTTTAGCTGTAAAGTGGTATTCGTTGTTGTGTTCGCTGTTAT GAATTAGCAAGACTATAGCACACTACTTTGGGAACACTCGACTCGTGTGCGGACCGTCTGTCCGACCGCGACCGGGGTTTA

		GTTGACAACTACGCGCGACCGGACAGTAACGTCCGGCAGTAGGAGATTTAAATTTTGACATAGTATTCCTCTAAGTATTGC<br>TACATCACAATAGTCCATAATAACATGTTTTATTTATACGTAATTAATTTTTAATTGGTCTCTACTGTCTCGTAACTGTACT<br>CAGGATGTATCCAGTCTATTGTCTGTAATTCAGTACTACAGCTTGGATGAATGGTTTTGAACAAGTTCGATTTCTGTGCAAA<br>TTCGGTAATTTATTTTTGGTTTTGAGCCTTCAGATATGTTGATATATGTCTTTTGCATATCTAGCATATTGGCTTTTTGGGGA<br>TTCAAAGTAGCACAGAGGTCGAACCTGTTGTAAATCACTAGATATGTCCTAGCCTTAGATCATATAGATAGTGCCTCAAGC<br>CTCAAAAACGGGTCAACTTTTTGTTACTAAATTATCCAATTAGCTGACTACATTTTTTAATGTTCTATATTGTACCTTGACG<br>TATCTATATTTAATTATTTAATATAAAATAAGCTTTTGCGCATTTAAAAGATGATTTATAAGATGAAATAAATAATGAAGT<br>TATAAAGGAGTCACTCATAACTATTTATCACGCAAAATTGTATGAAAAAGTTGCCCCATTGTTCTCGAAGCACTTGTAAGG<br>AGACGCTCTATTTATTTATTTATTTATTTATTTAACTCTTTATTGCACCATAAATAAAAATATACAATGACAAGTGAATAAT<br>TCAAATGAAATACAGTAACATAAATACAGATTGAAATATACACATAAAGGTACAAAAGGCGGTGTTATCGCTAGGGCAAT<br>CTCTTACAGACAACCTTTAGTTAAGGGAGAACAGATAGAAAAGGGTAGGTGGCGCAAGAGTGAATTTATAATTTACCTAT<br>ATAATCTAAGGTCTTGGTTTTTGGAACTTAATGAGGGAGACAGTGTGGCTGTCAGTCCGCTTCTATCGTGACACGATAGAG<br>GATTAATACCGGTATCACGACAAATAATATGTATTGAATGAGTGCTATCTTTCCACTCCGATACAGCAAATATTTTGAGTT<br>TTCTACCGATAGTGTTACTATGTTTTATAGTATAAATTATGTATATTTTCTCTGGTGTGTGTTCGAGTGAGCTGGAGGGAGA<br>GATATCCTATATTGTGAAGATAGTCGAGGCTATGTAAAGACACCAATATTATTTGTCGGCCCCCGTTATTAATGTTGGATA<br>ACCTGCAAATTGTTTTGGCGCTAACAACTGAACTTGTGTTTATCGCCTTATTGACTACGAGTGATGTTATTGTATACTATTT<br>TTGAAAGTTTTCACTTTAAATTCGTTAAATTCTAAATAATATGATTCGTATTGATTCTCATGTCGTTAATTATTAAAAATTTG<br>TTTTTATTCCCTTTGACGTAATGGTCGTCTCAATACACTTGTTAGTCGTGAAAATTAATAATATATCCTCCATGTATAAACC<br>CGACAGGTGGAACTGTATGACTCAAGATCAGAAATTGTTATAAACATGAATATATATTAGTTTTTTTTTTTTGCAAATCGTT<br>ACTAATAACGAATCACACCAGAGGGAGACTTTGTATAAAAGTTTGTTTAGGCACTTTTGCTGTGAATTTACAGGGTACAGA<br>TTAATACCTGAATTGTCAGGTATGGCAATGCATGGGGGGTATCATGGGCGAAACAGTACTCTGTTATGCCCATGGTACTGC<br>TCATGTCTATATGCGACGGTTACCACTTTCCATCAGGTGGGCCGTCAGCTAAGTGAAGACCATTCGAAGTTGCATAAATAA<br>AAAAAAAATCATTAATGACGGATCATTAATGACAGATTCATATACATTTTGCTGGTTTATGCAATACTTTGATGAGTACAT<br>TCACCATTTTGTATTTTTGTTTAGTATTATCAAGCAGGTGAGCAAATAAAGCTGCCTGCTATAGTTCCTGTGTTTACTGTCG<br>CGGTGTTTCATTGTGGGCCCATATTTTGTACAGTATTTAGAAATAAATACAAGCAGCACAGGAATAATAGACCAAATTTAT<br>TTACCGAGTGCTTTGAAACAAATATATTTTCCGTAAACGTCTCCATTCATCCAATAAAAGTTTGAAACGTTTGCTTGAAGTG<br>TTCAATGAAGCGTAAGACGCACATGAAATATGCGTCTCTCGTTTAATAAAATGATTGTTTTAGTATATATGTTCATGTTCTT<br>AGTTCTAAAACGATAATGTAATAATCTTAAATAATTTCATGGATTTATTTGTTTACAAGTATTAATGAAAGCCTAATATATC<br>ACATAATAATTCTATTACAAATTCAGAATTAATTTGAATTTCTTATTTGTAAGAAATGGGTATAAAATGTACCCGACAAGT<br>TTTGTTCTACATATTTTTTTTTAAGTGTAGAATTTCATTGTTTTTGTTGTGTATGTGTTGATGAGTGTGCTCGACCGGCGGCC<br>GTCGTAGGGCGGCGGTGGCGGTGGCGGTAGTGGCGGCGGCGGCGCCGCGCCGTGTGCCCGGTGTACTGTATGAAATGTCG<br>TGAATAATAAATGATTACTATATTTTGTACATATTGTGATAGTGTTAATAATTAAATGCTAATGATTTGAACAGACTTTCCT<br>TTCGCACAATTCACACTGACGTTTAAAAACAATGCCAGTCTATTTTCTATGTTCT
MSTRG.7751	Guanine nucleotide exchange factor	TAGATTGTGACAGCAGCGACTCAGGCCACCTTTACGAGAGTCTGGAGCCGCCTGCGCAGGCCGCGATGCCGCACCCGCCG<br>TTGCCCCTCGACGACGACTTCGACTCCTTCGACAGTGACTCCGATGATGACTACGACACTCACAAGAGTACGCAAGCGCCG<br>CCGACGCTGCCAGCATCGCGGTTGCCGTCCCCGCCTACCGAGAGAGGGCAGTACACGCTCACTAAAATCGCCAGCGCCGC<br>CCAGCGGAAGATGAGACAAATTAAAAGAAACCTCACTAAGAAGTATATAGTTGCAATGGACGGTAAATCTTTAACAAAA<br>ACATTTACAAACAATCAGAACAATACGTACGACACAACGAAAAAAACGAAGACGCCAATATACGCTAACTGTGAGAAGC<br>CAGAGATACAACACGTGTACAGCAATGTCAACTTTACCGACTCCCGGCCCGTACTCCATAAGACTGAGAATCCCGTGATA

		AAAGTCAACGGGACTTTTAAAGAGGAACTAAAGACTGTTATAGGGGAGAAGAGGCTTAGTAATGGGGACGTGCCGCCTCCTTTACCAGACAAGCCCCCTCCAGAGAAGCCCACGACCCCTACAAGTGAGAATAGCAAGAAGGACACTGGAACTCTGTCTAGGAAGACCTACTTCTCGTTCAAATCTAGATTCAGACGGGCGTCGTCGTTAGCGGTCGATATAAACACAGACGTGCCTAGTGCGTTGAAGATCACGAACTCAA
MSTRG.7775	WD repeat-containing protein 13-like	AGGCGGCACCAGCATTCTAGGCGGCAGGGTGTTGTCAATAGCGTGCGAATCAAGCGGCAGAATGTTTGGGCCGCTAACGATAAGGGCGTGATAGTGAGCTACCGGCTGGAGGGCGCGGGCGGCGCACTGAGCAAGCTGCGGCGCTGCAGCGTGGGCGGCGCGGCCGGCTGCGTCAGCTGGAGCCCCTGGCTGGCGCGCCACCCCGCGCTGCTCGTCAGCGCCGCCGACAACTCGCTCTATCTCTTCAGG
MSTRG.7778	Oxysterol-binding protein-related protein 1-like	CGCCAGGGTATACCGTTAACGTACGTTTTTTTTTAATGGAAAGGCCCATGCTAACAAGAATATTGCTAGCAATGGGGCTATTAACCTCAATTACTAATAATCCCTTCTCTCCTTACATTTGTTGGGTCGGCCAGTGCTAATTCATAATAGAATTACGTTCTGCAGGAGATAAAACACCGACCAATGAGGAGGAGGAATTCTGCCGTCGAAGGTCAGGCTCCTCGGAGTCCCTCCAGTCCCTGCACACCGCCATCAGCCCGGCCAGCTCCCGCGGGACCCTGGTCTCGCAAGGTGGTCACGTGTACAGAAACGCCAGGCCCTACAGGAAGACTACGAAACTAAGTTCGGACTGGTCAGATGATAGCGCATATAGTTATTAGTTATATGCACAAAAGCCTATATATTGTACTTAGATATCTCGCATTTACGTACACATGTTTATTTTACACACACGAGTACATGTATAATGTCTATTGATAGTATTTATAAATTGTATTGATCGATATGTCGATAGATACGATTTCGTTATCACACAAAATAAAAAAGTACAAGAAGCTGAATGTGCTGTCTCGTTCTATCTTATTTGGCAACGAGAAAGTGTCTCTAGATAGATTTGTACACTAATTGTCTATGGTGTCAGTTCGTGAATACGATCATAATATTTTGAG
MSTRG.7783	Kinesin-like protein unc-104	AGGTCGCCGAGCCAATAATTCTGAAGACCGGCTCCCGTGTGATCCTGGGCAAAAACCACGTGTTCCGTTTCACCCACCCTGGCCAGCGCCACGAGGAGGCCGTGTCCAATAAGGAACTCACTGACACTCCGGGCACTGGCGAGATCGAAGCAAGCAACACCGAAAATGTGGACTGGGACTTCGCTCAATGCGAACTGCTAGAAAAACAGGGTATTGATCTGAAAGCTGAAATGCAGAAGCGGCTCCTTGCCCTCGAGGAACAGTTCCGCAGAGAGAAGGAACACGCTGATCAGCAGTTTGAGGAGCAGCGAAAGAACTATGAGGCCCGTATCGACGCCCTACAACGTCAAGTGGAGGAGCAGAGTGTCACCATGTCAATGTACAGCTCCTACACACCAGAAGACTTCCACAACGACGAGGACATAT
MSTRG.7785	Protein croquemort-like	GCCGCGGAACCGCTGATGGAAGGCAAGGTGTGACGCAGGCGTGCTACTGCGCGGCGCAGCGGGACGCGGACTGCGTGCCGGCGGGCGCGCTGAACGTGTCGGCGTGCCGGCTGGGCGCGCCCGCCTTCGTGTCGCTGCCGCACCTGCTGCACGCCGCGCCGCACTACCGCGACCGCGTCGACGGCCTGCACCCCTTGCCTGAGCACAACTTCCGGCTGACGTTGGAGATGTTCACGGGCATGCCGCTGGCTGTGTCCGCTCAGCTGCAGATCAACTTCCTGGTGCGCCACTACGCCGGGATGAGGTATTTATAAGCCTGATTCACATTACTGGACCGCGATTAGGTCACGATCCGATCTCATTCTTT
MSTRG.7791	Uncharacterized protein LOC106130198 isoform X1	ATTCTGGTGGGGGTTAGCCCTCGGCATTGGAGCGGCAACCGCAACTGTGGCGGGTGGATTATATCTCTTGAGTAGAATCGAAGAAGTTGAGAGTAGACAAAATATACAAGACATCGAGAGACTCCCGCGAGCTCGCATTTTAAGTAGCACATCCGAATCGCATTTTTATGCTAGAGGATCAGACATCAACAGAGATAATGCACTAGAATTCGGGAGAATAGCTTTTTCTGATAGCTCAGCATCAGATCGACCAGTGATTAATAACTTAAACTCACTATTGGAAGACCTCTATGTGAGATTCATAGCGTTGGAGGATGACGATTTTGAGCTACATTACCAAGTATTCGATACTATATTTTCCACTATTCATAGAAATATGAAGGAGGTCGATTCATATTTTAATCGTTACTCGAGTACGGTAAGTAATTAATTGATACACAATATAGTCGTAAATATTGGCGAATTCAAGATTATTCTCTTATTAAAAAAAGTCGCATAAATGTAGACGTATCTGCTCCATAGCTCCGCTCCCGATCCTGGTATGAGTACTAGATAATTCTGGGGTAGTAACAGTTAATGGCCTATCTGCCCTCCTATCCGCCCTAAATACATTTATGGGTGAAGGGATATTTGAGTTTAGTAGCCCTATTGTTAGCAATATACCCTGGTAGATTGACCACCTTGCGGTCCTGGAAACCATTGTGCTTAATTCCTAGCGAAGCGAAGTCACGATGGGTTGACTGACCTTCTTTTCCATCCTCGCTTATTTTTCATTATTTGGTGTCCCGCCAGCGTATACAGATGGCGCAGGCGACGTTATCGACGTTATCATCGACATATACCCTTCTTTTTCATCCATCTTAAAACAGTACATCTATCATTGTTGTAGGCTGT

		GCGAATAAATAAGACTATCTCATTCACTCATTGAAATAAACTCCAACTGCCTTCCTCTCGACTTAAACTTAATTCCTCAAGC<br>CCTTATGTAATGTCTATAAACATCTCTAATCGTCTACCTGTAACATTACCAAATGAACAAAACCATAGAGTATTCATATCA<br>CAATTAAAACAACTATTAACTAAAAAATGTCATTACAATATAGATTAATATTTAAATGATAAAAATTTTGTAATAAAAAAA<br>ATAACGCATAATATTTAAATATTACTACCGGTTTAGTTGATAATACTAAAATTGATAATGACACTAATAAAAAGATATAAA<br>AATATAAAGAAAATAC
MSTRG.7803	Protein suppressor of white apricot	AGAAGAGGAGCTGAAGCGTCTCCACGCAGCTGGTTACGGCCAGGTGGGCTTCAATTATGAATCGACAGCGGAGCCCGCAC<br>AGCCGCCGCAGCCCGTCGAGGTCGATGAGCCGTTCGTGCCGACGCCTACATTCCGAGCGCTGCTGCCAGCAGACATGGTG<br>CTACCAGAGTCCATGAAGCAAAATGCAATAATAGAGAAGACCGCCAAGTTCATTGCTCTCCAAGGAGTTCAAATGGAGAT<br>ACTAATCAAGACGAAGCAGGGCGACAATCCGCAGTTCAAATTCCTCAACAAAGACTCTGTGTTGCATCCGTACTACACAG<br>CTCTGATAGCATTGGTGAAAGCTGAGAAGTGGCCAGAGANNNNNNNNNNAGTTATTGAAGAAAAACATGAACCTGAAGA<br>GTATTTACACCCTAGTTTGGCAACAGTTATTGAATCTGCACCATCAATACCAAGCATACACTACAAACCGTCAGCAGACTG<br>TGATTACACCATGCTGATATCGAAAATGCGAGGGGAAGCAACCCTTGACGAGGCATATACCCCGGAGTTGGCGCCGGGAG<br>AAGTGGCGCCTCCCGGGACTGAACCGTTACCGNNNNNNNNNNACGCGGAGATATCTCGCGCCCCAGTCATGTACAACAGA<br>GCTGGTGACGAGAACAACCATGG
MSTRG.7814	Golgi resident protein GCP60	AACGATTGAATCCAAAATAAACTTACTTTTTTTATAGTAAAGAGGAATTAATTTAATTTCTCGCGCACAGGTTTCGGTGTGT<br>ACTTTGAATGGGGCAAGTCGCCGACTACCGAGGTGACGATACAAGTGTCCGAGAGCGACGAAGAGGACGACCCCGACGA<br>AGATGGATACGACGACGAAGAGTATACGGTGTCGGGCAGCGGCGACCCAGAGCGCGGTGCGGGGGGCGGGCCTGGCGCC<br>ACGGGCGGCACCCGGCCGCTACTCAGCCTCGTCGTGTCCGTCTATCGCCGCGACTGCCACACTGAGGTATATGCTGGTTCT<br>CACACATACCCAGGCGAAGGAGTGTATTTACTTAAATTCGACAACACCTACAGTCTCTGGCGATCAAAAACATTATACTAC<br>AAAATATATTATACGCAGTAAACAAACTAAATTATAAGGAATCATTTATGAACATGACAAAATATAATAAAATGGAAATA<br>TTTATCGAGAAAAGGATGGTATGGCTGTGCTCGGCTTGGCGGAGACACTGCCGTGCCCCCTATTTGTGTTAGGTGTAAAT<br>GTCGCGCCCGCGGTGTCATGGTCTCAGATGCACTGGGCCGTCAATATTTTGCCCCCTTAACACTTAATTGCCCTATTCACCC<br>CGCGAATATTATGGTAGGTCCACTGGACTCCTAATTACCACTGACTCAAACTACACTTGTATTAGATTAAAAATATTAAAA<br>GTTGAACTCTGTTCCACGATCGAAAAAGTTTATATGTTTAATTTATTTTTTTAAAATGCACATCATCATATTGTTGAAATTG<br>CTCTGGTTTTAGTAATCTATTTATTTAATTATAAATTAGCCCACATCAACCCACTGCTGGGCATAGGCTTCCTTTTGGCGCC<br>ACTTGTAACCCACTGCTTCCCCGCTATCCGCGCTATGTCTTGCGACCGTCGATTTGGTGGTGTACCGACTGTTCTCTATCCC<br>TCTTGGGCACCATTCCATCTCACTCTCGGTCTACCGTCAAGTCCTCCTAGTCAAGTTTTAGTAACAATAGTATATATATACA<br>CACACATTGATATTCCCTTATGGTAAGGGACTACCAGAGCGAGACTTTGTACAAAAGTCGTTTGCTTGGGCATCTCTGTCC<br>CATTCGTGTTTCAACAGTGAAGCAGTTGTGTTTGCATGGCTGTGTTTCGGTTTGAAGGGTGGAATATGGCAGTGAATTTAC<br>AGGGTACAGAGGAATAACAGTCTGTCATTCAGGTGGGCCGTCAGCTTGTTTGCCATTCTAAGTTGCATAAAAAATGGGGC<br>GAGTGGACGCCTTAACACATTTTTTGCTGGTTTAACTGAGAAGCGGGGCAAAATTCGGGGGTTCAGTGGATCCGTGATCGG<br>TGTCGTCAGTATTTAAAAGTTTATTTAAATAGATAATAATTATGATGTGCAATGTTCTTAAAAACGTGTCTATGGTGTACTT<br>TTATTTAATGGAGATTAATGTATACAAATAAGTCCAGTAATATGAGATTTATTGGTGTATTTACGTGTAAATTATATGTATA<br>GTAAAGAATTTTAATATATAA
MSTRG.7819	Protein halfway	CTGAGGAAGTAAAGTTTTAGAATTGATATAAATTAATTATACTTTGCAGCCATGGCTCCAAGAGAATTTCAAAAACATACC<br>AGACTACAAAGGTTTGGAATGTGAAGACAACATGGGGCCGGTGGTCGAACTGATCGAGTCACGTGTCTGTCACACGCCTC<br>GCGATTGGACGGACTATATTTACTACATTATAGGTTTAGAAGTTTTAGTATTAGTTTTACTAATTAGTAAAGTGTCTTACGA<br>CTATTGGGTGTTTAAAACAGCCGGGTACTTGCCGTGGCCAGCCAATAAGATGCCGAGGTTGCCTTGTGATTGGTTGTGCGA<br>GTGAGATAGATGATAACCTCAATTTGGGAGGCAAAGACATATAATATATTCAGCCATAATATGTTGTTTTTTTTAATTCGT

AAAAAATATAGGGTTTAAAAAAATTAAAATGTTAATTTATTTACAATATTTGTTATTACGTTTAGTATGCATTAATCTATCT
ATCTAGTGGGTAACTACTTTAATAACCTTGCTAAGTTAAAAATATATATTACCTACTTAAAATTAGTTATTTAGAAATCAG
GAAGTTGTTTGTAAAACAAATTAGAGATCTCCTATTAATTATGTTGTACATTTTAAAAGGATTTAGTTAAGATATATGTATA
TAAATGTTGAAATATATGTAAGTGTAGATGTATTGTATTATTTCTTGGAAGAAGACTAAGTAAGTAAGTCACTAAAAAAAT
TGTATTATGTTTGGAATCTCCAAATAAATATTGTCTCGATTTTATGCGCTTATAATATGAGGCTAATTAAAGACGTATGCTA
ATACGTCTCGGTTTCGATAGAACTTGAAAAGATAACCAACTTTTGTTCTAAATAAAATATAGAACAAATTGAATCGATTAA
TCGATTCGTATCACTGGCTGATAGTCGATTCAATTATTCGATTGTTCATATTAAGTTCTGTTGAAACAGATTTCTTTTTGTGT
CTAAAACGTTGATTGGTAGGGTTTTTTGAATTTATGAATTTATCCATCATTGCTGGAGATAAATGGTGAGGTTAAGAATTT
ATTGTTAATCAAATTAGAAAATTATGTTACGTTGACGTCGTTATTTTTTTTTTGTGTAATAAAATTTTACCCCCTTATTGATA
AAAATGTTAAACCACATAAACCTATTTTAGCTATTGAACTGTTTTATCTCTTTCTTTTGTGCTTTTTCAATTCGAAAGTTAAT
AATAGTTAAAAGAGGTATGCAGGTTTTAATTTTTATTAATAAGAGAGTTAAAGTATGCGCAAATAGTAATGGCGTCGAATC
CGAAATGTCACTTTTCTAAACCATTGTATAACCTATTATTGTAATTTGTTAGTTTCGCAGAATTACTATTTTGTGAATTATCA
TTAACTAATTTTATAATAGGTTTAAAGATAGGTTTAGAGCATTGTTTTCCTAATTTTATGTCGCGACCAAATCGAATAATCC
AAATGAATTACTTAATCGAATAATTAGTAGTCGAATTAGTAAAGTCGCTCAATGTTACGTGAAACGAATCTTTCTATGTCT
TATTATTTGTTAACAGACTACGATTCTATTAAATAACAAACGCAAAATCCCTCCCCCACTTCTGACTAAGGATCGCGACTC
CCCGTTTGAAAGACGATGGTTTAGAGCAGTAATTCTCAACCACTGTGCCGCGGCCTATTTTTTGTGTGCCGCCAAATTTTAA
GTGTGCCGCCAAATTTTGCAAATATTAAAAATTTTTGTCATAAACTATTTATGCAGTGTGTTGTAGTAAGTAAATAATTTGT
ATCTTTTACTTTGTGTGCCGCCAAATTTTGAAATCGTTAAATATGTGCTGCAACAAAAAAAAAGGTCGAGAATCACTGGTTT
AGAGAAGTGATGTTTCAGAATCGACCCCATTGTCATTGCAAAATACTTAGTTGTTTTACACAGATCATACATTAACGATGA
TTTGTGAAAGAGATCTACATTGAAAGGCAATCATACACGTATATAGGTGTATTAATTTATTTATATGAGGCTTAACTTATA
TTCTATTGGAAAATCAAAGAATATTTAGTTTTTATTTTGTAGTGCCATATGGTTTAATTGGCTGGTAAAAATATATACTTAT
GTATTTATTTCATATTTATTTAAATGCATTACTGAATTTAATAAATAAATAAATAGTCTTTGATAGATTATAAATTAAAACA
GCGTAATCTAGTCTAAAATCTTAGGTACATTTTTAGGTTTTTTTTTTATACAACTTAGAACGGCAAACAAGCTGACGGCCCA
CCTGATGTAAAGTGATAACCGTCGCCTATAGACATGAGCAGTACCATGGACATAACAGAGTATACTGTTTCGCCCATGATA
CCCCCCTGCCCCCAATGTGTTGCCATTCCTGAGGACTCAGGTGTTATTCCTCTGTACCCAGTAAATTCACACCATATCCAAC
CCTTCAAACCGGAACACAGCCATGCAAACACAACTGCTTCACGGTTGAAACACGAATGGGACAGAGGTACCCAAGAAGG
TAGATACATATTTTAAATAGACTGAGTATATCAGACTATATTATACTACCAGCATAAATATATAAACTTAGAATACAAATG
TTTGTCTATTTGTATTTTTTTTAATGGGAGATAATGTAATTTTAGCTCCGTCTACGCATTGGTATAAGCTGGCGGAGCACCA
TTGCACCTCGATACTACAATGGTCAGCATTCATCAGCATTCATGCACCTTCTTCCAGCCAGCTCTATCCCAGGCCATATCCT
TTATCTCCCTATACGACATGACACCTAGTGTTTCCTTTATCTGTCCCATGTAGTTAATCCTCGGTCTTCCTCTTCCTCTTTTG
CCCTCTATCTTTCCTTCTACTATGCTCTTAAAAAAATCGTCTTGTCGTATAAGGTGGCCCAGCATTTTGCCTCTTCTATTCTT
TATTATTTCAACCAATGTTCTCTTCTCGTTTACTCTTTTCAGGACTTCCTCGTTCGTTAGTTTTTCTGTCCAGCTTATCCTTTC
CATTCTCCTCCATGTCCACATTTCGAATGCATCAAGCATCTTTTTTCCTTCTGCGTCATTGTCCATGTTTCACACTCATACAA
TGCTATGCTTCAAATACAAGTTTTTATTAATTTCTTAAGGATTATTTTGGATATTTTTGATGTAAATAGATGTTTTTTTGTAG
TCGTAGGCCTGTTTTGCGACCGTTATTCTAGTTTTAATATCCAATGTGCACCTAGCGTCCTCTGTGATTATACTATACCATTC
CATTATCATCCATTAAAAAAAAAACCTTTTTTTTTTATACAACTAAGAATGGCAAACAAGCTGACGGTCCACCAGATGGAA
AGTGGTAACCGTCGCCCATAGACATGAGCAGCACCATGGACATAACAAAGTATACCGTGATACCCCCCATGCGTTGCCAT
TCCCGAGGACTCAGGTGTTATTCCTCTGTACCCAGTAAATTCATGCCATATCCCACCCTTCAAACTGAAACACAGCCATGC
AAACACAACTGCTTCACGGTTGAAACACGAATG

MSTRG.7827	Structural maintenance of chromosomes protein 2	CAGTTCATAATAGTGTCGTTAAAAGACGGCATGTTCAATAACGCGAACGTGCTGTTCCGGACCAAGTTCGTGGACGGCAT<br>GTCGTCCGTACAGCGCACCGTCAACACGCGCCGATGATACGCGGCCGCACAACACTGCCTGCCACGGAATATTGGACCAT<br>ATGGCCTGTGCACACGAGGGGTAATGGGATAATTTGTGACGTGAAATAGATGTAGTGACAGACCTTAGGTGAATGGACGA<br>ACACCGTTGAACATTTTGTACACTAACATAAATACAGGGGTGGCCGAGTTGACTAGACCCAAGATCTGCTGTTTACTGACG<br>AAACCCATCATGCCATCTACGAATTACATACTTCGTGAAATCTCAAATGAAACAACATAGTTCAGTACATAACAAAATCGT<br>AGTGAGTGGCAAGAGCTCCACCGACAAGAGCGCAGCTCTTAAATGGGATAGATAGATAGATTCATTGAACATTTTTTTAT<br>AGTACAGATATGATAAACATGCTAACACAAATTGGGAATTGTTACCTATAAACATAAGCATTACAACACATACGCATCAT<br>GTAATTCGTTGATCTAATAATCGATTATCGATTGATCGATAGTGTTCACTTCTAAATATGGATTCGTTTATTTGATTGTTCA<br>AATCGATTTGAAACATACATTTTTTGATTGGTGATCAATGGTAAACAATGATACGGTTTATGTCGGTTAATCCCTCGAGCG<br>TTTATGCATCATACGTTGCGCTGTACCGAACAAAATGTGGTACCGGCTAATGCAGCAATGTCTGTTGCATAACTCGAATTT<br>GCAGCGTCATTTTCATTAAATTTATAATTAGGTTTTTCTAATCGAAATTTAGTAATTC
MSTRG.7828	Constitutive coactivator of PPAR-gamma-like protein 1	CTTGTCTTTGGCTTTGCCATAGACAAGGTAGGCAAGGCTGAAGGCAGAAATGAAATAATCTGAGGCAAATGGGTTGAGAA<br>AATTACAAATGGCTTCCGTTTTCGACTGTAGGCTATCATAATTTGCAGTTGGCATGGTTTTTTAGCTAAGACCATCAATCAT<br>TGTCGAGACGAACGTGTTCCATTCGATCGAGTCGCTATTTCGCTATCGTAACTAATTAGTCATGGGCATTCAAGATTTACA<br>AACATTTTTAGAAAACGAGGGAGTAGGGGTTGATCTATTTCGAATCGCTAGAAATCAAGCGGGCGGTTTTCGTCTAGTTCT<br>TGATGCAGAAGGCTGTTTAGACCGTCTCTATGGTGGATATTTTTCAGGT
MSTRG.7832	Myosin-Va isoform X1	GGCGGCGACAAGCACCTGGCGGCCACGAGCGCGCTGCTGGGCGTGGCGGCGGGCGAGCTGGCGCGCTGGCTGACGCACC<br>GGCGCATCGCGTCGGCGCGCGAGGTGATCGTGGCGCGGCTGGACGCGGCGCGCGCGGCGGCGGCGCGCGACGCGCTCGC<br>CAAGCGCATGTACGGCGAGCTGTTCGCGTGGCTCGTGCGCGCCGTCAACCGCGCCCTCCACACTGGACACGCCAAGAAGC<br>ATTTCATTGGCGTGCTGGATATATACGGGTTCGAGACCTTCGAGATCAACTCGTTCGAGCAGTTCTGCATCAACTACGCGA<br>ACGAGAAGCTACAGCAACAATTCAATTCGCATGTCTTCAAGTTAGAGCAGGATGAGTATATTAAGGAGGAGATCTCGTGG<br>AAGATGATCGACTTCTACGACAACCAGCCGTGTATAGACCTGATCGAGGACAAGCTGGGCGTGCTGGCGCTGTTGGACGA<br>GGAGTGCCGCGTGCCGCAGGGCTCTGACCAGGGCTTCGTGTCCAAGCTGCACGACAAGTGCGCCAAGTACCAGCACTTCA<br>CGAAGCCACGCTTCGGACGCTCTGCATTTATAATAAAACATTTCGCGGACCACGTGGAGTACCAATCAGGCGGTTTCCTGG<br>AGAAGAATCGGGACACGGTCTCCGAGGAGCAGCTGGAGTGTATAAAGTCGGCGACCAGCTGCCGCCTCATCCACACCATG<br>TTCGCTGACCAGGCCGACCAGACGGCCACCCTACCGCCACCTTCGAGGAGAAGGCAGCCCAACATGCCAATTGGCAGTCT<br>CACACAACCAGCCAAGCGGCAGTCGGCGCAGAAGCAGACGGTGGGCTCGCAGTTCCGGCAGTCGCTGTCGGCGCTGATGG<br>CGACACTGTCGGCCACCACGCCGCACTACGTGCGCTGCATCAAGCCCAACGACACCAAGCGCCCGTTCATGT
MSTRG.7854	Nuclear pore complex protein Nup98-Nup96	ATAACCTTCAATGCAGTTTCCAACCCACCTGCCAAGCGTGGCAACTATTGGCAAAACTCTTTTTATGGGGAAGGCTTATGT<br>CCAGCTGTGGACAGTTCACGGCTGAAATAATAATGAAAATATCTCCTTTTGAGACAATGCAATATTCTCGTATGTTTGCAT<br>TCAGAACGGTACCGGCCGCCGGCCGCTGGACCTGCGCTACGAGCTGCTGCGGGCCCGCGGGCAACAAGCGCGGCCCGTAC<br>TGCGACCCGCCGCCGCCTCGCCGCACCCGCTCGACTACGCCCTCTGCTTCCTCCTCGGGACCTGGTTTGGGAACCCTACAG<br>TGGAGAGCATCACGGGTGTAGCGGATCAGCTGGAAGCGTGCGGCCTCTGGCACCTCGCTGTACAGGCGCTGGCTTACCAC<br>CCTAATGACATAGCGTGAGTATATATTTTAGGACTTATGTAGTGAACCAGGCTTGTATCACTAGAATCTCGTAGCACCTGT<br>TGTGTTTTTTTTATACAACTTAGAATGGCAAACAAGCTGACGGTCCACCTGATGGAAAGTGGTAACCGTCGCCTATAGACA<br>CGAGCAGTACCATGGACATAACAGAGTATACTGTTTCGCCCATGATACCCCCCATGCGTTGCCATTCCTGAGGACTCAGGT<br>GTTATTCCTCTGTACCCAGTAAATTCACACCATATCCCACCCTTTAAATTGAAACACAGCC
MSTRG.786	Prostaglandin reductase 1-like	AGTATATTCATCGACACTAGTACAGCTAGCACGTTTATCAGTGTTGTAATATCTCTTCCTAAAATAAATACTTTTTTGATAT<br>ATTATTATAAAGTCAACAAATTCTACAATGGTCATAGCAAAAAAATACGTAGTGAAGAAATATTTCGAAGGTTTGCCGAA

		ACCTGAGGACTATGATATAGTTGAAGAAATTTTGCAACCAATAAAAGCTGGAGGGATTGTTGTAAGAGCTGAATGGATCA<br>GTGTAGATCCTTACCTTCGTGCATACAACAGAAGGACAGCAGTTCCTTATGATCAGTACAGTTACCAAGTCGGCGTGGTCG<br>AAGAAAGTAAAAACCCTGATTATCCAATTGGAACTAGAGTGGTTACTCATAAAGGATGGTCTAATTATACAGTTATATATC<br>CAAAAGAAATTACTGCAGAGGAGACTATATACAAGTTGCCTGATTTACGAGGTTTATCTAATTCATTGGGTGTTGGTGCTG<br>TTGGTATGCCAGGAGCAACAGCATACTTTGGATTTTTAGAACTTTGCAAGCCTAAACCTGGAGAAACAGTTGTCGTGACAG<br>GCGCTGCGGGAGCTGTTGGTTCCATTGTGGGTCAAATTGCGAAAATCAAAGGCTGCAAAGTGATAGGTTTCACTGGCTCA<br>GATGATAAAGTACAATGGCTGGAGAAAGAACTCGGATTCGATAAAGCTATTAATTATAAAACTGCGAATGTTGCTCAAGC<br>TCTGAAGGAAGCTGCCCCGAACGGAGTAGATTGCTATTTCGATAACGTAGGCGGCGATTTAAGCAGTGCAATAATATATC<br>AAATGAATTTGTTCGGACGCGTCGCCGTGTGCGGAAGTATTAGTGCATATAATGAAGATGTTAATAATACACCCAAGGCG<br>AATTTACTGCAACCAGCAATAGTAGCGAAACAATTAAGAATTGAAGGTTTTATAGTACACCGTTGGCGCAATCAATGGCC<br>AAGAGCATTTGCTGATATTGTTCAATGGATTGAAAGCGGGAAATTAGTAGCTCGGGAGCATGTCACAGAGGGTTTTGATA<br>ATATATTCAATGCATTTGTCGGAATGTTGCAGGGTGAAAATATTGGAAAAGCTGTAGTCAAAGTATAAATATAAATAAAA<br>CAATAAAACACAACACGACGGAGAAATGAATGACTTAGGAATGTAAGAAATAATATCATGTAATGACTTATTTTTATTTA<br>TATTATAAATTTATTGTAATA
MSTRG.7862	Uncharacterized protein LOC106125039	TTTGTGAATATAATTAATATAGATATAGAATGCTATCGTTGTATTTTACAAAACGTTGACTAAGTAATACTTTGTTGATTTC<br>GATGAAATTTTGTTATGTATTTATATTTTTATGCATTTACATTAATGGCATTTCTCATAGTTTTAATAGTTAAATGAAGGTTA<br>AACGTAATAGGGATTTTTGTTGCAATTTAACGACGTATCTCAGAAAGCATTGACTGTCTGGTGATGTGGTTAAAATTTACT<br>CTACTTGAGTAAGTTAGTTGATATGTCAACTGCATTTAGTAGTATTTCGGTTTGAAGGGTGGGGATATGGCGTGAATTTAC<br>TGGGTACAGAGGAATAACACCCGAGTCCTCAGGAATGGCAACGCATGGGGGGTATCATGGGCGAAACAGTATACTCTGTT<br>ATGTCCATGGTACTGCTCTTGTCTATAGGCGACGGTTACCACTTTCCATCAGGTGGGACGTCAGCTTGTTTGCCATTCTATG<br>TTGTATATAAAAAAAAATGTAGTAGCTTCTTTAATACTTTTCTACATGGAAATTAATTTGCCAACATCAAGACACAGTCAT<br>GCTACTCACTTCGAATTAAAAATATCTTTATAAGCTTTTATAAGCATGTACTATGTCGATAGGATTGTCAATTGTATTTGTC<br>CAATTCTAATTAGGCCATTCGAATGCAAATTGTTTGGAGTTGTGAGCTATCCCAGTATGTGTTTTATAGATCAAGCGATGTT<br>CTTATTGTGGTCCTTCGTGCCGGATGGGATAAATCTCATTTTACTATATAATTTCATGCTAATCTCCGAAAGCGCTCAACTG<br>ATTTTAATAAAATTTTGCAAATATGTATTGCTTGTTTTAACTTTAAAGATATTTTTTCCCCAATAAAGAAAAATGTACCTGG<br>TATGTGGCGGTGCAGGGTAAAACATTGGCGTATTAGTTTGTGGGTATTATGGGCTGGGTTAACTATAGTACTAAACTTGAT<br>ATTTGTTAAGTTTAAACTAATACGAAATAAATCTGTGACAGCTATTGTTTTTGTATTGCGGTTATTACTTTCTAGAAGATAC<br>ACGGACTCGTTCACTATTACCCTAGAATAATAGTTCCTAAAATCTATCTTATCTTCCAATCCTTATCTGCTAGTTATTTAGG<br>GTTGGTATAATATGTATTTTTTTTCATATAATACAAGGTTTTGGCTTTCCCTTTTACAATCCACCTGCCTGACATCAACTCCT<br>CGGGAATAAAAGCCTGTAAAATAAAACATAAAAAATATTTGCTTTTGTTTAATTTCCATCTCATTTATCTAATGTGTCTTGC<br>ATTTCTGTTTTGATTAAAACAGGGCTGTGTCTAATGTAGACAGTGTTGGTATTTCTTGTTCTTTTTAAATGTCAAGGGTAAA<br>AACTGGGTGCTGAATTTGATCTATACTGTATTGTTTGGCCATCTCCTATTGAGGGTCAGCGATCATCAGGGCTATCCTTACT<br>GTAGAGACTTCTCTCCCAAAGGATGTAGTACTCTATATTGTATTAGTAGTACATTAAACTACTGTAAGTAAAGCAAGGTCG<br>ACTCAGTGTGCAGTACCACATGGGTACATTTAGGGTACATTTTGACTAGTCATCATTTAGTCATTTCAGTCTTTACCTGTCC<br>ACTGCTGATAAGCCTTCCCCATAAGGGGAGTTTTGCCAGTAGTTGCCACGCTTGGCAAGCAAGTTAGCATTCGCAGTTAGG<br>CTTTTAGAGATGTTTCAAGAGTAACGCTGCTGCCCATCCCTCGTCCTTCCTTAGTCCACTCTTTTGACATCCACGGGAAAGG<br>ATGGGGTATCCTATTCTATACCGGGACCGCACAGCAAGTGACTAGTTATTATTTGTATTTATTGAAATATTAAAGTTGAGT<br>GGATTATTTGAAAATTTACCCACTTACAAGGTTAAAAATTAACCTTTTTCCAATTTTGAACGTCAAACTAGCTGACAGCTCT<br>TCTGGTCGGAAGTGATAACTGTCGCCCGTACACGTGTATGCTTCTATGAACATTACAAAATATTCTAAAGAACTACTTTAC

		GTGTTCATTTGAAGAAACTAATAGATTTACAGATTAGGAATTGAAGGAAACGTTATAGAGAAGTGTGGACTGAAAGACTA<br>TGTAGTAACAAAAATCGAAAAAGGAATGTTGCGTTGGTTTGGCCATTTGGAGAGGATGGATGAAAGAAGAATTACGAAA<br>GAGATTTATCGAGCAAGAATGAATGGAGATGTCGGTAGGGGCCGTCCTAGACGGACGTATGTCGACCAAACAGGCGACGT<br>GCTGAAGAAGGGCCAAATTAAAAGTACCTGTAACCGGCGAGCGTGTATGAAAAGATTGATGAATGTGGAGGAAGCGAAA<br>GACGTTTGTTTGAATCGAAGCATTTGGCATTCCATTGTTTCTGCCTACCCTAATGGGAGACAGGCGTGATAGATATGTATG<br>TATGTGTGTATGAATTGAAGGAATTAAATTTGCATCTTTCGGCTATATTATAAATGCTTTTAAAAGTATTTCCAAATGTTGA<br>GATAGCATTAAAAAATTGTTTTCAAACAGAGTTCTTCTGTTATATTAATGACACTTTCATTGTTATTCTTTAGATGTAGTTA<br>GCTTTTTTTTTTACAACACAGGGGGCAAACGAGCATGCGGCTCACTTGATGGTAACTGACTACCGCCGCCCATGATCACC<br>CACAACACCAGCGGCATTGCAGGTGTGCTGCCGGCCTTTTAAAAAGGAATATACTCTTTTCTTGAAGGTTATGTTGTCGTA<br>TCGGTTCGGAAATACCGCTGCTGGATGTTGATTCCAAAGAATGGTTGTGCGTGGAAGAAAGTGCCTTAGAAAACGCACGG<br>TGGGGGACCGCCACTCGTCCAAATGGTTGGGATGAAACTTTAGTTTGTAGCTATTGGTAAAAATAGGTATGGGTGGAAGG<br>AAGAGGGTCGATAAGGTGCAGGGTAGAGGTAAACTAATGCAGTGACTTTGACTTGATTAGAATTGTTTTGTTTAATTCCAC<br>TACCATAAGTATGCGTGCGATTGCTGCTAATATTTGAGGATTCGTTACTATTATTATTAATAGAATTTTTACTGTCGCTAGC<br>AACCAAATGTATTTTTTTAAATTTATTGTTTATTTCCAACTTGTGGTGCCACCTGGTGTCGTAATATAGAATTAGTTGTTGTG<br>ATGGTGACCGTACTCTAAACTGGTCGCTGGTCGGTCGTTCTGCATTCATAAATAATTATTTGCTAATATATATGTCAAAATA<br>CACTAGTTTTGTATAAAACAATGTTTAAATTCTATATGCAGTTCACGCTCTCATTACGAGTACTATTAGTCATCGATCTCCG<br>ACCATTATTCAGTCCACGCACATGTTGCGATACTTGTCTTTAAATGAAGCATCTTCAGGTTATTGTGATCGCTGTTTAAAGC<br>CTATTGATTGATATATAATATATTAAGCAAATGTTTTTTGTTTTTTTTTTTTTATATAGTGTGTTGTACTAACATATATGCCC<br>TCTCAGACTGCACGCGGAGTCCTCACTCATTTGTGGCTGTATCTAGCTATAAGTAGAGAATATTGTAAATAATCATCGGTT<br>TAGTATCGAATGTAGTTAAGGGGGGCTGACGAAAACTTTTTATAGCATAGCACAGGGATAACAGTTCAAAAACCGTCGAT<br>ATTATTCATAAAACTGGACAACTTGGTCACAGAAAGGTCGTTGAAAAATGGTTGAGGTTAGTAAAAAAAAAAAAAGTAAAT<br>AAATGCAC
MSTRG.7867	Hepatitis B virus X associated protein hbxa	GCAGAGAGCGCGCACTCGGGTGGTTGCCGGCGCTGCGGCAGCGGCGAACAGCCCGAATGGATCCTGCTGTGCGACCGCTG<br>CGACGCCGGCTACCATGCCTCCTGCTTAAAGCCGCTGCTGCTGCACGTCCCCGACGGCGACTGGTTCTGCCCGCCCTGCAA<br>TCACGATATGTTAATAGCATCCCTCGAAAAGGAGCTTAGTAACTATGACGAGTTGGTAACATCTGTAGAAGCAGAACGAG<br>CCCGCAAGAAACTAGAGGAGCCCGAGAAACAGAGTAGTGACGAGAAGGAGAACCAGGAGAAGGAGAACCAGGAGAAGG<br>AGAAAGAGGAAACTGAGGCATCTGGCAGCAGCTCGTGGTCCTCGTCGTCGGAGGAGGNNNN
MSTRG.7869	Remodeling and spacing factor 1	GCTAACCGCCCACCTGAAGGAAAGAGCTAACTGTCGCTTAAAGACATGAACCATTACTATAAATTTAACAGAGTATTCTGT<br>ATCGCCTATGGTACCCCTCATGTGTTACCATTTTTTAAGGTGAAGAAGGTGTTATGTCTCCAAACCCTGTAATTTCAACATG<br>TGTTGAATAGTCACAATTGATAATATGTCTATATATAGGAGGAGTATGTGGAACCTACGACGAGCGCGGGCAACCAGGGG<br>CGCGGTAAGGACATATCCACCATCATCGAGGCGGCCGAGGAGGAGAAACTGAAGGCTGAGAGGGAAGCTATCGAGCAGG<br>GTAAACCGATCGAAGTGAAGAAGATGCGTAAGAAGACGCGCCGAAAGCCGCGCAAACTGAACTCACTGGAAGTGCCTTC<br>CGAGGATGACGACGAGACCGACGAGGACTTCAAGGACACCGGTATGGAATCGTCATCAGAAGACATATCGTCATCCGCTG<br>AACGGGACAGCTCGAGTGACTCGGGATCGTCGGACTCGTTGCCTATTAGGCGAACAGGCAGAACTGATAAAAAAGGTAGC<br>ATTTTACATGTACGCATGCGGGGTATCATGGGCGAAACAGTATACTCTGTTATATGTCTGCTGTATAGGCGACGGTTATCA<br>CTTTCCATCAGGTGGGCCGTCAGCTTGTTTACCATTCTAAGTTGTGTATAAAAAACTTAACTCACATTCAGCACACCTCTTT<br>TTAGTTTGAGTAAATTCAAGCAAATATTTATTGCTAAATTGCTAATAATTCAT
MSTRG.7884	Uncharacterized protein	CCAAATGCTCGCTGAGCTGCCGCTGTACGACGTGAAGCTAGCCACTGCACCCCATTCCTGCACCAAGCCAACTCGTAGTGA<br>TGGAATTCAAATCGAACGTTCAGTTTAAATAGACGCGTGTAGTGTGTTATAGACAATACACTTGTCTATATGATCGTACAT

OBRU01_22018 TTTGTTACGCGTTTTTGTTATTACTATATTGTTACGCCTATTAACGGATATTCTGTCCCCGATCTTCCGTGTGTCGGCCACGC
GCACTCTACATGTATTGTTCGCCTAATGTGACGCGTTGTATGTTAGAATATGTGGACGTCGATGGATCCGTGCACGAATCA
CAAGTTTTTGCAAAAATAAACGGAACATTCTTGTCATGTCTCTGATCATAGAATATAAAAATGCTTCGATATACTCCCAGG
AAGATCCGCTTAATATGGCGCTAGTCTTACAGTAATATTAGATGTTATGCCAAATTAATTGTTGTTTAGTTGCGTTTGTTCC
TGGACTCGATGATAAGCAAAATTATCAATTCTAAATTTTTTATTTATTATTATTATTATTTACTATAGAGTAGAGGTCCAGT
GGCAATCGCCTAGATGCGGTAACGTGGTCATCGATACTGTGGCTCGAGGGCGTCCTCGCTTGCGACGCCCCCTGGCGGCCG
CAGTTCAAGCGGAGTGCAAGTGTAGTGCGGTTCGCGGCTGCAGAGCGGAATGGATGGTTCTGGACTAACAGTGCTGCACT
GTAATTCGATTTATAAATAAATCTTACGATTCATAAACAACATATAGGTGTCGCCTATAGTATAGCATATTTAACTGTCTGT
CTCTCGATGTACATAAGTTGTAATTTAAAGAGCTTCTTGGGATCTTCACAGGCCTCACTGAGCGACCTCACCGTTCCGAGG
ATTTCAAACGAGGGGGTCACAGCTTCACATTCCAGGATTACGTGAACGGCCGTTTCTCCGGGGTATTCGTTTCTTGTGTTGT
TTTGTGGTTGTTGTTGTTCATAGTTAGAGGTTAAAATGGACGTGGTGTAGTTTGCGCTGTTGTCCAGCCAAATCATTTAATA
GTGGCAGTTGATTATCAAAATTTTCTCTGACCAGTTTAAGAAATTTATGTTGGTCAGTCCCTAGGCTGTTGTTTGGGTAACT
CCATATTAACGGAGAATCGATCCGTCTATGGTCTGAGCATGATATTCTTTGGATACACCCTAGTCACAAATAGATTCCGAA
ATACCAACAGATGTAAGAGTTAAATCTATAATAGTTTGTGATCGAGCCTTTATAAAGGTACCCGTATGAATTGGACTGCGT
CCCCTTCACTTGTCATACTTTCAACTATGAGTTTTTCGTTCCTTAACGTCTACTTAGGTCTGGAATTATTGACGAACTCTGAA
AAGACCTGTTCTGTGGTTCCTAAAGCATCCAGCTGGATGCGAATTTCCTCTTTGACTGATCCTGCCTGTTCAAAGAATCCTG
CCAGATTTTGCGTTGCAGTCATTCCCGGTTGTAGTATGAGTCTTATATTTGGTGCGACATTATTTTGAGTGCTTTGTGCCAT
CAATACATCTCCACACACTCCGAGGCATGGAATCCCCTCAAATATCTTGTGTGTGTCCATATCGATCTGGGTAGCTAGATA
TCTTTCTATGCCTTGAGCTACCGTGACTGGCAGATTGTGGTCTGCTAGATATACCATTTTGCCGGTTTTATCTTTGACATGA
CCAACCGCTTTCAAATATAGATAGAGTGACTGCGGGACGGACAATTCCTGCTCATTCATCGCTTAGCAGTATTATATTTGA
AATGTGATCAGCGAGGCTTATACTCGTTTTGACTTTACATCTAATAATTTCGACCACGAAAATGAGGTTGAGTAATACATT
AACATCTTTTTTGACAGAGATTTGTTAATGTTTTTGTCATCTGTGCTTAGTGCGGAGTACAATACCTCCTCAGTCACATTTC
CCAAACGTGCGAGCGTTGGGGTGTGTTCGAGGTTGACTTGTAACTCTGACCGAGACGTCAACTCCAAATACGGAATTAGC
CCACATTGGGATGTTCTTCTGTGGAGTCGGCGTATTAGCCGCTTCGAGTTTAGTGTGCGGTGGAACCTTTTTTTCTATTAGG
CTTGACGACATTCTTTGGTTTGGGCGCCGGAGTCTTTTTTTCGACTTTGTCGGGATTAAAGTTCCCTGTTTTGTTTCTGTAAC
CAGGAGCGATCCTCTTTATTTCTTCGCTCGTGTATGGTTTTGGTGGTTGTCGCATTTTCAGATTAGCCTTTTTAGCGTTCAGC
GTAGATTAATCTGGGATGTACATAAGTTGTCGGCAGACCGTTTATAGCAGTGGTCGTACGAGGAGCCGTGCCCGCGTCGG
ACCGACTGATACGGTTTATGTAGAAACCTCCGGATGTCCGATATTAACCGATATCGTTTATATGAAGAGTTTGTGGAATAT
TACAAAAGTGTATATTTGTAAAGTGATCAACAAACCCATTATACGAGTCGCGGTTGGCGTCTGCTGCGCACCGTAATAAGT
TATTGTAGCGTTATTAGCACTTCGTTTTGTGGAACAATACGACGTGGCGAGCGCGCGGCCGCCGCCACCAGCCGCCACCTC
GCAGCGGTTCATTTTAAATGATGACATGACACGTGCCAGTCACATCGGCGCGTGGCATCGAGTTGGGAATGACGTTGTTGA
AAACGAAATGCTTTGAGTATTAAGATAGGGCTATTATCCGGCCGCTTAAATTGTGTGGTCTCTAGTTTAAACTAGCGAGGC
CTATTCATATTCTATAAAGCATTTCTAGAGATAAGTTTTAATAAGCCGACATTAATGGACTACTAATCTCGTCTATTTATAT
TTGTCGGCCGCACCCAAAATTGTATACTTATAATTTATTCGGGTGCATGTAAAATATTACTAAGTGAAAATTAGTGGAGCA
GTAAAAGAACGTAGCTATGTATACGTGTTTGCCACGTTCGTGGTTTAAAAAAAAGCTAGAGAAGACATTTGCACATTGAA
TATAAAAACTAGAACTAGAAACCAGTAGTTGAAAATGCTCAAACATAGGAGAAGGACCTTACATCCAACACAACTATGCG
GCTCAGTTTATAATCTATGACATCCTTGGACTAGTCCATATCCTTTGGTCACCATATTTGTGCTCCTTCGGCGAGAATGGGC
TCTCACCATTTCCTATTGTTTAAAGAGTTGCTGTCTTTGCATGTTATAAAATAGATTGCACGGTGCTCTATGGCACTCTTCA
TTGTCATCTGTTCCGCTCGTGGTGGATGTCGGCAATAACTCTCGAACTAGCTTTATTGAGAATATGGATGGGCCTTATTTAT

		CTAAAATAAGAAATTCCAAAGACTTAGTTTTGAGTATTTTTAGTGCTGTGTCTATTTGCGTGGTAACCACAGGGAGCACAC<br>ACAACAGAGGCACAACGATCTAATGTTTCTTTGTATCTATTTAATCTGTTTTCTTACAAGATCTGATTCTTCTACACGACTG<br>AGGTGCCAAACAAGCATACGGCCCACCTGATGGTGATTGCTTACCATCATTACCATAGCCTATGAACATCTGCAATATCAA<br>GGGACGCACGCGTTGCTGACCCTGAAGCAAGCTCTTTACTCCCCT
MSTRG.7904	Leucine-rich repeat serine/threonine-protein kinase 1	GCGCCGGAGGGCGACATGCTGGCGACGTTCGGGCGCGGCTACCGGCCGCTGCTGCAGCGGTACGCGCCCACGCCCACGCC<br>CACGCCCACGCCGTCAAATTCCACAAGCAACGCGGGCAACACGTACAGTGGCTTCTACTGCCTGCTGTGGCGGGCGCACC<br>ACTGGCTGCCCGACTAAGGCACGCGTCGCACGCCGCCAGGACTAGCGGCTCCACAGCCGCAGCGGACAGGGCGACCAGCT<br>GGTCAGGCTTGCACGCGTCTCGACAATTTGCATGACAGTTCGGGACACATTGTTGGACGGTTGGTAAAAGAAAGATTGGT<br>CGTGGTCGTGCTGTTGACATTAATGCCATGAAGCAGTTTTGCTCTCATGGCCATGTTTGTGGTGGGGTATGGCAATGAAGT<br>GGGATATGGTACAGAGACGTAACATAGTCCTAAGAAGTTGCAACACATGAGAGATACCATGATTATCAGAGTGATGTTCT<br>GTGATCCGTTATTCATGTGTATAGGCGAC
MSTRG.7905	Serine/threonine-protein kinase Genghis Khan-like	AGGTGCCGAGCAATGCTGGTGATCGGGCAGCGCTTAAAGCGCTCGAGCGTGAGAACGCGCTGCTCGCTCAGACTATCGCC<br>GAGTTGCGGGCCGGCGGGGAGCCCTCTAGTGATGATGTGATCCAACTGAAGGAGGAACTAGCGACGGTGACCAAGAGGA<br>ACGCGGACCTGGAGGCGCAGGTCAGGTGTTACGAGCAACTGAACAGCGGCATCACGGCACAGGACGTGCAAGCAATAAC<br>TGGCGTACCGCAGGAGACGGCGGCGCGGTTACGGGAGATGGAAACGTTGATCAACACTCTCACCGCGGACAAAGAGGAA<br>CTGGTGAAGGACAAAATGGATACGCTGGAGAAATTGACGCTGCAGG
MSTRG.7912	Coiled-coil domain-containing protein 22 homolog isoform X2	TAATGTTTCTTTTTCTTTTTTTTTTATAGAAATATTGATAATGGTGTTAAAAATATATGTGATTTGCCAGTGGAAACTATTAT<br>TGAATCCGCCTCTAAATGTTTGATTACCATTAATCAGTCATTAAAAGTGCCTATCAAATTACCAACAGGCATATCACACAG<br>AATAGAAGTGGCAGCACAAATAGCCTCCATTTGTAAGGACCTGGGTTACAAAAATGATGTTGGCTATCAAACATTTCTTTA<br>TTATAATGAAGCAGAATTACGACAAGTCTTTATGTTTCTTATTGAAAGATTACCAAATGAAGGAAAACAGAAAACAGCCG<br>ACGCAATAGATACAAATAGAAAATCATTACTTTTACGAAATATTAAATATAAAATTGGTGAAGAAATGAACTTGGTTTGG<br>ATTCCACCTTGTTGCAACCTTCCAAGTCAACAGAGTATTGGTGATTTTACATATTCCAAGG
MSTRG.7934	cAMP-responsive element-binding protein-like 2 isoform X1	AGTATTACAAATGGAATCTCGAGCTGGACCAGGGTCGGATACCCGAGGGTTTGGATGCGATGCTGCAAGAAATGGGGGTG<br>GTCAAACAGGAGGAAGCGAAACCCTTCTAGAGGGTTGATTTGGTACAGTTTAATATTATTTTGTTATAATCCCAACATATG<br>TCGTATCTATGCTTAGGTTTCCCCGTTCCTAACTTCGAGTTAAGTTGTAATTTTGTTATCGGTTTCAATATAATAAGACTTCA<br>TTATATTTATATTCTAAATGTGAGAATTTAGTGGGTGATAATTATCCATAGATTATGTAAAACAACAAAGTATGTTCAACA<br>ATCTCCGACTCAACTATCTCTCCATTTAAGTGCTATTTTTCTGTATACCCTCCGTATTGTATATGTGCTATTAGGTATGTAAA<br>TATTTGTTTACATGTAAGAATATATACATATATATTCATGCATATAAACCAATATATATGTTTTTATATATTAGAATCTATA<br>TACTAGTGCACCACCTACCCTTTGTCTTTTTCTCAATATCCTCTACCCAAAGGTTGTCTGGAAGAGATCGATAAGGCCACCT<br>TTTATATCACTTCTCGTTAAAATTAACTATATGTATTTATAAAGGGTGCAATAAAATGTAAATAAATAAGTATGTTCACGA<br>CTTTAAACTGTGGGGTAGTCAGAGTTACATTGTCGTCTGTTGCCGTTAATAATGTAGTTATCTAATTTTTTTTTATTTTATTT<br>CTTGTTTATATGTTAGTACTTTTTTAAATGGTTTTATATTGATTATAATGTAACGTTATATTTATACGATATGTTTAATATCA<br>CACTGTTGCCTAGCAATCACAGATAATGGCCGACTCTCTTCTTGTCATACTATTATTTATCAATTAATAAACATATAAAATA<br>ATGTATGCTATTTAATAAAGTATATTATTTTTATTAGAAGTGTCCGTAAATTCTATTTCTTATGTATATTTAAAAATAGATCT<br>CAATTTAATCCTCTTAATATGGAAGTGAATTGAAAATTGTAATAAAATAAGGATTCAAACATAACTAAAGTATTTAACACA<br>AGCTCAAGAGGTTTAAGTGGTACTAAAAGATTCAGTTTTTTTTAATGTATTTATGCATGCTAAAATAAATAAAAAAAAATC<br>AAAATATTGCATAATTCCAAACAAGTCTTGCCGTTTCCAACATATAAATTGCATAGCAAAAATATATCTTATAAAAATTTG<br>ATTATAGTCCAACTTAAACAGAAGAATACTTATATATTTTTATTTATTTTTTCATAGTATACAAAAAATCACGTTTACCATG<br>TAGTCAATCTTGAAGAAGAATATTATGAATAAAATTCATTATTTTACTAAGAATGCATATAATAAAATTATATGTATAATT

TTTTTTGAGCTTTATATTGCACATAGTGTATAAAATATCGCTGAAAATAATAATTTAACTCATTATAAATCTAATCATATAC
ATATATTTTTGAATATTCATTCAATGTTTATATAATATTTCGTCATTAAAAGATTTCATTATATTATATACTGTTTTTATATG
CTTTAACGATATTTTTTAAAATAAGTCTGTTGTAAAACAACTATATATGTAAGTAATATATTACTAAAAAATTTTAAGCTTA
ATGCTAATCGTAGATTTTATTTAATAATTCATAAGGTATATTTTTGTAGAGTGGTAATGTTTTTTTTTTTATTGAGTATGTGT
TTTTCAGCAAGGAAAAAATGTCAAAGCGTTTTATTCTAAACTTAGGTTTAATTAATTTTGATATTGTTTTGAACAAAGGAAA
AAATGTTAAAGCGATTTATTTTAAATTATCTATTATAGTAATTTAAAATTATTAATTTAAAATTAAGGTTTTAATAATAAGG
GGCTGGTTTTTCACTAGCGCCATATCAGTTGGATAGCATTTTATTTTTGACATATGGTAACATTGACTAAAAATAAAGGATT
AAACTATTTTACTACACACTACTTTACCTAGATGAAACGATAGTTGGTCACGACGTCGCTTGTAGAAAATAATTAATTTGA
ACACGGGTGAAAAAGCAGTTATAGTACATATAGGACATTTTGTGTGTTGATTGATTGGTACTATGTTCGCCCTTTCTAACA
GGGAGACGCATACTTTGTTTACAATTTCAAAAGTAGAATATTTATAAAACAAAATATACTTGTACATATTGCGTTCTGAAG
CGCCATTTCTCTACAACTATATCTTGATCTGATACCAACAGAATTTTGTCGTGTCTGCGATCAAACTCCTCTTCATACAAGG
TTGGCTGGAAAAAATCGCTTTTAGCGATAAGGCCGCCTATTGTTTTCTCAATGTAATTTATTGTAATTGTTTGTATTCATAA
CAAATATACTGATGCGAACTTTTGGAGGAAATTATACAAAGAGTATTTATTTATTTATCTATCTATTATAAATAAAATTTAT
AATAATCATGACTTTATTAGAAATTTAGTTCATATTTGTTCTTAAAGTATAGAGTTCGCTATATCAAATTGTGATTTTAAAA
TTAGAAATAGTTTTACAGAAATGGTGTCCGTTAATATTTAAGAAAATCCCTAATATATAAGAAAATTTACAAATTTCACAG
AAAACATTCAAATTCTGAATTTATTAAAAATATGAGGAAACTGAAAATATTATTTGTTTTAAATTCTTAATTTATTATTGTA
AAATTACTTCTTTATGTAACATAGAAACGGATATAGGTAAGTTAGAAATAATATAAAGTTAAAAATTTTAAGTAACTCTCT
GATGCTGAGTTGATGACCTTGAAACGTTTGAACAGATCTTCATAAATTGCAGCTAAGATCCATCTTGATCACGTCAGATTT
CAAAGGAAAAACCGCCGGTCGATCTATTCTTTTAGGAGATACAATGCCACAGACAGACATAGACACGTCAAACTTATAAC
AACCCTATTTTTGCGTCAGAGGTAAAAAATATCTGCAACTATTTTATACACGTCAGTTAAGAGTTATTGTTGGTTAACCGTT
TGTCTAATGAATATTTAGAAATACATATGTTGCCAGATTGTAAAATTGGCCTTAATGTTTTAATATGTAAAATGAATGCCA
AAATTGGCTTTAGCTTAGTTGGGTAAGTGCGATTCTATGTGAGGTATAAAATTCTGTAAAAATTAATAGTACTATCTATAG
ATAGTTATTTTGTATCTATAATACAAATTAAAGATTTTCTGTTGATTTATTAATTACGCTCTAAAAGAAAGTAAAGCAGCTC
TGATCTAAAATATAATAAAATCTAAAATAAGATAGCAGTTTGCAATTTTATATCTTTGATCACTTGATTAGGATTGTCATCG
TTTATTTCTCATTTATTTAATATGTATATATTGTATATTTACCTTCTTATTCATAAAAATATGAAAACCTGACATAAAGCTGT
TTTAGTAATTGAACTGTTTTGTCTCTTTCTTTAGTGCTATATTTCTAATTTAAAAGAAAGTGACAAAAGATCGTTTAGGTTT
GTAGGTTTTAATTTTTATGAATAAAGGTATACATATATATTTATTTATTTATGTAGGTTGTAATCATTTATGTATGTACAAT
GTATTATTGTATATTACAAATATAGATATATTGTTATGTAAATTATGTAAATACTTACTATTGTTGTTGACAAGTTCTGATT
TGCACTACTGCTGCTTAATACAGTCTGTGCCACTGTTGACTGGAGATCTCTTTTAGAGATAAGTTCACCTTTGCTCACATTG
TTTCTAATTGTTATTATTACTGTATGCTTTAGTGCAATAGATTATATATATTGCAACATTATTCTGCCTGACACACTAACAG
ATAAACCCAATACATATGGCATAATCATTGAATTGATAATATTTCGTATTTTAAAGGAATTTAATAAATTTTGATTACAATC
TCAGATAATTAGATGTTCACTTAGCATTTTTTTTATTGCAAGTTCTCTCTTTGTACAAAGCAAATAATGGAAATTTGCTAGT
CCGTTGTGTGCGGTGTTCCTTATGCGTGACAGAGGTAGGGGGAGGGGGGATGAGAGGGAGTTTTAATAACTATTCTCTTCC
ACGCTACACTAACTTGCAGCCCCGCAGACGTCTTATCCACCCGTTCCCACCAAAAATCATGTGATGTTTGAGACATGTTTG
TAATTATACAAGCTTCTATCGGCTAACAGGTATCAAACACACTCACTTTTACATTTATAATGTATGTAGTATGGATAACATT
CCGTGTATGTTTGGTGGAAACGTGTTGTTCCCAGTCTATATATACTTACGGTGTTACCTTGCCACCGCACACCGGACATTTG
CTGCGGAGAATCACGCATGGAGCTGATCGGCAGTAGCATTCGTTTTATAATCAAAATATTTAATTTCATTGGAGGTTTTAT
TTTAAAATATTAATTACTAAACTTTATAATGTAGTTTAGTCATGGTCGTAGCTAGAGTGGGGGGAAAGGCATTGAGTTGCA
ATGCCAAGTCACTTAATTATGGCTTCTGTCCTACCAAGAAGTTTTATTGCCCTTAAACTTGGCCCTATGGTCCCGAGTTCAG

		TGTTAGTTTTCATTAATCGTCTATAGAGCACGAGGTAGTTTTGGAGGCTTATTGAGATATAGGTATCATTTATTACTTAAT<br>TCTGTCATTACCTGCTTAATCCATTCAAGCCTTAACACCAAACAACTTAACACCAAAATTTAATATTTTTCAGCAATGTTTT<br>AGTTTAGACGTTGGACGTTTAATAAATGTTAAGAATATTTTATAATAATTTGTATTTGAAATAGTAGTTAAGTGTATATATA<br>TTAATAATGTGACCACGCCAGAAATACGAGTAATTGTAAATATATTAATACATGTAGATTACATCTATTATTATTTTATAAT<br>TATTATAATGTATATTAGACTTAAAAGAGATGCCGTTAAAGTGTATCTTATGCTGTAGTAGTCTTAGGTATTTCGACTAATT<br>TTAATGTGAATATACTGGGTGATTTTTTTACCATTAGAAAGATATGCCTTTTTTGTGTTAGTAAGAATGCAGTTCTTTATAA<br>TTTTTATACATCTTTATATACATACTGATATAGTAATTTATAGTAATGTTCCATTTGAATTAATACACTACATAGATTTCAA<br>AATATATTACTTTAGTTATAATAATATTTCAATTTAATCTAATCTAATTTATATATATATAACATAATTTAGAAGTGCGAAA<br>AAAAACGCAGAAAGAAAATCGGTGTATAAATATATTTAACCTAACAAAAAAAAAAAAATGTCAAAGCAAAAACATATTAA<br>AAAGAATATGAAAAAATATATATGCCAGCAAATATTAAAATCGAATCCGGTTATTTTTTTTAATAAAAAATTGGTTTGCTT<br>AATCTTGGTTTCTTTCCGTAGGGTAACAAATTCACTCCGTATAAATCTAATTTGTAATTTGTCACAAGATGACACTCGTCAA<br>CGAAGTGATATGATTAGCATTTTGTTTTCAAACAATTCTATATTGCCGTTACTTGAAAATGTAAGATTATGTACTTATAATG<br>TATACTAAAAATGAATAAATACGATTCGTCGGTAAGTACTTCGTTTCAGAACTGATTTTTTTTTTTTTATTTCCGTAATAGTC<br>CAACGAAACGAATTGGAATTTCGACAGGATGTTTATCATAAAAACTAGTAGTTATAGCCATGTGCAATACTGTGTAAATAT<br>CGTAAACTTAGTTTTCAGTTCACATGAACCTGCTGTATTTTACCATCTTACTAATTATTTAATTTGGTATCGACACTGTATG<br>GATAAAAAGTGTTTACAAGTTATTACACATGACTGTACCATATTCTGTACATAGTTATGTACATCATTGTACCGACAATTTG<br>ATTTCAAATCGTGAACATCCATCGACCTTTCAATTTTCAAGTAATGGCATATTAGTATAGGCTCGGTTTCACAGATTATTGA<br>TAAATTTCCCCATAGTATACTTGCCAGATAGCATATTCTTCTAAAAATGCCCGTATTTCTTACTAGACGAAAACACATCGAT<br>AAATAAAATGTAAATTTAGTAAATTTTTGTTTTATTTTTTTTATTAGTTTCATTTGTTTTTTGTCGTTCTAACTTTAATTTGCG<br>GTGTACTCCTGCGTTCAATTATTAGTTGGTAATTTTATTTATATTGAGTAGCTTCTTGTGGCCAAATAGTCGTATCTATAATT<br>ATAGTACAGTTTAGTGACAGTGGGGATGTCTGGGTTTATATTTTAATTAAATATTCCTTATTGATTACTTATCTATTTTTTTA<br>AAGCAATAGCGGTAAATATCCTTTTGAATCTGTGATTTTATCCACGTATAATGAATGTTATTAATATCTTTCAATATTTTGT<br>CGGCAATCCGCCTTTGTTTATGTACATTTAATGACTTTAATATACAGGTTGTCTATGAAATCATCAAGTTGTAAAAATATAA<br>ATTCATAATCCAATAAGTAACTCGTATTTAATTAGAATATGTTTGTGTATTCTCCGGTTTATATAATACAATGTACGCTGTA<br>CCAATACAGCGTTTACGTCGGTTTTACAGCCATCCTGTATATTGATACTTTTGTAATATATATTAATATTAATTATTTAGTTA<br>TTAA
MSTRG.7956	Zinc/cadmium resistance protein isoform X1	ACATCGACCCAGCGCTCAGTATAGTCCTAGTCATTCTTATCCTCGCGTCAGTATGGCCTTTGTTAAGAGAATCCTCGCTCAT<br>CTTGTTACAAACTGTGCCCACACATATCCAGGTGGACGCTATACAGAGGCGTTTGTTGGAGAAGGTGGACGGAGTGCTCG<br>CCGTCCACGAGTTCCACGTGTGGCAGTTGGCTGGGGACAGGATCATCGCTAGTGCGCATATTAGGTATGTTCTTTCAGTCA<br>GTATAGTTTGTCAGTCTAAATTTTTTTTGATGGGTTATCATCGAAGTTTTTTTATGGGTGATAATAGAAACATCATCATCAT<br>TATCGTCTCAGCCGTTAATTGTCCACTGCTGAATATAGTCCTTCCCCATAAGAGGGGATTTTGCCAGTAATTGCCACGCTTG<br>GCAGGAGGATTAGCAACGGCGGTTAGCTTTTGCAGATGTTTTAATAGGGACGTTGCTGCCGATCCCAAAACCATTAAGTTC<br>TCTCTTGCATCTTCGACACACACGGAAAAGAATGTGGTGGC
MSTRG.7974	Zinc transporter 2-like isoform X1	CAGTATACTCTGTTATGTACATGGTACTGCTCATGTCTATAGGCGACGGTTACCACTTTCCATCAGGTGGGCCGTCAGCTTG<br>TTTGCCATTCTAAGTTGTATAAAAAAAAGAGATTCTCAATTGATTTATCTCATAACTTCGTCAGTTGTGAACCGATTTGGAT<br>TTTTTTTATCTGAAAGTCTTTACTTACAGATTGGTCCCATTGAAGTTTTATCAAAATCGGTTCAGTAGTTTAGTTTTTAAATC<br>CAAATAACTAGTTTTACCACAATTGAAGTCGGTTTTTTTTGTGCGACAAAATGTTATAAGAAATACAAAACGTAACCATCG<br>TCTAGACTCCACTAACCCCCATGTCATTCAAAATAAGCAAAGCAACTTTACCTATTATCCATTATTCATTTCCCGCCAGCGT<br>AAACCAATAGGACACGCGGTATTTTTTTTTAATGGATGAAAATGGAATGGAGTAGCCCCGCCTGCGCTATTGATATACGCT

GGCAGGGCACCAGTGTGCGATGATTGGTGAAGATAAAGCAAGTCATTTAGCCCATCGTGACGAATTCACCTACAATTAAT
CATGGTGGTTTCCAGTCGTAACCACGTGGAGATCGACCTGTTCTGGGTTTATTTTTTATAAGCCCCTCCTCCCCCTCTGGGC
GTGCAACTAGTACCCGGATATTAAGTTCCAGGTTGTTTAGCTCTGGGTATATTGCTAATAGTGGGTCTGTTAAATTTCATCC
CTTCAATCTCTTTCCCCCCGGTATTACCATAAACTAAATGTGTTTGATCAGCTGAACTTATCCATTAACTTTACAGGCACCG
GCGTCAGTCCACAGAAGGTTCTAGAACAAGCCACAAAGCTAGTCCACGAGAAGTACAACTTTTTCGAAATGACACTTCAG
ATTGAAGAGTTTAACGACGGTATGGAAGCGTGCAGCCAGTGTAAGATGCCGCAGTCTTGAACGGGGAGTATTTATTAGAA
GGCTGATTATGAAGAAGTTAGAATGAGTAAGTTAACTGAGCGGCTGTAAACTTAGCGGCACCACGGATTAAGTTTAATTTT
AAATAATAAAGCATGTTTCTGATAATGTTGGCGATTGCTCTTTTAATTCGGAGATATTTTTCTTTTCTAGGTATTATAAGCC
GTAATTCTGCAATTTTTTTATGCAACTTAGAATGGCAAACAAGCTGTTGACCCACTTGATGGACAGTGGTAATCGTCGCCT
ATACACATGAGCAGTACCATGGACATAACAGAGTATACTGTTTCGCCCATGATACCCCCCATGCGTTGCAATTCTGCACCG
ATTTCGATATTATTTCTAGTTAGACTTTATTTTTTAAAATGGCCTCCAATCGATTAGTTAGTTCTACATTATATATTTTTTGC
ATTATCCAAAACTTTAACTAAAATTGAGTAGAGATAAACAAATGTCAATTGTATTTAATCATTTTAAATTTGAATAAGATT
TTTACATACCTTAGATGGATAGATAGATACTCTTTATTGTTTCCTTCAAAAAGATTACAATAGTAAACTTATTATAGACATA
AGCAATTACAAGAAATAATCACAATGAGAAAACAATGGGCGACCTTATCGCTAAAAGCGATCTCTTCCAGGCAACCTTTG
GATGGAGAGGAGTAATTATAAACTGATCGGAGGCACGATGAAAATTTTATTATTTCTAAATGTGGAGATGTGATACATGT
AATAATGTAAATGTATATCCCTACTCATTATTAGTGTTATAAAAGTGTTTAACAATTTTATATAAATGATATATATAAGTAA
AGTTAATAGGTTCTGATTTCACCAAAAGTAGTTTGTACCGAATAGTTCGAATTGAATATTTCATTTAATATTTAATAACTTA
TTCGTTTTACTAGTTACCTTTACAATTATTATTAAATTTAAGTAAAACCAATTTAGGATTTTTGCAGAGCATTATCAAATAT
GGTCCAACATTAGATATAATTATGTAGTTAAATTAAGGCCGCTAAGTTAGTGTATATATGCCCTCAAGATTATTCCAAAAT
ATAAAGTTACGGGTATATTCGTATTTTGTAACTTTATTATATACATATTCCTAGCACATCCTGATCAATTACTATGGTTAAC
ACATGTAATATATATATATATATAGTACTAAAAATTAGCAAATCTTTTTTTCAGGATATACAGGTAGATAGATAAACAATT
TACACAGGATGTGCTTAAGTTACTCACATATCAAATTTAATGCAAGAATTAGAGTTGCAAATAGTTTTGTGTTAAATATAA
TTATTAATTCTATCGTTATAAAATTTTGTTGTTTGTTTATTTATAATTTTTTTTTTGTATATAAACATTCAAATATTAATATTT
CCACAATGAGTTAGTAACAGTGAGTTGATTTTAAAGTAACTTTTATTTTTAATAATGTTAAATTTTTGCTCATGAAATAGTA
AAAGGTCGTGTGAAACAGGTTCTTAACTGCCAAATTTTAAATTATTATGCAATTTGCAATATCTTTTAATAATATTTAAAAA
TGGAACTTGTATGTACCCTGTGAGTTATTTAGTAGTGACAAATTTTGATTATTAATAGTCATTACTAAATTAAAGATACGCT
TTGTTCTTGATGATTACATATTACATTAAACAAAAATACGATTTAGTAATTGTTTATTATTATTATTATATAGTATTCCCGTA
GGTATTAAATTTTATTACAATCAAATATTCATTGTATGAAAGTATTCTTACAGCATTTATTTATACTAATATATACAGCTGA
GTATATTTTTTTGAAAATTGCATGTTATTCTGTGCAGTTGTTACACGTTTGTACATTATATTTTTTTTTTATTAATGATTTTCA
ACATTAATAATAAATAGTATATTTTACAGTAAATTTAATTTAAAAGGTAACATGCAGCCCATTTTACGGATCGCTTTAGAA
AGCTGTCAATTACATTAATGGTAAATTATTTTAAGATCCTGTAATGAACCATATTAATATGTGGTCCTTTTAGTCCCATTTA
AATGAAATTTATATTATTATAATATTGCATGATTTATAGTTTATAAGAGATGGGAATAAACAAATGGATTAACGTTCACAC
TAAGCTACACTGATAGAGATAAACTTATAAAAATAATAATATTACCCGCAGGCGGGGTTAACATTCCACTTTCATCCCATT
ATTCATCCCCCACTCGTTCTTTAGTTTGGATATCTCTTAACTTGCTATAAATGTAATAGTAATAATAAGAAATAGTAATAGA
TGGTAGTCACTTACCATCAGGGGAGCCGCATGCTCGTTTACCTCTGTGTTGAAAAAAAAAGAATTAATCCTTACTCCTCTT
CTCAAACTAAGCTCTACTCTTCTGTGATCTGGATATTTTGCTTGCAAATTGAACTCTCGCTTTTTACATACATTTAAATATGT
ACAGATGTTTTTTTATATAGAAGAGATAGCTACATATCATGCCATTTTACATGGCAAGCCAAAAAAGAATATAATGTTTTC
AGAGTTCATGTATGTGAGTCTCTATATTCCATCATAACTTCTGAACGCCTAAACAGATTTGCATACATGGGGTATTGTTAG
AGTCCTTACATCATTCAATTTTAGCATTATTAACTGCAAATTTCTTGATATTACAGCGAACGCTCTATATAAGCGACTCATT

		TGAACTAATTTCAATAAAAAAATTTTTATTGGGAAAAGTTGCTTATAGAGGAAAAAATGAGTATAAAAATATTTACCGTCT<br>GGAAAATATTTTTACTTATTTCTTTTACTAACTAAGCTGAGTAGAATAAATAAAATATAAAAAAATGTTGCTTATATAGAG<br>CGTTCACTGTATTTCCTTATATGGAAAAATTTGTGCTTATTAATTTACGGACCAAAACGTACATATTGTAAGATATACCAGT<br>GTGCCTTAGATAGTTAATTCTCTTTAATTCTTGTCTTAATTCTCAAATCTCTTATAAACAATTTATACCAATATGTTCACATT<br>AAGTGATTGCCGAAATGGGAATAATCTATTCCAGAAACGAATATTGTGTGATGATTTAATTTATATGTGTTACACCTAGTT<br>GTATTTAAGTATTCCGGAAATCCGAATTTATCCCAAGTATTCTAGTGTTATTTTTTAATGTAAAATATACAACGAAATACAT<br>TAATGTTTACAGCAAA
MSTRG.7986	Zinc finger and BTB domain-containing protein 41-like	GCAACACTGGGTAAAGCAGCCATTTTGTGGTGTCGTAGTTGGGTTGATTGTTCTTTATTAAATTTCGTCTATTTTACATGGA<br>AGTATTACTTTTTGTTGTGGTTTCGAAACCAATCAAAACAATAAACAAGTTTTGAAAGTAGTATTATCGTCAAACTAATTC<br>ATAGAAAATGGAGCAAGATGTCGATAATGGGTATATAAGTATCTGCAGTGCTTGCTTAAGTAGTGATCGTAATTTATTTTC<br>TATATGTAATTTACAACCAGTTTATGAAATGTTCAGGCTGCTTATGTATGATTTTGCGGGAGACCGGTATAACGAGGCTAT<br>GGCAGACGTGAAGCAGCTTGTGTGCTGGGAGTGTCTCGCCATTCTTAAAAAGTTTGCCCGTTTCAAGAAGCAGGTGCATAA<br>TGCTCAGGAACATCTAAGAGTCCTGGCCTTGAGCAGCACACAAGAG
MSTRG.8019	Talin-B-like	CAGTTAACAGCATCGTGTGTTAACGAGTCGCGTTGCCAGCCAGCATTAGTGTCTGCTGCAGACAAGCTGTCTGACGTAGCT<br>CGCGATTTAGAAGCTGGTATTGAACAAGTGGCTCAGGATCAACGGTATGCAGCCATTGGATCTAAGATAAAGAATCAACA<br>CCAGCTGCTGCAAGATGCGCTGGAAAAACTGAAGCGATCTTGTCAACCGGCAGTAAATAAAGGGCAAAGATTACAGTATG<br>TGAGAACTGCCCGAGAGATGAAGAATGCTCTGCAAGCTGCCCAACAAGAATTAGAAAAGCCAGTCGTGTTGCCAGTAAAA<br>GGTGAAGCAGCAGTTATCCGTCAGCTGCAACTAACCCAGCGGATGGCGCAGCTGAACTCAGCCATCGCCGCCCTCGTCGC<br>TGCCTCCTCTGATGCGAAGAATGTGGACTACACTGCAGCGGAGCAAGCTATCAGTACCATCAACGAATTATTGCCACAGG<br>TGGTCAAGGAGACGAGGGCTCTAAATGCAGGCAGTGACGAGAAATCTCAAGCTGCGCAGGCGGAGTTGTTAGCACTATGT<br>GACGCTGTGCGAGGAATTTCTGATGCTGTCGAGCAACAAGATTTGAAGGGTATAAACCAGTATGCATCGAAATTCTCAAA<br>CTCTTCAAACAAACTGATATTTGTTGTAAACCCACGAACAAATCTAGAGACGTCAAACCAGATTGTCGAATTGTCATCATC<br>GGCGTGTGAGCAGGCGTCTCAGTTAGTGGCAGAAGCACGGAAACTTTCTAAGACAGTCGGCGGTCAAGTTCATTTGGAAG<br>ACTGCTGTGAGAGAACGGCAA
MSTRG.8028	Adenosylhomocysteinase 2 isoform X1	CCAGGTGGATCACATAATTTGGCCTAATGGTAAACGTATAGTGTTGCTGGCGGAGGGACGGCTCGCCAACTTGTGCTGCTC<br>CTCGCTGCCGTCGTTCGTGGTGTCAGTCACGGCCGCCACGCAAGCCCTAGCGCTCATAGAGCTGTACAACGCACCGCAACA<br>TCGGTACAAGGCCGACGTGTATTTATTGCCGAAAAAAATGGACGAGTACGTAGCCAGTTTGCATCTGCCCACTTTCGACGC<br>GCATCTAACAGAGTTGACCGACGAGCAAGCCAAATATTTAGGTCTCAACAAGGTCGGCCCATTTAAACCTAATTATTACA<br>GATACTAGCATAAAATAGTATATCCAAATAACAATACGTGTAAAACAAGACACAATAGAAGGTGAAACGCAGCGATAAT<br>GAAGTTGTCACGTCAGAAACGCAACGGGTACGCACCGATAACGCACGGAAACGCAGTGAGAGTTATATATTGGTACTGAG<br>TTATATGTTGGTTGCGTTGCGTTAAGTTAAAACCAAACGGACTAAGTCACTTGTGTATTGTTAATTTTAGTATTGTGTAAGC<br>TTTATATAAACCGCTTTCGATGGTGTTCTGACAAAATAGCAGGCTTTGATATTGTTCACGAATGGTAATATATTGTAAGTGT<br>GTAAGTGATTAACTTTATAAGTTTTCGTAATGCACCAAGATTTAATTTTTGTATTAACAGATTTTACACAATTTTTTTTTAG<br>TACAATATATCACAAAATAGGCCTTAGGCGCTAGACTGTCGGACTTTGACGTTAATATTAGATAACACGGTCAAGTGGCTT<br>CAAATATGATACAAATACACATAAGAGCATAGAAATTTAAAATAATAGTTCCTCACTCTTCTACCAGTAGGAGAACGTAG<br>GAGTAGGAGGAAACGCCCTTAATAACATTTTGTCCTTATTATTTTGCCTTCAATAAAAAGAGATAAATCAACTTATACCTG<br>AAGGTTTAAAAACCCTGCCACACATAAATCATAAAGGGACTTATTTTGTATAAACCACGGATGTAAATGTACCACCGCGTT<br>AATATTACGCCTTCAATGCGAGAGGCATATTGAACTTGGCGAGATATGCAAGGTAAATCCTATTATTGATAAGTGTAAGAT<br>GGGCATTTTAACTCCACTGATGTGTGCCATAGATTCGTTAATGTCTTTTAAATTGGATTTTGAAACGACGACTAGAGAGAT

		GTTTTCAAATCGACTATTATTATTTTTTATGTTACTCGATTACGTCGTCAATATAGAACCGATTTTTAATTTATTTTTTGCG<br>ACTTTAATCCCGTGTAGATCTTTTGGAATCGATACAATCGAAATTCTTTTTGAGGTCGTTAGGGTTTAACTCCCGTGTTGAT<br>TCCATTTTGATTCTGTGACGATTGAAGCCGAAATTTTTCTTTGCGGAGCGAATCCTGCTTGTTGAAGGAAATTTACTTGTTG<br>ATACACGAGACGCTGTTTACCTATACTAATATTAGAAAGAGGAATAATTTTATTGTTTGTTTGTTTCTAATGGAATGAAATT<br>TTTTAGAAACTACTATAATTAAAAAAAAAAACACCCAATATAGAAAGCTTCGTTACCAGTGAGTAACTTATATTAGTTAATT<br>TTAAACTTTCACATTGTTTACATATATTTTAAATACACAATCGCGACTTAACGCGATGTAAAAGCCTTTACAGGTAAACAG<br>TACCTACATACCAGCTTCTTTATTTCCGACTTTTCCACATAGATTATATCACGTCGTGGTCACGACAGGACAGAAATGAGC<br>GGTGTCTTCGTCTAGCAGCCCTTGTAGACTGTTGTTAAGTTGACTTGAAGTTAAGTCCCGATTGTGTATTTCACATAACTTA<br>TATTATTTTCGAAAATTCTGTCCGTGCAAAGCCGGGTTGGATCGCTAGTCGATTATTAGAATATGAAAATGTATTTTATTTA<br>TTAATTTATAATTTGCAAAATTAAACATGTTAAAATAAAATAACGGAGCATAAAAGTAAAAAAACAAGATAATTCCAATG<br>TTTATTATTTTACATTAAAATATATAATCAGTATCGTGATATTACAGATATCCTGATATTCTATTGATATTGTCTTATTTGCT<br>TTTTTTTTTGTAATATAAAGCAAATTTTTTTTTATATTAAACTTACCAGGTTGAAGAATTTAATCTTTCGACAGCTCGATCT<br>GGTAGCTGGCGGAAGTTACTTTTTTTTTAATCCAGTATATTTTAAGATACTC
MSTRG.8035	Hypothetical protein TcasGA2_TC032951	GTTATGTCTATAAATGAAAGCGATTGGCTATTAAGTGTGCAATTACAGGACCCAAATATTTGCAACATTAGGAATATTTTG<br>ATGTCAGGGGATGCCGATGCGAATAAACAAATATTCCGTGAATATGAAATTCTAGGTAACAAAGTATACCGTAGAACCGA<br>ATACGGCAGACGTTGGTTGGTACCCAAACTGTGTATATGGCAAATAATTCGAGCCAATCATGACGATGTAGGTCATTTTGC<br>TGTTGATAAAACTGTAGATCGAATCCGTTCCAAATATTGGTTCCCACGACTGAAGAAAACGGTTTCCAAGTACATAAAAA<br>ACTGCTTGAACTGTATTTATTATAAAAGTGAACATGGAAAAAAACCAGGTCTCTTATATCCAATTCCTAAATATGCAAGAC<br>CTTTCCATACACTACATTTGGACCATTTGGGACCTTTTGTCAAGTCTACTAAACAAAATAGCTATTTGTTAGTAGCGGTTGA<br>TTCGTTTACAAAATTCGTATTTATATCGGCGGTTAAAAATACCAAAAGTAAGGTAGTGATAAATGAATTAAATAAAATATT<br>CAAAATATTTGGCAACCCTAAACGGTTAATATGTGATGCAGGTAGTGCTTATACATCAAAAATATTCACAAGATATTGTCA<br>AGAACGAAATATTAGACGACACATCATCGCCACTGCTATGCCACGCTCAAACGGACAAGTAGAGCGATATAATCGAACCA<br>TATTAGAAGCATTACGTAGCATGGGAGCAGACATAGACAACAACAAATGGGATCAACATACAACCAATATTCAACAAGG<br>AATTAACAGTACGATTAACAAAACTTCTGCTGCCATCCCAAGCGAAGTTTTTTTTGGTTACAGAATTCGCATGAATAGTGA<br>TAAAATTGCTGATGAACACGAGGAAAATGTGATTGACGTTACGGCATTAAGGGAAAAAGTGGACGAAAATATTAAACGA<br>GATGCTGAAAGGCAAAAAAATTCATTTGATTCAAAACGAAAAGAGGCACCTGGTTATAATGGTCCATTTCAGGTCACTGA<br>AGTTCTAGGTAAGGACAGGTATAAAATATCTGATATGAGGGGATCCGAAAGAAGCTC
MSTRG.8056	Ubiquitin carboxyl-terminal hydrolase 5	TGGAACCTTGTTAATGTTGGTCGCAGGTAGACCTGAGCTGCCTGCGGTCGCGCGGGCCGCAGCCGGGCGAAACTCAGCTG<br>CCCGACTCGGCGCCGGCCTCCGCCCCCGCGCCGCTCTACAACGACTCGCTGCTCTCAGACCTGTTGGAAATGGGATTCCCA<br>GTTGAAGCGTGCAAGAAGGCACTCTATTATACAAACAACTCTGGTATGGAAGCCGCCTCGAATTGGTTGATGGAGCACAT<br>GAACGACTGGGACTTCGCCAATAAATTCGAACCGCCAGAAACGCAGACTGCCGCAGGTGGGTGCAGCTCGCTGCCGCTGG<br>ACGAGGCGAGCGTGGAGCAGATCATGGGCATGGGGTTCGAGCGTGCGCAGGCGCAGCGCGCGCTGGCGGCGGCGGAGGG<br>CGACGTGGGCCGCGCCGTCGACTGGATCTTCAGCCACGCCGCCGAGCTGGACGCGCCCGCCGCCGCGCCGCCCGACCGCC<br>GCCTCGGCTGCCGCGACGGACCCGAGAGTCAGTTGGAATTCTTCTTTTTTACT
MSTRG.8065	Histone-lysine N-methyltransferase SETMAR-like	AGCTTTGAGTATTAAAACAAGGCAAATCTATAATGAAGTCATTACTGCATCTCAGGCGAACATCAATCTCGCGAAATTTGA<br>ACCTTCCTGAAGATAATTTGGACATGTTGGATCAAAAAGGATATTGAAAAGAAGAGTAAGACTGTGGACATTTCTCCGTA<br>TTATTTTGATATTAATAAAAAAATCTTTACGCGTCTGTTTATTTAGTTATCTTAAAAAAAGATTTAATTTCAGCTAATTACC<br>AACTAAAAAAAAAAATTGATGGTGGTATCTGCAGTGCATTACAATAACTTGGCGTGTTTAAAAGTTCATCATCTTGTTTAGA<br>ACACTTTTCTTTGTTTACACTATTTACACTAAACAAATTGCTGCATTAATCCACCTTCGTAGTTTCTTTACACTTTCAAATAC

		GAGCCCATTTTACTCTTAATTCTGTTAAATGTGTGATTCGCTATTTTTTTATCCACATTTAAAATATCATGTCCGATTTCATC<br>ATTACTCAGTCTTTCGCATGACCGCATAAAAAAAAAAAGTCAAGCGGAGTTAAGAGTCATTGATTACCTTTCTTTATCCATC<br>TCACACCATAGATCACATTTAAGCACAAAACTCGACACGTGATGCATAATCATTTATTTCATTTTCATCATCATTTATGGTA<br>GGCATGGAGTCCAACTTACACGAACACCAAATCTTTCTTGAAATAATATCATTGCATGCACAGTGATATTAGCACATTGCC<br>GAAAACAAAGTACAATTTCTAGATAATCTTAGATGCACTGTTGTACACACACGAAAAATGGCTAGGTTCACGTGTTATTAT<br>GAAAACAAACGCACAACGCGTCGACGTCAGATACGTAACTATTATGTACTTAAAAAAAAATTCTGAGGTCCACGTGAACT<br>GTGTCCTTGATGCGTCAATGAAATAACAGCACGATCAATGACAGCGCTAGACTGATTTGTCATTGGTAATAGGTAAGTTTC<br>CCGCGATGATCAAAGATTGGATTAAACGCATTTTTTTTATTTTATAGTCAATAGCGGCGTTTAAAGACTGTTCATTGTTATT<br>TAGAGTTACTCAAATACTATTGTAGTAGTGACTAAAGCGGCAACGACTCTTTATTTACACAGGATGAAACATTTAAATTTA<br>TTATGATTTTTTTTTTGTATGAAAAATTGTTTGATATTGGAGACACGAATTCTATCATTATCGTAACATATATATGTAATAC<br>TACGTGGGACACTCAGAAAGTTTTGCGATACTTAGAAAATGTTCTATAGGTAATTATAGTTTTATTACATCTTTTCAAAGTA<br>CTCTCCACCAGCCTCTATACAAAGTTTCATGCGTCAGAACCAGTTTTCGAAATACTTTTTCCAGTCCGCCGCAGACACCTCG<br>TTTTTCATTCACCAGCATGCTCGAACGCTAAGAGCGCATAATCCGGCGACGAAAATTGTATCCCTTTGAGCTCATTTTTTAT<br>TTTTGGGAATAAGAAGAAGTCACACGGTGCTAGATCAGGACTGTAAAGAGGGTGACTCAGGAGTTTGATCGGTGTGCTGT<br>CTAGAAAGTCCCTCGTTTTAATTGCAGTGTGGGCGGCCGCATTATTGTGGTGGAGCAGTAGCCCACGAAGCCCTGTGTTTG<br>GTCGCTTGTTGCTTACTACAGAGAAAACTTCTGCCAAGCATTTGATAGTATACCATTCGGCAGTAATGCTTCTTTGGTTTCG<br>TAAAGGAATTGACACCGAGTAGCCAGTCAAATTGAAAAAGCTCGCCATCAAAATCTTCCCACGCTTCTAGGTCGCTTTACT<br>TTGGTAGGCGTTGATTCGTTTTGAAACACCAAAACCGTTGACTGGCGCTTTGTTTCTGGATCGTATTGATACAAGTAAATCC<br>AGGATTTATCACCTGTAACGATGTCGTAAACACGTCGAGACGAGCCTGCTTCAAATTTGTCTATCATATCATGGCACCATT<br>CCACCATTGCATGCTTCTGAACATCGGTGAGGTTGTGCGGCACCCAGCGTGAAACTATCCTCCGCACCCGTAGATGCACGT<br>GTAGAATCTCGTGAAGGCTTTCTGATCCAATCTGCAGTATATGCTGACTCTCTTGGTATGTAACTAGTCTATTGTCTTGGAT<br>CAAATTTTTCACTACTAGCACGTTTTTTTCCGTAACAGCCGTCAAAGGGGGACCACTTCTTTCTTCATCTCCCAAAGTCAGT<br>CGACCGCGCCTTAATTCATTACACCAACGATACACGGGTACCTTAGAAGGGCCTTCATGCATGAATGCCATTTGAAGTCTA<br>TCGTACGATTCGTCCGGTTTCAGGCGACATTTAAAATTGTAAAAAATCATAGCGCGAAAATATTCTCGTCGCAAATCCATT<br>TTTCAATGAGACACGGTAATTTTAATTAGCTCGCAAAAAATGTGGAGGGAAATTTTGAATTTAGAACGTACTTATTTTTAC<br>TTATAAGTAATTATATTTTTAAATAAATTATTACTGGCCAGTTTCACATACATAAAAACTTTTTTTCGCAATACTTTCTGAG<br>TGACCTGGTATATCCCCTTAAAGGGAATTCAAAAATAACAATTATTGAACCTAAAAAAAAGCTAAACGCACCC
MSTRG.8068	Oxysterol-binding protein-related protein 9	GTCACCGCGCGTTGAAATGGGCGCCGAGTGTCGTGAGCTGCCGACCCCCACGCGACCGCTGCCGTCACTGGAGGACATGG<br>ACGCGCCCAGAGCGATGTCCCTCTTCGTACCTGACACATCGTACTCTTCTTCGGAGGGGGAGGACGATTTCTACGACGCGG<br>ACGACGAGCCCGCCGACATCCCCCAGCCTGGACCCAGGTGAGAATAGCTTAGTGCTTGCGACGCGGCAATCAACGAAGTT<br>AAGCAAC
MSTRG.8079	Hypothetical protein RR46_04589	TGACAGTAATTACTATTTACCTATATGAATCATATTTTACCTAATTTTTAAACACATAATACTATTTTGTTAATTGCATTCGC<br>TACAAAATAGCTAAATAGTAATATTACTCGTCTGAAGACCATAAACAGTAATCAAACCCACCGGAAATAACTTGTGAATA<br>TTGTGAGAATACTGCGCTACCATACCTAGAATAATACTTTAATTTCCTCATAAATCTACAAAACCACAATGCCTACACCAG<br>AGAAGAATGTTCTAAGTAATCCGGAGCAAATTCAGGAGCAACCCGTACGGGAAATAACACAAACTGATCATTTAAACAAA<br>AAGCTATTGACTTCCTTGTTTAACCGAATGAATGAAGTTGACGGTGGCGGAAACTCGTTACTGGCCAAGATGTTGGAGCCG<br>GACCCGGAGGAAGCAGAATGGGAAGACAGGGAAGGAGATAATTCACAAAAGTGATATTGTATTTTTAAGTCTGTGGAGT<br>AAACATACTAAATTAAAAATGAAACTGATACCTTTTCACTAAAGTGATAAAATTTTTTAATAAAATAACCATACTGAAAAC<br>ATACCAATAATGGTTGACTGGTAGAGAACACCATCCACTGTTAAATCCGCGAAATGTACATACAATTTCACTGTGCATTAA

		AGTTCGAATAAATACATTTATTCATTTTACAGTTATAGCTGTAATACAAAAAGTTTCTTATTTAAAGACACTAAAGTAATCCTATCATAGTTTGTAATGAAATCTTTTTAAATAAAACATATTAACATGATGAATATTAGACAGAATTGTATACTTGAATGTGAGGATGAAGGCAGGTCAGTAAAGGATGGACACACCTGCCTGATGTTTTAGTACACAAACATGAGCACCAGTGGAAGATAAAATGACGAGTATGAAGATTTTTACGGATATAATACTAAACTATTACAGCGACGCAACACCATTTAATACTGAACAAAATTGTTGCGTACCCGTTTTGTTCATAACAAAATTAAGTCCAAGCTTTGATTCGCCAAATTATTGCATTTCAACAACTATTCTCAATGGCATCTTATCAATAAATAACATACATAAAATAGGACATTGTAATCCTAATATTAACAACCTGCATCAAATTTAATAAACACAAAGAAAACTTGATTTTTTTTTTTAATGTCATAATATAACAGCTATATTATTTGTACATAAAATAGTATGTATATTTTACATGGGGACCGATAGAATTAACATTTGTGTAATGTCAGCAGATATGTGAAATAAAATAATTGTACAATTGATTGCGAGTGTATTTAGACCAAACGCTGGGTGGTCGAGACGGTGTCCGCTGCGCAACCTCGTAGCAATAAACCTGCAATCAAATATTCAATATATATAAATATCTATTTAGATACCATAAATAAATAAAAAAA
MSTRG.8082	CLIP-associating protein	GTTGAAGAAGATACGGTCGTTGCTGACGGCGAACGCGCACGTGCAGTACCCGACAGAGTTCGCGGCGCACCTCAAGGACCTGTCCGTGCCGTTCCTCGTCGTCATCAAGGACCTCCGCAGTCAGGTTGTCCGCGAGGCGTGCATCACCATAGCTCACATGGCCAAGGTTCTGAAGAACAAACTGGAACAGTTCAGTCTGTATATACTGCAGGAGCTCATAAACTTGATACAAAACG
MSTRG.8101	Uncharacterized protein LOC107037501	CAAATTCAGAGCAATCAAAAAAAAAGGTGATGTAGAGATTGGGCTGTATTTGATTTACACCATGTGCATGATGGGGAAGCGATCATGCCCATTCGGTGCAGGTGTTCGTTGAAATNNNNNNNNNNNGATTCGTAATCTACAAATATTGGTGTAAAATTTTCTGTTTGTATCCTTTCCTTTGCAGAACTTTTCCACGGTTTTCCAAAACGCCAATTAATTTTGCGGTACCATTTGCCTTTAGTCTGTAATATTNNNNNNNNNNNGCTCCTCCCATTTAGTTGTAACGATTTTATGTATTCCAATACTACAGTCTGAGTAACAAAGGGGTAAATCTTGAACACGAGCAGCACTTGAATTAATAATTTTAGCCAAAGTCTACTTGCTCATTCCCAAACACTCCAATATGTGAAGGGGTCCAGATGAAGATTATATCTTTTCCATATATAGTACAGAGATGATAATATTTATTACGAATTTCTTGAATTAAATAGTTTGATGATGAAATGAATGTGTTATTTTTTAATGCCATAAGAACACTCATGCTATCTGTAATTATAATCCATTTCGAATGTAGTACCTGTTTTTCTATGTATTCTAATGCTAAAAATATAGCTTTTGCTTCTGCTGTGTAAATACTAGCCAAAGTTGAAATTTTATAACCGTGACCAAATCTATTTGACAAATCGTAAAAAGCCACTGAGGTGCTAAGTGCATTTTTGGAACCATCTGTATATATCTGCCTGTAGTCAATAAATTCTGATAAAGTCTCATACATATCGT
MSTRG.8102	Uncharacterized protein LOC109042663	ACGATATGTATGAGACTTTATCAGAATTTATTGACTACAGGCAGATATATACAGATGGTTCCAAAAATGCACTTAGCACCTCAGTGGCTTTTTACGATTTGTCAAATAGATTTGGTCACGGTTATAAAATTTCAACTTTGGCTAGTATTTACACAGCAGAAGCAAAAGCTATATTTTTAGCATTAGAATACATAGAAAAACAGGTACTACATTCGAAATGGATTATAATTACAGATAGCATGAGTGTTCTTATGGCATTAAAAAATAACACATTCATTTCATCATCAAACTATTTAATTCAAGAAATTCGTAATAAATATTATCATCTCTGTACTATATATGGAAAAGATATAATCTTCATCTGGACCCCTTCACATATTGGAGTGTTTGGGAATGAGCAAGTAGACTTTGGCTAAAATTATTAATTCAAGTGCTGCTCGTGTTCAAGATTTACCCCTTTGTTACTCAGACTGTAGTATTGGAATACATAAAATCGTTACAACTAAATGGGAGGAGCNNNNNNNNNNNAATATTACAGACTAAAGGCAAATGGTACCGCAAAATTAATTGGCGTTTTGGAAAACCGTGGAAAAGTTCTGCAAAGGAAAGGATACAAACAGAAAATTTTACACCAATATTTGTAGATTACGAATCNNNNNNNNNNNATTTCAACGAACACCTGCACCGAATGGGCATGATCGCTTCCCCATCATGCACATGGTGTAAATCAAATACAGCCCAATCTCTACATCAC
MSTRG.8105	Hypothetical protein TcasGA2_TC034535	ACGCCTGTCACATCGACGATGTCAACACCCAGTAAATTAGCCTTCTCGACACCCGCTGCTGCATCTAAGGGTCCCGTTATGAAGAAAACCAGACCGAGATGCGGGTATTGTAAACAATTTGGTCATAAAACAGAAGGATGTCCGAAAGGACAGAGACAAAGAGGTAAGCAAGAGGGAAACACTAAGGAATATCCTCCACCTCCAACAATTACATGTTATGGCTGTGGTGCGCCGGGAGTGATCCGTTCAAATTGCCCCACGTGCAAGAAGCGGCCGACCATACCAGTAACGGCATCAACATCATCCACTGCTTTCCAATCGGTTGCTGCCTTGGGAAATAAGGATATAGACCCACGAGCACGCCCGAAACTTGATGTTGAGATATATGGTGAATGTGGTACCGCATTAATTGATACCGGTGCAAAACAATCTATTGCTAGTAAAAGTTTATATACATTTTTAAGAACTCGAAATCATGTGTT

		TAGTTTTGTAAATACTACTGTTAAATTAGCCGATGGCTCTAGTAAATTAATGAATGTTGCAACCACTAAGGTTGATGTAAA<br>GGTTCAAGGTATAATTATACCAACTGTGTTTATAGTATTACCCGATGCTACAGACTCCTTATTAGGAATGAACTTTATACA<br>GGAAGCTGGTATGATACTGAATTTTGAAAGGCACTTGTATACTCTTAAATCTGATTCTAGGCACTTCAACCTTAGGTATGA<br>AGCGATTGATCCGGTGGGTGAGATCGCAACAGCTTCAGCCATCATTCTTCGTGACGAGGAAGGTGCGTTCTTCTCAGCTTC<br>GGAACGGGAGTCATTTAACACTCTCCTGCATGACAATCGGGACATCTTCGAGCCAGGGGGAGCCCCTACAACGTTCGCAG<br>TCCACAGGATTGATACAGGTGAGCATGCTCCTGTAGCTGTACCGCCTTATCGTGTCTCGCCATGGAAGAAAGAAATTATGA<br>GGAAGGAGATAGAACGTATGTTAGAAGAGGAAATCATAGAAGAAGCAGAGTCTGAATGGGGATCTCCGGTAGTGCTGAT<br>TCCAAAGAAGTCGCCTGGTGAGTGGCGGTTTGCTATAGACTATAGAAACGTAAACGCCATCACAAAAACAGATAAGTATC<br>CATTGCCCGTCATTGATGACATCCTGTTTTCTACCAAGGCTGGTGCAGTCATGTCTACGATCGATTTAAAGTCGGGATACTG<br>GCAGATCGAGGTCCACCCCGATGACAGAGACAAGACGTCTTTTGTCATGCCTTTCGGTGTGTTCCGTTTCCGCCGCATGCC<br>GTTTGGGTTACGTAATGCGCCGGCAACCTTCCAAAGGATGATGGATCGTTTCAAGGCCGGACTAAAGGAGGTAACTATCG<br>TTGTATATCTTGATGATCTCCTTGTGATCTCCTCTGATCTGCAGTCGCACCTGCAGGACCTGCAACAGGTGATCGATCGACT<br>GCGCGTTTTTAACTTACGAGCCAATCGAGAGAAATGTGTATTTGGTCGTGAGAAGGTAAAGTATTTAGGTCACCTTATCTC<br>CGCGATAGGGATAGAGCCGGATCCATCCAAGGTGGATGCTATCGTAAATCGGGAACCGCCGGTGAATCTGAAGGAACTGA<br>AAACCTTCTTGCAAACCTGTTCTTGGTTCCGAAAATTTATTCCGGAGTTCTCAAAGGTGAGTGAACCACTTACAATGTTAA<br>CACGAAAAAAACAATCCTGGCTGTGGGAGGAACCGCAGAAGAGGGCTTTTGAAGAACTAAAGTCTCGCCTAGCTTCTGCC<br>CCGATTCTCCGCCAGCCCGCTTTCGACGAGCCGTTCATATTGAGGACTGATGCGAGTGCGTATGCCATAGGGGCCGTACTA<br>ATGCAGGGACCTGACCCCAAATCCGAACAATCTATTGAGTACGCCAGCCGATTACTCACCTGAGTACCAGTGCTGAGCGT<br>AATTATACCACTACGGAGCGGGAAGCTTTGGCCGTAGTATGGGCGTTAGAATAGTTCCGAGGTTATTTGGAGGGAGCCCA<br>GATACGTATCGGCACTGATCATCAACCACTGAAGTGGTTGTTCTCTCTGAAAACACCCAGTGGACGTCTGGCTCGTTGGGC<br>ACTGAAGATACAAGGTTTCAATCTAGATGTTGAATATACTCCCGGCAGGATCAACGTGGTTGCTGATACTCTTAGTCGTCC<br>TGCTGAGTCAACGAATTCTACATCCTGGAATCGAGCGCACGTACCTGAAACTGAAAGAAAGGTATTATTTCCCTAATATGA<br>AACAACTAGTTACGCAATATGTCAAGGCTTGTGAGCTATGCCAGAAGTATAAGCCTACCAACATGAAACGCGCAGGGTTA<br>TTACAAACCCCAACACCTCAGCAGCGTTTCGAAGTTTTGTCCATGGACCTGTTCGGACCCTTACCGGAAGGACCTAAAGGT<br>GAGCGCTGGATATTTTTAATTGAGGAC
MSTRG.8106	DDB1- and CUL4-associated factor 12 isoform X1	CACCCGCAGTTCCCCGACGAGGAGGCGGACGGCTTCAACCAGGTGAAGTACGTGCCGGCCATCTACACGCACTGCTACGA<br>CGACAGCGGCACGCGCATCTTCACCGCCGGCGGCCCGCTGCCCGCGCCGCTCGTCGGCAACTACGCGGGGCTCTGGCAGT<br>GACCCGGGGGACCGGCCTCCCCGCTTGATAAAATTATTTAAATATTATACTTATATCGTATTGCATCTCGCGGCTTTGCCCA<br>CAAACAGGCGACGACGATCGACGGTTCACCGCCAATAACTCCTGAAGGGGACAAAATGTATTATTACTGTCACTTATGGG<br>GTAGAGCTCCTAAATACCAGCTTGAACCCTTAATAAATTTGTAATAAATAAAAAAATAAGTCTTAGGAGACATTTTGCTAC<br>ACTGTACGTGTTCTACCGCTTGTACAATGGACTGTGCTCAGAGGAATCGTTCAATATGATGCCCACAGCCACTTTCCAGCA<br>CTGTACATCTCGCCACCGACTAGGAGTTCACTCCATAGCTTACAGTCAGAATGATAGCGTAGAACGCGGTTTCAGAAATAC<br>TTCTTCCCCCGAATGCTTTTTATCGAATGTCGAGGTTTTTCCAAGAGAACAGCATGGGGTTCTTCAAAAAAGGAGTAAAGA<br>GGTCAGCAACGTACGCGTAATAACCCCTTGTATTGCAGGCGTTCATAGGCTACAGTAACCGCTTACCCCTATACTTCTTTG<br>CCACATCTATGGTATAAAAAAAAAATACAAACATTTATCGAGCACAATTATTTATTAAGAAATTGTGTAACCCCTTTTATG<br>TACTTTCTTTTTTGTATATGTTTTGTTTTATTGCTTTAAATATATAAGAAAAGCTAATGGTGTTTGTGTGATTTAATTATTTTT<br>GATATAAATTCTCAATATATTAACATAAAAATGTCCAGGAGGTGTGTGCCTGTTAACCGTCGATGTCGTTCACGTATCAAA<br>TTCATATCCGTTCAACCATTTATGGTATAATTGAGTAAAAAAAACATCTTTACAAAATCACACATTTATTACACTGGAATA<br>TGAGTTTTTATACGCAGTCTAGAGAAAACGCCTTTGCCCCAAATAAAAAAAATAATATTGTATGAGTAATTAGCTTAAATA

ATTTTATATATACTTATTTGTAATTCCTCTTGGTTGTAATCTAGGAAATTTGTTTAAAAACTACAATAAAATAAGCTGTTAT
ACTAGGGTGCGTCCAGAATTTGTTGATATAAATAATATTATTTCTCAAACATGCTTGGAAATGTGAGCATTATTTTGACA
ATCTTCTTAGAGATAAATCAAAATTGCCGTACTGGCCAAATACATGCGGACAGCGGCCGGCAAAAGTTGTATAAAAATCC
ATATCGTCGTGTTTTGCGGCCAGATAAGTTACTATGTAAACTCATATCTGTCCTGTAATTTAAAAATGGAATGACCTTTTAC
TTCGAAACATGGCTACCAAGGAGGTAACTAATAAAAGGTTTTATTTAAATTTCGAACTGTCTTGCAAATAGAAAGCTGTTT
ATTGAAAAAAAAAACAAAGGAACATAATGGCGCGTGAAAAATTATTATTGTTCGTTATTCGTCATTGAAATTGAAAAGTAA
AATTGTGTTTTTGATTTCCGCGCATTGTTTGAGAAAAGTACTATACAAGCCTTGGCCACAACTAGGCATTGATGAACTCGC
GTATGTGTGCCTCGGCTATGCCTCGGCCCACATTTGCACCTACGTCTATCAATGCCTCAACTGCTGGCCGCTGCAGTAATGT
ACTATAGCATATGCCTTGTGATGGCTAATTGAATTTATTTAGTTTATTGTTACATGGATGATAATTATAATGGTAACCCTGG
CTGCAATAATTTTATTGTGGTCATTTTGATGGAAATTTTGGATTTATTTTCATATTAACTGTCCACTATTGAACATAAGCAC
CCATACACCATCTTTACCATTTAGAGGGTTTTACCAATAGTTGCCACGCTTGGCATTGCATTTCATCTCTACCTAGGACTTT
TTTTTAGTAACATTTTAGTCTGCCTAATAAATATTTCTTTTCATATTACAGTTTAACGTATAAATATTTGTCTGGACGCACCC
TTATTTAACTTATGTATGTACATATTACGCGATGGTGAACAGAAATCGTTTCAAATACTTTATATAACATTACATTATTTCT
GCCATGTAGTCGAGTACACATTGGGCCAGTGTTGGCCCCAAAATATAGTAGATTAACATCGAAATAAAATACATATGTATT
GTGTTTACATGTTTATTCTGTTTTTATGTTAACTTACAGTGCTGTACAATTATTTTTATATGTTTTGACATTTTATCTAATAGC
ACAATATTCTTTAAGATATGTTGCGCTCACCCTTACCTTTAGTTCGCTATTAATTCCTGACAATAACAGGAATTTTAAATAT
ATATTTTTTATATCCCAAAAATACAATTTATTTACTCAAATTATAATTGAATTTTCTTTAATATCTGTACTGATTATTATTTG
CAAATGTTATTAATGTATTATTTGTTAATATTGATTTGAAAATGATTTATGTATATATACTGTCATCAAACAATATTGAAAA
TTTATTTAATTGTATGTATTGGAATTCATTTATAAATAAGTATTACGGATACATCACAAGATTTCACGGTGGAATCAACCAT
AATAGTCTTAATACCCAACTGGACCCCAAAAACGGCCCGTTCACCCCACGAATAATTAGGTGGAACAAATTTCAACTTGCC
TCATAGTACAAGTGCATATCTGATATTAAATGTCAGATTTCAAATGTCATTAGTAGTTTTTTTTTTTTTTTTGGCTGATGTT
TGTATCCCAATATTGGTAACAAAAACATATTCAGACGGAACAAATGGACGCACTAAAAATATACCGCCTTATCATAAAAA
AAAGGTTGAAATTGTGTTCATTGTTGTCATGTCATGTCATTCATGTTTAAACATCTTGCTACAAAAGGATGTATAACATGTG
TGTATGTCTAAACAAAACAAAGTTAGTAGTGTATGTTTTTATTAAACTGACGTAACGATTTTTAATCGTTTAAAGTTACCTT
AGGCACTGTTTAGAGGAACAGTATTTTAAATATATTGTATAGTAAACTCAGAAAAGTTCACACCGCATTCGTTACCAATAA
TGGGGTCAGTGGGTTAATTAATATGGGTCTAACAAACAAGTTTTAGGGGTCCAGCGGTTGTGAAATGCCAAATAGTGCTG
ACTCTTCAGTCGATACGAAACCACGCAATTCTATTACAACGCAGTCTTGTCTGATACATTTGAACATTAGTTTTTTAATTAG
TAAATTTTCCATTATAATGTTAGGAGAGACGGCTGATATTGTATAGGATCGAATGACAGAGAGGGGTCAATGTAGACAAT
TGAAAAATAAATCTATCAATATTTTTAAGTTATGGTTGCATTAATTATATAAATTTTAGAGTTATTTAATTGTAAATAAAAG
GGAATAAGTCAAAATTTTCCATTTAGTCAGATCTTATTCTCCATAGAATTAACCAATCAATTCGACCCTATTTTTGTATTAC
ATATTTATTGGACATTTTATCGAGCTGTAATAATAATAATCATATAGTTCTCCCCCGTTTGACCTCACCGATCGCTTAGCCA
ATGTGCTATTGGAGTCGTTTGCGTGTAATGCCGTATTACCTCAGTATGACACGAGTGGTATAGTATAGCAACACTCGTCCG
TCTCGCTTTATCGGCTACTATTAGTGGTTGGCACGTCTCTGGCTGTAACACATTGGAGTGCGGACTGGATTTATAATTGTAA
AAGTTGCTTGTAAGGTTTTATCGGGACGTGCAAACCTCGTTAGTGATGTGTCGTGATAAAATCGTCAATTCGAGAACGGGC
GCGGGAGAATATTTTTTTTTGTTCTGGCAATACAAATTCTTTTGGCATTTGTAACCTTATTAAAGTATACAGTTTATAATTT
AATTCTAATGATTACATGATTCCTCGTCAACAAATAGGTAATTAATAAAAATTTATTTAATTTTTTTTTTTGCAATGATTC
ATAAAGTTCTAGAACGGTATTCGCACGTTCATTAAAAATTACGCATAAATTAAAAATAAAAATACATATGAAAAAATTTA
CGCATTGTTATTGCTTTTTAGAGTAAAAATGAGTTATGTAGATTACCTGCAATATTTATAAGAGATGGCTGTTTTCTTTGTA
ACTTAACTATAAAATCGAGCTCTTTTTTTTTAAATATATTTATGCGATTTATAACAAATAAGCGAGTGGATTATAAGATTAT

		TTTTTTGGGAATGCCTATTTTTTTTTTTAATGAATCGCTACTTTAAGCAACAAATTCTCCAAATCCGAAATTTTGTAAAAAA<br>AATACTATTCTTTGGAAAAAGGTCTAAAAATCGAGGGAATTATAACACTCGATTTTTGAAAAATTCGATTTTTTAGCCGGG<br>AAATTTCGAAAGAAATATCTATTAAATTTACCATCTATGGTGTCTACAGGTGACTGAAACATTCTTACAGATATTAAAGCA<br>ACAATAATTTGTAGCCTAATTGTTTTGACGACTTTATCACGAACCTCGTTTAAGTTTAGTGAACCAATATTATAATGTAATA<br>GATTAATTGGAGTATCCATTATGATTAATTGTATAAAGTGGCTAATTACGATATCAAGTGACAAGTGTTGGTGTGTTTTTAC<br>ATAATTATTATAATTTCTACAAATTATCCAAAAAAAAAAAATGGAGATTGTGACAAATAAAAGACTTGAATATGCCGATAT<br>GATTAA
MSTRG.8<br>111	Uncharacterized protein LOC106138579	GTCGTATCGGTTCTGGTAATACTGCTGCTGGAAGTTGATTCCAAAGAGTGGTTGTGCGTGGAAGAAAGATTTTGTTTTACA<br>TTTTATTTTATATAAATATTTTTTTTTTTACAATAGACGATAGCGCAGACTGGGTCACCATTCCATTGTCATAAAAATGCTC<br>TACAAGGAATTTTGAGTGAAACATTAATGTATATTTTCCAGCACCCTGATGCCCAAATCCTGCCAGACTTGGAGTCGGATG<br>AGGTGGACCCGCAATCGCGCTATGTGCAAGTGATCAACCCCGACAAGTCCGTTATGCAGCTGGATCTCCTCAACATGACTT<br>TGGTCAGATGCGACGACGGTTCGTACAAATTGGTGGCCAACGCGGAGACCACCAGCGACATACCGGCGGACTCGACCGTC<br>ACCTGCGTGCTGCAGTCCAGCGATAATGAAG
MSTRG.8<br>115	Uncharacterized protein LOC105384140	TTTAAATGTATGGAAATTATAAACTTTTAAAATTAGTAGTATTATATGTCAAAAGGAAACGTCTATTTGACTAAAAAAATG<br>GCAGAAATAGAAATTGGAATAACGGTACTGCGTGTAACAAATATTTTCGTTTTTATTTTTTTTTATAATCTTTTAATTTATA<br>AAAGATGAGTAATCCCACTGTGGAACAAGTAGATACCCTTCTGACATTTCTAGAAGAGCACCAGGAGTTAGCTCGTGGCC<br>GCTGTAGAAATGCTGAAGGGCGAACAAGATCCAACCAATTATGGGTTCGTCTCACAGACGAACTCAATGCATTGGGTGGA<br>TGTACTAAAACTGTGAAGCAGTGGCAGAAGGTATGGGCTGACAAAAAATATTTAACAAAAAAAGCTGCTGCTGCTGCTCG<br>AAGGTCTGCCAGAGCCACTGGGGGTGGGCCATCATCTGCTGAGCCTTTAAACCCCATACAAAAAAGAATATTGGCTGTTA<br>TGGGAGAAGGATTTGGGGAACCTCAGTCGGATGCATGTNNNNNNNNNNNTTCCAGAGGTNNNNNNNTCAGCAGATGAAGC<br>TCCATCACAAAATATAGAGCAGCAACAGCAGCAAAATCCCCAGCAACCGATGCAACATCACGGGTTCATATTTTCTAATG<br>CATAAATATAATTAAGACAAGTTGCCTTAGTTATATTTGTACATTTTCAAAACATTTCCGGCATTTCATAATGTTGATTGAA<br>ACTATTTAGAATGATCAAAACTTCAACGACTGCTACACGTCCAGAGCGACGACGCCGTCGAGCAGACGCCATTGGATCTG<br>AACGCAGTGTACGCAGGC
MSTRG.8<br>121	Sorting nexin-7-like	AGATATTCAAGAGCGATCACAGCGCCCTCAGCCTGTGGGGGTTGAGCAGCGCACTGTACGGCAGCGCGGACACCAAGCCA<br>GCGAACGGCGCCAGAGTGAAAGACCCAGAGTTCATAGCGGCGGCGGACTACTTGTTAGCGTTACAGCAGAAACTTACCAC<br>CCTGTGCGCGCTCACCAACAAACTGCACAAAGGATGCGCAGGCGTCGCCACTGAGCTCATGGGGTGAGTACTCTATTCGA<br>ATCATCGTCGCCATGCCAGCCCTTGACCAGCCCACTGATGGACATAGGCCTCACCCATAAGGGGAGTTTTGCCAGCAGTTG<br>CCACGCTTGGCTGGCGGATTGGCAGTTGCTGACTGGTTTTAATGATGTTTTAAAAGGGACGTTGTTGCCTGTTCCTCGATGA<br>TTAAACTGCTTTACTCGTCTCTTACGACACCCATGGGAAAGGAAGGGGTGACCCATTGTACGCCGGGACCCCACATTCTAT<br>GTTTTAGTGGACGATAATGGAATGGTAACCCTACCCGAGCTAACAGTATCTACACTAATATTATAAAGAGGAAAGATTTGT<br>ATTTTCGTGTGTATGTACGATAT
MSTRG.8<br>130	Hemicentin-2-like	AGGGCGTCCTCAACCGACCCTAACCTGGTTGATCAACAACAAGCCAGTGGAAGACCACACAGTCCTGAAGAATGATGGCA<br>AGGTCATCATCAGCAGGATCAGCGTAGGCGGAGCTGAAAGGAGTTGGCTGAACACCAGTATAAGGTGTCAAGCTACAAAT<br>ACTCCACTTTTGAGTCCTCATGAAAGGACCGCCAGAGTGGAGATGAGATTGCGTCCAACTTCAGTTATGATCACAAACAA<br>ACCAGTGCAGATGTCGGCCGACATTGAAACCGTGCTGGTTTGCATCTCGCATGGCAGTAGACCACCAGCACAGATCACTT<br>GGTACAGGGAGAACAGAAAATACACTAGAGGAAAG
MSTRG.8<br>136	Zinc finger protein 845-like	ATAAAATTTGATAAATTTATTAACTAATATTTCCAGATTCCTTATGTTAAACTTTACAGACTCAATAAAAAATGATTTACAGA<br>AGATAAAAAAAGACCAAAGAGATTATTCTAGTGGCAATAAAGTGATCATAGAAAAGGCACAACCAGTTGTGGTGTTAAA

		GCGAACCAAAATATTTGAATGCAAGAAATGTCGTTGTTGTTTTTAAGGGCCAAAGATTATATATTTCACAAATTGAAACA<br>TGCAACACCTATTGT
MSTRG.8138	5'-nucleotidase isoform X1	TGAGCACGTTCACGAAAGGCGAACAAATGGTACCAGTATTAAATGAAATAGGCACGCACTGCGCCGTATTTGGGAATCAT<br>GATTTTGATTTCGGTTTAGAAGTATTATCAGGCCTAGTCTCTCAGTGCAACTTCCCGTGGCTCATGTCCAATGTGATAGACA<br>ACGAAACTGGCCGACCACTCGGCGACGGGAAGATCACACATGCGTTGCTATGTAATGGCCACAAAATAGGATTAATAGGC<br>CTCGTAGAACAGGAATGGCTAGAGACCTTAGCAACAATAAATCCAGAGGAGGTCACATTTATTGACTTCTTACAAGCGGG<br>CACGAAGTTGGCTTCACAATTAAAACAGGAGGGTTGTGAATACGTAATAGCACTGACCCACATGAGGACCCCAAACGACA<br>TAAAGCTAGCTGAAGGCTGCACGGAGATTGACCTTATACTGGG
MSTRG.8140	Abrupt-like protein	AAACCATTAATAAAAAAAAAGCTTTGTTTACAACAGTTTCACAACAACAACAATATTTTCATGTATTTTTCTTCGTCTCATT<br>TTCATGTTAAATAAATATTAATTATCTGATATGACACTAACAAAATTTACTTTATCGTGGGATGCGTATCAGAAGAATTTCT<br>GCACTGGATTAGGATCACTGCAACAAAATGGAGAGTTTGTGGATATGACTCTGGCAGCGGATGGGCACCATGTGAGGGTG<br>CATCAGCTGG
MSTRG.8155	Uncharacterized protein LOC106113978	AGTCAGTCAGTCAGCTACCAATTTAAAATAAATTATTGGACCGAAATATTAAATGGTATGATAATTGGCCCATTTGAATTA<br>CCGGAGAATTTAACAGGGCAAAGCTATTTGGAGTTTCTTTAAAATGTTCTCCCCAATTTATTACAAGAGGTTTCCGAGGAG<br>CTGAAGGAATCGTATTGGCTGCAAAATGACGGTTGTCCTGCGCATTACTGGTTCGTGCATATTTAAATAGAAGGTATCCAA<br>ACAGATGTATCGGGCGTCTAGGGCCAATTCTGTGGCCTCCACGTTCACCGGATTTAAATCCACTGGATTTTTTTACTACTT<br>ATGATATATTTTGTTTCGTATTTGTTTTAGGACGGCCATAACCTTGAACTATAAGACGTTTCAAGGTTAACCATTAATTACC<br>GGACACCCTGTATACAGATTGGTCCAATAGAAATTTTATCAAAATCGGTTCAGCGGTTTAGTTTTTATATAAAAATAACTG<br>CTTTTGTCGCACAAGAGGTCTGTTTTATTGCGGGATACGATATTTTTTTTTATTACAATAACGGATTTACAGTTACTTTCTAA<br>GCTCGGCCAAGTCTGAAGTAAGTTATCATGTATATTTGTAAACATGTGTGCGTATCGGCATTATTACTTGTAATTAATTTGT<br>TTTTAAATAGAAATTAAGTGCTCGTTATATTTTTTTTTTTATAATTATTATTACTCGGGTATTTAATTTTAATATATAAACGA<br>TGTCTACATACATTGCAATCTGTATAGTTGTATGATTGTGTTTTTTTTTTTTTGGTGGCTGAAAATGGTATGGTAACCCCGCCT<br>GCGCTAACGGGATACGCTGGCGGAGCACTAGTGAAGGGTGATAGATGAGAATGAAAACAGGCCAGTTAGCCCATCATGA<br>TGAATTCATCAGGAATTAACTATGATAGTTTCCAGGGAGAACCGCGAGCAGGTCGACCTGGTTGGGTATATTGTTAGCAAT<br>GGGATTCTCAGTCTCCATTACTAACAATCCCTTTCCCCCCTGTATGATTGTGTTGATTTTGGGTGATTTTCAGCAGGTATCA<br>TGGGCGAAACAGTATGCTCTGTTATGTCCATGGTACTGCTCGTGTCTATAGGCGACGGTTGCCACTTTCCATCAGGTGGGC<br>CGTCAGCTTGTTTGCCATTCTAAGTTGTATAAAAAAAAAGTTGCGATTCATTTCTTATTTTGGTGAACTTTTCAGTATCGTGT<br>AAAGTTGATGTCCCTCGGAATATTTTTTTTTTATATTCTTTATTGCACTTATAAAAAGAGATACGGCGATTAGTTTAACAAC<br>TTGTTAGCAAGAGTGGGTTTGTAACTGAAAGAGATCTCTTTAAATAAAACAGTGTAAGTATATATATATTGTTGTACAAAT<br>ATGTACATATGTATTTATTTATAGAAAGATCATTAGTAGGCCCCCATTGCTAGTAATAGCGCAGGCGGGGATACCGTACCA<br>TTTTTACCCATTAAAAAAAATCACCCACACTTAAGCTCCATTTAATATAATAATAAATTATAATTCTAAATTATATATTTTT<br>ATTATTATAGTTTGTAAGGTAAGCTCAATGTTTTTTTTTTAACGTTGCAACGTTTGACTTGTGTTAATTTAAATTGTATATTT<br>TTTTTATTTTAATGTCATTAAAGTTGTTTATTTATTTATTTAAATTGTGATACATAGGTAACAATGGATGGTAAAACAAGAT<br>AAGTCCTCTACGATCAACCCCATATATCGAGGTATACAAATTTGCAATTATATAAGAAAAAACAAATTATATATATAAAA<br>AAATCAACAATGTCACATTTATAGTAAACAATTATAATATTATGGAAATTTATTATATTTATCTTCAAGGAATCATCATTGT<br>TAGATAAGATTGTAATGTATGTAGGCACGCTGATGTTTTTTGTACATATTTTATCAATTTCTATGTTGACTATTATTAATTA<br>ATGCAATAATTTCATTAGTATTGTGTTAAAGCTACTTAAAACACTGTCCACTTGTAGTTATGTAAATTGACTTTACTTAGTC<br>TATGGTTTCTGAATTAAGGCTTTTTTTTTTTAAAGAAACTGTCGAGGCAAAGGAAACATATTTGTTTCTAGGATATGTTCGG<br>TACTGTTTACGTTTGGATTTGTAAAATGGTCCGTGGGAAATTGGATTTTATAGTAATGGGACATTCCGTGATCTAGACTTGG

MSTRG.8159	Copper chaperone for superoxide dismutase	GTTATTGACATTCCGTGAATCGGGATTTGGTGGTTTTGAGTACTATGTCTACTTTTATGGGGACGTCTATGTTACATCAAAA<br>AAATGGCACGCTTACTAATATGTTAAGTCTGAAATAATGTATGTTACTAATATTTTAAACTATATTTTAACCGCGACTTCGC<br>CCGCGTGACTTGGGACATGTTTACTGATCGGTATAAAGCCTATGTCGTTCTCCATCCCATATAGTAAGTATATATCAAATTT<br>CATTGCGATTGGTTAAGCCGTTAAGACGCTAAGAGGGAATAAACAAACCTATTATCACATTCTTAGTAATATTATAAATGG<br>GAAAGTACGTTTGTTTGTTGACTTTTACGCTTATTATATGGAACGGAAGCAGACAAAGCCATGGGTGAAAGCTACTAAATC<br>AATAAAAGACATGTAAGTACTTGTAGTTATTGTAAGGTAGATTTATTGCGGATTCAATATTCAAAGTCGGGCGGCTCGAAT<br>TGTTGACGATCGGCGCCTTACCGTCCGCCTCGACTCACTGAAACTGCGCAGAGATGTTGCATCTCTGTGCATCTTTTACCGA<br>ATTTATCATGAGGAGTGTTCTGAGGAATTGTTCGGATTGATACCTGCTGCTGATTTTCACCACCGTACAACCCGCCACAAA<br>CTAAAGTTTCTAAGGAAAAGATTCTACATGACAACACAAATAGAGTACAAGACAACTTATCTTGGGAACCAATTACTCCG<br>AATGACAGACAAGACAGCTGACTGATAATATTGTTTATTTTGTTCAAAATTTTTAGGTTATTTATTTACCTACTTATCACTT<br>GATTATAAACTTGCAATTTTAATTTAATTTTTATTAATATCAAGTTGAATGAACAGTTTCAATTAATTAAAGATTACAGCGT<br>TTAAAGCCATGATTACTTCAAAGTTGGAAGTGTTGGTAGATTTTGGGCAAAACCCTGAACAGAAGGTATTGGATAAAACA<br>CTTTCAGAGCTGAAGTCTCAGGACGAAGTAAAAGAGGCACAATACAAAGATGGAGCCATTGTGGTTGAAACAACACTCCC<br>CAATGCGGTCATACTGGACATCGTTGCCAGAACGTCGGGCAAACGGACTGTGTTGCAAGGATTCGGAGGTGCATAATGTA<br>GAC
MSTRG.8178	Proteasome-associated protein ECM29 homolog	CAGCCATGTGAGCACAGCTGCTTCACGGCAGAAATACAGGTCAGACAGAGATACCTAAATAGACGACTTGTACAAAATCT<br>CTGATGGCATGTATGCGAGTGTTGTGAACACGAGATGTGCCGCTGGCGGCAGGCGCCGCGTTCGGGTTCCAGTCGATCGCT<br>CGCAGCGCGGGCGCGGCGCTGGCGCCGCAGCTGGGCCGCATCGTGCCGCGCCTGTTCCGCTACCGGTTCGACCCCACGCC<br>GCGCATACAGAACTCCATGGCCGCCATCTGGCACGCCCTCGTGCCCAACACGCATGCCACGGTACAAAAATATCATAAGG<br>AGATATTGGACGACTTGGTAGCGAACTTGACTAGCAACCAGTGGCGTGTAAGAATGTCTTGCTGTAACGCTCTCGCTGATT<br>TGTTGAGAGGTGCGGCGAGCCTGCAGAGTTCACTAGACCAGCTGCCGACAGTGTGGGCGCAGCTGTTCCGCGTCATGGAT<br>GATGTACACGAGGGTACTCGCAACGCTGCCACCTCCA
MSTRG.8184	Zinc finger protein 431-like	GCTTTTTGAAGGCAGTTCTGTAGGCGCCATATTGTCATTCAGAATCTCAGATGCAACACGCACAGAGTACCAATTTTAGAA<br>TAGAAAGCGATTGTTTTTAATAGAAGATATATAACTCCAAATTGATCTGTGTTTACTGTTTACTACGCGCGGTGTGATTGA<br>AAATCTAATTTGTTTTGAGTTTTGAAAGAGAATAATTATAAAAATGGAGGACATAGAAGCGTGCCGCGTCTGCTTATCAAC<br>GGACACTAAATTATTTAATATGTTTGAAAATCTACTTGTGGCGGCTTACGAAAATGTATCAGGTCTAAAGATGTCATCGAC<br>GGACGGGCTACCGCAGTACACATGCAGCTACTGCAGCACCATGCTGCTCAAGTACAACTCCTTCAGGGCCAACTGTCAGA<br>AGGCGAACAAACTTCTGAAGCTGCTCTCCAATCAGGATGAGGT
MSTRG.8185	Zinc finger protein 79-like	GTGTCAATGACCGCCATATTGTTTATTTTATTCAATTGTCAATACAATTGAGAGGCAAAGGAAATTCATTTGTCCAGTAATT<br>TGTTTGCGGAAGTATATTAATTTATTATTTTAATTTGTAATGGATAATATTGGTTTAAATACAGACGAAACAAAACCTGATT<br>TGGAGTCAGGAAGATCGATATGCCGGTGCTGTTTGACTACAGACAGACGGATGTCGAATGCGTCCATATACGAAGCTTTTT<br>TCCAAGATCTAGCTGGTGTTACTGTGTCAGAGTCTGACGGTCTACCGCAGTGGGTCTGCTACGTGTGCAGCAGGCTGCTGT<br>ACAAAGCAGTCCGCTTCAAGCACAAGCTGCTCAAAGCGCATAACCTTCTATACGAGTACCTCACCAGATGTGCGCCG
MSTRG.8195	Hypothetical protein	AAAAAAAAAAAAAAACAGGAATATCGCCTTTTAATTAAATCGAAACGTTAGTTCGTTTTAAATTTTCGCGTTGTTTCCACA<br>TGTTTTAAATACACAATCGGGACTTAACACGGTGTAAAAGCCTTTACAATAGTACCTACATATCTGCTTCTTAATTTCCGAT<br>GTTTCGACGTGGATTTAGAGCAGTGTGGATTCCATGAGTGGGATTTAAATAATGATGGGGCTTCACTTAATGATGGTGACA<br>ATATGAGGCTATTCGAGGAGACTGTGGGCTGCCAGACCAGAGGGAGACTTTGTACAAAAGTCGTTTGCTTGGACACCTCT<br>GTTCCTTTCATGTTTCTACTGTGAAGCAGTTGTGTTTACATGGCTGTGTTTCTGTTTGAAGGATGAAATGTGGTAGTGGATT<br>TATAGGGCACAGAGGAATAACACCTGAGTCCTCAGGAATGGCAACGCATGGGGGGTATCATGAACGTAACAGTATACTCT

		GTTATGTCCATGATACTGCTCATGTCTATAGGCGACGGTTACCACTTTCCATCAGGTGGACCGTCAGCTTGTTTGCCATACT<br>AAGTTGTATAAAAAAAGACGAGCACGCCGCTCACTTCAGTCCTGTTATTACCGAAACGTCAATAAAGAAGCAGGTATGTA<br>GGTACTATTTAGGTGTAAAAGCTTTTAAATCGCGTTAAGTTCCGATTTTTTATTTAAAATCGAAACATTGTTTTACTGTTAA<br>TATGTTGTGAAAGTGAAATTATCAGATATTTGAAGAAAATACTATTAATGTGTATTTATTAATTGCTTAAGTTTAAGGGGA<br>ATCGAAATGGTAGAAATAATTTTTAAGTGAGTACATAATGCGTTTATTCTCTGCTAATGACATTAATAAATTGTTATTTTTT<br>ATTTGTATCGCTCGTTGACCTTGAATGCCTTCAATTAACTGTAACATTACCTTTATTAATTTAATGTTACATTTATAATAATC<br>ACGCGTTACCCATACTTCAAACTACTAGAGTATAAAGGCCTAATGTTTTAATACCGAAACAGCGAATATAAGACTTTAACT<br>CCACTTCTAAAGATCTATTTTTGAATCTCGCCTCTATTTCTGTAAGGGTCAGTTCGCTGCACCCATTCTAAATGTTCTCTGCT<br>ATAATCTAACATATTATTTGCTTCAATTACATTCGTAACATTTTTTACTAGTTTCGTGTTTGTAAATCTTGTGTAATATCTCT<br>CTATATTACAATGTATAGCCCTCTATCTAGTCTCTGGATCGTATTTTCATAGACAATGTCTTTATTTACCAAAGACAATAAT<br>ATACTATGACAATTGAACAAATATGGTATACTACCTAAATTGGTACCTATTTATTCAATTATAAATTTTGCCTACAAACATA<br>TTTTTTCTCAAACATGCTGGGAAATGTGAGCATTATTTTGACAATCTTCTTCGAGATAAATCAAAATTGCCGTACTGGCCAG<br>ATACATGCGGGCAGCGGCCGACAAAAGTTGTATGAAAATTCATATCTGTCGTTTTGCGGCCAGATAAATTACTGTGTAAAC<br>TCATATCTGTCCTGTAATTTAAAAAAGGAATGACCTTTTACTTCAAAACATGGCTACCAAGGAGGTAACTAATAAAAGGTT<br>TTTTTTTTAAATTTCGAACTGGCATGCAAATAGAAAGTTGTTTATTGAAAAAAAAAAACAAACTTAAAAAGTAAAATTGTGT<br>TTTTGATTTCCGCGCATTGTTTGAGAAAAGTATTATACAAGCCTTGGCCACAACTAGGCATTGATGGACGGACGTGCAAAT<br>GTGGGCCGAGGCGTAGCCGAGGCACACATACGTGAGTCCATGCCTCAGTTGCTGGCCGCTGCAGTAATGTACTATTGTCTG<br>TAGAGTTTATTCATAAACAAAATCATATAATAACAACAAATTTATTCTTTTTTATAATCTTGCTATGTATGGGATACGTTAA<br>TTTTTAAATTGACTTTAACCTTGTCAGGTTTTTTTACAAACTTAGTATACTAGTTTGAAACCGAGATGACGTATGGCAAGT<br>TTTATAGTTATTTTGGAAATTGTCTATGCAGATACGAACGGATTTAATGTCTATATCCAAACGAACAAAAACCGACAAATA<br>ACTAATTGTAATTAGTTTTTTTTTAATAGATTTAGCTATTGTCTAAGTGTAAAGTAACAAATGCGTTTAACTAAACTGATTA<br>CCCTCTTGTACGCAAAAACATGAAGTTTCCATTAAAAATGTGTTACTATTTTTAACCAGAGGGAGACTTTGTACTCAAGCA<br>AACGACATTTGTAGGGAGACTTTGTCTGGTAAATAAATTATTAATATAAGGGGGTTATGACCTTAGTTGTATTATTAGGAT<br>CCATACCGATGCAAATTAGGCAGTTTTTATTATCAAATCTAATTAATACCTACCAATACGCATGTAACTATTGTTGATGGG<br>CAACCAATTGGGAATTATTATGATAAAATGCAACGGACTAATCGAACCTTGTTACAATTTAAAATAACGATTAAATTTAAG<br>TGCATTTTTATCAGAATTTAAATCGTCAAATAAGTCTGTTTCAACTAAATGCGTATCGATGTGTTCTTGGGGTGAAATAAA<br>GGAGATAGTTTTAGGAAGGAGTTAGGGAACAGTCTGAGATAAACGACGATGGATCCAACTCGGCTATTGTAAATTCAAAC<br>ATAAGTTTTACAGTATAGGTCGAACAAAATTATTTAGTTTTAATTAATCGACAAGAATATTCCTGTGAAGCAATATAAAAT<br>AAAAAAAAAAAAATGACTATCGCATCGTTCAAATACAAAAATAGAATATGCTTAAAAAAAAATAGTTTAATAAAAGTGATTC<br>CCAGAGTACCTATAAAATAACCTTGGATTATATAGATAGTGTCTCCCTCTATACAAATGCTTCGAGAAAACAGCTCAACTT<br>TTGCTTACAATTTTGCATGATAAATAGTTGAGTGTTTCCTTTATAACTTCTATATTTATTTTGTTTTGTTTTAATACGTAAAA<br>ACTCATTTTATTTATTATATAATTAAGTATAGATACGTCAATATACATTATAGATAGATAGATAGATAGATAATTTATTTAC<br>ATTACTTACAGCACACAAATTACAATACAAACATACATATAAAGGCACAGTAAAAGAGATAAAAGAATGAAAAACAAAA<br>AAAAAAAA
MSTRG.8<br>200	Pulmonary<br>surfactant-<br>associated protein<br>D-like	GATGGCACGTATACGTACAGAGTACTGGACTCAGTGGCGCCGCTAGAGGGCGGGTATTGTCGATGTGAAAGAGGGCCGAG<br>GGGTCCACCTGGCGCACCGGGCATGGAAGGGCCGAAGGGTGCGACGGGACCGCGCGGAGCTCGCGGGCCCAGGGGACCT<br>GAAGGGTCTTTGGACTTGATGCTATTGTTACTCGCAGACATGAGACATGACATCAACAATTTAGAAGGGCGTGTTTATAAT<br>AAAGGCGAAGT
MSTRG.8	Uncharacterized	TTTTAAGATATTTTTTTTAGCGACGCTTTTCAATCTCCTTAGGATATAAAAACGTTTGGAGAATTTGGAAAATACACTGTAT

204	protein LOC106137111	TTTCCAAAGGAGATATATCGTTTATGTATAAGTGGGCTACAATCACAAATTAAGTGCTGTGGCGTTTCTTTCTCATTGTGAC<br>AAAATCTGCAAACGCTTTTTGGGCGTAAGCAATTTATAACTGATCCTATATCATTGACAGACTTGATCATTGACAGGCTGA<br>CATTAATATATGTCACACATTAGATGAATAGTTAATATGAATCGTTTGGAAATCCTATTGCGAGCTACCAAAGTGGTCATT<br>TTAGACCTCTAATTATTTTACACTTCATCACGATCACATAATTATATGTACGTATTTTACACTGTTCAATTATCTATCGGTTA<br>TGGGCACAGATTAATTGTAGTTCCCAGGCCTTGTAGCGAACGAAGATGTCTTAGTGAGATAATTACTGTTCTTCGCCTTGT<br>CTTGTACTTGTAATGCGTCAGCATTGCAACTTGTAGAGTCTAGTACATCTCTGTTCACAATATAGGTACCTACAAATATACG<br>TACCTACTTACTTAATTGTTGTTGATATAGTGTTAGTACTTAATGACTAGTATACAGTCATGTTTTATGTGTTCTTTAACATT<br>ACAATTGTTATTTTTTATTTTTTATTTGATTTCTTTCTTAATTGTGTCATCCGGCTACTCCTATGAACAAGTTGAAGCGACTA<br>CTGCCGACTCTCTCGAGGAAGGATAAAGGCCCGCGTACACGCACATCGTGGTACGCTGGCTGTGGAGCCGACACACCATC<br>TGTGAACACCTCCACCGCATCCTGGT
MSTRG.8212	Nuclear pore complex protein Nup205	GGAGTACATGGAGCAGAGCCTGGTGAGCGTGCTGCAGCTGCTGCAGCGCGCGCTGGCGCTGGAGCGAGCGTTCGCCGCCG<br>CCGCCGCCGCCGCCGGCCGCGCGCTCGCCATCGTCGGCCTCTCCAAGCTCATACTAGAGCCAGAGGGCCCGGAGAACAGT<br>GACCGGCTGGTGACGTGCTGCAAGGTGCTGGCGCACACGGCCACGCTGGGCACCGCCGCCGCCCGCGCGGTGGCGCTGCT<br>ATCGCGTGCGCTGCAGCCACCCGCCGCCGCACGCCACCTGCTGGCAGCCGTCACGCAACGCCACTCGCTCGCCGCACAAG<br>TACGGCATGGATTTGTGGAATGCTTGGAAGCCGAGGAGTGGGCCCAAGGCGACGAGGGCGCGGCCATTCGCGAGGCGAA<br>AGAGGGCGTGATTGCGCTGCTGCTGCAGGTGCTACCAACCGCACCGCCCAATTTCGCGCACTTCCTTCTCGGATACCAGCT<br>TACAGAAGACGTGAGTCGCAGCGCTCTGCACGAGGCGGGCGCGGCGGGCAGCCCGCGCACGTGCCTGCATGCCGTACTCG<br>ACCTGCTGGACGACCACCTCGCGCGCGGCCCTCATCAGCACCACCAGCACCAGCAGCAGCAGCAGCATCGAGCTCCGCGA<br>GCCCCACGCCCCGCCGACAGGTGCGTACTCGCTGTGCCATTATTAGAC
MSTRG.8215	Uncharacterized protein LOC110382709	TGCTCTACGAAAGGGATATATCGGAGGCACCGTGGGCGCACGTAGTGAGCAATGCAGCTATCACGGAGTACCTAGCGCTG<br>CTGGTAGCGACACACGGCTGGGACATGGCCGCCCACCACTGGGACTTCACAACTATCACGCTCTGCTCGCTGTTCACCTCT<br>CTCGAACTCTCCAAGCAGCGATGGGGCTCCGTAAAGGTTGAACAAATCGCTCCTGTTAAATGTTACTACTAACATTGTTTT<br>CATTTGTACTGTTACTGAAGAGCTCCTAGTCTTGTTACTTTCCCACTTATTTTTACTAGTCAAAGTGTTACTGCACAACAAC<br>GATATTTACTCGCTAGTAGCTTCTAACTAGGGGGGAAAGGGATTGTTAGCAATGGAGTTTGGCAGCCCCATTGCTAGCAAT<br>ATGCCCTATCAGGTCGACGTCCACGTGGTTCCCCCTGGAAACCACCGTGAACAATTTTTGTCGAATTCGTCACGATAAGCT<br>AACTGATCTGCTTCATCCTCATCTATCATCCTTCACTGGTGCCCCGCCGGTATATACCGACAGCGTGGGTGACTATTTTATT<br>ATCACCCATAAAAAAAAAACTATGCTAGTTTTTTTTTGTACAAAATTTTACTAGAAACAGAG
MSTRG.8223	Cytosolic endo-beta-N-acetylglucosaminidase	GCTACTATCGTTACAGGTGTATTTCTATCTTCAATCTTAAGATATCGTTTGGACAGCATAATAACTAAGTGCTATTTATTGT<br>AGTTATTATGTCCAATATGTGAATACTTTACCTATCATCGTGATATCCGTTAGCCATATCATGGCATACCAACGTCTTGGGT<br>AAATTAGATTCATTGTGCCGGTCTATATCATCCGTATCCGGATCGAAATGACAAAATATATGTGGGTTATCAAGGATTACT<br>GCAGATTGTTTGTTTATCTCAATATTCTTTATTATTTTTTTACTATGTAACTGTAATTCTTTACATAAAGATCTCCACGAAGG<br>AGGACTTTCTAAAAATAATAATATTTCACTGTATGTATCGAGCGGTTTGCAGCGACAATAATGGCGGGCAATACGAACGT<br>GCGAAGCGTGAAGCATAAACAACTAAATTGATATGCCGGAATGCTAGAATATAAACTTCTAATAATGTACTTTAGAAAAT<br>AGGATACGTGTCAAGGTTTTTGCGCGTTTAGGCTTTGTTGTCGCTGATATGAATTATATAAATATTTATTAAACAAACTAAG<br>TTCAAATCAAATAGATTTTTGCTTGTACGCGCGTCAATATCGCTTGTAATCGAAACTCGACCGGTAGATATTTTGCCAGTG<br>ATAAAGATGTATAAACATAATGTGTTTACTCATTTATTTTATATATTCATATACCAACTCAGTTTATTCTCCGATTTTTCATA<br>ATTCTGGATTAAAAGTAATAAAATTTATAGTATATTTTTTTACAAGCCACTTGTTTGCACAATTTATTTATGGATTGTTATT<br>AATTACGTAGACGAAATAACAATAGAGGTTTCCGTATTCATAGATATTTCTAGAATGTAAGTAGGTATCATTAAATAAATA<br>CTTTAATTACCACTAGTAATGGAATATTTTACTAAACTGTGGGAGCCCAGTGCTCTAGTTTTATTTAGTTGCAATAATGTTT

		GTAGGTCAGCTCAGTGATAAGGGTTGCATTGTTCATTGGCATGATATTTCCGTTGGAGAACGGAAACTAGATCATAACTAT<br>AACTAAGTAAATGAGTGAACCCAAAATACAATGTTGCGGGATATGTTGATACGACGATATTGATAGTGACGAGGTAATAT<br>ATTTAAACTAACTAAAGACTAACAACAAATATGAATGATTTTATAATATACTAAATAAGTAACTAAGAAAATATAACAAA<br>CTAGCTGACGGAATACGACTACTACTACTAGAAGACGAACGGTGGAATGCGCGTGAGCTGCGCGCGTTACTGGACAGTAC<br>TTGGACATTGCAACATTACTTTATTTTTCTAAAATAAACGTAGCCTAAGTTACTTCTTATTACATCAGCTACCAGCCAATAA<br>AAGACCCGTCAAATCAGTCCAGCCATTTCAGAGATTAGCCGAAACAAACAGACAAAAATTGTAAAAATGTTATTTTGGTG<br>TATGTACAGTATATATATGTGTAGTAAAAAAACGGTTATTTCAATATTACAAACAGATACTCCAATTTTATTTATATGTATA<br>GATATGTTTGCATGGCTGTGATTCAAGGGTGGATGTGGCAGTGAAATTATAGGGCACAGCAGCATACACCTTAATTCGTAA<br>AAATGGCAACACATGTAGGGTATCATGGCCGACGGTTATCACTTTCCATCAGGTGATTTGCCATTGTAAGTTGCATAAATT<br>TTTTTATCTGTGCATTATAGTCATCAG
MSTRG.8253	Pyruvate dehydrogenase (acetyl-transferring) kinase	AGGCTCTATCAAACGAAGCCAACGAGTTCCTGCCAATCTTCAACAAGACATCGTCGAAGTTCTACAGGCCGGTGGCTCAG<br>CTCGCCGACTGGTCCGGTCCGGTCAACACGGCGGCACAGAGCAAGAACAAGGAGAAAATGTCACCGTTGTCTGCGCATAC<br>GCCGGTCTCGCATAGTCTATAGTGCGAGTCGGAGGAGTGAGACGTCACTCCTGAAACGCGCTAACGAAGTTTTTCATCACG<br>AAAGTATAAACATAGCCCTGTCTATAGTGTTCCGTAACAATCAATAGGATTAACAGATAATTAGAATAAGGATTTAAATAT<br>CGAATAAGTCGATCGATTCTGTGTTTAACAAATTCAATCGGGTATAATCGATTACGGTATCATCATCGTCATATTACTCGTT<br>AACTGCCCATTCTATGCCGGGCCTACAAGGTTTTCACCGTATTCAATAAATATTGGTTATATCGAAAATCAAATAAGATTT<br>TGTTTCTTAGTCGATATATAATCGTATCGATTGTACCGTATTATCGGTAATATTATACGTTACATTATTTGTGACCGATCTAT<br>CGAATTAACTCGATAACAAACGATTATTAGGCAGCACTACTTGTCTAAATGTTACAGTGATATTGTTGACAGAGTCTTGTA<br>TTTCAGTGTGATTGGTAGTTGATATTTTTAATATCTCTAATTTATCTACTCATAGGTATAGGATTAGATCCGAGTGAATTAG<br>TGTTGCAATCGATTTTAAGATTAATCGATTATGTTTAGACCAAATCGCTTAAGTTCTCGATTATGACCATACGAAGCTTTAT<br>AGATTCGATTTTATAACACTATAAGTGTAAGAACCATGCGGCCTATAAAGGACACAAAAAATTTTTTATCAATCATCGTA<br>TGAGAATTGTATTGAATCGAGTCATTGTAGATAATTGTCATCCCTAAACATTAAAATTTAAGATTTTAAAATTAGAATAGC<br>CACAGAATAAAAACGGTAGTGGAAGTGTCGTGTGCGCAGAGACGGGTTTCAAAGTCTTAACCCAATTCGCTCATTCTATA<br>CCCTATGTAAATATATATTCACACAAATATAGTGTGTAAGTAAGTCTGTGTGTGTGTAAACAGTTAATATGTATCTGATAC<br>AGCACAAAAATAGTCTAAAATTGCATAAAAGTTTATGAAACCAATCTCAATATACCAACCATAAGTCCTAGGCAAAATTT<br>GAGAGACATTTCAAATTTGGAACACAAGCAAAATTTGAGATATATAAATTTTTAATTAGGTCATATTGTCCCTATATTTGT<br>GGAACTTGAGAAAGATTTAAGAATTGCCTTTCTTCATATTATATATAATAACAACAATATTGTTTATAAGTATTATTAAGCC<br>ACGCCTAAGGTGTCACTATAAATGAATACAACTCTGATAGTTCCTCCCCCCCCTCGCCCCTCTTCATAGAAAGTCATGTCTT<br>TAAACAAGAAAAAAAACGTTTCGTTTTTCTTTACTACCACACCACACTGTTTTTATTCTGTGGTCTAAAAGAATTTGAAATT<br>TGGAACGCGCTGTAAAAATGTTTATTTTAGTAAAAATATATAGAAGCGCAATGTAGTTTAAGTAACTTGGATTCGTAGTCA<br>AACTACAGTGAGTACTAAATTATAAATAAAAAAAATTTCTTTTCCAGTTACAACCGATTTTGAAATTAGCATATATTAAAA<br>AACCAGTGGTTGTATTCAGTTAAAAAAATGAAAATGACAGTATGGCAATATTATTTTGAAACAAATTATATGAATAACAT<br>ATCCCAAGAAATGATGAGTTAAAAAACGTAATTTTTCATGTTCAAGTCCTGGACAAAAACAGCGCAATCTAGTCCCAAAA<br>CTAAGTATAACTTGTACTATGAGTACTAAACAATTGTTAAACATACTTAAATATATACTTACATACTCAATTTTCATCTAAA<br>TGAAAACAAATACGCCCTTATGTCAAATTGATTAAGAACAATTTTACTTTATCAAAATTAATCACTATATCTCTCTCGTTTG<br>CGCACACTAAATACGTATTTGTAGAAACGAGACGCCTTCTAAAGAGGAATGTAATGTAATTACTGCAGGTATTTTTTTTTT<br>AATATTTTCACCGATGTGATATTTATTTTAGGGTTGCGGTTGAATTATGGTTCACCGGAAATGGACTGATATAAGTGAGTTT<br>AGTGTTCTGCATAGTTTTGAATCGATATAGATTTTTTTTTTAGTGAAGACAGTAAAGAAAATTTTTACCATTAATTTGAGCA<br>GCCATTTTTATATAGCAACACTGTAAGTGACAGTTTTAAAATTGGAATATCACGCTACCGCCACATATTCATTTAAAAGTC

		TTTGTTTATTTTAAAAAGTCTTTAAAATTGCAATTAGTTACTCTGTGATATTATAAATTATCTACGAGATATTGACTAAAAG<br>ATTTATGAAATTCTTTGCATTTTATATACAATATTTTTTTTATCAGAATATTAAATTGGACTTAAATACCTAATTCAGATATT<br>ATTAGCAATCATTTTTTGTTTATTTTATCATACTGCCTTATCAAAATGACAATCTTGAGATAAATTTTGAATATCATTGAAA<br>AATACTATAAGACAACAAAAGTGTACTGAAATAGTAATACATTATACTTTAAAATTTATGTGGCAATGATTTTTTTATTCC<br>AAAATTATTGATTCAAAACTATGTCAACTAAAAAGCAAAGATATTAAAATTACTCTATTATTGTTGTTGATATAAAAAATA<br>TCATATTTTTTAGGAATGTTAGAATTATAATATTACAGTATGACTGTGCATGTGTATGTATGAGTGTTCATACAAAAAAAA<br>AATTATATCACTACTTATTAGGTTAATTGGTTGATATTACGACTAAATTATAGACAAGCCAAAATAATAAAACGACAATTT<br>AAATAATAGTTGTAAAAAAAAACCTTTATTTAATATGAAATAGTTGATATGAAAGCCCCATTACATACCTGTATAAATTTT<br>TTAATCTATAAAATGCTCATAGACAATTGCAAATGTATTGATGTATGTTGTATACACCTGTTTTTCTTTTTATGAAGTTTTG<br>AAGTCTTATAACCTCTCTGACTGCCAGTGTTATTTAAATATGTACTCCTTTTAACATTAATTGTGTTTGGCAACCAAAAAAT<br>TTATCTGTGTAGTTGTTTTGACCTGTGATTGTTGTTTTATCAGAATTTCTTTTTTACTTTAAATATTATATGTATTAACTTTCT<br>TCCTCGCCACAAATAATTATAATAAGTTCATTATTATTGTATACAGTTTATAATAAAATGCATTTGTATTAAATTATATATT<br>ACAATTATATATAAAAAAAAAAAATTAAAAACATACAGTTCGCGCCATTATACCAATATCCACAAGGTTTATATCACTATAC<br>TACAATATAGTTACGTATTTTCTATTACTTGTGTGGGGTGGCGCCCTCTGATAAAAGTTATTGGAATTTGACATGTTTATGT<br>ACACACCGAACATTAATATTATCCATGAATTATTTTTAAAAATGGAGAAGACAAAAATAGAATTGACTGGATTAACAATA<br>AAAAAAATTGTTACTTTATGCCTACTTATTTGTTGACATATGTTGTAATTTTTGTTTACTGAACTAAATTACTCACTTGCATT<br>TCATATACTTTGTTATTGTAAGTTTAATTCCAAAATCAATGTAAATGTGATTATAATTGTGTAGAGAAGATACATTTAATTT<br>TTACAAATAAAATAAAGTATTAAATCTT
MSTRG.8<br>274	Uncharacterized<br>protein<br>LOC106124473	GGTGGCGCTAGCCCCGAGCCCACCGGACCGGCGGTGTCAGTGGCCAACTCTTCGAGCCCAGCAGTGGACGGCGTGGGCGG<br>CGCGGGGGCGCTGGTGTGGGCCGTCACCGACATGTCGGCCGTGCCGCCCGGCGCGCTCGTCATCCACCCGCAGACGGGCC<br>GGCCGCTGACCAACCCGGACGGCAGCGTGTACCACTTCGATCCGGCGAATCCGCCGGTCCTCTACGACCCTAATATCTACG<br>TGCACAACGAGCAGAAGGT
MSTRG.8<br>278	Hypothetical<br>protein	TAAATGGACTATACATTGCATTTTTTTATTTACATTGATGATTAAGACATTTGTTTGAATATTTATTATTTATTTCTTTGACA<br>TTACATTGATATATATTTGAATAATTAATGTTAAAAATATCTTATTGATTTACATTACATTTTTTTTTAAATAGTGAAATGC<br>ATTTGATTTTTTGCAACACTGCGTTTCCATCAAAAGTTATACATGTATTATATTTAAATTTTAGTATTTTTGGCAAGCCTATA<br>TTTTTGTATATATAAGGTAATATCTATATTTTGAAATCGACTTCGGAGTTATGTTATTTATTTTAAACATATGATTCTACTTT<br>TATATTGAAATATTTTTTAAAGTCAATTTGTCTATGATACATCTTTTTATCTGTCGAAGTTTTTTAAATGTGTTACATTTTAA<br>AATAAGAATAGTGATTTTCTAGTCAATTTCGTCGTTTTGTCACATTTCTTTTTGCTGAGCTTGTATTTTGGTGGAAACGGAG<br>GGTAACTTTTAGACGGTTGTACTGTTTGAGGGAGAACTCGAGAGTTTGCGGAATTGTTTAGAGCTGAGGGCATTGATACTA<br>CTTTCTCTTTGGCTGAAACAATCAGTCGTGTAAATACATAACTTACATTAATCATACTTTTCGTTTTCCTTCATTATTAAACA<br>AATGTCTTAATATAGCTACAATATTAAGCCACTTATCAGATTATTGCACTCTTTTTAAAGTGCATATACTTGGGGTATTTAC<br>TACTATTTTTCAGTAATAGTTTTAAGATATTAATCATCAAAGAGAAATTATTACTTATAATTATATTGAAAGTATTGCCAGT<br>ATAAAAAAAAAAATAGTAGTAAACTATCTCAACATTATGGAAAATGTGCTTTGTGCCTGCCTGTTTGTGTTATTGAAGCCA<br>AAATGTCCATAGTTGATCCCGTAATCTAAGGTATACATAATTTGTAATTAAGTTGTAAAATCAAGTTTCAGGCTTAGTTCTC<br>GTAGTCCATGCAATGTTGTCTATGGTTATTATTATGGTTAATTCACAAACTGAAATTATATAGTAGCAATAATGTTTAATTT<br>TAATTTATAGTTACTAGTACGTTACTAATTTGTAGTTAGTACGTAGAACTGTTCAAAATCACCAATCAAATTGTATATATTG<br>GAATTAGTTTTATATAGGTTATGAATATTTTAATTTAATACTTTAAATAATTTGAGCGTGTGAATAATAGATAAAAAGCGT<br>ATATTATGGTGCATGTATGATATTATATAGGGTTTTTGTAACTTATTTGGTAAACAGTCTCGGTATATCTCTATCGTAATTA<br>GTAATTGAAGCTAGTTATGCAGGAAACAAACACATTACCCTGGATGACCTTATCACATGGTTGTTTCTTGCATTAACTGGT

GCACATTAAAAAAAAAAAATATATATATAAAAAGAGAATGCACAATAATAAAGGAATCATTTGACCCCAAAACATTTTTA
TCCTAGCAATTTGTTCATTTATGGACTGTTATTTAAGTAAATAACGACCAAGATTGTTTACTATATAAAAAGGATCAAGAC
TTTGTCAATGAGCAATCAGACTAATTAGGTATGTATTGTGTTTCCACTATGGAGCACAAGGATTTTATAACGAATGAAACG
ACAGTATACGCTTTAGGGAAGCATAGTGTGGACTTGTATATAGTGATAAAGTATAAACTAATAGTTTTTAAGTATATTTCC
ATGTGTATGGAAAAGCTATATAAATGTAAGAGATACAATTAACTAGTTTGGTTCGTGGTGAGCGTCCGTGAGTTGCGCTCT
TAAATGGAGCGTCGTTTGTACGGTCTATGATCTCTTCGTACTCGGTTTGTGTGATAGTGTATTTAATTATCAATAATATAGA
CGTTATTCACTTGCCTTAAGTAACAATTTTACTTGTCGTATACTCGCAAAACTATGACTAATTATATACGGAGATACAACGC
GCAAACGCAGCGTTCCGATTTGTATGATAGGTAATAAATATTAGTTATGCAGTTGTATAATTAATTTTATCATTAAGTATAT
ATACTATCGATTCTTTGTCCATTTCGCTTGTAGTTTTATAGACACACTAACAATGAATTAGATATTTAGGTGTATTTTTACTA
GATATTCGAACATGTTGGTCGCTTTATGTTAGCCTCAAATTTTATCAAAATTCCATTTATTTGTTACTCAGATAGAAATGGC
AGTATCCATTTTTATGTATCCGACATGAACTGTGCTTCACATTGTGTTATGTGAGCATAATTTTTTTCTAATTTAATAGACG
GACAAAATATTATAAATATATATATATATATAATTATTATTGATATTGACGATATTTTTATACATATAAAAAGACATCTCTT
TTCAATAGATTAGTGATAACACGATATACTTTTATTTCTTAGTGTCTACCATTATATTTTATTGATTACATATAGCGATAAG
CTTTTGTTTCGTTTTAGTGTAATTTAATAGTTTTAACGTCCATACATCACAGATATGAGCGAGCGCTGTACTGAAGTGGTTA
CCGACAGTTTTATTTCTCGTTCAGTACTTGGTGTGCACGTATTATACTCGCATAGTTTGTTAGATACTTACTAATAAGTTTG
CAATATATATATGCATTTCTTTGTATGCACTATAACACTTCTGAACTTCCGCCTAAGACTGACATAGGCAATTTTATTTGTT
ATTGTATATTAGGGTTTTATTTTTTATTTAACTCGCCTTATTGGATTATCAGCGGTGTTTTACAGTCAACTCTTGACATTATA
CAGCTGCTTATGATCATTGGTGGGGAGATGACATTGACGAGAAAAATCCCTATAAAAATGTGTTTGTCGTGCAGATTTCTT
ATTCGAGATTTTAAATGGCAGTTTAATAAGTAATTGATGTTTTTCAATTTGAAATTCGCTGAATTACCTTATTCATAGAAAT
TGTGGTCAAGTGAAATATTTCTTGGTATAACATCATCAGCATAATTTTTGGTGAAAATGGATCTTCTGCAAGTCTTAAACC
GTAACTCTGACACGGATTGTACGTAGAGATGTTTTGCTGATGATAGGGTTTATCCCATTGTCTTGTTAATGTACATAAATTA
TTCATTTATAAATGTAAACATTTATGCTTACAATACGTACAAACCTTTCTCGTTACATTAAAACCAGTCTCATAATTCGACG
TGGGTAATATCGATACCGCTTCGATATCTATATATTGGGTTTCTTTGAACACTGATGGCGAAAAAGGAGATCACCCATTTC
GATATTGCCTATATCGGTCAGATATCTATATTAATTTCGATAGGACTCTTTATTTTAATATTATATTTTGAACAACCGGTAT
ATGTTCATCATCGAGTTACAATATATAGATATCGCTATATTACCCATCACTACTAATAAGAGTTATTGAAGATTTCATTTAC
AAAGTTAATAAGTTTGCTGACTGTACAGATATATTTTGCTGACTCTAGTTCAATATGTATCCAATACGCTATGGAAGCGGC
ATCGTGATGCCAGACGATATTAGTAGTAGCTTGGGTTATGGGGTTTACTATTGTTTATTATTTATTAATTTTCAGTGCCCAT
TTCGAATAACTTTTGTAACTTATAAGCAATATCTGCTTTAGTTGTATAGTATGGTGTAAAATGAAGTTCTGTACCCACTATT
CCATATGGTAAGATGACGCTGTCACGCTTGAATAGAAGCGTTCCGAACACTATATATATATATATATAACATACATTATTT
ATTTCATGGTAAAATAAAATATTTTGTATATTTTTGCAAACGTGTTTATTGTTCATGCTGCATGTCATTTGTTAGTTACCTAC
AAAATATAAAATATGTATGCTCACGACTGTTAGTATTTACCAGCGATGGAACTGCGTTTGTTTCGTAGTAATATAGACAAA
TCGTATTTTTTCTATAGGTATGATCATGGTACGATATGATGTAGTGGGCACAGACTCTATATTTTTCTAATATAGTTTGCAC
GAATTATCTGCATCTCGCAAATGCCACTAGATATTTGAATTGTATATAAAACAAAGCGTTATAAAAATGGTATGTGTATGT
TTTTTTATAGTCATGTAATTTGGACTAGACACCAATGTATTGTTGATTATATACCAAAAACGTCAAAAAAGTGCATACTGT
AATTCGGGTAATATTATATTATATGATTTTTTCTTATTAATTATTTACACATTTTAAGTGATATTTTATCGTTATTATTTCTG
AGCTCTCGATATTTCGTTGTTACAAGCGTCCTGGTAAAAAAGTGTAAAGTTGTAAACAGCCGCGATCCAAGTGTCTTCTTT
TAATTTGTTTCCTTTTTTCGGTGCAACTTATTCATGCATTTTCTTCCAGCCAGCTCTATCCCAGGCCCATATCCTTGACCTCC
CTATACGATATGACACCTAGTGTTTCTTTTATCTGTCCCATGTAGTTATTCCTCTGTCTTCCTCTTCCTCTTTTGCCTTCTATT
TTTCCTTCTACTATGCCCTTAAAAAAATCGTCATGTCATATAAGATGGCCCAGCATTTTGCCTCTTCTATTCTCTATTCTAAC

		CAAGTGTAAAGCAATTAAGATTTTATGTTTAATATATATTTTAATGCATTCTTACGTGAAGCACTTTGTGTTTTTGTACAAA<br>AATATTAGTGGAAACGGCTACTGGAACACCTGTAAATGCTCCGTATTACTTCAACTATAAATAAATGTGCATATCCGTTTT<br>ATAAAGCTAGCTTCTTACATTGATCTCTTGTAATTTACTACTGCACGGTACCATATTGTATGGATTGTATCGTCACAATATA<br>ATAGGGAGCAAAATATATAGCAATGCATAGTGACTGTTAATTTTCCGCGGCGATATTTTGAACGTGCATTAATAATAATAA<br>TAATAAAAAAAAGTGGTAACATTTTTTATAAACAAAACAATAGTATATATTTTTCTCTCCTATAGTATCGTGATGATAAAA<br>TCGTACGAAATGATACCGTGATACGGTAACCTATAAAAACAAAAGCATCCGTTAGATTAGTTCAAATTGTCTAGTGCGAC<br>AAGTGGCATAACTATTCGCTCCCAAAGAACATACACACACGAAGTCATGTTATTTATTGACGCTAATTAGAACTAGTAGGT<br>ATATTGTTACACAATTAAATTATTTTTATTAGACTTTTATTGTGTTCTGTATATTAAGAATAATAATCAGTTGATGAATTTTT<br>TTTTGTAATTAAGTGAAAGCTCAAGTGGTACCTATTATTTATCCCAACACGAAAATTATGCGAAATATAGTCCTAAATTCA<br>GGGAGTTTTTATTTTAAGGATAATAAATAAATTAAGTCTGC
MSTRG.8<br>284	DDB1- and<br>CUL4-associated<br>factor 8-like	TCAAGGGCGTCGCGTTCTTCGGCCCCAACAGCGAGTACATCGTCTCCGGCTCGGACTGCTCCTACATTTACATATGGGAGA<br>AGAACTCCGAGTCCATAGTGCAGTGGATGCAGGGAGACGTTAACGGAGTTGTAAATTGTATAGAAGCGCATCCTCGTTGT<br>CCCGTCCTGGCGACAAGTGGGCTTGACAAGGACGTAAAGATCTGGATACCGAAACGAGAGGAGGACCCCGACTTTAAAG<br>GATTAGAACGGGTATGTATGGAGTTGGAATCTTATATATGAGGTGTCTGAACATTGATAGTTGTAAACATATATTATGGAA<br>TTATTTTTTACTGC
MSTRG.8<br>288	Tetratricopeptide<br>repeat protein 17	ACATGTTAGGTTTGCTAGTATAAGTTTGGTAGGATATTATAACAGACCGACAGTTGATTCGTAATTACAGATATTCGCACT<br>AATATTATAAACTAGTTCTCTCTTTTGCCATCCTGTTACATGACATCGTTTGTTTGACAATTAATAATTGTTCACAAAGGGT<br>TTGGGCCGTCTTTGCTGTCATAGATAATTTAACCCCCGGGACTGCTACTCGCTTTTATAATGTTGTGTCCATATGGAATTTT<br>AACACACCTAGGCAGTGTAAAATGAGGCCTTAACACCATATGGCTATCTATTATATTGTATGGATTATTATTGTACTACGA<br>CGGCGTTAAATGACTGTCGACAGAGGGCAGCACACTAGTTCGAATGTTAGAACTAAAGGATGACAACGTACAGCGTTCGT<br>CCGATCAAATGGACGATAGCATATTCGATGACTTCATAGACAATATGCCAGCTCAACGAACGATGAAACAGGCTCACGAA<br>CAGAATTATAAGACGATGTTGAAGGTTATGGACAGTGCTTTACAAGGATGCGGTCCGAGAGGGTGTAATGATATACAACC<br>GGAAGATTTAGCAATGAAAGAAGAGGACTGCACGTATCAACATTTACAGCTCGGATATTGGTTGCACATTTTATGTTTTAA<br>GCAACTACTCACTGACGCAAATTTGAAATTTCCATCGGAGATAGCATCGATGAACCCGTCGAATAAAAAGATACCGGAGT<br>GTCGTCTTTTCGAACCCTCTGAGGACTTCTACTTGGAGCGCGTGAGCCGCGTCGACACGGACGGCTGGGAGCCGGTGCTCA<br>GCCTCATGCACCAGTTCGCCGAGGTGTTCGACTTCTACGACTACG
MSTRG.8<br>290	Zinc finger<br>protein 37-like	GTGGAATGTTCCTTTTGAATATAGTTACACTGGCGACTTATGTCAATCAATGTTAGAAATTTTTTATTATTTAATTAATGTT<br>ATTCATACAGTAACAGTTTATAATTCAAGGATGTTTACTTTATTGAAATTGAAATAAAAATATAATTTAATTGAAAAGTGG<br>ATTAATGTCTTTAAATTATTATTCTTAGATTAAATTGGAAAAAAATTATTTATCCTAATCACAAATTCACAATATACCGGCA<br>TCAAATTTCTTATATGAATTGACAATGGTCAATTCTGAAAAAGTTAAAGCATGTAGACTGTGTCTTTTGATGGATGTCAGG<br>ATGATTGAAATCGATGCAAATCTGGAAAATAGTATGAAATCCATGTTAGGAAAAGACATGATCAACACAGATAGCTTTCC<br>ACCGTACATATGTTTTGAATGTGCTGCATATGTAAGAAAATTTAAAAAGTTTCGAGATAAATGTCTGATAGCTCAGTCAAT<br>TCTCTACACATTGTTGAAAAGCAAGAATAAA
MSTRG.8<br>319	Pre-mRNA-<br>splicing factor<br>ATP-dependent<br>RNA helicase<br>PRP16 like<br>protein	AGGAGAGTGTAGACCGCGTTCATCTACTCGTCCATAATATTGTGCCGCCGTTTCTTGATGGACGAATTGTGTTCACCAAGC<br>AGCCGGAGCCTGTGATACCGGTGAAAGATCCCACGTCCGACATGGCGATAAACGCTCGTAAAGGTTCGGCTCTAGTGAAA<br>GCATTTAGAGAGCAGAAGGAGAGACGGAAAGCACAGAAGAAACACTGGAAACTGGAAGGAACTAAAATTGGTAACATTA<br>TGGGTATTCAGAAGGAAAAAGAAGAATTGGAAGACGGCCCCACTAAAGAAGCCTACAAGTATGCCGAACATTTGGACA

MSTRG.8321	Uncharacterized protein LOC105286551	AAGTACGAGGTTTGTCCGGAAAGTACGTATAAAAGTTTTTAAAAATTTATTTTACAATTATTCAGGTAAATCAATTTTAT<br>CCCCTTCAAAGTACTCCCCCTGTGACATAATGCACTTGTGCCAACGCCGTTTCCACTGTGAGAAGGCTCCTTGAAAGTCCT<br>CTGGTTGTAGGTTTCTCAGCTGCCCCGTCGTAGCTTTTTTATCTCATTTATGTCCCCCAAATGCCGCCCCCGAAGTACCAA<br>TTTGGTTTTTGGGAACAAGAAGAAGTCACACGGGGCCAAATCCGGCGAATAGGGGGGGTGTTCGGTCACCGTAATGGAAT<br>TTTTACTCAAAAACTCATGGACAACAAGAGAGGTGTGGGCAGGGACATTGTCGTGGTGAAGAATCCAACGCCCCTCTCGC<br>GCCAATTCTGGACGAACTCGTGCCACACGAGCTTTGAGGCGTCTGAGCACATCGACGTAGAATTTTCCATCAACTGTCTGG<br>CCTGGAGGGACGAATTCTTGATGCTTTCTCGGCCCTCTCTGAATCTTTTTAGCCACTTGTGGACGGTTGGTTCCTTCACAGC<br>ATCATCCCCATAAACTGTTCTGAGATCGGCAAAAATTTCACGGCCATTCTTGCCGAGTTTCGAGAGAAACTTGATTACGAC<br>GCGCTGCTCTTCAACGGAAGCGGGCTCCATGCCGACGGGGTTTCGTTAAGAGGTAACTTCACAGATGGCGTGACAAGCCA<br>GTTCGCACCGCACGGCGGTCAGACCAGAACTGAAGCATTGTTAGGGACATAAGGAAGGGGTCCTGCGCTTACCTGGCCTC<br>CCCACTCCTCTTTCTCGCATTCCAGAACACTTTTATACGAACTTTCCGGACAGACCTCGTATTT
MSTRG.8323	NF-kappa-B essential modulator isoform X2	CATATGCCATACATCTGTCAAGAACGAGACGAAATAATCTCTTTTTTTTCTTTTCTTCCTTATATTTTCTTCGCTTCGCTCGC<br>GCAATTTACGCATGTAGCTTCATATTTTAATGTTATTATTCCATGATCCAATTTTTGTGTCATAATAGTGTACACAAGTTTA<br>ATAAACAAATCCGGCGATAGTGTGGTTGTTTCAACGTTGGAATTGTTACTGGTATAAATATGGACTCAATGTTAGATAATG<br>GTGATGATGAGTCATTCATTGTACTGGGTACATCCCCTGGCTCCAGTTTGGACTTGAAGTGTGAAGGCATAGTAAATGGCC<br>ATGGCTCCATTGACAAGTTACAAATTGAGGATGCAATGAAAGATTTGCCTGCTGAAGCAATGTCATCATTAAAAGCACATT<br>TCAAGCTTGGAGATAGTGTGAGTATTAAGGCATGCAAAAATTTTTAAATTATTAATTAGTTTATAAAACTTAATGTAATAA<br>TTAAGTTATTTTTCGAATTTTCTTACTCTTATTTTGATAAAAACATGATTGTAAGTGAAACATTCTAATTATTATGTCGATAT<br>TTTATTACTGATTTTACATTACATTACAATTTAAGATTACGCTGATACTTGTTATCTGGATCTTACGGAAGACTAGTTTAAC<br>AAACATGAGCGTCTTTAAAATGTTTTTATCAGGTTTATTTCCCTTTCGTTTTTGCCGTTACACTCACATTCTTTCATTGCCTA<br>CCATGCGTGGCAACTACTGACAAAACCCCCCAATGGTAGACCC
MSTRG.834	TATA-binding protein-associated factor 172	ACTCACCACTGAGGTGGGCGGACTGGGTTTGAACTTAACGGGCGCGGACACGGTGATCTTCGTGGAGCACGACTGGAACC<br>CGATGAAGGATCTGCAGGCGATGGACCGAGCACACCGCATCGGCCAGAAGAAAGTCGTCAACGTGTACCGACTCATCACC<br>AGGGACACGCTCGAGGAGAAGATTATGGGGCTACAAAAGTTCAAGCTACTAACGGCCAACACGGTGATCAGCAGTGAGA<br>ATGCAGCCTTAGAGACGATGGGTACTGAACAGCTACTAGATCTATTCCAGCTATCCAACGGGCAAAACAACCAGCCTCAA<br>CCAGGCTCCTCCAGTGGGAAGTCCATCCTCGACACACTGCCGGACCTGTGGGATGATAAACAGTATGAAGAGGAGTATGA<br>CATGTCAAACTTCATTAAAGGACTGAACAAAATGGGTGCATAATGTAACAAGGATTTTAAAAACAAATGGAGATTCAAAA<br>CAACTCATTAATATCCCACCGCTGGGCATCGGCCTCTCCTCTCGTGAGGAGCGTTGGCTTTCCACTACTAGGCTGATCTGTA<br>GTCAAAGATTTACTAATATTTTTGAAGATAAAATATCTGGCTTGCATCACAATTAACGTCGTTTATAAATTAAACAATGAT<br>ACAAGGGTCCATTCATACAGATTGGTATGGAGAAAAGAATAGATTCTGATCGTATATAAAACTCAGTTTTGGGTCATTGGA<br>ATAAGGTTATTTTTACATTTCACTTCTTTTAAATTTGCTCTCGTACGTTCTCATAAATAACAACAGAAGATCCCTCGAAGCT<br>GGTCTATGTGAATGGACACGAACTGGCTTGCACGTGTTGGCTGCTTCTTTGAAAGCCAATTTTTTTAGAATCAGTATTCTGT<br>TCTCTGTATAATCTCTATTTGGTTTTATATTTCTTAAATTCACTTACGTAACATATTGTTTCATTCGGAAGCCTTTTCATATG<br>GGAAATTTTATATTTTCGCTACTCATTATTTACACGAAATCGAGCTTTTATGTGACAGTATCGATCCGTAATATAGCTTTAA<br>ACGTGGTATTGAAATACGATGGATGAAATGTAAGGTCGTGTTCGAAATAGCAACCAAATTTTATTAGAAATTGGCTTTAAC<br>GTGAGTTTCTAACAAAATGGGAGCTAATTCGATTCTCTATGATCGATTAAGTATGAAGTTTTGGTAATTTGTTTAAATTTTG<br>GAACAGTAAAGAAGCTATAACTAGGTCAAGAACGTGAGATAACCAACTTGGAATTTTCTTCTGTATATGTATTTCTACTTA<br>TAATGATTGTTCTTATTAATTTACTAATCATCTACTTCATATATAGATTGTATCTATATATAATAAAAGGTAATGCTCGTAC<br>AAATACGTACGTAAAATCCTATAACATTATGATGTTACAGTAACCTAAAAGGCTCCATACACGTTCCGATAGTTGTAACAA

		TTCGTCTTTTGCACTTAAAATTTAACTGTAATTTATAACATTAATTTGTTTGACGTCAGGCTTCTTCTACATGGCAATTACGC<br>GTTCTACTAAATTATAAATTGCTTGAATGTGATCGAGAGTGACCTCTGACTGTCCCTCTTCTAATGGTATCTGTTATTAGTT<br>TAAAGTATTAAATAACAGTGATTGTAATGGTAACACACTATTTCTATTTTTTGTTTTGCTAAGAAATTTCTCGGCTGGCTTT<br>TGACCACAACAGAATTGGTGTTTTACTTTTTTGAGGGTAGAGTAGTAAATTGAGTTGTACGCATGAAAAGAAAAATTATGT<br>TAATTCTTTTATGCATTTTCCTCTTTTTCACGTAATATACTTTCTTCGCAGTGTGACCCTTTAATGTGATCAACCAAAAAGAA<br>TCTATCTATCTATCCATTTAAGAGCTGCGCTCTTGTCGGTGGAGCTCTTGCCACTCACTACGATTTTCAGCGAGTCTCTTCA<br>CCTCTTGATACGACACGACACCAACTCCTTCCTTGATCTGCCTGATGTAACTATGTCTCGGTCTCCCTCTCCCCCTTCTTCCT<br>TCTATCTTACCTTCTATTATAATGTTCATGAGTTCGTCGTGTCGTATCAAGTGTCCTAACATTTTTCCCCTTCTGTCCTCAAT<br>AGTTCTAATAATTTGTCGTTTTTCCTTAACCCTAAGCAGGACCTCCTCATTGCTAATTCGATCCCTCCAGCTAATTTTCTCCA<br>TCCTTCTCCAGCACCACATTTCAAAGGCTCTAAGTTTATCCATTTCCTTCTGAGTTAAAGACAATTATAATTTATCAAAAAG<br>AATATTTTTGATAAATTCATAAGTGATTTTTCATGTCGCTATATTTGAAAAGTTGAAAGGCCATATCCATATGGCGTTATGA<br>TATTTTGTCCACTTGTACATATATTTATTATTAGTGATTAATTTAAATTTCTAACTTTATTATAAACAACTCGATTATTATTG<br>TAATTTTGAAATATTAAAAGAGGTTAGTAACCAAATGAAAATAAATGATAAATTATTTGTTATTATTCTAATGCTATTTTTA<br>AAAAATATCACCGAGTCCATGTTATTTCTCGTAAGTTTATTCGTATTCCTGCAGTCAATTAAAATTAATGCATTCTTATGTA<br>TGTGCAGTTGGCTTCATAAAAATATTTAGTGGTTGGGTGTCATCATTCAGTGAGAAAAGGTGTACATGGTAAATGTATATT<br>ATTCATAAAAACTCCAAACGTCACGTAAAAATATTTTAGCTTAAAATCTGATTACAATTGTAATGTTGTTGAAAATATGAA<br>ACTGAAAAGTATTAAGGTATAATGATTACTAAGTGTTTACAGCTATAAAGGGACAAAAGTATTTAGTGTCCCTAATGATAA<br>TTATGACCATATTATTGTTCTGATTTTGAAGTAGCTGGAATGTACCCAATTTTATAACTTTAACAATGAATGGTAGGTTCCT<br>TGAGTAAGAAATACCTCAGATTTTGTTTGTTATATTGCTATCGATCGTTTCAGTTAATTTATTGTATTATTTAAGAGAGAAA<br>GTCATTGTTTTAGTCATTTTTATAGCGTTAGTGTACGCCGATAGTGTTGTTTTTATGTTTAATTTTGCTATTTTGTACCTGCTC<br>AAGTAAATGAAAATAGCATAATCCATTTCTTTTATATTTATAAATACAGTAATAAGTCTTTAAGTATGTATTTAGTTATAAG<br>ATATAGTATACACTTTTATAATTAAGTTTCTATATATCTAGTTATATATAATAAAATTAAAGAAAAAATGTTTTTTTCATTG<br>AATGGAAGTCTAATATGAATATAATGACAATTATATGTAAAATCACTTGAGAGAATTGAAAACTATTCTAATTTAAAAATA<br>TATAAACTTGTCCAATTCTATCTGGTGTATAATATAGATTGTTGTTAATGATAGACTTAGATATTCCAATCGACATCACGCA<br>AGTTACGCTTTCTGTTATTTATTTCATTCTGTTATATTGTGAACTTTGATTGGTACCAAATAAAGCGTGTTTTTGTAA
MSTRG.8 346	Uncharacterized protein LOC106710997	TTTGTGGGCCCCGATGAAGAGGATATAGTGAAAGCGAACGCTACATTGTACTTGCCTGATGGACAAGCTATTATCCTTACT<br>GATGAGTTCGTGCTAGAAACATACACGGTGATGAGGAACACCCTGAACTTGGTAACGCCGTTCCAAGTGGTGAAGGAACT<br>AAAGTCGGTGTACACAGTGGACATAACTATAGGCGAGAAGATCAACGTCTCTTGTGTCGCGCTGTTATCCAACGGGACTT<br>ATTGGCACGAGG
MSTRG.8 389	Endonuclease and reverse transcriptase-like protein	TTTCATAATTCATTGCATTCCGTTCACTCATTCACTCTCTCACTCACTTACTCGGTCAGTTCAGTTCTTTGTCGTGGATTGCG<br>CACGCGTCGATCGCAAACAAAACACGGCTGTTGTTTATTTTATTTTTTGCAAAACCGAATAACAAAATGACCGGGTCCACT<br>ATACCATATGACAAACGCCTGCAACACTAGGTATGTCACGTGCACGTTGCTGACCCTGCAGAAACCTTAATAAACCCTTTT<br>TGAAAGAACCCCATACTGTAGTCTCTACGGAAAACCTTGGCAAGGAGCTCATTCTACAGCCTAAACGTTCACGGAAGGAA<br>ACTCCTTTGAAACTGCACTGTACGTGACCATTTCGGTTCTAAGGCGTGAGGATGAATTTTCTGTCGATGGCCATCGGTGCG<br>ATGATAAAATCTGGTAATGGCCTATATCGTTCTCCATCCTAATATCGCATGTATACCATTTTGACTATTGATGGTTGAAGCC<br>ATTGAGGCAGAGGTAGACTTTATATGAAAGTCGTTTGCTTGGGTATCTCTGTCCCATTCGTGTTTCAACCGTAAAGCAGTT<br>GTATTTGCATGGCTGTGTTTTGGCTTGAAGGGTGGGATATGGCGTGAATTTAC
MSTRG.8 392	Lysine-specific demethylase lid	CAGCGACAGCGGCACCAAAGACGACATTAAAACGCGGTTCCGCAACCACGACGAATTATCTAAACAGAAAACAGGGTGT<br>GTCGTCGGGCTCCGGCCTGATGATGCGGAAGCACTACATGGCGCGACAGGAGCGCCGCAAACGAGGACTCGCAGCCTCCA

		CCAGGGGTAAACACGCGCGACAGTCCAGGCTGGGTGGCGGCGCTGGTAATACTGGTCGGCGGCGAGCAGCCGCACTAGC<br>GCCGCCGCCGCGACCGGCCCGGTGCACTGCTGTACCCCCCGCGCATCGATATACGAACATCCCACATTCACTGTCCCCTAC<br>AAAATTTGCACTTAAAACTGTCACTAATGTAATGTAATGTAGGTATCGGTGCCATGAGTGGAGTCGAAAATTGTTTTTCAT<br>TCGTGATACAGTTTATGAATTATTGTAACTTGAAAGATATGGAACTTTGTCCTTCCGATGTTAGGGACGTTACGTTTTGCGT<br>TAAGACATATAGAGGGCATTTGTCAAACATCACTGATTGGTTAGAAGCGAGTGCACGATCTGCTTGTATGCGATCTATTTT<br>CAATGTCGCTTGTTAGTGGCTTAATATTTATCTTGAGTATTTTTAATATCGATGATCGCAGGACTAATTTGTACGTCGGAAT<br>TAGGCACATTATATGGTCTTACTTCTAGAATTGTTAAGATCGTTGTACACATACCCGCAATAGAGTAATATGGAAACGTCA<br>TACAGTTTTTAATGACAAATTACATGATACATTGTTATTCAGCCATCCAGTGTGTTGCCCTATATGAAAAATTACATATTAT<br>TACCAGTGACAATTAGAGATATATTTATATAATAAACTGTAAATGTATAACGTACTGATTAACTGAAGTTAAATAAATGAG<br>AAATAAAGGAAATTGACTTTTAAATAAAAATGGTTTTTATAAATACACTATAGCTGGTCGCGATATCTTGGTTCGTATGTT<br>TAAATATGATAAAATGGCCAAGTTGTTTACTGTCTGTGTATGAAGCGTCCCATTAGTAATTAATAAACGTGTAAACAGAAT<br>TGTTACACTCTTTCCATGGGTCAAGTACTGATTTGGGGAGTTGAAATCTACAAAAAGTCAATTTGACATTTTAAATTTGATT<br>ATAACCACTATCAAGGGATTATTATTATAAATAATGACAGATATTGTATCTACATTTTAATGAAAGAGAAAAAAAAAAAC<br>GAGTAAAAGTGCCAGTTGCAATGCAAATTCTGGAACATTCATTTGGATCATTGTGAAATACATTGAATATGGATTTTTTTG<br>GATTTCAATTTTATGGACCTTTGACATTGATGCTTTTATAACGTGTTCTATATATGTATATGTAAAAATCGATCCTGATTGA<br>CTGGCTAATTCATATATCAACGTACAGCTGAAACTGCTGGTTTTTAAATAAAATTGAAAATAAAGCAGATCGGTTCTTTAT<br>ATAGTTTAGGTGAACAATATGAATTTTGATAATTCAACCCCTAAGGGGTTAAAAATGAGGGTTGCAATTTTGTATAGAATT<br>TTGTCACTAGAAGTTAAAATAAACACTTTAAATTTTAGTATAAGGTTTTTTGTTCAAAACAAAAATTAATATGTTTATTGAC<br>TTTTAAAATATTCACCCCTAAAGAGGTAAAAAATTTGTATGGGAGTTATAATTGAGGGTAGAAGTACAATACTTTAGTTTT<br>GAATTTTTCATATTAAAAATAAAATATTTTTCAGCATTTTTGGAGATTTCACCCCTAAAGAGATGAAATGCGGGATGAAAA<br>TTTGTATAGAAATTCTGTTATTTTTGTACTTAAAAGTATGAAAATTTGTTTTTTGACTTTTAATTAAAAACAAAACTTTTGTA<br>ACAGCTTTTAGAAATTCCATACCTTGGCGGAGTGAAATGGGGGCGATTTGGTTTAGGTGTAAATTTGTTAGTTGCAATATT<br>TACAATTGACTTTTGAGTCTTCCCTCTGAGGGAGTCAAGTAGAGAATGTAACTTTGCATGGAAGTCTGGTATTA
MSTRG.8448	Uncharacterized protein LOC110382642	TGAAAATATTCGCGGGTGTCAGCAAAAAGTTTAGCGGGTTGTTTTATTTCAATGTTTACAAAAGTTATCAGATTTCTTCTCA<br>TTCAGAATAAAAAAATCGAATTCTAAGAAAAATGTGGAGTTGTAATAAAATATGATACTATATATAAAACAAAACAATGAA<br>TCCCAATAAGAAAGATAATAGTGGTTGGATTTTATGTCCATCGAAAAGTTTTCCAGGCAAATTCTATTATTTTAATGTTACA<br>AATGGTGAGACCGCGTGGAGTTTGAACGATACTGATAAAAAAAAATGTAAGAAAGGACCATAATATAGAAAATATATCAG<br>ATAAATCCCATAATTATCCAGAACCAAAAAGTCCTCCAGCAGATTATCAAATACCTACTAGTTCACATGTGAATTATATGA<br>ACTATCAATATCCCCCACAAAAGCCTGTAAACCTAACGCAACCAACCCCAGTATTTGGCCAAGTTATCTTCCCCAAATATA<br>TTACAGAATCAGGTCCATATATGCAAAATGTTATATGGACTCCTATTCAACTTTCAACTCCAGTACTTGTAACCAGTACAC<br>CAATAAAACAAACAGTAAATCAAAAAACACAAACATTTGCAGAAGAAAACCATGCACATGTTCCACAGACCTGTGGTATT<br>CCTTTGTTTAAAAGGTTTAAGAACTTTGAAACACCCCTTCTAAGTTTTAATGATGAAAATAAAATAAAGGAGACAAATAAC<br>TTATTAAGCAACTTGACGAATGTATCAAATACTAAAATTGACAACCAAATCACAATTACTCAAGTAGTTGAACCAACAAC<br>CCCAG
MSTRG.8449	Uncharacterized protein LOC106140458	GACAAATTATTAATAATGTTGCACAGTAGTTCTGAAAACGGTAAACTGGACTCAAATATGCAGGCGAATGACTTTATGCA<br>AATTACAGGATGTTCTGTAATATTGTTAGAGAGCTTACAGGAGGGGGGGGAGGTAATTGTTAGTAATGCGGACTACCAGC<br>CCTATTGCTATCAATATACCCTGTCACCTTGACCTCCAAGTGGCCCCCCCTGGAAACCATCGTGTTTAATTCCAGGCGGTTT<br>ATCCAGATTGTGAAGCACTTTCGGAAGCGGAACAATTACTTATAAATTTGATACACAACATCCAAAAGCCTCCGTCACTCG<br>ACCAAAAAGATTTGGTACCACATTCAGTCCCCAGCCATAATTTAGAAGAAGAGACTGTAACACCAAGAAAATGTTTTACA

		AAAGAACCATCGAAGGTTCTGCAGTACCTGCAGAAACATTTCCCGCAGTGGAAGACAAATGTCGAGGTAAAAGAAACAC<br>CGAAAGAAGAAAGCTACACGATTAACAACGATACAAAAATAGTGCGTACATTCGGGAGAAATTTAAAAAATTTAACTATT<br>AATAACACGAAATCTCTGTCACTTGCCCAACCGGGCATAGACGATGCATATACAAGTACTAGGACAAACGAAACAAAAGT<br>AAAATGCCAAATAAATGACGTCATAATAATCAGTAATGAAACTAAACTTCCACAACAAGTAGACAGTACAGACAATAACA<br>ATTTTACAAAACTTGACGAGTCAAATTATACAAGACAATTAGAATGTGCAAATGTAGATAAAGAACATGATGTTAGTGAA<br>AGAGATGATGTTTATTTTAATACAGCCGTGCACGGTGACACTTCAAATGACGTTCTAAACGATGGACCTAAAGTAATAAA<br>AAATATAAGACTAATTGATGCATTCGAGGAGAAGTCAAAAATTCTGTGTAACCTAGAAGCTTTGGATTATAACACATTACC<br>ATTATCCAGTATAAGAAATGAAAGTTTCAGCATTGCAAATAATGTAAACGTAATTGACGATGATGACATAAATAAGGTTA<br>CAATAAATAATACTGATTCTGTTGTATTGAATAATGACAATGATTATACTACGAATAATAAATCCGCAATTGGAGACTCGG<br>ATACGAACAGAGACGATTGCACAAATGATAGTGGTTTCGAGAGTGAGAGTATACAGGCTTATTCATTGTTGAAAATATTTC<br>TAATCGAATTAAGCGGTTCTTTGAAATCAATTTATAAATTTATAGATGACAATATAGCAGAATTTAAATATGATGAAAACA<br>TTCAAGACGAGAAAAAACGGCATCTCCACAACAAAGCGTCACTCACGCACTGCCACCTTGCGAATATCATCGATAAGTTG<br>AAAGGTATAATTCAAAGAGAATCCAAGGAAAGCAGTGAACTAAAAGAACTGCTTATCAAGGCTGGAATACAAGCATCTA<br>CTGACAAACGAATGACGAGATACAGGTAACAAAATTTTTTTAACTCAAGTACATCAAGTACTTGAAAAACCTCAAAAAAT<br>TTGGCATTCTTTTTTATACCTTATTTTTTTCCTCTAGTGTATATATTTTGAATACGCTATTAGGGCTTAACCGGATGAGATTT<br>TTTTGCAGGCAAATAACAATAGTAATAATAATAAGTTCATTTTGCAAGAGCAATTCACCGATTAATC
MSTRG.8 513	Gastrula zinc finger protein XlCGF57.1-like	TGTCAATGTCATTCATTTTTCCATTAGAAATCTATACCTATTTTTTTATACCTACCTAATAAGTAAACTAAAACTTTTTTCTT<br>GTTTTGTTTAATAAATTGTGGATTTATAAAGGAATGAGACTTATTTAGTATGTCAGACACGTGGAATATGATGAGTTTGTG<br>CCGATGCTGCCACGCTGAAGGAAATTTCAAGAGTCTTGCTGTATCATACACATGCAGTGGAAGTGATGAAATATACTCTGT<br>TATGCTTAGGGACACTTTCAATTTAATTATAGCTCCAATACCGGGAGAAAGAGGGGCTTTCTCATACAGTATCTGTGATAC<br>ATGTGAGCCTCGGCTGCGAGATGCTGCATTGTTTAAACAACAAGTGGTTACCTGCGAACAGAAGTTCTCACAGTATTACAA<br>TAACCATGATTTCAAAG
MSTRG.8 544	Aminopeptidase N-like	CGGTGGTAGCGGAGCAAATGAACGGTTATGTGAAAGATTGTAAGCAATCATCTGTGAAAAAAATCGTATCGTCACTATTG<br>TGGTGTAAACGGGGCTTCTTCGATTTCAAATCGTTACATAAACTGCACTAAGTTAACTAAGTGACTGGCAGACGGCGCTTG<br>ACCGCCGGCTGAGTTTGTTGTGTTTGTGTAAAAAGTATTATTATTTATTTATCTAATTTTACGAACAAAAATAGTGTAACAT<br>AAAGTTAATAAGTCTATAAAGTTTAACCTGTAACAGCGATGGGCTGGAGTGTGCTACTATCGGTGTTGTACGCGGTCCAGT<br>TAGTCACCAGCCAGGAGTACTTGCTCCCTGGTGACATTCGTCCTACTCATTATGACGTTAAAGTGGTGTATGATATAGATC<br>CGACGACTAATTTTAGTTTCTATGGACTTGTTGATATAGCG
MSTRG.8 560	reverse transcriptase	AAAGAGTATATAACCGAAGTGCATTCTCTTATATGCACTTCATAAAAGCTACGTAAACCGACTGGGTCAATAATATATCAC<br>CAATCTAGACAAAAATCCTACTTTTCTCATTATATTAGCTAGCCTGTTGTTATGGAAACGAACAGGTTATAATTATACTATA<br>GTGTGTGATGCCGTTGACGACAAGGCTCGAAGGGCGCTTCGGCTAGCACCGGGGCGCTGGTACGCCCGGACGTTGACCTA<br>TCCCACATAGAAAGCACCCCCAATAAAAAGTCGAGCAGCACACAGTGGGTGTACAAGGCATAGCCCCCAAATGGGGCTCC<br>GTCGGCAACGCAAACGTTGAATATTTCGAAGGACCAGGTGCGTGAACACTACTATTGACGAGCCCGATGCCTCGACGGCA<br>CAATGATTCGACGTAGACGACACAACAATACACTGACGGCACGGTGACGAAACGGAGAAACACGATGATACGATGACTG<br>AGTGGTGAAGACCGATAAGGGGTGTGAAGAAGAATGACGATGAATGAAACGACAAATATCTTGAAGGATTCTGTGATAG<br>TGAAGCAAGGTACTAGCTTGCCGTTTCTTCTCTATTTCGCTTGGTCGGTCTTTTAGCTAATGAAGCTGAGAAACCTCGAAGC<br>CAATAAGCCAATGAACAGCGATCGAATGAGCGCTGTTTATATATGTACGGTCTGTGACTGGCCAGCAGACCAGTGGCAAA<br>CTATGTGCTTCGCCTTCTCTATCAATTTCTTTATTATTTTACAATCTGTTCATTTAGCAACATAGGATTTATGGCTAATGGTA<br>GGGCTACTTACTATTAACTATTCGCATACATGCTGCGCGCTACGCGGCGCAAGATAAATGAATAACCGGCATAAAACTCG

		ATTCGCAGTTTTATATGGCCCCTCTCTTCTTCCCCCTAAATATAGCTCGGATTAGCATCGCAGCCGATGAGAGCAGACGCA<br>CATTGCAGCGCTCCCGATACCAGCCGTCCGTACTACGAAAATCGTCGCTTCCATTTTGGTCTGCCCCTTTGCTGAGCAAGC<br>GAGACAAACAACGACCAGCTCTTCATCCCAGTAACACGCCTGTACAACTGTGTTATGCTCACAGGGCATTTCCCAGATCTA<br>TAGAAGATACGCAAAGTTATCACGCTGCCTAAGTCCGGCAAAAACGTATTGCAACTGGAATCTTACCGGCCAATAACATT<br>ATTGCAGCATATCTCGAAGGTGTTTGAGAGGCTGTTACTCAAACACATAACCGCCCACATCAAACCAAGAAGTGAACATT<br>TTCAACAAGTGAACAATTCGGGTTCAGAGCCGAACACTCAACCACACTGCAACTGACTAGGATACTGTACTGTAACAACA<br>GCTCTCAACAAAAGAAAATGCGCCGTTGCACTATTCATAGACATGGAAAAGACGTTCGACAGCGTCTGATACGGCGGTCT<br>GCTCTACAAGTTATCCATCTAGACGAACATGCCAAGAAGGCTCATCCTGGTAGTTATACGGCCGTATACATTTCTATACAC<br>GAGCCTGCTATGAATTTACAAAAATAGCAAGTGATGATGTATCTTTTATCATCTTTTTTGTTAAGGTGCAAACTAGGTATTA<br>ATAAACTCTAGTGAAATTTGAAAATAGAAAAAGAAAAATATTATTACTTTTATAAATTAAAATCAATAGACATCAGATAG<br>GTAATTCTTTAATAATACAATGAGAATGCACGTCATAAAACATACATTTAGAAAAGAGTAGCAG
MSTRG.8598	Huntingtin-like protein	GGGATTTAGACTGACAGATTGACTGAATTTTTAATTATAAATAAATAGACATTTTCGTTTACCTTGTATTTTCCTATTTTTCC<br>GATGATTTTTCTAATAAAATCGTGGCGATAATGCTAAAATTATTCAAATTAATCGACTGATCAAGAAAAAGCCATGAATTT<br>GTTAGAGAAGGCAGAAAAAGCATTGGAATATTTGAAAGCGAACGATGGATCTACGAAAAGTCATGATTTGCAATCGGCG<br>GCTGGTACATTAGGGCGATGTCTGGGAGCTTTAGGAAGCCGCCCGAATTTTGCAAAGCACTATGCAAATTTACTGCATACA<br>GCCATCCCTACTTTGCTCTCTCTGGCCAGTAACAATAGTG
MSTRG.8600	Uncharacterized protein LOC110374302	ATTAAATGTCACTGTTTCGTTTTGTACTTTTCGTTTCGCTAACTCTCGTTCTTGACGTTTGGTTTGTTTCGTCGTCGCATCAA<br>CACGTTTGACGACAAAATATTTTTAAGATGAGTATTTGAAACTAGTTTAAAATGAATTAATATTAGATAAAGAACAAGAA<br>CTTAACGCCCTTTAAAAAGCATTCTTTGTTACCTGTGAATGTTTACATATTACAACAAGTTTACGTGTCACGGACGTGTTCT<br>ATTTTTTTTTGTGTCGACGAGTGAACAAGTTTTCAAAATTGTGTGTTTCCTGACATCACAACACTAGTATTAAGTTTGCAAG<br>ACCTTGATTATTCAGATTTTCTTTTGAAAACGTCGTGTGAAGAAATAGGACAAAAGAAGACGCGGTTATGATCGACTCGTC<br>GAGAAATAATACAATGAATCTATCTCACGCGGGCTCTCACAAGCTGAATAGTTAGAAATTATCGTAAAAAGAGCAATCGA<br>AGCGAAAAACGGCGGTAAACAAACGATGTGACGTAACGCCATCTAGGGGGCGACGGTCGAACTTGTTCAAGTGAAAGTG<br>GTGCGGACCAAGATGAAGCTCAATCTATTTAAACGATCGATGATTTTCGCGACCTTCTTCGGGTCGTGCCTATGTATAGCA<br>TTGATTGTAGCGTCGCTGGGAACTACCCACTGGATAGACGCGAGGGCTCGAAGGACCTCGAACCCTCTCGAGTCAGAGGG<br>CAGAATCAGTTTTGGATTGTTCGAAGGTCACAAGGAGCTCAACTATGGGTACGGATGGAGAAACCATGACTTTAGTG
MSTRG.8602	Seminal fluid protein HACP040	CTGCGGCTTACCATGTGCACGCGCTCACGCGCGCACGAGTTCCCCGAGTGTTTCCGGCGACGAGCAGCGACAGCGCCGCC<br>GAGCGATACTCCCTCTTCCCACACCCGAGCACCACGTTTGAGATAATGACATTCGACCAAGTTCTACGTGGCAGCTACAAC<br>ACAACGGCCTGCGTTAAAGAGTATATGAATTGTATGGAAGTTATAAAAGCGGACTTAATATGTTTAGATGGCAGACTACA<br>GAACTCTGTTTGCGCCCATATACGAGATACGTGCACACAATATGCGGGTCAAAATTTTGATCGCGAATTACATATTTTCGA<br>ATCGGTGTGCGAAACTCCTGATGCCACCTAATTCAGTGTTGCTGTCCAAGTCCAAGCAACGTACTCTAACAATTAGAGTAT<br>TCATCGTCGAACAACAGCCGGCTCGGGCTGACGGATCATGTATTTATATTACGTGTAACGTAGCCATATAAATATTATTTT<br>AATCGTAATTTACCTTTATTTTTGTTTGTAATGTATGTAATTATGTACACAGTACGCACTGGGTGATCACGTTTCTATTCGCC<br>ATACAGTTGCGCTTGCACGACTGTTTCAGTTTGAAGGATACAGTAACAACGGGAGTTACTACTTTTAATTAGATAGGTACT<br>TATGGTTTTTAAAGCTTACGTCCATATTTATATTATTACATGTATCACATCTCCATATTTAGAACTAACAGAATTTTTCATCG<br>TGCCTCCCAACAAATTGTTTATAATTACTTCTCTCCATCCAAAGGTTTGGAAGAGATCGCTTTTAGCGATAACGTCGCCCGA<br>TGTTTTCTCATTGTGATTATTTCTTGTAATTGTTTATATCTAGAATAAGCTTACTATTATAATCTTTTGGAGGAAACAACGA<br>GTATCTATCTATCCATGTCAATTTTTATTCGATGTCTGTGTAAATAAGGGAAATTAATGAACAAGGTGAGACGCTGTGACG<br>TAAAGCGCTAAACGCCCCCTGAAGAAGGCTTCCGGATGGAGTCAAAACAGTTGTCCGAAACGACGAAAACACAATGAGTT

		CATTAGTCTCTCGTTATTACATAGACATCAGGTGACTATTGTATTTCACTTGTATTTTCTAATTTGTTATTATATATGTTTAT<br>GTTTTTGGTGCAATAAGGTGTTATATACTGCGCACATGGATTGTACTGTAAACAAGGGTTGTAACGTAAAAATCCTGATCA<br>TTAACTGACTTCAAAAAAGTAGGAGATTAAATATTGTTTATAAATATTGTTAACGCATGTAAGTACTCATTTATATTGGAA<br>TCAGAAATAAATGAAAACACAATTAATCGGAAA
MSTRG.8632	Uncharacterized protein LOC110381097	CAGCCCTGGTAAGACCCTGGAGAAGAAGGGCAGCGTGGCCGAGCTGGATGCACTGTGCGTGCCGGCCGCCTCCGCCAGCA<br>GGAAGAGCATCGCCGATCCCACCAGCGTGCAGCAGGCCATCAATGCTAGGGTAGCGGAGTCAGAACATTCCCCGATCCGC<br>CGCAAGCTCGTCCGCAACCAATCGGAGGGCTCCCCCAAGCCGGGCGACACGCTGTCGTCCGAGCTCCGCCGCCTGGTGGA<br>GGGGCCGGCTCGCAAACGGAACGAGTACGGCGTGATAGGCGATACGGCTTGCATGGGAGACCATAAGCCAATCTACCTCC<br>GCGTGATGCTGCAGAGCGACCGAGGTACCGTACCGTGTTGCGCCAGCGGCCTGCCGTGCGCCCTCTGCCTCAGCGCCGCCC<br>GCCTCCCCCGAAACCCCCGCCACCCCCTCGCACGACGACCCACCGATCCCGACCTTTACGCCAGTAAAAAAGTCAAAACC<br>CTCTTCTCCAGCCACCCTGACACCTTGACAGACGATCACAGACTGAAACGAACTCGAACCGGCTCCCTAAACGAGAAAAT<br>TCTCCGCATACCCGACGTCGAAATCACCACTGCCGATTATTCGAGTTACGACGGTGTATTCGTAAATGAAATCGATAACAC<br>GTTACTCTGCGTGCGACAGTGCTTAGATCCGTACACGCCGGAGAGTATCGACTCTCACACGCCCAACGTCGAGGATGCGTC<br>CAGTACTGAGGAGATCGCGGACGAGCTAGACGACGTTCTGGGGACGAGTATAATCGAGTCGAAATCGATTAAGTTGAATC<br>GAGACAGGAGCGTCTCTCCTACGCAATTAAAGCATAGACTCGATAAGTTACTCACCGACGATAGGTATAACGTACCTGAG<br>ATACAGAGGCTGAATAGTAGGGAGTCTTGCAAATCGGACGGTAGTAAAAAAGGGGGGTTGTGTTGTTTAGCTTTGAAATG<br>CTACGGGTTCTGCAGGCGGAGGGCGCCGAAGGTCAATTGCAGTTGTAGAGGCTTGAGTAACTGTTGCACCTCCTGAGCTTG<br>ACGTTTGTGATTCGAATTTCGAATGTTATTGACTTGTTGCGAACTCGATAGAAATCAAAACTCGAAATTTGAATTTTATTTT<br>TTCTCTAATCAGCTTTACGCCATAGACAATTATTAATTTGATTTTGATTTTTCATCGAGTCGTACGAGTGAGTGTTTCCACT<br>GTGTATTATTTTTTTCATTAAAATTTTTGTAGTGTATTGCATTGTGAACTTTTATAGTATTAACCGACCATTTTGTTGGTTTT<br>ACCGACCATGGTAATATTTACCATAGATATTTAGATGTGGTAATTCCTACCATTCAAATAACATTGACGATTATATGCATG<br>AATGTTGTAATCATTTGTATAACTTTAACTATATTATATCCATATCATTATTTAGACATTACACATGAAATAGCCAATATAT<br>GTGTATTATTTAGTACTAATCGTACGATGTAAGTTGGCCACTTAGAATGACGATCAGTCCGTCAATCAATCAATCATTTTGT<br>GTTGCCTAGTATTTTAATCATGAGTCCGCGATCACTAGCATCTAAACAAAGCCTCATGTCTTAAAAGCTAGATGCTCCCAA<br>TGATTTTAATGTGAGAAAGAATATGAATATCTCTAATGAATGCGAATGAGTGAAAAAGCTATATCATTCGTAGCCTTTCGT<br>TCATGCTTTCCCTTTCCTGCTCCCTACATTATACCATTGTTGATTTAGTTTTTAAAAATTGCTACTCCGTTATGTCATGTTCTC<br>CTGGTAGCCAATGTACATTACGCGCACTGTTAATGTTTTTGTATGTGCACTATTTTTATTATAATAAATGTAGTTTTATTAA<br>AGTATAATACACGTGCAAATGACAATAAAAATAATCATAGATGCATTATTTTTTCGTGTATATATTGAAGCAGCAATGTGT<br>AACGAATATTTATAAGGCACTCATTAGACGATCAGTCCAATCAAAATAGGTATTGTCCCTAATAATGTCTATAAAAACAAT<br>TTTTATATTATGTTATATTATAAATCCGCGTTGCATATTGACCTATATAAATGTTTTTTACTAATAAGGTTTTCTGAGGGACT<br>AAAACGCTTTTTTATACTCGTATAACCCGACCAATTAAAATACTAGTACTTTAATAGTGACTCCTCGACGCGAGATACCTA<br>TTGGACTCGGTTGGATATACGATATGAATGATAGTAATCATAGTTCATCTAAAGTGGCTATTTTGTACCTCTGATTATCTAA<br>TGAGTGTCTTAGCATGAGTGTTTAAAGTATCAGTATATTCGGAAATATTGTTTAAATTCTACTCTACATTTAAAGTATACTA<br>GTTATAAATTATTAAATTTATTATTAAGGTTGCTGGTCACTAAAAAAATCGGGCATGAAATTCTAGATGGCGCTGGAATCG<br>GGACTTGTGATTTTTGTTATTATTCTATAATTAAAGATAATTTAAAGTAAATTTTTTAATTGTCGAGTGGTTTTTATTATTAT<br>GCATGAATGTGAAGTAACAGATAATACGTGAACTTTTTCTAAACGAATTATAAACCACACAATCGCAATGTTTCTTACTAT<br>GTATGTTTTTTTACAAAGTTGTACAATACAAGAGAATAAAGACAAAAATTATGACAAATTATTACTATATCTGTAAGAAAT<br>AAATAAAATCACTCGCCAATTGTGAAATTTTGCTTCGAATTATGTTTAATTACAAGATAATCAACAAAAATTATAAGTCTC<br>GATTCTAGCGCCATCTAGAATTTCGTGTCTAATTTTGTTTAGTGATCAACCACCTTAATATGGTTTCAACTAGTCGATATCA

		GTTTCCATGTATATGAAATGGAGCTGATGGTATGTTAAGTCAATGCAAGCGGTTTAATCAAATTTAAAATAGGTTATTAAA<br>ATAGGTAATTCGATAGGTTATTATCCTTCGAAAGGGTAAAAGAATTGGTTGAGGTATTTTTATCATAAATTTTTATTTTATA<br>TTTAAAAAATGTTACCTATATAATATGTTAATAGTTCCTCATATAATATGTTGGTTTTCCATATATTTTTTTCTTATTTGAAT<br>CATTGATGATCATGGTGCCCCAGATGTGTGGATTTGATCCGGTCTAAAATGGCCATTTTGGGTGTTTAATAAAAAACCCAA<br>AGTAATTTAATCATTTGATAAATTTCTGCGAGAATCTGCTAAATTGCTAAAATTACTTGACACATTGTTTCTTTAATAATTC<br>TGTTGCATGTACTTTGACGTATCACCATTTTGTAGATTCTTGTAGAAAACTGTAACAGTTATTTCTTTGTTTCGTAAGTGGT<br>ATGATAATCATAATTGACAAATTAAATACTACTGCTTTTTTATGAAACATTTAATGTGGCCATTTTACGTACACATCTGTCA<br>GTAAATATACACAATTATAATAAAAAATCTGCAATACATATTTAAGATAAGATATATAGTTTTTATATAATTTTGATCCAA<br>ATTTCTCTCGGGTAAGTATTCAATTTCAGTGGTGGAGTAAGACGATAATGAATCAATATCAC
MSTRG.8646	Cecropin A	CATCATTCAACAGCATCGTTCCGTGATCGTTCTTCGAACGTTCATTTTTATTTCAAATTTCAAATATCGAATTTCGAAAATG<br>GATTTCTCTAAAATTTTTTTCTTCGTATTTGCCTGCATCGTAGCGCTGTCGACGGTTTCGGGTGCACCCAGCCCTAAATGGA<br>AATTTTTCAAAAAGATTGGAATCAGTGAATGCGGGGGACTCAGGATCATTCCTTATGGCAGTCTATGTGGGAGGCCATGTT<br>CAGCAGTGGACAGACAATGGCTGAGAAGAAGAAAAAGTATTAGTATATTAATTTTAATGATAATTCAAATATAATTCTTTT<br>TGTATTAAATATTAGACTATTTTTATTAAATATTACAAACATTTGATTTCGACGGTAGAATATTTTTGTCAAAGGATGCTGA<br>CAATTTCAATTTCATTTTGTATTCACCAAAATAAGAGAAGGAAAACTCAATATAACACCCTGTATTAATATTTTTAGTTAG<br>AGTTTTAATATATATCGTCTTTTCTTTTTGAAATCTCATTCTCAATAATGGATGTTAATATATTTTTTTTGTGAGGCGGTTAG<br>TGTTCCATATCATACGGAGAGATTGAGGGCAGCGGCCACGTCTGCGATGCTATGTACTGATAGTGCGCAGGCTGCTACAA<br>ACAAGC
MSTRG.8659	Fumarylacetoacetate hydrolase isoform B	CGTACGCTGACCATGTTCAACGTCAGTTCCCGGTTGAACCAATTTAGTGTCTGTTGTTTCTCAGTGCATGAAAGTGTGCCTT<br>TGTGATGTGGGATTGTCCTTAATTAAGGGAGGGTTTCCAGAACTCCAATATTCTGTATCTCGCTGCGGATAACGTCACCGG<br>GCTTCAGATACTCCGGGGGGCTCCGGTACATGCCGACGCCGCCTGGAGTGCCCGTTAGAATTATGTCCCCAGGGAGGAGG<br>GTCATCACCCTGGAAAATCAGGACACCGTTGACGCTGCACTTGATATCCAGGGTCTGAGGATCGCCGACTTCGTCGCTGGT<br>CACGATGTAAGGCCCAATGGGGCAAAAGGTGTCCATCGACTGTAAGAGACAGTCAAATATATAGAGAAATACTCGTATAT<br>GTACACAGGGTGAAATCGAACTCATTAGGAAACTTTCACAGGAGTCATCATCATCATGGACATCTCAATGTCCGCCTTATA<br>TTGTGCAGGCGGGCGAGGTAGGGGCCTCGGGGTAAACCCCTCT
MSTRG.870	Uncharacterized protein LOC106142343 isoform X1	GTTTGTCATTTTTGCTTATTTTGCTGTTATAGTTTTTCGAAGTATCTATCATCTGCCATAAATTGATATCTTTAAAATTTGAA<br>ATATCGTTTTTAGACATTAATATTATGGATTGTTTCCTCAATTCAATCTGTGACAATATTCTTCCATTCCATGTGACAATATT<br>AATAATTAAAAATAATATATAATCAGTGTCAATATAAATAATATAATTCTTCTAATTAGGTTCTTATTAGGACTAAAAACT<br>TTTCGATTATTATCTTCTATTCCTACTAGAATTTGTTGAAGTCAGTGGGAAGCCTAGGGCTTTTCTAGGCATAAAGACAATG<br>TGTTCTTGGACTAGTAAATATGCCATACTACGAGACCTATCAGAAGCACAGGGACATGTGCCCACGGAGATCAAGGGCAC<br>TGTTGGTGCTGAACCACCACCGACATTGCTGCTTACAGAAGAGACCGAGGAGGAATTTTTCGCCAACTGCATTGGAGCGG<br>GCGACTGCGTTGTGGGCAAACCGGACTTAGGACTAATTAGAAAAATGCTCGACATGGTAAAGGATGTTGATTCCACTTTA<br>CCGGAATCTGGTAATGGAGATGGTAAAAAAACAGTTATGTCTATACAAAAATTTAACGAACGTTGTCCTGACTTTGTGGCA<br>AAAAATGTGAATGTTAAGACTAACAATAAAACCGTCAGTACAAAGAAAATAGTGGAAAAAGATATAGACAACGACAAAA<br>AAGCAAAACCAAAGAAGGCGAGAAATGTTGCCTTAATTTCTTTACTGGACGCTTTAAATAAAAATGAAAATAAAGATCCA<br>TTGAACCTAATGAAACCCAATGATTTCATTGATAGTAATGTGAGCGAGTCTCTTGTGAAAGTCGATGATTGGCTAAATTCA<br>CAGGTCCAAAATGAATCCACAAATGGAAAAAAGGTGCCCAACTTAACACCAATTACTACTATGTTCAAGAGAAAAACTAA<br>TGTCGTAAAACAATCAAATGAAACTGCGAAATCTGTTGCAACAAAGAAAGAAAATTATACACCTTCTGGGTTAGCCACGG<br>AGTATTATAAAAAATATGTTGAGAAATCAAAATTAAATAATTGTATAAGAGAAG

MSTRG.8<br>758	Serine protease<br>HTRA2	TTTTACTATTTTCAGATGCCCACAGACATCGAGCATGGGATACTCGTGTGGAAGGTCATCATCGGATCACCAGCGTACAAT<br>GGTGGCCTGCAACCGGGTGATATAGTAACGCATATAAACGGTAAACCAGTGCACAGCGCCAGCGACATCTACATGATGTT<br>GGAGAACTCGAGCGGCAGTTTGCGTATCGACGTCGTTAGGGGGCGCCAGCGGATCACCCTTTCTGTCACACCTGAGATAC<br>ACTCGTAAACGTACTGTAGTAGGTGTTTCGATAATGTATTTTTTTTATGCCAACAAGGTTTAAAATGTACAGTTTACATAT<br>GGTGTTCTCATACGTACATGAAATGTTTTATAAATTTTTTTTTATACCACTGTGGTGGCAAACAAGCATACGGCCCACCTGA<br>TGGTAAGCGGTTACCGTAGCCTATGAACGTTTTATAAATTTAATGATATATGTACAAAAAATAATTCTTAAACATACAGAC<br>TTTTTTTTTTATTATACAACTTAGAATGGCAAACAAGCTGACGGCCCACCTGATGGAAAGTGGTAACCGTCGCCTATAGAC<br>ATAAGCAGTACCATGGACATAACAGAGTATACTGTTTCGCCCATGATACCCCCCATGCGTTGCCATTCCTGTGGACTCAGA<br>TGTTATTCCTCTGTACCCTGTAAATTCACGCCATATCCCACCCTTCAAACCGAAACACAGCTATGCAAATACAACTGCTTCA<br>CGGTAGAAACACGAATGAGACAGAGGTACTCAAGCAATCAACTTTTGTACAAAGTCTCCCTCTTGCAGGCAACTTGGATA<br>GAGAGGAGTAATTATAAACAGTTTGAAATTAAATTAATTAATTAATTTAAATGATCGATTTTATAATTTAATTGCTTTAGTT<br>TTAGATTATATAGACAGAGTGTCTCCACACAAATGCCTCGAAAAAAAAGCAGGTCTACTTATTCGTACAATTTCGTCACAA<br>TTTGGCGTGCATAATTTAAAATACATTGAAATAGTACATTACTGCAGCGGCCAGCAACTGAAGCATTGATGGACGAATGT<br>GCAAATGTGGCCCGAGGCGTAGCCGAGGCACATATACGTGAGTCCATCAATGCCTAGTTGTGGCCAAGGCTTATATAGTA<br>CTTTTCTCAAACAATGCGCAAAAATCAAAAACATAATTTTACTTTTTAAGTTCAATGAGGAATAACGAACAATAATATTTT<br>TTCACGTGCCATTATGTTCCTTTGATTTTTTTTTCAATAAACAGCTTTCTGTTTGCAAACCAGTTCGAAATTTAAATAAAACC<br>TTTTATTAGTTATCTCCTTGGTAGCCATGTTTCGAAGTAAAAGGTCATTCCATTTTTAAATTACAGGACAGATATGAGTTTA<br>CATAGTAATTTATCTGGCCGCAAAACACGACAGATATGGATTTTCATACAACTTTTGCCGCTGCCGCATGTATCTGGCCAG<br>TACGGCAATTTTGGTTTATCTCTAAAAAGATTGTCAAAATAATGCTCACATATCCAAGCATGTTTGAGAAAATGTATATTA<br>TATCAGTTTATTACTTCGCTCTCAAAAAGCAAAACACTGATATTTTGTTATGGTATGTGTATTAATATTGTATTGTATTTAA<br>TGTTTGTTATATATATTATAATGCAATATGCTGTTGGTGTTGCTATAAATAAATAAATATATATTGAAATAATAATATTCCC<br>GACTGATAGGTTTTAGTTTCTTTTGACGATATAAGAGGTGCTTGTATGTACAAATTATTACACGTAAAACTACCTATTTATT<br>GAGGACTACGGTGTTATTCCTCTGTACTCTATATTTTTTTTATGGGTGAAAATGGCATGGCAACCCTGCCTGCGCTGTCGGT<br>ATACACTGGCGAGGTACCAGTGAGAGATGGCAGGTGAGTATGAAAAGGCAGGTCAGTTAGCCCATCGTGACGAATTCAAC<br>GATAAATTAGTCACGATAGTTTCCAGAGGGAACCGCGTGGAGGTCGACCTGATAGGCTACATTGATAGCAATGGGGACTA<br>ACCTTCCCATTACTAACAATCCCTTCCCCCCAGGACTATGGTGCTATGCCTCTGTACCCTATAATGTGACTGCCACCCTTTC<br>TAATTCCACCGTTCAGGGGAGAAAGGCATTGTTAGCAAAGGAGTTTGATAGCCCCATTGCTAGCAATATGCCCTATTAGGT<br>CGACCTCCACGTGGTTCCCCCTGGAAACTATCGTGAACAATTCCTGTTGAATTCGTCACGATGGGCTAACTGACCTGCTTC<br>ATCCTCACGACAGATATGCATTTTCATACAACTTTTGCCGGCCGCTGCCCGCATGTATCTGGCCAGTATTTTATTAAGTGTT<br>ATAATAAAAATGTTAACTTTAAATGCAAATTAATGATACTGAATAAATATTGTAGGAAAGCATATAATTGTTTGGAATTGT<br>TGAATGGGTTTATTAGTTTGCGATTCAGCCATGGTTTTTGTATGTGTAAAATTGTTTTATTTTACCAAGACAATGTATTTAA<br>GTATTTTGGTATTGTTTTATGTAAGAAAAGGTAATTTTTTGTTAAGAGTTGTTCCAATAAAGTTGAGTATTTTTATAAAGCT<br>ATGTTTTTTTAAGCTATTGTATAGCTTTTATCGCGGGCCTTGAGCGCGGAGACCGAATGTAAAAATTCCGTAACG
MSTRG.8<br>759	Seipin	AGAATGTACCAATAACGGACGCGTACGTAGAGCTGCAGAGCCGGTACGCGCAGGTGTACTCGTGCGAGCTGCACGTGGAG<br>GCGCACTTCGCCGGCCTGCGCTACTATATGTACCACTGGCCGAAGATATCCGCGCTGATTGGTATAAGTTCGAACCTATTC<br>TTCGTATCACTGGTGTTCATACTAAGTTGGTATCACCTGCAAGACGGATTACCTGAATTTATAAAGGTATGCCATTTATTAC<br>AGAATATTTAATCTACCGTAATCTTCCTATCTATATCCTTTTATAGTACTACAGAT
MSTRG.8<br>760	G-protein coupled<br>receptor Mth2-	TATGAATACTGTACTTAATCATACATTTTGTGCTATTATTGCAATTATACTCGTATTTGGAATAAATTATAAATTTACCGTA<br>TTATTTATCAAAATGTCAAATCTCTTATAAATCTGTTTTACTCGTAGCTACTGAAGTGTTTTATCTTTTTCTTTTGCGCAAAT

	like	TTTTCAATTTGAAAGACAGTGACAAATCGGTCTAACAGTTAACATGTTTTTTTTTAATAATTAGTATAATGGAATAAAAT<br>GCCTGCATCATATTATACGCTGGCGAAGCACCAGTGAAAGATGACATGTGAGAATGAAAAGGCAGGTCAGTTAACCTATC<br>GTGATGAATACACCTGGAATTCAGCACGATGGTTTTCAGGGAGACCACGGGGAAGTCGACTTGACTGGGTATATTGCTAG<br>CAATGGGACATCCACATTACTAATAACACGTTTTAGTTTTTACGAATAAAAGGGTTAGACTTTGAAATGAAACATACAAAG<br>CACAGGACTGTACAAAGTGGCGCATTTTGGGCAAAGTCTATGTCCAACAGTGAACAGCTGTGGCCTGATGACGAGGAAAT<br>ATACACCACACGTCACCATAGGAACCTTCGATGAATAAACAGGGATACAACATCCAAATTGTACCCGCTCGTTGTTTAATC<br>ATAACACTTGTAATAGCGGTACACGGACAAACATACAAATGGGTGCGTTGTGTTTTCAACTTGTTTTATTTTCAACAATTTG<br>TTTCACCTATGCTTGGAGTTCGGCATTGATCAATGGTGACTTTGACATGTCAATTTGTGGACGAAAAAAATGTATATGGAA<br>GTGCTGCCCGGAAAATATGTATGTGAATAGAAAAAAATGCGCCCCGTACAAAGAAGCATTGAATTTCTCGGATTATCCCG<br>TGTTCGATGGCGATATGTATGAGAGTGGAAAGAAATTTTCGGAACTTTTCGCTTTTGTGCCGAACAAGTTTGATAACGAAT<br>CGTTCCGGAATGATTCCTACAACATGAACCGGCTGTTCAACGTTTACTTGATGACGGTAAGCTTTTTTTTT
MSTRG.8781	Glucuronyltransferase I	CAGGAAAGTGAGATTCTCAAATACTTCACAAAAACTGAAGACTTGGAGCCGCTCGCCGAAAACTGCACGAAAGTCTTGGT<br>ATGGCACACGCGTACACAAAAACCTTCCATCATCAGCCCGAGTAAGCTGAAACATCCAATACCTCCCGACAGTCATATTG<br>AAGTGTGAATTTAATTTGGCCGAGTTATTTGGTCTATAATAATAGGAGGCATCGCCCAGACATCATAGGGCAATGTTACCT<br>CAACGTACGTAACCTAAAATCGCATCTCAAAATACTTGAGATAGTCGACACTATTCGACTAACTTACTTATCGATCGATAT<br>AAGTGAATAATAATCGAATATATTATACAAGCAAGACAGGGTTACGATACTAGTGTTTGACACACTTTCGTAATAGTTAAA<br>TATCTATTTAAGAAAGGTATTAATGCGACTTAAATATATATGTTAGGTTGTAAGTAGTCCAGTGAAAAAAGGTTTAGAAAT<br>ATAATTTAATATGGCGATTAATAGCAAAGTATCATGAAACGTATTGTGGAAATAAAATATTCAAAGGTCACATGAATTTGC<br>TCATAATAGTTTTATTATTTATAATTTTATATAAACTTAATAATAAAATTAGAGTGTCATGATAAGATGAAATTTGATTTCA<br>CAATGCAGCCATAGAAAAATGTATCGTTAACTATTAAGAAACTTAGTAAAGATATTGTTTTGTAATATTAATGTTATAGAT<br>TTGAAAAATTCATGGAAGTGACAACTGCAGTAATCTTAGTATTTTTCTTTTGTTTTAAAATTTAAAATACATAGCAGAATTC<br>TGTTTGAATATTTCTGAACTGCCTTAAATTTACTACTGTATTGTATTATTAAAATTTTTAATTTAAATATTTCGGCCATTGTC<br>TTTTGTCAGAACTTGTACTGTTTCATGAATACGTACTATAGCTGAATGTAGGATTTTTATTACTTATACTATTTTTCCATTCC<br>TGTATCACAAATGTAAGTTCTAGAATAATTTGTTCCATTAACTTCAGTATTACTAATCAAATATGATACAATTGTAAATTAA<br>TTTTGTAAGTTGAGTTTATAAAAAAACTAGAATAGAACAATTAAAGTCAGCAGCGAACAAATGTAACAATCTTCAGATTA<br>TATATATACTAGTCTCTCATGGAACATAATACCTAATTATAAATTGTTTTATTTATTGAGTGTGTTATTAGAATGCAATTAT<br>TAGCAATCATACACGTAAGTAGTGAAATAATTAGTAAATTATGTTTTATTAAATTTTATACATTCATATGTTTATAGTTTAT<br>GTTCTTAGATTATATAGATAGAGTGTCTCCGTAGATATGCTTCGAGAAAAACGAGTCAACTTTTTCATACAACTTTGCGTG<br>ATAAATAGTTATTAGTGATTCCTTTATAACTTCTATATTTATTTTGTCTTATAGTTTTTGCATCTTTTTAATTCGTAAACATTC<br>AGTTTATTTATTAAATAATATGTCAAGATACATTATATGCGTATATATATAGAACACTGATAAAAAAACGAGGTAGCAAAT<br>TTAATAATTAGCTGATAAAAAGTCGACCCGTTTTTGAGGTTCGTTGCTGTATCTACGTAATCTAAGTTTATGTTATAGGTGG<br>TGGCTATAGGTTATCTTTATTCTATTCTTTATAATGTAGCAAAGAGAACGGGGTGCCAATGACCTCTTTTCACCTGAAAGGT<br>AATATTAATAATAATAAATATTATTTAGTAAAAATTATGTGAGAATTTCCATCTATATTTCTAGATGTCGATAAAATTTCAG<br>AAACGATTGAGCGAATATGGTCTCAATTGCATCGAGGTCTTATTTTAAATTAATATGTTTTCTTTCGCAATCTAAAATGGAC<br>TTTATTATTTCATTTTTTAAGTTTAAGTTACATTTGAATGATAAAATTTAAATTACTTTTTTCTATTAGTATAGTCAGTCTAT<br>GGCCATATTTGAATTCCTTCTGTTTTAATTAGTTTACAACGTCATCTAGTGGTATTAATAATTTGAATGAACAATGAAATTG<br>CTTTACAATATTTTCTAAGCCAATCTATGTAACATAATATTCAATGGAACGAGGACTTAAAATAGGTACAAAATTTCGATT<br>TAGGAGTCGTGTAACAAAATAGGTTACACAAATCCTAATATAATTCCTTTCATCTATGTATAGAGTTTAATAGATATTATG<br>CTTTAGAGGCGTAATATTAACGCAGTAGTTATTTCATTGGCATTGGCAATAATTTCATTTAATTTAAAATCAGTCATAATAC

TGTTCACGTAAAGTGACTTTTTAATTGTATTTGTATTACTTAAATGATATACTCACTATTTAATAATATTCCAAACCAATAT
TCCATTTGTAGCTATTTCACAGTCAGACAATCGTTCACCCATCGCTTCCAATCTTTTATGTAAATCGTCTATCCGAAAGGAA
AACTGACTGACAGATCAACAAACATGCAAGTTCCGCTAGTTCAGTAGTCTTTTCAATATTTACCCAATTCATCTTTCCATGT
TTTCTTTGATCTATTTTAATACAGTTCATTGTCAATCTTAAGATCTTAACCCTAAGGAATGAAATATCTAGACAAAGGATTA
AATTAATTTTGTTTAAAAACCTTCGATTTTTAAATGTATTTAATCTCGTTTTCATGTGAAATCGGCATCCTTTAATAATTACT
TAATATAGAATCACCCTCAAATCTAAACAATGTCACAAAGTGGATCGGGGGTCTACTCAGAAATAACAGATCCGAGTAAT
ACTCCATTTGTGTTGTGTGTGTGAGTTACACAGCTCTAAGGCTTACAAAACATTATACCTTAAATATGGTAGAAATAAAAA
AAAAATTGTAATGACAGACTGAATCTTATGAAGTTCATAGTAGAAAAATCGTATAAAAATGATGTTGATAAATATGATCTT
GAGTCATTTAAAACTTCTATAAAACCAGTCAAAGCTACAAACGGGTATATAATAAAAGTACAGCAAATAAATTAGACAGT
CGCGTGGTAATTTGCCAATAGATCAGTTTAATTTAAACAAAAATGTTGAATTTGTCATTTCATACTAAACATCCAAGGTCT
GACAAGGACAAATATATTTCCGCAGAATACTAAACAAGGAATGCAAGAATTAAACGTCTACATAGATAACAATTTATTTG
TTACAAAGCGCACACAAATACTAAAAACATTAATATATATATATAAGCCACTATAAAATGTAAGCGTATGCATGAGTATTT
CTGCTGTTTTAGTAAAGACGTTTAACTGTGAGTATTATTATTATTATTGATCGAAAAGAAGAAAATAAATGAGCTTATATC
TTACTTAATTGTATAAAAGATTACCTCATATTACATTAACAACACCAGAAGTGTTTAAAAAAGTATTATAACCTCTTCTCAT
TGCTATCTATGAATGAAATAAAGATGTTGAATTTTAATTATCTGGAGTCTGGGCTCAGTCAATGTACAGTATGTATAAAAA
AAAAGCAAATCCAAAACGTATGTCAATATAACTCATATTTTCTTAAAATTGCTATTATTTTGTTGGCACAAAATGGTTTCTT
AAACCCTGATACAATAATTTTCAAGCTACGTTAACTAGGTTATGAATATTACACCTACTATAAAAAGCATAATTTACAATT
ATAATTAGAAATATTCCTTTGTCTATCTGTAAACAATTATGATTATCATAGTATATATATCTTGTGTGAATCTAACAGTATT
TTTAAGCGACTTAGAATAGCAAATAAGCTGGCGACCCTGATTAAAAATGGTAACCGTCGTCTATAGATAGACATGAACAG
TACCATTTAATACAATTGAAACAAAGTCAAATATTGATAACTGTTGAAAACATGAGATTTCACAATTGTGCTTTTTAATTT
AGCTTTTATGTCTTATACAATTTATTTACTTTAATGTTCATTTCTTGTACTTGTAAAGGTAAAATTTGAGCAATAATTAATGT
ATTATAAAGAAATTCGTTACAACCAAAAAAAAAAAAAACTTTTAACGTTAGACTTAAGAACATATGTCATTTAACACAATA
TAACACATAAAAAATAACATTTTTGTATCAATTAATCACACTAATTTTAATATTCTTTTTGCCTCATATAATTTATTTACCTT
AAATGTTCATTTCTTGTACTTGTAAAGGTAAAATTTGAGCAATAATTTACGTATTATATAGATATTAATTATAACTTAAAAA
AAAAACATTTTACTTCAAAATTTGTCTTTAGAACATGTGCCATTTAACGCAATATGTAAAAAATTACATTTTTGCACCAAC
GAATTACACTATTTATTTAAATTGTTTTATATTTCAAATAATTCTCATATTATTTAAAATTTTAAGACATACTGTACGGTGA
CTGTGACCATAAAATAACCATCTTTAAAGTGTGACTGTTTGAGATACTTCAAACTTCATTTTGACACTGGGTTTTCTATATT
ATTTTTCTTGAAAGGTGAAACAATCTTAGGAATTGGCATGAACGAATGAGATGAATAATGAGAGTGTATAAAATAAAAGA
TTTGTAAAGGATAAAGACATTGTGTGTTTCCGTCTATCTACTTCGTATGGGACCTGGAATATAAATAATCGATCAAAGGTG
TACTCAGTGCTGACAAAGGTATAAAAGATTCCATATATTTTATATCTTGCAGTTATATAATAATGTAATTATTTGAGGTCAA
CGATTCCTTTAGCAGATTCTAAATACTGAAATATTTGAAGCGGTCTAAATATTTTTATTTTTAGTTATTAAAATATTTTTGTA
CTTATCAAAATATAAATTGTACAAATTCTTCCTTTAGTAAATTTCTTGTAAGTTTAAATTAATGATAAATGTATGACATAAG
TGAATAATTGTATTATACATATTATATCAATAGTAAATTATTTTCAATATATTTTGTTTGTATAAGATGTTGAGATGTTGTT
GCTGTGATTGTTGGCGCTTGATGGAGTTTTAATTTCGTTTTTATAAATGAGAGCCCTTGTACTCTTCCATTGGTTTACATATT
TTTTTTTTCTTAAATGTAAAATTTTATGATCAAACCTGGTTGCCATGCTATGAGGTTATGAAATGGATAAAAATTATAGTAT
AGGTCTATTATCGAAGTAGACTCGGACATTTTAAAACAGGATGTCGCACTTATCAGCTGTTATAGAACTAAAAATGATACA
AATGCAATAATATATAAATAACACAAAAACCTCAATTTATGTATTATTATTTGGATAAAAAGACGTTATGCTATTCAAACA
CTATACTATTTATTAATTTTTATAATCGACTTAAAAATTAAACTAAATCTATACAAAAAGGGCTTACGTAATAATTTAAATA
AATTTATTTGACTTTGAGAATTTTTGTTTTTCTTTGTAAATTTATTATGCATTTGACATTATTTTAGAAGGATAACTTTAGA

		ACAAATTAAGTAGTAGAAATATATAAATCACCACAGATCCACCATGAGATGTTTATCATAGCCTAAGATCAATATATATA<br>GGGTCGCCTGTAAAAAAGTACTGCAAGGAAGAGTTGTCTCCTGACAATTCAAAGATTCTCTTAATTCAAGGTAGGCAACT<br>GGGATGTATTAATGCAGTGTCGGGTGTGGTGTATTATATTTTAGCCCGTCTTTCATTTGTATCAATTGTCATTAAAAAGTTT<br>TATACCTGCAGAAATCAGCAGCTGGATCTTAGACTATCTGTTCCAGTTCTATCTACTTGAAATTATAATGATTAAATACTCA<br>TATGTTTAGTAGATACACTTAAGAATTTTATTAAAAAAAAACCATGTTGGTGTATCCATAATTGGTACATGCACCTTATTTA<br>TATCTATATTACAACATTGTTCTGAATTATTGTAAAAATTTAATTTATTTTATATGAACAAATATGTAATTGGATAAGTAGA<br>TACTGTTTAGAAATCAATATTAGAGATAATTAGCTAGGAGACTATGTGATGGATTTTTAGGTTTTTATTTCTATAATTTGTG<br>TAAATACTTGCATAATTTTACAATACAAGAACATAC
MSTRG.8787	EH domain-binding protein 1-like protein 1 isoform X1	GAGAGACTAGAATCCGGCTAAAGGACTAAATCTATACAGTATTGTGCAACTATGTTCGTTACTATTAATATTTTGTTAATA<br>TATCTGGTGATATATTATATGATATATGACATAAATATGTACATAGTAAGCAAGTATTATCTATCTATGTCTTTTTCGTTTT<br>ATCACCTATAACTTGACTCCTATGAACATTGAGTTGATGCGATTTGTTTGAGCTACAAATACCCTTGTATACTCATAGGCTT<br>CTTCATTGTTCTTGTTCACACATGGCCTATATTCTATTATTTATTTATTTTTTATAATACTAGACAGGTAAATAAGTACTTCA<br>GAGTAAATAGGATTCATAATTCGTATTATATGAATTTAAAATAAATGTAATGAATAATAACAATAATAATTCTTTATTTGA<br>AACAAAAACATTGGTTACAATATGACGCAGAAGTATAAAACTGTGTCGCGAACATGAACGCCCTCCTCCTCATTGCAGGA<br>TTCCAAAACGGACATTTAAAACGTATGGTAGCTAGTTACACCAACGAAGACATTTTTGTTATACCTAGCAACTAACTACAA<br>TACTAATGAACTACAATAATTTAGAAAAATTGAAAATTTAAATACAGTTGCCTACATGTTAACTTAAAAAAAAGTGTGTGT<br>GCGTAAATGTGTGTGTGTGTGTTTGTGTGTGTGTGCGTGCGTGCGTGTATGTATGTGTGTGTGTGTGAATTTGTGTATCAAG<br>AATAATTAGATCGTGGATAAGTCAGTTTACTGGATTAGTATTAAAATATCAATACACACAAAATATGAATTTACTTTTAAT<br>ATACAATATTAAAATAAGAAATGTATAATAATCATACAAAAAACACTAAAATAATAATATTAATAGCTAATAGAACATTG<br>GCCTGCTGTGAATCAAGCTTACATAGAATCATTGTAATGTATTCAAATGCTATATGAATACAATTTTTTTTTTCATTTCATT<br>CTCATCAACATGGCTTGCAAATTGTGTGTGCGTGTGCCGCCAACAACGTCTCATTGGAAAATGTCACGGACGCTTGTGATT<br>GGATGAAAACCGACGCTTCTGATTGGTCAATTTACTCTCCCGCTTTACTGTGATCGGTCGACGATTCCCGCGCTTTGGTTGA<br>CACATGAAACGCTTCGCTCTGATTGGCCGATGGGTATTGCCTCGTTTTGATTGGCTATTAGCAATTATTGTCTCGGCACGAT<br>GAGTTGAAAGAACGAGCGAGACAATTGCTTGAAGCTACTAAAAAGGAGGCGAGAGAAAAGGAGAGAAATAGAAGGCAA<br>AATTCCAAAGAGAAGGAAGAAGGGACGAGTGAGATTGCGAATGGACTTAGAAATGGGGAAATGAGTCCGAAGAAGCCA<br>ATGACCGAGGACGAGAGACAAGTAATGCTACGCGAAAGAGCGAGAAAGCTGATAGCGGATGCGAGAGCGGGCATTGTTA<br>GGTATGATATCCTTACTAATATTGCAAATAGGAAAGTTAGTTTGTTTGTTATATATATGGACAACCACTGCTGAATATTGG<br>CCTCCTCCAGAGACTGACATAAGGAACGGTCCTGAGCCACCTGCATCCACTGACTCCCTGCAATGCATTTGATATCGTCAC<br>TACATTACATAGAGGGGGGTTGTTTTCCACTGCGTTTACTTGTATGGGGTTGCCACTCAAGAAC
MSTRG.8791	Arrestin domain-containing protein 2-like	AGGAGTATCACAACTTCAGCCACAGGATTACACATTGACCTTCAAGTTTCAAATACCGCATTCAGCGCCATCTTCCTTCAA<br>AGGAAACCACGGATATGTGTGTTATCATATAGTCGCTTATATAGAGTTTCTGGACCCTCTGATGCCATGCGAGGAGGTGAT<br>GAGGGAGATTCAAGTCATTGCTCCTTATAGATTGTATAAAAGTCGTCTTAGTATAAATGAACCGATAACATTGATTTTCGA<br>TGAGATGTATTCATCTGGCTGTTTATGTGAGCCCAATCCCCTACACATCACCATAGTGCTGTCCCGGTCTGGATTCTGTCCT<br>GGAG
MSTRG.8804	Ferrochelatase	GGGCACGTCTCGCCCGGCCTTATCTCTTAATACATCAACGCTCGCAGGCGGGAGTACAGCAGATAGAGCGAGTCGCGACC<br>CCCAACGACCATCCCATATTCATCGCGGCGCTAGCTGATGTCGTGGCCAACCATCTGAAGAACGGCCCCAGGCTCAGCAA<br>TCAATTCCTTATCAGATGTCCGCACTGTGTCAGTCAAAGGTGTCAGTCGTCAAAG
MSTRG.8825	Regucalcin-like	GAAGCAGATCAGTGGACCCAAAGAAAGCCCATCGTGATGAATACAGCTGGAATTCACACGGTGGTTTCCAGGGGGAACA<br>GCGTGGAGGTCGACCTAATAGGGGTATATTGCTAGCACTAGTTGCCACACTTAGCAAACGTATTGGCAACCGCAGTTTGGC

		TTTTGGTGATGTTTTAAGAGGGACGCTGCTGCCCATCTCTCCACCATAAGCCCCCTTAGTCGCCTCTTACAACAGTCACGCG<br>AAAGGTAGAGATGGCCAGGACCTACACGGCTATGATTGAAAACGGGCATATGATTTGTCTTAACCCTTCTTTGCATGATTT<br>TTTTATTGATGAAATAAATCAAGGTGATATAATTCATGTTCAAAATTAAAGGAAAAATGGTTAAAGAAATAAATTTAACA<br>GCTATGTTTTTTGTTCTGCAATGGACATTAAAAAGGTGTCCAGACACGATCAACTTAAAGTGAACTTGTTCTTTTATATAAT<br>TTTTGAACATTCAATGTTATGATGGAAATTTCCACCATTACGCGAAGAAGGGTTAATACGTCAACTAGGAGTGTTTTTTTTT<br>TTAGTTTTCCTTCTTTTGAAAACGTGACGGGAAAAAAGGTATTTATTTGTATAACGTATTGAAATGTTTACACTTAACTGTT<br>TAAAATCTCTTTTAAAATTAATGTTTAAGCATTGTGTACACAATTTATCTGGCACATTAGTATAATCGTCTCTCAAATGACT<br>TTAGATTAGATGTGTAAATTGTAATTCAGTTGTAAACAGAGTTGAATGTTCAGAGCGTACACTGCATTCGCTCAAAACGTT<br>TTCAGAATTCATTTCTAACAATGGGACCGACGCTGAGGCAGGTGGTCGAGCCAGTGACGTTGGGCGAGGGTCCCCACTGG<br>GACGCCGAGGAACAAGCGCTGTACTTCATAAGCACCAATGATTACACCATAAATAAATATGTGCCAGCCAGCGGGAAACA<br>CACCACGTCCAAACTTGG
MSTRG.8843	Neuroblastoma-amplified sequence-like	CCTCACTGTTCTATTGAAGTTTAACACAACTGTCTGTGCGCAGAATAAAGGAGACGTTGTGGCCACTGATAGAGGGCCACG<br>ATCACAGCGCGCTGCTGAACCTGCTGTCTATACTTCGCGGTGTGGACGAGCGGCCTCTCATTGCCGGCCTCACCGCCGCCG<br>AACACATCAAGCTACTTAAGAAGGCCAAGGCCGCCTCTACAGAGCTGGACTATAAGCTGCTACTAGATCAGCCGTCGGCA<br>GAACAGTTCTCTGCGCACATGCTCGCGATACTGAAGCCGGAGACCATTGGAATGGTTTGCAAGTTCTTGCGCTCGCTGCCG<br>CCCACGTTCAAGCTACCCATCACCGTCAACGCGATATATACCATGTGGCTGACCAAACATTTCTTCAATGT
MSTRG.8867	Hypothetical protein KGM_210410A	TAAACGTGGCACTAACGCATCGAGTAACGAAACGCAAAACGTCAAAGTCGTCTGACGTGTTTAATTTTCTAATTTCCCAAT<br>AGTTTTTGAAAACGTTTGTGAAACATTGCTTACGTTTGTTTTATCAATACATCGATTCGATAAGCCATTATCGCTCATATGT<br>CCACGTAGCGAGTCTACCTAAACAGATTAAAAAAAATGCTGCGTGTAATATTTTTCGCTGCGATGCTGCAGTGTACGATAT<br>CACAACAATATCAACAGAAAGTAGCTCCGGGCGTTCCTCCGCAGAATTATCAGGTAACAGTTTGAGAAATAAACAAACAA<br>ACAACAAATGTATCATTAAATTGTAATATTTATATTATTAAC
MSTRG.887	KAT8 regulatory NSL complex subunit 1-like	GAGGTTTTGAAATGTGTATTCGTTGCTGGCAGACGTGTCGCCGTCGCTGCACATGGCGGCGCTGTGCCCGCGCGCGTGGTT<br>CCGGCCGCGGATGCTGCGCGGCCGCGTGTCGACCGCCGCCGCGCCCCGCGCCGCGCCCCGCCACCACCAGCACCAGCACC<br>AGCACCAGCATCCGCACAAGCACGCCAAGCGACCGCCCACCAAATTGAAGAGAGGAAGACCACCACTTTCTAGAAAAAT<br>AAGAGACAGAGAAAGGGATGAAGAAACATCAACACCAAGTCATGAAAG
MSTRG.8881	Hypothetical protein	TGCGTAAAACTAATCGTAACACTCGTAAATATGTGCTTAAATAAATGTAGTTGAAAATAATTACAGTAATATTTAAATGTT<br>TATTATTGAGAATTGTTAACCGACTTCAAAAAAGGAGGAGGTATATGTTTCACTGTTTATTTTTTTATGTATGTCCACCAAC<br>TTCTCCGTCAATTGTGGACCAATTTTAAAATTCTGTTTGTATTCGAAAGAGGTGGTCTCATTGTCATCGAGTCAGGATTTGA<br>AAATGGGCTCCCAAGGAAATCGAGGGCAAATGTCAAATTTTATAGGCACATGTGTGATGTTTGATATTTAATCAAAAGTTA<br>TTTAGATATTTACATCTGGTAGTCATCATCTTATGTTGCTTAGATGATGATGGTGATTTAAACACTATAGCCTCATATAGTC<br>AGTTAGTTGTTATCCTTACTTAAGTTATTCAGATGAGCTGATTATGGAAGGTTTGACTCCTTAACGGTTAGGATTTAGGGGA<br>TAATGCCTAGGATATTATAAGCACGTATAGACCTTTCTTAGGCTCTTTCGTTAGTTATTTGGGTGACTGCATCTGGGAGTGA<br>TCATTTCATGTTGCTGAGATGATGATGGAAGCCACAGGACTGTATCGGTAAACAAATCATCCTATTGGCGAGTGGGTTATC<br>CTGTCAGCGAGAGTTAAAGTTGTTGTTATATACAGTTTTACTATTACTAAAAATAAAAAGTTAAAAGCAGTAAATAAAATT<br>TATTTATTTATTTATTATTAATTCATTTTTAAACTAACATACAGTTCTGCAATGGTATAATGACAATTGGATTGTATTATAA<br>GATGTAGTTTTTAACTCACTTCTTACATATTATTTACACTAAAACAATAAAAATAAAGTTTAAAAAAATTAAACTGACTTC<br>AAAATTAATGTTGCAAAGCAAAGCAGTAAATAAACAGATACTGACTTTTAATTTGAAGGACAATATTTATTTAAAGTCAGT<br>GCCAGGCGCAAAGAGAAACAACAACAATAATTATGATGTATAAAGCATAGATTAATAACGAAATATAAATATAATGAAA<br>TACTTAAATATTATTGTTAATAGTTAAAGACCAACTTTTAAGCAACTGTATCTATCAACATAAGTAACATAACGCATCAAT

		TGGCACCAACTTCAAATGGATTTTGTTCTTTAAATTAAAAGTCAGTATCAGTTTTTATTTTTGTTTTTATAAAAGTTTTTTT<br>TTTATTAGATTAAAAAGTCACTCAAGGAATGTTCTGCCATAAAAAAAATTTTATCCCATACCTTAAAATCTTGATTTTGAAA<br>ATTTTTGCGCACACAGTTCTTATCAAGGTCATTATCTAAGAGGCGGAAAATGGATGTTTAAAGTTGGCGGCGCTTACGGCT<br>CATACTTCTGGTCTTAATCGTATTTAAGGGGTGGCCAAGTGGACTATACCATTTAGAAATGTCTAATAGCCCTCCAGGATG<br>ACGAACAAAGCTTTTTACTATGTGCAGATGTGTCTGAGTGTACATAAGTTACCTTTTTAAAAGGGCAAAATATGTACTCTC<br>GGACACGTCTCCCGACTCCCCCTGGAATCTTTATCACCTAGTATTGCAGATATCTTTACGCTACGGTAAGTGATTGTCACCA<br>TGCAGATAACTTGTTTGTCATATCAGTGGTATAAAAAAAAAACTTTAAATACAAATATGCACCAACATTTTTGTAGTTGAA<br>ATGTTTAAGTAAATTTTTTTTTGTCAAATACTAGCTGACCCCGTAAACGTTGTTTTTACCACTTATTGCGAAATATAAAAAT<br>CGCGATTAAAAGACAGGGGTTGATCGTAAAACGATGAAAATTTAAAGTTGTGTATATTTTTCAACGCTAAATCATAATAA<br>AAAGAAAAATGGAAATTTTTTTTTAAAAAATAAAATAAAAATTTTTTGGGGAGGGTCACCCTTATTATTGAGGGGTATGAA<br>AAATAGATGTTGGCCGATTCTCAGACCTACCCAATATGCACACAAAATTTCATGAAAATCGATAAAGCCGTTTCGGAGGA<br>GTTTGGTAACAAACACCGTGACACGAGAATTTTATATATAAGATTTGCCACTGTTTAATTTTGTGACATATTTTACTTTAGA<br>TTTTGTTTCCCACGACGGGTCGCAGGTCCGTTCGCTGTATTTACTTTTTTTAAATAAGATATTTATCATATAATATAAATATT<br>GATAAAAGATAGGTGCAAAGATTAAAGCCTAATAAAAACACTAGTAAAAGAGGCACGCTATTATACAAAATAAAGAAAA<br>TTATTATAATTTGGTAATCACTTATTGCGAATCATGTTTTATCAATACATATTATGATAACGTGTTTTTTTTTGTGTTTTTTTA<br>ATTTCCAATAATCTTCAAAAACATGATATATATTTAAAATAGTGATAATAATGAGTTACTAAGCGCAGTAATTATTCTTTA<br>CAGATTTTATTATTATGAATAAGATTTAGACTAATACTGAACGGAAACTTAATCCATCTTTGTCATTTACTCTTAATAAAGA<br>TTACAACTGTATTTAAAGGATTAGAGAATTGCCATTATGTCACTCTTATTATTAATTTATGTGTTGGCTGATTTAATATTAA<br>TTAATTTTTAATTAAAAAGTTCTACTTGTTTCCAAATTTAAAATGTTATTTCAACATTCAAGTAACAGTGTATAGAAATTTA<br>CTAATATTGGATATTGTTTTTAGGTGTTAAAAAGCCATATTATATTGCTATAGTATAATATGGTATCGAGTAAGTAAAACTT<br>TCTTTTTTCTACAGATTGTAGATTAGTTATTGTCTATATATTGTTTAAAAATGCTTGAATCACAGATAATATAATGATGTAC<br>TAGTACCTAAATATGTGTGATAATTTTTTAAAATCGTATTACAGTATTTTGTTGATTTTTTATTTGAATTTGTTTCGCTGTAT<br>GTAATACGATCGATTGGGAAATTCATACTGAGACTTTATTGCATGATCATGATTCTTGTTTACAGAACTACTAGAGGAGTT<br>CTATTAATTGTTGGTTTTATTTATTTTAGGGCATTTATTATTGTCATAGCCGTTATTCACTCATTCACCTATCTCACTAACTTT<br>AGAATGTACTTATTTGTATAGTTTAAGTGTACTTTAATTGTAATTCTAAGGGTCAACGCAGACTAACTGACTTGTGGTAAA<br>GGAAGTAAATCGGTCACAAGTCTTTTAACGTCTATCTTTGAGTCACAACACTCAAACAGTCGCAATTTCGAGAGTCGCCTT<br>TGTATATCAATTCAGCTGTAAGCTAATAGTAAATAAAAAGTTCATAAAATCCAGTTTTCCAATGCTGACATATTCTTTTTCC<br>GCTACTTGTTGGTTTGTGGTGGCCCTAATACGAGCATACTTATTACGTATATATCTTGCTTGTAGGTATTCTATAAATATTT<br>CTCATTATTGTATGCTATTCTTAAAAAGTGCCTTCTAGACATAAAATGCTTTTACACATAATTTTAATAAATTATAATAACA<br>TATATGTAATAATAATTTAATTATCTATTAATAATAGTATCTGTTTAATTTTCGATCAATCATATCATGGGTTATTGCCCAA<br>TATTATTTCTAATTCTGTTCATTCCTTTACGAGTACTTTACAGTATTTAAGAAATTGTTAAAACAAGCCATCAATTATG
MSTRG.8898	Integrin alpha-9	ATCGCTAAGAAATCTTACCAGCTAATACTTTATCGTTTCCAGCCTGCACGTCGGAGCGCCGAAAGCGAAGAATAGAAGTA<br>ACTCGTCAATAAGCACTGGATTAGTGTTTGTCTGTCCATTGAACGATTTGGATAGACATAACGTTACCTGCGCACCGTTAA<br>TCACAAATATGCGTATTACAAGAAATTTCTATGTAGACGACATGTGGTTTGGCGCGACCATGGGAGTAGTAAAGAAAGGA<br>AAGTTATGGAGCAAATTATTCAAACGGCTGACTAATAAATGTTAGATTAAAAAAGTTAGACATGAATAGTACCAT
MSTRG.8904	Uncharacterized protein LOC106138074	GTTATCGCGCTAATTTCCGATTGGTCGCCATTTGCGCCACGATTGCTGGCCAAGAAACTTTTTCAAAGAAGAGGTTACTTG<br>TTTTAATTTAATTGATAATTGTGCCTAAAAATATAGAAAAACAGTGTCAAAAACTCCGAAACATCTACCTATTTAATAGAA<br>GCACGAAAAGTGCGTTATATAACCTCCGAGAAGAATAGCAATGAAGATAGAAGAAAAACCCAAAAACAACTTTGCTACA<br>CTTATGAACACTGTGCCTCAAGTGATATATACACACACATTTAAGCACATTTAAGTAAATACCTCGATTATAGTCCGAGAG

		ACGAACAATAGTCCGGACGACGTACTAAACTCATCTCGGTTATAGTCCAAGAGACGGAACAGTATTTTTTTTTTTAATATC<br>TAAGTTTTTCATATAATTTGTAAGTGTATGGAGTAGGTATTAATCGGTCATCGTCATACAGTTGAGTGTTTAATAATGAAA<br>ATTCCGGATTCGACATCTAATGTTGGTGCTTACTATGGCAACGATGAGAATGGACAGTGGCAGCCAAACGGCGTGATGAT<br>AGAGAGCGGCGTTGGCGGAGGCGTGGTGGGCGGCGGCGACGCGGGCCCGCTGCGGCCGCCGCTGTACCCCGTACACCTGC<br>CGCGCGGACACATACCTGG
MSTRG.8940	Spastin	ATAGAGTTATATAAAAAAGGTATTTACGAGTTGGAACGGGGCATCGCCGTGGACTGCTGGGGCGGACGAGGGGACGCGT<br>GGCAGCGAGCACAGAGGCTCCACGACAAGATGAAGACCAACCTAGGGATGGCAAAGGACCGGCTCCATTTCCTCGCGAA<br>CCTAGTCGCCCTCAGTAAGTTGGGCGTTGAGAGTGAGCCAGAGCGCAGCGACAAAAAACCCGCGGAGTCACCTCTAAAGG<br>TGCGAAGGCCCTTAGAGAAGTCGAAGACGACGTTGCTCGCTCATACAGAAAGTAACAGTGGTCAAACGAAGCCACCGACT<br>GAAG
MSTRG.8945	Uncharacterized protein LOC106140429	CGCCAGCTCCGACGACGACGGCGACTGGCCCGAGCGCAGCTCCAGCGACGACTCGGACTCGCGTCCGCAGCCGCATCACG<br>CGCATCACGCCGCCAAGCGGAAAAAACTAACGAAGGTGAAGCGAAGCAGTCAAATAGACAGCAAAGACAAGGAAAAAG<br>AGGTATCGGTGAAGGAGGAGGCGCGCGAGGACGAGGAGACGTGCCGCGTGTGCAAGCTGCGGCTCGAGGCCAACCGCAA<br>GTACACGCACGAGAGGTTCCTCGTCTGCGCCACATGTAATTCTAAACAACACGCGGCATGCGTGCAACTGGCCGCCGCTA<br>GCGCGCGCCGCGCCCGCGAGTATGCGTGGCAGTGCCAGAGCTGCAAGGTGTGCGGCGTGTGTGGTCGACCCGCTGTTCCC<br>TCAGCCTCCGCGGACACCCCTCTCGTGTGCTGCGAACTGTGCGATCGTTCTTACCACGCGACCTGCATCGGCCGCGACGCG<br>CCGCCCTCAGG
MSTRG.8946	Phosphoglucomutase-2	TGACTCGATTGACATTCATCTTCTAAACTTTTTCTTAGTATATTTTATTGTTTGTGTTTGCGACGAGTTGTTGTCATTTTGTGT<br>TTTCATTGATTGCTTCACCAAAACTCTGATAATTAATTTATTTATTGATCGGAATATTTTTTCTTTCTCCTGTTTTTTATTTAC<br>ACTAAATTTATCGTTAAACTTTAATTCTATATAATACGAGTTCGATATTTTAATGAAACTTTTAAGTGCACATTGTTAATAT<br>CGAACTTATTGCTGGAAATCTCGTCAAAATGGTCACATGCCAAAGTGGAGACCATAAATTGGATGTAGCTGTTAATAATTG<br>GCTAAAGCACGACAAAAACCCAAAGACCATCCAAGAAGTAGAGGATGATATAGCCCATGCAAGATGGGACAAGCTAAGG<br>AAGACAATGCTGCGTAGACAGAAATTTGGCACGGCGGGCCTGCGTGGGCGCATGGGCGCTGGCTATATGTGCATGAATGA<br>TGTAGTCATATTGCAGACTGCCCAAGGTCTATGTTCATATGTACAGAAGCAGTGCAATCAGAGCCAACTACGCAATGGCAT<br>TGTGGTTGGATATGATGGGCGTTATAACAGTAAAAGGT
MSTRG.8964	Regulator of G-protein signaling 7	AAAGATTTTAATGGCAGAGATTTGACATTTATCTCGTATTCAAAATGTTAAAATTAAAACAAATAAGTGGTTTTTTCGATTT<br>TTATGAATAAAACATTATTGTTTTTCTAAGAAATAATAAAAAAAAGGTGTATAGTGGTTAATGTGGACTGGTTTTTCGACG<br>CTAGTCATAAAAACGAAAGGGTTTGAAATAATTATTTTTCGAAGAGTAAATCCAAAATATTTCGTTTAAAGTGCTTTTCTT<br>AGAACAAAGTATGATCTAATAAGTTAGAATTATCCTTATGTCGTTTATTAACCAGTAAAAATGTGTTTTTTAAGTGATCTGT<br>TAAATTATTTTCGTTCTAAAGTTCTTTAAGTATTTATTACGAAAATGATCTCGATACAATTAGATGTTTCGTTTTATTCGAAT<br>CTACTTTTAATACCAATAAACCTATGCATTAATTGAGAGACTAGAATATTGATTTGGGCATTGTGCAAAGCTATCATGATT<br>TCGTGTGTTTAAAAAAAATTATGAAGTGTTTTTGTGATTGTTGAACATTTTGGCGGTATTTTCGTTTGCTTTTATGGAGTGTC<br>TAAACTGTTTTTAAATTAAAATGGTTACGATGAACTCTTCGGAGAAGGCGGCACAGAAAGCCTGTCAGCAGGGTCCATCA<br>AGAGACAATTCAAAATATGTCAAAGAAGACAAAGAGAAAGAGAAGGATAGAGACAAAAAGGAGAATGCGACAAGTCTC<br>CCGCCAAGCGTGCAAGCTCTCATGTCCACTATACCCTCGCCGGAGGAGTCTCCCAACTACCTGATCTATAAAAAG
MSTRG.8967	Venom serine carboxypeptidase-like	CTTAGTGTATTTATTGTTTTTATTTGTTCCAAGTATATATGGAAGAACCGTACTAAACAACGATAAAGAAAATATAATTCC<br>ATTAAATGACACGAAAAATAACAAACTTTTATTGGATTTACCTGTATACAATTTGCCAAATGATACTGATATTAATTGCCA<br>ATCGAACGCTGTCGATATATCAAATAACATAGAGGAAAATAATGAACCTTGTGACCCTGTGCCTAAAGTTAATAATGGAA<br>CGGCGTTAATTCTAACACCGTATATAGAGGAAGGTCGGATCGAGGAGGCTCGGAATGCGTGCGCAGTAGATCCTCAAGTA

		TTCCTCGGCATCAGAAGTTATTCGGGTTTCTTCACTGTAAACAAGACATACAACTCGAATCTGTTCTTTTGGTACTTTCCCG<br>TGGAGAATAAAGCAGTGAATGAGACACCCTGGATAGTTTGGTTGCAAGGCGGTCCCGGAGCTTCGAGCATGACGGGGCTT<br>TTTGACGAGATCGGGCCCTTCAAAGTGGTCGACGGGACTTTGATACAGAATCCGTATACATGGCTTCAAAATCATTCTCTC<br>GTTTTCATCGAGAATCCGGTGGGCACTGGATATAGCTTCACTG
MSTRG.8972	Retrotransposable element	ACCTGGAAAACAATAACCAAACCAATGTCAGCCTCTTAGAAGCGGCGCCTTAAAGCCAAAGCGAAGGCGGTGGCACAAC<br>GTGCATTGGCAGCAGATGCTGCAAGGCAGATCGCAGGACCGAGTTCTCGCCCAGAGGAAACCAAACCAGTAGGAGAGGT<br>AACAATGGGCGGAACCTCGGCACCTGCGTCTGTCGGTGCGAGTGCTATGCCTTCCACAGACATACTCAAGTGCAACATGA<br>AACCAAGAGGTCGAAAGAAATCAGGTAACAACCAACAAAAACCAGAGCCGAGGGCGCAAAAGCGTGCACGTCCAGAAG<br>ATTCTGTAACACCGACTAGGGTGGGTAAAAAACCCAAAACTAACAAACCCCGAATGGTGAGAGGAACCGCTGCGTCGTAC<br>GCTGAAGCGGCCGCAACCTTCATCGCTAACGAGCTCTGCGTTGCTGTCATGTTGGAACCCTTCTCTGACATGACGCAAGAG<br>CAAGCTGAAGGTATTCGGCAAGAAATAGAAGGCAAGCTTCGGGATGAGCTCATGGTGGATATAAGGTTCCGAGGCAGAG<br>CACATTTTGCGGATGGCGTCCTCGAACCTGGTGCGAGGATGCTTTCACGCTAGCCTGGCTTAATCGGACTGTTCCAAACAT<br>GGCCTCACCGGTAGCTAATTCAAAATTAGTTGTCAGGCCACAATCAGCTATCCCTTAGAAAGTACCGTGTCTGCTTTTCAT<br>ACCAGGTCATACTGGCGATTCAGCTGCACTACGGAAGCTTTTGGCACGGCAGAACCGCAATTTGAACATCAACTCCTGGA<br>CACTGACATACGAGAGGGCCAGATCAGATCCGTTAGGAACCTGTCTGTTGTTCCAGGTGCCGGAAACGGATGCTCATCTGT<br>TAAAGGCCCAGGACAGGAAATTATATTACCTCATGGGAAACATTTATATTCGATTTCTGGATAAAGATGCTTCCCCACAAC<br>CCAATACAAACTCCACGCAACAACCGGAGGAGGCGCGAGCCTTGACGCGGAGCGCGACACCTAAGCCGACATTGTCGAT<br>GAAGGAAGCTGCGACCGTGCTTGGGCACGCGAAACCCCCGATTGCGATGGAGGTGCAAAATTCGCCTTCTCCGAGATCTA<br>TCTCGGATGAAGAGTGCTTTCCGGAAGGGGGGAGGGGAGAGTGAAACCAGCGATGGTGTCCTCTATTCCTCCCCTCTCCG<br>GGAAAACTAAAATCATACAGACCAA
MSTRG.8973	Uncharacterized protein LOC106098628	GTCTGAATGTAGTGTGTGTTTCGTAAAATATTTTTTTTCATTTATTTAAAAAAAAGGACAACCTTAACACGTCATCGCCGTG<br>TACTTGTTTTGAACATGCCACGAAAGAAGAATAACAATGCCACGACAGAAGCAAGAAAAAAAGCAAGGCGTGAATATGA<br>AAAGAGGAGACGTGCCAAAATAAAAGCAGATCCCGTTAAATACGAGGAAGCAAAAGAAAAAGAACGAGCACGGCGTCA<br>ACAAAGAAAGGAGCAAAAGAAAATACTATCAATAAAAGATTTAACACATCGTGATCAGAAGGCGCAACGAGAAAAATGG<br>CGTGAACGTACTAGAAAAAGTCGAGCATTAAAAAAATGCATAGAACGTGGGATGCAACTAATCAGGGAGAACACTCCTC<br>CGTCTGATTTGGAAATTAATGAAGCCCTGACGCCAGATCCAAGCGTCTCTTCACCAGTAAAACTGCGCGATACAACACCG<br>GAGGTTAGAAGAGACGATACACCTACAATATCACAACGAGGTGAAACTCCATCCTCCATCAGTTCTCGAGGCAGCGTAAT<br>AAGTAGACAAAGGCGCCAAGCGTTAAAAGAACACAAACAACGCAGGAAACTAACCGATGTTATAATTATGAATCAAAAA<br>GCAAAAATAAAAGTACAGCAGAAAGCAATACATACTCTTCAAGTTAAAATGAACAGGCTGATAAAGCAGGTTTCTCAAAT<br>ACCGATCGAAGCCAGGAAAAGACAAAGAGTTTTGAAAGATATTTGGTGTCGTCGTAAAATTTCAGTTCAAAAGTTTCTAG<br>AAAGTGAAGAAAATTCTATGATAAATCCTGGAGTGAAAGATACAATTACGCGGCATAAAATGAAGAAACAGAAAAGGTA<br>TATTACTGACACTATGGAAAATCTACAAAAAAAGTACAATGTCGTATCAGACCCAAAATTGCGTGTAAGTTATGCAAGTTT<br>TTGCCGTTTGCGTCCATTCTGGATTGTTAAGGCAAAATTAACTGATCGCGATACATGCTTGTGTGTACTTCACGACAATTTT<br>GATATGATGCTAAACAGATTAAAATCATTAAAAATCATACAAAATGGAGACACCGTAATAGAAAGTGTGGTATGTAATAC<br>TAATTCATATAATTGCATGAGTCGTAAATGCTCTCAGTGTCTTAACAAAATTCCTAAGTTTATGGCGTTCGAAAACGAAGG<br>CATTTCTTACCATCAATGGGTGAGCGAAAATGAAGAACGGGATATAAAAGGTAGTATCAAATCGATCAGAAGAATAATTA<br>AATGTAGACTTATAACTAACAAAAAGGGGCTAGTTGAAGCTTTTCTGGAAATTCTGCCAAGGTTTCTCCTGCACACTTTTA<br>ATATACGTAATCAATATCAAGCAATTAATCATATCAAGACTAATTTAGTTACAGGGGAAATTTTGGTAAGAATTGATTTTT<br>CTGAAAATTACGCATGCAAATATTTCTCAGAAATTCAATCTGTACATTTTGGAGCATCACGTCAGCAGGTGTCAATTCACA

		CAGGGGTTTTATATTACTTTTCGAATGTCACTGGACAAGTGGATACCATATCCTTTTGTTCTGTTTCTGCCTGCCTGCGTCAT<br>GATGCGGCCGCAGTACATGCTCATTTGAAGACAGCCTTAACAGCGTTAACGCATTTGATTGCACATGAAAATATTAAGGTG<br>CTACATATTGTTAGCGATGGCCCAAGCAGTCAATACAAAAACAAGAGCAATTTCTATCTTTTCACTCAACACCTTGTCCGT<br>ATATTGAATATTTCTGCAGCAACATGGAATTTTACTGAAACAGCACACGGTAAAGGTCCTGCAGATGGTGTCGGTGCGGCT<br>TTGAAAGGAGCTGCTAATAACCATGTCCTTAAAGGTAATGATATACCTGACTATAAAACTTTTGTCCAAGTTGTCAAAACC<br>AACTGCAACGTTTTGGTATATGAAGTAAGTGAGCAAGACATTAAAGAATCTGAAGCTTTGCTTCGAAATTCTGTTAAGATT<br>TTAAGTATACCAGGCACAAAGAATATGCATCAGTTAACATGGAATACTGAAGAACCCACAATGCTCAATATAAGGGATCT<br>TTCTTGTGCCTGCTGTAAAATAAAAGATAAATGTCCTCATTATCCCTGTTCAAGACGTCCGATAGTACGATTGACCATTGA<br>ACGTGATGCAGGATCAAATCTATACACGACTAATCCTAATCTGATTGAACCTATAATCGGTAACAATTCTTCTGAAGCTAA<br>TAAACGTAATTTGCAAGAAGGGACGTGGGTACTAGCCAAGTTTTCAGGAAAAAAGAATGTAGTTTCCCTTTTTGTAGGTCA<br>AATTGTTAGTTTAAATAACGACAAACCTGATGCACCTTACTATACCAAATTTACTAAGAAGAAGTATCATAACGTGATATC<br>AGGAGACGTAGTCTTTACTTGGAATAACAATGATTGTGCAGATATTGGCCCTGAAGACATTGTTTGTGTGTTGCAAGAACC<br>CACTCCCGGTAGGCGAGGAGAACTAATTTTCAAAATCGATTCCGATTTATTTAATCATCCCATTATGTAAAAGGTTTGAGG<br>CTTTTTACATAATTTGCAGGTGCATTGCAGTGATGATAGAGTATTGTACTACTACTTTTTATGTAAATGTACGATTTTACTT<br>CTTTGGTGAAAAAAAATCAATCCTTTAATTTTGTTGATTTTAAGTTTTTTTTTTCAGATGTTTAATAAGTGATATTGTGTTAT<br>TTTTTTTAGAAGTTTAACTTTCTTTCTAGAAGATTGACTTTTTAATTGTGTTC
MSTRG.8974	Rho GTPase activating protein 15	NNCACGGTGAAAAGTCCCATAGTTTACACTTATGCCAATTTATTTTCAATGCTTTTTCGCTAACAATATCGCCAAAAGAATT<br>TATGTTATATTGCACATATCTGTCTAAGGTGTTGAAGACATTCGTATTATAACTGCTTGGTATCGATTTAGCTAGATATATA<br>GCTATGGATAAGAACTCCATTTAATTTGATATAACATTTCAATTCTACAGAGACACAACTACTAACAGATCTTAAATTAGA<br>ACGATGTAGCAAGCACGACAACTTTAACTTATCATCGATGACGATAACATATCTACTTATCTTTCTCACACACAAAAATCA<br>TCATACAGTTCTCACATGTTGCATTCGACAGTTTGAATCGTGCACGTTTTGCATCAAAACATTCAGTGTCAATGCGATACA<br>GTGATATATTGTAATATTAAATCTAAAATTTGTGCAAAATTAAAAGGACTCCCTTGAAAGTCGGTGGCAGCAGCAATGAA<br>GATCTCAGCGACTCCACGGAAATATCCATTCAGAAGACACAAGTGAAGAGTAGATTGAAGAAATTCTTCGCTAAACGGCC<br>AGCTATGGAGGTCCTTGTCAAGAAAGGCATCTATAAAGGTTGGGATAGCTATTAAACTGGTGTGTGTTGCTTTAGCTATTT<br>ATAATGCACCATGGTTGGAGTGTATTTAATATATTAGAATAACATAGAATGGTTGGTGTACTCATATTATTTGATTTAAGA<br>ATAAACCTGTCTTTTATGTAGATATAAATGGCTTTTTCATCAGAGGTATACTAAAATTTGACGGCGTAGTGTAGTGTAGTG<br>CAATTATGATTTGTCAGTCTTAGCTACATTCTGCTTTTACTAACGACTACACAAAACCTGGAGAATCAATGAGAGAAGATA<br>CCTGGATATATAATTTAATGGTGTTTATTTGGAAATAACATTGGTGTGGTTTTATATCAATAATGCCAGAAGATTATAAAT<br>AGTTATATAATTTTATTTGGAAATATGGTGGAATACAAAAGTTAGTCATATATTACAAGGAATGTAGCTTGTTCAGATTAC<br>AGAGAAAGATAGATAGATAAGAGGAAATATAGATTTTGATCAAATCTAGAAATTGAAAAATATTTCGTGTTTATGAAGAA<br>AAACCGGGAACGCCAATCTCCAAAAACAGTGCATAGAATTAAGATGAATTGCTCATGAATGTTAAAAAGGATAAGGTTTA<br>TAATTCTCTTAACCCCTTGTTTCCTGTATATTAACAAGAAATGTCAATTCACTAATAGAAAATGCCAGGAAATTATAAATT<br>ATAATAATAATGTACAATGTAGATCTACAAACAATTTTGTGCGAAGTATGCTTCAGTGCTTCATTGCTATCGATGCAGAAG<br>AATCTGGAAAATCCTAAGACAATATCGAGAATCGCGATGTTGCAATATTGCTGGCTTTTGTAATTTTGCTTTTTTTTATTTT<br>AATGTATGTAAAATATTTTTTCTTTAATTTATTTATAGTGTTGTTTTATTTTTTTTATAACCTCTATCAGTCGTGAGTCGCTTA<br>ACGGTACGTGTACTATATGGTGTGAAAGATGGTCCATCTTTACAATAAGGTCATTTTGGCCAGAGATCGATGATCAAGGGC<br>TTTAGACTAAACCAAATGTTGATCAACGACCTTGGATACATGGTTGGAAATCAGATCAGGTTGATTTTTCACCATATAGTA<br>CTCGTTTTATTGCATAAAAATTTAACAACCGCGTAGCAAATGGGGAGATCTGTAGGTATACTCGCCCCACGTTAAAAAAAT<br>ATTACGTGTCGGATACAACCTGTCCCCATCCTAAGGGGTAGAGTCTTCGCCTCGTTCACGAAATTCAGTATCTATGTTGGA

MSTRG.8976	Uncharacterized protein LOC106142918	ATATTTAAAAGAGTCTGACGACTGAAAATTTCGACTTATTTTATTTTTATTTACGGTGGATGGTTTGTATATTTTGTAAATT<br>ATTTATTTATGTTTTTATTTAAATTTTATTTTAATATTTATACACTCTGTCACATATATTATAATTTTAACAAAGTATTAAAT<br>TAAAGTATATATTAATAGTTAGGTATATTGTGTCTTTTGTACATTGAGTTTTCTATTGTAAAACTAGTTTGGCATGCGTTTTG<br>GAGTGGTGTATGAGTGTGTTTATTTTGTTTAAACTAATGTCTCAATAAGATTGAGTAACAATAATGTTACTCAATTTTATTA<br>TGAGAATTATAAAGACGTGTGGACATGTCTGAATTATATTCATATCAAATATCAAAAATATTTTTTTCTCAAACATGCTTG<br>GAAATGTGAGCATTATTTTAACAATCTTCTTCGAGATAAATCAAAATTGCCGTACTGGGCAAGATACATGCGAGCAGCGG<br>CCGGCAAAAGTTGTATTTAAATCCATATCTGTCGTGTTTTGCGGCTAGATAAATTACTATTTAAACTCATATTTGTCCTGTA<br>ATTTAAAAATGAAATGACCTTTTACTTTGAAACATGGCTACCAAGGACGTAACTAATACAAAGTTTTATTTAAATTTCGAA<br>CTGGCTTGCAAATAGAAAGCTGTTTATTGAAAAAAAAAACAAAGGAACATTATGGCGCGTGAAAAATTATTATTGTTCGT<br>TATTCGTCATTAAACTTAAAAAGTAAAATTGTGTTTTTGATTTTCGCGCATTGTTTGAGAAAAGTACTATACAAGCCTTGGC<br>CACAACTAGGCATTGATGAACTCACTTATGTGTGCCTCGGCTACGCCTCGGTCCACATTTGTACGTTCGTTCATCAATGCCT<br>CAGTTGCTGGCCGCTGCAGTAATGTACTATTAACGGGTATCGAAAAAGGGTCTCCACAATTTTGATCTCATTATCGTCATA<br>TCAGCCTTTAGCTGTTCACTGCTGAATATAGGCCTCTTTTATTGGAGGGTTTTGCCAGTAGTTGCCACGCTTGGCAGGCGGA<br>TTGGCAACCGCAGTTAGGCTTTGGTGGTGTTTTAATAATTAAGTTGAGATAATTCTAAGGTTGACCCTTTTAAACCAATAA<br>AATATTGGTCTATGAAGTTAATTTATTATTAAACCAACCGTATGATAAAAGTCGAGTCACGAATCGTAAAAAATGAGAATT<br>CATCCTGATTGATTCGTGATTTTTCACGATTTACGAGAATTTGTCATATTTTAATATCGCTATACGCTAGTTGTACTATGTTT<br>GTGGATAAAACAATAAATTTTTTTAATGGGTGATAATGGAATGGTGGGGTTACCATACCCGCGCTATCGCTATGCACCGGC<br>GGGGCACCAGTGAAGGATGATAGGTGAGGATGTAGCAGGTCAGTTAGCCCATCGTGACGAATTCAACAAAAATTGTTCAC<br>GATGGTTTCCAGGGGGAACCATGTGGACCTCCAAGTAGTTTCGACCTAATAGGGCATATTGCTGACAATGGGGCTGTCAA<br>ACTCCATTGCCAACAATCCCTTTCCCCCCTAAAATAATCAATTACCTTATCTTACATCTCTATTATTGAACATAGGCCTTCC<br>CATAAAGGTAGGATTCGAACTTATTGCCACATAGGCTTGGTATTGTTCTGTTAAGAACTCTCTTCCTCTTACTCTACTCCTG<br>TATCTATTACATTGAATGCAATACCTCCTACTTTATGTTCTTTGTCACCCATTTCTCATCACCAATAATTTTCCAAGGCCCTT<br>TATAAATGCACATTTTAGGCATATCAGAGGGCCTACCATGAAATGTTTTCCGAAATTTCGGATCCGAACGAAACAAAAATT<br>TTATGCTAAAATTACATGATACGTACCGTTTAAAAATATTAAAAATGTTAAAATATCGTTCCTTTGAACTTTTATGGTAGGA<br>TTTATGACAAGCGAAATGTTAAGCTCCATTTACGGGTCGATTTCGGTACGAACAAAAACACGAAATTGAGCCCGAAATTTC<br>GAAATTTCATTTCATGGTAGACCCCCTGTTTTAATAATTAGAAGAACTCCCTACCCTAGGGTGACCAGACAAATTTTTGCT<br>AACCCATTGCATGTTCCAAGCATTAAAACAGCTCAGGATTTAAAACGCTGTTTGTTCTGGAGACATACCTTGAAAGGTGGA<br>ATAACATTCCAGTCTTACTGCCAATGGGGAAACGTTGGTAGGGACAAATGGAACTGATCTATATTTTTAAGGTGTAGTCGT<br>CTCTTGTAAGATATTGTCAAGATATAAGATCTACTGATAAGTAGTCAACGCAATATATTTTTACTCCGTGATCCGGAGATA<br>AACAATAGTTATCCGACCTTGTCGATTCGTTATAATACACGCTTTGTTGTAATTCACGATTTGTTGATGTGTGAGTGAAGAT<br>TCTCTCTAAATCTTCTAAAAACCTACATTAATTATCACAATGTTCTGTCACTTTCATTCAAATTAAGAATTAAGCATTTTTTT<br>AATTTATTGTAAAATCGTATATATATTGTGGTGGGTTGAGCTGACGTCATTATACAATTATAAAGTACAAAGCGTGCTCGA<br>GTTTGTTGTTGCAATAAATTATTAAATTTAATATAAATTGGGACTTCGTAAAGTGACGAATCAGTGGAATCGATCAAGATG<br>TCTCGTGAACTAATCGTATTATATATCGTATTGTTAGTTGCTGTCTCAGTTCACGGTAGACCTAGGAGCTACGTCTACAAAA<br>ATGCAACTATGCCAGAAAATTGCCAGAGGAATTATTGCTTCGACAAGAGTGATGAATATCCTTCTGCAGATGTAATAAAT<br>AGCATAGTGAACGATTTCAACATCACTGTACATAGTGCATACTTTAAACAACGCATAGGCTTTTGTAGCAGTGAAAATTCT<br>GATTGCGAATTAGATTCACACAACGAACCCATATATGAAATAATAGACGAGGCCGGAAATATTCGATATGTTGTACAAGC<br>AGAGAAGTTGAAACAACTCATCAATATTATTGAATGCATGTGAGTATTATATTAAATTTAGTGAAATTTTAAATACACAAT<br>CGGGATATACAGCATACGAGTACAAAAATACAC

MSTRG.8<br>981	G-protein coupled<br>receptor moody<br>isoform X1	CTTATTATTATTACATAAAACATGTTTATTGTTCCAGATTTCAAAATGGCCACATTTGACAGTATTATGAACAACACCAACG<br>ACGTAGATCCAGTAATGGGACCGAGTGAACTATCAAAATTCTCGCCAGGTCTCCTAACGTTTGCTGCTATAGTGACCGGCT<br>TCATCATGATGATTGGCCTCTTTGGTAACCTGCTCACTGTGGTCGCTCTTCTCAAATGTCCAAAAGTGCGGAATGTTGCCGC<br>GGCCTTTATAATCAGTCTTTGCATAGCGGACTTCCTATTCTGTGCAATGGTGTTACCGTTCGCCATATCAGGCTTCTGGACT<br>CGAACCTGGTCTCATGGGGGTGTGCTGTGCAAGCTGGTACCGTTCTTGCGGTACGGAAACGTTGGAGTATCTCTCCTGAGC<br>ATTGCCCTCATAACCTTGAATAGGTA
MSTRG.9<br>003	Prolow-density<br>lipoprotein<br>receptor-related<br>protein 1-like	GGACGAACCCTGCGACCACCGGTGCAGGAACACAGTGGGGAGCTTCGTGTGCTCGTGTGCGGACGGGTACCGCCTGCTGG<br>ACGACGGCGCCAGCTGCGCGCCCGTGTCGCCGGTGCCGGCGTCGCTGATCTTCACTAACCGGTACTACATCCGGCGCACGT<br>GGCTGCGGGCGGGCGCGGAGGCGACGTCGTTGCTGGTGCGCAATCTCACCAACGCCGTAGCCCTGGACCTACACTGGGCC<br>GCCGGCTGTCTCTACTGGAGC
MSTRG.9<br>030	Toll-like protein	CCGGCTCGCCGCTGCGCGCCCAGCACGTGGCCGGCCTGCGCGCGCTCGCCAGCTTGCGGCTCCTCGACGCCGCCGCCCAA<br>ACCACTGTGCCGTACGAAGCGCTGCAAGTATTGCCCTCCCTCACGGACTTCAGACTGCGAGGGGCTAACTTGACCCTCAGC<br>GAGGAGGGCTTCCAGTCCGGTGGCTCGCTGCAGCACGTGGAGCTGTCCAGTGACAATATCCGTGAACTGCCGGCCGGCGG<br>GTTCCGAGGGCTGGACAAGCTACCACAGCTCCACCTGTGGGATAATCTTATTGAACAACTCCATCCTGATGCGTTTCAAG<br>TCTGCCACTACCACTCCTACTGCACCGACCGGCTCAACCCCAACCTCTACGAGGACGGGAAGGTGTGCGTGTCTCTACTGG<br>GCACGTGGTCGGGACGCGGCGTGGAAGTGTGGGGCAAGGACAGCTCACTGCTGCAGGTCATAGTCTCCCTGCAGGGGCTC<br>ATACTCAACGCCGAGCCGTATTTCAACGAGGCCGGCTATGAGAAACAAAAGGGTACGCAGCAGGGCAAAGAGAACTCGC<br>GTATGTACAACGAAATGGTGCTCCTAAAACTGGTGCAGAGTATGACTAAGATGGTGATGAATCCACCGGAGCCGTTCCGC<br>ACCGAGATCCTCCAGCACATGCGGCAGCAGGCGGGCGCCCTCTGCCGGCGGCTCGAGGCACTAGTAGCGGAGCCTGCGCC<br>CGCGCCGCCCGACTACCCTCTCCTGCCTGCTTCGCGCGGTTTCTGCCTCACGCTTCGCTCCTCTCTCGAGTCTTTCCGCAAC<br>GCGCTTCGCCGCAACGATATCCACGTACCTCCATCCACCTTATAGCCGACCGAGTACCACCCCATAGATAGCACTAGTATT<br>TAATGTTGTTTTCGCGCCCATCGGCGTATATAAATCGAATCACGTATCACATACACCGTCACCCGACAGTCGAGCTAGTTT
MSTRG.9<br>056	E2/E3 hybrid<br>ubiquitin-protein<br>ligase UBE2O<br>isoform X1	CGGTTCCACACGCGAGACGTCTCTTTACAAATTATAAAAAAAATAAATCAATATTTGTAGCTATAGTATTTATTGTGATGT<br>ACTTTTCGACTATAGGAATATTATAGATGTTCGAGCCGAGCGTTATATCTAGTTATTATTTTCTACATTTCTTTTTTATTCAC<br>GATTTTAAATTGAAATTAATATTTCGATTTGTCATCGAGTATTATCGTGACGAGTGCATGGAAAACGTATTTGTTATTCATA<br>TTGTTAGCCAATATGAAAATAACCGGTTTGAATTATTGCTAAACCAAATTCTAAATGAAATGTTCGATCTTATGCGAATGA<br>TAGATTTATTAATTAATGAATAGATAATTAATAATATTTATTTATTGAAAAGTAATAGTACTTTTGATGCAACATCCAACGT<br>CGTAATTTTAGGTTTACGAGTACTTATTGAAATTATATTTTAAAATGGAAGCTTTTTGCTACGGTTAGTACTTCGCTACACT<br>CATGTTATTGCTGCAATAAAGACAGTTCATATAGTGTATAAATAGGATGTTCCTGTTAACTTATATTTCTACGGTCGTTGCG<br>GTACATTGTTTGCTAGTTATTTGTATGAGGAGTGTATTATGAATGTCTCATTAGTTTCCCTTTATGTATGTGTAATGTTATGA<br>ATAGATATGTTTTTTTTTTATGTTTGTTTTCTCCGCGATTGTATTATTTAATTGGGAATTGTAATTAAAAATTACAAAACAAA<br>ATCGTCCAGAGTTAAAATCAATACAGAAAATATTTTAGTATAGTAATTTAGTTGCTAATATTATCGGAATAACTTAGTCTC<br>AAAATTGTTTCTCATTACTGCTAAGGTTTTAAATTATTTTGTCATTCGAAATGAATTAGTTCAATAATGGTTTACACTTAGG<br>TACTAAAAAATCGTTATGCTGTGATCGGTCATTCGTATTAAGTACATAATAACACGTAAAAATATTTAGTCCGCAAATTCT<br>TCGTACCACTATAGTTTAGATGAACAACGTTATTATTCCAACAAAAACTAATTAAACTGTTACAACATTACTAGTGCGACT<br>TATCTAAAGCTTTCATACCTATATTGAAATAATTACACGTATCACAGACGAGTTATAGATGTCGCAATTACACCTATGTTA<br>CGTGTCACATTCGTTTCTCTCGTAGTCTCTAATAGCCAACTTGGTTACTAGTTAAACATTTGGGTAACCTTTATATTATACT<br>GAACAGCCAGGGATTTATTTTAAATAACATATTTACCAACATATAGTTGACGTGTTTTGCCCCACTGGCAATTTCTTTTGCT<br>TATTTTTTTACTCTGTATCTCCGACACTCATCTGCGTTACTCTCTCTCTGATTATAATTTTACATTAATACATAACTCTTACCT

		TTTTGTAGTTAATGTTTAATACCAAAATTAGTAATTCTACAGTTACTCTAACTGGCACTACTCCGAGCTCAAGAGTAACTTA<br>AAAACCCATTATTTTCGAAGCTCACTACGTCATCGTTGCAAGAGTTAACTACATTCAAATTTTTTTGGAAATTCATGACAA<br>AATATGTAGAAAAAGGTCTCATCCTGTTCTCCACCATCTCTGTTGCTTTAGAGATGATGTTTTTGAGTATTCACTAACAATA<br>TGACATTTGTAATTACAAAAGTATGGCACGCTTAGGTTTCACATTAGGTACTTGCGGCATCTATCTCTATTTGTCTGTGGCT<br>CAACTAGTGAGATAGTGAATGCAACATTTAGAATCTAATCTGGAAGAACGTTCCTTTTATCCGCTTCTAGTATATCCGACA<br>ATAATATTGAATATTGCGCGCGAAAAGCGCCGCGCGGCTGCACAAATACAATGTAACTTCATGCCAAAGTCAATTGAGCT<br>AAGAAAGGCTTTTGTCGCTTGTGTACGCGAGATAAGTGACAAC
MSTRG.9076	Hypothetical protein ALC62_13520	TGCCTGGTCCATAGAGTATTCCTTCCTCTTCCTCGTGAAACTCTTTTAAAGCAGATTGTTCAGCTCTTGAGTCAATCCTGGC<br>TTGTTTAACGACATCACTTACGCGCCGCTCAGAACGTAAGATTCGCTTTTCATCCCGTTTCTCAACATACGCTTGGGCTTCA<br>CGACCAACAGGGCATCCCATAATCGACATAATTTTTAAAATACCTTGAAAACCTTCATTAAAGATAATAACTGCTAAGTAT<br>CTAGGAATTTCAACAATTTTGGGACCAGAATGAAGATGTTTAGGAGCGAAAGTCCAAATCAAAGAATTCAAAGATTCGTT<br>ATTGTTTTGTGTCTCTGCTCCTAAACAACGAATTAATAATTCGTCTTTCGATAAATCTTCGAAAACCGGTTTGATAGCTTCT<br>TGGACTTGCGGAATTAGAGGTGGTTTATCGTGATGAAATTGGTCCAGTTCATTATTAGCTTCAGCCTTACGCCATTTACACC<br>AACTGTCTTCGCCTTCTGGACAAAATTGATGCTGAGGGTTTTGATCAGAAGAACATTTGTGATAGTACGTTGCCCAAACTT<br>CCTTTCTCATATCTTCAAGCGAATCTGGATGGCGTCGAATTGCTAATCCGTAGAATTTTGTAATTTCTTGGATAACTTTATC<br>AGTAAGTTTACCTGCTCCTTTTCCTCCTATTCCTTTATTGGTTTTATCCTTTTTAACAGCCCGAAGTCGTGTTCCCATCCTCTT<br>TTCTACGTGACCCACACATTCTTTTTTTGTTACCGCAGTATCTCCATACGGTTTAGTATCTAGGATCCCCTTGAAAGTTTTGC<br>TGTCACCGTCGCCAATGTATGTTAGATACTTTACGCCATATTTTTCTACAGACCTCAGAAACATTTTCGTGATGGCACTTAT<br>TTCCATTTTACCGGAACTTCCGGAATGGTTTATGGTGCATTCATCTACATGCGTTTCATACCACATTTCGTATTCTAAAGGA<br>TTTGTNNNNNNNNNNTCCACAAATTACATCCACCACAAAAACTACTCATGACCATGACATCTAAAACTTTCCCAGTGTACT<br>TGCCGATCAACGTAGATACGCCGAGCAGAGATGAAAAGCCTCTTTTCTTCCAAGTACCGTCACCTGACACAGTCAATTCAT<br>CCTGTGGTTTTCCTGATTTTTCATTCATAGTTTTTTCTTGCGTAACAGCGAATGCCAATAAAGTATCACACAGTGAAGATGT<br>AGCAATATGAATGTTTTCCAGTAGTGCAGTATAAGTAGAATTTGCAACACCCGAACACATGTCCATAAAGCTACAGAAAA<br>GATTTACTCCTTCTTGACCAACTCCCAATAATCGCATCGCAAATACAAACCGACGATTAACCTCAAATGCATTTCCGATCA<br>ACGGGCCTGAGTTTATAACTGTCTGTTTTTCACACTCACACGATAAAATAATTTTAAAGCCTAGGCCTCGGAAACTTGATC<br>GCGAAATAGACACGTCACTTTTACAAGTTTTACATATAACTATTTCAGAGATTGCAGCAAATACAGAAACAAACTCCAAA<br>ATGCAATATGCGAAATTCGAAGCGATGGGAACCTCCATGTTCATGTTATTTTTAAGTTTATTGGCCGAAGCTGATGTAAAT<br>ACCGTATCATTTTGGTGGGTAAAACGGTTTCCACTAAACTTTCTTTTTTTACTACGACCAGGATGAACATTTTC
MSTRG.9077	Uncharacterized protein LOC106743225 isoform X1	TGACTGTGTCAGGTGACGGTACTTGGAAGAAAAGAGGCTTTTCATCTCTGCTCGGCGTATCTACGTTGATCGGCAAGTACA<br>CTGGGAAAGTTTTAGATGTCATGGTCATGAGTAGTTTTTGTGGTGGATGTAATTTGTGGANNNNNNNNNNACAAATCCTTT<br>AGAATACGAAATGTGGTATGAAACGCATGTAGATGAATGCACCATAAACCATTCCGGAAGTTCCGGTAAAATGGAAATAA<br>GTGCCATCACGAAAATGTTTCTGAGGTCTGTAGAAAAATATGGCGTAAAGTATCTAACATACATTGGCGACGGTGACAGC<br>AAAACTTTCAAGGGGATCCTAGATACTAAACCGTATGGAGATACTGCGGTAACAAAAAAAGAATGTGTGGGTCACGTAGA<br>AAAGAGGATGGGAACACGACTTCGGGCTGTTAAAAAGGATAAAACCAATAAAGGAATAGGAGGAAAAGGAGCAGGTAA<br>ACTTACTGATAAAGTTATCCAAGAAATTACAAAATTCTACGGATTAGCAATTCGACGCCATCCAGATTCGCTTGAAGATAT<br>GAGAAAGGAAGTTTGGGCAACGTACTATCACAAATGTTCTTCTGATCAAAACCCTCAGCATCAATTTTGTCCAGAAGGCG<br>AAGACAGTTGGTGTAAATGGCGTAAGGCTGAAGCTAATAATGAACTGGACCAATTTCATCACGATAAACCACCTCTAATT<br>CCGCAAGTCCAAGAAGCTATCAAACCGGTTTTCGAAGATTTATCGAAAGACGAATTATTAATTCGTTGTTTAGGAGCAGAG<br>ACACAAAACAATAACGAATCTTTGAATTCTTTGATTTGGACTTTCGCTCCTAAACATCTTCATTCTGGTCCCAAAATTGTTG

MSTRG.9083	Rho-associated protein kinase	AAATTCCTAGATACTTAGCAGTTATTATCTTTAATGAAGGTTTTCAAGGTATTTTAAAAATTATGTCGATTATGGGATGCCC<br>TGTTGGTCGTGAAGCCCAAGCGTATGTTGAGAAACGGGATGAAAAGCGAATCTTACGTTCTGAGCGGCGCGTAAGTGATG<br>TCGTTAAACAAGCCAGGATTGACTCAAGAGCTGAACAATCTGCTTTAAAAGAGTTTCACGAGGAAGAGG<br>CAATTATGTCATTTACAGGCGGCGCAATTGACGCCATAGTGGACCAAATAAAAGGGGGCAAGTTCCATTTAAAGTCCACA<br>GAGCAAAACTTTATGGAACGCAAACCGCGGGAGGACCAGCCTGAAGCGGTAAAGGAAATGCTGCAAATCCTTGGTACAC<br>TACGCAAACGCAGGAGCTTCGCGAGAGAACCGAACACGTGACCAATGTGATCCATATGGAAAAAGATGATAGAAGATAT<br>AAAATTTACTGGATTTTGATAAATAATTATTTACTGGTTTACCAATATTGATAGCGGGTACCACAATTATTTGAACAAATTA<br>AATATATTTACAACTGATTGGTAGAAAGATATAGATAGCATTACATTGCTTACAGAGATAAGAATTGAGATGCTCAGTGC<br>ACTTTGTTGTATGTTTGAGAGCTGAAACTTTCATCTCAACATATACAATAAATAACTGTTGCAGGCTATCATAGGCGTATTT<br>AAGGATATATCTGTCTGGTGGGCCGTGCCCACCTCAAAATTGTCCTGTATTTACTCCAAGATCAAGAATGAAGCATTCAGC<br>TATGGATAAAAACGTGTTTGAGCACTTTTTTAAAGGGTTTGGCCCACTCTGGATAATATTACGTCAATGTAACCTATGCTG<br>GTAACACAAGAAATTCTCGTTCATTCACTCGTTCACTCGTTATTTACTAATAAAACTGCATACTAAAATGGATAAATAAAA<br>AATAATCCTTTTCTTCAGCGCAAATCAACTTATATATAGAATAGTCATTTTGCGATAACTATTTTATAAATGACTCTAAAAA<br>ACCGTCAGTCAGCGGTTTGCTTTACCTTTATAAAAACGAAGTGTGAAACAGATCTAATCTTAGAGATGGAGGTAGTCACAG<br>GGAAATACCGTTGTAATACTGAATTACGGCATTTTATATAAAATATATTTTGCCCTAAATAGGCGCCATCTCCTTTATTGAC<br>TTGGTATCTATCTATATAACTACGAATTGTCATACTAATTATATACATAACAGAATAATGGTCATTTACCTATATTTTTCCT<br>AACTATATTTTGTACTGAATATTAGTTTTGATATAAAAATATCTTATTACGAAATATAGTCTGTGTTTTAATTTTTATCAAA<br>CCAGATTTCTGATGGTAACATTTCTATTTCTAATATTTATAATATATGACAATGTTTCAATAGACTGAAAATTAAAAAAATA<br>TTTTGTATCAAATTAGCATTAATCCTTAATTTACACTACCGGACTACTCACATCGTCCATTATTTATGATAGTATAAACAAT<br>GCGTGTCTGCGGTATTCAGTCCACTGTGCCTAATCTCTTCTAGTGTTCCACTTCGTTACTCTTAGAGTCACCGTCTAACTGT<br>ATAAATTGTAAAACTGTGACTATACAAAGCAAGCATTTATGCAGTCTTAAATGAAGCAGTACATTCATGGACAAATAGTG<br>CCAATTTGACTGTTAATAAGTGATATACTTAGGTAGTAATTAATTATAATGAACGATGACTCATAATGCTAATATATAAAT<br>AAAAAAATATATTAAATTTATGTGGTAGCAAATATAATTAAGCTTCCATCGATAATAGAAGAATATTTCGTTGATAAATCT<br>GTTCTAATTAATTTTAAATCGAAACTTTAAATTTATAATCAAAACATATCATACCTAAATATGACCACTTATAGTGTATTAT<br>CTCCGTACAATATACTATTTCCATATCTACTTTTACTATCGATTTTCCTAAATGATATATATCTAATATAAATTCAATTTAGT<br>ACAATGTAAACCTGTCCTAGGCACTACTGGAATTGATGCTGCCATATCAAAAAAATATTGCCATTAGATAGTTAATTATAT<br>TTATACAATATTTCGAATAATTAGCATTTATATTTATATGAAATGTTGAATAATAGATATTTGTTAAATTTATACATGTACG<br>ATTGTAGGGTATAAAGTTATTATTATAATTATGTAATTTACACCTTTTATTAAATTTCTCGTATATAGCAAAACATTTTGGA<br>AATCTATATTAGATAGGTAATAGGTTACTAAGGCCTAAATATAAACTAAGTATATTTAATTGCGAATAAACATTCTTATAA<br>TTTGTCCTATATTATTTTTGTTAGAAATAAATTCCAACTATTTTTCATAGATGAATATCACAATTATAGAATTTTTTTTTTT<br>ATTATACGTGACTTAATTATGATTAAGATATTATTATCGACCGAATACTCCGTCTTATAAGACTTTAGTAATTATAAGATTC<br>TTATTATAACATATTTGTATAAAAATAAATTTTAAGTTTTGTATAAATATAATTATTTAGAATTTATTACATAGGTTTCCAT<br>GTTACGATGTTATTAATGAAAGAGTTACCTTTGGTGCCATTGATTTGTGTATTCTATGCATCAAAACTCAGCAAAAACGGT<br>CGAGGATTTTTGTAAAGTATTATGTAGGCTCTAAGTAACATTTACGTTTATTTTTAAATAAATGATTTATATCAAAGTATAT<br>TTACTGGGTTATTATTTACTGT
MSTRG.9089	UDP-glycosyltransferase 40D3	AGATTCTATTATTTGGTTTCTAAAGTAAACATTTTTTAAAGTAACTATAAAGGTTAAGTTCTTCCAGTTTGTCAGCTGTACT<br>GCATTGTCCATATATCTGGCTGTCTTCATTGAATCCTCATTGGATGATCCTCGAACTAATTGATGACGTGACGAGCCCAGC<br>ATACGTGCCAAGCTATAACAGTCTTACTGCACCCCCATTTACGTTCCAACAACGTGTGACCCAACTGTGGTATCAAATTCG<br>AGTTTATTACTTCAAACACTTTCAATATTCAATCGAAGAGCAAAAATTATACAGCCTACTGATTCCTTTCATGAGCAAGCG

		AGGAAGAAGTACTCCTGTACCAGCGTTAGAAGATA
MSTRG.909	Alkaline nuclease	AAATGTACTAATACAACGATAAAAACGCTGAACAAGTTATTTATTAACATCTCATTAAATAAAACTATGTATAACACTATA<br>ATTTGTGCATTTTATTTACATCTGTACAACTTACAATTTTTTTTAACTAAAACAGAATTTTACATTTGTTATATTTTTCAAT<br>GGGTTTGCAATTCTTAACATAACGTCGATAAATTTTTGTACTTGAGGCACACTTAATCTCAATAATGTGATCCTCACAAATC<br>CGGTGAACCTGCTATCATTGGATACTCTTCGGACAGCATGAGCCCATACTTTGAAATTTACTTGCGTAACTTATACTGACA<br>GGTTTTCTCACTTAATCTTTTAATAACCTACCATGTTTCATGGCCAATGGAATCTGGGCTATAAGAGTCCGATCACTCATTT<br>TACAACGNNN
MSTRG.9093	Myosin-Va	GATCGCGGACGCGGGCCTAGTGCTACGTATGCAGAACAGAATTTCGGCGCTGCAGAGCGAACTCTCCAGGACGACGAAGC<br>GCGCCGCTGATTTAGAGGAGCGACTTATGAATCGCATGGCGTCACCACCAAGCAACCCCAACATAGACAGGTTTAAAGTG<br>GAAGAGCTGGAAATTGAGAACAAGAAGCTGCGAGAGCACCTCGACAGGCTGCGAGCGGCTGGCGACAGCATGCTCATCT<br>CCAAGGAGGTTATAG
MSTRG.912	Hemocyte protease-1	TATTACAGCAGAGCTCACTGATCCAGCATGGCAAGATGTGATCTCCAAAGGTGGTATGCACATCGGCAGCGGAAGAACAA<br>AGCGGTTTGTTCAACTAAATGAAAGTCAGCCGAATATAGCATACCAAGAATGTGTATTGCCAAATGGCAGAGGAGGTCAT<br>TGTAGACATCTGCACTATTGCGTACAGGACGACTTCAAACAAAATATACTCAAGTTCACTCAGTACCTATGTCTTATTGAA<br>CAAAC
MSTRG.9128	Uncharacterized protein LOC110373625	ACGCATCAGAAATACCAACTCGGTTCGGCGCACGGCGACACATGCGCTTTTCTTTCAACATTGATTTATTGTAATTGTGTG<br>ATACCGTATAACCTGTTTAGATTTGTAAGAAAATCTAATTCCAATATAACACAGAGACGTAAATTACGCTTTGGAAGTAAT<br>AACGGATATGTTTACTTCGCAAGAACTAAAGAAATTTCTAGAACAAATATCTGAGAACGCGAAATTCGTAAAGTACGATT<br>TCGTGTCAGCGGAAAAAATTTACGATACAGTTGAGAAGTACGGTAGTAACCGACCAGTCGCAGGAGTACTACAACCAGTAT<br>GTCGGCAAATGGATACAAAAGTTCTCCGACTACACCGCCACCAACTGGATAGTGAGGAATTCATTTCCTAAAGTCAAACG<br>GATGCTGTTCCGCAAGGTGTTCACTTGCCACCGGTCGGGTTATAACAAGTCAAAGAGCGCCGATAGAACGCTCAATGTCG<br>AGTGTAAAGCAAAAATAGACTTCAAAATGAAGTTTATCAATAGAAACACCATAAGGAACGACGCGTACCTGAAGGATAA<br>TTTGAACATGTGCATCTTTATAAACTTCGTGCACTCCCACAAGGTGAGAGGCCGGGAGTCGTACAACTTATTAAAGAGCTC<br>GTCGGACACCGATCGGATTTTCTACGAGTTGTTCGACGATGGATACACACCGACCATGGCGAAAATGTACCATGAACTAA<br>CACTGATCGATAGACACGGGCACGGGTCCGAGGTTTTAATGGCGGCGAGTATAAACCCATCGATGCACCACGTACATTAC<br>GTACATAAAAAGTGGAAAAGTTTGGACAAAACGATTGGCACACCTGAAGAAGTTATGGCCAAAAAGAAGGAGAGTATCA<br>CCAAACTGGGCGGTCTGATGTGTCACGACAACCCTCAAGTTATCGTCGTCGTAACCCCTCTGATGAGGCGCGTAATCTCAG<br>AGTGTAGTTTGGATATTGTCATCGTGGAGTCAACGGTGACAAATATTCTTGTAACCTTCATGTACGTGCCGAGCAAAGTAG<br>GAGCTTTACCAATGGCTTGCGTGCTGCACACGGAACAAAACGAAGCCAGCTACAGTCAAGCTTTTTTCGCTGTAAAATTAC<br>TATTGGAAGATGAGTGCAGCAAAAGTTTCGAGCCGAAAACGCTGTTCGCGAACAATCTGCAGGTCCAAAGGAGCGCATTG<br>GAAGCTATCTATCCGAGTTCGAGGGTACTGGTCTCCAATTCGTCGATATGCTGCGATGTATGGGAATGGATATGTAAGGAC<br>ACCAATAAGATTGATCGCAAAAAGCGACACATCCTTATGACAATGTTCGACTCAATGTTTCGGGCTGATACTATGGAAACC<br>GCTGAGAAGTATTTCGACGTGTTAAGTAAAGACGAATATGTGAATGCGACACCGACATTGTTGGACTATGTCAATGAAAT<br>ATGGGGCCAATGTAGACATTGGTTTGTTGATCACGAGGAGGTCAAGGATAAACACATCGATTTATCTGTACGTCTGTTTAA<br>AGAACTAATTGTGAGTAAATGTAAGCCTTTCAACATAGGTGCAATGGTCGACGTCGCAACCAATATATTGGACAATTATTT<br>CCGCCAGATATTGCTAGCTTACGTGAAGGAGCAGGATGTTTTCAAGATGTATACAAAGTTTTTTAAAATGTGTAAAAGTGT<br>ATTAGAATTGGACTTTAAAAGAATTGGTGCTAATGAGTACAGGATGCAGCTCGAGTCGCCGGCATCGAAGAGCAAGTACA<br>TAAAATTCAGGACGGACACGTGGTGCTGCGACTGTGTTTACGGGAAGAGGGGACATTTCTGCGAACACCTGACGAGTATC<br>ATCAATTCGATGGAATCAAAGCTCAAAATAGCGGATTTGGGTGAAGATGAGAAATCGTTTATCGTTACAATAGCTGGGGA

		CGACAAATGTGTTAAGGAGCTAAAATTTGAGAATGTAGCTACTGAAATAATTCACGAGCACGGAGAATCGATAGATGACG<br>AATGCGACGACCAATCAATAAAATCATCGAAAGACAATGACGTTTATTGCTATGAAGAGATAAAAATAGAAAAGCTTGAG<br>AATACCGATCCTTTGAATGACGATGATAGGAATGACGAGCAAAACAGTGGACTAACTGCCGACTCCGAAGCTAGATCAGT<br>GGATGGACAATCAGAACTTCATAGAAATTACGGCAAGGCCTTGAGAGCGTTGAACGAAGAGTTTCGAAGGTTGAATAAGT<br>TATTCAAGGAGAATGCTAATACGTCCAACTTGAATACAGTGCAGCGGTTGGCGAGGGAGTTGAGCAAAATAAGGCCGATA<br>GAGAGAGTGAGTTTTGCTAATATGAGTATAGAGTTAAATTCCAATGCAAAAAAAAAGGAAACGTAACGATGATGACGATGA<br>TAATGATGATAATAATGATGATGATGATTAACCGTTTAGTTGATATTTTAAATATTTCAATTAAATGCTGATAACTAATTAT<br>TACTAAAGTTATACAATTTGCATATAAATATGTTCAACTTATTTGATGAATATAATATCATACCGTGCTATAGAGTGCAAT<br>ATCCATATATTGTAGTTACAATATATATATATTGACCCAATAACGGATATATCGGTTATCCAAAATTTCAAAATTCAAAAA<br>TCATTTATTCATGTAGGACATTTTAGCCGCTTATGGACGTCAAATTTTTACATTATAATTTTATTTTTTTCCCTACCGTCCAG<br>TTCCAATCCAATATATAATATAGCAGCCGATCAAAATTAATATAGATGTCCGACCAGTGTGTACAATAACGAAACGGATA<br>ATGACCTTTTTTTCACCATCAATGTTCAGAGAAAATCACCAGAAAGAGAC
MSTRG.9<br>150	Retinol dehydrogenase 14-like	CCGATAATAGCGAACGCCTTTTATCAATTGTCATTACATTATAAACACTGCTAAGATTAAAACGCAATGTTTGTTTAATTTC<br>TGCCGTTCGAATATAATTCAAAAATGTGGTTCCTGTGGGTGCTAGTTACTTTGTTGTTGATAAAAGTGTTGAATAAACTGTC<br>CAATGGAAAATGTTATTCTGATAATGTGATGTGTGGCCAAGTGGTGATAGTGACTGGAGCGAATAGCGGAATCGGGTACG<br>CCACAGCTTTGGAACTGGCCAGACGGGGCGCCAGGGTGATAGCGGCGTGCCGAGACGACGTTAAAGGGAGGACAGCAGT<br>GGAATCCATAATCAGAGCCACAAAAAACAAATCTGTCAAATACATACATCTAGATTTAAGCTCTTTGGCGTCTGTGAGAA<br>AATTCGTCGAAATATTCAAGAGTACGGAGGCTAAATTGGACGTATTGATTAATAATGCAGGCGCCAGTGGTATGGGGAAG<br>GAGAAAACAGCTGATGGCATCGTCAGGGATATGCAGGTGAACCACTTCGGTCCTTTCCTGCTGACTTTATTGCTGGTGCCT<br>ATTTTGAAAAAGTCCGCACCCAGCAGGGTAATTAATGTGTCCTCTGTTTTGCACAAGTTCGGTACCATAGAGAACATCAAT<br>GAGGAAGGCAGGTACGGGTACTTCCAGGTGTTTTGCAATAGTAAACTGTGTAATGTACTATTTAGCAACGAGTTGGCGAG<br>GAGACTGGAAGGCACAGGGGTGGACGTGAACAGCTTGCATCCCGGGCAGGTGAACACTTGTCTGTACAAGTCGACTGCCT<br>TCGAGAAACTGAGAAGTTTGATTTTGTACACTTTCTTCAAGAGTCCAGAGGAAGGCGCGCAGACTTCCCTGTATTTGGCTG<br>TATCGGATGAATGTGACCAAGTGACGGGCAAGTACTTCGCGGACTGCGAGGAGAGCAACATGTCTTTGAAGGCGCAAGAC<br>GAGTTACTGGCAAAGAGGTTGTGGAAGATGTCAGAGGAATTAGTGAGGATAAAGCCGGAGACCATCTGACTTGTTACATG<br>TGATAATTGTTATCATTTTGTTGGGCGAGGTCGCTGACCAATCTCTTTGATAAGATAAATATATCTGTCAAAAACATTTATG<br>TAACTAGATAGATAGAGTTTAATCCGGACTGTATTATTAATACCATTATCAAAATTATGATAATGATAATTCTTAAAAATA<br>GGCAAATTAATATAGATATTAGGAATGAAAAACAAAGCAAACGGAACCGTATCAATAATATCTTAGAGTTGATTTTCCCG<br>TCAACAAAACACATTGTGTTATGAAGCATTAAATTGTTATTGTAAGCTGCTACCTACCGGATGAAAGGTTAATAAAAATAT<br>ATACCATACGTTTGAGTCTAGTATATTTTTAATCCCTCATTTTTCAACGTTACACGAATGCATTCAGCTAAAAAACTGCCCT<br>CTATATTTTCACTCAGACACCCAGCGATTTATAAAAAAAAAAAAACGGGAATTAACAGGATTATTTAGGTAAACTATTATAG<br>TTTATTTGAAATAGCGTTTTGAGCAGATGACACCGACCATAGACATTAATGTAACACTGCCATATTTCCGCTTTTGAGGCA<br>TAATTTTTTTACCTACTCATTGCCAAATAGTATAAAAAAAACATTATCTCGTTATAGTGTGCAGTCGATAAATAAAGACAC<br>GATCATTATTGTGTGTAAAAAACTGACACAATTTGTATGATGTACATTTTCTCTCATGTCAATTATTATTCGCGGTTTAATC<br>AAGATAGCCGTACGCAAATAAACTTTTTAGTCCACAACAAGGAATTACATAGAAGGAATGCATAAAATAAAAACTGTAAA<br>TCATATGGTTGCCTCCTTTTTTCTTATGCAAATGTAATTAGTAAAAAGAGGACGAGAGATACACTATCAGTTGCTATTTTTC<br>ACAATTCATAGCAGGCTAGAGTGTATAGACAATGTTAGATATATCAAATGAATGAATGAATGAATGAGTATACTTTATTTG<br>CATTATACAATATAGGAAATAAACAAGACAATTTACNNNNNNNNNNNNNNNNNATTGTATAAGTACA
MSTRG.9	Bleomycin	AGGTGGCGGTAGACAAGAAATATGTGCCTGATGAAGTGCTACAAGTATTCAACCAAGAGCCGAAAGTTTTGCCAGCATGG

197	hydrolase isoform X2	GACCCCATGGGCACCCTGGCTTGTCCACTCTGCTCCAACGAATAACAAATACCTACTTAAATTTATAAGTATAGACGATGC<br>GTTATATACTTTTTTTTTCAATAATAGACAAGGGACTAACAGCTTTATTCATAAAAACTTAGAGCCTCCAATAGACCAAAT<br>TTTTACAATTAACGTTTTTTGTCACTTTCTTTCAAATTAAGAATCTAACTCGAAAGAAAGAGACAGGAGACAGTATTAAAC<br>ACGTTTTTACGAATACGGAAACTATTTTTCTAATGGTGGTACTTATAATAAATCCAGTGTGATGTAAGCAAATAATATCAT<br>TTCAAAATCCTCTCTTGCCTTTTTACTATTGACTTAGCTAATAGGGACCTTAGATTATGAATATAGAGTAGTAATTGGTAAT<br>CCGAAAGAGAATGAGCTCATTAGATTCATAAAAAAAATGTATAAAACTACGTTGTCTATACAATTGCAATAATTTTTTCTC<br>AAACGAGATTGAACTTATATATTTATAATTTTGAAATGATTAATGTAATGTAATAATATAACTTAGTATGTTAAGAACCTG<br>ACTGAATATTGGCAAAATGTGTTTTAATTAAATGTCGACCTAAAAGGCAACGACAATTGGGAACTATTTTGCTTAGAGCTT<br>TATTGGATTACTAGCGGTCCAATCTTGCTTTGCACGGGTTATTTTTCCCAGTAACATATAGTCTATTTTACTCGCTGATAAT<br>GTAGCTTTCTAAAGGTGAAAGAATTTATCAAATCGTTTAAGTACTGTTTCAAGTTTATCCATTACAAGCAATAAGAGACGG<br>GAATTAACGTCAATCATTTAAATAATTAATATTAAAGATAATTCCCCGTTTTTTTTTTTTTTTATATAAATAGCCGTGCAAC<br>GCGAAGTCTAAGTTTCTTAACTACGCAGTGAGACTTCCTTTTTTTTTTTTAATGAATCAATAATAAAGAAAATCTCATTTT<br>AGTTGTATTTTTTTTTTATCTGAAAAGTTATAGATACTAACAGATTGGCCCCACAGAAACGTTATCAAAATCAGTTAATAGT<br>TTAGTTTATAAATCAAAATAACTGCTTTTATCACAATTGAAGTCGAAATTTTTCTCAAACACAATCTTTATTGTTTTCAGGT<br>CGATATTCTAAAACTCGTTTTGCCAATTTTAAGACATGTCTATATTTTACCGAGCGTGTAACAGTCTATTTTGATACCTGTA<br>ATGATAATCATGTCTTAATCACAAATTTGTCATTACATATAATTATACAAACATTGTATGTTTATAAATTGTGTTCAATATT<br>CAGTTACCAAATAATTATATATCTATAAATATCGATGTGTTCATAGGTCTGTAGATATTCATAAAATATTGCAGTCAGCGT<br>GTTGAAGTACTCTACGATTACACTTTATCCATTCTATTACTTTTACGATGTAATTTATTCGCGTTATGTCCCAAAAATACTTT<br>TTAGTAGCAGTTTATTAAGTTCTTTTTAGTAAATTTCAGTTCAAACTACACTTTACGGTCTTATATTACCTTCTATGATAGAT<br>CTAATTTCGCGACACAGTTTTATACTTTTGCGGATGTCATCGTTTTTCAATAATATTGTAACCAATGTTTTTGTTCCAAATAC<br>ATTATTATTATTATGATAGATCTAATTAAAGAAACGCTGATTAGTAAAGTTTTTTTTTTATATAACTATGCCATCGAAAAAA<br>TAATAGAATGACCCAAGCAAATTTAGACGATTCCCAAATGCATCTAATTAATAGATTTGTTTTCGCTATTACATGCATCGT<br>GTATGCAGCGTTGTTATTTCTCATTTGTGCAGTGTTGAAAATATATCTACCGATAAACTCGTTTGTCGCTTTAGGTGTTACT<br>AGTTCATTATAATTTCCAAGTAACTTTTTCAGTATTTTATGTGTATTTAAAAAAACATCCTTTTAAATTTATTTATCTATGGG<br>TTTGTAATATATGGCAATATCTATATATTTTAGTTACAATATCTGTATATTGGCCCAATAACGAATATTTTAAATATCAGTT<br>ATCCAATATATAGTCGAAATGAGGAAGCCAATCGAAATTAATATACATATCCGACCGGTACGAGCGATATCAACATGGGT<br>GATTAACTTTTTTCCAAAATCAATATCCAATGAAACCAATATATAGATATTGGAGCAATACCAATTTCACCCATGCCTAAT<br>CTATTCCGTATCCCAATAGACAAAAAACCTCTGTCAGCATTCACATAAGTTTATAAAAAAAAAAAATATTTTTTTTATTAC<br>AA
MSTRG.9255	Uncharacterized protein LOC106708587	GTTGTTGTCTATGAAATGACGGAGTATTATTTCCATGTTTTTCCCGATGGCTCTGGGGGAGCACATGGAAGGCCATCCAC<br>GATTGTTAACTGCATATTTTTTTTCTGGAATTATCACTATACATCAACTCTTCCTCTGTCGAAGACCAAAAAGATCTGGGCG<br>GCCGCGTGCTGTTAGAACTACAAAGGCTGTTAATGCTATTAAAGCCAGAATTCGTCGAAACCCCATTAGGAAGCAAAAAA<br>TCTTATCGCGAGAAATGAACATTCCCGCTAGAACTTTGTCGCGTATTATAAAACAAAACCTGAAGCTGGGTGCTTATCGCC<br>GATATACAGGACATGCCCTAAATCAATCTTTACAATAAAAAAGAGAATGGATCGATCAAAACGTCTTCTGTCGCGATACG<br>CAGGTGAAAAGCACAGAAATATCCTCTTTACTGATGAAAACATTTTCATGATTGAAGAACACTACAATAAGCAAAATGAT<br>AAAGTGTACACTCACAGTTCTAAAGAAGCTGCTCAAGTGGTTGGAAAGGTACAACGTGGGCATCATCCTGCGTCAGTGAT<br>GGTTTGATGAGACGTGTCTTATCAAGGAATCACAAAACTACATTTTTGTGAAAAAGGAGTAAAAACTTCGGCCAAAGTGT<br>CAGTCTTGGATCGTATTGTGAAACTTCTCAGCAATACACTTTTTAAAAATATACCCTGGACTTTCCAGCAGGACTCTGCACC<br>TGGTTACAAACTACGAACTACTCAAGCCTAGCTTGAAACCAACGTTCTGGACTTTATAAGAGCTGAAGACTGGCCCTTTTC

		TATCCCAGACCTCAACCCTTTAGACTTCAAATTATAGCCAGTTTTAGAGAGCATGGCCTGCTCTAAACGACACGGCGACAT<br>TGAGTCTTTTAAAAAATCTTTGGAGCGGTCAGTAGCGAAATTTCCCGTTGGAAACAGTGCGTAAATCCATAGCTACGTGGC<br>TAGACAGATTAAAGGCCTGTATAAAAGACAAAGGTGGTCATTTCGAATAGAATATTTTTTGCTAAGACTTTAATAAATATG<br>TAGGTATTAAATTTTAGTAATATTACATAACGTCAAAAAAAAATGCATTTTCACTTTTAACAGAACTTATGACTTATTATTA<br>TATATGAACACTATTCATTTGGTCTTTATTGTTATGATATTATAATTACTTCAATAGATAAATAAATAATAAATACTGGCTA<br>ATATAAGTAAGTATACAATTTGCTTGCGCCAGAGCTTCTTCAGTACAATCTTATAAAGAGAATTGAAAAGAAGAATTTGAG<br>AATACACATCAAAATCCTATACACTTACCATTAAACATAAAGTTGGCCAAAGCTCCACATGGGTATATGGGGATGCCATTT<br>AAATAACTGTAAGGCTCACATTGAGCCGCAGGAGTTTCTGATATTTTACCTAATAATTGATTTTCATCTCTGTAAAAAAGA<br>CTTCATGTCGTTTAATGTCTTAGTAGACAAAAGCGATGGATTGCGACAACTGAGTTCAATAGTCATAATGTTCAAAAAAAT<br>AAATAAAGGAACCTCCACGGCACCTGTATTTAAAAACTGGTTTATTTCGAAGACTTTTACAAGAATGTTACAGTAGAATGC<br>CGAAATCTGTCATCACACATATAAAATGTCATAAAAAGAGCCCATTGCTTTTCTCTTTGTGATAAACAGAACAAAAAAAA<br>ATCTTATTTTCATTTTATAAATAACTACCGACCCGTTAAGTAAACCAATAAAAATAATCGTCAAAATCTGTTTACAATTTTA<br>TTTTTATTTTATTTTTAGGAACACATATTTCAATTCACATGAATTATTACTGAATAATTAATGTGAATTGAAAAACGCCTTC<br>CTATGGAAGTTGCGTAACAAAAATTTAATAATAAAAAAAAAGGAGAACTCACTTTGACTGACTGTAATTTGGCTGCTCGTAT<br>GAACTCAAATCATAGTATAGCGTTAACGGT
MSTRG.9286	Hypothetical protein RR48_00560	GAACTCACAATTGTACGAGAAACGCGACAACGAAGAATATCATGTGAATTACGGTACAGTTTCACTAATGTACGCGAACT<br>GGGCACGCGAATCAATGAGCACTGCTGTAGCGAACTATTAAACTCGCCAGTAAGTCGTCGTTGCGCCCGCGCAGCTGTTG<br>CATCTCGTCGCGCATCTGCGCACGTTCGCGGCTCGACAGCTCCTCTTGTTCCTCGGCGTGTAGACGACGTTTCTCGCTGTAC<br>ATCAACTCACTCTTCAGTTTGCGGATGTCATTTCGAGATCTGCGAACAAATAATTATGACGATATTTTACTGC
MSTRG.9294	Histone deacetylase 8	ATTTTAAGTTAAATATTGTAGAAAATAGTCATGTGTATTTAAAATAGAATTTTTGCTTTTTTTGTCTTACTACCTAATAGCA<br>GCATCAGAAATGTAAAATTTCTTTTGAAGCCAGCTTATTCGGGTGGCTGGAAGTCGTCGAGTTTGCATTTAAGATGATTGG<br>TCAGGACTTTCGAAAACAGTATAACGTATTTGCCTAATTGTAAGTAATATTGACATTATTTATGTGTGCAATATTGCCTATA<br>GTATGTAACTACTTTAAATATAATTTGGTTTTAAAAATATGAACAATTTTTTTTTGATAAGACTTACTTGGAAGTAATAGAA<br>AGGACTAGAAATTAAGAGAAAAATAATCTTTATTATATTAAGTGGATAAGTCAATGCTAATTATTATGGATAAACATAAA<br>GAGTCATGAAAATTTATAATTATTTTGGATCAGCAAATAAAACAAAAAATGTAGAGGTTTCCTTTGCCTAATTTGTTCCCTT<br>AGTCACCACTTATATTAATATGAAAAGGAGTACTAAAACATTGTAACCTCTCTTTCATCATACAGAGAAGATATCAGGAAA<br>ATATTCTTTTACCTCTGTGCATGATGCCAACCACCACACCAGTTAATGGCAACATTTGTAATGCCTAGCAATAAACACTTTG<br>CTGCTGTTATAGAACCACCTGCTAAGTTGGACACCAAATCAAACATGTTAGATACTGGTGGACAGTCATAACCTG
MSTRG.9329	Prophenoloxidase subunit 2	GTAGTTTCATTACAAGGCATCTAAGATATGGAATACCTTCACCCTTGAAAAATTGTAATAGTAAATTATCTCTTAAATATG<br>AATATAAAATCTATCTACTCAATGCGCAAAAGCTTGAATTACAAATATAGTGTTGTCTGTATAGTAATATGATTACCACTT<br>TCTATTGGATAGCCGTCTGATAATTAATACATAATTTTATTTGGCTGGAATATAATATAACTAAATAATAAATTTGTGATCC<br>TGATGGTTCAACGCGACCCTTAGATATTGATACATTGGGCAGCATGGTAGAAGCCAGTATCCTGTCCCCAAACCGGGAACT<br>CTACAGAAGCGTGCACAACAACGGCCACAGCTTCTCTACCTATGTCCATGATCCGAGTCACCGCTATCTTGTACGGATTTA<br>TTAAAATGTGTAATTTGATTATTTTGTAAATGAAGATCGATTCTACGAATTTGTAACACGATCAGTGTCGTGGAGTCATGG<br>CTCGGCTGACCTATTTGTCTATTTACATGCTAATTAATTAACTAGCCTTCGGCTCTATTGGAAATCTAAATCAAGTCGTTCA<br>TCGACAGAATAAAAAAAAATAGTAGCGTTCTCTTCGTTTATCAAGATATTTTATGACAAAACAATAATCTACAAAGTCTAA<br>TATAGATATACCGTTAGCGCAGGGGGGATTACTGTTCCATTATCATCCATTATTAAAAAAAACATGTAAAAAAATCTCTCC<br>ATACAACCACCTCAATATTCACATCTCAGTTGGATGAAATCATCTTTCATTCGTCACTTACTAGAATGCCTATGGGTTTTAG<br>TCCTAATTGCAATTTTGATATGATTGTGCAACTATTTTTTTTTTTATGGGCGAAAATAGTATGGTATCCCCGCCCGCGCTAA

CGGGATACGCCGGCGGAGCACCAGTGAAGGGTGATAGATGATAATGAAAACAGGCCAGTTAGCCCATCATGATGAATTTA
TCAGAAATTAACCATGATAGTTTCCAGAGGGAACCACGAGGAGGTCGACCTGGTTGGGTATATTGTTAGCAATGAGAATT
TTAGTCTCCATTACTAACAATCCCTTTCTCCCCGATTGTGCAACTATTGTATTTCTTGTTGGAAATAAACTACTCCAACCAG
ATAAGACCAAACAAAAGAAATTATAAATTAAAAAAGATAGTTTCAATGGTTTATACTCGTATAATGATTTTATGTGTTATT
ACCATCATATACAGATTGTCCCAGAAGTCGTCGTCAAGTCGAAATTAAGTAACAGGTTAAGTCATAATAGTTATCAAAAA
AAATTAAGTTTTTTTAAAATTAGTCATTTAAAAAGATCGAGAAAATCTTCACATGTGACGGTCTTTTCACTAGTTTCTATAG
ATATGTACTTTTATATTTGTTTTTTGTTTTGTAATCCCCAATACTTCTTTAAACTCTGGCCTAAAATTTAGTAAACTGCAACC
AAAAAATGATTACTTAGTTCCTCTTTAAGTGTCTATATTGAAAAAATTAAAATTTTCACCACCATACAACCCATCACAAAC
TAAAGTTCCATCCCAACCATCTGGATAAGTGGCGTTTTTTATAGCACTCTTCCACGCACAATCAGACTTTGGAAGTGACTTC
CAGTAGCAGTATTTTAGAACTGATACATCATAACCTCTAAGAGGATATTCCTTTTTAAAAGGCCGACAGCACACCTGCCAC
CTCAGTCCTCAAGGGCCGCGGTAGTCACTTACCATCAGGTTGCTGCATGCAGTTATAAAAAAAAAATTAGACAACTTTGGAA
AGTAACTTCAAAGCTCGGCACATTTAATAAGGATTTAAAAATGGTCGACAATTACTTATTTTCTTTCTTATATTGCAGACAT
TTTTTCATACTTCCTTACAAATGATTCTAATGTTCCCGTGGCTTTCCTTAAGGTCGTAAGTTTTAATTGGACTTATTAAACTC
TAATTGGAGTGAAACAAACAGACACACTTTAGGAAGTGTGAGTGAGACAAATAATGATGCTATTAAATTATTAAATTAAA
CATCTTTATTAAATACACAACAATTGTTTAACATCAATTATTTGAAATATAATACAGTGTTCAATCTGCATTCCTTCAACTG
CAATGTAAGCTCCACTTCTTAAAAAAGATCGGGTTGTCTGACAAGAGAATCTTCCGTTAACCAATTCAACTGCTTCCATTC
ATCTTTACCATAGCTCAGCTAAACTTTCAATTGGCTTTAAACAAACTTTTTCTTCATGGTCCCCCAGTAAAAAAATCTAATG
GATTTAAGTTGGGGAAAGTAGTACAATCTTATAGTCAGTCCGACCATAAGATTGTACTAGACCATCCCATCCATCTGCCTG
GGTATTCCTCATCAACATATTCTCTAACTGAGACGGCATACTGTGCTGTTAGAACAATATTTCACTGTGAATCATTTAATAT
TCCAGTCCACATGTTCACTTTAACAAAATACTGTGATCTGTCTTACTCACAAATTACAGTTATCATCCTATTTTTGTACCCC
ATGAAAACTGAAAAAGCAAAAGAAATACTTATTATAATAAATACAAAAATTAAACTATTTATATCTTACAATAAATAAAT
CAGGCCTATCATTTAGTATCATGTTGTTATCAAAATAACACATATTCGGCTCGTGTAAAATCTCATCATAAAGTTAAATTTA
TTATAAAGAATTAATAACAAGGAATTATTAATTGTGTATATCACCTTATTATACAGCATCACAGATCAGTTATTCCCTGCA
GAAAAAAAGATATCATATTTTAGACACCGTTTTGAATGACTAATAATTTCATATCATTTTCTTTTTTTCAGCCCTATCATTT
AATATCATGTGTTAATCATAATAACACATTTCGGCTCGTGTTTTTTGTCATCATAACAATTATGCAAAAAGTGGATACGGA
GGACGTAAATCGATAATTTACAGATACGATATTAACTGACCTAACCAAACATCCTTATCTTACCACTTGTAATTTTAAAGC
TTATCACAAAAGTAAATAGCAAATCGAAGAAAAGGTATCTTATTTAAGTAATAATAGATATGTAATTATCGTATTCATTTC
AGCTTACACAAATACAAAGAACTTTGGTGTAACACGTGAAAAACACATGGAATAAAAGCAACAAATTAACATTAATAGTA
ACCTATACAGGCAACAAAATTATGCTTTACATTAAGTAGATATTGATGAAGATAATTAGCGATAAATACATTTGGATTTAA
AATAATTAATTTCAGAATTATTATTATTATTAGACTTTCTTTACAAATGATAAGTTAAAAATTACCTCAATATCAAAGGAAT
AAAACGTAAAGTAACACATGTCGCTCGAGTTCGCTGGCGGTAAAAGTAGTGTTGCCTAATAATGATATGAATCGATTTATT
AATCGATAATTTTATTAACACAATGTGATTAAAGTTTAATTAATTTATTCTTTTGCGCACATTAAAATACAGGGTGGACTTA
CTGGCATTCTCTAGCAGCCAACTGTTACAATAAATATAAATTGCGGTGCATTGAACTAAATAAATTTATGAATATTATAAA
TAGTTTAATAGATAACACATATACATATAGATAACATTTATACATATAATTTAAAATAAATGAACATTATTATTAATTATT
ATTATTATTATCAATTATCCCAGAATCGGGTAACAGGCTGAGTCATAATAGTTACACCTTGTGACGGTCTTTTCACTCCTCT
GACTACAGATATGTAATCTTTTAACATTTTTATTTGTTTTGTAATTCTAATTACTTCATCTTTAAACTTTAGCCAAAAATTTA
AAGTCAACTACTCCAGGTTGGAAGTAAATGTCACTTTTTTACTCATCCAAAAATTCAAAATGGATACTTAACCTAATTTTG
ACTAGCTATACTGAAAAAAAAATGTCCTAAGTGTGTTTATTAAGTATATATATAATTAAAACCAGTTTCGAACATATTATTC
TTACTTACTTAAAAATGATTCTAATTTACCCTTGGCTTCCCTTAACGCCGTAACTTTTAAAATTCCAGTTGGAATGAGACGC

MSTRG.936	Nucleoporin-like protein 2	TACAAATCATAACAGGCCAAGTTTTTTTATGCAACTTATAGAAATGGCAGTACCATGGACAAAACAGAGTGTACTGTTAC<br>GCACATAATACCTCCATGTAGGGGGAAAGGGATTGTTAGTAATGGAGACTGTGAATCCCATTGCTAGCAATATACCCAAC<br>CAGGTCGACCTCTTTGTGGTTCTCCCTGGGAACTATCATGGTTAATTCCTGATGAATTCATCATGATGGGCTAACTGGCCTG<br>TTTTCATTCTCATCTATCAACCTTCACTGGTGTCACGTCAGCGTATCCCGTTAGTGCAAGCGGGGTTATCATACCATTTTCA<br>CCCATCAAAAAAAAACCTCCATGTGTGACCAAGTTGTGAATAAAAATCCATTCAGCTGCATTTTTATAATGGAATGTAGTA<br>AAGAATGCTGTCGACTTGAATTTTGTTGAATATGTAATAAACAATATTGATTTTAGACCAATATTTTGAAAACAGCTTTTTT<br>AGACAATGCAATCTCTGGCTGCAGTATTATTCAAGTCTAAGAACGATATTTACCATAAAAAGGACGGTTTTAGATGTAACA<br>TTTGAATGAGAATGTGAGTCTATGATGTTTTTAAAATAATTACTGGGTATGCTTACCATTGTACAATTGATAATTAACTGAA<br>ATATAATTAATATTGTAAACTTACCGTGATATGAATATTTCGACCCGTAAACATGTTCAAATCTACAATTCTGACCGTAAC<br>GGCAATAACCTTGTTGAAAATATTTACATACAACCATTATGTTTATTTATATTGTTCTTATATTATGTACTTTGTAAAATTA<br>ATACTCAATACATTTGAATGTTCTCAATTTATTATTTAATATATAAATATTAAACAATTCTAAATTTCAAATTTATTTTTCCA<br>ATCTTAAATTTATTTCAATAAATATATATTAATCACAAACTATTACTAATATATATTAATATTTGAAATAATCGTCTAAAAT<br>CTTTGATACAGAGGCATGAGAAAGTGACATTTGCTCACATGTCAATGTCAAAAGTCAACGGAATGAAAATATGAAGATAC<br>ATAAATACATAAATCCCTCTTCTAATCTAAAAGAAAATTTATTCAAGGGGAAATACCCATGCGCCACATTTATATGCAATG<br>TTTACTTAGAACCAAATGTAAAAATATAACTGCGTAGTTTCGTATTTTGACCGTTCCTTACAACGCGACTACAATAAGAAA<br>TTTGAATTTATTCTATAACATTTATTCAATTTGACGACCTATATTTATTGTTTTTAGACTCGCAAGTGCCGTGCTATCTTATT<br>TTGAATAGTGTAAAACTAAAATACAGTAAACATGTGCAATTGATGGTAGACGGAAAAATTAATGAACTGCACGTTTACGG<br>CCTCCACGCACTACTCGTGAGCATCATTGCTATAGGT
MSTRG.9362	Glucose dehydrogenase	ATAAAACCGTGAGCTTGGACGAACTGGATGCAGTGGTATTGAAGAAGCACGCATGTGCTGCGCCCATCGTGTGTTACATA<br>GATTTTCAATTAATTCAAGATTACAGCTGCAACTTAGTACCTACTTTGGTATTACAAATTTAAGCCATGAGAACTCCATACC<br>GTGGATCTACATTGACAACTATATGGATATTTTTTATTGCCTTAACGCCAACTATTATCCAATCCATTGAGCCAATATTATC<br>TTTCATGAATTTCATTCAAGAAGGTACTATAAATCACGAAAACGAACCACCTGATCAACAAGTACCTCTCTCAGAATACGA<br>CTTTATAGTCGTAGGAGCTGGTACAGCAGGTTGTGTTCTTGCAAATAGATTAACGGAGATTCACGATTGGAAAGTGTTACT<br>TGTAGAAGCTGGAGAAAATGAAAACTACCTTATGGATGTTCCTCTTTTTGCCCAGTATCTGCAAAATACGCCCGCGAACTG<br>G
MSTRG.9374	Phosphatidylethanolamine binding protein isoform X1	TGATTGACAGTTCGATAAGTGTACTGACGATTGTTACACTTTGGTCGCTTCATTTATTTTCTTATGGTAATTCGTCGGTGCC<br>GGTTCATTTTTTAGGTTCCTATTTCTTTCAATTGTAAAAGTTGTTGTATTTCTTGGCTTTTTACACAAACCGTGACTTCCAGT<br>TGGTTAAGGGTGCTAGCGAGAGCCATGTCCACGGTGGCGAAGGCGTTCGAAGCAAACCAAGTGGTGCCCGATGTAGTGTC<br>CAAGGCGCCCTCCGCTTTAGTAACGGTAAGACTGGAATATCTTATATTTTTTGTTTCTTATTCCTTTGTCCAGC
MSTRG.9390	Uncharacterized protein LOC106125039	ATCATATTAATCAGATGGAGGGTGTTTAATTAGCAAATCATTTTGTCTATGCATATTTCTAAAAGCCTATTCTGGACATGTT<br>ACAATTTTTATATAAACTTAATTAAAATATAAGCACATTTTCTCACTAACATATTTATAGTACTGTATATACAAATGAAATA<br>TACATTTCCGTTGTTCTCTTTTTAAATTACAATTTACAGAAAAATCTTTTTTTATATAATAAAAGCTTTTTATTCGCGGGATA<br>TATATTTTGTAAATATACCGCGAATGTAAAAACCGTGAATACAGAAAGCCGAGCTACTTATACAGCGGCAATAGTATTTA<br>AAACATACTTTAGGTATAGTTGTTTTTAAGTCTGATTAAAAGGAATAACATCTGAGTCCTCAGGAATGGCAACACATGGGG<br>GGGGTATCATGGGCGAAATAGTATACTCTATTATGTCCATGGTACTGCTCGTGTCTATAGGCGACGGTTACCACTTTCCAT<br>CAGGTGGGCCGTCAGCTTGTTTGCCATTCTAAGTTGTATAAAAAAAAATCCGCTTTATTAATAAATTTTTTTAATAGATGATA<br>ATGGAATGGTAACCGCGCCTGCGCCAACAGTATACGCTGGCATCATACCTTTCATCCCTCACTAGTGCACCAGTGAGGGAT<br>GATAGGTGAGCATATTGCTAGCAATGGGACTGGTAGTCCGCATTACTAACAGTCCCTTCCCCCTATTTTATATATTTGCTGA<br>CGCGTTTACAAAGTATACCGAATCTTTTTTATAATTATGCACCGTGAATAAAGAAAATTTTAATTCAGTGAATAGTGAAAA

		CGTGAATAAAGAGCTTTTACTGTATAAAAAAAAAAAACGAAAGACGTACACAGAAGGTATAGTGGCAGACTGTAACACTTG<br>TTTAGACGTTTCTAGTGATTGCTTTACACAAAGATGGTATAATTATTTTTGATGTACACATATCGAAAAGGTGGAAGAAAG<br>GTAAACACTAATAGGTATGCCATACCTATTGGTGTTTTCCTTTGAAAAAACCGATCACTGATGGAGAAAAACGGTACTTTT<br>CTGATATGAATTGCATTGCCTATTTTTCCAGTTTTCCAATTTATTTTTTCTCAAAGGTTCAATGTAAATGATTAATTAAAATA<br>GTTTTGTTTAAAGGATATATAACATATCAAATAATAATATCGACATATTAATTAGGACACAAACAAAACACGCAACTGAA<br>GTTATTGAGAGGTGCTTGAAAAACTACACTATTTCAAATACAATGACAGACACACAGATAACTCCCTTACACTATATGTAA<br>ATGATATAGCATACTTACTAAATCATGTTTTTTTTTTAATGGATGATAATTAAATGGTAACCCTGCCGTATGCGGTGCGCTC<br>CGTATACACTGGCAGGACACCAGTGAGGGATGATTGGTGAGGATCAAAAAACCATCATAACGAATTAAATGATATTTATA<br>TTAATGATAGTTTCCAGAGGGAACCGCGTAGAGGTCGACATGATTACCTATATTGATAGCAATGGGGACTAACTTTCCCAT<br>TACTAACAATCCCTTCTCCCAACTAAGTCATGTATCAACCGTTAACTGCTCACTGTTGAACATAACCTCCCCCATAAGGGG<br>AGGTTTGCTGATGAACTTTTGGTGATGTTTTAAGAGGGACGCTACTACCCATGCCTCGCCTTCCCTTATTCGCCTTTTACCG<br>GAAGGGATTGTTATCAATAGAGTTTGACAGCCCCATTGCTAGCAATATGCCCCTATCAGGTCGACCGCCACGTGGTTCCCC<br>CTGGAAACCATCGTGAACAATTCTTGTTGAATTCGTCACGATTGTAAATATACCTGACCTGCTTCATCCTCACCTATCATCC<br>TTCACTGGTGCCTCGCCGGTGTATACCGATAGCGCAAGTGGGATTACCATTACATTATCACCCATTAAAAAAAAAATATTC<br>GCCTTTTACAACACTCACGGGAAAGGAAGGGGTGACCTATTCTATGCTGAAACCTACACAGCTAAATATTATAGTGTGCTT<br>GCTAAATAATAAACACAAATGCCTTAAAAATGTAACAACACACTGCACATTTATATTAAAAAAAAAATTAATTAAAAAAA<br>TATTCAATTTAATGTCTTTGAAATAATTTAATAATTTATACATGGCAATAAAATGTATAGTATAAATAAAAATCAATTGCC<br>GGTTGTATGTACGTGTATTACTTGAAAACGGCCGCAAAATGGGGGCTAATTATTGTTTTTTTTTTATTGTATTTGCATGAAA<br>GAAAATGTTTTAAAATCGTAAAGAAAAGTAAAAAAAAAATCGGTTAATAAATGTCCATACAAAAATGTGAAAGATAGTG<br>AGGATTTGCTCTCGTTTTCTTCTTTCATAGTACAACTGTGCGAAGTCAGGCCGGACAGTTAATTCGAAGCAAAATGTAATT<br>AATATTATTACCTCTTAGATCATATAGATCATTATACAACTTAGAATGGCAAACAAGCTGACGGCCCACCTGATGGAAATT<br>GGTAACCGTCGCCTATAGACATGAGCAGTACCATGGACATAACAGAGTGTACTGTTTCGCCCGTGATACCCCCCATGCATT<br>GCCGTTCCTGAGGACTCAGGTGTTATTCCTCTATACCCTGCAAATTCACTGCCATATTCCACCCTTCAAACCGAAACACAG<br>CCATGCAAACACCACTGCTTCACGGTAGAAACACGGAGAGGTACCCAAGCAAACGACTTTTGTACGAAGTCTCCCTCTGG<br>TATTTTAGTTTTTAAATCAAAAGAACTAGTTTTGCTACAACTTTTCACCATCGTACAACCCGCCATAAACTAAAATTTCATC<br>CCAACCATCTGGACGAGTGGCGGTCCTCCACCGTGCGTTTTTCAAGGCACTTTCTCCCACGCACAACCACTCTTTGGAATC<br>AACTTCCAGCAGCAGTATTTCCGAACCGATACGACAACATAACCTTCAAGAAAAGAGCATATTGCTTCTTAAAAGGCCGG<br>CAGCACACCTGCAATGCCGTGTGTTGTGGGTGACCATGGGCGGCGGTAGTCACTTACCTTTAGGTGAGCCGCATGCTCGTT<br>TGCCCCCTGTGTTATATAAAAAAAAAAAAAAAACTAAAGTCGATATTTTTGTTGGGGCGAAATCTTTAATTATTCTTCTTCTA<br>AAATCGATGTACGAATTAATGGGCAAACAGTGTAATATAAAATTAATTATGCAATATTATTTCGGTACAAAACAATACTG<br>ATAAAATGTAAAATATGCACTCAAATTTGCAAGAGCGATAACAAGCAATGTGTTTGGTACCTAATGTATTATCAATACGTT<br>TTATTTTAATGGATGGTTTTATTTAAGGCATGATAGGTAAAGATGAAAGCAGGTCTGTTAGCCTATCGCGACGAATGTAAC<br>AAGTATTAGTCACAATTTCCAGGGGAAAACGCGTGGTGATCGACCTGACAGGGCTTACTAGCAATGGGGCAATTAGTCCC<br>CAATATTAACAATTCTTTTCTCCCATTATCAATACGTTAGGTAGGTATCCACCACGACGCCGATGGATGTTTACGAATTATT<br>TTTTATAAAATAATATTGTTGATATCAATGTGCTAGATTATTAATAATTTGTATTAATGGTAAAACGAC
MSTRG.9<br>391	Uncharacterized<br>protein<br>LOC106133236	GGAGTTATCTGTGTGTCTGTCATTGTATTTGAAATAGTGTAGTTTTTCAAGCACCTCTCAATAACTTCAGTTGCGTGTTTTGT<br>TTGTGTCCTAATTAATATGTCGATATTATTATTTGATATGTTATATATCCTTTAAACAAAACTATTTTAATTAATCATTTACA<br>TTGAACCTTTGAGAAAAAATAAATTGGAAAACTGGAAAAATAGGCAATGCAATTCATATCAGAAAAGTACCGTTTTTCTC<br>CATCAGTGATCGGTTTTTTCAAAGGAAAACACCAATAGGCGCAGCTCGAGCGCGGCGTGTGAGAGTAGCGCGGTGAAGAG

		CGCGGCGGCGAGCGCGACGAGCTGCGCGCGCACACAGCAGCTGGCGCGCCGCGCACGCCGCCGCCAGCCCCGCGTGC<br>CGCGCGCCCAGCGTCGCCGCCCACGGCCCGCGCCACCCCGCGCCGCCGATGCACGCGCCCGCCTGCACGTGAAGTTTTTA<br>TGCAATCTACATTTTTTATTTAATGAATCCCAAAATTATAAACTAGCAAATAATCCATCTAATTGAAAATGGCAATCGTCTC<br>TTAAAGACCTGAACGGCATCATGGATACTAAACAGGGGGTAGAAATCGCAATATCATAATCACCAGCGATGGAAACGAAT<br>GAACGGTGTATTCTGTGATGGATATCTGATGGTCTTGTTAATGTCTATATTCTATAGGCGACGATTACAAGATTATACTAG<br>AAAAAATAAACGTTTGTTCCCCAAGAAAAGTCTGCATTATATACTAATTTCAAGATCTCCGGCTATTATGACAGTAAACCA<br>ATGTGGTCGGGGACAGACCAACTGGGATCTGACCTGAAGTACGTCCCACAAGGCGGCACAAGCGCCCCAGGCGGCGGCG<br>GCGACGGCGGGCAGCGCCAGGTCAGTGCGGGCAGCGCGCCAACGCAGCAGCGCGAGCATCAGCGACGCGTGCGCCGCCG<br>CCCCGCCCGCCGCCAGCGCACCGCGCCGGCCCCGCGCCGCTGCCATGCTAAGAGTCGCCGCTGCCAATGCCTGCGAGTCG<br>GGACAGACGATTGTTAGTAGTAAGATCTGGTCCTAAATGAAAAGTACAGCGAGTTTTGCGTGCCAGGCAGTGTTGGATTG<br>CTCGAAAGATTACAAGCAGCGAAGATGGAAGAAGAGAATCCTACTCGGTCGACTACCACAACACCAGACGCGATAACTCT<br>TATCTGTGCACGTTCTTTATATTTCAGTTGTCCAGTTACCTTACTTATGTAATAAAATTATCTGCACATGGGAGATACTTAA<br>GAAAAATTAATATGAGTTTCTGTGACTGTATGTATATGTGTGTGTATGGCGTCCTAACAAGACCAATAGAAGCTAGAACAA<br>TATATATTTAAAGATTTGTTTGTCGGTTTGTATGTTTGTTTCGGCATCAGGCAAAAAATTGGATGCAAATTCTACAGTCATA<br>TTCCTGAACGATATGTTTTATGTAGGAGTGGTTTATTTAATTGATTTTTCTATTTCTGAAAAATCGCTAGTTGTTTAGTACCT<br>GTAAACAGCCAGCGCCGAACAGAGCTCTCCAGGCACAACTCCATCCCCCAACACAAACTCCGTACCACTGCGAATATTAG<br>GCAAGTTATAATTTCATATCTCCTTATTCAAGAGATAGATAGAGAATTACGTGACATTAGTCGTGTCCAAATTTCGGCTTG<br>CATTTTCCAATTATGAACCATATTTCTATGATCCATCCTTTAATTTAGTTTCTTAACGGCGTTTTTTAGCGGAAAGTCAAT<br>GAAAAAATTAAATTGGCTTAAAGTTTTAGTGTCAAGCAGATTTTTGAGTAATGTTGCCAGATCCAATTTGTTTTAAATCG<br>GTACATGAGGTCAAAAAATCGGGATTTAGGGTTGCATTAGGGACAGTAGACAATAAACAAGGGCTTTTTAACTCTGGGTT<br>CAATTTATTGCGTTGAAGTATAGTATTATATTAATTACCTAAATTAAATATTTAACTATTTATGTAAGAACATAACAAGTAA<br>TCAGTGTTGCTGGACTAGTAGAAATTATAAAATAAAATCGTACTTTAAGTGTAAATATACATAATACATAGTACTTAGCGT<br>AAATATACATTTTTTTATGAAAAAAACTTTTAATTTTTTGATTAATCGGGACATTTAGCGTCCCGTTCAGATACAAATCGG<br>GACGCGTCCCCAGACCCCTGAAAATCGGAACGTCCCGCACAAATCGGGACACCTGGCAACACTAATCTTGGGATATGTAA<br>ATCTAGAAAGTAAACAGCCACTAAGTTTTTCATTCTTATTAATCCTAAATGTGAAAGTGAGTTGATTCTTGGTGTTATAATG<br>GATTAATAAACTGTCCCAAAATATAGCGAACTATTTGGAATAGAGAAGGATATAGGCTAATCAGTCAATTATAGATGAAT<br>AACGTACTATTAACGCAAAAGTCGATTGCTTGGGTACCTCTGTCCCATTCGTGTTCCCACAGCCATGCAAGCAGTTGTGTTT<br>GCATGGCTGTATTTCGGTTTGAAGGGTGGGATATGGCGTGAATTTACTG
MSTRG.9440	Phosphatidylinositol 4-phosphate 5-kinase type-1 gamma-like isoform X1	GTTTATCGCTATTTTTCATTACAGCAACACAGTTACTATGAACGGTCGTGTCAGACGGATGCTTCGGTAGAGCATTTTCGCG<br>TAAACTAATTGATTTCTTCTAATTAAATTATAGTGAATCGTTGTGTTCGTGATGCAAGTGTTATGTGAGCCATGAGAATTAA<br>TATTGTGTATACATTTATTATAAAGACTTCAAAATAGGGGGTGTAACTAGAAAACAAAAATGGCTTCAGGCGACACGGAC<br>CATATCGAAGTAATGGATAGTTCCGCATCTAGAAAGACGGAAAACGCCACAGCACCGACTGAAACGGATGACGATGATA<br>ATGATAGAAGCTCAATCGCCGAAAAAAATGCACCGTACGACGTGAGTAACATGTTATATTATAATACGTAATATAATTTTA<br>ATTCATAATTATAACAAACGAAACTAGAATTCTCTGTATTGTAATAGGGAGAAAGTTGTCATAATTATTTAAAACTCTTAA<br>TTATTATTCGAGTTTCATTAACAATAGTATGCTGGGAGACTGCTAGATAACGTAGAAATTGTTTTACTGGTGATAACTAAT<br>ACAATGTACATGTATGTTTGTATTGTATGTTTGTATGCAATCATGTCCCACATGAATGCATTATCAATGAAATGGTGTCATT<br>GCCCAC
MSTRG.9470	Interference hedgehog-like	CGCTGCTCACGGGCGGCGTGCTCGGCGCCGCCGCCGTGCTCGTGCTGCTCGCGCTCACCTTCCTGCTGTGCCGCCGCGCCC<br>GCCGCCCCGCCGCCGACAAAGAAAAAGGATCAGTACCTGAAAGTGGTGGTGCCAACGGCTATATACCGGCCAAAGTGCCT

ATCACTATTACGGCAAATCCTATGCACGCCGAGGGAGGTGATGGTGGGGTAGAAATGTCTTTTATTCACAACAATAACACT
GGGAATGATGATACATTACCCCATTCCAGGAAGAACGGCCCAGCACGGCAATATGTGTGAGACGAAAAAAAGAGACTTG
AGGTCAGTAACCGCGAACAAATTAGGTGTTGCCACATTTTTTATTTTAGTAGAAATTGGCAATATTGTGGCGTATTATTAT
GAATAAATTGTGATACGAGTTTTGAATAAGTATAAATCATTAACACAGTGCGGGGACATGCAACAGTACAAAATTATTTA
ATTATAATGTAATTTAGCATGAATGTAAAATTGTGTGCCAATTGTGAAAAACCATTGTGTTTTCGGATCTGATCATCACGA
AGTTATAATTATTTACAATTAATGCTAATTAGCTATAAGATTTACAATTAATTTTATTTATTATTCAACTGCCTCTAATTCGT
AGAGATAGGTGTTTGTTATAAAGTAAAATAGATCTGATTATGTTTCCAATGGTACTAGTTAGATAGGTACGTGTGTAAGTT
TGTTACGCTTTGATTTATTATTATTTTATGTTATTTCAATATCAGAACTGTGCACTTGGAAGTTTTATATCTAACATAGGAGC
AAATTATTAGCAAAAATGTTTTACATGGCAATGTACTTAAAAGTATTCGTAAATTATCGTTGTTCTTTGAACTTGTTGTTTT
GAAGTTCTCAACAGCTTAATGTAAGGACTATAATGTATTTTTTAATTATCTAATACTTAATAGCTGCTATTTATTTGAAAGG
TTGTGATGGTGTCCATATGTATTGCATAAGGTTTTATTTTATCTATGAGTCTGTTGTCTGTGGTATCAACTGCGCCAACTTA
ATTAATTTATCGTAGAACGGAATGGCATTGGTACGACTTTATGATTTTATCGTCACGATATAGTAGGAATATATTTTACAAT
ATATAATAGTATTAGTAGGTAAATGTTTTAATTTTGCGTGCACGATAACATCAATTTGTGTATTTTTATGATATCGTGATAG
CACGATACACGGAATTTTTTTCCTATGAAAATTACTCAAAAAATAAAAGTCTTTATACCATCGCCTACATTTGGCCCCCAT
ATTATATCAAGACGATAAAATCGAGCAATGCTCTGTCGTGTTACGGTAAATAATAGAGGTACTTATTATCTGCCGTTCTCG
TGAGAATCTTTTTTTTAATGGATACCTCATCATGAAAATTATCTTTATTAAAAAAAATCAAGTTTTTTTTTATAATCCTAGC
GGTTTTTTATGTGTTTATATGTCAGTCACTTCTTTAATAGGTATATGATGAAGTCTTTTTCTATGAATAGATTTCTTCTAACA
TTTATTATGCAGAAGATATCTATACGAGTATATGTATATACAAATATTGACAGTGAAGTGTCAATAACTCAGTATTTTTTTA
AGAAATGGACGTTTTTTACGACTTTATAGTGGAAATAATATACTTTACGATGAATTCCGTATATGTATTTAATAGAATCTCG
CGAATAAGAATTTTATATTATTCTAGATAAACATCTCCTAGTAGGATAATTATTATGCAAATTGTAGGTTGATATATCGGC
ACACTTTCGATATGACCGTAAGTAATTCGTCAACTTCTCATGCAACAAGTTATTTTACAAAAGTATAAAAAGACATAAAAC
AAATCAAAAGATATAATGTGTATTCAAATTTGTAATATTTCTTTTATATTGAAGAATACCTTTATTTTTTATGTCACGAATA
TTTCGAATTCGTTGTGCGCCTACTTTTGACACTCCACAGTGTCAAAAGGTGTCAAATTTACTCACCTAATTTGACATTTGAC
AGTTTAAATTTGACACATTTTTTTTAAATCAAAACTTTACGTATTTTATTTCAGTGTTGCAAGTAATTTGATTAAAACAGTA
GATACATAAATGCAGAACTGTTACTTTATGTCATTTTTAATATCTTTCCATCGAAAACTGTTAGCAACGGAGTTACATGATC
GGCCTGTAGTTAAATGATCTTTCGTTTAATTCTAACATAAACTTTAAAATATATCTATCTCTATCTTACTTACTTACTTAAAT
TCCATCTTTTTAATATCTTGTCCTGTTTCCTGTTTGTTAGTGGCATTATGGTCAATACTAACACCCTAATTCTATCTTTTTAAT
GCTTCGTTCTATTTCTTGTTTATTAGCGGCACATTGGTCAATACTAGCTCTGCTCTAGCCTAAAAAATTAATGTAGGCTTAA
TTTATATGCAATAAAGGTTCACTTTATTTTAATTGTAAACTGCAACAAAATAACTGCATTTAGTGCACTCGTTTATTCTGAC
TCATGTTTAAGACTGAAGATGAAACTAATTTAATGTCATTATAGATATTGTGTATCAGTATAAATAATTATAAATATATTA
ATATACGTAATACTTTTTATTTCATTGACTTAATTAAATATAGACTGATTTTAATACTTAACAATTTTATTCGCTTATAAATT
TATAGATATACGAAATTGTACATATATTTTTTTAATTAATATTACTTCTTACAAAAAATTGACTTCAATTTTAACAAAAAAA
CGGTTATACTAATGATAATTTTATATTTAATAATTGTATTTTTATTAAGAAATATATACTAAGTCGAATTTAATATTTGAAG
TTGGTTAAAAGTACTAGCAACCCAGTCTGGTTGTGGTACGGTCAGTATCAAAATGCAAAAAAAAATCGATAAACATTAAG
ATATTCAATACTCCTTCACTCCATATCATACGTACGTCCTTGTATATATTCAACTAATTTTATCCTTAACTGTTATTCATTCA
ATTGGATATTTGATGGCTTAAACAAGGTGCAAAGGAATCTATCGATAGAATCGTTTCGGTCAAAACTCACGCGAGAAAGA
ATAAGACAACCTAAGTCGCTATCCCATTCTATTTCATGGACTCGCTACCAATAAAGATTCTATTGACAGGTTTGTTTGTACT
TTGTTTAGGGGCTTATTTATTGTTTATAACTTTGGAAATCTGCAGATATTCATCGATTTTTATGCTGATTGTACTTTGTGCTT
GTTGAGTATATTTTATACTTTGGCAAATTATTTTTCTAATATGTAACTTCACAATGTTATCGTTCTCATATTGTGATTTGTGA

		TTATTATGTTTAATACTTCTTATGAAAATTCAATTTACAGTCAAAATATTGTAC
MSTRG.9472	Microtubule-associated protein futsch-like	AAGCCGAGAAGCAGAATCCCAACAAAGCAGCAGCTCTGATGCACAATCTGAAGCAAGCCGAGAAGCAGAGTCACAACAA<br>AACAGCAGCTCTGAGGCACAGTCAGCTGCAAATGAAGATGAAATAGAACAATCTTCTAGTCCTGTGGGCCCACCAGTCAG<br>GCCAGGAACTGGTGGCAAATTGGCTGTGCAGCAAGGAAAGAAAGAAATCCTGCACGCATTGGAAACGAGTATGAACTTA<br>AAGATTCTAATACTACCAAACGGTCCAAAACAGAAGAACTGCTATTGTACCTGCGAGAGTGATGCCACACCAAAATTAGT<br>TCCTGTGAAGGACTTGCCAGCTGAAATAACGGCAATAGAATCCTAAATAAAAACTTTAAAAAATATTTAATAATAAAAAA<br>AAAATATAAATAGAATATTTTATATTTATGTGCATGGTCGTAGCTAGGAAGTCATCGAGGGGCAATGCCCTTCCTTAAAAG<br>GCCCAATGTCTTACCTAAAAATGAAAGGCCCAAGAAAATTAGACAGATTCGTCACTTAAATATGGCTTCTGCCCTACCTAA<br>AAGTTCTTTTGCCCTACCTTAAACTTGATTCTAGATACGGCCTTGTTTATGTGTAATTATTTTTCAACTTCTCTATTAACTAA<br>ATACATAAATTAAAATAATGTTAAACTATATAATATATACCTAAATATAGTTAATAAAACATAAAATAGTGTTATATGATA<br>TTAAAAAATATATTTTTAATAATAAAATGATAAGAAAC
MSTRG.9479	Hypothetical protein KGM_200187	AGATAATAAACTCACGTACTTATTTACCATCAATGTTCTAATATTAGTGCTAGCATTAAATTATAATCACTCATGCAGTGAT<br>ATGTTGAGTCGGGCTTTTTGGTTTTCAAGTGTGTCGAGTATTTATTAAACAAGAATCCTGCTTCGAACTCGAGGATTCGGAC<br>AAGGTATGGACCGTGAACCCGGCGTACGCGCACAATGTTTATTTGCCGTCGGATGATCCGAGGCCGGTATATAAGTCGGC<br>GGTATGAGCACCCGGCACCATTCGCGATTAACCGCCACCGCTGTGAGCTTCCGCAGAACGAGGCTACGCTGACCGCGAAC<br>TGATTGAACCAATTGCCACTTGCCACCCTTCCGCGATGATTTCCACACGTTTTCTCCTAATTGCTGTCCTTTTCCTAGCGGTT<br>GCGTACATTGAAGCCGCACCATTCTTTGATGCTATCTACGATGCAATCAATGGCAATGGAAGGAGATCTTATGGATATAGT<br>GGTTATGGATACCCGAATTACAACTATGGTTATAATGGATATAATAGTTACAACGGATACAGGAACGTGGAGAGAGCGCC<br>TCGGCAAGGAAAGACTTACAAGGAGATCTGCAGAGTCCACTACCCAGATGCGGCCGCCGTTCCTGGAACGGGGGGAATA<br>ATTTGTCCCTACTGAGGGTCAAGTCGCGTGTTCTACGCAATGGGGCGGCTGCTTCGGCGCCGCAATATCATGAGGATGGGC<br>TGATCTATATACAAGTACTTGAACTATAGACGAAAACTGTCATAGAAGCATGAAGTCTTGAATGTGTCTGCAGATAATCGA<br>CAATGTTTTGATGTATGTAATCTGAAAGAAGTAGCGATTTATAAGGATTCCGCTTTGGTCATTGGATGTCTGTTTAATTGCT<br>GTATATTGTTCAAGATTATAAGAAGAGGAAGATGAACAAGGATCAAAAATTCAGAAGTCAAATTATTGATCAACTGAAAT<br>TCATAATAGATGAGTTATTGTATTGGCTTGAGTTTTGAATCCTGTAAATGTTGAATTTGTTACAATCAAATAAACATTTGTA<br>ATTATGTTGATAATCAGACTAAACTGTTGTTTCTTCAAGCTAATTAGTGATGTTATGACAGAAATAGTGTTGTGCAGTGGA<br>ACAAACTTGTATAGATCGTCCCATCTGAGCCAAGCTGTGATGTTAAATTTGTGATATATCTATATGTGATATATCTCTGTTA<br>GTAATGAGAGATGTTACATTATTTATATATAGTATTTACGAGTTTATCGGCGGATGATAACCTTGTTGGTGGTAGTCTGTGT<br>ACTGTGGAGATAATTGTAGTTTCTCTTAATACATCGCCGGAACTGCCTAGAACACGCGATTTGAAAACTGTTGTGATTAAT<br>TTTAAATTAACATTATTGGCTAATTGTACTTATAAATTGCTATTGGTTGTAAATGTTAAAATTTTGTTTCCTTTAAGACTTTT<br>CTACTGCATGTTACCGCCAAATGTTACTGAATACCTAATGCAAGTAATTGTAAATAATAAATAAGGTTAAAATTAAATATA<br>TAGATAATATATCAATA
MSTRG.9485	BTB/POZ domain-containing protein 17	GGCGATGCCCGAAGTGCACTGACACCTGCGACTATCCTGAACCTAAGCACCTGTTAGGTACAAATCGGGACCAGTTACGG<br>ATACAAGTAGTAATAATGCCACTAACGGATTACTGCCACGTGGGTGTGACGGACTCTCTGCACGGATGACCACGTGCCAT<br>CCCGTTCGCACTCACAAGCGACGAGCCCGCGTAAAGTGAGACAGCACGCCCCAATGTGCCTCATATTGATGTATGCCAAA<br>ATGTTCATGAACAAGTAATAAAGGTGATTGGATAGGTTGGTTTCGATTTTACGAAAGAGCTAGATAATTGTCGTAGGTACA<br>ATGACAGTTGATTTCGCGGCAAAAAACTTCAACAAAACCATAGACATACTTGTGCAATAAAATTGTGGCATCTCATCGATC<br>AGTTGCCTTATTTATTTTATAAAAAAAAAAAAATGAAACAGTTCTTATTTTGAAATTTACCGCATCTATCATCTACAAGCTGC<br>AATACTGTTTTGTGATATAAATTGAAAATAAGAAGAAGAATTGAGAAATGAGGTATTATTAAGAAAATTATTAAAAAAAA<br>AATATTATGGATTTTTTTGTTAACTATTGTTTTAAATAGAATAGTATATTAATATTTTTGTCTTTTGATATAGTCTTTGGTCT

		TTGTGTTATGTTATTATTGATTTATGTATTAATTAACATATTTTGTTTAAGAATAAACAGCCTATGAGTGGCGCCATCTGTG<br>CTGAATGTTCAGCGGTATGACCATACAAGCCTTGCTTCCATAATCTCGGCTTCTACTAAAGTACATGTCATACACACATTTT<br>GTGTTTCTAATATGTATTTTTATAAAAATATTTTGGCATCCATATATGCGATCATGTTTTATACATGTTTCATATGTATAAAA<br>CTTATTATAAATACTACAAATATTTTACATGTACTTCTAATATGCATTATATGTTTGATAGATTATGAGTAAGATGTTACAC<br>AGTTAAAAACATTCATACCAGAGGGAGACTTTGTACAAAAGTCGATTGCTTGGGTACCTCTGTGCCATTCATGTTTCTACC<br>GTGAAGCAGTTGTGTTTGCATGGCTGTGGTTTGAAGGGTGAGATATGGCGTGAATTTACTGGGTACAGAGGAATAACACC<br>TGAGTCCTCAGGAATGGCAACGCATGGGGGGTATCATGGGTGAAACAGTATACTCTGTTATGTCCATGGTACTGCTCATGT<br>CTATAGGCGACGGTTACCACTTTCCATCAGGTGGGCCGTCAGCTTGTTTGCTATTCTACGTTGTATAAAAAAAATTCATGCC<br>AATGTATGGCAACATTTGAATTTATATATAATTTTCTTCTATAACTTTTAAATATTTATACCTACACAAGATCATAAAAATC<br>GATTAAAAGGTTCCTATAACCAGACTGCTAACTTGTTATTTGGGTCATTGAAACCGAATCTGCAATCTTTTTGGTTCTATTA<br>TTTCGACGTACATTGTTAAGTCCTCTAATCTTAAAAAAAAAATAGTAACAGTGATGTAATCAAAATATATTAATTTTCAAAG<br>CTAAACAATTTTTATTTCGTTTCCTATTTAAGAGCTGCGCTCTTGTCGGTGGAGCTCTTGCCACTCACTACGATTT
MSTRG.9507	Myotubularin-related protein 4 isoform X1	GGAATGGCAAAAACAAGATCGTGCGATAACCTTTTGGCTGAAGGGGAGAAAAGGACAACACAGCGGCGTAGTAGCGACC<br>CCAATATAGCGCCGGATGTTGCGAAACTGTCGTGTGGTGGTATGGTTGCTGGTGAGAGTGAGTCGAGGCGCGGAGCGCTT<br>ACCGACAGCTGCGTCGACAAATTGGCCGTACTACCAGTAGCAGTTGAAGAACAGCACCAAGTCGGCGCTCTTCATCCTGA<br>CCATTTTGAAAACGCCCAGAGATTACCGCGCGATATTGCAAGCGGTTCTTCTTCTTTGGAGAGGGAGCTAGGGGGCGCGG<br>CCGAGTCGGCCGACAGGGACGTAGTAGGGCCGGGGGCGGGTGCGGCCGGCACTAATGAAGACGTGGAGCGCGGCCTCGA<br>TGTGCAGGACGGCCCGTGCCGCATCCGGCATACGCCCGACGTCGAAACACCAGAGGAACAAGCGGTTTTTTTCTGCGACA<br>CGTATACTGATGTTATTGGGATGGCAGATGCGGCGAGAGAGTCCGGTCGCACCCGCCACATCAGTATCACGTGGCGTTCC<br>ATATCAGAATCAAGCAACCAGTCGAATCCTTCGTCGAGCGGGTTCG
MSTRG.9521	Glutathione S-transferase 2-like	GGAAAACACGAAAATTATTTTGTTTACAGTTAACATGGGGGGATTTCGTGTTCGCGGGAATGTACGATTATCTCAAGGCGA<br>TGCTGCAGTCCCCAGACTTGGATACAAAGTACCCGAGCTTCAAGAAGTTGCAGGATACAGTACTGTCCTTGCCTAAAGTGA<br>GGGAGTACGTCGCTGCTGCGCCACCTACTGAGTTGTAAGAGGTTTACAGTGGAGGAGTAAGGATTCTTTATTGTTACAGCT<br>GAGGTTTATTTGCTGTCTTAAATAATATGTAGATGGAGCGTCACAAAATGACCCATAAACGGTCAGCTAACTGTTGTTTAA<br>CATGTCGTTTATCTCTAGATTATTCTTGTTTTAGATCAGTCGTTCTAAAATCTAAAATTACACGGTGATCAAGTATTGTGCA<br>AATCGTCTGTGTAAGTAGCATTTAAATGTTTAATTTTTCAATATTTTGCACGCTATTTAAACAATGTGCCCTAAATGTGTGA<br>TTCTATAGCACTAATTAAGATCTGTTTCAAACATATTTATGCAAATAATATTATTAATATTACAATAAAATTACAAACAGA<br>ATAAAAAAAAAATATATAGATAACATAATAAATATAAGTAAATAAAATGAAGCTAAAAATCACAATTTAAAAGATCATTA<br>CTATATATTGCGCTTTTGAAAAGAAAAAAAAATGTTTTTTTTTTTAGCATTTATAGTTATGTTATTGATCTAATGTTGAATA<br>AGTCACAAATTAAGATGATATGCTCCATTCTTTATTCTAACAGCTTAATAAGTGTCTTATTTCTATAAATACTAAGAGACAC<br>GTAAACACCAATATATAAAGTTTTATTTGGGACCTGTCATCTTTAAACCAGGTAAAGAAAATTTATTTGAAAAGACTGCAA<br>CACGCTATGTTCTCATATTGACAGGGCCGTAGCTACCCCCATGTTTAAGGTAAGGCAAAAGAACTTTTTCGTAGGGCTGGA<br>GCTATAATTGACTTCGAAAAAGCAAATTGTCTAATTTCTTGGGACCTGTCATTGTAAGGTAGGGCATTGCCCGTCAATGC<br>CCCTCTTCCCTCTCAGCTACGGCCATGCGTATTGATTATTTAAGCTATTATTACCGGTATTTGTTTTCATATAATTTTATTAC<br>GTAACTATATAAGTATACCGTTTACAGAGTACTATATAAGTGTACCGTTTACAGAGTAATCCATGTTGTAGACTTTGAGAG<br>AGTATTTTGTTTACTGTTATATGCGTTACGTATATTTGAGTATTACATGAAAATTATACTATTTTTAGCAATATATTATTATA<br>AAAAAATAA
MSTRG.9580	Uncharacterized protein	ATAACGTGCATGCCATCTGTGCCAAAGTCAATTAAAACTGATGATATATCTGTTTCGTACATTTGGGTTCGACGGAACTGA<br>GGCCAAACAATTTCTGTAATGTGGCCTATAGCGTCATTTACCAATCCTTTTGTAACGTCAATGTTGGACCGCAACATTACTT

	LOC110382321	TTGCACCTACAAATATTTCAAGTACTGATGGTAAGCCTCCAGTCTTATTTATATTTGTTGATATTTTTTGTTTATTGTTACCA<br>TGTTGTTTCCACGATTAAGGGGCATTTTGACGGTCGACCCTCCCCCCGCAGTCCAGTAAAGGGGCAAGGCTTAATATACAA<br>CAAGATGCGCCCAATCTTGAGGGGGAAGCGGTTACGACATTACATTTTTATCCACCCTAATGCCATACAGTCCTCGGCACG<br>TTTTAACCTATTACCTTAACTTGGGTGGTCTAACTACCGCTTACTAACGAGGTATCTAATTACCTATTTTTTTTATATGCCCC<br>TCTAACGTCACACAATCTTAGACGCAACTGTTCTCAATACGACTAATCATGCCCCCACCTAATACGCCATTTTGTAACAGC<br>GTCCATCTTGAATTTGAAATTTTGACATAATCATCGTGTTTTACTAGTGTAGCCCTTTCATTTGATACCCATATTGCGGGGG<br>TTAAGGAAAAATATGTAATCCGCCATTTTGTATCGGCGGACATCTTAGATTTATAATGTTATTGATTTTATTATATTGTATT<br>GTCATCTAAATTAAATGTGTGTACCAAATTTCAGATCAATCTGACAACGGGAAGGTGCTTAAATTCCAATTTCTAATTTTG<br>ACCCAAATAAATAAATACCGTACAGGTCGAGCTAAATAAAACCGTTTAAAAA
MSTRG.9593	DD34D transposase	TTATAGTTACATATACGAGGTTTGTCCGGAAAGTACGTATAAAAGTTTTTTAAAAATTTTATTTTACAATTATTCAGGTAAA<br>TCAATTTTATCCCCTTCAAAGTACTCCCCCTGTGACATAATGCACTTGTGCCAACGCCGTTTCCACTGTGAGAAGGCTCCTT<br>GAAAGTCCTCTGGTTGTAGGTTTCTCAGCTGCCCCGTCGTAGCTTTTTTTATCTCATTTATGTCCCCCAAATGCCGCCCCCG<br>AAGTACCAATTTGGTTTTTGGGAACAAGAAGAAGTCACACGGGGCCAAATCCGGCGAATAGGGGGGGTGTTCGGTCACCG<br>TAATGGAATTTTTACTCAAAAACTCACGGACAACAAGAGAGGTGTGGGCAGGGGCATTGTCGTGGTGAAGAATCCAACGC<br>CCCTCTCGCGCCAATTCTGGACGAACTCGTGCCACACGAGCTTTAAGGCGTCTGAGCACATCGACGTAGAATTTTCCATCA<br>ACTGTCTGGCCTGAAGGGACGAATTCTTGATGGACAATCCCCCGAACATCGAAAAAAACAATCAACATTGTCTTGACATTT<br>GAGCGGGATTTCTTGATTTTTTTGTTCGTGGTTCCCCGGATGTCTTCCACTCTTTGCTTTCACGGCCATTCTTGCCGAGTTT<br>CGAGAGAAACTTGATTACGACGCGCTGCTCTTCAACGGAAGCGGGCTCCATGCCGACGGGGTTTCGTTAAGAGGTAACTT<br>CACAGATGGCGTGACAAGCCAGTTCGCACCGCACGGCGGTCAGACCAGAACTGAAGCATTGTTAGGGACATAAGGAAGG<br>GGTCCTGCGCTTACCTGGCCTCCCCACTCCTCTTTCTCGCATTCCAGAACACTTTTATACGAACTTTCCGGACAGACCTCGT<br>ATAGAT
MSTRG.9615	Retinol dehydrogenase 11-like	GCGTCATCGTAACAGCAGCATGTTGTTACTATCATTCATTATATTTAATATTATATTAGTATTTTTTATTTTCTTATACAATA<br>AATTAACACTAGGTATTTGTAAATGTAGCAGACATTTAGTTGGTAAAGTGGTTATTGTAACAGGTGGAAATACGGGCGTTG<br>GTTATGAAACAGCGAAAAATTTGGCAGAAAGAGGCGCCCGTGTTATTATAGCCTGCAGAAATGACCAACGCGGCAACACG<br>GCTCGGGAAAAAATCATCTCGGAAACCGGCAACCAGGATGTCCACTACCAAAAACTGGACCTTGCTTCTCTGAAATCTGT<br>AAGAGTTTTTGCTGAGAATATACTTAAAACCGAAAAGCGCCTAGATATACTTATAAACAACGCTGGAATTTATGGATCGG<br>AAAATGTAAAAACAGAGGATGGTTTAAATCTGGGGATGCAAGTCAACCACTTTGGACACTTTCTCCTAACCTGTTTACTGC<br>TTCCTCTTATCAAAACCTCAGCTCCTAGCCGTATTATAAACGTATCATCATTATCACATACCAGAGTTACTCTTGATCTAAA<br>TAACCTGAACTTTGAGAATGAAACTAAGGAAACTTACGATGTAAACAAAGTTTACGGAACTTCAAAACTATGCAACATCC<br>TAATGACGATTGAACTAGCTCGACAATTAAAAGGGACAAGAGTGACTGCGAATGCATTGAATCCAGGATTAGTCGACACA<br>AATATAATATCAGATGTTCCATTATTGAATCGTATTAGGCCGTTGATTAAACCATTCATGTTCAAGACAACATGGGAAGGT<br>GCGCAAACGTCAATCTACTTGGCTGTCTCCCCTGAGGTAGAAAACGTTAGTGGTTATTACTACAGCGATTGTCGACAAAAA<br>TCCACTTCTGCACAAGCTCAGGATACACGTCTTGCTAATGAACTGTGGAAAATATCAGAAAAACTAGTTAAATTAGAATG<br>ATCAGGTGTTATTCCTCTATATACCACTCTTCAAACCGAAATAACCATGCAAACATAACTGCTTCACGGTAAAAACACGAA<br>TGGGACAGAGGTACCCAAGCAAACGACTTTTGTATAGTCTCCCTC
MSTRG.9618	Zinc finger SWIM domain-containing protein 8-like	CTTCTCACATAATAATTCACAAACCGTGGAAAGGAATGAATTGTAAATATGTAGGTAAACTAAATAACAACTAACCTGTG<br>ATCCAGCTGACATGTGACGAATAAGTGCGACTTAACCAGTTTGCACTAACGCAATTATGAAGGCCCAGTGCATATAGACC<br>TAGCTGAAATGCAGCCATGTGTAAACCTCTGAAAAAGAATATAATAATTAAATATATATTATATGTACTTATAATACTCAT<br>TTCAATTTTTTTCTATTTCATTGTATATTATTTCAGTCTTATCACTTATTTTTTTAGGAACTAAGATTATATTACCTATGAGG

MSTRG.9668	Uncharacterized protein LOC106125039	GCCATGATGTTCTCTGGCGCAAGAAGTTTGAGTGAAGAGGCTGGTACTAGATGAGCCACCAGCTTTGAAAAGCACCGACT<br>TTGCAAGTTCAAACATAAAATGTGCTGAGGCTTCCGATGGTTGATTCGGTACGGACGGAGGACACAACTCTGATGCATTGC<br>TGGCCACATGTCACTTCCAATAAAAAGTGAACATTATTATTACCATGCTGGATGAGAGAATTGCCAAGACATTTTATTTTC<br>AAAGTTACAAATATGCAACCTGTCAGTTTAGATTACAAATATAGACAAAGAAATGAAAAATAGACGTAACAAAATTTAAT<br>AAAACTAACCGGCGGTGGCATGCACAGCTGCGCATGCGCCGGGTGCTGCGGATGTTGCTGGTGCTGTGGGTGTTGCGGAT<br>GCTGGGGATGCTGCTGGTGTTGAGGATAGGCTGTCGGCAGTGGAGCATCAGGCCTATACGCGGAGAGGCGCGGGTTGTTC<br>GTGTAGTACATGCTCACCATCGGCCCCTTAGTCAGCTGGTGGATTTCACGGTCCAACAGCTGCAAACAATATGTTTTATAA<br>GATTTTGTTCAAAAATAAATATTTGATTAAACAAAAACAACCGAAATTAGTTTAAAAATTACAAGGATATAATTTACTTAG<br>AAGCTAAGTTGTGGACTGTTACTATATTAAGGTAGCGCGATATACGTCAGATATAGCTGAAATTAAATGAACCATATTCTC<br>AATTGTTAGACTCAGTGTATAAAAAAAAATAACATAGATCATCATATTTGAATAATAGATTCATTGACTCTGTAATTTCAT<br>GATAGAGAAATGCCAATCTGAAAACTTTAAACTGAAAACAATTTTTGGCATTTGTCCAATGCTGAAAATAGGGCTCGCCC<br>ATCGATTACCATATGGCACGACCGCAAGGCAGTTTACCAGTAACTATAAATGACAGAAATTATGAAGACGATAATAACAT<br>ACCTTATTCATTATCCTGGCCAATTTAACAGGATCATCTTTGTAGAAAGAGAGCAATGTAAGCGCTAACTCTCCGCGCTGG<br>CGCCTAGTGCCCTCACATAATAATGGATGTTCCGCTTCAGACACATTTGCCTTAAGCCCTAAAGCAGCTACAGCTGCTTCG<br>AAACCCAAGGTTTCATCAGTGGGTACTCTTGCCTGGCAACATAATGGATAAGTTTGTAATGACGACATAAATGTAATGATG<br>CGCATTTTTTGTTTTTAAATAAAGTTAGAACTCACCGGATGTTTATTTTCTTTGTCAGTTGCCGATAAAACGAGGACATCAA<br>ACAAGAAACTTGCCAATGCAATAGGCAATAGAGCTGGGCCACGATTCCTATAATAATCAATTTAAATACATTAATTATAA<br>GGTCGAAAACAATACTATTATTATCCTTTTAAATTAAAATAATGAATATGATATAATTTTCGAATGTTCGGTGCGGCC<br>AAACAGGAAAGTTCTTTATACGATTATACTTTACTGTATGGAACACACACGCCAATAAGGAATAATTTAAGGTTCACTTTG<br>CTTGCTTTGGTTTGGTCAATCGATTTGCTTCTGAGAAAATCTTATATTTACATAAAGTATATAGTTATGTTGTCGTATCGGTT<br>CGGAAATACTGCTGCTGGAAGTTGATTCCAAAGTGTGGTTGTGCGTGGAAGAAAGTGCCTTGAAAAACGCACGGTGGAGG<br>ACCGCCACTCGTCCAGATGGTTGGGATGAAATTTTAGTTTATGGCGGGTTGTACGATGGTGAAAATCTGCAGCAGGTATCA<br>ATCCGAACAATTCTTCAGAGTACTCCCCATGATAAATTCGGTAGAAGATGCACAGAGATGCAACATCTCTACGCAGTTTCA<br>GTGGGTCGAGCCGGACAGTAAGACGCCGATCGTCAACAATTCGAGCTGCCCGACTTTGTATGGCCTAGTATAAGACGAAG<br>CGACC
MSTRG.9686	Hypothetical protein ALC56_13062	CATCAGGTGGGCCGTCAGCTTGTTTGATATACGAGGTTTGTCCGGAAAGTACGTATAAAAGTTTTTTAAAAATTTTATTTTA<br>CAATTATTCAGGTAAATCAATTTTATCCCCTTCAAAGTACTCCCCCTGTGACATAATGCACTTGTGCCAACGCCGTTTCCAC<br>TGTGAGAAGGCTCCTTGAAAGTCCTCTGGTTGTAGGTTTCTCAGCTGCCCCGTCGTAGCTTTTTTTATCTCATTTATGTCCCC<br>CAAATGCCGCCCCCGAAGTACCAATTTGGTTTTTGGGAACAAGAAGAAGTCACACGGGGCCAAATCCGGCGAATAGGGGG<br>GGTGTTCGGTCACCGTAATGGAATTTTTACTCAAAAACTCACGGACAACAAGAGAGGTGTGGGCAGGGGCATTGTCGTGG<br>TGAAGAATCCAACGCCCCTCTCGCGCCAATTCTGGACGAACTCGTGCCACACGAGCTTTGAGGCGTTAAACGACGGTCCCC<br>ATTAACACAAGTTCGAATTCTTTCCACGTTTTCGTCGGAATGTGAAGTGGAAGGACGTCCTGAACGTGGGTCATCTTGAAC<br>GCTTTCTCGGCCCTCTCTGAATCTTTTTAGCCACTTGTGGACGGTTGGTTCCTTCACAGCATCATCCCCATAAACTGTTCTG<br>AGATCGGCAAAAATTTCACGGCCATTCTTGCCGAGTTTCGAGAGAAACTTGATTACGACGCGCTGCTCTTCAACGGAAGCG<br>GGCTCCATGCCGACGGGGTTTCGTTAAGAGGTAACTTCACAGATGGCGTGACAAGCCAGTTCGCACCGCACGGCGGTCAG<br>ACCAGAACTGAAGCATTGTTAGGGACATAAGGAAGGGGTCCTGCGCTTACCTGGCCTCCCCACTCCTCTTTCTCGCATTCC<br>AGAACACTTTTATACGAACTTTCCGGACAGACCTCGTATATATATATATGATATTGTCGAATATAAGTCTATATTTAATTGA<br>TTGTACCTACCTACCTAACAACCTGGTAACAAATGTGCCTAAACCACAGCAACTTTGTCTAATAAGCAATAATAAGTAAAC<br>AAATTAGTTGAAAACTGCTGAATCAATATGGCAAATTTTAGTGTTTGTACCTATATAGTCCAATACAATAGTCCAATTAAG

		GGTTTTGGAAAAAAAAATGGAATATGGATAGAACAATGTCTGTCAAGTCGACTAATTGAAAAAAACAACATCGAATTCGAC<br>TCAGAATAAAAAAAAACCCTACGAATCTATTTATTGGGTTTAATTTGATTAATATACCTACGCTATAACTTAAACCTATTA<br>AATATATTATATATAATTACTTATAATGGATCATTTATCCATGCTGTGTCAATCTAATAATTTTGCCAAATTTGAAATCAAT<br>TTATTAAAAAAAATACTTTTTACCCAATGGCAGGTATGTAGGTATAGTACAATTTTTTAAACATTAATACATGTTTTTAAAT<br>GTTTAAGAAATATGTTTTAAGATTTAAAACAGTCTGGTTTAAATACTTAGAAATTAAATTGTACACAATAGCATTAAATTC<br>ATTTAATATCTAAATTCTGATAGAGCAAAACTAAAATATATACAATTTTCAATGATTGTTGATTATTATTTTGTTATACTGC<br>AATTTTTAATACTTTACTTTAGCTGGGAAAAAATATCCATATATCTTTACTTCATGAAATAAATTTAACGTCAAGCGAGTTC<br>AATATAAAATATGAAAGACGACAATCACGTTAATACTCGTACAAACACTATCACTGCAATAAAAACCAGTTTAGCCAAAG<br>ACACATTCAGGACTATCACTCCATAGATTTCAGTTTCAGGCTGTTTTTATAACTCGGTAGCAAACAGTTTGGAATCCCAAG<br>AATTGAAATAAAAAAAAAACTATCACTACACTTACATTGCTATTAAAAGTTATGTACATACTAACTTTTATTTGCGACTTC<br>GCGTAACCGGGATGTTTACTGGTCAGGATAAAAAGCAGCCTTCATATTTCACCATACCATATAGTACGTGTATACTAAGTT<br>TAATTGTAATTGGTTAAGCCATTAAGGCACTATAAGGTAACAAACAAACAAACTCACTTTCACATTTATAATATTAGTGAT<br>GTAATCAAATATAATACTTCAGGACAAAATTTTAAATATAAGAAACTTAACTCGTGAATATCATGTAGATACTGGCAATAG<br>ACACTACTTTCAGTAATAATACCATGTCTGAATTAATAGTTGTAAATATATACTCAAAACAATATTCATTGTCACAATTACT<br>CTATATATATAACTAATTCATTATTCTGTTTTACATTAGTCAACGGATTGCCCAGATTGCCCGCATTGCTTTATAGGCAGCT<br>TAAAAATTTATATGTAGGTATACCTTAAGGAGTACCTATACTCTGATATTTTTGGGTTAAGACAGTAATATACCCTTAACCT<br>AATAATATCTATTACTGTTGATATAATTTAATATAAGTACAATACTGTTAGCTAGAATTAAAATAGCTTATTTTAATTATAT<br>CTTTTATAAAGCAATTAACTAGGTAATAAAATAAGTATGTACGTATTAATAATATTTTTAATAAACTCCGTCATATTTAATG<br>AGTAAGTACATATACTATTGTTACGACACCATCTTAGAACACCATATTGTAAACTGTAGTAGGTACCTCCCTACTTACTCTA<br>AAGACAAAAAACATCAAATGTCTACGCACTGTCTCGATCGAAATAAAAATAACCTTTCTGTTGTTGCTACATACTATATGT<br>ATGTACATAATGATATAAATTTTTATTTATTATTATATGTCTATGATAAATAAATAAATAATTATATGTACCTATTATAGTG<br>ACTATTAGTTGTGGCACAGATGTGCGCGTCGCGCACTCTAATCACCAATTTTTGATTGCTGACTATACAATTATTTAGTAAA<br>ACTAATCGCAACATTAATATTTTGTACTTAGTTAAATTTACCTAGTAATGATAGCGAACACCCTCACGCATCGCCTTTGTAT<br>ATAAAAAAGAGTCTGCGATGACCGCCCGTTCATGTTTATATTGTGGC
MSTRG.969	Uncharacterized protein C1orf198 homolog	GATCTATCAGAAGAGAATCGCGGTCTCTTGTCATCAAGCCAGGCATCCTCTGGAACTGAATTCCAATCAACGGAAACATTG<br>ACCGACAGCGCACTACCAAAAACAGGTTATGACTTTTTAGACAACTGGTAAATATTGGAACATTTGCACCCGACGTTGGTG<br>CAATCGGCGTATTAAAATCTCAGCTACGGTCGGAAACTTGTCATATTATTTGTTAAGTTACGTGTCACATATATTAAGTTA<br>CATATTTAATTTTCAATTGTGAAAACGCAAAAATCTCGAATTTATATAAAGCAGGTTGTTTCGCATGGTTCCCTATGGGGTT<br>CTCCGCTAAATAATAAGTTATATAGGTGATATTGTAATAAGGAATCTCTTTTTATTGTCAAAATAGATTTTAGTGTTGTATA<br>ATGGAATTGAAACCACGTCTGAGCTATAAGTATACACTGGTGGGGCAACAGTGAGACAAGATTAAACCACGATGGTTTCC<br>AAAGGGGGACCGTTTGGAGTCGATCTGATAGGTTATTGTATTACTACGAACGGTATGTTGCTAGTATTGGGATTGTTAGTC<br>CCCATTGCTAACAATTCCTTTCCTCTCTTCACTATTCTGGTATATCTATTCCTAAAAAAATTATAGTACAGTTCTTAATGAGT<br>ACAGATAGAGAAATAGTTCGTGGGTCATTATAAAAGATAATAACAAATTGCAGATAGGTGTACTAGCCACAGATTAGCTA<br>ACGGATGTTTTTATGAATCAAGGCAAGGCACTGTTCCATCGTTACGGTATAACAGAAAAGCTAAATGTACCATATATATAA<br>TTTAAATTTCCAATATCGTTGTCGAAAATTAAGTTTTCATATTTTAGTTTACATACAATGGGGTCGACTCTGAGGCGCTAAT<br>CTTTCGACGTATTTCTAAAATTTAAATTACAATTTGATATGACAGCGAAGTCTTTAAAGCCCAATATAAATTAAATTTCTAA<br>TACATTTAGTAAAAAATATTATAAACTTAGTTTATAATGGATTATAAAGTGCTTTCAAGAAACTGTCAAATTGAAATTTTTT<br>TTTGATGGTTGAAAATAGTATGGTAATCTCGCTTGCGCTAACGGCATACACCGGCGGAGCACCAGTGAGGGGTGATAGAT<br>GAGAATAAAAACAGGCCAGTTAGCCCATCATGATGAATTCATCAGGAATTAACCATGATAGTTTCCAGGGGGAATCGCGA

		GGAGGTCGACCTGGTTGGTAAATTGAAGTATTAATTTTAGAGATGTAATTAGAAAATGGCGCTTCAAAATCTACCACATTT<br>TGATCAGTATCTGCTTTACGGGGAAAGTAGGATCGGGTATTAGGATCCATGCATTAATATCATTTGCGCAGTGCTGCTAGA<br>CGGGAGTACATCAACCAGCAGTCGGTAAACATGGCGCCCCCCGGGGGTGACAAAAGGCTAAAGCCGGCCCTGGTTTTTGC<br>TTTCTTATCACCTGTATCGTGGCGACACTACCATGTCATGCTACGCTATTACAAAAACGCTTAATTACGCCGTGAAAGTGG<br>CAGAATAGTTTGTATGAAAATTGCGTGTGATTTTCAATGTTCAATGAACTCACGGTGGGTTGAAACTGCGCTAGTAGATGA<br>TTCCTATGTAATGATATAAGCGAGTCGGCACTCGGCGCGCGTTCACGAGAAACTAATTCTATTGAATGTAAACTGTGTATT<br>AATTTTATTGGTTAAAATCTTATATTAAAATAGAAACTATTGTAGTATTTTAATGTAATAACTACCGATTTAATTTACGTGT<br>AATTAGATCGAAAAGACTCGACTAGTTTCGGAACCAATCCGCGATCCTTAGTCATGATAATTTTTATAGGGTACAGAGGAA<br>TAACACCTAAGTCCTCAGGAATGGCAACGCATGGGGGGTATCATGGGCGAAACAGTATCCTCTGTTATGTCCATAGTACTG<br>CTCATGTCTATAGGCGACGGTTACCACTTTCCATCAGGTGGGCCGTCAGTCAGCTTGTTTGTCATTCTAAGTCGTATAAAAA<br>AAATGAGCGACTTACCGTGTCAGACTCGTGCTCGCGTCACAGTCGCGCATGACTAAGGATCACGGATTGATTTCGAAACC<br>AGTCGAGCTTTGTCAATCTAATTACACTTAAATTAAACAGGTAATTATTGTATTATTATTGTTTTGCCTTGTGTTCATTTTTT<br>AACGATATCTATACACATTCACAGCGGGAATTTTGTGAACTTACTGTGTTTGTCGTTCCGGACAATTATCTGGACTTCATTC<br>GCAAAATCTTCAGAGGGCGTATAGCGTTGTGTCACTGTCTCGCCTCGCATTCTTGTATAAGAGAGTCATAAGTTCATAAAA<br>TTCCCGTTATGCGTATTATTGCAGTATTTGTCTTGTATTCACTCTTGCGCCACCTGCCCACTTCTATCTGTTCTCCTTTAACC<br>AAAAGTTGTCTGTAAGAAATTGCCCTAGCGATAAGACCGCCTTTTGTACCTTTATGTGTATATTTCAATCTGTATTTATGTT<br>ATTGTATTTCACTTGAATTATTCACTTGTCATTGTTTATTTTTATTTATGGTGCAACAAAGAATTAAATAAATAAATAAATA<br>TAATCCGCACCAATTTAAGGCAAATGTGAATAGGTTATTTGTTTACCCGCGAGCGCCATCTTAGGCCAGATCATTGCTTTTT<br>AACAGGTGAGAGACGTAATCAATCGCTGGAGTCTTGAATAATAATAAAAAAAAAATTATACTAATTGTGTGATTGTTTAT<br>GGTTGGTTGCTTTTGTTCTGTGTATTTATTAAATGGTGTAACATCCAATTATCGATCGATTAAGTTAATGAAACTTTAGTAT<br>TAGTTTTTTTGCCAAAAATAAAATATATTTGTCGGTTCCTTTTGTGATAATAGTAAATCTCATGTTGGAAGACTGCTTTTGT<br>ATTTTCTTATACTTATAAAAATCAATTTGTAAGCTAATTAGAAATAATTTGCTACCATAATTATCGTAGAGTTCGAAGTAAT<br>AAAGACTTTGTATCTCATAACTTGTTTTGATTGAAAACCCAAGTGTTTCTCAATAAATACACTGTAAGTACATTATTTTATT<br>GATAAAGAATCGTAGTTCAAGTTTTAAGAACATTTTTGGAAATATGAAAATAACATTTTCAATTACAAAAAAATACGTTTA<br>GCATTACGAAAATGATTATAATTCCAAATAATGATTTTATAATTATATTAAACCCAAAATTTACAAACTGTCAACGCTCCT<br>TTAATGGCATTATAGTAAACGGCTTAGATAGTAGAAGTAACGGATTTGTACTCTATCTGAGGTATCTTTTATATGTACTTGA<br>GATTTTTTTTTTGTTTTTATACAACGAACAGAAATTTGCATGCATTTATGTATTTCAATGTAAATTGTAAGATGTATTAGTTA<br>ACCAAATATAGTTTTATTGTATATATA
MSTRG.970	Ankyrin repeat domain-containing protein 50	AATTATTCAGTAGTTGCCATCAATTTGTCGAACGGTGTGGTAATGTGTTCTTTTAATTAAACAAATGTTAATTGAAAAATG<br>ACCTTTTATAATTAGAATTAATTAGTCATGAATGAAAGTGTTTAAATTTAATTAGTGTAGTACTCTTATAGTACTTATACAG<br>ATTATTCATTAAAAATTATCAGGTTGTCATTATGAAATTGGTTTAAAAGTTGAACAATATATATTAAAATATTTTACACATT<br>CATCACGCGTGTAAACCTAGGAAGTAGTGAACAGTGTGCCAAAGTTACAGGCGACCAGTCGACCTTAGAGACGATAATGC<br>CCGAGCTCATATCAGGATCGGGCGGGGCGGGCTGGCCGCGGGATCGCCGCGTCTTCAGGACTCTACTGGAGCTGAGTAGA<br>TCTGACTCTGTGACCGCTGAGAGTGATGCCGAGGAGATACGAGCGCTG
MSTRG.974	2-oxoisovalerate dehydrogenase subunit beta	TCAGCTTTTTTGCCATTCTAAGTTGTATAAAAAAAATGAAACCAATCAGGTCGTGGTGCCAAGAGGTCCGACCGCAGCCAA<br>GGGTCTGCTGCTGTCCTGTATACGCGAAAAGGACCCGTGCATATTCTTGGAACCCAAGATCTTGTACCGGTCTGCAACCGA<br>AGATGTACCAGTTGAAGATTACACACTGCCACTTGGCAAGGCACAGGTTTTGAGAGAAGGTGATGCAGCGACGCTGGTAG<br>CGTGGGGCACCCAGGTCCACGTACTCCTCGAGGTGGCTCAAAAGGCCCACGATGAGCTCGGCGTCAGCTGCGAAGTCATA<br>GATCTGCAGTCCATATTGCCGTGGGACGAGGAGACTGTTTGCAAT

MSTRG.9758	Uncharacterized protein LOC106142856 isoform X1	GACGGACGGGACTTCTTCGATGATGAGATAGCAGACCAGCCTGCACTTCTTTTTCGTAAAAACACGGACGGTAGCCCATCCAGCAAAGCAGTTGATTCTGAAGCCAACAGGCAGTTAAGGAGGTCAAGGGAACGAATTACATCAAGTCCGCTTGCACAAAGAAACCGAAAATTATGCGGTATCCGTAACGGCGCAACAGAACGCACCCCTTCCTTGGACACGCTGTCAAGTCTTGCATCTGATGACCTCATGATGGACAATGACCTAGCGCAATCCATCACCAGTCTACAAAGCGTTGATGATTATATTGAAAGGCTGGATAGCTCCCTACGCAGTGGACTAAACTCGTTGGACGAAAGGCAGCTACGTCAGGAGTTATCCTCATCCAAGACAGCGATACGCCAATGGAGTCAACTGCTTGAACAAAACAACATGCTGGACCGACAGCTCGCGGCAATGGCGCAGGGGAAGAGCGCGCCAGATTCGCTCACTGCCAGCAATACAAGTCTTTCCAACATCGG
MSTRG.981	Phosphatidylinositol 3-kinase 3 isoform X1	GCGAGCCCCCAGTGACAGAGGAGGGCGTCTGGCTAGAAGTGACGGCGGAGTGCCGCGTCGACCTGGTGCCGTTCTCCATTATTTTCTGCGTGCATAAACCTAGTCGAAAGTTTGGCGCGTTCTCGTACCGGAAACTCCGTGTGGCGGTGGTGAAGGCGGCACACGAGACGGAGCTGGCTGCTTCCGTGCTGGGGACCGTCAGACAGCCCAGCGATGCCGTCGAGGTGCTCAAAAGCTACGCAGTGGCGCATCGCTCCAGACGGACGACCCTCGCGCGGCTCGCAGAGAAATACGGGAACTCTTTGTTCATGGAGCCTATGGCCGAGGGTGGGTACCTGTTGAAGT
MSTRG.982	Uncharacterized protein LOC106139659	GGTATTCAACCCGCCCGGCCAGAATATGTCCAGGCAAACGGCACGCAATAGCAATCAGAACAACAACCGCGGCAACACTGGCGGCGGCGGCGGCGGCGGCGGACGGTACAATAACACGAACAATTACAACAGAAACAGAGCGAGCAACGACAGGCGGCCGCAGAAGCAGAATTCGCAAGAAGACGAAAACTGGGACAAGGGGGCGTTTACCAAACCAGAGTCCAAAGATGTCAAGCTCGCCCCATCGCCAAAGAATGCACCGAACTCGCCGAAAAGCCACTCCAACGTCCAGTTGAAAGCGAAAGAGGTTGAATCACCGAAAACACAAGAACCCACCAACCTGGTCAAGAATACGACCAGTAATGTGCCACAGACTAAGAATCCGAAGGAGCCAACCAAAGCGCCTGGTAGTTTGACCGACGTCACGCCTGGCAAAAAACCGTTGGTGCCTAAAAATTACCTGATAGAGCAGCTCTCAATCGGCGACACCTTCCTCCTCTCCGTGGAGAGTGCGGCTGCCGACTGCGCCACCAAGCGGCCCGGTTTCGTCTGTCTCTCCATGCACGAGTCGTACAACGACCAGTACATGAAACTCTGTGAAGATTACATCCTTGAATGCGAGGCGGACACCGT
MSTRG.9827	Histone-lysine N-methyltransferase 2C-like	AAACTGCATCCGAGGATAGTGGAGTTCAAAGTGTTGGCTTAGAGGGTCAGCAGTTCCGTCACCCGCTGCCTGTGGGCTTGCGGCCGCGGACGCCGCTCCAGCCTGGTCCTAGGCCTGATCATCAATTGATTATGCAACAACGCTTGATTGCAACTGACAATAGTCAAATACAACAGCAGACTATTGTACATACGACGCAACAGATCGTAGAGCAAAAAACGAATACTGCGCCACAAGGGAGTGCCCATGGAAATGACAATAAACAGGAATCCAGTCAAGAACATGGAACTGATGAAATGGATATGGGAGATATGGAAAAACTCGAACAAGACACGGGGAATATTGGCGAGGTGGACAATATCCTGACGGGCCTGGGCGGCGAGGACGATGAGGAGTTGCTGGAATCGCTGACGGCGGAGATCGGCGAGCAGTTCAACATCCTCGAGTACGCCGACCCCGAGCTGGCCGCGCTCAACGACGCCGAGCATCTGCTAGACGGGCTCGAGCTCGCCGAGGACAACATGCCCGAGCGACACGCCAAGCGCGAGCCTGAAGACGAGGCAGAACACAAACCGGACGTTAAAATTGATACAGATCTACATAAATCAGTGAAGAACATTGAGATAACAAAGACAGAGATGAAAACTGAAATGCAAGAAGTCAAGGCTGAGAATCAAGAACTAAAGTCAGACAGTACCCAGATTAGTGCAGTCAGTCAGATGTTTTGCAGAACAGATTCTGATGGAGTGCAGAGGGTCATTGCACAGAACCAGCAGATGACTTTACAGACGGCGATACAGTCGATAAAGGCGGAGGTGAAGCTGGAGGGCGCGCGGGACGACAAGCCGGAGGCGTGGCGGTCGCCCGTGTTCGCCGCCGGCGCCGCGCCGCGCGCGCCGCTGCCGCTGCAGCAGAGTTTGGGCCAGCGGCCAGTTCAGTTCAGCCAGGGTGGGCAGCAAGTGATGACCCAACAGGTGGTGCAGCGGCCACAGGTGCAGTTCTCGTCGCAACACATGCACTTTGGACAGGGTACGGCTGTGAGCTCGGTGGGCCCGAGCGCGGTGAGCGCGGTGAACACGCAGAGCA
MSTRG.9855	Uncharacterized protein LOC106125039	GTTACCTGTTAAATATACATATATATTTTTATATTTTCACCAGAGGGAGACTTTGTACAAAAGTCGATTGCTTGGGTACCTCTGTCGCATTCGTGTTTCTACCGTAAAGCAGTTGTGTTTGCATGACTGTGTTTCGGTTTGAAGGGTGGGATATGGCGTGAATTTACAGGGTACAGAGAAATAACACCTGAGTCCTCAGGAATGGCAACGCATGGGGGGTATCATGGGCGAAACAGTATACTCTGTTATGTCTATGGTACCGCTCATGTTTATAGGCGACGGTTAGCACTTTCCATCAGGTGGGACGTCAGCTTGTTTGCCATTCTAAGTTGTATAAAAAAAATGATATATGTTATTAATTGAGGTGTACTTACATTAAGACAATTTTTGTATAAATCCCTCTGACTT

MSTRG.9856	LIM and SH3 domain protein Lasp isoform X1	CGATTTTCAGAATCCCGACAAATGTTTGCGCAAGCCAAATATTTTTTGGCAGAAAAATACATGAAGAAATACAACTATATATGTAGAATTACAATGGTCTGTAAAACTTGTGGGAGACTGCACCCTAAACTAGATCAGAGATCTGTATCTCAGGAGTCCAGGCCCCCAACTGGAACACCATGCGTTTTTCAAGGCACTTTCTCCCACGCACAAGCACTCTTTGGAATCAACTTCCAGCAGCAGTATTTCCGAACCGATACGACAACATAATCTTTAAGAAAAGAGCATATTCCTTTTTAAAGGCCGGCAGCACACCTGCAATGCCGCTGGTGTTGTGGGTGATCATGGGCGGCGGTAGTCACTTACCATCAGGTGAGCCGCATGCTCGTTTGCCCCATGTGTTGTAAAAAAAAACCATGTAAAGTTTGCATTAAGGCTAAAATTAAAATTTGCGAAAACGAATATACAAAACAAAATAAAAGTATCATTCCGGGAAGGTGATTACATATCGAACGTGACCTCCATCGACGAGGGCTGGATGACGGGCCAGGTCCTTCGCACCGGACACACCGGCATGCTGCCCGCCAACTACGTGGAGCGGCTCTCGCAGCCGATCCAGTACTAACCACACTTCTAATTTGAAATTGTAATTAAAAAATAATTAAATATTTAAATAAACTGGGTAGAAGTGACATATGAATCGAGAATACCTCTAAAATGTATCACTCGTCCCTTCACACCGAGCATAGGTCATAGGGTTGTCACAAATTTATTCGGAATTGTACGATATATTAACGAAATATCGAGATGCAAGCTGATTTAGATCTTAAAATCATCTCACCAACGTTGCTATTTATTACAACGTTGTTATATTTATTTATTATTGGATTCACAGGAGTGTCACTTCTACTTAGATATTTAGTCTTGTTAAGGACGGGTAATATATAGCAATACCTATATACTGTAGTTACAATATCTATATATCAGTTTGATAAAGGATATCTTGAATATCAACTGTCTAATATATAATATCGAAGTGTCTGGAAATGAATGTACATACCCGACCAGTATGGGCGATATCGAAATAGGTATTGACCTTTATACCCAATATATAGATATTGGAACGGTACTGATATTACCCATGCCTATGACTTATGCTATAACTACACGACATACATATAAAATTAACATTAGATTCACATTCCGATACAAAACTACTCGTGTGAAGGTGTCGCCCTCTAGTGTTGAGTTTTTAAGCCGCCTTCGTTGTACGCGAAGGCCTCGGATGCGACTTTATCACAACAAAATTTTTAAATAAACCAAATGTAAATTTGTAAAGACTGGGAATCGATTATTATATATTTGAATGCGCTCTGCTGTCATCGAAATTTTAGCCTCTAATTGTGTATCTTGAGTTTTTTGTATATCAATGCTACACGATATAAATAGATAGATAGGTACTTTTTATTGTTTCCTTCGAAAGATCTATGATAATTTCAAATTATATATACATATATATAGCTTATATCATTATTTCAGTTTGAAGGGTGGGACATGGGCAGTGAATTTACAGGGTTCAGAGGAATAACACCTGAGTCCTCAGGAATGGCAACGCATGGGGGC
MSTRG.9867	Uncharacterized protein LOC106138072	CACTATTTAAACGTAAATATAACTGATTGGTTACATTTTACGGTGGGTGTAAACATGGCGGTTAGATTTCTCGTATTTCTTCCCTTTGCCATCGTATCAGCCCAACATCCCGCAGACTTATCAGATCTGAGACTTCCTGACACCAAATTGTGGATTGACAATGATAATGATTTTAATGCCATCCACGACAAAAACTCTAAAGAGGTAAAAATAGCTTCAGACTGGCTCTGGAAGTTAATGAAAGAGTCTAAATTCCAACTACCAGAAGGATCAGGCAACGAGGCACTAAGCCAGTACAACGGTCAGGAGTTAACAAAGCAAGATTACGATAGAATAAATAAAGTTTATATAGACAAATTAAAATTTAAACCGTGGCACAATGAAAAGAAATTGACATCCTCTTCCTCGAACGACGTACTAACATGGTTAGATAATGTTGATAAACCTAATGATCATAAACACGCCATAAAAGTATTTTGGGATCAGGACATGAGCGATAAACATTCTATTTGGAACACAAATTTAGGTAGAAATCAACATTGGCATCCAAACACTGTTGGAGATAAAGATGCCACTTCAAAAGGACAGACCATTGAAGAAATTGAATCTATAGAGGATAAACAGAAAGTAAATATCAATACAAATAAATTTGAACAATTGCCAACAACTAAAAAACCAATAAATAACGTAGCCATTTTTATTCCAAACATTATAAACGAAGAATCACCAAAAGCGACAAAGGACATTTCCCTTCAAAATAATAAAAAGAAAACTAAAAGACCGGATTTAGAAGACAATGAACCGGTATGGGACGTAAAGATATTAGCAAACGAAGTGGACTGGGATAAAAGAGGCGTTACAGGAAAACCGGTATGGGAAGAACATGGAGGTACCGGAAAATCTGTACAAGATATAACAAAACTGCAGGATCCTTTTAAATATATTAAACATAACGATTTAAACGAACCTGGACGTGATAAAATGATAAGATTAAAGCTTTTTGAATTAATCAAAATGACTAAAAATACTATAGACAATAAAATGAAAGGAATAGACGAAGTCCACGATTATTATAAGGAAAATAAATACTACAAAATCGGATACATAGTGAGCTATCTATTCAGAAGTATGGATCTTTTGGCGGATTTGTATAAATACATAGATGAAAACCATAATGAGTGGAGTACGCTGAAGATTCTAAATTCATATCAACAGATAAAGAAAGCCGAAGAACATTTGGCCAAGTCTTTAAATAAATTGACAGTGGCCGTTGGTAAAATGGAGAAGCAGATAATAAACAAGCCAGCTGCACATCTATCGACATCGGGAGCGTTTATGGTGAAACAAGCGCCACCAAAACCCATAGCGTGGTCTTACTACGATAACTAAAGCAGGG

MSTRG.9 879	Atypical protein kinase C isoform X4	TGCTGTCACCGCTATTTCCTATGAAATGTCAGTTTATCAGCTTATTGTTTTGTCAAACTTTTGTTGATATTACTTTAATACGA<br>TTAATAAAGTACAATTTGTTATTTTATACTTTTATCAATGGCAATCTAATCGCATTTTGTTACAAACAACGTGACAGTTTTG<br>TATTTTGAGGATATATCTTTAAAGGATTATTAAACCTTCGATACTGAATAACTGCCATGATGCCTACTCAGCTGGTGAACC<br>AGGATAACCATGCAAATGATGTGCGAGTGAAGACTGTATACAGCGGTGGTGTTATGATAACATACATCAAACATAACATC<br>ACCCTCGATGAGTTCACCCAGGAGATGGTGGCTATGTGTCGATTTCCACCAGATCAGGTG
MSTRG.9 883	Kinesin-like protein KIF16B	CTGTCAAGTGACAATATGTGTCAAAATTAAAAAACAAAAAAAAACCCCAAATCAAAATGTCAACTTGTTTTGTGGTGTCG<br>TATTTGTGTATTTAGATGTAATTTTATTAAAAACTAATACTATTGACAATATAATGTTCGACAATGGCTTCGGTAAAAGTGG<br>CTGTAAGGGTTCGCCCGTTCAATCAAAGAGAGAAGGACATGAACGCCAAATTGATTGTACAAATGGAGGGGAAGAAAAC<br>AAGATTACTCACAGTGAAA
MSTRG.9 891	O-acyltransferase like protein-like	TGATCATCAACAAGTCGCTGGTCGCCGCGCGCCTCGTGCCCACGCAGCTCGACAGGCTCACCGCGATCCTGGAGTGGTTCG<br>GCGTGGCGTTCGTGTCGTACCTGGCGGCGCTGCTGCTGGCGCTGCTGGTGGAGCTGCCCACGCAGCGCCTGCACCGCGCGC<br>TCAGCCAGCCGCCGCCGCCTGCACCCGCCGCCCACAAGAAGGACGCCGCCCAGTAGCGGCCTATAACTCGCCTACATCTG<br>GCACTACGGAACTATAGATCATACGGACGGCCGGTGATCGGTGTTAGGAATATACACTGGAATTAGGACACGCTAGCGGC<br>AGCGATCTCTAAACTTGAAGCCACGGAACACTAGAATTAACTATTAATTAATTAATTAATAGATTGAAAAAGGGACATTTT<br>GTGGTGGTTTATGGGAGAATGTTTGAGAATACAAAGTATTTACCTGCAAATTCCTATTTTAGGAACTGCTGCATTAGTACG<br>TTCATTATATTTTTGGGGTTACCAATCTTAATTACAGGCCTTACCCTTTTTTTCTTTTTTTTTTTTTCCGTGTGGAAAATCTTC<br>GGAAAGACCGTCCCATCTGCCGAAGGGAGGGCAGCTGGGGTGTGTGGGATTCGTACCCACTAAAAACCACACGACGTACC<br>TCGAGGCGCTTATGTTGGATCCCGGGTTCCCGTTTGTACGACTACCGAGTAACAGGCCTTACCCTGGTAAATCTTTATAAA<br>TAAGTAGATCACACGTATCTACCAGGTATCGTTAATTTAGAAAACTGTTATATAATTGAGAACTTTAGACAGGTTCAGAAA<br>GATTTATCTATCATAGTGTTTTGTATCGTTTAGTTACCACTAAACGTCTACAGACTTTGGTCTGAGTACATTTGATGAGTAG<br>GCTTTAAATACATTAATGTAGATATTATTTTTTATATATTATGTAAATAGATACCTACCTATTCGTGATGCATCAACGTACG<br>AACTGATACACCGACGTTTTACCCCATTTTCTATATACTTGTTCGTAATATCGATGTGTTAGACAATTTAAGGGATATCGCT<br>TGTAAAATGTATTTCTCCGAATAGTGATTTTAAACCAAGAGTTATTTATGGGGAAAAAAAACTATTGGACACGTATATTTT<br>ATGCATTATTTATGCAGAAAACAATTGTGGTAAATATTTCGTATATGCATTAAACATTTCCGGACGCAACATCCTTCTTTTC<br>CATTCATTCCATCAGGGAAGGAGGGTCCCTATCAAATCTGGGATCACATTGGGGGGTAGATGGACAAAATCAGTCTATAA<br>TCCAACTGCGCGTCGAGCTGGTCTAGTTCCGCTAAGATCACGAAAAAATCTCCGCCAGTTGCTTTAATTGACTCTTTTACTT<br>TAATTGATTCTGTCAACAATGTCACTTTTACTAACTGTATATTTTCACAATTCACCCTTCAGTTGATTGGCGAGCTACTTACT<br>AAAAGAAAAATACCTAATTTTCAGGGTAGACCTTTGTTTTAACATTTATAAAGTTGTATCTACCATTTATAATACTATTTAC<br>TACATATAGTACTGTAAATGATATTTTCATCGAATGAAAAAAGTGTTTATTTTATGCGACTTAGAATGGCGAACAAACTGA<br>CGGCCCACCTGATGGAAAATGGTAACCGTCGCCTATGGAAATGTTCATTGTTGGTATCGGTGGATTTATTTATCAATACAT<br>TTATATATTTAGAATTTATAAGTTTTACAAATGTGATAGCATGG
MSTRG.9 898	Acidic fibroblast growth factor intracellular-binding protein	AGTTATTAACAGCTGGTGGCTGTATAACTATTATGTGTGTGTGGCAGGCGATAATGGTGAGCTGGACGTACAGCTGCACGG<br>GCCCCGAGTACTACGACACGGAGATGGACCGCGAGTTCCTGCTGGAGCTGCGCGAGCTCAGGATCCTGCTCGACAAGGAG<br>AAGGAGCACAAACATTTAATCTGCATGAGACTAAAGCCGAAATTATTGGAGAAGTCGTATCAAGAGTTGGAATTGAACTT<br>TAGGTAAGGTGAATAGCTCTATATGTTTTAACATCGTTGATCAAGGTCAAGCGGTGTAGTATGAGGTCCCAAGATCGAATC<br>CCAGGCGTAAACCTTTCAATATTCATATAAATTATAAATTATCGGATCTGTTACATAATTAGTTATGTTTTATATATTTATT<br>AATTTAATATGTACTATGTATAGTTATTTAGAGAGACTACTACTACTACC
MSTRG.9 899	Hypothetical protein	AACAAATTTGTTACAAATTATTATATGACGGGAGGTAATTTTTATCTTCCTAAAAAACAAAAAAAGACTATTACTAGTTTA<br>CTAGCGTATAACATTTATGATCCCATTATAAGTAGTATAACGGATTTAATTAGTAATTATACTAATAATGTGACATGTGTTA

		ATTTAAACTAACATCTACGACATTGGTTAAGCCACAGGATATGGTCATTATTCATCAAAATATTCGTGGCTTCTCCAGCAA<br>AGAATTAGAATTAAGTCTTTTCTTAGAATCTAACAATGTACGGGTGTTATGTCTGACAGAACATTGGCTTAAACAATATGA<br>AATAATTTGTCCTTTTGAACATTATAATATAGCTAGTTTTTTTGCTAGGAACAATGCCATTCATGGCGGGTCAATTATTATT<br>TTAAAAAATGATGTAGAATATAAAAGCCGTCAAGACATTGTAAATATTTCTATTGAGCAAACAATTGAACTATCTTGCGTA<br>GAATTAGTTACTTGTATAATAATTTGCGTTTATCGTCCGCCATCGGCTTGTTTTGATTCTTTTGAACTGGC
MSTRG.9<br>9	Alpha-tocopherol transfer protein	GATCAGTTCTTCTCGGCGCGTGTTCAGAGTCGGACGTCTGTTGTGATAGTCGTCGCAAAGTTTTCCGTGTTCGGTTTCGAAA<br>TAATTATCAATAAAAATGTCACGTAGATTGACGAAGCGGTATGTGACGCAAGCTGACGAATACAAGTGCAGTTTGTCGGC<br>GGAGACACAAGCTATTGCACAAGAACAACTCAGGGAGACGGAAAACTCCAGGTCTCAGGCCCTCGCCGCGCTCAGAAAC<br>TGGCTGGAACAGAACCCCAAGTTCATGGC
MSTRG.9<br>904	General transcription factor IIH subunit 4-like	CTTTATGTTAACACCAGAAAAATCAAGCTCTAAATTTAAGGACACATTTTAAGCAAGCACATCATCCTGTTTGCCATTTTA<br>ATTTATAATTAAATTGATATATTCAATCATCATCATCATCTCAGCCTAATGACTGTCCACTGCTGATTGCGACGCTTGGCAG<br>GTGAATTGGCAACCGCAGTCAGGCTTTAATAATGTTTTAAGAGGGACGCTGCTGCCCATCCCTCGCCCTCCCTTAGTCGGC<br>TTTTATAACACCCACGGAATAGGAAGGGATGGCCTATTCTATGCCGGGACCACACGGCCACGGCCATATATATTATATACA<br>AAAATGAAGTGTATAAAACAGAAAGGTCATTGGCACTCTTTATTTTTACACCAACCCCACGAGGAACCGAGCGTGAAGAT<br>ATATGTCTGCGTAGATGCTAAACTCGGAGACGGGCGGTATCCGAGCGACATCCGTGCTGCCACCTACCGTCGTGGACCAG<br>ATACGGCTGTGGGAGACGGAGCGTAACCGGTTCACGTACACAGAGGGAGTTGTGTACAACCATTTCCTGTCACAAGCGGA<br>CTTCGCGGTGCTGCGCGACTACGCCAAGTCGCAGGGCGTGCTCACGTGGCACAGCGAGCGCGGCCGCACCATGGTGGTCA<br>CGCGCGCCGGCCACGACGACGTCAAGCGCTACTGGAAGAAGCACTCCAAGAGCTAGCCGCCGCGACACCCTCTTGTACCA<br>CTGAAAGCGTGAAAGAGCAAATCTCGATTATTCCACACTGTTCCATACATCCTCGCAAACTTTCAAGTTTATCACGGTAGT<br>AGGATATGTTCTTCCGCCTTTTTCCCCATGGAATAGGCAGAAACTGCCACTTGATTTTTTTTCCGTTTTATGCAACATAAA<br>ATGGCAAACAAGCTGACGGTCCACCTGATGGAAAGTGGTAGCCGTCGCCTATAGACACGAGCAGTACCATGGGCATAACA<br>GAGTATATTGTTTCGCCCATGATACCTCCCATATGTTGCCTGTACCCTGTAAATTCACTGCCATATTCCAACCTTTACACCG<br>AAACACAGCCATGCAAACAACTGCTTCATAGTACAAACACGAACGGGCAGCACACCTGCAATGCTGTTGATGTTGTGGGT<br>GACCATGGGCGGCGGTAGTCACTTACCATCAGGTGTTGTAAAAAAAAAATGCCCAAGCAACCGACTTTTGTACAAAGTCT<br>CCCTCTGGTGATTGATTTAACTTGATTGTCTCCTAACATATCTAACGACTACTATTTTTTTAAATAAAGACATGGCAACAT<br>TGTTTATGAATTGTGTCAAACAGAAAAACAGGTTATATAGAATTATTAATTTCTCTATTGTATACTCTGAAGTCAGCCAAA<br>TGTTGATACTTTTGTATTTATCGTAATATTATCTGTAAATATATTGATTATTTAATTTTA
MSTRG.9<br>915	Epidermal growth factor receptor substrate 15-like 1	AATCTCTGACTGCTATTGATTGGTCGACGAGGAGATGTGAAAAATGGCTGCCTTGCCCTCTCCTACTCAGGTGGCAGGGAC<br>CCACTGTGCTATATACGAAGCTTATTATCATCAAGTTGACCCCAATGGCACTGGGGCCATACAGGCTCCAGAGGCTGCGAG<br>GTTTCTCAAAAAGTCCCAACTTAGCGATGTTGTACTCAGCAAGATCTGGGATCTGTCCGATCCCACGGGGAAGGGCTACTT<br>GGACAGA
MSTRG.9<br>953	Protein raptor homolog	GCTGTGCCGCTTCCTGGCGCTGGGCGCGTGGGCGGTCCGCGCCGTGCTGCACGTTGGCATCTTCCCCTACATGCTCAAGCT<br>GCTGCAGGCCTCCGCGCACGACCTGCGCGCCTCCATGGTCTACATATGGGCCAAGATCATCGCTGTCGATCCGAGTTGTCA<br>GGTGGATTTGGTGAACGCGAAAGGCCACAAGTATTTTCTGTCTGTACTACAAGACCCTACAGTCGATTGCGAACACAGGA<br>CGCTAGCTGCATATGTTTTGGCTGGCATTGTGGATAATTATTCCGCTGGCCAAGAAGCGGCGTTGCAGGGCTCCATGATCT<br>CGATCTGCCTGGAGCAGCTGCAGCTATGTGGCAGCGGCAGCGGTGGCGGCAGCAGTGACGACGACGGTGCGGAGGGCAC<br>GGCGCTGCTGCAGCAGTGGCTTTGCATCGGCGTGGGCCGGCTGTGGCGCGGTTACGACGCCGCGCGCTGGGCCGGCGTCC<br>GGGACCTCGCGCACGAGAAGCTCTATCCGCTCCTGCACCACGACCATCCGGAGGTGCGTGCTGCGGGTGCGTTCGCGCTG<br>GGCACGTTCGTGGCGGCGGGCGGCGCGGGCGCGCGCACCGAGCACGCCAACGCGCTCGACCAGCAGGTGGCCATCGCGC

		TAGCCGCGCGCGCGCCGCAAGACGTCGCGCTCGTCGTACGCGCCGAGATCCTCGCGGCACTACAGTGGCCGGTATTGATA<br>TTCGAGCAGCATTTTATCGCAGTCTATATACAGGAGAGAATGAGGCGAAGGGACGGCGAACAGCGGCCGGACGTGGGGC<br>AGGACGCGCTGGTGCCGGCGCCGCGCGGCCCGCAGCCGCCGCCGCGCCACCCCTCCGCCGCGCTCCTCGCCAACACGCTC<br>ACCCT
MSTRG.998	Uncharacterized protein LOC106113188	AATTTCGCGAAATTATAAATTAAAATAATAAGAGATAGGTTAATATGAAAGGTGCTTCAGAATCGATCCCAGTTTCCTTTT<br>AAGCGTTTTATAATTGATCGTAGTACGATATAATATCGTACGATTTTACCGTCGCGATATAATATATATATTTTTTTTTCTG<br>CTTGCCTCATGGTCGACTGGTAAAGGACGCCATTAGATGTTAAGATTGTGACGGTAGAATCCGACGTAACGGTATTATTTC<br>ATGATATGTGAATTTATAAACGCGACCAACCACTATCCACTTTAGGTGTTTATATTATTTTTAAAGTGGACAAAGACTTCC<br>ATTTTTAGGAATTAAATGCGCTGTGATTCTCAAAGTGTTTATTTATTAATATAATAATATTTCTTGACTAAATATTGTGTGG<br>TATTTAAACATTTTTATAATTTATAAATATCATTTCGTCATTGATATAAAGAAAACACAAGATACACAATCGTACTAAAAA<br>TTGTGTACATCGAAAGCAAATATTTAACCAAATTATACACTACTTAATTAAAGTTCCATCCTAACCATCTGGACGAGTGGC<br>GGTCCTCCACCGTGCGTTTTTCAAGGCACTTTCTTCCACGCACAACCATTCTTTGGAATTAACTTCCAGCAGCAGTATTTCC<br>GAACCGATACGACATCGTAACCTTTAAGAAAAGAGCATATTCCTTTTTAAAAGGCCAGCATCACCTGCGATGTCTCCGGTG<br>TTGCGGGTGATAGGGTTTACTAATACCAGAGGGAGACTTTGTACAAAAGTCGATTGCTTGTGTACCTCTGTCTCATTTTTGT<br>TTCAACCGTGAAGCAGTTGTGTTTGCATGGCTGTGTTTCGGTTTGACGGGTGGGATATGGCGTGAATTTACTGGGTACAGA<br>GGAATAACACT
MSTRG.9982	FLYWCH-type zinc finger-containing protein 1-like	AAATAAAAAATACATTCAATAAATAAATACTTTTTTTTGTTGATTGAGAAAGGAATTATATTATTATTTTATTAGTAATTGT<br>AGTTGTAATAGTATAAATGTTGTTTTGACTTATTGCGCTGAACTCCAAATTTCACCAAGTGGCTAACGATTTTCCGAATCAA<br>CAGAATGTTATTTTTTTTTGGTATCAGTTGCAGAATTCTTGCCAACCGATAAAGGAAATTACAGGCTTCTATACGGTGGCC<br>ACACTTTCTGGTGTGACAAGTACAAACTGATGAAGGGGAAGCTGTGTCGTTGGTATTGTTCGAAGAAAAAACGCAAGCGC<br>TGTCCAGCGACTGTTCAGACTTTAAACTGCGAGATTATCGCTGTTCATGGCAGTCATTGCCATGAACTATGACTTCATTATC<br>ATCATCATCAGTCATTAACTGTCCAGGGTTGAACATAAGCCTTCTCCATAAGGGAAAAGTAGTTGTGACGCTTGCCAGACA<br>GGTTGACAACCACAATTATGCTTTGGTCACGTTTTAAGAGGAATGCTGCTGTCTATCTCTCGACCATAAGGTTCCCGCACTC<br>GTCTCTTACAACACTTATGGGAAAGGAAGGGATAGCCTAAGTTATGGTTACCGTGTGTAGTATTACTAGTAATTACGTGAG<br>GAAGGTGCCATCTAACGATGAGTTTGTAAATTGCCTTCACTGTGTAAAAAAATTTGCGACCCCTTTATTTATTATATATTTT<br>TAAAACCTTTATTACTACTTTTATGTAATATAAAATTGGTTTTAACTATAGGAAAATGATTTGCTCATGTCTGTTTGCAAT<br>TAAAATGAAAAACTTTTAAATAAAAAATAAAATCATTAATTCCTTATTGTTACTTTATTTTGTTATTGTTATCTGGATTTAA<br>TGTTTAATTTTCTAGTATTTATTTGTAAATTAAATATATTTTTATACACAGCTTGTTTATTGTATTGATCGCGACGAATTTAA<br>CAATAATTAATCACGATTGGAACCGCGTGTGTCAACCTGACAGTGTATATTGCTAGCAATGGGGATTAAAGTCCCCATTAC<br>TATCAACTTTTCTCCCACCCCTGTAAATACGAGTATTTATTATTAGCTTTTATTAGCGACTTCGTCCGTGTGAGACCGGGAT<br>ATGCTCAGGCTTGTGTTCGCGTTACAGCTGCTCACGATTAAGAGTCCCAGATAGTCCCAAAACTAGTCGAGCATTCTCGAT<br>TAAAATACGCGTAAGTCAATCCGGGTATTCTTACATTAAAATATAATGTGCACAATTATGAAACATTTATATAAAAAGGTT<br>AGAAGGTTTTTAATGGATGGAAATTGATCGGTAATCTTGTCTATGCTGATTTACGCACGCAGTATACTGAGGAATGAAGA<br>CGGGTCAATTGGCTTGTCGACAAATTTACTGAGAATTGACCACGTTGGTTTCCAAGAAG
MSTRG.9984	UDP-glucosyltransferase precursor	AGCCCATCCAAACTGTTTGTTCTTCATATCTCATGGTGGTTTGCTGTCATCCACTGAAGCCATACACTTTGGTGTGCCAACA<br>ATAGGCGTGCCAATATTCTTTGATCAATTCCTCAATGTAAACAGGGCTGTAACAAAAGGCTATGCTATCTATGTCCCATTG<br>ACTACCAACTTGCCGCATGATTTGAAGCCGGCTATTGAGACCATGTTGAGTGATTCGGGGTACAGAAGGAAAGCATGGGA<br>GCTCAGTAAAATATATCACGACCGGCCGGTATCCCCTGGGGCGGAGGTGGTGCACTGGGTTGAACACGTGATACGTACAG<br>GTGGCGCTCCGCACCTCCGCTCACCAGCGCTACATGTGCCTCTCTACCAGAAGTTCTACCTGGACCTAGCCGTTGT

MSTRG.9989	Insulin receptor substrate 1	CTCAACTACGCGGCGCTGGACCTGGAGCCGCGCGTGGCGCCGGCGGCCGCCGCGCCGCGCACCTACACGCAGATCGACTT<br>CCTGCGCAGCGAGAAGCTGCACGCCGCCGACGGCACGTAGGTACGCTGCGGCCATCGCCGCCCGGGGACGGCCCGCGGA<br>CGGCCCGGGGACCTCTCTGTGCCGAATTCGAAAATCCACCCCTGAAGCGAACCTTCCCGCTCGCCGCGAAGGAGGACTTT<br>GCTGATTTTAGTTTCGTGCGACCTCCCCGGCGGCCGTAGCGCAGCCCAGAGTTTGGACACCGAAGTTTGTGACGTGTTGAG<br>TTGTGCTGGAAACGAGCCGGATGATCGGAAATCGTATTAACAAAGTATACTCAAAACTGCGTTTGTGTGGCACCGTTGCCC<br>GTTTACCGTTTGTGATATCACGTCGGCCACGCATAGATGTGTGGTAGTAGTTCCTTTGCCTCGTTTTTATATAAGTACAGTT<br>TTAAAAGTTTTATTTAGATGATACGTTTCATTTTAAAAATTAATAGAACCGAAATTGATTTTATGATACATTATCACATTTT<br>TCTCAAAATTTTTAAACATTATTGATTGGCACGAGAACGTTTTATATAGAATACTGATTATTTTTTCTTATTATTATGTTAAC<br>ATATTAGATTTTTTTTCTATTATTCCCCTTTTTATTGTAATAAAAACAAAACATTAAAAAAAATACGGTACCTGTAACATCA<br>TTTTCTCATAACATAGTTTGTATATATACACTTTTAAGTTCTTAATCTACCTCATCTTTATCTAAAAAAAAAGAACAATTTA<br>CTTTTTTCTCGATTTAAATAAACCTAAGTCGAGGAAAGTTGCAAAAGATGAAAAAAAAATACTTAACATGTGTTAGCGGC<br>GAAATGTATGCAAGTCAAATGCTAAATTCACCATAGACAACAGTTGACTAATGTCTCTCATCCCATCTGGGGCAGTCTTAA<br>GAGCGTTCATACTAAACCTTACCGAAGTGTGGCAACAGTGCAGTTATCTGTCATATTTCAAATACAAGTAAATAATTGCAA<br>TGTTTCTAATTAATTGTAATCAAAATCTAATTATAATTAAACAATTTTCAGTATTTTCGATTATTTAAAAAGTGCTCTACTA<br>GTGAACTCACTAAATTTGGATTCTAATAGTAAAAAAGTATTCAAAAAATTCCCAAGCCAAAATATATAGAATAGGTGCAC<br>AATTTTTAGTGGTATTCTATGATTTACGTTTTAGTTTGCTAGCCAAGGCACTTAAATAATCGGAAATTTTGAAAATTTTGCA<br>ATTGTCTTAGAAAACTGACAGACGATTGCGACGTTGCAACACCTCGGTATGAACGCATAAACTTAAGCCTTAGAATACAT<br>AGATAGAGTGTCTCCACAGGAGTGCTTCGAGAAAAACGTGTTAACATTTTCATACAATTTTGCATGATATATTGTATTATA<br>ACTTTTATATTTGTTTTGTTTTATAATTTCTATATATTTTTTATGCGTACACACATTTTATTTATTAAATAACACGCCAAGAT<br>ACATTTTATGCGTAAATAGAATATTGATAAAAAACAAGTCAGCTAATTTGACAATTAGCTGAGACAAAAAGTTGAGCCGT<br>TCTTGAGGATCCTCTATGTATATAATGTAAGGCTTAAGTGTATTATTTTTATATGTTTATCTATTTATTACTATCTTCATACC<br>CGATAGGTATGACCTCATTTATAAAGAAATATTTATTTGTTTATTTATTTACCGTTTTCGTACTGGGTAGTCGTGAAGACAG<br>TTTAAAACGCTGCCTAATATTGAAAGTAGGAATCGAGTATGATTTGTTAATATGGTCTCTAGAGACACACACACGTGCTTA<br>CATTGTGTAACACGCCCAGTCTATCTTCCCTGGGCACGTCTCTGTCGTGTCTGCCGTCGCCTTGTATCTATGGGAATAGTCG<br>ATGCGGTGTGATGGTGTGGCAGGTACTGTGCGTACCATACGGGAATAATTTTATATATATATATATAATTAAACTAGTTTT<br>AGTGGCATTGCATGTAGTTTTTAATTTTTTTTTTTATTTTGAATACGTTTATATAATTATTTATTTATATCGACTATTGTCGCT<br>GGTAAGTTTAAATATTAAATAGATTTCAAAGTACAATACAATGCAAGTATTACATACAAAATGACATCACAAGTTAGTTCT<br>TATTAATGGTGTCGCGATGGGCGCCGTAGGCGTCACAGTTTACAGTGTAAATTGTAAATATGTATTACGTTATTTATTGTTC<br>TATTCATGTTTATAAGCAATGGTTATTTACTTTAAGTCCGACATAATTGGCGCCGCCAACTTGTTGCCATTCTGAGTTGTAT<br>AATTTTTTTATTTTTTTTTTTATTTTATGACGTGTTAGACCATTTAGGCTTGGGTATTACGGATATCGATCCAATATTTTTTTT<br>TGATGGGTGAAAATGGTATGGTAACCCCGCCTGCGCTAACGGGATACGCTGGCGGAGCACCAGTGAAGGGTGATAGATGA<br>GAATGAAAACAGGCCAGTTAGCCCATCATGATGAATTCATCAGGAATTAACCATGATATTTTCCAGGAAGAACCGCGAGG<br>AG
MSTRG.9991	Uncharacterized protein LOC106114390	CTGTAGTAGAATAGCGAGATGGGAGTGCACCTGCAACTTTTAATATCAATCTCTGTGCTTTTTCGGCCTCCACAGCTAAGC<br>TAAATTAACATACTATGTTAATTTAGCTTAGCTGAGTTAACAATGCTGTTAGCTTAGCTCAGTTAATAATGCTTTGTTTTTTT<br>AATGCATGATAATACAAAACTAACCTTGCCCACCCTTACGGTATACGCTGGTGGGATGCCGGTGAGGGATGATAGGCGAG<br>AATGAAAAGGCAGGTCAGTTAGCCCATCGAGATGAATACATCTGGAATTCAGTACAAAGGTTTCCAGAGGGACCATGTGG<br>AGATCAACCTGATAGAGGGTGTTTTACTAGCAGTGGGATTGGCAGTACAAATTATTAAATTCCCCCTCCCCCCATTCCTTTC<br>CCCCCAATAAAGCTATGTTAGTTTAGCCTGAGTTAACATGCTATGTAAGTTTAGCTTAGCTGAGTCAATAATACTATGTTA

GTAGAGCTTAGCTGAGCTAATAATGCTGTTTTGCTGTTATTCTAGTCTGATCTAATTATTGCGCTTGTGTTCAGGTATTAAG
CTGGATAACGAGTGCAATATAGTGTCAGTGGACCGCGCGTCGGCCGCGGGTCGTGCAGGGCTGCCCCTCGCGGGCCGCTG
GGCGCTCACCGAGGTCAACAACCGCCCGCTCACTCTGCTTAAGGGTGGAGAAGAAGAGATGAACCGTCTCGCCATGCACG
GCTCGGAGATCTCAATACTCATACAACCGGCGGCTCTAGTCAAGAAACTGCGTGCAGCCATCAAAGCAAACAAAACCCTC
ATGTCCATGAGATAAGGTTTAAATTCCTTTGGAGTTTGATGAGTGAATGAAGGAATGAAAGCTATCGGTCAGGTCAATGA
ACGATGTAATGACGTTTACACGATGGACGATTAGTTGTATTGTTTTGTTTTTTTTTTTTTTTTATCGCACAAGACACAAAAC
GCAAGTCTATTATTGGGAATAAGTTGAAATATTTGAAATGTCAAATTTAATTGATTTTTGGCTATTTTAAATTCAAATTTAC
AGTGATAGTTTAAAAATAATTTTATCCCGTGATTATTAATATGTTAAGAGTGTGTTTTTTTTTAATAATCAATCGGAAATAT
TGAAAATTGTTAACAAGTTGAAAAATTTAATTTTTGATATCTCTATGCACTGGGACACTGGATGGGAACCCATATCGCTAG
TTTATCGTAGTCCTGGCAACTGAGTTTCCAGTTACACCGCGAAAGGGTTTTTTTTTTGAAAAATTTTTCAACCTGTTAATA
TAATTTATGATATTTCAATGTAATAACTACCGGTTCAACTTAATTTCACGCCACAGCTGCCTACGATTAAGGGTTACGAATT
GATACTGAAACTAGTCGAGCTTTCTCGATATAATTACAGGTAAATCTAACCAGTAGTTATTATATTAAAATATAATATGTG
ATTACAAAACTTTGATAGTTATTTTTTATTTCCAAATGAAGAGTTAGACATATTTGAAATATTTTCTAAATAAACTATTCTA
TTGATTTTGAATGTTCACATACAATTTTGACGTTGCTTTACTCTCTTATAGTTATTTCCTGCCTTTTTGTATTAATATGGCGG
AGAGAAATTAAACACAACATGGCCGAATCACTAACCATAGACAAGAAGCAGTTACCCGCCAAATATATAGTTATTATAAA
AAAAAGCCGGTCAAGTCAGAATCGGACTCGTGCAACGAGGGTTCCGTACAAAATCATCAAGTTTATAATTTCTTTTTTGTG
AAATAACTACAAATTTTACTGTTTTCGGATTTTTTCCTTTACTTGTGCTAAGTGATGGTTTAAGAGGAACGCTGATGCTCAT
CTCTCGACTGTTAAGTTCCCTTAGTCGCTTACGACATCCACCAGTAAGGAATGAGTGGCCCATTCTATGCCGGGACCACAC
GGCAAGGCTGTCTTATGATTTTTTATTAAGATTCATTGGAGTTGTTTACGATAAGTTCTATTTCACAGATCTCTTGCACATG
TAATAAAAACACACAAGTATTATATAGGCTACGGTAATCGCTTACCATCAGGTGGGCCGTATGCTTGTTTGCCACCTCAGT
GGTTGATATGCAACTATTTGTTAAGCAATTTTGATTGTGATATATATATATACATATATATTTTATTATTAGAAAGGGTATT
TTTACATTTTACACATAACATTTTTACACTGTACTTTTATACTCTATGGCAATATACTATTTTTTATCTATAAGTGATACTAA
GAGTGGGCAATATATAGCAGTATCTATATATTTTAATTGCAATATTGATGCAATATATATCCAAAAAAAAAAATTGGATAA
CAGACTTTTAACATATTCGAAAATAGATATCCGACCGGTATAGGCGATATCGAAATGGGTGGTGACCTTTCCGCCATCGAT
ATCCAGAGGAAACCCAATATATAGATATCGGATTGGTATCCATTTTACCATTGCATTAGTGATATAACGCGAAGACTTCTT
ACCTACAGTTGTTCACATTCATGACTGTTTCTATATTTGCTTTCAAGGAAGCATCGCAGTCATGTGTCGGCCTAATATGTAG
TCGTGTTTGTACGCAGTTTGGTCAAGCTTTGAAAGTTCAATTTTTTTTAACCGGATAAAAAAACTAAGAGTTTGTATGTTCG
GCTCTATGTATTTTTCAAACCACTTCGAAAACATGGAAGTTTTTAATTCGATTGTGTTTTTATTTTATGTTTTGTTACTTTAT
AACTCTGTCAGTTGTAAACCGATTTGGAATTTCTTTTTTTTTTTTTCTAAAAAAGGGTATATTTACAGTTTGGTCCCGTAGG
ATTTTTATCAAAATTGAATTTTTTAAAAAATATACTTTTTTTAACAAATCCCATACCGTAGTTTCTAGGGAAAACTTCGGCA
GGTAGCTCATTACACAATTGAGCATTCACAGAAGAAAATTCCTTTAAAACCGCACTGTATTATTCCTATAAGAAGAAATAG
GCCCGGTAGGTGGCGCTAAGGAGCAAAATAACGTTTACCTGATTACCTAGCGAATAATAAATAGAAATACTGATTGCATA
AATTTACTTTACAATACTTTCATTGACCAAACTTGTACCTTGTCTACCATAAGAATAAATGTGCTGCGTAGAAATAACACCT
ATATACTTCATGGATGCGGCTATGCATCCAAAATGCATGATTTCCCTCTTCAGACCCTGCATAGGTCTGGAGAGATGCCAA
AAAATTACCATGTAACAATATGGCGCGACTCCAGCGACACACGTGCGACAGAGCGAGCCACGCGTCCTCAACGACGTCTG
TCACCCGCGAGCGCTGACTATCGACTGGCTTAGCTCGCAACAGCACGCGGGCCGGCCGCGCGACGTTCTTGTTATTATTAT
TGAATTCACGGGAGCGTTCATTCTACCCGCATATTTATTCTATTATTTGTACTATTTTTATATATACATTAATAAGTAAATC
AAACGGCAGCTAACATAGAAAGTAAGATTTGCAATACAAATATATATTTTAAAACTAAACGAAACAATATTTACTAGACT
TCTTTATATGAGAAACTGTGGGAATTCCAAAAAGTGGCTAAAAAACTGTTTGACTGATTTTTAAAGAAATATACAAGGATA

TATGCTTTTTGCATATTTGTACTAATATGTACACATATTTGTACTAATGAATAAATAAATAAATAAAAAATATATGTTTTTT
AAAACATTTTTTTGTAATCTACAAGTGCAATGATATAAGAAGTAAATACCTAAGTTATTAGTAATGGCGACGTGTAAGTTG
TACGGGCGGCACAGTGTATACACTTGTGTGTGTATGTCCAAGGTGCTGTACATGTCGACGGCCACCTTTTCATCAGGTGTA
CCGCCAGTTTCTTTGTCCTTTCCATTAAGAAGTACATTTTTATAAACTCCACTGATTTTTATAGGAAATTAATGTGAATTAA
TAATTTAATTTAATATTTCTTAATAATATAATGCGTGAAATGAAACGCAAAGAATTTCGGTTGTAATCGGGACCAAATTTT
GAAAAATTGTAAAATTAATTTAATAATCTTGGAGGATTCAACACGAGAGGGGCGACTCGAATGTTTTTTTCTACCGTAAGC
TTAGAAGAGTCGAATATTAGCTAAAACTACATGCATTTAGCCATAACCGCTTACCATCAGGTGGGCCGTATGCTTGTTTGC
CACCTTAGTGGTATAAAAAAAAATCGTGTCTTAAAGATTTGTTATTTGAAAGAATAGCTATCGAGTTGGAAATAAGTCATCT
ATCTTCGAATTTAAAACTTTAGTAAAGCGGATAGAATTAATTACAAAATCTACATAATTGTTTCAGCATAGACTCTGATCG
GTAATTGGTTAGGGGGACTGTACAAAAGTCGATAGCTTGGGTACCTCTGTCCCATTCGTGTTTCAACCGTGAAGCAGTTGT
GTTTGCACGGCTGTGTTTCGGTTTGAAGGGTGGGATATGGCGTGAATTACTGGGTACGGAGGAATAACACCTGAGCCCTCA
GGAATGGCAACGCATGGGGGGTATTTTTTTTTTAGTGGATAGCAATAGAATGGTAAACCCGCCTGCGTTTACGGTATATAC
ACGCTGGCGGGGCACCAGTGAGGGATGATAGGGGAGAACGAAAAGGCAGTTCAGTTAGTCCATCGTGATGAATTCACTTG
AAATCGTACACGATGGTTTCCAGGAGGGACCGCGAGGAGGTCGACCCGGCGGGGTGTATTGCTAGCAATGGGGCTACTAA
TCCCCATTTTTAATAATCATTTCTCTCTTGGAAGTTTTGTTTGTACGTGTTGTCTCCTCTCTGTGTTGAATTCTTGTTTTCATA
ATGGATCTCTATCGAATATCAATTAATTTATTTAAAAAAAATAATTCTGTATTTATTGGCA
