# Supplementary material for: Transcriptomic analyses reveal comprehensive responses of insect hemocytes to mycopathogen Beauveria bassiana, and fungal virulence-related cell wall protein assists pathogen to evade host cellular defense
Source: Virulence. 2020 Oct 5;11(1):1352–65. doi: 10.1080/21505594.2020.1827886 (PMC7549920; doi:10.1080/21505594.2020.1827886)
Supplement: Supplemental Material [file KVIR_A_1827886_SM8204.zip › Table S5.pdf]

Table S5 Differentially expressed genes of *Galleria mellonella* hemocytes challenged by *Beauveria bassiana* at 1 day post infection

Gene ID	Length	CK-R1	CK-R2	INT1d-R1	INT1d-R2	log_2(fold change)	P-value	FDR	Annotation
LOC113522728	1573	0	0	0	1.09	6.43	0.00	0.01	Tubulin beta chain
LOC113509611	432	10.79	12.44	397.31	429.15	4.95	0.00	0.00	Moricin-like peptide D
LOC113509614	407	18.73	18.58	618.47	648.84	4.86	0.00	0.00	Moricin-like peptide C1
MSTRG.3769	1793	0.11	0.08	2.45	2.69	4.59	0.00	0.00	Uncharacterized protein
LOC113509612	415	7.98	11.32	207.31	204.84	4.20	0.00	0.00	Moricin-like peptide C3
MSTRG.16508	304	0	1.25	10.62	21.71	4.13	0.00	0.03	Uncharacterized protein
LOC113509613	468	2.7	4.65	57.34	85.63	4.10	0.00	0.00	Moricin-like peptide C2
LOC113509868	1552	0.13	0.05	1.91	1.55	4.08	0.00	0.00	Uncharacterized protein LOC110377500
LOC113509609	514	14.56	15.88	257.95	319.5	4.08	0.00	0.00	Moricin-like peptide C5
LOC113521309	1673	0.43	0.4	6.1	6.51	3.82	0.00	0.00	Black
LOC113517529	2192	0.37	0.99	9.39	11.07	3.82	0.00	0.00	Tyrosine 3-monooxygenase
LOC113509694	2324	2.49	3.99	44.34	52.72	3.82	0.00	0.00	Transferrin precursor
LOC113515360	1125	0.06	0.14	2.19	1.08	3.80	0.00	0.00	Uncharacterized protein
LOC113523269	809	12.35	21.15	257.46	212.66	3.70	0.00	0.00	Gloverin-like
LOC113521568	1040	0.14	0.1	1.26	2.13	3.69	0.00	0.00	Uncharacterized protein LOC106132613
LOC113517566	10745	0.02	0	0.06	0.18	3.67	0.00	0.00	Titin
LOC113521399	1067	0.34	0.46	5.45	5.45	3.63	0.00	0.00	Collagen alpha-1(IX) chain-like isoform X1
LOC113511665	1342	0.05	0.4	3.61	2.12	3.57	0.00	0.00	Cardioacceleratory peptide receptor-like
MSTRG.8646	658	0	0.32	1.87	2.42	3.54	0.00	0.03	Cecropin A
LOC113516882	3917	0.05	0.12	0.95	0.99	3.47	0.00	0.00	Annulin-like isoform X1
LOC113516465	786	11.2	13.62	146.31	143.78	3.43	0.00	0.00	Uncharacterized protein LOC106132990
LOC113510528	1336	0.26	0.64	5.27	4.79	3.40	0.00	0.00	Collagen alpha-1(IX) chain-like isoform X1
LOC113519717	624	0.92	1.74	11.75	16.99	3.29	0.00	0.00	Uncharacterized protein LOC110373446
LOC113512533	1578.27	0.75	2.51	14.37	15.46	3.13	0.00	0.00	Protein yellow-like isoform X1
LOC113521401	1664	0.32	0.53	3.92	4.01	3.13	0.00	0.00	Collagen alpha-1(IX) chain-like isoform X1
LOC113515812	950	0.16	0.45	2.2	3.67	3.13	0.00	0.00	Uncharacterized protein LOC105398799
LOC113514266	373	25.16	45.41	376.2	346.9	3.11	0.00	0.00	Cecropin A
LOC113512071	1265	0.33	0.06	3.05	0.64	3.06	0.00	0.00	Lipase 3-like

LOC113523440	622	3.54	2.62	37.32	18.32	3.02	0.00	0.00	Antifungal peptide gallerimycin
LOC113515290	548	10.29	25.46	232.36	82.23	2.99	0.00	0.00	Lysozyme
LOC113513664	1161	19.73	26.36	173.26	210.88	2.96	0.00	0.00	Vitellogenin receptor
LOC113521027	1327	0.16	0.17	1.34	1.41	2.91	0.00	0.01	Laccase-4-like
LOC113509608	491	5.34	3.89	42.3	35.35	2.88	0.00	0.00	Moricin-like peptide A
LOC113521620	449.67	3.58	5.66	35.24	41.04	2.85	0.00	0.00	Uncharacterized protein LOC106132635
MSTRG.328	2801	0.83	1.29	5.65	9.59	2.76	0.00	0.00	Uncharacterized protein
LOC113514429	927	3.83	6.85	36.89	39.79	2.74	0.00	0.00	Corazonin
LOC113515831	1929	1.26	0.78	6.35	7.33	2.64	0.00	0.00	Chitinase 2
LOC113509117	1261.43	0.16	0	0.13	1.79	2.64	0.00	0.04	Uncharacterized protein LOC110375215 isoform X1
LOC113514951	1894	0.14	0.23	1.42	1	2.64	0.00	0.01	Glucose dehydrogenase
LOC113514955	909	0.51	0	2.03	1.68	2.63	0.00	0.03	Uncharacterized protein LOC106134169
LOC113519028	1342	0.1	0.46	2.29	1.39	2.62	0.00	0.01	4-coumarate--CoA ligase 1-like
LOC113509774	2162.02	0.38	0.29	2.13	2.4	2.59	0.00	0.00	Uncharacterized protein LOC106103079 isoform X2
MSTRG.9399	1248	0.5	0.13	1.52	2.69	2.57	0.00	0.01	Uncharacterized protein
LOC113510922	497	17.27	23.82	163.93	101.19	2.52	0.00	0.00	Protease inhibitor-like protein
LOC113518963	564.9	4.92	4.01	30.86	26.18	2.50	0.00	0.00	Zonadhesin-like isoform X1
LOC113514283	2739	0.69	0.67	3.27	4.26	2.37	0.00	0.00	Linear gramicidin synthase subunit D
LOC113513583	1703	0.5	0.39	1.39	3.56	2.36	0.00	0.01	Uncharacterized protein LOC110376907 isoform X1
MSTRG.11981	935	1.97	2.6	12.99	12.13	2.35	0.00	0.00	Uncharacterized protein LOC110371971
LOC113509570	5323	1.04	1.15	5.68	6.08	2.35	0.00	0.00	Uncharacterized protein DDB_G0277255 isoform X1
LOC113512385	3293	0.11	0.14	0.51	0.86	2.34	0.00	0.02	Endothelin-converting enzyme homolog
LOC113516725	580	26.01	93.75	347.6	308.15	2.32	0.00	0.00	Hypothetical protein RR48_09864
MSTRG.16298	759	4.68	5.92	31.96	24.83	2.30	0.00	0.00	Uncharacterized protein
MSTRG.3258	989	1.06	0.26	2.71	4.56	2.30	0.00	0.02	Uncharacterized protein LOC106140707
LOC113522527	1463.39	3.94	10.31	32.45	42.8	2.30	0.00	0.00	Serine protease inhibitor 6
LOC113523011	2301.91	66.7	94.04	437.76	398.95	2.29	0.00	0.00	Surface protein bspA-like
LOC113521574	955.34	47.18	65.34	261.93	334.5	2.29	0.00	0.00	Ejaculatory bulb-specific protein 3-like
MSTRG.9902	692	6.44	9.49	34.53	45.78	2.21	0.00	0.00	Uncharacterized protein
LOC113510012	2677	7.15	9.48	37.11	44.16	2.20	0.00	0.00	Cysteine dioxygenase type 1
LOC113515206	1759	9.55	21.41	102.26	48.77	2.20	0.00	0.00	Peptidoglycan-recognition protein-S
LOC113513838	6795	0.28	0.2	1.3	0.99	2.18	0.00	0.00	Uncharacterized protein LOC106140978

LOC113517675	3732	0.83	0.66	3.31	3.6	2.13	0.00	0.00	Uncharacterized threonine-rich GPI-anchored glycoprotein PJ4664.02
MSTRG.9506	1636	0.8	0.63	2.92	3.56	2.07	0.00	0.02	Uncharacterized protein
LOC113515683	5730	0.15	0.18	0.56	0.9	2.05	0.00	0.03	Uncharacterized protein LOC106143250 isoform X1
LOC113510107	1575	112.87	126.52	530.24	456.82	1.95	0.00	0.01	Hemolin
LOC113516003	1125	5.44	5.7	22.48	23.14	1.93	0.00	0.01	Serine protease inhibitor dipetalogastin-like
LOC113512455	1336.79	96.14	123.43	436.01	411.69	1.84	0.00	0.01	GATA Zinc finger domain-containing protein 14-like
LOC113518232	1512	8.17	7.5	29	30.42	1.83	0.00	0.02	Uncharacterized protein LOC106135508
LOC113521635	1838	1.65	2.48	6.81	8.17	1.77	0.00	0.04	Ejaculatory bulb-specific protein 3-like
LOC113518364	1873	6.99	7.42	22.34	29.19	1.74	0.00	0.03	Myrosinase 1-like isoform X2
LOC113520537	995	60.88	92.85	272.28	281.72	1.74	0.00	0.03	Uncharacterized protein LOC106132075
LOC113516369	5286	7.72	8.36	26.93	29.8	1.74	0.00	0.03	Uncharacterized protein LOC106135296
LOC113512095	998	10.97	11.26	37.33	41.04	1.71	0.00	0.04	fatty acid-binding protein, muscle-like
MSTRG.11855	1745	4.99	6.81	17.27	23.8	1.71	0.00	0.04	Uncharacterized protein
LOC113523611	990	619.1	768.48	2425.92	2233.59	1.64	0.00	0.05	6Tox
LOC113511557	812	78.93	90.88	32.91	24.48	-1.68	0.00	0.04	Glutathione S-transferase 1-like
LOC113516550	385	101.68	79.65	34.48	30.6	-1.72	0.00	0.05	Nucleolar GTP-binding protein 1
LOC113520900	773	73.59	114.07	35.99	25.55	-1.72	0.00	0.03	Uncharacterized protein LOC106133892
LOC113517478	1083	59.99	77.64	21.66	22.59	-1.74	0.00	0.03	IML1
LOC113521642	955	69.44	81.35	26.92	20.63	-1.77	0.00	0.02	Glutathione S-transferase 1-like
LOC113518306	4854	1.36	1.44	0.42	0.48	-1.80	0.00	0.05	Alpha-catulin isoform X1
LOC113509569	2465	13.3	12.68	3.4	4.23	-1.86	0.00	0.02	Carboxylesterase
LOC113518465	884	45.81	16.46	9.64	8.62	-1.88	0.00	0.02	Trypsin-like protein
LOC113519558	1177	10.39	14.48	3.71	3.21	-1.94	0.00	0.01	Uncharacterized protein LOC106142421
LOC113517432	2143	2.73	2.63	0.79	0.68	-1.95	0.00	0.03	Echinoderm microtubule-associated protein-like CG42247
LOC113520128	469	2044.61	2033.7	608.38	535.28	-2.02	0.00	0.00	Aminoacylase-1-like
LOC113516620	3510	6.24	7.33	1.66	1.85	-2.03	0.00	0.01	Junctophilin-1 isoform X1
LOC113517606	825	14.65	0	0.23	3.74	-2.03	0.00	0.03	Uncharacterized protein
LOC113519369	1431	3.02	2.6	0.61	0.86	-2.03	0.00	0.03	Protein takeout
LOC113522667	892	20.02	6.54	2.09	4.48	-2.14	0.00	0.01	Uncharacterized protein LOC106139316
LOC113513545	1236	3.12	3	0.6	0.88	-2.14	0.00	0.02	Echinoderm microtubule-associated protein-like CG42247

LOC113513259	1644	6.87	8.44	1.83	1.86	-2.14	0.00	0.00	Uncharacterized protein LOC110381499
LOC113510512	965	12.27	7.98	2.62	2.26	-2.16	0.00	0.01	Uncharacterized protein LOC106124870
LOC113515096	958	24.73	9.32	3.59	4.46	-2.19	0.00	0.00	Collagenase-like
MSTRG.1586	762	14.55	14.47	2.97	4.01	-2.20	0.00	0.00	Transmembrane protease serine
LOC113517147	533	982.98	1005.25	236.27	242.12	-2.21	0.00	0.00	Aminoacylase-1-like
LOC113513800	1950	663.02	536.49	131.25	140.71	-2.23	0.00	0.00	Uncharacterized family 31 glucosidase KIAA1161-like
LOC113512287	1754	1.44	1.99	0.48	0.29	-2.23	0.00	0.02	Mitochondrial carrier protein ymc
LOC113519055	2611	13.13	18.15	4.41	2.5	-2.26	0.00	0.00	Polyserase-2-like
LOC113522828	929	20.84	13.27	3.5	4.16	-2.26	0.00	0.00	Uncharacterized protein LOC110371440
LOC113513965	835	14.2	5.89	2.88	1.52	-2.29	0.00	0.00	Pancreatic triacylglycerol lipase-like
LOC113522683	956	37.13	18.28	4.38	7.8	-2.30	0.00	0.00	Uncharacterized protein LOC110371118
LOC113517602	3702	1.06	1.19	0.32	0.14	-2.34	0.00	0.01	Uncharacterized protein LOC106137997
MSTRG.13062	501	10.93	11.97	2.91	1.91	-2.39	0.00	0.01	Uncharacterized protein
LOC113522098	606	12.42	4.4	1.77	1.73	-2.40	0.00	0.01	Uncharacterized protein LOC106101275
LOC113514124	939	10.61	3.69	1.65	1.17	-2.44	0.00	0.00	Collagenase-like
LOC113518214	1972	2.13	2.14	0.19	0.62	-2.49	0.00	0.00	Uncharacterized protein LOC110373875 isoform X7
LOC113511649	2658	1.17	5.58	0.67	0.56	-2.53	0.00	0.00	Mucin-5AC-like isoform X1
LOC113522598	1425	10.92	2.78	1.45	1.04	-2.55	0.00	0.00	Uncharacterized protein LOC106139316
LOC113522572	842	37.69	18.43	5.01	5.13	-2.58	0.00	0.00	Uncharacterized protein LOC110371012
LOC113518649	1732	3.46	1.1	0.4	0.39	-2.62	0.00	0.00	Cytochrome P450 6B46
LOC113509439	1712	1.64	0.86	0.27	0.15	-2.64	0.00	0.01	Neutral ceramidase-like isoform X1
LOC113509096	1258	0.78	1.31	0.26	0.07	-2.65	0.00	0.05	Hypothetical protein RR46_05082
LOC113510707	560	5.78	2.55	0.69	0.75	-2.65	0.00	0.03	Uncharacterized protein LOC101747082
LOC113513404	330	406.31	400.14	77.94	77.46	-2.69	0.00	0.00	Aminoacylase-1-like
LOC113512906	3755	1.96	1.54	0.24	0.28	-2.82	0.00	0.00	Serine/arginine repetitive matrix protein 2
LOC113523533	1859	1.24	1.94	0.16	0.27	-2.96	0.00	0.00	Uncharacterized protein LOC106131316
LOC113516621	978	1.47	2.26	0.37	0.1	-3.01	0.00	0.01	Junctophilin-1
LOC113511343	493	11.55	9.92	1.81	0.66	-3.23	0.00	0.00	Aldo-keto reductase
LOC113520804	374	20.55	18.81	1.97	2.87	-3.25	0.00	0.00	Uncharacterized protein LOC105380498 isoform X2
LOC113522665	1110	1.63	0.73	0.08	0.17	-3.30	0.00	0.01	Uncharacterized protein LOC106139316
LOC113513300	1538	1.21	0.53	0.1	0	-3.99	0.00	0.00	Carboxypeptidase B-like
LOC113515168	4332	0.24	0.06	0	0.02	-4.02	0.00	0.03	Fatty acid synthase-like
LOC113513560	1068	1.85	0.85	0	0.09	-4.80	0.00	0.00	Chlorophyllide A binding protein isoform

									X1
LOC113518689	1588	0.79	1.26	0.05	0	-5.12	0.00	0.00	Acheron
MSTRG.30	394	5.02	1.94	0	0	-5.95	0.00	0.04	Uncharacterized protein
MSTRG.13731	249	23.45	27.79	0	0	-5.97	0.00	0.04	Protein takeout
LOC113519512	2311	0	0.47	0	0	-6.00	0.00	0.03	Uncharacterized transmembrane protein DDB_G0289901-like
LOC113510897	579	2.1	1	0	0	-6.04	0.00	0.03	Tetratricopeptide repeat protein 7B-like
LOC113512007	1223	1.49	28.14	0.14	0.3	-6.15	0.00	0.00	Fibroin light chain
