# Supplementary material for: Transcriptomic analyses reveal comprehensive responses of insect hemocytes to mycopathogen Beauveria bassiana, and fungal virulence-related cell wall protein assists pathogen to evade host cellular defense
Source: Virulence. 2020 Oct 5;11(1):1352–65. doi: 10.1080/21505594.2020.1827886 (PMC7549920; doi:10.1080/21505594.2020.1827886)
Supplement: Supplemental Material [file KVIR_A_1827886_SM8204.zip › Table S6.pdf]

Table S6 Differentially expressed genes of *Galleria mellonella* hemocytes challenged by *Beauveria bassiana* at 2 day post infection

Gene ID	Length	CK-R1	CK-R2	INT2d-R1	INT2d-R2	log_2(fold change)	P-value	FDR	Annotation
LOC113513385	787	0	0	120.81	98.72	12.96	0.00	0.00	Laccase-4-like
LOC113515460	1168	0	0	38.24	37.2	12.22	0.00	0.00	Carbonic anhydrase 7
LOC113516050	1413	0	0	21.06	23.6	11.82	0.00	0.00	Pancreatic secretory trypsin inhibitor-like
LOC113509613	468	2.7	4.65	7031.65	9651.76	11.24	0.00	0.00	Moricin-like peptide C2
LOC113517529	2192	0.37	0.99	1086.28	959.03	10.68	0.00	0.00	Tyrosine 3-monooxygenase
LOC113514009	1022	0	0	17.19	13.59	10.67	0.00	0.00	Serine protease gd-like
LOC113523572	879	0	0	16.46	15.65	10.42	0.00	0.00	Uncharacterized protein LOC106123314
LOC113516132	1511	0	0	8.24	6.63	10.34	0.00	0.00	Protein msta, isoform A
MSTRG.3769	1793	0.11	0.08	89.94	116.92	10.15	0.00	0.00	Uncharacterized protein
LOC113509612	415	7.98	11.32	8227.79	12317.65	10.13	0.00	0.00	Moricin-like peptide C3
LOC113509611	432	10.79	12.44	9993.08	13320.26	10.05	0.00	0.00	Moricin-like peptide D
LOC113523359	999	0	0	12.18	8.45	10.04	0.00	0.00	Proton-coupled amino acid transporter 4
MSTRG.10582	901	0	0	10.12	12.38	9.97	0.00	0.00	Uncharacterized protein
LOC113519717	624	0.92	1.74	1084.97	1249.96	9.88	0.00	0.00	Uncharacterized protein LOC110373446
LOC113509615	415	0	0	74.04	1.34	9.54	0.00	0.00	Moricin-like peptide B
LOC113518405	1240	0.06	0.06	50.17	39.59	9.50	0.00	0.00	Protein rolling stone-like isoform X1
MSTRG.10824	283	1.47	0	648.21	818.41	9.46	0.00	0.00	Uncharacterized protein
LOC113521027	1327	0.16	0.17	115.16	93.96	9.38	0.00	0.00	Laccase-4-like
LOC113521568	1040	0.14	0.1	63.75	58.5	9.09	0.00	0.00	Uncharacterized protein LOC106132613
LOC113515360	1125	0.06	0.14	45.75	59.48	9.02	0.00	0.00	Uncharacterized protein
LOC113514152	744	1.72	2.86	939	1113.81	8.93	0.00	0.00	Uncharacterized protein
LOC113521309	1673	0.43	0.4	159.94	191.17	8.85	0.00	0.00	Black
MSTRG.8976	599	0.16	0.94	233.11	235.46	8.83	0.00	0.00	Uncharacterized protein LOC106142918
LOC113522728	1573	0	0	1.5	3.21	8.77	0.00	0.00	Tubulin beta chain
LOC113509117	1261.43	0.16	0	62.01	59.06	8.75	0.00	0.00	Uncharacterized protein

									LOC110375215 isoform X1
LOC113512933	3490.68	0.06	0.02	13.33	14.26	8.68	0.00	0.00	Uncharacterized protein LOC106136478 isoform X1
LOC113509614	407	18.73	18.58	5531.09	8308.75	8.60	0.00	0.00	Moricin-like peptide C1
LOC113510643	1171	0	0	3.7	2.39	8.59	0.00	0.00	Alpha-tocopherol transfer protein-like
MSTRG.1877	569	0	0	10.07	8.14	8.57	0.00	0.00	Uncharacterized protein LOC106139762
MSTRG.3949	1028	0	0	2.59	4.02	8.47	0.00	0.00	Uncharacterized protein
LOC113512447	974	0.08	0	15.5	16.23	8.38	0.00	0.00	Uncharacterized protein
LOC113517540	573	0	0	6.55	8.24	8.29	0.00	0.00	Uncharacterized protein
LOC113521649	1184.85	0.1	0.43	67.62	68.75	8.09	0.00	0.00	CHH-like protein isoform X2
LOC113511506	438	0	0.73	96.07	98.01	8.05	0.00	0.00	L-ascorbate oxidase isoform X2
MSTRG.2571	650	0	0	4.58	4.2	7.87	0.00	0.00	Uncharacterized protein
MSTRG.7389	553	0.19	0.87	119	115.99	7.87	0.00	0.00	Uncharacterized protein
LOC113516882	3917	0.05	0.12	14.69	16.67	7.71	0.00	0.00	Annulin-like isoform X1
LOC113523448	568	0	0	7.07	2.58	7.65	0.00	0.00	Proton-coupled amino acid transporter 4
LOC113516877	1454	0	0.05	5.87	5.29	7.62	0.00	0.00	Protein tilB homolog
LOC113518655	977	0	0	2.45	1.44	7.60	0.00	0.00	Lipase member H-like isoform X2
LOC113519337	1435	0.19	0.85	86.58	93.91	7.58	0.00	0.00	Globin isoform X1
MSTRG.9902	692	6.44	9.49	1198.91	1494.98	7.52	0.00	0.00	Uncharacterized protein
LOC113516072	3316	0.11	0.12	19.31	18.74	7.48	0.00	0.00	Sialin-like isoform X1
LOC113516725	580	26.01	93.75	9324.31	10245.14	7.46	0.00	0.00	Hypothetical protein RR48_09864
LOC113512739	795	0	0	2.05	2.67	7.45	0.00	0.00	Uncharacterized protein LOC106135661
LOC113509608	491	5.34	3.89	679.62	839.6	7.44	0.00	0.00	Moricin-like peptide A
LOC113523468	2139	0	0	0.57	0.68	7.37	0.00	0.00	Nose resistant to fluoxetine protein 6-like
LOC113509868	1552	0.13	0.05	13.58	14.56	7.33	0.00	0.00	Uncharacterized protein LOC110377500
LOC113516003	1125	5.44	5.7	716.79	836.55	7.26	0.00	0.00	Serine protease inhibitor dipetalogastin-like
LOC113518757	3140	0.08	0.02	7.6	6.45	7.19	0.00	0.00	Amino acid transporter
LOC113515606	1002	0.45	0.93	88.42	80.72	7.06	0.00	0.00	Carbonic anhydrase 7-like
LOC113518215	3815	0.08	0.16	15.16	12.52	7.01	0.00	0.00	Nuclear pore complex protein Nup214-like isoform X1

LOC113515812	950	0.16	0.45	39.9	32.96	6.99	0.00	0.00	Uncharacterized protein LOC105398799
LOC113521574	955.34	47.18	65.34	5773.16	7231.38	6.97	0.00	0.00	Ejaculatory bulb-specific protein 3-like
LOC113518364	1873	6.99	7.42	784.11	840.34	6.95	0.00	0.00	Myrosinase 1-like isoform X2
LOC113523358	1875.47	0.04	0.05	5.16	3.69	6.89	0.00	0.00	Proton-coupled amino acid transporter 4-like
LOC113510868	3244	0.81	1.06	101.06	96.78	6.86	0.00	0.00	Uncharacterized protein OBRU01_00709
LOC113512634	553	0	0	4.26	1.59	6.86	0.00	0.00	Uncharacterized protein
LOC113509609	514	14.56	15.88	1435.56	1809.55	6.84	0.00	0.00	Moricin-like peptide C5
LOC113516722	3516	0	0	0.31	0.18	6.81	0.00	0.00	Protein eyes shut
LOC113509774	2162.02	0.38	0.29	34.49	33.27	6.78	0.00	0.00	Uncharacterized protein LOC106103079 isoform X2
LOC113518476	392	0	0	2.97	9.49	6.73	0.00	0.00	Uncharacterized protein
LOC113510638	3713	0.05	0	2.81	2.42	6.72	0.00	0.00	Bumetanide-sensitive sodium-(potassium)-chloride cotransporter
LOC113514283	2739	0.69	0.67	71.89	57.47	6.70	0.00	0.00	Linear gramicidin synthase subunit D
LOC113514053	1355	0.86	0.51	68.06	61.88	6.68	0.00	0.00	Uncharacterized protein LOC106142918
MSTRG.14089	2821	0	0.1	4.44	4.55	6.66	0.00	0.00	Ankycorbin
LOC113511039	2001.65	0.04	0.04	2.82	3.95	6.64	0.00	0.00	Solute carrier family 2, facilitated glucose transporter member 8-like
LOC113522083	1172	0	0	0.59	0.91	6.59	0.00	0.00	Sodium-independent sulfate anion transporter-like
LOC113518963	564.9	4.92	4.01	403.94	408.88	6.58	0.00	0.00	Zonadhesin-like isoform X1
LOC113516141	760.56	0	0	1	1.46	6.53	0.00	0.00	Protein drumstick
LOC113512533	1578.27	0.75	2.51	145.11	117.98	6.48	0.00	0.00	Protein yellow-like isoform X1
MSTRG.13498	382	0	0	2.15	9.16	6.48	0.00	0.00	Uncharacterized protein
LOC113522236	393	0	0	2.46	7.85	6.47	0.00	0.00	Cuticular protein RR-2 motif 134 precursor
LOC113519869	370	0	0	5.97	6.36	6.45	0.00	0.00	Serine protease gd-like
LOC113520073	1161	0.12	0.42	22.18	19.99	6.41	0.00	0.00	Uncharacterized protein LOC106136669 isoform X1
LOC113521502	784	0.21	0	9.42	10.03	6.39	0.00	0.00	Uncharacterized protein LOC106135000 isoform X1
LOC113518648	702	0	0	1.11	1.62	6.38	0.00	0.00	Lipase member H-like

MSTRG.8646	658	0	0.32	21.64	4.28	6.35	0.00	0.00	Cecropin A
LOC113511449	2098	0	0.07	3.21	2.18	6.34	0.00	0.00	Sodium-independent sulfate anion transporter-like
LOC113511874	1871	0	0.15	5.15	6.36	6.34	0.00	0.00	Rhomboid-related protein 2 isoform X1
LOC113512446	1138	0.13	0	5.42	5.7	6.34	0.00	0.00	Myophilin
LOC113518263	1144	0	0.07	3.34	2.68	6.29	0.00	0.00	Glycine-rich protein DOT1-like isoform X1
LOC113514337	2121.45	1.23	1.84	115.7	100.82	6.26	0.00	0.00	Facilitated trehalose transporter Tret1
LOC113515290	548	10.29	25.46	1717.7	828.82	6.24	0.00	0.00	Lysozyme
LOC113510911	426	0	0	1.17	5.8	6.24	0.00	0.00	Beta-1,3-glucan recognition protein
MSTRG.12598	1569.05	0	0.11	3.36	2.54	6.20	0.00	0.00	Uncharacterized protein LOC106125623
LOC113517406	3686	0.02	0.09	3.56	3.59	6.19	0.00	0.00	Guanylate cyclase 32E
LOC113517304	819	1.59	1.68	211.96	13.56	6.19	0.00	0.00	Proline-rich protein
LOC113518020	1160	0	0	0.47	0.64	6.14	0.00	0.00	Uncharacterized protein LOC101739749 isoform X1
LOC113521838	707	0	0	1.51	0.73	6.12	0.00	0.00	Uncharacterized protein LOC106132721 isoform X1
LOC113518076	861	0	0.1	4.14	3.55	6.08	0.00	0.00	Uncharacterized MFS-type transporter C09D4.1 isoform X2
LOC113520961	998	0	0	0.41	0.87	6.05	0.00	0.00	Tubulin Alpha-8 chain-like isoform X1
LOC113522527	1463.39	3.94	10.31	427.7	436.54	6.05	0.00	0.00	Serine protease inhibitor 6
LOC113514646	791	0	0	1.03	0.73	6.03	0.00	0.00	Proton-coupled amino acid transporter 2-like
LOC113516048	662	0	0	1.38	0.98	6.03	0.00	0.00	Uncharacterized protein LOC106131020
LOC113520078	1680	0	0	0.38	0.27	6.03	0.00	0.00	Adenylate cyclase type 2
LOC113510922	497	17.27	23.82	1294.51	1114.65	5.96	0.00	0.00	Protease inhibitor-like protein
LOC113519435	1427	0	0	0.21	0.55	5.96	0.00	0.00	Tubulin beta chain isoform X1
MSTRG.15246	283	0	0	14.73	11.95	5.94	0.00	0.00	Uncharacterized protein
LOC113517675	3732	0.83	0.66	44.72	35.82	5.89	0.00	0.00	Uncharacterized threonine-rich GPI-anchored glycoprotein PJ4664.02
LOC113512301	2287	0	0.03	1.24	0.72	5.88	0.00	0.00	Diapause hormone receptor
LOC113509994	958	1.03	1.79	74.01	76.88	5.86	0.00	0.00	Uncharacterized protein

									LOC101738673
MSTRG.7865	676	4.38	3.95	222.83	227.47	5.86	0.00	0.00	Uncharacterized protein
LOC113514450	1266	0	0	0.12	0.7	5.86	0.00	0.01	Uncharacterized protein LOC110378527 isoform X1
MSTRG.328	2801	0.83	1.29	58.55	53.12	5.86	0.00	0.00	Uncharacterized protein
LOC113514486	1343	0	0	0.33	0.41	5.84	0.00	0.01	Facilitated trehalose transporter Tret1
LOC113519601	4792	1.55	3.57	144.01	122.78	5.84	0.00	0.00	Protein phosphatase 1 regulatory subunit 12A
LOC113510107	1575	112.87	126.52	6363.02	5459.23	5.76	0.00	0.00	Hemolin
LOC113511685	2245.72	0	0.03	1.3	0.55	5.75	0.00	0.00	Pancreatic triacylglycerol lipase-like isoform X1
MSTRG.11335	1407	78.63	97.28	4116.49	4515.78	5.75	0.00	0.00	Uncharacterized protein
LOC113512893	409.65	14.95	32.59	1008.33	1400.39	5.75	0.00	0.00	Uncharacterized protein LOC106138361
LOC113515683	5730	0.15	0.18	9.32	7.02	5.75	0.00	0.00	Uncharacterized protein LOC106143250 isoform X1
LOC113522943	1011	0	0	0.16	0.86	5.74	0.00	0.01	Kallikrein-7-like
MSTRG.9041	1086	0.07	0.23	7.37	7.46	5.74	0.00	0.00	Uncharacterized protein
LOC113509237	799	0	0	0.79	0.6	5.72	0.00	0.01	DNA-binding protein D-ETS-6 isoform X1
LOC113518355	1127	0	0.07	2.43	1.7	5.72	0.00	0.00	Protein rolling stone-like
LOC113519931	1869	0.03	0.12	3.16	4.23	5.71	0.00	0.00	Protein yellow
LOC113515999	2514	0	0	0.26	0.06	5.70	0.00	0.02	Estradiol 17-beta-dehydrogenase 11 isoform X1
MSTRG.15148	436	0	0	3.66	0.78	5.70	0.00	0.02	Uncharacterized protein
LOC113522981	1929	12.91	13.19	621.96	608.87	5.70	0.00	0.00	Matrix metalloproteinase-14 isoform X1
LOC113510919	543	1.78	2.03	92.96	92.81	5.69	0.00	0.00	Lysozyme
LOC113516588	842	4.01	3.34	144.06	202.53	5.68	0.00	0.00	Tetraspanin
LOC113512706	325	0.8	0.94	81.57	19.75	5.68	0.00	0.00	Peptidoglycan recognition protein-like
LOC113512229	510	0.22	0	5.79	8.3	5.65	0.00	0.00	Uncharacterized protein LOC106139759
LOC113520441	4043	0.13	0.03	4.29	3.48	5.64	0.00	0.00	Proton-coupled amino acid transporter 1-like isoform X1
LOC113521397	3199	0	0.02	0.75	0.39	5.64	0.00	0.00	Collagen alpha-1(XVIII) chain-like

LOC113523044	577	0	0	0.2	2.09	5.62	0.00	0.02	Hypothetical protein KGM_204434
LOC113510411	1606	0	0	0.13	0.38	5.61	0.00	0.02	Uncharacterized protein LOC110380790
LOC113513784	1434	0.05	0.53	13.99	11.64	5.61	0.00	0.00	Membrane-bound alkaline phosphatase-like
LOC113523239	1265.54	9.66	12.77	496.73	472.21	5.60	0.00	0.00	GTP cyclohydrolase 1 isoform X1
LOC113512196	1678	17.27	34.2	1146.89	1114.03	5.59	0.00	0.00	Neutral Lipase
MSTRG.6746	280	1.55	0	60.32	42.13	5.54	0.00	0.00	Laccase-4-like
LOC113516037	1996	0	0.07	1.93	1.32	5.54	0.00	0.00	Uncharacterized protein LOC106143445
LOC113511279	1308	0.11	0	2.58	2.62	5.51	0.00	0.00	Facilitated trehalose transporter Tret1-like
LOC113509538	348	1.24	2.91	110.55	78.4	5.50	0.00	0.00	Low-density lipoprotein receptor 2-like
LOC113516293	1812	0	0	0.04	0.37	5.49	0.00	0.02	WEB family protein At1g65010, chloroplastic
LOC113509196	313	0	0	2.28	9.78	5.48	0.00	0.02	Uncharacterized protein LOC106136669 isoform X1
LOC113521829	1070	0	0	0.22	0.56	5.48	0.00	0.02	Uncharacterized protein LOC106716499
MSTRG.15912	922	0	0	0.27	0.68	5.48	0.00	0.02	Neuropeptide-like 3
LOC113516705	1164	0	0	0.27	0.43	5.47	0.00	0.02	Lipase 3-like
LOC113519221	493	0	0	1.36	1.44	5.47	0.00	0.02	Lipase member H-like isoform X1
LOC113519797	1656	0	0	0.21	0.23	5.47	0.00	0.02	Zinc finger protein 879-like
LOC113514938	3131	0	0	0.12	0.09	5.46	0.00	0.02	Uncharacterized protein LOC106139763
LOC113515419	1667	0	0	0.26	0.18	5.46	0.00	0.02	Myrosinase 1-like
LOC113523037	5570	0.04	0.03	2.13	1.02	5.44	0.00	0.00	Titin-like isoform X1
LOC113521034	1377	0.2	0.22	9.13	7.84	5.42	0.00	0.00	Inorganic phosphate cotransporter
LOC113523415	1629	0.04	0	0.96	1.13	5.41	0.00	0.00	Protein yellow-like
MSTRG.8654	2757	0.15	0.57	16.57	11.38	5.40	0.00	0.00	Uncharacterized protein
LOC113520329	1946	0	0.04	0.57	0.94	5.25	0.00	0.00	Prostaglandin E2 receptor EP2 subtype
LOC113514188	2796.03	0.76	0.45	23.19	17.56	5.24	0.00	0.00	cGMP-specific 3',5'-cyclic phosphodiesterase-like isoform X1
LOC113520633	2117.88	0.06	0	1.51	0.76	5.20	0.00	0.00	UNC93-like protein

MSTRG.9111	518	0.43	0.5	15.34	18.39	5.19	0.00	0.00	Uncharacterized protein
LOC113511046	2392	0.52	0.46	18.39	14.53	5.19	0.00	0.00	Transcription factor SOX-15
LOC113515031	3990	2.31	2.44	84.25	72.15	5.18	0.00	0.00	Proline dehydrogenase 1, mitochondrial
MSTRG.8275	1235	9.22	11.18	324.07	331.08	5.14	0.00	0.00	Uncharacterized protein
LOC113513664	1161	19.73	26.36	783.26	696.06	5.13	0.00	0.00	Vitellogenin receptor
LOC113521833	994	0	0.17	2.47	3.33	5.12	0.00	0.00	Odorant-binding protein A10
MSTRG.12258	1604	0.12	0.09	6.59	0.76	5.12	0.00	0.00	Uncharacterized protein
LOC113509209	1082	0.07	0	1.76	1.09	5.12	0.00	0.00	Sodium/calcium exchanger 1
LOC113509357	1342	2.35	1.96	71.94	64.87	5.12	0.00	0.00	Uncharacterized protein LOC110383549 isoform X1
LOC113520537	995	60.88	92.85	2206.09	2637.47	5.11	0.00	0.00	Uncharacterized protein LOC106132075
LOC113523190	662	0	0.16	4.13	1.79	5.10	0.00	0.00	Uncharacterized protein
LOC113509694	2324	2.49	3.99	109.15	93.67	5.10	0.00	0.00	Transferrin precursor
LOC113522517	1598	0.12	0.05	2.01	3.72	5.09	0.00	0.00	Tubulin beta-4B chain-like
LOC113514709	761	1.55	1.26	36.78	47.98	5.02	0.00	0.00	Hypothetical protein RR46_03464
LOC113510422	384	3.16	4.72	233.49	12.38	4.97	0.00	0.00	Gloverin
LOC113521739	2649	0	0.05	0.72	0.85	4.96	0.00	0.00	Wiskott-Aldrich syndrome protein family member 2-like
MSTRG.6381	570	1.08	0.21	14.24	24.55	4.93	0.00	0.00	Uncharacterized protein
LOC113513857	2221	0.96	1.11	29.96	26.6	4.91	0.00	0.00	Uncharacterized protein LOC106141788
LOC113514947	3706	0.02	0	0.24	0.33	4.89	0.00	0.00	Glucose dehydrogenase
LOC113514518	1015	1.32	0.5	30.39	20.02	4.89	0.00	0.00	cGMP-specific 3',5'-cyclic phosphodiesterase-like isoform X1
LOC113518075	1642	0.08	0.54	8.79	7.79	4.88	0.00	0.00	Hypothetical protein KGM_202228B
LOC113517530	3614.81	0.05	0	0.69	0.76	4.83	0.00	0.00	Patched domain-containing protein 3
LOC113510012	2677	7.15	9.48	199.3	224.33	4.81	0.00	0.00	Cysteine dioxygenase type 1
LOC113514846	1319	0.05	0.06	1.86	1.27	4.81	0.00	0.00	Calcitonin gene-related peptide type 1 receptor
MSTRG.11080	1017	0.07	0	1.04	1.44	4.80	0.00	0.00	Uncharacterized protein
MSTRG.12804	2070	0.24	0.31	7.58	6.46	4.79	0.00	0.00	Uncharacterized protein LOC106138099

LOC113516556	562	0	0.21	3.09	3.29	4.79	0.00	0.00	Cuticular protein RR-2 motif 59 precursor
LOC113514504	633	13.31	17.65	386.07	374.51	4.73	0.00	0.00	Uncharacterized protein
LOC113515473	1654	0	0.04	0.82	0.46	4.72	0.00	0.00	Protein enabled homolog isoform X1
LOC113510899	4012	0.66	0.58	15.46	14.16	4.71	0.00	0.00	Sodium-independent sulfate anion transporter
LOC113516706	1437	0.05	0	0.76	0.71	4.69	0.00	0.00	Uncharacterized protein LOC106142978
LOC113510941	547	0.19	0	3.7	2.55	4.69	0.00	0.00	Alpha-tocopherol transfer protein-like
MSTRG.14558	1369	3.69	8.64	129.73	154.78	4.66	0.00	0.00	Uncharacterized protein
LOC113523440	622	3.54	2.62	111.39	35.22	4.65	0.00	0.00	Antifungal peptide gallerimycin
LOC113516120	1726.17	0	0.14	1.77	1.16	4.63	0.00	0.00	Neuropeptide precursor protein isoform X1
MSTRG.9399	1248	0.5	0.13	8.27	6.41	4.61	0.00	0.00	Uncharacterized protein
LOC113513832	820	0.1	0	1.09	1.85	4.60	0.00	0.00	Serine protease 7-like
LOC113517365	1116	0.39	0	4.01	5.32	4.60	0.00	0.00	VMP32 protein
LOC113520988	370	0	0.59	7.16	8.9	4.58	0.00	0.00	Aminopeptidase N-like isoform X2
LOC113514027	1443	0.09	0.05	2.43	1.08	4.58	0.00	0.00	Mediator of RNA polymerase II transcription subunit 15-like
LOC113512420	1941	0.3	0.26	6.25	5.94	4.57	0.00	0.00	Gremlin-1-like isoform X1
MSTRG.14611	299	1.13	5.38	91.32	63.48	4.54	0.00	0.00	Uncharacterized protein
MSTRG.9506	1636	0.8	0.63	15.39	14.92	4.52	0.00	0.00	Uncharacterized protein
LOC113512073	1302	0.16	0.24	4.32	4.24	4.52	0.00	0.00	Venom acid phosphatase Acph-1-like
LOC113515831	1929	1.26	0.78	23.01	19.59	4.51	0.00	0.00	Chitinase 2
LOC113517566	10745	0.02	0	0.2	0.16	4.49	0.00	0.00	Titin
MSTRG.10254	1604	0.16	0.09	2.09	3.37	4.47	0.00	0.00	Uncharacterized protein
LOC113513999	2676.43	0.02	0.13	1.5	1.57	4.47	0.00	0.00	Proton-coupled folate transporter-like
MSTRG.675	320	0.85	0	13.47	12.21	4.47	0.00	0.00	Uncharacterized protein
LOC113512455	1336.79	96.14	123.43	2249.29	2235.57	4.46	0.00	0.00	GATA Zinc finger domain-containing protein 14-like
LOC113513334	500	0.93	0	12.62	8.95	4.45	0.00	0.00	Organic cation transporter protein-like isoform X1
LOC113511665	1342	0.05	0.4	6.27	2.78	4.44	0.00	0.00	Cardioacceleratory peptide

									receptor-like
LOC113513043	504	0.23	0	1.55	4.67	4.44	0.00	0.00	Uncharacterized protein
LOC113523293	1954	0.55	0.62	10.86	11.9	4.40	0.00	0.00	Pollen-specific Leucine-rich repeat extensin-like protein 1
LOC113521567	1369	0	0.17	1.79	1.56	4.40	0.00	0.00	Peptidyl-Alpha-hydroxyglycine Alpha-amidating lyase 2-like
LOC113509844	1722	359.71	518.68	7496.97	9302.79	4.39	0.00	0.00	Uncharacterized protein LOC106135184
LOC113522036	1353	0.05	0.06	0.88	1.35	4.34	0.00	0.00	Circadian clock-controlled protein-like
LOC113511774	2605	0	0.05	0.69	0.35	4.34	0.00	0.00	Seminal fluid protein CSSFP041
LOC113512294	3190	0.08	0.25	2.96	3.05	4.33	0.00	0.00	Inactive pancreatic Lipase-related protein 1-like isoform X4
LOC113509722	1401	0.05	0.05	0.95	1.18	4.33	0.00	0.00	Gustatory and odorant receptor 22
MSTRG.3258	989	1.06	0.26	11.27	13.51	4.31	0.00	0.00	Uncharacterized protein LOC106140707
LOC113513838	6795	0.28	0.2	5	3.49	4.28	0.00	0.00	Uncharacterized protein LOC106140978
LOC113523269	809	12.35	21.15	403.7	195.57	4.27	0.00	0.00	Gloverin-like
LOC113510565	930	0.17	0.19	3.7	2.89	4.27	0.00	0.00	CRAL-TRIO domain-containing protein
LOC113521719	2337	0.9	1.04	18.31	15.78	4.26	0.00	0.00	Hemocyte protein-glutamine gamma-glutamyltransferase-like
LOC113512554	345	14.08	18.76	275.92	339.59	4.25	0.00	0.00	Uncharacterized protein LOC110370740 isoform X2
LOC113521562	1714	0.04	0	0.16	0.7	4.24	0.00	0.00	Protein KBP homolog
LOC113511956	1931.3	0.03	0	0.18	0.57	4.24	0.00	0.00	Zonadhesin-like
LOC113510153	1196	1.42	0.6	17.29	17.66	4.23	0.00	0.00	E3 ubiquitin-protein ligase sinah isoform X1
LOC113521672	1341	0.26	0.12	4	2.6	4.21	0.00	0.00	UDP-glucose 4-epimerase-like
LOC113512071	1265	0.33	0.06	2.69	4.27	4.20	0.00	0.00	Lipase 3-like
LOC113515206	1759	9.55	21.41	313.39	207.32	4.20	0.00	0.00	Peptidoglycan-recognition protein-S
LOC113521712	651	0.14	0.16	2.68	3.02	4.17	0.00	0.00	Chemosensory protein
LOC113513204	923	153.32	184.92	2644.99	2866.49	4.15	0.00	0.00	Protein spaetzle
MSTRG.10296	1763	0.04	0.08	0.99	1.06	4.15	0.00	0.00	Uncharacterized protein
MSTRG.3742	1272.09	0.35	1.29	8.74	12.51	4.15	0.00	0.00	Uncharacterized protein

LOC113509871	614	0	0.18	1.4	2.04	4.14	0.00	0.00	Acetyltransferase ACT13
LOC113514207	2267	0.19	0.74	8.68	6.17	4.13	0.00	0.00	Aminopeptidase N-like isoform X2
LOC113513662	599	1.32	0.75	20.46	13.61	4.10	0.00	0.00	Serine protease 1-like
LOC113521620	449.67	3.58	5.66	62.34	89.93	4.08	0.00	0.00	Uncharacterized protein LOC106132635
LOC113513931	529	1.04	1.66	21.66	20.57	4.05	0.00	0.00	Uncharacterized protein
LOC113510451	1606	0	0.05	0.49	0.33	4.05	0.00	0.01	Lipase 3-like
MSTRG.8659	528	0.21	0.24	4.44	3.24	4.03	0.00	0.00	Fumarylacetoacetate hydrolase isoform B
LOC113519961	2467.67	19.39	16.96	317.39	223.2	4.03	0.00	0.00	Facilitated trehalose transporter Tret1 isoform X3
LOC113516784	2067	9.17	11.01	147.76	147.49	4.01	0.00	0.00	Buffy
LOC113514100	2111	11.76	12.89	193.05	162.95	3.99	0.00	0.00	Ferric-chelate reductase 1 homolog
LOC113518690	505	0	0.78	6.69	5.2	3.98	0.00	0.00	Gamma-glutamyltranspeptidase 1-like isoform X1
LOC113516161	2319	0	0.06	0.17	0.74	3.97	0.00	0.00	Uncharacterized protein LOC110373188
MSTRG.8877	1722	0.34	0.64	5.97	7.93	3.97	0.00	0.00	Uncharacterized protein
LOC113521572	756.43	1.55	1.73	20.05	28.03	3.96	0.00	0.00	Chemosensory protein 10
LOC113517115	1964	0.19	0.11	1.86	2.58	3.96	0.00	0.00	Uncharacterized protein LOC106143014
LOC113521491	1018	4.32	6.26	70.98	79.15	3.96	0.00	0.00	Uncharacterized protein
MSTRG.8544	446	0.6	0	5.86	4.77	3.95	0.00	0.00	Aminopeptidase N-like
LOC113514259	2649	1.36	1.63	25.7	16.36	3.94	0.00	0.00	ABC transporter G family member 23
LOC113523611	990	619.1	768.48	8837.07	10552.39	3.93	0.00	0.00	6Tox
LOC113520792	1440	0.98	1.11	16.21	12.99	3.93	0.00	0.00	G-protein coupled receptor Mth2
LOC113511994	2197	0.06	0.13	1.32	1.28	3.92	0.00	0.00	Uncharacterized protein LOC106138389 isoform X1
LOC113523442	432	300.54	476.31	5421.36	5594.39	3.90	0.00	0.00	Defensin
LOC113519028	1342	0.1	0.46	3.27	4.37	3.90	0.00	0.00	4-coumarate--CoA ligase 1-like
LOC113516369	5286	7.72	8.36	116.58	101.01	3.90	0.00	0.00	Uncharacterized protein LOC106135296
LOC113514407	4706	2.51	1.28	28.15	21.53	3.85	0.00	0.00	Multidrug resistance protein homolog 49
MSTRG.9855	1061.05	4.28	4.34	41.25	49.05	3.85	0.00	0.00	Uncharacterized protein LOC106125039

LOC113522123	458	4.82	5.54	69.65	68.97	3.82	0.00	0.00	Heat shock protein Hsp-12.2
LOC113516307	5358	0.01	0	0.07	0.11	3.81	0.00	0.02	Sperm flagellar protein
LOC113520503	5339	0.05	0.06	0.67	0.82	3.80	0.00	0.00	Dorsal-ventral patterning protein Sog
LOC113521541	873	0.09	0	0.89	0.63	3.80	0.00	0.02	15-hydroxyprostaglandin dehydrogenase
MSTRG.16508	304	0	1.25	9.1	11.18	3.80	0.00	0.02	Uncharacterized protein
LOC113518308	1933	59.79	74.45	844.3	841.19	3.79	0.00	0.00	Cystathionine gamma-lyase
LOC113512991	2242	0.06	0.03	0.57	0.61	3.77	0.00	0.00	Beta-amyloid-like protein isoform X1
LOC113522647	1350	0.2	0.17	2.75	2.05	3.76	0.00	0.00	Protein takeout
MSTRG.7153	1246	0.06	0.25	1.89	1.95	3.74	0.00	0.00	Uncharacterized protein
LOC113515146	2942.04	0.08	0	0.52	0.57	3.73	0.00	0.00	Protein orai-like isoform X1
LOC113519031	2294	0.08	0.03	0.67	0.78	3.72	0.00	0.00	Peroxidase-like isoform X2
LOC113514948	2304	0.03	0	0.15	0.28	3.72	0.00	0.02	Glucose dehydrogenase
LOC113516944	900	1.91	1.86	21.31	24	3.71	0.00	0.00	Cytochrome b5-like
LOC113514620	358	1.13	1.32	18.74	14.27	3.70	0.00	0.00	G-protein coupled receptor Mth2-like
LOC113518500	4265	0.1	0.02	0.7	0.7	3.70	0.00	0.00	Voltage-dependent calcium channel subunit Alpha-2/delta-4 isoform X1
LOC113513934	1400	0.05	0	0.53	0.22	3.69	0.00	0.02	Cuticle protein 16.8
LOC113514429	927	3.83	6.85	54.92	70.77	3.69	0.00	0.00	Corazonin
MSTRG.884	484	0.75	0.29	7.92	5.72	3.68	0.00	0.00	Uncharacterized protein
LOC113509570	5323	1.04	1.15	12.42	12.9	3.68	0.00	0.00	Uncharacterized protein DDB_G0277255 isoform X1
LOC113518265	620	2.17	1.06	21.1	17.9	3.67	0.00	0.00	Uncharacterized protein LOC110383784
MSTRG.2471	1226	0.4	0.26	4.67	3.25	3.67	0.00	0.00	Uncharacterized protein
LOC113510121	1878.41	9.36	12.36	133.77	113.29	3.67	0.00	0.00	Uncharacterized protein LOC106130105 isoform X1
LOC113509430	4001	11.35	15.44	148.65	158.28	3.66	0.00	0.00	Leucine-rich repeat-containing G-protein coupled receptor 5
LOC113514946	1387	17.68	19.32	227.16	198.26	3.65	0.00	0.00	Peptidoglycan recognition protein D
LOC113511560	884	2.31	3.11	30.42	31.98	3.65	0.00	0.00	27 kDa hemolymph protein-like
LOC113521813	1968	1.81	2.11	20.31	24.24	3.64	0.00	0.00	Uncharacterized protein LOC103309331

MSTRG.11622	717	0.12	0	0.13	1.71	3.63	0.00	0.03	Uncharacterized protein
LOC113512560	1271	50.54	55.86	594.77	596.3	3.62	0.00	0.00	Serine protease easter-like
MSTRG.10067	2263	0.44	0.56	5.51	5.61	3.61	0.00	0.00	G-protein coupled receptor Mth2-like
LOC113510025	2210	37.65	41.22	445.32	427.76	3.61	0.00	0.00	Transferrin-like
LOC113520181	783	91.3	104.76	1127.21	1061.73	3.60	0.00	0.00	NF-kappa-B inhibitor cactus
LOC113512558	850	0.19	0.32	1.54	4.27	3.60	0.00	0.00	Muscle-specific protein 20-like
LOC113516562	601	0.16	0	1.82	0.58	3.59	0.00	0.03	Ankyrin repeat domain-containing protein 65-like
LOC113516128	1756	1.63	1.45	16.9	16.69	3.58	0.00	0.00	Uncharacterized protein LOC110378115
LOC113523423	2400	0.03	0.03	0.33	0.33	3.57	0.00	0.00	Potassium channel subfamily K member 18-like isoform X1
LOC113509425	2031.61	3.58	3.06	34.14	34.29	3.57	0.00	0.00	Uncharacterized protein LOC106110582
LOC113520666	4002	4.06	4.89	51.27	44.82	3.56	0.00	0.00	Multidrug resistance protein 1A-like
LOC113509489	894	0.96	0.49	8.41	7.74	3.56	0.00	0.00	Organic cation transporter protein-like isoform X1
LOC113520692	1883	2.28	3.82	33.49	31.84	3.55	0.00	0.00	Tyrosine-protein phosphatase corkscrew-like
LOC113521399	1067	0.34	0.46	5.46	3.34	3.55	0.00	0.00	Collagen alpha-1(IX) chain-like isoform X1
LOC113513503	1693	0.19	0.3	3.26	2.09	3.54	0.00	0.00	Sodium/calcium exchanger 1
LOC113510700	2949.39	0.23	0.22	2.3	1.74	3.54	0.00	0.00	G protein-activated inward rectifier potassium channel 3-like isoform X2
LOC113509732	2250.47	48.81	67.76	677.19	556.77	3.54	0.00	0.00	Hexokinase type 2 isoform X1
LOC113519720	4971	0.07	0.09	0.87	0.83	3.51	0.00	0.00	Zinc finger protein 541
LOC113512108	3949	2.3	1.66	21.76	19.22	3.51	0.00	0.00	Uncharacterized protein LOC106139412
LOC113511049	1737.45	13.37	14.35	157.26	127.17	3.50	0.00	0.00	Glutathione synthetase-like isoform X3
MSTRG.1036	1886.7	16.43	20.6	164.74	167.97	3.50	0.00	0.00	Uncharacterized protein
LOC113522574	1698	0.04	0	0.17	0.35	3.50	0.01	0.04	MFS-type transporter SLC18B1-like
LOC113517068	4200.32	15.02	19.47	188.94	165.16	3.50	0.00	0.00	Integrin beta pat-3 precursor
LOC113515493	1809	42.28	45.63	441.21	457.65	3.49	0.00	0.00	Uncharacterized protein

									LOC106143241 isoform X4
LOC113516934	876	5.32	4.88	48.16	56.84	3.49	0.00	0.00	Uncharacterized protein LOC106143149
LOC113514256	1617	0.04	0.05	0.71	0.28	3.49	0.00	0.01	Fatty acyl-CoA reductase 1-like
LOC113509366	1840.87	366.22	426.04	4267.91	3782.93	3.48	0.00	0.00	Esterase FE4-like
LOC113509698	1923	2.06	4.4	32.43	33.28	3.48	0.00	0.00	Organic cation transporter protein-like
MSTRG.7703	549.56	26.63	32.18	292.69	313.15	3.48	0.00	0.00	6Tox
LOC113520242	770	87.65	83.94	849.98	901.42	3.47	0.00	0.00	Superoxide dismutase
LOC113509325	1596	51.95	65.84	613.43	579.91	3.47	0.00	0.00	Esterase FE4-like
MSTRG.9558	1391	0.15	0.22	2.17	1.64	3.47	0.00	0.00	Uncharacterized protein
LOC113523375	975	23.67	29.58	260.32	284.28	3.47	0.00	0.00	Uncharacterized protein LOC106131388 isoform X1
LOC113510528	1336	0.26	0.64	5.13	3.74	3.44	0.00	0.00	Collagen alpha-1(IX) chain-like isoform X1
MSTRG.16298	759	4.68	5.92	51.37	53.65	3.43	0.00	0.00	Uncharacterized protein
LOC113517800	1364	27.12	26.71	287.99	239.32	3.42	0.00	0.00	NF-kappa-B inhibitor cactus
LOC113512750	895	0.35	0.39	5.06	2.44	3.41	0.00	0.00	Serine protease 4 precursor
LOC113520000	849	0.75	0.75	7.94	6.92	3.41	0.00	0.00	Gamma-glutamyltranspeptidase 1-like isoform X1
LOC113516083	1336.11	0.1	0	0.26	0.85	3.40	0.00	0.01	Uncharacterized protein LOC110383028
LOC113511731	1113	0.45	1.24	6.85	9.33	3.39	0.00	0.00	Lambda-crystallin
LOC113518362	1374	20.37	25.35	211.54	222.51	3.38	0.00	0.00	Protein rolling stone-like
LOC113513902	804	0.72	0.23	5.93	3.46	3.36	0.00	0.00	G-protein coupled receptor Mth2-like
LOC113521358	3777	0.06	0.11	0.91	0.67	3.35	0.00	0.00	BAI1-associated protein 3
LOC113521401	1664	0.32	0.53	4.43	3.45	3.34	0.00	0.00	Collagen alpha-1(IX) chain-like isoform X1
LOC113516465	786	11.2	13.62	114.68	111.99	3.31	0.00	0.00	Uncharacterized protein LOC106132990
LOC113521819	891.14	3.39	3.83	35.19	30.54	3.31	0.00	0.00	Lipid phosphate phosphohydrolase 2-like
LOC113520443	1860	178.58	214.9	1746.37	1715.49	3.27	0.00	0.00	Antichymotrypsin-2-like
MSTRG.13104	1003	2.39	2.02	17.32	21.65	3.27	0.00	0.00	Unknown unsecreted protein
LOC113517371	5720	0.02	0.01	0.18	0.12	3.27	0.00	0.00	ATP-binding cassette sub-family C member Sur-like

LOC113514993	3751	24	28.96	226.47	230.23	3.25	0.00	0.00	Uncharacterized protein LOC105387960 isoform X1
LOC113520288	2387	0.05	0.29	1.6	1.34	3.24	0.00	0.00	Endochitinase
LOC113519738	2688	3.32	2.93	30.27	23.39	3.24	0.00	0.00	Zinc transporter ZIP1-like
LOC113511617	1353.51	135.13	181.67	1274.59	1433.69	3.23	0.00	0.00	Antichymotrypsin-2-like isoform X6
LOC113514266	373	25.16	45.41	415.28	225.3	3.22	0.00	0.00	Cecropin A
LOC113517095	2153.42	61.44	72.83	594.99	528.06	3.20	0.00	0.00	Aromatic-L-amino-acid decarboxylase isoform X1
LOC113511485	1186	0.66	0.4	4.94	4.15	3.20	0.00	0.00	Serine protease Bi-VSP-like isoform X1
LOC113521635	1838	1.65	2.48	18.1	16.42	3.20	0.00	0.00	Ejaculatory bulb-specific protein 3-like
LOC113514536	816	0.4	0.11	2.19	2.45	3.20	0.00	0.00	Protein toll-like
LOC113509735	3067	0.04	0.09	0.42	0.65	3.19	0.00	0.00	D-beta-hydroxybutyrate dehydrogenase, mitochondrial
LOC113520387	1693	0.08	0.09	0.75	0.67	3.17	0.00	0.00	Facilitated trehalose transporter Tret1-like
LOC113523482	2372	1.05	1.06	8.86	8.39	3.17	0.00	0.00	Heparan-Alpha-glucosaminide N-acetyltransferase
LOC113518892	1275	11.45	10.31	73.63	103.77	3.17	0.00	0.00	Venom peptide BmKAPI-like
LOC113516829	2418	0.05	0	0.3	0.18	3.14	0.00	0.03	Uncharacterized protein LOC106143591
LOC113515729	661	45.72	73.43	468.49	496.9	3.13	0.00	0.00	Uncharacterized protein
MSTRG.11542	473	33.21	26.3	227.35	262.85	3.13	0.00	0.00	Hypothetical protein RR48_14652
MSTRG.12036	1831	0.28	0.59	3.99	3	3.13	0.00	0.00	Uncharacterized protein
LOC113520587	1009	0.15	0.08	0.56	1.46	3.13	0.00	0.01	Uncharacterized protein LOC110374731
LOC113520736	1392	0.1	0.11	0.95	0.79	3.13	0.00	0.00	Uncharacterized protein LOC110380886 isoform X1
LOC113518906	2341	9.21	10.81	80.24	78.28	3.12	0.00	0.00	Uncharacterized protein
LOC113512008	1460	1.15	2.23	12.09	14.63	3.12	0.00	0.00	Senecionine N-oxygenase-like
LOC113522466	2969	6.05	4.5	44.64	38.43	3.12	0.00	0.00	Uncharacterized protein LOC106138412
LOC113512680	917	29.9	39.54	259.99	290.71	3.11	0.00	0.00	Uncharacterized protein
LOC113509382	1350.99	327.63	307.17	2769.37	2230.1	3.11	0.00	0.00	Venom carboxylesterase-6-like isoform X1
LOC113519387	4780.07	0.27	0.33	2.71	1.98	3.10	0.00	0.00	Latent-transforming growth factor

									beta-binding protein 4-like isoform X1
LOC113521377	1238	0.4	0.45	4.98	1.7	3.09	0.00	0.00	Alanine aminotransferase 1
LOC113516449	3740	12.19	13.52	106.66	92.13	3.09	0.00	0.00	Transmembrane protein 205
LOC113523474	1529	14.86	14.74	132.57	97.38	3.09	0.00	0.00	Venom dipeptidyl peptidase 4
LOC113515628	2124	0.06	0.1	0.9	0.38	3.08	0.00	0.00	Uncharacterized protein LOC106134633
MSTRG.4862	512	1.11	0.51	5.24	8.23	3.08	0.00	0.00	Myotubularin-related protein 10-A-like
LOC113515265	5865	0.01	0.01	0.05	0.12	3.07	0.00	0.03	Uncharacterized protein LOC105842577
LOC113517198	693	0.13	0.15	0.71	1.66	3.07	0.00	0.03	Uncharacterized protein LOC106116396 isoform X1
LOC113509550	2496	0.02	0.25	1.09	0.97	3.05	0.00	0.00	Uncharacterized protein LOC106129482
MSTRG.16406	223	12.3	0	89.36	34.43	3.04	0.00	0.03	Uncharacterized protein
LOC113513122	448	0	0.69	4.09	1.45	3.03	0.00	0.03	4-coumarate--CoA ligase 1-like
MSTRG.11855	1745	4.99	6.81	46.22	40.19	3.00	0.00	0.00	Uncharacterized protein
LOC113515498	1456	0.14	0	0.45	0.69	3.00	0.00	0.00	Serine protease gd
LOC113510062	1684	0.12	0.17	1.13	1.03	2.99	0.00	0.00	Uncharacterized protein LOC110374899
MSTRG.970	453	7.28	7.03	66.94	41.61	2.99	0.00	0.00	Ankyrin repeat domain-containing protein 50
LOC113523060	4030	4.51	3.84	34.09	25.79	2.98	0.00	0.00	Adenylate cyclase type 8
LOC113512041	4644	123.15	126.96	930.61	857.77	2.98	0.00	0.00	Hemicentin-1-like isoform X1
LOC113518566	1494	0.04	0.05	0.19	0.57	2.98	0.01	0.05	Serine proteinase stubble-like
LOC113516707	7032	0.02	0.03	0.19	0.19	2.96	0.00	0.00	Uncharacterized protein PFB0765w-like
LOC113517161	282	0	5.32	24.38	18.26	2.96	0.00	0.02	Coagulation factor-like protein 2
LOC113514427	964	0.79	0.89	5.33	6.59	2.95	0.00	0.00	Uncharacterized protein LOC106130714
LOC113512452	3281.03	0.4	0.45	2.88	3.03	2.93	0.00	0.00	Uncharacterized protein LOC106130842
MSTRG.15873	1711	2.13	3.12	16.69	19.62	2.92	0.00	0.00	Uncharacterized protein
LOC113519382	776	3.45	2.44	20.79	20.13	2.91	0.00	0.00	Uncharacterized protein LOC106137147
LOC113521614	1335	182.35	211.46	1213.58	1434.51	2.88	0.00	0.00	Dihydropteridine reductase

LOC113512948	2128	0.8	0.8	4.15	6.46	2.87	0.00	0.00	Uncharacterized protein
LOC113521636	583	274.64	452.68	2434.79	2503.92	2.87	0.00	0.00	Ejaculatory bulb-specific protein 3-like
LOC113512095	998	10.97	11.26	69.5	78.55	2.87	0.00	0.00	fatty acid-binding protein, muscle-like
LOC113522183	569	378.04	382.17	2503.3	2635.98	2.86	0.00	0.00	Cobatoxin-like protein
LOC113512252	891	1.67	1.59	10.09	11.66	2.86	0.00	0.00	Uncharacterized protein LOC106140275 isoform X1
MSTRG.7335	602	15.34	14.1	115.3	83.23	2.86	0.00	0.00	Venom dipeptidyl peptidase 4-like isoform X1
LOC113510566	519	1.08	1.24	8.49	7.48	2.85	0.00	0.00	Alpha-tocopherol transfer protein-like
LOC113522854	459	8.47	13.94	74.09	77.13	2.84	0.00	0.00	Glycine N-methyltransferase
LOC113523209	2258	0.28	0.62	3.13	2.7	2.84	0.00	0.00	23 kDa integral membrane protein-like
LOC113518433	1629	0	0.14	0.39	0.51	2.83	0.00	0.03	Uncharacterized protein LOC106137789
MSTRG.11424	1218	0.23	0.78	3.08	3.48	2.83	0.00	0.00	Uncharacterized protein
LOC113511912	1980	0	0.11	0.49	0.22	2.81	0.00	0.03	Vitamin K-dependent gamma-carboxylase
LOC113519603	753	191.1	217.03	1248.37	1384.23	2.81	0.00	0.00	Uncharacterized protein LOC106136193
LOC113515350	4022.08	26.2	25.69	195.56	132.48	2.81	0.00	0.00	Aminopeptidase N-like isoform X1
LOC113519018	6531	0.06	0.02	0.26	0.27	2.81	0.00	0.00	Uncharacterized protein LOC110375652
LOC113519756	2031.38	4.15	3.54	24.65	20.03	2.78	0.00	0.00	G-protein coupled receptor Mth2-like
MSTRG.5837	1674	1.96	2.72	13.45	15.55	2.77	0.00	0.00	Uncharacterized protein LOC106132472
MSTRG.1678	1271	13.14	12.5	89.61	68.99	2.76	0.00	0.00	Venom dipeptidyl peptidase 4-like
LOC113520845	3613	0.14	0.2	1.34	0.83	2.75	0.00	0.00	Multidrug resistance protein 1A-like
MSTRG.15180	1232.64	37.73	41.98	228.69	275.2	2.74	0.00	0.00	Uncharacterized protein
LOC113519032	1188	0.06	0.4	1.3	1.52	2.74	0.00	0.00	Uncharacterized protein LOC106137634
LOC113515213	7482	0.27	0.37	2.28	1.62	2.74	0.00	0.00	Tetratricopeptide repeat protein 28
LOC113519837	1086	0.54	0.23	2.7	2.1	2.73	0.00	0.00	Uncharacterized protein LOC105389589

LOC113519539	2902	0.1	0	0.25	0.43	2.72	0.00	0.01	Uncharacterized protein LOC106138567
MSTRG.5275	2371	0.16	0.38	1.81	1.41	2.71	0.00	0.00	Uncharacterized protein
LOC113514148	3212	0.07	0.17	0.83	0.62	2.71	0.00	0.00	Uncharacterized protein LOC106139738
LOC113509826	999	4.8	3.47	23.14	26.21	2.70	0.00	0.00	Uncharacterized protein LOC106104008
LOC113514198	768	71.69	93.8	431.39	556.59	2.70	0.00	0.00	Ras-related protein Rac1
LOC113513310	508	45.87	39.52	273.91	246.07	2.70	0.00	0.00	Beta-1,3-glucan recognition protein precursor
LOC113515392	1729	0.26	0.17	1.1	1.52	2.70	0.00	0.00	Trypsin beta-like
LOC113520749	1184	21.52	23.61	138.36	128.78	2.70	0.00	0.00	Facilitated trehalose transporter Tret1-like
LOC113513888	594	2.67	4.56	21.15	22.31	2.69	0.00	0.00	Uncharacterized protein LOC110381073
LOC113514838	469	0.27	0.62	2.74	2.92	2.69	0.01	0.04	Protein N-lysine methyltransferase METTL20-like
LOC113518034	1752	0.04	0.12	0.8	0.17	2.68	0.00	0.02	Uncharacterized protein LOC106137789
LOC113520402	1709	0.19	0.09	0.62	1.06	2.67	0.00	0.00	Spondin-2-like
LOC113513129	1433	2.41	1.27	11.14	10.18	2.66	0.00	0.00	Membrane-bound alkaline phosphatase-like
LOC113510374	1626	318.93	368.02	2169.96	1792.95	2.66	0.00	0.00	Esterase FE4 isoform X2
LOC113512844	1763	0.51	0.66	3.62	3.18	2.66	0.00	0.00	Uncharacterized protein LOC106708416
LOC113509269	2948	0.06	0.05	0.33	0.31	2.64	0.00	0.01	Fatty acyl-CoA reductase 1
LOC113511966	2253.64	31.41	29.33	222.63	216.67	2.63	0.00	0.00	Cystinosin homolog isoform X2
LOC113517728	3027	0.1	0.29	1.2	0.99	2.62	0.00	0.00	Uncharacterized protein LOC106139019
LOC113509387	2182	6.15	7.03	35.6	36.34	2.59	0.00	0.00	Uncharacterized protein LOC106140905 isoform X1
LOC113517777	1377	0.74	0.78	4.57	3.83	2.59	0.00	0.00	Zinc finger protein Gfi-1 isoform X1
LOC113514862	311	1.91	3.39	17.6	15.1	2.57	0.00	0.02	Mitochondrial glutamate carrier 1-like
MSTRG.4627	567	5.64	4.97	30.81	28.04	2.57	0.00	0.00	Hemicentin-1
LOC113517309	1888.33	0.38	0.58	2.67	2.39	2.55	0.00	0.00	NADPH oxidase 4-like
MSTRG.10207	1979	0.19	0.43	1.63	1.67	2.53	0.00	0.00	Uncharacterized protein

									LOC106134231
LOC113523210	2550	0.17	0.38	1.07	1.8	2.53	0.00	0.00	23 kDa integral membrane protein-like
MSTRG.10612	395	22.44	26.03	157.69	113.31	2.53	0.00	0.00	Transmembrane protein 205
LOC113519607	3088	0.04	0.09	0.3	0.39	2.53	0.00	0.01	Transient receptor potential channel pyrexia isoform X1
LOC113511563	3040.08	0.27	0.09	0.62	0.58	2.52	0.00	0.00	Extracellular serine/threonine protein CG31145 isoform X1
LOC113510109	2311	57.55	55.56	300.79	289.14	2.52	0.00	0.00	Suppressor of tumorigenicity 14 protein homolog
LOC113517076	1106	0.13	0.29	1.35	0.91	2.51	0.00	0.01	Visual pigment-like receptor peropsin
LOC113522488	3056.78	19.51	16.64	102.39	83.68	2.50	0.00	0.00	Inter-Alpha-trypsin inhibitor heavy chain H4-like
LOC113512388	1579	51.95	63.3	290.84	302.7	2.50	0.00	0.00	J domain-containing protein
LOC113521431	2894	2.01	1.67	9.58	9.32	2.50	0.00	0.00	Breast cancer anti-estrogen resistance protein 1
LOC113515807	2680	0.25	0.23	1.72	0.79	2.50	0.00	0.00	Irregular chiasm C-roughest protein-like isoform X2
MSTRG.14620	1657	132.63	159.49	776.16	717	2.49	0.00	0.00	Esterase B1-like isoform X1
LOC113514217	824	3.15	4.23	19.08	18.6	2.47	0.00	0.00	Uncharacterized protein LOC106130712
LOC113519628	3018	0.12	0.22	0.78	0.97	2.47	0.00	0.00	Uncharacterized protein LOC106136198
LOC113515407	689	0.26	0.29	1	1.98	2.47	0.01	0.04	Serine proteinase stubble
MSTRG.4280	866	9.71	15.1	63.1	62.71	2.46	0.00	0.00	Uncharacterized protein
LOC113523607	941	0.73	1.19	4.27	5.49	2.46	0.00	0.00	Uncharacterized protein
LOC113523011	2301.91	66.7	94.04	430.42	375.69	2.46	0.00	0.00	Surface protein bspA-like
LOC113518050	1554	0.13	0.1	0.83	0.35	2.46	0.00	0.02	Major facilitator superfamily domain-containing protein 12-like isoform X2
LOC113519768	1640.77	18.12	17.51	89.19	98.48	2.45	0.00	0.00	Calcium-binding mitochondrial carrier protein SCaMC-2
LOC113511948	5229.52	0	0.06	0.15	0.15	2.45	0.00	0.03	Transcriptional regulator ATRX-like isoform X1
LOC113522786	1993	1.43	0.97	6.82	5.17	2.45	0.00	0.00	Uncharacterized protein LOC106138639
LOC113509842	1254	0.22	0.19	1.15	0.97	2.45	0.00	0.01	Uncharacterized protein

									LOC106135203
LOC113520819	3306	0.29	0.28	1.35	1.5	2.44	0.00	0.00	MutS protein homolog 4-like isoform X2
LOC113514885	668	6.42	7.59	32.53	37.52	2.44	0.00	0.00	Glycine N-methyltransferase
LOC113523425	633	67.67	71.79	288.43	405.49	2.43	0.00	0.00	Defensin
LOC113510568	2173	6.24	5.31	24.38	31.82	2.43	0.00	0.00	Heat shock protein 68
AT056_gr02	1368	78.96	92.42	377.64	456.93	2.42	0.00	0.00	Uncharacterized protein
LOC113522333	1329	52.7	55.3	259.71	263.41	2.41	0.00	0.00	Growth arrest and DNA damage-inducible protein GADD45 alpha
LOC113509515	1539.41	4.99	7.24	34.06	24.54	2.41	0.00	0.00	Uncharacterized protein LOC106138752
LOC113520269	3047	119.52	127.36	570.27	617.83	2.41	0.00	0.00	Uncharacterized protein LOC106131445
LOC113515237	2671	4.05	5.65	26.11	20.6	2.40	0.00	0.00	4-methylmuconolactone transporter
LOC113513720	893	140.82	180.56	765.64	787.17	2.40	0.00	0.00	Serine protease 7-like
LOC113509767	2181	0.72	0.45	3.38	2.28	2.40	0.00	0.00	Organic cation transporter protein-like isoform X1
MSTRG.4513	532	14.61	19.49	75.29	90.65	2.38	0.00	0.00	Phosphoglycolate phosphatase 1B, chloroplastic-like
LOC113509813	804	109.92	134.12	535.95	629.76	2.38	0.00	0.00	Hypothetical protein RR46_13217
LOC113514718	2292	0.24	0.03	0.7	0.66	2.38	0.00	0.01	Uncharacterized protein LOC106130386
LOC113511510	1096	0.2	0.07	1.08	0.31	2.37	0.00	0.03	Uncharacterized protein LOC106130779
LOC113520493	705.29	9.77	9.24	49.41	52.08	2.35	0.00	0.00	Uncharacterized protein LOC110375908 isoform X4
LOC113520659	685	16.9	20.19	91.24	82.69	2.34	0.00	0.00	Facilitated trehalose transporter Tret1-like
LOC113518509	6328	0.35	0.51	1.87	2.09	2.34	0.00	0.00	Uncharacterized protein LOC106135505
MSTRG.8652	1223.53	0.36	0.45	2.37	2.88	2.33	0.00	0.00	Uncharacterized protein
MSTRG.4469	7365	3.82	3.92	19.98	15.35	2.33	0.00	0.00	Ankyrin repeat domain-containing protein 50 isoform X2
LOC113519422	642	4.67	1.99	12.74	18.2	2.32	0.00	0.00	Heat shock protein 24.3
LOC113511522	652	1.14	0.48	4.55	3.18	2.32	0.00	0.01	F-actin-methionine sulfoxide oxidase MICAL3 isoform X1
LOC113512015	2726	0.04	0.1	0.41	0.26	2.31	0.00	0.03	Facilitated trehalose transporter Tret1-2 homolog isoform X1

LOC113519587	1769	9.19	8.88	46.39	35.45	2.31	0.00	0.00	Serine protease HTRA2, mitochondrial-like
MSTRG.13222	3861	5.24	6.35	27.06	25.11	2.31	0.00	0.00	Golgin-45
LOC113523000	1785	0.51	0.69	2.9	2.51	2.30	0.00	0.00	Pseudouridine-metabolizing bifunctional protein C1861.05
LOC113515359	2483	0.12	0.2	0.7	0.74	2.30	0.00	0.00	Uncharacterized protein LOC106103767 isoform X2
LOC113517599	6463.96	13.01	14.03	72.9	47.64	2.29	0.00	0.00	Integrin Alpha-PS2
LOC113517978	739	4.3	4.34	19.39	19.42	2.28	0.00	0.00	Globin 1
LOC113523237	814	0.7	1.25	4.17	4.56	2.28	0.00	0.00	Serine/threonine-protein kinase MARK2 isoform X6
LOC113511295	3163	0.06	0.06	0.25	0.31	2.28	0.00	0.02	Prolow-density lipoprotein receptor-related protein 1
LOC113522134	585	1.03	1.17	4.77	5.28	2.28	0.00	0.00	Zwei Ig domain protein zig-8-like
LOC113518959	860	2.5	1.15	6.78	9.48	2.27	0.00	0.00	Glutathione S-transferase delta 4
LOC113518142	842	124.06	149.24	549.84	656.51	2.27	0.00	0.00	Mid1-interacting protein 1A
MSTRG.2506	462	1.39	1.28	5.68	6.72	2.26	0.00	0.01	Glypican-6 isoform X1
MSTRG.1343	1969	3.88	4.1	16.28	17.86	2.23	0.00	0.00	Uncharacterized protein LOC110374261
LOC113516144	5411	3.83	4.07	16.36	16.82	2.21	0.00	0.00	Uncharacterized protein LOC106710224
LOC113515596	1426	1.52	1.33	6.17	5.92	2.21	0.00	0.00	Uncharacterized protein LOC106143246
LOC113521752	2125	4.15	8.01	25.53	25.63	2.21	0.00	0.00	Uncharacterized protein LOC106132637
MSTRG.3030	897	121.81	131.93	531.19	533.73	2.20	0.00	0.00	Uncharacterized protein
MSTRG.2140	1975.81	0.13	0.36	1.15	0.89	2.19	0.00	0.01	Uncharacterized protein
LOC113516094	966	0.94	0.97	5.05	3.01	2.18	0.00	0.00	Sialin-like
LOC113509956	2032	0.84	0.38	2.43	2.66	2.18	0.00	0.00	Inverted formin-2 isoform X2
MSTRG.16473	366	19.91	15.9	79.89	77.91	2.17	0.00	0.00	Beta-1,3-glucan recognition protein precursor
LOC113516392	2171	0.87	1.14	3.56	4.56	2.16	0.00	0.00	Aromatic-L-amino-acid decarboxylase-like
MSTRG.5335	483	1.01	0.58	4.83	2.12	2.15	0.01	0.04	Uncharacterized protein
MSTRG.11410	974	0.16	0.79	2.37	1.44	2.15	0.00	0.01	Uncharacterized protein
LOC113518189	2401	0.83	0.64	2.76	3.14	2.15	0.00	0.00	Leucine-rich repeat neuronal protein 2-like

LOC113513801	2458.92	12.17	11.17	39.6	53.56	2.14	0.00	0.00	Suppressor of cytokine signaling 2-like isoform X2
LOC113521869	2871	0.13	0.05	0.46	0.27	2.13	0.00	0.02	Leucine-rich repeat-containing protein 49
MSTRG.5596	1584	48.49	53.07	203.41	200.36	2.13	0.00	0.00	Reverse transcriptase
LOC113523217	2206.39	16.72	17.44	75.84	59.86	2.12	0.00	0.00	Glycerol kinase-like isoform X1
LOC113515545	1393	4.49	6.04	22.92	18.95	2.12	0.00	0.00	Uncharacterized protein LOC106134731
LOC113513236	1351	15.47	12.72	60.01	51.68	2.12	0.00	0.00	Peptidyl-Alpha-hydroxyglycine Alpha-amidating lyase 1-like
LOC113522508	1381	51.63	66.59	214.64	252.68	2.12	0.00	0.00	HIG1 domain family member 1A, mitochondrial-like isoform X1
LOC113519574	1243	0.39	0.19	1.35	1.04	2.12	0.00	0.02	Immulectin-2
LOC113509379	528.58	17.29	22.31	80.45	81.11	2.11	0.00	0.00	Uncharacterized protein LOC106131730 isoform X1
LOC113511358	782	29.6	32.86	135.55	112.75	2.11	0.00	0.00	Protein canopy homolog 1
LOC113513109	420	19.68	23.13	85.75	89.97	2.10	0.00	0.00	Beta-1,3-glucan recognition protein precursor
LOC113519787	13893	0.05	0.06	0.25	0.19	2.10	0.00	0.00	Dynein beta chain, ciliary-like
LOC113515922	632	4.8	3.4	18.74	14.12	2.10	0.00	0.00	Uncharacterized protein
MSTRG.11278	1211	0.24	0.46	0.9	1.83	2.09	0.00	0.02	Uncharacterized protein
LOC113512385	3293	0.11	0.14	0.59	0.4	2.09	0.00	0.01	Endothelin-converting enzyme homolog
LOC113510373	1881	189.94	242.72	827.79	847.22	2.09	0.00	0.00	Carboxylesterase
LOC113514677	6076	1.13	0.93	4.93	3.04	2.08	0.00	0.00	Uncharacterized protein LOC110380322 isoform X2
LOC113512866	4177	53.96	56.23	222.39	201.69	2.08	0.00	0.00	Calcineurin B
LOC113521746	1936.47	1.15	2.59	7.04	7.39	2.08	0.00	0.00	Uncharacterized protein LOC106132632
LOC113514028	5614	0.49	0.84	2.64	2.5	2.08	0.00	0.00	Uncharacterized protein LOC106710656
LOC113517032	779	22.28	25.18	82.68	101.41	2.08	0.00	0.00	28S ribosomal protein S18b, mitochondrial
LOC113519654	1503	0.31	0.3	1.16	1.23	2.08	0.00	0.01	Senecionine N-oxygenase-like
LOC113517314	2624.28	0.97	0.81	3.44	3.33	2.08	0.00	0.00	Cytochrome b reductase 1-like isoform X2
LOC113518280	2679	1.05	0.87	3.62	3.75	2.08	0.00	0.00	Carbonic anhydrase 2
LOC113511419	738	116.27	139.58	481.98	510.69	2.08	0.00	0.00	G-protein coupled receptor Mth2-

									like isoform X2
LOC113512381	1790	0.9	0.97	3.63	3.58	2.07	0.00	0.00	Uridine phosphorylase 1 isoform X1
LOC113513817	627	6.08	9.49	34.64	26.13	2.07	0.00	0.00	Beta-1,3-glucan recognition protein
LOC113510923	349	17.82	16.56	74.88	68.16	2.07	0.00	0.00	Protease inhibitor1
LOC113516263	2672	0.69	0.51	2.72	1.89	2.07	0.00	0.00	Carboxylesterase 1E
LOC113515921	1197.22	10.95	9.99	46.53	34.43	2.06	0.00	0.00	Ankyrin repeat domain-containing protein 50 isoform X1
LOC113521822	1363	0.85	0.79	2.72	3.53	2.06	0.00	0.00	Sex peptide receptor-like
LOC113511323	1713	10.73	11.24	44.49	38.71	2.05	0.00	0.00	Acylcarnitine hydrolase-like
LOC113519795	2791	7.61	9.32	32.65	30.9	2.05	0.00	0.00	UDP-glucuronosyltransferase 1-1-like
LOC113513583	1703	0.5	0.39	0.99	2.34	2.04	0.00	0.01	Uncharacterized protein LOC110376907 isoform X1
LOC113514673	1416	0.48	0.59	2.49	1.55	2.04	0.00	0.00	Uncharacterized protein LOC106140704
LOC113516802	2983.38	24.01	26.19	91.6	90.07	2.02	0.00	0.00	Uncharacterized protein LOC106143158 isoform X1
LOC113514784	717	5.72	4.27	21.7	15.97	2.02	0.00	0.00	Neutral ceramidase
LOC113514672	1990	0.61	0.75	3.42	1.65	2.02	0.00	0.00	Uncharacterized protein LOC110380322 isoform X2
LOC113520028	889	187.53	240.08	719.34	863.95	2.02	0.00	0.00	Mesencephalic astrocyte-derived neurotrophic factor homolog
MSTRG.13956	964	1.73	1.95	7.56	6.13	2.01	0.00	0.00	Uncharacterized protein
LOC113521510	2124	0.41	0.3	1.7	0.95	2.01	0.00	0.01	WW domain-binding protein 11-like
LOC113515576	480	109.18	133.03	441.92	473.26	2.01	0.00	0.00	Cytochrome oxidase subunit 2(mitochondrion)
LOC113515351	2950	0.78	0.9	3.35	2.73	2.00	0.00	0.00	Aminopeptidase N
LOC113512426	1910	10.78	11.8	40.83	40.84	1.99	0.00	0.00	CMP-sialic acid transporter 1
LOC113517700	752	6.33	7.42	22.58	27.61	1.99	0.00	0.00	Uncharacterized protein LOC106129404
LOC113518800	5874	0.2	0.23	0.81	0.73	1.99	0.00	0.00	Chaoptin
LOC113515203	1926	9.15	9.79	32.87	35.25	1.98	0.00	0.00	Neurogenic locus notch homolog protein 1 isoform X1
LOC113522346	1128	3.13	2.59	11.03	9.68	1.98	0.00	0.00	G-protein coupled receptor moody-like isoform X1
MSTRG.11374	315	5.44	8.57	32.16	23.78	1.98	0.00	0.02	Sphingosine-1-phosphate

									phosphatase 1-like
LOC113512374	1932.7	2.94	2.48	10.82	8.67	1.98	0.00	0.00	Cytochrome P450 9e2-like
LOC113517538	1592	0.37	0.47	1.84	1.2	1.98	0.00	0.01	Uncharacterized protein LOC106138673 isoform X4
LOC113512639	1392	125.57	129.73	484.5	429.05	1.97	0.00	0.00	Serine protease easter-like
LOC113515542	429	59.22	60.62	192.74	253.04	1.97	0.00	0.00	Uncharacterized protein
LOC113511452	690	0.52	0.73	1.85	2.73	1.97	0.01	0.04	Uncharacterized protein OBRU01_13522
LOC113512044	2630	63.02	74.51	239.32	249.8	1.97	0.00	0.00	Hemicentin-1-like
LOC113512924	758	34.17	44.71	134.81	147.83	1.96	0.00	0.00	39S ribosomal protein L33, mitochondrial
LOC113511047	2660.17	0.07	0.22	0.39	0.53	1.96	0.00	0.03	A disintegrin and metalloproteinase with thrombospondin motifs 1 isoform X1
LOC113518143	3029	15.31	15.98	57.29	53.32	1.96	0.00	0.00	SHC-transforming protein 1 isoform X1
LOC113516792	842.1	84.72	94.31	299.94	334.04	1.96	0.00	0.00	Calcium load-activated calcium channel
LOC113517470	872	28.67	42.29	120.96	131.34	1.95	0.00	0.00	Alcohol dehydrogenase AD1
LOC113515993	827	110.32	138.32	393.96	488.57	1.95	0.00	0.00	Cytochrome c
LOC113518097	2553	1.59	1.38	5.3	5.12	1.95	0.00	0.00	Carboxypeptidase N subunit 2-like
LOC113522500	1863	2.52	2.98	10.1	9.21	1.95	0.00	0.00	Uncharacterized protein LOC106129400
LOC113523104	2677	526.04	594.21	2061.87	1837.57	1.94	0.00	0.00	Heat shock 70 kDa protein cognate 3 isoform X1
LOC113509584	530	1102.49	1215.07	3643.17	4585.76	1.93	0.00	0.00	JH-inducible protein
LOC113512770	1146	114.85	141.03	433.74	454.15	1.93	0.00	0.00	Chymotrypsinogen B-like
LOC113522449	1340.08	15.44	18.19	55.1	61.53	1.92	0.00	0.00	Cysteine-rich with EGF-like domain protein 2 isoform X1
MSTRG.7295	2361	4.26	4.69	17.16	13.72	1.92	0.00	0.00	Trehalase-like isoform X1
MSTRG.6737	299	3.4	20.16	32.31	55.92	1.92	0.00	0.01	Uncharacterized protein LOC106129042
LOC113509986	383	3.64	3.17	13.32	11.92	1.91	0.00	0.03	Neutral ceramidase
LOC113514834	404	20.28	21.68	63.74	87.11	1.91	0.00	0.00	Cytochrome c oxidase assembly factor 6 homolog
LOC113509500	403	3.93	1.82	8.2	13.09	1.91	0.00	0.02	von Willebrand factor A domain-containing protein 8 isoform X1
LOC113521183	3554.47	0.34	0.08	0.9	0.52	1.90	0.00	0.01	Potassium voltage-gated channel

									protein Shab isoform X1
LOC113513436	741	633.53	575.7	2311.56	1845.31	1.90	0.00	0.00	Venom carboxylesterase-6-like isoform X1
LOC113519444	5462.87	2.59	2.34	8.48	8.19	1.90	0.00	0.00	Solute carrier family 41 member 2-like isoform X1
LOC113518232	1512	8.17	7.5	24.31	28.74	1.90	0.00	0.00	Uncharacterized protein LOC106135508
LOC113523439	978	2.32	0.87	5.22	5.74	1.90	0.00	0.01	30.3 kDa protein
LOC113514316	3493.42	20.13	20.57	71.89	65.43	1.89	0.00	0.00	Serine/threonine-protein kinase pelle
LOC113510710	1260	4.1	5.79	15.32	18.11	1.89	0.00	0.00	Uncharacterized protein LOC110384236
LOC113518948	2403.37	370.95	389.35	1297.14	1261.77	1.89	0.00	0.00	Hypothetical protein RR46_03373
MSTRG.14756	3883.79	1.94	2.07	6.95	6.71	1.88	0.00	0.00	Uncharacterized protein
LOC113520065	823	0.79	1	3.56	2.53	1.88	0.00	0.02	Diamine acetyltransferase 2-like
LOC113522584	1075	0.61	0.77	1.63	2.99	1.87	0.00	0.02	DNA repair protein XRCC2
LOC113516157	3077	4.13	3.71	14.34	11.43	1.85	0.00	0.00	Insulin receptor substrate 1
LOC113520348	6644.22	0.39	0.44	1.53	1.17	1.84	0.00	0.00	Uncharacterized protein LOC106131594 isoform X4
LOC113515798	1402.05	546.2	646	1915.59	1962.95	1.84	0.00	0.00	Arginine kinase isoform X1
LOC113519959	769	8.96	2.96	21.09	18.24	1.84	0.00	0.00	Uncharacterized protein LOC105842437
LOC113520698	2487	45.53	52.82	165	154.58	1.84	0.00	0.00	Uncharacterized protein LOC106140812
LOC113516445	559	1.5	1.07	6.04	2.66	1.84	0.01	0.04	Alanine aminotransferase 1
MSTRG.15853	3144	0.59	0.67	2.35	1.74	1.83	0.00	0.00	Uncharacterized protein
MSTRG.1113	795	0.62	0.59	1.59	2.42	1.83	0.01	0.05	Uncharacterized protein
MSTRG.15392	960	0.55	1.25	2.59	3.22	1.82	0.00	0.02	Uncharacterized protein LOC106130466
LOC113518233	454	2.03	2	6.59	7.01	1.82	0.01	0.04	Serine/threonine-protein kinase 3 isoform X5
LOC113514387	647	31.55	30.23	87.68	112.47	1.81	0.00	0.00	28S ribosomal protein S25, mitochondrial
LOC113514898	1127	474.18	470.15	1615.35	1412.81	1.81	0.00	0.00	Venom carboxylesterase-6-like isoform X1
LOC113513755	1351	11.62	9.92	35.04	33.75	1.81	0.00	0.00	Follicle-specific yolk polypeptide-4
LOC113512976	1635	1.41	1.54	3.74	5.56	1.80	0.00	0.01	UDP-glucuronosyltransferase 1-6-like

LOC113518043	4350	5.67	3.42	15.68	12.86	1.79	0.00	0.00	Lysine-specific demethylase 6A isoform X1
LOC113509109	1505	13.64	18.22	48.55	52.13	1.79	0.00	0.00	Uncharacterized protein LOC106131182
LOC113513388	1990	149.73	111.66	459.76	364.54	1.79	0.00	0.00	Heat shock protein 90 cognate
LOC113513424	1830	1.23	1.15	3.08	4.38	1.79	0.00	0.01	Inverted formin-2 isoform X1
LOC113514654	828	2.93	2.1	7.06	8.89	1.78	0.00	0.01	Uncharacterized protein LOC106131814
LOC113516162	1321	5.63	6.92	20.82	18.5	1.78	0.00	0.00	Uncharacterized protein LOC106138810
LOC113521924	954	8.87	9.46	34.43	23.02	1.77	0.00	0.00	Lipid phosphate phosphohydrolase 1-like
LOC113519432	767	1.1	1.49	3.37	4.74	1.77	0.00	0.02	Protein lethal(2)essential for life-like
MSTRG.7643	2929	1.9	1.32	5.55	4.42	1.76	0.00	0.00	Uncharacterized protein
LOC113513308	525	145.38	168.14	510	485.26	1.76	0.00	0.00	Uncharacterized protein LOC106143374
LOC113509077	394	110.52	147.65	412.88	430.31	1.76	0.00	0.00	Uncharacterized protein
LOC113515353	1161	3.5	2.77	10.15	9.32	1.76	0.00	0.00	Aquaporin-like
LOC113514254	3966.94	2.19	2.62	7.66	7.14	1.76	0.00	0.00	Fatty acyl-CoA reductase 1
MSTRG.10058	348	113.58	131.64	435.52	381.7	1.76	0.00	0.00	Kunitz/Bovine pancreatic trypsin inhibitor domain protein
LOC113519988	1695	40.61	48.37	125.41	147.89	1.76	0.00	0.00	Phosducin-like protein
LOC113520434	6746.29	0.16	0.15	0.65	0.32	1.76	0.00	0.01	Uncharacterized protein LOC106104179 isoform X1
LOC113510223	545	4.71	4.25	16	12.36	1.75	0.00	0.01	Tafazzin homolog
LOC113521365	2920.52	87.31	96.96	315.91	236.11	1.75	0.00	0.00	3-phosphoinositide-dependent protein kinase 1 isoform X1
LOC113520620	2738	4.84	6.87	18.84	16.95	1.75	0.00	0.00	Solute carrier family 35 member B1 homolog
LOC113521910	1664	10.75	14.42	37.11	39.76	1.75	0.00	0.00	Cyclin-dependent kinases regulatory subunit
LOC113519816	2364	167.71	153.44	531.76	447.27	1.74	0.00	0.00	Unc-112-related protein-like
LOC113520849	2075	14.64	15.27	48.69	42.56	1.74	0.00	0.00	WAS/WASL-interacting protein family member 2-like isoform X1
MSTRG.6081	2096	0.81	0.81	2.54	2.42	1.74	0.00	0.01	Uncharacterized protein LOC105222572
LOC113512293	1127	20.7	25.18	66.83	72.88	1.74	0.00	0.00	Post-GPI attachment to proteins

									factor 2-like
LOC113522957	1947.12	123.75	141.4	404.31	394.07	1.73	0.00	0.00	Choline-phosphate cytidylyltransferase A-like isoform X2
MSTRG.9305	836	33.17	39.2	97.49	122.91	1.73	0.00	0.00	Uncharacterized protein
LOC113514343	3915.69	5.73	6.31	20.69	15.73	1.73	0.00	0.00	Mushroom body large-type Kenyon cell-specific protein 1 isoform X1
LOC113516936	3693.83	11.2	11.87	36.5	32.95	1.73	0.00	0.00	Uncharacterized protein LOC106099788 isoform X1
LOC113513318	736	5.84	3.44	17.85	10.66	1.73	0.00	0.01	Uncharacterized protein LOC106137281
MSTRG.3269	4293	4.17	3.89	13.52	10.72	1.73	0.00	0.00	Uncharacterized protein
MSTRG.5458	5000	1.24	0.69	3.03	2.76	1.72	0.00	0.00	Hypothetical protein KGM_200275B
LOC113515494	1243	4.28	8.49	15.96	22.4	1.72	0.00	0.00	tRNA (guanine-N(7)-)-methyltransferase non-catalytic subunit wuho
MSTRG.286	873	1.99	1.74	6.93	4.32	1.70	0.00	0.02	Centaurin-gamma-1A
LOC113520163	1137	407.35	310.51	1055.27	1070.26	1.70	0.00	0.00	Cation transport regulator-like protein 2
MSTRG.15084	352	20.28	17.46	66.86	53.88	1.69	0.00	0.01	Uncharacterized protein
LOC113521229	979	19.59	24.08	60.45	68.14	1.69	0.00	0.00	Prefoldin subunit 4
LOC113521931	3604	10.58	12.21	35.32	31.31	1.69	0.00	0.00	BAG family molecular chaperone regulator 2
MSTRG.14456	1396	3.8	5.15	11.14	14.96	1.68	0.00	0.01	Uncharacterized protein
LOC113514865	339	29.99	28.82	104.11	83.19	1.68	0.00	0.01	LIM and SH3 domain protein Lasp isoform X2
LOC113518216	7785	0.15	0.23	0.58	0.53	1.67	0.00	0.01	Mucin-5AC isoform X2
LOC113518758	1656	12.16	15.93	35.48	45.7	1.67	0.00	0.00	Uncharacterized protein LOC106122557
MSTRG.4195	723	7.45	5.03	14.4	22.08	1.67	0.00	0.01	Uncharacterized protein LOC106138234
LOC113516023	2397	10.75	10.44	32.55	28.67	1.67	0.00	0.00	Ribonuclease ZC3H12C isoform X1
LOC113511427	1132	5.91	3.65	16.3	11.47	1.66	0.00	0.01	Tyrosine-protein phosphatase 69D
LOC113523023	3879	4.59	2.78	11.97	9.2	1.66	0.00	0.01	GTPase-activating protein
LOC113509271	371	16.06	14.04	37.26	55.46	1.66	0.00	0.01	Adapter molecule Crk
LOC113518431	4159	0.13	0.29	0.56	0.62	1.66	0.00	0.03	Glutaminase

LOC113520096	342	19.81	15.5	51.5	59.23	1.66	0.00	0.02	Ester hydrolase C11orf54 homolog
LOC113521320	1147	0.75	0.63	1.7	2.31	1.66	0.01	0.04	Uncharacterized protein LOC106135015
LOC113519016	658	18.2	23.98	51.62	70.74	1.66	0.00	0.01	Uncharacterized protein C6orf203 homolog
LOC113518638	310	10.66	14.9	40.47	40.86	1.65	0.00	0.03	Uncharacterized protein
LOC113510138	1605	0.74	1.71	3.11	3.89	1.65	0.00	0.01	Hypothetical protein OBRU01_12387
MSTRG.14446	6906	6.71	6.73	21.24	17.15	1.65	0.00	0.01	Uncharacterized protein
MSTRG.14376	1458	2.91	3.01	9.56	7.41	1.65	0.00	0.01	Uncharacterized protein
LOC113519841	1702.99	0.54	0.86	1.91	2.07	1.65	0.00	0.02	SPARC-related modular calcium-binding protein 1
LOC113514216	1630	98.84	118.87	314.26	306.11	1.64	0.00	0.01	Uncharacterized protein LOC106106086
LOC113519838	9347	11.07	7.47	29.09	23.32	1.64	0.00	0.01	Myosin-I heavy chain-like
LOC113523460	3699	1.41	1.26	3.99	3.57	1.64	0.00	0.01	Uncharacterized protein LOC110370969
LOC113518067	1511	1.28	0.75	3.45	2.35	1.64	0.00	0.02	Cyclin E
LOC113522838	834.8	4.33	5.7	12.54	16.67	1.63	0.00	0.01	Small integral membrane protein 8
LOC113520679	705	7.38	10.77	22.16	29.6	1.63	0.00	0.01	Uncharacterized protein LOC106139261 isoform X1
LOC113520836	4205.91	8.17	8.5	24.18	22.3	1.63	0.00	0.01	Rho guanine nucleotide exchange factor 10-like protein
MSTRG.13915	1828	19.63	19.11	59.53	49.81	1.63	0.00	0.01	Uncharacterized protein
MSTRG.2751	2616	1.34	0.89	3.51	2.77	1.63	0.00	0.01	Zinc finger protein jing-like
MSTRG.14833	548	2.91	2.88	8.24	8.54	1.63	0.00	0.04	Uncharacterized protein
MSTRG.13220	592	696.02	889.89	2204.95	2327.43	1.62	0.00	0.01	Protein CREG1
LOC113509424	959	14.52	18.43	48.89	44.05	1.62	0.00	0.01	Uncharacterized protein OBRU01_26608
LOC113520837	2746	10.86	14.05	34.68	34.93	1.62	0.00	0.01	Uncharacterized protein LOC106130822
LOC113518671	1638	41.17	36.69	140.99	77.85	1.62	0.00	0.01	Titin
LOC113520844	4904.6	2.85	3.45	8.18	9.33	1.62	0.00	0.01	Inorganic phosphate cotransporter isoform X2
MSTRG.9856	1142	41.51	43.62	124.31	113.79	1.61	0.00	0.01	LIM and SH3 domain protein Lasp isoform X1
MSTRG.3287	544	17.53	16.17	46.64	49.16	1.61	0.00	0.01	Tryptase

LOC113517402	693	14.79	20.1	49.99	48.09	1.61	0.00	0.01	Serine hydrolase
LOC113519437	4693.48	6.23	5.9	18.87	14.66	1.60	0.00	0.01	IQ motif and SEC7 domain-containing protein 2 isoform X1
LOC113519555	1533	9.95	10.54	33.4	23.55	1.60	0.00	0.01	Krueppel-like factor 7 isoform X1
LOC113516393	521	211.16	294.8	626.01	805.58	1.60	0.00	0.01	Defensin-like protein precursor
LOC113522348	359	37.4	46.32	104.02	143.44	1.60	0.00	0.01	Mitochondrial import inner membrane translocase subunit Tim9-like
LOC113509748	1675	7.46	7.37	18.9	21.93	1.60	0.00	0.01	Methionine synthase reductase-like
LOC113518841	817	639.07	790.98	1956.05	2020.33	1.60	0.00	0.01	Protein CREG1
LOC113510575	2072	1.31	1.81	4.45	4.14	1.59	0.00	0.01	Uncharacterized protein LOC110380322 isoform X2
LOC113512485	543	213.72	161.4	518.01	534.78	1.59	0.00	0.01	Serine palmitoyltransferase 2
LOC113511749	1245	2.14	2.85	6.83	6.89	1.59	0.00	0.01	Uncharacterized protein LOC106133686
MSTRG.6685	1914	5.58	5.14	13.65	15.58	1.59	0.00	0.01	Uncharacterized protein
LOC113518176	3056.31	101.29	89.7	272.51	221.74	1.58	0.00	0.01	Dm0-like lamin
LOC113511421	2058	16.56	14.38	48.93	35.4	1.58	0.00	0.01	Protein yellow-like
MSTRG.2644	417	7.88	3.72	21.05	12.74	1.58	0.00	0.04	Uncharacterized protein
MSTRG.14322	364	31.47	41.72	98.46	114.4	1.58	0.00	0.01	Hypothetical protein RR46_08551
MSTRG.15052	940	1.88	0.74	4.9	2.37	1.58	0.01	0.04	Uncharacterized protein
LOC113521973	8757	38.32	37.66	124.61	81.45	1.58	0.00	0.01	Titin
MSTRG.12222	2605	12.69	11.35	33.68	31.34	1.57	0.00	0.01	Adapter molecule Crk isoform X1
LOC113522930	2298	4.15	3.07	10.71	8.79	1.57	0.00	0.01	Zinc finger protein 395
LOC113522768	2340	0.39	0.64	1.61	1.18	1.57	0.00	0.03	Glucose dehydrogenase
MSTRG.1873	5971.34	6.05	6.79	18.53	16.58	1.57	0.00	0.01	Ras-like protein 2
LOC113521322	2014	132.47	140.39	379.02	354.87	1.56	0.00	0.01	TPPP family protein CG45057-like
MSTRG.955	404	101.81	101.19	300.18	276.25	1.56	0.00	0.01	Uncharacterized protein
LOC113516020	1158.74	41.74	44.31	118.05	114.05	1.56	0.00	0.01	Dihydrolipoyllysine-residue acetyltransferase component of pyruvate dehydrogenase complex, mitochondrial isoform X1
LOC113519015	757	7.27	8.61	23.09	19.61	1.54	0.00	0.02	Pre-mRNA-splicing factor 18
MSTRG.11336	669	3.54	2.01	8.86	6.24	1.54	0.00	0.03	Gamma-1-syntrophin
LOC113522789	1568	2.58	3.23	7.78	7.66	1.54	0.00	0.02	FGFR1 oncogene partner-like
LOC113519438	728	2.85	2.69	5.23	9.6	1.54	0.00	0.03	Small heat shock protein 19.7

LOC113519511	1016	2.2	3.97	6.63	9.7	1.54	0.00	0.02	Uncharacterized protein LOC106129042
LOC113523377	2542	13.96	15.5	40.57	36.85	1.53	0.00	0.01	Bestrophin 1b
LOC113520220	2530	0.46	0.35	1.18	0.98	1.53	0.01	0.04	DE-cadherin
LOC113510280	2145	263.71	344.07	844.69	753.59	1.53	0.00	0.01	Acetylcholinesterase-like
LOC113519746	2868	45.72	40.7	116.19	110.05	1.53	0.00	0.01	Protein distal antenna-like
LOC113517347	751	23.45	17.42	63.67	45.15	1.53	0.00	0.01	LIM and SH3 domain protein 1 isoform X2
LOC113520850	4837.11	0.2	0.24	0.62	0.56	1.53	0.01	0.04	Serine/arginine repetitive matrix protein 1 isoform X2
LOC113523096	1622	119.97	138.81	317.27	360.91	1.53	0.00	0.01	Phosphoglycerate kinase
LOC113521317	1465	27.09	35.24	73.26	90.01	1.53	0.00	0.01	Glycerol-3-phosphate phosphatase
LOC113511593	1548	229.47	282.46	626.29	713.75	1.52	0.00	0.01	SH3 domain-binding glutamic acid-rich protein homolog
LOC113511018	3352	13.87	17.21	41.77	39.38	1.52	0.00	0.01	Tubulointerstitial nephritis antigen precursor
LOC113513003	4454	50.23	54.01	148.47	122.46	1.52	0.00	0.01	Hypoxia up-regulated protein 1
LOC113513616	617	20.63	28.4	58.67	70.92	1.52	0.00	0.01	DNA replication complex GINS protein PSF1-like
LOC113521362	630	43.41	57.15	114.55	149.79	1.51	0.00	0.01	Multifunctional methyltransferase subunit TRM112-like protein
LOC113518834	811.14	8.48	11.97	27.08	26.39	1.51	0.00	0.02	Mitochondrial pyruvate carrier 1
LOC113511606	892	1442.97	1865.1	3526.08	5028.85	1.50	0.00	0.01	27 kDa hemolymph protein
LOC113516241	267	176.05	166.95	557.71	462.42	1.50	0.00	0.02	Canopy-1 like protein
MSTRG.13326	1500	0.8	1.1	3.24	1.7	1.50	0.01	0.04	Uncharacterized protein
LOC113517403	671	13.28	11.06	30.49	32.94	1.50	0.00	0.02	Serine hydrolase
MSTRG.3045	1242	2.09	2.16	4.65	6.26	1.50	0.00	0.03	Uncharacterized protein
LOC113518369	2297	38.12	44.41	98.93	112.29	1.50	0.00	0.01	Uncharacterized protein C45G9.7
LOC113514877	600	148.44	135.87	374.99	365.77	1.49	0.00	0.01	Cation transport regulator-like protein 2
LOC113515081	1799.85	55.95	53.48	160.37	118.44	1.48	0.00	0.01	Ceramide synthase 6-like isoform X2
LOC113511175	471	113.36	141.03	291.12	374.84	1.48	0.00	0.02	Salivary cysteine-rich peptide precursor
LOC113516653	1335	172.98	219.39	461.42	534.67	1.48	0.00	0.01	DnaJ homolog shv
LOC113520067	2579	40.69	40.86	109.04	97.2	1.48	0.00	0.01	Transcription factor GATA-5-like isoform X1

LOC113510776	2344	13.49	14.42	40.27	30.32	1.47	0.00	0.02	Solute carrier family 22 member 21 like protein
LOC113516924	3143	12.12	11.07	30.44	27.85	1.47	0.00	0.02	Tribbles homolog 2-like
LOC113514911	1922	5.75	7.02	17.25	14.97	1.47	0.00	0.02	Prestin isoform X2
LOC113512998	1158	48.17	45.61	136.61	100.52	1.46	0.00	0.02	Hypoxia up-regulated protein 1
LOC113512883	3510.12	10.3	8.48	23.84	23.14	1.46	0.00	0.02	Uncharacterized protein LOC110384473 isoform X1
LOC113519823	3672	0.53	0.53	1.53	1.13	1.46	0.00	0.03	MATH and LRR domain-containing protein PFE0570w-like
LOC113515570	735	14.99	17.76	37.06	45.76	1.46	0.00	0.02	Coiled-coil domain-containing protein 58
LOC113521185	1969.15	1.64	0.63	1.57	3.77	1.46	0.00	0.03	Purine nucleoside phosphorylase-like isoform X1
LOC113520238	1458	4.48	4.72	14.96	8.21	1.46	0.00	0.02	UDP-glycosyltransferase UGT40L1
LOC113516119	1375	176.33	213.58	501.19	474.02	1.45	0.00	0.02	EF-hand domain-containing protein D2 homolog
LOC113517227	3979	10.46	11.87	27.27	28.21	1.45	0.00	0.02	Uncharacterized protein LOC106138607 isoform X1
LOC113511802	2550	94.74	114.72	240.32	279.61	1.45	0.00	0.02	Zonadhesin-like
LOC113518510	3866.87	18.63	18.65	47.36	44.79	1.45	0.00	0.02	Transcriptional coactivator YAP1-like isoform X1
LOC113514608	686	7.58	6.81	19.01	17.33	1.45	0.00	0.03	Solute carrier family 2, facilitated glucose transporter member 1-like
LOC113522643	2367	0.68	0.76	1.81	1.78	1.45	0.01	0.04	SET and MYND domain-containing protein 4
MSTRG.1217	330	37.69	62.99	139.56	132.66	1.45	0.00	0.03	Pleiotrophin-like protein precursor
LOC113509995	2534	11.1	6.78	26.98	17.33	1.45	0.00	0.02	Tyrosine-protein phosphatase 69D
LOC113523167	4971	0.43	0.68	1.56	1.2	1.44	0.00	0.03	Uncharacterized protein LOC106140814
LOC113516478	1631	203.97	216.53	539.87	499.8	1.44	0.00	0.02	Tubulin beta chain-like isoform X1
LOC113514371	2485	6.01	6.99	16.34	15.72	1.44	0.00	0.02	Uncharacterized protein LOC106133025
LOC113519740	593	112.02	115.16	252.58	317.35	1.44	0.00	0.02	Transcription initiation factor IIA subunit 2
LOC113509119	2032.72	7.1	6.86	16.1	17.86	1.44	0.00	0.02	Phospholipid-transporting ATPase IA
LOC113512416	3865	0.93	0.84	2.23	2.14	1.44	0.00	0.03	Phosphoinositide 3-kinase adapter protein 1 isoform X1

LOC113516872	2220	24.55	26.72	70.59	56.05	1.44	0.00	0.02	Large neutral amino acids transporter small subunit 2
LOC113517383	1035	23.32	28.77	53.58	74.65	1.43	0.00	0.02	Phosphatidylinositol N-acetylglucosaminyltransferase subunit P
LOC113510812	4781	2.32	2.19	5.9	5.15	1.43	0.00	0.02	Uncharacterized protein LOC110373838 isoform X4
LOC113509803	1572	19.26	25.57	49.92	60.23	1.43	0.00	0.02	Dehydrodolichyl diphosphate syntase complex subunit Nus1
LOC113521425	1400	6.65	8.89	17.41	20.79	1.43	0.00	0.02	Protein phosphatase 1 regulatory inhibitor subunit 16B
LOC113515943	1796	7.52	11.03	22.89	22.69	1.43	0.00	0.02	Group XIIA secretory phosphoLipase A2
LOC113514549	1811.23	39.27	36.73	105.62	80.02	1.43	0.00	0.02	Uncharacterized protein LOC110381915 isoform X1
LOC113513069	5694	24.1	23.76	64.06	52.73	1.43	0.00	0.02	Dual specificity tyrosine-phosphorylation-regulated kinase 1A isoform X1
LOC113515500	806	40.81	52.55	111.92	118.34	1.42	0.00	0.02	39S ribosomal protein L32, mitochondrial
LOC113515883	3148	7.07	8.68	19.18	19.23	1.42	0.00	0.02	Twinfilin
LOC113517327	2693	0.73	0.64	2.01	1.33	1.42	0.01	0.04	Nuclear receptor-binding protein homolog
MSTRG.3046	1354	14.72	10.35	34.78	26.47	1.42	0.00	0.02	Phospholipid-transporting ATPase VD
LOC113522936	2981.98	89.71	100.43	234.93	227.98	1.42	0.00	0.02	CTL-like protein 2 isoform X3
LOC113519999	1094	3.12	3.44	8.6	7.47	1.42	0.00	0.03	DAXX
LOC113515483	1150	54.71	68.37	142.66	155.98	1.41	0.00	0.02	Uncharacterized protein
LOC113511292	2017.75	8.36	8.16	22.75	18.82	1.41	0.00	0.02	Ceramide glucosyltransferase
LOC113520645	2302.21	29.97	31.24	68.93	67.83	1.41	0.00	0.02	Acetyl-coenzyme A transporter 1
LOC113510730	716	20.87	26.67	51.41	64.3	1.40	0.00	0.03	Uncharacterized protein C19orf52
LOC113509393	1475.75	225.93	275.14	538.8	666.21	1.40	0.00	0.02	Serine protease easter-like
LOC113510592	925	17.44	22.3	41.8	54.11	1.40	0.00	0.03	Cytochrome c oxidase assembly protein COX16 homolog, mitochondrial
LOC113520706	5118	2.73	2.06	6.21	5.25	1.40	0.00	0.03	Cyclin-dependent kinase 12 isoform X4
LOC113521451	4835	15.72	16.51	39.25	37.89	1.40	0.00	0.02	Uncharacterized protein

									LOC101736230
LOC113509717	957	38.04	49.8	99.2	112.66	1.40	0.00	0.02	Prefoldin subunit 3
LOC113513297	503	110.5	107.64	302.97	236.81	1.40	0.00	0.02	Tricarboxylate transport protein, mitochondrial
LOC113518809	1425	33.34	36.08	86.15	80.09	1.39	0.00	0.02	Pyruvate dehydrogenase E1 component subunit beta, mitochondrial
LOC113517271	1212	22.16	27.49	57.61	61.36	1.39	0.00	0.02	Serine/threonine-protein kinase Aurora-2
LOC113513022	5659	14.32	12.76	36.28	28.26	1.39	0.00	0.02	Uncharacterized protein LOC101746298 isoform X1
LOC113509711	1037	34.17	44.2	88.16	99.68	1.39	0.00	0.02	aminoacyl tRNA synthase complex-interacting multifunctional protein 1
LOC113523090	4528	46.43	43.43	117.15	96.74	1.39	0.00	0.02	Gibberellin 20 oxidase 2
LOC113518397	520	148.21	186.49	362.69	454.79	1.39	0.00	0.02	39S ribosomal protein L42, mitochondrial
LOC113516384	691	16.54	23.27	39.84	56.05	1.39	0.00	0.03	RNA-binding protein EIF1AD
LOC113509198	659	749.62	765.77	1731.86	1920.99	1.39	0.00	0.02	Uncharacterized protein LOC105389192
LOC113509677	1957	19.62	23.9	52	51.47	1.39	0.00	0.03	Asparagine synthetase
LOC113511796	545	564.58	727.77	1442.17	1696.76	1.38	0.00	0.02	Uncharacterized protein
LOC113519827	654	47.39	51.18	109.95	127.35	1.38	0.00	0.03	39S ribosomal protein L9, mitochondrial isoform X1
LOC113518350	3505.12	8.98	15.74	29.22	29.42	1.38	0.00	0.03	Uncharacterized protein LOC106138210
LOC113519620	2127.6	137.48	134.23	338.39	300.29	1.37	0.00	0.03	Transcription factor kayak isoform X7
MSTRG.15283	3302	6.64	5.86	16.64	12.77	1.37	0.00	0.03	Uncharacterized protein
MSTRG.8601	6734.13	29.98	24.34	64.99	62.18	1.37	0.00	0.03	Uncharacterized protein
LOC113518819	2314	9.44	8.81	21.62	21.21	1.37	0.00	0.03	NADP-dependent malic enzyme-like
LOC113519029	2199	109.51	116.32	252.42	277.33	1.37	0.00	0.03	Lachesin isoform X2
LOC113520423	551	18.64	17.96	46.1	41.98	1.37	0.00	0.04	Mitochondrial glutamate carrier 1
LOC113516452	1630	7.06	5.12	17.65	11.03	1.36	0.00	0.03	Neuroglian isoform X1
LOC113515482	1456	5.37	6.71	13.68	14.63	1.36	0.00	0.03	Phosphatidylinositol-glycan biosynthesis class F protein
LOC113523094	2939.26	53.99	57.5	136.75	123.41	1.36	0.00	0.03	Formin-binding protein 1-like

LOC113520669	3571	1.87	2.17	4.87	4.53	1.36	0.00	0.03	Uncharacterized protein LOC110374769
LOC113509438	483	82.54	71.5	219.93	152.68	1.36	0.00	0.03	Tricarboxylate transport protein, mitochondrial
LOC113516270	1070	14.16	14.87	34.25	33.78	1.36	0.00	0.03	Uncharacterized protein LOC106709791
LOC113522287	2557	5.89	5.26	14.73	11.27	1.36	0.00	0.03	Uncharacterized protein LOC106110164
LOC113514275	1363	21.27	19.74	48.03	47.69	1.36	0.00	0.03	Golgin subfamily A member 7
LOC113517492	1541.89	119.08	99.65	257.25	254.61	1.36	0.00	0.03	Inositol-trisphosphate 3-kinase A isoform X2
LOC113510131	2042	9.18	9.85	22.01	22.23	1.35	0.00	0.03	Tetraspanin-9-like
LOC113515552	2855.83	9.6	7.66	21.52	18.58	1.35	0.00	0.03	V-type proton ATPase 116 kDa subunit a isoform 1
LOC113514183	2268	91.74	108.29	231.12	233.53	1.35	0.00	0.03	Uncharacterized protein LOC101737344
MSTRG.5750	1591	1.58	2.85	4.59	5.7	1.35	0.01	0.04	Endonuclease-reverse transcriptase
LOC113513183	2447	8.03	9.12	22.21	17.71	1.35	0.00	0.03	Kinesin-like protein KIF20B
LOC113517774	1161	4.18	3.11	10.49	6.55	1.35	0.01	0.05	Uncharacterized protein LOC106127544
MSTRG.106	550	110.52	96.72	284.49	206.33	1.34	0.00	0.03	Asparagine--tRNA ligase, cytoplasmic
LOC113521742	2058	9.5	10.86	20.71	26.1	1.34	0.00	0.03	Cyclin-dependent kinases regulatory subunit
LOC113520498	2963	52.84	61.36	135.14	127.18	1.34	0.00	0.03	B-cell receptor-associated protein 31
LOC113519615	954	12.87	14.24	31.03	31.66	1.34	0.01	0.04	Uncharacterized protein LOC110379113
LOC113522759	1068.27	42.46	54.44	107.35	115.66	1.33	0.00	0.03	Surfeit locus protein 6 homolog
LOC113511698	3056	96.15	98.71	232.36	213.89	1.33	0.00	0.03	Innexin inx3
LOC113511934	1444	4.49	3.94	10.94	8.47	1.33	0.01	0.04	Atrial natriuretic peptide receptor 2
MSTRG.11349	1636	25.32	19.45	53.53	49.1	1.33	0.00	0.03	Uncharacterized protein
LOC113512947	753	6.99	10.21	18.33	21.49	1.33	0.01	0.04	Uncharacterized protein LOC106142568
LOC113519456	616	24.76	16.02	55.2	40.09	1.33	0.01	0.04	Transcriptional activator protein Pur-alpha isoform X4
LOC113516632	2393	13.44	15.36	33.11	32.69	1.33	0.00	0.03	Glucose-6-phosphate 1-dehydrogenase

LOC113515000	1409	72.33	99.54	193.68	199.3	1.33	0.00	0.03	Uncharacterized protein LOC106134174
LOC113511704	1761	229.21	243.48	528.87	546.46	1.32	0.00	0.03	Protein transport protein Sec61 subunit alpha isoform 2
LOC113517733	787	109.06	98.82	226.36	249.02	1.32	0.00	0.04	Cytochrome b5-like
LOC113517776	1207	5.03	5.72	12.51	11.97	1.32	0.01	0.05	Lysosomal thioesterase PPT2-A
LOC113510817	1180	34.61	43.42	97.97	79.41	1.31	0.01	0.04	Alpha-tocopherol transfer protein-like isoform X2
LOC113517211	3725.5	45.98	47.63	114.6	96.31	1.31	0.01	0.04	Nitric oxide synthase
LOC113523378	2070	4.95	5.48	9.99	13.42	1.31	0.01	0.04	Uncharacterized protein LOC106131292
LOC113518496	3070	69.01	68.11	168.12	140.52	1.31	0.01	0.04	Protein eiger
LOC113520821	2613	2.93	2.63	6.58	5.91	1.30	0.01	0.05	Transcription factor 2 isoform X4
LOC113511455	2161	14.82	16.4	34.52	35.47	1.30	0.01	0.04	Uncharacterized protein LOC106139975
LOC113510046	961	11.47	9.46	23.55	23.52	1.30	0.01	0.05	Phospholipid scramblase 2
LOC113509739	1235	32.14	42.28	80.11	86.6	1.30	0.01	0.04	Nucleolar protein 16
LOC113523605	2862	26.16	14.92	46.67	44.41	1.29	0.01	0.04	Eukaryotic translation initiation factor 4 gamma 2
LOC113521234	1523	159.58	188.4	385.03	391.81	1.29	0.01	0.04	Translocon-associated protein subunit alpha precursor
LOC113513625	1556	12.25	18.36	31.83	36.47	1.29	0.01	0.04	Uncharacterized protein
LOC113520589	853	189.44	223.39	417	506.84	1.29	0.01	0.04	Translocon-associated protein subunit gamma-like isoform X1
LOC113520711	3361	11.71	10.21	29.25	19.51	1.29	0.01	0.04	Valine--tRNA ligase isoform X1
LOC113518468	1466	44.81	54.44	105.55	114.87	1.29	0.01	0.04	Uncharacterized protein LOC110374098
LOC113519642	2927.78	133.62	138.15	311.38	290.57	1.28	0.01	0.04	Serine protease easter-like isoform X1
LOC113518491	3249	8.64	7.62	19.82	15.96	1.28	0.01	0.05	Beta-arrestin-1 isoform X1
LOC113514493	1355	74.34	55.06	166.1	119.31	1.27	0.01	0.04	Zinc transporter ZIP13 homolog
LOC113512633	6328.54	14.58	16	36.68	30.39	1.27	0.01	0.04	CUGBP Elav-like family member 1 isoform X5
LOC113514856	2383	12.67	11.64	29.34	23.99	1.27	0.01	0.05	Juvenile hormone esterase-like isoform X1
LOC113522996	4811	11.46	15.34	29.96	28.68	1.27	0.01	0.05	Golgin subfamily A member 4-like
MSTRG.1123	781	54.63	52.95	139.32	99.52	1.27	0.01	0.05	Uncharacterized protein

LOC113523539	1736	9.14	9.76	24.18	17.35	1.27	0.01	0.05	Glutamine-dependent NAD(+) synthetase
LOC113516464	889.11	275.13	333.18	615.96	717.47	1.26	0.01	0.05	Translocon-associated protein subunit beta
LOC113512993	3750	8.49	10.22	21.61	19.11	1.26	0.01	0.05	Fasciculation and elongation protein zeta-2
LOC113520619	1655	20.06	21.23	44.39	45.41	1.26	0.01	0.05	Uncharacterized protein LOC106129264
LOC113522456	1578	164.81	188.45	360.46	406.04	1.25	0.01	0.05	Ras-like GTP-binding protein RhoL
LOC113517945	1917	2995.98	2013.29	1051.78	855.99	-1.26	0.01	0.05	Prophenoloxidase
LOC113517354	875	299.88	344.25	127.36	119.97	-1.26	0.01	0.05	Sulfotransferase 1 family member D1-like
LOC113515218	517	216.23	204.42	74.31	88.44	-1.27	0.01	0.05	Apoptosis-inducing factor 3-like isoform X1
LOC113519225	844	79.5	87.51	29.44	34.01	-1.27	0.01	0.05	Sulfotransferase 1C4-like
LOC113518095	1232	27.57	34.37	11.29	12.21	-1.27	0.01	0.05	Trans-1,2-dihydrobenzene-1,2-diol dehydrogenase
LOC113521103	1313	131.9	120.62	49.75	45.33	-1.28	0.01	0.04	Uncharacterized protein LOC106130198 isoform X1
LOC113520664	1143.01	56.43	56.78	18.95	21.39	-1.29	0.01	0.04	Prostaglandin reductase 1-like
LOC113522531	1092	22.44	23.55	9.49	7.72	-1.29	0.01	0.05	Uncharacterized protein LOC106132906
LOC113511777	1606	21.69	20.03	7.83	7.63	-1.30	0.01	0.04	Meteorin-like protein
LOC113520762	2338	12.31	10.23	5.02	3.33	-1.30	0.01	0.04	F-box/LRR-repeat protein 4 isoform X1
LOC113516626	1093	86.01	98.35	30.39	37.99	-1.30	0.01	0.04	Alpha-tocopherol transfer protein-like
LOC113513841	1070	26.27	30.28	10.2	10.78	-1.30	0.01	0.04	Organic cation transporter protein-like
LOC113517016	1270.47	20.64	22.1	7.89	7.92	-1.30	0.01	0.05	Uncharacterized protein LOC106143663 isoform X1
LOC113511010	1724	2038.72	2139.23	686.47	849.8	-1.30	0.01	0.04	C1A cysteine protease precursor
MSTRG.4568	862	125.84	118.75	37.44	53.28	-1.30	0.01	0.04	Zinc finger CCHC domain-containing protein 24-like
LOC113520515	1847	423	426.12	162.41	149.92	-1.31	0.01	0.04	Uncharacterized protein LOC106132893 isoform X2
LOC113510094	1538	27.61	27.08	9.75	10.33	-1.31	0.01	0.04	Gastrula Zinc finger protein XlCGF57.1-like

LOC113512029	1705	9.07	15.42	4.55	4.45	-1.31	0.01	0.04	Uncharacterized protein LOC106135547
LOC113518015	1680	16.27	16.09	6.02	5.83	-1.31	0.01	0.04	Zinc finger protein OZF-like
MSTRG.3892	2751.46	33	34.76	11.55	14.03	-1.32	0.01	0.04	Uncharacterized protein
LOC113523301	5153.62	244.59	211.53	93.75	77.39	-1.32	0.00	0.03	Vascular endothelial growth factor receptor 1 isoform X1
LOC113517202	622.89	177.48	203.41	61.27	78.53	-1.32	0.01	0.04	Uncharacterized protein LOC110369978
LOC113518202	1522	83.32	90.43	27.86	35.39	-1.32	0.00	0.04	Hydroxymethylglutaryl-CoA lyase, mitochondrial
LOC113518761	980	76.87	83.95	24.85	33.89	-1.32	0.01	0.04	Kynurenine formamidase-like isoform X1
LOC113520552	1127	43.32	46.9	16.95	15.98	-1.32	0.01	0.04	Uncharacterized protein LOC106108538
LOC113509873	846	15.73	20.33	6.01	7.17	-1.32	0.01	0.05	Uncharacterized protein LOC106135995
LOC113520017	2249	47.27	39.63	17.63	13.93	-1.32	0.00	0.03	Glycosyltransferase 25 family member isoform X1
LOC113512351	4100.19	7.77	5.96	2.93	2.03	-1.33	0.01	0.04	Hemicentin-2
LOC113510862	992	102.8	105.36	41.61	34.37	-1.33	0.00	0.03	Zinc transporter ZIP3
LOC113511950	2122	89.13	83.9	31.15	31.34	-1.33	0.00	0.03	Pancreatic lipase-related protein 2-like
LOC113520319	1544	685.94	677.41	242.05	249.4	-1.34	0.00	0.03	Uncharacterized protein CG1785
LOC113510092	3013	23.58	24.44	8.47	8.77	-1.34	0.00	0.03	Zinc finger protein 100-like
LOC113517298	4936	27.47	25.57	10.64	9.69	-1.34	0.00	0.03	Ecdysone receptor isoform X1
LOC113510790	1393	65.59	60.5	24.35	21.03	-1.34	0.00	0.03	Endophilin-A isoform X5
LOC113519215	3276.85	10.81	10.49	3.63	3.79	-1.34	0.00	0.03	Uncharacterized protein LOC101735755 isoform X1
LOC113518106	3095	8.53	3.3	2.16	2.03	-1.35	0.01	0.04	Peroxisomal acyl-coenzyme A oxidase 1 isoform X1
LOC113514099	1745	36.03	40.21	13	14.24	-1.35	0.00	0.03	DNA polymerase epsilon subunit 2
LOC113519526	1246	29.09	32.66	11.46	10.65	-1.35	0.00	0.03	Zinc finger protein 525-like
LOC113515614	2231	8.52	12.55	4.57	2.97	-1.35	0.00	0.04	Fasciclin-1
LOC113518096	1845	128.88	126.29	47.61	43.13	-1.36	0.00	0.03	Facilitated trehalose transporter Tret1-like
LOC113512779	653	58.15	59.84	24.27	18.34	-1.36	0.00	0.03	Facilitated trehalose transporter Tret1-like isoform X1
LOC113521763	1062	19.63	22.42	5.94	8.97	-1.36	0.00	0.04	Trimeric intracellular cation

									channel type B
LOC113516356	429	2193.11	1661.81	664.1	762.78	-1.36	0.00	0.03	Sparc
LOC113513475	616	29.62	28.66	11.8	9.24	-1.36	0.01	0.04	Galactokinase-like
LOC113510675	762	1108.8	1192.35	381.29	439.08	-1.36	0.00	0.03	Ribosomal biogenesis protein RLP24
LOC113514381	1185	6.95	5.93	2.41	2.15	-1.36	0.01	0.05	Uncharacterized protein LOC106138920
LOC113516374	1254	1350.53	1319.57	444.71	497.82	-1.37	0.00	0.03	Prolylcarboxypeptidase
LOC113522896	1438	16.44	11.51	5.85	4.01	-1.37	0.00	0.03	Neurogenic locus Notch protein
LOC113515371	2174.5	119.8	121.23	43.36	41.23	-1.37	0.00	0.03	Cytochrome P450 monooxygenase CYP304F17
LOC113513725	636	830.09	666.02	267.27	265.81	-1.37	0.00	0.03	Nucleolar GTP-binding protein 1
MSTRG.9470	3405	15.11	16.25	6.37	4.63	-1.38	0.00	0.03	Interference hedgehog-like
LOC113510029	1432	13.78	14.11	6.24	3.6	-1.38	0.00	0.03	Programmed cell death protein 4
LOC113513595	1799	74.96	67.5	25.28	24.53	-1.38	0.00	0.03	N-acetylglucosaminidase
LOC113522101	734	2333.14	2526.87	800.59	917.29	-1.38	0.00	0.03	Nuclear protein 1
LOC113514150	1664	40.6	50.11	16.75	15.07	-1.38	0.00	0.03	Cytochrome P450 6B6-like
LOC113515147	1163	12.71	13.7	4.12	5.03	-1.38	0.00	0.03	Methyl-CpG-binding domain protein 4-like
LOC113511306	5172	1.76	1.35	0.48	0.59	-1.38	0.01	0.04	Uncharacterized protein LOC106139408
LOC113516488	374	341.98	344.22	117.43	137.41	-1.38	0.00	0.03	Membrane steroid binding protein
LOC113521781	1137	117.3	125.43	40.99	43.73	-1.39	0.00	0.03	Uncharacterized protein LOC106141718 isoform X1
LOC113517706	556	45.72	36.8	10.74	18.38	-1.39	0.00	0.03	5-methylcytosine rRNA methyltransferase NSUN4
LOC113510920	1280	5.21	4.82	1.53	1.94	-1.39	0.01	0.05	Microtubule-associated protein futsch-like
LOC113523541	11852.28	203.43	166.97	72.99	52.27	-1.39	0.00	0.02	Teneurin-3 isoform X1
LOC113517033	2609	3.1	2.45	1.27	0.65	-1.40	0.01	0.04	Venom dipeptidyl peptidase 4
LOC113510894	2383	2.91	2.57	1.29	0.6	-1.40	0.01	0.04	Uncharacterized protein LOC106133593 isoform X1
LOC113515116	2208	8368.43	7278.56	2666.76	2696.13	-1.40	0.00	0.02	Prophenoloxidase subunit 2
LOC113510995	424	221.47	187.56	79.2	67.93	-1.41	0.00	0.02	Nucleolar GTP-binding protein 1
LOC113512658	1502	4.5	3.95	1.4	1.49	-1.41	0.01	0.04	Uncharacterized protein LOC106114649
LOC113522844	1451.35	34.24	35.33	11.87	11.99	-1.41	0.00	0.02	Uncharacterized protein

LOC113518925	1024	85.33	95.41	32.16	30.04	-1.41	0.00	0.02	2-hydroxy-6-oxononadienedioate/2-hydroxy-6-oxononatrienedioate hydrolase isoform X1
LOC113523512	1880	11.24	11.39	4.54	3.2	-1.41	0.00	0.03	Uncharacterized protein
LOC113511898	1910	3.25	4.92	1.52	1.27	-1.41	0.01	0.04	Hypothetical protein KGM_210031
LOC113519881	1138	29.21	20.45	8.71	8.25	-1.42	0.00	0.03	Steroid hormone receptor ERR1 isoform X3
LOC113522801	4978	1.92	1.83	0.64	0.63	-1.42	0.00	0.03	Uncharacterized protein LOC109421171
MSTRG.13678	1084	1163.22	1103.91	362.75	411.58	-1.42	0.00	0.02	RNA exonuclease 4-like
LOC113521113	1387.27	73.83	60.09	25.27	20.46	-1.42	0.00	0.02	Protein cab-1 isoform X2
LOC113515318	1523	546.04	410.76	184.76	141.85	-1.42	0.00	0.02	Digestive cysteine proteinase 1
LOC113519736	599	39.69	49.23	12.24	18.47	-1.42	0.00	0.03	Ecdysteroid-regulated 16 kDa protein-like
LOC113516200	1181	7.65	5.26	2.34	1.99	-1.43	0.01	0.04	PR domain Zinc finger protein 16-like isoform X1
LOC113510161	1785.77	38.58	41.59	12.33	14.02	-1.43	0.00	0.02	Serine hydrolase-like protein
LOC113522361	871	13.36	16.92	5.66	4.65	-1.43	0.00	0.03	Zinc finger protein 57
LOC113521031	5722	2.55	1.75	0.86	0.59	-1.43	0.00	0.03	Ig-like and fibronectin type-III domain-containing protein T04A11.3 isoform X2
LOC113513453	847	41.44	51.59	15.21	16.42	-1.43	0.00	0.02	Bm8 interacting protein 2d-2
MSTRG.14140	805	6.73	9.45	3.35	2.14	-1.43	0.01	0.04	Autophagy-related protein 2 homolog A
LOC113515578	714	14.96	13.05	4.05	5.45	-1.44	0.00	0.04	Meiosis-specific nuclear structural protein 1-like isoform X2
LOC113513763	797	30.84	28.3	11.79	8.33	-1.44	0.00	0.02	Interference hedgehog-like
LOC113517246	3242.76	29.72	29.6	10.02	9.88	-1.44	0.00	0.02	TNF receptor-associated factor 4 isoform X1
LOC113517522	4088.21	100.76	82.23	33.07	28.04	-1.44	0.00	0.02	Receptor-type tyrosine-protein phosphatase N2 isoform X1
LOC113521224	1166	42.55	45.89	14.75	14.93	-1.44	0.00	0.02	E3 ubiquitin-protein ligase ZNRF1
LOC113516959	8640	0.75	0.83	0.28	0.24	-1.44	0.00	0.03	Myosin-VIIa isoform X1
LOC113517648	2581	55.91	48	17.61	17.03	-1.44	0.00	0.02	Leucine-rich repeat-containing G-protein coupled receptor 4
LOC113511027	3177.17	2.43	2.38	1.06	0.56	-1.45	0.00	0.03	Nucleolar protein 4-like
LOC113515181	1678	5.58	3.94	1.81	1.35	-1.45	0.00	0.03	Unconventional myosin-XVIIIa isoform X1

LOC113510662	1127.41	105.56	103.16	33.69	35.67	-1.45	0.00	0.02	Omega-amidase NIT2 isoform X1
LOC113519683	1733	43.38	39.53	13.6	13.88	-1.46	0.00	0.02	CXXC-type Zinc finger protein 1
LOC113519242	1940.76	170.1	147.67	50.66	54.43	-1.46	0.00	0.02	Cytochrome P450 9e2-like
LOC113515204	3479	83.65	68.89	28.83	21.69	-1.46	0.00	0.02	Small G protein signaling modulator 2-like
LOC113522442	2576	55.44	54.97	20.38	16.19	-1.46	0.00	0.02	Uncharacterized protein LOC106136992
LOC113519591	2375	12.72	12.92	4.34	4.11	-1.46	0.00	0.02	Uncharacterized protein LOC106141788
LOC113511011	1640	2.36	2.57	0.69	0.92	-1.47	0.01	0.05	Calbindin-32
LOC113511928	2551.81	1.97	1.87	0.63	0.64	-1.47	0.01	0.04	Polypyrimidine tract-binding protein 1 isoform X7
LOC113512851	894	117.14	91.96	28.59	40.32	-1.47	0.00	0.02	Uncharacterized protein LOC107172115
LOC113512275	1663	43.9	38.06	12.62	14.26	-1.47	0.00	0.02	Juvenile hormone epoxide hydrolase-like
LOC113518482	6282	15.42	14.96	5.41	4.56	-1.47	0.00	0.02	Uncharacterized protein LOC105842463 isoform X8
LOC113523275	2422	1.84	1.83	0.58	0.62	-1.48	0.01	0.04	Mitochondrial sodium/hydrogen exchanger 9B2 isoform X1
LOC113511437	1331	81.44	94.02	29.65	27.88	-1.48	0.00	0.02	Bacilysin biosynthesis oxidoreductase BacC-like
LOC113510453	329	101.43	96.07	36.94	33.58	-1.48	0.00	0.03	2-oxoisovalerate dehydrogenase subunit beta, mitochondrial
LOC113517557	2413	9.85	9.18	3.18	3.01	-1.48	0.00	0.02	Proton-coupled folate transporter isoform X1
LOC113517092	2224	90.71	93.59	29.7	30.28	-1.48	0.00	0.01	Homocysteine S-methyltransferase 1-like
LOC113511471	903	6.99	6.04	2.36	1.91	-1.48	0.01	0.04	Hexosaminidase D-like
LOC113509247	812	456.17	365.82	140.76	128.75	-1.49	0.00	0.01	Neurotransmitter gated ion channel
LOC113511638	4587	28.98	28.77	10.01	8.72	-1.49	0.00	0.01	Uncharacterized protein LOC110378979
LOC113515622	7845	1.03	1.32	0.45	0.32	-1.49	0.00	0.02	Uncharacterized protein LOC106134691
LOC113512684	1188	4.18	2.62	1.17	1.04	-1.49	0.01	0.05	Chloride channel protein 2 isoform X1
LOC113519771	1356	8.79	5	2.24	2.22	-1.49	0.00	0.02	Uncharacterized protein LOC105382408

MSTRG.1579	2219	195.75	174.98	63.63	56.47	-1.49	0.00	0.01	BCL2/adenovirus E1B 19 kDa protein-interacting protein 3 isoform X1
LOC113509337	5245	51.95	31.85	17.75	9.4	-1.49	0.00	0.01	Uncharacterized protein LOC106135284
LOC113519037	4827	1.81	1.16	0.27	0.68	-1.49	0.00	0.03	Uncharacterized protein LOC110379989 isoform X1
LOC113521952	651	144.56	165.1	50.69	50.94	-1.49	0.00	0.01	Aldehyde Dehydrogenase
LOC113513473	554	22.83	18.42	6.36	7.22	-1.49	0.00	0.03	Alpha-N-acetylgalactosaminidase-like
LOC113522775	1445	19.14	17.68	6.02	5.87	-1.49	0.00	0.02	Zinc finger protein 235-like
LOC113516004	2281.79	22.8	20.84	7.77	6.33	-1.50	0.00	0.01	PR domain Zinc finger protein 16-like isoform X1
LOC113516339	1094	8.97	7.93	3.54	1.92	-1.50	0.00	0.02	Uncharacterized oxidoreductase SSP0419-like
LOC113523092	4816.25	29.26	28.5	8.52	9.13	-1.50	0.00	0.01	Oxidation resistance protein 1 isoform X2
LOC113519227	1016	7.25	6.6	3.05	1.41	-1.50	0.00	0.03	Uncharacterized protein LOC106130388
LOC113512476	1699	38.11	44.01	12.84	13.5	-1.50	0.00	0.01	Mitochondrial aldehyde dehydrogenase
LOC113517643	4177	51.33	47.84	17.61	14.12	-1.51	0.00	0.01	BMP-binding endothelial regulator protein-like
LOC113522139	795	11.63	9.39	3.76	3.03	-1.51	0.00	0.03	5-oxoprolinase
MSTRG.10478	257	3141.77	3075.55	1007.35	1308.49	-1.51	0.00	0.01	Uncharacterized protein
LOC113522497	1363.61	813.83	919.68	270.38	286.06	-1.51	0.00	0.01	Uncharacterized protein LOC106138090
LOC113520135	765	20.92	19.92	6.89	6.3	-1.51	0.00	0.02	Zinc finger protein 816-like
LOC113515986	2466.14	116.84	110.16	38.51	35.32	-1.51	0.00	0.01	6-phosphofructo-2-kinase/fructose-2,6-bisphosphatase-like
LOC113512333	595	40.5	44.91	10.91	16.73	-1.51	0.00	0.02	Uncharacterized protein LOC106132298 isoform X1
MSTRG.9391	2559	15.07	14.2	4.37	4.91	-1.51	0.00	0.01	Uncharacterized protein LOC106133236
LOC113521268	1997	390.57	303.29	106.16	114.23	-1.51	0.00	0.01	Catalase
LOC113513360	639	56.2	33.39	15.12	13.85	-1.51	0.00	0.02	Uncharacterized protein LOC106135284
LOC113517644	1194	7.35	7.54	2.06	2.68	-1.52	0.00	0.02	15-hydroxyprostaglandin

									dehydrogenase
LOC113516667	2694	24.09	20.58	7	7.14	-1.52	0.00	0.01	Protein FAN-like
LOC113512738	486	127.11	123.41	43.7	38.45	-1.52	0.00	0.01	Triokinase/FMN cyclase-like
LOC113516036	838	4.9	6.73	1.68	2.02	-1.52	0.01	0.04	Uncharacterized protein LOC106138832
LOC113510042	1617	16.88	13.07	4.08	5.34	-1.53	0.00	0.01	UDP-glucuronosyltransferase 2A3-like
MSTRG.12744	719	541.48	494.08	176.78	154.28	-1.53	0.00	0.01	Lysosomal Pro-X carboxypeptidase
LOC113511363	772	6.62	7.12	3.22	1.14	-1.53	0.00	0.03	Uncharacterized protein LOC106129217
LOC113513259	1644	6.87	8.44	1.51	3.27	-1.53	0.00	0.02	Uncharacterized protein LOC110381499
MSTRG.7767	1187	2.93	4.5	1.36	0.97	-1.54	0.01	0.04	Uncharacterized protein
MSTRG.5902	723	5.13	6.74	2.61	1.17	-1.54	0.01	0.04	Hypothetical protein RR46_00006
LOC113511044	2479	12.94	10.16	3.71	3.49	-1.54	0.00	0.01	Long-chain fatty acid transport protein 1-like
LOC113515395	1688	2.86	3.61	0.66	1.38	-1.54	0.00	0.03	Cytochrome b5-related protein-like
LOC113512299	1443.27	40.1	32.54	11.8	10.99	-1.54	0.00	0.01	Protein decapentaplegic-like
LOC113509675	2019	2.17	3.07	1.05	0.58	-1.54	0.00	0.03	Spondin-2
LOC113518105	1799	2.58	3.88	0.68	1.3	-1.55	0.00	0.02	Ornithine aminotransferase, mitochondrial
LOC113521417	2961	0.67	1.03	0.22	0.31	-1.55	0.01	0.05	Fas-binding factor 1
LOC113517282	1475.84	5.9	7.22	3.33	0.55	-1.55	0.00	0.02	Argininosuccinate lyase isoform X1
LOC113516421	1499	116.56	120.35	36.8	36.83	-1.55	0.00	0.01	PRKCA-binding protein
LOC113511020	3092	2.75	2.82	1.05	0.67	-1.55	0.00	0.02	Uncharacterized protein LOC106135940
LOC113509072	4351.69	317.23	270.2	102.44	79.89	-1.56	0.00	0.01	Adhesive plaque matrix protein isoform X1
LOC113519349	1328	116.91	116.83	38.45	34.01	-1.56	0.00	0.01	Matrix metalloproteinase-25-like
MSTRG.10901	392	479.64	571.97	176.28	167.68	-1.56	0.00	0.01	Uncharacterized protein
MSTRG.2911	858.79	13	11.84	3.8	5.4	-1.56	0.00	0.02	THO complex subunit 2
LOC113522777	7139.49	15.34	10.33	4.56	3.32	-1.56	0.00	0.01	Neurogenic locus Notch protein isoform X3
LOC113522440	2428	0.96	1.24	0.51	0.15	-1.57	0.01	0.04	Uncharacterized protein OBRU01_10191
LOC113511075	671.62	12.42	18.75	3.14	6.46	-1.57	0.00	0.02	Uncharacterized protein
LOC113520694	3603.09	25.82	21.97	8.41	6.21	-1.57	0.00	0.01	Protein yippee-like CG15309

									isoform X1
LOC113521776	2684	15.56	15.71	4.99	4.55	-1.57	0.00	0.01	Translin-associated factor X-interacting protein 1-like isoform X2
MSTRG.16063	1997	71.51	71.15	21	22.33	-1.58	0.00	0.01	Uncharacterized protein
LOC113510928	785	122.92	116.8	38.76	34.85	-1.58	0.00	0.01	4-coumarate--CoA ligase 1-like
LOC113512055	1847.14	50.97	71.06	18.86	18.28	-1.58	0.00	0.01	Alpha-N-acetylgalactosaminidase isoform X1
LOC113514815	1015	37.52	34.09	13.68	8.26	-1.58	0.00	0.01	Interference hedgehog-like
LOC113511812	1578	4.95	5.61	1.45	1.74	-1.59	0.00	0.02	Zinc finger and BTB domain-containing protein 42
LOC113514026	800	15.13	13.37	4.62	4.08	-1.59	0.00	0.02	Hemicentin-2
LOC113516152	6405	30.41	25.44	9.32	7.49	-1.59	0.00	0.01	brefeldin A-inhibited guanine nucleotide-exchange protein 3
MSTRG.8387	941	43.26	50.8	13.06	15.43	-1.59	0.00	0.01	Uncharacterized protein
LOC113523523	4898	44.69	27.06	11.11	10.38	-1.59	0.00	0.01	MAP kinase-activating death domain protein
LOC113511336	813	17.78	18.39	5.83	5.15	-1.60	0.00	0.01	Hypothetical protein KGM_201149
LOC113516666	3133	66.55	57.44	20.07	17.19	-1.60	0.00	0.01	Monocarboxylate transporter 12
LOC113512615	10174	39.65	34.7	12.43	9.85	-1.60	0.00	0.01	Headcase protein
LOC113517925	837	12.13	9.67	3.79	2.8	-1.60	0.00	0.02	Zinc finger protein 624-like isoform X1
LOC113515380	1532.15	5.67	6.39	1.71	1.67	-1.61	0.00	0.01	Extensin isoform X3
LOC113510904	1434	5.7	4.45	1.23	1.8	-1.61	0.00	0.02	Acid sphingomyelinase-like phosphodiesterase 3a
LOC113518192	1721	68.83	77.8	21.86	21.89	-1.61	0.00	0.01	N-acetylgalactosamine kinase
MSTRG.15833	637.91	14.78	17.24	3.76	6.92	-1.61	0.00	0.01	Bifunctional coenzyme A synthase isoform X3
LOC113513277	2837	3.6	2.71	1.06	0.81	-1.61	0.00	0.01	Rho GTPase-activating protein 7 isoform X1
MSTRG.15546	2436	0.79	1.31	0.36	0.26	-1.61	0.01	0.04	Aminopeptidase M1
LOC113517959	575	72.55	54.91	16.97	21.63	-1.61	0.00	0.01	Calcium-dependent secretion activator
LOC113513185	1465	50.73	36.58	11.44	14.41	-1.61	0.00	0.01	Uncharacterized protein LOC106136069 isoform X1
LOC113517079	1068	4.53	4.79	1.42	1.35	-1.61	0.00	0.02	Tubulin polyglutamylase complex subunit 2 isoform X2
LOC113517119	2274	5.43	5.51	1.63	1.61	-1.62	0.00	0.01	Uncharacterized protein

									LOC106133560
LOC113510171	619	13.05	14.65	4.3	4.03	-1.62	0.00	0.02	Uncharacterized protein
LOC113510250	3864	4.27	2.5	0.97	1.02	-1.62	0.00	0.01	Neurexin-1
LOC113516168	2547	25.26	25.59	7.27	7.74	-1.62	0.00	0.01	SWI/SNF-related matrix-associated actin-dependent regulator of chromatin subfamily A-like protein 1
LOC113511922	1390	16.6	14.53	4.56	4.64	-1.62	0.00	0.01	Isovaleryl-CoA dehydrogenase, mitochondrial
LOC113517143	1401	44.69	48.16	17.09	10.42	-1.63	0.00	0.01	Tryptase-like
MSTRG.3523	521	18.03	23.03	6.73	5.63	-1.63	0.00	0.02	5-methylcytosine rRNA methyltransferase NSUN4
LOC113515506	5602.6	16.41	16.74	5.17	4.22	-1.64	0.00	0.01	Uncharacterized protein LOC110378016 isoform X1
LOC113514511	727	5.96	5.53	1.7	1.67	-1.64	0.00	0.03	Carbonyl reductase
LOC113512665	996	86.41	84.91	25.12	25.09	-1.64	0.00	0.01	Matrix metalloproteinase-25-like
LOC113513174	957	3.42	3.68	0.87	1.2	-1.64	0.00	0.03	Vacuolar protein sorting-associated protein 13C
LOC113509631	2127	10.83	8.27	2.68	2.86	-1.64	0.00	0.01	Carboxypeptidase N subunit 2-like
LOC113511629	1463	2.3	2.89	0.55	0.96	-1.65	0.00	0.03	Dual specificity protein phosphatase 14
LOC113509201	1774	2.04	1.35	0.43	0.55	-1.65	0.00	0.03	Caspase-3
LOC113519918	1371	1.84	2.91	0.97	0.4	-1.65	0.00	0.03	Sodium-dependent serotonin transporter
LOC113513214	1275.15	7.44	10.12	2.33	2.66	-1.65	0.00	0.01	Uncharacterized protein LOC110370951 isoform X2
LOC113510571	633	37.08	35.98	10.91	10.56	-1.65	0.00	0.01	Glutathione S-transferase epsilon 6 isoform X1
LOC113515686	1586	9.59	9.18	3.48	1.97	-1.65	0.00	0.01	Glutamate-gated chloride channel isoform X5
LOC113521566	2184	4.16	3.74	1.15	1.12	-1.66	0.00	0.01	RING finger protein nhl-1-like
LOC113516676	3286	8.08	7.46	2.24	2.24	-1.66	0.00	0.01	Uncharacterized protein LOC106130524
LOC113519915	1865	214.7	219.06	62.31	62.57	-1.66	0.00	0.00	Beta-hexosaminidase
MSTRG.6451	1063	8.21	9.02	2.33	2.64	-1.66	0.00	0.01	Uncharacterized protein
LOC113509200	1230	6.39	6.74	1.8	1.98	-1.66	0.00	0.01	Zinc finger protein 700-like
MSTRG.12967	2969	8.31	7.01	2.33	2.06	-1.66	0.00	0.01	Uncharacterized protein OBRU01_26429

LOC113523183	594	6.69	5.89	1.48	2.17	-1.66	0.01	0.04	EF-hand calcium-binding domain-containing protein 2 isoform X1
LOC113521899	620.86	261.87	194.53	65.84	64.35	-1.66	0.00	0.00	Uncharacterized protein LOC106139546
LOC113515681	1892	3.62	1.98	0.66	0.94	-1.67	0.00	0.02	Serum response factor homolog A
LOC113509113	1923.7	14.94	15.32	3.04	4.76	-1.67	0.00	0.01	Uncharacterized protein LOC106131089
LOC113522620	940	8.89	7.08	1.25	3.32	-1.67	0.00	0.01	Peritrophin-1-like
MSTRG.3168	544	16.35	18.19	6.82	3.28	-1.67	0.00	0.01	4-coumarate--CoA ligase 1-like
LOC113518207	2339	192.9	194.48	56.29	54.1	-1.67	0.00	0.00	Sialic acid synthase
MSTRG.4413	1358	32.81	37.74	10.98	9.2	-1.67	0.00	0.01	Gelsolin-like
LOC113515438	2510	0.81	1.32	0.29	0.31	-1.68	0.00	0.03	Ras-related and estrogen-regulated growth inhibitor-like protein
LOC113522757	1688	1.9	2.22	0.63	0.54	-1.68	0.00	0.02	Uncharacterized protein LOC106129350
MSTRG.474	1261	10.62	8.02	2.34	2.94	-1.68	0.00	0.01	NADP-dependent oxidoreductase
LOC113518767	864.51	78.76	71.05	25.41	19.53	-1.68	0.00	0.00	Hypothetical protein KGM_208267
LOC113512735	804	5.62	2.54	0.9	1.43	-1.68	0.00	0.03	Semaphorin-5A
LOC113520422	916	6.51	7.63	2.03	1.97	-1.69	0.00	0.01	RDH13
MSTRG.10169	412	396.02	331.83	102.06	113.18	-1.69	0.00	0.00	Uncharacterized protein LOC106134230
MSTRG.6397	1865	13.74	12.64	3.72	3.69	-1.69	0.00	0.01	Protein turtle-like
LOC113518306	4854	1.36	1.44	0.31	0.41	-1.70	0.00	0.01	Alpha-catulin isoform X1
LOC113517457	1334	7.83	7.3	1.79	2.44	-1.70	0.00	0.01	Fringe glycosyltransferase
LOC113516171	1275	93.15	71.77	19.83	26.16	-1.70	0.00	0.00	Uncharacterized protein LOC106720945
MSTRG.10774	1416	7.42	5.97	1.45	2.27	-1.71	0.00	0.01	Uncharacterized protein
LOC113516106	1434	7.12	7.15	2.4	1.58	-1.71	0.00	0.01	Monocyte to macrophage differentiation factor 2
LOC113509680	1449.67	68.27	54.59	17.78	17.61	-1.71	0.00	0.00	E3 SUMO-protein ligase EGR2 isoform X1
LOC113512481	2139	3.5	2.41	0.86	0.78	-1.71	0.00	0.01	WD repeat-containing protein on Y chromosome
LOC113517072	1257	23.93	20.92	6.57	5.91	-1.71	0.00	0.00	Uncharacterized protein LOC106143669
LOC113516550	385	101.68	79.65	27.24	26.23	-1.71	0.00	0.01	Nucleolar GTP-binding protein 1
LOC113522530	3888.25	0.95	1.47	0.3	0.44	-1.71	0.00	0.01	Regulating synaptic membrane

									exocytosis protein 2
LOC113522941	480	8.67	9.06	2.88	2.15	-1.71	0.00	0.03	Homeobox protein B-H1-like
LOC113522612	2247	1.7	1.78	0.54	0.42	-1.72	0.00	0.01	Aquaporin AQPAe.a
LOC113518530	5512	0.68	0.39	0.15	0.15	-1.72	0.00	0.02	Protein grainyhead isoform X1
LOC113519024	3942	3.37	4.03	0.92	1.12	-1.72	0.00	0.01	Microtubule-associated protein tau
LOC113509342	4290	96.92	72.55	26.96	19.74	-1.72	0.00	0.00	Uncharacterized protein LOC106135284
LOC113515726	3017.83	0.99	3.96	0.87	0.55	-1.72	0.00	0.01	Nuclear hormone receptor HR3
LOC113510031	884	8.98	10.94	2.72	2.79	-1.72	0.00	0.01	Uncharacterized protein
LOC113518766	1087	1208.9	1465.76	354.54	383.66	-1.73	0.00	0.00	Scolexin precursor
LOC113509439	1712	1.64	0.86	0.41	0.26	-1.73	0.00	0.03	Neutral ceramidase-like isoform X1
LOC113522888	699	17.37	16.66	4.33	5.05	-1.74	0.00	0.01	Uncharacterized protein LOC110383083
LOC113521152	1514	19.23	17.74	5.25	4.83	-1.74	0.00	0.00	Ataxin-2 homolog isoform X1
MSTRG.9128	2866	3.35	1.9	0.69	0.73	-1.74	0.00	0.01	Uncharacterized protein LOC110373625
LOC113520291	3023	13.43	12.79	3.92	3.19	-1.74	0.00	0.00	Glycoprotein 3-Alpha-L-fucosyltransferase A
LOC113523041	2157	0.96	0.98	0.22	0.3	-1.75	0.01	0.04	Uncharacterized protein LOC110371101
LOC113522299	2013	102.31	113.48	27.82	30.44	-1.75	0.00	0.00	Hypothetical protein KGM_205387
LOC113512935	1469	86.66	99.6	22.8	27.4	-1.75	0.00	0.00	Leucine-rich repeat-containing protein 58-like
LOC113511644	1662	14.75	18.42	4.91	4.05	-1.76	0.00	0.00	Protein FAM107B
LOC113519810	3234	8.07	6.93	2.48	1.56	-1.76	0.00	0.00	Myosin light chain kinase
MSTRG.3191	297	187.06	148.3	62.37	39.03	-1.76	0.00	0.01	CRAL-TRIO domain-containing protein
LOC113521698	3597	3.21	3.15	0.93	0.78	-1.76	0.00	0.00	Semaphorin-1A isoform X5
LOC113520464	1860	28.36	27.53	6.61	8.31	-1.76	0.00	0.00	Potassium voltage-gated channel protein Shaker
LOC113517164	774	68.25	64.56	21.35	14.65	-1.77	0.00	0.00	Calcium-dependent secretion activator isoform X3
LOC113515775	1810.7	13.85	14.44	3.88	3.71	-1.77	0.00	0.00	Delta(24)-sterol reductase-like
LOC113521929	2539.87	8.87	9.08	2.42	2.67	-1.77	0.00	0.00	Carbohydrate sulfotransferase 11 isoform X1
LOC113519265	2259	54.93	53.38	16.11	12.74	-1.77	0.00	0.00	Multiple C2 and transmembrane domain-containing protein 1

									isoform X5
LOC113518046	1414	12.56	12.29	3.12	3.49	-1.77	0.00	0.00	Lachesin-like
MSTRG.12943	1191	6.43	8.11	1.1	2.76	-1.77	0.00	0.01	Nucleolar pre-ribosomal-associated protein 1
MSTRG.4305	451	7.65	8.8	2.35	2.14	-1.77	0.01	0.04	Uncharacterized protein
LOC113515821	4106	8.35	6.64	1.88	2.07	-1.78	0.00	0.00	Serine/threonine-protein phosphatase 6 regulatory Ankyrin repeat subunit B
LOC113512401	3020	15.96	18.12	4.44	4.57	-1.78	0.00	0.00	Facilitated trehalose transporter Tret1-like isoform X1
LOC113521907	3023	70.95	74.38	17.57	20.77	-1.78	0.00	0.00	Uncharacterized protein LOC106132537
LOC113518214	1972	2.13	2.14	0.45	0.67	-1.78	0.00	0.01	Uncharacterized protein LOC110373875 isoform X7
LOC113521494	654	5.8	4.01	0.62	2	-1.78	0.00	0.03	Mpv17-like protein
MSTRG.10559	940	18.11	12.05	5.07	2.94	-1.79	0.00	0.00	Uncharacterized protein LOC106135284
LOC113509137	1083	8.21	9.17	2.49	2.11	-1.79	0.00	0.01	Ras-related and estrogen-regulated growth inhibitor
LOC113518820	16397	0.1	0.12	0.04	0.02	-1.79	0.00	0.03	Titin
LOC113516703	1520	3.13	4.94	1.01	1.11	-1.79	0.00	0.01	Krueppel homolog 1-like
LOC113512341	1235	4.6	6.07	1.36	1.44	-1.79	0.00	0.01	Alpha,Alpha-trehalose-phosphate synthase
LOC113513894	3484	20.25	2.46	2.71	3.14	-1.80	0.00	0.00	Fatty acid synthase
LOC113522739	1234	117.9	116.52	27.03	34.25	-1.80	0.00	0.00	Aldose 1-epimerase-like
LOC113517505	2322	7.04	7.3	2.05	1.69	-1.80	0.00	0.00	Leucine-rich repeat-containing protein 70
LOC113521173	2465	78.54	68.99	20.57	17.85	-1.80	0.00	0.00	Chitooligosaccharidolytic beta-N-acetylglucosaminidase-like
LOC113509440	1533	1.09	1.17	0.33	0.25	-1.80	0.01	0.04	Protein peste-like isoform X1
LOC113519022	1327	0.62	1.87	0.39	0.24	-1.81	0.01	0.05	Proton-coupled folate transporter-like
LOC113523298	2118	53.02	62.16	14.64	15.22	-1.81	0.00	0.00	Cytochrome P450 CYP12A2-like
LOC113519593	662	491.82	496.82	131.23	129.11	-1.81	0.00	0.00	Myrosinase 1-like
LOC113513186	4782	5.64	5.29	1.46	1.36	-1.81	0.00	0.00	Uncharacterized protein LOC106137033 isoform X1
LOC113510629	1146.69	54.52	61.59	13.57	16.57	-1.81	0.00	0.00	Krueppel-like factor 9
LOC113511315	1102	20.99	23.04	6.66	4.8	-1.81	0.00	0.00	Three prime repair exonuclease 2

LOC113517990	746	805.03	784.05	223.07	192.54	-1.82	0.00	0.00	Acetylcholine receptor subunit Alpha-type unc-38-like
LOC113511757	2076	367.91	263.94	80.9	81.46	-1.82	0.00	0.00	MAP kinase-interacting serine/threonine kinase
LOC113517042	5486	997.39	847.87	279.63	194.88	-1.82	0.00	0.00	Collagen alpha-5(IV) chain-like
LOC113520468	2081	73.48	67.14	20.02	16.09	-1.83	0.00	0.00	Lysosomal alpha-mannosidase-like isoform X1
MSTRG.15207	578	19.85	18.59	5.47	4.57	-1.83	0.00	0.01	Lysosomal alpha-mannosidase
LOC113512435	1529	199.14	180.11	57.01	40.37	-1.83	0.00	0.00	Synaptotagmin 1 isoform X1
LOC113515642	4388	24.81	22.58	6.95	5.14	-1.83	0.00	0.00	Tetratricopeptide repeat protein 21B-like
LOC113517138	2815	65.95	60.18	16.68	15.29	-1.84	0.00	0.00	Calcium-dependent secretion activator
MSTRG.9618	1937.69	1.91	0.89	0.37	0.34	-1.84	0.00	0.02	Zinc finger SWIM domain-containing protein 8-like
MSTRG.6504	469	7.79	10.48	2.13	2.59	-1.85	0.00	0.02	Uncharacterized protein
LOC113515960	3536	104.82	81.24	26.1	20.79	-1.85	0.00	0.00	Dyslexia-associated protein KIAA0319
LOC113521896	1530	11.37	10.47	3.16	2.36	-1.85	0.00	0.00	Ionotropic receptor IR64a
MSTRG.6118	971	100.63	100.77	24.66	26.35	-1.85	0.00	0.00	Uncharacterized protein
LOC113517827	519	16.65	13.83	2.67	5.16	-1.86	0.00	0.01	Alkaline phosphatase
LOC113513459	432	60.47	73.86	13.49	21.53	-1.86	0.00	0.00	Venom carboxylesterase-6-like
LOC113523131	1197	9.22	7.51	1.86	2.32	-1.86	0.00	0.00	Uncharacterized protein LOC106113363
LOC113510482	4274	69.46	55.1	19.42	11.67	-1.87	0.00	0.00	Uncharacterized protein LOC106136572
LOC113512115	897	2.79	3.15	0.86	0.61	-1.87	0.00	0.02	Uncharacterized protein LOC105561123
LOC113522141	1817	429.5	429.64	99.21	112.97	-1.88	0.00	0.00	Spermidine synthase
LOC113512773	321	742.43	687.5	204.57	185.1	-1.88	0.00	0.00	Myrosinase 1-like
LOC113522950	4225	9.75	8.81	2.65	1.92	-1.88	0.00	0.00	Multidrug resistance-associated protein 4-like
LOC113523219	3737	1.75	3.36	0.79	0.47	-1.89	0.00	0.00	Uncharacterized protein LOC106130432
LOC113514830	892	7.38	9.12	2.21	1.84	-1.90	0.00	0.00	Uncharacterized protein LOC106140625 isoform X1
LOC113512197	12606	1.29	1.03	0.25	0.31	-1.90	0.00	0.00	Dynein heavy chain 1, axonemal-like

LOC113511770	5620	2.13	2.02	0.55	0.45	-1.91	0.00	0.00	Uncharacterized protein PFB0145c
LOC113516077	827	9.5	11.4	2.57	2.51	-1.91	0.00	0.00	Uncharacterized protein LOC106134335
LOC113512396	1038	2.85	2.41	0.93	0.33	-1.91	0.00	0.01	Inactive pancreatic Lipase-related protein 1-like
MSTRG.1149	3470	1.46	1.08	0.35	0.26	-1.91	0.00	0.00	Uncharacterized protein
LOC113523522	751	16.76	7.94	3.36	2.65	-1.91	0.00	0.00	Uncharacterized protein
LOC113516507	2263	10.87	0.99	1.13	1.68	-1.92	0.00	0.00	Fatty acid synthase
LOC113514181	1741	6.61	5.45	1.78	1.12	-1.92	0.00	0.00	Hypothetical protein KGM_205793
LOC113514457	364	355.19	273.95	87.73	74.02	-1.93	0.00	0.00	BCL2/adenovirus E1B 19 kDa protein-interacting protein 3 isoform X1
LOC113519389	3819	15.27	14.35	4.02	3.04	-1.93	0.00	0.00	Forkhead box protein P3
MSTRG.16486	341	124.77	138.63	30.45	37.66	-1.93	0.00	0.00	Dihydroxyacetone kinase 2
LOC113517757	1564	5.39	6.15	0.83	1.91	-1.93	0.00	0.00	Uncharacterized protein LOC106143497
LOC113509757	1236	4.54	5.11	1.05	1.25	-1.93	0.00	0.00	Uncharacterized protein LOC106135696
LOC113517325	915	4.15	2.29	0.65	0.89	-1.93	0.00	0.02	Ankyrin repeat domain-containing protein 29
LOC113514878	801	10.89	9.05	2.25	2.52	-1.94	0.00	0.00	Polypeptide N-acetylgalactosaminyltransferase 2-like
LOC113510355	469	11.82	11.41	2.74	2.92	-1.94	0.00	0.01	Uncharacterized protein LOC101745796
LOC113520651	2030.14	10.95	9.82	2.73	2.22	-1.94	0.00	0.00	Uncharacterized protein LOC110375899 isoform X2
LOC113520364	1125	1177.55	1128.38	257.92	289.38	-1.94	0.00	0.00	Hydroxypyruvate isomerase
MSTRG.10085	729	3.56	3.22	1.04	0.56	-1.94	0.00	0.02	Uncharacterized protein
MSTRG.2773	1340	4.25	4.38	1.44	0.59	-1.95	0.00	0.00	Uncharacterized protein
MSTRG.13719	327	564.52	603.3	165.69	136.75	-1.95	0.00	0.00	Uncharacterized protein LOC110379302
LOC113517215	3163.78	9.04	8.78	2.07	2.1	-1.95	0.00	0.00	Cerebellar degeneration-related protein 2 isoform X3
LOC113519298	859	27.9	4.29	3.04	4.53	-1.95	0.00	0.00	Fatty acid synthase-like
LOC113519093	647	18.44	23.69	4.61	5.42	-1.95	0.00	0.00	Uncharacterized protein LOC106136406
LOC113516431	588	19.41	14.72	4.54	3.62	-1.95	0.00	0.00	Hemicentin-2

LOC113522635	1468	8.11	6.69	1.93	1.53	-1.96	0.00	0.00	Uncharacterized oxidoreductase SERP2049-like
MSTRG.12781	1356	4.75	4.68	1.15	1.05	-1.96	0.00	0.00	Neuroblastoma-amplified sequence-like
LOC113520217	348	68.89	90.91	23.74	16.63	-1.96	0.00	0.00	Membrane-associated progesterone receptor component 1-like
LOC113509934	818	13.45	19.82	3.05	4.76	-1.96	0.00	0.00	Uncharacterized protein LOC106135020
LOC113521556	620	139.63	101.19	32.77	24.47	-1.96	0.00	0.00	Apolipoprotein of lipid transfer particle-i/II
MSTRG.6172	934	2.06	1.58	0.27	0.57	-1.96	0.00	0.04	Uncharacterized protein
LOC113523146	318	19.19	24.71	4.26	6.84	-1.97	0.00	0.03	Lysosomal alpha-mannosidase-like isoform X1
LOC113521660	1423	7.47	9.04	2.37	1.48	-1.97	0.00	0.00	Alpha-N-acetylgalactosaminidase-like
LOC113517040	6201	875.45	773.81	224.39	159.02	-1.97	0.00	0.00	Collagen alpha-1(IV) chain
LOC113520794	3830	1.98	1.8	0.48	0.39	-1.97	0.00	0.00	Multidrug resistance protein 1A-like
LOC113511516	732	6.37	7.2	2.33	0.83	-1.97	0.00	0.00	Hypothetical protein KGM_209449
LOC113514062	388	17.95	14.24	5.11	2.72	-1.97	0.00	0.01	Unconventional myosin-Va
LOC113521986	2539	8.48	9.08	1.7	2.34	-1.98	0.00	0.00	Uncharacterized oxidoreductase YjmC
LOC113512391	1875	1.61	0.88	0.33	0.24	-1.98	0.00	0.01	Uncharacterized protein LOC106142706
LOC113513707	1513	1.15	0.6	0.14	0.25	-1.98	0.01	0.04	KRAB-A domain-containing protein 2-like
LOC113515034	1582	28.28	29.59	6.7	6.56	-1.99	0.00	0.00	Uncharacterized protein LOC106129906 isoform X1
LOC113517407	1352	3.5	3.25	0.66	0.88	-1.99	0.00	0.00	Sterol O-acyltransferase 2
LOC113510005	328	25.46	20.9	8.42	3	-1.99	0.00	0.02	Unconventional myosin-Va-like
LOC113518458	3727.89	7.31	5.73	1.96	1.07	-2.00	0.00	0.00	Solute carrier family 12 member 4 isoform X3
LOC113511409	440	20.61	20.86	7.14	2.66	-2.00	0.00	0.00	Hypothetical protein KGM_212946A
LOC113515396	3793	8.65	8.17	2.02	1.79	-2.00	0.00	0.00	Protein patched homolog 1-like
LOC113515253	1377	1.88	0.61	0.11	0.46	-2.00	0.00	0.02	Mucin-17-like
LOC113510961	963	5.52	4.36	0.77	1.47	-2.00	0.00	0.00	Lipopolysaccharide-induced tumor necrosis factor-alpha factor

									homolog
LOC113518335	7205	0.17	0.15	0.04	0.03	-2.01	0.01	0.04	Nuclear-pore anchor isoform X1
LOC113517686	494	1006.14	1011.37	228.68	240.73	-2.01	0.00	0.00	Acetylcholine receptor subunit Alpha-type unc-38-like
LOC113516401	2039	7.86	8.19	1.68	1.93	-2.01	0.00	0.00	Heat shock protein 68-like
LOC113522349	4291	233.72	202.32	61.94	36.55	-2.01	0.00	0.00	Nidogen-1
LOC113511682	1668.15	32.52	34.33	6.39	9.03	-2.02	0.00	0.00	Acetyltransferase
LOC113515367	794	3.02	5.29	1.37	0.49	-2.02	0.00	0.01	Diacylglycerol kinase 1
LOC113516587	546	8.8	6.91	2.4	1.16	-2.02	0.00	0.01	Short/branched chain specific acyl-CoA dehydrogenase, mitochondrial
LOC113515722	1600	1.4	1.11	0.22	0.33	-2.02	0.00	0.01	Tubulin beta chain-like
LOC113522562	636	320.2	240.86	68.38	58.99	-2.03	0.00	0.00	N-acetylglucosaminidase
MSTRG.11697	2463.58	4.88	5.65	1.23	1.07	-2.03	0.00	0.00	Uncharacterized protein OBRU01_03818
LOC113519004	1435	2.78	3.12	0.92	0.38	-2.04	0.00	0.00	Suppressor of lurcher protein 1-like
LOC113510143	1591.9	4.62	4.65	0.9	1.27	-2.04	0.00	0.00	Zinc finger protein 840
LOC113523353	1153	37.61	43.24	9.03	8.9	-2.05	0.00	0.00	LIM domain only protein 3 isoform X1
MSTRG.9867	1396	1.66	1.37	0.21	0.45	-2.05	0.00	0.01	Uncharacterized protein LOC106138072
LOC113509818	2011	136.45	126.82	30.85	26.98	-2.05	0.00	0.00	Regucalcin
LOC113518579	748	61.32	61.24	15.27	11.86	-2.06	0.00	0.00	GILT-like protein 2
LOC113523288	781	0.64	4.46	0.35	0.75	-2.06	0.00	0.02	Uncharacterized protein
LOC113519302	1017	5.57	4.21	1.28	0.85	-2.06	0.00	0.00	Semaphorin-5A
MSTRG.7296	1118	9.69	10.61	2.11	2.32	-2.06	0.00	0.00	Diamine acetyltransferase 2-like
LOC113518036	1819	10.96	8.56	2.11	2.12	-2.06	0.00	0.00	Phytanoyl-CoA dioxygenase domain-containing protein 1
LOC113514680	3022	30.26	31.64	7.2	6.24	-2.07	0.00	0.00	Insulin-like growth factor 1 receptor
LOC113517421	1931.58	32.19	36.03	7.74	7.08	-2.07	0.00	0.00	Uncharacterized protein LOC101735991 isoform X2
LOC113515169	479	50.23	4.99	4.35	8.01	-2.07	0.00	0.00	Fatty acid synthase-like
LOC113520813	1779	2	1.59	0.35	0.42	-2.07	0.00	0.01	Tyramine beta-hydroxylase
LOC113522676	1416	3.64	5.05	0.88	0.99	-2.07	0.00	0.00	Uncharacterized protein LOC106129334 isoform X2
LOC113515958	2512	74.55	68.01	16.14	14.66	-2.07	0.00	0.00	1,4-Alpha-glucan-branching enzyme isoform X2

LOC113513750	1409	3.32	3.63	0.99	0.5	-2.08	0.00	0.00	Acyl-coenzyme A dehydrogenase
LOC113521756	2294	3.2	3.41	0.76	0.66	-2.08	0.00	0.00	Leukotriene A-4 hydrolase isoform X1
LOC113519240	1553	6.24	7.88	1.76	1.28	-2.08	0.00	0.00	Autophagy-related protein 13 homolog
MSTRG.1301	3436	1.79	1.46	0.37	0.32	-2.08	0.00	0.00	Paired box protein and transposase domain containing protein
LOC113520094	1707	186.94	172.44	40.05	37.03	-2.09	0.00	0.00	Adenosine deaminase CECR1
MSTRG.9891	1752	1.56	0.96	0.4	0.13	-2.09	0.00	0.01	O-acyltransferase like protein-like
LOC113518649	1732	3.46	1.1	0.45	0.52	-2.09	0.00	0.00	Cytochrome P450 6B46
LOC113518573	4108	0.43	0.18	0.08	0.05	-2.09	0.00	0.03	Uncharacterized protein LOC110383802
LOC113518166	388.74	98.09	125.2	27.72	22.54	-2.09	0.00	0.00	Uncharacterized protein LOC106138152
LOC113515776	1475	8.42	5.42	1.53	1.42	-2.09	0.00	0.00	3-ketoacyl-CoA thiolase, mitochondrial-like
LOC113512748	1168	6.16	10.02	2.12	1.34	-2.09	0.00	0.00	Cytochrome P450 9e2-like
LOC113514900	1210	1.92	0.85	0.38	0.2	-2.09	0.00	0.02	Collagenase-like
LOC113515197	1275	23.12	22.39	5.56	4.16	-2.10	0.00	0.00	Rabphilin-3A
LOC113510939	596	22.27	17.76	3.5	5.1	-2.10	0.00	0.00	Venom carboxylesterase-6-like
LOC113513347	879	1.7	1.72	0.2	0.52	-2.10	0.00	0.03	Uncharacterized protein LOC110378737
LOC113512789	313	24.2	48.4	5.7	11.01	-2.11	0.00	0.01	Uncharacterized protein
LOC113511824	1587	43.51	46.11	9.65	9.27	-2.11	0.00	0.00	D-2-hydroxyglutarate dehydrogenase, mitochondrial-like
LOC113514458	1948	7.53	1.43	0.39	1.47	-2.11	0.00	0.00	Fatty-acyl CoA reductase 2
LOC113515461	2108	0.78	1.01	0.13	0.24	-2.11	0.00	0.01	Cytochrome P450 monooxygenase CYP304F17
LOC113514303	1485	5.42	5.53	0.88	1.41	-2.12	0.00	0.00	L-threonine ammonia-lyase-like
LOC113515525	859	76.66	81.94	15.41	18.02	-2.12	0.00	0.00	Uncharacterized protein LOC106134739
LOC113514317	3538	2.12	1.77	0.38	0.43	-2.12	0.00	0.00	Anoctamin-9
LOC113517597	1325	37.73	32.02	8.29	6.3	-2.13	0.00	0.00	Ras-related protein Rab-37 isoform X1
LOC113521642	955	69.44	81.35	13.84	17.7	-2.13	0.00	0.00	Glutathione S-transferase 1-like
LOC113517972	1426	128.49	132.9	28.54	26.07	-2.13	0.00	0.00	Serpin I2-like
LOC113515136	996	5.27	5.01	0.74	1.4	-2.13	0.00	0.00	Coiled-coil domain-containing

									protein 42 homolog
LOC113513462	1511	3.98	3.23	0.67	0.82	-2.13	0.00	0.00	Zinc finger protein rotund isoform X3
LOC113523382	941	2.12	1.48	0.36	0.38	-2.13	0.00	0.02	ATP-binding cassette sub-family G member 1-like
MSTRG.4673	649	3.05	2.49	0.33	0.83	-2.13	0.00	0.03	Uncharacterized protein LOC106129089 isoform X1
MSTRG.11123	1002	6.16	6.37	1.63	0.91	-2.14	0.00	0.00	Histone-lysine N-methyltransferase SETMAR-like
LOC113522241	1417	31.66	4.95	2.07	5.35	-2.14	0.00	0.00	Fatty acid synthase-like
LOC113512075	660	325.31	327.64	60.42	75.61	-2.14	0.00	0.00	Uncharacterized protein
LOC113509498	1052	1.68	2.21	0.38	0.41	-2.15	0.00	0.01	Kynurenine formamidase isoform X1
LOC113511112	1409	2.36	1.41	0.16	0.61	-2.15	0.00	0.01	Caspase-4
LOC113515727	1655	1457.61	749.2	292.53	160.63	-2.15	0.00	0.00	Hemocytin
LOC113513298	1339	639.13	689.4	122.21	148.78	-2.16	0.00	0.00	Serine protease inhibitor-like
LOC113523008	4464	1.05	1.19	0.21	0.24	-2.16	0.00	0.00	Multidrug resistance-associated protein lethal(2)03659 isoform X1
LOC113521028	408	35.69	39.1	6.59	9.35	-2.16	0.00	0.00	Uncharacterized protein LOC106133088
LOC113513537	1256	1.45	0.44	0.12	0.26	-2.16	0.01	0.04	Uncharacterized protein LOC106099929
MSTRG.7974	4179	19.25	21.4	4.06	4.15	-2.17	0.00	0.00	Zinc transporter 2-like isoform X1
LOC113522137	829	4.69	3.97	1.18	0.57	-2.17	0.00	0.00	Uncharacterized protein LOC106133593 isoform X1
LOC113509590	4380	14.58	14.64	3.09	2.81	-2.17	0.00	0.00	Protein toll-like
LOC113518797	1809	46.49	39.94	8.26	9.12	-2.17	0.00	0.00	Uncharacterized protein LOC106133123
LOC113518231	2711	4.69	5.35	1.36	0.75	-2.18	0.00	0.00	Uncharacterized protein LOC101738767
LOC113510787	9125	66.14	57.12	12.95	11.61	-2.18	0.00	0.00	Protein unc-13 homolog A
LOC113521705	849	2.92	1.92	0.41	0.55	-2.18	0.00	0.01	Actin cytoskeleton-regulatory complex protein PAN1-like
LOC113512403	2093	1.75	2	0.33	0.42	-2.19	0.00	0.00	ATP-binding cassette sub-family G member 4-like
LOC113514185	1828	0.81	0.99	0.23	0.12	-2.19	0.00	0.01	Uncharacterized protein
LOC113512683	1318	19.72	18	4.03	3.51	-2.19	0.00	0.00	Uncharacterized protein LOC106129086

LOC113521709	1048.04	87.83	95.23	16.07	19.34	-2.19	0.00	0.00	Ras-related protein Rab-23
LOC113520294	4131.67	1.54	2.17	0.28	0.46	-2.19	0.00	0.00	Protein trachealess isoform X1
LOC113517218	1705	7.88	6.49	1.53	1.32	-2.19	0.00	0.00	Aldehyde dehydrogenase family 7 member A1 homolog
LOC113516448	423	4059.15	2135.03	899.48	404.78	-2.19	0.00	0.00	Neurogenic locus Notch protein-like
MSTRG.6841	372	9.95	7.54	1.76	1.87	-2.19	0.00	0.04	Uncharacterized protein
LOC113512354	2038.92	94.12	91.57	19.45	16.92	-2.21	0.00	0.00	ATP-binding cassette sub-family G member 4-like isoform X2
LOC113518862	2182.85	3214.09	3237.49	652.7	617.87	-2.21	0.00	0.00	Carboxylesterase 5A
MSTRG.7690	399	10.1	8.89	1.88	2	-2.21	0.00	0.02	Aldose 1-epimerase
MSTRG.13762	402	12.26	13.74	0	5.42	-2.21	0.00	0.01	Plasma membrane calcium-transporting ATPase 1-like isoform X2
LOC113518480	2941.27	16.4	11.65	3.42	2.18	-2.21	0.00	0.00	Solute carrier organic anion transporter family member 3A1-like isoform X2
LOC113521631	2584	18.25	17.88	3.59	3.47	-2.22	0.00	0.00	Methylcrotonoyl-CoA carboxylase beta chain, mitochondrial
LOC113510258	862	10.79	12.29	2.82	1.72	-2.22	0.00	0.00	Hemicentin-2-like
MSTRG.15112	376	115.49	81.81	24.32	16.87	-2.22	0.00	0.00	Protein unc-13 homolog A-like
LOC113519224	1512	11.18	2.18	0.38	2.19	-2.22	0.00	0.00	Cytochrome P450 6B7-like
LOC113509423	1379.92	316.09	301.4	61.65	59.7	-2.22	0.00	0.00	InaD-like protein isoform X3
LOC113523417	2134	122.25	21.7	5.73	21.67	-2.22	0.00	0.00	Cytochrome P450 4g15
LOC113520906	1802	0.72	0.56	0.04	0.21	-2.23	0.00	0.03	Aminopeptidase W07G4.4
LOC113513387	1635	8.85	7.63	1.52	1.67	-2.23	0.00	0.00	Uncharacterized protein LOC106107480 isoform X1
MSTRG.12064	2371.24	1289.18	678.38	244.06	137.27	-2.23	0.00	0.00	Hemocytin
LOC113513586	1087	1.47	2.04	0.58	0.08	-2.23	0.00	0.01	Bardet-Biedl syndrome 1 protein
LOC113523363	1344.05	47.1	35.9	7.84	8.12	-2.24	0.00	0.00	Protein sprouty isoform X1
LOC113511182	5559	0.94	0.76	0.18	0.14	-2.24	0.00	0.00	ATP-binding cassette sub-family A member 3-like
LOC113522009	884	3.02	1.71	0.49	0.41	-2.24	0.00	0.01	Phosphatidate phosphatase like protein
LOC113514752	1520	6.32	6.07	1.57	0.81	-2.24	0.00	0.00	ATP-binding cassette sub-family G member 1-like
LOC113521263	1661	43.29	41.18	8.07	8.14	-2.24	0.00	0.00	Protein extra-macrochaetae

LOC113522459	1218	9.54	6.7	1.51	1.6	-2.24	0.00	0.00	Uncharacterized protein LOC106129346
LOC113513325	647	9.36	9.48	1.27	2.37	-2.25	0.00	0.00	Cytochrome P450 protein
LOC113517528	1078	185.49	166.17	35.01	32.58	-2.25	0.00	0.00	Ras-related protein Rap-2b
LOC113523568	1449	5.58	7.74	1.36	1.18	-2.25	0.00	0.00	Epoxide hydrolase 1-like
LOC113517896	445	16.71	10.49	2.77	2.58	-2.25	0.00	0.00	Polypeptide N-acetylgalactosaminyltransferase 2
LOC113509690	2161	9.82	7.27	1.73	1.5	-2.26	0.00	0.00	Potassium channel subfamily K member 1-like
LOC113509639	947	930.06	971.23	157.75	203.92	-2.26	0.00	0.00	Odorant binding protein
LOC113518044	2061	4.19	4.06	1.08	0.53	-2.26	0.00	0.00	Monocarboxylate transporter 5
LOC113510667	2266	1.27	1.54	0.18	0.35	-2.27	0.00	0.00	Solute carrier family 22 member 21 like protein
LOC113512546	1704	1.15	0.73	0.08	0.26	-2.28	0.00	0.01	Protein yellow
LOC113521590	1452	11.28	13.87	2.01	2.68	-2.28	0.00	0.00	Non-specific lipid-transfer protein-like
LOC113513582	303	83.41	102.57	18.49	19.86	-2.29	0.00	0.00	Glutathione S-transferase 1-like
LOC113513908	854	1.4	0.95	0.2	0.22	-2.30	0.01	0.04	Apyrase
LOC113516477	459	36.42	37.62	7.7	6.48	-2.30	0.00	0.00	Multiple C2 and transmembrane domain-containing protein 1 isoform X4
MSTRG.13181	4379	4.6	4.93	0.88	0.87	-2.30	0.00	0.00	DD34D transposase
LOC113510220	315	163.19	158.49	31.06	34.48	-2.30	0.00	0.00	Gelsolin-like
LOC113515914	794	2527.82	2379.51	454.72	459.39	-2.30	0.00	0.00	Uncharacterized protein LOC106142761
LOC113522808	2538	1.19	1.17	0.29	0.14	-2.31	0.00	0.00	Transient receptor potential cation channel subfamily V member 5
MSTRG.9472	762	5.43	3.51	0.61	1.04	-2.31	0.00	0.00	Microtubule-associated protein futsch-like
LOC113514722	577	1907.65	900.33	330.82	197.31	-2.31	0.00	0.00	Hemocytin
LOC113509086	1380	0.94	0.61	0.16	0.11	-2.31	0.01	0.04	Glutamate receptor ionotropic, delta-1-like
LOC113511207	4363	1.06	0.77	0.12	0.22	-2.31	0.00	0.00	Uncharacterized protein LOC106135765
LOC113515170	5086	17.69	1.83	1.18	2.29	-2.32	0.00	0.00	Fatty acid synthase-like
LOC113521184	2451	1.74	1.41	0.19	0.38	-2.32	0.00	0.00	Neuropeptide receptor A10
MSTRG.2273	928.54	303.03	325.2	54.41	59.89	-2.33	0.00	0.00	Uncharacterized protein LOC106137009 isoform X1

LOC113510563	1801	2961.54	1610.02	517.49	311.97	-2.33	0.00	0.00	Hemocytin
LOC113520670	586	36.32	44.04	7.04	7.7	-2.34	0.00	0.00	Antennal esterase CXE13
LOC113509885	1222	1.15	1.04	0.31	0.07	-2.34	0.00	0.01	Uncharacterized protein LOC106136933
MSTRG.12264	406	24.63	19.15	4.9	3.32	-2.34	0.00	0.00	Uncharacterized protein LOC110379302
MSTRG.9714	822	3.66	3.24	0.65	0.58	-2.34	0.00	0.00	Uncharacterized protein
LOC113519098	1741.36	47.08	44.56	9.04	7.4	-2.35	0.00	0.00	D-aspartate oxidase
LOC113510517	1154	4.15	0.21	0.2	0.57	-2.35	0.00	0.00	Acyl-CoA synthetase short-chain family member 3, mitochondrial
LOC113520534	2113	20.87	20.34	4.15	3.19	-2.35	0.00	0.00	Cytochrome P450 49a1
MSTRG.3190	1192	23.83	20.26	3.36	4.47	-2.36	0.00	0.00	Uncharacterized protein
LOC113515115	1302	21.78	20.79	3.57	3.99	-2.36	0.00	0.00	IAP-binding motif 1
MSTRG.14553	565	15.4	9.18	2.04	2.39	-2.36	0.00	0.00	Uncharacterized protein LOC106118616
LOC113509459	484	411.82	361.58	74.38	67.4	-2.36	0.00	0.00	Multiple epidermal growth factor-like domains protein 10
LOC113520021	957	3.1	2.06	0.26	0.65	-2.37	0.00	0.00	Zinc finger DNA binding protein
LOC113513807	1034	1.29	1.53	0.16	0.33	-2.37	0.00	0.02	Lipase member H-like isoform X2
LOC113522918	500	20.32	28.86	1.84	6.99	-2.37	0.00	0.00	Uncharacterized protein LOC106111100
LOC113523274	1609	358.14	426.97	64.15	73.68	-2.37	0.00	0.00	Uncharacterized protein
LOC113519558	1177	10.39	14.48	2.49	1.89	-2.37	0.00	0.00	Uncharacterized protein LOC106142421
LOC113520440	1376	20.08	22.58	3.33	4.12	-2.38	0.00	0.00	Aminomethyltransferase, mitochondrial
LOC113512517	1426	202.65	159.09	34.81	28.26	-2.39	0.00	0.00	Protein turtle
LOC113522200	444	10.69	3.52	1.05	1.48	-2.39	0.00	0.01	Uncharacterized protein LOC106101275
MSTRG.237	884	1.78	1.2	0.19	0.31	-2.40	0.00	0.03	Uncharacterized protein LOC105385187
MSTRG.4582	368	57.15	79.87	12.16	12.96	-2.40	0.00	0.00	Galactokinase-like
LOC113510166	1093	34.26	31.38	6.58	4.78	-2.40	0.00	0.00	Serine hydrolase-like protein
LOC113514820	873	15.85	17.79	1.68	4.11	-2.40	0.00	0.00	UDP-glycosyltransferase UGT46A3
LOC113521737	2762	95.68	88.73	14.8	16.76	-2.40	0.00	0.00	Uncharacterized protein LOC110379736

LOC113510248	790	1212.39	1089.45	187.69	210.49	-2.41	0.00	0.00	Venom protease
LOC113513674	1287	0.38	1.09	0.23	0	-2.41	0.00	0.03	Fibulin-2-like isoform X1
LOC113523050	1779	2.22	1.35	0.39	0.21	-2.41	0.00	0.00	4-coumarate--CoA ligase 1-like
LOC113510555	925	2407.27	1422.99	382.73	279.09	-2.41	0.00	0.00	Hemocytin
LOC113520128	469	2044.61	2033.7	307.78	412.99	-2.41	0.00	0.00	Aminoacylase-1-like
LOC113513007	1117	21.92	15.43	3.09	3.29	-2.41	0.00	0.00	G-protein coupled receptor moody
LOC113514205	2613	5.47	4.58	0.73	0.97	-2.41	0.00	0.00	Scavenger receptor class B member 1-like
LOC113522803	1299.03	44.2	42.39	6.97	8.61	-2.42	0.00	0.00	Zinc finger protein GLIS2-like
LOC113515641	2348	2.46	3.03	0.74	0.18	-2.42	0.00	0.00	Uncharacterized protein LOC106142856 isoform X1
LOC113516205	909	61.78	69.14	10.74	11.52	-2.43	0.00	0.00	Uncharacterized protein LOC101736819
LOC113513989	312	30.19	21.18	8.1	1.24	-2.43	0.00	0.01	Histone-lysine N-methyltransferase E(z) isoform X1
LOC113522881	700	45.42	20.78	3.2	8.01	-2.43	0.00	0.00	Uncharacterized protein
LOC113521492	1170	0.97	1.51	0.33	0.07	-2.43	0.00	0.01	GTP-binding protein Rhes
LOC113518529	3495	1.06	1.82	0.15	0.33	-2.43	0.00	0.00	Voltage-dependent calcium channel subunit Alpha-2/delta-3
LOC113520516	2405	50.9	55.25	9.64	8.23	-2.43	0.00	0.00	Sarcoplasmic calcium-binding protein
LOC113521464	666	1.37	1.56	0.15	0.32	-2.44	0.01	0.04	Uncharacterized protein LOC110370179
LOC113523354	1739.99	0.43	0.34	0.12	0	-2.44	0.01	0.04	Uncharacterized protein LOC110370996 isoform X1
LOC113515315	503	10.84	13.98	3.11	1.1	-2.44	0.00	0.00	Diamine acetyltransferase 2-like
LOC113512331	3518	144.65	117.61	24.31	19.52	-2.44	0.00	0.00	Hypothetical protein KGM_208540
LOC113515859	646	33.22	34.41	6.38	5.09	-2.44	0.00	0.00	Acyl-CoA synthetase family member 2, mitochondrial isoform X1
MSTRG.13907	430	1966.54	1071.31	351.97	182.84	-2.44	0.00	0.00	Hypothetical protein KGM_209630
LOC113512321	4233	2.14	1.94	0.36	0.33	-2.45	0.00	0.00	Xanthine dehydrogenase
LOC113516334	899	2.17	2.06	0.28	0.4	-2.46	0.00	0.01	Uncharacterized oxidoreductase SERP2049-like
LOC113523089	3174	1.7	2.12	0.39	0.24	-2.46	0.00	0.00	Neuroligin-4, Y-linked-like isoform X1
MSTRG.2454	793	3.44	2.36	0.46	0.49	-2.47	0.00	0.00	Uncharacterized protein

LOC113516430	818	1801.83	1562.2	287.45	269.98	-2.47	0.00	0.00	Uncharacterized protein LOC106142761
LOC113518889	1046	2.54	1.35	0.46	0.16	-2.47	0.00	0.00	Uncharacterized protein LOC106137666
LOC113522439	2504	1.26	1.46	0.19	0.25	-2.47	0.00	0.00	MFS-type transporter SLC18B1-like
LOC113516030	1584	3.51	0.56	0.27	0.39	-2.48	0.00	0.00	Cell wall integrity and stress response component 3-like
LOC113509256	3103	93.74	91.81	15.93	14.22	-2.48	0.00	0.00	Venus kinase receptor
LOC113509559	2521	2.12	2.14	0.26	0.42	-2.49	0.00	0.00	Uncharacterized protein LOC106142577 isoform X1
LOC113517570	1033	1.72	1.86	0.16	0.42	-2.49	0.00	0.01	Uncharacterized protein LOC110369958
LOC113511019	3678	0.47	0.11	0.02	0.07	-2.49	0.00	0.02	Transient receptor potential channel pyrexia-like
LOC113511087	3427	1.17	1.27	0.19	0.2	-2.50	0.00	0.00	Rhophilin-2-B isoform X2
LOC113515509	1967	0.39	1.02	0.07	0.15	-2.50	0.00	0.01	Katanin p60 ATPase-containing subunit A1-like
LOC113518439	2116	4.05	0.6	0.26	0.48	-2.50	0.00	0.00	Peroxisomal acyl-coenzyme A oxidase 1
MSTRG.9758	521	2.79	1.96	0.72	0	-2.51	0.01	0.04	Uncharacterized protein LOC106142856 isoform X1
LOC113510135	1052	0.7	0.87	0.08	0.16	-2.51	0.00	0.03	Serine hydrolase
LOC113520105	1203	51.56	54.31	7.91	9.03	-2.51	0.00	0.00	D-3-phosphoglycerate dehydrogenase
LOC113517147	533	982.98	1005.25	139.9	184.74	-2.51	0.00	0.00	Aminoacylase-1-like
LOC113513316	1969	3059.49	2076.92	440.66	377.77	-2.51	0.00	0.00	Hemocytin
LOC113516620	3510	6.24	7.33	1.06	1.09	-2.51	0.00	0.00	Junctophilin-1 isoform X1
LOC113515955	1263	400.33	447.44	66.31	69.23	-2.51	0.00	0.00	Uncharacterized protein LOC110373240
MSTRG.10659	1998	26.83	29.54	4.67	4.32	-2.51	0.00	0.00	Uncharacterized protein
LOC113520176	2476	3.56	3.41	0.62	0.49	-2.51	0.00	0.00	Facilitated trehalose transporter Tret1-2 homolog isoform X1
LOC113521718	939.71	24.84	25.04	3.83	4.1	-2.52	0.00	0.00	5-formyltetrahydrofolate cyclo-ligase-like isoform X1
LOC113520466	338	113.7	121.42	27.37	13.29	-2.52	0.00	0.00	3-hydroxyisobutyrate dehydrogenase, mitochondrial
MSTRG.912	246	3740.27	3199.62	636.79	662.81	-2.52	0.00	0.00	Hemocyte protease-1

LOC113522455	1629	8.81	11.07	1.22	1.91	-2.52	0.00	0.00	Uncharacterized protein LOC106136943
MSTRG.10620	388	960.41	890.3	178.42	132.85	-2.53	0.00	0.00	Protein shifted
MSTRG.5657	732	10.83	16.51	3.81	12.42	-2.53	0.00	0.00	Poly (ADP-ribose) polymerase
LOC113523571	3552	0.6	0.13	0.02	0.1	-2.53	0.00	0.01	Uncharacterized protein LOC110370405
LOC113516327	4941.43	26.86	25.27	4.07	3.95	-2.54	0.00	0.00	Homeobox protein cut isoform X2
LOC113518557	14025	0.07	0.08	0.01	0.01	-2.55	0.00	0.01	Dynein heavy chain 8, axonemal
LOC113509606	2333.49	22.52	21.43	3.58	3.4	-2.56	0.00	0.00	Monocarboxylate transporter 10-like
LOC113509642	1028	1.23	0.41	0.24	0	-2.56	0.00	0.03	Hemicentin-1-like
LOC113511069	1588	5.08	4.68	0.72	0.77	-2.57	0.00	0.00	Chondroadherin-like
MSTRG.1868	849	5.56	6.17	1.13	0.66	-2.57	0.00	0.00	Retrovirus-related pol polyprotein from transposon tnt 1-94
LOC113518775	4117	1.33	1.09	0.18	0.18	-2.57	0.00	0.00	Cubilin
LOC113514040	1160	1.91	1.32	0.33	0.14	-2.58	0.00	0.00	IQ and AAA domain-containing protein 1
LOC113512790	927	44.25	47.85	6.26	7.73	-2.59	0.00	0.00	Acyl-CoA synthetase family member 2, mitochondrial
LOC113517514	1219	1.96	0.65	0.25	0.13	-2.59	0.00	0.01	CD63 antigen-like
LOC113521583	4371	0.47	0.35	0.03	0.09	-2.59	0.00	0.00	Uncharacterized protein LOC106132743 isoform X1
LOC113521711	493	292.09	317.53	46.38	48.48	-2.59	0.00	0.00	Uncharacterized protein LOC106132549
LOC113513145	1138	5.43	5.33	1.03	0.58	-2.59	0.00	0.00	Zinc finger protein rotund isoform X3
LOC113513313	843	3.33	3.23	0.31	0.67	-2.60	0.00	0.00	Aldose reductase isoform X1
MSTRG.12634	665	34.99	37.59	4.72	6.3	-2.60	0.00	0.00	Microspherule protein 1-like
LOC113517645	1187.44	65.89	85.04	10.34	12.27	-2.61	0.00	0.00	Alcohol dehydrogenase AD1
LOC113512382	2818	0.43	0.24	0.05	0.05	-2.61	0.00	0.02	Synaptic vesicle glycoprotein 2C-like
LOC113518715	729	12.81	14.1	2.22	1.8	-2.61	0.00	0.00	Uncharacterized protein LOC110375196
LOC113512953	657	10.11	7	1.7	0.82	-2.62	0.00	0.00	MD-2-related lipid-recognition protein-like
MSTRG.2260	659	8.93	11.1	1.57	1.74	-2.63	0.00	0.00	Uncharacterized protein OBRU01_06617
MSTRG.5918	2269	23.43	27.48	3.65	3.79	-2.63	0.00	0.00	Protease inhibitor 5

LOC113515777	1769	6.3	6.87	1.07	0.84	-2.64	0.00	0.00	3-ketoacyl-CoA thiolase, mitochondrial-like isoform X1
LOC113521564	6008	9.94	8.9	1.67	1.04	-2.66	0.00	0.00	Down syndrome cell adhesion molecule-like protein Dscam2
LOC113516066	1805	4.79	4.94	0.7	0.7	-2.66	0.00	0.00	Sedoheptulokinase-like
LOC113516955	2572.5	0.42	0.41	0.02	0.24	-2.66	0.00	0.00	Uncharacterized protein LOC106099430
LOC113517602	3702	1.06	1.19	0.21	0.11	-2.66	0.00	0.00	Uncharacterized protein LOC106137997
LOC113515123	589	6.62	5.6	0.94	0.8	-2.67	0.00	0.00	Uncharacterized protein LOC106116870 isoform X1
LOC113510038	603	9.28	6.1	1.8	0.38	-2.67	0.00	0.00	Uncharacterized protein
LOC113510435	1809	3.71	3.45	0.23	0.78	-2.67	0.00	0.00	Inactive hydroxysteroid dehydrogenase-like protein 1 isoform X1
LOC113511647	545	3.73	5.82	1.1	0.23	-2.68	0.00	0.00	Bardet-Biedl syndrome 1 protein
MSTRG.8602	1334	25.64	33.84	4.08	4.35	-2.68	0.00	0.00	Seminal fluid protein HACP040
LOC113520941	3158	0.72	0.66	0.06	0.13	-2.68	0.00	0.00	Lysosomal alpha-glucosidase-like
LOC113522098	606	12.42	4.4	1.25	1.14	-2.68	0.00	0.00	Uncharacterized protein LOC106101275
LOC113520314	3461.74	1.12	0.64	0.14	0.11	-2.69	0.00	0.00	Ecdysone 20-monooxygenase
LOC113518198	3012	1.02	1.37	0.22	0.12	-2.69	0.00	0.00	Band 4.1-like protein 4
LOC113515857	424	0	6.33	0	0.84	-2.69	0.01	0.05	Collagen and calcium-binding EGF domain-containing protein 1-like isoform X2
LOC113511288	922	14.67	6.9	1.37	1.66	-2.69	0.00	0.00	Beta-1,3-galactosyltransferase 5-like
LOC113523144	966	6.83	5.76	0.94	0.82	-2.69	0.00	0.00	Oxidoreductase
LOC113517691	623	3.38	2.97	0.68	0.18	-2.70	0.00	0.00	Solute carrier family 23 member 2 isoform X2
LOC113518448	1188	17.78	17.33	2.92	2	-2.70	0.00	0.00	Fructose-bisphosphate aldolase-like
LOC113513526	1025	1.01	0.24	0	0.17	-2.70	0.01	0.05	Epidermal retinol dehydrogenase 2-like
LOC113518681	365	118.93	15.42	8.13	12	-2.71	0.00	0.00	Hypothetical protein OBRU01_15499
LOC113514448	1791	0.51	1.3	0.12	0.12	-2.71	0.00	0.00	Scavenger receptor class B member 1-like
LOC113509632	934	9.47	12.25	0.72	2.3	-2.71	0.00	0.00	Juvenile hormone binding protein

LOC113519522	489	29.4	27.77	6.08	2.06	-2.71	0.00	0.00	Zinc transporter 2-like isoform X1
MSTRG.7596	757	0.9	2.02	0.12	0.26	-2.71	0.00	0.02	Uncharacterized protein LOC106135670
LOC113515439	1604.03	195.78	202.14	28.1	27.05	-2.72	0.00	0.00	Retinal dehydrogenase 1-like
LOC113515484	2522	1.17	1.23	0.18	0.14	-2.72	0.00	0.00	V-type proton ATPase 116 kDa subunit a isoform 1-like
LOC113518403	1242	0.62	0.38	0.06	0.07	-2.73	0.01	0.05	Lachesin isoform X1
LOC113514606	726	2.63	3.51	0.39	0.42	-2.75	0.00	0.00	Insecticyanin-A
LOC113513545	1236	3.12	3	0.68	0.13	-2.76	0.00	0.00	Echinoderm microtubule-associated protein-like CG42247
MSTRG.7746	282	70.21	33.72	5.63	10.14	-2.76	0.00	0.00	Uncharacterized protein
LOC113520605	1538.45	64.28	53.51	7.83	7.82	-2.76	0.00	0.00	Uncharacterized protein
LOC113511769	3350	1.46	2.26	0.29	0.21	-2.77	0.00	0.00	PR domain Zinc finger protein 1
LOC113520120	1371	1.2	3.02	0.38	0.17	-2.77	0.00	0.00	Facilitated trehalose transporter Tret1-2 homolog
LOC113515608	1185	1.32	1.28	0.2	0.14	-2.77	0.00	0.01	Lachesin-like
LOC113521982	432	3.92	4.9	1.12	0	-2.77	0.00	0.01	Tctex1 domain-containing protein 1-A-like
LOC113509492	1142	0.88	0.28	0	0.15	-2.79	0.00	0.03	Uncharacterized protein LOC106712877
MSTRG.433	1115	1.71	1.14	0.21	0.15	-2.79	0.00	0.00	G-protein coupled receptor 112
LOC113513088	297	247.86	249.48	30.46	42.15	-2.80	0.00	0.00	Wnt inhibitory factor 1
LOC113519117	1582	28.03	29.46	4.12	3.38	-2.80	0.00	0.00	Cytochrome P450 9G3
MSTRG.6905	638	4.87	4.19	0.98	0.17	-2.81	0.00	0.00	G-protein coupled receptor moody
LOC113510744	2484	15.52	7.85	0.27	2.71	-2.81	0.00	0.00	Peritrophin 1
LOC113514174	844	1.43	1.18	0.21	0.11	-2.81	0.00	0.01	L-xylulose reductase-like
LOC113521930	1725	0.49	0.21	0.08	0	-2.81	0.00	0.03	Sodium/potassium/calcium exchanger Nckx30C
LOC113517432	2143	2.73	2.63	0.32	0.37	-2.81	0.00	0.00	Echinoderm microtubule-associated protein-like CG42247
LOC113518962	1239	41.17	39.4	5.65	4.84	-2.81	0.00	0.00	Lipid storage droplets surface-binding protein 1 isoform X1
LOC113510737	1266	7.21	8.06	1.08	0.89	-2.82	0.00	0.00	Venom protease-like
LOC113511683	940	0.73	0.83	0.09	0.09	-2.82	0.00	0.03	Uncharacterized protein C2orf47 homolog, mitochondrial-like
LOC113514269	1716	9.19	7.76	1.52	0.73	-2.82	0.00	0.00	Large neutral amino acids transporter small subunit 1

MSTRG.5472	1149	12.71	11.22	1.69	1.37	-2.83	0.00	0.00	Uncharacterized protein
LOC113515652	4515	1.41	3.46	0.47	0.15	-2.83	0.00	0.00	Tolloid-like protein 1
MSTRG.9516	374	108.12	109.99	15.54	14.11	-2.83	0.00	0.00	Uncharacterized protein
LOC113518463	1024	23.29	8.18	0.79	3.2	-2.83	0.00	0.00	Trypsin-like protein
LOC113514080	1320	2.34	3.58	0.1	0.72	-2.84	0.00	0.00	Cysteine and histidine-rich protein 1-like
LOC113523028	1347	1.17	1.14	0.17	0.12	-2.84	0.00	0.00	Uncharacterized protein LOC110384608
MSTRG.5341	1178	23.35	23.08	2.62	3.28	-2.84	0.00	0.00	Uncharacterized protein
LOC113517750	904	56.1	61.74	7.43	7.62	-2.84	0.00	0.00	UDP-glucuronosyltransferase 2A3-like
MSTRG.10395	457	46.77	39.98	6.16	5.17	-2.85	0.00	0.00	Eukaryotic translation initiation factor 4E-binding protein 2
LOC113521981	925	5.42	6.78	0.27	1.26	-2.85	0.00	0.00	Short-chain dehydrogenease/reductase
LOC113513641	469	0.81	4.62	0.3	0.32	-2.86	0.00	0.03	25 kDa silk glycoprotein Fibrohexamerin
MSTRG.5158	5008	2.45	2.89	0.39	0.28	-2.86	0.00	0.00	Uncharacterized protein LOC106135886
LOC113514149	1736	0.6	2.48	0.16	0.22	-2.86	0.00	0.00	Cytochrome P450 6B6-like
LOC113509901	8499	0.5	0.24	0.01	0.08	-2.87	0.00	0.00	Slit homolog 3 protein-like
LOC113516815	1663.32	11.78	11.2	1.42	1.43	-2.87	0.00	0.00	Nucleolar protein 10
LOC113514092	417	5.07	2.71	0	0.96	-2.87	0.00	0.03	Acyl-CoA-binding protein
LOC113512878	2231	2.16	1.92	0.27	0.23	-2.88	0.00	0.00	Semaphorin-2A-like
LOC113514552	1350	0	1.03	0.11	0	-2.88	0.00	0.03	Facilitated trehalose transporter Tret1-like
LOC113517584	904	3.02	2.62	0.19	0.5	-2.88	0.00	0.00	Uncharacterized protein LOC106713365
LOC113513336	444	47.66	39.03	6.97	4.08	-2.89	0.00	0.00	Fatty-acid amide hydrolase 2-B-like
LOC113520705	3380	6.26	6.09	0.8	0.71	-2.89	0.00	0.00	Nitrogen permease regulator 2-like protein
LOC113515907	220	232.98	195.12	33.45	28.39	-2.89	0.00	0.00	Chitooligosaccharidolytic beta-N-acetylglucosaminidase isoform X1
LOC113512508	1220	0.63	0.52	0.06	0.07	-2.89	0.00	0.03	Acyl-CoA Delta(11) desaturase
LOC113512940	721	6.88	5.47	0.8	0.71	-2.89	0.00	0.00	Uncharacterized protein LOC106136299
LOC113517233	4159	2.26	2.95	0.29	0.34	-2.90	0.00	0.00	Uncharacterized protein LOC106131872

MSTRG.13323	3068	3.83	4.85	0.51	0.54	-2.90	0.00	0.00	Synaptotagmin-5-like isoform X1
LOC113522219	590	68.54	68.7	9.58	7.4	-2.91	0.00	0.00	Biliverdin binding protein-1
MSTRG.9286	315	12.69	14.99	1.11	2.38	-2.92	0.00	0.01	Hypothetical protein RR48_00560
LOC113511860	4981	1.11	0.75	0.13	0.09	-2.92	0.00	0.00	Uncharacterized protein LOC110377235 isoform X5
LOC113517244	2590	1	0.58	0.1	0.08	-2.92	0.00	0.00	cGMP-dependent protein kinase, isozyme 1-like
LOC113518647	555	55.54	51.4	6.55	6.52	-2.92	0.00	0.00	Alkylglycerol monooxygenase-like
LOC113516007	2909	0.4	0.42	0.04	0.07	-2.93	0.00	0.00	Monocarboxylate transporter 4-like
MSTRG.5000	302	41.23	23.13	4.02	4.32	-2.94	0.00	0.00	Rabphilin-3A
LOC113511229	3570	0.3	0.04	0.04	0	-2.94	0.00	0.03	Uncharacterized protein LOC106135922
LOC113518224	1734	2.12	2.17	0.12	0.31	-2.94	0.00	0.00	Uncharacterized protein LOC106140166 isoform X1
LOC113523449	1136	1.08	1.91	0.14	0.22	-2.95	0.00	0.00	Uncharacterized protein
LOC113511309	278	56.14	89.6	8.08	10.94	-2.96	0.00	0.00	Hypothetical protein RR46_06210
LOC113519122	974	3.18	3.77	0.25	0.54	-2.98	0.00	0.00	Uncharacterized protein LOC106130390
LOC113513108	1210	1.63	1.31	0.19	0.13	-2.99	0.00	0.00	Mitochondrial sodium/hydrogen exchanger NHA2
LOC113509870	1791	10.76	11.07	1.21	1.25	-3.01	0.00	0.00	Alkaline phosphatase-like isoform X2
LOC113510286	3346	0.68	0.94	0.13	0.04	-3.02	0.00	0.00	Kinesin-like protein CG14535 isoform X1
LOC113512287	1754	1.44	1.99	0.04	0.34	-3.02	0.00	0.00	Mitochondrial carrier protein ymc
LOC113522454	1146	0.81	0.56	0.07	0.07	-3.03	0.00	0.01	Glia-derived nexin-like isoform X1
LOC113514869	861	1.85	1.15	0.2	0.11	-3.05	0.00	0.00	UDP-glucuronosyltransferase 2A3-like
LOC113515876	1058	24.31	22.3	3.25	1.85	-3.06	0.00	0.00	Juvenile hormone epoxide hydrolase-like
LOC113516290	685	7.08	6.68	0.72	0.77	-3.06	0.00	0.00	Uncharacterized protein LOC106143382
LOC113518374	1713.5	0.73	0.09	0.04	0.05	-3.07	0.00	0.01	Lipase 1
LOC113518285	4621	0.61	0.35	0.01	0.09	-3.07	0.00	0.00	Uncharacterized protein LOC101747221 isoform X1
LOC113511308	1122	1.8	2.39	0	0.45	-3.08	0.00	0.00	Lipase member H-B-like
LOC113516063	3222	2.8	0.02	0	0.3	-3.08	0.00	0.00	Frizzled-4

LOC113519226	730	822.98	795.49	72.98	102.49	-3.08	0.00	0.00	Uncharacterized protein LOC106720246
LOC113511849	1588	0.62	0.33	0	0.1	-3.08	0.00	0.01	Carboxypeptidase N subunit 2-like
LOC113521984	767	3.84	3.22	0.48	0.26	-3.08	0.00	0.00	Uncharacterized protein LOC105842600
LOC113516554	777	1.4	2.19	0.35	0	-3.09	0.00	0.00	Trypsin-like protein
LOC113520500	1248	16.3	2.52	0.3	1.68	-3.09	0.00	0.00	Acyl-CoA Delta(11) desaturase-like
LOC113510512	965	12.27	7.98	0.6	1.55	-3.09	0.00	0.00	Uncharacterized protein LOC106124870
MSTRG.3477	330	94.23	124.21	16.42	8.78	-3.10	0.00	0.00	3-hydroxyisobutyrate dehydrogenase, mitochondrial
LOC113514621	618	2.49	2.83	0	0.55	-3.10	0.00	0.01	N-acylneuraminate cytidylyltransferase
LOC113523473	784	4.99	3.12	0.35	0.5	-3.11	0.00	0.00	Synaptotagmin-5
MSTRG.1086	300	17.88	21.18	2.77	1.49	-3.11	0.00	0.00	Hemicentin-2
MSTRG.14617	563	4.84	5.48	0.66	0.51	-3.12	0.00	0.00	Uncharacterized protein LOC106684797
MSTRG.3959	576	4.42	5.43	0.59	0.42	-3.12	0.00	0.00	Cytochrome P450 9e2-like
LOC113514124	939	10.61	3.69	0.45	1.04	-3.12	0.00	0.00	Collagenase-like
LOC113509076	1078	17.01	13.84	1.47	1.69	-3.13	0.00	0.00	Uncharacterized protein LOC106131149
LOC113516110	1652	2.7	3.35	0.39	0.23	-3.14	0.00	0.00	Homogentisate 1,2-dioxygenase
LOC113517116	673	2.97	3.21	0.15	0.47	-3.14	0.00	0.00	B9 domain-containing protein 2
LOC113509251	468	16.2	14.25	0.92	2.28	-3.15	0.00	0.00	Uncharacterized protein
LOC113513980	1532	92.48	86.76	9.56	8.78	-3.15	0.00	0.00	Cytochrome P450 monooxygenase CYP9G18
MSTRG.7238	341	23.35	30.55	3.21	2.57	-3.16	0.00	0.00	Short/branched chain specific acyl-CoA dehydrogenase, mitochondrial
LOC113522572	842	37.69	18.43	0.1	5.56	-3.16	0.00	0.00	Uncharacterized protein LOC110371012
LOC113517249	747	2.74	1.29	0.13	0.27	-3.17	0.00	0.01	Uncharacterized protein
LOC113521779	1584	1.04	0.89	0.09	0.1	-3.17	0.00	0.00	Esterase FE4-like
LOC113523029	1761	411.88	385.89	41.3	39.25	-3.17	0.00	0.00	Pancreatic triacylglycerol lipase-like
LOC113523533	1859	1.24	1.94	0	0.32	-3.17	0.00	0.00	Uncharacterized protein LOC106131316
MSTRG.11997	764	2.65	1.25	0.24	0.13	-3.17	0.00	0.00	Uncharacterized protein LOC106139637

LOC113509292	770	23.22	19.1	2.63	1.66	-3.17	0.00	0.00	Alkylglycerol monooxygenase-like
LOC113510247	1163	6.69	7.04	0.8	0.57	-3.18	0.00	0.00	Acidic amino acid decarboxylase GADL1
LOC113510312	1873.05	13.9	15.69	1.34	1.72	-3.18	0.00	0.00	Carboxylesterase
LOC113512543	4022	11.9	12.39	1.17	1.25	-3.19	0.00	0.00	Xanthine dehydrogenase-like
LOC113521780	1764	0.96	1.2	0.08	0.13	-3.19	0.00	0.00	Esterase FE4-like
LOC113516939	1953	0.98	0.55	0.11	0.04	-3.19	0.00	0.00	Uncharacterized protein LOC106143151 isoform X1
LOC113513699	499	58.65	51.26	5.02	6.18	-3.19	0.00	0.00	Microsomal epoxide hydrolase
LOC113515816	950	492.5	588.49	52.26	55.75	-3.20	0.00	0.00	Uncharacterized protein LOC106130383
LOC113519055	2611	13.13	18.15	1.39	1.7	-3.20	0.00	0.00	Polyserase-2-like
LOC113509684	1708	0.73	1.5	0.17	0.04	-3.20	0.00	0.00	Uncharacterized protein LOC106136946
LOC113513404	330	406.31	400.14	43.78	42.92	-3.21	0.00	0.00	Aminoacylase-1-like
LOC113520429	960	6.26	4.82	0.35	0.74	-3.21	0.00	0.00	Uncharacterized abhydrolase domain-containing protein DDB_G0269086-like
LOC113518195	2151	3.36	0.43	0.06	0.3	-3.22	0.00	0.00	Peroxisomal acyl-coenzyme A oxidase 1
MSTRG.10275	378.36	4.97	7.26	0	1.28	-3.23	0.00	0.01	Azurocidin-like
LOC113511104	2465	0.43	0.51	0.03	0.06	-3.24	0.00	0.00	Collagen alpha-1(XXI) chain
MSTRG.13883	305	11.43	15.99	1.28	1.38	-3.24	0.00	0.01	Uncharacterized protein
LOC113514651	1043	3.54	4.07	0.23	0.49	-3.24	0.00	0.00	Alpha-tocopherol transfer protein-like
LOC113518704	1101	1.19	1.26	0.14	0.08	-3.24	0.00	0.00	Uncharacterized protein LOC106720002 isoform X1
LOC113519369	1431	3.02	2.6	0.26	0.27	-3.25	0.00	0.00	Protein takeout
LOC113512983	1925	2.75	2.38	0.18	0.31	-3.25	0.00	0.00	4-coumarate--CoA ligase 3
LOC113517875	393	15.6	15.17	2.46	0.52	-3.25	0.00	0.00	Fatty-acid amide hydrolase 2-A-like isoform X1
MSTRG.7565	810	5.54	2.96	0.55	0.24	-3.26	0.00	0.00	Uncharacterized protein LOC106133343 isoform X1
LOC113516941	3242	0.7	0.87	0.08	0.06	-3.27	0.00	0.00	Uncharacterized protein LOC106134039
LOC113516116	878	5.84	1.93	0.1	0.67	-3.27	0.00	0.00	Uncharacterized protein LOC106138839 isoform X1
LOC113518264	2827	2.34	2.58	0.19	0.27	-3.28	0.00	0.00	Uncharacterized protein

									LOC110378921
MSTRG.4261	602	2.12	2.23	0.18	0.19	-3.29	0.00	0.01	Facilitated trehalose transporter Tret1-like
LOC113518128	1064	6.13	1.63	0	0.72	-3.29	0.00	0.00	Pancreatic triacylglycerol lipase-like
LOC113521922	440	4.37	3.96	0.71	0	-3.29	0.00	0.01	Zinc finger protein 100-like
LOC113511941	537	2.22	0.69	0	0.24	-3.30	0.01	0.04	Uncharacterized protein
LOC113522760	1096	0.73	0.22	0	0.08	-3.30	0.01	0.04	Uncharacterized protein LOC106139325
LOC113515467	1471	1.1	0.1	0.06	0.05	-3.30	0.00	0.01	Odorant receptor 85b-like
LOC113510424	634	35.94	34.52	3.96	2.63	-3.30	0.00	0.00	Uncharacterized protein LOC110375326
LOC113513432	3479	1.77	1.17	0.17	0.1	-3.31	0.00	0.00	Uncharacterized protein LOC106137196
LOC113518915	482	2.28	1.45	0	0.3	-3.31	0.01	0.04	Uncharacterized protein
LOC113515515	1807	0.68	0.28	0	0.08	-3.31	0.00	0.00	Androgen-dependent TFPI-regulating protein-like
LOC113516303	744	2.3	0.78	0.13	0.13	-3.32	0.00	0.00	Leucine-rich repeat neuronal protein 3-like
LOC113511850	6041	0.82	0.39	0.03	0.08	-3.32	0.00	0.00	Uncharacterized protein LOC106133343 isoform X4
LOC113515564	2564	0.14	0.22	0	0.03	-3.33	0.01	0.04	Uncharacterized protein LOC106103590
LOC113516486	3207	0.07	0.21	0	0.02	-3.34	0.01	0.04	Apoptosis-stimulating of p53 protein 1
LOC113517853	1833	3.83	4.06	0.46	0.24	-3.34	0.00	0.00	Dipeptidase 1-like
MSTRG.13946	341	6.67	12.53	0	1.71	-3.35	0.00	0.00	Bifunctional ATP-dependent dihydroxyacetone kinase/FAD-AMP lyase (cyclizing)-like
LOC113517812	1115	1.56	0.22	0.07	0.08	-3.36	0.00	0.00	Leukocyte surface antigen CD53-like isoform X1
LOC113515849	958	3.89	5.29	0.52	0.28	-3.36	0.00	0.00	Uncharacterized protein LOC110378293
LOC113511028	1167	4.34	4.33	0.46	0.28	-3.37	0.00	0.00	Uncharacterized protein LOC106135978
LOC113513300	1538	1.21	0.53	0.14	0	-3.38	0.00	0.00	Carboxypeptidase B-like
LOC113509455	5197	0.87	0.43	0.1	0.01	-3.38	0.00	0.00	Hemicentin-1-like
LOC113520080	406	8.47	7.57	0.89	0.47	-3.39	0.00	0.00	Androgen-dependent TFPI-

									regulating protein-like
LOC113523317	3961	0.19	0.05	0	0.02	-3.39	0.00	0.03	Multidrug resistance protein 1A-like
LOC113519112	4752	0.16	0.03	0.01	0	-3.39	0.00	0.03	MATH and LRR domain-containing protein PFE0570w-like
LOC113515583	3559	1.74	0.51	0.04	0.15	-3.40	0.00	0.00	Membrane alanyl Aminopeptidase
LOC113512799	4700	0.42	0.1	0.03	0.01	-3.42	0.00	0.00	Niemann-Pick C1 protein-like
LOC113512262	497	1.42	2.44	0	0.28	-3.43	0.00	0.03	Uncharacterized protein LOC106138972
LOC113516769	632	0.9	1.53	0.17	0	-3.43	0.00	0.03	Uncharacterized protein LOC106136053
LOC113522958	531	1.03	2.36	0.23	0	-3.44	0.00	0.03	Centaurin-gamma-1A-like
LOC113520751	3993	1.03	1.18	0.1	0.08	-3.45	0.00	0.00	Uncharacterized protein LOC106132055
LOC113515096	958	24.73	9.32	0.61	2.21	-3.45	0.00	0.00	Collagenase-like
LOC113519094	459	40779.49	47065.56	3273.52	4289.16	-3.45	0.00	0.00	Anionic antimicrobial peptide 2
LOC113512295	1060	10.11	5.46	0.08	1.2	-3.46	0.00	0.00	Pancreatic lipase-related protein 2-like
LOC113515393	2054	2.16	0.45	0.07	0.14	-3.47	0.00	0.00	Trypsin beta-like
LOC113509364	2200	3.62	3	0.34	0.2	-3.47	0.00	0.00	Cytochrome P450 6B2-like
LOC113511640	2493	0.96	0.44	0.08	0.03	-3.47	0.00	0.00	luciferin 4-monooxygenase
LOC113522005	1849	3.29	2.96	0.15	0.36	-3.48	0.00	0.00	Esterase FE4
LOC113510707	560	5.78	2.55	0	0.66	-3.49	0.00	0.00	Uncharacterized protein LOC101747082
LOC113522928	432	7.52	7.16	0.75	0.4	-3.50	0.00	0.00	Uncharacterized protein
LOC113513808	306	11.27	6.06	1.26	0	-3.50	0.00	0.02	Unconventional myosin-Va-like
LOC113515865	1900	0.3	0.26	0.04	0	-3.51	0.00	0.02	Adhesion G-protein coupled receptor G2-like isoform X3
LOC113521987	2228	0.25	0.22	0.03	0	-3.51	0.00	0.02	Malate/L-sulfolactate dehydrogenase
LOC113515344	693	6.56	8.45	0.28	0.9	-3.52	0.00	0.00	Uncharacterized protein LOC106142844
LOC113510671	826	8.64	1.55	0.11	0.69	-3.54	0.00	0.00	Uncharacterized protein LOC101747082
LOC113521324	1678	2.89	0.61	0.05	0.22	-3.55	0.00	0.00	Alanine--glyoxylate aminotransferase 2, mitochondrial
LOC113513180	4250	4.12	3.33	0.27	0.3	-3.56	0.00	0.00	Protein TANC2 isoform X1

LOC113519512	2311	0	0.47	0.03	0	-3.56	0.00	0.02	Uncharacterized transmembrane protein DDB_G0289901-like
LOC113513939	1682	333.67	402.59	27.51	28.95	-3.57	0.00	0.00	Selenium-binding protein 1-A
LOC113522683	956	37.13	18.28	0.78	3.42	-3.57	0.00	0.00	Uncharacterized protein LOC110371118
LOC113521001	385	5.37	2.6	0	0.56	-3.58	0.00	0.02	Uncharacterized protein LOC106129135
MSTRG.12673	282	21.59	18.17	3.27	0	-3.59	0.00	0.02	Peroxisomal acyl-coenzyme A oxidase 1
LOC113522374	2005	0.51	0.53	0	0.07	-3.59	0.00	0.00	Tetratricopeptide repeat protein 30A
LOC113516617	1632	4.55	3.35	0.31	0.28	-3.60	0.00	0.00	Lectin3
LOC113516832	678	4.13	4.23	0.44	0.16	-3.60	0.00	0.00	Trypsin delta/gamma-like isoform X2
LOC113510881	3400	0.76	0.33	0.02	0.06	-3.61	0.00	0.00	Sodium-dependent nutrient amino acid transporter 1-like isoform X1
LOC113517478	1083	59.99	77.64	5.57	4.76	-3.61	0.00	0.00	IML1
LOC113516621	978	1.47	2.26	0.25	0	-3.62	0.00	0.00	Junctophilin-1
LOC113515485	675	10.47	4.26	0.44	0.63	-3.63	0.00	0.00	Myophilin-like isoform X1
LOC113521921	726	1.91	0.27	0.13	0	-3.65	0.00	0.02	Uncharacterized protein
LOC113514001	1409	3.71	2.44	0.16	0.28	-3.66	0.00	0.00	Cytochrome P450
LOC113520900	773	73.59	114.07	6.78	6.83	-3.66	0.00	0.00	Uncharacterized protein LOC106133892
LOC113512906	3755	1.96	1.54	0.12	0.13	-3.67	0.00	0.00	Serine/arginine repetitive matrix protein 2
LOC113515847	1279	1.9	1.65	0.18	0.06	-3.67	0.00	0.00	Uncharacterized oxidoreductase TM_0325-like
LOC113519789	1125	0.58	0.65	0	0.07	-3.68	0.00	0.01	1,5-anhydro-D-fructose reductase-like
MSTRG.13062	501	10.93	11.97	0.79	0.84	-3.68	0.00	0.00	Uncharacterized protein
LOC113509454	731	0.95	1.34	0	0.14	-3.68	0.00	0.01	Uncharacterized protein LOC106709162
MSTRG.12745	723	0.96	1.36	0	0.14	-3.68	0.00	0.01	Uncharacterized protein
LOC113522982	990	9.27	10.44	0.33	1.06	-3.69	0.00	0.00	Uncharacterized protein LOC106136966
LOC113523307	5127.76	6.72	6.53	0.29	0.64	-3.69	0.00	0.00	Uncharacterized protein LOC106131368 isoform X1
LOC113522899	1632	0.2	0.59	0	0.05	-3.70	0.00	0.01	Uncharacterized protein

									LOC106129443
LOC113519133	557	12.43	6.22	0.84	0.45	-3.70	0.00	0.00	Uncharacterized protein
LOC113522828	929	20.84	13.27	0.37	1.99	-3.72	0.00	0.00	Uncharacterized protein LOC110371440
LOC113520284	4832	0.23	0.2	0.01	0.01	-3.72	0.00	0.00	Titin-like isoform X1
LOC113511695	1823	31.65	39.5	2.56	2.28	-3.74	0.00	0.00	Protein yellow-like
LOC113513597	1673	0.51	0.26	0.04	0	-3.74	0.00	0.01	Myrosinase 1-like
LOC113520972	1225	2.58	1.42	0.06	0.2	-3.75	0.00	0.00	Myrosinase 1
LOC113516624	1771	1.35	1.23	0.12	0.04	-3.78	0.00	0.00	Myrosinase 1
LOC113510604	1101	4.61	0.22	0.07	0.23	-3.82	0.00	0.00	Pancreatic triacylglycerol lipase-like
LOC113522598	1425	10.92	2.78	0.41	0.44	-3.85	0.00	0.00	Uncharacterized protein LOC106139316
LOC113513560	1068	1.85	0.85	0	0.16	-3.86	0.00	0.00	Chlorophyllide A binding protein isoform X1
MSTRG.1586	762	14.55	14.47	1.06	0.76	-3.87	0.00	0.00	Transmembrane protease serine
LOC113513701	839	1.53	0.54	0.1	0	-3.88	0.00	0.01	Uncharacterized protein LOC106136208
LOC113509262	943	1.87	1.37	0.09	0.09	-3.88	0.00	0.00	low density lipoprotein receptor adapter protein 1
LOC113517017	2002	0.7	0.57	0.03	0.04	-3.88	0.00	0.00	Peritrophin-48-like
LOC113514403	1028	21.15	22.37	1.65	1	-3.89	0.00	0.00	ETS DNA-binding protein pokkuri
LOC113514842	308	11.95	10.61	0	1.32	-3.89	0.00	0.00	Solute carrier family 23 member 2 isoform X1
LOC113513965	835	14.2	5.89	0.21	1.01	-3.89	0.00	0.00	Pancreatic triacylglycerol lipase-like
LOC113509865	508	2.49	2.58	0	0.27	-3.89	0.00	0.00	Circadian clock-controlled protein-like
LOC113519703	3880	1.19	0.53	0.08	0.02	-3.90	0.00	0.00	Uncharacterized protein LOC106136222
LOC113522895	2682	0.43	4.37	0.07	0.21	-3.93	0.00	0.00	Mediator of DNA damage checkpoint protein 1
LOC113515168	4332	0.24	0.06	0.01	0	-3.94	0.00	0.00	Fatty acid synthase-like
LOC113517917	438	4.11	3.27	0	0.38	-3.95	0.00	0.00	Uncharacterized protein LOC106708587
LOC113522197	366	294.45	393.73	21.06	21.79	-3.96	0.00	0.00	D-arabinitol dehydrogenase 1-like isoform X1
LOC113510988	938	1.8	3.32	0.09	0.19	-3.98	0.00	0.00	Uncharacterized protein

									LOC106711668 isoform X1
LOC113517732	653	2.69	0.64	0	0.17	-3.99	0.00	0.00	Membrane alanyl Aminopeptidase-like isoform X1
LOC113512082	2021	1.95	3.13	0.1	0.18	-4.00	0.00	0.00	Uncharacterized protein LOC106135578
LOC113519571	850	1.22	1.06	0	0.11	-4.02	0.00	0.00	Serpin B5-like
LOC113509569	2465	13.3	12.68	0.83	0.62	-4.03	0.00	0.00	Carboxylesterase
LOC113517846	2118	2.54	1.81	0.06	0.17	-4.03	0.00	0.00	Uncharacterized protein LOC106134054
LOC113521632	2413	4.65	3.95	0.39	0.08	-4.04	0.00	0.00	Uncharacterized protein LOC105390299
LOC113512243	538	43.28	40.64	2.02	2.63	-4.06	0.00	0.00	D-arabinitol dehydrogenase 1-like
LOC113521723	1095	20.34	7.77	0.22	1.4	-4.06	0.00	0.00	Uncharacterized protein
LOC113520205	875	3.79	2.24	0	0.32	-4.07	0.00	0.00	Nucleoredoxin-like
LOC113510656	1107	0.72	0.96	0	0.08	-4.09	0.00	0.00	Pancreatic triacylglycerol lipase-like
MSTRG.16106	436	3.19	5.15	0.37	0	-4.10	0.00	0.00	Protein PRRC1-like
LOC113514178	1464	1.19	0.98	0.05	0.05	-4.12	0.00	0.00	Organic cation transporter protein-like
LOC113512968	1008	1.35	0.52	0.08	0	-4.12	0.00	0.00	Chymotrypsin-2-like
LOC113510598	1034	1.07	0.81	0	0.08	-4.13	0.00	0.00	Collagenase-like
LOC113515697	3463	1.79	0.58	0.06	0.06	-4.17	0.00	0.00	Aminopeptidase N-like
LOC113518465	884	45.81	16.46	0.87	2.17	-4.21	0.00	0.00	Trypsin-like protein
LOC113515584	4872	0.26	0.08	0	0.01	-4.27	0.00	0.00	Membrane alanyl Aminopeptidase
LOC113520054	1210	1.28	0.39	0	0.07	-4.27	0.00	0.00	GILT-like protein F37H8.5
LOC113513917	3009	0.4	0.18	0	0.02	-4.28	0.00	0.00	Indole-3-acetaldehyde oxidase-like
LOC113523629	1441	7.31	7.74	0.3	0.38	-4.31	0.00	0.00	LIM domain only protein 3
LOC113518462	878	1.98	0.71	0.1	0	-4.33	0.00	0.00	Trypsin-like protein
LOC113513733	443	33.17	38.89	1.4	1.87	-4.35	0.00	0.00	UDP-glucuronosyltransferase 2B1-like isoform X2
LOC113519946	1908.67	0.44	0.53	0	0.04	-4.36	0.00	0.00	Cationic amino acid transporter 2
LOC113517005	801	3.08	2.79	0	0.24	-4.38	0.00	0.00	Androgen-dependent TFPI-regulating protein-like isoform X1
LOC113522301	756	1.01	2.54	0.12	0	-4.39	0.00	0.00	Uncharacterized protein LOC106142083
LOC113510351	1944	0.82	0.22	0	0.04	-4.42	0.00	0.00	CYP6AB47
LOC113511557	812	78.93	90.88	3.32	3.88	-4.43	0.00	0.00	Glutathione S-transferase 1-like

LOC113516219	1911	1.04	0.04	0	0.04	-4.44	0.00	0.00	cell wall protein IFF6-like
LOC113520665	512	7.56	5.84	0.25	0.27	-4.46	0.00	0.00	Macrophage migration inhibitory factor homolog
LOC113512556	4728	0.35	0.4	0.01	0.01	-4.47	0.00	0.00	Cadherin-86C
LOC113511343	493	11.55	9.92	0.27	0.58	-4.48	0.00	0.00	Aldo-keto reductase
LOC113512007	1223	1.49	28.14	0.31	0.86	-4.52	0.00	0.00	Fibroin light chain
LOC113514532	1467	1.51	0.1	0	0.05	-4.57	0.00	0.00	Carboxypeptidase B-like
LOC113520899	1144	7.33	2.47	0.14	0.22	-4.60	0.00	0.00	Lipase member I-like
LOC113522904	554	12.17	12.14	0.42	0.45	-4.62	0.00	0.00	Uncharacterized protein LOC106127552
LOC113519287	637	2.81	2.69	0	0.17	-4.62	0.00	0.00	Uncharacterized protein LOC106130324
LOC113515885	1222	9.21	2.07	0.06	0.33	-4.67	0.00	0.00	Uncharacterized protein LOC110381014
LOC113509135	825	10.52	2.67	0.22	0.23	-4.69	0.00	0.00	Lipase 1-like
LOC113509365	1747	5.76	6.43	0.08	0.34	-4.69	0.00	0.00	Cytochrome P450 6B2-like
LOC113513463	3772	0.24	0.39	0.02	0	-4.73	0.00	0.00	Filamin-A isoform X1
LOC113520178	663	4.7	0.78	0	0.16	-4.73	0.00	0.00	Chymotrypsin BII-like
LOC113511822	861	4.34	2.4	0.1	0.11	-4.74	0.00	0.00	cAMP-specific 3',5'-cyclic phosphodiesterase, isoform I
MSTRG.15449	857	2.14	1.68	0	0.11	-4.77	0.00	0.00	Ubiquinone biosynthesis monooxygenase COQ6 like protein
LOC113516160	457	17.11	14.75	0.97	0	-4.82	0.00	0.00	Uncharacterized protein LOC106138812
LOC113517606	825	14.65	0	0.43	0	-4.85	0.00	0.00	Uncharacterized protein
LOC113521105	1473	1.28	0.77	0.05	0	-4.91	0.00	0.00	Guanine nucleotide-binding protein G(f) subunit alpha
LOC113515693	3625	0.66	0.13	0.02	0	-5.01	0.00	0.00	Membrane alanyl Aminopeptidase-like
LOC113517615	608	3.53	4.37	0.18	0	-5.03	0.00	0.00	Uncharacterized protein LOC106134414
MSTRG.13671	1683	1.18	0.75	0	0.06	-5.03	0.00	0.00	G-protein coupled receptor 158
LOC113514609	424	14.4	17.41	0.39	0.42	-5.07	0.00	0.00	Aldo-keto reductase AKR2E4-like
MSTRG.13930	361	22.47	5.76	0.65	0	-5.10	0.00	0.00	Carboxypeptidase
LOC113513511	394	12.14	12.14	0.49	0	-5.25	0.00	0.00	Uncharacterized protein C6orf105
LOC113510803	1559	0.13	0.33	0	0	-5.27	0.00	0.04	A disintegrin and metalloproteinase with thrombospondin motifs 7-like

LOC113522667	892	20.02	6.54	0.29	0.31	-5.30	0.00	0.00	Uncharacterized protein LOC106139316
LOC113517994	1476	0.45	0.05	0	0	-5.36	0.00	0.04	Facilitated trehalose transporter Tret1-2 homolog
LOC113519548	1016	0.66	0.17	0	0	-5.37	0.00	0.04	Brachyurin-like
LOC113521488	832	0.78	0.33	0	0	-5.38	0.00	0.04	Acidic Leucine-rich nuclear phosphoprotein 32-related protein
LOC113523362	1546	0.34	0.14	0	0	-5.38	0.00	0.04	Uncharacterized protein LOC106131717
LOC113515848	1042	9.21	9.98	0	0.41	-5.38	0.00	0.00	Uncharacterized protein LOC105841275
LOC113511809	3219	0.13	0.08	0	0	-5.38	0.00	0.04	Netrin receptor UNC5C-like
LOC113516350	928	0.58	0.37	0	0	-5.38	0.00	0.04	Proton-coupled folate transporter-like
MSTRG.10599	260	16.01	10.87	0	0	-5.38	0.00	0.04	Xanthine dehydrogenase-like
LOC113516210	687	0.78	0.74	0	0	-5.39	0.00	0.04	Troponin C-like isoform X2
LOC113517469	990	0.46	0.43	0	0	-5.39	0.00	0.04	15-hydroxyprostaglandin dehydrogenase
LOC113515172	5502	0.05	0.07	0	0	-5.40	0.00	0.04	Fatty acid synthase-like
MSTRG.14072	285	5.67	11.79	0	0	-5.40	0.00	0.04	Endonuclease-reverse transcriptase
MSTRG.7976	321	3.36	6.93	0	0	-5.40	0.00	0.04	Uncharacterized protein
LOC113515749	1440	0.14	0.42	0	0	-5.41	0.00	0.02	Glucose dehydrogenase
LOC113515696	3521	0.19	0.02	0	0	-5.49	0.00	0.02	Membrane alanyl Aminopeptidase-like isoform X1
LOC113509224	1365	0.45	0.17	0	0	-5.50	0.00	0.02	Uncharacterized protein
LOC113512086	1468	0.37	0.21	0	0	-5.50	0.00	0.02	Hypothetical protein RR46_14329
LOC113522400	681	1.06	0.6	0	0	-5.50	0.00	0.02	Uncharacterized protein
LOC113510738	1866	0.21	0.23	0	0	-5.52	0.00	0.02	Uncharacterized protein
LOC113517845	777	21.29	17.02	0.24	0.5	-5.53	0.00	0.00	Uncharacterized protein LOC110376544
LOC113522868	1305	0.16	0.54	0	0	-5.54	0.00	0.02	Innexin shaking-B
LOC113519020	749	1.36	0.13	0	0	-5.60	0.00	0.02	Proline-rich receptor-like protein kinase PERK11-like
LOC113521372	399	35.95	43.07	0.47	1	-5.60	0.00	0.00	Uncharacterized protein
LOC113512064	1410	0.48	0.16	0	0	-5.61	0.00	0.02	Uncharacterized protein LOC106135577
LOC113512282	844	0.86	0.43	0	0	-5.61	0.00	0.02	Na,K-ATPase beta-subunit

LOC113514043	789	0.84	0.59	0	0	-5.62	0.00	0.02	Nose resistant to fluoxetine protein 6-like
LOC113516117	1797	4.24	2.79	0.12	0	-5.64	0.00	0.00	Frizzled-10-like
LOC113519674	1097	0.2	0.75	0	0	-5.65	0.00	0.01	N-acetylneuraminate lyase-like
LOC113516107	1965	1.39	1.13	0	0.04	-5.69	0.00	0.00	Protein atonal homolog 8
LOC113515187	573	2.5	0	0	0	-5.69	0.00	0.01	PhosphoLipase A2
MSTRG.5549	422	4.51	0.4	0	0	-5.70	0.00	0.01	Uncharacterized PE-PGRS family protein PE_PGRS54-like isoform X1
LOC113521140	555	2.27	0.43	0	0	-5.71	0.00	0.01	Metal transporter CNNM4-like
LOC113510409	456	3.15	1.32	0	0	-5.81	0.00	0.01	Chloride channel protein 2 isoform X1
MSTRG.774	547	1.95	1.11	0	0	-5.82	0.00	0.01	Uncharacterized protein
LOC113510234	1340	0.61	0.23	0	0	-5.90	0.00	0.00	Organic cation transporter protein-like
LOC113518618	713	1.23	0.83	0	0	-5.91	0.00	0.00	Hemicentin-1-like
LOC113519524	1451	0.42	0.37	0	0	-5.92	0.00	0.00	Synaptic vesicle glycoprotein 2B-like
LOC113521538	1887	0.2	0.38	0	0	-5.94	0.00	0.00	Kelch-like protein 10
LOC113511167	1185	0.3	0.74	0	0	-5.94	0.00	0.00	RNA-binding protein fusilli isoform X2
LOC113514851	469	3.76	0.92	0	0	-5.98	0.00	0.00	Niemann-Pick type C2 protein Npc2-t01
LOC113511915	2061	0.34	0.21	0	0	-6.00	0.00	0.00	Adhesive plaque matrix protein-like isoform X1
LOC113522048	459	4.52	0.65	0	0	-6.05	0.00	0.00	CD9 antigen
LOC113517817	517	3.05	1	0	0	-6.07	0.00	0.00	Pancreatic triacylglycerol lipase-like
LOC113519377	1851	22.61	24.49	0.34	0.28	-6.10	0.00	0.00	Facilitated trehalose transporter Tret1-like
LOC113512534	900	0.69	0.98	0	0	-6.10	0.00	0.00	Pancreatic lipase-related protein 3-like
LOC113518749	774	0.54	1.59	0	0	-6.11	0.00	0.00	Hypothetical protein KGM_203921
LOC113514594	539	2.81	1.37	0	0	-6.22	0.00	0.00	Trypsin CFT-1-like
LOC113523010	2066	0.15	0.51	0	0	-6.27	0.00	0.00	Acetylcholinesterase 1
LOC113512473	1398	0.73	0.33	0	0	-6.29	0.00	0.00	Lipase 1-like isoform X2
LOC113513800	1950	663.02	536.49	7.38	6.32	-6.31	0.00	0.00	Uncharacterized family 31 glucosidase KIAA1161-like

LOC113514175	478	3.6	2.95	0	0	-6.49	0.00	0.00	Uncharacterized protein LOC106134414
LOC113511335	548	2.14	2.88	0	0	-6.51	0.00	0.00	Kynurenine formamidase isoform X1
LOC113513281	456	4.01	3.95	0	0	-6.61	0.00	0.00	Uncharacterized protein LOC106137863
LOC113521049	5019	0.19	0.14	0	0	-6.66	0.00	0.00	Voltage-dependent calcium channel type D subunit Alpha-1-like isoform X3
LOC113512858	2321	0.46	0.39	0	0	-6.81	0.00	0.00	4-coumarate--CoA ligase 3
LOC113515899	1670	0.94	0.31	0	0	-6.83	0.00	0.00	Cytochrome P450 6B6-like
LOC113519655	1374	0.99	0.67	0	0	-6.90	0.00	0.00	Carboxypeptidase A
LOC113522665	1110	1.63	0.73	0	0	-7.01	0.00	0.00	Uncharacterized protein LOC106139316
LOC113522052	428	11.04	1.16	0	0	-7.03	0.00	0.00	Fatty acid-binding protein
LOC113514771	1463	1.29	0.52	0	0	-7.13	0.00	0.00	Lipase 1-like
LOC113510498	676	2.41	3.19	0	0	-7.20	0.00	0.00	Aldo-keto reductase AKR2E4-like
MSTRG.7381	268	34.9	50.71	0	0	-7.24	0.00	0.00	Facilitated trehalose transporter Tret1-like
LOC113518689	1588	0.79	1.26	0	0	-7.45	0.00	0.00	Acheron
LOC113520040	893	3.83	1.8	0	0	-7.96	0.00	0.00	Carboxypeptidase B-like
LOC113522883	1999	1.49	0.89	0	0	-8.05	0.00	0.00	Innexin shaking-B
LOC113520804	374	20.55	18.81	0	0	-8.13	0.00	0.00	Uncharacterized protein LOC105380498 isoform X2
LOC113513466	1841	1.82	1.1	0	0	-8.21	0.00	0.00	Solute carrier family 22 member 6-like
