# Supplementary material for: Transcriptomic analyses reveal comprehensive responses of insect hemocytes to mycopathogen Beauveria bassiana, and fungal virulence-related cell wall protein assists pathogen to evade host cellular defense
Source: Virulence. 2020 Oct 5;11(1):1352–65. doi: 10.1080/21505594.2020.1827886 (PMC7549920; doi:10.1080/21505594.2020.1827886)
Supplement: Supplemental Material [file KVIR_A_1827886_SM8204.zip › Table S7.pdf]

Table S7 Differentially expressed genes of *Galleria mellonella* hemocytes challenged by *Beauveria bassiana* at 3 day post infection

Gene ID	Length	CK-R1	CK-R2	INT3d-R1	INT3d-R2	log_2(fold change)	P-value	FDR	Annotation
LOC113513385	787	0	0	389.44	347.6	14.58	0.00	0.00	Laccase-4-like
LOC113516050	1413	0	0	85.16	77.49	13.55	0.00	0.00	Pancreatic secretory trypsin inhibitor-like
LOC113515460	1168	0	0	101.91	96.68	13.49	0.00	0.00	Carbonic anhydrase 7
LOC113514009	1022	0	0	98.84	115.8	13.34	0.00	0.00	Serine protease gd-like
MSTRG.3769	1793	0.11	0.08	860.44	769.81	13.02	0.00	0.00	Uncharacterized protein
LOC113509613	468	2.7	4.65	23386.01	20428.85	12.52	0.00	0.00	Moricin-like peptide C2
LOC113509611	432	10.79	12.44	59278.67	54576.49	12.23	0.00	0.00	Moricin-like peptide D
LOC113509117	1261.43	0.16	0	702.12	689.27	12.17	0.00	0.00	Uncharacterized protein LOC110375215 isoform X1
LOC113509612	415	7.98	11.32	36284.01	31435.55	11.74	0.00	0.00	Moricin-like peptide C3
LOC113523572	879	0	0	36.73	44.05	11.62	0.00	0.00	Uncharacterized protein LOC106123314
LOC113519717	624	0.92	1.74	4339.51	3999.88	11.62	0.00	0.00	Uncharacterized protein LOC110373446
LOC113521027	1327	0.16	0.17	424.36	442.44	11.32	0.00	0.00	Laccase-4-like
LOC113517529	2192	0.37	0.99	1503.96	1697.06	11.23	0.00	0.00	Tyrosine 3-monooxygenase
LOC113515419	1667	0	0	11.77	13.62	11.16	0.00	0.00	Myrosinase 1-like
MSTRG.10582	901	0	0	27.22	19.9	10.90	0.00	0.00	Uncharacterized protein
LOC113523359	999	0	0	17.19	23.06	10.88	0.00	0.00	Proton-coupled amino acid transporter 4
MSTRG.15715	1898	0	0	9.85	8.03	10.88	0.00	0.00	Hypothetical protein KGM_203431
LOC113510329	1161.03	0	0	13.01	14.96	10.65	0.00	0.00	Uncharacterized protein LOC106130461
LOC113514152	744	1.72	2.86	3782.1	3245.56	10.60	0.00	0.00	Uncharacterized protein
LOC113515360	1125	0.06	0.14	165.71	169.67	10.58	0.00	0.00	Uncharacterized protein
LOC113512933	3490.68	0.06	0.02	52.89	60.66	10.56	0.00	0.00	Uncharacterized protein LOC106136478 isoform X1
LOC113510273	842	0.1	0	98.47	88.4	10.53	0.00	0.00	Uncharacterized protein LOC106143525
LOC113519449	681	0	0	27.69	27.74	10.51	0.00	0.00	Protein lethal(2)essential for life-like
LOC113509237	799	0	0	19.52	19.03	10.35	0.00	0.00	DNA-binding protein D-ETS-6 isoform X1

MSTRG.12915	927.45	0	0	13.67	16.97	10.35	0.00	0.00	Uncharacterized protein LOC110378268
LOC113518655	977	0	0	12.43	15.63	10.32	0.00	0.00	Lipase member H-like isoform X2
MSTRG.1877	569	0	0	31.86	30.55	10.22	0.00	0.00	Uncharacterized protein LOC106139762
MSTRG.3949	1028	0	0	13.51	10.1	10.17	0.00	0.00	Uncharacterized protein
LOC113521309	1673	0.43	0.4	440.31	499.42	10.16	0.00	0.00	Black
MSTRG.10824	283	1.47	0	1377.4	1036.01	10.09	0.00	0.00	Uncharacterized protein
MSTRG.8976	599	0.16	0.94	604.66	562.18	10.04	0.00	0.00	Uncharacterized protein LOC106142918
LOC113511506	438	0	0.73	397.97	357.26	9.91	0.00	0.00	L-ascorbate oxidase isoform X2
LOC113523468	2139	0	0	3.81	3.97	9.87	0.00	0.00	Nose resistant to fluoxetine protein 6-like
LOC113511039	2001.65	0.04	0.04	33.76	33.64	9.82	0.00	0.00	Solute carrier family 2, facilitated glucose transporter member 8-like
LOC113509614	407	18.73	18.58	18473.4	15293.95	9.78	0.00	0.00	Moricin-like peptide C1
LOC113516132	1511	0	0	5.96	4.84	9.76	0.00	0.00	Protein msta, isoform A
LOC113522236	393	0	0	55.61	54.24	9.75	0.00	0.00	Cuticular protein RR-2 motif 134 precursor
LOC113510643	1171	0	0	7.83	5.93	9.64	0.00	0.00	Alpha-tocopherol transfer protein-like
LOC113521649	1184.85	0.1	0.43	215.12	201.74	9.63	0.00	0.00	CHH-like protein isoform X2
MSTRG.7389	553	0.19	0.87	461.56	385.9	9.62	0.00	0.00	Uncharacterized protein
LOC113518405	1240	0.06	0.06	53.19	49.78	9.59	0.00	0.00	Protein rolling stone-like isoform X1
LOC113512461	1801	0	0	3.38	4.27	9.56	0.00	0.00	Limbic system-associated membrane protein-like
LOC113519869	370	0	0	57.86	55.74	9.52	0.00	0.00	Serine protease gd-like
LOC113518757	3140	0.08	0.02	35.7	39.94	9.51	0.00	0.00	Amino acid transporter
LOC113522123	458	4.82	5.54	3958.74	3648.67	9.49	0.00	0.00	Heat shock protein Hsp-12.2
LOC113509868	1552	0.13	0.05	71.71	62.09	9.47	0.00	0.00	Uncharacterized protein LOC110377500
LOC113518251	1093	0	0	4.62	7.9	9.37	0.00	0.00	Larval cuticle protein LCP-30-like
LOC113514947	3706	0.02	0	6.57	6.6	9.29	0.00	0.00	Glucose dehydrogenase
LOC113518215	3815	0.08	0.16	70.13	70.67	9.26	0.00	0.00	Nuclear pore complex protein Nup214-like isoform X1
LOC113519337	1435	0.19	0.85	311.3	296.24	9.23	0.00	0.00	Globin isoform X1
LOC113509615	415	0	0	21.09	41.3	9.15	0.00	0.00	Moricin-like peptide B

LOC113521568	1040	0.14	0.1	71.57	62.3	9.11	0.00	0.00	Uncharacterized protein LOC106132613
LOC113518263	1144	0	0.07	20.5	25.48	9.11	0.00	0.00	Glycine-rich protein DOT1-like isoform X1
LOC113512447	974	0.08	0	25.96	28.8	9.06	0.00	0.00	Uncharacterized protein
LOC113512533	1578.27	0.75	2.51	832.54	691.5	8.93	0.00	0.00	Protein yellow-like isoform X1
LOC113521833	994	0	0.17	42.93	43.35	8.90	0.00	0.00	Odorant-binding protein A10
LOC113509608	491	5.34	3.89	2316.31	2023.38	8.85	0.00	0.00	Moricin-like peptide A
LOC113515999	2514	0	0	1.48	1.62	8.81	0.00	0.00	Estradiol 17-beta-dehydrogenase 11 isoform X1
LOC113516882	3917	0.05	0.12	35.36	37.01	8.81	0.00	0.00	Annulin-like isoform X1
LOC113517566	10745	0.02	0	2.81	4.86	8.76	0.00	0.00	Titin
MSTRG.2571	650	0	0	10.23	7.56	8.76	0.00	0.00	Uncharacterized protein
LOC113516406	1609	0	0	2.74	2.27	8.76	0.00	0.00	Synaptic vesicle glycoprotein 2B-like
MSTRG.5201	709	0	0	8.53	6.37	8.72	0.00	0.00	Homologous-pairing protein 2 homolog
LOC113521722	2080	0	0	2.12	1.27	8.67	0.00	0.00	Protein dead ringer
LOC113521502	784	0.21	0	51.92	48.06	8.63	0.00	0.00	Uncharacterized protein LOC106135000 isoform X1
LOC113512634	553	0	0	8.6	13.12	8.61	0.00	0.00	Uncharacterized protein
LOC113511367	314	0	0	45.83	68.06	8.60	0.00	0.00	Uncharacterized protein
LOC113511874	1871	0	0.15	30.68	26.41	8.54	0.00	0.00	Rhomboid-related protein 2 isoform X1
LOC113509208	1793.3	0	0.05	8.3	8.9	8.52	0.00	0.00	UDP-glucuronosyltransferase 2B10-like
LOC113516072	3316	0.11	0.12	38.13	43.11	8.47	0.00	0.00	Sialin-like isoform X1
LOC113514709	761	1.55	1.26	517.61	480.82	8.47	0.00	0.00	Hypothetical protein RR46_03464
LOC113522083	1172	0	0	3.22	2.85	8.47	0.00	0.00	Sodium-independent sulfate anion transporter-like
LOC113514153	1983	0	0	1.61	1.46	8.41	0.00	0.00	Uncharacterized protein LOC101736974
LOC113512739	795	0	0	5.15	4.92	8.41	0.00	0.00	Uncharacterized protein LOC106135661
LOC113516877	1454	0	0.05	8.87	11.92	8.40	0.00	0.00	Protein tilB homolog
LOC113523448	568	0	0	10.03	7.61	8.39	0.00	0.00	Proton-coupled amino acid transporter 4
LOC113516141	760.56	0	0	5.34	5.27	8.39	0.00	0.00	Protein drumstick

LOC113519422	642	4.67	1.99	1142.72	1028.49	8.34	0.00	0.00	Heat shock protein 24.3
LOC113513999	2676.43	0.02	0.13	23.01	25.22	8.33	0.00	0.00	Proton-coupled folate transporter-like
LOC113517359	1397	0	0	2.33	2.1	8.33	0.00	0.00	Adenosine receptor A3
LOC113511774	2605	0	0.05	7.93	9.37	8.28	0.00	0.00	Seminal fluid protein CSSFP041
LOC113511685	2245.72	0	0.03	5.93	5.22	8.25	0.00	0.00	Pancreatic triacylglycerol lipase-like isoform X1
LOC113512903	2991	0	0.02	2.79	5.27	8.24	0.00	0.00	Uncharacterized protein LOC110369842
LOC113510833	637	0	0	4.35	8.39	8.22	0.00	0.00	Uncharacterized protein
LOC113511956	1931.3	0.03	0	6.45	6.37	8.21	0.00	0.00	Zonadhesin-like
LOC113521829	1070	0	0	2.42	3.3	8.20	0.00	0.00	Uncharacterized protein LOC106716499
LOC113516725	580	26.01	93.75	18202.66	15935.58	8.17	0.00	0.00	Hypothetical protein RR48_09864
LOC113521574	955.34	47.18	65.34	17045.24	14932.56	8.16	0.00	0.00	Ejaculatory bulb-specific protein 3-like
LOC113514486	1343	0	0	1.74	2.39	8.16	0.00	0.00	Facilitated trehalose transporter Tret1
LOC113521563	442	0	0	16.6	9.3	8.15	0.00	0.00	Chemosensory protein 6
LOC113515117	901	0	0	2.96	3.98	8.14	0.00	0.00	Pupal cuticle protein-like
LOC113514507	1092	0	0	3.87	1.4	8.13	0.00	0.00	Uncharacterized protein LOC110375939
LOC113522093	753	1.24	0.51	239.86	249.59	8.09	0.00	0.00	Cytochrome P450 6B6
LOC113509609	514	14.56	15.88	4202.32	3660.75	8.01	0.00	0.00	Moricin-like peptide C5
LOC113518959	860	2.5	1.15	502.73	419.71	7.98	0.00	0.00	Glutathione S-transferase delta 4
LOC113523190	662	0	0.16	25.67	19.35	7.92	0.00	0.00	Uncharacterized protein
LOC113509425	2031.61	3.58	3.06	769.19	755.51	7.90	0.00	0.00	Uncharacterized protein LOC106110582
LOC113521809	2430	0	0	0.7	1	7.89	0.00	0.00	Vascular endothelial growth factor receptor 1-like
LOC113511279	1308	0.11	0	15.85	13.61	7.89	0.00	0.00	Facilitated trehalose transporter Tret1-like
LOC113510638	3713	0.05	0	6.5	6.23	7.89	0.00	0.00	Bumetanide-sensitive sodium-(potassium)-chloride cotransporter
MSTRG.16142	1572	0	0	1.75	1.04	7.88	0.00	0.00	Uncharacterized protein
LOC113519931	1869	0.03	0.12	16.81	18.22	7.84	0.00	0.00	Protein yellow
LOC113516037	1996	0	0.07	8.13	9.02	7.83	0.00	0.00	Uncharacterized protein

									LOC106143445
LOC113509994	958	1.03	1.79	326.63	307.18	7.83	0.00	0.00	Uncharacterized protein LOC101738673
LOC113518076	861	0	0.1	14.06	13.79	7.82	0.00	0.00	Uncharacterized MFS-type transporter C09D4.1 isoform X2
LOC113515565	2037	0	0	0.7	1.26	7.81	0.00	0.00	Arylsulfatase B
LOC113520329	1946	0	0.04	4.37	5.03	7.76	0.00	0.00	Prostaglandin E2 receptor EP2 subtype
MSTRG.9902	692	6.44	9.49	1794.33	1555.15	7.73	0.00	0.00	Uncharacterized protein
MSTRG.6786	232	0	0	193.99	117.84	7.71	0.00	0.00	Uncharacterized protein
LOC113522092	635	1.93	1.52	355.94	344.53	7.65	0.00	0.00	Cytochrome CYP6AB14
LOC113518648	702	0	0	4.01	3.16	7.64	0.00	0.00	Lipase member H-like
LOC113521634	2420	0	0	0.48	0.94	7.63	0.00	0.00	ADAMTS-like protein 4
LOC113517530	3614.81	0.05	0	5.39	5.47	7.62	0.00	0.00	Patched domain-containing protein 3
LOC113520875	1439	0.05	0	5.74	6.36	7.61	0.00	0.00	Sodium-coupled neutral amino acid transporter 9 homolog isoform X1
LOC113515606	1002	0.45	0.93	134.18	130.75	7.61	0.00	0.00	Carbonic anhydrase 7-like
LOC113512446	1138	0.13	0	12.19	16.61	7.60	0.00	0.00	Myophilin
LOC113518364	1873	6.99	7.42	1371.9	1343.18	7.59	0.00	0.00	Myrosinase 1-like isoform X2
LOC113516003	1125	5.44	5.7	1114.29	982.86	7.58	0.00	0.00	Serine protease inhibitor dipetalogastin-like
LOC113510362	498.44	0	0	8.57	4.54	7.58	0.00	0.00	Uncharacterized protein
LOC113509295	754	0	0	3.44	2.67	7.57	0.00	0.00	Uncharacterized protein LOC106142916
LOC113521154	1656	0	0	1.2	0.93	7.57	0.00	0.00	Zinc finger protein 713-like
LOC113511449	2098	0	0.07	7.29	5.97	7.55	0.00	0.00	Sodium-independent sulfate anion transporter-like
LOC113520406	1280	0	0	1.37	1.47	7.54	0.00	0.00	Uncharacterized protein LOC106131448
MSTRG.14089	2821	0	0.1	8.95	7.91	7.46	0.00	0.00	Ankycorbin
LOC113513026	925	0.08	0	9.3	9.74	7.43	0.00	0.00	Hypothetical protein KGM_210399B
MSTRG.6746	280	1.55	0	194.97	205.38	7.41	0.00	0.00	Laccase-4-like
LOC113517275	2533	0.05	0	4.85	4.38	7.35	0.00	0.00	Cytochrome P450 18a1
MSTRG.15148	436	0	0	7.55	7.54	7.32	0.00	0.00	Uncharacterized protein
LOC113510868	3244	0.81	1.06	143.01	148.38	7.31	0.00	0.00	Uncharacterized protein OBRU01_00709
LOC113519432	767	1.1	1.49	220.52	188.49	7.31	0.00	0.00	Protein lethal(2)essential for life-like

MSTRG.4433	285	0	0	37.83	33.3	7.28	0.00	0.00	Synaptotagmin-16 isoform X1
LOC113519438	728	2.85	2.69	445.32	411.8	7.28	0.00	0.00	Small heat shock protein 19.7
MSTRG.1883	2880	0.21	0.26	32.7	39.08	7.27	0.00	0.00	CYP6AB46
LOC113519309	1611	0	0	0.57	1.21	7.27	0.00	0.00	Uncharacterized protein LOC105841534
LOC113512301	2287	0	0.03	2.74	2.71	7.24	0.00	0.00	Diapause hormone receptor
MSTRG.15912	922	0	0	2.34	1.25	7.24	0.00	0.00	Neuropeptide-like 3
MSTRG.8877	1722	0.34	0.64	71.15	69.05	7.19	0.00	0.00	Uncharacterized protein
MSTRG.12598	1569.05	0	0.11	6.41	7.54	7.19	0.00	0.00	Uncharacterized protein LOC106125623
LOC113516944	900	1.91	1.86	288.44	242.99	7.15	0.00	0.00	Cytochrome b5-like
LOC113518392	1029	0	0	1.37	1.52	7.15	0.00	0.00	Nuclear pore complex protein Nup214-like
LOC113520633	2117.88	0.06	0	3.83	5.84	7.14	0.00	0.00	UNC93-like protein
LOC113512706	325	0.8	0.94	183.89	107.13	7.12	0.00	0.00	Peptidoglycan recognition protein-like
LOC113513650	1222	0.06	0	5.01	5.47	7.11	0.00	0.00	Sugar transporter protein 2
LOC113511179	1128	0	0	0.81	1.66	7.10	0.00	0.00	Transcription factor AP-2-epsilon-like
MSTRG.6429	456	0	0	8.24	2.99	7.07	0.00	0.00	Uncharacterized protein
LOC113518963	564.9	4.92	4.01	619.92	599.97	7.06	0.00	0.00	Zonadhesin-like isoform X1
LOC113519221	493	0	0	3.44	5.89	7.05	0.00	0.00	Lipase member H-like isoform X1
LOC113523271	637	0	0	2.08	3.54	7.05	0.00	0.00	Neural/ectodermal development factor IMP-L2-like
LOC113513782	441	0	0	8.96	2.85	7.02	0.00	0.00	Alpha-tocopherol transfer protein-like
MSTRG.734	670	0	0	3.12	1.88	7.01	0.00	0.00	Uncharacterized protein
LOC113522388	2253	0	0	0.28	0.71	7.00	0.00	0.00	Uncharacterized protein LOC106141564
MSTRG.11591	945	0	0	0.82	2.11	7.00	0.00	0.00	Uncharacterized protein
LOC113520441	4043	0.13	0.03	10.28	10.95	6.98	0.00	0.00	Proton-coupled amino acid transporter 1-like isoform X1
LOC113521397	3199	0	0.02	1.16	1.86	6.93	0.00	0.00	Collagen alpha-1(XVIII) chain-like
MSTRG.9041	1086	0.07	0.23	18.74	17.76	6.93	0.00	0.00	Uncharacterized protein
LOC113516588	842	4.01	3.34	471.5	407.17	6.91	0.00	0.00	Tetraspanin
LOC113509557	1032	0	0	1.18	1.24	6.90	0.00	0.00	Circadian clock-controlled protein-like

LOC113510060	582	0	0	1.78	4.18	6.90	0.00	0.00	Nuclear factor interleukin-3-regulated protein-like
LOC113523415	1629	0.04	0	4.02	2.28	6.90	0.00	0.00	Protein yellow-like
LOC113514337	2121.45	1.23	1.84	169.38	191.86	6.89	0.00	0.00	Facilitated trehalose transporter Tret1
MSTRG.7865	676	4.38	3.95	481.27	486.74	6.86	0.00	0.00	Uncharacterized protein
LOC113522294	629	0	0	3.1	1.91	6.86	0.00	0.00	UDP-glucosyltransferase protein 3
LOC113515812	950	0.16	0.45	35.53	35.61	6.86	0.00	0.00	Uncharacterized protein LOC105398799
LOC113514283	2739	0.69	0.67	70.06	80.49	6.81	0.00	0.00	Linear gramicidin synthase subunit D
LOC113519028	1342	0.1	0.46	28.44	33.35	6.81	0.00	0.00	4-coumarate--CoA ligase 1-like
LOC113522196	1260	0	0	0.77	0.96	6.79	0.00	0.00	Uncharacterized protein
LOC113515290	548	10.29	25.46	2192.2	1683.61	6.77	0.00	0.00	Lysozyme
LOC113513832	820	0.1	0	7.56	6.8	6.77	0.00	0.00	Serine protease 7-like
LOC113517304	819	1.59	1.68	236.9	111.22	6.75	0.00	0.00	Proline-rich protein
LOC113514207	2267	0.19	0.74	42.96	54.06	6.74	0.00	0.00	Aminopeptidase N-like isoform X2
LOC113516722	3516	0	0	0.26	0.25	6.74	0.00	0.00	Protein eyes shut
LOC113513996	559	0	0	2.41	3.33	6.73	0.00	0.00	Cytochrome P450 4C1-like
LOC113515884	2169	0	0	0.36	0.5	6.73	0.00	0.00	Fatty acyl-CoA reductase 1-like
LOC113513857	2221	0.96	1.11	110.75	101.59	6.71	0.00	0.00	Uncharacterized protein LOC106141788
LOC113512893	409.65	14.95	32.59	2741.91	2298.77	6.71	0.00	0.00	Uncharacterized protein LOC106138361
LOC113518075	1642	0.08	0.54	27.6	35.52	6.71	0.00	0.00	Hypothetical protein KGM_202228B
LOC113509774	2162.02	0.38	0.29	35.82	32.92	6.69	0.00	0.00	Uncharacterized protein LOC106103079 isoform X2
LOC113510899	4012	0.66	0.58	54.85	70.12	6.68	0.00	0.00	Sodium-independent sulfate anion transporter
LOC113518566	1494	0.04	0.05	7.12	3.7	6.68	0.00	0.00	Serine proteinase stubble-like
MSTRG.6790	405	0	0	7.19	4.64	6.68	0.00	0.00	Uncharacterized protein
MSTRG.15794	384	0	0	7.24	6.69	6.68	0.00	0.00	Septin-4
LOC113513043	504	0.23	0	17.26	14.17	6.65	0.00	0.00	Uncharacterized protein
LOC113512294	3190	0.08	0.25	15.18	16.76	6.64	0.00	0.00	Inactive pancreatic Lipase-related protein 1-like isoform X4
LOC113517675	3732	0.83	0.66	67.16	76.6	6.62	0.00	0.00	Uncharacterized threonine-rich GPI-anchored glycoprotein PJ4664.02

LOC113518890	1326	0	0	1.18	0.26	6.62	0.00	0.00	Uncharacterized protein LOC106134782
LOC113519797	1656	0	0	0.7	0.39	6.62	0.00	0.00	Zinc finger protein 879-like
MSTRG.12993	436	0	0	4.61	4.61	6.61	0.00	0.00	Uncharacterized protein
LOC113509622	2787	0.11	0.27	19.01	16.69	6.59	0.00	0.00	Uncharacterized protein LOC106137572
LOC113522647	1350	0.2	0.17	17.16	19.65	6.59	0.00	0.00	Protein takeout
LOC113520988	370	0	0.59	27.91	40.6	6.55	0.00	0.00	Aminopeptidase N-like isoform X2
LOC113509835	4447	0	0	0.23	0.11	6.55	0.00	0.00	Uncharacterized protein LOC106135260
LOC113521572	756.43	1.55	1.73	166.71	145.04	6.55	0.00	0.00	Chemosensory protein 10
MSTRG.6381	570	1.08	0.21	63.27	65.71	6.54	0.00	0.00	Uncharacterized protein
LOC113512308	1512	0	0	0.48	0.65	6.54	0.00	0.00	Acyl-CoA desaturase HassNPVE
LOC113513802	1370	0	0	0.5	0.8	6.54	0.00	0.00	Neuronal PAS domain-containing protein 4A-like
LOC113523204	1524	0	0	0.39	0.75	6.54	0.00	0.00	Irregular chiasm C-roughest protein-like
LOC113523358	1875.47	0.04	0.05	3.6	3.82	6.53	0.00	0.00	Proton-coupled amino acid transporter 4-like
LOC113520073	1161	0.12	0.42	23.6	25.06	6.51	0.00	0.00	Uncharacterized protein LOC106136669 isoform X1
LOC113511665	1342	0.05	0.4	20.25	19.8	6.49	0.00	0.00	Cardioacceleratory peptide receptor-like
LOC113516083	1336.11	0.1	0	5.74	4.75	6.48	0.00	0.00	Uncharacterized protein LOC110383028
LOC113516326	1152	0	0	0.71	0.84	6.47	0.00	0.00	Cytochrome P450 monooxygenase CYP367B12
LOC113520116	2897	0	0	0.24	0.28	6.47	0.00	0.00	UDP-N-acetylglucosamine--peptide N-acetylglucosaminyltransferase 110 kDa subunit-like
LOC113520265	1662	0	0	0.4	0.58	6.47	0.00	0.00	Neuronal PAS domain-containing protein 4B-like
LOC113517103	1382	0	0	0.19	1.03	6.46	0.00	0.00	Venom acid phosphatase Acph-1-like
LOC113512229	510	0.22	0	12.47	13.81	6.43	0.00	0.00	Uncharacterized protein LOC106139759
LOC113517371	5720	0.02	0.01	1.24	1.68	6.42	0.00	0.00	ATP-binding cassette sub-family C member Sur-like

LOC113518426	1548	0	0	0.59	0.42	6.41	0.00	0.00	Uncharacterized protein LOC106137813
LOC113509263	1200	0	0	0.67	0.73	6.40	0.00	0.00	Uncharacterized protein LOC106139704
LOC113518505	1430	0	0	0.36	0.76	6.40	0.00	0.00	Uncharacterized protein LOC106129782
LOC113522981	1929	12.91	13.19	1078.27	1060.03	6.39	0.00	0.00	Matrix metalloproteinase-14 isoform X1
MSTRG.8101	778	0	0	0	2.59	6.39	0.00	0.00	Uncharacterized protein LOC107037501
MSTRG.6924	2078	0	0.07	2.55	3.38	6.37	0.00	0.00	Uncharacterized protein
MSTRG.11622	717	0.12	0	7.29	5.8	6.34	0.00	0.00	Uncharacterized protein
LOC113523209	2258	0.28	0.62	34.4	35.8	6.32	0.00	0.00	23 kDa integral membrane protein-like
LOC113520136	682	0	0	1.01	1.99	6.32	0.00	0.00	UDP-glucuronosyltransferase 2B10-like
LOC113510193	2518	0	0	0.12	0.42	6.32	0.00	0.00	Zinc finger protein basonuclin-2-like
LOC113520587	1009	0.15	0.08	11.05	8.9	6.31	0.00	0.00	Uncharacterized protein LOC110374731
MSTRG.884	484	0.75	0.29	47.53	43.34	6.31	0.00	0.00	Uncharacterized protein
LOC113523210	2550	0.17	0.38	19.78	22.54	6.30	0.00	0.00	23 kDa integral membrane protein-like
MSTRG.15163	2820.84	0.04	0.1	3.38	3.88	6.26	0.00	0.00	Uncharacterized protein
LOC113509570	5323	1.04	1.15	80.7	82.54	6.26	0.00	0.00	Uncharacterized protein DDB_G0277255 isoform X1
MSTRG.3430	862	0	0	1.37	0.55	6.25	0.00	0.00	Uncharacterized protein LOC105286551
LOC113509598	558	0	0	1.93	2.15	6.24	0.00	0.00	Odorant-binding protein 11
LOC113510442	674	0	0	1.22	1.69	6.24	0.00	0.00	Synaptotagmin-16 isoform X1
MSTRG.9399	1248	0.5	0.13	23.97	23.99	6.21	0.00	0.00	Uncharacterized protein
LOC113510700	2949.39	0.23	0.22	12.93	15.38	6.18	0.00	0.00	G protein-activated inward rectifier potassium channel 3-like isoform X2
LOC113518909	3313	0	0	0.29	0.07	6.17	0.00	0.00	Protein sidekick-1-like
LOC113521406	1002	0	0	0.85	0.65	6.16	0.00	0.00	Lipase member H-like isoform X2
MSTRG.15246	283	0	0	16.6	17.27	6.16	0.00	0.00	Uncharacterized protein
LOC113523160	1209	0	0	0.52	0.65	6.16	0.00	0.00	Neuropeptide receptor A6-B
LOC113521838	707	0	0	0.95	1.56	6.15	0.00	0.00	Uncharacterized protein LOC106132721 isoform X1

LOC113519601	4792	1.55	3.57	170.23	183.36	6.15	0.00	0.00	Protein phosphatase 1 regulatory subunit 12A
LOC113517406	3686	0.02	0.09	3.21	4.24	6.14	0.00	0.00	Guanylate cyclase 32E
MSTRG.8646	658	0	0.32	13.6	10.22	6.14	0.00	0.00	Cecropin A
LOC113511047	2660.17	0.07	0.22	8.95	10.12	6.14	0.00	0.00	A disintegrin and metalloproteinase with thrombospondin motifs 1 isoform X1
LOC113520537	995	60.88	92.85	5604.61	4956.82	6.13	0.00	0.00	Uncharacterized protein LOC106132075
LOC113517365	1116	0.39	0	16.85	12.08	6.12	0.00	0.00	VMP32 protein
LOC113509841	1970	0.03	0.04	2.68	2.58	6.12	0.00	0.00	Uncharacterized protein LOC106135211
LOC113510107	1575	112.87	126.52	8184.2	8053.19	6.11	0.00	0.00	Hemolin
MSTRG.8275	1235	9.22	11.18	695.35	686.87	6.11	0.00	0.00	Uncharacterized protein
LOC113514600	1160	0.06	0	2.33	3.27	6.11	0.00	0.00	Hypothetical protein RR48_04984
LOC113522527	1463.39	3.94	10.31	459.26	500.76	6.10	0.00	0.00	Serine protease inhibitor 6
LOC113521739	2649	0	0.05	2	1.73	6.10	0.00	0.00	Wiskott-Aldrich syndrome protein family member 2-like
LOC113511881	1266	0	0	0.56	0.48	6.07	0.00	0.00	Uncharacterized protein LOC106132083
LOC113516328	780	0	0	1.09	0.93	6.07	0.00	0.00	Uncharacterized oxidoreductase SSP0419-like
LOC113521130	1297	0	0	0.54	0.46	6.07	0.00	0.00	Uncharacterized protein
LOC113522179	1105	0	0	0.58	0.65	6.07	0.00	0.00	Irregular chiasm C-roughest protein-like
LOC113516174	901	0	0	0.66	0.97	6.06	0.00	0.00	Pancreatic lipase-related protein 2
LOC113512510	666	0	0	0.53	2.07	6.06	0.00	0.00	Homeobox protein goosecoid-like
LOC113521712	651	0.14	0.16	8.75	14	6.05	0.00	0.00	Chemosensory protein
MSTRG.5275	2371	0.16	0.38	18.57	16.16	6.04	0.00	0.00	Uncharacterized protein
LOC113513784	1434	0.05	0.53	18.65	16.71	5.98	0.00	0.00	Membrane-bound alkaline phosphatase-like
LOC113521092	1397	0	0	0.55	0.3	5.97	0.00	0.00	Cytochrome P450 6AB13
LOC113519141	504	0	0	1.79	2.36	5.97	0.00	0.00	Sodium-independent sulfate anion transporter isoform X1
LOC113522419	619	0	0	1.19	1.57	5.97	0.00	0.00	Fatty acyl-CoA reductase 1-like
MSTRG.7531	1002	0.07	0	2.93	3.15	5.95	0.00	0.00	Uncharacterized protein
LOC113519387	4780.07	0.27	0.33	14.64	21.24	5.94	0.00	0.00	Latent-transforming growth factor

									beta-binding protein 4-like isoform X1
LOC113513934	1400	0.05	0	2.57	1.32	5.93	0.00	0.00	Cuticle protein 16.8
MSTRG.8654	2757	0.15	0.57	19.74	23.31	5.93	0.00	0.00	Uncharacterized protein
LOC113510153	1196	1.42	0.6	64.61	57.79	5.92	0.00	0.00	E3 ubiquitin-protein ligase sinah isoform X1
LOC113523033	1281	0.27	0.06	9.44	11.23	5.88	0.00	0.00	Uncharacterized protein
LOC113523037	5570	0.04	0.03	2.11	2.46	5.88	0.00	0.00	Titin-like isoform X1
LOC113513334	500	0.93	0	27.55	34.85	5.87	0.00	0.00	Organic cation transporter protein-like isoform X1
LOC113520078	1680	0	0	0.44	0.19	5.87	0.00	0.00	Adenylate cyclase type 2
MSTRG.3195	325	0	0	9.91	4.51	5.87	0.00	0.00	Uncharacterized protein
LOC113512949	1278	0	0	0.48	0.4	5.86	0.00	0.00	Tetra-peptide repeat homeobox protein 1-like precursor
LOC113518712	376	0	0	4.52	3.91	5.86	0.00	0.00	Ubiquitin-like protein FUBI
MSTRG.9560	368	0	0	4.16	4.91	5.86	0.00	0.00	Uncharacterized protein
LOC113514650	899	0	0	0.44	0.97	5.86	0.00	0.00	Prostaglandin F synthase 1
LOC113517948	753	0	0	0.57	1.27	5.86	0.00	0.00	L-threonine ammonia-lyase-like
LOC113519720	4971	0.07	0.09	3.88	5.44	5.85	0.00	0.00	Zinc finger protein 541
LOC113518483	1395	0	0	0.12	0.66	5.85	0.00	0.00	Serine/arginine repetitive matrix protein 1-like
LOC113511731	1113	0.45	1.24	51.21	44.41	5.85	0.00	0.00	Lambda-crystallin
LOC113521034	1377	0.2	0.22	13.55	10.56	5.83	0.00	0.00	Inorganic phosphate cotransporter
LOC113512108	3949	2.3	1.66	101.74	115.78	5.81	0.00	0.00	Uncharacterized protein LOC106139412
LOC113515146	2942.04	0.08	0	2.02	3.02	5.81	0.00	0.00	Protein orai-like isoform X1
LOC113515447	1066	0.12	0	2.87	2.13	5.79	0.00	0.00	Uncharacterized protein LOC106134655 isoform X2
LOC113512558	850	0.19	0.32	12.3	16.4	5.78	0.00	0.00	Muscle-specific protein 20-like
LOC113521375	821	0	0	0.88	0.62	5.75	0.00	0.01	Cuticle protein 16.5, isoform B-like
LOC113516874	2007	0	0	0.24	0.23	5.75	0.00	0.01	Nuclear hormone receptor HR38
LOC113515610	5709	0	0	0.06	0.09	5.75	0.00	0.01	Serine protease nudel
LOC113516259	1403	0	0	0.24	0.48	5.75	0.00	0.01	Fatty-acid amide hydrolase 2
LOC113513085	420	0	0	1.39	4.18	5.74	0.00	0.01	Cytochrome P450 monooxygenase CYP367B12
LOC113513884	738	0	0	0.44	1.31	5.74	0.00	0.01	Hypothetical protein KGM_204941
LOC113514463	5976	0	0	0.04	0.11	5.74	0.00	0.01	Uncharacterized protein

									LOC106142077
LOC113513789	554	0	0	0.49	2.42	5.74	0.00	0.01	Superoxide dismutase
LOC113512420	1941	0.3	0.26	13.81	14.81	5.69	0.00	0.00	Gremlin-1-like isoform X1
MSTRG.8544	446	0.6	0	16.23	22.11	5.69	0.00	0.00	Aminopeptidase N-like
MSTRG.13104	1003	2.39	2.02	122.47	99.96	5.67	0.00	0.00	Unknown unsecreted protein
LOC113518265	620	2.17	1.06	67.07	100.16	5.66	0.00	0.00	Uncharacterized protein LOC110383784
LOC113520787	1363	0.05	0	1.77	1.55	5.65	0.00	0.00	Septin-2 isoform X1
LOC113522672	2715	0.02	0	0.73	0.74	5.65	0.00	0.00	Uncharacterized protein LOC106129314
LOC113512844	1763	0.51	0.66	28.05	29.73	5.64	0.00	0.00	Uncharacterized protein LOC106708416
LOC113512681	559	1.12	1.07	61.6	49.47	5.63	0.00	0.00	Salivary secreted peptide
LOC113513922	241	0	0	41.23	16.31	5.63	0.00	0.01	Pro-resilin-like
LOC113514646	791	0	0	0.93	0.52	5.63	0.00	0.01	Proton-coupled amino acid transporter 2-like
LOC113517221	3798	0	0	0.14	0.1	5.63	0.00	0.01	Potassium voltage-gated channel subfamily H member 8 isoform X1
LOC113513649	507	0	0	1.47	1.75	5.63	0.00	0.01	Carotenoid isomerooxygenase
LOC113516048	662	0	0	0.89	1.05	5.63	0.00	0.01	Uncharacterized protein LOC106131020
LOC113516895	1836	0	0	0.18	0.3	5.62	0.00	0.01	Maltase A1-like
LOC113518438	2136	0	0	0.15	0.25	5.62	0.00	0.01	Peroxisomal acyl-coenzyme A oxidase 1
LOC113512743	1527	0	0	0.16	0.43	5.62	0.00	0.01	Testis specific tektin
MSTRG.16294	282	0	0	6.34	17.59	5.62	0.00	0.01	Uncharacterized protein
LOC113510615	1787	0	0	0.09	0.4	5.62	0.00	0.01	Uncharacterized protein LOC106129672
LOC113514027	1443	0.09	0.05	3.18	4.49	5.60	0.00	0.00	Mediator of RNA polymerase II transcription subunit 15-like
LOC113514188	2796.03	0.76	0.45	27.04	29.03	5.59	0.00	0.00	cGMP-specific 3',5'-cyclic phosphodiesterase-like isoform X1
MSTRG.6923	377	0.48	0	14.95	17.64	5.57	0.00	0.00	Uncharacterized protein
LOC113515683	5730	0.15	0.18	7.47	7.98	5.57	0.00	0.00	Uncharacterized protein LOC106143250 isoform X1
LOC113520845	3613	0.14	0.2	8.03	8.13	5.55	0.00	0.00	Multidrug resistance protein 1A-like

MSTRG.11335	1407	78.63	97.28	4164.93	3820.39	5.53	0.00	0.00	Uncharacterized protein
LOC113510919	543	1.78	2.03	95.82	82.69	5.53	0.00	0.00	Lysozyme
LOC113511560	884	2.31	3.11	128.37	117.45	5.52	0.00	0.00	27 kDa hemolymph protein-like
LOC113520666	4002	4.06	4.89	203.17	196.55	5.52	0.00	0.00	Multidrug resistance protein 1A-like
LOC113509722	1401	0.05	0.05	3	2.21	5.51	0.00	0.00	Gustatory and odorant receptor 22
LOC113509446	461	0	0	2.55	1.09	5.50	0.00	0.01	Uncharacterized protein LOC106143208
LOC113513275	1003	0	0	0.66	0.28	5.50	0.00	0.01	Retinol dehydrogenase 13-like
MSTRG.7564	361	0	0	3.7	3.75	5.49	0.00	0.01	Protein amalgam-like
LOC113518469	2008	0	0	0.16	0.23	5.49	0.00	0.01	Uncharacterized protein LOC110374078
LOC113514058	1191	0	0	0.23	0.51	5.49	0.00	0.01	Voltage-dependent L-type calcium channel subunit beta-2 isoform X1
LOC113521483	1841	0	0	0.13	0.3	5.49	0.00	0.01	1-acylglycerophosphocholine O-acyltransferase 1
MSTRG.5836	285	0	0	6.01	14.57	5.49	0.00	0.01	Uncharacterized protein
LOC113520850	4837.11	0.2	0.24	9.54	9.94	5.47	0.00	0.00	Serine/arginine repetitive matrix protein 1 isoform X2
LOC113512196	1678	17.27	34.2	1178.74	1042.19	5.46	0.00	0.00	Neutral Lipase
MSTRG.11080	1017	0.07	0	2.32	1.91	5.45	0.00	0.00	Uncharacterized protein
LOC113523239	1265.54	9.66	12.77	481.26	476.94	5.45	0.00	0.00	GTP cyclohydrolase 1 isoform X1
LOC113521672	1341	0.26	0.12	8.26	8.14	5.42	0.00	0.00	UDP-glucose 4-epimerase-like
LOC113512073	1302	0.16	0.24	9.72	7.4	5.41	0.00	0.00	Venom acid phosphatase Acph-1-like
LOC113521620	449.67	3.58	5.66	211.15	191	5.40	0.00	0.00	Uncharacterized protein LOC106132635
LOC113510922	497	17.27	23.82	996.24	737.83	5.39	0.00	0.00	Protease inhibitor-like protein
LOC113515206	1759	9.55	21.41	660.61	605.15	5.39	0.00	0.00	Peptidoglycan-recognition protein-S
LOC113518433	1629	0	0.14	3.36	2.38	5.38	0.00	0.00	Uncharacterized protein LOC106137789
LOC113509871	614	0	0.18	3.84	4.98	5.38	0.00	0.00	Acetyltransferase ACT13
LOC113509357	1342	2.35	1.96	86.95	89.27	5.38	0.00	0.00	Uncharacterized protein LOC110383549 isoform X1
MSTRG.10254	1604	0.16	0.09	5.7	5.26	5.36	0.00	0.00	Uncharacterized protein
LOC113514053	1355	0.86	0.51	25.11	31.08	5.36	0.00	0.00	Uncharacterized protein LOC106142918
LOC113517412	1671	0.04	0.18	4.59	4.24	5.36	0.00	0.00	Cytochrome P450 CYP306A1

MSTRG.16406	223	12.3	0	417.74	242.92	5.35	0.00	0.00	Uncharacterized protein
LOC113516465	786	11.2	13.62	516.6	479.77	5.34	0.00	0.00	Uncharacterized protein LOC106132990
LOC113514518	1015	1.32	0.5	32.79	41.76	5.34	0.00	0.00	cGMP-specific 3',5'-cyclic phosphodiesterase-like isoform X1
LOC113521828	318	0.87	1.03	43.41	49.52	5.33	0.00	0.00	Uncharacterized protein
LOC113519032	1188	0.06	0.4	8.21	9.95	5.31	0.00	0.00	Uncharacterized protein LOC106137634
LOC113510568	2173	6.24	5.31	206.5	239.49	5.30	0.00	0.00	Heat shock protein 68
LOC113519436	662	3.74	4.24	167.68	145.55	5.30	0.00	0.00	Protein lethal(2)essential for life-like
LOC113519031	2294	0.08	0.03	2.22	2.46	5.29	0.00	0.00	Peroxidase-like isoform X2
LOC113523394	2276	0.03	0.03	1.21	1.31	5.29	0.00	0.00	Nose resistant to fluoxetine protein 6-like
LOC113521541	873	0.09	0	2.53	2.14	5.29	0.00	0.00	15-hydroxyprostaglandin dehydrogenase
LOC113512071	1265	0.33	0.06	7.92	7.88	5.26	0.00	0.00	Lipase 3-like
LOC113518355	1127	0	0.07	1.45	1.74	5.24	0.00	0.00	Protein rolling stone-like
LOC113513122	448	0	0.69	10.18	17.96	5.24	0.00	0.00	4-coumarate--CoA ligase 1-like
LOC113518500	4265	0.1	0.02	1.97	2.34	5.21	0.00	0.00	Voltage-dependent calcium channel subunit Alpha-2/delta-4 isoform X1
LOC113518101	1068.29	16.56	24.78	794.64	704.22	5.21	0.00	0.00	Uncharacterized protein LOC106133521
LOC113515031	3990	2.31	2.44	87.85	82.27	5.20	0.00	0.00	Proline dehydrogenase 1, mitochondrial
LOC113511046	2392	0.52	0.46	17.35	18.22	5.20	0.00	0.00	Transcription factor SOX-15
LOC113514927	823	0.1	0	2.63	2.09	5.18	0.00	0.00	Uncharacterized protein LOC106138571
MSTRG.3258	989	1.06	0.26	21.08	27.82	5.18	0.00	0.00	Uncharacterized protein LOC106140707
LOC113509209	1082	0.07	0	1.19	2	5.17	0.00	0.00	Sodium/calcium exchanger 1
LOC113521869	2871	0.13	0.05	2.79	3.62	5.16	0.00	0.00	Leucine-rich repeat-containing protein 49
LOC113515473	1654	0	0.04	0.75	1.07	5.13	0.00	0.00	Protein enabled homolog isoform X1
MSTRG.3742	1272.09	0.35	1.29	26.58	20.58	5.10	0.00	0.00	Uncharacterized protein
LOC113516076	1650.02	0.08	0.38	7.35	6.06	5.09	0.00	0.00	Sarcoplasmic calcium-binding protein 1
LOC113511771	966	0	0.09	1.69	1.85	5.09	0.00	0.00	Uncharacterized protein

									LOC110384167 isoform X2
LOC113517115	1964	0.19	0.11	4	6.53	5.08	0.00	0.00	Uncharacterized protein LOC106143014
LOC113520402	1709	0.19	0.09	5.09	4.5	5.07	0.00	0.00	Spondin-2-like
LOC113520366	972.36	0.09	0	1.55	1.83	5.06	0.00	0.00	Uncharacterized protein LOC110377214
LOC113522036	1353	0.05	0.06	2.23	1.68	5.04	0.00	0.00	Circadian clock-controlled protein-like
LOC113510528	1336	0.26	0.64	12.84	15.98	5.03	0.00	0.00	Collagen alpha-1(IX) chain-like isoform X1
MSTRG.675	320	0.85	0	19.96	20.48	5.02	0.00	0.00	Uncharacterized protein
LOC113520792	1440	0.98	1.11	37.93	27.94	5.00	0.00	0.00	G-protein coupled receptor Mth2
MSTRG.328	2801	0.83	1.29	32.9	32.64	4.99	0.00	0.00	Uncharacterized protein
LOC113511510	1096	0.2	0.07	4.27	5	4.98	0.00	0.00	Uncharacterized protein LOC106130779
LOC113523340	516	0.44	0.75	24.05	14.86	4.98	0.00	0.00	Hdd1-like protein
LOC113519423	603	0.65	0	11.76	11.32	4.98	0.00	0.00	Heat shock protein 24.3
LOC113518690	505	0	0.78	11.56	13.52	4.95	0.00	0.00	Gamma-glutamyltranspeptidase 1-like isoform X1
LOC113521401	1664	0.32	0.53	12	13.75	4.95	0.00	0.00	Collagen alpha-1(IX) chain-like isoform X1
MSTRG.14611	299	1.13	5.38	107.9	111.55	4.94	0.00	0.00	Uncharacterized protein
LOC113516440	3721.01	0.02	0	0.32	0.33	4.93	0.00	0.00	Mucin-5AC-like
LOC113520503	5339	0.05	0.06	1.96	1.47	4.90	0.00	0.00	Dorsal-ventral patterning protein Sog
LOC113520348	6644.22	0.39	0.44	11.32	12.69	4.90	0.00	0.00	Uncharacterized protein LOC106131594 isoform X4
LOC113510941	547	0.19	0	4.52	3.23	4.89	0.00	0.00	Alpha-tocopherol transfer protein-like
LOC113516213	2898	0.02	0	0.39	0.41	4.88	0.00	0.00	Uncharacterized protein LOC106142200
MSTRG.5837	1674	1.96	2.72	69.25	62.35	4.84	0.00	0.00	Uncharacterized protein LOC106132472
LOC113512452	3281.03	0.4	0.45	11.06	12.94	4.84	0.00	0.00	Uncharacterized protein LOC106130842
LOC113509411	1881	0.03	0	0.77	0.5	4.84	0.00	0.00	Chitinase 2
LOC113510451	1606	0	0.05	0.88	0.66	4.83	0.00	0.00	Lipase 3-like
LOC113512515	1816	0.07	0	1.25	1.09	4.82	0.00	0.00	Delta9-desaturase

LOC113517309	1888.33	0.38	0.58	13.61	12.42	4.81	0.00	0.00	NADPH oxidase 4-like
MSTRG.14456	1396	3.8	5.15	124.27	120.37	4.80	0.00	0.00	Uncharacterized protein
LOC113514256	1617	0.04	0.05	1.54	1.1	4.79	0.00	0.00	Fatty acyl-CoA reductase 1-like
MSTRG.4988	804	0	0.23	4.28	2.42	4.79	0.00	0.00	Uncharacterized protein
LOC113514762	512	0.22	0	2.88	5.42	4.78	0.00	0.00	Peroxisomal N(1)-acetyl-spermine/spermidine oxidase-like isoform X3
MSTRG.9111	518	0.43	0.5	15.44	11.68	4.78	0.00	0.00	Uncharacterized protein
LOC113515269	269	9.56	4.52	215.43	238.01	4.77	0.00	0.00	Diazepam binding inhibitor-like protein
LOC113523293	1954	0.55	0.62	15.26	15.98	4.75	0.00	0.00	Pollen-specific Leucine-rich repeat extensin-like protein 1
MSTRG.7153	1246	0.06	0.25	3.76	4.5	4.74	0.00	0.00	Uncharacterized protein
LOC113512008	1460	1.15	2.23	41.13	47.22	4.74	0.00	0.00	Senecionine N-oxygenase-like
LOC113516924	3143	12.12	11.07	296.21	307.6	4.74	0.00	0.00	Tribbles homolog 2-like
MSTRG.16169	380	0	0.54	9.36	8.18	4.73	0.00	0.00	Uncharacterized protein
LOC113511563	3040.08	0.27	0.09	3.59	2.7	4.73	0.00	0.00	Extracellular serine/threonine protein CG31145 isoform X1
LOC113521399	1067	0.34	0.46	9.81	11.13	4.69	0.00	0.00	Collagen alpha-1(IX) chain-like isoform X1
LOC113516776	4128	0.01	0	0.25	0.23	4.69	0.00	0.00	CD109 antigen-like
LOC113516120	1726.17	0	0.14	2.22	1.02	4.69	0.00	0.00	Neuropeptide precursor protein isoform X1
LOC113515392	1729	0.26	0.17	6.68	4.53	4.68	0.00	0.00	Trypsin beta-like
LOC113514950	1920	0.07	0.07	1.93	1.84	4.68	0.00	0.00	Glucose dehydrogenase
LOC113515807	2680	0.25	0.23	6.39	5.78	4.67	0.00	0.00	Irregular chiasm C-roughest protein-like isoform X2
LOC113520761	2241	0.03	0.06	1.3	1.09	4.66	0.00	0.00	G-protein coupled receptor Mth2-like isoform X1
LOC113509489	894	0.96	0.49	17.54	19.91	4.66	0.00	0.00	Organic cation transporter protein-like isoform X1
LOC113521449	766	0.55	0.12	10.62	7.41	4.66	0.00	0.00	Ribonuclease UK114-like
MSTRG.14558	1369	3.69	8.64	165.27	138.65	4.66	0.00	0.00	Uncharacterized protein
LOC113519417	1659	0.36	0	4.58	4.71	4.64	0.00	0.00	Facilitated trehalose transporter Tret1-2 homolog
LOC113513931	529	1.04	1.66	35.2	32.73	4.63	0.00	0.00	Uncharacterized protein
MSTRG.10296	1763	0.04	0.08	2.08	0.99	4.63	0.00	0.00	Uncharacterized protein

LOC113512991	2242	0.06	0.03	0.98	1.33	4.62	0.00	0.00	Beta-amyloid-like protein isoform X1
LOC113514259	2649	1.36	1.63	33.93	38.11	4.62	0.00	0.00	ABC transporter G family member 23
LOC113520780	3720	0.02	0.02	0.48	0.43	4.61	0.00	0.00	Dual oxidase
LOC113512554	345	14.08	18.76	433.17	398.24	4.58	0.00	0.00	Uncharacterized protein LOC110370740 isoform X2
MSTRG.9855	1061.05	4.28	4.34	82.69	77.94	4.55	0.00	0.00	Uncharacterized protein LOC106125039
LOC113520000	849	0.75	0.75	17.11	17.84	4.54	0.00	0.00	Gamma-glutamyltranspeptidase 1-like isoform X1
LOC113514948	2304	0.03	0	0.54	0.27	4.52	0.00	0.00	Glucose dehydrogenase
LOC113512296	885	0	0.1	1.35	1.32	4.51	0.00	0.00	Androgen-dependent TFPI-regulating protein-like
LOC113519419	2173	5.83	3.79	97.72	117.34	4.51	0.00	0.00	Heat shock protein 68-like isoform X1
LOC113519081	1312	0	0.06	0.73	0.84	4.51	0.00	0.00	Uncharacterized protein
LOC113516784	2067	9.17	11.01	213.82	235.44	4.51	0.00	0.00	Buffy
LOC113509694	2324	2.49	3.99	74.11	69.55	4.50	0.00	0.00	Transferrin precursor
LOC113520692	1883	2.28	3.82	67.64	67.78	4.50	0.00	0.00	Tyrosine-protein phosphatase corkscrew-like
LOC113513173	1014	0.15	0.17	3.54	3.83	4.49	0.00	0.00	Transcription initiation factor TFIID subunit 4 isoform X1
MSTRG.10067	2263	0.44	0.56	11.68	9.97	4.47	0.00	0.00	G-protein coupled receptor Mth2-like
MSTRG.13515	512	0.22	0	4.32	2.28	4.46	0.00	0.00	Vacuolar protein sorting-associated protein 13D
LOC113520521	4589	0.01	0	0.22	0.14	4.46	0.00	0.00	Protein bric-a-brac 1-like isoform X1
LOC113514309	1052.94	0.07	0	1.04	1.04	4.46	0.00	0.00	Sideroflexin-1
LOC113515831	1929	1.26	0.78	21.03	23.09	4.45	0.00	0.00	Chitinase 2
LOC113519837	1086	0.54	0.23	8.56	8.38	4.44	0.00	0.00	Uncharacterized protein LOC105389589
LOC113514407	4706	2.51	1.28	35.68	43.87	4.42	0.00	0.00	Multidrug resistance protein homolog 49
LOC113512750	895	0.35	0.39	7.2	8.8	4.40	0.00	0.00	Serine protease 4 precursor
LOC113510820	1670	0.04	0	0.49	0.58	4.39	0.00	0.00	Lachesin-like
LOC113515347	1040	0.07	0	0.9	1.06	4.39	0.00	0.00	Trypsin-1-like
LOC113517091	1766	0	0.04	0.65	0.36	4.39	0.00	0.00	Uncharacterized protein LOC106143133 isoform X1

LOC113519277	4131	1.13	1.07	19.56	21.03	4.37	0.00	0.00	ATP-binding cassette sub-family B member 3
LOC113516934	876	5.32	4.88	107.55	100.42	4.37	0.00	0.00	Uncharacterized protein LOC106143149
LOC113515232	3114	0.04	0.04	0.75	0.99	4.36	0.00	0.00	Bicaudal D-related protein homolog
LOC113521813	1968	1.81	2.11	39.67	38.72	4.35	0.00	0.00	Uncharacterized protein LOC103309331
LOC113511193	1995.69	0.35	0.58	9.51	9.65	4.35	0.00	0.00	Phosphoenolpyruvate carboxykinase
LOC113513902	804	0.72	0.23	9.08	10.95	4.35	0.00	0.00	G-protein coupled receptor Mth2-like
LOC113518906	2341	9.21	10.81	205.44	191.37	4.34	0.00	0.00	Uncharacterized protein
LOC113513791	617	182.08	170.76	3761.13	3346.38	4.34	0.00	0.00	Diazepam binding inhibitor protein
LOC113523607	941	0.73	1.19	18.47	20.02	4.33	0.00	0.00	Uncharacterized protein
LOC113510059	1517	0.04	0	0.5	0.65	4.32	0.00	0.00	Vacuolar ATP synthase subunit S1
LOC113522160	445	0.3	0	3.58	4.77	4.32	0.00	0.00	Uncharacterized protein LOC106130779
MSTRG.2103	694	0.13	0	1.47	1.93	4.32	0.00	0.00	Uncharacterized protein
LOC113511225	1812	0.04	0.08	1.52	0.87	4.32	0.00	0.00	Protein tyrosine phosphatase domain-containing protein 1-like
LOC113517705	975	0.23	0	1.77	3.46	4.32	0.00	0.00	Uncharacterized protein LOC105389318
LOC113523482	2372	1.05	1.06	20.34	20.64	4.31	0.00	0.00	Heparan-Alpha-glucosaminide N-acetyltransferase
MSTRG.4862	512	1.11	0.51	15.25	18.54	4.29	0.00	0.00	Myotubularin-related protein 10-A-like
MSTRG.1964	777.88	29.08	10.56	464.45	435.41	4.29	0.00	0.00	Uncharacterized protein
LOC113513450	1554	34.66	33.85	637.24	657.31	4.27	0.00	0.00	Protein charybde
LOC113515861	753	7.45	9.19	165.22	150.04	4.26	0.00	0.00	Protein lethal(2)essential for life-like
LOC113510138	1605	0.74	1.71	20.86	25.03	4.26	0.00	0.00	Hypothetical protein OBRU01_12387
LOC113511962	3335.94	0	0.02	0.21	0.22	4.25	0.00	0.00	Uncharacterized protein LOC110375307 isoform X1
LOC113516972	3642	0	0.02	0.2	0.2	4.25	0.00	0.00	Zinc transporter ZIP6
LOC113513838	6795	0.28	0.2	3.82	5.11	4.25	0.00	0.00	Uncharacterized protein LOC106140978
LOC113521358	3777	0.06	0.11	1.4	1.75	4.23	0.00	0.00	BAI1-associated protein 3
LOC113513899	803	2.97	4.51	73.72	64.96	4.23	0.00	0.00	Protein lethal(2)essential for life-like

LOC113516369	5286	7.72	8.36	143.18	150.86	4.23	0.00	0.00	Uncharacterized protein LOC106135296
LOC113517165	2243	5.79	4.5	89.76	97.96	4.22	0.00	0.00	GTP-binding protein 2-like isoform X1
LOC113523022	2511	0	0.06	0.62	0.45	4.21	0.00	0.00	Uncharacterized protein LOC101740673
LOC113519466	511	0.45	0	2.31	7.73	4.21	0.00	0.00	Uncharacterized protein
LOC113509551	786	7.93	4.9	120.35	115.52	4.21	0.00	0.00	GTP-binding protein 2-like
LOC113515359	2483	0.12	0.2	3.15	2.68	4.21	0.00	0.00	Uncharacterized protein LOC106103767 isoform X2
LOC113523114	743	16.81	10.29	239.53	257.4	4.21	0.00	0.00	Interferon-related developmental regulator 2
LOC113522488	3056.78	19.51	16.64	300.86	349.49	4.20	0.00	0.00	Inter-Alpha-trypsin inhibitor heavy chain H4-like
LOC113519477	1148	0.06	0	1.02	0.46	4.19	0.00	0.00	Facilitated trehalose transporter Tret1-like
LOC113522003	649	2.29	4.71	67.04	59.35	4.19	0.00	0.00	Protein lethal(2)essential for life-like isoform X1
LOC113522574	1698	0.04	0	0.48	0.43	4.19	0.00	0.00	MFS-type transporter SLC18B1-like
LOC113509698	1923	2.06	4.4	59.27	55.07	4.18	0.00	0.00	Organic cation transporter protein-like
LOC113518031	2424	0.03	0	0.16	0.44	4.18	0.00	0.00	A disintegrin and metalloproteinase with thrombospondin motifs 14-like
LOC113511548	1077	0.2	0.15	2.49	4.2	4.17	0.00	0.00	Uncharacterized protein LOC106132691
LOC113518308	1933	59.79	74.45	1175.67	1173.52	4.16	0.00	0.00	Cystathionine gamma-lyase
LOC113510213	689	14.93	10.44	219.24	231.65	4.16	0.00	0.00	GTP-binding protein 2-like
LOC113509844	1722	359.71	518.68	8246.71	7054.24	4.16	0.00	0.00	Uncharacterized protein LOC106135184
LOC113515229	3003	0.02	0.05	0.73	0.47	4.15	0.00	0.00	Vanin-like protein 1 isoform X1
LOC113518936	413	1.84	0	14.57	22.88	4.15	0.00	0.00	Uncharacterized protein
MSTRG.2140	1975.81	0.13	0.36	4.59	3.91	4.14	0.00	0.00	Uncharacterized protein
LOC113522438	1917	3.17	3.41	57.76	55.97	4.14	0.00	0.00	Innexin inx1
LOC113512234	468	7.29	2.17	81.29	92.45	4.14	0.00	0.00	Multidrug resistance-associated protein lethal(2)03659
LOC113521635	1838	1.65	2.48	36.79	34.41	4.14	0.00	0.00	Ejaculatory bulb-specific protein 3-like
LOC113511320	1414	0.05	0.11	1.21	1.65	4.14	0.00	0.00	Pancreatic lipase-related protein 2-

									like isoform X1
LOC113517025	2733	0.02	0.05	0.56	0.77	4.14	0.00	0.00	Uncharacterized protein LOC110380372
LOC113514885	668	6.42	7.59	117.53	125.99	4.13	0.00	0.00	Glycine N-methyltransferase
LOC113522854	459	8.47	13.94	200.56	192.06	4.11	0.00	0.00	Glycine N-methyltransferase
LOC113513897	836	36.16	39.31	672.49	613.77	4.11	0.00	0.00	Cytochrome P450 9e2-like
LOC113521719	2337	0.9	1.04	16.57	16.27	4.10	0.00	0.00	Hemocyte protein-glutamine gamma-glutamyltransferase-like
LOC113515841	1239	0.51	0.64	9.71	9.77	4.10	0.00	0.00	RNA polymerase II degradation factor 1-like
LOC113518672	740	0	0.13	1.18	1.45	4.10	0.00	0.00	Tkr
MSTRG.11341	607	0.32	0	3.09	3.46	4.09	0.00	0.00	Uncharacterized protein
LOC113515542	429	59.22	60.62	1123.29	956.36	4.09	0.00	0.00	Uncharacterized protein
MSTRG.12804	2070	0.24	0.31	4.49	4.79	4.09	0.00	0.00	Uncharacterized protein LOC106138099
LOC113510237	653	0.71	0.32	12.51	5.71	4.08	0.00	0.00	Protease inhibitor 4
LOC113516392	2171	0.87	1.14	16.79	16.31	4.08	0.00	0.00	Aromatic-L-amino-acid decarboxylase-like
LOC113518509	6328	0.35	0.51	7.27	6.78	4.06	0.00	0.00	Uncharacterized protein LOC106135505
LOC113519241	957	47.99	51.41	839.43	790.61	4.06	0.00	0.00	Cytochrome P450 protein
LOC113511049	1737.45	13.37	14.35	209.37	236.32	4.05	0.00	0.00	Glutathione synthetase-like isoform X3
LOC113523442	432	300.54	476.31	6439.63	6629.27	4.05	0.00	0.00	Defensin
LOC113517774	1161	4.18	3.11	65.22	52.71	4.04	0.00	0.00	Uncharacterized protein LOC106127544
LOC113523540	3219	0.04	0	0.33	0.37	4.00	0.00	0.00	Cytosolic carboxypeptidase 2
LOC113511136	3650	0.03	0.02	0.47	0.4	3.99	0.00	0.00	Uncharacterized protein LOC110379930
LOC113522466	2969	6.05	4.5	81.79	82	3.99	0.00	0.00	Uncharacterized protein LOC106138412
LOC113510025	2210	37.65	41.22	598.21	618.91	3.98	0.00	0.00	Transferrin-like
LOC113514504	633	13.31	17.65	201.63	281.24	3.97	0.00	0.00	Uncharacterized protein
LOC113510566	519	1.08	1.24	19.57	16.9	3.94	0.00	0.00	Alpha-tocopherol transfer protein-like
LOC113509585	493	92.43	90.65	1413.69	1419.34	3.94	0.00	0.00	Uncharacterized protein LOC101747015

LOC113521237	2552.85	0.1	0	0.68	0.91	3.94	0.00	0.00	C-type lectin 37Db isoform X2
LOC113520181	783	91.3	104.76	1476.61	1480.6	3.93	0.00	0.00	NF-kappa-B inhibitor cactus
LOC113513527	5289	0.01	0	0.04	0.17	3.93	0.00	0.01	Uncharacterized protein LOC103314804
LOC113513673	1662	0	0.04	0.3	0.48	3.93	0.00	0.01	Uncharacterized protein LOC106141535
LOC113520467	873	0.27	0.1	2.18	3.94	3.93	0.00	0.00	Protein jim lovell
LOC113521248	6048	0.02	0.03	0.33	0.45	3.93	0.00	0.00	Uncharacterized protein LOC106135118
LOC113518892	1275	11.45	10.31	172.99	147.47	3.91	0.00	0.00	Venom peptide BmKAPI-like
LOC113521491	1018	4.32	6.26	82.26	73.39	3.90	0.00	0.00	Uncharacterized protein
LOC113523494	1111	0.13	0	0.99	1.29	3.90	0.00	0.00	Tetraspanin-17
LOC113510422	384	3.16	4.72	48.27	75.4	3.90	0.00	0.00	Gloverin
LOC113521969	634	0.15	0.17	2.29	3.01	3.90	0.00	0.00	Uncharacterized protein
LOC113512455	1336.79	96.14	123.43	1617.74	1576.66	3.88	0.00	0.00	GATA Zinc finger domain-containing protein 14-like
LOC113510565	930	0.17	0.19	2.52	2.88	3.87	0.00	0.00	CRAL-TRIO domain-containing protein
LOC113515012	2550.18	0.16	0.21	2.25	2.87	3.87	0.00	0.00	Calponin homology domain-containing protein DDB_G0272472-like isoform X2
LOC113513458	1051	0.21	0	1.95	1.47	3.86	0.00	0.00	Uncharacterized protein LOC106141359
LOC113517068	4200.32	15.02	19.47	231.63	253.96	3.85	0.00	0.00	Integrin beta pat-3 precursor
LOC113513602	660	0	0.16	1.07	1.58	3.84	0.00	0.01	Uncharacterized protein LOC110376805 isoform X1
LOC113514993	3751	24	28.96	371.74	360.05	3.82	0.00	0.00	Uncharacterized protein LOC105387960 isoform X1
LOC113511534	832	15.83	12.5	187.76	206.83	3.81	0.00	0.00	Multidrug resistance-associated protein lethal(2)03659
LOC113514148	3212	0.07	0.17	1.31	2.04	3.81	0.00	0.00	Uncharacterized protein LOC106139738
LOC113522738	1647	1.59	2.01	25.45	23.97	3.80	0.00	0.00	Retinol dehydrogenase 11-like isoform X1
LOC113515237	2671	4.05	5.65	61.41	70.69	3.80	0.00	0.00	4-methylmuconolactone transporter
LOC113511994	2197	0.06	0.13	1.15	1.43	3.80	0.00	0.00	Uncharacterized protein LOC106138389 isoform X1
LOC113513129	1433	2.41	1.27	25.26	25.15	3.79	0.00	0.00	Membrane-bound alkaline

									phosphatase-like
LOC113513720	893	140.82	180.56	2299.75	2085.12	3.79	0.00	0.00	Serine protease 7-like
LOC113519295	531	0	0.47	3.2	3.7	3.78	0.00	0.00	Zonadhesin-like
LOC113519405	5172.16	0.03	0.03	0.44	0.4	3.78	0.00	0.00	Uncharacterized protein LOC110373911 isoform X6
LOC113519018	6531	0.06	0.02	0.57	0.57	3.78	0.00	0.00	Uncharacterized protein LOC110375652
LOC113517800	1364	27.12	26.71	348.29	375.81	3.78	0.00	0.00	NF-kappa-B inhibitor cactus
MSTRG.5530	695	0.13	0.29	4.08	1.93	3.78	0.00	0.00	Neural cell adhesion molecule 1-like
LOC113520065	823	0.79	1	15.66	8.73	3.77	0.00	0.00	Diamine acetyltransferase 2-like
LOC113517102	1371.72	0.29	0.13	2.41	2.58	3.77	0.00	0.00	Venom acid phosphatase Acph-1-like isoform X2
LOC113519768	1640.77	18.12	17.51	253.72	251.35	3.77	0.00	0.00	Calcium-binding mitochondrial carrier protein SCaMC-2
LOC113520001	347	103.45	134.48	1706.94	1688.31	3.76	0.00	0.00	Small heat shock protein
LOC113511966	2253.64	31.41	29.33	501.95	548.11	3.75	0.00	0.00	Cystinosin homolog isoform X2
LOC113522132	1292	0.05	0	0.47	0.46	3.75	0.00	0.01	Carboxypeptidase B-like isoform X1
LOC113513888	594	2.67	4.56	46.11	50.8	3.75	0.00	0.00	Uncharacterized protein LOC110381073
LOC113516706	1437	0.05	0	0.24	0.58	3.74	0.00	0.01	Uncharacterized protein LOC106142978
LOC113510950	2322	0.03	0.03	0.24	0.59	3.73	0.00	0.00	Uncharacterized protein LOC106131018
LOC113514100	2111	11.76	12.89	151.14	167.18	3.72	0.00	0.00	Ferric-chelate reductase 1 homolog
LOC113515628	2124	0.06	0.1	0.93	1.2	3.72	0.00	0.00	Uncharacterized protein LOC106134633
LOC113514368	319	1.72	7.11	52.36	72.05	3.72	0.00	0.00	Cecropin 2
MSTRG.9984	399	1.21	1.87	19.86	23.19	3.72	0.00	0.00	UDP-glucosyltransferase precursor
LOC113523106	1821	0.42	0.4	6.11	4.61	3.72	0.00	0.00	Cytochrome P450 6B5-like isoform X1
LOC113520493	705.29	9.77	9.24	132.75	155.46	3.72	0.00	0.00	Uncharacterized protein LOC110375908 isoform X4
LOC113523440	622	3.54	2.62	53.87	27.23	3.72	0.00	0.00	Antifungal peptide gallerimycin
LOC113514946	1387	17.68	19.32	233.84	242.26	3.71	0.00	0.00	Peptidoglycan recognition protein D
LOC113518671	1638	41.17	36.69	421.89	578.81	3.71	0.00	0.00	Titin
LOC113513204	923	153.32	184.92	2228.17	2131.63	3.71	0.00	0.00	Protein spaetzle
MSTRG.11625	782	0.32	0	2.71	1.99	3.70	0.00	0.00	Uncharacterized protein

MSTRG.320	1233	13.43	9.79	143.28	152.96	3.70	0.00	0.00	Multidrug resistance-associated protein lethal(2)03659
LOC113522500	1863	2.52	2.98	36.74	33.02	3.70	0.00	0.00	Uncharacterized protein LOC106129400
LOC113509550	2496	0.02	0.25	1.77	1.63	3.68	0.00	0.00	Uncharacterized protein LOC106129482
LOC113521973	8757	38.32	37.66	401.26	541.3	3.67	0.00	0.00	Titin
LOC113511946	4089	0.04	0	0.27	0.33	3.66	0.00	0.00	Uncharacterized protein LOC106142506
LOC113519792	925	0.58	0.47	5.81	7.46	3.64	0.00	0.00	Uncharacterized protein LOC105387048
LOC113514620	358	1.13	1.32	16.77	17	3.63	0.00	0.00	G-protein coupled receptor Mth2-like
LOC113516263	2672	0.69	0.51	6.61	7.93	3.62	0.00	0.00	Carboxylesterase 1E
LOC113517095	2153.42	61.44	72.83	807.81	799.93	3.61	0.00	0.00	Aromatic-L-amino-acid decarboxylase isoform X1
LOC113519448	1689	61.9	52.4	650.78	720.64	3.61	0.00	0.00	Nuclear factor interleukin-3-regulated protein
LOC113510698	1438	0.05	0.05	0.47	0.87	3.61	0.00	0.00	Extensin-like
LOC113517684	1296	0	0.12	0.61	0.92	3.61	0.00	0.00	Uncharacterized protein LOC106131541
LOC113509269	2948	0.06	0.05	0.59	0.75	3.60	0.00	0.00	Fatty acyl-CoA reductase 1
LOC113522463	6726	0.53	0.37	5.24	5.38	3.59	0.00	0.00	Mucin-5AC
LOC113523269	809	12.35	21.15	244.79	150.15	3.58	0.00	0.00	Gloverin-like
LOC113522295	541	11.15	14.76	157.89	152.92	3.58	0.00	0.00	Phospholipid scramblase 1-like
MSTRG.9506	1636	0.8	0.63	8.85	7.91	3.56	0.00	0.00	Uncharacterized protein
LOC113514170	1299	0.32	0.3	3.63	3.68	3.55	0.00	0.00	Zinc finger SWIM domain-containing protein 4-like
LOC113516393	521	211.16	294.8	3215.84	2729.59	3.55	0.00	0.00	Defensin-like protein precursor
LOC113523423	2400	0.03	0.03	0.36	0.35	3.55	0.00	0.00	Potassium channel subfamily K member 18-like isoform X1
LOC113522967	2205	0	0.06	0.46	0.31	3.55	0.00	0.00	Uncharacterized protein LOC110384273
LOC113515493	1809	42.28	45.63	502.27	504.36	3.55	0.00	0.00	Uncharacterized protein LOC106143241 isoform X4
LOC113522252	405	31.77	31.85	350.86	418.14	3.55	0.00	0.00	Multidrug resistance-associated protein lethal(2)03659
MSTRG.11855	1745	4.99	6.81	69.1	65.91	3.55	0.00	0.00	Uncharacterized protein
MSTRG.8130	355	0	1.36	7.85	9.56	3.54	0.00	0.00	Hemicentin-2-like

LOC113513037	3687	0.02	0.02	0.06	0.37	3.54	0.00	0.00	Xanthine dehydrogenase/oxidase-like
LOC113509323	574	0.18	0	2.29	0.45	3.54	0.01	0.03	Uncharacterized protein LOC106130856
MSTRG.12610	444	0.31	0	3.2	1.6	3.54	0.01	0.03	Uncharacterized protein
LOC113513010	2684	0.02	0	0.2	0.14	3.53	0.01	0.03	Uncharacterized protein LOC101736409
LOC113516017	1608	0.16	0.41	3.31	3.28	3.53	0.00	0.00	Zonadhesin-like
LOC113521767	996	0.08	0	0.48	0.65	3.53	0.01	0.03	Alpha-tocopherol transfer protein-like
MSTRG.5515	212	16.76	9.61	252.95	126.78	3.53	0.00	0.00	Multidrug resistance-associated protein lethal(2)03659
LOC113523296	2502	7.41	7.54	79.77	88.91	3.53	0.00	0.00	Uncharacterized protein LOC106131367
MSTRG.16508	304	0	1.25	8.82	9.1	3.52	0.01	0.03	Uncharacterized protein
LOC113515057	2128	0.12	0.27	2.01	2.32	3.52	0.00	0.00	Glucose dehydrogenase
LOC113509956	2032	0.84	0.38	6.35	7.58	3.51	0.00	0.00	Inverted formin-2 isoform X2
LOC113511322	2272.75	0.14	0.13	1.24	1.75	3.51	0.00	0.00	Sodium- and chloride-dependent transporter XTRP3 isoform X1
LOC113521567	1369	0	0.17	0.88	1.05	3.51	0.00	0.00	Peptidyl-Alpha-hydroxyglycine Alpha-amidating lyase 2-like
LOC113518938	949	0.16	0.18	2.24	1.8	3.50	0.00	0.00	Crossover junction endodeoxyribonuclease RuvC
LOC113512425	6008.91	10.66	7.42	92.29	105.86	3.49	0.00	0.00	Multidrug resistance-associated protein 1 isoform X1
LOC113514846	1319	0.05	0.06	0.59	0.77	3.48	0.00	0.00	Calcitonin gene-related peptide type 1 receptor
LOC113517198	693	0.13	0.15	1.48	1.94	3.48	0.00	0.00	Uncharacterized protein LOC106116396 isoform X1
LOC113515502	645.44	2.3	3.05	30.99	27.69	3.48	0.00	0.00	Whey acidic protein-like isoform X2
LOC113522333	1329	52.7	55.3	616.56	563.63	3.48	0.00	0.00	Growth arrest and DNA damage-inducible protein GADD45 alpha
LOC113522134	585	1.03	1.17	11.25	13.72	3.48	0.00	0.00	Zwei Ig domain protein zig-8-like
LOC113516139	1162	0.06	0.14	1.01	1.29	3.46	0.00	0.00	Enhancer of split malpha protein
LOC113518800	5874	0.2	0.23	2.45	2.16	3.46	0.00	0.00	Chaoptin
LOC113519607	3088	0.04	0.09	0.8	0.6	3.45	0.00	0.00	Transient receptor potential channel pyrexia isoform X1
LOC113514253	2974	2.56	1.51	22.73	20.74	3.45	0.00	0.00	Fatty acyl-CoA reductase 1-like

LOC113518443	1785	0.11	0	0.73	0.58	3.43	0.00	0.00	Sodium-coupled monocarboxylate transporter 1-like
LOC113512090	2884	0.08	0.05	0.55	0.9	3.43	0.00	0.00	GAS2-like protein 3 isoform X2
LOC113516829	2418	0.05	0	0.29	0.35	3.42	0.00	0.00	Uncharacterized protein LOC106143591
LOC113511844	2064	0.15	0	1.04	0.71	3.41	0.00	0.00	Histone-lysine N-methyltransferase PRDM9-like
LOC113509513	2545	0.1	0.05	1	0.65	3.41	0.00	0.00	Vanin-like protein 3
LOC113510053	774	0.76	0.61	8.11	6.62	3.41	0.00	0.00	Hypothetical protein KGM_202555
LOC113519628	3018	0.12	0.22	1.73	1.86	3.41	0.00	0.00	Uncharacterized protein LOC106136198
LOC113515990	397	0	0.48	3.81	2.19	3.40	0.01	0.04	Pancreatic secretory trypsin inhibitor-like
LOC113514814	1464	0	0.05	0.23	0.4	3.40	0.01	0.04	Serine proteinase stubble
LOC113522753	2412	0	0.03	0.13	0.22	3.40	0.01	0.04	Uncharacterized protein LOC106138721
LOC113513801	2458.92	12.17	11.17	117.25	121.23	3.39	0.00	0.00	Suppressor of cytokine signaling 2-like isoform X2
LOC113514254	3966.94	2.19	2.62	24.24	25.02	3.39	0.00	0.00	Fatty acyl-CoA reductase 1
LOC113520221	1014	0.29	0.17	2.6	2.37	3.38	0.00	0.00	DE-cadherin
LOC113510062	1684	0.12	0.17	1.42	1.62	3.38	0.00	0.00	Uncharacterized protein LOC110374899
LOC113509386	1479	5.58	5.05	55.8	52.34	3.37	0.00	0.00	Uncharacterized protein LOC110382337
LOC113512883	3510.12	10.3	8.48	92.55	96.73	3.37	0.00	0.00	Uncharacterized protein LOC110384473 isoform X1
MSTRG.7314	762	1	0.75	10.57	7.61	3.37	0.00	0.00	Uncharacterized protein
LOC113514215	3944.7	0.18	0.39	2.55	3.13	3.36	0.00	0.00	ABC transporter G family member 23 isoform X2
LOC113519961	2467.67	19.39	16.96	165.29	200.84	3.36	0.00	0.00	Facilitated trehalose transporter Tret1 isoform X3
LOC113515408	2722	2.27	2.46	22.93	24.28	3.35	0.00	0.00	Molybdenum cofactor sulfurase
LOC113511617	1353.51	135.13	181.67	1623.02	1536.77	3.35	0.00	0.00	Antichymotrypsin-2-like isoform X6
LOC113513424	1830	1.23	1.15	9.88	13.92	3.34	0.00	0.00	Inverted formin-2 isoform X1
LOC113513566	3798	0.02	0.02	0.14	0.23	3.34	0.00	0.01	Uncharacterized protein LOC106139737
LOC113516387	518	1.3	1.98	17.96	15.58	3.33	0.00	0.00	Apolipophorin-III
LOC113523011	2301.91	66.7	94.04	734.86	850.18	3.33	0.00	0.00	Surface protein bspA-like

LOC113515470	1720	0.38	0.38	4.43	3.16	3.33	0.00	0.00	Hypothetical protein RR46_01680
LOC113514551	800	25.52	29.19	284.79	258.37	3.33	0.00	0.00	FAD-linked sulfhydryl oxidase ALR
MSTRG.12036	1831	0.28	0.59	3.72	4.88	3.33	0.00	0.00	Uncharacterized protein
LOC113523375	975	23.67	29.58	280.47	245.07	3.33	0.00	0.00	Uncharacterized protein LOC106131388 isoform X1
MSTRG.6941	749	0.11	0.26	1.45	2.42	3.31	0.00	0.00	Hypothetical protein KGM_206686B
LOC113513679	2598	0.24	0.21	2.08	2.31	3.30	0.00	0.00	Fibroblast growth factor receptor 3-like isoform X1
LOC113520774	7277	7.93	6.31	63.36	73.07	3.29	0.00	0.00	Clustered mitochondria protein homolog
LOC113519738	2688	3.32	2.93	30.56	29.24	3.29	0.00	0.00	Zinc transporter ZIP1-like
LOC113515498	1456	0.14	0	1.22	0.28	3.29	0.00	0.00	Serine protease gd
LOC113515729	661	45.72	73.43	589.89	558.56	3.28	0.00	0.00	Uncharacterized protein
LOC113513324	1050	18.8	16.44	163.6	173.53	3.28	0.00	0.00	GTP-binding protein 1
LOC113509430	4001	11.35	15.44	129.88	123.78	3.28	0.00	0.00	Leucine-rich repeat-containing G-protein coupled receptor 5
LOC113511902	6108	0.02	0	0.11	0.1	3.27	0.00	0.01	Protocadherin-like wing polarity protein stan
LOC113514294	1241	0.17	0.06	1.21	1.18	3.27	0.00	0.00	Uncharacterized protein LOC106130786
LOC113509362	2465	0.63	0.34	4.75	4.45	3.27	0.00	0.00	Hyaluronidase isoform X2
LOC113517441	3611	1.28	1.57	13.39	13.37	3.26	0.00	0.00	Uncharacterized protein LOC106131879 isoform X1
LOC113520966	436	0.32	0.37	2.94	4.61	3.26	0.00	0.01	Hypothetical protein KGM_200275A
LOC113512560	1271	50.54	55.86	526.22	473.17	3.26	0.00	0.00	Serine protease easter-like
LOC113514091	790	10.59	7.47	79.84	91.07	3.25	0.00	0.00	Ribosomal protein S6 kinase-like 1
LOC113514718	2292	0.24	0.03	1.33	1.37	3.25	0.00	0.00	Uncharacterized protein LOC106130386
LOC113521819	891.14	3.39	3.83	31.64	34.32	3.24	0.00	0.00	Lipid phosphate phosphohydrolase 2-like
MSTRG.2506	462	1.39	1.28	11.25	14.82	3.22	0.00	0.00	Glypican-6 isoform X1
LOC113517216	640	0.15	0.33	2.06	2.59	3.20	0.00	0.00	Alkaline nuclease
LOC113515329	525	0.42	0	3.28	1.35	3.20	0.00	0.02	Cadherin-23
LOC113519196	1595	0.17	0.14	1.77	1.07	3.20	0.00	0.00	Cytochrome P450 6B46
LOC113516149	1234.25	0.13	0	0.65	0.56	3.19	0.00	0.02	Uncharacterized protein LOC106142225
MSTRG.6805	425	0.68	0	3.59	4.04	3.19	0.00	0.02	Uncharacterized protein

LOC113521625	1729	0.08	0.13	0.85	1.02	3.19	0.00	0.00	Diuretic hormone receptor isoform X1
LOC113510095	595	14.17	16.11	139.62	135.03	3.19	0.00	0.00	Prostamide/prostaglandin F synthase-like
MSTRG.8659	528	0.21	0.24	1.89	2.67	3.18	0.00	0.02	Fumarylacetoacetate hydrolase isoform B
LOC113511441	510	0.9	1.02	9.28	8.63	3.17	0.00	0.00	Insulin-related peptide binding protein precursor
LOC113511948	5229.52	0	0.06	0.21	0.34	3.17	0.00	0.00	Transcriptional regulator ATRX-like isoform X1
LOC113523167	4971	0.43	0.68	4.33	5.49	3.17	0.00	0.00	Uncharacterized protein LOC106140814
LOC113520749	1184	21.52	23.61	184.81	212.6	3.16	0.00	0.00	Facilitated trehalose transporter Tret1-like
MSTRG.13804	1732	0.26	0.25	2.03	2.58	3.16	0.00	0.00	Uncharacterized protein
LOC113509584	530	1102.49	1215.07	10870.36	9898.13	3.16	0.00	0.00	JH-inducible protein
LOC113519600	3587.5	0.15	0.15	0.99	1.66	3.16	0.00	0.00	C-1-tetrahydrofolate synthase, cytoplasmic isoform X2
LOC113517616	1164	1.96	3.17	23.29	21.35	3.15	0.00	0.00	UDP-glucuronosyltransferase 2B1-like
LOC113512894	1352	9.68	7.75	79.62	71.45	3.14	0.00	0.00	Prostaglandin reductase 1-like
MSTRG.2471	1226	0.4	0.26	2.84	3.04	3.14	0.00	0.00	Uncharacterized protein
LOC113514496	4323	0.1	0.09	0.75	0.88	3.13	0.00	0.00	Transmembrane channel-like protein 2 isoform X1
LOC113521121	1386	0.74	0.39	4.83	5.03	3.13	0.00	0.00	Facilitated trehalose transporter Tret1-like isoform X1
LOC113517314	2624.28	0.97	0.81	6.57	7.5	3.11	0.00	0.00	Cytochrome b reductase 1-like isoform X2
LOC113520498	2963	52.84	61.36	486.43	478.67	3.11	0.00	0.00	B-cell receptor-associated protein 31
LOC113509668	1115	0.19	0.29	2.13	2.08	3.10	0.00	0.00	Juvenile hormone binding protein
LOC113509826	999	4.8	3.47	30.95	39.43	3.10	0.00	0.00	Uncharacterized protein LOC106104008
LOC113516758	1713	0.61	1.58	9.48	8.84	3.10	0.00	0.00	Protein kinase DC2
LOC113510137	1221	0.06	0.06	0.58	0.57	3.10	0.00	0.02	Extradiol ring-cleavage dioxygenase-like
LOC113521975	11802	0	0.01	0.04	0.05	3.10	0.00	0.02	Dynein heavy chain 12, axonemal
LOC113510753	5859	7.41	6.32	54.51	60.15	3.10	0.00	0.00	Hypothetical protein KGM_203118
LOC113522448	3539	0.02	0.02	0.11	0.23	3.09	0.00	0.02	Uncharacterized protein

									LOC106130957
LOC113515164	1669	0.27	0.31	2.57	2.36	3.09	0.00	0.00	Rhomboid-related protein 1-like isoform X1
LOC113515627	1249	0.22	0.06	1.06	1.52	3.09	0.00	0.00	Protein amalgam-like
LOC113515402	2460.79	1.91	1.54	14.45	14.21	3.08	0.00	0.00	Tyrosine kinase receptor Cad96Ca isoform X1
LOC113516677	2644	1.27	0.31	7.56	5.7	3.08	0.00	0.00	Long-chain fatty acid transport protein 4-like isoform X1
LOC113521871	1416	6.27	3.5	39.27	41.91	3.08	0.00	0.00	Zinc finger SWIM domain-containing protein 5-like
LOC113509538	348	1.24	2.91	21.1	16.31	3.08	0.00	0.00	Low-density lipoprotein receptor 2-like
LOC113521825	1110	1.96	0.88	13.43	10.32	3.07	0.00	0.00	Uncharacterized protein
LOC113518043	4350	5.67	3.42	35.53	38.85	3.07	0.00	0.00	Lysine-specific demethylase 6A isoform X1
MSTRG.2751	2616	1.34	0.89	8.1	10.02	3.05	0.00	0.00	Zinc finger protein jing-like
LOC113515699	2234	0.42	0.72	4.99	4.19	3.04	0.00	0.00	Synaptic vesicle glycoprotein 2B-like isoform X2
MSTRG.6298	1376	17.16	23.69	169.35	159.83	3.04	0.00	0.00	Hypothetical protein ALC56_14032
LOC113509658	2374	0.08	0	0.39	0.32	3.04	0.00	0.00	Organic cation transporter protein-like
LOC113520220	2530	0.46	0.35	3.45	3.16	3.04	0.00	0.00	DE-cadherin
MSTRG.545	1064	0.34	0.78	3.83	5.2	3.03	0.00	0.00	Uncharacterized protein
MSTRG.15182	404	15.99	17.17	139.7	139.72	3.03	0.00	0.00	Uncharacterized protein
LOC113519756	2031.38	4.15	3.54	27.27	29.13	3.01	0.00	0.00	G-protein coupled receptor Mth2-like
LOC113511741	653	271.17	295.82	2417.48	2121.27	3.01	0.00	0.00	Uncharacterized protein
LOC113512346	2430.21	24.02	25.88	200.37	191.77	3.01	0.00	0.00	Sodium/hydrogen exchanger 9B2-like isoform X1
LOC113521636	583	274.64	452.68	3047.18	2771.25	3.01	0.00	0.00	Ejaculatory bulb-specific protein 3-like
LOC113514862	311	1.91	3.39	29.21	17.76	3.01	0.00	0.00	Mitochondrial glutamate carrier 1-like
LOC113517045	1732	0.08	0.13	0.76	0.88	3.00	0.00	0.00	Protein anon-37Cs
LOC113518806	6088	0.03	0.01	0.18	0.15	2.99	0.00	0.00	Protocadherin-15
LOC113517700	752	6.33	7.42	54.52	53.84	2.99	0.00	0.00	Uncharacterized protein LOC106129404
LOC113514911	1922	5.75	7.02	46.42	52.98	2.99	0.00	0.00	Prestin isoform X2

MSTRG.15873	1711	2.13	3.12	22.83	17.93	2.99	0.00	0.00	Uncharacterized protein
LOC113511598	866.39	0.7	0.99	6.41	7.4	2.99	0.00	0.00	Uncharacterized protein LOC106132252
LOC113523460	3699	1.41	1.26	9.2	11.5	2.98	0.00	0.00	Uncharacterized protein LOC110370969
LOC113520242	770	87.65	83.94	652.16	691.26	2.98	0.00	0.00	Superoxide dismutase
MSTRG.14401	805	3.36	4.38	33.03	26.83	2.97	0.00	0.00	Uncharacterized protein
LOC113509226	1348	12.97	13.04	92.62	104.26	2.95	0.00	0.00	Ribosomal protein S6 kinase delta-1-like isoform X2
MSTRG.11542	473	33.21	26.3	261.2	201.42	2.94	0.00	0.00	Hypothetical protein RR48_14652
LOC113516642	4865	0.12	0	0.55	0.4	2.94	0.00	0.00	Homeotic protein spalt-major-like isoform X1
LOC113517923	993	0.68	0.43	4.98	3.56	2.94	0.00	0.00	Protein phosphatase 2C T23F11.1 isoform X2
LOC113518881	595	1.67	0.76	7.7	11.39	2.93	0.00	0.00	RING finger and transmembrane domain-containing protein 2
LOC113518280	2679	1.05	0.87	7.48	6.88	2.93	0.00	0.00	Carbonic anhydrase 2
LOC113510121	1878.41	9.36	12.36	80.23	79.17	2.93	0.00	0.00	Uncharacterized protein LOC106130105 isoform X1
MSTRG.15229	541	0.2	0.68	3.08	3.81	2.93	0.00	0.00	AF4/FMR2 family member 3
MSTRG.970	453	7.28	7.03	46.83	64.52	2.92	0.00	0.00	Ankyrin repeat domain-containing protein 50
LOC113519084	967	0.08	0.35	2.58	0.68	2.92	0.00	0.00	Hypothetical protein KGM_213880
LOC113522221	2782	9.15	8.15	64.13	63.43	2.92	0.00	0.00	ATP-dependent RNA helicase DHX57
LOC113522824	980	0.15	0.26	1.36	1.81	2.91	0.00	0.00	Lipopolysaccharide-induced tumor necrosis factor-alpha factor-like
LOC113513793	1433	0.24	0.21	1.43	1.97	2.91	0.00	0.00	Peroxisomal N(1)-acetyl-spermine/spermidine oxidase-like isoform X4
LOC113519802	4341	0.03	0	0.05	0.18	2.91	0.01	0.04	Epidermal cell surface receptor
LOC113512490	969	10.17	10.14	71.01	79.36	2.91	0.00	0.00	ATP-binding cassette sub-family G member 1
LOC113509325	1596	51.95	65.84	420.16	446	2.91	0.00	0.00	Esterase FE4-like
LOC113517926	354	0.59	0.68	4.76	6.44	2.91	0.01	0.04	Phosphatidylinositol 4,5-bisphosphate 3-kinase catalytic subunit delta isoform
LOC113519444	5462.87	2.59	2.34	18.03	17.95	2.90	0.00	0.00	Solute carrier family 41 member 2-

									like isoform X1
MSTRG.16392	5581	4.81	3.33	28.18	31.21	2.90	0.00	0.00	Zinc finger SWIM domain-containing protein 4-like
LOC113515912	883	0.27	0.3	1.47	2.88	2.90	0.00	0.00	GTP-binding protein REM 1-like
MSTRG.3337	464.58	1.14	1.4	11.38	6.77	2.90	0.00	0.00	Uncharacterized protein
LOC113514899	529	0	0.48	1.34	2.39	2.90	0.01	0.04	Hemicentin-1-like
MSTRG.15255	314.06	4.05	0	11.92	14.39	2.90	0.00	0.00	Mediator of RNA polymerase 2 transcription subunit 26
LOC113515772	1340	0.56	0.29	4.78	1.58	2.90	0.00	0.00	Minus strand ecdysteroid 22-kinase
LOC113521320	1147	0.75	0.63	5.68	4.55	2.90	0.00	0.00	Uncharacterized protein LOC106135015
LOC113513503	1693	0.19	0.3	1.99	1.66	2.89	0.00	0.00	Sodium/calcium exchanger 1
LOC113523060	4030	4.51	3.84	27.8	32.48	2.89	0.00	0.00	Adenylate cyclase type 8
LOC113511452	690	0.52	0.73	4.46	4.88	2.88	0.00	0.00	Uncharacterized protein OBRU01_13522
LOC113519315	1289.54	197	186.98	1417.35	1533.37	2.88	0.00	0.00	L-lactate dehydrogenase
LOC113515705	5526.85	0.08	0	0.23	0.36	2.88	0.00	0.00	Uncharacterized protein LOC106720737 isoform X2
LOC113514145	1344	65.19	74.39	521.6	485.99	2.88	0.00	0.00	Mitochondrial fission 1 protein
LOC113511854	491	1.21	0.56	7.25	6.57	2.88	0.00	0.00	UPF0691 protein C9orf116
MSTRG.8652	1223.53	0.36	0.45	4.69	4.26	2.88	0.00	0.00	Uncharacterized protein
LOC113511100	252	8.2	6.48	55.01	74.41	2.88	0.00	0.00	GTP-binding protein 1
LOC113509785	963	7.27	8.07	54.79	55.96	2.87	0.00	0.00	Transposase
LOC113522413	1506	55.18	47.09	377.21	355.45	2.87	0.00	0.00	UDP-glycosyltransferase UGT44A2
MSTRG.12258	1604	0.12	0.09	0.41	1.21	2.87	0.00	0.00	Uncharacterized protein
LOC113518034	1752	0.04	0.12	0.37	0.82	2.87	0.00	0.01	Uncharacterized protein LOC106137789
MSTRG.25	552	4.98	3.71	31.08	32.68	2.86	0.00	0.00	ATP-binding cassette sub-family G member 1
LOC113521153	404	8.19	8.58	52.77	73.75	2.86	0.00	0.00	Methionine--tRNA ligase, cytoplasmic isoform X1
MSTRG.14756	3883.79	1.94	2.07	13.55	14.82	2.86	0.00	0.00	Uncharacterized protein
LOC113510374	1626	318.93	368.02	2333.63	2538.38	2.86	0.00	0.00	Esterase FE4 isoform X2
MSTRG.15853	3144	0.59	0.67	4.2	4.71	2.85	0.00	0.00	Uncharacterized protein
LOC113510614	3623.94	14.58	14.63	104.36	103.29	2.85	0.00	0.00	Elongation factor-like GTPase 1
LOC113510688	2638.97	9.6	6.14	55.33	55.75	2.85	0.00	0.00	E3 ubiquitin-protein ligase RNF126-B isoform X1

LOC113512252	891	1.67	1.59	10.71	12.57	2.84	0.00	0.00	Uncharacterized protein LOC106140275 isoform X1
MSTRG.3030	897	121.81	131.93	907.58	888.09	2.84	0.00	0.00	Uncharacterized protein
LOC113519844	2265	0.17	0.03	0.45	1.01	2.84	0.00	0.00	Hypothetical protein KGM_202689
LOC113514672	1990	0.61	0.75	3.9	5.64	2.83	0.00	0.00	Uncharacterized protein LOC110380322 isoform X2
LOC113514897	1295	0.21	0.48	2.36	2.51	2.83	0.00	0.00	Organic cation transporter
LOC113514822	617	0.31	0.18	2.6	1.18	2.83	0.00	0.02	Lipopolysaccharide-induced tumor necrosis factor-alpha factor
LOC113522536	1827	0.04	0.12	0.75	0.35	2.82	0.00	0.01	Argininosuccinate synthase
MSTRG.12523	639	0.15	0.33	2.82	0.74	2.82	0.00	0.02	Uncharacterized protein
LOC113523237	814	0.7	1.25	7.39	6.25	2.82	0.00	0.00	Serine/threonine-protein kinase MARK2 isoform X6
LOC113511175	471	113.36	141.03	904.07	900.43	2.81	0.00	0.00	Salivary cysteine-rich peptide precursor
MSTRG.14620	1657	132.63	159.49	936.48	1072.16	2.81	0.00	0.00	Esterase B1-like isoform X1
LOC113511912	1980	0	0.11	0.28	0.47	2.80	0.00	0.02	Vitamin K-dependent gamma-carboxylase
LOC113517161	282	0	5.32	12.67	28.58	2.80	0.00	0.02	Coagulation factor-like protein 2
LOC113517403	671	13.28	11.06	77.19	91.47	2.80	0.00	0.00	Serine hydrolase
LOC113521822	1363	0.85	0.79	6.26	5.01	2.80	0.00	0.00	Sex peptide receptor-like
LOC113523374	2156	47.73	50.71	351.06	316.7	2.80	0.00	0.00	Cytochrome P450 CYP12A2-like
LOC113523474	1529	14.86	14.74	98.55	102.11	2.79	0.00	0.00	Venom dipeptidyl peptidase 4
MSTRG.13027	1395	12.24	9.54	72.09	75.73	2.79	0.00	0.00	RING finger and transmembrane domain-containing protein 2
LOC113509667	4180	0.04	0.03	0.25	0.28	2.78	0.00	0.00	Toll-like receptor 7
LOC113521876	1644	0.44	0.45	2.57	3.49	2.78	0.00	0.00	Ionotropic receptor
LOC113514656	1085	8.13	10.21	57.05	66.81	2.78	0.00	0.00	ATP-dependent RNA helicase DHX57
MSTRG.11424	1218	0.23	0.78	3.36	3.42	2.78	0.00	0.00	Uncharacterized protein
LOC113518050	1554	0.13	0.1	0.48	1.1	2.78	0.00	0.00	Major facilitator superfamily domain-containing protein 12-like isoform X2
LOC113509827	1099	0.33	0.59	2.92	3.35	2.77	0.00	0.00	Uncharacterized protein LOC106143557
LOC113509243	792	9.72	7.79	54.83	64	2.77	0.00	0.00	Methionine--tRNA ligase, cytoplasmic
LOC113518240	1441	0	0.26	1.24	0.52	2.77	0.00	0.00	Mitochondrial enolase superfamily

									member 1-like
LOC113509813	804	109.92	134.12	855.08	787.61	2.77	0.00	0.00	Hypothetical protein RR46_13217
LOC113517713	1091	3.67	2.55	21.41	20.29	2.76	0.00	0.00	CD63 antigen-like
LOC113521650	2631	10.64	9.95	67.01	69.55	2.76	0.00	0.00	Uncharacterized protein LOC106139988
MSTRG.16298	759	4.68	5.92	36.29	34.81	2.76	0.00	0.00	Uncharacterized protein
LOC113520936	1521	4.39	4.04	29.73	26.12	2.76	0.00	0.00	Adhesive plaque matrix protein-like
LOC113520659	685	16.9	20.19	114.19	134.56	2.76	0.00	0.00	Facilitated trehalose transporter Tret1-like
LOC113515552	2855.83	9.6	7.66	52.29	61.38	2.75	0.00	0.00	V-type proton ATPase 116 kDa subunit a isoform 1
LOC113510696	2565	4.98	5.21	34.58	32.34	2.75	0.00	0.00	Monocarboxylate transporter 1-like
LOC113515213	7482	0.27	0.37	2.1	2.12	2.75	0.00	0.00	Tetratricopeptide repeat protein 28
LOC113523425	633	67.67	71.79	528.81	400.25	2.75	0.00	0.00	Defensin
LOC113517777	1377	0.74	0.78	5.62	4.46	2.75	0.00	0.00	Zinc finger protein Gfi-1 isoform X1
LOC113517402	693	14.79	20.1	111.69	119.87	2.74	0.00	0.00	Serine hydrolase
LOC113513022	5659	14.32	12.76	83.98	92.7	2.74	0.00	0.00	Uncharacterized protein LOC101746298 isoform X1
LOC113519914	477	0.78	0.59	4.71	5.02	2.74	0.00	0.00	Vacuolar protein sorting-associated protein 13D
LOC113521970	657	0.42	0.64	4.66	2.47	2.73	0.00	0.00	Zinc finger SWIM domain-containing protein 5-like
MSTRG.5335	483	1.01	0.58	6.21	4.88	2.72	0.00	0.00	Uncharacterized protein
LOC113522287	2557	5.89	5.26	35.45	36.11	2.71	0.00	0.00	Uncharacterized protein LOC106110164
LOC113521192	4422	2.6	2.91	16.83	18.44	2.71	0.00	0.00	Uncharacterized protein LOC106135037
LOC113509382	1350.99	327.63	307.17	2034.83	2045.64	2.71	0.00	0.00	Venom carboxylesterase-6-like isoform X1
LOC113523611	990	619.1	768.48	4612.39	4313.81	2.71	0.00	0.00	6Tox
LOC113517767	1040	0.14	0.16	1.26	0.79	2.71	0.00	0.01	Protein arginine N-methyltransferase 9
LOC113523334	502	12.51	16.43	102.36	87.55	2.71	0.00	0.00	Uncharacterized protein LOC106721094
LOC113518350	3505.12	8.98	15.74	72.41	85.33	2.71	0.00	0.00	Uncharacterized protein LOC106138210
MSTRG.4589	696	0.64	0	1.95	2.56	2.70	0.00	0.00	Uncharacterized transmembrane protein DDB_G0289901-like

LOC113522199	471	6.12	5.49	37.79	38.97	2.70	0.00	0.00	Uncharacterized protein LOC110380143
LOC113509732	2250.47	48.81	67.76	371.52	359.25	2.68	0.00	0.00	Hexokinase type 2 isoform X1
LOC113519987	1202	5.29	3.97	29.46	28.96	2.68	0.00	0.00	Uncharacterized protein
LOC113518097	2553	1.59	1.38	9.23	9.35	2.68	0.00	0.00	Carboxypeptidase N subunit 2-like
LOC113513628	449	6.54	4.79	33.47	41.14	2.68	0.00	0.00	Solute carrier family 26 member 6 isoform X1
LOC113522881	700	45.42	20.78	209.25	209.92	2.67	0.00	0.00	Uncharacterized protein
LOC113517559	1043	0.78	0.4	4.48	3.07	2.67	0.00	0.00	23 kDa integral membrane protein-like
MSTRG.1678	1271	13.14	12.5	76.62	83.29	2.67	0.00	0.00	Venom dipeptidyl peptidase 4-like
LOC113513952	870	0.18	0.1	1.04	0.91	2.66	0.01	0.04	Sulfotransferase isoform X1
LOC113517076	1106	0.13	0.29	1.32	1.38	2.66	0.00	0.00	Visual pigment-like receptor peropsin
LOC113511515	1894	2.81	1.94	15.57	13.83	2.66	0.00	0.00	Uncharacterized protein LOC106132000
LOC113516033	1196	0.06	0.13	0.52	0.73	2.65	0.01	0.04	Glyceraldehyde-3-phosphate dehydrogenase
LOC113510224	2296	1.68	0.52	5.83	7.85	2.65	0.00	0.00	Vacuolar protein sorting-associated protein 13D
LOC113513693	1415	0.05	0.11	0.3	0.71	2.65	0.01	0.04	Organic cation transporter protein-like isoform X1
LOC113520269	3047	119.52	127.36	758.37	752.14	2.65	0.00	0.00	Uncharacterized protein LOC106131445
LOC113512074	3492	11.56	12.15	74.16	70.54	2.64	0.00	0.00	AN1-type Zinc finger protein 2A-like
LOC113519197	1640	0.36	0.27	1.87	2.07	2.64	0.00	0.00	Cytochrome P450 6B46
LOC113513664	1161	19.73	26.36	138.89	143.32	2.64	0.00	0.00	Vitellogenin receptor
LOC113521573	906	1.03	2.62	10.78	11.53	2.64	0.00	0.00	Chemosensory protein 2
LOC113520377	2051	0.06	0.07	0.35	0.49	2.64	0.00	0.01	Uncharacterized protein LOC106131697
LOC113518143	3029	15.31	15.98	93.95	96.07	2.64	0.00	0.00	SHC-transforming protein 1 isoform X1
LOC113516128	1756	1.63	1.45	9.53	9.26	2.64	0.00	0.00	Uncharacterized protein LOC110378115
LOC113522449	1340.08	15.44	18.19	104.39	99.41	2.63	0.00	0.00	Cysteine-rich with EGF-like domain protein 2 isoform X1
LOC113512388	1579	51.95	63.3	365.64	331.5	2.63	0.00	0.00	J domain-containing protein
LOC113520698	2487	45.53	52.82	289.75	304.19	2.63	0.00	0.00	Uncharacterized protein

									LOC106140812
LOC113520443	1860	178.58	214.9	1180.19	1196.29	2.63	0.00	0.00	Antichymotrypsin-2-like
LOC113523547	884.15	81.07	78	488.12	470.13	2.62	0.00	0.00	Programmed cell death protein 6 isoform X1
LOC113510222	1066	0.55	0.15	1.56	2.89	2.62	0.00	0.00	Neuroendocrine convertase 1
MSTRG.7335	602	15.34	14.1	91.66	88.15	2.61	0.00	0.00	Venom dipeptidyl peptidase 4-like isoform X1
MSTRG.439	662	0.14	0.79	2.83	2.79	2.61	0.00	0.00	Uncharacterized protein
MSTRG.9558	1391	0.15	0.22	0.86	1.39	2.61	0.00	0.00	Uncharacterized protein
MSTRG.15234	506	14.13	12.76	81.14	84.57	2.61	0.00	0.00	Uncharacterized protein LOC106103208
MSTRG.11941	3108	8.76	8.76	49.17	54.89	2.60	0.00	0.00	Uncharacterized protein
LOC113512374	1932.7	2.94	2.48	16.2	16.02	2.60	0.00	0.00	Cytochrome P450 9e2-like
LOC113512920	1809	0.21	0	0.72	0.66	2.60	0.00	0.00	Organic solute transporter Alpha-like protein
LOC113519546	311	5.74	2.26	31.86	23.23	2.59	0.00	0.00	Lon protease homolog, mitochondrial isoform X1
LOC113517178	1647	0.12	0.09	0.95	0.34	2.59	0.00	0.01	Uncharacterized protein
LOC113519787	13893	0.05	0.06	0.29	0.37	2.59	0.00	0.00	Dynein beta chain, ciliary-like
LOC113521991	2329.2	0.09	0.05	0.39	0.16	2.58	0.01	0.05	Gonadotropin-releasing hormone II receptor isoform X3
LOC113515814	813	29.34	29.4	171.89	174.96	2.58	0.00	0.00	MAP kinase-activated protein kinase 2-like isoform X1
LOC113516140	827	0.2	0.11	0.87	1.1	2.57	0.01	0.05	Uncharacterized protein
LOC113511829	1886	0.31	0.11	1.28	1.25	2.57	0.00	0.00	PDZ and LIM domain protein 7 isoform X1
LOC113515418	2958	0.68	0.57	3.62	3.66	2.57	0.00	0.00	Retinal dehydrogenase 1-like
LOC113520202	833	0.1	0.22	0.74	1.21	2.57	0.01	0.05	Potassium channel subfamily K member 18-like
LOC113523354	1739.99	0.43	0.34	2.29	2.25	2.56	0.00	0.00	Uncharacterized protein LOC110370996 isoform X1
MSTRG.7017	1588.06	0.8	1.17	6.73	4.61	2.56	0.00	0.00	Uncharacterized protein LOC110372193
LOC113515795	1370	0.35	0.11	1.01	1.8	2.56	0.00	0.00	Uncharacterized protein
MSTRG.12820	386	4	4.65	28.5	24.52	2.55	0.00	0.00	G-protein coupled receptor moody-like isoform X1
LOC113509366	1840.87	366.22	426.04	2209.17	2328.02	2.55	0.00	0.00	Esterase FE4-like

LOC113515596	1426	1.52	1.33	8.73	7.71	2.55	0.00	0.00	Uncharacterized protein LOC106143246
LOC113518905	2986	0.57	1	5.31	3.59	2.55	0.00	0.00	Angiotensin-converting enzyme-like
MSTRG.7956	529	1.04	0.71	3.76	6.92	2.54	0.00	0.00	Zinc/cadmium resistance protein isoform X1
LOC113520283	1860	0.38	0.31	1.87	2.12	2.54	0.00	0.00	DE-cadherin
LOC113512868	2055	30.07	28.15	171.49	158.77	2.54	0.00	0.00	Uncharacterized protein LOC106135683 isoform X1
LOC113509574	1663	14.86	15.98	91.88	83.18	2.54	0.00	0.00	Hypothetical protein RR48_07415
LOC113510109	2311	57.55	55.56	303.08	338.56	2.54	0.00	0.00	Suppressor of tumorigenicity 14 protein homolog
LOC113511103	2042	53.67	59.08	328.65	306.45	2.53	0.00	0.00	CCAAT/enhancer-binding protein gamma
LOC113513039	3645	0.07	0	0.23	0.18	2.52	0.00	0.01	Xanthine dehydrogenase/oxidase-like
MSTRG.5458	5000	1.24	0.69	4.9	5.99	2.52	0.00	0.00	Hypothetical protein KGM_200275B
LOC113522860	673	2.02	1.38	9.99	9.66	2.52	0.00	0.00	Protein crumbs
LOC113520257	966	0.31	0	0.8	1.17	2.52	0.00	0.01	Hypothetical protein KGM_212484
LOC113521614	1335	182.35	211.46	1151.76	1053.98	2.51	0.00	0.00	Dihydropteridine reductase
LOC113509130	7504	0.06	0.05	0.43	0.2	2.51	0.00	0.00	Low-density lipoprotein receptor-related protein 4
LOC113514146	2478	0.1	0.11	0.63	0.58	2.51	0.00	0.00	Protein abrupt
LOC113519812	462	0.56	0.64	3.99	3.25	2.51	0.00	0.01	Uncharacterized protein LOC106136143
LOC113518388	3081	10.47	9.32	55.61	54.03	2.50	0.00	0.00	Facilitated trehalose transporter Tret1-like
LOC113509294	495	14.56	19.94	86.37	110.62	2.50	0.00	0.00	Methionine--tRNA ligase, cytoplasmic isoform X1
LOC113509840	740	0.35	0	1.62	0.58	2.50	0.01	0.03	Kv channel-interacting protein 4-like
LOC113516493	5450	0.09	0	0.31	0.19	2.50	0.00	0.00	Microtubule-associated protein futsch
LOC113519923	3664	1.46	1.62	7.77	9.22	2.50	0.00	0.00	Cationic amino acid transporter 2 isoform X1
LOC113511292	2017.75	8.36	8.16	44.22	47.45	2.50	0.00	0.00	Ceramide glucosyltransferase
MSTRG.4469	7365	3.82	3.92	17.82	24.65	2.49	0.00	0.00	Ankyrin repeat domain-containing protein 50 isoform X2
LOC113518702	864	2.11	0.93	8.12	8.98	2.49	0.00	0.00	Uncharacterized protein LOC110378268

LOC113514677	6076	1.13	0.93	4.7	6.6	2.48	0.00	0.00	Uncharacterized protein LOC110380322 isoform X2
MSTRG.1036	1886.7	16.43	20.6	92.48	76.24	2.48	0.00	0.00	Uncharacterized protein
LOC113523133	1071	13.19	8.62	59.95	59.95	2.48	0.00	0.00	Leucine rich repeat protein
LOC113521924	954	8.87	9.46	50.91	49.78	2.48	0.00	0.00	Lipid phosphate phosphohydrolase 1-like
LOC113522183	569	378.04	382.17	2177.92	2047.83	2.48	0.00	0.00	Cobatoxin-like protein
LOC113518445	2127.33	140.27	126.56	711.35	738.92	2.47	0.00	0.00	Sequestosome-1-like isoform X2
MSTRG.15052	940	1.88	0.74	7.54	6.99	2.47	0.00	0.00	Uncharacterized protein
LOC113522291	2081	0.18	0.27	1.07	1.41	2.47	0.00	0.00	Fibroblast growth factor 7
LOC113520233	8335	0.34	0.31	1.63	1.92	2.47	0.00	0.00	Tight junction protein ZO-1-like isoform X2
MSTRG.1768	319	3.44	1.02	7.14	21.98	2.46	0.00	0.01	Uncharacterized protein
LOC113518362	1374	20.37	25.35	126.63	120.27	2.46	0.00	0.00	Protein rolling stone-like
LOC113520836	4205.91	8.17	8.5	42.46	48.42	2.46	0.00	0.00	Rho guanine nucleotide exchange factor 10-like protein
LOC113511222	5007	0.02	0.04	0.12	0.23	2.45	0.00	0.01	Uncharacterized protein LOC106135801
MSTRG.11858	7785.36	5.02	5.61	26.66	30.29	2.45	0.00	0.00	Uncharacterized protein
LOC113509077	394	110.52	147.65	766.38	683.87	2.45	0.00	0.00	Uncharacterized protein
LOC113521510	2124	0.41	0.3	1.68	2.18	2.44	0.00	0.00	WW domain-binding protein 11-like
LOC113514266	373	25.16	45.41	217.36	179.41	2.44	0.00	0.00	Cecropin A
LOC113512680	917	29.9	39.54	202.38	168.99	2.44	0.00	0.00	Uncharacterized protein
LOC113518792	1956.8	21.98	27.12	132.71	128.84	2.44	0.00	0.00	ATP-dependent RNA helicase DDX18-like
LOC113511106	1366.69	11.54	12.13	59.56	66.18	2.44	0.00	0.00	Glutamate--cysteine ligase regulatory subunit
LOC113517978	739	4.3	4.34	19.66	26.88	2.44	0.00	0.00	Globin 1
MSTRG.3893	673.06	588.65	729.88	3676.84	3386.82	2.43	0.00	0.00	Uncharacterized protein
LOC113513140	3263	11.05	9.6	52.87	56.13	2.43	0.00	0.00	Protein shuttle craft-like
LOC113510485	2167	0.09	0.1	0.44	0.57	2.43	0.00	0.01	Protocadherin-like wing polarity protein stan
LOC113518142	842	124.06	149.24	750.68	700.28	2.43	0.00	0.00	Mid1-interacting protein 1A
LOC113514198	768	71.69	93.8	486.78	390.58	2.43	0.00	0.00	Ras-related protein Rac1
LOC113523495	3088	29.51	30.06	155.79	155.64	2.42	0.00	0.00	Cysteine--tRNA ligase, cytoplasmic isoform X1
LOC113520823	2586	0.12	0.05	0.48	0.47	2.42	0.00	0.01	Fibroblast growth factor receptor 2-

									like isoform X2
MSTRG.11278	1211	0.24	0.46	2.47	1.16	2.42	0.00	0.00	Uncharacterized protein
LOC113510846	1881	8.63	7.65	38	47.35	2.42	0.00	0.00	Sex-determining protein fem-1
LOC113513329	1585	1.25	0.23	2.94	5.03	2.42	0.00	0.00	Vacuolar protein sorting-associated protein 13D
LOC113520028	889	187.53	240.08	1198.84	1042.4	2.41	0.00	0.00	Mesencephalic astrocyte-derived neurotrophic factor homolog
LOC113521377	1238	0.4	0.45	2.5	1.96	2.41	0.00	0.00	Alanine aminotransferase 1
LOC113518948	2403.37	370.95	389.35	1956.5	1954.09	2.41	0.00	0.00	Hypothetical protein RR46_03373
LOC113514673	1416	0.48	0.59	2.71	2.89	2.41	0.00	0.00	Uncharacterized protein LOC106140704
LOC113522346	1128	3.13	2.59	15.8	13.96	2.40	0.00	0.00	G-protein coupled receptor moody-like isoform X1
LOC113521931	3604	10.58	12.21	57.09	60.49	2.40	0.00	0.00	BAG family molecular chaperone regulator 2
LOC113509814	3895.45	5.83	6.64	31.03	33.28	2.39	0.00	0.00	Axoneme-associated protein mst101(2) isoform X1
LOC113513388	1990	149.73	111.66	633.78	711.63	2.39	0.00	0.00	Heat shock protein 90 cognate
LOC113522957	1947.12	123.75	141.4	677.7	674.7	2.39	0.00	0.00	Choline-phosphate cytidylyltransferase A-like isoform X2
LOC113517754	1333	10.32	9.92	47.48	56.78	2.39	0.00	0.00	Ankyrin repeat and Zinc finger domain-containing protein 1-like
LOC113519144	15001	0.18	0.08	0.67	0.66	2.39	0.00	0.00	Microtubule-associated protein futsch
LOC113519383	1393.43	1.21	1.37	7.07	6.25	2.39	0.00	0.00	Protein THEM6-like
LOC113512298	1612.52	0.29	0.23	1.5	1.21	2.39	0.00	0.00	Serpin B8-like
LOC113516058	1662	0.16	0.09	0.6	0.73	2.38	0.00	0.01	Carboxypeptidase B-like
LOC113516094	966	0.94	0.97	4.67	5.26	2.38	0.00	0.00	Sialin-like
LOC113515545	1393	4.49	6.04	26.41	27.25	2.38	0.00	0.00	Uncharacterized protein LOC106134731
LOC113511819	964	3.62	1.69	12.37	15.05	2.37	0.00	0.00	Uncharacterized protein LOC106130173
LOC113520151	1524	0.13	0.05	0.83	0.16	2.37	0.00	0.02	Uncharacterized protein DDB_G0283357-like
LOC113509707	798	0.41	0	1.44	0.9	2.37	0.00	0.02	Uncharacterized protein LOC106139395
LOC113523104	2677	526.04	594.21	2779.11	2887.59	2.37	0.00	0.00	Heat shock 70 kDa protein cognate

									3 isoform X1
LOC113523555	1652	0.16	0.13	0.8	0.73	2.37	0.00	0.01	Proton-coupled amino acid transporter 4-like
LOC113523000	1785	0.51	0.69	2.87	3.21	2.37	0.00	0.00	Pseudouridine-metabolizing bifunctional protein C1861.05
LOC113523079	3231	0.13	0.15	0.79	0.61	2.37	0.00	0.00	Cytochrome P450 family 337 subfamily a polypeptide 1
LOC113519382	776	3.45	2.44	16.71	13.45	2.37	0.00	0.00	Uncharacterized protein LOC106137147
MSTRG.4513	532	14.61	19.49	96.42	78.62	2.36	0.00	0.00	Phosphoglycolate phosphatase 1B, chloroplastic-like
LOC113510353	539	0.6	0.23	1.55	3.07	2.36	0.01	0.03	Uncharacterized protein ZK1073.1 isoform X2
MSTRG.15909	827	0.28	0.48	1.12	2.69	2.35	0.00	0.01	Uncharacterized protein
MSTRG.534	262	8.77	2.6	43.91	26.31	2.35	0.00	0.02	Lethal(2) giant larvae protein isoform X1
LOC113521978	490.86	69.63	57.42	374.28	234.77	2.35	0.00	0.00	Uncharacterized protein
LOC113520996	615	24.21	25.35	125.64	126.26	2.35	0.00	0.00	Ankyrin repeat and Zinc finger domain-containing protein 1-like
LOC113514955	909	0.51	0	1.84	0.96	2.35	0.00	0.01	Uncharacterized protein LOC106134169
MSTRG.10481	686	0.26	0.89	4	1.81	2.35	0.00	0.01	Neural/ectodermal development factor IMP-L2-like
LOC113522784	994	119.29	111.62	559.48	596.17	2.35	0.00	0.00	Brinker
LOC113511124	1452	0.09	0.16	0.94	0.34	2.34	0.00	0.02	Dual specificity protein phosphatase 23-like
LOC113517599	6463.96	13.01	14.03	60.01	73.79	2.34	0.00	0.00	Integrin Alpha-PS2
LOC113510177	2945	1.64	1.08	7.41	6.12	2.34	0.00	0.00	Transferrin-like
MSTRG.1343	1969	3.88	4.1	18.75	20.76	2.34	0.00	0.00	Uncharacterized protein LOC110374261
LOC113518747	1407	4.92	4.83	25.96	22.22	2.33	0.00	0.00	Saccharopine dehydrogenase-like oxidoreductase
LOC113522116	1621	12.11	11.27	53.15	62.45	2.33	0.00	0.00	Ero1-like protein isoform X2
LOC113516163	2053	59.35	63.7	313.68	292.57	2.33	0.00	0.00	Hypothetical protein RR46_04746
LOC113510373	1881	189.94	242.72	1080	1049.3	2.33	0.00	0.00	Carboxylesterase
LOC113518534	1188	0.12	0.2	0.6	1.03	2.33	0.00	0.02	Mitochondrial import receptor subunit TOM40 homolog
LOC113509932	2082	23.02	25.45	121.35	116.96	2.33	0.00	0.00	Uncharacterized aarF domain-

									containing protein kinase 1
MSTRG.7639	805	0.71	0.58	3.11	3.43	2.33	0.00	0.00	Vacuolar protein sorting-associated protein 13D-like
LOC113516452	1630	7.06	5.12	28	31.91	2.32	0.00	0.00	Neuroglian isoform X1
LOC113522930	2298	4.15	3.07	17.03	18.37	2.32	0.00	0.00	Zinc finger protein 395
LOC113515088	601	46.93	59.21	270.42	255.17	2.31	0.00	0.00	Uncharacterized protein
LOC113521746	1936.47	1.15	2.59	9.49	8.66	2.31	0.00	0.00	Uncharacterized protein LOC106132632
LOC113514856	2383	12.67	11.64	57.92	60.01	2.31	0.00	0.00	Juvenile hormone esterase-like isoform X1
MSTRG.10207	1979	0.19	0.43	1.54	1.5	2.31	0.00	0.00	Uncharacterized protein LOC106134231
LOC113510847	912	5.11	3.55	19.09	23.47	2.31	0.00	0.00	Protein fem-1 homolog CG6966 isoform X3
LOC113515761	1956	0.13	0.15	0.61	0.76	2.31	0.00	0.01	Nose resistant to fluoxetine protein 6-like
LOC113510766	3218.8	22.68	22.03	108.9	107.36	2.31	0.00	0.00	Uncharacterized protein LOC110376441
LOC113520423	551	18.64	17.96	92.33	88.37	2.30	0.00	0.00	Mitochondrial glutamate carrier 1
LOC113509771	1100	5.54	5.96	28.38	27.18	2.30	0.00	0.00	Transposase
MSTRG.13222	3861	5.24	6.35	28.97	26.44	2.29	0.00	0.00	Golgin-45
MSTRG.160	275	8.47	2.01	38.51	22.61	2.29	0.00	0.02	Prostamide/prostaglandin F synthase-like
LOC113518908	2001	14.44	15.57	70.76	72.1	2.28	0.00	0.00	AarF domain-containing protein kinase 4
LOC113518661	2810	0.22	0.05	0.52	0.8	2.28	0.00	0.00	Toll-like receptor 6
LOC113516035	3236.8	1.15	0.96	4.47	5.56	2.28	0.00	0.00	Uncharacterized protein LOC106138830
LOC113513310	508	45.87	39.52	213.57	201.63	2.27	0.00	0.00	Beta-1,3-glucan recognition protein precursor
LOC113516936	3693.83	11.2	11.87	53.52	54.93	2.27	0.00	0.00	Uncharacterized protein LOC106099788 isoform X1
LOC113514618	577	1.06	0.6	2.49	5.81	2.27	0.00	0.01	Ankyrin repeat domain-containing protein 50
LOC113510502	302	3.26	3.86	13.63	25.03	2.27	0.00	0.02	Membrane-bound alkaline phosphatase
MSTRG.12030	619	1.24	0	3.38	2.94	2.25	0.00	0.01	Ionotropic receptor 4
MSTRG.13956	964	1.73	1.95	8.28	8.99	2.24	0.00	0.00	Uncharacterized protein

LOC113521752	2125	4.15	8.01	27.89	28.12	2.24	0.00	0.00	Uncharacterized protein LOC106132637
LOC113511485	1186	0.66	0.4	2.79	2.22	2.24	0.00	0.00	Serine protease Bi-VSP-like isoform X1
MSTRG.10427	3847.05	17.17	12.4	63.89	72.33	2.24	0.00	0.00	Uncharacterized protein
LOC113519252	1107	0.72	0.66	3.22	3.24	2.23	0.00	0.00	Exonuclease GOR-like isoform X1
LOC113520942	568	25.04	19.42	97.29	110.88	2.22	0.00	0.00	Ankyrin repeat and Zinc finger domain-containing protein 1-like
LOC113514257	3646	22.05	22.23	99.47	101.8	2.22	0.00	0.00	Peroxisomal biogenesis factor 19
LOC113518380	2739	12.36	12.79	59.13	55.18	2.22	0.00	0.00	FAST kinase domain-containing protein 3, mitochondrial-like
LOC113513726	931	6.2	8.21	33.94	32.03	2.22	0.00	0.00	Protein RRNAD1-like
LOC113520821	2613	2.93	2.63	12.32	12.99	2.22	0.00	0.00	Transcription factor 2 isoform X4
LOC113516157	3077	4.13	3.71	16.9	18.75	2.22	0.00	0.00	Insulin receptor substrate 1
LOC113514799	852	2.63	1.27	7.97	10.16	2.22	0.00	0.00	Required for meiotic nuclear division protein 1-like
LOC113515292	710	13.24	11.48	58.17	55.65	2.21	0.00	0.00	Required for meiotic nuclear division protein 1 homolog
MSTRG.3269	4293	4.17	3.89	17.16	19.4	2.21	0.00	0.00	Uncharacterized protein
LOC113509398	2536	0.07	0.11	0.34	0.51	2.21	0.00	0.01	ATP-sensitive inward rectifier potassium channel 1-like
MSTRG.7703	549.56	26.63	32.18	142.08	127.45	2.21	0.00	0.00	6Tox
MSTRG.16473	366	19.91	15.9	107.32	65.73	2.21	0.00	0.00	Beta-1,3-glucan recognition protein precursor
LOC113516513	1758	0.66	0.95	2.97	4.35	2.21	0.00	0.00	Uncharacterized protein LOC106133772
LOC113520818	947	47	47.8	223.35	206.51	2.20	0.00	0.00	Phosphoserine phosphatase
LOC113517426	1536	3.21	3.17	13.65	15.15	2.20	0.00	0.00	Phosphatidylinositol 4,5-bisphosphate 3-kinase catalytic subunit delta isoform
LOC113512123	607	0.48	0.55	0.82	4.07	2.20	0.00	0.02	Cytochrome P450 49a1
LOC113511358	782	29.6	32.86	133.07	150.2	2.20	0.00	0.00	Protein canopy homolog 1
LOC113513452	1004	24.15	30.4	131.07	112.66	2.18	0.00	0.00	Uncharacterized protein LOC106105275
LOC113516449	3740	12.19	13.52	56.6	57.56	2.18	0.00	0.00	Transmembrane protein 205
LOC113513223	412	1.48	2.57	7.34	11.77	2.18	0.00	0.01	Uncharacterized protein LOC110378268
LOC113509108	12015	0.11	0.07	0.35	0.43	2.18	0.00	0.00	Cubilin

LOC113521365	2920.52	87.31	96.96	378.35	405.33	2.18	0.00	0.00	3-phosphoinositide-dependent protein kinase 1 isoform X1
LOC113517176	717	1.34	0.55	3.57	5.19	2.18	0.00	0.00	Acetyl-CoA carboxylase
LOC113514491	2962	3.84	2.22	13.85	13.01	2.18	0.00	0.00	Actin-binding LIM protein 2
LOC113520632	1008	166.63	183.68	828.59	729.68	2.18	0.00	0.00	Eukaryotic translation initiation factor 6
LOC113515921	1197.22	10.95	9.99	42.15	52.46	2.18	0.00	0.00	Ankyrin repeat domain-containing protein 50 isoform X1
LOC113509713	918	32.21	37.76	168.41	142.96	2.18	0.00	0.00	Uncharacterized protein LOC106136999
LOC113512398	1562.25	7.53	8.03	33.26	36.41	2.18	0.00	0.00	Solute carrier family 52, riboflavin transporter, member 3-B isoform X2
LOC113509116	1325	2.65	2.22	10.81	10.83	2.17	0.00	0.00	Uncharacterized protein LOC110372303 isoform X2
LOC113516719	4451	0.74	0.88	3.4	3.75	2.17	0.00	0.00	Slit homolog 1 protein
LOC113519096	797	5.28	6.55	24.85	27.88	2.17	0.00	0.00	Uncharacterized protein LOC106104211
LOC113517222	6959.79	1.53	1.89	7.92	6.8	2.17	0.00	0.00	Uncharacterized protein LOC106133799
LOC113509404	1682	0.43	0.17	1.37	1.34	2.16	0.00	0.00	Uncharacterized protein LOC110371130
LOC113514777	846	2.46	0.64	5.41	8.49	2.15	0.00	0.00	Zinc finger DNA binding protein
MSTRG.7295	2361	4.26	4.69	17.94	20.82	2.15	0.00	0.00	Trehalase-like isoform X1
LOC113513616	617	20.63	28.4	110.7	104.8	2.14	0.00	0.00	DNA replication complex GINS protein PSF1-like
LOC113514183	2268	91.74	108.29	436.84	425.6	2.14	0.00	0.00	Uncharacterized protein LOC101737344
LOC113519823	3672	0.53	0.53	2.05	2.53	2.14	0.00	0.00	MATH and LRR domain-containing protein PFE0570w-like
LOC113518841	817	639.07	790.98	3171.44	3047.22	2.14	0.00	0.00	Protein CREG1
LOC113518778	928.12	0.89	0.13	1.65	2.27	2.14	0.00	0.01	Uncharacterized protein LOC106136399 precursor
MSTRG.13220	592	696.02	889.89	3518.7	3391.27	2.13	0.00	0.00	Protein CREG1
LOC113520835	5607.65	6.86	7.12	28.12	31.6	2.13	0.00	0.00	Integrin Alpha-PS1-like
MSTRG.6946	684	1.23	0	1.84	3.64	2.12	0.00	0.01	Ankyrin repeat and SOCS box protein 3-like
MSTRG.3852	5440	4.4	5.46	19.56	22.22	2.12	0.00	0.00	KAT8 regulatory NSL complex subunit 1-like

LOC113521007	1682	0.19	0.09	0.49	0.76	2.12	0.01	0.02	Alpha-1,3-mannosyl-glycoprotein 4-beta-N-acetylglucosaminyltransferase A-like isoform X1
LOC113522508	1381	51.63	66.59	264.48	237.07	2.11	0.00	0.00	HIG1 domain family member 1A, mitochondrial-like isoform X1
MSTRG.11410	974	0.16	0.79	1.77	2.22	2.11	0.00	0.01	Uncharacterized protein
LOC113517650	53087	0.01	0.01	0.04	0.07	2.11	0.00	0.00	Uncharacterized protein LOC110370027
LOC113509240	554	3.23	1.73	10.29	11.62	2.11	0.00	0.00	Lysine-specific demethylase 5A-like
LOC113516025	505	8	7.61	37.06	30.86	2.11	0.00	0.00	Histidine triad nucleotide-binding protein 2, mitochondrial-like
MSTRG.12417	590	0.51	0.77	2.39	3.22	2.11	0.01	0.02	Uncharacterized protein LOC110378902
MSTRG.14833	548	2.91	2.88	13.01	12.13	2.11	0.00	0.00	Uncharacterized protein
LOC113515404	1715	0.8	0.81	3.44	3.36	2.10	0.00	0.00	Alanine--glyoxylate aminotransferase 2-like
LOC113520153	1560	0.47	0.19	1.29	1.57	2.10	0.00	0.00	Plasma glutamate carboxypeptidase
LOC113516487	2392	3.48	2.21	10.34	12.54	2.10	0.00	0.00	Uncharacterized protein LOC110374577 isoform X1
LOC113521041	692	22.69	19.13	89.27	88.14	2.10	0.00	0.00	Lipoma HMGIC fusion partner-like 3 protein
LOC113509379	528.58	17.29	22.31	79.12	92.74	2.09	0.00	0.00	Uncharacterized protein LOC106131730 isoform X1
LOC113516253	780	0.43	0.48	2.58	1.33	2.09	0.00	0.02	Sialin
MSTRG.14446	6906	6.71	6.73	24.69	31.03	2.09	0.00	0.00	Uncharacterized protein
LOC113520285	2576.52	9.44	10.89	39.7	43.69	2.08	0.00	0.00	Uncharacterized protein LOC106131566 isoform X1
LOC113516707	7032	0.02	0.03	0.06	0.16	2.08	0.01	0.03	Uncharacterized protein PFB0765w-like
LOC113516451	5514	11.44	11.37	43.65	50.49	2.08	0.00	0.00	Neuroglian
LOC113517257	5025	0.49	0.62	2.34	2.23	2.08	0.00	0.00	Uncharacterized protein LOC106134103 isoform X1
MSTRG.14641	2565	1.37	1.21	4.18	6.49	2.08	0.00	0.00	Uncharacterized protein LOC106133263
LOC113515943	1796	7.52	11.03	39.78	36.72	2.08	0.00	0.00	Group XIIA secretory phosphoLipase A2
LOC113515350	4022.08	26.2	25.69	95.95	115.94	2.08	0.00	0.00	Aminopeptidase N-like isoform X1

LOC113518210	1587.12	48.9	54.02	229.78	194.45	2.07	0.00	0.00	Uncharacterized protein LOC110383836
LOC113520586	822	147.49	160.23	634.89	642.95	2.07	0.00	0.00	Coiled-coil-helix-coiled-coil-helix domain-containing protein 2, mitochondrial-like
LOC113522159	1073	11.52	11.36	45.84	48.84	2.07	0.00	0.00	Uncharacterized protein F09G8.5
LOC113518138	2783	17.51	16.88	66.99	74.23	2.07	0.00	0.00	Translation initiation factor eIF-2B subunit delta isoform X1
MSTRG.4627	567	5.64	4.97	24.82	19.89	2.07	0.00	0.00	Hemicentin-1
LOC113516060	2819.79	2.58	2.27	10.03	9.9	2.07	0.00	0.00	Zinc finger and BTB domain-containing protein 24-like isoform X3
LOC113522415	1196	0.53	0.4	1.72	2.19	2.07	0.00	0.01	AF4/FMR2 family member 1-like isoform X5
LOC113521335	1163	5.09	5.8	23.24	21.37	2.06	0.00	0.00	Mitochondrial deoxynucleotide carrier
LOC113515922	632	4.8	3.4	15.35	18.9	2.06	0.00	0.00	Uncharacterized protein
LOC113515813	1044	0.64	0.72	2.59	2.98	2.05	0.00	0.00	Uncharacterized protein LOC106141310
LOC113522222	549	5.81	4.19	18.7	23.18	2.05	0.00	0.00	CD63 antigen-like
LOC113513957	1735.79	6.37	6.44	26.48	25.27	2.05	0.00	0.00	Solute carrier family 25 member 35-like isoform X2
LOC113512866	4177	53.96	56.23	212.06	232.06	2.05	0.00	0.00	Calcineurin B
LOC113521599	1643	78.08	93.63	353.8	339.72	2.04	0.00	0.00	Serine--tRNA ligase, cytoplasmic
LOC113518216	7785	0.15	0.23	0.84	0.7	2.04	0.00	0.00	Mucin-5AC isoform X2
LOC113511577	5615	14.43	13.04	52	58.44	2.04	0.00	0.00	Cation-transporting ATPase 13A3 isoform X1
LOC113509748	1675	7.46	7.37	31.82	27.95	2.04	0.00	0.00	Methionine synthase reductase-like
LOC113510710	1260	4.1	5.79	20.33	19.53	2.04	0.00	0.00	Uncharacterized protein LOC110384236
MSTRG.259	568	0.73	0.83	3.73	2.77	2.04	0.01	0.03	Uncharacterized protein
LOC113521604	3357	21.55	15.64	71.75	77.14	2.03	0.00	0.00	Transcription factor Sox-12-like
LOC113523582	2608	21	19.7	79.93	83	2.03	0.00	0.00	Zinc/cadmium resistance protein
LOC113521368	10451	2.97	2.17	8.52	11.94	2.03	0.00	0.00	Zinc finger homeobox protein 3
LOC113509596	346	2.53	2.97	8.62	15.79	2.03	0.01	0.03	LYR motif-containing protein 2
LOC113518351	5113.7	17.52	15.8	59.97	70.67	2.02	0.00	0.00	Phosphatidate phosphatase LPIN2
LOC113521797	708	0.37	0.42	1.9	1.4	2.02	0.01	0.04	Hypothetical protein CDL32_26650
LOC113521607	1775.47	33.69	31.19	123.58	132.37	2.02	0.00	0.00	Acetylcholinesterase-like

LOC113513861	877	53.36	58.59	235.06	211.13	2.02	0.00	0.00	Phosphomannomutase 2
LOC113515494	1243	4.28	8.49	27.65	22.87	2.01	0.00	0.00	tRNA (guanine-N(7)-)-methyltransferase non-catalytic subunit wuho
LOC113518756	1276	268.34	299.18	1164.2	1081.67	2.01	0.00	0.00	Uncharacterized protein LOC101737963
LOC113521962	727	2.86	3.64	16.09	9.84	2.01	0.00	0.00	Uncharacterized protein
LOC113509252	3704	0.26	0.31	1.03	1.2	2.01	0.00	0.00	Uncharacterized protein LOC106137745
LOC113513276	703.9	1.32	0.18	1.98	3.46	2.00	0.00	0.01	Uncharacterized protein ZK1073.1 isoform X1
LOC113520844	4904.6	2.85	3.45	12.92	11.7	2.00	0.00	0.00	Inorganic phosphate cotransporter isoform X2
LOC113518416	2675	38.71	40.48	150.87	159.31	2.00	0.00	0.00	Uncharacterized protein LOC110375181
MSTRG.3449	278	673.41	667.23	3023.69	2920.16	2.00	0.00	0.00	Uncharacterized protein
LOC113514818	248	93.07	53.34	432.41	232.24	2.00	0.00	0.00	Uncharacterized protein
MSTRG.8981	434	0.65	3.72	9.76	7.64	1.99	0.00	0.01	G-protein coupled receptor moody isoform X1
LOC113518431	4159	0.13	0.29	0.94	0.66	1.99	0.00	0.00	Glutaminase
LOC113513109	420	19.68	23.13	86.69	86.79	1.98	0.00	0.00	Beta-1,3-glucan recognition protein precursor
LOC113516679	2564.94	0.19	0.19	0.74	0.73	1.98	0.00	0.01	Tyrosine-protein kinase transmembrane receptor Ror-like isoform X1
MSTRG.3046	1354	14.72	10.35	45.8	51.43	1.98	0.00	0.00	Phospholipid-transporting ATPase VD
MSTRG.7675	355	3.66	0	12.17	2.82	1.98	0.01	0.05	Uncharacterized protein
LOC113510759	923.13	12.91	8.35	41.04	40.52	1.98	0.00	0.00	Protein fem-1 homolog CG6966 isoform X1
LOC113517733	787	109.06	98.82	419.2	387.75	1.97	0.00	0.00	Cytochrome b5-like
LOC113514316	3493.42	20.13	20.57	75.94	80.17	1.97	0.00	0.00	Serine/threonine-protein kinase pelle
LOC113518684	1622	1.06	0.82	3.27	3.99	1.97	0.00	0.00	Thyrotroph embryonic factor isoform X5
LOC113513003	4454	50.23	54.01	183.1	213.86	1.96	0.00	0.00	Hypoxia up-regulated protein 1
MSTRG.9618	1937.69	1.91	0.89	6.05	4.05	1.96	0.00	0.00	Zinc finger SWIM domain-containing protein 8-like
MSTRG.8330	552	0.57	0.65	3.45	1.46	1.96	0.01	0.05	Uncharacterized protein

LOC113521431	2894	2.01	1.67	6.87	7.08	1.95	0.00	0.00	Breast cancer anti-estrogen resistance protein 1
LOC113514419	1015	0.22	0.25	0.74	1.09	1.95	0.01	0.05	Estrogen sulfotransferase
LOC113512140	3373.92	8.73	8.28	29.64	33.55	1.95	0.00	0.00	Uncharacterized protein LOC106143264
LOC113517327	2693	0.73	0.64	2.74	2.42	1.95	0.00	0.00	Nuclear receptor-binding protein homolog
LOC113511295	3163	0.06	0.06	0.14	0.33	1.94	0.01	0.05	Prolow-density lipoprotein receptor-related protein 1
LOC113510310	1650	0.4	0.18	1.35	0.88	1.94	0.00	0.01	Uncharacterized protein LOC110370405
LOC113521949	2619.89	22.21	17.94	57.36	74.44	1.94	0.00	0.00	Transcription factor HNF-4 homolog isoform X1
LOC113509803	1572	19.26	25.57	87.38	81.2	1.94	0.00	0.00	Dehydrodolichyl diphosphate syntase complex subunit Nus1
LOC113519864	1204	26.22	18.68	80.27	88.22	1.93	0.00	0.00	Bifunctional glutamate/proline--tRNA ligase
MSTRG.4830	2437	5.81	5.35	19.63	22.01	1.93	0.00	0.00	Uncharacterized protein
LOC113520654	1381	1.18	1.61	4.61	5.84	1.93	0.00	0.00	Protein hairy
LOC113518819	2314	9.44	8.81	31.87	35.73	1.92	0.00	0.00	NADP-dependent malic enzyme-like
LOC113514975	2445	25.28	26.87	99.39	93.17	1.92	0.00	0.00	X-ray repair cross-complementing protein 5-like
LOC113515424	4238	0.04	0.08	0.14	0.31	1.91	0.01	0.04	Chaoptin
LOC113519993	424	1.71	1.98	7.23	7.23	1.91	0.01	0.03	Facilitated trehalose transporter Tret1-like
LOC113517527	1642	2.36	2.47	9.32	8.46	1.91	0.00	0.00	F-box only protein 7-like
LOC113514951	1894	0.14	0.23	0.68	0.66	1.91	0.01	0.03	Glucose dehydrogenase
LOC113518086	4842.45	7.25	7.74	26.54	28.43	1.90	0.00	0.00	C-Maf-inducing protein-like
LOC113512315	1948.64	2.25	1.97	7.77	7.19	1.90	0.00	0.00	Carboxylesterase CXE26
LOC113519518	1115	5.18	2.77	13.26	16.18	1.90	0.00	0.00	Phosphatidylinositol 4,5-bisphosphate 3-kinase catalytic subunit delta isoform isoform X1
LOC113510506	619	40.56	35.65	126.58	157.94	1.90	0.00	0.00	Bifunctional glutamate/proline--tRNA ligase
LOC113515993	827	110.32	138.32	495.08	420.47	1.90	0.00	0.00	Cytochrome c
LOC113518414	5614.21	15.52	13.99	51.35	56.22	1.90	0.00	0.00	Solute carrier organic anion transporter family member 3A1 isoform X1

LOC113509159	637	3.85	3.86	14	14.73	1.90	0.00	0.00	Transmembrane protein 60
LOC113520445	6628.24	20.25	17.93	64.17	74.77	1.90	0.00	0.00	Activating signal cointegrator 1 complex subunit 3
LOC113517884	1323	2.66	2.93	10.18	10.27	1.90	0.00	0.00	Serine hydrolase-like protein
LOC113516653	1335	172.98	219.39	746.31	686.16	1.90	0.00	0.00	DnaJ homolog shv
LOC113521506	1265	5.18	6.63	22.24	20.86	1.90	0.00	0.00	Uncharacterized protein LOC106134940
LOC113518189	2401	0.83	0.64	3.37	1.96	1.89	0.00	0.00	Leucine-rich repeat neuronal protein 2-like
LOC113519988	1695	40.61	48.37	162.41	160.67	1.89	0.00	0.00	Phosducin-like protein
LOC113520711	3361	11.71	10.21	36.48	42.96	1.89	0.00	0.00	Valine--tRNA ligase isoform X1
LOC113510126	1635	3.38	3.39	13.57	10.98	1.89	0.00	0.00	F-box/WD repeat-containing protein 11
MSTRG.9305	836	33.17	39.2	149.33	114.44	1.89	0.00	0.00	Uncharacterized protein
LOC113509636	5789.77	16.02	18.49	57.5	65.95	1.88	0.00	0.00	Protein unc-13 homolog D isoform X4
LOC113523002	2016	0.09	0.32	0.55	0.93	1.88	0.01	0.02	EF-hand domain-containing protein 1-like
MSTRG.15283	3302	6.64	5.86	21.47	23.42	1.88	0.00	0.00	Uncharacterized protein
LOC113516838	578	0.53	2	3.61	5.57	1.88	0.01	0.02	Protein tramtrack, beta isoform-like isoform X2
LOC113514085	706	1	0.42	2.39	2.97	1.87	0.01	0.03	Uncharacterized protein LOC106130373
LOC113520620	2738	4.84	6.87	20.71	21.2	1.87	0.00	0.00	Solute carrier family 35 member B1 homolog
LOC113521313	2698	0.44	0.46	1.65	1.22	1.87	0.00	0.00	Glycogen-binding subunit 76A
LOC113523217	2206.39	16.72	17.44	58.64	63.8	1.87	0.00	0.00	Glycerol kinase-like isoform X1
LOC113510718	3379.26	13.77	11.92	42.07	49.68	1.87	0.00	0.00	RNA polymerase II elongation factor ELL
LOC113513445	583	19.37	11.02	57.51	53.5	1.87	0.00	0.00	TBC1 domain family member 20
LOC113517728	3027	0.1	0.29	0.65	0.73	1.86	0.00	0.01	Uncharacterized protein LOC106139019
LOC113510927	1539	1.51	1.26	4.63	5.32	1.86	0.00	0.00	Uncoordinated protein 58-like
MSTRG.4617	2266	3.11	3.92	11.87	13.07	1.86	0.00	0.00	Uncharacterized protein
LOC113523505	2802	0.17	0.17	0.49	0.74	1.86	0.00	0.01	Mucin-5AC-like isoform X1
LOC113513806	888	5.3	5.78	18.72	20.88	1.85	0.00	0.00	Phosphatidylinositol 4,5-bisphosphate 3-kinase catalytic subunit delta isoform

LOC113521852	2402	0.15	0.17	0.26	0.92	1.85	0.01	0.03	Follistatin-A
LOC113519816	2364	167.71	153.44	535.96	594.48	1.85	0.00	0.00	Unc-112-related protein-like
LOC113517000	1800	43.11	51.7	176.09	157.27	1.85	0.00	0.00	Protein slowmo
LOC113512948	2128	0.8	0.8	3.01	2.61	1.85	0.00	0.00	Uncharacterized protein
LOC113511522	652	1.14	0.48	3.45	2.51	1.84	0.01	0.03	F-actin-methionine sulfoxide oxidase MICAL3 isoform X1
LOC113519475	1023	6.32	4.75	18.01	20.97	1.83	0.00	0.00	Ankyrin-3
LOC113518193	3353.71	22.88	24.4	83.13	81.26	1.83	0.00	0.00	Dual specificity protein phosphatase 3-like isoform X1
MSTRG.7150	507	11.57	15.05	45.83	49.53	1.83	0.00	0.00	Uncharacterized protein
LOC113516023	2397	10.75	10.44	36.66	37.06	1.83	0.00	0.00	Ribonuclease ZC3H12C isoform X1
LOC113523076	1410	2.21	1.14	5.15	6.64	1.83	0.00	0.00	GC-rich sequence DNA-binding factor 1
LOC113517610	3043	0.42	0.27	0.85	1.55	1.83	0.00	0.00	Laccase-5
LOC113509515	1539.41	4.99	7.24	22.25	19.73	1.83	0.00	0.00	Uncharacterized protein LOC106138752
LOC113512873	3684.59	7.89	8.04	26.19	29.05	1.83	0.00	0.00	MAGUK p55 subfamily member 5 isoform X2
LOC113522304	1731.27	7.01	2.44	15.35	17.74	1.83	0.00	0.00	Fatty acid synthase
LOC113518876	1064	0.83	2.56	5.75	5.97	1.82	0.00	0.00	Uncharacterized protein LOC106136779
MSTRG.3045	1242	2.09	2.16	6.9	7.86	1.82	0.00	0.00	Uncharacterized protein
LOC113519016	658	18.2	23.98	73.53	74.33	1.82	0.00	0.00	Uncharacterized protein C6orf203 homolog
LOC113516001	2628	13.24	11.19	40.77	43.56	1.82	0.00	0.00	Activating signal cointegrator 1 complex subunit 2-like
LOC113517054	3371.46	28.94	28.58	98.93	106.49	1.82	0.00	0.00	Ral guanine nucleotide dissociation stimulator isoform X1
LOC113517898	1819	16.89	19.67	64.68	61.31	1.82	0.00	0.00	Uncharacterized protein OBRU01_11868
LOC113520663	2043	16.24	18.26	59.38	59.35	1.82	0.00	0.00	Hypothetical protein KGM_207817
LOC113512993	3750	8.49	10.22	33.03	31.19	1.81	0.00	0.00	Fasciculation and elongation protein zeta-2
LOC113510880	2974.94	298.43	334.11	1046.65	1087.09	1.81	0.00	0.00	Uncharacterized protein LOC106129699 isoform X2
LOC113523094	2939.26	53.99	57.5	185.66	196.95	1.81	0.00	0.00	Formin-binding protein 1-like
LOC113522563	771	59.3	53.88	206.43	186.43	1.81	0.00	0.00	Sulfiredoxin-1 isoform X1

LOC113519888	523	6.39	9.73	28.61	28.07	1.81	0.00	0.00	Tryptophanyl-tRNA synthetase
MSTRG.6685	1914	5.58	5.14	17.61	19.24	1.81	0.00	0.00	Uncharacterized protein
MSTRG.1453	2886.77	0.96	1.39	3.41	4.68	1.81	0.00	0.00	Uncharacterized protein
LOC113509588	1130	29.35	38.06	125.09	106.73	1.81	0.00	0.00	DNL-type Zinc finger protein-like
LOC113521964	900	0.95	1.27	3.63	4.09	1.81	0.00	0.01	Uncharacterized protein LOC105842592
LOC113512280	3264	2.54	0.91	4.92	6.92	1.81	0.00	0.00	Fatty acid synthase 2
LOC113515837	469	30.9	25.28	86.49	113.58	1.81	0.00	0.00	Bifunctional glutamate/proline--tRNA ligase
LOC113517157	355	19.12	14.24	51.04	73.31	1.80	0.00	0.00	Glutamine-dependent NAD(+) synthetase
LOC113522576	1382	11.84	14.41	46.01	43.75	1.80	0.00	0.00	Protein maternal effect lethal 26-like
LOC113515000	1409	72.33	99.54	296.08	291.18	1.80	0.00	0.00	Uncharacterized protein LOC106134174
LOC113512416	3865	0.93	0.84	3.13	2.92	1.80	0.00	0.00	Phosphoinositide 3-kinase adapter protein 1 isoform X1
LOC113511698	3056	96.15	98.71	318.75	344.97	1.80	0.00	0.00	Innexin inx3
LOC113509080	1268	12.58	8.09	34.51	36.32	1.80	0.00	0.00	Phospholipid-transporting ATPase VD
LOC113515573	922	1.17	0.47	3.08	2.71	1.80	0.00	0.02	Ras-related and estrogen-regulated growth inhibitor-like protein
LOC113521429	822	26	25.79	98.28	79.57	1.80	0.00	0.00	Anamorsin homolog
LOC113515438	2510	0.81	1.32	3.58	3.67	1.80	0.00	0.00	Ras-related and estrogen-regulated growth inhibitor-like protein
LOC113513004	3774	38.58	25.73	95.85	122.77	1.80	0.00	0.00	Sortilin-related receptor-like isoform X1
LOC113512998	1158	48.17	45.61	157.59	162.71	1.80	0.00	0.00	Hypoxia up-regulated protein 1
LOC113522767	1282	17.44	9.26	46.47	44.56	1.79	0.00	0.00	PAX3- and PAX7-binding protein 1
MSTRG.15649	1968.78	3.19	3.47	12.3	10.32	1.79	0.00	0.00	NFX1-type Zinc finger-containing protein 1-like
LOC113519654	1503	0.31	0.3	1.18	0.93	1.79	0.01	0.03	Senecionine N-oxygenase-like
LOC113509568	1023	28.34	28.57	94.02	99.71	1.79	0.00	0.00	Fat body acyl-CoA delta-9 desaturase
LOC113521082	391	11.56	5.47	23.96	38.46	1.79	0.00	0.01	Phosphatidylinositol 4,5-bisphosphate 3-kinase catalytic subunit delta isoform
MSTRG.1123	781	54.63	52.95	169.55	198.05	1.79	0.00	0.00	Uncharacterized protein
LOC113517545	3251	56.82	60.34	192.19	202.32	1.79	0.00	0.00	Prisilkin-39-like

MSTRG.16445	268	36.84	48.41	132.72	196.77	1.78	0.00	0.01	Methionine--tRNA ligase, cytoplasmic
LOC113514848	594	21.4	16.73	58.34	72.38	1.78	0.00	0.00	Bifunctional glutamate/proline--tRNA ligase-like
LOC113509492	1142	0.88	0.28	2.46	1.55	1.78	0.00	0.02	Uncharacterized protein LOC106712877
LOC113519367	1057	1.95	1.02	5.01	5.08	1.77	0.00	0.00	Protein SMG7-like isoform X2
LOC113520238	1458	4.48	4.72	16.64	14.16	1.77	0.00	0.00	UDP-glycosyltransferase UGT40L1
MSTRG.1708	3210.78	3.63	2.44	8.82	12.02	1.77	0.00	0.00	Uncharacterized protein
LOC113522311	438	9.79	10.91	43.53	28.15	1.77	0.00	0.00	Protein phosphatase 2C T23F11.1 isoform X1
LOC113523539	1736	9.14	9.76	30.59	32.26	1.76	0.00	0.00	Glutamine-dependent NAD(+) synthetase
LOC113519068	2480	0.32	0.03	0.75	0.46	1.75	0.01	0.03	Uncharacterized protein LOC110385045
LOC113520702	4264	0.18	0.14	0.54	0.52	1.75	0.00	0.02	Uncharacterized protein LOC106130581
LOC113518992	2181	7.39	4.97	18.94	21.83	1.75	0.00	0.00	E3 ubiquitin-protein ligase RNF19B-like isoform X1
LOC113518330	2082.89	0.65	0.56	2.01	1.97	1.75	0.00	0.01	Ras-related protein Rab-26 isoform X1
LOC113520612	2270	22.84	24.36	76.81	78.47	1.75	0.00	0.00	ATPase family AAA domain-containing protein 3
LOC113521317	1465	27.09	35.24	109.97	95.07	1.75	0.00	0.00	Glycerol-3-phosphate phosphatase
LOC113521241	1317	130.38	140.73	458.74	433.91	1.75	0.00	0.00	Laminin subunit alpha isoform X3
LOC113517467	2080	44.05	48.2	154.8	148.01	1.75	0.00	0.00	RNA polymerase I-specific transcription initiation factor RRN3 isoform X1
LOC113513928	600	6.08	3.73	13.26	19.94	1.75	0.00	0.00	Myotubularin-related protein 10-A-like
LOC113519620	2127.6	137.48	134.23	430.04	460.35	1.75	0.00	0.00	Transcription factor kayak isoform X7
MSTRG.14318	1595	6.71	6.47	20.71	22.62	1.75	0.00	0.00	Uncharacterized protein
LOC113522114	247	18.45	50.97	128.5	130.69	1.74	0.00	0.02	GrpE protein homolog, mitochondrial
MSTRG.1650	2579.59	15.57	10.68	32.74	41.27	1.74	0.00	0.00	Uncharacterized protein
LOC113513491	1506	25.04	25.99	81.26	85.99	1.74	0.00	0.00	Glycine receptor subunit Alpha-4-like isoform X3
LOC113514967	7681	0.27	0.15	0.92	0.44	1.74	0.00	0.00	Fatty acid synthase-like

LOC113519185	252	84.71	16.2	216.13	177.76	1.74	0.00	0.01	Long-chain-fatty-acid--CoA ligase 4 isoform X2
MSTRG.400	695	4.74	2.75	13.22	11.72	1.74	0.00	0.00	AF4/FMR2 family member 3-like isoform X4
LOC113514916	289	94.21	130.73	425.87	389.7	1.73	0.00	0.00	Glutathione-S-transferase-like protein
LOC113521856	573	2.68	2.84	7.8	10.65	1.73	0.00	0.01	Uncharacterized protein LOC101742184
LOC113516792	842.1	84.72	94.31	329.38	256.82	1.73	0.00	0.00	Calcium load-activated calcium channel
MSTRG.4654	4627	7.73	5.87	21.04	23	1.73	0.00	0.00	OTU domain-containing protein 7B
LOC113512758	560	2.42	0.85	4.8	6.4	1.73	0.01	0.02	Zinc finger protein 628-like
LOC113520317	1439.91	5.09	4.83	14.33	18.25	1.73	0.00	0.00	Transmembrane protein 198 isoform X1
LOC113511818	4775.77	10.35	9.28	30.58	32.26	1.73	0.00	0.00	Caskin-2 isoform X1
LOC113509968	1278	7.99	8.5	25.32	28.31	1.73	0.00	0.00	KAT8 regulatory NSL complex subunit 1-like
MSTRG.4280	866	9.71	15.1	41.14	39.55	1.72	0.00	0.00	Uncharacterized protein
LOC113516769	632	0.9	1.53	4.22	3.78	1.72	0.01	0.03	Uncharacterized protein LOC106136053
MSTRG.13915	1828	19.63	19.11	64.53	60.18	1.72	0.00	0.00	Uncharacterized protein
LOC113515413	2431	0.43	0.62	1.79	1.53	1.72	0.00	0.01	Sodium-dependent multivitamin transporter isoform X2
LOC113516115	4963	12.26	11.17	33.8	41.32	1.71	0.00	0.00	Inositol-3-phosphate synthase
MSTRG.7165	9860	0.97	0.86	2.77	3.06	1.71	0.00	0.00	Uncharacterized protein
LOC113522462	2617	3.65	4.18	12.27	12.77	1.71	0.00	0.00	Fizzy-related protein homolog
MSTRG.14725	2609	3.34	3.88	12.05	11	1.71	0.00	0.00	Uncharacterized protein
LOC113515631	4241	7.36	7.13	22.79	23.33	1.71	0.00	0.00	CD2-associated protein isoform X1
LOC113515203	1926	9.15	9.79	28.33	32.13	1.70	0.00	0.00	Neurogenic locus notch homolog protein 1 isoform X1
LOC113511307	1128	1.66	1.44	4.92	5.05	1.70	0.00	0.01	Oxysterol-binding protein-related protein 3-like
LOC113516693	2499	12.65	12.01	34.92	43.64	1.70	0.00	0.00	RNA pseudouridylate synthase domain-containing protein 2-like isoform X3
LOC113510002	3720	0.1	0.29	0.62	0.59	1.70	0.00	0.02	Serine-rich adhesin for platelets
LOC113514275	1363	21.27	19.74	68.51	62.2	1.70	0.00	0.00	Golgin subfamily A member 7
LOC113520837	2746	10.86	14.05	41.75	37.18	1.70	0.00	0.00	Uncharacterized protein

									LOC106130822
LOC113517694	546	22.89	19.4	61.75	76.04	1.70	0.00	0.00	SUN domain-containing ossification factor isoform X1
LOC113513044	2639	6.08	6.77	19.59	21.1	1.70	0.00	0.00	Zinc finger protein 436-like
LOC113512486	1433	93.29	98.49	287.7	320.16	1.69	0.00	0.00	Reticulon-3-like isoform X2
LOC113510386	754.57	3.88	1.1	7.16	9	1.69	0.00	0.01	Uncharacterized protein LOC106136737 isoform X2
LOC113518331	2426	0.2	0.17	0.64	0.56	1.69	0.01	0.03	Cationic amino acid transporter
MSTRG.8578	5051	0.76	1.17	2.4	3.67	1.68	0.00	0.00	Uncharacterized protein
LOC113515128	768	10.29	14.85	45.94	33.63	1.68	0.00	0.00	Uncharacterized protein LOC110371709
LOC113522525	851	0.94	0.21	2.38	1.4	1.68	0.01	0.04	Beta-carotene-binding protein
MSTRG.1387	6123	1.46	1.83	4.5	5.79	1.68	0.00	0.00	Tigger transposable element-derived protein 6-like
LOC113523377	2542	13.96	15.5	42.22	50.02	1.68	0.00	0.00	Bestrophin 1b
LOC113519887	2835	6.67	5.7	17.63	21.08	1.68	0.00	0.00	Acetyl-coenzyme A synthetase
LOC113518801	2030	146.97	155.61	473.73	471.73	1.68	0.00	0.00	Glutamate dehydrogenase
LOC113521759	1592	15.24	18.1	53.33	50.86	1.67	0.00	0.00	Activating signal cointegrator 1
MSTRG.15392	960	0.55	1.25	2.61	3.05	1.67	0.00	0.02	Uncharacterized protein LOC106130466
LOC113516334	899	2.17	2.06	7.6	5.72	1.67	0.00	0.01	Uncharacterized oxidoreductase SERP2049-like
LOC113523292	2992.84	51.31	42.53	129.02	162.43	1.67	0.00	0.00	Calnexin isoform X1
LOC113515259	5766	4.83	3.31	12.04	13.19	1.67	0.00	0.00	Uncharacterized protein LOC106132232
LOC113515508	1327.11	35.26	31.65	96.95	109.4	1.67	0.00	0.00	2-acylglycerol O-acyltransferase 2-A-like
LOC113519245	719	3.15	2.61	8.96	9.26	1.67	0.00	0.01	Protein FAM57A
LOC113517984	1830	1.73	0.95	5.14	3.2	1.67	0.00	0.01	Uncharacterized protein LOC108734152
AT056_gr02	1368	78.96	92.42	299.04	232.57	1.66	0.00	0.00	Uncharacterized protein
LOC113510874	4217	11.99	9.47	30.57	35.83	1.66	0.00	0.00	Furin-like protease 2 isoform X1
MSTRG.4875	3398.18	5.35	5.25	15.79	16.97	1.66	0.00	0.00	Uncharacterized protein
MSTRG.1873	5971.34	6.05	6.79	19.46	20.56	1.66	0.00	0.00	Ras-like protein 2
LOC113512924	758	34.17	44.71	132.83	112.62	1.66	0.00	0.00	39S ribosomal protein L33, mitochondrial
LOC113514427	964	0.79	0.89	2.69	2.54	1.65	0.01	0.03	Uncharacterized protein

									LOC106130714
LOC113513009	2981	0.49	0.5	1.02	2.04	1.65	0.00	0.01	Fasciclin-2 isoform X2
LOC113519360	983	1.15	0.78	3.01	3.04	1.65	0.00	0.02	23 kDa integral membrane protein-like
LOC113512577	2742	2.22	1.47	4.94	6.46	1.65	0.00	0.00	Dorsal 1b
LOC113517317	1390	4.21	1.65	9.01	9.17	1.65	0.00	0.00	Mediator of RNA polymerase II transcription subunit 13
LOC113518540	1608.03	26.09	27.79	78.76	81.42	1.65	0.00	0.00	Peptidoglycan-recognition protein LF-like isoform X1
LOC113509972	1505	0.98	0.85	2.97	2.68	1.65	0.00	0.01	Solute carrier family 25 member 35-like isoform X1
LOC113521220	1911	8.54	9.99	29.72	27.08	1.65	0.00	0.00	RNA exonuclease NEF-sp
LOC113511904	3370	0.16	0.32	0.82	0.63	1.64	0.01	0.02	Serine/threonine-protein kinase DDB_G0267686
LOC113519595	2114	4.44	3.28	11.5	12.1	1.64	0.00	0.00	RING finger and SPRY domain-containing protein 1-like
LOC113510817	1180	34.61	43.42	116.45	122.01	1.64	0.00	0.00	Alpha-tocopherol transfer protein-like isoform X2
LOC113513436	741	633.53	575.7	1798.12	1927.57	1.64	0.00	0.00	Venom carboxylesterase-6-like isoform X1
LOC113510014	474	40.14	29.78	108.61	112.09	1.64	0.00	0.00	Sortilin-related receptor isoform X1
LOC113518616	2434	1.3	1.97	4.98	4.92	1.64	0.00	0.01	Uncharacterized protein LOC106138515
LOC113523378	2070	4.95	5.48	17.12	14.53	1.63	0.00	0.00	Uncharacterized protein LOC106131292
LOC113511018	3352	13.87	17.21	44.15	49.97	1.63	0.00	0.00	Tubulointerstitial nephritis antigen precursor
LOC113510752	1807.85	323.26	374.96	1090.54	1186.06	1.63	0.00	0.00	Tropomyosin-2 isoform X2
LOC113522786	1993	1.43	0.97	4.21	3.09	1.63	0.00	0.01	Uncharacterized protein LOC106138639
MSTRG.11336	669	3.54	2.01	8.52	8.73	1.63	0.00	0.01	Gamma-1-syntrophin
LOC113510674	3930	17.04	18.84	53	55.35	1.63	0.00	0.00	Elongator complex protein 1 isoform X2
LOC113514898	1127	474.18	470.15	1318.02	1551.31	1.63	0.00	0.00	Venom carboxylesterase-6-like isoform X1
LOC113520864	524	57.91	41.65	139.21	170.73	1.63	0.00	0.00	Sortilin-related receptor-like
LOC113515770	2927	5.96	5.85	17.57	18.04	1.63	0.00	0.00	Uncharacterized protein LOC106114015

LOC113510993	852	5.72	5.82	16.43	18.8	1.62	0.00	0.01	Mitochondrial uncoupling protein Bmcp
LOC113520436	6287	10.01	7.67	24	29.2	1.62	0.00	0.00	Uncharacterized protein LOC110379888
LOC113509500	403	3.93	1.82	10.94	7.85	1.62	0.01	0.05	von Willebrand factor A domain-containing protein 8 isoform X1
MSTRG.14376	1458	2.91	3.01	7.86	10.06	1.62	0.00	0.01	Uncharacterized protein
LOC113519311	823	1.87	1.23	4.39	5.16	1.62	0.00	0.02	Myotubularin-related protein 10-B
LOC113513743	303	19.25	7.6	50.73	41.59	1.62	0.01	0.02	Kidney mitochondrial carrier protein 1
LOC113516264	2911.28	4.84	3.76	12.5	12.59	1.62	0.00	0.00	MOXD1 homolog 1-like
LOC113517036	3654	29.45	28.83	90.85	83.62	1.62	0.00	0.00	Protein unzipped
LOC113511140	1175	1.45	0.68	3.21	3.29	1.61	0.00	0.02	S-adenosylmethionine mitochondrial carrier protein homolog
LOC113515483	1150	54.71	68.37	189.84	179.12	1.61	0.00	0.00	Uncharacterized protein
LOC113523072	1628	2.22	1.91	5.8	6.56	1.61	0.00	0.01	Glypican-6 isoform X2
LOC113515836	1216	13.09	5.47	26.32	29.39	1.61	0.00	0.00	Uncharacterized protein LOC106137681
LOC113523023	3879	4.59	2.78	10.58	11.34	1.60	0.00	0.00	GTPase-activating protein
LOC113514906	758	75.49	64.91	193.92	228.86	1.60	0.00	0.00	Plexin domain-containing protein 2
LOC113510872	1272	3.17	0.49	4.49	6.61	1.60	0.00	0.01	Acyl-CoA Delta(11) desaturase-like
LOC113522477	1150	84.46	88.48	274.82	239.87	1.60	0.00	0.00	Uncharacterized protein LOC105388560
LOC113512639	1392	125.57	129.73	377.48	381.13	1.60	0.00	0.00	Serine protease easter-like
MSTRG.10058	348	113.58	131.64	427.08	353.56	1.60	0.00	0.00	Kunitz/Bovine pancreatic trypsin inhibitor domain protein
LOC113511427	1132	5.91	3.65	12.28	16.25	1.60	0.00	0.01	Tyrosine-protein phosphatase 69D
LOC113514144	741	14.22	13.61	34.6	48.89	1.59	0.00	0.01	Serine/threonine-protein kinase WNK3
LOC113515073	3514.14	17.91	19.77	56.23	53.96	1.59	0.00	0.00	Myb-binding protein 1A-like protein
LOC113515687	4643	0.21	0.14	0.46	0.6	1.59	0.01	0.02	Calcium-activated potassium channel slowpoke isoform X8
MSTRG.8904	663	22.12	15.83	53.51	60.33	1.59	0.00	0.01	Uncharacterized protein LOC106138074
LOC113522443	4370.9	16.69	15.32	43.33	50.82	1.59	0.00	0.00	Serine/threonine-protein kinase GA29083
MSTRG.11246	989	57.51	69.96	175.79	201.88	1.59	0.00	0.00	Uncharacterized protein

LOC113515997	1224	16.54	18.55	53.21	50.34	1.59	0.00	0.00	Protein FAM136A
LOC113519728	4346	33.07	33.22	97.94	96.43	1.59	0.00	0.00	N-acylneuraminate-9-phosphatase
LOC113521579	3578.82	61.17	62.17	181.29	178.9	1.59	0.00	0.00	1-acylglycerol-3-phosphate O-acyltransferase ABHD5-like isoform X2
LOC113517513	2918	36.33	39.38	109.29	112.41	1.58	0.00	0.00	ATP-binding cassette sub-family F member 3
LOC113522840	2452	50.64	59.29	162.15	159.28	1.58	0.00	0.00	Glycine--tRNA ligase
LOC113517150	1306	4.5	6.43	15.08	16.97	1.58	0.00	0.01	Uncharacterized protein LOC106110612
LOC113516055	2309	63.94	67.68	198.57	185.39	1.58	0.00	0.00	Serine/threonine-protein kinase RIO3
LOC113520822	4748	9.98	8.48	24.19	29.6	1.58	0.00	0.00	Tyrosine-protein phosphatase non-receptor type 4
LOC113522456	1578	164.81	188.45	528.36	502.23	1.58	0.00	0.00	Ras-like GTP-binding protein RhoL
LOC113520637	2274	11.05	12.71	33.57	35.61	1.57	0.00	0.00	Serine hydrolase
LOC113521110	535	2.24	4.41	7.35	12.48	1.57	0.01	0.03	E3 ubiquitin-protein ligase UBR3
LOC113511052	2350	57.5	61.18	175.2	170	1.57	0.00	0.00	Derlin-1
MSTRG.286	873	1.99	1.74	4.59	6.42	1.57	0.00	0.02	Centaurin-gamma-1A
LOC113517636	558	7.69	2.78	13.04	18.62	1.57	0.00	0.01	Protein outspread-like
LOC113513069	5694	24.1	23.76	65.44	73.22	1.57	0.00	0.00	Dual specificity tyrosine-phosphorylation-regulated kinase 1A isoform X1
LOC113515150	1039	1.71	1.84	4.68	5.73	1.57	0.00	0.02	Mevalonate kinase
LOC113522293	874	1.09	2.65	4.82	6.07	1.57	0.00	0.02	Solute carrier family 25 member 35-like isoform X1
LOC113523379	1546	17.85	17.15	46.75	55.03	1.57	0.00	0.01	Uncharacterized protein LOC106131151
MSTRG.16416	417	2.15	5.38	9.46	13.26	1.57	0.01	0.04	Max-binding protein MNT-like isoform X1
LOC113511400	520	43.3	41.33	119.45	131.87	1.56	0.00	0.01	Bifunctional glutamate/proline--tRNA ligase
LOC113511769	3350	1.46	2.26	5.36	5.38	1.56	0.00	0.01	PR domain Zinc finger protein 1
LOC113522480	2333	25.15	22.09	66.89	69.4	1.56	0.00	0.01	Glutamate--cysteine ligase catalytic subunit
LOC113522542	2759	34.6	24.15	75.35	94.26	1.56	0.00	0.01	Long-chain-fatty-acid--CoA ligase 4
LOC113516996	1183	12.9	13.91	40.15	37.5	1.56	0.00	0.01	MKRN2 opposite strand protein isoform X1

MSTRG.398	6044	4.26	3.85	10.5	12.81	1.56	0.00	0.01	Uncharacterized protein
LOC113519587	1769	9.19	8.88	27.61	24.48	1.56	0.00	0.01	Serine protease HTRA2, mitochondrial-like
LOC113521273	3787	16.19	18.04	50.47	47.75	1.56	0.00	0.01	FIT family protein CG10671
MSTRG.7529	604	3.41	2.03	7.9	8.21	1.55	0.01	0.03	Uncharacterized protein
LOC113520075	496	20.44	18.22	55.78	58.73	1.55	0.00	0.01	Beta-1,3-glucan recognition protein
LOC113513247	3052	17.64	16.89	48.8	49.87	1.55	0.00	0.01	Testis-expressed protein 2
LOC113515953	661	4.31	2.84	9.41	11.53	1.55	0.00	0.02	2-oxoglutarate/malate carrier protein
MSTRG.106	550	110.52	96.72	297.61	309.3	1.55	0.00	0.01	Asparagine--tRNA ligase, cytoplasmic
LOC113518829	2066	102.21	113.36	320.41	295.17	1.55	0.00	0.01	Glucosidase 2 subunit beta
LOC113519642	2927.78	133.62	138.15	374.02	402.67	1.55	0.00	0.01	Serine protease easter-like isoform X1
LOC113509314	1341	43.11	41.45	109.95	132.2	1.54	0.00	0.01	Asparagine--tRNA ligase, cytoplasmic
MSTRG.9989	2606	2.83	2.64	7.6	7.96	1.54	0.00	0.01	Insulin receptor substrate 1
MSTRG.12222	2605	12.69	11.35	34.07	34.15	1.54	0.00	0.01	Adapter molecule Crk isoform X1
LOC113510687	1882	54.58	53.62	150.66	156.76	1.54	0.00	0.01	Tumor necrosis factor, Alpha-induced protein 8-like protein 2 A
LOC113518820	16397	0.1	0.12	0.31	0.31	1.54	0.00	0.02	Titin
LOC113511386	1891	1.46	1.56	3.96	4.62	1.54	0.00	0.01	Spastin isoform X1
LOC113513823	2877.39	9.31	9.92	27.27	27.86	1.54	0.00	0.01	GPI inositol-deacylase
LOC113516615	3833.49	23.84	24.19	63.6	71.58	1.53	0.00	0.01	SUN domain-containing ossification factor
LOC113521229	979	19.59	24.08	65.41	58.43	1.53	0.00	0.01	Prefoldin subunit 4
LOC113515704	3268	22.81	20.9	60.5	62.53	1.53	0.00	0.01	Glycerol-3-phosphate dehydrogenase
LOC113521427	823	5.62	4.46	13.41	15.37	1.52	0.00	0.01	Mpv17-like protein 2
LOC113509403	5217.1	179.66	182.72	490.32	525.52	1.52	0.00	0.01	Uncharacterized protein LOC101742334 isoform X2
LOC113519808	6321	17.45	15.91	45.02	48.33	1.52	0.00	0.01	Protein pellino
LOC113510223	545	4.71	4.25	11.64	14.26	1.52	0.01	0.02	Tafazzin homolog
LOC113509793	570	3.97	3.08	10.2	10.07	1.51	0.01	0.02	Uncharacterized protein OBRU01_15671
LOC113517211	3725.5	45.98	47.63	128.67	132.36	1.51	0.00	0.01	Nitric oxide synthase
LOC113513377	497	29.81	23.28	87.44	65.17	1.51	0.00	0.01	Hypothetical protein KGM_213212
LOC113519476	828	9.29	7.51	19.25	28.26	1.51	0.00	0.01	Hypothetical protein 2 - silkworm

									transposon mag
LOC113512540	2304	5.73	4.55	12.53	16.13	1.51	0.00	0.01	Signal-induced proliferation-associated 1-like protein 1
LOC113516462	1757	17.24	14.3	43.73	44.24	1.51	0.00	0.01	Adenosine monophosphate-protein transferase FICD homolog
LOC113509350	1236	1.13	1.4	2.43	4.69	1.51	0.01	0.02	Uncharacterized protein LOC110382040
MSTRG.3286	3117	8.23	10.33	25.34	26.08	1.50	0.00	0.01	Organic cation transporter protein
LOC113515459	1825.72	1.03	1.36	3.93	2.82	1.50	0.00	0.02	Sodium-dependent multivitamin transporter
LOC113514764	745	4.47	0.91	8.32	7.03	1.50	0.00	0.02	Lysine-specific demethylase lid
LOC113515288	1147	0.87	2.39	5.44	3.55	1.50	0.01	0.02	Nose resistant to fluoxetine protein 6-like
LOC113510926	1708	16.23	9.43	33.49	37.54	1.50	0.00	0.01	Protein mahjong
LOC113522228	572	6.63	3.06	12.66	15.01	1.50	0.00	0.02	FERM domain-containing protein 5
LOC113513542	1764	4.34	5.12	13.41	12.69	1.50	0.00	0.01	Uncharacterized protein LOC106142389
LOC113518262	4177	1.54	1.8	4.74	4.43	1.49	0.00	0.01	Tyrosine-protein kinase Drl
LOC113514041	961	19.62	12.67	41.28	48.29	1.49	0.00	0.01	Nuclear protein localization protein 4 homolog
LOC113512465	1035	8.44	11.28	29.85	24.53	1.49	0.00	0.01	Transmembrane protein 177
MSTRG.2528	1650	1.26	1.01	2.34	4	1.49	0.00	0.02	PAX3- and PAX7-binding protein 1
LOC113512136	3699.47	2.43	3.05	7.05	7.91	1.49	0.00	0.01	Myosin light chain kinase, smooth muscle-like
LOC113512650	3158.81	123.32	133.99	337.58	365.99	1.48	0.00	0.01	Calphotin-like isoform X2
LOC113520819	3306	0.29	0.28	0.93	0.64	1.48	0.01	0.03	MutS protein homolog 4-like isoform X2
LOC113522789	1568	2.58	3.23	7.03	8.89	1.48	0.00	0.01	FGFR1 oncogene partner-like
LOC113523090	4528	46.43	43.43	118.49	126.59	1.48	0.00	0.01	Gibberellin 20 oxidase 2
LOC113519555	1533	9.95	10.54	27.86	28.17	1.48	0.00	0.01	Krueppel-like factor 7 isoform X1
LOC113512095	998	10.97	11.26	32.14	28.87	1.48	0.00	0.01	fatty acid-binding protein, muscle-like
LOC113517455	964	39.71	46.99	123.49	114.24	1.48	0.00	0.01	RNA-binding protein 18
LOC113514889	630	11.91	8.21	26.23	29.85	1.48	0.00	0.01	Phospholipid-transporting ATPase VD
LOC113511187	903	2.24	1.75	5.36	5.68	1.48	0.01	0.03	Hypothetical protein RR46_05501
MSTRG.6925	1544	1.64	1.41	4.47	3.87	1.48	0.00	0.02	Endonuclease-reverse transcriptase

LOC113522681	1589	22.68	23.7	59.58	66.53	1.47	0.00	0.01	DnaJ homolog subfamily C member 3
LOC113510765	744	9.31	11.7	24.28	33.49	1.47	0.00	0.01	Lon protease homolog, mitochondrial-like
MSTRG.10612	395	22.44	26.03	77.99	60.07	1.47	0.00	0.01	Transmembrane protein 205
LOC113511232	2411.78	64.48	65.6	165.06	182.73	1.47	0.00	0.01	BAG domain-containing protein Samui-like
LOC113515353	1161	3.5	2.77	7.14	9.95	1.46	0.00	0.02	Aquaporin-like
LOC113513982	830	41.13	41.16	102.44	121.87	1.46	0.00	0.01	Galectin-9
LOC113516019	1077	203.56	241.84	637.07	569.08	1.46	0.00	0.01	Calcyclin-binding protein
LOC113516282	664	24.27	25.18	69.93	65.38	1.46	0.00	0.01	COX assembly mitochondrial protein homolog
LOC113514685	1004	11.55	9.15	27.72	28.54	1.46	0.00	0.01	Mitochondrial fission process protein 1
LOC113514336	3092.83	21.44	24.54	66.05	56.16	1.46	0.00	0.01	Facilitated trehalose transporter Tret1-like
LOC113509250	448	6.88	2.41	14.48	12.1	1.46	0.01	0.04	Bumetanide-sensitive sodium-(potassium)-chloride cotransporter isoform X1
LOC113515444	1947	0.59	1.03	2.14	2.21	1.46	0.01	0.03	Zinc finger protein Elbow
LOC113510501	1721	42.64	37.94	106.63	110.58	1.46	0.00	0.01	Rho GTPase-activating protein 21-A-like isoform X1
LOC113518748	2965.14	104.66	105.69	295.61	323.8	1.46	0.00	0.01	Serine/threonine-protein kinase dyrk2 isoform X1
LOC113516266	2159	1.98	2.09	6.36	4.57	1.46	0.00	0.01	Hexamerin
LOC113514240	3755	0.22	0.32	0.77	0.68	1.46	0.01	0.04	Muscle M-line assembly protein unc-89-like
LOC113514217	824	3.15	4.23	11.26	8.71	1.46	0.00	0.02	Uncharacterized protein LOC106130712
LOC113512276	1236	1.02	1.47	2.79	3.92	1.46	0.01	0.03	Kinesin-like protein KIF19 isoform X1
LOC113513369	1367	5.15	4.38	12.61	13.06	1.45	0.00	0.01	Uncharacterized protein LOC106129477
LOC113510648	4640	39.92	37.03	99.7	106.04	1.45	0.00	0.01	Ubiquilin-1
LOC113513706	1436	1.51	0.58	2.43	3.24	1.45	0.01	0.03	Protein crumbs
MSTRG.12191	500	4.2	3.21	12.41	8.11	1.45	0.01	0.04	Uncharacterized protein
LOC113519799	951	1.85	1.36	5.08	3.59	1.45	0.01	0.03	Serine protease easter-like
LOC113518753	2222	17.68	18.79	48.9	48.33	1.45	0.00	0.01	Ras-related C3 botulinum toxin

									substrate 1
LOC113513848	2670	8.13	10.02	23.25	25.09	1.45	0.00	0.01	DNA-directed RNA polymerase III subunit RPC5
LOC113510575	2072	1.31	1.81	4.03	4.3	1.45	0.00	0.02	Uncharacterized protein LOC110380322 isoform X2
LOC113523403	5868	26.88	24.14	64.8	70.8	1.45	0.00	0.01	Phosphatidylcholine:ceramide cholinephosphotransferase 2-like isoform X1
LOC113512927	1354	4.6	4.72	12.76	12.16	1.45	0.00	0.01	Mitochondrial tRNA-specific 2-thiouridylase 1 isoform X1
LOC113516799	3692	0.5	0.49	1.15	1.47	1.44	0.01	0.03	Glucose transporter type 1 isoform X9
LOC113513501	1429	3.69	3.99	11.15	9.27	1.44	0.00	0.01	Atrial natriuretic peptide receptor 1 isoform X1
LOC113510876	882.53	3.61	4.3	8.9	12.19	1.44	0.00	0.02	Endoprotease FURIN
LOC113516162	1321	5.63	6.92	17.5	15.89	1.44	0.00	0.01	Uncharacterized protein LOC106138810
LOC113511934	1444	4.49	3.94	11.19	11.22	1.44	0.00	0.01	Atrial natriuretic peptide receptor 2
LOC113515946	2210	104.13	124.05	318.49	285.17	1.44	0.00	0.01	ADP-ribosylation factor 2
LOC113509757	1236	4.54	5.11	15.61	9.94	1.44	0.00	0.02	Uncharacterized protein LOC106135696
LOC113518632	836	13.98	12.52	35.62	35.19	1.43	0.00	0.01	Hsp70-binding protein 1
MSTRG.8891	2074	20.71	21.94	51.08	61.69	1.43	0.00	0.01	Uncharacterized protein
LOC113519469	19573	9.45	9.03	22.12	26.52	1.43	0.00	0.01	Uncharacterized protein LOC106135241 isoform X1
LOC113510044	645	35.62	25.3	79.24	84.43	1.43	0.00	0.01	Protein mahjong
LOC113515918	866	25.2	15.82	50.67	58.47	1.43	0.00	0.01	Protein mahjong isoform X1
LOC113514387	647	31.55	30.23	85.81	79.26	1.43	0.00	0.01	28S ribosomal protein S25, mitochondrial
LOC113516834	1323	205.66	159.42	460.39	502.8	1.43	0.00	0.01	Krueppel-like factor 10
LOC113515352	7022.74	8.31	6.46	17.53	21.26	1.42	0.00	0.01	Uncharacterized protein LOC106142867
LOC113523330	5421	9.03	9.85	24.16	25.08	1.42	0.00	0.01	Uncharacterized protein LOC106131362
LOC113512293	1127	20.7	25.18	64.58	55.69	1.42	0.00	0.01	Post-GPI attachment to proteins factor 2-like
LOC113521966	1996	3.63	1.86	7.04	7.34	1.42	0.00	0.02	Catenin Alpha-like
LOC113514606	726	2.63	3.51	9.73	6.43	1.41	0.01	0.03	Insecticyanin-A

LOC113513724	3978	22.44	25.39	63	61.2	1.41	0.00	0.01	Metalloprotease
LOC113509119	2032.72	7.1	6.86	16.4	18.82	1.41	0.00	0.01	Phospholipid-transporting ATPase IA
LOC113514690	3420	8.35	8.08	19.18	23.55	1.41	0.00	0.01	Phosphatidylinositol 4-kinase alpha isoform X1
LOC113520100	242	97.47	90.46	241.1	333.67	1.41	0.01	0.03	Bifunctional glutamate/proline--tRNA ligase-like
LOC113517313	6656	10.23	6	18.77	23.32	1.41	0.00	0.01	Protein outspread
LOC113510529	1855	2.39	2.02	5.67	5.83	1.41	0.00	0.02	Protein phosphatase 1 regulatory subunit 3B-B
LOC113516683	1597	2.82	3.35	9.33	6.64	1.41	0.00	0.02	JmjC domain-containing protein 4
LOC113522165	525	15.85	9.41	34.66	32.98	1.41	0.00	0.02	Lysine-specific demethylase lid
LOC113519773	1793	21.24	20.2	51.36	55.85	1.40	0.00	0.01	CncC
LOC113520262	3095	45.33	43.11	105.91	122.41	1.40	0.00	0.01	Uncharacterized protein LOC106131566 isoform X3
LOC113516109	955.9	3.16	2.35	7.71	7.52	1.40	0.01	0.03	Androgen-dependent TFPI-regulating protein-like
MSTRG.9856	1142	41.51	43.62	105.91	114.64	1.40	0.00	0.01	LIM and SH3 domain protein Lasp isoform X1
LOC113520485	1083.84	34.95	34.7	82.27	95.69	1.40	0.00	0.01	Ras-related protein Ral-a isoform X2
LOC113510592	925	17.44	22.3	53.17	49.73	1.40	0.00	0.02	Cytochrome c oxidase assembly protein COX16 homolog, mitochondrial
MSTRG.7593	1565	18.94	12.85	36.72	45.35	1.39	0.00	0.01	Uncharacterized protein
LOC113511401	355	51	44.75	113.86	152.99	1.39	0.00	0.02	Sortilin-related receptor-like
MSTRG.7609	732.24	8.27	5.76	14.65	22.85	1.39	0.00	0.02	E3 ubiquitin-protein ligase UBR4
LOC113513817	627	6.08	9.49	21.2	19.36	1.39	0.01	0.02	Beta-1,3-glucan recognition protein
LOC113516270	1070	14.16	14.87	39.44	35.47	1.39	0.00	0.02	Uncharacterized protein LOC106709791
LOC113511421	2058	16.56	14.38	37.01	42.49	1.39	0.00	0.01	Protein yellow-like
MSTRG.6498	1991	5.27	5.52	12.4	15.32	1.39	0.00	0.02	Uncharacterized protein
LOC113521066	7501	35.59	29.45	80.21	86.07	1.39	0.00	0.01	Uncharacterized protein LOC110372636 isoform X6
LOC113518345	1978	245.7	252.98	627.88	649.07	1.39	0.00	0.01	Protein disulfide-isomerase A6
LOC113519745	2644	29.53	35.27	82.76	82.81	1.39	0.00	0.01	Uncharacterized protein LOC110373384
LOC113523085	2740	68.44	73.99	181.56	182.15	1.39	0.00	0.01	Hypothetical protein KGM_208650

LOC113515576	480	109.18	133.03	329.44	308.32	1.38	0.00	0.02	Cytochrome oxidase subunit 2(mitochondrion)
LOC113511042	1535	18.87	23.64	53.27	55.46	1.38	0.00	0.02	Protein NDUFAF4 homolog
LOC113514881	593	49.8	65.78	156.41	142.89	1.38	0.00	0.02	Uncharacterized protein LOC106142650
LOC113517288	1080.77	29.94	30.96	79.31	65.99	1.38	0.00	0.02	Mpv17-like protein
LOC113519921	5508	1.08	0.42	1.96	1.87	1.38	0.01	0.02	E3 ubiquitin-protein ligase UBR3
LOC113513321	1468	1.19	2.42	4.21	4.96	1.38	0.01	0.03	Inorganic phosphate cotransporter isoform X2
LOC113519015	757	7.27	8.61	21.21	19.58	1.38	0.01	0.02	Pre-mRNA-splicing factor 18
MSTRG.1928	3708	3.44	2.51	7.25	7.87	1.38	0.00	0.02	Protein crumbs
LOC113511419	738	116.27	139.58	350.12	306.49	1.38	0.00	0.02	G-protein coupled receptor Mth2-like isoform X2
LOC113510661	3936	47.95	53.57	128.6	128.58	1.38	0.00	0.02	Isoleucine--tRNA ligase, cytoplasmic
LOC113516241	267	176.05	166.95	491.09	505.26	1.38	0.00	0.02	Canopy-1 like protein
LOC113510192	3471.68	23.6	22.29	54.54	61.84	1.37	0.00	0.02	Kinesin-like protein KIF3A isoform X1
MSTRG.11819	656	3.24	1.92	6.29	7.08	1.37	0.01	0.05	Uncharacterized protein
MSTRG.8601	6734.13	29.98	24.34	62.07	74.73	1.37	0.00	0.02	Uncharacterized protein
LOC113515023	1600	19.4	24.11	60.33	49.33	1.37	0.00	0.02	Serine--tRNA ligase, mitochondrial
LOC113516656	4389	16.64	18.91	45.22	44.08	1.36	0.00	0.02	Abhydrolase domain-containing protein 2
MSTRG.11854	1605	271.14	324.62	725.97	776.53	1.36	0.00	0.02	Uncharacterized protein
LOC113522591	2910.76	55.77	55.99	150.91	134.19	1.36	0.00	0.02	Multiple C2 and transmembrane domain-containing protein 1-like
LOC113513335	858	8.64	8.18	19.44	23.34	1.36	0.00	0.02	R3H domain-containing protein 2 isoform X1
LOC113518338	956.2	0.16	5.89	7.58	7.61	1.36	0.01	0.03	23 kDa glycoprotein Seroin
LOC113518369	2297	38.12	44.41	105.84	101.13	1.36	0.00	0.02	Uncharacterized protein C45G9.7
LOC113515165	834	3.1	3.17	6.26	9.64	1.36	0.01	0.04	Rhomboid-related protein 2-like
LOC113520358	835	34.87	44.62	100.43	100.21	1.36	0.00	0.02	Proteasome assembly chaperone 2
LOC113519838	9347	11.07	7.47	20.37	25.89	1.35	0.00	0.02	Myosin-I heavy chain-like
LOC113522856	483	161.3	180.76	453	428.03	1.35	0.00	0.02	Vesicle-trafficking protein SEC22b-B
LOC113509556	684	10.51	9.08	23.97	25.72	1.35	0.01	0.03	Transmembrane protein 135-like
MSTRG.15170	5820	2.75	2.11	5.83	6.26	1.35	0.00	0.02	Beta-1-syntrophin
LOC113521180	1409.07	15.51	19.15	36.57	41.58	1.35	0.00	0.02	Protein N-terminal asparagine amidohydrolase isoform X1

LOC113515108	1838	1.19	1.69	3.57	3.61	1.35	0.01	0.03	Uncharacterized protein LOC106129912
LOC113516645	1112	37.98	43.78	95.97	108.79	1.35	0.00	0.02	NADH dehydrogenase
MSTRG.8781	6071	4.73	5.09	11.98	12.41	1.35	0.00	0.02	Glucuronyltransferase I
LOC113516935	4157	7.72	5.68	15.1	18.24	1.35	0.00	0.02	Uncharacterized protein LOC106133603
LOC113509995	2534	11.1	6.78	20.59	23.86	1.34	0.00	0.02	Tyrosine-protein phosphatase 69D
LOC113509073	1519	26.71	29.22	77.92	60.98	1.34	0.00	0.02	Uncharacterized protein C6orf106 homolog
LOC113521536	11788	0.25	0.2	0.5	0.62	1.34	0.01	0.03	Low-density lipoprotein receptor-related protein 2
LOC113519083	4925	11.26	5.7	20.46	21.54	1.34	0.00	0.02	Mitogen-activated protein kinase kinase kinase 4
LOC113509440	1533	1.09	1.17	2.85	2.78	1.34	0.01	0.04	Protein peste-like isoform X1
LOC113511595	4528.84	73.77	65.15	155.11	180.56	1.34	0.00	0.02	2-oxoglutarate dehydrogenase, mitochondrial isoform X4
LOC113513096	1059	4.51	2.81	9.65	8.67	1.34	0.01	0.03	Bumetanide-sensitive sodium-(potassium)-chloride cotransporter isoform X1
LOC113519875	1378	9.6	10.74	23.96	26.59	1.34	0.00	0.02	Tryptophan--tRNA ligase, cytoplasmic
LOC113515500	806	40.81	52.55	121.53	111.67	1.34	0.00	0.02	39S ribosomal protein L32, mitochondrial
LOC113517194	1325	10.71	10.23	25.36	26.65	1.34	0.00	0.02	Lethal(2) giant larvae protein
MSTRG.12111	7576	2.43	2.42	5.38	6.6	1.34	0.00	0.02	Uncharacterized protein
LOC113512373	6419.33	3.73	2.99	7.1	9.26	1.34	0.00	0.02	Down syndrome cell adhesion molecule-like protein Dscam2
LOC113516166	4648	36.32	29.83	72.78	90.54	1.34	0.00	0.02	Niemann-Pick C1 protein-like
LOC113521118	2041	1.12	0.45	1.76	2.17	1.34	0.01	0.04	Hypothetical protein KGM_206502
LOC113520987	408	44.42	38.66	115.16	100.89	1.34	0.01	0.02	Acyl-CoA Delta(11) desaturase isoform X1
MSTRG.11162	1467.33	1.8	1.62	4.81	3.84	1.34	0.01	0.03	Uncharacterized protein
LOC113522593	2001	21.55	19.95	53.72	48.69	1.34	0.00	0.02	Neuronal membrane glycoprotein M6-a
LOC113521451	4835	15.72	16.51	39.6	39.65	1.33	0.00	0.02	Uncharacterized protein LOC101736230
LOC113517542	749.17	56.77	67.39	163.7	142.58	1.33	0.00	0.02	DNA-directed RNA polymerase III subunit RPC9-like isoform X1

LOC113510387	2363	18.59	19.68	49.93	44.26	1.33	0.00	0.02	Transcription factor Y subunit alpha isoform X2
LOC113509424	959	14.52	18.43	42.6	38.78	1.33	0.01	0.02	Uncharacterized protein OBRU01_26608
LOC113521863	2344	12.01	6.12	20.55	24.04	1.33	0.00	0.02	Flagellar attachment zone protein 1
LOC113514935	3005	3.54	3.7	9.29	8.44	1.33	0.01	0.02	DNA-directed RNA polymerase, mitochondrial isoform X1
LOC113511891	341	70.73	54.04	164.84	168.88	1.33	0.01	0.03	N-ethylmaleimide sensitive fusion protein
MSTRG.15252	3976	1.96	1.45	3.76	4.59	1.33	0.01	0.03	Uncharacterized protein
LOC113516974	1129.37	16.55	20.56	47.78	42.38	1.32	0.01	0.02	NADH dehydrogenase (ubiquinone) complex I, assembly factor 6 isoform X1
LOC113517643	4177	51.33	47.84	118.13	124.17	1.32	0.00	0.02	BMP-binding endothelial regulator protein-like
LOC113520421	556	32.68	34.44	92.16	75.49	1.32	0.01	0.02	DPH3
LOC113515377	979	39.65	45.99	112.28	98.22	1.32	0.00	0.02	Ommochrome-binding protein-like
MSTRG.9899	559	5.42	11.29	19.25	22.36	1.32	0.01	0.04	Hypothetical protein
LOC113522838	834.8	4.33	5.7	13.45	11.55	1.32	0.01	0.04	Small integral membrane protein 8
LOC113517569	4283.96	75.29	66.44	153.78	189.03	1.32	0.00	0.02	Sarco/endoplasmic reticulum calcium ATPase
LOC113511953	3101.48	67.22	78.07	177.08	177.63	1.32	0.00	0.02	Elongation of very long chain fatty acids protein AAEL008004 isoform X1
LOC113517316	1440	5.63	1.95	8.04	10.62	1.32	0.01	0.03	Mediator of RNA polymerase II transcription subunit 13
LOC113514391	3167.31	86.18	74.29	189.31	200.88	1.32	0.00	0.02	C-terminal-binding protein
LOC113519637	1273	3.5	4.43	10.76	8.56	1.31	0.01	0.03	Lipopolysaccharide-induced tumor necrosis factor-alpha factor homolog
LOC113514549	1811.23	39.27	36.73	87.87	97.03	1.31	0.00	0.02	Uncharacterized protein LOC110381915 isoform X1
LOC113512995	1256	4.72	5.75	10.8	14.81	1.31	0.01	0.03	Uncharacterized protein LOC106136292
LOC113512426	1910	10.78	11.8	29.53	25.37	1.31	0.01	0.02	CMP-sialic acid transporter 1
LOC113521052	924	27.49	24.6	58.65	69.1	1.31	0.01	0.02	Uncharacterized protein LOC106139651
LOC113513233	2152	4.93	1.87	6.35	10.24	1.31	0.01	0.03	Baculoviral IAP repeat-containing protein 6

LOC113519414	4088	3.06	2.62	5.89	7.86	1.31	0.01	0.03	Potassium channel subfamily K member 9-like
LOC113513308	525	145.38	168.14	395.5	383.34	1.31	0.01	0.02	Uncharacterized protein LOC106143374
MSTRG.5596	1584	48.49	53.07	128.13	117.75	1.31	0.01	0.02	Reverse transcriptase
LOC113518758	1656	12.16	15.93	32.91	35.01	1.30	0.01	0.02	Uncharacterized protein LOC106122557
LOC113521980	3817.7	174.02	172.98	403.72	433.48	1.30	0.01	0.02	Synaptic vesicle membrane protein VAT-1 homolog-like isoform X1
LOC113516614	4071	71.79	61.99	150.91	171.23	1.30	0.01	0.02	Tetratricopeptide repeat protein 14 homolog isoform X4
LOC113516119	1375	176.33	213.58	489.42	452.33	1.30	0.01	0.02	EF-hand domain-containing protein D2 homolog
LOC113511317	5137	2.71	1.9	6.01	5.09	1.30	0.01	0.03	Ras-responsive element-binding protein 1
LOC113522996	4811	11.46	15.34	31.8	32.59	1.30	0.01	0.02	Golgin subfamily A member 4-like
LOC113513781	690	11.91	10.12	23.29	30.71	1.30	0.01	0.03	Uncharacterized protein LOC110372689 isoform X4
LOC113518303	1651	16.81	13.48	33.18	39.98	1.30	0.01	0.02	Sorting nexin-17
LOC113519603	753	191.1	217.03	494.79	498.48	1.30	0.01	0.02	Uncharacterized protein LOC106136193
LOC113510611	2861	17.07	15.18	37.87	39.61	1.30	0.01	0.02	Glycosylated lysosomal membrane protein B-like
LOC113513163	2495	37.66	40.82	90.76	97.79	1.30	0.01	0.02	AFG3-like protein 2
LOC113517298	4936	27.47	25.57	61.7	68.7	1.30	0.01	0.02	Ecdysone receptor isoform X1
MSTRG.14322	364	31.47	41.72	92.8	94.68	1.29	0.01	0.03	Hypothetical protein RR46_08551
LOC113514659	1086	21.74	12.84	39.09	44.4	1.29	0.01	0.03	Protein mahjong
LOC113515482	1456	5.37	6.71	14.11	14.81	1.29	0.01	0.03	Phosphatidylinositol-glycan biosynthesis class F protein
LOC113517197	1362	2.71	1.52	4.24	5.94	1.28	0.01	0.04	Exportin-5
LOC113516020	1158.74	41.74	44.31	102.76	104.01	1.28	0.01	0.03	Dihydrolipoyllysine-residue acetyltransferase component of pyruvate dehydrogenase complex, mitochondrial isoform X1
LOC113509455	5197	0.87	0.43	1.75	1.34	1.28	0.01	0.04	Hemicentin-1-like
LOC113515680	3214	56.17	41.01	106.38	124.66	1.28	0.01	0.03	Disintegrin and metalloproteinase domain-containing protein 10
LOC113512885	1822	76.79	78.34	180.71	188.36	1.28	0.01	0.03	G-protein coupled receptor moody-

									like
LOC113517802	811	9.08	4.1	13.83	17.98	1.28	0.01	0.04	Fibrillin-1-like
LOC113511908	7198	5.61	5.53	11.8	14.55	1.28	0.01	0.03	DNA N6-methyl adenine demethylase
LOC113520279	2235	5.17	5.24	10.76	13.92	1.27	0.01	0.03	Msx2-interacting protein-like isoform X1
LOC113517799	1279	19.88	15.03	35.78	47.31	1.27	0.01	0.03	Phosphatidylinositol 4-kinase alpha
LOC113509486	530	16.59	9.47	30.78	32.86	1.27	0.01	0.04	Nodal modulator 1
MSTRG.15180	1232.64	37.73	41.98	106.15	89.44	1.27	0.01	0.03	Uncharacterized protein
LOC113509626	1651	14.75	19.02	39.93	39.73	1.27	0.01	0.03	Nuclear receptor-binding factor 2-like
LOC113522759	1068.27	42.46	54.44	125.54	103.43	1.27	0.01	0.03	Surfeit locus protein 6 homolog
LOC113516080	1924	21.92	20.8	50.42	50.23	1.27	0.01	0.03	Runt-related Transcription factor 3
LOC113520240	557	30.31	23.59	64.22	65.88	1.27	0.01	0.03	Eukaryotic translation initiation factor 2-alpha kinase-like
LOC113510218	3146	33.22	35.89	83.98	78.34	1.27	0.01	0.03	Ras-related protein Rab-18-B isoform X1
LOC113516632	2393	13.44	15.36	37.02	30.56	1.26	0.01	0.03	Glucose-6-phosphate 1-dehydrogenase
LOC113519999	1094	3.12	3.44	8.9	6.57	1.26	0.01	0.05	DAXX
LOC113509389	1668.2	39.7	32.43	77.91	90.16	1.26	0.01	0.03	Homocysteine-responsive endoplasmic reticulum-resident ubiquitin-like domain member 2 protein
LOC113520849	2075	14.64	15.27	31.84	38.39	1.26	0.01	0.03	WAS/WASL-interacting protein family member 2-like isoform X1
LOC113518176	3056.31	101.29	89.7	198.3	223.16	1.26	0.01	0.03	Dm0-like lamin
LOC113517439	939	250.84	261.03	589.39	619.21	1.26	0.01	0.03	CD63 antigen-like isoform X1
LOC113515355	1935	71.16	74.47	175.81	165.36	1.26	0.01	0.03	5-aminolevulinate synthase, nonspecific, mitochondrial
LOC113521578	2421	223.03	234.28	523.18	546.73	1.26	0.01	0.03	Transcription factor jun-D
LOC113516012	1490	11.06	13.29	30.65	26.4	1.26	0.01	0.03	Iron-sulfur cluster assembly 2 homolog, mitochondrial
LOC113519445	5368.37	7.37	7.19	15.61	18.32	1.26	0.01	0.03	Latrophilin Cirl
LOC113509393	1475.75	225.93	275.14	584.31	590.54	1.26	0.01	0.03	Serine protease easter-like
LOC113509109	1505	13.64	18.22	39.63	34.94	1.26	0.01	0.03	Uncharacterized protein LOC106131182

LOC113516075	715	206.48	260.47	597.12	505.54	1.26	0.01	0.03	Glutaredoxin-C4-like
LOC113519981	5222.69	13.56	13.54	31.53	31.75	1.25	0.01	0.03	C-MYC
LOC113521918	2007	165.19	162.57	364.51	400.96	1.25	0.01	0.03	Guanine nucleotide-binding protein G(o) subunit alpha isoform X1
LOC113511975	1671	6.39	6.29	15.98	13.63	1.25	0.01	0.03	Ribosome biogenesis protein TSR3 homolog
LOC113520458	582	23.42	16.97	44.56	51.91	1.25	0.01	0.04	Asparagine--tRNA ligase, cytoplasmic
LOC113511703	3456	22.28	20.24	47.22	51.65	1.25	0.01	0.03	Uncharacterized protein LOC106143543
LOC113515513	2118	38.27	35.1	88.33	82.21	1.25	0.01	0.03	Protein LMBR1L
MSTRG.8392	2020	3.92	2.75	6.55	9.01	1.25	0.01	0.04	Lysine-specific demethylase lid
LOC113517347	751	23.45	17.42	44.68	51.55	1.25	0.01	0.04	LIM and SH3 domain protein 1 isoform X2
LOC113511593	1548	229.47	282.46	613.31	576.1	1.25	0.01	0.03	SH3 domain-binding glutamic acid-rich protein homolog
LOC113515992	2055	2.34	1.66	4.06	5.25	1.25	0.01	0.04	Phosphatidylinositol 3,4,5-trisphosphate 3-phosphatase and dual-specificity protein phosphatase PTEN isoform X1
LOC113509852	4225	90.24	96.99	209.1	223.95	1.24	0.01	0.03	Inhibitor of apoptosis protein
LOC113521362	630	43.41	57.15	124.42	111.97	1.24	0.01	0.03	Multifunctional methyltransferase subunit TRM112-like protein
LOC113514222	3384	11.75	13.13	28.29	29.2	1.24	0.01	0.03	Uncharacterized protein LOC106140037
LOC113519521	1999	4.16	3.92	8.91	9.83	1.24	0.01	0.04	Protein PFC0760c-like isoform X2
LOC113509846	2020	16.14	18.29	41.29	38.29	1.24	0.01	0.03	Testis-expressed sequence 10 protein homolog
LOC113511473	934	20.66	20.05	48.38	46.39	1.24	0.01	0.04	Trehalase-2
LOC113511004	1501	6.24	5.96	12.13	16.18	1.24	0.01	0.04	Syntaxin-16
LOC113517470	872	28.67	42.29	92.85	71.87	1.24	0.01	0.03	Alcohol dehydrogenase AD1
LOC113520924	4754	9.8	8.48	19.43	22.7	1.24	0.01	0.03	La-related protein 1-like isoform X2
LOC113518098	1397	52.61	59.35	134.9	123.56	1.24	0.01	0.03	Immunoglobulin-binding protein 1
MSTRG.16346	2047	2.16	2.05	4.08	5.65	1.24	0.01	0.05	Uncharacterized protein
MSTRG.7576	1014	16.62	15.33	33.95	40.17	1.24	0.01	0.04	Phosphatidylinositol 4-kinase alpha isoform X1
LOC113512819	3273.3	14.53	16.11	35.66	34.16	1.24	0.01	0.03	Myb-like protein I

LOC113519316	1009	10.81	7.92	21.82	21.55	1.23	0.01	0.04	Uncharacterized protein LOC106129825
LOC113511455	2161	14.82	16.4	35.1	36.6	1.23	0.01	0.03	Uncharacterized protein LOC106139975
LOC113511128	2600	15.62	18.1	38.98	38.27	1.23	0.01	0.03	Uridine 5'-monophosphate synthase
LOC113520676	550	61.53	65.73	161.23	136.84	1.23	0.01	0.04	Transcription elongation factor SPT4 isoform X1
LOC113511286	3220	13.87	11.87	28.91	30.03	1.23	0.01	0.03	TBC1 domain family member 16 isoform X4
LOC113509215	3364.05	7.32	7.61	16.62	17.53	1.23	0.01	0.04	Lethal(2) giant larvae protein isoform X1
MSTRG.6831	7833	1.45	1.3	3.25	3	1.22	0.01	0.04	Uncharacterized protein
LOC113514493	1355	74.34	55.06	138.92	157.71	1.22	0.01	0.04	Zinc transporter ZIP13 homolog
LOC113515476	1530.68	27.35	32.52	73.38	62.13	1.22	0.01	0.04	Major facilitator superfamily domain-containing protein 9 isoform X1
LOC113513266	987	9.23	7.91	18.73	20.61	1.22	0.01	0.04	Lon protease homolog, mitochondrial isoform X1
LOC113522714	578	19.85	19.19	44.91	45.91	1.22	0.01	0.04	E3 ubiquitin-protein ligase RNF180-like
LOC113522666	1252	19.81	24.61	53.1	48.27	1.22	0.01	0.04	Protein archease-like
LOC113512093	1867	5.92	4.25	11.16	11.99	1.22	0.01	0.04	Hypothetical protein KGM_205821
MSTRG.11376	880	13.07	18.9	38.49	34.62	1.22	0.01	0.04	Uncharacterized protein
LOC113517032	779	22.28	25.18	57.6	51.21	1.22	0.01	0.04	28S ribosomal protein S18b, mitochondrial
LOC113523560	4870	17.42	18.58	41.25	40.25	1.21	0.01	0.04	Uncharacterized protein LOC106131089
LOC113509198	659	749.62	765.77	1724.58	1765.26	1.21	0.01	0.04	Uncharacterized protein LOC105389192
MSTRG.4535	7977	1.99	1.77	3.98	4.52	1.21	0.01	0.04	Uncharacterized protein
LOC113521225	3184	41.74	48.06	102.98	99.59	1.21	0.01	0.04	Neurofilament heavy polypeptide-like
MSTRG.248	3587	1.92	1.6	3.62	4.34	1.21	0.01	0.05	Uncharacterized protein
LOC113520286	5330.41	12.53	10.42	25	26.7	1.21	0.01	0.04	UPF0518 protein GF15501 isoform X1
LOC113518408	1360.31	540.88	580.08	1285.92	1257.95	1.21	0.01	0.04	Protein tyrosine phosphatase type IVA 1
LOC113512212	2478	11.25	7.18	18.92	22.67	1.20	0.01	0.04	Rho GTPase-activating protein 26
LOC113520110	1129	61.77	50.24	112.67	141.21	1.20	0.01	0.04	Niemann-Pick C1 protein-like

LOC113520645	2302.21	29.97	31.24	63.38	62.93	1.20	0.01	0.04	Acetyl-coenzyme A transporter 1
LOC113523187	1402	58.33	64.54	141.2	135	1.20	0.01	0.04	Porphobilinogen deaminase
LOC113522721	2764	47.65	45.76	100.23	109	1.20	0.01	0.04	ATP-binding cassette sub-family F member 1
LOC113523004	815.28	122.81	148.13	309.75	303.66	1.20	0.01	0.04	Stromal cell-derived factor 2
LOC113513670	1612.58	35.51	35.52	64.99	62.14	1.20	0.01	0.04	Sugar transporter SWEET1
LOC113516384	691	16.54	23.27	48.95	41.18	1.19	0.01	0.05	RNA-binding protein EIF1AD
LOC113518274	3670	8.51	8.03	17.65	19.32	1.19	0.01	0.04	Arf-GAP with SH3 domain, ANK repeat and PH domain-containing protein 1 isoform X1
LOC113523585	2885	29.97	31.55	68.02	69.32	1.19	0.01	0.04	Leucine--tRNA ligase, cytoplasmic
LOC113509711	1037	34.17	44.2	91.85	84.1	1.19	0.01	0.04	aminoacyl tRNA synthase complex-interacting multifunctional protein 1
LOC113516605	6222	6.08	3.47	10.03	11.24	1.19	0.01	0.04	Rho guanine nucleotide exchange factor 17 isoform X1
LOC113520410	645	22.73	24.48	54.99	51.79	1.19	0.01	0.05	Glutathione-specific gamma-glutamylcyclotransferase 2
MSTRG.8662	1386	6.58	7.79	15.12	16.95	1.19	0.01	0.05	Uncharacterized protein
LOC113514369	3680	9.08	10.2	21.14	21.65	1.18	0.01	0.04	Equilibrative nucleoside transporter 1
LOC113522889	5090	15.08	12.37	28.82	32.09	1.18	0.01	0.04	Protein SMG7-like isoform X2
LOC113517434	1098	20.96	26.5	52.64	53.32	1.18	0.01	0.04	Coenzyme Q-binding protein COQ10 homolog A, mitochondrial
LOC113518691	930	19.54	10.27	26.96	40.09	1.18	0.01	0.05	Baculoviral IAP repeat-containing protein 6
MSTRG.11349	1636	25.32	19.45	47.86	51.73	1.18	0.01	0.04	Uncharacterized protein
LOC113523605	2862	26.16	14.92	42.87	48.24	1.18	0.01	0.04	Eukaryotic translation initiation factor 4 gamma 2
LOC113517492	1541.89	119.08	99.65	235.38	252.95	1.18	0.01	0.04	Inositol-trisphosphate 3-kinase A isoform X2
LOC113523601	1490	9.44	8.69	18.02	22.31	1.18	0.01	0.05	Phospholipid-transporting ATPase IA isoform X2
LOC113511954	1580	8.34	9.7	21.26	18.71	1.18	0.01	0.05	Phosphatidylinositol N-acetylglucosaminyltransferase subunit Q
LOC113517007	3478	19.17	17.97	39.08	42.99	1.18	0.01	0.04	TBC1 domain family member 24 isoform X4
LOC113516097	2373	34.63	42.37	88.28	81.82	1.18	0.01	0.04	U3 small nucleolar RNA-associated

									protein 14 homolog A
LOC113510812	4781	2.32	2.19	4.73	5.22	1.18	0.01	0.05	Uncharacterized protein LOC110373838 isoform X4
LOC113517377	5289	8.51	5.09	14.37	15.62	1.18	0.01	0.05	Kinase D-interacting substrate of 220 kDa isoform X2
LOC113511545	654	29	41.23	89.84	67.11	1.18	0.01	0.05	Uncharacterized protein LOC106140795
LOC113510114	762	864.67	992.69	2122.58	2018.82	1.17	0.01	0.04	Phosphatidylethanolamine-binding protein isoform 1
LOC113518736	6037	5.15	4.8	10.09	11.78	1.17	0.01	0.05	Nucleolar pre-ribosomal-associated protein 1
LOC113521691	2027	8.88	7.05	17.22	17.88	1.17	0.01	0.05	Nicalin-1
LOC113510461	1911	55	59.21	127.02	124.15	1.17	0.01	0.05	Cysteine desulfurase, mitochondrial
LOC113514887	1058	26.67	35.12	72.18	64.21	1.17	0.01	0.05	Hypothetical protein RR46_05101
LOC113519229	1186	22.43	22.28	47.12	51.56	1.17	0.01	0.05	Protein SYS1 homolog
LOC113517458	541	431.13	506.61	1098.55	1004.39	1.16	0.01	0.05	Nuclear transport factor 2-like protein
LOC113521331	1675.87	72.86	81.63	179.82	158.5	1.16	0.01	0.05	Tether containing UBX domain for GLUT4
MSTRG.1232	6587	4.51	5.45	10.43	11.27	1.16	0.01	0.05	Uncharacterized protein LOC105380700
LOC113515970	657	2553.41	2921.44	1293.03	1149	-1.15	0.01	0.05	Ribosomal protein L11
LOC113516100	391	2037.27	2224.84	1043.42	928.86	-1.16	0.01	0.05	Ribosomal protein L37A
LOC113518514	4341	20.47	21.33	9.44	8.81	-1.16	0.01	0.05	DNA ligase 4
LOC113519166	2897	270.59	278.26	116.13	123.87	-1.16	0.01	0.05	Procollagen-lysine,2-oxoglutarate 5-dioxygenase 3 isoform X2
LOC113516826	2879	63.66	70.64	29.56	29.11	-1.16	0.01	0.05	Uncharacterized protein LOC106143636
LOC113513725	636	830.09	666.02	332.01	333.29	-1.16	0.01	0.05	Nucleolar GTP-binding protein 1
LOC113517603	1775	77.93	80.58	34.21	35.1	-1.16	0.01	0.05	4-aminobutyrate aminotransferase, mitochondrial
LOC113517580	3397	17.35	19.7	8.85	7.27	-1.16	0.01	0.05	DNA polymerase delta catalytic subunit
LOC113509617	547	2904.52	2896.62	1341.29	1239.24	-1.17	0.01	0.05	60S ribosomal protein L26
LOC113512002	1419	46.21	54.52	22.08	21.64	-1.17	0.01	0.05	RNA-binding protein 41-like
MSTRG.15840	884	3087.42	3072.95	1483.98	1195.64	-1.18	0.01	0.04	Cuticle protein CPH43
LOC113521764	1238.11	49.23	51.54	24.38	19.21	-1.18	0.01	0.05	Uncharacterized protein LOC106132753

LOC113509412	843	76.03	76.06	32.76	30.72	-1.18	0.01	0.05	GATA Zinc finger domain containing protein 1
LOC113516044	3865	81.19	92.37	37.43	37.38	-1.18	0.01	0.04	Histone-binding protein Caf1
LOC113517398	3190	41.87	28.34	15.25	14.94	-1.18	0.01	0.04	Nuclear pore complex protein Nup155
LOC113519622	3829	22.16	23.84	9.56	10.19	-1.18	0.01	0.04	Chromosome-associated kinesin KIF4 isoform X3
LOC113522379	690	64.49	60.3	19.82	34.78	-1.19	0.01	0.05	Uncharacterized protein K02A2.6-like
LOC113517323	3291	486.17	467.9	185.22	223.72	-1.19	0.01	0.04	Integrin Alpha-9-like
LOC113516250	2120	31.69	30.33	12.36	14.25	-1.19	0.01	0.04	Inositol polyphosphate multikinase
LOC113510145	2199.07	62.59	63.31	24.8	29.03	-1.19	0.01	0.04	Zinc finger protein 26-like
LOC113510318	5497	21.35	20.28	8.42	9.35	-1.19	0.01	0.04	Coronin-7 isoform X1
LOC113516180	2092	68.16	75.97	32.53	29.04	-1.19	0.01	0.04	Uncharacterized protein LOC106134226
LOC113515920	414	282.88	307.61	137.02	126.68	-1.20	0.01	0.04	Cytosolic non-specific dipeptidase
LOC113517593	2865	38.83	38.87	15.86	17.23	-1.20	0.01	0.04	Sodium- and chloride-dependent GABA transporter ine isoform X1
LOC113521749	1708	173.87	184.88	77.61	75.36	-1.20	0.01	0.04	Selenide, water dikinase
LOC113510668	2087	12.28	11.67	4.94	5.23	-1.20	0.01	0.04	Vacuolar protein sorting-associated protein 33B
LOC113520381	1739	15.51	16.24	6.59	6.89	-1.21	0.01	0.04	E3 ubiquitin-protein ligase TRAIP-like
LOC113512653	2683	21.83	22.38	8.5	10.23	-1.21	0.01	0.04	Chromatin assembly factor 1 subunit B
LOC113516813	1330	68.17	76.37	32.88	28.5	-1.21	0.01	0.04	GDP-fucose protein O-fucosyltransferase 1-like
LOC113519934	2450	21.51	23.36	10.9	8.07	-1.21	0.01	0.04	UDP-xylose and UDP-N-acetylglucosamine transporter isoform X1
LOC113512330	2488	27.97	31.72	12.86	12.39	-1.21	0.01	0.04	T-cell activation inhibitor, mitochondrial
LOC113519687	2756.6	64.46	61.36	25.79	27.16	-1.21	0.01	0.04	Protein UBASH3A homolog isoform X1
LOC113521276	1391	21.18	22.05	9.56	8.74	-1.21	0.01	0.04	RNA-binding protein 40-like
LOC113509291	5073	7.81	7.93	3.05	3.58	-1.21	0.01	0.04	Hypothetical protein KGM_207016
LOC113519846	927	26.87	46.53	18.34	12.7	-1.21	0.01	0.04	N-acetylneuraminate lyase-like
LOC113511631	577	2818.56	3093.08	1357.48	1178.7	-1.22	0.01	0.04	60S ribosomal protein L22-like

LOC113512343	1655	196.71	213.72	91.01	81.72	-1.22	0.01	0.04	Endoplasmic reticulum lectin 1 isoform X2
LOC113513413	1378	117	119.24	58.16	41.23	-1.22	0.01	0.04	Uncharacterized protein OBRU01_00994
LOC113522555	3208	5.3	6.28	2	2.86	-1.22	0.01	0.04	GTPase-activating Rap/Ran-GAP domain-like protein 3
LOC113520135	765	20.92	19.92	8.12	9.22	-1.22	0.01	0.05	Zinc finger protein 816-like
LOC113521144	1500	12.67	11.08	4.55	5.44	-1.22	0.01	0.04	Uncharacterized protein LOC106142945
LOC113518084	2033	14.55	9.62	5.02	5.12	-1.22	0.01	0.04	Structural maintenance of chromosomes protein 6-like
LOC113523465	3395	47.15	51.44	20.06	21.15	-1.22	0.01	0.04	Transient receptor potential cation channel protein painless
LOC113511154	2913.01	13.54	13.71	5.75	5.63	-1.22	0.01	0.04	Uncharacterized protein LOC106102871
LOC113520509	1053	26.42	28.11	10.87	12.03	-1.23	0.01	0.04	Protein lin-37 homolog isoform X2
LOC113517016	1270.47	20.64	22.1	7.25	10.41	-1.23	0.01	0.04	Uncharacterized protein LOC106143663 isoform X1
LOC113513429	3900	10.34	9.44	4.09	4.15	-1.23	0.01	0.04	Intraflagellar transport protein 140 homolog
LOC113512603	2457	3.97	5.67	2.27	1.72	-1.23	0.01	0.05	Uncharacterized protein OBRU01_14198
LOC113514345	1540.64	12.15	13.91	5.59	5.26	-1.23	0.01	0.04	Nitrilase homolog 1 isoform X1
LOC113515939	4863	13.87	10.49	5.38	4.72	-1.23	0.01	0.03	Ski oncogene
LOC113520491	4723	10.2	10.76	3.78	4.8	-1.23	0.01	0.04	DNA (cytosine-5)-methyltransferase PliMCI-like
LOC113521147	1892	10.57	12.66	5.03	4.62	-1.24	0.01	0.04	Glucose dehydrogenase
LOC113521736	3992.39	11.08	11.8	4.89	4.59	-1.24	0.01	0.04	Growth arrest-specific protein 1-like
LOC113523252	1188	23.04	22.7	11.3	7.66	-1.24	0.01	0.04	Three-prime repair exonuclease 1-like
LOC113510074	7787	9.78	9.83	4.09	4	-1.24	0.01	0.03	Zinc finger protein 62 homolog isoform X9
LOC113522677	1715.87	33.26	37.14	15.1	14.05	-1.24	0.01	0.03	von Willebrand factor A domain-containing protein 9
LOC113516367	595	34.67	25.77	13.26	12.24	-1.24	0.01	0.04	Atlastin-like
LOC113517324	1249	44.91	46.05	18.79	18.93	-1.24	0.01	0.03	Uncharacterized protein LOC106137858
LOC113519604	1552	13.74	13.27	5.39	5.79	-1.24	0.01	0.04	Solute carrier family 35 member C2

LOC113521655	1084	97.58	116.7	50.58	38.19	-1.24	0.01	0.03	tRNA selenocysteine 1-associated protein 1-like
LOC113517379	4920	8.95	6.5	2.98	3.38	-1.24	0.01	0.03	Rotatin-like
LOC113517854	7796	2.64	2.36	0.98	1.06	-1.24	0.01	0.04	Anion exchange protein 2 isoform X3
LOC113516123	4218	92.81	81.83	33.45	38.53	-1.24	0.01	0.03	Uncharacterized protein LOC106142239
LOC113512044	2630	63.02	74.51	28.06	28.6	-1.25	0.01	0.03	Hemicentin-1-like
LOC113519854	3064	19.06	8.72	5.04	6.41	-1.25	0.01	0.03	Protein serrate
LOC113512011	598	2555.53	2828.84	1180.82	1077.49	-1.25	0.01	0.03	Ribosomal protein S16
LOC113516654	828	8757.38	9684.63	4021.41	3636.38	-1.25	0.01	0.03	Translationally-controlled tumor protein homolog
LOC113515283	1286	614.51	623.48	265.99	244.81	-1.25	0.01	0.03	Uncharacterized protein LOC106136012 isoform X1
MSTRG.4266	3289	2.77	2.03	1.04	0.93	-1.25	0.01	0.05	Uncharacterized protein
LOC113518164	5607	8.87	9.8	3.74	3.92	-1.25	0.01	0.03	Uncharacterized protein LOC106138152
LOC113522138	743	26.95	19.41	8.5	10.8	-1.25	0.01	0.04	Cyclin dependent kinase 4
LOC113516922	2001	9.2	10.86	3.83	4.4	-1.25	0.01	0.04	Pyruvate dehydrogenase phosphatase regulatory subunit, mitochondrial isoform X1
LOC113519218	350	2585.71	2645.15	1243.84	1064.91	-1.25	0.01	0.03	60S ribosomal protein L38
LOC113512453	3589.83	4184.03	3214.23	1385.84	1641.96	-1.26	0.01	0.03	Uncharacterized protein LOC106130912
LOC113510634	1464.56	624.19	594.3	232.71	269.53	-1.26	0.01	0.03	Cuticlin-2 isoform X1
LOC113509992	1327	106.13	125.23	46.12	48.92	-1.26	0.01	0.03	RAB6-interacting golgin
LOC113517215	3163.78	9.04	8.78	3.83	3.42	-1.26	0.01	0.03	Cerebellar degeneration-related protein 2 isoform X3
LOC113522679	868.4	63.29	76.49	30.73	27.13	-1.26	0.01	0.03	Glutathione S-transferase zeta 2
LOC113515973	2084	78.31	77.5	34.24	29.39	-1.26	0.01	0.03	SET and MYND domain-containing protein 4-like
LOC113519691	2719	27.08	27.77	12.04	10.34	-1.26	0.01	0.03	Hemicentin-1
LOC113509407	1774	15.12	19.1	7.21	6.77	-1.26	0.01	0.03	Uncharacterized protein DDB_G0291812
LOC113509501	1901	17.61	13.95	5.72	7.19	-1.26	0.01	0.03	Uncharacterized protein LOC106124574 isoform X1
LOC113517982	626	41.3	53.12	20.29	18.84	-1.26	0.01	0.04	Cancer-related nucleoside-triphosphatase homolog

MSTRG.11014	5806	4.25	4.82	1.53	2.16	-1.26	0.01	0.03	Uncharacterized protein
MSTRG.9390	3637	12.99	10.77	4.83	4.86	-1.26	0.01	0.03	Uncharacterized protein LOC106125039
LOC113516771	3771	11.11	9.86	3.92	4.61	-1.26	0.01	0.03	WD repeat-containing protein 35
LOC113513419	1234	10.18	9.46	4.16	3.86	-1.26	0.01	0.04	Protein regulator of cytokinesis 1 isoform X1
LOC113518246	500	38.08	48.1	21.2	14.72	-1.26	0.01	0.04	6-pyruvoyl tetrahydrobiopterin synthase
LOC113511672	1755	12.2	15.57	5.4	5.91	-1.27	0.01	0.03	Protein anon-73B1
LOC113521864	1075	14.21	11.1	5.42	4.88	-1.27	0.01	0.04	ATP-dependent (S)-NAD(P)H-hydrate dehydratase-like
LOC113516277	2026	129.09	133.99	53.31	53.14	-1.27	0.01	0.03	Fatty-acid amide hydrolase 2-B-like
MSTRG.6349	2660	143.84	142.33	57.44	58.16	-1.27	0.01	0.03	Protein phosphatase 1 regulatory subunit 14B isoform X3
LOC113522512	2943	25.12	20.76	8.96	9.57	-1.27	0.01	0.03	Gamma-tubulin complex component 3 isoform X1
LOC113517980	1118	366.31	402.3	158.6	153.2	-1.28	0.01	0.03	Enoyl-CoA hydratase, mitochondrial
MSTRG.5166	3764	5.78	5.95	2.47	2.25	-1.28	0.01	0.03	Uncharacterized protein LOC106135993
LOC113519049	1460	8.25	9.64	3.49	3.75	-1.28	0.01	0.04	Uncharacterized protein
LOC113514022	1293	12.16	9.22	3.65	5.02	-1.28	0.01	0.04	Sn1-specific diacylglycerol Lipase beta-like
LOC113515659	817	17.75	20.28	8.74	6.71	-1.28	0.01	0.04	Uncharacterized protein LOC106130344
LOC113510788	1425	4.75	5.23	2.15	1.87	-1.28	0.01	0.05	Phorbol ester/diacylglycerol-binding protein unc-13-like
LOC113519817	1611	33.89	37.56	14.17	14.65	-1.28	0.01	0.03	Uncharacterized protein LOC110377460
LOC113519418	879	13.72	13.46	5.12	5.91	-1.28	0.01	0.04	Nuclease HARBI1
LOC113511054	1568	206.54	201.35	82.75	81.61	-1.28	0.01	0.03	Protein LTV1 homolog isoform X1
LOC113511287	1757	13.55	10.12	4.28	5.26	-1.28	0.01	0.03	5-oxoprolinase
LOC113518301	2080.49	7.5	8.9	3.64	2.98	-1.28	0.01	0.03	Serine/threonine-protein kinase greatwall isoform X1
LOC113516300	688	63.63	76.02	30.14	26.62	-1.29	0.01	0.03	Tail-anchored protein insertion receptor WRB-like
LOC113514411	4519.69	57.88	53.58	22.42	24.64	-1.29	0.01	0.03	Leucine-rich repeat flightless-interacting protein 2 isoform X1
LOC113514990	2757	26.45	20.6	8.97	9.88	-1.29	0.01	0.03	Protein I'm not dead yet

LOC113509985	1024	212.25	214.28	86.19	85.74	-1.29	0.01	0.03	Aldose reductase isoform X1
LOC113514905	558	121.4	129.53	51.68	51.08	-1.29	0.01	0.03	Protein D7-like
LOC113523151	945	54.5	62.01	22.67	24.32	-1.29	0.01	0.03	Cyclin-dependent kinase 1
LOC113510744	2484	15.52	7.85	4.55	4.81	-1.29	0.01	0.03	Peritrophin 1
LOC113516550	385	101.68	79.65	30.52	47.03	-1.29	0.01	0.03	Nucleolar GTP-binding protein 1
LOC113523008	4464	1.05	1.19	0.4	0.5	-1.29	0.01	0.05	Multidrug resistance-associated protein lethal(2)03659 isoform X1
LOC113522055	2259	48.41	44.27	17.4	19.68	-1.29	0.01	0.03	Retrotransposable element
LOC113519293	587	21.35	18.26	7.45	8.66	-1.29	0.01	0.04	Synapse-associated protein of 47 kDa isoform X3
LOC113514137	1083	129.41	143.08	53.7	55.39	-1.30	0.01	0.02	Vacuolar protein sorting-associated protein 33A
MSTRG.7848	1002	17.71	19.96	7.85	7.22	-1.30	0.01	0.03	Uncharacterized protein
LOC113510932	2559.42	37.52	36.86	12.75	16.86	-1.30	0.01	0.02	Spermatogenesis-associated protein 20 isoform X1
LOC113510675	762	1108.8	1192.35	494.57	429.31	-1.30	0.01	0.02	Ribosomal biogenesis protein RLP24
LOC113523321	1493	190.79	214.1	84.05	77.08	-1.30	0.01	0.02	Insect cytokine precursor uENF2
LOC113516407	746	248.58	172.32	76.76	92.74	-1.30	0.01	0.02	Talin-1-like isoform X1
LOC113518791	1060	132.32	130	51.24	53.5	-1.30	0.01	0.02	MOB kinase activator-like 1
LOC113516519	832	69.04	59.44	21.08	30.48	-1.30	0.01	0.03	Serine protease snake-like
LOC113516045	1324	165.52	128.92	54.19	62.91	-1.30	0.01	0.02	S-adenosylmethionine decarboxylase proenzyme
LOC113521146	1106	8.12	8.19	3.48	3	-1.30	0.01	0.04	Uncharacterized protein LOC110380962 isoform X2
LOC113513100	875	105.08	99.03	40.41	40.97	-1.31	0.01	0.02	Uncharacterized protein LOC110378060 isoform X2
LOC113521102	1347	127.43	136.47	55.08	49.45	-1.31	0.01	0.02	Gastrula Zinc finger protein XlCGF17.1-like
LOC113522390	3657	286.67	301.05	110.68	120.98	-1.31	0.01	0.02	Ceramide phosphoethanolamine synthase
LOC113521278	1439	63.44	64.45	25.49	25.08	-1.31	0.01	0.02	Arf-GAP with dual PH domain-containing protein 1-like isoform X2
LOC113519160	749	22.75	18.66	8.83	7.68	-1.31	0.01	0.03	Retinoblastoma-binding protein 5 homolog
LOC113522425	449	102.59	115.6	52.16	36.87	-1.31	0.01	0.03	Partner of xrn-2 protein 1-like
LOC113512729	1818	2.87	2.95	1.03	1.26	-1.31	0.01	0.04	Uncharacterized protein LOC106713194

LOC113521608	762	20.49	17.29	6.34	8.72	-1.31	0.01	0.03	Uncharacterized protein LOC106132646 isoform X2
LOC113509605	2928	15.29	16.82	6.23	6.38	-1.31	0.01	0.02	Protein inturned
LOC113514268	1475	64.74	83.7	29.31	29.2	-1.31	0.01	0.02	Homeobox protein MSX-2
LOC113523315	1786	26.22	30.71	10.67	11.75	-1.31	0.01	0.02	Arylsulfatase I-like
LOC113519668	2830	16.93	17.1	5.72	7.63	-1.32	0.01	0.02	Beta-mannosidase-like isoform X1
LOC113512105	1179	15.07	18.04	6.54	6.48	-1.32	0.01	0.03	Uncharacterized protein LOC105391666
LOC113515469	1475.34	5.74	6.35	2.68	2.07	-1.32	0.01	0.04	Substance-P receptor-like
LOC113515060	2071	100.83	103.56	40.2	39.79	-1.32	0.00	0.02	Venom serine carboxypeptidase
LOC113509730	2063	314.9	241.73	108.9	108.98	-1.32	0.00	0.02	Lysosomal aspartic protease
LOC113518692	694	363.08	306.78	105.05	161.59	-1.32	0.00	0.02	Uncharacterized protein LOC106136531
MSTRG.8560	1756	2.51	3.61	1.12	1.27	-1.32	0.01	0.04	Reverse transcriptase
LOC113521279	2006	20.84	23.19	8.2	9.01	-1.32	0.01	0.02	DNA replication factor Cdt1
LOC113523136	441	35.72	32.18	11.81	15.86	-1.32	0.01	0.04	2-oxoisovalerate dehydrogenase subunit beta, mitochondrial isoform X1
LOC113509755	1025	54.5	58.13	23.28	20.91	-1.32	0.01	0.02	DNA fragmentation factor subunit alpha
LOC113511017	1894	10.99	8.47	3.66	3.95	-1.32	0.01	0.03	5-oxoprolinase
MSTRG.9374	328.47	147.59	163.59	69.84	60.15	-1.33	0.01	0.03	Phosphatidylethanolamine binding protein isoform X1
LOC113520775	1618	34.08	39.61	14	14.77	-1.33	0.00	0.02	Ubiquitin carboxyl-terminal hydrolase calypso
LOC113518572	2841	2.48	4.68	1.21	1.57	-1.33	0.01	0.03	Acetylcholinesterase
LOC113514128	268	226.87	165.96	82.95	92.6	-1.33	0.01	0.04	Laminin A
LOC113512840	2485.13	106.92	98.28	38.38	41.57	-1.33	0.00	0.02	Speckle targeted PIP5K1A-regulated poly(A) polymerase-like
LOC113511602	3214	18.89	19.1	6.78	7.98	-1.33	0.00	0.02	Sulfatase-modifying factor 1
LOC113522502	2824	56.03	54.2	21.79	20.96	-1.33	0.00	0.02	Carbohydrate sulfotransferase 4-like
LOC113512912	1854	77.81	75.86	30.05	29.64	-1.33	0.00	0.02	Methylmalonate-semialdehyde dehydrogenase
MSTRG.8065	2597	3.21	3.38	1.39	1.15	-1.33	0.01	0.03	Histone-lysine N-methyltransferase SETMAR-like
LOC113520795	931	19.27	19.02	7.65	7.29	-1.33	0.01	0.03	Alcohol dehydrogenase
LOC113517141	3548	13.57	12.42	4.82	5.21	-1.34	0.00	0.02	Tyrosine-protein kinase PR2

LOC113517246	3242.76	29.72	29.6	10.62	12.09	-1.34	0.00	0.02	TNF receptor-associated factor 4 isoform X1
LOC113516985	3070	542.25	588.11	215.28	221.85	-1.34	0.00	0.02	Integrin beta-PS-like
LOC113522845	1094	18.87	18.7	6.72	7.89	-1.34	0.01	0.02	Hypothetical protein KGM_202170
LOC113517588	1838	4.52	1.65	0.97	1.42	-1.34	0.01	0.04	Uncharacterized protein LOC106109243
MSTRG.13265	1645	14.21	17.4	5.68	6.53	-1.34	0.01	0.02	Uncharacterized protein
LOC113516980	1595	15.24	15.68	5.42	6.52	-1.34	0.01	0.02	Zinc finger protein 131-like isoform X2
LOC113518516	2199	150.39	113.28	43.15	58.7	-1.34	0.00	0.02	Peroxisomal acyl-coenzyme A oxidase 1
MSTRG.11460	2178	12.21	9.46	3.73	4.63	-1.34	0.01	0.02	Uncharacterized protein LOC106137858
LOC113522510	2147	10.39	10.22	3.97	3.95	-1.35	0.01	0.02	Division abnormally delayed protein
LOC113517596	1201	11.77	15.56	6.02	4.51	-1.35	0.01	0.02	Uncharacterized protein LOC106139924
LOC113511679	2158	1.5	1.59	0.59	0.61	-1.35	0.01	0.05	Sodium channel protein Nach-like
LOC113515158	937	71.3	54.52	22.01	26.75	-1.35	0.00	0.02	GPI transamidase component PIG-S
LOC113521169	1741	76.74	82.36	31.19	29.9	-1.35	0.00	0.02	Serine protease K12H4.7 isoform X1
LOC113516124	2892	40.61	30.92	13.19	14.23	-1.35	0.00	0.02	Uncharacterized protein LOC106142239
LOC113509125	899	6.76	6.84	3.08	2.16	-1.35	0.01	0.04	Troponin I isoform X2
LOC113518314	1410	22.32	20.06	8.73	7.53	-1.35	0.00	0.02	UPF0489 protein C5orf22 homolog
LOC113512911	2182	3.85	4.58	1.77	1.45	-1.35	0.01	0.03	Gastrula Zinc finger protein XlCGF57.1-like
LOC113512913	667	269.41	308.68	116.43	107.72	-1.35	0.00	0.02	39S ribosomal protein L49, mitochondrial
LOC113515982	1684	81.49	86.99	34.5	29.83	-1.36	0.00	0.02	Glyoxylate reductase/hydroxypyruvate reductase
LOC113522932	1794	485.8	526.1	201.76	184.23	-1.36	0.00	0.02	Uncharacterized protein LOC105383334
LOC113516864	1858	26.25	28.07	10.92	9.77	-1.36	0.00	0.02	Cell division cycle protein 23
MSTRG.13777	375	56.28	63.29	22.79	25.64	-1.36	0.01	0.03	Uncharacterized protein
LOC113519576	753	7.67	6.76	2.58	2.96	-1.36	0.01	0.05	Autophagy-related protein 2 homolog B
LOC113523214	1682	29.24	25.85	9.45	11.57	-1.36	0.00	0.02	Uncharacterized protein LOC106125798

LOC113515147	1163	12.71	13.7	4.61	5.38	-1.36	0.01	0.02	Methyl-CpG-binding domain protein 4-like
LOC113512614	3231	50.61	48.52	17.98	19.64	-1.36	0.00	0.02	Chitinase-3-like protein 2
LOC113513912	776	40.62	37.39	16.71	13.18	-1.36	0.00	0.02	Short-chain specific acyl-CoA dehydrogenase, mitochondrial-like isoform X1
LOC113512394	1654	9.12	9.89	3.89	3.32	-1.37	0.01	0.02	Progestin and adipoQ receptor family member 3
LOC113510171	619	13.05	14.65	4.78	5.89	-1.37	0.01	0.03	Uncharacterized protein
LOC113512613	695	318.98	326.5	124.71	122.99	-1.37	0.00	0.02	Uncharacterized protein LOC101739854 isoform X1
LOC113510542	1345	22.49	24.77	8.94	8.99	-1.37	0.00	0.02	Deoxynucleoside kinase-like
LOC113519667	1871	291.77	313.67	118.42	110.66	-1.37	0.00	0.02	RNA helicase Mov10l1 isoform X2
LOC113513094	1806	4.97	3.61	1.35	1.89	-1.38	0.01	0.03	Gamma-tubulin complex component 6
LOC113518256	2363.66	43.31	48.64	18.13	16.51	-1.38	0.00	0.02	Long-chain-fatty-acid--CoA ligase ACSBG2 isoform X2
LOC113517079	1068	4.53	4.79	1.47	2.04	-1.38	0.01	0.04	Tubulin polyglutamylase complex subunit 2 isoform X2
LOC113509765	2622.85	87.19	83.45	31.41	32.69	-1.38	0.00	0.02	Delta(14)-sterol reductase
MSTRG.11342	2069	223.2	224.22	82.75	85.4	-1.38	0.00	0.02	Uncharacterized protein
LOC113515320	858	146.25	164.78	56.55	61.12	-1.38	0.00	0.02	DNA-directed RNA polymerase III subunit RPC7-like
LOC113518398	607	189.96	231.19	82.05	78.48	-1.38	0.00	0.02	ARL14 effector protein
LOC113511991	622	8.94	8.57	2.96	3.7	-1.38	0.01	0.04	Oxidoreductase GLYR1 homolog isoform X1
LOC113515043	1545	16.05	17.36	6.02	6.51	-1.38	0.00	0.02	Uncharacterized protein
LOC113510111	1400	15.24	17.57	6.79	5.5	-1.38	0.00	0.02	Hypothetical protein KGM_213196
LOC113511619	3411	1.23	1.16	0.41	0.49	-1.39	0.01	0.04	ATP-binding cassette sub-family G member 8
LOC113514739	1124	6.99	6.06	2.67	2.22	-1.39	0.01	0.03	Kinesin-like protein KIF18A
LOC113516036	838	4.9	6.73	2.56	1.79	-1.39	0.01	0.04	Uncharacterized protein LOC106138832
LOC113510207	960	18.94	23.58	7.32	8.65	-1.39	0.00	0.02	Fidgetin-like protein 1
LOC113510031	884	8.98	10.94	3.61	3.87	-1.39	0.01	0.03	Uncharacterized protein
LOC113517287	4323	271.32	281.66	99.19	106.86	-1.39	0.00	0.01	Failed axon connections
LOC113518173	743	204.3	230.74	86.2	77.78	-1.39	0.00	0.01	Alpha carbonic anhydrase 8-like

LOC113509839	4063	88.56	58.7	24.13	30.69	-1.39	0.00	0.01	Protein toll-like
LOC113510092	3013	23.58	24.44	7.75	10.11	-1.39	0.00	0.01	Zinc finger protein 100-like
MSTRG.374	508	94.51	97.38	28.52	45.34	-1.39	0.00	0.02	Serine protease HP21 precursor
LOC113514891	902	7.87	6.63	2.74	2.69	-1.39	0.01	0.03	Protein real-time
LOC113509083	2437.45	80.56	77.13	29.04	30.43	-1.40	0.00	0.01	High mobility group protein DSP1-like
LOC113510810	2507	3.42	2.51	1.08	1.11	-1.40	0.01	0.03	Sodium-independent sulfate anion transporter-like
LOC113513757	709	8.31	4.21	2.21	2.49	-1.40	0.01	0.05	Group XV phosphoLipase A2-like isoform X1
LOC113514238	1318.82	50.76	56.22	20.61	18.95	-1.40	0.00	0.01	THO complex subunit 3
MSTRG.5170	6009	1.35	1.3	0.55	0.42	-1.40	0.01	0.02	Uncharacterized protein LOC106135953 isoform X1
LOC113518799	2989	21.79	17.71	7.26	7.3	-1.41	0.00	0.01	DNA replication licensing factor Mcm3 isoform X2
LOC113513288	1237	117.31	125.78	46.2	43.78	-1.41	0.00	0.01	E3 ubiquitin-protein ligase sina-like isoform X1
LOC113514379	2998	57.3	62.38	23.26	20.73	-1.41	0.00	0.01	Uncharacterized protein LOC106133038
LOC113521060	7174	249.59	204.73	67.56	99.53	-1.41	0.00	0.01	Talin-B
LOC113523600	1114	62.99	64.93	25.33	21.9	-1.41	0.00	0.01	Vesicle-associated membrane protein 7
LOC113510344	513	632.06	499.78	199.98	228.91	-1.41	0.00	0.01	Aldehyde dehydrogenase, mitochondrial
LOC113514267	776	1386.06	1570.34	564.66	533.1	-1.41	0.00	0.01	Bicaudal
MSTRG.936	301.01	4.05	2.57	1.26	17.04	-1.41	0.01	0.03	Nucleoporin-like protein 2
LOC113522864	1145	8.83	11.55	3.56	3.94	-1.41	0.00	0.02	Outer kinetochore Dsn1
MSTRG.8598	364	35.73	24.9	10.07	13.84	-1.42	0.01	0.04	Huntingtin-like
LOC113509938	1482.78	57.14	63.86	21.44	22.94	-1.42	0.00	0.01	Uncharacterized protein LOC110377493 isoform X2
MSTRG.9841	688	6.64	8.55	2.82	2.78	-1.42	0.01	0.04	Uncharacterized protein
LOC113510735	1297	312.09	346.24	122.75	118.62	-1.42	0.00	0.01	Venom protease-like
LOC113517059	1274	12.94	15.23	5.92	4.38	-1.42	0.00	0.02	Uncharacterized protein LOC110371035
LOC113519758	1804	20.19	20.7	7.12	7.83	-1.42	0.00	0.01	PH domain-containing protein DDB_G0287875-like
LOC113511719	399	3894.72	4167.88	1635.84	1461.36	-1.42	0.00	0.01	60S ribosomal protein L37

LOC113510072	1065	8.19	10.86	3.13	3.83	-1.42	0.00	0.02	Zinc finger protein 28 homolog
LOC113513473	554	22.83	18.42	7.84	7.51	-1.43	0.01	0.02	Alpha-N-acetylgalactosaminidase-like
MSTRG.6023	312	650.92	619.93	268.34	241.06	-1.43	0.00	0.01	Uncharacterized protein
LOC113519026	9914	2.11	1.64	0.57	0.79	-1.43	0.00	0.01	Protein unc-80 homolog
LOC113513356	759	406.54	408.96	143.05	156.67	-1.43	0.00	0.01	Uncharacterized protein LOC106130098
LOC113523502	2007	172.34	191.82	69.01	63.11	-1.43	0.00	0.01	Uncharacterized protein LOC106131056
LOC113516390	449	17.54	9.23	5.45	4.66	-1.43	0.01	0.05	Superoxide dismutase
LOC113510783	1268	258.12	201.07	76.99	90.25	-1.43	0.00	0.01	Aldehyde dehydrogenase, mitochondrial
MSTRG.3514	511	157.39	111.24	40.74	59.86	-1.43	0.00	0.01	Talin-1-like
LOC113521717	3178	6.68	7.97	2.83	2.45	-1.44	0.00	0.01	Uncharacterized protein DDB_G0286901
LOC113518245	1675	45.09	46.65	15.1	18.11	-1.44	0.00	0.01	Tubulin gamma-1 chain
MSTRG.14604	1190	8.99	7.84	3.08	3.02	-1.44	0.00	0.02	DNA-mediated transposase
LOC113510790	1393	65.59	60.5	24.07	21.48	-1.44	0.00	0.01	Endophilin-A isoform X5
LOC113515202	436	84.33	93.48	34.4	32.27	-1.44	0.00	0.01	Vacuolar protein sorting-associated protein 33A
LOC113514823	468	14.31	18.9	6.69	5.6	-1.44	0.01	0.03	Inactive peptidyl-prolyl cis-trans isomerase shutdown-like
LOC113517531	1725	10.98	13.14	4.51	4.17	-1.44	0.00	0.01	Uncharacterized protein LOC106138558
LOC113518797	1809	46.49	39.94	13.33	17.88	-1.44	0.00	0.01	Uncharacterized protein LOC106133123
LOC113522095	385	378.93	373.25	111.32	176.68	-1.45	0.00	0.01	Uncharacterized protein LOC106136531
LOC113514079	1737	2.13	4.16	1.51	0.74	-1.45	0.01	0.02	Uncharacterized protein LOC110374962
LOC113515364	1127	101.66	100.13	35.28	37.44	-1.45	0.00	0.01	ATP-dependent (S)-NAD(P)H-hydrate dehydratase
LOC113518180	5189	13.58	14.04	4.66	5.22	-1.45	0.00	0.01	Myosin-IIIb-like isoform X2
LOC113512678	1161	49.36	44.77	17.08	16.79	-1.45	0.00	0.01	Clavesin-2
LOC113509794	883	746.15	816.88	286.3	277.61	-1.45	0.00	0.01	Ribosome biogenesis protein NSA2 homolog
LOC113509623	1149	513.61	570.39	191.48	198.34	-1.45	0.00	0.01	Uncharacterized protein OBRU01_17125

MSTRG.12967	2969	8.31	7.01	2.66	2.82	-1.45	0.00	0.01	Uncharacterized protein OBRU01_26429
LOC113517495	1238.98	50.12	52.45	20.56	16.19	-1.45	0.00	0.01	Multiple inositol polyphosphate phosphatase 1-like
LOC113521954	668	10.24	12.85	5.41	2.92	-1.45	0.01	0.02	RecQ-mediated genome instability protein 2-like
LOC113516461	2652	26.06	26.61	10.67	8.09	-1.45	0.00	0.01	Scm-like with four MBT domains protein 2
LOC113520595	1210.64	28.71	33.24	10.59	11.58	-1.46	0.00	0.01	Target of rapamycin complex subunit lst8
LOC113515375	3935	14.11	15.24	4.95	5.49	-1.46	0.00	0.01	Protein timeless homolog isoform X2
MSTRG.4044	958	298.23	296.84	103.62	109.79	-1.46	0.00	0.01	Uncharacterized protein
LOC113513977	1287	11.86	13.64	4.9	4.19	-1.46	0.00	0.01	Protein Spindly
LOC113514269	1716	9.19	7.76	2.63	3.41	-1.46	0.00	0.01	Large neutral amino acids transporter small subunit 1
LOC113513282	722	7.11	9	2.3	3.47	-1.46	0.01	0.03	Transcription termination factor 2 isoform X2
LOC113520797	4330	239.25	219.15	74.05	88.43	-1.46	0.00	0.01	Matrix metalloproteinase-14-like
LOC113511091	2736	9.1	8.03	2.8	3.25	-1.47	0.00	0.01	Protein CIP2A homolog
MSTRG.9439	889	26.48	23.8	7.83	10.07	-1.47	0.00	0.01	Uncharacterized protein
LOC113510405	448	14.05	12.72	4.7	5.07	-1.47	0.01	0.04	ATP-dependent RNA helicase DHX34
LOC113522771	3075	31.85	35.61	11.37	12.36	-1.47	0.00	0.01	Rab-like protein 6
LOC113519683	1733	43.38	39.53	15.02	14.2	-1.47	0.00	0.01	CXXC-type Zinc finger protein 1
LOC113514440	1691	5.14	4.95	1.56	1.99	-1.47	0.00	0.02	Uncharacterized protein LOC106138911 isoform X1
LOC113512070	1520	31.3	34.03	10.17	12.86	-1.48	0.00	0.01	Replication factor C subunit 2
LOC113513006	3020	20.76	17.92	6.43	7.15	-1.48	0.00	0.01	Hormone-sensitive Lipase isoform X1
MSTRG.10478	257	3141.77	3075.55	1369.63	1129.19	-1.48	0.00	0.01	Uncharacterized protein
LOC113512666	361	23.01	35.21	13.32	8.25	-1.48	0.01	0.03	Adenosine kinase
LOC113515268	4453	3.04	3.33	1.01	1.22	-1.48	0.00	0.01	Zinc finger protein 91-like
LOC113516195	443	1404.97	1540.46	601.96	469.35	-1.48	0.00	0.01	Cytochrome c oxidase subunit 7C, mitochondrial
LOC113509548	2781	4.77	5.01	1.62	1.8	-1.48	0.00	0.01	NAD kinase 2, mitochondrial
LOC113521757	1811	17.68	18.84	6.86	5.91	-1.48	0.00	0.01	Uncharacterized protein LOC106140333

LOC113517477	1466.5	18.44	17.42	6.46	6.1	-1.48	0.00	0.01	Uncharacterized protein LOC106131834 isoform X1
LOC113521223	3539	13.87	15.52	5.05	5.21	-1.48	0.00	0.01	FK506-binding protein 5-like
MSTRG.2858	1164	23.95	25.26	9.59	7.65	-1.48	0.00	0.01	otopetrin-2-like
LOC113517475	1964	21.16	24.99	8.65	7.56	-1.48	0.00	0.01	FGGY carbohydrate kinase domain-containing protein
MSTRG.6005	1610	286.5	307.15	105.19	102.54	-1.48	0.00	0.01	Uncharacterized protein
LOC113520264	5189	5.14	4.63	1.56	1.85	-1.49	0.00	0.01	Neuralized-like protein 4
LOC113514033	3113	4.75	4.92	1.51	1.87	-1.49	0.00	0.01	Uncharacterized protein LOC110381450 isoform X1
LOC113516004	2281.79	22.8	20.84	7.83	7.38	-1.49	0.00	0.01	PR domain Zinc finger protein 16-like isoform X1
MSTRG.10470	2204	9.09	11.73	4.25	3	-1.49	0.00	0.01	Uncharacterized protein
MSTRG.14312	9191	7.12	6.38	2.05	2.65	-1.49	0.00	0.01	Uncharacterized protein LOC110378431
MSTRG.8019	823	188.07	146.56	45.73	72.54	-1.49	0.00	0.01	Talin-B-like
LOC113513174	957	3.42	3.68	0.6	1.88	-1.49	0.01	0.03	Vacuolar protein sorting-associated protein 13C
LOC113516446	1620	54.2	54.48	18.53	19.35	-1.49	0.00	0.01	N(4)-(Beta-N-acetylglucosaminyl)-L-asparaginase-like
LOC113521596	2154	2.57	1.7	0.66	0.82	-1.49	0.01	0.02	Bardet-Biedl syndrome 2 protein homolog
LOC113520322	2072	1632.82	1773.7	581.06	603.97	-1.49	0.00	0.01	Hypothetical protein KGM_209029
LOC113522775	1445	19.14	17.68	5.88	6.96	-1.49	0.00	0.01	Zinc finger protein 235-like
LOC113523476	1788	15.16	12.39	3.55	6.04	-1.49	0.00	0.01	Axoneme-associated protein mst101(2)
LOC113511921	3761.57	51.06	45.91	15.93	17.77	-1.50	0.00	0.01	Bifunctional 3'-phosphoadenosine 5'-phosphosulfate synthase
MSTRG.3191	297	187.06	148.3	65.5	64.39	-1.50	0.00	0.01	CRAL-TRIO domain-containing protein
LOC113515743	1810	33.02	36.63	11.62	12.53	-1.50	0.00	0.01	Uncharacterized protein LOC106136462
LOC113513171	652	34.41	36.77	10.72	14.32	-1.50	0.00	0.01	Adenosine kinase
LOC113509200	1230	6.39	6.74	1.95	2.61	-1.50	0.00	0.02	Zinc finger protein 700-like
MSTRG.7710	411	30.52	34.87	10.34	13.33	-1.50	0.00	0.02	Uncharacterized protein
LOC113517722	551	55.36	46	14.85	21.05	-1.50	0.00	0.01	P protein-like
LOC113510094	1538	27.61	27.08	9.38	9.53	-1.50	0.00	0.01	Gastrula Zinc finger protein

									XlCGF57.1-like
LOC113516168	2547	25.26	25.59	8.46	9.05	-1.50	0.00	0.01	SWI/SNF-related matrix-associated actin-dependent regulator of chromatin subfamily A-like protein 1
LOC113518669	2461	12.55	11.31	4.67	3.54	-1.51	0.00	0.01	Transmembrane channel-like protein 7 isoform X1
LOC113511743	1592	1.9	1.19	0.68	0.41	-1.51	0.01	0.04	Substance-K receptor-like isoform X2
LOC113514921	1460.25	41.27	45.42	16.11	13.28	-1.51	0.00	0.01	Aminoacylase-1-like
LOC113510168	736	30.5	38.75	12.35	11.7	-1.51	0.00	0.01	Uncharacterized protein LOC105383361
LOC113520563	2440.47	17.75	15.82	5.7	5.84	-1.51	0.00	0.01	USP6 N-terminal-like protein
LOC113509169	2292	23.65	24.12	8.23	8.16	-1.51	0.00	0.01	CD151 antigen-like
MSTRG.14999	2016	5.98	6.43	2.02	2.24	-1.51	0.00	0.01	Uncharacterized protein
LOC113514133	942	5.61	7.43	2.27	2.22	-1.51	0.00	0.02	Hypothetical protein KGM_206883
LOC113514348	2032	7.73	6.61	2.55	2.37	-1.51	0.00	0.01	Importin subunit Alpha-1-like
LOC113519903	3013.31	19.96	19.04	7.07	6.18	-1.51	0.00	0.01	Hemicentin-1-like
LOC113523025	5137	21.88	22.62	8.06	7.14	-1.51	0.00	0.01	Carboxylesterase
MSTRG.1763	721	12.19	15.03	3.69	5.74	-1.51	0.00	0.01	DNA-directed RNA polymerase III subunit RPC4-like
LOC113518015	1680	16.27	16.09	5.73	5.36	-1.51	0.00	0.01	Zinc finger protein OZF-like
LOC113515654	2670	26.46	21.99	7.8	8.78	-1.51	0.00	0.01	Geranylgeranyl pyrophosphate synthase
LOC113520195	900	46.48	43.05	17.15	13.69	-1.51	0.00	0.01	Retinoblastoma-binding protein 5 homolog isoform X1
LOC113513535	1122	29.37	29.95	9.5	10.89	-1.52	0.00	0.01	Structural maintenance of chromosomes protein 4
LOC113513585	1425.2	35.39	43.91	13.58	12.69	-1.52	0.00	0.01	Hypothetical protein CAPTEDRAFT_227268
LOC113523483	2379	1.8	1.81	0.59	0.64	-1.52	0.00	0.02	Golgin subfamily B member 1-like isoform X3
LOC113512027	1889.27	146.72	139.9	42.03	47.02	-1.52	0.00	0.01	Uncharacterized protein LOC110379767
MSTRG.8243	1239	4.02	3.18	0.79	1.68	-1.52	0.00	0.02	Uncharacterized protein
LOC113510916	1524	30.06	31.37	10.36	10.61	-1.52	0.00	0.01	Uncharacterized protein C630.12
LOC113523609	2084	732.17	417.84	177.69	213.12	-1.53	0.00	0.01	Spondin-1
LOC113522135	980	13.94	15.19	4.58	5.35	-1.53	0.00	0.01	Uncharacterized protein LOC106134417

MSTRG.15833	637.91	14.78	17.24	5.82	5.94	-1.53	0.00	0.01	Bifunctional coenzyme A synthase isoform X3
LOC113521791	669	30364.96	36013.57	12129.54	10660.95	-1.53	0.00	0.01	Uncharacterized protein
LOC113523275	2422	1.84	1.83	0.67	0.56	-1.53	0.00	0.02	Mitochondrial sodium/hydrogen exchanger 9B2 isoform X1
LOC113522799	2268	14.44	14.13	4.53	5.13	-1.53	0.00	0.01	Uncharacterized protein LOC110374636
LOC113516059	1661	12.51	11.88	4.02	4.22	-1.53	0.00	0.01	Origin recognition complex subunit 4
LOC113518354	1444	13.22	14.86	4.89	4.6	-1.53	0.00	0.01	Nucleoside diphosphate kinase 7
LOC113523175	1344	3.37	3.39	0.71	1.57	-1.53	0.00	0.02	Tyrosine-protein kinase-like otk
LOC113516579	568	11.98	7.44	2.1	4.61	-1.53	0.01	0.03	Protein O-mannosyl-transferase 2 isoform X2
MSTRG.5544	2077	3.99	5.41	1.04	2.13	-1.54	0.00	0.01	E3 ubiquitin-protein ligase HECTD1
LOC113510478	1001	315.93	316.82	100.94	113.59	-1.54	0.00	0.01	Apolipoprotein D
LOC113517407	1352	3.5	3.25	0.96	1.31	-1.54	0.00	0.02	Sterol O-acyltransferase 2
MSTRG.12888	725	29.68	31.56	10.06	10.79	-1.54	0.00	0.01	Vacuolar protein sorting-associated protein 16 homolog
LOC113512454	1709	1047.42	957.52	308.45	367.58	-1.54	0.00	0.01	Uncharacterized protein LOC105340758 isoform X1
LOC113509482	860	73.2	65.29	20.78	26.25	-1.54	0.00	0.01	Uncharacterized protein
LOC113522731	550.97	1073.92	1173.9	392.46	378.72	-1.54	0.00	0.01	Aquaporin AQPAn.G isoform X1
LOC113515162	703	32.04	24.9	9.29	10.08	-1.54	0.00	0.01	GPI transamidase component PIG-S
LOC113518121	979.99	1856.81	2159.36	705.73	676.71	-1.54	0.00	0.01	Elongation factor 1-delta isoform X1
LOC113522547	1418	51.36	56.28	16.8	19.36	-1.55	0.00	0.01	O-glucosyltransferase rumi homolog
LOC113515583	3559	1.74	0.51	0.48	0.27	-1.55	0.00	0.02	Membrane alanyl Aminopeptidase
LOC113516833	1245	6.97	8.09	1.92	3.12	-1.55	0.00	0.01	Protein msta, isoform B-like isoform X1
MSTRG.4164	2991	2.24	1.9	0.58	0.79	-1.56	0.00	0.01	Uncharacterized protein
LOC113518685	1474	73.27	84.7	28.42	24.12	-1.56	0.00	0.01	Serine protease snake
LOC113513184	1115	6.48	6.13	1.64	2.56	-1.56	0.00	0.01	Kinesin-like protein KIF20B
LOC113518072	18869	2.25	1.79	0.67	0.66	-1.56	0.00	0.01	Muscle M-line assembly protein unc-89
MSTRG.5604	4955	8.77	11.74	3.21	3.56	-1.56	0.00	0.01	Uncharacterized protein LOC106114752
MSTRG.7386	2279	14.05	14.21	4.16	5.2	-1.56	0.00	0.01	Uncharacterized protein
LOC113518342	1833	140.72	140.83	47.43	45.79	-1.56	0.00	0.01	Hydroxyacid-oxoacid transhydrogenase, mitochondrial

									isoform X1
LOC113511122	867	96.13	103.55	34.81	31.63	-1.57	0.00	0.01	Ubiquitin-conjugating enzyme E2 T-like
LOC113513127	676	16.34	13.96	3.59	6.56	-1.57	0.00	0.01	Hypothetical protein RR48_08923
LOC113514409	1434	10.18	11.02	3.62	3.37	-1.57	0.00	0.01	Uncharacterized protein LOC106136549
LOC113511074	1998.43	94.47	92.23	29.37	31.99	-1.57	0.00	0.00	Uncharacterized protein LOC106135838 isoform X3
LOC113515270	1267	10.5	7.73	2.36	3.66	-1.57	0.00	0.01	Unconventional myosin-Va
LOC113520017	2249	47.27	39.63	14.1	14.44	-1.57	0.00	0.00	Glycosyltransferase 25 family member isoform X1
LOC113517537	2021	1.57	0.81	0.36	0.42	-1.58	0.01	0.03	Uncharacterized protein LOC106131769
LOC113509137	1083	8.21	9.17	2.64	3.08	-1.58	0.00	0.01	Ras-related and estrogen-regulated growth inhibitor
LOC113520351	1654	29.43	36.22	10.14	11.41	-1.58	0.00	0.01	Histone acetyltransferase type B catalytic subunit
LOC113516540	476	10.14	6.85	3.04	2.69	-1.58	0.01	0.04	Uncharacterized protein LOC106129968
LOC113512735	804	5.62	2.54	0.91	1.78	-1.58	0.01	0.03	Semaphorin-5A
LOC113510949	897	23.53	27.43	8.62	8.12	-1.58	0.00	0.01	THAP domain-containing protein 4-like
MSTRG.7116	1794	63.17	73.31	23.08	21.47	-1.58	0.00	0.00	Hypothetical protein RR46_09733
LOC113521232	5600	25.43	25.58	7.46	9.16	-1.58	0.00	0.00	Epidermal growth factor receptor isoform X1
LOC113517202	622.89	177.48	203.41	70.17	60.11	-1.59	0.00	0.00	Uncharacterized protein LOC110369978
LOC113518668	595	28.34	20.28	7.48	8.65	-1.59	0.00	0.01	Phosphatidate phosphatase PPAPDC1B
LOC113513562	2809	34.57	37.67	11.31	12.15	-1.59	0.00	0.00	Uncharacterized protein LOC106142695
LOC113522710	1251.69	33.29	33.32	10.76	11.02	-1.59	0.00	0.00	Replication factor C subunit 4
LOC113514004	2498	3.92	3.27	1.18	1.15	-1.59	0.00	0.01	Hypothetical protein RR48_10059
MSTRG.10707	589	9.68	5.98	1.74	3.44	-1.59	0.00	0.02	Homeodomain-interacting protein kinase 2 isoform X1
LOC113513268	752	30.02	37.21	11.94	9.61	-1.59	0.00	0.01	Centromere protein S-like
LOC113513352	968	7.83	8.48	2.58	2.72	-1.60	0.00	0.01	Protein regulator of cytokinesis 1-like

LOC113514287	1915.49	9.6	8.13	2.72	2.99	-1.60	0.00	0.01	Uncharacterized protein LOC106130694 isoform X1
LOC113515399	1357	14.93	15.71	4.33	5.6	-1.60	0.00	0.01	Inositol polyphosphate 1-phosphatase
LOC113518815	3995	1202.25	971.67	349.18	351.07	-1.60	0.00	0.00	NADP-dependent malic enzyme-like isoform X2
LOC113515614	2231	8.52	12.55	3.84	2.92	-1.60	0.00	0.01	Fasciclin-1
LOC113516444	510	8.07	6.92	2.9	2.01	-1.61	0.01	0.03	Pyruvate dehydrogenase phosphatase regulatory subunit, mitochondrial
MSTRG.3598	2417	9.65	6.18	2.48	2.61	-1.61	0.00	0.01	Uncharacterized protein
LOC113517820	2007	26.28	28.68	8.79	8.85	-1.61	0.00	0.00	Cell division cycle protein 20 homolog
LOC113518576	3217.29	2.47	1.68	0.57	0.73	-1.61	0.00	0.01	Uncharacterized protein LOC106133529 isoform X1
LOC113521734	1311	77.31	65.91	25.17	20.84	-1.61	0.00	0.00	Protein PRRC1-like
LOC113521414	1774	18.44	18.45	6.29	5.52	-1.61	0.00	0.00	Condensin complex subunit 2
LOC113518288	5080	6.73	7.15	2.22	2.2	-1.61	0.00	0.00	Ras GTPase-activating-like protein IQGAP1
LOC113516620	3510	6.24	7.33	2.09	2.22	-1.62	0.00	0.00	Junctophilin-1 isoform X1
LOC113509396	2216	91.61	94.61	29.96	29.44	-1.62	0.00	0.00	Juvenile hormone epoxide hydrolase-like
LOC113515894	1603	9.39	11.19	2.9	3.65	-1.62	0.00	0.01	ATP-dependent RNA helicase DHX34
LOC113510715	952	3.37	3.62	1.42	0.8	-1.62	0.01	0.03	Hemicentin-2-like
LOC113509111	966.65	251.88	283.98	85.27	86.05	-1.62	0.00	0.00	Uncharacterized protein LOC106139573
LOC113515129	1723	14.36	11.2	4.04	4.09	-1.62	0.00	0.00	Cytochrome P450 6B5-like
LOC113514226	2516	29.74	30.97	8.46	10.8	-1.63	0.00	0.00	DNA replication licensing factor Mcm6
LOC113522635	1468	8.11	6.69	2.83	1.86	-1.63	0.00	0.01	Uncharacterized oxidoreductase SERP2049-like
LOC113514261	414	416.47	482.3	173.21	123.78	-1.63	0.00	0.00	U6 snRNA-associated Sm-like protein LSm5
LOC113518106	3095	8.53	3.3	2.35	1.38	-1.63	0.00	0.00	Peroxisomal acyl-coenzyme A oxidase 1 isoform X1
LOC113518851	1210	46.57	55.09	16.56	15.69	-1.63	0.00	0.00	Iodotyrosine dehalogenase 1
LOC113523542	2412	9.47	11.89	3.19	3.55	-1.63	0.00	0.00	Uncharacterized protein PFB0145c-

									like
LOC113511638	4587	28.98	28.77	8.7	9.49	-1.63	0.00	0.00	Uncharacterized protein LOC110378979
LOC113520993	800	48.47	38.49	10.85	16.94	-1.63	0.00	0.00	P protein-like
LOC113513611	537	8.08	8.07	2.35	2.84	-1.63	0.01	0.02	Uncharacterized protein OBRU01_06696
LOC113516752	1657.09	30.81	30.86	10.63	7.59	-1.63	0.00	0.00	Apterous A isoform X3
LOC113512377	1821	4.78	4.41	1.07	1.83	-1.63	0.00	0.01	Organic cation transporter protein-like
LOC113511027	3177.17	2.43	2.38	0.8	0.74	-1.63	0.00	0.01	Nucleolar protein 4-like
LOC113512996	2895	4.17	4.86	1.53	1.31	-1.64	0.00	0.01	DNA repair protein REV1
LOC113520356	865	93.24	89.61	32.6	25.35	-1.64	0.00	0.00	Deoxyuridine 5'-triphosphate nucleotidohydrolase
LOC113516521	688	158.48	119.99	37.51	51.6	-1.64	0.00	0.00	Talin-1-like
LOC113522127	3042	1.94	1.76	0.7	0.46	-1.64	0.00	0.01	Uncharacterized protein LOC105389520
LOC113512450	1435	149.68	135.42	42.55	47.2	-1.64	0.00	0.00	ATP-binding cassette sub-family G member 4-like isoform X1
MSTRG.12093	918	22.01	13.22	3.74	7.44	-1.64	0.00	0.01	Mitochondrial aldehyde dehydrogenase
LOC113510960	808.69	15.42	12.38	3.99	4.8	-1.64	0.00	0.01	Uncharacterized protein LOC106129749
LOC113518711	1827	8.78	9.15	2.88	2.73	-1.64	0.00	0.00	UDP-N-acetylglucosamine pyrophosphorylase
LOC113517453	2139	1.59	1.22	0.41	0.47	-1.64	0.00	0.02	Sodium-coupled monocarboxylate transporter 1
LOC113515715	841	37.47	35.08	10.56	12.38	-1.64	0.00	0.00	Transcription factor CP2 isoform X5
LOC113511964	2279	114.25	121.87	36.58	37.26	-1.64	0.00	0.00	Transcription cofactor vestigial-like protein 4
LOC113511497	514	12.13	12.85	5.14	2.83	-1.64	0.00	0.02	Sn1-specific diacylglycerol Lipase beta-like
LOC113521103	1313	131.9	120.62	50.47	28.51	-1.64	0.00	0.00	Uncharacterized protein LOC106130198 isoform X1
LOC113518201	697	13.89	15.15	4.06	5.11	-1.65	0.00	0.01	Transmembrane protein 138-like
LOC113516200	1181	7.65	5.26	1.67	2.34	-1.65	0.00	0.01	PR domain Zinc finger protein 16-like isoform X1
LOC113515693	3625	0.66	0.13	0.12	0.12	-1.65	0.01	0.04	Membrane alanyl Aminopeptidase-like

LOC113516240	2854	5.47	5.49	1.47	1.93	-1.66	0.00	0.00	Chromosome transmission fidelity protein 18 homolog
LOC113509183	865.37	25.58	13.87	6.28	6.05	-1.66	0.00	0.00	Uncharacterized protein
LOC113509343	2671	22.33	21.78	6.53	7.1	-1.66	0.00	0.00	Structural maintenance of chromosomes protein 4
LOC113522675	1599.86	451.69	436.73	137.02	137.81	-1.66	0.00	0.00	Uncharacterized protein LOC106118229
LOC113513798	306	628.02	501.95	168.37	220.69	-1.67	0.00	0.00	Protein croquemort-like isoform X1
LOC113522657	4292	4.84	5.41	1.48	1.67	-1.67	0.00	0.00	Fanconi anemia group D2 protein
LOC113519526	1246	29.09	32.66	9.14	9.91	-1.67	0.00	0.00	Zinc finger protein 525-like
LOC113523449	1136	1.08	1.91	0.59	0.31	-1.67	0.01	0.04	Uncharacterized protein
LOC113522785	1950	6.25	7.23	1.81	2.33	-1.67	0.00	0.00	Dual specificity protein kinase TTK
LOC113520601	2058	10.68	9.06	2.94	3.13	-1.67	0.00	0.00	Rac GTPase-activating protein 1-like isoform X2
LOC113515557	1651	6.28	8.88	2.25	2.39	-1.67	0.00	0.00	G2/mitotic-specific cyclin-B
LOC113509559	2521	2.12	2.14	0.58	0.72	-1.67	0.00	0.01	Uncharacterized protein LOC106142577 isoform X1
LOC113515217	1883	147.17	143.8	43.09	46.12	-1.67	0.00	0.00	Disulfide oxidoreductase
MSTRG.14290	1805	1.22	1.73	0.59	0.31	-1.68	0.00	0.02	Uncharacterized protein
MSTRG.11687	1834	1.37	2.09	0.62	0.43	-1.68	0.00	0.02	Uncharacterized protein
MSTRG.10169	412	396.02	331.83	110.05	124.54	-1.68	0.00	0.00	Uncharacterized protein LOC106134230
LOC113516223	734	23.83	27.09	8.52	7.2	-1.68	0.00	0.00	Protein MIS12 homolog
LOC113512525	1779	105.35	93.2	27.7	33.13	-1.68	0.00	0.00	ATP-binding cassette sub-family G member 4-like isoform X2
MSTRG.7832	952	7.13	5.33	2.03	1.79	-1.68	0.00	0.01	Unconventional myosin-Va isoform X1
LOC113516710	3210.7	278.3	296.29	87.74	87.17	-1.68	0.00	0.00	Alpha-tocopherol transfer protein-like isoform X1
MSTRG.2884	1914	10.41	10.48	3.4	2.94	-1.68	0.00	0.00	Uncharacterized protein
LOC113512481	2139	3.5	2.41	1.11	0.69	-1.68	0.00	0.01	WD repeat-containing protein on Y chromosome
LOC113519401	960.62	3.41	3.4	0.17	1.77	-1.68	0.00	0.02	Protein pangolin, isoforms A/H/I/S isoform X3
LOC113521896	1530	11.37	10.47	2.96	3.7	-1.68	0.00	0.00	Ionotropic receptor IR64a
LOC113519053	2271	50.04	48.07	14.81	15.02	-1.69	0.00	0.00	Vacuolar protein sorting-associated protein 16 homolog

LOC113515417	1190	13.04	14.34	4.59	3.75	-1.69	0.00	0.00	Aldo-keto reductase AKR2E4-like isoform X5
LOC113522693	4600.19	24.85	26.64	7.51	8.11	-1.69	0.00	0.00	DNA polymerase alpha catalytic subunit
LOC113518480	2941.27	16.4	11.65	3.69	4.79	-1.69	0.00	0.00	Solute carrier organic anion transporter family member 3A1-like isoform X2
LOC113515554	1863	5.14	7.35	1.91	1.86	-1.69	0.00	0.00	Zinc finger protein Noc
LOC113520600	1445	24.65	25.66	9.12	6.09	-1.69	0.00	0.00	BMP and activin membrane-bound inhibitor homolog
LOC113515131	648	8.48	7.01	2.2	2.53	-1.69	0.00	0.01	Uncharacterized protein LOC110371738
LOC113510337	691	4.26	3.81	0.99	1.46	-1.70	0.01	0.03	Glutathione S-transferase
MSTRG.9030	321	27.72	10.9	3.48	9.51	-1.70	0.01	0.04	Protein toll-like
LOC113515181	1678	5.58	3.94	1.47	1.39	-1.70	0.00	0.01	Unconventional myosin-XVIIIa isoform X1
LOC113522496	2195.42	45.77	41.75	11.74	14.6	-1.70	0.00	0.00	Kynurenine aminotransferase
LOC113510671	826	8.64	1.55	2.12	0.98	-1.70	0.00	0.01	Uncharacterized protein LOC101747082
LOC113521024	1120	25.12	32.71	9.36	8.04	-1.70	0.00	0.00	Uncharacterized protein LOC106133696
LOC113511083	1900	2.36	1.85	0.72	0.54	-1.71	0.00	0.01	Facilitated trehalose transporter Tret1-like
LOC113513108	1210	1.63	1.31	0.59	0.29	-1.71	0.01	0.03	Mitochondrial sodium/hydrogen exchanger NHA2
LOC113509367	2225	28.76	33.4	8.62	10	-1.71	0.00	0.00	Ommochrome-binding protein-like
LOC113517164	774	68.25	64.56	14.99	25.12	-1.72	0.00	0.00	Calcium-dependent secretion activator isoform X3
LOC113515952	1306	242.88	258.44	76.89	72.75	-1.72	0.00	0.00	Glyoxylate reductase/hydroxypyruvate reductase-like isoform X1
LOC113520523	2461	59.24	57.13	17.75	16.76	-1.72	0.00	0.00	Replication protein A 70 kDa DNA-binding subunit
LOC113510306	1506	0.89	1.2	0.39	0.22	-1.72	0.01	0.03	Neural cell adhesion molecule 2-like
LOC113516667	2694	24.09	20.58	6.81	6.42	-1.72	0.00	0.00	Protein FAN-like
LOC113517325	915	4.15	2.29	1.18	0.74	-1.72	0.00	0.02	Ankyrin repeat domain-containing protein 29
LOC113519350	1091	10.87	12.46	3.46	3.46	-1.73	0.00	0.00	Hypothetical protein KGM_206883

LOC113512972	1651	48.33	51.7	15.26	14.27	-1.73	0.00	0.00	Speckle targeted PIP5K1A-regulated poly(A) polymerase isoform X2
MSTRG.10017	768	2.77	3.79	0.95	0.96	-1.73	0.01	0.03	Uncharacterized protein LOC106138419
MSTRG.5431	1677	5.7	5.35	1.57	1.68	-1.73	0.00	0.00	Uncharacterized protein
LOC113520319	1544	685.94	677.41	195.31	205.94	-1.74	0.00	0.00	Uncharacterized protein CG1785
LOC113518309	1158	82.04	87.26	25.24	24.62	-1.74	0.00	0.00	Uncharacterized protein LOC105393001
LOC113511207	4363	1.06	0.77	0.3	0.23	-1.74	0.00	0.01	Uncharacterized protein LOC106135765
LOC113521665	7646	10.14	10.94	3.06	3.09	-1.74	0.00	0.00	Centromere protein F-like
LOC113514651	1043	3.54	4.07	1.16	1.05	-1.75	0.00	0.01	Alpha-tocopherol transfer protein-like
LOC113518457	2528	99.15	88.42	26.06	28.55	-1.75	0.00	0.00	Protein AF-10-like isoform X1
LOC113515170	5086	17.69	1.83	4.2	1.45	-1.75	0.00	0.00	Fatty acid synthase-like
LOC113518224	1734	2.12	2.17	1.04	0.28	-1.75	0.00	0.02	Uncharacterized protein LOC106140166 isoform X1
LOC113514176	2801	81.94	51.55	17.93	20.83	-1.75	0.00	0.00	Laminin A chain
LOC113520363	1527	34.57	39.24	11.38	10.04	-1.75	0.00	0.00	Aurora kinase B-like
LOC113518420	678	3.06	3.32	0.68	1.17	-1.75	0.01	0.03	Uncharacterized protein LOC110381236 isoform X2
LOC113518531	13445	0.61	0.59	0.17	0.17	-1.75	0.00	0.00	E3 ubiquitin-protein ligase MYCBP2
LOC113517409	4434	0.26	0.25	0.03	0.11	-1.76	0.01	0.05	Transmembrane protein 132B
LOC113520938	949	13.28	13.34	3.36	4.4	-1.76	0.00	0.00	Uncharacterized protein LOC105842306
MSTRG.6905	638	4.87	4.19	2.26	0.37	-1.76	0.01	0.03	G-protein coupled receptor moody
MSTRG.2911	858.79	13	11.84	3.28	4.07	-1.76	0.00	0.00	THO complex subunit 2
LOC113521605	5599.58	19.8	17.1	5.39	5.24	-1.76	0.00	0.00	Transient receptor potential cation channel trpm isoform X8
LOC113509102	481	24459.11	27725.01	8791.14	6716.09	-1.76	0.00	0.00	Uncharacterized protein LOC110371017
LOC113517271	1212	22.16	27.49	6.75	7.61	-1.76	0.00	0.00	Serine/threonine-protein kinase Aurora-2
MSTRG.5071	3811	1.65	1.57	0.41	0.51	-1.76	0.00	0.00	Uncharacterized protein LOC110379667
LOC113509107	2260	15.6	17.35	5.14	4.33	-1.76	0.00	0.00	Iduronate 2-sulfatase
LOC113520474	1261	4.76	4.35	1.33	1.3	-1.76	0.00	0.01	Uncharacterized protein

									LOC106130527 isoform X1
LOC113520762	2338	12.31	10.23	2.67	3.81	-1.77	0.00	0.00	F-box/LRR-repeat protein 4 isoform X1
LOC113512029	1705	9.07	15.42	4.48	2.54	-1.77	0.00	0.00	Uncharacterized protein LOC106135547
LOC113516171	1275	93.15	71.77	24.01	23.55	-1.77	0.00	0.00	Uncharacterized protein LOC106720945
LOC113517479	1515	4.45	4.31	0.89	1.63	-1.77	0.00	0.00	Polycomb
LOC113513738	6215	9.19	9.27	2.33	2.95	-1.77	0.00	0.00	Uncharacterized protein LOC110381944
LOC113514576	5692	31.62	31.23	8.5	9.45	-1.77	0.00	0.00	Complexin
LOC113521283	2502	12.02	10.97	3.34	3.23	-1.77	0.00	0.00	Guanylate kinase-associated protein mars isoform X1
LOC113513314	1087.7	16.8	16.95	4.53	5.55	-1.77	0.00	0.00	Tumor protein p63-regulated gene 1-like protein isoform X2
LOC113516859	1740	37.99	32.72	9.21	11.02	-1.78	0.00	0.00	Acid trehalase-like protein 1
LOC113523215	15327.2	0.09	0.11	0.03	0.03	-1.78	0.01	0.03	Ryanodien receptor
LOC113522361	871	13.36	16.92	4.15	4.52	-1.78	0.00	0.00	Zinc finger protein 57
MSTRG.4880	453	9.61	12.72	5.71	0.76	-1.78	0.00	0.02	Uncharacterized protein LOC106136113
MSTRG.16509	1748	3.02	3.16	0.72	1.05	-1.78	0.00	0.01	Chromosome transmission fidelity protein 18 homolog
LOC113518925	1024	85.33	95.41	28.64	22.92	-1.78	0.00	0.00	2-hydroxy-6-oxononadienedioate/2-hydroxy-6-oxononatrienedioate hydrolase isoform X1
LOC113511516	732	6.37	7.2	2.1	1.77	-1.78	0.00	0.01	Hypothetical protein KGM_209449
LOC113523100	3211.52	20.66	17.96	5.94	4.74	-1.78	0.00	0.00	Uncharacterized protein LOC106136948 isoform X1
MSTRG.1832	590	8.63	9.43	3.04	2.14	-1.78	0.00	0.01	Uncharacterized protein
LOC113509701	1235	78.99	84.46	23.65	22.85	-1.79	0.00	0.00	Replication factor C subunit 3
LOC113513306	521	7.94	6.37	1.66	2.47	-1.79	0.00	0.02	NAD kinase 2, mitochondrial
LOC113519073	951	5.22	3.62	1.12	1.39	-1.79	0.00	0.01	Cyclin-dependent kinase 2
LOC113518311	2406	14.93	15.88	4.69	4.01	-1.79	0.00	0.00	DNA helicase MCM8
LOC113514632	714	14.71	16.24	4.37	4.45	-1.79	0.00	0.00	Fidgetin-like protein 1
LOC113522063	835	13.81	20.84	4.9	4.93	-1.79	0.00	0.00	RNA pseudouridylate synthase domain-containing protein 1-like
LOC113517028	3054	44.37	50.1	12.28	14.32	-1.80	0.00	0.00	Centrosomin

LOC113511655	1495	2.91	2.47	0.9	0.61	-1.80	0.00	0.01	KRAB-A domain-containing protein 2-like
LOC113514087	1733	8.26	6.53	2.13	2.03	-1.80	0.00	0.00	Cryptochrome-1 isoform X2
LOC113519788	2223	25.47	29.3	7.71	7.66	-1.80	0.00	0.00	DNA replication licensing factor Mcm5
LOC113511682	1668.15	32.52	34.33	9.65	9.7	-1.80	0.00	0.00	Acetyltransferase
LOC113513510	1423	17.51	18.13	4.38	5.62	-1.80	0.00	0.00	Diacylglycerol kinase epsilon isoform X2
LOC113517807	1605	116.72	87.56	24.12	33.27	-1.80	0.00	0.00	Laminin subunit gamma-1
LOC113517014	1427	1.23	0.91	0.3	0.29	-1.81	0.01	0.03	Uncharacterized protein LOC106143120
LOC113522198	558	238.86	252.85	82.34	57.29	-1.81	0.00	0.00	Ras-related protein Rab-18-B isoform X1
LOC113514281	1234.9	185.7	190.78	55.12	50.05	-1.81	0.00	0.00	HMG box-containing protein 4
MSTRG.7518	405	23.25	24.67	9.25	4.64	-1.81	0.00	0.01	Protein espinas isoform X3
MSTRG.6511	477	9.31	9.49	1.68	3.68	-1.81	0.00	0.02	Hypothetical protein RR46_14024
MSTRG.8449	1675	0.9	0.7	0.3	0.14	-1.82	0.01	0.03	Uncharacterized protein LOC106140458
LOC113521663	2687	18.36	13.34	4.23	4.57	-1.82	0.00	0.00	DNA replication licensing factor MCM4
LOC113510051	1185	3.71	2.49	0.98	0.74	-1.82	0.00	0.01	Hypothetical protein RR48_06950
LOC113522992	1228.41	340.96	344.32	93.79	97.32	-1.82	0.00	0.00	1-acyl-sn-glycerol-3-phosphate acyltransferase Alpha-like
LOC113522139	795	11.63	9.39	2.51	3.37	-1.82	0.00	0.00	5-oxoprolinase
MSTRG.4848	701	36.61	37.6	11.11	9.66	-1.82	0.00	0.00	Uncharacterized protein LOC106143306
LOC113513289	421	18.16	16.94	3.22	6.92	-1.82	0.00	0.01	TBC1 domain family member 4 isoform X1
LOC113520146	1074	33.68	38.33	10.76	9.16	-1.83	0.00	0.00	Hypothetical protein KGM_206883
LOC113520754	1050	5.82	7.59	2.31	1.39	-1.83	0.00	0.00	Uncharacterized protein LOC110374696
LOC113513860	902	63.63	72.85	17.75	20.09	-1.83	0.00	0.00	Phytanoyl-CoA dioxygenase, peroxisomal-like
LOC113515218	517	216.23	204.42	60.31	58.37	-1.83	0.00	0.00	Apoptosis-inducing factor 3-like isoform X1
LOC113511378	639	2.94	2.5	0.75	0.74	-1.83	0.01	0.05	Zinc finger protein 648-like isoform X5
LOC113517282	1475.84	5.9	7.22	0.3	3.25	-1.84	0.00	0.00	Argininosuccinate lyase isoform X1

LOC113521494	654	5.8	4.01	2.17	0.53	-1.84	0.00	0.02	Mpv17-like protein
LOC113511104	2465	0.43	0.51	0.16	0.09	-1.84	0.01	0.05	Collagen alpha-1(XXI) chain
MSTRG.7934	6630	79.39	75.21	20.78	21.31	-1.84	0.00	0.00	cAMP-responsive element-binding protein-like 2 isoform X1
LOC113518896	1072	1287.39	1431.67	405.34	338.48	-1.84	0.00	0.00	Apolipoprotein D
LOC113509644	2825	12.7	14.45	3.9	3.37	-1.85	0.00	0.00	Uncharacterized protein LOC106137340 isoform X1
LOC113509602	1789	6.31	5.42	2.05	1.11	-1.85	0.00	0.00	Leucine zipper tumor suppressor 2 homolog isoform X1
LOC113518258	572	84.72	147.6	36.6	27.29	-1.85	0.00	0.00	Uncharacterized protein
LOC113511203	844	2.95	1.93	0.97	0.36	-1.85	0.00	0.02	Hypothetical protein RR46_07971
LOC113523265	2287	1.58	1.68	0.34	0.53	-1.85	0.00	0.01	Protein bric-a-brac 2-like isoform X2
LOC113520328	2317	1358.28	1532.48	389.3	392.19	-1.85	0.00	0.00	Uncharacterized protein LOC106131668
LOC113521028	408	35.69	39.1	11.06	10.09	-1.85	0.00	0.00	Uncharacterized protein LOC106133088
LOC113517597	1325	37.73	32.02	9.96	8.91	-1.86	0.00	0.00	Ras-related protein Rab-37 isoform X1
MSTRG.1009	1031	1.73	2.67	0.82	0.36	-1.86	0.00	0.02	Uncharacterized protein
LOC113521784	704	3.64	3.41	0.8	1.1	-1.86	0.00	0.02	DNA repair protein RAD52 homolog isoform X2
LOC113518005	1335	20.49	20.26	4.61	6.41	-1.86	0.00	0.00	Glucose-1-phosphatase-like
LOC113512799	4700	0.42	0.1	0.06	0.08	-1.87	0.01	0.03	Niemann-Pick C1 protein-like
LOC113513141	1679.92	180.74	182.46	50.66	46.71	-1.87	0.00	0.00	Cytochrome P450 9e2-like
LOC113514381	1185	6.95	5.93	1.97	1.48	-1.87	0.00	0.00	Uncharacterized protein LOC106138920
LOC113512371	2241	1.65	2.28	0.67	0.38	-1.87	0.00	0.00	Uncharacterized protein LOC106142703
LOC113522631	2306	227.85	224.22	56.72	64.07	-1.87	0.00	0.00	Beta-1,4-glucuronyltransferase 1
LOC113518482	6282	15.42	14.96	3.97	4.1	-1.88	0.00	0.00	Uncharacterized protein LOC105842463 isoform X8
LOC113512130	7014	10.23	10.85	2.66	2.94	-1.88	0.00	0.00	Protein abnormal spindle
LOC113513243	311	229.47	266.76	71.69	73.78	-1.88	0.00	0.00	Glutathione S-transferase sigma 5
LOC113510785	2468	8.04	8.18	2.61	1.69	-1.88	0.00	0.00	Centrosomal protein of 120 kDa-like
LOC113517270	1362	568.39	550.17	157.09	140.6	-1.88	0.00	0.00	Isocitrate dehydrogenase
LOC113514086	256	144.75	145.17	42.99	45.17	-1.88	0.00	0.01	Talin-B
MSTRG.3184	2944	5.04	5	1.5	1.16	-1.88	0.00	0.00	Uncharacterized protein

LOC113512275	1663	43.9	38.06	10.72	11.05	-1.88	0.00	0.00	Juvenile hormone epoxide hydrolase-like
LOC113512897	1960	98.13	84.79	22.85	25.69	-1.88	0.00	0.00	Fumarate hydratase, mitochondrial
LOC113511315	1102	20.99	23.04	6.32	5.37	-1.89	0.00	0.00	Three prime repair exonuclease 2
LOC113517925	837	12.13	9.67	2.69	3.12	-1.89	0.00	0.00	Zinc finger protein 624-like isoform X1
LOC113520349	1144.34	1413.8	1523.6	402.78	375.82	-1.89	0.00	0.00	Uncharacterized protein LOC106131507
LOC113520159	579	3086.15	2600.27	713.57	819.04	-1.89	0.00	0.00	Lysosome-associated membrane glycoprotein 2-like
MSTRG.5918	2269	23.43	27.48	7.05	6.3	-1.90	0.00	0.00	Protease inhibitor 5
LOC113519685	2651	5.06	5.54	1.33	1.44	-1.90	0.00	0.00	Uncharacterized protein LOC106129641
LOC113514317	3538	2.12	1.77	0.4	0.62	-1.90	0.00	0.00	Anoctamin-9
LOC113520840	1777	1583.55	1746.48	451.05	416.45	-1.91	0.00	0.00	High mobility group protein D
MSTRG.16063	1997	71.51	71.15	20	17.06	-1.91	0.00	0.00	Uncharacterized protein
LOC113511922	1390	16.6	14.53	4.51	3.56	-1.92	0.00	0.00	Isovaleryl-CoA dehydrogenase, mitochondrial
LOC113512156	3825	7.32	6.63	1.73	1.85	-1.93	0.00	0.00	Trichohyalin-like
MSTRG.1579	2219	195.75	174.98	47.35	48.1	-1.93	0.00	0.00	BCL2/adenovirus E1B 19 kDa protein-interacting protein 3 isoform X1
LOC113510666	1244	70.78	55.13	15.34	17.22	-1.93	0.00	0.00	Fructose-1,6-bisphosphatase
MSTRG.6595	229	1202.6	1001.53	407.66	248.12	-1.93	0.00	0.00	Uncharacterized protein
LOC113514563	1026	15.27	16.63	3.84	4.39	-1.93	0.00	0.00	Transmembrane protein 80-like
LOC113515821	4106	8.35	6.64	1.93	1.91	-1.93	0.00	0.00	Serine/threonine-protein phosphatase 6 regulatory Ankyrin repeat subunit B
LOC113514250	1149	56.26	66.56	16.13	15.48	-1.93	0.00	0.00	Proline synthase co-transcribed bacterial homolog protein
LOC113509276	822	4.25	3.01	0.88	0.99	-1.93	0.00	0.01	Immune-related Hdd13
LOC113518649	1732	3.46	1.1	0.47	0.69	-1.93	0.00	0.00	Cytochrome P450 6B46
LOC113523213	614	16.87	17.73	3.03	5.97	-1.93	0.00	0.00	Inactive tyrosine-protein kinase 7
LOC113513036	601	341.34	383.48	96.98	91.74	-1.93	0.00	0.00	Ecdysteroid-regulated 16 kDa protein-like
LOC113512947	753	6.99	10.21	2.3	2.12	-1.94	0.00	0.00	Uncharacterized protein LOC106142568

LOC113510250	3864	4.27	2.5	0.81	0.92	-1.94	0.00	0.00	Neurexin-1
LOC113523050	1779	2.22	1.35	0.46	0.45	-1.94	0.00	0.00	4-coumarate--CoA ligase 1-like
LOC113513731	759	18.28	18.9	4.68	4.87	-1.94	0.00	0.00	7,8-dihydro-8-oxoguanine triphosphatase-like
LOC113519810	3234	8.07	6.93	2.26	1.54	-1.94	0.00	0.00	Myosin light chain kinase
LOC113520555	1958	36.38	39.52	9.7	9.59	-1.94	0.00	0.00	Cytochrome P450 9e2-like
LOC113522357	1143	12.99	10.03	2.62	3.26	-1.94	0.00	0.00	Kinesin-like protein KIF18A
LOC113521544	1414	46.41	50.44	11.91	12.76	-1.94	0.00	0.00	Palmitoyl-protein thioesterase 1
LOC113522905	540	1248.5	1134.32	307.42	312.86	-1.95	0.00	0.00	Aquaporin AQPAn.G isoform X1
LOC113519796	2077	45.12	47.09	11.16	12.24	-1.95	0.00	0.00	N-Alpha-acetyltransferase 40
LOC113510299	825	33.53	43.42	9.99	9.68	-1.95	0.00	0.00	Uncharacterized protein LOC106136821
LOC113514597	636	51.17	78.77	16.51	16.82	-1.95	0.00	0.00	Uncharacterized protein
LOC113512980	3687	7.68	6.69	1.74	1.89	-1.95	0.00	0.00	Uncharacterized protein LOC106121753 isoform X1
LOC113519291	1208	5.08	3.88	1.25	1.01	-1.95	0.00	0.00	Transcription termination factor 2 isoform X2
LOC113517017	2002	0.7	0.57	0.08	0.23	-1.95	0.01	0.03	Peritrophin-48-like
LOC113520016	889.69	43.42	45.29	10.37	12.49	-1.96	0.00	0.00	Dihydrofolate reductase
LOC113513215	641	8.05	7.31	2.25	1.66	-1.96	0.00	0.00	Cytosolic 10-formyltetrahydrofolate dehydrogenase
LOC113522951	989	1.6	1.46	0.19	0.57	-1.96	0.01	0.03	Sarcosine dehydrogenase, mitochondrial
LOC113517648	2581	55.91	48	12.13	14.02	-1.96	0.00	0.00	Leucine-rich repeat-containing G-protein coupled receptor 4
MSTRG.9521	1312	159.94	167.97	40.94	41.78	-1.96	0.00	0.00	Glutathione S-transferase 2-like
LOC113520841	6653	89.93	74.51	20.55	20.52	-1.97	0.00	0.00	NFX1-type Zinc finger-containing protein 1-like
LOC113517249	747	2.74	1.29	0.44	0.57	-1.97	0.01	0.04	Uncharacterized protein
LOC113518962	1239	41.17	39.4	9.92	10.33	-1.97	0.00	0.00	Lipid storage droplets surface-binding protein 1 isoform X1
LOC113513508	1247	14.3	15.4	3.96	3.46	-1.97	0.00	0.00	G2/mitotic-specific cyclin-B3 isoform X1
LOC113511646	1927	55.59	63.7	15.59	14.16	-1.97	0.00	0.00	Uncharacterized protein LOC106133717
MSTRG.3078	1046	1.34	1.91	0.54	0.26	-1.97	0.00	0.02	Uncharacterized protein LOC106132165

LOC113516126	2456	0.4	0.37	0.03	0.15	-1.97	0.01	0.05	Uncharacterized transporter slc-17.2-like
LOC113512863	1006	999.21	1057.65	264.25	250.95	-1.97	0.00	0.00	Uncharacterized protein LOC101744658
LOC113517138	2815	65.95	60.18	15.14	16.25	-1.97	0.00	0.00	Calcium-dependent secretion activator
MSTRG.1611	1091	10	10.06	2.61	2.39	-1.97	0.00	0.00	Aldehyde dehydrogenase family 1 member L1
LOC113510048	6835	4.24	3.3	0.9	0.96	-1.98	0.00	0.00	Uncharacterized protein LOC110381761
LOC113509675	2019	2.17	3.07	0.83	0.46	-1.98	0.00	0.00	Spondin-2
LOC113523301	5153.62	244.59	211.53	54.08	63.32	-1.98	0.00	0.00	Vascular endothelial growth factor receptor 1 isoform X1
MSTRG.2627	713.25	168.16	159.23	36.01	47.08	-1.98	0.00	0.00	Calpain-A-like isoform X3
LOC113515642	4388	24.81	22.58	5.78	5.91	-1.98	0.00	0.00	Tetratricopeptide repeat protein 21B-like
LOC113523512	1880	11.24	11.39	2.71	2.91	-1.98	0.00	0.00	Uncharacterized protein
MSTRG.13866	1241	1.64	1.52	0.28	0.49	-1.98	0.00	0.01	Uncharacterized protein
LOC113515722	1600	1.4	1.11	0.26	0.36	-1.98	0.00	0.01	Tubulin beta chain-like
LOC113516800	1950	0.62	0.78	0.16	0.2	-1.99	0.00	0.01	Uncharacterized protein LOC110375454
LOC113518166	388.74	98.09	125.2	27.53	30.39	-1.99	0.00	0.00	Uncharacterized protein LOC106138152
MSTRG.10901	392	479.64	571.97	148.87	124.15	-1.99	0.00	0.00	Uncharacterized protein
LOC113513186	4782	5.64	5.29	1.45	1.22	-1.99	0.00	0.00	Uncharacterized protein LOC106137033 isoform X1
LOC113517959	575	72.55	54.91	17.09	14.86	-1.99	0.00	0.00	Calcium-dependent secretion activator
LOC113511759	1934	65.99	69.36	16.48	16.74	-1.99	0.00	0.00	D-2-hydroxyglutarate dehydrogenase, mitochondrial-like
MSTRG.7567	513	14.17	11.89	2.29	4.26	-2.00	0.00	0.00	Uncharacterized protein
LOC113514252	1593	35.05	34.35	8.36	8.67	-2.00	0.00	0.00	Targeting protein for Xklp2-like
MSTRG.9268	4207.8	36.84	40.87	8.61	10.25	-2.01	0.00	0.00	Uncharacterized protein
LOC113521957	1135	3.1	2.49	1.12	0.23	-2.01	0.00	0.00	Uncharacterized protein LOC106711175 isoform X1
MSTRG.15013	1764	145.18	161.86	36.45	38.02	-2.01	0.00	0.00	Uncharacterized protein
MSTRG.8223	1733	2.41	2.44	0.61	0.55	-2.01	0.00	0.00	Cytosolic endo-beta-N-acetylglucosaminidase

LOC113518833	2122.36	94.05	107.47	24.6	24.27	-2.01	0.00	0.00	TAF5-like RNA polymerase II p300/CBP-associated factor-associated factor 65 kDa subunit 5L
LOC113510572	668	4808.21	4833.72	1138.16	1227.88	-2.02	0.00	0.00	Uncharacterized protein
LOC113518995	1749	17.67	19.84	4.91	4.15	-2.02	0.00	0.00	Protein zwilch isoform X1
LOC113510571	633	37.08	35.98	9.56	8.29	-2.02	0.00	0.00	Glutathione S-transferase epsilon 6 isoform X1
LOC113515083	3479.94	8.68	7.2	2.23	1.57	-2.02	0.00	0.00	Fanconi anemia group J protein homolog isoform X1
LOC113515980	917	1.27	1.43	0.21	0.42	-2.02	0.01	0.04	Troponin C
LOC113517047	5812	1.44	1.49	0.3	0.4	-2.03	0.00	0.00	Neural-cadherin isoform X4
LOC113516716	1479	13.25	14.94	2.86	3.91	-2.03	0.00	0.00	DNA primase large subunit
LOC113514675	784	70.65	69.66	17.13	16.81	-2.03	0.00	0.00	N-acetyltransferase 6
LOC113521944	986	31.85	37.19	7.83	8.71	-2.04	0.00	0.00	Glutathione S-transferase
LOC113515136	996	5.27	5.01	0.95	1.49	-2.04	0.00	0.00	Coiled-coil domain-containing protein 42 homolog
LOC113509700	1108	108.07	119.83	31.04	23.11	-2.05	0.00	0.00	Methylthioribulose-1-phosphate dehydratase
LOC113518191	1735	9.41	7.15	2.12	1.8	-2.05	0.00	0.00	PDZ domain-containing protein 2-like
LOC113513387	1635	8.85	7.63	1.92	1.98	-2.05	0.00	0.00	Uncharacterized protein LOC106107480 isoform X1
LOC113511647	545	3.73	5.82	0.76	1.5	-2.05	0.00	0.01	Bardet-Biedl syndrome 1 protein
LOC113515509	1967	0.39	1.02	0.24	0.08	-2.05	0.00	0.02	Katanin p60 ATPase-containing subunit A1-like
LOC113510066	641	88.96	101.28	22.27	23.41	-2.05	0.00	0.00	Uncharacterized protein LOC110382578
LOC113514610	975	78.26	93.82	20.23	20.68	-2.05	0.00	0.00	Uncharacterized protein LOC106134395
MSTRG.6462	1616	4.9	5.22	1.19	1.21	-2.05	0.00	0.00	Menin
LOC113520069	707	25.28	29.33	5.24	7.8	-2.05	0.00	0.00	Uncharacterized protein
LOC113509351	871	46.89	30.1	7.95	10.4	-2.05	0.00	0.00	Ommochrome-binding protein-like
LOC113509976	2206	0.79	0.57	0.18	0.14	-2.05	0.00	0.01	Facilitated trehalose transporter Tret1-2-like
MSTRG.7962	476	15.33	9.83	3.38	2.69	-2.06	0.00	0.00	Uncharacterized protein
LOC113512475	1637	1786.8	1976.59	459.46	421.25	-2.06	0.00	0.00	Aldehyde dehydrogenase X, mitochondrial-like
LOC113517673	515	1849.7	1568.53	391.85	429.21	-2.07	0.00	0.00	Lysosome-associated membrane

									glycoprotein 1
LOC113513347	879	1.7	1.72	0.23	0.56	-2.07	0.01	0.02	Uncharacterized protein LOC110378737
LOC113521037	1516	10.26	9.75	2.5	2.17	-2.07	0.00	0.00	Protein nessun dorma isoform X3
LOC113513362	1550	37.3	29.86	8.69	6.96	-2.07	0.00	0.00	Apyrase-like
LOC113511336	813	17.78	18.39	4.72	3.76	-2.07	0.00	0.00	Hypothetical protein KGM_201149
LOC113512946	1212.58	24	22.31	5.28	5.53	-2.07	0.00	0.00	Uncharacterized protein LOC106136501
LOC113521763	1062	19.63	22.42	5.77	4.02	-2.07	0.00	0.00	Trimeric intracellular cation channel type B
LOC113516846	22986	0.39	0.24	0.02	0.12	-2.08	0.00	0.00	Titin
LOC113515395	1688	2.86	3.61	0.68	0.81	-2.08	0.00	0.00	Cytochrome b5-related protein-like
LOC113517225	2198.25	125.14	139.67	35.2	25.93	-2.08	0.00	0.00	Uncharacterized protein LOC106128213
LOC113515756	1403	12.25	7.24	2.01	2.5	-2.08	0.00	0.00	Pachytene checkpoint protein 2 homolog
LOC113519242	1940.76	170.1	147.67	38.95	34.43	-2.08	0.00	0.00	Cytochrome P450 9e2-like
LOC113514457	364	355.19	273.95	69.06	87.4	-2.08	0.00	0.00	BCL2/adenovirus E1B 19 kDa protein-interacting protein 3 isoform X1
LOC113511664	1704	0.96	0.73	0.34	0.05	-2.08	0.00	0.02	Uncharacterized protein LOC110375286
MSTRG.3420	5996	62.67	56.58	11.84	15.62	-2.08	0.00	0.00	Laminin subunit gamma-1
LOC113516106	1434	7.12	7.15	1.31	1.97	-2.09	0.00	0.00	Monocyte to macrophage differentiation factor 2
LOC113518339	2088	13.09	15.19	3.15	3.34	-2.09	0.00	0.00	G2/mitotic-specific cyclin-B
LOC113517415	1241	5.7	8.83	1.92	1.39	-2.10	0.00	0.00	Uncharacterized protein LOC106134838 isoform X2
LOC113514853	1361	18.94	17.7	4.1	4.27	-2.10	0.00	0.00	Uncharacterized protein LOC110381924
MSTRG.202	421	7.68	8.06	1.84	1.84	-2.10	0.00	0.01	Cuticular protein RR-1 motif 54 precursor
LOC113509259	3357	140.56	105.67	26.08	30.09	-2.10	0.00	0.00	Uncharacterized protein LOC101742154 isoform X1
LOC113516626	1093	86.01	98.35	22.7	19.5	-2.10	0.00	0.00	Alpha-tocopherol transfer protein-like
MSTRG.9150	2175.03	14.55	13.62	2.92	3.64	-2.10	0.00	0.00	Retinol dehydrogenase 14-like
MSTRG.12981	556	331.37	391.71	91.68	76.45	-2.10	0.00	0.00	Mitotic-spindle organizing protein 1

LOC113514209	5090	37.67	34.45	7.45	8.94	-2.10	0.00	0.00	P protein-like isoform X2
LOC113520515	1847	423	426.12	96.22	96.92	-2.11	0.00	0.00	Uncharacterized protein LOC106132893 isoform X2
LOC113521776	2684	15.56	15.71	3.78	3.29	-2.11	0.00	0.00	Translin-associated factor X-interacting protein 1-like isoform X2
LOC113512326	2694	10.8	11.83	2.45	2.67	-2.11	0.00	0.00	Immunoglobulin superfamily containing Leucine-rich repeat protein-like
LOC113510707	560	5.78	2.55	0.96	0.95	-2.11	0.00	0.01	Uncharacterized protein LOC101747082
LOC113515986	2466.14	116.84	110.16	24.24	27.39	-2.11	0.00	0.00	6-phosphofructo-2-kinase/fructose-2,6-bisphosphatase-like
LOC113522005	1849	3.29	2.96	0.79	0.64	-2.11	0.00	0.00	Esterase FE4
MSTRG.7791	1235	26.18	26.58	7.1	4.84	-2.11	0.00	0.00	Uncharacterized protein LOC106130198 isoform X1
LOC113512854	3236.35	28.89	41.09	7.21	8.58	-2.11	0.00	0.00	Uncharacterized protein LOC106136339
LOC113512870	4006.9	8.38	6.95	1.42	2.03	-2.11	0.00	0.00	Tubulin-specific chaperone cofactor E-like protein isoform X1
LOC113514062	388	17.95	14.24	3.51	4.12	-2.12	0.00	0.01	Unconventional myosin-Va
LOC113517896	445	16.71	10.49	2.39	3.97	-2.12	0.00	0.00	Polypeptide N-acetylgalactosaminyltransferase 2
LOC113512228	728	2.02	2.69	0	1.04	-2.12	0.00	0.02	Uncharacterized protein LOC106129208
LOC113522340	3447	0.24	0.25	0.04	0.06	-2.12	0.01	0.04	Uncharacterized protein K02A2.6-like
LOC113522801	4978	1.92	1.83	0.35	0.49	-2.12	0.00	0.00	Uncharacterized protein LOC109421171
LOC113512048	1701	1	1.16	0.14	0.33	-2.13	0.00	0.01	Uncharacterized protein LOC106716864
LOC113519240	1553	6.24	7.88	1.99	1.16	-2.13	0.00	0.00	Autophagy-related protein 13 homolog
LOC113510204	1324	537.18	586.2	136.46	115.37	-2.13	0.00	0.00	Uncharacterized protein
LOC113519918	1371	1.84	2.91	0.25	0.8	-2.13	0.00	0.00	Sodium-dependent serotonin transporter
LOC113511832	2445	0.25	0.48	0.1	0.06	-2.13	0.01	0.04	Adenylate kinase 7
LOC113509468	530	211.91	185.75	44.16	46.64	-2.14	0.00	0.00	Protein singed
LOC113517522	4088.21	100.76	82.23	18.7	21.83	-2.14	0.00	0.00	Receptor-type tyrosine-protein

									phosphatase N2 isoform X1
LOC113510631	3043	7.76	6.74	1.47	1.75	-2.14	0.00	0.00	Uncharacterized protein LOC106129727
LOC113513087	548	4.47	3.32	0.5	1.24	-2.14	0.00	0.02	PhosphoLipase C beta 1
LOC113520465	1424	160.42	173.26	38.76	35.42	-2.14	0.00	0.00	Alpha-tubulin
MSTRG.6841	372	9.95	7.54	2.67	1.35	-2.14	0.01	0.03	Uncharacterized protein
LOC113513507	628	56.23	48.87	9.7	13.96	-2.15	0.00	0.00	Geranylgeranyl diphosphate synthase
LOC113523364	2162	59.08	53.84	12.56	12.36	-2.15	0.00	0.00	G1/S-specific cyclin-D3-like
LOC113519024	3942	3.37	4.03	0.62	1.01	-2.15	0.00	0.00	Microtubule-associated protein tau
LOC113521004	2708	35.03	36.12	7.96	7.71	-2.15	0.00	0.00	Chaoptin-like
LOC113512587	1705	1.57	1.93	0.48	0.28	-2.15	0.00	0.00	Sodium-dependent nutrient amino acid transporter 1-like isoform X1
LOC113521492	1170	0.97	1.51	0.31	0.23	-2.15	0.00	0.02	GTP-binding protein Rhes
LOC113513883	299	124.77	133.08	31.73	31.17	-2.15	0.00	0.00	2-oxoisovalerate dehydrogenase subunit alpha, mitochondrial
LOC113510312	1873.05	13.9	15.69	3.07	3.41	-2.15	0.00	0.00	Carboxylesterase
LOC113521929	2539.87	8.87	9.08	2.19	1.6	-2.16	0.00	0.00	Carbohydrate sulfotransferase 11 isoform X1
LOC113514441	1058	1.18	0.86	0.35	0.09	-2.16	0.01	0.03	BTB/POZ domain-containing protein
LOC113517200	904	1.47	1.07	0.44	0.11	-2.16	0.01	0.03	Uncharacterized protein LOC106131819
MSTRG.9472	762	5.43	3.51	1.13	0.83	-2.16	0.00	0.00	Microtubule-associated protein futsch-like
MSTRG.1742	2331	7.47	6.97	1.64	1.5	-2.16	0.00	0.00	PiggyBac transposable element-derived protein 4-like
LOC113522803	1299.03	44.2	42.39	8.9	10.71	-2.16	0.00	0.00	Zinc finger protein GLIS2-like
MSTRG.11462	1970	6.24	6.82	1.14	1.71	-2.17	0.00	0.00	Endonuclease and reverse transcriptase-like protein
LOC113512232	1479	0.64	0.71	0.29	0	-2.17	0.01	0.03	5-hydroxytryptamine receptor 1A-like
LOC113517089	4355	96.15	92.26	21.29	19.48	-2.17	0.00	0.00	Long-chain fatty acid transport protein 4-like
MSTRG.13268	789	2.31	2.02	0.53	0.39	-2.17	0.00	0.01	Uncharacterized protein
LOC113510482	4274	69.46	55.1	11.69	15.29	-2.17	0.00	0.00	Uncharacterized protein LOC106136572
LOC113511144	3488	34.31	41.81	8.26	8.2	-2.17	0.00	0.00	Protein toll

LOC113515677	5776.89	48.36	44.07	10.2	9.73	-2.17	0.00	0.00	Uncharacterized protein LOC110383954 isoform X1
LOC113515641	2348	2.46	3.03	0.57	0.62	-2.18	0.00	0.00	Uncharacterized protein LOC106142856 isoform X1
LOC113516939	1953	0.98	0.55	0.25	0.08	-2.18	0.00	0.01	Uncharacterized protein LOC106143151 isoform X1
LOC113511950	2122	89.13	83.9	20.93	16.45	-2.18	0.00	0.00	Pancreatic lipase-related protein 2-like
MSTRG.353	344	33.2	24.49	5.77	7.45	-2.18	0.00	0.00	Gelsolin-like
LOC113511622	1180	130.09	133.32	28.73	28.4	-2.18	0.00	0.00	Proliferating cell nuclear antigen
LOC113516066	1805	4.79	4.94	1.22	0.88	-2.18	0.00	0.00	Sedoheptulokinase-like
MSTRG.13262	2150	8.45	7.54	1.65	1.79	-2.18	0.00	0.00	Uncharacterized protein
LOC113519163	2741	14.78	17.49	3.27	3.68	-2.18	0.00	0.00	Kinesin-like protein KIF23
LOC113518463	1024	23.29	8.18	3.67	3.15	-2.18	0.00	0.00	Trypsin-like protein
LOC113517010	2603	2.19	2.19	0.53	0.4	-2.19	0.00	0.00	Alpha-tocopherol transfer protein-like
LOC113514274	2191	1.06	1.64	0.36	0.21	-2.19	0.00	0.00	Rho-related BTB domain-containing protein 1
LOC113519372	4009	3.65	3.71	0.79	0.77	-2.19	0.00	0.00	Calcium/calmodulin-dependent protein kinase kinase 2 isoform X3
LOC113521718	939.71	24.84	25.04	5.19	5.47	-2.19	0.00	0.00	5-formyltetrahydrofolate cyclo-ligase-like isoform X1
LOC113516110	1652	2.7	3.35	0.45	0.83	-2.20	0.00	0.00	Homogentisate 1,2-dioxygenase
LOC113520629	1656	1.66	2.76	0.25	0.68	-2.20	0.00	0.00	Uncharacterized protein OBRU01_00156
LOC113520126	972	62.78	74.2	14.59	14.68	-2.20	0.00	0.00	Baculoviral IAP repeat-containing protein 5
LOC113519860	3139	4.91	5.27	1.18	0.96	-2.21	0.00	0.00	Muscle M-line assembly protein unc-89-like
LOC113511521	1524	3.24	3	0.77	0.54	-2.21	0.00	0.00	Troponin T
LOC113516466	878	7713.82	8114.44	1794.64	1564.9	-2.21	0.00	0.00	Alpha-crystallin
MSTRG.7313	844	28.72	21.05	5.55	4.97	-2.22	0.00	0.00	Protein AF-10 isoform X9
LOC113519366	660	12.26	14.38	2.14	3.5	-2.22	0.00	0.00	G2/mitotic-specific cyclin-B3
LOC113516538	441	67.39	77.24	15.48	15.86	-2.23	0.00	0.00	Uncharacterized protein
LOC113512289	1358.73	14.25	15.01	3.02	3.17	-2.23	0.00	0.00	Isoaspartyl peptidase/L-asparaginase CG7860
LOC113511069	1588	5.08	4.68	1.1	0.92	-2.23	0.00	0.00	Chondroadherin-like

LOC113522778	1550	38.93	36.9	7.51	8.28	-2.23	0.00	0.00	Multifunctional protein ADE2
LOC113511632	1023	407.61	455.25	90.9	88.92	-2.24	0.00	0.00	Uncharacterized protein
LOC113513273	978	0.7	0.78	0.2	0.1	-2.24	0.01	0.05	Olfactory receptor 36
LOC113521907	3023	70.95	74.38	15.05	14.97	-2.24	0.00	0.00	Uncharacterized protein LOC106132537
LOC113520013	656	29.27	42.62	8.81	6.2	-2.24	0.00	0.00	Calponin homology domain-containing protein DDB_G0272472-like
LOC113512391	1875	1.61	0.88	0.22	0.29	-2.24	0.00	0.00	Uncharacterized protein LOC106142706
LOC113518557	14025	0.07	0.08	0.01	0.02	-2.25	0.00	0.02	Dynein heavy chain 8, axonemal
LOC113512793	1747	1.19	1.09	0.14	0.32	-2.25	0.00	0.00	Cytochrome P450 301a1, mitochondrial
LOC113514763	1860	1.55	1.82	0.35	0.34	-2.25	0.00	0.00	Uncharacterized protein LOC106137312
LOC113523522	751	16.76	7.94	2.31	2.83	-2.25	0.00	0.00	Uncharacterized protein
LOC113517345	2214.29	35.85	41.49	8.44	7.45	-2.25	0.00	0.00	Protein claret segregational-like
LOC113509096	1258	0.78	1.31	0.21	0.21	-2.25	0.00	0.01	Hypothetical protein RR46_05082
LOC113520164	424	13.37	7.91	2.26	2.26	-2.26	0.00	0.00	Programmed cell death 4a
LOC113510774	1089	7.89	11.37	2.37	1.57	-2.26	0.00	0.00	Uncharacterized protein
MSTRG.3523	521	18.03	23.03	4.16	4.4	-2.26	0.00	0.00	5-methylcytosine rRNA methyltransferase NSUN4
LOC113517261	2150.3	20.36	17.57	4.01	3.87	-2.26	0.00	0.00	Beta-1,3-glucosyltransferase
LOC113513336	444	47.66	39.03	8.81	9.59	-2.26	0.00	0.00	Fatty-acid amide hydrolase 2-B-like
LOC113522607	2297	26.9	26.01	5.72	5.05	-2.26	0.00	0.00	DNA replication licensing factor Mcm7
LOC113522241	1417	31.66	4.95	4.88	2.59	-2.26	0.00	0.00	Fatty acid synthase-like
MSTRG.4121	828	6.85	5.2	1.12	1.34	-2.26	0.00	0.00	Uncharacterized protein LOC106103906
LOC113514430	1391	0.54	0.71	0.12	0.12	-2.26	0.01	0.05	Serine/threonine-protein kinase
LOC113513641	469	0.81	4.62	0.35	0.7	-2.27	0.01	0.05	25 kDa silk glycoprotein Fibrohexamerin
LOC113516832	678	4.13	4.23	1.02	0.67	-2.27	0.00	0.00	Trypsin delta/gamma-like isoform X2
LOC113518394	1675	11.6	14.22	2.16	3.07	-2.27	0.00	0.00	Protein-cysteine N-palmitoyltransferase Rasp
LOC113514901	486	50.89	36.4	6.77	11.53	-2.27	0.00	0.00	Protein singed
LOC113509770	1078	19.65	23.8	4.8	4.02	-2.27	0.00	0.00	Ribonuclease H2 subunit A-like

LOC113522941	480	8.67	9.06	1.66	1.98	-2.28	0.00	0.00	Homeobox protein B-H1-like
LOC113522998	3303	0.22	0.37	0.09	0.02	-2.28	0.01	0.02	Zinc finger protein Dzip1
MSTRG.4292	614	5.57	6.39	1.06	1.37	-2.29	0.00	0.00	Uncharacterized protein LOC106141537
LOC113517119	2274	5.43	5.51	1.07	1.11	-2.29	0.00	0.00	Uncharacterized protein LOC106133560
LOC113510662	1127.41	105.56	103.16	21.24	20.53	-2.29	0.00	0.00	Omega-amidase NIT2 isoform X1
LOC113510198	1823	10.75	10.52	2.36	1.87	-2.30	0.00	0.00	TBC1 domain family member 4 isoform X1
MSTRG.9714	822	3.66	3.24	0.38	0.99	-2.30	0.00	0.00	Uncharacterized protein
LOC113514150	1664	40.6	50.11	10.16	7.84	-2.30	0.00	0.00	Cytochrome P450 6B6-like
LOC113514005	868	3.93	2.99	0.12	1.25	-2.30	0.00	0.00	Uncharacterized protein LOC101742758 isoform X1
MSTRG.5657	732	10.83	16.51	0.15	1.03	-2.30	0.00	0.01	Poly (ADP-ribose) polymerase
LOC113513612	638	4.87	2.68	0.57	0.93	-2.30	0.00	0.01	Serine-enriched protein
LOC113510598	1034	1.07	0.81	0.27	0.09	-2.31	0.01	0.04	Collagenase-like
LOC113510551	1328	3066.67	3166.84	634.66	600.2	-2.31	0.00	0.00	Cathepsin B-like cysteine proteinase
LOC113517595	1622	80.75	90.2	17.28	16.52	-2.31	0.00	0.00	Tissue Alpha-L-fucosidase-like precursor
LOC113510029	1432	13.78	14.11	2.38	3.14	-2.31	0.00	0.00	Programmed cell death protein 4
LOC113512935	1469	86.66	99.6	19.2	17.57	-2.31	0.00	0.00	Leucine-rich repeat-containing protein 58-like
LOC113522283	1765	21.19	22.52	3.93	4.69	-2.31	0.00	0.00	Uncharacterized protein LOC101743779
LOC113518202	1522	83.32	90.43	18.33	15.91	-2.31	0.00	0.00	Hydroxymethylglutaryl-CoA lyase, mitochondrial
LOC113515195	774	1.19	0.98	0.14	0.27	-2.31	0.01	0.05	Trypsin, alkaline C-like
LOC113512684	1188	4.18	2.62	0.3	1.03	-2.31	0.00	0.00	Chloride channel protein 2 isoform X1
LOC113519951	491	110.24	107.3	24.27	19.72	-2.31	0.00	0.00	Glutathione S-transferase sigma 5
LOC113511437	1331	81.44	94.02	17.92	16.63	-2.32	0.00	0.00	Bacilysin biosynthesis oxidoreductase BacC-like
LOC113515487	1241	24.32	22.29	4.42	4.74	-2.32	0.00	0.00	Uncharacterized protein LOC106142908 isoform X3
LOC113521139	336	9.86	14.07	1.93	2.95	-2.33	0.00	0.02	ATP-binding cassette sub-family G member 1-like
LOC113512962	5802	0.13	0.2	0.02	0.04	-2.33	0.00	0.02	Protein stoned-B-like

LOC113510005	328	25.46	20.9	7.43	2.17	-2.33	0.00	0.00	Unconventional myosin-Va-like
LOC113511228	477	1.55	3.86	0.34	0.67	-2.33	0.01	0.04	Uncharacterized protein
LOC113513707	1513	1.15	0.6	0.28	0.05	-2.33	0.00	0.01	KRAB-A domain-containing protein 2-like
LOC113522219	590	68.54	68.7	16.29	10.72	-2.34	0.00	0.00	Biliverdin binding protein-1
LOC113520694	3603.09	25.82	21.97	5.07	4.12	-2.34	0.00	0.00	Protein yippee-like CG15309 isoform X1
LOC113515204	3479	83.65	68.89	14.65	14.6	-2.35	0.00	0.00	Small G protein signaling modulator 2-like
LOC113521779	1584	1.04	0.89	0.16	0.21	-2.35	0.00	0.01	Esterase FE4-like
MSTRG.6451	1063	8.21	9.02	2.18	1.11	-2.35	0.00	0.00	Uncharacterized protein
LOC113513462	1511	3.98	3.23	0.56	0.82	-2.35	0.00	0.00	Zinc finger protein rotund isoform X3
LOC113520651	2030.14	10.95	9.82	1.94	2.03	-2.36	0.00	0.00	Uncharacterized protein LOC110375899 isoform X2
LOC113511756	947.16	11.43	16.09	2.07	3.21	-2.36	0.00	0.00	Hypothetical protein RR46_01282
LOC113521640	3161.57	27.32	22.3	4.82	4.63	-2.36	0.00	0.00	Hexosaminidase
LOC113518767	864.51	78.76	71.05	17.11	18.74	-2.36	0.00	0.00	Hypothetical protein KGM_208267
LOC113522009	884	3.02	1.71	0.45	0.44	-2.36	0.00	0.00	Phosphatidate phosphatase like protein
LOC113511360	822	14.04	13.18	2.01	3.2	-2.36	0.00	0.00	Uncharacterized protein LOC106139170
LOC113522290	2339	4.62	3.4	0.83	0.68	-2.36	0.00	0.00	Outer kinetochore Ndc80
LOC113517989	577	11.81	12.84	2.04	2.68	-2.37	0.00	0.00	Zinc finger protein 43-like
LOC113510737	1266	7.21	8.06	1.32	1.56	-2.37	0.00	0.00	Venom protease-like
LOC113513361	382	24.3	16	4.29	3.71	-2.38	0.00	0.00	Trypsin-like
LOC113514594	539	2.81	1.37	0.26	0.51	-2.38	0.01	0.04	Trypsin CFT-1-like
MSTRG.495	226	154.1	138.83	17.18	45.41	-2.38	0.00	0.00	Uncharacterized protein
LOC113511087	3427	1.17	1.27	0.24	0.21	-2.38	0.00	0.00	Rhophilin-2-B isoform X2
LOC113518620	1130	74.63	73.69	12.87	15.11	-2.38	0.00	0.00	Glucosylceramidase-like
LOC113513030	3156	56.38	45.4	9.21	9.87	-2.38	0.00	0.00	1-phosphatidylinositol 4,5-bisphosphate phosphodiesterase isoform X1
LOC113513259	1644	6.87	8.44	0.96	1.92	-2.38	0.00	0.00	Uncharacterized protein LOC110381499
MSTRG.10267	222	80.97	51.29	2.64	27.95	-2.38	0.01	0.04	Uncharacterized protein
LOC113513160	643	7.28	7.43	1.12	1.65	-2.38	0.00	0.00	Mitotic spindle assembly checkpoint protein MAD2A

LOC113513185	1465	50.73	36.58	7.46	8.93	-2.39	0.00	0.00	Uncharacterized protein LOC106136069 isoform X1
MSTRG.12050	2756	258.46	248.08	49.01	46.16	-2.39	0.00	0.00	Uncharacterized protein
LOC113511062	1851	51.62	52.37	10.23	9.21	-2.39	0.00	0.00	Flap endonuclease 1
LOC113513732	1758	8.41	8.83	1.63	1.59	-2.39	0.00	0.00	Uncharacterized protein LOC106124838
LOC113514719	322	34.84	57.71	10.3	8.21	-2.39	0.00	0.00	Protein phosphatase 1 regulatory subunit 14B isoform X1
LOC113512299	1443.27	40.1	32.54	6.77	6.85	-2.40	0.00	0.00	Protein decapentaplegic-like
LOC113519227	1016	7.25	6.6	1.48	1.05	-2.40	0.00	0.00	Uncharacterized protein LOC106130388
LOC113515211	1653	1.15	0.76	0.2	0.15	-2.40	0.00	0.00	Sodium channel protein Nach-like isoform X1
LOC113523568	1449	5.58	7.74	1.11	1.32	-2.42	0.00	0.00	Epoxide hydrolase 1-like
LOC113521049	5019	0.19	0.14	0.03	0.03	-2.42	0.01	0.02	Voltage-dependent calcium channel type D subunit Alpha-1-like isoform X3
LOC113519591	2375	12.72	12.92	2.2	2.46	-2.42	0.00	0.00	Uncharacterized protein LOC106141788
LOC113516676	3286	8.08	7.46	1.03	1.81	-2.43	0.00	0.00	Uncharacterized protein LOC106130524
LOC113521417	2961	0.67	1.03	0.18	0.13	-2.43	0.00	0.00	Fas-binding factor 1
LOC113519793	3745.67	300.84	273.08	52.97	52.99	-2.43	0.00	0.00	ETS-like protein pointed isoform X3
LOC113510944	349	9.22	8.64	0.84	2.55	-2.43	0.01	0.02	Uncharacterized protein DDB_G0282133
LOC113520101	2760	139.22	141.77	24.54	26.5	-2.43	0.00	0.00	Uncharacterized family 31 glucosidase KIAA1161 isoform X1
LOC113512600	1525	52.34	44.66	8.98	8.66	-2.43	0.00	0.00	UNC93-like protein MFSD11 isoform X2
LOC113511798	802	191.83	202.39	36.21	36.06	-2.43	0.00	0.00	Ras-related protein Rab-3 isoform X1
LOC113509901	8499	0.5	0.24	0.08	0.06	-2.44	0.00	0.00	Slit homolog 3 protein-like
LOC113517293	805	89.93	119.69	16.45	21.74	-2.44	0.00	0.00	REPAT31
LOC113513263	1071	92.25	86.04	16.33	15.9	-2.44	0.00	0.00	2-oxoisovalerate dehydrogenase subunit alpha, mitochondrial isoform X2
LOC113518006	839	5.18	5.52	0.49	1.43	-2.44	0.00	0.00	2-oxoglutarate dehydrogenase E1 component DHKTD1 homolog,

									mitochondrial
LOC113521811	2744	1.82	1.6	0.36	0.24	-2.45	0.00	0.00	Uncharacterized protein LOC106137262
MSTRG.10131	2567	4	4.16	0.69	0.76	-2.45	0.00	0.00	Zinc finger BED domain-containing protein 1-like
LOC113513548	1322	2002.95	2299.87	399.37	372.27	-2.45	0.00	0.00	Venom protease-like
LOC113514820	873	15.85	17.79	3.1	2.93	-2.45	0.00	0.00	UDP-glycosyltransferase UGT46A3
LOC113519168	714	5.52	3.19	0.62	0.92	-2.46	0.00	0.00	Fanconi anemia group I protein homolog
LOC113520869	1971	17.35	17.22	2.88	3.25	-2.46	0.00	0.00	GPI mannosyltransferase 4
LOC113516666	3133	66.55	57.44	10.9	11.05	-2.46	0.00	0.00	Monocarboxylate transporter 12
LOC113518715	729	12.81	14.1	2.42	2.38	-2.47	0.00	0.00	Uncharacterized protein LOC110375196
LOC113518804	2219.64	17.2	21.45	3.41	3.39	-2.47	0.00	0.00	Peptide transporter family 1 isoform X1
LOC113513370	657	29.76	33.11	4.66	6.53	-2.48	0.00	0.00	Uncharacterized protein LOC106134371
LOC113517218	1705	7.88	6.49	1.16	1.36	-2.48	0.00	0.00	Aldehyde dehydrogenase family 7 member A1 homolog
LOC113509684	1708	0.73	1.5	0.19	0.19	-2.48	0.00	0.00	Uncharacterized protein LOC106136946
LOC113516173	812	1.41	1.59	0.26	0.25	-2.48	0.00	0.02	Ecdysis triggering hormone precursor
MSTRG.6118	971	100.63	100.77	18.17	17.22	-2.48	0.00	0.00	Uncharacterized protein
LOC113517567	759	49.7	64.4	9.92	10.17	-2.49	0.00	0.00	DNA replication complex GINS protein PSF2
LOC113515168	4332	0.24	0.06	0.05	0	-2.49	0.01	0.04	Fatty acid synthase-like
LOC113514099	1745	36.03	40.21	7.4	5.81	-2.50	0.00	0.00	DNA polymerase epsilon subunit 2
LOC113518195	2151	3.36	0.43	0.4	0.25	-2.50	0.00	0.00	Peroxisomal acyl-coenzyme A oxidase 1
LOC113510667	2266	1.27	1.54	0.31	0.17	-2.50	0.00	0.00	Solute carrier family 22 member 21 like protein
LOC113518306	4854	1.36	1.44	0.22	0.22	-2.50	0.00	0.00	Alpha-catulin isoform X1
LOC113511820	935	1.97	1.67	0.1	0.51	-2.50	0.00	0.01	Protein lin-28 homolog
LOC113510367	787	161.69	160.1	28.02	28.17	-2.50	0.00	0.00	Uncharacterized family 31 glucosidase KIAA1161 isoform X1
MSTRG.12813	457	4.85	1.64	0.75	0.37	-2.50	0.01	0.04	Uncharacterized protein LOC106107297 isoform X1

LOC113519454	2137	2.77	2.58	0.41	0.51	-2.51	0.00	0.00	Chloride channel protein 2 isoform X1
LOC113515461	2108	0.78	1.01	0.23	0.07	-2.51	0.00	0.00	Cytochrome P450 monooxygenase CYP304F17
LOC113522851	1502	2.32	3.6	0.56	0.44	-2.52	0.00	0.00	Gustatory receptor for sugar taste 64f-like
LOC113509632	934	9.47	12.25	1.67	2.04	-2.52	0.00	0.00	Juvenile hormone binding protein
MSTRG.9757	337.13	5.9	3.75	1.35	1.86	-2.52	0.00	0.02	Uncharacterized protein
LOC113511898	1910	3.25	4.92	0.8	0.58	-2.53	0.00	0.00	Hypothetical protein KGM_210031
LOC113522098	606	12.42	4.4	1.65	1.22	-2.53	0.00	0.00	Uncharacterized protein LOC106101275
LOC113509106	1304	2.2	2.82	0.4	0.52	-2.54	0.00	0.00	Delta-sarcoglycan-like isoform X1
LOC113520070	471	17.83	16.18	2.77	3.1	-2.54	0.00	0.00	Uncharacterized protein DDB_G0282133
LOC113523183	594	6.69	5.89	0.64	1.48	-2.54	0.00	0.00	EF-hand calcium-binding domain-containing protein 2 isoform X1
LOC113510042	1617	16.88	13.07	1.89	3.15	-2.54	0.00	0.00	UDP-glucuronosyltransferase 2A3-like
LOC113515169	479	50.23	4.99	5.33	4.31	-2.54	0.00	0.00	Fatty acid synthase-like
LOC113514878	801	10.89	9.05	1.44	1.92	-2.54	0.00	0.00	Polypeptide N-acetylgalactosaminyltransferase 2-like
LOC113509686	2157	5.07	6.31	1.35	0.54	-2.55	0.00	0.00	Glutamate receptor ionotropic, delta-2
MSTRG.456	850.47	1413.91	1235.44	211.95	229.82	-2.56	0.00	0.00	Uncharacterized protein
LOC113518916	1351.87	920.62	972.7	158.05	152.54	-2.57	0.00	0.00	Kynurenine formamidase
MSTRG.7478	364	188.26	192.39	26.62	40.79	-2.57	0.00	0.00	Glutathione S-transferase 2-like
LOC113512333	595	40.5	44.91	8.34	5.91	-2.57	0.00	0.00	Uncharacterized protein LOC106132298 isoform X1
LOC113522844	1451.35	34.24	35.33	6.02	5.43	-2.57	0.00	0.00	Uncharacterized protein
LOC113513807	1034	1.29	1.53	0.18	0.27	-2.58	0.00	0.01	Lipase member H-like isoform X2
LOC113523523	4898	44.69	27.06	5.03	6.73	-2.58	0.00	0.00	MAP kinase-activating death domain protein
LOC113510773	2523	25.67	32.1	4.87	4.57	-2.58	0.00	0.00	Uncharacterized protein
LOC113521135	828	1.37	0.99	0.25	0.12	-2.58	0.01	0.03	Inorganic phosphate cotransporter
LOC113520664	1143.01	56.43	56.78	9.57	10	-2.58	0.00	0.00	Prostaglandin reductase 1-like
LOC113515197	1275	23.12	22.39	2.68	4.78	-2.58	0.00	0.00	Rabphilin-3A

LOC113519008	2448	0.3	0.31	0.06	0.03	-2.59	0.01	0.03	Protein toll-like
LOC113509441	794	11.24	12	1.72	2.08	-2.59	0.00	0.00	Separin isoform X1
LOC113518612	522	2.57	4.39	0.55	0.55	-2.59	0.00	0.01	Paired box protein Pax-1
LOC113510920	1280	5.21	4.82	0.75	0.87	-2.59	0.00	0.00	Microtubule-associated protein futsch-like
LOC113518775	4117	1.33	1.09	0.18	0.21	-2.59	0.00	0.00	Cubilin
LOC113522927	1205	0.7	0.26	0.07	0.07	-2.59	0.01	0.04	Uncharacterized protein LOC106129317
LOC113519549	487	20.51	18.12	4.17	2.23	-2.59	0.00	0.00	Uncharacterized protein LOC106134838 isoform X1
LOC113509113	1923.7	14.94	15.32	2.49	2.12	-2.59	0.00	0.00	Uncharacterized protein LOC106131089
MSTRG.7498	513	2.66	2.78	0	0.85	-2.59	0.00	0.02	Unconventional myosin-Va
LOC113513295	736	4.56	3.7	0.74	0.58	-2.59	0.00	0.00	Uncharacterized protein LOC110378785
LOC113512507	1638	0.36	0.63	0.05	0.1	-2.60	0.00	0.02	Glutamate receptor ionotropic, delta-1-like
MSTRG.14617	563	4.84	5.48	0.24	1.47	-2.60	0.00	0.00	Uncharacterized protein LOC106684797
LOC113518663	899	0.52	1.67	0.33	0	-2.61	0.01	0.03	Crustacean cardioactive peptide precursor
LOC113512738	486	127.11	123.41	20.32	21.15	-2.61	0.00	0.00	Triokinase/FMN cyclase-like
LOC113521184	2451	1.74	1.41	0.25	0.25	-2.61	0.00	0.00	Neuropeptide receptor A10
LOC113518046	1414	12.56	12.29	1.57	2.42	-2.61	0.00	0.00	Lachesin-like
LOC113521275	2638	2.21	2.11	0.15	0.54	-2.61	0.00	0.00	Carboxylesterase
LOC113519302	1017	5.57	4.21	0.93	0.64	-2.61	0.00	0.00	Semaphorin-5A
LOC113516955	2572.5	0.42	0.41	0.2	0	-2.62	0.00	0.00	Uncharacterized protein LOC106099430
LOC113513951	1103	1.38	0.22	0.17	0.08	-2.62	0.01	0.03	Fatty-acyl CoA reductase 1
LOC113510881	3400	0.76	0.33	0.02	0.15	-2.62	0.00	0.00	Sodium-dependent nutrient amino acid transporter 1-like isoform X1
LOC113515034	1582	28.28	29.59	4.52	4.68	-2.62	0.00	0.00	Uncharacterized protein LOC106129906 isoform X1
LOC113514181	1741	6.61	5.45	0.89	1.01	-2.63	0.00	0.00	Hypothetical protein KGM_205793
LOC113522203	2004	0.51	0.28	0.12	0	-2.63	0.01	0.03	Leucine-rich repeat-containing G-protein coupled receptor 6-like isoform X1
MSTRG.10541	687	4.3	4.14	0.67	0.65	-2.63	0.00	0.00	Uncharacterized protein

LOC113512772	661	12.92	12.61	1.78	2.27	-2.64	0.00	0.00	Uncharacterized protein LOC110371712
LOC113515420	1322	64.21	71.26	11.37	9.89	-2.64	0.00	0.00	Phosphoserine aminotransferase
LOC113522918	500	20.32	28.86	3.03	4.81	-2.65	0.00	0.00	Uncharacterized protein LOC106111100
LOC113509312	566	4.02	3.54	0	1.16	-2.65	0.00	0.00	Serine-enriched protein isoform X1
LOC113519389	3819	15.27	14.35	2.26	2.35	-2.65	0.00	0.00	Forkhead box protein P3
LOC113520021	957	3.1	2.06	0.2	0.59	-2.65	0.00	0.00	Zinc finger DNA binding protein
LOC113510546	1592	22.67	22.11	3.4	3.57	-2.65	0.00	0.00	TBC1 domain family member 4 isoform X1
MSTRG.474	1261	10.62	8.02	1.54	1.36	-2.65	0.00	0.00	NADP-dependent oxidoreductase
LOC113517391	4787	160.19	154.79	21.84	27.01	-2.65	0.00	0.00	Peroxidasin isoform X1
MSTRG.16106	436	3.19	5.15	0.85	0.43	-2.66	0.01	0.03	Protein PRRC1-like
LOC113512396	1038	2.85	2.41	0.36	0.44	-2.66	0.00	0.00	Inactive pancreatic Lipase-related protein 1-like
LOC113515775	1810.7	13.85	14.44	2.1	2.23	-2.66	0.00	0.00	Delta(24)-sterol reductase-like
LOC113510161	1785.77	38.58	41.59	5.97	6.23	-2.67	0.00	0.00	Serine hydrolase-like protein
LOC113519736	599	39.69	49.23	8.66	5.21	-2.67	0.00	0.00	Ecdysteroid-regulated 16 kDa protein-like
LOC113515578	714	14.96	13.05	2.34	2	-2.67	0.00	0.00	Meiosis-specific nuclear structural protein 1-like isoform X2
LOC113514621	618	2.49	2.83	0.6	0.2	-2.67	0.00	0.01	N-acylneuraminate cytidylyltransferase
LOC113515209	4551	11.61	7.73	1.39	1.57	-2.67	0.00	0.00	Citron Rho-interacting kinase-like
LOC113521205	1065	0.89	0.31	0.09	0.09	-2.67	0.01	0.04	Chondroadherin-like
LOC113514830	892	7.38	9.12	0.67	1.86	-2.68	0.00	0.00	Uncharacterized protein LOC106140625 isoform X1
LOC113523041	2157	0.96	0.98	0.18	0.11	-2.68	0.00	0.00	Uncharacterized protein LOC110371101
LOC113510286	3346	0.68	0.94	0.04	0.2	-2.68	0.00	0.00	Kinesin-like protein CG14535 isoform X1
LOC113518403	1242	0.62	0.38	0.14	0	-2.68	0.01	0.03	Lachesin isoform X1
LOC113516429	510	4.26	1.54	0.58	0.29	-2.68	0.00	0.02	ATP-dependent RNA helicase SUV3 homolog, mitochondrial
LOC113518497	3204	0.17	0.17	0.02	0.02	-2.69	0.01	0.03	Ankyrin repeat and MYND domain-containing protein 1
LOC113519243	1116	0.58	0.58	0.08	0.08	-2.69	0.01	0.03	Trypsin 5G1-like isoform X1

LOC113520422	916	6.51	7.63	1.07	1.05	-2.70	0.00	0.00	RDH13
LOC113512790	927	44.25	47.85	7.06	6.82	-2.71	0.00	0.00	Acyl-CoA synthetase family member 2, mitochondrial
MSTRG.4449	1461	1.34	1.81	0.23	0.23	-2.71	0.00	0.00	Uncharacterized protein
LOC113519349	1328	116.91	116.83	13.72	21.34	-2.71	0.00	0.00	Matrix metalloproteinase-25-like
LOC113523144	966	6.83	5.76	1.09	0.78	-2.71	0.00	0.00	Oxidoreductase
LOC113513490	1945	8.11	6.35	1.27	0.89	-2.71	0.00	0.00	Glucose dehydrogenase
LOC113510301	2013	18.63	21.26	2.62	3.33	-2.71	0.00	0.00	Uncharacterized protein LOC106131008
LOC113521982	432	3.92	4.9	0	1.29	-2.71	0.00	0.01	Tctex1 domain-containing protein 1-A-like
LOC113514263	4031	0.7	0.41	0.02	0.14	-2.72	0.00	0.00	ABC transporter G family member 20-like
LOC113523307	5127.76	6.72	6.53	0.93	1.04	-2.72	0.00	0.00	Uncharacterized protein LOC106131368 isoform X1
LOC113521009	929	75.97	79.51	11.24	11.84	-2.73	0.00	0.00	Papilin-like
LOC113520459	276	169.7	166.12	33.06	22.17	-2.73	0.00	0.00	Alpha-tubulin
LOC113522950	4225	9.75	8.81	1.34	1.38	-2.73	0.00	0.00	Multidrug resistance-associated protein 4-like
LOC113512401	3020	15.96	18.12	2.79	2.2	-2.73	0.00	0.00	Facilitated trehalose transporter Tret1-like isoform X1
LOC113512981	471	6.65	2.75	0.69	0.69	-2.74	0.00	0.01	Echinoderm microtubule-associated protein-like 2 isoform X1
LOC113519416	1684	22.4	24.98	3.47	3.48	-2.74	0.00	0.00	Hypothetical protein KGM_208609A
LOC113522730	3104	45.46	48.15	6.31	7.39	-2.74	0.00	0.00	Uncharacterized protein LOC106138999
LOC113515515	1807	0.68	0.28	0.13	0	-2.74	0.00	0.02	Androgen-dependent TFPI-regulating protein-like
LOC113520740	1069	4.8	4.63	0.69	0.68	-2.74	0.00	0.00	Broad-complex core protein isoform 6
LOC113516187	2047	0.56	0.28	0.12	0	-2.74	0.00	0.02	Protein KIAA0556-like
MSTRG.6172	934	2.06	1.58	0.31	0.2	-2.74	0.00	0.00	Uncharacterized protein
MSTRG.8967	608	4.17	4.37	1.03	0.2	-2.75	0.00	0.00	Venom serine carboxypeptidase-like
LOC113517143	1401	44.69	48.16	7.09	6.45	-2.75	0.00	0.00	Tryptase-like
LOC113515236	2072	11.54	8.86	1.8	1.16	-2.75	0.00	0.00	Uncharacterized protein LOC106133236
LOC113515318	1523	546.04	410.76	63.6	75.82	-2.75	0.00	0.00	Digestive cysteine proteinase 1

LOC113514174	844	1.43	1.18	0.36	0	-2.75	0.00	0.02	L-xylulose reductase-like
LOC113510258	862	10.79	12.29	1.05	2.3	-2.76	0.00	0.00	Hemicentin-2-like
LOC113511192	1427	0.62	0.27	0.06	0.06	-2.76	0.01	0.03	Uncharacterized protein LOC106718755
LOC113521930	1725	0.49	0.21	0.05	0.05	-2.76	0.01	0.03	Sodium/potassium/calcium exchanger Nckx30C
MSTRG.11697	2463.58	4.88	5.65	0.96	0.46	-2.76	0.00	0.00	Uncharacterized protein OBRU01_03818
LOC113519172	430	27.78	33.56	4.79	4.35	-2.76	0.00	0.00	Apyrase-like
LOC113517557	2413	9.85	9.18	1.29	1.45	-2.76	0.00	0.00	Proton-coupled folate transporter isoform X1
MSTRG.6537	727	10.28	11.73	1.97	1.19	-2.77	0.00	0.00	Hypothetical protein RR46_13991
LOC113515958	2512	74.55	68.01	9.98	10.49	-2.77	0.00	0.00	1,4-Alpha-glucan-branching enzyme isoform X2
MSTRG.12821	858	14.51	13.24	2.1	1.87	-2.78	0.00	0.00	Trypsin-like
MSTRG.981	345	16.64	24.76	4.36	1.77	-2.78	0.00	0.00	Phosphatidylinositol 3-kinase 3 isoform X1
LOC113511254	1698	3.08	2.33	0.24	0.52	-2.78	0.00	0.00	Uncharacterized protein LOC106138012
LOC113523028	1347	1.17	1.14	0.26	0.06	-2.78	0.00	0.00	Uncharacterized protein LOC110384608
LOC113519506	1344	3.78	4.82	0.77	0.44	-2.78	0.00	0.00	Uncharacterized protein LOC101746861
LOC113511256	3858	2.24	1.79	0.21	0.36	-2.78	0.00	0.00	Uncharacterized protein LOC106138012
LOC113522777	7139.49	15.34	10.33	1.69	1.95	-2.78	0.00	0.00	Neurogenic locus Notch protein isoform X3
LOC113521660	1423	7.47	9.04	1.86	0.47	-2.79	0.00	0.00	Alpha-N-acetylgalactosaminidase-like
MSTRG.6397	1865	13.74	12.64	2.17	1.56	-2.79	0.00	0.00	Protein turtle-like
LOC113522896	1438	16.44	11.51	1.48	2.49	-2.79	0.00	0.00	Neurogenic locus Notch protein
LOC113517116	673	2.97	3.21	0.34	0.51	-2.79	0.00	0.00	B9 domain-containing protein 2
LOC113521781	1137	117.3	125.43	17.55	16.86	-2.79	0.00	0.00	Uncharacterized protein LOC106141718 isoform X1
LOC113517354	875	299.88	344.25	49.8	41.54	-2.80	0.00	0.00	Sulfotransferase 1 family member D1-like
LOC113520122	742	719.56	758.83	101.8	108.4	-2.80	0.00	0.00	von Willebrand factor D and EGF domain-containing protein

LOC113518095	1232	27.57	34.37	5.08	3.8	-2.80	0.00	0.00	Trans-1,2-dihydrobenzene-1,2-diol dehydrogenase
LOC113511822	861	4.34	2.4	0.7	0.23	-2.81	0.00	0.00	cAMP-specific 3',5'-cyclic phosphodiesterase, isoform I
LOC113520105	1203	51.56	54.31	6.67	8.12	-2.81	0.00	0.00	D-3-phosphoglycerate dehydrogenase
MSTRG.7690	399	10.1	8.89	1.07	1.62	-2.82	0.00	0.00	Aldose 1-epimerase
LOC113515506	5602.6	16.41	16.74	2.34	2.18	-2.82	0.00	0.00	Uncharacterized protein LOC110378016 isoform X1
MSTRG.15546	2436	0.79	1.31	0.1	0.19	-2.82	0.00	0.00	Aminopeptidase M1
LOC113515371	2174.5	119.8	121.23	15.98	17.45	-2.82	0.00	0.00	Cytochrome P450 monooxygenase CYP304F17
LOC113509413	1750	4.08	3.62	0.28	0.77	-2.83	0.00	0.00	Hydroxylysine kinase
LOC113522200	444	10.69	3.52	0.4	1.6	-2.83	0.00	0.00	Uncharacterized protein LOC106101275
LOC113509885	1222	1.15	1.04	0.29	0	-2.83	0.00	0.00	Uncharacterized protein LOC106136933
LOC113514464	1263	91.73	126.34	15.88	14.16	-2.83	0.00	0.00	Uncharacterized protein
MSTRG.10659	1998	26.83	29.54	2.88	4.88	-2.83	0.00	0.00	Uncharacterized protein
LOC113518198	3012	1.02	1.37	0.1	0.22	-2.84	0.00	0.00	Band 4.1-like protein 4
LOC113509247	812	456.17	365.82	58.55	54.83	-2.84	0.00	0.00	Neurotransmitter gated ion channel
LOC113514178	1464	1.19	0.98	0.17	0.11	-2.84	0.00	0.00	Organic cation transporter protein-like
LOC113523219	3737	1.75	3.36	0.44	0.25	-2.85	0.00	0.00	Uncharacterized protein LOC106130432
LOC113509251	468	16.2	14.25	2.46	1.75	-2.85	0.00	0.00	Uncharacterized protein
LOC113519071	1578	0.42	0.42	0.05	0.05	-2.85	0.00	0.02	Arabinose-proton symporter-like
LOC113514680	3022	30.26	31.64	3.76	4.62	-2.85	0.00	0.00	Insulin-like growth factor 1 receptor
MSTRG.11123	1002	6.16	6.37	0.93	0.74	-2.85	0.00	0.00	Histone-lysine N-methyltransferase SETMAR-like
LOC113515396	3793	8.65	8.17	1.08	1.18	-2.86	0.00	0.00	Protein patched homolog 1-like
LOC113509631	2127	10.83	8.27	1.15	1.42	-2.86	0.00	0.00	Carboxypeptidase N subunit 2-like
LOC113512971	2192.98	739.86	710.29	94.23	100.52	-2.86	0.00	0.00	Iron/zinc purple acid phosphatase-like protein
MSTRG.12790	465	1.37	4.4	0.71	0	-2.87	0.00	0.02	Uncharacterized protein
LOC113511860	4981	1.11	0.75	0.12	0.13	-2.87	0.00	0.00	Uncharacterized protein LOC110377235 isoform X5

LOC113509792	802	1.64	1.39	0	0.38	-2.87	0.00	0.00	Rootletin
LOC113520534	2113	20.87	20.34	2.62	2.89	-2.87	0.00	0.00	Cytochrome P450 49a1
LOC113515321	326	587.88	670.25	98.95	83.49	-2.88	0.00	0.00	Uncharacterized protein LOC106134903
LOC113522141	1817	429.5	429.64	57.3	57.25	-2.88	0.00	0.00	Spermidine synthase
LOC113522497	1363.61	813.83	919.68	119.56	111.53	-2.88	0.00	0.00	Uncharacterized protein LOC106138090
LOC113513145	1138	5.43	5.33	0.64	0.78	-2.88	0.00	0.00	Zinc finger protein rotund isoform X3
MSTRG.14076	455	4.9	6.62	0.75	0.75	-2.88	0.00	0.00	Uncharacterized protein
LOC113514511	727	5.96	5.53	1.21	0.3	-2.88	0.00	0.00	Carbonyl reductase
LOC113513432	3479	1.77	1.17	0.26	0.13	-2.89	0.00	0.00	Uncharacterized protein LOC106137196
LOC113514771	1463	1.29	0.52	0.17	0.06	-2.89	0.00	0.00	Lipase 1-like
LOC113512779	653	58.15	59.84	5.98	9.82	-2.89	0.00	0.00	Facilitated trehalose transporter Tret1-like isoform X1
LOC113522133	1005	40.49	38.91	4.71	5.81	-2.89	0.00	0.00	Facilitated trehalose transporter Tret1-like isoform X1
LOC113523146	318	19.19	24.71	1.21	4.95	-2.89	0.00	0.00	Lysosomal alpha-mannosidase-like isoform X1
LOC113516431	588	19.41	14.72	1.53	3.02	-2.90	0.00	0.00	Hemicentin-2
LOC113517217	1580	4.4	2.82	0.42	0.52	-2.90	0.00	0.00	Kynurenine 3-monooxygenase
LOC113522888	699	17.37	16.66	1.78	2.7	-2.90	0.00	0.00	Uncharacterized protein LOC110383083
LOC113509262	943	1.87	1.37	0.31	0.1	-2.90	0.00	0.00	low density lipoprotein receptor adapter protein 1
LOC113520291	3023	13.43	12.79	1.66	1.76	-2.90	0.00	0.00	Glycoprotein 3-Alpha-L-fucosyltransferase A
LOC113519661	1499	20.76	22.77	3.15	2.53	-2.91	0.00	0.00	Protein NDRG3-like
LOC113519762	977	6.96	11.77	1.37	1.05	-2.91	0.00	0.00	Elongation of very long chain fatty acids protein AAEL008004-like
LOC113514303	1485	5.42	5.53	0.51	0.89	-2.93	0.00	0.00	L-threonine ammonia-lyase-like
LOC113511777	1606	21.69	20.03	2.59	2.78	-2.93	0.00	0.00	Meteorin-like protein
LOC113515885	1222	9.21	2.07	1.24	0.21	-2.93	0.00	0.00	Uncharacterized protein LOC110381014
LOC113520813	1779	2	1.59	0.23	0.22	-2.93	0.00	0.00	Tyramine beta-hydroxylase
LOC113509934	818	13.45	19.82	1.9	2.36	-2.94	0.00	0.00	Uncharacterized protein LOC106135020

LOC113510046	961	11.47	9.46	1.1	1.57	-2.94	0.00	0.00	Phospholipid scramblase 2
LOC113512197	12606	1.29	1.03	0.12	0.17	-2.95	0.00	0.00	Dynein heavy chain 1, axonemal-like
MSTRG.8239	745	3.44	5.84	0.29	0.86	-2.95	0.00	0.00	Uncharacterized protein
LOC113523410	2792	1.16	1.25	0.14	0.16	-2.96	0.00	0.00	Zinc transporter 1
LOC113512382	2818	0.43	0.24	0	0.08	-2.96	0.00	0.00	Synaptic vesicle glycoprotein 2C-like
LOC113517382	1193	130.46	153.69	18.58	17.22	-2.96	0.00	0.00	Uncharacterized protein LOC106109383
LOC113516021	1481	48.38	60.53	7.36	6.3	-2.96	0.00	0.00	Fumarylacetoacetase
LOC113523401	6023	161.91	149.94	17.98	21	-2.96	0.00	0.00	Laminin subunit beta-1
LOC113521874	483	6.29	6.92	1.64	0	-2.97	0.00	0.00	Uncharacterized protein LOC110382845
MSTRG.8387	941	43.26	50.8	5.16	6.57	-2.98	0.00	0.00	Uncharacterized protein
LOC113523541	11852.3	203.43	166.97	19.81	24.83	-2.98	0.00	0.00	Teneurin-3 isoform X1
MSTRG.8602	1334	25.64	33.84	3.64	3.75	-2.99	0.00	0.00	Seminal fluid protein HACP040
LOC113520094	1707	186.94	172.44	22.65	21.74	-2.99	0.00	0.00	Adenosine deaminase CECR1
LOC113513586	1087	1.47	2.04	0.08	0.33	-2.99	0.00	0.00	Bardet-Biedl syndrome 1 protein
LOC113515960	3536	104.82	81.24	10.42	12.47	-2.99	0.00	0.00	Dyslexia-associated protein KIAA0319
MSTRG.16486	341	124.77	138.63	16.39	18.56	-2.99	0.00	0.00	Dihydroxyacetone kinase 2
LOC113512351	4100.19	7.77	5.96	0.64	1.07	-2.99	0.00	0.00	Hemicentin-2
LOC113515341	3094	33.25	35.46	4.33	4	-3.01	0.00	0.00	A disintegrin and metalloproteinase with thrombospondin motifs 3-like
LOC113512665	996	86.41	84.91	8.96	11.95	-3.01	0.00	0.00	Matrix metalloproteinase-25-like
LOC113522374	2005	0.51	0.53	0.08	0.04	-3.01	0.00	0.00	Tetratricopeptide repeat protein 30A
MSTRG.7565	810	5.54	2.96	0.9	0.13	-3.01	0.00	0.00	Uncharacterized protein LOC106133343 isoform X1
LOC113518374	1713.5	0.73	0.09	0.09	0	-3.02	0.00	0.01	Lipase 1
LOC113517644	1194	7.35	7.54	1.05	0.73	-3.03	0.00	0.00	15-hydroxyprostaglandin dehydrogenase
MSTRG.7296	1118	9.69	10.61	1.47	0.96	-3.03	0.00	0.00	Diamine acetyltransferase 2-like
LOC113514205	2613	5.47	4.58	0.74	0.46	-3.03	0.00	0.00	Scavenger receptor class B member 1-like
LOC113519815	754	2.14	2.8	0.29	0.28	-3.03	0.00	0.00	Homeobox protein MSH-D-like
LOC113519224	1512	11.18	2.18	0.89	0.71	-3.03	0.00	0.00	Cytochrome P450 6B7-like
LOC113519878	7682	189.69	124.64	17.07	20.41	-3.03	0.00	0.00	Papilin like protein
LOC113519639	1637	25.1	28.73	2.73	3.7	-3.03	0.00	0.00	Zinc transporter ZIP1-like

LOC113519265	2259	54.93	53.38	6.25	6.67	-3.03	0.00	0.00	Multiple C2 and transmembrane domain-containing protein 1 isoform X5
LOC113510435	1809	3.71	3.45	0.54	0.31	-3.04	0.00	0.00	Inactive hydroxysteroid dehydrogenase-like protein 1 isoform X1
MSTRG.2846	1105	175.73	189.37	21.46	22.22	-3.04	0.00	0.00	Uncharacterized protein
LOC113522644	792	3.55	2.13	0.4	0.26	-3.04	0.00	0.00	Uncharacterized oxidoreductase SERP2049-like
LOC113521952	651	144.56	165.1	18.58	18.66	-3.04	0.00	0.00	Aldehyde Dehydrogenase
LOC113509069	971	219.66	146.06	20.84	22.35	-3.06	0.00	0.00	Papilin-like
LOC113521152	1514	19.23	17.74	1.89	2.44	-3.06	0.00	0.00	Ataxin-2 homolog isoform X1
LOC113520464	1860	28.36	27.53	3.3	3.22	-3.07	0.00	0.00	Potassium voltage-gated channel protein Shaker
LOC113519323	951	2.25	0.45	0	0.3	-3.08	0.00	0.00	Retrotransposable element Tf2 155 kDa protein type 2
LOC113513537	1256	1.45	0.44	0.14	0.07	-3.08	0.00	0.00	Uncharacterized protein LOC106099929
MSTRG.11269	3864	1.39	1.1	0.13	0.15	-3.08	0.00	0.00	G-protein coupled receptor 158 isoform X3
LOC113517457	1334	7.83	7.3	1.1	0.64	-3.08	0.00	0.00	Fringe glycosyltransferase
LOC113519225	844	79.5	87.51	11.1	8.29	-3.08	0.00	0.00	Sulfotransferase 1C4-like
LOC113510172	1344	2.2	1.78	0.32	0.13	-3.09	0.00	0.00	Gamma-butyrobetaine dioxygenase
MSTRG.7238	341	23.35	30.55	6.38	0	-3.10	0.00	0.00	Short/branched chain specific acyl-CoA dehydrogenase, mitochondrial
LOC113516635	1563.19	54.22	60.22	6.4	6.61	-3.10	0.00	0.00	Uncharacterized protein LOC110375481 isoform X2
LOC113512983	1925	2.75	2.38	0.38	0.2	-3.10	0.00	0.00	4-coumarate--CoA ligase 3
MSTRG.4413	1358	32.81	37.74	3.94	4.1	-3.10	0.00	0.00	Gelsolin-like
LOC113512112	1011	1.26	0.5	0.09	0.09	-3.10	0.00	0.01	Carbonic anhydrase-related protein 10 isoform X1
LOC113520804	374	20.55	18.81	2.63	1.99	-3.11	0.00	0.00	Uncharacterized protein LOC105380498 isoform X2
LOC113513007	1117	21.92	15.43	2.29	1.92	-3.12	0.00	0.00	G-protein coupled receptor moody
LOC113519571	850	1.22	1.06	0	0.23	-3.12	0.00	0.01	Serpin B5-like
LOC113518192	1721	68.83	77.8	8.1	8.37	-3.12	0.00	0.00	N-acetylgalactosamine kinase
LOC113521090	613	290.24	362.2	38.68	35.55	-3.13	0.00	0.00	Uncharacterized protein LOC106709604

LOC113509076	1078	17.01	13.84	1.46	2.01	-3.13	0.00	0.00	Uncharacterized protein LOC106131149
LOC113518128	1064	6.13	1.63	0.35	0.51	-3.13	0.00	0.00	Pancreatic triacylglycerol lipase-like
LOC113513475	616	29.62	28.66	3.41	3.17	-3.13	0.00	0.00	Galactokinase-like
LOC113522459	1218	9.54	6.7	0.95	0.86	-3.13	0.00	0.00	Uncharacterized protein LOC106129346
LOC113512072	1994.87	15.7	17.01	1.89	1.72	-3.14	0.00	0.00	Uncharacterized protein LOC106140671
LOC113522457	1826.5	92.22	95.02	10.26	10.4	-3.14	0.00	0.00	Serine/threonine-protein kinase polo isoform X2
LOC113518105	1799	2.58	3.88	0.36	0.4	-3.14	0.00	0.00	Ornithine aminotransferase, mitochondrial
LOC113515777	1769	6.3	6.87	0.51	0.95	-3.14	0.00	0.00	3-ketoacyl-CoA thiolase, mitochondrial-like isoform X1
MSTRG.4582	368	57.15	79.87	6.24	9.81	-3.15	0.00	0.00	Galactokinase-like
LOC113520294	4131.67	1.54	2.17	0.18	0.23	-3.15	0.00	0.00	Protein trachealess isoform X1
LOC113514532	1467	1.51	0.1	0.06	0.11	-3.15	0.00	0.00	Carboxypeptidase B-like
MSTRG.13323	3068	3.83	4.85	0.49	0.46	-3.15	0.00	0.00	Synaptotagmin-5-like isoform X1
LOC113509256	3103	93.74	91.81	10.11	10.29	-3.15	0.00	0.00	Venus kinase receptor
LOC113521263	1661	43.29	41.18	4.82	4.46	-3.15	0.00	0.00	Protein extra-macrochaetae
LOC113521631	2584	18.25	17.88	2	1.95	-3.16	0.00	0.00	Methylcrotonoyl-CoA carboxylase beta chain, mitochondrial
LOC113514635	720	1.21	0.41	0	0.15	-3.16	0.01	0.04	LIM/homeobox protein Lhx3 isoform X2
LOC113514026	800	15.13	13.37	1.96	1.16	-3.16	0.00	0.00	Hemicentin-2
LOC113514043	789	0.84	0.59	0	0.13	-3.17	0.01	0.04	Nose resistant to fluoxetine protein 6-like
LOC113521062	2717	0.18	0.13	0	0.03	-3.17	0.01	0.04	Protein boule-like
LOC113521590	1452	11.28	13.87	1.64	1.09	-3.17	0.00	0.00	Non-specific lipid-transfer protein-like
LOC113519932	2099.69	1120.77	997.62	110.59	118.37	-3.17	0.00	0.00	Uncharacterized protein LOC105388803 isoform X3
LOC113511182	5559	0.94	0.76	0.1	0.08	-3.17	0.00	0.00	ATP-binding cassette sub-family A member 3-like
MSTRG.1027	562	3.89	2.95	0.48	0.24	-3.18	0.00	0.00	Acyl-CoA-binding domain-containing protein 5 isoform X6
LOC113511649	2658	1.17	5.58	0.38	0.34	-3.19	0.00	0.00	Mucin-5AC-like isoform X1
MSTRG.10275	378.36	4.97	7.26	0	1.41	-3.19	0.00	0.00	Azurocidin-like

LOC113512556	4728	0.35	0.4	0.05	0.03	-3.19	0.00	0.00	Cadherin-86C
LOC113516152	6405	30.41	25.44	3.08	2.86	-3.20	0.00	0.00	brefeldin A-inhibited guanine nucleotide-exchange protein 3
LOC113512476	1699	38.11	44.01	4.69	4.01	-3.20	0.00	0.00	Mitochondrial aldehyde dehydrogenase
LOC113514061	369	18.89	12.5	0	3.47	-3.21	0.00	0.00	Tyrosine-protein kinase-like otk
LOC113521113	1387.27	73.83	60.09	6	8.1	-3.22	0.00	0.00	Protein cab-1 isoform X2
LOC113512725	3377	364.23	394.72	44.31	35.03	-3.22	0.00	0.00	Tartan/capricious-like protein
LOC113517478	1083	59.99	77.64	6.98	7.5	-3.22	0.00	0.00	IML1
LOC113519755	3833	0.56	0.38	0.06	0.04	-3.23	0.00	0.00	Uncharacterized protein LOC106136237
LOC113519582	1562	17.54	8.4	1.28	1.41	-3.23	0.00	0.00	M-phase inducer phosphatase-like
LOC113511745	4251.05	0.21	0.15	0	0.03	-3.24	0.00	0.00	Blood vessel epicardial substance
LOC113518264	2827	2.34	2.58	0.14	0.37	-3.24	0.00	0.00	Uncharacterized protein LOC110378921
MSTRG.4261	602	2.12	2.23	0	0.41	-3.24	0.00	0.00	Facilitated trehalose transporter Tret1-like
LOC113517696	1619	0.45	0.64	0.1	0	-3.25	0.00	0.00	Glucosylceramidase-like isoform X1
LOC113515484	2522	1.17	1.23	0.06	0.18	-3.25	0.00	0.00	V-type proton ATPase 116 kDa subunit a isoform 1-like
LOC113521173	2465	78.54	68.99	6.92	8.23	-3.25	0.00	0.00	Chitooligosaccharidolytic beta-N-acetylglucosaminidase-like
MSTRG.1868	849	5.56	6.17	0.96	0.23	-3.25	0.00	0.00	Retrovirus-related pol polyprotein from transposon tnt 1-94
LOC113514953	729	2.85	1.75	0.3	0.15	-3.25	0.00	0.00	Glucose dehydrogenase
LOC113522883	1999	1.49	0.89	0.2	0.04	-3.25	0.00	0.00	Innexin shaking-B
LOC113516374	1254	1350.53	1319.57	138.6	136.14	-3.25	0.00	0.00	Prolylcarboxypeptidase
LOC113511941	537	2.22	0.69	0	0.26	-3.26	0.01	0.03	Uncharacterized protein
LOC113521376	523	5.04	0.49	0.28	0.27	-3.26	0.00	0.00	Lipase
MSTRG.329	4384	7.12	3.15	0.49	0.56	-3.26	0.00	0.00	Uncharacterized protein
LOC113519494	504	4.82	4.2	0.3	0.59	-3.26	0.00	0.00	Prohormone-3 isoform X1
LOC113512075	660	325.31	327.64	32.93	33.99	-3.28	0.00	0.00	Uncharacterized protein
LOC113521737	2762	95.68	88.73	9.23	9.32	-3.28	0.00	0.00	Uncharacterized protein LOC110379736
LOC113516421	1499	116.56	120.35	11.47	12.42	-3.28	0.00	0.00	PRKCA-binding protein
LOC113515564	2564	0.14	0.22	0.03	0	-3.28	0.01	0.03	Uncharacterized protein LOC106103590

LOC113516663	996	50.33	36.43	3.81	4.95	-3.28	0.00	0.00	Inner centromere protein isoform X1
LOC113511850	6041	0.82	0.39	0.1	0.02	-3.28	0.00	0.00	Uncharacterized protein LOC106133343 isoform X4
LOC113516133	597	81.5	82.49	9.76	6.92	-3.29	0.00	0.00	Cytidine deaminase-like
LOC113517608	967	1.02	1.15	0.1	0.1	-3.30	0.00	0.00	Uncharacterized protein LOC106133773
LOC113509873	846	15.73	20.33	1.92	1.65	-3.30	0.00	0.00	Uncharacterized protein LOC106135995
LOC113510424	634	35.94	34.52	3.05	3.95	-3.32	0.00	0.00	Uncharacterized protein LOC110375326
LOC113521986	2539	8.48	9.08	0.76	0.95	-3.32	0.00	0.00	Uncharacterized oxidoreductase YjmC
LOC113513582	303	83.41	102.57	13.43	6.16	-3.33	0.00	0.00	Glutathione S-transferase 1-like
LOC113518439	2116	4.05	0.6	0.45	0	-3.33	0.00	0.00	Peroxisomal acyl-coenzyme A oxidase 1
LOC113521031	5722	2.55	1.75	0.22	0.2	-3.34	0.00	0.00	Ig-like and fibronectin type-III domain-containing protein T04A11.3 isoform X2
LOC113509965	621	2.01	2.46	0	0.39	-3.36	0.00	0.00	Uncharacterized protein LOC110375196
LOC113510038	603	9.28	6.1	0.83	0.62	-3.36	0.00	0.00	Uncharacterized protein
LOC113518657	1719	1.67	1.4	0.1	0.19	-3.37	0.00	0.00	Uncharacterized protein LOC106130394
LOC113521268	1997	390.57	303.29	31.28	34.51	-3.37	0.00	0.00	Catalase
LOC113521951	708	0.99	0.98	0.16	0	-3.37	0.00	0.02	Calphotin-like
LOC113510862	992	102.8	105.36	10.55	9.2	-3.37	0.00	0.00	Zinc transporter ZIP3
MSTRG.3959	576	4.42	5.43	0.23	0.67	-3.37	0.00	0.00	Cytochrome P450 9e2-like
LOC113510220	315	163.19	158.49	11.3	21.94	-3.38	0.00	0.00	Gelsolin-like
LOC113523089	3174	1.7	2.12	0.19	0.16	-3.39	0.00	0.00	Neuroligin-4, Y-linked-like isoform X1
LOC113513270	390	34.93	18.52	4.02	1.16	-3.39	0.00	0.00	TBC1 domain family member 4 isoform X1
LOC113515889	558	6.19	9.62	1.21	0.24	-3.40	0.00	0.00	2-oxoglutarate dehydrogenase E1 component DHKTD1 homolog, mitochondrial
MSTRG.7767	1187	2.93	4.5	0.6	0.07	-3.40	0.00	0.00	Uncharacterized protein
LOC113521756	2294	3.2	3.41	0.24	0.37	-3.40	0.00	0.00	Leukotriene A-4 hydrolase isoform X1

LOC113512435	1529	199.14	180.11	17.35	17.64	-3.41	0.00	0.00	Synaptotagmin 1 isoform X1
LOC113512403	2093	1.75	2	0.15	0.18	-3.41	0.00	0.00	ATP-binding cassette sub-family G member 4-like
LOC113515296	6337	3.56	3.22	0.3	0.32	-3.42	0.00	0.00	RNA-binding protein orb2
LOC113518207	2339	192.9	194.48	17.33	18.07	-3.42	0.00	0.00	Sialic acid synthase
LOC113517474	2610	1.25	1.69	0.06	0.2	-3.42	0.00	0.00	Neuronal PAS domain-containing protein 2-like
LOC113511640	2493	0.96	0.44	0.09	0.03	-3.42	0.00	0.00	luciferin 4-monooxygenase
MSTRG.9391	2559	15.07	14.2	1.47	1.19	-3.42	0.00	0.00	Uncharacterized protein LOC106133236
LOC113510166	1093	34.26	31.38	2.69	3.29	-3.43	0.00	0.00	Serine hydrolase-like protein
LOC113516941	3242	0.7	0.87	0.05	0.09	-3.43	0.00	0.00	Uncharacterized protein LOC106134039
LOC113515859	646	33.22	34.41	1.29	4.91	-3.43	0.00	0.00	Acyl-CoA synthetase family member 2, mitochondrial isoform X1
LOC113512773	321	742.43	687.5	63.75	78.45	-3.44	0.00	0.00	Myrosinase 1-like
LOC113516617	1632	4.55	3.35	0.46	0.25	-3.44	0.00	0.00	Lectin3
LOC113517875	393	15.6	15.17	1.12	1.69	-3.44	0.00	0.00	Fatty-acid amide hydrolase 2-A-like isoform X1
MSTRG.2260	659	8.93	11.1	1.8	0.18	-3.45	0.00	0.00	Uncharacterized protein OBRU01_06617
MSTRG.10774	1416	7.42	5.97	0.48	0.71	-3.45	0.00	0.00	Uncharacterized protein
LOC113511379	1422	13.95	14.61	1.2	1.35	-3.45	0.00	0.00	Zinc finger protein 628-like isoform X8
MSTRG.3892	2751.46	33	34.76	3.95	2.88	-3.45	0.00	0.00	Uncharacterized protein
LOC113510234	1340	0.61	0.23	0	0.06	-3.45	0.00	0.01	Organic cation transporter protein-like
MSTRG.30	394	5.02	1.94	0	0.56	-3.45	0.00	0.01	Uncharacterized protein
LOC113513808	306	11.27	6.06	1.43	0	-3.45	0.00	0.01	Unconventional myosin-Va-like
LOC113515849	958	3.89	5.29	0.5	0.3	-3.46	0.00	0.00	Uncharacterized protein LOC110378293
LOC113522301	756	1.01	2.54	0.29	0	-3.47	0.00	0.00	Uncharacterized protein LOC106142083
MSTRG.1301	3436	1.79	1.46	0.2	0.09	-3.48	0.00	0.00	Paired box protein and transposase domain containing protein
LOC113522153	2502	95.25	88.01	7.46	8.63	-3.48	0.00	0.00	Uncharacterized protein LOC106133695
LOC113521538	1887	0.2	0.38	0	0.04	-3.48	0.00	0.01	Kelch-like protein 10

MSTRG.12744	719	541.48	494.08	46.49	45.25	-3.48	0.00	0.00	Lysosomal Pro-X carboxypeptidase
MSTRG.7954	1200	4.77	5.5	0.3	0.58	-3.49	0.00	0.00	Uncharacterized protein
MSTRG.15207	578	19.85	18.59	1.58	1.78	-3.49	0.00	0.00	Lysosomal alpha-mannosidase
LOC113515955	1263	400.33	447.44	41.23	32.41	-3.50	0.00	0.00	Uncharacterized protein LOC110373240
LOC113521621	1148	0.12	0.98	0	0.08	-3.50	0.00	0.01	Protein amalgam-like
MSTRG.9128	2866	3.35	1.9	0.29	0.16	-3.50	0.00	0.00	Uncharacterized protein LOC110373625
LOC113522739	1234	117.9	116.52	9.47	10.8	-3.50	0.00	0.00	Aldose 1-epimerase-like
LOC113519512	2311	0	0.47	0	0.03	-3.50	0.00	0.01	Uncharacterized transmembrane protein DDB_G0289901-like
LOC113509072	4351.69	317.23	270.2	22.76	28.15	-3.51	0.00	0.00	Adhesive plaque matrix protein isoform X1
MSTRG.9516	374	108.12	109.99	7.88	11.94	-3.52	0.00	0.00	Uncharacterized protein
LOC113509423	1379.92	316.09	301.4	25.29	28.61	-3.52	0.00	0.00	InaD-like protein isoform X3
LOC113517233	4159	2.26	2.95	0.23	0.21	-3.52	0.00	0.00	Uncharacterized protein LOC106131872
MSTRG.9470	3405	15.11	16.25	1.21	1.44	-3.53	0.00	0.00	Interference hedgehog-like
LOC113509487	1872	5.28	4.89	0.35	0.51	-3.53	0.00	0.00	Uncharacterized protein LOC106140707
LOC113509690	2161	9.82	7.27	0.47	0.96	-3.54	0.00	0.00	Potassium channel subfamily K member 1-like
LOC113520516	2405	50.9	55.25	3.66	5.3	-3.54	0.00	0.00	Sarcoplasmic calcium-binding protein
LOC113521001	385	5.37	2.6	0	0.6	-3.54	0.00	0.01	Uncharacterized protein LOC106129135
LOC113512287	1754	1.44	1.99	0.19	0.09	-3.55	0.00	0.00	Mitochondrial carrier protein ymc
LOC113511011	1640	2.36	2.57	0.2	0.2	-3.56	0.00	0.00	Calbindin-32
LOC113511075	671.62	12.42	18.75	1.72	0.85	-3.56	0.00	0.00	Uncharacterized protein
LOC113518918	614	6862.36	5316.8	476.55	550.94	-3.56	0.00	0.00	Sparc
LOC113517645	1187.44	65.89	85.04	7.03	5.46	-3.57	0.00	0.00	Alcohol dehydrogenase AD1
LOC113509680	1449.67	68.27	54.59	5.35	5.1	-3.58	0.00	0.00	E3 SUMO-protein ligase EGR2 isoform X1
LOC113512007	1223	1.49	28.14	1.31	1.06	-3.60	0.00	0.00	Fibroin light chain
MSTRG.13671	1683	1.18	0.75	0	0.14	-3.60	0.00	0.00	G-protein coupled receptor 158
LOC113513835	1300	0.85	0.12	0.07	0	-3.60	0.00	0.01	Cyclic nucleotide-gated cation channel beta-3-like

LOC113517570	1033	1.72	1.86	0.09	0.18	-3.61	0.00	0.00	Uncharacterized protein LOC110369958
LOC113520741	552	9.77	5.89	0.99	0.24	-3.62	0.00	0.00	MDS1 and EVI1 complex locus protein EVI1-like
LOC113519593	662	491.82	496.82	38.95	40.44	-3.63	0.00	0.00	Myrosinase 1-like
LOC113519330	865	0.97	0.72	0	0.11	-3.63	0.00	0.01	G-protein coupled receptor 179
LOC113514936	521	8.58	5.64	0.28	0.82	-3.63	0.00	0.00	Protein KIAA0556-like
MSTRG.13181	4379	4.6	4.93	0.4	0.35	-3.63	0.00	0.00	DD34D transposase
LOC113519789	1125	0.58	0.65	0	0.08	-3.64	0.00	0.01	1,5-anhydro-D-fructose reductase-like
LOC113509454	731	0.95	1.34	0.15	0	-3.64	0.00	0.01	Uncharacterized protein LOC106709162
LOC113515876	1058	24.31	22.3	1.23	2.41	-3.65	0.00	0.00	Juvenile hormone epoxide hydrolase-like
LOC113516169	926	28.16	28.56	2.64	1.76	-3.66	0.00	0.00	Glyoxylate reductase/hydroxypyruvate reductase-like
MSTRG.11997	764	2.65	1.25	0	0.28	-3.67	0.00	0.00	Uncharacterized protein LOC106139637
LOC113520751	3993	1.03	1.18	0.15	0.02	-3.67	0.00	0.00	Uncharacterized protein LOC106132055
MSTRG.9891	1752	1.56	0.96	0.09	0.09	-3.67	0.00	0.00	O-acyltransferase like protein-like
LOC113520906	1802	0.72	0.56	0.05	0.04	-3.67	0.00	0.00	Aminopeptidase W07G4.4
MSTRG.13678	1084	1163.22	1103.91	91.12	83.28	-3.67	0.00	0.00	RNA exonuclease 4-like
LOC113523274	1609	358.14	426.97	29.58	30.35	-3.68	0.00	0.00	Uncharacterized protein
MSTRG.11809	2134	1.74	2.22	0.07	0.22	-3.69	0.00	0.00	Uncharacterized protein
LOC113513597	1673	0.51	0.26	0.05	0	-3.70	0.00	0.01	Myrosinase 1-like
LOC113522440	2428	0.96	1.24	0.06	0.1	-3.70	0.00	0.00	Uncharacterized protein OBRU01_10191
LOC113512508	1220	0.63	0.52	0.07	0	-3.70	0.00	0.00	Acyl-CoA Delta(11) desaturase
MSTRG.4246	349	6.76	5.76	0.84	0	-3.70	0.00	0.00	Rotatin-like
LOC113520705	3380	6.26	6.09	0.44	0.48	-3.71	0.00	0.00	Nitrogen permease regulator 2-like protein
LOC113522665	1110	1.63	0.73	0	0.16	-3.71	0.00	0.00	Uncharacterized protein LOC106139316
LOC113519751	1638.68	737.63	674.15	51.55	53.7	-3.72	0.00	0.00	Uncharacterized protein LOC106136278
LOC113522052	428	11.04	1.16	0	0.88	-3.73	0.00	0.00	Fatty acid-binding protein

LOC113513051	1701	0.69	0.73	0	0.09	-3.73	0.00	0.00	Parapinopsin-like
LOC113510247	1163	6.69	7.04	0.62	0.38	-3.73	0.00	0.00	Acidic amino acid decarboxylase GADL1
LOC113510512	965	12.27	7.98	0.7	0.78	-3.74	0.00	0.00	Uncharacterized protein LOC106124870
LOC113513699	499	58.65	51.26	6.08	2.11	-3.74	0.00	0.00	Microsomal epoxide hydrolase
LOC113517602	3702	1.06	1.19	0.06	0.1	-3.75	0.00	0.00	Uncharacterized protein LOC106137997
LOC113521899	620.86	261.87	194.53	17.34	14.83	-3.76	0.00	0.00	Uncharacterized protein LOC106139546
LOC113522895	2682	0.43	4.37	0.11	0.22	-3.76	0.00	0.00	Mediator of DNA damage checkpoint protein 1
LOC113516356	429	2193.11	1661.81	134.83	155.38	-3.77	0.00	0.00	Sparc
LOC113513595	1799	74.96	67.5	4.34	5.78	-3.79	0.00	0.00	N-acetylglucosaminidase
LOC113517647	2094	19.22	14.84	1.17	1.22	-3.79	0.00	0.00	Sodium-dependent dopamine transporter
LOC113522226	2615	12.03	11.78	1	0.66	-3.80	0.00	0.00	Uncharacterized protein LOC106136638
LOC113515776	1475	8.42	5.42	0.46	0.5	-3.80	0.00	0.00	3-ketoacyl-CoA thiolase, mitochondrial-like
LOC113516327	4941.43	26.86	25.27	1.74	1.86	-3.80	0.00	0.00	Homeobox protein cut isoform X2
LOC113518465	884	45.81	16.46	2.59	1.77	-3.81	0.00	0.00	Trypsin-like protein
LOC113523029	1761	411.88	385.89	26.42	28.96	-3.82	0.00	0.00	Pancreatic triacylglycerol lipase-like
LOC113523473	784	4.99	3.12	0.13	0.4	-3.84	0.00	0.00	Synaptotagmin-5
LOC113519703	3880	1.19	0.53	0.08	0.04	-3.84	0.00	0.00	Uncharacterized protein LOC106136222
LOC113521709	1048.04	87.83	95.23	4.89	5.49	-3.84	0.00	0.00	Ras-related protein Rab-23
MSTRG.2773	1340	4.25	4.38	0.19	0.38	-3.85	0.00	0.00	Uncharacterized protein
LOC113517801	862	0.92	1.15	0	0.11	-3.86	0.00	0.00	Uncharacterized family 31 glucosidase KIAA1161-like isoform X1
LOC113511224	3933	0.14	0.2	0	0.02	-3.86	0.00	0.00	Sodium/hydrogen exchanger 10-like
LOC113516107	1965	1.39	1.13	0.04	0.12	-3.87	0.00	0.00	Protein atonal homolog 8
LOC113510787	9125	66.14	57.12	3.72	4.43	-3.88	0.00	0.00	Protein unc-13 homolog A
LOC113517147	533	982.98	1005.25	59.27	75.17	-3.89	0.00	0.00	Aminoacylase-1-like
MSTRG.14256	1488	5.68	3.9	0.51	0.11	-3.89	0.00	0.00	Uncharacterized protein
MSTRG.12809	300	19	6.62	1.56	0	-3.90	0.00	0.00	Uncharacterized protein

LOC113519133	557	12.43	6.22	0.73	0.48	-3.90	0.00	0.00	Uncharacterized protein
MSTRG.12810	409	5.66	3.05	0.5	0	-3.90	0.00	0.00	Uncharacterized protein
LOC113521583	4371	0.47	0.35	0	0.05	-3.91	0.00	0.00	Uncharacterized protein LOC106132743 isoform X1
LOC113517072	1257	23.93	20.92	1.82	1.1	-3.91	0.00	0.00	Uncharacterized protein LOC106143669
LOC113515525	859	76.66	81.94	5.17	5.08	-3.93	0.00	0.00	Uncharacterized protein LOC106134739
MSTRG.9867	1396	1.66	1.37	0	0.18	-3.93	0.00	0.00	Uncharacterized protein LOC106138072
LOC113521642	955	69.44	81.35	5.36	4.26	-3.94	0.00	0.00	Glutathione S-transferase 1-like
LOC113520176	2476	3.56	3.41	0.28	0.15	-3.95	0.00	0.00	Facilitated trehalose transporter Tret1-2 homolog isoform X1
LOC113520552	1127	43.32	46.9	3.15	2.53	-3.96	0.00	0.00	Uncharacterized protein LOC106108538
LOC113521632	2413	4.65	3.95	0.42	0.13	-3.96	0.00	0.00	Uncharacterized protein LOC105390299
LOC113510629	1146.69	54.52	61.59	3.65	3.63	-3.97	0.00	0.00	Krueppel-like factor 9
LOC113520128	469	2044.61	2033.7	151.26	111.49	-3.97	0.00	0.00	Aminoacylase-1-like
LOC113523069	759	1.78	0.93	0	0.14	-3.97	0.00	0.00	Uncharacterized protein LOC110378344
LOC113523382	941	2.12	1.48	0	0.2	-3.98	0.00	0.00	ATP-binding cassette sub-family G member 1-like
LOC113512748	1168	6.16	10.02	0.54	0.45	-3.98	0.00	0.00	Cytochrome P450 9e2-like
LOC113513750	1409	3.32	3.63	0.06	0.36	-3.98	0.00	0.00	Acyl-coenzyme A dehydrogenase
LOC113522928	432	7.52	7.16	0	0.86	-3.98	0.00	0.00	Uncharacterized protein
LOC113510781	588	1.87	2.32	0	0.22	-3.99	0.00	0.00	Uncharacterized protein OBRU01_02162
LOC113517584	904	3.02	2.62	0.33	0	-4.00	0.00	0.00	Uncharacterized protein LOC106713365
LOC113520364	1125	1177.55	1128.38	73.88	67.69	-4.00	0.00	0.00	Hydroxypyruvate isomerase
LOC113521105	1473	1.28	0.77	0.11	0	-4.00	0.00	0.00	Guanine nucleotide-binding protein G(f) subunit alpha
LOC113509569	2465	13.3	12.68	0.59	0.97	-4.00	0.00	0.00	Carboxylesterase
LOC113520468	2081	73.48	67.14	3.89	4.65	-4.01	0.00	0.00	Lysosomal alpha-mannosidase-like isoform X1
LOC113522757	1688	1.9	2.22	0	0.24	-4.01	0.00	0.00	Uncharacterized protein LOC106129350

MSTRG.5158	5008	2.45	2.89	0.16	0.16	-4.03	0.00	0.00	Uncharacterized protein LOC106135886
LOC113520500	1248	16.3	2.52	0.78	0.35	-4.03	0.00	0.00	Acyl-CoA Delta(11) desaturase-like
LOC113511309	278	56.14	89.6	4.55	4.74	-4.03	0.00	0.00	Hypothetical protein RR46_06210
MSTRG.6504	469	7.79	10.48	1.05	0	-4.03	0.00	0.00	Uncharacterized protein
LOC113513404	330	406.31	400.14	25.88	26.46	-4.03	0.00	0.00	Aminoacylase-1-like
LOC113514175	478	3.6	2.95	0.33	0	-4.04	0.00	0.00	Uncharacterized protein LOC106134414
LOC113511695	1823	31.65	39.5	2.22	2	-4.04	0.00	0.00	Protein yellow-like
LOC113510000	1941	0.43	0.41	0.04	0	-4.04	0.00	0.00	Ecdysone oxidase
LOC113513325	647	9.36	9.48	0.55	0.54	-4.04	0.00	0.00	Cytochrome P450 protein
MSTRG.5540	2041	38.47	40.77	2.93	1.75	-4.05	0.00	0.00	Zinc finger CCHC domain-containing protein 24-like
LOC113513763	797	30.84	28.3	1.58	1.94	-4.05	0.00	0.00	Interference hedgehog-like
LOC113520670	586	36.32	44.04	2.2	2.61	-4.05	0.00	0.00	Antennal esterase CXE13
MSTRG.13883	305	11.43	15.99	1.45	0	-4.05	0.00	0.00	Uncharacterized protein
LOC113521711	493	292.09	317.53	16.87	19.85	-4.06	0.00	0.00	Uncharacterized protein LOC106132549
LOC113512354	2038.92	94.12	91.57	6.05	4.61	-4.07	0.00	0.00	ATP-binding cassette sub-family G member 4-like isoform X2
LOC113509818	2011	136.45	126.82	7.54	7.71	-4.08	0.00	0.00	Regucalcin
LOC113509606	2333.49	22.52	21.43	1.23	1.37	-4.08	0.00	0.00	Monocarboxylate transporter 10-like
LOC113509135	825	10.52	2.67	0.12	0.61	-4.10	0.00	0.00	Lipase 1-like
LOC113517518	1773	4263.76	4465	289.78	207.64	-4.10	0.00	0.00	Cathepsin K-like
LOC113523409	1096.89	4874.19	5326.85	328.08	257.62	-4.10	0.00	0.00	Hemolymph lipopolysaccharide-binding protein
LOC113523533	1859	1.24	1.94	0.04	0.13	-4.10	0.00	0.00	Uncharacterized protein LOC106131316
MSTRG.5472	1149	12.71	11.22	0.63	0.69	-4.13	0.00	0.00	Uncharacterized protein
MSTRG.15112	376	115.49	81.81	1.94	9.77	-4.14	0.00	0.00	Protein unc-13 homolog A-like
LOC113509573	1492	1.75	1.51	0.17	0	-4.15	0.00	0.00	Neuronal acetylcholine receptor subunit beta-2-like
LOC113514815	1015	37.52	34.09	1.77	2.18	-4.15	0.00	0.00	Interference hedgehog-like
LOC113516205	909	61.78	69.14	4.23	2.95	-4.16	0.00	0.00	Uncharacterized protein LOC101736819
MSTRG.13946	341	6.67	12.53	0.91	0	-4.17	0.00	0.00	Bifunctional ATP-dependent dihydroxyacetone kinase/FAD-AMP

									lyase (cyclizing)-like
LOC113511028	1167	4.34	4.33	0.15	0.3	-4.17	0.00	0.00	Uncharacterized protein LOC106135978
LOC113512321	4233	2.14	1.94	0.14	0.08	-4.17	0.00	0.00	Xanthine dehydrogenase
LOC113522808	2538	1.19	1.17	0.06	0.06	-4.18	0.00	0.00	Transient receptor potential cation channel subfamily V member 5
LOC113512953	657	10.11	7	0.54	0.35	-4.20	0.00	0.00	MD-2-related lipid-recognition protein-like
MSTRG.15154	302	16.28	15.42	0	1.56	-4.21	0.00	0.00	Myosin-11-like
LOC113515096	958	24.73	9.32	0.8	0.99	-4.21	0.00	0.00	Collagenase-like
LOC113519558	1177	10.39	14.48	0.92	0.37	-4.23	0.00	0.00	Uncharacterized protein LOC106142421
LOC113509639	947	930.06	971.23	51.75	47.8	-4.23	0.00	0.00	Odorant binding protein
LOC113513917	3009	0.4	0.18	0	0.02	-4.25	0.00	0.00	Indole-3-acetaldehyde oxidase-like
LOC113513841	1070	26.27	30.28	1.38	1.52	-4.25	0.00	0.00	Organic cation transporter protein-like
LOC113514001	1409	3.71	2.44	0.12	0.18	-4.27	0.00	0.00	Cytochrome P450
LOC113523363	1344.05	47.1	35.9	2.04	2.14	-4.27	0.00	0.00	Protein sprouty isoform X1
LOC113512055	1847.14	50.97	71.06	2.45	3.72	-4.27	0.00	0.00	Alpha-N-acetylgalactosaminidase isoform X1
MSTRG.3477	330	94.23	124.21	6.21	5.29	-4.31	0.00	0.00	3-hydroxyisobutyrate dehydrogenase, mitochondrial
LOC113512908	562	2.96	2.74	0	0.24	-4.31	0.00	0.00	Promoting protein precursor
LOC113514149	1736	0.6	2.48	0.09	0.05	-4.32	0.00	0.00	Cytochrome P450 6B6-like
MSTRG.14364	2212	5.15	4.38	0.11	0.35	-4.34	0.00	0.00	Uncharacterized protein LOC106107598 isoform X1
LOC113517005	801	3.08	2.79	0.26	0	-4.34	0.00	0.00	Androgen-dependent TFPI-regulating protein-like isoform X1
MSTRG.4568	862	125.84	118.75	7.15	5.05	-4.34	0.00	0.00	Zinc finger CCHC domain-containing protein 24-like
MSTRG.12781	1356	4.75	4.68	0.19	0.25	-4.35	0.00	0.00	Neuroblastoma-amplified sequence-like
LOC113513511	394	12.14	12.14	0	1.12	-4.35	0.00	0.00	Uncharacterized protein C6orf105
LOC113516815	1663.32	11.78	11.2	0.3	0.77	-4.36	0.00	0.00	Nucleolar protein 10
LOC113520120	1371	1.2	3.02	0	0.18	-4.37	0.00	0.00	Facilitated trehalose transporter Tret1-2 homolog
LOC113515914	794	2527.82	2379.51	109.91	124.51	-4.37	0.00	0.00	Uncharacterized protein LOC106142761

MSTRG.5000	302	41.23	23.13	1.51	1.56	-4.38	0.00	0.00	Rabphilin-3A
MSTRG.4912	635	3.27	1.52	0.19	0	-4.39	0.00	0.00	Uncharacterized protein
LOC113517505	2322	7.04	7.3	0.18	0.49	-4.39	0.00	0.00	Leucine-rich repeat-containing protein 70
LOC113514869	861	1.85	1.15	0.12	0	-4.40	0.00	0.00	UDP-glucuronosyltransferase 2A3-like
MSTRG.12264	406	24.63	19.15	0.51	1.54	-4.40	0.00	0.00	Uncharacterized protein LOC110379302
LOC113522299	2013	102.31	113.48	5.35	4.53	-4.42	0.00	0.00	Hypothetical protein KGM_205387
LOC113514752	1520	6.32	6.07	0.33	0.22	-4.44	0.00	0.00	ATP-binding cassette sub-family G member 1-like
LOC113512543	4022	11.9	12.39	0.48	0.61	-4.44	0.00	0.00	Xanthine dehydrogenase-like
LOC113519122	974	3.18	3.77	0.1	0.19	-4.45	0.00	0.00	Uncharacterized protein LOC106130390
LOC113521324	1678	2.89	0.61	0.1	0.05	-4.47	0.00	0.00	Alanine--glyoxylate aminotransferase 2, mitochondrial
LOC113514609	424	14.4	17.41	1.36	0	-4.48	0.00	0.00	Aldo-keto reductase AKR2E4-like
LOC113513459	432	60.47	73.86	3.44	2.58	-4.48	0.00	0.00	Venom carboxylesterase-6-like
LOC113522976	1666	2.44	2.34	0.1	0.1	-4.50	0.00	0.00	Elongation factor 1-alpha 2
LOC113517606	825	14.65	0	0.25	0.37	-4.51	0.00	0.00	Uncharacterized protein
LOC113521984	767	3.84	3.22	0.14	0.14	-4.51	0.00	0.00	Uncharacterized protein LOC105842600
LOC113513910	1513	90.93	109.77	4.28	4.29	-4.52	0.00	0.00	Uncharacterized protein LOC101744654
MSTRG.13762	402	12.26	13.74	1.05	0	-4.52	0.00	0.00	Plasma membrane calcium-transporting ATPase 1-like isoform X2
LOC113510939	596	22.27	17.76	1.07	0.63	-4.52	0.00	0.00	Venom carboxylesterase-6-like
LOC113522562	636	320.2	240.86	13.66	10.47	-4.53	0.00	0.00	N-acetylglucosaminidase
LOC113520284	4832	0.23	0.2	0	0.01	-4.54	0.00	0.00	Titin-like isoform X1
LOC113519171	1113	1.17	1.17	0	0.08	-4.54	0.00	0.00	Facilitated trehalose transporter Tret1
LOC113511129	1622	77.01	91.43	3.27	3.79	-4.54	0.00	0.00	Protein toll
LOC113519004	1435	2.78	3.12	0.12	0.12	-4.55	0.00	0.00	Suppressor of lurcher protein 1-like
MSTRG.14365	1466	75.04	70.93	3.64	2.43	-4.55	0.00	0.00	Uncharacterized protein LOC106107598 isoform X1
LOC113520899	1144	7.33	2.47	0.4	0	-4.56	0.00	0.00	Lipase member I-like

LOC113511824	1587	43.51	46.11	1.94	1.74	-4.57	0.00	0.00	D-2-hydroxyglutarate dehydrogenase, mitochondrial-like
MSTRG.13062	501	10.93	11.97	0.6	0.3	-4.57	0.00	0.00	Uncharacterized protein
LOC113509888	3575.4	955.21	1041.23	43.03	38.03	-4.59	0.00	0.00	Toll-like receptor 3
MSTRG.5434	3563	2.51	2.25	0.13	0.06	-4.60	0.00	0.00	Reverse transcriptase
MSTRG.7746	282	70.21	33.72	4.22	0	-4.61	0.00	0.00	Uncharacterized protein
LOC113511409	440	20.61	20.86	0.82	0.82	-4.61	0.00	0.00	Hypothetical protein KGM_212946A
LOC113519098	1741.36	47.08	44.56	1.53	2.16	-4.63	0.00	0.00	D-aspartate oxidase
LOC113511010	1724	2038.72	2139.23	92.3	71.88	-4.64	0.00	0.00	C1A cysteine protease precursor
LOC113512940	721	6.88	5.47	0	0.45	-4.65	0.00	0.00	Uncharacterized protein LOC106136299
LOC113517990	746	805.03	784.05	28.99	33.06	-4.66	0.00	0.00	Acetylcholine receptor subunit Alpha-type unc-38-like
LOC113513463	3772	0.24	0.39	0.02	0	-4.67	0.00	0.00	Filamin-A isoform X1
LOC113516290	685	7.08	6.68	0.33	0.16	-4.68	0.00	0.00	Uncharacterized protein LOC106143382
LOC113516430	818	1801.83	1562.2	62.08	67.75	-4.68	0.00	0.00	Uncharacterized protein LOC106142761
LOC113513560	1068	1.85	0.85	0.09	0	-4.68	0.00	0.00	Chlorophyllide A binding protein isoform X1
LOC113521564	6008	9.94	8.9	0.35	0.36	-4.68	0.00	0.00	Down syndrome cell adhesion molecule-like protein Dscam2
LOC113517042	5486	997.39	847.87	33.6	36.38	-4.69	0.00	0.00	Collagen alpha-5(IV) chain-like
LOC113514403	1028	21.15	22.37	0.93	0.8	-4.69	0.00	0.00	ETS DNA-binding protein pokkuri
LOC113520900	773	73.59	114.07	3.31	3.79	-4.70	0.00	0.00	Uncharacterized protein LOC106133892
LOC113513300	1538	1.21	0.53	0	0.05	-4.72	0.00	0.00	Carboxypeptidase B-like
MSTRG.15449	857	2.14	1.68	0.12	0	-4.73	0.00	0.00	Ubiquinone biosynthesis monooxygenase COQ6 like protein
LOC113512683	1318	19.72	18	0.79	0.58	-4.74	0.00	0.00	Uncharacterized protein LOC106129086
LOC113510498	676	2.41	3.19	0.17	0	-4.74	0.00	0.00	Aldo-keto reductase AKR2E4-like
LOC113517845	777	21.29	17.02	0.96	0.4	-4.77	0.00	0.00	Uncharacterized protein LOC110376544
LOC113518862	2182.85	3214.09	3237.49	116.83	114.51	-4.77	0.00	0.00	Carboxylesterase 5A
LOC113512331	3518	144.65	117.61	4.06	5.22	-4.79	0.00	0.00	Hypothetical protein KGM_208540
LOC113519093	647	18.44	23.69	0.74	0.73	-4.80	0.00	0.00	Uncharacterized protein

									LOC106136406
LOC113517528	1078	185.49	166.17	6.51	5.7	-4.82	0.00	0.00	Ras-related protein Rap-2b
LOC113516587	546	8.8	6.91	0.5	0	-4.82	0.00	0.00	Short/branched chain specific acyl-CoA dehydrogenase, mitochondrial
LOC113519117	1582	28.03	29.46	0.79	1.18	-4.83	0.00	0.00	Cytochrome P450 9G3
LOC113519915	1865	214.7	219.06	6.84	7.91	-4.85	0.00	0.00	Beta-hexosaminidase
LOC113509870	1791	10.76	11.07	0.64	0.09	-4.86	0.00	0.00	Alkaline phosphatase-like isoform X2
LOC113515848	1042	9.21	9.98	0.36	0.26	-4.88	0.00	0.00	Uncharacterized protein LOC105841275
LOC113513980	1532	92.48	86.76	3.18	2.57	-4.93	0.00	0.00	Cytochrome P450 monooxygenase CYP9G18
LOC113522442	2576	55.44	54.97	2.07	1.46	-4.93	0.00	0.00	Uncharacterized protein LOC106136992
LOC113515816	950	492.5	588.49	18.73	15.76	-4.94	0.00	0.00	Uncharacterized protein LOC106130383
LOC113514080	1320	2.34	3.58	0.13	0.06	-4.95	0.00	0.00	Cysteine and histidine-rich protein 1-like
LOC113522904	554	12.17	12.14	0.49	0.24	-4.95	0.00	0.00	Uncharacterized protein LOC106127552
LOC113510219	515	5.71	5.02	0.28	0	-4.97	0.00	0.00	Meiosis-specific nuclear structural protein 1-like
LOC113517945	1917	2995.98	2013.29	72.95	82.13	-4.98	0.00	0.00	Prophenoloxidase
LOC113518689	1588	0.79	1.26	0.05	0	-4.98	0.00	0.00	Acheron
MSTRG.12064	2371.24	1289.18	678.38	22.81	37.58	-5.00	0.00	0.00	Hemocytin
LOC113516077	827	9.5	11.4	0.5	0.12	-5.00	0.00	0.00	Uncharacterized protein LOC106134335
LOC113520466	338	113.7	121.42	2.83	4.8	-5.00	0.00	0.00	3-hydroxyisobutyrate dehydrogenase, mitochondrial
LOC113514448	1791	0.51	1.3	0	0.04	-5.00	0.00	0.00	Scavenger receptor class B member 1-like
LOC113515652	4515	1.41	3.46	0.05	0.1	-5.01	0.00	0.00	Tolloid-like protein 1
LOC113511283	2366	1.23	1.15	0.03	0.03	-5.01	0.00	0.00	Trypsin proteinase T2b precursor
MSTRG.7974	4179	19.25	21.4	0.72	0.5	-5.01	0.00	0.00	Zinc transporter 2-like isoform X1
LOC113509459	484	411.82	361.58	11.07	12.94	-5.02	0.00	0.00	Multiple epidermal growth factor-like domains protein 10
LOC113514124	939	10.61	3.69	0.21	0.2	-5.03	0.00	0.00	Collagenase-like
LOC113517686	494	1006.14	1011.37	31.09	30.87	-5.03	0.00	0.00	Acetylcholine receptor subunit

									Alpha-type unc-38-like
LOC113510563	1801	2961.54	1610.02	52.58	84.2	-5.04	0.00	0.00	Hemocytin
LOC113522982	990	9.27	10.44	0.1	0.47	-5.04	0.00	0.00	Uncharacterized protein LOC106136966
LOC113515727	1655	1457.61	749.2	25.8	39.86	-5.05	0.00	0.00	Hemocytin
LOC113518647	555	55.54	51.4	0.98	2.17	-5.07	0.00	0.00	Alkylglycerol monooxygenase-like
LOC113519226	730	822.98	795.49	25.33	22.08	-5.08	0.00	0.00	Uncharacterized protein LOC106720246
LOC113517853	1833	3.83	4.06	0.18	0.04	-5.08	0.00	0.00	Dipeptidase 1-like
LOC113514722	577	1907.65	900.33	29.66	52.77	-5.10	0.00	0.00	Hemocytin
LOC113515116	2208	8368.43	7278.56	222.87	211.81	-5.14	0.00	0.00	Prophenoloxidase subunit 2
LOC113521981	925	5.42	6.78	0.21	0.1	-5.15	0.00	0.00	Short-chain dehydrogenease/reductase
LOC113512517	1426	202.65	159.09	5.08	4.85	-5.16	0.00	0.00	Protein turtle
MSTRG.10620	388	960.41	890.3	30.38	22.94	-5.16	0.00	0.00	Protein shifted
LOC113516448	423	4059.15	2135.03	74.11	104.19	-5.16	0.00	0.00	Neurogenic locus Notch protein-like
MSTRG.2454	793	3.44	2.36	0	0.13	-5.17	0.00	0.00	Uncharacterized protein
MSTRG.3190	1192	23.83	20.26	0.68	0.51	-5.17	0.00	0.00	Uncharacterized protein
LOC113512823	984	0.69	0.09	0	0	-5.22	0.01	0.03	Uncharacterized protein LOC106136292
LOC113517766	1002	0.67	0.08	0	0	-5.22	0.01	0.03	Hypothetical protein KGM_201298
LOC113519530	556	1.7	0.22	0	0	-5.22	0.01	0.03	Uncharacterized protein LOC108019554
LOC113520446	348	5.59	0.73	0	0	-5.22	0.01	0.03	Dynein light chain Tctex-type 1-like
LOC113511289	2502	0.2	0.06	0	0	-5.22	0.01	0.03	Uncharacterized protein LOC106140597
LOC113513421	786	0.74	0.36	0	0	-5.23	0.01	0.03	Larval cuticle protein F1-like
LOC113514156	1383	0.34	0.17	0	0	-5.23	0.01	0.03	Nose resistant to fluoxetine protein 6-like
MSTRG.3401	270	13.05	6.65	0	0	-5.23	0.01	0.03	Fatty-acid amide hydrolase 2-B-like
LOC113521780	1764	0.96	1.2	0.05	0	-5.23	0.00	0.00	Esterase FE4-like
LOC113511345	678	0.67	0.76	0	0	-5.24	0.01	0.03	Uncharacterized protein LOC106127048 isoform X1
LOC113510555	925	2407.27	1422.99	41.12	59.16	-5.24	0.00	0.00	Hemocytin
LOC113521050	856	0.37	0.63	0	0	-5.24	0.01	0.03	BTB/POZ domain-containing protein KCTD1
LOC113510803	1559	0.13	0.33	0	0	-5.25	0.01	0.03	A disintegrin and metalloproteinase

									with thrombospondin motifs 7-like
MSTRG.13719	327	564.52	603.3	9.67	23.08	-5.26	0.00	0.00	Uncharacterized protein LOC110379302
LOC113513316	1969	3059.49	2076.92	50.95	80.76	-5.26	0.00	0.00	Hemocytin
LOC113522667	892	20.02	6.54	0.45	0.22	-5.26	0.00	0.00	Uncharacterized protein LOC106139316
LOC113520665	512	7.56	5.84	0.29	0	-5.27	0.00	0.00	Macrophage migration inhibitory factor homolog
LOC113513965	835	14.2	5.89	0.37	0.12	-5.28	0.00	0.00	Pancreatic triacylglycerol lipase-like
LOC113513298	1339	639.13	689.4	16.82	16.31	-5.30	0.00	0.00	Serine protease inhibitor-like
LOC113517040	6201	875.45	773.81	20.64	19.83	-5.31	0.00	0.00	Collagen alpha-1(IV) chain
LOC113510988	938	1.8	3.32	0	0.1	-5.33	0.00	0.00	Uncharacterized protein LOC106711668 isoform X1
LOC113510904	1434	5.7	4.45	0	0.23	-5.33	0.00	0.00	Acid sphingomyelinase-like phosphodiesterase 3a
LOC113515681	1892	3.62	1.98	0.09	0.04	-5.34	0.00	0.00	Serum response factor homolog A
LOC113517972	1426	128.49	132.9	3.53	2.75	-5.35	0.00	0.00	Serpin I2-like
LOC113517994	1476	0.45	0.05	0	0	-5.35	0.00	0.02	Facilitated trehalose transporter Tret1-2 homolog
MSTRG.912	246	3740.27	3199.62	113.54	76.56	-5.36	0.00	0.00	Hemocyte protease-1
LOC113509480	776	0.86	0.37	0	0	-5.36	0.00	0.02	Uncharacterized protein LOC110378812
LOC113521488	832	0.78	0.33	0	0	-5.36	0.00	0.02	Acidic Leucine-rich nuclear phosphoprotein 32-related protein
LOC113523362	1546	0.34	0.14	0	0	-5.36	0.00	0.02	Uncharacterized protein LOC106131717
LOC113515344	693	6.56	8.45	0.33	0	-5.36	0.00	0.00	Uncharacterized protein LOC106142844
LOC113511809	3219	0.13	0.08	0	0	-5.36	0.00	0.02	Netrin receptor UNC5C-like
LOC113516350	928	0.58	0.37	0	0	-5.36	0.00	0.02	Proton-coupled folate transporter-like
LOC113519035	741	0.81	0.52	0	0	-5.36	0.00	0.02	Uncharacterized protein LOC106137625
MSTRG.10599	260	16.01	10.87	0	0	-5.36	0.00	0.02	Xanthine dehydrogenase-like
LOC113521606	765	0.66	0.62	0	0	-5.37	0.00	0.02	Enkurin isoform X2
MSTRG.12319	506	1.37	1.3	0	0	-5.37	0.00	0.02	Trypsin-like protein
LOC113518448	1188	17.78	17.33	0.15	0.66	-5.37	0.00	0.00	Fructose-bisphosphate aldolase-like

LOC113512906	3755	1.96	1.54	0	0.08	-5.38	0.00	0.00	Serine/arginine repetitive matrix protein 2
LOC113517026	791	0.42	0.83	0	0	-5.38	0.00	0.02	Sodium- and chloride-dependent glycine transporter 1-like
MSTRG.14072	285	5.67	11.79	0	0	-5.38	0.00	0.02	Endonuclease-reverse transcriptase
LOC113511308	1122	1.8	2.39	0	0.08	-5.39	0.00	0.00	Lipase member H-B-like
LOC113515907	220	232.98	195.12	0	9.85	-5.40	0.00	0.00	Chitooligosaccharidolytic beta-N-acetylglucosaminidase isoform X1
LOC113520605	1538.45	64.28	53.51	1.35	1.38	-5.40	0.00	0.00	Uncharacterized protein
LOC113519377	1851	22.61	24.49	0.48	0.6	-5.41	0.00	0.00	Facilitated trehalose transporter Tret1-like
LOC113513939	1682	333.67	402.59	7.64	9.08	-5.43	0.00	0.00	Selenium-binding protein 1-A
LOC113520205	875	3.79	2.24	0	0.11	-5.43	0.00	0.00	Nucleoredoxin-like
LOC113522598	1425	10.92	2.78	0.12	0.18	-5.45	0.00	0.00	Uncharacterized protein LOC106139316
LOC113509224	1365	0.45	0.17	0	0	-5.48	0.00	0.01	Uncharacterized protein
LOC113516703	1520	3.13	4.94	0.11	0.05	-5.48	0.00	0.00	Krueppel homolog 1-like
LOC113512121	448	2.39	1.38	0	0	-5.48	0.00	0.01	Uncharacterized protein LOC110372977
LOC113513088	297	247.86	249.48	4.91	6.78	-5.50	0.00	0.00	Wnt inhibitory factor 1
LOC113516624	1771	1.35	1.23	0.05	0	-5.51	0.00	0.00	Myrosinase 1
MSTRG.10395	457	46.77	39.98	0.37	1.48	-5.51	0.00	0.00	Eukaryotic translation initiation factor 4E-binding protein 2
LOC113518490	969	0.16	0.88	0	0	-5.51	0.00	0.01	Aquaporin PIP2-7 isoform X2
LOC113518579	748	61.32	61.24	1.16	1.43	-5.53	0.00	0.00	GILT-like protein 2
LOC113518681	365	118.93	15.42	1.43	1.44	-5.57	0.00	0.00	Hypothetical protein OBRU01_15499
MSTRG.13907	430	1966.54	1071.31	29.15	36.1	-5.58	0.00	0.00	Hypothetical protein KGM_209630
LOC113510604	1101	4.61	0.22	0.08	0	-5.58	0.00	0.00	Pancreatic triacylglycerol lipase-like
LOC113512282	844	0.86	0.43	0	0	-5.59	0.00	0.01	Na,K-ATPase beta-subunit
LOC113516937	1167	0.55	0.27	0	0	-5.59	0.00	0.01	Alpha-tocopherol transfer protein-like isoform X2
LOC113517621	516	1.75	1.25	0	0	-5.60	0.00	0.01	Uncharacterized protein LOC106141905
LOC113510248	790	1212.39	1089.45	24.92	21.85	-5.60	0.00	0.00	Venom protease
LOC113522232	791	0.52	0.95	0	0	-5.61	0.00	0.01	Uncharacterized protein LOC106104845

LOC113509292	770	23.22	19.1	0.14	0.68	-5.62	0.00	0.00	Alkylglycerol monooxygenase-like
LOC113519050	485	0.5	3.14	0	0	-5.63	0.00	0.01	Glutathione S-transferase unclassified 1
LOC113515187	573	2.5	0	0	0	-5.68	0.00	0.01	PhosphoLipase A2
LOC113510992	681	1.59	0.3	0	0	-5.69	0.00	0.01	Uncharacterized protein LOC106136019
LOC113522760	1096	0.73	0.22	0	0	-5.69	0.00	0.01	Uncharacterized protein LOC106139325
MSTRG.15168	930	0.66	0.56	0	0	-5.71	0.00	0.01	Retinoid-inducible serine carboxypeptidase-like
LOC113511557	812	78.93	90.88	1.93	1.25	-5.71	0.00	0.00	Glutathione S-transferase 1-like
LOC113519055	2611	13.13	18.15	0.24	0.32	-5.77	0.00	0.00	Polyserase-2-like
LOC113518231	2711	4.69	5.35	0	0.19	-5.77	0.00	0.00	Uncharacterized protein LOC101738767
LOC113519112	4752	0.16	0.03	0	0	-5.78	0.00	0.00	MATH and LRR domain-containing protein PFE0570w-like
LOC113510409	456	3.15	1.32	0	0	-5.79	0.00	0.00	Chloride channel protein 2 isoform X1
MSTRG.774	547	1.95	1.11	0	0	-5.80	0.00	0.00	Uncharacterized protein
LOC113512622	654	0.85	1.44	0	0	-5.82	0.00	0.00	Sodium-dependent nutrient amino acid transporter 1-like isoform X1
LOC113519522	489	29.4	27.77	0.32	0.63	-5.82	0.00	0.00	Zinc transporter 2-like isoform X1
LOC113513733	443	33.17	38.89	0.4	0.8	-5.82	0.00	0.00	UDP-glucuronosyltransferase 2B1-like isoform X2
LOC113511343	493	11.55	9.92	0.31	0	-5.83	0.00	0.00	Aldo-keto reductase
LOC113515439	1604.03	195.78	202.14	3.81	2.99	-5.85	0.00	0.00	Retinal dehydrogenase 1-like
LOC113515902	975	0.93	0.35	0	0	-5.88	0.00	0.00	O-acyltransferase like protein-like
MSTRG.5341	1178	23.35	23.08	0.3	0.45	-5.89	0.00	0.00	Uncharacterized protein
LOC113519524	1451	0.42	0.37	0	0	-5.90	0.00	0.00	Synaptic vesicle glycoprotein 2B-like
MSTRG.13731	249	23.45	27.79	0	0	-5.90	0.00	0.00	Protein takeout
LOC113522349	4291	233.72	202.32	2.73	4.28	-5.93	0.00	0.00	Nidogen-1
LOC113509365	1747	5.76	6.43	0.19	0	-5.93	0.00	0.00	Cytochrome P450 6B2-like
LOC113515857	424	0	6.33	0	0	-5.94	0.00	0.00	Collagen and calcium-binding EGF domain-containing protein 1-like isoform X2
LOC113520087	1744	0	0.67	0	0	-5.94	0.00	0.00	Ubiquitin carboxyl-terminal hydrolase 7-like isoform X1

LOC113514851	469	3.76	0.92	0	0	-5.96	0.00	0.00	Niemann-Pick type C2 protein Npc2-t01
MSTRG.2273	928.54	303.03	325.2	4.79	4.84	-5.97	0.00	0.00	Uncharacterized protein LOC106137009 isoform X1
MSTRG.12673	282	21.59	18.17	0	0	-5.98	0.00	0.00	Peroxisomal acyl-coenzyme A oxidase 1
LOC113521921	726	1.91	0.27	0	0	-6.04	0.00	0.00	Uncharacterized protein
LOC113517817	517	3.05	1	0	0	-6.05	0.00	0.00	Pancreatic triacylglycerol lipase-like
LOC113515324	249	32.25	24.31	0	0	-6.06	0.00	0.00	Cytochrome b5
LOC113512534	900	0.69	0.98	0	0	-6.07	0.00	0.00	Pancreatic lipase-related protein 3-like
MSTRG.12745	723	0.96	1.36	0	0	-6.07	0.00	0.00	Uncharacterized protein
LOC113518749	774	0.54	1.59	0	0	-6.08	0.00	0.00	Hypothetical protein KGM_203921
LOC113515315	503	10.84	13.98	0.3	0	-6.10	0.00	0.00	Diamine acetyltransferase 2-like
LOC113514092	417	5.07	2.71	0	0	-6.13	0.00	0.00	Acyl-CoA-binding protein
LOC113515644	2094	0.27	0.34	0	0	-6.15	0.00	0.00	Xaa-Pro Aminopeptidase 1
LOC113517575	1333	0.36	0.7	0	0	-6.16	0.00	0.00	Heparan sulfate glucosamine 3-O-sulfotransferase 1
LOC113517628	717	1.22	1.38	0	0	-6.22	0.00	0.00	Hypothetical protein KGM_202129
LOC113513111	785	0.74	1.56	0	0	-6.23	0.00	0.00	Uncharacterized protein LOC106122485
LOC113517750	904	56.1	61.74	0.66	0.86	-6.24	0.00	0.00	UDP-glucuronosyltransferase 2A3-like
LOC113519369	1431	3.02	2.6	0.06	0	-6.26	0.00	0.00	Protein takeout
LOC113512243	538	43.28	40.64	1.04	0	-6.26	0.00	0.00	D-arabinitol dehydrogenase 1-like
LOC113512473	1398	0.73	0.33	0	0	-6.27	0.00	0.00	Lipase 1-like isoform X2
LOC113522118	1711	0.57	0.26	0	0	-6.27	0.00	0.00	Uncharacterized protein LOC106107037
LOC113509865	508	2.49	2.58	0	0	-6.29	0.00	0.00	Circadian clock-controlled protein-like
MSTRG.12634	665	34.99	37.59	0.53	0.35	-6.30	0.00	0.00	Microspherule protein 1-like
LOC113519474	399	3.49	6.05	0	0	-6.36	0.00	0.00	Uncharacterized protein OBRU01_08063
LOC113509364	2200	3.62	3	0	0.07	-6.37	0.00	0.00	Cytochrome P450 6B2-like
LOC113512118	863	0.55	1.66	0	0	-6.37	0.00	0.00	Cilia- and flagella-associated protein 97-like
LOC113518214	1972	2.13	2.14	0	0.04	-6.41	0.00	0.00	Uncharacterized protein

									LOC110373875 isoform X7
LOC113520971	1623	0.81	0.18	0	0	-6.45	0.00	0.00	Myrosinase 1
LOC113514657	869	1.09	1.23	0	0	-6.48	0.00	0.00	Cytochrome P450 9G3
LOC113510656	1107	0.72	0.96	0	0	-6.48	0.00	0.00	Pancreatic triacylglycerol lipase-like
LOC113511274	1540	0.39	0.73	0	0	-6.49	0.00	0.00	Metabolite transport protein CsbC
MSTRG.7596	757	0.9	2.02	0	0	-6.49	0.00	0.00	Uncharacterized protein LOC106135670
LOC113522197	366	294.45	393.73	3.53	4.29	-6.49	0.00	0.00	D-arabinitol dehydrogenase 1-like isoform X1
LOC113512968	1008	1.35	0.52	0	0	-6.51	0.00	0.00	Chymotrypsin-2-like
LOC113511288	922	14.67	6.9	0	0.21	-6.51	0.00	0.00	Beta-1,3-galactosyltransferase 5-like
LOC113516477	459	36.42	37.62	0.37	0.37	-6.52	0.00	0.00	Multiple C2 and transmembrane domain-containing protein 1 isoform X4
LOC113517096	1779	0.47	0.49	0	0	-6.53	0.00	0.00	Hypothetical protein KGM_214113
LOC113512878	2231	2.16	1.92	0.04	0	-6.55	0.00	0.00	Semaphorin-2A-like
LOC113517846	2118	2.54	1.81	0.04	0	-6.55	0.00	0.00	Uncharacterized protein LOC106134054
LOC113521961	633	2.84	1.19	0	0	-6.57	0.00	0.00	Vegetative cell wall protein gp1-like isoform X1
LOC113513281	456	4.01	3.95	0	0	-6.59	0.00	0.00	Uncharacterized protein LOC106137863
LOC113520054	1210	1.28	0.39	0	0	-6.67	0.00	0.00	GILT-like protein F37H8.5
MSTRG.9286	315	12.69	14.99	0	0	-6.70	0.00	0.00	Hypothetical protein RR48_00560
LOC113512082	2021	1.95	3.13	0	0.04	-6.70	0.00	0.00	Uncharacterized protein LOC106135578
LOC113519946	1908.67	0.44	0.53	0	0	-6.75	0.00	0.00	Cationic amino acid transporter 2
LOC113522572	842	37.69	18.43	0.12	0.36	-6.78	0.00	0.00	Uncharacterized protein LOC110371012
LOC113512858	2321	0.46	0.39	0	0	-6.79	0.00	0.00	4-coumarate--CoA ligase 3
LOC113519655	1374	0.99	0.67	0	0	-6.88	0.00	0.00	Carboxypeptidase A
MSTRG.1086	300	17.88	21.18	0	0	-6.89	0.00	0.00	Hemicentin-2
MSTRG.4673	649	3.05	2.49	0	0	-7.09	0.00	0.00	Uncharacterized protein LOC106129089 isoform X1
LOC113520178	663	4.7	0.78	0	0	-7.13	0.00	0.00	Chymotrypsin BII-like
LOC113520080	406	8.47	7.57	0	0	-7.17	0.00	0.00	Androgen-dependent TFPI-regulating protein-like

LOC113517421	1931.58	32.19	36.03	0.23	0.24	-7.18	0.00	0.00	Uncharacterized protein LOC101735991 isoform X2
LOC113522828	929	20.84	13.27	0.21	0	-7.20	0.00	0.00	Uncharacterized protein LOC110371440
MSTRG.7381	268	34.9	50.71	0	0	-7.21	0.00	0.00	Facilitated trehalose transporter Tret1-like
MSTRG.433	1115	1.71	1.14	0	0	-7.27	0.00	0.00	G-protein coupled receptor 112
LOC113522683	956	37.13	18.28	0.2	0.1	-7.40	0.00	0.00	Uncharacterized protein LOC110371118
LOC113517615	608	3.53	4.37	0	0	-7.41	0.00	0.00	Uncharacterized protein LOC106134414
MSTRG.1586	762	14.55	14.47	0	0.14	-7.45	0.00	0.00	Transmembrane protease serine
MSTRG.13930	361	22.47	5.76	0	0	-7.49	0.00	0.00	Carboxypeptidase
LOC113519094	459	40779.49	47065.56	238.53	215.2	-7.61	0.00	0.00	Anionic antimicrobial peptide 2
LOC113519174	653	3.69	4.66	0	0	-7.67	0.00	0.00	Deoxycytidylate deaminase
LOC113509439	1712	1.64	0.86	0	0	-7.84	0.00	0.00	Neutral ceramidase-like isoform X1
LOC113520972	1225	2.58	1.42	0	0	-7.93	0.00	0.00	Myrosinase 1
LOC113520040	893	3.83	1.8	0	0	-7.95	0.00	0.00	Carboxypeptidase B-like
LOC113513466	1841	1.82	1.1	0	0	-8.19	0.00	0.00	Solute carrier family 22 member 6-like
LOC113516160	457	17.11	14.75	0	0	-8.59	0.00	0.00	Uncharacterized protein LOC106138812
LOC113520429	960	6.26	4.82	0	0	-8.92	0.00	0.00	Uncharacterized abhydrolase domain-containing protein DDB_G0269086-like
LOC113521372	399	35.95	43.07	0	0	-9.38	0.00	0.00	Uncharacterized protein
LOC113516117	1797	4.24	2.79	0	0	-9.41	0.00	0.00	Frizzled-10-like
LOC113513800	1950	663.02	536.49	0.62	0.48	-10.05	0.00	0.00	Uncharacterized family 31 glucosidase KIAA1161-like
