# Supplementary material for: Transcriptomic analyses reveal comprehensive responses of insect hemocytes to mycopathogen Beauveria bassiana, and fungal virulence-related cell wall protein assists pathogen to evade host cellular defense
Source: Virulence. 2020 Oct 5;11(1):1352–65. doi: 10.1080/21505594.2020.1827886 (PMC7549920; doi:10.1080/21505594.2020.1827886)
Supplement: Supplemental Material [file KVIR_A_1827886_SM8204.zip › Table S8.pdf]

Table S8 Enrichment analyses of Gene ontology (GO) assignments for the differentially expressed genes (DEGs) of *Galleria mellonella* hemocytes against *Beauveria bassiana*

Up/down-regulated DEGs	GO class	GO ID	GO term	Hit* number	Background number**	P Value	Q Value	Gene IDs
**1 day post infection**								
Up	Biological Process	GO:0003013	circulatory system process	1	1	0.00	0.03	LOC113514429
	Biological Process	GO:0003015	heart process	1	1	0.00	0.03	LOC113514429
	Biological Process	GO:0006584	catecholamine metabolic process	1	1	0.00	0.03	LOC113517529
	Biological Process	GO:0008015	blood circulation	1	1	0.00	0.03	LOC113514429
	Biological Process	GO:0008016	regulation of heart contraction	1	1	0.00	0.03	LOC113514429
	Biological Process	GO:0008592	regulation of Toll signaling pathway	1	1	0.00	0.03	LOC113515812
	Biological Process	GO:0009712	catechol-containing compound metabolic process	1	1	0.00	0.03	LOC113517529
	Biological Process	GO:0009713	catechol-containing compound biosynthetic process	1	1	0.00	0.03	LOC113517529
	Biological Process	GO:0021551	central nervous system morphogenesis	1	1	0.00	0.03	LOC113515812
	Biological Process	GO:0021556	central nervous system formation	1	1	0.00	0.03	LOC113515812
	Biological	GO:0042	catecholamine	1	1	0.00	0.03	LOC113517529

	Process	423	biosynthetic process					
	Biological Process	GO:0045823	positive regulation of heart contraction	1	1	0.00	0.03	LOC113514429
	Biological Process	GO:0060047	heart contraction	1	1	0.00	0.03	LOC113514429
	Biological Process	GO:1903522	regulation of blood circulation	1	1	0.00	0.03	LOC113514429
	Biological Process	GO:1903524	positive regulation of blood circulation	1	1	0.00	0.03	LOC113514429
	Biological Process	GO:0044057	regulation of system process	1	2	0.01	0.05	LOC113514429
	Cellular Component	GO:0005576	extracellular region	4	101	0.00	0.03	LOC113514429,LOC113522527,LOC113509694,LOC113515812
	Cellular Component	GO:0005615	extracellular space	2	31	0.00	0.03	LOC113522527,LOC113509694
	Cellular Component	GO:0044421	extracellular region part	2	38	0.01	0.05	LOC113522527,LOC113509694
	Molecular Function	GO:0004068	aspartate 1-decarboxylase activity	1	1	0.00	0.03	LOC113521309
	Molecular Function	GO:0004511	tyrosine 3-monooxygenase activity	1	1	0.00	0.03	LOC113517529
	Molecular Function	GO:0005121	Toll binding	1	1	0.00	0.03	LOC113515812
	Molecular Function	GO:0005102	receptor binding	2	29	0.00	0.03	LOC113514429,LOC113515812
	Molecular Function	GO:0005000	vasopressin receptor activity	1	2	0.01	0.05	LOC113511665
Down	Biological Process	GO:0006520	cellular amino acid metabolic process	3	74	0.00	0.02	LOC113517147,LOC113520128,LOC113513404

	Molecular Function	GO:0004046	aminoacylase activity	3	3	0.00	0.00	LOC113517147,LOC113520128,LOC113513404
	Molecular Function	GO:0016811	hydrolase activity, acting on carbon-nitrogen (but not peptide) bonds, in linear amides	3	10	0.00	0.00	LOC113517147,LOC113520128,LOC113513404
	Molecular Function	GO:0016810	hydrolase activity, acting on carbon-nitrogen (but not peptide) bonds	3	21	0.00	0.00	LOC113517147,LOC113520128,LOC113513404
	Molecular Function	GO:0008237	metallopeptidase activity	3	43	0.00	0.01	LOC113517147,LOC113520128,LOC113513404

2 days post infection

Up	Cellular Component	GO:0005576	extracellular region	18	101	0.00	0.00	MSTRG.5837,LOC113514429,LOC113520288,LOC113511560,LOC113511606,LOC113518496,LOC113516120,LOC113522527,LOC113512196,LOC113522981,LOC113511175,MSTRG.16473,LOC113520443,LOC113509694,LOC113517115,LOC113521649,LOC113515812,LOC113513310
Down	Biological Process	GO:0055114	oxidation-reduction process	46	303	0.00	0.00	LOC113523417,LOC113514609,LOC113521987,LOC113515958,LOC113515172,LOC113515168,LOC113519242,LOC113517945,LOC113522197,MSTRG.474,LOC113513917,LOC113515775,LOC113518439,LOC113520105,LOC113515170,MSTRG.7238,LOC113511922,MSTRG.3959,LOC113512543,LOC113515169,LOC113520466,LOC113514001,LOC113512321,LOC113513750,LOC113521268,LOC113518195,MSTRG.3477,LOC113512748,LOC113512243,LOC113520534,LOC113513325,MSTRG.10599,LOC113520500,LOC113518647,LOC113522241,LOC113520813,LOC113513980,LOC113516110,LOC113516587,LOC113519117,L

								OC113512476,LOC113520314,LOC113509292,LOC113509365,LOC113517469,LOC113520664
	Biological Process	GO:0044710	single-organism metabolic process	60	529	0.00	0.00	LOC113523417,LOC113517147,LOC113514609,LOC113521987,LOC113515958,LOC113515172,LOC113515168,LOC113519242,MSTRG.16486,LOC113517945,LOC113518207,LOC113522197,MSTRG.474,LOC113513917,LOC113520128,LOC113509684,LOC113515775,MSTRG.13946,LOC113518439,LOC113520105,LOC113514771,LOC113515170,MSTRG.7238,LOC113511922,MSTRG.3959,LOC113520466,LOC113512543,LOC113515169,LOC113514001,LOC113512321,LOC113513750,LOC113521268,LOC113518195,MSTRG.3477,LOC113512243,LOC113512748,LOC113520534,LOC113513325,MSTRG.10599,LOC113520500,LOC113518647,LOC113522241,LOC113510247,LOC113520813,LOC113513980,LOC113512473,LOC113516110,LOC113516587,LOC113522739,LOC113520291,LOC113518192,LOC113513404,LOC113519117,LOC113520314,LOC113512476,LOC113509292,LOC113509365,LOC113515395,LOC113517469,LOC113520664
	Biological Process	GO:0044262	cellular carbohydrate metabolic process	4	6	0.00	0.01	MSTRG.13946,MSTRG.16486,LOC113518192,LOC113515958
	Biological Process	GO:0005975	carbohydrate metabolic process	14	80	0.00	0.01	LOC113515907,LOC113515885,LOC113522739,LOC113518192,LOC113515958,LOC113519593,LOC113512055,LOC113513537,MSTRG.13946,LOC113521173,LOC113521660,MSTRG.16486,LOC113512773,LOC113518207
	Biological Process	GO:0006869	lipid transport	4	9	0.00	0.03	LOC113509337,MSTRG.10559,LOC113521556,LOC113509342
	Biological Process	GO:0010876	lipid localization	4	10	0.00	0.04	LOC113509337,MSTRG.10559,LOC113521556,LOC113509342
	Biological Process	GO:0006030	chitin metabolic process	6	24	0.00	0.04	LOC113515907,LOC113522676,LOC113521488,LOC113522098,LOC113517017,LOC113522620
	Biological	GO:0016	organic acid catabolic	5	17	0.00	0.04	MSTRG.3477,LOC113518439,LOC113520466,LOC11

	Process	054	process					3516110,LOC113518195
	Biological Process	GO:0044723	single-organism carbohydrate metabolic process	6	24	0.00	0.04	MSTRG.13946,MSTRG.16486,LOC113522739,LOC113518207,LOC113518192,LOC113515958
	Biological Process	GO:0046395	carboxylic acid catabolic process	5	17	0.00	0.04	MSTRG.3477,LOC113518439,LOC113520466,LOC113516110,LOC113518195
	Biological Process	GO:0005976	polysaccharide metabolic process	2	2	0.00	0.04	LOC113515907,LOC113515958
	Biological Process	GO:0006071	glycerol metabolic process	2	2	0.00	0.04	MSTRG.13946,MSTRG.16486
	Biological Process	GO:0006508	proteolysis	23	207	0.00	0.04	LOC113518766,LOC113517147,LOC113516554,LOC113515696,LOC113510248,LOC113518462,LOC113520040,LOC113512968,LOC113520906,LOC113510598,LOC113511112,LOC113514080,LOC113515697,LOC113521756,LOC113520128,LOC113517143,LOC113513404,MSTRG.912,LOC113518463,LOC113516374,MSTRG.15546,LOC113514900,LOC113510737
	Biological Process	GO:0006573	valine metabolic process	2	2	0.00	0.04	MSTRG.3477,LOC113520466
	Biological Process	GO:0006574	valine catabolic process	2	2	0.00	0.04	MSTRG.3477,LOC113520466
	Biological Process	GO:0009083	branched-chain amino acid catabolic process	2	2	0.00	0.04	MSTRG.3477,LOC113520466
	Biological Process	GO:0019400	alditol metabolic process	2	2	0.00	0.04	MSTRG.13946,MSTRG.16486
	Biological Process	GO:0046835	carbohydrate phosphorylation	2	2	0.00	0.04	MSTRG.13946,LOC113518192
	Biological Process	GO:0006040	amino sugar metabolic process	6	26	0.00	0.05	LOC113515907,LOC113522676,LOC113521488,LOC113522098,LOC113517017,LOC113522620
	Biological Process	GO:1901071	glucosamine-containing compound metabolic process	6	26	0.00	0.05	LOC113515907,LOC113522676,LOC113521488,LOC113522098,LOC113517017,LOC113522620

	Cellular Component	GO:0005576	extracellular region	17	101	0.00	0.01	LOC113519571,LOC113512299,LOC113522497,LOC113517017,LOC113517972,LOC113513298,LOC113517945,LOC113523041,LOC113522676,LOC113516356,LOC113521488,LOC113522098,LOC113512007,MSTRG.13946,LOC113520094,LOC113513641,LOC113522620
	Cellular Component	GO:0005615	extracellular space	7	31	0.00	0.04	LOC113523041,MSTRG.13946,LOC113517972,LOC113520094,LOC113513298,LOC113519571,LOC113516356
	Molecular Function	GO:0003824	catalytic activity	147	1554	0.00	0.00	LOC113515147,LOC113520040,LOC113518105,LOC113513336,LOC113517750,LOC113511315,LOC113521718,LOC113520906,LOC113521105,LOC113521756,LOC113520105,LOC113520670,LOC113510928,LOC113518766,LOC113510248,LOC113516334,LOC113512243,LOC113510312,MSTRG.10599,LOC113517875,LOC113510517,MSTRG.912,LOC113512476,LOC113521981,LOC113520664,LOC113513459,LOC113523417,LOC113514820,LOC113510166,LOC113510571,LOC113519810,LOC113510042,LOC113511112,MSTRG.474,LOC113511182,LOC113515776,LOC113518439,LOC113511922,LOC113516554,LOC113515885,LOC113511557,LOC113515696,LOC113520466,LOC113515169,LOC113517615,LOC113513750,LOC113518195,LOC113520534,LOC113510598,LOC113513582,LOC113520500,LOC113522241,LOC113521631,LOC113509492,LOC113522141,LOC113516587,LOC113510939,LOC113514174,LOC113520291,LOC113518192,LOC113516955,LOC113521224,LOC113513404,LOC113514099,LOC113514175,LOC113518463,LOC113510453,LOC113521660,LOC113517354,LOC113514609,LOC113514869,LOC113520364,LOC113515172,LOC113515958,LOC113521987,LOC113513537,LOC113519242,LOC113511437,LOC113518207,LOC113522635,LOC113522197,LOC113515697,LOC113518095,LOC113509684,LOC113520128,MSTRG.13678,

								LOC113522918,LOC113514771,LOC113510161,LOC113521779,MSTRG.7238,LOC113515907,MSTRG.3959,LOC113512543,LOC113514001,LOC113516339,LOC113521780,LOC113519593,LOC113519020,LOC113520794,LOC113512748,LOC113512968,LOC113523041,LOC113518647,LOC113514080,LOC113513980,LOC113520813,LOC113512473,LOC113516110,LOC113522739,LOC113512055,LOC113520314,LOC113521173,LOC113512773,LOC113517147,LOC113517457,LOC113515777,LOC113518462,LOC113515168,MSTRG.16486,LOC113517945,LOC113521590,LOC113523050,LOC113513917,LOC113517143,LOC113515775,MSTRG.13946,LOC113515170,LOC113516374,LOC113512321,LOC113521268,LOC113521642,MSTRG.3477,LOC113513325,LOC113511629,LOC113512275,LOC113510247,LOC113513526,LOC113518889,LOC113519117,LOC113520094,LOC113509292,LOC113509365,LOC113517469,MSTRG.15546,LOC113514900,LOC113510737
	Molecular Function	GO:0016491	oxidoreductase activity	53	360	0.00	0.00	LOC113523417,LOC113514609,LOC113521987,LOC113515172,LOC113515168,LOC113519242,LOC113517945,LOC113511437,LOC113522635,LOC113522197,MSTRG.474,LOC113518095,LOC113513917,LOC113515775,LOC113518439,LOC113520105,LOC113515170,MSTRG.7238,LOC113511922,MSTRG.3959,LOC113520466,LOC113512543,LOC113515169,LOC113514001,LOC113512321,LOC113513750,LOC113516339,LOC113521268,LOC113516334,LOC113518195,MSTRG.3477,LOC113512243,LOC113512748,LOC113520534,LOC113513325,MSTRG.10599,LOC113520500,LOC113518647,LOC113522241,LOC113520813,LOC113513980,LOC113513526,LOC113514174,LOC113516110,LOC113516587,LOC113519117,LOC113520314,LOC113512476,LOC113521981,LOC113509292,LOC113509365,LOC113517469,LOC113520664

	Molecular Function	GO:0005506	iron ion binding	17	82	0.00	0.00	LOC113523417,MSTRG.3959,LOC113512543,LOC113514001,LOC113512321,LOC113519242,LOC113512748,LOC113520534,LOC113513325,MSTRG.10599,LOC113518647,LOC113513980,LOC113513917,LOC113519117,LOC113520314,LOC113509365,LOC113509292
	Molecular Function	GO:0046914	transition metal ion binding	38	316	0.00	0.00	LOC113523417,LOC113520040,LOC113518015,LOC113519242,LOC113520906,LOC113517925,LOC113522197,MSTRG.474,LOC113515697,LOC113521756,LOC113510092,LOC113513917,LOC113521922,LOC113515696,MSTRG.3959,LOC113512543,LOC113514001,LOC113512321,MSTRG.4568,LOC113512748,LOC113512243,LOC113513325,LOC113520534,MSTRG.10599,LOC113510143,MSTRG.9128,LOC113518647,LOC113514080,LOC113519227,LOC113513980,LOC113520813,LOC113521224,LOC113519117,LOC113520314,LOC113509292,LOC113509365,LOC113520664,MSTRG.15546
	Molecular Function	GO:0003995	acyl-CoA dehydrogenase activity	6	12	0.00	0.00	MSTRG.7238,LOC113511922,LOC113518439,LOC113516587,LOC113513750,LOC113518195
	Molecular Function	GO:0050662	coenzyme binding	15	76	0.00	0.00	MSTRG.7238,LOC113511922,LOC113520466,LOC113512543,LOC113516587,LOC113513917,LOC113514092,LOC113513750,LOC113512321,LOC113518195,MSTRG.3477,LOC113515775,LOC113518439,LOC113520105,MSTRG.10599
	Molecular Function	GO:0050660	flavin adenine dinucleotide binding	11	45	0.00	0.00	MSTRG.7238,LOC113511922,LOC113512543,LOC113516587,LOC113513917,LOC113513750,LOC113512321,LOC113518195,LOC113515775,LOC113518439,MSTRG.10599
	Molecular Function	GO:0048037	cofactor binding	17	103	0.00	0.01	MSTRG.7238,LOC113511922,LOC113520466,LOC113512543,LOC113513750,LOC113512321,LOC113518195,MSTRG.3477,LOC113518105,MSTRG.10599,LOC113510247,LOC113513917,LOC113516587,LOC113

								514092,LOC113515775,LOC113518439,LOC113520105
	Molecular Function	GO:0004046	aminoacylase activity	3	3	0.00	0.01	LOC113517147,LOC113520128,LOC113513404
	Molecular Function	GO:0004854	xanthine dehydrogenase activity	3	3	0.00	0.01	LOC113512543,MSTRG.10599,LOC113512321
	Molecular Function	GO:0016705	oxidoreductase activity, acting on paired donors, with incorporation or reduction of molecular oxygen	14	77	0.00	0.01	LOC113523417,LOC113520500,MSTRG.3959,LOC113520813,LOC113513980,LOC113514001,LOC113519117,LOC113520314,LOC113512748,LOC113519242,LOC113520534,LOC113509365,LOC113513325,LOC113517945
	Molecular Function	GO:0016726	oxidoreductase activity, acting on CH or CH2 groups, NAD or NADP as acceptor	3	3	0.00	0.01	LOC113512543,MSTRG.10599,LOC113512321
	Molecular Function	GO:0004497	monooxygenase activity	12	64	0.00	0.01	LOC113523417,MSTRG.3959,LOC113520813,LOC113513980,LOC113514001,LOC113519117,LOC113520314,LOC113512748,LOC113519242,LOC113509365,LOC113513325,LOC113517945
	Molecular Function	GO:0005319	lipid transporter activity	4	7	0.00	0.01	LOC113509337,MSTRG.10559,LOC113521556,LOC113509342
	Molecular Function	GO:0070011	peptidase activity, acting on L-amino acid peptides	23	177	0.00	0.01	LOC113518766,LOC113517147,LOC113516554,LOC113515696,LOC113510248,LOC113518462,LOC113520040,LOC113512968,LOC113520906,LOC113510598,LOC113511112,LOC113515697,LOC113521756,LOC113520128,LOC113517143,LOC113513404,MSTRG.912,LOC113522918,LOC113518463,LOC113516374,MSTRG.15546,LOC113514900,LOC113510737
	Molecular Function	GO:0043169	cation binding	56	598	0.00	0.01	LOC113523417,LOC113517147,LOC113522803,LOC113522777,LOC113520040,LOC113515958,LOC113516703,LOC113518015,LOC113519242,LOC113521718,LOC113520906,LOC113511011,LOC113517945,LO

								C113517925,LOC113522197,MSTRG.474,LOC113515697,LOC113521756,LOC113516356,LOC113510092,LOC113513917,LOC113520128,LOC113522349,LOC113521922,LOC113510629,LOC113513462,LOC113515696,MSTRG.3959,LOC113512543,LOC113514001,LOC113512321,LOC113521268,MSTRG.4568,LOC113512243,LOC113512748,LOC113520534,LOC113513325,MSTRG.10599,LOC113522896,LOC113510143,MSTRG.9128,LOC113518647,LOC113513145,LOC113514080,LOC113519227,LOC113520813,LOC113513980,LOC113521224,LOC113513404,LOC113519117,LOC113520314,LOC113520516,LOC113509292,LOC113509365,LOC113520664,MSTRG.15546
	Molecular Function	GO:0016787	hydrolase activity	55	591	0.00	0.02	LOC113517147,LOC113510166,LOC113518462,LOC113520040,LOC113515958,LOC113515172,LOC113515168,LOC113513537,LOC113511315,LOC113520906,LOC113521105,LOC113511112,LOC113515697,LOC113521756,LOC113511182,LOC113520128,LOC113517143,LOC113509684,MSTRG.13678,LOC113522918,LOC113514771,LOC113516374,LOC113515170,LOC113520670,LOC113510161,LOC113521779,LOC113518766,LOC113515907,LOC113516554,LOC113515696,LOC113515885,LOC113510248,LOC113515169,LOC113521780,LOC113519593,LOC113520794,LOC113512968,LOC113510312,LOC113510598,LOC113511629,LOC113512275,LOC113522241,LOC113512473,LOC113510939,LOC113513404,LOC113512055,MSTRG.912,LOC113521173,LOC113518463,LOC113521660,LOC113512773,LOC113513459,MSTRG.15546,LOC113510737,LOC113514900
	Molecular Function	GO:0031177	phosphopantetheine binding	5	13	0.00	0.02	LOC113522241,LOC113515169,LOC113515170,LOC113515172,LOC113515168
	Molecular Function	GO:0072341	modified amino acid binding	5	13	0.00	0.02	LOC113522241,LOC113515169,LOC113515170,LOC113515172,LOC113515168
	Molecular	GO:0046	metal ion binding	55	596	0.00	0.02	LOC113523417,LOC113517147,LOC113522803,LOC

	Function	872						113522777,LOC113520040,LOC113516703,LOC113518015,LOC113519242,LOC113521718,LOC113520906,LOC113511011,LOC113517945,LOC113517925,LOC113522197,MSTRG.474,LOC113515697,LOC113521756,LOC113516356,LOC113510092,LOC113513917,LOC113520128,LOC113522349,LOC113521922,LOC113510629,LOC113513462,LOC113515696,MSTRG.3959,LOC113512543,LOC113514001,LOC113512321,LOC113521268,MSTRG.4568,LOC113512243,LOC113512748,LOC113520534,LOC113513325,MSTRG.10599,LOC113522896,LOC113510143,MSTRG.9128,LOC113518647,LOC113513145,LOC113514080,LOC113519227,LOC113520813,LOC113513980,LOC113521224,LOC113513404,LOC113519117,LOC113520314,LOC113520516,LOC113509292,LOC113509365,LOC113520664,MSTRG.15546
	Molecular Function	GO:0008237	metallopeptidase activity	9	43	0.00	0.02	LOC113517147,LOC113515696,LOC113515697,LOC113521756,LOC113520040,LOC113520128,LOC113513404,LOC113520906,MSTRG.15546
	Molecular Function	GO:0008233	peptidase activity	23	189	0.00	0.03	LOC113518766,LOC113517147,LOC113516554,LOC113515696,LOC113510248,LOC113518462,LOC113520040,LOC113512968,LOC113520906,LOC113510598,LOC113511112,LOC113515697,LOC113521756,LOC113520128,LOC113517143,LOC113513404,MSTRG.912,LOC113522918,LOC113518463,LOC113516374,MSTRG.15546,LOC113514900,LOC113510737
	Molecular Function	GO:0004553	hydrolase activity, hydrolyzing O-glycosyl compounds	7	28	0.00	0.03	LOC113519593,LOC113515907,LOC113512055,LOC113521173,LOC113521660,LOC113512773,LOC113515958
	Molecular Function	GO:0016627	oxidoreductase activity, acting on the CH-CH group of donors	6	21	0.00	0.03	MSTRG.7238,LOC113511922,LOC113518439,LOC113516587,LOC113513750,LOC113518195
	Molecular	GO:0020	heme binding	12	75	0.00	0.04	LOC113523417,MSTRG.3959,LOC113513980,LOC11

	Function	037						3514001,LOC113521268,LOC113519117,LOC113520314,LOC113512748,LOC113519242,LOC113520534,LOC113509365,LOC113513325
	Molecular Function	GO:0016614	oxidoreductase activity, acting on CH-OH group of donors	9	47	0.00	0.04	LOC113520466,LOC113512543,LOC113513917,LOC113512321,MSTRG.3477,LOC113515775,LOC113517469,LOC113520105,MSTRG.10599
	Molecular Function	GO:0046906	tetrapyrrole binding	12	76	0.00	0.04	LOC113523417,MSTRG.3959,LOC113513980,LOC113514001,LOC113521268,LOC113519117,LOC113520314,LOC113512748,LOC113519242,LOC113520534,LOC113509365,LOC113513325
	Molecular Function	GO:0016725	oxidoreductase activity, acting on CH or CH2 groups	3	5	0.00	0.04	LOC113512543,MSTRG.10599,LOC113512321
	Molecular Function	GO:0008238	exopeptidase activity	7	32	0.00	0.04	LOC113515696,LOC113522918,LOC113515697,LOC113520906,LOC113520040,LOC113516374,MSTRG.15546
	Molecular Function	GO:0016798	hydrolase activity, acting on glycosyl bonds	7	32	0.00	0.04	LOC113519593,LOC113515907,LOC113512055,LOC113521173,LOC113521660,LOC113512773,LOC113515958
	Molecular Function	GO:0004371	glycerone kinase activity	2	2	0.00	0.04	MSTRG.13946,MSTRG.16486
	Molecular Function	GO:0004855	xanthine oxidase activity	2	2	0.00	0.04	LOC113512543,MSTRG.10599
	Molecular Function	GO:0008442	3-hydroxyisobutyrate dehydrogenase activity	2	2	0.00	0.04	MSTRG.3477,LOC113520466
	Molecular Function	GO:0016727	oxidoreductase activity, acting on CH or CH2 groups, oxygen as acceptor	2	2	0.00	0.04	LOC113512543,MSTRG.10599
	Molecular Function	GO:0033218	amide binding	5	18	0.00	0.04	LOC113522241,LOC113515169,LOC113515170,LOC113515172,LOC113515168

**3 days post infection**								
Up	Biological Process	GO:0006418	tRNA aminoacylation for protein translation	13	24	0.00	0.00	LOC113509243,LOC113522840,LOC113520458,LOC113510506,MSTRG.16445,LOC113515023,LOC113519888,LOC113519875,LOC113521153,MSTRG.106,LOC113521599,LOC113510661,LOC113509294
	Biological Process	GO:0043038	amino acid activation	13	25	0.00	0.00	LOC113509243,LOC113522840,LOC113520458,LOC113510506,MSTRG.16445,LOC113515023,LOC113519888,LOC113519875,LOC113521153,MSTRG.106,LOC113521599,LOC113510661,LOC113509294
	Biological Process	GO:0043039	tRNA aminoacylation	13	25	0.00	0.00	LOC113509243,LOC113522840,LOC113520458,LOC113510506,MSTRG.16445,LOC113515023,LOC113519888,LOC113519875,LOC113521153,MSTRG.106,LOC113521599,LOC113510661,LOC113509294
	Biological Process	GO:0006399	tRNA metabolic process	15	42	0.00	0.00	LOC113509243,LOC113522840,LOC113520458,LOC113510506,MSTRG.16445,LOC113515023,LOC113519888,LOC113515494,LOC113519875,LOC113521153,MSTRG.106,LOC113521599,LOC113510661,LOC113509294,LOC113510674
	Biological Process	GO:0034660	ncRNA metabolic process	17	60	0.00	0.00	LOC113522840,LOC113520458,LOC113519875,LOC113521599,LOC113516097,LOC113509243,LOC113510506,MSTRG.16445,LOC113515023,LOC113519888,LOC113515494,LOC113521153,LOC113511975,LOC113510661,MSTRG.106,LOC113510674,LOC113509294
	Biological Process	GO:0019752	carboxylic acid metabolic process	28	133	0.00	0.00	LOC113523239,LOC113522840,LOC113520458,LOC113518819,LOC113518438,LOC113519315,LOC113517095,LOC113511953,LOC113510872,LOC113519887,LOC113519875,LOC113521599,LOC113511731,LOC113509243,LOC113516392,LOC113510506,LOC113518801,MSTRG.16445,LOC113521309,LOC113515023,LOC113519888,LOC113521153,LOC113517529,MSTRG.106,LOC113519600,LOC113510661,LOC113509294,LOC113516033

	Biological Process	GO:0043436	oxoacid metabolic process	28	135	0.00	0.00	LOC113523239,LOC113522840,LOC113520458,LOC113518819,LOC113518438,LOC113519315,LOC113517095,LOC113511953,LOC113510872,LOC113519887,LOC113519875,LOC113521599,LOC113511731,LOC113509243,LOC113516392,LOC113510506,LOC113518801,MSTRG.16445,LOC113521309,LOC113515023,LOC113519888,LOC113521153,LOC113517529,MSTRG.106,LOC113519600,LOC113510661,LOC113509294,LOC113516033
	Biological Process	GO:0006082	organic acid metabolic process	28	136	0.00	0.00	LOC113523239,LOC113522840,LOC113520458,LOC113518819,LOC113518438,LOC113519315,LOC113517095,LOC113511953,LOC113510872,LOC113519887,LOC113519875,LOC113521599,LOC113511731,LOC113509243,LOC113516392,LOC113510506,LOC113518801,MSTRG.16445,LOC113521309,LOC113515023,LOC113519888,LOC113521153,LOC113517529,MSTRG.106,LOC113519600,LOC113510661,LOC113509294,LOC113516033
	Biological Process	GO:0006431	methionyl-tRNA aminoacylation	4	4	0.00	0.01	LOC113509243,MSTRG.16445,LOC113521153,LOC113509294
	Biological Process	GO:0044710	single-organism metabolic process	72	529	0.00	0.02	LOC113523239,LOC113522840,LOC113515355,LOC113519315,LOC113510872,LOC113514967,LOC113512008,LOC113509243,LOC113512894,LOC113510223,LOC113516632,LOC113515704,LOC113511953,LOC113514650,LOC113515993,LOC113519887,LOC113523090,LOC113515576,LOC113518034,LOC113513789,LOC113515482,LOC113518801,LOC113518280,LOC113517211,LOC113512123,LOC113522563,LOC113518841,LOC113517529,LOC113520780,LOC113511193,LOC113515418,LOC113520458,LOC113518438,LOC113517095,LOC113513649,LOC113519875,LOC113517426,LOC113511731,LOC113516115,LOC113510506,LOC113521309,LOC113515023,LOC113521672,LOC113514551,LOC113509252,LOC113518433,LOC113519654,MSTRG.106,LOC113516033,LOC113515150,LOC113514951,LOC113518819,LOC11351112

								8,LOC113517610,LOC113519241,LOC113521599,LOC113513897,LOC113517157,LOC113516392,LOC113516645,MSTRG.16445,LOC113512374,LOC113517150,LOC113517275,LOC113519888,LOC113522116,LOC113523415,LOC113523106,LOC113521153,LOC113510661,LOC113519600,LOC113509294
	Biological Process	GO:0006520	cellular amino acid metabolic process	17	74	0.00	0.02	LOC113522840,LOC113520458,LOC113517095,LOC113519875,LOC113521599,LOC113509243,LOC113516392,LOC113510506,LOC113518801,MSTRG.16445,LOC113515023,LOC113519888,LOC113517529,LOC113521153,LOC113510661,MSTRG.106,LOC113509294
	Biological Process	GO:0045087	innate immune response	5	8	0.00	0.02	MSTRG.16473,LOC113514368,LOC113515812,LOC113513310,LOC113512706
	Biological Process	GO:0044281	small molecule metabolic process	37	230	0.00	0.03	LOC113523239,LOC113522840,LOC113520458,LOC113518438,LOC113519315,LOC113517095,LOC113510872,LOC113519875,LOC113511731,LOC113509243,LOC113516115,LOC113510506,LOC113521309,LOC113521672,LOC113515023,LOC113509252,MSTRG.106,LOC113516033,LOC113516632,LOC113518819,LOC113511953,LOC113511128,LOC113519887,LOC113521599,LOC113517157,LOC113516392,LOC113518280,LOC113518801,MSTRG.16445,LOC113517150,LOC113519888,LOC113517529,LOC113521153,LOC113510661,LOC113519600,LOC113511193,LOC113509294
	Cellular Component	GO:0005576	extracellular region	20	101	0.00	0.04	MSTRG.5837,LOC113512298,LOC113514368,LOC113511560,LOC113516120,LOC113522527,LOC113512196,LOC113522981,LOC113515502,LOC113511175,LOC113517091,MSTRG.16473,LOC113520443,LOC113509694,LOC113517115,LOC113509263,LOC113509835,LOC113521649,LOC113515812,LOC113513310
	Molecular Function	GO:0004812	aminoacyl-tRNA ligase activity	13	25	0.00	0.00	LOC113509243,LOC113522840,LOC113520458,LOC113510506,MSTRG.16445,LOC113515023,LOC113519888,LOC113519875,LOC113521153,MSTRG.106,LOC113521599,LOC113510661,LOC113509294

	Molecular Function	GO:0016875	ligase activity, forming carbon-oxygen bonds	13	25	0.00	0.00	LOC113509243,LOC113522840,LOC113520458,LOC113510506,MSTRG.16445,LOC113515023,LOC113519888,LOC113519875,LOC113521153,MSTRG.106,LOC113521599,LOC113510661,LOC113509294
	Molecular Function	GO:0016876	ligase activity, forming aminoacyl-tRNA and related compounds	13	25	0.00	0.00	LOC113509243,LOC113522840,LOC113520458,LOC113510506,MSTRG.16445,LOC113515023,LOC113519888,LOC113519875,LOC113521153,MSTRG.106,LOC113521599,LOC113510661,LOC113509294
	Molecular Function	GO:0004825	methionine-tRNA ligase activity	4	4	0.00	0.01	LOC113509243,MSTRG.16445,LOC113521153,LOC113509294
	Molecular Function	GO:0016831	carboxy-lyase activity	5	8	0.00	0.02	LOC113516392,LOC113511128,LOC113517095,LOC113521309,LOC113511193
	Molecular Function	GO:0016874	ligase activity	17	77	0.00	0.03	LOC113522840,LOC113520458,LOC113519887,LOC113519875,LOC113521599,LOC113517157,LOC113509243,LOC113510506,MSTRG.16445,LOC113515023,LOC113516259,LOC113519888,LOC113521153,LOC113519600,LOC113510661,MSTRG.106,LOC113509294
Down	Biological Process	GO:0044710	single-organism metabolic process	92	529	0.00	0.00	LOC113514609,LOC113516021,LOC113515958,LOC113519242,LOC113512912,LOC113518207,LOC113522197,LOC113515420,LOC113520016,LOC113516133,LOC113520128,LOC113509684,LOC113515417,LOC113522951,LOC113520356,LOC113510783,LOC113520105,LOC113513030,LOC113514771,LOC113517595,MSTRG.7238,LOC113510172,MSTRG.3959,LOC113512543,LOC113514001,LOC113515364,LOC113512243,LOC113512748,LOC113512666,MSTRG.10599,LOC113509396,LOC113522778,LOC113518647,LOC113520813,LOC113513980,LOC113522679,LOC113516110,LOC113512473,LOC113522739,LOC113512454,LOC113512476,LOC113509259,LOC113520664,LOC113517147,LOC113513171,LOC113513087,LOC113515168,LOC113522992,LOC113512793,LOC113515399,MSTRG.16486,LOC113517945,LOC113517270,M

								STRG.474,LOC113513490,LOC113513917,LOC113515775,MSTRG.13946,LOC113514657,LOC113518439,LOC113513141,LOC113515170,LOC113511922,LOC113510344,LOC113520466,LOC113515169,LOC113513750,LOC113512321,LOC113521268,LOC113518195,MSTRG.3477,LOC113509700,LOC113516390,LOC113520534,LOC113513325,LOC113515982,LOC113520500,LOC113517217,LOC113522241,LOC113510247,LOC113516587,LOC113520291,LOC113518192,LOC113513404,LOC113520555,LOC113519117,LOC113519854,LOC113509365,LOC113509292,LOC113512453,LOC113515395,MSTRG.12093
	Biological Process	GO:0055114	oxidation-reduction process	60	303	0.00	0.00	LOC113514609,LOC113515958,LOC113515168,LOC113512793,LOC113519242,LOC113512912,LOC113517945,LOC113517270,LOC113522197,MSTRG.474,LOC113513490,LOC113513917,LOC113520016,LOC113515417,LOC113522951,LOC113515775,LOC113518439,LOC113514657,LOC113510783,LOC113520105,LOC113513141,LOC113515170,MSTRG.7238,LOC113511922,LOC113510172,LOC113510344,MSTRG.3959,LOC113520466,LOC113512543,LOC113515169,LOC113514001,LOC113512321,LOC113513750,LOC113521268,LOC113518195,MSTRG.3477,LOC113512243,LOC113512748,LOC113516390,LOC113520534,LOC113513325,MSTRG.10599,LOC113509396,LOC113515982,LOC113520500,LOC113518647,LOC113517217,LOC113522241,LOC113520813,LOC113513980,LOC113516587,LOC113516110,LOC113520555,LOC113519117,LOC113519854,LOC113512476,LOC113509292,LOC113509365,LOC113520664,MSTRG.12093
	Biological Process	GO:0006260	DNA replication	13	37	0.00	0.01	LOC113517567,LOC113511622,LOC113513268,LOC113516716,LOC113509701,LOC113511062,LOC113514099,LOC113522710,LOC113520523,LOC113518799,LOC113522607,LOC113517580,LOC113512070
	Biological	GO:0006261	DNA-dependent	6	9	0.00	0.02	LOC113514099,LOC113511062,LOC113518799,LOC

	Process		DNA replication					113522607,LOC113513268,LOC113516716
	Molecular Function	GO:0003824	catalytic activity	230	1554	0.00	0.00	LOC113515147,MSTRG.3401,LOC113516021,LOC113510916,LOC113520040,LOC113513548,LOC113520363,LOC113518711,LOC113523213,LOC113518105,LOC113513336,LOC113517750,LOC113511315,LOC113521718,LOC113520906,LOC113520459,LOC113521105,LOC113521756,LOC113518514,LOC113520016,LOC113516133,LOC113515417,LOC113522951,LOC113520356,LOC113510783,LOC113520491,LOC113518246,LOC113520105,LOC113518799,LOC113513030,LOC113520670,LOC113517595,LOC113510666,LOC113511017,LOC113510172,LOC113510248,LOC113512243,LOC113510337,LOC113512666,LOC113510312,MSTRG.10599,LOC113515341,LOC113518005,LOC113509396,LOC113522679,LOC113513860,LOC113520351,LOC113517875,MSTRG.12319,LOC113516716,MSTRG.912,LOC113516367,LOC113512476,LOC113521981,LOC113520664,LOC113521640,LOC113513459,LOC113514820,LOC113512614,LOC113510166,LOC113510571,LOC113513087,LOC113512450,LOC113511062,LOC113512823,LOC113519810,LOC113509111,LOC113521944,LOC113517270,LOC113522496,LOC113510042,MSTRG.474,LOC113513490,LOC113515776,LOC113510551,LOC113511182,LOC113520869,LOC113518439,LOC113517580,LOC113523025,LOC113511922,LOC113515885,LOC113511557,LOC113522976,LOC113520466,LOC113515169,LOC113522153,LOC113517615,LOC113513750,LOC113518195,LOC113510542,LOC113520534,LOC113513582,LOC113510598,LOC113520500,LOC113522241,LOC113517980,LOC113521631,LOC113522141,LOC113516587,LOC113514174,LOC113510939,LOC113519951,LOC113520291,LOC113518192,LOC113516955,LOC113513404,LOC113514099,LOC113519854,LOC113514175,LOC113518463,LOC113521660,LOC113

517354,MSTRG.12093,LOC113519073,LOC11352245
7,LOC113514609,LOC113511203,LOC113514869,LO
C113520364,LOC113511129,LOC113515958,LOC113
513537,LOC113519242,LOC113512912,LOC1135182
07,LOC113511437,LOC113522635,LOC113522197,L
OC113518095,LOC113515420,LOC113517518,LOC1
13520128,LOC113509684,MSTRG.13678,LOC113522
918,LOC113519050,LOC113514771,LOC113510161,
LOC113521779,LOC113516195,MSTRG.7238,LOC11
3515907,MSTRG.3959,LOC113512543,LOC11351400
1,LOC113515364,LOC113521780,LOC113519593,LO
C113515320,LOC113523315,LOC113512748,LOC113
512968,LOC113518791,LOC113517475,LOC1135230
41,LOC113522778,LOC113518647,LOC113513883,L
OC113514080,LOC113510735,LOC113520813,LOC1
13513980,LOC113512473,LOC113516110,LOC11352
2739,LOC113516519,LOC113512055,MSTRG.9521,L
OC113521173,LOC113512840,LOC113509259,LOC1
13512773,LOC113515060,LOC113517345,LOC11352
2138,LOC113517147,LOC113522644,LOC113517457
,LOC113515777,LOC113518685,LOC113513171,LO
C113514675,LOC113515168,LOC113522992,LOC113
512793,LOC113513263,MSTRG.16486,MSTRG.1763,
LOC113521590,LOC113517945,LOC113523050,LOC
113513917,LOC113517143,LOC113515775,MSTRG.1
3946,LOC113514657,LOC113523151,LOC113513141
,LOC113509770,LOC113522607,LOC113515170,LO
C113516374,LOC113510344,LOC113512321,LOC113
521642,LOC113521268,MSTRG.374,LOC113521544,
MSTRG.3477,LOC113509700,LOC113516390,LOC11
3513325,LOC113512275,MSTRG.7478,LOC11351598
2,LOC113516446,LOC113516813,LOC113517217,LO
C113510247,LOC113513731,LOC113520555,LOC113
519117,LOC113520094,LOC113509292,LOC1135093
65,LOC113520465,LOC113509413,MSTRG.15546,L

								OC113510737
	Molecular Function	GO:0016491	oxidoreductase activity	70	360	0.00	0.00	LOC113514609,LOC113511203,LOC113519242,LOC113512912,LOC113511437,LOC113522635,LOC113522197,LOC113518095,LOC113520016,LOC113515417,LOC113522951,LOC113510783,LOC113520105,LOC113516195,MSTRG.7238,LOC113510172,MSTRG.3959,LOC113512543,LOC113514001,LOC113512243,LOC113512748,MSTRG.10599,LOC113509396,LOC113518647,LOC113513883,LOC113520813,LOC113513980,LOC113516110,LOC113513860,LOC113512476,LOC113521981,LOC113520664,LOC113522644,LOC113515168,LOC113512793,LOC113513263,LOC113517945,LOC113517270,MSTRG.474,LOC113513490,LOC113513917,LOC113515775,LOC113514657,LOC113518439,LOC113513141,LOC113515170,LOC113511922,LOC113510344,LOC113515169,LOC113520466,LOC113513750,LOC113512321,LOC113518195,LOC113521268,MSTRG.3477,LOC113516390,LOC113513325,LOC113520534,LOC113515982,LOC113517217,LOC113520500,LOC113522241,LOC113514174,LOC113516587,LOC113519117,LOC113520555,LOC113519854,LOC113509365,LOC113509292,MSTRG.12093
	Molecular Function	GO:0050662	coenzyme binding	20	76	0.00	0.02	MSTRG.7238,LOC113511922,LOC113520466,LOC113512543,LOC113513750,LOC113512321,LOC113518195,MSTRG.3477,MSTRG.10599,LOC113517270,LOC113515982,LOC113517217,LOC113513490,LOC113513917,LOC113516587,LOC113514092,LOC113520016,LOC113515775,LOC113518439,LOC113520105

*Hit number: the number of DEG sorted in the indicated category.

**Background number: the number of all genes sorted in the indicated category.
