# Supplementary material for: Transcriptomic analyses reveal comprehensive responses of insect hemocytes to mycopathogen Beauveria bassiana, and fungal virulence-related cell wall protein assists pathogen to evade host cellular defense
Source: Virulence. 2020 Oct 5;11(1):1352–65. doi: 10.1080/21505594.2020.1827886 (PMC7549920; doi:10.1080/21505594.2020.1827886)
Supplement: Supplemental Material [file KVIR_A_1827886_SM8204.zip › Table S9.pdf]

Table S9 Enrichment analyses of the KEGG pathways for the differentially expressed genes (DEGs) of *Galleria mellonella* hemocytes against *Beauveria bassiana*

Up/down regulated DEGs	Pathway hierarchy @ level 1	Pathway hierarchy @ level 2	KEGG Pathway	Pathway ID	Gene Number*	Background number**	P Value	Q Value	Gene IDs
**1 day post infection**									
Up	Metabolism	Metabolism of other amino acids	Taurine and hypotaurine metabolism	ko00430	2	12	0.000137	0.003159	LOC113521309,LOC113510012
Down	Metabolism	Amino acid metabolism	Arginine biosynthesis	ko00220	3	33	8.30E-05	0.000995	LOC113513404,LOC113520128,LOC113517147
	Metabolism	Overview	2-Oxocarboxylic acid metabolism	ko01210	3	33	8.30E-05	0.000995	LOC113520128,LOC113517147,LOC113513404
	Metabolism	Overview	Biosynthesis of amino acids	ko01230	3	90	1.62E-03	0.012968	LOC113520128,LOC113517147,LOC113513404
**2 days post infection**									
Up	Genetic Information Processing	Folding, sorting and degradation	Protein processing in endoplasmic	ko04141	16	153	5.25E-04	0.046918	LOC113519422,LOC113511704,LOC113523104,LOC113522123,LOC113513003,LOC113510568,LOC113520589,LOC113520498,LOC113516464,LOC113519432,LOC113516653,LOC113521234,LOC113512998,LOC11351943

			reticulum						8,LOC113513388,LOC113521931
	Metabolism	Lipid metabolism	Sphingolipid metabolism	ko00600	8	40	1.82E-04	0.024338	LOC113514784,LOC113511292,LOC113521924,MSTRG.11374,LOC113512485,LOC113521819,LOC113515081,LOC113509986
	Organismal Systems	Digestive system	Fat digestion and absorption	ko04975	7	27	8.10E-05	0.021716	LOC113514677,LOC113521924,LOC113511685,LOC113510575,LOC113521819,LOC113510121,LOC113515943
	Organismal Systems	Nervous system	Neurotrophin signaling pathway	ko04722	10	73	7.43E-04	0.049786	LOC113511948,LOC113514198,LOC113522456,LOC113509271,LOC113514316,LOC113516157,LOC113518143,MSTRG.12222,LOC113521365,LOC113520692
Down	Cellular Processes	Transport and catabolism	Lysosome	ko04142	21	152	9.74E-05	1.88E-03	LOC113513595,LOC113512799,LOC113517812,LOC113515907,LOC113522562,LOC113520941,LOC113519736,LOC113515484,LOC113519915,LOC113520468,LOC113510961,LOC113521173,LOC113514851,LOC113513473,LOC113512473,LOC113512055,LOC113523146,LOC113512953,LOC113511409,MSTRG.15207,LOC113521660
	Environmental Information Processing	Signaling molecules and interaction	ECM-receptor interaction	ko04512	10	48	2.52E-04	4.00E-03	LOC113517040,LOC113510563,LOC113513316,MSTRG.13907,LOC113517042,LOC113519524,MSTRG.12064,LOC113510555,LOC113515727,LOC113511104
	Metabolism	Amino acid metabolism	Valine, leucine and isoleucine degradation	ko00280	16	57	4.61E-08	6.22E-06	LOC113511922,LOC113513750,LOC113511682,LOC113518202,MSTRG.3477,LOC113520466,LOC113515777,MSTRG.7238,LOC113510453,LOC113521631,LOC113516587,LOC113515439,LOC113517218,LOC113521324,LOC113512476,LOC113515776
	Metabolism	Biosynthesis of other secondary metabolite	Caffeine metabolism	ko00232	4	11	2.29E-03	2.69E-02	LOC113512321,MSTRG.10599,LOC113513917,LOC113512543

		s							
	Metabolism	Carbohydrate metabolism	Amino sugar and nucleotide sugar metabolism	ko00520	13	58	1.32E-05	6.11E-04	LOC113515907,LOC113522562,LOC113522137,LOC113513595,LOC113518207,LOC113513475,LOC113521173,LOC113510894,LOC113514621,MSTRG.4582,LOC113519674,LOC113518704,LOC113519915
	Metabolism	Carbohydrate metabolism	Galactose metabolism	ko00052	12	56	4.58E-05	1.24E-03	LOC113511343,LOC113513475,MSTRG.7690,LOC113520941,LOC113514609,LOC113522739,LOC113510498,LOC113516624,LOC113519789,MSTRG.4582,LOC113521660,LOC113513313
	Metabolism	Carbohydrate metabolism	Fructose and mannose metabolism	ko00051	10	43	9.49E-05	1.88E-03	LOC113512738,LOC113511343,MSTRG.16486,LOC113515986,LOC113514609,LOC113518448,LOC113510498,LOC113513313,MSTRG.13946,LOC113519789
	Metabolism	Carbohydrate metabolism	Pentose and glucuronate interconversions	ko00040	13	85	7.81E-04	1.14E-02	LOC113513313,LOC113518095,LOC113513733,LOC113514174,LOC113517750,LOC113519789,LOC113514820,LOC113510498,LOC113510042,LOC113514609,LOC113514175,LOC113514869,LOC113511343
	Metabolism	Energy metabolism	Methane metabolism	ko00680	6	25	2.07E-03	2.54E-02	MSTRG.13946,MSTRG.16486,LOC113518573,LOC113520105,LOC113512738,LOC113518448
	Metabolism	Glycan biosynthesis and metabolism	Glycosphingolipid biosynthesis - globo and isoglobo series	ko00603	8	12	3.55E-08	6.22E-06	LOC113519915,LOC113521660,LOC113521173,LOC113512055,LOC113513595,LOC113522562,LOC113515907,LOC113513473
	Metabolism	Glycan	Other	ko00511	10	32	5.55E-	3.75E-	LOC113513595,LOC113515907,LOC113522

		biosynthesis and metabolism	glycan degradation				06	04	562,LOC113523146,MSTRG.15207,LOC113511409,LOC113520468,LOC113511471,LOC113519915,LOC113521173
	Metabolism	Glycan biosynthesis and metabolism	Glycosphingolipid biosynthesis - ganglio series	ko00604	5	8	2.56E-05	9.34E-04	LOC113522562,LOC113515907,LOC113513595,LOC113521173,LOC113519915
	Metabolism	Glycan biosynthesis and metabolism	Glycosphingolipid biosynthesis - lacto and neolacto series	ko00601	3	5	1.58E-03	2.13E-02	LOC113511288,MSTRG.7746,LOC113523131
	Metabolism	Glycan biosynthesis and metabolism	Glycosaminoglycan degradation	ko00531	5	18	2.46E-03	2.77E-02	LOC113513595,LOC113522562,LOC113515907,LOC113519915,LOC113521173
	Metabolism	Glycan biosynthesis and metabolism	Various types of N-glycan biosynthesis	ko00513	8	44	2.62E-03	2.83E-02	LOC113522562,LOC113522299,LOC113515907,LOC113513595,LOC113521173,LOC113520291,LOC113511471,LOC113519915
	Metabolism	Lipid metabolism	Fatty acid degradation	ko00071	12	54	3.11E-05	9.34E-04	LOC113513750,LOC113518439,MSTRG.12673,MSTRG.7238,LOC113516587,LOC113518195,LOC113518106,LOC113517218,LOC113515439,LOC113515777,LOC113515776,LOC113512476
	Metabolism	Lipid metabolism	Glycerolipid metabolis	ko00561	14	71	2.92E-05	9.34E-04	LOC113510498,LOC113515439,LOC113517218,LOC113515367,LOC113512476,LOC113521660,LOC113519789,LOC113522009,MST

			m						RG.13946,LOC113513313,LOC113511343,LOC113512738,MSTRG.16486,LOC113514609
	Metabolism	Lipid metabolism	Fatty acid biosynthesis	ko00061	8	37	8.05E-04	1.14E-02	LOC113515170,LOC113515172,LOC113516507,LOC113515169,LOC113522241,LOC113519298,LOC113513894,LOC113515168
	Metabolism	Metabolism of other amino acids	Glutathione metabolism	ko00480	14	77	7.49E-05	1.84E-03	LOC113522139,LOC113515583,LOC113522141,LOC113510571,LOC113519287,LOC113515696,LOC113515584,LOC113521642,LOC113515693,LOC113511557,LOC113520906,LOC113517732,LOC113515697,LOC113513582
	Metabolism	Overview	Fatty acid metabolism	ko01212	19	94	7.51E-07	6.76E-05	LOC113512508,LOC113518195,LOC113515172,LOC113515777,LOC113515168,MSTRG.12673,LOC113513750,LOC113513894,LOC113522241,LOC113515169,LOC113516507,LOC113518106,LOC113515170,LOC113515776,MSTRG.7238,LOC113518439,LOC113519298,LOC113520500,LOC113516587
	Metabolism	Xenobiotics biodegradation and metabolism	Metabolism of xenobiotics by cytochrome P450	ko00980	12	86	2.75E-03	2.85E-02	LOC113513733,LOC113517750,LOC113518095,LOC113514820,LOC113514511,LOC113510571,LOC113511557,LOC113521642,LOC113510042,LOC113513582,LOC113514869,LOC113514175
	Organismal Systems	Digestive system	Protein digestion and absorption	ko04974	13	75	2.22E-04	3.75E-03	LOC113516832,LOC113518766,MSTRG.12744,LOC113517033,LOC113516374,LOC113516554,LOC113518463,LOC113512282,LOC113517040,LOC113511104,LOC113518462,LOC113518465,LOC113517042
	Organismal Systems	Endocrine system	Renin-angiotensin system	ko04614	10	35	1.36E-05	6.11E-04	LOC113515693,LOC113517732,LOC113515697,MSTRG.12744,LOC113515583,LOC113522801,LOC113519287,LOC113516374,LOC113515696,LOC113515584

	Organismal Systems	Endocrine system	PPAR signaling pathway	ko03320	10	61	1.81E-03	2.33E-02	LOC113521590,LOC113518195,LOC113522052,LOC113518106,LOC113512508,LOC113520500,LOC113514092,MSTRG.12673,LOC113518439,LOC113511044
	Organismal Systems	Immune system	Hematopoietic cell lineage	ko04640	7	21	9.35E-05	1.88E-03	LOC113519287,LOC113515696,LOC113515697,LOC113515584,LOC113515583,LOC113517732,LOC113515693
	Organismal Systems	Immune system	Complement and coagulation cascades	ko04610	6	16	1.44E-04	2.59E-03	LOC113513316,LOC113510555,LOC113515727,MSTRG.13907,MSTRG.12064,LOC113510563
**3 days post infection**									
Up	Cellular Processes	Cell growth and death	Ferroptosis	ko04216	6	18	4.09E-03	4.04E-02	LOC113511106,LOC113520065,LOC113522480,LOC113520366,LOC113519185,LOC113522542
	Environmental Information Processing	Membrane transport	ABC transporters	ko02010	13	56	1.32E-03	2.37E-02	LOC113512425,LOC113514407,LOC113514677,LOC113520666,MSTRG.5515,MSTRG.320,LOC113519277,LOC113510575,LOC113511534,LOC113520845,LOC113512234,LOC113522252,LOC113514673
	Environmental Information Processing	Signal transduction	VEGF signaling pathway	ko04370	10	27	7.73E-05	3.94E-03	LOC113512866,LOC113518753,LOC113517426,LOC113522456,LOC113515814,LOC113519518,LOC113517926,LOC113514198,LOC113521082,LOC113513806
	Environmental Information Processing	Signal transduction	Jak-STAT signaling pathway	ko04630	9	32	1.75E-03	2.60E-02	LOC113519981,LOC113516487,LOC113513801,LOC113517426,LOC113520692,LOC113521082,LOC113513806,LOC113517926,LOC113519518
	Environmental Information Processing	Signal transduction	Ras signaling pathway	ko04014	19	102	2.02E-03	2.60E-02	LOC113517426,LOC113513679,LOC113519518,LOC113523023,LOC113517054,LOC113518143,LOC113521082,LOC113520692,LOC113518753,MSTRG.1873,LOC113522456,LOC113515943,LOC113510121,LOC113521192,LOC113517926,LOC113514198,LOC113514

									153,LOC113513806,LOC113520485
	Genetic Information Processing	Folding, sorting and degradation	Protein processing in endoplasmic reticulum	ko04141	31	153	1.68E-05	1.71E-03	LOC113519436,LOC113522123,LOC113519432,LOC113520001,LOC113514600,LOC113518632,LOC113513899,LOC113522116,LOC113513388,LOC113523104,LOC113513003,LOC113510568,LOC113510648,LOC113522003,LOC113520498,LOC113514041,LOC113520240,LOC113516653,LOC113519449,LOC113512998,LOC113518829,LOC113519419,LOC113515861,LOC113511052,LOC113519423,LOC113519438,LOC113523292,LOC113521931,LOC113519422,LOC113518345,LOC113522681
	Genetic Information Processing	Translation	Aminoacyl-tRNA biosynthesis	ko00970	21	60	3.00E-08	9.19E-06	LOC113519875,LOC113519888,LOC113509243,LOC113510661,LOC113520458,LOC113520100,LOC113515837,LOC113519864,LOC113511400,LOC113523495,LOC113522840,LOC113521153,LOC113509314,LOC113514848,LOC113523585,LOC113520711,MSTRG.16445,LOC113510506,LOC113509294,LOC113521599,MSTRG.106
	Human Diseases	Cancers	Central carbon metabolism in cancer	ko05230	12	40	1.59E-04	6.94E-03	LOC113515992,LOC113513679,LOC113519981,LOC113516799,LOC113509732,LOC113517426,LOC113521082,LOC113513806,LOC113516632,LOC113518431,LOC113519518,LOC113517926
	Human Diseases	Cancers	Colorectal cancer	ko05210	11	40	6.81E-04	1.89E-02	LOC113521578,LOC113519518,LOC113517926,LOC113514198,LOC113513806,LOC113521082,LOC113515993,LOC113522456,LOC113518753,LOC113517426,LOC113519981
	Human Diseases	Cancers	Renal cell carcinoma	ko05211	12	49	1.22E-03	2.37E-02	LOC113521578,LOC113519518,LOC113517926,LOC113514198,LOC113520692,LOC113513806,LOC113521082,LOC113518753,LOC113522456,LOC113517426,MSTRG.12222,LOC113516799

	Human Diseases	Cancers	Endometrial cancer	ko05213	9	37	5.12E-03	4.75E-02	LOC113517926,LOC113519518,LOC113521365,LOC113521082,LOC113513806,LOC113521966,LOC113517426,LOC113519981,LOC113515992
	Human Diseases	Endocrine and metabolic diseases	Insulin resistance	ko04931	17	77	4.74E-04	1.61E-02	LOC113510880,LOC113521313,LOC113517426,LOC113520692,LOC113521082,LOC113510529,LOC113511948,LOC113519518,LOC113516157,LOC113516799,LOC113515992,LOC113520116,LOC113516677,LOC113521365,LOC113511193,LOC113513806,LOC113517926
	Human Diseases	Endocrine and metabolic diseases	Type II diabetes mellitus	ko04930	9	33	2.21E-03	2.60E-02	LOC113513801,LOC113517426,LOC113509732,LOC113516157,LOC113511948,LOC113517926,LOC113519518,LOC113521082,LOC113513806
	Human Diseases	Infectious diseases	Kaposi's sarcoma-associated herpesvirus infection	ko05167	13	63	4.02E-03	4.04E-02	LOC113519981,LOC113512866,LOC113515993,LOC113522456,LOC113518753,LOC113517426,LOC113521082,LOC113513806,LOC113515814,LOC113519518,LOC113517926,LOC113521578,LOC113514198
	Human Diseases	Neurodegenerative diseases	Amyotrophic lateral sclerosis (ALS)	ko05014	10	41	3.16E-03	3.57E-02	LOC113517211,LOC113511052,LOC113518534,LOC113514198,LOC113513789,LOC113518753,LOC113520242,LOC113522456,LOC113512866,LOC113515993
	Metabolism	Biosynthesis of other secondary metabolites	Betalain biosynthesis	ko00965	4	7	1.95E-03	2.60E-02	LOC113517095,LOC113510138,LOC113510137,LOC113516392
	Metabolism	Metabolism of other amino acids	Selenocompound metabolism	ko00450	5	11	1.83E-03	2.60E-02	LOC113521153,LOC113518308,LOC113509294,MSTRG.16445,LOC113509243
	Metabolism	Metabolism of	Porphyrin and	ko00860	15	76	3.26E-03	3.57E-02	LOC113510506,LOC113517616,LOC113522294,LOC113520238,LOC113520100,MSTRG

		cofactors and vitamins	chlorophyll metabolism						.9984,LOC113511400,LOC113515837,LOC113519864,LOC113523187,LOC113514848,LOC113509208,LOC113522413,LOC113515355,LOC113520136
	Organismal Systems	Aging	Longevity regulating pathway - multiple species	ko04213	24	83	2.04E-07	3.12E-05	LOC113519419,LOC113517426,LOC113519449,LOC113513789,LOC113519518,LOC113519422,LOC113521082,LOC113519438,LOC113519423,LOC113515861,LOC113513899,LOC113520242,LOC113514600,LOC113520001,LOC113519432,LOC113522123,LOC113519436,LOC113516157,LOC113523060,LOC113520078,LOC113517926,LOC113522003,LOC113513806,LOC113510568
	Organismal Systems	Aging	Longevity regulating pathway - worm	ko04212	19	100	1.59E-03	2.60E-02	LOC113517426,LOC113515884,LOC113509568,LOC113512308,LOC113512515,LOC113514256,LOC113519518,LOC113521082,LOC113515992,LOC113514253,LOC113516157,LOC113510872,LOC113517926,LOC113514254,LOC113522480,LOC113513806,LOC113509269,LOC113520987,LOC113522419
	Organismal Systems	Development	Osteoclast differentiation	ko04380	11	41	8.55E-04	2.18E-02	LOC113519518,LOC113521578,LOC113517926,LOC113514198,LOC113521082,LOC113513806,LOC113512866,LOC113518753,LOC113517426,LOC113522456,LOC113518445
	Organismal Systems	Digestive system	Bile secretion	ko04976	13	56	1.32E-03	2.37E-02	LOC113520845,LOC113512234,LOC113516799,LOC113511534,LOC113522252,LOC113519277,LOC113512920,MSTRG.320,MSTRG.5515,LOC113520666,LOC113514407,LOC113523060,LOC113520078
	Organismal Systems	Digestive system	Fat digestion and absorption	ko04975	8	27	2.18E-03	2.60E-02	LOC113514677,LOC113521924,LOC113511685,LOC113521819,LOC113515508,LOC113515943,LOC113510121,LOC113510575
	Organismal Systems	Endocrine system	Regulation of lipolysis	ko04923	11	37	3.25E-04	1.24E-02	LOC113521579,LOC113517426,LOC113516157,LOC113519518,LOC113517926,LOC113

			in adipocyte						523060,LOC113520078,LOC113513501,LOC113511934,LOC113521082,LOC113513806
	Organismal Systems	Endocrine system	PPAR signaling pathway	ko03320	14	61	9.80E-04	2.30E-02	LOC113513791,LOC113512515,LOC113518438,LOC113515269,LOC113512308,LOC113509568,LOC113519185,LOC113522542,LOC113510872,LOC113523217,LOC113520987,LOC113516677,LOC113521365,LOC113511193
	Organismal Systems	Endocrine system	Estrogen signaling pathway	ko04915	15	78	4.23E-03	4.04E-02	LOC113521918,LOC113519419,LOC113513388,LOC113517426,LOC113510880,LOC113511948,LOC113517926,LOC113519518,LOC113521578,LOC113523060,LOC113520078,LOC113518143,LOC113510568,LOC113521082,LOC113513806
	Organismal Systems	Endocrine system	Prolactin signaling pathway	ko04917	9	36	4.21E-03	4.04E-02	LOC113516487,LOC113517426,LOC113513801,LOC113513806,LOC113521082,LOC113517529,LOC113518143,LOC113519518,LOC113517926
	Organismal Systems	Immune system	Natural killer cell mediated cytotoxicity	ko04650	11	30	3.77E-05	2.88E-03	LOC113517926,LOC113519518,LOC113514198,LOC113518143,LOC113520692,LOC113521082,LOC113513806,LOC113512866,LOC113518753,LOC113517426,LOC113522456
	Organismal Systems	Immune system	B cell receptor signaling pathway	ko04662	10	34	6.65E-04	1.89E-02	LOC113514198,LOC113517926,LOC113519518,LOC113521578,LOC113521082,LOC113513806,LOC113518753,LOC113522456,LOC113517426,LOC113512866
	Organismal Systems	Immune system	Fc epsilon RI signaling pathway	ko04664	9	30	1.05E-03	2.30E-02	LOC113522456,LOC113517426,LOC113518753,LOC113513806,LOC113521365,LOC113521082,LOC113517926,LOC113519518,LOC113514198
	Organismal Systems	Immune system	Fc gamma R-mediated phagocytos	ko04666	13	59	2.18E-03	2.60E-02	LOC113517926,LOC113519518,LOC113511948,LOC113521924,LOC113514198,LOC113518274,LOC113513806,LOC113521082,LOC113521819,LOC113517426,LOC113518753,L

			is						OC113522456,MSTRG.12222
	Organismal Systems	Immune system	Toll-like receptor signaling pathway	ko04620	10	38	1.71E-03	2.60E-02	LOC113521578,LOC113519518,LOC113517926,LOC113514198,LOC113514316,LOC113521082,LOC113513806,LOC113518753,LOC113522456,LOC113517426
	Organismal Systems	Nervous system	Neurotrophin signaling pathway	ko04722	18	73	7.26E-05	3.94E-03	LOC113516157,MSTRG.12222,LOC113518753,LOC113522456,LOC113513806,LOC113521365,LOC113514316,LOC113514198,LOC113521578,LOC113517926,LOC113515814,LOC113517426,LOC113517377,LOC113521082,LOC113520692,LOC113518143,LOC113519518,LOC113511948
Down	Cellular Processes	Cell growth and death	Cell cycle	ko04110	22	93	6.24E-05	1.45E-03	LOC113522457,LOC113517820,LOC113512772,LOC113509441,LOC113516059,LOC113513160,LOC113518339,LOC113514226,LOC113519073,LOC113516864,LOC113522785,LOC113523151,LOC113518799,LOC113519366,LOC113523364,LOC113515557,LOC113511622,LOC113519788,LOC113522607,LOC113522138,LOC113521663,LOC113513508
	Cellular Processes	Cell growth and death	Cell cycle - yeast	ko04111	16	74	1.71E-03	1.93E-02	LOC113518799,LOC113519788,LOC113509343,LOC113522785,LOC113518791,LOC113522607,LOC113521663,LOC113509441,LOC113517820,LOC113512772,LOC113513535,LOC113521414,LOC113514226,LOC113516864,LOC113516059,LOC113513160
	Cellular Processes	Cellular community - eukaryotes	Focal adhesion	ko04510	25	131	7.47E-04	8.76E-03	LOC113514086,LOC113514176,LOC113517042,LOC113521232,LOC113516407,LOC113514128,MSTRG.3420,LOC113519810,LOC113521060,LOC113515727,LOC113523301,LOC113517807,LOC113511104,LOC113516985,LOC113523401,LOC113510563,LOC113522095,LOC113517040,LOC113523364,LOC113513463,LOC113513316,MSTRG.12064,MSTRG.13907,LOC113516521,LOC113510555

	Cellular Processes	Transport and catabolism	Lysosome	ko04142	36	152	3.05E-07	1.55E-05	LOC113511602,LOC113521640,LOC113509107,LOC113523146,LOC113512453,LOC113511409,LOC113522226,LOC113521376,LOC113521660,LOC113512454,LOC113513757,LOC113514851,LOC113512799,LOC113512908,LOC113515907,LOC113517696,LOC113512473,LOC113516446,LOC113519915,LOC113521173,MSTRG.15207,LOC113520468,LOC113510551,LOC113519736,LOC113519668,LOC113521544,LOC113512055,LOC113513473,LOC113518620,LOC113509730,LOC113515484,LOC113517673,LOC113513036,LOC113512953,LOC113522562,LOC113513595
	Environmental Information Processing	Signaling molecules and interaction	ECM-receptor interaction	ko04512	16	48	6.43E-06	2.80E-04	LOC113510563,LOC113516985,LOC113523401,LOC113511104,LOC113515727,LOC113517807,MSTRG.13907,LOC113510555,LOC113513316,MSTRG.12064,LOC113517040,LOC113519524,LOC113514176,LOC113517042,LOC113514128,MSTRG.3420
	Genetic Information Processing	Replication and repair	DNA replication	ko03030	17	40	5.58E-08	4.26E-06	LOC113511622,LOC113519788,LOC113518799,LOC113520523,LOC113517477,LOC113522710,LOC113514099,LOC113521663,LOC113522607,LOC113509701,LOC113511062,LOC113522693,LOC113512070,LOC113517580,LOC113514226,LOC113509770,LOC113516716
	Human Diseases	Cancers	Chemical carcinogenesis	ko05204	21	87	6.66E-05	1.45E-03	LOC113509111,LOC113514175,LOC113514511,LOC113510042,LOC113509573,MSTRG.9521,LOC113514869,LOC113519050,LOC113521944,MSTRG.7478,LOC113522496,LOC113519951,LOC113517750,LOC113514820,LOC113510337,LOC113521642,LOC113511557,LOC113513582,LOC113513733,LOC113513243,LOC113510571
	Human	Infectious	Amoebiasi	ko05146	14	62	2.12E-	2.23E-	LOC113514176,LOC113514086,LOC113517

	Diseases	diseases	s				03	02	807,LOC113517042,LOC113513030,LOC113523401,LOC113513087,MSTRG.3420,LOC113522095,LOC113517040,LOC113514128,LOC113513298,LOC113517972,LOC113521060
	Human Diseases	Neurodegenerative diseases	Prion diseases	ko05020	7	21	2.78E-03	2.83E-02	LOC113522896,LOC113517807,LOC113509680,LOC113514176,MSTRG.3420,LOC113522777,LOC113516390
	Metabolism	Amino acid metabolism	Valine, leucine and isoleucine degradation	ko00280	27	57	2.65E-13	8.08E-11	LOC113521631,LOC113513263,LOC113511682,MSTRG.12093,LOC113515439,LOC113515777,LOC113516587,LOC113518202,LOC113517218,LOC113513750,MSTRG.7238,LOC113510783,LOC113511922,LOC113517603,LOC113521324,LOC113512475,LOC113523136,LOC113520466,LOC113515776,LOC113513883,LOC113511991,LOC113517980,LOC113513912,LOC113512476,MSTRG.3477,LOC113510344,LOC113512912
	Metabolism	Amino acid metabolism	Histidine metabolism	ko00340	8	20	3.41E-04	5.66E-03	LOC113517218,LOC113510344,LOC113512476,LOC113515920,LOC113510783,MSTRG.12093,LOC113515439,LOC113512475
	Metabolism	Amino acid metabolism	Tryptophan metabolism	ko00380	12	41	3.71E-04	5.66E-03	LOC113512475,MSTRG.12093,LOC113515439,LOC113510783,LOC113512476,LOC113510344,LOC113521268,LOC113517217,LOC113520629,LOC113517218,LOC113522496,LOC113517980
	Metabolism	Amino acid metabolism	Arginine and proline metabolism	ko00330	12	42	4.74E-04	6.29E-03	LOC113522141,LOC113516045,LOC113510783,LOC113518105,LOC113515920,LOC113515315,LOC113515439,MSTRG.12093,LOC113512475,LOC113517218,LOC113510344,LOC113512476
	Metabolism	Amino acid metabolis	Lysine degradation	ko00310	15	73	3.97E-03	3.67E-02	LOC113512475,MSTRG.12093,LOC113515439,LOC113510783,LOC113510172,LOC113520017,LOC113509413,LOC113512476,LOC1

		m							13519166,LOC113520629,LOC113510344,LOC113517218,LOC113511192,MSTRG.11123,LOC113517980
	Metabolism	Carbohydrate metabolism	Fructose and mannose metabolism	ko00051	12	43	6.00E-04	7.32E-03	LOC113512738,LOC113510498,LOC113519789,LOC113510666,MSTRG.13946,LOC113518448,LOC113511343,LOC113514609,MSTRG.16486,LOC113515986,LOC113515417,LOC113509985
	Metabolism	Carbohydrate metabolism	Amino sugar and nucleotide sugar metabolism	ko00520	13	58	3.24E-03	3.19E-02	LOC113513595,LOC113518711,LOC113519846,LOC113515907,LOC113518207,LOC113522562,MSTRG.4582,LOC113513475,LOC113521640,LOC113512614,LOC113514621,LOC113521173,LOC113519915
	Metabolism	Carbohydrate metabolism	Pyruvate metabolism	ko00620	12	53	4.21E-03	3.78E-02	LOC113512476,LOC113510344,LOC113518815,LOC113515952,LOC113517218,LOC113515982,LOC113515439,MSTRG.12093,LOC113512475,LOC113516169,LOC113512897,LOC113510783
	Metabolism	Glycan biosynthesis and metabolism	Other glycan degradation	ko00511	17	32	7.56E-10	1.15E-07	MSTRG.8223,LOC113516446,LOC113513595,LOC113514033,LOC113517696,LOC113515907,LOC113522562,LOC113517595,LOC113518620,LOC113519668,LOC113511409,LOC113523146,MSTRG.15207,LOC113520468,LOC113521640,LOC113521173,LOC113519915
	Metabolism	Glycan biosynthesis and metabolism	Glycosphingolipid biosynthesis - globo and isoglobo series	ko00603	9	12	1.28E-07	7.78E-06	LOC113513595,LOC113522562,LOC113515907,LOC113521640,LOC113521173,LOC113513473,LOC113512055,LOC113519915,LOC113521660
	Metabolism	Glycan biosynthesi	Glycosphingolipid	ko00604	6	8	1.98E-05	6.05E-04	LOC113519915,LOC113515907,LOC113521173,LOC113522562,LOC113521640,LOC113

		s and metabolism	biosynthesis - ganglio series						513595
	Metabolism	Glycan biosynthesis and metabolism	Glycosaminoglycan degradation	ko00531	8	18	1.41E-04	2.69E-03	LOC113515907,LOC113522562,LOC113522226,LOC113513595,LOC113519915,LOC113521173,LOC113521640,LOC113509107
	Metabolism	Glycan biosynthesis and metabolism	Other types of O-glycan biosynthesis	ko00514	6	15	1.95E-03	2.12E-02	LOC113517261,LOC113516813,LOC113520017,LOC113516579,LOC113517457,LOC113522547
	Metabolism	Lipid metabolism	Fatty acid degradation	ko00071	21	54	1.03E-08	1.05E-06	LOC113510344,LOC113512476,LOC113513912,LOC113517980,LOC113518106,LOC113515776,LOC113518516,LOC113518256,LOC113512475,LOC113510783,MSTRG.7238,LOC113520629,LOC113513750,LOC113518439,LOC113517218,LOC113515777,LOC113516587,MSTRG.12673,LOC113515439,MSTRG.12093,LOC113518195
	Metabolism	Lipid metabolism	Glycerolipid metabolism	ko00561	20	71	8.63E-06	3.29E-04	MSTRG.12093,LOC113515439,LOC113519789,LOC113522992,LOC113511343,LOC113521660,LOC113515417,LOC113517218,MSTRG.16486,LOC113510498,LOC113512475,MSTRG.13946,LOC113514609,LOC113510783,LOC113512738,LOC113522009,LOC113512476,LOC113510344,LOC113509985,LOC113513510
	Metabolism	Metabolism of other amino acids	beta-Alanine metabolism	ko00410	12	31	1.66E-05	5.62E-04	MSTRG.12093,LOC113515439,LOC113512475,LOC113522141,LOC113510783,LOC113517603,LOC113515920,LOC113510344,LOC113512912,LOC113512476,LOC113517980,LOC113517218
	Metabolism	Metabolis	Glutathion	ko00480	20	77	3.20E-	8.89E-	LOC113519050,LOC113521944,MSTRG.747

		m of other amino acids	e metabolism				05	04	8,LOC113520906,LOC113519951,MSTRG.9521,LOC113509111,LOC113515693,LOC113511017,LOC113517270,LOC113513582,LOC113511557,LOC113513243,LOC113511287,LOC113510571,LOC113515583,LOC113522139,LOC113522141,LOC113521642,LOC113510337
	Metabolism	Metabolism of terpenoids and polyketides	Limonene and pinene degradation	ko00903	6	9	5.46E-05	1.39E-03	LOC113510783,LOC113510344,LOC113512476,LOC113512475,LOC113515439,MSTRG.12093
	Metabolism	Metabolism of terpenoids and polyketides	Insect hormone biosynthesis	ko00981	10	30	3.58E-04	5.66E-03	LOC113509396,LOC113512476,LOC113513699,LOC113510344,LOC113510783,LOC113512475,LOC113512275,MSTRG.12093,LOC113515439,LOC113515876
	Metabolism	Overview	Fatty acid metabolism	ko01212	20	94	5.97E-04	7.32E-03	LOC113515170,LOC113518195,LOC113520500,LOC113516587,MSTRG.12673,LOC113515777,LOC113515169,MSTRG.7238,LOC113521544,LOC113513750,LOC113518439,LOC113512508,LOC113518516,LOC113515776,LOC113522241,LOC113518256,LOC113517980,LOC113513912,LOC113515168,LOC113518106
	Metabolism	Xenobiotics biodegradation and metabolism	Chloroalkane and chloroalkene degradation	ko00625	6	10	1.25E-04	2.55E-03	LOC113512476,MSTRG.12093,LOC113512475,LOC113515439,LOC113510344,LOC113510783
	Metabolism	Xenobiotics	Metabolism of	ko00980	20	86	1.71E-04	3.07E-03	LOC113518095,LOC113510042,LOC113514175,LOC113514511,LOC113509111,MSTRG

		biodegradation and metabolism	xenobiotics by cytochrome P450						.9521,LOC113519951,LOC113514869,LOC113521944,MSTRG.7478,LOC113519050,LOC113510337,LOC113514820,LOC113521642,LOC113517750,LOC113510571,LOC113513243,LOC113513733,LOC113511557,LOC113513582
	Metabolism	Xenobiotics biodegradation and metabolism	Drug metabolism - cytochrome P450	ko00982	18	78	3.95E-04	5.74E-03	LOC113509111,LOC113510042,LOC113514175,MSTRG.9521,LOC113519951,LOC113514869,LOC113519050,MSTRG.7478,LOC113521944,LOC113510337,LOC113514820,LOC113521642,LOC113517750,LOC113510571,LOC113511557,LOC113513582,LOC113513243,LOC113513733
	Metabolism	Xenobiotics biodegradation and metabolism	Styrene degradation	ko00643	3	4	3.42E-03	3.26E-02	LOC113516021,LOC113522679,LOC113516110
	Organismal Systems	Immune system	Complement and coagulation cascades	ko04610	7	16	4.23E-04	5.86E-03	LOC113515727,MSTRG.12064,LOC113513316,LOC113510555,LOC113510563,MSTRG.13907,MSTRG.12821

*Gene number: the number of DEG sorted in the indicated category.

**Background number: the number of all genes sorted in the indicated category.
